# Selective inhibitors of JAK1 targeting an isoform-restricted allosteric cysteine

**DOI:** 10.1038/s41589-022-01098-0

**Published:** 2022-09-12

**Authors:** Madeline E. Kavanagh, Benjamin D. Horning, Roli Khattri, Nilotpal Roy, Justine P. Lu, Landon R. Whitby, Elva Ye, Jaclyn C. Brannon, Albert Parker, Joel M. Chick, Christie L. Eissler, Ashley J. Wong, Joe L. Rodriguez, Socorro Rodiles, Kim Masuda, John R. Teijaro, Gabriel M. Simon, Matthew P. Patricelli, Benjamin F. Cravatt

**Affiliations:** 1 Department of Chemistry, Scripps Research, La Jolla, CA, USA; 2 Vividion Therapeutics, San Diego, CA, USA; 3 Department of Immunology and Microbial Science, Scripps Research, La Jolla, CA, USA

## Abstract

The Janus tyrosine kinase (JAK) family of non-receptor tyrosine kinases includes four isoforms (JAK1, JAK2, JAK3, and TYK2) and is responsible for signal transduction downstream of diverse cytokine receptors. JAK inhibitors have emerged as important therapies for immun(onc)ological disorders, but their use is limited by undesirable side effects presumed to arise from poor isoform selectivity, a common challenge for inhibitors targeting the ATP-binding pocket of kinases. Here we describe the chemical proteomic discovery of a druggable allosteric cysteine present in the non-catalytic pseudokinase domain of JAK1 (C817) and TYK2 (C838), but absent from JAK2 or JAK3. Electrophilic compounds selectively engaging this site block JAK1-dependent *trans*-phosphorylation and cytokine signaling, while appearing to act largely as ‘silent’ ligands for TYK2. Importantly, the allosteric JAK1 inhibitors do not impair JAK2-dependent cytokine signaling and are inactive in cells expressing a C817A JAK1 mutant. Our findings thus reveal an allosteric approach for inhibiting JAK1 with unprecedented isoform selectivity.

Dysregulated cytokine signaling is central to the pathology of a wide range of diseases, including autoimmune and inflammatory conditions, cardiovascular, gastrointestinal and neurodegenerative diseases, and cancer^1,2^. More than 50 different cytokines signal through a family of non-receptor Janus tyrosine kinases (JAKs), which, in humans, consists of JAK1, JAK2, JAK3, and TYK2^1,2^. JAKs associate with the intracellular tail of specific cytokine receptors and are activated by receptor-induced dimerization to phosphorylate themselves in *trans*, the receptor, and downstream signaling proteins, including the STAT family of transcription factors. The specific combination of JAK enzymes and STAT transcription factors that are activated by a given cytokine is cell-type and context-dependent, allowing the JAK–STAT system to regulate diverse biological and disease processes^2^.

The key role of JAK–STAT pathways in immunology and cancer has motivated the pursuit of JAK inhibitors, and many pan-JAK inhibitors have been described^1,3^. These compounds have provided preclinical and clinical evidence that inhibiting JAK–STAT signaling can alleviate aberrant cytokine responses and have established JAKs as important therapeutic targets^1,3^. All of the FDA-approved JAK inhibitors act by an orthosteric mechanism, meaning that they bind to the conserved ATP pocket of the kinase domain, and, even though individual compounds have differing relative selectivity profiles across the JAK family, each inhibits more than one JAK isoform with moderate-to-high potency (half-maximal inhibitory concentration (IC_50_) < 1 μM)^3,4^. This lack of selectivity has important translational implications, as there is growing concern over an array of adverse side effects caused by JAK inhibitors^1,3,4^, including dose-limiting cytope-nias thought to be due to inhibition of JAK2-mediated growth factor receptor signaling^5^ and increased risk of cardiovascular events, prompting the FDA to place a ‘black box’ warning on JAK inhibitors indicated for chronic conditions such as rheumatoid arthritis^6^.

Isoform-selective JAK inhibitors as potential next-generation therapeutics have been pursued by multiple strategies. Covalent inhibitors of JAK3, such as ritlecitinib, have been developed that target a cysteine (C909) uniquely found in the activation loop of this kinase in comparison to other JAKs^7^. While this approach achieves specificity for JAK3 over other JAKs, ritlecitinib cross-reacts with TEC family kinases, which share a cysteine at an equivalent position. JAKs are distinguished from many other kinases by having an additional non-catalytic pseudokinase (JH2) domain, and compounds binding the ATP pocket of the JH2 domain of TYK2 have been found to inhibit this kinase with remarkable functional selec-tivity over other JAKs^8^. One of these agents, BMS-986165 (deuc-ravacitinib), is in late-stage clinical development for autoimmune disorders^9,10^.

In contrast to the progress made on isoform-restricted JAK3 and TYK2 inhibitors, selective JAK1 inhibitors are still lacking. Although some orthosteric JAK1 inhibitors display improved isoform selectivity, these compounds (for example, abrocitinib and filgotinib) still generally show substantial cross-reactivity with JAK2^11–13^. The generation of highly selective inhibitors of JAK1 is an important objective, as several lines of evidence indicate that blockade of this kinase contributes to the efficacy of pan-JAK inhibitors in chronic autoimmune disorders. For instance, gain-of-function *JAK1* mutations promote multi-organ immune dysregulation^14^, while deleterious mutations cause severe immunosuppression in humans^2^. Additionally, JAK1 plays essential and non-redundant roles downstream of class II, γc, and gp130 cytokines^15^, many of which are dysregulated in inflammatory diseases^1,2^. Nonetheless, the precise contribution of JAK1 to homeostatic immune function and disease remains only partly understood owing to a lack of genetic models and selective chemical tools. *JAK1* deletion is perinatal lethal to mice^15^, and consequently, much of our understanding of JAK1 biology has relied on studies with conditional knockout mice lacking JAK1 in specific cell types^16,17^, JAK1-deficient human cell lines^18^ and/or non-selective orthosteric inhibitors^19^.

Here we describe the chemical proteomic discovery of a ligand-able allosteric cysteine in the pseudokinase domain of JAK1 (C817) and TYK2 (C838) but absent from JAK2 and JAK3. An advanced electrophilic compound that engages this allosteric cysteine with high potency and proteome-wide selectivity blocks JAK1 signaling in human cancer cell lines and primary immune cells, while sparing JAK2-dependent pathways. Mechanistic studies indicate that the allosteric inhibition is mediated, at least in part, by disruption of JAK1 *trans*-phosphorylation in cells. Integrating our findings with previous work on allosteric TYK2 inhibitors, such as BMS-986165, points to the potential for leveraging multiple druggable pockets in the pseudokinase domain of JAKs to develop inhibitors with unprecedented isoform selectivity.

## Results

### Discovery of a ligandable allosteric cysteine in JAK1/TYK2

**Extended Data Fig. 1:**
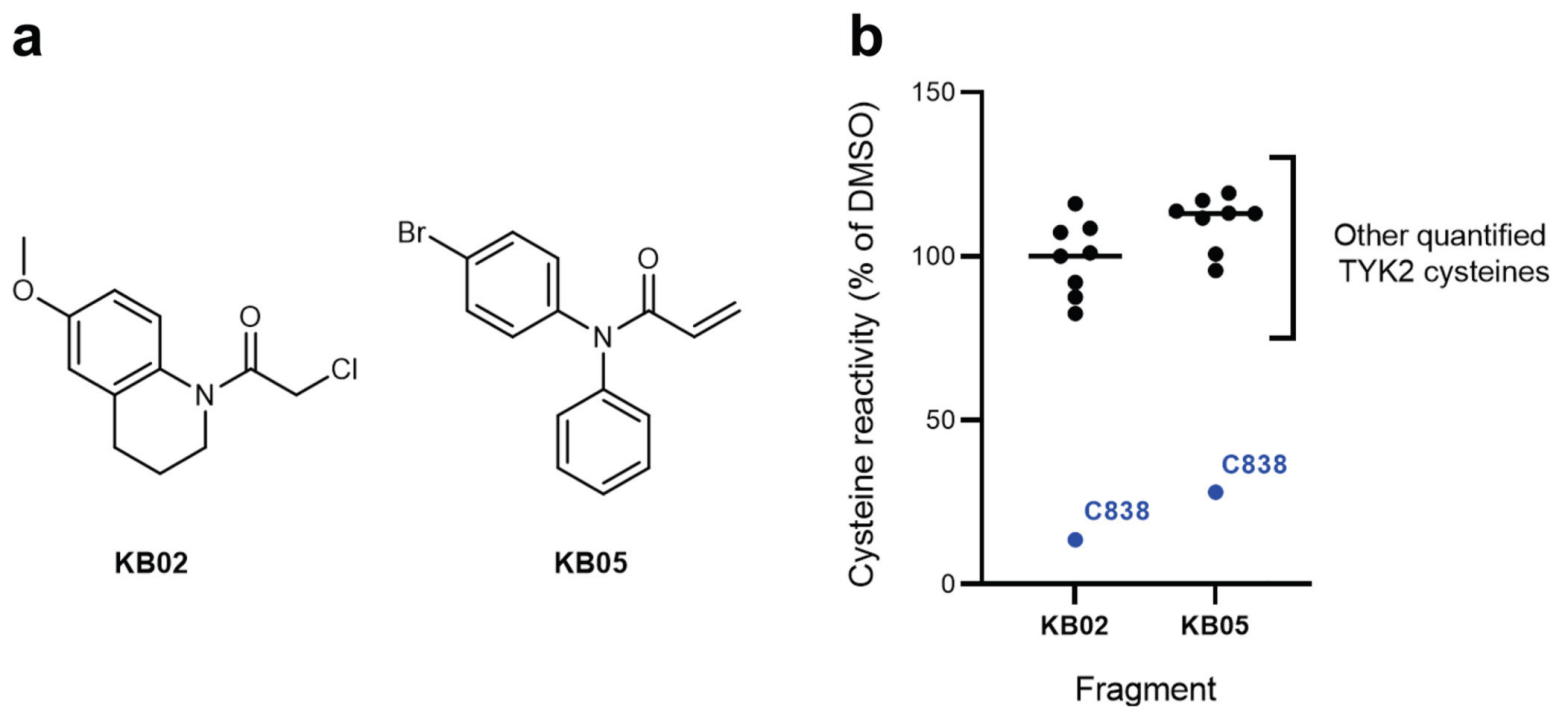
Discovery of a ligandable cysteine in the JAK1/TYK2 pseudokinase domain. **a**, Chemical structures of broadly reactive electrophilic fragments KB02 and KB05 evaluated previously for covalent reactivity with cysteines in the human T-cell proteome (Vinogradova, E. V. *et al*, *Cell* **182**, 1009-1026 e29 (2020), **b**, Relative MS3 signal intensity values for all quantified IA-DTB-labeled, cysteine-containing peptides in TYK2 in KB02-or KB05-treated T cells compared to DMSO-treated T cells. The KB02- and KB05-liganded cysteine in TYK2 (C838) is highlighted in blue. Horizontal black bars indicate the median signal intensity for all other quantified TYK2 cysteines. Data are mean values combined from soluble and particulate proteomic of n **=** 2 (KB02) or n **=** 3 (KB05) independent replicates analyzed over 2 MS-ABPP experiments.

**Fig. 1:**
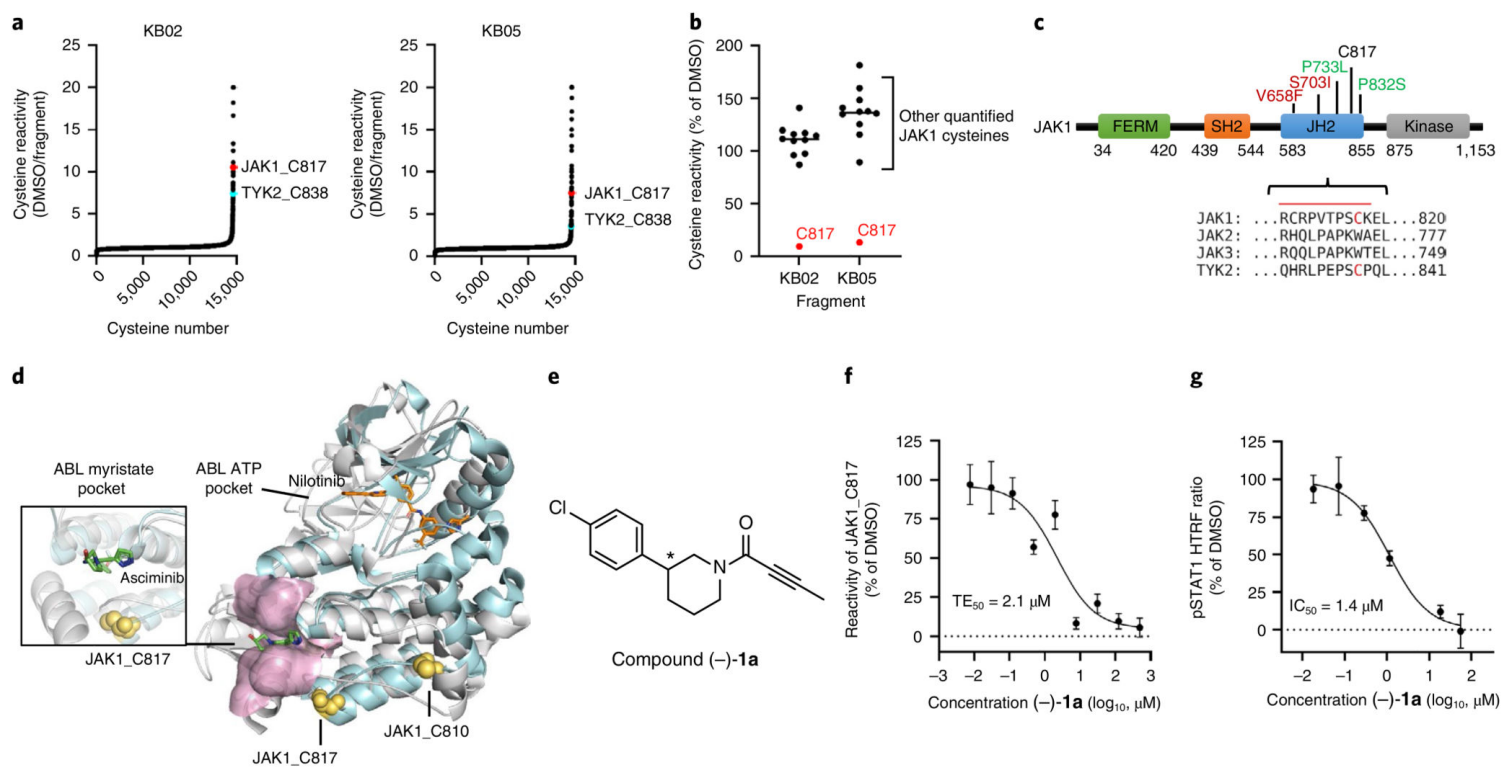
Discovery of a ligandable cysteine in the JAK1/TYK2 pseudokinase domain. **a**, Competition ratios of IA-DTB-labeled and enriched cysteine-containing peptides quantified in MS-ABPP experiments performed using proteomes from human T cells treated in situ with the cysteine-reactive small-molecule fragments KB02 and KB05 (50 **μ**M, 1 h) or DMSO control. **b**, Relative MS3 signal intensity values for all quantified lA-DTB-labeled, cysteine-containing peptides in JAK1 in KB02- or KB05-treated T cells as compared to DMSO-treated T cells. The KB02- and KB05-liganded cysteine C817 is highlighted in red. Horizontal black bars indicate the median signal intensity for all other quantified JAK1 cysteines. **a**,**b**, Data are mean values combined from soluble and particulate proteomes of *n = 2* (KB02) or *n* **=** 3 (KB05) independent replicates analyzed over 2 MS-ABPP experiments as described previously^20^. **c**, Top, domain structure of JAK1, noting C817 and select gain (red)- or loss (green)-of-function mutations in the pseudokinase (JH2) domain. Below, partial amino acid sequence alignment of human JAK family proteins. Electrophilic fragment-liganded cysteines in JAK1 (C817) and TYK2 (C838) are in red. Red line indicates the tryptic peptide containing JAK1_C817. **d**, Overlay of the X-ray crystal structures of the JAK1 JH2 domain (Protein Data Bank (PDB) 4L00) and ABL kinase domain (PDB 5MO4), highlighting the proximity of JAK1 C817 (yellow spheres) to the ABL myristate-binding pocket (pink). The allosteric ABL inhibitor asciminib (green) and orthosteric inhibitor nilotinib (orange) are show in stick representations.**e**, Structure of compound (–)-**1a** (asterisk indicates a single stereoisomer, absolute configuration not assigned). **f**, Concentration-dependent engagement of JAK1_C817 by (–)-**1a** determined by targeted MS-ABPP experiments (1-h compound treatment of human PBMC or Jurkat cell proteome). TE, target engagement. **g**, Concentration-dependent inhibition of IFN**α**-stimulated STAT1 phosphorylation (pSTAT1) by (–)-**1a** in human PBMCs. Cells were treated with (-)-**1a** for 2 h followed by 100ng ml^−1^ of IFN**α** for 30 min, lysed, and pSTAT1 signals measured by HTRF. **f**,**g**, Data are mean **±** s.d. from *n* **=** 3 (**f**) or *n* **=** 2 (**g**) independent experiments.

Previous activity-based protein profiling (ABPP) studies assessing the interactions of electrophilic small-molecule fragments with cysteines in human T cells uncovered a ligandable cysteine shared by JAK1 (C817) and TYK2 (C838)^20^. Both cysteines were substantially engaged by chloroacetamide (KB02) and acrylamide (KB05) frag-ments^20^ (Extended Data Fig. 1a), as determined by mass spectrometry (MS)-ABPP that monitored electrophile-dependent changes in iodoacetamide-desthiobiotin (IA-DTB) reactivity of >10,000 cysteines in the human T-cell proteome (Fig. 1a). The IA-DTB reactivity of other quantified JAK1 and TYK2 cysteines was unaffected by KB02 or KB05 (Fig. 1b and Extended Data Fig. 1b).

JAK1_C817 and TYK2_C838 are located in the JH2 pseudokinase domain (Fig. 1c), which regulates kinase activity of the JH1 domain through allosteric mechanisms and is a hotspot for gain-and loss-of-function mutations^2,21^ (Fig. 1c). We noted that other JAK family members—JAK2 and JAK3—did not share the ligand-able cysteine (Fig. 1c). A closer examination of the JAK1 JH2 crystal structure in comparison to other kinase structures revealed that C817 is proximal to a pocket in the kinase domain of ABL that binds an auto-inhibitory *N*-terminal lipid (myristoylation) modificatio^22^ (Fig. 1d). This pocket in ABL is targeted by the allosteric inhibitor asciminib, which stabilizes the inactive conformation of the kinase and has recently been approved for the treatment of chronic myeloid leukemia^23,24^. Even though asciminib is a reversible inhibitor, and JAK1 and TYK2 are not themselves known to be myristoylated, the proximity of C817/C838 to a pocket that has been exploited to create allosteric drugs for another kinase encouraged us to further characterize the potential functional impact of electrophilic compounds targeting these cysteines.

### Optimization of covalent allosteric JAK1 inhibitors

**Extended Data Fig. 2:**
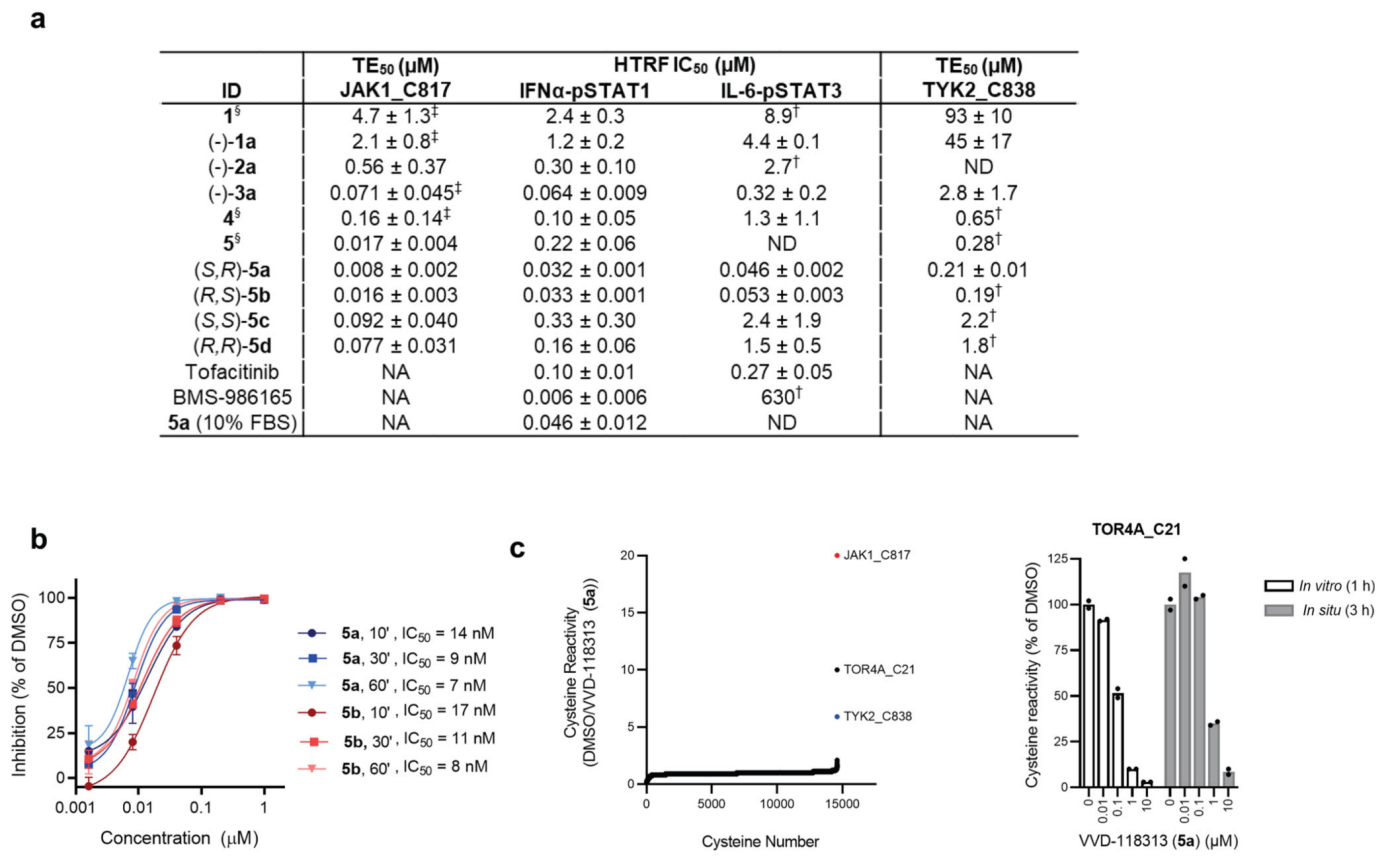
Chemical optimization, characterization and proteome-wide reactivity of VVD-118313 (5a). **a**, Engagement and inhibitory activity of covalent ligands targeting JAK1-C817. Engagement (TE_50_, **μ**M, 1 h, *in vitro*) for JAK1-C817 or TYK2-C838 determined by targeted TMT-ABPP in human cell lysates. Data are mean values **±** S.D. from n **=** 2-3 independent experiments with the exception of values marked with ^†^, which were from a single experiment. JAK1 inhibition (IC_50_) determined using HTRF assays measuring IFN**α** (100 ng/mL, 30 min)-stimulated STAT1 phosphorylation or IL-6 (25 ng/mL, 30 min)-stimulated STAT3 phosphorylation in human PBMCs pretreated with compounds for 2 h. Compounds were tested as single stereoisomers except where noted ^§^. Absolute configuration not assigned for (-)-**1a**, (-)-**2a** and (-)-**3a**. For **5a**, JAK1 inhibition following IFN**α** stimulation was also measured in PBMCs cultured in media supplemented with 10% v/v fetal bovine serum (FBS). Data are mean values **±** S.D. from n **=** 2 independent experiments except where noted (^‡^n **=** 3, ^†^n **=** 1). ND - not determined. NA - not applicable for a non-covalent orthosteric inhibitor. **b**, IC_50_ values for JAK1_C817 engagement by VVD-118313 (**5a**) and enantiomer **5b** in Jurkat T-cell lysate at 10, 30 and 60 minutes. JAK1-C817 engagement was measured by targeted MS-ABPP, where iodoacetamide desthiobiotin (200 **μ**M) was added to cell lysates at the indicated timepoints after incubation with **5a** or **5b**. Data are mean values **±** S.D. of n **=** 2 independent experiments. TE_50_ values were estimated by fitting data to a 4PL model and are reported as 95% confidence interval. **c**, *Left*, Global cysteine reactivity profile for VVD-118313 (1 **μ**M, 1 h, *in vitro*) in primary human PBMC lysates (2 mg/mL proteome). Data represent mean ratio values (DMSO/VVD-118313) for IA-DTB-labeled, cysteine-containing peptides quantified from n **=** 2 replicate cell treatments analyzed in a single MS-ABPP experiment. Ratio values for JAK1-C817 (red) and TYK2-C838 (blue) are highlighted. Quantified cysteines with ratios ⁥ 4 (⁥ 75% engagement) are marked. *Right*, Concentration-dependent reactivity profile for VVD-118313 reactivity with TOR4A_C21 in human PBMCs (0.01-10 **μ**M, 3 h, *in situ*) or PBMC lysates (0.01-10 **μ**M, 1 h, *in vitro*). Bars show mean values from VVD-118313-treated samples shown as a percentage of DMSO-treated samples from n **=** 2 replicate cell treatments analyzed in a single MS-ABPP experiment.

**Fig. 2:**
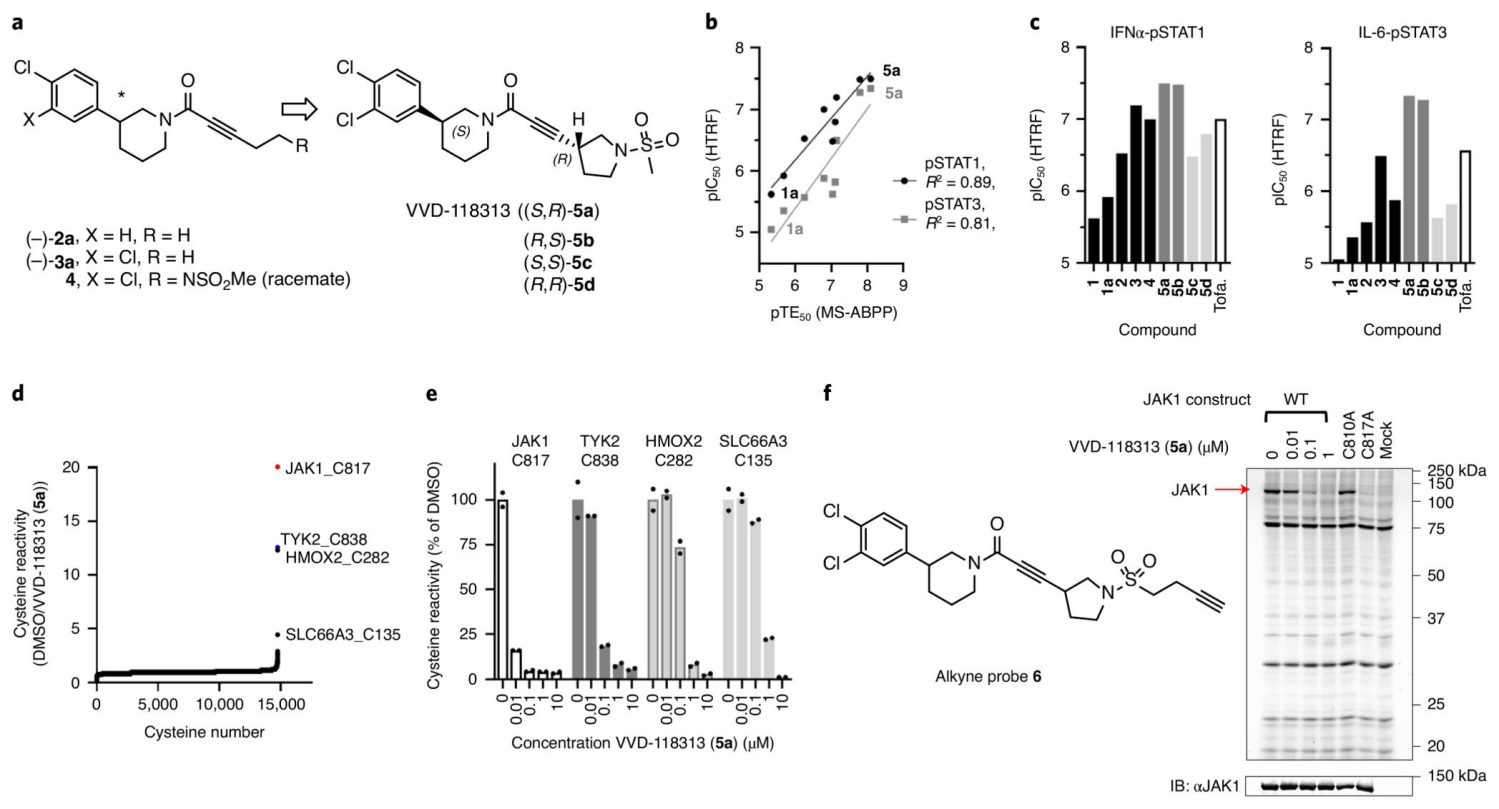
Optimization of covalent allosteric JAK1 inhibitors. **a**, Structures of VVD-118313 (compound **5a**), stereoisomers (**5b**–**d**), and key precursors ((–)- **2a**, (–)-**3a**, and **4**). Compounds (–)-**2a**, (–)-**3a**, and **5a**–**d** were tested as single stereoisomers (asterisk indicates absolute configuration not assigned). **b**, Correlation between engagement of JAK1_C817 (pTE_50_) determined for compounds as described in Fig. 1f and inhibition of cytokine-induced STAT1/3 phosphorylation (pSTAT1/pSTAT3; HTRF pIC_50_) determined for compounds tested as described in Fig. 1g and Extended Data Fig. 2a. *R*^2^ values comparing pTE_50_ values to pIC_50_ values for inhibition of pSTAT1 (black circles) and pSTAT3 (gray squares) were determined by linear regression. **c**, pIC_50_ values for inhibition of IFN**α**-stimulated pSTAT1 and IL-6-stimulated pSTAT3 for representative compounds determined as described in Fig. 1g and Extended Data Fig. 2a. Tofa., tofacitinib. **b**,**c**, Data are mean **–**log-transformed values from *n* = 2 independent experiments. **d**, Global cysteine reactivity profile for VVD-118313 (**5a**) (1 **μ**M, 3 h, in situ) in primary human PBMCs. Data represent mean ratio values (DMSO/VVD-118313) for lA-DTB-labeled, cysteine-containing peptides quantified from *n* **=** 2 replicate cell treatment experiments analyzed in a single MS-ABPP experiment. Ratio values for JAK1_C817 (red) and TYK2_C838 (blue) are highlighted. Quantified cysteines with ratios ≥ 4 (≥75% engagement) are marked. **e**, Concentration-dependent reactivity profiles for cysteines engaged by VVD-118313 in human PBMCs (0.01-10 **μ**M, 3h, in situ). Bars show mean values from VVD-118313-treated cells as a percentage of DMSO-treated cells from *n* **=** 2 replicate cell treatment experiments analyzed in a single MS-ABPP experiment. **f**, Left, structure of alkyne probe **6**. Right, gel-ABPP experiment showing labeling of recombinant WT-JAK1 and C810A-JAK1, but not C817A-JAK1, expressed in 22Rv1 cells with alkyne probe **6** (0.1 **μ**M, 2 h, in situ). The labeling of WT-JAK1 is blocked by pre-treatment with VVD-118313 (0.01-1 **μ**M, 2 h, in situ). Bottom, western blot showing JAK1 expression in-gel ABPP experiment. Data are from a single experiment representative of *n* **=** 2 independent experiments.

We pursued the discovery of more potent and selective covalent ligands for JAK1_C817/TYK2_C838 by screening an internal library of electrophilic compounds using a targeted MS-ABPP assay (Supplementary Table 1). This approach furnished an attractive piperidine butyn-amide fragment hit (–)-**1a** (Fig. 1e) that showed target engagement (TE_50_) values of 2.1 μM and 45 μM for JAK1-C817 and TYK2-C838, respectively (Fig. 1f and Extended Data Fig. 2a). Using a homogeneous time-resolved fluorescence (HTRF) assay in human peripheral blood mononuclear cells (PBMCs), we also found that (–)-**1a** inhibited IFNα-stimulated STAT1 phosphorylation—a JAK1/TYK2-dependent cytokine pathway—with an IC_50_ value of 1.4 μM (Fig. 1g and Extended Data Fig. 2a). The corresponding racemate of (–)-**1a** (compound **1**) was roughly twofold less active in both HTRF and TE assays (Extended Data Fig. 2a). We next synthesized a focused library of (–)-**1a** analogs (Fig. 2a) and screened these compounds for: (i) engagement of JAK1_C817 and TYK2_C838 (TE_50_) in human cell proteomes by targeted MS-ABPP; and (ii) functional activity (IC_50_) on JAK1-dependent signaling pathways (interferon α (IFNα)-STAT1 and interleukin 6 (IL-6)-STAT3) in human PBMCs. We iteratively improved compounds by three orders of magnitude and observed a strong correlation (*R*^2^ values in the range of 0.81–0.89) between TE_50_ values for JAK1_C817 engagement and IC_50_ values for blocking JAK1-dependent STAT phosphorylation (Fig. 2b,c and Extended Data Fig. 2a). Key modifications included extension of the alkynamide group (for example, compound (–)-**2a**), addition of a second chlorine on the phenyl ring (for example, compound (–)-**3a**), incorporation of a methanesulfonamide group to the alkynamide (for example, compound **4**), and subsequent cyclization to a pyrrolidine sulfonamide (for example, compound **5**). The tested compounds generally showed more than tenfold greater potency for engagement of JAK1_C817 as compared to TYK2_C838 (Extended Data Fig. 2a). Separation of the stereo-isomers of compound **5** revealed that the enantiomers (*S*,*R*)**-5a** and (*R*,*S*)-**5b** were substantially more potent than the corresponding diastereomers (**5c** and **5d**) (Fig. 2c and Extended Data Fig. 2a). A more extended analysis of the kinetics of JAK1-C817 engagement revealed that **5a** and **5b** showed time-dependent decreases in TE_50_ values, as expected for covalent ligands, and these TE_50_ values were equivalent for the two enantiomeric compounds at each preincubation time point (Extended Data Fig. 2b). We finally confirmed that **5a** inhibited IFNα-stimulated STAT1 phosphorylation with a similar potency in both serum-free medium or medium supplemented with 10% fetal bovine serum (FBS) (IC_50_ values of 32 and 46 nM, respectively; Extended Data Fig. 2a). On the basis of these data, we selected **5a**, referred to hereafter as VVD-118313, as our lead compound for further functional characterization of allosteric JAK1 inhibition.

### VVD-118313 selectively inhibits JAK1 by engagement of C817

We next evaluated the broader proteomic reactivity of VVD-118313 by untargeted MS-ABPP in human PBMCs. Across >14,000 quantified cysteines, JAK1_C817 was the most potently engaged site by VVD-118313, followed by TYK2_C838, with both cysteines showing near-complete blockade in their IA-DTB reactivity in cells treated with 0.1 μM VVD-118313 for 3 h (Fig. 2d,e and Supplementary Dataset 1). Two additional cysteines (HMOX2-C282, SLC66A3-C135) were engaged by VVD-118313 when tested at a tenfold higher concentration (1 μM; Fig. 2d,e). Similar results were obtained in MS-ABPP experiments that analyzed the in vitro proteome-wide reactivity of VVD-118313 in PBMC lysates, in which JAK1_C817 was again the most potently engaged cysteine, followed by TOR4A_C21, a site that was also engaged in situ, albeit more weakly, and TYK2-C838 (Extended Data Fig. 2c and Supplementary Dataset 1). Taken together, these chemical proteomic data support that VVD-118313 is a potent and selective covalent ligand for JAK1_C817.

To test whether VVD-118313 inhibits JAK1 through engagement of C817, we recombinantly expressed wild-type (WT)-JAK1 and a C817A-JAK1 mutant in the 22Rv1 human prostate cancer cell line, which lacks endogenous JAK1 expression owing to a frameshift mutation in the *JAK1* gene^25^. We also evaluated a C810A-JAK1 mutant, as although C810 is not conserved in TYK2, the JAK1 tryptic peptide quantified in our MS-ABPP experiments contained both C810 and C817 (Fig. 1c). We first treated 22Rv1 cells expressing the JAK1 variants with an alkynylated analog of VVD-118313 (alkyne probe **6** (0.1 μM, 2 h); Fig. 2f) and, after cell lysis, detected **6**-labeled proteins by copper-catalyzed azide–alkyne cycloaddition (CuAAC)^26^ with a rhodamine (Rh)-azide reporter group, followed by SDS-PAGE and in-gel fluorescence scanning. Alkyne probe **6** reacted with WT- and C810A-JAK1, but not C817A-JAK1, and the labeling of WT-JAK1 was blocked in a concentration-dependent manner by pre-treatment with VVD-118313 (Fig. 2f). We interpret these data to indicate that VVD-118313 site-specifically engages JAK1 at C817.

**Extended Data Fig. 3:**
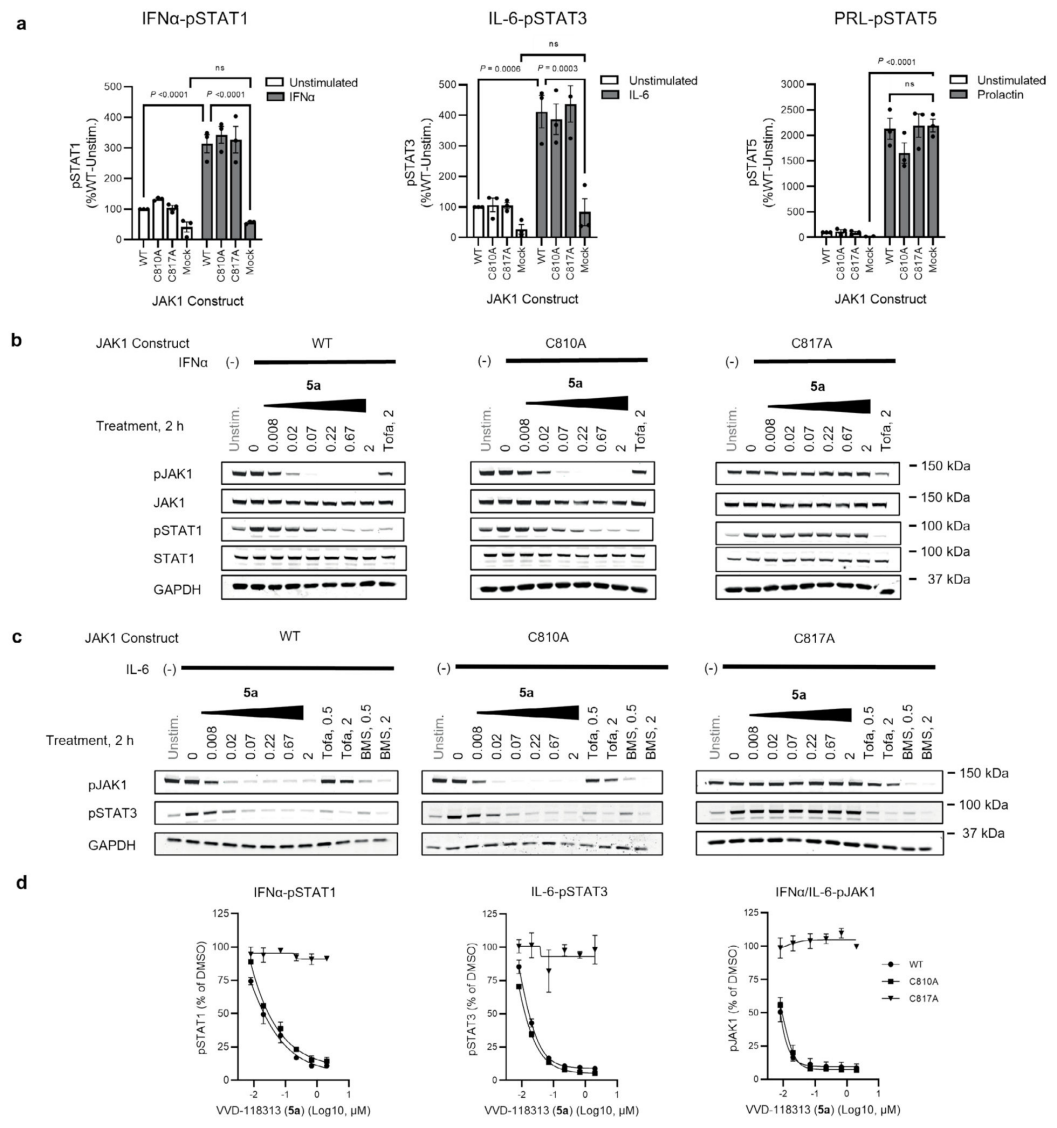
Characterization of VVD-118313 inhibitory activity against JAK1 in 22Rv1 cells. **a**, Quantification of western blotting data measuring cytokine-stimulated STAT phosphorylation in 22Rv1 cells expressing WT-, C810A-, or C817A-JAK1 variants compared to mock-transfected 22Rv1 cells (see Fig. 3b for representative western blots). Cells were treated with IFN**α** (100 ng/mL, 30 min), IL-6 (50 ng/mL, 30 min) or prolactin (PRL, 500 ng/mL, 15 min) after which the indicated phosphorylated STATs (pSTATs) were measured. Signal intensities were normalized to unstimulated 22Rv1 cells expressing WT-JAK1. Data are mean values **±** S.E.M. from n **=** 3 independent experiments. Significance was determined by two-way ANOVA with Tukey’s post hoc test and reported for select comparisons. IFN**α** and IL-6-stimulated STAT1/3 phosphorylation was significantly enhanced by expression of any of the three JAK1 variants (P**<** 0.0001), while prolactin-stimulated STAT5 phosphorylation was unaffected by JAK1 expression. **b**, **c**, Western blots showing concentration-dependent effects of VVD-118313 (**5a**) on IFN**α**-stimulated STAT1 phosphorylation (**b**) and IL-6-stimulated STAT3 phosphorylation (**c**) in 22Rv1 cells expressing WT-, C810A-, or C817A-JAK1 variants. Blots are representative of n **=** 2 independent experiments. **d**, Quantification of concentration-dependent effects of VVD-118313 (**5a**) on IFN**α**-stimulated pSTAT1 (left), IL-6-stimulated pSTAT3 (middle), and pJAK1 (integrated from both IFN**α**- and IL-6-stimulations) in 22Rv1 cells expressing WT-JAK1. Data are mean values **±** S.E.M. from n **=** 2 (pSTAT1, pSTAT3) or n **=** 3 (pJAK1) independent experiments.

**Fig. 3:**
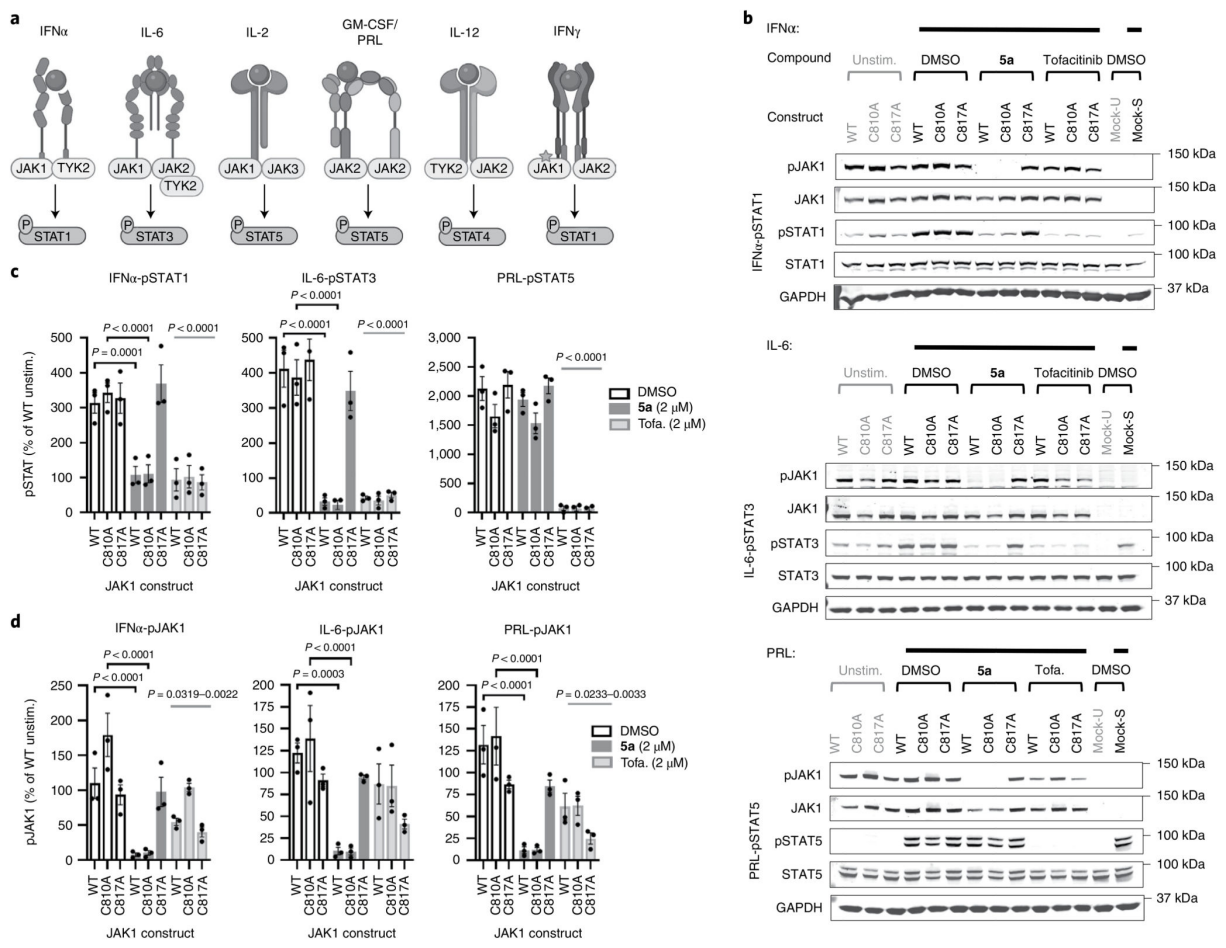
VVD-118313 inhibits JAK1 through engagement of C817. **a**, Representative cytokine signaling pathways involving different JAK family members (asterisk indicates that JAK1 serves a scaffolding role for IFN**γ**-STAT1 signaling)^33^. **b**, Western blots showing effects of VVD-118313 (**5a**) and the pan-JAK inhibitor tofacitinib on JAK1 phosphorylation (pJAK1; Y1034/Y1035 phosphorylation detected with (D7N4Z) Rabbit monoclonal antibody #74129, CST) and IFN**α**-stimulated STAT1 (JAK1-dependent), IL-6-stimulated STAT3 (JAK1-dependent), and PRL-stimulated STAT5 (JAK2-dependent) phosphorylation in 22RvI cells expressing WT-, C810A-, or C817A-JAK1. Cells were treated with compounds (2 **μ**M) for 2 h and then stimulated with IFN**α** (100 ng ml^−1^, 30min), IL-6 (50 ngml^−1^, 30min) or PRL (500 ngml^−1^, 15 min) before analysis. Blots are representative of *n* **=** 3 independent experiments used for quantification. Unstim., unstimulated control. **c**,**d**, Quantification of pSTAT1/3/5 (**c**) and pJAK1 (**d**) signals from **b**. Signal intensities were normalized relative to the unstimulated WT-JAK1-transfected control for each experiment. Data are mean**±** s.e.m. from *n* **=** 3 independent experiments. Significance was determined by two-way ANOVA with Dunnett’s post hoc test and reported relative to stimulated, DMSO-treated control of respective JAK1 construct.

JAK kinases, in various combinations, mediate STAT phosphorylation downstream of different cytokine receptors^3^ (Fig. 3a). We selected a representative subset of these pathways that exhibit JAK1-dependence (IFNα-STAT1 and IL-6-STAT3) or JAK2-dependence (prolactin (PRL)-STAT5)^12^ to evaluate the functional effects and selectivity of VVD-118313. We first verified that recombinant WT-JAK1 and the C810A and C817A mutants equivalently rectified intrinsic defects in IFNα and IL-6 signaling in parental 22Rv1 cells^27,28^, as reflected by the greater IFNα- or IL-6-stimulated STAT1/3 phosphorylation in cells expressing these JAK1 variants as compared to mock-transfected cells (Fig. 3b and Extended Data Fig. 3a). We also noted that all of the JAK1 variants displayed a similar degree of constitutive phosphorylation of the JAK1 kinase domain activation loop (Y1034/Y1035), which was not further increased by cytokine treatment (Fig. 3b). VVD-118313 (2 μM, 2h) blocked IFNα-simulated STAT1 and IL-6-stimulated STAT3 phosphorylation in WT- or C810A-JAK1-expressing 22Rv1 cells, but not in C817A-JAK1-expressing cells (Fig. 3b,c). By contrast, the orthosteric JAK inhibitor tofacitinib equivalently blocked IFNα-simulated STAT1 phosphorylation in cells expressing WT-, C810A-, or C817A-JAK1 (Fig. 3b,c). Interestingly, VVD-118313 also completely blocked the constitutive phosphorylation of WT- and C810A-JAK1 but did not affect the phosphoryla-tion of C817A-JAK1 (Fig. 3b,d). By contrast, tofacitinib only partly (~50%) reduced phosphorylation of all JAK1 variants (Fig. 3b,d). VVD-118313 and tofacitinib further differed in their effects on JAK2-mediated signaling, where VVD-118313 was inactive, while tofacitinib fully inhibited PRL-induced STAT5 phosphorylation (Fig. 3b,c). Concentration-dependent analyses revealed that VVD-118313 maximally inhibited IFNα-STAT1 and IL-6-STAT3 phosphorylation (>80% in each case) in WT- or C810A-JAK1-expressing 22Rv1 cells at ~0.2 μM, while showing negligible impact (<10%) in cells expressing C817A-JAK1 up to 2 μM (Extended Data Fig. 3b–d). VVD-118313 inhibited WT- and C810A-JAK1 phosphorylation with even greater potency than STAT1/STAT3 phosphorylation, showing maximal activity (>90% blockade) at 0.05 μM (Extended Data Fig. 3d), which could indicate that only a small fraction of residually phosphorylated and activated recombinant JAK1 is required to support signal transduction in IFNα/IL-6-stimulated 22Rv1 cells. Together, these data indicate that VVD-118313 acts as a selective allosteric inhibitor of JAK1 through covalent engagement of C817.

**Extended Data Fig. 4:**
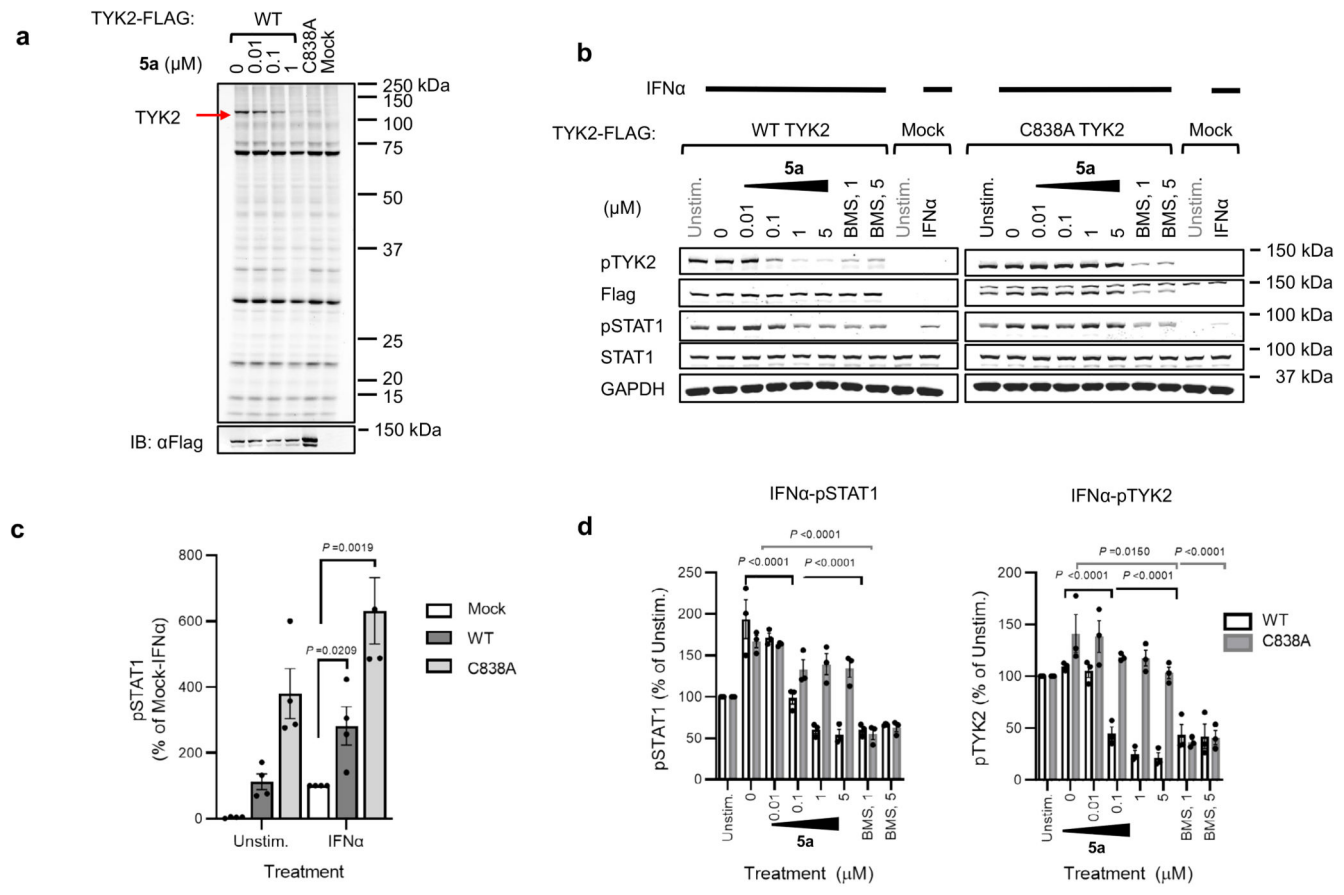
Engagement of TYK2_C838 and inhibition of TYK2-dependent signaling in 22Rv1 cells. **a**, Gel-ABPP experiment showing labeling of recombinant WT-TYK2, but not C838A-TYK2, expressed in 22Rv1 cells by alkyne probe **6** (0.1 **μ**M, 2 h*, in situ*). The labeling of WT-TYK2 was blocked by pretreatment with VVD-118313 (**5a**) (0.01-1 **μ**M, 2 h, *in situ*). We noted that the C838A-TYK2 mutant consistently expressed at higher levels than WT-TYK2, as revealed by the anti-TYK2 immunoblot (bottom). Data are from a single experiment representative of n **=** 2 independent experiments.**b**, Western blots showing concentration-dependent effects of VVD-118313 (**5a**; 0.01 - 5**μ**M, 2 h) and BMS- 986165 (BMS, 1 or 5**μ** M, 2 h) on TYK2 phosphorylation (pTYK2) and IFN**α**-stimulated STAT1 phosphorylation in 22Rv1 cells expressing recombinant WT-TYK2 or a C838A-TYK2 mutant. Blots representative of n **=** 3 independent experiments. **c**, Quantification of IFN**α**-stimulated STAT1 phosphorylation in TYK2 (WT or C838A)-transfected 22Rv1 cells compared to mock-transfected cells. Signal intensities were normalized to IFN**α**-treated (100 ng/mL, 30 min) mock-transfected cells. Data are mean values **±** S.E.M. from n **=** 4 independent experiments. Significance was determined using a two-tailed Student’s *t*-test. **d**, Quantification of pSTAT1 (left) and pTYK2 (right) signals normalized to unstimulated control cells expressing WT-TYK or C838A-TYK2. Data are mean values **±** S.E.M. from n **=** 3 independent experiments. Significance was determined by two-way ANOVA with Dunnett’s post-hoc test. P-values are only shown for the lowest concentration of each compound to inhibit pSTAT1 or pTYK2 S.I. ≥ 50%.

**Extended Data Fig. 5:**
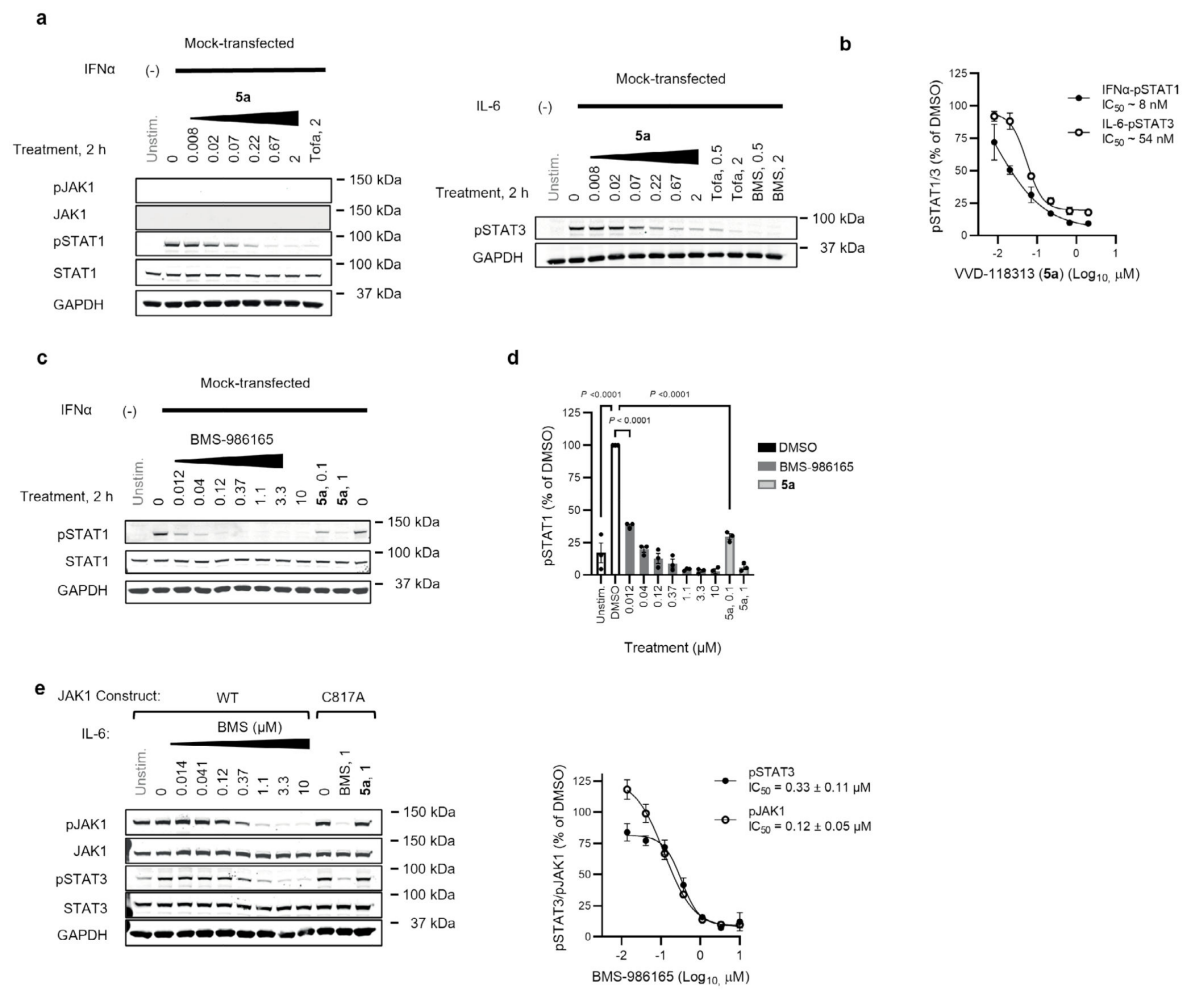
Allosteric JAK1 and TYK2 inhibitors block endogenous signaling in 22Rv1s and JAK1 phosphorylation. **a-d**, Western blots(**a, c**) and quantification (**b, d**) of the effect of VVD-118313 (**5a**), tofacitinib (Tofa), and BMS-986165 (BMS) on IFN**α**-stimulated STAT1 and IL-6-stimulated STAT3 phosphorylation in mock-transfected 22Rv1 cells, which lack JAK1. Unstim, unstimulated controls. **b, d**, Quantification of pSTAT1 signals shown as a percent of the stimulated DMSO-treated control cells for each assay. Data are mean values **±** S.E.M.. from n **=** 2 (**a, b**), or n **=** 3 (**c, d**) independent experiments. Significance was determined by one-way ANOVA with Tukey’s post-hoc test and shown for the lowest concentration to inhibit pSTAT1 S.I. ≥ 50%. **e**, *Left*, Western blots showing concentration dependent effects of BMS-986165 (BMS) on IL-6-stimulated STAT3 phosphorylation and JAK1 phosphorylation in 22Rv1 cells transfected with WT or C817A-JAK1. *Right*, Quantification of pSTAT3 and pJAK1 signal intensity. Western blots are representative, and data are mean values **±** S.E.M., from n **=** 3 independent experiments.

Alkyne probe **6** also reacted with recombinantly expressed WT-TYK2, but not a C838A-TYK2 mutant, in 22Rv1 cells, and pre-treatment with VVD-118313 blocked probe **6** reactivity with WT-TYK2 (Extended Data Fig. 4a). 22Rv1 cells expressing WT- or C838A-TYK2 displayed increased IFNα-induced STAT1 phosphorylation as compared to untransfected 22Rv1 cells (Extended Data Fig. 4b,c), and VVD-118313 (0.01–5 μM, 2 h) blocked this increase in STAT1 phosphorylation in WT-TYK2, but not C838A-TYK2 expressing cells. By contrast, the TYK2 inhibitor BMS-986165 blocked IFNα-dependent STAT1 phosphorylation in both cell populations (Extended Data Fig. 4b, d). We further noted that VVD-118313 and BMS-986165 blocked the weaker IFNα/IL-6-stimulated STAT1/STAT3 phosphorylation in untransfected 22Rv1 cells (Extended Data Fig. 5a–d), suggesting that these pathways are mediated by endogenous TYK2. Similar to what we observed for JAK1, VVD-118313 inhibited phosphorylation of the activation loop of WT-, but not C838A-TYK2 (Extended Data Fig. 4b,d). BMS- 986165 also suppressed TYK2 phosphorylation (Extended Data Fig. 4b,d), as well as JAK1 phosphorylation (Extended Data Fig. 5e), consistent with the documented cross-reactivity of this compound with JAK1 at high-nanomolar concentration^8^; however, the effects of BMS-986165 were independent of TYK2_C838 (Extended Data Fig. 4b,d) and JAK1_C817 (Extended Data Fig. 5e). These data support that VVD-118313 can site-specifically inhibit the signaling of TYK2, at least in the context of a cell line where JAK1 is absent.

### Selective inhibition of JAK1 in primary immune cells

**Extended Data Fig. 6:**
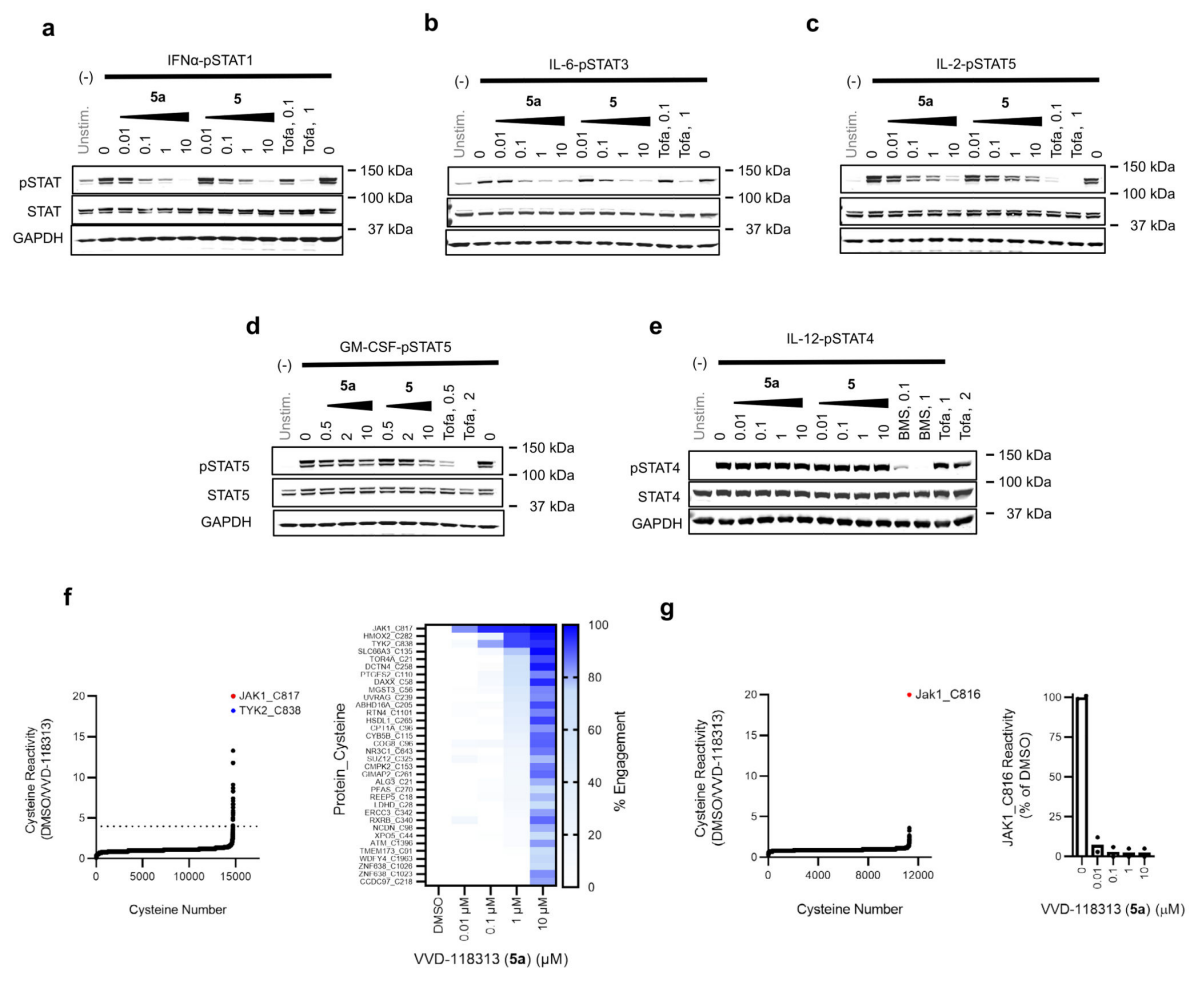
VVD-118313 functional activity and proteomic selectivity in primary immune cells. **a-e**, Western blots corresponding to Fig. 4a-e, showing effects of VVD-118313 (**5a**), stereoisomeric mixture **5**, and tofacitinib (Tofa) on JAK-STAT signaling pathways in human PBMCs and PHA-P/IL-2 generated T-blasts. Cells were treated with compounds at the indicated concentrations for 2 h prior to stimulation with IFN**α** (**a**; 100 ng/mL, 30 min), IL-6 (**b**; 25 ng/mL, 30 min), IL-2 (**c**; 20 U/mL, 15 min), GM-CSF (**d**; 0.5mg/mL, 15 min), or IL-12 (**e**; 12.5ng/mL, 15 min). Blots are representative of n **=** 3 (IL-6, IL-2, IL-12) or n **=** 4 (IFN**α**, GM-CSF) independent experiments. **f**, *Left*, Global cysteine reactivity profile for VVD-118313 (**5a**; 10 **μ**M, 3 h, *in situ*) in primary human PBMCs. Reactivity values for JAK1-C817 (red) and TYK2-C838 (blue) are highlighted, and dashed horizontal line marks boundary for **>** 75% engagement by VVD-118313 at 10**μ**M. *Right*, heat map showing the reactivity profiles for cysteines in PBMCs treated with the indicated concentrations of VVD-118313. Only cysteines that were engaged **>**75% by VVD-118313 at 10 **μ**M are shown. Data in both panels represent mean ratio values (DMSO/VVD- 118313) for IA-DTB-labeled, cysteine-containing peptides quantified from n **=** 2 replicate cell treatments analyzed in a single MS-ABPP experiment. **g**, *Left*, Global cysteine reactivity profile for VVD-118313 (1 **μ**M, 1 h, *in vitro*) in mouse splenocyte lysates. Jak1-C816 shown in red. *Right*, Reactivity of JAK1-C816 in mouse splenocyte lysates treated with the indicated concentrations of VVD-118313 (**5a**; 1 h). Data in both panels represent mean ratio values (DMSO/ VVD-118313) for IA-DTB-labeled, cysteine-containing peptides quantified from n **=** 2 replicate cell treatments analyzed in a single MS-ABPP experiment.

**Fig. 4:**
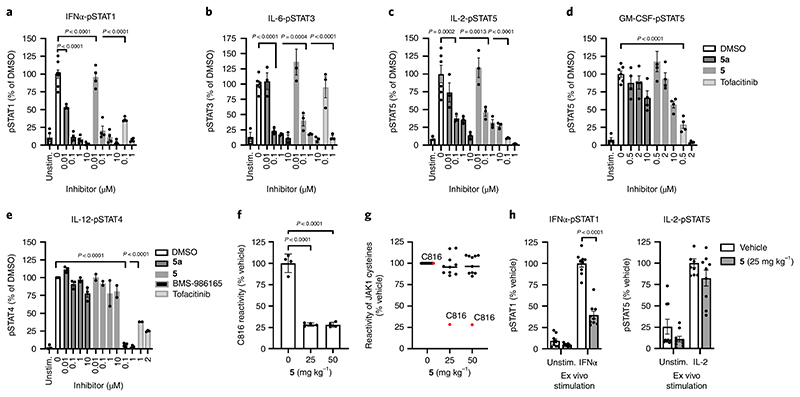
VVD-118313 selectively inhibits JAK1 signaling in primary human immune cells and mice. **a**-**d**, JAK-STAT pathway activity (pSTAT signals measured by western blotting) in human PBMCs treated with the indicated compounds for 2 h before stimulation with IFN**α** (**a**; 100 ng ml^−1^, 30 min), IL-6 (**b**; 25 ngml^−1^, 30min), IL-2 (**c**; 20U ml^−1^, 15 min), or GM-CSF (**d**; 0.5 mgml^−1^, 15 min). Data are mean**±** s.e.m. from *n* **=** 3 (IL-6, IL-2) or *n* **=** 4 (IFN**α**, GM-CSF) independent experiments. Significance determined by one-way ANOVA with Dunnett’s post hoc test. *P* values are only shown for the lowest concentration of each compound that displayed significance for inhibition of pSTAT. **e**, STAT4 phosphorylation in phytohemagglutinin (PHA-P)/ IL-2-generated PBMC-derived T-blasts treated with the indicated compounds for 2h before stimulation with IL-12 (12.5ng ml^−1^, 15 min). Data are mean**±**s.e.m. from *n* **=** 3 independent experiments, except for compound **5**, where data are from *n* **=** 2 experiments. Significance was determined as for (**a**-**d**). See Extended Data Fig. 6a-e for representative western blots of data quantified in panels **a**-**e**. **f**,**g**, Reactivity profiles for JAK1-C816 (**f**) and all quantified JAK1 cysteines (**g**) from spleen proteomic lysates of mice treated with vehicle (dose 0 mg kg^−1^) or compound **5** (25 or 50 mgkg^−1^, subcutaneous (s.c.), 2 **×**4h). Data are mean**±** s.d. from *n* **=** 4 animals/group analyzed in a single targeted MS-ABPP experiment. In **g**, bars represent median reactivity values for all JAK1 cysteines other than C816. **h**, Ex vivo cytokine stimulation of splenocytes from mice treated with vehicle or **5** (25 mg kg^−1^, s.c., 2 **×**4h); IFN**α** (100 ng ml^−1^, 30 min) or IL-2 (20U ml^−1^, 15 min). Data are mean**±**s.e.m. from *n* **=** 3 independent experiments, each containing *n* **=** 3 mice per treatment group. Significance determined by two-way ANOVA with Šidák’s post hoc test. See Extended Data Fig. 8c for representative western blots of data quantified in **h**.

Both VVD-118313 (**5a**) and its mixture of stereoisomers (compound **5**) potently inhibited JAK1-dependent IFNα-pSTAT1, IL-6-pSTAT3, and IL-2-pSTAT5 pathways in human PBMCs, while sparing JAK2- and TYK2/JAK2-dependent signaling (Fig. 4a–e and Extended Data Fig. 6a–e). At concentrations of 0.1 and 1 μM—where VVD-118313 fully engaged JAK1-C817 in human PBMCs (Fig. 2d,e)— the compound near-completely blocked IFNα-stimulated STAT1 and IL-6-stimulated STAT3 phosphorylation (>85% inhibition at 0.1 μM), and partially blocked IL-2-stimulated STAT5 phosphorylation (~70% inhibition at 0.1–1 μM) (Fig. 4a–c), while having no effect on JAK2-dependent GM-CSF stimulated STAT5 phosphorylation (Fig. 4d). When tested at 10 μM—a 100-fold greater concentration than that required to fully engage JAK1-C817—VVD-118313 showed modest inhibitory effects on GM-CSF/JAK2-STAT5 phosphorylation (Fig. 4d). We interpret these data to reflect an off-target activity, as several additional cysteines in the human PBMC proteome were substantially engaged by VVD-118313 at 10 μM (Extended Data Fig. 6f and Supplementary Dataset 1). Compound **5** behaved similarly to VVD-118313, with the expected reduction in potency (Fig. 4a–d) that matched the TE_50_ values measured by MS-ABPP (Extended Data Fig. 2a). The pan-JAK inhibitor tofaci-tinib blocked all of the evaluated cytokine-JAK/STAT pathways at 1–2 μM (Fig. 4a–d). Finally, we found that TYK2/JAK2-dependent IL-12-STAT4 signaling in human PBMC-derived T-blasts was inhibited by both BMS-986165 and tofacitinib, but not VVD-118313 (Fig. 4e). This result differed from the inhibitory activity displayed by VVD-118313 in *JAK1*-null 22Rv1 cells, in which the compound suppressed TYK2-dependent STAT1 phosphorylation and suggests that, under more physiological settings, VVD-118313 does not act as a functional antagonist of TYK2.

**Extended Data Fig. 7:**
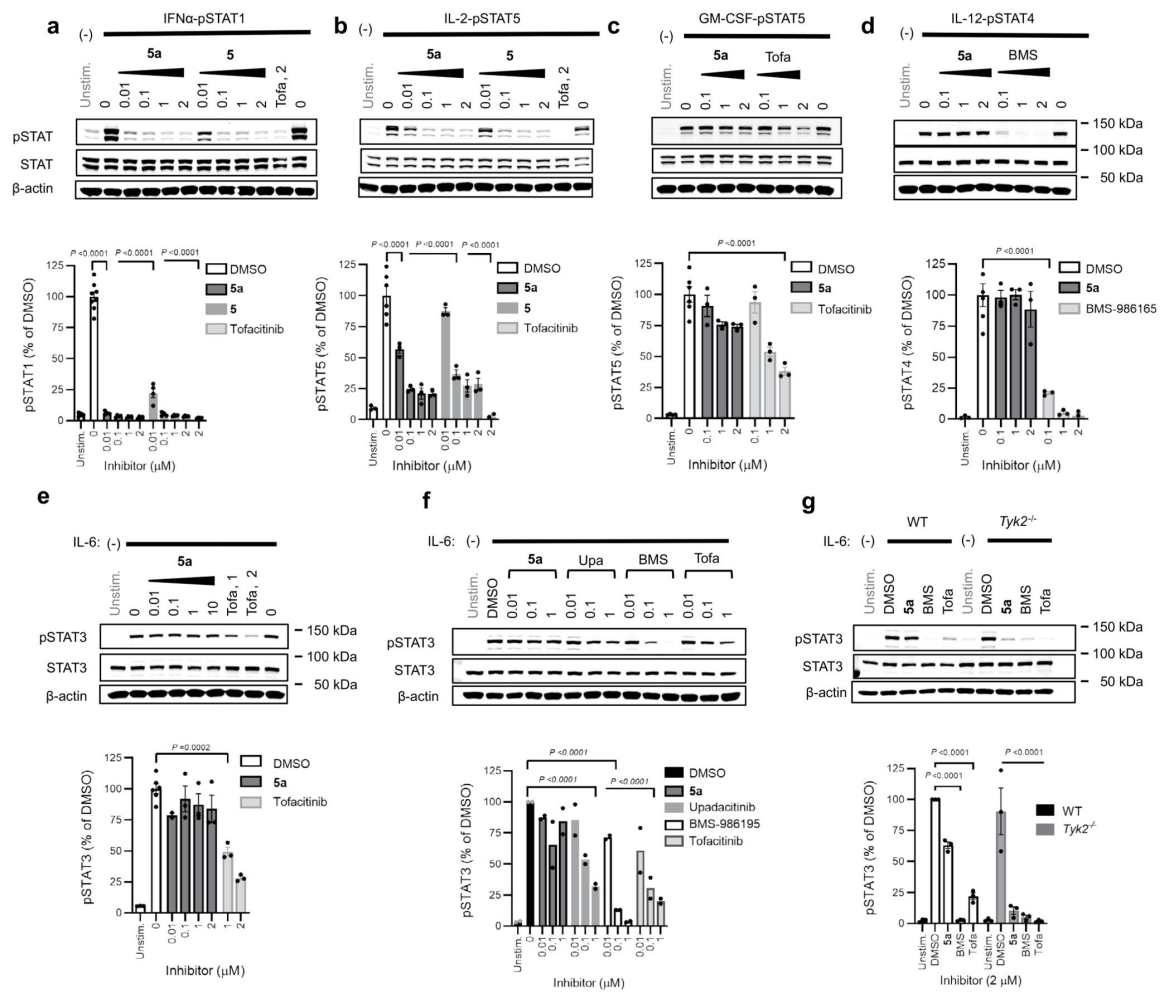
Characterization of the inhibitory activity of VVD-118313 in mouse splenocytes. **a-e**, *Top*, Western blots showing concentration-dependent effects of VVD-118313 (**5a**) and/or compound **5** on IFN**α**-stimulated STAT1 phosphorylation (**a**), IL-2-stimulated STAT5 phosphorylation (**b**), GM-CSF-stimulated STAT5 phosphorylation (**c**), IL-12-stimulated STAT4 phosphorylation (**d**), and IL-6-stimulated STAT3 phosphorylation (**e**) in mouse splenocytes. Tofacitinib (Tofa) and BMS-986165 were also tested where indicated. Splenocytes were treated with compounds at indicated concentration for 2 hours prior to stimulation with IFN**α** (100 ng/mL, 30 min), IL-2 (20 U/mL, 15 min), GM-CSF (10 mg/mL, 15 min), IL-12 (12.5 ng/mL, 15 min) or IL-6 (10 ng/mL, 30 min). *Bottom*, Quantification of pSTAT signals. Signal intensities were normalized relative to stimulated DMSO-treated controls in each assay. Data are mean values **±** S.E.M. from n **=** 3 (IL-2, GM-CSF, IL-12, IL-6) or n **=** 4 (IFN**α**) biologically independent experiments. Significance determined by one-way-ANOVA with Dunnett’s post-hoc test. P-values are shown for the lowest concentration of compound to inhibit S.I.≥ 50%.. **f**, *Top*, Western blot showing effects of a panel of JAK inhibitors on IL-6-stimulated STAT3 phosphorylation in mouse splenocytes. *Bottom*, quantification of pSTAT3 signals performed and analyzed as described in **a-e**. Data are mean values **±** S.E.M. from n **=** 2 independent experiments. **g**, *Top*, Western blot showing effect of JAK inhibitors on IL-6 stimulated STAT3 phosphorylation in splenocytes from *Tyk2*-null mice (*Tyk2^-/-^*) or matched wildtype (WT, *Tyk2^fl/fl^*) mice^1^. *Bottom*, Quantification of pSTAT3 signal intensity normalized to the stimulated DMSO control of WT splenocytes. Data are mean values **±** S.E.M. from n **=** 3 mice. Significance of inhibition relative to DMSO-treatment controls was determined by two-way ANOVA with Tukey’s post-hoc test.

We next evaluated whether the covalent allosteric inhibitors were capable of engaging and inhibiting JAK1 in vivo. We first confirmed by MS-ABPP that VVD-118313 potently and selectively engaged (>90% inhibition at 0.01 μM, 1 h) C816 of mouse JAK1 (the corresponding residue to human JAK1-C817) in splenocyte lysates (Extended Data Fig. 6g and Supplementary Dataset 1). Mouse TYK2 was not targeted by VVD-118313 because this protein possesses a serine residue (S858) in the position corresponding to human TYK2_C838. As in human immune cells, VVD-118313 and compound **5** inhibited both IFNα-dependent STAT1 and IL-2-dependent STAT5 phosphorylation in mouse splenocytes at 0.01–0.1 μM, while sparing GM-CSF-STAT5 and IL-12-STAT4 signaling (Extended Data Fig. 7a–d). One unexpected observation was that VVD-118313 did not inhibit IL-6-STAT3 signaling in mouse splenocytes (Extended Data Fig. 7e), which contrasted with the robust inhibition of this pathway observed in human PBMCs (Fig. 4b). Tofacitinib, upadacitinib, and BMS-986165 all suppressed IL-6-stimulated STAT3 phosphorylation in mouse splenocytes, with BMS-986165 showing the greatest potency (Extended Data Fig. 7f).

This result was initially surprising, as TYK2 knockout (*Tyk2*^−/−^) mice do not show defects in IL-6 signaling^29^. However, as we found that VVD-118313 fully blocked IL-6-dependent STAT3 phosphorylation in *Tyk2*^−/−^ splenocytes^30^ (Extended Data Fig. 7g), we hypothesize that IL-6-stimulated phosphorylation of STAT3 is primarily dependent on TYK2 in WT mouse splenocytes, but, JAK1 likely compensates in *Tyk2*^−/−^ splenocytes to maintain near-wild-type levels of IL-6-STAT3 signaling.

**Extended Data Fig. 8:**
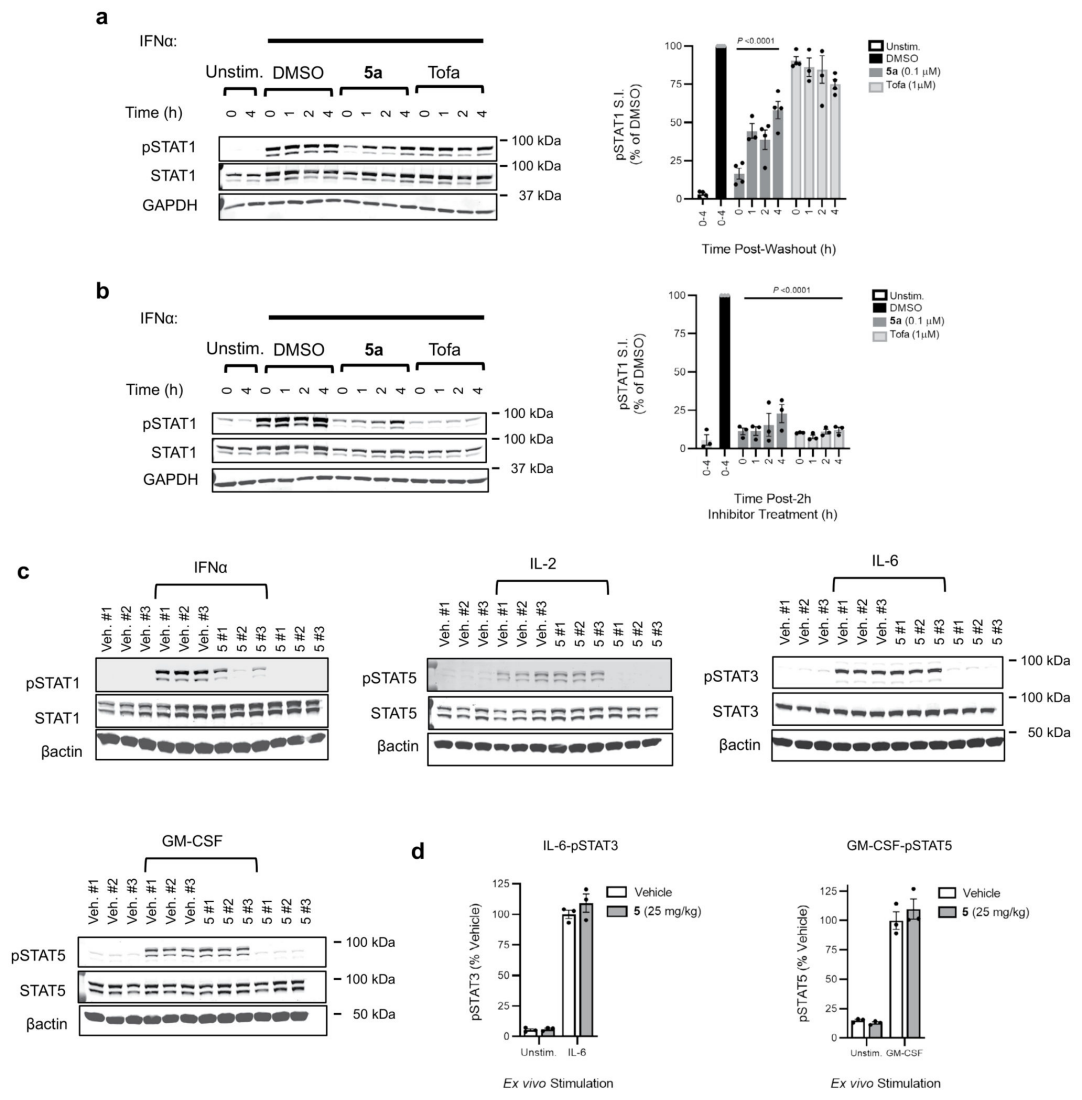
VVD-118313 inhibits JAK1-dependent signaling *ex vivo*. **a**, *Left*, Representative western blots showing recovery of JAK1-mediated STAT1-phosphorylation in human PBMCs that were treated with **5a** (0.1 **μ**M) or tofacitinib (1 **μ**M) for 2 h, then compounds were removed by washing and PBMCs were stimulated with IFN**α** (100 ng/mL, 30 min) at the indicated timepoint post washout. *Right*, Quantification of pSTAT1 signal intensity normalized to DMSO-treated control. Data are mean values **±** S.E.M. from n **=** 4 independent experiments. Significance determined by two-way-ANOVA with Tukey’s post-hoc test. **b**, *Left*, Representative western blots, and *right*, quantification of pSTAT1 signal intensity from equivalent experiment to that described in (**a**), except that media was not exchanged after the first 2 h. Data are mean values **±** S.E.M from n **=** 3 independent experiments. Significance relative to stimulated DMSO-treated control at each time point determined by two-way-ANOVA with Tukey’s post-hoc test. For **a** and **b**, T **=** 0 refers to the time of the washout step performed in **a**. **c**, Western blots containing the results quantified in Fig. 4h, which represents *ex vivo* cytokine-stimulated STAT phosphorylation assays performed in splenocytes from mice treated with vehicle or compound **5** (25 mg/kg, 2 **×**4 h). Splenocytes were stimulated with IFN**α** (1000 U/mL, 30 min), IL-2 (20 U/mL, 15 min), IL-6 (10 ng/mL, 30 min) or GM-CSF (10 ng/mL) prior to analysis of indicated STAT phosphorylation signals. #1-3 correspond to n **=** 3 individual mice per treatment groups. Blots are representative of n **=** 3 (IFN**α**, IL-2) or n **=** 1 (GM-CSF, IL-6) independent experiments. **d**, Quantification of e*x vivo* stimulation of splenocytes from mice treated with vehicle or **5** (25 mg/kg, s.c., 2 **×**4h) with IL-6 (10 ng/mL, 30 min) or GM-CSF (10 ng/mL, 15 min). Data are mean values **±** S.E.M., from n **=** 3 mice analyzed in one experiment.

We next performed in vivo studies using compound **5**, because of the comparable functional activity of the mixture of stereoisomers in primary immune cells to VVD-118313 (Fig. 4a–d) and the relative ease of synthesis. Initial pharmacokinetic studies revealed that compound **5** exhibited a short half-life (0.36 h) and rapid clearance in mice (112 ml min kg^−1^) (Supplementary Data Table 2). Nonetheless, we hypothesized that the covalent mechanism of action of the compound may overcome these suboptimal pharmacokinetic properties to still enable substantial engagement of JAK1_C816 in vivo. ABPP-MS analysis of spleen tissue from mice that were subcutaneously administered compound 5 (25 or 50 mg kg^−1^) in two doses over an 8 h period, revealed 75% engagement of JAK1_C816 at both concentrations, while other JAK1 cysteines were unaffected in their reactivity (Fig. 4f,g). As JAK kinases have moderately short half lives^31,32^, we speculated that JAK1 turnover may contribute to the incomplete engagement of C816 in mouse spleen. Consistent with this conclusion, washout studies in human PBMCs demonstrated that IFNα-stimulated STAT1 phosphorylation (>50%) substantially recovered within 4 h of removing VVD-118313 by exchange of the culture medium (Extended Data Fig. 8a,b). Despite incomplete engagement of JAK1_C816, splenocytes from mice treated with compound **5** showed substantial impairments in IFNα-stimulated STAT1 phosphorylation as compared to vehicle-treated mice (Fig. 4h and Extended Data Fig. 8c). By contrast, IL-2-dependent STAT5 phosphorylation, which was only partially blocked by VVD-118313 or compound **5** when immune cells were treated with compound in vitro (Fig. 4c and Extended Data Fig. 7b), was not substantially altered in splenocytes from mice treated with compound **5** (Fig. 4h and Extended Data Fig. 8c), suggesting that insufficient JAK1 engagement occurred in vivo to impact this pathway. Finally, consistent with our cultured immune cell studies, IL-6-STAT3 and GM-CSF-STAT5 signaling were unaffected in splenocytes isolated from mice treated with compound **5** (Extended Data Fig. 8c,d).

Taken together, our data indicate that covalent ligands engaging human JAK1_C817 (or mouse JAK1-C816) selectively disrupt JAK1-dependent cytokine signaling in human and mouse immune cells and can serve as chemical probes for both cellular and in vivo studies.

### Engagement of C817 blocks JAK1 *trans*-phosphorylation

**Extended Data Fig. 9:**
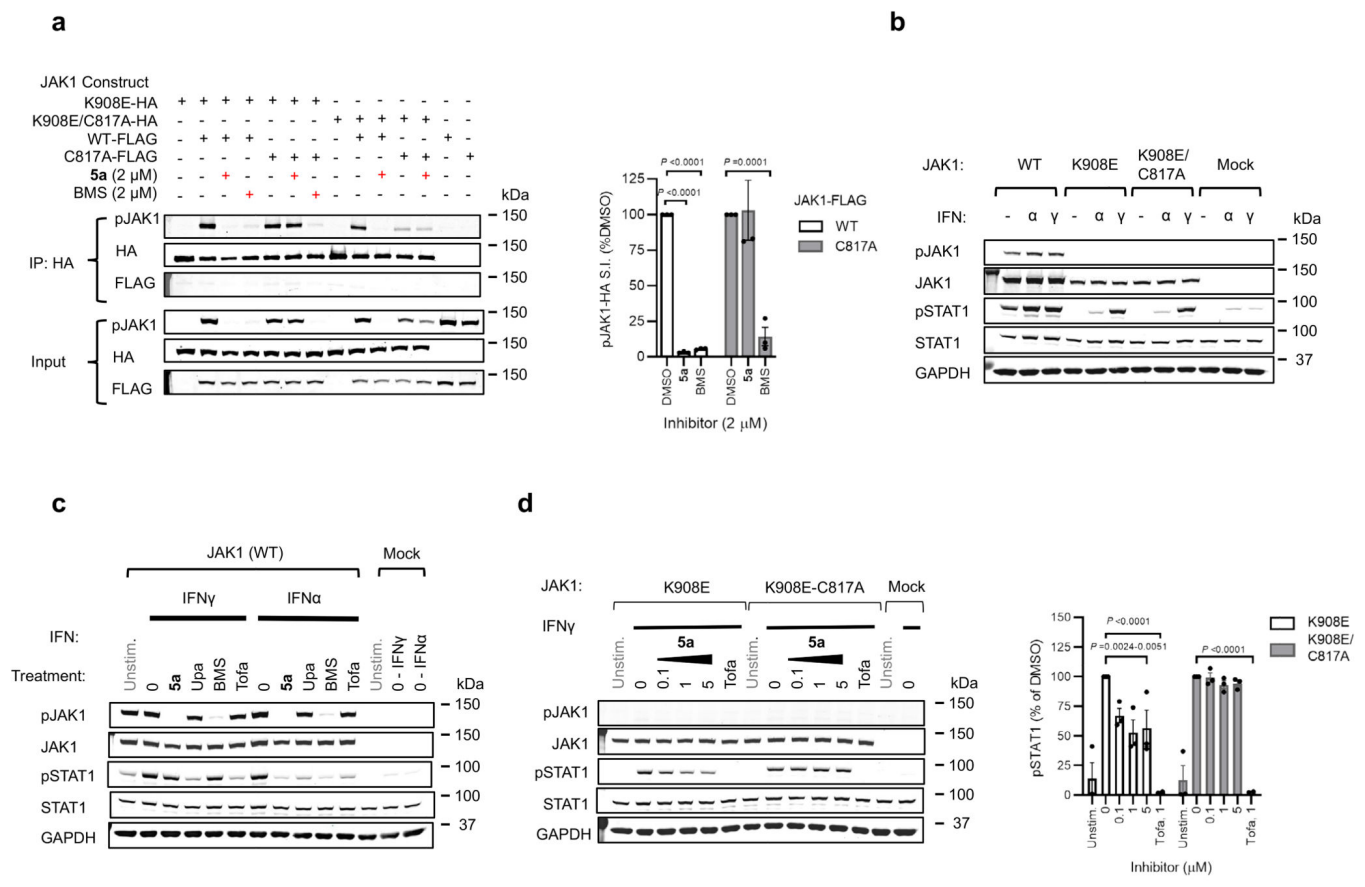
Mechanistic properties of allosteric JAK1 inhibitors. **a**, *Left*, Western blots measuring effects of VVD-118313 (**5a**) and BMS-986165 (BMS) (2 **μ**M, 2 h) on JAK1 phosphorylation (pJAK1) from anti-HA immunoprecipitations (IPs) of HA-tagged kinase dead (K908E) JAK1 (WT or C817A mutant) expressed in 22Rv1 cells alongside catalytically active FLAG-tagged JAK1 (WT or C817A mutant). *Right*, quantification of pJAK1 data, where pJAK1 signals in HA-immunoprecipitation eluent were normalized as a % of the respective DMSO-treated controls. Data are mean values **±** S.E.M. from n **=** 3 independent experiments. Significance determined by two-way ANOVA with Dunnett’s post-hoc test. **b**, Western blots showing that both K908E- and K908E/C817A-JAK1 mutants support IFN**γ**-stimulated (50 ng/mL, 30 min), but not IFN**α**-stimulated (100 ng/mL, 30 min) STAT1 phosphorylation (pSTAT1) in 22Rv1 cells. Blots are representative of n **=** 3 independent experiments. **c**, Westerns blots showing the effects of DMSO, VVD-118313 (**5a**), upadacitinib (Upa), BMS-986165 (BMS) or tofacitinib (all 1 **μ**M, 2 h) on IFN**α** (100 ng/mL, 30 min) or IFN**γ** (50 ng/mL, 30 min)-stimulated STAT1 phosphorylation in WT-JAK1 transfected 22Rv1 cells. Blots are representative of n **=** 3 independent experiments. **d**, *Left*, western blots showing the effects of VVD-118313 (**5a**; 0.1-5**μ**M, 2 h) and tofacitinib (Tofa; 1 **μ**M, 2 h) on IFN**γ**-dependent STAT1 phosphorylation (pSTAT1) in 22Rv1 cells expressing K908E-JAK1-HA or K908E/C817A-JAK1-HA. *Right*, quantification of western blot data. Data are mean values **±** S.E.M. from n **=** 3 independent experiments. Significance determined by two-way ANOVA with Dunnett’s post-hoc test.

**Fig. 5:**
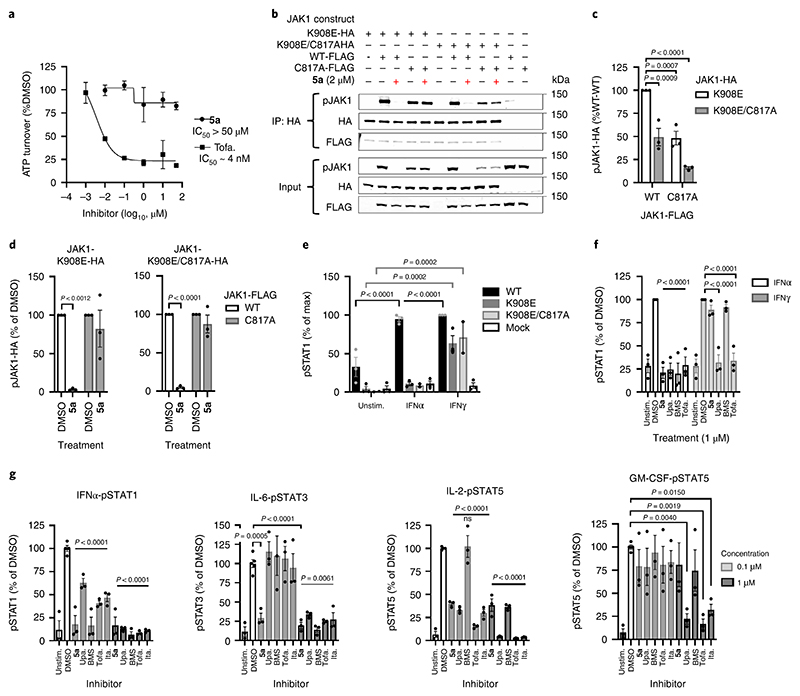
Mechanistic properties of allosteric JAK1 inhibitors. **a**, Substrate assay with recombinant purified JAK1 (residues 438–1154) treated with DMSO, VVD-118313 (**5a**) or tofacitinib (Tofa) (0.001-50 **μ**M, 30min) before addition of an IRS-1 peptide substrate (0.2**μ**g ml^−1^) and ATP (50**μ**M, 1h). Data are mean**±**s.d. from *n* **=** 2 independent experiments. **b**, Western blots measuring JAK1 phosphorylation (pJAK1) from anti-HA immunoprecipitations (IPs) of HA-tagged kinase-dead (K908E) JAK1 (WT or C817A mutant) expressed in 22Rv1 cells alongside catalytically active FLAG-tagged JAK1 (WT or C817A mutant). Blots are representative of *n* **=** 3 experiments. **c**, Quantification of pJAK1 signals from anti-HA IPs from DMSO-treated cells co-expressing the indicated combinations JAK1 constructs. Data are normalized to signals in 22Rv1 cells expressing K908E-JAK1-HA and WT-JAK1-FLAG. **d**, Quantification of pJAK1 signals from anti-HA IPs of 22Rv1 cells treated with either DMSO or VVD-118313 (**5a**; 2**μ**M, 2h) and expressing the indicated combination of JAK1 constructs. Data are normalized to signals in DMSO-treated control cells. **e**, Quantification of STAT1 phosphorylation (pSTAT1) in IFN**α** (100 ng ml^−1^, 30 min) or IFN**γ**-stimulated (50 ngml^−1^, 30min) 22Rv1 cells expressing the indicated JAK1 constructs. pSTAT1 signals were normalized to the maximum signal (IFN**γ**-stimulated WT-JAK1 transfected cells). **f**, Inhibition of IFN**α** (100 ngml^−1^, 30 min)- or IFN**γ** (50 ng ml^−1^, 30 min)-stimulated STAT1 phosphorylation in WT-JAK1 transfected 22Rv1 cells treated with DMSO, VVD-118313 (**5a**), upadacitinib (Upa.), BMS-986165 (BMS) or tofacitinib (1 **μ**M, 2h). **c**-**f**, Data are mean**±** s.e.m. from *n* **=** 3 independent experiments. Significance was determined by two-way ANOVA with Dunnett’s post hoc test (**c**,**d**,**f**) or with Tukey’s post hoc test (**e**). See Extended Data Fig. 9b,c for representative western blots of data quantified in **e**,**f**. **g**, Effects of VVD-118313 (**5a**) and other JAK inhibitors on the indicated cytokine-stimulated pSTAT pathways in human PBMCs as determined by western blotting (see Extended Data Fig. 10a for representative western blots). Data are mean**±** s.e.m. from *n* **=** 2 (IL-6) or *n* **=** 3 (IFN**α**, IL-2, GM-CSF) independent experiments. Significance determined by one-way ANOVA with Šidák’s post hoc test. Ita., itacitinib.

The more extensive blockade of JAK1 phosphorylation by covalent allo-steric inhibitors as compared to orthosteric inhibitors (Fig. 3d and Extended Data Fig. 3b–d) pointed to distinct mechanisms of action for each class of compounds. Also consistent with this premise, VVD-118313 did not inhibit the catalytic activity of recombinant purified JAK1 (amino acids 438–1154, J01–11G, SignalChem) in a peptide substrate assay (VA7207, Promega), whereas tofacitinib displayed a strong inhibitory effect (Fig. 5a). We explored the potential mechanistic basis for blockade of JAK1 phosphorylation by VVD-118313 by evaluating this compound in 22Rv1 cells co-expressing differentially epitope-tagged catalytically active (FLAG-tagged WT or C817A) or inactive (HA-tagged K908E or C817A/K908E) variants of JAK1. We first found that, in the absence of VVD-118313, individually expressed catalytically active WT- and C817A-JAK1-FLAG were robustly auto-phosphorylated in 22Rv1 cells, while the K908E- and K908E/C817A-JAK1-HA variants showed no evidence of phosphorylation in the absence of a co-transfected active JAK1 construct (Fig. 5b). Co-expression with either JAK1-FLAG variant (WT or C817A) led to clear *trans*-phosphorylation of either inactive JAK1-HA variant (K908E or C817A/K908E), although the magnitude of this *trans*-phosphorylation activity was noticeably higher in cells expressing WT-JAK1-FLAG versus C817A-JAK1-FLAG and weakest in cells co-expressing both C817A-JAK1-FLAG and C817A/K908E-JAK1-HA (Fig. 5b,c). VVD-118313 (2 μM, 2 h) completely blocked *trans*-phosphorylation of either inactive JAK1-HA variant (K908E or C817A/K908E) in cells expressing active WT-JAK1-FLAG, but not C817A-JAK1-FLAG (Fig. 5b,d). We also found that BMS-986165 blocked JAK1 *trans-*phosphorylation in a C817-independent manner consistent with the functional activity of the compound on JAK1-dependent cytokine signaling at >0.5 μM (Extended Data Fig. 9a). We interpret these data to indicate that the inhibition of JAK1 *trans*-phosphorylation is relevant to the allosteric mechanism of action of VVD-118313 and that this effect requires the presence of C817 on the donor (phosphorylating), but not the recipient (phosphorylated) JAK1 variant.

### VVD-118313 has a distinct functional profile

Both JAK1 and JAK2 participate in the IFNγ-STAT1 pathway, but only the catalytic activity of JAK2 is required for STAT1 phosphorylation, while the JAK1 pseudokinase domain serves a scaffolding function^33^. We verified that IFNγ signaling in 22Rv1 cells required the expression of recombinant JAK1, but not JAK1 catalytic activity, as reflected in the greater IFNγ-stimulated phosphorylation of STAT1 in cells expressing either WT or kinase-dead (K908E, K908E/C817A) versions of JAK1 as compared to mock-transfected cells (Fig. 5e and Extended Data Fig. 9b). These profiles contrasted with IFNα-mediated STAT1 phosphorylation, which was only supported by WT-JAK1, but not the K908E-JAK1 mutants (Fig. 5e and Extended Data Fig. 9b). Neither VVD-118313 nor the TYK2 inhibitor BMS-986165 altered IFNγ-stimulated STAT1 phosphorylation in 22Rv1 cells, while tofacitinib and upadacitinib completely inhibited this process (Fig. 5f and Extended Data Fig. 9c). Curiously, however, VVD-118313 produced a modest ~40% blockade of IFNγ-stimulated STAT1 phosphorylation in K908E-JAK1-expressing 22Rv1 cells, and this effect was not observed in K908E/C817A-JAK1-expressing 22Rv1 cells, which were otherwise responsive to tofacitinib (Extended Data Fig. 9d). Although, it remains unclear why VVD-118313 partly suppresses IFNγ-stimulated STAT1 phosphorylation mediated by K908E-JAK1, but not WT-JAK1, it is possible that these two JAK1 variants have distinct conformations that are differentially responsive to allosteric inhibitors targeting C817. Regardless, these results suggest that VVD-118313 has only a limited impact on the scaffolding function of JAK1 in IFNγ signaling.

**Extended Data Fig. 10:**
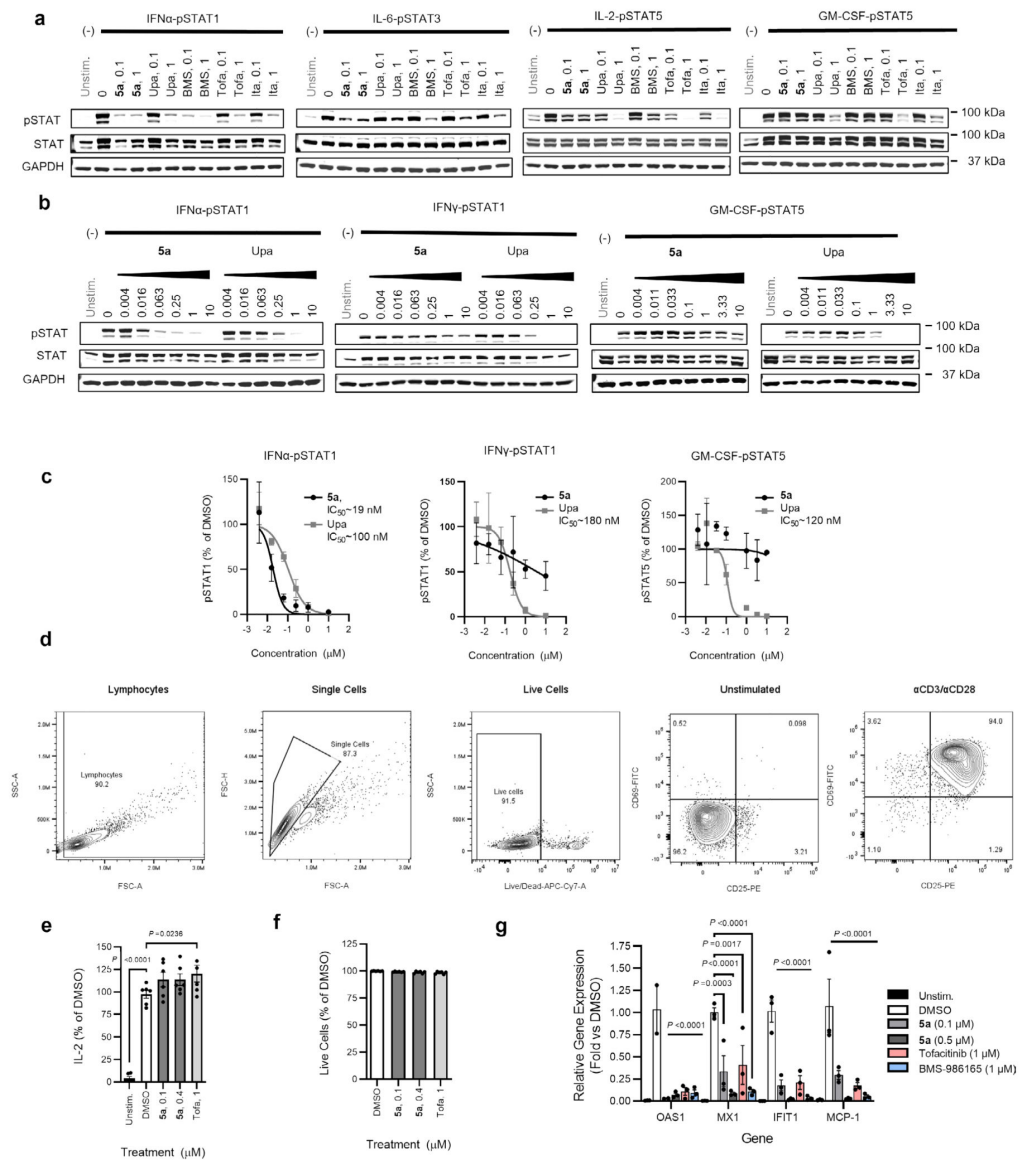
Distinct pharmacological profile and effects of VVD-118313 on JAK/STAT-dependent immune cell processes. **a**, Western blots related to Fig. 5g showing effects of the indicated JAK inhibitors on the indicated cytokine-STAT phosphorylation pathways. Human PBMCs were treated with the compounds - VVD-118313 (**5a**), upadactinib (Upa), BMS-986165 (BMS), tofacitinib (Tofa. and itacitinib (Ita) - at the indicated concentrations (**μ**M) for 2 h and then stimulated with IFN**α** (100 ng/mL, 309min), IL-6 (25ng/mL, 30 min), IL-2 (20 U/mL**<** 15 min) or GM-CSF (0.5ng/mL, 15 minutes). Blots are representative of n **=** 2 (IL-6) or n **=** 3 (IFN**α**, IL-2, GM-CSF) independent experiments. **b**, Western blots showing concentration-dependent effects of VVD-118313 (**5a**) and upadacitinib (Upa) on IFN**α**-stimulated STAT1, IFN**γ**-stimulated STAT1, and GM-CSF-stimulated STAT5 phosphorylation in human PBMCs. Blots are representative of n **=** 2 independent experiments. **c**, Quantification of the concentration-dependent effects of VVD-118313 (**5a**) or upadacitinib (Upa) on pSTAT signals related to (**b**). Data were normalized to the DMSO-treated cytokine-stimulated control in each assay. Dose-response curves are mean values **±** S.D. from n **=** 2 independent experiments. IC_50_ values were estimated by fitting data to a 4PL model. **d**, Flow cytometry plots showing gating strategy for the quantification of CD25 **+** and CD69 **+** T-cells in Fig. 6a, **b**. **e**, Quantification of secreted IL-2 from T cells treated with the indicated concentrations of VVD-118313 (**5a**) or tofacitinib and stimulated with **α**CD3/**α**CD28 (5/2 **μ**g/mL) for 24 h. Data are mean values **±** S.E.M. from n **=** 3 independent experiments and are normalized as a percent of the DMSO-treated stimulated cells from each donor. Significance was determined by two-way ANOVA with Dunnett’s post-hoc test. **f**, Proportion of single cell lymphocyte population staining negative with Near IR Live/Dead cell viability stain. Data are normalized as a percent of the DMSO-treated control are mean values **±** S.E.M. of n **=** 3 biological replicates. **g**, RT-PCR analysis of the expression of the indicated interferon-stimulated genes in PBMCs treated with VVD-118313 (0.1, 0.5 **μ**M), tofacitinib (1 **μ**M) or BMS-986165 (1 **μ**M) for 2 h followed by IFN**α** (100 ng/mL, 16h). Gene expression values were normalized to GAPDH and are reported as fold-change relative to DMSO-treated stimulated control (**ΔΔ**Ct). Data are mean values **±** S.E.M from n **=** 3 independent experiments. Significance determined by two-way ANOVA with Tukey’s post-hoc test.

To further explore the distinct pharmacological profile of VVD-118313, we compared the compound to a set of orthosteric JAK inhibitors (tofacitinib, upadacitinib or itacitinib) in a panel of cytokine-induced STAT phosphorylation assays in human PBMCs. These experiments illuminated a unique pharmacological profile for VVD-118313 that we interpret to reflect the specific contributions of JAK1 to each cytokine signaling pathway, where robust activity of VVD-118313 pointed to pathways that show strong dependence on JAK1 catalytic function for STAT phosphorylation (IFNα-STAT1 and IL-6-STAT3), partial activity of VVD-118313 indicated pathways with a shared dependency on multiple JAK isoforms (for example, JAK1/JAK3-dependent IL-2-STAT5 pathway), and minimal activity of VVD-118313 reflected pathways that are independent of JAK1 (for example, JAK2-dependent GM-CSF-STAT5 and TYK2/JAK2-dependent IL-12-STAT4 pathway) (Fig. 5g and Extended Data Fig. 10a–c). As expected, the orthosteric JAK inhibitors showed pan-activity across the cytokine-STAT phosphorylation assays, while BMS-986165 displayed greatest potency in suppressing IFNα-STAT1 signaling, followed by IL-6-STAT3 and IL-2-STAT5 signaling, and was inactive against GM-CSF-STAT5 signaling (Fig. 5g and Extended Data Fig. 10a). We should note that some studies have pointed to a more dominant role for JAK1 in IL-2 signaling^19,34^, and it is therefore also possible that VVD-118313 shows differential engagement or functional effects on JAK1 in specific cytokine receptor complexes (for example, IFNα-STAT1/IL-6-STAT3 versus IL-2-STAT5).

### VVD-118313 inhibits T-cell activation and cytokine induction

**Fig. 6:**
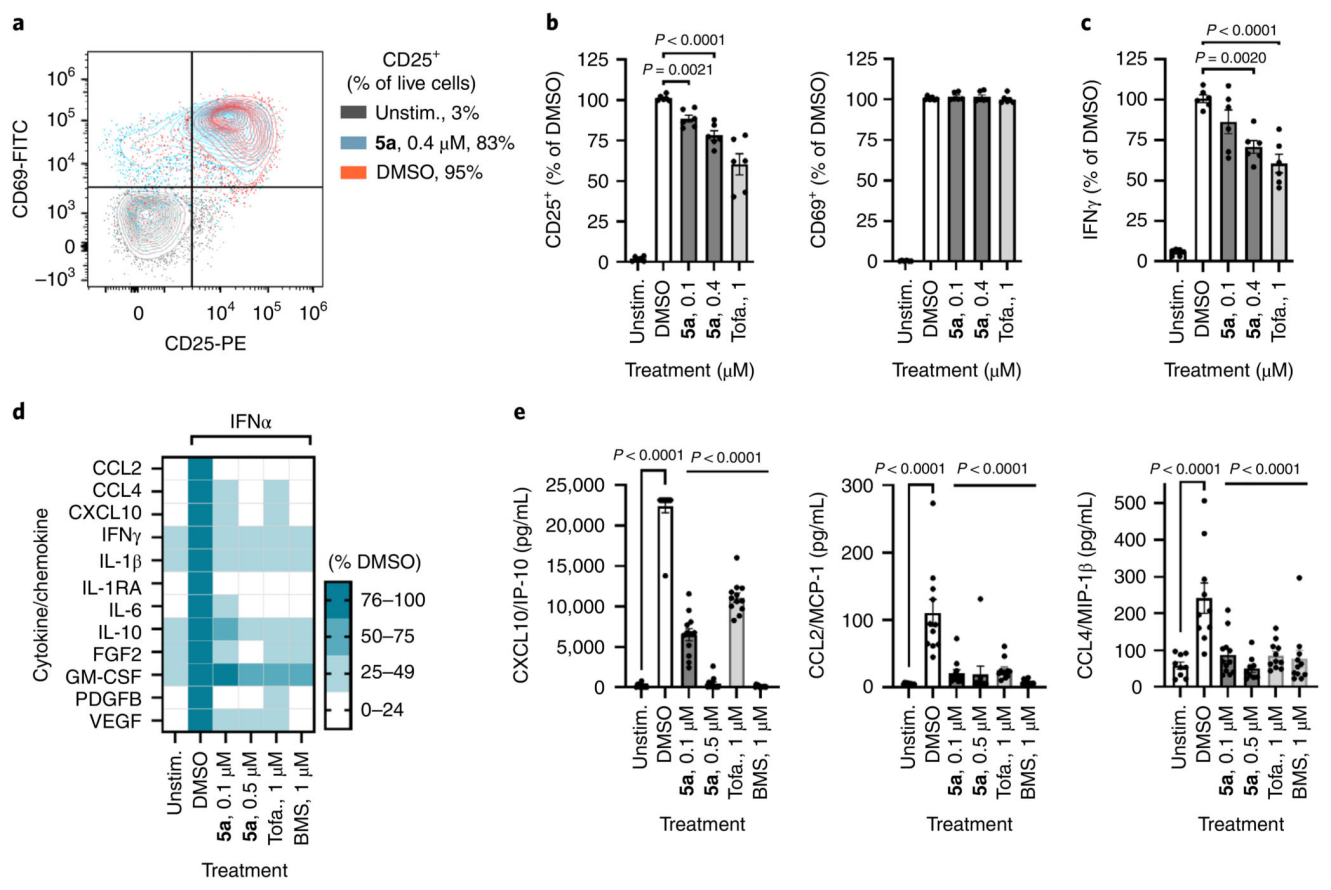
Effects of VVD-118313 on JAK/STAT-dependent immune cell processes. **a**, Representative flow cytometry plot showing CD25 and CD69 expression on unstimulated or **α**CD3/**α**CD28-stimulated primary human T cells treated with DMSO or VVD-118313 (**5a**, 0.4 **μ**M) for 24h. **b**, Quantification of CD25^+^ and CD69^+^ T cells stimulated with **α**CD3/**α**CD28 and treated with the indicated concentration of VVD-118313 or tofacitinib. Data are mean**±**s.e.m. of *n* **=** 3 biological replicates and are normalized as a proportion of the DMSO-treated stimulated cells from each donor. Significance relative to stimulated, DMSO-treated control was determined by two-way ANOVA with Dunnett’s post hoc test. **c**, Quantification of IFN**γ** in T-cell medium measured by ELISA. Data are mean**±**s.e.m. of *n* **=** 3 biological replicates. Cell treatment and data analysis performed as described in **a**,**b**. **d**, Heat map of cytokine/chemokine expression in culture medium from human PBMCs treated with DMSO, VVD-118313 (**5a**, 0.1 or 0.5 **μ**M), tofacitinib (1 **μ**M), or BMS-986165 (1 **μ**M) for 2h, then stimulated with IFN**α** (100 ngml^-1^, 16h). Cytokines were quantified using a 27-plex immunoassay kit and data are shown for cytokines induced by IFN**α** treatment at least twofold versus unstimulated controls across *n* **=** 3 biological replicates. Data are expressed as a proportion of stimulated DMSO-treated cells from *n* **=** 3-4 technical replicates and *n* **=** 3 independent biological experiments. **e**, Quantification of select cytokines/chemokines in PBMC medium treated as described in **d**. Cytokine concentrations are expressed as pg ml^−1^, calculated from standard curves and are mean**±**s.e.m. of *n* **=** 3-4 technical replicates and *n* **=** 3 independent biological donors. Significance relative to stimulated, DMSO-treated control was determined by one-way ANOVA with Dunnett’s post hoc test.

We finally evaluated VVD-118313 in models of immune cell activation and inflammatory responses that have been shown to be sensitive to other JAK inhibitors^35^. We first found that VVD-118313 partially inhibited the activation of human T cells co-stimulated with αCD3/αCD28, as reflected by a reduction in the proportion of CD25^+^ T cells (Fig. 6a,b and Extended Data Fig. 10d). As seen in previous studies using orthosteric JAK inhibitors^35–37^, VVD-118313 also partially blocked the secretion of the Th1-polarizing cytokine IFNγ (Fig. 6c), and slightly increased the production of IL-2 (Extended Data Fig. 10e). The effects of VVD-118313 were qualitatively similar to, but less pronounced than those of tofacitinib (Fig. 6b,c and Extended Data Fig. 10e). Neither compound affected viability of T cells (Extended Data Fig. 10f) or the early-stage activation marker CD69 (Fig. 6b), which aligns with a model where JAK inhibitors spare signaling mediated by T-cell receptors and instead block the secondary action of the IL-2-STAT5 pathway^35,38^. However, we cannot exclude an alternative mechanism in which the compounds directly perturb activation of T cells.

We next assessed the consequences of isoform-restricted JAK1 inhibition on the production of pro-inflammatory cytokines and chemokines (Fig. 6d,e), and the induction of interferon-stimulated genes (Extended Data Fig. 10g), in human PBMCs treated with IFNα (100 ng ml^−1^, 16 h). VVD-118313 near-completely suppressed the induction of several pro-inflammatory chemokines, including CCL2/MCP-1, CXCL10/IP-10, and CCL4/MIP-1β^39^ (Fig. 6d,e), as well as the expression of angiogenic and mitogenic factors, such as VEGF, FGF2, and PDG-FB^39^ (Fig. 6e,f). VVD-118313 also blocked the induction of several interferon-stimulated genes (for example, *OAS1*, *MX1*, *IFIT1*, *CCL2*) (Extended Data Fig. 10g). Similar pharmacological effects were observed with tofacitinib and BMS-986165 (Fig. 6d,e and Extended Data Fig. 10g).

These data support that isoform-restricted allosteric JAK1 inhibitors can impair immune cell activation and functional responses relevant to autoimmune and inflammatory processes.

## Discussion

Despite the potential benefits afforded by allosteric over orthosteric kinase inhibitors, including not only improved selectivity owing to interactions with less conserved pockets, but also avoidance of direct ATP competition for binding, the identification of ligandable and functional allosteric sites remains challenging^40,41^. Allostery is often context dependent and, therefore, may not be detected in more conventional high-throughput assays with purified kinases and simple peptide substrates, especially if these assays only use truncated catalytic domains. Existing allosteric kinase inhibitors have largely been discovered serendipitously or with detailed knowledge of endogenous regulatory mechanisms^40,41^. Here we have shown that chemical proteomics offers a distinct way to discover allosteric inhibitors of kinases.

Our initial mechanistic studies indicate that VVD-118313 may inhibit JAK1 by blocking *trans*-phosphorylation of the activation loop of this kinase. This effect was much stronger for VVD-118313 as compared to orthosteric JAK inhibitors, and we even observed some attenuation of JAK1 *trans*-phosphorylation for the C817A mutant (Fig. 5b,c). Our data thus point to a strong potential for allosteric regulation of JAK1 phosphorylation by the VVD-118313-binding pocket. Considering this pocket mirrors the myristate-binding pocket of ABL^22^, it is tempting to speculate that endogenous metabolites might also bind to JAK1 at this site to regulate kinase activity. Indeed, recent studies suggest that electrophilic metabolites derived from tricarboxylic acid cycle intermediates may modify JAK1_C816 in mouse macrophages^42^. We also wonder how many additional kinases may possess this ligandable pocket and prove amenable to a similar mode of allosteric small-molecule regulation.

The remarkable proteome-wide selectivity displayed by VVD-118313 for JAK_C817 across more than 14,000 quantified cysteines in human and mouse immune cell proteomes supports the broader utility of this compound as a cellular probe to investigate the specific biological functions of JAK1. Indeed, using VVD-118313, we discovered that JAK1 makes differential contributions to IL-6-STAT3 signaling in human PBMCs versus mouse spleno-cytes, a finding that may have been obscured in past experiments with JAK1 inhibitors owing to their lack of isoform selectivity. We also found that the TYK2 inhibitor BMS-986165 was noticeably more potent in blocking IL-6-STAT3 signaling in mouse spleno-cytes in comparison to human PBMCs (Extended Data Fig. 7f and Fig. 5g); results that contrasted with reports that IL-6-STAT3 signaling is unperturbed in *Tyk2*^−/−^ mice^29,43^. By demonstrating that VVD-118313 potently inhibited STAT3 phosphorylation in spleno-cytes from *Tyk2*^−/−^ mice^30^, we provide evidence that JAK1 may compensate for the chronic genetic loss of TYK2 (Extended Data Fig. 7g). Thus, by using a combination of allosteric inhibitors with high isoform selectivity, we have provided evidence for species and/ or immune cell-type differences in the relative contributions of JAK family members to an important cytokine signaling pathway. VVD-118313 should also help to illuminate JAK1 contributions to other signaling pathways, including, for instance, PI3K/AKT, and MAPK/ERK/p38 kinase signaling^44,45^.

Projecting forward, while VVD-118313 was capable of inhibiting JAK1 in mice, the full utility of this chemical probe for in vivo studies would benefit from improvements in its pharmacokinetic properties. One challenge that will need to be overcome is the short half-life of JAK1, which prevents covalent inhibitors from benefiting from a prolonged pharmacodynamic effect observed with slow-turnover protein targets^46^. Nonetheless, covalent chemical probes that are active in vivo and drug candidates have been developed for other JAK family members (for example, JAK3) that have similarly short half lives by optimization of the metabolic stability of these compounds^31^. We also wonder if further exploration of the SAR might uncover compounds that show greater functional activity for TYK2, which could provide an additional class of useful chemical probes that act as dual allosteric JAK1/TYK2 inhibitors. From a translational perspective, covalent allosteric JAK1 inhibitors may circumvent some of the systemic toxicities associated with pan-JAK inhibition in humans^1^. However, it is also possible that selective inhibition of JAK1 may sacrifice a proportion of the efficacy observed with pan-JAK inhibitors^1^. Nonetheless, we are encouraged by the strong pharmacological effects displayed by VVD-118313 in a range of functional assays performed in human PBMCs, where the compound was found to match the activity of pan-JAK inhibitors in suppressing multiple human cytokine pathways (for example, IFNα, IL-6) as measured by phospho-STAT signaling and/or cytokine/chemokine production. Considering that selective inhibitors of JAK3 and TYK2 have demonstrated sufficient pharmacological activity to progress to Phase 3 clinical trials for immune disorders^9,10,47^, we are optimistic that isoform-restricted JAK1 inhibitors may have similar translational potential.

Finally, we believe that our findings provide another compelling example of the utility of chemical proteomics for discovering small molecules that act by unconventional mechanisms^20,48–50^. By evaluating compounds against thousands of sites on endogenously expressed proteins, chemical proteomic platforms like MS-ABPP can uncover ligandable pockets that may be missed by more conventional assays. Nonetheless, chemical proteomics is still principally a binding assay and interpreting how newly discovered small-molecule interactions affect the functions of proteins can be technically challenging. Here we benefited from the availability of robust cell-based activity assays for JAK1 and, in particular, structural information that emphasized the potential functionality of a conserved pocket adjacent to the covalently liganded C817 resi-due^22^. As the structures of more full-length proteins are solved or accurately predicted, the integration of this information with global small-molecule interaction maps furnished by chemical proteomics should facilitate the discovery of additional cryptic functional and druggable allosteric pockets on a broad range of proteins.

## Methods

### Antibodies, cytokines and inhibitors

For western blotting protocols phospho-JAK1 (Tyr1034/1035) (D7N4Z) (#74129), phospho-TYK2 (Tyr1054/1055) (D7T8A) (#68790), TYK2 (D4I5T) (#14193), phospho-STAT1 (Tyr701) (58D6) (#9167), phospho-STAT3 (Tyr705) (D3A7) XP (#9145), phospho-STAT4 (Tyr693) (D2E4) (#4134), phospho-STAT5 (Tyr694) (C11C5) (#9359), STAT1 (D1K9Y) (#14994), STAT3 (79D7) (#4904), STAT4 (C46B10) (#2653), STAT5 (D2O6Y) (#94205), HA-Tag (C29F4) (#3724) and β-actin (13E5) (#4970) rabbit monoclonal antibodies were obtained from Cell Signaling Technologies. Anti-JAK1 antibody (#610231, BD Transduction Laboratories), anti-Flag M2 antibody (#F1804, Sigma) and anti-GAPDH (#sc-47724, Santa Cruz Biotechnology) mouse antibodies were sourced as indicated. Secondary IRDye antibodies for western blot: 800CW Goat anti-Mouse IgG (#926-32210), 800CW Donkey anti-Rabbit IgG (#926-32213), 680LT Goat anti-Mouse IgG (#926-68020) and 680LT Donkey anti-Rabbit IgG (#926-68023) were purchased from Li-Cor. For flow cytometry, FITC anti-human CD69 (#310904) and PE anti-human CD25 (#302606) were obtained from Biolegend.

Commercial JAK inhibitors BMS-986165 (HY-117287, MedChemExpress), tofacitinib (S2789, Selleckchem), upadacitinib (NC1927829, Fisher Scientific) and itacitinib (INCB39110) (501948171, Selleckchem) were obtained from commercial vendors.

Recombinant human IFNα (#11101-2) and mouse IFNα (#12100-1) were purchased from PBL Assay Sciences. Recombinant IL-2 was purchased from Hoffman-La Roche (TECIN Teceleukin, Bulk Ro 23-6019). All other cytokines were purchased from R&D Biosystems: recombinant human IFNγ (#285-IF-100/ CF), IL-6 (#206-IL-010/CF), IL-12 (#219-IL/CF), GM-CSF (#7954-GM-010/CF), prolactin (#682-PL-050), and recombinant mouse IL-6 (#406-ML-005/CF), IL-12 (#419-ML-010/CF), GM-CSF (#415-ML-005/CF).

Cell lysates for western blotting were prepared using mPER mammalian protein extraction reagent (78501, ThermoFisher), unless otherwise noted. All lysis buffers were supplemented with cOmplete EDTA-free Protease Inhibitor Cocktail tablets (#11873580001, Roche) and PhosSTOP phosphatase inhibitor cocktail tablets (#4906837001, Roche). Protein concentration in cell lysates were determined using DC assay reagents (#5000113, #5000114, Bio-Rad), normalizing to a bovine serum albumin standard curve.

### DNA constructs and transfection

pCMV6-JAK1-Myc-Flag (RC213878) and pCMV6-TYK2-Myc-Flag (RC204351) vectors were obtained from Origene. Mutant JAK1 (C810A, C817A, K908E, K908E/C817A) and TYK2 (C838A) constructs were generated by site-directed mutagenesis, and HA-tagged constructs were generated by epitope-tag insertion using the Q5 Site-Directed Mutagenesis kit (New England BioLabs, E0552S). All constructs were confirmed by Sanger sequencing (Azenta). Transfections were performed using polyethylenimine (PEI-MAX, Polysciences, #24765).

### Cell lines, primary cells, mice

22Rv1 human prostate cancer cells (#CRL-2505) and Jurkat human T cells (Clone E6-1, #TIB-152) were purchased from the American Type Culture Collection and cultured at 37 °C and 5% CO_2_ in complete RPMI-1640 (supplemented with L-glutamine (2 mM), penicillin (100 U ml^−1^), streptomycin (100 μg ml^−1^) and 10% vol/vol fetal bovine serum (FBS)). 22Rv1 cells were passaged every 3 days using trypsin and seeded into new 6–12-well tissue culture plates at the stated densities for assays. 22Rv1 cell treatments were performed in serum-free RPMI.

Human peripheral blood mononuclear cells (PBMCs) for in vitro MS-ABPP experiments were isolated from leukopaks (AllCells, # LP,FR, 10B). PBMCs for functional experiments and in situ MS-ABPP experiments were obtained from healthy donors (aged 18–50 years) recruited through the Scripps Normal Blood Donor service by informed consent and used according to protocols approved by the Scripps Research Institute Institutional Review Board (protocol #IRB-187252). PBMCs were isolated from heparinized blood by Ficoll gradient (Lymphoprep, #7861, Stem Cell Technologies) and red blood cells were lysed with 1× Red Blood Cell Lysis buffer (#00-4300-54, eBiosciences). Purified PBMCs were then washed with Dulbecco’s phosphate-buffered saline (DPBS) (1×) and RPMI (1×), before being resuspended in serum-free RPMI (supplemented with L-glutamine (2 mM), penicillin (100 U ml^−1^), and streptomycin (100 μg ml^−1^)), or complete RPMI containing 10% vol/vol FBS (FB-01, Omega Scientific) depending on the assay protocol. PBMC-derived T-blasts for IL-12 phospho-STAT assays were generated by stimulating freshly isolated PBMCs with phytohaemagglutinin (PHA-P, 10 μg ml^−1^) in complete RPMI for 72 h, followed by IL-2 (100 U ml^−1^) in fresh RPMI for 24 h. Cells were then washed and rested overnight in serum-free RPMI before performing IL-12-phospho-STAT4 assays. T cells for αCD3/αCD28 activation assays were isolated by negative selection from freshly purified PBMCs using an EasySep T-cell isolation kit (#17951, Stem Cell Technologies) according to manufacturer instructions.

Tyk2-null mice (*Tyk2*^−/−^*, Tyk2^ΔCMV^*) were generated as previously described^30^ by crossing *Tyk2^fl^*^/*fl*^ mice to B6.C-Tg(CMV-Cre) mice on C57BL/6 N background. Tissues from Tyk2^−/−^ mice were obtained by approved MTA between Scripps Research and M. Müller and B. Strobl at the University of Veterinary Medicine, Vienna. All other animals used in this study were adult (8–12 weeks) C57BL/6 mice, except for pharmacokinetic studies, where male CD-1 (ICR) mice were used. Mice were maintained under pathogen-free conditions on a standard diet at an ambient temperature 20–26 °C, relative humidity (30–70%), 12-h light/dark cycle; handled in accordance with requirements of the National Institutes of Health and the Institutional Animal Care and Use Committee at The Scripps Research Institute. Splenocytes were isolated from fresh spleens by passing through a 70-μm sieve (#130-110-916, Miltenyi Biotec). Debris and red blood cells were removed by lysis in 1× RBC Lysis buffer (#00-4300-54, eBiosciences), followed by sequential washes with DPBS (×2), and serum-free RPMI (×1). Splenocytes were then resuspended at 6 × 10^6^ cells per milliliter in serum-free RPMI (supplemented with L-glutamine (2 mM), penicillin (100 U ml^−1^), and streptomycin (100 μg ml^−1^)) for phospho-STAT assays.

### Gel-based ABPP fluorescence

22Rv1 cells were seeded (2.5 million per 10-cm plate) in complete RPMI medium (10% vol/vol FBS) 24 h before PEI transfection with JAK1/TYK2 constructs (5 μg per plate). Twenty four hours after transfection, cells were serum starved overnight, then treated with dimethyl sulfoxide (DMSO) or **5a** (0.01, 0.1 or 1 μM) for 2 h at 37 °C, followed by alkyne **6** (0.1 μM) for 2 h at 37 °C. After treatments, cells were harvested by scraping in cold DPBS, centrifuged (1,400*g*, 2 min), pellets were washed with DPBS (1×) and then frozen at −80 °C until use.

To label alkyne **6** with a rhodamine reporter tag, cells were thawed on ice, resuspended in DPBS (350 μl) DPBS containing complete protease inhibitors, and lysed by sonication (2 × 8 pulses). The protein concentration of whole-cell lysates was normalized to 1.2 mg ml^−1^ and 50 μl was used for CuAAC reactions. The CuAAC reaction mixture was prepared using a 1:1:1:3 ratio of rhodamine azide (1.25 mM in DMSO; 25 μM final), 50 mM CuSO_4_ (aq., 1 mM final), 50 mM TCEP (aq., 1 mM final) and 1.7 mM tris(benzyltriazolylmethyl)amine (4:1 *t*-BuOH/ DMSO; 100 μM final). Lysates were treated with 6 μl Rh-CuAAC reaction mixtures, vortexed and incubated for 1 h at room temperature (vortexing 1–2×). Reactions were then quenched with 18 μl 4× Laemmli sample buffer and either frozen at −20 °C or immediately resolved on 10% Tris-glycine polyacrylamide gels, loading 20 μg protein per lane. Rhodamine fluorescence was detected using a Bio-Rad Imager flatbed scanner and images were processed in manufacturer software.

### Phospho-STAT western blot assays in 22Rv1 cells

22Rv1 cells (0.3 × 10^6^ cells per well) were seeded in 12-well plates in complete RPMI (10% vol/vol FBS). After 24 h, cells were transfected with JAK1/TYK2 constructs (500–1,500 ng DNA per well) using PEI-max (24765-1, Polysciences) diluted in OptiMEM (31985062, Gibco) and incubated at 37 °C for 24 h. Medium was then replaced, and cells were serum starved overnight before compound treatments. Cells were treated with DMSO or JAK inhibitors in serum-free RPMI for 2 h at 37 °C, then stimulated with IFNα (100 ng ml^−1^, 30 min), IFNγ (50 ng ml^−1^, 30 min), IL-6 (50 ng ml^−1^, 30 min) or prolactin (PRL) (500 ng ml^−1^, 15 min). Medium was removed, cells were washed with DPBS (1×) and lysed in mPER buffer (150 μl), supplemented with protease and phosphatase inhibitors for 15 min at room temperature. Lysates were collected, cleared by centrifugation (5 min, 16,000*g*, 4 °C) and supernatants were combined with 4× Laemmli sample buffer for western blotting.

### Phospho-STAT western blot assays in primary immune cells

Cytokines were used at a concentration that generated ∼50–90% of the maximal cytokine-induced signal in each assay (EC_50–90_). Duration of stimulation was selected on the basis of literature-reported kinetics of maximum STAT phosphorylation or are consistent with conditions used for the characterization of existing JAK inhibitors. Freshly isolated PBMCs (or PHA/IL-2 activated T-blasts for IL-12 assays) were resuspended in serum-free RPMI (6 million cells per milliliter) and treated with DMSO or JAK inhibitors for 2 h at 37 °C, then stimulated with cytokines as follows: IFNα (100 ng ml^−1^, 30 min), IFNγ (50 ng ml^−1^, 30 min), IL-6 (25 ng ml^−1^, 30 min), IL-2 (20 U ml^−1^, 15 min), GM-CSF (0.5 ng ml^−1^, 15 min) or IL-12 (12.5 ng ml^−1^, 15 min). Cells were then pelleted by centrifugation (1.5 min, 16,000*g*), medium was removed, and cells were lysed in 90 μl mPER buffer, containing protease and phosphatase inhibitors for 15–20 min at room temperature. Lysates were cleared by centrifugation (5 min, 16,000*g*, 4 °C) and supernatant were combined with 4× Laemmli sample buffer for western blotting.

Western blot assays with splenocytes were performed as described above, with the following cytokine concentrations: murine IFNα (1,000 U ml^−1^, 30 min), IL-6 (10 ng ml^−1^, 30 min), IL-2 (20 U ml^–1^, 15 min), GM-CSF (10 ng ml^−1^, 15 min) or IL-12 (12.5 ng ml^−1^, 15 min). Murine IL-12 assays were performed on freshly isolated splenocytes and did not require in vitro activation prior to assays to stimulate receptor expression.

## JAK1 half-life assays.

### Compound washout

freshly isolated PBMCs were resuspended in serum-free RPMI (6 million cells per milliliter) and treated with DMSO, VVD-118313 (0.1 μM) or tofacitinib (1 μM) for 2 h at 37 °C. Medium was then replaced by centrifuging cells (650*g*, 5 min) and washing 2 × 1 ml with serum-free RPMI. Cells were resuspended in serum-free RPMI and then stimulated at the indicated time points with IFNα (100 ng ml^−1^, 30 min). Cells were then harvested and prepared for analysis by western blot as described above.

Duration of action: in an alternate procedure, PBMCs were treated as described above, except that medium was not exchanged after the first 2 h.

### In vivo compound treatment

Compound **5** was formulated at 2.5 mg ml^−1^ in 5% DMSO/20% hydroxy-propyl-β-cyclodextrin for all in vivo experiments. Adult (8–12 weeks), age and sex matched mice (*n* = 3) were administered two doses of **5** (25 mg kg^−1^) or 5% vol/vol DMSO vehicle by subcutaneous injection at 4-h intervals. Mice were killed 4 h after the second dose according to approved protocols.

### Ex vivo phospho-STAT assays

Splenocytes from mice treated with compound **5** or DMSO vehicle were isolated as described above and seeded at 6 × 10^6^ cells per milliliter in serum-free RPMI for ex vivo stimulation with IFNα (1,000 U ml^−1^, 30 min), IL-6 (10 ng ml^−1^, 30 min), IL-2 (20 U ml^−1^, 15 min) or GM-CSF (10 ng ml^−1^, 15 min). Cells were collected by centrifugation, lysed with mPER buffer supplemented with protease and phosphatase inhibitors (60 μl, room temperature, 10–20 min), cleared (5 min, 16,000*g*, 4 °C) and then supernatants were combined with 4× Laemmli sample buffer to prepare western blotting samples.

### Western blotting

Samples in 1× Laemmli sample buffer were boiled for 5–10 min at 95 °C, then resolved by electrophoresis on either 10% or 4–20% Novex WedgeWell Tris-Glycine mini-gels (XP00105BOX, XP04205BOX, Invitrogen), and transferred to nitrocellulose (45004011, Amersham). Membranes were blocked with 5% milk in Tris-buffered saline (20 mM Tris-HCl 7.6, 150 mM NaCl) supplemented with 0.1% Tween-20 (TBST) buffer for 1 h at room temperature and then probed overnight at 4 °C with primary antibodies (1:1,000) in 5% BSA/TBST. Membranes were washed 3 ×5 min with TBST, then probed for 1 h at room temperature with IRDye secondary antibodies (1:10,000) in 5% BSA/TBST, washed a further 3 ×5 min with TBST and then visualized using the Odyssey Infrared Imaging System (Li-Cor Biosciences). Densitometry was performed using Odyssey software, subtracting any background fluorescence and normalizing channels as a percentage of the relevant control channels in each experiment.

### HTRF phospho-STAT assays

HTRF assays to detect IFNα-stimulated STAT1 (Tyr701) phosphorylation and IL-6-stimulated STAT3 (Tyr705) phosphorylation in human PBMCs were performed using CisBio assay kits #63ADK026PEG and #62AT3PEG. Assays were performed using a modified version of the manufacturer’s two-plate assay protocol, except that phospho-total protein lysis buffer #2 (64KL2FDF) was used for both pSTAT1 and pSTAT3 assays. In brief, stock compound plates were prepared by a seven-point serial dilution of compounds in 100% DMSO. Working plates were prepared immediately before assays by diluting stock plates in RPMI (0 or 10% vol/vol FBS) to give working solutions containing 0.2% vol/vol DMSO. Freshly isolated PBMCs (25 × 10^6^ cells per milliliter) were resuspended in RPMI (0% or 10% FBS) and aliquoted (40 μl) in duplicate in 96-well microplates (655098, Greiner Bio-one). Cells were treated with diluted compounds (40 μl) for 2 h at 37 °C (0.1% vol/vol final DMSO, 1 nM–2 μM, 10 μM or 50 μM final compound concentration) and then stimulated with diluted cytokines (20 μl) to give a final concentration of IFNα (100 ng ml^−1^) or IL-6 (25 ng ml^−1^). After 30 min at 37 °C, cells were lysed using 33 μl phospho-total protein lysis buffer 2 supplemented with phospho-peptide blocking regent (4% vol/vol) and incubated at room temperature for 45 min. After pipetting to homogenize lysates, 16 μl was transferred to 384-well white microplates (784075, Greiner Bio-one) and treated with a premixed solution of anti-pSTAT d2:Eu Cryptate antibodies (1:1, 4 μl). Solutions were covered with a plate sealer, incubated overnight at room temperature, and then fluorescence intensity was measured using a BMG PHERAstar plate reader, with excitation set to 337 nm, and emission wavelengths at 665 nm and 620 nm.

HTRF ratios were calculated as (signal 665 nm/signal 620 nm) × 10^4^. The basal HTRF ratio of unstimulated DMSO-treated controls (two per assay plate) was subtracted from cytokine treated samples and the data were normalized as a percentage of mean HTRF ratio of the cytokine-stimulated DMSO-treated controls (five per assay plate). At least two dose–response experiments were performed per compound. IC_50_ values were estimated by fitting data to a four-parameter logistic model in Graphpad Prism v.9.3.1, and the mean ± s.d. of a minimum of two dose–response experiments, except where noted in Extended Data Fig. 2a.

### In vitro kinase assay

JAK1 biochemical activity assays were performed in 384-well microplates (784075, Greiner) using the Promega JAK1 Kinase Enzyme System (VA7207) and ADP Glo Kinase Assay Kit (V6930). Assay conditions were optimized to quantify initial reaction rates and performed according to manufacturer instructions. Before assays, compounds were prepared as working solutions containing 5% vol/vol DMSO and added to assays to give a final concentration of 1% vol/vol DMSO. In brief, 30 ng recombinant GST-JAK1 protein (residues 438–1,154) was incubated with DMSO or VVD-118313 (**5a**) or tofacitinib in 1× kinase assay buffer (supplemented with 50 μM dithithreitol (DTT)) for 30 min at room temperature before the addition of 0.2 μg ml^−1^ IRS-1 peptide and 50 μM ATP. Kinase assays were incubated at room temperature for 60 min, then quenched with 5 μl ADP Glo reagent (40 min, rate), followed by 5 μl Kinase Detection Reagent (60 min, room temperature). ATP conversion was quantified by luminescence using a CLARIOstar (BMG Labtech) plate reader and data were normalized as a percent of the maximum (DMSO-treated) response. IC_50_ values were calculated by fitting data to a four-parameter logistic model in GraphPad Prism (v.9.3.1) software.

### *Trans*-phosphorylation assay

22Rv1 cells (0.6 × 10^6^ cells per well) were seeded into 6-well TC plates in complete RPMI medium 24 h before transfection with HA-tagged kinase-dead (K908E) JAK1 (WT or C817A) and Flag-tagged catalytically active JAK1 (WT or C817A) constructs (1:1 ratio, 3 μg total DNA). Twenty four hours after transfection, medium was replaced and cells were serum starved overnight and then treated with DMSO, **5a**, BMS-986165 or tofacitinib (2 μM) for 2 h at 37 °C. Cells were then washed with cold DPBS and lysed on ice for 30 min in 400 μl immunoprecipitation (IP) buffer (50 mM Tris, pH 8, 150 mM NaCl, 1% NP-40, 1 mM EDTA), supplemented with protease and phosphatase inhibitors. Lysates were collected, cleared (10,000*g*, 10 min, 4 °C), the protein concentration of supernatants was normalized to ~1.5 mg ml^−1^, and ~480 μg was aliquoted to LoBind SafeLock 1.5-ml Eppendorf tubes for HA-tag immunoprecipitation. Remaining supernatant was combined with 4× Laemmli sample buffer and used to quantify IP input. Anti-HA agarose (26181, Pierce) was equilibrated with IP buffer (3× washes, 1 min, 2,000*g*), and 7.5 μl per sample of packed resin was used to immunoprecipitate HA-tagged K908E-JAK1 (2 h, 4 °C). Immunoprecipitated proteins were collected by pelleting resin (2 min, 2,000*g*, 4 °C), washing 3 × 500 μL with IP buffer, and then eluting proteins using 50 μl 2× Laemmli sample buffer. Samples were boiled (5–10 min) and then resolved on 10% or 4–20% Tris-glycine gels and transferred to nitrocellulose as described above.

The effect of C817A mutation on *trans*-phosphorylation efficiency was calculated by normalizing the phospho-JAK1 signal intensity of DMSO-treated samples to transfection conditions where both kinase-dead (K908E) and active JAK1 constructs contained the native C817. As *trans*-phosphorylation efficiency was lower for C817A mutants, phospho-JAK1 signal intensity from compound-treated samples was normalized to the respective DMSO-treated sample for a given pair of JAK1 constructs. All *trans*-phosphorylation assays were performed in at least triplicate.

### In vivo pharmacokinetic analysis

Male CD-1 (ICR) mice (*n* = 3 per group) were dosed by intravenous (i.v.) or subcutaneous (s.c.) administration with compound **5**, prepared in 5% DMSO/95% (20% 2-hydroxypropyl-β-cyclodextrin). Plasma samples for pharmacokinetic analysis were collected in a composite manner with three animals per time point. The plasma concentration of compound **5** was measured by a liquid chromatography–tandem mass spectrometry (LC–MS/MS) method using positive electrospray ionization in multiple reaction monitoring mode. Plasma samples were extracted by protein precipitation using acetonitrile containing an internal standard. After vortexing and centrifugation, the supernatant was injected into an API6500 (AB SCIEX) liquid chromatography–tandem mass spectrometry system for quantification. Pharmacokinetic parameters were calculated by non-compartmental analysis of the plasma concentration–time profiles and reported as mean values in Extended Data Table 1.

### Multiplex immunoassay

Freshly isolated human PBMCs in complete RPMI (containing 10% vol/vol FBS) were seeded into 96-well suspension cell plates (250,000 per well) and treated for 2 h with DMSO or compounds, before stimulation with IFNα (100 ng ml^−1^). After 16 h, cells were pelleted (1,000*g*, 15 min, 4 °C) and medium was transferred without dilution to prepared multiplex immunoassay plates for cytokine analysis. The concentration of human cytokines in cell medium from IFNα-stimulated PBMCs was measured using a Bio-Plex Pro Human Cytokine 27-plex Assay kit (M500KCAF0Y, Bio-Rad) according to manufacturer instructions. Data were collected on Luminex instrument. Cytokine concentrations were estimated in xPONENT software (v.4.2.1324.0) from standard curves prepared on each plate and are expressed as picogram per milliliter (pg ml^−1^). Experiments were performed using 3 independent blood donors and 3–4 replicates per donor.

### Real-time PCR

Suppression of interferon-stimulated gene expression in IFNα-stimulated PBMCs was quantified by PCR with reverse transcription (RT–PCR). Freshly isolated PBMCs in complete RPMI (containing 10% vol/vol FBS) were seeded into 12-well TC plates (1.5–3 million per well), and treated for 2 h with DMSO or compounds, before stimulation with IFNα (100 mg ml^−1^). After 16 h, cells were harvested and washed with DPBS. RNA was isolated using a RNeasy Mini Kit (74106, Qiagen) and converted to cDNA by iScript Reverse Transcription (1708841, Bio-Rad). RT–PCR reactions were performed using SYBR Select Master Mix (4472908, Applied Biosystems) and the following primers: *OAS1*, forward 5′-TGAGGTCCAGGCTCCACGCT-3′, reverse 5′-GCAGGTCG GTGCACTCCTCG-3′; *MX1*, forward 5′- GATGATCAAAGGGATGTGGC-3′, reverse 5′-AGCTCGGCAACAGACTCTTC-3′; *IFIT1*, forward 5′-CAGAACGG CTGCCTAATTT-3′, reverse 5′-GGCCTTTCAGGTGTTTCAC-3′; *MCP-1*/*CCL2*, forward 5′-CAATAGGAAGATCTCAGTGC-3′, reverse 5′-GTGTTCAAGTCT TCGGAGTT-3′; *GAPDH*, forward 5′-CCATGGAGAAGGCTGGGG-3′, reverse 5′-CAAAGTTGTCATGGATGACC-3′. Data were collected on an 7900HT Fast Real-Time PCR system (Applied Biosciences) and *C*t values were calculated in SDS software (v2.4.1). Ct values were normalized to GAPDH and reported as a fold-change relative to IFNα-stimulated DMSO-treated controls using the 2^- ΔΔCT^ method.

### T-cell activation analysis by flow cytometry and ELISA

Freshly isolated human T cells were resuspended in complete RPMI (containing 10% vol/vol FBS) at 3 million cells per milliliter and 100 μ l (300,000 cells) was transferred to 96-well suspension cell plates that had been pre-coated overnight at 4 °C with α CD3 (OKT3) (BE0001-2, Bio X-cell) (5 μg ml^−1^) and αCD28 (9.3) (BE0248, Bio X-cell) (2 μ g ml^-1^) antibodies. Working solutions of JAK inhibitors were prepared by 100× dilution of DMSO compound stocks in complete RPMI and then 100 μl diluted compounds was transferred to assay plates. T cells were incubated at 37 °C for 24 h and then cells and medium were transferred to round-bottomed 96-well plates for cell-surface staining and flow cytometry. Cells were pelleted (600*g*, 3 min) and medium was transferred to a separate plate and frozen at –80 °C before analysis of secreted IFNγ, using a DuoSet ELISA kit (DY285B-05, R&D Biosystems). Enzyme-linked immunoabsorbent assays (ELISAs) were performed according to manufacturer guidelines using a 1:50–1:100 dilution of the collected medium. Data were recorded using a ClarioStar microplate reader (v.5.40 R3, BMG) and analyzed in MARS data analysis software (v.3.32) and Microsoft Excel v.2205.

Cells were washed 1 × 200 μl cold DPBS and then stained with Fixable Near IR stain (Invitrogen, #L34976) for cell viability analysis (1:1,000) and antibodies against CD69 and CD25 cells surface markers (1:200) in DPBS for 30 min at 4 °C. Cells were washed 1 × 200 μl cold DPBS then fixed in 4% vol/vol paraformaldehyde in DPBS for 30 min at 4 °C. Cells were washed 1 × 200 μl cold DPBS, then resuspended in 150 μl FACS buffer (2% BSA, 1 mM EDTA in DPBS). Flow cytometry data were collected using a Novocyte 3000 instrument (Aglient) and analyzed using FlowJo (v.10.8.1, BD Life Sciences).

### Additional software, data analysis and statistics

Quantitative data are expressed as mean ± s.e.m. in bar charts and as mean ± s.d. in dose–response curves used to calculate IC_50_ values. In western blot quantification, pSTAT or pJAK signal intensity was normalized as a percent of the cytokine-stimulated DMSO-treated sample on the same membrane, or the respective WT control in recombinant 22Rv1 experiments. Statistical analysis was performed in Graphpad Prism Software v.9.3.1 using one-way analysis of variance (ANOVA) or two-way ANOVA with either Dunnett’s, Turkey’s or Šidák’s post hoc test as noted in the corresponding figure legend. Significant *P* values are only noted for the lowest concentration of compound to inhibit STAT/JAK phosphorylation ≥50%, unless otherwise noted. All higher concentrations tested produced a similarly significant effect. IC_50_ values were estimated by fitting data using a four-parameter logistic model (GraphPad Prism, v.9.3.1). All other data processing was performed using Microsoft Excel v.2205. Structures of JAK1, TYK2 and ABL were prepared using PyMOL (v.4.6, Schrodinger). Additional figures were created using BioRender (https://biorender.com).

### Proteome-wide cysteine ligandability profiling by MS-ABPP

#### In vitro treatments

PBMCs isolated from a single blood donor and flash frozen were thawed on ice and resuspended in DPBS, containing protease inhibitors, and lysed by sonication on ice (3 × 10 pulses, 40% output). Protein concentration in whole-cell lysate was estimated using a DC protein assay (Bio-Rad), normalized to 2 mg ml^−1^ and then 500 μl was aliquoted to 10× LoBind SafeLock 1.5-ml Eppendorf tubes. Duplicate samples were treated with 5 μl DMSO or compound **5a** (final concentration 0.01, 0.1, 1 and 10 μM), vortexed and incubated for 1 h at room temperature. Samples were then processed according to the cysteine MS-ABPP procedure described below. Freshly isolated splenocytes (red blood cells removed) from 6 adult C57BL/6 mice were washed with DPBS (3×), then resuspended in DPBS containing protease inhibitors and processed as above for PBMCs, except that protein concentration was normalized to 1.7 mg ml^−1^.

#### In situ treatments

Freshly isolated PBMCs from a single blood donor were resuspended at 5 million cells per milliliter in serum-free RPMI and 40 million cells were aliquoted to 10× tissue culture flasks. Cells were treated in duplicate with DMSO or compound **5a** (0.01, 0.1, 1, or 10 μM), incubated for 2 h at 37 °C, then harvested. Cell pellets were washed 2–3× DPBS, then frozen at -80 °C until processing. Frozen pellets were thawed on ice, resuspended in DPBS containing protease inhibitors and lysed by sonication 3 × 10 pulses, 40% output. The proteome concentration of each sample was normalized to 2 mg ml^−1^ and then 500 μl was taken forward for labeling with IA-DTB.

#### Cysteine ABPP-MS

Samples were treated with 5 μl 10 mM IA-DTB (in DMSO), vortexed and incubated for 1 h at room temperature. Protein was then precipitated by the addition of 600 μl ice-cold MeOH, 200 μl CHCl_3_ and 100 μl H_2_O, vortexed, and centrifuged (10 min, 10,000*g*, 4 °C). Without disrupting the protein disk, both top and bottom layers of solvent were aspirated, and the protein disk was washed with 1 ml ice-cold MeOH, vortexed, and centrifuged (10 min, 16,000*g*, 4 °C). Solvent was aspirated and the pellets were allowed to air dry (5–10 min, room temperature). Pellets were then resuspended in 90 μl reducing buffer (9 M urea, 10 mM DTT, 50 mM triethylammonium bicarbonate buffer (TEAB), pH 8.5) and heated at 65 °C for 15 min, vortexing several times to solubilize proteins. Solutions were allowed to cool briefly, spun down, and then alkylated by treatment with 10 μl 500 mM iodoacetamide and incubated for 30 min, 37 °C with shaking. Samples were then diluted with 300 μl 50 mM TEAB, pH 8.5 to reach final concentration of 2 M urea, and probe sonicated (10 pulses) to ensure homogenous resuspension of all proteins. Samples were then digested overnight at 37 °C using 1 μg trypsin (resuspended at 0.25 μg μl^−1^ in trypsin resuspension buffer supplemented with 25 mM CaCl_2_) (V5111, Promega). Desthiobiotin-labeled peptides were enriched from digested samples using 25 μl packed streptavidin agarose resin per sample. Streptavidin was initially washed 3× with enrichment buffer (50 mM TEAB pH 8.5, 150 mM NaCl, 0.2% NP-40), and then 300 μl resuspended agarose was added per sample. Samples were rotated at room temperature for 2–3 h, and then pelleted by centrifugation (2,000*g*, 2 min). Samples were transferred to BioSpin columns using 2 × 500 μ l wash buffer (50 mM TEAB, pH 8.5, 150 mM NaCl, 0.1% NP-40), then washed 2 × 1 ml wash buffer, 3 × 1 ml DPBS, 3 × 1 ml H_2_O. Peptides were eluted by gravity into LoBind 1.5-ml Eppendorf tubes using 2 × 200 μl 50% acetonitrile with 0.1% formic acid and evaporated to dryness in a SpeedVac vacuum concentrator. Dried samples were then resuspended in 100 μl 70% 200 mM EPPS (pH 8), containing 30% acetonitrile, vortexed, and water bath sonicated (5 min). Samples were treated with 3 μl a resuspended 10-plex tandem mass tag (TMT) (20 μg μl^−1^ in dry acetonitrile) and incubated at room temperature for 1 h with intermittent vortexing. Reactions were then quenched by the addition of 5 μl hydroxylamine (5% vol/vol in water, 15 min, room temperature), acidified with 5 μl formic acid, combined and dried using a SpeedVac.

Samples were resuspended in 500 μl buffer A (5% MeCN, 0.1% formic acid), supplemented with 20 μl additional formic acid, and desalted using Sep-Pak C18 cartridge (WAT054955, Waters). In brief, cartridges were conditioned 3 × 1 ml 100% MeCN, equilibrated 3 × 1 ml buffer A and then samples were flowed through 2× under ambient pressure. Samples were washed 3 × 1 ml buffer A, then eluted into LoBind Eppendorf tubes using 1 ml buffer B (80% MeCN, 0.1% formic acid), and evaporated to dryness in a SpeedVac.

Samples were resuspended in 500 μl buffer A and fractionated by high-performance liquid chromatography using a capillary column (ZORBAX 300 Extend-C18, 3.5 μm), a gradient of 0–80% vol/vol buffer B in buffer A (buffer A: 10 mM aqueous NH_4_HCO_3_; buffer B: acetonitrile) at a flow rate of 0.5 ml min^−1^ over 100 min. Peptides were eluted as 1-ml fractions into 96-well (deep-well) plates (Aglient), which contained 20 μl 20% formic acid per well, and then evaporated to dryness using a SpeedVac. Peptides were resuspended in 80% MeCN, 0.1% formic acid buffer (100 μl per well) and columns were combined to give 12 fractions. Samples were dried using SpeedVac, then resuspended in 8 μl 5% acetonitrile, 0.1% formic acid for mass spectrometry.

#### Tandem mass tag LC–MS/MS analysis

Samples were analyzed by LC–MS/MS using an Orbitrap Fusion Tribrid Mass Spectrometer (Thermo Scientific) coupled to an UltiMate 3000 Series Rapid Separation liquid chromatography system and autosampler (Thermo Scientific Dionex). The peptides were eluted onto a capillary column (75-μm inner diameter fused silica, packed with C18 (Waters, Acquity BEH C18, 1.7 μm, 25 cm)) or an EASY-Spray HPLC column (Thermo ES902, ES903) using an Acclaim PepMap 100 (Thermo 164535) loading column, and separated at a flow rate of 0.25 μl min^−1^. Data was acquired using an MS3-based TMT method. In brief, the scan sequence began with an MS1 master scan (Orbitrap analysis, resolution 120,000, 400–1,700 *m*/*z*, RF lens 60%, automatic gain control (AGC) target 2E5, maximum injection time 50 ms, centroid mode) with dynamic exclusion enabled (repeat count 1, duration 15 s). The top ten precursors were then selected for MS2/MS3 analysis. MS2 analysis consisted of quadrupole isolation (isolation window 0.7) of precursor ion followed by collision-induced dissociation in the ion trap (AGC 1.8 × 10^4^, normalized collision energy 35%, maximum injection time 120 ms). Following the acquisition of each MS2 spectrum, synchronous precursor selection enabled the selection of up to 10 MS2 fragment ions for MS3 analysis. MS3 precursors were fragmented by higher energy collisional dissociation and analyzed using the Orbitrap (collision energy 55%, AGC 1.5 × 10^5^, maximum injection time 120 ms, resolution was 50,000). For MS3 analysis, we used charge-state-dependent isolation windows. For charge state *z* = 2, the MS isolation window was set at 1.2; for *z* = 3–6, the MS isolation window was set at 0.7. RAW files were uploaded to Integrated Proteomics Pipeline (IP2) and converted to MS2 and MS3 files. Files were searched using the ProLuCID algorithm (publicly available at http://fields.scripps.edu/downloads.php) using a reverse concatenated, non-redundant variant of the Human UniProt database (release 2016) or Mouse UniProt database (release 2017). Cysteine residues were searched with a static modification for carboxyamidomethylation (+57.02146 Da) and a dynamic modification for IA-DTB labeling (+398.25292 Da) (maximum 2 differential modifications per peptide). N-termini and lysine residues were also searched with a static modification corresponding to the respective TMT (+229.1629 Da). Peptides were required to be at least six amino acids long, to be fully tryptic (K or R cleavage sites), and to have a maximum of two mis-cleavage sites. ProLuCID data was filtered through DTASelect (v.2.0) to achieve a peptide false-positive rate below 1%. The MS3-based peptide quantification was performed with reporter ion mass tolerance set to 20 p.p.m. with Integrated Proteomics Pipeline (IP2).

#### Data processing

Cysteine engagement (DMSO versus inhibitor) was calculated for each peptide–spectra match by dividing each TMT reporter ion intensity by the average intensity for the channels corresponding to DMSO treatment.

Peptide–spectra matches were then grouped on the basis of protein ID and residue number (for example, JAK1_C817). Data were filtered to exclude peptides with summed reporter ion intensities for the DMSO channels <10,000, a coefficient of variation for DMSO channels >0.5, non-tryptic or reverse peptide sequences. Cysteine engagement is reported as either a percentage of peptide signal intensity in DMSO-treated channels, or a competition ratio (signal intensity DMSO/signal intensity compound) and are the mean value of the two replicate channels in each TMT 10-plex sample. Competition ratios ≥4 (corresponding to a ≥75% loss in peptide signal intensity as compared to DMSO) were considered significantly engaged by inhibitors.

#### In vitro and in vivo target engagement

In vitro TE_50_ values for JAK1_C817 and TYK2-C838 were obtained by treating 500 μg cell lysate generated from primary human PBMCs (AllCells), Jurkat T cells (ATCC, #TIB-152), or MDA-MB-468 breast cancer cells (ATCC, #HTB-132), with DMSO or compound for 1 h at room temperature (with the exception of experiments to determine the kinetics of JAK1_C817 engagement by **5a** and **5b**, where lysates were treated with compound for 10, 30 or 60 min), followed by the addition of 200 μM IA-DTB (in DMSO) for 1 hr at room temperature. Samples were then precipitated by the addition of 8x ice-cold acetone and incubated at -80 °C for 2 h. Protein was then pelleted by centrifugation (4,200 RPM, 45 min, 4 °C). The pelleted material was resuspended in 50 mM ammonium bicarbonate buffer containing 9 M Urea, and proteins were reduced and alkylated by the addition of DTT (10 mM) and iodoacetamide (30 mM). Following reduction and alkylation, samples were exchanged into 2 M urea (Zeba spin desalting plates, Thermo Fisher) and digested with trypsin. IA-DTB-labeled peptides were isolated with by enrichment with streptavidin agarose resin. Target engagement was determined from isolated peptides by parallel reaction monitoring (PRM) or tTMT as described below.

*In vivo* engagement of JAK1 C817 by compound **5** was determined from whole spleen lysate of mice (n = 4 per group) that had been treated subcutaneously with 2 doses of compound **5** (25 mg kg^-1^) or DMSO vehicle (prepared as 5% DMSO/20% hydroxy-propyl-β-cyclodextrin). Mice were killed 4 h after the second dose. Harvested spleens were homogenized in 700 μl PBS by bead beating (1.4 mM ceramic beads, Omni Bead Ruptor Elite) for 30 s. Samples were further homogenized by sonication in a water bath and clarified by centrifugation (1,000*g* for 10 min). Protein concentration was determined and 1.5 mg lysate was treated with 200 μM IA-DTB (in DMSO) for 1 h at room temperature, and then samples were processed and analyzed by PRM as described below.

#### Parallel reaction monitoring

Peptides were eluted from an EASY-Spray C18 loading column (5-μm particle size, 100 μm × 2 cm; Fisher Scientific, DX164564) and resolved on a custom analytical column (2-μM particle size, 75 μm x 15 cm) using a Dionex Ultimate 3000 nano-LC (Thermo Fisher Scientific). Peptides were separated over an 11-min gradient of 6–33% acetonitrile (0.1% vol/vol formic acid) and analyzed by PRM on a Q-Exactive instrument (Thermo Fisher Scientific). Selected precursor ions targeting JAK1-C817 (amino acids 810–818, +3 charge state) and TYK2_C838 (amino acids 833–860, +3 charge state) were isolated and fragmented by high-energy collision-induced dissociation and fragments were detected in the Orbitrap at 17,500 resolution. The resulting PRM data were analyzed using Skyline (v.21.1.0.278) and quantification was performed by summing the peak areas corresponding to six fragment ions from each peptide. The peptides and fragment ions were pre-selected from in-house reference spectral libraries acquired in data-dependent acquisition mode to identify authentic spectra for each peptide.

#### Targeted TMT

Targeted TMT measurements were collected using an Orbitrap LumosTribrid Mass Spectrometer (Thermo Scientific) coupled to an UltiMate 3000 Series Rapid Separation LC system and autosampler (Thermo Scientific Dionex). Peptides were eluted onto a custom C18 capillary analytical column (75-μm inner diameter fused silica, packed with Acclaim PepMap C18 resin (Thermo Scientific)) using an Acclaim PepMap 100 (Thermo 164535) loading column, and separated at a flow rate of 1.0 μl min^−1^. Data were acquired using a specific MS3-based TMT method targeting peptides containing JAK1_C817 (amino acids 810–818, +3 charge state) and TYK2_C838 (amino acids 830–860, +5 charge state), where MS2 peptide fragmentation is triggered upon detection of the peptide precursor ion. Subsequent MS3 analysis was then performed using pre-selected peptide fragment ions that were isolated for fragmentation using synchronous precursor selection. RAW files were converted to MZXML format and searched with the SEQUEST algorithm using the MassPike software package. TMT quantitation was performed with a filter requiring at least ten summed signal-to-noise for control channels.

#### Reporting summary

Further information on research design is available in the Nature Research Reporting Summary linked to this article.

## Extended Data

## Supplementary Material

Proteomics Table 1

Proteomics Table 2

Proteomics Table 3

Source data

Supplementary Data 2

Supplementary Information

## Data Availability

Proteomics datasets profiling cysteine reactivity relevant to Figs. 2d,e and 4f,g, Extended Data Figs. 2c and 6f,g, have been deposited to the ProteomeXchange Consortium via the PRIDE partner repository with the dataset identifier PXD031384. Small-molecule crystal structure of **13a** has been deposited in Cambridge Crystallographic Data Center with accession number 2169712 (https:// www.ccdc.cam.ac.uk/). The human Uniprot database (2016 release) and mouse Uniprot database (2017 release) used for proteomic searches can be accessed at https://www.uniprot.org/. All other data is available in the Source Data and Supplementary Data files that accompany this manuscript. Source data are provided with this paper.
