# Supplementary material for: Selective inhibitors of JAK1 targeting an isoform-restricted allosteric cysteine
Source: Nat Chem Biol. Author manuscript; Available in PMC 2023 Jul 18. (PMC7614775; doi:10.1038/s41589-022-01098-0)
Supplement: Proteomics Table 1 [file EMS178742-supplement-Proteomics_Table_1.pdf]

Protein	Uniprot Accession	Cysteine Number	%Engagement				Engagement Ratio (DMSO/VVD-118313)			
			0.01 µM	0.1 µM	1 µM	10 µM	0.01 µM	0.1 µM	1 µM	10 µM
JAK1	P23458	817	84	96	96	97	6.3	20.0	20.0	20.0
HMOX2	P30519	282	-3	27	92	98	1.0	1.4	12.5	20.0
TYK2	P29597	838	9	82	92	95	1.1	5.4	12.5	18.2
SLC66A3	Q8N755	135	-1	12	78	99	1.0	1.1	4.4	20.0
TOR4A	Q9NXH8	21	-18	-4	65	92	0.9	1.0	2.9	11.8
DCTN4	Q9UJW0	258	-3	3	63	98	1.0	1.0	2.7	20.0
ARFGAP2	Q8N6H7	97	-55	67	62	-48	0.6	3.0	2.6	0.7
PTGES2	Q9H7Z7	110	-7	13	61	85	0.9	1.1	2.5	6.7
DAXX	Q9UER7	58	-7	12	59	96	0.9	1.1	2.4	20.0
NR4A1	P22736	551	-63	52	54	-24	0.6	2.1	2.2	0.8
PID1	Q7Z2X4	91	-42	69	53	-73	0.7	3.2	2.1	0.6
TARBP1	Q13395	202	6	14	52	67	1.1	1.2	2.1	3.0
MGST3	O14880	56	1	7	52	85	1.0	1.1	2.1	6.7
CLTC	Q00610	617	-26	57	52	-41	0.8	2.3	2.1	0.7
UVRAG	Q9P2Y5	239	-3	-6	49	84	1.0	0.9	2.0	6.3
ZNF200	P98182	389	-42	67	49	-37	0.7	3.0	2.0	0.7
PSD4	Q8NDX1	933	-28	60	48	-76	0.8	2.5	1.9	0.6
C11orf54	Q9H0W9	149	5	38	47	-4	1.0	1.6	1.9	1.0
EML3	Q32P44	825	-55	52	46	-19	0.6	2.1	1.9	0.8
VPS37A	Q8NEZ2	325	-60	71	46	-89	0.6	3.4	1.9	0.5
POLR2A	P24928	13	-43	53	46	-13	0.7	2.1	1.8	0.9
CTDSPL2	Q05D32	381	-29	59	46	-28	0.8	2.4	1.8	0.8
CAPZA2	P47755	95	10	20	45	45	1.1	1.3	1.8	1.8
DOK1	Q99704	70	12	6	44	14	1.1	1.1	1.8	1.2
HNRNPA1	P09651	43	-41	69	44	-27	0.7	3.2	1.8	0.8
EML4	Q9HC35	820	-59	58	44	-41	0.6	2.4	1.8	0.7
MED19	A0JLT2	163	-56	23	43	-12	0.6	1.3	1.8	0.9
EXOC5	O00471	194	-2	45	43	-14	1.0	1.8	1.8	0.9
IRF2BP2	Q7Z5L9	65	-36	47	43	-17	0.7	1.9	1.8	0.9
NEK7	Q8TDX7	298	-26	62	43	-29	0.8	2.6	1.8	0.8
HSDL2	Q6YN16	166	-17	66	43	-35	0.9	2.9	1.8	0.7
FARSB	Q9NSD9	151	-36	54	42	-34	0.7	2.2	1.7	0.7
ABHD16A	O95870	205	4	7	41	92	1.0	1.1	1.7	11.8
RTN4	Q9NQC3	1101	3	-15	41	85	1.0	0.9	1.7	6.7
VDAC2	P45880	210	1	14	41	70	1.0	1.2	1.7	3.3
LRRC47	Q8N1G4	367	-47	47	41	-43	0.7	1.9	1.7	0.7
ARAP1	Q96P48	638	-28	59	41	-67	0.8	2.4	1.7	0.6
ZAP70	P43403	84	-7	49	41	-30	0.9	2.0	1.7	0.8
IL4I1	Q96RQ9	252	-38	55	41	-51	0.7	2.2	1.7	0.7
SRC	P12931	188	-3	15	40	58	1.0	1.2	1.7	2.4
HERC3	Q15034	321	-33	58	40	9	0.8	2.4	1.7	1.1
FGD3	Q5JSP0	43	-58	49	40	-51	0.6	2.0	1.7	0.7
DEF6	Q9H4E7	94	-3	17	40	-53	1.0	1.2	1.7	0.7
PRPF8	Q6P2Q9	435	-44	54	40	-15	0.7	2.2	1.7	0.9
TBL2	Q9Y4P3	375	-18	49	39	-19	0.9	2.0	1.6	0.8
PDLIM1	O00151	307	-21	47	39	-254	0.8	1.9	1.6	0.3
C20orf27	Q9GZN8	156	6	25	37	28	1.1	1.3	1.6	1.4
HSDL1	Q3SXM5	265	-7	4	37	93	0.9	1.0	1.6	13.3
PROSC	O94903	261	11	31	36	39	1.1	1.4	1.6	1.6
UMPS	P11172	174	0	56	36	-28	1.0	2.2	1.6	0.8
ACYP2	P14621	22	28	37	36	55	1.4	1.6	1.6	2.2
FLAD1	Q8NFF5	236	-37	51	36	-10	0.7	2.0	1.6	0.9
CCDC88C	Q9P219	1194	2	58	36	-13	1.0	2.4	1.6	0.9

NLE1	Q9NVX2	280	-42	46	36	-28	0.7	1.9	1.6	0.8
COMMD4	Q9H0A8	107	-38	37	36	-38	0.7	1.6	1.6	0.7
GSTP1	P09211	102	22	26	35	29	1.3	1.4	1.5	1.4
ZBED1	O96006	76	-11	52	35	-21	0.9	2.1	1.5	0.8
COPA	P53621	254	-43	33	35	-60	0.7	1.5	1.5	0.6
PNPO	Q9NVS9	156	1	13	34	58	1.0	1.1	1.5	2.4
HN1L	Q9H910	118	-8	51	34	-17	0.9	2.0	1.5	0.9
ZNF512B	Q96KM6	756	-43	48	34	-20	0.7	1.9	1.5	0.8
ZNF22	P17026	141	11	38	34	-91	1.1	1.6	1.5	0.5
EIF2B4	Q9UI10	444	-32	41	34	-16	0.8	1.7	1.5	0.9
FAM120A	Q9NZB2	279	-32	53	34	-22	0.8	2.1	1.5	0.8
HCLS1	P14317	470	-14	61	34	-45	0.9	2.5	1.5	0.7
NEDD4	P46934	1286	-20	47	34	-47	0.8	1.9	1.5	0.7
SMURF2	Q9HAU4	716	-20	47	34	-47	0.8	1.9	1.5	0.7
ZNF512	Q96ME7	430	-48	56	34	-57	0.7	2.2	1.5	0.6
CPT1A	P50416	96	-2	-6	33	83	1.0	0.9	1.5	5.9
PTGDS	P41222	65	8	16	33	65	1.1	1.2	1.5	2.9
TPI1	P60174	104	32	36	33	57	1.5	1.6	1.5	2.3
EPRS	P07814	1497	2	15	33	43	1.0	1.2	1.5	1.8
ALDH3A2	P51648	50	-3	14	33	33	1.0	1.2	1.5	1.5
IRS2	Q9Y4H2	409	-6	30	33	20	0.9	1.4	1.5	1.2
NBEAL2	Q6ZNJ1	1224	4	13	32	74	1.0	1.1	1.5	3.8
RPIA	P49247	178	21	19	32	57	1.3	1.2	1.5	2.3
TPI1	P60174	79	26	30	32	47	1.3	1.4	1.5	1.9
PCK2	Q16822	425	10	-4	32	31	1.1	1.0	1.5	1.4
DDA1	Q9BW61	25	-38	49	32	-19	0.7	1.9	1.5	0.8
GALK1	P51570	203	-13	34	32	-20	0.9	1.5	1.5	0.8
TPI1	P60174	164	35	31	32	41	1.5	1.4	1.5	1.7
ANKIB1	Q9P2G1	715	22	23	32	23	1.3	1.3	1.5	1.3
PES1	O00541	153	-25	41	32	21	0.8	1.7	1.5	1.3
HNRNPLL	Q8WVV9	235	27	12	32	19	1.4	1.1	1.5	1.2
MIS18BP1	Q6P0N0	890	-34	45	32	5	0.7	1.8	1.5	1.1
ZSWIM8	A7E2V4	215	-45	49	32	-32	0.7	2.0	1.5	0.8
DUSP11	O75319	189	-58	26	32	-40	0.6	1.4	1.5	0.7
C11orf54	Q9H0W9	249	33	24	31	46	1.5	1.3	1.4	1.8
WDFY4	Q6ZS81	233	-18	47	31	2	0.8	1.9	1.4	1.0
TRERF1	Q96PN7	876	-27	56	31	-33	0.8	2.3	1.4	0.8
PANK4	Q9NVE7	513	-45	57	31	-50	0.7	2.3	1.4	0.7
TRIM21	P19474	103	-46	56	31	-77	0.7	2.2	1.4	0.6
ALOX5	P09917	100	19	28	30	57	1.2	1.4	1.4	2.3
TXN	P10599	73	10	20	30	53	1.1	1.3	1.4	2.1
MRPL38	Q96DV4	290	23	35	30	40	1.3	1.5	1.4	1.7
MX1	P20591	533	-33	50	30	-17	0.8	2.0	1.4	0.9
VDAC2	P45880	227	3	5	30	72	1.0	1.0	1.4	3.6
SNRPD2	P62316	46	13	23	30	37	1.1	1.3	1.4	1.6
MTA2	O94776	44	6	10	30	29	1.1	1.1	1.4	1.4
TRRAP	Q9Y4A5	3075	-40	47	30	-26	0.7	1.9	1.4	0.8
PREP	P48147	532	38	32	29	33	1.6	1.5	1.4	1.5
UNC13D	Q70J99	505	15	7	29	-4	1.2	1.1	1.4	1.0
ADCK4	Q96D53	508	-32	48	29	-21	0.8	1.9	1.4	0.8
RAD21	O60216	35	-14	57	29	-25	0.9	2.3	1.4	0.8
CARD11	Q9BXL7	539	-54	33	29	-37	0.6	1.5	1.4	0.7
FLNA	P21333	1157	-25	51	29	-61	0.8	2.0	1.4	0.6
PDE5A	O76074	68	-16	52	29	-298	0.9	2.1	1.4	0.3
PTER	Q96BW5	43	21	23	29	50	1.3	1.3	1.4	2.0

RPL30	P62888	52	10	15	29	44	1.1	1.2	1.4	1.8
NHP2	Q9NX24	125	19	20	29	40	1.2	1.2	1.4	1.7
CDK5RAP3	Q96JB5	165	28	25	29	6	1.4	1.3	1.4	1.1
CYB5B	O43169	115	-14	-13	28	89	0.9	0.9	1.4	8.7
TESC	Q96BS2	213	2	-7	28	62	1.0	0.9	1.4	2.6
FAHD1	Q6P587	129	15	25	28	54	1.2	1.3	1.4	2.2
PROSC	O94903	86	7	19	28	44	1.1	1.2	1.4	1.8
PEPD	P12955	482	-18	35	28	23	0.9	1.5	1.4	1.3
USP9X	Q93008	864	18	6	28	13	1.2	1.1	1.4	1.1
FGD3	Q5JSP0	184	6	38	28	-13	1.1	1.6	1.4	0.9
TAF6	P49848	141	-41	45	28	-40	0.7	1.8	1.4	0.7
THOC6	Q86W42	35	-53	43	28	-84	0.7	1.8	1.4	0.5
ENO1	P06733	339	21	26	28	40	1.3	1.3	1.4	1.7
ZBTB24	O43167	408	18	-1	28	22	1.2	1.0	1.4	1.3
ZBTB24	O43167	411	18	-1	28	22	1.2	1.0	1.4	1.3
ARHGAP9	Q9BRR9	562	17	13	28	14	1.2	1.1	1.4	1.2
DDX5	P17844	89	-24	39	28	-20	0.8	1.6	1.4	0.8
HIP1R	O75146	105	-3	45	28	-37	1.0	1.8	1.4	0.7
TPI1	P60174	124	23	24	27	46	1.3	1.3	1.4	1.9
AP3D1	O14617	137	30	27	27	25	1.4	1.4	1.4	1.3
SLAIN2	Q9P270	306	12	21	27	18	1.1	1.3	1.4	1.2
SNRPD3	P62318	20	-6	25	27	10	0.9	1.3	1.4	1.1
IDH3B	O43837	232	15	23	27	8	1.2	1.3	1.4	1.1
CDK5RAP1	Q96SZ6	538	11	28	27	6	1.1	1.4	1.4	1.1
CAND1	Q86VP6	356	14	14	27	2	1.2	1.2	1.4	1.0
CAD	P27708	1455	-33	33	27	-17	0.8	1.5	1.4	0.9
ARHGAP30	Q7Z6I6	965	-42	40	27	-26	0.7	1.7	1.4	0.8
CAPN7	Q9Y6W3	197	-22	36	27	-27	0.8	1.6	1.4	0.8
ETHE1	O95571	189	2	15	27	44	1.0	1.2	1.4	1.8
ATP13A1	Q9HD20	530	18	10	27	-4	1.2	1.1	1.4	1.0
MED1	Q15648	135	5	-3	27	-17	1.0	1.0	1.4	0.9
EML4	Q9HC35	516	-38	38	27	-22	0.7	1.6	1.4	0.8
FAM213B	Q8TBF2	34	4	32	27	-24	1.0	1.5	1.4	0.8
CDC16	Q13042	79	-17	48	27	-42	0.9	1.9	1.4	0.7
ZMYM2	Q9UBW7	663	-31	38	26	-13	0.8	1.6	1.4	0.9
PHF5A	Q7RTV0	11	-3	33	26	-125	1.0	1.5	1.4	0.4
POLR2B	P30876	177	20	11	26	64	1.3	1.1	1.3	2.7
SASH3	O75995	152	20	28	26	58	1.2	1.4	1.3	2.4
TPI1	P60174	255	27	25	26	42	1.4	1.3	1.3	1.7
RPL3	P39023	336	4	16	26	38	1.0	1.2	1.3	1.6
RNASEH2A	O75792	181	30	20	26	19	1.4	1.2	1.3	1.2
SYTL1	Q8IYJ3	290	-36	45	25	-22	0.7	1.8	1.3	0.8
ITGAM	P11215	494	12	11	25	43	1.1	1.1	1.3	1.8
ITGAX	P20702	495	12	11	25	43	1.1	1.1	1.3	1.8
ALB	P02768	58	16	16	25	20	1.2	1.2	1.3	1.2
RPS26	P62854	77	-49	42	25	-62	0.7	1.7	1.3	0.6
LCN2	P80188	107	16	21	24	61	1.2	1.3	1.3	2.5
APEX1	P27695	310	18	12	24	42	1.2	1.1	1.3	1.7
NFX1	Q12986	892	17	9	24	26	1.2	1.1	1.3	1.3
METAP2	P50579	436	-2	23	24	7	1.0	1.3	1.3	1.1
IDH3A	P50213	359	9	20	24	0	1.1	1.3	1.3	1.0
IRF2BPL	Q9H1B7	63	-25	28	24	-7	0.8	1.4	1.3	0.9
PDDC1	Q8NB37	94	-35	36	24	-27	0.7	1.6	1.3	0.8
COG8	Q96MW5	96	11	13	24	89	1.1	1.1	1.3	9.1
PIGQ	Q9BRB3	107	13	20	24	23	1.1	1.3	1.3	1.3

MDN1	Q9NU22	4517	11	2	24	19	1.1	1.0	1.3	1.2
PTPN12	Q05209	242	40	30	24	12	1.7	1.4	1.3	1.1
CTR9	Q6PD62	363	32	1	24	10	1.5	1.0	1.3	1.1
WDFY4	Q6ZS81	234	-22	31	24	6	0.8	1.4	1.3	1.1
POLR2A	P24928	184	-26	31	24	-1	0.8	1.4	1.3	1.0
GAS7	O60861	424	-28	30	24	-7	0.8	1.4	1.3	0.9
ZBTB9	Q96C00	452	-26	31	24	-8	0.8	1.4	1.3	0.9
LPXN	O60711	155	-29	36	24	-38	0.8	1.6	1.3	0.7
HELZ2	Q9BYK8	1867	-21	28	24	-49	0.8	1.4	1.3	0.7
NUP62	P37198	506	2	5	23	37	1.0	1.0	1.3	1.6
TRIM22	Q8IYM9	59	3	7	23	30	1.0	1.1	1.3	1.4
RACK1	P63244	286	10	13	23	5	1.1	1.1	1.3	1.1
SAAL1	Q96ER3	430	-7	18	23	3	0.9	1.2	1.3	1.0
TGFB1I1	O43294	275	30	16	23	-7	1.4	1.2	1.3	0.9
EML3	Q32P44	307	-23	41	23	-15	0.8	1.7	1.3	0.9
USP4	Q13107	856	20	1	23	41	1.3	1.0	1.3	1.7
ANKRD12	Q6UB98	638	22	14	23	37	1.3	1.2	1.3	1.6
CPSF3	Q9UKF6	527	18	11	23	31	1.2	1.1	1.3	1.4
SH2B1	Q9NRF2	323	-3	-4	23	22	1.0	1.0	1.3	1.3
USP19	O94966	833	1	-13	23	20	1.0	0.9	1.3	1.3
CARD11	Q9BXL7	1060	14	5	23	7	1.2	1.0	1.3	1.1
IPO9	Q96P70	545	19	18	23	6	1.2	1.2	1.3	1.1
ASCC2	Q9H1I8	165	-5	18	23	2	1.0	1.2	1.3	1.0
CASP1	P29466	331	-5	28	23	-18	1.0	1.4	1.3	0.9
PXK	Q7Z7A4	570	-12	25	23	-19	0.9	1.3	1.3	0.8
EIF5B	O60841	853	21	-1	23	-30	1.3	1.0	1.3	0.8
CRLF3	Q8IUI8	218	3	0	22	55	1.0	1.0	1.3	2.2
RAN	P62826	112	14	17	22	38	1.2	1.2	1.3	1.6
PITPNC1	Q9UKF7	136	4	10	22	25	1.0	1.1	1.3	1.3
SUGP2	Q8IX01	476	6	22	22	14	1.1	1.3	1.3	1.2
ANKFY1	Q9P2R3	995	5	5	22	-1	1.0	1.0	1.3	1.0
POLR2I	P36954	52	-35	18	22	-5	0.7	1.2	1.3	1.0
TBRG4	Q969Z0	335	22	6	22	-27	1.3	1.1	1.3	0.8
ANKRD44	Q8N8A2	615	-25	34	22	-70	0.8	1.5	1.3	0.6
HMOX2	P30519	265	-9	-3	22	73	0.9	1.0	1.3	3.6
PROSC	O94903	15	4	11	22	53	1.0	1.1	1.3	2.1
IL16	Q14005	975	14	21	22	43	1.2	1.3	1.3	1.8
MYCBP2	O75592	3354	7	5	22	28	1.1	1.0	1.3	1.4
HBD	P02042	113	29	24	22	28	1.4	1.3	1.3	1.4
RAC2	P15153	105	5	36	22	-105	1.1	1.6	1.3	0.5
POLR2B	P30876	172	-9	2	21	62	0.9	1.0	1.3	2.6
NRDE2	Q9H7Z3	203	21	-6	21	52	1.3	0.9	1.3	2.1
PDLIM7	Q9NR12	400	17	15	21	48	1.2	1.2	1.3	1.9
TRMT112	Q9UI30	100	-37	11	21	47	0.7	1.1	1.3	1.9
CDC42	P60953	18	6	20	21	35	1.1	1.2	1.3	1.5
RPS16	P62249	25	4	19	21	12	1.0	1.2	1.3	1.1
CDK5RAP3	Q96JB5	136	10	24	21	0	1.1	1.3	1.3	1.0
SAMD9	Q5K651	420	-7	34	21	-20	0.9	1.5	1.3	0.8
AKAP9	Q99996	882	4	3	21	45	1.0	1.0	1.3	1.8
ENO1	P06733	337	20	23	21	38	1.2	1.3	1.3	1.6
SAFB2	Q14151	449	16	19	21	23	1.2	1.2	1.3	1.3
SRP14	P37108	56	8	5	21	20	1.1	1.1	1.3	1.3
AAK1	Q2M2I8	193	-15	29	21	-2	0.9	1.4	1.3	1.0
APMAP	Q9HDC9	149	51	33	20	56	2.0	1.5	1.3	2.3
HTATSF1	O43719	471	4	41	20	46	1.0	1.7	1.3	1.9

MAPK9	P45984	222	21	2	20	31	1.3	1.0	1.3	1.4
PDXDC1	Q6P996	456	14	-7	20	24	1.2	0.9	1.3	1.3
CLIP1	P30622	752	24	29	20	23	1.3	1.4	1.3	1.3
SYNE1	Q8NF91	4436	-3	4	20	21	1.0	1.0	1.3	1.3
ALS2	Q96Q42	1454	24	9	20	21	1.3	1.1	1.3	1.3
Uncharacteriz	H0Y626	178	-5	1	20	21	1.0	1.0	1.3	1.3
PSMD1	Q99460	571	14	-15	20	18	1.2	0.9	1.3	1.2
DBT	P11182	333	24	9	20	15	1.3	1.1	1.3	1.2
PMVK	Q15126	38	32	20	20	10	1.5	1.2	1.3	1.1
NKIRAS2	Q9NYR9	122	12	7	20	8	1.1	1.1	1.3	1.1
PXN	P49023	476	15	19	20	4	1.2	1.2	1.3	1.0
OBSCN	Q5VST9	4919	5	15	20	-1	1.1	1.2	1.3	1.0
SNX3	O60493	140	3	16	20	-2	1.0	1.2	1.3	1.0
CKAP4	Q07065	100	-3	-2	20	73	1.0	1.0	1.2	3.6
ZNF185	O15231	466	-1	14	20	48	1.0	1.2	1.2	1.9
PDIA4	P13667	209	-12	-22	20	39	0.9	0.8	1.2	1.6
RP2	O75695	110	23	36	20	31	1.3	1.6	1.2	1.4
FCGRT	P55899	182	3	3	20	23	1.0	1.0	1.2	1.3
SCP2	P22307	307	32	39	20	21	1.5	1.6	1.2	1.3
MARCH6	O60337	28	29	-7	20	20	1.4	0.9	1.2	1.2
TARBP2	Q15633	263	8	-6	20	8	1.1	0.9	1.2	1.1
RAB3GAP1	Q15042	783	16	4	20	7	1.2	1.0	1.2	1.1
GIMAP7	Q8NHV1	78	18	15	20	5	1.2	1.2	1.2	1.1
PRPF31	Q8WWY3	299	19	16	20	-8	1.2	1.2	1.2	0.9
HDAC3	O15379	279	-12	27	20	-17	0.9	1.4	1.2	0.9
TRAFD1	O14545	135	-15	31	20	-25	0.9	1.4	1.2	0.8
SQRDL	Q9Y6N5	379	-8	-6	19	59	0.9	0.9	1.2	2.4
TMC6	Q7Z403	199	-7	-2	19	56	0.9	1.0	1.2	2.3
PTGER2	P43116	346	10	24	19	39	1.1	1.3	1.2	1.6
GM2A	P17900	138	0	10	19	14	1.0	1.1	1.2	1.2
ARFGEF2	Q9Y6D5	36	5	6	19	10	1.0	1.1	1.2	1.1
RAVER1	Q8IY67	297	6	14	19	8	1.1	1.2	1.2	1.1
UQCRC1	P31930	347	2	8	19	7	1.0	1.1	1.2	1.1
GSK3A	P49840	398	11	15	19	-2	1.1	1.2	1.2	1.0
FAM129A	Q9BZQ8	41	12	13	19	-2	1.1	1.1	1.2	1.0
HEATR5B	Q9P2D3	1254	4	11	19	-7	1.0	1.1	1.2	0.9
PTBP3	O95758	249	24	-10	19	-12	1.3	0.9	1.2	0.9
RBM4	Q9BWF3	89	-12	38	19	-15	0.9	1.6	1.2	0.9
NR3C1	P04150	643	3	-7	19	87	1.0	0.9	1.2	7.4
GPAT4	Q86UL3	325	7	-2	19	66	1.1	1.0	1.2	2.9
ENO1	P06733	389	16	30	19	36	1.2	1.4	1.2	1.6
PITHD1	Q9GZP4	14	4	8	19	33	1.0	1.1	1.2	1.5
SMCHD1	A6NHR9	1235	10	7	19	24	1.1	1.1	1.2	1.3
UBE2Z	Q9H832	286	16	15	19	12	1.2	1.2	1.2	1.1
PPP2R5C	Q13362	50	10	26	19	10	1.1	1.4	1.2	1.1
PPP2R5D	Q14738	126	10	26	19	10	1.1	1.4	1.2	1.1
KDM1B	Q8NB78	600	20	-9	19	7	1.3	0.9	1.2	1.1
ACAP1	Q15027	474	31	8	19	2	1.4	1.1	1.2	1.0
SEC24C	P53992	447	-15	-3	19	-2	0.9	1.0	1.2	1.0
CAMK1	Q14012	179	8	10	19	-6	1.1	1.1	1.2	0.9
UNC45A	Q9H3U1	420	17	4	19	-6	1.2	1.0	1.2	0.9
ARHGAP26	Q9UNA1	396	19	22	19	-7	1.2	1.3	1.2	0.9
PRKAA1	Q13131	238	1	-6	19	-7	1.0	0.9	1.2	0.9
CLIC1	O00299	191	-30	41	19	-19	0.8	1.7	1.2	0.8
ZAP70	P43403	78	-26	25	19	-26	0.8	1.3	1.2	0.8

TLN1	Q9Y490	1671	18	9	19	-49	1.2	1.1	1.2	0.7
CERK	Q8TCT0	400	-5	-3	18	72	1.0	1.0	1.2	3.5
FAM160B1	Q5W0V3	304	-15	-1	18	54	0.9	1.0	1.2	2.2
SPAG9	O60271	788	13	16	18	32	1.1	1.2	1.2	1.5
CTBP2	P56545	243	19	-2	18	31	1.2	1.0	1.2	1.4
METAP2	P50579	263	18	8	18	16	1.2	1.1	1.2	1.2
GTF3A	Q92664	62	13	5	18	15	1.1	1.1	1.2	1.2
IRF8	Q02556	223	18	10	18	11	1.2	1.1	1.2	1.1
EURL	Q9NYK6	73	-49	23	18	-1	0.7	1.3	1.2	1.0
SRP68	Q9UHB9	344	8	-1	18	-5	1.1	1.0	1.2	1.0
SUCLA2	Q9P2R7	152	9	14	18	-9	1.1	1.2	1.2	0.9
VPS28	Q9UK41	60	-5	-11	18	-10	1.0	0.9	1.2	0.9
RFTN1	Q14699	211	-83	-2	18	-106	0.5	1.0	1.2	0.5
LIMS1	P48059	138	-19	36	18	-253	0.8	1.6	1.2	0.3
CCDC97	Q96F63	78	-1	-4	18	59	1.0	1.0	1.2	2.4
DAXX	Q9UER7	131	5	6	18	48	1.0	1.1	1.2	1.9
ETHE1	O95571	170	-8	7	18	47	0.9	1.1	1.2	1.9
BTAF1	O14981	939	8	22	18	37	1.1	1.3	1.2	1.6
PREP	P48147	57	8	12	18	30	1.1	1.1	1.2	1.4
MTAP	Q13126	55	25	19	18	27	1.3	1.2	1.2	1.4
NASP	P49321	84	16	22	18	23	1.2	1.3	1.2	1.3
KLC1	Q07866	390	17	3	18	19	1.2	1.0	1.2	1.2
KLC4	Q9NSK0	388	17	3	18	19	1.2	1.0	1.2	1.2
FAM129C	Q86XR2	62	-7	3	18	19	0.9	1.0	1.2	1.2
LRSAM1	Q6UWE0	205	20	17	18	18	1.2	1.2	1.2	1.2
GGNBP2	Q9H3C7	262	12	19	18	16	1.1	1.2	1.2	1.2
WDFY4	Q6ZS81	242	-11	23	18	11	0.9	1.3	1.2	1.1
OTUD4	Q01804	903	-1	8	18	10	1.0	1.1	1.2	1.1
RRP8	O43159	320	6	6	18	10	1.1	1.1	1.2	1.1
RALGPS2	Q86X27	76	14	-4	18	10	1.2	1.0	1.2	1.1
UBR4	Q5T4S7	4916	-9	7	18	4	0.9	1.1	1.2	1.0
RPS5	P46782	172	9	15	18	1	1.1	1.2	1.2	1.0
DDX19A	Q9NUU7	224	16	4	18	-11	1.2	1.0	1.2	0.9
DDX19B	Q9UMR2	225	16	4	18	-11	1.2	1.0	1.2	0.9
GIMAP1-GIMA	A0A087WTJ2	104	-46	44	18	-50	0.7	1.8	1.2	0.7
GIMAP1	Q8WWP7	104	-46	44	18	-50	0.7	1.8	1.2	0.7
SUZ12	Q15022	325	13	4	17	79	1.1	1.0	1.2	4.8
PREP	P48147	255	10	9	17	46	1.1	1.1	1.2	1.8
ANKRD13D	Q6ZTN6	160	10	19	17	27	1.1	1.2	1.2	1.4
NOSIP	Q9Y314	47	6	17	17	22	1.1	1.2	1.2	1.3
PAX2	Q02962	64	5	-2	17	13	1.0	1.0	1.2	1.1
RPL4	P36578	125	1	8	17	12	1.0	1.1	1.2	1.1
TRAPPC10	P48553	155	15	-3	17	6	1.2	1.0	1.2	1.1
HDAC6	Q9UBN7	128	12	9	17	1	1.1	1.1	1.2	1.0
FBXO7	Q9Y3I1	359	13	11	17	-1	1.1	1.1	1.2	1.0
SNX5	Q9Y5X3	397	11	13	17	-1	1.1	1.1	1.2	1.0
XPO5	Q9HAV4	1157	12	4	17	-3	1.1	1.0	1.2	1.0
FLII	Q13045	232	-1	-17	17	-3	1.0	0.9	1.2	1.0
TNPO2	O14787	287	17	11	17	-5	1.2	1.1	1.2	1.0
DALRD3	Q5D0E6	156	-20	33	17	-6	0.8	1.5	1.2	0.9
FAM105A	Q9NUU6	100	6	-38	17	-13	1.1	0.7	1.2	0.9
PARP10	Q53GL7	757	-39	37	17	-40	0.7	1.6	1.2	0.7
DNAH1	Q9P2D7	2225	-56	19	17	-50	0.6	1.2	1.2	0.7
EVX2	Q03828	219	7	8	17	67	1.1	1.1	1.2	3.0
MPEG1	Q2M385	534	-4	-8	17	66	1.0	0.9	1.2	2.9

ETHE1	O95571	219	7	9	17	51	1.1	1.1	1.2	2.0
ADCK2	Q7Z695	624	13	11	17	30	1.1	1.1	1.2	1.4
LYPLA1	O75608	144	9	2	17	25	1.1	1.0	1.2	1.3
RRP12	Q5JTH9	673	16	5	17	25	1.2	1.1	1.2	1.3
COPA	P53621	85	1	-1	17	22	1.0	1.0	1.2	1.3
FAM129B	Q96TA1	154	13	6	17	22	1.1	1.1	1.2	1.3
H2AFY	O75367	297	14	9	17	17	1.2	1.1	1.2	1.2
EXOC8	Q8IYI6	521	12	8	17	16	1.1	1.1	1.2	1.2
PELP1	Q8IZL8	594	-1	-8	17	16	1.0	0.9	1.2	1.2
GFPT1	Q06210	264	2	0	17	15	1.0	1.0	1.2	1.2
RPS21	P63220	56	-2	19	17	11	1.0	1.2	1.2	1.1
VAC14	Q08AM6	114	11	-7	17	10	1.1	0.9	1.2	1.1
CSE1L	P55060	344	7	18	17	6	1.1	1.2	1.2	1.1
CCT4	P50991	120	11	16	17	6	1.1	1.2	1.2	1.1
DOCK8	Q8NF50	391	8	4	17	6	1.1	1.0	1.2	1.1
RPS21	P63220	17	-5	14	17	6	1.0	1.2	1.2	1.1
DNAJC13	O75165	2110	8	13	17	2	1.1	1.1	1.2	1.0
PCBP2	Q15366	158	20	7	17	-1	1.2	1.1	1.2	1.0
FEN1	P39748	163	27	8	17	-5	1.4	1.1	1.2	1.0
MMS19	Q96T76	386	13	15	17	-7	1.1	1.2	1.2	0.9
GSTM3	P21266	208	27	8	17	-7	1.4	1.1	1.2	0.9
RPS17	P08708	35	6	18	17	-8	1.1	1.2	1.2	0.9
UNC13D	Q70J99	992	8	18	17	-15	1.1	1.2	1.2	0.9
STRIP1	Q5VSL9	798	-24	33	17	-18	0.8	1.5	1.2	0.9
CMPK2	Q5EBM0	153	-7	-7	16	86	0.9	0.9	1.2	7.1
TMEM256-PLS	I3L3X5	125	4	2	16	68	1.0	1.0	1.2	3.1
KDM2B	Q8NHM5	770	26	10	16	34	1.3	1.1	1.2	1.5
IRF2BP1	Q8IU81	239	0	13	16	34	1.0	1.1	1.2	1.5
RAN	P62826	120	8	12	16	30	1.1	1.1	1.2	1.4
COG2	Q14746	287	3	12	16	23	1.0	1.1	1.2	1.3
MUT	P22033	742	19	-9	16	18	1.2	0.9	1.2	1.2
RRP8	O43159	317	5	16	16	17	1.1	1.2	1.2	1.2
FAM76B	Q5HYJ3	106	15	-12	16	11	1.2	0.9	1.2	1.1
TBCE	Q15813	301	27	-3	16	10	1.4	1.0	1.2	1.1
NBAS	A2RRP1	1769	-14	-10	16	8	0.9	0.9	1.2	1.1
ARHGAP1	Q07960	91	11	13	16	7	1.1	1.1	1.2	1.1
CHM	P24386	364	-3	-6	16	3	1.0	0.9	1.2	1.0
EIF2B4	Q9UI10	437	-20	33	16	-20	0.8	1.5	1.2	0.8
THEM6	Q8WUY1	104	-21	38	16	-56	0.8	1.6	1.2	0.6
CMIP	Q8IY22	455	16	5	16	-90	1.2	1.1	1.2	0.5
RAC1	P63000	105	1	25	16	-106	1.0	1.3	1.2	0.5
GIMAP2	Q9UG22	261	-3	-1	16	88	1.0	1.0	1.2	8.3
ALG3	Q92685	21	-2	4	16	80	1.0	1.0	1.2	5.0
ZC3H8	Q8N5P1	46	3	-5	16	51	1.0	1.0	1.2	2.0
RBM12B	Q8IXT5	919	15	12	16	35	1.2	1.1	1.2	1.5
FXR1	P51114	157	-11	5	16	34	0.9	1.1	1.2	1.5
NELFCD	Q8IXH7	417	23	9	16	31	1.3	1.1	1.2	1.4
CASP2	P42575	173	19	-8	16	30	1.2	0.9	1.2	1.4
EXOSC10	Q01780	612	5	-4	16	24	1.1	1.0	1.2	1.3
NLRP2	Q9NX02	1008	9	20	16	21	1.1	1.2	1.2	1.3
RTEL1-TNFRSF	F6WH68	20	22	-4	16	16	1.3	1.0	1.2	1.2
ITPKC	Q96DU7	564	-2	4	16	15	1.0	1.0	1.2	1.2
ADSS	P30520	182	2	19	16	13	1.0	1.2	1.2	1.1
VTA1	Q9NP79	155	18	13	16	13	1.2	1.1	1.2	1.1
SCFD1	Q8WVM8	623	18	15	16	9	1.2	1.2	1.2	1.1

KPNA4	O00629	191	10	9	16	2	1.1	1.1	1.2	1.0
RFC5	P40937	73	11	22	16	0	1.1	1.3	1.2	1.0
IDH3G	P51553	235	7	16	16	-3	1.1	1.2	1.2	1.0
SULT1A1	P50225	287	15	-1	16	-3	1.2	1.0	1.2	1.0
MINK1	Q8N4C8	269	17	4	16	-3	1.2	1.0	1.2	1.0
SAMHD1	Q9Y3Z3	573	14	9	16	-6	1.2	1.1	1.2	0.9
BCL11B	Q9C0K0	833	-20	16	16	-9	0.8	1.2	1.2	0.9
BCL11A	Q9H165	779	-20	16	16	-9	0.8	1.2	1.2	0.9
SNRNP70	P08621	39	1	25	16	-99	1.0	1.3	1.2	0.5
PSME1	Q06323	22	0	-2	15	68	1.0	1.0	1.2	3.1
HERC2	O95714	914	-7	-7	15	35	0.9	0.9	1.2	1.5
GLS	O94925	525	4	3	15	25	1.0	1.0	1.2	1.3
SSRP1	Q08945	343	16	-5	15	22	1.2	1.0	1.2	1.3
PPP2R3A	Q06190	640	10	-2	15	21	1.1	1.0	1.2	1.3
TNPO1	Q92973	153	9	4	15	19	1.1	1.0	1.2	1.2
AKAP10	O43572	475	14	17	15	9	1.2	1.2	1.2	1.1
UBE2Z	Q9H832	243	14	7	15	5	1.2	1.1	1.2	1.0
RNF213	Q63HN8	3539	9	14	15	4	1.1	1.2	1.2	1.0
DNAJC13	O75165	1490	20	-2	15	2	1.3	1.0	1.2	1.0
LPXN	O60711	152	-28	32	15	-28	0.8	1.5	1.2	0.8
FUBP1	Q96AE4	332	-8	-10	15	-72	0.9	0.9	1.2	0.6
PFAS	O15067	270	-9	4	15	75	0.9	1.0	1.2	4.0
CAT	P04040	377	7	8	15	34	1.1	1.1	1.2	1.5
CDC42	P60953	81	-8	11	15	33	0.9	1.1	1.2	1.5
POLD1	P28340	1011	10	11	15	31	1.1	1.1	1.2	1.4
CLPTM1	O96005	308	9	-4	15	26	1.1	1.0	1.2	1.4
RPS3	P23396	134	8	8	15	24	1.1	1.1	1.2	1.3
TCERG1	O14776	1062	10	11	15	22	1.1	1.1	1.2	1.3
RPL32	P62910	91	6	14	15	19	1.1	1.2	1.2	1.2
DEF6	Q9H4E7	244	6	2	15	16	1.1	1.0	1.2	1.2
ZNFX1	Q9P2E3	872	-5	26	15	13	1.0	1.3	1.2	1.1
CSE1L	P55060	842	12	9	15	4	1.1	1.1	1.2	1.0
IST1	P53990	125	16	17	15	2	1.2	1.2	1.2	1.0
ILKAP	Q9H0C8	367	15	8	15	1	1.2	1.1	1.2	1.0
PDCD4	Q53EL6	433	22	12	15	-1	1.3	1.1	1.2	1.0
RPS6KA5	O75582	631	-7	15	15	-1	0.9	1.2	1.2	1.0
SETX	Q7Z333	2451	-12	18	15	-2	0.9	1.2	1.2	1.0
KBTBD6	Q86V97	375	0	-7	15	-2	1.0	0.9	1.2	1.0
GIMAP7	Q8NHV1	160	19	5	15	-13	1.2	1.0	1.2	0.9
RNF213	Q63HN8	2868	-1	10	15	-25	1.0	1.1	1.2	0.8
REEP5	Q00765	18	-4	5	14	79	1.0	1.1	1.2	4.8
TBC1D10C	Q8IV04	305	8	10	14	35	1.1	1.1	1.2	1.5
CAPZA1	P52907	157	23	-8	14	28	1.3	0.9	1.2	1.4
ACSS2	Q9NR19	75	13	24	14	21	1.1	1.3	1.2	1.3
ZNF512B	Q96KM6	129	28	13	14	20	1.4	1.1	1.2	1.3
COG7	P83436	419	-1	22	14	19	1.0	1.3	1.2	1.2
AGTPBP1	Q9UPW5	179	8	11	14	18	1.1	1.1	1.2	1.2
ZRSR2	Q15696	326	8	-1	14	16	1.1	1.0	1.2	1.2
RBM28	Q9NW13	490	-2	8	14	16	1.0	1.1	1.2	1.2
CORO7-PAM1	A0A0A6YYL4	691	9	5	14	15	1.1	1.0	1.2	1.2
ZMYM2	Q9UBW7	495	6	-4	14	15	1.1	1.0	1.2	1.2
RPL4	P36578	250	3	25	14	11	1.0	1.3	1.2	1.1
CDC42	P60953	157	1	9	14	7	1.0	1.1	1.2	1.1
HK3	P52790	190	3	18	14	6	1.0	1.2	1.2	1.1
USP24	Q9UPU5	1628	3	33	14	5	1.0	1.5	1.2	1.0

SH3GLB2	Q9NR46	267	7	5	14	3	1.1	1.1	1.2	1.0
DNM2	P50570	427	4	18	14	-1	1.0	1.2	1.2	1.0
LRRC57	Q8N9N7	82	11	11	14	-3	1.1	1.1	1.2	1.0
RPS8	P62241	100	-8	8	14	-10	0.9	1.1	1.2	0.9
OCIAD2	Q56VL3	137	17	13	14	-21	1.2	1.1	1.2	0.8
NCOR2	Q9Y618	2179	-19	13	14	-22	0.8	1.1	1.2	0.8
INF2	Q27J81	758	13	7	14	-76	1.1	1.1	1.2	0.6
PREP	P48147	526	21	17	14	47	1.3	1.2	1.2	1.9
CASP7	P55210	290	0	-5	14	38	1.0	1.0	1.2	1.6
ASRGL1	Q7L266	162	6	14	14	37	1.1	1.2	1.2	1.6
PPWD1	Q96BP3	512	6	13	14	31	1.1	1.1	1.2	1.4
RASA4	O43374	657	-8	16	14	30	0.9	1.2	1.2	1.4
TMEM214	Q6NUQ4	529	11	-1	14	27	1.1	1.0	1.2	1.4
MON2	Q7Z3U7	1453	0	12	14	24	1.0	1.1	1.2	1.3
ARHGEF10L	Q9HCE6	767	-8	1	14	23	0.9	1.0	1.2	1.3
RBCK1	Q9BYM8	305	7	4	14	23	1.1	1.0	1.2	1.3
FCGR2B	P31994	261	-3	3	14	22	1.0	1.0	1.2	1.3
RPS5	P46782	66	-3	15	14	21	1.0	1.2	1.2	1.3
THG1L	Q9NWX6	279	10	4	14	18	1.1	1.0	1.2	1.2
NFKBIA	P25963	152	-5	6	14	17	1.0	1.1	1.2	1.2
HERC5	Q9UII4	585	5	4	14	12	1.0	1.0	1.2	1.1
PPP2R5C	Q13362	408	11	-2	14	9	1.1	1.0	1.2	1.1
GALK2	Q01415	303	12	4	14	8	1.1	1.0	1.2	1.1
FAM129A	Q9BZQ8	194	15	14	14	5	1.2	1.2	1.2	1.1
SYNE1	Q8NF91	6090	10	-15	14	5	1.1	0.9	1.2	1.1
PDCD6IP	Q8WUM4	76	10	8	14	3	1.1	1.1	1.2	1.0
PSMD5	Q16401	64	13	8	14	2	1.1	1.1	1.2	1.0
DUSP19	Q8WTR2	209	6	12	14	-1	1.1	1.1	1.2	1.0
NCBP1	Q09161	332	9	11	14	-3	1.1	1.1	1.2	1.0
PDCD6IP	Q8WUM4	691	6	17	14	-4	1.1	1.2	1.2	1.0
ARCN1	P48444	94	10	1	14	-11	1.1	1.0	1.2	0.9
DMXL2	Q8TDJ6	1578	-9	30	14	-12	0.9	1.4	1.2	0.9
FERMT3	Q86UX7	128	18	-1	14	-36	1.2	1.0	1.2	0.7
DNM1L	O00429	431	2	9	14	-48	1.0	1.1	1.2	0.7
METTL3	Q86U44	483	-51	47	14	-69	0.7	1.9	1.2	0.6
MTMR12	Q9C0I1	694	-10	17	14	-123	0.9	1.2	1.2	0.4
LDHD	Q86WU2	28	-9	-6	13	75	0.9	0.9	1.1	4.0
ETFDH	Q16134	561	6	4	13	35	1.1	1.0	1.1	1.5
ARRB2	P32121	252	7	12	13	35	1.1	1.1	1.1	1.5
DHRS1	Q96LJ7	177	12	-3	13	34	1.1	1.0	1.1	1.5
IMPDH1	P20839	327	15	13	13	26	1.2	1.1	1.1	1.4
PLSCR1	O15162	239	18	18	13	21	1.2	1.2	1.1	1.3
HDHD2	Q9H0R4	244	3	6	13	21	1.0	1.1	1.1	1.3
CNP	P09543	49	19	1	13	16	1.2	1.0	1.1	1.2
ZFYVE26	Q68DK2	2407	-1	-1	13	16	1.0	1.0	1.1	1.2
STIP1	P31948	403	9	-5	13	11	1.1	1.0	1.1	1.1
ATP5H	O75947	101	16	6	13	11	1.2	1.1	1.1	1.1
RNF114	Q9Y508	64	-3	-6	13	11	1.0	0.9	1.1	1.1
POLR2G	P62487	38	13	1	13	9	1.1	1.0	1.1	1.1
RPL32	P62910	96	1	15	13	6	1.0	1.2	1.1	1.1
RABGAP1L	Q5R372	202	10	10	13	6	1.1	1.1	1.1	1.1
RABL2A	Q9UBK7	26	12	9	13	4	1.1	1.1	1.1	1.0
ARMC6	Q6NXE6	118	12	20	13	2	1.1	1.3	1.1	1.0
STAT6	P42226	452	7	4	13	1	1.1	1.0	1.1	1.0
HK3	P52790	409	-4	9	13	-2	1.0	1.1	1.1	1.0

RAB20	Q9NX57	131	17	-4	13	-4	1.2	1.0	1.1	1.0
CDC16	Q13042	544	-12	14	13	-5	0.9	1.2	1.1	1.0
UBA5	Q9GZZ9	181	21	5	13	-10	1.3	1.0	1.1	0.9
PDE5A	O76074	81	10	28	13	-176	1.1	1.4	1.1	0.4
PLA2G2F	Q9BZM2	48	-8	-13	13	60	0.9	0.9	1.1	2.5
PLA2G2F	Q9BZM2	46	-8	-13	13	60	0.9	0.9	1.1	2.5
SP100	P23497	468	4	17	13	56	1.0	1.2	1.1	2.3
DUSP7	Q16829	256	3	9	13	54	1.0	1.1	1.1	2.2
FXYD7	P58549	63	5	-2	13	35	1.1	1.0	1.1	1.5
ETHE1	O95571	98	1	6	13	23	1.0	1.1	1.1	1.3
UBAP1	Q9NZ09	81	-5	10	13	21	1.0	1.1	1.1	1.3
EXOC6	Q8TAG9	28	10	14	13	21	1.1	1.2	1.1	1.3
RHOT2	Q8IXI1	543	12	2	13	19	1.1	1.0	1.1	1.2
LRRC41	Q15345	520	8	-14	13	18	1.1	0.9	1.1	1.2
RABL3	Q5HYI8	180	19	20	13	17	1.2	1.2	1.1	1.2
RPL10	P27635	80	8	5	13	16	1.1	1.1	1.1	1.2
ZFYVE16	Q7Z3T8	1009	10	-3	13	12	1.1	1.0	1.1	1.1
EPHX2	P34913	74	-2	-3	13	12	1.0	1.0	1.1	1.1
Uncharacteriz	A0A0C4DFX4	848	-3	1	13	10	1.0	1.0	1.1	1.1
WDR92	Q96MX6	70	-9	-10	13	8	0.9	0.9	1.1	1.1
AP3D1	O14617	39	9	7	13	7	1.1	1.1	1.1	1.1
CAD	P27708	280	-17	23	13	6	0.9	1.3	1.1	1.1
DUSP23	Q9BVJ7	95	14	-6	13	0	1.2	0.9	1.1	1.0
UBR4	Q5T4S7	953	-16	-7	13	0	0.9	0.9	1.1	1.0
WDR24	Q96S15	474	2	-14	13	-1	1.0	0.9	1.1	1.0
SHC1	P29353	196	9	8	13	-2	1.1	1.1	1.1	1.0
PXN	P49023	564	2	4	13	-3	1.0	1.0	1.1	1.0
RANGAP1	P46060	169	15	6	13	-5	1.2	1.1	1.1	1.0
PUM1	Q14671	977	4	11	13	-7	1.0	1.1	1.1	0.9
PUM2	Q8TB72	857	4	11	13	-7	1.0	1.1	1.1	0.9
CLTC	Q00610	909	7	0	13	-8	1.1	1.0	1.1	0.9
TELO2	Q9Y4R8	222	7	0	13	-10	1.1	1.0	1.1	0.9
SMTN	P53814	541	11	7	13	-40	1.1	1.1	1.1	0.7
LNPEP	Q9UIQ6	103	15	3	12	42	1.2	1.0	1.1	1.7
ABCB1	P08183	1074	-2	25	12	31	1.0	1.3	1.1	1.4
RFC1	P35251	752	7	15	12	31	1.1	1.2	1.1	1.4
RPAIN	Q86UA6	122	24	4	12	26	1.3	1.0	1.1	1.3
XPO4	Q9C0E2	492	-9	2	12	25	0.9	1.0	1.1	1.3
LIG4	P49917	807	3	2	12	24	1.0	1.0	1.1	1.3
SLC25A13	Q9UJS0	503	12	-7	12	23	1.1	0.9	1.1	1.3
ATP6V0D1	P61421	244	8	3	12	22	1.1	1.0	1.1	1.3
TAF1	P21675	1364	19	-20	12	22	1.2	0.8	1.1	1.3
HRH1	P35367	302	15	7	12	21	1.2	1.1	1.1	1.3
SLFN11	Q7Z7L1	126	4	5	12	21	1.0	1.0	1.1	1.3
ARHGDIA	P52565	79	17	6	12	20	1.2	1.1	1.1	1.3
DCAF13	Q9NV06	120	6	13	12	17	1.1	1.1	1.1	1.2
BAZ1A	Q9NRL2	547	6	7	12	16	1.1	1.1	1.1	1.2
IKBKG	Q9Y6K9	167	18	14	12	15	1.2	1.2	1.1	1.2
ALKBH2	Q6NS38	192	6	1	12	14	1.1	1.0	1.1	1.2
GFOD2	Q3B7J2	377	14	1	12	12	1.2	1.0	1.1	1.1
TRIM25	Q14258	174	5	-5	12	9	1.0	1.0	1.1	1.1
RASAL3	Q86YV0	614	7	-4	12	6	1.1	1.0	1.1	1.1
PACS2	Q86VP3	858	-1	1	12	4	1.0	1.0	1.1	1.0
FASN	P49327	1118	1	12	12	4	1.0	1.1	1.1	1.0
CLASP2	O75122	1179	3	-6	12	2	1.0	0.9	1.1	1.0

ZC3HC1	Q86WB0	156	6	10	12	1	1.1	1.1	1.1	1.0
RPS20	P60866	70	3	12	12	-1	1.0	1.1	1.1	1.0
PLEC	Q15149	3295	-10	26	12	-5	0.9	1.3	1.1	1.0
NR4A1	P22736	566	1	-1	12	-7	1.0	1.0	1.1	0.9
NR4A2	P43354	566	1	-1	12	-7	1.0	1.0	1.1	0.9
CAND1	Q86VP6	179	4	-28	12	-8	1.0	0.8	1.1	0.9
CAND1	Q86VP6	954	5	5	12	-9	1.1	1.1	1.1	0.9
UTRN	P46939	2602	15	13	12	-9	1.2	1.1	1.1	0.9
ATRX	P46100	1814	-33	50	12	-21	0.8	2.0	1.1	0.8
IL7R	P16871	369	4	20	12	46	1.0	1.3	1.1	1.8
YDJC	A8MPS7	182	4	11	12	35	1.0	1.1	1.1	1.5
TNPO1	Q92973	142	-10	10	12	34	0.9	1.1	1.1	1.5
CDC42	P60953	105	7	4	12	25	1.1	1.0	1.1	1.3
MANBA	O00462	800	4	-3	12	23	1.0	1.0	1.1	1.3
CSDE1	O75534	730	6	7	12	20	1.1	1.1	1.1	1.2
C5orf45	Q6NTE8	52	-22	21	12	19	0.8	1.3	1.1	1.2
PPP4C	P60510	52	11	4	12	19	1.1	1.0	1.1	1.2
CFHR4	Q92496	567	-21	-11	12	18	0.8	0.9	1.1	1.2
IPO9	Q96P70	864	8	19	12	17	1.1	1.2	1.1	1.2
NCF2	P19878	40	7	0	12	16	1.1	1.0	1.1	1.2
SMG1	Q96Q15	3401	6	1	12	16	1.1	1.0	1.1	1.2
VPS8	Q8N3P4	974	1	1	12	15	1.0	1.0	1.1	1.2
EIF2S3	P41091	269	6	-2	12	14	1.1	1.0	1.1	1.2
HNRNPR	O43390	99	-6	10	12	13	0.9	1.1	1.1	1.1
SYNCRIP	O60506	96	-6	10	12	13	0.9	1.1	1.1	1.1
ARF6	P62330	155	2	17	12	10	1.0	1.2	1.1	1.1
TTLL12	Q14166	612	4	5	12	7	1.0	1.0	1.1	1.1
GCFC2	P16383	336	10	2	12	6	1.1	1.0	1.1	1.1
DYNC1LI2	O43237	104	6	8	12	1	1.1	1.1	1.1	1.0
DYNC1LI1	Q9Y6G9	117	6	8	12	1	1.1	1.1	1.1	1.0
MRPS18A	Q9NVS2	108	9	-1	12	1	1.1	1.0	1.1	1.0
CAPNS1	P04632	232	-3	2	12	-6	1.0	1.0	1.1	0.9
REPS2	Q8NFH8	380	-5	-28	12	-6	1.0	0.8	1.1	0.9
VWF	P04275	257	-6	-11	12	-11	0.9	0.9	1.1	0.9
TRUB1	Q8WWH5	269	5	-8	12	-11	1.1	0.9	1.1	0.9
KDM2A	Q9Y2K7	582	9	3	12	-12	1.1	1.0	1.1	0.9
PCBP1	Q15365	158	11	5	12	-13	1.1	1.0	1.1	0.9
FAM160B1	Q5W0V3	298	-3	-11	11	39	1.0	0.9	1.1	1.6
APEX1	P27695	208	11	8	11	35	1.1	1.1	1.1	1.5
GMPR2	Q9P2T1	186	3	14	11	29	1.0	1.2	1.1	1.4
CCT3	P49368	40	-3	13	11	29	1.0	1.1	1.1	1.4
C9orf114	Q5T280	139	15	10	11	24	1.2	1.1	1.1	1.3
INTS6	Q9UL03	211	7	-7	11	23	1.1	0.9	1.1	1.3
IMPA1	P29218	184	32	9	11	20	1.5	1.1	1.1	1.3
EIF3CL	B5ME19	79	21	0	11	20	1.3	1.0	1.1	1.2
MVP	Q14764	59	9	0	11	20	1.1	1.0	1.1	1.2
NOA1	Q8NC60	393	19	10	11	19	1.2	1.1	1.1	1.2
GGNBP2	Q9H3C7	243	8	19	11	17	1.1	1.2	1.1	1.2
KPNA1	P52294	210	17	9	11	16	1.2	1.1	1.1	1.2
CEP192	Q8TEP8	1586	8	-2	11	16	1.1	1.0	1.1	1.2
SMARCA5	O60264	165	5	4	11	14	1.1	1.0	1.1	1.2
RPL4	P36578	208	-1	3	11	14	1.0	1.0	1.1	1.2
UTP6	Q9NYH9	175	-3	22	11	12	1.0	1.3	1.1	1.1
RGPD3	A6NKT7	537	26	1	11	10	1.3	1.0	1.1	1.1
RANBP2	P49792	536	26	1	11	10	1.3	1.0	1.1	1.1

RGPD6	Q99666	537	26	1	11	10	1.3	1.0	1.1	1.1
RPL18A	Q02543	64	-11	-2	11	10	0.9	1.0	1.1	1.1
PDCD6IP	Q8WUM4	40	11	11	11	9	1.1	1.1	1.1	1.1
DDX3X	O00571	341	16	7	11	9	1.2	1.1	1.1	1.1
DDX3Y	O15523	339	16	7	11	9	1.2	1.1	1.1	1.1
CUL2	Q13617	385	12	-3	11	9	1.1	1.0	1.1	1.1
PCF11	O94913	102	13	7	11	8	1.1	1.1	1.1	1.1
PRKCB	P05771	70	-1	-3	11	8	1.0	1.0	1.1	1.1
PRKCA	P17252	70	-1	-3	11	8	1.0	1.0	1.1	1.1
DET1	Q7L5Y6	482	16	16	11	5	1.2	1.2	1.1	1.0
CHD3	Q12873	1464	18	3	11	3	1.2	1.0	1.1	1.0
CAP1	Q01518	356	-1	15	11	2	1.0	1.2	1.1	1.0
UBE3B	Q7Z3V4	979	-4	-2	11	0	1.0	1.0	1.1	1.0
EPRS	P07814	1301	11	5	11	-1	1.1	1.0	1.1	1.0
HSPD1	P10809	447	7	12	11	-2	1.1	1.1	1.1	1.0
SNX12	Q9UMY4	141	5	6	11	-3	1.1	1.1	1.1	1.0
PRKDC	P78527	1128	7	-8	11	-4	1.1	0.9	1.1	1.0
ARFGAP2	Q8N6H7	26	7	5	11	-5	1.1	1.0	1.1	1.0
UNC13D	Q70J99	112	5	5	11	-7	1.1	1.1	1.1	0.9
WAPL	Q7Z5K2	891	10	13	11	-11	1.1	1.1	1.1	0.9
NDRG3	Q9UGV2	290	20	-1	11	-11	1.2	1.0	1.1	0.9
KDM5A	P29375	690	-4	-6	11	-18	1.0	0.9	1.1	0.8
DIAPH1	O60610	267	1	6	11	-21	1.0	1.1	1.1	0.8
RAB11FIP3	O75154	564	26	17	11	-82	1.4	1.2	1.1	0.6
ERCC3	P19447	342	2	-6	11	82	1.0	0.9	1.1	5.6
NUDT8	Q8WV74	207	-5	-13	11	60	1.0	0.9	1.1	2.5
SMCHD1	A6NHR9	897	5	14	11	43	1.0	1.2	1.1	1.8
CDA	P32320	65	13	-10	11	41	1.1	0.9	1.1	1.7
FBXO42	Q6P3S6	302	1	4	11	37	1.0	1.0	1.1	1.6
RNH1	P13489	273	12	-2	11	36	1.1	1.0	1.1	1.6
PMF1-BGLAP	U3KQ54	61	10	6	11	32	1.1	1.1	1.1	1.5
HDAC7	Q8WUI4	566	9	-2	11	30	1.1	1.0	1.1	1.4
EIF3I	Q13347	81	4	2	11	28	1.0	1.0	1.1	1.4
KDM4B	O94953	694	-3	-9	11	28	1.0	0.9	1.1	1.4
ACAA2	P42765	128	-7	2	11	26	0.9	1.0	1.1	1.4
SPATA13	Q96N96	384	8	12	11	25	1.1	1.1	1.1	1.3
RRAGC	Q9HB90	52	22	-25	11	23	1.3	0.8	1.1	1.3
MTUS1	Q9ULD2	1240	-2	7	11	23	1.0	1.1	1.1	1.3
PRKRA	O75569	163	6	12	11	21	1.1	1.1	1.1	1.3
FUBP3	Q96I24	366	-4	3	11	20	1.0	1.0	1.1	1.2
RPL28	P46779	13	-4	15	11	19	1.0	1.2	1.1	1.2
ESR2	Q92731	224	-11	15	11	19	0.9	1.2	1.1	1.2
SH3GLB1	Q9Y371	121	6	13	11	18	1.1	1.1	1.1	1.2
UNK	Q9C0B0	788	11	1	11	17	1.1	1.0	1.1	1.2
NFKBIE	O00221	345	3	-5	11	17	1.0	1.0	1.1	1.2
CNOT4	O95628	175	0	-9	11	17	1.0	0.9	1.1	1.2
FAM65A	Q6ZS17	987	-8	14	11	16	0.9	1.2	1.1	1.2
SYNE2	Q8WXH0	1844	16	-6	11	16	1.2	0.9	1.1	1.2
CSE1L	P55060	387	10	10	11	14	1.1	1.1	1.1	1.2
PTPN7	P35236	300	6	2	11	14	1.1	1.0	1.1	1.2
MAP2K6	P52564	294	28	16	11	13	1.4	1.2	1.1	1.1
ATP9B	O43861	626	15	3	11	12	1.2	1.0	1.1	1.1
S100A11	P31949	13	5	15	11	10	1.0	1.2	1.1	1.1
CUL2	Q13617	465	6	-6	11	9	1.1	0.9	1.1	1.1
TRIO	O75962	2709	-3	5	11	7	1.0	1.1	1.1	1.1

PLEC	Q15149	3667	7	9	11	6	1.1	1.1	1.1	1.1
ETFA	P13804	109	10	3	11	6	1.1	1.0	1.1	1.1
SMC5	Q8IY18	176	7	5	11	5	1.1	1.1	1.1	1.1
MRRF	Q96E11	154	2	-8	11	5	1.0	0.9	1.1	1.1
DYNC1H1	Q14204	2454	-18	4	11	5	0.9	1.0	1.1	1.0
AIMP2	Q13155	168	9	4	11	4	1.1	1.0	1.1	1.0
PFDN5	Q99471	49	-1	0	11	3	1.0	1.0	1.1	1.0
ZCCHC3	Q9NUD5	179	7	-8	11	2	1.1	0.9	1.1	1.0
IPO5	O00410	110	13	9	11	-1	1.1	1.1	1.1	1.0
UBOX5	O94941	340	-9	-3	11	-3	0.9	1.0	1.1	1.0
KPNA4	O00629	228	2	14	11	-5	1.0	1.2	1.1	1.0
TRIM28	Q13263	209	10	12	11	-9	1.1	1.1	1.1	0.9
ACSL4	O60488	602	-20	-6	11	-12	0.8	0.9	1.1	0.9
PRPF6	O94906	901	6	9	11	-13	1.1	1.1	1.1	0.9
AUP1	Q9Y679	212	2	-4	11	-16	1.0	1.0	1.1	0.9
TLN1	Q9Y490	750	8	6	11	-70	1.1	1.1	1.1	0.6
TLN1	Q9Y490	719	18	16	11	-91	1.2	1.2	1.1	0.5
ATF7IP	Q6VMQ6	1228	22	9	10	41	1.3	1.1	1.1	1.7
RANBP3	Q9H6Z4	116	7	3	10	41	1.1	1.0	1.1	1.7
NCAPH	Q15003	418	13	6	10	38	1.1	1.1	1.1	1.6
CPNE3	O75131	249	-5	-1	10	32	1.0	1.0	1.1	1.5
SLC15A4	Q8N697	37	-3	3	10	30	1.0	1.0	1.1	1.4
ZNF699	Q32M78	626	17	-4	10	26	1.2	1.0	1.1	1.4
BIN1	O00499	47	16	13	10	26	1.2	1.1	1.1	1.3
TRPV2	Q9Y5S1	332	17	8	10	24	1.2	1.1	1.1	1.3
CD2AP	Q9Y5K6	595	12	3	10	23	1.1	1.0	1.1	1.3
PLCG1	P19174	247	6	11	10	23	1.1	1.1	1.1	1.3
PDIA6	Q15084	55	11	-4	10	20	1.1	1.0	1.1	1.3
BIRC6	Q9NR09	4183	-3	5	10	19	1.0	1.0	1.1	1.2
RPF2	Q9H7B2	135	12	7	10	16	1.1	1.1	1.1	1.2
REL	Q04864	195	-1	2	10	15	1.0	1.0	1.1	1.2
ETHE1	O95571	34	-4	0	10	15	1.0	1.0	1.1	1.2
SMARCA5	O60264	527	8	11	10	14	1.1	1.1	1.1	1.2
GNL2	Q13823	362	-3	20	10	13	1.0	1.2	1.1	1.1
TPR	P12270	75	9	-11	10	12	1.1	0.9	1.1	1.1
RPS5	P46782	155	4	13	10	11	1.0	1.1	1.1	1.1
MTR	Q99707	400	18	-2	10	10	1.2	1.0	1.1	1.1
NME7	Q9Y5B8	228	21	11	10	10	1.3	1.1	1.1	1.1
CUL3	Q13618	522	8	1	10	9	1.1	1.0	1.1	1.1
ATXN10	Q9UBB4	134	19	10	10	8	1.2	1.1	1.1	1.1
SH3BP1	Q9Y3L3	56	-4	8	10	7	1.0	1.1	1.1	1.1
OGDH	Q02218	566	-4	6	10	7	1.0	1.1	1.1	1.1
SH3GLB2	Q9NR46	252	6	2	10	7	1.1	1.0	1.1	1.1
FNTA	P49354	341	4	-17	10	7	1.0	0.9	1.1	1.1
DDX6	P26196	184	19	11	10	5	1.2	1.1	1.1	1.1
DCTN4	Q9UJW0	30	-2	-4	10	4	1.0	1.0	1.1	1.0
RGPD3	A6NKT7	1109	2	-6	10	4	1.0	0.9	1.1	1.0
RANBP2	P49792	2084	2	-6	10	4	1.0	0.9	1.1	1.0
RGPD6	Q99666	1108	2	-6	10	4	1.0	0.9	1.1	1.0
TNPO2	O14787	805	13	-9	10	2	1.1	0.9	1.1	1.0
TNPO1	Q92973	806	13	-9	10	2	1.1	0.9	1.1	1.0
PTBP1	P26599	251	4	6	10	1	1.0	1.1	1.1	1.0
DIAPH1	O60610	437	14	5	10	1	1.2	1.0	1.1	1.0
PMVK	Q15126	92	8	2	10	-2	1.1	1.0	1.1	1.0
TRADD	Q15628	138	3	4	10	-5	1.0	1.0	1.1	1.0

COG2	Q14746	358	14	8	10	-5	1.2	1.1	1.1	1.0
LRBA	P50851	2820	7	12	10	-10	1.1	1.1	1.1	0.9
IDH3A	P50213	331	11	12	10	-11	1.1	1.1	1.1	0.9
FLYWCH1	Q4VC44	478	-14	12	10	-14	0.9	1.1	1.1	0.9
TPM4	P67936	154	3	11	10	-15	1.0	1.1	1.1	0.9
CDC27	P30260	71	-1	39	10	-18	1.0	1.6	1.1	0.8
HMHA1	Q92619	809	6	-13	10	-27	1.1	0.9	1.1	0.8
VEZF1	Q14119	319	-32	29	10	-34	0.8	1.4	1.1	0.7
ZBTB7A	O95365	468	-8	13	10	-35	0.9	1.1	1.1	0.7
SPTBN1	Q01082	624	22	-12	10	-44	1.3	0.9	1.1	0.7
FERMT3	Q86UX7	452	12	-2	10	-70	1.1	1.0	1.1	0.6
ARIH1	Q9Y4X5	161	3	-6	10	73	1.0	0.9	1.1	3.7
TAP2	Q03519	641	1	-3	10	72	1.0	1.0	1.1	3.5
BRAT1	Q6PJG6	513	3	-6	10	58	1.0	0.9	1.1	2.4
AHNAK	Q09666	5382	6	-2	10	44	1.1	1.0	1.1	1.8
ZMYND19	Q96E35	200	1	-4	10	41	1.0	1.0	1.1	1.7
ETHE1	O95571	80	-12	2	10	40	0.9	1.0	1.1	1.7
POC5	Q8NA72	360	1	-4	10	37	1.0	1.0	1.1	1.6
SLC25A6	P12236	57	15	12	10	34	1.2	1.1	1.1	1.5
PTBP1	P26599	23	2	7	10	34	1.0	1.1	1.1	1.5
LSM7	Q9UK45	85	8	-3	10	34	1.1	1.0	1.1	1.5
ACTL7A	Q9Y615	318	2	-13	10	33	1.0	0.9	1.1	1.5
NFS1	Q9Y697	158	-4	2	10	31	1.0	1.0	1.1	1.4
ECM29	Q5VYK3	1390	14	0	10	29	1.2	1.0	1.1	1.4
EIF2AK1	Q9BQI3	494	-4	-9	10	28	1.0	0.9	1.1	1.4
EIF2S1	P05198	218	2	9	10	24	1.0	1.1	1.1	1.3
CEP41	Q9BYV8	171	12	2	10	24	1.1	1.0	1.1	1.3
ALDH9A1	P49189	443	4	-19	10	19	1.0	0.8	1.1	1.2
COG4	Q9H9E3	102	3	9	10	19	1.0	1.1	1.1	1.2
ZNF267	Q14586	419	6	7	10	19	1.1	1.1	1.1	1.2
NDUFV1	P49821	286	-6	4	10	18	0.9	1.0	1.1	1.2
GLMN	Q92990	218	4	-14	10	18	1.0	0.9	1.1	1.2
DNAJA2	O60884	280	5	7	10	16	1.1	1.1	1.1	1.2
RLF	Q13129	453	-3	2	10	16	1.0	1.0	1.1	1.2
HS1BP3	Q53T59	118	7	1	10	14	1.1	1.0	1.1	1.2
LDAH	Q9H6V9	274	13	11	10	12	1.1	1.1	1.1	1.1
TBCK	Q8TEA7	353	2	11	10	12	1.0	1.1	1.1	1.1
METAP1	P53582	194	-1	-5	10	12	1.0	1.0	1.1	1.1
LRRC57	Q8N9N7	128	8	7	10	11	1.1	1.1	1.1	1.1
SRPRA	P08240	621	8	5	10	11	1.1	1.0	1.1	1.1
RPS28	P62857	27	-3	13	10	10	1.0	1.1	1.1	1.1
ZNF624	Q9P2J8	542	4	-5	10	10	1.0	1.0	1.1	1.1
CPNE2	Q96FN4	116	10	15	10	8	1.1	1.2	1.1	1.1
MFF	Q9GZY8	209	2	-13	10	8	1.0	0.9	1.1	1.1
RIMBP3B	A6NNM3	1428	-2	-6	10	7	1.0	0.9	1.1	1.1
RIMBP3B	A6NNM3	1418	-2	-6	10	7	1.0	0.9	1.1	1.1
ITPRIP	Q8IWB1	228	-7	17	10	5	0.9	1.2	1.1	1.1
ZNF786	Q8N393	734	6	3	10	5	1.1	1.0	1.1	1.1
HBB	P68871	113	7	4	10	5	1.1	1.0	1.1	1.0
MARCH5	Q9NX47	65	-1	4	10	2	1.0	1.0	1.1	1.0
PTPN7	P35236	204	0	14	10	1	1.0	1.2	1.1	1.0
PDCD6IP	Q8WUM4	122	4	6	10	1	1.0	1.1	1.1	1.0
TBC1D5	Q92609	192	14	3	10	-3	1.2	1.0	1.1	1.0
CCT7	Q99832	370	1	4	10	-5	1.0	1.0	1.1	1.0
STXBP2	Q15833	353	-2	5	10	-10	1.0	1.0	1.1	0.9

MBD1	Q9UIS9	353	5	13	10	-11	1.0	1.1	1.1	0.9
DIAPH1	O60610	314	9	12	10	-16	1.1	1.1	1.1	0.9
RNGTT	O60942	97	-42	14	10	-25	0.7	1.2	1.1	0.8
WDR59	Q6PJI9	163	-30	-12	10	-25	0.8	0.9	1.1	0.8
UBE2O	Q9C0C9	1099	2	16	10	-26	1.0	1.2	1.1	0.8
FYCO1	Q9BQS8	848	-29	25	10	-36	0.8	1.3	1.1	0.7
FERMT3	Q86UX7	439	-1	5	10	-62	1.0	1.1	1.1	0.6
EDC4	Q6P2E9	299	-61	24	10	-102	0.6	1.3	1.1	0.5
NCAPD3	P42695	631	10	-8	9	73	1.1	0.9	1.1	3.6
RELB	Q01201	109	5	-1	9	54	1.0	1.0	1.1	2.2
EPHA4	P54764	649	1	11	9	41	1.0	1.1	1.1	1.7
C19orf68	Q86XI8	403	8	-11	9	40	1.1	0.9	1.1	1.7
ARL11	Q969Q4	180	3	8	9	33	1.0	1.1	1.1	1.5
SECISBP2	Q96T21	291	-1	20	9	25	1.0	1.3	1.1	1.3
DOCK8	Q8NF50	1836	3	14	9	24	1.0	1.2	1.1	1.3
TMEM154	Q6P9G4	164	10	14	9	23	1.1	1.2	1.1	1.3
ZNF428	Q96B54	115	3	-2	9	21	1.0	1.0	1.1	1.3
CAT	P04040	460	-1	7	9	19	1.0	1.1	1.1	1.2
SAFB	Q15424	448	-3	1	9	19	1.0	1.0	1.1	1.2
DDX55	Q8NHQ9	389	-5	5	9	19	1.0	1.0	1.1	1.2
SH2D3C	Q8N5H7	599	11	2	9	17	1.1	1.0	1.1	1.2
SORD	Q00796	130	22	-7	9	17	1.3	0.9	1.1	1.2
GTF3C5	Q9Y5Q8	411	14	22	9	15	1.2	1.3	1.1	1.2
NUDCD1	Q96RS6	513	8	1	9	14	1.1	1.0	1.1	1.2
MBD1	Q9UIS9	28	-4	4	9	13	1.0	1.0	1.1	1.1
RASGRP4	Q8TDF6	122	11	1	9	12	1.1	1.0	1.1	1.1
RPS14	P62263	31	15	-17	9	12	1.2	0.9	1.1	1.1
RPS15A	P62244	72	-6	6	9	11	0.9	1.1	1.1	1.1
TBCK	Q8TEA7	772	8	1	9	11	1.1	1.0	1.1	1.1
USP28	Q96RU2	575	28	16	9	10	1.4	1.2	1.1	1.1
API5	Q9BZZ5	234	8	26	9	9	1.1	1.3	1.1	1.1
ZW10	O43264	650	16	19	9	9	1.2	1.2	1.1	1.1
ZBTB1	Q9Y2K1	450	7	2	9	9	1.1	1.0	1.1	1.1
TSPAN32	Q96QS1	275	-2	-7	9	9	1.0	0.9	1.1	1.1
ARHGAP9	Q9BRR9	548	10	5	9	8	1.1	1.1	1.1	1.1
IKBKAP	O95163	1033	6	9	9	8	1.1	1.1	1.1	1.1
ZW10	O43264	588	11	2	9	8	1.1	1.0	1.1	1.1
ERI3	O43414	258	12	-32	9	7	1.1	0.8	1.1	1.1
DRAP1	Q14919	73	6	7	9	6	1.1	1.1	1.1	1.1
PRKD3	O94806	719	5	12	9	5	1.0	1.1	1.1	1.1
PRKD1	Q15139	726	5	12	9	5	1.0	1.1	1.1	1.1
PRKD2	Q9BZL6	694	5	12	9	5	1.0	1.1	1.1	1.1
ATP13A1	Q9HD20	862	10	7	9	5	1.1	1.1	1.1	1.1
ARHGAP4	P98171	102	13	-6	9	5	1.1	0.9	1.1	1.1
AKAP13	Q12802	2397	-1	-4	9	3	1.0	1.0	1.1	1.0
VTA1	Q9NP79	38	13	6	9	2	1.1	1.1	1.1	1.0
PDS5A	Q29RF7	589	7	5	9	-1	1.1	1.1	1.1	1.0
DYNC1H1	Q14204	1888	11	1	9	-2	1.1	1.0	1.1	1.0
NELFCD	Q8IXH7	398	8	-1	9	-2	1.1	1.0	1.1	1.0
DR1	Q01658	42	10	1	9	-4	1.1	1.0	1.1	1.0
QKI	Q96PU8	119	3	2	9	-4	1.0	1.0	1.1	1.0
RFC4	P35249	177	6	-3	9	-5	1.1	1.0	1.1	1.0
TIMM17B	O60830	17	1	-3	9	-6	1.0	1.0	1.1	0.9
GGPS1	O95749	247	9	4	9	-7	1.1	1.0	1.1	0.9
CEP152	O94986	818	-4	-11	9	-12	1.0	0.9	1.1	0.9

PEX1	O43933	935	1	-3	9	-13	1.0	1.0	1.1	0.9
ECM29	Q5VYK3	1459	3	14	9	-18	1.0	1.2	1.1	0.8
CDK5	Q00535	157	-4	2	9	-21	1.0	1.0	1.1	0.8
RPS26	P62854	74	-50	27	9	-79	0.7	1.4	1.1	0.6
VPS18	Q9P253	806	1	0	9	62	1.0	1.0	1.1	2.6
GNPAT	O15228	54	-3	-3	9	56	1.0	1.0	1.1	2.2
CD79A	P11912	119	-6	-2	9	36	0.9	1.0	1.1	1.6
MAP4K1	Q92918	698	9	11	9	33	1.1	1.1	1.1	1.5
CD180	Q99467	419	4	11	9	27	1.0	1.1	1.1	1.4
CTU1	Q7Z7A3	147	-45	-25	9	27	0.7	0.8	1.1	1.4
BRF1	Q92994	184	16	13	9	26	1.2	1.1	1.1	1.4
BOP1	Q14137	373	13	10	9	24	1.1	1.1	1.1	1.3
HERC2	O95714	3864	-3	-5	9	22	1.0	1.0	1.1	1.3
SRP9	P49458	39	6	5	9	21	1.1	1.0	1.1	1.3
RPL11	P62913	21	4	12	9	19	1.0	1.1	1.1	1.2
ATP5A1	P25705	294	2	8	9	19	1.0	1.1	1.1	1.2
EIF3I	Q13347	76	3	-1	9	19	1.0	1.0	1.1	1.2
STAT1	P42224	174	7	-1	9	18	1.1	1.0	1.1	1.2
OSBPL9	Q96SU4	173	11	17	9	17	1.1	1.2	1.1	1.2
RASA2	Q15283	314	5	-3	9	16	1.0	1.0	1.1	1.2
MAP2K2	P36507	211	2	-9	9	16	1.0	0.9	1.1	1.2
MAP2K1	Q02750	207	2	-9	9	16	1.0	0.9	1.1	1.2
DDX24	Q9GZR7	467	8	11	9	16	1.1	1.1	1.1	1.2
ECHDC1	Q9NTX5	133	8	-9	9	15	1.1	0.9	1.1	1.2
USP9Y	O00507	1773	21	-2	9	14	1.3	1.0	1.1	1.2
USP9X	Q93008	1771	21	-2	9	14	1.3	1.0	1.1	1.2
N4BP1	O75113	56	1	3	9	13	1.0	1.0	1.1	1.1
SMG8	Q8ND04	261	-4	-3	9	13	1.0	1.0	1.1	1.1
RPS3A	P61247	201	9	10	9	12	1.1	1.1	1.1	1.1
AP1G2	O75843	354	11	16	9	11	1.1	1.2	1.1	1.1
GART	P22102	237	1	-1	9	11	1.0	1.0	1.1	1.1
SERPINB6	P35237	350	12	7	9	10	1.1	1.1	1.1	1.1
MCM6	Q14566	721	1	3	9	10	1.0	1.0	1.1	1.1
ANXA7	P20073	298	4	10	9	9	1.0	1.1	1.1	1.1
ATAD2	Q6PL18	501	15	2	9	8	1.2	1.0	1.1	1.1
ATAD2B	Q9ULI0	475	15	2	9	8	1.2	1.0	1.1	1.1
UBE2Z	Q9H832	100	-2	-3	9	7	1.0	1.0	1.1	1.1
FAM114A2	Q9NRY5	388	11	16	9	6	1.1	1.2	1.1	1.1
HIP1	O00291	63	14	13	9	6	1.2	1.1	1.1	1.1
CCT3	P49368	398	13	12	9	6	1.1	1.1	1.1	1.1
RANBP2	P49792	2407	9	5	9	6	1.1	1.1	1.1	1.1
WDFY4	Q6ZS81	264	9	-4	9	6	1.1	1.0	1.1	1.1
ABR	Q12979	201	4	6	9	5	1.0	1.1	1.1	1.1
USP32	Q8NFA0	641	16	17	9	5	1.2	1.2	1.1	1.0
STK11IP	Q8N1F8	929	-12	11	9	5	0.9	1.1	1.1	1.0
HPS3	Q969F9	915	1	2	9	5	1.0	1.0	1.1	1.0
MOB3A	Q96BX8	88	4	6	9	4	1.0	1.1	1.1	1.0
RNF214	Q8ND24	149	-14	-9	9	4	0.9	0.9	1.1	1.0
KPNA4	O00629	325	16	8	9	1	1.2	1.1	1.1	1.0
EIF3K	Q9UBQ5	85	12	3	9	1	1.1	1.0	1.1	1.0
LACE1	Q8WV93	72	18	10	9	-2	1.2	1.1	1.1	1.0
ARFGEF2	Q9Y6D5	76	9	5	9	-2	1.1	1.0	1.1	1.0
PIGQ	Q9BRB3	67	-15	-1	9	-2	0.9	1.0	1.1	1.0
LAS1L	Q9Y4W2	456	-16	12	9	-3	0.9	1.1	1.1	1.0
FAM114A2	Q9NRY5	438	15	7	9	-3	1.2	1.1	1.1	1.0

COPB1	P53618	616	9	2	9	-4	1.1	1.0	1.1	1.0
APAF1	O14727	317	9	3	9	-6	1.1	1.0	1.1	0.9
PML	P29590	204	-2	4	9	-7	1.0	1.0	1.1	0.9
LRBA	P50851	832	4	7	9	-8	1.0	1.1	1.1	0.9
COG3	Q96JB2	334	5	3	9	-8	1.1	1.0	1.1	0.9
SENP8	Q96LD8	64	4	5	9	-10	1.0	1.1	1.1	0.9
VTA1	Q9NP79	87	12	11	9	-11	1.1	1.1	1.1	0.9
DIAPH1	O60610	1005	12	-1	9	-17	1.1	1.0	1.1	0.9
CTNND1	O60716	450	-17	11	9	-22	0.9	1.1	1.1	0.8
RASSF4	Q9H2L5	249	10	-18	9	-22	1.1	0.9	1.1	0.8
UBE2M	P61081	47	-13	17	9	-25	0.9	1.2	1.1	0.8
SEPT1	Q8WYJ6	136	-22	12	9	-32	0.8	1.1	1.1	0.8
SLFN14	P0C7P3	176	27	24	9	-116	1.4	1.3	1.1	0.5
RXRB	P28702	340	17	-27	8	88	1.2	0.8	1.1	8.3
NCDN	Q9UBB6	98	-6	-5	8	80	0.9	1.0	1.1	5.0
MSRB2	Q9Y3D2	105	-43	11	8	72	0.7	1.1	1.1	3.5
MTAP	Q13126	145	8	11	8	52	1.1	1.1	1.1	2.1
ZNF644	Q9H582	152	14	-6	8	52	1.2	0.9	1.1	2.1
CCR6	P51684	348	-6	-12	8	43	0.9	0.9	1.1	1.8
ITPA	Q9BY32	146	18	5	8	36	1.2	1.0	1.1	1.6
RPS2	P15880	229	0	11	8	29	1.0	1.1	1.1	1.4
ANKFY1	Q9P2R3	1060	-1	-2	8	27	1.0	1.0	1.1	1.4
RNMT	O43148	356	7	-3	8	26	1.1	1.0	1.1	1.4
GSTCD	Q8NEC7	140	10	-5	8	26	1.1	1.0	1.1	1.4
SEC14L1	Q92503	258	3	7	8	25	1.0	1.1	1.1	1.3
CUX1	P39880	1338	2	3	8	25	1.0	1.0	1.1	1.3
URB2	Q14146	1294	-1	9	8	23	1.0	1.1	1.1	1.3
TEX10	Q9NXF1	774	24	18	8	23	1.3	1.2	1.1	1.3
TRAPPC10	P48553	1080	11	3	8	21	1.1	1.0	1.1	1.3
RPS6KA1	Q15418	552	4	-2	8	20	1.0	1.0	1.1	1.3
RPL14	P50914	42	3	1	8	18	1.0	1.0	1.1	1.2
FTO	Q9C0B1	326	0	-1	8	17	1.0	1.0	1.1	1.2
BTLA	Q7Z6A9	286	-12	2	8	17	0.9	1.0	1.1	1.2
NUP160	Q12769	1309	3	-4	8	16	1.0	1.0	1.1	1.2
LRRC40	Q9H9A6	264	8	6	8	14	1.1	1.1	1.1	1.2
ALDH7A1	P49419	478	3	-1	8	14	1.0	1.0	1.1	1.2
NCF2	P19878	45	20	-7	8	13	1.2	0.9	1.1	1.1
TNIP2	Q8NFZ5	427	7	-4	8	12	1.1	1.0	1.1	1.1
pk	D4Q8H0	285	0	2	8	12	1.0	1.0	1.1	1.1
APRT	P07741	140	-4	-17	8	11	1.0	0.9	1.1	1.1
CLGN	O14967	511	-1	7	8	10	1.0	1.1	1.1	1.1
POLD3	Q15054	129	2	7	8	10	1.0	1.1	1.1	1.1
NDUFA5	Q16718	17	3	3	8	10	1.0	1.0	1.1	1.1
PDXDC1	Q6P996	425	13	7	8	9	1.1	1.1	1.1	1.1
PRKCQ	Q04759	332	11	17	8	9	1.1	1.2	1.1	1.1
CASP10	Q92851	43	20	0	8	9	1.3	1.0	1.1	1.1
SHC1	P29353	248	6	-11	8	6	1.1	0.9	1.1	1.1
INTS8	Q75QN2	701	19	2	8	5	1.2	1.0	1.1	1.0
ACAT2	Q9BWD1	92	9	-3	8	4	1.1	1.0	1.1	1.0
UBXN6	Q9BZV1	125	10	-9	8	4	1.1	0.9	1.1	1.0
PSMC2	P35998	236	3	10	8	4	1.0	1.1	1.1	1.0
FPGT	O14772	519	7	-9	8	4	1.1	0.9	1.1	1.0
PHF1	O43189	91	11	-9	8	3	1.1	0.9	1.1	1.0
DNAJC13	O75165	2082	11	5	8	2	1.1	1.1	1.1	1.0
DDX50	Q9BQ39	417	7	4	8	2	1.1	1.0	1.1	1.0

ATP6V1B2	P21281	289	1	16	8	2	1.0	1.2	1.1	1.0
LRSAM1	Q6UWE0	193	12	4	8	2	1.1	1.0	1.1	1.0
MYO1G	B0I1T2	143	2	-1	8	1	1.0	1.0	1.1	1.0
PDHA1	P08559	273	17	-6	8	1	1.2	0.9	1.1	1.0
RFC2	P35250	88	9	9	8	0	1.1	1.1	1.1	1.0
GAPVD1	Q14C86	1253	15	8	8	-1	1.2	1.1	1.1	1.0
CARS2	Q9HA77	526	-11	-7	8	-2	0.9	0.9	1.1	1.0
GCA	P28676	182	8	1	8	-4	1.1	1.0	1.1	1.0
SUPV3L1	Q8IYB8	575	10	1	8	-5	1.1	1.0	1.1	1.0
CMYA5	Q8N3K9	3290	-2	-3	8	-6	1.0	1.0	1.1	0.9
QRICH1	Q2TAL8	611	-3	2	8	-7	1.0	1.0	1.1	0.9
BTAF1	O14981	1310	-13	9	8	-10	0.9	1.1	1.1	0.9
STAT6	P42226	228	5	17	8	-11	1.0	1.2	1.1	0.9
LIMD1	Q9UGP4	585	8	3	8	-11	1.1	1.0	1.1	0.9
MRPL38	Q96DV4	143	-3	7	8	-12	1.0	1.1	1.1	0.9
PRKCB	P05771	71	8	5	8	-16	1.1	1.1	1.1	0.9
PRKCA	P17252	71	8	5	8	-16	1.1	1.1	1.1	0.9
THOC3	Q96J01	65	-13	21	8	-21	0.9	1.3	1.1	0.8
NAE1	Q13564	294	-5	-25	8	-23	1.0	0.8	1.1	0.8
PLA2R1	Q13018	515	7	6	8	-50	1.1	1.1	1.1	0.7
PUF60	Q9UHX1	255	-7	-1	8	-61	0.9	1.0	1.1	0.6
XPO5	Q9HAV4	44	-3	2	8	75	1.0	1.0	1.1	4.0
NT5DC1	Q5TFE4	119	-6	-15	8	64	0.9	0.9	1.1	2.8
GPAT3	Q53EU6	306	3	-6	8	53	1.0	0.9	1.1	2.1
FAM160B1	Q5W0V3	306	-5	-5	8	50	1.0	1.0	1.1	2.0
BCAS3	Q9H6U6	258	18	2	8	39	1.2	1.0	1.1	1.6
FAM45A	Q8TCE6	135	12	12	8	38	1.1	1.1	1.1	1.6
RXRA	P19793	432	-1	0	8	33	1.0	1.0	1.1	1.5
RXRB	P28702	503	-1	0	8	33	1.0	1.0	1.1	1.5
DDX46	Q7L014	367	13	3	8	32	1.1	1.0	1.1	1.5
CYC1	P08574	271	6	-8	8	31	1.1	0.9	1.1	1.4
SERPINB8	P50452	364	-1	6	8	28	1.0	1.1	1.1	1.4
RGS3	P49796	814	6	-1	8	27	1.1	1.0	1.1	1.4
SERPINB9	P50453	370	10	1	8	26	1.1	1.0	1.1	1.4
EIF4G2	P78344	253	2	12	8	25	1.0	1.1	1.1	1.3
LRSAM1	Q6UWE0	23	-12	-10	8	25	0.9	0.9	1.1	1.3
MTR	Q99707	798	-3	3	8	24	1.0	1.0	1.1	1.3
RAE1	P78406	173	11	12	8	18	1.1	1.1	1.1	1.2
RPL11	P62913	25	6	7	8	18	1.1	1.1	1.1	1.2
ATP1A1	P05023	464	4	6	8	18	1.0	1.1	1.1	1.2
RAVER1	Q8IY67	224	5	7	8	17	1.0	1.1	1.1	1.2
XPO7	Q9UIA9	606	2	7	8	16	1.0	1.1	1.1	1.2
ATRX	P46100	223	11	3	8	16	1.1	1.0	1.1	1.2
ZNF428	Q96B54	114	-3	2	8	16	1.0	1.0	1.1	1.2
DCAF5	Q96JK2	711	6	7	8	16	1.1	1.1	1.1	1.2
RPS14	P62263	85	1	15	8	15	1.0	1.2	1.1	1.2
NUP155	O75694	704	3	13	8	15	1.0	1.1	1.1	1.2
PRKDC	P78527	1432	10	6	8	14	1.1	1.1	1.1	1.2
CTBS	Q01459	93	5	13	8	14	1.0	1.1	1.1	1.2
LRCH3	Q96II8	531	-1	13	8	13	1.0	1.1	1.1	1.1
TBC1D10C	Q8IV04	111	10	11	8	13	1.1	1.1	1.1	1.1
EMC4	Q5J8M3	69	3	13	8	12	1.0	1.1	1.1	1.1
VPS53	Q5VIR6	242	4	-3	8	12	1.0	1.0	1.1	1.1
RPL7	P18124	186	2	3	8	12	1.0	1.0	1.1	1.1
CTBP2	P56545	60	8	-1	8	12	1.1	1.0	1.1	1.1

WDSUB1	Q8N9V3	410	-17	19	8	11	0.9	1.2	1.1	1.1
WNK1	Q9H4A3	352	1	8	8	11	1.0	1.1	1.1	1.1
KDM6A	O15550	384	11	-1	8	11	1.1	1.0	1.1	1.1
AGAP2	Q99490	548	4	2	8	10	1.0	1.0	1.1	1.1
IPO8	O15397	749	3	-2	8	10	1.0	1.0	1.1	1.1
IPO7	O95373	749	3	-2	8	10	1.0	1.0	1.1	1.1
ADH5	P11766	111	4	21	8	9	1.0	1.3	1.1	1.1
PRKAA1	Q13131	185	-3	7	8	8	1.0	1.1	1.1	1.1
YWHAQ	P27348	94	14	-2	8	8	1.2	1.0	1.1	1.1
DOCK5	Q9H7D0	841	0	-9	8	8	1.0	0.9	1.1	1.1
SPECC1	Q5M775	470	5	16	8	7	1.1	1.2	1.1	1.1
IDH3A	P50213	222	11	0	8	7	1.1	1.0	1.1	1.1
COIL	P38432	80	-1	-5	8	7	1.0	1.0	1.1	1.1
ZNF512	Q96ME7	174	3	-3	8	6	1.0	1.0	1.1	1.1
USP9X	Q93008	1061	2	6	8	6	1.0	1.1	1.1	1.1
PTGES3	Q15185	40	8	4	8	5	1.1	1.0	1.1	1.1
NBAS	A2RRP1	862	10	12	8	5	1.1	1.1	1.1	1.0
ZC3H7A	Q8IWR0	506	2	9	8	5	1.0	1.1	1.1	1.0
DNM1L	O00429	644	14	7	8	5	1.2	1.1	1.1	1.0
AHSA1	O95433	56	17	-7	8	5	1.2	0.9	1.1	1.0
CLTC	Q00610	39	3	-7	8	5	1.0	0.9	1.1	1.0
NELFCD	Q8IXH7	397	4	5	8	4	1.0	1.1	1.1	1.0
NFIC	P08651	163	5	-5	8	4	1.1	1.0	1.1	1.0
PRPF38A	Q8NAV1	72	-2	-7	8	4	1.0	0.9	1.1	1.0
IPO5	O00410	473	6	1	8	4	1.1	1.0	1.1	1.0
PPID	Q08752	282	-3	-2	8	3	1.0	1.0	1.1	1.0
RAB4A	P20338	28	6	5	8	3	1.1	1.1	1.1	1.0
ZNF512	Q96ME7	192	10	9	8	2	1.1	1.1	1.1	1.0
LRRC47	Q8N1G4	249	-2	5	8	1	1.0	1.0	1.1	1.0
SHOC2	Q9UQ13	260	4	3	8	-1	1.0	1.0	1.1	1.0
CSE1L	P55060	272	10	2	8	-2	1.1	1.0	1.1	1.0
XPO5	Q9HAV4	977	13	19	8	-3	1.1	1.2	1.1	1.0
POLR2D	O15514	104	-20	26	8	-3	0.8	1.3	1.1	1.0
MRPS30	Q9NP92	292	7	12	8	-4	1.1	1.1	1.1	1.0
VPS13A	Q96RL7	1608	12	2	8	-5	1.1	1.0	1.1	1.0
ARHGAP25	P42331	183	10	3	8	-5	1.1	1.0	1.1	1.0
ITPR2	Q14571	1929	22	5	8	-6	1.3	1.0	1.1	0.9
ITPR3	Q14573	1881	22	5	8	-6	1.3	1.0	1.1	0.9
ITPR1	Q14643	1985	22	5	8	-6	1.3	1.0	1.1	0.9
COPB1	P53618	102	6	-2	8	-6	1.1	1.0	1.1	0.9
LIMD1	Q9UGP4	472	3	-1	8	-18	1.0	1.0	1.1	0.9
RNF31	Q96EP0	237	-24	17	8	-21	0.8	1.2	1.1	0.8
ARIH2	O95376	188	5	3	8	-24	1.1	1.0	1.1	0.8
RASGRP2	Q7LDG7	296	2	7	8	-25	1.0	1.1	1.1	0.8
ATM	Q13315	1396	-9	-17	7	83	0.9	0.9	1.1	5.7
HERPUD1	Q15011	86	15	13	7	44	1.2	1.1	1.1	1.8
NUDT16L1	Q9BRJ7	171	-2	11	7	43	1.0	1.1	1.1	1.7
RING1	Q06587	279	8	-1	7	40	1.1	1.0	1.1	1.7
PLBD1	Q6P4A8	474	0	5	7	39	1.0	1.1	1.1	1.6
GLE1	Q53GS7	287	-2	-6	7	35	1.0	0.9	1.1	1.5
POLR2A	P24928	1287	29	3	7	34	1.4	1.0	1.1	1.5
BRAT1	Q6PJG6	228	-9	5	7	34	0.9	1.1	1.1	1.5
MED23	Q9ULK4	40	7	3	7	30	1.1	1.0	1.1	1.4
PITPNM1	O00562	668	-2	-8	7	29	1.0	0.9	1.1	1.4
SMC3	Q9UQE7	1134	8	16	7	28	1.1	1.2	1.1	1.4

GCDH	Q92947	115	6	-2	7	28	1.1	1.0	1.1	1.4
ZNF280C	Q8ND82	94	-14	6	7	24	0.9	1.1	1.1	1.3
GPX4	P36969	175	-9	3	7	24	0.9	1.0	1.1	1.3
SNX18	Q96RF0	259	-1	7	7	23	1.0	1.1	1.1	1.3
FBXO11	Q86XK2	581	-14	2	7	23	0.9	1.0	1.1	1.3
HBB	P68871	94	-3	-3	7	23	1.0	1.0	1.1	1.3
BMP2K	Q9NSY1	197	0	13	7	22	1.0	1.1	1.1	1.3
MYO9B	Q13459	1876	23	7	7	20	1.3	1.1	1.1	1.2
PLAA	Q9Y263	27	-1	2	7	20	1.0	1.0	1.1	1.2
RPL37A	P61513	39	7	4	7	19	1.1	1.0	1.1	1.2
EIF3F	A0A0D9SEZ9	195	3	5	7	18	1.0	1.1	1.1	1.2
PDCD4	Q53EL6	447	0	1	7	18	1.0	1.0	1.1	1.2
AHDC1	Q5TGY3	147	5	-11	7	18	1.0	0.9	1.1	1.2
USP48	Q86UV5	691	-8	2	7	17	0.9	1.0	1.1	1.2
RRBP1	Q9P2E9	1038	1	-4	7	16	1.0	1.0	1.1	1.2
TPT1	P13693	28	7	-5	7	14	1.1	1.0	1.1	1.2
XIAP	P98170	202	15	5	7	14	1.2	1.1	1.1	1.2
ZNF276	Q8N554	533	8	6	7	12	1.1	1.1	1.1	1.1
COG1	Q8WTW3	625	3	8	7	12	1.0	1.1	1.1	1.1
UBA3	Q8TBC4	82	9	11	7	11	1.1	1.1	1.1	1.1
LAS1L	Q9Y4W2	474	10	18	7	10	1.1	1.2	1.1	1.1
MATR3	A8MXP9	851	3	13	7	10	1.0	1.1	1.1	1.1
FAM98C	Q17RN3	287	13	-2	7	10	1.1	1.0	1.1	1.1
NKRF	O15226	386	-10	-3	7	10	0.9	1.0	1.1	1.1
GCN1	Q92616	55	8	8	7	9	1.1	1.1	1.1	1.1
PTPN23	Q9H3S7	65	9	1	7	9	1.1	1.0	1.1	1.1
CASP1	P29466	362	6	-8	7	9	1.1	0.9	1.1	1.1
IPO11	Q9UI26	262	2	2	7	9	1.0	1.0	1.1	1.1
HNRNPUL1	Q9BUJ2	391	3	1	7	8	1.0	1.0	1.1	1.1
VWA8	A3KMH1	416	-2	-7	7	7	1.0	0.9	1.1	1.1
SMCHD1	A6NHR9	444	-4	19	7	6	1.0	1.2	1.1	1.1
TRMT1	Q9NXH9	376	14	12	7	6	1.2	1.1	1.1	1.1
IQGAP1	P46940	151	11	9	7	6	1.1	1.1	1.1	1.1
IRF9	Q00978	241	12	1	7	6	1.1	1.0	1.1	1.1
TTC4	O95801	374	4	-6	7	5	1.0	0.9	1.1	1.1
NEK9	Q8TD19	375	-7	-5	7	3	0.9	1.0	1.1	1.0
IPO13	O94829	838	-1	4	7	3	1.0	1.0	1.1	1.0
OAS3	Q9Y6K5	444	2	4	7	3	1.0	1.0	1.1	1.0
WDFY4	Q6ZS81	835	10	5	7	1	1.1	1.0	1.1	1.0
DLG4	P78352	445	7	2	7	1	1.1	1.0	1.1	1.0
PDCD6IP	Q8WUM4	127	8	21	7	-2	1.1	1.3	1.1	1.0
TRIM24	O15164	134	-1	-3	7	-4	1.0	1.0	1.1	1.0
RPL3	P39023	114	-1	-4	7	-4	1.0	1.0	1.1	1.0
PLA2G15	Q8NCC3	355	-2	-12	7	-4	1.0	0.9	1.1	1.0
RIC8A	Q9NPQ8	341	12	10	7	-4	1.1	1.1	1.1	1.0
DICER1	Q9UPY3	699	8	3	7	-4	1.1	1.0	1.1	1.0
RRP12	Q5JTH9	799	2	4	7	-5	1.0	1.0	1.1	1.0
TREM1	Q9NP99	69	2	-5	7	-6	1.0	1.0	1.1	0.9
HADHB	P55084	435	6	-19	7	-6	1.1	0.8	1.1	0.9
MRI1	Q9BV20	199	1	-11	7	-10	1.0	0.9	1.1	0.9
ARHGAP4	P98171	527	6	0	7	-11	1.1	1.0	1.1	0.9
SEPT1	Q8WYJ6	137	-9	8	7	-13	0.9	1.1	1.1	0.9
SP3	Q02447	600	8	-2	7	-13	1.1	1.0	1.1	0.9
IQGAP1	P46940	45	16	5	7	-14	1.2	1.1	1.1	0.9
TRIM22	Q8IYM9	97	14	-2	7	-14	1.2	1.0	1.1	0.9

DDX19A	Q9NUU7	313	-2	-4	7	-15	1.0	1.0	1.1	0.9
DDX19B	Q9UMR2	314	-2	-4	7	-15	1.0	1.0	1.1	0.9
TRIOBP	Q9H2D6	2280	-1	-19	7	-19	1.0	0.8	1.1	0.8
MED24	O75448	336	-21	-2	7	-41	0.8	1.0	1.1	0.7
CAD	P27708	758	-2	-3	7	70	1.0	1.0	1.1	3.3
PEPD	P12955	158	9	4	7	39	1.1	1.0	1.1	1.6
ELAC2	Q9BQ52	51	4	9	7	38	1.0	1.1	1.1	1.6
FAM102B	Q5T8I3	282	6	8	7	36	1.1	1.1	1.1	1.6
GPATCH1	Q9BRR8	839	18	15	7	34	1.2	1.2	1.1	1.5
ANKRD50	Q9ULJ7	639	13	5	7	33	1.1	1.0	1.1	1.5
DDX60L	Q5H9U9	284	11	4	7	32	1.1	1.0	1.1	1.5
JMJD7	P0C870	52	7	-12	7	31	1.1	0.9	1.1	1.4
USP34	Q70CQ2	1380	-3	2	7	31	1.0	1.0	1.1	1.4
DDX17	Q92841	199	-1	-7	7	30	1.0	0.9	1.1	1.4
STUB1	Q9UNE7	48	8	11	7	27	1.1	1.1	1.1	1.4
CCNL2	Q96S94	82	11	11	7	26	1.1	1.1	1.1	1.3
HNRNPK	P61978	145	-1	4	7	25	1.0	1.0	1.1	1.3
ESYT1	Q9BSJ8	370	15	11	7	24	1.2	1.1	1.1	1.3
GSTCD	Q8NEC7	150	9	11	7	24	1.1	1.1	1.1	1.3
IRF1	P10914	53	13	2	7	22	1.1	1.0	1.1	1.3
CAND1	Q86VP6	571	9	3	7	21	1.1	1.0	1.1	1.3
PES1	O00541	391	7	7	7	20	1.1	1.1	1.1	1.2
CD4	P01730	445	-1	-5	7	19	1.0	1.0	1.1	1.2
BCCIP	Q9P287	275	6	15	7	18	1.1	1.2	1.1	1.2
INPP5D	Q92835	49	7	-1	7	18	1.1	1.0	1.1	1.2
PCMTD2	Q9NV79	223	-1	-10	7	18	1.0	0.9	1.1	1.2
FRYL	O94915	2160	7	13	7	18	1.1	1.1	1.1	1.2
CSE1L	P55060	939	2	4	7	18	1.0	1.0	1.1	1.2
HNRNPL	P14866	452	-2	4	7	17	1.0	1.0	1.1	1.2
ZC3H13	Q5T200	1549	-6	-5	7	17	0.9	1.0	1.1	1.2
YDJC	A8MPS7	212	1	8	7	17	1.0	1.1	1.1	1.2
TBC1D2B	Q9UPU7	38	-2	2	7	16	1.0	1.0	1.1	1.2
HNRNPL	P14866	260	2	4	7	16	1.0	1.0	1.1	1.2
ERN1	O75460	572	-9	-8	7	16	0.9	0.9	1.1	1.2
MDN1	Q9NU22	43	-5	5	7	15	1.0	1.1	1.1	1.2
PLXNB2	O15031	1408	13	5	7	14	1.1	1.1	1.1	1.2
KANK1	Q14678	633	3	-5	7	14	1.0	1.0	1.1	1.2
EIF3I	Q13347	99	-5	-2	7	13	1.0	1.0	1.1	1.1
EARS2	Q5JPH6	341	5	-10	7	13	1.1	0.9	1.1	1.1
LPP	Q93052	569	9	15	7	13	1.1	1.2	1.1	1.1
DYNC1H1	Q14204	2594	3	-2	7	13	1.0	1.0	1.1	1.1
ANKRD13A	Q8IZ07	141	-1	9	7	12	1.0	1.1	1.1	1.1
SUPT6H	Q7KZ85	1435	21	5	7	11	1.3	1.0	1.1	1.1
PDCD11	Q14690	333	-2	-5	7	11	1.0	1.0	1.1	1.1
ACSL3	O95573	450	6	8	7	10	1.1	1.1	1.1	1.1
TMA16	Q96EY4	75	7	4	7	9	1.1	1.0	1.1	1.1
TNFAIP2	Q03169	512	13	-4	7	8	1.1	1.0	1.1	1.1
NFX1	Q12986	190	-10	-1	7	8	0.9	1.0	1.1	1.1
DAZAP1	Q96EP5	85	-6	4	7	7	0.9	1.0	1.1	1.1
PLEKHO1	Q53GL0	269	-11	-7	7	7	0.9	0.9	1.1	1.1
SYNE1	Q8NF91	2598	5	-17	7	5	1.1	0.9	1.1	1.1
ATG4C	Q96DT6	111	15	3	7	5	1.2	1.0	1.1	1.0
C16orf62	Q7Z3J2	277	1	-4	7	2	1.0	1.0	1.1	1.0
GAB2	Q9UQC2	303	-16	-8	7	2	0.9	0.9	1.1	1.0
CCT7	Q99832	450	7	3	7	1	1.1	1.0	1.1	1.0

GCN1	Q92616	246	13	1	7	1	1.1	1.0	1.1	1.0
RIC8A	Q9NPQ8	79	12	11	7	0	1.1	1.1	1.1	1.0
AP1G1	O43747	400	-1	-7	7	-1	1.0	0.9	1.1	1.0
GSDMD	P57764	38	11	2	7	-1	1.1	1.0	1.1	1.0
IPO5	O00410	348	9	1	7	-1	1.1	1.0	1.1	1.0
EXOC6	Q8TAG9	532	-2	-6	7	-1	1.0	0.9	1.1	1.0
RPS29	P62273	39	-3	-2	7	-2	1.0	1.0	1.1	1.0
ARFGEF2	Q9Y6D5	995	4	7	7	-2	1.0	1.1	1.1	1.0
MYCBP2	O75592	3225	-1	-13	7	-5	1.0	0.9	1.1	1.0
RHOG	P84095	18	-1	-8	7	-6	1.0	0.9	1.1	0.9
CCT8	P50990	148	10	-5	7	-6	1.1	1.0	1.1	0.9
NFATC2	Q13469	569	1	3	7	-7	1.0	1.0	1.1	0.9
SLC30A1	Q9Y6M5	433	8	3	7	-8	1.1	1.0	1.1	0.9
BANK1	Q8NDB2	98	0	-5	7	-10	1.0	1.0	1.1	0.9
Uncharacteriz	G3V4G9	95	6	16	7	-11	1.1	1.2	1.1	0.9
HMHA1	Q92619	781	9	16	7	-12	1.1	1.2	1.1	0.9
TCEB1	Q15369	74	3	-8	7	-13	1.0	0.9	1.1	0.9
IQGAP2	Q13576	1183	8	-10	7	-16	1.1	0.9	1.1	0.9
PCBP1	Q15365	163	22	12	7	-17	1.3	1.1	1.1	0.9
RSF1	Q96T23	944	-5	-9	7	-18	1.0	0.9	1.1	0.9
FCHO1	O14526	647	-14	14	7	-19	0.9	1.2	1.1	0.8
TLN1	Q9Y490	1023	4	-2	7	-59	1.0	1.0	1.1	0.6
GON4L	Q3T8J9	789	-2	-2	6	62	1.0	1.0	1.1	2.6
YY1AP1	Q9H869	266	-2	-2	6	62	1.0	1.0	1.1	2.6
LRRC47	Q8N1G4	390	3	-6	6	36	1.0	0.9	1.1	1.6
IRS2	Q9Y4H2	802	4	-14	6	35	1.0	0.9	1.1	1.5
FHL3	Q13643	150	-13	3	6	34	0.9	1.0	1.1	1.5
CX3CR1	P49238	286	16	-10	6	31	1.2	0.9	1.1	1.4
CX3CR1	P49238	287	16	-10	6	31	1.2	0.9	1.1	1.4
KMT2B	Q9UMN6	1306	-8	7	6	31	0.9	1.1	1.1	1.4
RHOH	Q15669	165	13	3	6	30	1.1	1.0	1.1	1.4
LYZ	P61626	146	0	4	6	30	1.0	1.0	1.1	1.4
BRAT1	Q6PJG6	366	1	-4	6	29	1.0	1.0	1.1	1.4
GAPDH	P04406	247	2	3	6	27	1.0	1.0	1.1	1.4
USP24	Q9UPU5	1362	8	6	6	27	1.1	1.1	1.1	1.4
AKAP8L	Q9ULX6	211	-5	22	6	26	1.0	1.3	1.1	1.3
TRMT2A	Q8IZ69	538	1	1	6	24	1.0	1.0	1.1	1.3
FAM126A	Q9BYI3	300	-6	6	6	23	0.9	1.1	1.1	1.3
SFSWAP	Q12872	220	-8	1	6	22	0.9	1.0	1.1	1.3
ARID4A	P29374	107	1	-13	6	21	1.0	0.9	1.1	1.3
DNAJB1	P25685	269	10	-5	6	20	1.1	1.0	1.1	1.3
CEP57	Q86XR8	399	18	11	6	17	1.2	1.1	1.1	1.2
STAU2	Q9NUL3	11	18	1	6	15	1.2	1.0	1.1	1.2
KIF1BP	Q96EK5	371	3	-3	6	15	1.0	1.0	1.1	1.2
PMPCB	O75439	485	9	6	6	14	1.1	1.1	1.1	1.2
HUWE1	Q7Z6Z7	624	5	-4	6	14	1.1	1.0	1.1	1.2
BPTF	Q12830	1969	5	0	6	12	1.0	1.0	1.1	1.1
KPNA6	O60684	357	3	-3	6	12	1.0	1.0	1.1	1.1
KPNA1	P52294	359	3	-3	6	12	1.0	1.0	1.1	1.1
DCUN1D3	Q8IWE4	66	7	8	6	12	1.1	1.1	1.1	1.1
TAF4B	Q92750	160	2	-11	6	12	1.0	0.9	1.1	1.1
MED16	Q9Y2X0	545	-14	10	6	11	0.9	1.1	1.1	1.1
ARFGEF1	Q9Y6D6	1526	0	-2	6	11	1.0	1.0	1.1	1.1
NOL11	Q9H8H0	67	-6	9	6	10	0.9	1.1	1.1	1.1
PLEC	Q15149	4071	5	4	6	10	1.0	1.0	1.1	1.1

APOBEC3G	Q9HC16	321	11	6	6	9	1.1	1.1	1.1	1.1
UBE4A	Q14139	710	-5	-10	6	9	1.0	0.9	1.1	1.1
HBD	P02042	94	9	-6	6	8	1.1	0.9	1.1	1.1
UBE2V2	Q15819	69	29	-5	6	8	1.4	1.0	1.1	1.1
RPA3	P35244	81	0	2	6	7	1.0	1.0	1.1	1.1
ARFGEF1	Q9Y6D6	1729	11	-7	6	7	1.1	0.9	1.1	1.1
UBE2Z	Q9H832	261	4	5	6	6	1.0	1.0	1.1	1.1
RING1	Q06587	84	9	-1	6	6	1.1	1.0	1.1	1.1
RNF2	Q99496	87	9	-1	6	6	1.1	1.0	1.1	1.1
ATXN10	Q9UBB4	95	-3	12	6	5	1.0	1.1	1.1	1.0
CCT3	P49368	455	2	3	6	5	1.0	1.0	1.1	1.0
SRRT	Q9BXP5	441	-2	-5	6	5	1.0	1.0	1.1	1.0
METAP2	P50579	290	3	15	6	4	1.0	1.2	1.1	1.0
CYP20A1	Q6UW02	331	13	8	6	3	1.1	1.1	1.1	1.0
ARFGEF2	Q9Y6D5	1273	9	-9	6	3	1.1	0.9	1.1	1.0
ARFGEF1	Q9Y6D6	1326	9	-9	6	3	1.1	0.9	1.1	1.0
FRY	Q5TBA9	1809	6	4	6	3	1.1	1.0	1.1	1.0
WDR43	Q15061	315	9	-8	6	2	1.1	0.9	1.1	1.0
ANXA7	P20073	413	11	12	6	1	1.1	1.1	1.1	1.0
DOCK7	Q96N67	2125	2	-9	6	1	1.0	0.9	1.1	1.0
KAT8	Q9H7Z6	442	13	-13	6	1	1.1	0.9	1.1	1.0
GIMAP1	Q8WWP7	181	15	-7	6	1	1.2	0.9	1.1	1.0
NPM1	P06748	275	5	0	6	0	1.0	1.0	1.1	1.0
RPA1	P27694	500	5	11	6	-1	1.0	1.1	1.1	1.0
ZNF276	Q8N554	136	0	-10	6	-1	1.0	0.9	1.1	1.0
PHF12	Q96QT6	335	2	-7	6	-1	1.0	0.9	1.1	1.0
EXOC2	Q96KP1	586	0	10	6	-2	1.0	1.1	1.1	1.0
ARFGEF1	Q9Y6D6	1050	6	7	6	-2	1.1	1.1	1.1	1.0
BOP1	Q14137	532	-9	5	6	-2	0.9	1.0	1.1	1.0
HNRNPF	P52597	290	-4	-6	6	-2	1.0	0.9	1.1	1.0
CAP1	Q01518	416	7	11	6	-3	1.1	1.1	1.1	1.0
RSF1	Q96T23	129	-7	-12	6	-3	0.9	0.9	1.1	1.0
XPO1	O14980	164	2	1	6	-4	1.0	1.0	1.1	1.0
MED17	Q9NVC6	304	-4	-7	6	-4	1.0	0.9	1.1	1.0
ARHGEF6	Q15052	319	9	-2	6	-9	1.1	1.0	1.1	0.9
CAMK2G	Q13555	290	8	6	6	-11	1.1	1.1	1.1	0.9
CXorf38	Q8TB03	77	14	11	6	-12	1.2	1.1	1.1	0.9
WAPL	Q7Z5K2	964	4	-1	6	-15	1.0	1.0	1.1	0.9
CTCF	P49711	500	12	2	6	-21	1.1	1.0	1.1	0.8
NOL10	Q9BSC4	16	5	-5	6	43	1.0	1.0	1.1	1.8
POLRMT	O00411	726	7	-5	6	38	1.1	1.0	1.1	1.6
SRD5A3	Q9H8P0	66	0	6	6	37	1.0	1.1	1.1	1.6
CCDC97	Q96F63	122	13	-6	6	33	1.1	0.9	1.1	1.5
EIF2S3	P41091	348	-1	-9	6	31	1.0	0.9	1.1	1.4
TANGO6	Q9C0B7	184	-5	-11	6	30	1.0	0.9	1.1	1.4
INTS12	Q96CB8	379	7	3	6	30	1.1	1.0	1.1	1.4
TCEA2	Q15560	135	6	0	6	29	1.1	1.0	1.1	1.4
RNF214	Q8ND24	655	15	-23	6	28	1.2	0.8	1.1	1.4
RPL18	Q07020	134	4	-5	6	27	1.0	1.0	1.1	1.4
APEX1	P27695	65	10	4	6	26	1.1	1.0	1.1	1.4
NOP56	O00567	112	-7	-12	6	26	0.9	0.9	1.1	1.4
ENTHD2	Q96N21	493	4	4	6	25	1.0	1.0	1.1	1.3
EZH1	Q92800	423	-6	1	6	24	0.9	1.0	1.1	1.3
DOK1	Q99704	308	-18	6	6	24	0.9	1.1	1.1	1.3
BRAP	Q7Z569	473	16	8	6	23	1.2	1.1	1.1	1.3

CHD6	Q8TD26	172	8	-4	6	22	1.1	1.0	1.1	1.3
BUB3	O43684	270	5	2	6	21	1.0	1.0	1.1	1.3
HK2	P52789	628	11	3	6	20	1.1	1.0	1.1	1.3
NMD3	Q96D46	214	11	1	6	20	1.1	1.0	1.1	1.3
DARS2	Q6PI48	449	10	2	6	20	1.1	1.0	1.1	1.2
USP34	Q70CQ2	2931	4	-1	6	19	1.0	1.0	1.1	1.2
PRPS1	P60891	41	2	3	6	17	1.0	1.0	1.1	1.2
RCSD1	Q6JBY9	381	22	-9	6	17	1.3	0.9	1.1	1.2
PDE3B	Q13370	35	-6	-3	6	16	0.9	1.0	1.1	1.2
ERP44	Q9BS26	301	3	6	6	15	1.0	1.1	1.1	1.2
CLIP1	P30622	150	-1	1	6	14	1.0	1.0	1.1	1.2
VPS29	Q9UBQ0	36	10	-3	6	14	1.1	1.0	1.1	1.2
DCTD	P32321	83	7	3	6	13	1.1	1.0	1.1	1.1
SRP72	O76094	323	25	20	6	12	1.3	1.2	1.1	1.1
CPNE3	O75131	385	-2	6	6	12	1.0	1.1	1.1	1.1
Uncharacteriz	K7ESF4	129	7	-5	6	12	1.1	1.0	1.1	1.1
PDCD2L	Q9BRP1	100	7	-5	6	12	1.1	1.0	1.1	1.1
VAV2	P52735	202	-14	18	6	10	0.9	1.2	1.1	1.1
MED23	Q9ULK4	238	-13	-4	6	10	0.9	1.0	1.1	1.1
RNF31	Q96EP0	911	-5	8	6	9	1.0	1.1	1.1	1.1
RACK1	P63244	249	-6	2	6	9	0.9	1.0	1.1	1.1
LRMP	Q12912	264	11	10	6	9	1.1	1.1	1.1	1.1
CPOX	P36551	127	1	6	6	8	1.0	1.1	1.1	1.1
TRIM22	Q8IYM9	35	13	0	6	8	1.1	1.0	1.1	1.1
GIMAP8	Q8ND71	166	8	10	6	7	1.1	1.1	1.1	1.1
USP15	Q9Y4E8	127	3	-6	6	7	1.0	0.9	1.1	1.1
MTPAP	Q9NVV4	82	-1	10	6	7	1.0	1.1	1.1	1.1
ITPR2	Q14571	420	12	8	6	6	1.1	1.1	1.1	1.1
DOCK2	Q92608	41	10	-5	6	3	1.1	1.0	1.1	1.0
ZMYND8	Q9ULU4	196	-2	-16	6	3	1.0	0.9	1.1	1.0
DMXL2	Q8TDJ6	920	-8	-16	6	2	0.9	0.9	1.1	1.0
CRBN	Q96SW2	205	11	4	6	2	1.1	1.0	1.1	1.0
SEPT11	Q9NVA2	41	0	9	6	1	1.0	1.1	1.1	1.0
TRPV2	Q9Y5S1	362	22	-4	6	1	1.3	1.0	1.1	1.0
HK3	P52790	399	27	5	6	-1	1.4	1.0	1.1	1.0
HCFC1	P51610	326	-7	1	6	-1	0.9	1.0	1.1	1.0
COPG2	Q9UBF2	296	10	-1	6	-2	1.1	1.0	1.1	1.0
SQSTM1	Q13501	145	0	-5	6	-2	1.0	1.0	1.1	1.0
SCAF4	O95104	102	8	10	6	-3	1.1	1.1	1.1	1.0
ARAP1	Q96P48	774	-5	-2	6	-3	1.0	1.0	1.1	1.0
SEC24C	P53992	376	-1	15	6	-4	1.0	1.2	1.1	1.0
KPNA3	O00505	417	3	-1	6	-5	1.0	1.0	1.1	1.0
GIMAP7	Q8NHV1	164	11	11	6	-6	1.1	1.1	1.1	0.9
CSNK1G1	Q9HCP0	110	-2	10	6	-6	1.0	1.1	1.1	0.9
NELFB	Q8WX92	269	-4	15	6	-6	1.0	1.2	1.1	0.9
SRSF7	Q16629	109	4	-4	6	-7	1.0	1.0	1.1	0.9
IRAK3	Q9Y616	539	7	1	6	-8	1.1	1.0	1.1	0.9
GMIP	Q9P107	571	4	9	6	-9	1.0	1.1	1.1	0.9
ATXN10	Q9UBB4	382	4	-5	6	-9	1.0	1.0	1.1	0.9
RNF213	Q63HN8	3093	4	-1	6	-12	1.0	1.0	1.1	0.9
COPG2	Q9UBF2	446	14	6	6	-13	1.2	1.1	1.1	0.9
FERMT3	Q86UX7	407	13	4	6	-13	1.1	1.0	1.1	0.9
CKAP5	Q14008	493	7	5	6	-15	1.1	1.0	1.1	0.9
FLNA	P21333	444	9	0	6	-15	1.1	1.0	1.1	0.9
USP24	Q9UPU5	1516	2	9	6	-16	1.0	1.1	1.1	0.9

VKORC1L1	Q8N0U8	58	-3	-8	6	-23	1.0	0.9	1.1	0.8
EPB41	P11171	480	-4	-16	6	-25	1.0	0.9	1.1	0.8
EPB41L3	Q9Y2J2	380	-4	-16	6	-25	1.0	0.9	1.1	0.8
SP4	Q02446	627	-8	1	6	-26	0.9	1.0	1.1	0.8
SAR1A	Q9NR31	102	5	12	6	-37	1.0	1.1	1.1	0.7
GMPR	P36959	186	11	-7	6	-90	1.1	0.9	1.1	0.5
PRAF2	O60831	28	-10	-8	5	48	0.9	0.9	1.1	1.9
CCR7	P32248	358	2	3	5	45	1.0	1.0	1.1	1.8
ADCK4	Q96D53	347	6	2	5	38	1.1	1.0	1.1	1.6
TRABD	Q9H4I3	184	4	-5	5	36	1.0	1.0	1.1	1.6
ALPK1	Q96QP1	104	10	5	5	34	1.1	1.0	1.1	1.5
CTC1	Q2NKJ3	128	13	5	5	28	1.1	1.0	1.1	1.4
HLA-DOB	P13765	273	-7	-10	5	26	0.9	0.9	1.1	1.4
MED16	Q9Y2X0	30	-7	2	5	26	0.9	1.0	1.1	1.3
NUP160	Q12769	480	11	10	5	25	1.1	1.1	1.1	1.3
MYO1G	B0I1T2	788	2	-1	5	25	1.0	1.0	1.1	1.3
BLMH	Q13867	73	1	5	5	24	1.0	1.0	1.1	1.3
PIGH	Q14442	11	-3	1	5	24	1.0	1.0	1.1	1.3
CAMK4	Q16566	208	9	7	5	23	1.1	1.1	1.1	1.3
HMCES	Q96FZ2	131	10	15	5	23	1.1	1.2	1.1	1.3
CCT7	Q99832	29	-1	7	5	22	1.0	1.1	1.1	1.3
AHCYL1	O43865	292	-1	2	5	22	1.0	1.0	1.1	1.3
CASP7	P55210	116	5	-1	5	22	1.0	1.0	1.1	1.3
LRRK2	Q5S007	695	14	15	5	21	1.2	1.2	1.1	1.3
PREP	P48147	25	1	7	5	21	1.0	1.1	1.1	1.3
ABHD5	Q8WTS1	299	20	4	5	21	1.2	1.0	1.1	1.3
ZZEF1	O43149	2466	11	13	5	21	1.1	1.1	1.1	1.3
POLR2A	P24928	1245	2	-1	5	21	1.0	1.0	1.1	1.3
PAX2	Q02962	52	-9	-3	5	21	0.9	1.0	1.1	1.3
AAR2	Q9Y312	156	2	9	5	20	1.0	1.1	1.1	1.3
UHRF2	Q96PU4	42	4	-1	5	20	1.0	1.0	1.1	1.2
RPL14	P50914	54	-1	12	5	19	1.0	1.1	1.1	1.2
ANAPC4	Q9UJX5	7	4	8	5	19	1.0	1.1	1.1	1.2
EXD2	Q9NVH0	423	-6	4	5	18	0.9	1.0	1.1	1.2
XPO5	Q9HAV4	941	12	-8	5	18	1.1	0.9	1.1	1.2
NPEPL1	Q8NDH3	504	-1	7	5	17	1.0	1.1	1.1	1.2
ZYG11B	Q9C0D3	735	1	7	5	16	1.0	1.1	1.1	1.2
NOP2	P46087	463	1	0	5	14	1.0	1.0	1.1	1.2
ARL6IP4	Q66PJ3	220	-3	9	5	14	1.0	1.1	1.1	1.2
CTSW	P56202	152	7	4	5	13	1.1	1.0	1.1	1.1
GMPS	P49915	631	4	3	5	13	1.0	1.0	1.1	1.1
TCAF2	A6NFQ2	576	8	-6	5	13	1.1	0.9	1.1	1.1
UBAP1	Q9NZ09	434	-10	1	5	12	0.9	1.0	1.1	1.1
KPNA3	O00505	325	12	8	5	12	1.1	1.1	1.1	1.1
EFL1	Q7Z2Z2	402	1	5	5	12	1.0	1.0	1.1	1.1
RPL8	P62917	115	-2	4	5	11	1.0	1.0	1.1	1.1
DR1	Q01658	43	23	-1	5	11	1.3	1.0	1.1	1.1
RPS20	P60866	36	-4	8	5	11	1.0	1.1	1.1	1.1
RPS3A	P61247	139	4	3	5	11	1.0	1.0	1.1	1.1
FYCO1	Q9BQS8	882	6	6	5	9	1.1	1.1	1.1	1.1
SAMHD1	Q9Y3Z3	205	10	8	5	9	1.1	1.1	1.1	1.1
HERC4	Q5GLZ8	1025	15	-1	5	8	1.2	1.0	1.1	1.1
NOP58	Q9Y2X3	205	5	-4	5	8	1.0	1.0	1.1	1.1
CXXC1	Q9P0U4	566	3	8	5	8	1.0	1.1	1.1	1.1
KMT2D	O14686	273	-5	1	5	8	1.0	1.0	1.1	1.1

KMT2C	Q8NEZ4	388	-5	1	5	8	1.0	1.0	1.1	1.1
PI4KA	P42356	1898	13	-8	5	8	1.1	0.9	1.1	1.1
AGPS	O00116	413	7	6	5	7	1.1	1.1	1.1	1.1
PGP	A6NDG6	243	5	2	5	7	1.1	1.0	1.1	1.1
STAT6	P42226	272	13	4	5	6	1.1	1.0	1.1	1.1
DHX30	Q7L2E3	786	-1	-1	5	6	1.0	1.0	1.1	1.1
RFC3	P40938	32	0	10	5	4	1.0	1.1	1.1	1.0
PRR14L	Q5THK1	1640	-2	8	5	4	1.0	1.1	1.1	1.0
MAP3K3	Q99759	430	1	2	5	4	1.0	1.0	1.1	1.0
MAP3K2	Q9Y2U5	424	1	2	5	4	1.0	1.0	1.1	1.0
UQCRC1	P31930	69	-10	-9	5	4	0.9	0.9	1.1	1.0
CUL5	Q93034	404	-8	-1	5	3	0.9	1.0	1.1	1.0
IDH3G	P51553	148	5	2	5	3	1.0	1.0	1.1	1.0
ZRANB2	O95218	74	-4	7	5	-1	1.0	1.1	1.1	1.0
COPG1	Q9Y678	129	13	3	5	-1	1.1	1.0	1.1	1.0
AIP	O00170	90	4	2	5	-1	1.0	1.0	1.1	1.0
MDN1	Q9NU22	1400	-17	-8	5	-2	0.9	0.9	1.1	1.0
CCNT2	O60583	26	5	1	5	-3	1.0	1.0	1.1	1.0
IQGAP1	P46940	781	9	3	5	-3	1.1	1.0	1.1	1.0
GMIP	Q9P107	529	-5	0	5	-3	1.0	1.0	1.1	1.0
MROH1	Q8NDA8	186	-13	-1	5	-5	0.9	1.0	1.1	1.0
HUWE1	Q7Z6Z7	29	5	-5	5	-6	1.0	1.0	1.1	0.9
HARS2	P49590	456	4	1	5	-7	1.0	1.0	1.1	0.9
MAP2K3	P46734	305	7	6	5	-8	1.1	1.1	1.1	0.9
COG4	Q9H9E3	589	12	4	5	-8	1.1	1.0	1.1	0.9
PRKCQ	Q04759	193	6	2	5	-10	1.1	1.0	1.1	0.9
PRKCD	Q05655	192	6	2	5	-10	1.1	1.0	1.1	0.9
UBR4	Q5T4S7	3807	8	-3	5	-15	1.1	1.0	1.1	0.9
TYMP	P19971	293	6	-5	5	-19	1.1	1.0	1.1	0.8
AFMID	Q63HM1	28	2	-3	5	-36	1.0	1.0	1.1	0.7
TLN1	Q9Y490	1199	8	2	5	-81	1.1	1.0	1.1	0.6
LGALSL	Q3ZCW2	101	15	2	5	-179	1.2	1.0	1.1	0.4
RNF40	O75150	950	18	3	5	32	1.2	1.0	1.0	1.5
LIMD1	Q9UGP4	148	-6	-8	5	31	0.9	0.9	1.0	1.4
PEPD	P12955	58	-6	-1	5	31	0.9	1.0	1.0	1.4
GLYR1	Q49A26	416	16	11	5	30	1.2	1.1	1.0	1.4
EPRS	P07814	336	1	-3	5	28	1.0	1.0	1.0	1.4
AP2A2	O94973	330	6	6	5	27	1.1	1.1	1.0	1.4
AP2A1	O95782	331	6	6	5	27	1.1	1.1	1.0	1.4
RABGGTA	Q92696	342	5	2	5	27	1.0	1.0	1.0	1.4
MCM6	Q14566	302	9	7	5	26	1.1	1.1	1.0	1.3
ODF2	Q5BJF6	136	3	1	5	25	1.0	1.0	1.0	1.3
DECR1	Q16698	86	15	-3	5	25	1.2	1.0	1.0	1.3
TRANK1	O15050	1790	17	17	5	24	1.2	1.2	1.0	1.3
MKL1	Q969V6	326	7	-9	5	23	1.1	0.9	1.0	1.3
FKBP8	Q14318	227	-2	-10	5	22	1.0	0.9	1.0	1.3
WDR11	Q9BZH6	1080	4	3	5	20	1.0	1.0	1.0	1.2
TRRAP	Q9Y4A5	567	5	-1	5	20	1.0	1.0	1.0	1.2
CBX6	O95503	386	-7	-3	5	19	0.9	1.0	1.0	1.2
UFL1	O94874	708	-2	10	5	18	1.0	1.1	1.0	1.2
DLG1	Q12959	378	2	2	5	18	1.0	1.0	1.0	1.2
USP13	Q92995	826	4	8	5	16	1.0	1.1	1.0	1.2
MTFR1	Q15390	145	6	-1	5	16	1.1	1.0	1.0	1.2
WDR89	Q96FK6	82	10	6	5	16	1.1	1.1	1.0	1.2
MIOS	Q9NXC5	148	7	-5	5	15	1.1	1.0	1.0	1.2

LANCL1	O43813	363	8	0	5	15	1.1	1.0	1.0	1.2
NLRC3	Q7RTR2	195	6	-17	5	15	1.1	0.9	1.0	1.2
AP3M1	Q9Y2T2	236	-17	1	5	14	0.9	1.0	1.0	1.2
CXorf38	Q8TB03	300	2	1	5	13	1.0	1.0	1.0	1.1
RPL11	P62913	150	-4	9	5	12	1.0	1.1	1.0	1.1
SNRNP200	O75643	1359	12	6	5	12	1.1	1.1	1.0	1.1
NUDT2	P50583	143	7	1	5	12	1.1	1.0	1.0	1.1
ZC3H11A	O75152	22	4	-6	5	12	1.0	0.9	1.0	1.1
TTC39C	Q8N584	463	11	8	5	12	1.1	1.1	1.0	1.1
AK2	P54819	232	4	-4	5	12	1.0	1.0	1.0	1.1
PI4KA	P42356	1824	-10	5	5	11	0.9	1.1	1.0	1.1
RPA2	P15927	219	1	-5	5	11	1.0	1.0	1.0	1.1
ATG3	Q9NT62	81	11	3	5	11	1.1	1.0	1.0	1.1
SENP6	Q9GZR1	607	1	-1	5	11	1.0	1.0	1.0	1.1
GBP5	Q96PP8	394	1	-2	5	11	1.0	1.0	1.0	1.1
PEBP1	P30086	133	-3	-3	5	11	1.0	1.0	1.0	1.1
PITRM1	Q5JRX3	619	-13	-6	5	11	0.9	0.9	1.0	1.1
GNAI2	P04899	352	3	6	5	10	1.0	1.1	1.0	1.1
MICAL1	Q8TDZ2	82	-8	-2	5	10	0.9	1.0	1.0	1.1
GPC1	P35052	20	-18	-3	5	8	0.9	1.0	1.0	1.1
RHPN2	Q8IUC4	657	7	20	5	7	1.1	1.2	1.0	1.1
UFL1	O94874	372	1	10	5	7	1.0	1.1	1.0	1.1
TRMT2A	Q8IZ69	365	0	-1	5	7	1.0	1.0	1.0	1.1
SUCLG1	P53597	60	0	-2	5	6	1.0	1.0	1.0	1.1
FASN	P49327	1459	-21	-4	5	6	0.8	1.0	1.0	1.1
TMF1	P82094	518	9	0	5	6	1.1	1.0	1.0	1.1
COPS7A	Q9UBW8	110	2	1	5	5	1.0	1.0	1.0	1.1
FAM98C	Q17RN3	340	-2	-3	5	5	1.0	1.0	1.0	1.1
GNAS	Q5JWF2	1008	6	-7	5	5	1.1	0.9	1.0	1.1
SGF29	Q96ES7	287	8	-15	5	5	1.1	0.9	1.0	1.0
PCBP2	Q15366	109	8	1	5	3	1.1	1.0	1.0	1.0
SEC24C	P53992	910	-2	0	5	3	1.0	1.0	1.0	1.0
STAT3	P40763	108	4	3	5	3	1.0	1.0	1.0	1.0
RAB3GAP2	Q9H2M9	896	6	-5	5	3	1.1	1.0	1.0	1.0
PPP6R2	O75170	451	-7	1	5	2	0.9	1.0	1.0	1.0
FGD2	Q7Z6J4	484	-12	-7	5	1	0.9	0.9	1.0	1.0
RFC5	P40937	152	7	10	5	0	1.1	1.1	1.0	1.0
FAM129A	Q9BZQ8	516	13	8	5	0	1.1	1.1	1.0	1.0
GAS7	O60861	318	-11	6	5	-1	0.9	1.1	1.0	1.0
PPP2R1A	P30153	174	5	3	5	-1	1.0	1.0	1.0	1.0
RPS14	P62263	54	13	-1	5	-2	1.1	1.0	1.0	1.0
SF1	Q15637	292	1	10	5	-2	1.0	1.1	1.0	1.0
INTS2	Q9H0H0	33	3	5	5	-2	1.0	1.0	1.0	1.0
FARSB	Q9NSD9	362	6	3	5	-2	1.1	1.0	1.0	1.0
GCN1	Q92616	2015	11	0	5	-3	1.1	1.0	1.0	1.0
FLOT2	Q14254	88	-1	18	5	-4	1.0	1.2	1.0	1.0
WDR11	Q9BZH6	411	-3	12	5	-4	1.0	1.1	1.0	1.0
PDE12	Q6L8Q7	321	15	-14	5	-4	1.2	0.9	1.0	1.0
DDX5	P17844	354	3	5	5	-5	1.0	1.0	1.0	1.0
MYO9B	Q13459	1662	14	14	5	-6	1.2	1.2	1.0	0.9
SERPINB8	P50452	98	-10	10	5	-6	0.9	1.1	1.0	0.9
DYNC1H1	Q14204	3147	11	-4	5	-7	1.1	1.0	1.0	0.9
LRRK2	Q5S007	804	4	3	5	-8	1.0	1.0	1.0	0.9
VKORC1L1	Q8N0U8	50	12	10	5	-9	1.1	1.1	1.0	0.9
DIAPH1	O60610	964	11	2	5	-11	1.1	1.0	1.0	0.9

PDHA1	P08559	261	-6	-6	5	-12	0.9	0.9	1.0	0.9
SH3GL1	Q99961	96	-4	-7	5	-17	1.0	0.9	1.0	0.9
DYNC1H1	Q14204	2639	-8	4	5	-22	0.9	1.0	1.0	0.8
TRIM28	Q13263	68	-13	-29	5	-23	0.9	0.8	1.0	0.8
CNST	Q6PJW8	192	21	-10	5	-49	1.3	0.9	1.0	0.7
EHD4	Q9H223	175	-2	-1	4	54	1.0	1.0	1.0	2.2
CTSB	P07858	105	5	0	4	45	1.1	1.0	1.0	1.8
INTS4	Q96HW7	926	-30	18	4	41	0.8	1.2	1.0	1.7
TRAPPC4	Q9Y296	195	7	4	4	38	1.1	1.0	1.0	1.6
EEFSEC	P57772	55	6	4	4	38	1.1	1.0	1.0	1.6
TRMT61A	Q96FX7	209	-5	-3	4	34	1.0	1.0	1.0	1.5
RFTN1	Q14699	129	-10	-2	4	32	0.9	1.0	1.0	1.5
TANGO6	Q9C0B7	350	0	0	4	30	1.0	1.0	1.0	1.4
IMPDH1	P20839	331	5	5	4	27	1.1	1.1	1.0	1.4
PLEKHM1	Q9Y4G2	555	14	8	4	24	1.2	1.1	1.0	1.3
AGFG1	P52594	29	-3	14	4	22	1.0	1.2	1.0	1.3
CCDC84	Q86UT8	16	-8	11	4	22	0.9	1.1	1.0	1.3
CTU1	Q7Z7A3	302	4	1	4	22	1.0	1.0	1.0	1.3
MED1	Q15648	489	7	-7	4	22	1.1	0.9	1.0	1.3
SEC24D	O94855	853	-2	2	4	21	1.0	1.0	1.0	1.3
INTS1	Q8N201	1866	11	-2	4	20	1.1	1.0	1.0	1.3
URGCP	Q8TCY9	466	14	11	4	20	1.2	1.1	1.0	1.2
PARP4	Q9UKK3	1488	-1	9	4	20	1.0	1.1	1.0	1.2
SYNE1	Q8NF91	2649	14	1	4	20	1.2	1.0	1.0	1.2
DDT	P30046	24	6	-13	4	19	1.1	0.9	1.0	1.2
ATP11C	Q8NB49	200	11	4	4	18	1.1	1.0	1.0	1.2
PADI4	Q9UM07	225	5	8	4	17	1.0	1.1	1.0	1.2
SMCR8	Q8TEV9	406	15	0	4	17	1.2	1.0	1.0	1.2
NAXD	Q8IW45	82	2	4	4	16	1.0	1.0	1.0	1.2
LSM4	Q9Y4Z0	45	-11	-2	4	16	0.9	1.0	1.0	1.2
CHN2	P52757	91	8	8	4	15	1.1	1.1	1.0	1.2
STAG1	Q8WVM7	644	8	-4	4	15	1.1	1.0	1.0	1.2
ZFR	Q96KR1	844	4	4	4	14	1.0	1.0	1.0	1.2
SENP1	Q9P0U3	60	1	-1	4	14	1.0	1.0	1.0	1.2
CD7	P09564	142	-12	-10	4	14	0.9	0.9	1.0	1.2
NOL8	Q76FK4	259	-2	22	4	13	1.0	1.3	1.0	1.1
GNB1L	Q9BYB4	175	-7	5	4	13	0.9	1.1	1.0	1.1
Uncharacteriz	V9GY48	86	6	11	4	13	1.1	1.1	1.0	1.1
LARP7	Q4G0J3	148	-16	3	4	13	0.9	1.0	1.0	1.1
EPHA1	P21709	969	9	-3	4	13	1.1	1.0	1.0	1.1
ATG2B	Q96BY7	243	-7	-4	4	13	0.9	1.0	1.0	1.1
TRMT10A	Q8TBZ6	37	1	2	4	12	1.0	1.0	1.0	1.1
MCM4	P33991	605	7	2	4	12	1.1	1.0	1.0	1.1
TREX1	Q9NSU2	90	4	0	4	11	1.0	1.0	1.0	1.1
MYO1A	Q9UBC5	122	-3	-7	4	10	1.0	0.9	1.0	1.1
LRCH1	Q9Y2L9	251	2	-4	4	9	1.0	1.0	1.0	1.1
UCHL5	Q9Y5K5	191	7	-14	4	9	1.1	0.9	1.0	1.1
MACF1	Q9UPN3	6754	5	4	4	8	1.1	1.0	1.0	1.1
RPRD1B	Q9NQG5	234	9	3	4	8	1.1	1.0	1.0	1.1
CWF19L1	Q69YN2	176	7	-1	4	8	1.1	1.0	1.0	1.1
PITPNB	P48739	94	3	-11	4	7	1.0	0.9	1.0	1.1
PITPNA	Q00169	94	3	-11	4	7	1.0	0.9	1.0	1.1
AARS	P49588	671	5	0	4	7	1.0	1.0	1.0	1.1
CDYL2	Q8N8U2	423	17	11	4	6	1.2	1.1	1.0	1.1
SLC25A5	P05141	257	-1	4	4	6	1.0	1.0	1.0	1.1

AP1G2	O75843	32	1	-2	4	6	1.0	1.0	1.0	1.1
ZEB2	O60315	586	12	-2	4	6	1.1	1.0	1.0	1.1
COPB1	P53618	888	-2	-8	4	6	1.0	0.9	1.0	1.1
CCT5	P48643	377	4	-1	4	5	1.0	1.0	1.0	1.0
ARHGAP30	Q7Z6I6	94	0	-3	4	5	1.0	1.0	1.0	1.0
CASP9	P55211	272	6	-8	4	5	1.1	0.9	1.0	1.0
NCBP1	Q09161	36	7	3	4	4	1.1	1.0	1.0	1.0
MED14	O60244	635	3	-2	4	3	1.0	1.0	1.0	1.0
MTMR3	Q13615	188	3	-12	4	2	1.0	0.9	1.0	1.0
PDCD4	Q53EL6	350	8	2	4	1	1.1	1.0	1.0	1.0
RNF20	Q5VTR2	383	5	-1	4	1	1.1	1.0	1.0	1.0
TPP2	P29144	150	5	1	4	1	1.1	1.0	1.0	1.0
TGFBRAP1	Q8WUH2	818	14	31	4	0	1.2	1.4	1.0	1.0
EPS15L1	Q9UBC2	470	1	4	4	-1	1.0	1.0	1.0	1.0
SERPINB9	P50453	108	6	-2	4	-1	1.1	1.0	1.0	1.0
DDX17	Q92841	431	5	17	4	-3	1.1	1.2	1.0	1.0
RPLP0	P05388	226	6	7	4	-3	1.1	1.1	1.0	1.0
SRGAP2	A2RUF3	34	-11	-7	4	-3	0.9	0.9	1.0	1.0
FRYL	O94915	1118	16	-26	4	-4	1.2	0.8	1.0	1.0
ADGRG1	Q9Y653	434	-14	-20	4	-5	0.9	0.8	1.0	1.0
DTWD2	Q8NBA8	143	-13	-8	4	-6	0.9	0.9	1.0	0.9
RBCK1	Q9BYM8	285	11	-14	4	-6	1.1	0.9	1.0	0.9
DOCK9	Q9BZ29	94	2	1	4	-8	1.0	1.0	1.0	0.9
LUC7L2	A0A0A6YYJ8	259	8	1	4	-8	1.1	1.0	1.0	0.9
LUC7L	Q9NQ29	193	8	1	4	-8	1.1	1.0	1.0	0.9
L3HYPDH	Q96EM0	39	-7	-5	4	-10	0.9	1.0	1.0	0.9
NUP98	P52948	1735	-1	10	4	-10	1.0	1.1	1.0	0.9
EFL1	Q7Z2Z2	831	-2	-4	4	-10	1.0	1.0	1.0	0.9
UBE3C	Q15386	140	1	-10	4	-12	1.0	0.9	1.0	0.9
FLNB	O75369	1617	6	-11	4	-12	1.1	0.9	1.0	0.9
RFC2	P35250	255	4	8	4	-12	1.0	1.1	1.0	0.9
DENND4B	O75064	815	2	11	4	-14	1.0	1.1	1.0	0.9
ZYX	Q15942	537	3	14	4	-15	1.0	1.2	1.0	0.9
EHD4	Q9H223	141	11	-4	4	-16	1.1	1.0	1.0	0.9
PRKCB	P05771	502	2	-7	4	-21	1.0	0.9	1.0	0.8
PRKCA	P17252	499	2	-7	4	-21	1.0	0.9	1.0	0.8
UBAP1	Q9NZ09	45	-12	-8	4	-21	0.9	0.9	1.0	0.8
APBB1IP	Q7Z5R6	400	-18	-17	4	-21	0.8	0.9	1.0	0.8
FLYWCH1	Q4VC44	173	-34	46	4	-38	0.7	1.8	1.0	0.7
F13A1	P00488	410	-5	3	4	-62	1.0	1.0	1.0	0.6
SH3BGRL2	Q9UJC5	76	15	-5	4	-79	1.2	1.0	1.0	0.6
SOS2	Q07890	403	-7	-4	4	54	0.9	1.0	1.0	2.2
CD97	P48960	802	9	-2	4	43	1.1	1.0	1.0	1.8
RARA	P10276	203	2	-20	4	43	1.0	0.8	1.0	1.8
ZDHHC18	Q9NUE0	375	14	-9	4	38	1.2	0.9	1.0	1.6
PTGDR2	Q9Y5Y4	357	4	3	4	36	1.0	1.0	1.0	1.6
DPYD	Q12882	1025	6	-1	4	33	1.1	1.0	1.0	1.5
IGKC	A0A087X1V9	239	-16	-15	4	31	0.9	0.9	1.0	1.4
TRMT2A	Q8IZ69	224	3	6	4	23	1.0	1.1	1.0	1.3
DOPEY1	Q5JWR5	1652	24	-17	4	23	1.3	0.9	1.0	1.3
VPS16	Q9H269	529	2	6	4	22	1.0	1.1	1.0	1.3
MRPL21	Q7Z2W9	203	6	11	4	22	1.1	1.1	1.0	1.3
RGS19	P49795	73	2	-2	4	22	1.0	1.0	1.0	1.3
CHUK	O15111	371	3	-3	4	21	1.0	1.0	1.0	1.3
SUMF2	Q8NBJ7	290	5	15	4	20	1.0	1.2	1.0	1.3

CAND1	Q86VP6	413	14	6	4	20	1.2	1.1	1.0	1.2
SNX6	Q9UNH7	264	6	2	4	20	1.1	1.0	1.0	1.2
NSUN2	Q08J23	93	6	0	4	19	1.1	1.0	1.0	1.2
HDLBP	Q00341	855	6	-1	4	19	1.1	1.0	1.0	1.2
PPTC7	Q8NI37	124	-22	-5	4	19	0.8	1.0	1.0	1.2
HERC2	O95714	1410	2	1	4	18	1.0	1.0	1.0	1.2
ASRGL1	Q7L266	176	-6	0	4	17	0.9	1.0	1.0	1.2
DCTN2	Q13561	256	9	8	4	17	1.1	1.1	1.0	1.2
UPF1	Q92900	374	4	8	4	17	1.0	1.1	1.0	1.2
FKBP8	Q14318	274	-2	11	4	16	1.0	1.1	1.0	1.2
USP28	Q96RU2	203	2	1	4	16	1.0	1.0	1.0	1.2
APBB1IP	Q7Z5R6	355	-8	-1	4	16	0.9	1.0	1.0	1.2
CENPC	Q03188	291	19	7	4	16	1.2	1.1	1.0	1.2
DIAPH2	O60879	862	13	3	4	16	1.1	1.0	1.0	1.2
TYK2	P29597	1140	8	0	4	16	1.1	1.0	1.0	1.2
CIAO1	O76071	52	4	5	4	15	1.0	1.0	1.0	1.2
PRPF19	Q9UMS4	298	12	-6	4	15	1.1	0.9	1.0	1.2
SPTAN1	Q13813	466	-3	-4	4	14	1.0	1.0	1.0	1.2
RACK1	P63244	138	5	3	4	14	1.1	1.0	1.0	1.2
TP53BP1	Q12888	1933	-5	10	4	13	1.0	1.1	1.0	1.1
PPIP5K2	O43314	663	-10	-10	4	13	0.9	0.9	1.0	1.1
TRAPPC10	P48553	833	11	7	4	12	1.1	1.1	1.0	1.1
CCAR1	Q8IX12	974	1	2	4	12	1.0	1.0	1.0	1.1
IPO7	O95373	90	4	2	4	12	1.0	1.0	1.0	1.1
GNL3	Q9BVP2	156	-2	-3	4	12	1.0	1.0	1.0	1.1
RGP1	Q92546	314	9	7	4	11	1.1	1.1	1.0	1.1
AIMP2	Q13155	143	7	4	4	11	1.1	1.0	1.0	1.1
ZNF12	P17014	537	-8	3	4	11	0.9	1.0	1.0	1.1
DDX54	Q8TDD1	149	-11	-6	4	11	0.9	0.9	1.0	1.1
REL	Q04864	250	-4	5	4	10	1.0	1.1	1.0	1.1
RPS12	P25398	69	-2	-1	4	10	1.0	1.0	1.0	1.1
LYPLA2	O95372	171	4	2	4	9	1.0	1.0	1.0	1.1
LAMTOR5	O43504	66	1	-2	4	9	1.0	1.0	1.0	1.1
NUP50	Q9UKX7	165	4	2	4	8	1.0	1.0	1.0	1.1
RBM26	Q5T8P6	53	8	-9	4	8	1.1	0.9	1.0	1.1
UHRF1BP1L	A0JNW5	81	-2	9	4	8	1.0	1.1	1.0	1.1
IGFBP5	P24593	100	-5	-1	4	8	1.0	1.0	1.0	1.1
IKBKB	O14920	299	6	-3	4	8	1.1	1.0	1.0	1.1
NUP160	Q12769	916	4	2	4	7	1.0	1.0	1.0	1.1
IPO5	O00410	682	14	0	4	7	1.2	1.0	1.0	1.1
TSSC1	Q53HC9	95	10	-2	4	7	1.1	1.0	1.0	1.1
RPLP0	P05388	27	1	6	4	7	1.0	1.1	1.0	1.1
MAP4K1	Q92918	494	-14	-11	4	7	0.9	0.9	1.0	1.1
TRIM14	Q14142	24	9	13	4	6	1.1	1.1	1.0	1.1
DPP9	Q86TI2	184	9	9	4	6	1.1	1.1	1.0	1.1
MCM3	P25205	123	1	4	4	5	1.0	1.0	1.0	1.1
NUP205	Q92621	1032	2	7	4	5	1.0	1.1	1.0	1.0
TRAF6	Q9Y4K3	85	2	3	4	5	1.0	1.0	1.0	1.0
FCHO1	O14526	113	2	1	4	5	1.0	1.0	1.0	1.0
ANKFY1	Q9P2R3	843	6	4	4	4	1.1	1.0	1.0	1.0
SF3A3	Q12874	103	5	8	4	4	1.1	1.1	1.0	1.0
C7orf26	Q96N11	145	19	10	4	3	1.2	1.1	1.0	1.0
HUWE1	Q7Z6Z7	1879	10	9	4	3	1.1	1.1	1.0	1.0
HEATR5A	Q86XA9	516	12	0	4	3	1.1	1.0	1.0	1.0
TBCB	Q99426	39	23	5	4	2	1.3	1.1	1.0	1.0

ZNF143	P52747	329	7	-3	4	2	1.1	1.0	1.0	1.0
PHF23	Q9BUL5	381	-5	-12	4	2	1.0	0.9	1.0	1.0
ACTR5	Q9H9F9	48	-5	-25	4	2	1.0	0.8	1.0	1.0
FXR2	P51116	87	4	-2	4	2	1.0	1.0	1.0	1.0
MAPRE1	Q15691	228	2	10	4	1	1.0	1.1	1.0	1.0
PPP1R21	Q6ZMI0	200	2	5	4	1	1.0	1.0	1.0	1.0
ANXA7	P20073	285	0	2	4	1	1.0	1.0	1.0	1.0
GRWD1	Q9BQ67	235	-1	-1	4	1	1.0	1.0	1.0	1.0
KMT2D	O14686	1909	2	-8	4	1	1.0	0.9	1.0	1.0
FOXP4	Q8IVH2	342	-8	-14	4	1	0.9	0.9	1.0	1.0
RALBP1	Q15311	411	3	-3	4	1	1.0	1.0	1.0	1.0
CPT1A	P50416	304	4	-2	4	-1	1.0	1.0	1.0	1.0
CHTF18	Q8WVB6	373	8	-7	4	-1	1.1	0.9	1.0	1.0
WDR6	Q9NNW5	26	5	4	4	-2	1.1	1.0	1.0	1.0
ACTN4	O43707	499	-2	-3	4	-2	1.0	1.0	1.0	1.0
POLR2A	P24928	1407	1	-7	4	-3	1.0	0.9	1.0	1.0
GLS	O94925	203	10	-2	4	-6	1.1	1.0	1.0	0.9
DNAJC11	Q9NVH1	235	-4	-14	4	-6	1.0	0.9	1.0	0.9
VWA5A	O00534	743	-8	4	4	-6	0.9	1.0	1.0	0.9
ESYT2	A0FGR8	181	-2	-1	4	-6	1.0	1.0	1.0	0.9
MPP7	Q5T2T1	249	-1	-10	4	-7	1.0	0.9	1.0	0.9
ZNF267	Q14586	531	7	-10	4	-8	1.1	0.9	1.0	0.9
ASUN	Q9NVM9	560	-15	-8	4	-9	0.9	0.9	1.0	0.9
HK3	P52790	840	9	1	4	-11	1.1	1.0	1.0	0.9
COPG1	Q9Y678	280	16	-2	4	-15	1.2	1.0	1.0	0.9
COPG1	Q9Y678	440	-12	5	4	-16	0.9	1.1	1.0	0.9
CASP1	P29466	169	6	-1	4	-16	1.1	1.0	1.0	0.9
DLAT	P10515	488	5	6	4	-17	1.0	1.1	1.0	0.9
BTK	Q06187	464	10	-9	4	-18	1.1	0.9	1.0	0.8
ASUN	Q9NVM9	331	9	-1	4	-19	1.1	1.0	1.0	0.8
DLD	P09622	477	4	25	4	-20	1.0	1.3	1.0	0.8
TNPO1	Q92973	620	-2	-9	4	-24	1.0	0.9	1.0	0.8
MYO1G	B0I1T2	831	-2	3	4	-51	1.0	1.0	1.0	0.7
FHL2	Q14192	132	2	-3	4	-67	1.0	1.0	1.0	0.6
NOP58	Q9Y2X3	106	-3	-1	4	-75	1.0	1.0	1.0	0.6
HLA-C	P10321	350	2	-4	3	54	1.0	1.0	1.0	2.2
GCLC	P48506	491	7	-7	3	48	1.1	0.9	1.0	1.9
APOOL	Q6UXV4	74	5	6	3	42	1.1	1.1	1.0	1.7
RALGAPB	Q86X10	344	-7	-6	3	37	0.9	0.9	1.0	1.6
BLK	P51451	319	-13	-7	3	37	0.9	0.9	1.0	1.6
NAA10	P41227	194	7	-1	3	36	1.1	1.0	1.0	1.6
BRAT1	Q6PJG6	361	-11	-6	3	35	0.9	0.9	1.0	1.5
DMXL1	Q9Y485	1419	6	8	3	34	1.1	1.1	1.0	1.5
DPYD	Q12882	684	5	0	3	33	1.1	1.0	1.0	1.5
C17orf59	Q96GS4	296	-8	-19	3	27	0.9	0.8	1.0	1.4
RPTOR	Q8N122	713	1	0	3	26	1.0	1.0	1.0	1.4
CTSH	P09668	138	-9	1	3	24	0.9	1.0	1.0	1.3
PALD1	Q9ULE6	750	11	2	3	24	1.1	1.0	1.0	1.3
PTBP3	O95758	55	0	0	3	23	1.0	1.0	1.0	1.3
ANKRD17	O75179	1746	11	-1	3	23	1.1	1.0	1.0	1.3
COMT	P21964	241	14	5	3	22	1.2	1.1	1.0	1.3
CSTF2	P33240	150	0	2	3	22	1.0	1.0	1.0	1.3
SLFN5	Q08AF3	369	-2	5	3	21	1.0	1.0	1.0	1.3
KCNAB2	Q13303	248	2	1	3	21	1.0	1.0	1.0	1.3
SFXN4	Q6P4A7	70	9	5	3	20	1.1	1.0	1.0	1.3

RBKS	Q9H477	59	12	14	3	20	1.1	1.2	1.0	1.2
ADK	P55263	143	3	5	3	20	1.0	1.0	1.0	1.2
MAST2	Q6P0Q8	930	-9	5	3	20	0.9	1.0	1.0	1.2
MPO	P05164	398	-11	-5	3	20	0.9	1.0	1.0	1.2
EPX	P11678	370	-11	-5	3	20	0.9	1.0	1.0	1.2
LANCL2	Q9NS86	121	9	-5	3	20	1.1	1.0	1.0	1.2
IPO4	Q8TEX9	735	2	-5	3	19	1.0	1.0	1.0	1.2
PLEKHA2	Q9HB19	81	-21	-6	3	19	0.8	0.9	1.0	1.2
ARID2	Q68CP9	1785	-2	-16	3	19	1.0	0.9	1.0	1.2
KLC1	Q07866	456	6	-2	3	18	1.1	1.0	1.0	1.2
CYBB	P04839	537	2	-4	3	18	1.0	1.0	1.0	1.2
RCHY1	Q96PM5	136	-13	-10	3	17	0.9	0.9	1.0	1.2
EIF2B5	Q13144	618	4	3	3	17	1.0	1.0	1.0	1.2
CNN2	Q99439	61	2	2	3	16	1.0	1.0	1.0	1.2
IGHD	A0A0A0MS09	15	-10	-5	3	16	0.9	1.0	1.0	1.2
DIS3L	Q8TF46	918	12	0	3	15	1.1	1.0	1.0	1.2
PTPN6	P29350	171	4	4	3	15	1.0	1.0	1.0	1.2
C2orf49	Q9BVC5	10	7	-1	3	15	1.1	1.0	1.0	1.2
VAPB	O95292	173	10	11	3	14	1.1	1.1	1.0	1.2
RPL37A	P61513	60	4	-2	3	14	1.0	1.0	1.0	1.2
PLP2	Q04941	16	8	-9	3	14	1.1	0.9	1.0	1.2
CBR3	O75828	143	3	-12	3	14	1.0	0.9	1.0	1.2
SMAD2	Q15796	81	14	6	3	14	1.2	1.1	1.0	1.2
COPB1	P53618	635	8	2	3	14	1.1	1.0	1.0	1.2
STRBP	Q96SI9	83	-4	1	3	14	1.0	1.0	1.0	1.2
HAGH	Q16775	219	3	-7	3	14	1.0	0.9	1.0	1.2
STAT2	P52630	323	2	0	3	13	1.0	1.0	1.0	1.1
DMXL1	Q9Y485	2916	4	-5	3	13	1.0	1.0	1.0	1.1
INPP5D	Q92835	43	9	-8	3	12	1.1	0.9	1.0	1.1
ELAC2	Q9BQ52	670	-2	5	3	11	1.0	1.0	1.0	1.1
MRPS31	Q92665	356	6	0	3	11	1.1	1.0	1.0	1.1
DOCK11	Q5JSL3	615	2	4	3	11	1.0	1.0	1.0	1.1
KTN1	Q86UP2	303	5	-5	3	11	1.0	1.0	1.0	1.1
GNL3	Q9BVP2	251	9	-2	3	10	1.1	1.0	1.0	1.1
DNTTIP1	Q9H147	192	-3	3	3	10	1.0	1.0	1.0	1.1
ERCC2	P18074	116	14	9	3	9	1.2	1.1	1.0	1.1
EXOC6B	Q9Y2D4	32	8	-3	3	9	1.1	1.0	1.0	1.1
GARS	P41250	616	-3	3	3	8	1.0	1.0	1.0	1.1
TRRAP	Q9Y4A5	429	11	2	3	8	1.1	1.0	1.0	1.1
SRPK2	P78362	535	11	-5	3	7	1.1	1.0	1.0	1.1
SRPK1	Q96SB4	502	11	-5	3	7	1.1	1.0	1.0	1.1
RPS26	P62854	26	-5	6	3	5	1.0	1.1	1.0	1.1
NSL1	Q96IY1	136	6	-3	3	5	1.1	1.0	1.0	1.1
NBEAL2	Q6ZNJ1	1619	5	3	3	5	1.1	1.0	1.0	1.0
UHRF1BP1L	A0JNW5	647	-14	2	3	4	0.9	1.0	1.0	1.0
PLAA	Q9Y263	605	4	-2	3	4	1.0	1.0	1.0	1.0
COPS7B	Q9H9Q2	110	-1	4	3	3	1.0	1.0	1.0	1.0
ADCK3	Q8NI60	641	2	-1	3	2	1.0	1.0	1.0	1.0
VPS18	Q9P253	713	1	-13	3	1	1.0	0.9	1.0	1.0
NUP133	Q8WUM0	641	4	1	3	1	1.0	1.0	1.0	1.0
RANGAP1	P46060	274	4	-1	3	1	1.0	1.0	1.0	1.0
FMR1	Q06787	584	-1	-15	3	1	1.0	0.9	1.0	1.0
ERCC2	P18074	663	-6	15	3	0	0.9	1.2	1.0	1.0
ZYX	Q15942	447	5	12	3	-1	1.0	1.1	1.0	1.0
CLASP2	O75122	221	6	5	3	-1	1.1	1.0	1.0	1.0

TNRC6C	Q9HCJ0	481	-6	-3	3	-1	0.9	1.0	1.0	1.0
DSCR3	O14972	137	2	-8	3	-1	1.0	0.9	1.0	1.0
ASNA1	O43681	53	-2	-4	3	-3	1.0	1.0	1.0	1.0
TBCEL	Q5QJ74	14	2	-7	3	-3	1.0	0.9	1.0	1.0
OGT	O15294	962	-10	4	3	-3	0.9	1.0	1.0	1.0
IDH3A	P50213	127	1	-8	3	-3	1.0	0.9	1.0	1.0
FLII	Q13045	1257	6	1	3	-4	1.1	1.0	1.0	1.0
UTP20	O75691	2058	1	-4	3	-4	1.0	1.0	1.0	1.0
ACSL1	P33121	132	-6	-9	3	-4	0.9	0.9	1.0	1.0
PDS5A	Q29RF7	92	-6	-12	3	-4	0.9	0.9	1.0	1.0
PDS5B	Q9NTI5	82	-6	-12	3	-4	0.9	0.9	1.0	1.0
GOT1	P17174	46	7	-2	3	-5	1.1	1.0	1.0	1.0
ACSL3	O95573	573	-4	3	3	-7	1.0	1.0	1.0	0.9
EFL1	Q7Z2Z2	23	1	8	3	-7	1.0	1.1	1.0	0.9
FAM63A	Q8N5J2	115	9	6	3	-8	1.1	1.1	1.0	0.9
KDM2A	Q9Y2K7	577	8	0	3	-8	1.1	1.0	1.0	0.9
XPO1	O14980	498	25	0	3	-11	1.3	1.0	1.0	0.9
NBEAL2	Q6ZNJ1	822	6	-2	3	-14	1.1	1.0	1.0	0.9
HNRNPL	P14866	581	3	-21	3	-14	1.0	0.8	1.0	0.9
PRKCB	P05771	572	-3	-15	3	-17	1.0	0.9	1.0	0.9
SP4	Q02446	625	2	-7	3	-19	1.0	0.9	1.0	0.8
ZYX	Q15942	444	-1	8	3	-39	1.0	1.1	1.0	0.7
ATG7	O95352	368	-2	3	3	-43	1.0	1.0	1.0	0.7
TLN1	Q9Y490	709	2	2	3	-73	1.0	1.0	1.0	0.6
TLN1	Q9Y490	1486	-25	-10	3	-81	0.8	0.9	1.0	0.6
FHL2	Q14192	150	16	11	3	-90	1.2	1.1	1.0	0.5
G6PC3	Q9BUM1	269	-11	-11	3	60	0.9	0.9	1.0	2.5
TAF6L	Q9Y6J9	230	-9	-4	3	47	0.9	1.0	1.0	1.9
SAMM50	Q9Y512	457	-7	-11	3	45	0.9	0.9	1.0	1.8
HLA-C	P10321	345	-1	-4	3	40	1.0	1.0	1.0	1.7
APBA2	Q99767	272	17	-5	3	38	1.2	1.0	1.0	1.6
U2AF1	Q01081	169	2	-6	3	37	1.0	0.9	1.0	1.6
ARID4A	P29374	25	3	3	3	36	1.0	1.0	1.0	1.6
ARID4B	Q4LE39	25	3	3	3	36	1.0	1.0	1.0	1.6
TAP2	Q03519	213	9	-6	3	35	1.1	0.9	1.0	1.5
SIT1	Q9Y3P8	126	-3	1	3	33	1.0	1.0	1.0	1.5
FAM168B	A1KXE4	63	1	-4	3	32	1.0	1.0	1.0	1.5
PREP	P48147	601	11	6	3	31	1.1	1.1	1.0	1.4
POLR2A	P24928	981	-3	-2	3	30	1.0	1.0	1.0	1.4
SRGAP2	A2RUF3	98	-19	-6	3	30	0.8	0.9	1.0	1.4
NAA25	Q14CX7	381	3	-5	3	28	1.0	1.0	1.0	1.4
TLR2	O60603	640	-10	-4	3	27	0.9	1.0	1.0	1.4
CD8A	P01732	217	2	2	3	26	1.0	1.0	1.0	1.4
FBXO11	Q86XK2	113	13	-2	3	26	1.1	1.0	1.0	1.3
AKNA	Q7Z591	1244	-6	-4	3	24	0.9	1.0	1.0	1.3
ARHGAP27	Q6ZUM4	703	5	8	3	23	1.1	1.1	1.0	1.3
SYMPK	Q92797	848	-14	5	3	22	0.9	1.1	1.0	1.3
RNASEH2B	Q5TBB1	125	7	13	3	22	1.1	1.1	1.0	1.3
UBE2L6	O14933	86	9	-4	3	22	1.1	1.0	1.0	1.3
SH3TC1	Q8TE82	75	13	6	3	20	1.1	1.1	1.0	1.3
AP5B1	Q2VPB7	800	1	-10	3	20	1.0	0.9	1.0	1.2
IPO5	O00410	944	4	6	3	19	1.0	1.1	1.0	1.2
DNMT1	P26358	896	-3	2	3	19	1.0	1.0	1.0	1.2
RIN2	Q8WYP3	408	-6	-4	3	19	0.9	1.0	1.0	1.2
AARS	P49588	901	-5	-16	3	19	1.0	0.9	1.0	1.2

GHDC	Q8N2G8	377	-13	-6	3	19	0.9	0.9	1.0	1.2
AMDHD2	Q9Y303	18	-10	0	3	18	0.9	1.0	1.0	1.2
TAP1	Q03518	722	6	-4	3	18	1.1	1.0	1.0	1.2
CHMP5	Q9NZZ3	20	7	4	3	18	1.1	1.0	1.0	1.2
CDYL	Q9Y232	515	11	5	3	17	1.1	1.1	1.0	1.2
CTU1	Q7Z7A3	299	2	-6	3	17	1.0	0.9	1.0	1.2
RACK1	P63244	240	16	17	3	16	1.2	1.2	1.0	1.2
C9orf114	Q5T280	151	3	-4	3	16	1.0	1.0	1.0	1.2
OSBPL10	Q9BXB5	33	-10	-8	3	16	0.9	0.9	1.0	1.2
SRRM2	Q9UQ35	956	-1	-4	3	16	1.0	1.0	1.0	1.2
HERC4	Q5GLZ8	708	20	10	3	15	1.2	1.1	1.0	1.2
IPO5	O00410	733	16	11	3	15	1.2	1.1	1.0	1.2
SIPA1	Q96FS4	732	6	5	3	14	1.1	1.0	1.0	1.2
DDX46	Q7L014	501	-2	-3	3	14	1.0	1.0	1.0	1.2
ALDH3A2	P51648	425	13	-4	3	14	1.1	1.0	1.0	1.2
AP3D1	O14617	574	2	7	3	13	1.0	1.1	1.0	1.1
PUM1	Q14671	980	17	21	3	12	1.2	1.3	1.0	1.1
PUM2	Q8TB72	860	17	21	3	12	1.2	1.3	1.0	1.1
RHOT1	Q8IXI2	350	6	8	3	12	1.1	1.1	1.0	1.1
JAK1	P23458	716	16	3	3	12	1.2	1.0	1.0	1.1
COPA	P53621	245	-4	2	3	12	1.0	1.0	1.0	1.1
METTL3	Q86U44	294	-5	0	3	12	1.0	1.0	1.0	1.1
HELB	Q8NG08	1041	-8	-4	3	12	0.9	1.0	1.0	1.1
MAN2C1	Q9NTJ4	29	0	-9	3	12	1.0	0.9	1.0	1.1
SMCHD1	A6NHR9	1899	6	-6	3	11	1.1	0.9	1.0	1.1
GRK6	P43250	425	3	6	3	10	1.0	1.1	1.0	1.1
EEFSEC	P57772	442	-1	2	3	10	1.0	1.0	1.0	1.1
UTRN	P46939	3094	9	-1	3	9	1.1	1.0	1.0	1.1
RPS4X	P62701	181	1	5	3	8	1.0	1.1	1.0	1.1
DIS3	Q9Y2L1	194	2	1	3	8	1.0	1.0	1.0	1.1
ARL1	P40616	80	-6	6	3	8	0.9	1.1	1.0	1.1
ADAR	P55265	773	5	-1	3	8	1.0	1.0	1.0	1.1
UBE4A	Q14139	465	19	-1	3	6	1.2	1.0	1.0	1.1
PRPS1	P60891	265	0	-3	3	6	1.0	1.0	1.0	1.1
URB2	Q14146	205	-12	-1	3	6	0.9	1.0	1.0	1.1
NELFCD	Q8IXH7	293	7	4	3	5	1.1	1.0	1.0	1.1
TIPRL	O75663	75	14	1	3	5	1.2	1.0	1.0	1.1
NELFB	Q8WX92	141	6	0	3	5	1.1	1.0	1.0	1.1
PLD4	Q96BZ4	313	5	-6	3	5	1.1	0.9	1.0	1.0
ALKBH4	Q9NXW9	267	-14	5	3	4	0.9	1.1	1.0	1.0
RNASEH2A	O75792	24	-2	-2	3	4	1.0	1.0	1.0	1.0
ARCN1	P48444	286	1	7	3	4	1.0	1.1	1.0	1.0
UTRN	P46939	1627	8	2	3	4	1.1	1.0	1.0	1.0
GCN1	Q92616	1235	-4	9	3	3	1.0	1.1	1.0	1.0
MSH6	P52701	765	9	7	3	3	1.1	1.1	1.0	1.0
SLC25A1	P53007	262	-2	6	3	3	1.0	1.1	1.0	1.0
GSTK1	Q9Y2Q3	176	8	3	3	3	1.1	1.0	1.0	1.0
UNC45A	Q9H3U1	663	8	1	3	3	1.1	1.0	1.0	1.0
CPSF2	Q9P2I0	577	3	-2	3	3	1.0	1.0	1.0	1.0
IRF2BP2	Q7Z5L9	16	-6	-13	3	3	0.9	0.9	1.0	1.0
IRF2BPL	Q9H1B7	14	-6	-13	3	3	0.9	0.9	1.0	1.0
ELMO1	Q92556	561	7	8	3	2	1.1	1.1	1.0	1.0
SPTBN1	Q01082	1970	-4	-1	3	2	1.0	1.0	1.0	1.0
PHF20	Q9BVI0	59	-2	-2	3	1	1.0	1.0	1.0	1.0
SPTAN1	Q13813	2233	-2	-3	3	1	1.0	1.0	1.0	1.0

UBE3A	Q05086	843	7	8	3	1	1.1	1.1	1.0	1.0
CARD9	Q9H257	402	1	8	3	1	1.0	1.1	1.0	1.0
SH3GLB2	Q9NR46	172	-2	-3	3	1	1.0	1.0	1.0	1.0
TRIM5	Q9C035	106	9	-5	3	0	1.1	1.0	1.0	1.0
GSS	P48637	409	6	-4	3	-1	1.1	1.0	1.0	1.0
STK24	Q9Y6E0	375	8	3	3	-2	1.1	1.0	1.0	1.0
IVD	P26440	352	-1	3	3	-2	1.0	1.0	1.0	1.0
BRAP	Q7Z569	110	-6	5	3	-4	0.9	1.1	1.0	1.0
IQGAP2	Q13576	1449	1	-2	3	-4	1.0	1.0	1.0	1.0
PI4KA	P42356	1774	-2	-2	3	-4	1.0	1.0	1.0	1.0
MYO1G	B0I1T2	618	4	-3	3	-4	1.0	1.0	1.0	1.0
COPS6	Q7L5N1	143	1	-4	3	-4	1.0	1.0	1.0	1.0
PAXIP1	Q6ZW49	671	7	3	3	-4	1.1	1.0	1.0	1.0
SUCLA2	Q9P2R7	430	9	4	3	-5	1.1	1.0	1.0	1.0
RAP1GDS1	P52306	144	2	-14	3	-5	1.0	0.9	1.0	1.0
PLEKHF2	Q9H8W4	21	-3	-15	3	-6	1.0	0.9	1.0	0.9
BCAT2	O15382	345	1	-22	3	-6	1.0	0.8	1.0	0.9
DDX19A	Q9NUU7	164	2	3	3	-6	1.0	1.0	1.0	0.9
MON2	Q7Z3U7	609	18	-1	3	-6	1.2	1.0	1.0	0.9
SLK	Q9H2G2	993	5	-22	3	-6	1.1	0.8	1.0	0.9
CDK5RAP3	Q96JB5	150	10	13	3	-7	1.1	1.1	1.0	0.9
NUDT5	Q9UKK9	76	1	-11	3	-7	1.0	0.9	1.0	0.9
FCF1	Q9Y324	154	-4	2	3	-9	1.0	1.0	1.0	0.9
CLASP1	Q7Z460	8	1	-2	3	-9	1.0	1.0	1.0	0.9
MGA	Q8IWI9	1047	3	-24	3	-10	1.0	0.8	1.0	0.9
TRIO	O75962	2909	6	7	3	-10	1.1	1.1	1.0	0.9
DDX19A	Q9NUU7	166	8	2	3	-11	1.1	1.0	1.0	0.9
CCAR2	Q8N163	387	3	1	3	-11	1.0	1.0	1.0	0.9
GATAD2B	Q8WXI9	420	6	-5	3	-11	1.1	1.0	1.0	0.9
ATP2A2	P16615	447	13	8	3	-12	1.1	1.1	1.0	0.9
ATP2A3	Q93084	447	13	8	3	-12	1.1	1.1	1.0	0.9
NHLRC2	Q8NBF2	609	6	-2	3	-12	1.1	1.0	1.0	0.9
FAM65A	Q6ZS17	1173	2	-12	3	-12	1.0	0.9	1.0	0.9
ZFYVE16	Q7Z3T8	1429	-13	8	3	-14	0.9	1.1	1.0	0.9
ANO6	Q4KMQ2	105	3	-1	3	-15	1.0	1.0	1.0	0.9
RASAL3	Q86YV0	533	11	-2	3	-18	1.1	1.0	1.0	0.9
RAB18	Q9NP72	155	-18	9	3	-18	0.9	1.1	1.0	0.8
RBM22	Q9NW64	179	13	-9	3	-24	1.1	0.9	1.0	0.8
GBP4	Q96PP9	421	2	-10	3	-26	1.0	0.9	1.0	0.8
MIA-RAB4B	W4VSR3	7	-15	-17	3	-38	0.9	0.9	1.0	0.7
TLN1	Q9Y490	2243	10	6	3	-69	1.1	1.1	1.0	0.6
TMEM173	Q86WV6	91	-13	-4	2	75	0.9	1.0	1.0	4.0
IL2RB	P14784	280	-1	6	2	47	1.0	1.1	1.0	1.9
HELZ	P42694	100	3	-14	2	36	1.0	0.9	1.0	1.6
PRPF40A	O75400	39	-2	4	2	33	1.0	1.0	1.0	1.5
MYO1G	B0I1T2	793	11	-2	2	29	1.1	1.0	1.0	1.4
GMEB1	Q9Y692	274	6	2	2	29	1.1	1.0	1.0	1.4
FTH1	P02794	91	-2	-15	2	28	1.0	0.9	1.0	1.4
TATDN2	Q93075	65	-11	-10	2	28	0.9	0.9	1.0	1.4
MINOS1	Q5TGZ0	13	4	5	2	27	1.0	1.0	1.0	1.4
NFIC	P08651	88	10	-1	2	26	1.1	1.0	1.0	1.4
ZDHHC24	Q6UX98	242	-2	4	2	25	1.0	1.0	1.0	1.3
SEMA4D	Q92854	783	-3	3	2	25	1.0	1.0	1.0	1.3
AGFG2	O95081	107	9	-10	2	24	1.1	0.9	1.0	1.3
CRBN	Q96SW2	318	3	6	2	24	1.0	1.1	1.0	1.3

ACO2	Q99798	451	-3	-5	2	24	1.0	1.0	1.0	1.3
FAM118A	Q9NWS6	291	8	3	2	23	1.1	1.0	1.0	1.3
ZDHHC4	Q9NPG8	337	-3	-2	2	22	1.0	1.0	1.0	1.3
MTHFD1L	Q6UB35	779	1	3	2	22	1.0	1.0	1.0	1.3
RAD50	Q92878	53	12	-3	2	22	1.1	1.0	1.0	1.3
SRRM2	Q9UQ35	785	6	-2	2	20	1.1	1.0	1.0	1.3
THADA	Q6YHU6	1452	-13	1	2	20	0.9	1.0	1.0	1.2
TECPR1	Q7Z6L1	736	9	1	2	19	1.1	1.0	1.0	1.2
POLR3E	Q9NVU0	85	-2	3	2	19	1.0	1.0	1.0	1.2
VDAC2	P45880	103	11	2	2	18	1.1	1.0	1.0	1.2
RIN3	Q8TB24	484	8	1	2	18	1.1	1.0	1.0	1.2
CAMK1D	Q8IU85	270	2	14	2	17	1.0	1.2	1.0	1.2
DDX41	Q9UJV9	568	12	6	2	17	1.1	1.1	1.0	1.2
ZNHIT3	Q15649	30	1	3	2	17	1.0	1.0	1.0	1.2
PFKP	Q01813	411	-7	-7	2	17	0.9	0.9	1.0	1.2
WDR81	Q562E7	765	-4	5	2	17	1.0	1.0	1.0	1.2
MEPCE	Q7L2J0	429	6	-5	2	17	1.1	1.0	1.0	1.2
UBA1	P22314	1039	2	-3	2	16	1.0	1.0	1.0	1.2
USP9Y	O00507	674	12	-7	2	16	1.1	0.9	1.0	1.2
USP9X	Q93008	673	12	-7	2	16	1.1	0.9	1.0	1.2
PID1	Q7Z2X4	77	3	-6	2	16	1.0	0.9	1.0	1.2
SUPT6H	Q7KZ85	1169	-3	-9	2	16	1.0	0.9	1.0	1.2
ZC3H13	Q5T200	57	6	6	2	15	1.1	1.1	1.0	1.2
LAGE3	Q14657	23	10	-11	2	15	1.1	0.9	1.0	1.2
CSDE1	O75534	42	3	0	2	15	1.0	1.0	1.0	1.2
SYMPK	Q92797	859	-3	-2	2	15	1.0	1.0	1.0	1.2
IFI16	Q16666	637	3	-3	2	15	1.0	1.0	1.0	1.2
NUDCD2	Q8WVJ2	45	3	-5	2	15	1.0	1.0	1.0	1.2
HTATSF1	O43719	186	-5	4	2	14	1.0	1.0	1.0	1.2
STIP1	P31948	461	4	-2	2	14	1.0	1.0	1.0	1.2
GNB1L	Q9BYB4	109	9	3	2	14	1.1	1.0	1.0	1.2
PDS5A	Q29RF7	1084	6	2	2	14	1.1	1.0	1.0	1.2
PRRC2B	Q5JSZ5	1121	-8	-15	2	14	0.9	0.9	1.0	1.2
OVCA2	Q8WZ82	152	-5	-4	2	13	1.0	1.0	1.0	1.1
N4BP2L2	Q92802	578	-2	-8	2	13	1.0	0.9	1.0	1.1
PRKDC	P78527	630	14	10	2	13	1.2	1.1	1.0	1.1
ARPC1B	O15143	26	3	-2	2	13	1.0	1.0	1.0	1.1
UTP18	Q9Y5J1	507	5	-8	2	13	1.1	0.9	1.0	1.1
RPL18A	Q02543	16	-1	7	2	12	1.0	1.1	1.0	1.1
ELP2	Q6IA86	68	-1	4	2	12	1.0	1.0	1.0	1.1
UQCRC2	P22695	192	5	3	2	12	1.0	1.0	1.0	1.1
HDLBP	Q00341	636	-2	3	2	12	1.0	1.0	1.0	1.1
GDPD1	Q8N9F7	126	8	-3	2	12	1.1	1.0	1.0	1.1
SMARCC1	Q92922	119	7	-18	2	11	1.1	0.9	1.0	1.1
USP34	Q70CQ2	741	-7	1	2	11	0.9	1.0	1.0	1.1
ZMYM2	Q9UBW7	801	-11	-4	2	11	0.9	1.0	1.0	1.1
NUP98	P52948	1476	17	-1	2	10	1.2	1.0	1.0	1.1
SKI	P12755	449	5	-8	2	10	1.1	0.9	1.0	1.1
DOK2	O60496	217	7	9	2	10	1.1	1.1	1.0	1.1
NTPCR	Q9BSD7	184	-6	-2	2	10	0.9	1.0	1.0	1.1
RPAP1	Q9BWH6	1039	-11	-3	2	10	0.9	1.0	1.0	1.1
WBP2	Q969T9	80	5	-7	2	10	1.1	0.9	1.0	1.1
ADRBK1	P25098	340	2	3	2	9	1.0	1.0	1.0	1.1
ADRBK2	P35626	340	2	3	2	9	1.0	1.0	1.0	1.1
TBC1D4	O60343	316	-18	9	2	9	0.8	1.1	1.0	1.1

SYTL3	Q4VX76	221	-2	-10	2	8	1.0	0.9	1.0	1.1
MIS12	Q9H081	133	-3	-4	2	7	1.0	1.0	1.0	1.1
CRYZL1	O95825	302	5	1	2	7	1.1	1.0	1.0	1.1
HCFC2	Q9Y5Z7	673	-2	2	2	6	1.0	1.0	1.0	1.1
IPO9	Q96P70	606	-4	-1	2	6	1.0	1.0	1.0	1.1
CLIC3	O95833	219	2	-3	2	6	1.0	1.0	1.0	1.1
AIM1	Q9Y4K1	1404	-3	-10	2	6	1.0	0.9	1.0	1.1
STAMBP	O95630	264	13	-1	2	6	1.1	1.0	1.0	1.1
LARS	Q9P2J5	546	7	-4	2	6	1.1	1.0	1.0	1.1
NCF2	P19878	165	-2	-8	2	6	1.0	0.9	1.0	1.1
LIMD2	Q9BT23	88	1	-8	2	6	1.0	0.9	1.0	1.1
COPG1	Q9Y678	44	2	1	2	5	1.0	1.0	1.0	1.1
LAP3	P28838	376	-3	-10	2	5	1.0	0.9	1.0	1.1
MYO1G	B0I1T2	97	3	3	2	5	1.0	1.0	1.0	1.0
Uncharacteriz	A0A087WZG4	394	0	0	2	5	1.0	1.0	1.0	1.0
PTK2B	Q14289	180	8	9	2	4	1.1	1.1	1.0	1.0
DDX17	Q92841	319	7	6	2	4	1.1	1.1	1.0	1.0
ACAP1	Q15027	328	8	8	2	3	1.1	1.1	1.0	1.0
EFL1	Q7Z2Z2	124	-2	-9	2	3	1.0	0.9	1.0	1.0
SF3A3	Q12874	437	-11	-9	2	3	0.9	0.9	1.0	1.0
RUVBL1	Q9Y265	49	13	-6	2	3	1.1	0.9	1.0	1.0
SUPV3L1	Q8IYB8	175	13	4	2	2	1.1	1.0	1.0	1.0
THEMIS2	Q5TEJ8	62	4	3	2	2	1.0	1.0	1.0	1.0
COPG1	Q9Y678	258	9	2	2	2	1.1	1.0	1.0	1.0
OAS2	P29728	180	1	-2	2	2	1.0	1.0	1.0	1.0
RUFY1	Q96T51	184	-3	-4	2	1	1.0	1.0	1.0	1.0
PHF1	O43189	265	-9	9	2	1	0.9	1.1	1.0	1.0
TIA1	P31483	33	-8	-15	2	1	0.9	0.9	1.0	1.0
PPP2R1A	P30153	154	8	7	2	0	1.1	1.1	1.0	1.0
PPP2R1B	P30154	166	8	7	2	0	1.1	1.1	1.0	1.0
PLEKHF1	Q96S99	183	6	-6	2	0	1.1	0.9	1.0	1.0
TRIM38	O00635	237	3	8	2	-1	1.0	1.1	1.0	1.0
CAND1	Q86VP6	802	5	6	2	-1	1.1	1.1	1.0	1.0
PPM1A	P35813	314	2	6	2	-1	1.0	1.1	1.0	1.0
EXOC2	Q96KP1	719	1	1	2	-1	1.0	1.0	1.0	1.0
XPO6	Q96QU8	159	-3	-4	2	-1	1.0	1.0	1.0	1.0
SYNE2	Q8WXH0	39	9	-1	2	-2	1.1	1.0	1.0	1.0
MRPS18C	Q9Y3D5	90	-13	1	2	-2	0.9	1.0	1.0	1.0
SH3GL1	Q99961	147	5	0	2	-3	1.0	1.0	1.0	1.0
ITPK1	Q13572	156	-1	1	2	-3	1.0	1.0	1.0	1.0
ZNF451	Q9Y4E5	724	3	13	2	-4	1.0	1.1	1.0	1.0
SLFN5	Q08AF3	439	24	19	2	-4	1.3	1.2	1.0	1.0
PPFIBP2	Q8ND30	145	1	-9	2	-4	1.0	0.9	1.0	1.0
ECI1	P42126	173	3	-12	2	-5	1.0	0.9	1.0	1.0
SACM1L	Q9NTJ5	344	8	-4	2	-5	1.1	1.0	1.0	1.0
LIG3	P49916	729	1	-4	2	-6	1.0	1.0	1.0	0.9
ANKRD44	Q8N8A2	834	-11	-7	2	-7	0.9	0.9	1.0	0.9
MAP3K2	Q9Y2U5	512	3	-9	2	-7	1.0	0.9	1.0	0.9
VAC14	Q08AM6	719	2	-21	2	-7	1.0	0.8	1.0	0.9
ZFPL1	O95159	56	12	4	2	-8	1.1	1.0	1.0	0.9
TBC1D13	Q9NVG8	131	7	-3	2	-8	1.1	1.0	1.0	0.9
TXLNA	P40222	245	0	-10	2	-9	1.0	0.9	1.0	0.9
TXLNG	Q9NUQ3	212	0	-10	2	-9	1.0	0.9	1.0	0.9
ARID4A	P29374	849	6	7	2	-10	1.1	1.1	1.0	0.9
PHF14	O94880	871	8	6	2	-10	1.1	1.1	1.0	0.9

VPS50	Q96JG6	324	3	-5	2	-11	1.0	1.0	1.0	0.9
PTPN23	Q9H3S7	425	-18	8	2	-11	0.9	1.1	1.0	0.9
HMHA1	Q92619	136	3	-9	2	-12	1.0	0.9	1.0	0.9
DHRS13	Q6UX07	128	9	7	2	-13	1.1	1.1	1.0	0.9
CDKAL1	Q5VV42	109	0	-15	2	-13	1.0	0.9	1.0	0.9
RPL11	P62913	72	-4	-2	2	-15	1.0	1.0	1.0	0.9
LENG8	Q96PV6	686	-14	-3	2	-15	0.9	1.0	1.0	0.9
TBL2	Q9Y4P3	108	-18	-22	2	-17	0.9	0.8	1.0	0.9
CUL2	Q13617	266	2	-11	2	-21	1.0	0.9	1.0	0.8
SBF1	O95248	600	11	-2	2	-24	1.1	1.0	1.0	0.8
PARP1	P09874	56	-3	-13	2	-41	1.0	0.9	1.0	0.7
ZFP64	Q9NTW7	146	3	-4	2	-62	1.0	1.0	1.0	0.6
TLN1	Q9Y490	1927	6	-2	2	-105	1.1	1.0	1.0	0.5
PDE5A	O76074	447	1	-2	2	-140	1.0	1.0	1.0	0.4
DOK3	Q7L591	402	8	15	2	45	1.1	1.2	1.0	1.8
OSBP	P22059	343	4	-6	2	40	1.0	0.9	1.0	1.7
SEC24D	O94855	1021	5	-8	2	38	1.0	0.9	1.0	1.6
IBA57	Q5T440	259	2	4	2	38	1.0	1.0	1.0	1.6
WHSC1L1	Q9BZ95	627	-8	-4	2	36	0.9	1.0	1.0	1.6
DHCR24	Q15392	252	-2	-1	2	30	1.0	1.0	1.0	1.4
XRCC4	Q13426	128	12	8	2	29	1.1	1.1	1.0	1.4
IPO7	O95373	757	5	1	2	28	1.1	1.0	1.0	1.4
IMPDH1	P20839	215	1	-2	2	28	1.0	1.0	1.0	1.4
NPLOC4	Q8TAT6	355	1	4	2	27	1.0	1.0	1.0	1.4
GIMAP2	Q9UG22	175	2	-22	2	27	1.0	0.8	1.0	1.4
MACF1	Q9UPN3	7344	10	-10	2	26	1.1	0.9	1.0	1.3
AKAP11	Q9UKA4	497	18	11	2	25	1.2	1.1	1.0	1.3
GDPGP1	Q6ZNW5	64	4	14	2	24	1.0	1.2	1.0	1.3
NFXL1	Q6ZNB6	775	11	-4	2	24	1.1	1.0	1.0	1.3
COG3	Q96JB2	349	-2	-2	2	23	1.0	1.0	1.0	1.3
PRKCH	P24723	399	-6	-8	2	23	0.9	0.9	1.0	1.3
SNUPN	O95149	199	6	0	2	22	1.1	1.0	1.0	1.3
TRPV2	Q9Y5S1	722	-4	-10	2	21	1.0	0.9	1.0	1.3
RRP8	O43159	451	1	-3	2	20	1.0	1.0	1.0	1.2
POLA1	P09884	1458	5	4	2	19	1.0	1.0	1.0	1.2
TOP2B	Q02880	426	8	-4	2	19	1.1	1.0	1.0	1.2
IMPDH2	P12268	339	10	-1	2	18	1.1	1.0	1.0	1.2
MICAL1	Q8TDZ2	95	3	6	2	17	1.0	1.1	1.0	1.2
CD4	P01730	447	-3	-4	2	17	1.0	1.0	1.0	1.2
HECTD1	Q9ULT8	369	-2	-5	2	17	1.0	1.0	1.0	1.2
PAPOLA	P51003	118	13	8	2	17	1.1	1.1	1.0	1.2
PAPOLG	Q9BWT3	117	13	8	2	17	1.1	1.1	1.0	1.2
ADCY7	P51828	1050	9	5	2	16	1.1	1.0	1.0	1.2
PWP1	Q13610	329	9	2	2	16	1.1	1.0	1.0	1.2
GART	P22102	291	17	-2	2	16	1.2	1.0	1.0	1.2
MYCBP2	O75592	4540	-5	-4	2	16	1.0	1.0	1.0	1.2
TYK2	P29597	936	0	6	2	15	1.0	1.1	1.0	1.2
CTSW	P56202	284	13	1	2	15	1.1	1.0	1.0	1.2
FGD3	Q5JSP0	608	7	-1	2	15	1.1	1.0	1.0	1.2
DDX1	Q92499	122	-10	-15	2	15	0.9	0.9	1.0	1.2
ECHS1	P30084	213	-7	-8	2	14	0.9	0.9	1.0	1.2
OTUB1	Q96FW1	212	6	6	2	14	1.1	1.1	1.0	1.2
TRIM56	Q9BRZ2	44	-7	-1	2	14	0.9	1.0	1.0	1.2
XPO1	O14980	34	14	-2	2	14	1.2	1.0	1.0	1.2
TEP1	Q99973	149	-8	-3	2	14	0.9	1.0	1.0	1.2

CSNK2B-LY6G!	N0E472	109	-4	-6	2	14	1.0	0.9	1.0	1.2
STAG2	Q8N3U4	711	-14	-20	2	14	0.9	0.8	1.0	1.2
STK17A	Q9UEE5	62	2	6	2	13	1.0	1.1	1.0	1.1
KBTBD11	O94819	537	-2	3	2	13	1.0	1.0	1.0	1.1
ASUN	Q9NVM9	556	-4	2	2	13	1.0	1.0	1.0	1.1
SEPHS1	P49903	31	3	-1	2	13	1.0	1.0	1.0	1.1
SCCPDH	Q8NBX0	238	-3	-6	2	13	1.0	0.9	1.0	1.1
NUP210	Q8TEM1	543	-1	-8	2	13	1.0	0.9	1.0	1.1
MX2	P20592	164	6	1	2	13	1.1	1.0	1.0	1.1
RANGAP1	P46060	573	5	1	2	13	1.1	1.0	1.0	1.1
PTBP1	P26599	250	4	4	2	12	1.0	1.0	1.0	1.1
LZTR1	Q8N653	342	13	1	2	12	1.1	1.0	1.0	1.1
CCT3	P49368	372	-9	-12	2	12	0.9	0.9	1.0	1.1
HGH1	Q9BTY7	138	-2	9	2	12	1.0	1.1	1.0	1.1
VWA5A	O00534	163	9	2	2	12	1.1	1.0	1.0	1.1
PRPF4	O43172	399	6	-3	2	12	1.1	1.0	1.0	1.1
HBP1	O60381	243	-7	-5	2	12	0.9	1.0	1.0	1.1
PRMT9	Q6P2P2	290	-7	-6	2	12	0.9	0.9	1.0	1.1
PARP4	Q9UKK3	1687	4	-13	2	12	1.0	0.9	1.0	1.1
ILVBL	A1L0T0	568	-5	-6	2	11	1.0	0.9	1.0	1.1
ENOPH1	Q9UHY7	202	-7	-7	2	11	0.9	0.9	1.0	1.1
NOP2	P46087	487	-6	2	2	11	0.9	1.0	1.0	1.1
STRBP	Q96SI9	142	-7	-9	2	11	0.9	0.9	1.0	1.1
GTF2A2	P52657	98	1	-3	2	10	1.0	1.0	1.0	1.1
TRIO	O75962	2136	10	0	2	9	1.1	1.0	1.0	1.1
WDR11	Q9BZH6	1000	-1	-2	2	9	1.0	1.0	1.0	1.1
BOLA1	Q9Y3E2	20	2	-7	2	9	1.0	0.9	1.0	1.1
FCHSD1	Q86WN1	471	-17	-9	2	9	0.9	0.9	1.0	1.1
COG7	P83436	174	5	8	2	9	1.1	1.1	1.0	1.1
TNPO2	O14787	93	6	-9	2	9	1.1	0.9	1.0	1.1
SACS	Q9NZJ4	2856	-7	-13	2	9	0.9	0.9	1.0	1.1
GABPA	Q06546	338	2	5	2	8	1.0	1.1	1.0	1.1
HTT	P42858	942	-13	1	2	8	0.9	1.0	1.0	1.1
GAK	O14976	472	-9	-1	2	8	0.9	1.0	1.0	1.1
UTP11	Q9Y3A2	201	-5	-4	2	8	1.0	1.0	1.0	1.1
USP16	Q9Y5T5	726	4	9	2	8	1.0	1.1	1.0	1.1
CACYBP	Q9HB71	173	0	-2	2	8	1.0	1.0	1.0	1.1
OPLAH	O14841	408	-21	-4	2	8	0.8	1.0	1.0	1.1
HIP1	O00291	613	-1	-12	2	8	1.0	0.9	1.0	1.1
PEX6	Q13608	761	10	4	2	7	1.1	1.0	1.0	1.1
CYLD	Q9NQC7	934	-7	-3	2	7	0.9	1.0	1.0	1.1
PAFAH1B2	P68402	206	7	-8	2	7	1.1	0.9	1.0	1.1
EPS15	P42566	419	-1	4	2	7	1.0	1.0	1.0	1.1
BANK1	Q8NDB2	332	6	6	2	6	1.1	1.1	1.0	1.1
LTA4H	P09960	141	4	5	2	6	1.0	1.0	1.0	1.1
METAP2	P50579	448	9	3	2	6	1.1	1.0	1.0	1.1
SPR	P35270	159	-4	2	2	6	1.0	1.0	1.0	1.1
ARHGAP4	P98171	569	3	-3	2	6	1.0	1.0	1.0	1.1
GBF1	Q92538	1392	-6	-3	2	5	0.9	1.0	1.0	1.0
UPP1	Q16831	71	-5	6	2	4	1.0	1.1	1.0	1.0
FAM76B	Q5HYJ3	110	19	5	2	4	1.2	1.0	1.0	1.0
NBEAL2	Q6ZNJ1	1661	-3	1	2	4	1.0	1.0	1.0	1.0
NCDN	Q9UBB6	469	4	-13	2	4	1.0	0.9	1.0	1.0
SHMT1	P34896	204	4	-4	2	4	1.0	1.0	1.0	1.0
CCDC47	Q96A33	214	12	-8	2	3	1.1	0.9	1.0	1.0

ZMYM3	Q14202	527	2	-10	2	3	1.0	0.9	1.0	1.0
HSPA4	P34932	245	3	-7	2	3	1.0	0.9	1.0	1.0
RGS14	O43566	304	-4	6	2	2	1.0	1.1	1.0	1.0
ACAT2	Q9BWD1	65	-7	-15	2	2	0.9	0.9	1.0	1.0
CCT3	P49368	173	10	3	2	2	1.1	1.0	1.0	1.0
RASA2	Q15283	354	2	1	2	2	1.0	1.0	1.0	1.0
PIK3C2B	O00750	384	10	-16	2	2	1.1	0.9	1.0	1.0
SMC5	Q8IY18	91	-19	-29	2	2	0.8	0.8	1.0	1.0
ITPR1	Q14643	1468	5	9	2	1	1.1	1.1	1.0	1.0
SAAL1	Q96ER3	248	1	-1	2	1	1.0	1.0	1.0	1.0
UBXN11	Q5T124	55	-10	-17	2	1	0.9	0.9	1.0	1.0
NLRP1	Q9C000	1257	2	10	2	1	1.0	1.1	1.0	1.0
PDS5B	Q9NTI5	333	0	6	2	1	1.0	1.1	1.0	1.0
RPS12	P25398	56	2	-3	2	1	1.0	1.0	1.0	1.0
ANKRD27	Q96NW4	39	2	-6	2	1	1.0	0.9	1.0	1.0
RRP12	Q5JTH9	288	18	9	2	0	1.2	1.1	1.0	1.0
CPSF4	O95639	110	1	3	2	0	1.0	1.0	1.0	1.0
SMARCC2	Q8TAQ2	635	-9	-2	2	0	0.9	1.0	1.0	1.0
SMARCC1	Q92922	657	-9	-2	2	0	0.9	1.0	1.0	1.0
DNM2	P50570	27	-2	-21	2	0	1.0	0.8	1.0	1.0
RAVER1	Q8IY67	161	3	-7	2	-1	1.0	0.9	1.0	1.0
PIK3CD	O00329	672	-7	-11	2	-1	0.9	0.9	1.0	1.0
ATM	Q13315	2801	7	8	2	-2	1.1	1.1	1.0	1.0
VPS11	Q9H270	890	-7	-7	2	-2	0.9	0.9	1.0	1.0
EDRF1	Q3B7T1	918	1	-8	2	-2	1.0	0.9	1.0	1.0
KANSL1	A0A0G2JNT7	588	-14	-12	2	-2	0.9	0.9	1.0	1.0
EP300	Q09472	369	-3	-4	2	-2	1.0	1.0	1.0	1.0
METTL16	Q86W50	57	-2	-9	2	-2	1.0	0.9	1.0	1.0
WDFY3	Q8IZQ1	575	1	4	2	-3	1.0	1.0	1.0	1.0
MRPL3	P09001	78	-19	-22	2	-3	0.8	0.8	1.0	1.0
DNAJA2	O60884	146	-3	7	2	-4	1.0	1.1	1.0	1.0
CPSF4	O95639	162	-3	-10	2	-4	1.0	0.9	1.0	1.0
TBP	P20226	262	-10	-10	2	-5	0.9	0.9	1.0	1.0
ZCCHC6	Q5VYS8	417	-5	-2	2	-5	1.0	1.0	1.0	1.0
ARHGAP17	Q68EM7	305	0	-7	2	-6	1.0	0.9	1.0	0.9
COPG2	Q9UBF2	440	8	14	2	-7	1.1	1.2	1.0	0.9
KIF13A	Q9H1H9	81	-3	8	2	-7	1.0	1.1	1.0	0.9
RIN3	Q8TB24	909	8	5	2	-8	1.1	1.1	1.0	0.9
FLII	Q13045	808	-7	1	2	-8	0.9	1.0	1.0	0.9
TRIM56	Q9BRZ2	131	8	-1	2	-8	1.1	1.0	1.0	0.9
APPL1	Q9UKG1	551	-2	-5	2	-8	1.0	1.0	1.0	0.9
ROCK2	O75116	330	4	1	2	-9	1.0	1.0	1.0	0.9
ROCK1	Q13464	314	4	1	2	-9	1.0	1.0	1.0	0.9
FASN	P49327	1127	-6	6	2	-10	0.9	1.1	1.0	0.9
ARHGAP4	P98171	34	-3	-3	2	-11	1.0	1.0	1.0	0.9
FASTKD1	Q53R41	446	7	-7	2	-12	1.1	0.9	1.0	0.9
IKBKB	O14920	115	7	6	2	-13	1.1	1.1	1.0	0.9
RQCD1	Q92600	91	4	-1	2	-15	1.0	1.0	1.0	0.9
KDM2A	Q9Y2K7	585	-2	-17	2	-15	1.0	0.9	1.0	0.9
TOR1AIP1	Q5JTV8	496	-15	3	2	-18	0.9	1.0	1.0	0.8
RAB18	Q9NP72	160	-19	3	2	-19	0.8	1.0	1.0	0.8
UNC13D	Q70J99	667	3	-6	2	-19	1.0	0.9	1.0	0.8
FAM118B	Q9BPY3	126	22	14	2	-72	1.3	1.2	1.0	0.6
ZFP36	P26651	147	-10	-1	2	-74	0.9	1.0	1.0	0.6
FHL1	Q13642	71	7	4	2	-81	1.1	1.0	1.0	0.6

SYTL4	Q96C24	66	11	-4	2	-122	1.1	1.0	1.0	0.5
SDPR	O95810	136	6	0	2	-152	1.1	1.0	1.0	0.4
CYBB	P04839	86	-8	-8	1	55	0.9	0.9	1.0	2.2
TXNIP	Q9H3M7	170	-10	-11	1	41	0.9	0.9	1.0	1.7
STK11IP	Q8N1F8	133	-12	-1	1	38	0.9	1.0	1.0	1.6
CRYM	Q14894	284	-2	-3	1	37	1.0	1.0	1.0	1.6
RIF1	Q5UIP0	1594	28	-18	1	37	1.4	0.9	1.0	1.6
PNPLA6	Q8IY17	982	1	-7	1	35	1.0	0.9	1.0	1.5
GPKOW	Q92917	137	-6	5	1	32	0.9	1.1	1.0	1.5
HNRNPLL	Q8WVV9	84	1	4	1	32	1.0	1.0	1.0	1.5
TRAF5	O00463	116	21	4	1	31	1.3	1.0	1.0	1.4
PFAS	O15067	1287	14	-3	1	30	1.2	1.0	1.0	1.4
RGP1	Q92546	90	2	5	1	29	1.0	1.1	1.0	1.4
MMS22L	Q6ZRQ5	1068	3	-1	1	28	1.0	1.0	1.0	1.4
KIF4B	Q2VIQ3	269	4	-5	1	28	1.0	1.0	1.0	1.4
VCPIP1	Q96JH7	1178	-6	-9	1	28	0.9	0.9	1.0	1.4
NBR1	Q14596	433	4	-1	1	27	1.0	1.0	1.0	1.4
HK2	P52789	909	-1	-5	1	27	1.0	1.0	1.0	1.4
SMARCAD1	Q9H4L7	772	-5	-13	1	27	1.0	0.9	1.0	1.4
RAVER2	Q9HCJ3	362	12	13	1	26	1.1	1.1	1.0	1.4
ALMS1	Q8TCU4	3799	9	-3	1	26	1.1	1.0	1.0	1.4
SQRDL	Q9Y6N5	337	0	-9	1	26	1.0	0.9	1.0	1.4
SMC5	Q8IY18	726	4	7	1	26	1.0	1.1	1.0	1.3
EPX	P11678	291	-11	-5	1	26	0.9	1.0	1.0	1.3
HLA-C	P04222	283	-3	-13	1	26	1.0	0.9	1.0	1.3
HLA-C	P30510	283	-3	-13	1	26	1.0	0.9	1.0	1.3
HLA-A	Q09160	283	-3	-13	1	26	1.0	0.9	1.0	1.3
PDPR	Q8NCN5	85	5	6	1	25	1.1	1.1	1.0	1.3
ENOSF1	Q7L5Y1	307	-2	-10	1	25	1.0	0.9	1.0	1.3
STARD7	Q9NQZ5	302	-3	3	1	25	1.0	1.0	1.0	1.3
ATRX	P46100	2404	8	-1	1	24	1.1	1.0	1.0	1.3
DENND2D	Q9H6A0	159	-1	-8	1	24	1.0	0.9	1.0	1.3
DNAJC25-GNG	A0A024R161	129	4	3	1	23	1.0	1.0	1.0	1.3
SBNO1	A3KN83	445	-6	2	1	23	0.9	1.0	1.0	1.3
SCAF8	Q9UPN6	514	-1	1	1	23	1.0	1.0	1.0	1.3
NUB1	Q9Y5A7	299	3	-3	1	23	1.0	1.0	1.0	1.3
PPIP5K2	O43314	532	4	-2	1	22	1.0	1.0	1.0	1.3
ATP13A1	Q9HD20	336	-12	-11	1	22	0.9	0.9	1.0	1.3
BOD1L1	Q8NFC6	1663	-7	10	1	21	0.9	1.1	1.0	1.3
AKR7A2	O43488	156	10	-5	1	21	1.1	1.0	1.0	1.3
QRICH1	Q2TAL8	701	14	-9	1	21	1.2	0.9	1.0	1.3
RFTN1	Q14699	551	-6	0	1	19	0.9	1.0	1.0	1.2
REL	Q04864	143	7	-2	1	19	1.1	1.0	1.0	1.2
BCL2L11	O43521	12	6	-15	1	19	1.1	0.9	1.0	1.2
CCT5	P48643	493	8	0	1	18	1.1	1.0	1.0	1.2
YTHDC2	Q9H6S0	25	7	2	1	17	1.1	1.0	1.0	1.2
DRAP1	Q14919	54	3	0	1	17	1.0	1.0	1.0	1.2
RSL1D1	O76021	47	-1	-5	1	17	1.0	1.0	1.0	1.2
BPTF	Q12830	1548	13	-6	1	17	1.1	0.9	1.0	1.2
RPP30	P78346	180	1	-15	1	17	1.0	0.9	1.0	1.2
ADD2	P35612	210	3	-13	1	17	1.0	0.9	1.0	1.2
AKAP12	Q02952	1479	2	-19	1	16	1.0	0.8	1.0	1.2
TMEM259	Q4ZIN3	129	7	-7	1	16	1.1	0.9	1.0	1.2
RRP12	Q5JTH9	839	-3	2	1	15	1.0	1.0	1.0	1.2
DDI2	Q5TDH0	361	-2	2	1	15	1.0	1.0	1.0	1.2

MBD1	Q9UIS9	57	8	-3	1	15	1.1	1.0	1.0	1.2
CUL3	Q13618	156	6	-2	1	15	1.1	1.0	1.0	1.2
MMAB	Q96EY8	132	3	9	1	14	1.0	1.1	1.0	1.2
EIF3I	Q13347	144	1	-5	1	14	1.0	1.0	1.0	1.2
UQCRC1	P31930	154	-2	-12	1	14	1.0	0.9	1.0	1.2
EXOSC9	Q06265	45	11	-17	1	14	1.1	0.9	1.0	1.2
ERBB2IP	Q96RT1	464	4	1	1	14	1.0	1.0	1.0	1.2
OSTC	Q9NRP0	14	4	0	1	14	1.0	1.0	1.0	1.2
FTSJ1	Q9UET6	238	-8	-1	1	14	0.9	1.0	1.0	1.2
NUP160	Q12769	529	6	-1	1	14	1.1	1.0	1.0	1.2
UAP1L1	Q3KQV9	254	-2	-6	1	14	1.0	0.9	1.0	1.2
FOXN3	O00409	469	-3	-7	1	14	1.0	0.9	1.0	1.2
HNRNPL	P14866	261	0	5	1	13	1.0	1.0	1.0	1.1
HAUS8	Q9BT25	182	-7	2	1	13	0.9	1.0	1.0	1.1
EIF6	P56537	11	4	0	1	13	1.0	1.0	1.0	1.1
CARD19	Q96LW7	63	2	7	1	13	1.0	1.1	1.0	1.1
TBL1X	O60907	401	3	1	1	13	1.0	1.0	1.0	1.1
GFM1	Q96RP9	153	15	-3	1	13	1.2	1.0	1.0	1.1
Uncharacteriz	H3BRB1	415	5	12	1	12	1.0	1.1	1.0	1.1
DDX60L	Q5H9U9	1671	-1	8	1	12	1.0	1.1	1.0	1.1
ARHGAP9	Q9BRR9	693	14	6	1	12	1.2	1.1	1.0	1.1
NXT1	Q9UKK6	14	2	3	1	12	1.0	1.0	1.0	1.1
MT-ND3	P03897	39	3	-3	1	12	1.0	1.0	1.0	1.1
AGO2	Q9UKV8	66	-1	-5	1	12	1.0	1.0	1.0	1.1
TGFBI	Q15582	65	4	-7	1	12	1.0	0.9	1.0	1.1
GATB	O75879	170	6	-2	1	11	1.1	1.0	1.0	1.1
SEPHS1	P49903	337	4	-3	1	11	1.0	1.0	1.0	1.1
PPIL1	Q9Y3C6	133	4	-1	1	11	1.0	1.0	1.0	1.1
PCNA	P12004	81	-1	-2	1	11	1.0	1.0	1.0	1.1
RBM25	P49756	83	-1	-2	1	11	1.0	1.0	1.0	1.1
RBBP7	Q16576	116	-2	-4	1	11	1.0	1.0	1.0	1.1
BTN3A3	O00478	512	5	-11	1	11	1.0	0.9	1.0	1.1
GAPVD1	Q14C86	275	6	-12	1	11	1.1	0.9	1.0	1.1
KMT2D	O14686	5314	12	-1	1	10	1.1	1.0	1.0	1.1
LPCAT1	Q8NF37	330	6	-6	1	10	1.1	0.9	1.0	1.1
SNRPN	P63162	43	8	1	1	9	1.1	1.0	1.0	1.1
LGALS9B	Q3B8N2	74	5	-4	1	9	1.1	1.0	1.0	1.1
ABTB1	Q969K4	62	-8	9	1	9	0.9	1.1	1.0	1.1
PML	P29590	151	2	4	1	9	1.0	1.0	1.0	1.1
WDR81	Q562E7	1226	4	-2	1	9	1.0	1.0	1.0	1.1
CAMSAP1	Q5T5Y3	395	8	11	1	8	1.1	1.1	1.0	1.1
PRKDC	P78527	3347	7	1	1	8	1.1	1.0	1.0	1.1
LRRC59	Q96AG4	48	1	-2	1	8	1.0	1.0	1.0	1.1
KIAA1429	Q69YN4	907	2	4	1	7	1.0	1.0	1.0	1.1
EIF2AK2	P19525	121	7	-2	1	7	1.1	1.0	1.0	1.1
TRAFD1	O14545	267	-10	-13	1	7	0.9	0.9	1.0	1.1
MTCH2	Q9Y6C9	79	2	-6	1	7	1.0	0.9	1.0	1.1
AASDH	Q4L235	369	2	-7	1	7	1.0	0.9	1.0	1.1
SRM	P19623	25	3	5	1	6	1.0	1.0	1.0	1.1
AGTPBP1	Q9UPW5	1046	-4	-9	1	6	1.0	0.9	1.0	1.1
DMXL1	Q9Y485	2532	-1	1	1	6	1.0	1.0	1.0	1.1
HSPD1	P10809	442	-3	-1	1	6	1.0	1.0	1.0	1.1
GCA	P28676	181	5	-3	1	5	1.1	1.0	1.0	1.1
RPL37A	P61513	57	0	-7	1	5	1.0	0.9	1.0	1.0
FKBP15	Q5T1M5	845	-9	7	1	4	0.9	1.1	1.0	1.0

TRIM27	P14373	359	2	-1	1	4	1.0	1.0	1.0	1.0
BRPF1	P55201	570	-15	6	1	4	0.9	1.1	1.0	1.0
USP48	Q86UV5	290	8	8	1	3	1.1	1.1	1.0	1.0
PIKFYVE	Q9Y2I7	192	6	4	1	3	1.1	1.0	1.0	1.0
SLC25A5	P05141	57	-22	-10	1	3	0.8	0.9	1.0	1.0
SLC25A4	P12235	57	-22	-10	1	3	0.8	0.9	1.0	1.0
ZBTB1	Q9Y2K1	160	-12	-11	1	3	0.9	0.9	1.0	1.0
RBM39	Q14498	478	-5	11	1	3	1.0	1.1	1.0	1.0
KPNA4	O00629	417	9	8	1	3	1.1	1.1	1.0	1.0
CDK13	Q14004	987	-3	-2	1	3	1.0	1.0	1.0	1.0
LARS	Q9P2J5	248	8	-5	1	3	1.1	1.0	1.0	1.0
DIS3L2	Q8IYB7	312	9	-3	1	2	1.1	1.0	1.0	1.0
INPP5B	P32019	990	13	7	1	1	1.1	1.1	1.0	1.0
PDHA1	P08559	41	-13	-3	1	1	0.9	1.0	1.0	1.0
TNPO1	Q92973	103	-8	-5	1	1	0.9	1.0	1.0	1.0
UBR4	Q5T4S7	4146	6	14	1	0	1.1	1.2	1.0	1.0
DDX58	O95786	278	4	3	1	0	1.0	1.0	1.0	1.0
FLNA	P21333	2479	-1	3	1	0	1.0	1.0	1.0	1.0
ACACA	Q13085	1396	-1	7	1	-1	1.0	1.1	1.0	1.0
WAPL	Q7Z5K2	675	-10	-3	1	-1	0.9	1.0	1.0	1.0
SURF4	O15260	32	6	-4	1	-1	1.1	1.0	1.0	1.0
PLXNC1	O60486	1178	-7	-9	1	-1	0.9	0.9	1.0	1.0
SPAG9	O60271	443	6	-11	1	-1	1.1	0.9	1.0	1.0
RRP12	Q5JTH9	763	2	-15	1	-1	1.0	0.9	1.0	1.0
PITPNM1	O00562	928	3	15	1	-3	1.0	1.2	1.0	1.0
ATG7	O95352	550	-25	-25	1	-3	0.8	0.8	1.0	1.0
DPF2	Q92785	276	2	-3	1	-4	1.0	1.0	1.0	1.0
CAP1	Q01518	375	4	-1	1	-5	1.0	1.0	1.0	1.0
ARFGEF1	Q9Y6D6	1685	8	3	1	-6	1.1	1.0	1.0	0.9
SCYL2	Q6P3W7	511	-5	2	1	-6	1.0	1.0	1.0	0.9
APPL1	Q9UKG1	616	-4	-2	1	-7	1.0	1.0	1.0	0.9
FAM175B	Q15018	224	-2	-5	1	-7	1.0	1.0	1.0	0.9
APAF1	O14727	115	5	6	1	-8	1.0	1.1	1.0	0.9
FLNA	P21333	1453	1	1	1	-9	1.0	1.0	1.0	0.9
ANKFY1	Q9P2R3	675	-8	-2	1	-9	0.9	1.0	1.0	0.9
AGO3	Q9H9G7	283	-1	2	1	-10	1.0	1.0	1.0	0.9
AGO4	Q9HCK5	272	-1	2	1	-10	1.0	1.0	1.0	0.9
AGO2	Q9UKV8	282	-1	2	1	-10	1.0	1.0	1.0	0.9
AGO1	Q9UL18	280	-1	2	1	-10	1.0	1.0	1.0	0.9
SQSTM1	Q13501	113	-2	-9	1	-10	1.0	0.9	1.0	0.9
FAM160B1	Q5W0V3	624	1	6	1	-10	1.0	1.1	1.0	0.9
BTK	Q06187	337	3	2	1	-10	1.0	1.0	1.0	0.9
ABI1	Q8IZP0	33	-4	-5	1	-12	1.0	1.0	1.0	0.9
PDLIM5	Q96HC4	479	-10	-13	1	-12	0.9	0.9	1.0	0.9
CHORDC1	Q9UHD1	59	-16	5	1	-12	0.9	1.1	1.0	0.9
RANGAP1	P46060	141	23	2	1	-12	1.3	1.0	1.0	0.9
AIMP2	Q13155	306	3	5	1	-13	1.0	1.0	1.0	0.9
PDCD4	Q53EL6	227	4	8	1	-16	1.0	1.1	1.0	0.9
MRPL14	Q6P1L8	57	3	6	1	-17	1.0	1.1	1.0	0.9
DHRS4	Q9BTZ2	88	-19	-7	1	-17	0.8	0.9	1.0	0.9
CSK	P41240	299	-20	-6	1	-22	0.8	0.9	1.0	0.8
CRYZ	Q08257	45	-24	5	1	-24	0.8	1.0	1.0	0.8
SERPINB2	P05120	405	-6	-20	1	-27	0.9	0.8	1.0	0.8
DNM1L	O00429	345	9	3	1	-32	1.1	1.0	1.0	0.8
FHL1	Q13642	40	1	15	1	-45	1.0	1.2	1.0	0.7

DHRS13	Q6UX07	174	-7	-34	1	54	0.9	0.7	1.0	2.2
USP16	Q9Y5T5	205	14	-15	1	53	1.2	0.9	1.0	2.1
PLSCR1	O15162	148	3	-1	1	52	1.0	1.0	1.0	2.1
NFU1	Q9UMS0	213	-5	-5	1	47	1.0	1.0	1.0	1.9
TARBP1	Q13395	483	-1	-3	1	40	1.0	1.0	1.0	1.7
TBL3	Q12788	696	-9	-4	1	39	0.9	1.0	1.0	1.6
FSCN1	Q16658	397	3	-11	1	39	1.0	0.9	1.0	1.6
PPIA	P62937	52	2	-12	1	37	1.0	0.9	1.0	1.6
USP28	Q96RU2	1058	-3	-11	1	35	1.0	0.9	1.0	1.5
PDDC1	Q8NB37	179	11	26	1	33	1.1	1.4	1.0	1.5
VCP	P55072	522	6	-4	1	32	1.1	1.0	1.0	1.5
PLCG1	P19174	1088	-8	-11	1	30	0.9	0.9	1.0	1.4
CPPED1	Q9BRF8	54	-2	5	1	30	1.0	1.0	1.0	1.4
WDR81	Q562E7	215	3	4	1	30	1.0	1.0	1.0	1.4
LARP7	Q4G0J3	272	3	2	1	30	1.0	1.0	1.0	1.4
HGH1	Q9BTY7	51	3	-4	1	29	1.0	1.0	1.0	1.4
NBEAL1	Q6ZS30	810	-4	-11	1	29	1.0	0.9	1.0	1.4
SPATA5	Q8NB90	459	9	-15	1	29	1.1	0.9	1.0	1.4
PPIA	P62937	115	3	3	1	28	1.0	1.0	1.0	1.4
RBM6	P78332	1057	-4	-5	1	27	1.0	1.0	1.0	1.4
RALGAPA2	Q2PPJ7	378	21	10	1	24	1.3	1.1	1.0	1.3
PCNT	O95613	1252	8	4	1	24	1.1	1.0	1.0	1.3
LIG3	P49916	929	3	-3	1	23	1.0	1.0	1.0	1.3
DUSP23	Q9BVJ7	53	-10	-17	1	23	0.9	0.9	1.0	1.3
FASN	P49327	1558	-1	-2	1	22	1.0	1.0	1.0	1.3
PDS5A	Q29RF7	430	-4	-1	1	22	1.0	1.0	1.0	1.3
NCL	P19338	543	6	-3	1	22	1.1	1.0	1.0	1.3
SLC2A9	Q9NRM0	36	-3	-10	1	22	1.0	0.9	1.0	1.3
METTL9	Q9H1A3	215	-11	10	1	21	0.9	1.1	1.0	1.3
SSSCA1	O60232	53	10	3	1	21	1.1	1.0	1.0	1.3
DPYD	Q12882	49	3	2	1	21	1.0	1.0	1.0	1.3
TNRC6C	Q9HCJ0	1637	2	-8	1	21	1.0	0.9	1.0	1.3
PAFAH1B3	Q15102	205	4	0	1	20	1.0	1.0	1.0	1.3
C14orf159	Q7Z3D6	265	-9	0	1	20	0.9	1.0	1.0	1.3
PARP10	Q53GL7	124	8	7	1	20	1.1	1.1	1.0	1.2
ATE1	O95260	429	13	-3	1	19	1.1	1.0	1.0	1.2
ATPAF1	Q5TC12	194	-13	4	1	19	0.9	1.0	1.0	1.2
ZNF638	Q14966	1947	-2	-2	1	19	1.0	1.0	1.0	1.2
ANKLE2	Q86XL3	844	18	-16	1	18	1.2	0.9	1.0	1.2
GNL3L	Q9NVN8	152	-1	16	1	18	1.0	1.2	1.0	1.2
PRPSAP1	Q14558	19	3	1	1	18	1.0	1.0	1.0	1.2
FLNB	O75369	991	3	-5	1	18	1.0	1.0	1.0	1.2
Uncharacteriz	V9GY48	208	5	5	1	17	1.1	1.1	1.0	1.2
UFC1	Q9Y3C8	116	0	-4	1	17	1.0	1.0	1.0	1.2
IRAK3	Q9Y616	509	12	-16	1	17	1.1	0.9	1.0	1.2
BROX	Q5VW32	288	-16	-12	1	16	0.9	0.9	1.0	1.2
RPL34	P49207	83	0	1	1	15	1.0	1.0	1.0	1.2
TMEM173	Q86WV6	148	-7	-3	1	15	0.9	1.0	1.0	1.2
TTLL12	Q14166	98	6	-4	1	15	1.1	1.0	1.0	1.2
COPB1	P53618	623	7	-5	1	15	1.1	1.0	1.0	1.2
NCF4	Q15080	84	2	3	1	14	1.0	1.0	1.0	1.2
FASTKD2	Q9NYY8	377	-6	-2	1	14	0.9	1.0	1.0	1.2
PPP2R2A	P63151	262	-1	-6	1	14	1.0	0.9	1.0	1.2
GCDH	Q92947	232	-2	4	1	14	1.0	1.0	1.0	1.2
EIF6	P56537	15	-1	0	1	14	1.0	1.0	1.0	1.2

ZMYM3	Q14202	675	4	-1	1	14	1.0	1.0	1.0	1.2
LRRK2	Q5S007	2492	1	-5	1	14	1.0	1.0	1.0	1.2
TRIM14	Q14142	75	4	7	1	13	1.0	1.1	1.0	1.1
LRRK2	Q5S007	696	2	2	1	13	1.0	1.0	1.0	1.1
HDGF	P51858	12	-4	4	1	13	1.0	1.0	1.0	1.1
NAPRT	Q6XQN6	463	-1	-2	1	13	1.0	1.0	1.0	1.1
APOBEC3C	Q9NRW3	76	-1	-5	1	13	1.0	1.0	1.0	1.1
PLEC	Q15149	4245	-3	-2	1	12	1.0	1.0	1.0	1.1
NUBP2	Q9Y5Y2	177	9	-6	1	12	1.1	0.9	1.0	1.1
FAM129B	Q96TA1	334	3	-3	1	12	1.0	1.0	1.0	1.1
PIK3CD	O00329	438	3	7	1	11	1.0	1.1	1.0	1.1
PRKCI	P41743	507	21	-2	1	11	1.3	1.0	1.0	1.1
HAAO	P46952	281	1	-8	1	11	1.0	0.9	1.0	1.1
PCMT1	P22061	102	7	-1	1	11	1.1	1.0	1.0	1.1
SMARCD2	Q92925	365	1	-1	1	11	1.0	1.0	1.0	1.1
ACLY	P53396	728	6	-3	1	11	1.1	1.0	1.0	1.1
DENND4C	Q5VZ89	809	4	11	1	10	1.0	1.1	1.0	1.1
KIAA1429	Q69YN4	1008	-8	7	1	10	0.9	1.1	1.0	1.1
TRIP12	Q14669	1538	-1	3	1	10	1.0	1.0	1.0	1.1
FAM49A	Q9H0Q0	83	10	0	1	10	1.1	1.0	1.0	1.1
VPS4B	O75351	240	3	-4	1	10	1.0	1.0	1.0	1.1
GOLGA2	Q08379	984	7	-8	1	10	1.1	0.9	1.0	1.1
RARRES3	Q9UL19	72	-1	-19	1	10	1.0	0.8	1.0	1.1
THNSL1	Q8IYQ7	163	-5	3	1	10	1.0	1.0	1.0	1.1
TRIM25	Q14258	168	-4	-1	1	10	1.0	1.0	1.0	1.1
ANKS1A	Q92625	849	21	4	1	9	1.3	1.0	1.0	1.1
PTK2B	Q14289	545	2	-5	1	9	1.0	1.0	1.0	1.1
RPSA	P08865	163	-3	1	1	9	1.0	1.0	1.0	1.1
GIMAP7	Q8NHV1	165	9	15	1	7	1.1	1.2	1.0	1.1
EPRS	P07814	105	7	7	1	7	1.1	1.1	1.0	1.1
RABGEF1	Q9UJ41	621	6	-3	1	7	1.1	1.0	1.0	1.1
WDR59	Q6PJI9	654	2	-4	1	7	1.0	1.0	1.0	1.1
AGL	P35573	399	11	3	1	7	1.1	1.0	1.0	1.1
ZC3HC1	Q86WB0	149	3	-1	1	7	1.0	1.0	1.0	1.1
CHD3	Q12873	500	9	6	1	6	1.1	1.1	1.0	1.1
SND1	Q7KZF4	31	-1	2	1	6	1.0	1.0	1.0	1.1
NUP160	Q12769	929	4	-3	1	6	1.0	1.0	1.0	1.1
ZBTB7A	O95365	34	0	-9	1	6	1.0	0.9	1.0	1.1
WDR1	O75083	225	-6	-3	1	6	0.9	1.0	1.0	1.1
TTC33	Q6PID6	215	3	13	1	5	1.0	1.1	1.0	1.0
RPLP0	P05388	119	2	-3	1	5	1.0	1.0	1.0	1.0
DNAJC13	O75165	331	0	-1	1	4	1.0	1.0	1.0	1.0
PMS2	P54278	202	18	12	1	4	1.2	1.1	1.0	1.0
USP24	Q9UPU5	1556	-5	2	1	4	1.0	1.0	1.0	1.0
ASPH	Q12797	660	13	6	1	3	1.1	1.1	1.0	1.0
U2AF2	P26368	464	-1	-4	1	3	1.0	1.0	1.0	1.0
ANKRD27	Q96NW4	899	1	-8	1	3	1.0	0.9	1.0	1.0
ZC3H11A	O75152	80	11	-4	1	3	1.1	1.0	1.0	1.0
KMT2A	Q03164	3909	7	-9	1	3	1.1	0.9	1.0	1.0
MX2	P20592	100	2	4	1	2	1.0	1.0	1.0	1.0
MX1	P20591	52	2	4	1	2	1.0	1.0	1.0	1.0
IFI16	Q16666	727	2	9	1	2	1.0	1.1	1.0	1.0
GLE1	Q53GS7	131	7	-5	1	2	1.1	1.0	1.0	1.0
RPS2	P15880	188	-7	8	1	1	0.9	1.1	1.0	1.0
METAP2	P50579	121	0	-5	1	1	1.0	1.0	1.0	1.0

AAK1	Q2M2I8	288	-1	-6	1	1	1.0	0.9	1.0	1.0
SCAI	Q8N9R8	63	5	-14	1	1	1.1	0.9	1.0	1.0
PTPN6	P29350	361	-7	4	1	0	0.9	1.0	1.0	1.0
GBP5	Q96PP8	233	5	2	1	0	1.1	1.0	1.0	1.0
FLNA	P21333	2601	2	-1	1	0	1.0	1.0	1.0	1.0
LRRK2	Q5S007	749	-4	-1	1	0	1.0	1.0	1.0	1.0
KIF1C	O43896	198	5	-13	1	0	1.0	0.9	1.0	1.0
SAMM50	Q9Y512	65	-3	1	1	-1	1.0	1.0	1.0	1.0
SF3A3	Q12874	274	-8	7	1	-1	0.9	1.1	1.0	1.0
KPNA6	O60684	253	-4	3	1	-1	1.0	1.0	1.0	1.0
CHMP6	Q96FZ7	110	15	-25	1	-2	1.2	0.8	1.0	1.0
YWHAZ	P63104	25	4	3	1	-3	1.0	1.0	1.0	1.0
SEPT2	Q15019	114	3	-5	1	-3	1.0	1.0	1.0	1.0
HNRNPK	P61978	184	3	-15	1	-3	1.0	0.9	1.0	1.0
DOCK2	Q92608	297	9	-3	1	-3	1.1	1.0	1.0	1.0
AP2A2	O94973	353	17	-1	1	-4	1.2	1.0	1.0	1.0
AP2A1	O95782	354	17	-1	1	-4	1.2	1.0	1.0	1.0
HSH2D	Q96JZ2	125	-15	7	1	-6	0.9	1.1	1.0	0.9
HGH1	Q9BTY7	227	10	13	1	-7	1.1	1.1	1.0	0.9
DBT	P11182	279	-8	-8	1	-7	0.9	0.9	1.0	0.9
RNF213	Q63HN8	3981	7	-8	1	-8	1.1	0.9	1.0	0.9
NUP153	P49790	667	15	-12	1	-8	1.2	0.9	1.0	0.9
HNRNPH2	P55795	290	-5	-24	1	-10	1.0	0.8	1.0	0.9
HEATR6	Q6AI08	476	13	5	1	-11	1.1	1.0	1.0	0.9
TRIM28	Q13263	232	10	1	1	-11	1.1	1.0	1.0	0.9
MYH9	P35579	740	7	4	1	-12	1.1	1.0	1.0	0.9
DGKG	P49619	399	10	-6	1	-12	1.1	0.9	1.0	0.9
ZFR	Q96KR1	899	0	18	1	-14	1.0	1.2	1.0	0.9
MYO1G	B0I1T2	655	5	0	1	-15	1.0	1.0	1.0	0.9
SMC1A	Q14683	1073	-4	2	1	-16	1.0	1.0	1.0	0.9
TRAF2	Q12933	129	8	-10	1	-16	1.1	0.9	1.0	0.9
NSF	P46459	250	7	5	1	-22	1.1	1.0	1.0	0.8
ATP5EP2	Q5VTU8	19	3	-10	1	-23	1.0	0.9	1.0	0.8
ZYX	Q15942	504	-9	18	1	-28	0.9	1.2	1.0	0.8
RPSA	P08865	148	-30	-1	1	-31	0.8	1.0	1.0	0.8
PRKCB	P05771	386	2	-8	1	-34	1.0	0.9	1.0	0.7
PRKCA	P17252	383	2	-8	1	-34	1.0	0.9	1.0	0.7
SPTB	P11277	1552	0	-5	1	-54	1.0	1.0	1.0	0.7
TLN1	Q9Y490	956	4	0	1	-77	1.0	1.0	1.0	0.6
FHOD1	Q9Y613	650	-1	-1	1	-79	1.0	1.0	1.0	0.6
NEXN	Q0ZGT2	224	-1	-28	1	-82	1.0	0.8	1.0	0.6
VCL	P18206	1053	1	-10	1	-95	1.0	0.9	1.0	0.5
UBA7	P41226	599	-5	-6	0	47	1.0	0.9	1.0	1.9
CTSB	P07858	108	14	5	0	40	1.2	1.0	1.0	1.7
GEMIN4	P57678	683	10	-9	0	40	1.1	0.9	1.0	1.7
TBC1D24	Q9ULP9	29	3	5	0	37	1.0	1.0	1.0	1.6
PPOX	P50336	183	5	-2	0	35	1.1	1.0	1.0	1.5
NOP56	O00567	142	-1	0	0	34	1.0	1.0	1.0	1.5
HERC3	Q15034	333	-10	-13	0	34	0.9	0.9	1.0	1.5
CEP85	Q6P2H3	281	-5	-6	0	33	1.0	0.9	1.0	1.5
CDCA7L	Q96GN5	422	-10	-8	0	33	0.9	0.9	1.0	1.5
PSMD5	Q16401	412	8	-3	0	32	1.1	1.0	1.0	1.5
USP9Y	O00507	843	14	-14	0	31	1.2	0.9	1.0	1.4
TBC1D10A	Q9BXI6	381	-5	-4	0	30	1.0	1.0	1.0	1.4
QRSL1	Q9H0R6	512	4	-5	0	29	1.0	1.0	1.0	1.4

RECQL5	O94762	434	-16	-6	0	28	0.9	0.9	1.0	1.4
CDK4	P11802	135	2	-7	0	27	1.0	0.9	1.0	1.4
MRPS16	Q9Y3D3	26	0	-4	0	25	1.0	1.0	1.0	1.3
NFKBIE	O00221	457	6	-8	0	25	1.1	0.9	1.0	1.3
UIMC1	Q96RL1	582	2	-7	0	24	1.0	0.9	1.0	1.3
P4HB	P07237	397	-5	-2	0	23	1.0	1.0	1.0	1.3
HIP1R	O75146	650	11	14	0	22	1.1	1.2	1.0	1.3
HNRNPU	Q00839	335	0	2	0	22	1.0	1.0	1.0	1.3
DDX59	Q5T1V6	414	-5	2	0	22	1.0	1.0	1.0	1.3
VPS18	Q9P253	776	5	-10	0	22	1.0	0.9	1.0	1.3
RBCK1	Q9BYM8	411	11	-14	0	22	1.1	0.9	1.0	1.3
MYO1G	B0I1T2	472	-3	-9	0	21	1.0	0.9	1.0	1.3
MSH2	P43246	697	5	9	0	21	1.1	1.1	1.0	1.3
CPNE3	O75131	506	13	-2	0	21	1.1	1.0	1.0	1.3
P4HB	P07237	53	0	-12	0	21	1.0	0.9	1.0	1.3
NCKAP1L	P55160	338	-2	-22	0	20	1.0	0.8	1.0	1.3
EVI5L	Q96CN4	637	17	0	0	19	1.2	1.0	1.0	1.2
TYK2	P29597	291	-2	-1	0	19	1.0	1.0	1.0	1.2
THOC6	Q86W42	314	3	-4	0	19	1.0	1.0	1.0	1.2
MOSPD2	Q8NHP6	471	-16	-8	0	19	0.9	0.9	1.0	1.2
FNDC3B	Q53EP0	442	-5	-26	0	19	1.0	0.8	1.0	1.2
THOP1	P52888	253	1	3	0	19	1.0	1.0	1.0	1.2
ELP2	Q6IA86	204	5	3	0	19	1.0	1.0	1.0	1.2
UBR5	O95071	739	5	2	0	19	1.0	1.0	1.0	1.2
FLNB	O75369	2501	-2	-1	0	18	1.0	1.0	1.0	1.2
PWWP2B	Q6NUJ5	39	2	1	0	18	1.0	1.0	1.0	1.2
SARNP	P82979	20	8	-1	0	18	1.1	1.0	1.0	1.2
RNH1	P13489	409	-5	-5	0	18	1.0	1.0	1.0	1.2
TRPC5	Q9UL62	248	11	6	0	17	1.1	1.1	1.0	1.2
LPXN	O60711	332	5	3	0	17	1.0	1.0	1.0	1.2
MYO1F	O00160	517	13	2	0	17	1.1	1.0	1.0	1.2
CCDC50	Q8IVM0	85	-6	-2	0	17	0.9	1.0	1.0	1.2
TRIM38	O00635	58	-8	-5	0	17	0.9	1.0	1.0	1.2
RANBP6	O60518	48	-1	-8	0	17	1.0	0.9	1.0	1.2
POLA2	Q14181	222	-6	2	0	16	0.9	1.0	1.0	1.2
KIAA0100	Q14667	180	-3	-1	0	16	1.0	1.0	1.0	1.2
LNPEP	Q9UIQ6	35	-7	-4	0	16	0.9	1.0	1.0	1.2
ILVBL	A1L0T0	354	6	-8	0	16	1.1	0.9	1.0	1.2
ILKAP	Q9H0C8	301	-5	1	0	15	1.0	1.0	1.0	1.2
TPR	P12270	1068	4	0	0	15	1.0	1.0	1.0	1.2
STX12	Q86Y82	29	5	-5	0	15	1.0	1.0	1.0	1.2
UBE2N	P61088	87	6	-7	0	15	1.1	0.9	1.0	1.2
RANBP2	P49792	707	-5	-10	0	15	1.0	0.9	1.0	1.2
PARN	O95453	317	-13	-16	0	15	0.9	0.9	1.0	1.2
SEC24A	O95486	388	3	8	0	14	1.0	1.1	1.0	1.2
UBA2	Q9UBT2	30	3	2	0	14	1.0	1.0	1.0	1.2
RBM33	Q96EV2	726	-4	7	0	14	1.0	1.1	1.0	1.2
HNRNPU	Q00839	295	-2	2	0	14	1.0	1.0	1.0	1.2
PPP2R4	Q15257	199	6	-2	0	14	1.1	1.0	1.0	1.2
BORCS7-ASM1	A0A0B4J1R7	28	2	-3	0	14	1.0	1.0	1.0	1.2
RBBP9	O75884	127	-7	-4	0	14	0.9	1.0	1.0	1.2
CFL1	P23528	147	7	5	0	13	1.1	1.0	1.0	1.1
BTAF1	O14981	914	2	-2	0	13	1.0	1.0	1.0	1.1
HERC2	O95714	349	4	-4	0	13	1.0	1.0	1.0	1.1
GPATCH8	Q9UKJ3	569	9	2	0	13	1.1	1.0	1.0	1.1

PCMT1	P22061	95	3	-4	0	13	1.0	1.0	1.0	1.1
EIF3B	P55884	302	-34	-11	0	13	0.7	0.9	1.0	1.1
RPL30	P62888	92	2	8	0	12	1.0	1.1	1.0	1.1
PIAS4	Q8N2W9	299	12	4	0	12	1.1	1.0	1.0	1.1
ZMYM2	Q9UBW7	704	4	-9	0	12	1.0	0.9	1.0	1.1
ZNF346	Q9UL40	79	2	0	0	11	1.0	1.0	1.0	1.1
N4BP2	Q86UW6	1438	-12	-7	0	11	0.9	0.9	1.0	1.1
GSTP1	P09211	48	2	-1	0	10	1.0	1.0	1.0	1.1
SEC24B	O95487	1175	-2	-6	0	10	1.0	0.9	1.0	1.1
TRIM22	Q8IYM9	140	5	-1	0	10	1.0	1.0	1.0	1.1
ZMYM2	Q9UBW7	587	-1	-5	0	10	1.0	1.0	1.0	1.1
TNPO3	Q9Y5L0	912	11	7	0	9	1.1	1.1	1.0	1.1
MYCBP2	O75592	2006	1	-1	0	9	1.0	1.0	1.0	1.1
PTPN9	P43378	230	-6	-5	0	9	0.9	1.0	1.0	1.1
UROD	P06132	59	9	-8	0	9	1.1	0.9	1.0	1.1
HDAC6	Q9UBN7	572	2	4	0	9	1.0	1.0	1.0	1.1
EIF3D	O15371	195	-1	-5	0	9	1.0	1.0	1.0	1.1
BCL11B	Q9C0K0	808	5	3	0	8	1.0	1.0	1.0	1.1
BCL11A	Q9H165	754	5	3	0	8	1.0	1.0	1.0	1.1
MTMR3	Q13615	564	11	1	0	8	1.1	1.0	1.0	1.1
VCP	P55072	105	3	-2	0	8	1.0	1.0	1.0	1.1
ARHGAP35	Q9NRY4	559	5	-2	0	8	1.1	1.0	1.0	1.1
COASY	Q13057	140	3	-4	0	8	1.0	1.0	1.0	1.1
NOP56	O00567	211	-3	-5	0	8	1.0	1.0	1.0	1.1
KDM2B	Q8NHM5	716	-6	-8	0	8	0.9	0.9	1.0	1.1
AGO3	Q9H9G7	328	-8	-9	0	8	0.9	0.9	1.0	1.1
AGO4	Q9HCK5	317	-8	-9	0	8	0.9	0.9	1.0	1.1
AGO2	Q9UKV8	327	-8	-9	0	8	0.9	0.9	1.0	1.1
AGO1	Q9UL18	325	-8	-9	0	8	0.9	0.9	1.0	1.1
KIF1C	O43896	350	-9	9	0	8	0.9	1.1	1.0	1.1
IFIH1	Q9BYX4	510	7	6	0	8	1.1	1.1	1.0	1.1
RASSF1	Q9NS23	106	8	6	0	8	1.1	1.1	1.0	1.1
PABPN1	Q86U42	205	0	1	0	8	1.0	1.0	1.0	1.1
HNRNPUL2-BS	H3BQZ7	293	-2	-1	0	8	1.0	1.0	1.0	1.1
LRPPRC	P42704	1043	-7	-16	0	8	0.9	0.9	1.0	1.1
SCFD2	Q8WU76	507	18	12	0	7	1.2	1.1	1.0	1.1
HSPH1	Q92598	34	-3	4	0	7	1.0	1.0	1.0	1.1
PIK3CG	P48736	395	-6	1	0	7	0.9	1.0	1.0	1.1
AFF1	P51825	892	13	-3	0	7	1.1	1.0	1.0	1.1
MYH14	Q7Z406	401	12	9	0	7	1.1	1.1	1.0	1.1
PKM	P14618	424	3	4	0	7	1.0	1.0	1.0	1.1
SEC31A	O94979	458	-1	2	0	7	1.0	1.0	1.0	1.1
HUWE1	Q7Z6Z7	1891	1	-3	0	7	1.0	1.0	1.0	1.1
SHOC2	Q9UQ13	238	4	-4	0	7	1.0	1.0	1.0	1.1
TMEM104	Q8NE00	455	3	-7	0	7	1.0	0.9	1.0	1.1
IPO4	Q8TEX9	708	5	3	0	6	1.1	1.0	1.0	1.1
ADPRHL2	Q9NX46	132	-9	1	0	6	0.9	1.0	1.0	1.1
AGPS	O00116	226	2	-2	0	6	1.0	1.0	1.0	1.1
PDXDC1	Q6P996	481	-2	-6	0	6	1.0	0.9	1.0	1.1
PTRHD1	Q6GMV3	52	12	-8	0	6	1.1	0.9	1.0	1.1
MIA3	Q5JRA6	1486	5	4	0	5	1.1	1.0	1.0	1.1
WAC	Q9BTA9	615	13	1	0	5	1.1	1.0	1.0	1.1
MACF1	Q9UPN3	3315	9	-1	0	5	1.1	1.0	1.0	1.1
PEX6	Q13608	167	-4	-12	0	5	1.0	0.9	1.0	1.1
ADRBK1	P25098	154	2	-19	0	5	1.0	0.8	1.0	1.1

SART3	Q15020	341	5	-6	0	5	1.1	0.9	1.0	1.0
NAPA	P54920	42	7	-6	0	5	1.1	0.9	1.0	1.0
RAP1GDS1	P52306	85	-1	-10	0	5	1.0	0.9	1.0	1.0
ITGB8	P26012	481	-3	-11	0	4	1.0	0.9	1.0	1.0
ITGB8	P26012	491	-3	-11	0	4	1.0	0.9	1.0	1.0
MYO1F	O00160	856	-2	-1	0	4	1.0	1.0	1.0	1.0
ZFR	Q96KR1	856	2	-1	0	4	1.0	1.0	1.0	1.0
TNPO1	Q92973	467	4	-3	0	4	1.0	1.0	1.0	1.0
MALT1	Q9UDY8	464	2	2	0	3	1.0	1.0	1.0	1.0
TRIM38	O00635	188	9	3	0	3	1.1	1.0	1.0	1.0
IFT27	Q9BW83	8	-6	-4	0	3	0.9	1.0	1.0	1.0
TXNRD2	Q9NNW7	168	3	-8	0	3	1.0	0.9	1.0	1.0
PRPF38A	Q8NAV1	158	7	2	0	2	1.1	1.0	1.0	1.0
NUP205	Q92621	141	0	2	0	2	1.0	1.0	1.0	1.0
CNOT1	A5YKK6	1687	15	-4	0	2	1.2	1.0	1.0	1.0
NUP98	P52948	1590	9	-8	0	2	1.1	0.9	1.0	1.0
BEND3	Q5T5X7	32	1	-13	0	2	1.0	0.9	1.0	1.0
SRI	P30626	162	1	-14	0	2	1.0	0.9	1.0	1.0
AP2A1	O95782	397	7	-2	0	2	1.1	1.0	1.0	1.0
SCFD2	Q8WU76	79	5	-4	0	2	1.1	1.0	1.0	1.0
SEC31A	O94979	689	1	-15	0	2	1.0	0.9	1.0	1.0
ZC3HC1	Q86WB0	112	1	-2	0	1	1.0	1.0	1.0	1.0
CLIC6	Q96NY7	487	-4	-7	0	1	1.0	0.9	1.0	1.0
EPB41L3	Q9Y2J2	124	-5	-12	0	1	1.0	0.9	1.0	1.0
CPSF2	Q9P2I0	621	1	-4	0	1	1.0	1.0	1.0	1.0
PACS2	Q86VP3	541	-3	6	0	0	1.0	1.1	1.0	1.0
RIPK1	Q13546	256	11	-2	0	0	1.1	1.0	1.0	1.0
OXCT1	P55809	235	-2	-6	0	0	1.0	0.9	1.0	1.0
MPHOSPH8	Q99549	693	2	-8	0	0	1.0	0.9	1.0	1.0
HSP90AA1	P07900	420	8	-13	0	0	1.1	0.9	1.0	1.0
SETDB1	Q15047	781	-7	-14	0	0	0.9	0.9	1.0	1.0
MTA2	O94776	391	8	4	0	-1	1.1	1.0	1.0	1.0
NMD3	Q96D46	218	14	-1	0	-1	1.2	1.0	1.0	1.0
UBE2M	P61081	65	1	-2	0	-1	1.0	1.0	1.0	1.0
VMAC	Q2NL98	108	5	-2	0	-1	1.0	1.0	1.0	1.0
ZCCHC11	Q5TAX3	572	5	-4	0	-2	1.0	1.0	1.0	1.0
ZW10	O43264	404	1	-6	0	-2	1.0	0.9	1.0	1.0
DICER1	Q9UPY3	1641	11	18	0	-3	1.1	1.2	1.0	1.0
IQGAP1	P46940	1534	2	5	0	-3	1.0	1.1	1.0	1.0
GIMAP8	Q8ND71	599	-1	4	0	-4	1.0	1.0	1.0	1.0
ITSN2	Q9NZM3	530	-4	3	0	-4	1.0	1.0	1.0	1.0
SERPINB6	P35237	100	-4	3	0	-4	1.0	1.0	1.0	1.0
PFKL	P17858	114	0	0	0	-4	1.0	1.0	1.0	1.0
GSPT1	P15170	237	-4	-11	0	-4	1.0	0.9	1.0	1.0
EHD1	Q9H4M9	138	-3	4	0	-5	1.0	1.0	1.0	1.0
MED6	O75586	45	1	-4	0	-5	1.0	1.0	1.0	1.0
LRPPRC	P42704	208	1	-13	0	-5	1.0	0.9	1.0	1.0
FASN	P49327	2024	-8	-4	0	-6	0.9	1.0	1.0	0.9
SRP54	P61011	136	11	-4	0	-6	1.1	1.0	1.0	0.9
CHFR	Q96EP1	431	1	-15	0	-6	1.0	0.9	1.0	0.9
EPRS	P07814	660	-4	11	0	-7	1.0	1.1	1.0	0.9
HSPA1B	P0DMV9	306	-3	-5	0	-7	1.0	1.0	1.0	0.9
HSPA6	P17066	308	-3	-5	0	-7	1.0	1.0	1.0	0.9
HNRNPK	P61978	205	11	-17	0	-7	1.1	0.9	1.0	0.9
NDUFA10	E7ESZ7	183	4	-8	0	-7	1.0	0.9	1.0	0.9

MRE11A	P49959	336	-9	-15	0	-7	0.9	0.9	1.0	0.9
BIN2	Q9UBW5	205	6	-5	0	-8	1.1	1.0	1.0	0.9
STAT5A	P42229	508	-4	-2	0	-9	1.0	1.0	1.0	0.9
STAT5B	P51692	508	-4	-2	0	-9	1.0	1.0	1.0	0.9
PSMD1	Q99460	112	1	-20	0	-9	1.0	0.8	1.0	0.9
HDLBP	Q00341	104	-3	-4	0	-10	1.0	1.0	1.0	0.9
STK24	Q9Y6E0	394	-8	-4	0	-10	0.9	1.0	1.0	0.9
RUVBL1	Q9Y265	206	-3	-7	0	-10	1.0	0.9	1.0	0.9
NBAS	A2RRP1	1634	1	-8	0	-11	1.0	0.9	1.0	0.9
FLNA	P21333	1165	4	10	0	-11	1.0	1.1	1.0	0.9
UBE2O	Q9C0C9	598	5	-19	0	-11	1.0	0.8	1.0	0.9
PAN3	Q58A45	506	5	-6	0	-12	1.0	0.9	1.0	0.9
HMHA1	Q92619	324	4	-1	0	-13	1.0	1.0	1.0	0.9
EML2	O95834	301	-13	-12	0	-13	0.9	0.9	1.0	0.9
SCYL3	Q8IZE3	327	-22	-2	0	-14	0.8	1.0	1.0	0.9
TRIM21	P19474	92	-7	8	0	-14	0.9	1.1	1.0	0.9
THUMPD3	Q9BV44	239	3	-3	0	-16	1.0	1.0	1.0	0.9
MON2	Q7Z3U7	212	-3	-4	0	-16	1.0	1.0	1.0	0.9
PPP1CA	P62136	245	6	-6	0	-16	1.1	0.9	1.0	0.9
RBM22	Q9NW64	74	1	-15	0	-20	1.0	0.9	1.0	0.8
FLNA	P21333	1402	33	3	0	-21	1.5	1.0	1.0	0.8
BAG5	Q9UL15	360	-38	-10	0	-21	0.7	0.9	1.0	0.8
FLNB	O75369	26	6	2	0	-22	1.1	1.0	1.0	0.8
FLNA	P21333	53	6	2	0	-22	1.1	1.0	1.0	0.8
FLNC	Q14315	46	6	2	0	-22	1.1	1.0	1.0	0.8
RNF40	O75150	210	-2	-7	0	-22	1.0	0.9	1.0	0.8
HYOU1	Q9Y4L1	352	4	-6	0	-31	1.0	0.9	1.0	0.8
GCLM	P48507	35	-18	-3	0	-37	0.8	1.0	1.0	0.7
TLN1	Q9Y490	732	14	11	0	-40	1.2	1.1	1.0	0.7
TLN1	Q9Y490	1661	10	6	0	-66	1.1	1.1	1.0	0.6
SYTL4	Q96C24	477	-7	7	0	-197	0.9	1.1	1.0	0.3
DGKA	P23743	277	20	32	-1	54	1.2	1.5	1.0	2.2
PLXND1	Q9Y4D7	1363	-12	5	-1	53	0.9	1.1	1.0	2.1
PELI2	Q9HAT8	284	-12	-7	-1	51	0.9	0.9	1.0	2.0
NOTCH1	P46531	2168	4	-10	-1	45	1.0	0.9	1.0	1.8
APEX1	P27695	93	9	-7	-1	44	1.1	0.9	1.0	1.8
EPX	P11678	455	-12	-8	-1	43	0.9	0.9	1.0	1.8
WAPL	Q7Z5K2	1170	5	-1	-1	41	1.1	1.0	1.0	1.7
ARHGAP9	Q9BRR9	210	-4	-12	-1	41	1.0	0.9	1.0	1.7
MXI1	P50539	100	-8	-3	-1	37	0.9	1.0	1.0	1.6
SCAF4	O95104	545	2	-6	-1	34	1.0	0.9	1.0	1.5
RCSD1	Q6JBY9	181	5	-1	-1	32	1.1	1.0	1.0	1.5
ATM	Q13315	2092	2	-7	-1	30	1.0	0.9	1.0	1.4
NO66	Q9H6W3	150	11	10	-1	29	1.1	1.1	1.0	1.4
MBNL1	Q9NR56	200	5	-1	-1	29	1.0	1.0	1.0	1.4
BANP	Q8N9N5	74	7	-6	-1	27	1.1	0.9	1.0	1.4
FNBP1	Q96RU3	511	7	-8	-1	26	1.1	0.9	1.0	1.4
HEATR6	Q6AI08	846	2	5	-1	26	1.0	1.1	1.0	1.3
BLMH	Q13867	164	-1	-3	-1	26	1.0	1.0	1.0	1.3
UBE3B	Q7Z3V4	998	3	10	-1	25	1.0	1.1	1.0	1.3
CAP1	Q01518	427	-1	7	-1	25	1.0	1.1	1.0	1.3
PHF10	Q8WUB8	134	6	3	-1	24	1.1	1.0	1.0	1.3
ARRB2	P32121	126	1	-4	-1	24	1.0	1.0	1.0	1.3
BANK1	Q8NDB2	18	-7	-10	-1	24	0.9	0.9	1.0	1.3
SCP2	P22307	71	-1	-1	-1	23	1.0	1.0	1.0	1.3

MKRN2	Q9H000	337	4	5	-1	22	1.0	1.0	1.0	1.3
SNX20	Q7Z614	222	-3	-5	-1	22	1.0	1.0	1.0	1.3
IQSEC2	Q5JU85	571	-9	-18	-1	22	0.9	0.8	1.0	1.3
SLC25A20	O43772	155	3	0	-1	22	1.0	1.0	1.0	1.3
VPS36	Q86VN1	271	-6	-1	-1	22	0.9	1.0	1.0	1.3
HSP90AB1	P08238	589	-10	-11	-1	21	0.9	0.9	1.0	1.3
PXN	P49023	535	3	9	-1	20	1.0	1.1	1.0	1.3
SUN1	O94901	657	1	5	-1	20	1.0	1.1	1.0	1.3
SUN2	Q9UH99	563	1	5	-1	20	1.0	1.1	1.0	1.3
PRMT2	P55345	76	15	-7	-1	20	1.2	0.9	1.0	1.3
ZNF8	P17098	383	3	3	-1	20	1.0	1.0	1.0	1.2
DDX24	Q9GZR7	599	3	1	-1	20	1.0	1.0	1.0	1.2
DPYD	Q12882	671	3	3	-1	19	1.0	1.0	1.0	1.2
TDRKH	Q9Y2W6	109	-2	-2	-1	19	1.0	1.0	1.0	1.2
NIPBL	Q6KC79	1876	7	-16	-1	19	1.1	0.9	1.0	1.2
PAXBP1	Q9Y5B6	870	-8	21	-1	18	0.9	1.3	1.0	1.2
GLYR1	Q49A26	486	-2	5	-1	18	1.0	1.0	1.0	1.2
PAG1	Q9NWQ8	355	3	2	-1	18	1.0	1.0	1.0	1.2
RAC2	P15153	178	4	-5	-1	18	1.0	1.0	1.0	1.2
TBC1D4	O60343	74	-9	-6	-1	18	0.9	0.9	1.0	1.2
Uncharacteriz	H0YHG0	333	3	-3	-1	17	1.0	1.0	1.0	1.2
NPC1	O15118	1261	8	5	-1	16	1.1	1.0	1.0	1.2
ZWILCH	Q9H900	7	3	-3	-1	16	1.0	1.0	1.0	1.2
JAK3	P52333	1105	0	-4	-1	16	1.0	1.0	1.0	1.2
DCAF12	Q5T6F0	380	-19	-11	-1	16	0.8	0.9	1.0	1.2
PFKL	P17858	170	-4	2	-1	16	1.0	1.0	1.0	1.2
ASH2L	Q9UBL3	362	4	-1	-1	16	1.0	1.0	1.0	1.2
NMI	Q13287	230	-1	-2	-1	16	1.0	1.0	1.0	1.2
SMARCD2	Q92925	355	3	-5	-1	16	1.0	1.0	1.0	1.2
GOLPH3	Q9H4A6	108	3	-4	-1	15	1.0	1.0	1.0	1.2
APOBEC3C	Q9NRW3	70	1	-4	-1	15	1.0	1.0	1.0	1.2
VPS11	Q9H270	660	-1	7	-1	15	1.0	1.1	1.0	1.2
SNRNP40	Q96DI7	168	0	-4	-1	14	1.0	1.0	1.0	1.2
WDR43	Q15061	307	9	-7	-1	14	1.1	0.9	1.0	1.2
MCM3	P25205	119	3	6	-1	14	1.0	1.1	1.0	1.2
PLEKHF2	Q9H8W4	219	-2	2	-1	14	1.0	1.0	1.0	1.2
RPRD2	Q5VT52	903	3	5	-1	13	1.0	1.1	1.0	1.1
HNRNPLL	Q8WVV9	405	-1	0	-1	13	1.0	1.0	1.0	1.1
SEC24C	P53992	881	10	6	-1	13	1.1	1.1	1.0	1.1
RCC2	Q9P258	144	1	5	-1	13	1.0	1.0	1.0	1.1
HNRNPU	Q00839	497	1	-4	-1	13	1.0	1.0	1.0	1.1
LIMD2	Q9BT23	43	-5	-6	-1	13	1.0	0.9	1.0	1.1
VPRBP	Q9Y4B6	1070	-5	8	-1	12	1.0	1.1	1.0	1.1
NNT	Q13423	936	4	-5	-1	12	1.0	1.0	1.0	1.1
CLIC1	O00299	59	5	-14	-1	12	1.1	0.9	1.0	1.1
CDK11B	J3QR44	440	-4	3	-1	12	1.0	1.0	1.0	1.1
CNOT1	A5YKK6	624	5	2	-1	12	1.0	1.0	1.0	1.1
TMC8	Q8IU68	705	3	-19	-1	12	1.0	0.8	1.0	1.1
GFER	P55789	165	5	-4	-1	11	1.0	1.0	1.0	1.1
URB2	Q14146	120	-10	-17	-1	11	0.9	0.9	1.0	1.1
ANKRD17	O75179	1158	0	4	-1	11	1.0	1.0	1.0	1.1
ANKHD1	Q8IWZ3	1130	0	4	-1	11	1.0	1.0	1.0	1.1
WDR11	Q9BZH6	1071	11	1	-1	11	1.1	1.0	1.0	1.1
LMNB1	P20700	110	0	7	-1	10	1.0	1.1	1.0	1.1
DDX39A	O00148	223	3	5	-1	10	1.0	1.0	1.0	1.1

GIMAP4	Q9NUV9	187	2	4	-1	10	1.0	1.0	1.0	1.1
G3BP1	Q13283	73	10	3	-1	10	1.1	1.0	1.0	1.1
SETD1A	O15047	1649	-4	-1	-1	10	1.0	1.0	1.0	1.1
INPP5D	Q92835	217	-1	-4	-1	10	1.0	1.0	1.0	1.1
DUT	P33316	222	10	-1	-1	10	1.1	1.0	1.0	1.1
BTAF1	O14981	1542	-8	-9	-1	10	0.9	0.9	1.0	1.1
CRLF3	Q8IUI8	95	15	-3	-1	9	1.2	1.0	1.0	1.1
RACK1	P63244	182	-3	-4	-1	9	1.0	1.0	1.0	1.1
TTC39C	Q8N584	517	-1	-4	-1	9	1.0	1.0	1.0	1.1
ZMAT2	Q96NC0	82	8	15	-1	9	1.1	1.2	1.0	1.1
EDC4	Q6P2E9	81	11	6	-1	9	1.1	1.1	1.0	1.1
ITPR3	Q14573	2144	11	1	-1	9	1.1	1.0	1.0	1.1
RAB43	Q86YS6	157	2	-2	-1	9	1.0	1.0	1.0	1.1
C12orf4	Q9NQ89	25	-5	-4	-1	9	1.0	1.0	1.0	1.1
STIP1	P31948	339	11	-8	-1	9	1.1	0.9	1.0	1.1
AKAP1	Q92667	102	4	-15	-1	9	1.0	0.9	1.0	1.1
PRKCB	P05771	50	1	-20	-1	9	1.0	0.8	1.0	1.1
PRKCA	P17252	50	1	-20	-1	9	1.0	0.8	1.0	1.1
FGD2	Q7Z6J4	43	2	-26	-1	9	1.0	0.8	1.0	1.1
RNF213	Q63HN8	3008	-1	5	-1	8	1.0	1.0	1.0	1.1
ANKRD27	Q96NW4	20	-1	4	-1	8	1.0	1.0	1.0	1.1
RPL27A	P46776	144	-2	1	-1	8	1.0	1.0	1.0	1.1
CASP1	P29466	364	3	-3	-1	8	1.0	1.0	1.0	1.1
ARID1A	O14497	2163	1	-6	-1	8	1.0	0.9	1.0	1.1
ARID1B	Q8NFD5	2114	1	-6	-1	8	1.0	0.9	1.0	1.1
HNRNPDL	O14979	177	0	-9	-1	8	1.0	0.9	1.0	1.1
METTL7A	Q9H8H3	79	-1	-13	-1	8	1.0	0.9	1.0	1.1
KCNAB2	Q13303	212	1	5	-1	8	1.0	1.0	1.0	1.1
OAS1	P00973	38	10	2	-1	8	1.1	1.0	1.0	1.1
MRPL44	Q9H9J2	53	-4	-4	-1	8	1.0	1.0	1.0	1.1
EPB41L2	O43491	232	-5	-4	-1	8	1.0	1.0	1.0	1.1
TECR	Q9NZ01	18	-5	-9	-1	8	1.0	0.9	1.0	1.1
USO1	O60763	478	23	14	-1	7	1.3	1.2	1.0	1.1
SLFN5	Q08AF3	495	11	11	-1	7	1.1	1.1	1.0	1.1
ILKAP	Q9H0C8	190	7	3	-1	7	1.1	1.0	1.0	1.1
PDS5A	Q29RF7	1079	3	-4	-1	7	1.0	1.0	1.0	1.1
DFFB	O76075	306	12	-15	-1	7	1.1	0.9	1.0	1.1
ZNF221	A0A087WT08	492	-16	-26	-1	7	0.9	0.8	1.0	1.1
ZC3H4	Q9UPT8	433	6	0	-1	7	1.1	1.0	1.0	1.1
WASF2	Q9Y6W5	27	-5	-3	-1	7	1.0	1.0	1.0	1.1
PSMG4	Q5JS54	55	-7	-9	-1	6	0.9	0.9	1.0	1.1
DGKA	P23743	253	1	1	-1	6	1.0	1.0	1.0	1.1
EPHA4	P54764	760	4	-2	-1	6	1.0	1.0	1.0	1.1
RBM19	Q9Y4C8	77	-3	-2	-1	6	1.0	1.0	1.0	1.1
EVI5	O60447	641	-1	-9	-1	6	1.0	0.9	1.0	1.1
PTPN11	Q06124	318	-5	-11	-1	6	1.0	0.9	1.0	1.1
EML4	Q9HC35	811	2	2	-1	5	1.0	1.0	1.0	1.1
KBTBD11	O94819	99	15	0	-1	5	1.2	1.0	1.0	1.1
TRAF6	Q9Y4K3	349	1	-8	-1	5	1.0	0.9	1.0	1.1
SLFN5	Q08AF3	207	-6	-9	-1	5	0.9	0.9	1.0	1.1
SETDB1	Q15047	1226	3	-9	-1	5	1.0	0.9	1.0	1.0
PSME4	Q14997	1100	0	-4	-1	4	1.0	1.0	1.0	1.0
VPS39	Q96JC1	358	9	-12	-1	4	1.1	0.9	1.0	1.0
HNRNPL	P14866	151	5	-13	-1	4	1.1	0.9	1.0	1.0
STXBP3	O00186	90	-6	3	-1	4	0.9	1.0	1.0	1.0

VBP1	P61758	113	-1	-2	-1	4	1.0	1.0	1.0	1.0
CNBP	P62633	161	3	-9	-1	4	1.0	0.9	1.0	1.0
EXOSC10	Q01780	554	-2	5	-1	3	1.0	1.0	1.0	1.0
PLCG2	P16885	791	7	1	-1	3	1.1	1.0	1.0	1.0
SF3A3	Q12874	145	5	-6	-1	3	1.0	0.9	1.0	1.0
CASP4	P49662	109	-9	1	-1	2	0.9	1.0	1.0	1.0
PARG	Q86W56	963	-2	-9	-1	2	1.0	0.9	1.0	1.0
SEC61A1	P61619	13	7	5	-1	1	1.1	1.0	1.0	1.0
ANKFY1	Q9P2R3	34	-3	5	-1	1	1.0	1.0	1.0	1.0
DDB1	Q16531	173	-9	-6	-1	1	0.9	0.9	1.0	1.0
GCLC	P48506	613	-1	-5	-1	1	1.0	1.0	1.0	1.0
HNRNPUL2-BS	H3BQZ7	452	2	1	-1	0	1.0	1.0	1.0	1.0
ABCD3	P28288	472	2	-3	-1	0	1.0	1.0	1.0	1.0
FLII	Q13045	119	4	5	-1	-1	1.0	1.0	1.0	1.0
NUP98	P52948	1492	-7	-10	-1	-1	0.9	0.9	1.0	1.0
RNF213	Q63HN8	2536	-5	7	-1	-1	1.0	1.1	1.0	1.0
ARAF	P10398	549	1	4	-1	-1	1.0	1.0	1.0	1.0
PNPLA8	Q9NP80	559	-2	-5	-1	-2	1.0	1.0	1.0	1.0
UBR4	Q5T4S7	260	1	11	-1	-2	1.0	1.1	1.0	1.0
MACF1	Q9UPN3	1336	-4	4	-1	-2	1.0	1.0	1.0	1.0
ARRB1	P49407	140	6	2	-1	-3	1.1	1.0	1.0	1.0
HSPH1	Q92598	658	-3	1	-1	-3	1.0	1.0	1.0	1.0
CNOT1	A5YKK6	1420	-2	-2	-1	-3	1.0	1.0	1.0	1.0
UIMC1	Q96RL1	298	1	-5	-1	-3	1.0	1.0	1.0	1.0
L3HYPDH	Q96EM0	205	8	3	-1	-4	1.1	1.0	1.0	1.0
USO1	O60763	303	5	2	-1	-4	1.1	1.0	1.0	1.0
ALAD	P13716	203	1	-5	-1	-5	1.0	1.0	1.0	1.0
SCAF4	O95104	54	4	11	-1	-5	1.0	1.1	1.0	1.0
SCAF8	Q9UPN6	54	4	11	-1	-5	1.0	1.1	1.0	1.0
SMAP2	Q8WU79	28	-12	-8	-1	-5	0.9	0.9	1.0	1.0
IQGAP1	P46940	148	-5	-8	-1	-6	1.0	0.9	1.0	0.9
WDR11	Q9BZH6	364	11	8	-1	-7	1.1	1.1	1.0	0.9
GCN1	Q92616	1595	-2	7	-1	-8	1.0	1.1	1.0	0.9
SCYL1	Q96KG9	512	6	4	-1	-8	1.1	1.0	1.0	0.9
TBC1D2	Q9BYX2	496	7	6	-1	-8	1.1	1.1	1.0	0.9
C7orf60	Q1RMZ1	174	-7	-18	-1	-8	0.9	0.9	1.0	0.9
NSUN4	Q96CB9	258	-7	2	-1	-9	0.9	1.0	1.0	0.9
INTS2	Q9H0H0	48	-6	-7	-1	-9	0.9	0.9	1.0	0.9
FUBP3	Q96I24	109	-7	-13	-1	-9	0.9	0.9	1.0	0.9
FRYL	O94915	2369	-3	-11	-1	-9	1.0	0.9	1.0	0.9
LRCH4	O75427	134	-6	0	-1	-10	0.9	1.0	1.0	0.9
PPP2R5C	Q13362	334	11	-8	-1	-11	1.1	0.9	1.0	0.9
PPP2R5D	Q14738	410	11	-8	-1	-11	1.1	0.9	1.0	0.9
PPP2R5B	Q15173	365	11	-8	-1	-11	1.1	0.9	1.0	0.9
PPP2R5E	Q16537	351	11	-8	-1	-11	1.1	0.9	1.0	0.9
CTNND1	O60716	394	9	5	-1	-11	1.1	1.1	1.0	0.9
EIF5	P55010	122	10	11	-1	-13	1.1	1.1	1.0	0.9
RASGRP2	Q7LDG7	548	-6	3	-1	-13	0.9	1.0	1.0	0.9
STK39	Q9UEW8	82	-1	0	-1	-13	1.0	1.0	1.0	0.9
ACAP1	Q15027	338	4	-2	-1	-13	1.0	1.0	1.0	0.9
KIAA1033	Q2M389	1031	-15	-21	-1	-13	0.9	0.8	1.0	0.9
TUBGCP3	Q96CW5	844	-2	-1	-1	-14	1.0	1.0	1.0	0.9
APAF1	O14727	258	6	4	-1	-15	1.1	1.0	1.0	0.9
AP1B1	Q10567	921	-3	-12	-1	-18	1.0	0.9	1.0	0.8
MCU	Q8NE86	97	6	8	-1	-21	1.1	1.1	1.0	0.8

ZYX	Q15942	553	-6	2	-1	-22	0.9	1.0	1.0	0.8
EEF1E1	O43324	147	1	-8	-1	-22	1.0	0.9	1.0	0.8
FASN	P49327	1141	9	-6	-1	-24	1.1	0.9	1.0	0.8
MYO1G	B0I1T2	965	1	3	-1	-25	1.0	1.0	1.0	0.8
RNF40	O75150	986	5	0	-1	-26	1.1	1.0	1.0	0.8
STAM	Q92783	85	-33	-4	-1	-28	0.8	1.0	1.0	0.8
TNFAIP2	Q03169	356	1	7	-1	-35	1.0	1.1	1.0	0.7
ADCY10	Q96PN6	570	-2	-1	-1	-37	1.0	1.0	1.0	0.7
PCIF1	Q9H4Z3	255	16	-5	-1	-51	1.2	1.0	1.0	0.7
LIMS1	P48059	184	11	-1	-1	-63	1.1	1.0	1.0	0.6
LIMS2	Q7Z4I7	189	11	-1	-1	-63	1.1	1.0	1.0	0.6
UBASH3B	Q8TF42	240	-20	-25	-1	-65	0.8	0.8	1.0	0.6
TLN1	Q9Y490	1363	8	-3	-1	-88	1.1	1.0	1.0	0.5
EGR4	Q05215	23	-9	-5	-1	-102	0.9	1.0	1.0	0.5
DISC1	Q9NRI5	684	-8	-1	-1	54	0.9	1.0	1.0	2.2
TEP1	Q99973	171	-7	0	-1	53	0.9	1.0	1.0	2.1
TLR2	O60603	713	-4	-5	-1	52	1.0	1.0	1.0	2.1
PNPLA7	Q6ZV29	1231	-4	-5	-1	47	1.0	1.0	1.0	1.9
MCTP2	Q6DN12	497	-8	-12	-1	44	0.9	0.9	1.0	1.8
NAA25	Q14CX7	339	-32	-1	-1	42	0.8	1.0	1.0	1.7
FBP1	P09467	39	7	0	-1	40	1.1	1.0	1.0	1.7
GPCPD1	Q9NPB8	640	-5	4	-1	36	1.0	1.0	1.0	1.6
RNASEL	Q05823	479	-34	-5	-1	35	0.7	1.0	1.0	1.5
FKBP2	P26885	42	-7	-6	-1	35	0.9	0.9	1.0	1.5
SPATA2	Q9UM82	41	1	-10	-1	35	1.0	0.9	1.0	1.5
TCP11L2	Q8N4U5	451	1	-4	-1	32	1.0	1.0	1.0	1.5
PPOX	P50336	258	-3	-9	-1	32	1.0	0.9	1.0	1.5
PUS3	Q9BZE2	420	-7	-10	-1	32	0.9	0.9	1.0	1.5
HNRNPA3	P51991	85	0	-7	-1	30	1.0	0.9	1.0	1.4
THADA	Q6YHU6	1585	5	-2	-1	30	1.1	1.0	1.0	1.4
MEPCE	Q7L2J0	54	-6	-3	-1	30	0.9	1.0	1.0	1.4
ANKS1A	Q92625	114	1	-5	-1	30	1.0	1.0	1.0	1.4
CYFIP1	Q7L576	346	16	4	-1	28	1.2	1.0	1.0	1.4
KDM4C	Q9H3R0	455	3	-9	-1	28	1.0	0.9	1.0	1.4
LANCL2	Q9NS86	49	-1	5	-1	28	1.0	1.0	1.0	1.4
MTA1	Q13330	68	10	1	-1	28	1.1	1.0	1.0	1.4
ARHGEF2	Q92974	383	9	1	-1	27	1.1	1.0	1.0	1.4
PLOD3	O60568	691	-3	7	-1	25	1.0	1.1	1.0	1.3
IDH3B	O43837	185	-2	3	-1	24	1.0	1.0	1.0	1.3
EIF3L	Q9Y262	417	5	-1	-1	24	1.1	1.0	1.0	1.3
MCM3AP	O60318	1377	5	-1	-1	23	1.0	1.0	1.0	1.3
SCLT1	Q96NL6	292	3	-11	-1	23	1.0	0.9	1.0	1.3
IVD	P26440	258	2	3	-1	22	1.0	1.0	1.0	1.3
PABPC1	P11940	339	3	0	-1	21	1.0	1.0	1.0	1.3
PABPC4	Q13310	339	3	0	-1	21	1.0	1.0	1.0	1.3
SLC25A20	O43772	136	2	2	-1	21	1.0	1.0	1.0	1.3
TRIM22	Q8IYM9	441	2	-2	-1	21	1.0	1.0	1.0	1.3
AFTPH	Q6ULP2	250	12	4	-1	20	1.1	1.0	1.0	1.3
GNB2	P62879	271	17	3	-1	20	1.2	1.0	1.0	1.3
XPOT	O43592	693	-2	-5	-1	20	1.0	1.0	1.0	1.3
ENTHD2	Q96N21	31	-6	-5	-1	20	0.9	1.0	1.0	1.2
RABGGTA	Q92696	354	4	5	-1	19	1.0	1.1	1.0	1.2
ESYT1	Q9BSJ8	522	0	-1	-1	19	1.0	1.0	1.0	1.2
RNF213	Q63HN8	2229	1	4	-1	19	1.0	1.0	1.0	1.2
PSTPIP1	O43586	180	15	-3	-1	19	1.2	1.0	1.0	1.2

MED17	Q9NVC6	241	12	4	-1	18	1.1	1.0	1.0	1.2
SERPINA1	A0A024R6I7	256	3	1	-1	17	1.0	1.0	1.0	1.2
RPA4	Q13156	49	-9	-1	-1	17	0.9	1.0	1.0	1.2
NSA2	O95478	193	-7	-6	-1	17	0.9	0.9	1.0	1.2
KLHDC3	Q9BQ90	102	-6	11	-1	17	0.9	1.1	1.0	1.2
CASP4	P49662	258	-3	-1	-1	17	1.0	1.0	1.0	1.2
EHMT1	Q9H9B1	575	4	12	-1	16	1.0	1.1	1.0	1.2
HP1BP3	Q5SSJ5	359	-9	2	-1	16	0.9	1.0	1.0	1.2
SLC25A6	P12236	257	5	-1	-1	16	1.0	1.0	1.0	1.2
SLC25A4	P12235	257	5	-1	-1	16	1.0	1.0	1.0	1.2
NEK6	Q9HC98	64	7	-5	-1	16	1.1	1.0	1.0	1.2
UBR4	Q5T4S7	2688	11	6	-1	16	1.1	1.1	1.0	1.2
SDHA	P31040	89	2	-8	-1	16	1.0	0.9	1.0	1.2
CUL4B	Q13620	292	3	4	-1	15	1.0	1.0	1.0	1.2
PRKDC	P78527	90	6	-2	-1	15	1.1	1.0	1.0	1.2
LMNA	P02545	522	-7	-5	-1	15	0.9	1.0	1.0	1.2
TSPYL1	Q9H0U9	135	-2	-13	-1	15	1.0	0.9	1.0	1.2
RARS	P54136	312	-5	-19	-1	15	1.0	0.8	1.0	1.2
SYNJ1	O43426	374	5	10	-1	14	1.0	1.1	1.0	1.2
SMARCC1	Q92922	520	1	-1	-1	14	1.0	1.0	1.0	1.2
USP5	P45974	219	-9	-5	-1	14	0.9	1.0	1.0	1.2
METAP2	P50579	135	6	-9	-1	14	1.1	0.9	1.0	1.2
DAZAP1	Q96EP5	63	-2	-6	-1	14	1.0	0.9	1.0	1.2
WDR1	O75083	194	-3	-9	-1	14	1.0	0.9	1.0	1.2
ARHGAP35	Q9NRY4	562	0	-12	-1	14	1.0	0.9	1.0	1.2
ZMYM3	Q14202	743	-1	9	-1	13	1.0	1.1	1.0	1.1
ALDH9A1	P49189	484	3	3	-1	13	1.0	1.0	1.0	1.1
CCT2	P78371	395	9	-3	-1	13	1.1	1.0	1.0	1.1
ADGRG4	Q8IZF6	2414	1	-4	-1	13	1.0	1.0	1.0	1.1
HK1	P19367	158	1	-8	-1	13	1.0	0.9	1.0	1.1
ERH	P84090	33	2	-21	-1	13	1.0	0.8	1.0	1.1
PHGDH	O43175	19	-5	-1	-1	12	1.0	1.0	1.0	1.1
EPPK1	A0A087X1U6	927	-9	-9	-1	12	0.9	0.9	1.0	1.1
ZNF189	O75820	525	13	-1	-1	12	1.1	1.0	1.0	1.1
MMTAG2	Q9BU76	179	-1	-7	-1	12	1.0	0.9	1.0	1.1
NUP85	Q9BW27	230	2	-2	-1	11	1.0	1.0	1.0	1.1
CDC37	Q16543	64	-14	-14	-1	11	0.9	0.9	1.0	1.1
THEMIS	Q8N1K5	41	-12	-8	-1	11	0.9	0.9	1.0	1.1
DGKZ	Q13574	905	-3	-3	-1	10	1.0	1.0	1.0	1.1
ANXA1	P04083	343	5	-2	-1	10	1.1	1.0	1.0	1.1
MRPL39	Q9NYK5	133	-1	-3	-1	10	1.0	1.0	1.0	1.1
RNF7	Q9UBF6	61	-4	-5	-1	10	1.0	1.0	1.0	1.1
KIAA0100	Q14667	1055	4	-2	-1	9	1.0	1.0	1.0	1.1
EIF4G2	P78344	282	4	4	-1	9	1.0	1.0	1.0	1.1
UBA6	A0AVT1	546	8	2	-1	9	1.1	1.0	1.0	1.1
CCDC88B	A6NC98	150	9	1	-1	9	1.1	1.0	1.0	1.1
LARS	Q9P2J5	554	4	0	-1	9	1.0	1.0	1.0	1.1
BOD1L1	Q8NFC6	1170	-12	-3	-1	9	0.9	1.0	1.0	1.1
XPO1	O14980	723	-8	-4	-1	9	0.9	1.0	1.0	1.1
ARAF	P10398	538	-3	-5	-1	9	1.0	1.0	1.0	1.1
WDFY1	Q8IWB7	401	-4	-5	-1	9	1.0	1.0	1.0	1.1
ZNF512	Q96ME7	413	-3	-11	-1	9	1.0	0.9	1.0	1.1
NIPBL	Q6KC79	2557	1	4	-1	8	1.0	1.0	1.0	1.1
ERCC1	P07992	274	-7	-1	-1	8	0.9	1.0	1.0	1.1
DYNC1H1	Q14204	3940	3	-3	-1	8	1.0	1.0	1.0	1.1

CPNE8	Q86YQ8	156	-6	-3	-1	8	0.9	1.0	1.0	1.1
CRYBG3	Q68DQ2	2402	8	-3	-1	8	1.1	1.0	1.0	1.1
PADI4	Q9UM07	434	-2	-2	-1	8	1.0	1.0	1.0	1.1
NISCH	Q9Y2I1	185	3	-2	-1	8	1.0	1.0	1.0	1.1
SARS2	Q9NP81	425	2	-5	-1	8	1.0	1.0	1.0	1.1
CCT4	P50991	221	0	-6	-1	8	1.0	0.9	1.0	1.1
PREX1	Q8TCU6	1651	5	-7	-1	8	1.1	0.9	1.0	1.1
COMT	P21964	207	5	11	-1	7	1.0	1.1	1.0	1.1
MLLT1	Q03111	42	1	1	-1	7	1.0	1.0	1.0	1.1
TBC1D9B	Q66K14	494	-12	-2	-1	7	0.9	1.0	1.0	1.1
KBTBD4	Q9NVX7	455	1	-4	-1	7	1.0	1.0	1.0	1.1
RDH14	Q9HBH5	74	-5	-6	-1	7	1.0	0.9	1.0	1.1
TPP2	P29144	342	1	-6	-1	7	1.0	0.9	1.0	1.1
GOLGA8K	D6RF30	264	2	-8	-1	7	1.0	0.9	1.0	1.1
XRCC1	P18887	615	-5	-10	-1	7	1.0	0.9	1.0	1.1
MYCBP2	O75592	4181	-7	19	-1	7	0.9	1.2	1.0	1.1
PPIG	Q13427	33	3	-2	-1	7	1.0	1.0	1.0	1.1
GOLGA2	Q08379	356	4	0	-1	6	1.0	1.0	1.0	1.1
ZNF408	Q9H9D4	366	3	-5	-1	6	1.0	1.0	1.0	1.1
CCT4	P50991	379	1	3	-1	6	1.0	1.0	1.0	1.1
DNAJC13	O75165	2181	0	0	-1	6	1.0	1.0	1.0	1.1
RILPL1	Q5EBL4	47	-11	-8	-1	6	0.9	0.9	1.0	1.1
GNAI2	P04899	112	-9	-8	-1	5	0.9	0.9	1.0	1.1
PDP1	Q9P0J1	132	0	-23	-1	5	1.0	0.8	1.0	1.1
GNL1	P36915	410	-3	4	-1	5	1.0	1.0	1.0	1.0
PSMA5	P28066	76	-12	-16	-1	5	0.9	0.9	1.0	1.0
MVK	Q03426	339	2	-1	-1	4	1.0	1.0	1.0	1.0
TFB2M	Q9H5Q4	383	-3	-16	-1	4	1.0	0.9	1.0	1.0
MRM3	Q9HC36	290	-4	-19	-1	4	1.0	0.8	1.0	1.0
EIF2S2	P20042	226	-1	-5	-1	4	1.0	1.0	1.0	1.0
LMNB2	Q03252	212	-10	-5	-1	4	0.9	1.0	1.0	1.0
CHST11	Q9NPF2	128	-3	-7	-1	4	1.0	0.9	1.0	1.0
RGS14	O43566	183	4	-9	-1	4	1.0	0.9	1.0	1.0
SMPD4	Q9NXE4	737	-12	-24	-1	3	0.9	0.8	1.0	1.0
PHKB	Q93100	734	9	7	-1	2	1.1	1.1	1.0	1.0
DOPEY1	Q5JWR5	2388	0	-6	-1	2	1.0	0.9	1.0	1.0
LANCL1	O43813	98	-8	-7	-1	2	0.9	0.9	1.0	1.0
FAM96B	Q9Y3D0	158	-4	-12	-1	2	1.0	0.9	1.0	1.0
MIB2	Q96AX9	972	-3	-12	-1	2	1.0	0.9	1.0	1.0
SRRT	Q9BXP5	479	11	-13	-1	2	1.1	0.9	1.0	1.0
GBF1	Q92538	1110	3	1	-1	2	1.0	1.0	1.0	1.0
HNRNPLL	Q8WVV9	357	14	-6	-1	2	1.2	0.9	1.0	1.0
RNF213	Q63HN8	700	-7	-10	-1	2	0.9	0.9	1.0	1.0
MYO1G	B0I1T2	979	-5	1	-1	1	1.0	1.0	1.0	1.0
SGTA	O43765	148	6	0	-1	1	1.1	1.0	1.0	1.0
NUB1	Q9Y5A7	400	-18	1	-1	1	0.8	1.0	1.0	1.0
KLC1	Q07866	236	3	-2	-1	-1	1.0	1.0	1.0	1.0
KLC4	Q9NSK0	234	3	-2	-1	-1	1.0	1.0	1.0	1.0
AP3B1	O00203	507	-4	-1	-1	-1	1.0	1.0	1.0	1.0
NUP88	Q99567	595	9	-4	-1	-1	1.1	1.0	1.0	1.0
CRBN	Q96SW2	188	4	-6	-1	-1	1.0	0.9	1.0	1.0
NUP155	O75694	917	-2	-8	-1	-1	1.0	0.9	1.0	1.0
PDCD6IP	Q8WUM4	231	5	2	-1	-2	1.1	1.0	1.0	1.0
CCT7	Q99832	511	-2	-2	-1	-2	1.0	1.0	1.0	1.0
CRIM1	Q9NZV1	291	5	16	-1	-3	1.1	1.2	1.0	1.0

CRIM1	Q9NZV1	303	5	16	-1	-3	1.1	1.2	1.0	1.0
CNDP2	Q96KP4	205	0	4	-1	-3	1.0	1.0	1.0	1.0
CHD4	Q14839	493	-6	3	-1	-3	0.9	1.0	1.0	1.0
RPUSD2	Q8IZ73	246	6	2	-1	-3	1.1	1.0	1.0	1.0
ALDH1L2	Q3SY69	707	13	-2	-1	-3	1.1	1.0	1.0	1.0
MICAL3	Q7RTP6	1760	8	-3	-1	-3	1.1	1.0	1.0	1.0
C11orf68	Q9H3H3	173	14	-16	-1	-3	1.2	0.9	1.0	1.0
CLASP1	Q7Z460	453	5	7	-1	-4	1.1	1.1	1.0	1.0
DYNC1H1	Q14204	4438	5	-8	-1	-4	1.1	0.9	1.0	1.0
RELA	Q04206	216	-11	-1	-1	-5	0.9	1.0	1.0	1.0
PLEKHO2	Q8TD55	135	5	-23	-1	-5	1.1	0.8	1.0	1.0
VWA8	A3KMH1	724	-5	-4	-1	-7	1.0	1.0	1.0	0.9
FLII	Q13045	241	2	-4	-1	-7	1.0	1.0	1.0	0.9
pk	D4Q8H0	150	-5	-16	-1	-7	1.0	0.9	1.0	0.9
COPS6	Q7L5N1	299	5	3	-1	-8	1.1	1.0	1.0	0.9
MTHFD1L	Q6UB35	906	3	-2	-1	-8	1.0	1.0	1.0	0.9
TTC37	Q6PGP7	352	-4	-3	-1	-8	1.0	1.0	1.0	0.9
RILPL2	Q969X0	161	-4	1	-1	-9	1.0	1.0	1.0	0.9
ETFB	P38117	66	-3	-19	-1	-9	1.0	0.8	1.0	0.9
CYFIP1	Q7L576	1087	-13	-11	-1	-11	0.9	0.9	1.0	0.9
CYFIP2	Q96F07	1111	-13	-11	-1	-11	0.9	0.9	1.0	0.9
RIN1	Q13671	284	4	2	-1	-12	1.0	1.0	1.0	0.9
RABGAP1	Q9Y3P9	250	1	-7	-1	-12	1.0	0.9	1.0	0.9
USP9Y	O00507	684	-3	2	-1	-12	1.0	1.0	1.0	0.9
USP9X	Q93008	683	-3	2	-1	-12	1.0	1.0	1.0	0.9
GAPVD1	Q14C86	70	6	2	-1	-13	1.1	1.0	1.0	0.9
ZAP70	P43403	254	19	-27	-1	-14	1.2	0.8	1.0	0.9
ANKRD17	O75179	242	2	-26	-1	-16	1.0	0.8	1.0	0.9
IQGAP2	Q13576	548	6	-9	-1	-17	1.1	0.9	1.0	0.9
USP24	Q9UPU5	995	2	-19	-1	-18	1.0	0.8	1.0	0.9
SNX4	O95219	172	-6	-6	-1	-18	0.9	0.9	1.0	0.8
DOCK5	Q9H7D0	1532	-20	-9	-1	-19	0.8	0.9	1.0	0.8
PARP1	P09874	298	4	-11	-1	-19	1.0	0.9	1.0	0.8
SPG11	Q96JI7	1890	-17	-14	-1	-19	0.9	0.9	1.0	0.8
MBNL2	Q5VZF2	19	-5	-21	-1	-22	1.0	0.8	1.0	0.8
MBNL1	Q9NR56	19	-5	-21	-1	-22	1.0	0.8	1.0	0.8
ESRRA	P11474	46	19	3	-1	-29	1.2	1.0	1.0	0.8
SNRPF	P62306	66	-3	7	-1	-36	1.0	1.1	1.0	0.7
CMPK2	Q5EBM0	287	-2	-3	-1	-41	1.0	1.0	1.0	0.7
FHOD1	Q9Y613	31	6	-5	-1	-42	1.1	1.0	1.0	0.7
GRPEL1	Q9HAV7	108	4	-5	-1	-48	1.0	1.0	1.0	0.7
VCL	P18206	737	3	-5	-1	-69	1.0	1.0	1.0	0.6
C15orf39	Q6ZRI6	367	-9	-7	-2	53	0.9	0.9	1.0	2.1
NCAPD2	Q15021	767	-9	-10	-2	47	0.9	0.9	1.0	1.9
PNPLA2	Q96AD5	412	-4	-5	-2	46	1.0	1.0	1.0	1.8
MRPS12	O15235	64	-5	-15	-2	43	1.0	0.9	1.0	1.7
NVL	O15381	626	-1	2	-2	41	1.0	1.0	1.0	1.7
MTR	Q99707	812	-4	-7	-2	40	1.0	0.9	1.0	1.7
OCIAD2	Q56VL3	27	-4	1	-2	39	1.0	1.0	1.0	1.6
LSP1	P33241	170	2	-12	-2	36	1.0	0.9	1.0	1.6
PGPEP1	Q9NXJ5	149	-5	-9	-2	33	1.0	0.9	1.0	1.5
NEDD9	Q14511	475	5	-7	-2	32	1.0	0.9	1.0	1.5
ATXN10	Q9UBB4	283	6	-3	-2	30	1.1	1.0	1.0	1.4
ELP4	Q96EB1	218	0	-4	-2	30	1.0	1.0	1.0	1.4
NME2	P22392	109	17	-20	-2	30	1.2	0.8	1.0	1.4

HEATR3	Q7Z4Q2	57	-3	-6	-2	29	1.0	0.9	1.0	1.4
HSPA9	P38646	66	11	6	-2	28	1.1	1.1	1.0	1.4
SPATA5L1	Q9BVQ7	509	4	5	-2	28	1.0	1.0	1.0	1.4
PSMD9	O00233	216	-8	-15	-2	28	0.9	0.9	1.0	1.4
KDM3A	Q9Y4C1	1140	3	-4	-2	27	1.0	1.0	1.0	1.4
ANXA11	P50995	226	4	-5	-2	26	1.0	1.0	1.0	1.4
NLRX1	Q86UT6	331	5	7	-2	26	1.0	1.1	1.0	1.3
VHL	P40337	77	8	-4	-2	26	1.1	1.0	1.0	1.3
NUP107	P57740	78	-1	-4	-2	25	1.0	1.0	1.0	1.3
NUFIP2	Q7Z417	234	-1	2	-2	25	1.0	1.0	1.0	1.3
TTC33	Q6PID6	55	-2	-7	-2	24	1.0	0.9	1.0	1.3
BCKDK	O14874	111	4	0	-2	24	1.0	1.0	1.0	1.3
RABEP1	Q15276	350	-7	-3	-2	24	0.9	1.0	1.0	1.3
JMJD7	P0C870	47	0	-22	-2	24	1.0	0.8	1.0	1.3
PPIA	P62937	161	6	5	-2	23	1.1	1.0	1.0	1.3
NOP58	Q9Y2X3	139	1	7	-2	22	1.0	1.1	1.0	1.3
NCAPD2	Q15021	286	-2	4	-2	21	1.0	1.0	1.0	1.3
NSUN5	Q96P11	343	10	-5	-2	21	1.1	1.0	1.0	1.3
DFFB	O76075	194	4	-11	-2	21	1.0	0.9	1.0	1.3
NBEAL2	Q6ZNJ1	121	1	-3	-2	20	1.0	1.0	1.0	1.3
IRF3	Q14653	222	-1	-5	-2	20	1.0	1.0	1.0	1.3
RPRD2	Q5VT52	1071	2	-8	-2	20	1.0	0.9	1.0	1.3
SVBP	Q8N300	58	4	-4	-2	19	1.0	1.0	1.0	1.2
PRKDC	P78527	1499	-8	-12	-2	19	0.9	0.9	1.0	1.2
LRWD1	Q9UFC0	454	5	-1	-2	19	1.1	1.0	1.0	1.2
ANKFY1	Q9P2R3	742	8	-3	-2	19	1.1	1.0	1.0	1.2
CD27	P26842	258	5	1	-2	18	1.1	1.0	1.0	1.2
RIN1	Q13671	623	-2	-10	-2	18	1.0	0.9	1.0	1.2
DAGLB	Q8NCG7	661	6	-1	-2	17	1.1	1.0	1.0	1.2
RBBP4	Q09028	138	-3	-4	-2	17	1.0	1.0	1.0	1.2
ZNF207	O43670	16	-14	0	-2	17	0.9	1.0	1.0	1.2
GRIPAP1	Q4V328	243	3	0	-2	17	1.0	1.0	1.0	1.2
BLK	P51451	373	-12	-2	-2	17	0.9	1.0	1.0	1.2
PPP4R2	Q9NY27	30	1	-4	-2	16	1.0	1.0	1.0	1.2
FOXP3	Q9BZS1	232	-9	-16	-2	16	0.9	0.9	1.0	1.2
GBP2	P32456	394	10	4	-2	16	1.1	1.0	1.0	1.2
DRG1	Q9Y295	243	2	-4	-2	16	1.0	1.0	1.0	1.2
NTHL1	P78549	118	5	-5	-2	16	1.0	1.0	1.0	1.2
UBE2I	P63279	138	5	-8	-2	16	1.1	0.9	1.0	1.2
ADARB1	P78563	674	2	-1	-2	15	1.0	1.0	1.0	1.2
IMPDH2	P12268	331	1	-4	-2	15	1.0	1.0	1.0	1.2
ENOSF1	Q7L5Y1	330	-2	-8	-2	15	1.0	0.9	1.0	1.2
RPS27A	P62979	149	4	8	-2	15	1.0	1.1	1.0	1.2
GIMAP1-GIMA	A0A087WTJ2	98	4	8	-2	15	1.0	1.1	1.0	1.2
GIMAP1	Q8WWP7	98	4	8	-2	15	1.0	1.1	1.0	1.2
TSR2	Q969E8	17	7	-1	-2	15	1.1	1.0	1.0	1.2
DHX30	Q7L2E3	606	-2	-1	-2	15	1.0	1.0	1.0	1.2
SEC62	Q99442	32	-6	-1	-2	15	0.9	1.0	1.0	1.2
SIN3A	Q96ST3	551	-3	-4	-2	15	1.0	1.0	1.0	1.2
GDE1	Q9NZC3	127	12	8	-2	14	1.1	1.1	1.0	1.2
POLD1	P28340	428	-1	5	-2	14	1.0	1.0	1.0	1.2
HPS3	Q969F9	707	9	-2	-2	14	1.1	1.0	1.0	1.2
PSMA7	O14818	63	-4	-6	-2	14	1.0	0.9	1.0	1.2
LIG1	P18858	895	1	-11	-2	14	1.0	0.9	1.0	1.2
STIM2	Q9P246	679	-8	-14	-2	14	0.9	0.9	1.0	1.2

NFKBIB	Q15653	142	-1	6	-2	14	1.0	1.1	1.0	1.2
LPCAT2	Q7L5N7	326	-1	6	-2	14	1.0	1.1	1.0	1.2
CNOT2	Q9NZN8	504	15	1	-2	14	1.2	1.0	1.0	1.2
LSP1	P33241	283	4	-5	-2	14	1.0	1.0	1.0	1.2
ZZEF1	O43149	466	-9	-6	-2	14	0.9	0.9	1.0	1.2
OSBPL1A	Q9BXW6	412	3	3	-2	13	1.0	1.0	1.0	1.1
EHBP1L1	Q8N3D4	1135	5	0	-2	13	1.0	1.0	1.0	1.1
NUMA1	Q14980	735	6	-3	-2	13	1.1	1.0	1.0	1.1
EEFSEC	P57772	289	3	-5	-2	13	1.0	1.0	1.0	1.1
UBA2	Q9UBT2	432	0	-5	-2	13	1.0	1.0	1.0	1.1
VPS4A	Q9UN37	359	-3	-10	-2	13	1.0	0.9	1.0	1.1
TANC2	Q9HCD6	1355	-1	-13	-2	13	1.0	0.9	1.0	1.1
SRPRB	Q9Y5M8	246	11	4	-2	13	1.1	1.0	1.0	1.1
RNF40	O75150	69	7	-7	-2	13	1.1	0.9	1.0	1.1
RBM12B	Q8IXT5	204	1	1	-2	12	1.0	1.0	1.0	1.1
CTSC	P53634	331	1	-8	-2	12	1.0	0.9	1.0	1.1
COL4A3BP	Q9Y5P4	172	-5	-8	-2	12	1.0	0.9	1.0	1.1
DYRK1B	Q9Y463	264	-2	-1	-2	12	1.0	1.0	1.0	1.1
PGM2	Q96G03	573	2	-4	-2	12	1.0	1.0	1.0	1.1
PCK2	Q16822	92	7	-21	-2	12	1.1	0.8	1.0	1.1
PRKCQ	Q04759	17	6	9	-2	11	1.1	1.1	1.0	1.1
RABGGTA	Q92696	532	-6	3	-2	11	0.9	1.0	1.0	1.1
ARMC1	Q9NVT9	69	-3	-1	-2	11	1.0	1.0	1.0	1.1
CRYBG3	Q68DQ2	647	-8	-6	-2	11	0.9	0.9	1.0	1.1
ZNF331	Q9NQX6	366	-7	-21	-2	11	0.9	0.8	1.0	1.1
BLNK	Q8WV28	271	3	10	-2	10	1.0	1.1	1.0	1.1
FRYL	O94915	372	4	4	-2	10	1.0	1.0	1.0	1.1
PLSCR1	O15162	254	1	1	-2	10	1.0	1.0	1.0	1.1
PRKD2	Q9BZL6	615	11	-4	-2	10	1.1	1.0	1.0	1.1
VPS11	Q9H270	586	-4	-4	-2	10	1.0	1.0	1.0	1.1
GTF2I	P78347	903	-2	10	-2	10	1.0	1.1	1.0	1.1
PHKB	Q93100	348	-1	4	-2	10	1.0	1.0	1.0	1.1
MACROD1	Q9BQ69	199	6	-5	-2	10	1.1	1.0	1.0	1.1
R3HCC1	Q9Y3T6	402	-13	-9	-2	10	0.9	0.9	1.0	1.1
ARF4	P18085	62	-3	7	-2	9	1.0	1.1	1.0	1.1
MYCBP2	O75592	4600	-3	-2	-2	9	1.0	1.0	1.0	1.1
PPM1G	O15355	13	-9	-5	-2	9	0.9	1.0	1.0	1.1
DCP2	Q8IU60	140	17	-7	-2	9	1.2	0.9	1.0	1.1
RFTN1	Q14699	433	-4	-9	-2	9	1.0	0.9	1.0	1.1
OTUB1	Q96FW1	91	-5	-10	-2	9	1.0	0.9	1.0	1.1
PPP4R2	Q9NY27	134	10	11	-2	9	1.1	1.1	1.0	1.1
THADA	Q6YHU6	588	-4	3	-2	9	1.0	1.0	1.0	1.1
ZBTB14	O43829	215	-11	-6	-2	9	0.9	0.9	1.0	1.1
NUMA1	Q14980	65	3	-6	-2	9	1.0	0.9	1.0	1.1
EED	O75530	324	-5	-20	-2	9	1.0	0.8	1.0	1.1
SRSF1	Q07955	148	-4	4	-2	8	1.0	1.0	1.0	1.1
SRP9	P49458	48	2	-5	-2	8	1.0	1.0	1.0	1.1
POP1	Q99575	705	1	-17	-2	8	1.0	0.9	1.0	1.1
CHERP	Q8IWX8	190	18	3	-2	7	1.2	1.0	1.0	1.1
PPP1R9B	Q96SB3	270	-5	1	-2	7	1.0	1.0	1.0	1.1
EIF2A	Q9BY44	80	2	-8	-2	7	1.0	0.9	1.0	1.1
NFATC3	Q12968	388	-4	-9	-2	7	1.0	0.9	1.0	1.1
ARHGEF1	Q92888	594	5	-2	-2	6	1.0	1.0	1.0	1.1
SSH1	Q8WYL5	762	-1	-6	-2	6	1.0	0.9	1.0	1.1
GOLGA3	Q08378	1431	-9	4	-2	5	0.9	1.0	1.0	1.1

PPIH	O43447	131	6	3	-2	5	1.1	1.0	1.0	1.1
GMIP	Q9P107	274	-1	-1	-2	5	1.0	1.0	1.0	1.1
NIPBL	Q6KC79	1940	2	-2	-2	5	1.0	1.0	1.0	1.1
HECTD3	Q5T447	487	-4	-6	-2	5	1.0	0.9	1.0	1.1
MATR3	A8MXP9	230	-4	-9	-2	5	1.0	0.9	1.0	1.1
FCHO1	O14526	84	4	-9	-2	5	1.0	0.9	1.0	1.1
FNDC3A	Q9Y2H6	1124	1	-15	-2	5	1.0	0.9	1.0	1.1
GCOM1	H8Y6P7	662	6	-22	-2	5	1.1	0.8	1.0	1.1
EIF3E	P60228	345	3	2	-2	5	1.0	1.0	1.0	1.0
SETD1A	O15047	1695	-3	-2	-2	5	1.0	1.0	1.0	1.0
AHCYL1	O43865	293	-4	-4	-2	5	1.0	1.0	1.0	1.0
SEC22A	Q96IW7	111	-4	-4	-2	5	1.0	1.0	1.0	1.0
RUFY1	Q96T51	667	-24	-11	-2	5	0.8	0.9	1.0	1.0
FOXJ2	Q9P0K8	42	-1	-8	-2	4	1.0	0.9	1.0	1.0
GCN1	Q92616	1535	3	-3	-2	3	1.0	1.0	1.0	1.0
SMARCC2	Q8TAQ2	495	-3	3	-2	3	1.0	1.0	1.0	1.0
SGF29	Q96ES7	74	12	8	-2	2	1.1	1.1	1.0	1.0
TEP1	Q99973	360	-11	-1	-2	2	0.9	1.0	1.0	1.0
EXOC7	Q9UPT5	83	-1	-6	-2	2	1.0	0.9	1.0	1.0
GIGYF1	O75420	944	-7	-5	-2	1	0.9	1.0	1.0	1.0
DPP3	Q9NY33	654	4	-17	-2	1	1.0	0.9	1.0	1.0
USP34	Q70CQ2	1768	9	-21	-2	1	1.1	0.8	1.0	1.0
ZMYM2	Q9UBW7	724	-3	-5	-2	0	1.0	1.0	1.0	1.0
LRCH3	Q96II8	236	-2	-3	-2	-1	1.0	1.0	1.0	1.0
THUMPD3	Q9BV44	426	-9	-3	-2	-1	0.9	1.0	1.0	1.0
DNAJC13	O75165	1432	3	-9	-2	-1	1.0	0.9	1.0	1.0
PKN1	Q16512	762	-3	-1	-2	-1	1.0	1.0	1.0	1.0
PKN2	Q16513	804	-3	-1	-2	-1	1.0	1.0	1.0	1.0
GPS1	Q13098	251	-7	-7	-2	-1	0.9	0.9	1.0	1.0
DYNC1LI1	Q9Y6G9	247	-9	4	-2	-2	0.9	1.0	1.0	1.0
PCK2	Q16822	306	2	-6	-2	-2	1.0	0.9	1.0	1.0
LRPPRC	P42704	930	1	-10	-2	-2	1.0	0.9	1.0	1.0
TOP2B	Q02880	476	-7	-11	-2	-3	0.9	0.9	1.0	1.0
CPSF4	O95639	41	-2	-4	-2	-3	1.0	1.0	1.0	1.0
TRIM28	Q13263	65	10	-7	-2	-3	1.1	0.9	1.0	1.0
DTX3L	Q8TDB6	417	2	12	-2	-4	1.0	1.1	1.0	1.0
INPPL1	O15357	689	-5	8	-2	-4	1.0	1.1	1.0	1.0
EPG5	Q9HCE0	486	-9	-3	-2	-4	0.9	1.0	1.0	1.0
RPS12	P25398	92	-5	-5	-2	-4	1.0	1.0	1.0	1.0
LRCH4	O75427	213	-4	-9	-2	-4	1.0	0.9	1.0	1.0
TMED8	Q6PL24	161	-1	-1	-2	-4	1.0	1.0	1.0	1.0
RECQL	P46063	475	0	-3	-2	-4	1.0	1.0	1.0	1.0
FGD3	Q5JSP0	284	0	-7	-2	-4	1.0	0.9	1.0	1.0
ANKRD54	Q6NXT1	152	-1	-17	-2	-5	1.0	0.9	1.0	1.0
FLII	Q13045	337	7	-1	-2	-5	1.1	1.0	1.0	1.0
TTF2	Q9UNY4	522	-7	-11	-2	-5	0.9	0.9	1.0	1.0
PRMT9	Q6P2P2	476	-8	-19	-2	-6	0.9	0.8	1.0	0.9
HINT3	Q9NQE9	66	5	-5	-2	-8	1.1	1.0	1.0	0.9
DDX60L	Q5H9U9	1563	12	-1	-2	-8	1.1	1.0	1.0	0.9
DDX60	Q8IY21	1579	12	-1	-2	-8	1.1	1.0	1.0	0.9
NPLOC4	Q8TAT6	130	-18	6	-2	-9	0.9	1.1	1.0	0.9
HEATR6	Q6AI08	426	7	10	-2	-9	1.1	1.1	1.0	0.9
ARHGAP30	Q7Z6I6	40	7	-6	-2	-9	1.1	0.9	1.0	0.9
LRBA	P50851	2740	9	-9	-2	-9	1.1	0.9	1.0	0.9
TMPO	P42166	629	-1	2	-2	-12	1.0	1.0	1.0	0.9

AP5Z1	O43299	775	5	-12	-2	-12	1.0	0.9	1.0	0.9
WRNIP1	Q96S55	502	4	1	-2	-13	1.0	1.0	1.0	0.9
TFCP2	Q12800	395	-7	-1	-2	-14	0.9	1.0	1.0	0.9
PTK2B	Q14289	352	-4	3	-2	-15	1.0	1.0	1.0	0.9
DHX38	Q92620	618	-7	-8	-2	-15	0.9	0.9	1.0	0.9
PPP2R1A	P30153	310	1	2	-2	-16	1.0	1.0	1.0	0.9
STAG2	Q8N3U4	632	13	-1	-2	-16	1.1	1.0	1.0	0.9
PLPPR2	Q96GM1	207	8	9	-2	-16	1.1	1.1	1.0	0.9
TREML1	Q86YW5	196	2	-9	-2	-18	1.0	0.9	1.0	0.9
BRAT1	Q6PJG6	308	3	-3	-2	-18	1.0	1.0	1.0	0.8
KDM2A	Q9Y2K7	609	7	-17	-2	-19	1.1	0.9	1.0	0.8
GTF2A2	P52657	68	-3	-4	-2	-19	1.0	1.0	1.0	0.8
LRPPRC	P42704	130	2	-5	-2	-23	1.0	1.0	1.0	0.8
DSTN	P60981	46	8	-11	-2	-24	1.1	0.9	1.0	0.8
UPF2	Q9HAU5	578	-1	-2	-2	-24	1.0	1.0	1.0	0.8
PRKAR2B	P31323	211	-5	0	-2	-26	1.0	1.0	1.0	0.8
CDC42BPB	Q9Y5S2	784	6	-6	-2	-26	1.1	0.9	1.0	0.8
MYH10	P35580	678	-4	-5	-2	-31	1.0	1.0	1.0	0.8
MGLL	Q99685	242	4	1	-2	-62	1.0	1.0	1.0	0.6
COPA	P53621	1148	10	2	-2	-78	1.1	1.0	1.0	0.6
ANK1	P16157	278	-1	-4	-2	-89	1.0	1.0	1.0	0.5
HEATR3	Q7Z4Q2	266	-12	-11	-2	65	0.9	0.9	1.0	2.8
EXOSC6	Q5RKV6	117	-3	-10	-2	48	1.0	0.9	1.0	1.9
ATAD3B	Q5T9A4	634	-7	-7	-2	45	0.9	0.9	1.0	1.8
PCIF1	Q9H4Z3	29	4	-18	-2	43	1.0	0.9	1.0	1.8
ACTR5	Q9H9F9	265	-7	-8	-2	42	0.9	0.9	1.0	1.7
PDE3B	Q13370	297	7	2	-2	41	1.1	1.0	1.0	1.7
AQP3	Q92482	11	4	6	-2	37	1.0	1.1	1.0	1.6
SLC27A1	Q6PCB7	392	7	-2	-2	36	1.1	1.0	1.0	1.6
ZNF780B	Q9Y6R6	450	10	-3	-2	35	1.1	1.0	1.0	1.5
ZNF598	Q86UK7	32	2	-1	-2	33	1.0	1.0	1.0	1.5
ZNF84	P51523	293	-25	-14	-2	32	0.8	0.9	1.0	1.5
PPA1	Q15181	242	6	1	-2	30	1.1	1.0	1.0	1.4
ERI1	Q8IV48	75	-3	-4	-2	30	1.0	1.0	1.0	1.4
EXOSC10	Q01780	852	-1	4	-2	30	1.0	1.0	1.0	1.4
PLEC	Q15149	730	5	-10	-2	30	1.1	0.9	1.0	1.4
KARS	Q15046	209	6	-1	-2	29	1.1	1.0	1.0	1.4
RERE	Q9P2R6	1144	-5	-6	-2	29	1.0	0.9	1.0	1.4
FMNL1	O95466	1090	1	4	-2	28	1.0	1.0	1.0	1.4
PKM	P14618	474	-1	-4	-2	27	1.0	1.0	1.0	1.4
ABCB8	Q9NUT2	323	12	4	-2	26	1.1	1.0	1.0	1.4
HNRNPM	P52272	114	3	-5	-2	26	1.0	1.0	1.0	1.4
CECR1	Q9NZK5	408	-7	-10	-2	26	0.9	0.9	1.0	1.4
SART1	O43290	560	-1	-5	-2	26	1.0	1.0	1.0	1.3
CDK9	P50750	85	4	-4	-2	25	1.0	1.0	1.0	1.3
PREX1	Q8TCU6	879	12	4	-2	24	1.1	1.0	1.0	1.3
SWAP70	Q9UH65	261	-6	-7	-2	24	0.9	0.9	1.0	1.3
SPG20	Q8N0X7	504	-5	-1	-2	24	1.0	1.0	1.0	1.3
SZT2	Q5T011	2962	-7	-7	-2	24	0.9	0.9	1.0	1.3
SEC24D	O94855	1028	2	-3	-2	23	1.0	1.0	1.0	1.3
SCLY	Q96I15	377	-14	-3	-2	23	0.9	1.0	1.0	1.3
RECQL4	A0A087WZ30	749	8	-1	-2	22	1.1	1.0	1.0	1.3
MRFAP1	Q9Y605	97	-9	-4	-2	22	0.9	1.0	1.0	1.3
RNF114	Q9Y508	8	-4	-7	-2	22	1.0	0.9	1.0	1.3
SYTL3	Q4VX76	66	14	7	-2	21	1.2	1.1	1.0	1.3

SDR39U1	Q9NRG7	78	12	4	-2	21	1.1	1.0	1.0	1.3
HDGFRP2	A0A087WT54	16	1	1	-2	21	1.0	1.0	1.0	1.3
FLNB	O75369	1952	-4	-7	-2	21	1.0	0.9	1.0	1.3
FECH	P22830	196	-3	-33	-2	21	1.0	0.8	1.0	1.3
GALK1	P51570	182	14	-2	-2	20	1.2	1.0	1.0	1.3
GON4L	Q3T8J9	813	2	-21	-2	20	1.0	0.8	1.0	1.3
YY1AP1	Q9H869	290	2	-21	-2	20	1.0	0.8	1.0	1.3
SLC25A22	Q9H936	271	-3	18	-2	20	1.0	1.2	1.0	1.2
DARS	P14868	349	6	5	-2	20	1.1	1.0	1.0	1.2
HNRNPUL2-BS	H3BQZ7	602	5	-4	-2	20	1.1	1.0	1.0	1.2
DCUN1D4	Q92564	253	-2	-2	-2	19	1.0	1.0	1.0	1.2
RNF213	Q63HN8	4407	0	-3	-2	19	1.0	1.0	1.0	1.2
RNF14	Q9UBS8	262	4	-5	-2	19	1.0	1.0	1.0	1.2
FAM208B	Q5VWN6	623	-3	-13	-2	19	1.0	0.9	1.0	1.2
ZMYM4	Q5VZL5	467	-1	2	-2	18	1.0	1.0	1.0	1.2
UBE4A	Q14139	79	-1	-6	-2	18	1.0	0.9	1.0	1.2
SYNE1	Q8NF91	8076	5	-9	-2	18	1.0	0.9	1.0	1.2
ORC3	Q9UBD5	621	-5	2	-2	17	1.0	1.0	1.0	1.2
C12orf29	Q8N999	302	1	-10	-2	17	1.0	0.9	1.0	1.2
ATN1	P54259	777	-2	-13	-2	17	1.0	0.9	1.0	1.2
ELP2	Q6IA86	487	4	6	-2	17	1.0	1.1	1.0	1.2
PPP1R11	O60927	60	-5	5	-2	17	1.0	1.1	1.0	1.2
RNF113A	O15541	282	12	2	-2	17	1.1	1.0	1.0	1.2
HSPA6	P17066	624	-10	-7	-2	17	0.9	0.9	1.0	1.2
PTPRC	P08575	1259	-1	-1	-2	16	1.0	1.0	1.0	1.2
MYBBP1A	Q9BQG0	1046	3	7	-2	15	1.0	1.1	1.0	1.2
RNASEH2C	Q8TDP1	67	-4	3	-2	15	1.0	1.0	1.0	1.2
WDR55	Q9H6Y2	306	-9	-8	-2	15	0.9	0.9	1.0	1.2
EPRS	P07814	1076	3	-9	-2	15	1.0	0.9	1.0	1.2
RANBP2	P49792	3071	7	4	-2	15	1.1	1.0	1.0	1.2
LGALS1	P09382	43	1	-6	-2	15	1.0	0.9	1.0	1.2
OSBPL7	Q9BZF2	466	-3	-8	-2	15	1.0	0.9	1.0	1.2
MSH3	P20585	763	-5	5	-2	14	1.0	1.1	1.0	1.2
ATP6V1A	P38606	532	-14	-7	-2	14	0.9	0.9	1.0	1.2
FTO	Q9C0B1	397	13	-3	-2	14	1.1	1.0	1.0	1.2
SMPD4	Q9NXE4	125	-2	-9	-2	14	1.0	0.9	1.0	1.2
ITGB2	P05107	400	3	5	-2	13	1.0	1.0	1.0	1.1
CYTH4	Q9UIA0	49	7	-2	-2	13	1.1	1.0	1.0	1.1
ORC3	Q9UBD5	440	8	-3	-2	13	1.1	1.0	1.0	1.1
ZNF280C	Q8ND82	734	-8	-2	-2	13	0.9	1.0	1.0	1.1
ARL8B	Q9NVJ2	159	-2	-3	-2	13	1.0	1.0	1.0	1.1
ZC3H13	Q5T200	42	3	0	-2	12	1.0	1.0	1.0	1.1
POT1	Q9NUX5	329	-3	-4	-2	12	1.0	1.0	1.0	1.1
TRAF1	Q13077	41	1	-12	-2	12	1.0	0.9	1.0	1.1
PGM1	P36871	374	6	-14	-2	12	1.1	0.9	1.0	1.1
ASMTL	O95671	441	3	2	-2	12	1.0	1.0	1.0	1.1
LCMT1	Q9UIC8	250	2	-2	-2	12	1.0	1.0	1.0	1.1
BBX	Q8WY36	712	-5	-6	-2	12	1.0	0.9	1.0	1.1
TBC1D1	Q86TI0	67	20	15	-2	11	1.2	1.2	1.0	1.1
C17orf75	Q9HAS0	288	-14	3	-2	11	0.9	1.0	1.0	1.1
NIPBL	Q6KC79	661	-4	-6	-2	11	1.0	0.9	1.0	1.1
HMGB1	P09429	106	-1	-9	-2	11	1.0	0.9	1.0	1.1
ILF3	Q12906	116	-8	-14	-2	11	0.9	0.9	1.0	1.1
UBA1	P22314	179	-5	-16	-2	11	1.0	0.9	1.0	1.1
FBXO38	Q6PIJ6	670	3	5	-2	11	1.0	1.0	1.0	1.1

GNA13	Q14344	37	-5	-9	-2	11	1.0	0.9	1.0	1.1
CRBN	Q96SW2	366	-3	7	-2	10	1.0	1.1	1.0	1.1
RPL8	P62917	90	-5	1	-2	10	1.0	1.0	1.0	1.1
MYO1G	B0I1T2	942	4	0	-2	10	1.0	1.0	1.0	1.1
PRKAR2A	P13861	101	-9	-1	-2	10	0.9	1.0	1.0	1.1
ELAC2	Q9BQ52	136	-8	-4	-2	10	0.9	1.0	1.0	1.1
TIPRL	O75663	87	-5	-6	-2	10	1.0	0.9	1.0	1.1
AP1G1	O43747	160	10	5	-2	10	1.1	1.1	1.0	1.1
FAM98B	Q52LJ0	216	4	3	-2	10	1.0	1.0	1.0	1.1
EIF1AD	Q8N9N8	89	1	-4	-2	10	1.0	1.0	1.0	1.1
PDXDC1	Q6P996	361	-2	-7	-2	10	1.0	0.9	1.0	1.1
CIAPIN1	Q6FI81	92	-7	-11	-2	10	0.9	0.9	1.0	1.1
MBNL2	Q5VZF2	43	-2	-2	-2	9	1.0	1.0	1.0	1.1
MBNL1	Q9NR56	43	-2	-2	-2	9	1.0	1.0	1.0	1.1
PLAA	Q9Y263	584	-4	-18	-2	9	1.0	0.8	1.0	1.1
TRAF3IP3	Q9Y228	135	3	-22	-2	9	1.0	0.8	1.0	1.1
TDRD7	Q8NHU6	673	12	11	-2	9	1.1	1.1	1.0	1.1
TOMM70	O94826	475	1	2	-2	9	1.0	1.0	1.0	1.1
PPP2R1A	P30153	377	2	-2	-2	9	1.0	1.0	1.0	1.1
MACF1	Q9UPN3	5049	10	-2	-2	9	1.1	1.0	1.0	1.1
SETD1B	Q9UPS6	1908	5	-6	-2	9	1.1	0.9	1.0	1.1
DRG2	P55039	241	4	-13	-2	8	1.0	0.9	1.0	1.1
MBNL2	Q5VZF2	231	-2	3	-2	8	1.0	1.0	1.0	1.1
MBNL1	Q9NR56	234	-2	3	-2	8	1.0	1.0	1.0	1.1
ARL8B	Q9NVJ2	158	3	-2	-2	8	1.0	1.0	1.0	1.1
PRKDC	P78527	25	-4	-3	-2	8	1.0	1.0	1.0	1.1
ZNF628	Q5EBL2	540	8	-3	-2	8	1.1	1.0	1.0	1.1
ZNF628	Q5EBL2	543	8	-3	-2	8	1.1	1.0	1.0	1.1
USP34	Q70CQ2	856	-11	-24	-2	8	0.9	0.8	1.0	1.1
PIK3AP1	Q6ZUJ8	358	3	7	-2	7	1.0	1.1	1.0	1.1
ANKRD44	Q8N8A2	122	-7	2	-2	7	0.9	1.0	1.0	1.1
SBF1	O95248	1638	-6	-1	-2	7	0.9	1.0	1.0	1.1
VAT1	Q99536	50	-6	-2	-2	7	0.9	1.0	1.0	1.1
PSMD4	P55036	37	2	-4	-2	7	1.0	1.0	1.0	1.1
UNC45A	Q9H3U1	426	1	-5	-2	7	1.0	1.0	1.0	1.1
GAR1	Q9NY12	80	5	-7	-2	7	1.1	0.9	1.0	1.1
USP24	Q9UPU5	802	1	-9	-2	7	1.0	0.9	1.0	1.1
ALDH9A1	P49189	355	8	-6	-2	7	1.1	0.9	1.0	1.1
PSMD3	O43242	483	9	2	-2	6	1.1	1.0	1.0	1.1
TBC1D24	Q9ULP9	161	-2	-2	-2	6	1.0	1.0	1.0	1.1
NLRP1	Q9C000	837	0	-4	-2	6	1.0	1.0	1.0	1.1
MAEA	Q7L5Y9	333	-6	-8	-2	6	0.9	0.9	1.0	1.1
STAC3	Q96MF2	106	-7	-18	-2	6	0.9	0.8	1.0	1.1
RCBTB1	Q8NDN9	72	1	-26	-2	6	1.0	0.8	1.0	1.1
EIF4E3	Q8N5X7	69	-1	-4	-2	6	1.0	1.0	1.0	1.1
EGLN1	Q9GZT9	201	-1	-8	-2	6	1.0	0.9	1.0	1.1
MGA	Q8IWI9	1056	-9	-12	-2	6	0.9	0.9	1.0	1.1
PRKDC	P78527	1176	-7	-23	-2	6	0.9	0.8	1.0	1.1
CDK4	P11802	78	9	-6	-2	5	1.1	0.9	1.0	1.1
NUP205	Q92621	1275	3	-7	-2	5	1.0	0.9	1.0	1.1
TNFAIP8	O95379	186	1	-10	-2	5	1.0	0.9	1.0	1.1
KDM1A	O60341	573	4	3	-2	5	1.0	1.0	1.0	1.0
ITPRIP	Q8IWB1	257	2	-4	-2	5	1.0	1.0	1.0	1.0
ABCF2	Q9UG63	388	-1	-1	-2	4	1.0	1.0	1.0	1.0
UBA1	P22314	278	0	-3	-2	4	1.0	1.0	1.0	1.0

DENR	O43583	132	-4	-7	-2	4	1.0	0.9	1.0	1.0
TJAP1	Q5JTD0	172	5	-9	-2	4	1.0	0.9	1.0	1.0
MRPL44	Q9H9J2	96	0	-7	-2	4	1.0	0.9	1.0	1.0
ARPC1B	O15143	134	14	-9	-2	4	1.2	0.9	1.0	1.0
DUS2	Q9NX74	116	-5	-19	-2	4	1.0	0.8	1.0	1.0
ZADH2	Q8N4Q0	218	-6	12	-2	3	0.9	1.1	1.0	1.0
CAPG	P40121	77	3	-1	-2	3	1.0	1.0	1.0	1.0
UBE2Z	Q9H832	263	9	-7	-2	3	1.1	0.9	1.0	1.0
MRPS14	O60783	91	-18	-8	-2	3	0.8	0.9	1.0	1.0
SRR	Q9GZT4	217	11	3	-2	3	1.1	1.0	1.0	1.0
ENY2	Q9NPA8	40	0	-2	-2	3	1.0	1.0	1.0	1.0
TRAF6	Q9Y4K3	139	-7	-5	-2	3	0.9	1.0	1.0	1.0
SFPQ	P23246	431	-11	5	-2	2	0.9	1.0	1.0	1.0
GCN1	Q92616	2179	5	4	-2	2	1.1	1.0	1.0	1.0
SEC24B	O95487	562	-5	-2	-2	2	1.0	1.0	1.0	1.0
DOCK5	Q9H7D0	1223	7	-3	-2	2	1.1	1.0	1.0	1.0
ACOX1	Q15067	449	2	-9	-2	2	1.0	0.9	1.0	1.0
PRKDC	P78527	478	3	-1	-2	1	1.0	1.0	1.0	1.0
CAD	P27708	183	2	-2	-2	1	1.0	1.0	1.0	1.0
TRIM24	O15164	246	14	-3	-2	1	1.2	1.0	1.0	1.0
PKM	P14618	152	3	-3	-2	1	1.0	1.0	1.0	1.0
TRIM33	Q9UPN9	299	14	-3	-2	1	1.2	1.0	1.0	1.0
ALDOC	P09972	290	2	-6	-2	1	1.0	0.9	1.0	1.0
OSBPL2	Q9H1P3	122	-5	-6	-2	1	1.0	0.9	1.0	1.0
HLA-DRB1	P04229	146	-11	-33	-2	1	0.9	0.8	1.0	1.0
MYCBP2	O75592	2243	-8	-14	-2	1	0.9	0.9	1.0	1.0
PLEC	Q15149	4254	-1	-5	-2	0	1.0	1.0	1.0	1.0
TYMP	P19971	136	-8	-17	-2	0	0.9	0.9	1.0	1.0
GCOM1	H8Y6P7	479	-1	-8	-2	-1	1.0	0.9	1.0	1.0
INTS1	Q8N201	643	12	-10	-2	-1	1.1	0.9	1.0	1.0
ELAC2	Q9BQ52	441	-14	5	-2	-1	0.9	1.1	1.0	1.0
MSH3	P20585	166	10	5	-2	-2	1.1	1.1	1.0	1.0
PML	P29590	227	-4	-7	-2	-2	1.0	0.9	1.0	1.0
INPP5D	Q92835	736	8	9	-2	-3	1.1	1.1	1.0	1.0
LRPPRC	P42704	113	3	7	-2	-3	1.0	1.1	1.0	1.0
CTPS1	P17812	218	-9	1	-2	-3	0.9	1.0	1.0	1.0
YWHAH	Q04917	112	3	-1	-2	-3	1.0	1.0	1.0	1.0
PHF14	O94880	388	-6	-11	-2	-3	0.9	0.9	1.0	1.0
COPG1	Q9Y678	296	5	6	-2	-3	1.0	1.1	1.0	1.0
RPS8	P62241	174	-8	1	-2	-4	0.9	1.0	1.0	1.0
WIPI2	Q9Y4P8	334	-5	-10	-2	-4	1.0	0.9	1.0	1.0
TNK2	Q07912	434	-13	-5	-2	-4	0.9	1.0	1.0	1.0
GAPVD1	Q14C86	741	1	-9	-2	-4	1.0	0.9	1.0	1.0
PTAR1	Q7Z6K3	381	-2	2	-2	-5	1.0	1.0	1.0	1.0
NUB1	Q9Y5A7	317	7	-6	-2	-5	1.1	0.9	1.0	1.0
DHX36	Q9H2U1	977	2	-8	-2	-5	1.0	0.9	1.0	1.0
HECTD4	Q9Y4D8	3985	4	-4	-2	-6	1.0	1.0	1.0	0.9
MTA2	O94776	495	2	-5	-2	-6	1.0	1.0	1.0	0.9
MROH1	Q8NDA8	1263	-2	2	-2	-6	1.0	1.0	1.0	0.9
FAM160B1	Q5W0V3	574	-5	-2	-2	-7	1.0	1.0	1.0	0.9
MYO5A	Q9Y4I1	684	-7	-14	-2	-7	0.9	0.9	1.0	0.9
CEP350	Q5VT06	179	-2	-1	-2	-8	1.0	1.0	1.0	0.9
DDX42	Q86XP3	382	14	6	-2	-9	1.2	1.1	1.0	0.9
CORO7-PAM1	A0A0A6YYL4	695	-7	-10	-2	-10	0.9	0.9	1.0	0.9
RAB3GAP1	Q15042	322	0	-14	-2	-10	1.0	0.9	1.0	0.9

TES	Q9UGI8	294	6	-6	-2	-11	1.1	0.9	1.0	0.9
UBN1	Q9NPG3	59	-5	-2	-2	-11	1.0	1.0	1.0	0.9
SRPK1	Q96SB4	647	1	2	-2	-13	1.0	1.0	1.0	0.9
NAP1L1	P55209	88	-13	5	-2	-13	0.9	1.0	1.0	0.9
ZNF451	Q9Y4E5	940	9	2	-2	-14	1.1	1.0	1.0	0.9
FLNA	P21333	1723	8	4	-2	-15	1.1	1.0	1.0	0.9
TAF8	Q7Z7C8	80	2	-12	-2	-20	1.0	0.9	1.0	0.8
CHERP	Q8IWX8	246	7	1	-2	-20	1.1	1.0	1.0	0.8
ACTN1	P12814	480	-4	1	-2	-29	1.0	1.0	1.0	0.8
EHHADH	Q08426	227	-11	-7	-2	-29	0.9	0.9	1.0	0.8
ROCK2	O75116	649	-4	-7	-2	-31	1.0	0.9	1.0	0.8
RASGRP4	Q8TDF6	532	-4	-31	-2	-34	1.0	0.8	1.0	0.7
NAE1	Q13564	483	-1	-22	-2	-51	1.0	0.8	1.0	0.7
PPP1R14A	Q96A00	100	15	4	-2	-74	1.2	1.0	1.0	0.6
WDFY4	Q6ZS81	1963	-8	-12	-3	76	0.9	0.9	1.0	4.1
ZNF638	Q14966	1026	-24	-17	-3	75	0.8	0.9	1.0	4.0
KDM3B	Q7LBC6	295	8	-3	-3	45	1.1	1.0	1.0	1.8
SIGIRR	Q6IA17	238	-4	-4	-3	45	1.0	1.0	1.0	1.8
DHX37	Q8IY37	903	4	10	-3	39	1.0	1.1	1.0	1.6
Uncharacteriz	F5H5T6	83	1	-6	-3	39	1.0	0.9	1.0	1.6
LRRC41	Q15345	607	-4	2	-3	35	1.0	1.0	1.0	1.5
STAP1	Q9ULZ2	269	-12	1	-3	35	0.9	1.0	1.0	1.5
PPME1	Q9Y570	312	-9	-11	-3	35	0.9	0.9	1.0	1.5
DGKG	P49619	357	-4	-2	-3	33	1.0	1.0	1.0	1.5
KMT2A	Q03164	1575	2	-3	-3	33	1.0	1.0	1.0	1.5
TMC6	Q7Z403	751	10	4	-3	32	1.1	1.0	1.0	1.5
XRN1	Q8IZH2	657	9	1	-3	32	1.1	1.0	1.0	1.5
TNFAIP3	P21580	627	-5	-6	-3	32	1.0	0.9	1.0	1.5
SRPRA	P08240	25	18	20	-3	31	1.2	1.2	1.0	1.4
MYBBP1A	Q9BQG0	614	3	9	-3	30	1.0	1.1	1.0	1.4
NISCH	Q9Y2I1	168	-8	6	-3	28	0.9	1.1	1.0	1.4
HRAS	P01112	186	8	3	-3	27	1.1	1.0	1.0	1.4
ATPAF1	Q5TC12	27	-7	-3	-3	26	0.9	1.0	1.0	1.4
PARP16	Q8N5Y8	52	3	-7	-3	26	1.0	0.9	1.0	1.4
DOK2	O60496	110	-3	-11	-3	26	1.0	0.9	1.0	1.4
DDX23	Q9BUQ8	677	6	5	-3	25	1.1	1.0	1.0	1.3
HSPA4	P34932	376	1	-12	-3	25	1.0	0.9	1.0	1.3
HSPH1	Q92598	376	1	-12	-3	25	1.0	0.9	1.0	1.3
GRM7	Q14831	249	-2	18	-3	23	1.0	1.2	1.0	1.3
FLCN	Q8NFG4	335	-2	1	-3	23	1.0	1.0	1.0	1.3
SLC25A1	P53007	70	-1	-4	-3	23	1.0	1.0	1.0	1.3
SART1	O43290	645	-9	-6	-3	23	0.9	0.9	1.0	1.3
WARS	P23381	274	-4	-6	-3	23	1.0	0.9	1.0	1.3
KIF21B	O75037	298	-3	-9	-3	23	1.0	0.9	1.0	1.3
MAU2	Q9Y6X3	47	9	10	-3	23	1.1	1.1	1.0	1.3
RBM39	Q14498	303	2	-5	-3	23	1.0	1.0	1.0	1.3
RGPD3	A6NKT7	349	-3	-5	-3	23	1.0	1.0	1.0	1.3
RANBP2	P49792	348	-3	-5	-3	23	1.0	1.0	1.0	1.3
SETX	Q7Z333	1915	-5	-6	-3	22	1.0	0.9	1.0	1.3
IARS2	Q9NSE4	521	-6	-12	-3	22	0.9	0.9	1.0	1.3
ZMYM3	Q14202	314	-4	4	-3	21	1.0	1.0	1.0	1.3
ZBTB33	Q86T24	431	-6	-6	-3	21	0.9	0.9	1.0	1.3
PIK3R1	P27986	146	2	-7	-3	21	1.0	0.9	1.0	1.3
TRRAP	Q9Y4A5	1868	6	-12	-3	21	1.1	0.9	1.0	1.3
DDX27	Q96GQ7	261	-1	-8	-3	20	1.0	0.9	1.0	1.3

GTF3C2	Q8WUA4	291	-9	1	-3	20	0.9	1.0	1.0	1.2
PARG	Q86W56	603	7	-4	-3	20	1.1	1.0	1.0	1.2
RNF113A	O15541	15	-1	-9	-3	20	1.0	0.9	1.0	1.2
DGKA	P23743	707	2	-4	-3	19	1.0	1.0	1.0	1.2
SIRT1	Q96EB6	67	5	-21	-3	19	1.1	0.8	1.0	1.2
UTP15	Q8TED0	385	-3	-1	-3	18	1.0	1.0	1.0	1.2
MARS	P56192	38	-7	-13	-3	18	0.9	0.9	1.0	1.2
NLRP1	Q9C000	1310	4	-1	-3	17	1.0	1.0	1.0	1.2
SMG1	Q96Q15	254	8	-10	-3	17	1.1	0.9	1.0	1.2
DNPEP	Q9ULA0	447	-12	-48	-3	17	0.9	0.7	1.0	1.2
KDM3A	Q9Y4C1	251	-3	-12	-3	16	1.0	0.9	1.0	1.2
ATAD2	Q6PL18	1156	11	-22	-3	16	1.1	0.8	1.0	1.2
WDR45B	Q5MNZ6	63	-6	5	-3	16	0.9	1.0	1.0	1.2
TWF2	Q6IBS0	67	0	1	-3	16	1.0	1.0	1.0	1.2
FECH	P22830	395	1	-1	-3	16	1.0	1.0	1.0	1.2
GBP3	Q9H0R5	394	9	-2	-3	16	1.1	1.0	1.0	1.2
ZHX3	Q9H4I2	11	-3	-2	-3	16	1.0	1.0	1.0	1.2
JARID2	Q92833	1210	2	-2	-3	16	1.0	1.0	1.0	1.2
HIRA	P54198	763	-6	-6	-3	16	0.9	0.9	1.0	1.2
WDR45	Q9Y484	343	19	-10	-3	16	1.2	0.9	1.0	1.2
RAB27A	P51159	188	-7	1	-3	15	0.9	1.0	1.0	1.2
SPG7	Q9UQ90	353	3	-7	-3	15	1.0	0.9	1.0	1.2
TOP2B	Q02880	191	-8	-8	-3	15	0.9	0.9	1.0	1.2
VPS51	Q9UID3	421	2	-10	-3	15	1.0	0.9	1.0	1.2
PECR	Q9BY49	191	5	9	-3	15	1.1	1.1	1.0	1.2
SEPT1	Q8WYJ6	293	-2	4	-3	15	1.0	1.0	1.0	1.2
DHRS7	Q9Y394	233	6	3	-3	15	1.1	1.0	1.0	1.2
HUWE1	Q7Z6Z7	471	5	0	-3	15	1.1	1.0	1.0	1.2
C12orf4	Q9NQ89	55	10	-1	-3	15	1.1	1.0	1.0	1.2
FBXW5	Q969U6	277	6	-4	-3	15	1.1	1.0	1.0	1.2
RBPJ	Q06330	397	-7	1	-3	14	0.9	1.0	1.0	1.2
GRB2	P62993	32	6	1	-3	14	1.1	1.0	1.0	1.2
BECN1	Q14457	353	4	-2	-3	14	1.0	1.0	1.0	1.2
UPF1	Q92900	209	-2	-4	-3	14	1.0	1.0	1.0	1.2
ERCC5	P28715	12	11	-6	-3	14	1.1	0.9	1.0	1.2
ASUN	Q9NVM9	349	-3	-7	-3	14	1.0	0.9	1.0	1.2
SPG11	Q96JI7	2291	4	15	-3	13	1.0	1.2	1.0	1.1
SNRNP200	O75643	238	-2	0	-3	13	1.0	1.0	1.0	1.1
TNKS1BP1	Q9C0C2	1114	9	-11	-3	13	1.1	0.9	1.0	1.1
AKR1C3	P42330	242	0	1	-3	13	1.0	1.0	1.0	1.1
METAP1	P53582	14	-2	-1	-3	12	1.0	1.0	1.0	1.1
MTOR	P42345	713	4	-6	-3	12	1.0	0.9	1.0	1.1
ARHGAP24	Q8N264	327	0	-11	-3	12	1.0	0.9	1.0	1.1
RPS8	P62241	182	-7	-5	-3	12	0.9	1.0	1.0	1.1
FBL	P22087	268	-3	-6	-3	12	1.0	0.9	1.0	1.1
POLD1	P28340	713	5	-8	-3	12	1.0	0.9	1.0	1.1
OXSM	Q9NWU1	415	5	-9	-3	12	1.0	0.9	1.0	1.1
INTS9	Q9NV88	578	-6	-7	-3	11	0.9	0.9	1.0	1.1
ACTRT1	Q8TDG2	182	-9	5	-3	10	0.9	1.0	1.0	1.1
RIPK3	Q9Y572	234	-3	-3	-3	10	1.0	1.0	1.0	1.1
TYMP	P19971	361	6	-11	-3	10	1.1	0.9	1.0	1.1
PDLIM2	Q96JY6	289	-10	8	-3	10	0.9	1.1	1.0	1.1
UBA3	Q8TBC4	139	1	-2	-3	10	1.0	1.0	1.0	1.1
SURF2	Q15527	93	14	-7	-3	10	1.2	0.9	1.0	1.1
ZAP70	P43403	618	6	-4	-3	9	1.1	1.0	1.0	1.1

SBF1	O95248	209	0	-6	-3	9	1.0	0.9	1.0	1.1
POLR1A	O95602	937	3	-7	-3	9	1.0	0.9	1.0	1.1
QARS	P47897	298	-3	-10	-3	9	1.0	0.9	1.0	1.1
KCTD12	Q96CX2	50	-5	-2	-3	9	1.0	1.0	1.0	1.1
CDKN1C	A0A0G2JPX0	295	-6	-5	-3	9	0.9	1.0	1.0	1.1
MEFV	O15553	773	-9	-6	-3	9	0.9	0.9	1.0	1.1
TTLL12	Q14166	572	5	-10	-3	9	1.1	0.9	1.0	1.1
CBL	P22681	840	-3	6	-3	8	1.0	1.1	1.0	1.1
DNMT1	P26358	751	-2	2	-3	8	1.0	1.0	1.0	1.1
TARS2	Q9BW92	233	7	1	-3	8	1.1	1.0	1.0	1.1
CTCF	P49711	275	-2	-4	-3	8	1.0	1.0	1.0	1.1
SBF1	O95248	986	-2	-6	-3	8	1.0	0.9	1.0	1.1
MKI67	P46013	2706	-16	-6	-3	8	0.9	0.9	1.0	1.1
POLA2	Q14181	198	-1	-10	-3	8	1.0	0.9	1.0	1.1
NARFL	Q9H6Q4	74	6	-13	-3	8	1.1	0.9	1.0	1.1
UBR1	Q8IWV7	993	1	1	-3	8	1.0	1.0	1.0	1.1
ACAA2	P42765	92	-1	-1	-3	8	1.0	1.0	1.0	1.1
TES	Q9UGI8	238	2	-1	-3	8	1.0	1.0	1.0	1.1
UBA1	P22314	632	3	-3	-3	8	1.0	1.0	1.0	1.1
CCDC93	Q567U6	282	3	-4	-3	8	1.0	1.0	1.0	1.1
MEFV	O15553	144	-13	-6	-3	7	0.9	0.9	1.0	1.1
THOC7	Q6I9Y2	90	4	-8	-3	7	1.0	0.9	1.0	1.1
DOCK2	Q92608	1605	1	-8	-3	7	1.0	0.9	1.0	1.1
SYNE1	Q8NF91	8380	4	-9	-3	7	1.0	0.9	1.0	1.1
EED	O75530	401	-2	-12	-3	7	1.0	0.9	1.0	1.1
ACLY	P53396	764	-1	-12	-3	7	1.0	0.9	1.0	1.1
DPYD	Q12882	322	-2	3	-3	7	1.0	1.0	1.0	1.1
LUC7L3	O95232	40	3	-1	-3	7	1.0	1.0	1.0	1.1
GIGYF2	Q6Y7W6	573	2	-6	-3	7	1.0	0.9	1.0	1.1
SEPT1	Q8WYJ6	260	5	-8	-3	7	1.0	0.9	1.0	1.1
RIOK3	O14730	171	-2	-9	-3	7	1.0	0.9	1.0	1.1
FBXO30	Q8TB52	562	2	5	-3	6	1.0	1.0	1.0	1.1
CASP1	P29466	397	-2	1	-3	6	1.0	1.0	1.0	1.1
GLS	O94925	287	10	-1	-3	6	1.1	1.0	1.0	1.1
BRAT1	Q6PJG6	720	-8	-1	-3	6	0.9	1.0	1.0	1.1
RAC2	P15153	157	4	-6	-3	6	1.0	0.9	1.0	1.1
RAC1	P63000	157	4	-6	-3	6	1.0	0.9	1.0	1.1
HDAC3	O15379	218	4	-8	-3	6	1.0	0.9	1.0	1.1
KARS	Q15046	427	2	-11	-3	6	1.0	0.9	1.0	1.1
CAPN2	P17655	640	0	-2	-3	6	1.0	1.0	1.0	1.1
HNRNPL	P14866	472	-4	-7	-3	6	1.0	0.9	1.0	1.1
GANAB	Q14697	41	6	3	-3	5	1.1	1.0	1.0	1.1
HECTD4	Q9Y4D8	1488	-12	3	-3	5	0.9	1.0	1.0	1.1
E2F8	A0AVK6	444	4	-2	-3	5	1.0	1.0	1.0	1.1
STAT3	P40763	542	0	-2	-3	5	1.0	1.0	1.0	1.1
MARC2	Q969Z3	299	-5	-5	-3	5	1.0	1.0	1.0	1.1
NTN5	Q8WTR8	418	-1	-8	-3	5	1.0	0.9	1.0	1.1
ABCF1	Q8NE71	655	-1	-6	-3	5	1.0	0.9	1.0	1.0
ITCH	Q96J02	57	-9	3	-3	4	0.9	1.0	1.0	1.0
RANBP2	P49792	1787	-1	-1	-3	4	1.0	1.0	1.0	1.0
UPF1	Q92900	683	1	-3	-3	4	1.0	1.0	1.0	1.0
JAK1	P23458	169	-3	-3	-3	4	1.0	1.0	1.0	1.0
KDM3A	Q9Y4C1	953	-14	-3	-3	3	0.9	1.0	1.0	1.0
TPR	P12270	1127	2	-12	-3	3	1.0	0.9	1.0	1.0
CTC1	Q2NKJ3	28	-9	-23	-3	3	0.9	0.8	1.0	1.0

SKAP1	Q86WV1	177	6	-4	-3	3	1.1	1.0	1.0	1.0
MBD1	Q9UIS9	208	8	-9	-3	3	1.1	0.9	1.0	1.0
HNRNPUL1	Q9BUJ2	487	2	2	-3	2	1.0	1.0	1.0	1.0
CLIC5	Q9NZA1	191	1	-2	-3	2	1.0	1.0	1.0	1.0
RUBCN	Q92622	739	-7	-5	-3	2	0.9	1.0	1.0	1.0
NUBP1	P53384	277	-10	4	-3	2	0.9	1.0	1.0	1.0
EEA1	Q15075	1377	13	-3	-3	2	1.1	1.0	1.0	1.0
TRIM28	Q13263	124	-2	-6	-3	2	1.0	0.9	1.0	1.0
SETD1A	O15047	1702	-6	-8	-3	1	0.9	0.9	1.0	1.0
DTX3L	Q8TDB6	406	5	-9	-3	1	1.1	0.9	1.0	1.0
ATG16L1	Q676U5	145	10	3	-3	1	1.1	1.0	1.0	1.0
WRNIP1	Q96S55	272	6	-4	-3	1	1.1	1.0	1.0	1.0
STK11	Q15831	151	-2	-9	-3	1	1.0	0.9	1.0	1.0
FCHSD2	O94868	354	0	-9	-3	0	1.0	0.9	1.0	1.0
ITSN2	Q9NZM3	592	-8	2	-3	-1	0.9	1.0	1.0	1.0
MAPT	P10636	639	-7	-7	-3	-1	0.9	0.9	1.0	1.0
WDR43	Q15061	341	5	-11	-3	-1	1.1	0.9	1.0	1.0
FNDC3B	Q53EP0	1025	-5	-19	-3	-1	1.0	0.8	1.0	1.0
EIF3B	P55884	700	9	3	-3	-1	1.1	1.0	1.0	1.0
PSMC1	P62191	399	-6	2	-3	-1	0.9	1.0	1.0	1.0
ORC3	Q9UBD5	363	7	2	-3	-2	1.1	1.0	1.0	1.0
OSBP	P22059	224	-2	-6	-3	-2	1.0	0.9	1.0	1.0
EXOC1	Q9NV70	27	-3	4	-3	-2	1.0	1.0	1.0	1.0
NDRG3	Q9UGV2	166	-2	-2	-3	-2	1.0	1.0	1.0	1.0
MYO1F	O00160	402	-6	-9	-3	-2	0.9	0.9	1.0	1.0
MDN1	Q9NU22	309	4	-4	-3	-3	1.0	1.0	1.0	1.0
MYOF	Q9NZM1	1392	5	-17	-3	-3	1.0	0.9	1.0	1.0
SYNE1	Q8NF91	8196	-7	1	-3	-3	0.9	1.0	1.0	1.0
SYNE2	Q8WXH0	6333	-7	1	-3	-3	0.9	1.0	1.0	1.0
SLFN5	Q08AF3	48	3	-5	-3	-4	1.0	1.0	1.0	1.0
PRKAR1A	P10644	362	0	-6	-3	-4	1.0	0.9	1.0	1.0
NAT10	Q9H0A0	517	12	-2	-3	-4	1.1	1.0	1.0	1.0
SNRNP200	O75643	516	-10	5	-3	-5	0.9	1.1	1.0	1.0
DIAPH1	O60610	164	0	-9	-3	-5	1.0	0.9	1.0	1.0
RAF1	P04049	637	9	-22	-3	-5	1.1	0.8	1.0	1.0
UFL1	O94874	143	2	-2	-3	-5	1.0	1.0	1.0	1.0
SMARCA5	O60264	259	-1	-3	-3	-5	1.0	1.0	1.0	1.0
KDM1B	Q8NB78	62	-2	-9	-3	-6	1.0	0.9	1.0	0.9
DSTN	P60981	135	12	4	-3	-6	1.1	1.0	1.0	0.9
Uncharacteriz	A0A0C4DFX4	2069	6	-3	-3	-6	1.1	1.0	1.0	0.9
Uncharacteriz	E9PCH4	572	-1	-5	-3	-7	1.0	1.0	1.0	0.9
USP14	P54578	257	-1	4	-3	-8	1.0	1.0	1.0	0.9
IDH3A	P50213	351	-10	0	-3	-8	0.9	1.0	1.0	0.9
FNBP1	Q96RU3	248	-6	5	-3	-8	0.9	1.1	1.0	0.9
NBEAL2	Q6ZNJ1	390	-2	-17	-3	-8	1.0	0.9	1.0	0.9
RBPJ	Q06330	123	4	-8	-3	-9	1.0	0.9	1.0	0.9
SRI	P30626	163	-5	-13	-3	-9	1.0	0.9	1.0	0.9
NAT10	Q9H0A0	194	1	4	-3	-9	1.0	1.0	1.0	0.9
MTPAP	Q9NVV4	299	-4	-8	-3	-10	1.0	0.9	1.0	0.9
AFF4	Q9UHB7	889	5	-10	-3	-10	1.0	0.9	1.0	0.9
COBLL1	Q53SF7	686	-13	-9	-3	-11	0.9	0.9	1.0	0.9
PARP1	P09874	24	0	-2	-3	-12	1.0	1.0	1.0	0.9
DOCK7	Q96N67	1944	-3	-8	-3	-12	1.0	0.9	1.0	0.9
CAAP1	Q9H8G2	152	2	-7	-3	-13	1.0	0.9	1.0	0.9
AARS2	Q5JTZ9	750	-11	-29	-3	-14	0.9	0.8	1.0	0.9

JADE2	Q9NQC1	246	-2	5	-3	-14	1.0	1.0	1.0	0.9
COPG1	Q9Y678	516	7	-7	-3	-14	1.1	0.9	1.0	0.9
INTS1	Q8N201	867	0	-5	-3	-16	1.0	1.0	1.0	0.9
DIAPH1	O60610	796	4	-4	-3	-18	1.0	1.0	1.0	0.9
ATP2A3	Q93084	404	6	0	-3	-19	1.1	1.0	1.0	0.8
MYH9	P35579	694	-8	10	-3	-21	0.9	1.1	1.0	0.8
MYH10	P35580	701	-8	10	-3	-21	0.9	1.1	1.0	0.8
MYH14	Q7Z406	718	-8	10	-3	-21	0.9	1.1	1.0	0.8
COPB1	P53618	235	10	-23	-3	-22	1.1	0.8	1.0	0.8
OCIAD2	Q56VL3	134	11	-3	-3	-23	1.1	1.0	1.0	0.8
HDAC4	P56524	777	7	-11	-3	-24	1.1	0.9	1.0	0.8
NIN	Q8N4C6	956	-20	12	-3	-25	0.8	1.1	1.0	0.8
TRAF3IP3	Q9Y228	315	2	-9	-3	-27	1.0	0.9	1.0	0.8
MICU1	Q9BPX6	275	-10	5	-3	-27	0.9	1.1	1.0	0.8
SAV1	Q9H4B6	266	0	-3	-3	-32	1.0	1.0	1.0	0.8
ACTN4	O43707	60	-3	0	-3	-37	1.0	1.0	1.0	0.7
ACTN1	P12814	41	-3	0	-3	-37	1.0	1.0	1.0	0.7
UNC13D	Q70J99	370	4	1	-3	-44	1.0	1.0	1.0	0.7
TLN1	Q9Y490	1478	24	13	-3	-52	1.3	1.1	1.0	0.7
ZC3HAV1L	Q96H79	15	-29	-20	-3	-58	0.8	0.8	1.0	0.6
HVCN1	Q96D96	249	8	-8	-3	-221	1.1	0.9	1.0	0.3
NLRC5	Q86WI3	16	-1	-3	-3	49	1.0	1.0	1.0	1.9
TXNIP	Q9H3M7	120	-10	-5	-3	49	0.9	1.0	1.0	1.9
STK11IP	Q8N1F8	633	-7	-5	-3	47	0.9	1.0	1.0	1.9
SEC22A	Q96IW7	33	-14	-1	-3	41	0.9	1.0	1.0	1.7
IPO8	O15397	757	1	0	-3	38	1.0	1.0	1.0	1.6
ZC3H7B	Q9UGR2	956	3	-3	-3	37	1.0	1.0	1.0	1.6
POLR3B	Q9NW08	161	10	-1	-3	35	1.1	1.0	1.0	1.5
RRP1B	Q14684	286	-13	-9	-3	35	0.9	0.9	1.0	1.5
IARS	P41252	1088	8	-2	-3	34	1.1	1.0	1.0	1.5
MBNL1	Q9NR56	193	-3	-3	-3	34	1.0	1.0	1.0	1.5
PRPF8	Q6P2Q9	772	7	2	-3	33	1.1	1.0	1.0	1.5
PRKDC	P78527	373	3	-4	-3	32	1.0	1.0	1.0	1.5
ASB13	Q8WXK3	148	15	-4	-3	31	1.2	1.0	1.0	1.4
PLCL2	Q9UPR0	90	-4	-12	-3	31	1.0	0.9	1.0	1.4
PUS1	Q9Y606	260	2	-5	-3	30	1.0	1.0	1.0	1.4
LOC10272415	A0A0B4J2E5	24	2	-7	-3	29	1.0	0.9	1.0	1.4
RPL10A	P62906	164	7	-1	-3	28	1.1	1.0	1.0	1.4
DPYSL2	Q16555	504	6	-3	-3	28	1.1	1.0	1.0	1.4
PHIP	Q8WWQ0	130	6	-4	-3	28	1.1	1.0	1.0	1.4
HSP90AA1	P07900	597	-11	-5	-3	27	0.9	1.0	1.0	1.4
CAMK2B	Q13554	273	3	-3	-3	27	1.0	1.0	1.0	1.4
MDC1	Q14676	26	10	2	-3	26	1.1	1.0	1.0	1.4
RNH1	P13489	220	3	-1	-3	26	1.0	1.0	1.0	1.4
RABEP2	Q9H5N1	115	-2	-1	-3	26	1.0	1.0	1.0	1.3
TUBGCP5	A0A0G2JSA7	670	-8	-4	-3	26	0.9	1.0	1.0	1.3
IREB2	P48200	178	19	-3	-3	25	1.2	1.0	1.0	1.3
ZNF346	Q9UL40	68	-9	1	-3	25	0.9	1.0	1.0	1.3
AKAP13	Q12802	934	-2	-4	-3	25	1.0	1.0	1.0	1.3
ZNF701	Q9NV72	493	11	-4	-3	24	1.1	1.0	1.0	1.3
RAD54L2	Q9Y4B4	601	-5	-6	-3	23	1.0	0.9	1.0	1.3
MYD88	Q99836	280	5	1	-3	23	1.1	1.0	1.0	1.3
AKAP9	Q99996	1006	-9	1	-3	23	0.9	1.0	1.0	1.3
GTPBP6	H0Y2S1	394	-13	-1	-3	23	0.9	1.0	1.0	1.3
TXNDC12	O95881	66	-2	-8	-3	23	1.0	0.9	1.0	1.3

SUGP2	Q8IX01	540	-1	-9	-3	23	1.0	0.9	1.0	1.3
IL7R	P16871	287	2	-6	-3	22	1.0	0.9	1.0	1.3
RPS27A	P62979	126	-1	2	-3	22	1.0	1.0	1.0	1.3
DAZAP2	Q15038	132	-3	-1	-3	22	1.0	1.0	1.0	1.3
SCAPER	Q9BY12	304	-5	-10	-3	22	1.0	0.9	1.0	1.3
MCMBP	Q9BTE3	396	-10	-13	-3	22	0.9	0.9	1.0	1.3
TRIO	O75962	1717	-2	4	-3	21	1.0	1.0	1.0	1.3
PIAS1	O75925	335	-6	-5	-3	21	0.9	1.0	1.0	1.3
ZNFX1	Q9P2E3	1076	-18	-8	-3	21	0.9	0.9	1.0	1.3
HCFC1	P51610	89	-1	-5	-3	21	1.0	1.0	1.0	1.3
KBTBD11	O94819	555	-16	-1	-3	20	0.9	1.0	1.0	1.3
GLIPR2	Q9H4G4	32	-4	-5	-3	20	1.0	1.0	1.0	1.2
MCM3AP	O60318	439	-4	-5	-3	20	1.0	1.0	1.0	1.2
NEURL1	O76050	225	2	-9	-3	20	1.0	0.9	1.0	1.2
ZMIZ1	Q9ULJ6	17	-7	7	-3	19	0.9	1.1	1.0	1.2
SDCBP	O00560	118	6	-2	-3	19	1.1	1.0	1.0	1.2
EIF5B	O60841	749	-10	-4	-3	19	0.9	1.0	1.0	1.2
FYTTD1	Q96QD9	242	-7	-6	-3	19	0.9	0.9	1.0	1.2
UNK	Q9C0B0	791	-7	-1	-3	18	0.9	1.0	1.0	1.2
TXNDC5	Q8NBS9	350	-4	-6	-3	18	1.0	0.9	1.0	1.2
KAT6A	Q92794	773	5	0	-3	17	1.1	1.0	1.0	1.2
TRPM7	Q96QT4	29	-2	4	-3	17	1.0	1.0	1.0	1.2
CLTC	Q00610	934	3	2	-3	17	1.0	1.0	1.0	1.2
EPRS	P07814	697	-7	-5	-3	16	0.9	1.0	1.0	1.2
ARV1	Q9H2C2	30	-11	-7	-3	16	0.9	0.9	1.0	1.2
ADCY7	P51828	895	-6	-7	-3	16	0.9	0.9	1.0	1.2
NUP188	Q5SRE5	1152	4	-1	-3	16	1.0	1.0	1.0	1.2
DNA2	P51530	103	-4	-2	-3	16	1.0	1.0	1.0	1.2
ANP32E	Q9BTT0	87	-7	-7	-3	16	0.9	0.9	1.0	1.2
NSUN5	Q96P11	362	1	-8	-3	16	1.0	0.9	1.0	1.2
ACAA1	P09110	177	6	4	-3	15	1.1	1.0	1.0	1.2
ZMYM4	Q5VZL5	517	-10	-35	-3	15	0.9	0.7	1.0	1.2
ZNF516	Q92618	1016	-14	-9	-3	15	0.9	0.9	1.0	1.2
ATF7IP	Q6VMQ6	451	-3	-12	-3	15	1.0	0.9	1.0	1.2
ANAPC5	Q9UJX4	86	5	7	-3	14	1.0	1.1	1.0	1.2
INPP5D	Q92835	672	3	0	-3	14	1.0	1.0	1.0	1.2
RNF25	Q96BH1	159	-6	-2	-3	14	0.9	1.0	1.0	1.2
POU2F2	P09086	346	-5	-10	-3	14	1.0	0.9	1.0	1.2
TDRD3	Q9H7E2	98	-2	-1	-3	14	1.0	1.0	1.0	1.2
SCRN3	Q0VDG4	241	5	-2	-3	14	1.1	1.0	1.0	1.2
MYO1F	O00160	105	15	-12	-3	14	1.2	0.9	1.0	1.2
ZNF267	Q14586	272	2	2	-3	13	1.0	1.0	1.0	1.1
RNF2	Q99496	72	-5	2	-3	13	1.0	1.0	1.0	1.1
RDH14	Q9HBH5	97	10	2	-3	13	1.1	1.0	1.0	1.1
UTRN	P46939	2185	2	-4	-3	13	1.0	1.0	1.0	1.1
HSP90AA1	P07900	598	-16	-7	-3	13	0.9	0.9	1.0	1.1
RABEPK	Q7Z6M1	115	-3	7	-3	13	1.0	1.1	1.0	1.1
KDM3B	Q7LBC6	1126	-2	-4	-3	13	1.0	1.0	1.0	1.1
DOCK7	Q96N67	457	-14	-12	-3	13	0.9	0.9	1.0	1.1
RBPJ	Q06330	252	-5	-14	-3	13	1.0	0.9	1.0	1.1
SLC7A6OS	Q96CW6	62	12	-2	-3	12	1.1	1.0	1.0	1.1
ACAP1	Q15027	64	4	-4	-3	12	1.0	1.0	1.0	1.1
EXOSC1	Q9Y3B2	15	2	-5	-3	12	1.0	1.0	1.0	1.1
ARHGEF40	Q8TER5	183	0	-9	-3	12	1.0	0.9	1.0	1.1
RECQL	P46063	478	-13	-3	-3	12	0.9	1.0	1.0	1.1

MECR	Q9BV79	263	20	-4	-3	12	1.2	1.0	1.0	1.1
EPC2	Q52LR7	420	-4	-6	-3	12	1.0	0.9	1.0	1.1
C12orf57	Q99622	73	-2	-8	-3	12	1.0	0.9	1.0	1.1
MYSM1	Q5VVJ2	200	-2	-9	-3	12	1.0	0.9	1.0	1.1
TARBP2	Q15633	282	-2	-2	-3	11	1.0	1.0	1.0	1.1
SLC9A1	P19634	794	3	-3	-3	11	1.0	1.0	1.0	1.1
FUK	Q8N0W3	1080	-6	-6	-3	11	0.9	0.9	1.0	1.1
RB1CC1	Q8TDY2	81	6	-10	-3	11	1.1	0.9	1.0	1.1
VPS35	Q96QK1	253	8	-1	-3	11	1.1	1.0	1.0	1.1
PPP2R5A	Q15172	72	2	-1	-3	11	1.0	1.0	1.0	1.1
MARCH1	Q8TCQ1	241	2	-3	-3	11	1.0	1.0	1.0	1.1
STAT1	P42224	155	-1	-5	-3	11	1.0	1.0	1.0	1.1
SMAD2	Q15796	70	4	-6	-3	11	1.0	0.9	1.0	1.1
CCDC127	Q96BQ5	144	3	-6	-3	11	1.0	0.9	1.0	1.1
Uncharacteriz	G3V599	1163	-10	4	-3	10	0.9	1.0	1.0	1.1
MPHOSPH8	Q99549	753	1	4	-3	10	1.0	1.0	1.0	1.1
PSMD1	Q99460	806	1	1	-3	10	1.0	1.0	1.0	1.1
MTMR3	Q13615	1152	1	-4	-3	10	1.0	1.0	1.0	1.1
RASSF1	Q9NS23	341	-4	-4	-3	10	1.0	1.0	1.0	1.1
MCM3	P25205	360	-6	-6	-3	10	0.9	0.9	1.0	1.1
SACS	Q9NZJ4	2976	-6	-9	-3	10	0.9	0.9	1.0	1.1
ATOX1	O00244	41	1	-15	-3	10	1.0	0.9	1.0	1.1
PTBP3	O95758	248	7	5	-3	10	1.1	1.1	1.0	1.1
RANBP2	P49792	1749	1	2	-3	10	1.0	1.0	1.0	1.1
DYNC1H1	Q14204	3389	2	0	-3	10	1.0	1.0	1.0	1.1
SBF1	O95248	1550	2	-2	-3	10	1.0	1.0	1.0	1.1
ESD	P10768	56	-2	-2	-3	10	1.0	1.0	1.0	1.1
RPS27A	P62979	121	-4	-3	-3	10	1.0	1.0	1.0	1.1
POLDIP3	Q9BY77	303	-10	-3	-3	10	0.9	1.0	1.0	1.1
CGGBP1	Q9UFW8	43	4	-4	-3	10	1.0	1.0	1.0	1.1
SCAF11	Q99590	478	-9	-5	-3	10	0.9	1.0	1.0	1.1
ARL4C	P56559	85	-12	10	-3	9	0.9	1.1	1.0	1.1
IPO5	O00410	687	-1	-4	-3	9	1.0	1.0	1.0	1.1
CSDE1	O75534	680	0	-5	-3	9	1.0	1.0	1.0	1.1
RIC1	Q4ADV7	818	-2	-8	-3	9	1.0	0.9	1.0	1.1
MYCBP2	O75592	4634	-2	-11	-3	9	1.0	0.9	1.0	1.1
RPS12	P25398	106	-3	-5	-3	9	1.0	1.0	1.0	1.1
RNASEK-C17o	H0YIS7	107	-12	-18	-3	9	0.9	0.8	1.0	1.1
HSPA4	P34932	34	-5	4	-3	8	1.0	1.0	1.0	1.1
ALDH5A1	P51649	340	-6	1	-3	8	0.9	1.0	1.0	1.1
TES	Q9UGI8	239	-3	-1	-3	8	1.0	1.0	1.0	1.1
RRP12	Q5JTH9	261	5	-3	-3	8	1.1	1.0	1.0	1.1
UBR4	Q5T4S7	779	6	-6	-3	8	1.1	0.9	1.0	1.1
EZH1	Q92800	597	-8	-12	-3	8	0.9	0.9	1.0	1.1
NFKBIB	Q15653	121	15	-15	-3	8	1.2	0.9	1.0	1.1
EGLN1	Q9GZT9	43	2	-3	-3	8	1.0	1.0	1.0	1.1
SORD	Q00796	106	-6	-6	-3	8	0.9	0.9	1.0	1.1
NFE2	Q16621	283	-16	-6	-3	8	0.9	0.9	1.0	1.1
DNTTIP2	Q5QJE6	116	-1	-11	-3	8	1.0	0.9	1.0	1.1
HSD17B12	Q53GQ0	166	1	-13	-3	8	1.0	0.9	1.0	1.1
TRA2B	P62995	118	-8	-5	-3	7	0.9	1.0	1.0	1.1
TARS2	Q9BW92	322	2	-6	-3	7	1.0	0.9	1.0	1.1
SNU13	P55769	30	-1	-9	-3	7	1.0	0.9	1.0	1.1
BOLA2B	Q9H3K6	59	-2	-13	-3	7	1.0	0.9	1.0	1.1
Uncharacteriz	U3KPZ7	49	10	-15	-3	7	1.1	0.9	1.0	1.1

UBE2G2	P60604	89	-12	-17	-3	7	0.9	0.9	1.0	1.1
RAD18	Q9NS91	64	-9	-7	-3	7	0.9	0.9	1.0	1.1
LETM1	O95202	379	-4	-13	-3	7	1.0	0.9	1.0	1.1
CPNE1	Q99829	53	-5	-2	-3	6	1.0	1.0	1.0	1.1
MIEF1	Q9NQG6	111	4	-8	-3	6	1.0	0.9	1.0	1.1
INTS4	Q96HW7	44	2	-9	-3	6	1.0	0.9	1.0	1.1
KIAA1551	Q9HCM1	1638	-1	-14	-3	6	1.0	0.9	1.0	1.1
COPA	P53621	975	-3	-2	-3	5	1.0	1.0	1.0	1.1
UBXN6	Q9BZV1	347	7	-5	-3	5	1.1	1.0	1.0	1.1
GIT2	Q14161	235	2	-1	-3	5	1.0	1.0	1.0	1.0
GIT1	Q9Y2X7	235	2	-1	-3	5	1.0	1.0	1.0	1.0
LYPLAL1	Q5VWZ2	12	-1	-4	-3	5	1.0	1.0	1.0	1.0
SNX9	Q9Y5X1	427	1	0	-3	4	1.0	1.0	1.0	1.0
DAGLB	Q8NCG7	519	9	-4	-3	4	1.1	1.0	1.0	1.0
ATP6V1B2	P21281	112	-3	-6	-3	4	1.0	0.9	1.0	1.0
CHD9	Q3L8U1	784	1	-8	-3	4	1.0	0.9	1.0	1.0
AP2A1	O95782	970	2	1	-3	4	1.0	1.0	1.0	1.0
ARL6IP6	Q8N6S5	48	3	-3	-3	4	1.0	1.0	1.0	1.0
PLCG2	P16885	496	-1	-12	-3	4	1.0	0.9	1.0	1.0
RFX5	P48382	126	3	-14	-3	4	1.0	0.9	1.0	1.0
IKBKE	Q14164	545	0	-3	-3	3	1.0	1.0	1.0	1.0
TROVE2	P10155	38	3	-9	-3	3	1.0	0.9	1.0	1.0
PHKB	Q93100	280	-1	-7	-3	3	1.0	0.9	1.0	1.0
PDCL3	Q9H2J4	154	5	8	-3	2	1.0	1.1	1.0	1.0
PTPRC	P08575	398	-10	-1	-3	2	0.9	1.0	1.0	1.0
GAPVD1	Q14C86	1396	6	-14	-3	2	1.1	0.9	1.0	1.0
OPA1	O60313	551	-6	2	-3	1	0.9	1.0	1.0	1.0
THOC6	Q86W42	279	2	2	-3	1	1.0	1.0	1.0	1.0
SNU13	P55769	93	2	0	-3	1	1.0	1.0	1.0	1.0
ERCC2	P18074	489	-3	-2	-3	1	1.0	1.0	1.0	1.0
SAMHD1	Q9Y3Z3	350	-6	-8	-3	1	0.9	0.9	1.0	1.0
DOCK11	Q5JSL3	633	6	-10	-3	1	1.1	0.9	1.0	1.0
PPP6C	O00743	265	3	4	-3	1	1.0	1.0	1.0	1.0
STK38	Q15208	362	-5	-7	-3	1	1.0	0.9	1.0	1.0
ALDH1A1	P00352	370	11	-1	-3	0	1.1	1.0	1.0	1.0
RRAGA	Q7L523	159	2	-1	-3	0	1.0	1.0	1.0	1.0
SIPA1	Q96FS4	634	7	-2	-3	0	1.1	1.0	1.0	1.0
RRP8	O43159	332	-8	-7	-3	0	0.9	0.9	1.0	1.0
DDX42	Q86XP3	281	3	-7	-3	0	1.0	0.9	1.0	1.0
TCF25	Q9BQ70	216	-7	6	-3	-1	0.9	1.1	1.0	1.0
COPS4	Q9BT78	378	4	-1	-3	-1	1.0	1.0	1.0	1.0
CKAP5	Q14008	1203	-7	-3	-3	-1	0.9	1.0	1.0	1.0
USP24	Q9UPU5	1859	5	-11	-3	-1	1.0	0.9	1.0	1.0
TTF2	Q9UNY4	553	-1	-7	-3	-1	1.0	0.9	1.0	1.0
CUL4A	Q13619	483	-2	-10	-3	-1	1.0	0.9	1.0	1.0
CUL4B	Q13620	637	-2	-10	-3	-1	1.0	0.9	1.0	1.0
GNL3L	Q9NVN8	89	-2	-11	-3	-1	1.0	0.9	1.0	1.0
ZBTB7A	O95365	123	2	-17	-3	-1	1.0	0.9	1.0	1.0
MYO1F	O00160	584	-4	-2	-3	-2	1.0	1.0	1.0	1.0
PCCA	P05165	111	-12	-9	-3	-2	0.9	0.9	1.0	1.0
C10orf11	Q9H2I8	106	14	-2	-3	-3	1.2	1.0	1.0	1.0
RABEP2	Q9H5N1	79	11	-6	-3	-3	1.1	0.9	1.0	1.0
TATDN1	Q6P1N9	123	7	-6	-3	-3	1.1	0.9	1.0	1.0
PPP2R1A	P30153	329	-5	-19	-3	-3	1.0	0.8	1.0	1.0
IARS	P41252	433	-7	13	-3	-3	0.9	1.1	1.0	1.0

GIT2	Q14161	14	7	-6	-3	-3	1.1	0.9	1.0	1.0
USP7	Q93009	711	3	-12	-3	-3	1.0	0.9	1.0	1.0
MATR3	A8MXP9	869	3	-18	-3	-3	1.0	0.8	1.0	1.0
PHF6	Q8IWS0	28	-4	-10	-3	-4	1.0	0.9	1.0	1.0
MYOF	Q9NZM1	1467	-24	-11	-3	-4	0.8	0.9	1.0	1.0
STXBP2	Q15833	110	-2	-13	-3	-4	1.0	0.9	1.0	1.0
FLII	Q13045	289	-5	1	-3	-4	1.0	1.0	1.0	1.0
GNB1	P62873	149	1	-17	-3	-4	1.0	0.9	1.0	1.0
DLD	P09622	80	-6	5	-3	-5	0.9	1.1	1.0	1.0
HMHA1	Q92619	413	-9	-1	-3	-5	0.9	1.0	1.0	1.0
ADNP	Q9H2P0	624	8	-4	-3	-5	1.1	1.0	1.0	1.0
PDIA3	P30101	85	-6	-6	-3	-5	0.9	0.9	1.0	1.0
NADSYN1	Q6IA69	686	-7	-6	-3	-5	0.9	0.9	1.0	1.0
RHOF	Q9HBH0	131	9	-5	-3	-5	1.1	1.0	1.0	1.0
ATP6V1A	P38606	277	1	2	-3	-6	1.0	1.0	1.0	0.9
HMHA1	Q92619	739	8	0	-3	-6	1.1	1.0	1.0	0.9
PDDC1	Q8NB37	154	-5	-7	-3	-6	1.0	0.9	1.0	0.9
VPS13C	Q709C8	2177	-5	7	-3	-9	1.0	1.1	1.0	0.9
LPP	Q93052	593	-3	8	-3	-10	1.0	1.1	1.0	0.9
GCN1	Q92616	1362	4	-4	-3	-10	1.0	1.0	1.0	0.9
PAPOLG	Q9BWT3	604	6	-8	-3	-10	1.1	0.9	1.0	0.9
PLEKHF2	Q9H8W4	129	11	6	-3	-11	1.1	1.1	1.0	0.9
DEAF1	A0A0J9YWD6	120	-6	-7	-3	-12	0.9	0.9	1.0	0.9
ATG3	Q9NT62	264	-5	-18	-3	-13	1.0	0.8	1.0	0.9
GEMIN4	P57678	784	-7	-22	-3	-13	0.9	0.8	1.0	0.9
PARP1	P09874	162	0	-8	-3	-14	1.0	0.9	1.0	0.9
OPTN	Q96CV9	555	16	-12	-3	-14	1.2	0.9	1.0	0.9
RASGRF2	O14827	904	-21	1	-3	-15	0.8	1.0	1.0	0.9
ZZEF1	O43149	1786	-15	6	-3	-16	0.9	1.1	1.0	0.9
ITPR1	Q14643	530	9	-13	-3	-16	1.1	0.9	1.0	0.9
HNRNPH1	P31943	290	-6	-8	-3	-18	0.9	0.9	1.0	0.8
PPP6R2	O75170	82	-11	-8	-3	-19	0.9	0.9	1.0	0.8
CHORDC1	Q9UHD1	211	-10	-1	-3	-19	0.9	1.0	1.0	0.8
FLNA	P21333	717	2	-2	-3	-19	1.0	1.0	1.0	0.8
KIF2A	O00139	334	-5	1	-3	-21	1.0	1.0	1.0	0.8
EIF2AK4	Q9P2K8	454	-7	-14	-3	-24	0.9	0.9	1.0	0.8
CHAMP1	Q96JM3	787	-9	-11	-3	-25	0.9	0.9	1.0	0.8
GIPC3	Q8TF64	209	2	-9	-3	-28	1.0	0.9	1.0	0.8
ARHGAP30	Q7Z6I6	20	-4	-19	-3	-30	1.0	0.8	1.0	0.8
FASN	P49327	1548	-67	-22	-3	-39	0.6	0.8	1.0	0.7
ZC3HAV1L	Q96H79	202	6	-16	-3	-44	1.1	0.9	1.0	0.7
RINL	Q6ZS11	421	-4	-29	-3	-59	1.0	0.8	1.0	0.6
ILK	Q13418	428	4	-3	-3	-116	1.0	1.0	1.0	0.5
TGFB1I1	O43294	334	-3	-6	-3	-146	1.0	0.9	1.0	0.4
SLC25A19	Q9HC21	268	3	-12	-4	59	1.0	0.9	1.0	2.4
CELF2	O95319	86	-3	-6	-4	48	1.0	0.9	1.0	1.9
CELF1	Q92879	62	-3	-6	-4	48	1.0	0.9	1.0	1.9
DCUN1D4	Q92564	219	-5	-10	-4	48	1.0	0.9	1.0	1.9
APRT	P07741	83	-1	4	-4	33	1.0	1.0	1.0	1.5
CFLAR	O15519	393	-1	-7	-4	33	1.0	0.9	1.0	1.5
DNAJC7	Q99615	225	-1	-1	-4	32	1.0	1.0	1.0	1.5
AAAS	Q9NRG9	368	4	-3	-4	30	1.0	1.0	1.0	1.4
BAG5	Q9UL15	213	-16	2	-4	30	0.9	1.0	1.0	1.4
PGK1	P00558	380	-4	-5	-4	30	1.0	1.0	1.0	1.4
CAMLG	P49069	123	-2	-12	-4	30	1.0	0.9	1.0	1.4

USP7	Q93009	315	2	-4	-4	29	1.0	1.0	1.0	1.4
SEC23A	Q15436	432	-1	-7	-4	29	1.0	0.9	1.0	1.4
HCCS	P53701	66	-3	-8	-4	29	1.0	0.9	1.0	1.4
HIPK1	Q86Z02	1024	7	15	-4	29	1.1	1.2	1.0	1.4
GSK3A	P49840	281	-1	-7	-4	28	1.0	0.9	1.0	1.4
GSK3B	P49841	218	-1	-7	-4	28	1.0	0.9	1.0	1.4
TAF5L	O75529	15	-10	-3	-4	28	0.9	1.0	1.0	1.4
TCEA3	O75764	105	5	-3	-4	28	1.0	1.0	1.0	1.4
GCLC	P48506	553	2	2	-4	27	1.0	1.0	1.0	1.4
ITPRIPL2	Q3MIP1	371	-2	-16	-4	27	1.0	0.9	1.0	1.4
CMPK1	P30085	20	-7	11	-4	26	0.9	1.1	1.0	1.4
KMT2E	Q8IZD2	1146	-6	-12	-4	26	0.9	0.9	1.0	1.4
ST20-MTHFS	A0A0A6YYL1	54	-4	-15	-4	26	1.0	0.9	1.0	1.3
CEP57	Q86XR8	285	-5	-5	-4	25	1.0	1.0	1.0	1.3
HSP90B1	P14625	645	-12	-7	-4	25	0.9	0.9	1.0	1.3
FGL2	Q14314	39	7	-10	-4	25	1.1	0.9	1.0	1.3
CERK	Q8TCT0	50	2	-4	-4	24	1.0	1.0	1.0	1.3
TMEM209	Q96SK2	158	10	-9	-4	24	1.1	0.9	1.0	1.3
ATP2A2	P16615	635	3	-9	-4	24	1.0	0.9	1.0	1.3
ATP2A3	Q93084	636	3	-9	-4	24	1.0	0.9	1.0	1.3
DMXL1	Q9Y485	681	-8	-8	-4	24	0.9	0.9	1.0	1.3
THTPA	Q9BU02	228	0	-4	-4	23	1.0	1.0	1.0	1.3
FES	P07332	689	10	2	-4	23	1.1	1.0	1.0	1.3
NPAT	Q14207	978	4	-3	-4	23	1.0	1.0	1.0	1.3
CUL9	Q8IWT3	2177	2	-12	-4	22	1.0	0.9	1.0	1.3
NFRKB	Q6P4R8	487	-2	-5	-4	22	1.0	1.0	1.0	1.3
EPC1	Q9H2F5	797	2	-6	-4	22	1.0	0.9	1.0	1.3
GRWD1	Q9BQ67	66	2	-5	-4	21	1.0	1.0	1.0	1.3
CTC1	Q2NKJ3	837	-7	3	-4	21	0.9	1.0	1.0	1.3
IFIT1	P09914	138	1	10	-4	20	1.0	1.1	1.0	1.3
ALDH6A1	Q02252	317	-8	6	-4	20	0.9	1.1	1.0	1.3
RNF213	Q63HN8	3554	6	-3	-4	20	1.1	1.0	1.0	1.3
GLYR1	Q49A26	413	23	-6	-4	20	1.3	0.9	1.0	1.3
CUTA	O60888	96	4	1	-4	19	1.0	1.0	1.0	1.2
STK17A	Q9UEE5	31	-7	-14	-4	19	0.9	0.9	1.0	1.2
IRAK3	Q9Y616	399	0	-3	-4	19	1.0	1.0	1.0	1.2
ARHGDIB	P52566	76	6	-1	-4	18	1.1	1.0	1.0	1.2
GPX4	P36969	134	5	-6	-4	18	1.1	0.9	1.0	1.2
OASL	Q15646	188	-3	-6	-4	18	1.0	0.9	1.0	1.2
WDFY4	Q6ZS81	1569	-8	-6	-4	18	0.9	0.9	1.0	1.2
SARS2	Q9NP81	64	-4	-8	-4	18	1.0	0.9	1.0	1.2
SP100	P23497	270	0	-13	-4	18	1.0	0.9	1.0	1.2
SP110	Q9HB58	468	-3	5	-4	18	1.0	1.1	1.0	1.2
NCF1	P14598	378	-10	2	-4	18	0.9	1.0	1.0	1.2
C9orf114	Q5T280	239	-5	0	-4	18	1.0	1.0	1.0	1.2
MRPS18B	Q9Y676	108	-10	-14	-4	18	0.9	0.9	1.0	1.2
VAV1	P15498	369	2	-4	-4	17	1.0	1.0	1.0	1.2
NCOR1	O75376	1274	-2	-5	-4	17	1.0	1.0	1.0	1.2
ATXN7	O15265	839	-13	3	-4	17	0.9	1.0	1.0	1.2
CHD4	Q14839	1827	-34	-4	-4	17	0.7	1.0	1.0	1.2
ANXA6	P08133	59	1	-4	-4	17	1.0	1.0	1.0	1.2
PTRH1	Q86Y79	20	-8	-6	-4	17	0.9	0.9	1.0	1.2
RPL8	P62917	114	-2	1	-4	16	1.0	1.0	1.0	1.2
GTF3C1	Q12789	1681	-4	1	-4	16	1.0	1.0	1.0	1.2
C11orf73	Q53FT3	4	6	-3	-4	16	1.1	1.0	1.0	1.2

PIK3CD	O00329	132	4	-19	-4	16	1.0	0.8	1.0	1.2
MRPS18A	Q9NVS2	186	-1	2	-4	16	1.0	1.0	1.0	1.2
AP1G2	O75843	180	-5	-8	-4	16	1.0	0.9	1.0	1.2
IRF2BP2	Q7Z5L9	533	3	1	-4	15	1.0	1.0	1.0	1.2
IRF2BP1	Q8IU81	530	3	1	-4	15	1.0	1.0	1.0	1.2
IRF2BPL	Q9H1B7	742	3	1	-4	15	1.0	1.0	1.0	1.2
RHOC	P08134	107	-3	-5	-4	15	1.0	1.0	1.0	1.2
RHOA	P61586	107	-3	-5	-4	15	1.0	1.0	1.0	1.2
MEPCE	Q7L2J0	522	-1	-6	-4	15	1.0	0.9	1.0	1.2
NCAPH2	Q6IBW4	309	-5	-14	-4	15	1.0	0.9	1.0	1.2
RIF1	Q5UIP0	1904	5	-19	-4	15	1.0	0.8	1.0	1.2
LRRK2	Q5S007	1526	7	7	-4	15	1.1	1.1	1.0	1.2
C17orf80	Q9BSJ5	602	-12	-1	-4	15	0.9	1.0	1.0	1.2
BIN2	Q9UBW5	496	4	-3	-4	15	1.0	1.0	1.0	1.2
TYK2	P29597	536	-2	-6	-4	15	1.0	0.9	1.0	1.2
ASPSCR1	Q9BZE9	174	-6	8	-4	14	0.9	1.1	1.0	1.2
PML	P29590	60	-5	5	-4	14	1.0	1.1	1.0	1.2
MADD	Q8WXG6	202	-4	2	-4	14	1.0	1.0	1.0	1.2
PXN	P49023	405	-1	-4	-4	14	1.0	1.0	1.0	1.2
MED25	Q71SY5	506	-12	-5	-4	14	0.9	1.0	1.0	1.2
DDX20	Q9UHI6	577	3	-7	-4	14	1.0	0.9	1.0	1.2
C7orf26	Q96N11	157	1	-8	-4	14	1.0	0.9	1.0	1.2
RAP1GDS1	P52306	26	9	-9	-4	14	1.1	0.9	1.0	1.2
ARHGEF2	Q92974	335	3	7	-4	14	1.0	1.1	1.0	1.2
KDM3B	Q7LBC6	1451	-7	4	-4	14	0.9	1.0	1.0	1.2
DCK	P27707	59	1	-13	-4	14	1.0	0.9	1.0	1.2
U2SURP	O15042	320	-4	8	-4	13	1.0	1.1	1.0	1.1
TCP1	P17987	147	1	1	-4	13	1.0	1.0	1.0	1.1
OSBPL3	Q9H4L5	520	3	-2	-4	13	1.0	1.0	1.0	1.1
PROSER1	Q86XN7	518	-1	-4	-4	13	1.0	1.0	1.0	1.1
LYPLA1	O75608	169	2	-7	-4	13	1.0	0.9	1.0	1.1
PTBP3	O95758	68	-1	-8	-4	13	1.0	0.9	1.0	1.1
RELB	Q01201	144	-8	6	-4	13	0.9	1.1	1.0	1.1
CSDE1	O75534	506	-2	-3	-4	13	1.0	1.0	1.0	1.1
TMCO4	Q5TGY1	537	1	-7	-4	13	1.0	0.9	1.0	1.1
HINT1	P49773	38	5	-10	-4	13	1.1	0.9	1.0	1.1
PIK3C2A	O00443	317	16	-10	-4	13	1.2	0.9	1.0	1.1
GNPAT	O15228	650	-14	-14	-4	13	0.9	0.9	1.0	1.1
BCR	P11274	240	9	10	-4	12	1.1	1.1	1.0	1.1
MTG2	Q9H4K7	206	2	6	-4	12	1.0	1.1	1.0	1.1
ATL3	Q6DD88	429	3	5	-4	12	1.0	1.1	1.0	1.1
C20orf27	Q9GZN8	124	1	-4	-4	12	1.0	1.0	1.0	1.1
HSF1	Q00613	103	-4	-6	-4	12	1.0	0.9	1.0	1.1
CD14	P08571	187	-16	-8	-4	12	0.9	0.9	1.0	1.1
ZDHHC7	Q9NXF8	136	-4	-9	-4	12	1.0	0.9	1.0	1.1
HSPD1	P10809	237	-6	-1	-4	11	0.9	1.0	1.0	1.1
RAD50	Q92878	221	3	-3	-4	11	1.0	1.0	1.0	1.1
IRF2BP2	Q7Z5L9	19	-5	-5	-4	11	1.0	1.0	1.0	1.1
IRF2BPL	Q9H1B7	17	-5	-5	-4	11	1.0	1.0	1.0	1.1
FCF1	Q9Y324	134	-1	-6	-4	11	1.0	0.9	1.0	1.1
SORL1	Q92673	1112	-4	-15	-4	11	1.0	0.9	1.0	1.1
CYTH1	Q15438	94	4	7	-4	11	1.0	1.1	1.0	1.1
OPA1	O60313	375	-4	-3	-4	11	1.0	1.0	1.0	1.1
MYO1F	O00160	788	-12	2	-4	10	0.9	1.0	1.0	1.1
GMPS	P49915	489	2	-1	-4	10	1.0	1.0	1.0	1.1

SRSF9	Q13242	80	-1	-4	-4	10	1.0	1.0	1.0	1.1
PABPC1	P11940	132	2	-6	-4	10	1.0	0.9	1.0	1.1
CMAS	Q8NFW8	394	2	-6	-4	10	1.0	0.9	1.0	1.1
RABGEF1	Q9UJ41	507	-13	-9	-4	10	0.9	0.9	1.0	1.1
CCT8	P50990	149	8	-6	-4	10	1.1	0.9	1.0	1.1
KMT2D	O14686	3543	5	-8	-4	10	1.0	0.9	1.0	1.1
KAT6B	Q8WYB5	751	-5	-9	-4	10	1.0	0.9	1.0	1.1
KAT6A	Q92794	540	-5	-9	-4	10	1.0	0.9	1.0	1.1
IL18	Q14116	74	6	18	-4	9	1.1	1.2	1.0	1.1
PDK2	Q15119	45	0	-4	-4	9	1.0	1.0	1.0	1.1
RIOK3	O14730	346	-2	-7	-4	9	1.0	0.9	1.0	1.1
RAI1	Q7Z5J4	1729	-2	-9	-4	9	1.0	0.9	1.0	1.1
MAP2K4	P45985	158	-1	2	-4	9	1.0	1.0	1.0	1.1
MIA3	Q5JRA6	1513	-2	1	-4	9	1.0	1.0	1.0	1.1
DST	Q03001	5610	-5	-1	-4	9	1.0	1.0	1.0	1.1
STAT4	Q14765	378	-1	-6	-4	9	1.0	0.9	1.0	1.1
BAZ2A	Q9UIF9	917	-6	-8	-4	9	0.9	0.9	1.0	1.1
VPRBP	Q9Y4B6	1098	-1	-17	-4	9	1.0	0.9	1.0	1.1
LYN	P07948	203	-5	-7	-4	8	1.0	0.9	1.0	1.1
FARSA	Q9Y285	493	1	-7	-4	8	1.0	0.9	1.0	1.1
PLEC	Q15149	1136	-15	5	-4	8	0.9	1.1	1.0	1.1
NUP88	Q99567	447	-9	2	-4	8	0.9	1.0	1.0	1.1
KDM2A	Q9Y2K7	463	-9	1	-4	8	0.9	1.0	1.0	1.1
MRPL14	Q6P1L8	90	-2	-2	-4	8	1.0	1.0	1.0	1.1
SP140	Q13342	756	0	-4	-4	8	1.0	1.0	1.0	1.1
SP140L	Q9H930	469	0	-4	-4	8	1.0	1.0	1.0	1.1
RPP38	P78345	159	-17	-4	-4	8	0.9	1.0	1.0	1.1
TARS2	Q9BW92	506	-2	-13	-4	8	1.0	0.9	1.0	1.1
EXOC4	Q96A65	522	-12	-15	-4	8	0.9	0.9	1.0	1.1
RPL9P9	P32969	134	-6	-1	-4	7	0.9	1.0	1.0	1.1
EFR3A	Q14156	767	3	-2	-4	7	1.0	1.0	1.0	1.1
ALDOA	P04075	135	-6	-6	-4	7	0.9	0.9	1.0	1.1
ALDOC	P09972	135	-6	-6	-4	7	0.9	0.9	1.0	1.1
MACF1	Q9UPN3	6646	3	-6	-4	7	1.0	0.9	1.0	1.1
VWA9	Q96SY0	193	2	-7	-4	7	1.0	0.9	1.0	1.1
ASPSCR1	Q9BZE9	48	-5	-10	-4	7	1.0	0.9	1.0	1.1
RNH1	P13489	387	2	1	-4	7	1.0	1.0	1.0	1.1
OAS3	Q9Y6K5	574	5	-3	-4	7	1.0	1.0	1.0	1.1
PRDX5	P30044	204	5	2	-4	6	1.1	1.0	1.0	1.1
MAN2C1	Q9NTJ4	322	1	-12	-4	6	1.0	0.9	1.0	1.1
TDRD7	Q8NHU6	568	-2	2	-4	6	1.0	1.0	1.0	1.1
PLEC	Q15149	3336	-6	-2	-4	6	0.9	1.0	1.0	1.1
CLTC	Q00610	1528	1	-4	-4	6	1.0	1.0	1.0	1.1
SMARCA2	P51531	968	-11	-4	-4	6	0.9	1.0	1.0	1.1
RASSF4	Q9H2L5	33	-2	-5	-4	6	1.0	1.0	1.0	1.1
TP53BP1	Q12888	101	0	-6	-4	6	1.0	0.9	1.0	1.1
MAP2K6	P52564	109	-17	-11	-4	6	0.9	0.9	1.0	1.1
RFC4	P35249	48	-4	3	-4	5	1.0	1.0	1.0	1.1
PPP1R7	Q15435	112	3	1	-4	5	1.0	1.0	1.0	1.1
NBN	O60934	119	-4	-3	-4	5	1.0	1.0	1.0	1.1
PSMD7	P51665	116	3	-4	-4	5	1.0	1.0	1.0	1.1
NUP93	Q8N1F7	392	5	-13	-4	5	1.0	0.9	1.0	1.1
EEF2	P13639	136	-5	6	-4	5	1.0	1.1	1.0	1.0
STOML2	Q9UJZ1	167	1	-11	-4	5	1.0	0.9	1.0	1.0
NAPRT	Q6XQN6	477	6	-13	-4	5	1.1	0.9	1.0	1.0

UBXN2B	Q14CS0	41	-5	-19	-4	5	1.0	0.8	1.0	1.0
SLC30A1	Q9Y6M5	447	1	1	-4	4	1.0	1.0	1.0	1.0
IKZF1	Q13422	254	5	-5	-4	4	1.0	1.0	1.0	1.0
RABGAP1L	B7ZAP0	115	2	-9	-4	4	1.0	0.9	1.0	1.0
THNSL1	Q8IYQ7	532	-15	-9	-4	4	0.9	0.9	1.0	1.0
NUP155	O75694	844	3	-10	-4	4	1.0	0.9	1.0	1.0
CD3EAP	O15446	107	1	-11	-4	4	1.0	0.9	1.0	1.0
PDS5A	Q29RF7	583	-2	-6	-4	4	1.0	0.9	1.0	1.0
PPP4R1	Q8TF05	645	4	14	-4	3	1.0	1.2	1.0	1.0
PPAT	Q06203	348	-6	4	-4	3	0.9	1.0	1.0	1.0
GAK	O14976	595	-1	-3	-4	3	1.0	1.0	1.0	1.0
TRAPPC1	Q9Y5R8	115	-4	-8	-4	3	1.0	0.9	1.0	1.0
UBE2D2	P62837	85	-2	-12	-4	3	1.0	0.9	1.0	1.0
DPM3	Q9P2X0	67	-9	-12	-4	3	0.9	0.9	1.0	1.0
WNK1	Q9H4A3	459	-5	-1	-4	3	1.0	1.0	1.0	1.0
MIPEP	Q99797	281	7	-17	-4	3	1.1	0.9	1.0	1.0
THOP1	P52888	246	4	-23	-4	3	1.0	0.8	1.0	1.0
BACH1	O14867	621	-3	-1	-4	2	1.0	1.0	1.0	1.0
MYH10	P35580	1238	12	-1	-4	2	1.1	1.0	1.0	1.0
OSBPL8	Q9BZF1	531	5	-3	-4	2	1.1	1.0	1.0	1.0
EIF2S2	P20042	284	1	-9	-4	2	1.0	0.9	1.0	1.0
SFSWAP	Q12872	42	-15	-19	-4	2	0.9	0.8	1.0	1.0
ARPC3	O15145	162	-6	-4	-4	2	0.9	1.0	1.0	1.0
CSDE1	O75534	645	6	-5	-4	2	1.1	1.0	1.0	1.0
BPGM	P07738	23	-3	-3	-4	1	1.0	1.0	1.0	1.0
EIF5	P55010	59	-2	-5	-4	1	1.0	1.0	1.0	1.0
PBRM1	Q86U86	1047	-6	-6	-4	1	0.9	0.9	1.0	1.0
EIF5B	O60841	635	-5	-3	-4	1	1.0	1.0	1.0	1.0
FGD3	Q5JSP0	655	-2	-3	-4	1	1.0	1.0	1.0	1.0
PANK4	Q9NVE7	537	-8	3	-4	0	0.9	1.0	1.0	1.0
ZBTB7B	O15156	34	4	-2	-4	0	1.0	1.0	1.0	1.0
HEATR5B	Q9P2D3	870	10	2	-4	-1	1.1	1.0	1.0	1.0
GTF3C2	Q8WUA4	503	-5	-19	-4	-1	1.0	0.8	1.0	1.0
TKFC	Q3LXA3	13	25	5	-4	-1	1.3	1.1	1.0	1.0
ABCF1	Q8NE71	741	-1	-6	-4	-1	1.0	0.9	1.0	1.0
RANGAP1	P46060	303	5	-1	-4	-2	1.1	1.0	1.0	1.0
CNP	P09543	111	9	-7	-4	-2	1.1	0.9	1.0	1.0
DTX3L	Q8TDB6	250	3	-11	-4	-2	1.0	0.9	1.0	1.0
SZT2	Q5T011	2453	-9	-14	-4	-2	0.9	0.9	1.0	1.0
ZDHHC13	Q8IUH4	51	6	-17	-4	-2	1.1	0.9	1.0	1.0
CCT8	P50990	430	-3	-5	-4	-2	1.0	1.0	1.0	1.0
ARID1B	Q8NFD5	1919	-7	-9	-4	-2	0.9	0.9	1.0	1.0
PXN	P49023	546	7	5	-4	-3	1.1	1.1	1.0	1.0
AAK1	Q2M2I8	250	1	-7	-4	-3	1.0	0.9	1.0	1.0
BMP2K	Q9NSY1	254	1	-7	-4	-3	1.0	0.9	1.0	1.0
SLC27A1	Q6PCB7	640	-3	-11	-4	-3	1.0	0.9	1.0	1.0
ARRB1	P49407	242	9	-2	-4	-3	1.1	1.0	1.0	1.0
RBL2	Q08999	120	-8	-11	-4	-3	0.9	0.9	1.0	1.0
TRMT2A	Q8IZ69	392	-2	2	-4	-4	1.0	1.0	1.0	1.0
BAG6	P46379	345	-8	9	-4	-4	0.9	1.1	1.0	1.0
EWSR1	Q01844	524	10	2	-4	-4	1.1	1.0	1.0	1.0
EPB41L2	O43491	424	-8	1	-4	-4	0.9	1.0	1.0	1.0
MARS	P56192	441	-4	-18	-4	-4	1.0	0.9	1.0	1.0
TOP2B	Q02880	237	13	-13	-4	-5	1.1	0.9	1.0	1.0
TNKS2	Q9H2K2	269	2	-13	-4	-5	1.0	0.9	1.0	1.0

NAMPT	P43490	401	1	4	-4	-5	1.0	1.0	1.0	1.0
PSMC6	P62333	193	5	4	-4	-5	1.1	1.0	1.0	1.0
VPS28	Q9UK41	96	3	-4	-4	-5	1.0	1.0	1.0	1.0
IARS	P41252	87	4	-3	-4	-6	1.0	1.0	1.0	0.9
RAVER1	Q8IY67	251	-13	-21	-4	-6	0.9	0.8	1.0	0.9
TRIM25	Q14258	186	-3	8	-4	-6	1.0	1.1	1.0	0.9
PSMC5	P62195	209	6	-4	-4	-6	1.1	1.0	1.0	0.9
CCDC88C	Q9P219	1007	8	-8	-4	-6	1.1	0.9	1.0	0.9
DMAP1	Q9NPF5	199	1	-9	-4	-6	1.0	0.9	1.0	0.9
ANKFY1	Q9P2R3	749	5	-19	-4	-6	1.1	0.8	1.0	0.9
MTM1	Q13496	191	2	4	-4	-7	1.0	1.0	1.0	0.9
WDFY1	Q8IWB7	344	-4	-6	-4	-7	1.0	0.9	1.0	0.9
LYN	P07948	419	-9	-11	-4	-7	0.9	0.9	1.0	0.9
ACSL3	O95573	503	-11	-3	-4	-7	0.9	1.0	1.0	0.9
UBN1	Q9NPG3	467	-5	-5	-4	-7	1.0	1.0	1.0	0.9
HNRNPK	P61978	185	-6	-12	-4	-8	0.9	0.9	1.0	0.9
SEC23IP	Q9Y6Y8	467	-2	-6	-4	-10	1.0	0.9	1.0	0.9
PRKCH	P24723	493	7	-14	-4	-10	1.1	0.9	1.0	0.9
BAZ1A	Q9NRL2	537	-2	-18	-4	-10	1.0	0.8	1.0	0.9
TMEM55B	Q86T03	125	-7	2	-4	-11	0.9	1.0	1.0	0.9
FKBP8	Q14318	295	-3	-6	-4	-12	1.0	0.9	1.0	0.9
MRPL16	Q9NX20	167	-17	-21	-4	-13	0.9	0.8	1.0	0.9
PDCD11	Q14690	599	3	-9	-4	-14	1.0	0.9	1.0	0.9
RBM22	Q9NW64	71	1	3	-4	-14	1.0	1.0	1.0	0.9
FLNA	P21333	2107	-4	-11	-4	-15	1.0	0.9	1.0	0.9
EEF1B2	P24534	161	25	-21	-4	-16	1.3	0.8	1.0	0.9
PATL1	Q86TB9	563	4	-5	-4	-17	1.0	1.0	1.0	0.9
RTEL1-TNFRSF	F6WH68	623	2	-2	-4	-20	1.0	1.0	1.0	0.8
MAP1S	Q66K74	51	-6	1	-4	-20	0.9	1.0	1.0	0.8
ANKRD44	Q8N8A2	547	-5	-12	-4	-21	1.0	0.9	1.0	0.8
HMHA1	Q92619	747	-2	2	-4	-21	1.0	1.0	1.0	0.8
FLNA	P21333	1018	3	-4	-4	-22	1.0	1.0	1.0	0.8
NFATC3	Q12968	591	12	-7	-4	-23	1.1	0.9	1.0	0.8
EEA1	Q15075	1402	3	-3	-4	-28	1.0	1.0	1.0	0.8
CHAMP1	Q96JM3	770	8	-7	-4	-29	1.1	0.9	1.0	0.8
CKAP5	Q14008	1317	-9	-23	-4	-31	0.9	0.8	1.0	0.8
ARPC1B	O15143	227	-5	-42	-4	-31	1.0	0.7	1.0	0.8
MYH9	P35579	671	-3	-5	-4	-33	1.0	1.0	1.0	0.8
CSRP1	P21291	122	-2	11	-4	-40	1.0	1.1	1.0	0.7
SNAP23	O00161	87	3	-4	-4	-43	1.0	1.0	1.0	0.7
TLN1	Q9Y490	1045	2	4	-4	-56	1.0	1.0	1.0	0.6
TLN1	Q9Y490	2196	-5	-3	-4	-58	1.0	1.0	1.0	0.6
IFIT5	Q13325	124	-1	-1	-4	-65	1.0	1.0	1.0	0.6
PROSER2	Q86WR7	367	-8	1	-4	-71	0.9	1.0	1.0	0.6
DNM3	Q9UQ16	27	-16	-2	-4	-88	0.9	1.0	1.0	0.5
CECR1	Q9NZK5	134	-9	-4	-4	41	0.9	1.0	1.0	1.7
TNFAIP2	Q03169	45	-6	-9	-4	38	0.9	0.9	1.0	1.6
AKAP17A	Q02040	563	-3	-9	-4	38	1.0	0.9	1.0	1.6
IKBKAP	O95163	213	5	-7	-4	35	1.0	0.9	1.0	1.5
C19orf35	Q6ZS72	318	-4	-14	-4	33	1.0	0.9	1.0	1.5
RASSF1	Q9NS23	40	-2	-5	-4	32	1.0	1.0	1.0	1.5
GNPAT	O15228	66	-2	-3	-4	31	1.0	1.0	1.0	1.4
IARS	P41252	400	6	8	-4	31	1.1	1.1	1.0	1.4
GRB2	P62993	198	-3	-8	-4	31	1.0	0.9	1.0	1.4
MYBBP1A	Q9BQG0	623	-7	-6	-4	30	0.9	0.9	1.0	1.4

DDX1	Q92499	110	0	2	-4	30	1.0	1.0	1.0	1.4
MDH2	P40926	93	-8	-6	-4	30	0.9	0.9	1.0	1.4
KANK3	Q6NY19	546	-11	0	-4	29	0.9	1.0	1.0	1.4
PISD	Q9UG56	181	1	-5	-4	29	1.0	1.0	1.0	1.4
MCL1	Q07820	16	-8	-8	-4	28	0.9	0.9	1.0	1.4
ALOX5	P09917	265	2	-8	-4	28	1.0	0.9	1.0	1.4
ARPC1A	Q92747	279	2	-4	-4	27	1.0	1.0	1.0	1.4
CYBB	P04839	72	-13	-2	-4	26	0.9	1.0	1.0	1.4
TFAP4	Q01664	29	1	-11	-4	26	1.0	0.9	1.0	1.4
BCAT2	O15382	342	6	-1	-4	25	1.1	1.0	1.0	1.3
CMAS	Q8NFW8	432	2	-2	-4	24	1.0	1.0	1.0	1.3
GAPVD1	Q14C86	176	14	1	-4	24	1.2	1.0	1.0	1.3
PPP1CA	P62136	127	7	-12	-4	23	1.1	0.9	1.0	1.3
PPP1CB	P62140	126	7	-12	-4	23	1.1	0.9	1.0	1.3
STIM2	Q9P246	426	3	-1	-4	23	1.0	1.0	1.0	1.3
PRF1	P14222	73	6	-3	-4	23	1.1	1.0	1.0	1.3
CSDE1	O75534	464	8	-3	-4	22	1.1	1.0	1.0	1.3
SKP1	P63208	120	3	-9	-4	22	1.0	0.9	1.0	1.3
CBX5	P45973	133	7	-4	-4	22	1.1	1.0	1.0	1.3
MMS22L	Q6ZRQ5	111	6	-5	-4	22	1.1	1.0	1.0	1.3
SETX	Q7Z333	602	-8	-10	-4	22	0.9	0.9	1.0	1.3
LIPT1	Q9Y234	337	0	-12	-4	22	1.0	0.9	1.0	1.3
DAXX	Q9UER7	245	-2	-31	-4	21	1.0	0.8	1.0	1.3
SSH2	Q76I76	1210	1	-7	-4	21	1.0	0.9	1.0	1.3
RNASEL	Q05823	639	-8	-8	-4	21	0.9	0.9	1.0	1.3
AHNAK	Q09666	108	4	-11	-4	21	1.0	0.9	1.0	1.3
KDM4B	O94953	235	0	-7	-4	20	1.0	0.9	1.0	1.3
HERPUD1	Q15011	68	0	-17	-4	20	1.0	0.9	1.0	1.3
FTO	Q9C0B1	346	-1	5	-4	20	1.0	1.0	1.0	1.2
SETD2	Q9BYW2	900	-2	-1	-4	20	1.0	1.0	1.0	1.2
SYNE2	Q8WXH0	90	6	-5	-4	20	1.1	1.0	1.0	1.2
ADO	Q96SZ5	239	-1	-6	-4	20	1.0	0.9	1.0	1.2
ZDHHC18	Q9NUE0	156	4	-8	-4	20	1.0	0.9	1.0	1.2
SYNE2	Q8WXH0	1235	5	-3	-4	19	1.0	1.0	1.0	1.2
ACAD9	Q9H845	613	-1	-6	-4	19	1.0	0.9	1.0	1.2
WDR37	Q9Y2I8	417	10	4	-4	19	1.1	1.0	1.0	1.2
SDAD1	Q9NVU7	87	-5	-5	-4	19	1.0	1.0	1.0	1.2
EDC3	Q96F86	353	-1	-6	-4	19	1.0	0.9	1.0	1.2
SELPLG	Q14242	364	-15	-12	-4	19	0.9	0.9	1.0	1.2
MRPL37	Q9BZE1	104	2	6	-4	18	1.0	1.1	1.0	1.2
AGAP2	Q99490	811	2	-3	-4	18	1.0	1.0	1.0	1.2
AHNAK	Q09666	2806	-1	-1	-4	18	1.0	1.0	1.0	1.2
PDCD11	Q14690	361	0	-2	-4	18	1.0	1.0	1.0	1.2
USP4	Q13107	475	2	-7	-4	18	1.0	0.9	1.0	1.2
FNBP1	Q96RU3	70	5	2	-4	17	1.1	1.0	1.0	1.2
PRKDC	P78527	1229	-8	-13	-4	17	0.9	0.9	1.0	1.2
IRF5	Q13568	272	-4	-1	-4	17	1.0	1.0	1.0	1.2
ALKBH4	Q9NXW9	26	1	-3	-4	17	1.0	1.0	1.0	1.2
PDS5A	Q29RF7	508	-9	1	-4	16	0.9	1.0	1.0	1.2
ZAP70	P43403	346	3	-2	-4	16	1.0	1.0	1.0	1.2
CEP63	Q96MT8	534	4	-10	-4	16	1.0	0.9	1.0	1.2
LST1	O00453	90	-5	-12	-4	16	1.0	0.9	1.0	1.2
SPOP	O43791	361	-1	-14	-4	16	1.0	0.9	1.0	1.2
C1orf174	Q8IYL3	124	-4	-17	-4	16	1.0	0.9	1.0	1.2
PXK	Q7Z7A4	112	3	3	-4	16	1.0	1.0	1.0	1.2

SEPT9	Q9UHD8	531	-4	1	-4	16	1.0	1.0	1.0	1.2
VAT1	Q99536	324	-6	-4	-4	16	0.9	1.0	1.0	1.2
GMFB	P60983	96	14	-1	-4	15	1.2	1.0	1.0	1.2
SASH3	O75995	351	1	-4	-4	15	1.0	1.0	1.0	1.2
SPRYD3	Q8NCJ5	229	2	-5	-4	15	1.0	1.0	1.0	1.2
PHF3	Q92576	885	-1	-9	-4	15	1.0	0.9	1.0	1.2
FLNB	O75369	1158	-5	7	-4	15	1.0	1.1	1.0	1.2
BRD1	O95696	937	1	5	-4	15	1.0	1.1	1.0	1.2
MGEA5	O60502	896	-2	-6	-4	15	1.0	0.9	1.0	1.2
CDKAL1	Q5VV42	214	2	13	-4	14	1.0	1.1	1.0	1.2
EFL1	Q7Z2Z2	646	6	1	-4	14	1.1	1.0	1.0	1.2
CHERP	Q8IWX8	69	-2	-5	-4	14	1.0	1.0	1.0	1.2
SP110	Q9HB58	283	-4	-5	-4	14	1.0	1.0	1.0	1.2
MEPCE	Q7L2J0	419	1	-5	-4	14	1.0	1.0	1.0	1.2
TMEM209	Q96SK2	295	-3	-2	-4	14	1.0	1.0	1.0	1.2
WDR75	Q8IWA0	444	4	-6	-4	14	1.0	0.9	1.0	1.2
PCNA	P12004	135	-14	-10	-4	14	0.9	0.9	1.0	1.2
MAP7D3	Q8IWC1	572	-7	4	-4	13	0.9	1.0	1.0	1.1
SPATA5L1	Q9BVQ7	580	-3	3	-4	13	1.0	1.0	1.0	1.1
RBMS1	P29558	221	6	-3	-4	13	1.1	1.0	1.0	1.1
RNF213	Q63HN8	4348	7	-6	-4	13	1.1	0.9	1.0	1.1
PRDM2	Q13029	1204	-4	-12	-4	13	1.0	0.9	1.0	1.1
BIRC6	Q9NR09	752	-8	-3	-4	13	0.9	1.0	1.0	1.1
KLHL36	Q8N4N3	254	-4	-6	-4	13	1.0	0.9	1.0	1.1
STAT3	P40763	718	4	-18	-4	13	1.0	0.9	1.0	1.1
ARHGEF40	Q8TER5	1233	-12	-2	-4	12	0.9	1.0	1.0	1.1
BAZ1B	Q9UIG0	338	-17	-13	-4	12	0.9	0.9	1.0	1.1
SNRNP200	O75643	576	6	1	-4	11	1.1	1.0	1.0	1.1
ACAA1	P09110	123	-9	1	-4	11	0.9	1.0	1.0	1.1
THOP1	P52888	175	7	-4	-4	11	1.1	1.0	1.0	1.1
EPB41	P11171	179	2	-5	-4	11	1.0	1.0	1.0	1.1
GTF2B	Q00403	223	-10	-6	-4	11	0.9	0.9	1.0	1.1
RNF213	Q63HN8	1510	-4	-12	-4	11	1.0	0.9	1.0	1.1
VPS8	Q8N3P4	286	-4	1	-4	11	1.0	1.0	1.0	1.1
AKAP17A	Q02040	95	1	-4	-4	11	1.0	1.0	1.0	1.1
CKB	P12277	283	-5	-11	-4	11	1.0	0.9	1.0	1.1
ARHGEF1	Q92888	595	0	-1	-4	10	1.0	1.0	1.0	1.1
STAT6	P42226	355	2	-6	-4	10	1.0	0.9	1.0	1.1
RNPEP	Q9H4A4	85	-11	-12	-4	10	0.9	0.9	1.0	1.1
SSC5D	A1L4H1	810	-11	-1	-4	10	0.9	1.0	1.0	1.1
SSC5D	A1L4H1	815	-11	-1	-4	10	0.9	1.0	1.0	1.1
TUBGCP3	Q96CW5	497	1	-3	-4	10	1.0	1.0	1.0	1.1
GPCPD1	Q9NPB8	83	-1	-7	-4	10	1.0	0.9	1.0	1.1
RPAP2	Q8IXW5	105	-2	-8	-4	10	1.0	0.9	1.0	1.1
OAS2	P29728	108	2	-8	-4	10	1.0	0.9	1.0	1.1
LTN1	O94822	981	-2	-9	-4	10	1.0	0.9	1.0	1.1
CPNE3	O75131	202	6	-14	-4	10	1.1	0.9	1.0	1.1
UBE3C	Q15386	1051	-3	3	-4	9	1.0	1.0	1.0	1.1
NCAPH	Q15003	239	-4	1	-4	9	1.0	1.0	1.0	1.1
PHF1	O43189	327	2	-1	-4	9	1.0	1.0	1.0	1.1
NOL9	Q5SY16	648	-2	-4	-4	9	1.0	1.0	1.0	1.1
CCT2	P78371	535	2	-5	-4	9	1.0	1.0	1.0	1.1
HEATR1	Q9H583	1138	11	-7	-4	9	1.1	0.9	1.0	1.1
NOXA1	Q86UR1	384	-11	8	-4	9	0.9	1.1	1.0	1.1
PLEC	Q15149	4454	0	-3	-4	9	1.0	1.0	1.0	1.1

CBR1	P16152	122	3	-3	-4	8	1.0	1.0	1.0	1.1
TAP2	Q03519	353	3	-7	-4	8	1.0	0.9	1.0	1.1
GABPA	Q06546	61	-17	-15	-4	8	0.9	0.9	1.0	1.1
FARS2	O95363	334	-6	-19	-4	8	0.9	0.8	1.0	1.1
GSTZ1	O43708	205	-5	2	-4	8	1.0	1.0	1.0	1.1
BRE	Q9NXR7	53	-1	2	-4	8	1.0	1.0	1.0	1.1
TPMT	P51580	216	-3	-4	-4	8	1.0	1.0	1.0	1.1
STAG2	Q8N3U4	176	-3	-8	-4	8	1.0	0.9	1.0	1.1
JAK1	P23458	944	-6	-16	-4	8	0.9	0.9	1.0	1.1
PRPF18	Q99633	336	5	1	-4	7	1.1	1.0	1.0	1.1
PRDM10	Q9NQV6	562	4	-7	-4	7	1.0	0.9	1.0	1.1
TRAFD1	O14545	109	0	-1	-4	7	1.0	1.0	1.0	1.1
SPTAN1	Q13813	1314	-6	-3	-4	7	0.9	1.0	1.0	1.1
MOV10	Q9HCE1	16	1	-4	-4	7	1.0	1.0	1.0	1.1
RARS	P54136	369	-1	-7	-4	7	1.0	0.9	1.0	1.1
REL	Q04864	304	-14	-20	-4	7	0.9	0.8	1.0	1.1
CTSC	P53634	136	-3	2	-4	6	1.0	1.0	1.0	1.1
USP7	Q93009	90	-2	-2	-4	6	1.0	1.0	1.0	1.1
PRPF6	O94906	913	2	-4	-4	6	1.0	1.0	1.0	1.1
RAD50	Q92878	48	-1	-4	-4	6	1.0	1.0	1.0	1.1
RAD1	O60671	148	-14	-7	-4	6	0.9	0.9	1.0	1.1
TRIP12	Q14669	332	-6	-10	-4	6	0.9	0.9	1.0	1.1
GLTP	Q9NZD2	36	-3	-14	-4	6	1.0	0.9	1.0	1.1
PML	P29590	140	-8	1	-4	6	0.9	1.0	1.0	1.1
ATP6V1C1	P21283	15	-9	-1	-4	6	0.9	1.0	1.0	1.1
CCT6A	P40227	282	6	-4	-4	6	1.1	1.0	1.0	1.1
VDAC1	P21796	232	4	-5	-4	6	1.0	1.0	1.0	1.1
CMAS	Q8NFW8	405	-4	-11	-4	6	1.0	0.9	1.0	1.1
SETX	Q7Z333	555	-10	-31	-4	6	0.9	0.8	1.0	1.1
RAI1	Q7Z5J4	40	-9	1	-4	5	0.9	1.0	1.0	1.1
TNFAIP2	Q03169	429	6	-4	-4	5	1.1	1.0	1.0	1.1
RAP1GAP2	Q684P5	228	1	-5	-4	5	1.0	1.0	1.0	1.1
TAOK1	Q7L7X3	730	2	-6	-4	5	1.0	0.9	1.0	1.1
TAOK3	Q9H2K8	727	2	-6	-4	5	1.0	0.9	1.0	1.1
RDH13	Q8NBN7	201	-4	-8	-4	5	1.0	0.9	1.0	1.1
RPL10	P27635	49	-4	-1	-4	5	1.0	1.0	1.0	1.0
SEC24B	O95487	555	-4	-9	-4	5	1.0	0.9	1.0	1.0
CUL9	Q8IWT3	512	-8	-12	-4	5	0.9	0.9	1.0	1.0
SPATA13	Q96N96	419	-4	8	-4	4	1.0	1.1	1.0	1.0
PDXDC1	Q6P996	491	1	-1	-4	4	1.0	1.0	1.0	1.0
LPXN	O60711	214	-5	-2	-4	4	1.0	1.0	1.0	1.0
CDKN2AIP	Q9NXV6	24	-13	7	-4	4	0.9	1.1	1.0	1.0
ZC3HAV1	Q7Z2W4	831	1	-2	-4	4	1.0	1.0	1.0	1.0
GBP3	Q9H0R5	405	-2	-2	-4	4	1.0	1.0	1.0	1.0
PDLIM2	Q96JY6	310	-2	-5	-4	4	1.0	1.0	1.0	1.0
AKT2	P31751	77	3	7	-4	3	1.0	1.1	1.0	1.0
ALDH16A1	Q8IZ83	59	-9	1	-4	3	0.9	1.0	1.0	1.0
EIF6	P56537	152	-17	-7	-4	3	0.9	0.9	1.0	1.0
IFI35	P80217	193	0	-8	-4	3	1.0	0.9	1.0	1.0
DSTN	P60981	147	1	-1	-4	3	1.0	1.0	1.0	1.0
NFKB1	P19838	261	-2	-8	-4	3	1.0	0.9	1.0	1.0
CHML	P26374	320	2	-8	-4	2	1.0	0.9	1.0	1.0
KIAA1429	Q69YN4	33	-10	-10	-4	2	0.9	0.9	1.0	1.0
CNOT10	Q9H9A5	327	0	-12	-4	2	1.0	0.9	1.0	1.0
PLEKHF2	Q9H8W4	186	-3	-5	-4	2	1.0	1.0	1.0	1.0

MAP4K1	Q92918	334	-1	-7	-4	2	1.0	0.9	1.0	1.0
EPRS	P07814	1448	2	-8	-4	2	1.0	0.9	1.0	1.0
DYNC1H1	Q14204	792	-2	11	-4	1	1.0	1.1	1.0	1.0
LRPPRC	P42704	571	-1	-3	-4	1	1.0	1.0	1.0	1.0
MBOAT7	Q96N66	280	-2	-4	-4	1	1.0	1.0	1.0	1.0
CREBBP	Q92793	409	-1	-5	-4	1	1.0	1.0	1.0	1.0
NBEAL2	Q6ZNJ1	245	-12	-30	-4	1	0.9	0.8	1.0	1.0
BAZ1A	Q9NRL2	799	5	-1	-4	1	1.0	1.0	1.0	1.0
ELMO1	Q92556	622	-5	-2	-4	1	1.0	1.0	1.0	1.0
Uncharacteriz	E9PLN8	43	1	-4	-4	1	1.0	1.0	1.0	1.0
ROCK1	Q13464	231	-12	-6	-4	1	0.9	0.9	1.0	1.0
FSD1L	Q9BXM9	250	-8	-7	-4	1	0.9	0.9	1.0	1.0
ZC3HAV1	Q7Z2W4	162	-8	-11	-4	1	0.9	0.9	1.0	1.0
MLLT4	P55196	1684	-9	-12	-4	1	0.9	0.9	1.0	1.0
ARID1B	Q8NFD5	1934	1	7	-4	0	1.0	1.1	1.0	1.0
WDR91	A4D1P6	418	10	6	-4	0	1.1	1.1	1.0	1.0
CCDC88C	Q9P219	1321	2	5	-4	0	1.0	1.1	1.0	1.0
SLFN5	Q08AF3	489	0	-5	-4	0	1.0	1.0	1.0	1.0
FAM175A	Q6UWZ7	281	-5	-5	-4	0	1.0	1.0	1.0	1.0
TPP2	P29144	28	1	-7	-4	0	1.0	0.9	1.0	1.0
NUDT2	P50583	123	3	5	-4	-1	1.0	1.0	1.0	1.0
LPXN	O60711	199	-2	-5	-4	-1	1.0	1.0	1.0	1.0
ZMYM2	Q9UBW7	494	0	12	-4	-1	1.0	1.1	1.0	1.0
PCCB	P05166	517	3	-2	-4	-1	1.0	1.0	1.0	1.0
PPP4R1	Q8TF05	663	6	-7	-4	-2	1.1	0.9	1.0	1.0
UBR2	Q8IWV8	1619	9	-9	-4	-2	1.1	0.9	1.0	1.0
PRKDC	P78527	1904	-9	-22	-4	-2	0.9	0.8	1.0	1.0
WDR5	P61964	205	-1	-8	-4	-3	1.0	0.9	1.0	1.0
RAD50	Q92878	990	1	-14	-4	-3	1.0	0.9	1.0	1.0
ZMYM3	Q14202	480	3	-2	-4	-3	1.0	1.0	1.0	1.0
SNRPN	P63162	19	-1	10	-4	-4	1.0	1.1	1.0	1.0
GMIP	Q9P107	537	-3	7	-4	-4	1.0	1.1	1.0	1.0
SH3BP1	Q9Y3L3	205	5	-1	-4	-4	1.1	1.0	1.0	1.0
CLTC	Q00610	824	-5	-9	-4	-4	1.0	0.9	1.0	1.0
ABCF2	Q9UG63	586	3	-9	-4	-4	1.0	0.9	1.0	1.0
C18orf8	Q96DM3	25	-5	-36	-4	-4	1.0	0.7	1.0	1.0
ZC3HAV1	Q7Z2W4	15	-3	-8	-4	-5	1.0	0.9	1.0	1.0
CDC42SE1	Q9NRR8	10	-10	-13	-4	-5	0.9	0.9	1.0	1.0
LANCL1	O43813	108	12	-2	-4	-5	1.1	1.0	1.0	1.0
CSTF3	Q12996	536	-8	-5	-4	-5	0.9	1.0	1.0	1.0
DNMT1	P26358	409	1	-7	-4	-5	1.0	0.9	1.0	1.0
CCT8	P50990	136	0	-7	-4	-5	1.0	0.9	1.0	1.0
GRK5	P34947	54	4	-7	-4	-5	1.0	0.9	1.0	1.0
INPP5K	Q9BT40	213	-4	-7	-4	-6	1.0	0.9	1.0	0.9
ACSL4	O60488	494	4	1	-4	-6	1.0	1.0	1.0	0.9
CHD3	Q12873	502	13	-3	-4	-6	1.1	1.0	1.0	0.9
GAPVD1	Q14C86	71	9	-3	-4	-6	1.1	1.0	1.0	0.9
PDHA1	P08559	218	-7	-9	-4	-6	0.9	0.9	1.0	0.9
RNF213	Q63HN8	3261	-6	-8	-4	-7	0.9	0.9	1.0	0.9
USP24	Q9UPU5	172	10	-3	-4	-7	1.1	1.0	1.0	0.9
GOLGB1	Q14789	2664	-2	-10	-4	-7	1.0	0.9	1.0	0.9
TRAF5	O00463	521	-20	-11	-4	-8	0.8	0.9	1.0	0.9
PARP11	Q9NR21	208	-17	-2	-4	-9	0.9	1.0	1.0	0.9
LDHB	P07195	36	-1	-23	-4	-9	1.0	0.8	1.0	0.9
RPRD2	Q5VT52	88	5	7	-4	-9	1.1	1.1	1.0	0.9

PPP1R3E	Q9H7J1	131	-10	2	-4	-9	0.9	1.0	1.0	0.9
UBE2O	Q9C0C9	375	3	0	-4	-9	1.0	1.0	1.0	0.9
SLC30A1	Q9Y6M5	437	-12	-15	-4	-9	0.9	0.9	1.0	0.9
MALT1	Q9UDY8	91	-1	-11	-4	-10	1.0	0.9	1.0	0.9
FYCO1	Q9BQS8	1110	-9	-14	-4	-10	0.9	0.9	1.0	0.9
RASA3	Q14644	678	1	-13	-4	-10	1.0	0.9	1.0	0.9
UPF2	Q9HAU5	929	-21	-29	-4	-11	0.8	0.8	1.0	0.9
HGS	O14964	212	-9	-10	-4	-11	0.9	0.9	1.0	0.9
EFTUD2	Q15029	144	0	4	-4	-13	1.0	1.0	1.0	0.9
LRCH1	Q9Y2L9	242	-4	-18	-4	-13	1.0	0.9	1.0	0.9
MYH9	P35579	896	-1	-6	-4	-14	1.0	0.9	1.0	0.9
NMNAT3	Q96T66	134	2	-2	-4	-14	1.0	1.0	1.0	0.9
DOCK8	Q8NF50	121	-8	-14	-4	-14	0.9	0.9	1.0	0.9
SLC25A24	Q6NUK1	330	-3	-6	-4	-16	1.0	0.9	1.0	0.9
PTPN12	Q05209	164	-5	-6	-4	-17	1.0	0.9	1.0	0.9
VPS13C	Q709C8	1613	15	-2	-4	-19	1.2	1.0	1.0	0.8
LRCH3	Q96II8	740	-4	-13	-4	-19	1.0	0.9	1.0	0.8
AP3M1	Q9Y2T2	29	3	-13	-4	-25	1.0	0.9	1.0	0.8
PEX7	O00628	34	-8	-12	-4	-26	0.9	0.9	1.0	0.8
NLRX1	Q86UT6	481	9	-10	-4	-29	1.1	0.9	1.0	0.8
CTCF	P49711	497	-4	-15	-4	-33	1.0	0.9	1.0	0.8
CLIC4	Q9Y696	100	-12	-14	-4	-34	0.9	0.9	1.0	0.7
INF2	Q27J81	898	8	-7	-4	-76	1.1	0.9	1.0	0.6
TLN1	Q9Y490	1087	1	2	-4	-93	1.0	1.0	1.0	0.5
TLN1	Q9Y490	1939	5	-5	-4	-102	1.1	1.0	1.0	0.5
RETSAT	Q6NUM9	547	-17	-11	-5	69	0.9	0.9	1.0	3.2
SETD1A	O15047	243	-3	11	-5	57	1.0	1.1	1.0	2.3
CADPS2	Q86UW7	1052	-9	-14	-5	52	0.9	0.9	1.0	2.1
FKBP8	Q14318	378	1	-12	-5	48	1.0	0.9	1.0	1.9
UBR4	Q5T4S7	3721	10	4	-5	39	1.1	1.0	1.0	1.6
ARHGEF7	Q14155	490	-11	-6	-5	36	0.9	0.9	1.0	1.6
ACAP1	Q15027	657	-1	-6	-5	36	1.0	0.9	1.0	1.6
CNTRL	Q7Z7A1	639	8	-5	-5	35	1.1	1.0	1.0	1.5
ALDH9A1	P49189	288	-3	2	-5	34	1.0	1.0	1.0	1.5
WDR33	Q9C0J8	996	-1	-5	-5	33	1.0	1.0	1.0	1.5
CDA	P32320	53	-3	1	-5	33	1.0	1.0	1.0	1.5
SENP5	Q96HI0	326	-1	-8	-5	33	1.0	0.9	1.0	1.5
LAS1L	Q9Y4W2	173	-2	7	-5	32	1.0	1.1	1.0	1.5
OSBPL1A	Q9BXW6	387	-3	-9	-5	31	1.0	0.9	1.0	1.4
TNS3	Q68CZ2	842	-6	-2	-5	31	0.9	1.0	1.0	1.4
PTPN6	P29350	480	3	4	-5	30	1.0	1.0	1.0	1.4
APBA2	Q99767	483	3	-5	-5	27	1.0	1.0	1.0	1.4
SMARCA2	P51531	91	-4	-9	-5	27	1.0	0.9	1.0	1.4
CPTP	Q5TA50	163	-4	4	-5	26	1.0	1.0	1.0	1.4
TRMT1L	Q7Z2T5	656	-6	3	-5	26	0.9	1.0	1.0	1.4
PSMC4	P43686	210	3	2	-5	26	1.0	1.0	1.0	1.4
ASXL1	Q8IXJ9	687	-10	-2	-5	26	0.9	1.0	1.0	1.4
USP48	Q86UV5	850	2	-6	-5	26	1.0	0.9	1.0	1.3
NR3C2	P08235	910	-22	9	-5	25	0.8	1.1	1.0	1.3
ACSL5	Q9ULC5	93	-2	-11	-5	25	1.0	0.9	1.0	1.3
PLAA	Q9Y263	26	-2	-5	-5	25	1.0	1.0	1.0	1.3
SPTLC1	O15269	192	0	-9	-5	25	1.0	0.9	1.0	1.3
UHRF2	Q96PU4	704	-10	-17	-5	25	0.9	0.9	1.0	1.3
RASGRP4	Q8TDF6	237	-4	-1	-5	24	1.0	1.0	1.0	1.3
HSPH1	Q92598	650	9	-1	-5	24	1.1	1.0	1.0	1.3

VPS11	Q9H270	568	3	-1	-5	24	1.0	1.0	1.0	1.3
GALK1	P51570	322	6	2	-5	23	1.1	1.0	1.0	1.3
CHRAC1	Q9NRG0	55	0	-4	-5	23	1.0	1.0	1.0	1.3
MTHFD1	P11586	147	0	-4	-5	23	1.0	1.0	1.0	1.3
IQSEC1	Q6DN90	359	-3	-11	-5	23	1.0	0.9	1.0	1.3
SIMC1	Q8NDZ2	430	-13	-17	-5	23	0.9	0.9	1.0	1.3
SLF2	Q8IX21	244	-5	-8	-5	22	1.0	0.9	1.0	1.3
BBX	Q8WY36	938	-2	-5	-5	21	1.0	1.0	1.0	1.3
ZNF483	Q8TF39	416	-5	-17	-5	21	1.0	0.9	1.0	1.3
ATP6V1B2	P21281	162	7	3	-5	21	1.1	1.0	1.0	1.3
DPH2	Q9BQC3	251	-2	-6	-5	21	1.0	0.9	1.0	1.3
NUP54	Q7Z3B4	180	6	-2	-5	20	1.1	1.0	1.0	1.2
PPP1R11	O60927	62	-3	-2	-5	20	1.0	1.0	1.0	1.2
SNAPIN	O95295	66	2	-2	-5	20	1.0	1.0	1.0	1.2
NUP85	Q9BW27	51	-4	-4	-5	20	1.0	1.0	1.0	1.2
INO80E	Q8NBZ0	179	-5	-5	-5	20	1.0	1.0	1.0	1.2
ALDH16A1	Q8IZ83	28	0	-5	-5	20	1.0	1.0	1.0	1.2
SLC30A1	Q9Y6M5	469	-2	-2	-5	19	1.0	1.0	1.0	1.2
GEMIN4	P57678	317	18	10	-5	19	1.2	1.1	1.0	1.2
STXBP3	O00186	49	6	-2	-5	19	1.1	1.0	1.0	1.2
GLUD1	P00367	112	-1	-15	-5	18	1.0	0.9	1.0	1.2
CXorf38	Q8TB03	12	3	-7	-5	18	1.0	0.9	1.0	1.2
HLA-C	P04222	188	2	-22	-5	18	1.0	0.8	1.0	1.2
HLA-B	P30464	188	2	-22	-5	18	1.0	0.8	1.0	1.2
MSH2	P43246	843	7	-2	-5	17	1.1	1.0	1.0	1.2
PSMC6	P62333	170	-2	-2	-5	17	1.0	1.0	1.0	1.2
GAPDHS	O14556	319	2	-9	-5	17	1.0	0.9	1.0	1.2
CORO7-PAM1	A0A0A6YYL4	34	5	-1	-5	17	1.0	1.0	1.0	1.2
ZFP62	Q8NB50	321	-13	-12	-5	17	0.9	0.9	1.0	1.2
PLCG1	P19174	881	-4	-12	-5	17	1.0	0.9	1.0	1.2
PDS5A	Q29RF7	327	-2	0	-5	16	1.0	1.0	1.0	1.2
PADI2	Q9Y2J8	167	3	-6	-5	16	1.0	0.9	1.0	1.2
UBR4	Q5T4S7	4487	9	-3	-5	16	1.1	1.0	1.0	1.2
NEURL4	Q96JN8	57	-3	-4	-5	16	1.0	1.0	1.0	1.2
TAP1	Q03518	795	1	-5	-5	16	1.0	1.0	1.0	1.2
POLR1A	O95602	1289	4	-7	-5	16	1.0	0.9	1.0	1.2
L3MBTL3	Q96JM7	233	-1	-8	-5	16	1.0	0.9	1.0	1.2
WDR46	O15213	172	6	1	-5	15	1.1	1.0	1.0	1.2
ANXA11	P50995	294	2	-5	-5	15	1.0	1.0	1.0	1.2
PIGH	Q14442	166	12	-6	-5	15	1.1	0.9	1.0	1.2
SETD2	Q9BYW2	1471	-28	-7	-5	15	0.8	0.9	1.0	1.2
PDLIM5	Q96HC4	213	-2	-8	-5	15	1.0	0.9	1.0	1.2
NCOA4	Q13772	244	2	-7	-5	15	1.0	0.9	1.0	1.2
LTA4H	P09960	26	-2	1	-5	14	1.0	1.0	1.0	1.2
TNPO3	Q9Y5L0	908	2	-2	-5	14	1.0	1.0	1.0	1.2
PPP2R2A	P63151	398	2	-3	-5	14	1.0	1.0	1.0	1.2
TNFAIP3	P21580	158	3	-4	-5	14	1.0	1.0	1.0	1.2
MTA1	Q13330	229	-2	-7	-5	14	1.0	0.9	1.0	1.2
MTA3	Q9BTC8	212	-2	-7	-5	14	1.0	0.9	1.0	1.2
COX20	Q5RI15	29	-2	-9	-5	14	1.0	0.9	1.0	1.2
ZAP70	P43403	249	15	2	-5	14	1.2	1.0	1.0	1.2
NEK7	Q8TDX7	53	0	-1	-5	14	1.0	1.0	1.0	1.2
ZZEF1	O43149	2084	7	-3	-5	14	1.1	1.0	1.0	1.2
TGFBR2	P37173	533	1	-2	-5	13	1.0	1.0	1.0	1.1
ITSN2	Q9NZM3	907	-3	-6	-5	13	1.0	0.9	1.0	1.1

SART3	Q15020	670	-5	1	-5	13	1.0	1.0	1.0	1.1
CCS	O14618	244	5	-1	-5	13	1.1	1.0	1.0	1.1
PAPOLA	P51003	36	-5	-5	-5	13	1.0	1.0	1.0	1.1
RNMT	O43148	206	2	-6	-5	13	1.0	0.9	1.0	1.1
MRPL39	Q9NYK5	335	0	-7	-5	13	1.0	0.9	1.0	1.1
RANBP2	P49792	1196	8	-3	-5	12	1.1	1.0	1.0	1.1
LOC10272415	A0A0B4J2E5	716	-6	-6	-5	12	0.9	0.9	1.0	1.1
TTC14	Q96N46	371	9	-1	-5	12	1.1	1.0	1.0	1.1
ACAA2	P42765	107	-1	-2	-5	12	1.0	1.0	1.0	1.1
SSRP1	Q08945	200	-9	-3	-5	12	0.9	1.0	1.0	1.1
RELA	Q04206	120	-6	-2	-5	11	0.9	1.0	1.0	1.1
EIF2S3	P41091	105	-4	-5	-5	11	1.0	1.0	1.0	1.1
STK39	Q9UEW8	525	-2	-2	-5	11	1.0	1.0	1.0	1.1
NADSYN1	Q6IA69	428	-6	-3	-5	11	0.9	1.0	1.0	1.1
TRIP12	Q14669	35	-6	-6	-5	11	0.9	0.9	1.0	1.1
HPRT1	P00492	106	-1	-8	-5	11	1.0	0.9	1.0	1.1
RRP7A	Q9Y3A4	8	-6	-1	-5	10	0.9	1.0	1.0	1.1
IFI16	Q16666	679	1	-3	-5	10	1.0	1.0	1.0	1.1
C5orf51	A6NDU8	179	-1	-4	-5	10	1.0	1.0	1.0	1.1
PIK3CD	O00329	474	-3	-6	-5	10	1.0	0.9	1.0	1.1
CCT4	P50991	252	5	-3	-5	10	1.0	1.0	1.0	1.1
ADO	Q96SZ5	18	-26	-5	-5	10	0.8	1.0	1.0	1.1
PABPC4	Q13310	132	1	-6	-5	10	1.0	0.9	1.0	1.1
UEVLD	Q8IX04	319	1	-14	-5	10	1.0	0.9	1.0	1.1
SYNE1	Q8NF91	2036	-2	-15	-5	10	1.0	0.9	1.0	1.1
MAT2B	Q9NZL9	297	-3	6	-5	9	1.0	1.1	1.0	1.1
RANBP2	P49792	1296	0	0	-5	9	1.0	1.0	1.0	1.1
SEC23IP	Q9Y6Y8	604	-4	-3	-5	9	1.0	1.0	1.0	1.1
GABPB1	Q06547	331	-2	-8	-5	9	1.0	0.9	1.0	1.1
OSBPL3	Q9H4L5	515	-5	3	-5	9	1.0	1.0	1.0	1.1
ANKRD10	Q9NXR5	73	-6	1	-5	9	0.9	1.0	1.0	1.1
DDX39A	O00148	164	1	-4	-5	9	1.0	1.0	1.0	1.1
FBXO42	Q6P3S6	403	-10	-9	-5	9	0.9	0.9	1.0	1.1
PTPN11	Q06124	104	-2	-3	-5	8	1.0	1.0	1.0	1.1
RAB7A	P51149	143	-2	-6	-5	8	1.0	0.9	1.0	1.1
NUMB	P49757	165	-7	-8	-5	8	0.9	0.9	1.0	1.1
SYNE2	Q8WXH0	5212	-1	-12	-5	8	1.0	0.9	1.0	1.1
SPTLC2	O15270	204	-11	-18	-5	8	0.9	0.9	1.0	1.1
PFKFB2	O60825	105	0	-2	-5	8	1.0	1.0	1.0	1.1
SMARCA4	P51532	423	3	-5	-5	8	1.0	1.0	1.0	1.1
POGZ	Q7Z3K3	986	-3	-5	-5	8	1.0	1.0	1.0	1.1
NAA50	Q9GZZ1	60	9	-6	-5	8	1.1	0.9	1.0	1.1
BAG1	Q99933	330	-3	-12	-5	8	1.0	0.9	1.0	1.1
RAVER1	Q8IY67	255	-5	-16	-5	8	1.0	0.9	1.0	1.1
AKAP9	Q99996	1966	-16	4	-5	7	0.9	1.0	1.0	1.1
SPTLC1	O15269	438	-5	-1	-5	7	1.0	1.0	1.0	1.1
TES	Q9UGI8	22	2	-2	-5	7	1.0	1.0	1.0	1.1
CSRP1	P21291	167	1	-2	-5	7	1.0	1.0	1.0	1.1
POLR3A	O14802	960	2	-4	-5	7	1.0	1.0	1.0	1.1
USP40	Q9NVE5	500	5	-6	-5	7	1.1	0.9	1.0	1.1
RCC2	Q9P258	209	2	-10	-5	7	1.0	0.9	1.0	1.1
IPCEF1	Q8WWN9	347	5	-11	-5	7	1.0	0.9	1.0	1.1
CCDC109B	Q9NWR8	147	9	-15	-5	7	1.1	0.9	1.0	1.1
JMJD4	Q9H9V9	375	16	-24	-5	7	1.2	0.8	1.0	1.1
EPRS	P07814	1377	-7	-3	-5	7	0.9	1.0	1.0	1.1

HK3	P52790	634	1	-4	-5	7	1.0	1.0	1.0	1.1
GNB1	P62873	271	6	-4	-5	7	1.1	1.0	1.0	1.1
CTBP1	Q13363	54	3	5	-5	6	1.0	1.0	1.0	1.1
LUC7L3	O95232	58	-4	-6	-5	6	1.0	0.9	1.0	1.1
EPC1	Q9H2F5	22	-14	-9	-5	6	0.9	0.9	1.0	1.1
RAB8B	Q92930	123	-3	4	-5	6	1.0	1.0	1.0	1.1
NBAS	A2RRP1	406	4	-1	-5	6	1.0	1.0	1.0	1.1
DNMT3A	Q9Y6K1	710	3	-1	-5	6	1.0	1.0	1.0	1.1
RANBP2	P49792	3122	2	-5	-5	6	1.0	1.0	1.0	1.1
ECI2	O75521	312	4	-10	-5	6	1.0	0.9	1.0	1.1
KMT2B	Q9UMN6	353	-3	-24	-5	6	1.0	0.8	1.0	1.1
PPP4R1	Q8TF05	78	6	3	-5	5	1.1	1.0	1.0	1.1
AAK1	Q2M2I8	270	10	2	-5	5	1.1	1.0	1.0	1.1
SNX6	Q9UNH7	149	4	0	-5	5	1.0	1.0	1.0	1.1
EEA1	Q15075	1134	-4	-6	-5	5	1.0	0.9	1.0	1.1
PAIP1	Q9H074	470	1	-9	-5	5	1.0	0.9	1.0	1.1
IPO4	Q8TEX9	726	14	9	-5	5	1.2	1.1	1.0	1.0
RRBP1	Q9P2E9	933	6	3	-5	5	1.1	1.0	1.0	1.0
PHKA2	P46019	681	0	2	-5	5	1.0	1.0	1.0	1.0
FNBP1	Q96RU3	130	0	-3	-5	5	1.0	1.0	1.0	1.0
FAM101B	Q8N5W9	186	-1	-5	-5	5	1.0	1.0	1.0	1.0
CBFB	Q13951	48	1	-6	-5	5	1.0	0.9	1.0	1.0
NUP214	P35658	298	-3	-7	-5	4	1.0	0.9	1.0	1.0
RANBP10	Q6VN20	243	2	-9	-5	4	1.0	0.9	1.0	1.0
C14orf93	Q9H972	186	-1	-10	-5	4	1.0	0.9	1.0	1.0
SSU72	Q9NP77	111	1	10	-5	4	1.0	1.1	1.0	1.0
PGLS	O95336	237	4	-2	-5	4	1.0	1.0	1.0	1.0
ZNF185	O15231	317	-3	-6	-5	4	1.0	0.9	1.0	1.0
HCK	P08631	234	4	-10	-5	4	1.0	0.9	1.0	1.0
NFATC2IP	Q8NCF5	232	2	-16	-5	4	1.0	0.9	1.0	1.0
ZNF501	Q96CX3	176	-8	-25	-5	4	0.9	0.8	1.0	1.0
MTCH2	Q9Y6C9	49	1	-14	-5	3	1.0	0.9	1.0	1.0
PHF1	O43189	139	4	0	-5	3	1.0	1.0	1.0	1.0
SPG21	Q9NZD8	204	-3	-4	-5	3	1.0	1.0	1.0	1.0
MCM6	Q14566	180	1	-6	-5	3	1.0	0.9	1.0	1.0
ILKAP	Q9H0C8	325	2	-6	-5	3	1.0	0.9	1.0	1.0
GMIP	Q9P107	336	2	-6	-5	3	1.0	0.9	1.0	1.0
CAPG	P40121	290	-8	-7	-5	3	0.9	0.9	1.0	1.0
GIGYF1	O75420	177	-17	-9	-5	3	0.9	0.9	1.0	1.0
SAMHD1	Q9Y3Z3	522	-10	-15	-5	3	0.9	0.9	1.0	1.0
MYD88	Q99836	233	6	2	-5	2	1.1	1.0	1.0	1.0
PDXDC1	Q6P996	490	7	-4	-5	2	1.1	1.0	1.0	1.0
MPRIP	Q6WCQ1	831	-11	-8	-5	2	0.9	0.9	1.0	1.0
TRIM25	Q14258	110	-2	-9	-5	2	1.0	0.9	1.0	1.0
NBEAL2	Q6ZNJ1	482	-9	-10	-5	2	0.9	0.9	1.0	1.0
PAFAH1B2	P68402	35	7	-11	-5	2	1.1	0.9	1.0	1.0
CLASP2	O75122	1184	-1	11	-5	2	1.0	1.1	1.0	1.0
CLASP1	Q7Z460	1428	-1	11	-5	2	1.0	1.1	1.0	1.0
CNOT1	A5YKK6	1541	-1	-7	-5	2	1.0	0.9	1.0	1.0
PPM1M	Q96MI6	7	-22	-8	-5	2	0.8	0.9	1.0	1.0
ADD1	P35611	430	-8	-10	-5	2	0.9	0.9	1.0	1.0
MFN1	Q8IWA4	428	-13	-11	-5	2	0.9	0.9	1.0	1.0
RNMT	O43148	225	-2	-16	-5	2	1.0	0.9	1.0	1.0
RNF2	Q99496	75	-4	2	-5	1	1.0	1.0	1.0	1.0
ELMO2	Q96JJ3	615	-5	-1	-5	1	1.0	1.0	1.0	1.0

FAM65A	Q6ZS17	925	5	-6	-5	1	1.1	0.9	1.0	1.0
USP48	Q86UV5	557	-8	-6	-5	1	0.9	0.9	1.0	1.0
SGPP1	Q9BX95	399	3	-6	-5	1	1.0	0.9	1.0	1.0
TFIP11	Q9UBB9	391	-12	-9	-5	1	0.9	0.9	1.0	1.0
CISD3	P0C7P0	109	2	-11	-5	1	1.0	0.9	1.0	1.0
MACF1	Q9UPN3	2387	-2	-2	-5	1	1.0	1.0	1.0	1.0
METTL13	Q8N6R0	391	5	-2	-5	1	1.1	1.0	1.0	1.0
RABGAP1	Q9Y3P9	433	6	-3	-5	1	1.1	1.0	1.0	1.0
RNF213	Q63HN8	2524	-11	-7	-5	1	0.9	0.9	1.0	1.0
SKIV2L2	P42285	297	2	-5	-5	0	1.0	1.0	1.0	1.0
KLF4	O43474	83	-19	-12	-5	0	0.8	0.9	1.0	1.0
ALPK1	Q96QP1	512	3	8	-5	-1	1.0	1.1	1.0	1.0
MTMR6	Q9Y217	214	7	-4	-5	-1	1.1	1.0	1.0	1.0
RAB3GAP1	Q15042	522	-3	-9	-5	-1	1.0	0.9	1.0	1.0
BRE	Q9NXR7	129	1	1	-5	-1	1.0	1.0	1.0	1.0
BUD13	Q9BRD0	348	2	-3	-5	-1	1.0	1.0	1.0	1.0
DGKA	P23743	645	-2	-5	-5	-1	1.0	1.0	1.0	1.0
EPB41	P11171	224	-9	-5	-5	-1	0.9	1.0	1.0	1.0
LMNB1	P20700	198	-8	-7	-5	-1	0.9	0.9	1.0	1.0
PNKP	Q96T60	353	1	-8	-5	-1	1.0	0.9	1.0	1.0
EPPK1	A0A087X1U6	2404	-23	-9	-5	-1	0.8	0.9	1.0	1.0
BDH1	Q02338	115	-5	-5	-5	-2	1.0	1.0	1.0	1.0
FAM208A	Q9UK61	1421	-3	2	-5	-2	1.0	1.0	1.0	1.0
RTTN	Q86VV8	35	-20	-14	-5	-2	0.8	0.9	1.0	1.0
SCFD1	Q8WVM8	485	-5	-16	-5	-2	1.0	0.9	1.0	1.0
ACSL5	Q9ULC5	69	-4	7	-5	-3	1.0	1.1	1.0	1.0
EIF3H	O15372	327	-2	4	-5	-3	1.0	1.0	1.0	1.0
LARP7	Q4G0J3	438	-12	3	-5	-3	0.9	1.0	1.0	1.0
CRELD1	Q96HD1	278	4	-8	-5	-3	1.0	0.9	1.0	1.0
ILF3	Q12906	203	-1	12	-5	-3	1.0	1.1	1.0	1.0
ALDH16A1	Q8IZ83	39	3	-5	-5	-3	1.0	1.0	1.0	1.0
NELFB	Q8WX92	265	3	3	-5	-4	1.0	1.0	1.0	1.0
SPG11	Q96JI7	1996	4	-4	-5	-4	1.0	1.0	1.0	1.0
AGTPBP1	Q9UPW5	971	-2	6	-5	-4	1.0	1.1	1.0	1.0
EHMT2	Q96KQ7	596	8	-16	-5	-4	1.1	0.9	1.0	1.0
NAP1L1	P55209	132	1	9	-5	-5	1.0	1.1	1.0	1.0
SUCLA2	Q9P2R7	158	-1	1	-5	-5	1.0	1.0	1.0	1.0
FGD3	Q5JSP0	563	4	-3	-5	-5	1.0	1.0	1.0	1.0
CBL	P22681	353	-8	-12	-5	-5	0.9	0.9	1.0	1.0
POLR2B	P30876	837	14	3	-5	-6	1.2	1.0	1.0	0.9
WDFY1	Q8IWB7	317	-5	-1	-5	-6	1.0	1.0	1.0	0.9
ISOC2	Q96AB3	84	-3	-1	-5	-6	1.0	1.0	1.0	0.9
C19orf25	Q9UFG5	87	-9	-5	-5	-6	0.9	1.0	1.0	0.9
COPB1	P53618	284	1	2	-5	-7	1.0	1.0	1.0	0.9
ACSS1	Q9NUB1	242	-4	-4	-5	-7	1.0	1.0	1.0	0.9
ZMYM4	Q5VZL5	531	0	-6	-5	-7	1.0	0.9	1.0	0.9
PLEK	P08567	59	3	-4	-5	-7	1.0	1.0	1.0	0.9
TARDBP	G3V162	244	-2	-16	-5	-7	1.0	0.9	1.0	0.9
RASA3	Q14644	662	-1	-7	-5	-8	1.0	0.9	1.0	0.9
AGPS	O00116	349	5	-4	-5	-8	1.0	1.0	1.0	0.9
DDX39B	Q13838	300	5	11	-5	-9	1.0	1.1	1.0	0.9
VPS54	Q9P1Q0	785	-21	-14	-5	-9	0.8	0.9	1.0	0.9
GTF2B	Q00403	15	9	2	-5	-11	1.1	1.0	1.0	0.9
FAM118B	Q9BPY3	93	-2	-1	-5	-11	1.0	1.0	1.0	0.9
TAGLN2	P37802	124	-8	-3	-5	-12	0.9	1.0	1.0	0.9

DBNL	Q9UJU6	97	-3	-24	-5	-12	1.0	0.8	1.0	0.9
PGGT1B	P53609	369	-7	-33	-5	-13	0.9	0.8	1.0	0.9
HMGCL	P35914	141	-6	1	-5	-14	0.9	1.0	1.0	0.9
TAGLN2	P37802	63	11	-16	-5	-14	1.1	0.9	1.0	0.9
MRPL10	Q7Z7H8	180	-7	1	-5	-14	0.9	1.0	1.0	0.9
FBXO30	Q8TB52	570	-8	-5	-5	-15	0.9	1.0	1.0	0.9
GTF2I	P78347	80	-1	-4	-5	-16	1.0	1.0	1.0	0.9
ANKRD44	Q8N8A2	408	6	13	-5	-18	1.1	1.1	1.0	0.8
CASP1	P29466	136	-7	-7	-5	-18	0.9	0.9	1.0	0.8
RAB29	O14966	127	-7	-1	-5	-20	0.9	1.0	1.0	0.8
PTPN6	P29350	382	-10	-12	-5	-22	0.9	0.9	1.0	0.8
MROH1	Q8NDA8	505	-3	-2	-5	-22	1.0	1.0	1.0	0.8
RIPK2	O43353	324	-14	-34	-5	-27	0.9	0.7	1.0	0.8
ROCK2	O75116	1257	4	-4	-5	-31	1.0	1.0	1.0	0.8
TRAP1	Q12931	261	2	-1	-5	-34	1.0	1.0	1.0	0.7
VCL	P18206	325	-28	-14	-5	-34	0.8	0.9	1.0	0.7
RASGRP2	Q7LDG7	536	5	4	-5	-36	1.1	1.0	1.0	0.7
POLR2B	P30876	958	-8	-17	-5	-47	0.9	0.9	1.0	0.7
TLN1	Q9Y490	236	3	-6	-5	-50	1.0	0.9	1.0	0.7
DCAF10	Q5QP82	102	1	-3	-5	-78	1.0	1.0	1.0	0.6
KIAA1211	Q6ZU35	860	-4	-19	-5	-171	1.0	0.8	1.0	0.4
TGFB1I1	O43294	416	0	4	-5	-179	1.0	1.0	1.0	0.4
PNPLA6	Q8IY17	482	-7	-1	-5	67	0.9	1.0	1.0	3.0
ZCCHC8	Q6NZY4	393	-9	-11	-5	55	0.9	0.9	1.0	2.2
DIP2A	Q14689	6	-8	-8	-5	47	0.9	0.9	1.0	1.9
VEZT	Q9HBM0	242	1	-8	-5	45	1.0	0.9	1.0	1.8
IGHG3	A0A087WVW2	158	-4	-6	-5	45	1.0	0.9	1.0	1.8
ZC3H4	Q9UPT8	1302	-3	-6	-5	42	1.0	0.9	1.0	1.7
ATF7IP	Q6VMQ6	608	-3	-6	-5	41	1.0	0.9	1.0	1.7
RNF25	Q96BH1	360	10	3	-5	35	1.1	1.0	1.0	1.5
CAMKK2	Q96RR4	223	-8	-8	-5	34	0.9	0.9	1.0	1.5
PLCG1	P19174	1109	-14	-24	-5	34	0.9	0.8	1.0	1.5
SHTN1	A0MZ66	442	1	-6	-5	32	1.0	0.9	1.0	1.5
RPS6KA2	Q15349	220	5	6	-5	31	1.0	1.1	1.0	1.4
CELF2	O95319	85	-9	-13	-5	31	0.9	0.9	1.0	1.4
CELF1	Q92879	61	-9	-13	-5	31	0.9	0.9	1.0	1.4
CUL2	Q13617	103	3	-18	-5	31	1.0	0.8	1.0	1.4
BAZ2A	Q9UIF9	726	-9	0	-5	29	0.9	1.0	1.0	1.4
HLA-A	P01892	363	3	-11	-5	29	1.0	0.9	1.0	1.4
HLA-A	Q09160	363	3	-11	-5	29	1.0	0.9	1.0	1.4
RPS3	P23396	97	0	3	-5	28	1.0	1.0	1.0	1.4
SPTAN1	Q13813	1622	1	-5	-5	28	1.0	1.0	1.0	1.4
NAA16	Q6N069	58	4	-6	-5	27	1.0	0.9	1.0	1.4
NAA15	Q9BXJ9	58	4	-6	-5	27	1.0	0.9	1.0	1.4
SCLY	Q96I15	22	4	-7	-5	27	1.0	0.9	1.0	1.4
NUP160	Q12769	65	15	-6	-5	25	1.2	0.9	1.0	1.3
ALDH18A1	P54886	88	4	-7	-5	25	1.0	0.9	1.0	1.3
IVNS1ABP	Q9Y6Y0	143	-4	1	-5	25	1.0	1.0	1.0	1.3
ZFYVE27	Q5T4F4	165	-2	-13	-5	25	1.0	0.9	1.0	1.3
THNSL1	Q8IYQ7	277	3	5	-5	24	1.0	1.0	1.0	1.3
LNPEP	Q9UIQ6	305	2	-4	-5	23	1.0	1.0	1.0	1.3
CDC42EP4	Q9H3Q1	313	-4	-7	-5	23	1.0	0.9	1.0	1.3
PDHA1	P08559	94	3	13	-5	23	1.0	1.1	1.0	1.3
ADAR	P55265	1224	-5	-1	-5	23	1.0	1.0	1.0	1.3
EIF5B	O60841	1092	-1	-4	-5	23	1.0	1.0	1.0	1.3

ORC3	Q9UBD5	561	10	-7	-5	22	1.1	0.9	1.0	1.3
MED16	Q9Y2X0	790	7	-8	-5	22	1.1	0.9	1.0	1.3
CARD11	Q9BXL7	427	5	-1	-5	21	1.0	1.0	1.0	1.3
SLK	Q9H2G2	358	1	-3	-5	21	1.0	1.0	1.0	1.3
WDR11	Q9BZH6	83	10	1	-5	20	1.1	1.0	1.0	1.3
OTULIN	Q96BN8	17	8	-1	-5	20	1.1	1.0	1.0	1.3
SYNE1	Q8NF91	3049	4	-3	-5	20	1.0	1.0	1.0	1.3
CX3CR1	P49238	221	-7	-28	-5	20	0.9	0.8	1.0	1.3
NKTR	P30414	393	-7	-9	-5	20	0.9	0.9	1.0	1.2
KHNYN	O15037	46	2	2	-5	19	1.0	1.0	1.0	1.2
SAC3D1	A6NKF1	84	-4	-9	-5	19	1.0	0.9	1.0	1.2
ZMYND8	Q9ULU4	567	-2	-10	-5	19	1.0	0.9	1.0	1.2
MCMBP	Q9BTE3	636	-5	-2	-5	18	1.0	1.0	1.0	1.2
ZNF668	Q96K58	126	-15	-14	-5	18	0.9	0.9	1.0	1.2
LAS1L	Q9Y4W2	169	-14	-16	-5	18	0.9	0.9	1.0	1.2
MIPEP	Q99797	142	3	5	-5	17	1.0	1.0	1.0	1.2
AGL	P35573	767	5	-5	-5	17	1.1	1.0	1.0	1.2
MAP4K1	Q92918	484	-1	-7	-5	17	1.0	0.9	1.0	1.2
MEMO1	Q9Y316	88	-15	-7	-5	17	0.9	0.9	1.0	1.2
UBR4	Q5T4S7	1080	7	4	-5	17	1.1	1.0	1.0	1.2
CHD1L	Q86WJ1	135	3	-10	-5	17	1.0	0.9	1.0	1.2
SCP2	P22307	94	1	-13	-5	17	1.0	0.9	1.0	1.2
TRIM21	P19474	463	-17	2	-5	16	0.9	1.0	1.0	1.2
MPP1	Q00013	179	-6	-7	-5	16	0.9	0.9	1.0	1.2
KLHL36	Q8N4N3	263	4	-7	-5	16	1.0	0.9	1.0	1.2
GCN1	Q92616	932	1	1	-5	16	1.0	1.0	1.0	1.2
PBRM1	Q86U86	951	-1	-5	-5	15	1.0	1.0	1.0	1.2
NAA30	Q147X3	195	-5	-7	-5	15	1.0	0.9	1.0	1.2
RNF34	Q969K3	65	-12	-10	-5	15	0.9	0.9	1.0	1.2
RNF34	Q969K3	286	-12	-4	-5	15	0.9	1.0	1.0	1.2
DPYD	Q12882	52	-1	-6	-5	15	1.0	0.9	1.0	1.2
PNKP	Q96T60	516	0	-8	-5	15	1.0	0.9	1.0	1.2
FNIP1	Q8TF40	744	2	-18	-5	15	1.0	0.9	1.0	1.2
PTPN2	P17706	330	-9	2	-5	14	0.9	1.0	1.0	1.2
TBC1D9B	Q66K14	444	-7	-1	-5	14	0.9	1.0	1.0	1.2
EIF2B3	Q9NR50	64	-3	-4	-5	14	1.0	1.0	1.0	1.2
SUZ12	Q15022	531	-7	-7	-5	14	0.9	0.9	1.0	1.2
DCK	P27707	45	-6	-9	-5	14	0.9	0.9	1.0	1.2
DNAJC14	Q6Y2X3	52	-8	-21	-5	14	0.9	0.8	1.0	1.2
MAML2	Q8IZL2	275	1	-5	-5	14	1.0	1.0	1.0	1.2
PARP1	P09874	845	-1	-7	-5	14	1.0	0.9	1.0	1.2
LAT2	Q9GZY6	142	-7	-9	-5	14	0.9	0.9	1.0	1.2
SUPT20H	Q8NEM7	298	3	-14	-5	14	1.0	0.9	1.0	1.2
AP3M1	Q9Y2T2	288	-1	-5	-5	13	1.0	1.0	1.0	1.1
GRSF1	Q12849	476	-1	-5	-5	13	1.0	1.0	1.0	1.1
SENP2	Q9HC62	158	1	-8	-5	13	1.0	0.9	1.0	1.1
SURF6	O75683	189	0	-13	-5	13	1.0	0.9	1.0	1.1
SUGT1	Q9Y2Z0	49	5	8	-5	13	1.0	1.1	1.0	1.1
RFC3	P40938	11	-5	5	-5	13	1.0	1.0	1.0	1.1
LSM7	Q9UK45	76	-11	3	-5	13	0.9	1.0	1.0	1.1
CUTC	Q9NTM9	248	-8	1	-5	13	0.9	1.0	1.0	1.1
CNOT2	Q9NZN8	175	-1	-3	-5	13	1.0	1.0	1.0	1.1
NUMA1	Q14980	1136	5	-4	-5	13	1.0	1.0	1.0	1.1
TRA2A	Q13595	120	4	-8	-5	13	1.0	0.9	1.0	1.1
HIBCH	Q6NVY1	45	3	-5	-5	12	1.0	1.0	1.0	1.1

TBC1D2	Q9BYX2	504	-23	11	-5	12	0.8	1.1	1.0	1.1
LTA4H	P09960	136	-5	1	-5	12	1.0	1.0	1.0	1.1
ALDH3A2	P51648	214	-10	-3	-5	12	0.9	1.0	1.0	1.1
EED	O75530	182	2	-4	-5	12	1.0	1.0	1.0	1.1
YARS	P54577	250	-3	-4	-5	12	1.0	1.0	1.0	1.1
PTGES3	Q15185	76	3	-4	-5	12	1.0	1.0	1.0	1.1
NDUFS8	O00217	121	6	-8	-5	12	1.1	0.9	1.0	1.1
TAF4	O00268	1022	7	-10	-5	12	1.1	0.9	1.0	1.1
CHAMP1	Q96JM3	194	3	-11	-5	12	1.0	0.9	1.0	1.1
SENP3-EIF4A1	A0A087X0R7	52	-14	-18	-5	12	0.9	0.8	1.0	1.1
EIF3CL	B5ME19	620	-6	-2	-5	11	0.9	1.0	1.0	1.1
AGO2	Q9UKV8	188	-5	-4	-5	11	1.0	1.0	1.0	1.1
CAMK2G	Q13555	273	3	-5	-5	11	1.0	1.0	1.0	1.1
HADHA	P40939	349	-2	-10	-5	11	1.0	0.9	1.0	1.1
MIER1	Q8N108	263	-3	5	-5	10	1.0	1.0	1.0	1.1
BOLA2B	Q9H3K6	31	-8	-5	-5	10	0.9	1.0	1.0	1.1
SIPA1L1	O43166	1037	-8	-7	-5	10	0.9	0.9	1.0	1.1
NUP205	Q92621	1662	-3	-7	-5	10	1.0	0.9	1.0	1.1
HMGB1	P09429	23	1	-9	-5	10	1.0	0.9	1.0	1.1
HMGB2	P26583	23	1	-9	-5	10	1.0	0.9	1.0	1.1
ALKBH3	Q96Q83	201	-5	-9	-5	10	1.0	0.9	1.0	1.1
MCM3	P25205	148	1	-18	-5	10	1.0	0.9	1.0	1.1
DDX20	Q9UHI6	98	-3	3	-5	9	1.0	1.0	1.0	1.1
TRAF3IP3	Q9Y228	35	-8	-4	-5	9	0.9	1.0	1.0	1.1
CDC42SE1	Q9NRR8	11	-7	-5	-5	9	0.9	1.0	1.0	1.1
PPP1R3D	O95685	41	5	-12	-5	9	1.0	0.9	1.0	1.1
UBR1	Q8IWV7	1603	2	-2	-5	9	1.0	1.0	1.0	1.1
CCDC22	O60826	441	-2	-2	-5	9	1.0	1.0	1.0	1.1
IAH1	Q2TAA2	145	2	-8	-5	9	1.0	0.9	1.0	1.1
FBXL3	Q9UKT7	78	-1	-9	-5	9	1.0	0.9	1.0	1.1
ADSS	P30520	58	-5	-14	-5	9	1.0	0.9	1.0	1.1
EEF1D	P29692	217	-14	-4	-5	8	0.9	1.0	1.0	1.1
UNK	Q9C0B0	308	-4	-5	-5	8	1.0	1.0	1.0	1.1
METTL3	Q86U44	375	0	-7	-5	8	1.0	0.9	1.0	1.1
NAMPT	P43490	397	6	-3	-5	8	1.1	1.0	1.0	1.1
GNL3L	Q9NVN8	278	-1	-4	-5	8	1.0	1.0	1.0	1.1
EFTUD2	Q15029	148	0	-9	-5	8	1.0	0.9	1.0	1.1
ABI3	Q9P2A4	137	3	-10	-5	8	1.0	0.9	1.0	1.1
LIME1	Q9H400	280	1	-10	-5	8	1.0	0.9	1.0	1.1
CCT2	P78371	289	-1	-2	-5	7	1.0	1.0	1.0	1.1
DOCK11	Q5JSL3	797	3	-4	-5	7	1.0	1.0	1.0	1.1
PELO	Q9BRX2	258	2	-5	-5	7	1.0	1.0	1.0	1.1
C20orf27	Q9GZN8	131	-3	-7	-5	7	1.0	0.9	1.0	1.1
PARP9	Q8IXQ6	82	-4	-11	-5	7	1.0	0.9	1.0	1.1
FAM65A	Q6ZS17	997	-1	1	-5	7	1.0	1.0	1.0	1.1
GLB1	P16278	393	5	-2	-5	7	1.1	1.0	1.0	1.1
PARP14	Q460N5	1274	-5	-5	-5	7	1.0	1.0	1.0	1.1
CSNK1G3	Q9Y6M4	51	2	-8	-5	7	1.0	0.9	1.0	1.1
DLG5	Q8TDM6	1207	-29	-1	-5	6	0.8	1.0	1.0	1.1
ANPEP	P15144	223	-15	-7	-5	6	0.9	0.9	1.0	1.1
SKIV2L	Q15477	1014	-2	-8	-5	6	1.0	0.9	1.0	1.1
ARMC8	Q8IUR7	661	1	-10	-5	6	1.0	0.9	1.0	1.1
OPTN	Q96CV9	334	2	9	-5	6	1.0	1.1	1.0	1.1
OAS2	P29728	381	1	6	-5	6	1.0	1.1	1.0	1.1
SRP68	Q9UHB9	173	-5	5	-5	6	1.0	1.0	1.0	1.1

HNRNPLL	Q8WVV9	505	-5	-2	-5	6	1.0	1.0	1.0	1.1
PDIA3	P30101	57	-8	-4	-5	6	0.9	1.0	1.0	1.1
EPN1	Q9Y6I3	96	7	-4	-5	6	1.1	1.0	1.0	1.1
OVCA2	Q8WZ82	64	-1	-6	-5	6	1.0	0.9	1.0	1.1
RAC1	P63000	178	2	-6	-5	6	1.0	0.9	1.0	1.1
ACADSB	P45954	261	-5	-8	-5	6	1.0	0.9	1.0	1.1
RNF31	Q96EP0	59	-6	-1	-5	5	0.9	1.0	1.0	1.1
ATP6V1B2	P21281	207	-5	-4	-5	5	1.0	1.0	1.0	1.1
GPD1L	Q8N335	216	10	-5	-5	5	1.1	1.0	1.0	1.1
TRMT10C	Q7L0Y3	78	-6	-8	-5	5	0.9	0.9	1.0	1.1
MTG1	Q9BT17	262	-5	-10	-5	5	1.0	0.9	1.0	1.1
TBCC	Q15814	184	-15	-11	-5	5	0.9	0.9	1.0	1.1
Uncharacteriz	F8W031	28	-9	-15	-5	5	0.9	0.9	1.0	1.1
UNC119B	A6NIH7	117	7	9	-5	5	1.1	1.1	1.0	1.0
PURB	Q96QR8	238	-3	-3	-5	5	1.0	1.0	1.0	1.0
ATG12	O94817	134	-1	-4	-5	5	1.0	1.0	1.0	1.0
TBCE	Q15813	371	1	-5	-5	5	1.0	1.0	1.0	1.0
RTN3	O95197	582	-2	-6	-5	5	1.0	0.9	1.0	1.0
RPL9P9	P32969	74	-5	-6	-5	5	1.0	0.9	1.0	1.0
CIAO1	O76071	234	2	-8	-5	5	1.0	0.9	1.0	1.0
MGA	Q8IWI9	2974	7	-9	-5	5	1.1	0.9	1.0	1.0
EVI5L	Q96CN4	342	11	5	-5	4	1.1	1.1	1.0	1.0
GSKIP	Q9P0R6	59	-8	3	-5	4	0.9	1.0	1.0	1.0
VPS13C	Q709C8	1372	-2	1	-5	4	1.0	1.0	1.0	1.0
ARRB1	P49407	150	5	-1	-5	4	1.0	1.0	1.0	1.0
GIMAP1-GIMA	A0A087WTJ2	442	-6	-7	-5	4	0.9	0.9	1.0	1.0
VAT1	Q99536	86	-6	-8	-5	4	0.9	0.9	1.0	1.0
AKAP13	Q12802	1232	3	-8	-5	4	1.0	0.9	1.0	1.0
GMPPA	Q96IJ6	389	-5	-9	-5	4	1.0	0.9	1.0	1.0
KDSR	Q06136	245	-1	-9	-5	4	1.0	0.9	1.0	1.0
NRDC	O43847	673	-4	-10	-5	4	1.0	0.9	1.0	1.0
PPCDC	Q96CD2	7	4	-12	-5	4	1.0	0.9	1.0	1.0
NOP56	O00567	52	-4	-13	-5	4	1.0	0.9	1.0	1.0
ACAA2	P42765	287	1	3	-5	4	1.0	1.0	1.0	1.0
SECISBP2L	Q93073	683	-1	-1	-5	4	1.0	1.0	1.0	1.0
PPFIBP2	Q8ND30	446	-2	-6	-5	4	1.0	0.9	1.0	1.0
SUGT1	Q9Y2Z0	88	6	-13	-5	4	1.1	0.9	1.0	1.0
RPL34	P49207	49	-10	-13	-5	4	0.9	0.9	1.0	1.0
RACK1	P63244	168	-7	-16	-5	4	0.9	0.9	1.0	1.0
TGM2	P21980	230	-1	-4	-5	3	1.0	1.0	1.0	1.0
EEF1G	P26641	194	-7	-7	-5	3	0.9	0.9	1.0	1.0
RANBP2	P49792	1424	6	-8	-5	3	1.1	0.9	1.0	1.0
PTPRC	P08575	1167	-7	-12	-5	3	0.9	0.9	1.0	1.0
CPT2	P23786	84	-8	-21	-5	3	0.9	0.8	1.0	1.0
FKBP15	Q5T1M5	809	-6	-1	-5	3	0.9	1.0	1.0	1.0
RAB2B	Q8WUD1	21	3	-1	-5	3	1.0	1.0	1.0	1.0
UBXN6	Q9BZV1	210	1	-1	-5	3	1.0	1.0	1.0	1.0
HNRNPUL2-BS	H3BQZ7	405	2	-5	-5	3	1.0	1.0	1.0	1.0
SAMD9	Q5K651	721	1	-17	-5	3	1.0	0.9	1.0	1.0
CLTC	Q00610	1257	8	-2	-5	2	1.1	1.0	1.0	1.0
RNH1	P13489	96	-8	-5	-5	2	0.9	1.0	1.0	1.0
FMNL1	O95466	300	-1	-14	-5	2	1.0	0.9	1.0	1.0
CAD	P27708	2161	-12	-4	-5	2	0.9	1.0	1.0	1.0
PIK3CD	O00329	90	-3	-5	-5	2	1.0	1.0	1.0	1.0
PDCD6IP	Q8WUM4	90	-3	-5	-5	2	1.0	1.0	1.0	1.0

RNF213	Q63HN8	1064	-9	-11	-5	2	0.9	0.9	1.0	1.0
TBC1D13	Q9NVG8	145	2	-14	-5	2	1.0	0.9	1.0	1.0
HOOK1	Q9UJC3	432	-11	1	-5	1	0.9	1.0	1.0	1.0
EIF3J	O75822	207	-5	-1	-5	1	1.0	1.0	1.0	1.0
GLRX3	O76003	229	-2	-4	-5	1	1.0	1.0	1.0	1.0
RNF215	Q9Y6U7	239	-8	-5	-5	1	0.9	1.0	1.0	1.0
SP140	Q13342	744	9	-1	-5	1	1.1	1.0	1.0	1.0
SP140L	Q9H930	457	9	-1	-5	1	1.1	1.0	1.0	1.0
OLA1	Q9NTK5	55	-2	-4	-5	0	1.0	1.0	1.0	1.0
ZFYVE26	Q68DK2	60	-4	-5	-5	0	1.0	1.0	1.0	1.0
ARID1B	Q8NFD5	2074	-3	-5	-5	0	1.0	1.0	1.0	1.0
VPS13C	Q709C8	2159	-7	-8	-5	0	0.9	0.9	1.0	1.0
NCKAP5L	Q9HCH0	57	-21	-9	-5	0	0.8	0.9	1.0	1.0
TRAPPC8	Q9Y2L5	1163	-8	-21	-5	0	0.9	0.8	1.0	1.0
UBE4A	Q14139	398	0	3	-5	-1	1.0	1.0	1.0	1.0
SLC27A4	Q6P1M0	232	4	-2	-5	-1	1.0	1.0	1.0	1.0
Uncharacteriz	E7EQ34	18	1	-9	-5	-1	1.0	0.9	1.0	1.0
NOL6	Q9H6R4	265	-5	-5	-5	-1	1.0	1.0	1.0	1.0
ACTR6	Q9GZN1	28	1	-8	-5	-2	1.0	0.9	1.0	1.0
GRPEL1	Q9HAV7	124	2	-14	-5	-2	1.0	0.9	1.0	1.0
TACC3	Q9Y6A5	828	-8	-5	-5	-2	0.9	1.0	1.0	1.0
CHID1	Q9BWS9	68	8	-15	-5	-2	1.1	0.9	1.0	1.0
C9orf142	Q9BUH6	80	2	-9	-5	-3	1.0	0.9	1.0	1.0
IMPDH2	P12268	468	-5	-19	-5	-3	1.0	0.8	1.0	1.0
PTPRC	P08575	851	8	-20	-5	-3	1.1	0.8	1.0	1.0
NONO	Q15233	208	-7	3	-5	-3	0.9	1.0	1.0	1.0
VAPA	Q9P0L0	60	1	-4	-5	-3	1.0	1.0	1.0	1.0
KIAA1429	Q69YN4	1170	-5	-23	-5	-4	1.0	0.8	1.0	1.0
THOC1	Q96FV9	156	-15	-26	-5	-4	0.9	0.8	1.0	1.0
TRIM32	Q13049	464	-1	-7	-5	-4	1.0	0.9	1.0	1.0
GTF3C1	Q12789	1008	-1	-7	-5	-4	1.0	0.9	1.0	1.0
COPG1	Q9Y678	813	-10	-10	-5	-4	0.9	0.9	1.0	1.0
MED16	Q9Y2X0	801	-5	-3	-5	-5	1.0	1.0	1.0	1.0
SLC15A4	Q8N697	299	6	-6	-5	-5	1.1	0.9	1.0	1.0
SPECC1	Q5M775	355	-17	-8	-5	-5	0.9	0.9	1.0	1.0
NUMA1	Q14980	299	-3	-5	-5	-5	1.0	1.0	1.0	1.0
NUP155	O75694	974	-2	-5	-5	-5	1.0	1.0	1.0	1.0
RPS3	P23396	119	-8	-7	-5	-5	0.9	0.9	1.0	1.0
INTS1	Q8N201	1833	-2	-20	-5	-5	1.0	0.8	1.0	1.0
VARS	P26640	444	0	2	-5	-6	1.0	1.0	1.0	0.9
ATG2B	Q96BY7	891	-20	-11	-5	-6	0.8	0.9	1.0	0.9
KTI12	Q96EK9	93	-3	-1	-5	-6	1.0	1.0	1.0	0.9
DHRS4	Q9BTZ2	209	-5	-6	-5	-6	1.0	0.9	1.0	0.9
HPS4	Q9NQG7	535	-1	-6	-5	-7	1.0	0.9	1.0	0.9
EIF1B	O60739	69	-4	-6	-5	-7	1.0	0.9	1.0	0.9
EIF1	P41567	69	-4	-6	-5	-7	1.0	0.9	1.0	0.9
ORC5	O43913	237	10	-7	-5	-7	1.1	0.9	1.0	0.9
TEX2	Q8IWB9	1114	-8	-7	-5	-7	0.9	0.9	1.0	0.9
HUWE1	Q7Z6Z7	19	1	-7	-5	-7	1.0	0.9	1.0	0.9
VPS18	Q9P253	741	-4	-6	-5	-7	1.0	0.9	1.0	0.9
GOLGA4	Q13439	1729	-29	-14	-5	-8	0.8	0.9	1.0	0.9
TNIP1	Q15025	171	7	-3	-5	-8	1.1	1.0	1.0	0.9
AKAP9	Q99996	2137	-8	-13	-5	-8	0.9	0.9	1.0	0.9
PTK2B	Q14289	310	-9	3	-5	-9	0.9	1.0	1.0	0.9
ATP6V1A	P38606	254	2	-2	-5	-9	1.0	1.0	1.0	0.9

PRMT9	Q6P2P2	747	5	-6	-5	-9	1.0	0.9	1.0	0.9
DENND3	A2RUS2	101	-4	-7	-5	-9	1.0	0.9	1.0	0.9
RRBP1	Q9P2E9	1128	-3	-4	-5	-9	1.0	1.0	1.0	0.9
INPP4A	Q96PE3	540	-3	-10	-5	-10	1.0	0.9	1.0	0.9
NEU1	Q99519	287	2	2	-5	-10	1.0	1.0	1.0	0.9
EML4	Q9HC35	314	-5	-5	-5	-11	1.0	1.0	1.0	0.9
SLMAP	Q14BN4	406	-3	-16	-5	-11	1.0	0.9	1.0	0.9
TOP2B	Q02880	125	6	-1	-5	-11	1.1	1.0	1.0	0.9
VPS35	Q96QK1	653	-7	-7	-5	-11	0.9	0.9	1.0	0.9
LRMP	Q12912	436	-7	-14	-5	-13	0.9	0.9	1.0	0.9
RASAL3	Q86YV0	447	2	-6	-5	-13	1.0	0.9	1.0	0.9
DNM1L	O00429	361	2	-1	-5	-14	1.0	1.0	1.0	0.9
WDR33	Q9C0J8	249	1	2	-5	-17	1.0	1.0	1.0	0.9
H2AFY	O75367	276	3	-9	-5	-18	1.0	0.9	1.0	0.9
DNMBP	Q6XZF7	1134	5	-5	-5	-19	1.0	1.0	1.0	0.8
ANKRD44	Q8N8A2	420	-9	-7	-5	-20	0.9	0.9	1.0	0.8
DYNC1H1	Q14204	1999	-5	-1	-5	-21	1.0	1.0	1.0	0.8
RABEP2	Q9H5N1	372	-7	-13	-5	-23	0.9	0.9	1.0	0.8
PREX1	Q8TCU6	234	-4	-11	-5	-24	1.0	0.9	1.0	0.8
DLAT	P10515	586	-4	2	-5	-26	1.0	1.0	1.0	0.8
NUDCD1	Q96RS6	402	3	-26	-5	-28	1.0	0.8	1.0	0.8
PHF5A	Q7RTV0	33	-14	-12	-5	-29	0.9	0.9	1.0	0.8
MYBBP1A	Q9BQG0	890	-9	-11	-5	-35	0.9	0.9	1.0	0.7
ADRM1	Q16186	121	15	-1	-5	-36	1.2	1.0	1.0	0.7
PSTPIP2	Q9H939	221	3	-3	-5	-43	1.0	1.0	1.0	0.7
ACTN1	P12814	370	14	-8	-5	-56	1.2	0.9	1.0	0.6
CSRP1	P21291	37	5	-3	-5	-64	1.0	1.0	1.0	0.6
LIMS1	P48059	275	2	-15	-5	-88	1.0	0.9	1.0	0.5
COX11	Q9Y6N1	219	3	2	-5	-113	1.0	1.0	1.0	0.5
KMO	O15229	468	-15	-6	-6	42	0.9	0.9	0.9	1.7
RNF130	Q86XS8	320	6	-16	-6	41	1.1	0.9	0.9	1.7
TPP2	P29144	967	-7	-7	-6	39	0.9	0.9	0.9	1.6
XPC	Q01831	377	0	-8	-6	33	1.0	0.9	0.9	1.5
EPHX2	P34913	141	4	-13	-6	33	1.0	0.9	0.9	1.5
RNF213	Q63HN8	614	-8	-6	-6	31	0.9	0.9	0.9	1.4
PAPSS1	O43252	53	-4	-9	-6	31	1.0	0.9	0.9	1.4
PAPSS2	O95340	43	-4	-9	-6	31	1.0	0.9	0.9	1.4
NSF	P46459	264	1	-3	-6	31	1.0	1.0	0.9	1.4
PMS2	P54278	591	-3	-9	-6	29	1.0	0.9	0.9	1.4
ACAP2	Q15057	691	-3	-9	-6	29	1.0	0.9	0.9	1.4
RPP40	O75818	219	-9	-7	-6	28	0.9	0.9	0.9	1.4
ARAF	P10398	59	-9	-9	-6	28	0.9	0.9	0.9	1.4
EIF2B4	Q9UI10	69	-6	-3	-6	28	0.9	1.0	0.9	1.4
ALDH9A1	P49189	289	-4	-8	-6	28	1.0	0.9	0.9	1.4
ITK	Q08881	289	-4	-9	-6	28	1.0	0.9	0.9	1.4
PSMD9	O00233	59	-3	-2	-6	27	1.0	1.0	0.9	1.4
TRIM21	P19474	54	-8	-9	-6	27	0.9	0.9	0.9	1.4
ADCY7	P51828	904	-9	-11	-6	27	0.9	0.9	0.9	1.4
HIVEP2	P31629	2329	-7	-4	-6	25	0.9	1.0	0.9	1.3
WDR6	Q9NNW5	688	-4	-7	-6	25	1.0	0.9	0.9	1.3
TSSC4	Q9Y5U2	99	-1	-15	-6	25	1.0	0.9	0.9	1.3
STAT2	P52630	529	-6	0	-6	24	0.9	1.0	0.9	1.3
SEMA4A	Q9H3S1	719	-4	-14	-6	24	1.0	0.9	0.9	1.3
NFAM1	Q8NET5	233	-6	-14	-6	23	0.9	0.9	0.9	1.3
CTSH	P09668	102	-13	-17	-6	23	0.9	0.9	0.9	1.3

SGK3	Q96BR1	322	2	5	-6	23	1.0	1.1	0.9	1.3
KANSL1	A0A0G2JNT7	539	-4	-4	-6	23	1.0	1.0	0.9	1.3
NADK2	Q4G0N4	58	-3	-5	-6	23	1.0	1.0	0.9	1.3
HNRNPA3	P51991	94	-1	-6	-6	22	1.0	0.9	0.9	1.3
MBD2	Q9UBB5	359	-4	6	-6	21	1.0	1.1	0.9	1.3
CPSF4	O95639	55	-1	-1	-6	21	1.0	1.0	0.9	1.3
USP48	Q86UV5	39	-5	-7	-6	21	1.0	0.9	0.9	1.3
GTF2H1	P32780	506	-1	-8	-6	21	1.0	0.9	0.9	1.3
VAMP5	O95183	9	-5	9	-6	20	1.0	1.1	0.9	1.3
RRP12	Q5JTH9	102	-2	0	-6	20	1.0	1.0	0.9	1.3
ANKRD17	O75179	644	-3	-11	-6	20	1.0	0.9	0.9	1.3
SYNE2	Q8WXH0	5348	-6	-14	-6	20	0.9	0.9	0.9	1.3
RPL23	P62829	125	-9	-19	-6	20	0.9	0.8	0.9	1.3
SRSF3	P84103	6	3	-5	-6	20	1.0	1.0	0.9	1.2
GCN1	Q92616	939	-1	-2	-6	19	1.0	1.0	0.9	1.2
KMT2D	O14686	58	1	-2	-6	19	1.0	1.0	0.9	1.2
DDX27	Q96GQ7	259	5	-3	-6	19	1.1	1.0	0.9	1.2
SYNE1	Q8NF91	6460	-2	8	-6	19	1.0	1.1	0.9	1.2
CEP128	Q6ZU80	915	-3	-7	-6	19	1.0	0.9	0.9	1.2
HNRNPM	P52272	694	-4	-7	-6	18	1.0	0.9	0.9	1.2
NOL6	Q9H6R4	486	-3	-12	-6	18	1.0	0.9	0.9	1.2
MBNL1	Q9NR56	27	-4	-14	-6	18	1.0	0.9	0.9	1.2
CLUH	O75153	200	-2	-16	-6	18	1.0	0.9	0.9	1.2
DNAAF5	Q86Y56	173	-12	9	-6	17	0.9	1.1	0.9	1.2
NUP205	Q92621	1297	4	-1	-6	17	1.0	1.0	0.9	1.2
NECAP1	Q8NC96	162	-7	-3	-6	17	0.9	1.0	0.9	1.2
ERO1A	Q96HE7	208	-2	-7	-6	17	1.0	0.9	0.9	1.2
PTPN11	Q06124	567	12	8	-6	17	1.1	1.1	0.9	1.2
ACAP1	Q15027	501	6	2	-6	17	1.1	1.0	0.9	1.2
SERPINB9	P50453	259	2	-1	-6	17	1.0	1.0	0.9	1.2
NFKB1	P19838	925	0	-9	-6	17	1.0	0.9	0.9	1.2
MAPKAPK2	P49137	98	0	-1	-6	16	1.0	1.0	0.9	1.2
SMCHD1	A6NHR9	59	1	-4	-6	16	1.0	1.0	0.9	1.2
NAT10	Q9H0A0	658	0	-10	-6	16	1.0	0.9	0.9	1.2
GNAI3	P08754	351	-6	4	-6	15	0.9	1.0	0.9	1.2
LCP1	P13796	618	6	-3	-6	15	1.1	1.0	0.9	1.2
TMA16	Q96EY4	162	0	-5	-6	15	1.0	1.0	0.9	1.2
RBM26	Q5T8P6	25	-3	-14	-6	15	1.0	0.9	0.9	1.2
CLIC3	O95833	187	8	6	-6	14	1.1	1.1	0.9	1.2
TAF6	P49848	130	-2	-3	-6	14	1.0	1.0	0.9	1.2
MVD	P53602	160	-2	-7	-6	14	1.0	0.9	0.9	1.2
HCFC1	P51610	1895	-4	0	-6	14	1.0	1.0	0.9	1.2
ZNF638	Q14966	747	-2	-2	-6	14	1.0	1.0	0.9	1.2
CRBN	Q96SW2	287	0	-7	-6	13	1.0	0.9	0.9	1.1
MSH6	P52701	88	-1	-2	-6	13	1.0	1.0	0.9	1.1
DDX60L	Q5H9U9	1031	5	-6	-6	13	1.1	0.9	0.9	1.1
RPAP1	Q9BWH6	185	9	-10	-6	13	1.1	0.9	0.9	1.1
ZCCHC6	Q5VYS8	690	-4	5	-6	12	1.0	1.0	0.9	1.1
INO80B-WBP1	J3KQ70	334	-5	4	-6	12	1.0	1.0	0.9	1.1
TRMT1L	Q7Z2T5	132	-3	2	-6	12	1.0	1.0	0.9	1.1
POLDIP3	Q9BY77	338	-6	2	-6	12	0.9	1.0	0.9	1.1
GLE1	Q53GS7	528	-1	1	-6	12	1.0	1.0	0.9	1.1
ATF7IP	Q6VMQ6	955	4	1	-6	12	1.0	1.0	0.9	1.1
CES1	P23141	390	-7	-3	-6	12	0.9	1.0	0.9	1.1
ZMYM3	Q14202	454	4	-4	-6	12	1.0	1.0	0.9	1.1

HAT1	O14929	168	-2	-4	-6	12	1.0	1.0	0.9	1.1
COIL	P38432	425	11	-9	-6	12	1.1	0.9	0.9	1.1
CUL3	Q13618	298	-6	-7	-6	12	0.9	0.9	0.9	1.1
MACF1	Q9UPN3	4325	-12	-17	-6	12	0.9	0.9	0.9	1.1
MTMR6	Q9Y217	569	8	2	-6	11	1.1	1.0	0.9	1.1
SKIV2L2	P42285	110	-5	-10	-6	11	1.0	0.9	0.9	1.1
BAZ1B	Q9UIG0	497	2	1	-6	11	1.0	1.0	0.9	1.1
AHCYL1	O43865	272	2	-1	-6	11	1.0	1.0	0.9	1.1
POLA1	P09884	1403	-5	-4	-6	11	1.0	1.0	0.9	1.1
SNX6	Q9UNH7	348	2	-4	-6	11	1.0	1.0	0.9	1.1
VAV1	P15498	794	-3	-4	-6	11	1.0	1.0	0.9	1.1
CCDC25	Q86WR0	83	3	-4	-6	11	1.0	1.0	0.9	1.1
VAV3	Q9UKW4	800	-3	-4	-6	11	1.0	1.0	0.9	1.1
KDM3B	Q7LBC6	1470	1	-5	-6	11	1.0	1.0	0.9	1.1
SRRT	Q9BXP5	490	-9	-10	-6	11	0.9	0.9	0.9	1.1
SBF2	Q86WG5	1540	-3	-12	-6	11	1.0	0.9	0.9	1.1
ABCE1	P61221	88	1	1	-6	10	1.0	1.0	0.9	1.1
HERC1	Q15751	1941	-8	-1	-6	10	0.9	1.0	0.9	1.1
HADHB	P55084	458	-3	-4	-6	10	1.0	1.0	0.9	1.1
DCTPP1	Q9H773	162	-9	-4	-6	10	0.9	1.0	0.9	1.1
TCEA1	P23193	263	-6	-8	-6	10	0.9	0.9	0.9	1.1
TCEA2	Q15560	261	-6	-8	-6	10	0.9	0.9	0.9	1.1
SLC38A7	Q9NVC3	30	3	-8	-6	10	1.0	0.9	0.9	1.1
GLS	O94925	283	0	-14	-6	10	1.0	0.9	0.9	1.1
DNAJC13	O75165	12	6	-1	-6	10	1.1	1.0	0.9	1.1
DNAJC30	Q96LL9	73	3	-4	-6	10	1.0	1.0	0.9	1.1
FBXO30	Q8TB52	592	-3	-6	-6	10	1.0	0.9	0.9	1.1
GATAD2B	Q8WXI9	409	-4	-6	-6	10	1.0	0.9	0.9	1.1
DDX5	P17844	234	-5	-7	-6	10	1.0	0.9	0.9	1.1
RBM28	Q9NW13	71	3	-8	-6	10	1.0	0.9	0.9	1.1
STK26	Q9P289	410	1	4	-6	9	1.0	1.0	0.9	1.1
EXOSC3	Q9NQT5	184	5	2	-6	9	1.0	1.0	0.9	1.1
MACF1	Q9UPN3	222	-2	2	-6	9	1.0	1.0	0.9	1.1
RAB34	Q9BZG1	116	-9	1	-6	9	0.9	1.0	0.9	1.1
ANAPC7	Q9UJX3	259	-6	-4	-6	9	0.9	1.0	0.9	1.1
SNRNP200	O75643	428	-10	-10	-6	9	0.9	0.9	0.9	1.1
GART	P22102	134	-3	-11	-6	9	1.0	0.9	0.9	1.1
CLTC	Q00610	926	-3	-1	-6	9	1.0	1.0	0.9	1.1
EXOSC4	Q9NPD3	97	-7	-3	-6	9	0.9	1.0	0.9	1.1
C7orf50	Q9BRJ6	107	-3	-5	-6	9	1.0	1.0	0.9	1.1
APAF1	O14727	450	-4	-6	-6	9	1.0	0.9	0.9	1.1
ZBTB40	Q9NUA8	234	-3	-10	-6	9	1.0	0.9	0.9	1.1
EP400	Q96L91	2013	-4	6	-6	8	1.0	1.1	0.9	1.1
PSMD11	O00231	289	-3	3	-6	8	1.0	1.0	0.9	1.1
DDB2	Q92466	364	0	-9	-6	8	1.0	0.9	0.9	1.1
CXXC1	Q9P0U4	380	-6	-10	-6	8	0.9	0.9	0.9	1.1
NADSYN1	Q6IA69	309	6	-13	-6	8	1.1	0.9	0.9	1.1
KDM3B	Q7LBC6	1212	-3	-20	-6	8	1.0	0.8	0.9	1.1
STK39	Q9UEW8	99	6	10	-6	8	1.1	1.1	0.9	1.1
NBAS	A2RRP1	1255	4	-1	-6	8	1.0	1.0	0.9	1.1
ERCC5	P28715	326	-13	-2	-6	8	0.9	1.0	0.9	1.1
INTS3	Q68E01	843	-2	-2	-6	8	1.0	1.0	0.9	1.1
PLEC	Q15149	3008	2	-3	-6	8	1.0	1.0	0.9	1.1
DDX56	Q9NY93	518	-6	-4	-6	8	0.9	1.0	0.9	1.1
NXF1	Q9UBU9	252	-1	-5	-6	8	1.0	1.0	0.9	1.1

G6PD	P11413	232	-2	-6	-6	8	1.0	0.9	0.9	1.1
NIT2	Q9NQR4	153	-2	-7	-6	8	1.0	0.9	0.9	1.1
CUL5	Q93034	112	-1	-9	-6	8	1.0	0.9	0.9	1.1
MAD1L1	Q9Y6D9	201	-8	-1	-6	7	0.9	1.0	0.9	1.1
RPS2	P15880	182	2	-4	-6	7	1.0	1.0	0.9	1.1
MTMR1	Q13613	170	8	-5	-6	7	1.1	1.0	0.9	1.1
ANAPC1	Q9H1A4	1630	-5	7	-6	7	1.0	1.1	0.9	1.1
GTF2F2	P13984	116	4	6	-6	7	1.0	1.1	0.9	1.1
TBCD	Q9BTW9	1192	-9	1	-6	7	0.9	1.0	0.9	1.1
XAB2	Q9HCS7	98	3	1	-6	7	1.0	1.0	0.9	1.1
ZAP70	P43403	222	6	-8	-6	7	1.1	0.9	0.9	1.1
EIF1	P41567	94	8	-15	-6	7	1.1	0.9	0.9	1.1
NUP205	Q92621	698	-2	3	-6	6	1.0	1.0	0.9	1.1
CBR1	P16152	227	4	0	-6	6	1.0	1.0	0.9	1.1
ARHGAP27	Q6ZUM4	121	-2	-2	-6	6	1.0	1.0	0.9	1.1
MCM7	P33993	482	2	-4	-6	6	1.0	1.0	0.9	1.1
RPL18A	Q02543	109	-6	-4	-6	6	0.9	1.0	0.9	1.1
GNB1	P62873	204	-5	-7	-6	6	1.0	0.9	0.9	1.1
LRRK2	Q5S007	1770	-7	-10	-6	6	0.9	0.9	0.9	1.1
GRAP	Q13588	161	-11	-11	-6	6	0.9	0.9	0.9	1.1
LIMK1	P53667	208	-3	-13	-6	6	1.0	0.9	0.9	1.1
SBDS	Q9Y3A5	31	4	-16	-6	6	1.0	0.9	0.9	1.1
HSP90AB1	P08238	366	-1	-2	-6	6	1.0	1.0	0.9	1.1
PRG2	P13727	147	-6	-3	-6	6	0.9	1.0	0.9	1.1
URB1	O60287	1900	-6	-8	-6	6	0.9	0.9	0.9	1.1
OSBPL8	Q9BZF1	209	5	-11	-6	6	1.0	0.9	0.9	1.1
OSBPL5	Q9H0X9	187	5	-11	-6	6	1.0	0.9	0.9	1.1
EIF4G1	Q04637	662	-13	-12	-6	6	0.9	0.9	0.9	1.1
TBC1D13	Q9NVG8	36	-11	-13	-6	6	0.9	0.9	0.9	1.1
DYNC1H1	Q14204	867	4	4	-6	5	1.0	1.0	0.9	1.1
NCAPD3	P42695	867	-3	-3	-6	5	1.0	1.0	0.9	1.1
CCT3	P49368	366	-1	-3	-6	5	1.0	1.0	0.9	1.1
GOLGB1	Q14789	3070	-5	-4	-6	5	1.0	1.0	0.9	1.1
PUS7	Q96PZ0	443	-10	-4	-6	5	0.9	1.0	0.9	1.1
SGTA	O43765	153	-2	-8	-6	5	1.0	0.9	0.9	1.1
IPO9	Q96P70	90	-1	-9	-6	5	1.0	0.9	0.9	1.1
BAG1	Q99933	213	-5	-9	-6	5	1.0	0.9	0.9	1.1
GTF3C1	Q12789	544	-11	-12	-6	5	0.9	0.9	0.9	1.1
DARS2	Q6PI48	108	0	5	-6	5	1.0	1.1	0.9	1.0
STIP1	P31948	370	-9	4	-6	5	0.9	1.0	0.9	1.0
ATG2B	Q96BY7	1597	-6	-1	-6	5	0.9	1.0	0.9	1.0
SHMT2	P34897	412	-1	-14	-6	5	1.0	0.9	0.9	1.0
RBBP9	O75884	163	-4	0	-6	4	1.0	1.0	0.9	1.0
ADK	P55263	353	-4	-2	-6	4	1.0	1.0	0.9	1.0
SYNE2	Q8WXH0	3096	8	-3	-6	4	1.1	1.0	0.9	1.0
METAP1	P53582	40	-2	-5	-6	4	1.0	1.0	0.9	1.0
GSTO1	P78417	32	-10	-7	-6	4	0.9	0.9	0.9	1.0
PLEC	Q15149	3110	2	-12	-6	4	1.0	0.9	0.9	1.0
ORC3	Q9UBD5	483	-2	-14	-6	4	1.0	0.9	0.9	1.0
RPS26	P62854	23	-10	7	-6	4	0.9	1.1	0.9	1.0
ZC3HAV1	Q7Z2W4	168	-3	2	-6	4	1.0	1.0	0.9	1.0
RPL37	P61927	37	-6	1	-6	4	0.9	1.0	0.9	1.0
SMAP2	Q8WU79	31	-1	-2	-6	4	1.0	1.0	0.9	1.0
PRPF4	O43172	441	-1	-3	-6	4	1.0	1.0	0.9	1.0
ZMYND8	Q9ULU4	321	13	-4	-6	4	1.1	1.0	0.9	1.0

AP1M2	Q9Y6Q5	241	-4	-4	-6	4	1.0	1.0	0.9	1.0
GMPPB	Q9Y5P6	245	5	-5	-6	4	1.1	1.0	0.9	1.0
GCC2	Q8IWJ2	416	-5	-16	-6	4	1.0	0.9	0.9	1.0
VPS13C	Q709C8	2184	-6	-3	-6	3	0.9	1.0	0.9	1.0
PDCD11	Q14690	694	2	-4	-6	3	1.0	1.0	0.9	1.0
PSTPIP1	O43586	213	-2	-6	-6	3	1.0	0.9	0.9	1.0
IARS2	Q9NSE4	819	-11	-9	-6	3	0.9	0.9	0.9	1.0
KHSRP	Q92945	296	1	-11	-6	3	1.0	0.9	0.9	1.0
MPP7	Q5T2T1	340	-1	-11	-6	3	1.0	0.9	0.9	1.0
TMEM173	Q86WV6	309	6	0	-6	3	1.1	1.0	0.9	1.0
TRAPPC8	Q9Y2L5	583	-13	-2	-6	3	0.9	1.0	0.9	1.0
PHF3	Q92576	402	-1	-5	-6	3	1.0	1.0	0.9	1.0
ANXA1	P04083	270	-4	-6	-6	3	1.0	0.9	0.9	1.0
PACS2	Q86VP3	831	1	-21	-6	3	1.0	0.8	0.9	1.0
KDM1A	O60341	360	-8	-4	-6	2	0.9	1.0	0.9	1.0
SMARCC2	Q8TAQ2	486	-16	-6	-6	2	0.9	0.9	0.9	1.0
SMARCC1	Q92922	511	-16	-6	-6	2	0.9	0.9	0.9	1.0
FGD4	Q96M96	234	3	-7	-6	2	1.0	0.9	0.9	1.0
METTL7A	Q9H8H3	92	-7	-7	-6	2	0.9	0.9	0.9	1.0
ARFRP1	Q13795	125	3	-10	-6	2	1.0	0.9	0.9	1.0
ANXA4	P09525	315	6	-22	-6	2	1.1	0.8	0.9	1.0
SIRT6	Q8N6T7	18	-3	7	-6	2	1.0	1.1	0.9	1.0
HGS	O14964	166	12	-1	-6	2	1.1	1.0	0.9	1.0
INTS1	Q8N201	611	8	-4	-6	2	1.1	1.0	0.9	1.0
PMS1	P54277	457	-12	-5	-6	2	0.9	1.0	0.9	1.0
EPRS	P07814	1487	-1	-10	-6	2	1.0	0.9	0.9	1.0
USP9Y	O00507	1729	-1	-13	-6	2	1.0	0.9	0.9	1.0
USP9X	Q93008	1727	-1	-13	-6	2	1.0	0.9	0.9	1.0
TRIM14	Q14142	151	-12	-48	-6	2	0.9	0.7	0.9	1.0
SUCLG2	Q96I99	162	-2	-9	-6	1	1.0	0.9	0.9	1.0
WWP2	O00308	802	0	-9	-6	1	1.0	0.9	0.9	1.0
ITCH	Q96J02	835	0	-9	-6	1	1.0	0.9	0.9	1.0
GNB1L	Q9BYB4	116	-12	-12	-6	1	0.9	0.9	0.9	1.0
MAVS	Q7Z434	46	4	-2	-6	1	1.0	1.0	0.9	1.0
FMNL1	O95466	69	-4	-6	-6	1	1.0	0.9	0.9	1.0
RAP1A	P62834	139	-2	-6	-6	1	1.0	0.9	0.9	1.0
MTAP	Q13126	131	-5	-7	-6	1	1.0	0.9	0.9	1.0
RNH1	P13489	30	-6	-16	-6	1	0.9	0.9	0.9	1.0
AGO3	Q9H9G7	58	-6	-18	-6	1	0.9	0.9	0.9	1.0
TRIM24	O15164	78	12	2	-6	0	1.1	1.0	0.9	1.0
MED15	Q96RN5	588	-10	2	-6	0	0.9	1.0	0.9	1.0
PSMC6	P62333	83	6	-2	-6	0	1.1	1.0	0.9	1.0
IPO5	O00410	915	1	-4	-6	0	1.0	1.0	0.9	1.0
H2AFY	O75367	286	-2	-7	-6	0	1.0	0.9	0.9	1.0
SMARCB1	Q12824	350	-5	1	-6	-1	1.0	1.0	0.9	1.0
DYNC1H1	Q14204	978	-3	-7	-6	-1	1.0	0.9	0.9	1.0
WDR4	P57081	137	-2	-8	-6	-1	1.0	0.9	0.9	1.0
SEC24C	P53992	635	-4	-8	-6	-1	1.0	0.9	0.9	1.0
HPS3	Q969F9	439	-11	-9	-6	-1	0.9	0.9	0.9	1.0
DDX5	P17844	200	-1	-4	-6	-1	1.0	1.0	0.9	1.0
DDX17	Q92841	277	-1	-4	-6	-1	1.0	1.0	0.9	1.0
HSPA1B	P0DMV9	17	-6	-12	-6	-1	0.9	0.9	0.9	1.0
KBTBD8	Q8NFY9	490	17	-15	-6	-1	1.2	0.9	0.9	1.0
MSTO1	Q9BUK6	485	-29	-15	-6	-1	0.8	0.9	0.9	1.0
VDR	P11473	76	-17	-20	-6	-1	0.9	0.8	0.9	1.0

MYBBP1A	Q9BQG0	884	2	-6	-6	-2	1.0	0.9	0.9	1.0
TRIM56	Q9BRZ2	41	6	-12	-6	-2	1.1	0.9	0.9	1.0
APPL1	Q9UKG1	570	-6	-13	-6	-2	0.9	0.9	0.9	1.0
UTRN	P46939	3097	-6	-1	-6	-2	0.9	1.0	0.9	1.0
TEX264	Q9Y6I9	165	1	-6	-6	-2	1.0	0.9	0.9	1.0
RSBN1	Q5VWQ0	646	-4	-6	-6	-2	1.0	0.9	0.9	1.0
MED13	Q9UHV7	245	-10	-8	-6	-2	0.9	0.9	0.9	1.0
NDRG1	Q92597	289	3	1	-6	-3	1.0	1.0	0.9	1.0
ARHGEF6	Q15052	514	-10	-1	-6	-3	0.9	1.0	0.9	1.0
MARS	P56192	12	-3	-14	-6	-3	1.0	0.9	0.9	1.0
FAM120A	Q9NZB2	53	-7	-11	-6	-3	0.9	0.9	0.9	1.0
SPATA13	Q96N96	361	-5	3	-6	-4	1.0	1.0	0.9	1.0
TASP1	Q9H6P5	351	-5	0	-6	-4	1.0	1.0	0.9	1.0
DCTN1	Q14203	636	-3	-4	-6	-4	1.0	1.0	0.9	1.0
GOLGB1	Q14789	1569	2	-5	-6	-4	1.0	1.0	0.9	1.0
MLH1	P40692	142	-2	-19	-6	-4	1.0	0.8	0.9	1.0
WDR37	Q9Y2I8	146	-5	-10	-6	-4	1.0	0.9	0.9	1.0
BECN1	Q14457	391	-3	9	-6	-5	1.0	1.1	0.9	1.0
AP4M1	O00189	235	6	-2	-6	-5	1.1	1.0	0.9	1.0
SPECC1	Q5M775	633	-23	-8	-6	-5	0.8	0.9	0.9	1.0
GPD1L	Q8N335	104	8	-8	-6	-5	1.1	0.9	0.9	1.0
NCOA5	Q9HCD5	200	-6	-10	-6	-5	0.9	0.9	0.9	1.0
TESMIN	Q9Y4I5	377	1	-11	-6	-5	1.0	0.9	0.9	1.0
TESMIN	Q9Y4I5	382	1	-11	-6	-5	1.0	0.9	0.9	1.0
LRRC14	Q15048	15	-16	-16	-6	-6	0.9	0.9	0.9	0.9
NAP1L4	Q99733	77	-10	1	-6	-6	0.9	1.0	0.9	0.9
RFFL	Q8WZ73	50	0	-14	-6	-6	1.0	0.9	0.9	0.9
PGD	P52209	289	3	-5	-6	-7	1.0	1.0	0.9	0.9
SYNE3	Q6ZMZ3	651	-4	-16	-6	-7	1.0	0.9	0.9	0.9
DYSF	O75923	1685	-14	-14	-6	-7	0.9	0.9	0.9	0.9
TSC22D2	O75157	63	3	-28	-6	-7	1.0	0.8	0.9	0.9
LRRC40	Q9H9A6	54	-3	-1	-6	-8	1.0	1.0	0.9	0.9
CMIP	Q8IY22	363	-2	4	-6	-9	1.0	1.0	0.9	0.9
CHML	P26374	582	-6	-1	-6	-9	0.9	1.0	0.9	0.9
EP300	Q09472	393	0	-12	-6	-9	1.0	0.9	0.9	0.9
TRAPPC9	Q96Q05	141	-11	-21	-6	-9	0.9	0.8	0.9	0.9
CLTC	Q00610	778	2	-1	-6	-9	1.0	1.0	0.9	0.9
YWHAE	P62258	98	-2	-8	-6	-9	1.0	0.9	0.9	0.9
BRCC3	P46736	228	-5	-10	-6	-9	1.0	0.9	0.9	0.9
ASCC3	Q8N3C0	1874	-5	-10	-6	-10	1.0	0.9	0.9	0.9
DHX37	Q8IY37	680	-5	-7	-6	-10	1.0	0.9	0.9	0.9
C2CD5	Q86YS7	831	2	-9	-6	-11	1.0	0.9	0.9	0.9
UBR4	Q5T4S7	1274	-2	10	-6	-12	1.0	1.1	0.9	0.9
TUBGCP2	Q9BSJ2	463	9	-15	-6	-13	1.1	0.9	0.9	0.9
FLNA	P21333	2582	-2	-7	-6	-13	1.0	0.9	0.9	0.9
ARHGAP10	A1A4S6	82	-14	3	-6	-14	0.9	1.0	0.9	0.9
DYNC1H1	Q14204	3573	-1	-5	-6	-14	1.0	1.0	0.9	0.9
DR1	Q01658	58	9	-2	-6	-15	1.1	1.0	0.9	0.9
CKAP5	Q14008	619	-1	-1	-6	-16	1.0	1.0	0.9	0.9
DIS3	Q9Y2L1	483	7	-12	-6	-16	1.1	0.9	0.9	0.9
DNMT1	P26358	414	4	-20	-6	-16	1.0	0.8	0.9	0.9
FLNA	P21333	205	-9	-20	-6	-17	0.9	0.8	0.9	0.9
ARHGEF7	Q14155	448	-9	2	-6	-21	0.9	1.0	0.9	0.8
SNAP23	O00161	79	-6	-7	-6	-26	0.9	0.9	0.9	0.8
MYH9	P35579	816	2	4	-6	-27	1.0	1.0	0.9	0.8

MYH10	P35580	823	2	4	-6	-27	1.0	1.0	0.9	0.8
MYH14	Q7Z406	840	2	4	-6	-27	1.0	1.0	0.9	0.8
FLNA	P21333	810	-4	3	-6	-28	1.0	1.0	0.9	0.8
TLN1	Q9Y490	2161	-3	0	-6	-33	1.0	1.0	0.9	0.8
GTPBP2	Q9BX10	450	3	-5	-6	-39	1.0	1.0	0.9	0.7
CYFIP1	Q7L576	1241	-9	-34	-6	-43	0.9	0.7	0.9	0.7
FHL1	Q13642	150	6	-7	-6	-44	1.1	0.9	0.9	0.7
RAB28	P51157	31	16	3	-6	-45	1.2	1.0	0.9	0.7
TLN1	Q9Y490	1392	-8	9	-6	-59	0.9	1.1	0.9	0.6
VCL	P18206	313	2	-1	-6	-60	1.0	1.0	0.9	0.6
TLN1	Q9Y490	29	-4	-6	-6	-71	1.0	0.9	0.9	0.6
LIMS1	P48059	272	1	-3	-6	-73	1.0	1.0	0.9	0.6
TLN1	Q9Y490	1509	-1	-17	-6	-114	1.0	0.9	0.9	0.5
PDLIM1	O00151	263	1	5	-6	-136	1.0	1.1	0.9	0.4
SAE1	Q9UBE0	342	0	-7	-6	37	1.0	0.9	0.9	1.6
SMG9	Q9H0W8	380	-18	-20	-6	36	0.9	0.8	0.9	1.6
ARL2BP	Q9Y2Y0	149	-2	-8	-6	36	1.0	0.9	0.9	1.6
APOBEC3G	Q9HC16	243	2	-13	-6	34	1.0	0.9	0.9	1.5
TGFBR2	P37173	368	-18	-10	-6	32	0.9	0.9	0.9	1.5
NCAPD3	P42695	541	6	3	-6	31	1.1	1.0	0.9	1.4
RALGAPA2	Q2PPJ7	495	-7	-7	-6	31	0.9	0.9	0.9	1.4
PSMD10	O75832	107	0	-2	-6	30	1.0	1.0	0.9	1.4
SRRM2	Q9UQ35	1016	-1	-7	-6	28	1.0	0.9	0.9	1.4
HERC1	Q15751	56	-7	-10	-6	28	0.9	0.9	0.9	1.4
MVB12A	Q96EY5	33	-1	-6	-6	27	1.0	0.9	0.9	1.4
PXN	P49023	358	-13	-15	-6	27	0.9	0.9	0.9	1.4
C3orf38	Q5JPI3	308	-2	-5	-6	27	1.0	1.0	0.9	1.4
DDX58	O95786	268	-8	-6	-6	27	0.9	0.9	0.9	1.4
CEP76	Q8TAP6	624	7	-14	-6	27	1.1	0.9	0.9	1.4
RNF31	Q96EP0	473	-6	-14	-6	26	0.9	0.9	0.9	1.3
EGLN1	Q9GZT9	127	2	-5	-6	24	1.0	1.0	0.9	1.3
IDH1	O75874	379	0	-7	-6	24	1.0	0.9	0.9	1.3
PRKDC	P78527	1364	3	-2	-6	24	1.0	1.0	0.9	1.3
SYNE2	Q8WXH0	2480	3	-11	-6	24	1.0	0.9	0.9	1.3
CCDC88A	Q3V6T2	1729	7	-6	-6	23	1.1	0.9	0.9	1.3
DHRS1	Q96LJ7	256	-8	-12	-6	23	0.9	0.9	0.9	1.3
FN3KRP	Q9HA64	24	2	-13	-6	23	1.0	0.9	0.9	1.3
BCR	P11274	609	24	6	-6	23	1.3	1.1	0.9	1.3
MDN1	Q9NU22	2890	-8	-9	-6	23	0.9	0.9	0.9	1.3
DYSF	O75923	1621	-3	-14	-6	23	1.0	0.9	0.9	1.3
HERC3	Q15034	552	-2	-14	-6	23	1.0	0.9	0.9	1.3
KMT5C	Q86Y97	111	2	-2	-6	22	1.0	1.0	0.9	1.3
CEP350	Q5VT06	2716	1	-9	-6	22	1.0	0.9	0.9	1.3
ACAD9	Q9H845	271	-11	-11	-6	22	0.9	0.9	0.9	1.3
ZHX2	Q9Y6X8	759	-9	8	-6	21	0.9	1.1	0.9	1.3
RPL7A	P62424	199	5	-12	-6	21	1.1	0.9	0.9	1.3
TXNDC5	Q8NBS9	217	-11	-10	-6	20	0.9	0.9	0.9	1.3
SEC24C	P53992	78	3	2	-6	20	1.0	1.0	0.9	1.2
PAN2	Q504Q3	798	-7	-6	-6	20	0.9	0.9	0.9	1.2
PTGES3	Q15185	75	-4	-8	-6	19	1.0	0.9	0.9	1.2
ACOT11	Q8WXI4	498	-7	-9	-6	19	0.9	0.9	0.9	1.2
STAT2	P52630	676	-4	-13	-6	19	1.0	0.9	0.9	1.2
C12orf29	Q8N999	294	-6	9	-6	19	0.9	1.1	0.9	1.2
QTRTD1	Q9H974	324	2	-2	-6	19	1.0	1.0	0.9	1.2
ZNF836	Q6ZNA1	652	4	-3	-6	19	1.0	1.0	0.9	1.2

EML3	Q32P44	420	3	-4	-6	19	1.0	1.0	0.9	1.2
MEFV	O15553	639	-7	-8	-6	19	0.9	0.9	0.9	1.2
HUS1	O60921	44	6	-3	-6	18	1.1	1.0	0.9	1.2
CRKL	P46109	249	-1	-9	-6	18	1.0	0.9	0.9	1.2
GORAB	Q5T7V8	354	14	-10	-6	18	1.2	0.9	0.9	1.2
RPUSD3	Q6P087	147	1	-10	-6	18	1.0	0.9	0.9	1.2
TRIM33	Q9UPN9	447	-5	-2	-6	18	1.0	1.0	0.9	1.2
HADHA	P40939	747	-11	-16	-6	18	0.9	0.9	0.9	1.2
ATRIP	Q8WXE1	585	-4	-7	-6	17	1.0	0.9	0.9	1.2
VARS	P26640	663	1	-8	-6	17	1.0	0.9	0.9	1.2
TRIM33	Q9UPN9	150	-4	-12	-6	17	1.0	0.9	0.9	1.2
VAV1	P15498	83	1	7	-6	17	1.0	1.1	0.9	1.2
INTS3	Q68E01	80	0	-5	-6	17	1.0	1.0	0.9	1.2
OFD1	O75665	503	-1	-7	-6	17	1.0	0.9	0.9	1.2
OXR1	Q8N573	410	-12	-7	-6	17	0.9	0.9	0.9	1.2
KIAA0355	O15063	502	-7	-15	-6	17	0.9	0.9	0.9	1.2
CDK19	Q9BWU1	349	-5	-18	-6	17	1.0	0.9	0.9	1.2
TOMM40	O96008	86	4	8	-6	16	1.0	1.1	0.9	1.2
ZAP70	P43403	117	0	-6	-6	16	1.0	0.9	0.9	1.2
PSMD1	Q99460	898	-6	-6	-6	16	0.9	0.9	0.9	1.2
CMTR1	Q8N1G2	9	-1	-1	-6	16	1.0	1.0	0.9	1.2
NAPRT	Q6XQN6	533	4	-1	-6	16	1.0	1.0	0.9	1.2
ZFYVE19	Q96K21	104	-7	-17	-6	16	0.9	0.9	0.9	1.2
PRMT9	Q6P2P2	683	-5	-2	-6	15	1.0	1.0	0.9	1.2
ANKRD54	Q6NXT1	230	-5	-4	-6	15	1.0	1.0	0.9	1.2
ARAP3	Q8WWN8	1022	-10	-8	-6	15	0.9	0.9	0.9	1.2
ZC3H4	Q9UPT8	439	-6	-2	-6	15	0.9	1.0	0.9	1.2
MAP2K1	Q02750	277	-3	-5	-6	15	1.0	1.0	0.9	1.2
SEC13	P55735	299	-3	-7	-6	15	1.0	0.9	0.9	1.2
CUL3	Q13618	464	-2	-8	-6	15	1.0	0.9	0.9	1.2
SIPA1L3	O60292	18	-13	-8	-6	15	0.9	0.9	0.9	1.2
FLNA	P21333	1353	-6	-10	-6	15	0.9	0.9	0.9	1.2
JAK1	P23458	731	-1	-10	-6	15	1.0	0.9	0.9	1.2
CNTRL	Q7Z7A1	1535	-8	2	-6	14	0.9	1.0	0.9	1.2
MGEA5	O60502	596	3	-4	-6	14	1.0	1.0	0.9	1.2
SPATA20	Q8TB22	778	-3	-8	-6	14	1.0	0.9	0.9	1.2
RBM33	Q96EV2	285	-5	-13	-6	14	1.0	0.9	0.9	1.2
HDAC1	Q13547	273	-1	-2	-6	14	1.0	1.0	0.9	1.2
ZNF69	Q9UC07	4	-5	-2	-6	14	1.0	1.0	0.9	1.2
ZNF69	Q9UC07	3	-5	-2	-6	14	1.0	1.0	0.9	1.2
CMAS	Q8NFW8	422	5	-4	-6	14	1.0	1.0	0.9	1.2
SEC31A	O94979	704	-3	-9	-6	14	1.0	0.9	0.9	1.2
PSIP1	O75475	204	1	-9	-6	14	1.0	0.9	0.9	1.2
ABCE1	P61221	65	-18	-13	-6	14	0.9	0.9	0.9	1.2
IRF2BP1	Q8IU81	207	14	6	-6	13	1.2	1.1	0.9	1.1
CTR9	Q6PD62	231	0	0	-6	13	1.0	1.0	0.9	1.1
FMNL1	O95466	939	7	-8	-6	13	1.1	0.9	0.9	1.1
ASMTL	O95671	333	-3	-9	-6	13	1.0	0.9	0.9	1.1
SRP68	Q9UHB9	562	1	-1	-6	13	1.0	1.0	0.9	1.1
ARRB2	P32121	17	-2	-5	-6	13	1.0	1.0	0.9	1.1
CAPZB	P47756	147	-2	-6	-6	13	1.0	0.9	0.9	1.1
POLR3F	Q9H1D9	221	-2	-12	-6	13	1.0	0.9	0.9	1.1
FLNA	P21333	2102	-2	-13	-6	13	1.0	0.9	0.9	1.1
PA2G4	Q9UQ80	149	4	-27	-6	13	1.0	0.8	0.9	1.1
NIPBL	Q6KC79	52	-3	-5	-6	12	1.0	1.0	0.9	1.1

PSME4	Q14997	1001	-1	-7	-6	12	1.0	0.9	0.9	1.1
NUMA1	Q14980	658	-2	-7	-6	12	1.0	0.9	0.9	1.1
NME2	P22392	145	13	-10	-6	12	1.1	0.9	0.9	1.1
CMSS1	Q9BQ75	121	-9	3	-6	12	0.9	1.0	0.9	1.1
ZMYM2	Q9UBW7	612	3	2	-6	12	1.0	1.0	0.9	1.1
PCNA	P12004	162	2	-17	-6	12	1.0	0.9	0.9	1.1
MAP2K7	O14733	176	-5	-26	-6	12	1.0	0.8	0.9	1.1
ADAR	P55265	1036	-9	1	-6	11	0.9	1.0	0.9	1.1
OTULIN	Q96BN8	347	2	-4	-6	11	1.0	1.0	0.9	1.1
SAMD9	Q5K651	1085	0	-6	-6	11	1.0	0.9	0.9	1.1
FUBP1	Q96AE4	148	-8	-9	-6	11	0.9	0.9	0.9	1.1
PIP4K2C	Q8TBX8	104	-6	-13	-6	11	0.9	0.9	0.9	1.1
RPA1	P27694	503	-3	1	-6	11	1.0	1.0	0.9	1.1
SDHA	P31040	654	11	0	-6	11	1.1	1.0	0.9	1.1
BTK	Q06187	481	5	-1	-6	11	1.1	1.0	0.9	1.1
PAN2	Q504Q3	263	14	-6	-6	11	1.2	0.9	0.9	1.1
EHBP1L1	Q8N3D4	1364	-8	-10	-6	11	0.9	0.9	0.9	1.1
VPS29	Q9UBQ0	41	2	-10	-6	11	1.0	0.9	0.9	1.1
SETD1B	Q9UPS6	1961	-21	-20	-6	11	0.8	0.8	0.9	1.1
USP9X	Q93008	540	2	-4	-6	10	1.0	1.0	0.9	1.1
RNF213	Q63HN8	3794	9	-6	-6	10	1.1	0.9	0.9	1.1
SEC24D	O94855	848	1	-7	-6	10	1.0	0.9	0.9	1.1
PAPSS1	O43252	360	4	-9	-6	10	1.0	0.9	0.9	1.1
KMT5C	Q86Y97	185	-3	-10	-6	10	1.0	0.9	0.9	1.1
HSPE1-MOB4	S4R3N1	155	-16	-10	-6	10	0.9	0.9	0.9	1.1
VRK1	Q99986	50	1	-4	-6	10	1.0	1.0	0.9	1.1
PAF1	Q8N7H5	31	1	-5	-6	10	1.0	1.0	0.9	1.1
TEP1	Q99973	2275	-9	-5	-6	10	0.9	1.0	0.9	1.1
SCAF1	Q9H7N4	362	-5	-16	-6	10	1.0	0.9	0.9	1.1
KMT2A	Q03164	2065	4	-7	-6	9	1.0	0.9	0.9	1.1
FAM129A	Q9BZQ8	680	2	-8	-6	9	1.0	0.9	0.9	1.1
ATG7	O95352	298	-3	-12	-6	9	1.0	0.9	0.9	1.1
VDAC2	P45880	47	0	-3	-6	9	1.0	1.0	0.9	1.1
PSMD8	P48556	112	2	-6	-6	9	1.0	0.9	0.9	1.1
TDG	Q13569	276	-14	-7	-6	9	0.9	0.9	0.9	1.1
CRYL1	Q9Y2S2	125	3	-3	-6	8	1.0	1.0	0.9	1.1
ZMYM3	Q14202	715	7	-5	-6	8	1.1	1.0	0.9	1.1
CSNK1G1	Q9HCP0	52	-1	-6	-6	8	1.0	0.9	0.9	1.1
BPNT1	O95861	243	-4	-9	-6	8	1.0	0.9	0.9	1.1
PPP2R1B	P30154	389	-14	-7	-6	8	0.9	0.9	0.9	1.1
DHX9	Q08211	12	-4	-8	-6	8	1.0	0.9	0.9	1.1
ANAPC1	Q9H1A4	988	-6	-10	-6	8	0.9	0.9	0.9	1.1
ADNP	Q9H2P0	79	-3	-4	-6	7	1.0	1.0	0.9	1.1
GIT2	Q14161	122	-3	-8	-6	7	1.0	0.9	0.9	1.1
ZZEF1	O43149	162	20	16	-6	7	1.3	1.2	0.9	1.1
FYCO1	Q9BQS8	293	-2	-3	-6	7	1.0	1.0	0.9	1.1
KMT2B	Q9UMN6	268	-8	-3	-6	7	0.9	1.0	0.9	1.1
EIF3G	O75821	139	-9	-4	-6	7	0.9	1.0	0.9	1.1
SIRT5	Q9NXA8	181	-6	-11	-6	7	0.9	0.9	0.9	1.1
TARS	P26639	107	5	-2	-6	6	1.1	1.0	0.9	1.1
UBXN1	Q04323	214	-4	-7	-6	6	1.0	0.9	0.9	1.1
TRRAP	Q9Y4A5	1874	6	-8	-6	6	1.1	0.9	0.9	1.1
SLC9A3R1	O14745	206	2	-9	-6	6	1.0	0.9	0.9	1.1
UBA6	A0AVT1	449	-4	-16	-6	6	1.0	0.9	0.9	1.1
MCM5	P33992	221	-8	-1	-6	6	0.9	1.0	0.9	1.1

AGPS	O00116	214	-2	-1	-6	6	1.0	1.0	0.9	1.1
TACC1	O75410	219	3	-4	-6	6	1.0	1.0	0.9	1.1
GMPS	P49915	456	-2	-9	-6	6	1.0	0.9	0.9	1.1
ICE1	Q9Y2F5	1817	-14	-9	-6	6	0.9	0.9	0.9	1.1
MACF1	Q9UPN3	1025	-5	-13	-6	6	1.0	0.9	0.9	1.1
RANBP3	Q9H6Z4	384	2	7	-6	5	1.0	1.1	0.9	1.1
PPA2	Q9H2U2	302	1	0	-6	5	1.0	1.0	0.9	1.1
OTULIN	Q96BN8	129	3	-1	-6	5	1.0	1.0	0.9	1.1
SERPINB8	P50452	348	-5	-2	-6	5	1.0	1.0	0.9	1.1
SERPINB9	P50453	350	-5	-2	-6	5	1.0	1.0	0.9	1.1
GNB1	P62873	294	-4	-4	-6	5	1.0	1.0	0.9	1.1
RNF213	Q63HN8	3979	-1	-5	-6	5	1.0	1.0	0.9	1.0
DYNC1H1	Q14204	633	-1	-6	-6	5	1.0	0.9	0.9	1.0
SART1	O43290	674	-6	-7	-6	5	0.9	0.9	0.9	1.0
AP3B1	O00203	893	2	-7	-6	5	1.0	0.9	0.9	1.0
CREBBP	Q92793	380	-8	-11	-6	5	0.9	0.9	0.9	1.0
CNOT10	Q9H9A5	633	-4	8	-6	4	1.0	1.1	0.9	1.0
PGM2L1	Q6PCE3	520	5	1	-6	4	1.0	1.0	0.9	1.0
MAP2K2	P36507	384	-6	-11	-6	4	0.9	0.9	0.9	1.0
NIP7	Q9Y221	36	0	-11	-6	4	1.0	0.9	0.9	1.0
USP16	Q9Y5T5	662	-21	-13	-6	4	0.8	0.9	0.9	1.0
NUP88	Q99567	391	-1	-8	-6	4	1.0	0.9	0.9	1.0
QKI	Q96PU8	35	-5	-8	-6	4	1.0	0.9	0.9	1.0
INO80	Q9ULG1	108	-10	-10	-6	4	0.9	0.9	0.9	1.0
TEP1	Q99973	2524	-4	-18	-6	4	1.0	0.9	0.9	1.0
BIRC6	Q9NR09	396	3	4	-6	3	1.0	1.0	0.9	1.0
RQCD1	Q92600	99	-13	3	-6	3	0.9	1.0	0.9	1.0
AARS	P49588	773	-2	-5	-6	3	1.0	1.0	0.9	1.0
ADD3	Q9UEY8	73	-2	-5	-6	3	1.0	1.0	0.9	1.0
NUP160	Q12769	369	-14	-9	-6	3	0.9	0.9	0.9	1.0
BUD31	P41223	119	-4	-16	-6	3	1.0	0.9	0.9	1.0
HMGCL	P35914	323	-1	-4	-6	3	1.0	1.0	0.9	1.0
DOCK8	Q8NF50	846	-13	-10	-6	3	0.9	0.9	0.9	1.0
ZW10	O43264	124	-8	-13	-6	3	0.9	0.9	0.9	1.0
TRIP12	Q14669	1411	-5	-15	-6	3	1.0	0.9	0.9	1.0
STRN	O43815	765	-5	-2	-6	2	1.0	1.0	0.9	1.0
PLEKHA2	Q9HB19	332	-1	-2	-6	2	1.0	1.0	0.9	1.0
USP5	P45974	838	5	-8	-6	2	1.0	0.9	0.9	1.0
COPB2	P35606	351	10	-1	-6	2	1.1	1.0	0.9	1.0
TCEB1	Q15369	11	-2	-7	-6	2	1.0	0.9	0.9	1.0
CD14	P08571	306	-6	-11	-6	2	0.9	0.9	0.9	1.0
LPXN	O60711	211	-7	2	-6	1	0.9	1.0	0.9	1.0
LDAH	Q9H6V9	261	-2	-3	-6	1	1.0	1.0	0.9	1.0
PLXNC1	O60486	1217	7	-4	-6	1	1.1	1.0	0.9	1.0
PLCG2	P16885	849	-4	-15	-6	1	1.0	0.9	0.9	1.0
CAPN7	Q9Y6W3	242	5	-15	-6	1	1.0	0.9	0.9	1.0
GOLGA1	Q92805	450	-10	-16	-6	1	0.9	0.9	0.9	1.0
TLE3	Q04726	26	8	2	-6	1	1.1	1.0	0.9	1.0
ALDH4A1	P30038	348	-19	-1	-6	1	0.8	1.0	0.9	1.0
HNRNPUL1	Q9BUJ2	377	2	-4	-6	1	1.0	1.0	0.9	1.0
NAA60	Q9H7X0	19	6	-4	-6	1	1.1	1.0	0.9	1.0
ADCK4	Q96D53	479	-4	-11	-6	1	1.0	0.9	0.9	1.0
SLTM	Q9NWH9	441	-6	-13	-6	1	0.9	0.9	0.9	1.0
UQCRC1	P31930	380	-8	-2	-6	0	0.9	1.0	0.9	1.0
FBXO4	Q9UKT5	147	-3	-6	-6	0	1.0	0.9	0.9	1.0

HGS	O14964	190	0	-9	-6	-1	1.0	0.9	0.9	1.0
BRD1	O95696	393	-7	-9	-6	-1	0.9	0.9	0.9	1.0
CORO7-PAM1	A0A0A6YYL4	790	11	-12	-6	-1	1.1	0.9	0.9	1.0
PRKDC	P78527	223	1	-19	-6	-1	1.0	0.8	0.9	1.0
PLRG1	O43660	272	-4	1	-6	-1	1.0	1.0	0.9	1.0
HMHA1	Q92619	732	-2	-2	-6	-1	1.0	1.0	0.9	1.0
ATP2A2	P16615	997	-9	-4	-6	-1	0.9	1.0	0.9	1.0
ZNF800	Q2TB10	293	-5	-9	-6	-1	1.0	0.9	0.9	1.0
EIF3B	P55884	515	-3	3	-6	-2	1.0	1.0	0.9	1.0
ESD	P10768	176	3	-8	-6	-2	1.0	0.9	0.9	1.0
UBE2K	P61086	170	4	-13	-6	-2	1.0	0.9	0.9	1.0
SYNE1	Q8NF91	7765	3	-14	-6	-2	1.0	0.9	0.9	1.0
LRRC16A	Q5VZK9	1106	-1	-15	-6	-2	1.0	0.9	0.9	1.0
CYLD	Q9NQC7	106	2	-8	-6	-2	1.0	0.9	0.9	1.0
ANKRD44	Q8N8A2	334	2	6	-6	-3	1.0	1.1	0.9	1.0
PTPN1	P18031	121	0	1	-6	-3	1.0	1.0	0.9	1.0
BPNT1	O95861	249	-2	-10	-6	-3	1.0	0.9	0.9	1.0
PAK1IP1	Q9NWT1	298	-1	-14	-6	-3	1.0	0.9	0.9	1.0
PRPF8	Q6P2Q9	2116	-1	1	-6	-3	1.0	1.0	0.9	1.0
ACTB	P60709	285	-2	-3	-6	-3	1.0	1.0	0.9	1.0
SAMHD1	Q9Y3Z3	51	-4	-6	-6	-3	1.0	0.9	0.9	1.0
CKAP5	Q14008	1768	-4	-11	-6	-3	1.0	0.9	0.9	1.0
GRIPAP1	Q4V328	744	0	-16	-6	-3	1.0	0.9	0.9	1.0
EPPK1	A0A087X1U6	2225	0	-4	-6	-4	1.0	1.0	0.9	1.0
HUWE1	Q7Z6Z7	1892	7	-2	-6	-4	1.1	1.0	0.9	1.0
RPS3A	P61247	96	-9	-3	-6	-4	0.9	1.0	0.9	1.0
MCM6	Q14566	91	4	-3	-6	-4	1.0	1.0	0.9	1.0
ELOF1	P60002	29	8	-3	-6	-4	1.1	1.0	0.9	1.0
PTK2B	Q14289	89	-4	-5	-6	-4	1.0	1.0	0.9	1.0
OAS1	P00973	331	-5	-9	-6	-4	1.0	0.9	0.9	1.0
TRAPPC8	Q9Y2L5	1341	-3	-10	-6	-4	1.0	0.9	0.9	1.0
NPL	Q9BXD5	185	-6	-13	-6	-4	0.9	0.9	0.9	1.0
LIN9	Q5TKA1	476	-3	-4	-6	-5	1.0	1.0	0.9	1.0
KLHL36	Q8N4N3	308	-18	-8	-6	-5	0.9	0.9	0.9	1.0
UBE3A	Q05086	83	-10	-16	-6	-5	0.9	0.9	0.9	1.0
FASTKD3	Q14CZ7	378	-2	-6	-6	-6	1.0	0.9	0.9	0.9
ATG5	Q9H1Y0	223	-4	-12	-6	-6	1.0	0.9	0.9	0.9
TNIP1	Q15025	212	-4	-1	-6	-7	1.0	1.0	0.9	0.9
FYB	O15117	519	-3	-5	-6	-7	1.0	1.0	0.9	0.9
AMPD2	Q01433	123	3	-6	-6	-7	1.0	0.9	0.9	0.9
RPA2	P15927	49	-3	-7	-6	-7	1.0	0.9	0.9	0.9
TANK	Q92844	20	1	-10	-6	-7	1.0	0.9	0.9	0.9
DGKA	P23743	222	6	-6	-6	-8	1.1	0.9	0.9	0.9
TAF5	Q15542	632	6	-10	-6	-9	1.1	0.9	0.9	0.9
PRKAR2A	P13861	359	-8	-12	-6	-9	0.9	0.9	0.9	0.9
FYN	P06241	404	5	2	-6	-9	1.0	1.0	0.9	0.9
CCAR1	Q8IX12	465	-4	-2	-6	-9	1.0	1.0	0.9	0.9
CORO1B	Q9BR76	41	-3	-11	-6	-9	1.0	0.9	0.9	0.9
CPSF4	O95639	124	-4	-4	-6	-10	1.0	1.0	0.9	0.9
RP2	O75695	131	7	-5	-6	-10	1.1	1.0	0.9	0.9
GPD2	P43304	385	-5	4	-6	-10	1.0	1.0	0.9	0.9
HUWE1	Q7Z6Z7	699	6	-3	-6	-10	1.1	1.0	0.9	0.9
PTPRE	P23469	266	-33	-18	-6	-10	0.8	0.9	0.9	0.9
SF3B1	O75533	796	2	1	-6	-11	1.0	1.0	0.9	0.9
TRANK1	O15050	924	6	-1	-6	-11	1.1	1.0	0.9	0.9

HK3	P52790	892	-5	-12	-6	-11	1.0	0.9	0.9	0.9
SPATA2L	Q8IUW3	26	4	11	-6	-11	1.0	1.1	0.9	0.9
NUMB	P49757	37	-13	1	-6	-11	0.9	1.0	0.9	0.9
ALDH7A1	P49419	522	4	-1	-6	-11	1.0	1.0	0.9	0.9
COPB1	P53618	390	-2	-5	-6	-12	1.0	1.0	0.9	0.9
ARFGEF1	Q9Y6D6	1503	-3	-6	-6	-13	1.0	0.9	0.9	0.9
NOC2L	Q9Y3T9	567	0	-2	-6	-13	1.0	1.0	0.9	0.9
TBC1D2	Q9BYX2	49	-7	-8	-6	-14	0.9	0.9	0.9	0.9
GBF1	Q92538	1707	-8	-5	-6	-16	0.9	1.0	0.9	0.9
RPL36A-HNRN	H7BZ11	88	-6	-9	-6	-16	0.9	0.9	0.9	0.9
ARFGAP3	Q9NP61	28	-11	-25	-6	-17	0.9	0.8	0.9	0.9
TRIP6	Q15654	310	-3	-4	-6	-18	1.0	1.0	0.9	0.9
SAMHD1	Q9Y3Z3	177	-2	-9	-6	-18	1.0	0.9	0.9	0.9
ZCCHC11	Q5TAX3	394	-12	-7	-6	-18	0.9	0.9	0.9	0.8
ZNF267	Q14586	450	9	-17	-6	-18	1.1	0.9	0.9	0.8
PNKD	Q8N490	209	6	9	-6	-19	1.1	1.1	0.9	0.8
AGER	Q15109	99	-3	-16	-6	-19	1.0	0.9	0.9	0.8
ALDH6A1	Q02252	368	-3	-14	-6	-21	1.0	0.9	0.9	0.8
ECHDC1	Q9NTX5	268	-3	-8	-6	-22	1.0	0.9	0.9	0.8
MATR3	A8MXP9	854	-9	-9	-6	-22	0.9	0.9	0.9	0.8
FLNA	P21333	649	6	-9	-6	-22	1.1	0.9	0.9	0.8
SCAF4	O95104	616	-3	-13	-6	-22	1.0	0.9	0.9	0.8
FLNA	P21333	478	4	-7	-6	-25	1.0	0.9	0.9	0.8
PSMC3	P17980	240	-2	-2	-6	-27	1.0	1.0	0.9	0.8
IQGAP2	Q13576	575	5	-10	-6	-28	1.1	0.9	0.9	0.8
FLNA	P21333	483	-3	-8	-6	-31	1.0	0.9	0.9	0.8
SNAP23	O00161	85	4	-9	-6	-35	1.0	0.9	0.9	0.7
TLN1	Q9Y490	1953	3	-4	-6	-53	1.0	1.0	0.9	0.7
UPF2	Q9HAU5	1107	28	-15	-6	-54	1.4	0.9	0.9	0.6
LIMS1	P48059	255	5	-5	-6	-74	1.0	1.0	0.9	0.6
LIMS1	P48059	240	6	-8	-6	-92	1.1	0.9	0.9	0.5
LIMS1	P48059	59	3	-19	-6	-95	1.0	0.8	0.9	0.5
FRMD4B	Q9Y2L6	527	-4	-8	-6	-98	1.0	0.9	0.9	0.5
ALOX12	P18054	89	5	-5	-6	-162	1.0	1.0	0.9	0.4
MED16	Q9Y2X0	539	-8	-17	-7	62	0.9	0.9	0.9	2.6
ENG	P17813	516	-7	-33	-7	45	0.9	0.8	0.9	1.8
RALGAPA2	Q2PPJ7	169	18	1	-7	43	1.2	1.0	0.9	1.7
PNPLA6	Q8IY17	1199	0	-11	-7	41	1.0	0.9	0.9	1.7
ZNF830	Q96NB3	314	2	21	-7	40	1.0	1.3	0.9	1.7
SEC24D	O94855	1022	-2	-7	-7	39	1.0	0.9	0.9	1.6
SLC12A9	Q9BXP2	911	-5	-17	-7	39	1.0	0.9	0.9	1.6
TBL1XR1	Q9BZK7	434	-4	-6	-7	37	1.0	0.9	0.9	1.6
SPAST	Q9UBP0	171	-2	3	-7	35	1.0	1.0	0.9	1.5
ACOX3	O15254	220	-7	-15	-7	34	0.9	0.9	0.9	1.5
TXNL1	O43396	37	-7	-17	-7	34	0.9	0.9	0.9	1.5
SCAF4	O95104	506	-7	-11	-7	33	0.9	0.9	0.9	1.5
CCDC109B	Q9NWR8	324	10	-13	-7	33	1.1	0.9	0.9	1.5
KMT2D	O14686	2249	-9	-6	-7	32	0.9	0.9	0.9	1.5
MSH3	P20585	252	8	-9	-7	32	1.1	0.9	0.9	1.5
ACLY	P53396	633	0	-10	-7	32	1.0	0.9	0.9	1.5
ABCB8	Q9NUT2	483	-4	-11	-7	32	1.0	0.9	0.9	1.5
CRYBG3	Q68DQ2	1278	-13	-10	-7	31	0.9	0.9	0.9	1.4
ATP6V1G1	O75348	69	-6	-8	-7	30	0.9	0.9	0.9	1.4
SART3	Q15020	780	0	1	-7	29	1.0	1.0	0.9	1.4
ACSL1	P33121	108	-3	-7	-7	29	1.0	0.9	0.9	1.4

DNMT3A	Q9Y6K1	666	1	-2	-7	27	1.0	1.0	0.9	1.4
SCRIB	A0A0G2JPP5	496	3	-2	-7	26	1.0	1.0	0.9	1.4
RUNX3	Q13761	22	-2	-5	-7	26	1.0	1.0	0.9	1.3
ZBTB1	Q9Y2K1	448	1	-6	-7	24	1.0	0.9	0.9	1.3
TBCE	Q15813	152	-5	-8	-7	24	1.0	0.9	0.9	1.3
TERF2	Q15554	207	0	1	-7	23	1.0	1.0	0.9	1.3
LRRK2	Q5S007	2384	-6	-2	-7	23	0.9	1.0	0.9	1.3
TNS3	Q68CZ2	85	-1	-2	-7	23	1.0	1.0	0.9	1.3
HMGCL	P35914	174	-5	-5	-7	23	1.0	1.0	0.9	1.3
CASP6	P55212	68	2	-9	-7	23	1.0	0.9	0.9	1.3
PDP1	Q9P0J1	149	-2	-8	-7	22	1.0	0.9	0.9	1.3
RASGRP2	Q7LDG7	398	-11	-11	-7	22	0.9	0.9	0.9	1.3
TMPO	P42166	684	1	-3	-7	21	1.0	1.0	0.9	1.3
QARS	P47897	657	-5	-10	-7	21	1.0	0.9	0.9	1.3
TNS3	Q68CZ2	1241	-1	3	-7	21	1.0	1.0	0.9	1.3
SBF1	O95248	1374	4	-11	-7	21	1.0	0.9	0.9	1.3
MESDC1	Q9H1K6	331	10	3	-7	20	1.1	1.0	0.9	1.3
ANKIB1	Q9P2G1	640	4	2	-7	20	1.0	1.0	0.9	1.3
P4HB	P07237	312	-15	-8	-7	20	0.9	0.9	0.9	1.3
DDX59	Q5T1V6	453	-2	-12	-7	20	1.0	0.9	0.9	1.3
GMPR2	Q9P2T1	348	-3	-1	-7	20	1.0	1.0	0.9	1.2
NLRP1	Q9C000	264	-4	-4	-7	20	1.0	1.0	0.9	1.2
AGO4	Q9HCK5	262	5	-6	-7	20	1.0	0.9	0.9	1.2
AGO1	Q9UL18	270	5	-6	-7	20	1.0	0.9	0.9	1.2
NARS	O43776	537	4	-8	-7	20	1.0	0.9	0.9	1.2
DIDO1	Q9BTC0	1079	5	-1	-7	19	1.1	1.0	0.9	1.2
NFKBIA	P25963	156	-11	-3	-7	19	0.9	1.0	0.9	1.2
SP140	Q13342	792	-1	-4	-7	19	1.0	1.0	0.9	1.2
SP140L	Q9H930	505	-1	-4	-7	19	1.0	1.0	0.9	1.2
C14orf166	Q9Y224	69	-3	-5	-7	18	1.0	1.0	0.9	1.2
GNLY	P22749	138	-2	-8	-7	18	1.0	0.9	0.9	1.2
ARAP3	Q8WWN8	1051	-11	-10	-7	18	0.9	0.9	0.9	1.2
ZNF292	O60281	430	-2	-1	-7	18	1.0	1.0	0.9	1.2
FXR1	P51114	211	3	-1	-7	18	1.0	1.0	0.9	1.2
HNRNPM	P52272	676	3	-2	-7	18	1.0	1.0	0.9	1.2
EXOSC7	Q15024	199	-4	-7	-7	18	1.0	0.9	0.9	1.2
ATP2B4	P23634	721	-13	-10	-7	18	0.9	0.9	0.9	1.2
EP300	Q09472	1183	0	0	-7	17	1.0	1.0	0.9	1.2
CREBBP	Q92793	1219	0	0	-7	17	1.0	1.0	0.9	1.2
INO80D	Q53TQ3	416	0	-3	-7	17	1.0	1.0	0.9	1.2
MYBBP1A	Q9BQG0	1031	-6	-5	-7	17	0.9	1.0	0.9	1.2
ECM29	Q5VYK3	449	-1	-6	-7	17	1.0	0.9	0.9	1.2
LPXN	O60711	376	0	-6	-7	17	1.0	0.9	0.9	1.2
SAFB2	Q14151	672	-3	-9	-7	17	1.0	0.9	0.9	1.2
SRSF11	Q05519	455	1	-5	-7	16	1.0	1.0	0.9	1.2
USP22	Q9UPT9	171	5	-5	-7	16	1.0	1.0	0.9	1.2
UBA2	Q9UBT2	158	-9	-12	-7	16	0.9	0.9	0.9	1.2
PCCA	P05165	155	-1	3	-7	16	1.0	1.0	0.9	1.2
IFI30	P13284	237	-5	-4	-7	16	1.0	1.0	0.9	1.2
GANC	Q8TET4	114	2	-4	-7	16	1.0	1.0	0.9	1.2
SIPA1	Q96FS4	446	4	-1	-7	15	1.0	1.0	0.9	1.2
DGKZ	Q13574	793	-6	-7	-7	15	0.9	0.9	0.9	1.2
PIK3CD	O00329	219	-8	-7	-7	15	0.9	0.9	0.9	1.2
RGS14	O43566	496	-9	4	-7	15	0.9	1.0	0.9	1.2
CNTROB	Q8N137	533	7	-2	-7	15	1.1	1.0	0.9	1.2

COMMD7	Q86VX2	198	-3	-4	-7	15	1.0	1.0	0.9	1.2
RPS6KA4	O75676	257	-9	-6	-7	15	0.9	0.9	0.9	1.2
OAS2	P29728	668	1	-12	-7	15	1.0	0.9	0.9	1.2
PHB	P35232	69	0	-12	-7	15	1.0	0.9	0.9	1.2
STBD1	O95210	168	-6	-13	-7	14	0.9	0.9	0.9	1.2
BOD1L1	Q8NFC6	74	-8	-14	-7	14	0.9	0.9	0.9	1.2
HTATSF1	O43719	462	8	1	-7	14	1.1	1.0	0.9	1.2
SRBD1	Q8N5C6	120	2	-4	-7	14	1.0	1.0	0.9	1.2
TRAPPC8	Q9Y2L5	1074	9	-7	-7	14	1.1	0.9	0.9	1.2
KCTD5	Q9NXV2	28	-13	-3	-7	13	0.9	1.0	0.9	1.1
MRGBP	Q9NV56	170	-1	-1	-7	13	1.0	1.0	0.9	1.1
UBR5	O95071	730	3	-3	-7	13	1.0	1.0	0.9	1.1
EPPK1	A0A087X1U6	1888	-15	-4	-7	13	0.9	1.0	0.9	1.1
ZC3H11A	O75152	636	-6	-5	-7	13	0.9	1.0	0.9	1.1
AHCTF1	Q8WYP5	521	-4	-5	-7	13	1.0	1.0	0.9	1.1
METTL13	Q8N6R0	90	-2	-11	-7	13	1.0	0.9	0.9	1.1
TCF20	Q9UGU0	1641	-25	-16	-7	13	0.8	0.9	0.9	1.1
TNKS1BP1	Q9C0C2	716	11	3	-7	12	1.1	1.0	0.9	1.1
SIRT1	Q96EB6	380	-8	1	-7	12	0.9	1.0	0.9	1.1
UHRF1BP1L	A0JNW5	1235	-8	-6	-7	12	0.9	0.9	0.9	1.1
LAS1L	Q9Y4W2	306	-5	-8	-7	12	1.0	0.9	0.9	1.1
NASP	P49321	708	-2	-15	-7	12	1.0	0.9	0.9	1.1
SLAIN1	Q8ND83	449	2	3	-7	12	1.0	1.0	0.9	1.1
ARAP1	Q96P48	900	0	-5	-7	12	1.0	1.0	0.9	1.1
MAPRE3	Q9UPY8	182	-5	-6	-7	12	1.0	0.9	0.9	1.1
DMXL1	Q9Y485	543	-3	-8	-7	12	1.0	0.9	0.9	1.1
TRAPPC10	P48553	696	5	-9	-7	12	1.0	0.9	0.9	1.1
TCAF1	Q9Y4C2	487	-3	-9	-7	12	1.0	0.9	0.9	1.1
USP5	P45974	532	-8	-13	-7	12	0.9	0.9	0.9	1.1
PPP2R1A	P30153	317	3	-7	-7	11	1.0	0.9	0.9	1.1
CCT8	P50990	244	-5	-9	-7	11	1.0	0.9	0.9	1.1
SATB1	Q01826	529	-2	-9	-7	11	1.0	0.9	0.9	1.1
EFTUD2	Q15029	166	0	-9	-7	11	1.0	0.9	0.9	1.1
COPB2	P35606	190	-7	-12	-7	11	0.9	0.9	0.9	1.1
ADA	P00813	75	1	-1	-7	11	1.0	1.0	0.9	1.1
BABAM1	Q9NWV8	222	-1	-6	-7	11	1.0	0.9	0.9	1.1
HNRNPU	Q00839	562	-2	-8	-7	11	1.0	0.9	0.9	1.1
DIS3	Q9Y2L1	533	-8	-11	-7	11	0.9	0.9	0.9	1.1
ZHX3	Q9H4I2	335	-4	-11	-7	11	1.0	0.9	0.9	1.1
EDC4	Q6P2E9	384	-6	-14	-7	11	0.9	0.9	0.9	1.1
OAS3	Q9Y6K5	386	0	-16	-7	11	1.0	0.9	0.9	1.1
CCT6A	P40227	406	-2	13	-7	10	1.0	1.1	0.9	1.1
BLOC1S3	Q6QNY0	180	-1	-3	-7	10	1.0	1.0	0.9	1.1
KCNA3	P22001	556	-5	-4	-7	10	1.0	1.0	0.9	1.1
NMRK1	Q9NWW6	194	-6	-4	-7	10	0.9	1.0	0.9	1.1
SCML2	Q9UQR0	559	-5	-4	-7	10	1.0	1.0	0.9	1.1
PSMB10	P40306	83	-6	-11	-7	10	0.9	0.9	0.9	1.1
SYNJ1	O43426	799	1	11	-7	10	1.0	1.1	0.9	1.1
LPXN	O60711	340	-3	5	-7	10	1.0	1.1	0.9	1.1
PLEKHF1	Q96S99	21	-3	-2	-7	10	1.0	1.0	0.9	1.1
EFHD2	Q96C19	172	-4	-6	-7	10	1.0	0.9	0.9	1.1
ZNF280D	Q6N043	487	-5	-8	-7	10	1.0	0.9	0.9	1.1
MAP3K4	Q9Y6R4	596	-7	2	-7	9	0.9	1.0	0.9	1.1
PHGDH	O43175	18	-3	-4	-7	9	1.0	1.0	0.9	1.1
MED28	Q9H204	93	-2	-5	-7	9	1.0	1.0	0.9	1.1

CIAPIN1	Q6FI81	116	-1	-10	-7	9	1.0	0.9	0.9	1.1
CORO1A	P31146	40	-2	-12	-7	9	1.0	0.9	0.9	1.1
ZNF292	O60281	55	-5	-15	-7	9	1.0	0.9	0.9	1.1
MACF1	Q9UPN3	5346	2	3	-7	9	1.0	1.0	0.9	1.1
POLR1B	Q9H9Y6	307	-2	-4	-7	9	1.0	1.0	0.9	1.1
MRPS27	Q92552	49	3	-8	-7	9	1.0	0.9	0.9	1.1
HMCES	Q96FZ2	39	-1	-9	-7	9	1.0	0.9	0.9	1.1
GEMIN2	O14893	34	2	-17	-7	9	1.0	0.9	0.9	1.1
KRI1	Q8N9T8	591	-9	-19	-7	9	0.9	0.8	0.9	1.1
HK2	P52789	158	-4	-21	-7	9	1.0	0.8	0.9	1.1
ARHGEF6	Q15052	25	-3	-4	-7	8	1.0	1.0	0.9	1.1
TBCE	Q15813	141	-9	-4	-7	8	0.9	1.0	0.9	1.1
ECHS1	P30084	62	0	-5	-7	8	1.0	1.0	0.9	1.1
SEC23A	Q15436	74	-5	-14	-7	8	1.0	0.9	0.9	1.1
SEC23B	Q15437	74	-5	-14	-7	8	1.0	0.9	0.9	1.1
ZAP70	P43403	510	-7	-22	-7	8	0.9	0.8	0.9	1.1
cDNA	B4DLN1	45	-7	4	-7	8	0.9	1.0	0.9	1.1
RNH1	P13489	75	-3	-5	-7	8	1.0	1.0	0.9	1.1
KIAA2013	Q8IYS2	185	13	-5	-7	8	1.1	1.0	0.9	1.1
DGKA	P23743	95	-1	-6	-7	8	1.0	0.9	0.9	1.1
UAP1L1	Q3KQV9	57	-8	-7	-7	8	0.9	0.9	0.9	1.1
TBC1D1	Q86TI0	161	-4	-7	-7	8	1.0	0.9	0.9	1.1
CAPN2	P17655	341	8	-8	-7	8	1.1	0.9	0.9	1.1
PLCL2	Q9UPR0	425	-2	-3	-7	7	1.0	1.0	0.9	1.1
PGM3	O95394	200	-2	-4	-7	7	1.0	1.0	0.9	1.1
SMCHD1	A6NHR9	1856	0	-4	-7	7	1.0	1.0	0.9	1.1
RPS4X	P62701	41	-4	-4	-7	7	1.0	1.0	0.9	1.1
CCDC25	Q86WR0	150	-5	-11	-7	7	1.0	0.9	0.9	1.1
PDE12	Q6L8Q7	119	-10	-15	-7	7	0.9	0.9	0.9	1.1
NAIF1	Q69YI7	61	1	-23	-7	7	1.0	0.8	0.9	1.1
MED7	O43513	197	-14	13	-7	7	0.9	1.1	0.9	1.1
HNRNPUL2-BS	H3BQZ7	518	-3	-3	-7	7	1.0	1.0	0.9	1.1
DNPEP	Q9ULA0	413	-7	-5	-7	7	0.9	1.0	0.9	1.1
WDR11	Q9BZH6	1029	11	-7	-7	7	1.1	0.9	0.9	1.1
AKAP9	Q99996	1156	-5	-10	-7	7	1.0	0.9	0.9	1.1
SYMPK	Q92797	969	-11	-12	-7	7	0.9	0.9	0.9	1.1
THEMIS2	Q5TEJ8	110	-2	-13	-7	7	1.0	0.9	0.9	1.1
BDH1	Q02338	221	-1	-15	-7	7	1.0	0.9	0.9	1.1
ASUN	Q9NVM9	406	4	7	-7	6	1.0	1.1	0.9	1.1
EXOC1	Q9NV70	114	-1	5	-7	6	1.0	1.0	0.9	1.1
WDSUB1	Q8N9V3	316	-1	-3	-7	6	1.0	1.0	0.9	1.1
BTRC	Q9Y297	206	3	-5	-7	6	1.0	1.0	0.9	1.1
PIP4K2A	P48426	94	1	-8	-7	6	1.0	0.9	0.9	1.1
PIP4K2B	P78356	99	1	-8	-7	6	1.0	0.9	0.9	1.1
SMCHD1	A6NHR9	1286	-2	-12	-7	6	1.0	0.9	0.9	1.1
ZNF251	Q9BRH9	410	4	-13	-7	6	1.0	0.9	0.9	1.1
ZNF251	Q9BRH9	407	4	-13	-7	6	1.0	0.9	0.9	1.1
STARD3	Q14849	291	-10	-16	-7	6	0.9	0.9	0.9	1.1
ASH2L	Q9UBL3	120	-7	-22	-7	6	0.9	0.8	0.9	1.1
NPRL3	Q12980	278	3	-6	-7	6	1.0	0.9	0.9	1.1
XIAP	P98170	12	-8	-7	-7	6	0.9	0.9	0.9	1.1
ARHGEF2	Q92974	306	-2	-2	-7	5	1.0	1.0	0.9	1.1
YWHAQ	P27348	134	0	-5	-7	5	1.0	1.0	0.9	1.1
KAT6B	Q8WYB5	252	-15	3	-7	5	0.9	1.0	0.9	1.0
LYST	Q99698	20	-6	-8	-7	5	0.9	0.9	0.9	1.0

KIAA1429	Q69YN4	781	-4	-10	-7	5	1.0	0.9	0.9	1.0
THOC2	Q8NI27	996	-18	-19	-7	5	0.9	0.8	0.9	1.0
NEK9	Q8TD19	506	5	8	-7	4	1.1	1.1	0.9	1.0
NLRP1	Q9C000	11	5	1	-7	4	1.1	1.0	0.9	1.0
STAT5A	P42229	101	-3	-1	-7	4	1.0	1.0	0.9	1.0
NUP88	Q99567	561	0	-4	-7	4	1.0	1.0	0.9	1.0
SART3	Q15020	486	-1	-7	-7	4	1.0	0.9	0.9	1.0
PDPR	Q8NCN5	357	2	-8	-7	4	1.0	0.9	0.9	1.0
FPGT	O14772	401	-22	-19	-7	4	0.8	0.8	0.9	1.0
PDS5B	Q9NTI5	571	-3	-6	-7	4	1.0	0.9	0.9	1.0
SEPT6	Q14141	14	-3	-7	-7	4	1.0	0.9	0.9	1.0
PPP1CA	P62136	171	-5	-10	-7	4	1.0	0.9	0.9	1.0
PPP1CB	P62140	170	-5	-10	-7	4	1.0	0.9	0.9	1.0
POP1	Q99575	530	-4	-3	-7	3	1.0	1.0	0.9	1.0
ACSL1	P33121	297	-4	-4	-7	3	1.0	1.0	0.9	1.0
LRRC45	Q96CN5	385	-14	-5	-7	3	0.9	1.0	0.9	1.0
METAP1	P53582	36	-2	-7	-7	3	1.0	0.9	0.9	1.0
RSBN1	Q5VWQ0	282	6	-13	-7	3	1.1	0.9	0.9	1.0
RPN1	P04843	545	-7	-4	-7	3	0.9	1.0	0.9	1.0
ATP5A1	P25705	244	-3	-4	-7	3	1.0	1.0	0.9	1.0
PLEC	Q15149	965	2	-12	-7	3	1.0	0.9	0.9	1.0
FBXO7	Q9Y3I1	286	-1	-15	-7	3	1.0	0.9	0.9	1.0
SHARPIN	Q9H0F6	275	1	3	-7	2	1.0	1.0	0.9	1.0
C10orf12	Q8N655	1236	-11	-21	-7	2	0.9	0.8	0.9	1.0
TAB3	Q8N5C8	564	-2	8	-7	2	1.0	1.1	0.9	1.0
UPF1	Q92900	237	-6	5	-7	2	0.9	1.1	0.9	1.0
TRIP12	Q14669	156	-12	-1	-7	2	0.9	1.0	0.9	1.0
EIF3D	O15371	258	-4	-9	-7	2	1.0	0.9	0.9	1.0
ZMYND8	Q9ULU4	138	3	-5	-7	1	1.0	1.0	0.9	1.0
ZBTB1	Q9Y2K1	238	3	-5	-7	1	1.0	1.0	0.9	1.0
LRMP	Q12912	223	-4	-8	-7	1	1.0	0.9	0.9	1.0
UBA7	P41226	145	-5	-1	-7	1	1.0	1.0	0.9	1.0
PSMA6	P60900	161	-8	-6	-7	1	0.9	0.9	0.9	1.0
FPGT	O14772	531	20	-7	-7	1	1.3	0.9	0.9	1.0
FAM175B	Q15018	237	1	-10	-7	1	1.0	0.9	0.9	1.0
CBR1	P16152	150	3	-2	-7	0	1.0	1.0	0.9	1.0
SENP7	Q9BQF6	125	-10	-6	-7	0	0.9	0.9	0.9	1.0
DDX3Y	O15523	126	-15	-10	-7	0	0.9	0.9	0.9	1.0
HSPA8	P11142	603	-7	-24	-7	0	0.9	0.8	0.9	1.0
STRN	O43815	740	-4	1	-7	-1	1.0	1.0	0.9	1.0
FAM65B	Q9Y4F9	820	-4	-7	-7	-1	1.0	0.9	0.9	1.0
CASP1	P29466	285	-6	-9	-7	-1	0.9	0.9	0.9	1.0
XPO6	Q96QU8	547	4	-40	-7	-1	1.0	0.7	0.9	1.0
STX11	O75558	102	2	-3	-7	-1	1.0	1.0	0.9	1.0
AQR	O60306	449	-2	-7	-7	-1	1.0	0.9	0.9	1.0
IQSEC1	Q6DN90	636	-12	-13	-7	-1	0.9	0.9	0.9	1.0
RALYL	Q86SE5	51	-8	-14	-7	-1	0.9	0.9	0.9	1.0
CLIC6	Q96NY7	641	-19	-19	-7	-1	0.8	0.8	0.9	1.0
CLIC5	Q9NZA1	345	-19	-19	-7	-1	0.8	0.8	0.9	1.0
ACSL4	O60488	595	-21	-23	-7	-1	0.8	0.8	0.9	1.0
CRACR2A	Q9BSW2	220	-1	-3	-7	-2	1.0	1.0	0.9	1.0
BMS1	Q14692	101	0	-8	-7	-2	1.0	0.9	0.9	1.0
RHOG	P84095	157	-4	-11	-7	-2	1.0	0.9	0.9	1.0
HSP90AA1	P07900	374	2	-15	-7	-2	1.0	0.9	0.9	1.0
UQCRC1	P31930	453	0	-12	-7	-2	1.0	0.9	0.9	1.0

RAPGEF1	Q13905	958	-2	-12	-7	-3	1.0	0.9	0.9	1.0
GTF3C1	Q12789	1999	-1	-12	-7	-3	1.0	0.9	0.9	1.0
MAP3K3	Q99759	518	5	-22	-7	-3	1.0	0.8	0.9	1.0
CA5B	Q9Y2D0	149	-2	-3	-7	-3	1.0	1.0	0.9	1.0
CLIP1	P30622	1022	3	-4	-7	-3	1.0	1.0	0.9	1.0
CLASP2	O75122	129	2	-9	-7	-3	1.0	0.9	0.9	1.0
TTC5	Q8N0Z6	439	-2	-10	-7	-4	1.0	0.9	0.9	1.0
DLGAP4	Q9Y2H0	823	-7	-13	-7	-4	0.9	0.9	0.9	1.0
SMCHD1	A6NHR9	1710	-4	-4	-7	-4	1.0	1.0	0.9	1.0
OSBPL8	Q9BZF1	255	-9	-6	-7	-4	0.9	0.9	0.9	1.0
SEPT6	Q14141	42	-6	-10	-7	-4	0.9	0.9	0.9	1.0
USP9Y	O00507	160	5	-16	-7	-4	1.0	0.9	0.9	1.0
USP9X	Q93008	159	5	-16	-7	-4	1.0	0.9	0.9	1.0
COPA	P53621	1191	-5	-4	-7	-5	1.0	1.0	0.9	1.0
ACTR3	P61158	189	-6	-5	-7	-5	0.9	1.0	0.9	1.0
FLAD1	Q8NFF5	409	-3	-10	-7	-5	1.0	0.9	0.9	1.0
TRIO	O75962	2224	-13	4	-7	-5	0.9	1.0	0.9	1.0
NAPA	P54920	66	0	-2	-7	-5	1.0	1.0	0.9	1.0
GLG1	Q92896	515	-7	-13	-7	-5	0.9	0.9	0.9	1.0
NT5C3A	Q9H0P0	73	1	-4	-7	-6	1.0	1.0	0.9	0.9
G3BP2	Q9UN86	73	-8	-7	-7	-6	0.9	0.9	0.9	0.9
KIF5C	O60282	296	2	-10	-7	-6	1.0	0.9	0.9	0.9
KIF5B	P33176	294	2	-10	-7	-6	1.0	0.9	0.9	0.9
RPS27L	Q71UM5	77	-6	-6	-7	-7	0.9	0.9	0.9	0.9
FBXL15	Q9H469	139	-6	-8	-7	-7	0.9	0.9	0.9	0.9
PPP1R21	Q6ZMI0	468	-1	1	-7	-7	1.0	1.0	0.9	0.9
PAK1	Q13153	411	2	-8	-7	-7	1.0	0.9	0.9	0.9
IFI16	Q16666	737	12	-3	-7	-8	1.1	1.0	0.9	0.9
PIK3R6	Q5UE93	713	-10	-11	-7	-8	0.9	0.9	0.9	0.9
TRIM28	Q13263	88	-12	-25	-7	-8	0.9	0.8	0.9	0.9
HDAC1	Q13547	284	-5	-6	-7	-9	1.0	0.9	0.9	0.9
SETX	Q7Z333	1959	8	-2	-7	-9	1.1	1.0	0.9	0.9
PIK3CB	P42338	116	1	-11	-7	-9	1.0	0.9	0.9	0.9
TES	Q9UGI8	164	-6	-13	-7	-9	0.9	0.9	0.9	0.9
GSK3B	P49841	76	-2	-2	-7	-10	1.0	1.0	0.9	0.9
PPP2R1A	P30153	148	1	-3	-7	-10	1.0	1.0	0.9	0.9
PPP2R1B	P30154	160	1	-3	-7	-10	1.0	1.0	0.9	0.9
MDN1	Q9NU22	1867	-9	-8	-7	-10	0.9	0.9	0.9	0.9
RASA3	Q14644	67	-21	-9	-7	-11	0.8	0.9	0.9	0.9
CPSF2	Q9P2I0	765	-9	-13	-7	-11	0.9	0.9	0.9	0.9
EWSR1	Q01844	543	-5	-4	-7	-12	1.0	1.0	0.9	0.9
TBC1D15	Q8TC07	24	-4	-32	-7	-13	1.0	0.8	0.9	0.9
RAB18	Q9NP72	110	-3	-7	-7	-13	1.0	0.9	0.9	0.9
PCBP1	Q15365	201	-5	-8	-7	-14	1.0	0.9	0.9	0.9
TBXAS1	P24557	418	4	-8	-7	-14	1.0	0.9	0.9	0.9
RHOF	Q9HBH0	121	1	-1	-7	-17	1.0	1.0	0.9	0.9
BAZ1B	Q9UIG0	1231	-14	-33	-7	-17	0.9	0.8	0.9	0.9
HSD17B12	Q53GQ0	215	9	-8	-7	-18	1.1	0.9	0.9	0.8
PADI2	Q9Y2J8	647	-1	-6	-7	-22	1.0	0.9	0.9	0.8
TRIM28	Q13263	229	-4	-7	-7	-24	1.0	0.9	0.9	0.8
RANBP2	P49792	1499	-20	-2	-7	-25	0.8	1.0	0.9	0.8
TUBA4A	P68366	129	12	-5	-7	-30	1.1	1.0	0.9	0.8
VCL	P18206	85	-6	-10	-7	-39	0.9	0.9	0.9	0.7
C5orf51	A6NDU8	120	7	-47	-7	-44	1.1	0.7	0.9	0.7
ANK1	P16157	476	-1	-5	-7	-65	1.0	1.0	0.9	0.6

RABGAP1	Q9Y3P9	155	-3	-6	-7	-71	1.0	0.9	0.9	0.6
MGLL	Q99685	201	7	1	-7	-80	1.1	1.0	0.9	0.6
LIMS1	P48059	222	5	-2	-7	-108	1.1	1.0	0.9	0.5
LIMS2	Q7Z4I7	227	5	-2	-7	-108	1.1	1.0	0.9	0.5
MYLK	Q15746	1339	7	2	-7	-162	1.1	1.0	0.9	0.4
MGLL	Q99685	208	-5	-9	-7	-167	1.0	0.9	0.9	0.4
LMF2	Q9BU23	659	-8	-3	-7	66	0.9	1.0	0.9	2.9
KEAP1	Q14145	288	-6	-1	-7	58	0.9	1.0	0.9	2.4
PAFAH2	Q99487	72	-15	-1	-7	57	0.9	1.0	0.9	2.3
TXNIP	Q9H3M7	36	-4	-8	-7	43	1.0	0.9	0.9	1.8
ATAD1	Q8NBU5	137	0	-6	-7	35	1.0	0.9	0.9	1.5
DDX1	Q92499	638	1	-7	-7	34	1.0	0.9	0.9	1.5
DDX17	Q92841	166	-13	-10	-7	34	0.9	0.9	0.9	1.5
ENOSF1	Q7L5Y1	67	-5	-9	-7	33	1.0	0.9	0.9	1.5
CAMK4	Q16566	202	-4	2	-7	32	1.0	1.0	0.9	1.5
IFT74	Q96LB3	53	-3	-3	-7	32	1.0	1.0	0.9	1.5
MAP4K4	O95819	883	2	-1	-7	31	1.0	1.0	0.9	1.4
OAS1	P00973	54	7	-12	-7	31	1.1	0.9	0.9	1.4
HLA-A	P01892	188	-4	-8	-7	28	1.0	0.9	0.9	1.4
HLA-C	P10321	188	-4	-8	-7	28	1.0	0.9	0.9	1.4
HLA-C	P30510	188	-4	-8	-7	28	1.0	0.9	0.9	1.4
HLA-B	P30479	188	-4	-8	-7	28	1.0	0.9	0.9	1.4
DOCK11	Q5JSL3	1190	9	-8	-7	27	1.1	0.9	0.9	1.4
C3AR1	Q16581	468	-9	-29	-7	27	0.9	0.8	0.9	1.4
EPPK1	A0A087X1U6	1380	-5	-11	-7	26	1.0	0.9	0.9	1.3
TRIM22	Q8IYM9	416	3	-3	-7	25	1.0	1.0	0.9	1.3
ACIN1	Q9UKV3	546	-2	-10	-7	25	1.0	0.9	0.9	1.3
CTSH	P09668	327	-4	-8	-7	24	1.0	0.9	0.9	1.3
CD86	P42081	322	1	-11	-7	24	1.0	0.9	0.9	1.3
STAT2	P52630	74	-7	-2	-7	24	0.9	1.0	0.9	1.3
CEACAM4	O75871	234	-3	-11	-7	24	1.0	0.9	0.9	1.3
NUDT8	Q8WV74	224	-5	1	-7	23	1.0	1.0	0.9	1.3
DDX3X	O00571	317	-3	-4	-7	23	1.0	1.0	0.9	1.3
DDX3Y	O15523	315	-3	-4	-7	23	1.0	1.0	0.9	1.3
DYSF	O75923	1578	-1	2	-7	22	1.0	1.0	0.9	1.3
ZBTB1	Q9Y2K1	532	3	-3	-7	22	1.0	1.0	0.9	1.3
ZC3HAV1	Q7Z2W4	521	-5	-6	-7	22	1.0	0.9	0.9	1.3
RASA4	O43374	396	-9	-16	-7	22	0.9	0.9	0.9	1.3
COL4A3BP	Q9Y5P4	70	-4	-7	-7	22	1.0	0.9	0.9	1.3
KANSL2	Q9H9L4	117	4	-9	-7	22	1.0	0.9	0.9	1.3
HPS5	Q9UPZ3	377	-27	-10	-7	22	0.8	0.9	0.9	1.3
ELP3	Q9H9T3	227	1	6	-7	21	1.0	1.1	0.9	1.3
GPCPD1	Q9NPB8	642	-7	-1	-7	21	0.9	1.0	0.9	1.3
RPL12	P30050	141	-7	-5	-7	21	0.9	1.0	0.9	1.3
LSM12	Q3MHD2	20	-17	7	-7	21	0.9	1.1	0.9	1.3
MAF1	Q9H063	34	9	-7	-7	21	1.1	0.9	0.9	1.3
CAST	P20810	241	-1	-13	-7	21	1.0	0.9	0.9	1.3
NCF2	P19878	291	-6	-17	-7	21	0.9	0.9	0.9	1.3
TOP1	P11387	504	-11	-19	-7	21	0.9	0.8	0.9	1.3
NADK	O95544	23	-7	14	-7	20	0.9	1.2	0.9	1.3
ACAA1	P09110	218	-4	-6	-7	20	1.0	0.9	0.9	1.3
WDFY3	Q8IZQ1	667	0	13	-7	20	1.0	1.1	0.9	1.2
NT5C	Q8TCD5	166	-14	-3	-7	20	0.9	1.0	0.9	1.2
ZMYM3	Q14202	432	-4	-4	-7	20	1.0	1.0	0.9	1.2
APOBR	Q0VD83	257	1	-11	-7	20	1.0	0.9	0.9	1.2

DHX29	Q7Z478	670	1	-11	-7	20	1.0	0.9	0.9	1.2
FRYL	O94915	888	-4	3	-7	19	1.0	1.0	0.9	1.2
ZNF136	P52737	291	2	-4	-7	19	1.0	1.0	0.9	1.2
THYN1	Q9P016	89	-4	-6	-7	19	1.0	0.9	0.9	1.2
UBR4	Q5T4S7	2554	-4	-8	-7	19	1.0	0.9	0.9	1.2
PDCD4	Q53EL6	288	2	-9	-7	19	1.0	0.9	0.9	1.2
PPA1	Q15181	254	3	0	-7	19	1.0	1.0	0.9	1.2
YLPM1	P49750	1772	-1	-9	-7	19	1.0	0.9	0.9	1.2
MAP2K1	Q02750	376	2	-10	-7	19	1.0	0.9	0.9	1.2
SPTBN1	Q01082	1900	-1	-5	-7	18	1.0	1.0	0.9	1.2
ANKRD54	Q6NXT1	265	-7	-3	-7	18	0.9	1.0	0.9	1.2
IPO4	Q8TEX9	269	-1	-5	-7	18	1.0	1.0	0.9	1.2
CPSF6	Q16630	159	-2	-6	-7	18	1.0	0.9	0.9	1.2
PPFIBP2	Q8ND30	361	-12	5	-7	17	0.9	1.0	0.9	1.2
LETM1	O95202	552	-4	-5	-7	17	1.0	1.0	0.9	1.2
RCC2	Q9P258	428	-3	-5	-7	17	1.0	1.0	0.9	1.2
CHORDC1	Q9UHD1	86	1	-6	-7	17	1.0	0.9	0.9	1.2
ZFAND3	Q9H8U3	35	-2	0	-7	17	1.0	1.0	0.9	1.2
SETX	Q7Z333	511	8	-2	-7	17	1.1	1.0	0.9	1.2
PPA2	Q9H2U2	171	0	-7	-7	17	1.0	0.9	0.9	1.2
HINT1	P49773	84	-9	-12	-7	17	0.9	0.9	0.9	1.2
U2SURP	O15042	65	14	-9	-7	16	1.2	0.9	0.9	1.2
UPF1	Q92900	188	3	0	-7	16	1.0	1.0	0.9	1.2
TEC	P42680	449	3	-6	-7	16	1.0	0.9	0.9	1.2
GTF2I	P78347	215	-2	-7	-7	16	1.0	0.9	0.9	1.2
SEC23B	Q15437	40	16	-8	-7	16	1.2	0.9	0.9	1.2
HIST2H3PS2	Q5TEC6	111	22	12	-7	15	1.3	1.1	0.9	1.2
SH2D1A	O60880	124	-2	0	-7	15	1.0	1.0	0.9	1.2
TBC1D8	O95759	24	-28	-10	-7	15	0.8	0.9	0.9	1.2
CMTM7	Q96FZ5	12	-12	-11	-7	15	0.9	0.9	0.9	1.2
LRP1	Q07954	3847	-5	-32	-7	15	1.0	0.8	0.9	1.2
IRF9	Q00978	329	-5	-2	-7	15	1.0	1.0	0.9	1.2
RANBP2	P49792	3032	0	-5	-7	15	1.0	1.0	0.9	1.2
ADAR	P55265	909	-2	-5	-7	15	1.0	1.0	0.9	1.2
NCOA3	Q9Y6Q9	716	8	-5	-7	15	1.1	1.0	0.9	1.2
DUS1L	Q6P1R4	213	-1	-8	-7	15	1.0	0.9	0.9	1.2
LRRC41	Q15345	297	2	-4	-7	14	1.0	1.0	0.9	1.2
TTF2	Q9UNY4	759	-10	-9	-7	14	0.9	0.9	0.9	1.2
PTK2B	Q14289	44	-2	-10	-7	14	1.0	0.9	0.9	1.2
SUPT6H	Q7KZ85	1281	0	-10	-7	14	1.0	0.9	0.9	1.2
FTSJ3	Q8IY81	577	-5	-12	-7	14	1.0	0.9	0.9	1.2
ACAP3	Q96P50	373	4	7	-7	14	1.0	1.1	0.9	1.2
SMAP2	Q8WU79	196	-4	-5	-7	14	1.0	1.0	0.9	1.2
CNKSR1	Q969H4	42	-13	-8	-7	14	0.9	0.9	0.9	1.2
MORC3	Q14149	307	2	0	-7	13	1.0	1.0	0.9	1.1
ZC3H11A	O75152	209	-4	-5	-7	13	1.0	1.0	0.9	1.1
ATM	Q13315	2021	-2	-5	-7	13	1.0	1.0	0.9	1.1
BCL7B	Q9BQE9	189	-5	-7	-7	13	1.0	0.9	0.9	1.1
TIGAR	Q9NQ88	114	-2	-8	-7	13	1.0	0.9	0.9	1.1
FLCN	Q8NFG4	215	-25	-16	-7	13	0.8	0.9	0.9	1.1
UBA2	Q9UBT2	173	2	-1	-7	13	1.0	1.0	0.9	1.1
PPP3CB	P16298	477	-2	-3	-7	13	1.0	1.0	0.9	1.1
TNFAIP3	P21580	612	3	-6	-7	13	1.0	0.9	0.9	1.1
TAPBP	O15533	115	-7	-8	-7	13	0.9	0.9	0.9	1.1
PNO1	Q9NRX1	64	1	-9	-7	13	1.0	0.9	0.9	1.1

PPP1R3D	Q95685	60	-9	-2	-7	12	0.9	1.0	0.9	1.1
WDFY4	Q6ZS81	1665	-6	-6	-7	12	0.9	0.9	0.9	1.1
ATP1A1	P05023	705	-4	-8	-7	12	1.0	0.9	0.9	1.1
ATP1A3	P13637	695	-4	-8	-7	12	1.0	0.9	0.9	1.1
PMPCA	Q10713	225	-4	-9	-7	12	1.0	0.9	0.9	1.1
COPB1	P53618	684	-7	2	-7	12	0.9	1.0	0.9	1.1
DHX37	Q8IY37	766	-16	0	-7	12	0.9	1.0	0.9	1.1
PPP1CA	P62136	158	-3	-4	-7	12	1.0	1.0	0.9	1.1
PPP1CB	P62140	157	-3	-4	-7	12	1.0	1.0	0.9	1.1
FADD	Q13158	98	-8	-17	-7	12	0.9	0.9	0.9	1.1
MCTP2	Q6DN12	212	-13	3	-7	11	0.9	1.0	0.9	1.1
RPS3A	P61247	111	-1	2	-7	11	1.0	1.0	0.9	1.1
APPL1	Q9UKG1	549	5	1	-7	11	1.1	1.0	0.9	1.1
NUP88	Q99567	713	1	-5	-7	11	1.0	1.0	0.9	1.1
C9orf64	Q5T6V5	227	-4	-9	-7	11	1.0	0.9	0.9	1.1
TUBGCP2	Q9BSJ2	469	-1	-11	-7	11	1.0	0.9	0.9	1.1
IRAK3	Q9Y616	581	-2	-18	-7	11	1.0	0.8	0.9	1.1
NAPRT	Q6XQN6	484	-10	-1	-7	11	0.9	1.0	0.9	1.1
ITPKC	Q96DU7	434	-11	-4	-7	11	0.9	1.0	0.9	1.1
SDHC	Q99643	107	-2	-5	-7	11	1.0	1.0	0.9	1.1
WDR7	Q9Y4E6	289	-8	-6	-7	11	0.9	0.9	0.9	1.1
USP5	P45974	195	1	-6	-7	11	1.0	0.9	0.9	1.1
MED19	A0JLT2	62	-6	-11	-7	11	0.9	0.9	0.9	1.1
ZNF408	Q9H9D4	685	-18	-12	-7	11	0.9	0.9	0.9	1.1
KANSL1	A0A0G2JNT7	1050	-9	-14	-7	11	0.9	0.9	0.9	1.1
NTPCR	Q9BSD7	101	-17	-3	-7	10	0.9	1.0	0.9	1.1
TXNRD2	Q9NNW7	54	-1	-3	-7	10	1.0	1.0	0.9	1.1
NDUFS1	P28331	554	-1	-4	-7	10	1.0	1.0	0.9	1.1
COPB1	P53618	143	5	-6	-7	10	1.0	0.9	0.9	1.1
UHRF1	Q96T88	497	10	-9	-7	10	1.1	0.9	0.9	1.1
SPTAN1	Q13813	315	-2	-9	-7	10	1.0	0.9	0.9	1.1
SRBD1	Q8N5C6	571	-3	-9	-7	10	1.0	0.9	0.9	1.1
IQGAP1	P46940	660	1	-13	-7	10	1.0	0.9	0.9	1.1
EFL1	Q7Z2Z2	921	1	-19	-7	10	1.0	0.8	0.9	1.1
SDHA	P31040	191	-10	11	-7	10	0.9	1.1	0.9	1.1
ARF5	P84085	62	-1	-5	-7	10	1.0	1.0	0.9	1.1
PPA1	Q15181	274	-2	-6	-7	10	1.0	0.9	0.9	1.1
FOXK1	P85037	665	5	-6	-7	10	1.0	0.9	0.9	1.1
CHCHD3	Q9NX63	112	-6	-12	-7	10	0.9	0.9	0.9	1.1
PSMD11	O00231	202	0	2	-7	9	1.0	1.0	0.9	1.1
IRF2BP1	Q8IU81	15	1	0	-7	9	1.0	1.0	0.9	1.1
MAP2K7	O14733	260	-9	-2	-7	9	0.9	1.0	0.9	1.1
OSBPL1A	Q9BXW6	297	-3	-3	-7	9	1.0	1.0	0.9	1.1
COMTD1	Q86VU5	45	8	-4	-7	9	1.1	1.0	0.9	1.1
CDK12	Q9NYV4	862	-8	-5	-7	9	0.9	1.0	0.9	1.1
HSP90AB1	P08238	590	-5	-4	-7	9	1.0	1.0	0.9	1.1
BUB3	O43684	129	0	-5	-7	9	1.0	1.0	0.9	1.1
UGP2	Q16851	123	1	-5	-7	9	1.0	1.0	0.9	1.1
PDCD11	Q14690	1283	-16	-6	-7	9	0.9	0.9	0.9	1.1
SMARCE1	Q969G3	274	-2	-7	-7	9	1.0	0.9	0.9	1.1
DCP2	Q8IU60	73	-2	-8	-7	9	1.0	0.9	0.9	1.1
GIMAP7	Q8NHV1	195	-1	-11	-7	9	1.0	0.9	0.9	1.1
GEMIN5	Q8TEQ6	441	2	-13	-7	9	1.0	0.9	0.9	1.1
ZHX2	Q9Y6X8	11	-6	-15	-7	9	0.9	0.9	0.9	1.1
RBL2	Q08999	635	2	2	-7	8	1.0	1.0	0.9	1.1

GMEB2	Q9UKD1	171	6	-8	-7	8	1.1	0.9	0.9	1.1
IL17RA	Q96F46	632	-9	-13	-7	8	0.9	0.9	0.9	1.1
KMT5B	Q4FZB7	275	9	-18	-7	8	1.1	0.9	0.9	1.1
FAM98C	Q17RN3	92	0	-23	-7	8	1.0	0.8	0.9	1.1
HECTD1	Q9ULT8	2071	-12	1	-7	8	0.9	1.0	0.9	1.1
PIK3CG	P48736	817	-3	-1	-7	8	1.0	1.0	0.9	1.1
SIPA1	Q96FS4	755	-8	-10	-7	8	0.9	0.9	0.9	1.1
PUS7	Q96PZ0	38	0	-10	-7	8	1.0	0.9	0.9	1.1
ARPC1B	O15143	70	4	-10	-7	8	1.0	0.9	0.9	1.1
SH3BP1	Q9Y3L3	102	3	-11	-7	8	1.0	0.9	0.9	1.1
MED12	Q93074	1651	1	-11	-7	8	1.0	0.9	0.9	1.1
MTMR14	Q8NCE2	252	-4	5	-7	7	1.0	1.0	0.9	1.1
SETD1A	O15047	1648	-4	-7	-7	7	1.0	0.9	0.9	1.1
TARBP1	Q13395	1556	-12	-10	-7	7	0.9	0.9	0.9	1.1
ME2	P23368	185	-2	-21	-7	7	1.0	0.8	0.9	1.1
TMOD3	Q9NYL9	231	-8	2	-7	7	0.9	1.0	0.9	1.1
MSTO1	Q9BUK6	46	6	-1	-7	7	1.1	1.0	0.9	1.1
PSMA4	P25789	107	-4	-5	-7	7	1.0	1.0	0.9	1.1
NUP62	P37198	475	-7	-10	-7	7	0.9	0.9	0.9	1.1
PI4KA	P42356	1902	-4	-10	-7	7	1.0	0.9	0.9	1.1
EFR3A	Q14156	237	-15	2	-7	6	0.9	1.0	0.9	1.1
FNBP1	Q96RU3	609	-3	-4	-7	6	1.0	1.0	0.9	1.1
HNRNPLL	Q8WVV9	464	0	-6	-7	6	1.0	0.9	0.9	1.1
HCCS	P53701	178	-3	-8	-7	6	1.0	0.9	0.9	1.1
ZNF672	Q499Z4	24	3	-8	-7	6	1.0	0.9	0.9	1.1
RNF219	Q5W0B1	321	-9	-10	-7	6	0.9	0.9	0.9	1.1
CORO1A	P31146	345	-2	-10	-7	6	1.0	0.9	0.9	1.1
HNRNPD	Q14103	126	-4	-15	-7	6	1.0	0.9	0.9	1.1
IKBKAP	O95163	456	-5	4	-7	6	1.0	1.0	0.9	1.1
JAK3	P52333	909	-2	-3	-7	6	1.0	1.0	0.9	1.1
KMT2C	Q8NEZ4	473	-4	-3	-7	6	1.0	1.0	0.9	1.1
PACS1	Q6VY07	616	-2	-7	-7	6	1.0	0.9	0.9	1.1
PTPN6	P29350	102	-4	-9	-7	6	1.0	0.9	0.9	1.1
TRANK1	O15050	1514	1	-11	-7	6	1.0	0.9	0.9	1.1
PARG	Q86W56	711	-7	-1	-7	5	0.9	1.0	0.9	1.1
DFFA	O00273	47	-14	-5	-7	5	0.9	1.0	0.9	1.1
AK2	P54819	92	-8	-5	-7	5	0.9	1.0	0.9	1.1
HSD17B10	Q99714	58	0	-9	-7	5	1.0	0.9	0.9	1.1
PAPSS2	O95340	350	1	-14	-7	5	1.0	0.9	0.9	1.1
MDH1	P40925	154	-4	-15	-7	5	1.0	0.9	0.9	1.1
NUDT3	O95989	25	-4	0	-7	5	1.0	1.0	0.9	1.0
NUDT4	Q9NZJ9	25	-4	0	-7	5	1.0	1.0	0.9	1.0
DICER1	Q9UPY3	199	4	-2	-7	5	1.0	1.0	0.9	1.0
POGZ	Q7Z3K3	1267	1	-4	-7	5	1.0	1.0	0.9	1.0
PTPRC	P08575	760	-4	-5	-7	5	1.0	1.0	0.9	1.0
RB1CC1	Q8TDY2	1236	1	-7	-7	5	1.0	0.9	0.9	1.0
GOLGA7	Q7Z5G4	24	-4	-8	-7	5	1.0	0.9	0.9	1.0
POLR3D	P05423	316	-16	-13	-7	5	0.9	0.9	0.9	1.0
NUMA1	Q14980	1729	-11	-16	-7	5	0.9	0.9	0.9	1.0
WDR48	Q8TAF3	342	-3	-16	-7	5	1.0	0.9	0.9	1.0
SLFN11	Q7Z7L1	760	-27	-18	-7	5	0.8	0.9	0.9	1.0
RNGTT	O60942	419	-14	-4	-7	4	0.9	1.0	0.9	1.0
VPS16	Q9H269	184	-4	-10	-7	4	1.0	0.9	0.9	1.0
MED20	Q9H944	120	2	-10	-7	4	1.0	0.9	0.9	1.0
PREX1	Q8TCU6	963	-7	-15	-7	4	0.9	0.9	0.9	1.0

PPP2R4	Q15257	103	-6	-16	-7	4	0.9	0.9	0.9	1.0
SON	P18583	1551	7	-24	-7	4	1.1	0.8	0.9	1.0
SYNE2	Q8WXH0	5586	6	-7	-7	4	1.1	0.9	0.9	1.0
PUM1	Q14671	1007	2	-26	-7	4	1.0	0.8	0.9	1.0
EIF2S2	P20042	305	-1	-2	-7	3	1.0	1.0	0.9	1.0
PAPOLG	Q9BWT3	530	-1	-2	-7	3	1.0	1.0	0.9	1.0
DUT	P33316	166	1	-7	-7	3	1.0	0.9	0.9	1.0
SMG7	Q92540	345	-2	-15	-7	3	1.0	0.9	0.9	1.0
XPO1	O14980	99	0	6	-7	3	1.0	1.1	0.9	1.0
CDC5L	Q99459	769	-9	-13	-7	3	0.9	0.9	0.9	1.0
STRN	O43815	665	-9	9	-7	2	0.9	1.1	0.9	1.0
RPL10A	P62906	66	-5	-4	-7	2	1.0	1.0	0.9	1.0
TRIM21	P19474	359	1	-7	-7	2	1.0	0.9	0.9	1.0
TRAF2	Q12933	287	-11	-13	-7	2	0.9	0.9	0.9	1.0
FAM117A	Q9C073	79	-8	-15	-7	2	0.9	0.9	0.9	1.0
SETD1A	O15047	1638	-6	-4	-7	2	0.9	1.0	0.9	1.0
ABR	Q12979	158	5	-5	-7	2	1.1	1.0	0.9	1.0
UXS1	Q8NBZ7	230	-4	-9	-7	2	1.0	0.9	0.9	1.0
MTM1	Q13496	53	-1	-10	-7	2	1.0	0.9	0.9	1.0
EML2	O95834	395	-8	-18	-7	2	0.9	0.9	0.9	1.0
UROD	P06132	308	-10	-18	-7	2	0.9	0.9	0.9	1.0
HSPA4	P34932	146	-8	-9	-7	1	0.9	0.9	0.9	1.0
LRRC40	Q9H9A6	438	3	1	-7	1	1.0	1.0	0.9	1.0
SART3	Q15020	729	-6	-2	-7	1	0.9	1.0	0.9	1.0
SUCLG1	P53597	172	-3	-8	-7	1	1.0	0.9	0.9	1.0
ZNF407	Q9C0G0	1753	-3	-14	-7	1	1.0	0.9	0.9	1.0
GARS	P41250	471	-7	-21	-7	1	0.9	0.8	0.9	1.0
PDIA3	P30101	60	-8	-1	-7	0	0.9	1.0	0.9	1.0
URI1	O94763	44	4	-3	-7	0	1.0	1.0	0.9	1.0
NUP88	Q99567	608	-2	-6	-7	0	1.0	0.9	0.9	1.0
ZC3HAV1	Q7Z2W4	82	-2	-7	-7	0	1.0	0.9	0.9	1.0
GTF2E2	P29084	208	-5	-4	-7	-1	1.0	1.0	0.9	1.0
TFCP2	Q12800	453	-4	-6	-7	-1	1.0	0.9	0.9	1.0
ODR4	Q5SWX8	275	-2	-7	-7	-1	1.0	0.9	0.9	1.0
FTO	Q9C0B1	104	-1	-12	-7	-1	1.0	0.9	0.9	1.0
NR3C1	P04150	457	9	6	-7	-1	1.1	1.1	0.9	1.0
NR3C2	P08235	639	9	6	-7	-1	1.1	1.1	0.9	1.0
KIF2A	O00139	406	8	5	-7	-1	1.1	1.1	0.9	1.0
ALAD	P13716	223	-8	-3	-7	-1	0.9	1.0	0.9	1.0
CCDC91	Q7Z6B0	424	-8	-8	-7	-1	0.9	0.9	0.9	1.0
JMJD7	P0C870	132	-5	-8	-7	-1	1.0	0.9	0.9	1.0
HSPA4	P34932	270	0	-26	-7	-1	1.0	0.8	0.9	1.0
AP2B1	P63010	123	0	-8	-7	-2	1.0	0.9	0.9	1.0
AP1B1	Q10567	123	0	-8	-7	-2	1.0	0.9	0.9	1.0
UTY	O14607	381	-21	-9	-7	-2	0.8	0.9	0.9	1.0
SMARCC2	Q8TAQ2	80	2	-12	-7	-2	1.0	0.9	0.9	1.0
PPP6R3	Q5H9R7	830	-12	-13	-7	-2	0.9	0.9	0.9	1.0
BPNT1	O95861	59	6	6	-7	-2	1.1	1.1	0.9	1.0
NMRK1	Q9NWW6	125	-4	-4	-7	-2	1.0	1.0	0.9	1.0
MIF4GD	A9UHW6	49	-5	0	-7	-3	1.0	1.0	0.9	1.0
ARFGEF2	Q9Y6D5	1617	5	-1	-7	-3	1.1	1.0	0.9	1.0
CRNKL1	Q9BZJ0	640	-8	-13	-7	-3	0.9	0.9	0.9	1.0
MAEA	Q7L5Y9	302	-8	-18	-7	-3	0.9	0.9	0.9	1.0
VPS11	Q9H270	44	4	-1	-7	-3	1.0	1.0	0.9	1.0
ACTR10	Q9NZ32	27	-22	-4	-7	-3	0.8	1.0	0.9	1.0

CPSF2	Q9P2I0	763	-6	-6	-7	-3	0.9	0.9	0.9	1.0
PCNT	O95613	2705	0	-9	-7	-3	1.0	0.9	0.9	1.0
PRDX1	Q06830	83	-7	-14	-7	-3	0.9	0.9	0.9	1.0
THEMIS2	Q5TEJ8	146	-5	-4	-7	-4	1.0	1.0	0.9	1.0
SF3B1	O75533	933	-5	-12	-7	-4	1.0	0.9	0.9	1.0
GART	P22102	298	-10	-4	-7	-4	0.9	1.0	0.9	1.0
MMAB	Q96EY8	119	3	-11	-7	-4	1.0	0.9	0.9	1.0
ARFGEF2	Q9Y6D5	1147	-1	-3	-7	-5	1.0	1.0	0.9	1.0
ARFGEF1	Q9Y6D6	1200	-1	-3	-7	-5	1.0	1.0	0.9	1.0
TLR1	Q15399	528	-17	-4	-7	-5	0.9	1.0	0.9	1.0
DCLRE1B	Q9H816	149	2	-6	-7	-5	1.0	0.9	0.9	1.0
TRIM28	Q13263	224	2	-6	-7	-5	1.0	0.9	0.9	1.0
RBBP5	Q15291	212	2	-6	-7	-5	1.0	0.9	0.9	1.0
HERC6	Q8IVU3	557	1	-12	-7	-5	1.0	0.9	0.9	1.0
PLEC	Q15149	1156	1	-13	-7	-5	1.0	0.9	0.9	1.0
VPS18	Q9P253	574	2	4	-7	-5	1.0	1.0	0.9	1.0
EIF3K	Q9UBQ5	190	5	-1	-7	-5	1.0	1.0	0.9	1.0
RASA3	Q14644	58	-1	-2	-7	-5	1.0	1.0	0.9	1.0
ACAP1	Q15027	10	8	-4	-7	-5	1.1	1.0	0.9	1.0
PDS5A	Q29RF7	486	-9	-3	-7	-6	0.9	1.0	0.9	0.9
BCAS3	Q9H6U6	139	-10	-4	-7	-6	0.9	1.0	0.9	0.9
FASN	P49327	1992	4	-14	-7	-6	1.0	0.9	0.9	0.9
CYFIP2	Q96F07	1265	11	-18	-7	-6	1.1	0.8	0.9	0.9
TBL1X	O60907	446	-6	11	-7	-6	0.9	1.1	0.9	0.9
RINT1	Q6NUQ1	720	-5	-3	-7	-6	1.0	1.0	0.9	0.9
HCFC1	P51610	135	-12	-12	-7	-6	0.9	0.9	0.9	0.9
MFSD6	Q6ZSS7	743	-2	-6	-7	-7	1.0	0.9	0.9	0.9
NCBP2-AS2	Q69YL0	63	4	1	-7	-7	1.0	1.0	0.9	0.9
ANKLE2	Q86XL3	203	6	-9	-7	-9	1.1	0.9	0.9	0.9
DSTN	P60981	23	-1	-2	-7	-9	1.0	1.0	0.9	0.9
AP1G1	O43747	353	-1	-3	-7	-9	1.0	1.0	0.9	0.9
PDE12	Q6L8Q7	268	-28	-9	-7	-9	0.8	0.9	0.9	0.9
ILVBL	A1L0T0	608	-12	-14	-7	-9	0.9	0.9	0.9	0.9
CKAP5	Q14008	604	-3	0	-7	-10	1.0	1.0	0.9	0.9
DIAPH2	O60879	655	0	-13	-7	-10	1.0	0.9	0.9	0.9
SDCBP	O00560	239	-20	-4	-7	-10	0.8	1.0	0.9	0.9
POLR2A	P24928	451	13	-8	-7	-11	1.1	0.9	0.9	0.9
RANBP2	P49792	1626	-3	-5	-7	-12	1.0	1.0	0.9	0.9
PBRM1	Q86U86	1163	-15	-22	-7	-12	0.9	0.8	0.9	0.9
MGMT	P16455	24	1	-5	-7	-12	1.0	1.0	0.9	0.9
CASP6	P55212	277	-3	-5	-7	-13	1.0	1.0	0.9	0.9
ANKFY1	Q9P2R3	499	-16	-5	-7	-13	0.9	1.0	0.9	0.9
COPB2	P35606	741	13	10	-7	-13	1.1	1.1	0.9	0.9
WDFY4	Q6ZS81	2226	-16	-2	-7	-14	0.9	1.0	0.9	0.9
ACBD3	Q9H3P7	168	-10	-3	-7	-15	0.9	1.0	0.9	0.9
FAM160A2	Q8N612	522	-6	-17	-7	-15	0.9	0.9	0.9	0.9
FLII	Q13045	560	8	-4	-7	-16	1.1	1.0	0.9	0.9
MICU1	Q9BPX6	83	-3	-12	-7	-18	1.0	0.9	0.9	0.9
DPYD	Q12882	385	5	0	-7	-18	1.1	1.0	0.9	0.8
CSRP1	P21291	40	-4	1	-7	-19	1.0	1.0	0.9	0.8
DCTN3	O75935	140	8	-4	-7	-23	1.1	1.0	0.9	0.8
AIM1	Q9Y4K1	1477	-12	-10	-7	-23	0.9	0.9	0.9	0.8
DOCK8	Q8NF50	590	7	-17	-7	-24	1.1	0.9	0.9	0.8
MKRN1	Q9UHC7	214	-11	-11	-7	-26	0.9	0.9	0.9	0.8
RASGRP2	Q7LDG7	532	-3	-2	-7	-28	1.0	1.0	0.9	0.8

NDRG2	Q9UN36	321	10	-27	-7	-31	1.1	0.8	0.9	0.8
SIPA1	Q96FS4	632	4	-4	-7	-38	1.0	1.0	0.9	0.7
INTS1	Q8N201	2151	-4	-8	-7	-45	1.0	0.9	0.9	0.7
THOC1	Q96FV9	445	-19	-16	-7	-58	0.8	0.9	0.9	0.6
FHL1	Q13642	132	4	-10	-7	-61	1.0	0.9	0.9	0.6
CTCF	P49711	577	20	-17	-7	-67	1.2	0.9	0.9	0.6
DLD	P09622	484	-16	-18	-7	-77	0.9	0.9	0.9	0.6
DMTN	Q08495	336	-2	-11	-7	-78	1.0	0.9	0.9	0.6
ABCB7	O75027	747	1	-10	-8	46	1.0	0.9	0.9	1.9
DNMBP	Q6XZF7	691	-12	-20	-8	45	0.9	0.8	0.9	1.8
FBXL6	Q8N531	368	-25	-27	-8	43	0.8	0.8	0.9	1.8
INPPL1	O15357	926	-7	-9	-8	42	0.9	0.9	0.9	1.7
CASK	O14936	633	-8	-10	-8	40	0.9	0.9	0.9	1.7
PRDM10	Q9NQV6	249	-1	-5	-8	37	1.0	1.0	0.9	1.6
PCNX1	Q96RV3	1621	-10	-3	-8	34	0.9	1.0	0.9	1.5
PRKDC	P78527	1507	-2	1	-8	34	1.0	1.0	0.9	1.5
TMEM209	Q96SK2	367	4	11	-8	33	1.0	1.1	0.9	1.5
DENND1A	Q8TEH3	338	-10	-9	-8	32	0.9	0.9	0.9	1.5
ZNF687	Q8N1G0	672	0	3	-8	32	1.0	1.0	0.9	1.5
BLMH	Q13867	40	-3	-4	-8	31	1.0	1.0	0.9	1.4
PPA2	Q9H2U2	44	-2	-11	-8	31	1.0	0.9	0.9	1.4
GPATCH8	Q9UKJ3	1161	-2	-21	-8	31	1.0	0.8	0.9	1.4
ALDH2	P05091	319	-6	-29	-8	31	0.9	0.8	0.9	1.4
PRRC2A	P48634	437	4	-4	-8	30	1.0	1.0	0.9	1.4
BTF3	P20290	22	-5	-13	-8	30	1.0	0.9	0.9	1.4
KLRD1	Q13241	89	-4	-9	-8	30	1.0	0.9	0.9	1.4
NEK9	Q8TD19	808	-8	-8	-8	28	0.9	0.9	0.9	1.4
MBNL2	Q5VZF2	197	7	4	-8	27	1.1	1.0	0.9	1.4
TANGO6	Q9C0B7	286	-5	-15	-8	27	1.0	0.9	0.9	1.4
FBXO34	Q9NWN3	290	-8	-22	-8	27	0.9	0.8	0.9	1.4
GAK	O14976	87	-4	-2	-8	26	1.0	1.0	0.9	1.3
ZNF592	Q92610	777	14	-1	-8	25	1.2	1.0	0.9	1.3
APOBEC3B	Q9UH17	247	-7	-5	-8	25	0.9	1.0	0.9	1.3
PXN	P49023	582	6	-5	-8	25	1.1	1.0	0.9	1.3
STK17B	O94768	372	-6	-14	-8	25	0.9	0.9	0.9	1.3
HLTF	Q14527	461	-3	-8	-8	25	1.0	0.9	0.9	1.3
PABPC1	P11940	128	1	-8	-8	24	1.0	0.9	0.9	1.3
PABPC4	Q13310	128	1	-8	-8	24	1.0	0.9	0.9	1.3
CS	O75390	101	-3	-15	-8	23	1.0	0.9	0.9	1.3
CRYBG3	Q68DQ2	2290	-8	9	-8	23	0.9	1.1	0.9	1.3
IGLC2	A0A075B6K9	105	-10	-3	-8	23	0.9	1.0	0.9	1.3
CDA	P32320	8	-7	-10	-8	22	0.9	0.9	0.9	1.3
URB2	Q14146	1280	-4	-12	-8	22	1.0	0.9	0.9	1.3
SIGIRR	Q6IA17	403	0	-8	-8	22	1.0	0.9	0.9	1.3
ZBTB18	Q99592	13	-17	-17	-8	22	0.9	0.9	0.9	1.3
BAZ2A	Q9UIF9	1037	9	7	-8	21	1.1	1.1	0.9	1.3
MPI	P34949	289	-1	-2	-8	21	1.0	1.0	0.9	1.3
PDHB	P11177	169	-1	-7	-8	21	1.0	0.9	0.9	1.3
COMMD6	Q7Z4G1	35	1	-8	-8	21	1.0	0.9	0.9	1.3
TIPRL	O75663	14	2	0	-8	21	1.0	1.0	0.9	1.3
SRSF3	P84103	10	-1	-7	-8	21	1.0	0.9	0.9	1.3
HNRNPU	Q00839	648	3	-5	-8	20	1.0	1.0	0.9	1.3
FTO	Q9C0B1	171	-1	-7	-8	20	1.0	0.9	0.9	1.3
CLEC16A	Q2KHT3	588	-6	-21	-8	20	0.9	0.8	0.9	1.3
ANKRD44	Q8N8A2	967	-2	-2	-8	20	1.0	1.0	0.9	1.2

SLC25A20	O43772	283	3	-9	-8	20	1.0	0.9	0.9	1.2
ARAF	P10398	58	-6	-17	-8	20	0.9	0.9	0.9	1.2
LRCH4	O75427	283	-9	-20	-8	20	0.9	0.8	0.9	1.2
RFC1	P35251	665	19	18	-8	19	1.2	1.2	0.9	1.2
SAMD9	Q5K651	1364	-1	4	-8	19	1.0	1.0	0.9	1.2
CYFIP1	Q7L576	1088	1	-9	-8	19	1.0	0.9	0.9	1.2
CYFIP2	Q96F07	1112	1	-9	-8	19	1.0	0.9	0.9	1.2
SRBD1	Q8N5C6	502	2	-10	-8	19	1.0	0.9	0.9	1.2
HMGXB3	Q12766	680	-1	-14	-8	19	1.0	0.9	0.9	1.2
SMURF2	Q9HAU4	706	-8	-8	-8	18	0.9	0.9	0.9	1.2
PRDX5	P30044	100	-7	-9	-8	18	0.9	0.9	0.9	1.2
TCIRG1	Q13488	317	-8	-3	-8	18	0.9	1.0	0.9	1.2
IWS1	Q96ST2	749	-1	-5	-8	18	1.0	1.0	0.9	1.2
MED14	O60244	621	-1	-9	-8	18	1.0	0.9	0.9	1.2
UBR4	Q5T4S7	4049	-8	-13	-8	18	0.9	0.9	0.9	1.2
VPS18	Q9P253	421	-3	-5	-8	17	1.0	1.0	0.9	1.2
SMARCA2	P51531	1401	-5	5	-8	17	1.0	1.1	0.9	1.2
RRP12	Q5JTH9	317	3	-3	-8	17	1.0	1.0	0.9	1.2
PER1	O15534	978	18	-2	-8	16	1.2	1.0	0.9	1.2
NANP	Q8TBE9	67	4	-4	-8	16	1.0	1.0	0.9	1.2
DDX27	Q96GQ7	248	-3	-5	-8	16	1.0	1.0	0.9	1.2
NARS	O43776	511	1	-10	-8	16	1.0	0.9	0.9	1.2
YTHDF2	Q9Y5A9	482	0	-13	-8	16	1.0	0.9	0.9	1.2
NTMT1	Q9BV86	64	-26	-1	-8	16	0.8	1.0	0.9	1.2
STK35	Q8TDR2	235	2	-4	-8	16	1.0	1.0	0.9	1.2
MAT2B	Q9NZL9	58	10	-9	-8	16	1.1	0.9	0.9	1.2
PLEC	Q15149	950	-2	-12	-8	16	1.0	0.9	0.9	1.2
UPF3B	Q9BZI7	453	-9	5	-8	15	0.9	1.0	0.9	1.2
TCEB3	Q14241	568	-11	3	-8	15	0.9	1.0	0.9	1.2
TBCK	Q8TEA7	702	-2	-9	-8	15	1.0	0.9	0.9	1.2
BRD4	O60885	674	-12	-3	-8	15	0.9	1.0	0.9	1.2
PARN	O95453	169	-4	-3	-8	15	1.0	1.0	0.9	1.2
MYO9B	Q13459	1279	-4	-3	-8	15	1.0	1.0	0.9	1.2
MTF1	Q14872	129	-6	-6	-8	15	0.9	0.9	0.9	1.2
THOC2	Q8NI27	981	9	-6	-8	15	1.1	0.9	0.9	1.2
GRHPR	Q9UBQ7	29	12	-7	-8	15	1.1	0.9	0.9	1.2
BIRC2	Q13490	45	-10	-8	-8	15	0.9	0.9	0.9	1.2
STRN4	Q9NRL3	17	5	-9	-8	15	1.1	0.9	0.9	1.2
CENPH	Q9H3R5	35	-5	-11	-8	15	1.0	0.9	0.9	1.2
DDX6	P26196	102	-5	-6	-8	14	1.0	0.9	0.9	1.2
LGALS1	P09382	89	-2	-8	-8	14	1.0	0.9	0.9	1.2
NPEPPS	P55786	66	-1	-9	-8	14	1.0	0.9	0.9	1.2
ZNF808	Q8N4W9	466	4	-11	-8	14	1.0	0.9	0.9	1.2
UBN1	Q9NPG3	837	-3	-11	-8	14	1.0	0.9	0.9	1.2
POLR2G	P62487	106	-11	10	-8	14	0.9	1.1	0.9	1.2
SH3BP5L	Q7L8J4	109	14	1	-8	14	1.2	1.0	0.9	1.2
Uncharacteriz	F5H5P2	231	1	-7	-8	14	1.0	0.9	0.9	1.2
HADH	Q16836	211	3	-11	-8	14	1.0	0.9	0.9	1.2
CALML4	Q96GE6	82	-16	15	-8	13	0.9	1.2	0.9	1.1
MX1	P20591	42	-3	1	-8	13	1.0	1.0	0.9	1.1
SF3A1	Q15459	244	-2	-11	-8	13	1.0	0.9	0.9	1.1
SEC24D	O94855	371	-10	-13	-8	13	0.9	0.9	0.9	1.1
SEC24C	P53992	433	-10	-13	-8	13	0.9	0.9	0.9	1.1
SF3B6	Q9Y3B4	83	-6	-18	-8	13	0.9	0.8	0.9	1.1
PPP6C	O00743	192	-14	-27	-8	13	0.9	0.8	0.9	1.1

UBE2L6	O14933	102	-5	-3	-8	13	1.0	1.0	0.9	1.1
ZW10	O43264	39	1	-5	-8	13	1.0	1.0	0.9	1.1
FES	P07332	781	-3	-5	-8	13	1.0	1.0	0.9	1.1
HNRNPM	P52272	653	-2	-9	-8	13	1.0	0.9	0.9	1.1
TANK	Q92844	183	15	-9	-8	13	1.2	0.9	0.9	1.1
MYO1G	B0I1T2	503	-9	-9	-8	12	0.9	0.9	0.9	1.1
SURF2	Q15527	127	4	-10	-8	12	1.0	0.9	0.9	1.1
THEMIS2	Q5TEJ8	410	-4	8	-8	12	1.0	1.1	0.9	1.1
BCL11A	Q9H165	221	15	6	-8	12	1.2	1.1	0.9	1.1
GGNBP2	Q9H3C7	236	5	6	-8	12	1.1	1.1	0.9	1.1
REXO1	Q8N1G1	694	13	-2	-8	12	1.1	1.0	0.9	1.1
TTC1	Q99614	149	-1	-3	-8	12	1.0	1.0	0.9	1.1
BPHL	Q86WA6	234	-2	-4	-8	12	1.0	1.0	0.9	1.1
PIAS4	Q8N2W9	326	-1	-5	-8	12	1.0	1.0	0.9	1.1
TNPO3	Q9Y5L0	793	16	-27	-8	12	1.2	0.8	0.9	1.1
GBE1	Q04446	81	-5	4	-8	11	1.0	1.0	0.9	1.1
ACAP1	Q15027	320	1	-3	-8	11	1.0	1.0	0.9	1.1
MNDA	P41218	61	3	-5	-8	11	1.0	1.0	0.9	1.1
ARHGEF6	Q15052	57	10	-9	-8	11	1.1	0.9	0.9	1.1
CARS	P49589	27	-6	-9	-8	11	0.9	0.9	0.9	1.1
PSMA6	P60900	154	-6	-2	-8	11	0.9	1.0	0.9	1.1
RECQL	P46063	49	2	-2	-8	11	1.0	1.0	0.9	1.1
ANXA11	P50995	501	2	-2	-8	11	1.0	1.0	0.9	1.1
ADAMTS18	Q8TE60	1204	-10	-3	-8	11	0.9	1.0	0.9	1.1
RPAP1	Q9BWH6	195	-3	2	-8	10	1.0	1.0	0.9	1.1
ACP1	P24666	18	8	-3	-8	10	1.1	1.0	0.9	1.1
NAIF1	Q69YI7	266	1	-6	-8	10	1.0	0.9	0.9	1.1
SUPT3H	O75486	268	-1	-10	-8	10	1.0	0.9	0.9	1.1
RALGAPA1	Q6GYQ0	293	2	-14	-8	10	1.0	0.9	0.9	1.1
RPP14	O95059	31	-4	-16	-8	10	1.0	0.9	0.9	1.1
MILR1	Q7Z6M3	38	-13	-29	-8	10	0.9	0.8	0.9	1.1
PRKCD	Q05655	127	1	0	-8	10	1.0	1.0	0.9	1.1
POC5	Q8NA72	494	-11	0	-8	10	0.9	1.0	0.9	1.1
EFL1	Q7Z2Z2	953	0	-2	-8	10	1.0	1.0	0.9	1.1
LASP1	Q14847	29	4	-4	-8	10	1.0	1.0	0.9	1.1
RGPD3	A6NKT7	141	-5	-4	-8	10	1.0	1.0	0.9	1.1
RANBP2	P49792	141	-5	-4	-8	10	1.0	1.0	0.9	1.1
RGPD6	Q99666	141	-5	-4	-8	10	1.0	1.0	0.9	1.1
ZMYND11	Q15326	73	-5	-5	-8	10	1.0	1.0	0.9	1.1
ALDH5A1	P51649	110	6	-8	-8	10	1.1	0.9	0.9	1.1
MCM5	P33992	482	-7	1	-8	9	0.9	1.0	0.9	1.1
NUMA1	Q14980	1673	-5	-1	-8	9	1.0	1.0	0.9	1.1
HADHA	P40939	470	-1	-2	-8	9	1.0	1.0	0.9	1.1
NCF1	P14598	111	-7	-4	-8	9	0.9	1.0	0.9	1.1
TRNT1	Q96Q11	373	0	-5	-8	9	1.0	1.0	0.9	1.1
MAP1S	Q66K74	342	-3	-10	-8	9	1.0	0.9	0.9	1.1
ATAD2B	Q9ULI0	458	7	-19	-8	9	1.1	0.8	0.9	1.1
TUBB6	Q9BUF5	12	1	-3	-8	9	1.0	1.0	0.9	1.1
ACADVL	P49748	237	-4	-4	-8	9	1.0	1.0	0.9	1.1
SKIV2L	Q15477	913	-1	-7	-8	9	1.0	0.9	0.9	1.1
ASPSCR1	Q9BZE9	127	4	-7	-8	9	1.0	0.9	0.9	1.1
UBXN7	O94888	160	-1	-9	-8	9	1.0	0.9	0.9	1.1
OAS2	P29728	691	-6	-9	-8	9	0.9	0.9	0.9	1.1
NDUFS1	P28331	64	-5	-10	-8	9	1.0	0.9	0.9	1.1
NUMB	P49757	176	-1	-10	-8	9	1.0	0.9	0.9	1.1

EPHX4	Q8IUS5	287	-11	-20	-8	9	0.9	0.8	0.9	1.1
COPB1	P53618	248	10	7	-8	8	1.1	1.1	0.9	1.1
COPA	P53621	673	3	-2	-8	8	1.0	1.0	0.9	1.1
SEC13	P55735	245	-1	-3	-8	8	1.0	1.0	0.9	1.1
SECISBP2L	Q93073	743	1	-4	-8	8	1.0	1.0	0.9	1.1
SECISBP2	Q96T21	698	1	-4	-8	8	1.0	1.0	0.9	1.1
NIPBL	Q6KC79	2151	4	-6	-8	8	1.0	0.9	0.9	1.1
DDX3X	O00571	298	-6	-10	-8	8	0.9	0.9	0.9	1.1
DDX3Y	O15523	296	-6	-10	-8	8	0.9	0.9	0.9	1.1
AGK	Q53H12	72	-1	-11	-8	8	1.0	0.9	0.9	1.1
HARS	P12081	379	3	-6	-8	8	1.0	0.9	0.9	1.1
HK1	P19367	813	2	-6	-8	8	1.0	0.9	0.9	1.1
SGTA	O43765	129	-16	-17	-8	8	0.9	0.9	0.9	1.1
PPA2	Q9H2U2	161	3	-1	-8	7	1.0	1.0	0.9	1.1
DDX46	Q7L014	590	-1	-2	-8	7	1.0	1.0	0.9	1.1
RBM34	P42696	196	-5	-3	-8	7	1.0	1.0	0.9	1.1
DTYMK	P23919	31	2	-4	-8	7	1.0	1.0	0.9	1.1
IDNK	Q5T6J7	105	-1	-4	-8	7	1.0	1.0	0.9	1.1
PI4KA	P42356	1832	-9	-13	-8	7	0.9	0.9	0.9	1.1
DYNC1H1	Q14204	4121	-3	-13	-8	7	1.0	0.9	0.9	1.1
TNPO3	Q9Y5L0	312	5	-18	-8	7	1.0	0.8	0.9	1.1
NSUN5	Q96P11	146	-11	-3	-8	7	0.9	1.0	0.9	1.1
ETFDH	Q16134	248	8	-9	-8	7	1.1	0.9	0.9	1.1
ZZEF1	O43149	1069	-2	-16	-8	7	1.0	0.9	0.9	1.1
WDR81	Q562E7	76	-11	-17	-8	7	0.9	0.9	0.9	1.1
THAP12	O43422	230	-5	-4	-8	6	1.0	1.0	0.9	1.1
DFFA	O00273	38	4	-13	-8	6	1.0	0.9	0.9	1.1
UBR1	Q8IWV7	477	-2	3	-8	6	1.0	1.0	0.9	1.1
PRSS1	P07477	196	7	-2	-8	6	1.1	1.0	0.9	1.1
MAT2B	Q9NZL9	231	8	-3	-8	6	1.1	1.0	0.9	1.1
FBXO22	Q8NEZ5	228	8	-5	-8	6	1.1	1.0	0.9	1.1
PSMB2	P49721	91	-11	-9	-8	6	0.9	0.9	0.9	1.1
MAP3K4	Q9Y6R4	326	2	-9	-8	6	1.0	0.9	0.9	1.1
VCP	P55072	184	-9	-11	-8	6	0.9	0.9	0.9	1.1
SPTAN1	Q13813	2120	-14	-1	-8	5	0.9	1.0	0.9	1.1
PTK2B	Q14289	298	-3	-5	-8	5	1.0	1.0	0.9	1.1
GTPBP6	H0Y2S1	305	-3	-16	-8	5	1.0	0.9	0.9	1.1
EP300	Q09472	1753	-8	1	-8	5	0.9	1.0	0.9	1.0
CREBBP	Q92793	1790	-8	1	-8	5	0.9	1.0	0.9	1.0
GAPVD1	Q14C86	568	-6	-5	-8	5	0.9	1.0	0.9	1.0
PDCD6IP	Q8WUM4	512	0	-5	-8	5	1.0	1.0	0.9	1.0
PAFAH1B1	P43034	184	2	-12	-8	5	1.0	0.9	0.9	1.0
DNPEP	Q9ULA0	144	-8	-4	-8	4	0.9	1.0	0.9	1.0
PAICS	P22234	81	2	-9	-8	4	1.0	0.9	0.9	1.0
IL32	P24001	227	-14	-9	-8	4	0.9	0.9	0.9	1.0
ARHGAP27	Q6ZUM4	830	9	3	-8	4	1.1	1.0	0.9	1.0
SAAL1	Q96ER3	366	-2	-6	-8	4	1.0	0.9	0.9	1.0
UNK	Q9C0B0	675	-2	-9	-8	4	1.0	0.9	0.9	1.0
ADK	P55263	160	3	-13	-8	4	1.0	0.9	0.9	1.0
SAP30BP	Q9UHR5	172	3	-3	-8	3	1.0	1.0	0.9	1.0
EIF2D	P41214	489	-7	-6	-8	3	0.9	0.9	0.9	1.0
LPCAT2	Q7L5N7	509	-3	-7	-8	3	1.0	0.9	0.9	1.0
TUFM	P49411	222	-14	-14	-8	3	0.9	0.9	0.9	1.0
NUP205	Q92621	1028	-12	-20	-8	3	0.9	0.8	0.9	1.0
SRP72	O76094	54	-3	-1	-8	3	1.0	1.0	0.9	1.0

NCKAP1L	P55160	632	4	-1	-8	3	1.0	1.0	0.9	1.0
NAA35	Q5VZE5	409	-3	-2	-8	3	1.0	1.0	0.9	1.0
RPS4Y2	Q8TD47	41	-13	-6	-8	3	0.9	0.9	0.9	1.0
THYN1	Q9P016	118	4	-6	-8	3	1.0	0.9	0.9	1.0
NUP133	Q8WUM0	530	-6	-15	-8	3	0.9	0.9	0.9	1.0
CXorf21	Q9HAI6	103	-5	0	-8	2	1.0	1.0	0.9	1.0
SCYL1	Q96KG9	241	-2	-1	-8	2	1.0	1.0	0.9	1.0
LRSAM1	Q6UWE0	75	-4	-3	-8	2	1.0	1.0	0.9	1.0
DOCK10	Q96BY6	1508	8	-3	-8	2	1.1	1.0	0.9	1.0
MACF1	Q9UPN3	3407	1	-3	-8	2	1.0	1.0	0.9	1.0
NDUFS1	P28331	92	-5	-6	-8	2	1.0	0.9	0.9	1.0
IFIT5	Q13325	476	-3	-9	-8	2	1.0	0.9	0.9	1.0
AKAP9	Q99996	1794	4	3	-8	2	1.0	1.0	0.9	1.0
CCT3	P49368	213	-1	-4	-8	2	1.0	1.0	0.9	1.0
RNF14	Q9UBS8	350	2	-9	-8	2	1.0	0.9	0.9	1.0
PPP6R1	Q9UPN7	172	-6	-9	-8	2	0.9	0.9	0.9	1.0
NDUFV1	P49821	125	-10	-16	-8	2	0.9	0.9	0.9	1.0
UBA1	P22314	481	6	-18	-8	2	1.1	0.9	0.9	1.0
GOLPH3	Q9H4A6	280	-1	-1	-8	1	1.0	1.0	0.9	1.0
TMEM201	Q5SNT2	167	7	-2	-8	1	1.1	1.0	0.9	1.0
SAP18	O00422	26	0	-3	-8	1	1.0	1.0	0.9	1.0
UPP1	Q16831	89	-13	-3	-8	1	0.9	1.0	0.9	1.0
MORC2	Q9Y6X9	962	-3	-9	-8	1	1.0	0.9	0.9	1.0
ADCY7	P51828	1068	-8	-9	-8	1	0.9	0.9	0.9	1.0
TM9SF3	Q9HD45	428	-10	-10	-8	1	0.9	0.9	0.9	1.0
RNF113A	O15541	296	13	9	-8	1	1.1	1.1	0.9	1.0
COPS5	Q92905	218	1	3	-8	1	1.0	1.0	0.9	1.0
EEA1	Q15075	346	4	-9	-8	1	1.0	0.9	0.9	1.0
CWF19L1	Q69YN2	160	3	-1	-8	0	1.0	1.0	0.9	1.0
PEX1	O43933	1002	-17	-4	-8	0	0.9	1.0	0.9	1.0
INTS7	Q9NVH2	374	9	-8	-8	0	1.1	0.9	0.9	1.0
GNB2	P62879	148	0	-15	-8	0	1.0	0.9	0.9	1.0
GNB4	Q9HAV0	148	0	-15	-8	0	1.0	0.9	0.9	1.0
CDC16	Q13042	194	-9	-18	-8	0	0.9	0.9	0.9	1.0
TBC1D31	Q96DN5	1004	-8	-4	-8	-1	0.9	1.0	0.9	1.0
EP300	Q09472	1250	-7	-9	-8	-1	0.9	0.9	0.9	1.0
GDI1	P31150	317	-2	-18	-8	-1	1.0	0.9	0.9	1.0
PSMA3	P25788	42	-9	-2	-8	-1	0.9	1.0	0.9	1.0
RICTOR	Q6R327	218	-6	-10	-8	-1	0.9	0.9	0.9	1.0
SETX	Q7Z333	2384	-8	-14	-8	-1	0.9	0.9	0.9	1.0
RNF31	Q96EP0	871	-7	2	-8	-2	0.9	1.0	0.9	1.0
HNRNPU	Q00839	408	-9	-3	-8	-2	0.9	1.0	0.9	1.0
UNK	Q9C0B0	782	1	-5	-8	-2	1.0	1.0	0.9	1.0
PURA	Q00577	272	2	-8	-8	-2	1.0	0.9	0.9	1.0
ETF1	P62495	335	1	-1	-8	-2	1.0	1.0	0.9	1.0
AGK	Q53H12	43	-12	-8	-8	-2	0.9	0.9	0.9	1.0
SHMT1	P34896	68	4	-9	-8	-2	1.0	0.9	0.9	1.0
MACF1	Q9UPN3	3085	3	-16	-8	-2	1.0	0.9	0.9	1.0
KMT2C	Q8NEZ4	4345	2	-15	-8	-3	1.0	0.9	0.9	1.0
HSD17B10	Q99714	214	-4	-6	-8	-3	1.0	0.9	0.9	1.0
SLX4	Q8IY92	1109	-14	-16	-8	-3	0.9	0.9	0.9	1.0
TRAF6	Q9Y4K3	366	2	-6	-8	-4	1.0	0.9	0.9	1.0
TRMU	O75648	48	8	-10	-8	-4	1.1	0.9	0.9	1.0
BPTF	Q12830	882	-1	-4	-8	-4	1.0	1.0	0.9	1.0
VPS51	Q9UID3	190	-9	-11	-8	-4	0.9	0.9	0.9	1.0

THAP12	O43422	207	-20	2	-8	-5	0.8	1.0	0.9	1.0
THOC2	Q8NI27	931	-11	-1	-8	-5	0.9	1.0	0.9	1.0
ZNF512B	Q96KM6	515	-4	5	-8	-6	1.0	1.0	0.9	0.9
HSD17B10	Q99714	91	0	-5	-8	-6	1.0	1.0	0.9	0.9
PFKP	Q01813	112	1	-6	-8	-6	1.0	0.9	0.9	0.9
G6PD	P11413	446	-3	-19	-8	-6	1.0	0.8	0.9	0.9
U2SURP	O15042	918	4	-6	-8	-6	1.0	0.9	0.9	0.9
TRAPPC6B	Q86SZ2	123	-20	-15	-8	-6	0.8	0.9	0.9	0.9
HPS1	Q92902	180	1	-8	-8	-7	1.0	0.9	0.9	0.9
MVP	Q14764	572	4	-20	-8	-7	1.0	0.8	0.9	0.9
LONP1	P36776	520	7	-3	-8	-7	1.1	1.0	0.9	0.9
ABHD5	Q8WTS1	140	-1	-5	-8	-7	1.0	1.0	0.9	0.9
ATP6V1G1	O75348	104	-2	-7	-8	-7	1.0	0.9	0.9	0.9
MCCC1	Q96RQ3	595	-10	-16	-8	-7	0.9	0.9	0.9	0.9
SAMHD1	Q9Y3Z3	341	1	-2	-8	-8	1.0	1.0	0.9	0.9
NBAS	A2RRP1	1594	1	-6	-8	-8	1.0	0.9	0.9	0.9
TBC1D2	Q9BYX2	651	-17	-15	-8	-8	0.9	0.9	0.9	0.9
CSK	P41240	31	-4	-17	-8	-8	1.0	0.9	0.9	0.9
USP9Y	O00507	1810	-10	-12	-8	-8	0.9	0.9	0.9	0.9
USP9X	Q93008	1808	-10	-12	-8	-8	0.9	0.9	0.9	0.9
PI4KA	P42356	1131	-11	0	-8	-9	0.9	1.0	0.9	0.9
CPT1A	P50416	742	-13	-9	-8	-9	0.9	0.9	0.9	0.9
HNRNPU	Q00839	391	4	-14	-8	-9	1.0	0.9	0.9	0.9
MCTP2	Q6DN12	865	-7	-21	-8	-9	0.9	0.8	0.9	0.9
TWF2	Q6IBS0	141	-2	-29	-8	-9	1.0	0.8	0.9	0.9
NFATC1	O95644	125	2	3	-8	-9	1.0	1.0	0.9	0.9
MRPS11	P82912	112	0	-14	-8	-9	1.0	0.9	0.9	0.9
CTU1	Q7Z7A3	237	8	-5	-8	-10	1.1	1.0	0.9	0.9
NHLRC2	Q8NBF2	716	3	-8	-8	-10	1.0	0.9	0.9	0.9
PHRF1	Q9P1Y6	585	-1	-9	-8	-10	1.0	0.9	0.9	0.9
ACTN4	O43707	879	-1	-15	-8	-10	1.0	0.9	0.9	0.9
ACTN1	P12814	860	-1	-15	-8	-10	1.0	0.9	0.9	0.9
ARID1B	Q8NFD5	1093	-1	-19	-8	-10	1.0	0.8	0.9	0.9
SKAP1	Q86WV1	133	2	-1	-8	-11	1.0	1.0	0.9	0.9
PREX1	Q8TCU6	1543	-2	-13	-8	-11	1.0	0.9	0.9	0.9
CCT5	P48643	181	-2	-17	-8	-11	1.0	0.9	0.9	0.9
CORO1C	Q9ULV4	456	4	-5	-8	-12	1.0	1.0	0.9	0.9
MTHFD1	P11586	408	-19	-10	-8	-12	0.8	0.9	0.9	0.9
RAC2	P15153	6	-4	-10	-8	-13	1.0	0.9	0.9	0.9
CDC42	P60953	6	-4	-10	-8	-13	1.0	0.9	0.9	0.9
RAC1	P63000	6	-4	-10	-8	-13	1.0	0.9	0.9	0.9
RHOG	P84095	6	-4	-10	-8	-13	1.0	0.9	0.9	0.9
U2AF1	Q01081	18	-8	-16	-8	-13	0.9	0.9	0.9	0.9
MYH9	P35579	1437	-1	-7	-8	-13	1.0	0.9	0.9	0.9
ADPRH	P54922	129	-7	-12	-8	-13	0.9	0.9	0.9	0.9
DTYMK	P23919	163	-4	-3	-8	-14	1.0	1.0	0.9	0.9
KIAA0513	O60268	317	-4	-6	-8	-14	1.0	0.9	0.9	0.9
PCBP1	Q15365	109	3	-1	-8	-14	1.0	1.0	0.9	0.9
PDS5A	Q29RF7	350	-6	-7	-8	-14	0.9	0.9	0.9	0.9
PDS5B	Q9NTI5	340	-6	-7	-8	-14	0.9	0.9	0.9	0.9
KDM2A	Q9Y2K7	675	-8	-15	-8	-14	0.9	0.9	0.9	0.9
P4HB	P07237	343	3	-34	-8	-14	1.0	0.7	0.9	0.9
AIMP1	Q12904	161	-18	-22	-8	-15	0.8	0.8	0.9	0.9
VPS13C	Q709C8	2440	-10	-24	-8	-15	0.9	0.8	0.9	0.9
MYL12B	O14950	109	-13	-15	-8	-15	0.9	0.9	0.9	0.9

CLTC	Q00610	736	0	-7	-8	-18	1.0	0.9	0.9	0.8
OSBPL9	Q96SU4	720	-11	-18	-8	-18	0.9	0.9	0.9	0.8
MMS19	Q96T76	331	-3	-7	-8	-19	1.0	0.9	0.9	0.8
EWSR1	Q01844	540	-4	-9	-8	-19	1.0	0.9	0.9	0.8
MIOS	Q9NXC5	748	-8	-31	-8	-20	0.9	0.8	0.9	0.8
TRIM56	Q9BRZ2	169	-1	9	-8	-21	1.0	1.1	0.9	0.8
PLA2G4A	P47712	726	-15	-4	-8	-22	0.9	1.0	0.9	0.8
KPNA6	O60684	427	-17	3	-8	-24	0.9	1.0	0.9	0.8
RNF213	Q63HN8	2092	11	-14	-8	-24	1.1	0.9	0.9	0.8
AGO3	Q9H9G7	345	-9	-22	-8	-24	0.9	0.8	0.9	0.8
AGO4	Q9HCK5	334	-9	-22	-8	-24	0.9	0.8	0.9	0.8
AGO2	Q9UKV8	344	-9	-22	-8	-24	0.9	0.8	0.9	0.8
AGO1	Q9UL18	342	-9	-22	-8	-24	0.9	0.8	0.9	0.8
ATP2A3	Q93084	581	-1	-16	-8	-24	1.0	0.9	0.9	0.8
HMHA1	Q92619	469	-1	-5	-8	-25	1.0	1.0	0.9	0.8
POLR2B	P30876	1119	-8	-9	-8	-31	0.9	0.9	0.9	0.8
EHBP1L1	Q8N3D4	1141	-1	-16	-8	-32	1.0	0.9	0.9	0.8
MYH9	P35579	931	-8	-6	-8	-33	0.9	0.9	0.9	0.8
ACTN1	P12814	774	-7	-4	-8	-46	0.9	1.0	0.9	0.7
FHL1	Q13642	92	2	-1	-8	-79	1.0	1.0	0.9	0.6
DBN1	Q16643	98	-13	-9	-8	-88	0.9	0.9	0.9	0.5
TUFM	P49411	127	-10	-15	-8	-91	0.9	0.9	0.9	0.5
TC2N	Q8N9U0	158	-15	-18	-8	-93	0.9	0.9	0.9	0.5
TUBB1	Q9H4B7	340	2	-1	-8	-194	1.0	1.0	0.9	0.3
SYNE2	Q8WXH0	6462	-10	-5	-8	54	0.9	1.0	0.9	2.2
NUDT6	P53370	44	-13	-7	-8	50	0.9	0.9	0.9	2.0
SMNDC1	O75940	214	-3	-13	-8	45	1.0	0.9	0.9	1.8
ATF7IP	Q6VMQ6	612	-6	-16	-8	36	0.9	0.9	0.9	1.6
MTG1	Q9BT17	23	6	-8	-8	35	1.1	0.9	0.9	1.5
FNDC3A	Q9Y2H6	1005	7	-8	-8	33	1.1	0.9	0.9	1.5
IL17RA	Q96F46	703	-5	-9	-8	33	1.0	0.9	0.9	1.5
MROH1	Q8NDA8	1394	5	-1	-8	31	1.0	1.0	0.9	1.4
MACF1	Q9UPN3	4388	-1	-2	-8	30	1.0	1.0	0.9	1.4
DHX30	Q7L2E3	1183	-4	-1	-8	30	1.0	1.0	0.9	1.4
MYO1F	O00160	807	2	-12	-8	29	1.0	0.9	0.9	1.4
EPM2AIP1	Q7L775	337	-8	-3	-8	28	0.9	1.0	0.9	1.4
GAK	O14976	145	-4	-6	-8	28	1.0	0.9	0.9	1.4
VPS51	Q9UID3	81	-10	-7	-8	28	0.9	0.9	0.9	1.4
AIM1	Q9Y4K1	481	-4	7	-8	27	1.0	1.1	0.9	1.4
HMHA1	Q92619	599	-2	-6	-8	26	1.0	0.9	0.9	1.4
HSPA2	P54652	191	20	10	-8	26	1.3	1.1	0.9	1.3
MED12	Q93074	1188	-8	-7	-8	25	0.9	0.9	0.9	1.3
HNRNPH1	P31943	22	-7	-2	-8	25	0.9	1.0	0.9	1.3
SKIV2L	Q15477	719	-4	-17	-8	25	1.0	0.9	0.9	1.3
HNRNPR	O43390	214	-1	-15	-8	24	1.0	0.9	0.9	1.3
HSP90AB1	P08238	521	8	-3	-8	24	1.1	1.0	0.9	1.3
RCBTB1	Q8NDN9	177	-6	-12	-8	24	0.9	0.9	0.9	1.3
ANKIB1	Q9P2G1	359	3	-12	-8	23	1.0	0.9	0.9	1.3
NPAT	Q14207	1172	-1	-10	-8	22	1.0	0.9	0.9	1.3
SMURF2	Q9HAU4	743	-6	-3	-8	22	0.9	1.0	0.9	1.3
CBX4	O00257	185	-1	-6	-8	22	1.0	0.9	0.9	1.3
GLTSCR1	Q9NZM4	1382	-3	-5	-8	21	1.0	1.0	0.9	1.3
COMT	P21964	238	-4	-7	-8	21	1.0	0.9	0.9	1.3
ELMO1	Q92556	411	-10	-7	-8	21	0.9	0.9	0.9	1.3
ELMO2	Q96JJ3	404	-10	-7	-8	21	0.9	0.9	0.9	1.3

AKR7A2	O43488	16	-11	-22	-8	20	0.9	0.8	0.9	1.3
NOL9	Q5SY16	637	7	8	-8	20	1.1	1.1	0.9	1.2
RSAD1	Q9HA92	74	-6	-3	-8	20	0.9	1.0	0.9	1.2
ZAP70	P43403	596	15	-10	-8	20	1.2	0.9	0.9	1.2
TAPBP	O15533	91	-2	-36	-8	20	1.0	0.7	0.9	1.2
RNPC3	Q96LT9	156	10	-1	-8	19	1.1	1.0	0.9	1.2
SLC38A10	Q9HBR0	301	-2	-4	-8	19	1.0	1.0	0.9	1.2
CTSC	P53634	337	3	-14	-8	19	1.0	0.9	0.9	1.2
TRIM14	Q14142	291	-7	5	-8	19	0.9	1.0	0.9	1.2
HACE1	Q8IYU2	508	8	1	-8	19	1.1	1.0	0.9	1.2
PARK7	Q99497	53	0	-9	-8	19	1.0	0.9	0.9	1.2
XPO4	Q9C0E2	723	2	-10	-8	19	1.0	0.9	0.9	1.2
EPHX2	P34913	423	-11	-12	-8	19	0.9	0.9	0.9	1.2
MSL1	Q68DK7	221	9	-12	-8	18	1.1	0.9	0.9	1.2
SMUG1	Q53HV7	131	-1	14	-8	18	1.0	1.2	0.9	1.2
EHBP1L1	Q8N3D4	346	-4	1	-8	18	1.0	1.0	0.9	1.2
DNAJC7	Q99615	58	-5	-1	-8	18	1.0	1.0	0.9	1.2
BIRC3	Q13489	28	-5	-5	-8	18	1.0	1.0	0.9	1.2
NADSYN1	Q6IA69	627	-4	-9	-8	18	1.0	0.9	0.9	1.2
ZNF318	Q5VUA4	1740	8	-13	-8	18	1.1	0.9	0.9	1.2
PGD	P52209	422	-14	-19	-8	17	0.9	0.8	0.9	1.2
HMGCS1	Q01581	224	-10	-5	-8	17	0.9	1.0	0.9	1.2
ZNF709	Q8N972	375	-13	-7	-8	17	0.9	0.9	0.9	1.2
ZNF564	Q8TBZ8	404	-13	-7	-8	17	0.9	0.9	0.9	1.2
RFC1	P35251	607	-7	-2	-8	16	0.9	1.0	0.9	1.2
TECPR1	Q7Z6L1	462	2	-2	-8	16	1.0	1.0	0.9	1.2
MIS12	Q9H081	104	1	-5	-8	16	1.0	1.0	0.9	1.2
CHD3	Q12873	1997	2	-13	-8	16	1.0	0.9	0.9	1.2
RUBCN	Q92622	58	-7	-18	-8	16	0.9	0.8	0.9	1.2
PRF1	P14222	497	-19	-22	-8	16	0.8	0.8	0.9	1.2
PPA1	Q15181	114	3	6	-8	16	1.0	1.1	0.9	1.2
RPP30	P78346	257	3	1	-8	16	1.0	1.0	0.9	1.2
ERI3	O43414	285	4	-3	-8	16	1.0	1.0	0.9	1.2
PBRM1	Q86U86	1228	-5	-5	-8	16	1.0	1.0	0.9	1.2
SDCCAG8	Q86SQ7	604	4	-9	-8	16	1.0	0.9	0.9	1.2
BRAT1	Q6PJG6	422	-4	-29	-8	16	1.0	0.8	0.9	1.2
RPL5	P46777	100	-5	-1	-8	15	1.0	1.0	0.9	1.2
AHNAK	Q09666	1967	-7	-7	-8	15	0.9	0.9	0.9	1.2
RABEP1	Q15276	263	6	-7	-8	15	1.1	0.9	0.9	1.2
IRF5	Q13568	44	-3	-9	-8	15	1.0	0.9	0.9	1.2
TADA3	O75528	7	5	-17	-8	15	1.0	0.9	0.9	1.2
PUM1	Q14671	234	-3	-10	-8	15	1.0	0.9	0.9	1.2
MIOS	Q9NXC5	799	-6	3	-8	14	0.9	1.0	0.9	1.2
CREB1	P16220	337	3	-3	-8	14	1.0	1.0	0.9	1.2
OAS2	P29728	652	5	-5	-8	14	1.0	1.0	0.9	1.2
EXOC5	O00471	111	-14	-7	-8	14	0.9	0.9	0.9	1.2
TNK2	Q07912	843	-3	-2	-8	14	1.0	1.0	0.9	1.2
CASP8	Q14790	345	-6	-3	-8	14	0.9	1.0	0.9	1.2
UBA3	Q8TBC4	28	-6	-5	-8	14	0.9	1.0	0.9	1.2
GLCCI1	Q86VQ1	461	-13	-8	-8	14	0.9	0.9	0.9	1.2
SMS	P52788	337	-7	-11	-8	14	0.9	0.9	0.9	1.2
CCND3	P30281	47	-1	-12	-8	14	1.0	0.9	0.9	1.2
ZFP64	Q9NTW7	147	-10	-16	-8	14	0.9	0.9	0.9	1.2
SMARCA2	P51531	906	-8	7	-8	13	0.9	1.1	0.9	1.1
NEK1	Q96PY6	276	-3	1	-8	13	1.0	1.0	0.9	1.1

WDFY3	Q8IZQ1	1975	9	-2	-8	13	1.1	1.0	0.9	1.1
TNPO1	Q92973	862	-1	-3	-8	13	1.0	1.0	0.9	1.1
EIF4ENIF1	Q9NRA8	767	-9	-6	-8	13	0.9	0.9	0.9	1.1
DHX40	Q8IX18	641	-1	-8	-8	13	1.0	0.9	0.9	1.1
PYCRL	Q53H96	129	-10	-10	-8	13	0.9	0.9	0.9	1.1
UROS	P10746	143	-3	-12	-8	13	1.0	0.9	0.9	1.1
KLHL11	Q9NVR0	570	-10	-12	-8	13	0.9	0.9	0.9	1.1
CEP192	Q8TEP8	286	3	-2	-8	13	1.0	1.0	0.9	1.1
CARD11	Q9BXL7	971	4	6	-8	12	1.0	1.1	0.9	1.1
TMEM55B	Q86T03	147	-4	-8	-8	12	1.0	0.9	0.9	1.1
TMEM214	Q6NUQ4	468	2	-10	-8	12	1.0	0.9	0.9	1.1
HDAC10	Q969S8	403	-4	6	-8	12	1.0	1.1	0.9	1.1
TIMM21	Q9BVV7	192	3	-5	-8	12	1.0	1.0	0.9	1.1
Uncharacteriz	G3V4G9	75	-3	-6	-8	12	1.0	0.9	0.9	1.1
HECTD1	Q9ULT8	1855	2	-9	-8	12	1.0	0.9	0.9	1.1
ATP6V1A	P38606	138	-2	-10	-8	12	1.0	0.9	0.9	1.1
PLCL2	Q9UPR0	1095	-4	-14	-8	12	1.0	0.9	0.9	1.1
HGS	O14964	185	-1	-3	-8	11	1.0	1.0	0.9	1.1
ACBD3	Q9H3P7	129	-5	-5	-8	11	1.0	1.0	0.9	1.1
ANXA11	P50995	384	-5	-5	-8	11	1.0	1.0	0.9	1.1
L3MBTL3	Q96JM7	682	-4	-6	-8	11	1.0	0.9	0.9	1.1
CTR9	Q6PD62	171	-6	-13	-8	11	0.9	0.9	0.9	1.1
TNFAIP3	P21580	674	-26	-17	-8	11	0.8	0.9	0.9	1.1
RIN3	Q8TB24	931	-7	-4	-8	11	0.9	1.0	0.9	1.1
TAF2	Q6P1X5	732	-5	-5	-8	11	1.0	1.0	0.9	1.1
PIAS4	Q8N2W9	164	3	-9	-8	11	1.0	0.9	0.9	1.1
HSDL2	Q6YN16	218	3	11	-8	10	1.0	1.1	0.9	1.1
DPYD	Q12882	202	-6	3	-8	10	0.9	1.0	0.9	1.1
JRK	Q86XJ5	428	1	-3	-8	10	1.0	1.0	0.9	1.1
DHX15	O43143	190	-3	-4	-8	10	1.0	1.0	0.9	1.1
PHGDH	O43175	295	-2	-5	-8	10	1.0	1.0	0.9	1.1
NOP58	Q9Y2X3	439	-3	-8	-8	10	1.0	0.9	0.9	1.1
AHCTF1	Q8WYP5	354	-6	-8	-8	10	0.9	0.9	0.9	1.1
SSB	P05455	245	-9	-10	-8	10	0.9	0.9	0.9	1.1
CARD6	Q9BX69	872	-14	-24	-8	10	0.9	0.8	0.9	1.1
SRP72	O76094	349	-1	-6	-8	10	1.0	0.9	0.9	1.1
MCRS1	Q96EZ8	421	2	-6	-8	10	1.0	0.9	0.9	1.1
SMARCAD1	Q9H4L7	568	-7	-10	-8	10	0.9	0.9	0.9	1.1
CCZ1B	P86790	471	6	6	-8	9	1.1	1.1	0.9	1.1
TRRAP	Q9Y4A5	2449	-9	4	-8	9	0.9	1.0	0.9	1.1
ATP1A1	P05023	374	-6	-2	-8	9	0.9	1.0	0.9	1.1
ATP1A3	P13637	364	-6	-2	-8	9	0.9	1.0	0.9	1.1
AKR1B1	P15121	299	-5	-6	-8	9	1.0	0.9	0.9	1.1
TBCK	Q8TEA7	737	-4	-6	-8	9	1.0	0.9	0.9	1.1
SLC27A1	Q6PCB7	442	-4	-17	-8	9	1.0	0.9	0.9	1.1
CLUH	O75153	1196	-5	-3	-8	9	1.0	1.0	0.9	1.1
BAZ1B	Q9UIG0	156	2	-6	-8	9	1.0	0.9	0.9	1.1
RAD50	Q92878	157	-35	-12	-8	9	0.7	0.9	0.9	1.1
IPCEF1	Q8WWN9	368	-2	-2	-8	8	1.0	1.0	0.9	1.1
MYO18A	Q92614	1155	1	-2	-8	8	1.0	1.0	0.9	1.1
CCDC88A	Q3V6T2	1244	-4	-8	-8	8	1.0	0.9	0.9	1.1
CHM	P24386	276	-1	-9	-8	8	1.0	0.9	0.9	1.1
TRIP12	Q14669	1276	6	-9	-8	8	1.1	0.9	0.9	1.1
XPO5	Q9HAV4	736	3	3	-8	8	1.0	1.0	0.9	1.1
PDS5A	Q29RF7	1093	-1	-3	-8	8	1.0	1.0	0.9	1.1

TCF20	Q9UGU0	1328	-16	-6	-8	8	0.9	0.9	0.9	1.1
KDM3B	Q7LBC6	569	-2	-8	-8	8	1.0	0.9	0.9	1.1
CHD4	Q14839	495	-9	-3	-8	7	0.9	1.0	0.9	1.1
NHLRC1	Q6VVB1	262	-3	-3	-8	7	1.0	1.0	0.9	1.1
GNL3	Q9BVP2	234	-6	-6	-8	7	0.9	0.9	0.9	1.1
MAP4	P27816	535	-7	-6	-8	7	0.9	0.9	0.9	1.1
TRAFD1	O14545	482	-3	-7	-8	7	1.0	0.9	0.9	1.1
SERGEF	Q9UGK8	189	1	-8	-8	7	1.0	0.9	0.9	1.1
LRBA	P50851	2675	-7	6	-8	7	0.9	1.1	0.9	1.1
KHSRP	Q92945	379	-2	-5	-8	7	1.0	1.0	0.9	1.1
RSL1D1	O76021	211	-12	-7	-8	7	0.9	0.9	0.9	1.1
SAFB2	Q14151	224	-2	-9	-8	7	1.0	0.9	0.9	1.1
DCAF13	Q9NV06	87	-5	-9	-8	7	1.0	0.9	0.9	1.1
PCGF5	Q86SE9	145	-13	-9	-8	7	0.9	0.9	0.9	1.1
FUBP1	Q96AE4	132	-8	-11	-8	7	0.9	0.9	0.9	1.1
ALDH2	P05091	66	-5	-12	-8	7	1.0	0.9	0.9	1.1
PTPRC	P08575	608	-3	-4	-8	6	1.0	1.0	0.9	1.1
PRKDC	P78527	3187	-5	-5	-8	6	1.0	1.0	0.9	1.1
TBC1D1	Q86TI0	1160	2	-6	-8	6	1.0	0.9	0.9	1.1
CCT5	P48643	253	-3	-8	-8	6	1.0	0.9	0.9	1.1
ACIN1	Q9UKV3	1083	-5	-10	-8	6	1.0	0.9	0.9	1.1
AP2A1	O95782	941	4	-15	-8	6	1.0	0.9	0.9	1.1
TAF2	Q6P1X5	127	-1	6	-8	6	1.0	1.1	0.9	1.1
RAB34	Q9BZG1	38	-2	-3	-8	6	1.0	1.0	0.9	1.1
NUMA1	Q14980	1367	-5	-5	-8	6	1.0	1.0	0.9	1.1
GFM1	Q96RP9	514	3	-6	-8	6	1.0	0.9	0.9	1.1
ZC3H11A	O75152	431	-6	-14	-8	6	0.9	0.9	0.9	1.1
RPS15A	P62244	30	-5	-4	-8	5	1.0	1.0	0.9	1.1
PLEKHA2	Q9HB19	191	-6	-11	-8	5	0.9	0.9	0.9	1.1
FGFR1OP2	Q9NVK5	198	2	-13	-8	5	1.0	0.9	0.9	1.1
SEC23IP	Q9Y6Y8	457	-7	1	-8	5	0.9	1.0	0.9	1.0
TRANK1	O15050	1947	3	-2	-8	5	1.0	1.0	0.9	1.0
CRYZL1	O95825	184	-7	-2	-8	5	0.9	1.0	0.9	1.0
MYD88	Q99836	274	1	-4	-8	5	1.0	1.0	0.9	1.0
CTDP1	Q9Y5B0	73	4	-7	-8	5	1.0	0.9	0.9	1.0
URB2	Q14146	570	-6	-8	-8	5	0.9	0.9	0.9	1.0
CKMT2	P17540	317	-13	-9	-8	5	0.9	0.9	0.9	1.0
HSPA9	P38646	317	-5	-3	-8	4	1.0	1.0	0.9	1.0
UBR1	Q8IWV7	180	-12	-3	-8	4	0.9	1.0	0.9	1.0
CLTC	Q00610	753	-4	-6	-8	4	1.0	0.9	0.9	1.0
PTPN7	P35236	62	-13	-6	-8	4	0.9	0.9	0.9	1.0
PIK3R4	Q99570	899	-11	-13	-8	4	0.9	0.9	0.9	1.0
NSUN6	Q8TEA1	120	-8	-21	-8	4	0.9	0.8	0.9	1.0
NF1	P21359	1711	4	1	-8	4	1.0	1.0	0.9	1.0
SCML4	Q8N228	403	3	-9	-8	4	1.0	0.9	0.9	1.0
ATP6V1A	P38606	240	-3	-10	-8	4	1.0	0.9	0.9	1.0
CKAP5	Q14008	637	-18	-10	-8	4	0.8	0.9	0.9	1.0
USP3	Q9Y6I4	157	-7	-10	-8	4	0.9	0.9	0.9	1.0
PIK3R1	P27986	656	1	-5	-8	3	1.0	1.0	0.9	1.0
PLEC	Q15149	3017	1	-6	-8	3	1.0	0.9	0.9	1.0
Uncharacteriz	H3BMM5	121	-6	-8	-8	3	0.9	0.9	0.9	1.0
MED13	Q9UHV7	1756	-4	2	-8	3	1.0	1.0	0.9	1.0
COMMD3-BM	R4GMX3	309	-5	-3	-8	3	1.0	1.0	0.9	1.0
CORO7-PAM1	A0A0A6YYL4	325	7	-5	-8	3	1.1	1.0	0.9	1.0
ANXA6	P08133	552	4	-8	-8	3	1.0	0.9	0.9	1.0

RAF1	P04049	588	-2	-10	-8	3	1.0	0.9	0.9	1.0
LIMA1	Q9UHB6	164	9	-27	-8	3	1.1	0.8	0.9	1.0
LIMK1	P53667	390	-7	3	-8	2	0.9	1.0	0.9	1.0
XPNPEP1	Q9NQW7	309	-2	0	-8	2	1.0	1.0	0.9	1.0
MX1	P20591	322	-1	-14	-8	2	1.0	0.9	0.9	1.0
ZCCHC8	Q6NZY4	633	9	4	-8	2	1.1	1.0	0.9	1.0
FLII	Q13045	1265	-2	-3	-8	2	1.0	1.0	0.9	1.0
MICAL1	Q8TDZ2	711	-3	-4	-8	2	1.0	1.0	0.9	1.0
PRKCD	Q05655	344	-3	-8	-8	2	1.0	0.9	0.9	1.0
C16orf62	Q7Z3J2	588	-6	-9	-8	2	0.9	0.9	0.9	1.0
DDX23	Q9BUQ8	791	4	-9	-8	2	1.0	0.9	0.9	1.0
GTF3C1	Q12789	1363	8	-12	-8	2	1.1	0.9	0.9	1.0
FAM96A	Q9H5X1	90	-3	-20	-8	2	1.0	0.8	0.9	1.0
HSD17B11	Q8NBQ5	217	1	-3	-8	1	1.0	1.0	0.9	1.0
THNSL1	Q8IYQ7	81	-5	-4	-8	1	1.0	1.0	0.9	1.0
HSP90AB1	P08238	564	1	-6	-8	1	1.0	0.9	0.9	1.0
DCTN1	Q14203	791	1	-12	-8	1	1.0	0.9	0.9	1.0
EIF4G3	O43432	982	0	-20	-8	1	1.0	0.8	0.9	1.0
NTAN1	Q96AB6	118	-22	-27	-8	1	0.8	0.8	0.9	1.0
SEC24A	O95486	452	-4	-1	-8	1	1.0	1.0	0.9	1.0
SEC24B	O95487	626	-4	-1	-8	1	1.0	1.0	0.9	1.0
ADAR	P55265	649	0	-8	-8	1	1.0	0.9	0.9	1.0
HARS2	P49590	380	2	-11	-8	1	1.0	0.9	0.9	1.0
FAM65B	Q9Y4F9	147	1	-15	-8	1	1.0	0.9	0.9	1.0
HADHA	P40939	550	-4	-3	-8	0	1.0	1.0	0.9	1.0
PYROXD1	Q8WU10	122	-10	-6	-8	0	0.9	0.9	0.9	1.0
POLR1A	O95602	1699	-3	-10	-8	0	1.0	0.9	0.9	1.0
NDUFA7	O95182	55	-7	-11	-8	0	0.9	0.9	0.9	1.0
UBE2O	Q9C0C9	230	6	11	-8	-1	1.1	1.1	0.9	1.0
COPA	P53621	380	8	7	-8	-1	1.1	1.1	0.9	1.0
VPS13C	Q709C8	2395	-8	0	-8	-1	0.9	1.0	0.9	1.0
RPS8	P62241	72	-10	-1	-8	-1	0.9	1.0	0.9	1.0
CEP131	Q9UPN4	218	1	-7	-8	-1	1.0	0.9	0.9	1.0
MAP2K3	P46734	207	-6	-1	-8	-1	0.9	1.0	0.9	1.0
MAP2K6	P52564	196	-6	-1	-8	-1	0.9	1.0	0.9	1.0
ESYT1	Q9BSJ8	995	2	-6	-8	-1	1.0	0.9	0.9	1.0
NANS	Q9NR45	19	-10	-10	-8	-1	0.9	0.9	0.9	1.0
PPCDC	Q96CD2	173	4	-11	-8	-1	1.0	0.9	0.9	1.0
EIF4A2	Q14240	135	-8	-15	-8	-1	0.9	0.9	0.9	1.0
LRP1	Q07954	2404	-8	-16	-8	-1	0.9	0.9	0.9	1.0
GNAI2	P04899	140	-13	-18	-8	-1	0.9	0.9	0.9	1.0
BMS1	Q14692	734	6	-8	-8	-2	1.1	0.9	0.9	1.0
RNH1	P13489	362	-2	-10	-8	-2	1.0	0.9	0.9	1.0
TBC1D1	Q86TI0	75	-3	6	-8	-2	1.0	1.1	0.9	1.0
MTMR12	Q9C0I1	236	-1	-7	-8	-2	1.0	0.9	0.9	1.0
SPATS2	Q86XZ4	61	-14	-8	-8	-2	0.9	0.9	0.9	1.0
EXOSC8	Q96B26	89	-6	-10	-8	-2	0.9	0.9	0.9	1.0
SSRP1	Q08945	139	-1	-12	-8	-2	1.0	0.9	0.9	1.0
ZW10	O43264	346	-7	2	-8	-3	0.9	1.0	0.9	1.0
C4orf27	Q9NWY4	152	-5	-7	-8	-3	1.0	0.9	0.9	1.0
OAS2	P29728	523	-6	1	-8	-3	0.9	1.0	0.9	1.0
STAT3	P40763	687	-3	-9	-8	-3	1.0	0.9	0.9	1.0
FGD3	Q5JSP0	558	-5	-11	-8	-3	1.0	0.9	0.9	1.0
EIF3B	P55884	384	-6	-13	-8	-4	0.9	0.9	0.9	1.0
CAP1	Q01518	93	2	-8	-8	-4	1.0	0.9	0.9	1.0

ESYT2	A0FGR8	810	-13	-14	-8	-4	0.9	0.9	0.9	1.0
UBE2B	P63146	88	-1	-18	-8	-4	1.0	0.9	0.9	1.0
DSTN	P60981	163	-1	-6	-8	-5	1.0	0.9	0.9	1.0
ME2	P23368	274	-9	-9	-8	-5	0.9	0.9	0.9	1.0
CORO1A	P31146	51	-2	-25	-8	-5	1.0	0.8	0.9	1.0
RAB37	Q96AX2	22	-2	-8	-8	-5	1.0	0.9	0.9	1.0
ANKMY2	Q8IV38	390	-7	-11	-8	-6	0.9	0.9	0.9	0.9
LPCAT2	Q7L5N7	223	3	-16	-8	-6	1.0	0.9	0.9	0.9
HADHA	P40939	145	-4	-16	-8	-6	1.0	0.9	0.9	0.9
NSF	P46459	11	-1	-17	-8	-6	1.0	0.9	0.9	0.9
KPNA6	O60684	467	1	-9	-8	-7	1.0	0.9	0.9	0.9
AKAP13	Q12802	536	-13	-15	-8	-7	0.9	0.9	0.9	0.9
ARID2	Q68CP9	82	-5	-11	-8	-7	1.0	0.9	0.9	0.9
RBCK1	Q9BYM8	30	-14	7	-8	-8	0.9	1.1	0.9	0.9
POLR1A	O95602	613	-9	-20	-8	-8	0.9	0.8	0.9	0.9
WDR1	O75083	170	2	-7	-8	-8	1.0	0.9	0.9	0.9
XPR1	Q9UBH6	188	-7	-11	-8	-8	0.9	0.9	0.9	0.9
DTNB	O60941	246	-14	-12	-8	-8	0.9	0.9	0.9	0.9
PAPSS1	O43252	165	-2	-5	-8	-9	1.0	1.0	0.9	0.9
TMEM57	Q8N5G2	315	-1	-14	-8	-9	1.0	0.9	0.9	0.9
SEC24C	P53992	383	-8	-4	-8	-9	0.9	1.0	0.9	0.9
SAE1	Q9UBE0	146	-2	-8	-8	-10	1.0	0.9	0.9	0.9
MYH9	P35579	790	-5	-1	-8	-10	1.0	1.0	0.9	0.9
UTRN	P46939	539	-5	-3	-8	-10	1.0	1.0	0.9	0.9
DBNL	Q9UJU6	67	8	-9	-8	-10	1.1	0.9	0.9	0.9
CKAP5	Q14008	144	10	1	-8	-11	1.1	1.0	0.9	0.9
ALG2	Q9H553	70	-16	-16	-8	-11	0.9	0.9	0.9	0.9
DOCK11	Q5JSL3	1663	-11	-26	-8	-11	0.9	0.8	0.9	0.9
C17orf62	Q9BQA9	165	-17	6	-8	-11	0.9	1.1	0.9	0.9
WDFY1	Q8IWB7	322	-8	-2	-8	-11	0.9	1.0	0.9	0.9
CASP1	P29466	270	-8	-5	-8	-11	0.9	1.0	0.9	0.9
APOBR	Q0VD83	895	6	-8	-8	-11	1.1	0.9	0.9	0.9
KCMF1	Q9P0J7	12	5	-13	-8	-11	1.0	0.9	0.9	0.9
MAD1L1	Q9Y6D9	134	-24	-21	-8	-11	0.8	0.8	0.9	0.9
YARS	P54577	519	-11	-25	-8	-12	0.9	0.8	0.9	0.9
SEC24C	P53992	886	-7	8	-8	-12	0.9	1.1	0.9	0.9
SBF1	O95248	895	-7	-8	-8	-12	0.9	0.9	0.9	0.9
PATZ1	Q9HBE1	41	-9	-9	-8	-13	0.9	0.9	0.9	0.9
ACSL5	Q9ULC5	70	-9	-2	-8	-14	0.9	1.0	0.9	0.9
RNF213	Q63HN8	2943	-1	-2	-8	-14	1.0	1.0	0.9	0.9
AGPS	O00116	58	-2	-3	-8	-17	1.0	1.0	0.9	0.9
GNPAT	O15228	73	6	-4	-8	-18	1.1	1.0	0.9	0.9
PML	P29590	129	-7	-9	-8	-18	0.9	0.9	0.9	0.8
CORO1C	Q9ULV4	39	-2	-11	-8	-21	1.0	0.9	0.9	0.8
SKIV2L2	P42285	650	-5	-18	-8	-21	1.0	0.9	0.9	0.8
DTX4	Q9Y2E6	498	-14	-14	-8	-22	0.9	0.9	0.9	0.8
ANO6	Q4KMQ2	261	-14	-10	-8	-22	0.9	0.9	0.9	0.8
PRPF19	Q9UMS4	230	-20	-30	-8	-23	0.8	0.8	0.9	0.8
CTBP2	P56545	140	7	-24	-8	-24	1.1	0.8	0.9	0.8
MAVS	Q7Z434	133	-15	-5	-8	-24	0.9	1.0	0.9	0.8
UTP20	O75691	1794	-10	-4	-8	-26	0.9	1.0	0.9	0.8
FLNA	P21333	574	-1	-6	-8	-28	1.0	0.9	0.9	0.8
ROCK2	O75116	314	2	5	-8	-29	1.0	1.0	0.9	0.8
RAB30	Q15771	168	-13	-18	-8	-30	0.9	0.9	0.9	0.8
MYH9	P35579	569	-1	-9	-8	-30	1.0	0.9	0.9	0.8

MYH10	P35580	576	-1	-9	-8	-30	1.0	0.9	0.9	0.8
SP2	Q02086	505	-4	-11	-8	-30	1.0	0.9	0.9	0.8
LRCH3	Q96II8	116	-16	-1	-8	-32	0.9	1.0	0.9	0.8
MAML1	Q92585	957	-26	-46	-8	-32	0.8	0.7	0.9	0.8
ROCK2	O75116	804	-6	-2	-8	-36	0.9	1.0	0.9	0.7
NCK2	O43639	297	7	-6	-8	-40	1.1	0.9	0.9	0.7
LRRFIP2	Q9Y608	435	8	-14	-8	-43	1.1	0.9	0.9	0.7
PPP1R21	Q6ZMI0	696	9	-18	-8	-60	1.1	0.8	0.9	0.6
LTBP1	Q14766	581	-1	-5	-8	-66	1.0	1.0	0.9	0.6
ARHGAP18	Q8N392	323	1	-4	-8	-88	1.0	1.0	0.9	0.5
CTTN	Q14247	246	4	1	-8	-134	1.0	1.0	0.9	0.4
BRAT1	Q6PJG6	185	4	6	-9	74	1.0	1.1	0.9	3.8
PSTPIP1	O43586	305	-5	-8	-9	54	1.0	0.9	0.9	2.2
SESN3	P58005	17	-1	-7	-9	51	1.0	0.9	0.9	2.0
GANC	Q8TET4	890	37	15	-9	42	1.6	1.2	0.9	1.7
SKI	P12755	435	-1	-12	-9	40	1.0	0.9	0.9	1.7
MMS19	Q96T76	819	-3	-9	-9	39	1.0	0.9	0.9	1.6
DTWD2	Q8NBA8	226	-22	3	-9	38	0.8	1.0	0.9	1.6
CYLD	Q9NQC7	129	-2	-9	-9	36	1.0	0.9	0.9	1.6
ERCC5	P28715	529	0	-6	-9	36	1.0	0.9	0.9	1.6
XPC	Q01831	60	2	-7	-9	35	1.0	0.9	0.9	1.5
RPAP1	Q9BWH6	84	-24	-20	-9	34	0.8	0.8	0.9	1.5
RASGRP1	O95267	80	-8	-19	-9	34	0.9	0.8	0.9	1.5
ARID5A	Q03989	130	-4	-1	-9	33	1.0	1.0	0.9	1.5
PSMB10	P40306	17	-15	-14	-9	32	0.9	0.9	0.9	1.5
AHNAK	Q09666	1833	-4	-10	-9	31	1.0	0.9	0.9	1.4
CEP152	O94986	1417	-5	7	-9	31	1.0	1.1	0.9	1.4
RPS6KA5	O75582	214	16	5	-9	30	1.2	1.1	0.9	1.4
PRKDC	P78527	1164	-4	-6	-9	30	1.0	0.9	0.9	1.4
IRF9	Q00978	319	-7	-8	-9	30	0.9	0.9	0.9	1.4
ALKBH5	Q6P6C2	267	-2	-7	-9	29	1.0	0.9	0.9	1.4
PPP6R2	O75170	511	-6	-10	-9	29	0.9	0.9	0.9	1.4
HSPA4	P34932	380	-5	-17	-9	28	1.0	0.9	0.9	1.4
HSPH1	Q92598	380	-5	-17	-9	28	1.0	0.9	0.9	1.4
RPS6KA3	P51812	229	3	-7	-9	28	1.0	0.9	0.9	1.4
TRAPPC8	Q9Y2L5	265	-11	9	-9	27	0.9	1.1	0.9	1.4
IKZF3	Q9UKT9	434	-6	-9	-9	27	0.9	0.9	0.9	1.4
STRIP1	Q5VSL9	769	-8	-9	-9	26	0.9	0.9	0.9	1.4
ADRM1	Q16186	88	-1	-5	-9	25	1.0	1.0	0.9	1.3
PTK2B	Q14289	650	5	7	-9	24	1.0	1.1	0.9	1.3
KMT2D	O14686	414	-1	-1	-9	24	1.0	1.0	0.9	1.3
SPG21	Q9NZD8	44	-6	-3	-9	24	0.9	1.0	0.9	1.3
E4F1	Q66K89	361	-2	-10	-9	24	1.0	0.9	0.9	1.3
CTC1	Q2NKJ3	584	-27	-19	-9	24	0.8	0.8	0.9	1.3
HEATR3	Q7Z4Q2	20	-7	-1	-9	24	0.9	1.0	0.9	1.3
CUL7	Q14999	1569	-9	-4	-9	24	0.9	1.0	0.9	1.3
CABIN1	Q9Y6J0	717	-2	-18	-9	24	1.0	0.9	0.9	1.3
Uncharacteriz	U3KQE9	70	7	-6	-9	23	1.1	0.9	0.9	1.3
ENTHD2	Q96N21	479	-9	-1	-9	22	0.9	1.0	0.9	1.3
PHF1	O43189	518	-14	-4	-9	22	0.9	1.0	0.9	1.3
PRPF8	Q6P2Q9	1896	-2	-7	-9	22	1.0	0.9	0.9	1.3
KDM3A	Q9Y4C1	390	6	-11	-9	22	1.1	0.9	0.9	1.3
FUK	Q8N0W3	1081	-1	0	-9	21	1.0	1.0	0.9	1.3
ADRBK1	P25098	120	3	-5	-9	21	1.0	1.0	0.9	1.3
EVI2B	P34910	253	-5	-10	-9	21	1.0	0.9	0.9	1.3

DKC1	O60832	74	-6	-15	-9	21	0.9	0.9	0.9	1.3
RBM42	Q9BTD8	385	7	4	-9	20	1.1	1.0	0.9	1.3
ATP5C1	P36542	103	2	-28	-9	20	1.0	0.8	0.9	1.3
SCRIB	A0A0G2JPP5	864	-8	-4	-9	20	0.9	1.0	0.9	1.2
OSBPL5	Q9H0X9	14	-2	-1	-9	19	1.0	1.0	0.9	1.2
CEP162	Q5TB80	1014	2	2	-9	19	1.0	1.0	0.9	1.2
NLRC5	Q86WI3	1838	-8	-4	-9	19	0.9	1.0	0.9	1.2
CSNK2A2	P19784	336	-3	-6	-9	19	1.0	0.9	0.9	1.2
POLE4	Q9NR33	84	-6	-11	-9	19	0.9	0.9	0.9	1.2
PDIA3	P30101	406	-2	-12	-9	18	1.0	0.9	0.9	1.2
NPEPL1	Q8NDH3	189	-1	1	-9	18	1.0	1.0	0.9	1.2
RAD1	O60671	239	6	-8	-9	18	1.1	0.9	0.9	1.2
TRAF3IP3	Q9Y228	105	-7	-23	-9	18	0.9	0.8	0.9	1.2
MTHFD1	P11586	143	-4	-9	-9	17	1.0	0.9	0.9	1.2
ZFYVE19	Q96K21	83	6	-1	-9	17	1.1	1.0	0.9	1.2
APEX1	P27695	296	1	-12	-9	17	1.0	0.9	0.9	1.2
MTA2	O94776	209	-3	-7	-9	16	1.0	0.9	0.9	1.2
PANK4	Q9NVE7	155	-4	-12	-9	16	1.0	0.9	0.9	1.2
PI4KB	Q9UBF8	52	-6	-12	-9	16	0.9	0.9	0.9	1.2
TBC1D5	Q92609	96	4	-15	-9	16	1.0	0.9	0.9	1.2
PRKCZ	Q05513	107	3	-4	-9	16	1.0	1.0	0.9	1.2
MBD1	Q9UIS9	525	-10	-9	-9	16	0.9	0.9	0.9	1.2
MGST1	P10620	50	-2	-4	-9	15	1.0	1.0	0.9	1.2
KLHL6	Q8WZ60	252	6	-7	-9	15	1.1	0.9	0.9	1.2
PLEC	Q15149	3299	-4	-9	-9	15	1.0	0.9	0.9	1.2
NUDCD1	Q96RS6	111	12	-10	-9	15	1.1	0.9	0.9	1.2
CD2AP	Q9Y5K6	540	2	-11	-9	15	1.0	0.9	0.9	1.2
IKZF1	Q13422	394	9	4	-9	15	1.1	1.0	0.9	1.2
SORBS3	O60504	482	-3	-6	-9	15	1.0	0.9	0.9	1.2
SRRM2	Q9UQ35	1480	0	-6	-9	15	1.0	0.9	0.9	1.2
OLA1	Q9NTK5	187	2	-7	-9	15	1.0	0.9	0.9	1.2
SLC26A6	Q9BXS9	628	-2	-8	-9	15	1.0	0.9	0.9	1.2
PDCD4	Q53EL6	275	-3	-8	-9	15	1.0	0.9	0.9	1.2
ANKRD39	Q53RE8	173	-2	-10	-9	15	1.0	0.9	0.9	1.2
PPTC7	Q8NI37	57	-6	-16	-9	15	0.9	0.9	0.9	1.2
SSBP3	Q9BWW4	80	-24	-27	-9	15	0.8	0.8	0.9	1.2
CAPN2	P17655	82	3	-5	-9	14	1.0	1.0	0.9	1.2
MARC2	Q969Z3	272	-14	-6	-9	14	0.9	0.9	0.9	1.2
PPP6R1	Q9UPN7	326	5	-7	-9	14	1.0	0.9	0.9	1.2
NARS	O43776	438	-6	-8	-9	14	0.9	0.9	0.9	1.2
CPSF3	Q9UKF6	498	-8	-8	-9	14	0.9	0.9	0.9	1.2
STK26	Q9P289	392	2	5	-9	14	1.0	1.1	0.9	1.2
PHGDH	O43175	369	-13	-3	-9	14	0.9	1.0	0.9	1.2
PARP9	Q8IXQ6	728	0	-5	-9	14	1.0	1.0	0.9	1.2
FANCD2	Q9BXW9	1369	-13	-7	-9	14	0.9	0.9	0.9	1.2
MAP2K4	P45985	266	-6	-5	-9	13	0.9	1.0	0.9	1.1
RAE1	P78406	106	0	-6	-9	13	1.0	0.9	0.9	1.1
SUPT6H	Q7KZ85	336	-9	4	-9	13	0.9	1.0	0.9	1.1
TOR1AIP1	Q5JTV8	424	-11	-4	-9	13	0.9	1.0	0.9	1.1
RENBP	P51606	423	-2	-4	-9	13	1.0	1.0	0.9	1.1
NAA15	Q9BXJ9	721	-2	-6	-9	13	1.0	0.9	0.9	1.1
ELP2	Q6IA86	746	-4	-7	-9	13	1.0	0.9	0.9	1.1
TBCD	Q9BTW9	234	6	-8	-9	13	1.1	0.9	0.9	1.1
RNASEH2C	Q8TDP1	34	3	3	-9	12	1.0	1.0	0.9	1.1
RIN3	Q8TB24	942	-2	-2	-9	12	1.0	1.0	0.9	1.1

BLOC1S5-TXNI	H3BN57	113	-1	-4	-9	12	1.0	1.0	0.9	1.1
HNRNPK	P61978	132	0	-6	-9	12	1.0	0.9	0.9	1.1
TMLHE	Q9NVH6	393	4	-7	-9	12	1.0	0.9	0.9	1.1
SUGT1	Q9Y2Z0	62	-1	-7	-9	12	1.0	0.9	0.9	1.1
IDH2	P48735	418	-1	-11	-9	12	1.0	0.9	0.9	1.1
SUPT6H	Q7KZ85	939	2	-25	-9	12	1.0	0.8	0.9	1.1
GALNT6	Q8NCL4	141	-1	0	-9	12	1.0	1.0	0.9	1.1
NAPRT	Q6XQN6	385	3	-5	-9	12	1.0	1.0	0.9	1.1
LDHA	P00338	131	-12	-7	-9	12	0.9	0.9	0.9	1.1
UBR4	Q5T4S7	3075	-3	-7	-9	12	1.0	0.9	0.9	1.1
MTFR1L	Q9H019	52	-11	-12	-9	12	0.9	0.9	0.9	1.1
PI4KA	P42356	246	-7	-6	-9	11	0.9	0.9	0.9	1.1
CCT7	Q99832	310	-1	-13	-9	11	1.0	0.9	0.9	1.1
GCN1	Q92616	648	-9	-18	-9	11	0.9	0.9	0.9	1.1
OTUD4	Q01804	489	19	-18	-9	11	1.2	0.8	0.9	1.1
RBPJ	Q06330	313	2	2	-9	11	1.0	1.0	0.9	1.1
SEPT6	Q14141	47	1	-2	-9	11	1.0	1.0	0.9	1.1
PTPRC	P08575	787	-6	-4	-9	11	0.9	1.0	0.9	1.1
DGKA	P23743	101	-6	-6	-9	11	0.9	0.9	0.9	1.1
BAZ1A	Q9NRL2	970	-7	-11	-9	11	0.9	0.9	0.9	1.1
ESYT1	Q9BSJ8	635	1	-23	-9	11	1.0	0.8	0.9	1.1
PFAS	O15067	1027	-3	-1	-9	10	1.0	1.0	0.9	1.1
BTAF1	O14981	109	-7	-10	-9	10	0.9	0.9	0.9	1.1
PRDX6	P30041	91	-6	-11	-9	10	0.9	0.9	0.9	1.1
ZFC3H1	O60293	1068	-3	-14	-9	10	1.0	0.9	0.9	1.1
RPS27A	P62979	145	-11	5	-9	10	0.9	1.0	0.9	1.1
ZNF148	Q9UQR1	14	-6	0	-9	10	0.9	1.0	0.9	1.1
COL4A3BP	Q9Y5P4	65	-4	-6	-9	10	1.0	0.9	0.9	1.1
INTS7	Q9NVH2	352	2	-8	-9	10	1.0	0.9	0.9	1.1
AHNAK	Q09666	5502	-3	-9	-9	10	1.0	0.9	0.9	1.1
UBN2	Q6ZU65	966	-7	-10	-9	10	0.9	0.9	0.9	1.1
NUMA1	Q14980	160	-4	-13	-9	10	1.0	0.9	0.9	1.1
PARP1	P09874	429	-5	-15	-9	10	1.0	0.9	0.9	1.1
MYO9B	Q13459	62	-5	0	-9	9	1.0	1.0	0.9	1.1
NUP205	Q92621	922	1	-5	-9	9	1.0	1.0	0.9	1.1
DUSP22	Q9NRW4	124	0	-6	-9	9	1.0	0.9	0.9	1.1
PGAM5	Q96HS1	168	1	-8	-9	9	1.0	0.9	0.9	1.1
CAMK2D	Q13557	273	-8	-10	-9	9	0.9	0.9	0.9	1.1
KDM3B	Q7LBC6	1124	-16	7	-9	9	0.9	1.1	0.9	1.1
SND1	Q7KZF4	560	-3	-2	-9	9	1.0	1.0	0.9	1.1
PDXK	O00764	273	-3	-3	-9	9	1.0	1.0	0.9	1.1
XRCC5	P13010	493	-1	-6	-9	9	1.0	0.9	0.9	1.1
YEATS4	O95619	210	2	-6	-9	9	1.0	0.9	0.9	1.1
RNH1	P13489	313	-4	-7	-9	9	1.0	0.9	0.9	1.1
TGFBR2	P37173	483	-12	-9	-9	9	0.9	0.9	0.9	1.1
VPS16	Q9H269	490	-2	-10	-9	9	1.0	0.9	0.9	1.1
MYOF	Q9NZM1	275	-2	-10	-9	9	1.0	0.9	0.9	1.1
CNOT1	A5YKK6	1706	-4	-12	-9	9	1.0	0.9	0.9	1.1
LYAR	Q9NX58	33	3	13	-9	8	1.0	1.1	0.9	1.1
OSGEPL1	Q9H4B0	390	-2	-2	-9	8	1.0	1.0	0.9	1.1
CDK2AP2	O75956	116	5	-6	-9	8	1.1	0.9	0.9	1.1
TKFC	Q3LXA3	404	-6	-8	-9	8	0.9	0.9	0.9	1.1
TRIM27	P14373	393	-5	-10	-9	8	1.0	0.9	0.9	1.1
ACAT1	P24752	126	-3	-10	-9	8	1.0	0.9	0.9	1.1
DNAAF5	Q86Y56	138	-5	-10	-9	8	1.0	0.9	0.9	1.1

U2AF2	P26368	429	1	-11	-9	8	1.0	0.9	0.9	1.1
DCAF7	P61962	109	-11	-16	-9	8	0.9	0.9	0.9	1.1
DYNC1H1	Q14204	4570	-9	-22	-9	8	0.9	0.8	0.9	1.1
TXLNA	P40222	523	-2	-7	-9	8	1.0	0.9	0.9	1.1
DOCK11	Q5JSL3	1878	1	-8	-9	8	1.0	0.9	0.9	1.1
PARVG	Q9HBI0	314	-5	-9	-9	8	1.0	0.9	0.9	1.1
WDR89	Q96FK6	16	2	-1	-9	7	1.0	1.0	0.9	1.1
TRAPPC11	Q7Z392	162	1	-7	-9	7	1.0	0.9	0.9	1.1
GMEB1	Q9Y692	174	8	-10	-9	7	1.1	0.9	0.9	1.1
AHCTF1	Q8WYP5	313	-8	-5	-9	7	0.9	1.0	0.9	1.1
GATAD2A	Q86YP4	426	-9	-6	-9	7	0.9	0.9	0.9	1.1
ZSWIM8	A7E2V4	507	-11	-8	-9	7	0.9	0.9	0.9	1.1
TBC1D13	Q9NVG8	282	-2	-8	-9	7	1.0	0.9	0.9	1.1
MCM5	P33992	219	-5	-10	-9	7	1.0	0.9	0.9	1.1
PLOD1	Q02809	680	-6	-2	-9	6	0.9	1.0	0.9	1.1
ADSL	P30566	399	6	-5	-9	6	1.1	1.0	0.9	1.1
CARM1	Q86X55	420	5	-6	-9	6	1.0	0.9	0.9	1.1
ZZEF1	O43149	1026	-1	-7	-9	6	1.0	0.9	0.9	1.1
ELF2	Q15723	591	-2	-8	-9	6	1.0	0.9	0.9	1.1
FUK	Q8N0W3	582	-3	-11	-9	6	1.0	0.9	0.9	1.1
THEM6	Q8WUY1	168	1	-12	-9	6	1.0	0.9	0.9	1.1
KHSRP	Q92945	176	1	-4	-9	6	1.0	1.0	0.9	1.1
ZNF672	Q499Z4	350	-6	-5	-9	6	0.9	1.0	0.9	1.1
GMIP	Q9P107	895	-6	-5	-9	6	0.9	1.0	0.9	1.1
DBNL	Q9UJU6	127	2	-9	-9	6	1.0	0.9	0.9	1.1
INPP5B	P32019	362	-1	-10	-9	6	1.0	0.9	0.9	1.1
IRF2BP1	Q8IU81	280	-2	-12	-9	6	1.0	0.9	0.9	1.1
BTBD11	A6QL63	844	-5	-13	-9	6	1.0	0.9	0.9	1.1
CMPK2	Q5EBM0	189	-1	-3	-9	5	1.0	1.0	0.9	1.1
GOLGA4	Q13439	1085	-4	-7	-9	5	1.0	0.9	0.9	1.1
GNL2	Q13823	336	-6	-12	-9	5	0.9	0.9	0.9	1.1
DGKG	P49619	413	-8	-13	-9	5	0.9	0.9	0.9	1.1
SYNE2	Q8WXH0	2212	-6	-14	-9	5	0.9	0.9	0.9	1.1
SND1	Q7KZF4	96	0	-18	-9	5	1.0	0.9	0.9	1.1
OXCT1	P55809	456	-7	1	-9	5	0.9	1.0	0.9	1.0
EXOSC9	Q06265	46	-7	-4	-9	5	0.9	1.0	0.9	1.0
MYCBP2	O75592	1478	-4	-5	-9	5	1.0	1.0	0.9	1.0
RPS12	P25398	108	3	-6	-9	5	1.0	0.9	0.9	1.0
RUVBL2	Q9Y230	413	-13	-13	-9	5	0.9	0.9	0.9	1.0
OAS2	P29728	307	-9	-16	-9	5	0.9	0.9	0.9	1.0
CMPK2	Q5EBM0	243	-9	-18	-9	5	0.9	0.8	0.9	1.0
POLG2	Q9UHN1	377	-1	-5	-9	4	1.0	1.0	0.9	1.0
ARHGAP25	P42331	258	10	-7	-9	4	1.1	0.9	0.9	1.0
INPP5D	Q92835	138	-3	-7	-9	4	1.0	0.9	0.9	1.0
AARS2	Q5JTZ9	609	-5	-7	-9	4	1.0	0.9	0.9	1.0
ANAPC7	Q9UJX3	329	-2	-9	-9	4	1.0	0.9	0.9	1.0
TRIM33	Q9UPN9	943	0	-9	-9	4	1.0	0.9	0.9	1.0
LARS	Q9P2J5	573	-12	-1	-9	4	0.9	1.0	0.9	1.0
DYNC1H1	Q14204	4510	-4	-5	-9	4	1.0	1.0	0.9	1.0
FAM102A	Q5T9C2	119	-10	-6	-9	4	0.9	0.9	0.9	1.0
BAZ1B	Q9UIG0	1420	3	-6	-9	4	1.0	0.9	0.9	1.0
TRAPPC9	Q96Q05	935	-10	-12	-9	4	0.9	0.9	0.9	1.0
SUPT4H1	P63272	19	1	-13	-9	4	1.0	0.9	0.9	1.0
ZFYVE16	Q7Z3T8	269	-5	-19	-9	4	1.0	0.8	0.9	1.0
EIF5A2	Q9GZV4	73	-1	-20	-9	4	1.0	0.8	0.9	1.0

ITPRIP	Q8IWB1	255	1	-7	-9	3	1.0	0.9	0.9	1.0
LAP3	P28838	445	3	-9	-9	3	1.0	0.9	0.9	1.0
GNB2	P62879	149	2	-15	-9	3	1.0	0.9	0.9	1.0
GNB4	Q9HAV0	149	2	-15	-9	3	1.0	0.9	0.9	1.0
GTF3C2	Q8WUA4	608	2	-4	-9	3	1.0	1.0	0.9	1.0
STAG2	Q8N3U4	915	-3	-6	-9	3	1.0	0.9	0.9	1.0
STAG1	Q8WVM7	919	-3	-6	-9	3	1.0	0.9	0.9	1.0
CPNE3	O75131	54	-1	-7	-9	3	1.0	0.9	0.9	1.0
MAST3	O60307	363	-2	-8	-9	3	1.0	0.9	0.9	1.0
NIPBL	Q6KC79	1795	-7	-8	-9	3	0.9	0.9	0.9	1.0
CDC123	O75794	212	-13	-8	-9	3	0.9	0.9	0.9	1.0
SLFN5	Q08AF3	627	1	-9	-9	3	1.0	0.9	0.9	1.0
COPA	P53621	1185	-10	-11	-9	3	0.9	0.9	0.9	1.0
MVD	P53602	386	0	-12	-9	3	1.0	0.9	0.9	1.0
SPC25	Q9HBM1	27	-16	-15	-9	3	0.9	0.9	0.9	1.0
CSTF3	Q12996	646	-1	2	-9	2	1.0	1.0	0.9	1.0
SRBD1	Q8N5C6	912	-3	-1	-9	2	1.0	1.0	0.9	1.0
ARMC8	Q8IUR7	275	-6	-5	-9	2	0.9	1.0	0.9	1.0
NTN1	O95631	70	-2	-9	-9	2	1.0	0.9	0.9	1.0
NTN1	O95631	78	-2	-9	-9	2	1.0	0.9	0.9	1.0
CLIC3	O95833	22	-8	-17	-9	2	0.9	0.9	0.9	1.0
ZC3H13	Q5T200	1643	0	-17	-9	2	1.0	0.9	0.9	1.0
XRN2	Q9H0D6	29	-15	-18	-9	2	0.9	0.8	0.9	1.0
CYHR1	Q6ZMK1	279	-5	-1	-9	2	1.0	1.0	0.9	1.0
TNPO1	Q92973	297	5	10	-9	1	1.1	1.1	0.9	1.0
EML2	O95834	53	-5	0	-9	1	1.0	1.0	0.9	1.0
AP1B1	Q10567	57	5	-1	-9	1	1.1	1.0	0.9	1.0
SEPT7	Q16181	204	2	-9	-9	1	1.0	0.9	0.9	1.0
IQGAP1	P46940	494	-5	-11	-9	1	1.0	0.9	0.9	1.0
MTHFD1	P11586	691	-13	-15	-9	1	0.9	0.9	0.9	1.0
L3HYPDH	Q96EM0	139	-3	-2	-9	1	1.0	1.0	0.9	1.0
PRKD2	Q9BZL6	84	-1	-7	-9	1	1.0	0.9	0.9	1.0
GLYR1	Q49A26	303	-12	-9	-9	1	0.9	0.9	0.9	1.0
TCAF2	A6NFQ2	401	-9	-9	-9	1	0.9	0.9	0.9	1.0
NMNAT1	Q9HAN9	111	0	-10	-9	1	1.0	0.9	0.9	1.0
BZW1	Q7L1Q6	96	7	-10	-9	1	1.1	0.9	0.9	1.0
TRABD	Q9H4I3	108	-6	-32	-9	1	0.9	0.8	0.9	1.0
HDLBP	Q00341	948	-7	-5	-9	0	0.9	1.0	0.9	1.0
DDX52	Q9Y2R4	536	-6	-6	-9	0	0.9	0.9	0.9	1.0
SMCHD1	A6NHR9	1433	-4	-7	-9	0	1.0	0.9	0.9	1.0
UPF1	Q92900	213	-2	-10	-9	0	1.0	0.9	0.9	1.0
COG5	Q9UP83	173	-8	-18	-9	0	0.9	0.9	0.9	1.0
PKM	P14618	423	-1	-1	-9	-1	1.0	1.0	0.9	1.0
COG4	Q9H9E3	25	-10	-1	-9	-1	0.9	1.0	0.9	1.0
TTF1	Q15361	73	-5	-7	-9	-1	1.0	0.9	0.9	1.0
TRAPPC10	P48553	1126	-9	-9	-9	-1	0.9	0.9	0.9	1.0
LUC7L3	O95232	43	-11	-15	-9	-1	0.9	0.9	0.9	1.0
ARHGEF6	Q15052	182	-5	-11	-9	-1	1.0	0.9	0.9	1.0
UPF3B	Q9BZI7	319	-8	-13	-9	-1	0.9	0.9	0.9	1.0
HACE1	Q8IYU2	481	-4	-6	-9	-2	1.0	0.9	0.9	1.0
MAP2K4	P45985	379	2	-8	-9	-2	1.0	0.9	0.9	1.0
ZFYVE21	Q9BQ24	88	-7	-4	-9	-2	0.9	1.0	0.9	1.0
DOCK11	Q5JSL3	492	0	-8	-9	-2	1.0	0.9	0.9	1.0
HOMEZ	Q8IX15	378	-3	-11	-9	-2	1.0	0.9	0.9	1.0
PTK2B	Q14289	562	-2	-3	-9	-3	1.0	1.0	0.9	1.0

C8orf82	Q6P1X6	98	-7	-5	-9	-3	0.9	1.0	0.9	1.0
GARS	P41250	466	-10	-11	-9	-3	0.9	0.9	0.9	1.0
METAP1	P53582	9	-1	-11	-9	-3	1.0	0.9	0.9	1.0
RUVBL2	Q9Y230	227	-11	-12	-9	-3	0.9	0.9	0.9	1.0
FAM126B	Q8IXS8	7	-4	-13	-9	-3	1.0	0.9	0.9	1.0
FAM65B	Q9Y4F9	821	6	6	-9	-3	1.1	1.1	0.9	1.0
SF1	Q15637	279	0	2	-9	-3	1.0	1.0	0.9	1.0
PSMD2	Q13200	251	-3	-4	-9	-3	1.0	1.0	0.9	1.0
TAF1	P21675	1066	-2	-5	-9	-3	1.0	1.0	0.9	1.0
PLAA	Q9Y263	263	7	-12	-9	-3	1.1	0.9	0.9	1.0
CEP70	Q8NHQ1	215	2	-8	-9	-4	1.0	0.9	0.9	1.0
FBL	P22087	99	-1	-9	-9	-4	1.0	0.9	0.9	1.0
CROCC	Q5TZA2	515	-10	-10	-9	-4	0.9	0.9	0.9	1.0
COQ10B	Q9H8M1	108	2	-3	-9	-4	1.0	1.0	0.9	1.0
YWHAG	P61981	112	-2	-4	-9	-4	1.0	1.0	0.9	1.0
PITRM1	Q5JRX3	556	-5	-8	-9	-4	1.0	0.9	0.9	1.0
DDX60	Q8IY21	1224	-11	4	-9	-5	0.9	1.0	0.9	1.0
CCAR2	Q8N163	754	1	-5	-9	-5	1.0	1.0	0.9	1.0
CKAP5	Q14008	441	9	-9	-9	-5	1.1	0.9	0.9	1.0
YWHAE	P62258	97	-4	-5	-9	-5	1.0	1.0	0.9	1.0
TPP2	P29144	787	-4	-15	-9	-5	1.0	0.9	0.9	1.0
CLIC1	O00299	24	-9	-6	-9	-6	0.9	0.9	0.9	0.9
PHF14	O94880	662	2	-11	-9	-6	1.0	0.9	0.9	0.9
IKBKG	Q9Y6K9	76	8	1	-9	-6	1.1	1.0	0.9	0.9
PARP1	P09874	295	-11	-18	-9	-6	0.9	0.8	0.9	0.9
CTU1	Q7Z7A3	290	-4	1	-9	-7	1.0	1.0	0.9	0.9
DOK3	Q7L591	144	-9	-3	-9	-7	0.9	1.0	0.9	0.9
TRIM33	Q9UPN9	145	1	-5	-9	-7	1.0	1.0	0.9	0.9
ASPHD2	Q6ICH7	116	-10	-4	-9	-7	0.9	1.0	0.9	0.9
BRK1	Q8WUW1	43	-2	-7	-9	-7	1.0	0.9	0.9	0.9
NUP88	Q99567	155	10	-14	-9	-7	1.1	0.9	0.9	0.9
PHF1	O43189	93	-6	-17	-9	-7	0.9	0.9	0.9	0.9
EIF3A	Q14152	78	-6	3	-9	-8	0.9	1.0	0.9	0.9
ADH5	P11766	100	-2	-5	-9	-8	1.0	1.0	0.9	0.9
TUBG2	Q9NRH3	13	-4	-8	-9	-8	1.0	0.9	0.9	0.9
TBP	P20226	221	1	-12	-9	-8	1.0	0.9	0.9	0.9
AARS	P49588	947	-21	-14	-9	-8	0.8	0.9	0.9	0.9
AKT1S1	Q96B36	61	12	12	-9	-9	1.1	1.1	0.9	0.9
EIF5	P55010	138	4	-4	-9	-9	1.0	1.0	0.9	0.9
RASA3	Q14644	206	-2	-9	-9	-9	1.0	0.9	0.9	0.9
INPP4B	O15327	278	-2	-9	-9	-10	1.0	0.9	0.9	0.9
CORO1B	Q9BR76	332	7	-12	-9	-10	1.1	0.9	0.9	0.9
LRCH4	O75427	102	8	-4	-9	-10	1.1	1.0	0.9	0.9
WDR26	Q9H7D7	338	16	-9	-9	-10	1.2	0.9	0.9	0.9
ALG2	Q9H553	59	-14	-17	-9	-10	0.9	0.9	0.9	0.9
HECTD1	Q9ULT8	2545	-4	-6	-9	-11	1.0	0.9	0.9	0.9
PPIL2	Q13356	387	-9	-14	-9	-11	0.9	0.9	0.9	0.9
KIF2A	O00139	420	3	-5	-9	-11	1.0	1.0	0.9	0.9
ALKBH1	Q13686	371	3	-14	-9	-11	1.0	0.9	0.9	0.9
DYNC1H1	Q14204	2076	5	-15	-9	-13	1.0	0.9	0.9	0.9
CLTC	Q00610	918	-2	-8	-9	-13	1.0	0.9	0.9	0.9
RGPD3	A6NKT7	1167	-9	-8	-9	-13	0.9	0.9	0.9	0.9
RANBP2	P49792	2142	-9	-8	-9	-13	0.9	0.9	0.9	0.9
RGPD6	Q99666	1166	-9	-8	-9	-13	0.9	0.9	0.9	0.9
UTRN	P46939	2877	-5	-10	-9	-13	1.0	0.9	0.9	0.9

CARD9	Q9H257	168	-10	-15	-9	-16	0.9	0.9	0.9	0.9
TBL1XR1	Q9BZK7	383	-4	-2	-9	-16	1.0	1.0	0.9	0.9
TUBA3D	Q13748	347	-5	-8	-9	-17	1.0	0.9	0.9	0.9
TUBA1A	Q71U36	347	-5	-8	-9	-17	1.0	0.9	0.9	0.9
ZYX	Q15942	540	-10	2	-9	-17	0.9	1.0	0.9	0.9
SACM1L	Q9NTJ5	389	-1	-2	-9	-17	1.0	1.0	0.9	0.9
BCR	P11274	753	-4	-9	-9	-17	1.0	0.9	0.9	0.9
CHTF18	Q8WVB6	542	-8	-11	-9	-19	0.9	0.9	0.9	0.8
MPP7	Q5T2T1	462	6	-17	-9	-21	1.1	0.9	0.9	0.8
CRBN	Q96SW2	234	-9	10	-9	-22	0.9	1.1	0.9	0.8
COPE	O14579	34	-8	-7	-9	-22	0.9	0.9	0.9	0.8
ROCK2	O75116	607	-1	-4	-9	-27	1.0	1.0	0.9	0.8
CLIC4	Q9Y696	234	-2	-13	-9	-28	1.0	0.9	0.9	0.8
KIAA0513	O60268	149	-10	3	-9	-29	0.9	1.0	0.9	0.8
SUN2	Q9UH99	705	-14	-12	-9	-36	0.9	0.9	0.9	0.7
TNPO3	Q9Y5L0	634	9	-16	-9	-43	1.1	0.9	0.9	0.7
CALCOCO1	Q9P1Z2	57	-8	-10	-9	-45	0.9	0.9	0.9	0.7
GAPVD1	Q14C86	1160	15	-19	-9	-46	1.2	0.8	0.9	0.7
LIMS1	P48059	181	1	-6	-9	-59	1.0	0.9	0.9	0.6
LIMS2	Q7Z4I7	186	1	-6	-9	-59	1.0	0.9	0.9	0.6
TRIM14	Q14142	42	-23	-6	-9	-61	0.8	0.9	0.9	0.6
ILK	Q13418	422	2	-12	-9	-87	1.0	0.9	0.9	0.5
APOBEC3C	Q9NRW3	65	20	-1	-9	-168	1.3	1.0	0.9	0.4
CIC	Q96RK0	1420	-3	-10	-9	56	1.0	0.9	0.9	2.2
ZC3HAV1	Q7Z2W4	518	-37	-2	-9	53	0.7	1.0	0.9	2.1
UBR3	Q6ZT12	1858	2	-3	-9	43	1.0	1.0	0.9	1.7
STARD7	Q9NQZ5	362	6	7	-9	41	1.1	1.1	0.9	1.7
ABCB7	O75027	750	-9	-8	-9	40	0.9	0.9	0.9	1.7
NISCH	Q9Y2I1	894	-14	-6	-9	35	0.9	0.9	0.9	1.5
NHSL2	Q5HYW2	679	-6	-3	-9	35	0.9	1.0	0.9	1.5
PRKDC	P78527	232	-3	-9	-9	35	1.0	0.9	0.9	1.5
PLP2	Q04941	12	-20	-27	-9	34	0.8	0.8	0.9	1.5
ATM	Q13315	2991	-4	-12	-9	32	1.0	0.9	0.9	1.5
TRANK1	O15050	1319	-3	-9	-9	30	1.0	0.9	0.9	1.4
KIN	O60870	212	-10	-15	-9	30	0.9	0.9	0.9	1.4
ARMC10	Q8N2F6	59	-7	-11	-9	30	0.9	0.9	0.9	1.4
RNH1	P13489	277	-3	-10	-9	28	1.0	0.9	0.9	1.4
HERC5	Q9UII4	994	-10	-18	-9	28	0.9	0.8	0.9	1.4
NIPBL	Q6KC79	419	4	3	-9	27	1.0	1.0	0.9	1.4
HK3	P52790	915	-1	-7	-9	27	1.0	0.9	0.9	1.4
ZFP41	A0A0G2JH32	186	3	4	-9	27	1.0	1.0	0.9	1.4
HNRNPU	Q00839	450	-2	-1	-9	27	1.0	1.0	0.9	1.4
RNF213	Q63HN8	2918	4	-1	-9	26	1.0	1.0	0.9	1.4
PISD	Q9UG56	300	-3	-5	-9	26	1.0	1.0	0.9	1.4
INTS2	Q9H0H0	1164	2	-8	-9	26	1.0	0.9	0.9	1.4
PKN1	Q16512	442	2	-3	-9	26	1.0	1.0	0.9	1.3
CCDC85B	Q15834	82	-2	-16	-9	26	1.0	0.9	0.9	1.3
PRMT1	Q99873	350	-7	-7	-9	25	0.9	0.9	0.9	1.3
AHCYL1	O43865	106	-2	-15	-9	23	1.0	0.9	0.9	1.3
ELMO1	Q92556	726	-9	-4	-9	22	0.9	1.0	0.9	1.3
HPCAL1	P37235	185	-1	-8	-9	22	1.0	0.9	0.9	1.3
BRD7	Q9NPI1	367	2	-11	-9	22	1.0	0.9	0.9	1.3
SAE1	Q9UBE0	214	-11	-10	-9	21	0.9	0.9	0.9	1.3
DEF6	Q9H4E7	267	-1	-7	-9	20	1.0	0.9	0.9	1.3
ZBTB21	Q9ULJ3	283	-5	-14	-9	20	1.0	0.9	0.9	1.2

TIMELESS	Q9UNS1	1126	3	3	-9	19	1.0	1.0	0.9	1.2
TFAM	Q00059	246	3	-4	-9	19	1.0	1.0	0.9	1.2
PARK7	Q99497	106	-3	-10	-9	19	1.0	0.9	0.9	1.2
WASL	O00401	431	-3	-11	-9	19	1.0	0.9	0.9	1.2
PXN	P49023	585	0	2	-9	18	1.0	1.0	0.9	1.2
NUDCD1	Q96RS6	376	-3	-8	-9	18	1.0	0.9	0.9	1.2
IREB2	P48200	137	-15	-10	-9	18	0.9	0.9	0.9	1.2
ANKHD1	Q8IWZ3	615	-5	-10	-9	18	1.0	0.9	0.9	1.2
PRRC2C	Q9Y520	620	-10	-11	-9	18	0.9	0.9	0.9	1.2
NABP2	Q9BQ15	81	-7	-6	-9	18	0.9	0.9	0.9	1.2
PDIA4	P13667	555	-6	-11	-9	18	0.9	0.9	0.9	1.2
HPS6	Q86YV9	692	-9	1	-9	17	0.9	1.0	0.9	1.2
TSN	Q15631	225	1	-6	-9	17	1.0	0.9	0.9	1.2
EZH2	Q15910	503	-4	-6	-9	17	1.0	0.9	0.9	1.2
EZH1	Q92800	504	-4	-6	-9	17	1.0	0.9	0.9	1.2
ZNHIT6	Q9NWK9	211	-5	-12	-9	17	1.0	0.9	0.9	1.2
ZFYVE26	Q68DK2	715	-2	-3	-9	17	1.0	1.0	0.9	1.2
ARID4B	Q4LE39	1113	-4	-3	-9	17	1.0	1.0	0.9	1.2
ADAMTS2	O95450	680	-4	-5	-9	17	1.0	1.0	0.9	1.2
ITK	Q08881	339	-7	-5	-9	17	0.9	1.0	0.9	1.2
PPP2R5C	Q13362	513	-7	-7	-9	16	0.9	0.9	0.9	1.2
SIPA1	Q96FS4	811	-3	-12	-9	16	1.0	0.9	0.9	1.2
EPG5	Q9HCE0	53	-5	-5	-9	16	1.0	1.0	0.9	1.2
KYNU	Q16719	45	-4	-10	-9	16	1.0	0.9	0.9	1.2
ATM	Q13315	532	-12	-6	-9	15	0.9	0.9	0.9	1.2
PRMT5	O14744	22	-7	-7	-9	15	0.9	0.9	0.9	1.2
WDR81	Q562E7	643	-11	-9	-9	15	0.9	0.9	0.9	1.2
PSMB8	P28062	174	11	-20	-9	15	1.1	0.8	0.9	1.2
PPP6R3	Q5H9R7	844	-6	-4	-9	15	0.9	1.0	0.9	1.2
MKL1	Q969V6	508	-2	-2	-9	14	1.0	1.0	0.9	1.2
TES	Q9UGI8	196	1	-7	-9	14	1.0	0.9	0.9	1.2
USP10	Q14694	40	-7	-12	-9	14	0.9	0.9	0.9	1.2
MSH2	P43246	822	-2	-13	-9	14	1.0	0.9	0.9	1.2
ADAM15	Q13444	830	-8	-15	-9	14	0.9	0.9	0.9	1.2
EZH2	Q15910	14	-4	-17	-9	14	1.0	0.9	0.9	1.2
CNTRL	Q7Z7A1	1784	-1	-7	-9	14	1.0	0.9	0.9	1.2
DTX3L	Q8TDB6	219	-4	-8	-9	14	1.0	0.9	0.9	1.2
TES	Q9UGI8	29	-4	-10	-9	14	1.0	0.9	0.9	1.2
PALLD	Q8WX93	964	-2	-5	-9	13	1.0	1.0	0.9	1.1
POM121	Q96HA1	268	5	-9	-9	13	1.0	0.9	0.9	1.1
SCAF8	Q9UPN6	1170	-3	-10	-9	13	1.0	0.9	0.9	1.1
RBBP6	Q7Z6E9	1566	-3	-10	-9	13	1.0	0.9	0.9	1.1
NCOA5	Q9HCD5	301	-7	-18	-9	13	0.9	0.9	0.9	1.1
CXorf21	Q9HAI6	117	-11	-25	-9	13	0.9	0.8	0.9	1.1
IRGQ	Q8WZA9	373	4	15	-9	13	1.0	1.2	0.9	1.1
MARK3	P27448	510	-12	1	-9	13	0.9	1.0	0.9	1.1
DHX36	Q9H2U1	135	-11	-3	-9	13	0.9	1.0	0.9	1.1
TRMT2A	Q8IZ69	243	-13	-6	-9	13	0.9	0.9	0.9	1.1
SDHAF3	Q9NRP4	80	-6	-13	-9	13	0.9	0.9	0.9	1.1
ICAM3	P32942	407	-5	-15	-9	13	1.0	0.9	0.9	1.1
IFIT3	O14879	365	-5	-7	-9	12	1.0	0.9	0.9	1.1
PSMC1	P62191	58	-6	-8	-9	12	0.9	0.9	0.9	1.1
RUVBL1	Q9Y265	94	-8	-9	-9	12	0.9	0.9	0.9	1.1
PSMD13	Q9UNM6	357	-3	-11	-9	12	1.0	0.9	0.9	1.1
GBF1	Q92538	1173	4	-1	-9	12	1.0	1.0	0.9	1.1

NDUFAF4	Q9P032	87	-5	-4	-9	12	1.0	1.0	0.9	1.1
SNX6	Q9UNH7	398	3	-5	-9	12	1.0	1.0	0.9	1.1
RRBP1	Q9P2E9	1216	3	-8	-9	12	1.0	0.9	0.9	1.1
RHBDF2	Q6PJF5	181	-13	-14	-9	12	0.9	0.9	0.9	1.1
SF3B3	Q15393	547	-24	-6	-9	11	0.8	0.9	0.9	1.1
CMPK2	Q5EBM0	119	-12	-16	-9	11	0.9	0.9	0.9	1.1
NSD1	Q96L73	2022	-5	-21	-9	11	1.0	0.8	0.9	1.1
F8A3	P23610	110	1	1	-9	11	1.0	1.0	0.9	1.1
ZMYM2	Q9UBW7	708	-6	0	-9	11	0.9	1.0	0.9	1.1
GATAD1	Q8WUU5	179	-5	-5	-9	11	1.0	1.0	0.9	1.1
STK10	O94804	947	-3	-6	-9	11	1.0	0.9	0.9	1.1
TOE1	Q96GM8	371	4	2	-9	10	1.0	1.0	0.9	1.1
STAG2	Q8N3U4	347	-1	-1	-9	10	1.0	1.0	0.9	1.1
GTSF1	Q8WW33	51	-8	-3	-9	10	0.9	1.0	0.9	1.1
ANKRD17	O75179	1622	-5	-12	-9	10	1.0	0.9	0.9	1.1
ASB1	Q9Y576	36	-6	15	-9	10	0.9	1.2	0.9	1.1
PACS1	Q6VY07	116	1	4	-9	10	1.0	1.0	0.9	1.1
U2SURP	O15042	624	7	0	-9	10	1.1	1.0	0.9	1.1
TRAPPC5	Q8IUR0	139	3	-2	-9	10	1.0	1.0	0.9	1.1
MAP7D1	Q3KQU3	382	-15	-3	-9	10	0.9	1.0	0.9	1.1
GIMAP8	Q8ND71	321	-2	-6	-9	10	1.0	0.9	0.9	1.1
COMT	P21964	223	3	-7	-9	10	1.0	0.9	0.9	1.1
MBD4	O95243	254	-12	-2	-9	9	0.9	1.0	0.9	1.1
ZFAND1	Q8TCF1	184	1	-3	-9	9	1.0	1.0	0.9	1.1
MBD1	Q9UIS9	237	-12	-3	-9	9	0.9	1.0	0.9	1.1
GPX4	P36969	102	-10	-4	-9	9	0.9	1.0	0.9	1.1
PITPNC1	Q9UKF7	142	-4	-4	-9	9	1.0	1.0	0.9	1.1
DTX3	Q8N9I9	272	-4	-12	-9	9	1.0	0.9	0.9	1.1
UBR5	O95071	2267	-2	1	-9	9	1.0	1.0	0.9	1.1
SLFN11	Q7Z7L1	884	-6	-2	-9	9	0.9	1.0	0.9	1.1
SLFN5	Q08AF3	342	1	-3	-9	9	1.0	1.0	0.9	1.1
TMLHE	Q9NVH6	103	-2	-3	-9	9	1.0	1.0	0.9	1.1
PIK3CD	O00329	366	2	-6	-9	9	1.0	0.9	0.9	1.1
PPA1	Q15181	270	-4	-8	-9	9	1.0	0.9	0.9	1.1
ISG20L2	Q9H9L3	168	-9	-3	-9	8	0.9	1.0	0.9	1.1
AHCTF1	Q8WYP5	1131	1	-6	-9	8	1.0	0.9	0.9	1.1
Uncharacteriz	U3KPZ7	785	-12	-9	-9	8	0.9	0.9	0.9	1.1
TAF12	Q16514	143	1	-10	-9	8	1.0	0.9	0.9	1.1
MCM3AP	O60318	1238	-3	-10	-9	8	1.0	0.9	0.9	1.1
DTX3L	Q8TDB6	175	-4	-13	-9	8	1.0	0.9	0.9	1.1
RPL27A	P46776	70	-10	-4	-9	8	0.9	1.0	0.9	1.1
UPP1	Q16831	80	-7	-4	-9	8	0.9	1.0	0.9	1.1
NUBP2	Q9Y5Y2	269	-4	-7	-9	8	1.0	0.9	0.9	1.1
FARSB	Q9NSD9	195	-1	-8	-9	8	1.0	0.9	0.9	1.1
DBR1	Q9UK59	339	1	-8	-9	8	1.0	0.9	0.9	1.1
EIF2S2	P20042	281	2	-9	-9	8	1.0	0.9	0.9	1.1
BZW1	Q7L1Q6	35	0	-9	-9	8	1.0	0.9	0.9	1.1
PSMA4	P25789	163	-2	-16	-9	8	1.0	0.9	0.9	1.1
VCPIP1	Q96JH7	816	-5	-19	-9	8	1.0	0.8	0.9	1.1
STAT6	P42226	356	-2	9	-9	7	1.0	1.1	0.9	1.1
TBC1D2B	Q9UPU7	536	4	1	-9	7	1.0	1.0	0.9	1.1
QTRTD1	Q9H974	312	-10	-1	-9	7	0.9	1.0	0.9	1.1
SQRDL	Q9Y6N5	127	-8	-6	-9	7	0.9	0.9	0.9	1.1
GCN1	Q92616	2255	0	-9	-9	7	1.0	0.9	0.9	1.1
FAM120A	Q9NZB2	14	-2	-11	-9	7	1.0	0.9	0.9	1.1

PGLS	O95336	78	-11	-13	-9	7	0.9	0.9	0.9	1.1
SPTBN1	Q01082	964	-2	-14	-9	7	1.0	0.9	0.9	1.1
LAP3	P28838	462	0	4	-9	7	1.0	1.0	0.9	1.1
XAB2	Q9HCS7	86	4	-1	-9	7	1.0	1.0	0.9	1.1
PTPN11	Q06124	259	-1	-3	-9	7	1.0	1.0	0.9	1.1
TNFAIP3	P21580	677	-2	-6	-9	7	1.0	0.9	0.9	1.1
SNRNP200	O75643	502	2	-6	-9	7	1.0	0.9	0.9	1.1
SUCLG1	P53597	181	-4	-8	-9	7	1.0	0.9	0.9	1.1
PAK4	O96013	58	-8	-8	-9	7	0.9	0.9	0.9	1.1
SAFB	Q15424	225	-1	-8	-9	7	1.0	0.9	0.9	1.1
IDH2	P48735	308	-2	-9	-9	7	1.0	0.9	0.9	1.1
CEP350	Q5VT06	1018	-4	-15	-9	7	1.0	0.9	0.9	1.1
TBC1D2	Q9BYX2	528	9	-3	-9	6	1.1	1.0	0.9	1.1
GAPDH	P04406	152	-4	-9	-9	6	1.0	0.9	0.9	1.1
NIN	Q8N4C6	647	1	-14	-9	6	1.0	0.9	0.9	1.1
EEF2	P13639	290	-9	-14	-9	6	0.9	0.9	0.9	1.1
IQSEC1	Q6DN90	906	-8	-17	-9	6	0.9	0.9	0.9	1.1
GNL3	Q9BVP2	158	-5	2	-9	6	1.0	1.0	0.9	1.1
SUPT16H	Q9Y5B9	323	-8	1	-9	6	0.9	1.0	0.9	1.1
SNX1	Q13596	318	4	-1	-9	6	1.0	1.0	0.9	1.1
ARID1A	O14497	1105	1	-5	-9	6	1.0	1.0	0.9	1.1
NUP214	P35658	1003	-4	-12	-9	6	1.0	0.9	0.9	1.1
NAGA	P17050	343	-6	-12	-9	6	0.9	0.9	0.9	1.1
HPRT1	P00492	206	-8	-26	-9	6	0.9	0.8	0.9	1.1
EXOC1	Q9NV70	650	-11	5	-9	5	0.9	1.0	0.9	1.1
SPATA5	Q8NB90	291	-6	-1	-9	5	0.9	1.0	0.9	1.1
XPC	Q01831	680	2	-3	-9	5	1.0	1.0	0.9	1.1
LSM14A	Q8ND56	361	-2	-5	-9	5	1.0	1.0	0.9	1.1
PROSER1	Q86XN7	171	-5	-6	-9	5	1.0	0.9	0.9	1.1
KDM5A	P29375	1248	2	-22	-9	5	1.0	0.8	0.9	1.1
KDM5D	Q9BY66	1267	2	-22	-9	5	1.0	0.8	0.9	1.1
TEP1	Q99973	531	-1	1	-9	5	1.0	1.0	0.9	1.0
RPS11	P62280	131	-6	-10	-9	5	0.9	0.9	0.9	1.0
UHRF2	Q96PU4	671	-6	-10	-9	5	0.9	0.9	0.9	1.0
PNPLA6	Q8IY17	1221	13	-21	-9	5	1.1	0.8	0.9	1.0
PDE4DIP	Q5VU43	271	-14	-3	-9	4	0.9	1.0	0.9	1.0
ARHGEF7	Q14155	721	-4	-3	-9	4	1.0	1.0	0.9	1.0
COASY	Q13057	144	-7	-6	-9	4	0.9	0.9	0.9	1.0
PNP	P00491	31	-1	-8	-9	4	1.0	0.9	0.9	1.0
PRKDC	P78527	3683	-8	-11	-9	4	0.9	0.9	0.9	1.0
PRKCSH	P14314	471	-11	-23	-9	4	0.9	0.8	0.9	1.0
MED1	Q15648	373	4	12	-9	4	1.0	1.1	0.9	1.0
PPME1	Q9Y570	347	0	-1	-9	4	1.0	1.0	0.9	1.0
SAT2	Q96F10	14	-11	-4	-9	4	0.9	1.0	0.9	1.0
BPNT1	O95861	42	-7	-7	-9	4	0.9	0.9	0.9	1.0
OPA3	Q9H6K4	164	-5	-7	-9	4	1.0	0.9	0.9	1.0
TES	Q9UGI8	46	-8	-7	-9	4	0.9	0.9	0.9	1.0
GART	P22102	41	4	-8	-9	4	1.0	0.9	0.9	1.0
PGD	P52209	402	-8	-9	-9	4	0.9	0.9	0.9	1.0
PPP2R1B	P30154	306	-6	-9	-9	4	0.9	0.9	0.9	1.0
ATXN7	O15265	692	-9	-24	-9	4	0.9	0.8	0.9	1.0
SYNE2	Q8WXH0	2768	10	-2	-9	3	1.1	1.0	0.9	1.0
CAMKMT	Q7Z624	25	6	-7	-9	3	1.1	0.9	0.9	1.0
WDR46	O15213	515	-5	11	-9	3	1.0	1.1	0.9	1.0
SPECC1	Q5M775	493	-5	-4	-9	3	1.0	1.0	0.9	1.0

ARPC2	O15144	120	-3	-5	-9	3	1.0	1.0	0.9	1.0
RASSF1	Q9NS23	102	-10	-9	-9	3	0.9	0.9	0.9	1.0
TTC37	Q6PGP7	329	1	-10	-9	3	1.0	0.9	0.9	1.0
PTPRC	P08575	752	-6	-1	-9	2	0.9	1.0	0.9	1.0
FASN	P49327	1448	-7	-3	-9	2	0.9	1.0	0.9	1.0
PHF5A	Q7RTV0	49	-3	-5	-9	2	1.0	1.0	0.9	1.0
FAM160B1	Q5W0V3	99	0	-19	-9	2	1.0	0.8	0.9	1.0
DDX42	Q86XP3	339	-5	12	-9	2	1.0	1.1	0.9	1.0
C16orf70	Q9BSU1	222	-6	-4	-9	2	0.9	1.0	0.9	1.0
RBM33	Q96EV2	1093	-4	-5	-9	2	1.0	1.0	0.9	1.0
PTPN23	Q9H3S7	628	-5	-8	-9	2	1.0	0.9	0.9	1.0
PLCG2	P16885	937	-2	-9	-9	2	1.0	0.9	0.9	1.0
HTT	P42858	1708	-2	-9	-9	2	1.0	0.9	0.9	1.0
SYNE2	Q8WXH0	1151	-6	-9	-9	2	0.9	0.9	0.9	1.0
SIRT1	Q96EB6	502	-8	-11	-9	2	0.9	0.9	0.9	1.0
ZNF780A	O75290	458	-19	-16	-9	2	0.8	0.9	0.9	1.0
RHOH	Q15669	130	1	-16	-9	2	1.0	0.9	0.9	1.0
SPAG1	Q07617	657	-11	-16	-9	2	0.9	0.9	0.9	1.0
PPP1R21	Q6ZMI0	516	-5	-8	-9	1	1.0	0.9	0.9	1.0
AGFG1	P52594	89	-3	-10	-9	1	1.0	0.9	0.9	1.0
FLNB	O75369	706	2	-11	-9	1	1.0	0.9	0.9	1.0
ABTB1	Q969K4	72	-5	-12	-9	1	1.0	0.9	0.9	1.0
RBBP4	Q09028	167	3	-19	-9	1	1.0	0.8	0.9	1.0
GDI2	P50395	282	1	-23	-9	1	1.0	0.8	0.9	1.0
ACSS1	Q9NUB1	422	5	0	-9	1	1.0	1.0	0.9	1.0
ROCK1	Q13464	714	-3	-6	-9	1	1.0	0.9	0.9	1.0
CNOT7	Q9UIV1	18	0	-6	-9	1	1.0	0.9	0.9	1.0
CLIC1	O00299	89	3	-11	-9	1	1.0	0.9	0.9	1.0
RRS1	Q15050	52	-13	-7	-9	0	0.9	0.9	0.9	1.0
NVL	O15381	431	5	-9	-9	0	1.1	0.9	0.9	1.0
RPL36A-HNRN	H7BZ11	77	-3	-10	-9	0	1.0	0.9	0.9	1.0
PDXDC1	Q6P996	135	3	-5	-9	-1	1.0	1.0	0.9	1.0
POLR1B	Q9H9Y6	1061	1	-7	-9	-1	1.0	0.9	0.9	1.0
RABGEF1	Q9UJ41	442	7	-10	-9	-1	1.1	0.9	0.9	1.0
ALG11	Q2TAA5	274	-5	13	-9	-1	1.0	1.1	0.9	1.0
PTRH1	Q86Y79	147	-4	-4	-9	-1	1.0	1.0	0.9	1.0
TBL1XR1	Q9BZK7	465	10	-7	-9	-1	1.1	0.9	0.9	1.0
CNST	Q6PJW8	408	-5	-13	-9	-1	1.0	0.9	0.9	1.0
NCBP1	Q09161	483	5	3	-9	-2	1.1	1.0	0.9	1.0
ARRB1	P49407	269	-2	1	-9	-2	1.0	1.0	0.9	1.0
ZZEF1	O43149	716	7	-1	-9	-2	1.1	1.0	0.9	1.0
BAG5	Q9UL15	166	-3	-3	-9	-2	1.0	1.0	0.9	1.0
PPP6R2	O75170	762	-9	-7	-9	-2	0.9	0.9	0.9	1.0
VDAC3	Q9Y277	65	-1	-8	-9	-2	1.0	0.9	0.9	1.0
TBC1D13	Q9NVG8	387	-2	-9	-9	-2	1.0	0.9	0.9	1.0
THOC2	Q8NI27	1064	-8	-11	-9	-2	0.9	0.9	0.9	1.0
RSBN1L	Q6PCB5	595	-13	-29	-9	-2	0.9	0.8	0.9	1.0
IARS2	Q9NSE4	883	-5	-6	-9	-2	1.0	0.9	0.9	1.0
PKM	P14618	358	-6	-6	-9	-2	0.9	0.9	0.9	1.0
SPEN	Q96T58	1134	-32	-25	-9	-2	0.8	0.8	0.9	1.0
SLFN5	Q08AF3	258	-2	-6	-9	-3	1.0	0.9	0.9	1.0
MAP3K5	Q99683	928	-4	-8	-9	-3	1.0	0.9	0.9	1.0
ERC1	X6RLX0	258	-9	-8	-9	-3	0.9	0.9	0.9	1.0
RNF213	Q63HN8	680	-1	-12	-9	-3	1.0	0.9	0.9	1.0
CPVL	Q9H3G5	320	-2	-14	-9	-3	1.0	0.9	0.9	1.0

DOCK8	Q8NF50	521	4	-7	-9	-3	1.0	0.9	0.9	1.0
ARPC5	O15511	45	-5	-11	-9	-3	1.0	0.9	0.9	1.0
KAT7	O95251	418	-7	-15	-9	-3	0.9	0.9	0.9	1.0
KAT6B	Q8WYB5	801	-7	-15	-9	-3	0.9	0.9	0.9	1.0
KAT8	Q9H7Z6	260	-7	-15	-9	-3	0.9	0.9	0.9	1.0
RING1	Q06587	69	-11	-18	-9	-3	0.9	0.8	0.9	1.0
SNRNP200	O75643	1278	-11	-8	-9	-4	0.9	0.9	0.9	1.0
DMXL2	Q8TDJ6	1897	-13	-5	-9	-4	0.9	1.0	0.9	1.0
GSK3A	P49840	262	-9	-11	-9	-4	0.9	0.9	0.9	1.0
GSK3B	P49841	199	-9	-11	-9	-4	0.9	0.9	0.9	1.0
MYL6	P60660	32	-10	-9	-9	-5	0.9	0.9	0.9	1.0
ROCK1	Q13464	741	-10	-12	-9	-5	0.9	0.9	0.9	1.0
C10orf12	Q8N655	658	-17	-13	-9	-5	0.9	0.9	0.9	1.0
CRIPT	Q9P021	73	-10	-16	-9	-5	0.9	0.9	0.9	1.0
TAPT1	Q6NXT6	562	-8	-4	-9	-5	0.9	1.0	0.9	1.0
EML4	Q9HC35	617	-3	-6	-9	-5	1.0	0.9	0.9	1.0
CMPK2	Q5EBM0	40	0	-9	-9	-5	1.0	0.9	0.9	1.0
TYK2	P29597	736	-38	-9	-9	-5	0.7	0.9	0.9	1.0
PHF14	O94880	874	1	-15	-9	-5	1.0	0.9	0.9	1.0
CFP	P27918	360	-14	2	-9	-6	0.9	1.0	0.9	0.9
OCIAD2	Q56VL3	130	9	-3	-9	-6	1.1	1.0	0.9	0.9
ACAT1	P24752	413	1	-5	-9	-6	1.0	1.0	0.9	0.9
SYNE2	Q8WXH0	2935	1	-5	-9	-6	1.0	1.0	0.9	0.9
DSTN	P60981	39	12	-12	-9	-6	1.1	0.9	0.9	0.9
GMIP	Q9P107	599	-4	1	-9	-6	1.0	1.0	0.9	0.9
COQ10B	Q9H8M1	152	3	-1	-9	-6	1.0	1.0	0.9	0.9
MYO9A	B2RTY4	1149	7	-6	-9	-6	1.1	0.9	0.9	0.9
TLE4	Q04727	32	-6	-6	-9	-6	0.9	0.9	0.9	0.9
TECPR2	O15040	226	-3	-8	-9	-6	1.0	0.9	0.9	0.9
ZMYM4	Q5VZL5	752	-3	-17	-9	-6	1.0	0.9	0.9	0.9
ANKS1A	Q92625	965	-7	-6	-9	-7	0.9	0.9	0.9	0.9
RENBP	P51606	249	3	-11	-9	-7	1.0	0.9	0.9	0.9
RHOC	P08134	20	-6	-8	-9	-7	0.9	0.9	0.9	0.9
RHOA	P61586	20	-6	-8	-9	-7	0.9	0.9	0.9	0.9
ANAPC7	Q9UJX3	509	-6	-3	-9	-8	0.9	1.0	0.9	0.9
MED1	Q15648	302	-5	-8	-9	-8	1.0	0.9	0.9	0.9
RAP1GAP2	Q684P5	444	-7	-9	-9	-8	0.9	0.9	0.9	0.9
HSP90AA1	P07900	572	-4	-11	-9	-8	1.0	0.9	0.9	0.9
ACTB	P60709	272	3	-18	-9	-8	1.0	0.9	0.9	0.9
C16orf62	Q7Z3J2	315	-5	-5	-9	-8	1.0	1.0	0.9	0.9
PTK2B	Q14289	972	-16	-23	-9	-8	0.9	0.8	0.9	0.9
TAF10	Q12962	174	0	-6	-9	-9	1.0	0.9	0.9	0.9
SORBS3	O60504	521	-3	2	-9	-9	1.0	1.0	0.9	0.9
RAB2B	Q8WUD1	154	-3	-3	-9	-9	1.0	1.0	0.9	0.9
LAS1L	Q9Y4W2	504	-8	-8	-9	-9	0.9	0.9	0.9	0.9
FKBP15	Q5T1M5	828	-3	-10	-9	-9	1.0	0.9	0.9	0.9
EIF4A1	P60842	134	5	-29	-9	-11	1.1	0.8	0.9	0.9
LRCH1	Q9Y2L9	276	-9	-3	-9	-11	0.9	1.0	0.9	0.9
UBE2O	Q9C0C9	341	-7	-10	-9	-12	0.9	0.9	0.9	0.9
SCFD2	Q8WU76	54	-12	-16	-9	-12	0.9	0.9	0.9	0.9
PPP4R1	Q8TF05	174	-3	-11	-9	-12	1.0	0.9	0.9	0.9
INPP4B	O15327	534	5	-6	-9	-13	1.0	0.9	0.9	0.9
PML	P29590	57	-10	-6	-9	-13	0.9	0.9	0.9	0.9
ATP2C1	P98194	49	-5	-9	-9	-14	1.0	0.9	0.9	0.9
UBE4B	O95155	1164	1	-12	-9	-14	1.0	0.9	0.9	0.9

CLPB	Q9H078	572	-4	-13	-9	-14	1.0	0.9	0.9	0.9
BAZ1B	Q9UIG0	1045	-21	-10	-9	-15	0.8	0.9	0.9	0.9
FASN	P49327	2468	-2	-22	-9	-16	1.0	0.8	0.9	0.9
NBEAL2	Q6ZNJ1	1814	-5	-13	-9	-17	1.0	0.9	0.9	0.9
NPLOC4	Q8TAT6	188	6	-18	-9	-17	1.1	0.8	0.9	0.9
PARP1	P09874	256	0	-11	-9	-18	1.0	0.9	0.9	0.8
ERN1	O75460	605	5	-7	-9	-19	1.0	0.9	0.9	0.8
GDI2	P50395	414	-1	-9	-9	-20	1.0	0.9	0.9	0.8
MFN2	O95140	281	-7	-6	-9	-20	0.9	0.9	0.9	0.8
UBE2O	Q9C0C9	1040	4	-9	-9	-22	1.0	0.9	0.9	0.8
NFU1	Q9UMS0	210	-5	-20	-9	-22	1.0	0.8	0.9	0.8
ACTR1B	P42025	222	-15	-8	-9	-24	0.9	0.9	0.9	0.8
MARS	P56192	66	10	-20	-9	-24	1.1	0.8	0.9	0.8
RAP1GAP2	Q684P5	443	-9	-22	-9	-24	0.9	0.8	0.9	0.8
TRAF6	Q9Y4K3	105	-3	-23	-9	-26	1.0	0.8	0.9	0.8
PNKD	Q8N490	329	7	3	-9	-29	1.1	1.0	0.9	0.8
PLEC	Q15149	1098	15	-25	-9	-31	1.2	0.8	0.9	0.8
ZYX	Q15942	562	-8	2	-9	-31	0.9	1.0	0.9	0.8
CSRP1	P21291	58	-5	-5	-9	-33	1.0	1.0	0.9	0.8
ZYX	Q15942	384	6	-11	-9	-34	1.1	0.9	0.9	0.7
DUSP3	P51452	124	2	-5	-9	-35	1.0	1.0	0.9	0.7
LASP1	Q14847	20	20	-15	-9	-36	1.3	0.9	0.9	0.7
FHOD1	Q9Y613	373	-3	-7	-9	-38	1.0	0.9	0.9	0.7
PSTPIP2	Q9H939	90	-1	-8	-9	-48	1.0	0.9	0.9	0.7
FOSB	P53539	222	4	4	-9	-117	1.0	1.0	0.9	0.5
APPBP2	Q92624	54	-4	-1	-10	43	1.0	1.0	0.9	1.8
SRD5A3	Q9H8P0	57	-13	-4	-10	42	0.9	1.0	0.9	1.7
BRAT1	Q6PJG6	28	-8	-18	-10	42	0.9	0.9	0.9	1.7
BRAT1	Q6PJG6	54	0	4	-10	40	1.0	1.0	0.9	1.7
BTG2	P78543	29	-12	-20	-10	38	0.9	0.8	0.9	1.6
ACADS	P16219	246	3	-16	-10	35	1.0	0.9	0.9	1.5
LSM14A	Q8ND56	85	-14	-5	-10	34	0.9	1.0	0.9	1.5
HAAO	P46952	23	-5	-15	-10	33	1.0	0.9	0.9	1.5
ATAD2	Q6PL18	484	-10	-1	-10	33	0.9	1.0	0.9	1.5
OAS3	Q9Y6K5	83	3	-10	-10	31	1.0	0.9	0.9	1.4
PRG2	P13727	104	-1	-6	-10	29	1.0	0.9	0.9	1.4
SEC23B	Q15437	434	-1	-16	-10	29	1.0	0.9	0.9	1.4
RBM15B	Q8NDT2	323	0	-2	-10	29	1.0	1.0	0.9	1.4
GAPVD1	Q14C86	374	-7	-3	-10	28	0.9	1.0	0.9	1.4
ELMOD2	Q8IZ81	285	3	-9	-10	27	1.0	0.9	0.9	1.4
ALOX5	P09917	301	-11	-19	-10	27	0.9	0.8	0.9	1.4
SOS1	Q07889	405	-3	-9	-10	26	1.0	0.9	0.9	1.4
CORO7-PAM1	A0A0A6YYL4	505	-2	-7	-10	26	1.0	0.9	0.9	1.3
SRSF5	Q13243	63	-1	-10	-10	26	1.0	0.9	0.9	1.3
TTC3	P53804	1159	-6	-13	-10	26	0.9	0.9	0.9	1.3
EHMT2	Q96KQ7	743	-5	-13	-10	25	1.0	0.9	0.9	1.3
FLNB	O75369	1081	-1	-7	-10	24	1.0	0.9	0.9	1.3
FLNC	Q14315	1103	-1	-7	-10	24	1.0	0.9	0.9	1.3
CDA	P32320	14	-5	-8	-10	23	1.0	0.9	0.9	1.3
MED13L	Q71F56	1256	-6	-8	-10	23	0.9	0.9	0.9	1.3
NIPBL	Q6KC79	1971	6	2	-10	22	1.1	1.0	0.9	1.3
TDRKH	Q9Y2W6	160	-5	-12	-10	22	1.0	0.9	0.9	1.3
MED12	Q93074	444	-18	-16	-10	22	0.8	0.9	0.9	1.3
MILR1	Q7Z6M3	297	-4	-4	-10	21	1.0	1.0	0.9	1.3
ECM29	Q5VYK3	1183	-13	-21	-10	21	0.9	0.8	0.9	1.3

MYNN	Q9NPC7	371	-10	6	-10	21	0.9	1.1	0.9	1.3
ITPR3	Q14573	2668	-4	-13	-10	21	1.0	0.9	0.9	1.3
POLD1	P28340	837	1	0	-10	20	1.0	1.0	0.9	1.2
TNIP2	Q8NFZ5	21	-4	2	-10	19	1.0	1.0	0.9	1.2
MAPK12	P53778	165	1	-4	-10	19	1.0	1.0	0.9	1.2
WDR1	O75083	507	-5	-11	-10	19	1.0	0.9	0.9	1.2
TMEM39B	Q9GZU3	67	-1	-9	-10	18	1.0	0.9	0.9	1.2
TRMT112	Q9UI30	33	-1	-5	-10	18	1.0	1.0	0.9	1.2
INTS10	Q9NVR2	122	-2	-10	-10	18	1.0	0.9	0.9	1.2
TRAPPC10	P48553	373	-24	-23	-10	18	0.8	0.8	0.9	1.2
AKR7A2	O43488	132	0	-2	-10	17	1.0	1.0	0.9	1.2
SPTB	P11277	183	2	-10	-10	17	1.0	0.9	0.9	1.2
SPTBN1	Q01082	183	2	-10	-10	17	1.0	0.9	0.9	1.2
ZC3HC1	Q86WB0	500	8	6	-10	17	1.1	1.1	0.9	1.2
ATM	Q13315	1821	1	-4	-10	17	1.0	1.0	0.9	1.2
DOPEY2	Q9Y3R5	1329	1	-5	-10	17	1.0	1.0	0.9	1.2
HACL1	Q9UJ83	261	-11	-13	-10	17	0.9	0.9	0.9	1.2
PNN	Q9H307	249	-5	-1	-10	16	1.0	1.0	0.9	1.2
RANBP2	P49792	2659	-3	-3	-10	16	1.0	1.0	0.9	1.2
SLC25A20	O43772	58	-6	-14	-10	16	0.9	0.9	0.9	1.2
NSD1	Q96L73	2273	5	-23	-10	16	1.1	0.8	0.9	1.2
ORC3	Q9UBD5	159	-11	-24	-10	16	0.9	0.8	0.9	1.2
ZBTB33	Q86T24	505	-1	-6	-10	16	1.0	0.9	0.9	1.2
DCAF7	P61962	61	9	4	-10	15	1.1	1.0	0.9	1.2
DNMT1	P26358	62	-4	-1	-10	15	1.0	1.0	0.9	1.2
PRKAR1A	P10644	18	-4	-12	-10	15	1.0	0.9	0.9	1.2
PPIG	Q13427	310	1	-6	-10	15	1.0	0.9	0.9	1.2
MED17	Q9NVC6	15	-7	-8	-10	15	0.9	0.9	0.9	1.2
TMPO	P42167	363	-4	-10	-10	15	1.0	0.9	0.9	1.2
SEC23B	Q15437	767	2	-10	-10	15	1.0	0.9	0.9	1.2
GAB3	Q8WWW8	336	1	-12	-10	15	1.0	0.9	0.9	1.2
NFKBIB	Q15653	240	-8	-14	-10	15	0.9	0.9	0.9	1.2
ITPA	Q9BY32	33	-5	-21	-10	15	1.0	0.8	0.9	1.2
ELMSAN1	Q6PJG2	16	-10	-4	-10	14	0.9	1.0	0.9	1.2
DNTTIP2	Q5QJE6	618	1	-6	-10	14	1.0	0.9	0.9	1.2
PES1	O00541	272	0	-7	-10	14	1.0	0.9	0.9	1.2
AARSD1	Q9BTE6	22	1	-13	-10	14	1.0	0.9	0.9	1.2
UTY	O14607	76	1	2	-10	14	1.0	1.0	0.9	1.2
KDM6A	O15550	79	1	2	-10	14	1.0	1.0	0.9	1.2
FAS	P25445	304	-1	-7	-10	14	1.0	0.9	0.9	1.2
CAMK2D	Q13557	290	6	-7	-10	14	1.1	0.9	0.9	1.2
DIDO1	Q9BTC0	350	6	-7	-10	14	1.1	0.9	0.9	1.2
CAMK2A	Q9UQM7	289	6	-7	-10	14	1.1	0.9	0.9	1.2
TIGAR	Q9NQ88	161	-6	-9	-10	14	0.9	0.9	0.9	1.2
CD7	P09564	219	-7	-12	-10	14	0.9	0.9	0.9	1.2
LCP1	P13796	164	-2	-12	-10	14	1.0	0.9	0.9	1.2
MAPK9	P45984	177	-9	-4	-10	13	0.9	1.0	0.9	1.1
RAB10	P61026	24	-3	-7	-10	13	1.0	0.9	0.9	1.1
NR2C2	P49116	204	-3	-13	-10	13	1.0	0.9	0.9	1.1
GNB1L	Q9BYB4	29	-26	-14	-10	13	0.8	0.9	0.9	1.1
BACH1	O14867	107	2	1	-10	13	1.0	1.0	0.9	1.1
THUMPD1	Q9NXG2	31	-6	-3	-10	13	0.9	1.0	0.9	1.1
PPIP5K2	O43314	900	-1	-11	-10	13	1.0	0.9	0.9	1.1
SYTL1	Q8IYJ3	187	-1	1	-10	12	1.0	1.0	0.9	1.1
SQRDL	Q9Y6N5	201	-12	-4	-10	12	0.9	1.0	0.9	1.1

BOP1	Q14137	108	-6	-7	-10	12	0.9	0.9	0.9	1.1
TRRAP	Q9Y4A5	1879	-4	-9	-10	12	1.0	0.9	0.9	1.1
CD2BP2	O95400	234	-3	-10	-10	12	1.0	0.9	0.9	1.1
ARHGAP9	Q9BRR9	239	-4	1	-10	12	1.0	1.0	0.9	1.1
SPAG7	O75391	191	1	-2	-10	12	1.0	1.0	0.9	1.1
DDX24	Q9GZR7	832	-5	-3	-10	12	1.0	1.0	0.9	1.1
PTPRO	Q16827	855	-7	-4	-10	12	0.9	1.0	0.9	1.1
LTN1	O94822	595	3	-8	-10	12	1.0	0.9	0.9	1.1
SNRNP200	O75643	1127	-11	-7	-10	11	0.9	0.9	0.9	1.1
LRCH3	Q96II8	676	-2	-8	-10	11	1.0	0.9	0.9	1.1
GPATCH8	Q9UKJ3	508	-3	-14	-10	11	1.0	0.9	0.9	1.1
EXOSC9	Q06265	9	-5	-5	-10	11	1.0	1.0	0.9	1.1
NUP35	Q8NFH5	255	-13	-5	-10	11	0.9	1.0	0.9	1.1
VPS39	Q96JC1	681	-4	-10	-10	11	1.0	0.9	0.9	1.1
EEFSEC	P57772	406	-2	-14	-10	11	1.0	0.9	0.9	1.1
SP140	Q13342	478	3	4	-10	10	1.0	1.0	0.9	1.1
CBX8	Q9HC52	261	1	-4	-10	10	1.0	1.0	0.9	1.1
R3HCC1L	Q7Z5L2	665	-4	-7	-10	10	1.0	0.9	0.9	1.1
SLFN5	Q08AF3	114	-6	-13	-10	10	0.9	0.9	0.9	1.1
LARP1	Q6PKG0	238	4	5	-10	10	1.0	1.1	0.9	1.1
CPOX	P36551	198	-2	-7	-10	10	1.0	0.9	0.9	1.1
PUS7L	Q9H0K6	640	-12	-7	-10	10	0.9	0.9	0.9	1.1
EDC3	Q96F86	499	-3	-9	-10	10	1.0	0.9	0.9	1.1
ABI3	Q9P2A4	145	-2	-9	-10	10	1.0	0.9	0.9	1.1
CLNS1A	P54105	73	5	-10	-10	10	1.0	0.9	0.9	1.1
TBL2	Q9Y4P3	44	-2	-11	-10	10	1.0	0.9	0.9	1.1
ALKBH4	Q9NXW9	126	-9	-16	-10	10	0.9	0.9	0.9	1.1
WDR91	A4D1P6	246	-5	-5	-10	9	1.0	1.0	0.9	1.1
TRAF3IP3	Q9Y228	380	-1	-9	-10	9	1.0	0.9	0.9	1.1
MBD1	Q9UIS9	274	-3	-18	-10	9	1.0	0.9	0.9	1.1
OSBPL8	Q9BZF1	266	-10	-1	-10	9	0.9	1.0	0.9	1.1
TTC37	Q6PGP7	1162	-7	-2	-10	9	0.9	1.0	0.9	1.1
PITPNB	P48739	187	1	-4	-10	9	1.0	1.0	0.9	1.1
KMT2B	Q9UMN6	2540	-10	-14	-10	9	0.9	0.9	0.9	1.1
MBD1	Q9UIS9	70	-3	-16	-10	9	1.0	0.9	0.9	1.1
ROGDI	Q9GZN7	66	-7	-3	-10	8	0.9	1.0	0.9	1.1
TERF2IP	Q9NYB0	54	-10	-4	-10	8	0.9	1.0	0.9	1.1
BLVRA	P53004	204	4	-5	-10	8	1.0	1.0	0.9	1.1
BIRC6	Q9NR09	566	-1	-12	-10	8	1.0	0.9	0.9	1.1
CTNNA1	P35221	116	-6	-12	-10	8	0.9	0.9	0.9	1.1
NPEPPS	P55786	190	-2	-12	-10	8	1.0	0.9	0.9	1.1
CA8	P35219	200	-1	-16	-10	8	1.0	0.9	0.9	1.1
TRAF2	Q12933	171	2	-3	-10	8	1.0	1.0	0.9	1.1
XRN1	Q8IZH2	893	-4	-8	-10	8	1.0	0.9	0.9	1.1
ACSF2	Q96CM8	64	-7	-13	-10	8	0.9	0.9	0.9	1.1
FRY	Q5TBA9	2815	-9	-21	-10	8	0.9	0.8	0.9	1.1
MACF1	Q9UPN3	4825	-9	1	-10	7	0.9	1.0	0.9	1.1
AKAP13	Q12802	65	-9	-8	-10	7	0.9	0.9	0.9	1.1
SKA3	Q8IX90	8	-4	-8	-10	7	1.0	0.9	0.9	1.1
AQR	O60306	28	-5	-10	-10	7	1.0	0.9	0.9	1.1
NDUFA4	O00483	44	-5	-11	-10	7	1.0	0.9	0.9	1.1
SQSTM1	Q13501	26	-7	-12	-10	7	0.9	0.9	0.9	1.1
AHCY	P23526	421	-2	-13	-10	7	1.0	0.9	0.9	1.1
DPYD	Q12882	324	-2	-13	-10	7	1.0	0.9	0.9	1.1
ATIC	P31939	325	6	-3	-10	7	1.1	1.0	0.9	1.1

SAMHD1	Q9Y3Z3	15	-8	-3	-10	7	0.9	1.0	0.9	1.1
AK5	Q9Y6K8	245	-3	-17	-10	7	1.0	0.9	0.9	1.1
LRMP	Q12912	72	-9	-10	-10	6	0.9	0.9	0.9	1.1
NO66	Q9H6W3	613	-2	-15	-10	6	1.0	0.9	0.9	1.1
SCRN1	Q12765	290	-2	-20	-10	6	1.0	0.8	0.9	1.1
PPP6R1	Q9UPN7	455	0	-6	-10	6	1.0	0.9	0.9	1.1
RALGAPB	Q86X10	992	4	-8	-10	6	1.0	0.9	0.9	1.1
LIMK1	P53667	297	-2	-9	-10	6	1.0	0.9	0.9	1.1
IPO7	O95373	401	-1	-9	-10	6	1.0	0.9	0.9	1.1
UNC45A	Q9H3U1	488	0	5	-10	5	1.0	1.0	0.9	1.1
JAK1	P23458	445	11	-3	-10	5	1.1	1.0	0.9	1.1
PSMA1	P25786	148	-3	-5	-10	5	1.0	1.0	0.9	1.1
KPNB1	Q14974	223	15	-6	-10	5	1.2	0.9	0.9	1.1
GDPD1	Q8N9F7	124	11	-7	-10	5	1.1	0.9	0.9	1.1
FGR	P09769	415	-9	-8	-10	5	0.9	0.9	0.9	1.1
KIF13B	Q9NQT8	1234	-9	-9	-10	5	0.9	0.9	0.9	1.1
VIM	P08670	328	-4	-9	-10	5	1.0	0.9	0.9	1.1
EEF2	P13639	369	-8	-9	-10	5	0.9	0.9	0.9	1.1
XPOT	O43592	650	0	-11	-10	5	1.0	0.9	0.9	1.1
CELF2	O95319	161	6	-13	-10	5	1.1	0.9	0.9	1.1
RALGAPA2	Q2PPJ7	1662	-5	-13	-10	5	1.0	0.9	0.9	1.1
KIAA0391	O15091	568	-10	-4	-10	5	0.9	1.0	0.9	1.0
ATRX	P46100	1718	-10	-8	-10	5	0.9	0.9	0.9	1.0
ARRB2	P32121	141	-3	-10	-10	5	1.0	0.9	0.9	1.0
FGD2	Q7Z6J4	89	-1	-10	-10	5	1.0	0.9	0.9	1.0
PTPN23	Q9H3S7	218	-4	-11	-10	5	1.0	0.9	0.9	1.0
UBE2L6	O14933	98	3	-17	-10	5	1.0	0.9	0.9	1.0
ARPC1B	O15143	346	-2	-3	-10	4	1.0	1.0	0.9	1.0
POC1B	Q8TC44	306	1	-2	-10	4	1.0	1.0	0.9	1.0
UTRN	P46939	447	-2	-3	-10	4	1.0	1.0	0.9	1.0
NUP50	Q9UKX7	181	7	-6	-10	4	1.1	0.9	0.9	1.0
NONO	Q15233	145	-6	-10	-10	4	0.9	0.9	0.9	1.0
CEP95	Q96GE4	678	5	-11	-10	4	1.0	0.9	0.9	1.0
OPTN	Q96CV9	472	-2	-15	-10	4	1.0	0.9	0.9	1.0
WDR81	Q562E7	130	1	-31	-10	4	1.0	0.8	0.9	1.0
XRCC2	O43543	217	0	2	-10	3	1.0	1.0	0.9	1.0
MYCBP2	O75592	1131	-14	0	-10	3	0.9	1.0	0.9	1.0
RFC4	P35249	141	6	-4	-10	3	1.1	1.0	0.9	1.0
ACTR3	P61158	34	-4	-19	-10	3	1.0	0.8	0.9	1.0
GOLGB1	Q14789	3213	-4	-20	-10	3	1.0	0.8	0.9	1.0
LARS2	Q15031	167	3	-7	-10	3	1.0	0.9	0.9	1.0
KMT2A	Q03164	2051	-4	-7	-10	3	1.0	0.9	0.9	1.0
KMT2B	Q9UMN6	1760	-4	-7	-10	3	1.0	0.9	0.9	1.0
HCFC1	P51610	353	0	-10	-10	3	1.0	0.9	0.9	1.0
IAH1	Q2TAA2	137	0	-10	-10	3	1.0	0.9	0.9	1.0
TRMT2A	Q8IZ69	486	1	-16	-10	3	1.0	0.9	0.9	1.0
ANKFY1	Q9P2R3	389	-8	-26	-10	3	0.9	0.8	0.9	1.0
ALKBH8	Q96BT7	296	-7	-14	-10	2	0.9	0.9	0.9	1.0
RNH1	P13489	209	-6	-15	-10	2	0.9	0.9	0.9	1.0
DDX39B	Q13838	165	-5	-4	-10	2	1.0	1.0	0.9	1.0
ROCK1	Q13464	1206	-5	-11	-10	2	1.0	0.9	0.9	1.0
KHNYN	O15037	427	7	-17	-10	2	1.1	0.9	0.9	1.0
STRAP	Q9Y3F4	305	-6	-5	-10	1	0.9	1.0	0.9	1.0
TUFM	P49411	290	-1	-5	-10	1	1.0	1.0	0.9	1.0
Uncharacteriz	A0A0J9YVX5	171	2	-6	-10	1	1.0	0.9	0.9	1.0

CLTC	Q00610	491	-8	-10	-10	1	0.9	0.9	0.9	1.0
ACO2	Q99798	126	-19	-12	-10	1	0.8	0.9	0.9	1.0
FAM98B	Q52LJ0	295	-7	-6	-10	1	0.9	0.9	0.9	1.0
ROCK1	Q13464	1070	-3	-14	-10	1	1.0	0.9	0.9	1.0
PGM1	P36871	160	-12	-14	-10	0	0.9	0.9	0.9	1.0
TP53BP1	Q12888	1178	-8	-14	-10	0	0.9	0.9	0.9	1.0
IFI16	Q16666	191	-6	-2	-10	-1	0.9	1.0	0.9	1.0
PDK1	Q15118	71	-5	-8	-10	-1	1.0	0.9	0.9	1.0
TPMT	P51580	234	-4	-9	-10	-1	1.0	0.9	0.9	1.0
SPTAN1	Q13813	1930	-14	-10	-10	-1	0.9	0.9	0.9	1.0
SEC62	Q99442	55	-3	-11	-10	-1	1.0	0.9	0.9	1.0
PARP14	Q460N5	933	7	-6	-10	-1	1.1	0.9	0.9	1.0
PSMA7	O14818	91	-3	-8	-10	-1	1.0	0.9	0.9	1.0
CAPN2	P17655	301	-11	-10	-10	-1	0.9	0.9	0.9	1.0
FBXL12	Q9NXK8	212	14	-11	-10	-1	1.2	0.9	0.9	1.0
AP2A2	O94973	932	-5	-11	-10	-2	1.0	0.9	0.9	1.0
MIIP	Q5JXC2	215	-15	-12	-10	-2	0.9	0.9	0.9	1.0
MED30	Q96HR3	34	-3	-17	-10	-2	1.0	0.9	0.9	1.0
GUK1	Q16774	98	-6	-18	-10	-2	0.9	0.8	0.9	1.0
GPRIN3	Q6ZVF9	434	4	-9	-10	-2	1.0	0.9	0.9	1.0
RASAL3	Q86YV0	896	-6	-9	-10	-2	0.9	0.9	0.9	1.0
EFNB1	P98172	314	-14	-1	-10	-3	0.9	1.0	0.9	1.0
RBM22	Q9NW64	269	-3	-2	-10	-3	1.0	1.0	0.9	1.0
BAG6	P46379	349	-9	-7	-10	-3	0.9	0.9	0.9	1.0
RPAP3	Q9H6T3	190	-13	-7	-10	-3	0.9	0.9	0.9	1.0
RAB11FIP1	Q6WKZ4	318	-13	-14	-10	-3	0.9	0.9	0.9	1.0
PARP10	Q53GL7	494	-21	-5	-10	-4	0.8	1.0	0.9	1.0
SSFA2	P28290	766	-10	-14	-10	-4	0.9	0.9	0.9	1.0
POGZ	Q7Z3K3	987	-6	-8	-10	-4	0.9	0.9	0.9	1.0
ABCE1	P61221	201	-13	-10	-10	-4	0.9	0.9	0.9	1.0
MRPS18B	Q9Y676	128	-11	-13	-10	-4	0.9	0.9	0.9	1.0
USP7	Q93009	799	-4	-13	-10	-4	1.0	0.9	0.9	1.0
FAM65B	Q9Y4F9	983	-4	-13	-10	-4	1.0	0.9	0.9	1.0
PAF1	Q8N7H5	218	-13	-24	-10	-4	0.9	0.8	0.9	1.0
ATPAF1	Q5TC12	321	-12	-3	-10	-5	0.9	1.0	0.9	1.0
NUBP2	Q9Y5Y2	199	13	-6	-10	-5	1.1	0.9	0.9	1.0
NAA16	Q6N069	259	-18	-13	-10	-5	0.9	0.9	0.9	1.0
INTS1	Q8N201	1770	-1	-7	-10	-6	1.0	0.9	0.9	0.9
DOCK8	Q8NF50	259	-14	-21	-10	-6	0.9	0.8	0.9	0.9
KMT2A	Q03164	1536	-1	-5	-10	-7	1.0	1.0	0.9	0.9
RNF213	Q63HN8	2132	-5	-9	-10	-7	1.0	0.9	0.9	0.9
FLNA	P21333	2476	6	-2	-10	-8	1.1	1.0	0.9	0.9
RAB31	Q13636	49	-3	-18	-10	-8	1.0	0.8	0.9	0.9
WDFY1	Q8IWB7	290	-14	-7	-10	-9	0.9	0.9	0.9	0.9
PIKFYVE	Q9Y2I7	1677	-14	-9	-10	-9	0.9	0.9	0.9	0.9
EXOSC7	Q15024	85	-4	-11	-10	-9	1.0	0.9	0.9	0.9
PRKCA	P17252	583	1	-17	-10	-9	1.0	0.9	0.9	0.9
CNOT1	A5YKK6	890	6	3	-10	-9	1.1	1.0	0.9	0.9
PPP1CB	P62140	61	3	-7	-10	-9	1.0	0.9	0.9	0.9
HDAC3	O15379	123	-14	0	-10	-10	0.9	1.0	0.9	0.9
DUSP3	P51452	171	5	-5	-10	-10	1.0	1.0	0.9	0.9
ATP2A3	Q93084	614	-18	-9	-10	-10	0.9	0.9	0.9	0.9
UBR4	Q5T4S7	3765	-8	-18	-10	-10	0.9	0.8	0.9	0.9
RHOG	P84095	22	-6	-8	-10	-11	0.9	0.9	0.9	0.9
WDR26	Q9H7D7	238	-12	-10	-10	-11	0.9	0.9	0.9	0.9

NANS	Q9NR45	184	2	-12	-10	-11	1.0	0.9	0.9	0.9
OSBPL8	Q9BZF1	494	-30	-13	-10	-11	0.8	0.9	0.9	0.9
NFYC	Q13952	345	-21	-36	-10	-12	0.8	0.7	0.9	0.9
BCAS2	O75934	132	-4	-13	-10	-13	1.0	0.9	0.9	0.9
ACAP2	Q15057	321	14	-5	-10	-14	1.2	1.0	0.9	0.9
POLR1E	Q9GZS1	143	-12	2	-10	-14	0.9	1.0	0.9	0.9
SUPT5H	O00267	740	-2	-30	-10	-14	1.0	0.8	0.9	0.9
RPP40	O75818	291	-10	-5	-10	-15	0.9	1.0	0.9	0.9
MACF1	Q9UPN3	777	2	6	-10	-15	1.0	1.1	0.9	0.9
THEMIS2	Q5TEJ8	395	1	-28	-10	-15	1.0	0.8	0.9	0.9
DDAH2	O95865	262	-17	-18	-10	-16	0.9	0.8	0.9	0.9
Uncharacteriz	A0A087WZG4	300	5	-11	-10	-16	1.0	0.9	0.9	0.9
EDC3	Q96F86	137	2	-8	-10	-17	1.0	0.9	0.9	0.9
GDI1	P31150	394	-19	-29	-10	-19	0.8	0.8	0.9	0.8
RNF213	Q63HN8	1985	-10	-19	-10	-19	0.9	0.8	0.9	0.8
DARS2	Q6PI48	590	-4	-8	-10	-20	1.0	0.9	0.9	0.8
MYH9	P35579	91	2	-11	-10	-20	1.0	0.9	0.9	0.8
ZNF330	Q9Y3S2	186	-1	-7	-10	-22	1.0	0.9	0.9	0.8
ATG7	O95352	406	-2	-7	-10	-23	1.0	0.9	0.9	0.8
UBR4	Q5T4S7	1662	6	-16	-10	-23	1.1	0.9	0.9	0.8
YY1	P25490	298	-8	-16	-10	-29	0.9	0.9	0.9	0.8
RAB30	Q15771	93	6	-2	-10	-32	1.1	1.0	0.9	0.8
ARHGAP6	O43182	769	-7	-16	-10	-48	0.9	0.9	0.9	0.7
TLN1	Q9Y490	1202	1	-14	-10	-57	1.0	0.9	0.9	0.6
FHOD1	Q9Y613	43	-9	-12	-10	-62	0.9	0.9	0.9	0.6
FHL1	Q13642	191	3	-8	-10	-76	1.0	0.9	0.9	0.6
ILK	Q13418	239	-1	-5	-10	-80	1.0	1.0	0.9	0.6
GALM	Q96C23	247	-11	-19	-10	-88	0.9	0.8	0.9	0.5
ZNF638	Q14966	1023	-17	-19	-10	84	0.9	0.8	0.9	6.3
HECTD4	Q9Y4D8	594	-13	-23	-10	61	0.9	0.8	0.9	2.6
ZNF561	Q8N587	17	-6	-13	-10	44	0.9	0.9	0.9	1.8
HELZ	P42694	101	-7	-11	-10	41	0.9	0.9	0.9	1.7
TAP1	Q03518	239	0	-9	-10	40	1.0	0.9	0.9	1.7
HLA-C	P04222	345	-6	-13	-10	37	0.9	0.9	0.9	1.6
HLA-C	P30510	345	-6	-13	-10	37	0.9	0.9	0.9	1.6
NLRP2	Q9NX02	143	-7	-8	-10	34	0.9	0.9	0.9	1.5
USP34	Q70CQ2	3326	-13	-20	-10	33	0.9	0.8	0.9	1.5
TRPV2	Q9Y5S1	205	14	-1	-10	32	1.2	1.0	0.9	1.5
NGLY1	Q96IV0	309	-8	-10	-10	30	0.9	0.9	0.9	1.4
DNTTIP1	Q9H147	286	-3	-10	-10	30	1.0	0.9	0.9	1.4
EEFSEC	P57772	130	-5	-11	-10	30	1.0	0.9	0.9	1.4
EIF2B5	Q13144	571	4	-13	-10	29	1.0	0.9	0.9	1.4
ANAPC16	Q96DE5	55	-7	-6	-10	27	0.9	0.9	0.9	1.4
EHMT2	Q96KQ7	126	-24	-17	-10	27	0.8	0.9	0.9	1.4
NCOR1	O75376	2056	-13	-13	-10	27	0.9	0.9	0.9	1.4
CECR5	Q9BXW7	392	-12	-4	-10	26	0.9	1.0	0.9	1.4
SLFN11	Q7Z7L1	150	-3	-4	-10	26	1.0	1.0	0.9	1.4
MYOF	Q9NZM1	1425	-4	-5	-10	26	1.0	1.0	0.9	1.4
DNMT1	P26358	1476	-5	-12	-10	26	1.0	0.9	0.9	1.3
NVL	O15381	309	-11	-12	-10	26	0.9	0.9	0.9	1.3
DDX1	Q92499	111	-14	-8	-10	25	0.9	0.9	0.9	1.3
MGMT	P16455	150	-4	-20	-10	25	1.0	0.8	0.9	1.3
SMC5	Q8IY18	582	2	-6	-10	24	1.0	0.9	0.9	1.3
SMARCD1	Q96GM5	460	-4	-5	-10	24	1.0	1.0	0.9	1.3
TCAF2	A6NFQ2	275	1	-10	-10	24	1.0	0.9	0.9	1.3

VPS18	Q9P253	780	4	-17	-10	24	1.0	0.9	0.9	1.3
PML	P29590	389	1	-5	-10	23	1.0	1.0	0.9	1.3
ARID2	Q68CP9	711	-7	-8	-10	23	0.9	0.9	0.9	1.3
FAM208A	Q9UK61	690	-5	-15	-10	23	1.0	0.9	0.9	1.3
PAXBP1	Q9Y5B6	597	-8	-11	-10	22	0.9	0.9	0.9	1.3
TNS3	Q68CZ2	1251	-5	-10	-10	22	1.0	0.9	0.9	1.3
DMXL1	Q9Y485	259	-8	-19	-10	22	0.9	0.8	0.9	1.3
SPTLC1	O15269	133	1	-5	-10	20	1.0	1.0	0.9	1.2
SACS	Q9NZJ4	2514	2	-5	-10	20	1.0	1.0	0.9	1.2
BAZ2A	Q9UIF9	795	1	-5	-10	19	1.0	1.0	0.9	1.2
MPHOSPH8	Q99549	799	-8	-10	-10	19	0.9	0.9	0.9	1.2
RTN1	Q16799	471	1	-9	-10	18	1.0	0.9	0.9	1.2
RAB7A	P51149	83	-1	-10	-10	18	1.0	0.9	0.9	1.2
TRMO	Q9BU70	275	2	-16	-10	18	1.0	0.9	0.9	1.2
UBR5	O95071	2094	-6	-4	-10	18	0.9	1.0	0.9	1.2
PDLIM5	Q96HC4	467	-2	-6	-10	18	1.0	0.9	0.9	1.2
DIS3L	Q8TF46	124	9	-9	-10	17	1.1	0.9	0.9	1.2
PADI2	Q9Y2J8	338	-1	-9	-10	17	1.0	0.9	0.9	1.2
GNL3L	Q9NVN8	322	-8	3	-10	17	0.9	1.0	0.9	1.2
SLFN13	Q68D06	104	-7	2	-10	17	0.9	1.0	0.9	1.2
SLFN11	Q7Z7L1	102	-7	2	-10	17	0.9	1.0	0.9	1.2
MINA	Q8IUF8	19	-3	-8	-10	17	1.0	0.9	0.9	1.2
VBP1	P61758	8	-4	-12	-10	17	1.0	0.9	0.9	1.2
SRPRB	Q9Y5M8	100	2	-6	-10	16	1.0	0.9	0.9	1.2
ADD1	P35611	68	4	-8	-10	16	1.0	0.9	0.9	1.2
SKP1	P63208	160	-4	-9	-10	16	1.0	0.9	0.9	1.2
SSRP1	Q08945	207	4	4	-10	16	1.0	1.0	0.9	1.2
FCHO1	O14526	371	-8	-6	-10	16	0.9	0.9	0.9	1.2
CAD	P27708	1296	-9	-6	-10	16	0.9	0.9	0.9	1.2
FLYWCH2	Q96CP2	64	-3	-9	-10	16	1.0	0.9	0.9	1.2
SLC17A4	Q9Y2C5	342	-7	-9	-10	16	0.9	0.9	0.9	1.2
ALDH9A1	P49189	267	-8	-10	-10	16	0.9	0.9	0.9	1.2
DNAJA1	P31689	149	-1	-10	-10	16	1.0	0.9	0.9	1.2
APLF	Q8IW19	111	-4	-4	-10	15	1.0	1.0	0.9	1.2
USP9Y	O00507	1238	-3	-9	-10	15	1.0	0.9	0.9	1.2
METTL25	Q8N6Q8	96	3	-9	-10	15	1.0	0.9	0.9	1.2
USP9X	Q93008	1237	-3	-9	-10	15	1.0	0.9	0.9	1.2
ATAD1	Q8NBU5	310	-7	-10	-10	15	0.9	0.9	0.9	1.2
ZC3H12A	Q5D1E8	306	16	-13	-10	15	1.2	0.9	0.9	1.2
EIF2B3	Q9NR50	281	-3	-5	-10	15	1.0	1.0	0.9	1.2
MYCBP2	O75592	3326	-1	-14	-10	15	1.0	0.9	0.9	1.2
ELF2	Q15723	470	-12	-22	-10	15	0.9	0.8	0.9	1.2
RSF1	Q96T23	118	3	-1	-10	14	1.0	1.0	0.9	1.2
DDX21	Q9NR30	378	-2	-6	-10	14	1.0	0.9	0.9	1.2
FRY	Q5TBA9	2009	-6	-7	-10	14	0.9	0.9	0.9	1.2
PPP1R3D	O95685	291	-13	-9	-10	14	0.9	0.9	0.9	1.2
PPP2R5D	Q14738	17	-8	-2	-10	14	0.9	1.0	0.9	1.2
CCNH	P51946	244	-9	-6	-10	14	0.9	0.9	0.9	1.2
GMDS	O60547	336	-8	-14	-10	14	0.9	0.9	0.9	1.2
TRRAP	Q9Y4A5	2691	-2	1	-10	13	1.0	1.0	0.9	1.1
SYTL3	Q4VX76	258	-22	-4	-10	13	0.8	1.0	0.9	1.1
TRANK1	O15050	1041	-4	-7	-10	13	1.0	0.9	0.9	1.1
WDR45	Q9Y484	148	-3	-12	-10	13	1.0	0.9	0.9	1.1
HELZ2	Q9BYK8	2617	7	1	-10	13	1.1	1.0	0.9	1.1
TRABD	Q9H4I3	287	-4	-5	-10	13	1.0	1.0	0.9	1.1

GLRX	P35754	83	-1	-11	-10	13	1.0	0.9	0.9	1.1
GATAD2A	Q86YP4	417	-4	-11	-10	12	1.0	0.9	0.9	1.1
CBX3	Q13185	177	-5	-13	-10	12	1.0	0.9	0.9	1.1
KDM4B	O94953	309	-17	-3	-10	12	0.9	1.0	0.9	1.1
DHX15	O43143	774	6	-8	-10	12	1.1	0.9	0.9	1.1
POLR2B	P30876	1155	-6	-11	-10	12	0.9	0.9	0.9	1.1
TRIP6	Q15654	181	-5	-19	-10	12	1.0	0.8	0.9	1.1
ANKRD50	Q9ULJ7	122	1	9	-10	11	1.0	1.1	0.9	1.1
FLNB	O75369	1326	-3	-8	-10	11	1.0	0.9	0.9	1.1
CCDC94	Q9BW85	275	-8	-11	-10	11	0.9	0.9	0.9	1.1
HNRNPUL2-BS	H3BQZ7	308	-5	-12	-10	11	1.0	0.9	0.9	1.1
VPS41	P49754	638	-15	-18	-10	11	0.9	0.8	0.9	1.1
TMEM173	Q86WV6	206	4	-2	-10	11	1.0	1.0	0.9	1.1
USP9X	Q93008	842	1	-14	-10	11	1.0	0.9	0.9	1.1
TUBGCP3	Q96CW5	18	3	1	-10	10	1.0	1.0	0.9	1.1
ZZEF1	O43149	2546	-6	-6	-10	10	0.9	0.9	0.9	1.1
LPXN	O60711	379	-6	-6	-10	10	0.9	0.9	0.9	1.1
PTK2B	Q14289	899	-6	-9	-10	10	0.9	0.9	0.9	1.1
TRAPPC11	Q7Z392	481	-7	-10	-10	10	0.9	0.9	0.9	1.1
NFKB2	Q00653	114	-4	-12	-10	10	1.0	0.9	0.9	1.1
ZNHIT2	Q9UHR6	188	-1	-14	-10	10	1.0	0.9	0.9	1.1
HAUS1	Q96CS2	156	-2	-3	-10	10	1.0	1.0	0.9	1.1
EDC4	Q6P2E9	1054	-10	-6	-10	10	0.9	0.9	0.9	1.1
PDE12	Q6L8Q7	180	-6	-7	-10	10	0.9	0.9	0.9	1.1
PRKCD	Q05655	393	15	-8	-10	10	1.2	0.9	0.9	1.1
NUDC	Q9Y266	188	0	-8	-10	10	1.0	0.9	0.9	1.1
ZZEF1	O43149	69	-8	-9	-10	10	0.9	0.9	0.9	1.1
ANP32A	P39687	87	-7	-11	-10	10	0.9	0.9	0.9	1.1
URB2	Q14146	702	-2	2	-10	9	1.0	1.0	0.9	1.1
FMR1	Q06787	77	-4	-1	-10	9	1.0	1.0	0.9	1.1
DIS3	Q9Y2L1	213	-5	-9	-10	9	1.0	0.9	0.9	1.1
MAP2K6	P52564	216	-4	-12	-10	9	1.0	0.9	0.9	1.1
ARHGAP25	P42331	292	-1	-13	-10	9	1.0	0.9	0.9	1.1
APOBR	Q0VD83	844	-4	-5	-10	9	1.0	1.0	0.9	1.1
MAP2K6	P52564	38	-1	-8	-10	9	1.0	0.9	0.9	1.1
NXF1	Q9UBU9	373	-5	-10	-10	9	1.0	0.9	0.9	1.1
TRPV2	Q9Y5S1	281	-4	-10	-10	9	1.0	0.9	0.9	1.1
ACSL1	P33121	311	-3	7	-10	8	1.0	1.1	0.9	1.1
OASL	Q15646	181	-14	1	-10	8	0.9	1.0	0.9	1.1
MED1	Q15648	443	-4	-3	-10	8	1.0	1.0	0.9	1.1
VDAC2	P45880	76	-1	-6	-10	8	1.0	0.9	0.9	1.1
GIGYF2	Q6Y7W6	938	-6	-10	-10	8	0.9	0.9	0.9	1.1
SEC24D	O94855	573	-11	-11	-10	8	0.9	0.9	0.9	1.1
ELMSAN1	Q6PJG2	442	-4	-14	-10	8	1.0	0.9	0.9	1.1
IL17RB	Q9NRM6	87	11	-14	-10	8	1.1	0.9	0.9	1.1
BRAP	Q7Z569	47	-4	-3	-10	8	1.0	1.0	0.9	1.1
DDX17	Q92841	447	-6	-7	-10	8	0.9	0.9	0.9	1.1
CHAMP1	Q96JM3	119	-1	-9	-10	8	1.0	0.9	0.9	1.1
CTBS	Q01459	281	-19	-10	-10	8	0.8	0.9	0.9	1.1
ACOT11	Q8WXI4	198	-2	-13	-10	8	1.0	0.9	0.9	1.1
PPTC7	Q8NI37	41	9	-17	-10	8	1.1	0.9	0.9	1.1
RGS3	P49796	1135	3	-4	-10	7	1.0	1.0	0.9	1.1
LPIN1	Q14693	843	-6	-4	-10	7	0.9	1.0	0.9	1.1
PSMG1	O95456	169	-1	-7	-10	7	1.0	0.9	0.9	1.1
IFIT2	P09913	54	-14	-18	-10	7	0.9	0.8	0.9	1.1

FAM98C	Q17RN3	98	-8	-19	-10	7	0.9	0.8	0.9	1.1
INPP5D	Q92835	385	1	7	-10	7	1.0	1.1	0.9	1.1
DGKZ	Q13574	265	4	-7	-10	7	1.0	0.9	0.9	1.1
DAZAP1	Q96EP5	124	-13	-15	-10	7	0.9	0.9	0.9	1.1
ZNF808	Q8N4W9	382	10	-28	-10	7	1.1	0.8	0.9	1.1
RNF7	Q9UBF6	64	-18	1	-10	6	0.9	1.0	0.9	1.1
HMHA1	Q92619	278	-8	0	-10	6	0.9	1.0	0.9	1.1
WIPI2	Q9Y4P8	328	-5	-10	-10	6	1.0	0.9	0.9	1.1
NLRX1	Q86UT6	644	-18	-16	-10	6	0.9	0.9	0.9	1.1
POGZ	Q7Z3K3	547	-13	-18	-10	6	0.9	0.9	0.9	1.1
ATRX	P46100	1595	-21	-27	-10	6	0.8	0.8	0.9	1.1
RLF	Q13129	400	-6	6	-10	6	0.9	1.1	0.9	1.1
INPP5D	Q92835	4	-3	-6	-10	6	1.0	0.9	0.9	1.1
IKBKG	Q9Y6K9	95	-2	-8	-10	6	1.0	0.9	0.9	1.1
KDM2A	Q9Y2K7	840	-9	-10	-10	6	0.9	0.9	0.9	1.1
ZKSCAN1	P17029	63	-9	-10	-10	6	0.9	0.9	0.9	1.1
C5orf51	A6NDU8	244	2	-17	-10	6	1.0	0.9	0.9	1.1
HADHA	P40939	97	-2	-7	-10	5	1.0	0.9	0.9	1.1
DDX51	Q8N8A6	402	-13	-8	-10	5	0.9	0.9	0.9	1.1
HAUS3	Q68CZ6	271	-1	-12	-10	5	1.0	0.9	0.9	1.1
Uncharacteriz	F5H5P2	212	22	7	-10	5	1.3	1.1	0.9	1.0
UCHL5	Q9Y5K5	88	7	6	-10	5	1.1	1.1	0.9	1.0
PPP2R5D	Q14738	484	3	2	-10	5	1.0	1.0	0.9	1.0
SND1	Q7KZF4	440	0	1	-10	5	1.0	1.0	0.9	1.0
GIMAP4	Q9NUV9	220	-5	-4	-10	5	1.0	1.0	0.9	1.0
TEX264	Q9Y6I9	68	5	-5	-10	5	1.0	1.0	0.9	1.0
NLN	Q9BYT8	256	-5	-6	-10	5	1.0	0.9	0.9	1.0
LYN	P07948	219	3	-7	-10	5	1.0	0.9	0.9	1.0
EEF1A1	P68104	234	-5	-9	-10	5	1.0	0.9	0.9	1.0
STIP1	P31948	26	-5	-15	-10	5	1.0	0.9	0.9	1.0
PPP2CA	P67775	266	1	-4	-10	4	1.0	1.0	0.9	1.0
MCTS1	Q9ULC4	14	-2	-6	-10	4	1.0	0.9	0.9	1.0
TUBA4A	P68366	315	4	-9	-10	4	1.0	0.9	0.9	1.0
TUBA1A	Q71U36	315	4	-9	-10	4	1.0	0.9	0.9	1.0
SND1	Q7KZF4	549	-5	-9	-10	4	1.0	0.9	0.9	1.0
SENP7	Q9BQF6	274	-9	-9	-10	4	0.9	0.9	0.9	1.0
DOCK10	Q96BY6	1997	1	-9	-10	4	1.0	0.9	0.9	1.0
PXK	Q7Z7A4	196	-5	-11	-10	4	1.0	0.9	0.9	1.0
IMMT	Q16891	603	-4	-12	-10	4	1.0	0.9	0.9	1.0
STAG2	Q8N3U4	535	-4	-12	-10	4	1.0	0.9	0.9	1.0
NLRC5	Q86WI3	678	8	-32	-10	4	1.1	0.8	0.9	1.0
XAB2	Q9HCS7	66	1	-9	-10	4	1.0	0.9	0.9	1.0
RNF146	Q9NTX7	354	-12	-12	-10	4	0.9	0.9	0.9	1.0
GPX4	P36969	93	-9	-13	-10	4	0.9	0.9	0.9	1.0
AKT2	P31751	124	-2	6	-10	3	1.0	1.1	0.9	1.0
PRMT9	Q6P2P2	175	-1	-4	-10	3	1.0	1.0	0.9	1.0
YLPM1	P49750	580	-18	-5	-10	3	0.9	1.0	0.9	1.0
HECTD1	Q9ULT8	2579	-2	-7	-10	3	1.0	0.9	0.9	1.0
SART3	Q15020	472	-3	-10	-10	3	1.0	0.9	0.9	1.0
GTF2B	Q00403	168	-13	-13	-10	3	0.9	0.9	0.9	1.0
ELMO2	Q96JJ3	550	-23	-15	-10	3	0.8	0.9	0.9	1.0
PPP1R12A	O14974	553	-5	-5	-10	3	1.0	1.0	0.9	1.0
ACTR3	P61158	8	-1	-13	-10	3	1.0	0.9	0.9	1.0
EP300	Q09472	364	-4	-2	-10	2	1.0	1.0	0.9	1.0
ACAP2	Q15057	407	-5	-4	-10	2	1.0	1.0	0.9	1.0

ADRM1	Q16186	80	-4	-6	-10	2	1.0	0.9	0.9	1.0
HMHA1	Q92619	783	-5	-7	-10	2	1.0	0.9	0.9	1.0
HAUS7	Q99871	50	-14	-8	-10	2	0.9	0.9	0.9	1.0
ARAF	P10398	597	-5	-9	-10	2	1.0	0.9	0.9	1.0
NBEAL2	Q6ZNJ1	2335	-6	-13	-10	2	0.9	0.9	0.9	1.0
POLDIP3	Q9BY77	301	-15	-19	-10	2	0.9	0.8	0.9	1.0
HSD17B11	Q8NBQ5	215	-6	-3	-10	2	0.9	1.0	0.9	1.0
HGS	O14964	215	-6	-8	-10	2	0.9	0.9	0.9	1.0
ADRBK2	P35626	501	-6	-8	-10	2	0.9	0.9	0.9	1.0
PPP4R1	Q8TF05	566	0	-9	-10	2	1.0	0.9	0.9	1.0
LINGO3	P0C6S8	379	17	-9	-10	2	1.2	0.9	0.9	1.0
ARIH2	O95376	161	0	-10	-10	2	1.0	0.9	0.9	1.0
TXNRD1	Q16881	339	-3	-10	-10	2	1.0	0.9	0.9	1.0
TES	Q9UGI8	412	1	-2	-10	1	1.0	1.0	0.9	1.0
PARP1	P09874	311	4	-2	-10	1	1.0	1.0	0.9	1.0
DNAJC2	Q99543	394	-10	-9	-10	1	0.9	0.9	0.9	1.0
KIF1C	O43896	975	-8	-13	-10	1	0.9	0.9	0.9	1.0
ACAP2	Q15057	468	0	-2	-10	0	1.0	1.0	0.9	1.0
COQ5	Q5HYK3	244	-19	-5	-10	0	0.8	1.0	0.9	1.0
TRIM4	Q9C037	293	-9	-13	-10	0	0.9	0.9	0.9	1.0
STRAP	Q9Y3F4	152	-2	-13	-10	0	1.0	0.9	0.9	1.0
NCBP1	Q09161	320	2	3	-10	-1	1.0	1.0	0.9	1.0
MEFV	O15553	355	-19	-1	-10	-1	0.8	1.0	0.9	1.0
AHCYL1	O43865	373	-1	-7	-10	-1	1.0	0.9	0.9	1.0
NUP133	Q8WUM0	1037	-14	-9	-10	-1	0.9	0.9	0.9	1.0
NDUFA10	E7ESZ7	67	-9	-13	-10	-1	0.9	0.9	0.9	1.0
VARS	P26640	41	-6	-13	-10	-1	0.9	0.9	0.9	1.0
POLR2A	P24928	641	5	1	-10	-1	1.0	1.0	0.9	1.0
ACAT1	P24752	119	2	-5	-10	-2	1.0	1.0	0.9	1.0
GNB1	P62873	25	-8	-7	-10	-2	0.9	0.9	0.9	1.0
CLTC	Q00610	1260	3	-7	-10	-2	1.0	0.9	0.9	1.0
NOP14	P78316	755	-1	-14	-10	-2	1.0	0.9	0.9	1.0
OGFR	Q9NZT2	330	-2	-6	-10	-2	1.0	0.9	0.9	1.0
RNH1	P13489	191	1	-8	-10	-2	1.0	0.9	0.9	1.0
ACSL3	O95573	85	3	-9	-10	-2	1.0	0.9	0.9	1.0
LRP1B	Q9NZR2	3560	-5	-10	-10	-2	1.0	0.9	0.9	1.0
TMEM214	Q6NUQ4	464	2	-15	-10	-2	1.0	0.9	0.9	1.0
HCFC1	P51610	149	-8	-3	-10	-3	0.9	1.0	0.9	1.0
RNF213	Q63HN8	1736	-3	-16	-10	-3	1.0	0.9	0.9	1.0
RAB3GAP1	Q15042	693	-5	-21	-10	-3	1.0	0.8	0.9	1.0
APBB1IP	Q7Z5R6	304	7	-4	-10	-3	1.1	1.0	0.9	1.0
EXOC4	Q96A65	957	2	-9	-10	-3	1.0	0.9	0.9	1.0
GUSB	P08236	644	-7	-11	-10	-3	0.9	0.9	0.9	1.0
PADI4	Q9UM07	645	13	-15	-10	-3	1.1	0.9	0.9	1.0
AIMP2	Q13155	205	-4	-10	-10	-4	1.0	0.9	0.9	1.0
ATP2B4	P23634	537	-2	-15	-10	-4	1.0	0.9	0.9	1.0
BRIX1	Q8TDN6	52	0	-8	-10	-4	1.0	0.9	0.9	1.0
ZBTB3	Q9H5J0	483	-3	-29	-10	-4	1.0	0.8	0.9	1.0
ALS2	Q96Q42	664	-4	2	-10	-5	1.0	1.0	0.9	1.0
PRDX4	Q13162	51	-4	-10	-10	-5	1.0	0.9	0.9	1.0
HERC1	Q15751	4811	3	-10	-10	-5	1.0	0.9	0.9	1.0
USP24	Q9UPU5	1580	-3	-11	-10	-5	1.0	0.9	0.9	1.0
ZFC3H1	O60293	1379	3	-9	-10	-5	1.0	0.9	0.9	1.0
ARFGAP1	Q8N6T3	96	-10	-11	-10	-5	0.9	0.9	0.9	1.0
CCDC88A	Q3V6T2	524	-2	0	-10	-6	1.0	1.0	0.9	0.9

TNFAIP3	P21580	736	-10	-15	-10	-6	0.9	0.9	0.9	0.9
RANBP2	P49792	1502	-16	-2	-10	-6	0.9	1.0	0.9	0.9
ZC3H15	Q8WU90	105	-1	-5	-10	-6	1.0	1.0	0.9	0.9
MCCC2	Q9HCC0	267	-6	-5	-10	-6	0.9	1.0	0.9	0.9
RPA1	P27694	323	5	-6	-10	-6	1.0	0.9	0.9	0.9
HK1	P19367	886	-10	-9	-10	-6	0.9	0.9	0.9	0.9
ALG11	Q2TAA5	345	1	-13	-10	-6	1.0	0.9	0.9	0.9
SCYL1	Q96KG9	309	-3	-4	-10	-7	1.0	1.0	0.9	0.9
IPO5	O00410	1057	-5	-12	-10	-7	1.0	0.9	0.9	0.9
EWSR1	Q01844	529	1	-11	-10	-7	1.0	0.9	0.9	0.9
EEA1	Q15075	1382	-3	-12	-10	-7	1.0	0.9	0.9	0.9
CORO7-PAM1	A0A0A6YYL4	287	-6	-13	-10	-7	0.9	0.9	0.9	0.9
RAD50	Q92878	325	-3	-9	-10	-8	1.0	0.9	0.9	0.9
ICE1	Q9Y2F5	58	-13	-7	-10	-8	0.9	0.9	0.9	0.9
RAB4B	P61018	23	-4	-12	-10	-8	1.0	0.9	0.9	0.9
MYH9	P35579	1379	-1	-1	-10	-9	1.0	1.0	0.9	0.9
KIF13B	Q9NQT8	26	3	-3	-10	-9	1.0	1.0	0.9	0.9
NEK9	Q8TD19	556	-3	-8	-10	-9	1.0	0.9	0.9	0.9
NSD1	Q96L73	573	-13	-14	-10	-9	0.9	0.9	0.9	0.9
PYGB	P11216	808	-21	-16	-10	-9	0.8	0.9	0.9	0.9
CDK12	Q9NYV4	1009	-6	-17	-10	-9	0.9	0.9	0.9	0.9
TTC37	Q6PGP7	305	-18	-23	-10	-9	0.9	0.8	0.9	0.9
ARAP1	Q96P48	984	-8	-6	-10	-10	0.9	0.9	0.9	0.9
AKAP8	O43823	395	-4	-21	-10	-10	1.0	0.8	0.9	0.9
INTS3	Q68E01	731	7	-9	-10	-11	1.1	0.9	0.9	0.9
RTCA	O00442	28	-5	-11	-10	-11	1.0	0.9	0.9	0.9
UTRN	P46939	3023	-7	-13	-10	-11	0.9	0.9	0.9	0.9
CTPS1	P17812	362	-1	-17	-10	-11	1.0	0.9	0.9	0.9
SSH1	Q8WYL5	920	7	-7	-10	-11	1.1	0.9	0.9	0.9
RPS11	P62280	116	-5	-16	-10	-12	1.0	0.9	0.9	0.9
RNF213	Q63HN8	978	-4	-5	-10	-12	1.0	1.0	0.9	0.9
LIN9	Q5TKA1	435	-1	-11	-10	-12	1.0	0.9	0.9	0.9
PPP4R1	Q8TF05	261	-2	1	-10	-13	1.0	1.0	0.9	0.9
CLTC	Q00610	436	-5	-14	-10	-13	1.0	0.9	0.9	0.9
PSMD8	P48556	173	-2	0	-10	-14	1.0	1.0	0.9	0.9
ARFGAP3	Q9NP61	25	-1	-5	-10	-14	1.0	1.0	0.9	0.9
PLEC	Q15149	1405	12	-9	-10	-14	1.1	0.9	0.9	0.9
MYO9B	Q13459	863	-3	-24	-10	-15	1.0	0.8	0.9	0.9
HECTD4	Q9Y4D8	1636	4	-3	-10	-16	1.0	1.0	0.9	0.9
DGKA	P23743	246	-3	-7	-10	-16	1.0	0.9	0.9	0.9
CLCN3	P51790	733	-4	-10	-10	-17	1.0	0.9	0.9	0.9
MMAA	Q8IVH4	100	-4	-12	-10	-17	1.0	0.9	0.9	0.9
L2HGDH	Q9H9P8	272	4	2	-10	-18	1.0	1.0	0.9	0.9
THUMPD3	Q9BV44	434	-13	-12	-10	-18	0.9	0.9	0.9	0.8
FLNA	P21333	1865	-3	-12	-10	-19	1.0	0.9	0.9	0.8
SF3B3	Q15393	1156	1	-20	-10	-20	1.0	0.8	0.9	0.8
GBF1	Q92538	1588	-6	-20	-10	-22	0.9	0.8	0.9	0.8
CDA	P32320	31	-2	-18	-10	-23	1.0	0.9	0.9	0.8
CLIP2	Q9UDT6	390	1	-6	-10	-24	1.0	0.9	0.9	0.8
ZBTB7A	O95365	472	-8	-10	-10	-27	0.9	0.9	0.9	0.8
SLC9A1	P19634	561	-1	-13	-10	-27	1.0	0.9	0.9	0.8
FLNA	P21333	1260	0	-5	-10	-30	1.0	1.0	0.9	0.8
INF2	Q27J81	1029	-16	-7	-10	-30	0.9	0.9	0.9	0.8
CTC1	Q2NKJ3	479	-1	-12	-10	-36	1.0	0.9	0.9	0.7
ADH5	P11766	174	-8	-11	-10	-44	0.9	0.9	0.9	0.7

TES	Q9UGI8	327	19	-25	-10	-46	1.2	0.8	0.9	0.7
APPL2	Q8NEU8	412	-7	-11	-10	-51	0.9	0.9	0.9	0.7
ANK1	P16157	943	-3	-13	-10	-62	1.0	0.9	0.9	0.6
ZFP36	P26651	109	-7	-1	-10	-82	0.9	1.0	0.9	0.6
SARG	Q9BW04	338	5	6	-10	-102	1.0	1.1	0.9	0.5
TLN1	Q9Y490	2442	-3	-10	-10	-102	1.0	0.9	0.9	0.5
TNFAIP3	P21580	54	-12	-11	-11	43	0.9	0.9	0.9	1.7
DDX10	Q13206	323	-13	-7	-11	41	0.9	0.9	0.9	1.7
HLA-C	P04222	364	-5	-14	-11	38	1.0	0.9	0.9	1.6
HLA-C	P30510	364	-5	-14	-11	38	1.0	0.9	0.9	1.6
NUS1	Q96E22	77	-5	-18	-11	36	1.0	0.9	0.9	1.6
BRF2	Q9HAW0	168	-3	-14	-11	35	1.0	0.9	0.9	1.5
ALOX5	P09917	417	-3	-8	-11	35	1.0	0.9	0.9	1.5
ZMYM3	Q14202	636	5	-15	-11	33	1.1	0.9	0.9	1.5
PSMD2	Q13200	779	-3	-12	-11	32	1.0	0.9	0.9	1.5
GAB2	Q9UQC2	406	-18	-19	-11	32	0.8	0.8	0.9	1.5
BAG1	Q99933	272	-1	-4	-11	31	1.0	1.0	0.9	1.4
SRSF1	Q07955	16	3	-2	-11	31	1.0	1.0	0.9	1.4
AHCYL1	O43865	327	-5	-7	-11	29	1.0	0.9	0.9	1.4
C18orf32	Q8TCD1	71	-3	-8	-11	28	1.0	0.9	0.9	1.4
HTT	P42858	1998	-13	-9	-11	27	0.9	0.9	0.9	1.4
HPS3	Q969F9	56	10	-1	-11	26	1.1	1.0	0.9	1.4
UBA6	A0AVT1	347	-6	-12	-11	26	0.9	0.9	0.9	1.4
BLOC1S3	Q6QNY0	168	-10	-6	-11	26	0.9	0.9	0.9	1.3
DHX40	Q8IX18	42	0	-8	-11	26	1.0	0.9	0.9	1.3
COLGALT1	Q8NBJ5	412	0	-12	-11	25	1.0	0.9	0.9	1.3
NUDT16L1	Q9BRJ7	88	-9	-12	-11	25	0.9	0.9	0.9	1.3
PRPSAP2	O60256	31	2	0	-11	24	1.0	1.0	0.9	1.3
ZNF451	Q9Y4E5	730	-2	-9	-11	24	1.0	0.9	0.9	1.3
MMS19	Q96T76	549	-1	-1	-11	24	1.0	1.0	0.9	1.3
UBE2L3	P68036	86	-3	-11	-11	24	1.0	0.9	0.9	1.3
LRRK2	Q5S007	1618	-11	-14	-11	24	0.9	0.9	0.9	1.3
MRPS12	O15235	132	-10	-3	-11	23	0.9	1.0	0.9	1.3
RAD54L2	Q9Y4B4	616	-5	-6	-11	23	1.0	0.9	0.9	1.3
HPS3	Q969F9	39	1	-8	-11	23	1.0	0.9	0.9	1.3
LAS1L	Q9Y4W2	316	-8	-10	-11	23	0.9	0.9	0.9	1.3
CBX4	O00257	424	-7	-13	-11	23	0.9	0.9	0.9	1.3
DSN1	Q9H410	65	-12	-4	-11	22	0.9	1.0	0.9	1.3
FAAH	O00519	166	-10	-9	-11	22	0.9	0.9	0.9	1.3
UBLCP1	Q8WVY7	154	-6	2	-11	22	0.9	1.0	0.9	1.3
TRIM14	Q14142	237	1	-4	-11	22	1.0	1.0	0.9	1.3
OTUD4	Q01804	686	-4	-11	-11	22	1.0	0.9	0.9	1.3
TBC1D1	Q86TI0	548	-7	-12	-11	22	0.9	0.9	0.9	1.3
FBXO30	Q8TB52	725	-2	-10	-11	21	1.0	0.9	0.9	1.3
LYST	Q99698	2168	-8	-30	-11	21	0.9	0.8	0.9	1.3
KMT2D	O14686	1424	5	-7	-11	20	1.1	0.9	0.9	1.3
WNK1	Q9H4A3	547	-13	-10	-11	20	0.9	0.9	0.9	1.3
BUB1	O43683	973	2	-8	-11	20	1.0	0.9	0.9	1.2
PAPD4	Q6PIY7	225	-3	-8	-11	20	1.0	0.9	0.9	1.2
HNRNPD	Q14103	226	-2	-16	-11	20	1.0	0.9	0.9	1.2
PRKDC	P78527	1791	3	-2	-11	19	1.0	1.0	0.9	1.2
AHNAK	Q09666	2162	-6	-8	-11	19	0.9	0.9	0.9	1.2
DDX5	P17844	170	-10	-13	-11	19	0.9	0.9	0.9	1.2
DDX17	Q92841	247	-10	-13	-11	19	0.9	0.9	0.9	1.2
RBM15B	Q8NDT2	128	-1	-14	-11	19	1.0	0.9	0.9	1.2

MAP3K1	Q13233	193	-10	-8	-11	18	0.9	0.9	0.9	1.2
ZNFX1	Q9P2E3	1822	-15	-12	-11	18	0.9	0.9	0.9	1.2
KDM3A	Q9Y4C1	753	-15	-13	-11	18	0.9	0.9	0.9	1.2
DCPS	Q96C86	37	-1	-7	-11	18	1.0	0.9	0.9	1.2
SPG11	Q96JI7	1914	-5	-28	-11	17	1.0	0.8	0.9	1.2
ZNF644	Q9H582	507	-10	-8	-11	17	0.9	0.9	0.9	1.2
LYSMD2	Q8IV50	106	-4	-10	-11	17	1.0	0.9	0.9	1.2
CSNK2B-LY6G!	N0E472	23	2	11	-11	16	1.0	1.1	0.9	1.2
DR1	Q01658	94	-4	-5	-11	16	1.0	1.0	0.9	1.2
ATF2	P15336	79	-5	-10	-11	16	1.0	0.9	0.9	1.2
GINS3	Q9BRX5	188	2	-11	-11	16	1.0	0.9	0.9	1.2
USP16	Q9Y5T5	618	-13	-9	-11	16	0.9	0.9	0.9	1.2
PIK3R6	Q5UE93	496	-12	-10	-11	16	0.9	0.9	0.9	1.2
SATB1	Q01826	173	-2	-5	-11	15	1.0	1.0	0.9	1.2
GPCPD1	Q9NPB8	205	-6	-11	-11	15	0.9	0.9	0.9	1.2
EPG5	Q9HCE0	1201	-2	2	-11	15	1.0	1.0	0.9	1.2
POLM	Q9NP87	409	-4	-7	-11	15	1.0	0.9	0.9	1.2
PIKFYVE	Q9Y2I7	1970	-4	-5	-11	14	1.0	1.0	0.9	1.2
TKT	P29401	362	2	-7	-11	14	1.0	0.9	0.9	1.2
THEMIS2	Q5TEJ8	545	-5	-8	-11	14	1.0	0.9	0.9	1.2
IFIT2	P09913	289	-3	-11	-11	14	1.0	0.9	0.9	1.2
PPIP5K2	O43314	488	-6	-13	-11	14	0.9	0.9	0.9	1.2
KAT2B	Q92831	690	-4	-13	-11	14	1.0	0.9	0.9	1.2
GLOD4	Q9HC38	221	-8	-13	-11	14	0.9	0.9	0.9	1.2
HNRNPH1	P31943	267	-9	-23	-11	14	0.9	0.8	0.9	1.2
HNRNPH2	P55795	267	-9	-23	-11	14	0.9	0.8	0.9	1.2
ORC1	Q13415	469	-6	7	-11	14	0.9	1.1	0.9	1.2
LYZ	P61626	83	-7	-4	-11	14	0.9	1.0	0.9	1.2
MANBA	O00462	748	-4	-11	-11	14	1.0	0.9	0.9	1.2
IVNS1ABP	Q9Y6Y0	274	1	-12	-11	14	1.0	0.9	0.9	1.2
CTDP1	Q9Y5B0	277	-2	-15	-11	14	1.0	0.9	0.9	1.2
JAK3	P52333	1048	3	-21	-11	14	1.0	0.8	0.9	1.2
TRAPPC9	Q96Q05	317	2	-3	-11	13	1.0	1.0	0.9	1.1
HPS5	Q9UPZ3	707	-3	-9	-11	13	1.0	0.9	0.9	1.1
RERE	Q9P2R6	41	4	5	-11	13	1.0	1.0	0.9	1.1
SENP7	Q9BQF6	221	-5	-1	-11	13	1.0	1.0	0.9	1.1
ZNF318	Q5VUA4	1559	-10	-2	-11	13	0.9	1.0	0.9	1.1
CNTRL	Q7Z7A1	2100	-4	-5	-11	13	1.0	1.0	0.9	1.1
TLK1	Q9UKI8	81	1	-8	-11	13	1.0	0.9	0.9	1.1
C9orf142	Q9BUH6	180	-6	-9	-11	13	0.9	0.9	0.9	1.1
ALDH16A1	Q8IZ83	350	-7	-12	-11	13	0.9	0.9	0.9	1.1
TYMP	P19971	138	-3	-7	-11	12	1.0	0.9	0.9	1.1
KDM5A	P29375	692	-15	-9	-11	12	0.9	0.9	0.9	1.1
DHX8	Q14562	637	-12	-11	-11	12	0.9	0.9	0.9	1.1
ISY1	Q9ULR0	36	1	-14	-11	12	1.0	0.9	0.9	1.1
PPP4R2	Q9NY27	22	-5	-8	-11	12	1.0	0.9	0.9	1.1
UROS	P10746	14	-1	-6	-11	11	1.0	0.9	0.9	1.1
MVB12A	Q96EY5	231	-9	-7	-11	11	0.9	0.9	0.9	1.1
DDX27	Q96GQ7	321	-4	-5	-11	11	1.0	1.0	0.9	1.1
ZNF276	Q8N554	425	-7	-7	-11	11	0.9	0.9	0.9	1.1
ZC3H13	Q5T200	1592	-3	-11	-11	11	1.0	0.9	0.9	1.1
NHP2	Q9NX24	18	-7	-12	-11	11	0.9	0.9	0.9	1.1
LTN1	O94822	723	-9	-21	-11	11	0.9	0.8	0.9	1.1
PRKDC	P78527	2342	-7	-7	-11	10	0.9	0.9	0.9	1.1
SPG7	Q9UQ90	403	-9	-8	-11	10	0.9	0.9	0.9	1.1

ELP5	Q8TE02	151	11	-12	-11	10	1.1	0.9	0.9	1.1
PGLS	O95336	32	-4	-13	-11	10	1.0	0.9	0.9	1.1
CXorf56	Q9H5V9	11	-4	-14	-11	10	1.0	0.9	0.9	1.1
C9orf78	Q9NZ63	145	-7	-1	-11	10	0.9	1.0	0.9	1.1
RASGRP4	Q8TDF6	578	2	-9	-11	10	1.0	0.9	0.9	1.1
pk	D4Q8H0	22	-12	-11	-11	10	0.9	0.9	0.9	1.1
EP400	Q96L91	694	-8	-14	-11	10	0.9	0.9	0.9	1.1
TARDBP	G3V162	50	-4	-5	-11	9	1.0	1.0	0.9	1.1
CCZ1B	P86790	358	1	-6	-11	9	1.0	0.9	0.9	1.1
CPNE8	Q86YQ8	155	-12	-8	-11	9	0.9	0.9	0.9	1.1
ACTR3	P61158	235	-6	-9	-11	9	0.9	0.9	0.9	1.1
EXOC3	O60645	161	-6	-10	-11	9	0.9	0.9	0.9	1.1
TYW1B	A0A087WZB2	169	-3	-12	-11	9	1.0	0.9	0.9	1.1
GTF2H3	Q13889	257	-6	-13	-11	9	0.9	0.9	0.9	1.1
EFL1	Q7Z2Z2	474	-5	-16	-11	9	1.0	0.9	0.9	1.1
FRY	Q5TBA9	1193	-6	-1	-11	9	0.9	1.0	0.9	1.1
EDC4	Q6P2E9	90	8	-8	-11	9	1.1	0.9	0.9	1.1
DIAPH1	O60610	1227	-5	-11	-11	9	1.0	0.9	0.9	1.1
RIF1	Q5UIP0	312	-8	-15	-11	9	0.9	0.9	0.9	1.1
DDX56	Q9NY93	185	9	-3	-11	8	1.1	1.0	0.9	1.1
SMCHD1	A6NHR9	1656	-5	-6	-11	8	1.0	0.9	0.9	1.1
HCFC1	P51610	352	-3	-9	-11	8	1.0	0.9	0.9	1.1
SMARCC2	Q8TAQ2	145	-9	-10	-11	8	0.9	0.9	0.9	1.1
RECQL5	O94762	987	-4	-26	-11	8	1.0	0.8	0.9	1.1
CPSF2	Q9P2I0	294	-12	-2	-11	8	0.9	1.0	0.9	1.1
NUP153	P49790	404	-6	-3	-11	8	0.9	1.0	0.9	1.1
SRP54	P61011	36	-5	-5	-11	8	1.0	1.0	0.9	1.1
KLHL36	Q8N4N3	523	-2	-8	-11	8	1.0	0.9	0.9	1.1
NIPBL	Q6KC79	2035	-2	-8	-11	8	1.0	0.9	0.9	1.1
EPPK1	A0A087X1U6	406	-6	-13	-11	8	0.9	0.9	0.9	1.1
GAB3	Q8WWW8	376	10	-19	-11	8	1.1	0.8	0.9	1.1
UBR1	Q8IWV7	1369	-14	-20	-11	8	0.9	0.8	0.9	1.1
DDB1	Q16531	732	-4	0	-11	7	1.0	1.0	0.9	1.1
SLFN5	Q08AF3	610	4	-1	-11	7	1.0	1.0	0.9	1.1
METTL18	O95568	48	2	-4	-11	7	1.0	1.0	0.9	1.1
UBTF	P17480	68	8	-5	-11	7	1.1	1.0	0.9	1.1
WAPL	Q7Z5K2	906	-2	-10	-11	7	1.0	0.9	0.9	1.1
SSNA1	O43805	18	-6	-10	-11	7	0.9	0.9	0.9	1.1
RPA3	P35244	26	-9	-11	-11	7	0.9	0.9	0.9	1.1
KTN1	Q86UP2	1105	-7	-13	-11	7	0.9	0.9	0.9	1.1
STRN	O43815	316	1	-18	-11	7	1.0	0.9	0.9	1.1
AGO2	Q9UKV8	272	-14	-21	-11	7	0.9	0.8	0.9	1.1
COG1	Q8WTW3	72	-7	-1	-11	7	0.9	1.0	0.9	1.1
TMPO	P42166	518	-5	-2	-11	7	1.0	1.0	0.9	1.1
DOCK9	Q9BZ29	628	-5	-8	-11	7	1.0	0.9	0.9	1.1
GBF1	Q92538	403	-2	-8	-11	7	1.0	0.9	0.9	1.1
HNRNPU	Q00839	607	-4	-9	-11	7	1.0	0.9	0.9	1.1
CLIC2	O15247	30	-9	-10	-11	7	0.9	0.9	0.9	1.1
EWSR1	Q01844	384	1	-11	-11	7	1.0	0.9	0.9	1.1
PRKD2	Q9BZL6	217	-3	-12	-11	7	1.0	0.9	0.9	1.1
PIK3CD	O00329	382	3	2	-11	6	1.0	1.0	0.9	1.1
SDHA	P31040	189	-15	-3	-11	6	0.9	1.0	0.9	1.1
ZNF740	Q8NDX6	182	-11	-17	-11	6	0.9	0.9	0.9	1.1
WDR76	Q9H967	527	-1	-5	-11	6	1.0	1.0	0.9	1.1
PIK3AP1	Q6ZUJ8	147	-6	-7	-11	6	0.9	0.9	0.9	1.1

RARS	P54136	32	-9	-8	-11	6	0.9	0.9	0.9	1.1
PDK3	Q15120	41	-4	-12	-11	5	1.0	0.9	0.9	1.1
SMAD2	Q15796	380	-4	-13	-11	5	1.0	0.9	0.9	1.1
C2CD4B	A6NLJ0	259	-7	-13	-11	5	0.9	0.9	0.9	1.1
RSL1D1	O76021	96	-9	-13	-11	5	0.9	0.9	0.9	1.1
BLM	P54132	120	6	-14	-11	5	1.1	0.9	0.9	1.1
EPS15	P42566	657	-14	-17	-11	5	0.9	0.9	0.9	1.1
CPSF2	Q9P2I0	335	-7	-18	-11	5	0.9	0.8	0.9	1.1
PIK3CB	P42338	745	-19	-21	-11	5	0.8	0.8	0.9	1.1
PPP2R1A	P30153	390	-3	-4	-11	5	1.0	1.0	0.9	1.0
PPP2R1B	P30154	402	-3	-4	-11	5	1.0	1.0	0.9	1.0
EPRS	P07814	856	-5	-10	-11	5	1.0	0.9	0.9	1.0
CXXC1	Q9P0U4	420	-7	-14	-11	5	0.9	0.9	0.9	1.0
HCFC2	Q9Y5Z7	346	-6	-16	-11	5	0.9	0.9	0.9	1.0
MACF1	Q9UPN3	3173	6	-22	-11	5	1.1	0.8	0.9	1.0
SLC25A22	Q9H936	246	-3	10	-11	4	1.0	1.1	0.9	1.0
CHAF1A	Q13111	465	-9	-5	-11	4	0.9	1.0	0.9	1.0
DDX23	Q9BUQ8	692	-1	-6	-11	4	1.0	0.9	0.9	1.0
EML2	O95834	163	3	-11	-11	4	1.0	0.9	0.9	1.0
TPMT	P51580	70	-3	-18	-11	4	1.0	0.9	0.9	1.0
GLTSCR1L	Q6AI39	618	-13	-4	-11	4	0.9	1.0	0.9	1.0
PPME1	Q9Y570	238	-3	-6	-11	4	1.0	0.9	0.9	1.0
BLOC1S2	Q6QNY1	41	-9	-7	-11	4	0.9	0.9	0.9	1.0
LYST	Q99698	1354	-13	-8	-11	4	0.9	0.9	0.9	1.0
EXOC2	Q96KP1	541	-4	-9	-11	4	1.0	0.9	0.9	1.0
SMARCC2	Q8TAQ2	136	1	-3	-11	3	1.0	1.0	0.9	1.0
CYFIP1	Q7L576	98	-9	-3	-11	3	0.9	1.0	0.9	1.0
CYFIP2	Q96F07	98	-9	-3	-11	3	0.9	1.0	0.9	1.0
RNPEP	Q9H4A4	151	-7	-10	-11	3	0.9	0.9	0.9	1.0
MYO1D	O94832	353	7	-11	-11	3	1.1	0.9	0.9	1.0
UBA6	A0AVT1	721	0	-12	-11	3	1.0	0.9	0.9	1.0
ACP2	P11117	349	-9	-14	-11	3	0.9	0.9	0.9	1.0
PARP4	Q9UKK3	1293	-11	-19	-11	3	0.9	0.8	0.9	1.0
SEC13	P55735	187	-6	-5	-11	3	0.9	1.0	0.9	1.0
TRIP4	Q15650	86	-4	-6	-11	3	1.0	0.9	0.9	1.0
SMC1A	Q14683	1210	-4	-7	-11	3	1.0	0.9	0.9	1.0
MAP1S	Q66K74	440	-6	-10	-11	3	0.9	0.9	0.9	1.0
ZFPL1	O95159	230	2	7	-11	2	1.0	1.1	0.9	1.0
CELF1	Q92879	119	-6	-5	-11	2	0.9	1.0	0.9	1.0
LUZP1	Q86V48	969	-6	-16	-11	2	0.9	0.9	0.9	1.0
ZC3HAV1	Q7Z2W4	88	-5	-7	-11	2	1.0	0.9	0.9	1.0
VPS8	Q8N3P4	1371	-2	-8	-11	2	1.0	0.9	0.9	1.0
HSPA4	P34932	417	-3	-12	-11	2	1.0	0.9	0.9	1.0
LARP4B	Q92615	633	-2	-15	-11	2	1.0	0.9	0.9	1.0
ACRBP	Q8NEB7	361	-4	-26	-11	2	1.0	0.8	0.9	1.0
ACRBP	Q8NEB7	375	-4	-26	-11	2	1.0	0.8	0.9	1.0
ACRBP	Q8NEB7	378	-4	-26	-11	2	1.0	0.8	0.9	1.0
TNS3	Q68CZ2	615	-11	-6	-11	1	0.9	0.9	0.9	1.0
VPS13C	Q709C8	2925	-1	-7	-11	1	1.0	0.9	0.9	1.0
HNRNPUL1	Q9BUJ2	532	-18	-9	-11	1	0.8	0.9	0.9	1.0
CCDC88C	Q9P219	1871	-7	-10	-11	1	0.9	0.9	0.9	1.0
ADK	P55263	140	-13	-5	-11	1	0.9	1.0	0.9	1.0
NUMA1	Q14980	375	-6	-6	-11	1	0.9	0.9	0.9	1.0
PDS5B	Q9NTI5	732	-4	-9	-11	1	1.0	0.9	0.9	1.0
MKLN1	Q9UL63	582	-2	-14	-11	1	1.0	0.9	0.9	1.0

PDE3B	Q13370	394	-6	-15	-11	1	0.9	0.9	0.9	1.0
Uncharacteriz	G3V599	682	-1	-8	-11	0	1.0	0.9	0.9	1.0
G6PD	P11413	13	-5	-9	-11	0	1.0	0.9	0.9	1.0
STK38	Q15208	234	-7	-9	-11	0	0.9	0.9	0.9	1.0
CERS5	Q8N5B7	124	3	-10	-11	0	1.0	0.9	0.9	1.0
RBM25	P49756	132	-16	-12	-11	0	0.9	0.9	0.9	1.0
FAM175A	Q6UWZ7	67	-10	-14	-11	0	0.9	0.9	0.9	1.0
ODR4	Q5SWX8	141	-2	-17	-11	0	1.0	0.9	0.9	1.0
MROH1	Q8NDA8	1376	-10	-24	-11	0	0.9	0.8	0.9	1.0
AP2A2	O94973	491	-7	-12	-11	-1	0.9	0.9	0.9	1.0
TBC1D15	Q8TC07	184	-8	-20	-11	-1	0.9	0.8	0.9	1.0
ITPRIP	Q8IWB1	268	-2	-1	-11	-1	1.0	1.0	0.9	1.0
BOLA3	Q53S33	59	-3	-4	-11	-1	1.0	1.0	0.9	1.0
ASNA1	O43681	55	-3	-7	-11	-1	1.0	0.9	0.9	1.0
ACLY	P53396	20	-4	-8	-11	-1	1.0	0.9	0.9	1.0
WDR26	Q9H7D7	345	-15	-10	-11	-1	0.9	0.9	0.9	1.0
CCDC106	Q9BWC9	184	3	5	-11	-2	1.0	1.1	0.9	1.0
BOD1L1	Q8NFC6	2164	-8	-9	-11	-2	0.9	0.9	0.9	1.0
AKAP10	O43572	466	-12	-11	-11	-2	0.9	0.9	0.9	1.0
TCAF2	A6NFQ2	278	-2	-14	-11	-2	1.0	0.9	0.9	1.0
CHD3	Q12873	1131	-8	-16	-11	-2	0.9	0.9	0.9	1.0
CHD4	Q14839	1121	-8	-16	-11	-2	0.9	0.9	0.9	1.0
MKRN1	Q9UHC7	305	-17	-17	-11	-2	0.9	0.9	0.9	1.0
RNF213	Q63HN8	2424	-8	2	-11	-3	0.9	1.0	0.9	1.0
ATG4B	Q9Y4P1	74	-3	-2	-11	-3	1.0	1.0	0.9	1.0
OPA1	O60313	786	-7	-3	-11	-3	0.9	1.0	0.9	1.0
ACOX1	Q15067	199	-5	-3	-11	-3	1.0	1.0	0.9	1.0
GMIP	Q9P107	335	-5	-13	-11	-3	1.0	0.9	0.9	1.0
ALDOA	P04075	178	-13	-15	-11	-3	0.9	0.9	0.9	1.0
ALDOC	P09972	178	-13	-15	-11	-3	0.9	0.9	0.9	1.0
RABGGTA	Q92696	516	-11	-17	-11	-3	0.9	0.9	0.9	1.0
IDH2	P48735	113	-15	-4	-11	-3	0.9	1.0	0.9	1.0
INO80E	Q8NBZ0	31	-8	-6	-11	-3	0.9	0.9	0.9	1.0
TMPO	P42166	658	3	-10	-11	-3	1.0	0.9	0.9	1.0
RASAL3	Q86YV0	546	-9	-6	-11	-4	0.9	0.9	0.9	1.0
ATM	Q13315	2704	-2	-10	-11	-4	1.0	0.9	0.9	1.0
FAM193A	P78312	1237	-9	-11	-11	-4	0.9	0.9	0.9	1.0
THOC5	Q13769	613	-6	-29	-11	-4	0.9	0.8	0.9	1.0
LRBA	P50851	2017	3	-14	-11	-4	1.0	0.9	0.9	1.0
LPP	Q93052	566	-5	-4	-11	-5	1.0	1.0	0.9	1.0
UBA6	A0AVT1	178	-10	-13	-11	-5	0.9	0.9	0.9	1.0
STX16-NPEPL1	H3BU86	279	-4	-14	-11	-5	1.0	0.9	0.9	1.0
FECH	P22830	360	-7	-8	-11	-6	0.9	0.9	0.9	0.9
DIS3L	Q8TF46	312	-23	-8	-11	-6	0.8	0.9	0.9	0.9
TUBA4A	P68366	376	-7	-10	-11	-6	0.9	0.9	0.9	0.9
TUBA3D	Q13748	376	-7	-10	-11	-6	0.9	0.9	0.9	0.9
TUBA1A	Q71U36	376	-7	-10	-11	-6	0.9	0.9	0.9	0.9
JAK3	P52333	839	2	-10	-11	-6	1.0	0.9	0.9	0.9
DOCK8	Q8NF50	361	-4	-19	-11	-6	1.0	0.8	0.9	0.9
DGKZ	Q13574	713	-7	-7	-11	-6	0.9	0.9	0.9	0.9
RPL12	P30050	162	-4	-13	-11	-6	1.0	0.9	0.9	0.9
SYAP1	Q96A49	283	2	-13	-11	-6	1.0	0.9	0.9	0.9
CAND1	Q86VP6	131	1	-30	-11	-6	1.0	0.8	0.9	0.9
AP2B1	P63010	57	2	1	-11	-7	1.0	1.0	0.9	0.9
MBD4	O95243	82	-5	-4	-11	-7	1.0	1.0	0.9	0.9

ZNF185	O15231	681	-9	-9	-11	-7	0.9	0.9	0.9	0.9
ARHGEF6	Q15052	66	-6	-16	-11	-7	0.9	0.9	0.9	0.9
RABGAP1	Q9Y3P9	1026	-3	-6	-11	-7	1.0	0.9	0.9	0.9
ARHGAP17	Q68EM7	43	-31	-18	-11	-7	0.8	0.9	0.9	0.9
MDH1	P40925	251	-9	-21	-11	-7	0.9	0.8	0.9	0.9
RNH1	P13489	266	-7	-31	-11	-7	0.9	0.8	0.9	0.9
TYK2	P29597	965	-7	-5	-11	-8	0.9	1.0	0.9	0.9
FAM129B	Q96TA1	194	20	-7	-11	-8	1.2	0.9	0.9	0.9
ACTR10	Q9NZ32	37	-11	-11	-11	-8	0.9	0.9	0.9	0.9
BANK1	Q8NDB2	672	-16	-24	-11	-8	0.9	0.8	0.9	0.9
DIS3L2	Q8IYB7	739	3	-32	-11	-8	1.0	0.8	0.9	0.9
ACLY	P53396	229	3	-17	-11	-8	1.0	0.9	0.9	0.9
NEK9	Q8TD19	626	-8	4	-11	-9	0.9	1.0	0.9	0.9
CKAP5	Q14008	1113	-8	-6	-11	-9	0.9	0.9	0.9	0.9
TUBA4A	P68366	347	-6	-10	-11	-9	0.9	0.9	0.9	0.9
FMNL1	O95466	233	8	-11	-11	-9	1.1	0.9	0.9	0.9
DPF2	Q92785	298	-3	-5	-11	-9	1.0	1.0	0.9	0.9
ANK3	Q12955	1409	-10	-11	-11	-9	0.9	0.9	0.9	0.9
MTERF4	Q7Z6M4	182	-15	-13	-11	-9	0.9	0.9	0.9	0.9
HNRNPUL2-BS	H3BQZ7	408	-12	-5	-11	-10	0.9	1.0	0.9	0.9
PACSIN2	Q9UNF0	44	10	5	-11	-11	1.1	1.1	0.9	0.9
GCN1	Q92616	1781	-9	-3	-11	-11	0.9	1.0	0.9	0.9
MYH9	P35579	988	-1	-6	-11	-11	1.0	0.9	0.9	0.9
UBA7	P41226	680	-3	-18	-11	-11	1.0	0.8	0.9	0.9
CLIC3	O95833	174	-15	-4	-11	-11	0.9	1.0	0.9	0.9
FES	P07332	372	6	5	-11	-12	1.1	1.0	0.9	0.9
PTBP3	O95758	500	-5	-7	-11	-12	1.0	0.9	0.9	0.9
LATS2	Q9NRM7	868	-9	20	-11	-13	0.9	1.3	0.9	0.9
PRKD2	Q9BZL6	425	-13	-5	-11	-13	0.9	1.0	0.9	0.9
LPP	Q93052	524	-4	-4	-11	-14	1.0	1.0	0.9	0.9
IARS	P41252	1001	-4	-8	-11	-14	1.0	0.9	0.9	0.9
RPAP2	Q8IXW5	185	-8	-24	-11	-14	0.9	0.8	0.9	0.9
RANBP2	P49792	1629	-9	-5	-11	-15	0.9	1.0	0.9	0.9
IRF2BP2	Q7Z5L9	521	-4	-9	-11	-15	1.0	0.9	0.9	0.9
IRF2BPL	Q9H1B7	730	-4	-9	-11	-15	1.0	0.9	0.9	0.9
ZNF512	Q96ME7	35	-14	-2	-11	-15	0.9	1.0	0.9	0.9
AAK1	Q2M2I8	87	1	-3	-11	-16	1.0	1.0	0.9	0.9
SDCBP	O00560	166	-23	-10	-11	-16	0.8	0.9	0.9	0.9
Uncharacteriz	A0A087WZG4	438	0	-36	-11	-17	1.0	0.7	0.9	0.9
PCIF1	Q9H4Z3	626	5	-20	-11	-17	1.0	0.8	0.9	0.9
MBNL1	Q9NR56	185	-7	-4	-11	-18	0.9	1.0	0.9	0.9
MYH9	P35579	789	-4	-7	-11	-18	1.0	0.9	0.9	0.9
NUP93	Q8N1F7	397	-9	-18	-11	-18	0.9	0.8	0.9	0.9
CORO7-PAM1	A0A0A6YYL4	75	-9	-3	-11	-20	0.9	1.0	0.9	0.8
SACM1L	Q9NTJ5	445	-11	-9	-11	-20	0.9	0.9	0.9	0.8
PTPN1	P18031	32	-3	-12	-11	-21	1.0	0.9	0.9	0.8
CORO1C	Q9ULV4	420	-7	-8	-11	-21	0.9	0.9	0.9	0.8
AGTPBP1	Q9UPW5	305	25	-17	-11	-21	1.3	0.9	0.9	0.8
PHF5A	Q7RTV0	46	2	-4	-11	-22	1.0	1.0	0.9	0.8
FLNA	P21333	210	4	-11	-11	-23	1.0	0.9	0.9	0.8
FLNA	P21333	1997	-8	-9	-11	-25	0.9	0.9	0.9	0.8
CLIC4	Q9Y696	35	-5	-4	-11	-26	1.0	1.0	0.9	0.8
CNST	Q6PJW8	317	-3	-4	-11	-38	1.0	1.0	0.9	0.7
FERMT3	Q86UX7	235	-3	-3	-11	-51	1.0	1.0	0.9	0.7
FN3K	Q9H479	24	-1	-9	-11	-56	1.0	0.9	0.9	0.6

TLN1	Q9Y490	286	-3	3	-11	-60	1.0	1.0	0.9	0.6
PLEK	P08567	155	0	-20	-11	-63	1.0	0.8	0.9	0.6
LIN7C	Q9NUP9	47	-7	-26	-11	-70	0.9	0.8	0.9	0.6
TJP2	Q9UDY2	601	4	-8	-11	-82	1.0	0.9	0.9	0.5
PTGES3	Q15185	58	12	-13	-11	-95	1.1	0.9	0.9	0.5
TUBB1	Q9H4B7	315	8	-3	-11	-139	1.1	1.0	0.9	0.4
TUBB1	Q9H4B7	12	4	-5	-11	-172	1.0	1.0	0.9	0.4
MPND	Q8N594	457	-1	-13	-11	-213	1.0	0.9	0.9	0.3
BAX	Q07812	126	-12	-21	-11	59	0.9	0.8	0.9	2.4
FBXL3	Q9UKT7	137	-5	-21	-11	45	1.0	0.8	0.9	1.8
BHLHE40	O14503	260	-14	-23	-11	33	0.9	0.8	0.9	1.5
TRABD	Q9H4I3	366	-3	-13	-11	31	1.0	0.9	0.9	1.4
FCMR	O60667	318	-5	-9	-11	31	1.0	0.9	0.9	1.4
TBC1D9B	Q66K14	289	-13	-25	-11	29	0.9	0.8	0.9	1.4
TTC4	O95801	160	1	-6	-11	28	1.0	0.9	0.9	1.4
ZBTB21	Q9ULJ3	129	3	-7	-11	28	1.0	0.9	0.9	1.4
ALMS1	Q8TCU4	2601	-13	-10	-11	28	0.9	0.9	0.9	1.4
ZZEF1	O43149	2312	2	-11	-11	27	1.0	0.9	0.9	1.4
GTF2H2	Q13888	299	-3	-11	-11	27	1.0	0.9	0.9	1.4
ARID2	Q68CP9	382	2	-1	-11	25	1.0	1.0	0.9	1.3
PSMF1	Q92530	185	5	-26	-11	25	1.0	0.8	0.9	1.3
CACTIN	Q8WUQ7	699	-16	-17	-11	25	0.9	0.9	0.9	1.3
MYBBP1A	Q9BQG0	942	-1	-19	-11	25	1.0	0.8	0.9	1.3
SUN1	O94901	526	-6	-8	-11	24	0.9	0.9	0.9	1.3
NEK9	Q8TD19	890	-11	-12	-11	24	0.9	0.9	0.9	1.3
DCXR	Q7Z4W1	138	-4	-12	-11	24	1.0	0.9	0.9	1.3
HOPX	Q9BPY8	68	-18	-15	-11	24	0.9	0.9	0.9	1.3
ABL1	P00519	1100	3	-5	-11	23	1.0	1.0	0.9	1.3
SHKBP1	Q8TBC3	550	-11	-6	-11	23	0.9	0.9	0.9	1.3
CD7	P09564	230	-6	-7	-11	22	0.9	0.9	0.9	1.3
PARP10	Q53GL7	768	-5	-9	-11	22	1.0	0.9	0.9	1.3
CDK13	Q14004	701	-9	-1	-11	22	0.9	1.0	0.9	1.3
CDK12	Q9NYV4	723	-9	-1	-11	22	0.9	1.0	0.9	1.3
USP4	Q13107	702	-3	-8	-11	22	1.0	0.9	0.9	1.3
CRYL1	Q9Y2S2	182	2	-12	-11	22	1.0	0.9	0.9	1.3
RELA	Q04206	105	4	-8	-11	21	1.0	0.9	0.9	1.3
BANP	Q8N9N5	435	-4	-12	-11	21	1.0	0.9	0.9	1.3
CMAS	Q8NFW8	364	1	-7	-11	20	1.0	0.9	0.9	1.2
PARP4	Q9UKK3	360	5	-8	-11	20	1.0	0.9	0.9	1.2
ARFGAP1	Q8N6T3	351	-6	-16	-11	20	0.9	0.9	0.9	1.2
HERC3	Q15034	882	-2	-8	-11	19	1.0	0.9	0.9	1.2
AKAP12	Q02952	470	-10	-8	-11	19	0.9	0.9	0.9	1.2
PELP1	Q8IZL8	71	-8	-11	-11	19	0.9	0.9	0.9	1.2
GTF3C1	Q12789	42	-20	-16	-11	19	0.8	0.9	0.9	1.2
KMT2D	O14686	5142	4	-6	-11	18	1.0	0.9	0.9	1.2
GOLGA8O	A0A0J9YX86	241	9	-17	-11	18	1.1	0.9	0.9	1.2
EDC3	Q96F86	91	2	-20	-11	18	1.0	0.8	0.9	1.2
RARS2	Q5T160	576	1	-8	-11	17	1.0	0.9	0.9	1.2
SZT2	Q5T011	1421	2	-3	-11	17	1.0	1.0	0.9	1.2
GLRX5	Q86SX6	67	-3	-6	-11	17	1.0	0.9	0.9	1.2
MRPS14	O60783	83	-12	-9	-11	17	0.9	0.9	0.9	1.2
DPYSL2	Q16555	248	-11	-12	-11	17	0.9	0.9	0.9	1.2
CAPN1	P07384	384	-4	-12	-11	17	1.0	0.9	0.9	1.2
CAPN2	P17655	374	-4	-12	-11	17	1.0	0.9	0.9	1.2
WDR92	Q96MX6	29	-2	-6	-11	16	1.0	0.9	0.9	1.2

NAT1	P18440	148	-9	-14	-11	16	0.9	0.9	0.9	1.2
NVL	O15381	192	3	-9	-11	16	1.0	0.9	0.9	1.2
EPRS	P07814	692	-7	-9	-11	16	0.9	0.9	0.9	1.2
RAP2A	P10114	140	6	5	-11	15	1.1	1.0	0.9	1.2
ZNF598	Q86UK7	456	10	-2	-11	15	1.1	1.0	0.9	1.2
SGK3	Q96BR1	13	-5	-10	-11	15	1.0	0.9	0.9	1.2
PTPRE	P23469	132	-8	-1	-11	15	0.9	1.0	0.9	1.2
ANXA2	P07355	133	-2	-4	-11	14	1.0	1.0	0.9	1.2
FAM208A	Q9UK61	149	-2	-5	-11	14	1.0	1.0	0.9	1.2
ARNTL	O00327	269	-3	-8	-11	14	1.0	0.9	0.9	1.2
AHCTF1	Q8WYP5	1261	-7	-9	-11	14	0.9	0.9	0.9	1.2
MDN1	Q9NU22	1011	-5	-12	-11	14	1.0	0.9	0.9	1.2
IQSEC1	Q6DN90	214	-4	-13	-11	14	1.0	0.9	0.9	1.2
SMCHD1	A6NHR9	458	-1	-3	-11	14	1.0	1.0	0.9	1.2
SIPA1L3	O60292	620	-3	-8	-11	14	1.0	0.9	0.9	1.2
MDN1	Q9NU22	1332	-8	-13	-11	14	0.9	0.9	0.9	1.2
YLPM1	P49750	1035	-7	-13	-11	14	0.9	0.9	0.9	1.2
SRRT	Q9BXP5	715	-10	-13	-11	14	0.9	0.9	0.9	1.2
ANXA6	P08133	669	-4	-14	-11	14	1.0	0.9	0.9	1.2
NEMP1	O14524	434	-16	-4	-11	13	0.9	1.0	0.9	1.1
THNSL1	Q8IYQ7	324	-1	-8	-11	13	1.0	0.9	0.9	1.1
DAGLB	Q8NCG7	658	-5	-10	-11	13	1.0	0.9	0.9	1.1
PRKDC	P78527	795	1	-11	-11	13	1.0	0.9	0.9	1.1
PDCD11	Q14690	535	5	-12	-11	13	1.0	0.9	0.9	1.1
BAZ1B	Q9UIG0	1205	6	-12	-11	13	1.1	0.9	0.9	1.1
THOC1	Q96FV9	49	-2	-1	-11	13	1.0	1.0	0.9	1.1
MON1B	Q7L1V2	297	-10	-7	-11	13	0.9	0.9	0.9	1.1
AMPD2	Q01433	263	-9	-10	-11	13	0.9	0.9	0.9	1.1
ATOH7	Q8N100	29	13	-18	-11	13	1.1	0.9	0.9	1.1
FRYL	O94915	1107	5	-19	-11	13	1.0	0.8	0.9	1.1
NCK1	P16333	266	-8	-1	-11	12	0.9	1.0	0.9	1.1
HIBCH	Q6NVY1	95	-4	-5	-11	12	1.0	1.0	0.9	1.1
HCK	P08631	12	-12	-8	-11	12	0.9	0.9	0.9	1.1
FUBP3	Q96I24	460	-8	-8	-11	12	0.9	0.9	0.9	1.1
RABEPK	Q7Z6M1	29	-7	-10	-11	12	0.9	0.9	0.9	1.1
NUP155	O75694	1344	-9	-12	-11	12	0.9	0.9	0.9	1.1
SRI	P30626	194	14	17	-11	12	1.2	1.2	0.9	1.1
TOMM40	O96008	76	-6	-7	-11	12	0.9	0.9	0.9	1.1
MX1	P20591	336	-8	-7	-11	12	0.9	0.9	0.9	1.1
NMRAL1	Q9HBL8	154	-2	-11	-11	12	1.0	0.9	0.9	1.1
SP140L	Q9H930	191	-3	-11	-11	12	1.0	0.9	0.9	1.1
CHD6	Q8TD26	1555	-4	-3	-11	11	1.0	1.0	0.9	1.1
EDC4	Q6P2E9	510	5	-4	-11	11	1.0	1.0	0.9	1.1
EFHD2	Q96C19	53	3	-4	-11	11	1.0	1.0	0.9	1.1
FOXJ2	Q9P0K8	499	-6	-5	-11	11	0.9	1.0	0.9	1.1
SGPP1	Q9BX95	76	-10	-12	-11	11	0.9	0.9	0.9	1.1
ALDH9A1	P49189	45	-6	-16	-11	11	0.9	0.9	0.9	1.1
BABAM1	Q9NWV8	91	-11	-5	-11	11	0.9	1.0	0.9	1.1
GLUD1	P00367	172	-11	-9	-11	11	0.9	0.9	0.9	1.1
NCF2	P19878	514	-6	-10	-11	11	0.9	0.9	0.9	1.1
ADK	P55263	106	8	-24	-11	11	1.1	0.8	0.9	1.1
EPS15	P42566	782	-2	-9	-11	10	1.0	0.9	0.9	1.1
RNPC3	Q96LT9	420	-7	-9	-11	10	0.9	0.9	0.9	1.1
MYD88	Q99836	203	-2	-14	-11	10	1.0	0.9	0.9	1.1
USP48	Q86UV5	986	-1	-1	-11	10	1.0	1.0	0.9	1.1

MRPL33	O75394	29	1	-3	-11	10	1.0	1.0	0.9	1.1
ADAR	P55265	622	-2	-6	-11	10	1.0	0.9	0.9	1.1
TROVE2	P10155	71	-4	-7	-11	10	1.0	0.9	0.9	1.1
PPAT	Q06203	100	-2	-8	-11	10	1.0	0.9	0.9	1.1
MKI67	P46013	3199	-8	-14	-11	10	0.9	0.9	0.9	1.1
VPS13B	Q7Z7G8	3481	-5	-21	-11	10	1.0	0.8	0.9	1.1
KLF12	Q9Y4X4	349	2	-1	-11	9	1.0	1.0	0.9	1.1
PRKDC	P78527	1312	-1	-2	-11	9	1.0	1.0	0.9	1.1
RAD21	O60216	78	1	-5	-11	9	1.0	1.0	0.9	1.1
RECQL	P46063	606	-5	-9	-11	9	1.0	0.9	0.9	1.1
MAPKAP1	Q9BPZ7	149	-7	-10	-11	9	0.9	0.9	0.9	1.1
TNFAIP3	P21580	590	-4	-16	-11	9	1.0	0.9	0.9	1.1
STXBP3	O00186	114	4	-11	-11	9	1.0	0.9	0.9	1.1
AKAP11	Q9UKA4	1587	-15	-14	-11	9	0.9	0.9	0.9	1.1
MCCC1	Q96RQ3	129	11	11	-11	8	1.1	1.1	0.9	1.1
AP3M1	Q9Y2T2	10	7	1	-11	8	1.1	1.0	0.9	1.1
C15orf41	Q9Y2V0	277	1	-1	-11	8	1.0	1.0	0.9	1.1
C7orf43	Q8WVR3	418	-7	-7	-11	8	0.9	0.9	0.9	1.1
CARD6	Q9BX69	975	-10	-8	-11	8	0.9	0.9	0.9	1.1
DHX30	Q7L2E3	346	-6	-11	-11	8	0.9	0.9	0.9	1.1
DOK1	Q99704	463	-9	-11	-11	8	0.9	0.9	0.9	1.1
CLIC2	O15247	114	-5	-13	-11	8	1.0	0.9	0.9	1.1
BZW2	Q9Y6E2	270	-1	-13	-11	8	1.0	0.9	0.9	1.1
ZZEF1	O43149	1304	1	-14	-11	8	1.0	0.9	0.9	1.1
TACC1	O75410	174	-7	-17	-11	8	0.9	0.9	0.9	1.1
FUBP3	Q96I24	125	-12	-6	-11	7	0.9	0.9	0.9	1.1
ZCCHC11	Q5TAX3	994	1	-8	-11	7	1.0	0.9	0.9	1.1
PHF12	Q96QT6	613	-7	-10	-11	7	0.9	0.9	0.9	1.1
PLCG1	P19174	646	-8	2	-11	7	0.9	1.0	0.9	1.1
HNRNPU	Q00839	289	1	-4	-11	7	1.0	1.0	0.9	1.1
PPP1R18	Q6NYC8	611	-6	-8	-11	7	0.9	0.9	0.9	1.1
RPTOR	Q8N122	283	-12	-8	-11	7	0.9	0.9	0.9	1.1
ATE1	O95260	138	-4	-9	-11	7	1.0	0.9	0.9	1.1
PLEC	Q15149	530	-3	-11	-11	7	1.0	0.9	0.9	1.1
VRK1	Q99986	205	-14	-13	-11	7	0.9	0.9	0.9	1.1
NT5C2	P49902	336	-14	-15	-11	7	0.9	0.9	0.9	1.1
RNF214	Q8ND24	696	-2	3	-11	6	1.0	1.0	0.9	1.1
MYO9B	Q13459	1701	-1	-4	-11	6	1.0	1.0	0.9	1.1
RAB3GAP1	Q15042	218	-9	-4	-11	6	0.9	1.0	0.9	1.1
DCAF6	Q58WW2	593	-3	-6	-11	6	1.0	0.9	0.9	1.1
PPP2R1A	P30153	294	-5	-8	-11	6	1.0	0.9	0.9	1.1
GMPS	P49915	523	-5	-9	-11	6	1.0	0.9	0.9	1.1
FKBP5	Q13451	394	1	-9	-11	6	1.0	0.9	0.9	1.1
ZMYM3	Q14202	451	9	10	-11	6	1.1	1.1	0.9	1.1
HUWE1	Q7Z6Z7	1401	-9	-9	-11	6	0.9	0.9	0.9	1.1
GNL3	Q9BVP2	131	-3	-12	-11	6	1.0	0.9	0.9	1.1
UNC45A	Q9H3U1	853	-4	-23	-11	6	1.0	0.8	0.9	1.1
KDELR2	P33947	29	-9	-2	-11	5	0.9	1.0	0.9	1.1
TROVE2	P10155	141	-13	-3	-11	5	0.9	1.0	0.9	1.1
PRKDC	P78527	974	-8	-18	-11	5	0.9	0.9	0.9	1.1
RAPGEF1	Q13905	817	-18	2	-11	5	0.8	1.0	0.9	1.0
CACNA2D2	Q9NY47	1065	-1	-1	-11	5	1.0	1.0	0.9	1.0
HSPE1-MOB4	S4R3N1	170	2	-4	-11	5	1.0	1.0	0.9	1.0
PCNT	O95613	430	-3	-10	-11	5	1.0	0.9	0.9	1.0
CASP8	Q14790	360	-1	-12	-11	5	1.0	0.9	0.9	1.0

HPRT1	P00492	23	1	-14	-11	5	1.0	0.9	0.9	1.0
EEF1B2	P24534	50	-3	-16	-11	5	1.0	0.9	0.9	1.0
CTIF	O43310	145	-5	-22	-11	5	1.0	0.8	0.9	1.0
BRAF	P15056	173	-15	-5	-11	4	0.9	1.0	0.9	1.0
PLEC	Q15149	4574	-7	-5	-11	4	0.9	1.0	0.9	1.0
PSMB8	P28062	120	-1	-7	-11	4	1.0	0.9	0.9	1.0
KRIT1	O00522	134	-10	-9	-11	4	0.9	0.9	0.9	1.0
XPA	P23025	153	0	-9	-11	4	1.0	0.9	0.9	1.0
CAPZB	P47756	206	-6	-10	-11	4	0.9	0.9	0.9	1.0
ZC3H14	Q6PJT7	261	-5	-13	-11	4	1.0	0.9	0.9	1.0
DOCK11	Q5JSL3	371	3	-15	-11	4	1.0	0.9	0.9	1.0
NAXE	Q8NCW5	127	-2	-1	-11	4	1.0	1.0	0.9	1.0
CDK12	Q9NYV4	850	9	-4	-11	4	1.1	1.0	0.9	1.0
FDFT1	P37268	374	-12	-8	-11	4	0.9	0.9	0.9	1.0
IQCB1	Q15051	125	-3	-9	-11	4	1.0	0.9	0.9	1.0
WDR7	Q9Y4E6	326	3	-9	-11	4	1.0	0.9	0.9	1.0
CDK5	Q00535	290	-4	-15	-11	4	1.0	0.9	0.9	1.0
AP1B1	Q10567	866	-13	-18	-11	4	0.9	0.9	0.9	1.0
SEPT1	Q8WYJ6	102	-6	-7	-11	3	0.9	0.9	0.9	1.0
ARHGEF2	Q92974	715	-7	-10	-11	3	0.9	0.9	0.9	1.0
RELA	Q04206	109	-8	-12	-11	3	0.9	0.9	0.9	1.0
UTP20	O75691	2571	-4	-14	-11	3	1.0	0.9	0.9	1.0
PTPRE	P23469	186	-8	-16	-11	3	0.9	0.9	0.9	1.0
KDM5A	P29375	345	-8	-18	-11	3	0.9	0.9	0.9	1.0
TRANK1	O15050	867	-3	-2	-11	3	1.0	1.0	0.9	1.0
LIMA1	Q9UHB6	316	-6	-6	-11	3	0.9	0.9	0.9	1.0
R3HDM1	Q15032	304	-6	-10	-11	3	0.9	0.9	0.9	1.0
BAG5	Q9UL15	157	-7	-16	-11	3	0.9	0.9	0.9	1.0
ZYX	Q15942	492	4	-4	-11	2	1.0	1.0	0.9	1.0
SYNE1	Q8NF91	7251	-10	-6	-11	2	0.9	0.9	0.9	1.0
GNB1	P62873	317	-12	-6	-11	2	0.9	0.9	0.9	1.0
GNB2	P62879	317	-12	-6	-11	2	0.9	0.9	0.9	1.0
TACC3	Q9Y6A5	749	-9	-6	-11	2	0.9	0.9	0.9	1.0
SCRN1	Q12765	324	-6	-7	-11	2	0.9	0.9	0.9	1.0
USP10	Q14694	456	4	-12	-11	2	1.0	0.9	0.9	1.0
MROH1	Q8NDA8	1329	8	-17	-11	2	1.1	0.9	0.9	1.0
NUBP2	Q9Y5Y2	72	-4	-18	-11	2	1.0	0.9	0.9	1.0
AMT	P48728	367	-1	2	-11	2	1.0	1.0	0.9	1.0
MAP2K3	P46734	227	-6	-1	-11	2	0.9	1.0	0.9	1.0
XYLB	O75191	161	-21	-2	-11	2	0.8	1.0	0.9	1.0
CDC73	Q6P1J9	145	-3	-10	-11	2	1.0	0.9	0.9	1.0
RNF213	Q63HN8	438	-6	-11	-11	2	0.9	0.9	0.9	1.0
SEPT1	Q8WYJ6	99	-11	-13	-11	2	0.9	0.9	0.9	1.0
HELZ	P42694	14	14	-1	-11	1	1.2	1.0	0.9	1.0
RPS6	P62753	12	-6	-4	-11	1	0.9	1.0	0.9	1.0
TPMT	P51580	165	1	-5	-11	1	1.0	1.0	0.9	1.0
UBE3C	Q15386	1018	1	-8	-11	1	1.0	0.9	0.9	1.0
TYK2	P29597	278	-1	-9	-11	1	1.0	0.9	0.9	1.0
OXR1	Q8N573	299	-3	-10	-11	1	1.0	0.9	0.9	1.0
PLEC	Q15149	4494	-3	-11	-11	1	1.0	0.9	0.9	1.0
UBAP1	Q9NZ09	161	-9	-12	-11	1	0.9	0.9	0.9	1.0
GATM	P50440	50	-12	-13	-11	1	0.9	0.9	0.9	1.0
ANKRD12	Q6UB98	511	-11	-15	-11	1	0.9	0.9	0.9	1.0
PHF5A	Q7RTV0	61	1	1	-11	0	1.0	1.0	0.9	1.0
DCTN1	Q14203	888	-1	-2	-11	0	1.0	1.0	0.9	1.0

SEC23A	Q15436	180	3	-18	-11	0	1.0	0.9	0.9	1.0
STX16-NPEPL1	H3BU86	150	-9	-4	-11	-1	0.9	1.0	0.9	1.0
PRKCQ	Q04759	303	-3	-9	-11	-1	1.0	0.9	0.9	1.0
TAB2	Q9NYJ8	608	-7	-10	-11	-1	0.9	0.9	0.9	1.0
ACIN1	Q9UKV3	1223	-8	-11	-11	-1	0.9	0.9	0.9	1.0
DOCK8	Q8NF50	685	3	-11	-11	-1	1.0	0.9	0.9	1.0
RASA2	Q15283	436	-1	-13	-11	-1	1.0	0.9	0.9	1.0
MIC13	Q5XKP0	60	-7	-4	-11	-1	0.9	1.0	0.9	1.0
GDI2	P50395	202	-10	-9	-11	-1	0.9	0.9	0.9	1.0
RAD23B	P54727	390	-12	-12	-11	-1	0.9	0.9	0.9	1.0
ATG9A	Q7Z3C6	764	-6	-21	-11	-1	0.9	0.8	0.9	1.0
GDI2	P50395	317	-4	-9	-11	-2	1.0	0.9	0.9	1.0
EDC3	Q96F86	47	-9	-10	-11	-2	0.9	0.9	0.9	1.0
MRPS9	P82933	233	2	-12	-11	-2	1.0	0.9	0.9	1.0
SBF1	O95248	1642	-7	-13	-11	-2	0.9	0.9	0.9	1.0
TUBA4A	P68366	316	-4	-18	-11	-2	1.0	0.9	0.9	1.0
TUBA1A	Q71U36	316	-4	-18	-11	-2	1.0	0.9	0.9	1.0
NANS	Q9NR45	287	-8	-6	-11	-2	0.9	0.9	0.9	1.0
LRRC58	Q96CX6	296	-3	-7	-11	-2	1.0	0.9	0.9	1.0
MFN2	O95140	521	13	-2	-11	-3	1.1	1.0	0.9	1.0
ANXA1	P04083	324	0	-4	-11	-3	1.0	1.0	0.9	1.0
DCP1B	Q8IZD4	369	-10	-7	-11	-3	0.9	0.9	0.9	1.0
USP19	O94966	362	-15	-14	-11	-3	0.9	0.9	0.9	1.0
LAP3	P28838	335	-7	-17	-11	-3	0.9	0.9	0.9	1.0
RHOT2	Q8IXI1	185	-20	-26	-11	-3	0.8	0.8	0.9	1.0
EIF2S1	P05198	70	2	-10	-11	-4	1.0	0.9	0.9	1.0
ZNF341	Q9BYN7	756	-8	-11	-11	-4	0.9	0.9	0.9	1.0
DNPEP	Q9ULA0	327	-5	-26	-11	-4	1.0	0.8	0.9	1.0
HLA-DQB1	Q5SU54	149	-3	2	-11	-4	1.0	1.0	0.9	1.0
VRK2	Q86Y07	194	-2	-2	-11	-4	1.0	1.0	0.9	1.0
CORO7-PAM1	A0A0A6YYL4	656	-5	-11	-11	-4	1.0	0.9	0.9	1.0
UBXN2A	P68543	51	-11	-8	-11	-5	0.9	0.9	0.9	1.0
ACTB	P60709	217	0	-8	-11	-5	1.0	0.9	0.9	1.0
POTEE	Q6S8J3	917	0	-8	-11	-5	1.0	0.9	0.9	1.0
TRAPPC8	Q9Y2L5	943	-4	-8	-11	-5	1.0	0.9	0.9	1.0
RAB3GAP1	Q15042	678	-10	-9	-11	-5	0.9	0.9	0.9	1.0
JAK3	P52333	227	0	-11	-11	-5	1.0	0.9	0.9	1.0
SF3B5	Q9BWJ5	76	-8	-15	-11	-5	0.9	0.9	0.9	1.0
NEURL4	Q96JN8	1368	-4	-5	-11	-5	1.0	1.0	0.9	1.0
DCXR	Q7Z4W1	244	-9	-7	-11	-5	0.9	0.9	0.9	1.0
TROVE2	P10155	102	0	-8	-11	-5	1.0	0.9	0.9	1.0
NLRC5	Q86WI3	797	-10	-14	-11	-5	0.9	0.9	0.9	1.0
DOK2	O60496	36	-10	-16	-11	-5	0.9	0.9	0.9	1.0
SP1	P08047	604	2	-18	-11	-5	1.0	0.8	0.9	1.0
AMPD2	Q01433	230	-6	-1	-11	-6	0.9	1.0	0.9	0.9
RNF213	Q63HN8	3609	1	-8	-11	-6	1.0	0.9	0.9	0.9
PSME1	Q06323	101	-5	-4	-11	-6	1.0	1.0	0.9	0.9
INPP5B	P32019	678	6	-12	-11	-6	1.1	0.9	0.9	0.9
ACTR1B	P42025	34	-11	-14	-11	-7	0.9	0.9	0.9	0.9
ACTR1A	P61163	34	-11	-14	-11	-7	0.9	0.9	0.9	0.9
LPP	Q93052	572	-12	2	-11	-7	0.9	1.0	0.9	0.9
SND1	Q7KZF4	152	-8	-9	-11	-7	0.9	0.9	0.9	0.9
FIBP	O43427	262	-12	-10	-11	-7	0.9	0.9	0.9	0.9
LASP1	Q14847	35	-5	-10	-11	-7	1.0	0.9	0.9	0.9
ARAP3	Q8WWN8	1215	-6	-6	-11	-8	0.9	0.9	0.9	0.9

ADH5	P11766	97	-4	-10	-11	-8	1.0	0.9	0.9	0.9
CFL1	P23528	39	0	-11	-11	-8	1.0	0.9	0.9	0.9
TBCK	Q8TEA7	871	3	-18	-11	-8	1.0	0.9	0.9	0.9
USP8	P40818	575	-6	-6	-11	-9	0.9	0.9	0.9	0.9
GPX1	P07203	156	-9	-10	-11	-9	0.9	0.9	0.9	0.9
OAS3	Q9Y6K5	350	-9	-18	-11	-9	0.9	0.8	0.9	0.9
DDX42	Q86XP3	346	8	-3	-11	-10	1.1	1.0	0.9	0.9
SNIP1	Q8TAD8	299	3	-25	-11	-10	1.0	0.8	0.9	0.9
PEX1	O43933	746	-10	-11	-11	-10	0.9	0.9	0.9	0.9
MCCC2	Q9HCC0	392	-2	-20	-11	-10	1.0	0.8	0.9	0.9
TRIM33	Q9UPN9	291	-4	-25	-11	-10	1.0	0.8	0.9	0.9
RAB31	Q13636	9	-7	-9	-11	-11	0.9	0.9	0.9	0.9
SEC24D	O94855	780	-8	-3	-11	-12	0.9	1.0	0.9	0.9
APAF1	O14727	158	-2	-4	-11	-12	1.0	1.0	0.9	0.9
SLAIN2	Q9P270	152	-6	-11	-11	-12	0.9	0.9	0.9	0.9
TACC1	O75410	794	-8	-1	-11	-12	0.9	1.0	0.9	0.9
USP15	Q9Y4E8	791	-10	-25	-11	-12	0.9	0.8	0.9	0.9
ZYX	Q15942	433	-3	-5	-11	-13	1.0	1.0	0.9	0.9
NUP93	Q8N1F7	569	0	-15	-11	-13	1.0	0.9	0.9	0.9
PPP1R21	Q6ZMI0	351	3	-12	-11	-13	1.0	0.9	0.9	0.9
DGKA	P23743	238	-4	-2	-11	-14	1.0	1.0	0.9	0.9
IRF3	Q14653	371	7	-6	-11	-15	1.1	0.9	0.9	0.9
RPS6KB2	Q9UBS0	66	-18	5	-11	-15	0.9	1.0	0.9	0.9
RIOK1	Q9BRS2	264	-21	-22	-11	-16	0.8	0.8	0.9	0.9
CLK3	P49761	294	10	-7	-11	-17	1.1	0.9	0.9	0.9
FES	P07332	221	1	-12	-11	-17	1.0	0.9	0.9	0.9
DENND1C	Q8IV53	52	-10	-4	-11	-18	0.9	1.0	0.9	0.8
ACOT9	Q9Y305	299	-5	-3	-11	-21	1.0	1.0	0.9	0.8
ZYX	Q15942	409	-6	7	-11	-28	0.9	1.1	0.9	0.8
MYO9B	Q13459	152	12	3	-11	-32	1.1	1.0	0.9	0.8
SEC24C	P53992	493	-20	-19	-11	-32	0.8	0.8	0.9	0.8
ZNFX1	Q9P2E3	1302	-18	-11	-11	-41	0.9	0.9	0.9	0.7
GTPBP2	Q9BX10	321	-9	-6	-11	-45	0.9	0.9	0.9	0.7
VCL	P18206	545	-19	-17	-11	-73	0.8	0.9	0.9	0.6
INF2	Q27J81	332	0	-4	-11	-76	1.0	1.0	0.9	0.6
CASS4	Q9NQ75	741	5	-25	-11	-81	1.0	0.8	0.9	0.6
PLEK	P08567	102	-1	-6	-11	-84	1.0	0.9	0.9	0.5
NLRP1	Q9C000	649	-1	-11	-12	46	1.0	0.9	0.9	1.9
C15orf39	Q6ZRI6	892	-7	-12	-12	45	0.9	0.9	0.9	1.8
PAFAH1B2	P68402	188	-12	-21	-12	38	0.9	0.8	0.9	1.6
HNRNPU	Q00839	453	5	-3	-12	38	1.1	1.0	0.9	1.6
PRG2	P13727	201	-2	-15	-12	34	1.0	0.9	0.9	1.5
ASCC3	Q8N3C0	2101	-1	-2	-12	31	1.0	1.0	0.9	1.4
CLCC1	Q96S66	550	4	-8	-12	30	1.0	0.9	0.9	1.4
PIK3CA	P42336	242	-2	-9	-12	29	1.0	0.9	0.9	1.4
TRMT1	Q9NXH9	621	-11	-12	-12	29	0.9	0.9	0.9	1.4
SPATA5L1	Q9BVQ7	309	-13	-14	-12	29	0.9	0.9	0.9	1.4
BACH2	Q9BYV9	370	-14	-24	-12	29	0.9	0.8	0.9	1.4
GCLC	P48506	295	-1	-18	-12	28	1.0	0.8	0.9	1.4
PPAT	Q06203	207	-1	-3	-12	27	1.0	1.0	0.9	1.4
ATXN10	Q9UBB4	356	-5	-22	-12	26	1.0	0.8	0.9	1.4
MDN1	Q9NU22	2158	-8	2	-12	26	0.9	1.0	0.9	1.3
TNPO3	Q9Y5L0	511	-1	-16	-12	25	1.0	0.9	0.9	1.3
SYNE1	Q8NF91	5598	3	-12	-12	24	1.0	0.9	0.9	1.3
CARD9	Q9H257	454	-6	-14	-12	24	0.9	0.9	0.9	1.3

ASPHD2	Q6ICH7	200	1	0	-12	23	1.0	1.0	0.9	1.3
PRKCB	P05771	217	-21	-21	-12	23	0.8	0.8	0.9	1.3
CDK19	Q9BWU1	25	9	5	-12	23	1.1	1.1	0.9	1.3
INTS1	Q8N201	1534	-5	-5	-12	23	1.0	1.0	0.9	1.3
ADAMTS3	O15072	455	6	2	-12	22	1.1	1.0	0.9	1.3
CHD2	O14647	415	3	-4	-12	22	1.0	1.0	0.9	1.3
SLC19A1	P41440	220	-2	-14	-12	21	1.0	0.9	0.9	1.3
AKAP12	Q02952	1139	-11	-13	-12	21	0.9	0.9	0.9	1.3
ZNF331	Q9NQX6	282	-8	-12	-12	20	0.9	0.9	0.9	1.3
HAT1	O14929	120	-6	-15	-12	20	0.9	0.9	0.9	1.3
FAM192A	Q9GZU8	187	-5	-11	-12	20	1.0	0.9	0.9	1.2
AGO3	Q9H9G7	491	0	-2	-12	19	1.0	1.0	0.9	1.2
AGO4	Q9HCK5	482	0	-2	-12	19	1.0	1.0	0.9	1.2
AGO2	Q9UKV8	490	0	-2	-12	19	1.0	1.0	0.9	1.2
AGO1	Q9UL18	488	0	-2	-12	19	1.0	1.0	0.9	1.2
MRPS6	P82932	105	-3	-20	-12	19	1.0	0.8	0.9	1.2
UNC13D	Q70J99	276	-14	-6	-12	19	0.9	0.9	0.9	1.2
PIK3CD	O00329	991	-3	-12	-12	19	1.0	0.9	0.9	1.2
PFAS	O15067	606	-14	-12	-12	19	0.9	0.9	0.9	1.2
UIMC1	Q96RL1	257	2	-8	-12	18	1.0	0.9	0.9	1.2
HPS6	Q86YV9	408	0	-21	-12	18	1.0	0.8	0.9	1.2
ABHD10	Q9NUJ1	239	-2	8	-12	18	1.0	1.1	0.9	1.2
C18orf8	Q96DM3	333	1	-3	-12	17	1.0	1.0	0.9	1.2
FBXO22	Q8NEZ5	378	1	-9	-12	17	1.0	0.9	0.9	1.2
GLUD1	P00367	376	-4	-16	-12	17	1.0	0.9	0.9	1.2
ERCC4	Q92889	560	-12	-17	-12	17	0.9	0.9	0.9	1.2
MED29	Q9NX70	170	-2	-8	-12	17	1.0	0.9	0.9	1.2
ANXA5	P08758	316	-7	-12	-12	17	0.9	0.9	0.9	1.2
MSL3	Q8N5Y2	188	-9	-3	-12	16	0.9	1.0	0.9	1.2
NELFA	Q9H3P2	471	-5	-9	-12	16	1.0	0.9	0.9	1.2
CTDP1	Q9Y5B0	429	-1	-16	-12	16	1.0	0.9	0.9	1.2
SMARCA2	P51531	1175	-6	-6	-12	15	0.9	0.9	0.9	1.2
SMARCA4	P51532	1205	-6	-6	-12	15	0.9	0.9	0.9	1.2
NUP50	Q9UKX7	151	-4	-14	-12	15	1.0	0.9	0.9	1.2
TTLL12	Q14166	361	-12	-15	-12	15	0.9	0.9	0.9	1.2
SNTB2	Q13425	391	-10	-20	-12	15	0.9	0.8	0.9	1.2
PDS5B	Q9NTI5	317	-4	1	-12	15	1.0	1.0	0.9	1.2
RNF213	Q63HN8	981	1	-10	-12	15	1.0	0.9	0.9	1.2
ATAD2B	Q9ULI0	928	-10	-12	-12	15	0.9	0.9	0.9	1.2
SP140	Q13342	97	-8	-17	-12	15	0.9	0.9	0.9	1.2
MBD3L2	Q8NHZ7	159	-1	-5	-12	14	1.0	1.0	0.9	1.2
DDX31	Q9H8H2	660	-2	-8	-12	14	1.0	0.9	0.9	1.2
HCFC1	P51610	1886	-3	-8	-12	14	1.0	0.9	0.9	1.2
OSGEP	Q9NPF4	265	-6	-9	-12	14	0.9	0.9	0.9	1.2
ACSS1	Q9NUB1	151	-4	-12	-12	14	1.0	0.9	0.9	1.2
NDUFS1	P28331	727	-4	-13	-12	14	1.0	0.9	0.9	1.2
PML	P29590	212	-7	-21	-12	14	0.9	0.8	0.9	1.2
RIN1	Q13671	733	-12	-5	-12	14	0.9	1.0	0.9	1.2
RPS6KA1	Q15418	432	-3	-9	-12	14	1.0	0.9	0.9	1.2
HELZ2	Q9BYK8	2131	-4	-11	-12	14	1.0	0.9	0.9	1.2
ANKLE2	Q86XL3	91	-1	5	-12	13	1.0	1.0	0.9	1.1
HAT1	O14929	299	0	-8	-12	13	1.0	0.9	0.9	1.1
PRKRA	O75569	106	-6	-14	-12	13	0.9	0.9	0.9	1.1
LEF1	Q9UJU2	321	6	2	-12	13	1.1	1.0	0.9	1.1
HDAC9	Q9UKV0	534	13	0	-12	13	1.1	1.0	0.9	1.1

RPP38	P78345	135	-3	-2	-12	13	1.0	1.0	0.9	1.1
AKAP9	Q99996	3525	-6	-7	-12	13	0.9	0.9	0.9	1.1
AAMP	Q13685	216	-11	-7	-12	13	0.9	0.9	0.9	1.1
ZBTB11	O95625	197	-8	-9	-12	13	0.9	0.9	0.9	1.1
ATG2A	Q2TAZ0	1577	-1	-20	-12	13	1.0	0.8	0.9	1.1
NIPBL	Q6KC79	1754	-7	-21	-12	13	0.9	0.8	0.9	1.1
MLYCD	O95822	360	-11	8	-12	12	0.9	1.1	0.9	1.1
PSMB10	P40306	70	-4	-8	-12	12	1.0	0.9	0.9	1.1
VPS13C	Q709C8	3707	-4	-9	-12	12	1.0	0.9	0.9	1.1
CRYZ	Q08257	145	6	-11	-12	12	1.1	0.9	0.9	1.1
HERC1	Q15751	1388	-13	-14	-12	12	0.9	0.9	0.9	1.1
MUT	P22033	471	-7	-7	-12	12	0.9	0.9	0.9	1.1
NAA15	Q9BXJ9	238	0	-9	-12	12	1.0	0.9	0.9	1.1
SP140	Q13342	686	-2	-11	-12	11	1.0	0.9	0.9	1.1
SP140L	Q9H930	399	-2	-11	-12	11	1.0	0.9	0.9	1.1
UQCRC1	P31930	268	-2	-12	-12	11	1.0	0.9	0.9	1.1
TP53BP1	Q12888	1159	-1	-19	-12	11	1.0	0.8	0.9	1.1
BMF	Q96LC9	139	-11	-19	-12	11	0.9	0.8	0.9	1.1
STAT1	P42224	492	-9	-5	-12	11	0.9	1.0	0.9	1.1
SRRM1	Q8IYB3	33	-3	-7	-12	11	1.0	0.9	0.9	1.1
DNAJC21	Q5F1R6	444	2	-11	-12	11	1.0	0.9	0.9	1.1
MACF1	Q9UPN3	877	-4	-15	-12	11	1.0	0.9	0.9	1.1
PNPLA6	Q8IY17	199	-7	-16	-12	11	0.9	0.9	0.9	1.1
TOMM40	O96008	74	-28	-16	-12	11	0.8	0.9	0.9	1.1
LAMP1	P11279	338	3	-6	-12	10	1.0	0.9	0.9	1.1
TBXAS1	P24557	220	-14	-7	-12	10	0.9	0.9	0.9	1.1
TRIM56	Q9BRZ2	104	-1	-7	-12	10	1.0	0.9	0.9	1.1
UBE2R2	Q712K3	191	-3	-11	-12	10	1.0	0.9	0.9	1.1
MTHFSD	Q2M296	361	-1	-14	-12	10	1.0	0.9	0.9	1.1
GFM1	Q96RP9	723	-5	-17	-12	10	1.0	0.9	0.9	1.1
HMOX2	P30519	127	-10	-23	-12	10	0.9	0.8	0.9	1.1
NDUFV2	P19404	225	-11	-6	-12	10	0.9	0.9	0.9	1.1
PYCARD	Q9ULZ3	173	-4	-9	-12	10	1.0	0.9	0.9	1.1
PHIP	Q8WWQ0	1612	-3	-9	-12	10	1.0	0.9	0.9	1.1
ERCC4	Q92889	143	-11	-17	-12	10	0.9	0.9	0.9	1.1
TCP1	P17987	385	-11	-3	-12	9	0.9	1.0	0.9	1.1
SF1	Q15637	171	-8	-5	-12	9	0.9	1.0	0.9	1.1
TRAPPC10	P48553	916	-3	-6	-12	9	1.0	0.9	0.9	1.1
DDX58	O95786	680	-5	-7	-12	9	1.0	0.9	0.9	1.1
ABCF3	Q9NUQ8	102	-6	-7	-12	9	0.9	0.9	0.9	1.1
RPA1	P27694	476	0	-12	-12	9	1.0	0.9	0.9	1.1
TKT	P29401	386	-5	-5	-12	9	1.0	1.0	0.9	1.1
RPA1	P27694	486	1	-10	-12	9	1.0	0.9	0.9	1.1
ACSF3	Q4G176	399	-4	-10	-12	9	1.0	0.9	0.9	1.1
PDLIM2	Q96JY6	160	6	-11	-12	9	1.1	0.9	0.9	1.1
PCNT	O95613	2059	-4	-24	-12	9	1.0	0.8	0.9	1.1
CBX8	Q9HC52	142	-10	-8	-12	8	0.9	0.9	0.9	1.1
CDKN2AIP	Q9NXV6	516	-8	-12	-12	8	0.9	0.9	0.9	1.1
CDC27	P30260	137	3	-14	-12	8	1.0	0.9	0.9	1.1
AGFG2	O95081	50	-1	-1	-12	8	1.0	1.0	0.9	1.1
ZC3H13	Q5T200	1591	-5	-9	-12	8	1.0	0.9	0.9	1.1
ATM	Q13315	2770	-4	-10	-12	8	1.0	0.9	0.9	1.1
NUP188	Q5SRE5	1283	-2	-11	-12	8	1.0	0.9	0.9	1.1
RABEP2	Q9H5N1	482	-4	-11	-12	8	1.0	0.9	0.9	1.1
SMC2	O95347	326	-15	-13	-12	8	0.9	0.9	0.9	1.1

SYNE2	Q8WXH0	2520	-7	-15	-12	8	0.9	0.9	0.9	1.1
GTF3C1	Q12789	853	-5	-16	-12	8	1.0	0.9	0.9	1.1
ITSN2	Q9NZM3	1389	3	-5	-12	7	1.0	1.0	0.9	1.1
GAPDH	P04406	156	-5	-11	-12	7	1.0	0.9	0.9	1.1
TBC1D2B	Q9UPU7	651	-10	-12	-12	7	0.9	0.9	0.9	1.1
APIP	Q96GX9	147	-5	-6	-12	7	1.0	0.9	0.9	1.1
QRICH1	Q2TAL8	713	-5	-7	-12	7	1.0	0.9	0.9	1.1
NANS	Q9NR45	180	-1	-11	-12	7	1.0	0.9	0.9	1.1
LRRK2	Q5S007	1187	-10	-12	-12	7	0.9	0.9	0.9	1.1
PGM2L1	Q6PCE3	303	-10	-15	-12	7	0.9	0.9	0.9	1.1
LCP1	P13796	336	-8	-16	-12	7	0.9	0.9	0.9	1.1
THADA	Q6YHU6	1393	-15	-17	-12	7	0.9	0.9	0.9	1.1
SPEN	Q96T58	877	-11	-6	-12	6	0.9	0.9	0.9	1.1
EEF2	P13639	41	-1	-8	-12	6	1.0	0.9	0.9	1.1
NBN	O60934	285	-4	-14	-12	6	1.0	0.9	0.9	1.1
DNMT1	P26358	1339	-6	-16	-12	6	0.9	0.9	0.9	1.1
NT5C2	P49902	181	-5	-6	-12	6	1.0	0.9	0.9	1.1
SEPT1	Q8WYJ6	231	-3	-7	-12	6	1.0	0.9	0.9	1.1
ZNF800	Q2TB10	49	-2	-9	-12	6	1.0	0.9	0.9	1.1
PPP2R2D	Q66LE6	404	2	-12	-12	6	1.0	0.9	0.9	1.1
TBC1D1	Q86TI0	766	-1	-12	-12	6	1.0	0.9	0.9	1.1
L3MBTL2	Q969R5	408	-10	-14	-12	6	0.9	0.9	0.9	1.1
CCNL2	Q96S94	52	-6	-15	-12	6	0.9	0.9	0.9	1.1
YDJC	A8MPS7	18	-8	-24	-12	6	0.9	0.8	0.9	1.1
GGACT	Q9BVM4	84	2	-4	-12	5	1.0	1.0	0.9	1.1
PDIA3	P30101	92	2	-5	-12	5	1.0	1.0	0.9	1.1
MACF1	Q9UPN3	443	0	-7	-12	5	1.0	0.9	0.9	1.1
ZZEF1	O43149	1441	1	-9	-12	5	1.0	0.9	0.9	1.1
SOS1	Q07889	980	-1	-10	-12	5	1.0	0.9	0.9	1.1
SOS2	Q07890	978	-1	-10	-12	5	1.0	0.9	0.9	1.1
PSMD1	Q99460	633	-14	-22	-12	5	0.9	0.8	0.9	1.1
PPP1R37	O75864	375	1	2	-12	5	1.0	1.0	0.9	1.0
TRIM65	Q6PJ69	174	-3	-9	-12	5	1.0	0.9	0.9	1.0
C3orf38	Q5JPI3	259	-13	-13	-12	5	0.9	0.9	0.9	1.0
PIK3R1	P27986	498	-5	-14	-12	5	1.0	0.9	0.9	1.0
DFFA	O00273	165	0	-9	-12	4	1.0	0.9	0.9	1.0
PSMB1	P20618	224	8	-12	-12	4	1.1	0.9	0.9	1.0
GCLC	P48506	152	-5	-12	-12	4	1.0	0.9	0.9	1.0
LRRC59	Q96AG4	59	-1	-13	-12	4	1.0	0.9	0.9	1.0
SSH2	Q76I76	890	-6	-14	-12	4	0.9	0.9	0.9	1.0
THOC2	Q8NI27	1236	-8	-14	-12	4	0.9	0.9	0.9	1.0
HTATIP2	Q9BUP3	172	-5	-10	-12	4	1.0	0.9	0.9	1.0
RNF213	Q63HN8	4000	-8	-10	-12	4	0.9	0.9	0.9	1.0
SERPINB2	P05120	79	-3	-12	-12	4	1.0	0.9	0.9	1.0
NLRP1	Q9C000	894	-5	-14	-12	4	1.0	0.9	0.9	1.0
TAF1	P21675	786	-11	-16	-12	4	0.9	0.9	0.9	1.0
TFPT	P0C1Z6	89	-45	-5	-12	3	0.7	1.0	0.9	1.0
PYCRL	Q53H96	49	-15	-5	-12	3	0.9	1.0	0.9	1.0
PPTC7	Q8NI37	278	-5	-6	-12	3	1.0	0.9	0.9	1.0
NLRC5	Q86WI3	741	-3	-8	-12	3	1.0	0.9	0.9	1.0
COPG1	Q9Y678	706	-13	-11	-12	3	0.9	0.9	0.9	1.0
NAPRT	Q6XQN6	48	-11	-11	-12	3	0.9	0.9	0.9	1.0
SNX30	Q5VWJ9	103	-11	-14	-12	3	0.9	0.9	0.9	1.0
PGM2L1	Q6PCE3	348	-6	-14	-12	3	0.9	0.9	0.9	1.0
ZC3H11A	O75152	559	-1	-15	-12	3	1.0	0.9	0.9	1.0

SEC16A	O15027	1619	-2	-5	-12	3	1.0	1.0	0.9	1.0
GEMIN4	P57678	927	-5	-9	-12	3	1.0	0.9	0.9	1.0
CEP70	Q8NHQ1	138	1	-9	-12	3	1.0	0.9	0.9	1.0
ZFYVE26	Q68DK2	66	-9	-9	-12	3	0.9	0.9	0.9	1.0
NCBP1	Q09161	44	-6	-11	-12	3	0.9	0.9	0.9	1.0
PRMT1	Q99873	240	-2	-11	-12	3	1.0	0.9	0.9	1.0
ARID1A	O14497	1983	-12	-11	-12	3	0.9	0.9	0.9	1.0
PIK3R1	P27986	167	-2	-13	-12	3	1.0	0.9	0.9	1.0
BBX	Q8WY36	682	-6	-14	-12	3	0.9	0.9	0.9	1.0
ICT1	Q14197	82	-8	-15	-12	3	0.9	0.9	0.9	1.0
ZNF696	Q9H7X3	136	-7	-21	-12	3	0.9	0.8	0.9	1.0
RLF	Q13129	227	-9	0	-12	2	0.9	1.0	0.9	1.0
INPP5D	Q92835	1088	-7	-13	-12	2	0.9	0.9	0.9	1.0
CEP135	Q66GS9	316	-11	-14	-12	2	0.9	0.9	0.9	1.0
IKBKG	Q9Y6K9	131	-6	2	-12	2	0.9	1.0	0.9	1.0
C2CD5	Q86YS7	449	-3	-5	-12	2	1.0	1.0	0.9	1.0
EIF5	P55010	102	-4	-8	-12	2	1.0	0.9	0.9	1.0
C7orf43	Q8WVR3	302	-20	-8	-12	2	0.8	0.9	0.9	1.0
RICTOR	Q6R327	1317	5	-12	-12	2	1.0	0.9	0.9	1.0
OGFR	Q9NZT2	417	-5	-16	-12	2	1.0	0.9	0.9	1.0
TIAM1	Q13009	226	-12	-17	-12	2	0.9	0.9	0.9	1.0
ACOT2	P49753	401	-9	-4	-12	1	0.9	1.0	0.9	1.0
EIF3D	O15371	196	3	-12	-12	1	1.0	0.9	0.9	1.0
ITPR2	Q14571	1879	-16	-13	-12	1	0.9	0.9	0.9	1.0
TBCA	O75347	67	-10	-17	-12	1	0.9	0.9	0.9	1.0
GALNT2	Q10471	229	-17	-19	-12	1	0.9	0.8	0.9	1.0
Uncharacteriz	K7ELQ4	61	-9	-8	-12	1	0.9	0.9	0.9	1.0
ELF2	Q15723	348	-13	-15	-12	1	0.9	0.9	0.9	1.0
TES	Q9UGI8	416	-1	-4	-12	0	1.0	1.0	0.9	1.0
LRCH3	Q96II8	261	-6	-6	-12	-1	0.9	0.9	0.9	1.0
EIF2S3	P41091	101	-4	-12	-12	-1	1.0	0.9	0.9	1.0
MROH1	Q8NDA8	637	-5	-15	-12	-1	1.0	0.9	0.9	1.0
ZFAND5	O76080	76	0	-8	-12	-1	1.0	0.9	0.9	1.0
OGT	O15294	323	-5	-11	-12	-1	1.0	0.9	0.9	1.0
LYRM7	Q5U5X0	97	-6	-11	-12	-1	0.9	0.9	0.9	1.0
BLVRA	P53004	281	-1	-16	-12	-1	1.0	0.9	0.9	1.0
DOK3	Q7L591	323	-10	-10	-12	-2	0.9	0.9	0.9	1.0
EMC8	O43402	24	-10	-11	-12	-2	0.9	0.9	0.9	1.0
NAPA	P54920	103	-11	-16	-12	-2	0.9	0.9	0.9	1.0
ARHGAP31	Q2M1Z3	122	-7	-8	-12	-2	0.9	0.9	0.9	1.0
FLNB	O75369	2537	-7	-9	-12	-2	0.9	0.9	0.9	1.0
RALBP1	Q15311	451	3	-14	-12	-2	1.0	0.9	0.9	1.0
BRE	Q9NXR7	34	-9	1	-12	-3	0.9	1.0	0.9	1.0
CEP170	Q5SW79	967	-9	0	-12	-3	0.9	1.0	0.9	1.0
ARL14EP	Q8N8R7	231	-4	-1	-12	-3	1.0	1.0	0.9	1.0
LRCH4	O75427	105	-5	-2	-12	-3	1.0	1.0	0.9	1.0
RNF213	Q63HN8	1614	-8	-3	-12	-3	0.9	1.0	0.9	1.0
KMT2B	Q9UMN6	1447	-17	-15	-12	-3	0.9	0.9	0.9	1.0
ACAA1	P09110	381	-3	-8	-12	-3	1.0	0.9	0.9	1.0
PRKCB	P05771	586	-3	-13	-12	-3	1.0	0.9	0.9	1.0
IMMP1L	Q96LU5	128	-14	-26	-12	-3	0.9	0.8	0.9	1.0
CARS	P49589	405	-1	-5	-12	-4	1.0	1.0	0.9	1.0
SNW1	Q13573	250	-4	-8	-12	-4	1.0	0.9	0.9	1.0
ADRBK2	P35626	120	-5	-13	-12	-4	1.0	0.9	0.9	1.0
G6PD	P11413	294	0	-12	-12	-4	1.0	0.9	0.9	1.0

LYST	Q99698	1827	-17	-19	-12	-4	0.9	0.8	0.9	1.0
EIF2S2	P20042	309	-7	-2	-12	-5	0.9	1.0	0.9	1.0
FKBP4	Q02790	396	-11	-15	-12	-5	0.9	0.9	0.9	1.0
KIFAP3	Q92845	78	-3	-24	-12	-5	1.0	0.8	0.9	1.0
LRCH4	O75427	224	-6	-6	-12	-5	0.9	0.9	0.9	1.0
LYAR	Q9NX58	51	-7	-7	-12	-5	0.9	0.9	0.9	1.0
RNF213	Q63HN8	2412	-8	-9	-12	-5	0.9	0.9	0.9	1.0
CAMSAP1	Q5T5Y3	848	-4	-16	-12	-5	1.0	0.9	0.9	1.0
PPP2R2D	Q66LE6	268	-18	-18	-12	-5	0.9	0.8	0.9	1.0
PNKP	Q96T60	405	2	-5	-12	-6	1.0	1.0	0.9	0.9
FGD3	Q5JSP0	541	-5	-6	-12	-6	1.0	0.9	0.9	0.9
PPM1B	O75688	163	3	-7	-12	-6	1.0	0.9	0.9	0.9
DGKZ	Q13574	993	2	-2	-12	-6	1.0	1.0	0.9	0.9
GCN1	Q92616	1275	-8	-13	-12	-6	0.9	0.9	0.9	0.9
PPP1CB	P62140	154	-5	-4	-12	-7	1.0	1.0	0.9	0.9
PPP1CA	P62136	155	-5	-4	-12	-7	1.0	1.0	0.9	0.9
ME2	P23368	441	-15	-15	-12	-7	0.9	0.9	0.9	0.9
GAMT	Q14353	220	-11	-20	-12	-7	0.9	0.8	0.9	0.9
PFAS	O15067	336	-16	-33	-12	-7	0.9	0.8	0.9	0.9
SMARCAL1	Q9NZC9	508	-9	-16	-12	-7	0.9	0.9	0.9	0.9
DENND5A	Q6IQ26	1077	-1	-18	-12	-7	1.0	0.9	0.9	0.9
SSFA2	P28290	901	-10	-25	-12	-7	0.9	0.8	0.9	0.9
DARS	P14868	259	-1	-5	-12	-8	1.0	1.0	0.9	0.9
OSBPL10	Q9BXB5	615	-2	-10	-12	-8	1.0	0.9	0.9	0.9
EVI5	O60447	479	-12	-23	-12	-8	0.9	0.8	0.9	0.9
RNF213	Q63HN8	1464	-7	-9	-12	-8	0.9	0.9	0.9	0.9
DYNC1H1	Q14204	3712	1	2	-12	-9	1.0	1.0	0.9	0.9
WDFY4	Q6ZS81	1696	-7	-17	-12	-9	0.9	0.9	0.9	0.9
GORASP2	Q9H8Y8	173	-4	-17	-12	-9	1.0	0.9	0.9	0.9
KDM5A	P29375	558	5	-3	-12	-9	1.0	1.0	0.9	0.9
ECI2	O75521	380	-10	-10	-12	-9	0.9	0.9	0.9	0.9
PTRH2	Q9Y3E5	111	-11	-12	-12	-9	0.9	0.9	0.9	0.9
FAM91A1	Q658Y4	750	-3	-17	-12	-9	1.0	0.9	0.9	0.9
EPRS	P07814	1480	-12	-2	-12	-10	0.9	1.0	0.9	0.9
CUL1	Q13616	355	3	-7	-12	-10	1.0	0.9	0.9	0.9
ETFA	P13804	155	-8	-8	-12	-10	0.9	0.9	0.9	0.9
POLA2	Q14181	392	9	-17	-12	-10	1.1	0.9	0.9	0.9
TUBA1A	Q71U36	129	5	-18	-12	-10	1.0	0.9	0.9	0.9
TRIP6	Q15654	328	-5	-12	-12	-12	1.0	0.9	0.9	0.9
WAPL	Q7Z5K2	160	-11	-20	-12	-12	0.9	0.8	0.9	0.9
PRDX1	Q06830	173	-5	-15	-12	-13	1.0	0.9	0.9	0.9
IKBKG	Q9Y6K9	54	1	-1	-12	-13	1.0	1.0	0.9	0.9
ACADSB	P45954	175	-1	-2	-12	-13	1.0	1.0	0.9	0.9
APAF1	O14727	8	-5	2	-12	-14	1.0	1.0	0.9	0.9
BAG5	Q9UL15	191	6	-21	-12	-15	1.1	0.8	0.9	0.9
NUMB	P49757	160	-5	-16	-12	-15	1.0	0.9	0.9	0.9
G6PD	P11413	385	-5	-12	-12	-16	1.0	0.9	0.9	0.9
KIAA0196	Q12768	969	-15	-13	-12	-16	0.9	0.9	0.9	0.9
ARID1A	O14497	1968	8	-11	-12	-16	1.1	0.9	0.9	0.9
CWC22	Q9HCG8	253	-1	-11	-12	-16	1.0	0.9	0.9	0.9
ADAR	P55265	499	-6	-15	-12	-16	0.9	0.9	0.9	0.9
THOP1	P52888	231	-6	-21	-12	-17	0.9	0.8	0.9	0.9
MED17	Q9NVC6	363	-3	-7	-12	-17	1.0	0.9	0.9	0.9
RANBP2	P49792	581	25	-8	-12	-19	1.3	0.9	0.9	0.8
PRKD2	Q9BZL6	180	-8	-5	-12	-19	0.9	1.0	0.9	0.8

SEC31A	O94979	173	-27	-23	-12	-20	0.8	0.8	0.9	0.8
TARDBP	G3V162	173	-7	-9	-12	-20	0.9	0.9	0.9	0.8
ZNF185	O15231	542	-11	-24	-12	-20	0.9	0.8	0.9	0.8
ANO6	Q4KMQ2	250	-6	-16	-12	-21	0.9	0.9	0.9	0.8
CDC16	Q13042	133	-1	-23	-12	-21	1.0	0.8	0.9	0.8
GBP4	Q96PP9	172	-18	-5	-12	-21	0.8	1.0	0.9	0.8
MPND	Q8N594	63	-28	-4	-12	-24	0.8	1.0	0.9	0.8
STRN4	Q9NRL3	713	-10	-13	-12	-24	0.9	0.9	0.9	0.8
MYH10	P35580	95	4	-16	-12	-27	1.0	0.9	0.9	0.8
WDR44	Q5JSH3	716	-6	-6	-12	-28	0.9	0.9	0.9	0.8
ACTN4	O43707	351	-7	-11	-12	-29	0.9	0.9	0.9	0.8
ACTN1	P12814	332	-7	-11	-12	-29	0.9	0.9	0.9	0.8
ITPR2	Q14571	2087	13	-11	-12	-35	1.1	0.9	0.9	0.7
PDE3A	Q14432	526	-26	-12	-12	-38	0.8	0.9	0.9	0.7
RIF1	Q5UIP0	1732	-6	-5	-12	-39	0.9	1.0	0.9	0.7
PARP14	Q460N5	849	-19	-13	-12	-44	0.8	0.9	0.9	0.7
SLFN14	P0C7P3	668	14	5	-12	-54	1.2	1.1	0.9	0.6
TLN1	Q9Y490	243	-14	-16	-12	-59	0.9	0.9	0.9	0.6
SP1	P08047	633	-18	-19	-12	-66	0.9	0.8	0.9	0.6
DBN1	Q16643	96	-1	-8	-12	-69	1.0	0.9	0.9	0.6
SMAD2	Q15796	312	9	-12	-12	-70	1.1	0.9	0.9	0.6
CDC42BPB	Q9Y5S2	654	-29	5	-12	-76	0.8	1.1	0.9	0.6
PES1	O00541	361	-1	-6	-12	-81	1.0	0.9	0.9	0.6
TUBB6	Q9BUF5	354	-2	-6	-12	-167	1.0	0.9	0.9	0.4
TUBB1	Q9H4B7	354	-2	-6	-12	-167	1.0	0.9	0.9	0.4
PREX1	Q8TCU6	1266	-7	-13	-12	48	0.9	0.9	0.9	1.9
FAM98C	Q17RN3	36	-7	-12	-12	41	0.9	0.9	0.9	1.7
STAT2	P52630	284	-4	-11	-12	40	1.0	0.9	0.9	1.7
GOLGA3	Q08378	1403	-6	-17	-12	37	0.9	0.9	0.9	1.6
NAA16	Q6N069	322	4	-9	-12	35	1.0	0.9	0.9	1.5
ADRBK1	P25098	208	-8	-6	-12	33	0.9	0.9	0.9	1.5
ADRBK2	P35626	208	-8	-6	-12	33	0.9	0.9	0.9	1.5
EP400	Q96L91	162	-13	-15	-12	29	0.9	0.9	0.9	1.4
CABIN1	Q9Y6J0	589	4	-10	-12	28	1.0	0.9	0.9	1.4
MGMT	P16455	145	-9	-14	-12	27	0.9	0.9	0.9	1.4
ZMYM1	Q5SVZ6	989	10	-8	-12	25	1.1	0.9	0.9	1.3
SLC25A40	Q8TBP6	314	-2	-14	-12	24	1.0	0.9	0.9	1.3
PRKCI	P41743	117	-5	15	-12	24	1.0	1.2	0.9	1.3
BOP1	Q14137	404	-3	-11	-12	24	1.0	0.9	0.9	1.3
DNMT3A	Q9Y6K1	554	2	-12	-12	24	1.0	0.9	0.9	1.3
LHPP	Q9H008	88	5	7	-12	23	1.0	1.1	0.9	1.3
RNF213	Q63HN8	4570	2	-3	-12	23	1.0	1.0	0.9	1.3
RNASEL	Q05823	301	-4	-13	-12	23	1.0	0.9	0.9	1.3
DFNA5	O60443	45	2	-6	-12	23	1.0	0.9	0.9	1.3
NUP205	Q92621	1073	-2	-6	-12	23	1.0	0.9	0.9	1.3
LRRK2	Q5S007	2025	-5	-9	-12	23	1.0	0.9	0.9	1.3
HECTD1	Q9ULT8	936	-3	-4	-12	22	1.0	1.0	0.9	1.3
NARFL	Q9H6Q4	179	-5	-7	-12	21	1.0	0.9	0.9	1.3
ARID4B	Q4LE39	198	12	-15	-12	21	1.1	0.9	0.9	1.3
FAM49A	Q9H0Q0	273	13	-14	-12	21	1.1	0.9	0.9	1.3
WDR82	Q6UXN9	50	6	-3	-12	20	1.1	1.0	0.9	1.3
HDAC7	Q8WUI4	904	1	-8	-12	20	1.0	0.9	0.9	1.3
NFATC1	O95644	263	-8	-6	-12	20	0.9	0.9	0.9	1.2
PASK	Q96RG2	991	-8	-8	-12	20	0.9	0.9	0.9	1.2
GIT1	Q9Y2X7	576	-6	-10	-12	20	0.9	0.9	0.9	1.2

BAZ2B	Q9UIF8	776	-1	-4	-12	19	1.0	1.0	0.9	1.2
CBX1	P83916	156	-17	2	-12	18	0.9	1.0	0.9	1.2
DOCK10	Q96BY6	238	-14	-14	-12	18	0.9	0.9	0.9	1.2
Uncharacterize	A0A0C4DFX4	1865	-9	-12	-12	18	0.9	0.9	0.9	1.2
CHUK	O15111	406	-7	-9	-12	17	0.9	0.9	0.9	1.2
PSTPIP1	O43586	13	-6	-17	-12	17	0.9	0.9	0.9	1.2
DCLRE1C	Q96SD1	435	-11	-23	-12	17	0.9	0.8	0.9	1.2
CBWD1	Q9BRT8	394	-1	2	-12	17	1.0	1.0	0.9	1.2
SDE2	Q6IQ49	406	0	-2	-12	17	1.0	1.0	0.9	1.2
TBC1D22B	Q9NU19	144	-12	-11	-12	17	0.9	0.9	0.9	1.2
DHX15	O43143	226	-5	-5	-12	16	1.0	1.0	0.9	1.2
BBX	Q8WY36	150	-2	-7	-12	16	1.0	0.9	0.9	1.2
LY9	Q9HBG7	627	2	-9	-12	16	1.0	0.9	0.9	1.2
GCFC2	P16383	320	3	-10	-12	16	1.0	0.9	0.9	1.2
CREBRF	Q8IUR6	186	-2	-13	-12	16	1.0	0.9	0.9	1.2
NAT10	Q9H0A0	505	-1	-16	-12	16	1.0	0.9	0.9	1.2
CTC1	Q2NKJ3	482	-3	-17	-12	16	1.0	0.9	0.9	1.2
RUFY1	Q96T51	8	-6	-8	-12	15	0.9	0.9	0.9	1.2
RCSD1	Q6JBY9	49	-4	-10	-12	15	1.0	0.9	0.9	1.2
RCC2	Q9P258	337	-9	-12	-12	15	0.9	0.9	0.9	1.2
MARCH6	O60337	52	-5	-11	-12	15	1.0	0.9	0.9	1.2
HNRNPF	P52597	267	-1	-11	-12	15	1.0	0.9	0.9	1.2
AKNA	Q7Z591	289	-10	-12	-12	15	0.9	0.9	0.9	1.2
CHD6	Q8TD26	1940	-10	-14	-12	15	0.9	0.9	0.9	1.2
HUWE1	Q7Z6Z7	3658	3	-2	-12	14	1.0	1.0	0.9	1.2
PLEKHA1	Q9HB21	389	3	-7	-12	14	1.0	0.9	0.9	1.2
SRBD1	Q8N5C6	397	-5	-8	-12	14	1.0	0.9	0.9	1.2
ARHGEF6	Q15052	553	-6	-19	-12	14	0.9	0.8	0.9	1.2
CHPF2	Q9P2E5	393	-2	-8	-12	14	1.0	0.9	0.9	1.2
ATM	Q13315	384	-3	-13	-12	14	1.0	0.9	0.9	1.2
MORC3	Q14149	15	-9	-10	-12	13	0.9	0.9	0.9	1.1
SMG5	Q9UPR3	109	-19	-12	-12	13	0.8	0.9	0.9	1.1
AAMP	Q13685	220	-5	3	-12	13	1.0	1.0	0.9	1.1
PDE3B	Q13370	1082	-5	-2	-12	13	1.0	1.0	0.9	1.1
RSRC2	Q7L4I2	382	-2	-2	-12	13	1.0	1.0	0.9	1.1
ZNF14	P17017	458	-15	-9	-12	13	0.9	0.9	0.9	1.1
MCM4	P33991	328	5	-20	-12	13	1.1	0.8	0.9	1.1
TWISTNB	Q3B726	193	4	1	-12	12	1.0	1.0	0.9	1.1
MACF1	Q9UPN3	1722	-2	-2	-12	12	1.0	1.0	0.9	1.1
SIK3	Q9Y2K2	597	-11	-8	-12	12	0.9	0.9	0.9	1.1
PYHIN1	Q6K0P9	119	-2	-11	-12	12	1.0	0.9	0.9	1.1
TRIM25	Q14258	475	-8	-14	-12	12	0.9	0.9	0.9	1.1
LEMD3	Q9Y2U8	754	-5	-21	-12	12	1.0	0.8	0.9	1.1
SYNE1	Q8NF91	3027	-2	1	-12	12	1.0	1.0	0.9	1.1
KIAA0196	Q12768	385	-6	-7	-12	11	0.9	0.9	0.9	1.1
VCPIP1	Q96JH7	127	4	-8	-12	11	1.0	0.9	0.9	1.1
SRSF3	P84103	74	-7	-3	-12	11	0.9	1.0	0.9	1.1
GBP2	P32456	233	-9	-5	-12	11	0.9	1.0	0.9	1.1
NECAP2	Q9NVZ3	133	-5	-5	-12	11	1.0	1.0	0.9	1.1
FOXK1	P85037	439	-4	-7	-12	11	1.0	0.9	0.9	1.1
NQO2	P16083	223	-10	-9	-12	11	0.9	0.9	0.9	1.1
BRAF	P15056	748	1	-11	-12	11	1.0	0.9	0.9	1.1
UTP20	O75691	616	3	-1	-12	10	1.0	1.0	0.9	1.1
PPP6R3	Q5H9R7	172	-3	-7	-12	10	1.0	0.9	0.9	1.1
PELP1	Q8IZL8	237	-5	-9	-12	10	1.0	0.9	0.9	1.1

KIAA0430	Q9Y4F3	654	-13	-10	-12	10	0.9	0.9	0.9	1.1
STIP1	P31948	420	7	16	-12	10	1.1	1.2	0.9	1.1
MAP7D3	Q8IWC1	264	-9	-2	-12	10	0.9	1.0	0.9	1.1
EARS2	Q5JPH6	386	3	-3	-12	10	1.0	1.0	0.9	1.1
DEPDC5	O75140	498	1	-7	-12	10	1.0	0.9	0.9	1.1
SH3TC1	Q8TE82	941	-13	-8	-12	10	0.9	0.9	0.9	1.1
CARS2	Q9HA77	387	-5	-10	-12	10	1.0	0.9	0.9	1.1
NUP205	Q92621	925	-4	-12	-12	10	1.0	0.9	0.9	1.1
DYNC1H1	Q14204	1977	1	-13	-12	10	1.0	0.9	0.9	1.1
WDR82	Q6UXN9	195	15	11	-12	9	1.2	1.1	0.9	1.1
PITPNM1	O00562	259	-10	-1	-12	9	0.9	1.0	0.9	1.1
TRANK1	O15050	882	1	-4	-12	9	1.0	1.0	0.9	1.1
MCM5	P33992	207	-5	-5	-12	9	1.0	1.0	0.9	1.1
GIMAP2	Q9UG22	227	-7	-6	-12	9	0.9	0.9	0.9	1.1
HCFC1	P51610	227	-12	-7	-12	9	0.9	0.9	0.9	1.1
NMRK1	Q9NWW6	72	-7	-15	-12	9	0.9	0.9	0.9	1.1
SEC23IP	Q9Y6Y8	814	-4	-19	-12	9	1.0	0.8	0.9	1.1
CHEK2	O96017	539	-13	-20	-12	9	0.9	0.8	0.9	1.1
BACH2	Q9BYV9	238	-12	-23	-12	9	0.9	0.8	0.9	1.1
DDX24	Q9GZR7	620	1	-5	-12	9	1.0	1.0	0.9	1.1
NCBP1	Q09161	477	-7	-8	-12	9	0.9	0.9	0.9	1.1
LCP1	P13796	140	-8	-9	-12	9	0.9	0.9	0.9	1.1
PLS3	P13797	143	-8	-9	-12	9	0.9	0.9	0.9	1.1
RIC1	Q4ADV7	434	-11	-11	-12	9	0.9	0.9	0.9	1.1
SRP54	P61011	118	2	-3	-12	8	1.0	1.0	0.9	1.1
METTL13	Q8N6R0	273	5	-6	-12	8	1.1	0.9	0.9	1.1
PKM	P14618	326	1	-11	-12	8	1.0	0.9	0.9	1.1
GOLGA3	Q08378	769	-3	-11	-12	8	1.0	0.9	0.9	1.1
RIN2	Q8WYP3	802	-8	-11	-12	8	0.9	0.9	0.9	1.1
ERAP1	Q9NZ08	193	-9	-12	-12	8	0.9	0.9	0.9	1.1
MFHAS1	Q9Y4C4	1040	-11	1	-12	8	0.9	1.0	0.9	1.1
PLRG1	O43660	264	-4	-8	-12	8	1.0	0.9	0.9	1.1
RRBP1	Q9P2E9	1323	-6	-9	-12	8	0.9	0.9	0.9	1.1
ADAP2	Q9NPF8	282	-9	-10	-12	8	0.9	0.9	0.9	1.1
NUDT4	Q9NZJ9	131	1	-10	-12	8	1.0	0.9	0.9	1.1
USP4	Q13107	624	-8	-12	-12	8	0.9	0.9	0.9	1.1
CORO1A	P31146	332	-4	-15	-12	8	1.0	0.9	0.9	1.1
PCF11	O94913	1534	-6	-2	-12	7	0.9	1.0	0.9	1.1
MCM3AP	O60318	1269	-6	-6	-12	7	0.9	0.9	0.9	1.1
PDCD11	Q14690	1154	-6	-10	-12	7	0.9	0.9	0.9	1.1
CDKN2AIP	Q9NXV6	178	-10	-11	-12	7	0.9	0.9	0.9	1.1
SIPA1	Q96FS4	307	0	-12	-12	7	1.0	0.9	0.9	1.1
PFKM	P08237	709	-1	3	-12	7	1.0	1.0	0.9	1.1
ILKAP	Q9H0C8	312	-2	-2	-12	7	1.0	1.0	0.9	1.1
TCEA1	P23193	212	-8	-7	-12	7	0.9	0.9	0.9	1.1
GNL1	P36915	412	-14	-8	-12	7	0.9	0.9	0.9	1.1
NEK9	Q8TD19	623	-9	-10	-12	7	0.9	0.9	0.9	1.1
ADAR	P55265	392	-9	-17	-12	7	0.9	0.9	0.9	1.1
PRKDC	P78527	4061	-7	-18	-12	7	0.9	0.9	0.9	1.1
SMYD5	Q6GMV2	123	1	1	-12	6	1.0	1.0	0.9	1.1
MED13	Q9UHV7	681	-11	-4	-12	6	0.9	1.0	0.9	1.1
PHF3	Q92576	1616	-2	-7	-12	6	1.0	0.9	0.9	1.1
PGP	A6NDG6	297	-14	-8	-12	6	0.9	0.9	0.9	1.1
RABL6	H0Y4Z8	172	-15	1	-12	6	0.9	1.0	0.9	1.1
FCHSD1	Q86WN1	88	-14	-3	-12	6	0.9	1.0	0.9	1.1

PCBP1	Q15365	54	-5	-6	-12	6	1.0	0.9	0.9	1.1
MAST3	O60307	541	-2	-8	-12	6	1.0	0.9	0.9	1.1
UGP2	Q16851	276	-10	-8	-12	6	0.9	0.9	0.9	1.1
DTWD1	Q8N5C7	293	-20	-20	-12	6	0.8	0.8	0.9	1.1
CAND1	Q86VP6	1007	-5	-4	-12	5	1.0	1.0	0.9	1.1
PCBP2	Q15366	54	-5	-6	-12	5	1.0	0.9	0.9	1.1
NUP214	P35658	186	-1	-8	-12	5	1.0	0.9	0.9	1.1
CNP	P09543	158	-6	-11	-12	5	0.9	0.9	0.9	1.1
KIAA1551	Q9HCM1	650	-8	-4	-12	5	0.9	1.0	0.9	1.0
RNGTT	O60942	375	-2	-7	-12	5	1.0	0.9	0.9	1.0
OSBPL11	Q9BXB4	34	-12	-7	-12	5	0.9	0.9	0.9	1.0
DCUN1D1	Q96GG9	115	-5	-8	-12	5	1.0	0.9	0.9	1.0
SAFB2	Q14151	361	-6	-11	-12	5	0.9	0.9	0.9	1.0
SAFB	Q15424	362	-6	-11	-12	5	0.9	0.9	0.9	1.0
CSTF2T	Q9H0L4	150	-7	-12	-12	5	0.9	0.9	0.9	1.0
ACTR3	P61158	408	-10	-13	-12	5	0.9	0.9	0.9	1.0
PIK3C2A	O00443	669	3	-15	-12	5	1.0	0.9	0.9	1.0
SPTBN1	Q01082	604	-4	-11	-12	4	1.0	0.9	0.9	1.0
UBR2	Q8IWV8	1360	-9	-12	-12	4	0.9	0.9	0.9	1.0
XPO7	Q9UIA9	43	-8	-15	-12	4	0.9	0.9	0.9	1.0
ZC3HAV1	Q7Z2W4	645	-2	-8	-12	4	1.0	0.9	0.9	1.0
DNAJC11	Q9NVH1	494	-4	-11	-12	4	1.0	0.9	0.9	1.0
IPO4	Q8TEX9	732	11	-13	-12	4	1.1	0.9	0.9	1.0
SZT2	Q5T011	1948	4	-14	-12	4	1.0	0.9	0.9	1.0
DCXR	Q7Z4W1	51	-12	-18	-12	4	0.9	0.9	0.9	1.0
NSDHL	Q15738	86	-2	-3	-12	3	1.0	1.0	0.9	1.0
COPA	P53621	514	-1	-9	-12	3	1.0	0.9	0.9	1.0
GOLGA4	Q13439	1862	-1	-14	-12	3	1.0	0.9	0.9	1.0
AKAP9	Q99996	2022	4	-19	-12	3	1.0	0.8	0.9	1.0
AKAP13	Q12802	653	-7	-9	-12	2	0.9	0.9	0.9	1.0
PGD	P52209	171	-8	-12	-12	2	0.9	0.9	0.9	1.0
GNAI2	P04899	326	-12	-24	-12	2	0.9	0.8	0.9	1.0
GNAI3	P08754	325	-12	-24	-12	2	0.9	0.8	0.9	1.0
WHSC1L1	Q9BZ95	173	-7	-26	-12	2	0.9	0.8	0.9	1.0
CDC40	O60508	378	-12	-15	-12	2	0.9	0.9	0.9	1.0
CNTRL	Q7Z7A1	1781	-6	-23	-12	2	0.9	0.8	0.9	1.0
SAMD9	Q5K651	975	8	-24	-12	2	1.1	0.8	0.9	1.0
RELA	Q04206	206	-4	-4	-12	1	1.0	1.0	0.9	1.0
GABPA	Q06546	421	-9	-8	-12	1	0.9	0.9	0.9	1.0
ACTR3	P61158	12	1	-8	-12	1	1.0	0.9	0.9	1.0
IL16	Q14005	1016	-7	-11	-12	1	0.9	0.9	0.9	1.0
WDFY4	Q6ZS81	2218	-7	-15	-12	1	0.9	0.9	0.9	1.0
TRMT1L	Q7Z2T5	419	-8	-16	-12	1	0.9	0.9	0.9	1.0
SEC24C	P53992	875	-10	2	-12	1	0.9	1.0	0.9	1.0
DICER1	Q9UPY3	251	-6	-6	-12	1	0.9	0.9	0.9	1.0
MBD4	O95243	88	1	-12	-12	1	1.0	0.9	0.9	1.0
PLCB2	Q00722	1001	-7	-13	-12	1	0.9	0.9	0.9	1.0
DBR1	Q9UK59	8	-4	-6	-12	0	1.0	0.9	0.9	1.0
DDX3X	O00571	128	-16	-9	-12	0	0.9	0.9	0.9	1.0
RNF213	Q63HN8	1748	-3	-13	-12	0	1.0	0.9	0.9	1.0
POLR2B	P30876	1093	-8	-6	-12	-1	0.9	0.9	0.9	1.0
BIRC6	Q9NR09	2892	-5	-11	-12	-1	1.0	0.9	0.9	1.0
VPS13B	Q7Z7G8	2209	-1	-14	-12	-1	1.0	0.9	0.9	1.0
PRPF6	O94906	429	-17	-26	-12	-1	0.9	0.8	0.9	1.0
NBEAL1	Q6ZS30	2129	-5	2	-12	-1	1.0	1.0	0.9	1.0

NEK7	Q8TDX7	247	-1	-5	-12	-1	1.0	1.0	0.9	1.0
BCCIP	Q9P287	141	3	-10	-12	-1	1.0	0.9	0.9	1.0
GPD2	P43304	45	-10	-12	-12	-1	0.9	0.9	0.9	1.0
IP6K1	Q92551	248	-11	-12	-12	-1	0.9	0.9	0.9	1.0
RNH1	P13489	248	14	-26	-12	-1	1.2	0.8	0.9	1.0
BRE	Q9NXR7	44	-7	-3	-12	-2	0.9	1.0	0.9	1.0
DDX21	Q9NR30	291	-4	-4	-12	-2	1.0	1.0	0.9	1.0
VCP	P55072	572	-8	-10	-12	-2	0.9	0.9	0.9	1.0
BLOC1S4	Q9NUP1	210	-20	-14	-12	-2	0.8	0.9	0.9	1.0
FRG1	Q14331	159	1	-4	-12	-2	1.0	1.0	0.9	1.0
PAICS	P22234	295	-10	-15	-12	-2	0.9	0.9	0.9	1.0
LSS	P48449	609	-3	-17	-12	-2	1.0	0.9	0.9	1.0
DYNLL2	Q96FJ2	56	-5	-10	-12	-3	1.0	0.9	0.9	1.0
FRMD8	Q9BZ67	246	-11	-11	-12	-3	0.9	0.9	0.9	1.0
VDAC3	Q9Y277	229	-1	-13	-12	-3	1.0	0.9	0.9	1.0
TCEAL1	Q15170	88	-8	-14	-12	-3	0.9	0.9	0.9	1.0
ESD	P10768	181	-15	-17	-12	-4	0.9	0.9	0.9	1.0
TDRD7	Q8NHU6	662	-10	-24	-12	-4	0.9	0.8	0.9	1.0
DDB1	Q16531	378	-6	-11	-12	-4	0.9	0.9	0.9	1.0
UBASH3A	P57075	607	-10	-14	-12	-4	0.9	0.9	0.9	1.0
FLNA	P21333	1912	9	2	-12	-5	1.1	1.0	0.9	1.0
BPTF	Q12830	1501	-19	-5	-12	-5	0.8	1.0	0.9	1.0
PDS5A	Q29RF7	742	-6	-6	-12	-5	0.9	0.9	0.9	1.0
MRPS25	P82663	139	-9	-10	-12	-5	0.9	0.9	0.9	1.0
MTMR10	Q9NXD2	564	-7	-13	-12	-5	0.9	0.9	0.9	1.0
NAPG	Q99747	193	-12	-17	-12	-5	0.9	0.9	0.9	1.0
CTBP1	Q13363	134	-3	-21	-12	-6	1.0	0.8	0.9	0.9
TATDN1	Q6P1N9	253	-4	-24	-12	-6	1.0	0.8	0.9	0.9
VDAC3	Q9Y277	36	-3	-17	-12	-6	1.0	0.9	0.9	0.9
CHUK	O15111	658	-9	-19	-12	-6	0.9	0.8	0.9	0.9
AP3B1	O00203	215	5	-25	-12	-7	1.1	0.8	0.9	0.9
PYGB	P11216	437	4	-8	-12	-7	1.0	0.9	0.9	0.9
DMAP1	Q9NPF5	381	-18	-15	-12	-7	0.8	0.9	0.9	0.9
AP5M1	Q9H0R1	18	-18	-21	-12	-8	0.8	0.8	0.9	0.9
DTX3L	Q8TDB6	309	-8	-1	-12	-8	0.9	1.0	0.9	0.9
PTPN7	P35236	228	-11	-8	-12	-8	0.9	0.9	0.9	0.9
ICAM3	P32942	423	-10	-12	-12	-8	0.9	0.9	0.9	0.9
RING1	Q06587	87	-6	-3	-12	-9	0.9	1.0	0.9	0.9
RNF2	Q99496	90	-6	-3	-12	-9	0.9	1.0	0.9	0.9
EPRS	P07814	1309	-3	-9	-12	-9	1.0	0.9	0.9	0.9
NFKB1	P19838	118	11	-5	-12	-9	1.1	1.0	0.9	0.9
STRN4	Q9NRL3	337	-8	-16	-12	-9	0.9	0.9	0.9	0.9
HECTD3	Q5T447	112	-3	-16	-12	-9	1.0	0.9	0.9	0.9
TAOK3	Q9H2K8	795	1	-17	-12	-9	1.0	0.9	0.9	0.9
TAZ	Q16635	122	-4	-10	-12	-10	1.0	0.9	0.9	0.9
VPS39	Q96JC1	844	-7	-4	-12	-11	0.9	1.0	0.9	0.9
PFKFB3	Q16875	102	-2	-12	-12	-11	1.0	0.9	0.9	0.9
PFKFB4	Q16877	106	-2	-12	-12	-11	1.0	0.9	0.9	0.9
L2HGDH	Q9H9P8	376	-14	-12	-12	-11	0.9	0.9	0.9	0.9
NLN	Q9BYT8	153	2	-5	-12	-12	1.0	1.0	0.9	0.9
SMC1A	Q14683	1115	-3	-12	-12	-12	1.0	0.9	0.9	0.9
PTK2B	Q14289	463	-8	-14	-12	-12	0.9	0.9	0.9	0.9
OAS3	Q9Y6K5	449	-7	-16	-12	-12	0.9	0.9	0.9	0.9
PIK3R6	Q5UE93	446	-10	-8	-12	-13	0.9	0.9	0.9	0.9
PSMC5	P62195	112	-7	-7	-12	-13	0.9	0.9	0.9	0.9

KPNB1	Q14974	543	7	-9	-12	-13	1.1	0.9	0.9	0.9
CHUK	O15111	59	4	-16	-12	-13	1.0	0.9	0.9	0.9
GART	P22102	1005	-11	-18	-12	-13	0.9	0.8	0.9	0.9
MKNK1	Q9BUB5	101	1	-3	-12	-14	1.0	1.0	0.9	0.9
FAM76B	Q5HYJ3	90	-16	-5	-12	-14	0.9	1.0	0.9	0.9
TSC1	Q92574	822	3	-8	-12	-14	1.0	0.9	0.9	0.9
AHCYL1	O43865	317	-1	-14	-12	-15	1.0	0.9	0.9	0.9
U2AF1	Q01081	163	-6	-9	-12	-17	0.9	0.9	0.9	0.9
TRMT2A	Q8IZ69	260	-22	-30	-12	-18	0.8	0.8	0.9	0.9
WDR44	Q5JSH3	701	-6	-15	-12	-18	0.9	0.9	0.9	0.8
AP2B1	P63010	380	4	-19	-12	-18	1.0	0.8	0.9	0.8
AP1B1	Q10567	380	4	-19	-12	-18	1.0	0.8	0.9	0.8
IPO5	O00410	229	-9	-8	-12	-19	0.9	0.9	0.9	0.8
SKAP2	O75563	185	0	-12	-12	-19	1.0	0.9	0.9	0.8
PKM	P14618	49	-12	-11	-12	-20	0.9	0.9	0.9	0.8
PJA1	Q8NG27	241	9	-31	-12	-20	1.1	0.8	0.9	0.8
CDC37L1	Q7L3B6	233	-14	10	-12	-21	0.9	1.1	0.9	0.8
ALAD	P13716	124	-20	-21	-12	-22	0.8	0.8	0.9	0.8
PAIP1	Q9H074	358	-11	-22	-12	-22	0.9	0.8	0.9	0.8
GIMAP1-GIMA	A0A087WTJ2	375	11	-11	-12	-25	1.1	0.9	0.9	0.8
ACTN4	O43707	173	-3	-11	-12	-26	1.0	0.9	0.9	0.8
ACTN1	P12814	154	-3	-11	-12	-26	1.0	0.9	0.9	0.8
TBXAS1	P24557	183	-1	-9	-12	-26	1.0	0.9	0.9	0.8
LIMD2	Q9BT23	64	-9	-36	-12	-27	0.9	0.7	0.9	0.8
SMAD2	Q15796	149	-1	-15	-12	-31	1.0	0.9	0.9	0.8
ACBD3	Q9H3P7	487	1	-8	-12	-37	1.0	0.9	0.9	0.7
NLRC5	Q86WI3	984	-3	-20	-12	-37	1.0	0.8	0.9	0.7
ARFGEF2	Q9Y6D5	1450	-10	-18	-12	-40	0.9	0.8	0.9	0.7
GAPVD1	Q14C86	1129	-8	-24	-12	-42	0.9	0.8	0.9	0.7
PHF6	Q8IWS0	212	18	-12	-12	-45	1.2	0.9	0.9	0.7
PROS1	P07225	88	14	-6	-12	-59	1.2	0.9	0.9	0.6
ACTN1	P12814	180	-5	-7	-12	-67	1.0	0.9	0.9	0.6
C1orf198	Q9H425	73	-3	-5	-12	-69	1.0	1.0	0.9	0.6
LIMS1	P48059	300	-3	-8	-12	-72	1.0	0.9	0.9	0.6
LIMS2	Q7Z4I7	305	-3	-8	-12	-72	1.0	0.9	0.9	0.6
PDLIM7	Q9NR12	388	4	-10	-12	-84	1.0	0.9	0.9	0.5
NDUFA10	E7ESZ7	112	4	-46	-12	-134	1.0	0.7	0.9	0.4
RAB27B	O00194	188	5	-13	-12	-165	1.0	0.9	0.9	0.4
AKR1A1	P14550	200	-3	3	-13	41	1.0	1.0	0.9	1.7
OGT	O15294	620	-4	-8	-13	37	1.0	0.9	0.9	1.6
THOP1	P52888	682	-12	-12	-13	36	0.9	0.9	0.9	1.6
MADD	Q8WXG6	1489	-10	-12	-13	35	0.9	0.9	0.9	1.5
ANKRD11	Q6UB99	646	-12	-7	-13	33	0.9	0.9	0.9	1.5
MCMBP	Q9BTE3	325	-6	-15	-13	32	0.9	0.9	0.9	1.5
SGK3	Q96BR1	308	-6	-17	-13	31	0.9	0.9	0.9	1.4
PGPEP1	Q9NXJ5	206	-11	-9	-13	31	0.9	0.9	0.9	1.4
DHX9	Q08211	415	1	-6	-13	29	1.0	0.9	0.9	1.4
ZNF746	Q6NUN9	155	6	-12	-13	29	1.1	0.9	0.9	1.4
CELF1	Q92879	150	0	-7	-13	28	1.0	0.9	0.9	1.4
FAM65A	Q6ZS17	896	-6	4	-13	27	0.9	1.0	0.9	1.4
MED12	Q93074	997	-8	-5	-13	26	0.9	1.0	0.9	1.4
RTTN	Q86VV8	753	-3	-3	-13	26	1.0	1.0	0.9	1.3
TRIM65	Q6PJ69	320	-9	-11	-13	25	0.9	0.9	0.9	1.3
MICAL1	Q8TDZ2	837	-12	-3	-13	24	0.9	1.0	0.9	1.3
EIF3CL	B5ME19	444	-7	-10	-13	24	0.9	0.9	0.9	1.3

S100A10	P60903	62	-5	-12	-13	24	1.0	0.9	0.9	1.3
NOTCH2	Q04721	2085	5	-4	-13	23	1.0	1.0	0.9	1.3
DDHD2	O94830	207	-12	-6	-13	23	0.9	0.9	0.9	1.3
INPP5D	Q92835	902	5	-6	-13	23	1.1	0.9	0.9	1.3
ATM	Q13315	536	-14	-1	-13	22	0.9	1.0	0.9	1.3
GTF3C1	Q12789	286	-1	-11	-13	22	1.0	0.9	0.9	1.3
PML	P29590	213	-8	-16	-13	22	0.9	0.9	0.9	1.3
NCOR1	O75376	2403	22	-7	-13	22	1.3	0.9	0.9	1.3
NARF	Q9UHQ1	99	-3	-22	-13	22	1.0	0.8	0.9	1.3
SIGIRR	Q6IA17	174	3	-8	-13	21	1.0	0.9	0.9	1.3
SH3RF1	Q7Z6J0	658	5	12	-13	21	1.0	1.1	0.9	1.3
NUP153	P49790	1129	-11	-5	-13	21	0.9	1.0	0.9	1.3
AVEN	Q9NQS1	193	-7	-10	-13	21	0.9	0.9	0.9	1.3
RXRB	P28702	30	-2	-6	-13	20	1.0	0.9	0.9	1.3
CARMIL2	Q6F5E8	746	-9	-13	-13	20	0.9	0.9	0.9	1.2
PLCB2	Q00722	1169	-3	-9	-13	19	1.0	0.9	0.9	1.2
CAPN2	P17655	240	2	-16	-13	19	1.0	0.9	0.9	1.2
MALT1	Q9UDY8	71	-6	-9	-13	19	0.9	0.9	0.9	1.2
LDHB	P07195	294	2	-14	-13	19	1.0	0.9	0.9	1.2
NUP155	O75694	874	2	-19	-13	19	1.0	0.8	0.9	1.2
DGCR14	Q96DF8	263	-13	-4	-13	18	0.9	1.0	0.9	1.2
SMCR8	Q8TEV9	350	1	-12	-13	18	1.0	0.9	0.9	1.2
MAPK10	P53779	283	3	-1	-13	17	1.0	1.0	0.9	1.2
RXRB	P28702	19	-9	-5	-13	17	0.9	1.0	0.9	1.2
ZNF445	P59923	215	-1	-11	-13	17	1.0	0.9	0.9	1.2
COG1	Q8WTW3	513	-8	-13	-13	17	0.9	0.9	0.9	1.2
MAT2B	Q9NZL9	17	-2	-24	-13	17	1.0	0.8	0.9	1.2
DGKE	P52429	420	4	-5	-13	16	1.0	1.0	0.9	1.2
HECTD1	Q9ULT8	254	-7	-8	-13	16	0.9	0.9	0.9	1.2
EPRS	P07814	337	1	-9	-13	16	1.0	0.9	0.9	1.2
SPATA5	Q8NB90	81	0	0	-13	16	1.0	1.0	0.9	1.2
RAD17	O75943	141	2	-6	-13	15	1.0	0.9	0.9	1.2
RLF	Q13129	78	5	-8	-13	15	1.0	0.9	0.9	1.2
USP16	Q9Y5T5	191	-9	-12	-13	15	0.9	0.9	0.9	1.2
BRPF1	P55201	515	16	-15	-13	15	1.2	0.9	0.9	1.2
SOS2	Q07890	1255	-6	13	-13	15	0.9	1.1	0.9	1.2
UPF2	Q9HAU5	944	-8	-15	-13	15	0.9	0.9	0.9	1.2
UBASH3A	P57075	371	8	-5	-13	14	1.1	1.0	0.9	1.2
DENND4C	Q5VZ89	850	-6	-13	-13	14	0.9	0.9	0.9	1.2
TMCC3	Q9ULS5	180	-1	-13	-13	14	1.0	0.9	0.9	1.2
TCAF1	Q9Y4C2	488	2	-14	-13	14	1.0	0.9	0.9	1.2
IRAK1	P51617	498	-5	-3	-13	14	1.0	1.0	0.9	1.2
CARMIL2	Q6F5E8	562	-6	-6	-13	14	0.9	0.9	0.9	1.2
FNBP4	Q8N3X1	673	-10	-7	-13	14	0.9	0.9	0.9	1.2
RBL2	Q08999	368	-13	-8	-13	14	0.9	0.9	0.9	1.2
HUWE1	Q7Z6Z7	1832	-7	-9	-13	14	0.9	0.9	0.9	1.2
KANSL1	A0A0G2JNT7	84	-3	-10	-13	14	1.0	0.9	0.9	1.2
CTSC	P53634	448	-3	-15	-13	14	1.0	0.9	0.9	1.2
SERPINB10	P48595	68	-7	-1	-13	13	0.9	1.0	0.9	1.1
ADCK4	Q96D53	147	-9	-3	-13	13	0.9	1.0	0.9	1.1
TRIM14	Q14142	20	-6	-7	-13	13	0.9	0.9	0.9	1.1
MYO5A	Q9Y4I1	535	-3	-2	-13	13	1.0	1.0	0.9	1.1
MDH2	P40926	212	-6	-8	-13	13	0.9	0.9	0.9	1.1
PCYT2	Q99447	180	-11	-9	-13	13	0.9	0.9	0.9	1.1
NCOA2	Q15596	22	-3	-11	-13	13	1.0	0.9	0.9	1.1

BCLAF1	Q9NYF8	688	-5	-4	-13	12	1.0	1.0	0.9	1.1
MED15	Q96RN5	618	-8	-7	-13	12	0.9	0.9	0.9	1.1
CYFIP2	Q96F07	427	-1	-8	-13	12	1.0	0.9	0.9	1.1
CAST	P20810	381	-9	-11	-13	12	0.9	0.9	0.9	1.1
MAPKAPK3	Q16644	379	-4	-13	-13	12	1.0	0.9	0.9	1.1
SARM1	Q6SZW1	311	-12	-14	-13	12	0.9	0.9	0.9	1.1
RNF146	Q9NTX7	57	0	-4	-13	12	1.0	1.0	0.9	1.1
DECR2	Q9NUI1	22	-14	-6	-13	12	0.9	0.9	0.9	1.1
PRR12	Q9ULL5	307	-7	-7	-13	12	0.9	0.9	0.9	1.1
TMEM189-UB	I3L0A0	367	-1	-8	-13	12	1.0	0.9	0.9	1.1
BAG4	O95429	193	1	-9	-13	12	1.0	0.9	0.9	1.1
RNF213	Q63HN8	4258	0	-12	-13	12	1.0	0.9	0.9	1.1
CHD3	Q12873	405	-7	-15	-13	12	0.9	0.9	0.9	1.1
RAF1	P04049	95	-8	-15	-13	12	0.9	0.9	0.9	1.1
C6orf106	Q9H6K1	18	-8	-5	-13	11	0.9	1.0	0.9	1.1
XPO1	O14980	528	2	-6	-13	11	1.0	0.9	0.9	1.1
HNRNPH1	P31943	34	-1	-7	-13	11	1.0	0.9	0.9	1.1
HNRNPH2	P55795	34	-1	-7	-13	11	1.0	0.9	0.9	1.1
UBQLN4	Q9NRR5	29	-5	-8	-13	11	1.0	0.9	0.9	1.1
SEC61B	P60468	39	-6	-10	-13	11	0.9	0.9	0.9	1.1
VPS11	Q9H270	317	2	-10	-13	11	1.0	0.9	0.9	1.1
IFT27	Q9BW83	166	-5	-11	-13	11	1.0	0.9	0.9	1.1
FNBP4	Q8N3X1	347	2	-11	-13	11	1.0	0.9	0.9	1.1
RBL2	Q08999	1118	-8	-12	-13	11	0.9	0.9	0.9	1.1
UIMC1	Q96RL1	121	-8	-12	-13	11	0.9	0.9	0.9	1.1
BTRC	Q9Y297	212	-2	-12	-13	11	1.0	0.9	0.9	1.1
SENP7	Q9BQF6	934	-2	-20	-13	11	1.0	0.8	0.9	1.1
C5orf22	Q49AR2	215	2	8	-13	11	1.0	1.1	0.9	1.1
UPF3A	Q9H1J1	420	-12	-4	-13	11	0.9	1.0	0.9	1.1
ABHD12	Q8N2K0	52	-9	-6	-13	11	0.9	0.9	0.9	1.1
ARCN1	P48444	441	-7	-6	-13	11	0.9	0.9	0.9	1.1
FAM105A	Q9NUU6	177	-8	-10	-13	11	0.9	0.9	0.9	1.1
MAPK6	Q16659	42	-14	-15	-13	11	0.9	0.9	0.9	1.1
CARD6	Q9BX69	185	-8	-16	-13	11	0.9	0.9	0.9	1.1
CBFA2T3	O75081	319	-7	-18	-13	11	0.9	0.8	0.9	1.1
RPS6KA3	P51812	579	0	-10	-13	10	1.0	0.9	0.9	1.1
RPS6KA1	Q15418	575	0	-10	-13	10	1.0	0.9	0.9	1.1
WIPF1	O43516	446	-6	-5	-13	10	0.9	1.0	0.9	1.1
TMEM63A	O94886	791	-2	-5	-13	10	1.0	1.0	0.9	1.1
COQ3	Q9NZJ6	358	-2	-8	-13	10	1.0	0.9	0.9	1.1
MAPKAPK2	P49137	224	-2	-9	-13	10	1.0	0.9	0.9	1.1
TRDMT1	O14717	287	-6	-16	-13	10	0.9	0.9	0.9	1.1
SCAF11	Q99590	566	-11	-16	-13	10	0.9	0.9	0.9	1.1
PRF1	P14222	377	-8	-22	-13	10	0.9	0.8	0.9	1.1
UVRAG	Q9P2Y5	186	-7	-4	-13	9	0.9	1.0	0.9	1.1
COPA	P53621	580	-4	-7	-13	9	1.0	0.9	0.9	1.1
SMYD5	Q6GMV2	194	-3	-8	-13	9	1.0	0.9	0.9	1.1
TANGO6	Q9C0B7	15	-4	-12	-13	9	1.0	0.9	0.9	1.1
HDAC2	Q92769	274	2	-13	-13	9	1.0	0.9	0.9	1.1
CDK2	P24941	177	-8	-13	-13	9	0.9	0.9	0.9	1.1
UCHL3	P15374	95	7	-6	-13	9	1.1	0.9	0.9	1.1
SYNE1	Q8NF91	6415	-14	-13	-13	9	0.9	0.9	0.9	1.1
RALGAPB	Q86X10	327	-8	-15	-13	9	0.9	0.9	0.9	1.1
CPSF1	Q10570	1020	-2	-2	-13	8	1.0	1.0	0.9	1.1
DDX19A	Q9NUU7	392	1	-9	-13	8	1.0	0.9	0.9	1.1

DDX19B	Q9UMR2	393	1	-9	-13	8	1.0	0.9	0.9	1.1
PNPLA8	Q9NP80	714	-9	-7	-13	8	0.9	0.9	0.9	1.1
SEC63	Q9UGP8	295	-9	-11	-13	8	0.9	0.9	0.9	1.1
ALDOA	P04075	290	-15	-14	-13	8	0.9	0.9	0.9	1.1
KMT2C	Q8NEZ4	4643	-3	-15	-13	8	1.0	0.9	0.9	1.1
SP100	P23497	96	-12	-8	-13	7	0.9	0.9	0.9	1.1
CDKAL1	Q5VV42	138	0	-9	-13	7	1.0	0.9	0.9	1.1
ZMYM2	Q9UBW7	1268	-12	-18	-13	7	0.9	0.8	0.9	1.1
CORO1A	P31146	192	-6	-6	-13	7	0.9	0.9	0.9	1.1
MAPK9	P45984	163	-6	-7	-13	7	0.9	0.9	0.9	1.1
MAPK10	P53779	201	-6	-7	-13	7	0.9	0.9	0.9	1.1
MACF1	Q9UPN3	2175	-8	-10	-13	7	0.9	0.9	0.9	1.1
PSMA4	P25789	115	-10	-11	-13	7	0.9	0.9	0.9	1.1
POLD2	P49005	83	-6	-12	-13	7	0.9	0.9	0.9	1.1
LRSAM1	Q6UWE0	397	-6	-16	-13	7	0.9	0.9	0.9	1.1
DUSP23	Q9BVJ7	67	-3	-2	-13	6	1.0	1.0	0.9	1.1
DOCK8	Q8NF50	1471	-17	-13	-13	6	0.9	0.9	0.9	1.1
KDSR	Q06136	121	3	-5	-13	6	1.0	1.0	0.9	1.1
MMAA	Q8IVH4	184	-6	-9	-13	6	0.9	0.9	0.9	1.1
ROCK1	Q13464	1281	-3	-12	-13	6	1.0	0.9	0.9	1.1
TSR1	Q2NL82	126	-8	-12	-13	6	0.9	0.9	0.9	1.1
LIG3	P49916	261	-2	-13	-13	6	1.0	0.9	0.9	1.1
CARD8	Q9Y2G2	44	-24	-16	-13	6	0.8	0.9	0.9	1.1
RABEP1	Q15276	533	-8	-3	-13	5	0.9	1.0	0.9	1.1
RANBP1	P43487	132	-2	-12	-13	5	1.0	0.9	0.9	1.1
CRLF3	Q8IUI8	336	-1	-15	-13	5	1.0	0.9	0.9	1.1
EDC4	Q6P2E9	838	-7	-18	-13	5	0.9	0.9	0.9	1.1
SPTAN1	Q13813	956	-2	-23	-13	5	1.0	0.8	0.9	1.1
INO80	Q9ULG1	1011	-16	2	-13	5	0.9	1.0	0.9	1.0
NAT10	Q9H0A0	105	0	-9	-13	5	1.0	0.9	0.9	1.0
TMEM209	Q96SK2	301	-7	-11	-13	5	0.9	0.9	0.9	1.0
MACF1	Q9UPN3	3227	-2	-12	-13	5	1.0	0.9	0.9	1.0
ERLIN2	O94905	262	-4	-19	-13	5	1.0	0.8	0.9	1.0
FNDC3B	Q53EP0	558	2	2	-13	4	1.0	1.0	0.9	1.0
VARS	P26640	917	-3	-7	-13	4	1.0	0.9	0.9	1.0
NARFL	Q9H6Q4	300	4	-10	-13	4	1.0	0.9	0.9	1.0
MSL2	Q9HCI7	107	-4	-8	-13	3	1.0	0.9	0.9	1.0
HNRNPU	Q00839	594	-4	-9	-13	3	1.0	0.9	0.9	1.0
FAM118B	Q9BPY3	319	-8	-11	-13	3	0.9	0.9	0.9	1.0
ERCC5	P28715	1021	4	-21	-13	3	1.0	0.8	0.9	1.0
DIDO1	Q9BTC0	1196	-13	-24	-13	3	0.9	0.8	0.9	1.0
RTF1	Q92541	599	-9	-8	-13	3	0.9	0.9	0.9	1.0
SRRM2	Q9UQ35	1509	-16	-9	-13	3	0.9	0.9	0.9	1.0
TOP1	P11387	630	-1	-10	-13	3	1.0	0.9	0.9	1.0
UBN1	Q9NPG3	765	-8	-17	-13	3	0.9	0.9	0.9	1.0
GIT1	Q9Y2X7	122	-5	-5	-13	2	1.0	1.0	0.9	1.0
LACTB2	Q53H82	100	-2	-7	-13	2	1.0	0.9	0.9	1.0
Uncharacteriz	G3V3G9	427	-3	-9	-13	2	1.0	0.9	0.9	1.0
ELMSAN1	Q6PJG2	502	-15	-10	-13	2	0.9	0.9	0.9	1.0
ALDH3A2	P51648	220	-6	-12	-13	2	0.9	0.9	0.9	1.0
SMAP2	Q8WU79	51	-4	8	-13	2	1.0	1.1	0.9	1.0
TRMT61B	Q9BVS5	396	-5	-11	-13	2	1.0	0.9	0.9	1.0
AUP1	Q9Y679	391	-6	-22	-13	2	0.9	0.8	0.9	1.0
TNKS1BP1	Q9C0C2	136	-18	-7	-13	1	0.9	0.9	0.9	1.0
NPRL3	Q12980	141	4	-8	-13	1	1.0	0.9	0.9	1.0

TRIP13	Q15645	14	-8	-13	-13	1	0.9	0.9	0.9	1.0
BOD1L1	Q8NFC6	2438	-5	-17	-13	1	1.0	0.9	0.9	1.0
CD3EAP	O15446	150	-14	-19	-13	1	0.9	0.8	0.9	1.0
BIRC6	Q9NR09	777	-6	-20	-13	1	0.9	0.8	0.9	1.0
PHF5A	Q7RTV0	40	-5	-7	-13	1	1.0	0.9	0.9	1.0
HNMT	P50135	82	-4	-8	-13	1	1.0	0.9	0.9	1.0
MAPKAPK3	Q16644	61	-11	-15	-13	1	0.9	0.9	0.9	1.0
EFTUD2	Q15029	674	-9	-8	-13	0	0.9	0.9	0.9	1.0
INTS1	Q8N201	969	-5	-9	-13	0	1.0	0.9	0.9	1.0
COPG1	Q9Y678	446	-4	-9	-13	0	1.0	0.9	0.9	1.0
PRPF6	O94906	604	-9	-15	-13	0	0.9	0.9	0.9	1.0
CSE1L	P55060	85	-3	-16	-13	0	1.0	0.9	0.9	1.0
DNAJC7	Q99615	175	-8	-17	-13	0	0.9	0.9	0.9	1.0
FAM175A	Q6UWZ7	186	-5	-3	-13	-1	1.0	1.0	0.9	1.0
RAB1A	P62820	126	-12	-11	-13	-1	0.9	0.9	0.9	1.0
RUBCN	Q92622	749	-5	-12	-13	-1	1.0	0.9	0.9	1.0
CRACR2A	Q9BSW2	272	9	-14	-13	-1	1.1	0.9	0.9	1.0
HADHA	P40939	322	-7	-9	-13	-1	0.9	0.9	0.9	1.0
TAF9B	Q9HBM6	121	-7	-11	-13	-1	0.9	0.9	0.9	1.0
SYMPK	Q92797	686	4	-11	-13	-1	1.0	0.9	0.9	1.0
HECTD3	Q5T447	143	0	-14	-13	-1	1.0	0.9	0.9	1.0
TBCB	Q99426	216	4	-15	-13	-1	1.0	0.9	0.9	1.0
PHF3	Q92576	843	-15	-17	-13	-1	0.9	0.9	0.9	1.0
FASTKD1	Q53R41	70	-21	-17	-13	-1	0.8	0.9	0.9	1.0
LUC7L2	A0A0A6YYJ8	414	-13	-5	-13	-2	0.9	1.0	0.9	1.0
BAHD1	Q8TBE0	703	-2	-10	-13	-2	1.0	0.9	0.9	1.0
AHCTF1	Q8WYP5	693	2	-25	-13	-2	1.0	0.8	0.9	1.0
ATG5	Q9H1Y0	19	2	-4	-13	-2	1.0	1.0	0.9	1.0
GIMAP1-GIMA	A0A087WTJ2	66	-4	-8	-13	-2	1.0	0.9	0.9	1.0
GIMAP1	Q8WWP7	66	-4	-8	-13	-2	1.0	0.9	0.9	1.0
TMEM192	Q8IY95	266	-1	-15	-13	-2	1.0	0.9	0.9	1.0
DHX9	Q08211	469	-4	-16	-13	-2	1.0	0.9	0.9	1.0
LENG9	Q96B70	219	-17	-26	-13	-2	0.9	0.8	0.9	1.0
KTN1	Q86UP2	736	-8	-7	-13	-3	0.9	0.9	0.9	1.0
RPS11	P62280	60	-12	-8	-13	-3	0.9	0.9	0.9	1.0
OGDH	Q02218	331	4	-9	-13	-3	1.0	0.9	0.9	1.0
NEDD1	Q8NHV4	64	-6	-13	-13	-3	0.9	0.9	0.9	1.0
CCDC77	Q9BR77	164	-10	-14	-13	-3	0.9	0.9	0.9	1.0
TARS	P26639	656	-9	-15	-13	-3	0.9	0.9	0.9	1.0
DOCK7	Q96N67	193	-9	-15	-13	-3	0.9	0.9	0.9	1.0
SUN2	Q9UH99	601	-8	-17	-13	-3	0.9	0.9	0.9	1.0
POLR1A	O95602	185	-8	-17	-13	-3	0.9	0.9	0.9	1.0
UBA3	Q8TBC4	367	-19	-18	-13	-3	0.8	0.9	0.9	1.0
JARID2	Q92833	162	-19	-19	-13	-3	0.8	0.8	0.9	1.0
ACO2	Q99798	592	-6	-31	-13	-3	0.9	0.8	0.9	1.0
ATP11B	Q9Y2G3	605	-9	-7	-13	-4	0.9	0.9	0.9	1.0
PRPS1	P60891	165	-16	-7	-13	-4	0.9	0.9	0.9	1.0
MAST4	O15021	1512	-4	-8	-13	-4	1.0	0.9	0.9	1.0
ELL	P55199	121	1	-10	-13	-4	1.0	0.9	0.9	1.0
UHRF1BP1	Q6BDS2	173	4	-15	-13	-4	1.0	0.9	0.9	1.0
USP7	Q93009	702	1	-23	-13	-4	1.0	0.8	0.9	1.0
TBL2	Q9Y4P3	367	-5	-6	-13	-4	1.0	0.9	0.9	1.0
MYO18A	Q92614	811	-5	-14	-13	-4	1.0	0.9	0.9	1.0
TECPR2	O15040	282	-15	-24	-13	-4	0.9	0.8	0.9	1.0
SRP68	Q9UHB9	525	1	-2	-13	-5	1.0	1.0	0.9	1.0

MAP3K4	Q9Y6R4	1322	-8	-5	-13	-5	0.9	1.0	0.9	1.0
COL4A3BP	Q9Y5P4	185	-9	-7	-13	-5	0.9	0.9	0.9	1.0
TUBGCP5	A0A0G2JSA7	574	-22	-12	-13	-5	0.8	0.9	0.9	1.0
CCZ1B	P86790	65	-7	3	-13	-5	0.9	1.0	0.9	1.0
PRG2	P13727	220	-8	-13	-13	-5	0.9	0.9	0.9	1.0
FLNA	P21333	2543	-7	-15	-13	-5	0.9	0.9	0.9	1.0
MAF1	Q9H063	48	-5	-15	-13	-5	1.0	0.9	0.9	1.0
XPO5	Q9HAV4	419	-8	-10	-13	-6	0.9	0.9	0.9	0.9
UPF1	Q92900	531	-16	-21	-13	-6	0.9	0.8	0.9	0.9
ISG20L2	Q9H9L3	220	-17	-58	-13	-6	0.9	0.6	0.9	0.9
FLNA	P21333	2293	3	-8	-13	-6	1.0	0.9	0.9	0.9
CTNND1	O60716	618	-6	-9	-13	-6	0.9	0.9	0.9	0.9
AP2B1	P63010	95	-6	-2	-13	-7	0.9	1.0	0.9	0.9
AP1B1	Q10567	95	-6	-2	-13	-7	0.9	1.0	0.9	0.9
ZMYM3	Q14202	656	-8	-18	-13	-7	0.9	0.8	0.9	0.9
Uncharacteriz	G3V599	666	-7	-19	-13	-7	0.9	0.8	0.9	0.9
ATM	Q13315	755	-4	-13	-13	-7	1.0	0.9	0.9	0.9
NCF2	P19878	499	-11	-19	-13	-7	0.9	0.8	0.9	0.9
POLR2A	P24928	602	-15	-5	-13	-8	0.9	1.0	0.9	0.9
GRIPAP1	Q4V328	104	-2	-8	-13	-8	1.0	0.9	0.9	0.9
ATP13A1	Q9HD20	723	-2	-10	-13	-8	1.0	0.9	0.9	0.9
BTBD9	Q96Q07	318	11	-9	-13	-9	1.1	0.9	0.9	0.9
PRKDC	P78527	3781	-22	-24	-13	-9	0.8	0.8	0.9	0.9
SPTLC2	O15270	188	-12	-10	-13	-10	0.9	0.9	0.9	0.9
TBL2	Q9Y4P3	335	-29	-22	-13	-10	0.8	0.8	0.9	0.9
CAPNS1	P04632	144	-17	-6	-13	-11	0.9	0.9	0.9	0.9
RNF213	Q63HN8	1643	-3	-13	-13	-12	1.0	0.9	0.9	0.9
LRRC16A	Q5VZK9	790	2	-11	-13	-12	1.0	0.9	0.9	0.9
HSD17B4	P51659	189	-2	-14	-13	-12	1.0	0.9	0.9	0.9
USP7	Q93009	488	-9	-21	-13	-13	0.9	0.8	0.9	0.9
SCYL2	Q6P3W7	496	-1	-14	-13	-14	1.0	0.9	0.9	0.9
SPG11	Q96JI7	1866	-4	-30	-13	-15	1.0	0.8	0.9	0.9
DDX5	P17844	221	-16	-11	-13	-15	0.9	0.9	0.9	0.9
DDX17	Q92841	298	-16	-11	-13	-15	0.9	0.9	0.9	0.9
CHD8	Q9HCK8	2168	-6	-11	-13	-16	0.9	0.9	0.9	0.9
AP1G1	O43747	539	-19	-13	-13	-18	0.8	0.9	0.9	0.9
MGA	Q8IWI9	355	-12	-29	-13	-18	0.9	0.8	0.9	0.8
FRMD8	Q9BZ67	206	-1	-13	-13	-19	1.0	0.9	0.9	0.8
POLR2B	P30876	984	4	-5	-13	-21	1.0	1.0	0.9	0.8
CLK1	P49759	84	-4	-10	-13	-22	1.0	0.9	0.9	0.8
TBCD	Q9BTW9	773	-8	-4	-13	-23	0.9	1.0	0.9	0.8
TUBA4A	P68366	54	-5	-9	-13	-23	1.0	0.9	0.9	0.8
SNAP23	O00161	112	-7	-13	-13	-24	0.9	0.9	0.9	0.8
XRCC1	P18887	20	16	-8	-13	-28	1.2	0.9	0.9	0.8
KBTBD8	Q8NFY9	181	-8	-18	-13	-35	0.9	0.9	0.9	0.7
ILK	Q13418	346	-6	-7	-13	-44	0.9	0.9	0.9	0.7
WDR7	Q9Y4E6	1387	12	-4	-13	-67	1.1	1.0	0.9	0.6
SRC	P12931	248	-10	-14	-13	-108	0.9	0.9	0.9	0.5
GTF3C3	Q9Y5Q9	607	-6	-13	-13	41	0.9	0.9	0.9	1.7
DENND5A	Q6IQ26	39	-24	-20	-13	38	0.8	0.8	0.9	1.6
FAM129B	Q96TA1	466	-8	-5	-13	37	0.9	1.0	0.9	1.6
MTCH1	Q9NZJ7	385	-4	-12	-13	37	1.0	0.9	0.9	1.6
GGA2	Q9UJY4	429	-14	-11	-13	36	0.9	0.9	0.9	1.6
SS18L1	O75177	47	-5	-8	-13	34	1.0	0.9	0.9	1.5
QARS	P47897	358	-4	-11	-13	31	1.0	0.9	0.9	1.4

ZNF174	Q15697	232	-6	-24	-13	31	0.9	0.8	0.9	1.4
FDXR	P22570	353	-27	-18	-13	28	0.8	0.9	0.9	1.4
BRMS1L	Q5PSV4	275	3	1	-13	26	1.0	1.0	0.9	1.4
SLAMF7	Q9NQ25	275	-3	-7	-13	26	1.0	0.9	0.9	1.3
ZNF598	Q86UK7	33	-4	-2	-13	25	1.0	1.0	0.9	1.3
HECTD4	Q9Y4D8	1084	-9	-10	-13	25	0.9	0.9	0.9	1.3
SLFN5	Q08AF3	846	-13	-13	-13	25	0.9	0.9	0.9	1.3
EPS15	P42566	824	0	-16	-13	25	1.0	0.9	0.9	1.3
HSPA8	P11142	17	-13	-17	-13	25	0.9	0.9	0.9	1.3
RPS6KA1	Q15418	223	0	-11	-13	24	1.0	0.9	0.9	1.3
TSEN15	Q8WW01	13	-4	-11	-13	24	1.0	0.9	0.9	1.3
OAS3	Q9Y6K5	73	-5	-4	-13	23	1.0	1.0	0.9	1.3
FASTK	Q14296	212	-8	-13	-13	23	0.9	0.9	0.9	1.3
TOPBP1	Q92547	1099	-3	3	-13	23	1.0	1.0	0.9	1.3
ARMC5	Q96C12	894	-2	-15	-13	22	1.0	0.9	0.9	1.3
LTV1	Q96GA3	309	0	-31	-13	22	1.0	0.8	0.9	1.3
BCL11B	Q9C0K0	186	-11	-14	-13	21	0.9	0.9	0.9	1.3
NELFCD	Q8IXH7	169	-7	-11	-13	21	0.9	0.9	0.9	1.3
GSR	P00390	377	2	-17	-13	21	1.0	0.9	0.9	1.3
PHGDH	O43175	234	3	4	-13	20	1.0	1.0	0.9	1.3
SYNE2	Q8WXH0	568	-1	-8	-13	20	1.0	0.9	0.9	1.3
EIF2AK4	Q9P2K8	1255	-9	-16	-13	20	0.9	0.9	0.9	1.2
ATM	Q13315	74	4	-6	-13	19	1.0	0.9	0.9	1.2
PREX1	Q8TCU6	746	9	-6	-13	19	1.1	0.9	0.9	1.2
ITGB2	P05107	615	1	-30	-13	19	1.0	0.8	0.9	1.2
CTC1	Q2NKJ3	908	0	-3	-13	19	1.0	1.0	0.9	1.2
NLRC5	Q86WI3	1113	5	-33	-13	19	1.1	0.8	0.9	1.2
TRAPPC8	Q9Y2L5	978	-5	-5	-13	18	1.0	1.0	0.9	1.2
VPRBP	Q9Y4B6	784	1	-6	-13	18	1.0	0.9	0.9	1.2
DOCK8	Q8NF50	2091	-3	-10	-13	18	1.0	0.9	0.9	1.2
TMCO6	Q96DC7	14	-2	1	-13	17	1.0	1.0	0.9	1.2
DOCK11	Q5JSL3	160	-2	-7	-13	17	1.0	0.9	0.9	1.2
SEC13	P55735	234	-3	-6	-13	17	1.0	0.9	0.9	1.2
PPP2R3C	Q969Q6	17	-7	-14	-13	16	0.9	0.9	0.9	1.2
HENMT1	Q5T8I9	313	-3	-16	-13	16	1.0	0.9	0.9	1.2
MIOS	Q9NXC5	276	-4	-17	-13	16	1.0	0.9	0.9	1.2
TACC1	O75410	78	-7	-14	-13	15	0.9	0.9	0.9	1.2
RASA2	Q15283	170	11	-5	-13	15	1.1	1.0	0.9	1.2
APC	P25054	2664	-5	-13	-13	15	1.0	0.9	0.9	1.2
GHDC	Q8N2G8	502	-10	-13	-13	15	0.9	0.9	0.9	1.2
PNPLA6	Q8IY17	1212	-6	-7	-13	14	0.9	0.9	0.9	1.2
RNF213	Q63HN8	1007	3	-11	-13	14	1.0	0.9	0.9	1.2
GET4	Q7L5D6	160	0	-13	-13	14	1.0	0.9	0.9	1.2
HK3	P52790	612	-7	-13	-13	14	0.9	0.9	0.9	1.2
SYNCRIP	O60506	211	1	-24	-13	14	1.0	0.8	0.9	1.2
ZFYVE19	Q96K21	128	4	2	-13	13	1.0	1.0	0.9	1.1
USP14	P54578	277	-2	-8	-13	13	1.0	0.9	0.9	1.1
NUP88	Q99567	454	-8	-4	-13	13	0.9	1.0	0.9	1.1
GRAP2	O75791	244	-3	-6	-13	12	1.0	0.9	0.9	1.1
MCM3AP	O60318	1240	2	-6	-13	11	1.0	0.9	0.9	1.1
MAPKAPK3	Q16644	203	-5	-8	-13	11	1.0	0.9	0.9	1.1
KMT2D	O14686	5230	-12	-12	-13	11	0.9	0.9	0.9	1.1
RFC1	P35251	822	-6	-20	-13	11	0.9	0.8	0.9	1.1
ITGB1BP1	O14713	60	8	18	-13	11	1.1	1.2	0.9	1.1
MIB1	Q86YT6	88	7	5	-13	11	1.1	1.0	0.9	1.1

SETX	Q7Z333	1227	-8	0	-13	11	0.9	1.0	0.9	1.1
RAP1GDS1	P52306	29	-6	-6	-13	11	0.9	0.9	0.9	1.1
UPF2	Q9HAU5	25	-15	-16	-13	11	0.9	0.9	0.9	1.1
CHFR	Q96EP1	603	-11	-5	-13	10	0.9	1.0	0.9	1.1
TP53BP1	Q12888	1375	-9	-10	-13	10	0.9	0.9	0.9	1.1
RNF213	Q63HN8	3192	-1	-12	-13	10	1.0	0.9	0.9	1.1
TCERG1	O14776	535	-8	-16	-13	10	0.9	0.9	0.9	1.1
MRPL4	Q9BYD3	311	-4	4	-13	10	1.0	1.0	0.9	1.1
RAB44	Q7Z6P3	584	-3	-4	-13	10	1.0	1.0	0.9	1.1
MYCBP2	O75592	3152	-1	-9	-13	10	1.0	0.9	0.9	1.1
METTL7A	Q9H8H3	96	-7	-12	-13	10	0.9	0.9	0.9	1.1
LTA4H	P09960	147	-12	-14	-13	10	0.9	0.9	0.9	1.1
SAP30	O75446	184	-10	-15	-13	10	0.9	0.9	0.9	1.1
KMT2A	Q03164	2159	2	-17	-13	10	1.0	0.9	0.9	1.1
MON1B	Q7L1V2	349	2	-18	-13	10	1.0	0.9	0.9	1.1
TRIM28	Q13263	628	-6	-28	-13	10	0.9	0.8	0.9	1.1
NUBP1	P53384	31	-19	-1	-13	9	0.8	1.0	0.9	1.1
CHD1	O14646	376	-1	-2	-13	9	1.0	1.0	0.9	1.1
SEPT9	Q9UHD8	248	-3	-10	-13	9	1.0	0.9	0.9	1.1
PIBF1	Q8WXW3	199	-9	-13	-13	9	0.9	0.9	0.9	1.1
SP110	Q9HB58	435	-11	-19	-13	9	0.9	0.8	0.9	1.1
PIN1	Q13526	113	-7	-8	-13	9	0.9	0.9	0.9	1.1
OTUD6B	Q8N6M0	172	-8	-9	-13	9	0.9	0.9	0.9	1.1
ZMYM2	Q9UBW7	608	-4	-13	-13	9	1.0	0.9	0.9	1.1
KLHDC4	Q8TBB5	373	-8	-16	-13	9	0.9	0.9	0.9	1.1
RAD21	O60216	392	-5	-11	-13	8	1.0	0.9	0.9	1.1
CTDP1	Q9Y5B0	700	-1	-12	-13	8	1.0	0.9	0.9	1.1
NIN	Q8N4C6	1912	-4	-12	-13	8	1.0	0.9	0.9	1.1
LTN1	O94822	869	-11	-19	-13	8	0.9	0.8	0.9	1.1
PSMD9	O00233	81	13	-3	-13	8	1.1	1.0	0.9	1.1
ISG15	P05161	78	5	-3	-13	8	1.1	1.0	0.9	1.1
GDAP2	Q9NXN4	348	-3	-12	-13	8	1.0	0.9	0.9	1.1
FAM117B	Q6P1L5	513	-21	-12	-13	8	0.8	0.9	0.9	1.1
RNF213	Q63HN8	1429	-7	-12	-13	8	0.9	0.9	0.9	1.1
STK39	Q9UEW8	450	-6	-12	-13	8	0.9	0.9	0.9	1.1
ZMYM2	Q9UBW7	754	-7	3	-13	7	0.9	1.0	0.9	1.1
NUMB	P49757	611	-2	-4	-13	7	1.0	1.0	0.9	1.1
HECTD1	Q9ULT8	1995	-9	-15	-13	7	0.9	0.9	0.9	1.1
PTPN9	P43378	506	-5	-34	-13	7	1.0	0.7	0.9	1.1
UBE2F	Q969M7	50	1	-9	-13	7	1.0	0.9	0.9	1.1
ZNF598	Q86UK7	610	-14	-13	-13	7	0.9	0.9	0.9	1.1
NIPBL	Q6KC79	573	-7	-13	-13	7	0.9	0.9	0.9	1.1
RNF31	Q96EP0	702	-10	-17	-13	7	0.9	0.9	0.9	1.1
FAM117B	Q6P1L5	198	-10	-19	-13	7	0.9	0.8	0.9	1.1
PC	P11498	622	0	-4	-13	6	1.0	1.0	0.9	1.1
RSBN1L	Q6PCB5	495	2	-4	-13	6	1.0	1.0	0.9	1.1
KMT2A	Q03164	990	-8	-6	-13	6	0.9	0.9	0.9	1.1
ARID1A	O14497	1827	-10	-7	-13	6	0.9	0.9	0.9	1.1
INPPL1	O15357	1187	-7	-11	-13	6	0.9	0.9	0.9	1.1
MDN1	Q9NU22	4041	-7	-13	-13	6	0.9	0.9	0.9	1.1
PDCD2	Q16342	125	-3	-19	-13	6	1.0	0.8	0.9	1.1
TALDO1	P37837	250	-1	-21	-13	6	1.0	0.8	0.9	1.1
SLC25A16	P16260	311	-6	-3	-13	6	0.9	1.0	0.9	1.1
GAK	O14976	508	-15	-4	-13	6	0.9	1.0	0.9	1.1
TOP2B	Q02880	271	-2	-7	-13	6	1.0	0.9	0.9	1.1

HEATR6	Q6AI08	38	-5	-13	-13	6	1.0	0.9	0.9	1.1
SQSTM1	Q13501	131	-4	-3	-13	5	1.0	1.0	0.9	1.1
PARP14	Q460N5	509	-8	-13	-13	5	0.9	0.9	0.9	1.1
DHX8	Q14562	123	-12	-16	-13	5	0.9	0.9	0.9	1.1
CAPZA1	P52907	124	-6	-19	-13	5	0.9	0.8	0.9	1.1
AKAP10	O43572	110	-6	-9	-13	5	0.9	0.9	0.9	1.0
UBE3A	Q05086	198	-13	-14	-13	5	0.9	0.9	0.9	1.0
RYR3	Q15413	2227	-12	-8	-13	4	0.9	0.9	0.9	1.0
HMGB2	P26583	106	1	-36	-13	4	1.0	0.7	0.9	1.0
ZNF141	Q15928	55	-7	12	-13	4	0.9	1.1	0.9	1.0
PRKDC	P78527	1742	2	1	-13	4	1.0	1.0	0.9	1.0
C11orf68	Q9H3H3	199	-8	-9	-13	4	0.9	0.9	0.9	1.0
SAMHD1	Q9Y3Z3	80	-6	-10	-13	4	0.9	0.9	0.9	1.0
PHC2	Q8IXK0	673	-7	-17	-13	4	0.9	0.9	0.9	1.0
VPS18	Q9P253	445	-2	4	-13	3	1.0	1.0	0.9	1.0
STIM2	Q9P246	313	-7	-7	-13	3	0.9	0.9	0.9	1.0
KMT5A	Q9NQR1	124	-19	-13	-13	3	0.8	0.9	0.9	1.0
NEDD9	Q14511	18	-8	-14	-13	3	0.9	0.9	0.9	1.0
CABIN1	Q9Y6J0	1679	-12	-16	-13	3	0.9	0.9	0.9	1.0
GEMIN6	Q8WXD5	91	-4	-19	-13	3	1.0	0.8	0.9	1.0
GTF3C1	Q12789	1520	-3	-3	-13	3	1.0	1.0	0.9	1.0
KLC1	Q07866	114	-6	-10	-13	3	0.9	0.9	0.9	1.0
KANK1	Q14678	907	-13	-11	-13	3	0.9	0.9	0.9	1.0
NUDT4	Q9NZJ9	147	-9	-15	-13	3	0.9	0.9	0.9	1.0
C14orf166	Q9Y224	19	-5	-15	-13	3	1.0	0.9	0.9	1.0
ADAT3	Q96EY9	13	-20	-20	-13	3	0.8	0.8	0.9	1.0
KBTBD11	O94819	485	-6	-7	-13	2	0.9	0.9	0.9	1.0
KIF13A	Q9H1H9	127	0	-12	-13	2	1.0	0.9	0.9	1.0
OTUB1	Q96FW1	23	-16	-12	-13	2	0.9	0.9	0.9	1.0
OGDH	Q02218	395	-3	-15	-13	2	1.0	0.9	0.9	1.0
APEH	P13798	292	-1	-17	-13	2	1.0	0.9	0.9	1.0
ABRACL	Q9P1F3	39	-3	-8	-13	2	1.0	0.9	0.9	1.0
IQGAP2	Q13576	276	2	-9	-13	2	1.0	0.9	0.9	1.0
CREBL2	O60519	63	8	9	-13	1	1.1	1.1	0.9	1.0
RRP1	P56182	274	-10	-7	-13	1	0.9	0.9	0.9	1.0
PARP10	Q53GL7	434	2	-7	-13	1	1.0	0.9	0.9	1.0
EEF2	P13639	728	-7	-11	-13	1	0.9	0.9	0.9	1.0
ANXA6	P08133	96	-6	-14	-13	1	0.9	0.9	0.9	1.0
BAZ1A	Q9NRL2	764	-9	-15	-13	1	0.9	0.9	0.9	1.0
PYGB	P11216	373	-8	-20	-13	1	0.9	0.8	0.9	1.0
IL6R	P08887	25	-7	-1	-13	1	0.9	1.0	0.9	1.0
THOC2	Q8NI27	1045	-2	-2	-13	1	1.0	1.0	0.9	1.0
ARAP1	Q96P48	995	-10	-4	-13	1	0.9	1.0	0.9	1.0
DDHD2	O94830	470	-19	-24	-13	1	0.8	0.8	0.9	1.0
SETD1B	Q9UPS6	1007	-10	19	-13	0	0.9	1.2	0.9	1.0
IBA57	Q5T440	170	-4	-7	-13	0	1.0	0.9	0.9	1.0
PPP5C	P53041	77	2	-9	-13	0	1.0	0.9	0.9	1.0
SEPT2	Q15019	111	-4	-11	-13	0	1.0	0.9	0.9	1.0
IRAK3	Q9Y616	410	-7	-12	-13	0	0.9	0.9	0.9	1.0
DDX1	Q92499	231	-10	-7	-13	-1	0.9	0.9	0.9	1.0
PCBP1	Q15365	194	2	-11	-13	-1	1.0	0.9	0.9	1.0
PDS5A	Q29RF7	1116	-6	-14	-13	-1	0.9	0.9	0.9	1.0
FAM185A	Q8N0U4	284	-4	-8	-13	-1	1.0	0.9	0.9	1.0
NRAS	P01111	118	-9	-11	-13	-1	0.9	0.9	0.9	1.0
STK16	O75716	84	-8	-21	-13	-1	0.9	0.8	0.9	1.0

MBNL2	Q5VZF2	53	-3	-7	-13	-2	1.0	0.9	0.9	1.0
MBNL1	Q9NR56	53	-3	-7	-13	-2	1.0	0.9	0.9	1.0
FBXW4	P57775	64	-10	-14	-13	-2	0.9	0.9	0.9	1.0
TAOK2	Q9UL54	623	-4	-14	-13	-2	1.0	0.9	0.9	1.0
NEDD9	Q14511	437	-13	5	-13	-3	0.9	1.1	0.9	1.0
DOCK8	Q8NF50	170	-14	-7	-13	-3	0.9	0.9	0.9	1.0
UPF1	Q92900	186	-7	-15	-13	-3	0.9	0.9	0.9	1.0
PMM1	Q92871	57	4	-16	-13	-3	1.0	0.9	0.9	1.0
LRCH3	Q96II8	277	4	-7	-13	-4	1.0	0.9	0.9	1.0
LIMD2	Q9BT23	40	-9	-12	-13	-4	0.9	0.9	0.9	1.0
LACTB	P83111	476	-4	-14	-13	-4	1.0	0.9	0.9	1.0
LNP	Q9C0E8	149	-8	-16	-13	-4	0.9	0.9	0.9	1.0
PFKFB3	Q16875	155	1	-11	-13	-4	1.0	0.9	0.9	1.0
CORO7-PAM1	A0A0A6YYL4	389	-6	-13	-13	-5	0.9	0.9	0.9	1.0
PPP4R3A	Q6IN85	90	-2	-13	-13	-5	1.0	0.9	0.9	1.0
RALY	Q9UKM9	51	-18	-33	-13	-5	0.9	0.8	0.9	1.0
GLMN	Q92990	375	-13	-16	-13	-5	0.9	0.9	0.9	1.0
SH3BP1	Q9Y3L3	338	5	-10	-13	-6	1.1	0.9	0.9	0.9
GIMAP1	Q8WWP7	186	-8	-14	-13	-6	0.9	0.9	0.9	0.9
PARP10	Q53GL7	981	-13	-21	-13	-6	0.9	0.8	0.9	0.9
BIRC6	Q9NR09	946	4	-4	-13	-7	1.0	1.0	0.9	0.9
SEPT7	Q16181	280	-7	-6	-13	-7	0.9	0.9	0.9	0.9
RBM47	A0AV96	273	-8	-13	-13	-7	0.9	0.9	0.9	0.9
TLR1	Q15399	686	13	-15	-13	-7	1.1	0.9	0.9	0.9
FLNA	P21333	2160	-5	-19	-13	-7	1.0	0.8	0.9	0.9
SIRT7	Q9NRC8	266	1	-5	-13	-7	1.0	1.0	0.9	0.9
THOC2	Q8NI27	743	-3	-13	-13	-7	1.0	0.9	0.9	0.9
RASGRP4	Q8TDF6	33	-24	-19	-13	-8	0.8	0.8	0.9	0.9
PTPN2	P17706	278	-8	-25	-13	-8	0.9	0.8	0.9	0.9
LRCH1	Q9Y2L9	292	-2	-10	-13	-9	1.0	0.9	0.9	0.9
HSPA9	P38646	366	-7	-16	-13	-9	0.9	0.9	0.9	0.9
CASP9	P55211	287	-5	-14	-13	-9	1.0	0.9	0.9	0.9
ABCD2	Q9UBJ2	635	7	-14	-13	-9	1.1	0.9	0.9	0.9
ARHGEF2	Q92974	573	0	-6	-13	-10	1.0	0.9	0.9	0.9
PPP2R5E	Q16537	106	-24	-15	-13	-10	0.8	0.9	0.9	0.9
TACC1	O75410	715	-9	-16	-13	-10	0.9	0.9	0.9	0.9
FLNB	O75369	660	-14	-15	-13	-11	0.9	0.9	0.9	0.9
AK6	D6RDH4	50	-1	-17	-13	-11	1.0	0.9	0.9	0.9
ARAP1	Q96P48	376	-2	-8	-13	-11	1.0	0.9	0.9	0.9
VASP	P50552	64	-4	-17	-13	-11	1.0	0.9	0.9	0.9
IKBKB	O14920	114	7	-2	-13	-12	1.1	1.0	0.9	0.9
NUMA1	Q14980	1937	-3	-20	-13	-12	1.0	0.8	0.9	0.9
PGP	A6NDG6	217	-19	-31	-13	-12	0.8	0.8	0.9	0.9
SUPV3L1	Q8IYB8	418	-15	-32	-13	-12	0.9	0.8	0.9	0.9
RNF213	Q63HN8	2620	3	-11	-13	-13	1.0	0.9	0.9	0.9
GGPS1	O95749	205	-10	-8	-13	-14	0.9	0.9	0.9	0.9
SRM	P19623	209	-1	2	-13	-15	1.0	1.0	0.9	0.9
CTCF	P49711	504	5	-5	-13	-16	1.0	1.0	0.9	0.9
FNDC3A	Q9Y2H6	211	-13	-19	-13	-18	0.9	0.8	0.9	0.9
BPTF	Q12830	445	-3	-16	-13	-18	1.0	0.9	0.9	0.8
MBOAT2	Q6ZWT7	505	-10	-14	-13	-19	0.9	0.9	0.9	0.8
MAP2K4	P45985	246	1	-8	-13	-19	1.0	0.9	0.9	0.8
RACK1	P63244	153	-11	-6	-13	-20	0.9	0.9	0.9	0.8
COMT	P21964	145	-9	-13	-13	-20	0.9	0.9	0.9	0.8
RIPK1	Q13546	34	-7	-21	-13	-20	0.9	0.8	0.9	0.8

TRIM21	P19474	273	-1	-28	-13	-21	1.0	0.8	0.9	0.8
LIMD2	Q9BT23	67	-2	-34	-13	-25	1.0	0.7	0.9	0.8
LONP1	P36776	682	-3	-13	-13	-31	1.0	0.9	0.9	0.8
CEBPD	P49716	268	13	-17	-13	-35	1.1	0.9	0.9	0.7
CTNND1	O60716	429	-3	-7	-13	-36	1.0	0.9	0.9	0.7
SF1	Q15637	282	4	-3	-13	-39	1.0	1.0	0.9	0.7
INF2	Q27J81	971	-8	-12	-13	-44	0.9	0.9	0.9	0.7
PIK3CD	O00329	500	5	-12	-13	-54	1.0	0.9	0.9	0.7
TCF20	Q9UGU0	951	18	-18	-13	-60	1.2	0.9	0.9	0.6
DICER1	Q9UPY3	686	-1	-19	-13	-70	1.0	0.8	0.9	0.6
GP1BA	P07359	81	1	-7	-13	-338	1.0	0.9	0.9	0.2
TBC1D2B	Q9UPU7	726	-14	-40	-14	47	0.9	0.7	0.9	1.9
KAT8	Q9H7Z6	416	-4	-17	-14	36	1.0	0.9	0.9	1.6
NCAPH2	Q6IBW4	342	-32	-22	-14	30	0.8	0.8	0.9	1.4
FLCN	Q8NFG4	131	-1	-14	-14	29	1.0	0.9	0.9	1.4
MYCBP2	O75592	2662	-6	-10	-14	29	0.9	0.9	0.9	1.4
NOL8	Q76FK4	578	-7	-11	-14	27	0.9	0.9	0.9	1.4
TADA1	Q96BN2	335	-7	-10	-14	26	0.9	0.9	0.9	1.3
LIMK1	P53667	349	-10	-13	-14	26	0.9	0.9	0.9	1.3
DTWD2	Q8NBA8	220	-19	-7	-14	25	0.8	0.9	0.9	1.3
KDM8	Q8N371	164	2	-8	-14	25	1.0	0.9	0.9	1.3
ACSL4	O60488	420	-2	-9	-14	25	1.0	0.9	0.9	1.3
OTUD6B	Q8N6M0	292	2	8	-14	24	1.0	1.1	0.9	1.3
TRIO	O75962	1713	-8	-4	-14	23	0.9	1.0	0.9	1.3
SULT1B1	O43704	22	-2	-3	-14	23	1.0	1.0	0.9	1.3
LRBA	P50851	289	-7	-7	-14	23	0.9	0.9	0.9	1.3
NBEA	Q8NFP9	315	-7	-7	-14	23	0.9	0.9	0.9	1.3
FAM21C	Q9Y4E1	826	-13	-12	-14	23	0.9	0.9	0.9	1.3
GPX1	P07203	78	-1	-13	-14	22	1.0	0.9	0.9	1.3
CHD6	Q8TD26	1938	-5	-7	-14	22	1.0	0.9	0.9	1.3
TBL3	Q12788	358	-15	-12	-14	21	0.9	0.9	0.9	1.3
ITGB2	P05107	447	-20	-14	-14	21	0.8	0.9	0.9	1.3
ERO1A	Q96HE7	35	3	-25	-14	20	1.0	0.8	0.9	1.3
DALRD3	Q5D0E6	468	-6	-13	-14	20	0.9	0.9	0.9	1.2
PDS5A	Q29RF7	14	-5	-9	-14	19	1.0	0.9	0.9	1.2
DCP1B	Q8IZD4	291	-1	-20	-14	19	1.0	0.8	0.9	1.2
TP53BP2	Q13625	290	-9	-9	-14	18	0.9	0.9	0.9	1.2
ZNF331	Q9NQX6	85	-11	14	-14	18	0.9	1.2	0.9	1.2
TRIM26	Q12899	353	8	-1	-14	18	1.1	1.0	0.9	1.2
ADD2	P35612	201	2	-11	-14	18	1.0	0.9	0.9	1.2
JOSD1	Q15040	36	-5	-16	-14	18	1.0	0.9	0.9	1.2
SOAT1	P35610	92	-4	-12	-14	17	1.0	0.9	0.9	1.2
CPSF1	Q10570	1135	-4	-12	-14	17	1.0	0.9	0.9	1.2
WWP2	O00308	186	-8	-12	-14	17	0.9	0.9	0.9	1.2
HLA-E	P13747	185	-6	-13	-14	17	0.9	0.9	0.9	1.2
ATRX	P46100	1789	-6	-21	-14	17	0.9	0.8	0.9	1.2
ATAD2B	Q9ULI0	53	-10	-4	-14	16	0.9	1.0	0.9	1.2
TIGAR	Q9NQ88	215	-7	-16	-14	16	0.9	0.9	0.9	1.2
CERS5	Q8N5B7	369	-7	-7	-14	16	0.9	0.9	0.9	1.2
ARID1A	O14497	1981	2	2	-14	15	1.0	1.0	0.9	1.2
HTT	P42858	1441	-6	-14	-14	15	0.9	0.9	0.9	1.2
MPHOSPH10	O00566	27	0	-4	-14	15	1.0	1.0	0.9	1.2
UBR2	Q8IWV8	1717	-8	-13	-14	15	0.9	0.9	0.9	1.2
AVEN	Q9NQS1	251	1	-16	-14	15	1.0	0.9	0.9	1.2
LOC10272415	A0A0B4J2E5	508	-5	-8	-14	14	1.0	0.9	0.9	1.2

PPP1R18	Q6NYC8	276	0	-8	-14	14	1.0	0.9	0.9	1.2
PHAX	Q9H814	51	-5	-13	-14	14	1.0	0.9	0.9	1.2
TIAM1	Q13009	1046	-13	-18	-14	14	0.9	0.9	0.9	1.2
RAD54L2	Q9Y4B4	841	-17	-18	-14	14	0.9	0.8	0.9	1.2
TCF12	Q99081	129	-2	-9	-14	14	1.0	0.9	0.9	1.2
ANAPC2	Q9UJX6	221	-8	-11	-14	14	0.9	0.9	0.9	1.2
MED17	Q9NVC6	488	-9	-15	-14	14	0.9	0.9	0.9	1.2
SP4	Q02446	55	-7	-5	-14	13	0.9	1.0	0.9	1.1
MYO1F	O00160	101	-4	-10	-14	13	1.0	0.9	0.9	1.1
R3HCC1L	Q7Z5L2	147	-13	-13	-14	13	0.9	0.9	0.9	1.1
NFATC2	Q13469	588	-2	1	-14	13	1.0	1.0	0.9	1.1
TBX21	Q9UL17	207	-2	-5	-14	13	1.0	1.0	0.9	1.1
ARHGEF2	Q92974	478	-5	-10	-14	13	1.0	0.9	0.9	1.1
DTWD2	Q8NBA8	252	-21	-11	-14	13	0.8	0.9	0.9	1.1
PLCL1	Q15111	33	-6	-1	-14	12	0.9	1.0	0.9	1.1
RNH1	P13489	85	-12	-14	-14	12	0.9	0.9	0.9	1.1
FGD3	Q5JSP0	583	-1	-14	-14	12	1.0	0.9	0.9	1.1
TBC1D2	Q9BYX2	686	-12	-22	-14	12	0.9	0.8	0.9	1.1
XRCC5	P13010	339	-7	0	-14	12	0.9	1.0	0.9	1.1
ACAP2	Q15057	53	-2	-1	-14	12	1.0	1.0	0.9	1.1
PMPCA	Q10713	142	12	-6	-14	12	1.1	0.9	0.9	1.1
PIKFYVE	Q9Y2I7	723	-8	-7	-14	12	0.9	0.9	0.9	1.1
SIDT2	Q8NBJ9	335	-2	-13	-14	12	1.0	0.9	0.9	1.1
WDR43	Q15061	380	-11	-21	-14	12	0.9	0.8	0.9	1.1
SEC31A	O94979	644	10	2	-14	11	1.1	1.0	0.9	1.1
AKAP12	Q02952	1521	-7	-6	-14	11	0.9	0.9	0.9	1.1
THEMIS2	Q5TEJ8	290	-11	-7	-14	11	0.9	0.9	0.9	1.1
SYNE2	Q8WXH0	5930	-4	-9	-14	11	1.0	0.9	0.9	1.1
TRMT2A	Q8IZ69	270	-5	-10	-14	11	1.0	0.9	0.9	1.1
ACBD5	Q5T8D3	170	-12	-15	-14	11	0.9	0.9	0.9	1.1
LSP1	P33241	109	-6	-19	-14	11	0.9	0.8	0.9	1.1
VPS13C	Q709C8	2588	-8	-1	-14	11	0.9	1.0	0.9	1.1
KIF1B	O60333	1771	-20	-6	-14	11	0.8	0.9	0.9	1.1
SYNE2	Q8WXH0	6161	0	-6	-14	11	1.0	0.9	0.9	1.1
NUP153	P49790	1065	-11	-13	-14	11	0.9	0.9	0.9	1.1
RIN2	Q8WYP3	138	-3	-26	-14	11	1.0	0.8	0.9	1.1
VCPIP1	Q96JH7	219	-7	-8	-14	10	0.9	0.9	0.9	1.1
ADCK1	Q86TW2	386	12	-9	-14	10	1.1	0.9	0.9	1.1
BTAF1	O14981	936	-14	-5	-14	10	0.9	1.0	0.9	1.1
LPCAT2	Q7L5N7	4	-12	-6	-14	10	0.9	0.9	0.9	1.1
IARS	P41252	526	-2	-7	-14	10	1.0	0.9	0.9	1.1
HSPBP1	Q9NZL4	22	-9	-13	-14	10	0.9	0.9	0.9	1.1
ARIH1	Q9Y4X5	327	-4	-5	-14	9	1.0	1.0	0.9	1.1
EXOSC3	Q9NQT5	215	-7	-7	-14	9	0.9	0.9	0.9	1.1
PIGQ	Q9BRB3	69	-8	-11	-14	9	0.9	0.9	0.9	1.1
PSMA1	P25786	85	-13	-15	-14	9	0.9	0.9	0.9	1.1
METTL16	Q86W50	253	-5	-5	-14	9	1.0	1.0	0.9	1.1
SETX	Q7Z333	788	-12	-19	-14	9	0.9	0.8	0.9	1.1
XPO6	Q96QU8	324	-13	-4	-14	8	0.9	1.0	0.9	1.1
HIVEP2	P31629	1500	-15	-7	-14	8	0.9	0.9	0.9	1.1
PLEKHA2	Q9HB19	232	-3	-7	-14	8	1.0	0.9	0.9	1.1
EHMT2	Q96KQ7	1115	-2	-8	-14	8	1.0	0.9	0.9	1.1
SYNE2	Q8WXH0	489	20	-10	-14	8	1.3	0.9	0.9	1.1
ATG3	Q9NT62	182	-6	-10	-14	8	0.9	0.9	0.9	1.1
MYSM1	Q5VVJ2	440	3	-1	-14	8	1.0	1.0	0.9	1.1

NFATC3	Q12968	358	-9	-13	-14	8	0.9	0.9	0.9	1.1
PHKB	Q93100	736	-22	-14	-14	8	0.8	0.9	0.9	1.1
PHF3	Q92576	1771	-9	-16	-14	8	0.9	0.9	0.9	1.1
INTS1	Q8N201	1755	-5	-25	-14	8	1.0	0.8	0.9	1.1
RARRES3	Q9UL19	113	-9	0	-14	7	0.9	1.0	0.9	1.1
PAK1IP1	Q9NWT1	288	-9	-5	-14	7	0.9	1.0	0.9	1.1
NFKB1	P19838	87	-8	-7	-14	7	0.9	0.9	0.9	1.1
FRY	Q5TBA9	336	-16	-7	-14	7	0.9	0.9	0.9	1.1
MDM1	Q8TC05	405	-7	-7	-14	7	0.9	0.9	0.9	1.1
SPECC1L-ADOI	F8WAN1	79	3	-10	-14	7	1.0	0.9	0.9	1.1
TP53BP1	Q12888	1703	-9	-21	-14	7	0.9	0.8	0.9	1.1
STBD1	O95210	108	-10	-21	-14	7	0.9	0.8	0.9	1.1
RB1CC1	Q8TDY2	839	10	-1	-14	7	1.1	1.0	0.9	1.1
TAPBPL	Q9BX59	448	-19	-2	-14	7	0.8	1.0	0.9	1.1
GPAT3	Q53EU6	266	-4	-3	-14	7	1.0	1.0	0.9	1.1
GPAT4	Q86UL3	285	-4	-3	-14	7	1.0	1.0	0.9	1.1
CEP135	Q66GS9	615	-5	-8	-14	7	1.0	0.9	0.9	1.1
PDCD6IP	Q8WUM4	250	7	-18	-14	7	1.1	0.8	0.9	1.1
CD3EAP	O15446	86	-6	-4	-14	6	0.9	1.0	0.9	1.1
DOCK8	Q8NF50	143	-6	-7	-14	6	0.9	0.9	0.9	1.1
SPATA5	Q8NB90	318	-33	4	-14	6	0.8	1.0	0.9	1.1
MAPK9	P45984	6	-12	3	-14	6	0.9	1.0	0.9	1.1
TSR3	Q9UJK0	124	0	-4	-14	6	1.0	1.0	0.9	1.1
C9orf64	Q5T6V5	170	14	-10	-14	6	1.2	0.9	0.9	1.1
HSDL2	Q6YN16	71	-12	-10	-14	6	0.9	0.9	0.9	1.1
NDUFS1	P28331	564	1	-15	-14	6	1.0	0.9	0.9	1.1
NOCT	Q9UK39	302	-1	-6	-14	5	1.0	0.9	0.9	1.1
SPHKAP	Q2M3C7	259	-4	-7	-14	5	1.0	0.9	0.9	1.1
UBR4	Q5T4S7	4272	1	-10	-14	5	1.0	0.9	0.9	1.1
VPS53	Q5VIR6	365	-8	-12	-14	5	0.9	0.9	0.9	1.1
PAXIP1	Q6ZW49	740	2	-13	-14	5	1.0	0.9	0.9	1.1
HNRNPL	P14866	404	-7	-20	-14	5	0.9	0.8	0.9	1.1
SMC5	Q8IY18	393	-16	-20	-14	5	0.9	0.8	0.9	1.1
EEF1A1	P68104	411	-8	-7	-14	5	0.9	0.9	0.9	1.0
ILF3	Q12906	295	0	-14	-14	5	1.0	0.9	0.9	1.0
USO1	O60763	678	-2	-3	-14	4	1.0	1.0	0.9	1.0
PHF3	Q92576	561	-9	-6	-14	4	0.9	0.9	0.9	1.0
OAS1	P00973	25	-7	-10	-14	4	0.9	0.9	0.9	1.0
EEF2	P13639	651	-3	-10	-14	4	1.0	0.9	0.9	1.0
NFIC	P08651	104	-12	-13	-14	4	0.9	0.9	0.9	1.0
NPEPPS	P55786	339	-11	-13	-14	4	0.9	0.9	0.9	1.0
CTSW	P56202	153	-28	-9	-14	4	0.8	0.9	0.9	1.0
DOCK8	Q8NF50	186	3	-10	-14	4	1.0	0.9	0.9	1.0
DLAT	P10515	291	-10	-10	-14	4	0.9	0.9	0.9	1.0
ZRANB2	O95218	88	0	-12	-14	4	1.0	0.9	0.9	1.0
CPSF6	Q16630	476	0	-12	-14	4	1.0	0.9	0.9	1.0
EMSY	Q7Z589	194	-11	-12	-14	4	0.9	0.9	0.9	1.0
WDR82	Q6UXN9	287	-4	-13	-14	4	1.0	0.9	0.9	1.0
AGO1	Q9UL18	64	-4	-14	-14	4	1.0	0.9	0.9	1.0
SACM1L	Q9NTJ5	23	3	-2	-14	3	1.0	1.0	0.9	1.0
DHX9	Q08211	242	-3	-12	-14	3	1.0	0.9	0.9	1.0
PFKL	P17858	708	-15	-13	-14	3	0.9	0.9	0.9	1.0
HSPA1B	P0DMV9	603	-11	-24	-14	3	0.9	0.8	0.9	1.0
MLKL	Q8NB16	184	-7	-5	-14	3	0.9	1.0	0.9	1.0
INTS6	Q9UL03	219	-9	-12	-14	3	0.9	0.9	0.9	1.0

ARHGAP25	P42331	154	-8	-6	-14	2	0.9	0.9	0.9	1.0
LOC10272402	A0A0B4J2D5	153	-9	-7	-14	2	0.9	0.9	0.9	1.0
FAM126A	Q9BYI3	401	-4	-14	-14	2	1.0	0.9	0.9	1.0
CDYL2	Q8N8U2	288	-9	-17	-14	2	0.9	0.9	0.9	1.0
SLK	Q9H2G2	1212	9	-5	-14	2	1.1	1.0	0.9	1.0
RPS19BP1	Q86WX3	104	-2	-12	-14	2	1.0	0.9	0.9	1.0
CTPS1	P17812	491	-4	-13	-14	2	1.0	0.9	0.9	1.0
SEC13	P55735	31	-13	-9	-14	1	0.9	0.9	0.9	1.0
ATP2C1	P98194	162	-3	-20	-14	1	1.0	0.8	0.9	1.0
FDFT1	P37268	6	7	-4	-14	1	1.1	1.0	0.9	1.0
GSDMD	P57764	56	-9	-9	-14	1	0.9	0.9	0.9	1.0
CCM2	Q9BSQ5	170	-10	-9	-14	1	0.9	0.9	0.9	1.0
PDE4DIP	Q5VU43	617	-9	-11	-14	1	0.9	0.9	0.9	1.0
STK25	O00506	357	-10	-4	-14	0	0.9	1.0	0.9	1.0
RNH1	P13489	216	-6	-17	-14	0	0.9	0.9	0.9	1.0
PASK	Q96RG2	228	-2	-8	-14	-1	1.0	0.9	0.9	1.0
ATP6V1A	P38606	394	-22	-11	-14	-1	0.8	0.9	0.9	1.0
CENPT	Q96BT3	550	-4	-17	-14	-1	1.0	0.9	0.9	1.0
SCAF11	Q99590	450	-12	-18	-14	-1	0.9	0.9	0.9	1.0
YRDC	Q86U90	99	-9	-25	-14	-1	0.9	0.8	0.9	1.0
ARHGAP25	P42331	302	-5	-9	-14	-1	1.0	0.9	0.9	1.0
ZNF512B	Q96KM6	786	-1	-12	-14	-1	1.0	0.9	0.9	1.0
JADE2	Q9NQC1	254	-1	-12	-14	-1	1.0	0.9	0.9	1.0
PLEC	Q15149	3821	-14	-16	-14	-1	0.9	0.9	0.9	1.0
USP8	P40818	809	-14	-5	-14	-2	0.9	1.0	0.9	1.0
AGPAT5	Q9NUQ2	252	-2	-21	-14	-2	1.0	0.8	0.9	1.0
SMCHD1	A6NHR9	492	-1	0	-14	-3	1.0	1.0	0.9	1.0
RAB29	O14966	120	-11	-11	-14	-3	0.9	0.9	0.9	1.0
U2AF2	P26368	442	-6	-11	-14	-3	0.9	0.9	0.9	1.0
ATE1	O95260	72	12	1	-14	-3	1.1	1.0	0.9	1.0
ATP5SL	Q9NW81	184	-6	-14	-14	-3	0.9	0.9	0.9	1.0
CLASP1	Q7Z460	1212	-11	-14	-14	-3	0.9	0.9	0.9	1.0
FAF2	Q96CS3	349	-5	-17	-14	-3	1.0	0.9	0.9	1.0
CCT4	P50991	295	1	-8	-14	-4	1.0	0.9	0.9	1.0
SARM1	Q6SZW1	482	-6	-16	-14	-4	0.9	0.9	0.9	1.0
TAF6	P49848	460	2	5	-14	-5	1.0	1.1	0.9	1.0
FAM120A	Q9NZB2	1088	-15	-4	-14	-5	0.9	1.0	0.9	1.0
PHKB	Q93100	1066	-17	-8	-14	-5	0.9	0.9	0.9	1.0
DGCR8	Q8WYQ5	430	-5	-13	-14	-5	1.0	0.9	0.9	1.0
FBXW2	Q9UKT8	327	-10	-14	-14	-5	0.9	0.9	0.9	1.0
VEZF1	Q14119	292	-10	-15	-14	-5	0.9	0.9	0.9	1.0
PFKFB4	Q16877	197	-4	-16	-14	-5	1.0	0.9	0.9	1.0
EP400	Q96L91	1820	-4	-2	-14	-5	1.0	1.0	0.9	1.0
HTT	P42858	664	-2	-8	-14	-5	1.0	0.9	0.9	1.0
OSBPL3	Q9H4L5	203	-3	-12	-14	-5	1.0	0.9	0.9	1.0
RBM14-RBM4	A0A0A0MSL8	90	4	-11	-14	-6	1.0	0.9	0.9	0.9
IDH2	P48735	154	-15	-14	-14	-6	0.9	0.9	0.9	0.9
SCRN1	Q12765	188	2	-21	-14	-6	1.0	0.8	0.9	0.9
BYSL	Q13895	300	-8	-22	-14	-6	0.9	0.8	0.9	0.9
ZNF330	Q9Y3S2	192	-25	-7	-14	-6	0.8	0.9	0.9	0.9
SART3	Q15020	821	-8	-15	-14	-6	0.9	0.9	0.9	0.9
ETFA	P13804	53	-10	-18	-14	-6	0.9	0.9	0.9	0.9
PFN1	P07737	71	-3	0	-14	-7	1.0	1.0	0.9	0.9
UBA2	Q9UBT2	444	-6	-8	-14	-7	0.9	0.9	0.9	0.9
FHOD1	Q9Y613	502	-4	-8	-14	-7	1.0	0.9	0.9	0.9

C1orf174	Q8IYL3	61	-15	-9	-14	-7	0.9	0.9	0.9	0.9
TUBB2B	Q9BVA1	354	-10	-12	-14	-7	0.9	0.9	0.9	0.9
CLMN	Q96JQ2	353	-6	-13	-14	-7	0.9	0.9	0.9	0.9
AGL	P35573	608	11	-19	-14	-7	1.1	0.8	0.9	0.9
RNF14	Q9UBS8	417	-5	-19	-14	-7	1.0	0.8	0.9	0.9
CEBPZ	Q03701	706	-10	-13	-14	-7	0.9	0.9	0.9	0.9
UROS	P10746	241	-17	-15	-14	-8	0.9	0.9	0.9	0.9
WDR1	O75083	382	-14	-16	-14	-8	0.9	0.9	0.9	0.9
ADD3	Q9UEY8	286	-16	-8	-14	-8	0.9	0.9	0.9	0.9
PLAA	Q9Y263	193	-10	-16	-14	-9	0.9	0.9	0.9	0.9
ATG14	Q6ZNE5	330	-16	-25	-14	-9	0.9	0.8	0.9	0.9
AP2A1	O95782	492	-6	-12	-14	-10	0.9	0.9	0.9	0.9
MMS22L	Q6ZRQ5	597	-1	-9	-14	-10	1.0	0.9	0.9	0.9
CAPN2	P17655	577	2	-11	-14	-10	1.0	0.9	0.9	0.9
ALDOA	P04075	73	-8	-26	-14	-10	0.9	0.8	0.9	0.9
ATP6V1C1	P21283	376	-2	-27	-14	-11	1.0	0.8	0.9	0.9
TRANK1	O15050	242	0	-23	-14	-12	1.0	0.8	0.9	0.9
CEP44	Q9C0F1	260	-4	-10	-14	-13	1.0	0.9	0.9	0.9
CENPC	Q03188	78	1	-13	-14	-13	1.0	0.9	0.9	0.9
OPA1	O60313	801	1	-5	-14	-14	1.0	1.0	0.9	0.9
RAP1GAP2	Q684P5	185	-1	2	-14	-15	1.0	1.0	0.9	0.9
EP400	Q96L91	1775	-10	-16	-14	-15	0.9	0.9	0.9	0.9
SMARCB1	Q12824	167	-10	-12	-14	-15	0.9	0.9	0.9	0.9
VCPIP1	Q96JH7	397	-4	-12	-14	-16	1.0	0.9	0.9	0.9
TOR4A	Q9NXH8	413	-1	-17	-14	-16	1.0	0.9	0.9	0.9
PLEKHO2	Q8TD55	388	-10	-5	-14	-17	0.9	1.0	0.9	0.9
SKIL	P12757	495	6	-12	-14	-18	1.1	0.9	0.9	0.9
RHOC	P08134	159	-4	-7	-14	-18	1.0	0.9	0.9	0.8
KCTD18	Q6PI47	56	-4	-8	-14	-19	1.0	0.9	0.9	0.8
ACO2	Q99798	385	-5	-7	-14	-21	1.0	0.9	0.9	0.8
DENND1B	Q6P3S1	52	-6	-8	-14	-21	0.9	0.9	0.9	0.8
TLK2	Q86UE8	737	-34	-13	-14	-22	0.7	0.9	0.9	0.8
PML	P29590	189	-4	-12	-14	-24	1.0	0.9	0.9	0.8
SMAD2	Q15796	161	-1	-8	-14	-26	1.0	0.9	0.9	0.8
PELP1	Q8IZL8	522	-13	-18	-14	-26	0.9	0.9	0.9	0.8
DDX6	P26196	324	-19	-4	-14	-32	0.8	1.0	0.9	0.8
PSTPIP2	Q9H939	65	0	-8	-14	-34	1.0	0.9	0.9	0.7
DAPP1	Q9UN19	227	-4	-9	-14	-34	1.0	0.9	0.9	0.7
TPM4	P67936	247	-2	-15	-14	-44	1.0	0.9	0.9	0.7
TNKS1BP1	Q9C0C2	1175	3	-24	-14	-45	1.0	0.8	0.9	0.7
ERO1A	Q96HE7	166	14	-18	-14	-50	1.2	0.8	0.9	0.7
ZZEF1	O43149	728	-12	-8	-14	-55	0.9	0.9	0.9	0.6
GUCY1A3	Q02108	15	-16	-7	-14	-56	0.9	0.9	0.9	0.6
SCAF11	Q99590	1020	36	-10	-14	-101	1.6	0.9	0.9	0.5
BAG3	O95817	151	4	-2	-14	49	1.0	1.0	0.9	2.0
DIP2B	Q9P265	1019	2	1	-14	40	1.0	1.0	0.9	1.7
SPATA5	Q8NB90	672	-5	-8	-14	35	1.0	0.9	0.9	1.5
EXOSC7	Q15024	238	-7	-18	-14	31	0.9	0.9	0.9	1.4
MYO9B	Q13459	2028	0	-12	-14	30	1.0	0.9	0.9	1.4
TTC9	Q92623	30	-5	-13	-14	30	1.0	0.9	0.9	1.4
RBL2	Q08999	579	-7	-14	-14	29	0.9	0.9	0.9	1.4
SLC25A11	Q02978	184	-4	-6	-14	27	1.0	0.9	0.9	1.4
BRAT1	Q6PJG6	326	-8	-6	-14	25	0.9	0.9	0.9	1.3
CDC42EP2	O14613	92	-5	10	-14	24	1.0	1.1	0.9	1.3
HNRNPH1	P31943	122	-12	-22	-14	24	0.9	0.8	0.9	1.3

HNRNPH2	P55795	122	-12	-22	-14	24	0.9	0.8	0.9	1.3
ERCC6	Q03468	61	-13	-4	-14	23	0.9	1.0	0.9	1.3
MOCS2	O96007	182	0	-24	-14	23	1.0	0.8	0.9	1.3
ERLIN1	O75477	310	-15	-11	-14	22	0.9	0.9	0.9	1.3
STK40	Q8N2I9	218	2	-15	-14	22	1.0	0.9	0.9	1.3
AMMECR1	Q9Y4X0	175	-15	-29	-14	22	0.9	0.8	0.9	1.3
CAMSAP1	Q5T5Y3	1020	-6	5	-14	21	0.9	1.0	0.9	1.3
PIK3R5	Q8WYR1	460	-8	-11	-14	20	0.9	0.9	0.9	1.3
FDPS	P14324	183	1	-8	-14	20	1.0	0.9	0.9	1.2
SDHA	P31040	190	-13	19	-14	19	0.9	1.2	0.9	1.2
UBA6	A0AVT1	455	-2	-13	-14	19	1.0	0.9	0.9	1.2
LRRC58	Q96CX6	309	-27	-17	-14	19	0.8	0.9	0.9	1.2
DOCK2	Q92608	730	-8	-9	-14	18	0.9	0.9	0.9	1.2
LOC10272402	A0A0B4J2D5	176	-5	-10	-14	18	1.0	0.9	0.9	1.2
PCM1	Q15154	783	-5	-17	-14	18	1.0	0.9	0.9	1.2
ZC2HC1A	Q96GY0	316	-2	-13	-14	18	1.0	0.9	0.9	1.2
NUP205	Q92621	921	4	0	-14	17	1.0	1.0	0.9	1.2
SETD1A	O15047	1514	3	-19	-14	17	1.0	0.8	0.9	1.2
SNX8	Q9Y5X2	200	-8	-10	-14	17	0.9	0.9	0.9	1.2
ATP5O	P48047	141	-14	-16	-14	17	0.9	0.9	0.9	1.2
LEMD3	Q9Y2U8	785	-13	-12	-14	16	0.9	0.9	0.9	1.2
MAP4K4	O95819	496	-12	-13	-14	16	0.9	0.9	0.9	1.2
SMC1A	Q14683	933	-18	-9	-14	16	0.9	0.9	0.9	1.2
PELP1	Q8IZL8	600	2	4	-14	15	1.0	1.0	0.9	1.2
TRRAP	Q9Y4A5	555	-6	-2	-14	15	0.9	1.0	0.9	1.2
RDH13	Q8NBN7	30	-6	-7	-14	15	0.9	0.9	0.9	1.2
NTMT1	Q9BV86	195	-8	-7	-14	15	0.9	0.9	0.9	1.2
MYSM1	Q5VVJ2	822	2	-8	-14	15	1.0	0.9	0.9	1.2
PRPF4B	Q13523	962	-2	-11	-14	15	1.0	0.9	0.9	1.2
AHNAK	Q09666	1900	-7	-14	-14	15	0.9	0.9	0.9	1.2
AKAP9	Q99996	1687	0	-15	-14	15	1.0	0.9	0.9	1.2
PDS5A	Q29RF7	532	-6	-15	-14	14	0.9	0.9	0.9	1.2
DTNBP1	Q96EV8	302	-12	-17	-14	14	0.9	0.9	0.9	1.2
RARRES3	Q9UL19	73	-7	-11	-14	14	0.9	0.9	0.9	1.2
ALDH2	P05091	386	-4	-5	-14	13	1.0	1.0	0.9	1.1
IKBKB	O14920	412	-13	-11	-14	13	0.9	0.9	0.9	1.1
ZW10	O43264	568	7	-8	-14	13	1.1	0.9	0.9	1.1
TRIP12	Q14669	1959	-4	-9	-14	13	1.0	0.9	0.9	1.1
MED15	Q96RN5	660	-3	-9	-14	13	1.0	0.9	0.9	1.1
XPO4	Q9C0E2	868	-14	-11	-14	13	0.9	0.9	0.9	1.1
PRR14L	Q5THK1	2061	-12	-6	-14	12	0.9	0.9	0.9	1.1
POR	P16435	363	0	-12	-14	12	1.0	0.9	0.9	1.1
SAMM50	Q9Y512	421	1	1	-14	12	1.0	1.0	0.9	1.1
LRRFIP1	Q32MZ4	644	-6	-14	-14	12	0.9	0.9	0.9	1.1
TRAF3IP3	Q9Y228	42	-16	-4	-14	11	0.9	1.0	0.9	1.1
PDCD11	Q14690	81	-7	-9	-14	11	0.9	0.9	0.9	1.1
ATM	Q13315	1509	-5	-12	-14	11	1.0	0.9	0.9	1.1
RNF169	Q8NCN4	28	-15	-21	-14	11	0.9	0.8	0.9	1.1
ZNHIT2	Q9UHR6	30	3	17	-14	11	1.0	1.2	0.9	1.1
METAP1	P53582	25	15	-1	-14	11	1.2	1.0	0.9	1.1
MAP2K3	P46734	29	-10	-11	-14	11	0.9	0.9	0.9	1.1
AMPD3	Q01432	47	-8	-13	-14	11	0.9	0.9	0.9	1.1
PRRC2C	Q9Y520	481	-8	-13	-14	11	0.9	0.9	0.9	1.1
ASXL2	Q76L83	1225	-5	-14	-14	11	1.0	0.9	0.9	1.1
XPNPEP1	Q9NQW7	279	-2	-19	-14	11	1.0	0.8	0.9	1.1

RPS27A	P62979	144	-13	-4	-14	10	0.9	1.0	0.9	1.1
POLR3A	O14802	261	-22	-15	-14	10	0.8	0.9	0.9	1.1
GTF3C1	Q12789	1977	-14	-12	-14	10	0.9	0.9	0.9	1.1
SRRT	Q9BXP5	412	-5	-12	-14	10	1.0	0.9	0.9	1.1
SRGAP2	A2RUF3	1020	-5	-15	-14	9	1.0	0.9	0.9	1.1
SETD2	Q9BYW2	1125	-9	-15	-14	9	0.9	0.9	0.9	1.1
RUFY1	Q96T51	107	2	-23	-14	9	1.0	0.8	0.9	1.1
STAG1	Q8WVM7	179	-10	-7	-14	9	0.9	0.9	0.9	1.1
MTCH2	Q9Y6C9	296	-7	-7	-14	9	0.9	0.9	0.9	1.1
MLXIP	Q9HAP2	107	0	-12	-14	9	1.0	0.9	0.9	1.1
RTCA	O00442	153	6	-15	-14	9	1.1	0.9	0.9	1.1
SSH2	Q76I76	777	-11	-21	-14	9	0.9	0.8	0.9	1.1
C1orf123	Q9NWV4	102	-12	-22	-14	9	0.9	0.8	0.9	1.1
PRRC2C	Q9Y520	223	-6	-11	-14	8	0.9	0.9	0.9	1.1
ABCF2	Q9UG63	411	-3	-17	-14	8	1.0	0.9	0.9	1.1
WDR81	Q562E7	198	-10	-17	-14	8	0.9	0.9	0.9	1.1
PARP16	Q8N5Y8	169	-4	-18	-14	8	1.0	0.8	0.9	1.1
VPS26B	Q4G0F5	334	-1	-11	-14	8	1.0	0.9	0.9	1.1
XRN2	Q9H0D6	736	-7	-16	-14	8	0.9	0.9	0.9	1.1
DOCK9	Q9BZ29	613	-13	-8	-14	7	0.9	0.9	0.9	1.1
DPP8	Q6V1X1	611	1	-13	-14	7	1.0	0.9	0.9	1.1
CCDC88B	A6NC98	238	-18	-15	-14	7	0.8	0.9	0.9	1.1
ASCC3	Q8N3C0	1535	-19	-15	-14	7	0.8	0.9	0.9	1.1
CRYBG3	Q68DQ2	772	-6	-3	-14	7	0.9	1.0	0.9	1.1
LUC7L2	A0A0A6YYJ8	125	-5	-10	-14	7	1.0	0.9	0.9	1.1
CUL3	Q13618	636	-5	-11	-14	7	1.0	0.9	0.9	1.1
AHSA1	O95433	301	5	-2	-14	6	1.1	1.0	0.9	1.1
RBM5	P52756	766	-5	-8	-14	6	1.0	0.9	0.9	1.1
SMC1A	Q14683	1180	-14	-14	-14	6	0.9	0.9	0.9	1.1
SPTAN1	Q13813	2441	0	-25	-14	6	1.0	0.8	0.9	1.1
FAM65B	Q9Y4F9	35	-15	-5	-14	5	0.9	1.0	0.9	1.1
VCP	P55072	535	-5	-9	-14	5	1.0	0.9	0.9	1.1
PBRM1	Q86U86	450	-7	-15	-14	5	0.9	0.9	0.9	1.1
ASUN	Q9NVM9	119	3	-17	-14	5	1.0	0.9	0.9	1.1
CHML	P26374	278	-9	-19	-14	5	0.9	0.8	0.9	1.1
PHF11	Q9UIL8	311	11	3	-14	5	1.1	1.0	0.9	1.0
CCDC88B	A6NC98	1222	-5	-13	-14	5	1.0	0.9	0.9	1.0
Uncharacteriz	A0A0C4DFX4	643	-4	-19	-14	5	1.0	0.8	0.9	1.0
PHF3	Q92576	276	-10	-22	-14	5	0.9	0.8	0.9	1.0
PPP5C	P53041	11	-8	-7	-14	4	0.9	0.9	0.9	1.0
ZNF816	Q0VGE8	551	-6	-8	-14	4	0.9	0.9	0.9	1.0
UGDH	O60701	276	-12	-10	-14	4	0.9	0.9	0.9	1.0
PFKFB2	O60825	158	-8	-9	-14	4	0.9	0.9	0.9	1.0
NUP88	Q99567	460	-14	-10	-14	4	0.9	0.9	0.9	1.0
ZNF106	Q9H2Y7	457	-7	-24	-14	4	0.9	0.8	0.9	1.0
CSK	P41240	290	-4	-9	-14	3	1.0	0.9	0.9	1.0
MAP4K1	Q92918	335	-11	-11	-14	3	0.9	0.9	0.9	1.0
RPN1	P04843	477	1	-11	-14	3	1.0	0.9	0.9	1.0
CGGBP1	Q9UFW8	92	-7	-14	-14	3	0.9	0.9	0.9	1.0
MTCH2	Q9Y6C9	297	1	-14	-14	3	1.0	0.9	0.9	1.0
TTI2	Q6NXR4	36	-14	-20	-14	3	0.9	0.8	0.9	1.0
MAP3K3	Q99759	411	-6	-7	-14	3	0.9	0.9	0.9	1.0
CTR9	Q6PD62	533	-10	-11	-14	3	0.9	0.9	0.9	1.0
NDUFAB1	O14561	140	-8	-15	-14	3	0.9	0.9	0.9	1.0
BOD1L1	Q8NFC6	1618	-5	-20	-14	3	1.0	0.8	0.9	1.0

NOP9	Q86U38	123	-4	-5	-14	2	1.0	1.0	0.9	1.0
Uncharacteriz	A0A087WZG4	534	-2	-6	-14	2	1.0	0.9	0.9	1.0
ECM29	Q5VYK3	1257	3	-2	-14	2	1.0	1.0	0.9	1.0
NSRP1	Q9H0G5	171	-9	-11	-14	2	0.9	0.9	0.9	1.0
INTS2	Q9H0H0	988	-15	-21	-14	2	0.9	0.8	0.9	1.0
TBC1D15	Q8TC07	686	-9	-6	-14	1	0.9	0.9	0.9	1.0
DARS	P14868	334	-12	-11	-14	1	0.9	0.9	0.9	1.0
RASSF2	P50749	251	-7	-11	-14	1	0.9	0.9	0.9	1.0
ACTL6A	O96019	423	-10	-13	-14	1	0.9	0.9	0.9	1.0
CENPB	P07199	65	-6	-16	-14	1	0.9	0.9	0.9	1.0
INPP5D	Q92835	1050	-2	-16	-14	1	1.0	0.9	0.9	1.0
GNPNAT1	Q96EK6	128	-4	-21	-14	1	1.0	0.8	0.9	1.0
SH2B2	A0A0A0MTI2	366	-25	-30	-14	1	0.8	0.8	0.9	1.0
MYO9B	Q13459	1857	-5	-5	-14	1	1.0	1.0	0.9	1.0
ATRN	O75882	1077	4	-7	-14	1	1.0	0.9	0.9	1.0
GABPA	Q06546	388	10	-8	-14	1	1.1	0.9	0.9	1.0
ASCC3	Q8N3C0	208	-5	-9	-14	1	1.0	0.9	0.9	1.0
NARS	O43776	452	-11	-13	-14	1	0.9	0.9	0.9	1.0
MIF	P14174	81	-4	-17	-14	1	1.0	0.9	0.9	1.0
GPHN	Q9NQX3	293	-16	-1	-14	0	0.9	1.0	0.9	1.0
PLEC	Q15149	3493	2	-4	-14	0	1.0	1.0	0.9	1.0
PAF1	Q8N7H5	36	-6	-5	-14	0	0.9	1.0	0.9	1.0
PHF3	Q92576	1673	-4	-10	-14	0	1.0	0.9	0.9	1.0
IL16	Q14005	1011	-9	-20	-14	0	0.9	0.8	0.9	1.0
AKR1B1	P15121	81	6	-13	-14	-1	1.1	0.9	0.9	1.0
ERI3	O43414	245	-7	-20	-14	-1	0.9	0.8	0.9	1.0
PRPS1	P60891	91	-12	-8	-14	-1	0.9	0.9	0.9	1.0
PRKDC	P78527	123	-8	-9	-14	-1	0.9	0.9	0.9	1.0
IFIT3	O14879	39	2	-11	-14	-1	1.0	0.9	0.9	1.0
RABGGTB	P53611	40	-3	-13	-14	-1	1.0	0.9	0.9	1.0
BUD31	P41223	105	-15	-34	-14	-1	0.9	0.7	0.9	1.0
PPM1B	O75688	172	-8	-11	-14	-2	0.9	0.9	0.9	1.0
GSPT1	P15170	387	-5	-6	-14	-2	1.0	0.9	0.9	1.0
LASP1	Q14847	53	0	-8	-14	-2	1.0	0.9	0.9	1.0
ZFYVE26	Q68DK2	262	-10	-10	-14	-2	0.9	0.9	0.9	1.0
DMXL1	Q9Y485	551	-6	-11	-14	-2	0.9	0.9	0.9	1.0
MCTS1	Q9ULC4	113	-4	-13	-14	-2	1.0	0.9	0.9	1.0
BRMS1	Q9HCU9	136	-4	2	-14	-3	1.0	1.0	0.9	1.0
CDC40	O60508	569	-4	-13	-14	-3	1.0	0.9	0.9	1.0
LGALS12	Q96DT0	87	-9	-11	-14	-3	0.9	0.9	0.9	1.0
MAP3K7	O43318	51	7	-14	-14	-3	1.1	0.9	0.9	1.0
PCBD2	Q9H0N5	109	3	-17	-14	-3	1.0	0.9	0.9	1.0
CYB5R1	Q9UHQ9	208	-14	-7	-14	-4	0.9	0.9	0.9	1.0
TEX264	Q9Y6I9	182	-32	-13	-14	-4	0.8	0.9	0.9	1.0
SERPINB9	P50453	98	-3	-2	-14	-5	1.0	1.0	0.9	1.0
SPECC1L-ADO	F8WAN1	162	-7	-4	-14	-5	0.9	1.0	0.9	1.0
RAB3GAP1	Q15042	873	-2	-7	-14	-5	1.0	0.9	0.9	1.0
CLTC	Q00610	1205	-1	-9	-14	-5	1.0	0.9	0.9	1.0
NGLY1	Q96IV0	197	5	-45	-14	-5	1.1	0.7	0.9	1.0
SGTB	Q96EQ0	67	-2	-9	-14	-6	1.0	0.9	0.9	0.9
DOK2	O60496	132	-5	-10	-14	-6	1.0	0.9	0.9	0.9
CISD2	Q8N5K1	92	-3	-10	-14	-6	1.0	0.9	0.9	0.9
DARS2	Q6PI48	152	-14	-12	-14	-6	0.9	0.9	0.9	0.9
AKAP11	Q9UKA4	1501	-5	-12	-14	-6	1.0	0.9	0.9	0.9
NUP205	Q92621	1216	-3	-11	-14	-6	1.0	0.9	0.9	0.9

SH3RF1	Q7Z6J0	107	-13	-12	-14	-6	0.9	0.9	0.9	0.9
FAM117B	Q6P1L5	496	-13	-12	-14	-7	0.9	0.9	0.9	0.9
PARG	Q86W56	155	-6	-23	-14	-7	0.9	0.8	0.9	0.9
NUP160	Q12769	1187	-3	-15	-14	-8	1.0	0.9	0.9	0.9
CASP6	P55212	264	-2	-8	-14	-8	1.0	0.9	0.9	0.9
NCOR1	O75376	943	-15	-12	-14	-8	0.9	0.9	0.9	0.9
RNF213	Q63HN8	1156	-5	-9	-14	-9	1.0	0.9	0.9	0.9
GDI1	P31150	202	-11	-12	-14	-10	0.9	0.9	0.9	0.9
CLIC1	O00299	178	-8	-14	-14	-10	0.9	0.9	0.9	0.9
CAPN1	P07384	590	-1	-5	-14	-10	1.0	1.0	0.9	0.9
ZC3H7B	Q9UGR2	142	-14	-11	-14	-10	0.9	0.9	0.9	0.9
CUL4A	Q13619	94	1	-11	-14	-11	1.0	0.9	0.9	0.9
PARP14	Q460N5	522	-6	-18	-14	-11	0.9	0.8	0.9	0.9
KIAA0100	Q14667	1979	2	-6	-14	-11	1.0	0.9	0.9	0.9
ACOT7	O00154	288	-2	2	-14	-12	1.0	1.0	0.9	0.9
Uncharacteriz	A0A0B4J203	216	-3	-10	-14	-12	1.0	0.9	0.9	0.9
WDR1	O75083	438	-11	-10	-14	-12	0.9	0.9	0.9	0.9
GNAI3	P08754	139	-10	-24	-14	-12	0.9	0.8	0.9	0.9
WDR1	O75083	325	-10	-23	-14	-12	0.9	0.8	0.9	0.9
MPG	P29372	222	-8	-10	-14	-13	0.9	0.9	0.9	0.9
ABCF1	Q8NE71	758	-7	-10	-14	-13	0.9	0.9	0.9	0.9
APEH	P13798	30	-27	-10	-14	-14	0.8	0.9	0.9	0.9
LASP1	Q14847	32	-6	-15	-14	-14	0.9	0.9	0.9	0.9
VPS18	Q9P253	22	-12	-22	-14	-15	0.9	0.8	0.9	0.9
RIN3	Q8TB24	60	-13	-21	-14	-16	0.9	0.8	0.9	0.9
ARMCX3	Q9UH62	52	8	-13	-14	-16	1.1	0.9	0.9	0.9
CNBP	P62633	150	-29	-44	-14	-16	0.8	0.7	0.9	0.9
DYSF	O75923	1420	1	-4	-14	-17	1.0	1.0	0.9	0.9
ASAP1	Q9ULH1	557	2	-12	-14	-17	1.0	0.9	0.9	0.9
GNB4	Q9HAV0	204	-15	-12	-14	-18	0.9	0.9	0.9	0.9
ADD3	Q9UEY8	245	3	-10	-14	-18	1.0	0.9	0.9	0.8
HK1	P19367	823	-13	-12	-14	-18	0.9	0.9	0.9	0.8
PDS5B	Q9NTI5	573	-16	-13	-14	-19	0.9	0.9	0.9	0.8
CAPN1	P07384	677	6	-32	-14	-19	1.1	0.8	0.9	0.8
PFKFB3	Q16875	440	-3	-13	-14	-23	1.0	0.9	0.9	0.8
RNF213	Q63HN8	1325	-12	-18	-14	-23	0.9	0.9	0.9	0.8
FLNA	P21333	631	-4	-7	-14	-25	1.0	0.9	0.9	0.8
FLNC	Q14315	626	-4	-7	-14	-25	1.0	0.9	0.9	0.8
CORO1C	Q9ULV4	343	-6	-24	-14	-25	0.9	0.8	0.9	0.8
LRCH4	O75427	179	-8	-11	-14	-27	0.9	0.9	0.9	0.8
DNM1L	O00429	367	-3	-22	-14	-28	1.0	0.8	0.9	0.8
ABHD16A	O95870	248	-23	-34	-14	-35	0.8	0.7	0.9	0.7
NCK2	O43639	144	-23	-4	-14	-39	0.8	1.0	0.9	0.7
NCK1	P16333	139	-23	-4	-14	-39	0.8	1.0	0.9	0.7
MAVS	Q7Z434	283	-5	-11	-14	-43	1.0	0.9	0.9	0.7
DUSP12	Q9UNI6	115	-20	-5	-14	-59	0.8	1.0	0.9	0.6
SPTBN1	Q01082	619	-3	-35	-14	-72	1.0	0.7	0.9	0.6
PLEK	P08567	160	-3	-14	-14	-75	1.0	0.9	0.9	0.6
PDLIM7	Q9NR12	311	-4	-2	-14	-82	1.0	1.0	0.9	0.5
TLN1	Q9Y490	1506	1	-29	-14	-96	1.0	0.8	0.9	0.5
SKI	P12755	10	-10	-23	-15	42	0.9	0.8	0.9	1.7
IKBKB	O14920	464	-16	-9	-15	35	0.9	0.9	0.9	1.5
ICE1	Q9Y2F5	2094	-14	-13	-15	34	0.9	0.9	0.9	1.5
PDE3B	Q13370	321	-2	-3	-15	34	1.0	1.0	0.9	1.5
UBR4	Q5T4S7	140	-2	-11	-15	33	1.0	0.9	0.9	1.5

DYSF	O75923	933	-10	-17	-15	31	0.9	0.9	0.9	1.4
MUM1	Q2TAK8	548	1	-3	-15	30	1.0	1.0	0.9	1.4
LRRC8A	Q8IWT6	768	5	-13	-15	30	1.0	0.9	0.9	1.4
IMPA1	P29218	141	-4	-14	-15	30	1.0	0.9	0.9	1.4
ARL6IP4	Q66PJ3	410	-9	-8	-15	30	0.9	0.9	0.9	1.4
SHMT2	P34897	80	-10	-19	-15	30	0.9	0.8	0.9	1.4
SENP7	Q9BQF6	85	-8	-14	-15	27	0.9	0.9	0.9	1.4
HNRNPR	O43390	292	-8	-12	-15	27	0.9	0.9	0.9	1.4
SYNCRIP	O60506	289	-8	-12	-15	27	0.9	0.9	0.9	1.4
SYNE2	Q8WXH0	553	-7	-9	-15	26	0.9	0.9	0.9	1.3
PFKL	P17858	351	-1	-3	-15	22	1.0	1.0	0.9	1.3
CHORDC1	Q9UHD1	162	-4	-22	-15	22	1.0	0.8	0.9	1.3
GTF3C5	Q9Y5Q8	75	-7	-2	-15	21	0.9	1.0	0.9	1.3
PEX1	O43933	1185	-8	-13	-15	21	0.9	0.9	0.9	1.3
PIAS1	O75925	481	-3	-7	-15	20	1.0	0.9	0.9	1.3
SLC27A3	Q5K4L6	426	-5	-16	-15	20	1.0	0.9	0.9	1.3
SP100	P23497	289	0	-7	-15	19	1.0	0.9	0.9	1.2
SKIV2L	Q15477	247	-13	-15	-15	19	0.9	0.9	0.9	1.2
PCNT	O95613	2614	-7	-8	-15	18	0.9	0.9	0.9	1.2
BAG5	Q9UL15	327	-6	-10	-15	18	0.9	0.9	0.9	1.2
NLRC3	Q7RTR2	561	-8	-3	-15	17	0.9	1.0	0.9	1.2
RETSAT	Q6NUM9	534	-13	-17	-15	17	0.9	0.9	0.9	1.2
ADCK4	Q96D53	293	-28	-34	-15	17	0.8	0.7	0.9	1.2
HADHA	P40939	110	-9	-8	-15	17	0.9	0.9	0.9	1.2
AIM1	Q9Y4K1	1574	8	8	-15	16	1.1	1.1	0.9	1.2
CCNYL1	Q8N7R7	335	-6	-13	-15	16	0.9	0.9	0.9	1.2
ANXA4	P09525	108	-9	-15	-15	16	0.9	0.9	0.9	1.2
RABEP2	Q9H5N1	422	-7	-17	-15	16	0.9	0.9	0.9	1.2
CIR1	Q86X95	10	2	-7	-15	16	1.0	0.9	0.9	1.2
NCAPD2	Q15021	596	1	-9	-15	16	1.0	0.9	0.9	1.2
XPO5	Q9HAV4	706	-13	-16	-15	16	0.9	0.9	0.9	1.2
RNF213	Q63HN8	3084	-6	-14	-15	15	0.9	0.9	0.9	1.2
PUM2	Q8TB72	169	-4	-20	-15	15	1.0	0.8	0.9	1.2
GPATCH8	Q9UKJ3	795	-1	-18	-15	15	1.0	0.8	0.9	1.2
ALOX5	P09917	450	3	-21	-15	15	1.0	0.8	0.9	1.2
IKBKAP	O95163	453	-23	6	-15	14	0.8	1.1	0.9	1.2
RABGAP1	Q9Y3P9	533	1	2	-15	14	1.0	1.0	0.9	1.2
NSUN2	Q08J23	502	-1	-7	-15	14	1.0	0.9	0.9	1.2
MARC1	Q5VT66	273	-13	-7	-15	14	0.9	0.9	0.9	1.2
CPSF1	Q10570	1044	1	-9	-15	14	1.0	0.9	0.9	1.2
LRRFIP1	Q32MZ4	14	-4	-13	-15	14	1.0	0.9	0.9	1.2
PDE4DIP	Q5VU43	603	-9	-12	-15	14	0.9	0.9	0.9	1.2
FAM21A	Q641Q2	594	-8	-14	-15	14	0.9	0.9	0.9	1.2
SRRM2	Q9UQ35	872	-8	-2	-15	13	0.9	1.0	0.9	1.1
ZC3H12A	Q5D1E8	223	0	-3	-15	13	1.0	1.0	0.9	1.1
NFXL1	Q6ZNB6	823	-6	-10	-15	13	0.9	0.9	0.9	1.1
RETSAT	Q6NUM9	105	-2	-12	-15	13	1.0	0.9	0.9	1.1
NUP155	O75694	373	-13	-17	-15	13	0.9	0.9	0.9	1.1
TNRC6C	Q9HCJ0	868	-7	-25	-15	13	0.9	0.8	0.9	1.1
ARHGAP30	Q7Z6I6	1021	0	-7	-15	13	1.0	0.9	0.9	1.1
HUWE1	Q7Z6Z7	2721	-14	-12	-15	13	0.9	0.9	0.9	1.1
IFIT3	O14879	239	-2	-4	-15	12	1.0	1.0	0.9	1.1
NEK4	P51957	466	1	-12	-15	12	1.0	0.9	0.9	1.1
XRCC6	P12956	398	-9	-5	-15	12	0.9	1.0	0.9	1.1
SIPA1L1	O43166	105	-5	-10	-15	12	1.0	0.9	0.9	1.1

POLR3E	Q9NVU0	456	-5	-12	-15	12	1.0	0.9	0.9	1.1
KIAA0391	O15091	367	5	-14	-15	12	1.0	0.9	0.9	1.1
SON	P18583	2070	-9	-15	-15	12	0.9	0.9	0.9	1.1
SIRT7	Q9NRC8	79	6	-5	-15	11	1.1	1.0	0.9	1.1
NDUFV1	P49821	255	-10	-11	-15	11	0.9	0.9	0.9	1.1
RANBP6	O60518	31	-17	-13	-15	11	0.9	0.9	0.9	1.1
ARHGEF6	Q15052	738	-3	-5	-15	11	1.0	1.0	0.9	1.1
ABCB8	Q9NUT2	462	2	-6	-15	11	1.0	0.9	0.9	1.1
EIF3M	Q7L2H7	175	-3	-10	-15	11	1.0	0.9	0.9	1.1
WARS	P23381	309	-10	-12	-15	11	0.9	0.9	0.9	1.1
KRI1	Q8N9T8	531	-7	-2	-15	10	0.9	1.0	0.9	1.1
TBC1D4	O60343	1277	-8	-14	-15	10	0.9	0.9	0.9	1.1
SLFN13	Q68D06	884	7	9	-15	10	1.1	1.1	0.9	1.1
FBXO18	Q8NFZ0	302	-5	-2	-15	10	1.0	1.0	0.9	1.1
NEDD9	Q14511	281	-3	-10	-15	10	1.0	0.9	0.9	1.1
ATM	Q13315	2323	-3	-12	-15	10	1.0	0.9	0.9	1.1
SPG11	Q96JI7	1019	3	-1	-15	9	1.0	1.0	0.9	1.1
LTN1	O94822	237	-5	-18	-15	9	1.0	0.8	0.9	1.1
S100A8	P05109	42	-9	-20	-15	9	0.9	0.8	0.9	1.1
NUMA1	Q14980	2009	-10	-8	-15	9	0.9	0.9	0.9	1.1
ZNF687	Q8N1G0	91	-13	-9	-15	9	0.9	0.9	0.9	1.1
ACLY	P53396	742	-5	-12	-15	9	1.0	0.9	0.9	1.1
IRF2BP1	Q8IU81	363	-12	-13	-15	9	0.9	0.9	0.9	1.1
UBR5	O95071	2314	1	-15	-15	9	1.0	0.9	0.9	1.1
WDR20	Q8TBZ3	514	5	-2	-15	8	1.0	1.0	0.9	1.1
AKAP8	O43823	173	-8	-4	-15	8	0.9	1.0	0.9	1.1
TRMT2A	Q8IZ69	463	-14	-6	-15	8	0.9	0.9	0.9	1.1
CENPC	Q03188	800	-9	-8	-15	8	0.9	0.9	0.9	1.1
GNLY	P22749	43	-7	-8	-15	8	0.9	0.9	0.9	1.1
PPIG	Q13427	10	-19	-9	-15	8	0.8	0.9	0.9	1.1
WDFY3	Q8IZQ1	3027	0	-11	-15	8	1.0	0.9	0.9	1.1
SH2D3C	Q8N5H7	317	-9	-13	-15	8	0.9	0.9	0.9	1.1
MAT2A	P31153	56	-6	-14	-15	8	0.9	0.9	0.9	1.1
CWF19L1	Q69YN2	511	-8	-6	-15	7	0.9	0.9	0.9	1.1
TRAFD1	O14545	476	-8	-9	-15	7	0.9	0.9	0.9	1.1
RABIF	P47224	106	-7	-10	-15	7	0.9	0.9	0.9	1.1
MTOR	P42345	1214	-7	-8	-15	7	0.9	0.9	0.9	1.1
PRPF4	O43172	299	-1	-10	-15	7	1.0	0.9	0.9	1.1
SLIRP	Q9GZT3	48	9	-11	-15	7	1.1	0.9	0.9	1.1
TRIP11	Q15643	212	-2	-16	-15	7	1.0	0.9	0.9	1.1
WIZ	O95785	1429	-13	-18	-15	7	0.9	0.8	0.9	1.1
GABPA	Q06546	22	-1	-1	-15	6	1.0	1.0	0.9	1.1
CDC37L1	Q7L3B6	67	-8	-4	-15	6	0.9	1.0	0.9	1.1
MCM3AP	O60318	981	-3	-10	-15	6	1.0	0.9	0.9	1.1
PRKDC	P78527	3837	-5	-10	-15	6	1.0	0.9	0.9	1.1
PRKDC	P78527	1335	-1	-12	-15	6	1.0	0.9	0.9	1.1
TBC1D14	Q9P2M4	219	-34	-15	-15	6	0.7	0.9	0.9	1.1
MAP3K2	Q9Y2U5	405	-2	-7	-15	6	1.0	0.9	0.9	1.1
PAXIP1	Q6ZW49	985	-8	-15	-15	6	0.9	0.9	0.9	1.1
ADAR	P55265	630	-1	-16	-15	6	1.0	0.9	0.9	1.1
TPRN	Q4KMQ1	536	6	-24	-15	6	1.1	0.8	0.9	1.1
ATP13A1	Q9HD20	711	3	-1	-15	5	1.0	1.0	0.9	1.1
TBK1	Q9UHD2	423	0	-8	-15	5	1.0	0.9	0.9	1.1
CRTC2	Q53ET0	515	-10	-8	-15	5	0.9	0.9	0.9	1.1
DOCK11	Q5JSL3	1906	-3	-10	-15	5	1.0	0.9	0.9	1.1

SH3GL1	Q99961	277	-3	-10	-15	5	1.0	0.9	0.9	1.1
CNBP	P62633	140	-5	-12	-15	5	1.0	0.9	0.9	1.1
KPNB1	Q14974	228	-9	-18	-15	5	0.9	0.8	0.9	1.1
CSRP2BP	Q9H8E8	547	-7	-4	-15	5	0.9	1.0	0.9	1.0
USP9Y	O00507	1735	4	-5	-15	5	1.0	1.0	0.9	1.0
USP9X	Q93008	1733	4	-5	-15	5	1.0	1.0	0.9	1.0
SAMD9	Q5K651	1538	-2	-7	-15	5	1.0	0.9	0.9	1.0
FAM98C	Q17RN3	302	-11	-10	-15	5	0.9	0.9	0.9	1.0
ASH2L	Q9UBL3	581	-9	-11	-15	5	0.9	0.9	0.9	1.0
DOK3	Q7L591	493	-17	-15	-15	5	0.9	0.9	0.9	1.0
PCM1	Q15154	1717	-7	-19	-15	5	0.9	0.8	0.9	1.0
CAMK1D	Q8IU85	354	13	-2	-15	4	1.1	1.0	0.9	1.0
BCL11B	Q9C0K0	81	-8	-11	-15	4	0.9	0.9	0.9	1.0
FGR	P09769	273	-5	-12	-15	4	1.0	0.9	0.9	1.0
PGK1	P00558	50	-7	-12	-15	4	0.9	0.9	0.9	1.0
DENND4A	Q7Z401	1137	-6	-12	-15	4	0.9	0.9	0.9	1.0
ANAPC7	Q9UJX3	131	-10	-12	-15	4	0.9	0.9	0.9	1.0
VPS13C	Q709C8	2872	-7	-13	-15	4	0.9	0.9	0.9	1.0
PTPRC	P08575	309	-10	-15	-15	4	0.9	0.9	0.9	1.0
NOL11	Q9H8H0	578	-10	-28	-15	4	0.9	0.8	0.9	1.0
PHKB	Q93100	559	-13	-7	-15	4	0.9	0.9	0.9	1.0
SNX27	Q96L92	432	-17	-10	-15	4	0.9	0.9	0.9	1.0
PCNT	O95613	2576	-12	-14	-15	4	0.9	0.9	0.9	1.0
TOR4A	Q9NXH8	374	-22	-20	-15	4	0.8	0.8	0.9	1.0
SRPRB	Q9Y5M8	73	-4	0	-15	3	1.0	1.0	0.9	1.0
RBCK1	Q9BYM8	323	-16	-10	-15	3	0.9	0.9	0.9	1.0
AKAP9	Q99996	1493	-2	-22	-15	3	1.0	0.8	0.9	1.0
VPS72	Q15906	292	-18	-26	-15	3	0.9	0.8	0.9	1.0
AP2B1	P63010	241	4	-3	-15	3	1.0	1.0	0.9	1.0
AP1B1	Q10567	241	4	-3	-15	3	1.0	1.0	0.9	1.0
MORC3	Q14149	671	-11	-5	-15	3	0.9	1.0	0.9	1.0
ZC3HC1	Q86WB0	125	-13	-6	-15	3	0.9	0.9	0.9	1.0
TANGO2	Q6ICL3	231	-8	5	-15	2	0.9	1.0	0.9	1.0
WWC3	Q9ULE0	988	-7	-9	-15	2	0.9	0.9	0.9	1.0
TEP1	Q99973	2053	-6	-8	-15	2	0.9	0.9	0.9	1.0
COPA	P53621	453	-2	-9	-15	2	1.0	0.9	0.9	1.0
MORC2	Q9Y6X9	270	-8	-20	-15	2	0.9	0.8	0.9	1.0
BRD8	Q9H0E9	142	-8	-10	-15	1	0.9	0.9	0.9	1.0
RNF213	Q63HN8	1788	1	-12	-15	1	1.0	0.9	0.9	1.0
C17orf59	Q96GS4	176	-7	-15	-15	1	0.9	0.9	0.9	1.0
LSM14B	Q9BX40	310	-18	-37	-15	1	0.9	0.7	0.9	1.0
RPRD2	Q5VT52	222	1	-10	-15	1	1.0	0.9	0.9	1.0
ZC3HAV1	Q7Z2W4	78	-7	-9	-15	0	0.9	0.9	0.9	1.0
DOCK10	Q96BY6	2164	-11	-12	-15	0	0.9	0.9	0.9	1.0
NUP58	Q9BVL2	252	-12	-13	-15	0	0.9	0.9	0.9	1.0
RPAP3	Q9H6T3	519	-5	-15	-15	0	1.0	0.9	0.9	1.0
GOT2	P00505	187	-11	-23	-15	0	0.9	0.8	0.9	1.0
PDIA3	P30101	409	-7	-23	-15	0	0.9	0.8	0.9	1.0
FAM129A	Q9BZQ8	409	2	-5	-15	-1	1.0	1.0	0.9	1.0
MDH1	P40925	137	-5	-7	-15	-1	1.0	0.9	0.9	1.0
MCM3AP	O60318	641	-7	-9	-15	-1	0.9	0.9	0.9	1.0
BPTF	Q12830	1746	-1	0	-15	-1	1.0	1.0	0.9	1.0
KMT2A	Q03164	2074	-4	-3	-15	-1	1.0	1.0	0.9	1.0
LLGL1	Q15334	505	-2	-12	-15	-1	1.0	0.9	0.9	1.0
BOD1L1	Q8NFC6	2384	-11	-24	-15	-1	0.9	0.8	0.9	1.0

EIF4G3	O43432	461	0	4	-15	-2	1.0	1.0	0.9	1.0
DEF6	Q9H4E7	253	-7	-14	-15	-2	0.9	0.9	0.9	1.0
ZFP91	Q96JP5	182	-10	-15	-15	-2	0.9	0.9	0.9	1.0
GCC2	Q8IWJ2	399	-5	-18	-15	-2	1.0	0.8	0.9	1.0
ACOT2	P49753	76	-8	-2	-15	-2	0.9	1.0	0.9	1.0
POTEJ	P0CG39	928	2	-3	-15	-2	1.0	1.0	0.9	1.0
POTEE	Q6S8J3	965	2	-3	-15	-2	1.0	1.0	0.9	1.0
SMARCA2	P51531	399	-7	-11	-15	-2	0.9	0.9	0.9	1.0
ASMTL	O95671	274	-12	-13	-15	-2	0.9	0.9	0.9	1.0
SH3BP1	Q9Y3L3	388	-16	6	-15	-3	0.9	1.1	0.9	1.0
THOC2	Q8NI27	804	-17	-16	-15	-3	0.9	0.9	0.9	1.0
PPP1R3D	O95685	97	-7	-18	-15	-3	0.9	0.9	0.9	1.0
MTMR14	Q8NCE2	131	-20	-21	-15	-3	0.8	0.8	0.9	1.0
PPM1M	Q96MI6	9	-14	-10	-15	-3	0.9	0.9	0.9	1.0
RIPK2	O43353	380	-10	-11	-15	-3	0.9	0.9	0.9	1.0
SLC22A16	Q86VW1	320	-3	-14	-15	-3	1.0	0.9	0.9	1.0
SCAF11	Q99590	553	-5	-20	-15	-3	1.0	0.8	0.9	1.0
SMAD2	Q15796	129	-2	-7	-15	-4	1.0	0.9	0.9	1.0
C3	P01024	1158	-16	-8	-15	-4	0.9	0.9	0.9	1.0
DHX9	Q08211	1099	-1	-9	-15	-4	1.0	0.9	0.9	1.0
PHF12	Q96QT6	534	-7	-13	-15	-4	0.9	0.9	0.9	1.0
CARD11	Q9BXL7	718	-8	-20	-15	-4	0.9	0.8	0.9	1.0
ZBTB1	Q9Y2K1	193	-5	-27	-15	-4	1.0	0.8	0.9	1.0
RANBP2	P49792	1566	-5	0	-15	-4	1.0	1.0	0.9	1.0
RIOK3	O14730	287	-8	-11	-15	-4	0.9	0.9	0.9	1.0
SYK	P43405	593	-6	-8	-15	-5	0.9	0.9	0.9	1.0
PINX1	Q96BK5	265	-6	-14	-15	-5	0.9	0.9	0.9	1.0
MTHFD1	P11586	863	-6	-10	-15	-5	0.9	0.9	0.9	1.0
SDHA	P31040	287	-23	-34	-15	-5	0.8	0.7	0.9	1.0
ANXA4	P09525	198	-11	-13	-15	-6	0.9	0.9	0.9	0.9
DDX21	Q9NR30	445	4	-6	-15	-6	1.0	0.9	0.9	0.9
POTEE	Q6S8J3	957	-4	-3	-15	-7	1.0	1.0	0.9	0.9
MCM3	P25205	263	-4	-14	-15	-7	1.0	0.9	0.9	0.9
POLDIP2	Q9Y2S7	143	-1	-16	-15	-7	1.0	0.9	0.9	0.9
UTRN	P46939	3076	-14	-21	-15	-7	0.9	0.8	0.9	0.9
TOP3B	O95985	190	3	-19	-15	-8	1.0	0.8	0.9	0.9
FAM192A	Q9GZU8	44	-12	-7	-15	-8	0.9	0.9	0.9	0.9
TUBB2B	Q9BVA1	239	-9	-19	-15	-9	0.9	0.8	0.9	0.9
DLGAP4	Q9Y2H0	726	-18	-22	-15	-9	0.9	0.8	0.9	0.9
SLFN5	Q08AF3	14	-11	-11	-15	-9	0.9	0.9	0.9	0.9
RAD17	O75943	326	-15	-16	-15	-10	0.9	0.9	0.9	0.9
DXO	O77932	108	-3	1	-15	-11	1.0	1.0	0.9	0.9
SNX2	O60749	332	3	-6	-15	-11	1.0	0.9	0.9	0.9
ISCU	Q9H1K1	69	-4	-7	-15	-11	1.0	0.9	0.9	0.9
PPP6R1	Q9UPN7	37	-4	-11	-15	-11	1.0	0.9	0.9	0.9
INPP5D	Q92835	506	-9	-16	-15	-11	0.9	0.9	0.9	0.9
NR4A2	P43354	190	-20	-16	-15	-12	0.8	0.9	0.9	0.9
LTN1	O94822	1131	-10	-25	-15	-13	0.9	0.8	0.9	0.9
PPA2	Q9H2U2	180	-4	-25	-15	-13	1.0	0.8	0.9	0.9
ISCU	Q9H1K1	95	-13	-10	-15	-15	0.9	0.9	0.9	0.9
MFN1	Q8IWA4	418	-24	-34	-15	-15	0.8	0.7	0.9	0.9
PPAN	Q9NQ55	432	-5	-20	-15	-16	1.0	0.8	0.9	0.9
INO80	Q9ULG1	673	-9	-8	-15	-17	0.9	0.9	0.9	0.9
NSF	P46459	568	-1	-10	-15	-19	1.0	0.9	0.9	0.8
TTC27	Q6P3X3	299	-3	-15	-15	-19	1.0	0.9	0.9	0.8

CORO1C	Q9ULV4	330	-9	-16	-15	-22	0.9	0.9	0.9	0.8
ATG14	Q6ZNE5	17	-12	-21	-15	-23	0.9	0.8	0.9	0.8
MCU	Q8NE86	191	-6	-7	-15	-26	0.9	0.9	0.9	0.8
FIG4	Q92562	839	-12	-9	-15	-26	0.9	0.9	0.9	0.8
CNOT2	Q9NZN8	408	-8	-35	-15	-28	0.9	0.7	0.9	0.8
TOR4A	Q9NXH8	239	-1	-18	-15	-29	1.0	0.9	0.9	0.8
ERP44	Q9BS26	92	-14	-29	-15	-30	0.9	0.8	0.9	0.8
SYNE2	Q8WXH0	1432	-8	-21	-15	-32	0.9	0.8	0.9	0.8
CHD4	Q14839	1468	12	-15	-15	-37	1.1	0.9	0.9	0.7
TAOK1	Q7L7X3	798	-21	-9	-15	-42	0.8	0.9	0.9	0.7
CNST	Q6PJW8	268	-7	-21	-15	-44	0.9	0.8	0.9	0.7
RREB1	Q92766	1248	-9	-26	-15	-46	0.9	0.8	0.9	0.7
PTK2	Q05397	459	-4	-9	-15	-65	1.0	0.9	0.9	0.6
MATK	P42679	16	-12	-39	-15	-67	0.9	0.7	0.9	0.6
FHL1	Q13642	212	-5	-8	-15	-68	1.0	0.9	0.9	0.6
TLN1	Q9Y490	1353	-3	-11	-15	-73	1.0	0.9	0.9	0.6
SLC37A1	P57057	250	-5	-15	-15	-86	1.0	0.9	0.9	0.5
ITGAL	P20701	1154	6	-8	-15	42	1.1	0.9	0.9	1.7
WDR74	Q6RFH5	134	-6	-27	-15	36	0.9	0.8	0.9	1.6
IFIT3	O14879	343	-4	-4	-15	33	1.0	1.0	0.9	1.5
HTT	P42858	777	-4	-16	-15	31	1.0	0.9	0.9	1.4
AHCTF1	Q8WYP5	722	-2	-20	-15	31	1.0	0.8	0.9	1.4
OARD1	Q9Y530	24	0	-4	-15	30	1.0	1.0	0.9	1.4
UBASH3A	P57075	435	-8	0	-15	25	0.9	1.0	0.9	1.3
BOD1L1	Q8NFC6	1947	-6	-16	-15	24	0.9	0.9	0.9	1.3
MAFK	O60675	68	-10	-10	-15	23	0.9	0.9	0.9	1.3
CABIN1	Q9Y6J0	207	-5	-6	-15	22	1.0	0.9	0.9	1.3
SUPT16H	Q9Y5B9	574	-8	-9	-15	22	0.9	0.9	0.9	1.3
DENND1B	Q6P3S1	356	-4	-2	-15	21	1.0	1.0	0.9	1.3
NHSL2	Q5HYW2	85	-2	-3	-15	20	1.0	1.0	0.9	1.3
TRIP11	Q15643	726	-2	-11	-15	20	1.0	0.9	0.9	1.3
PJA2	O43164	48	-15	-22	-15	20	0.9	0.8	0.9	1.2
SMCHD1	A6NHR9	505	-9	-9	-15	18	0.9	0.9	0.9	1.2
ADAR	P55265	125	-2	-23	-15	18	1.0	0.8	0.9	1.2
LMO2	P25791	130	-22	-23	-15	18	0.8	0.8	0.9	1.2
DNMT1	P26358	41	1	-13	-15	18	1.0	0.9	0.9	1.2
ZZEF1	O43149	2229	3	-17	-15	17	1.0	0.9	0.9	1.2
APBA3	O96018	500	3	-6	-15	16	1.0	0.9	0.9	1.2
LAGE3	Q14657	113	4	-9	-15	16	1.0	0.9	0.9	1.2
FUK	Q8N0W3	787	-3	-15	-15	16	1.0	0.9	0.9	1.2
EZR	P15311	117	-11	-6	-15	15	0.9	0.9	0.9	1.2
GBF1	Q92538	1430	-3	-9	-15	15	1.0	0.9	0.9	1.2
SEH1L	Q96EE3	77	-18	-13	-15	15	0.9	0.9	0.9	1.2
PML	P29590	479	-12	-19	-15	15	0.9	0.8	0.9	1.2
PHKG2	P15735	294	-17	-5	-15	15	0.9	1.0	0.9	1.2
DCTN2	Q13561	240	4	1	-15	14	1.0	1.0	0.9	1.2
ZNF350	Q9GZX5	332	-10	-8	-15	14	0.9	0.9	0.9	1.2
THAP11	Q96EK4	48	-2	-19	-15	14	1.0	0.8	0.9	1.2
IPMK	Q8NFU5	373	-14	-18	-15	13	0.9	0.9	0.9	1.1
INPP4A	Q96PE3	854	-6	-9	-15	13	0.9	0.9	0.9	1.1
PGM2	Q96G03	510	-1	-15	-15	13	1.0	0.9	0.9	1.1
LRRK2	Q5S007	1123	-5	-6	-15	12	1.0	0.9	0.9	1.1
MDH2	P40926	275	-12	-15	-15	12	0.9	0.9	0.9	1.1
NOP9	Q86U38	242	1	-4	-15	12	1.0	1.0	0.9	1.1
EEF1G	P26641	266	2	-10	-15	12	1.0	0.9	0.9	1.1

MAP3K6	O95382	803	-13	-22	-15	12	0.9	0.8	0.9	1.1
PRR12	Q9ULL5	173	2	-7	-15	11	1.0	0.9	0.9	1.1
RBM15	Q96T37	926	-12	-11	-15	11	0.9	0.9	0.9	1.1
BOD1L1	Q8NFC6	285	-6	-13	-15	11	0.9	0.9	0.9	1.1
MYSM1	Q5VVJ2	445	-11	-3	-15	11	0.9	1.0	0.9	1.1
ARHGAP26	Q9UNA1	441	3	-5	-15	11	1.0	1.0	0.9	1.1
ESD	P10768	45	-7	-9	-15	11	0.9	0.9	0.9	1.1
AP4E1	Q9UPM8	1119	-12	-12	-15	11	0.9	0.9	0.9	1.1
PLPP6	Q8IY26	55	-5	-15	-15	11	1.0	0.9	0.9	1.1
MED13	Q9UHV7	402	5	-15	-15	11	1.0	0.9	0.9	1.1
TRIM25	Q14258	498	-5	-16	-15	11	1.0	0.9	0.9	1.1
MACF1	Q9UPN3	666	2	-17	-15	11	1.0	0.9	0.9	1.1
ALDH16A1	Q8IZ83	467	1	-10	-15	10	1.0	0.9	0.9	1.1
PNN	Q9H307	439	-6	-10	-15	10	0.9	0.9	0.9	1.1
WARS	P23381	225	-8	0	-15	10	0.9	1.0	0.9	1.1
VPS13D	Q5THJ4	3795	-9	-15	-15	10	0.9	0.9	0.9	1.1
WIZ	O95785	1279	3	-8	-15	9	1.0	0.9	0.9	1.1
MALT1	Q9UDY8	441	-8	-9	-15	9	0.9	0.9	0.9	1.1
SCRN3	Q0VDG4	143	-10	-12	-15	9	0.9	0.9	0.9	1.1
XPOT	O43592	522	-6	-6	-15	9	0.9	0.9	0.9	1.1
AKAP13	Q12802	972	-5	-8	-15	9	1.0	0.9	0.9	1.1
STAT1	P42224	108	3	-8	-15	9	1.0	0.9	0.9	1.1
SNX27	Q96L92	519	-8	-9	-15	9	0.9	0.9	0.9	1.1
KMT5A	Q9NQR1	186	-4	-9	-15	9	1.0	0.9	0.9	1.1
GNB2	P62879	204	-5	-11	-15	9	1.0	0.9	0.9	1.1
NHEJ1	Q9H9Q4	74	-5	-11	-15	9	1.0	0.9	0.9	1.1
EPRS	P07814	381	-4	-6	-15	8	1.0	0.9	0.9	1.1
MSN	P26038	117	-7	-14	-15	8	0.9	0.9	0.9	1.1
CREBBP	Q92793	1212	-5	-17	-15	8	1.0	0.9	0.9	1.1
MMS19	Q96T76	794	10	-21	-15	8	1.1	0.8	0.9	1.1
DENND4A	Q7Z401	1561	-4	18	-15	8	1.0	1.2	0.9	1.1
DNAJB1	P25685	267	4	-17	-15	8	1.0	0.9	0.9	1.1
RB1CC1	Q8TDY2	853	-6	1	-15	7	0.9	1.0	0.9	1.1
Uncharacteriz	A0A087WZG4	664	-10	-13	-15	7	0.9	0.9	0.9	1.1
NPRL2	Q8WTW4	348	-3	-16	-15	7	1.0	0.9	0.9	1.1
MSL1	Q68DK7	125	-6	-18	-15	7	0.9	0.9	0.9	1.1
MKLN1	Q9UL63	411	-8	-14	-15	7	0.9	0.9	0.9	1.1
MARCH7	Q9H992	394	-10	-2	-15	6	0.9	1.0	0.9	1.1
CNOT10	Q9H9A5	504	-5	-4	-15	6	1.0	1.0	0.9	1.1
DUS3L	Q96G46	396	-4	-7	-15	6	1.0	0.9	0.9	1.1
TTC4	O95801	63	-3	-19	-15	6	1.0	0.8	0.9	1.1
ZNF101	Q8IZC7	321	-7	-23	-15	6	0.9	0.8	0.9	1.1
YTHDC2	Q9H6S0	460	20	0	-15	6	1.3	1.0	0.9	1.1
USP22	Q9UPT9	44	-4	-1	-15	6	1.0	1.0	0.9	1.1
XPO1	O14980	119	-6	-4	-15	6	0.9	1.0	0.9	1.1
DOCK11	Q5JSL3	592	6	-11	-15	6	1.1	0.9	0.9	1.1
PTPRC	P08575	320	-10	-16	-15	6	0.9	0.9	0.9	1.1
GBAS	O75323	85	1	-18	-15	6	1.0	0.9	0.9	1.1
RBM48	Q5RL73	258	-27	-9	-15	5	0.8	0.9	0.9	1.1
COG8	Q96MW5	595	-3	-10	-15	5	1.0	0.9	0.9	1.1
HDAC3	O15379	94	-13	-22	-15	5	0.9	0.8	0.9	1.1
SNAPC1	Q16533	256	-9	-6	-15	5	0.9	0.9	0.9	1.0
KLC4	Q9NSK0	113	-8	-11	-15	5	0.9	0.9	0.9	1.0
HDGF	P51858	108	-6	-11	-15	5	0.9	0.9	0.9	1.0
OSTF1	Q92882	185	1	-12	-15	5	1.0	0.9	0.9	1.0

NUDT7	P0C024	72	-4	-12	-15	5	1.0	0.9	0.9	1.0
PFAS	O15067	318	-1	-17	-15	5	1.0	0.9	0.9	1.0
GTF2H1	P32780	246	-7	-20	-15	5	0.9	0.8	0.9	1.0
ATG3	Q9NT62	50	-9	-3	-15	4	0.9	1.0	0.9	1.0
ZNF451	Q9Y4E5	133	-7	-9	-15	4	0.9	0.9	0.9	1.0
IP6K1	Q92551	221	-6	-11	-15	4	0.9	0.9	0.9	1.0
ABCD2	Q9UBJ2	515	-7	-11	-15	4	0.9	0.9	0.9	1.0
SYNE2	Q8WXH0	5758	-2	-12	-15	4	1.0	0.9	0.9	1.0
BCAS3	Q9H6U6	836	-7	-4	-15	4	0.9	1.0	0.9	1.0
PPP1R11	O60927	61	-13	-4	-15	4	0.9	1.0	0.9	1.0
MPO	P05164	180	7	-7	-15	3	1.1	0.9	0.9	1.0
STK17B	O94768	190	-1	-10	-15	3	1.0	0.9	0.9	1.0
TRAFD1	O14545	93	-6	-11	-15	3	0.9	0.9	0.9	1.0
SART3	Q15020	182	-15	-11	-15	3	0.9	0.9	0.9	1.0
ARHGAP24	Q8N264	75	-11	-16	-15	3	0.9	0.9	0.9	1.0
GPS1	Q13098	67	-22	-19	-15	3	0.8	0.8	0.9	1.0
SMG5	Q9UPR3	39	10	-29	-15	3	1.1	0.8	0.9	1.0
MACF1	Q9UPN3	5131	-2	-2	-15	3	1.0	1.0	0.9	1.0
TDP1	Q9NUW8	135	5	-4	-15	3	1.0	1.0	0.9	1.0
GTF3C4	Q9UKN8	129	-2	-8	-15	3	1.0	0.9	0.9	1.0
KIAA0196	Q12768	21	3	-10	-15	3	1.0	0.9	0.9	1.0
HTATSF1	O43719	512	-8	-11	-15	3	0.9	0.9	0.9	1.0
HDAC4	P56524	194	-15	-13	-15	3	0.9	0.9	0.9	1.0
RPLP1	P05386	61	-7	-14	-15	3	0.9	0.9	0.9	1.0
NUP153	P49790	593	-6	-19	-15	3	0.9	0.8	0.9	1.0
ATRX	P46100	618	-12	-9	-15	2	0.9	0.9	0.9	1.0
SMARCD1	Q96GM5	492	-9	-13	-15	2	0.9	0.9	0.9	1.0
NIF3L1	Q9GZT8	254	-5	-13	-15	2	1.0	0.9	0.9	1.0
SH3BP5	O60239	438	-10	-21	-15	2	0.9	0.8	0.9	1.0
PRPSAP2	O60256	135	-1	-10	-15	2	1.0	0.9	0.9	1.0
PSMB8	P28062	160	-4	-11	-15	2	1.0	0.9	0.9	1.0
GSR	P00390	107	-4	-12	-15	1	1.0	0.9	0.9	1.0
PREB	Q9HCU5	128	-9	-12	-15	1	0.9	0.9	0.9	1.0
CCDC51	Q96ER9	316	-4	-15	-15	1	1.0	0.9	0.9	1.0
TUBGCP6	Q96RT7	487	-9	-15	-15	1	0.9	0.9	0.9	1.0
MPP1	Q00013	94	-4	-5	-15	1	1.0	1.0	0.9	1.0
NUDCD1	Q96RS6	32	-20	-11	-15	1	0.8	0.9	0.9	1.0
ACTL6A	O96019	206	-5	-17	-15	1	1.0	0.9	0.9	1.0
RANBP2	P49792	815	5	-15	-15	0	1.0	0.9	0.9	1.0
HCFC2	Q9Y5Z7	199	-4	-17	-15	0	1.0	0.9	0.9	1.0
PSMD14	O00487	238	-1	-18	-15	0	1.0	0.9	0.9	1.0
PAPD4	Q6PIY7	374	-2	3	-15	-1	1.0	1.0	0.9	1.0
GTF3C4	Q9UKN8	116	-4	-5	-15	-1	1.0	1.0	0.9	1.0
XRN2	Q9H0D6	296	0	-18	-15	-1	1.0	0.9	0.9	1.0
BRD7	Q9NPI1	338	-6	-21	-15	-1	0.9	0.8	0.9	1.0
ZZEF1	O43149	2553	-10	5	-15	-1	0.9	1.1	0.9	1.0
SH3BP2	P78314	267	1	-5	-15	-1	1.0	1.0	0.9	1.0
CHMP1A	Q9HD42	44	-12	-8	-15	-1	0.9	0.9	0.9	1.0
VPS35	Q96QK1	640	-1	-13	-15	-1	1.0	0.9	0.9	1.0
ITPR1	Q14643	850	5	-22	-15	-1	1.0	0.8	0.9	1.0
ABCD3	P28288	477	-10	-12	-15	-2	0.9	0.9	0.9	1.0
RHOT1	Q8IXI2	175	-1	-12	-15	-2	1.0	0.9	0.9	1.0
ACAA2	P42765	179	-12	-15	-15	-2	0.9	0.9	0.9	1.0
ST13	P50502	209	-7	-18	-15	-2	0.9	0.8	0.9	1.0
CDC5L	Q99459	96	-30	-23	-15	-2	0.8	0.8	0.9	1.0

OGFR	Q9NZT2	87	-2	-8	-15	-2	1.0	0.9	0.9	1.0
TYK2	P29597	966	-5	-11	-15	-2	1.0	0.9	0.9	1.0
FLYWCH2	Q96CP2	132	-15	-13	-15	-2	0.9	0.9	0.9	1.0
UNK	Q9C0B0	696	-10	-13	-15	-2	0.9	0.9	0.9	1.0
TJAP1	Q5JTD0	196	-4	-6	-15	-3	1.0	0.9	0.9	1.0
ATP2A2	P16615	349	2	-8	-15	-3	1.0	0.9	0.9	1.0
ATP2A3	Q93084	349	2	-8	-15	-3	1.0	0.9	0.9	1.0
IGSF6	O95976	83	-19	-20	-15	-3	0.8	0.8	0.9	1.0
YWHAH	Q04917	97	-17	-1	-15	-4	0.9	1.0	0.9	1.0
GNA13	Q14344	318	-3	-13	-15	-4	1.0	0.9	0.9	1.0
LIMD1	Q9UGP4	222	-14	-16	-15	-4	0.9	0.9	0.9	1.0
SMCHD1	A6NHR9	1018	-10	-17	-15	-4	0.9	0.9	0.9	1.0
TARBP2	Q15633	294	-22	-16	-15	-4	0.8	0.9	0.9	1.0
EPS15	P42566	586	-20	-21	-15	-4	0.8	0.8	0.9	1.0
RAP1GDS1	P52306	117	0	-23	-15	-4	1.0	0.8	0.9	1.0
GIMAP2	Q9UG22	180	-3	-24	-15	-4	1.0	0.8	0.9	1.0
ZC3HAV1	Q7Z2W4	272	-11	-12	-15	-5	0.9	0.9	0.9	1.0
ANKRD17	O75179	1690	-7	-19	-15	-5	0.9	0.8	0.9	1.0
KIF21B	O75037	1398	-9	-27	-15	-5	0.9	0.8	0.9	1.0
RNF126	Q9BV68	32	-3	1	-15	-5	1.0	1.0	0.9	1.0
NRDC	O43847	965	-4	-13	-15	-6	1.0	0.9	0.9	0.9
DOCK2	Q92608	1258	-22	-15	-15	-6	0.8	0.9	0.9	0.9
KANSL1	A0A0G2JNT7	599	-9	-17	-15	-6	0.9	0.9	0.9	0.9
CBR3	O75828	227	-3	-17	-15	-6	1.0	0.9	0.9	0.9
BMP2K	Q9NSY1	32	-22	-15	-15	-6	0.8	0.9	0.9	0.9
SND1	Q7KZF4	736	-8	-12	-15	-7	0.9	0.9	0.9	0.9
PARP4	Q9UKK3	1499	-14	-15	-15	-7	0.9	0.9	0.9	0.9
LONP2	Q86WA8	405	-11	-4	-15	-7	0.9	1.0	0.9	0.9
TDRD7	Q8NHU6	77	-13	-10	-15	-7	0.9	0.9	0.9	0.9
PKN1	Q16512	662	1	-8	-15	-8	1.0	0.9	0.9	0.9
INPP1	P49441	10	-9	-7	-15	-8	0.9	0.9	0.9	0.9
ESYT2	A0FGR8	611	-3	-16	-15	-8	1.0	0.9	0.9	0.9
NUBP1	P53384	235	-9	-16	-15	-8	0.9	0.9	0.9	0.9
RANBP2	P49792	2982	-1	-19	-15	-9	1.0	0.8	0.9	0.9
ATP2A2	P16615	344	-5	-7	-15	-9	1.0	0.9	0.9	0.9
ATP2A3	Q93084	344	-5	-7	-15	-9	1.0	0.9	0.9	0.9
DENND2D	Q9H6A0	162	-9	-11	-15	-9	0.9	0.9	0.9	0.9
CAND1	Q86VP6	942	14	3	-15	-10	1.2	1.0	0.9	0.9
FASN	P49327	2202	-8	-13	-15	-10	0.9	0.9	0.9	0.9
FUK	Q8N0W3	802	-7	-33	-15	-10	0.9	0.8	0.9	0.9
SH2B3	Q9UQQ2	450	-3	-19	-15	-10	1.0	0.8	0.9	0.9
PRKCD	Q05655	28	-14	-21	-15	-10	0.9	0.8	0.9	0.9
KIFC1	Q9BW19	509	-8	-14	-15	-11	0.9	0.9	0.9	0.9
MSH3	P20585	370	-12	-40	-15	-11	0.9	0.7	0.9	0.9
TTC37	Q6PGP7	705	-21	-5	-15	-12	0.8	1.0	0.9	0.9
LRBA	P50851	1704	-11	-6	-15	-12	0.9	0.9	0.9	0.9
CACUL1	Q86Y37	94	-23	-11	-15	-12	0.8	0.9	0.9	0.9
CLTB	P09497	199	-14	-18	-15	-13	0.9	0.8	0.9	0.9
RBM22	Q9NW64	84	8	-12	-15	-13	1.1	0.9	0.9	0.9
INPP4A	Q96PE3	367	2	-6	-15	-14	1.0	0.9	0.9	0.9
PLXNB2	O15031	1484	-2	-11	-15	-14	1.0	0.9	0.9	0.9
CDC40	O60508	366	-7	-11	-15	-14	0.9	0.9	0.9	0.9
CSK	P41240	223	-6	-15	-15	-14	0.9	0.9	0.9	0.9
NUP153	P49790	678	-11	-5	-15	-14	0.9	1.0	0.9	0.9
ADH5	P11766	282	5	-11	-15	-15	1.1	0.9	0.9	0.9

DHX16	O60231	450	-5	-26	-15	-15	1.0	0.8	0.9	0.9
PHKB	Q93100	434	-14	-19	-15	-17	0.9	0.8	0.9	0.9
ADH5	P11766	170	4	-11	-15	-18	1.0	0.9	0.9	0.9
AP2B1	P63010	144	-11	-18	-15	-20	0.9	0.9	0.9	0.8
AP1B1	Q10567	144	-11	-18	-15	-20	0.9	0.9	0.9	0.8
BCAS3	Q9H6U6	192	-16	-19	-15	-20	0.9	0.8	0.9	0.8
HNRNPUL2-BS	H3BQZ7	538	-1	-1	-15	-22	1.0	1.0	0.9	0.8
NAMPT	P43490	39	-4	-9	-15	-23	1.0	0.9	0.9	0.8
GATM	P50440	252	-12	-35	-15	-23	0.9	0.7	0.9	0.8
AKR1A1	P14550	46	7	-13	-15	-24	1.1	0.9	0.9	0.8
RABGAP1	Q9Y3P9	582	-6	-16	-15	-24	0.9	0.9	0.9	0.8
DENND4A	Q7Z401	35	-14	-10	-15	-25	0.9	0.9	0.9	0.8
L3MBTL2	Q969R5	110	-7	-22	-15	-28	0.9	0.8	0.9	0.8
ARAF	P10398	192	-43	-25	-15	-29	0.7	0.8	0.9	0.8
RHOF	Q9HBH0	30	-2	-10	-15	-30	1.0	0.9	0.9	0.8
SIPA1	Q96FS4	340	1	-19	-15	-30	1.0	0.8	0.9	0.8
PFKP	Q01813	641	2	-3	-15	-35	1.0	1.0	0.9	0.7
PPA1	Q15181	113	3	-5	-15	-43	1.0	1.0	0.9	0.7
FCAR	P24071	49	-11	23	-15	-45	0.9	1.3	0.9	0.7
CDC42BPB	Q9Y5S2	1517	-8	-12	-15	-49	0.9	0.9	0.9	0.7
ADCK1	Q86TW2	94	4	-6	-15	-52	1.0	0.9	0.9	0.7
PREX1	Q8TCU6	720	1	-15	-15	-57	1.0	0.9	0.9	0.6
ASAP2	O43150	88	-8	-13	-15	-167	0.9	0.9	0.9	0.4
IQSEC1	Q6DN90	744	-25	-24	-16	52	0.8	0.8	0.9	2.1
MVD	P53602	108	15	-8	-16	47	1.2	0.9	0.9	1.9
ARHGAP30	Q7Z6I6	449	-14	-12	-16	41	0.9	0.9	0.9	1.7
NUBP1	P53384	25	-9	-15	-16	36	0.9	0.9	0.9	1.6
RFFL	Q8WZ73	273	12	-10	-16	34	1.1	0.9	0.9	1.5
SP100	P23497	16	-13	-10	-16	33	0.9	0.9	0.9	1.5
DDB2	Q92466	253	6	-8	-16	28	1.1	0.9	0.9	1.4
MLYCD	O95822	206	-1	-10	-16	28	1.0	0.9	0.9	1.4
NAA15	Q9BXJ9	322	2	-11	-16	27	1.0	0.9	0.9	1.4
TMEM57	Q8N5G2	502	-9	-11	-16	26	0.9	0.9	0.9	1.4
PLCB2	Q00722	945	-7	-13	-16	26	0.9	0.9	0.9	1.4
PML	P29590	207	-7	-18	-16	24	0.9	0.8	0.9	1.3
SCML4	Q8N228	211	-12	-19	-16	24	0.9	0.8	0.9	1.3
SLC43A2	Q8N370	295	-7	-18	-16	23	0.9	0.9	0.9	1.3
NCALD	P61601	185	-9	-24	-16	23	0.9	0.8	0.9	1.3
CLP1	Q92989	311	-16	-11	-16	23	0.9	0.9	0.9	1.3
GLIPR2	Q9H4G4	63	-12	-16	-16	22	0.9	0.9	0.9	1.3
CNBP	P62633	54	-29	4	-16	22	0.8	1.0	0.9	1.3
ATRX	P46100	605	-3	-13	-16	22	1.0	0.9	0.9	1.3
C15orf39	Q6ZRI6	831	-19	-2	-16	21	0.8	1.0	0.9	1.3
PIK3IP1	Q96FE7	228	-2	-7	-16	21	1.0	0.9	0.9	1.3
NFKBIL1	Q9UBC1	82	-16	-12	-16	21	0.9	0.9	0.9	1.3
PRKCD	Q05655	459	11	11	-16	20	1.1	1.1	0.9	1.2
SYNE1	Q8NF91	8703	4	-27	-16	20	1.0	0.8	0.9	1.2
GYS1	P13807	699	-8	-15	-16	19	0.9	0.9	0.9	1.2
SSSCA1	O60232	125	-1	-3	-16	18	1.0	1.0	0.9	1.2
RAB5C	P51148	64	-6	-6	-16	17	0.9	0.9	0.9	1.2
TBC1D31	Q96DN5	916	-11	-7	-16	16	0.9	0.9	0.9	1.2
LGALS9B	Q3B8N2	313	1	-11	-16	16	1.0	0.9	0.9	1.2
QTRTD1	Q9H974	151	7	-15	-16	16	1.1	0.9	0.9	1.2
TRANK1	O15050	2766	2	-5	-16	15	1.0	1.0	0.9	1.2
CAD	P27708	897	-9	-12	-16	15	0.9	0.9	0.9	1.2

MTHFD1	P11586	152	-9	-13	-16	15	0.9	0.9	0.9	1.2
RANBP2	P49792	2791	-1	-13	-16	15	1.0	0.9	0.9	1.2
KAT6B	Q8WYB5	837	-14	-32	-16	15	0.9	0.8	0.9	1.2
KAT6A	Q92794	626	-14	-32	-16	15	0.9	0.8	0.9	1.2
SOS2	Q07890	1064	-3	-34	-16	15	1.0	0.7	0.9	1.2
SCAF1	Q9H7N4	950	-3	-11	-16	14	1.0	0.9	0.9	1.2
ZBTB1	Q9Y2K1	227	-8	-21	-16	14	0.9	0.8	0.9	1.2
FOXN2	P32314	413	-11	-2	-16	14	0.9	1.0	0.9	1.2
HUWE1	Q7Z6Z7	3375	-9	-6	-16	14	0.9	0.9	0.9	1.2
TACC3	Q9Y6A5	426	-12	-14	-16	14	0.9	0.9	0.9	1.2
CHD8	Q9HCK8	1780	2	-6	-16	13	1.0	0.9	0.9	1.1
NOL6	Q9H6R4	1034	-10	-18	-16	13	0.9	0.8	0.9	1.1
EZH1	Q92800	11	-11	-11	-16	13	0.9	0.9	0.9	1.1
COG5	Q9UP83	64	-5	-12	-16	13	1.0	0.9	0.9	1.1
LAS1L	Q9Y4W2	256	-2	-14	-16	13	1.0	0.9	0.9	1.1
VEZF1	Q14119	327	-10	-18	-16	12	0.9	0.9	0.9	1.1
RANBP3	Q9H6Z4	203	-6	-19	-16	12	0.9	0.8	0.9	1.1
DHX9	Q08211	1029	-7	-10	-16	12	0.9	0.9	0.9	1.1
HIRA	P54198	745	-11	-13	-16	12	0.9	0.9	0.9	1.1
CMPK2	Q5EBM0	76	-10	-26	-16	12	0.9	0.8	0.9	1.1
ANKFY1	Q9P2R3	460	-10	-17	-16	11	0.9	0.9	0.9	1.1
PUM1	Q14671	1179	-15	2	-16	11	0.9	1.0	0.9	1.1
BMS1	Q14692	327	-4	-3	-16	11	1.0	1.0	0.9	1.1
CBL	P22681	508	-9	-12	-16	11	0.9	0.9	0.9	1.1
NOC2L	Q9Y3T9	585	-2	-12	-16	11	1.0	0.9	0.9	1.1
ACD	Q96AP0	406	-5	-15	-16	11	1.0	0.9	0.9	1.1
ACD	Q96AP0	508	-5	-16	-16	10	1.0	0.9	0.9	1.1
ZNF609	O15014	1290	-5	-12	-16	10	1.0	0.9	0.9	1.1
GPSM1	Q86YR5	497	-7	-13	-16	10	0.9	0.9	0.9	1.1
RGPD3	A6NKT7	1602	-7	-14	-16	10	0.9	0.9	0.9	1.1
RANBP2	P49792	2577	-7	-14	-16	10	0.9	0.9	0.9	1.1
RGPD6	Q99666	1601	-7	-14	-16	10	0.9	0.9	0.9	1.1
ATRX	P46100	450	-4	-5	-16	9	1.0	1.0	0.9	1.1
TNKS1BP1	Q9C0C2	1324	10	12	-16	9	1.1	1.1	0.9	1.1
AHCTF1	Q8WYP5	1628	-3	-10	-16	9	1.0	0.9	0.9	1.1
PAXBP1	Q9Y5B6	621	-6	-10	-16	9	0.9	0.9	0.9	1.1
DIDO1	Q9BTC0	1449	-6	-12	-16	9	0.9	0.9	0.9	1.1
HEATR5B	Q9P2D3	1896	-9	-16	-16	9	0.9	0.9	0.9	1.1
CEP76	Q8TAP6	89	-10	-26	-16	9	0.9	0.8	0.9	1.1
HIRA	P54198	219	3	-1	-16	8	1.0	1.0	0.9	1.1
ASCC3	Q8N3C0	1906	-2	-16	-16	8	1.0	0.9	0.9	1.1
RICTOR	Q6R327	1576	-5	-2	-16	8	1.0	1.0	0.9	1.1
DOCK2	Q92608	607	1	-10	-16	8	1.0	0.9	0.9	1.1
AKAP9	Q99996	1688	-5	-15	-16	8	1.0	0.9	0.9	1.1
EPRS	P07814	744	-14	-18	-16	8	0.9	0.8	0.9	1.1
GBA2	Q9HCG7	867	-11	-24	-16	8	0.9	0.8	0.9	1.1
KNTC1	P50748	683	2	-30	-16	8	1.0	0.8	0.9	1.1
SETX	Q7Z333	997	-13	-3	-16	7	0.9	1.0	0.9	1.1
ZAP70	P43403	102	-7	-17	-16	7	0.9	0.9	0.9	1.1
ATG7	O95352	524	-7	-10	-16	7	0.9	0.9	0.9	1.1
NFKB2	Q00653	57	-4	-16	-16	7	1.0	0.9	0.9	1.1
UBR4	Q5T4S7	5121	-8	-16	-16	7	0.9	0.9	0.9	1.1
PCM1	Q15154	1585	-21	-18	-16	7	0.8	0.9	0.9	1.1
RNPEP	Q9H4A4	130	-19	-20	-16	7	0.8	0.8	0.9	1.1
TXNDC5	Q8NBS9	254	-12	4	-16	6	0.9	1.0	0.9	1.1

ZNF318	Q5VUA4	2168	-4	-8	-16	6	1.0	0.9	0.9	1.1
TBC1D2	Q9BYX2	469	-9	-10	-16	6	0.9	0.9	0.9	1.1
LARS	Q9P2J5	1093	-3	-11	-16	6	1.0	0.9	0.9	1.1
SYNE1	Q8NF91	3677	2	-11	-16	6	1.0	0.9	0.9	1.1
ANP32E	Q9BTT0	123	-4	-18	-16	6	1.0	0.9	0.9	1.1
LRRK2	Q5S007	1266	-17	-26	-16	6	0.9	0.8	0.9	1.1
RBL2	Q08999	274	-14	-6	-16	5	0.9	0.9	0.9	1.1
RNF169	Q8NCN4	293	-3	-7	-16	5	1.0	0.9	0.9	1.1
NCOA7	Q8NI08	439	-6	-10	-16	5	0.9	0.9	0.9	1.1
DOPEY2	Q9Y3R5	2199	-9	-13	-16	5	0.9	0.9	0.9	1.1
ATXN1L	P0C7T5	619	-2	-15	-16	5	1.0	0.9	0.9	1.1
TSTA3	Q13630	116	-2	-15	-16	5	1.0	0.9	0.9	1.1
RNF166	Q96A37	127	-3	-20	-16	5	1.0	0.8	0.9	1.1
PPIA	P62937	62	-6	-14	-16	5	0.9	0.9	0.9	1.0
GSR	P00390	102	-8	-17	-16	5	0.9	0.9	0.9	1.0
PCBP1	Q15365	293	-2	-18	-16	5	1.0	0.8	0.9	1.0
CEP164	Q9UPV0	626	-29	-27	-16	4	0.8	0.8	0.9	1.0
PPWD1	Q96BP3	185	7	6	-16	4	1.1	1.1	0.9	1.0
NCAPG	Q9BPX3	177	-6	-2	-16	4	0.9	1.0	0.9	1.0
C16orf62	Q7Z3J2	925	-1	-5	-16	4	1.0	1.0	0.9	1.0
UBA2	Q9UBT2	185	-6	-13	-16	4	0.9	0.9	0.9	1.0
TRANK1	O15050	19	-10	-23	-16	4	0.9	0.8	0.9	1.0
SYNE2	Q8WXH0	923	1	-1	-16	3	1.0	1.0	0.9	1.0
SASH1	O94885	1120	-14	-1	-16	3	0.9	1.0	0.9	1.0
LRRC47	Q8N1G4	224	-12	-11	-16	3	0.9	0.9	0.9	1.0
ISCU	Q9H1K1	130	-1	-13	-16	3	1.0	0.9	0.9	1.0
SUPT16H	Q9Y5B9	889	-9	-16	-16	3	0.9	0.9	0.9	1.0
GZMH	P20718	49	-1	-19	-16	3	1.0	0.8	0.9	1.0
RPL12	P30050	17	-15	-8	-16	2	0.9	0.9	0.9	1.0
MORF4L2	Q15014	165	-3	-15	-16	2	1.0	0.9	0.9	1.0
RQCD1	Q92600	252	-4	-15	-16	2	1.0	0.9	0.9	1.0
CDAN1	Q8IWY9	1071	-16	-35	-16	2	0.9	0.7	0.9	1.0
DOCK10	Q96BY6	1310	-6	-7	-16	2	0.9	0.9	0.9	1.0
NEK6	Q9HC98	258	-6	-8	-16	2	0.9	0.9	0.9	1.0
PDIA5	Q14554	231	-9	-15	-16	2	0.9	0.9	0.9	1.0
LRRC16A	Q5VZK9	51	3	-7	-16	1	1.0	0.9	0.9	1.0
RASSF4	Q9H2L5	236	-8	-13	-16	1	0.9	0.9	0.9	1.0
MED31	Q9Y3C7	93	1	-10	-16	1	1.0	0.9	0.9	1.0
EP300	Q09472	1738	-13	-17	-16	1	0.9	0.9	0.9	1.0
CREBBP	Q92793	1775	-13	-17	-16	1	0.9	0.9	0.9	1.0
ATXN2	Q99700	892	-6	-12	-16	0	0.9	0.9	0.9	1.0
WDFY4	Q6ZS81	582	-7	-14	-16	0	0.9	0.9	0.9	1.0
RAI1	Q7Z5J4	239	2	-14	-16	0	1.0	0.9	0.9	1.0
KLC1	Q07866	436	-1	-17	-16	0	1.0	0.9	0.9	1.0
ANKRD12	Q6UB98	1754	-1	-13	-16	-1	1.0	0.9	0.9	1.0
GIMAP8	Q8ND71	214	-8	-5	-16	-1	0.9	1.0	0.9	1.0
TAPT1	Q6NXT6	474	-3	-8	-16	-1	1.0	0.9	0.9	1.0
TARSL2	A2RTX5	62	-7	-13	-16	-1	0.9	0.9	0.9	1.0
CRACR2A	Q9BSW2	291	-7	-18	-16	-1	0.9	0.9	0.9	1.0
TGFBRAP1	Q8WUH2	33	-4	-25	-16	-1	1.0	0.8	0.9	1.0
GCLM	P48507	72	-17	-26	-16	-1	0.9	0.8	0.9	1.0
HCK	P08631	7	-8	-36	-16	-1	0.9	0.7	0.9	1.0
AFG3L2	Q9Y4W6	402	-12	-16	-16	-2	0.9	0.9	0.9	1.0
ADSL	P30566	304	-11	-2	-16	-2	0.9	1.0	0.9	1.0
CPSF3L	Q5TA45	542	4	-8	-16	-2	1.0	0.9	0.9	1.0

NUP98	P52948	1068	-10	-14	-16	-2	0.9	0.9	0.9	1.0
GFPT1	Q06210	254	-11	-15	-16	-2	0.9	0.9	0.9	1.0
Uncharacteriz	G3V3G9	426	-18	-9	-16	-3	0.9	0.9	0.9	1.0
ARIH1	Q9Y4X5	357	-12	-22	-16	-3	0.9	0.8	0.9	1.0
HSPH1	Q92598	245	1	-9	-16	-3	1.0	0.9	0.9	1.0
DDB1	Q16531	128	-2	-19	-16	-3	1.0	0.8	0.9	1.0
TBC1D1	Q86TI0	604	-13	-20	-16	-3	0.9	0.8	0.9	1.0
PEX11B	O96011	153	-6	-23	-16	-3	0.9	0.8	0.9	1.0
Uncharacteriz	A0A0A6YYC8	129	-11	-6	-16	-4	0.9	0.9	0.9	1.0
ANKRD16	Q6P6B7	231	-9	-13	-16	-4	0.9	0.9	0.9	1.0
GOLGA4	Q13439	1771	-11	-16	-16	-4	0.9	0.9	0.9	1.0
RAD21	O60216	585	-11	-12	-16	-4	0.9	0.9	0.9	1.0
ARID1A	O14497	2043	-7	-13	-16	-4	0.9	0.9	0.9	1.0
FRYL	O94915	560	-5	-3	-16	-5	1.0	1.0	0.9	1.0
CHD9	Q3L8U1	2584	2	-11	-16	-5	1.0	0.9	0.9	1.0
NUBP2	Q9Y5Y2	54	-5	-22	-16	-5	1.0	0.8	0.9	1.0
ACTR2	P61160	20	-14	-10	-16	-5	0.9	0.9	0.9	1.0
HTT	P42858	2971	-7	-14	-16	-5	0.9	0.9	0.9	1.0
ARAP1	Q96P48	1310	-6	-9	-16	-6	0.9	0.9	0.9	0.9
TCP1	P17987	397	-2	-26	-16	-6	1.0	0.8	0.9	0.9
PLCD1	P51178	367	5	-5	-16	-7	1.0	1.0	0.9	0.9
WHSC1	O96028	1144	-15	-7	-16	-7	0.9	0.9	0.9	0.9
NMT2	O60551	485	2	-16	-16	-7	1.0	0.9	0.9	0.9
IGFBPL1	Q8WX77	151	-4	-17	-16	-7	1.0	0.9	0.9	0.9
SEC24A	O95486	455	-7	-10	-16	-8	0.9	0.9	0.9	0.9
SEC24B	O95487	629	-7	-10	-16	-8	0.9	0.9	0.9	0.9
NUP98	P52948	1027	-11	-18	-16	-8	0.9	0.8	0.9	0.9
NUMA1	Q14980	80	-6	-41	-16	-8	0.9	0.7	0.9	0.9
ACAP2	Q15057	415	-15	-15	-16	-8	0.9	0.9	0.9	0.9
RNF213	Q63HN8	4012	-13	-19	-16	-9	0.9	0.8	0.9	0.9
ZC3HAV1	Q7Z2W4	581	6	-8	-16	-10	1.1	0.9	0.9	0.9
GAB3	Q8WWW8	295	-20	-9	-16	-10	0.8	0.9	0.9	0.9
CKAP5	Q14008	1500	-13	-5	-16	-10	0.9	1.0	0.9	0.9
FLNA	P21333	1920	7	-6	-16	-10	1.1	0.9	0.9	0.9
LPP	Q93052	364	-8	-12	-16	-10	0.9	0.9	0.9	0.9
RAB14	P61106	26	-1	-15	-16	-10	1.0	0.9	0.9	0.9
UTP18	Q9Y5J1	241	-15	-2	-16	-11	0.9	1.0	0.9	0.9
DDX21	Q9NR30	682	-9	-23	-16	-11	0.9	0.8	0.9	0.9
HMGCL	P35914	307	-9	-13	-16	-12	0.9	0.9	0.9	0.9
THUMPD1	Q9NXG2	169	-10	-32	-16	-12	0.9	0.8	0.9	0.9
PAPSS1	O43252	83	-7	-4	-16	-12	0.9	1.0	0.9	0.9
SVIL	O95425	1586	-17	-4	-16	-12	0.9	1.0	0.9	0.9
GPATCH8	Q9UKJ3	402	0	-7	-16	-12	1.0	0.9	0.9	0.9
PFKP	Q01813	718	-15	-11	-16	-12	0.9	0.9	0.9	0.9
L2HGDH	Q9H9P8	179	-3	-11	-16	-12	1.0	0.9	0.9	0.9
ZFC3H1	O60293	241	-7	-15	-16	-13	0.9	0.9	0.9	0.9
HEATR5B	Q9P2D3	518	-18	-10	-16	-13	0.8	0.9	0.9	0.9
RNF126	Q9BV68	15	-7	-19	-16	-13	0.9	0.8	0.9	0.9
SVIL	O95425	671	-9	6	-16	-14	0.9	1.1	0.9	0.9
CCAR1	Q8IX12	579	-18	-13	-16	-14	0.8	0.9	0.9	0.9
AP2B1	P63010	391	-12	-9	-16	-14	0.9	0.9	0.9	0.9
AP1B1	Q10567	391	-12	-9	-16	-14	0.9	0.9	0.9	0.9
MACF1	Q9UPN3	523	-24	-2	-16	-15	0.8	1.0	0.9	0.9
SPTLC2	O15270	19	-17	-14	-16	-15	0.9	0.9	0.9	0.9
ZC3H3	Q8IXZ2	338	-1	-29	-16	-15	1.0	0.8	0.9	0.9

DTYMK	P23919	117	-6	-5	-16	-15	0.9	1.0	0.9	0.9
MUTYH	Q9UIF7	514	-2	-7	-16	-15	1.0	0.9	0.9	0.9
STAT5B	P51692	688	-9	-17	-16	-15	0.9	0.9	0.9	0.9
DPH6	Q7L8W6	88	-6	-7	-16	-16	0.9	0.9	0.9	0.9
PSMC4	P43686	379	-13	-11	-16	-16	0.9	0.9	0.9	0.9
PYGB	P11216	326	-6	-11	-16	-17	0.9	0.9	0.9	0.9
DPF2	Q92785	273	8	-20	-16	-17	1.1	0.8	0.9	0.9
FLII	Q13045	576	-7	4	-16	-18	0.9	1.0	0.9	0.8
NUMA1	Q14980	961	-16	-19	-16	-19	0.9	0.8	0.9	0.8
INTS1	Q8N201	246	-5	-11	-16	-20	1.0	0.9	0.9	0.8
ACSF2	Q96CM8	77	-9	-26	-16	-20	0.9	0.8	0.9	0.8
DOK3	Q7L591	230	-18	-21	-16	-21	0.8	0.8	0.9	0.8
POMP	Q9Y244	37	17	-20	-16	-25	1.2	0.8	0.9	0.8
CBL	P22681	659	-5	-27	-16	-27	1.0	0.8	0.9	0.8
MYO1C	O00159	802	-5	0	-16	-28	1.0	1.0	0.9	0.8
FAM65C	Q96MK2	796	6	-6	-16	-30	1.1	0.9	0.9	0.8
RAP1A	P62834	118	-2	-10	-16	-30	1.0	0.9	0.9	0.8
POTEJ	P0CG39	935	-6	-38	-16	-33	0.9	0.7	0.9	0.8
POTEE	Q6S8J3	972	-6	-38	-16	-33	0.9	0.7	0.9	0.8
MYO5A	Q9Y4I1	660	-4	-15	-16	-47	1.0	0.9	0.9	0.7
ARIH1	Q9Y4X5	269	-2	-4	-16	-54	1.0	1.0	0.9	0.6
THBS1	P07996	930	8	-29	-16	-55	1.1	0.8	0.9	0.6
FNBP1L	Q5T0N5	69	-21	-28	-16	-66	0.8	0.8	0.9	0.6
ALOX12	P18054	96	-5	-14	-16	-106	1.0	0.9	0.9	0.5
TESK1	Q15569	59	-11	-16	-16	-158	0.9	0.9	0.9	0.4
USP43	Q70EL4	118	-2	-14	-16	-179	1.0	0.9	0.9	0.4
TNFAIP3	P21580	57	-19	-15	-16	40	0.8	0.9	0.9	1.7
BCKDHB	P21953	235	-6	-2	-16	38	0.9	1.0	0.9	1.6
DHX37	Q8IY37	1026	-1	-9	-16	37	1.0	0.9	0.9	1.6
MGME1	Q9BQP7	79	-8	-17	-16	36	0.9	0.9	0.9	1.6
ERP29	P30040	157	-7	-24	-16	32	0.9	0.8	0.9	1.5
LYST	Q99698	2223	-10	-14	-16	31	0.9	0.9	0.9	1.4
CD5	P06127	342	-1	-16	-16	31	1.0	0.9	0.9	1.4
DIP2A	Q14689	252	-5	-18	-16	28	1.0	0.8	0.9	1.4
STK11IP	Q8N1F8	149	-9	-11	-16	27	0.9	0.9	0.9	1.4
RABL6	H0Y4Z8	492	-9	-18	-16	27	0.9	0.9	0.9	1.4
TTC38	Q5R3I4	53	-6	-12	-16	27	0.9	0.9	0.9	1.4
SPR	P35270	234	4	-6	-16	26	1.0	0.9	0.9	1.3
RASSF4	Q9H2L5	92	-9	-13	-16	26	0.9	0.9	0.9	1.3
NLRP12	P59046	150	6	1	-16	25	1.1	1.0	0.9	1.3
BRAF	P15056	194	-10	-4	-16	24	0.9	1.0	0.9	1.3
UHMK1	Q8TAS1	43	-13	-4	-16	24	0.9	1.0	0.9	1.3
GIGYF1	O75420	583	6	-22	-16	24	1.1	0.8	0.9	1.3
ZC3HAV1	Q7Z2W4	527	-7	-7	-16	24	0.9	0.9	0.9	1.3
C15orf39	Q6ZRI6	417	-9	-23	-16	23	0.9	0.8	0.9	1.3
CNOT1	A5YKK6	2359	-4	-7	-16	22	1.0	0.9	0.9	1.3
TMF1	P82094	158	-9	-10	-16	22	0.9	0.9	0.9	1.3
TAF6	P49848	235	-9	-13	-16	22	0.9	0.9	0.9	1.3
SON	P18583	92	-13	-15	-16	21	0.9	0.9	0.9	1.3
UQCRH	P07919	37	-22	-22	-16	21	0.8	0.8	0.9	1.3
STAT2	P52630	174	-4	-10	-16	20	1.0	0.9	0.9	1.3
MAEA	Q7L5Y9	61	2	-2	-16	20	1.0	1.0	0.9	1.2
GZMH	P20718	170	1	-8	-16	20	1.0	0.9	0.9	1.2
SETD1B	Q9UPS6	931	-8	-14	-16	19	0.9	0.9	0.9	1.2
AKAP13	Q12802	418	-11	-12	-16	19	0.9	0.9	0.9	1.2

RXRA	P19793	138	-12	-6	-16	18	0.9	0.9	0.9	1.2
BOLA1	Q9Y3E2	18	5	-10	-16	18	1.1	0.9	0.9	1.2
IMP3	Q9NV31	107	-11	-13	-16	18	0.9	0.9	0.9	1.2
XPO5	Q9HAV4	1131	-4	-14	-16	18	1.0	0.9	0.9	1.2
ZMYM2	Q9UBW7	823	-7	-21	-16	18	0.9	0.8	0.9	1.2
GLRX	P35754	26	-4	-10	-16	17	1.0	0.9	0.9	1.2
GTF3C1	Q12789	320	-8	-3	-16	16	0.9	1.0	0.9	1.2
CDK6	Q00534	306	3	6	-16	16	1.0	1.1	0.9	1.2
DHX58	Q96C10	322	5	-7	-16	16	1.1	0.9	0.9	1.2
MLH1	P40692	233	-7	-9	-16	16	0.9	0.9	0.9	1.2
PPM1G	O15355	241	-6	-20	-16	16	0.9	0.8	0.9	1.2
LEMD3	Q9Y2U8	346	14	-1	-16	14	1.2	1.0	0.9	1.2
DGKQ	P52824	357	-6	-2	-16	14	0.9	1.0	0.9	1.2
KIAA1429	Q69YN4	353	-11	-12	-16	14	0.9	0.9	0.9	1.2
ATXN2L	Q8WWM7	628	1	4	-16	13	1.0	1.0	0.9	1.1
RAI1	Q7Z5J4	649	-3	-5	-16	13	1.0	1.0	0.9	1.1
HDLBP	Q00341	940	-5	-13	-16	13	1.0	0.9	0.9	1.1
CLASP2	O75122	528	-12	-20	-16	13	0.9	0.8	0.9	1.1
MAN2C1	Q9NTJ4	40	-8	-29	-16	13	0.9	0.8	0.9	1.1
RNPEPL1	Q9HAU8	51	-6	-13	-16	12	0.9	0.9	0.9	1.1
GSG2	Q8TF76	300	-5	-12	-16	12	1.0	0.9	0.9	1.1
Uncharacteriz	G3V3G9	349	-23	-16	-16	12	0.8	0.9	0.9	1.1
NBEAL2	Q6ZNJ1	712	-3	-6	-16	11	1.0	0.9	0.9	1.1
CXorf21	Q9HAI6	58	-9	-10	-16	11	0.9	0.9	0.9	1.1
DHX57	Q6P158	1369	-10	-12	-16	11	0.9	0.9	0.9	1.1
TINF2	Q9BSI4	332	-9	-8	-16	11	0.9	0.9	0.9	1.1
CYTIP	O60759	284	-15	0	-16	10	0.9	1.0	0.9	1.1
CYP1B1	Q16678	92	-11	-12	-16	10	0.9	0.9	0.9	1.1
ATG2B	Q96BY7	1750	-4	-14	-16	10	1.0	0.9	0.9	1.1
TUBB2B	Q9BVA1	129	-8	-16	-16	9	0.9	0.9	0.9	1.1
AGTPBP1	Q9UPW5	558	-8	-17	-16	9	0.9	0.9	0.9	1.1
ABHD17A	Q96GS6	230	-10	-1	-16	9	0.9	1.0	0.9	1.1
LAPTM5	Q13571	218	-12	-12	-16	9	0.9	0.9	0.9	1.1
GOLGA2	Q08379	427	-1	-17	-16	9	1.0	0.9	0.9	1.1
XPO1	O14980	1070	-7	-8	-16	8	0.9	0.9	0.9	1.1
CSTF2T	Q9H0L4	441	-2	-18	-16	8	1.0	0.9	0.9	1.1
LIMD1	Q9UGP4	305	-3	-6	-16	8	1.0	0.9	0.9	1.1
SRSF4	Q08170	115	-8	-7	-16	8	0.9	0.9	0.9	1.1
DIS3L2	Q8IYB7	146	-4	-8	-16	8	1.0	0.9	0.9	1.1
TNRC6B	Q9UPQ9	600	-7	-10	-16	8	0.9	0.9	0.9	1.1
FAM102A	Q5T9C2	151	-2	-11	-16	8	1.0	0.9	0.9	1.1
LSM14A	Q8ND56	375	-4	-15	-16	8	1.0	0.9	0.9	1.1
ELP6	Q0PNE2	218	-5	-15	-16	8	1.0	0.9	0.9	1.1
ZNF512	Q96ME7	540	-8	-17	-16	8	0.9	0.9	0.9	1.1
HNRNPA3	P51991	64	-12	-9	-16	7	0.9	0.9	0.9	1.1
Uncharacteriz	K7ESF4	111	-6	-6	-16	7	0.9	0.9	0.9	1.1
PDCD2L	Q9BRP1	82	-6	-6	-16	7	0.9	0.9	0.9	1.1
AGAP2	Q99490	915	3	-11	-16	7	1.0	0.9	0.9	1.1
ZDHHC6	Q9H6R6	398	-8	-9	-16	6	0.9	0.9	0.9	1.1
SUGP2	Q8IX01	107	-11	-12	-16	6	0.9	0.9	0.9	1.1
PRKDC	P78527	1183	-10	-15	-16	6	0.9	0.9	0.9	1.1
ANKMY2	Q8IV38	277	-3	-15	-16	6	1.0	0.9	0.9	1.1
ZNF106	Q9H2Y7	643	-1	-19	-16	6	1.0	0.8	0.9	1.1
HPCAL1	P37235	38	-9	-19	-16	6	0.9	0.8	0.9	1.1
NUP214	P35658	728	-6	-22	-16	6	0.9	0.8	0.9	1.1

DENND4A	Q7Z401	117	-9	-12	-16	6	0.9	0.9	0.9	1.1
NR3C1	P04150	367	-13	-4	-16	5	0.9	1.0	0.9	1.1
SRGAP2	A2RUF3	39	-7	-7	-16	5	0.9	0.9	0.9	1.1
WDFY1	Q8IWB7	142	-7	-11	-16	5	0.9	0.9	0.9	1.1
SPG11	Q96JI7	526	-20	-11	-16	5	0.8	0.9	0.9	1.1
YWHAQ	P27348	237	-10	-19	-16	5	0.9	0.8	0.9	1.1
PDCD11	Q14690	510	-6	-9	-16	5	0.9	0.9	0.9	1.0
CBX5	P45973	160	-12	-9	-16	5	0.9	0.9	0.9	1.0
CBX3	Q13185	160	-12	-9	-16	5	0.9	0.9	0.9	1.0
NUP188	Q5SRE5	585	-7	-12	-16	5	0.9	0.9	0.9	1.0
ANXA6	P08133	114	-10	-13	-16	5	0.9	0.9	0.9	1.0
ANP32B	Q92688	123	-11	-17	-16	5	0.9	0.9	0.9	1.0
C2orf76	Q3KRA6	111	-3	-24	-16	5	1.0	0.8	0.9	1.0
ZC3H3	Q8IXZ2	603	-9	-16	-16	4	0.9	0.9	0.9	1.0
USP34	Q70CQ2	1486	-14	-1	-16	4	0.9	1.0	0.9	1.0
ETS1	P14921	99	-10	-6	-16	4	0.9	0.9	0.9	1.0
ARID1A	O14497	1874	-6	-11	-16	4	0.9	0.9	0.9	1.0
THOP1	P52888	434	-16	-24	-16	4	0.9	0.8	0.9	1.0
DNM2	P50570	607	-8	-10	-16	3	0.9	0.9	0.9	1.0
PHC3	Q8NDX5	816	-6	-15	-16	3	0.9	0.9	0.9	1.0
NOP56	O00567	384	-7	-16	-16	3	0.9	0.9	0.9	1.0
ARID1B	Q8NFD5	1141	-25	-16	-16	3	0.8	0.9	0.9	1.0
UIMC1	Q96RL1	601	-10	-17	-16	3	0.9	0.9	0.9	1.0
EXOC5	O00471	254	-2	26	-16	3	1.0	1.3	0.9	1.0
MRPL24	Q96A35	190	-11	-14	-16	3	0.9	0.9	0.9	1.0
RHOC	P08134	16	-9	-15	-16	3	0.9	0.9	0.9	1.0
RHOA	P61586	16	-9	-15	-16	3	0.9	0.9	0.9	1.0
TRAF2	Q12933	112	-4	-15	-16	3	1.0	0.9	0.9	1.0
NCKAP5L	Q9HCH0	604	-11	-15	-16	3	0.9	0.9	0.9	1.0
CTC1	Q2NKJ3	736	6	-8	-16	2	1.1	0.9	0.9	1.0
LAP3	P28838	145	-4	-13	-16	2	1.0	0.9	0.9	1.0
PITRM1	Q5JRX3	119	-11	-18	-16	2	0.9	0.9	0.9	1.0
MRPL50	Q8N5N7	52	-10	-11	-16	2	0.9	0.9	0.9	1.0
ZNF592	Q92610	353	-20	-10	-16	1	0.8	0.9	0.9	1.0
NDUFS6	O75380	87	-8	-12	-16	1	0.9	0.9	0.9	1.0
BTRC	Q9Y297	495	-11	-14	-16	1	0.9	0.9	0.9	1.0
LEMD3	Q9Y2U8	254	1	-17	-16	1	1.0	0.9	0.9	1.0
CNOT7	Q9UIV1	67	-1	-4	-16	1	1.0	1.0	0.9	1.0
PIN1	Q13526	57	-12	-5	-16	1	0.9	1.0	0.9	1.0
ABI3	Q9P2A4	33	-2	-8	-16	1	1.0	0.9	0.9	1.0
TBCK	Q8TEA7	386	-17	-22	-16	1	0.9	0.8	0.9	1.0
NACC1	Q96RE7	416	-13	-4	-16	0	0.9	1.0	0.9	1.0
AIMP2	Q13155	23	-12	-9	-16	0	0.9	0.9	0.9	1.0
PHC3	Q8NDX5	746	-9	-11	-16	0	0.9	0.9	0.9	1.0
BIRC6	Q9NR09	4750	-15	-16	-16	0	0.9	0.9	0.9	1.0
RPL23	P62829	28	-11	-17	-16	0	0.9	0.9	0.9	1.0
ULK1	O75385	1003	-47	-33	-16	0	0.7	0.8	0.9	1.0
FAM110A	Q9BQ89	209	8	-5	-16	-1	1.1	1.0	0.9	1.0
TRIM25	Q14258	506	2	-9	-16	-1	1.0	0.9	0.9	1.0
FOXO4	P98177	243	-12	-10	-16	-1	0.9	0.9	0.9	1.0
MACF1	Q9UPN3	1953	-9	-13	-16	-1	0.9	0.9	0.9	1.0
MYO5A	Q9Y4I1	1008	-12	-15	-16	-1	0.9	0.9	0.9	1.0
PLEKHM2	Q8IWE5	551	-5	-20	-16	-1	1.0	0.8	0.9	1.0
PPP4R1	Q8TF05	224	-16	-7	-16	-1	0.9	0.9	0.9	1.0
LDHA	P00338	163	-4	-11	-16	-1	1.0	0.9	0.9	1.0

LDHB	P07195	164	-4	-11	-16	-1	1.0	0.9	0.9	1.0
RAB3D	O95716	184	2	-16	-16	-1	1.0	0.9	0.9	1.0
DNTTIP2	Q5QJE6	296	2	-17	-16	-1	1.0	0.9	0.9	1.0
FARSB	Q9NSD9	548	-15	-8	-16	-2	0.9	0.9	0.9	1.0
ETV3	P41162	81	-14	-32	-16	-2	0.9	0.8	0.9	1.0
SDE2	Q6IQ49	415	-7	2	-16	-2	0.9	1.0	0.9	1.0
DHRS7	Q9Y394	147	3	-2	-16	-2	1.0	1.0	0.9	1.0
SF3B3	Q15393	1179	-4	-6	-16	-2	1.0	0.9	0.9	1.0
PDIA4	P13667	558	-11	-9	-16	-2	0.9	0.9	0.9	1.0
PSMB10	P40306	82	-12	-17	-16	-2	0.9	0.9	0.9	1.0
WDR7	Q9Y4E6	336	-6	-20	-16	-2	0.9	0.8	0.9	1.0
RPA1	P27694	481	-9	-21	-16	-2	0.9	0.8	0.9	1.0
GMEB2	Q9UKD1	323	-4	-11	-16	-3	1.0	0.9	0.9	1.0
PA2G4	Q9UQ80	296	-4	-19	-16	-3	1.0	0.8	0.9	1.0
LRRC40	Q9H9A6	23	-8	-10	-16	-3	0.9	0.9	0.9	1.0
WDCP	Q9H6R7	302	-8	-16	-16	-4	0.9	0.9	0.9	1.0
GSS	P48637	294	-9	-18	-16	-4	0.9	0.9	0.9	1.0
WDR81	Q562E7	464	-15	-21	-16	-4	0.9	0.8	0.9	1.0
MOB3A	Q96BX8	186	-6	-24	-16	-4	0.9	0.8	0.9	1.0
ILVBL	A1L0T0	315	-11	-4	-16	-4	0.9	1.0	0.9	1.0
NF1	P21359	124	-7	-11	-16	-4	0.9	0.9	0.9	1.0
METTL13	Q8N6R0	226	-10	-16	-16	-4	0.9	0.9	0.9	1.0
RASSF4	Q9H2L5	153	-12	-21	-16	-4	0.9	0.8	0.9	1.0
TBL2	Q9Y4P3	148	-39	-12	-16	-5	0.7	0.9	0.9	1.0
ACTR2	P61160	11	-9	-12	-16	-5	0.9	0.9	0.9	1.0
BCOR	Q6W2J9	1311	-35	-21	-16	-5	0.7	0.8	0.9	1.0
FAM120A	Q9NZB2	919	-8	-44	-16	-5	0.9	0.7	0.9	1.0
LRBA	P50851	950	-6	-18	-16	-5	0.9	0.9	0.9	1.0
UQCRC2	P22695	125	-6	17	-16	-6	0.9	1.2	0.9	0.9
MAP3K1	Q13233	1012	0	-13	-16	-6	1.0	0.9	0.9	0.9
PDE12	Q6L8Q7	277	-13	-19	-16	-6	0.9	0.8	0.9	0.9
ACAP2	Q15057	414	-10	-19	-16	-7	0.9	0.8	0.9	0.9
ZNF592	Q92610	790	-2	-21	-16	-7	1.0	0.8	0.9	0.9
INO80	Q9ULG1	1001	2	0	-16	-7	1.0	1.0	0.9	0.9
LRBA	P50851	1843	-1	-8	-16	-7	1.0	0.9	0.9	0.9
COMT	P21964	83	-13	-2	-16	-8	0.9	1.0	0.9	0.9
CRIP1	P50238	7	-6	-2	-16	-8	0.9	1.0	0.9	0.9
PAXIP1	Q6ZW49	136	-13	-1	-16	-8	0.9	1.0	0.9	0.9
CRYZL1	O95825	100	-9	-13	-16	-8	0.9	0.9	0.9	0.9
DGKQ	P52824	25	-6	-18	-16	-8	0.9	0.9	0.9	0.9
DPYSL2	Q16555	179	-10	-11	-16	-9	0.9	0.9	0.9	0.9
RNF34	Q969K3	287	-16	-11	-16	-9	0.9	0.9	0.9	0.9
PYGO2	Q9BRQ0	350	-23	-13	-16	-9	0.8	0.9	0.9	0.9
CCAR1	Q8IX12	373	-14	-13	-16	-9	0.9	0.9	0.9	0.9
SETMAR	Q53H47	394	-3	-9	-16	-10	1.0	0.9	0.9	0.9
FGD2	Q7Z6J4	489	-9	-10	-16	-10	0.9	0.9	0.9	0.9
RHOT1	Q8IXI2	522	-10	-17	-16	-10	0.9	0.9	0.9	0.9
CPT1A	P50416	613	-9	-19	-16	-10	0.9	0.8	0.9	0.9
CDK11B	J3QR44	432	-5	-5	-16	-11	1.0	1.0	0.9	0.9
BCKDHB	P21953	316	-10	-21	-16	-11	0.9	0.8	0.9	0.9
MED23	Q9ULK4	225	-18	-8	-16	-12	0.9	0.9	0.9	0.9
APBB1IP	Q7Z5R6	401	-12	-12	-16	-12	0.9	0.9	0.9	0.9
CKAP5	Q14008	1946	-14	-11	-16	-12	0.9	0.9	0.9	0.9
ARHGEF1	Q92888	815	-8	-15	-16	-12	0.9	0.9	0.9	0.9
ETFA	P13804	68	-9	-22	-16	-12	0.9	0.8	0.9	0.9

AGL	P35573	957	-11	-13	-16	-13	0.9	0.9	0.9	0.9
SLAIN2	Q9P270	165	-5	-16	-16	-13	1.0	0.9	0.9	0.9
Uncharacteriz	E9PCH4	1343	-11	-17	-16	-13	0.9	0.9	0.9	0.9
FARS2	O95363	344	5	-9	-16	-13	1.0	0.9	0.9	0.9
HSPA9	P38646	487	-11	-20	-16	-13	0.9	0.8	0.9	0.9
HDAC1	Q13547	261	2	-10	-16	-14	1.0	0.9	0.9	0.9
CYFIP1	Q7L576	428	-12	-28	-16	-14	0.9	0.8	0.9	0.9
FASN	P49327	1759	-17	-15	-16	-15	0.9	0.9	0.9	0.9
PLAA	Q9Y263	116	-29	-4	-16	-15	0.8	1.0	0.9	0.9
UBE2O	Q9C0C9	182	-6	-6	-16	-15	0.9	0.9	0.9	0.9
KLHL22	Q53GT1	22	-6	-19	-16	-16	0.9	0.8	0.9	0.9
DNAJB1	P25685	179	-7	-20	-16	-16	0.9	0.8	0.9	0.9
NAPA	P54920	84	-4	-12	-16	-16	1.0	0.9	0.9	0.9
EPC2	Q52LR7	446	-15	-16	-16	-16	0.9	0.9	0.9	0.9
EPC1	Q9H2F5	451	-15	-16	-16	-16	0.9	0.9	0.9	0.9
RNASEL	Q05823	395	-4	-1	-16	-17	1.0	1.0	0.9	0.9
MOCS3	O95396	95	-20	-23	-16	-17	0.8	0.8	0.9	0.9
SACM1L	Q9NTJ5	392	-1	-5	-16	-22	1.0	1.0	0.9	0.8
GTPBP1	O00178	360	-20	-37	-16	-23	0.8	0.7	0.9	0.8
MYCBP2	O75592	114	-15	42	-16	-25	0.9	1.7	0.9	0.8
PPIG	Q13427	174	-9	-15	-16	-25	0.9	0.9	0.9	0.8
VPS13A	Q96RL7	1626	-12	-14	-16	-33	0.9	0.9	0.9	0.8
RAVER1	Q8IY67	87	-15	-26	-16	-35	0.9	0.8	0.9	0.7
EML3	Q32P44	310	0	-22	-16	-40	1.0	0.8	0.9	0.7
DDX58	O95786	869	12	-23	-16	-43	1.1	0.8	0.9	0.7
PUM1	Q14671	1085	10	-40	-16	-56	1.1	0.7	0.9	0.6
PUM2	Q8TB72	965	10	-40	-16	-56	1.1	0.7	0.9	0.6
ITK	Q08881	143	0	-8	-16	-67	1.0	0.9	0.9	0.6
PDLIM7	Q9NR12	282	-33	-26	-16	-83	0.8	0.8	0.9	0.5
LIMS1	P48059	100	-6	-2	-16	-99	0.9	1.0	0.9	0.5
LIMS1	P48059	281	-9	-14	-16	-117	0.9	0.9	0.9	0.5
PDE5A	O76074	220	-1	-20	-16	-160	1.0	0.8	0.9	0.4
ATAD2	Q6PL18	463	-3	-14	-17	34	1.0	0.9	0.9	1.5
ATAD2B	Q9ULI0	437	-3	-14	-17	34	1.0	0.9	0.9	1.5
ATXN10	Q9UBB4	354	-13	-23	-17	31	0.9	0.8	0.9	1.4
CDK7	P50613	305	11	4	-17	24	1.1	1.0	0.9	1.3
RBM28	Q9NW13	97	-9	-13	-17	23	0.9	0.9	0.9	1.3
ACAD9	Q9H845	327	-10	-7	-17	22	0.9	0.9	0.9	1.3
SPATA13	Q96N96	599	-5	-13	-17	22	1.0	0.9	0.9	1.3
TXLNG	Q9NUQ3	480	1	-10	-17	21	1.0	0.9	0.9	1.3
PFKL	P17858	653	6	-9	-17	21	1.1	0.9	0.9	1.3
PFKP	Q01813	664	6	-9	-17	21	1.1	0.9	0.9	1.3
TBC1D17	Q9HA65	112	-8	-17	-17	20	0.9	0.9	0.9	1.3
KDM5D	Q9BY66	714	12	-6	-17	19	1.1	0.9	0.9	1.2
LDHA	P00338	293	-2	-11	-17	19	1.0	0.9	0.9	1.2
NBEAL2	Q6ZNJ1	1491	-7	-12	-17	19	0.9	0.9	0.9	1.2
LMF2	Q9BU23	696	-8	-14	-17	19	0.9	0.9	0.9	1.2
AGO3	Q9H9G7	493	-3	-14	-17	19	1.0	0.9	0.9	1.2
AGO4	Q9HCK5	484	-3	-14	-17	19	1.0	0.9	0.9	1.2
AGO2	Q9UKV8	492	-3	-14	-17	19	1.0	0.9	0.9	1.2
AGO1	Q9UL18	490	-3	-14	-17	19	1.0	0.9	0.9	1.2
ITGAL	P20701	771	-4	2	-17	19	1.0	1.0	0.9	1.2
MIA3	Q5JRA6	1899	-9	-9	-17	19	0.9	0.9	0.9	1.2
JARID2	Q92833	522	-8	-10	-17	19	0.9	0.9	0.9	1.2
TANK	Q92844	218	-10	-15	-17	19	0.9	0.9	0.9	1.2

MX2	P20592	707	0	-10	-17	18	1.0	0.9	0.9	1.2
FAM208A	Q9UK61	761	-19	-21	-17	17	0.8	0.8	0.9	1.2
GNL3	Q9BVP2	280	-4	-5	-17	17	1.0	1.0	0.9	1.2
NCAPG2	Q86XI2	184	-9	-9	-17	16	0.9	0.9	0.9	1.2
EMSY	Q7Z589	971	-13	-10	-17	16	0.9	0.9	0.9	1.2
GOLGA4	Q13439	1340	4	-14	-17	16	1.0	0.9	0.9	1.2
AKAP1	Q92667	147	-10	-27	-17	15	0.9	0.8	0.9	1.2
RHOA	P61586	159	0	-9	-17	15	1.0	0.9	0.9	1.2
NAA15	Q9BXJ9	214	6	-8	-17	14	1.1	0.9	0.9	1.2
AGL	P35573	234	-21	-14	-17	14	0.8	0.9	0.9	1.2
RBBP9	O75884	39	1	-16	-17	14	1.0	0.9	0.9	1.2
KIF1C	O43896	685	-9	-8	-17	14	0.9	0.9	0.9	1.2
RBL2	Q08999	415	22	-27	-17	14	1.3	0.8	0.9	1.2
CAD	P27708	1889	-8	-13	-17	13	0.9	0.9	0.9	1.1
pk	D4Q8H0	231	7	-14	-17	13	1.1	0.9	0.9	1.1
LENG8	Q96PV6	547	-4	-17	-17	13	1.0	0.9	0.9	1.1
ODF2	Q5BJF6	243	-4	-18	-17	13	1.0	0.9	0.9	1.1
KDM4A	O75164	560	-8	-11	-17	12	0.9	0.9	0.9	1.1
BCAS3	Q9H6U6	16	-14	-11	-17	12	0.9	0.9	0.9	1.1
RASA2	Q15283	129	-1	-6	-17	12	1.0	0.9	0.9	1.1
XRCC4	Q13426	130	-4	-16	-17	12	1.0	0.9	0.9	1.1
TDG	Q13569	233	-6	-17	-17	12	0.9	0.9	0.9	1.1
CTR9	Q6PD62	196	-8	-2	-17	11	0.9	1.0	0.9	1.1
CCDC88B	A6NC98	1382	-15	-8	-17	11	0.9	0.9	0.9	1.1
PANK4	Q9NVE7	699	-8	-12	-17	11	0.9	0.9	0.9	1.1
CORO1A	P31146	195	-12	-14	-17	11	0.9	0.9	0.9	1.1
KMT2E	Q8IZD2	513	-2	-17	-17	11	1.0	0.9	0.9	1.1
SIN3B	O75182	1056	-3	-8	-17	10	1.0	0.9	0.9	1.1
GNL3L	Q9NVN8	518	2	-8	-17	10	1.0	0.9	0.9	1.1
NUP93	Q8N1F7	422	-1	-10	-17	10	1.0	0.9	0.9	1.1
PRPF39	Q86UA1	450	-4	-19	-17	10	1.0	0.8	0.9	1.1
POLR2A	P24928	1430	-11	-3	-17	10	0.9	1.0	0.9	1.1
FASN	P49327	1828	-13	-6	-17	10	0.9	0.9	0.9	1.1
CIC	Q96RK0	336	-9	-18	-17	10	0.9	0.9	0.9	1.1
PXN	P49023	108	-8	-9	-17	9	0.9	0.9	0.9	1.1
TNFAIP3	P21580	767	-9	-12	-17	9	0.9	0.9	0.9	1.1
TRAPPC11	Q7Z392	658	-3	-12	-17	9	1.0	0.9	0.9	1.1
UBN1	Q9NPG3	420	-14	-14	-17	9	0.9	0.9	0.9	1.1
ATAD1	Q8NBU5	359	-4	-17	-17	9	1.0	0.9	0.9	1.1
SDHA	P31040	536	-2	-12	-17	9	1.0	0.9	0.9	1.1
MTM1	Q13496	113	-9	-18	-17	9	0.9	0.8	0.9	1.1
MSANTD2	Q6P1R3	99	-15	3	-17	8	0.9	1.0	0.9	1.1
ATRX	P46100	1531	-7	-15	-17	8	0.9	0.9	0.9	1.1
NAA15	Q9BXJ9	817	-8	2	-17	8	0.9	1.0	0.9	1.1
MAST4	O15021	1800	-4	-8	-17	8	1.0	0.9	0.9	1.1
GPX1	P07203	202	-12	-11	-17	8	0.9	0.9	0.9	1.1
RCC2	Q9P258	271	-3	-16	-17	8	1.0	0.9	0.9	1.1
SPG11	Q96JI7	838	-10	-17	-17	8	0.9	0.9	0.9	1.1
RTTN	Q86VV8	1502	-12	-8	-17	7	0.9	0.9	0.9	1.1
COPG1	Q9Y678	169	-8	-8	-17	7	0.9	0.9	0.9	1.1
ATM	Q13315	2112	-1	-13	-17	7	1.0	0.9	0.9	1.1
ANP32B	Q92688	27	-9	-16	-17	7	0.9	0.9	0.9	1.1
NDUFV1	P49821	238	-7	-19	-17	7	0.9	0.8	0.9	1.1
HNRNPA2B1	P22626	50	-15	-20	-17	7	0.9	0.8	0.9	1.1
LCK	P06239	465	15	6	-17	7	1.2	1.1	0.9	1.1

SCAF1	Q9H7N4	675	-5	-12	-17	7	1.0	0.9	0.9	1.1
CHD4	Q14839	1594	-7	-8	-17	6	0.9	0.9	0.9	1.1
EIF2S3	P41091	434	-5	-13	-17	6	1.0	0.9	0.9	1.1
ARID2	Q68CP9	1512	-6	-15	-17	6	0.9	0.9	0.9	1.1
ARHGAP25	P42331	524	-7	-7	-17	6	0.9	0.9	0.9	1.1
DHX30	Q7L2E3	461	-4	1	-17	5	1.0	1.0	0.9	1.1
NEK9	Q8TD19	878	-5	-11	-17	5	1.0	0.9	0.9	1.1
WDR33	Q9C0J8	120	-9	-13	-17	5	0.9	0.9	0.9	1.1
SRSF6	Q13247	121	-9	-11	-17	5	0.9	0.9	0.9	1.0
UBN1	Q9NPG3	711	-6	-13	-17	5	0.9	0.9	0.9	1.0
PSME3	P61289	92	-7	-14	-17	5	0.9	0.9	0.9	1.0
PRDX3	P30048	229	-4	-18	-17	5	1.0	0.9	0.9	1.0
DIDO1	Q9BTC0	455	-14	-7	-17	4	0.9	0.9	0.9	1.0
DDX60	Q8IY21	517	-7	-8	-17	4	0.9	0.9	0.9	1.0
EP300	Q09472	1790	-11	-8	-17	4	0.9	0.9	0.9	1.0
CREBBP	Q92793	1827	-11	-8	-17	4	0.9	0.9	0.9	1.0
SRPRA	P08240	253	-3	-10	-17	4	1.0	0.9	0.9	1.0
GAR1	Q9NY12	86	-8	-11	-17	4	0.9	0.9	0.9	1.0
MCM3AP	O60318	1285	-13	-17	-17	4	0.9	0.9	0.9	1.0
KBTBD11	O94819	421	-6	-25	-17	4	0.9	0.8	0.9	1.0
ZFAND3	Q9H8U3	118	-2	2	-17	4	1.0	1.0	0.9	1.0
NLRP3	Q96P20	196	-14	-14	-17	4	0.9	0.9	0.9	1.0
DBR1	Q9UK59	9	-9	-14	-17	4	0.9	0.9	0.9	1.0
MCF2L	O15068	951	-32	-17	-17	4	0.8	0.9	0.9	1.0
TKT	P29401	225	-6	-6	-17	3	0.9	0.9	0.9	1.0
PRRC2A	P48634	486	-8	-11	-17	3	0.9	0.9	0.9	1.0
AGFG2	O95081	39	-10	-12	-17	3	0.9	0.9	0.9	1.0
ENTHD2	Q96N21	302	-17	-21	-17	3	0.9	0.8	0.9	1.0
IRAK4	Q9NWZ3	13	-13	-4	-17	3	0.9	1.0	0.9	1.0
AAK1	Q2M2I8	319	-4	-10	-17	3	1.0	0.9	0.9	1.0
RANBP2	P49792	50	-2	-11	-17	3	1.0	0.9	0.9	1.0
RGPD6	Q99666	50	-2	-11	-17	3	1.0	0.9	0.9	1.0
GPBAR1	Q8TDU6	207	-8	-9	-17	2	0.9	0.9	0.9	1.0
MFHAS1	Q9Y4C4	179	-20	-15	-17	2	0.8	0.9	0.9	1.0
DDB2	Q92466	322	-4	-17	-17	2	1.0	0.9	0.9	1.0
RNPEP	Q9H4A4	181	-16	-22	-17	2	0.9	0.8	0.9	1.0
PSMA1	P25786	92	-6	-14	-17	2	0.9	0.9	0.9	1.0
WTAP	Q15007	270	-8	-16	-17	2	0.9	0.9	0.9	1.0
SUPT5H	O00267	626	-5	-12	-17	1	1.0	0.9	0.9	1.0
RIN1	Q13671	639	-4	-16	-17	1	1.0	0.9	0.9	1.0
GLTSCR1	Q9NZM4	1083	-2	-17	-17	1	1.0	0.9	0.9	1.0
DHX34	Q14147	32	-13	-18	-17	1	0.9	0.8	0.9	1.0
ANKRD40	Q6AI12	209	-20	-14	-17	0	0.8	0.9	0.9	1.0
FXR2	P51116	282	-12	-14	-17	0	0.9	0.9	0.9	1.0
TNFAIP8	O95379	165	-1	-23	-17	0	1.0	0.8	0.9	1.0
SP110	Q9HB58	461	-15	-35	-17	0	0.9	0.7	0.9	1.0
DNAJB14	Q8TBM8	178	0	-11	-17	-1	1.0	0.9	0.9	1.0
ANP32A	P39687	123	-10	-14	-17	-1	0.9	0.9	0.9	1.0
GNB2	P62879	25	-4	-21	-17	-1	1.0	0.8	0.9	1.0
MAST4	O15021	2377	-33	-26	-17	-1	0.8	0.8	0.9	1.0
TBCB	Q99426	51	-3	-4	-17	-1	1.0	1.0	0.9	1.0
PRMT2	P55345	75	-1	-9	-17	-1	1.0	0.9	0.9	1.0
LRMP	Q12912	355	-3	-12	-17	-1	1.0	0.9	0.9	1.0
CAPN1	P07384	351	-2	-13	-17	-1	1.0	0.9	0.9	1.0
STX5	Q13190	68	-11	-14	-17	-1	0.9	0.9	0.9	1.0

IRF8	Q02556	306	-11	-15	-17	-1	0.9	0.9	0.9	1.0
FAM102A	Q5T9C2	175	-10	-15	-17	-1	0.9	0.9	0.9	1.0
PDCD11	Q14690	557	-17	-16	-17	-1	0.9	0.9	0.9	1.0
FAM101B	Q8N5W9	81	-7	-14	-17	-2	0.9	0.9	0.9	1.0
NANS	Q9NR45	283	-12	-22	-17	-2	0.9	0.8	0.9	1.0
HCFC1	P51610	1139	-9	-13	-17	-2	0.9	0.9	0.9	1.0
ZNF276	Q8N554	18	-2	-8	-17	-3	1.0	0.9	0.9	1.0
RABEP2	Q9H5N1	327	3	-12	-17	-3	1.0	0.9	0.9	1.0
POR	P16435	566	-1	-19	-17	-3	1.0	0.8	0.9	1.0
TCF20	Q9UGU0	1188	-6	-23	-17	-3	0.9	0.8	0.9	1.0
HUS1	O60921	200	-6	-9	-17	-3	0.9	0.9	0.9	1.0
DEF6	Q9H4E7	279	-7	-11	-17	-4	0.9	0.9	0.9	1.0
NFKB1	P19838	61	-5	-16	-17	-4	1.0	0.9	0.9	1.0
OSBPL5	Q9H0X9	233	-20	-12	-17	-4	0.8	0.9	0.9	1.0
CD36	P16671	272	-2	-18	-17	-4	1.0	0.9	0.9	1.0
OXCT1	P55809	504	-16	-22	-17	-4	0.9	0.8	0.9	1.0
BANK1	Q8NDB2	780	-35	-26	-17	-5	0.7	0.8	0.9	1.0
LAP3	P28838	129	-6	-12	-17	-5	0.9	0.9	0.9	1.0
DIAPH2	O60879	484	-12	-16	-17	-5	0.9	0.9	0.9	1.0
GTPBP1	O00178	662	-1	-12	-17	-6	1.0	0.9	0.9	0.9
TUBB6	Q9BUF5	303	-15	-10	-17	-6	0.9	0.9	0.9	0.9
TUBB2B	Q9BVA1	303	-15	-10	-17	-6	0.9	0.9	0.9	0.9
WDR70	Q9NW82	229	-7	-12	-17	-6	0.9	0.9	0.9	0.9
TRPM7	Q96QT4	1484	-12	-19	-17	-6	0.9	0.8	0.9	0.9
NLN	Q9BYT8	272	-6	-22	-17	-6	0.9	0.8	0.9	0.9
MYO18A	Q92614	1699	2	-14	-17	-7	1.0	0.9	0.9	0.9
FAM193A	P78312	594	-18	-28	-17	-7	0.9	0.8	0.9	0.9
FIG4	Q92562	489	-17	-9	-17	-7	0.9	0.9	0.9	0.9
DHX30	Q7L2E3	309	-4	-22	-17	-7	1.0	0.8	0.9	0.9
ADNP	Q9H2P0	75	-17	-12	-17	-8	0.9	0.9	0.9	0.9
STK19	P49842	230	-14	-10	-17	-8	0.9	0.9	0.9	0.9
ZYX	Q15942	436	-20	-15	-17	-8	0.8	0.9	0.9	0.9
TARS	P26639	343	-8	-27	-17	-8	0.9	0.8	0.9	0.9
PMS2	P54278	653	-8	-10	-17	-9	0.9	0.9	0.9	0.9
PPP3R1	P63098	154	6	-13	-17	-9	1.1	0.9	0.9	0.9
MTPN	P58546	45	-5	-17	-17	-9	1.0	0.9	0.9	0.9
RIPK1	Q13546	656	-17	-35	-17	-9	0.9	0.7	0.9	0.9
CDC42BPB	Q9Y5S2	120	4	-8	-17	-10	1.0	0.9	0.9	0.9
MAD1L1	Q9Y6D9	662	-3	-14	-17	-11	1.0	0.9	0.9	0.9
CBL	P22681	567	-12	-16	-17	-11	0.9	0.9	0.9	0.9
DARS	P14868	130	-10	-7	-17	-12	0.9	0.9	0.9	0.9
SAE1	Q9UBE0	133	-20	-21	-17	-12	0.8	0.8	0.9	0.9
DNM1L	O00429	505	6	-7	-17	-12	1.1	0.9	0.9	0.9
NMD3	Q96D46	250	-4	-18	-17	-13	1.0	0.9	0.9	0.9
COG5	Q9UP83	664	1	-21	-17	-13	1.0	0.8	0.9	0.9
GBE1	Q04446	221	-7	-22	-17	-13	0.9	0.8	0.9	0.9
ZYX	Q15942	415	-21	-23	-17	-13	0.8	0.8	0.9	0.9
NAT10	Q9H0A0	489	-30	-8	-17	-14	0.8	0.9	0.9	0.9
RALGAPB	Q86X10	1096	-24	-9	-17	-14	0.8	0.9	0.9	0.9
AKAP2	Q9Y2D5	205	-8	-16	-17	-14	0.9	0.9	0.9	0.9
LYPLA2	O95372	213	-11	-31	-17	-15	0.9	0.8	0.9	0.9
CHURC1-FNTB	B4DL54	333	3	16	-17	-16	1.0	1.2	0.9	0.9
ERCC5	P28715	550	-2	-19	-17	-16	1.0	0.8	0.9	0.9
DENND3	A2RUS2	71	-7	-7	-17	-20	0.9	0.9	0.9	0.8
PDK3	Q15120	191	11	-15	-17	-21	1.1	0.9	0.9	0.8

ACAP2	Q15057	329	-5	-16	-17	-21	1.0	0.9	0.9	0.8
SYK	P43405	259	-3	-5	-17	-24	1.0	1.0	0.9	0.8
CKAP5	Q14008	1795	-8	-16	-17	-24	0.9	0.9	0.9	0.8
MYO18A	Q92614	1199	-12	-4	-17	-27	0.9	1.0	0.9	0.8
ABCF2	Q9UG63	186	-10	-38	-17	-44	0.9	0.7	0.9	0.7
CAPNS1	P04632	190	-1	-8	-17	-45	1.0	0.9	0.9	0.7
TUBB1	Q9H4B7	303	-3	2	-17	-212	1.0	1.0	0.9	0.3
SH3TC1	Q8TE82	1324	4	-13	-17	51	1.0	0.9	0.9	2.0
GLIPR1	P48060	192	-8	-8	-17	43	0.9	0.9	0.9	1.7
AGPS	O00116	565	1	-10	-17	31	1.0	0.9	0.9	1.4
ZNF430	Q9H8G1	17	-7	-19	-17	31	0.9	0.8	0.9	1.4
RB1	P06400	438	-9	-10	-17	29	0.9	0.9	0.9	1.4
PI4KA	P42356	19	-20	-1	-17	28	0.8	1.0	0.9	1.4
STK11	Q15831	418	-10	-20	-17	26	0.9	0.8	0.9	1.3
USP32	Q8NFA0	1320	2	-12	-17	25	1.0	0.9	0.9	1.3
UBR4	Q5T4S7	2222	-3	-12	-17	24	1.0	0.9	0.9	1.3
THUMPD3	Q9BV44	505	-13	-14	-17	24	0.9	0.9	0.9	1.3
VPS13C	Q709C8	1098	-12	-2	-17	23	0.9	1.0	0.9	1.3
BIRC6	Q9NR09	381	3	-6	-17	22	1.0	0.9	0.9	1.3
RTN1	Q16799	678	-2	-7	-17	22	1.0	0.9	0.9	1.3
PGRMC2	O15173	159	-12	-8	-17	22	0.9	0.9	0.9	1.3
SETDB1	Q15047	53	17	-25	-17	21	1.2	0.8	0.9	1.3
TBC1D24	Q9ULP9	223	-3	-5	-17	21	1.0	1.0	0.9	1.3
SETD1B	Q9UPS6	1698	-9	-7	-17	20	0.9	0.9	0.9	1.2
MTMR14	Q8NCE2	429	-5	-10	-17	19	1.0	0.9	0.9	1.2
MEPCE	Q7L2J0	244	-9	-24	-17	19	0.9	0.8	0.9	1.2
NUB1	Q9Y5A7	270	-9	-11	-17	18	0.9	0.9	0.9	1.2
TAF3	Q5VWG9	431	3	-17	-17	18	1.0	0.9	0.9	1.2
ZNF189	O75820	610	-8	-31	-17	18	0.9	0.8	0.9	1.2
PMPCA	Q10713	466	-7	-10	-17	17	0.9	0.9	0.9	1.2
SARS	P49591	162	-8	-10	-17	17	0.9	0.9	0.9	1.2
SUGP2	Q8IX01	417	-8	-11	-17	17	0.9	0.9	0.9	1.2
LOC10272402	A0A0B4J2D5	177	-2	-12	-17	16	1.0	0.9	0.9	1.2
MDN1	Q9NU22	1394	-8	-10	-17	16	0.9	0.9	0.9	1.2
ZNF490	Q9ULM2	317	-8	-13	-17	15	0.9	0.9	0.9	1.2
PRPF38B	Q5VTL8	113	-13	-15	-17	15	0.9	0.9	0.9	1.2
HPS5	Q9UPZ3	727	-12	7	-17	15	0.9	1.1	0.9	1.2
MARCH7	Q9H992	604	-3	-9	-17	14	1.0	0.9	0.9	1.2
TOLLIP	Q9H0E2	229	-3	2	-17	14	1.0	1.0	0.9	1.2
ZNF337	Q9Y3M9	82	-21	-13	-17	14	0.8	0.9	0.9	1.2
AVEN	Q9NQS1	169	-3	-24	-17	14	1.0	0.8	0.9	1.2
TSPYL1	Q9H0U9	160	-25	-12	-17	13	0.8	0.9	0.9	1.1
NSUN2	Q08J23	221	0	-5	-17	12	1.0	1.0	0.9	1.1
TDRD7	Q8NHU6	692	-9	-8	-17	12	0.9	0.9	0.9	1.1
KMT2D	O14686	4424	-10	-17	-17	12	0.9	0.9	0.9	1.1
SNRK	Q9NRH2	408	-8	-13	-17	11	0.9	0.9	0.9	1.1
TTC38	Q5R3I4	10	-6	-13	-17	11	0.9	0.9	0.9	1.1
BOLA2B	Q9H3K6	33	-3	-4	-17	11	1.0	1.0	0.9	1.1
ZMYM2	Q9UBW7	750	-7	-14	-17	11	0.9	0.9	0.9	1.1
AHCY	P23526	195	-3	-11	-17	10	1.0	0.9	0.9	1.1
CBLL1	Q75N03	73	-11	-12	-17	10	0.9	0.9	0.9	1.1
TOE1	Q96GM8	80	-8	-17	-17	10	0.9	0.9	0.9	1.1
ZC3H12A	Q5D1E8	328	-9	-1	-17	10	0.9	1.0	0.9	1.1
MSN	P26038	284	-3	-12	-17	10	1.0	0.9	0.9	1.1
C1orf50	Q9BV19	110	0	-9	-17	9	1.0	0.9	0.9	1.1

UBR7	Q8N806	260	-8	-12	-17	9	0.9	0.9	0.9	1.1
CNTRL	Q7Z7A1	1451	-7	-14	-17	9	0.9	0.9	0.9	1.1
TBCD	Q9BTW9	850	-5	-13	-17	9	1.0	0.9	0.9	1.1
BMS1	Q14692	725	-2	-8	-17	8	1.0	0.9	0.9	1.1
C14orf159	Q7Z3D6	418	-14	-9	-17	8	0.9	0.9	0.9	1.1
ZCCHC11	Q5TAX3	20	-8	-10	-17	8	0.9	0.9	0.9	1.1
SRD5A3	Q9H8P0	238	-3	-12	-17	8	1.0	0.9	0.9	1.1
WDR81	Q562E7	974	-1	-14	-17	8	1.0	0.9	0.9	1.1
UPP1	Q16831	17	-12	-16	-17	7	0.9	0.9	0.9	1.1
DDX18	Q9NVP1	407	-17	-16	-17	7	0.9	0.9	0.9	1.1
SREK1	Q8WXA9	494	-6	-23	-17	7	0.9	0.8	0.9	1.1
KLC1	Q07866	559	-6	-13	-17	7	0.9	0.9	0.9	1.1
NASP	P49321	254	-5	-14	-17	7	1.0	0.9	0.9	1.1
ZNF592	Q92610	463	-11	-15	-17	7	0.9	0.9	0.9	1.1
RRM1	P23921	492	-1	-13	-17	6	1.0	0.9	0.9	1.1
TCEA1	P23193	271	-19	-16	-17	6	0.8	0.9	0.9	1.1
TCEA2	Q15560	269	-19	-16	-17	6	0.8	0.9	0.9	1.1
LDB1	Q86U70	273	-11	-10	-17	6	0.9	0.9	0.9	1.1
DPP4	P27487	551	-11	-11	-17	6	0.9	0.9	0.9	1.1
ZFAND6	Q6FIF0	163	-11	-11	-17	6	0.9	0.9	0.9	1.1
ZNF451	Q9Y4E5	441	-8	-25	-17	6	0.9	0.8	0.9	1.1
C15orf40	Q8WUR7	104	-10	-11	-17	5	0.9	0.9	0.9	1.1
PREX1	Q8TCU6	52	-5	-16	-17	5	1.0	0.9	0.9	1.1
HSP90B1	P14625	576	-1	-19	-17	5	1.0	0.8	0.9	1.1
CHD2	O14647	365	11	-20	-17	5	1.1	0.8	0.9	1.1
SMC2	O95347	800	-5	-13	-17	5	1.0	0.9	0.9	1.0
RNF213	Q63HN8	876	-13	-16	-17	5	0.9	0.9	0.9	1.0
COPS7B	Q9H9Q2	240	-7	-16	-17	5	0.9	0.9	0.9	1.0
GOLGA2	Q08379	205	-6	-12	-17	4	0.9	0.9	0.9	1.0
DNMT1	P26358	1499	1	-16	-17	4	1.0	0.9	0.9	1.0
FNIP1	Q8TF40	608	-4	-30	-17	4	1.0	0.8	0.9	1.0
BOD1L1	Q8NFC6	686	-14	-11	-17	4	0.9	0.9	0.9	1.0
VPS39	Q96JC1	235	-6	-16	-17	4	0.9	0.9	0.9	1.0
HPS5	Q9UPZ3	228	-13	-7	-17	3	0.9	0.9	0.9	1.0
ZNF131	P52739	235	-7	-15	-17	3	0.9	0.9	0.9	1.0
GCFC2	P16383	595	-5	-18	-17	3	1.0	0.8	0.9	1.0
NFKB2	Q00653	891	-8	-11	-17	2	0.9	0.9	0.9	1.0
APOBR	Q0VD83	149	-9	-17	-17	2	0.9	0.9	0.9	1.0
PHGDH	O43175	281	-16	-26	-17	2	0.9	0.8	0.9	1.0
ARFGAP3	Q9NP61	312	-12	-12	-17	2	0.9	0.9	0.9	1.0
USP15	Q9Y4E8	264	-8	-12	-17	2	0.9	0.9	0.9	1.0
KMT2E	Q8IZD2	815	-10	-18	-17	2	0.9	0.8	0.9	1.0
DOCK9	Q9BZ29	69	-16	-6	-17	1	0.9	0.9	0.9	1.0
USP47	Q96K76	767	-6	-5	-17	1	0.9	1.0	0.9	1.0
ATIC	P31939	434	-10	-15	-17	1	0.9	0.9	0.9	1.0
DENND1C	Q8IV53	412	-3	-16	-17	1	1.0	0.9	0.9	1.0
CACUL1	Q86Y37	362	-11	-20	-17	1	0.9	0.8	0.9	1.0
TMEM30A	Q9NV96	17	-4	-9	-17	0	1.0	0.9	0.9	1.0
ZNFX1	Q9P2E3	112	-27	-18	-17	0	0.8	0.9	0.9	1.0
PINX1	Q96BK5	145	-10	-19	-17	0	0.9	0.8	0.9	1.0
IL7R	P16871	306	-8	-20	-17	0	0.9	0.8	0.9	1.0
DYNC1H1	Q14204	2712	-3	-11	-17	-1	1.0	0.9	0.9	1.0
PSMG1	O95456	157	-17	-20	-17	-1	0.9	0.8	0.9	1.0
MBD3	O95983	215	-3	-4	-17	-2	1.0	1.0	0.9	1.0
DNMT1	P26358	1125	-9	-15	-17	-2	0.9	0.9	0.9	1.0

ZNF276	Q8N554	472	-2	-16	-17	-2	1.0	0.9	0.9	1.0
ALDOA	P04075	339	-7	-17	-17	-2	0.9	0.9	0.9	1.0
ALDOA	P04075	240	-2	-9	-17	-2	1.0	0.9	0.9	1.0
SH3KBP1	Q96B97	103	-3	-10	-17	-2	1.0	0.9	0.9	1.0
CNOT1	A5YKK6	219	-9	-11	-17	-2	0.9	0.9	0.9	1.0
PGK1	P00558	316	-13	-12	-17	-2	0.9	0.9	0.9	1.0
HLA-A	P01892	283	4	-16	-17	-2	1.0	0.9	0.9	1.0
HLA-B	P30479	283	4	-16	-17	-2	1.0	0.9	0.9	1.0
HLA-B	P30464	283	4	-16	-17	-2	1.0	0.9	0.9	1.0
TBC1D15	Q8TC07	197	-11	-17	-17	-2	0.9	0.9	0.9	1.0
MAP2K7	O14733	280	-10	-11	-17	-3	0.9	0.9	0.9	1.0
FABP5	Q01469	120	-14	-19	-17	-3	0.9	0.8	0.9	1.0
ADSS	P30520	338	-12	-9	-17	-3	0.9	0.9	0.9	1.0
AKR1A1	P14550	134	-2	-17	-17	-3	1.0	0.9	0.9	1.0
HNRNPU	Q00839	389	-4	-8	-17	-4	1.0	0.9	0.9	1.0
ARHGEF11	O15085	1047	-8	-23	-17	-4	0.9	0.8	0.9	1.0
SEPT6	Q14141	269	-24	-17	-17	-4	0.8	0.9	0.9	1.0
TRIM59	Q8IWR1	314	-12	-18	-17	-4	0.9	0.9	0.9	1.0
TCP1	P17987	357	-5	-5	-17	-5	1.0	1.0	0.9	1.0
CBWD1	Q9BRT8	107	-14	-7	-17	-5	0.9	0.9	0.9	1.0
ZC3H7A	Q8IWR0	123	-12	-2	-17	-6	0.9	1.0	0.9	0.9
MLH1	P40692	77	-6	-12	-17	-6	0.9	0.9	0.9	0.9
TNFAIP3	P21580	733	-14	2	-17	-6	0.9	1.0	0.9	0.9
FAM160B1	Q5W0V3	156	-8	-12	-17	-7	0.9	0.9	0.9	0.9
UNC45A	Q9H3U1	384	-11	-11	-17	-7	0.9	0.9	0.9	0.9
LYPLA2	O95372	56	-3	-18	-17	-7	1.0	0.9	0.9	0.9
XRN1	Q8IZH2	16	-16	-21	-17	-7	0.9	0.8	0.9	0.9
HUWE1	Q7Z6Z7	3361	-8	-15	-17	-8	0.9	0.9	0.9	0.9
USP10	Q14694	254	-15	-25	-17	-8	0.9	0.8	0.9	0.9
SUZ12	Q15022	201	1	2	-17	-8	1.0	1.0	0.9	0.9
AKR7A2	O43488	214	-8	-20	-17	-8	0.9	0.8	0.9	0.9
METTL2A	Q96IZ6	171	-21	-29	-17	-9	0.8	0.8	0.9	0.9
CCNT2	O60583	36	-7	-5	-17	-9	0.9	1.0	0.9	0.9
WTAP	Q15007	90	-5	-23	-17	-10	1.0	0.8	0.9	0.9
BDP1	A6H8Y1	789	-13	-42	-17	-10	0.9	0.7	0.9	0.9
TCP1	P17987	296	-6	-4	-17	-10	0.9	1.0	0.9	0.9
GFM2	Q969S9	248	-22	-30	-17	-11	0.8	0.8	0.9	0.9
ASAP1	Q9ULH1	248	-7	-13	-17	-12	0.9	0.9	0.9	0.9
ZSWIM8	A7E2V4	186	3	0	-17	-13	1.0	1.0	0.9	0.9
ACOT7	O00154	100	-4	-3	-17	-13	1.0	1.0	0.9	0.9
C2CD2L	O14523	385	-15	-17	-17	-13	0.9	0.9	0.9	0.9
SEPT11	Q9NVA2	268	-8	-15	-17	-14	0.9	0.9	0.9	0.9
SEPT10	Q9P0V9	293	-8	-15	-17	-14	0.9	0.9	0.9	0.9
DOCK8	Q8NF50	932	-34	-19	-17	-14	0.7	0.8	0.9	0.9
LIMD1	Q9UGP4	431	-7	-18	-17	-15	0.9	0.8	0.9	0.9
TTC14	Q96N46	634	-5	-9	-17	-15	1.0	0.9	0.9	0.9
RNF213	Q63HN8	418	-9	-8	-17	-16	0.9	0.9	0.9	0.9
GLS	O94925	266	2	-11	-17	-17	1.0	0.9	0.9	0.9
DSTN	P60981	80	7	-8	-17	-17	1.1	0.9	0.9	0.9
ASNA1	O43681	289	-8	-13	-17	-17	0.9	0.9	0.9	0.9
PLEK	P08567	295	-6	-16	-17	-17	0.9	0.9	0.9	0.9
ACAP2	Q15057	339	1	-13	-17	-18	1.0	0.9	0.9	0.9
ACAP3	Q96P50	341	1	-13	-17	-18	1.0	0.9	0.9	0.9
EXTL3	O43909	74	-31	-25	-17	-18	0.8	0.8	0.9	0.8
TXNL1	O43396	135	-11	-6	-17	-19	0.9	0.9	0.9	0.8

METAP1	P53582	292	13	-13	-17	-19	1.1	0.9	0.9	0.8
PIN4	Q9Y237	45	-16	-21	-17	-20	0.9	0.8	0.9	0.8
FLNB	O75369	455	-3	-20	-17	-22	1.0	0.8	0.9	0.8
RARS	P54136	115	-8	-18	-17	-22	0.9	0.9	0.9	0.8
NSD1	Q96L73	1586	-11	-18	-17	-23	0.9	0.9	0.9	0.8
NSD1	Q96L73	1583	-11	-18	-17	-23	0.9	0.9	0.9	0.8
ZYX	Q15942	473	-19	-17	-17	-23	0.8	0.9	0.9	0.8
GSTO1	P78417	192	-8	-18	-17	-26	0.9	0.8	0.9	0.8
CORO1C	Q9ULV4	23	-12	-15	-17	-26	0.9	0.9	0.9	0.8
FLNA	P21333	623	-4	-12	-17	-33	1.0	0.9	0.9	0.8
FLNC	Q14315	618	-4	-12	-17	-33	1.0	0.9	0.9	0.8
DBN1	Q16643	613	4	-16	-17	-34	1.0	0.9	0.9	0.7
UNC13D	Q70J99	255	6	-6	-17	-35	1.1	0.9	0.9	0.7
RNF31	Q96EP0	572	5	-39	-17	-36	1.0	0.7	0.9	0.7
USP31	Q70CQ4	465	-3	-17	-17	-39	1.0	0.9	0.9	0.7
THBD	P07204	554	-14	-13	-17	-54	0.9	0.9	0.9	0.7
THBS1	P07996	910	-4	-23	-17	-63	1.0	0.8	0.9	0.6
CCSER2	Q9H7U1	751	-16	-20	-17	-74	0.9	0.8	0.9	0.6
FHL1	Q13642	129	4	-8	-17	-76	1.0	0.9	0.9	0.6
POLR2B	P30876	221	-6	-13	-17	-88	0.9	0.9	0.9	0.5
MAP3K11	Q16584	327	-15	-18	-18	38	0.9	0.8	0.9	1.6
GSDMD	P57764	268	-10	-15	-18	37	0.9	0.9	0.9	1.6
LYZ	P61626	48	-20	-17	-18	35	0.8	0.9	0.9	1.5
SAMM50	Q9Y512	237	1	-8	-18	33	1.0	0.9	0.9	1.5
MPND	Q8N594	241	4	-10	-18	33	1.0	0.9	0.9	1.5
PGK1	P00558	367	-5	-12	-18	29	1.0	0.9	0.9	1.4
CNBP	P62633	97	-2	-26	-18	29	1.0	0.8	0.9	1.4
USP24	Q9UPU5	116	-9	3	-18	28	0.9	1.0	0.9	1.4
GFPT1	Q06210	55	-5	-13	-18	28	1.0	0.9	0.9	1.4
AGPAT1	Q99943	218	-4	-29	-18	27	1.0	0.8	0.9	1.4
MAP3K6	O95382	232	-10	-17	-18	25	0.9	0.9	0.9	1.3
CDKN1B	P46527	99	-9	-17	-18	25	0.9	0.9	0.9	1.3
SENP7	Q9BQF6	539	-5	-16	-18	23	1.0	0.9	0.9	1.3
LRRFIP1	Q32MZ4	334	-7	-10	-18	22	0.9	0.9	0.9	1.3
ACP6	Q9NPH0	267	-12	-11	-18	22	0.9	0.9	0.9	1.3
VRK3	Q8IV63	191	-4	-13	-18	21	1.0	0.9	0.9	1.3
RBMXL1	Q96E39	338	-7	-11	-18	20	0.9	0.9	0.9	1.2
ARRB2	P32121	270	-9	-7	-18	19	0.9	0.9	0.9	1.2
DCP2	Q8IU60	158	0	-10	-18	19	1.0	0.9	0.9	1.2
TEP1	Q99973	2000	-8	-15	-18	18	0.9	0.9	0.9	1.2
GRAMD1B	Q3KR37	210	-8	-15	-18	18	0.9	0.9	0.9	1.2
SRXN1	Q9BYN0	99	-12	-16	-18	18	0.9	0.9	0.9	1.2
CDK7	P50613	312	-2	-6	-18	17	1.0	0.9	0.9	1.2
CHST14	Q8NCH0	159	-6	-9	-18	17	0.9	0.9	0.9	1.2
ANXA2	P07355	335	6	-21	-18	17	1.1	0.8	0.9	1.2
PIK3R1	P27986	670	-14	-23	-18	17	0.9	0.8	0.9	1.2
ANKRD50	Q9ULJ7	516	8	-26	-18	17	1.1	0.8	0.9	1.2
LRPPRC	P42704	848	14	5	-18	16	1.2	1.0	0.9	1.2
MSL3	Q8N5Y2	358	4	-14	-18	16	1.0	0.9	0.9	1.2
LAT2	Q9GZY6	160	-17	-20	-18	16	0.9	0.8	0.9	1.2
CAPRIN1	Q14444	226	-1	-14	-18	16	1.0	0.9	0.9	1.2
RMI1	Q9H9A7	268	-15	-19	-18	16	0.9	0.8	0.9	1.2
ARHGAP25	P42331	448	-15	-15	-18	15	0.9	0.9	0.9	1.2
TTC13	Q8NBP0	110	-28	-27	-18	15	0.8	0.8	0.9	1.2
SLC43A2	Q8N370	392	-1	-10	-18	14	1.0	0.9	0.9	1.2

ZNF292	O60281	377	-4	-12	-18	14	1.0	0.9	0.9	1.2
CHD4	Q14839	475	-27	4	-18	14	0.8	1.0	0.9	1.2
SLC27A3	Q5K4L6	112	-5	-8	-18	14	1.0	0.9	0.9	1.2
GRK4	P32298	202	-5	-13	-18	13	1.0	0.9	0.9	1.1
GRK5	P34947	201	-5	-13	-18	13	1.0	0.9	0.9	1.1
GRK6	P43250	201	-5	-13	-18	13	1.0	0.9	0.9	1.1
ATG7	O95352	182	-2	-1	-18	13	1.0	1.0	0.9	1.1
PLCB2	Q00722	1164	4	-4	-18	12	1.0	1.0	0.9	1.1
SLFN12	Q8IYM2	287	-6	-13	-18	12	0.9	0.9	0.9	1.1
PYURF	Q96I23	60	-21	-26	-18	12	0.8	0.8	0.9	1.1
SREBF1	P36956	656	-5	-15	-18	12	1.0	0.9	0.9	1.1
SAMD9	Q5K651	979	-8	-13	-18	11	0.9	0.9	0.9	1.1
ZNF501	Q96CX3	116	-9	-13	-18	11	0.9	0.9	0.9	1.1
CAPN15	O75808	123	-19	-20	-18	11	0.8	0.8	0.9	1.1
EIF4G1	Q04637	1265	4	-5	-18	11	1.0	1.0	0.9	1.1
ATP13A2	Q9NQ11	365	-10	-9	-18	11	0.9	0.9	0.9	1.1
NEPRO	Q6NW34	492	-9	-26	-18	11	0.9	0.8	0.9	1.1
IVNS1ABP	Q9Y6Y0	454	-9	-13	-18	10	0.9	0.9	0.9	1.1
DOCK8	Q8NF50	197	-7	-10	-18	10	0.9	0.9	0.9	1.1
NDUFV1	P49821	206	-15	-12	-18	10	0.9	0.9	0.9	1.1
WARS	P23381	305	-17	-16	-18	9	0.9	0.9	0.9	1.1
KIF21A	Q7Z4S6	299	-5	-17	-18	9	1.0	0.9	0.9	1.1
ECI2	O75521	77	-8	-7	-18	9	0.9	0.9	0.9	1.1
GCDH	Q92947	289	-6	2	-18	8	0.9	1.0	0.9	1.1
RPS6KA5	O75582	731	-4	-13	-18	8	1.0	0.9	0.9	1.1
LAS1L	Q9Y4W2	690	-8	-15	-18	8	0.9	0.9	0.9	1.1
CLP1	Q92989	231	-13	-17	-18	8	0.9	0.9	0.9	1.1
NBAS	A2RRP1	807	-9	-9	-18	8	0.9	0.9	0.9	1.1
XRCC5	P13010	157	-8	-12	-18	8	0.9	0.9	0.9	1.1
HLA-DQB1	Q5SU54	111	-12	-15	-18	8	0.9	0.9	0.9	1.1
WHSC1L1	Q9BZ95	1292	-17	-15	-18	8	0.9	0.9	0.9	1.1
PLEC	Q15149	317	-19	-2	-18	7	0.8	1.0	0.9	1.1
ARHGAP25	P42331	89	2	-11	-18	7	1.0	0.9	0.9	1.1
RAPH1	Q70E73	1045	2	-8	-18	7	1.0	0.9	0.9	1.1
ING3	Q9NXR8	187	-11	-13	-18	7	0.9	0.9	0.9	1.1
CBX5	P45973	59	-14	-14	-18	7	0.9	0.9	0.9	1.1
ARFGAP3	Q9NP61	241	-12	-14	-18	7	0.9	0.9	0.9	1.1
KDM3B	Q7LBC6	1357	-11	-16	-18	7	0.9	0.9	0.9	1.1
PELO	Q9BRX2	68	-10	-20	-18	7	0.9	0.8	0.9	1.1
LSP1	P33241	36	-13	-21	-18	7	0.9	0.8	0.9	1.1
PFKFB2	O60825	415	-10	-5	-18	6	0.9	1.0	0.9	1.1
DENND4C	Q5VZ89	719	0	-9	-18	6	1.0	0.9	0.9	1.1
ZFYVE26	Q68DK2	1871	-4	-10	-18	6	1.0	0.9	0.9	1.1
CARD6	Q9BX69	90	-14	-11	-18	6	0.9	0.9	0.9	1.1
SHTN1	A0MZ66	550	-3	-13	-18	6	1.0	0.9	0.9	1.1
LIME1	Q9H400	270	-16	-16	-18	6	0.9	0.9	0.9	1.1
PHIP	Q8WWQ0	1020	-8	9	-18	6	0.9	1.1	0.9	1.1
WDR7	Q9Y4E6	234	-1	-19	-18	6	1.0	0.8	0.9	1.1
RNH1	P13489	380	-17	-6	-18	5	0.9	0.9	0.9	1.1
RB1CC1	Q8TDY2	831	-6	-12	-18	5	0.9	0.9	0.9	1.1
WDR11	Q9BZH6	297	-15	-6	-18	5	0.9	0.9	0.9	1.0
DOCK10	Q96BY6	1092	-2	-8	-18	5	1.0	0.9	0.9	1.0
RASA1	P20936	576	-11	-16	-18	5	0.9	0.9	0.9	1.0
THOC2	Q8NI27	1518	-6	-16	-18	5	0.9	0.9	0.9	1.0
NUB1	Q9Y5A7	52	-12	-19	-18	5	0.9	0.8	0.9	1.0

GNPNAT1	Q96EK6	157	-2	-7	-18	4	1.0	0.9	0.9	1.0
RPP30	P78346	87	-7	-11	-18	4	0.9	0.9	0.9	1.0
MROH1	Q8NDA8	888	-6	-15	-18	4	0.9	0.9	0.9	1.0
PPP4R1	Q8TF05	222	-4	-15	-18	4	1.0	0.9	0.9	1.0
RAB27A	P51159	123	-3	-9	-18	4	1.0	0.9	0.9	1.0
RAB39A	Q14964	41	-8	-12	-18	4	0.9	0.9	0.9	1.0
EML4	Q9HC35	82	-8	-14	-18	4	0.9	0.9	0.9	1.0
GNL1	P36915	607	-12	-17	-18	4	0.9	0.9	0.9	1.0
ZNF761	A0A087WXT7	217	-8	-18	-18	4	0.9	0.9	0.9	1.0
ZNF845	Q96IR2	217	-8	-18	-18	4	0.9	0.9	0.9	1.0
RANBP2	P49792	1335	-8	-22	-18	4	0.9	0.8	0.9	1.0
MSH6	P52701	615	-6	-12	-18	3	0.9	0.9	0.9	1.0
CAMKK2	Q96RR4	397	-10	-13	-18	3	0.9	0.9	0.9	1.0
TADA3	O75528	255	-8	-16	-18	3	0.9	0.9	0.9	1.0
BCL11B	Q9C0K0	88	-8	-17	-18	3	0.9	0.9	0.9	1.0
IRAK3	Q9Y616	572	-17	-18	-18	3	0.9	0.9	0.9	1.0
NRF1	Q16656	229	-13	-20	-18	3	0.9	0.8	0.9	1.0
UBR7	Q8N806	374	-8	-22	-18	3	0.9	0.8	0.9	1.0
PPM1G	O15355	351	-17	-10	-18	3	0.9	0.9	0.9	1.0
THOC2	Q8NI27	1044	-17	-13	-18	3	0.9	0.9	0.9	1.0
CORO1A	P31146	24	7	-23	-18	3	1.1	0.8	0.9	1.0
THEMIS2	Q5TEJ8	453	-10	-4	-18	2	0.9	1.0	0.9	1.0
FARS2	O95363	413	-10	-11	-18	2	0.9	0.9	0.9	1.0
AKAP11	Q9UKA4	1435	-2	-15	-18	2	1.0	0.9	0.9	1.0
MYO9B	Q13459	1169	-8	-16	-18	2	0.9	0.9	0.9	1.0
SCML4	Q8N228	324	-24	-21	-18	2	0.8	0.8	0.9	1.0
DDX59	Q5T1V6	345	-7	-11	-18	1	0.9	0.9	0.9	1.0
DHX9	Q08211	777	-3	-13	-18	1	1.0	0.9	0.9	1.0
ANKHD1	Q8IWZ3	643	-5	-19	-18	1	1.0	0.8	0.9	1.0
ZZEF1	O43149	1287	-3	-24	-18	1	1.0	0.8	0.9	1.0
KDM3B	Q7LBC6	509	-1	-5	-18	1	1.0	1.0	0.9	1.0
LIN37	Q96GY3	28	-10	-12	-18	1	0.9	0.9	0.9	1.0
OAS3	Q9Y6K5	850	-21	-14	-18	1	0.8	0.9	0.9	1.0
NIT2	Q9NQR4	146	-10	-15	-18	1	0.9	0.9	0.9	1.0
PLEKHF1	Q96S99	186	-9	-21	-18	1	0.9	0.8	0.9	1.0
PARG	Q86W56	397	-14	-25	-18	1	0.9	0.8	0.9	1.0
STK39	Q9UEW8	237	-4	-7	-18	0	1.0	0.9	0.9	1.0
TTF2	Q9UNY4	512	1	-12	-18	0	1.0	0.9	0.9	1.0
C2CD5	Q86YS7	867	-7	-13	-18	0	0.9	0.9	0.9	1.0
FRMD8	Q9BZ67	191	-14	-14	-18	0	0.9	0.9	0.9	1.0
ATIC	P31939	101	-7	-15	-18	0	0.9	0.9	0.9	1.0
CLK1	P49759	200	-4	-4	-18	-1	1.0	1.0	0.9	1.0
JMJD1C	Q15652	1448	-8	-5	-18	-1	0.9	1.0	0.9	1.0
SIK3	Q9Y2K2	1119	-10	-9	-18	-1	0.9	0.9	0.9	1.0
CAD	P27708	73	-12	-17	-18	-1	0.9	0.9	0.9	1.0
SMAD2	Q15796	41	-4	-8	-18	-2	1.0	0.9	0.9	1.0
SLC7A6OS	Q96CW6	27	-11	-24	-18	-2	0.9	0.8	0.9	1.0
PFKFB4	Q16877	159	-8	-6	-18	-2	0.9	0.9	0.9	1.0
MPI	P34949	11	-4	-11	-18	-3	1.0	0.9	0.9	1.0
TARS	P26639	630	-12	-17	-18	-3	0.9	0.9	0.9	1.0
SP1	P08047	68	-25	-23	-18	-3	0.8	0.8	0.9	1.0
GBF1	Q92538	685	-4	-14	-18	-3	1.0	0.9	0.9	1.0
TRAPPC12	Q8WVT3	731	-8	-14	-18	-3	0.9	0.9	0.9	1.0
AKAP9	Q99996	3585	-7	-17	-18	-3	0.9	0.9	0.9	1.0
ALMS1	Q8TCU4	2394	-9	-18	-18	-3	0.9	0.9	0.9	1.0

PPP5C	P53041	404	-13	-14	-18	-4	0.9	0.9	0.9	1.0
KMT2E	Q8IZD2	1013	-11	-24	-18	-4	0.9	0.8	0.9	1.0
RABGAP1	Q9Y3P9	476	3	-10	-18	-5	1.0	0.9	0.9	1.0
LGALS1	P09382	61	-8	-14	-18	-5	0.9	0.9	0.9	1.0
KIAA0430	Q9Y4F3	549	-14	-28	-18	-5	0.9	0.8	0.9	1.0
FLII	Q13045	46	-10	-9	-18	-6	0.9	0.9	0.9	0.9
RBBP5	Q15291	258	-8	-11	-18	-6	0.9	0.9	0.9	0.9
TWF2	Q6IBS0	275	9	4	-18	-6	1.1	1.0	0.9	0.9
RECQL	P46063	321	-9	-17	-18	-6	0.9	0.9	0.9	0.9
TMLHE	Q9NVH6	51	-16	-25	-18	-6	0.9	0.8	0.9	0.9
SMN2	Q16637	289	-19	-10	-18	-7	0.8	0.9	0.9	0.9
WWC3	Q9ULE0	741	-5	-23	-18	-7	1.0	0.8	0.9	0.9
RPS6KC1	Q96S38	905	-9	-12	-18	-8	0.9	0.9	0.9	0.9
COPA	P53621	522	-3	-10	-18	-9	1.0	0.9	0.9	0.9
PASK	Q96RG2	15	-2	-11	-18	-9	1.0	0.9	0.9	0.9
PCM1	Q15154	582	-14	-17	-18	-10	0.9	0.9	0.9	0.9
SCYL3	Q8IZE3	553	-2	-19	-18	-10	1.0	0.8	0.9	0.9
NCKAP1L	P55160	1032	-4	-25	-18	-10	1.0	0.8	0.9	0.9
POP1	Q99575	745	-8	-12	-18	-10	0.9	0.9	0.9	0.9
SP100	P23497	629	-8	-13	-18	-10	0.9	0.9	0.9	0.9
NBN	O60934	487	-8	-20	-18	-11	0.9	0.8	0.9	0.9
ACTR1A	P61163	222	-3	-13	-18	-11	1.0	0.9	0.9	0.9
CS	O75390	211	-11	-19	-18	-11	0.9	0.8	0.9	0.9
ARPC1B	O15143	162	-10	-15	-18	-12	0.9	0.9	0.9	0.9
ARPC1A	Q92747	162	-10	-15	-18	-12	0.9	0.9	0.9	0.9
MED25	Q71SY5	429	-10	-20	-18	-12	0.9	0.8	0.9	0.9
SENP7	Q9BQF6	316	-8	-12	-18	-13	0.9	0.9	0.9	0.9
LPP	Q93052	465	-9	-16	-18	-13	0.9	0.9	0.9	0.9
PLEKHG3	A1L390	967	-29	-17	-18	-13	0.8	0.9	0.9	0.9
GOLGA2	Q08379	794	-18	-22	-18	-13	0.9	0.8	0.9	0.9
USP47	Q96K76	1138	0	-14	-18	-14	1.0	0.9	0.9	0.9
CBLL1	Q75N03	158	-11	-23	-18	-14	0.9	0.8	0.9	0.9
ZNF627	Q7L945	290	-14	-15	-18	-14	0.9	0.9	0.9	0.9
LSM2	Q9Y333	26	-7	-8	-18	-15	0.9	0.9	0.9	0.9
EML3	Q32P44	615	-8	-21	-18	-15	0.9	0.8	0.9	0.9
MED10	Q9BTT4	54	-9	-13	-18	-15	0.9	0.9	0.9	0.9
ZFAND5	O76080	168	-12	-7	-18	-16	0.9	0.9	0.9	0.9
TLE4	Q04727	712	-4	-9	-18	-16	1.0	0.9	0.9	0.9
ARHGEF12	Q9NZN5	1325	-20	-8	-18	-17	0.8	0.9	0.9	0.9
Uncharacteriz	E9PLN8	51	-13	-10	-18	-18	0.9	0.9	0.9	0.8
YY1	P25490	355	25	-18	-18	-18	1.3	0.9	0.9	0.8
SYNE2	Q8WXH0	1509	-5	-10	-18	-20	1.0	0.9	0.9	0.8
ANKRD44	Q8N8A2	921	-9	2	-18	-22	0.9	1.0	0.9	0.8
ATG9A	Q7Z3C6	433	-15	-17	-18	-22	0.9	0.9	0.9	0.8
ASCC2	Q9H1I8	276	-8	-7	-18	-23	0.9	0.9	0.9	0.8
HCFC1	P51610	1872	-10	-17	-18	-23	0.9	0.9	0.9	0.8
USP32	Q8NFA0	1387	-1	-37	-18	-25	1.0	0.7	0.9	0.8
ARHGAP30	Q7Z6I6	89	-12	-21	-18	-28	0.9	0.8	0.9	0.8
RAD17	O75943	252	-26	7	-18	-28	0.8	1.1	0.9	0.8
MYH9	P35579	917	-20	-14	-18	-34	0.8	0.9	0.9	0.7
ATP2A2	P16615	498	2	-14	-18	-42	1.0	0.9	0.9	0.7
ASCC3	Q8N3C0	1315	-5	-21	-18	-43	1.0	0.8	0.9	0.7
CSRP1	P21291	25	-7	-24	-18	-46	0.9	0.8	0.9	0.7
C5AR1	P21730	188	-21	-19	-18	-55	0.8	0.8	0.9	0.6
MPP1	Q00013	242	-5	-8	-18	-62	1.0	0.9	0.9	0.6

ZFP36	P26651	67	-12	-19	-18	-71	0.9	0.8	0.9	0.6
PTPN1	P18031	226	-8	-1	-18	-73	0.9	1.0	0.9	0.6
INF2	Q27J81	713	-17	-16	-18	-101	0.9	0.9	0.9	0.5
PDLIM1	O00151	73	5	-3	-18	-103	1.0	1.0	0.9	0.5
MRPL54	Q6P161	62	-20	-16	-18	45	0.8	0.9	0.8	1.8
LRWD1	Q9UFC0	384	-8	-6	-18	29	0.9	0.9	0.8	1.4
CCAR2	Q8N163	644	-9	-2	-18	28	0.9	1.0	0.8	1.4
ILF2	Q12905	37	-10	-17	-18	24	0.9	0.9	0.8	1.3
FBXO38	Q6PIJ6	401	1	-21	-18	23	1.0	0.8	0.8	1.3
BANP	Q8N9N5	48	-9	-17	-18	22	0.9	0.9	0.8	1.3
AHCY	P23526	228	-14	-11	-18	22	0.9	0.9	0.8	1.3
KEAP1	Q14145	622	8	-1	-18	21	1.1	1.0	0.8	1.3
ANXA2	P07355	262	-3	-12	-18	20	1.0	0.9	0.8	1.3
NUP160	Q12769	605	-2	-15	-18	20	1.0	0.9	0.8	1.3
PRKCE	Q02156	352	-12	-13	-18	20	0.9	0.9	0.8	1.2
CUL1	Q13616	594	6	-22	-18	20	1.1	0.8	0.8	1.2
MX2	P20592	580	-5	-10	-18	19	1.0	0.9	0.8	1.2
PLCG2	P16885	624	-10	-11	-18	18	0.9	0.9	0.8	1.2
RGPD6	Q99666	220	1	-7	-18	17	1.0	0.9	0.8	1.2
HINT3	Q9NQE9	32	-16	-11	-18	17	0.9	0.9	0.8	1.2
MDH2	P40926	89	-4	-16	-18	16	1.0	0.9	0.8	1.2
DOCK9	Q9BZ29	1292	-6	1	-18	16	0.9	1.0	0.8	1.2
ABHD14B	Q96IU4	190	-8	-14	-18	15	0.9	0.9	0.8	1.2
HCCS	P53701	46	-7	-17	-18	14	0.9	0.9	0.8	1.2
TEP1	Q99973	692	-8	-18	-18	14	0.9	0.9	0.8	1.2
PBRM1	Q86U86	1224	-4	-15	-18	13	1.0	0.9	0.8	1.1
CTBP2	P56545	44	-4	-9	-18	13	1.0	0.9	0.8	1.1
CTBP1	Q13363	38	-4	-9	-18	13	1.0	0.9	0.8	1.1
AREL1	O15033	382	-9	-14	-18	13	0.9	0.9	0.8	1.1
ADAM17	P78536	365	-9	-18	-18	13	0.9	0.9	0.8	1.1
SEC23A	Q15436	61	-1	-14	-18	12	1.0	0.9	0.8	1.1
RB1CC1	Q8TDY2	925	1	-14	-18	12	1.0	0.9	0.8	1.1
ATP9B	O43861	1143	-18	-29	-18	12	0.9	0.8	0.8	1.1
SPATA13	Q96N96	520	-4	-5	-18	12	1.0	1.0	0.8	1.1
IDH1	O75874	73	-14	-6	-18	12	0.9	0.9	0.8	1.1
USP47	Q96K76	856	-4	-10	-18	12	1.0	0.9	0.8	1.1
CDAN1	Q8IWY9	1213	9	-11	-18	12	1.1	0.9	0.8	1.1
MBIP	Q9NS73	243	-4	-12	-18	12	1.0	0.9	0.8	1.1
LENG8	Q96PV6	529	-1	-13	-18	12	1.0	0.9	0.8	1.1
ALG13	Q9NP73	86	-16	-20	-18	12	0.9	0.8	0.8	1.1
MECP2	P51608	413	-1	-22	-18	12	1.0	0.8	0.8	1.1
RABGAP1L	Q5R372	248	-7	-11	-18	11	0.9	0.9	0.8	1.1
PSMG1	O95456	68	-1	-19	-18	11	1.0	0.8	0.8	1.1
PLEKHA5	Q9HAU0	916	-10	-22	-18	11	0.9	0.8	0.8	1.1
CDC37L1	Q7L3B6	35	-7	-9	-18	11	0.9	0.9	0.8	1.1
MAP4K1	Q92918	428	-18	-14	-18	11	0.8	0.9	0.8	1.1
RANBP2	P49792	3221	-7	-18	-18	11	0.9	0.8	0.8	1.1
CARS	P49589	335	-15	-24	-18	11	0.9	0.8	0.8	1.1
NR3C1	P04150	463	-7	-6	-18	10	0.9	0.9	0.8	1.1
NR3C2	P08235	645	-7	-6	-18	10	0.9	0.9	0.8	1.1
RNASEL	Q05823	407	-3	-8	-18	10	1.0	0.9	0.8	1.1
LANCL2	Q9NS86	169	-2	-10	-18	10	1.0	0.9	0.8	1.1
RNF123	Q5XPI4	1180	-8	-12	-18	10	0.9	0.9	0.8	1.1
UBAP1	Q9NZ09	243	-2	-13	-18	10	1.0	0.9	0.8	1.1
MGA	Q8IWI9	823	-9	-16	-18	10	0.9	0.9	0.8	1.1

ZNF14	P17017	234	-22	-22	-18	10	0.8	0.8	0.8	1.1
EHHADH	Q08426	559	-13	-1	-18	10	0.9	1.0	0.8	1.1
PTCD3	Q96EY7	139	-7	-11	-18	10	0.9	0.9	0.8	1.1
LANCL2	Q9NS86	187	-12	-17	-18	10	0.9	0.9	0.8	1.1
ACAD10	Q6JQN1	197	-29	-10	-18	9	0.8	0.9	0.8	1.1
ATRX	P46100	1760	-3	-11	-18	9	1.0	0.9	0.8	1.1
GTPBP1	O00178	313	-10	-25	-18	9	0.9	0.8	0.8	1.1
ZC3H13	Q5T200	1666	-7	-8	-18	9	0.9	0.9	0.8	1.1
CCSAP	Q6IQ19	26	-9	-16	-18	9	0.9	0.9	0.8	1.1
FES	P07332	785	-16	-19	-18	9	0.9	0.8	0.8	1.1
OAS2	P29728	361	4	1	-18	8	1.0	1.0	0.8	1.1
MAU2	Q9Y6X3	252	-4	-6	-18	8	1.0	0.9	0.8	1.1
INTS10	Q9NVR2	387	-12	-11	-18	8	0.9	0.9	0.8	1.1
TNFAIP3	P21580	483	-5	-11	-18	7	1.0	0.9	0.8	1.1
PLEKHO2	Q8TD55	295	-13	-14	-18	7	0.9	0.9	0.8	1.1
FMNL1	O95466	45	-8	-14	-18	7	0.9	0.9	0.8	1.1
TRANK1	O15050	383	-17	-16	-18	7	0.9	0.9	0.8	1.1
VPS13C	Q709C8	1094	-9	-17	-18	7	0.9	0.9	0.8	1.1
ATRX	P46100	324	-13	-20	-18	7	0.9	0.8	0.8	1.1
GTF3C1	Q12789	317	-2	-14	-18	7	1.0	0.9	0.8	1.1
UBXN4	Q92575	155	-13	-1	-18	6	0.9	1.0	0.8	1.1
ZFYVE1	Q9HBF4	74	-6	-4	-18	6	0.9	1.0	0.8	1.1
FCHO1	O14526	889	1	-7	-18	6	1.0	0.9	0.8	1.1
PCNT	O95613	2830	-7	-14	-18	6	0.9	0.9	0.8	1.1
PMS2	P54278	216	-5	-17	-18	6	1.0	0.9	0.8	1.1
MGEA5	O60502	631	-17	1	-18	6	0.9	1.0	0.8	1.1
ARL3	P36405	118	-5	-10	-18	6	1.0	0.9	0.8	1.1
EEF2	P13639	751	-6	-13	-18	6	0.9	0.9	0.8	1.1
CCT7	Q99832	364	-14	-15	-18	6	0.9	0.9	0.8	1.1
C5orf45	Q6NTE8	300	-7	-16	-18	6	0.9	0.9	0.8	1.1
IMPDH1	P20839	468	13	-5	-18	5	1.1	1.0	0.8	1.1
RUFY1	Q96T51	81	-7	-20	-18	5	0.9	0.8	0.8	1.1
AKAP17A	Q02040	579	-1	-11	-18	5	1.0	0.9	0.8	1.0
Uncharacteriz	K7ESF4	46	-11	-14	-18	5	0.9	0.9	0.8	1.0
DDX24	Q9GZR7	380	-10	-11	-18	4	0.9	0.9	0.8	1.0
AGFG1	P52594	32	-11	-15	-18	4	0.9	0.9	0.8	1.0
PBRM1	Q86U86	1257	-3	-19	-18	4	1.0	0.8	0.8	1.0
LRP1	Q07954	2605	-3	2	-18	4	1.0	1.0	0.8	1.0
SH2D3C	Q8N5H7	452	-7	-13	-18	4	0.9	0.9	0.8	1.0
CFL1	P23528	139	0	-14	-18	4	1.0	0.9	0.8	1.0
HPS3	Q969F9	254	-9	-15	-18	4	0.9	0.9	0.8	1.0
FAM120A	Q9NZB2	669	-6	-18	-18	4	0.9	0.8	0.8	1.0
TTC5	Q8N0Z6	133	-4	-23	-18	4	1.0	0.8	0.8	1.0
CYFIP2	Q96F07	423	-1	-17	-18	3	1.0	0.9	0.8	1.0
NFATC1	O95644	383	-5	-18	-18	3	1.0	0.8	0.8	1.0
ATRX	P46100	335	-3	-18	-18	3	1.0	0.8	0.8	1.0
TBC1D2	Q9BYX2	102	-3	2	-18	3	1.0	1.0	0.8	1.0
LRSAM1	Q6UWE0	418	-24	-5	-18	3	0.8	1.0	0.8	1.0
MED26	O95402	356	-6	-12	-18	3	0.9	0.9	0.8	1.0
MACROD1	Q9BQ69	186	-1	-13	-18	3	1.0	0.9	0.8	1.0
NIPBL	Q6KC79	56	-4	-18	-18	3	1.0	0.9	0.8	1.0
SRGAP2	A2RUF3	486	-30	-42	-18	3	0.8	0.7	0.8	1.0
CWC27	Q6UX04	164	-13	1	-18	2	0.9	1.0	0.8	1.0
RAB21	Q9UL25	29	-1	-6	-18	2	1.0	0.9	0.8	1.0
RDH14	Q9HBH5	148	-16	-24	-18	2	0.9	0.8	0.8	1.0

MTMR3	Q13615	11	-12	-6	-18	2	0.9	0.9	0.8	1.0
EHBP1L1	Q8N3D4	23	-5	-7	-18	2	1.0	0.9	0.8	1.0
GOLGB1	Q14789	1055	-7	-8	-18	2	0.9	0.9	0.8	1.0
ANKRD13A	Q8IZ07	540	-11	-14	-18	2	0.9	0.9	0.8	1.0
LCP1	P13796	283	-15	-19	-18	2	0.9	0.8	0.8	1.0
SF3B1	O75533	1244	-13	-20	-18	2	0.9	0.8	0.8	1.0
CELF2	O95319	143	0	-3	-18	1	1.0	1.0	0.8	1.0
IL16	Q14005	1004	-7	-10	-18	1	0.9	0.9	0.8	1.0
ATG16L2	Q8NAA4	267	-15	-30	-18	1	0.9	0.8	0.8	1.0
NPLOC4	Q8TAT6	403	1	-21	-18	1	1.0	0.8	0.8	1.0
MTOR	P42345	674	0	-12	-18	0	1.0	0.9	0.8	1.0
PML	P29590	338	-12	-13	-18	0	0.9	0.9	0.8	1.0
LZTR1	Q8N653	427	-14	-8	-18	-1	0.9	0.9	0.8	1.0
KHNYN	O15037	157	-7	-16	-18	-1	0.9	0.9	0.8	1.0
PPFIA2	O75334	143	-7	-7	-18	-2	0.9	0.9	0.8	1.0
AGO2	Q9UKV8	462	-5	-18	-18	-2	1.0	0.9	0.8	1.0
C10orf12	Q8N655	62	-8	3	-18	-2	0.9	1.0	0.8	1.0
PCNT	O95613	2185	4	-8	-18	-2	1.0	0.9	0.8	1.0
IFIT3	O14879	283	-9	-14	-18	-2	0.9	0.9	0.8	1.0
ATXN2L	Q8WWM7	58	-12	-16	-18	-2	0.9	0.9	0.8	1.0
DICER1	Q9UPY3	1604	0	-13	-18	-3	1.0	0.9	0.8	1.0
CWF19L1	Q69YN2	288	-7	-14	-18	-3	0.9	0.9	0.8	1.0
CRYBG3	Q68DQ2	394	2	-17	-18	-3	1.0	0.9	0.8	1.0
STAT1	P42224	324	-13	-18	-18	-3	0.9	0.8	0.8	1.0
STAT4	Q14765	323	-13	-18	-18	-3	0.9	0.8	0.8	1.0
TRIP12	Q14669	710	-6	-23	-18	-3	0.9	0.8	0.8	1.0
ESD	P10768	11	-21	-7	-18	-3	0.8	0.9	0.8	1.0
PPP1R18	Q6NYC8	397	-11	-9	-18	-3	0.9	0.9	0.8	1.0
ZNF592	Q92610	201	-4	-15	-18	-3	1.0	0.9	0.8	1.0
TRIM27	P14373	365	-11	-18	-18	-4	0.9	0.8	0.8	1.0
ZNF516	Q92618	398	-7	-14	-18	-4	0.9	0.9	0.8	1.0
ACAD9	Q9H845	507	-14	-25	-18	-4	0.9	0.8	0.8	1.0
ZC3HAV1	Q7Z2W4	721	-2	-13	-18	-5	1.0	0.9	0.8	1.0
SMARCA4	P51532	1359	-15	-15	-18	-5	0.9	0.9	0.8	1.0
ZNF609	O15014	178	-12	-18	-18	-5	0.9	0.9	0.8	1.0
RPRD1A	Q96P16	151	-10	-29	-18	-5	0.9	0.8	0.8	1.0
ZNF318	Q5VUA4	2040	-14	-36	-18	-5	0.9	0.7	0.8	1.0
GAK	O14976	599	-12	-17	-18	-5	0.9	0.9	0.8	1.0
GMIP	Q9P107	957	-13	-20	-18	-5	0.9	0.8	0.8	1.0
MACF1	Q9UPN3	4206	-5	-23	-18	-5	1.0	0.8	0.8	1.0
RAB11FIP4	Q86YS3	517	-5	-24	-18	-5	1.0	0.8	0.8	1.0
TECPR2	O15040	14	-12	-17	-18	-6	0.9	0.9	0.8	0.9
GART	P22102	93	4	-4	-18	-6	1.0	1.0	0.8	0.9
DCTN4	Q9UJW0	33	5	-6	-18	-7	1.1	0.9	0.8	0.9
HECTD1	Q9ULT8	1368	-9	-9	-18	-7	0.9	0.9	0.8	0.9
HK1	P19367	628	-1	-15	-18	-7	1.0	0.9	0.8	0.9
VAMP7	P51809	21	10	-15	-18	-7	1.1	0.9	0.8	0.9
KANSL3	Q9P2N6	161	-9	-20	-18	-7	0.9	0.8	0.8	0.9
UBR4	Q5T4S7	2619	-9	-21	-18	-7	0.9	0.8	0.8	0.9
PDIA3	P30101	244	-7	-18	-18	-7	0.9	0.8	0.8	0.9
CCS	O14618	227	-8	-11	-18	-8	0.9	0.9	0.8	0.9
LRWD1	Q9UFC0	281	-1	-12	-18	-8	1.0	0.9	0.8	0.9
UBE3B	Q7Z3V4	399	-10	-8	-18	-9	0.9	0.9	0.8	0.9
CTIF	O43310	501	-6	-13	-18	-9	0.9	0.9	0.8	0.9
IGHMBP2	P38935	191	-11	-21	-18	-9	0.9	0.8	0.8	0.9

ANKLE2	Q86XL3	350	-12	-10	-18	-9	0.9	0.9	0.8	0.9
ARAP3	Q8WWN8	1199	-19	-18	-18	-9	0.8	0.8	0.8	0.9
APBB1IP	Q7Z5R6	381	-6	-14	-18	-10	0.9	0.9	0.8	0.9
EPRS	P07814	92	1	-22	-18	-10	1.0	0.8	0.8	0.9
PTK2B	Q14289	61	-5	3	-18	-10	1.0	1.0	0.8	0.9
XAF1	Q6GPH4	173	-10	-16	-18	-10	0.9	0.9	0.8	0.9
MTMR3	Q13615	534	-9	-17	-18	-11	0.9	0.9	0.8	0.9
HPS1	Q92902	561	-10	4	-18	-11	0.9	1.0	0.8	0.9
ABCC2	Q92887	631	-8	-15	-18	-11	0.9	0.9	0.8	0.9
INPP4B	O15327	206	1	-12	-18	-12	1.0	0.9	0.8	0.9
CNN2	Q99439	175	-13	-9	-18	-13	0.9	0.9	0.8	0.9
VPS11	Q9H270	231	-10	-19	-18	-13	0.9	0.8	0.8	0.9
CRIP1	P50238	28	-5	-15	-18	-14	1.0	0.9	0.8	0.9
DDX39A	O00148	197	1	-15	-18	-15	1.0	0.9	0.8	0.9
RNH1	P13489	102	-29	-6	-18	-15	0.8	0.9	0.8	0.9
UBTF	P17480	328	0	-17	-18	-18	1.0	0.9	0.8	0.9
COMT	P21964	119	-8	-24	-18	-18	0.9	0.8	0.8	0.9
ST7	Q9NRC1	133	-18	-19	-18	-19	0.9	0.8	0.8	0.8
SMAD4	Q13485	123	-4	6	-18	-20	1.0	1.1	0.8	0.8
BCL9L	Q86UU0	68	-7	-17	-18	-20	0.9	0.9	0.8	0.8
NOXA1	Q86UR1	451	-6	-19	-18	-20	0.9	0.8	0.8	0.8
APOO	Q9BUR5	78	-17	-6	-18	-23	0.9	0.9	0.8	0.8
C1QBP	Q07021	186	-23	-7	-18	-24	0.8	0.9	0.8	0.8
COG3	Q96JB2	564	3	-30	-18	-35	1.0	0.8	0.8	0.7
ATXN2	Q99700	301	-16	-8	-18	-38	0.9	0.9	0.8	0.7
TCF20	Q9UGU0	498	-12	-24	-18	-41	0.9	0.8	0.8	0.7
MYO5A	Q9Y4I1	513	-9	-14	-18	-46	0.9	0.9	0.8	0.7
DYNLT3	P51808	8	2	-13	-18	-54	1.0	0.9	0.8	0.7
RREB1	Q92766	329	-3	-12	-19	33	1.0	0.9	0.8	1.5
JAK2	O60674	243	-14	-13	-19	32	0.9	0.9	0.8	1.5
ETS1	P14921	31	3	-3	-19	29	1.0	1.0	0.8	1.4
NUBP1	P53384	22	-15	-18	-19	29	0.9	0.8	0.8	1.4
LACC1	Q8IV20	284	-7	-26	-19	28	0.9	0.8	0.8	1.4
NCAPG2	Q86XI2	564	-9	-16	-19	26	0.9	0.9	0.8	1.4
RASSF5	Q8WWW0	225	1	-12	-19	23	1.0	0.9	0.8	1.3
FCHO1	O14526	571	-13	-18	-19	23	0.9	0.9	0.8	1.3
SLC41A3	Q96GZ6	14	2	-8	-19	22	1.0	0.9	0.8	1.3
WDR24	Q96S15	607	-16	1	-19	21	0.9	1.0	0.8	1.3
MYCBP2	O75592	273	-15	-20	-19	20	0.9	0.8	0.8	1.3
DHX32	Q7L7V1	316	-11	-5	-19	19	0.9	1.0	0.8	1.2
LANCL1	O43813	300	1	-11	-19	19	1.0	0.9	0.8	1.2
CRACR2A	Q9BSW2	341	-1	-5	-19	18	1.0	1.0	0.8	1.2
ATP6V0D1	P61421	39	22	-11	-19	18	1.3	0.9	0.8	1.2
LRRFIP1	Q32MZ4	726	-8	-22	-19	18	0.9	0.8	0.8	1.2
COMMD7	Q86VX2	200	-10	-8	-19	18	0.9	0.9	0.8	1.2
CASP9	P55211	172	-13	-21	-19	18	0.9	0.8	0.8	1.2
CNBP	P62633	171	-2	-26	-19	18	1.0	0.8	0.8	1.2
PHF12	Q96QT6	292	-17	-26	-19	18	0.9	0.8	0.8	1.2
N4BP1	O75113	82	-4	-9	-19	17	1.0	0.9	0.8	1.2
FRY	Q5TBA9	2723	-8	-18	-19	17	0.9	0.8	0.8	1.2
PAXIP1	Q6ZW49	293	-3	-10	-19	16	1.0	0.9	0.8	1.2
SARS2	Q9NP81	66	-15	-9	-19	15	0.9	0.9	0.8	1.2
NLRC5	Q86WI3	783	-8	-13	-19	15	0.9	0.9	0.8	1.2
SEC31A	O94979	669	-3	-13	-19	15	1.0	0.9	0.8	1.2
CHD3	Q12873	879	-8	-10	-19	14	0.9	0.9	0.8	1.2

FAM63A	Q8N5J2	94	-9	-14	-19	14	0.9	0.9	0.8	1.2
PXN	P49023	290	-15	-6	-19	13	0.9	0.9	0.8	1.1
MICALL1	Q8N3F8	597	-11	-23	-19	13	0.9	0.8	0.8	1.1
TBC1D8	O95759	543	-11	-7	-19	13	0.9	0.9	0.8	1.1
WDR77	Q9BQA1	65	0	-16	-19	13	1.0	0.9	0.8	1.1
MTMR3	Q13615	826	-7	-20	-19	13	0.9	0.8	0.8	1.1
ZNF615	Q8N8J6	638	-6	-23	-19	13	0.9	0.8	0.8	1.1
MED9	Q9NWA0	139	-13	-7	-19	12	0.9	0.9	0.8	1.1
RASGRF2	O14827	814	-9	-17	-19	12	0.9	0.9	0.8	1.1
AKAP8L	Q9ULX6	128	-5	-19	-19	12	1.0	0.8	0.8	1.1
WDR44	Q5JSH3	581	-12	-27	-19	12	0.9	0.8	0.8	1.1
NLRC5	Q86WI3	1362	-5	-16	-19	12	1.0	0.9	0.8	1.1
TXNDC11	Q6PKC3	344	-5	-15	-19	11	1.0	0.9	0.8	1.1
MNDA	P41218	274	-1	-12	-19	11	1.0	0.9	0.8	1.1
GRK6	P43250	138	-14	-18	-19	11	0.9	0.9	0.8	1.1
RANBP2	P49792	2696	-16	-6	-19	10	0.9	0.9	0.8	1.1
CRTC1	Q6UUV9	131	9	12	-19	10	1.1	1.1	0.8	1.1
XRCC5	P13010	346	-6	-3	-19	9	0.9	1.0	0.8	1.1
ZNF319	Q9P2F9	451	2	-14	-19	9	1.0	0.9	0.8	1.1
CYBB	P04839	371	-7	-15	-19	9	0.9	0.9	0.8	1.1
SLFN11	Q7Z7L1	51	2	-16	-19	9	1.0	0.9	0.8	1.1
BOD1L1	Q8NFC6	2599	-4	-9	-19	9	1.0	0.9	0.8	1.1
C19orf47	Q8N9M1	123	-5	-19	-19	9	1.0	0.8	0.8	1.1
CIAO1	O76071	212	-3	-9	-19	8	1.0	0.9	0.8	1.1
NOSIP	Q9Y314	185	-15	-13	-19	8	0.9	0.9	0.8	1.1
ZNF592	Q92610	284	-17	-10	-19	8	0.9	0.9	0.8	1.1
ZBTB34	Q8NCN2	436	-14	-11	-19	8	0.9	0.9	0.8	1.1
SETX	Q7Z333	688	-7	-17	-19	8	0.9	0.9	0.8	1.1
ARHGEF1	Q92888	911	-6	-15	-19	7	0.9	0.9	0.8	1.1
FRYL	O94915	288	-10	-8	-19	7	0.9	0.9	0.8	1.1
SPATA5L1	Q9BVQ7	732	1	-10	-19	7	1.0	0.9	0.8	1.1
USP39	Q53GS9	105	-12	-13	-19	7	0.9	0.9	0.8	1.1
LRRC8A	Q8IWT6	776	-5	-20	-19	7	1.0	0.8	0.8	1.1
DIS3L2	Q8IYB7	216	-11	-22	-19	7	0.9	0.8	0.8	1.1
LRP1	Q07954	1812	-3	-27	-19	7	1.0	0.8	0.8	1.1
MVB12A	Q96EY5	90	-7	-6	-19	6	0.9	0.9	0.8	1.1
MTFR1L	Q9H019	37	-2	-16	-19	6	1.0	0.9	0.8	1.1
Uncharacteriz	A0A0G2JPG4	111	-11	-17	-19	6	0.9	0.9	0.8	1.1
GLRX	P35754	23	-8	-23	-19	6	0.9	0.8	0.8	1.1
TACC3	Q9Y6A5	242	-3	-15	-19	6	1.0	0.9	0.8	1.1
CIZ1	Q9ULV3	848	-20	-21	-19	6	0.8	0.8	0.8	1.1
ATP5S	Q99766	111	-19	-5	-19	5	0.8	1.0	0.8	1.1
IQSEC2	Q5JU85	720	-8	-17	-19	5	0.9	0.9	0.8	1.1
EEF1A1	P68104	31	-14	-3	-19	5	0.9	1.0	0.8	1.0
SETDB1	Q15047	92	-12	-13	-19	5	0.9	0.9	0.8	1.0
ERMP1	Q7Z2K6	43	-1	-15	-19	5	1.0	0.9	0.8	1.0
ARCN1	P48444	479	-14	-18	-19	5	0.9	0.8	0.8	1.0
RAPGEF1	Q13905	474	-8	-19	-19	5	0.9	0.8	0.8	1.0
SART3	Q15020	537	-8	-10	-19	4	0.9	0.9	0.8	1.0
RALGAPA1	Q6GYQ0	257	-3	-13	-19	4	1.0	0.9	0.8	1.0
SMARCA2	P51531	861	1	-19	-19	4	1.0	0.8	0.8	1.0
SMARCA4	P51532	891	1	-19	-19	4	1.0	0.8	0.8	1.0
ZNF180	Q9UJW8	619	-2	-19	-19	4	1.0	0.8	0.8	1.0
RECQL	P46063	414	3	-10	-19	3	1.0	0.9	0.8	1.0
CCDC88C	Q9P219	1467	-9	-11	-19	3	0.9	0.9	0.8	1.0

ZBTB2	Q8N680	296	2	-11	-19	3	1.0	0.9	0.8	1.0
TRAF1	Q13077	169	15	-1	-19	3	1.2	1.0	0.8	1.0
NEDD1	Q8NHV4	66	-13	-9	-19	3	0.9	0.9	0.8	1.0
TGFBRAP1	Q8WUH2	826	-11	-13	-19	3	0.9	0.9	0.8	1.0
DNMT1	P26358	1071	-6	-15	-19	3	0.9	0.9	0.8	1.0
WDTC1	Q8N5D0	431	-14	-19	-19	3	0.9	0.8	0.8	1.0
PRPF8	Q6P2Q9	547	-16	-17	-19	2	0.9	0.9	0.8	1.0
ATAD2B	Q9ULI0	100	-12	-13	-19	2	0.9	0.9	0.8	1.0
ACD	Q96AP0	466	-12	-23	-19	2	0.9	0.8	0.8	1.0
SCRIB	A0A0G2JPP5	1637	-5	-8	-19	1	1.0	0.9	0.8	1.0
SEC24C	P53992	1083	-10	-16	-19	1	0.9	0.9	0.8	1.0
ATXN2L	Q8WWM7	135	-10	-16	-19	1	0.9	0.9	0.8	1.0
SENP6	Q9GZR1	379	-4	-11	-19	1	1.0	0.9	0.8	1.0
SYNJ2	O15056	795	-13	-14	-19	1	0.9	0.9	0.8	1.0
VPRBP	Q9Y4B6	1113	-4	-12	-19	0	1.0	0.9	0.8	1.0
SAMD1	E9PIW9	277	-5	-13	-19	0	1.0	0.9	0.8	1.0
DARS	P14868	76	-19	-7	-19	-1	0.8	0.9	0.8	1.0
MDH2	P40926	285	-13	-17	-19	-1	0.9	0.9	0.8	1.0
SAMD9L	Q8IVG5	724	-8	-19	-19	-1	0.9	0.8	0.8	1.0
ANKLE2	Q86XL3	286	-4	-15	-19	-1	1.0	0.9	0.8	1.0
VPS41	P49754	125	-11	-18	-19	-1	0.9	0.9	0.8	1.0
UBR4	Q5T4S7	3430	-10	-25	-19	-1	0.9	0.8	0.8	1.0
ACSF3	Q4G176	88	4	-14	-19	-2	1.0	0.9	0.8	1.0
CCDC88C	Q9P219	460	-8	-18	-19	-2	0.9	0.9	0.8	1.0
ACLY	P53396	845	-5	-7	-19	-2	1.0	0.9	0.8	1.0
PHF3	Q92576	1709	-9	-10	-19	-2	0.9	0.9	0.8	1.0
GAPVD1	Q14C86	293	-9	-14	-19	-2	0.9	0.9	0.8	1.0
MCMBP	Q9BTE3	200	-8	-17	-19	-2	0.9	0.9	0.8	1.0
TRMT61A	Q96FX7	165	-14	-14	-19	-3	0.9	0.9	0.8	1.0
COTL1	Q14019	10	-4	-23	-19	-3	1.0	0.8	0.8	1.0
STAT5B	P51692	101	-2	-6	-19	-5	1.0	0.9	0.8	1.0
GLB1	P16278	426	2	-14	-19	-5	1.0	0.9	0.8	1.0
RGL2	O15211	508	-2	6	-19	-6	1.0	1.1	0.8	0.9
ZCCHC11	Q5TAX3	966	-1	-2	-19	-6	1.0	1.0	0.8	0.9
SHTN1	A0MZ66	565	-13	-18	-19	-6	0.9	0.9	0.8	0.9
MYCBP2	O75592	4520	-21	-12	-19	-6	0.8	0.9	0.8	0.9
SP100	P23497	238	-5	-26	-19	-7	1.0	0.8	0.8	0.9
CASP10	Q92851	16	-1	-32	-19	-7	1.0	0.8	0.8	0.9
SERPINB2	P05120	161	-12	-12	-19	-7	0.9	0.9	0.8	0.9
HTT	P42858	1808	-7	-27	-19	-7	0.9	0.8	0.8	0.9
Uncharacteriz	A0A087WZG4	240	-1	-14	-19	-8	1.0	0.9	0.8	0.9
XPO7	Q9UIA9	123	-3	-8	-19	-8	1.0	0.9	0.8	0.9
WWC3	Q9ULE0	334	-41	-37	-19	-8	0.7	0.7	0.8	0.9
TNS3	Q68CZ2	25	6	-17	-19	-9	1.1	0.9	0.8	0.9
CAD	P27708	2092	-14	-23	-19	-9	0.9	0.8	0.8	0.9
VPS13C	Q709C8	3723	-8	-9	-19	-9	0.9	0.9	0.8	0.9
CBLB	Q13191	895	-3	-13	-19	-9	1.0	0.9	0.8	0.9
ASPH	Q12797	384	-5	-12	-19	-10	1.0	0.9	0.8	0.9
GPHN	Q9NQX3	26	-1	-14	-19	-11	1.0	0.9	0.8	0.9
SMG8	Q8ND04	72	-7	-17	-19	-11	0.9	0.9	0.8	0.9
NUMA1	Q14980	1930	-7	-18	-19	-11	0.9	0.8	0.8	0.9
MAVS	Q7Z434	33	3	-1	-19	-11	1.0	1.0	0.8	0.9
DOCK9	Q9BZ29	683	-9	-18	-19	-12	0.9	0.8	0.8	0.9
BDH2	Q9BUT1	176	-8	-10	-19	-12	0.9	0.9	0.8	0.9
AKAP13	Q12802	407	-12	-6	-19	-13	0.9	0.9	0.8	0.9

RNH1	P13489	38	-22	-30	-19	-13	0.8	0.8	0.8	0.9
TUBA4A	P68366	295	-7	-24	-19	-14	0.9	0.8	0.8	0.9
TUBA3D	Q13748	295	-7	-24	-19	-14	0.9	0.8	0.8	0.9
TUBA1A	Q71U36	295	-7	-24	-19	-14	0.9	0.8	0.8	0.9
CASK	O14936	684	7	-12	-19	-14	1.1	0.9	0.8	0.9
CCS	O14618	246	-3	-15	-19	-15	1.0	0.9	0.8	0.9
MYO18A	Q92614	1022	-1	-3	-19	-16	1.0	1.0	0.8	0.9
CMPK2	Q5EBM0	85	-10	-12	-19	-17	0.9	0.9	0.8	0.9
CNBP	P62633	57	-9	-31	-19	-17	0.9	0.8	0.8	0.9
SH3BP2	P78314	260	-7	-8	-19	-18	0.9	0.9	0.8	0.9
CNN2	Q99439	164	-9	-19	-19	-19	0.9	0.8	0.8	0.8
CFL1	P23528	80	-1	-24	-19	-21	1.0	0.8	0.8	0.8
PRKDC	P78527	3014	-18	-20	-19	-21	0.9	0.8	0.8	0.8
IFI16	Q16666	351	-12	-20	-19	-24	0.9	0.8	0.8	0.8
STXBP5	Q5T5C0	933	-1	-16	-19	-28	1.0	0.9	0.8	0.8
TBC1D4	O60343	45	-24	-16	-19	-29	0.8	0.9	0.8	0.8
KDM6B	O15054	349	-10	-12	-19	-33	0.9	0.9	0.8	0.8
MYH9	P35579	172	-9	-12	-19	-36	0.9	0.9	0.8	0.7
GPD2	P43304	188	-11	-37	-19	-41	0.9	0.7	0.8	0.7
RARS	P54136	150	6	-25	-19	-48	1.1	0.8	0.8	0.7
GNAQ	P50148	144	-11	-26	-19	-68	0.9	0.8	0.8	0.6
LIMS1	P48059	97	-7	-4	-19	-76	0.9	1.0	0.8	0.6
FHL1	Q13642	10	-9	-12	-19	-80	0.9	0.9	0.8	0.6
RANBP2	P49792	188	5	-11	-19	-80	1.1	0.9	0.8	0.6
RGPD6	Q99666	188	5	-11	-19	-80	1.1	0.9	0.8	0.6
PLS3	P13797	33	-6	-22	-19	-152	0.9	0.8	0.8	0.4
VWF	P04275	1927	22	-14	-19	-166	1.3	0.9	0.8	0.4
MYLK	Q15746	1305	-5	-14	-19	-170	1.0	0.9	0.8	0.4
UBR5	O95071	2084	-22	0	-19	33	0.8	1.0	0.8	1.5
HNRNPF	P52597	122	-10	-21	-19	30	0.9	0.8	0.8	1.4
FAM8A1	Q9UBU6	38	-9	-11	-19	29	0.9	0.9	0.8	1.4
RXRB	P28702	191	0	-16	-19	27	1.0	0.9	0.8	1.4
CELF2	O95319	174	-3	-7	-19	26	1.0	0.9	0.8	1.3
POLRMT	O00411	119	-9	-13	-19	23	0.9	0.9	0.8	1.3
NUP98	P52948	1312	-7	-19	-19	18	0.9	0.8	0.8	1.2
DPYSL4	O14531	522	-14	-18	-19	18	0.9	0.9	0.8	1.2
RNF213	Q63HN8	1677	4	-18	-19	17	1.0	0.8	0.8	1.2
HDHD3	Q9BSH5	109	-4	-7	-19	17	1.0	0.9	0.8	1.2
AKNA	Q7Z591	818	-10	-10	-19	16	0.9	0.9	0.8	1.2
SLC2A13	Q96QE2	39	-12	-28	-19	15	0.9	0.8	0.8	1.2
NIFK	Q9BYG3	269	9	-1	-19	15	1.1	1.0	0.8	1.2
TTC37	Q6PGP7	1349	-16	-7	-19	14	0.9	0.9	0.8	1.2
RASGRP2	Q7LDG7	186	-15	-21	-19	14	0.9	0.8	0.8	1.2
RIPK1	Q13546	53	-5	-10	-19	13	1.0	0.9	0.8	1.1
IK	Q13123	263	-8	-25	-19	13	0.9	0.8	0.8	1.1
TEX10	Q9NXF1	454	-2	-15	-19	13	1.0	0.9	0.8	1.1
RNH1	P13489	152	-6	-13	-19	12	0.9	0.9	0.8	1.1
TPRKB	Q9Y3C4	167	1	-2	-19	12	1.0	1.0	0.8	1.1
PSTPIP1	O43586	259	-4	-22	-19	12	1.0	0.8	0.8	1.1
POM121	Q96HA1	307	-6	-16	-19	11	0.9	0.9	0.8	1.1
PAPSS1	O43252	207	-1	-2	-19	11	1.0	1.0	0.8	1.1
PRKDC	P78527	3293	-2	-11	-19	11	1.0	0.9	0.8	1.1
DHX40	Q8IX18	33	-2	-13	-19	11	1.0	0.9	0.8	1.1
PPP2R4	Q15257	202	-2	-13	-19	11	1.0	0.9	0.8	1.1
RPP38	P78345	80	-9	-13	-19	10	0.9	0.9	0.8	1.1

PRF1	P14222	381	-9	-12	-19	10	0.9	0.9	0.8	1.1
SCLY	Q96I15	359	-17	-24	-19	10	0.9	0.8	0.8	1.1
PCF11	O94913	1518	-13	-7	-19	9	0.9	0.9	0.8	1.1
RFX1	P22670	794	-6	-13	-19	9	0.9	0.9	0.8	1.1
MNDA	P41218	385	0	-17	-19	9	1.0	0.9	0.8	1.1
ZAP70	P43403	96	-10	-13	-19	9	0.9	0.9	0.8	1.1
EEF1A1	P68104	370	-10	-14	-19	9	0.9	0.9	0.8	1.1
INTS9	Q9NV88	446	-18	2	-19	8	0.8	1.0	0.8	1.1
TMEM199	Q8N511	124	-10	-6	-19	8	0.9	0.9	0.8	1.1
NIT1	Q86X76	203	-11	-16	-19	8	0.9	0.9	0.8	1.1
FDX1	P10109	155	-10	-19	-19	8	0.9	0.8	0.8	1.1
FAM60A	Q9NP50	173	-7	-12	-19	7	0.9	0.9	0.8	1.1
BPTF	Q12830	1357	-15	-19	-19	7	0.9	0.8	0.8	1.1
NBN	O60934	478	-1	-8	-19	7	1.0	0.9	0.8	1.1
CNOT3	O75175	600	-9	-15	-19	7	0.9	0.9	0.8	1.1
WAPL	Q7Z5K2	94	-12	-1	-19	6	0.9	1.0	0.8	1.1
TSC22D2	O75157	158	8	-6	-19	6	1.1	0.9	0.8	1.1
MED8	Q96G25	31	-11	-11	-19	6	0.9	0.9	0.8	1.1
KIAA1551	Q9HCM1	717	-5	-14	-19	6	1.0	0.9	0.8	1.1
AKR1A1	P14550	260	-12	-17	-19	6	0.9	0.9	0.8	1.1
PPIP5K2	O43314	547	-17	-17	-19	6	0.9	0.9	0.8	1.1
PPIG	Q13427	549	-10	-15	-19	6	0.9	0.9	0.8	1.1
EEF2	P13639	812	-14	-20	-19	5	0.9	0.8	0.8	1.1
CUL1	Q13616	149	-6	-9	-19	5	0.9	0.9	0.8	1.0
EEF1A1	P68104	363	-14	-15	-19	5	0.9	0.9	0.8	1.0
JOSD2	Q8TAC2	24	-8	-7	-19	4	0.9	0.9	0.8	1.0
TAF4	O00268	867	-9	-22	-19	4	0.9	0.8	0.8	1.0
PTPN12	Q05209	470	-4	-10	-19	3	1.0	0.9	0.8	1.0
GPHN	Q9NQX3	284	-8	-14	-19	3	0.9	0.9	0.8	1.0
IRF2	P14316	83	-15	-10	-19	3	0.9	0.9	0.8	1.0
MTMR3	Q13615	81	-1	-11	-19	3	1.0	0.9	0.8	1.0
MACF1	Q9UPN3	2854	-9	-25	-19	3	0.9	0.8	0.8	1.0
RB1CC1	Q8TDY2	603	-14	-19	-19	2	0.9	0.8	0.8	1.0
EEF2	P13639	693	-8	-20	-19	2	0.9	0.8	0.8	1.0
MSH6	P52701	1337	-9	-9	-19	2	0.9	0.9	0.8	1.0
AKAP11	Q9UKA4	1232	-18	-16	-19	1	0.8	0.9	0.8	1.0
RPL34	P49207	46	-16	-20	-19	1	0.9	0.8	0.8	1.0
GAK	O14976	691	-1	-10	-19	0	1.0	0.9	0.8	1.0
SYNE2	Q8WXH0	1806	-4	-14	-19	-1	1.0	0.9	0.8	1.0
Uncharacteriz	V9GY48	263	-11	-21	-19	-1	0.9	0.8	0.8	1.0
FITM2	Q8N6M3	251	-8	-22	-19	-1	0.9	0.8	0.8	1.0
ZFC3H1	O60293	1405	-10	-17	-19	-2	0.9	0.9	0.8	1.0
ZNF644	Q9H582	202	-7	-21	-19	-2	0.9	0.8	0.8	1.0
PFAS	O15067	587	-22	-16	-19	-3	0.8	0.9	0.8	1.0
ATP1A1	P05023	249	-4	-11	-19	-4	1.0	0.9	0.8	1.0
TANK	Q92844	75	-3	-10	-19	-4	1.0	0.9	0.8	1.0
CHD6	Q8TD26	1307	-7	-13	-19	-4	0.9	0.9	0.8	1.0
CHD8	Q9HCK8	1655	-7	-13	-19	-4	0.9	0.9	0.8	1.0
SLFN5	Q08AF3	268	0	-19	-19	-4	1.0	0.8	0.8	1.0
POLD1	P28340	360	-4	-3	-19	-5	1.0	1.0	0.8	1.0
IRGQ	Q8WZA9	340	-13	-15	-19	-5	0.9	0.9	0.8	1.0
GPATCH8	Q9UKJ3	639	-5	-19	-19	-5	1.0	0.8	0.8	1.0
CAST	P20810	328	-14	-19	-19	-5	0.9	0.8	0.8	1.0
SNX29	Q8TEQ0	44	-1	-14	-19	-7	1.0	0.9	0.8	0.9
SZT2	Q5T011	3172	35	-3	-19	-7	1.5	1.0	0.8	0.9

MTSS1	O43312	9	-4	-4	-19	-7	1.0	1.0	0.8	0.9
RRN3	Q9NYV6	552	-15	-21	-19	-7	0.9	0.8	0.8	0.9
ZBTB48	P10074	265	-6	-24	-19	-8	0.9	0.8	0.8	0.9
ARID4A	P29374	889	-19	-11	-19	-8	0.8	0.9	0.8	0.9
NCKAP1L	P55160	612	-7	-10	-19	-9	0.9	0.9	0.8	0.9
GBF1	Q92538	661	-2	-13	-19	-9	1.0	0.9	0.8	0.9
SUCLG2	Q96I99	255	3	-18	-19	-9	1.0	0.9	0.8	0.9
TARS	P26639	261	-13	0	-19	-10	0.9	1.0	0.8	0.9
SPECC1	Q5M775	740	-13	-9	-19	-10	0.9	0.9	0.8	0.9
DHX15	O43143	750	-13	-14	-19	-10	0.9	0.9	0.8	0.9
CDC37	Q16543	336	-6	-17	-19	-10	0.9	0.9	0.8	0.9
ALDH18A1	P54886	612	-3	-3	-19	-11	1.0	1.0	0.8	0.9
FLOT1	O75955	34	-13	-20	-19	-11	0.9	0.8	0.8	0.9
GMEB2	Q9UKD1	110	-6	-20	-19	-11	0.9	0.8	0.8	0.9
GMEB1	Q9Y692	113	-6	-20	-19	-11	0.9	0.8	0.8	0.9
UFL1	O94874	32	-11	-7	-19	-11	0.9	0.9	0.8	0.9
MACF1	Q9UPN3	2787	2	-24	-19	-11	1.0	0.8	0.8	0.9
BAX	Q07812	62	7	-5	-19	-12	1.1	1.0	0.8	0.9
DIAPH2	O60879	159	-10	-13	-19	-12	0.9	0.9	0.8	0.9
FDPS	P14324	333	-16	-17	-19	-12	0.9	0.9	0.8	0.9
GDI1	P31150	282	-3	-20	-19	-12	1.0	0.8	0.8	0.9
TAP2	Q03519	540	-2	-25	-19	-12	1.0	0.8	0.8	0.9
HTT	P42858	1961	-16	-6	-19	-13	0.9	0.9	0.8	0.9
UROD	P06132	294	2	-32	-19	-13	1.0	0.8	0.8	0.9
NXF1	Q9UBU9	528	-2	-7	-19	-13	1.0	0.9	0.8	0.9
PPP6C	O00743	129	4	-11	-19	-13	1.0	0.9	0.8	0.9
C2orf69	Q8N8R5	219	3	-14	-19	-17	1.0	0.9	0.8	0.9
APBB1	O00213	672	-3	-17	-19	-18	1.0	0.9	0.8	0.9
IMPDH1	P20839	246	-15	-11	-19	-19	0.9	0.9	0.8	0.8
PI4K2A	Q9BTU6	124	-15	-25	-19	-23	0.9	0.8	0.8	0.8
SETDB1	Q15047	1286	-8	-24	-19	-29	0.9	0.8	0.8	0.8
CTSS	P25774	170	-5	-22	-19	-35	1.0	0.8	0.8	0.7
HDAC6	Q9UBN7	523	-25	-35	-19	-36	0.8	0.7	0.8	0.7
RASA3	Q14644	144	-1	-20	-19	-48	1.0	0.8	0.8	0.7
WDR44	Q5JSH3	642	-7	-6	-19	-48	0.9	0.9	0.8	0.7
TES	Q9UGI8	331	29	-16	-19	-59	1.4	0.9	0.8	0.6
ROCK2	O75116	766	-12	-7	-19	-62	0.9	0.9	0.8	0.6
PSME2	Q9UL46	91	-3	4	-19	-113	1.0	1.0	0.8	0.5
RAP1GAP2	Q684P5	617	-9	-19	-20	44	0.9	0.8	0.8	1.8
DENND1C	Q8IV53	174	-19	-20	-20	44	0.8	0.8	0.8	1.8
PDE3B	Q13370	311	-7	-24	-20	41	0.9	0.8	0.8	1.7
IQSEC1	Q6DN90	890	0	-15	-20	37	1.0	0.9	0.8	1.6
GSPT2	Q8IYD1	93	0	-15	-20	34	1.0	0.9	0.8	1.5
LAS1L	Q9Y4W2	140	-14	-15	-20	34	0.9	0.9	0.8	1.5
USP9X	Q93008	1212	-23	1	-20	29	0.8	1.0	0.8	1.4
ZC3H12D	A2A288	64	-7	-12	-20	27	0.9	0.9	0.8	1.4
PARP14	Q460N5	557	-12	-27	-20	25	0.9	0.8	0.8	1.3
HARS2	P49590	84	1	1	-20	22	1.0	1.0	0.8	1.3
GPC4	O75487	257	-9	-24	-20	20	0.9	0.8	0.8	1.3
SMPD2	O60906	304	-20	-22	-20	20	0.8	0.8	0.8	1.2
RPAP2	Q8IXW5	358	-13	-12	-20	19	0.9	0.9	0.8	1.2
SPECC1L-ADOI	F8WAN1	395	-10	-30	-20	19	0.9	0.8	0.8	1.2
PDE4A	P27815	218	-5	-13	-20	17	1.0	0.9	0.8	1.2
TXN	P10599	32	-13	-17	-20	17	0.9	0.9	0.8	1.2
AKR1C3	P42330	188	-5	-7	-20	16	1.0	0.9	0.8	1.2

COPG1	Q9Y678	97	-1	11	-20	15	1.0	1.1	0.8	1.2
MNDA	P41218	371	-2	-6	-20	14	1.0	0.9	0.8	1.2
AP1G2	O75843	555	3	-20	-20	14	1.0	0.8	0.8	1.2
TRAF2	Q12933	124	-12	-22	-20	14	0.9	0.8	0.8	1.2
GON4L	Q3T8J9	2140	-9	1	-20	13	0.9	1.0	0.8	1.1
DRG1	Q9Y295	195	-9	-12	-20	13	0.9	0.9	0.8	1.1
DIEXF	Q68CQ4	642	-2	-16	-20	13	1.0	0.9	0.8	1.1
APOBEC3G	Q9HC16	261	7	-17	-20	13	1.1	0.9	0.8	1.1
INTS9	Q9NV88	471	-13	-11	-20	13	0.9	0.9	0.8	1.1
AKT1S1	Q96B36	44	-8	-16	-20	12	0.9	0.9	0.8	1.1
CLP1	Q92989	338	-12	-9	-20	11	0.9	0.9	0.8	1.1
KDM3A	Q9Y4C1	304	-14	-21	-20	11	0.9	0.8	0.8	1.1
ANKLE1	Q8NAG6	517	-7	-1	-20	10	0.9	1.0	0.8	1.1
PURB	Q96QR8	273	2	-7	-20	10	1.0	0.9	0.8	1.1
WDFY3	Q8IZQ1	1600	-16	-19	-20	10	0.9	0.8	0.8	1.1
BAZ2B	Q9UIF8	212	1	-5	-20	9	1.0	1.0	0.8	1.1
TMF1	P82094	318	-5	-18	-20	9	1.0	0.8	0.8	1.1
LRWD1	Q9UFC0	249	-23	-15	-20	9	0.8	0.9	0.8	1.1
SCAF11	Q99590	506	-8	-15	-20	9	0.9	0.9	0.8	1.1
DIS3	Q9Y2L1	799	-6	-17	-20	9	0.9	0.9	0.8	1.1
GPATCH4	Q5T3I0	370	-13	-19	-20	8	0.9	0.8	0.8	1.1
ACBD6	Q9BR61	267	-10	-10	-20	8	0.9	0.9	0.8	1.1
YWHAZ	P63104	94	-11	-12	-20	8	0.9	0.9	0.8	1.1
BRD1	O95696	805	-5	-22	-20	8	1.0	0.8	0.8	1.1
TDRD7	Q8NHU6	1029	6	-1	-20	7	1.1	1.0	0.8	1.1
MTERF4	Q7Z6M4	69	-6	-11	-20	7	0.9	0.9	0.8	1.1
UBR4	Q5T4S7	3930	-39	-11	-20	7	0.7	0.9	0.8	1.1
HEATR3	Q7Z4Q2	585	-8	-4	-20	7	0.9	1.0	0.8	1.1
ATM	Q13315	2246	-5	-9	-20	7	1.0	0.9	0.8	1.1
ERCC6	Q03468	1464	-8	-17	-20	7	0.9	0.9	0.8	1.1
SMYD5	Q6GMV2	101	-11	-19	-20	6	0.9	0.8	0.8	1.1
SYNE3	Q6ZMZ3	74	-10	-11	-20	5	0.9	0.9	0.8	1.1
EPG5	Q9HCE0	472	-16	-26	-20	5	0.9	0.8	0.8	1.1
RRM1	P23921	254	-6	-3	-20	5	0.9	1.0	0.8	1.0
CCNL2	Q96S94	190	-6	-29	-20	5	0.9	0.8	0.8	1.0
ARHGAP30	Q7Z6I6	407	-12	-50	-20	5	0.9	0.7	0.8	1.0
PHKB	Q93100	76	-13	-11	-20	4	0.9	0.9	0.8	1.0
UBN1	Q9NPG3	548	-16	-12	-20	4	0.9	0.9	0.8	1.0
DST	Q03001	5080	-6	-19	-20	4	0.9	0.8	0.8	1.0
RIF1	Q5UIP0	1865	0	8	-20	3	1.0	1.1	0.8	1.0
TRANK1	O15050	905	-3	-6	-20	3	1.0	0.9	0.8	1.0
PLCB2	Q00722	1162	-11	-16	-20	3	0.9	0.9	0.8	1.0
OTUD1	Q5VV17	146	-23	-2	-20	3	0.8	1.0	0.8	1.0
CAPZB	P47756	62	-11	-17	-20	2	0.9	0.9	0.8	1.0
Uncharacteriz	H0YHG0	52	-16	-8	-20	2	0.9	0.9	0.8	1.0
DNAJC14	Q6Y2X3	422	-16	-8	-20	2	0.9	0.9	0.8	1.0
RPL10A	P62906	74	-12	-12	-20	2	0.9	0.9	0.8	1.0
HAUS8	Q9BT25	354	-15	-18	-20	2	0.9	0.8	0.8	1.0
FBXO7	Q9Y3I1	36	-2	-1	-20	1	1.0	1.0	0.8	1.0
GRHPR	Q9UBQ7	216	-7	-7	-20	1	0.9	0.9	0.8	1.0
SPG20	Q8N0X7	405	-3	-8	-20	1	1.0	0.9	0.8	1.0
TRIM38	O00635	339	-12	-26	-20	1	0.9	0.8	0.8	1.0
TNRC6B	Q9UPQ9	557	-14	-20	-20	0	0.9	0.8	0.8	1.0
ADAM10	O14672	173	-15	-21	-20	-1	0.9	0.8	0.8	1.0
FASTKD5	Q7L8L6	670	-14	-21	-20	-1	0.9	0.8	0.8	1.0

VAV1	P15498	652	-11	-4	-20	-2	0.9	1.0	0.8	1.0
TPR	P12270	224	-12	-9	-20	-2	0.9	0.9	0.8	1.0
EEF2	P13639	567	-6	-10	-20	-2	0.9	0.9	0.8	1.0
PGAM1	P18669	153	-10	-14	-20	-2	0.9	0.9	0.8	1.0
GNB1	P62873	148	-7	-30	-20	-2	0.9	0.8	0.8	1.0
CAAP1	Q9H8G2	226	4	-4	-20	-2	1.0	1.0	0.8	1.0
PPP6R2	O75170	366	3	-5	-20	-2	1.0	1.0	0.8	1.0
GOLGB1	Q14789	1462	-6	-15	-20	-2	0.9	0.9	0.8	1.0
STT3B	Q8TCJ2	715	-6	-27	-20	-2	0.9	0.8	0.8	1.0
SCML2	Q9UQR0	688	-4	-12	-20	-3	1.0	0.9	0.8	1.0
THADA	Q6YHU6	635	4	-10	-20	-3	1.0	0.9	0.8	1.0
GBF1	Q92538	1766	-19	-27	-20	-4	0.8	0.8	0.8	1.0
ADAT1	Q9BUB4	186	-7	-27	-20	-4	0.9	0.8	0.8	1.0
NEDD9	Q14511	525	-9	-11	-20	-4	0.9	0.9	0.8	1.0
NR2C1	P13056	200	-22	-21	-20	-4	0.8	0.8	0.8	1.0
CIAPIN1	Q6FI81	288	-4	-14	-20	-5	1.0	0.9	0.8	1.0
NADSYN1	Q6IA69	531	-15	-20	-20	-5	0.9	0.8	0.8	1.0
KCNAB2	Q13303	301	-16	-21	-20	-5	0.9	0.8	0.8	1.0
SCLT1	Q96NL6	390	-9	-20	-20	-6	0.9	0.8	0.8	0.9
WDR11	Q9BZH6	1035	-2	-9	-20	-6	1.0	0.9	0.8	0.9
ARRB2	P32121	243	-5	-14	-20	-7	1.0	0.9	0.8	0.9
PTPRC	P08575	432	-20	-19	-20	-7	0.8	0.8	0.8	0.9
KRI1	Q8N9T8	521	-4	-34	-20	-7	1.0	0.7	0.8	0.9
MAPKAPK2	P49137	114	-4	-17	-20	-9	1.0	0.9	0.8	0.9
WAC	Q9BTA9	150	-15	-21	-20	-9	0.9	0.8	0.8	0.9
AMPD2	Q01433	107	-7	-12	-20	-10	0.9	0.9	0.8	0.9
NIF3L1	Q9GZT8	213	-8	-18	-20	-10	0.9	0.9	0.8	0.9
MYO1F	O00160	445	-18	-23	-20	-10	0.9	0.8	0.8	0.9
HABP4	Q5JVS0	236	-17	-16	-20	-10	0.9	0.9	0.8	0.9
NLRC5	Q86WI3	698	-8	-6	-20	-11	0.9	0.9	0.8	0.9
RAI1	Q7Z5J4	594	-3	-34	-20	-11	1.0	0.7	0.8	0.9
DOK1	Q99704	224	-5	-19	-20	-12	1.0	0.8	0.8	0.9
CEP55	Q53EZ4	236	-13	-17	-20	-13	0.9	0.9	0.8	0.9
UBASH3B	Q8TF42	367	-8	-14	-20	-13	0.9	0.9	0.8	0.9
POLR2B	P30876	892	-6	-13	-20	-14	0.9	0.9	0.8	0.9
LRBA	P50851	1458	-14	-6	-20	-15	0.9	0.9	0.8	0.9
MAP3K5	Q99683	835	-11	-17	-20	-15	0.9	0.9	0.8	0.9
TNRC6B	Q9UPQ9	97	-8	-18	-20	-15	0.9	0.9	0.8	0.9
TANGO2	Q6ICL3	228	-4	-26	-20	-16	1.0	0.8	0.8	0.9
ANKRD12	Q6UB98	1885	-25	-47	-20	-17	0.8	0.7	0.8	0.9
EGLN1	Q9GZT9	42	12	12	-20	-20	1.1	1.1	0.8	0.8
GPX1	P07203	115	-12	-9	-20	-21	0.9	0.9	0.8	0.8
TBXAS1	P24557	309	-4	-10	-20	-22	1.0	0.9	0.8	0.8
LAMTOR2	Q9Y2Q5	76	1	-21	-20	-24	1.0	0.8	0.8	0.8
IMPACT	Q9P2X3	226	-7	-16	-20	-24	0.9	0.9	0.8	0.8
POR	P16435	434	-7	-11	-20	-25	0.9	0.9	0.8	0.8
LRRC16A	Q5VZK9	1181	-4	-13	-20	-25	1.0	0.9	0.8	0.8
HPCAL4	Q9UM19	187	-5	-1	-20	32	1.0	1.0	0.8	1.5
HCCS	P53701	35	-10	-16	-20	26	0.9	0.9	0.8	1.4
CAPN15	O75808	101	-8	-14	-20	26	0.9	0.9	0.8	1.3
ERO1A	Q96HE7	37	-9	-19	-20	23	0.9	0.8	0.8	1.3
ARMC8	Q8IUR7	578	-6	-9	-20	22	0.9	0.9	0.8	1.3
SIVA1	O15304	6	-8	-10	-20	22	0.9	0.9	0.8	1.3
SLF2	Q8IX21	135	-8	-16	-20	22	0.9	0.9	0.8	1.3
ZNF446	Q9NWS9	33	-10	-21	-20	22	0.9	0.8	0.8	1.3

NEK9	Q8TD19	892	-11	-23	-20	22	0.9	0.8	0.8	1.3
SYNE2	Q8WXH0	3515	3	-30	-20	21	1.0	0.8	0.8	1.3
RALGAPA2	Q2PPJ7	1484	23	-36	-20	21	1.3	0.7	0.8	1.3
ALMS1	Q8TCU4	436	10	-4	-20	19	1.1	1.0	0.8	1.2
TCEAL3	Q969E4	44	-30	-29	-20	18	0.8	0.8	0.8	1.2
RAD50	Q92878	1302	-10	-12	-20	17	0.9	0.9	0.8	1.2
BOLA1	Q9Y3E2	126	-6	-7	-20	17	0.9	0.9	0.8	1.2
SRBD1	Q8N5C6	294	-22	-18	-20	17	0.8	0.8	0.8	1.2
PTPN23	Q9H3S7	1466	-2	-12	-20	15	1.0	0.9	0.8	1.2
AMBRA1	Q9C0C7	571	-5	-26	-20	15	1.0	0.8	0.8	1.2
SYNE2	Q8WXH0	2994	-3	-6	-20	14	1.0	0.9	0.8	1.2
IKBKAP	O95163	341	-6	-15	-20	14	0.9	0.9	0.8	1.2
MED26	O95402	271	-13	-2	-20	12	0.9	1.0	0.8	1.1
ZNF696	Q9H7X3	74	-5	-15	-20	12	1.0	0.9	0.8	1.1
GTF3C1	Q12789	1704	-13	0	-20	11	0.9	1.0	0.8	1.1
AKAP9	Q99996	3067	-8	-8	-20	11	0.9	0.9	0.8	1.1
SYNE1	Q8NF91	1234	-10	-23	-20	10	0.9	0.8	0.8	1.1
RRAGA	Q7L523	219	-12	-27	-20	10	0.9	0.8	0.8	1.1
ZBTB21	Q9ULJ3	152	-20	-12	-20	10	0.8	0.9	0.8	1.1
HUWE1	Q7Z6Z7	3239	0	-12	-20	10	1.0	0.9	0.8	1.1
ATP6V1H	Q9UI12	85	-7	-7	-20	9	0.9	0.9	0.8	1.1
ZNF638	Q14966	879	5	-2	-20	9	1.1	1.0	0.8	1.1
HMHA1	Q92619	1020	-3	-9	-20	9	1.0	0.9	0.8	1.1
ADSL	P30566	27	2	-7	-20	8	1.0	0.9	0.8	1.1
TTC38	Q5R3I4	367	-11	-15	-20	8	0.9	0.9	0.8	1.1
ERAP2	Q6P179	210	-9	-18	-20	8	0.9	0.8	0.8	1.1
WHSC1L1	Q9BZ95	495	-8	-2	-20	8	0.9	1.0	0.8	1.1
MCRS1	Q96EZ8	84	-17	-13	-20	8	0.9	0.9	0.8	1.1
ZZZ3	Q8IYH5	24	-9	-16	-20	8	0.9	0.9	0.8	1.1
SNRNP200	O75643	133	-6	-8	-20	7	0.9	0.9	0.8	1.1
TTF1	Q15361	708	0	-7	-20	6	1.0	0.9	0.8	1.1
AKAP11	Q9UKA4	1010	-6	-9	-20	6	0.9	0.9	0.8	1.1
HARS	P12081	507	-9	-13	-20	6	0.9	0.9	0.8	1.1
SMARCA4	P51532	936	-30	-17	-20	6	0.8	0.9	0.8	1.1
CWF19L2	Q2TBE0	214	-15	-22	-20	6	0.9	0.8	0.8	1.1
SMARCD2	Q92925	405	-9	-7	-20	6	0.9	0.9	0.8	1.1
EEF2	P13639	466	-15	-12	-20	6	0.9	0.9	0.8	1.1
LRRC14	Q15048	221	-22	4	-20	5	0.8	1.0	0.8	1.1
HK3	P52790	18	-9	-6	-20	5	0.9	0.9	0.8	1.1
BANF1	O75531	80	-5	-26	-20	5	1.0	0.8	0.8	1.1
BLOC1S4	Q9NUP1	211	-16	-13	-20	5	0.9	0.9	0.8	1.0
GALE	Q14376	264	6	-22	-20	5	1.1	0.8	0.8	1.0
AP4M1	O00189	431	-5	-14	-20	4	1.0	0.9	0.8	1.0
RAVER1	Q8IY67	239	-2	-16	-20	4	1.0	0.9	0.8	1.0
SOS2	Q07890	506	-3	-19	-20	4	1.0	0.8	0.8	1.0
MTM1	Q13496	482	-6	-11	-20	3	0.9	0.9	0.8	1.0
OSBPL5	Q9H0X9	253	-8	-13	-20	2	0.9	0.9	0.8	1.0
MEFV	O15553	226	-11	-18	-20	2	0.9	0.8	0.8	1.0
KDM6A	O15550	797	-9	-22	-20	2	0.9	0.8	0.8	1.0
C19orf35	Q6ZS72	107	-20	15	-20	2	0.8	1.2	0.8	1.0
ACADVL	P49748	156	-15	-21	-20	2	0.9	0.8	0.8	1.0
WDR41	Q9HAD4	131	-5	-21	-20	2	1.0	0.8	0.8	1.0
KDM6A	O15550	4	-18	-5	-20	1	0.9	1.0	0.8	1.0
DCK	P27707	9	-13	-17	-20	1	0.9	0.9	0.8	1.0
CRKL	P46109	44	-8	-11	-20	1	0.9	0.9	0.8	1.0

AHR	P35869	639	-12	-31	-20	1	0.9	0.8	0.8	1.0
ARHGEF1	Q92888	537	-11	-14	-20	0	0.9	0.9	0.8	1.0
AKR1B1	P15121	200	-2	-11	-20	-1	1.0	0.9	0.8	1.0
SRSF9	Q13242	138	-2	-10	-20	-1	1.0	0.9	0.8	1.0
COIL	P38432	126	-9	-16	-20	-1	0.9	0.9	0.8	1.0
HUWE1	Q7Z6Z7	3635	-18	-22	-20	-1	0.9	0.8	0.8	1.0
NLRP3	Q96P20	150	-17	-27	-20	-1	0.9	0.8	0.8	1.0
ZNF490	Q9ULM2	485	-8	-17	-20	-2	0.9	0.9	0.8	1.0
FAM49B	Q9NUQ9	10	5	-24	-20	-2	1.1	0.8	0.8	1.0
RMI1	Q9H9A7	366	7	-3	-20	-2	1.1	1.0	0.8	1.0
RBBP5	Q15291	233	-9	-15	-20	-2	0.9	0.9	0.8	1.0
EIF2S1	P05198	199	-10	-31	-20	-2	0.9	0.8	0.8	1.0
FAM65B	Q9Y4F9	218	0	-22	-20	-3	1.0	0.8	0.8	1.0
CHD9	Q3L8U1	1705	-11	-21	-20	-3	0.9	0.8	0.8	1.0
ING1	Q9UK53	216	-7	-17	-20	-4	0.9	0.9	0.8	1.0
MAVS	Q7Z434	20	-5	-1	-20	-5	1.0	1.0	0.8	1.0
PRKCI	P41743	400	6	-11	-20	-5	1.1	0.9	0.8	1.0
PRKCZ	Q05513	398	6	-11	-20	-5	1.1	0.9	0.8	1.0
FLNB	O75369	2115	-7	-14	-20	-5	0.9	0.9	0.8	1.0
ITGB2	P05107	420	-15	-16	-20	-5	0.9	0.9	0.8	1.0
RAB5C	P51148	213	-12	-21	-20	-5	0.9	0.8	0.8	1.0
PTPRC	P08575	1070	5	-23	-20	-5	1.1	0.8	0.8	1.0
SEC24C	P53992	816	0	-11	-20	-5	1.0	0.9	0.8	1.0
HECTD3	Q5T447	494	5	-15	-20	-5	1.0	0.9	0.8	1.0
RASSF4	Q9H2L5	145	-14	-13	-20	-6	0.9	0.9	0.8	0.9
POLR3E	Q9NVU0	70	-23	-16	-20	-6	0.8	0.9	0.8	0.9
ZNF646	O15015	150	8	-18	-20	-6	1.1	0.9	0.8	0.9
GTF3C2	Q8WUA4	622	-4	-15	-20	-7	1.0	0.9	0.8	0.9
POLM	Q9NP87	369	-11	-16	-20	-7	0.9	0.9	0.8	0.9
SLA	Q13239	76	2	-30	-20	-7	1.0	0.8	0.8	0.9
CNN2	Q99439	215	-17	-7	-20	-8	0.9	0.9	0.8	0.9
NBAS	A2RRP1	256	-10	-9	-20	-10	0.9	0.9	0.8	0.9
KDM3A	Q9Y4C1	467	-16	-19	-20	-10	0.9	0.8	0.8	0.9
ARAP3	Q8WWN8	1193	-4	-23	-20	-10	1.0	0.8	0.8	0.9
HDDC3	Q8N4P3	133	-14	-3	-20	-10	0.9	1.0	0.8	0.9
COPB2	P35606	56	-8	-9	-20	-10	0.9	0.9	0.8	0.9
ANKHD1	Q8IWZ3	1965	-11	-15	-20	-11	0.9	0.9	0.8	0.9
CARMIL2	Q6F5E8	472	-10	-16	-20	-11	0.9	0.9	0.8	0.9
RB1CC1	Q8TDY2	282	5	-16	-20	-12	1.1	0.9	0.8	0.9
SMG7	Q92540	537	-28	-27	-20	-14	0.8	0.8	0.8	0.9
ZRSR2	Q15696	302	-3	-29	-20	-14	1.0	0.8	0.8	0.9
QSER1	Q2KHR3	283	-19	-19	-20	-15	0.8	0.8	0.8	0.9
CAPZA2	P47755	111	-11	-18	-20	-16	0.9	0.9	0.8	0.9
BRF1	Q92994	430	-5	-30	-20	-16	1.0	0.8	0.8	0.9
ZNF609	O15014	345	-13	-19	-20	-16	0.9	0.8	0.8	0.9
NEK9	Q8TD19	909	-18	-9	-20	-17	0.8	0.9	0.8	0.9
NFKB2	Q00653	83	-13	-34	-20	-19	0.9	0.7	0.8	0.8
GNAI2	P04899	255	-4	-29	-20	-19	1.0	0.8	0.8	0.8
GNAI3	P08754	254	-4	-29	-20	-19	1.0	0.8	0.8	0.8
AP4E1	Q9UPM8	976	0	-31	-20	-23	1.0	0.8	0.8	0.8
SKIL	P12757	73	-23	-25	-20	-24	0.8	0.8	0.8	0.8
ACTB	P60709	257	1	-23	-20	-24	1.0	0.8	0.8	0.8
LSS	P48449	471	-22	-22	-20	-25	0.8	0.8	0.8	0.8
CARMIL2	Q6F5E8	502	-14	-20	-20	-30	0.9	0.8	0.8	0.8
DUS3L	Q96G46	315	-22	-36	-20	-32	0.8	0.7	0.8	0.8

ATP2A2	P16615	471	-3	-24	-20	-33	1.0	0.8	0.8	0.8
ROCK2	O75116	428	-6	-18	-20	-34	0.9	0.9	0.8	0.7
APOBEC3D	Q96AK3	261	4	-12	-20	-41	1.0	0.9	0.8	0.7
RDH11	Q8TC12	203	2	-3	-20	-68	1.0	1.0	0.8	0.6
HELZ	P42694	1784	-7	-29	-21	31	0.9	0.8	0.8	1.4
PPOX	P50336	167	-26	-14	-21	23	0.8	0.9	0.8	1.3
PRR12	Q9ULL5	244	2	-16	-21	22	1.0	0.9	0.8	1.3
RSBN1L	Q6PCB5	818	-16	-22	-21	22	0.9	0.8	0.8	1.3
UPF3A	Q9H1J1	315	-15	-25	-21	20	0.9	0.8	0.8	1.2
SMARCAL1	Q9NZC9	108	-6	-8	-21	19	0.9	0.9	0.8	1.2
KCTD5	Q9NXV2	25	-13	-22	-21	17	0.9	0.8	0.8	1.2
FAM65B	Q9Y4F9	519	-9	-15	-21	16	0.9	0.9	0.8	1.2
APOBEC3C	Q9NRW3	130	-7	-18	-21	15	0.9	0.9	0.8	1.2
TBC1D4	O60343	1286	-4	-19	-21	11	1.0	0.8	0.8	1.1
KIF21B	O75037	1152	-3	-23	-21	11	1.0	0.8	0.8	1.1
CTSC	P53634	258	-14	-7	-21	10	0.9	0.9	0.8	1.1
XIAP	P98170	351	-26	-20	-21	10	0.8	0.8	0.8	1.1
SOD1	P00441	147	-11	-14	-21	9	0.9	0.9	0.8	1.1
MRPL37	Q9BZE1	177	-14	-16	-21	9	0.9	0.9	0.8	1.1
GLCCI1	Q86VQ1	207	-4	-18	-21	9	1.0	0.9	0.8	1.1
PPP2CA	P67775	20	10	-16	-21	9	1.1	0.9	0.8	1.1
BTBD10	Q9BSF8	143	-3	-8	-21	8	1.0	0.9	0.8	1.1
CTCF	P49711	155	-7	-16	-21	8	0.9	0.9	0.8	1.1
NIPBL	Q6KC79	2374	-4	-13	-21	8	1.0	0.9	0.8	1.1
MKI67	P46013	1285	-10	-24	-21	8	0.9	0.8	0.8	1.1
ABR	Q12979	346	-10	-7	-21	7	0.9	0.9	0.8	1.1
TOP1	P11387	505	-15	-23	-21	7	0.9	0.8	0.8	1.1
PNKP	Q96T60	308	-15	-29	-21	7	0.9	0.8	0.8	1.1
AP2M1	Q96CW1	212	-16	-15	-21	7	0.9	0.9	0.8	1.1
USP7	Q93009	961	-9	-9	-21	6	0.9	0.9	0.8	1.1
GAK	O14976	1142	-16	-16	-21	5	0.9	0.9	0.8	1.1
MVB12B	Q9H7P6	313	-12	-10	-21	5	0.9	0.9	0.8	1.0
LBR	Q14739	591	-4	-14	-21	5	1.0	0.9	0.8	1.0
ALKBH5	Q6P6C2	378	-4	-23	-21	5	1.0	0.8	0.8	1.0
CAST	P20810	413	-11	-9	-21	4	0.9	0.9	0.8	1.0
PHIP	Q8WWQ0	28	-15	-13	-21	4	0.9	0.9	0.8	1.0
TP53BP1	Q12888	1040	-6	3	-21	3	0.9	1.0	0.8	1.0
NPAT	Q14207	1059	-10	-24	-21	3	0.9	0.8	0.8	1.0
FAM120B	Q96EK7	37	0	2	-21	3	1.0	1.0	0.8	1.0
ARHGEF6	Q15052	119	-13	-15	-21	3	0.9	0.9	0.8	1.0
GRASP	Q7Z6J2	246	-25	-16	-21	3	0.8	0.9	0.8	1.0
INPPL1	O15357	1121	-8	-12	-21	2	0.9	0.9	0.8	1.0
ZNF264	O43296	64	-12	-25	-21	2	0.9	0.8	0.8	1.0
ORC4	O43929	421	-35	-19	-21	1	0.7	0.8	0.8	1.0
MTF1	Q14872	634	-12	-24	-21	1	0.9	0.8	0.8	1.0
TRMT1L	Q7Z2T5	320	-9	-19	-21	0	0.9	0.8	0.8	1.0
CHD1	O14646	411	-8	-21	-21	-1	0.9	0.8	0.8	1.0
TBC1D5	Q92609	706	-9	-17	-21	-1	0.9	0.9	0.8	1.0
RRAGA	Q7L523	157	-21	-18	-21	-1	0.8	0.9	0.8	1.0
DPP3	Q9NY33	509	-12	-21	-21	-1	0.9	0.8	0.8	1.0
NBAS	A2RRP1	1453	-16	-9	-21	-2	0.9	0.9	0.8	1.0
ARAP2	Q8WZ64	1635	-19	-12	-21	-2	0.8	0.9	0.8	1.0
RPL4	P36578	96	-6	-16	-21	-2	0.9	0.9	0.8	1.0
TRAF3IP3	Q9Y228	256	-7	-17	-21	-2	0.9	0.9	0.8	1.0
BAK1	Q16611	14	-16	-25	-21	-2	0.9	0.8	0.8	1.0

FABP5	Q01469	127	-9	-19	-21	-3	0.9	0.8	0.8	1.0
ARAP1	Q96P48	1357	-12	-25	-21	-3	0.9	0.8	0.8	1.0
DOCK2	Q92608	1117	-2	-12	-21	-4	1.0	0.9	0.8	1.0
ALDOA	P04075	202	-12	-13	-21	-4	0.9	0.9	0.8	1.0
ALDOC	P09972	202	-12	-13	-21	-4	0.9	0.9	0.8	1.0
ERAL1	O75616	150	-11	-7	-21	-4	0.9	0.9	0.8	1.0
AK2	P54819	40	-15	-16	-21	-4	0.9	0.9	0.8	1.0
LARP1	Q6PKG0	864	-15	-18	-21	-4	0.9	0.8	0.8	1.0
SMC1A	Q14683	523	-5	-19	-21	-4	1.0	0.8	0.8	1.0
SPICE1	Q8N0Z3	453	-11	3	-21	-5	0.9	1.0	0.8	1.0
CIAPIN1	Q6FI81	274	-5	-10	-21	-5	1.0	0.9	0.8	1.0
MORC3	Q14149	800	-28	-28	-21	-5	0.8	0.8	0.8	1.0
SEC16A	O15027	1115	-15	-19	-21	-6	0.9	0.8	0.8	0.9
DENND3	A2RUS2	499	-19	-14	-21	-8	0.8	0.9	0.8	0.9
WDTC1	Q8N5D0	236	-5	-28	-21	-8	1.0	0.8	0.8	0.9
TCF20	Q9UGU0	868	-13	-15	-21	-9	0.9	0.9	0.8	0.9
RIN3	Q8TB24	881	-4	-18	-21	-10	1.0	0.8	0.8	0.9
NDUFV1	P49821	187	-28	-21	-21	-10	0.8	0.8	0.8	0.9
EP400	Q96L91	1368	12	-7	-21	-10	1.1	0.9	0.8	0.9
FAM120A	Q9NZB2	825	-4	-16	-21	-10	1.0	0.9	0.8	0.9
RIPK2	O43353	414	-14	-16	-21	-11	0.9	0.9	0.8	0.9
PLCB2	Q00722	1149	-12	-8	-21	-12	0.9	0.9	0.8	0.9
VAV3	Q9UKW4	187	-3	-8	-21	-12	1.0	0.9	0.8	0.9
THNSL1	Q8IYQ7	242	2	-13	-21	-12	1.0	0.9	0.8	0.9
PRPF4	O43172	237	-1	-32	-21	-12	1.0	0.8	0.8	0.9
SSPO	A0A096LNW2	3840	-14	-36	-21	-14	0.9	0.7	0.8	0.9
ATP2A2	P16615	364	-14	-12	-21	-15	0.9	0.9	0.8	0.9
ATP2A3	Q93084	364	-14	-12	-21	-15	0.9	0.9	0.8	0.9
RPL22	P35268	25	-12	-17	-21	-16	0.9	0.9	0.8	0.9
OLA1	Q9NTK5	75	-8	-30	-21	-16	0.9	0.8	0.8	0.9
CWF19L1	Q69YN2	11	-9	-26	-21	-16	0.9	0.8	0.8	0.9
BAZ1B	Q9UIG0	1213	0	-22	-21	-17	1.0	0.8	0.8	0.9
CASP7	P55210	186	-3	-15	-21	-17	1.0	0.9	0.8	0.9
KSR1	Q8IVT5	777	-28	-26	-21	-19	0.8	0.8	0.8	0.8
FLNA	P21333	733	6	-33	-21	-21	1.1	0.8	0.8	0.8
RBM22	Q9NW64	48	-13	-17	-21	-23	0.9	0.9	0.8	0.8
PANK4	Q9NVE7	104	10	-13	-21	-24	1.1	0.9	0.8	0.8
KPNB1	Q14974	158	-14	-43	-21	-25	0.9	0.7	0.8	0.8
PYCRL	Q53H96	235	-37	-34	-21	-29	0.7	0.7	0.8	0.8
GCN1	Q92616	2558	-9	-15	-21	-36	0.9	0.9	0.8	0.7
DAPP1	Q9UN19	54	-12	-23	-21	-39	0.9	0.8	0.8	0.7
EIF2AK3	Q9NZJ5	598	-34	-44	-21	-39	0.7	0.7	0.8	0.7
ARIH1	Q9Y4X5	281	-1	-17	-21	-43	1.0	0.9	0.8	0.7
RASGRF2	O14827	850	-23	-5	-21	-45	0.8	1.0	0.8	0.7
ARHGAP21	Q5T5U3	19	-9	-8	-21	-71	0.9	0.9	0.8	0.6
FHL1	Q13642	209	-7	-13	-21	-99	0.9	0.9	0.8	0.5
SRC	P12931	280	-9	-20	-21	-110	0.9	0.8	0.8	0.5
TLN1	Q9Y490	116	1	-12	-21	-115	1.0	0.9	0.8	0.5
CTTN	Q14247	112	-14	-35	-21	-154	0.9	0.7	0.8	0.4
AHDC1	Q5TGY3	1540	-2	-25	-21	47	1.0	0.8	0.8	1.9
ALOX5	P09917	419	-7	-19	-21	40	0.9	0.8	0.8	1.7
RENBP	P51606	390	-5	-15	-21	32	1.0	0.9	0.8	1.5
BACH1	O14867	224	-13	-17	-21	28	0.9	0.9	0.8	1.4
SBNO2	Q9Y2G9	501	-14	-17	-21	26	0.9	0.9	0.8	1.4
TAF6L	Q9Y6J9	122	11	-2	-21	26	1.1	1.0	0.8	1.3

ARID5A	Q03989	248	-7	-18	-21	24	0.9	0.8	0.8	1.3
CD99	P14209	154	-12	-21	-21	23	0.9	0.8	0.8	1.3
HAT1	O14929	101	-2	-7	-21	20	1.0	0.9	0.8	1.3
RNPC3	Q96LT9	14	-8	-22	-21	20	0.9	0.8	0.8	1.3
VPS52	Q8N1B4	220	-6	-9	-21	18	0.9	0.9	0.8	1.2
MIS12	Q9H081	69	-23	-20	-21	17	0.8	0.8	0.8	1.2
STOM	P27105	87	11	0	-21	17	1.1	1.0	0.8	1.2
PDCD1	Q15116	93	-18	-23	-21	17	0.8	0.8	0.8	1.2
CYBB	P04839	428	-5	-13	-21	16	1.0	0.9	0.8	1.2
SUGP2	Q8IX01	381	-8	-9	-21	15	0.9	0.9	0.8	1.2
DENND6A	Q8IWF6	108	-13	-6	-21	14	0.9	0.9	0.8	1.2
SYMPK	Q92797	578	-7	-8	-21	14	0.9	0.9	0.8	1.2
Uncharacteriz	A0A087WZG4	633	-16	-19	-21	14	0.9	0.8	0.8	1.2
MCM3AP	O60318	1945	-14	-18	-21	13	0.9	0.8	0.8	1.1
AGAP2	Q99490	916	9	-2	-21	12	1.1	1.0	0.8	1.1
EEFSEC	P57772	422	-15	-19	-21	12	0.9	0.8	0.8	1.1
TBC1D1	Q86TI0	701	-1	-10	-21	10	1.0	0.9	0.8	1.1
NCOA7	Q8NI08	487	-10	-18	-21	10	0.9	0.9	0.8	1.1
GARS	P41250	211	-6	-12	-21	10	0.9	0.9	0.8	1.1
CUX1	P39880	802	-7	-16	-21	9	0.9	0.9	0.8	1.1
KBTBD2	Q8IY47	145	-2	-17	-21	8	1.0	0.9	0.8	1.1
AKAP11	Q9UKA4	1338	-8	-18	-21	8	0.9	0.8	0.8	1.1
RPS6KA3	P51812	436	-6	-7	-21	7	0.9	0.9	0.8	1.1
RNF31	Q96EP0	722	-3	-19	-21	6	1.0	0.8	0.8	1.1
DDX51	Q8N8A6	187	1	-21	-21	5	1.0	0.8	0.8	1.1
THOP1	P52888	350	-4	-10	-21	5	1.0	0.9	0.8	1.0
NOL11	Q9H8H0	333	-7	-11	-21	4	0.9	0.9	0.8	1.0
RRP1B	Q14684	583	-4	-16	-21	4	1.0	0.9	0.8	1.0
EFTUD2	Q15029	780	-10	-13	-21	4	0.9	0.9	0.8	1.0
DHX9	Q08211	773	-14	-18	-21	4	0.9	0.8	0.8	1.0
HELZ2	Q9BYK8	723	-19	-28	-21	3	0.8	0.8	0.8	1.0
SLFN5	Q08AF3	362	-3	-6	-21	2	1.0	0.9	0.8	1.0
PIKFYVE	Q9Y2I7	1663	-2	-9	-21	2	1.0	0.9	0.8	1.0
SH3BGRL3	Q9H299	71	-7	-32	-21	2	0.9	0.8	0.8	1.0
DEPDC5	O75140	481	1	-16	-21	1	1.0	0.9	0.8	1.0
ADAT1	Q9BUB4	48	-7	-17	-21	1	0.9	0.9	0.8	1.0
STRAP	Q9Y3F4	340	-5	-11	-21	0	1.0	0.9	0.8	1.0
ODF2	Q5BJF6	80	-19	-19	-21	0	0.8	0.8	0.8	1.0
URI1	O94763	167	-16	-17	-21	-1	0.9	0.9	0.8	1.0
TARDBP	G3V162	39	-7	-21	-21	-1	0.9	0.8	0.8	1.0
ADRBK2	P35626	103	-5	-24	-21	-1	1.0	0.8	0.8	1.0
DDX58	O95786	738	-6	-7	-21	-1	0.9	0.9	0.8	1.0
HUWE1	Q7Z6Z7	3372	-17	-8	-21	-1	0.9	0.9	0.8	1.0
RSF1	Q96T23	562	-10	-17	-21	-2	0.9	0.9	0.8	1.0
ENTHD2	Q96N21	287	-11	-19	-21	-2	0.9	0.8	0.8	1.0
NSUN2	Q08J23	599	7	-12	-21	-2	1.1	0.9	0.8	1.0
APIP	Q96GX9	16	-19	-27	-21	-2	0.8	0.8	0.8	1.0
AKAP13	Q12802	2685	-7	-14	-21	-3	0.9	0.9	0.8	1.0
ADD1	P35611	525	-16	-14	-21	-3	0.9	0.9	0.8	1.0
BOD1L1	Q8NFC6	2570	-13	-12	-21	-4	0.9	0.9	0.8	1.0
NAPRT	Q6XQN6	101	-11	-11	-21	-4	0.9	0.9	0.8	1.0
UBAP1	Q9NZ09	408	-10	-25	-21	-4	0.9	0.8	0.8	1.0
CARD6	Q9BX69	937	-14	-17	-21	-5	0.9	0.9	0.8	1.0
MLST8	Q9BVC4	298	-11	-18	-21	-5	0.9	0.8	0.8	1.0
NVL	O15381	687	-22	-10	-21	-5	0.8	0.9	0.8	1.0

COMMD9	Q9P000	160	-9	-21	-21	-5	0.9	0.8	0.8	1.0
XRCC5	P13010	296	-12	-23	-21	-5	0.9	0.8	0.8	1.0
USP4	Q13107	461	-25	-19	-21	-7	0.8	0.8	0.8	0.9
USP15	Q9Y4E8	448	-25	-19	-21	-7	0.8	0.8	0.8	0.9
NRBF2	Q96F24	155	-12	-15	-21	-8	0.9	0.9	0.8	0.9
ZCCHC6	Q5VYS8	911	-4	-11	-21	-8	1.0	0.9	0.8	0.9
AOAH	P28039	160	-13	-23	-21	-9	0.9	0.8	0.8	0.9
SVIL	O95425	178	-16	-16	-21	-9	0.9	0.9	0.8	0.9
BACH1	O14867	263	-20	-16	-21	-9	0.8	0.9	0.8	0.9
GID4	Q8IVV7	90	-14	-18	-21	-9	0.9	0.9	0.8	0.9
C19orf54	Q5BKX5	226	-12	-5	-21	-10	0.9	1.0	0.8	0.9
HSP90AA1	P07900	481	-10	-16	-21	-10	0.9	0.9	0.8	0.9
INPP4A	Q96PE3	230	-3	-18	-21	-12	1.0	0.8	0.8	0.9
POTEJ	P0CG39	920	-7	-6	-21	-13	0.9	0.9	0.8	0.9
UBR4	Q5T4S7	1962	-26	-32	-21	-14	0.8	0.8	0.8	0.9
SDE2	Q6IQ49	150	13	-24	-21	-14	1.1	0.8	0.8	0.9
TTC1	Q99614	8	-12	-22	-21	-15	0.9	0.8	0.8	0.9
GCC2	Q8IWJ2	284	-11	-26	-21	-15	0.9	0.8	0.8	0.9
RARA	P10276	91	-6	-16	-21	-17	0.9	0.9	0.8	0.9
WDHD1	O75717	716	-1	-20	-21	-18	1.0	0.8	0.8	0.8
RFX1	P22670	461	-11	-8	-21	-19	0.9	0.9	0.8	0.8
GAMT	Q14353	91	-14	-16	-21	-21	0.9	0.9	0.8	0.8
RAPGEF1	Q13905	351	-15	-23	-21	-21	0.9	0.8	0.8	0.8
ZNF217	O75362	674	-19	-19	-21	-22	0.8	0.8	0.8	0.8
KSR1	Q8IVT5	62	-14	-9	-21	-22	0.9	0.9	0.8	0.8
COPB1	P53618	189	11	-3	-21	-23	1.1	1.0	0.8	0.8
RAB3D	O95716	137	-7	-12	-21	-23	0.9	0.9	0.8	0.8
DICER1	Q9UPY3	1122	-21	-21	-21	-26	0.8	0.8	0.8	0.8
DHX32	Q7L7V1	329	-30	-35	-21	-26	0.8	0.7	0.8	0.8
UTRN	P46939	2098	12	-17	-21	-34	1.1	0.9	0.8	0.7
CALR	P27797	163	9	-21	-21	-36	1.1	0.8	0.8	0.7
CTSS	P25774	213	-28	-23	-21	-42	0.8	0.8	0.8	0.7
UACA	Q9BZF9	980	-6	-12	-21	-46	0.9	0.9	0.8	0.7
F13A1	P00488	315	-29	-13	-21	-130	0.8	0.9	0.8	0.4
DUSP6	Q16828	353	-10	-9	-22	40	0.9	0.9	0.8	1.7
ARID5A	Q03989	235	-8	-14	-22	29	0.9	0.9	0.8	1.4
NFATC3	Q12968	271	-9	3	-22	27	0.9	1.0	0.8	1.4
ERO1A	Q96HE7	241	-16	-31	-22	25	0.9	0.8	0.8	1.3
IL4R	P24394	509	-14	-9	-22	22	0.9	0.9	0.8	1.3
KEAP1	Q14145	297	-16	-17	-22	22	0.9	0.9	0.8	1.3
AHR	P35869	101	2	-9	-22	21	1.0	0.9	0.8	1.3
SETD2	Q9BYW2	495	-14	-37	-22	21	0.9	0.7	0.8	1.3
NEDD1	Q8NHV4	314	-20	-26	-22	19	0.8	0.8	0.8	1.2
RAD50	Q92878	133	-10	-4	-22	16	0.9	1.0	0.8	1.2
FLCN	Q8NFG4	134	-17	-23	-22	16	0.9	0.8	0.8	1.2
HAVCR2	Q8TDQ0	296	-12	-26	-22	15	0.9	0.8	0.8	1.2
KIF1B	O60333	1810	-5	-8	-22	15	1.0	0.9	0.8	1.2
WDR7	Q9Y4E6	1103	-10	-18	-22	14	0.9	0.9	0.8	1.2
SRPK1	Q96SB4	188	-22	-18	-22	12	0.8	0.8	0.8	1.1
GTF3C4	Q9UKN8	268	-3	-11	-22	12	1.0	0.9	0.8	1.1
LRRC41	Q15345	123	-12	-17	-22	11	0.9	0.9	0.8	1.1
CASP8	Q14790	131	-12	-25	-22	10	0.9	0.8	0.8	1.1
SUGP2	Q8IX01	183	-18	-19	-22	10	0.8	0.8	0.8	1.1
WAPL	Q7Z5K2	344	-9	-11	-22	9	0.9	0.9	0.8	1.1
HSPBP1	Q9NZL4	313	-4	-14	-22	9	1.0	0.9	0.8	1.1

WDR70	Q9NW82	448	-17	-20	-22	8	0.9	0.8	0.8	1.1
SYNE1	Q8NF91	7334	-4	-15	-22	8	1.0	0.9	0.8	1.1
FN3KRP	Q9HA64	11	-13	-19	-22	8	0.9	0.8	0.8	1.1
ITGB2	P05107	598	-22	-22	-22	8	0.8	0.8	0.8	1.1
UVRAG	Q9P2Y5	112	-9	-11	-22	7	0.9	0.9	0.8	1.1
RASGRP1	O95267	237	-7	-18	-22	6	0.9	0.8	0.8	1.1
TNFRSF1B	P20333	293	0	-20	-22	6	1.0	0.8	0.8	1.1
OSBP	P22059	344	-12	-30	-22	6	0.9	0.8	0.8	1.1
EIF3D	O15371	19	-13	-10	-22	6	0.9	0.9	0.8	1.1
TMPO	P42166	341	-10	-17	-22	6	0.9	0.9	0.8	1.1
HNRNPUL2-BS	H3BQZ7	57	-18	-20	-22	5	0.8	0.8	0.8	1.1
SDCCAG8	Q86SQ7	649	-8	-10	-22	5	0.9	0.9	0.8	1.0
ZMYM3	Q14202	524	-18	-17	-22	5	0.9	0.9	0.8	1.0
ENTHD2	Q96N21	463	-21	-17	-22	5	0.8	0.9	0.8	1.0
SETD1B	Q9UPS6	1812	-16	-17	-22	5	0.9	0.9	0.8	1.0
CEP350	Q5VT06	1432	-11	-20	-22	5	0.9	0.8	0.8	1.0
ABCB8	Q9NUT2	454	1	-4	-22	4	1.0	1.0	0.8	1.0
FAM53B	Q14153	271	1	-11	-22	4	1.0	0.9	0.8	1.0
MKI67	P46013	903	-12	-18	-22	4	0.9	0.9	0.8	1.0
WAPL	Q7Z5K2	133	-7	-11	-22	3	0.9	0.9	0.8	1.0
C18orf25	Q96B23	57	-16	-18	-22	3	0.9	0.8	0.8	1.0
GREB1L	Q9C091	1609	1	9	-22	3	1.0	1.1	0.8	1.0
KSR1	Q8IVT5	166	-15	-8	-22	2	0.9	0.9	0.8	1.0
CYP4F12	Q9HCS2	402	-11	-19	-22	2	0.9	0.8	0.8	1.0
ABTB1	Q969K4	222	-7	-23	-22	2	0.9	0.8	0.8	1.0
RAI1	Q7Z5J4	1516	-23	-22	-22	1	0.8	0.8	0.8	1.0
C1orf43	Q9BWL3	97	0	-17	-22	1	1.0	0.9	0.8	1.0
DHX57	Q6P158	453	-6	-19	-22	1	0.9	0.8	0.8	1.0
HCK	P08631	48	-4	-35	-22	1	1.0	0.7	0.8	1.0
CDK13	Q14004	840	-16	-9	-22	-1	0.9	0.9	0.8	1.0
ASCC3	Q8N3C0	366	2	-11	-22	-2	1.0	0.9	0.8	1.0
CD38	P28907	296	-19	-18	-22	-2	0.8	0.9	0.8	1.0
C2CD2L	O14523	115	-9	3	-22	-2	0.9	1.0	0.8	1.0
PLEC	Q15149	992	-9	-18	-22	-3	0.9	0.8	0.8	1.0
PIK3C2A	O00443	514	-16	-18	-22	-3	0.9	0.8	0.8	1.0
CEBPB	P17676	184	-11	-18	-22	-3	0.9	0.8	0.8	1.0
PTK2B	Q14289	677	-10	-12	-22	-4	0.9	0.9	0.8	1.0
EPC1	Q9H2F5	566	-13	-19	-22	-5	0.9	0.8	0.8	1.0
ENGASE	Q8NFI3	113	-10	-13	-22	-6	0.9	0.9	0.8	0.9
DARS	P14868	203	-6	-29	-22	-6	0.9	0.8	0.8	0.9
PRPF19	Q9UMS4	351	-13	-30	-22	-6	0.9	0.8	0.8	0.9
CBLB	Q13191	686	-19	-6	-22	-7	0.8	0.9	0.8	0.9
DDX21	Q9NR30	537	-32	-20	-22	-8	0.8	0.8	0.8	0.9
AAR2	Q9Y312	181	-17	-6	-22	-8	0.9	0.9	0.8	0.9
MACF1	Q9UPN3	887	-3	-17	-22	-8	1.0	0.9	0.8	0.9
AKT2	P31751	311	-20	-19	-22	-8	0.8	0.8	0.8	0.9
MYO9A	B2RTY4	1541	-32	-28	-22	-9	0.8	0.8	0.8	0.9
CEP95	Q96GE4	645	-16	-22	-22	-9	0.9	0.8	0.8	0.9
RECQL5	O94762	627	-11	-24	-22	-9	0.9	0.8	0.8	0.9
ACAP2	Q15057	417	-13	-10	-22	-10	0.9	0.9	0.8	0.9
HMHA1	Q92619	731	-6	-13	-22	-11	0.9	0.9	0.8	0.9
ARL6IP4	Q66PJ3	243	-7	-18	-22	-12	0.9	0.8	0.8	0.9
USP28	Q96RU2	733	-11	-57	-22	-12	0.9	0.6	0.8	0.9
ODF2	Q5BJF6	279	-3	-40	-22	-12	1.0	0.7	0.8	0.9
ANKRD10	Q9NXR5	215	-8	-17	-22	-13	0.9	0.9	0.8	0.9

UBR5	Q95071	1291	-13	-35	-22	-13	0.9	0.7	0.8	0.9
TONSL	Q96HA7	762	-6	-8	-22	-14	0.9	0.9	0.8	0.9
MPRIP	Q6WCQ1	957	-11	-18	-22	-14	0.9	0.8	0.8	0.9
ALDH16A1	Q8IZ83	249	-22	-23	-22	-15	0.8	0.8	0.8	0.9
PGD	P52209	170	-20	-30	-22	-16	0.8	0.8	0.8	0.9
RASGRP4	Q8TDF6	590	-18	-7	-22	-16	0.9	0.9	0.8	0.9
FMNL1	O95466	787	-17	-14	-22	-16	0.9	0.9	0.8	0.9
WDR33	Q9C0J8	220	-11	-17	-22	-16	0.9	0.9	0.8	0.9
MARK3	P27448	46	-17	-13	-22	-17	0.9	0.9	0.8	0.9
RUFY1	Q96T51	351	-14	-13	-22	-19	0.9	0.9	0.8	0.8
RPL17-C18orf:	A0A0A6YYL6	57	-13	-15	-22	-21	0.9	0.9	0.8	0.8
VPS35	Q96QK1	673	-3	-29	-22	-23	1.0	0.8	0.8	0.8
SPG11	Q96JI7	1427	-34	-46	-22	-28	0.7	0.7	0.8	0.8
LRRC8D	Q7L1W4	218	-17	-6	-22	-30	0.9	0.9	0.8	0.8
LIMS1	P48059	164	10	-22	-22	-572	1.1	0.8	0.8	0.1
HNRNPUL1	Q9BUJ2	258	4	-6	-22	-799	1.0	0.9	0.8	0.1
FAM120A	Q9NZB2	531	-5	-18	-22	31	1.0	0.8	0.8	1.4
OSGEPL1	Q9H4B0	295	-3	8	-22	28	1.0	1.1	0.8	1.4
AIP	O00170	122	-6	-14	-22	26	0.9	0.9	0.8	1.3
NFATC2	Q13469	256	-7	-26	-22	23	0.9	0.8	0.8	1.3
TTC38	Q5R3I4	380	-11	-20	-22	22	0.9	0.8	0.8	1.3
DHX36	Q9H2U1	284	2	2	-22	20	1.0	1.0	0.8	1.3
TNFAIP3	P21580	657	2	-9	-22	15	1.0	0.9	0.8	1.2
UTP14A	Q9BVJ6	522	-9	-11	-22	14	0.9	0.9	0.8	1.2
GPAM	Q9HCL2	813	-17	-25	-22	14	0.9	0.8	0.8	1.2
DCAF5	Q96JK2	812	-21	-32	-22	14	0.8	0.8	0.8	1.2
PPP1R3G	B7ZBB8	124	-27	-16	-22	14	0.8	0.9	0.8	1.2
TMEM189-UB	I3L0A0	294	2	-6	-22	12	1.0	0.9	0.8	1.1
ZC3HC1	Q86WB0	406	-14	-14	-22	11	0.9	0.9	0.8	1.1
ZFP91	Q96JP5	520	-10	-20	-22	11	0.9	0.8	0.8	1.1
MYO18A	Q92614	1080	-23	-21	-22	10	0.8	0.8	0.8	1.1
IARS	P41252	350	-18	-27	-22	10	0.9	0.8	0.8	1.1
ERAP1	Q9NZ08	806	-6	-10	-22	9	0.9	0.9	0.8	1.1
MPHOSPH8	Q99549	802	4	-16	-22	9	1.0	0.9	0.8	1.1
FBXL15	Q9H469	244	-29	-15	-22	8	0.8	0.9	0.8	1.1
RCAN3	Q9UKA8	239	-7	-20	-22	8	0.9	0.8	0.8	1.1
AKAP13	Q12802	732	-19	-24	-22	8	0.8	0.8	0.8	1.1
ERAP2	Q6P179	557	-2	-22	-22	7	1.0	0.8	0.8	1.1
MAP3K4	Q9Y6R4	521	-5	-17	-22	7	1.0	0.9	0.8	1.1
MCM3	P25205	134	-16	3	-22	6	0.9	1.0	0.8	1.1
SETX	Q7Z333	2038	-7	-18	-22	6	0.9	0.8	0.8	1.1
ATG2B	Q96BY7	1667	-7	-11	-22	6	0.9	0.9	0.8	1.1
ARPC4-TTLL3	A0A0A6YYG9	21	-3	-15	-22	6	1.0	0.9	0.8	1.1
EIF3M	Q7L2H7	134	-8	-15	-22	6	0.9	0.9	0.8	1.1
UBE3B	Q7Z3V4	75	-6	-14	-22	5	0.9	0.9	0.8	1.1
TAX1BP1	Q86VP1	350	-7	-9	-22	5	0.9	0.9	0.8	1.0
EEFSEC	P57772	426	-8	-22	-22	5	0.9	0.8	0.8	1.0
GCC2	Q8IWJ2	297	-10	-7	-22	4	0.9	0.9	0.8	1.0
FKBP15	Q5T1M5	1031	-6	-12	-22	4	0.9	0.9	0.8	1.0
ORAOV1	Q8WV07	70	-13	-23	-22	4	0.9	0.8	0.8	1.0
SMARCAL1	Q9NZC9	62	-3	-25	-22	4	1.0	0.8	0.8	1.0
ZFR	Q96KR1	525	-4	-25	-22	4	1.0	0.8	0.8	1.0
TBC1D9B	Q66K14	839	-3	-14	-22	3	1.0	0.9	0.8	1.0
ZHX2	Q9Y6X8	738	-10	-16	-22	3	0.9	0.9	0.8	1.0
SLFN5	Q08AF3	601	-9	-33	-22	3	0.9	0.8	0.8	1.0

KAT6A	Q92794	508	-7	-12	-22	2	0.9	0.9	0.8	1.0
HPS5	Q9UPZ3	431	-12	-19	-22	2	0.9	0.8	0.8	1.0
IAH1	Q2TAA2	150	4	-13	-22	2	1.0	0.9	0.8	1.0
RNFT1	Q5M7Z0	73	-13	-16	-22	1	0.9	0.9	0.8	1.0
IFI16	Q16666	366	-11	-9	-22	1	0.9	0.9	0.8	1.0
ZNF3	P17036	267	-14	-21	-22	1	0.9	0.8	0.8	1.0
SRPK1	Q96SB4	455	-12	-24	-22	1	0.9	0.8	0.8	1.0
FNDC3A	Q9Y2H6	1022	-8	5	-22	-1	0.9	1.1	0.8	1.0
TP53BP1	Q12888	513	-13	-13	-22	-1	0.9	0.9	0.8	1.0
ZNF185	O15231	615	-9	-20	-22	-1	0.9	0.8	0.8	1.0
CNTN5	O94779	458	19	-4	-22	-1	1.2	1.0	0.8	1.0
KHNYN	O15037	221	-17	-19	-22	-2	0.9	0.8	0.8	1.0
PIGA	P37287	28	-5	-20	-22	-3	1.0	0.8	0.8	1.0
ZZZ3	Q8IYH5	268	-9	-24	-22	-3	0.9	0.8	0.8	1.0
HLA-DRB1	P04229	108	-15	-29	-22	-4	0.9	0.8	0.8	1.0
GNL3L	Q9NVN8	490	-8	-10	-22	-4	0.9	0.9	0.8	1.0
ZC3H12D	A2A288	177	-11	-21	-22	-4	0.9	0.8	0.8	1.0
FPGT	O14772	395	-15	-32	-22	-4	0.9	0.8	0.8	1.0
EML4	Q9HC35	610	-13	-7	-22	-5	0.9	0.9	0.8	1.0
SRRM2	Q9UQ35	1029	-9	-21	-22	-5	0.9	0.8	0.8	1.0
FAM101B	Q8N5W9	86	-13	-21	-22	-5	0.9	0.8	0.8	1.0
PDE12	Q6L8Q7	108	-12	-22	-22	-5	0.9	0.8	0.8	1.0
JMJD1C	Q15652	508	-16	-15	-22	-6	0.9	0.9	0.8	0.9
ASCC3	Q8N3C0	742	-6	-20	-22	-6	0.9	0.8	0.8	0.9
CAPN2	P17655	405	-5	-21	-22	-7	1.0	0.8	0.8	0.9
ZC3H11A	O75152	588	-10	-24	-22	-7	0.9	0.8	0.8	0.9
BRD8	Q9H0E9	64	2	-24	-22	-7	1.0	0.8	0.8	0.9
NR2C2	P49116	414	8	-12	-22	-7	1.1	0.9	0.8	0.9
RUFY1	Q96T51	320	-14	-32	-22	-9	0.9	0.8	0.8	0.9
TRRAP	Q9Y4A5	949	-5	-7	-22	-9	1.0	0.9	0.8	0.9
GANAB	Q14697	47	-16	-15	-22	-9	0.9	0.9	0.8	0.9
GMPS	P49915	554	11	-24	-22	-9	1.1	0.8	0.8	0.9
UQCRC1	P31930	410	-19	-14	-22	-10	0.8	0.9	0.8	0.9
ZNF761	A0A087WXT7	24	-18	-16	-22	-10	0.9	0.9	0.8	0.9
ZNF845	Q96IR2	24	-18	-16	-22	-10	0.9	0.9	0.8	0.9
ZNF701	Q9NV72	90	-18	-16	-22	-10	0.9	0.9	0.8	0.9
RNF213	Q63HN8	2633	-5	-15	-22	-10	1.0	0.9	0.8	0.9
PAPSS1	O43252	78	-14	-21	-22	-10	0.9	0.8	0.8	0.9
CEP192	Q8TEP8	1805	-16	-27	-22	-11	0.9	0.8	0.8	0.9
UBE2O	Q9C0C9	400	-10	-13	-22	-11	0.9	0.9	0.8	0.9
SYNE2	Q8WXH0	3154	7	-23	-22	-11	1.1	0.8	0.8	0.9
PPP1R12C	Q9BZL4	494	-3	-4	-22	-12	1.0	1.0	0.8	0.9
PRPF3	O43395	651	-29	-40	-22	-12	0.8	0.7	0.8	0.9
TRIP11	Q15643	1722	-17	-22	-22	-12	0.9	0.8	0.8	0.9
GALK1	P51570	243	1	-19	-22	-13	1.0	0.8	0.8	0.9
MAP3K5	Q99683	1360	-2	-11	-22	-14	1.0	0.9	0.8	0.9
IRF7	Q92985	458	-20	-37	-22	-14	0.8	0.7	0.8	0.9
LRCH3	Q96II8	102	-8	6	-22	-16	0.9	1.1	0.8	0.9
TXNRD1	Q16881	214	-10	-20	-22	-17	0.9	0.8	0.8	0.9
TXNRD2	Q9NNW7	91	-10	-20	-22	-17	0.9	0.8	0.8	0.9
HSP90AA1	P07900	529	10	-19	-22	-18	1.1	0.8	0.8	0.8
ARHGEF1	Q92888	892	-10	-16	-22	-19	0.9	0.9	0.8	0.8
FAHD2B	Q6P2I3	119	-6	-20	-22	-19	0.9	0.8	0.8	0.8
ILF3	Q12906	278	-24	-23	-22	-19	0.8	0.8	0.8	0.8
ANKRD44	Q8N8A2	854	-12	-16	-22	-21	0.9	0.9	0.8	0.8

CTR9	Q6PD62	817	-13	-16	-22	-22	0.9	0.9	0.8	0.8
RAB10	P61026	124	-14	-23	-22	-22	0.9	0.8	0.8	0.8
RAB33A	Q14088	197	-19	-24	-22	-28	0.8	0.8	0.8	0.8
TBXAS1	P24557	479	-18	-24	-22	-29	0.9	0.8	0.8	0.8
MYH10	P35580	176	-8	-18	-22	-37	0.9	0.8	0.8	0.7
MYH14	Q7Z406	196	-8	-18	-22	-37	0.9	0.8	0.8	0.7
NT5DC1	Q5TFE4	179	7	-15	-22	-37	1.1	0.9	0.8	0.7
ITGAL	P20701	119	-11	-6	-22	-38	0.9	0.9	0.8	0.7
GGA2	Q9UJY4	343	-3	-15	-22	-50	1.0	0.9	0.8	0.7
ZFP36	P26651	253	-21	-34	-22	-55	0.8	0.7	0.8	0.6
DHX36	Q9H2U1	611	4	-16	-22	-56	1.0	0.9	0.8	0.6
WDR61	Q9GZS3	266	1	-20	-22	-56	1.0	0.8	0.8	0.6
HSF1	Q00613	153	2	-18	-23	32	1.0	0.8	0.8	1.5
ARHGAP30	Q7Z6I6	640	-17	-25	-23	30	0.9	0.8	0.8	1.4
PARP14	Q460N5	906	-9	-19	-23	29	0.9	0.8	0.8	1.4
TERF1	P54274	118	-13	-22	-23	27	0.9	0.8	0.8	1.4
PDPK1	O15530	21	-18	-4	-23	26	0.9	1.0	0.8	1.3
BRAT1	Q6PJG6	487	-19	-24	-23	24	0.8	0.8	0.8	1.3
KMT2E	Q8IZD2	923	-6	-7	-23	24	0.9	0.9	0.8	1.3
EML4	Q9HC35	368	6	-3	-23	20	1.1	1.0	0.8	1.3
CNOT6L	Q96LI5	449	1	-7	-23	20	1.0	0.9	0.8	1.3
ATM	Q13315	198	-4	-11	-23	20	1.0	0.9	0.8	1.3
RARA	P10276	174	-10	-14	-23	16	0.9	0.9	0.8	1.2
PARP9	Q8IXQ6	251	16	-2	-23	14	1.2	1.0	0.8	1.2
TRRAP	Q9Y4A5	2241	-12	-13	-23	14	0.9	0.9	0.8	1.2
FBXO9	Q9UK97	68	-26	-30	-23	13	0.8	0.8	0.8	1.1
NBR1	Q14596	669	-7	-4	-23	12	0.9	1.0	0.8	1.1
RNF40	O75150	890	2	-28	-23	12	1.0	0.8	0.8	1.1
ZBED1	O96006	38	-23	-18	-23	11	0.8	0.9	0.8	1.1
MTA3	Q9BTC8	532	-13	-18	-23	11	0.9	0.8	0.8	1.1
VWA5A	O00534	649	-5	-28	-23	11	1.0	0.8	0.8	1.1
CCP110	O43303	600	-4	7	-23	10	1.0	1.1	0.8	1.1
KARS	Q15046	496	-7	-12	-23	10	0.9	0.9	0.8	1.1
INPP5D	Q92835	956	-7	-18	-23	10	0.9	0.9	0.8	1.1
IMPACT	Q9P2X3	195	-13	-21	-23	10	0.9	0.8	0.8	1.1
PSMD12	O00232	255	-10	-14	-23	9	0.9	0.9	0.8	1.1
MTMR3	Q13615	867	-8	-15	-23	8	0.9	0.9	0.8	1.1
RPRD1B	Q9NQG5	100	-13	-18	-23	8	0.9	0.9	0.8	1.1
HARS	P12081	509	-15	-23	-23	8	0.9	0.8	0.8	1.1
ZC3H12D	A2A288	367	-10	-24	-23	8	0.9	0.8	0.8	1.1
HERC2	O95714	1525	6	-14	-23	6	1.1	0.9	0.8	1.1
SP140	Q13342	224	-13	-19	-23	6	0.9	0.8	0.8	1.1
GNB2	P62879	294	-19	-34	-23	6	0.8	0.7	0.8	1.1
PI4K2A	Q9BTU6	320	-5	-25	-23	6	1.0	0.8	0.8	1.1
FANCB	Q8NB91	78	-8	-35	-23	5	0.9	0.7	0.8	1.1
ASCC3	Q8N3C0	324	-9	-17	-23	5	0.9	0.9	0.8	1.0
LIMK2	P53671	317	-12	-8	-23	4	0.9	0.9	0.8	1.0
AIP	O00170	208	-10	-14	-23	4	0.9	0.9	0.8	1.0
AKNA	Q7Z591	1078	-16	-16	-23	4	0.9	0.9	0.8	1.0
BMP2K	Q9NSY1	912	-9	-18	-23	4	0.9	0.9	0.8	1.0
PITRM1	Q5JRX3	241	-11	-23	-23	4	0.9	0.8	0.8	1.0
PCNT	O95613	563	-1	-13	-23	4	1.0	0.9	0.8	1.0
RHOH	Q15669	108	-15	-15	-23	4	0.9	0.9	0.8	1.0
ELP4	Q96EB1	412	-17	-14	-23	3	0.9	0.9	0.8	1.0
SMARCA2	P51531	1296	-8	-15	-23	2	0.9	0.9	0.8	1.0

SH2D3C	Q8N5H7	377	-17	-19	-23	2	0.9	0.8	0.8	1.0
PSMD4	P55036	58	-7	-26	-23	2	0.9	0.8	0.8	1.0
SMU1	Q2TAY7	383	-6	-14	-23	1	0.9	0.9	0.8	1.0
GRK6	P43250	474	-10	-21	-23	1	0.9	0.8	0.8	1.0
RANBP9	Q96S59	610	-11	-19	-23	-1	0.9	0.8	0.8	1.0
BOD1L1	Q8NFC6	2386	-14	-15	-23	-1	0.9	0.9	0.8	1.0
GBF1	Q92538	613	-7	-16	-23	-1	0.9	0.9	0.8	1.0
UPP1	Q16831	225	-9	-17	-23	-1	0.9	0.9	0.8	1.0
RBBP7	Q16576	166	-17	-28	-23	-1	0.9	0.8	0.8	1.0
HTATSF1	O43719	480	-10	-16	-23	-2	0.9	0.9	0.8	1.0
TRAPPC11	Q7Z392	615	-3	-2	-23	-2	1.0	1.0	0.8	1.0
PI4K2B	Q8TCG2	446	-26	-19	-23	-2	0.8	0.8	0.8	1.0
NOL8	Q76FK4	517	-1	-12	-23	-3	1.0	0.9	0.8	1.0
TACC1	O75410	514	-8	-17	-23	-4	0.9	0.9	0.8	1.0
SPG11	Q96JI7	2436	5	-25	-23	-4	1.0	0.8	0.8	1.0
LRCH4	O75427	247	-18	-20	-23	-4	0.9	0.8	0.8	1.0
AP1G2	O75843	539	-16	-12	-23	-5	0.9	0.9	0.8	1.0
TRRAP	Q9Y4A5	3555	-9	-20	-23	-5	0.9	0.8	0.8	1.0
GCNT1	Q02742	217	-21	-3	-23	-5	0.8	1.0	0.8	1.0
KMT2A	Q03164	2783	-11	-24	-23	-5	0.9	0.8	0.8	1.0
PIK3CB	P42338	287	2	-17	-23	-6	1.0	0.9	0.8	0.9
RANBP2	P49792	2710	-6	-12	-23	-7	0.9	0.9	0.8	0.9
CARMIL2	Q6F5E8	1339	-13	-18	-23	-8	0.9	0.9	0.8	0.9
ANGEL2	Q5VTE6	139	-22	-20	-23	-8	0.8	0.8	0.8	0.9
SPTY2D1	Q68D10	535	2	-19	-23	-8	1.0	0.8	0.8	0.9
ITGB2	P05107	549	-13	-24	-23	-10	0.9	0.8	0.8	0.9
MCM4	P33991	212	-12	-27	-23	-10	0.9	0.8	0.8	0.9
PRKAR1A	P10644	39	-4	-17	-23	-10	1.0	0.9	0.8	0.9
RNF169	Q8NCN4	337	-16	-22	-23	-12	0.9	0.8	0.8	0.9
CRBN	Q96SW2	441	-14	-24	-23	-13	0.9	0.8	0.8	0.9
NLRC3	Q7RTR2	224	5	4	-23	-13	1.0	1.0	0.8	0.9
LCK	P06239	217	-9	-41	-23	-15	0.9	0.7	0.8	0.9
NAT10	Q9H0A0	255	6	-8	-23	-16	1.1	0.9	0.8	0.9
DPF2	Q92785	24	-11	-22	-23	-19	0.9	0.8	0.8	0.8
INPP5K	Q9BT40	280	-6	-24	-23	-20	0.9	0.8	0.8	0.8
WDFY1	Q8IWB7	347	-3	-10	-23	-25	1.0	0.9	0.8	0.8
LIG3	P49916	94	-9	-26	-23	-32	0.9	0.8	0.8	0.8
AP3D1	O14617	208	-26	-24	-23	-38	0.8	0.8	0.8	0.7
TAF15	Q92804	365	-9	1	-23	-43	0.9	1.0	0.8	0.7
PARD3B	Q8TEW8	777	-16	-27	-23	-66	0.9	0.8	0.8	0.6
ZNF24	P17028	189	13	-36	-23	-75	1.1	0.7	0.8	0.6
PF4V1	P10720	70	-18	-32	-23	-85	0.9	0.8	0.8	0.5
LIMS2	Q7Z4I7	105	-17	-21	-23	-91	0.9	0.8	0.8	0.5
GCLC	P48506	501	1	-3	-23	37	1.0	1.0	0.8	1.6
GZMB	P10144	209	-7	-36	-23	34	0.9	0.7	0.8	1.5
TRIM33	Q9UPN9	582	-8	-15	-23	33	0.9	0.9	0.8	1.5
UBA2	Q9UBT2	161	-7	-21	-23	23	0.9	0.8	0.8	1.3
KBTBD4	Q9NVX7	68	-8	-22	-23	23	0.9	0.8	0.8	1.3
CDC23	Q9UJX2	500	-18	-28	-23	22	0.8	0.8	0.8	1.3
VDAC2	P45880	13	-7	-9	-23	19	0.9	0.9	0.8	1.2
UBE2O	Q9C0C9	314	-11	-16	-23	19	0.9	0.9	0.8	1.2
POM121	Q96HA1	217	1	-10	-23	18	1.0	0.9	0.8	1.2
UTY	O14607	511	-7	-25	-23	17	0.9	0.8	0.8	1.2
Uncharacteriz	G3V599	1114	-14	-19	-23	17	0.9	0.8	0.8	1.2
TANGO6	Q9C0B7	360	-9	-2	-23	16	0.9	1.0	0.8	1.2

CEBPB	P17676	248	-7	-21	-23	16	0.9	0.8	0.8	1.2
TSR2	Q969E8	114	-3	-8	-23	13	1.0	0.9	0.8	1.1
ARID1A	O14497	336	8	-14	-23	13	1.1	0.9	0.8	1.1
DOCK11	Q5JSL3	1204	-7	-21	-23	12	0.9	0.8	0.8	1.1
GBP5	Q96PP8	184	-10	-22	-23	12	0.9	0.8	0.8	1.1
RNF169	Q8NCN4	563	-7	-11	-23	12	0.9	0.9	0.8	1.1
DHX15	O43143	307	-4	-13	-23	11	1.0	0.9	0.8	1.1
PPHLN1	Q8NEY8	309	-8	-15	-23	9	0.9	0.9	0.8	1.1
CCDC88C	Q9P219	1973	-6	-9	-23	8	0.9	0.9	0.8	1.1
TARSL2	A2RTX5	49	-10	-34	-23	8	0.9	0.7	0.8	1.1
CIZ1	Q9ULV3	252	-8	-21	-23	8	0.9	0.8	0.8	1.1
ATP6V0A2	Q9Y487	165	-27	-8	-23	7	0.8	0.9	0.8	1.1
ECM29	Q5VYK3	1712	6	-9	-23	7	1.1	0.9	0.8	1.1
TMEM23	D3DWC4	50	-12	-17	-23	7	0.9	0.9	0.8	1.1
DUS2	Q9NX74	475	-17	-23	-23	6	0.9	0.8	0.8	1.1
ZNF217	O75362	901	1	-5	-23	5	1.0	1.0	0.8	1.1
RAP1GAP2	Q684P5	584	-12	-14	-23	5	0.9	0.9	0.8	1.1
PAFAH1B1	P43034	356	-7	-10	-23	4	0.9	0.9	0.8	1.0
PIK3R4	Q99570	693	-6	-17	-23	4	0.9	0.9	0.8	1.0
Uncharacteriz	A0A0A6YYC8	86	-6	-14	-23	3	0.9	0.9	0.8	1.0
ANKFY1	Q9P2R3	716	-34	-50	-23	3	0.7	0.7	0.8	1.0
STAT3	P40763	259	-14	-13	-23	2	0.9	0.9	0.8	1.0
ALMS1	Q8TCU4	2878	-17	-20	-23	2	0.9	0.8	0.8	1.0
EDC4	Q6P2E9	976	-2	-22	-23	2	1.0	0.8	0.8	1.0
WDR91	A4D1P6	351	-8	-26	-23	2	0.9	0.8	0.8	1.0
MYSM1	Q5VVJ2	169	-11	-20	-23	1	0.9	0.8	0.8	1.0
HAUS6	Q7Z4H7	743	-7	-25	-23	1	0.9	0.8	0.8	1.0
HDAC3	O15379	268	-13	4	-23	-1	0.9	1.0	0.8	1.0
INPP4A	Q96PE3	286	-8	-12	-23	-2	0.9	0.9	0.8	1.0
STX17	P56962	290	-8	-14	-23	-2	0.9	0.9	0.8	1.0
TNS3	Q68CZ2	928	-20	-26	-23	-2	0.8	0.8	0.8	1.0
OPTN	Q96CV9	239	-5	8	-23	-2	1.0	1.1	0.8	1.0
PPIH	O43447	47	-45	-29	-23	-2	0.7	0.8	0.8	1.0
COIL	P38432	170	-5	-23	-23	-3	1.0	0.8	0.8	1.0
ASZ1	Q8WWH4	131	-95	-45	-23	-3	0.5	0.7	0.8	1.0
GRIPAP1	Q4V328	581	-13	-16	-23	-4	0.9	0.9	0.8	1.0
POGK	Q9P215	218	-13	-19	-23	-4	0.9	0.8	0.8	1.0
TRIM33	Q9UPN9	849	-9	-22	-23	-4	0.9	0.8	0.8	1.0
ADCK4	Q96D53	415	-5	-15	-23	-5	1.0	0.9	0.8	1.0
PA2G4	Q9UQ80	179	-9	-20	-23	-5	0.9	0.8	0.8	1.0
SLC27A1	Q6PCB7	406	-11	-11	-23	-6	0.9	0.9	0.8	0.9
GNPAT	O15228	544	-10	-18	-23	-6	0.9	0.9	0.8	0.9
QTRT1	Q9BXR0	23	-12	-36	-23	-6	0.9	0.7	0.8	0.9
GPSM3	Q9Y4H4	116	-13	-15	-23	-6	0.9	0.9	0.8	0.9
PPM1G	O15355	164	-6	-20	-23	-6	0.9	0.8	0.8	0.9
FAM208B	Q5VWN6	1534	-10	-15	-23	-7	0.9	0.9	0.8	0.9
GNPDA1	P46926	48	-6	-17	-23	-7	0.9	0.9	0.8	0.9
GNPDA2	Q8TDQ7	48	-6	-17	-23	-7	0.9	0.9	0.8	0.9
ZNF414	Q96IQ9	117	-12	-7	-23	-8	0.9	0.9	0.8	0.9
SMN2	Q16637	60	-6	-19	-23	-8	0.9	0.8	0.8	0.9
MORC3	Q14149	632	-12	-7	-23	-8	0.9	0.9	0.8	0.9
ARAP1	Q96P48	123	-9	-22	-23	-8	0.9	0.8	0.8	0.9
BBX	Q8WY36	606	-26	-25	-23	-8	0.8	0.8	0.8	0.9
GSR	P00390	278	0	-9	-23	-9	1.0	0.9	0.8	0.9
NR3C1	P04150	431	-1	-23	-23	-10	1.0	0.8	0.8	0.9

TUBGCP5	A0A0G2JSA7	580	-13	-1	-23	-10	0.9	1.0	0.8	0.9
RNF20	Q5VTR2	905	-24	-18	-23	-11	0.8	0.8	0.8	0.9
TTC1	Q99614	28	-12	-17	-23	-12	0.9	0.9	0.8	0.9
YARS	P54577	501	-13	-13	-23	-14	0.9	0.9	0.8	0.9
UBE2D2	P62837	111	-5	-27	-23	-14	1.0	0.8	0.8	0.9
TMPO	P42166	280	-10	-22	-23	-15	0.9	0.8	0.8	0.9
STRN	O43815	587	-15	-29	-23	-15	0.9	0.8	0.8	0.9
CTNNA1	P35221	772	-12	-20	-23	-15	0.9	0.8	0.8	0.9
IKBKAP	O95163	1025	-10	-19	-23	-16	0.9	0.8	0.8	0.9
LCP1	P13796	206	-9	-21	-23	-16	0.9	0.8	0.8	0.9
SMCHD1	A6NHR9	1982	-12	-22	-23	-17	0.9	0.8	0.8	0.9
PPP1CB	P62140	244	4	-15	-23	-18	1.0	0.9	0.8	0.8
MYCBP2	O75592	3856	-11	-7	-23	-21	0.9	0.9	0.8	0.8
CYLD	Q9NQC7	712	-6	-16	-23	-22	0.9	0.9	0.8	0.8
TRIM65	Q6PJ69	500	-4	-17	-23	-22	1.0	0.9	0.8	0.8
NPEPL1	Q8NDH3	357	-7	-37	-23	-23	0.9	0.7	0.8	0.8
MTO1	Q9Y2Z2	315	-13	-16	-23	-25	0.9	0.9	0.8	0.8
CBLB	Q13191	523	-1	-4	-23	-27	1.0	1.0	0.8	0.8
CYFIP2	Q96F07	531	-12	-21	-23	-30	0.9	0.8	0.8	0.8
ITGB1	P05556	636	-2	-17	-23	-31	1.0	0.9	0.8	0.8
IKZF5	Q9H5V7	409	-3	-32	-23	-38	1.0	0.8	0.8	0.7
CABIN1	Q9Y6J0	1908	-3	-11	-23	-46	1.0	0.9	0.8	0.7
HEXIM2	Q96MH2	80	-11	-26	-23	-53	0.9	0.8	0.8	0.7
PAPOLA	P51003	293	-78	-54	-23	-70	0.6	0.7	0.8	0.6
ZNF124	Q15973	220	-8	-34	-23	-103	0.9	0.7	0.8	0.5
TXN2	Q99757	90	-18	-32	-24	38	0.8	0.8	0.8	1.6
MRPL20	Q9BYC9	93	-22	-20	-24	32	0.8	0.8	0.8	1.5
CD86	P42081	282	-4	2	-24	28	1.0	1.0	0.8	1.4
FOXK1	P85037	404	-20	-24	-24	25	0.8	0.8	0.8	1.3
SUGP2	Q8IX01	656	-10	-23	-24	20	0.9	0.8	0.8	1.3
NOP16	Q9Y3C1	36	-12	-37	-24	20	0.9	0.7	0.8	1.2
MLLT6	A0A087WW39	571	-12	-13	-24	17	0.9	0.9	0.8	1.2
KDM1B	Q8NB78	234	-16	-17	-24	17	0.9	0.9	0.8	1.2
TP53BP1	Q12888	986	-17	-18	-24	16	0.9	0.9	0.8	1.2
GIMAP2	Q9UG22	88	-1	-9	-24	16	1.0	0.9	0.8	1.2
BACH1	O14867	435	-10	-34	-24	16	0.9	0.7	0.8	1.2
HAUS3	Q68CZ6	249	-10	-11	-24	14	0.9	0.9	0.8	1.2
MTMR3	Q13615	915	-24	-28	-24	13	0.8	0.8	0.8	1.1
SIN3A	Q96ST3	1167	-8	-11	-24	11	0.9	0.9	0.8	1.1
SMARCAD1	Q9H4L7	861	-1	-17	-24	11	1.0	0.9	0.8	1.1
RIN1	Q13671	355	-21	-19	-24	11	0.8	0.8	0.8	1.1
RIPK3	Q9Y572	365	-15	-19	-24	11	0.9	0.8	0.8	1.1
ASPSCR1	Q9BZE9	225	-11	-27	-24	9	0.9	0.8	0.8	1.1
ZFYVE16	Q7Z3T8	292	-1	-17	-24	8	1.0	0.9	0.8	1.1
KYNU	Q16719	327	-8	-19	-24	8	0.9	0.8	0.8	1.1
OAS2	P29728	237	-14	-23	-24	8	0.9	0.8	0.8	1.1
SYNE1	Q8NF91	7793	5	6	-24	7	1.1	1.1	0.8	1.1
PCK2	Q16822	151	-3	-25	-24	7	1.0	0.8	0.8	1.1
RPL36	Q9Y3U8	48	-2	-12	-24	7	1.0	0.9	0.8	1.1
C2orf69	Q8N8R5	43	-4	4	-24	6	1.0	1.0	0.8	1.1
SQSTM1	Q13501	27	-4	-9	-24	6	1.0	0.9	0.8	1.1
AGL	P35573	126	2	-16	-24	6	1.0	0.9	0.8	1.1
PDE3B	Q13370	410	-16	-18	-24	6	0.9	0.9	0.8	1.1
MAP3K8	P41279	398	-19	-23	-24	6	0.8	0.8	0.8	1.1
MAD2L1BP	Q15013	186	-20	-19	-24	6	0.8	0.8	0.8	1.1

ATM	Q13315	669	-10	-21	-24	4	0.9	0.8	0.8	1.0
IKZF3	Q9UKT9	281	16	-12	-24	2	1.2	0.9	0.8	1.0
THOC2	Q8NI27	1016	-13	-19	-24	2	0.9	0.8	0.8	1.0
INPPL1	O15357	405	-7	-22	-24	2	0.9	0.8	0.8	1.0
RARS	P54136	34	-13	-13	-24	1	0.9	0.9	0.8	1.0
ARID2	Q68CP9	1549	-2	-13	-24	1	1.0	0.9	0.8	1.0
PNPLA6	Q8IY17	409	-16	-15	-24	1	0.9	0.9	0.8	1.0
HERC4	Q5GLZ8	175	-1	-10	-24	0	1.0	0.9	0.8	1.0
SMU1	Q2TAY7	448	-5	-13	-24	0	1.0	0.9	0.8	1.0
TONSL	Q96HA7	283	-18	-24	-24	0	0.9	0.8	0.8	1.0
PARP4	Q9UKK3	1019	-6	-3	-24	-1	0.9	1.0	0.8	1.0
PHRF1	Q9P1Y6	658	-24	-25	-24	-1	0.8	0.8	0.8	1.0
EDC3	Q96F86	413	-18	-7	-24	-1	0.8	0.9	0.8	1.0
TRANK1	O15050	646	-13	-19	-24	-1	0.9	0.8	0.8	1.0
ECHS1	P30084	111	-15	-22	-24	-1	0.9	0.8	0.8	1.0
CLIC1	O00299	223	2	-5	-24	-2	1.0	1.0	0.8	1.0
ISCA2	Q86U28	79	-2	-25	-24	-2	1.0	0.8	0.8	1.0
CDC42EP3	Q9UKI2	145	-11	-17	-24	-3	0.9	0.9	0.8	1.0
NUP153	P49790	753	-11	-24	-24	-3	0.9	0.8	0.8	1.0
CTSS	P25774	126	-4	-17	-24	-3	1.0	0.9	0.8	1.0
Uncharacteriz	A0A0A6YYC8	144	-13	-27	-24	-4	0.9	0.8	0.8	1.0
SSFA2	P28290	823	-10	-16	-24	-4	0.9	0.9	0.8	1.0
ZBTB14	O43829	124	-12	-16	-24	-5	0.9	0.9	0.8	1.0
RNF213	Q63HN8	3766	-14	-17	-24	-5	0.9	0.9	0.8	1.0
ERH	P84090	28	-6	-22	-24	-5	0.9	0.8	0.8	1.0
CASP3	P42574	264	-15	-26	-24	-6	0.9	0.8	0.8	0.9
BRD7	Q9NPI1	574	1	-20	-24	-7	1.0	0.8	0.8	0.9
NSL1	Q96IY1	264	-27	-23	-24	-7	0.8	0.8	0.8	0.9
ERN1	O75460	109	-33	-39	-24	-7	0.8	0.7	0.8	0.9
ESYT1	Q9BSJ8	611	-15	-46	-24	-7	0.9	0.7	0.8	0.9
AP5B1	Q2VPB7	732	-25	-10	-24	-8	0.8	0.9	0.8	0.9
ARID2	Q68CP9	1271	-23	-39	-24	-8	0.8	0.7	0.8	0.9
BCL10	O95999	57	-12	-23	-24	-8	0.9	0.8	0.8	0.9
CUL4A	Q13619	633	-5	-28	-24	-8	1.0	0.8	0.8	0.9
CUL4B	Q13620	787	-5	-28	-24	-8	1.0	0.8	0.8	0.9
MTFR1	Q15390	49	-13	-13	-24	-9	0.9	0.9	0.8	0.9
BIRC6	Q9NR09	1420	-10	-17	-24	-9	0.9	0.9	0.8	0.9
MGEA5	O60502	663	-6	-11	-24	-9	0.9	0.9	0.8	0.9
KIAA1033	Q2M389	604	-12	-15	-24	-9	0.9	0.9	0.8	0.9
PRPF4	O43172	263	-18	-27	-24	-10	0.8	0.8	0.8	0.9
TLR1	Q15399	530	-26	-36	-24	-10	0.8	0.7	0.8	0.9
PSMG2	Q969U7	178	-15	-9	-24	-10	0.9	0.9	0.8	0.9
BRCC3	P46736	240	4	-21	-24	-10	1.0	0.8	0.8	0.9
POLG	P54098	1077	-8	-16	-24	-11	0.9	0.9	0.8	0.9
PRKAB1	Q9Y478	223	-7	-20	-24	-11	0.9	0.8	0.8	0.9
NSMCE2	Q96MF7	215	-11	-7	-24	-12	0.9	0.9	0.8	0.9
ZBP1	Q9H171	327	-19	-26	-24	-12	0.8	0.8	0.8	0.9
DAXX	Q9UER7	720	-3	-52	-24	-12	1.0	0.7	0.8	0.9
MAP2K5	Q13163	300	-20	-15	-24	-13	0.8	0.9	0.8	0.9
GSDMD	P57764	191	-1	-34	-24	-13	1.0	0.7	0.8	0.9
WDR47	O94967	322	7	-22	-24	-14	1.1	0.8	0.8	0.9
TMF1	P82094	448	-16	-25	-24	-14	0.9	0.8	0.8	0.9
BSDC1	Q9NW68	151	-27	-33	-24	-14	0.8	0.8	0.8	0.9
NTPCR	Q9BSD7	110	-1	-12	-24	-15	1.0	0.9	0.8	0.9
BCL9	O00512	64	-6	-19	-24	-15	0.9	0.8	0.8	0.9

DCTN1	Q14203	1252	-16	-18	-24	-16	0.9	0.8	0.8	0.9
ZMYM1	Q5SVZ6	438	-9	4	-24	-17	0.9	1.0	0.8	0.9
HUWE1	Q7Z6Z7	4341	-6	-31	-24	-17	0.9	0.8	0.8	0.9
XRN2	Q9H0D6	21	-13	-34	-24	-19	0.9	0.7	0.8	0.8
DUS3L	Q96G46	124	4	-25	-24	-20	1.0	0.8	0.8	0.8
RPS23	P62266	90	-12	-19	-24	-22	0.9	0.8	0.8	0.8
RUFY1	Q96T51	544	-29	-19	-24	-26	0.8	0.8	0.8	0.8
SMG1	Q96Q15	3587	-19	-21	-24	-26	0.8	0.8	0.8	0.8
CRIPT	Q9P021	76	-11	-43	-24	-28	0.9	0.7	0.8	0.8
SDE2	Q6IQ49	298	8	-14	-24	-31	1.1	0.9	0.8	0.8
APAF1	O14727	538	14	-32	-24	-47	1.2	0.8	0.8	0.7
CORO1A	P31146	285	17	-34	-24	-49	1.2	0.7	0.8	0.7
RAP1B	P61224	118	-8	-16	-24	-51	0.9	0.9	0.8	0.7
MMRN1	Q13201	765	-18	-7	-24	-383	0.8	0.9	0.8	0.2
RRP1B	Q14684	155	-17	-24	-24	49	0.9	0.8	0.8	2.0
NIPBL	Q6KC79	304	-6	-21	-24	35	0.9	0.8	0.8	1.5
C15orf39	Q6ZRI6	426	-21	-7	-24	30	0.8	0.9	0.8	1.4
IMPDH2	P12268	140	-13	-15	-24	26	0.9	0.9	0.8	1.4
RASA2	Q15283	567	-12	-21	-24	23	0.9	0.8	0.8	1.3
MMTAG2	Q9BU76	58	-12	-20	-24	20	0.9	0.8	0.8	1.3
SIRT1	Q96EB6	574	-7	-18	-24	19	0.9	0.8	0.8	1.2
HSPBP1	Q9NZL4	204	-12	-20	-24	19	0.9	0.8	0.8	1.2
HELZ2	Q9BYK8	1545	-7	-10	-24	16	0.9	0.9	0.8	1.2
ACIN1	Q9UKV3	691	-10	-15	-24	16	0.9	0.9	0.8	1.2
AIM1	Q9Y4K1	503	-16	-22	-24	14	0.9	0.8	0.8	1.2
GORAB	Q5T7V8	156	-18	-16	-24	14	0.9	0.9	0.8	1.2
RIPK1	Q13546	325	-9	-18	-24	13	0.9	0.8	0.8	1.1
RAPGEF1	Q13905	328	-4	-15	-24	12	1.0	0.9	0.8	1.1
KIR2DL2	P43627	169	-13	-22	-24	12	0.9	0.8	0.8	1.1
MSRA	Q9UJ68	220	-33	-24	-24	12	0.8	0.8	0.8	1.1
CHEK2	O96017	385	-6	-11	-24	11	0.9	0.9	0.8	1.1
KIAA0753	Q2KHM9	53	-13	-17	-24	11	0.9	0.9	0.8	1.1
TACC3	Q9Y6A5	206	-2	-12	-24	10	1.0	0.9	0.8	1.1
RAD50	Q92878	1201	0	18	-24	7	1.0	1.2	0.8	1.1
CHEK2	O96017	231	-7	-13	-24	7	0.9	0.9	0.8	1.1
SURF6	O75683	19	-7	-25	-24	6	0.9	0.8	0.8	1.1
TOP2B	Q02880	754	-10	-10	-24	6	0.9	0.9	0.8	1.1
RSBN1L	Q6PCB5	280	-11	-31	-24	6	0.9	0.8	0.8	1.1
XPNPEP3	Q9NQH7	492	-20	-27	-24	5	0.8	0.8	0.8	1.1
SCAF11	Q99590	346	-9	-19	-24	4	0.9	0.8	0.8	1.0
PRRC2C	Q9Y520	177	-20	-23	-24	3	0.8	0.8	0.8	1.0
ARHGEF1	Q92888	752	-6	-12	-24	2	0.9	0.9	0.8	1.0
OGFR	Q9NZT2	443	-18	-19	-24	2	0.9	0.8	0.8	1.0
TMEM175	Q9BSA9	32	-11	-11	-24	1	0.9	0.9	0.8	1.0
ZC3H12D	A2A288	517	-22	-10	-24	1	0.8	0.9	0.8	1.0
UBR1	Q8IWV7	350	-19	-18	-24	1	0.8	0.8	0.8	1.0
HIST1H3J	P68431	97	-14	-7	-24	0	0.9	0.9	0.8	1.0
CBLB	Q13191	607	-15	-21	-24	0	0.9	0.8	0.8	1.0
HUWE1	Q7Z6Z7	1252	-1	-24	-24	-1	1.0	0.8	0.8	1.0
CEP97	Q8IW35	484	-22	-31	-24	-1	0.8	0.8	0.8	1.0
CREBRF	Q8IUR6	265	-8	-23	-24	-1	0.9	0.8	0.8	1.0
TACC1	O75410	467	-12	-16	-24	-3	0.9	0.9	0.8	1.0
LARP4B	Q92615	679	-7	-26	-24	-3	0.9	0.8	0.8	1.0
PARP8	Q8N3A8	367	-5	-16	-24	-4	1.0	0.9	0.8	1.0
RPS8	P62241	71	-8	-18	-24	-4	0.9	0.8	0.8	1.0

RAB11FIP1	Q6WKZ4	1007	-8	-30	-24	-5	0.9	0.8	0.8	1.0
MRPL43	Q8N983	70	-1	-16	-24	-6	1.0	0.9	0.8	0.9
HDAC3	O15379	104	8	-9	-24	-6	1.1	0.9	0.8	0.9
NUP210	Q8TEM1	76	-15	-16	-24	-6	0.9	0.9	0.8	0.9
ARHGAP4	P98171	855	-16	-20	-24	-7	0.9	0.8	0.8	0.9
NCOR1	O75376	2322	-6	-24	-24	-8	0.9	0.8	0.8	0.9
LPIN2	Q92539	827	-11	6	-24	-8	0.9	1.1	0.8	0.9
PSMC2	P35998	377	-18	-21	-24	-8	0.9	0.8	0.8	0.9
NDUFA10	E7ESZ7	253	-18	-12	-24	-10	0.8	0.9	0.8	0.9
MAP7D1	Q3KQU3	361	2	-30	-24	-10	1.0	0.8	0.8	0.9
EPC2	Q52LR7	543	-26	-35	-24	-10	0.8	0.7	0.8	0.9
STT3A	P46977	637	-7	-29	-24	-12	0.9	0.8	0.8	0.9
UPF1	Q92900	165	-9	-17	-24	-13	0.9	0.9	0.8	0.9
SYK	P43405	597	-1	4	-24	-13	1.0	1.0	0.8	0.9
GAR1	Q9NY12	88	-14	-19	-24	-13	0.9	0.8	0.8	0.9
ATM	Q13315	2286	-12	-23	-24	-13	0.9	0.8	0.8	0.9
KIF13B	Q9NQT8	651	-5	-11	-24	-14	1.0	0.9	0.8	0.9
DOCK2	Q92608	1203	-6	-15	-24	-14	0.9	0.9	0.8	0.9
DLGAP4	Q9Y2H0	736	-11	-26	-24	-14	0.9	0.8	0.8	0.9
ACIN1	Q9UKV3	733	-19	-27	-24	-14	0.8	0.8	0.8	0.9
APOBR	Q0VD83	129	-19	-27	-24	-15	0.8	0.8	0.8	0.9
AKAP13	Q12802	683	-16	-15	-24	-15	0.9	0.9	0.8	0.9
PMPCA	Q10713	140	-1	-23	-24	-16	1.0	0.8	0.8	0.9
NUS1	Q96E22	164	-3	5	-24	-16	1.0	1.0	0.8	0.9
NBAS	A2RRP1	1777	-14	-34	-24	-16	0.9	0.7	0.8	0.9
MAGOHB	Q96A72	133	-6	-4	-24	-17	0.9	1.0	0.8	0.9
AES	Q08117	47	-9	-12	-24	-17	0.9	0.9	0.8	0.9
ANKRD44	Q8N8A2	914	-9	-14	-24	-17	0.9	0.9	0.8	0.9
TTC27	Q6P3X3	749	-14	-23	-24	-18	0.9	0.8	0.8	0.8
PRKCQ	Q04759	14	-7	-16	-24	-26	0.9	0.9	0.8	0.8
TBXAS1	P24557	505	-15	-24	-24	-32	0.9	0.8	0.8	0.8
TELO2	Q9Y4R8	568	18	-23	-24	-58	1.2	0.8	0.8	0.6
ITGB2	P05107	559	-10	-23	-25	34	0.9	0.8	0.8	1.5
ACSL3	O95573	652	-7	-18	-25	29	0.9	0.8	0.8	1.4
SART3	Q15020	375	-28	-24	-25	27	0.8	0.8	0.8	1.4
CYLD	Q9NQC7	655	-6	-17	-25	23	0.9	0.9	0.8	1.3
SWAP70	Q9UH65	40	-13	-16	-25	22	0.9	0.9	0.8	1.3
AIP	O00170	121	-4	-15	-25	21	1.0	0.9	0.8	1.3
DENND1C	Q8IV53	349	-14	-8	-25	21	0.9	0.9	0.8	1.3
ZZEF1	O43149	2037	-7	-23	-25	17	0.9	0.8	0.8	1.2
PPP2R4	Q15257	91	-19	-18	-25	17	0.8	0.9	0.8	1.2
NFATC2	Q13469	231	-13	-18	-25	16	0.9	0.8	0.8	1.2
NDC1	Q9BTX1	468	-2	-13	-25	15	1.0	0.9	0.8	1.2
TSC22D3	Q99576	112	-21	-17	-25	15	0.8	0.9	0.8	1.2
RPS6KB2	Q9UBS0	348	-15	-18	-25	15	0.9	0.9	0.8	1.2
UPF3A	Q9H1J1	52	-12	-17	-25	14	0.9	0.9	0.8	1.2
N4BP1	O75113	454	-6	-8	-25	10	0.9	0.9	0.8	1.1
BIRC6	Q9NR09	1236	-23	-44	-25	9	0.8	0.7	0.8	1.1
NOL7	Q9UMY1	149	-11	-14	-25	8	0.9	0.9	0.8	1.1
DDX55	Q8NHQ9	437	-15	-19	-25	8	0.9	0.8	0.8	1.1
LARP4B	Q92615	590	-14	-16	-25	6	0.9	0.9	0.8	1.1
WDR43	Q15061	372	-12	-10	-25	6	0.9	0.9	0.8	1.1
CHUK	O15111	30	-16	-18	-25	6	0.9	0.9	0.8	1.1
CAST	P20810	408	-11	-23	-25	5	0.9	0.8	0.8	1.1
CLPX	O76031	538	-16	-13	-25	5	0.9	0.9	0.8	1.0

ATM	Q13315	489	5	-9	-25	4	1.1	0.9	0.8	1.0
MTF1	Q14872	638	-12	-9	-25	4	0.9	0.9	0.8	1.0
PPIB	P23284	202	-7	-15	-25	4	0.9	0.9	0.8	1.0
EPRS	P07814	1148	-18	-15	-25	3	0.8	0.9	0.8	1.0
SENP1	Q9P0U3	222	-20	-18	-25	2	0.8	0.8	0.8	1.0
HIVEP2	P31629	641	-2	-12	-25	2	1.0	0.9	0.8	1.0
RHOC	P08134	83	-29	-34	-25	2	0.8	0.7	0.8	1.0
RHOA	P61586	83	-29	-34	-25	2	0.8	0.7	0.8	1.0
ARHGAP4	P98171	330	-14	-43	-25	1	0.9	0.7	0.8	1.0
SYNE2	Q8WXH0	2993	-9	-4	-25	1	0.9	1.0	0.8	1.0
GLYR1	Q49A26	242	-2	-9	-25	1	1.0	0.9	0.8	1.0
ITGAX	P20702	639	-15	-34	-25	0	0.9	0.7	0.8	1.0
Uncharacteriz	G3V3G9	328	-5	-8	-25	-1	1.0	0.9	0.8	1.0
TRIO	O75962	1207	-3	-18	-25	-1	1.0	0.8	0.8	1.0
PSMD10	O75832	180	-13	-19	-25	-1	0.9	0.8	0.8	1.0
ETFB	P38117	71	-5	-20	-25	-1	1.0	0.8	0.8	1.0
MAP4	P27816	635	-12	-18	-25	-1	0.9	0.9	0.8	1.0
RCC1	P18754	93	-11	-17	-25	-2	0.9	0.9	0.8	1.0
ITGA4	P13612	183	-15	-27	-25	-2	0.9	0.8	0.8	1.0
GEMIN2	O14893	63	-6	-15	-25	-3	0.9	0.9	0.8	1.0
ERCC4	Q92889	176	-28	-19	-25	-4	0.8	0.8	0.8	1.0
GSTM2	P28161	174	-15	-13	-25	-4	0.9	0.9	0.8	1.0
LYSMD3	Q7Z3D4	122	-11	-18	-25	-5	0.9	0.8	0.8	1.0
TUBB2B	Q9BVA1	127	-10	-25	-25	-6	0.9	0.8	0.8	0.9
TKT	P29401	133	-13	-15	-25	-6	0.9	0.9	0.8	0.9
EML4	Q9HC35	311	10	-21	-25	-6	1.1	0.8	0.8	0.9
SCP2	P22307	495	-6	-26	-25	-6	0.9	0.8	0.8	0.9
CNTRL	Q7Z7A1	1375	-14	-25	-25	-7	0.9	0.8	0.8	0.9
PHRF1	Q9P1Y6	878	-9	-17	-25	-7	0.9	0.9	0.8	0.9
MTHFSD	Q2M296	267	-12	-25	-25	-7	0.9	0.8	0.8	0.9
TIAM1	Q13009	1006	-17	-21	-25	-8	0.9	0.8	0.8	0.9
TEP1	Q99973	111	-9	-9	-25	-8	0.9	0.9	0.8	0.9
NRDC	O43847	60	-13	-25	-25	-8	0.9	0.8	0.8	0.9
PRKDC	P78527	4106	-14	-5	-25	-9	0.9	1.0	0.8	0.9
TBC1D1	Q86TI0	676	-13	-17	-25	-9	0.9	0.9	0.8	0.9
AKAP9	Q99996	2777	-10	-28	-25	-9	0.9	0.8	0.8	0.9
DDX51	Q8N8A6	338	-19	-34	-25	-9	0.8	0.7	0.8	0.9
MKRN1	Q9UHC7	186	-8	-10	-25	-10	0.9	0.9	0.8	0.9
SENP1	Q9P0U3	164	-10	-1	-25	-11	0.9	1.0	0.8	0.9
BCL10	O95999	119	-19	-2	-25	-12	0.8	1.0	0.8	0.9
EPG5	Q9HCE0	430	-4	-19	-25	-13	1.0	0.8	0.8	0.9
EIF1B	O60739	94	-12	-25	-25	-14	0.9	0.8	0.8	0.9
TRIM25	Q14258	70	-18	3	-25	-15	0.8	1.0	0.8	0.9
PPM1F	P49593	315	-9	-11	-25	-16	0.9	0.9	0.8	0.9
CORO7-PAM1	A0A0A6YYL4	187	-2	-13	-25	-16	1.0	0.9	0.8	0.9
RRAGC	Q9HB90	358	8	-17	-25	-17	1.1	0.9	0.8	0.9
REPS2	Q8NFH8	523	-10	-30	-25	-18	0.9	0.8	0.8	0.9
FUS	P35637	444	-10	-10	-25	-19	0.9	0.9	0.8	0.8
CHD9	Q3L8U1	1257	-16	-24	-25	-20	0.9	0.8	0.8	0.8
CHD8	Q9HCK8	1208	-16	-24	-25	-20	0.9	0.8	0.8	0.8
SLC25A46	Q96AG3	239	-24	-10	-25	-21	0.8	0.9	0.8	0.8
NCOA3	Q9Y6Q9	22	-15	-21	-25	-21	0.9	0.8	0.8	0.8
PPP6R3	Q5H9R7	605	-16	-31	-25	-23	0.9	0.8	0.8	0.8
MYO9B	Q13459	234	-35	-7	-25	-34	0.7	0.9	0.8	0.7
THBS1	P07996	318	1	-16	-25	-46	1.0	0.9	0.8	0.7

EMILIN1	Q9Y6C2	353	-10	-13	-25	-47	0.9	0.9	0.8	0.7
LTBP1	Q14766	702	6	-17	-25	-354	1.1	0.9	0.8	0.2
RRP12	Q5JTH9	125	-13	-19	-25	23	0.9	0.8	0.8	1.3
UTP6	Q9NYH9	370	-7	-19	-25	22	0.9	0.8	0.8	1.3
BACH2	Q9BYV9	420	-6	-30	-25	22	0.9	0.8	0.8	1.3
MBNL1	Q9NR56	34	-2	-17	-25	16	1.0	0.9	0.8	1.2
PNMA3	Q9UL41	130	-20	-9	-25	16	0.8	0.9	0.8	1.2
NEK4	P51957	664	-22	-13	-25	15	0.8	0.9	0.8	1.2
MECP2	P51608	429	-15	-23	-25	14	0.9	0.8	0.8	1.2
PKNOX1	P55347	326	-2	-15	-25	13	1.0	0.9	0.8	1.1
SRRM2	Q9UQ35	2116	-6	-16	-25	11	0.9	0.9	0.8	1.1
ZCCHC2	Q9C0B9	305	-22	-30	-25	11	0.8	0.8	0.8	1.1
ZMYM2	Q9UBW7	1331	-13	-22	-25	11	0.9	0.8	0.8	1.1
DVL3	Q92997	344	-4	-45	-25	10	1.0	0.7	0.8	1.1
ZNF587	Q96SQ5	366	-12	-23	-25	9	0.9	0.8	0.8	1.1
SSH2	Q76I76	687	-3	-21	-25	9	1.0	0.8	0.8	1.1
LUZP1	Q86V48	594	-13	-25	-25	9	0.9	0.8	0.8	1.1
WAC	Q9BTA9	553	-5	-38	-25	8	1.0	0.7	0.8	1.1
RPS6KA2	Q15349	429	2	-12	-25	7	1.0	0.9	0.8	1.1
KIF1B	O60333	1216	-9	-13	-25	7	0.9	0.9	0.8	1.1
DDX60	Q8IY21	1051	-6	-16	-25	5	0.9	0.9	0.8	1.1
MARS2	Q96GW9	425	-40	-29	-25	5	0.7	0.8	0.8	1.0
ZEB2	O60315	140	-18	-13	-25	4	0.9	0.9	0.8	1.0
AHR	P35869	300	-7	-25	-25	4	0.9	0.8	0.8	1.0
DHX36	Q9H2U1	234	-10	-7	-25	3	0.9	0.9	0.8	1.0
DOK1	Q99704	389	-9	-19	-25	3	0.9	0.8	0.8	1.0
TRIM38	O00635	335	-17	-24	-25	3	0.9	0.8	0.8	1.0
RAD50	Q92878	1296	-5	-18	-25	3	1.0	0.8	0.8	1.0
TCP1	P17987	236	-13	-12	-25	2	0.9	0.9	0.8	1.0
NSUN2	Q08J23	271	-9	-14	-25	2	0.9	0.9	0.8	1.0
SLC31A1	O15431	189	-19	-25	-25	2	0.8	0.8	0.8	1.0
SMU1	Q2TAY7	298	-11	-13	-25	0	0.9	0.9	0.8	1.0
MAST2	Q6P0Q8	96	-18	-22	-25	0	0.9	0.8	0.8	1.0
PYCRL	Q53H96	266	-16	-21	-25	-1	0.9	0.8	0.8	1.0
CHPT1	Q8WUD6	386	-12	-27	-25	-1	0.9	0.8	0.8	1.0
SYNE1	Q8NF91	8617	-1	-13	-25	-2	1.0	0.9	0.8	1.0
FXR2	P51116	270	-9	-21	-25	-3	0.9	0.8	0.8	1.0
TIAM1	Q13009	1061	-4	-3	-25	-4	1.0	1.0	0.8	1.0
SEC16A	O15027	1633	-14	-19	-25	-4	0.9	0.8	0.8	1.0
RNF213	Q63HN8	3330	-3	-19	-25	-4	1.0	0.8	0.8	1.0
KMT2D	O14686	369	-12	-23	-25	-5	0.9	0.8	0.8	1.0
ANKRD17	O75179	2063	-11	-7	-25	-6	0.9	0.9	0.8	0.9
ARHGAP31	Q2M1Z3	509	-12	-15	-25	-6	0.9	0.9	0.8	0.9
WDR91	A4D1P6	366	-13	-20	-25	-6	0.9	0.8	0.8	0.9
EEF2	P13639	591	-14	-20	-25	-7	0.9	0.8	0.8	0.9
JAK1	P23458	988	-17	-16	-25	-7	0.9	0.9	0.8	0.9
CPSF1	Q10570	324	-8	-19	-25	-8	0.9	0.8	0.8	0.9
BACH1	O14867	581	-13	-22	-25	-8	0.9	0.8	0.8	0.9
PACS2	Q86VP3	519	-8	-6	-25	-9	0.9	0.9	0.8	0.9
AFF4	Q9UHB7	315	-1	-20	-25	-9	1.0	0.8	0.8	0.9
INHBA	P08476	322	-20	-31	-25	-9	0.8	0.8	0.8	0.9
INHBA	P08476	321	-20	-31	-25	-9	0.8	0.8	0.8	0.9
VPS18	Q9P253	433	3	-7	-25	-9	1.0	0.9	0.8	0.9
PARK7	Q99497	46	-12	-17	-25	-11	0.9	0.9	0.8	0.9
UHRF1BP1L	A0JNW5	476	-12	-22	-25	-11	0.9	0.8	0.8	0.9

BOD1L1	Q8NFC6	1729	-4	-24	-25	-11	1.0	0.8	0.8	0.9
HLA-F	A0A0G2JPA3	185	-22	-9	-25	-11	0.8	0.9	0.8	0.9
RAI1	Q7Z5J4	8	-15	-19	-25	-12	0.9	0.8	0.8	0.9
LARP4B	Q92615	654	-9	-21	-25	-12	0.9	0.8	0.8	0.9
ANKRD17	O75179	58	-22	-24	-25	-12	0.8	0.8	0.8	0.9
CDK17	Q00537	233	-9	-23	-25	-14	0.9	0.8	0.8	0.9
KDM6B	O15054	1602	-27	-5	-25	-14	0.8	1.0	0.8	0.9
PRKDC	P78527	2435	-5	-26	-25	-14	1.0	0.8	0.8	0.9
UBE2O	Q9C0C9	406	-10	-18	-25	-15	0.9	0.9	0.8	0.9
VPS39	Q96JC1	487	-1	-24	-25	-15	1.0	0.8	0.8	0.9
EIF4ENIF1	Q9NRA8	125	6	-48	-25	-15	1.1	0.7	0.8	0.9
POC5	Q8NA72	208	-2	-12	-25	-16	1.0	0.9	0.8	0.9
ATAD2B	Q9ULI0	360	-14	-40	-25	-17	0.9	0.7	0.8	0.9
RNF213	Q63HN8	4111	10	-16	-25	-18	1.1	0.9	0.8	0.8
XPO7	Q9UIA9	244	-17	-23	-25	-18	0.9	0.8	0.8	0.8
GSPT1	P15170	464	-5	-6	-25	-19	1.0	0.9	0.8	0.8
GSPT2	Q8IYD1	593	-5	-6	-25	-19	1.0	0.9	0.8	0.8
PGM1	P36871	101	-14	-20	-25	-20	0.9	0.8	0.8	0.8
PCYT1A	P49585	346	-23	-8	-25	-20	0.8	0.9	0.8	0.8
SIPA1L1	O43166	327	-24	-28	-25	-22	0.8	0.8	0.8	0.8
ANKRD44	Q8N8A2	704	-11	-20	-25	-23	0.9	0.8	0.8	0.8
GTF2B	Q00403	219	-10	-16	-25	-27	0.9	0.9	0.8	0.8
AP2B1	P63010	857	-10	-42	-25	-27	0.9	0.7	0.8	0.8
KATNB1	Q9BVA0	250	-5	-26	-25	-33	1.0	0.8	0.8	0.8
NLRX1	Q86UT6	155	-4	-18	-25	-36	1.0	0.8	0.8	0.7
POLR2C	P19387	236	-14	-18	-25	-151	0.9	0.8	0.8	0.4
RECQL5	O94762	664	-5	-1	-26	25	1.0	1.0	0.8	1.3
PLEKHG3	A1L390	359	5	-16	-26	22	1.1	0.9	0.8	1.3
NUP93	Q8N1F7	522	-11	-20	-26	18	0.9	0.8	0.8	1.2
ITM2B	Q9Y287	38	-3	-16	-26	17	1.0	0.9	0.8	1.2
TRAPPC12	Q8WVT3	160	-13	-16	-26	17	0.9	0.9	0.8	1.2
MFSD5	Q6N075	33	-21	-9	-26	16	0.8	0.9	0.8	1.2
MARK3	P27448	213	-21	-28	-26	16	0.8	0.8	0.8	1.2
MARK2	Q7KZI7	210	-21	-28	-26	16	0.8	0.8	0.8	1.2
MARK4	Q96L34	216	-21	-28	-26	16	0.8	0.8	0.8	1.2
HDLBP	Q00341	683	-12	-7	-26	15	0.9	0.9	0.8	1.2
TXNDC17	Q9BRA2	43	-22	-28	-26	14	0.8	0.8	0.8	1.2
C17orf75	Q9HAS0	182	-20	-8	-26	13	0.8	0.9	0.8	1.1
PML	P29590	484	-13	-27	-26	12	0.9	0.8	0.8	1.1
UBE2O	Q9C0C9	913	-4	-32	-26	11	1.0	0.8	0.8	1.1
PBRM1	Q86U86	1059	-8	4	-26	11	0.9	1.0	0.8	1.1
ATP11A	P98196	502	-2	-8	-26	11	1.0	0.9	0.8	1.1
SMARCC1	Q92922	845	12	-12	-26	11	1.1	0.9	0.8	1.1
SNRNP200	O75643	742	-8	-4	-26	10	0.9	1.0	0.8	1.1
TRAF1	Q13077	38	-9	-14	-26	10	0.9	0.9	0.8	1.1
REL	Q04864	524	-13	-22	-26	9	0.9	0.8	0.8	1.1
RNMT	O43148	95	-18	-37	-26	9	0.8	0.7	0.8	1.1
ZNF654	Q8IZM8	380	-21	-12	-26	7	0.8	0.9	0.8	1.1
PCNT	O95613	2014	-9	-24	-26	7	0.9	0.8	0.8	1.1
ASPSCR1	Q9BZE9	224	-13	-28	-26	7	0.9	0.8	0.8	1.1
DDX42	Q86XP3	333	-14	-21	-26	7	0.9	0.8	0.8	1.1
PANK4	Q9NVE7	150	-5	-14	-26	5	1.0	0.9	0.8	1.0
RSBN1	Q5VWQ0	736	-13	-27	-26	5	0.9	0.8	0.8	1.0
NR3C1	P04150	302	-9	-19	-26	4	0.9	0.8	0.8	1.0
KCTD20	Q7Z5Y7	369	-2	-17	-26	3	1.0	0.9	0.8	1.0

SNX27	Q96L92	434	10	-1	-26	2	1.1	1.0	0.8	1.0
PSMA7	O14818	70	-8	-16	-26	2	0.9	0.9	0.8	1.0
CCDC102A	Q96A19	402	-14	-18	-26	2	0.9	0.8	0.8	1.0
TACC3	Q9Y6A5	236	-18	-17	-26	2	0.9	0.9	0.8	1.0
HOOK2	Q96ED9	317	-23	-18	-26	2	0.8	0.9	0.8	1.0
GCA	P28676	76	-20	-11	-26	1	0.8	0.9	0.8	1.0
WDFY1	Q8IWB7	189	-2	-22	-26	1	1.0	0.8	0.8	1.0
STAT1	P42224	255	-7	-33	-26	1	0.9	0.8	0.8	1.0
MCEE	Q96PE7	166	-2	-21	-26	0	1.0	0.8	0.8	1.0
LANCL1	O43813	264	-25	-14	-26	-1	0.8	0.9	0.8	1.0
JCHAIN	P01591	131	-28	-23	-26	-1	0.8	0.8	0.8	1.0
AIM1	Q9Y4K1	976	-3	-23	-26	-1	1.0	0.8	0.8	1.0
GNE	Q9Y223	183	3	-14	-26	-2	1.0	0.9	0.8	1.0
NUP188	Q5SRE5	1433	3	-18	-26	-2	1.0	0.8	0.8	1.0
GHDC	Q8N2G8	82	-6	-50	-26	-2	0.9	0.7	0.8	1.0
AKAP8	O43823	631	4	-9	-26	-3	1.0	0.9	0.8	1.0
PPP6R3	Q5H9R7	836	-17	-30	-26	-3	0.9	0.8	0.8	1.0
PFKFB3	Q16875	412	-18	-14	-26	-3	0.8	0.9	0.8	1.0
PFKFB4	Q16877	415	-18	-14	-26	-3	0.8	0.9	0.8	1.0
ARHGAP9	Q9BRR9	666	1	-2	-26	-5	1.0	1.0	0.8	1.0
HEXIM2	Q96MH2	246	-18	-33	-26	-6	0.8	0.8	0.8	0.9
LUZP1	Q86V48	138	-35	-37	-26	-6	0.7	0.7	0.8	0.9
CTSG	P08311	142	-11	-20	-26	-6	0.9	0.8	0.8	0.9
TRRAP	Q9Y4A5	2876	-16	-24	-26	-6	0.9	0.8	0.8	0.9
PRG2	P13727	107	-8	-30	-26	-6	0.9	0.8	0.8	0.9
TLK1	Q9UKI8	600	-2	-9	-26	-7	1.0	0.9	0.8	0.9
POLD2	P49005	398	-26	-14	-26	-7	0.8	0.9	0.8	0.9
TP53BP1	Q12888	1926	-7	-19	-26	-7	0.9	0.8	0.8	0.9
CBL	P22681	372	12	6	-26	-7	1.1	1.1	0.8	0.9
CBLB	Q13191	364	12	6	-26	-7	1.1	1.1	0.8	0.9
SETX	Q7Z333	133	-3	2	-26	-8	1.0	1.0	0.8	0.9
ZNF638	Q14966	652	-13	-26	-26	-8	0.9	0.8	0.8	0.9
ARHGAP26	Q9UNA1	82	-1	-6	-26	-9	1.0	0.9	0.8	0.9
SPECC1	Q5M775	578	-20	-22	-26	-10	0.8	0.8	0.8	0.9
PDCD7	Q8N8D1	159	8	-30	-26	-10	1.1	0.8	0.8	0.9
TMPO	P42166	330	-10	-23	-26	-11	0.9	0.8	0.8	0.9
VAV1	P15498	31	-10	-18	-26	-12	0.9	0.9	0.8	0.9
EEA1	Q15075	704	-18	-33	-26	-12	0.8	0.8	0.8	0.9
PRKCQ	Q04759	322	-6	-15	-26	-14	0.9	0.9	0.8	0.9
BACH1	O14867	184	-22	-24	-26	-14	0.8	0.8	0.8	0.9
CDC34	P49427	191	-18	-30	-26	-14	0.8	0.8	0.8	0.9
GNPDA2	Q8TDQ7	239	-17	-20	-26	-15	0.9	0.8	0.8	0.9
DDX52	Q9Y2R4	59	-11	-58	-26	-15	0.9	0.6	0.8	0.9
TARS	P26639	254	-23	-17	-26	-15	0.8	0.9	0.8	0.9
CDK6	Q00534	83	-10	-17	-26	-16	0.9	0.9	0.8	0.9
PSMD2	Q13200	448	-19	-26	-26	-16	0.8	0.8	0.8	0.9
NCKAP1L	P55160	617	-21	-14	-26	-17	0.8	0.9	0.8	0.9
NADK	O95544	402	-7	-27	-26	-17	0.9	0.8	0.8	0.9
RFC1	P35251	916	-8	-26	-26	-17	0.9	0.8	0.8	0.9
MED29	Q9NX70	131	-7	-27	-26	-19	0.9	0.8	0.8	0.8
R3HCC1	Q9Y3T6	134	-17	-24	-26	-20	0.9	0.8	0.8	0.8
TMEM209	Q96SK2	422	-3	-7	-26	-22	1.0	0.9	0.8	0.8
TACC1	O75410	587	-16	-30	-26	-23	0.9	0.8	0.8	0.8
NADK	O95544	69	-15	-72	-26	-25	0.9	0.6	0.8	0.8
DPF2	Q92785	295	-13	-30	-26	-31	0.9	0.8	0.8	0.8

MTMR12	Q9C0I1	67	-15	-21	-26	-41	0.9	0.8	0.8	0.7
POLR2B	P30876	357	4	-5	-26	-44	1.0	1.0	0.8	0.7
ZFP36	P26651	249	-15	-12	-26	-59	0.9	0.9	0.8	0.6
BCCIP	Q9P287	216	-8	-10	-26	-71	0.9	0.9	0.8	0.6
LIMS1	P48059	303	-8	-18	-26	-83	0.9	0.9	0.8	0.5
LIMS2	Q7Z4I7	308	-8	-18	-26	-83	0.9	0.9	0.8	0.5
CDKN1A	P38936	13	-25	-33	-26	-116	0.8	0.8	0.8	0.5
APOL3	O95236	399	9	-23	-26	-144	1.1	0.8	0.8	0.4
MMRN1	Q13201	238	-1	-15	-26	-163	1.0	0.9	0.8	0.4
CTSD	P07339	329	-18	-35	-26	29	0.9	0.7	0.8	1.4
TMX4	Q9H1E5	326	-6	15	-26	26	0.9	1.2	0.8	1.4
CDA	P32320	59	-3	-14	-26	22	1.0	0.9	0.8	1.3
CYP1B1	Q16678	470	-16	-24	-26	21	0.9	0.8	0.8	1.3
ALDH3A2	P51648	241	-16	-42	-26	21	0.9	0.7	0.8	1.3
SMU1	Q2TAY7	312	2	-24	-26	20	1.0	0.8	0.8	1.2
TRRAP	Q9Y4A5	3535	-8	-22	-26	19	0.9	0.8	0.8	1.2
PEX5	P50542	11	-5	-14	-26	16	1.0	0.9	0.8	1.2
PCNT	O95613	860	-13	-12	-26	15	0.9	0.9	0.8	1.2
ZNF609	O15014	1120	-5	4	-26	14	1.0	1.0	0.8	1.2
CEP350	Q5VT06	211	-10	-8	-26	14	0.9	0.9	0.8	1.2
ZFYVE28	Q9HCC9	49	-11	-13	-26	13	0.9	0.9	0.8	1.1
PTPRE	P23469	287	-12	-21	-26	12	0.9	0.8	0.8	1.1
PYHIN1	Q6K0P9	361	-11	-11	-26	11	0.9	0.9	0.8	1.1
SPG20	Q8N0X7	499	-17	-13	-26	9	0.9	0.9	0.8	1.1
NMT2	O60551	104	-19	-28	-26	8	0.8	0.8	0.8	1.1
GZMA	P12544	105	-11	-27	-26	8	0.9	0.8	0.8	1.1
KEAP1	Q14145	257	-3	-30	-26	7	1.0	0.8	0.8	1.1
TLDC1	Q6P9B6	13	-9	-8	-26	7	0.9	0.9	0.8	1.1
ZNF101	Q8IZC7	352	-3	-14	-26	6	1.0	0.9	0.8	1.1
PIK3R5	Q8WYR1	813	-14	-9	-26	6	0.9	0.9	0.8	1.1
MORC3	Q14149	797	-11	-15	-26	5	0.9	0.9	0.8	1.1
UNC93B1	Q9H1C4	583	-18	-26	-26	5	0.8	0.8	0.8	1.1
ACACA	Q13085	1297	2	-11	-26	5	1.0	0.9	0.8	1.0
AKAP9	Q99996	1417	-7	-20	-26	5	0.9	0.8	0.8	1.0
PARN	O95453	543	-2	-20	-26	4	1.0	0.8	0.8	1.0
WDR81	Q562E7	207	-21	-25	-26	3	0.8	0.8	0.8	1.0
BCL7C	Q8WUZ0	211	-10	-28	-26	2	0.9	0.8	0.8	1.0
OGDH	Q02218	283	7	12	-26	1	1.1	1.1	0.8	1.0
EPC1	Q9H2F5	135	24	0	-26	1	1.3	1.0	0.8	1.0
HK2	P52789	813	-2	-17	-26	1	1.0	0.9	0.8	1.0
MEPCE	Q7L2J0	153	-7	-4	-26	1	0.9	1.0	0.8	1.0
NFX1	Q12986	92	-17	-11	-26	1	0.9	0.9	0.8	1.0
CTBP2	P56545	18	-14	-17	-26	1	0.9	0.9	0.8	1.0
RARS2	Q5T160	74	8	-18	-26	0	1.1	0.9	0.8	1.0
RPA1	P27694	200	1	-6	-26	-1	1.0	0.9	0.8	1.0
ANAPC2	Q9UJX6	224	-18	-32	-26	-1	0.8	0.8	0.8	1.0
DNAJC1	Q96KC8	380	-2	-28	-26	-2	1.0	0.8	0.8	1.0
DOPEY1	Q5JWR5	1158	-24	-30	-26	-2	0.8	0.8	0.8	1.0
TRMT2B	Q96GJ1	451	-3	-19	-26	-3	1.0	0.8	0.8	1.0
DDX47	Q9H0S4	226	-1	-18	-26	-3	1.0	0.8	0.8	1.0
IKBKE	Q14164	626	-20	-12	-26	-4	0.8	0.9	0.8	1.0
RBX1	P62877	94	-14	-29	-26	-4	0.9	0.8	0.8	1.0
UTP20	O75691	1757	-13	-22	-26	-5	0.9	0.8	0.8	1.0
DEK	P35659	161	-9	-35	-26	-5	0.9	0.7	0.8	1.0
DCAF13	Q9NV06	215	-15	-39	-26	-6	0.9	0.7	0.8	0.9

CSK	P41240	122	-6	-18	-26	-9	0.9	0.9	0.8	0.9
TACC1	O75410	114	-10	-22	-26	-10	0.9	0.8	0.8	0.9
PAPOLA	P51003	677	-2	-14	-26	-11	1.0	0.9	0.8	0.9
RSF1	Q96T23	404	-11	-27	-26	-12	0.9	0.8	0.8	0.9
ITGA4	P13612	198	-11	-23	-26	-16	0.9	0.8	0.8	0.9
ADGRE1	Q14246	322	-34	-32	-26	-17	0.7	0.8	0.8	0.9
EIF4G3	O43432	1411	-12	-20	-26	-19	0.9	0.8	0.8	0.8
VWA5A	O00534	624	-17	-9	-26	-20	0.9	0.9	0.8	0.8
USP4	Q13107	649	3	-44	-26	-20	1.0	0.7	0.8	0.8
EAF1	Q96JC9	86	-18	-34	-26	-22	0.8	0.7	0.8	0.8
PSMG2	Q969U7	168	-19	-21	-26	-24	0.8	0.8	0.8	0.8
STXBP5	Q5T5C0	697	-17	-19	-26	-28	0.9	0.8	0.8	0.8
NUP98	P52948	1711	19	-35	-26	-44	1.2	0.7	0.8	0.7
IRAK2	O43187	244	-50	-25	-26	-60	0.7	0.8	0.8	0.6
SAYSD1	Q9NPB0	159	-52	-47	-26	-106	0.7	0.7	0.8	0.5
MYLK	Q15746	1307	-5	-23	-26	-184	1.0	0.8	0.8	0.4
HERC4	Q5GLZ8	60	29	-28	-26	-193	1.4	0.8	0.8	0.3
TAPBP	O15533	440	1	-15	-27	44	1.0	0.9	0.8	1.8
POLE	Q07864	1612	-3	-23	-27	24	1.0	0.8	0.8	1.3
ZNF609	O15014	1338	-8	-35	-27	22	0.9	0.7	0.8	1.3
PRPF6	O94906	807	2	-2	-27	21	1.0	1.0	0.8	1.3
XRCC5	P13010	235	-7	-21	-27	20	0.9	0.8	0.8	1.2
FRY	Q5TBA9	2719	-17	-9	-27	19	0.9	0.9	0.8	1.2
KIAA1551	Q9HCM1	798	-15	-11	-27	16	0.9	0.9	0.8	1.2
RIN1	Q13671	223	-14	-16	-27	14	0.9	0.9	0.8	1.2
NLRC4	Q9NPP4	517	-6	-20	-27	13	0.9	0.8	0.8	1.1
STAT5A	P42229	126	-21	-19	-27	12	0.8	0.8	0.8	1.1
PASK	Q96RG2	128	-3	-10	-27	12	1.0	0.9	0.8	1.1
MTERF4	Q7Z6M4	76	-15	-10	-27	11	0.9	0.9	0.8	1.1
MKLN1	Q9UL63	435	-18	-11	-27	11	0.9	0.9	0.8	1.1
RPS6	P62753	100	-20	-27	-27	11	0.8	0.8	0.8	1.1
FAM98B	Q52LJ0	93	-11	-26	-27	9	0.9	0.8	0.8	1.1
BIRC6	Q9NR09	2123	-7	-12	-27	8	0.9	0.9	0.8	1.1
LANCL1	O43813	133	-32	-27	-27	8	0.8	0.8	0.8	1.1
PSAT1	Q9Y617	224	-2	-19	-27	6	1.0	0.8	0.8	1.1
PGK1	P00558	99	-2	-18	-27	6	1.0	0.8	0.8	1.1
GMDS	O60547	237	-8	-16	-27	5	0.9	0.9	0.8	1.1
PARP14	Q460N5	1530	-15	-16	-27	5	0.9	0.9	0.8	1.0
OSBPL11	Q9BXB4	353	-7	-6	-27	4	0.9	0.9	0.8	1.0
PGK1	P00558	108	-1	-12	-27	3	1.0	0.9	0.8	1.0
RNH1	P13489	199	-12	-14	-27	3	0.9	0.9	0.8	1.0
CAND1	Q86VP6	1134	-10	-18	-27	2	0.9	0.9	0.8	1.0
NIPBL	Q6KC79	279	-13	-21	-27	1	0.9	0.8	0.8	1.0
IMPA2	O14732	90	-1	-26	-27	1	1.0	0.8	0.8	1.0
LRWD1	Q9UFC0	449	-18	-11	-27	-1	0.9	0.9	0.8	1.0
SUGP2	Q8IX01	970	-11	-15	-27	-1	0.9	0.9	0.8	1.0
SP100	P23497	248	-12	-29	-27	-2	0.9	0.8	0.8	1.0
LENG9	Q96B70	316	-28	-30	-27	-2	0.8	0.8	0.8	1.0
SPAST	Q9UBP0	220	-26	-6	-27	-4	0.8	0.9	0.8	1.0
CHM	P24386	580	-15	-14	-27	-4	0.9	0.9	0.8	1.0
PCM1	Q15154	171	-16	-29	-27	-4	0.9	0.8	0.8	1.0
METTL16	Q86W50	276	-18	-30	-27	-4	0.8	0.8	0.8	1.0
RIPK3	Q9Y572	436	-17	-21	-27	-7	0.9	0.8	0.8	0.9
ZNF318	Q5VUA4	1860	-19	-29	-27	-7	0.8	0.8	0.8	0.9
TRAF1	Q13077	37	-15	-29	-27	-7	0.9	0.8	0.8	0.9

ATAD2	Q6PL18	1261	-26	-57	-27	-8	0.8	0.6	0.8	0.9
CENPC	Q03188	70	-22	-21	-27	-9	0.8	0.8	0.8	0.9
GLOD4	Q9HC38	197	-13	-42	-27	-9	0.9	0.7	0.8	0.9
THAP12	O43422	644	-23	-13	-27	-11	0.8	0.9	0.8	0.9
HEATR6	Q6AI08	360	-26	-28	-27	-11	0.8	0.8	0.8	0.9
FAM120B	Q96EK7	392	-13	-19	-27	-11	0.9	0.8	0.8	0.9
ELANE	P08246	187	-1	-23	-27	-15	1.0	0.8	0.8	0.9
ALDH1A1	P00352	303	-20	-15	-27	-18	0.8	0.9	0.8	0.8
KPNA4	O00629	57	-12	-21	-27	-18	0.9	0.8	0.8	0.8
SPTBN1	Q01082	1389	-5	-23	-27	-19	1.0	0.8	0.8	0.8
PIK3CB	P42338	765	-13	-6	-27	-22	0.9	0.9	0.8	0.8
SP100	P23497	373	-10	-19	-27	-24	0.9	0.8	0.8	0.8
PRRC2C	Q9Y520	2340	-15	-23	-27	-24	0.9	0.8	0.8	0.8
CEP152	O94986	1407	-20	-39	-27	-24	0.8	0.7	0.8	0.8
HECTD3	Q5T447	823	-14	-34	-27	-29	0.9	0.7	0.8	0.8
NT5C3A	Q9H0P0	103	3	-13	-27	-37	1.0	0.9	0.8	0.7
FLNB	O75369	604	-12	-34	-27	-37	0.9	0.7	0.8	0.7
BTG1	P62324	149	-2	-16	-27	-47	1.0	0.9	0.8	0.7
TMX1	Q9H3N1	106	-6	-19	-27	-48	0.9	0.8	0.8	0.7
GP5	P40197	35	3	-17	-27	-105	1.0	0.9	0.8	0.5
SESN3	P58005	411	-22	-33	-27	34	0.8	0.8	0.8	1.5
KLF2	Q9Y5W3	159	-16	-26	-27	30	0.9	0.8	0.8	1.4
DMXL2	Q8TDJ6	1788	-13	-17	-27	17	0.9	0.9	0.8	1.2
BANF1	O75531	77	-12	-3	-27	16	0.9	1.0	0.8	1.2
COG7	P83436	505	-17	-19	-27	16	0.9	0.8	0.8	1.2
NOP56	O00567	472	-3	-15	-27	14	1.0	0.9	0.8	1.2
SETX	Q7Z333	1229	-13	-22	-27	13	0.9	0.8	0.8	1.1
CENPI	Q92674	752	-19	-31	-27	13	0.8	0.8	0.8	1.1
ITGB2	P05107	445	1	-16	-27	12	1.0	0.9	0.8	1.1
PITPNM1	O00562	889	-17	-17	-27	12	0.9	0.9	0.8	1.1
TRIM56	Q9BRZ2	338	-19	-20	-27	10	0.8	0.8	0.8	1.1
PDCL	Q13371	81	-16	-30	-27	7	0.9	0.8	0.8	1.1
KAT6A	Q92794	393	-12	-24	-27	4	0.9	0.8	0.8	1.0
BTBD11	A6QL63	783	-11	-17	-27	3	0.9	0.9	0.8	1.0
EIF4G1	Q04637	1516	-7	-18	-27	3	0.9	0.8	0.8	1.0
PSAP	P07602	33	-6	-26	-27	3	0.9	0.8	0.8	1.0
SCML2	Q9UQR0	350	-11	-27	-27	3	0.9	0.8	0.8	1.0
IPO4	Q8TEX9	95	-3	-35	-27	0	1.0	0.7	0.8	1.0
KDM3A	Q9Y4C1	695	-8	-18	-27	-1	0.9	0.9	0.8	1.0
MAP3K11	Q16584	828	-12	-18	-27	-1	0.9	0.8	0.8	1.0
TOR1A	O14656	50	-26	-38	-27	-3	0.8	0.7	0.8	1.0
AFG3L2	Q9Y4W6	313	-5	-4	-27	-4	1.0	1.0	0.8	1.0
RCAN3	Q9UKA8	173	-13	-21	-27	-4	0.9	0.8	0.8	1.0
SP100	P23497	309	-6	-22	-27	-4	0.9	0.8	0.8	1.0
DOCK8	Q8NF50	522	6	-15	-27	-5	1.1	0.9	0.8	1.0
RSBN1	Q5VWQ0	318	-14	-31	-27	-5	0.9	0.8	0.8	1.0
ZNF761	A0A087WXT7	735	-23	-10	-27	-8	0.8	0.9	0.8	0.9
SAMSN1	Q9NSI8	165	-1	-18	-27	-8	1.0	0.9	0.8	0.9
CYTH4	Q9UIA0	329	-14	-19	-27	-9	0.9	0.8	0.8	0.9
BACH1	O14867	140	-20	-24	-27	-9	0.8	0.8	0.8	0.9
RAB14	P61106	40	-7	-19	-27	-9	0.9	0.8	0.8	0.9
EHBP1L1	Q8N3D4	422	-19	-24	-27	-10	0.8	0.8	0.8	0.9
TCP11L1	Q9NUJ3	495	-19	-23	-27	-11	0.8	0.8	0.8	0.9
PPCDC	Q96CD2	166	-17	-29	-27	-11	0.9	0.8	0.8	0.9
KAT6A	Q92794	723	-28	-21	-27	-11	0.8	0.8	0.8	0.9

TCEB2	Q15370	60	-12	-17	-27	-12	0.9	0.9	0.8	0.9
NIT2	Q9NQR4	44	-18	-21	-27	-12	0.8	0.8	0.8	0.9
ZNF512	Q96ME7	171	-16	-32	-27	-12	0.9	0.8	0.8	0.9
DEPDC5	O75140	1053	-16	-23	-27	-12	0.9	0.8	0.8	0.9
METTL7B	Q6UX53	96	-13	-27	-27	-13	0.9	0.8	0.8	0.9
KMT2E	Q8IZD2	839	-19	-24	-27	-14	0.8	0.8	0.8	0.9
TRPV2	Q9Y5S1	349	-11	-26	-27	-17	0.9	0.8	0.8	0.9
AOAH	P28039	249	-14	-31	-27	-21	0.9	0.8	0.8	0.8
NELFA	Q9H3P2	141	-19	-7	-27	-22	0.8	0.9	0.8	0.8
RCSD1	Q6JBY9	155	-11	-20	-27	-25	0.9	0.8	0.8	0.8
PDK4	Q16654	49	-19	-19	-27	-33	0.8	0.8	0.8	0.8
PXN	P49023	417	-15	-14	-27	-34	0.9	0.9	0.8	0.7
PXN	P49023	420	-15	-14	-27	-34	0.9	0.9	0.8	0.7
POLR2B	P30876	945	-13	-8	-27	-35	0.9	0.9	0.8	0.7
TST	Q16762	248	-1	-23	-27	-41	1.0	0.8	0.8	0.7
THBS1	P07996	270	-13	-17	-27	-43	0.9	0.9	0.8	0.7
TREML1	Q86YW5	275	-11	-24	-27	-120	0.9	0.8	0.8	0.5
BACH1	O14867	646	-13	-26	-28	58	0.9	0.8	0.8	2.4
SLC25A28	Q96A46	49	-19	-8	-28	36	0.8	0.9	0.8	1.6
PPP3CB	P16298	162	-9	-25	-28	23	0.9	0.8	0.8	1.3
PPP3CA	Q08209	153	-9	-25	-28	23	0.9	0.8	0.8	1.3
UTY	O14607	4	10	-36	-28	16	1.1	0.7	0.8	1.2
TRAF1	Q13077	57	-29	-14	-28	10	0.8	0.9	0.8	1.1
ACTR2	P61160	221	-10	-17	-28	10	0.9	0.9	0.8	1.1
CTSB	P07858	319	-12	-33	-28	7	0.9	0.8	0.8	1.1
CEP170	Q5SW79	235	-7	-20	-28	5	0.9	0.8	0.8	1.1
STAT3	P40763	712	-31	-45	-28	5	0.8	0.7	0.8	1.0
EOMES	O95936	640	-12	-19	-28	4	0.9	0.8	0.8	1.0
SNX5	Q9Y5X3	347	-4	-22	-28	4	1.0	0.8	0.8	1.0
UBE4B	O95155	581	-24	-22	-28	2	0.8	0.8	0.8	1.0
IDH1	O75874	269	-19	-18	-28	0	0.8	0.9	0.8	1.0
TRMT1L	Q7Z2T5	239	-13	-22	-28	-2	0.9	0.8	0.8	1.0
RANBP10	Q6VN20	501	-6	-16	-28	-3	0.9	0.9	0.8	1.0
FTSJ1	Q9UET6	278	-13	-30	-28	-3	0.9	0.8	0.8	1.0
R3HCC1L	Q7Z5L2	469	-10	-27	-28	-4	0.9	0.8	0.8	1.0
ZDHHC13	Q8IUH4	28	-17	-28	-28	-4	0.9	0.8	0.8	1.0
CEP44	Q9C0F1	28	-16	-16	-28	-5	0.9	0.9	0.8	1.0
ITSN2	Q9NZM3	1307	-7	-21	-28	-5	0.9	0.8	0.8	1.0
LIMD1	Q9UGP4	401	-20	-33	-28	-6	0.8	0.8	0.8	0.9
LRRK2	Q5S007	925	-29	-7	-28	-7	0.8	0.9	0.8	0.9
AES	Q08117	26	-12	-11	-28	-7	0.9	0.9	0.8	0.9
ESYT1	Q9BSJ8	604	-28	-12	-28	-7	0.8	0.9	0.8	0.9
UROS	P10746	264	-11	-15	-28	-7	0.9	0.9	0.8	0.9
ZFYVE16	Q7Z3T8	863	-11	-18	-28	-7	0.9	0.8	0.8	0.9
AKAP13	Q12802	2142	-20	-34	-28	-7	0.8	0.7	0.8	0.9
CHM	P24386	564	-9	-18	-28	-8	0.9	0.9	0.8	0.9
OSBPL2	Q9H1P3	402	-11	6	-28	-8	0.9	1.1	0.8	0.9
IRF8	Q02556	410	-14	-15	-28	-9	0.9	0.9	0.8	0.9
KIF15	Q9NS87	576	-13	-22	-28	-9	0.9	0.8	0.8	0.9
ZNF302	Q9NR11	274	-16	-30	-28	-9	0.9	0.8	0.8	0.9
LMNA	P02545	591	-14	-12	-28	-10	0.9	0.9	0.8	0.9
RNF213	Q63HN8	4937	-10	-13	-28	-10	0.9	0.9	0.8	0.9
AKAP11	Q9UKA4	216	-16	-26	-28	-16	0.9	0.8	0.8	0.9
TRAF5	O00463	98	-7	-20	-28	-17	0.9	0.8	0.8	0.9
CORO7-PAM1	A0A0A6YYL4	42	-3	-15	-28	-22	1.0	0.9	0.8	0.8

MTIF2	P46199	646	-13	-24	-28	-22	0.9	0.8	0.8	0.8
SLFN5	Q08AF3	303	-4	-26	-28	-27	1.0	0.8	0.8	0.8
SLFN12	Q8IYM2	312	-4	-26	-28	-27	1.0	0.8	0.8	0.8
PFKFB3	Q16875	193	-1	-17	-28	-29	1.0	0.9	0.8	0.8
GTPBP2	Q9BX10	457	-1	-3	-28	-34	1.0	1.0	0.8	0.7
SMARCAL1	Q9NZC9	388	-35	-13	-28	-156	0.7	0.9	0.8	0.4
DUSP6	Q16828	218	2	-1	-28	37	1.0	1.0	0.8	1.6
DGUOK	Q16854	87	11	-21	-28	33	1.1	0.8	0.8	1.5
VAC14	Q08AM6	516	-8	-10	-28	26	0.9	0.9	0.8	1.4
SLC27A1	Q6PCB7	394	12	10	-28	22	1.1	1.1	0.8	1.3
E2F4	Q16254	88	-11	-26	-28	21	0.9	0.8	0.8	1.3
USP24	Q9UPU5	2160	-7	-21	-28	13	0.9	0.8	0.8	1.1
DDB2	Q92466	66	-10	-26	-28	13	0.9	0.8	0.8	1.1
PRMT1	Q99873	109	-5	-19	-28	11	1.0	0.8	0.8	1.1
RGPD3	A6NKT7	220	1	-8	-28	11	1.0	0.9	0.8	1.1
RANBP2	P49792	220	1	-8	-28	11	1.0	0.9	0.8	1.1
NUP153	P49790	66	-13	-23	-28	8	0.9	0.8	0.8	1.1
SNRPE	P62304	46	3	-20	-28	6	1.0	0.8	0.8	1.1
NEDD9	Q14511	615	-1	-13	-28	5	1.0	0.9	0.8	1.1
ZNF324B	Q6AW86	198	-6	-38	-28	5	0.9	0.7	0.8	1.1
ELP3	Q9H9T3	456	-7	-8	-28	3	0.9	0.9	0.8	1.0
RABEP1	Q15276	718	-7	-10	-28	2	0.9	0.9	0.8	1.0
WDCP	Q9H6R7	472	0	-4	-28	1	1.0	1.0	0.8	1.0
KMT2E	Q8IZD2	723	-22	-15	-28	0	0.8	0.9	0.8	1.0
LARP1	Q6PKG0	1054	-12	-15	-28	-1	0.9	0.9	0.8	1.0
PEX19	P40855	128	-14	-21	-28	-4	0.9	0.8	0.8	1.0
ATG2A	Q2TAZ0	1458	-27	-23	-28	-4	0.8	0.8	0.8	1.0
SAMSN1	Q9NSI8	196	-6	-19	-28	-5	0.9	0.8	0.8	1.0
ME2	P23368	198	-14	-22	-28	-5	0.9	0.8	0.8	1.0
RAB12	Q6IQ22	68	-11	-11	-28	-6	0.9	0.9	0.8	0.9
UNG	P13051	290	-7	-28	-28	-6	0.9	0.8	0.8	0.9
RNF169	Q8NCN4	132	-17	-20	-28	-7	0.9	0.8	0.8	0.9
FAM102B	Q5T8I3	201	-15	-27	-28	-7	0.9	0.8	0.8	0.9
PIK3C2A	O00443	418	-19	-15	-28	-7	0.8	0.9	0.8	0.9
SECISBP2L	Q93073	544	-9	-27	-28	-8	0.9	0.8	0.8	0.9
CIAPIN1	Q6FI81	249	-18	-11	-28	-8	0.8	0.9	0.8	0.9
VPS28	Q9UK41	103	-23	-5	-28	-10	0.8	1.0	0.8	0.9
RPP40	O75818	49	-13	-15	-28	-10	0.9	0.9	0.8	0.9
RIF1	Q5UIP0	2169	-14	-20	-28	-15	0.9	0.8	0.8	0.9
APAF1	O14727	203	-2	-10	-28	-20	1.0	0.9	0.8	0.8
MT-CO2	P00403	196	-19	-37	-28	-21	0.8	0.7	0.8	0.8
ITGB2	P05107	472	-27	-41	-28	-21	0.8	0.7	0.8	0.8
TGFB1	P01137	285	-8	-22	-28	-26	0.9	0.8	0.8	0.8
PIAS4	Q8N2W9	50	-28	-42	-28	-28	0.8	0.7	0.8	0.8
COTL1	Q14019	52	-2	-18	-28	-29	1.0	0.9	0.8	0.8
C17orf59	Q96GS4	242	-13	-14	-28	-40	0.9	0.9	0.8	0.7
ATP2A3	Q93084	471	-25	-39	-28	-60	0.8	0.7	0.8	0.6
GTPBP2	Q9BX10	249	-7	-12	-28	-82	0.9	0.9	0.8	0.6
MMRN1	Q13201	245	-5	-8	-28	-164	1.0	0.9	0.8	0.4
MATK	P42679	266	-24	-27	-29	26	0.8	0.8	0.8	1.4
FAM168A	Q92567	75	-8	-10	-29	20	0.9	0.9	0.8	1.3
SEMA6B	Q9H3T3	116	-5	-22	-29	20	1.0	0.8	0.8	1.2
RSF1	Q96T23	1436	-7	-18	-29	17	0.9	0.8	0.8	1.2
DYNC1LI2	O43237	191	1	-7	-29	14	1.0	0.9	0.8	1.2
MCM3AP	O60318	1309	-5	-23	-29	13	1.0	0.8	0.8	1.1

VPS72	Q15906	163	-13	-14	-29	12	0.9	0.9	0.8	1.1
NR3C1	P04150	622	-16	-26	-29	9	0.9	0.8	0.8	1.1
ZKSCAN8	Q15776	289	-23	-23	-29	8	0.8	0.8	0.8	1.1
HK2	P52789	794	-10	-21	-29	7	0.9	0.8	0.8	1.1
PREX1	Q8TCU6	37	-10	-28	-29	7	0.9	0.8	0.8	1.1
GCOM1	H8Y6P7	512	-6	-38	-29	7	0.9	0.7	0.8	1.1
SH3TC1	Q8TE82	956	-13	-30	-29	6	0.9	0.8	0.8	1.1
PLEKHA5	Q9HAU0	891	2	-32	-29	6	1.0	0.8	0.8	1.1
ZEB2	O60315	539	-8	-12	-29	5	0.9	0.9	0.8	1.1
AP3D1	O14617	1103	-14	-17	-29	4	0.9	0.9	0.8	1.0
KIAA1551	Q9HCM1	962	-17	-19	-29	3	0.9	0.8	0.8	1.0
TDP1	Q9NUW8	48	-13	-22	-29	-1	0.9	0.8	0.8	1.0
ANKRD17	O75179	1260	-21	-21	-29	-1	0.8	0.8	0.8	1.0
ANKHD1	Q8IWZ3	1232	-21	-21	-29	-1	0.8	0.8	0.8	1.0
FAM98B	Q52LJ0	63	-20	-24	-29	-3	0.8	0.8	0.8	1.0
CNN2	Q99439	240	-7	-26	-29	-4	0.9	0.8	0.8	1.0
TBCB	Q99426	83	-14	-19	-29	-7	0.9	0.8	0.8	0.9
ARHGEF6	Q15052	763	-13	-20	-29	-7	0.9	0.8	0.8	0.9
TKT	P29401	206	-19	-15	-29	-8	0.8	0.9	0.8	0.9
PPP4C	P60510	130	-8	-18	-29	-8	0.9	0.9	0.8	0.9
PPP2CA	P67775	133	-8	-18	-29	-8	0.9	0.9	0.8	0.9
CYFIP2	Q96F07	346	2	-16	-29	-8	1.0	0.9	0.8	0.9
TRIM33	Q9UPN9	786	-13	-17	-29	-8	0.9	0.9	0.8	0.9
IKBKG	Q9Y6K9	347	-21	-18	-29	-9	0.8	0.8	0.8	0.9
MYCBP2	O75592	2913	-15	-13	-29	-9	0.9	0.9	0.8	0.9
IFIT5	Q13325	137	-3	-24	-29	-10	1.0	0.8	0.8	0.9
SPAG1	Q07617	552	-4	-6	-29	-11	1.0	0.9	0.8	0.9
GNAI2	P04899	66	-17	-33	-29	-11	0.9	0.8	0.8	0.9
CAMK2D	Q13557	428	-20	-15	-29	-12	0.8	0.9	0.8	0.9
CCDC117	Q8IWD4	81	-21	-16	-29	-15	0.8	0.9	0.8	0.9
USP9Y	O00507	148	-19	-31	-29	-16	0.8	0.8	0.8	0.9
USP9X	Q93008	147	-19	-31	-29	-16	0.8	0.8	0.8	0.9
SEC63	Q9UGP8	490	-17	-29	-29	-16	0.9	0.8	0.8	0.9
HUWE1	Q7Z6Z7	3259	-12	-38	-29	-17	0.9	0.7	0.8	0.9
GOLGB1	Q14789	2233	-27	-32	-29	-20	0.8	0.8	0.8	0.8
FAM208A	Q9UK61	1260	-21	-37	-29	-20	0.8	0.7	0.8	0.8
CNST	Q6PJW8	439	-15	-17	-29	-21	0.9	0.9	0.8	0.8
SLC26A2	P50443	594	-19	-29	-29	-23	0.8	0.8	0.8	0.8
RAB35	Q15286	110	-14	-16	-29	-28	0.9	0.9	0.8	0.8
USP24	Q9UPU5	153	3	-20	-29	-28	1.0	0.8	0.8	0.8
AGFG2	O95081	30	-16	-29	-29	-28	0.9	0.8	0.8	0.8
NPLOC4	Q8TAT6	208	-16	-16	-29	-30	0.9	0.9	0.8	0.8
WDR81	Q562E7	1653	-27	-34	-29	-35	0.8	0.7	0.8	0.7
FRMD4B	Q9Y2L6	929	-17	-3	-29	-100	0.9	1.0	0.8	0.5
MYO1F	O00160	577	33	-11	-29	58	1.5	0.9	0.8	2.4
COX17	Q14061	23	-20	-28	-29	36	0.8	0.8	0.8	1.6
ATG2A	Q2TAZ0	11	-20	-12	-29	25	0.8	0.9	0.8	1.3
ZNHIT2	Q9UHR6	57	-26	-19	-29	20	0.8	0.8	0.8	1.3
RASAL3	Q86YV0	1005	-6	-21	-29	20	0.9	0.8	0.8	1.2
GSK3A	P49840	170	1	-13	-29	19	1.0	0.9	0.8	1.2
GSK3B	P49841	107	1	-13	-29	19	1.0	0.9	0.8	1.2
TTC38	Q5R3I4	28	-10	-23	-29	17	0.9	0.8	0.8	1.2
NRDE2	Q9H7Z3	781	-21	-32	-29	14	0.8	0.8	0.8	1.2
NXF1	Q9UBU9	328	-6	-15	-29	14	0.9	0.9	0.8	1.2
ARHGEF40	Q8TER5	1282	-17	-20	-29	13	0.9	0.8	0.8	1.1

CFAP20	Q9Y6A4	160	-18	-30	-29	12	0.9	0.8	0.8	1.1
CAD	P27708	1636	-9	-19	-29	8	0.9	0.8	0.8	1.1
ZNF77	Q15935	522	-4	-19	-29	6	1.0	0.8	0.8	1.1
RTFDC1	Q9BY42	262	-13	-19	-29	6	0.9	0.8	0.8	1.1
GIMAP8	Q8ND71	75	-12	-23	-29	5	0.9	0.8	0.8	1.1
PHACTR2	O75167	400	-17	-13	-29	4	0.9	0.9	0.8	1.0
EPG5	Q9HCE0	1865	-28	-12	-29	3	0.8	0.9	0.8	1.0
SETX	Q7Z333	1277	-11	-15	-29	3	0.9	0.9	0.8	1.0
HLA-C	P04222	125	-10	-24	-29	2	0.9	0.8	0.8	1.0
HLA-B	P30479	125	-10	-24	-29	2	0.9	0.8	0.8	1.0
HLA-B	P30464	125	-10	-24	-29	2	0.9	0.8	0.8	1.0
SEPHS1	P49903	71	-13	-58	-29	1	0.9	0.6	0.8	1.0
KDM3A	Q9Y4C1	479	-7	-21	-29	0	0.9	0.8	0.8	1.0
CAPN2	P17655	39	-6	-6	-29	-1	0.9	0.9	0.8	1.0
PPP1R3D	O95685	93	-10	-33	-29	-1	0.9	0.8	0.8	1.0
TRIM65	Q6PJ69	112	-24	-3	-29	-2	0.8	1.0	0.8	1.0
UBE3C	Q15386	158	-16	-17	-29	-2	0.9	0.9	0.8	1.0
TGS1	Q96RS0	573	-11	-18	-29	-2	0.9	0.9	0.8	1.0
SPCS2	Q15005	26	-13	-29	-29	-2	0.9	0.8	0.8	1.0
CTSG	P08311	207	-10	-50	-29	-2	0.9	0.7	0.8	1.0
ERCC6L2	Q5T890	1514	-41	-18	-29	-2	0.7	0.9	0.8	1.0
ISCA2	Q86U28	146	6	-25	-29	-3	1.1	0.8	0.8	1.0
GTF3C2	Q8WUA4	828	0	-12	-29	-6	1.0	0.9	0.8	0.9
FRG1	Q14331	205	-12	-21	-29	-6	0.9	0.8	0.8	0.9
GOLGB1	Q14789	3144	-25	-24	-29	-6	0.8	0.8	0.8	0.9
GEMIN7	Q9H840	44	-15	-22	-29	-8	0.9	0.8	0.8	0.9
WDR41	Q9HAD4	99	-28	-26	-29	-8	0.8	0.8	0.8	0.9
RASGRP4	Q8TDF6	502	-23	-15	-29	-9	0.8	0.9	0.8	0.9
HDAC1	Q13547	408	-13	-27	-29	-9	0.9	0.8	0.8	0.9
CBL	P22681	572	-8	-25	-29	-10	0.9	0.8	0.8	0.9
USP24	Q9UPU5	1287	-28	-29	-29	-10	0.8	0.8	0.8	0.9
IRF2BP2	Q7Z5L9	530	-10	-4	-29	-11	0.9	1.0	0.8	0.9
IRF2BP1	Q8IU81	527	-10	-4	-29	-11	0.9	1.0	0.8	0.9
IRF2BPL	Q9H1B7	739	-10	-4	-29	-11	0.9	1.0	0.8	0.9
SPECC1	Q5M775	598	-4	-12	-29	-12	1.0	0.9	0.8	0.9
TBCCD1	Q9NVR7	298	-13	-25	-29	-13	0.9	0.8	0.8	0.9
USP10	Q14694	94	-18	-20	-29	-14	0.8	0.8	0.8	0.9
KIF1B	O60333	356	16	-2	-29	-14	1.2	1.0	0.8	0.9
PLEKHA1	Q9HB21	163	-11	-22	-29	-14	0.9	0.8	0.8	0.9
FLNB	O75369	450	-20	-24	-29	-15	0.8	0.8	0.8	0.9
JMJD6	Q6NYC1	101	-18	-17	-29	-17	0.8	0.9	0.8	0.9
SEC16A	O15027	1273	-24	-33	-29	-21	0.8	0.8	0.8	0.8
PFN1	P07737	128	-15	-22	-29	-22	0.9	0.8	0.8	0.8
PPP6R1	Q9UPN7	82	4	-5	-29	-26	1.0	1.0	0.8	0.8
USF1	P22415	248	-9	-18	-29	-28	0.9	0.8	0.8	0.8
AKAP13	Q12802	1666	-10	-34	-29	-29	0.9	0.7	0.8	0.8
LIMD1	Q9UGP4	521	-27	-22	-29	-34	0.8	0.8	0.8	0.7
EXOSC5	Q9NQT4	33	-17	-29	-29	-35	0.9	0.8	0.8	0.7
BACH1	O14867	557	-4	-22	-29	-36	1.0	0.8	0.8	0.7
RGS1	Q08116	131	-4	-17	-29	-78	1.0	0.9	0.8	0.6
G6B	O95866	53	0	-23	-29	-229	1.0	0.8	0.8	0.3
CCDC97	Q96F63	218	-10	-33	-30	82	0.9	0.8	0.8	5.4
QARS	P47897	456	-6	-4	-30	24	0.9	1.0	0.8	1.3
NUAK2	Q9H093	351	-20	-24	-30	20	0.8	0.8	0.8	1.3
ZNF618	Q5T7W0	619	-18	-28	-30	19	0.9	0.8	0.8	1.2

NEIL1	Q96FI4	136	-20	-27	-30	17	0.8	0.8	0.8	1.2
NAT10	Q9H0A0	499	-10	-9	-30	16	0.9	0.9	0.8	1.2
DLG5	Q8TDM6	1917	-6	-18	-30	12	0.9	0.9	0.8	1.1
FBXL4	Q9UKA2	547	-23	-2	-30	10	0.8	1.0	0.8	1.1
BBX	Q8WY36	265	-4	-16	-30	10	1.0	0.9	0.8	1.1
DUSP10	Q9Y6W6	218	2	-14	-30	9	1.0	0.9	0.8	1.1
ZNF8	P17098	94	-13	-33	-30	8	0.9	0.8	0.8	1.1
PIP4K2B	P78356	30	-13	-8	-30	6	0.9	0.9	0.8	1.1
BANF1	O75531	85	-12	-18	-30	5	0.9	0.8	0.8	1.0
PLIN3	O60664	39	-7	-28	-30	5	0.9	0.8	0.8	1.0
EP300	Q09472	1621	-13	-10	-30	3	0.9	0.9	0.8	1.0
KIF21B	O75037	697	-11	-20	-30	2	0.9	0.8	0.8	1.0
PPIL2	Q13356	15	-8	-20	-30	1	0.9	0.8	0.8	1.0
RNF214	Q8ND24	139	-15	-26	-30	1	0.9	0.8	0.8	1.0
ZCCHC6	Q5VYS8	343	-18	-5	-30	0	0.9	1.0	0.8	1.0
ELF2	Q15723	307	-17	-16	-30	0	0.9	0.9	0.8	1.0
AKAP1	Q92667	438	-15	-27	-30	-1	0.9	0.8	0.8	1.0
MAPK14	Q16539	162	-11	-17	-30	-1	0.9	0.9	0.8	1.0
PDS5B	Q9NTI5	1069	-6	-15	-30	-2	0.9	0.9	0.8	1.0
NUP210	Q8TEM1	767	-17	-22	-30	-2	0.9	0.8	0.8	1.0
CEP95	Q96GE4	351	-16	-9	-30	-4	0.9	0.9	0.8	1.0
DDX54	Q8TDD1	73	-21	-29	-30	-4	0.8	0.8	0.8	1.0
MGA	Q8IWI9	474	-19	-26	-30	-5	0.8	0.8	0.8	1.0
NOSIP	Q9Y314	8	-12	-21	-30	-7	0.9	0.8	0.8	0.9
FTSJ1	Q9UET6	295	-22	-26	-30	-8	0.8	0.8	0.8	0.9
WIPI2	Q9Y4P8	393	-11	-35	-30	-9	0.9	0.7	0.8	0.9
SETDB2	Q96T68	574	-11	-17	-30	-10	0.9	0.9	0.8	0.9
TRIM65	Q6PJ69	106	-12	-17	-30	-10	0.9	0.9	0.8	0.9
APIP	Q96GX9	11	-42	-10	-30	-12	0.7	0.9	0.8	0.9
C4orf27	Q9NWY4	29	-4	-19	-30	-12	1.0	0.8	0.8	0.9
PGAM1	P18669	55	-6	-27	-30	-12	0.9	0.8	0.8	0.9
LRBA	P50851	2655	0	-12	-30	-14	1.0	0.9	0.8	0.9
MON1B	Q7L1V2	87	-27	-43	-30	-17	0.8	0.7	0.8	0.9
FAM193B	Q96PV7	873	-16	-28	-30	-18	0.9	0.8	0.8	0.9
SASH1	O94885	406	-17	-25	-30	-24	0.9	0.8	0.8	0.8
NCAPG2	Q86XI2	776	-37	-20	-30	-33	0.7	0.8	0.8	0.8
AGPS	O00116	404	-10	-14	-30	-36	0.9	0.9	0.8	0.7
RPAP1	Q9BWH6	586	-8	-16	-30	-38	0.9	0.9	0.8	0.7
TREML1	Q86YW5	297	-14	-30	-30	-86	0.9	0.8	0.8	0.5
CASS4	Q9NQ75	26	-29	-22	-30	-109	0.8	0.8	0.8	0.5
GUCY1A3	Q02108	176	-9	-18	-30	-175	0.9	0.8	0.8	0.4
STOML1	Q9UBI4	32	-9	6	-30	27	0.9	1.1	0.8	1.4
KCTD20	Q7Z5Y7	82	-10	-12	-30	18	0.9	0.9	0.8	1.2
PSMD1	Q99460	703	-14	-11	-30	12	0.9	0.9	0.8	1.1
ATG2A	Q2TAZ0	1270	7	-14	-30	12	1.1	0.9	0.8	1.1
THAP4	Q8WY91	269	-13	-18	-30	12	0.9	0.8	0.8	1.1
ZFP62	Q8NB50	43	-20	-19	-30	11	0.8	0.8	0.8	1.1
ANKLE2	Q86XL3	254	-14	-28	-30	4	0.9	0.8	0.8	1.0
ZMYM2	Q9UBW7	734	-4	1	-30	3	1.0	1.0	0.8	1.0
NUP153	P49790	68	-24	-13	-30	3	0.8	0.9	0.8	1.0
SLFN5	Q08AF3	875	-11	-14	-30	2	0.9	0.9	0.8	1.0
INPP4A	Q96PE3	464	-10	-3	-30	-2	0.9	1.0	0.8	1.0
DCAF5	Q96JK2	503	-18	-32	-30	-3	0.8	0.8	0.8	1.0
S100PBP	Q96BU1	156	-18	-22	-30	-4	0.8	0.8	0.8	1.0
PGM2	Q96G03	86	-13	-17	-30	-6	0.9	0.9	0.8	0.9

DOCK5	Q9H7D0	627	-18	-17	-30	-6	0.8	0.9	0.8	0.9
NRBP2	Q9NSY0	285	-18	-18	-30	-7	0.8	0.9	0.8	0.9
IKBKB	O14920	99	3	-7	-30	-13	1.0	0.9	0.8	0.9
PBRM1	Q86U86	1233	-19	-19	-30	-13	0.8	0.8	0.8	0.9
EIF2S1	P05198	98	-19	-24	-30	-15	0.8	0.8	0.8	0.9
SLC25A3	Q00325	237	-14	-27	-30	-15	0.9	0.8	0.8	0.9
FABP5	Q01469	67	-15	-17	-30	-17	0.9	0.9	0.8	0.9
ATOX1	O00244	12	-10	-9	-30	-18	0.9	0.9	0.8	0.9
PYCR2	Q96C36	262	-18	-21	-30	-19	0.8	0.8	0.8	0.8
PPP2CA	P67775	269	-19	-14	-30	-22	0.8	0.9	0.8	0.8
MESDC2	Q14696	180	-19	-12	-30	-34	0.8	0.9	0.8	0.7
THBS1	P07996	813	-6	-22	-30	-74	0.9	0.8	0.8	0.6
PADI2	Q9Y2J8	629	-32	-18	-31	15	0.8	0.8	0.8	1.2
PABPC4	Q13310	472	12	-16	-31	14	1.1	0.9	0.8	1.2
ITGB2	P05107	483	-25	-11	-31	11	0.8	0.9	0.8	1.1
NAGK	Q9UJ70	268	-11	-26	-31	10	0.9	0.8	0.8	1.1
UTP18	Q9Y5J1	90	4	-9	-31	8	1.0	0.9	0.8	1.1
ALDH5A1	P51649	502	-17	-11	-31	8	0.9	0.9	0.8	1.1
MTMR3	Q13615	922	-17	-29	-31	6	0.9	0.8	0.8	1.1
SARS	P49591	398	-13	-24	-31	6	0.9	0.8	0.8	1.1
CENPC	Q03188	612	-9	-37	-31	2	0.9	0.7	0.8	1.0
AP3M1	Q9Y2T2	209	-14	6	-31	-1	0.9	1.1	0.8	1.0
MICU2	Q8IYU8	144	-16	-16	-31	-3	0.9	0.9	0.8	1.0
ITGB2	P05107	198	-12	-30	-31	-4	0.9	0.8	0.8	1.0
TRIM41	Q8WV44	180	-26	-15	-31	-6	0.8	0.9	0.8	0.9
QTRTD1	Q9H974	174	-8	-18	-31	-6	0.9	0.8	0.8	0.9
ITFG2	Q969R8	438	-12	-26	-31	-6	0.9	0.8	0.8	0.9
RIF1	Q5UIP0	1718	-10	-23	-31	-7	0.9	0.8	0.8	0.9
AKAP13	Q12802	709	-11	-16	-31	-8	0.9	0.9	0.8	0.9
SAAL1	Q96ER3	25	-26	-29	-31	-8	0.8	0.8	0.8	0.9
C4orf27	Q9NWY4	17	-16	-23	-31	-9	0.9	0.8	0.8	0.9
ZBTB40	Q9NUA8	119	-11	-18	-31	-10	0.9	0.9	0.8	0.9
PNLIP	P16233	20	-10	-24	-31	-13	0.9	0.8	0.8	0.9
PNLIP	P16233	26	-10	-24	-31	-13	0.9	0.8	0.8	0.9
VARS	P26640	479	-18	-30	-31	-13	0.9	0.8	0.8	0.9
PPP1R18	Q6NYC8	396	-20	-27	-31	-14	0.8	0.8	0.8	0.9
GOT2	P00505	106	-15	-39	-31	-15	0.9	0.7	0.8	0.9
RNF146	Q9NTX7	185	-22	-30	-31	-17	0.8	0.8	0.8	0.9
NFKB2	Q00653	432	-11	-18	-31	-17	0.9	0.8	0.8	0.9
CHKB	Q9Y259	28	-13	-12	-31	-19	0.9	0.9	0.8	0.8
MKI67	P46013	2206	-4	-17	-31	-20	1.0	0.9	0.8	0.8
CTSG	P08311	49	-11	-27	-31	-31	0.9	0.8	0.8	0.8
ISCA2	Q86U28	144	-26	-37	-31	-35	0.8	0.7	0.8	0.7
METTL6	Q8TCB7	256	-11	-5	-31	14	0.9	1.0	0.8	1.2
SMARCAD1	Q9H4L7	951	-10	-27	-31	14	0.9	0.8	0.8	1.2
ARPP21	Q9UBL0	302	-11	-9	-31	10	0.9	0.9	0.8	1.1
CUL1	Q13616	170	-3	-17	-31	10	1.0	0.9	0.8	1.1
NLRC3	Q7RTR2	936	-3	-9	-31	9	1.0	0.9	0.8	1.1
ITPR2	Q14571	579	6	-1	-31	7	1.1	1.0	0.8	1.1
PHF3	Q92576	1631	-14	-11	-31	7	0.9	0.9	0.8	1.1
TMEM33	P57088	224	-20	-26	-31	4	0.8	0.8	0.8	1.0
UTY	O14607	528	-3	-10	-31	2	1.0	0.9	0.8	1.0
ITGB2	P05107	191	-13	-26	-31	-1	0.9	0.8	0.8	1.0
SVIL	O95425	26	-27	-39	-31	-2	0.8	0.7	0.8	1.0
SP110	Q9HB58	327	-6	-24	-31	-3	0.9	0.8	0.8	1.0

DUS3L	Q96G46	282	-22	-22	-31	-4	0.8	0.8	0.8	1.0
SMYD5	Q6GMV2	136	-15	-27	-31	-4	0.9	0.8	0.8	1.0
MLLT3	P42568	320	-17	-28	-31	-5	0.9	0.8	0.8	1.0
RAB11FIP1	Q6WKZ4	1028	-14	-28	-31	-6	0.9	0.8	0.8	0.9
RPS6KB1	P23443	90	-12	-29	-31	-9	0.9	0.8	0.8	0.9
APBA2	Q99767	319	-12	-21	-31	-9	0.9	0.8	0.8	0.9
PRKD3	O94806	29	-11	-14	-31	-10	0.9	0.9	0.8	0.9
CCDC9	Q9Y3X0	233	-22	-21	-31	-10	0.8	0.8	0.8	0.9
MACF1	Q9UPN3	2994	-14	-13	-31	-12	0.9	0.9	0.8	0.9
FAM53B	Q14153	181	-20	-30	-31	-16	0.8	0.8	0.8	0.9
PPP2CA	P67775	50	-17	-10	-31	-18	0.9	0.9	0.8	0.9
NFATC2	Q13469	355	2	-23	-31	-19	1.0	0.8	0.8	0.8
SVIL	O95425	1101	-20	-19	-31	-21	0.8	0.8	0.8	0.8
AP4B1	Q9Y6B7	584	-13	-14	-31	-25	0.9	0.9	0.8	0.8
NR4A3	Q92570	420	-18	-30	-31	-40	0.9	0.8	0.8	0.7
NLRC5	Q86WI3	1190	-4	-2	-31	-45	1.0	1.0	0.8	0.7
CARMIL2	Q6F5E8	698	-12	-26	-31	-58	0.9	0.8	0.8	0.6
TLN1	Q9Y490	1434	-12	-11	-31	-138	0.9	0.9	0.8	0.4
PKHD1L1	Q86WI1	1236	-15	-30	-31	-161	0.9	0.8	0.8	0.4
NLRC5	Q86WI3	1219	-30	-24	-32	26	0.8	0.8	0.8	1.3
NCOA4	Q13772	368	-11	-20	-32	15	0.9	0.8	0.8	1.2
SUGP2	Q8IX01	947	-20	-23	-32	11	0.8	0.8	0.8	1.1
EXOSC7	Q15024	34	-7	-16	-32	8	0.9	0.9	0.8	1.1
TATDN1	Q6P1N9	150	1	-9	-32	8	1.0	0.9	0.8	1.1
URB2	Q14146	931	-13	-21	-32	5	0.9	0.8	0.8	1.0
JARID2	Q92833	206	-14	-16	-32	3	0.9	0.9	0.8	1.0
LGMN	Q99538	219	2	-29	-32	2	1.0	0.8	0.8	1.0
NCOA5	Q9HCD5	137	-12	-17	-32	-1	0.9	0.9	0.8	1.0
NUP153	P49790	681	-12	3	-32	-1	0.9	1.0	0.8	1.0
EP400	Q96L91	1761	-24	-5	-32	-4	0.8	1.0	0.8	1.0
POLA1	P09884	592	6	-24	-32	-7	1.1	0.8	0.8	0.9
DOCK2	Q92608	1408	-3	-9	-32	-8	1.0	0.9	0.8	0.9
ITGAM	P11215	638	-19	-35	-32	-9	0.8	0.7	0.8	0.9
ZNF318	Q5VUA4	475	-21	-34	-32	-11	0.8	0.7	0.8	0.9
REXO2	Q9Y3B8	137	-4	-28	-32	-12	1.0	0.8	0.8	0.9
THAP12	O43422	68	-14	-9	-32	-13	0.9	0.9	0.8	0.9
METTL7B	Q6UX53	203	-18	-28	-32	-13	0.9	0.8	0.8	0.9
INTS8	Q75QN2	385	3	-17	-32	-14	1.0	0.9	0.8	0.9
GCC2	Q8IWJ2	92	-22	-32	-32	-14	0.8	0.8	0.8	0.9
DAXX	Q9UER7	664	-15	-31	-32	-16	0.9	0.8	0.8	0.9
BOD1L1	Q8NFC6	1554	-23	-28	-32	-17	0.8	0.8	0.8	0.9
TBC1D2	Q9BYX2	419	-14	-13	-32	-17	0.9	0.9	0.8	0.9
HSPBP1	Q9NZL4	175	-21	-18	-32	-22	0.8	0.8	0.8	0.8
BACH1	O14867	683	-5	-23	-32	-41	1.0	0.8	0.8	0.7
ARHGEF6	Q15052	418	11	-11	-32	-44	1.1	0.9	0.8	0.7
SNRNP48	Q6IEG0	132	-9	-20	-32	-47	0.9	0.8	0.8	0.7
MRM2	Q9UI43	79	-10	-60	-32	-78	0.9	0.6	0.8	0.6
MACF1	Q9UPN3	2062	-11	-7	-32	15	0.9	0.9	0.8	1.2
ITGB2	P05107	470	-18	-38	-32	15	0.9	0.7	0.8	1.2
DIS3L	Q8TF46	482	-18	-26	-32	12	0.8	0.8	0.8	1.1
Uncharacteriz	E9PCH4	1611	-22	7	-32	7	0.8	1.1	0.8	1.1
APIP	Q96GX9	12	-10	-27	-32	6	0.9	0.8	0.8	1.1
GPATCH2	Q9NW75	59	-16	-39	-32	6	0.9	0.7	0.8	1.1
SYNE1	Q8NF91	8052	-8	-21	-32	4	0.9	0.8	0.8	1.0
AKAP13	Q12802	1394	-18	-28	-32	3	0.8	0.8	0.8	1.0

PPP2R2A	P63151	334	-16	-36	-32	3	0.9	0.7	0.8	1.0
PPP2R2D	Q66LE6	340	-16	-36	-32	3	0.9	0.7	0.8	1.0
EFL1	Q7Z2Z2	934	-14	-12	-32	2	0.9	0.9	0.8	1.0
BPTF	Q12830	1241	-6	-19	-32	1	0.9	0.8	0.8	1.0
SMC6	Q96SB8	854	-21	-41	-32	-3	0.8	0.7	0.8	1.0
ZNF710	Q8N1W2	91	-12	-14	-32	-4	0.9	0.9	0.8	1.0
DLG5	Q8TDM6	1736	-17	-38	-32	-8	0.9	0.7	0.8	0.9
ANKRD10	Q9NXR5	123	-14	-15	-32	-8	0.9	0.9	0.8	0.9
KDM3A	Q9Y4C1	337	-8	-18	-32	-8	0.9	0.9	0.8	0.9
ARHGAP30	Q7Z6I6	874	-15	-23	-32	-9	0.9	0.8	0.8	0.9
AFTPH	Q6ULP2	511	-17	-27	-32	-9	0.9	0.8	0.8	0.9
MPG	P29372	61	-31	-34	-32	-12	0.8	0.7	0.8	0.9
DIS3L2	Q8IYB7	196	-13	-24	-32	-13	0.9	0.8	0.8	0.9
SPCS2	Q15005	17	-19	-25	-32	-14	0.8	0.8	0.8	0.9
CD93	Q9NPY3	104	-23	-25	-32	-14	0.8	0.8	0.8	0.9
SUFU	Q9UMX1	292	-9	-28	-32	-16	0.9	0.8	0.8	0.9
TAP2	Q03519	362	-24	-33	-32	-16	0.8	0.8	0.8	0.9
EEF2KMT	Q96G04	72	-24	-13	-32	-17	0.8	0.9	0.8	0.9
SYNE1	Q8NF91	7341	-16	-39	-32	-17	0.9	0.7	0.8	0.9
SLFN5	Q08AF3	302	-13	-25	-32	-19	0.9	0.8	0.8	0.8
SLFN12	Q8IYM2	311	-13	-25	-32	-19	0.9	0.8	0.8	0.8
RIN3	Q8TB24	43	-18	-13	-32	-35	0.9	0.9	0.8	0.7
LPCAT1	Q8NF37	216	-17	-31	-32	-54	0.9	0.8	0.8	0.7
HELZ2	Q9BYK8	103	2	-7	-32	-125	1.0	0.9	0.8	0.4
HELZ2	Q9BYK8	91	2	-7	-32	-125	1.0	0.9	0.8	0.4
RUNX2	Q13950	350	-7	-2	-33	38	0.9	1.0	0.8	1.6
APOBR	Q0VD83	535	-45	-23	-33	9	0.7	0.8	0.8	1.1
BMS1	Q14692	556	-19	-26	-33	7	0.8	0.8	0.8	1.1
CNBP	P62633	158	-32	-39	-33	6	0.8	0.7	0.8	1.1
POLK	Q9UBT6	545	0	-24	-33	4	1.0	0.8	0.8	1.0
SARS	P49591	395	-12	-33	-33	3	0.9	0.8	0.8	1.0
SARS	P49591	438	-4	-25	-33	-1	1.0	0.8	0.8	1.0
HK2	P52789	368	-14	-20	-33	-1	0.9	0.8	0.8	1.0
SMU1	Q2TAY7	416	-18	-23	-33	-1	0.9	0.8	0.8	1.0
CALR	P27797	105	-7	-29	-33	-3	0.9	0.8	0.8	1.0
DNAJC11	Q9NVH1	518	-19	-34	-33	-3	0.8	0.7	0.8	1.0
MCF2L	O15068	223	-19	-31	-33	-6	0.8	0.8	0.8	0.9
GSK3B	P49841	14	-18	-22	-33	-6	0.9	0.8	0.8	0.9
ACOT2	P49753	75	-16	-22	-33	-8	0.9	0.8	0.8	0.9
ADAM10	O14672	435	-22	-33	-33	-9	0.8	0.8	0.8	0.9
LYSMD3	Q7Z3D4	174	-25	-17	-33	-13	0.8	0.9	0.8	0.9
NIFK	Q9BYG3	237	-15	-30	-33	-14	0.9	0.8	0.8	0.9
HELZ	P42694	1055	-28	-43	-33	-16	0.8	0.7	0.8	0.9
IFIT5	Q13325	429	-6	-17	-33	-16	0.9	0.9	0.8	0.9
ECHDC2	Q86YB7	180	-17	-29	-33	-17	0.9	0.8	0.8	0.9
FPGT	O14772	411	-25	-28	-33	-18	0.8	0.8	0.8	0.9
IFIT5	Q13325	142	-6	-30	-33	-18	0.9	0.8	0.8	0.8
GOLGB1	Q14789	681	-26	-32	-33	-22	0.8	0.8	0.8	0.8
ANKRD17	O75179	210	-19	-33	-33	-24	0.8	0.8	0.8	0.8
ANKHD1	Q8IWZ3	181	-19	-33	-33	-24	0.8	0.8	0.8	0.8
SLC7A6OS	Q96CW6	166	-28	-34	-33	-39	0.8	0.7	0.8	0.7
TBC1D2B	Q9UPU7	554	-13	-25	-33	-43	0.9	0.8	0.8	0.7
MARCKSL1	P49006	134	-15	-22	-33	22	0.9	0.8	0.8	1.3
UPP1	Q16831	162	-10	-28	-33	20	0.9	0.8	0.8	1.3
IFI16	Q16666	356	-5	-15	-33	5	1.0	0.9	0.8	1.1

LEMD2	Q8NC56	253	-9	-25	-33	4	0.9	0.8	0.8	1.0
ATXN7L3B	Q96GX2	75	-11	-7	-33	3	0.9	0.9	0.8	1.0
UBIAD1	Q9Y5Z9	31	-14	-29	-33	3	0.9	0.8	0.8	1.0
ESCO1	Q5FWF5	362	1	-9	-33	1	1.0	0.9	0.8	1.0
DENND1C	Q8IV53	594	-18	-34	-33	1	0.9	0.7	0.8	1.0
DNTTIP1	Q9H147	156	-13	-30	-33	-1	0.9	0.8	0.8	1.0
ZNF318	Q5VUA4	1681	-13	-23	-33	-1	0.9	0.8	0.8	1.0
RNMT	O43148	73	-13	-26	-33	-2	0.9	0.8	0.8	1.0
PSMD5	Q16401	290	-5	-3	-33	-2	1.0	1.0	0.8	1.0
CIAPIN1	Q6FI81	237	-15	-20	-33	-3	0.9	0.8	0.8	1.0
AIM1	Q9Y4K1	541	-25	-31	-33	-3	0.8	0.8	0.8	1.0
TAF7	Q15545	72	-15	-21	-33	-4	0.9	0.8	0.8	1.0
ADPGK	Q9BRR6	178	-4	-10	-33	-5	1.0	0.9	0.8	1.0
ZNF34	Q8IZ26	292	-25	-18	-33	-5	0.8	0.9	0.8	1.0
RMND5A	Q9H871	308	-17	-23	-33	-6	0.9	0.8	0.8	0.9
LEO1	Q8WVC0	530	-16	-31	-33	-6	0.9	0.8	0.8	0.9
ARHGAP30	Q7Z6I6	954	-20	-23	-33	-7	0.8	0.8	0.8	0.9
DOCK8	Q8NF50	939	-15	-20	-33	-8	0.9	0.8	0.8	0.9
LIN54	Q6MZP7	571	-10	-21	-33	-8	0.9	0.8	0.8	0.9
TP53BP1	Q12888	772	-13	-22	-33	-10	0.9	0.8	0.8	0.9
ZCCHC6	Q5VYS8	800	-27	-27	-33	-11	0.8	0.8	0.8	0.9
CD163	Q86VB7	309	-34	-40	-33	-12	0.7	0.7	0.8	0.9
PPP6R3	Q5H9R7	299	-25	-34	-33	-17	0.8	0.7	0.8	0.9
TCEAL4	Q96EI5	34	-19	-31	-33	-18	0.8	0.8	0.8	0.9
RALGAPA2	Q2PPJ7	775	-18	-24	-33	-19	0.9	0.8	0.8	0.8
CDK5	Q00535	269	-23	-16	-33	-20	0.8	0.9	0.8	0.8
CNOT1	A5YKK6	1932	-42	-35	-33	-20	0.7	0.7	0.8	0.8
JAK3	P52333	16	-22	-33	-33	-21	0.8	0.8	0.8	0.8
ARHGEF7	Q14155	560	3	-18	-33	-22	1.0	0.8	0.8	0.8
RIN3	Q8TB24	35	-7	-30	-33	-23	0.9	0.8	0.8	0.8
NKIRAS2	Q9NYR9	78	-20	-40	-33	-23	0.8	0.7	0.8	0.8
RNF113A	O15541	299	-28	-40	-33	-34	0.8	0.7	0.8	0.7
CTNND1	O60716	692	-17	-9	-33	-35	0.9	0.9	0.8	0.7
PUF60	Q9UHX1	487	-16	-8	-34	17	0.9	0.9	0.7	1.2
SSFA2	P28290	703	3	-7	-34	13	1.0	0.9	0.7	1.1
NCOA3	Q9Y6Q9	627	-7	-11	-34	12	0.9	0.9	0.7	1.1
CAD	P27708	1374	-5	-21	-34	12	1.0	0.8	0.7	1.1
GPATCH11	Q8N954	69	-16	-21	-34	4	0.9	0.8	0.7	1.0
AGTPBP1	Q9UPW5	1164	-18	-24	-34	4	0.9	0.8	0.7	1.0
PYGL	P06737	79	-13	-18	-34	3	0.9	0.8	0.7	1.0
ITPKB	P27987	493	-9	-22	-34	3	0.9	0.8	0.7	1.0
KIT	P10721	714	-5	-22	-34	2	1.0	0.8	0.7	1.0
ALAS1	P13196	124	-9	-22	-34	-2	0.9	0.8	0.7	1.0
ATM	Q13315	564	-11	-30	-34	-2	0.9	0.8	0.7	1.0
NADK	O95544	79	-13	-34	-34	-3	0.9	0.7	0.7	1.0
PPP6R1	Q9UPN7	795	-22	-31	-34	-5	0.8	0.8	0.7	1.0
CNN2	Q99439	274	-13	-37	-34	-5	0.9	0.7	0.7	1.0
RASGRP4	Q8TDF6	343	-24	-4	-34	-6	0.8	1.0	0.7	0.9
RARS2	Q5T160	11	-16	-15	-34	-6	0.9	0.9	0.7	0.9
UIMC1	Q96RL1	577	-15	-22	-34	-8	0.9	0.8	0.7	0.9
PREX1	Q8TCU6	1173	-15	-19	-34	-9	0.9	0.8	0.7	0.9
SQSTM1	Q13501	289	-22	-21	-34	-10	0.8	0.8	0.7	0.9
DICER1	Q9UPY3	1621	-22	-22	-34	-10	0.8	0.8	0.7	0.9
ARL8B	Q9NVJ2	164	-9	-29	-34	-11	0.9	0.8	0.7	0.9
VASP	P50552	334	-13	-17	-34	-15	0.9	0.9	0.7	0.9

ZNF8	P17098	427	-10	-33	-34	-16	0.9	0.8	0.7	0.9
SYNRG	Q9UMZ2	1020	-5	-40	-34	-16	1.0	0.7	0.7	0.9
NDRG3	Q9UGV2	359	-28	-13	-34	-17	0.8	0.9	0.7	0.9
DENND1C	Q8IV53	585	-13	-46	-34	-17	0.9	0.7	0.7	0.9
ALKBH4	Q9NXW9	204	-24	-36	-34	-18	0.8	0.7	0.7	0.9
ELMO1	Q92556	438	-5	-15	-34	-25	1.0	0.9	0.7	0.8
PTPN1	P18031	344	-7	-31	-34	-27	0.9	0.8	0.7	0.8
KDM6B	O15054	972	-20	-29	-34	-34	0.8	0.8	0.7	0.7
ITGA5	P08648	192	-2	-25	-34	22	1.0	0.8	0.7	1.3
CASP3	P42574	184	-2	-24	-34	10	1.0	0.8	0.7	1.1
PARP10	Q53GL7	123	-5	-1	-34	6	1.0	1.0	0.7	1.1
TXNRD1	Q16881	515	-2	-12	-34	1	1.0	0.9	0.7	1.0
GOT2	P00505	272	4	-25	-34	-4	1.0	0.8	0.7	1.0
NMT1	P30419	483	-18	-16	-34	-4	0.9	0.9	0.7	1.0
SEC16A	O15027	1515	-10	-25	-34	-6	0.9	0.8	0.7	0.9
C1orf122	Q6ZSJ8	61	-13	-15	-34	-7	0.9	0.9	0.7	0.9
PKDREJ	Q9NTG1	1119	-13	-25	-34	-8	0.9	0.8	0.7	0.9
PUM2	Q8TB72	983	-2	-20	-34	-10	1.0	0.8	0.7	0.9
PPM1B	O75688	209	-23	-27	-34	-10	0.8	0.8	0.7	0.9
KANK3	Q6NY19	19	-22	2	-34	-12	0.8	1.0	0.7	0.9
CEP170	Q5SW79	776	6	-13	-34	-16	1.1	0.9	0.7	0.9
GNPAT	O15228	668	-22	-26	-34	-16	0.8	0.8	0.7	0.9
ARID2	Q68CP9	1319	-13	-26	-34	-17	0.9	0.8	0.7	0.9
ALMS1	Q8TCU4	4005	-32	-32	-34	-18	0.8	0.8	0.7	0.8
FRAT1	Q92837	257	-19	-16	-34	-20	0.8	0.9	0.7	0.8
SAP30BP	Q9UHR5	127	-12	-38	-34	-25	0.9	0.7	0.7	0.8
TYMP	P19971	280	-16	-8	-34	-29	0.9	0.9	0.7	0.8
SEC16A	O15027	1648	-4	-29	-34	-30	1.0	0.8	0.7	0.8
NOL8	Q76FK4	556	-32	-11	-34	-31	0.8	0.9	0.7	0.8
NCOA3	Q9Y6Q9	31	-20	-40	-34	-31	0.8	0.7	0.7	0.8
PDE12	Q6L8Q7	42	-15	-28	-34	-33	0.9	0.8	0.7	0.8
FEM1B	Q9UK73	465	-25	-27	-35	18	0.8	0.8	0.7	1.2
POLK	Q9UBT6	8	-13	-21	-35	13	0.9	0.8	0.7	1.1
MAEA	Q7L5Y9	195	-16	-17	-35	5	0.9	0.9	0.7	1.1
EP400	Q96L91	879	-9	-38	-35	5	0.9	0.7	0.7	1.1
MED12	Q93074	83	-11	-34	-35	2	0.9	0.7	0.7	1.0
PLCG2	P16885	1082	-23	-19	-35	-7	0.8	0.8	0.7	0.9
PCK2	Q16822	230	-3	-17	-35	-10	1.0	0.9	0.7	0.9
CWF19L1	Q69YN2	87	-29	-29	-35	-11	0.8	0.8	0.7	0.9
SEH1L	Q96EE3	167	-22	-29	-35	-12	0.8	0.8	0.7	0.9
PHF3	Q92576	627	-19	-32	-35	-14	0.8	0.8	0.7	0.9
GMIP	Q9P107	958	-16	-30	-35	-15	0.9	0.8	0.7	0.9
RNPEP	Q9H4A4	254	-14	-21	-35	-16	0.9	0.8	0.7	0.9
CEP350	Q5VT06	1999	-17	-33	-35	-17	0.9	0.8	0.7	0.9
TYW3	Q6IPR3	71	-14	-31	-35	-19	0.9	0.8	0.7	0.8
UIMC1	Q96RL1	691	-19	-25	-35	-23	0.8	0.8	0.7	0.8
RNASEL	Q05823	727	-4	-32	-35	-23	1.0	0.8	0.7	0.8
RHOF	Q9HBH0	162	-11	-17	-35	-25	0.9	0.9	0.7	0.8
ARHGEF40	Q8TER5	1148	-28	-29	-35	-26	0.8	0.8	0.7	0.8
OPA1	O60313	853	-10	-17	-35	-30	0.9	0.9	0.7	0.8
FAM91A1	Q658Y4	191	-8	1	-35	7	0.9	1.0	0.7	1.1
HK1	P19367	368	-14	-41	-35	4	0.9	0.7	0.7	1.0
ACADVL	P49748	477	-22	-35	-35	3	0.8	0.7	0.7	1.0
OTULIN	Q96BN8	47	-14	-39	-35	1	0.9	0.7	0.7	1.0
FAM208A	Q9UK61	538	-21	-17	-35	-1	0.8	0.9	0.7	1.0

ZNF512B	Q96KM6	58	-14	-41	-35	-1	0.9	0.7	0.7	1.0
AKAP8	O43823	139	-15	-45	-35	-1	0.9	0.7	0.7	1.0
CDKN1B	P46527	29	-27	-26	-35	-2	0.8	0.8	0.7	1.0
DDX60L	Q5H9U9	823	-3	-31	-35	-2	1.0	0.8	0.7	1.0
DDX58	O95786	829	-5	-27	-35	-2	1.0	0.8	0.7	1.0
GATAD2B	Q8WXI9	308	-23	-5	-35	-3	0.8	1.0	0.7	1.0
ZNF407	Q9C0G0	1394	-17	-38	-35	-4	0.9	0.7	0.7	1.0
RANBP3	Q9H6Z4	249	-19	4	-35	-10	0.8	1.0	0.7	0.9
XPO4	Q9C0E2	54	-19	-21	-35	-10	0.8	0.8	0.7	0.9
RPS6KC1	Q96S38	733	-13	-27	-35	-12	0.9	0.8	0.7	0.9
UCKL1	Q9NWZ5	35	-18	-37	-35	-16	0.8	0.7	0.7	0.9
ETFB	P38117	42	3	-15	-35	-17	1.0	0.9	0.7	0.9
TGFB1	P01137	355	-34	-66	-35	-20	0.7	0.6	0.7	0.8
ZNF628	Q5EBL2	252	-8	-30	-35	-20	0.9	0.8	0.7	0.8
ZMYM4	Q5VZL5	1232	-34	-4	-35	-38	0.7	1.0	0.7	0.7
PLIN3	O60664	60	-24	-26	-35	-40	0.8	0.8	0.7	0.7
TXNRD1	Q16881	209	-13	-22	-35	-102	0.9	0.8	0.7	0.5
TXNRD2	Q9NNW7	86	-13	-22	-35	-102	0.9	0.8	0.7	0.5
USP16	Q9Y5T5	509	-8	-31	-36	8	0.9	0.8	0.7	1.1
IMPDH2	P12268	173	-14	-24	-36	7	0.9	0.8	0.7	1.1
SARS	P49591	300	-10	-28	-36	3	0.9	0.8	0.7	1.0
BANF1	O75531	67	-14	-22	-36	2	0.9	0.8	0.7	1.0
CTSW	P56202	352	-14	-28	-36	2	0.9	0.8	0.7	1.0
HECTD1	Q9ULT8	1389	-10	-28	-36	-2	0.9	0.8	0.7	1.0
ESD	P10768	243	-9	-42	-36	-2	0.9	0.7	0.7	1.0
CMSS1	Q9BQ75	55	-9	-32	-36	-4	0.9	0.8	0.7	1.0
BCR	P11274	395	-24	-25	-36	-5	0.8	0.8	0.7	1.0
ANKFY1	Q9P2R3	712	-1	-42	-36	-7	1.0	0.7	0.7	0.9
SMCR8	Q8TEV9	644	-5	-14	-36	-9	1.0	0.9	0.7	0.9
PLCB2	Q00722	1108	-35	-28	-36	-17	0.7	0.8	0.7	0.9
ITPK1	Q13572	391	-26	-54	-36	-21	0.8	0.6	0.7	0.8
KSR1	Q8IVT5	157	-32	-7	-36	-22	0.8	0.9	0.7	0.8
RNF31	Q96EP0	885	-36	-30	-36	-24	0.7	0.8	0.7	0.8
BCL6	P41182	296	-31	-21	-36	-27	0.8	0.8	0.7	0.8
DCUN1D1	Q96GG9	29	-23	-49	-36	-42	0.8	0.7	0.7	0.7
TJAP1	Q5JTD0	76	-20	-33	-36	-45	0.8	0.8	0.7	0.7
RCCD1	A6NED2	139	-25	-23	-36	15	0.8	0.8	0.7	1.2
ITGB2	P05107	497	-13	-32	-36	12	0.9	0.8	0.7	1.1
NAB2	Q15742	481	-25	-14	-36	8	0.8	0.9	0.7	1.1
SEPW1	P63302	37	-6	3	-36	5	0.9	1.0	0.7	1.1
DEK	P35659	89	-7	-19	-36	5	0.9	0.8	0.7	1.0
NUP54	Q7Z3B4	171	-17	-28	-36	2	0.9	0.8	0.7	1.0
GTF3C1	Q12789	1981	-16	-41	-36	-2	0.9	0.7	0.7	1.0
RNF213	Q63HN8	4737	-7	-40	-36	-6	0.9	0.7	0.7	0.9
TCF25	Q9BQ70	83	-16	-31	-36	-8	0.9	0.8	0.7	0.9
NPAT	Q14207	551	-23	-21	-36	-10	0.8	0.8	0.7	0.9
CYTIP	O60759	210	0	-19	-36	-11	1.0	0.8	0.7	0.9
CIAPIN1	Q6FI81	251	-24	-24	-36	-16	0.8	0.8	0.7	0.9
BACH2	Q9BYV9	340	-20	-27	-36	-17	0.8	0.8	0.7	0.9
NR3C1	P04150	287	-18	-27	-36	-18	0.8	0.8	0.7	0.9
ERO1A	Q96HE7	104	-15	-33	-36	-18	0.9	0.8	0.7	0.9
BOD1L1	Q8NFC6	1742	-18	-22	-36	-23	0.8	0.8	0.7	0.8
MAP3K7	O43318	527	-11	-34	-36	-33	0.9	0.7	0.7	0.8
TBC1D2B	Q9UPU7	387	-15	-16	-36	-41	0.9	0.9	0.7	0.7
ZYX	Q15942	467	-17	-27	-36	-43	0.9	0.8	0.7	0.7

PSME1	Q06323	106	-4	-1	-36	-123	1.0	1.0	0.7	0.4
VWF	P04275	1940	4	-29	-36	-193	1.0	0.8	0.7	0.3
MMRN1	Q13201	315	-2	-21	-36	-229	1.0	0.8	0.7	0.3
IKBKB	O14920	179	-21	-30	-37	36	0.8	0.8	0.7	1.6
ITGB2	P05107	541	-15	-31	-37	11	0.9	0.8	0.7	1.1
BRF1	Q92994	175	-16	-14	-37	10	0.9	0.9	0.7	1.1
IQSEC1	Q6DN90	260	-32	-26	-37	7	0.8	0.8	0.7	1.1
DDB1	Q16531	725	-13	-50	-37	-10	0.9	0.7	0.7	0.9
THOC5	Q13769	208	-9	-11	-37	-11	0.9	0.9	0.7	0.9
ATF7IP	Q6VMQ6	93	-18	-37	-37	-11	0.9	0.7	0.7	0.9
ZZEF1	O43149	369	-11	-16	-37	-13	0.9	0.9	0.7	0.9
CANX	P27824	160	-12	-45	-37	-17	0.9	0.7	0.7	0.9
C15orf38-AP3	A0A0A6YYH1	183	-32	-17	-37	-21	0.8	0.9	0.7	0.8
AGAP2	Q99490	579	-7	-23	-37	-21	0.9	0.8	0.7	0.8
FAM65B	Q9Y4F9	596	-15	-34	-37	-21	0.9	0.7	0.7	0.8
HLCS	P50747	289	-13	-25	-37	-25	0.9	0.8	0.7	0.8
FHL1	Q13642	224	-10	-13	-37	-94	0.9	0.9	0.7	0.5
RCC2	Q9P258	305	-6	-13	-37	13	0.9	0.9	0.7	1.1
HBP1	O60381	383	-4	-28	-37	13	1.0	0.8	0.7	1.1
RANBP3	Q9H6Z4	228	-12	4	-37	5	0.9	1.0	0.7	1.0
WDFY4	Q6ZS81	545	-18	-23	-37	4	0.8	0.8	0.7	1.0
MTHFR	P42898	14	-22	-40	-37	4	0.8	0.7	0.7	1.0
CDK5RAP1	Q96SZ6	183	-41	-32	-37	2	0.7	0.8	0.7	1.0
ASCC3	Q8N3C0	310	-22	-27	-37	-10	0.8	0.8	0.7	0.9
GEMIN5	Q8TEQ6	806	-13	-25	-37	-11	0.9	0.8	0.7	0.9
UBE2O	Q9C0C9	370	-21	-32	-37	-12	0.8	0.8	0.7	0.9
RPL10	P27635	195	-17	-28	-37	-21	0.9	0.8	0.7	0.8
ERO1A	Q96HE7	99	-16	-35	-37	-24	0.9	0.7	0.7	0.8
AKAP13	Q12802	2163	-13	-45	-37	-35	0.9	0.7	0.7	0.7
GPD2	P43304	270	-19	-25	-37	-45	0.8	0.8	0.7	0.7
CNBP	P62633	111	2	-39	-37	-46	1.0	0.7	0.7	0.7
CASS4	Q9NQ75	80	-7	-35	-37	-169	0.9	0.7	0.7	0.4
C10orf99	Q6UWK7	60	15	-19	-37	-1436	1.2	0.8	0.7	0.1
ENO1	P06733	119	1	-14	-38	8	1.0	0.9	0.7	1.1
OAS1	P00973	109	12	6	-38	-3	1.1	1.1	0.7	1.0
MKI67	P46013	3241	-2	-11	-38	-7	1.0	0.9	0.7	0.9
PCK2	Q16822	63	-31	-23	-38	-12	0.8	0.8	0.7	0.9
RRAGC	Q9HB90	377	-17	-55	-38	-24	0.9	0.6	0.7	0.8
AKAP13	Q12802	2101	-13	-31	-38	-26	0.9	0.8	0.7	0.8
IRGQ	Q8WZA9	152	9	-16	-38	21	1.1	0.9	0.7	1.3
PWWP2B	Q6NUJ5	410	1	-26	-38	8	1.0	0.8	0.7	1.1
PTEN	A0A0U1RR63	244	-8	-20	-38	-5	0.9	0.8	0.7	1.0
PCNT	O95613	2729	-25	-27	-38	-11	0.8	0.8	0.7	0.9
PEAK1	Q9H792	1335	-24	-25	-38	-19	0.8	0.8	0.7	0.8
PPP6R2	O75170	880	-18	-36	-38	-21	0.8	0.7	0.7	0.8
DLL1	O00548	204	-16	-38	-38	-33	0.9	0.7	0.7	0.8
LYPLA1	O75608	173	-15	-23	-39	11	0.9	0.8	0.7	1.1
JAK1	P23458	424	-15	-37	-39	-4	0.9	0.7	0.7	1.0
FAM120A	Q9NZB2	1103	-22	-29	-39	-9	0.8	0.8	0.7	0.9
DOLPP1	Q86YN1	86	-22	-29	-39	-10	0.8	0.8	0.7	0.9
TANK	Q92844	318	-20	-25	-39	-12	0.8	0.8	0.7	0.9
ERO1A	Q96HE7	131	-19	-35	-39	-27	0.8	0.7	0.7	0.8
UBE2F	Q969M7	116	-19	-25	-39	-33	0.8	0.8	0.7	0.8
RB1	P06400	853	-9	-9	-39	-36	0.9	0.9	0.7	0.7
FGL2	Q14314	194	-27	-55	-39	-36	0.8	0.6	0.7	0.7

SAMSN1	Q9NSI8	334	-16	-36	-39	-55	0.9	0.7	0.7	0.6
PDE3A	Q14432	315	-16	-25	-39	-167	0.9	0.8	0.7	0.4
UBOX5	O94941	99	-10	-13	-39	8	0.9	0.9	0.7	1.1
TUFM	P49411	147	-108	-26	-39	6	0.5	0.8	0.7	1.1
CCDC86	Q9H6F5	116	-26	-26	-39	1	0.8	0.8	0.7	1.0
ZNF687	Q8N1G0	339	-20	-13	-39	-1	0.8	0.9	0.7	1.0
URB1	O60287	541	-11	-18	-39	-9	0.9	0.8	0.7	0.9
CHEK1	O14757	48	-28	-38	-39	-14	0.8	0.7	0.7	0.9
PDCL	Q13371	35	-11	-17	-39	-17	0.9	0.9	0.7	0.9
NCOR1	O75376	946	-19	-48	-39	-17	0.8	0.7	0.7	0.9
RPL36A-HNRN	H7BZ11	72	-13	-32	-39	-39	0.9	0.8	0.7	0.7
LDLRAP1	Q5SW96	199	0	-27	-39	-47	1.0	0.8	0.7	0.7
VPS11	Q9H270	266	-10	-30	-40	30	0.9	0.8	0.7	1.4
KIAA1671	Q9BY89	1590	-15	-34	-40	9	0.9	0.7	0.7	1.1
XRCC5	P13010	249	-15	-40	-40	3	0.9	0.7	0.7	1.0
BIRC6	Q9NR09	1417	-5	-17	-40	1	1.0	0.9	0.7	1.0
HIRA	P54198	752	-11	-27	-40	0	0.9	0.8	0.7	1.0
USP35	Q9P2H5	62	-7	-28	-40	0	0.9	0.8	0.7	1.0
SLCO5A1	Q9H2Y9	795	-47	-38	-40	-3	0.7	0.7	0.7	1.0
ITGB2	P05107	612	-10	-26	-40	-5	0.9	0.8	0.7	1.0
KRI1	Q8N9T8	667	-22	-35	-40	-6	0.8	0.7	0.7	0.9
UTRN	P46939	2839	6	-22	-40	-18	1.1	0.8	0.7	0.9
RBM33	Q96EV2	1098	-30	-33	-40	-23	0.8	0.8	0.7	0.8
AKAP13	Q12802	1838	-16	-36	-40	-31	0.9	0.7	0.7	0.8
ITGA2B	P08514	177	-18	-19	-40	-33	0.8	0.8	0.7	0.8
PYHIN1	Q6K0P9	366	-6	-30	-40	30	0.9	0.8	0.7	1.4
RAP2B	P61225	140	6	-19	-40	14	1.1	0.8	0.7	1.2
Uncharacteriz	E9PCH4	116	-16	-21	-40	3	0.9	0.8	0.7	1.0
OAS3	Q9Y6K5	648	-31	-21	-40	-3	0.8	0.8	0.7	1.0
CDKN1B	P46527	148	-37	-38	-40	-9	0.7	0.7	0.7	0.9
WDR75	Q8IWA0	397	-5	-39	-40	-10	1.0	0.7	0.7	0.9
MAPK1	P28482	65	-22	-50	-40	-12	0.8	0.7	0.7	0.9
ZNF24	P17028	233	-17	-23	-40	-13	0.9	0.8	0.7	0.9
CD97	P48960	495	-14	-44	-40	-18	0.9	0.7	0.7	0.9
C18orf21	Q32NC0	175	-15	-52	-40	-18	0.9	0.7	0.7	0.9
RFXAP	O00287	185	-23	-28	-40	-19	0.8	0.8	0.7	0.8
TBC1D5	Q92609	676	-27	-45	-40	-20	0.8	0.7	0.7	0.8
MTMR12	Q9C0I1	152	-23	-32	-40	-22	0.8	0.8	0.7	0.8
PSMA6	P60900	115	-13	-35	-40	-22	0.9	0.7	0.7	0.8
ATXN7L3B	Q96GX2	70	-24	-31	-40	-24	0.8	0.8	0.7	0.8
DHX16	O60231	486	-7	-34	-40	-24	0.9	0.7	0.7	0.8
OAS3	Q9Y6K5	1070	-7	-36	-40	-25	0.9	0.7	0.7	0.8
TES	Q9UGI8	349	13	-26	-40	-57	1.1	0.8	0.7	0.6
RNF213	Q63HN8	4009	10	3	-40	-66	1.1	1.0	0.7	0.6
AKR1B1	P15121	45	17	-41	-40	-182	1.2	0.7	0.7	0.4
LTBP1	Q14766	704	-8	-16	-40	-274	0.9	0.9	0.7	0.3
HSH2D	Q96JZ2	190	-33	-25	-41	-9	0.8	0.8	0.7	0.9
SCFD2	Q8WU76	404	-34	-38	-41	-12	0.7	0.7	0.7	0.9
GP5	P40197	472	-28	-35	-41	-72	0.8	0.7	0.7	0.6
SIK3	Q9Y2K2	63	-17	-28	-41	3	0.9	0.8	0.7	1.0
PRMT1	Q99873	262	-10	-29	-41	1	0.9	0.8	0.7	1.0
FLII	Q13045	264	-14	-34	-41	-2	0.9	0.7	0.7	1.0
PYHIN1	Q6K0P9	163	-23	-32	-41	-18	0.8	0.8	0.7	0.9
NCKIPSD	Q9NZQ3	412	-16	-11	-41	-64	0.9	0.9	0.7	0.6
TGFB1I1	O43294	91	-13	-27	-41	-176	0.9	0.8	0.7	0.4

LTBP1	Q14766	559	-10	-32	-41	-283	0.9	0.8	0.7	0.3
CCNI	Q14094	272	-30	-52	-42	25	0.8	0.7	0.7	1.3
PCNT	O95613	2446	-28	-20	-42	8	0.8	0.8	0.7	1.1
NRDC	O43847	62	-25	-9	-42	6	0.8	0.9	0.7	1.1
MAP1S	Q66K74	598	-32	-13	-42	-8	0.8	0.9	0.7	0.9
GOLGB1	Q14789	3197	-13	-29	-42	-13	0.9	0.8	0.7	0.9
SOWAHD	A6NJG2	25	-22	-19	-42	-17	0.8	0.8	0.7	0.9
RPL24	P83731	36	-29	-32	-42	-20	0.8	0.8	0.7	0.8
ZC2HC1A	Q96GY0	276	-25	-43	-42	-39	0.8	0.7	0.7	0.7
NEK9	Q8TD19	260	-19	-24	-42	12	0.8	0.8	0.7	1.1
LEMD2	Q8NC56	261	-20	-32	-42	-4	0.8	0.8	0.7	1.0
TROVE2	P10155	119	-33	-34	-42	-16	0.8	0.7	0.7	0.9
NIN	Q8N4C6	1692	-25	-11	-42	-17	0.8	0.9	0.7	0.9
MAP7D1	Q3KQU3	373	-25	-25	-42	-20	0.8	0.8	0.7	0.8
PPM1K	Q8N3J5	97	-25	-34	-42	-21	0.8	0.7	0.7	0.8
CTSG	P08311	186	-35	-52	-42	-46	0.7	0.7	0.7	0.7
CXCR1	P25024	277	-22	-49	-43	-2	0.8	0.7	0.7	1.0
SELL	P14151	147	-27	-42	-43	-6	0.8	0.7	0.7	0.9
MMADHC	Q9H3L0	19	-18	-19	-43	29	0.9	0.8	0.7	1.4
DCXR	Q7Z4W1	58	-21	-46	-43	-12	0.8	0.7	0.7	0.9
RNF213	Q63HN8	1134	3	-30	-43	-13	1.0	0.8	0.7	0.9
UBA6	A0AVT1	473	-10	-15	-43	-16	0.9	0.9	0.7	0.9
TRAPPC10	P48553	1130	-22	-13	-43	-17	0.8	0.9	0.7	0.9
TRIM24	O15164	73	-26	-17	-43	-17	0.8	0.9	0.7	0.9
DNAJB14	Q8TBM8	77	-26	-38	-43	-18	0.8	0.7	0.7	0.9
AFAP1	Q8N556	713	-22	-27	-43	-30	0.8	0.8	0.7	0.8
CTSB	P07858	93	-20	-34	-44	8	0.8	0.7	0.7	1.1
CEP164	Q9UPV0	810	-18	-5	-44	-11	0.9	1.0	0.7	0.9
AKAP13	Q12802	1677	-14	-28	-44	-15	0.9	0.8	0.7	0.9
MGEA5	O60502	878	-38	-8	-44	-17	0.7	0.9	0.7	0.9
RNF135	Q8IUD6	101	-13	-29	-44	-17	0.9	0.8	0.7	0.9
UBE2Q1	Q7Z7E8	65	-23	-30	-44	-19	0.8	0.8	0.7	0.8
C10orf12	Q8N655	172	-43	-30	-44	-26	0.7	0.8	0.7	0.8
YTHDC2	Q9H6S0	1265	-27	-27	-44	-30	0.8	0.8	0.7	0.8
SPAG9	O60271	1155	-18	-26	-44	-1	0.9	0.8	0.7	1.0
GTF2F1	P35269	130	-37	-14	-44	-4	0.7	0.9	0.7	1.0
TRPM2	O94759	1467	-43	-61	-44	-4	0.7	0.6	0.7	1.0
GORASP2	Q9H8Y8	192	-12	-10	-44	-6	0.9	0.9	0.7	0.9
DENND1C	Q8IV53	781	-26	-43	-44	-6	0.8	0.7	0.7	0.9
COMMD4	Q9H0A8	11	-27	-25	-44	-7	0.8	0.8	0.7	0.9
SRR	Q9GZT4	113	-21	-30	-44	-11	0.8	0.8	0.7	0.9
SMAD4	Q13485	523	-10	-31	-44	-14	0.9	0.8	0.7	0.9
CNST	Q6PJW8	292	-8	-27	-44	-32	0.9	0.8	0.7	0.8
RCE1	Q9Y256	314	-22	-34	-45	-4	0.8	0.7	0.7	1.0
ICE2	Q659A1	963	-31	-50	-45	-17	0.8	0.7	0.7	0.9
WIPI2	Q9Y4P8	70	-25	-27	-45	2	0.8	0.8	0.7	1.0
DGCR8	Q8WYQ5	91	-1	-15	-45	-17	1.0	0.9	0.7	0.9
DMXL1	Q9Y485	1760	-21	-29	-45	-25	0.8	0.8	0.7	0.8
NEK1	Q96PY6	1223	-57	-36	-45	-28	0.6	0.7	0.7	0.8
GSG1	Q2KHT4	284	-15	-34	-45	-34	0.9	0.7	0.7	0.7
ABHD16A	O95870	7	-9	-26	-45	-39	0.9	0.8	0.7	0.7
SPOCK2	Q92563	246	-5	-45	-46	15	1.0	0.7	0.7	1.2
FAM204A	Q9H8W3	192	-31	-54	-46	-11	0.8	0.6	0.7	0.9
GOT2	P00505	295	-18	-36	-46	-32	0.8	0.7	0.7	0.8
RPL24	P83731	6	-20	-39	-46	-33	0.8	0.7	0.7	0.8

TXLNG	Q9NUQ3	127	-12	-22	-46	15	0.9	0.8	0.7	1.2
FOXJ2	Q9P0K8	145	-6	-48	-46	10	0.9	0.7	0.7	1.1
ZNF609	O15014	239	-14	-32	-46	2	0.9	0.8	0.7	1.0
MAP1S	Q66K74	588	-31	-20	-46	-26	0.8	0.8	0.7	0.8
TXNRD1	Q16881	625	-20	-54	-46	-43	0.8	0.6	0.7	0.7
ZHX2	Q9Y6X8	681	-16	-28	-47	-7	0.9	0.8	0.7	0.9
ZNF561	Q8N587	243	-24	-23	-47	-13	0.8	0.8	0.7	0.9
DDX31	Q9H8H2	825	-19	-47	-47	-16	0.8	0.7	0.7	0.9
CAST	P20810	661	-9	-18	-47	-17	0.9	0.8	0.7	0.9
ITGAL	P20701	129	-38	-8	-47	-95	0.7	0.9	0.7	0.5
CMC4	P56277	12	-76	-43	-47	16	0.6	0.7	0.7	1.2
MAP7D1	Q3KQU3	251	-29	-37	-47	-30	0.8	0.7	0.7	0.8
HBG1	P69891	94	-16	-33	-48	-17	0.9	0.8	0.7	0.9
RPL3	P39023	253	-18	-43	-48	-29	0.8	0.7	0.7	0.8
PHF20	Q9BVI0	1010	-33	-13	-48	-3	0.8	0.9	0.7	1.0
ZNF649	Q9BS31	360	-41	-52	-48	-3	0.7	0.7	0.7	1.0
PTGR1	Q14914	239	15	-27	-48	-12	1.2	0.8	0.7	0.9
POP1	Q99575	358	-20	-39	-48	-23	0.8	0.7	0.7	0.8
TRIP11	Q15643	1329	-32	-39	-48	-26	0.8	0.7	0.7	0.8
TRIM56	Q9BRZ2	514	-40	-55	-48	-30	0.7	0.6	0.7	0.8
GTF3C3	Q9Y5Q9	810	-14	-30	-49	12	0.9	0.8	0.7	1.1
ABHD12	Q8N2K0	15	-24	-11	-49	1	0.8	0.9	0.7	1.0
ZNF276	Q8N554	57	-18	-45	-49	-28	0.9	0.7	0.7	0.8
DDX17	Q92841	584	-18	-40	-49	8	0.9	0.7	0.7	1.1
KDM3A	Q9Y4C1	497	2	-13	-50	-6	1.0	0.9	0.7	0.9
RPL10	P27635	105	-25	-38	-50	-18	0.8	0.7	0.7	0.8
CBLL1	Q75N03	130	-10	-30	-50	-21	0.9	0.8	0.7	0.8
IRAK3	Q9Y616	420	-27	-31	-51	-2	0.8	0.8	0.7	1.0
PHPT1	Q9NRX4	69	-13	-28	-51	-13	0.9	0.8	0.7	0.9
GEMIN5	Q8TEQ6	1421	-27	-49	-51	-14	0.8	0.7	0.7	0.9
GP5	P40197	427	-14	-60	-51	-74	0.9	0.6	0.7	0.6
FCN1	O00602	111	-31	-59	-52	26	0.8	0.6	0.7	1.4
GOT2	P00505	274	-7	-22	-52	-19	0.9	0.8	0.7	0.8
L3MBTL3	Q96JM7	169	-37	-28	-52	-21	0.7	0.8	0.7	0.8
AMACR	Q9UHK6	117	-5	-1	-52	-32	1.0	1.0	0.7	0.8
ERO1A	Q96HE7	94	-6	-14	-52	-42	0.9	0.9	0.7	0.7
POLR2B	P30876	622	-3	-18	-52	-206	1.0	0.9	0.7	0.3
PRRC2B	Q5JSZ5	1346	-20	-46	-52	-25	0.8	0.7	0.7	0.8
FGD6	Q6ZV73	463	-12	-41	-52	-42	0.9	0.7	0.7	0.7
DDX58	O95786	490	-13	-28	-53	2	0.9	0.8	0.7	1.0
DESI1	Q6ICB0	108	-38	-36	-53	1	0.7	0.7	0.7	1.0
DMXL2	Q8TDJ6	1833	-12	-40	-53	-9	0.9	0.7	0.7	0.9
KLRB1	Q12918	74	-13	-26	-53	-15	0.9	0.8	0.7	0.9
ZCCHC6	Q5VYS8	70	-31	-48	-53	-15	0.8	0.7	0.7	0.9
PIK3R4	Q99570	195	-19	-33	-53	-28	0.8	0.8	0.7	0.8
EIF4EBP2	Q13542	35	-26	-38	-53	-22	0.8	0.7	0.7	0.8
CD37	P11049	217	-39	-45	-53	-65	0.7	0.7	0.7	0.6
RAP1B	P61224	141	-32	-30	-53	-72	0.8	0.8	0.7	0.6
PBXIP1	Q96AQ6	588	1	-31	-54	12	1.0	0.8	0.7	1.1
INO80	Q9ULG1	13	-22	-33	-55	-3	0.8	0.8	0.6	1.0
LCP1	P13796	42	-16	-43	-55	-31	0.9	0.7	0.6	0.8
ZC3H12A	Q5D1E8	103	-21	-34	-56	-27	0.8	0.7	0.6	0.8
TREM1	Q9NP99	163	7	-16	-56	-40	1.1	0.9	0.6	0.7
PTPN12	Q05209	235	13	-42	-56	0	1.1	0.7	0.6	1.0
PURB	Q96QR8	17	-33	-58	-56	-18	0.8	0.6	0.6	0.8

PGM2	Q96G03	484	-15	-49	-56	-21	0.9	0.7	0.6	0.8
TRIM6-TRIM3	B2RNG4	593	3	-3	-56	-44	1.0	1.0	0.6	0.7
PCYT1A	P49585	73	-22	-36	-57	0	0.8	0.7	0.6	1.0
CD6	P30203	170	-28	-45	-58	16	0.8	0.7	0.6	1.2
CANX	P27824	194	-20	-47	-58	-13	0.8	0.7	0.6	0.9
CKB	P12277	254	-19	-30	-58	-19	0.8	0.8	0.6	0.8
IGHG3	A0A087WVW	255	-45	-76	-58	-40	0.7	0.6	0.6	0.7
ZCCHC6	Q5VYS8	1413	-26	-62	-59	-28	0.8	0.6	0.6	0.8
INPP5K	Q9BT40	173	-6	-8	-59	-42	0.9	0.9	0.6	0.7
BDH1	Q02338	63	-42	-47	-59	-10	0.7	0.7	0.6	0.9
NPRL2	Q8WTW4	96	-7	-37	-59	-29	0.9	0.7	0.6	0.8
CHD8	Q9HCK8	1980	-21	-38	-61	-34	0.8	0.7	0.6	0.7
GZMB	P10144	142	-22	-52	-62	-14	0.8	0.7	0.6	0.9
PPBP	P02775	89	-8	-50	-62	-38	0.9	0.7	0.6	0.7
CBR3	O75828	150	-25	-16	-62	-32	0.8	0.9	0.6	0.8
WDR44	Q5JSH3	140	-16	-36	-62	-61	0.9	0.7	0.6	0.6
IKBKB	O14920	524	-11	-41	-63	-32	0.9	0.7	0.6	0.8
FAM120B	Q96EK7	644	-21	-48	-63	-41	0.8	0.7	0.6	0.7
LTBP1	Q14766	568	-35	-56	-63	-318	0.7	0.6	0.6	0.2
AMBRA1	Q9C0C7	501	-15	-14	-64	-49	0.9	0.9	0.6	0.7
STIM1	Q13586	49	-13	-43	-65	-24	0.9	0.7	0.6	0.8
PNPLA6	Q8IY17	1354	-18	-27	-66	-12	0.9	0.8	0.6	0.9
ZBTB20	Q9HC78	311	-20	-52	-66	-17	0.8	0.7	0.6	0.9
THOC2	Q8NI27	986	-4	-13	-66	-46	1.0	0.9	0.6	0.7
RGS2	P41220	199	-19	-29	-66	-57	0.8	0.8	0.6	0.6
HEXA	P06865	58	-31	-78	-67	-28	0.8	0.6	0.6	0.8
TOR4A	Q9NXH8	62	-15	-33	-67	-114	0.9	0.8	0.6	0.5
BTN3A3	O00478	166	-23	-73	-68	-27	0.8	0.6	0.6	0.8
BTN3A2	P78410	166	-23	-73	-68	-27	0.8	0.6	0.6	0.8
CLGN	O14967	185	-29	-54	-70	-26	0.8	0.6	0.6	0.8
LTBP1	Q14766	361	-18	-57	-71	-212	0.9	0.6	0.6	0.3
STAU2	Q9NUL3	491	-18	-56	-72	-6	0.9	0.6	0.6	0.9
IGHG3	A0A087WVW	264	-66	-52	-72	-45	0.6	0.7	0.6	0.7
PLS3	P13797	104	-25	-55	-73	-46	0.8	0.6	0.6	0.7
PPME1	Q9Y570	381	-23	-51	-73	-61	0.8	0.7	0.6	0.6
SRRM2	Q9UQ35	2130	-54	-21	-74	-132	0.6	0.8	0.6	0.4
PPBP	P02775	105	-28	-59	-77	-111	0.8	0.6	0.6	0.5
CD93	Q9NPY3	300	-31	-59	-80	-20	0.8	0.6	0.6	0.8
FGA	P02671	47	-15	-102	-81	-194	0.9	0.5	0.6	0.3
PRAM1	Q96QH2	706	-54	-61	-82	-3	0.7	0.6	0.6	1.0
IGHG3	A0A087WVW	270	-43	-63	-82	-47	0.7	0.6	0.5	0.7
PQLC3	Q8N755	157	1	-17	-85	-25	1.0	0.9	0.5	0.8
ERAP1	Q9NZ08	498	-40	-62	-88	-8	0.7	0.6	0.5	0.9
SUN1	O94901	63	-16	-68	-88	-50	0.9	0.6	0.5	0.7
LCP1	P13796	101	-27	-67	-89	-57	0.8	0.6	0.5	0.6
MDM4	O15151	362	-36	-63	-90	-64	0.7	0.6	0.5	0.6
CXorf38	Q8TB03	31	-39	-74	-93	-244	0.7	0.6	0.5	0.3
PPME1	Q9Y570	386	-47	-74	-97	-60	0.7	0.6	0.5	0.6
CTSZ	Q9UBR2	179	-38	-91	-99	-56	0.7	0.5	0.5	0.6
CTSB	P07858	211	-50	-102	-106	-40	0.7	0.5	0.5	0.7
LTBP1	Q14766	430	-19	-81	-108	-357	0.8	0.6	0.5	0.2
ABCC1	P33527	1439	-19	-33	-111	-89	0.8	0.8	0.5	0.5
OSBPL3	Q9H4L5	337	12	8	-122	-12	1.1	1.1	0.5	0.9
THG1L	Q9NWX6	47	-62	-81	-127	1	0.6	0.6	0.4	1.0
CD38	P28907	287	-37	-108	-147	4	0.7	0.5	0.4	1.0

CLU	P10909	285	-68	-117	-176	-278	0.6	0.5	0.4	0.3
CTSD	P07339	117	-128	-239	-258	-80	0.4	0.3	0.3	0.6
CD48	P09326	193	-90	-201	-269	-41	0.5	0.3	0.3	0.7
CD48	P09326	196	-93	-208	-275	-48	0.5	0.3	0.3	0.7
ITPK1	Q13572	403	-5	-44	-364	-299	1.0	0.7	0.2	0.3
