# Supplementary material for: Selective inhibitors of JAK1 targeting an isoform-restricted allosteric cysteine
Source: Nat Chem Biol. Author manuscript; Available in PMC 2023 Jul 18. (PMC7614775; doi:10.1038/s41589-022-01098-0)
Supplement: Proteomics Table 2 [file EMS178742-supplement-Proteomics_Table_2.pdf]

Protein	Uniprot Accession	Cysteine Number	%Engagement				Engagement Ratio (DMSO/VVD-118313)			
			0.01 µM	0.1 µM	1 µM	10 µM	0.01 µM	0.1 µM	1 µM	10 µM
JAK1	P23458	817	81	96	97	98	5.1	20.0	20.0	20.0
TOR4A	Q9NXH8	21	9	49	90	97	1.1	1.9	10.0	20.0
TYK2	P29597	838	-16	32	83	96	0.9	1.5	5.9	20.0
RRP12	Q5JTH9	31	9	38	52	35	1.1	1.6	2.1	1.5
PPP2R2D	Q66LE6	404	5	28	49	52	1.1	1.4	1.9	2.1
MGST3	O14880	56	5	5	41	69	1.0	1.1	1.7	3.2
ZNF490	Q9ULM2	364	24	30	40	-148	1.3	1.4	1.7	0.4
DDX41	Q9UJV9	568	-8	29	39	3	0.9	1.4	1.6	1.0
MTMR9	Q96QG7	30	34	30	38	14	1.5	1.4	1.6	1.2
PDE1C	Q14123	344	-22	28	38	-79	0.8	1.4	1.6	0.6
DDAH2	O95865	176	31	32	37	30	1.4	1.5	1.6	1.4
SMC3	Q9UQE7	1134	28	-7	37	0	1.4	0.9	1.6	1.0
C14orf159	Q7Z3D6	418	5	2	36	5	1.1	1.0	1.6	1.1
ARHGAP30	Q7Z6I6	407	10	21	36	-2	1.1	1.3	1.6	1.0
CX3CR1	P49238	287	12	8	35	16	1.1	1.1	1.5	1.2
CX3CR1	P49238	286	12	8	35	16	1.1	1.1	1.5	1.2
CEP78	Q5JTW2	225	8	44	34	-4	1.1	1.8	1.5	1.0
ADARB1	P78563	674	37	24	34	20	1.6	1.3	1.5	1.2
AFTPH	Q6ULP2	659	9	-6	34	3	1.1	0.9	1.5	1.0
AAK1	Q2M2I8	250	40	-5	33	29	1.7	1.0	1.5	1.4
BMP2K	Q9NSY1	254	40	-5	33	29	1.7	1.0	1.5	1.4
SCFD1	Q8WVM8	623	35	2	33	34	1.5	1.0	1.5	1.5
MRPS27	Q92552	101	17	23	32	-1	1.2	1.3	1.5	1.0
PI4KA	P42356	95	35	22	32	27	1.5	1.3	1.5	1.4
FLAD1	Q8NFF5	537	22	5	32	22	1.3	1.1	1.5	1.3
TFCP2	Q12800	72	14	3	32	69	1.2	1.0	1.5	3.2
PGPEP1	Q9NXJ5	206	8	12	31	9	1.1	1.1	1.4	1.1
KLHL42	Q9P2K6	15	-5	14	31	-7	1.0	1.2	1.4	0.9
INPP5D	Q92835	1088	23	24	31	11	1.3	1.3	1.4	1.1
UBR4	Q5T4S7	3864	-5	20	30	25	1.0	1.3	1.4	1.3
TBC1D22A	Q8WUA7	151	-14	15	29	-4	0.9	1.2	1.4	1.0
ZAR1L	A6NP61	311	-15	-27	29	-28	0.9	0.8	1.4	0.8
PRPF39	Q86UA1	450	20	6	28	16	1.2	1.1	1.4	1.2
PPP1R12A	O14974	48	20	5	28	12	1.2	1.1	1.4	1.1
SPATA5	Q8NB90	459	-4	3	28	14	1.0	1.0	1.4	1.2
KIAA2013	Q8IYS2	185	23	12	27	14	1.3	1.1	1.4	1.2
Uncharacteri	A0A096LPA9	109	21	13	27	17	1.3	1.1	1.4	1.2
Uncharacteri	A0A096LPA9	117	21	13	27	17	1.3	1.1	1.4	1.2
UBA6	A0AVT1	473	10	-8	27	38	1.1	0.9	1.4	1.6
NOL9	Q5SY16	637	19	-3	27	-1	1.2	1.0	1.4	1.0
CTU1	Q7Z7A3	299	2	18	27	2	1.0	1.2	1.4	1.0
ETS1	P14921	31	20	8	26	-6	1.3	1.1	1.4	0.9
CTCF	P49711	275	6	8	26	-1	1.1	1.1	1.4	1.0
KPNA3	O00505	191	3	16	26	18	1.0	1.2	1.4	1.2
TYK2	P29597	936	19	9	26	-12	1.2	1.1	1.3	0.9
IRAK1	P51617	498	15	-4	26	-17	1.2	1.0	1.3	0.9
BCL9	O00512	64	10	12	26	3	1.1	1.1	1.3	1.0
HARS2	P49590	84	19	1	25	27	1.2	1.0	1.3	1.4
PQLC3	Q8N755	135	3	1	25	88	1.0	1.0	1.3	8.0
STAT3	P40763	426	0	-9	25	-14	1.0	0.9	1.3	0.9
LTBP1	Q14766	582	-1	-7	25	53	1.0	0.9	1.3	2.1

CAPZA1	P52907	157	33	-25	25	14	1.5	0.8	1.3	1.2
TP53BP1	Q12888	101	14	3	25	-17	1.2	1.0	1.3	0.9
GSR	P00390	134	9	-3	25	11	1.1	1.0	1.3	1.1
XPO7	Q9UIA9	194	26	-22	24	27	1.4	0.8	1.3	1.4
DENND1C	Q8IV53	349	19	-1	24	-5	1.2	1.0	1.3	1.0
GLTP	Q9NZD2	36	18	5	24	6	1.2	1.1	1.3	1.1
ERP44	Q9BS26	301	15	-24	24	6	1.2	0.8	1.3	1.1
SYNE2	Q8WXH0	4241	4	16	24	27	1.0	1.2	1.3	1.4
OAS3	Q9Y6K5	1069	3	-5	24	21	1.0	1.0	1.3	1.3
REEP5	Q00765	18	-4	-13	24	30	1.0	0.9	1.3	1.4
XPOT	O43592	676	28	10	24	22	1.4	1.1	1.3	1.3
CDA	P32320	99	22	22	24	10	1.3	1.3	1.3	1.1
GBF1	Q92538	1173	21	14	24	10	1.3	1.2	1.3	1.1
KPNA1	P52294	429	17	-7	24	18	1.2	0.9	1.3	1.2
ARFGEF2	Q9Y6D5	933	14	2	24	-1	1.2	1.0	1.3	1.0
TRADD	Q15628	135	22	16	23	7	1.3	1.2	1.3	1.1
ARFGEF2	Q9Y6D5	1279	18	20	23	14	1.2	1.2	1.3	1.2
ARFGEF1	Q9Y6D6	1332	18	20	23	14	1.2	1.2	1.3	1.2
RASA1	P20936	828	17	-7	23	9	1.2	0.9	1.3	1.1
RNF216	Q9NWF9	628	16	-4	23	0	1.2	1.0	1.3	1.0
SERPINB8	P50452	368	7	-12	23	22	1.1	0.9	1.3	1.3
FMR1NB	Q8N0W7	145	5	15	23	-1	1.0	1.2	1.3	1.0
NME7	Q9Y5B8	228	5	14	23	-14	1.0	1.2	1.3	0.9
SARG	Q9BW04	212	24	13	23	7	1.3	1.1	1.3	1.1
RUFY1	Q96T51	8	19	14	23	12	1.2	1.2	1.3	1.1
MAP3K6	O95382	16	11	13	23	5	1.1	1.1	1.3	1.0
ZC3HAV1	Q7Z2W4	518	-16	-22	23	-39	0.9	0.8	1.3	0.7
CCNL2	Q96S94	82	25	9	22	21	1.3	1.1	1.3	1.3
HERC4	Q5GLZ8	1025	25	6	22	15	1.3	1.1	1.3	1.2
PBRM1	Q86U86	1228	21	16	22	16	1.3	1.2	1.3	1.2
ZZEF1	O43149	2466	18	13	22	2	1.2	1.1	1.3	1.0
RNF213	Q63HN8	4348	9	-15	22	6	1.1	0.9	1.3	1.1
LRRC8A	Q8IWT6	768	-8	11	22	15	0.9	1.1	1.3	1.2
LAPTM5	Q13571	13	-10	20	22	12	0.9	1.3	1.3	1.1
DOCK8	Q8NF50	1481	34	-13	22	9	1.5	0.9	1.3	1.1
ZNF319	Q9P2F9	307	26	7	22	8	1.3	1.1	1.3	1.1
SRI	P30626	194	23	10	22	12	1.3	1.1	1.3	1.1
CNP	P09543	252	14	-7	22	17	1.2	0.9	1.3	1.2
SEC24D	O94855	819	14	14	22	8	1.2	1.2	1.3	1.1
UVRAG	Q9P2Y5	239	2	-1	22	88	1.0	1.0	1.3	8.3
IRAK3	Q9Y616	255	-11	-2	22	9	0.9	1.0	1.3	1.1
RBM26	Q5T8P6	53	23	3	21	12	1.3	1.0	1.3	1.1
PSMC5	P62195	363	20	19	21	23	1.2	1.2	1.3	1.3
NDOR1	Q9UHB4	507	19	15	21	12	1.2	1.2	1.3	1.1
HERC1	Q15751	1228	8	-1	21	-6	1.1	1.0	1.3	0.9
DIP2B	Q9P265	333	1	9	21	32	1.0	1.1	1.3	1.5
AGO3	Q9H9G7	565	-20	16	21	-9	0.8	1.2	1.3	0.9
AGO1	Q9UL18	562	-20	16	21	-9	0.8	1.2	1.3	0.9
EWSR1	Q01844	529	17	4	21	16	1.2	1.0	1.3	1.2
CASP8	Q14790	131	14	11	21	-31	1.2	1.1	1.3	0.8
RALGAPB	Q86X10	992	13	1	21	3	1.1	1.0	1.3	1.0
BTG2	P78543	29	11	5	21	5	1.1	1.0	1.3	1.1
NCAPD2	Q15021	767	8	1	21	6	1.1	1.0	1.3	1.1

INO80	Q9ULG1	977	6	-3	21	1	1.1	1.0	1.3	1.0
DCTN4	Q9UJW0	258	-3	-3	21	79	1.0	1.0	1.3	4.8
LTA4H	P09960	543	25	1	20	26	1.3	1.0	1.3	1.4
HNRNPK	P61978	205	23	-6	20	1	1.3	0.9	1.3	1.0
CAPN2	P17655	39	18	-30	20	19	1.2	0.8	1.3	1.2
SNX6	Q9UNH7	398	18	-6	20	-3	1.2	0.9	1.3	1.0
XRN2	Q9H0D6	547	16	5	20	4	1.2	1.0	1.3	1.0
TRIP12	Q14669	190	13	24	20	5	1.1	1.3	1.3	1.1
PPID	Q08752	282	4	-2	20	10	1.0	1.0	1.3	1.1
NACC1	Q96RE7	85	3	-11	20	-18	1.0	0.9	1.3	0.8
HSDL1	Q3SXM5	265	2	-9	20	77	1.0	0.9	1.3	4.3
CSE1L	P55060	853	-33	-23	20	-53	0.8	0.8	1.3	0.7
LYPLA2	O95372	171	19	8	20	14	1.2	1.1	1.2	1.2
NAA30	Q147X3	195	16	-8	20	1	1.2	0.9	1.2	1.0
PLOD3	O60568	691	9	12	20	7	1.1	1.1	1.2	1.1
SYNE2	Q8WXH0	1806	6	24	20	19	1.1	1.3	1.2	1.2
GCC2	Q8IWJ2	826	31	5	19	17	1.4	1.0	1.2	1.2
RABGGTA	Q92696	99	30	-21	19	14	1.4	0.8	1.2	1.2
CLTB	P09497	199	23	7	19	13	1.3	1.1	1.2	1.1
FYCO1	Q9BQS8	913	21	9	19	-1	1.3	1.1	1.2	1.0
MMS19	Q96T76	285	18	7	19	7	1.2	1.1	1.2	1.1
GSK3A	P49840	398	14	0	19	14	1.2	1.0	1.2	1.2
DENND1C	Q8IV53	781	14	-3	19	-3	1.2	1.0	1.2	1.0
NBEAL2	Q6ZNJ1	1205	10	5	19	36	1.1	1.0	1.2	1.6
PANX1	Q96RD7	136	8	6	19	2	1.1	1.1	1.2	1.0
VTA1	Q9NP79	56	3	15	19	17	1.0	1.2	1.2	1.2
HMOX2	P30519	282	0	-4	19	81	1.0	1.0	1.2	5.3
WDR48	Q8TAF3	225	34	9	19	6	1.5	1.1	1.2	1.1
C1orf122	Q6ZSJ8	61	24	13	19	-1	1.3	1.1	1.2	1.0
DDB2	Q92466	253	22	16	19	19	1.3	1.2	1.2	1.2
ZCCHC11	Q5TAX3	394	18	22	19	12	1.2	1.3	1.2	1.1
RNF213	Q63HN8	2235	18	17	19	20	1.2	1.2	1.2	1.3
RERE	Q9P2R6	1144	13	-16	19	-12	1.1	0.9	1.2	0.9
SUGP2	Q8IX01	947	2	11	19	2	1.0	1.1	1.2	1.0
PTPN7	P35236	300	-8	-7	19	15	0.9	0.9	1.2	1.2
VDAC2	P45880	210	-12	-8	19	50	0.9	0.9	1.2	2.0
DLST	P36957	66	34	1	18	24	1.5	1.0	1.2	1.3
ANKRD28	O15084	201	31	8	18	8	1.4	1.1	1.2	1.1
MTPAP	Q9NVV4	242	25	0	18	5	1.3	1.0	1.2	1.1
NMD3	Q96D46	71	24	5	18	11	1.3	1.1	1.2	1.1
IL7R	P16871	306	22	3	18	15	1.3	1.0	1.2	1.2
TPP2	P29144	710	20	19	18	2	1.3	1.2	1.2	1.0
SIPA1L3	O60292	1775	18	13	18	-1	1.2	1.1	1.2	1.0
HK3	P52790	409	16	2	18	21	1.2	1.0	1.2	1.3
NOL11	Q9H8H0	368	14	-14	18	40	1.2	0.9	1.2	1.7
USP16	Q9Y5T5	631	8	0	18	13	1.1	1.0	1.2	1.1
POP1	Q99575	559	7	5	18	5	1.1	1.0	1.2	1.1
MPND	Q8N594	457	3	-3	18	-11	1.0	1.0	1.2	0.9
HACE1	Q8IYU2	441	0	8	18	6	1.0	1.1	1.2	1.1
ZNF428	Q96B54	114	-8	-13	18	-3	0.9	0.9	1.2	1.0
AAMDC	Q9H7C9	122	-8	-18	18	-11	0.9	0.9	1.2	0.9
RFC1	P35251	767	-12	0	18	-2	0.9	1.0	1.2	1.0
FLNC	Q14315	52	-16	16	18	22	0.9	1.2	1.2	1.3

PDE3B	Q13370	52	-18	-22	18	-18	0.9	0.8	1.2	0.9
MED17	Q9NVC6	241	32	9	18	22	1.5	1.1	1.2	1.3
UBR4	Q5T4S7	4146	21	-3	18	29	1.3	1.0	1.2	1.4
TCAF1	Q9Y4C2	576	20	-25	18	6	1.3	0.8	1.2	1.1
GEMIN5	Q8TEQ6	867	20	6	18	20	1.2	1.1	1.2	1.2
AVEN	Q9NQS1	326	20	-8	18	12	1.2	0.9	1.2	1.1
PBRM1	Q86U86	69	19	-37	18	-20	1.2	0.7	1.2	0.8
SENP8	Q96LD8	64	12	13	18	22	1.1	1.1	1.2	1.3
BAG4	O95429	193	12	-5	18	2	1.1	1.0	1.2	1.0
DHX9	Q08211	940	10	10	18	8	1.1	1.1	1.2	1.1
MPHOSPH8	Q99549	689	10	-7	18	-1	1.1	0.9	1.2	1.0
STK17A	Q9UEE5	315	9	-4	18	2	1.1	1.0	1.2	1.0
CTU1	Q7Z7A3	147	7	0	18	19	1.1	1.0	1.2	1.2
AARS2	Q5JTZ9	749	6	11	18	4	1.1	1.1	1.2	1.0
HNRNPUL2-B	H3BQZ7	452	5	9	18	7	1.0	1.1	1.2	1.1
Transmembr	E9PSI1	654	5	-5	18	-2	1.0	1.0	1.2	1.0
CHD4	Q14839	467	-19	-19	18	-45	0.8	0.8	1.2	0.7
TRPV2	Q9Y5S1	134	35	1	17	16	1.5	1.0	1.2	1.2
MRPL14	Q6P1L8	90	20	8	17	5	1.2	1.1	1.2	1.0
PIAS4	Q8N2W9	50	19	14	17	-4	1.2	1.2	1.2	1.0
HSPH1	Q92598	290	18	14	17	11	1.2	1.2	1.2	1.1
RP2	O75695	130	18	-20	17	5	1.2	0.8	1.2	1.1
PAPD4	Q6PIY7	374	17	1	17	5	1.2	1.0	1.2	1.1
SERGEF	Q9UGK8	184	16	5	17	3	1.2	1.1	1.2	1.0
ALPK1	Q96QP1	104	15	20	17	18	1.2	1.3	1.2	1.2
NUB1	Q9Y5A7	269	13	16	17	26	1.1	1.2	1.2	1.3
CLIC4	Q9Y696	189	13	-15	17	9	1.1	0.9	1.2	1.1
RAD54L2	Q9Y4B4	616	12	-2	17	3	1.1	1.0	1.2	1.0
GSTP1	P09211	102	10	11	17	27	1.1	1.1	1.2	1.4
KMT2B	Q9UMN6	1306	8	18	17	8	1.1	1.2	1.2	1.1
IARS	P41252	336	4	-16	17	6	1.0	0.9	1.2	1.1
MBD4	O95243	549	3	-16	17	-16	1.0	0.9	1.2	0.9
TMEM129	A0AVI4	354	3	-8	17	-13	1.0	0.9	1.2	0.9
MIEF1	Q9NQG6	359	-1	-27	17	2	1.0	0.8	1.2	1.0
NFATC2IP	Q8NCF5	232	-1	-5	17	-8	1.0	1.0	1.2	0.9
TLN1	Q9Y490	2243	31	-4	17	19	1.4	1.0	1.2	1.2
GOLGB1	Q14789	1964	20	5	17	-5	1.3	1.0	1.2	1.0
UBR4	Q5T4S7	1458	19	23	17	1	1.2	1.3	1.2	1.0
ALG11	Q2TAA5	345	19	3	17	10	1.2	1.0	1.2	1.1
HTT	P42858	1998	17	13	17	13	1.2	1.1	1.2	1.1
COPB1	P53618	466	16	10	17	18	1.2	1.1	1.2	1.2
RANBP3	Q9H6Z4	203	14	6	17	-1	1.2	1.1	1.2	1.0
RPL4	P36578	125	14	5	17	-1	1.2	1.0	1.2	1.0
RASGRP2	Q7LDG7	20	14	-16	17	-6	1.2	0.9	1.2	0.9
EMILIN1	Q9Y6C2	302	12	14	17	16	1.1	1.2	1.2	1.2
SYNRG	Q9UMZ2	1134	9	-2	17	13	1.1	1.0	1.2	1.1
AKAP13	Q12802	1399	6	-10	17	-13	1.1	0.9	1.2	0.9
PARP4	Q9UKK3	1051	5	15	17	12	1.1	1.2	1.2	1.1
ZC3H4	Q9UPT8	1302	5	7	17	7	1.1	1.1	1.2	1.1
ITGB2	P05107	534	1	-3	17	-21	1.0	1.0	1.2	0.8
SLFN5	Q08AF3	846	-5	6	17	-9	1.0	1.1	1.2	0.9
PDE3B	Q13370	297	-12	19	17	12	0.9	1.2	1.2	1.1
TAF5L	O75529	431	-22	7	17	-31	0.8	1.1	1.2	0.8

FAF2	Q96CS3	32	29	-4	16	6	1.4	1.0	1.2	1.1
PWWP2B	Q6NUJ5	307	23	15	16	15	1.3	1.2	1.2	1.2
LTBP1	Q14766	581	22	14	16	2	1.3	1.2	1.2	1.0
PRKDC	P78527	1432	22	-48	16	1	1.3	0.7	1.2	1.0
PUS7	Q96PZ0	443	15	13	16	10	1.2	1.1	1.2	1.1
GCA	P28676	76	15	-44	16	-14	1.2	0.7	1.2	0.9
QTRTD1	Q9H974	356	14	3	16	7	1.2	1.0	1.2	1.1
TUBGCP3	Q96CW5	497	14	-9	16	4	1.2	0.9	1.2	1.0
DNAJC13	O75165	261	13	7	16	8	1.1	1.1	1.2	1.1
RPRD1B	Q9NQG5	100	11	-7	16	21	1.1	0.9	1.2	1.3
SMAD5	Q99717	65	9	1	16	6	1.1	1.0	1.2	1.1
ZEB2	O60315	586	7	3	16	-5	1.1	1.0	1.2	1.0
ACADVL	P49748	607	7	-2	16	-6	1.1	1.0	1.2	0.9
EPPK1	A0A087X1U6	1380	6	-7	16	-2	1.1	0.9	1.2	1.0
ZC3HAV1L	Q96H79	163	5	6	16	9	1.0	1.1	1.2	1.1
RAVER1	Q8IY67	222	5	-18	16	-11	1.0	0.8	1.2	0.9
ATP9B	O43861	626	3	20	16	-7	1.0	1.3	1.2	0.9
ZMYM3	Q14202	335	2	4	16	3	1.0	1.0	1.2	1.0
DAXX	Q9UER7	58	-4	-10	16	48	1.0	0.9	1.2	1.9
UBXN6	Q9BZV1	125	-7	-3	16	10	0.9	1.0	1.2	1.1
PSMD14	O00487	238	-8	3	16	7	0.9	1.0	1.2	1.1
RPAP1	Q9BWH6	84	-18	-7	16	-24	0.9	0.9	1.2	0.8
SLC25A3	Q00325	136	30	7	16	9	1.4	1.1	1.2	1.1
SURF6	O75683	19	29	11	16	4	1.4	1.1	1.2	1.0
PDK1	Q15118	240	23	18	16	11	1.3	1.2	1.2	1.1
PEA15	Q15121	27	19	4	16	12	1.2	1.0	1.2	1.1
SH3BP1	Q9Y3L3	102	19	8	16	-1	1.2	1.1	1.2	1.0
SDR9C7	Q8NEX9	56	17	25	16	18	1.2	1.3	1.2	1.2
WDR43	Q15061	380	15	-10	16	16	1.2	0.9	1.2	1.2
NUP98	P52948	1727	14	6	16	9	1.2	1.1	1.2	1.1
WDFY4	Q6ZS81	2436	12	6	16	1	1.1	1.1	1.2	1.0
AP3D1	O14617	348	8	-16	16	15	1.1	0.9	1.2	1.2
SNX4	O95219	172	7	12	16	18	1.1	1.1	1.2	1.2
PLCG1	P19174	247	6	8	16	-2	1.1	1.1	1.2	1.0
TPP1	O14773	365	5	-4	16	5	1.1	1.0	1.2	1.0
FLNA	P21333	733	3	-29	16	-4	1.0	0.8	1.2	1.0
ODF2	Q5BJF6	80	-4	-5	16	11	1.0	1.0	1.2	1.1
ZC3HAV1	Q7Z2W4	513	-11	-34	16	-29	0.9	0.7	1.2	0.8
FDXR	P22570	152	-16	-24	16	-6	0.9	0.8	1.2	0.9
NOSIP	Q9Y314	223	-17	5	16	18	0.9	1.0	1.2	1.2
AFF4	Q9UHB7	889	25	-5	15	-3	1.3	1.0	1.2	1.0
TYMP	P19971	136	24	15	15	20	1.3	1.2	1.2	1.3
ANK1	P16157	943	23	12	15	4	1.3	1.1	1.2	1.0
SRRT	Q9BXP5	479	21	12	15	26	1.3	1.1	1.2	1.3
G3BP2	Q9UN86	73	21	-4	15	19	1.3	1.0	1.2	1.2
ZCCHC6	Q5VYS8	1008	19	-11	15	2	1.2	0.9	1.2	1.0
KPNA3	O00505	325	18	3	15	1	1.2	1.0	1.2	1.0
NMRK1	Q9NWW6	72	18	-10	15	-2	1.2	0.9	1.2	1.0
DIAPH1	O60610	267	17	9	15	18	1.2	1.1	1.2	1.2
INTS10	Q9NVR2	387	17	6	15	-8	1.2	1.1	1.2	0.9
STRIP2	Q9ULQ0	222	17	4	15	21	1.2	1.0	1.2	1.3
UBE3C	Q15386	158	16	13	15	-3	1.2	1.1	1.2	1.0
KIAA0930	Q6ICG6	23	13	-1	15	2	1.1	1.0	1.2	1.0

ZNF428	Q96B54	111	11	10	15	3	1.1	1.1	1.2	1.0
NFKBIE	O00221	457	10	-17	15	2	1.1	0.9	1.2	1.0
RHOH	Q15669	165	9	6	15	6	1.1	1.1	1.2	1.1
DYSF	O75923	1369	9	-1	15	18	1.1	1.0	1.2	1.2
POLE3	Q9NRF9	51	8	2	15	0	1.1	1.0	1.2	1.0
SENP7	Q9BQF6	85	7	-6	15	-6	1.1	0.9	1.2	0.9
PIK3R4	Q99570	84	7	-18	15	-12	1.1	0.8	1.2	0.9
PTPN1	P18031	226	7	-1	15	12	1.1	1.0	1.2	1.1
TRAPPC8	Q9Y2L5	1341	6	10	15	-7	1.1	1.1	1.2	0.9
RAB33A	Q14088	200	3	9	15	-4	1.0	1.1	1.2	1.0
DNAJA1	P31689	150	3	2	15	-19	1.0	1.0	1.2	0.8
NADK	O95544	79	-1	0	15	-13	1.0	1.0	1.2	0.9
ARAP1	Q96P48	803	-8	-26	15	-1	0.9	0.8	1.2	1.0
PDE3B	Q13370	410	31	4	15	5	1.4	1.0	1.2	1.1
PRKDC	P78527	223	22	-29	15	25	1.3	0.8	1.2	1.3
FERMT3	Q86UX7	128	22	-13	15	3	1.3	0.9	1.2	1.0
SMARCC1	Q92922	119	22	-22	15	20	1.3	0.8	1.2	1.3
BIRC6	Q9NR09	4443	21	7	15	18	1.3	1.1	1.2	1.2
DGKD	Q16760	422	21	-4	15	1	1.3	1.0	1.2	1.0
IQGAP2	Q13576	42	20	5	15	15	1.2	1.1	1.2	1.2
BUB1	O43683	973	18	12	15	12	1.2	1.1	1.2	1.1
HIST1H3J	P68431	97	17	4	15	-5	1.2	1.0	1.2	1.0
CLASP2	O75122	1179	17	-10	15	2	1.2	0.9	1.2	1.0
PPP1R21	Q6ZMI0	516	16	-4	15	10	1.2	1.0	1.2	1.1
GNE	Q9Y223	303	15	-2	15	-10	1.2	1.0	1.2	0.9
YES1	P07947	287	14	5	15	6	1.2	1.0	1.2	1.1
CYFIP1	Q7L576	1088	13	14	15	7	1.1	1.2	1.2	1.1
CYFIP2	Q96F07	1112	13	14	15	7	1.1	1.2	1.2	1.1
DOCK11	Q5JSL3	1933	13	-9	15	-5	1.1	0.9	1.2	1.0
TMX4	Q9H1E5	326	12	-17	15	1	1.1	0.9	1.2	1.0
SRBD1	Q8N5C6	397	12	33	15	20	1.1	1.5	1.2	1.3
STT3B	Q8TCJ2	715	10	-21	15	16	1.1	0.8	1.2	1.2
NSUN4	Q96CB9	197	10	0	15	5	1.1	1.0	1.2	1.0
MIOS	Q9NXC5	748	9	4	15	-16	1.1	1.0	1.2	0.9
EDC4	Q6P2E9	81	9	-12	15	-8	1.1	0.9	1.2	0.9
MFN1	Q8IWA4	428	7	-6	15	-5	1.1	0.9	1.2	1.0
MADD	Q8WXG6	202	7	1	15	7	1.1	1.0	1.2	1.1
DDX39B	Q13838	300	7	-5	15	-4	1.1	1.0	1.2	1.0
COX5B	P10606	116	6	-9	15	5	1.1	0.9	1.2	1.0
XRN1	Q8IZH2	1450	4	5	15	11	1.0	1.0	1.2	1.1
CASP7	P55210	290	2	3	15	17	1.0	1.0	1.2	1.2
PFKP	Q01813	718	-1	-6	15	-15	1.0	0.9	1.2	0.9
PBLD	P30039	79	-9	14	15	10	0.9	1.2	1.2	1.1
LRBA	P50851	1228	-11	-2	15	-29	0.9	1.0	1.2	0.8
SESTD1	Q86VW0	139	38	27	14	-11	1.6	1.4	1.2	0.9
EFL1	Q7Z2Z2	402	36	2	14	16	1.6	1.0	1.2	1.2
ECM29	Q5VYK3	1712	32	-5	14	19	1.5	1.0	1.2	1.2
PLEKHM1	Q9Y4G2	935	25	1	14	17	1.3	1.0	1.2	1.2
Uncharacteri	U3KPZ7	49	24	3	14	4	1.3	1.0	1.2	1.0
Uncharacteri	A0A0C4DFX4	643	20	1	14	-15	1.3	1.0	1.2	0.9
INTS1	Q8N201	358	19	0	14	2	1.2	1.0	1.2	1.0
HSPBP1	Q9NZL4	175	18	22	14	34	1.2	1.3	1.2	1.5
RNASEL	Q05823	639	18	9	14	-2	1.2	1.1	1.2	1.0

SHMT1	P34896	96	18	-1	14	19	1.2	1.0	1.2	1.2
PDS5A	Q29RF7	486	17	11	14	14	1.2	1.1	1.2	1.2
SKIV2L2	P42285	297	17	-1	14	3	1.2	1.0	1.2	1.0
SETX	Q7Z333	2187	17	15	14	21	1.2	1.2	1.2	1.3
WDR26	Q9H7D7	338	17	-10	14	17	1.2	0.9	1.2	1.2
UBE2K	P61086	170	16	-1	14	9	1.2	1.0	1.2	1.1
INF2	Q27J81	758	15	2	14	6	1.2	1.0	1.2	1.1
TELO2	Q9Y4R8	222	14	10	14	-3	1.2	1.1	1.2	1.0
DBR1	Q9UK59	371	13	-8	14	-3	1.1	0.9	1.2	1.0
HDLBP	Q00341	855	12	-14	14	4	1.1	0.9	1.2	1.0
SP140	Q13342	708	12	0	14	-15	1.1	1.0	1.2	0.9
SP140L	Q9H930	421	12	0	14	-15	1.1	1.0	1.2	0.9
ZNF276	Q8N554	436	12	-1	14	-8	1.1	1.0	1.2	0.9
PSMC1	P62191	399	12	-5	14	1	1.1	1.0	1.2	1.0
NLRC3	Q7RTR2	859	11	-4	14	-5	1.1	1.0	1.2	1.0
NUP205	Q92621	1715	10	-21	14	2	1.1	0.8	1.2	1.0
CTSS	P25774	213	9	10	14	-13	1.1	1.1	1.2	0.9
ZNF638	Q14966	1930	8	3	14	-8	1.1	1.0	1.2	0.9
ERP44	Q9BS26	189	8	-10	14	3	1.1	0.9	1.2	1.0
MTA1	Q13330	229	7	9	14	8	1.1	1.1	1.2	1.1
MYCBP2	O75592	3354	6	8	14	20	1.1	1.1	1.2	1.2
RBM6	P78332	1057	5	5	14	12	1.1	1.0	1.2	1.1
HERC1	Q15751	4811	5	7	14	10	1.0	1.1	1.2	1.1
MDN1	Q9NU22	43	4	6	14	-7	1.0	1.1	1.2	0.9
ECM29	Q5VYK3	1070	4	-22	14	9	1.0	0.8	1.2	1.1
RFC1	P35251	822	3	9	14	5	1.0	1.1	1.2	1.0
Integrin	H3BM21	646	1	20	14	2	1.0	1.3	1.2	1.0
RNF213	Q63HN8	4737	0	13	14	14	1.0	1.1	1.2	1.2
SPTB	P11277	2012	-1	-4	14	-27	1.0	1.0	1.2	0.8
POLR1A	O95602	112	-3	3	14	-10	1.0	1.0	1.2	0.9
ZYX	Q15942	436	-6	-12	14	-5	0.9	0.9	1.2	1.0
GOLGA2	Q08379	984	-7	-1	14	16	0.9	1.0	1.2	1.2
NVL	O15381	626	-8	-11	14	61	0.9	0.9	1.2	2.6
IRS2	Q9Y4H2	801	-10	2	14	-12	0.9	1.0	1.2	0.9
LRRFIP1	Q32MZ4	726	-30	-4	14	-9	0.8	1.0	1.2	0.9
INTS6	Q9UL03	211	27	27	14	17	1.4	1.4	1.2	1.2
PCNT	O95613	2059	27	1	14	10	1.4	1.0	1.2	1.1
EIF3CL	B5ME19	79	27	-25	14	11	1.4	0.8	1.2	1.1
IQGAP1	P46940	151	26	1	14	10	1.4	1.0	1.2	1.1
C12orf29	Q8N999	302	21	-1	14	3	1.3	1.0	1.2	1.0
RPL10A	P62906	164	20	4	14	12	1.2	1.0	1.2	1.1
SACM1L	Q9NTJ5	389	18	7	14	7	1.2	1.1	1.2	1.1
IPO5	O00410	733	17	2	14	16	1.2	1.0	1.2	1.2
NUP153	P49790	1129	16	4	14	-14	1.2	1.0	1.2	0.9
C2CD5	Q86YS7	142	16	-4	14	-4	1.2	1.0	1.2	1.0
RANBP10	Q6VN20	501	16	10	14	-5	1.2	1.1	1.2	1.0
ZMYM3	Q14202	314	16	1	14	5	1.2	1.0	1.2	1.1
DNPH1	O43598	26	14	4	14	23	1.2	1.0	1.2	1.3
SNAP23	O00161	85	14	1	14	15	1.2	1.0	1.2	1.2
SH3GL1	Q99961	96	14	-4	14	6	1.2	1.0	1.2	1.1
PHGDH	O43175	234	14	-3	14	11	1.2	1.0	1.2	1.1
CAPN2	P17655	405	12	6	14	12	1.1	1.1	1.2	1.1
CSE1L	P55060	344	11	-10	14	-9	1.1	0.9	1.2	0.9

SP140	Q13342	756	11	0	14	7	1.1	1.0	1.2	1.1
SP140L	Q9H930	469	11	0	14	7	1.1	1.0	1.2	1.1
RIN2	Q8WYP3	802	10	5	14	-7	1.1	1.1	1.2	0.9
ZNF8	P17098	383	9	-2	14	-5	1.1	1.0	1.2	1.0
TJP2	Q9UDY2	601	8	2	14	1	1.1	1.0	1.2	1.0
DOCK8	Q8NF50	590	8	-3	14	-11	1.1	1.0	1.2	0.9
ZBTB7A	O95365	34	8	-11	14	5	1.1	0.9	1.2	1.0
TRIM14	Q14142	237	8	8	14	-2	1.1	1.1	1.2	1.0
CCT3	P49368	398	8	0	14	11	1.1	1.0	1.2	1.1
GATB	O75879	228	7	-3	14	-7	1.1	1.0	1.2	0.9
LPCAT1	Q8NF37	443	5	8	14	24	1.1	1.1	1.2	1.3
RPRD1A	Q96P16	100	4	-13	14	-41	1.0	0.9	1.2	0.7
MAF1	Q9H063	48	2	-2	14	-2	1.0	1.0	1.2	1.0
TRAPPC8	Q9Y2L5	265	2	-14	14	-26	1.0	0.9	1.2	0.8
NADSYN1	Q6IA69	309	1	3	14	-13	1.0	1.0	1.2	0.9
USP34	Q70CQ2	3193	-1	-4	14	-30	1.0	1.0	1.2	0.8
SMARCA2	P51531	399	-1	3	14	-8	1.0	1.0	1.2	0.9
ANKFY1	Q9P2R3	1159	-3	-8	14	15	1.0	0.9	1.2	1.2
EIF4E	P06730	170	-4	-11	14	2	1.0	0.9	1.2	1.0
EPRS	P07814	1448	-6	4	14	-1	0.9	1.0	1.2	1.0
CDK10	Q15131	142	-9	-6	14	-22	0.9	0.9	1.2	0.8
CPSF4	O95639	156	-21	-9	14	10	0.8	0.9	1.2	1.1
OAS2	P29728	180	24	-12	13	7	1.3	0.9	1.1	1.1
OSTF1	Q92882	114	23	-17	13	11	1.3	0.9	1.1	1.1
BAZ1B	Q9UIG0	1435	20	-45	13	18	1.3	0.7	1.1	1.2
IPO9	Q96P70	864	18	-4	13	19	1.2	1.0	1.1	1.2
DDX17	Q92841	319	18	-19	13	18	1.2	0.8	1.1	1.2
GCLC	P48506	295	15	-5	13	-1	1.2	1.0	1.1	1.0
MICALL1	Q8N3F8	214	11	11	13	-15	1.1	1.1	1.1	0.9
NELFCD	Q8IXH7	293	10	0	13	6	1.1	1.0	1.1	1.1
MALT1	Q9UDY8	464	7	4	13	9	1.1	1.0	1.1	1.1
TRIO	O75962	2222	7	-7	13	-3	1.1	0.9	1.1	1.0
VCAN	P13611	3339	7	13	13	4	1.1	1.1	1.1	1.0
ATRX	P46100	1595	6	1	13	9	1.1	1.0	1.1	1.1
NARS	O43776	537	6	-5	13	4	1.1	1.0	1.1	1.0
NELFA	Q9H3P2	44	5	1	13	-5	1.0	1.0	1.1	1.0
SNX9	Q9Y5X1	502	5	-8	13	0	1.0	0.9	1.1	1.0
C9orf142	Q9BUH6	24	4	4	13	-1	1.0	1.0	1.1	1.0
KANSL3	Q9P2N6	161	4	3	13	-4	1.0	1.0	1.1	1.0
NHLRC2	Q8NBF2	716	3	-1	13	-5	1.0	1.0	1.1	1.0
TOP2B	Q02880	204	2	-2	13	1	1.0	1.0	1.1	1.0
RNH1	P13489	142	-2	5	13	4	1.0	1.1	1.1	1.0
DTNBP1	Q96EV8	302	-6	-13	13	-13	0.9	0.9	1.1	0.9
TRIM4	Q9C037	397	-9	-25	13	4	0.9	0.8	1.1	1.0
RNF213	Q63HN8	1916	-9	16	13	16	0.9	1.2	1.1	1.2
EHBP1L1	Q8N3D4	221	-10	-6	13	16	0.9	0.9	1.1	1.2
TTC32	Q5I0X7	40	-10	-22	13	-25	0.9	0.8	1.1	0.8
JAK3	P52333	1040	-12	-1	13	-10	0.9	1.0	1.1	0.9
SNX30	Q5VWJ9	375	-18	-4	13	-25	0.9	1.0	1.1	0.8
HGH1	Q9BTY7	138	36	12	13	16	1.6	1.1	1.1	1.2
WNK1	Q9H4A3	352	27	-18	13	11	1.4	0.8	1.1	1.1
CUL4A	Q13619	633	25	3	13	9	1.3	1.0	1.1	1.1
CUL4B	Q13620	787	25	3	13	9	1.3	1.0	1.1	1.1

COG4	Q9H9E3	102	24	13	13	13	1.3	1.1	1.1	1.1
PPP2R5C	Q13362	49	24	5	13	13	1.3	1.0	1.1	1.1
PPP2R5D	Q14738	125	24	5	13	13	1.3	1.0	1.1	1.1
KARS	Q15046	434	22	0	13	21	1.3	1.0	1.1	1.3
TAF6	P49848	460	22	3	13	11	1.3	1.0	1.1	1.1
PPP1R3D	O95685	93	22	-4	13	20	1.3	1.0	1.1	1.3
USP14	P54578	415	21	17	13	15	1.3	1.2	1.1	1.2
LAP3	P28838	462	21	-2	13	0	1.3	1.0	1.1	1.0
ZFYVE16	Q7Z3T8	1463	20	19	13	13	1.3	1.2	1.1	1.1
HUWE1	Q7Z6Z7	612	20	-16	13	14	1.2	0.9	1.1	1.2
GPATCH1	Q9BRR8	839	19	17	13	16	1.2	1.2	1.1	1.2
PPID	Q08752	275	19	2	13	16	1.2	1.0	1.1	1.2
GP5	P40197	425	19	-3	13	17	1.2	1.0	1.1	1.2
NCDN	Q9UBB6	415	18	14	13	11	1.2	1.2	1.1	1.1
TRABD	Q9H4I3	366	18	-5	13	13	1.2	1.0	1.1	1.1
MTHFD1L	Q6UB35	302	17	-6	13	-16	1.2	0.9	1.1	0.9
ZNF746	Q6NUN9	385	17	-21	13	-4	1.2	0.8	1.1	1.0
PLEKHM2	Q8IWE5	735	16	17	13	8	1.2	1.2	1.1	1.1
TNRC18	O15417	2288	16	0	13	13	1.2	1.0	1.1	1.1
CSE1L	P55060	842	16	-2	13	7	1.2	1.0	1.1	1.1
CRLF3	Q8IUI8	313	14	6	13	8	1.2	1.1	1.1	1.1
ARHGEF6	Q15052	319	14	3	13	17	1.2	1.0	1.1	1.2
KIAA0196	Q12768	187	10	-7	13	17	1.1	0.9	1.1	1.2
P2RX7	Q99572	493	9	-6	13	13	1.1	0.9	1.1	1.1
DYNC1H1	Q14204	4644	9	-17	13	-3	1.1	0.9	1.1	1.0
CLTC	Q00610	436	9	-9	13	-4	1.1	0.9	1.1	1.0
TAF1	P21675	1066	8	-8	13	15	1.1	0.9	1.1	1.2
AP1G2	O75843	32	8	-11	13	10	1.1	0.9	1.1	1.1
HERC2	O95714	3864	8	-5	13	2	1.1	1.0	1.1	1.0
DNM1L	O00429	431	8	-16	13	-1	1.1	0.9	1.1	1.0
MRPS18A	Q9NVS2	186	7	-3	13	9	1.1	1.0	1.1	1.1
IPO5	O00410	560	7	-15	13	-19	1.1	0.9	1.1	0.8
DIDO1	Q9BTC0	350	6	0	13	-12	1.1	1.0	1.1	0.9
PIGA	P37287	28	6	-1	13	5	1.1	1.0	1.1	1.0
HBD	P02042	94	6	-2	13	0	1.1	1.0	1.1	1.0
NRBP1	Q9UHY1	307	6	-18	13	-16	1.1	0.8	1.1	0.9
ZFAND5	O76080	168	5	3	13	-3	1.1	1.0	1.1	1.0
PLXNB3	Q9ULL4	1363	5	9	13	-3	1.0	1.1	1.1	1.0
LPCAT1	Q8NF37	330	5	-1	13	5	1.0	1.0	1.1	1.0
NCDN	Q9UBB6	469	4	4	13	17	1.0	1.0	1.1	1.2
BIRC3	Q13489	28	3	-1	13	2	1.0	1.0	1.1	1.0
GCN1	Q92616	2558	3	-5	13	-5	1.0	1.0	1.1	1.0
LDHD	Q86WU2	28	-1	-5	13	11	1.0	1.0	1.1	1.1
RPAIN	Q86UA6	175	-5	-15	13	-10	1.0	0.9	1.1	0.9
PDDC1	Q8NB37	179	-6	-5	13	-10	0.9	1.0	1.1	0.9
VPS18	Q9P253	522	-8	-2	13	6	0.9	1.0	1.1	1.1
IFIH1	Q9BYX4	17	-9	-4	13	-11	0.9	1.0	1.1	0.9
HAUS6	Q7Z4H7	743	-9	-1	13	5	0.9	1.0	1.1	1.1
ANKRD39	Q53RE8	173	-11	-11	13	-11	0.9	0.9	1.1	0.9
FBXL20	Q96IG2	205	35	-3	12	12	1.5	1.0	1.1	1.1
FBXL2	Q9UKC9	192	35	-3	12	12	1.5	1.0	1.1	1.1
GOLPH3	Q9H4A6	92	32	3	12	27	1.5	1.0	1.1	1.4
OARD1	Q9Y530	38	26	8	12	30	1.3	1.1	1.1	1.4

DCAKD	Q8WVC6	135	25	14	12	19	1.3	1.2	1.1	1.2
DR1	Q01658	58	19	6	12	7	1.2	1.1	1.1	1.1
STK40	Q8N2I9	218	18	4	12	6	1.2	1.0	1.1	1.1
CTC1	Q2NKJ3	1181	17	-5	12	-8	1.2	1.0	1.1	0.9
PLIN3	O60664	341	16	-16	12	4	1.2	0.9	1.1	1.0
RHOF	Q9HBH0	131	15	2	12	-6	1.2	1.0	1.1	0.9
PPOX	P50336	167	15	11	12	29	1.2	1.1	1.1	1.4
TMEM173	Q86WV6	148	15	-6	12	-3	1.2	0.9	1.1	1.0
TBC1D5	Q92609	192	14	-4	12	8	1.2	1.0	1.1	1.1
PTPN23	Q9H3S7	65	13	1	12	8	1.1	1.0	1.1	1.1
KPNB1	Q14974	765	13	-28	12	-1	1.1	0.8	1.1	1.0
URB2	Q14146	1294	12	7	12	4	1.1	1.1	1.1	1.0
GBA	P04062	165	12	20	12	6	1.1	1.3	1.1	1.1
HPS3	Q969F9	439	12	-6	12	-4	1.1	0.9	1.1	1.0
TRAPPC9	Q96Q05	141	12	-17	12	-7	1.1	0.9	1.1	0.9
PPP2R5E	Q16537	106	11	8	12	8	1.1	1.1	1.1	1.1
FAM65B	Q9Y4F9	519	11	4	12	-10	1.1	1.0	1.1	0.9
CORO7-PAM:	A0A0A6YYL4	363	11	-6	12	8	1.1	0.9	1.1	1.1
AP3B1	O00203	507	11	-2	12	6	1.1	1.0	1.1	1.1
RASGRP2	Q7LDG7	398	10	0	12	-7	1.1	1.0	1.1	0.9
MT-ND3	P03897	39	9	-2	12	15	1.1	1.0	1.1	1.2
TTLL12	Q14166	126	8	-3	12	5	1.1	1.0	1.1	1.0
EXOSC3	Q9NQT5	67	8	-8	12	3	1.1	0.9	1.1	1.0
SCYL1	Q96KG9	512	7	-19	12	-11	1.1	0.8	1.1	0.9
IPO4	Q8TEX9	400	7	5	12	5	1.1	1.1	1.1	1.1
TRIP12	Q14669	710	7	3	12	-8	1.1	1.0	1.1	0.9
RPLP0	P05388	226	6	-4	12	-4	1.1	1.0	1.1	1.0
TRIM28	Q13263	152	6	18	12	3	1.1	1.2	1.1	1.0
WHAMM	Q8TF30	179	5	7	12	-1	1.0	1.1	1.1	1.0
ERN1	O75460	715	5	1	12	-3	1.0	1.0	1.1	1.0
DDX54	Q8TDD1	586	3	3	12	-4	1.0	1.0	1.1	1.0
KIF5C	O60282	296	-1	5	12	1	1.0	1.1	1.1	1.0
KIF5B	P33176	294	-1	5	12	1	1.0	1.1	1.1	1.0
CELF2	O95319	85	-1	-17	12	-4	1.0	0.9	1.1	1.0
CELF1	Q92879	61	-1	-17	12	-4	1.0	0.9	1.1	1.0
PHF5A	Q7RTV0	33	-1	-11	12	1	1.0	0.9	1.1	1.0
EML4	Q9HC35	638	-2	-36	12	-44	1.0	0.7	1.1	0.7
PIK3CG	P48736	435	-2	-24	12	-4	1.0	0.8	1.1	1.0
TTC4	O95801	63	-3	-13	12	-10	1.0	0.9	1.1	0.9
SOD1	P00441	147	-3	1	12	0	1.0	1.0	1.1	1.0
ZNF414	Q96IQ9	116	-4	-7	12	2	1.0	0.9	1.1	1.0
POMP	Q9Y244	37	-4	-17	12	2	1.0	0.9	1.1	1.0
UQCRC1	P31930	347	-6	-11	12	-6	0.9	0.9	1.1	0.9
ARSA	P15289	294	-15	-10	12	2	0.9	0.9	1.1	1.0
HNRNPDL	O14979	277	40	-10	12	3	1.7	0.9	1.1	1.0
ATF6B	Q99941	501	35	-4	12	3	1.5	1.0	1.1	1.0
NIF3L1	Q9GZT8	254	32	8	12	10	1.5	1.1	1.1	1.1
METAP2	P50579	416	29	9	12	0	1.4	1.1	1.1	1.0
GLS	O94925	287	28	5	12	13	1.4	1.1	1.1	1.1
GAK	O14976	599	24	-14	12	-1	1.3	0.9	1.1	1.0
CCT4	P50991	450	23	1	12	10	1.3	1.0	1.1	1.1
SRP68	Q9UHB9	344	23	0	12	12	1.3	1.0	1.1	1.1
GCA	P28676	182	22	7	12	15	1.3	1.1	1.1	1.2

WARS	P23381	225	22	-19	12	7	1.3	0.8	1.1	1.1
PSMD5	Q16401	290	21	3	12	6	1.3	1.0	1.1	1.1
TLN1	Q9Y490	1661	20	2	12	11	1.2	1.0	1.1	1.1
TMF1	P82094	158	19	2	12	0	1.2	1.0	1.1	1.0
UNC13D	Q70J99	505	18	3	12	-2	1.2	1.0	1.1	1.0
PTPN9	P43378	230	18	-2	12	3	1.2	1.0	1.1	1.0
NME2	P22392	109	18	-26	12	5	1.2	0.8	1.1	1.0
ANKRD16	Q6P6B7	231	17	23	12	-2	1.2	1.3	1.1	1.0
MRPS31	Q92665	356	17	-7	12	-6	1.2	0.9	1.1	0.9
ZBTB7B	O15156	55	16	10	12	8	1.2	1.1	1.1	1.1
RPL34	P49207	83	15	6	12	10	1.2	1.1	1.1	1.1
PPP2CB	P62714	20	15	-20	12	-8	1.2	0.8	1.1	0.9
GTF3C1	Q12789	1704	15	11	12	19	1.2	1.1	1.1	1.2
TMEM209	Q96SK2	301	14	4	12	6	1.2	1.0	1.1	1.1
ALOX12	P18054	96	14	1	12	4	1.2	1.0	1.1	1.0
KIF5B	P33176	174	14	-5	12	17	1.2	1.0	1.1	1.2
BTAF1	O14981	1310	13	6	12	3	1.1	1.1	1.1	1.0
RABGAP1	Q9Y3P9	582	13	-6	12	-10	1.1	0.9	1.1	0.9
GTF3C1	Q12789	1999	12	-7	12	3	1.1	0.9	1.1	1.0
PTK2B	Q14289	180	11	2	12	2	1.1	1.0	1.1	1.0
ARIH1	Q9Y4X5	357	11	3	12	8	1.1	1.0	1.1	1.1
SZT2	Q5T011	2962	11	1	12	3	1.1	1.0	1.1	1.0
TP53BP1	Q12888	513	10	6	12	-1	1.1	1.1	1.1	1.0
IQSEC1	Q6DN90	214	10	-9	12	-17	1.1	0.9	1.1	0.9
TRUB1	Q8WWH5	269	8	-14	12	-21	1.1	0.9	1.1	0.8
BYSL	Q13895	381	8	-14	12	23	1.1	0.9	1.1	1.3
RECQL	P46063	493	7	-27	12	16	1.1	0.8	1.1	1.2
MRPS18C	Q9Y3D5	90	6	7	12	-5	1.1	1.1	1.1	1.0
TRIM14	Q14142	20	6	-2	12	-3	1.1	1.0	1.1	1.0
EHD4	Q9H223	141	5	-5	12	-12	1.1	1.0	1.1	0.9
TUBA4A	P68366	129	5	-7	12	-6	1.1	0.9	1.1	0.9
FBXL2	Q9UKC9	351	5	4	12	4	1.0	1.0	1.1	1.0
DCTN4	Q9UJW0	70	4	2	12	17	1.0	1.0	1.1	1.2
LZTR1	Q8N653	106	4	-2	12	12	1.0	1.0	1.1	1.1
SHPK	Q9UHJ6	38	-2	-6	12	1	1.0	0.9	1.1	1.0
KIF1B	O60333	600	-4	-17	12	-7	1.0	0.9	1.1	0.9
TUBGCP4	Q9UGJ1	162	42	29	11	9	1.7	1.4	1.1	1.1
CKAP5	Q14008	441	34	-4	11	15	1.5	1.0	1.1	1.2
ASAP1	Q9ULH1	685	29	9	11	9	1.4	1.1	1.1	1.1
AHCYL1	O43865	326	25	0	11	8	1.3	1.0	1.1	1.1
XAB2	Q9HCS7	695	25	-4	11	4	1.3	1.0	1.1	1.0
PTK2B	Q14289	352	24	-12	11	2	1.3	0.9	1.1	1.0
COQ9	O75208	153	23	7	11	19	1.3	1.1	1.1	1.2
DIAPH1	O60610	164	19	3	11	25	1.2	1.0	1.1	1.3
PPP4R1	Q8TF05	241	18	5	11	7	1.2	1.0	1.1	1.1
INTS8	Q75QN2	701	18	-10	11	9	1.2	0.9	1.1	1.1
ENDOV	Q8N8Q3	270	17	-5	11	-2	1.2	1.0	1.1	1.0
SCML4	Q8N228	211	17	9	11	-6	1.2	1.1	1.1	0.9
ARHGAP21	Q5T5U3	19	16	6	11	7	1.2	1.1	1.1	1.1
EPRS	P07814	105	16	-5	11	17	1.2	1.0	1.1	1.2
SUPT4H1	P63272	16	16	2	11	17	1.2	1.0	1.1	1.2
SEC23B	Q15437	434	16	1	11	11	1.2	1.0	1.1	1.1
HOOK3	Q86VS8	129	16	-1	11	13	1.2	1.0	1.1	1.1

CD14	P08571	187	16	-4	11	17	1.2	1.0	1.1	1.2
LYPLA1	O75608	144	15	-5	11	9	1.2	1.0	1.1	1.1
PACS2	Q86VP3	517	14	8	11	16	1.2	1.1	1.1	1.2
PACSIN2	Q9UNF0	38	14	7	11	13	1.2	1.1	1.1	1.1
NUP98	P52948	1068	13	-2	11	-7	1.1	1.0	1.1	0.9
IFIH1	Q9BYX4	510	13	-6	11	11	1.1	0.9	1.1	1.1
CLPX	O76031	586	13	-10	11	-21	1.1	0.9	1.1	0.8
DYNC1H1	Q14204	4438	13	-12	11	-4	1.1	0.9	1.1	1.0
APAF1	O14727	569	12	-6	11	9	1.1	0.9	1.1	1.1
NUBP2	Q9Y5Y2	202	12	40	11	20	1.1	1.7	1.1	1.3
DCAF7	P61962	61	12	-4	11	4	1.1	1.0	1.1	1.0
CEP152	O94986	1417	11	-30	11	-4	1.1	0.8	1.1	1.0
TARBP1	Q13395	202	11	10	11	46	1.1	1.1	1.1	1.9
NDUFS2	O75306	326	11	8	11	13	1.1	1.1	1.1	1.1
SEC61A1	P61619	13	11	-6	11	3	1.1	0.9	1.1	1.0
HGS	O14964	166	11	-13	11	-8	1.1	0.9	1.1	0.9
RRP7A	Q9Y3A4	8	10	-3	11	-10	1.1	1.0	1.1	0.9
FAM126A	Q9BYI3	401	9	6	11	-3	1.1	1.1	1.1	1.0
TECPR1	Q7Z6L1	462	8	7	11	-6	1.1	1.1	1.1	0.9
VPRBP	Q9Y4B6	69	8	-7	11	15	1.1	0.9	1.1	1.2
MOV10	Q9HCE1	16	7	1	11	-6	1.1	1.0	1.1	0.9
USP4	Q13107	475	6	-10	11	53	1.1	0.9	1.1	2.1
LRSAM1	Q6UWE0	205	4	-10	11	5	1.0	0.9	1.1	1.1
ARHGAP27	Q6ZUM4	830	4	1	11	3	1.0	1.0	1.1	1.0
HEATR5A	Q86XA9	841	3	-1	11	12	1.0	1.0	1.1	1.1
NSUN2	Q08J23	758	2	7	11	-4	1.0	1.1	1.1	1.0
UBE2O	Q9C0C9	598	2	4	11	-3	1.0	1.0	1.1	1.0
DNAJA2	O60884	280	2	-25	11	21	1.0	0.8	1.1	1.3
NAGK	Q9UJ70	217	1	-20	11	9	1.0	0.8	1.1	1.1
RUFY1	Q96T51	544	0	4	11	-9	1.0	1.0	1.1	0.9
TEP1	Q99973	171	0	-1	11	6	1.0	1.0	1.1	1.1
WDR59	Q6PJI9	654	-1	-6	11	-2	1.0	0.9	1.1	1.0
HERC2	O95714	914	-3	-9	11	42	1.0	0.9	1.1	1.7
PHKB	Q93100	348	-4	1	11	-3	1.0	1.0	1.1	1.0
PSTPIP1	O43586	259	-5	-15	11	-5	1.0	0.9	1.1	1.0
PXN	P49023	358	-16	-1	11	4	0.9	1.0	1.1	1.0
MARCKSL1	P49006	134	-34	-14	11	-19	0.7	0.9	1.1	0.8
PEBP1	P30086	133	-247	-140	11	18	0.3	0.4	1.1	1.2
PAXIP1	Q6ZW49	997	23	3	11	-1	1.3	1.0	1.1	1.0
DDB1	Q16531	903	21	-13	11	6	1.3	0.9	1.1	1.1
SSU72	Q9NP77	101	20	4	11	6	1.2	1.0	1.1	1.1
SYNE2	Q8WXH0	587	19	11	11	-1	1.2	1.1	1.1	1.0
ADRBK1	P25098	634	19	-16	11	-5	1.2	0.9	1.1	1.0
TRRAP	Q9Y4A5	1133	18	4	11	7	1.2	1.0	1.1	1.1
SH3GL1	Q99961	311	18	-5	11	4	1.2	1.0	1.1	1.0
ILK	Q13418	428	17	-7	11	4	1.2	0.9	1.1	1.0
PTGES3	Q15185	40	17	-13	11	1	1.2	0.9	1.1	1.0
DHX9	Q08211	1099	17	-17	11	7	1.2	0.9	1.1	1.1
C10orf76	Q5T2E6	80	15	5	11	5	1.2	1.0	1.1	1.1
NOC2L	Q9Y3T9	567	15	4	11	8	1.2	1.0	1.1	1.1
DDB2	Q92466	254	14	12	11	7	1.2	1.1	1.1	1.1
SNRNP200	O75643	516	14	6	11	9	1.2	1.1	1.1	1.1
CDK7	P50613	305	14	-7	11	15	1.2	0.9	1.1	1.2

SETX	Q7Z333	2384	14	-2	11	13	1.2	1.0	1.1	1.1
FHL1	Q13642	132	13	-1	11	6	1.1	1.0	1.1	1.1
VPS51	Q9UID3	81	12	9	11	9	1.1	1.1	1.1	1.1
LZTR1	Q8N653	342	12	4	11	-4	1.1	1.0	1.1	1.0
ZNF428	Q96B54	93	11	4	11	11	1.1	1.0	1.1	1.1
SP1	P08047	606	11	-2	11	-2	1.1	1.0	1.1	1.0
RPS5	P46782	172	11	-1	11	10	1.1	1.0	1.1	1.1
INPP5D	Q92835	49	11	-5	11	7	1.1	1.0	1.1	1.1
RASAL3	Q86YV0	533	10	-3	11	4	1.1	1.0	1.1	1.0
SMPD4	Q9NXE4	659	10	-14	11	-14	1.1	0.9	1.1	0.9
CEP128	Q6ZU80	819	10	-5	11	-10	1.1	1.0	1.1	0.9
RALGAPB	Q86X10	242	10	-5	11	20	1.1	1.0	1.1	1.2
POGZ	Q7Z3K3	986	9	-5	11	-13	1.1	1.0	1.1	0.9
APBB1IP	Q7Z5R6	355	9	1	11	-4	1.1	1.0	1.1	1.0
CTDP1	Q9Y5B0	429	9	-15	11	1	1.1	0.9	1.1	1.0
NCOR1	O75376	474	9	-19	11	-3	1.1	0.8	1.1	1.0
PLEK	P08567	155	8	-6	11	17	1.1	0.9	1.1	1.2
PPP1R7	Q15435	112	8	-9	11	4	1.1	0.9	1.1	1.0
APAF1	O14727	761	7	9	11	0	1.1	1.1	1.1	1.0
PRDX5	P30044	204	6	-3	11	14	1.1	1.0	1.1	1.2
THOC2	Q8NI27	981	5	-5	11	-5	1.1	1.0	1.1	1.0
FNDC3A	Q9Y2H6	211	5	-6	11	-3	1.1	0.9	1.1	1.0
SLC26A6	Q9BXS9	565	3	2	11	3	1.0	1.0	1.1	1.0
NISCH	Q9Y2I1	848	2	-1	11	-2	1.0	1.0	1.1	1.0
ATAD5	Q96QE3	1628	2	-9	11	-12	1.0	0.9	1.1	0.9
GNB4	Q9HAV0	271	1	-1	11	10	1.0	1.0	1.1	1.1
ACAP1	Q15027	657	-1	3	11	4	1.0	1.0	1.1	1.0
DNAJC21	Q5F1R6	3	-1	-18	11	-1	1.0	0.9	1.1	1.0
C17orf59	Q96GS4	176	-3	8	11	2	1.0	1.1	1.1	1.0
BTLA	Q7Z6A9	286	-3	-14	11	-22	1.0	0.9	1.1	0.8
TBXAS1	P24557	78	-13	-16	11	-7	0.9	0.9	1.1	0.9
FHL3	Q13643	153	-19	-3	11	9	0.8	1.0	1.1	1.1
NTAN1	Q96AB6	118	-21	-1	11	-19	0.8	1.0	1.1	0.8
EPHX2	P34913	309	27	15	10	18	1.4	1.2	1.1	1.2
RFC5	P40937	73	25	1	10	3	1.3	1.0	1.1	1.0
CCNY	Q8ND76	238	23	5	10	9	1.3	1.0	1.1	1.1
ZFP36	P26651	249	22	13	10	-2	1.3	1.1	1.1	1.0
RARS	P54136	115	22	1	10	21	1.3	1.0	1.1	1.3
DTX3L	Q8TDB6	564	20	6	10	-7	1.2	1.1	1.1	0.9
ARHGEF6	Q15052	57	19	7	10	10	1.2	1.1	1.1	1.1
HSPBP1	Q9NZL4	313	19	-1	10	13	1.2	1.0	1.1	1.1
RUVBL1	Q9Y265	49	18	-4	10	6	1.2	1.0	1.1	1.1
RFK	Q969G6	8	18	-10	10	19	1.2	0.9	1.1	1.2
SUZ12	Q15022	494	17	6	10	10	1.2	1.1	1.1	1.1
THOC2	Q8NI27	996	17	4	10	24	1.2	1.0	1.1	1.3
VPS11	Q9H270	568	17	-9	10	14	1.2	0.9	1.1	1.2
ADK	P55263	140	15	0	10	5	1.2	1.0	1.1	1.0
PCBP1	Q15365	194	15	8	10	3	1.2	1.1	1.1	1.0
TTC4	O95801	374	14	9	10	2	1.2	1.1	1.1	1.0
KPNB1	Q14974	543	14	4	10	18	1.2	1.0	1.1	1.2
PUS1	Q9Y606	202	14	-10	10	2	1.2	0.9	1.1	1.0
GOLGA3	Q08378	1403	14	1	10	15	1.2	1.0	1.1	1.2
FCGRT	P55899	182	13	13	10	13	1.1	1.1	1.1	1.1

COPS4	Q9BT78	70	12	-14	10	-9	1.1	0.9	1.1	0.9
MVB12A	Q96EY5	90	12	14	10	0	1.1	1.2	1.1	1.0
LRPPRC	P42704	848	11	-36	10	3	1.1	0.7	1.1	1.0
LARS	Q9P2J5	83	10	13	10	5	1.1	1.1	1.1	1.1
INTS1	Q8N201	969	10	-6	10	23	1.1	0.9	1.1	1.3
GTF3C4	Q9UKN8	93	10	-15	10	23	1.1	0.9	1.1	1.3
GEMIN5	Q8TEQ6	973	10	6	10	-3	1.1	1.1	1.1	1.0
PLEC	Q15149	3008	10	-6	10	2	1.1	0.9	1.1	1.0
TTLL12	Q14166	563	9	-21	10	14	1.1	0.8	1.1	1.2
MIIP	Q5JXC2	215	8	2	10	13	1.1	1.0	1.1	1.1
HBS1L	Q9Y450	469	8	-11	10	-2	1.1	0.9	1.1	1.0
SP140	Q13342	224	8	-13	10	-25	1.1	0.9	1.1	0.8
PPP2R4	Q15257	91	7	7	10	5	1.1	1.1	1.1	1.0
ADSL	P30566	113	6	6	10	6	1.1	1.1	1.1	1.1
ALDH16A1	Q8IZ83	350	6	2	10	11	1.1	1.0	1.1	1.1
TRPV2	Q9Y5S1	156	6	-9	10	10	1.1	0.9	1.1	1.1
NDUFV1	P49821	286	6	-20	10	-11	1.1	0.8	1.1	0.9
RBM33	Q96EV2	1093	5	4	10	-8	1.0	1.0	1.1	0.9
SRP14	P37108	56	5	-4	10	1	1.0	1.0	1.1	1.0
SLC15A4	Q8N697	269	3	-2	10	14	1.0	1.0	1.1	1.2
BCAS2	O75934	132	3	-5	10	1	1.0	1.0	1.1	1.0
SCP2	P22307	94	3	3	10	1	1.0	1.0	1.1	1.0
BAIAP3	O94812	554	3	-5	10	-10	1.0	1.0	1.1	0.9
PTAR1	Q7Z6K3	37	2	2	10	10	1.0	1.0	1.1	1.1
RPS6	P62753	100	2	-8	10	-5	1.0	0.9	1.1	1.0
SLC25A20	O43772	136	2	-14	10	43	1.0	0.9	1.1	1.7
BAZ1A	Q9NRL2	799	2	-18	10	-7	1.0	0.8	1.1	0.9
GGCT	O75223	42	-1	-19	10	0	1.0	0.8	1.1	1.0
PHF3	Q92576	276	-2	0	10	-10	1.0	1.0	1.1	0.9
CARD6	Q9BX69	640	-4	21	10	-4	1.0	1.3	1.1	1.0
PITRM1	Q5JRX3	619	-4	-12	10	1	1.0	0.9	1.1	1.0
PHC3	Q8NDX5	746	-6	-8	10	-16	0.9	0.9	1.1	0.9
ELP6	Q0PNE2	218	-7	-2	10	11	0.9	1.0	1.1	1.1
STXBP2	Q15833	158	-10	24	10	19	0.9	1.3	1.1	1.2
ARHGAP25	P42331	258	37	2	10	27	1.6	1.0	1.1	1.4
PUM1	Q14671	977	28	6	10	25	1.4	1.1	1.1	1.3
PUM2	Q8TB72	857	28	6	10	25	1.4	1.1	1.1	1.3
PPP2R4	Q15257	334	28	-6	10	13	1.4	0.9	1.1	1.1
P4HB	P07237	343	26	-64	10	11	1.3	0.6	1.1	1.1
YWHAZ	P63104	94	23	4	10	24	1.3	1.0	1.1	1.3
UBR1	Q8IWV7	279	23	-17	10	1	1.3	0.9	1.1	1.0
PGPEP1	Q9NXJ5	99	20	4	10	-5	1.2	1.0	1.1	1.0
LRRC16A	Q5VZK9	464	19	-7	10	-4	1.2	0.9	1.1	1.0
PLCG2	P16885	849	18	-7	10	13	1.2	0.9	1.1	1.1
MATR3	A8MXP9	552	18	4	10	0	1.2	1.0	1.1	1.0
PAPD4	Q6PIY7	167	18	-6	10	-11	1.2	0.9	1.1	0.9
ZFYVE26	Q68DK2	1534	17	18	10	2	1.2	1.2	1.1	1.0
CYFIP2	Q96F07	531	16	-1	10	-2	1.2	1.0	1.1	1.0
BLVRA	P53004	281	16	-24	10	21	1.2	0.8	1.1	1.3
SEPHS2	Q99611	387	16	9	10	17	1.2	1.1	1.1	1.2
SMARCD1	Q96GM5	460	15	5	10	5	1.2	1.1	1.1	1.0
RNASEH2B	Q5TBB1	125	15	-10	10	18	1.2	0.9	1.1	1.2
GLMN	Q92990	36	15	-1	10	3	1.2	1.0	1.1	1.0

INTS1	Q8N201	643	14	9	10	-3	1.2	1.1	1.1	1.0
KIAA1033	Q2M389	99	14	-1	10	4	1.2	1.0	1.1	1.0
PRPF39	Q86UA1	413	14	-6	10	-2	1.2	0.9	1.1	1.0
C12orf4	Q9NQ89	55	13	5	10	12	1.1	1.1	1.1	1.1
ARHGAP27	Q6ZUM4	697	13	5	10	12	1.1	1.0	1.1	1.1
IQGAP2	Q13576	548	13	2	10	7	1.1	1.0	1.1	1.1
SUPV3L1	Q8IYB8	175	13	-6	10	15	1.1	0.9	1.1	1.2
BTBD9	Q96Q07	159	12	8	10	17	1.1	1.1	1.1	1.2
EPG5	Q9HCE0	486	12	-10	10	10	1.1	0.9	1.1	1.1
AP2A1	O95782	283	12	-17	10	2	1.1	0.9	1.1	1.0
NBEAL2	Q6ZNJ1	1224	11	-2	10	49	1.1	1.0	1.1	2.0
SPG11	Q96JI7	1424	11	-6	10	-20	1.1	0.9	1.1	0.8
UBE2Z	Q9H832	261	10	1	10	3	1.1	1.0	1.1	1.0
SIRT2	Q8IXJ6	164	10	-18	10	-1	1.1	0.9	1.1	1.0
TBC1D10C	Q8IV04	305	10	5	10	9	1.1	1.1	1.1	1.1
ZNF646	O15015	150	10	-4	10	10	1.1	1.0	1.1	1.1
TOP3B	O95985	190	9	-15	10	20	1.1	0.9	1.1	1.2
GDPGP1	Q6ZNW5	64	9	-10	10	-8	1.1	0.9	1.1	0.9
PLCG1	P19174	1109	8	-2	10	4	1.1	1.0	1.1	1.0
TTI2	Q6NXR4	339	8	-6	10	7	1.1	0.9	1.1	1.1
C9orf64	Q5T6V5	227	8	2	10	1	1.1	1.0	1.1	1.0
CMIP	Q8IY22	572	7	-5	10	-6	1.1	1.0	1.1	0.9
ARHGAP35	Q9NRY4	562	7	-2	10	-4	1.1	1.0	1.1	1.0
GMIP	Q9P107	895	7	-2	10	-6	1.1	1.0	1.1	0.9
HBB	P68871	113	7	-3	10	3	1.1	1.0	1.1	1.0
SLFN14	P0C7P3	893	7	-12	10	-11	1.1	0.9	1.1	0.9
HEATR5B	Q9P2D3	1254	6	-3	10	16	1.1	1.0	1.1	1.2
TCERG1	O14776	535	5	-7	10	3	1.1	0.9	1.1	1.0
DUT	P33316	222	5	-11	10	-2	1.1	0.9	1.1	1.0
CAPN2	P17655	498	5	-13	10	-3	1.1	0.9	1.1	1.0
VPS51	Q9UID3	226	4	5	10	-3	1.0	1.1	1.1	1.0
ARHGEF6	Q15052	514	4	-7	10	-10	1.0	0.9	1.1	0.9
C7orf43	Q8WVR3	369	4	-8	10	-24	1.0	0.9	1.1	0.8
AGFG2	O95081	39	3	1	10	-12	1.0	1.0	1.1	0.9
PACSIN2	Q9UNF0	44	3	2	10	9	1.0	1.0	1.1	1.1
AAK1	Q2M2I8	156	2	-2	10	16	1.0	1.0	1.1	1.2
EPC1	Q9H2F5	566	1	-1	10	-10	1.0	1.0	1.1	0.9
UBE2O	Q9C0C9	1099	0	5	10	-6	1.0	1.0	1.1	0.9
TNFAIP8	O95379	165	-1	10	10	-1	1.0	1.1	1.1	1.0
TNPO3	Q9Y5L0	530	-1	-1	10	25	1.0	1.0	1.1	1.3
USP16	Q9Y5T5	657	-2	-12	10	-8	1.0	0.9	1.1	0.9
RPS8	P62241	71	-3	-7	10	1	1.0	0.9	1.1	1.0
SLC25A20	O43772	283	-3	6	10	48	1.0	1.1	1.1	1.9
IPP	Q9Y573	312	-4	-2	10	9	1.0	1.0	1.1	1.1
CUL4A	Q13619	241	-6	-13	10	-1	0.9	0.9	1.1	1.0
SIRT1	Q96EB6	67	-8	12	10	-8	0.9	1.1	1.1	0.9
LRCH3	Q96II8	116	-9	-14	10	-3	0.9	0.9	1.1	1.0
SF3B6	Q9Y3B4	83	-13	1	10	-1	0.9	1.0	1.1	1.0
YY1	P25490	303	-16	-6	10	3	0.9	0.9	1.1	1.0
FAM118B	Q9BPY3	319	-21	-23	10	-35	0.8	0.8	1.1	0.7
CAND1	Q86VP6	92	30	-16	9	2	1.4	0.9	1.1	1.0
NCF2	P19878	45	29	1	9	24	1.4	1.0	1.1	1.3
DDX42	Q86XP3	346	28	5	9	13	1.4	1.1	1.1	1.1

GNPAT	O15228	138	26	2	9	22	1.4	1.0	1.1	1.3
DHX30	Q7L2E3	765	26	2	9	9	1.4	1.0	1.1	1.1
ATXN10	Q9UBB4	92	23	-4	9	4	1.3	1.0	1.1	1.0
HK3	P52790	840	23	-8	9	13	1.3	0.9	1.1	1.1
NAT10	Q9H0A0	517	22	4	9	11	1.3	1.0	1.1	1.1
PPP6R1	Q9UPN7	37	21	-10	9	3	1.3	0.9	1.1	1.0
RABEPK	Q7Z6M1	115	21	1	9	1	1.3	1.0	1.1	1.0
ZCCHC8	Q6NZY4	262	20	-5	9	3	1.2	1.0	1.1	1.0
COQ10A	Q96MF6	161	20	-12	9	6	1.2	0.9	1.1	1.1
USP9X	Q93008	1061	19	5	9	9	1.2	1.1	1.1	1.1
EXOSC10	Q01780	554	18	-5	9	6	1.2	1.0	1.1	1.1
SMARCC2	Q8TAQ2	91	18	-4	9	14	1.2	1.0	1.1	1.2
GTF3C1	Q12789	1681	17	-2	9	-7	1.2	1.0	1.1	0.9
IPO5	O00410	682	17	-2	9	12	1.2	1.0	1.1	1.1
PIK3CD	O00329	672	17	-4	9	11	1.2	1.0	1.1	1.1
MAP3K2	Q9Y2U5	424	16	5	9	6	1.2	1.0	1.1	1.1
KMT2A	Q03164	990	16	-11	9	3	1.2	0.9	1.1	1.0
TRIM24	O15164	134	15	8	9	0	1.2	1.1	1.1	1.0
ASNA1	O43681	289	15	-3	9	9	1.2	1.0	1.1	1.1
FAM45A	Q8TCE6	135	15	-19	9	-3	1.2	0.8	1.1	1.0
NUP214	P35658	186	14	0	9	-4	1.2	1.0	1.1	1.0
CDK5RAP3	Q96JB5	165	14	-12	9	-3	1.2	0.9	1.1	1.0
ARHGEF6	Q15052	530	14	-12	9	-4	1.2	0.9	1.1	1.0
PDXDC1	Q6P996	490	12	-1	9	8	1.1	1.0	1.1	1.1
RCHY1	Q96PM5	164	12	8	9	-10	1.1	1.1	1.1	0.9
MIS12	Q9H081	53	12	-1	9	7	1.1	1.0	1.1	1.1
TRMT61B	Q9BVS5	396	12	-1	9	-3	1.1	1.0	1.1	1.0
CTBP1	Q13363	118	11	14	9	21	1.1	1.2	1.1	1.3
ZNF267	Q14586	419	11	5	9	11	1.1	1.1	1.1	1.1
GTSF1	Q8WW33	51	10	-3	9	-17	1.1	1.0	1.1	0.9
SART3	Q15020	341	10	-7	9	14	1.1	0.9	1.1	1.2
AP2A2	O94973	396	10	-8	9	16	1.1	0.9	1.1	1.2
UBN1	Q9NPG3	837	9	4	9	15	1.1	1.0	1.1	1.2
FIG4	Q92562	427	9	5	9	-6	1.1	1.1	1.1	0.9
FARSA	Q9Y285	493	9	-2	9	5	1.1	1.0	1.1	1.1
GSTM3	P21266	208	9	-6	9	-4	1.1	0.9	1.1	1.0
CIAPIN1	Q6FI81	116	8	-5	9	5	1.1	1.0	1.1	1.0
GP5	P40197	35	8	-6	9	-5	1.1	0.9	1.1	1.0
CAPN7	Q9Y6W3	329	8	3	9	5	1.1	1.0	1.1	1.1
INPP4A	Q96PE3	275	7	14	9	-8	1.1	1.2	1.1	0.9
HDLBP	Q00341	636	7	2	9	8	1.1	1.0	1.1	1.1
COPG2	Q9UBF2	446	7	-5	9	8	1.1	1.0	1.1	1.1
PTPRC	P08575	320	7	-24	9	8	1.1	0.8	1.1	1.1
ZNF836	Q6ZNA1	652	7	1	9	-4	1.1	1.0	1.1	1.0
HIRIP3	Q9BW71	436	6	4	9	-2	1.1	1.0	1.1	1.0
AP2A1	O95782	267	6	-6	9	13	1.1	0.9	1.1	1.1
ZNF638	Q14966	1947	5	5	9	2	1.1	1.0	1.1	1.0
IMP3	Q9NV31	98	5	-3	9	4	1.1	1.0	1.1	1.0
FBN1	P35555	377	5	-13	9	10	1.0	0.9	1.1	1.1
FBN1	P35555	360	5	-13	9	10	1.0	0.9	1.1	1.1
CNST	Q6PJW8	192	4	14	9	4	1.0	1.2	1.1	1.0
LIMD2	Q9BT23	66	3	13	9	17	1.0	1.1	1.1	1.2
DMXL2	Q8TDJ6	1897	3	-2	9	-4	1.0	1.0	1.1	1.0

PDLIM7	Q9NR12	388	2	-1	9	4	1.0	1.0	1.1	1.0
TLR4	O00206	192	2	-7	9	-10	1.0	0.9	1.1	0.9
PHKB	Q93100	923	2	7	9	-5	1.0	1.1	1.1	1.0
ATAD1	Q8NBU5	137	1	-8	9	-2	1.0	0.9	1.1	1.0
ILKAP	Q9H0C8	367	0	-25	9	18	1.0	0.8	1.1	1.2
MTF2	Q9Y483	252	-1	-8	9	-5	1.0	0.9	1.1	1.0
OSBPL3	Q9H4L5	203	-1	7	9	-10	1.0	1.1	1.1	0.9
TRAF3	Q13114	105	-1	5	9	-2	1.0	1.0	1.1	1.0
EOMES	O95936	337	-2	1	9	5	1.0	1.0	1.1	1.1
ATP13A1	Q9HD20	755	-2	1	9	1	1.0	1.0	1.1	1.0
SEC24D	O94855	848	-2	-10	9	-4	1.0	0.9	1.1	1.0
ZZEF1	O43149	1026	-3	7	9	-4	1.0	1.1	1.1	1.0
H6PD	O95479	393	-3	-46	9	-19	1.0	0.7	1.1	0.8
AHR	P35869	639	-4	-9	9	-18	1.0	0.9	1.1	0.8
DHRS13	Q6UX07	174	-4	17	9	5	1.0	1.2	1.1	1.1
G6B	O95866	35	-6	-20	9	-14	0.9	0.8	1.1	0.9
VDAC2	P45880	227	-6	-13	9	59	0.9	0.9	1.1	2.4
RNFT1	Q5M7Z0	90	-13	-9	9	-17	0.9	0.9	1.1	0.9
EIF2A	Q9BY44	366	-13	2	9	16	0.9	1.0	1.1	1.2
ADRBK2	P35626	681	-18	0	9	-8	0.9	1.0	1.1	0.9
INPP5B	P32019	499	31	-5	9	13	1.4	1.0	1.1	1.1
STXBP2	Q15833	365	31	-25	9	3	1.4	0.8	1.1	1.0
HNRNPR	O43390	214	26	0	9	11	1.3	1.0	1.1	1.1
KIF5B	P33176	65	23	-3	9	15	1.3	1.0	1.1	1.2
PIEZO2	Q9H5I5	2312	23	5	9	-7	1.3	1.0	1.1	0.9
PCYOX1	Q9UHG3	242	22	6	9	30	1.3	1.1	1.1	1.4
NPEPPS	P55786	887	22	-8	9	11	1.3	0.9	1.1	1.1
ZNF592	Q92610	790	20	-1	9	2	1.3	1.0	1.1	1.0
XPO5	Q9HAV4	1157	20	10	9	8	1.2	1.1	1.1	1.1
SEC23IP	Q9Y6Y8	467	19	1	9	4	1.2	1.0	1.1	1.0
Uncharacteri	A0A0A6YYC8	86	19	-5	9	14	1.2	1.0	1.1	1.2
IQGAP2	Q13576	1183	18	-14	9	3	1.2	0.9	1.1	1.0
PFDN5	Q99471	49	17	-5	9	7	1.2	1.0	1.1	1.1
COPG2	Q9UBF2	387	17	1	9	3	1.2	1.0	1.1	1.0
COPG1	Q9Y678	387	17	1	9	3	1.2	1.0	1.1	1.0
ISCA1	Q9BUE6	121	16	14	9	-3	1.2	1.2	1.1	1.0
TMEM57	Q8N5G2	502	16	10	9	1	1.2	1.1	1.1	1.0
PDK3	Q15120	191	16	12	9	12	1.2	1.1	1.1	1.1
GNB1L	Q9BYB4	152	16	4	9	1	1.2	1.0	1.1	1.0
CD3EAP	O15446	107	16	-14	9	2	1.2	0.9	1.1	1.0
SEC14L1	Q92503	299	15	6	9	12	1.2	1.1	1.1	1.1
PDCD6IP	Q8WUM4	76	15	-14	9	8	1.2	0.9	1.1	1.1
EPHA4	P54764	649	15	1	9	-2	1.2	1.0	1.1	1.0
AP2A1	O95782	941	15	-5	9	9	1.2	1.0	1.1	1.1
ARL3	P36405	118	14	3	9	-13	1.2	1.0	1.1	0.9
FAM114A2	Q9NRY5	388	14	-7	9	6	1.2	0.9	1.1	1.1
FLII	Q13045	337	14	9	9	5	1.2	1.1	1.1	1.0
ZNF512	Q96ME7	540	14	3	9	-5	1.2	1.0	1.1	1.0
GIMAP7	Q8NHV1	78	14	-8	9	-1	1.2	0.9	1.1	1.0
ADAMTS2	O95450	680	13	0	9	-8	1.1	1.0	1.1	0.9
PPP1CA	P62136	155	13	-10	9	12	1.1	0.9	1.1	1.1
TTLL12	Q14166	370	13	5	9	29	1.1	1.0	1.1	1.4
PPFIBP2	Q8ND30	446	13	3	9	-16	1.1	1.0	1.1	0.9

SAMD9	Q5K651	721	12	-12	9	-1	1.1	0.9	1.1	1.0
OGT	O15294	417	12	-15	9	-3	1.1	0.9	1.1	1.0
CSE1L	P55060	272	12	-15	9	-10	1.1	0.9	1.1	0.9
ECM29	Q5VYK3	1594	11	0	9	-2	1.1	1.0	1.1	1.0
CTPS1	P17812	216	11	-3	9	5	1.1	1.0	1.1	1.0
IVNS1ABP	Q9Y6Y0	39	11	-5	9	3	1.1	1.0	1.1	1.0
CX3CR1	P49238	221	10	2	9	10	1.1	1.0	1.1	1.1
PPP1R3D	O95685	26	10	-4	9	-7	1.1	1.0	1.1	0.9
KIAA1429	Q69YN4	907	10	-8	9	4	1.1	0.9	1.1	1.0
LOC1027241!	A0A0B4J2E5	24	9	0	9	-5	1.1	1.0	1.1	1.0
TARSL2	A2RTX5	74	9	5	9	4	1.1	1.0	1.1	1.0
SPG11	Q96JI7	1019	9	2	9	13	1.1	1.0	1.1	1.1
UBR5	O95071	2084	9	2	9	3	1.1	1.0	1.1	1.0
PLCL2	Q9UPR0	794	9	-13	9	-3	1.1	0.9	1.1	1.0
CCDC88B	A6NC98	150	8	-3	9	9	1.1	1.0	1.1	1.1
MUT	P22033	742	8	-6	9	14	1.1	0.9	1.1	1.2
KDM3A	Q9Y4C1	751	8	-16	9	16	1.1	0.9	1.1	1.2
TAPT1	Q6NXT6	562	7	6	9	-4	1.1	1.1	1.1	1.0
LAMTOR5	O43504	66	7	-2	9	6	1.1	1.0	1.1	1.1
COPA	P53621	921	7	-3	9	5	1.1	1.0	1.1	1.1
WAPL	Q7Z5K2	776	7	-11	9	-12	1.1	0.9	1.1	0.9
FAM98C	Q17RN3	302	6	2	9	0	1.1	1.0	1.1	1.0
PRKDC	P78527	3781	5	-3	9	6	1.1	1.0	1.1	1.1
IL18	Q14116	74	5	-4	9	-4	1.1	1.0	1.1	1.0
THOC5	Q13769	613	5	-4	9	6	1.1	1.0	1.1	1.1
ZMYND11	Q15326	347	5	-40	9	0	1.1	0.7	1.1	1.0
ITGB7	P26010	624	4	-15	9	14	1.0	0.9	1.1	1.2
ITGB7	P26010	645	4	-15	9	14	1.0	0.9	1.1	1.2
ITGB7	P26010	621	4	-15	9	14	1.0	0.9	1.1	1.2
ITGB7	P26010	641	4	-15	9	14	1.0	0.9	1.1	1.2
TBCE	Q15813	301	4	-6	9	5	1.0	0.9	1.1	1.0
MYO9B	Q13459	2028	3	6	9	-12	1.0	1.1	1.1	0.9
TAF2	Q6P1X5	690	3	1	9	-5	1.0	1.0	1.1	1.0
LIMS1	P48059	252	3	-11	9	-17	1.0	0.9	1.1	0.9
TRIP4	Q15650	173	3	-22	9	13	1.0	0.8	1.1	1.1
HNRNPH1	P31943	290	2	-2	9	14	1.0	1.0	1.1	1.2
ECM29	Q5VYK3	1183	2	-4	9	45	1.0	1.0	1.1	1.8
PTGR1	Q14914	239	2	-6	9	5	1.0	0.9	1.1	1.1
UBE3A	Q05086	49	2	-17	9	32	1.0	0.9	1.1	1.5
SLC27A3	Q5K4L6	112	2	7	9	2	1.0	1.1	1.1	1.0
AGO2	Q9UKV8	272	2	5	9	-5	1.0	1.0	1.1	1.0
EIF2B3	Q9NR50	285	2	0	9	5	1.0	1.0	1.1	1.1
NCKAP5L	Q9HCH0	783	2	-7	9	2	1.0	0.9	1.1	1.0
MYCBP2	O75592	4540	1	-5	9	14	1.0	1.0	1.1	1.2
KMT2D	O14686	3543	1	-6	9	-13	1.0	0.9	1.1	0.9
RASA3	Q14644	678	1	5	9	13	1.0	1.1	1.1	1.1
SMARCD2	Q92925	405	0	1	9	-2	1.0	1.0	1.1	1.0
SPECC1	Q5M775	470	0	-7	9	-8	1.0	0.9	1.1	0.9
NOL6	Q9H6R4	1034	-3	-2	9	-9	1.0	1.0	1.1	0.9
CNN2	Q99439	240	-3	1	9	-17	1.0	1.0	1.1	0.9
EPG5	Q9HCE0	430	-4	-14	9	-13	1.0	0.9	1.1	0.9
AKAP13	Q12802	934	-5	-15	9	-18	1.0	0.9	1.1	0.8
PRKAA1	Q13131	321	-5	-7	9	9	1.0	0.9	1.1	1.1

SEC24C	P53992	875	-6	-3	9	4	0.9	1.0	1.1	1.0
GART	P22102	291	-6	-19	9	12	0.9	0.8	1.1	1.1
BAG5	Q9UL15	213	-7	-2	9	0	0.9	1.0	1.1	1.0
QARS	P47897	657	-7	-9	9	-6	0.9	0.9	1.1	0.9
PPP6R3	Q5H9R7	836	-13	-9	9	-14	0.9	0.9	1.1	0.9
SPATA5	Q8NB90	291	-15	-13	9	-33	0.9	0.9	1.1	0.8
RTN4	Q9NQC3	1101	-16	-6	9	63	0.9	0.9	1.1	2.7
CYFIP1	Q7L576	1087	-17	-10	9	-26	0.9	0.9	1.1	0.8
CYFIP2	Q96F07	1111	-17	-10	9	-26	0.9	0.9	1.1	0.8
PELO	Q9BRX2	175	-21	-3	9	-16	0.8	1.0	1.1	0.9
TWF2	Q6IBS0	141	-23	1	9	-13	0.8	1.0	1.1	0.9
CTBS	Q01459	281	-28	-39	9	-17	0.8	0.7	1.1	0.9
UBA1	P22314	481	32	-5	8	19	1.5	1.0	1.1	1.2
GPSM1	Q86YR5	497	29	31	8	15	1.4	1.4	1.1	1.2
TNRC6B	Q9UPQ9	557	25	16	8	-5	1.3	1.2	1.1	1.0
RCCD1	A6NED2	139	24	8	8	5	1.3	1.1	1.1	1.0
RPL7A	P62424	174	24	7	8	-28	1.3	1.1	1.1	0.8
TRPS1	Q9UHF7	326	22	-12	8	-11	1.3	0.9	1.1	0.9
FASN	P49327	2024	21	8	8	11	1.3	1.1	1.1	1.1
IRF8	Q02556	299	20	-4	8	-9	1.2	1.0	1.1	0.9
AIP	O00170	121	19	0	8	5	1.2	1.0	1.1	1.1
THUMPD1	Q9NXG2	169	19	-4	8	1	1.2	1.0	1.1	1.0
CDK5	Q00535	83	18	4	8	0	1.2	1.0	1.1	1.0
SLC25A5	P05141	257	18	2	8	18	1.2	1.0	1.1	1.2
TGFB1I1	O43294	452	16	2	8	1	1.2	1.0	1.1	1.0
HBS1L	Q9Y450	504	16	-1	8	-4	1.2	1.0	1.1	1.0
MYO1G	B0I1T2	472	16	-7	8	11	1.2	0.9	1.1	1.1
NAT10	Q9H0A0	255	15	9	8	24	1.2	1.1	1.1	1.3
MYO9B	Q13459	863	15	-3	8	-2	1.2	1.0	1.1	1.0
GIT2	Q14161	14	15	-18	8	14	1.2	0.8	1.1	1.2
LRWD1	Q9UFC0	88	15	8	8	-11	1.2	1.1	1.1	0.9
ZBTB7B	O15156	34	15	-4	8	-4	1.2	1.0	1.1	1.0
DNAJC14	Q6Y2X3	52	15	-6	8	-25	1.2	0.9	1.1	0.8
OPHN1	O60890	260	14	-6	8	4	1.2	0.9	1.1	1.0
GSPT2	Q8IYD1	93	14	-12	8	2	1.2	0.9	1.1	1.0
URB2	Q14146	205	14	-19	8	9	1.2	0.8	1.1	1.1
FANCD2	Q9BXW9	729	13	8	8	15	1.1	1.1	1.1	1.2
INTS4	Q96HW7	751	13	-22	8	-6	1.1	0.8	1.1	0.9
GLO1	Q04760	139	13	-56	8	-6	1.1	0.6	1.1	0.9
TRIM22	Q8IYM9	35	12	-4	8	7	1.1	1.0	1.1	1.1
PRPF6	O94906	901	12	-12	8	-14	1.1	0.9	1.1	0.9
CAND1	Q86VP6	356	12	-16	8	5	1.1	0.9	1.1	1.0
KIF21B	O75037	1152	12	9	8	-5	1.1	1.1	1.1	1.0
DDX10	Q13206	323	12	6	8	3	1.1	1.1	1.1	1.0
IDH3G	P51553	236	12	-3	8	11	1.1	1.0	1.1	1.1
VEZF1	Q14119	319	12	-8	8	13	1.1	0.9	1.1	1.1
CCT4	P50991	120	11	10	8	17	1.1	1.1	1.1	1.2
COPG2	Q9UBF2	296	11	-7	8	-2	1.1	0.9	1.1	1.0
EDC3	Q96F86	353	11	-6	8	2	1.1	0.9	1.1	1.0
TBL1X	O60907	446	10	5	8	-2	1.1	1.1	1.1	1.0
ACIN1	Q9UKV3	1083	10	2	8	0	1.1	1.0	1.1	1.0
ANKFY1	Q9P2R3	1060	10	-2	8	29	1.1	1.0	1.1	1.4
BTAF1	O14981	1651	10	-20	8	-1	1.1	0.8	1.1	1.0

HECTD1	Q9ULT8	369	9	1	8	6	1.1	1.0	1.1	1.1
C1orf198	Q9H425	228	8	10	8	-1	1.1	1.1	1.1	1.0
ARHGAP1	Q07960	91	8	0	8	3	1.1	1.0	1.1	1.0
COG3	Q96JB2	697	8	-7	8	-2	1.1	0.9	1.1	1.0
POLR2A	P24928	641	8	-11	8	-5	1.1	0.9	1.1	1.0
CSK	P41240	299	8	-16	8	10	1.1	0.9	1.1	1.1
SSRP1	Q08945	207	8	-10	8	6	1.1	0.9	1.1	1.1
P4HA1	P13674	528	8	-20	8	7	1.1	0.8	1.1	1.1
DEPDC5	O75140	498	7	6	8	12	1.1	1.1	1.1	1.1
LONP1	P36776	682	7	-5	8	1	1.1	1.0	1.1	1.0
BANK1	Q8NDB2	672	7	-7	8	-13	1.1	0.9	1.1	0.9
HK2	P52789	628	6	3	8	2	1.1	1.0	1.1	1.0
CSE1L	P55060	387	6	-2	8	13	1.1	1.0	1.1	1.1
SKP1	P63208	120	6	-3	8	-1	1.1	1.0	1.1	1.0
RNF123	Q5XPI4	977	5	-17	8	-20	1.1	0.9	1.1	0.8
ERCC3	P19447	342	5	-1	8	31	1.0	1.0	1.1	1.4
PLEC	Q15149	3110	4	3	8	-5	1.0	1.0	1.1	1.0
CLASP2	O75122	528	4	-2	8	-6	1.0	1.0	1.1	0.9
PARG	Q86W56	603	4	2	8	6	1.0	1.0	1.1	1.1
CTCF	P49711	271	4	-1	8	-9	1.0	1.0	1.1	0.9
NAPRT	Q6XQN6	533	3	1	8	2	1.0	1.0	1.1	1.0
FAM118A	Q9NWS6	291	3	-2	8	-3	1.0	1.0	1.1	1.0
CUL2	Q13617	465	3	-10	8	3	1.0	0.9	1.1	1.0
ZMYM3	Q14202	1326	3	3	8	13	1.0	1.0	1.1	1.1
THOC5	Q13769	507	2	4	8	-15	1.0	1.0	1.1	0.9
PHF3	Q92576	1771	2	2	8	-9	1.0	1.0	1.1	0.9
PAPOLA	P51003	293	2	-8	8	-3	1.0	0.9	1.1	1.0
EXOC4	Q96A65	522	2	-1	8	13	1.0	1.0	1.1	1.1
RICTOR	Q6R327	218	2	-2	8	-2	1.0	1.0	1.1	1.0
TBCK	Q8TEA7	54	2	-16	8	17	1.0	0.9	1.1	1.2
SH3BP1	Q9Y3L3	388	2	-35	8	-6	1.0	0.7	1.1	0.9
ENG	P17813	516	1	-5	8	-9	1.0	1.0	1.1	0.9
SYNE1	Q8NF91	7765	1	-11	8	-5	1.0	0.9	1.1	1.0
VKORC1L1	Q8N0U8	50	0	-8	8	-10	1.0	0.9	1.1	0.9
ANKFY1	Q9P2R3	389	-1	15	8	15	1.0	1.2	1.1	1.2
RPL37A	P61513	42	-1	-8	8	1	1.0	0.9	1.1	1.0
BRCC3	P46736	240	-2	2	8	6	1.0	1.0	1.1	1.1
ANKRD27	Q96NW4	166	-2	-16	8	-18	1.0	0.9	1.1	0.9
DOCK8	Q8NF50	305	-2	-17	8	-2	1.0	0.9	1.1	1.0
UBE3A	Q05086	198	-3	-2	8	-9	1.0	1.0	1.1	0.9
PLCH2	O75038	1137	-4	0	8	-12	1.0	1.0	1.1	0.9
DGKA	P23743	253	-4	-2	8	1	1.0	1.0	1.1	1.0
TRIM33	Q9UPN9	582	-4	2	8	-13	1.0	1.0	1.1	0.9
ORC3	Q9UBD5	711	-5	-2	8	-9	1.0	1.0	1.1	0.9
ECI2	O75521	282	-5	1	8	4	1.0	1.0	1.1	1.0
NFKBIA	P25963	156	-7	-11	8	-20	0.9	0.9	1.1	0.8
DDX3X	O00571	468	-11	-19	8	-17	0.9	0.8	1.1	0.9
DDX3Y	O15523	466	-11	-19	8	-17	0.9	0.8	1.1	0.9
MIB2	Q96AX9	152	-12	-10	8	2	0.9	0.9	1.1	1.0
NEDD9	Q14511	475	-14	3	8	-7	0.9	1.0	1.1	0.9
C11orf54	Q9H0W9	249	-14	-22	8	-1	0.9	0.8	1.1	1.0
CRIPT	Q9P021	73	-16	0	8	-18	0.9	1.0	1.1	0.8
PDS5B	Q9NTI5	83	27	-2	8	7	1.4	1.0	1.1	1.1

PDS5A	Q29RF7	93	27	-2	8	7	1.4	1.0	1.1	1.1
SYNE2	Q8WXH0	2480	26	15	8	4	1.3	1.2	1.1	1.0
EEF1B2	P24534	161	24	-1	8	16	1.3	1.0	1.1	1.2
RBM17	Q96I25	302	23	1	8	14	1.3	1.0	1.1	1.2
NCF2	P19878	40	21	-1	8	9	1.3	1.0	1.1	1.1
MBD4	O95243	324	21	-1	8	5	1.3	1.0	1.1	1.0
FUS	P35637	444	21	-10	8	-2	1.3	0.9	1.1	1.0
RNASEH2C	Q8TDP1	34	20	-9	8	9	1.2	0.9	1.1	1.1
ASUN	Q9NVM9	331	20	-15	8	-9	1.2	0.9	1.1	0.9
UGGT1	Q9NYU2	1386	19	1	8	23	1.2	1.0	1.1	1.3
CNOT1	A5YKK6	1687	19	-11	8	-8	1.2	0.9	1.1	0.9
CIC	Q96RK0	1420	18	-3	8	2	1.2	1.0	1.1	1.0
APAF1	O14727	426	18	-4	8	10	1.2	1.0	1.1	1.1
FOCAD	Q5VW36	522	17	-12	8	1	1.2	0.9	1.1	1.0
SMARCC1	Q92922	108	16	-9	8	6	1.2	0.9	1.1	1.1
LRCH3	Q96II8	277	16	-4	8	-9	1.2	1.0	1.1	0.9
CCT5	P48643	377	15	7	8	14	1.2	1.1	1.1	1.2
LCP1	P13796	618	15	-3	8	11	1.2	1.0	1.1	1.1
IPO11	Q9UI26	262	15	-4	8	1	1.2	1.0	1.1	1.0
RAB3GAP2	Q9H2M9	792	15	5	8	11	1.2	1.0	1.1	1.1
GNB2	P62879	294	15	-3	8	10	1.2	1.0	1.1	1.1
ADCK1	Q86TW2	386	15	-7	8	17	1.2	0.9	1.1	1.2
IPO5	O00410	473	15	-9	8	11	1.2	0.9	1.1	1.1
PRG2	P13727	104	14	-5	8	-9	1.2	1.0	1.1	0.9
NCAPG2	Q86XI2	776	14	-7	8	13	1.2	0.9	1.1	1.1
INTS2	Q9H0H0	48	14	-14	8	15	1.2	0.9	1.1	1.2
IDH3G	P51553	235	14	-3	8	10	1.2	1.0	1.1	1.1
RNF213	Q63HN8	3261	14	-15	8	-9	1.2	0.9	1.1	0.9
FDPS	P14324	183	13	-5	8	8	1.1	1.0	1.1	1.1
PCBP1	Q15365	158	13	-8	8	1	1.1	0.9	1.1	1.0
ALDOC	P09972	290	13	-13	8	-2	1.1	0.9	1.1	1.0
IPO5	O00410	944	12	9	8	15	1.1	1.1	1.1	1.2
UBR5	O95071	1291	12	6	8	13	1.1	1.1	1.1	1.1
CHD3	Q12873	1997	12	1	8	-6	1.1	1.0	1.1	0.9
ACTR10	Q9NZ32	215	12	-4	8	-2	1.1	1.0	1.1	1.0
CUL2	Q13617	266	12	-20	8	23	1.1	0.8	1.1	1.3
SRRM2	Q9UQ35	785	12	3	8	-11	1.1	1.0	1.1	0.9
HAVCR2	Q8TDQ0	296	12	-27	8	1	1.1	0.8	1.1	1.0
SAMHD1	Q9Y3Z3	350	11	4	8	-3	1.1	1.0	1.1	1.0
TNFAIP3	P21580	483	11	-1	8	-1	1.1	1.0	1.1	1.0
NUP188	Q5SRE5	1283	11	-9	8	8	1.1	0.9	1.1	1.1
TLN1	Q9Y490	29	11	-12	8	3	1.1	0.9	1.1	1.0
HAAO	P46952	23	11	4	8	-1	1.1	1.0	1.1	1.0
MANBA	O00462	800	11	-4	8	12	1.1	1.0	1.1	1.1
SHC1	P29353	212	11	-5	8	-13	1.1	1.0	1.1	0.9
FBXL6	Q8N531	368	10	-2	8	-10	1.1	1.0	1.1	0.9
ATP2C1	P98194	409	10	-7	8	5	1.1	0.9	1.1	1.0
TRIM28	Q13263	124	10	-1	8	7	1.1	1.0	1.1	1.1
INO80	Q9ULG1	816	10	-1	8	-4	1.1	1.0	1.1	1.0
ZEB2	O60315	140	10	-1	8	-9	1.1	1.0	1.1	0.9
F8A3	P23610	69	10	-3	8	3	1.1	1.0	1.1	1.0
DCP2	Q8IU60	73	10	-4	8	6	1.1	1.0	1.1	1.1
DSCR3	O14972	243	10	-10	8	-3	1.1	0.9	1.1	1.0

EXOSC7	Q15024	204	9	-18	8	-16	1.1	0.9	1.1	0.9
TBK1	Q9UHD2	423	9	4	8	6	1.1	1.0	1.1	1.1
SEC24C	P53992	910	9	-5	8	11	1.1	1.0	1.1	1.1
CUL4B	Q13620	264	9	-44	8	13	1.1	0.7	1.1	1.1
UNC45A	Q9H3U1	420	8	-8	8	-6	1.1	0.9	1.1	0.9
ERCC4	Q92889	143	8	4	8	-7	1.1	1.0	1.1	0.9
ZMAT2	Q96NC0	82	7	8	8	7	1.1	1.1	1.1	1.1
MCM3AP	O60318	1240	7	1	8	10	1.1	1.0	1.1	1.1
TLN1	Q9Y490	1434	7	1	8	-1	1.1	1.0	1.1	1.0
TTC37	Q6PGP7	705	7	-5	8	4	1.1	1.0	1.1	1.0
AKAP11	Q9UKA4	1010	7	-9	8	-9	1.1	0.9	1.1	0.9
NUP205	Q92621	1073	7	9	8	10	1.1	1.1	1.1	1.1
SKAP1	Q86WV1	133	7	2	8	15	1.1	1.0	1.1	1.2
BOLA2B	Q9H3K6	33	7	-6	8	5	1.1	0.9	1.1	1.1
EIF2B5	Q13144	618	7	-11	8	0	1.1	0.9	1.1	1.0
RPL3	P39023	336	6	-3	8	5	1.1	1.0	1.1	1.1
CAPN2	P17655	341	6	-13	8	-4	1.1	0.9	1.1	1.0
DOCK8	Q8NF50	170	5	4	8	-2	1.1	1.0	1.1	1.0
GPSM3	Q9Y4H4	116	5	0	8	-10	1.1	1.0	1.1	0.9
GCLC	P48506	491	5	-3	8	5	1.1	1.0	1.1	1.1
KMT2D	O14686	369	5	-4	8	2	1.0	1.0	1.1	1.0
WRAP73	Q9P2S5	122	4	8	8	-13	1.0	1.1	1.1	0.9
TTC9	Q92623	30	4	1	8	-6	1.0	1.0	1.1	0.9
PITRM1	Q5JRX3	313	4	-3	8	16	1.0	1.0	1.1	1.2
SETDB1	Q15047	1286	4	-3	8	7	1.0	1.0	1.1	1.1
LY75	O60449	566	4	-3	8	-7	1.0	1.0	1.1	0.9
SEPHS1	P49903	71	3	8	8	9	1.0	1.1	1.1	1.1
UBA5	Q9GZZ9	181	3	-13	8	-7	1.0	0.9	1.1	0.9
NEDD9	Q14511	615	3	4	8	-8	1.0	1.0	1.1	0.9
SART1	O43290	645	3	1	8	4	1.0	1.0	1.1	1.0
VPS18	Q9P253	433	2	-6	8	6	1.0	0.9	1.1	1.1
PI4KA	P42356	1388	1	-12	8	18	1.0	0.9	1.1	1.2
FBXL4	Q9UKA2	547	1	17	8	1	1.0	1.2	1.1	1.0
FOXK1	P85037	439	1	-1	8	2	1.0	1.0	1.1	1.0
TRIP12	Q14669	156	-1	-10	8	-7	1.0	0.9	1.1	0.9
MCM5	P33992	355	-2	-15	8	5	1.0	0.9	1.1	1.0
IARS	P41252	526	-2	3	8	2	1.0	1.0	1.1	1.0
PREX1	Q8TCU6	720	-3	-15	8	3	1.0	0.9	1.1	1.0
LPP	Q93052	465	-4	-5	8	-10	1.0	1.0	1.1	0.9
TARBP2	Q15633	295	-7	-2	8	12	0.9	1.0	1.1	1.1
ATOX1	O00244	41	-8	-3	8	-11	0.9	1.0	1.1	0.9
KIAA1551	Q9HCM1	650	-9	-25	8	6	0.9	0.8	1.1	1.1
VAV1	P15498	369	-15	1	8	4	0.9	1.0	1.1	1.0
TBC1D2B	Q9UPU7	726	-16	1	8	1	0.9	1.0	1.1	1.0
CYFIP2	Q96F07	346	-19	-9	8	-19	0.8	0.9	1.1	0.8
PSMD1	Q99460	660	29	5	7	14	1.4	1.1	1.1	1.2
BAZ1A	Q9NRL2	537	28	-6	7	10	1.4	0.9	1.1	1.1
EXOC5	O00471	254	27	-8	7	-17	1.4	0.9	1.1	0.9
DDX3X	O00571	341	23	2	7	10	1.3	1.0	1.1	1.1
DDX3Y	O15523	339	23	2	7	10	1.3	1.0	1.1	1.1
ZC3HAV1L	Q96H79	202	21	9	7	19	1.3	1.1	1.1	1.2
CARMIL2	Q6F5E8	472	21	-3	7	3	1.3	1.0	1.1	1.0
PPP6R2	O75170	451	20	-3	7	18	1.3	1.0	1.1	1.2

SERPINB6	P35237	370	20	-30	7	-11	1.3	0.8	1.1	0.9
ATPAF1	Q5TC12	27	20	12	7	11	1.2	1.1	1.1	1.1
LRCH1	Q9Y2L9	161	20	-6	7	16	1.2	0.9	1.1	1.2
GPATCH8	Q9UKJ3	639	19	11	7	-11	1.2	1.1	1.1	0.9
TCEB1	Q15369	74	19	-8	7	11	1.2	0.9	1.1	1.1
ARHGAP25	P42331	292	19	-26	7	7	1.2	0.8	1.1	1.1
KIAA0196	Q12768	1064	18	-8	7	-1	1.2	0.9	1.1	1.0
TBXAS1	P24557	309	18	8	7	-10	1.2	1.1	1.1	0.9
PPP2R5A	Q15172	72	17	3	7	10	1.2	1.0	1.1	1.1
APBB1	O00213	672	17	13	7	-1	1.2	1.1	1.1	1.0
TUBGCP3	Q96CW5	388	17	-6	7	5	1.2	0.9	1.1	1.1
GIT2	Q14161	149	17	-26	7	0	1.2	0.8	1.1	1.0
GIT1	Q9Y2X7	149	17	-26	7	0	1.2	0.8	1.1	1.0
ALDH1L2	Q3SY69	728	16	3	7	13	1.2	1.0	1.1	1.1
INTS6L	Q5JSJ4	219	16	-6	7	7	1.2	0.9	1.1	1.1
INTS6	Q9UL03	219	16	-6	7	7	1.2	0.9	1.1	1.1
AGPAT1	Q99943	218	16	-6	7	-7	1.2	0.9	1.1	0.9
FAM105A	Q9NUU6	226	16	-4	7	10	1.2	1.0	1.1	1.1
FERMT3	Q86UX7	407	15	-7	7	2	1.2	0.9	1.1	1.0
ETFA	P13804	109	15	6	7	20	1.2	1.1	1.1	1.2
HSP90AB1	P08238	521	15	-5	7	4	1.2	1.0	1.1	1.0
VTA1	Q9NP79	38	15	-7	7	1	1.2	0.9	1.1	1.0
SIK3	Q9Y2K2	63	14	6	7	4	1.2	1.1	1.1	1.0
CUL3	Q13618	156	14	3	7	9	1.2	1.0	1.1	1.1
MYH9	P35579	740	14	-12	7	10	1.2	0.9	1.1	1.1
HEATR1	Q9H583	182	14	-13	7	9	1.2	0.9	1.1	1.1
GCNT1	Q02742	217	14	20	7	13	1.2	1.2	1.1	1.1
RFC5	P40937	152	14	-2	7	13	1.2	1.0	1.1	1.1
PPP2R5C	Q13362	50	14	-2	7	16	1.2	1.0	1.1	1.2
PPP2R5D	Q14738	126	14	-2	7	16	1.2	1.0	1.1	1.2
AP4B1	Q9Y6B7	18	14	-5	7	-2	1.2	1.0	1.1	1.0
UPF1	Q92900	657	14	-12	7	-25	1.2	0.9	1.1	0.8
SNRNP40	Q96DI7	52	13	14	7	3	1.1	1.2	1.1	1.0
IRAK3	Q9Y616	399	13	-5	7	10	1.1	1.0	1.1	1.1
WAPL	Q7Z5K2	94	13	-12	7	-8	1.1	0.9	1.1	0.9
UNC13D	Q70J99	667	13	13	7	18	1.1	1.1	1.1	1.2
SAE1	Q9UBE0	342	13	-2	7	5	1.1	1.0	1.1	1.1
CMIP	Q8IY22	455	13	-6	7	-1	1.1	0.9	1.1	1.0
PPA1	Q15181	242	12	4	7	3	1.1	1.0	1.1	1.0
PLAU	P00749	217	12	-4	7	9	1.1	1.0	1.1	1.1
PLAU	P00749	225	12	-4	7	9	1.1	1.0	1.1	1.1
CKAP5	Q14008	1203	12	-3	7	21	1.1	1.0	1.1	1.3
USP34	Q70CQ2	214	12	-9	7	-21	1.1	0.9	1.1	0.8
PDHA1	P08559	181	12	-51	7	-5	1.1	0.7	1.1	1.0
INPP5K	Q9BT40	280	11	6	7	21	1.1	1.1	1.1	1.3
STAT3	P40763	367	11	-1	7	4	1.1	1.0	1.1	1.0
CRBN	Q96SW2	310	11	-4	7	4	1.1	1.0	1.1	1.0
COPS6	Q7L5N1	266	11	-9	7	6	1.1	0.9	1.1	1.1
PRKDC	P78527	90	11	-1	7	9	1.1	1.0	1.1	1.1
ZMYM3	Q14202	451	11	-14	7	-2	1.1	0.9	1.1	1.0
EFR3A	Q14156	767	10	2	7	2	1.1	1.0	1.1	1.0
TRIM25	Q14258	70	10	2	7	-5	1.1	1.0	1.1	1.0
PRDM10	Q9NQV6	249	10	0	7	-7	1.1	1.0	1.1	0.9

FAM129B	Q96TA1	512	10	-3	7	3	1.1	1.0	1.1	1.0
RNF213	Q63HN8	1748	10	-7	7	1	1.1	0.9	1.1	1.0
RRP12	Q5JTH9	288	10	-20	7	-9	1.1	0.8	1.1	0.9
ATG7	O95352	182	10	12	7	4	1.1	1.1	1.1	1.0
JAK1	P23458	716	10	-10	7	4	1.1	0.9	1.1	1.0
ZHX3	Q9H4I2	335	9	-4	7	-10	1.1	1.0	1.1	0.9
ARHGAP4	P98171	527	9	-10	7	1	1.1	0.9	1.1	1.0
GTF2B	Q00403	168	8	-2	7	-6	1.1	1.0	1.1	0.9
RASGRP4	Q8TDF6	122	8	-11	7	8	1.1	0.9	1.1	1.1
SULT1A3	P0DMM9	287	8	-4	7	-4	1.1	1.0	1.1	1.0
REL	Q04864	143	7	8	7	-5	1.1	1.1	1.1	1.0
LRBA	P50851	1704	7	6	7	8	1.1	1.1	1.1	1.1
C16orf62	Q7Z3J2	20	7	-1	7	5	1.1	1.0	1.1	1.0
LRRC14	Q15048	15	7	-3	7	1	1.1	1.0	1.1	1.0
VASP	P50552	334	7	-3	7	2	1.1	1.0	1.1	1.0
VPS54	Q9P1Q0	886	7	-4	7	18	1.1	1.0	1.1	1.2
LCP1	P13796	42	7	-3	7	-2	1.1	1.0	1.1	1.0
ARID1B	Q8NFD5	2074	7	-13	7	-11	1.1	0.9	1.1	0.9
NIFK	Q9BYG3	237	6	3	7	-8	1.1	1.0	1.1	0.9
TARBP1	Q13395	483	6	0	7	-12	1.1	1.0	1.1	0.9
RNF31	Q96EP0	59	6	-4	7	-4	1.1	1.0	1.1	1.0
CYP20A1	Q6UW02	331	6	-10	7	6	1.1	0.9	1.1	1.1
TIGAR	Q9NQ88	215	6	-12	7	8	1.1	0.9	1.1	1.1
RANBP2	P49792	3071	6	3	7	3	1.1	1.0	1.1	1.0
CCZ1B	P86790	471	6	-12	7	-4	1.1	0.9	1.1	1.0
CKAP4	Q07065	100	5	-3	7	21	1.1	1.0	1.1	1.3
VWF	P04275	2570	5	-8	7	7	1.1	0.9	1.1	1.1
KTI12	Q96EK9	93	5	-8	7	-7	1.1	0.9	1.1	0.9
MBD1	Q9UIS9	357	5	10	7	18	1.0	1.1	1.1	1.2
UVRAG	Q9P2Y5	112	5	5	7	2	1.0	1.0	1.1	1.0
SAAL1	Q96ER3	366	4	4	7	10	1.0	1.0	1.1	1.1
TARDBP	G3V162	244	4	-9	7	-5	1.0	0.9	1.1	1.0
RAVER1	Q8IY67	239	4	5	7	3	1.0	1.0	1.1	1.0
NCALD	P61601	185	4	-3	7	33	1.0	1.0	1.1	1.5
PDXDC1	Q6P996	481	4	-4	7	5	1.0	1.0	1.1	1.1
BRPF1	P55201	515	4	-10	7	0	1.0	0.9	1.1	1.0
ZZEF1	O43149	1069	4	-11	7	-5	1.0	0.9	1.1	1.0
USP34	Q70CQ2	856	3	-4	7	-13	1.0	1.0	1.1	0.9
ANKHD1	Q8IWZ3	1601	3	-6	7	-6	1.0	0.9	1.1	0.9
PTK2B	Q14289	545	3	-13	7	22	1.0	0.9	1.1	1.3
PMS1	P54277	457	3	9	7	6	1.0	1.1	1.1	1.1
WHSC1L1	Q9BZ95	627	3	-1	7	-5	1.0	1.0	1.1	1.0
PLEC	Q15149	3017	3	-4	7	-2	1.0	1.0	1.1	1.0
STAMBPL1	Q96FJ0	276	2	-2	7	9	1.0	1.0	1.1	1.1
ADRBK2	P35626	103	2	-2	7	-21	1.0	1.0	1.1	0.8
PPP4R1	Q8TF05	78	2	-4	7	12	1.0	1.0	1.1	1.1
HLA-C	P04222	364	2	-6	7	1	1.0	0.9	1.1	1.0
HLA-C	P30510	364	2	-6	7	1	1.0	0.9	1.1	1.0
SRSF1	Q07955	16	2	-7	7	7	1.0	0.9	1.1	1.1
PHF6	Q8IWS0	85	1	6	7	-8	1.0	1.1	1.1	0.9
WASL	O00401	431	1	8	7	-8	1.0	1.1	1.1	0.9
NLRC5	Q86WI3	1190	1	-4	7	-13	1.0	1.0	1.1	0.9
VPS4A	Q9UN37	359	1	-5	7	6	1.0	1.0	1.1	1.1

ELAC2	Q9BQ52	51	1	-5	7	-11	1.0	1.0	1.1	0.9
TPP2	P29144	967	0	-7	7	-1	1.0	0.9	1.1	1.0
PHF6	Q8IWS0	28	0	-10	7	-7	1.0	0.9	1.1	0.9
UBN1	Q9NPG3	38	-1	8	7	-13	1.0	1.1	1.1	0.9
EDC4	Q6P2E9	976	-1	6	7	-5	1.0	1.1	1.1	1.0
NCBP1	Q09161	409	-2	6	7	-1	1.0	1.1	1.1	1.0
ALDH1A1	P00352	276	-2	4	7	-11	1.0	1.0	1.1	0.9
ACAA2	P42765	128	-2	-15	7	1	1.0	0.9	1.1	1.0
STAT1	P42224	174	-2	2	7	5	1.0	1.0	1.1	1.1
PCCB	P05166	269	-3	-35	7	-16	1.0	0.7	1.1	0.9
CCDC88A	Q3V6T2	1729	-5	3	7	-1	1.0	1.0	1.1	1.0
PSMC2	P35998	236	-5	-25	7	19	1.0	0.8	1.1	1.2
C16orf70	Q9BSU1	385	-6	10	7	1	0.9	1.1	1.1	1.0
DIP2A	Q14689	6	-6	5	7	4	0.9	1.0	1.1	1.0
RNF31	Q96EP0	885	-12	-11	7	-15	0.9	0.9	1.1	0.9
NLRC3	Q7RTR2	361	-12	-13	7	8	0.9	0.9	1.1	1.1
NMD3	Q96D46	20	-17	-1	7	-17	0.9	1.0	1.1	0.9
RCC2	Q9P258	280	-22	-9	7	-5	0.8	0.9	1.1	1.0
IFI16	Q16666	737	29	-6	7	15	1.4	0.9	1.1	1.2
LDHB	P07195	36	29	6	7	2	1.4	1.1	1.1	1.0
DUSP7	Q16829	256	26	30	7	34	1.3	1.4	1.1	1.5
OSGEP	Q9NPF4	110	24	20	7	5	1.3	1.3	1.1	1.1
DR1	Q01658	43	24	0	7	-1	1.3	1.0	1.1	1.0
FGD4	Q96M96	234	24	-7	7	4	1.3	0.9	1.1	1.0
TXNDC5	Q8NBS9	381	23	3	7	10	1.3	1.0	1.1	1.1
UBA3	Q8TBC4	82	22	-3	7	21	1.3	1.0	1.1	1.3
ARHGEF6	Q15052	418	21	-3	7	10	1.3	1.0	1.1	1.1
NFKBIB	Q15653	142	21	-15	7	8	1.3	0.9	1.1	1.1
DDX54	Q8TDD1	149	20	-8	7	18	1.3	0.9	1.1	1.2
KLRD1	Q13241	89	18	7	7	5	1.2	1.1	1.1	1.1
NAA50	Q9GZZ1	79	16	-12	7	12	1.2	0.9	1.1	1.1
PLAA	Q9Y263	208	16	-24	7	-1	1.2	0.8	1.1	1.0
NOS1	P29475	437	16	10	7	-4	1.2	1.1	1.1	1.0
G6PD	P11413	446	16	8	7	-2	1.2	1.1	1.1	1.0
USP40	Q9NVE5	810	16	-4	7	-21	1.2	1.0	1.1	0.8
PDE4DIP	Q5VU43	271	15	7	7	10	1.2	1.1	1.1	1.1
AGK	Q53H12	43	15	-1	7	5	1.2	1.0	1.1	1.1
FAM98A	Q8NCA5	126	14	-2	7	4	1.2	1.0	1.1	1.0
SMC2	O95347	585	14	2	7	10	1.2	1.0	1.1	1.1
PDS5A	Q29RF7	508	14	0	7	7	1.2	1.0	1.1	1.1
TRAPPC10	P48553	372	13	0	7	-3	1.1	1.0	1.1	1.0
DDX27	Q96GQ7	248	13	-3	7	4	1.1	1.0	1.1	1.0
MKLN1	Q9UL63	582	13	-4	7	2	1.1	1.0	1.1	1.0
AP2A2	O94973	330	13	-8	7	5	1.1	0.9	1.1	1.1
AP2A1	O95782	331	13	-8	7	5	1.1	0.9	1.1	1.1
RANGAP1	P46060	169	13	-9	7	1	1.1	0.9	1.1	1.0
RIN3	Q8TB24	906	13	-9	7	-1	1.1	0.9	1.1	1.0
ZNF609	O15014	345	13	6	7	-16	1.1	1.1	1.1	0.9
IKBKB	O14920	179	13	4	7	12	1.1	1.0	1.1	1.1
HK3	P52790	399	13	-21	7	1	1.1	0.8	1.1	1.0
AKAP13	Q12802	1232	12	4	7	1	1.1	1.0	1.1	1.0
SLC25A11	Q02978	184	12	2	7	6	1.1	1.0	1.1	1.1
ANKRD50	Q9ULJ7	639	12	-6	7	-6	1.1	0.9	1.1	0.9

BCR	P11274	1074	12	-10	7	-5	1.1	0.9	1.1	1.0
NSL1	Q96IY1	136	12	-13	7	1	1.1	0.9	1.1	1.0
MROH1	Q8NDA8	186	12	-14	7	28	1.1	0.9	1.1	1.4
CTBP2	P56545	18	11	13	7	-19	1.1	1.1	1.1	0.8
GCN1	Q92616	1362	11	7	7	-4	1.1	1.1	1.1	1.0
RPS6KA5	O75582	731	11	5	7	-4	1.1	1.1	1.1	1.0
EPHX2	P34913	141	11	-6	7	12	1.1	0.9	1.1	1.1
UBR4	Q5T4S7	122	10	8	7	2	1.1	1.1	1.1	1.0
PPTC7	Q8NI37	41	10	0	7	1	1.1	1.0	1.1	1.0
HEATR5B	Q9P2D3	1896	10	-2	7	-5	1.1	1.0	1.1	1.0
NT5C	Q8TCD5	166	9	10	7	13	1.1	1.1	1.1	1.1
EIF3B	P55884	302	9	2	7	-5	1.1	1.0	1.1	1.0
SYNE1	Q8NF91	4021	9	-5	7	-1	1.1	1.0	1.1	1.0
CTPS1	P17812	299	9	-8	7	3	1.1	0.9	1.1	1.0
NSD1	Q96L73	1312	9	-9	7	-10	1.1	0.9	1.1	0.9
PRPF6	O94906	739	8	3	7	4	1.1	1.0	1.1	1.0
MBD1	Q9UIS9	28	8	1	7	5	1.1	1.0	1.1	1.1
VPS36	Q86VN1	271	8	0	7	-2	1.1	1.0	1.1	1.0
FAM63A	Q8N5J2	115	8	-1	7	11	1.1	1.0	1.1	1.1
FHL2	Q14192	254	8	-13	7	-15	1.1	0.9	1.1	0.9
TRANK1	O15050	1947	8	-18	7	7	1.1	0.8	1.1	1.1
CBX8	Q9HC52	142	7	2	7	-12	1.1	1.0	1.1	0.9
CCDC97	Q96F63	17	7	-5	7	-14	1.1	1.0	1.1	0.9
RIC8A	Q9NPQ8	341	7	-6	7	4	1.1	0.9	1.1	1.0
DOK3	Q7L591	230	7	-21	7	1	1.1	0.8	1.1	1.0
KIFAP3	Q92845	78	7	2	7	-14	1.1	1.0	1.1	0.9
ARHGAP9	Q9BRR9	542	7	-8	7	11	1.1	0.9	1.1	1.1
DBNL	Q9UJU6	97	6	9	7	0	1.1	1.1	1.1	1.0
BPTF	Q12830	882	6	-13	7	-4	1.1	0.9	1.1	1.0
RAVER1	Q8IY67	251	5	1	7	10	1.1	1.0	1.1	1.1
CARD6	Q9BX69	463	5	-2	7	-1	1.1	1.0	1.1	1.0
TTC1	Q99614	149	5	5	7	5	1.0	1.0	1.1	1.1
C17orf75	Q9HAS0	288	5	-2	7	1	1.0	1.0	1.1	1.0
NOP58	Q9Y2X3	139	4	-3	7	-5	1.0	1.0	1.1	1.0
THOP1	P52888	246	4	-6	7	8	1.0	0.9	1.1	1.1
SIK3	Q9Y2K2	165	4	1	7	6	1.0	1.0	1.1	1.1
TAP2	Q03519	641	4	-1	7	29	1.0	1.0	1.1	1.4
C17orf59	Q96GS4	296	4	-11	7	-3	1.0	0.9	1.1	1.0
SH3PXD2A	Q5TCZ1	749	3	13	7	-5	1.0	1.1	1.1	1.0
VPS8	Q8N3P4	564	3	-2	7	-1	1.0	1.0	1.1	1.0
ALDH6A1	Q02252	368	3	-4	7	9	1.0	1.0	1.1	1.1
ICT1	Q14197	82	3	2	7	0	1.0	1.0	1.1	1.0
SEC24D	O94855	1028	3	-5	7	2	1.0	1.0	1.1	1.0
SYK	P43405	543	3	-5	7	-5	1.0	1.0	1.1	1.0
RAI1	Q7Z5J4	1553	3	-18	7	-16	1.0	0.8	1.1	0.9
CDKN1C	A0A0G2JPX0	295	2	-9	7	4	1.0	0.9	1.1	1.0
TRIM24	O15164	73	2	-15	7	3	1.0	0.9	1.1	1.0
NCAPD3	P42695	631	2	-1	7	3	1.0	1.0	1.1	1.0
VPS18	Q9P253	806	2	-10	7	34	1.0	0.9	1.1	1.5
L3HYPDH	Q96EM0	205	1	-5	7	-12	1.0	1.0	1.1	0.9
ZMYM3	Q14202	675	1	-5	7	-24	1.0	1.0	1.1	0.8
ANKRD54	Q6NXT1	137	1	-6	7	-10	1.0	0.9	1.1	0.9
CLASP1	Q7Z460	173	1	-10	7	-2	1.0	0.9	1.1	1.0

TRIP11	Q15643	212	0	-3	7	-4	1.0	1.0	1.1	1.0
ARHGAP17	Q68EM7	305	0	-7	7	-6	1.0	0.9	1.1	0.9
FLNB	O75369	450	0	-24	7	12	1.0	0.8	1.1	1.1
LMF2	Q9BU23	696	-1	5	7	-5	1.0	1.0	1.1	1.0
TBC1D17	Q9HA65	112	-1	-16	7	-3	1.0	0.9	1.1	1.0
FAM65B	Q9Y4F9	596	-2	-6	7	-5	1.0	0.9	1.1	1.0
APLF	Q8IW19	111	-3	0	7	-5	1.0	1.0	1.1	1.0
MEPCE	Q7L2J0	429	-3	0	7	-5	1.0	1.0	1.1	1.0
EP300	Q09472	1183	-3	-3	7	4	1.0	1.0	1.1	1.0
CREBBP	Q92793	1219	-3	-3	7	4	1.0	1.0	1.1	1.0
ENGASE	Q8NFI3	113	-3	-4	7	2	1.0	1.0	1.1	1.0
UEVLD	Q8IX04	319	-3	-5	7	1	1.0	1.0	1.1	1.0
DCTN1	Q14203	791	-3	-17	7	7	1.0	0.9	1.1	1.1
PPP6R3	Q5H9R7	844	-4	-1	7	2	1.0	1.0	1.1	1.0
WAC	Q9BTA9	553	-5	5	7	-6	1.0	1.0	1.1	0.9
GRIPAP1	Q4V328	550	-5	-6	7	-16	1.0	0.9	1.1	0.9
HCFC2	Q9Y5Z7	673	-5	-13	7	-8	1.0	0.9	1.1	0.9
RDH14	Q9HBH5	74	-6	1	7	11	0.9	1.0	1.1	1.1
SLFN13	Q68D06	631	-7	-6	7	-9	0.9	0.9	1.1	0.9
SLFN11	Q7Z7L1	631	-7	-6	7	-9	0.9	0.9	1.1	0.9
INTS1	Q8N201	1440	-9	-27	7	14	0.9	0.8	1.1	1.2
SAMHD1	Q9Y3Z3	522	-9	-2	7	-12	0.9	1.0	1.1	0.9
RNASEK-C17(	H0YIS7	107	-10	1	7	-4	0.9	1.0	1.1	1.0
SYTL4	Q96C24	83	-11	-24	7	-15	0.9	0.8	1.1	0.9
BAK1	Q16611	166	-12	-3	7	20	0.9	1.0	1.1	1.2
TIRAP	P58753	157	-13	-4	7	-8	0.9	1.0	1.1	0.9
IPO4	Q8TEX9	95	-13	-15	7	2	0.9	0.9	1.1	1.0
COMMD3-BN	R4GMX3	309	-13	-22	7	-9	0.9	0.8	1.1	0.9
NUDT4	Q9NZJ9	147	-14	-16	7	-14	0.9	0.9	1.1	0.9
TRPS1	Q9UHF7	567	-23	-13	7	-8	0.8	0.9	1.1	0.9
MRPL21	Q7Z2W9	203	-27	-24	7	3	0.8	0.8	1.1	1.0
CTSG	P08311	207	-47	-13	7	-10	0.7	0.9	1.1	0.9
IKBKE	Q14164	545	40	23	6	7	1.7	1.3	1.1	1.1
MBD1	Q9UIS9	338	38	15	6	19	1.6	1.2	1.1	1.2
RASSF1	Q9NS23	40	27	13	6	17	1.4	1.1	1.1	1.2
FCN1	O00602	111	26	4	6	7	1.4	1.0	1.1	1.1
MINK1	Q8N4C8	269	26	-6	6	10	1.4	0.9	1.1	1.1
ARHGEF6	Q15052	66	26	-13	6	-1	1.3	0.9	1.1	1.0
KYAT3	Q6YP21	416	24	-5	6	15	1.3	1.0	1.1	1.2
ARID1A	O14497	1968	23	11	6	20	1.3	1.1	1.1	1.3
MON1B	Q7L1V2	87	22	5	6	8	1.3	1.1	1.1	1.1
BTBD11	A6QL63	554	21	7	6	9	1.3	1.1	1.1	1.1
CBR1	P16152	122	19	7	6	15	1.2	1.1	1.1	1.2
PDCD6IP	Q8WUM4	122	19	-13	6	1	1.2	0.9	1.1	1.0
SMARCA4	P51532	998	18	-6	6	12	1.2	0.9	1.1	1.1
SRP72	O76094	87	18	-13	6	11	1.2	0.9	1.1	1.1
NR3C1	P04150	457	18	-2	6	1	1.2	1.0	1.1	1.0
NR3C2	P08235	639	18	-2	6	1	1.2	1.0	1.1	1.0
SKAP2	O75563	186	17	-6	6	11	1.2	0.9	1.1	1.1
DDX60L	Q5H9U9	1652	17	-9	6	-11	1.2	0.9	1.1	0.9
DDX60L	Q5H9U9	1671	17	-9	6	-11	1.2	0.9	1.1	0.9
CAND1	Q86VP6	1007	16	-2	6	5	1.2	1.0	1.1	1.0
VPS35	Q96QK1	253	16	-6	6	12	1.2	0.9	1.1	1.1

NELFB	Q8WX92	235	16	-15	6	17	1.2	0.9	1.1	1.2
NUP160	Q12769	605	15	-5	6	-3	1.2	1.0	1.1	1.0
BACH1	O14867	683	15	-16	6	-16	1.2	0.9	1.1	0.9
RNF213	Q63HN8	3192	15	3	6	11	1.2	1.0	1.1	1.1
UBE2Z	Q9H832	286	15	-10	6	-6	1.2	0.9	1.1	0.9
DAPP1	Q9UN19	229	14	8	6	18	1.2	1.1	1.1	1.2
PSMA5	P28066	165	14	6	6	11	1.2	1.1	1.1	1.1
PSMD6	Q15008	188	14	4	6	13	1.2	1.0	1.1	1.1
EHD1	Q9H4M9	138	14	-2	6	3	1.2	1.0	1.1	1.0
CSNK1A1L	Q8N752	150	13	-12	6	11	1.1	0.9	1.1	1.1
ZC3H7B	Q9UGR2	138	13	0	6	12	1.1	1.0	1.1	1.1
UNC45A	Q9H3U1	663	13	-7	6	-5	1.1	0.9	1.1	1.0
ZW10	O43264	686	13	-12	6	-10	1.1	0.9	1.1	0.9
PPP5C	P53041	404	12	-3	6	6	1.1	1.0	1.1	1.1
STXBP3	O00186	501	12	6	6	3	1.1	1.1	1.1	1.0
STK10	O94804	728	12	-4	6	17	1.1	1.0	1.1	1.2
RASAL3	Q86YV0	447	12	-6	6	3	1.1	0.9	1.1	1.0
DYNC1H1	Q14204	867	12	-6	6	5	1.1	0.9	1.1	1.1
HECTD1	Q9ULT8	2545	11	10	6	9	1.1	1.1	1.1	1.1
GNL3	Q9BVP2	280	11	-14	6	-11	1.1	0.9	1.1	0.9
PRKDC	P78527	232	11	-18	6	13	1.1	0.9	1.1	1.1
TBL2	Q9Y4P3	240	11	1	6	7	1.1	1.0	1.1	1.1
ACIN1	Q9UKV3	691	11	-1	6	-15	1.1	1.0	1.1	0.9
PSMD1	Q99460	112	11	-33	6	9	1.1	0.8	1.1	1.1
TCP11L1	Q9NUJ3	495	10	4	6	3	1.1	1.0	1.1	1.0
FHL1	Q13642	129	10	4	6	-2	1.1	1.0	1.1	1.0
YWHAQ	P27348	25	10	0	6	14	1.1	1.0	1.1	1.2
LRPPRC	P42704	484	10	-5	6	4	1.1	1.0	1.1	1.0
GNB4	Q9HAV0	25	10	-7	6	6	1.1	0.9	1.1	1.1
LCP2	Q13094	524	10	-7	6	0	1.1	0.9	1.1	1.0
POLA2	Q14181	222	10	-2	6	4	1.1	1.0	1.1	1.0
DICER1	Q9UPY3	686	10	-6	6	-3	1.1	0.9	1.1	1.0
EEA1	Q15075	1402	9	5	6	28	1.1	1.0	1.1	1.4
MRPL16	Q9NX20	167	9	-12	6	5	1.1	0.9	1.1	1.0
TBC1D13	Q9NVG8	145	9	8	6	8	1.1	1.1	1.1	1.1
FES	P07332	626	9	0	6	19	1.1	1.0	1.1	1.2
COG1	Q8WTW3	87	9	-2	6	9	1.1	1.0	1.1	1.1
RNF213	Q63HN8	3084	8	-1	6	10	1.1	1.0	1.1	1.1
NUP153	P49790	664	8	-19	6	1	1.1	0.8	1.1	1.0
STUB1	Q9UNE7	199	8	4	6	8	1.1	1.0	1.1	1.1
TYK2	P29597	291	8	-1	6	8	1.1	1.0	1.1	1.1
GAB3	Q8WWW8	376	8	-2	6	-22	1.1	1.0	1.1	0.8
ELOF1	P60002	29	8	-4	6	-3	1.1	1.0	1.1	1.0
INF2	Q27J81	101	8	-14	6	7	1.1	0.9	1.1	1.1
ACSL1	P33121	336	7	2	6	6	1.1	1.0	1.1	1.1
WDR46	O15213	515	7	1	6	-2	1.1	1.0	1.1	1.0
SENP1	Q9P0U3	164	7	-5	6	5	1.1	1.0	1.1	1.0
IST1	P53990	125	7	-10	6	4	1.1	0.9	1.1	1.0
FAM120B	Q96EK7	37	7	2	6	-7	1.1	1.0	1.1	0.9
CYLD	Q9NQC7	655	7	-15	6	-7	1.1	0.9	1.1	0.9
FKBP5	Q13451	394	6	5	6	11	1.1	1.1	1.1	1.1
CRBN	Q96SW2	188	6	5	6	-2	1.1	1.0	1.1	1.0
N4BP1	O75113	82	6	-1	6	5	1.1	1.0	1.1	1.0

RAB3GAP1	Q15042	678	6	2	6	-11	1.1	1.0	1.1	0.9
NCBP1	Q09161	616	6	-3	6	-7	1.1	1.0	1.1	0.9
DOCK8	Q8NF50	1471	6	-6	6	2	1.1	0.9	1.1	1.0
FAM129B	Q96TA1	334	6	-7	6	-1	1.1	0.9	1.1	1.0
C4B	P0C0L5	1010	5	8	6	2	1.1	1.1	1.1	1.0
VWA5A	O00534	163	5	-3	6	1	1.1	1.0	1.1	1.0
DENND1C	Q8IV53	585	5	-4	6	-11	1.1	1.0	1.1	0.9
KMT2D	O14686	5314	5	-6	6	-9	1.1	0.9	1.1	0.9
TNS3	Q68CZ2	25	5	10	6	2	1.0	1.1	1.1	1.0
PHF6	Q8IWS0	128	5	6	6	12	1.0	1.1	1.1	1.1
MDN1	Q9NU22	1400	5	-7	6	10	1.0	0.9	1.1	1.1
NPAT	Q14207	1172	5	-15	6	-15	1.0	0.9	1.1	0.9
TRIM14	Q14142	198	4	13	6	-13	1.0	1.1	1.1	0.9
CRBN	Q96SW2	441	4	-2	6	-1	1.0	1.0	1.1	1.0
NLRC5	Q86WI3	1219	4	-5	6	3	1.0	1.0	1.1	1.0
KPNA5	O15131	238	4	-14	6	-6	1.0	0.9	1.1	0.9
KPNA6	O60684	238	4	-14	6	-6	1.0	0.9	1.1	0.9
KPNA1	P52294	240	4	-14	6	-6	1.0	0.9	1.1	0.9
RAP1GDS1	P52306	85	4	1	6	8	1.0	1.0	1.1	1.1
OVCA2	Q8WZ82	152	4	-1	6	-7	1.0	1.0	1.1	0.9
RABGGTB	P53611	40	4	-3	6	3	1.0	1.0	1.1	1.0
PTBP3	O95758	55	4	-6	6	1	1.0	0.9	1.1	1.0
ACLY	P53396	728	4	-6	6	5	1.0	0.9	1.1	1.0
SYNE1	Q8NF91	6460	4	-19	6	-19	1.0	0.8	1.1	0.8
PAIP1	Q9H074	470	3	7	6	3	1.0	1.1	1.1	1.0
RPL37A	P61513	60	3	-2	6	1	1.0	1.0	1.1	1.0
BAZ1A	Q9NRL2	547	3	-6	6	9	1.0	0.9	1.1	1.1
GSTP1	P09211	48	3	-8	6	21	1.0	0.9	1.1	1.3
RNF146	Q9NTX7	354	3	-1	6	-2	1.0	1.0	1.1	1.0
SHC1	P29353	196	3	-9	6	-6	1.0	0.9	1.1	0.9
CLASP2	O75122	221	2	-1	6	2	1.0	1.0	1.1	1.0
MASTL	Q96GX5	455	2	-5	6	-9	1.0	1.0	1.1	0.9
RABGAP1	Q9Y3P9	1026	2	-11	6	-5	1.0	0.9	1.1	1.0
SKIV2L	Q15477	247	1	-2	6	-12	1.0	1.0	1.1	0.9
SMG1	Q96Q15	3401	1	-15	6	-2	1.0	0.9	1.1	1.0
SRBD1	Q8N5C6	294	1	-19	6	-5	1.0	0.8	1.1	1.0
PBRM1	Q86U86	1224	1	4	6	-2	1.0	1.0	1.1	1.0
ACSL3	O95573	450	1	3	6	7	1.0	1.0	1.1	1.1
POLG	P54098	1077	1	-1	6	-8	1.0	1.0	1.1	0.9
MACF1	Q9UPN3	4388	1	-6	6	-8	1.0	0.9	1.1	0.9
SYMPK	Q92797	848	-1	7	6	-2	1.0	1.1	1.1	1.0
C16orf13	Q96S19	63	-1	-23	6	26	1.0	0.8	1.1	1.3
MYCBP2	O75592	3326	-2	7	6	-6	1.0	1.1	1.1	0.9
PLS3	P13797	104	-2	-6	6	-19	1.0	0.9	1.1	0.8
NUDT7	P0C024	72	-2	-11	6	2	1.0	0.9	1.1	1.0
ITPR1	Q14643	1468	-3	-4	6	-3	1.0	1.0	1.1	1.0
PCNA	P12004	62	-3	-7	6	8	1.0	0.9	1.1	1.1
BROX	Q5VW32	288	-3	-33	6	-1	1.0	0.8	1.1	1.0
CDC123	O75794	159	-3	0	6	-10	1.0	1.0	1.1	0.9
NFATC2	Q13469	231	-3	-7	6	-15	1.0	0.9	1.1	0.9
GCLC	P48506	142	-3	-10	6	3	1.0	0.9	1.1	1.0
LRWD1	Q9UFC0	522	-3	-10	6	-21	1.0	0.9	1.1	0.8
RABGAP1	Q9Y3P9	476	-4	-7	6	-5	1.0	0.9	1.1	1.0

PRDM10	Q9NQV6	654	-4	8	6	-8	1.0	1.1	1.1	0.9
TUBB2B	Q9BVA1	129	-5	1	6	-5	1.0	1.0	1.1	1.0
NELL1	Q92832	515	-5	-9	6	8	1.0	0.9	1.1	1.1
NELL1	Q92832	523	-5	-9	6	8	1.0	0.9	1.1	1.1
POLR2B	P30876	1122	-6	5	6	-15	0.9	1.1	1.1	0.9
PDCD2	Q16342	125	-6	-14	6	14	0.9	0.9	1.1	1.2
NUP85	Q9BW27	511	-6	-16	6	-16	0.9	0.9	1.1	0.9
PANK4	Q9NVE7	104	-7	-19	6	-6	0.9	0.8	1.1	0.9
SCAMP4	Q969E2	20	-8	-2	6	-4	0.9	1.0	1.1	1.0
PRDM10	Q9NQV6	562	-10	-9	6	-8	0.9	0.9	1.1	0.9
ATG3	Q9NT62	264	-10	-2	6	-3	0.9	1.0	1.1	1.0
HNRNPL	P14866	581	-12	3	6	4	0.9	1.0	1.1	1.0
NAPRT	Q6XQN6	101	-13	-7	6	6	0.9	0.9	1.1	1.1
COPE	O14579	212	-22	-14	6	-20	0.8	0.9	1.1	0.8
DDX6	P26196	184	30	4	6	5	1.4	1.0	1.1	1.1
DYNC1H1	Q14204	3712	25	-9	6	2	1.3	0.9	1.1	1.0
DDX42	Q86XP3	382	24	3	6	13	1.3	1.0	1.1	1.1
GNL1	P36915	294	24	-14	6	10	1.3	0.9	1.1	1.1
SLC25A13	Q9UJS0	503	22	6	6	22	1.3	1.1	1.1	1.3
FAM120B	Q96EK7	392	22	4	6	0	1.3	1.0	1.1	1.0
NEK6	Q9HC98	258	20	-4	6	-2	1.3	1.0	1.1	1.0
WDFY4	Q6ZS81	264	19	17	6	-6	1.2	1.2	1.1	0.9
KIF21B	O75037	203	19	-7	6	10	1.2	0.9	1.1	1.1
DERA	Q9Y315	118	19	-9	6	8	1.2	0.9	1.1	1.1
IFIT2	P09913	54	18	-3	6	14	1.2	1.0	1.1	1.2
DDX39A	O00148	299	18	-5	6	13	1.2	1.0	1.1	1.1
VPS28	Q9UK41	70	18	-9	6	4	1.2	0.9	1.1	1.0
GPATCH8	Q9UKJ3	508	17	-3	6	-1	1.2	1.0	1.1	1.0
ALS2	Q96Q42	664	16	7	6	1	1.2	1.1	1.1	1.0
G6PD	P11413	232	16	-4	6	5	1.2	1.0	1.1	1.1
PEX19	P40855	128	16	4	6	-17	1.2	1.0	1.1	0.9
ARHGAP9	Q9BRR9	693	16	-6	6	2	1.2	0.9	1.1	1.0
POLR2B	P30876	1119	16	-8	6	4	1.2	0.9	1.1	1.0
ATM	Q13315	2704	16	-11	6	1	1.2	0.9	1.1	1.0
CHERP	Q8IWX8	246	15	2	6	12	1.2	1.0	1.1	1.1
ITGAL	P20701	1154	15	5	6	-6	1.2	1.1	1.1	0.9
EIF3K	Q9UBQ5	85	15	-13	6	8	1.2	0.9	1.1	1.1
HSPA1B	P0DMV9	306	15	-31	6	5	1.2	0.8	1.1	1.1
HSPA6	P17066	308	15	-31	6	5	1.2	0.8	1.1	1.1
TBCD	Q9BTW9	457	14	2	6	7	1.2	1.0	1.1	1.1
MCF2L	O15068	223	14	-2	6	-24	1.2	1.0	1.1	0.8
INPP5D	Q92835	736	14	-10	6	-5	1.2	0.9	1.1	1.0
RRBP1	Q9P2E9	1216	14	10	6	-9	1.2	1.1	1.1	0.9
TLN1	Q9Y490	1671	14	2	6	9	1.2	1.0	1.1	1.1
GALK1	P51570	322	14	1	6	7	1.2	1.0	1.1	1.1
TRAPPC11	Q7Z392	41	13	4	6	14	1.1	1.0	1.1	1.2
IPO8	O15397	757	13	-4	6	6	1.1	1.0	1.1	1.1
RASA2	Q15283	430	13	-8	6	7	1.1	0.9	1.1	1.1
SEPT5	Q99719	300	12	8	6	12	1.1	1.1	1.1	1.1
PNO1	Q9NRX1	226	12	-4	6	6	1.1	1.0	1.1	1.1
GTF3C4	Q9UKN8	116	12	-10	6	-7	1.1	0.9	1.1	0.9
SIGLEC14	Q08ET2	345	12	-15	6	-14	1.1	0.9	1.1	0.9
HSDL2	Q6YN16	218	12	-3	6	-9	1.1	1.0	1.1	0.9

RPS6KA1	Q15418	552	12	-8	6	3	1.1	0.9	1.1	1.0
CPOX	P36551	127	12	-10	6	-4	1.1	0.9	1.1	1.0
VCL	P18206	1053	11	-3	6	6	1.1	1.0	1.1	1.1
ATM	Q13315	1736	11	-18	6	8	1.1	0.8	1.1	1.1
ARNT	P27540	256	10	15	6	20	1.1	1.2	1.1	1.2
BCL11B	Q9C0K0	464	10	12	6	-5	1.1	1.1	1.1	1.0
CRIP1	P50238	28	10	2	6	7	1.1	1.0	1.1	1.1
RIN1	Q13671	733	10	1	6	-5	1.1	1.0	1.1	1.0
TANGO6	Q9C0B7	350	10	-5	6	15	1.1	1.0	1.1	1.2
SNRNP200	O75643	1580	10	-6	6	-1	1.1	0.9	1.1	1.0
MEFV	O15553	144	10	-7	6	-28	1.1	0.9	1.1	0.8
MON1B	Q7L1V2	349	10	9	6	6	1.1	1.1	1.1	1.1
TOP2B	Q02880	125	10	-6	6	1	1.1	0.9	1.1	1.0
WDR89	Q96FK6	82	10	-13	6	-11	1.1	0.9	1.1	0.9
FASN	P49327	1141	10	-21	6	2	1.1	0.8	1.1	1.0
RANBP1	P43487	99	9	4	6	14	1.1	1.0	1.1	1.2
TYK2	P29597	544	9	4	6	6	1.1	1.0	1.1	1.1
RXRB	P28702	340	9	-5	6	7	1.1	1.0	1.1	1.1
PNKP	Q96T60	353	9	-6	6	-15	1.1	0.9	1.1	0.9
LRCH4	O75427	213	9	-11	6	1	1.1	0.9	1.1	1.0
TTC4	O95801	160	8	1	6	-4	1.1	1.0	1.1	1.0
CCT3	P49368	40	8	-1	6	-2	1.1	1.0	1.1	1.0
EPRS	P07814	1301	8	-5	6	8	1.1	1.0	1.1	1.1
TRAF7	Q6Q0C0	426	8	-21	6	-7	1.1	0.8	1.1	0.9
TCF20	Q9UGU0	1188	8	16	6	-10	1.1	1.2	1.1	0.9
VMAC	Q2NL98	108	8	1	6	-9	1.1	1.0	1.1	0.9
UBR1	Q8IWV7	1577	7	13	6	9	1.1	1.1	1.1	1.1
CDK5	Q00535	157	7	3	6	-17	1.1	1.0	1.1	0.9
C16orf62	Q7Z3J2	277	7	-13	6	-3	1.1	0.9	1.1	1.0
GTF3C5	Q9Y5Q8	242	7	-19	6	0	1.1	0.8	1.1	1.0
YRDC	Q86U90	99	7	4	6	-5	1.1	1.0	1.1	1.0
IQSEC1	Q6DN90	906	7	1	6	1	1.1	1.0	1.1	1.0
SGTB	Q96EQ0	67	7	-1	6	-5	1.1	1.0	1.1	1.0
NAA15	Q9BXJ9	322	7	-8	6	7	1.1	0.9	1.1	1.1
ZC3HC1	Q86WB0	149	7	-10	6	0	1.1	0.9	1.1	1.0
NAA25	Q14CX7	339	7	-11	6	46	1.1	0.9	1.1	1.8
SLC25A4	P12235	160	7	-11	6	27	1.1	0.9	1.1	1.4
C16orf87	Q6PH81	27	6	-3	6	-8	1.1	1.0	1.1	0.9
PXN	P49023	582	6	-4	6	-2	1.1	1.0	1.1	1.0
GEMIN5	Q8TEQ6	441	6	-5	6	5	1.1	1.0	1.1	1.0
CXXC1	Q9P0U4	592	6	-7	6	-1	1.1	0.9	1.1	1.0
CASP2	P42575	320	5	4	6	-7	1.1	1.0	1.1	0.9
ARIH1	Q9Y4X5	161	5	-3	6	2	1.1	1.0	1.1	1.0
MED1	Q15648	424	5	-5	6	11	1.1	1.0	1.1	1.1
SPG11	Q96JI7	1996	5	-8	6	-12	1.1	0.9	1.1	0.9
ARID1A	O14497	336	5	6	6	-8	1.0	1.1	1.1	0.9
PFKL	P17858	351	5	0	6	10	1.0	1.0	1.1	1.1
THBS1	P07996	836	5	-8	6	12	1.0	0.9	1.1	1.1
YARS	P54577	250	5	-12	6	1	1.0	0.9	1.1	1.0
TXNL4B	Q9NX01	38	4	5	6	12	1.0	1.1	1.1	1.1
RAE1	P78406	175	4	-4	6	11	1.0	1.0	1.1	1.1
BOD1L1	Q8NFC6	749	4	-6	6	-15	1.0	0.9	1.1	0.9
RNF213	Q63HN8	680	4	-7	6	-8	1.0	0.9	1.1	0.9

BAG1	Q99933	213	4	1	6	1	1.0	1.0	1.1	1.0
SPTAN1	Q13813	2233	4	-6	6	-11	1.0	0.9	1.1	0.9
PPP1R21	Q6ZMI0	468	3	2	6	4	1.0	1.0	1.1	1.0
CBLL1	Q75N03	158	3	-11	6	-21	1.0	0.9	1.1	0.8
HNRNPF	P52597	267	3	1	6	-2	1.0	1.0	1.1	1.0
C18orf8	Q96DM3	635	3	-3	6	1	1.0	1.0	1.1	1.0
SLC38A10	Q9HBR0	301	3	-10	6	-6	1.0	0.9	1.1	0.9
KIF13B	Q9NQT8	82	3	-14	6	0	1.0	0.9	1.1	1.0
EARS2	Q5JPH6	140	3	-15	6	-9	1.0	0.9	1.1	0.9
ZZEF1	O43149	2961	2	7	6	0	1.0	1.1	1.1	1.0
ASCC3	Q8N3C0	1874	2	-9	6	-3	1.0	0.9	1.1	1.0
Uncharacteri	A0A0C4DFX4	1865	2	-10	6	-13	1.0	0.9	1.1	0.9
PTBP3	O95758	249	2	-3	6	-1	1.0	1.0	1.1	1.0
OAS3	Q9Y6K5	574	2	-13	6	-5	1.0	0.9	1.1	1.0
HPCAL1	P37235	185	1	-2	6	27	1.0	1.0	1.1	1.4
DHRS1	Q96LJ7	256	1	-4	6	-2	1.0	1.0	1.1	1.0
OAT	P04181	150	1	-10	6	15	1.0	0.9	1.1	1.2
THOP1	P52888	682	1	-10	6	-11	1.0	0.9	1.1	0.9
SMARCA5	O60264	165	1	-10	6	2	1.0	0.9	1.1	1.0
PRKDC	P78527	1229	1	1	6	-3	1.0	1.0	1.1	1.0
PSTPIP2	Q9H939	65	1	-3	6	4	1.0	1.0	1.1	1.0
HBS1L	Q9Y450	491	1	-5	6	5	1.0	1.0	1.1	1.0
LLGL1	Q15334	947	1	-15	6	-15	1.0	0.9	1.1	0.9
PDCD11	Q14690	330	0	2	6	13	1.0	1.0	1.1	1.1
RABEP2	Q9H5N1	79	-1	4	6	1	1.0	1.0	1.1	1.0
SACS	Q9NZJ4	2514	-1	0	6	2	1.0	1.0	1.1	1.0
SUPT6H	Q7KZ85	1169	-1	-1	6	11	1.0	1.0	1.1	1.1
OASL	Q15646	181	-1	-9	6	-2	1.0	0.9	1.1	1.0
VASP	P50552	64	-2	-7	6	4	1.0	0.9	1.1	1.0
TRIM21	P19474	273	-2	4	6	-1	1.0	1.0	1.1	1.0
ANKRD11	Q6UB99	646	-2	-4	6	-3	1.0	1.0	1.1	1.0
HNRNPF	P52597	290	-2	-8	6	5	1.0	0.9	1.1	1.1
PUF60	Q9UHX1	487	-3	3	6	-10	1.0	1.0	1.1	0.9
HK2	P52789	909	-3	-7	6	-9	1.0	0.9	1.1	0.9
PDCD6IP	Q8WUM4	250	-4	-2	6	7	1.0	1.0	1.1	1.1
TIAL1	Q01085	205	-4	-13	6	-3	1.0	0.9	1.1	1.0
RIN3	Q8TB24	976	-5	-3	6	-4	1.0	1.0	1.1	1.0
CAST	P20810	408	-5	-11	6	-13	1.0	0.9	1.1	0.9
ATP5C1	P36542	103	-5	4	6	13	1.0	1.0	1.1	1.1
DUSP23	Q9BVJ7	67	-6	-8	6	-5	0.9	0.9	1.1	1.0
POLD1	P28340	428	-6	-10	6	3	0.9	0.9	1.1	1.0
PCID2	Q5JVF3	83	-7	-23	6	-13	0.9	0.8	1.1	0.9
FAM160B1	Q5W0V3	609	-8	-19	6	2	0.9	0.8	1.1	1.0
ABCB7	O75027	747	-8	-20	6	-8	0.9	0.8	1.1	0.9
METAP2	P50579	263	-8	-21	6	-4	0.9	0.8	1.1	1.0
CCP110	O43303	436	-8	-42	6	-15	0.9	0.7	1.1	0.9
FAM129A	Q9BZQ8	680	-9	1	6	-4	0.9	1.0	1.1	1.0
RBBP5	Q15291	126	-9	-1	6	-14	0.9	1.0	1.1	0.9
ACAD9	Q9H845	507	-10	3	6	-10	0.9	1.0	1.1	0.9
GUCY1A3	Q02108	176	-14	10	6	-3	0.9	1.1	1.1	1.0
RFTN1	Q14699	129	-14	-13	6	-15	0.9	0.9	1.1	0.9
TBC1D15	Q8TC07	24	-15	10	6	5	0.9	1.1	1.1	1.1
TRAFD1	O14545	267	-16	-3	6	-17	0.9	1.0	1.1	0.9

MYO1F	O00160	788	-20	-11	6	13	0.8	0.9	1.1	1.1
PREP	P48147	264	-22	-53	6	-20	0.8	0.7	1.1	0.8
FKBP4	Q02790	342	29	-19	5	7	1.4	0.8	1.1	1.1
HECTD1	Q9ULT8	146	28	16	5	3	1.4	1.2	1.1	1.0
GAPVD1	Q14C86	176	27	-10	5	10	1.4	0.9	1.1	1.1
IMPACT	Q9P2X3	284	22	8	5	14	1.3	1.1	1.1	1.2
IVD	P26440	251	21	14	5	-7	1.3	1.2	1.1	0.9
IKZF1	Q13422	203	20	-1	5	-1	1.3	1.0	1.1	1.0
CDC23	Q9UJX2	324	20	-7	5	4	1.3	0.9	1.1	1.0
ARID1B	Q8NFD5	1093	20	12	5	-15	1.2	1.1	1.1	0.9
MVP	Q14764	572	20	-15	5	11	1.2	0.9	1.1	1.1
ARCN1	P48444	94	18	-15	5	2	1.2	0.9	1.1	1.0
ETFA	P13804	68	17	6	5	9	1.2	1.1	1.1	1.1
FAM129A	Q9BZQ8	194	17	-7	5	-11	1.2	0.9	1.1	0.9
PIK3CD	O00329	627	17	-7	5	6	1.2	0.9	1.1	1.1
ERAP1	Q9NZ08	806	17	-8	5	16	1.2	0.9	1.1	1.2
ECM29	Q5VYK3	1638	16	-10	5	2	1.2	0.9	1.1	1.0
WBP4	O75554	13	16	4	5	4	1.2	1.0	1.1	1.0
SARG	Q9BW04	574	15	0	5	0	1.2	1.0	1.1	1.0
ADAR	P55265	304	15	-19	5	10	1.2	0.8	1.1	1.1
KIAA1429	Q69YN4	781	15	4	5	9	1.2	1.0	1.1	1.1
NAIP	Q13075	1264	15	-1	5	-4	1.2	1.0	1.1	1.0
HECTD3	Q5T447	143	14	6	5	-1	1.2	1.1	1.1	1.0
GEMIN4	P57678	683	14	-1	5	2	1.2	1.0	1.1	1.0
POLR2B	P30876	622	14	-12	5	12	1.2	0.9	1.1	1.1
BTK	Q06187	337	14	-5	5	8	1.2	1.0	1.1	1.1
CYB5R1	Q9UHQ9	208	14	-11	5	20	1.2	0.9	1.1	1.2
ZNF77	Q15935	391	13	5	5	20	1.1	1.1	1.1	1.3
ADCK3	Q8NI60	641	13	0	5	-9	1.1	1.0	1.1	0.9
EIF3F	A0A0D9SEZ9	195	13	-4	5	18	1.1	1.0	1.1	1.2
NBEAL2	Q6ZNJ1	1619	13	-7	5	10	1.1	0.9	1.1	1.1
ERCC2	P18074	663	13	-7	5	5	1.1	0.9	1.1	1.1
PSMD1	Q99460	104	13	-60	5	23	1.1	0.6	1.1	1.3
DGKA	P23743	101	13	2	5	5	1.1	1.0	1.1	1.1
DHX9	Q08211	469	13	-16	5	3	1.1	0.9	1.1	1.0
VTA1	Q9NP79	87	12	-17	5	-4	1.1	0.9	1.1	1.0
TXNDC9	O14530	94	12	2	5	10	1.1	1.0	1.1	1.1
COPG1	Q9Y678	296	12	-8	5	1	1.1	0.9	1.1	1.0
COPB1	P53618	284	12	-9	5	3	1.1	0.9	1.1	1.0
COPB1	P53618	102	12	-12	5	22	1.1	0.9	1.1	1.3
LETM1	O95202	379	11	8	5	25	1.1	1.1	1.1	1.3
MRVI1	Q9Y6F6	642	11	-6	5	-11	1.1	0.9	1.1	0.9
SLC25A16	P16260	311	11	-20	5	-9	1.1	0.8	1.1	0.9
FBXO7	Q9Y3I1	286	11	-2	5	3	1.1	1.0	1.1	1.0
AKAP13	Q12802	1414	11	-14	5	-8	1.1	0.9	1.1	0.9
RNF2	Q99496	72	10	-4	5	0	1.1	1.0	1.1	1.0
RFC5	P40937	155	10	-5	5	4	1.1	1.0	1.1	1.0
XPO5	Q9HAV4	221	10	11	5	16	1.1	1.1	1.1	1.2
HPS3	Q969F9	39	10	-3	5	-3	1.1	1.0	1.1	1.0
PTK2B	Q14289	44	10	-3	5	-13	1.1	1.0	1.1	0.9
TCEB2	Q15370	60	10	-8	5	5	1.1	0.9	1.1	1.1
KDM4B	O94953	235	10	-9	5	8	1.1	0.9	1.1	1.1
PLCL2	Q9UPR0	1095	10	-10	5	19	1.1	0.9	1.1	1.2

PIK3C2B	O00750	151	9	6	5	4	1.1	1.1	1.1	1.0
MAP3K4	Q9Y6R4	596	9	-1	5	-2	1.1	1.0	1.1	1.0
SAMD9	Q5K651	1085	9	-9	5	6	1.1	0.9	1.1	1.1
NUP98	P52948	1492	9	-13	5	-9	1.1	0.9	1.1	0.9
PSMC3	P17980	240	9	8	5	7	1.1	1.1	1.1	1.1
USP9Y	O00507	693	9	-2	5	2	1.1	1.0	1.1	1.0
USP9X	Q93008	692	9	-2	5	2	1.1	1.0	1.1	1.0
MTAP	Q13126	131	9	-4	5	-5	1.1	1.0	1.1	1.0
MED16	Q9Y2X0	584	8	7	5	-4	1.1	1.1	1.1	1.0
DDHD2	O94830	470	8	2	5	-1	1.1	1.0	1.1	1.0
CARD11	Q9BXL7	743	8	-1	5	-8	1.1	1.0	1.1	0.9
ARHGEF7	Q14155	349	8	4	5	-2	1.1	1.0	1.1	1.0
DNAJC7	Q99615	175	8	0	5	12	1.1	1.0	1.1	1.1
MX1	P20591	52	8	-5	5	-2	1.1	1.0	1.1	1.0
MX2	P20592	100	8	-5	5	-2	1.1	1.0	1.1	1.0
NASP	P49321	84	8	-9	5	-4	1.1	0.9	1.1	1.0
KAT7	O95251	418	7	-2	5	15	1.1	1.0	1.1	1.2
KAT6B	Q8WYB5	801	7	-2	5	15	1.1	1.0	1.1	1.2
KAT8	Q9H7Z6	260	7	-2	5	15	1.1	1.0	1.1	1.2
ATM	Q13315	2092	7	-2	5	-1	1.1	1.0	1.1	1.0
PDCD11	Q14690	535	7	-4	5	-4	1.1	1.0	1.1	1.0
DYNC1H1	Q14204	978	7	-5	5	-2	1.1	1.0	1.1	1.0
DDX39A	O00148	238	7	7	5	4	1.1	1.1	1.1	1.0
BCAS3	Q9H6U6	442	7	4	5	9	1.1	1.0	1.1	1.1
EDC4	Q6P2E9	384	7	3	5	-8	1.1	1.0	1.1	0.9
ATIC	P31939	363	7	0	5	5	1.1	1.0	1.1	1.0
COQ3	Q9NZJ6	358	7	-2	5	-11	1.1	1.0	1.1	0.9
KRI1	Q8N9T8	591	7	-8	5	-7	1.1	0.9	1.1	0.9
MX1	P20591	322	7	-13	5	6	1.1	0.9	1.1	1.1
ARHGEF2	Q92974	573	6	3	5	1	1.1	1.0	1.1	1.0
PYGO2	Q9BRQ0	350	6	1	5	6	1.1	1.0	1.1	1.1
WDR81	Q562E7	130	6	-4	5	-6	1.1	1.0	1.1	0.9
PIKFYVE	Q9Y2I7	183	6	-4	5	-9	1.1	1.0	1.1	0.9
NELFB	Q8WX92	265	6	-5	5	-32	1.1	1.0	1.1	0.8
LOC1027241!	A0A0B4J2E5	508	6	-9	5	-12	1.1	0.9	1.1	0.9
LSM14B	Q9BX40	310	6	-10	5	1	1.1	0.9	1.1	1.0
AP2A2	O94973	353	6	-13	5	2	1.1	0.9	1.1	1.0
AP2A1	O95782	354	6	-13	5	2	1.1	0.9	1.1	1.0
TNRC6C	Q9HCJ0	1637	6	6	5	4	1.1	1.1	1.1	1.0
ZZEF1	O43149	2229	6	3	5	-8	1.1	1.0	1.1	0.9
DNPEP	Q9ULA0	447	6	-7	5	12	1.1	0.9	1.1	1.1
RPL18	Q07020	134	6	-9	5	1	1.1	0.9	1.1	1.0
FARSB	Q9NSD9	498	6	-15	5	11	1.1	0.9	1.1	1.1
GPN2	Q9H9Y4	262	5	-5	5	-7	1.1	1.0	1.1	0.9
GNL3	Q9BVP2	156	5	-11	5	1	1.1	0.9	1.1	1.0
PPP1R10	Q96QC0	463	5	-12	5	7	1.1	0.9	1.1	1.1
FASN	P49327	2468	5	-12	5	-3	1.1	0.9	1.1	1.0
LRRK2	Q5S007	2139	5	9	5	-10	1.0	1.1	1.1	0.9
ZNF609	O15014	1290	5	1	5	4	1.0	1.0	1.1	1.0
DOK2	O60496	110	5	1	5	-7	1.0	1.0	1.1	0.9
ALDH7A1	P49419	70	5	1	5	9	1.0	1.0	1.1	1.1
CDC42EP4	Q9H3Q1	313	4	-2	5	-1	1.0	1.0	1.1	1.0
RPL8	P62917	114	4	-6	5	3	1.0	0.9	1.1	1.0

LTN1	O94822	751	4	-6	5	-2	1.0	0.9	1.1	1.0
HTT	P42858	664	4	-8	5	-5	1.0	0.9	1.1	1.0
PARG	Q86W56	155	4	2	5	-9	1.0	1.0	1.1	0.9
NUS1	Q96E22	77	4	2	5	-19	1.0	1.0	1.1	0.8
MMAA	Q8IVH4	100	4	-1	5	3	1.0	1.0	1.1	1.0
PCBP2	Q15366	109	4	-1	5	1	1.0	1.0	1.1	1.0
IL7R	P16871	349	3	2	5	-1	1.0	1.0	1.1	1.0
FAM101B	Q8N5W9	86	3	1	5	-2	1.0	1.0	1.1	1.0
RAB3GAP2	Q9H2M9	896	3	-9	5	5	1.0	0.9	1.1	1.0
PSMD6	Q15008	266	3	-10	5	-9	1.0	0.9	1.1	0.9
ZEB2	O60315	539	3	1	5	-4	1.0	1.0	1.1	1.0
EIF6	P56537	11	3	-5	5	7	1.0	1.0	1.1	1.1
NARFL	Q9H6Q4	71	2	1	5	-14	1.0	1.0	1.1	0.9
FASN	P49327	2202	2	1	5	-2	1.0	1.0	1.1	1.0
RALGAPA1	Q6GYQ0	293	1	5	5	-12	1.0	1.0	1.1	0.9
CEP44	Q9C0F1	260	1	-6	5	-9	1.0	0.9	1.1	0.9
TRAFD1	O14545	109	1	-10	5	-7	1.0	0.9	1.1	0.9
NUP93	Q8N1F7	392	1	-33	5	2	1.0	0.8	1.1	1.0
TNPO1	Q92973	862	1	-2	5	-4	1.0	1.0	1.1	1.0
GCDH	Q92947	115	1	-10	5	1	1.0	0.9	1.1	1.0
ITGB2	P05107	598	1	-23	5	-16	1.0	0.8	1.1	0.9
HDAC10	Q969S8	403	0	-9	5	2	1.0	0.9	1.1	1.0
RAD50	Q92878	53	0	-9	5	-1	1.0	0.9	1.1	1.0
DNAJC25-GN	A0A024R161	129	-1	-3	5	5	1.0	1.0	1.1	1.0
PHF1	O43189	518	-1	-5	5	9	1.0	1.0	1.1	1.1
TANK	Q92844	318	-1	-3	5	-11	1.0	1.0	1.1	0.9
EPHA4	P54764	760	-1	-6	5	6	1.0	0.9	1.1	1.1
EPHB1	P54762	758	-1	-6	5	6	1.0	0.9	1.1	1.1
HERC2	O95714	1410	-1	-7	5	-21	1.0	0.9	1.1	0.8
TRIM33	Q9UPN9	153	-2	1	5	2	1.0	1.0	1.1	1.0
DDX1	Q92499	110	-2	-5	5	-2	1.0	1.0	1.1	1.0
WDR46	O15213	172	-2	2	5	-1	1.0	1.0	1.1	1.0
CNP	P09543	49	-2	-5	5	-2	1.0	1.0	1.1	1.0
ISYNA1	Q9NPH2	235	-3	-16	5	-18	1.0	0.9	1.1	0.9
PRKDC	P78527	111	-3	0	5	4	1.0	1.0	1.1	1.0
RPL24	P83731	6	-4	-12	5	-4	1.0	0.9	1.1	1.0
PSMA1	P25786	148	-4	-14	5	-14	1.0	0.9	1.1	0.9
KSR1	Q8IVT5	166	-4	-3	5	-2	1.0	1.0	1.1	1.0
HLA-A	P16190	363	-4	-3	5	-14	1.0	1.0	1.1	0.9
HLA-A	P30457	363	-4	-3	5	-14	1.0	1.0	1.1	0.9
FYTTD1	Q96QD9	242	-5	-4	5	-7	1.0	1.0	1.1	0.9
HEATR3	Q7Z4Q2	266	-5	-8	5	-3	1.0	0.9	1.1	1.0
IARS2	Q9NSE4	521	-5	-9	5	1	1.0	0.9	1.1	1.0
CDAN1	Q8IWY9	1213	-6	-5	5	-10	0.9	1.0	1.1	0.9
RNF213	Q63HN8	2229	-6	-21	5	-16	0.9	0.8	1.1	0.9
SMURF2	Q9HAU4	151	-7	6	5	-20	0.9	1.1	1.1	0.8
AKAP1	Q92667	147	-7	-7	5	-18	0.9	0.9	1.1	0.8
INF2	Q27J81	38	-7	-11	5	-2	0.9	0.9	1.1	1.0
RASAL3	Q86YV0	605	-7	-11	5	-7	0.9	0.9	1.1	0.9
RABGAP1	Q9Y3P9	533	-9	5	5	-17	0.9	1.1	1.1	0.9
GMIP	Q9P107	274	-10	-35	5	-7	0.9	0.7	1.1	0.9
VPS50	Q96JG6	472	-12	-2	5	12	0.9	1.0	1.1	1.1
FAM63A	Q8N5J2	94	-13	-16	5	-11	0.9	0.9	1.1	0.9

CTPS1	P17812	362	-13	2	5	-14	0.9	1.0	1.1	0.9
ITFG2	Q969R8	438	-14	4	5	-11	0.9	1.0	1.1	0.9
CKB	P12277	283	-15	-15	5	2	0.9	0.9	1.1	1.0
SYNCRIP	O60506	211	31	-9	5	9	1.4	0.9	1.0	1.1
LRPPRC	P42704	952	27	-10	5	23	1.4	0.9	1.0	1.3
RNH1	P13489	273	27	4	5	8	1.4	1.0	1.0	1.1
YWHAH	Q04917	97	26	-10	5	16	1.3	0.9	1.0	1.2
USP9X	Q93008	2293	23	-14	5	4	1.3	0.9	1.0	1.0
MORC2	Q9Y6X9	269	21	-1	5	8	1.3	1.0	1.0	1.1
MED15	Q96RN5	588	19	12	5	-9	1.2	1.1	1.0	0.9
UBE4A	Q14139	465	19	-3	5	-4	1.2	1.0	1.0	1.0
PRKDC	P78527	1455	19	-104	5	5	1.2	0.5	1.0	1.1
NOL11	Q9H8H0	286	19	-12	5	0	1.2	0.9	1.0	1.0
CDA	P32320	102	18	-5	5	-2	1.2	1.0	1.0	1.0
TTC39C	Q8N584	97	18	-21	5	-6	1.2	0.8	1.0	0.9
ACOT2	P49753	75	18	-1	5	15	1.2	1.0	1.0	1.2
FAM175B	Q15018	237	18	-13	5	-14	1.2	0.9	1.0	0.9
MLYCD	O95822	360	18	-22	5	-12	1.2	0.8	1.0	0.9
BIRC6	Q9NR09	2910	17	7	5	21	1.2	1.1	1.0	1.3
CNTRL	Q7Z7A1	639	16	7	5	7	1.2	1.1	1.0	1.1
PPP2R1A	P30153	310	16	-14	5	6	1.2	0.9	1.0	1.1
CAMKK2	Q96RR4	223	16	-6	5	-12	1.2	0.9	1.0	0.9
GIMAP1	Q8WWP7	181	16	-24	5	14	1.2	0.8	1.0	1.2
HENMT1	Q5T8I9	223	15	7	5	-1	1.2	1.1	1.0	1.0
EZR	P15311	117	15	-1	5	-3	1.2	1.0	1.0	1.0
HSPA9	P38646	366	15	-2	5	11	1.2	1.0	1.0	1.1
EML4	Q9HC35	368	15	-13	5	7	1.2	0.9	1.0	1.1
ZNF598	Q86UK7	456	15	-4	5	-3	1.2	1.0	1.0	1.0
HDAC9	Q9UKV0	534	15	-5	5	5	1.2	1.0	1.0	1.1
ATP13A1	Q9HD20	862	14	2	5	6	1.2	1.0	1.0	1.1
RELB	Q01201	109	14	1	5	5	1.2	1.0	1.0	1.1
GCN1	Q92616	246	14	-10	5	7	1.2	0.9	1.0	1.1
PDCD4	Q53EL6	227	13	-6	5	6	1.1	0.9	1.0	1.1
PPP2R1A	P30153	148	13	4	5	17	1.1	1.0	1.0	1.2
PPP2R1B	P30154	160	13	4	5	17	1.1	1.0	1.0	1.2
PDHA1	P08559	218	13	1	5	11	1.1	1.0	1.0	1.1
MYO9B	Q13459	234	13	1	5	-23	1.1	1.0	1.0	0.8
MAN2C1	Q9NTJ4	29	13	-1	5	9	1.1	1.0	1.0	1.1
NSUN5	Q96P11	41	13	-8	5	7	1.1	0.9	1.0	1.1
BDH1	Q02338	221	12	2	5	3	1.1	1.0	1.0	1.0
MAEA	Q7L5Y9	378	12	-4	5	2	1.1	1.0	1.0	1.0
NR3C1	P04150	622	12	4	5	0	1.1	1.0	1.0	1.0
KPNA3	O00505	228	12	-22	5	-16	1.1	0.8	1.0	0.9
KIF4A	O95239	269	11	6	5	-11	1.1	1.1	1.0	0.9
CHUK	O15111	371	11	5	5	0	1.1	1.1	1.0	1.0
DIAPH1	O60610	796	11	4	5	7	1.1	1.0	1.0	1.1
TAF8	Q7Z7C8	80	11	-2	5	1	1.1	1.0	1.0	1.0
MBD1	Q9UIS9	70	11	-18	5	-22	1.1	0.9	1.0	0.8
HMGCS1	Q01581	268	11	7	5	-12	1.1	1.1	1.0	0.9
ZMYM4	Q5VZL5	1198	11	4	5	-1	1.1	1.0	1.0	1.0
ITGB1	P05556	636	11	-7	5	-5	1.1	0.9	1.0	1.0
CBR3	O75828	227	10	3	5	1	1.1	1.0	1.0	1.0
ATP5A1	P25705	294	10	3	5	12	1.1	1.0	1.0	1.1

ANPEP	P15144	223	10	-4	5	12	1.1	1.0	1.0	1.1
XPO1	O14980	528	10	-5	5	-1	1.1	1.0	1.0	1.0
DPP8	Q6V1X1	611	10	-11	5	-17	1.1	0.9	1.0	0.9
AGO2	Q9UKV8	188	9	3	5	-2	1.1	1.0	1.0	1.0
PPFIBP2	Q8ND30	145	9	-1	5	5	1.1	1.0	1.0	1.0
PTPN6	P29350	480	9	-8	5	6	1.1	0.9	1.0	1.1
DIAPH1	O60610	437	9	-39	5	-1	1.1	0.7	1.0	1.0
MROH1	Q8NDA8	1233	9	5	5	3	1.1	1.1	1.0	1.0
ALOX5	P09917	265	9	-3	5	-6	1.1	1.0	1.0	0.9
RABL2A	Q9UBK7	26	8	0	5	2	1.1	1.0	1.0	1.0
CCT5	P48643	493	8	-1	5	7	1.1	1.0	1.0	1.1
MAP4K1	Q92918	335	8	-1	5	-4	1.1	1.0	1.0	1.0
STK16	O75716	84	8	-7	5	1	1.1	0.9	1.0	1.0
PARK7	Q99497	46	8	-7	5	1	1.1	0.9	1.0	1.0
LIG3	P49916	94	8	-18	5	3	1.1	0.9	1.0	1.0
IRF3	Q14653	267	8	8	5	3	1.1	1.1	1.0	1.0
BLK	P51451	319	8	5	5	8	1.1	1.0	1.0	1.1
C1orf50	Q9BV19	110	8	4	5	6	1.1	1.0	1.0	1.1
FYB	O15117	778	8	-2	5	-19	1.1	1.0	1.0	0.8
RERE	Q9P2R6	510	8	-3	5	8	1.1	1.0	1.0	1.1
RGP1	Q92546	90	8	-4	5	2	1.1	1.0	1.0	1.0
INTS1	Q8N201	246	8	-9	5	13	1.1	0.9	1.0	1.1
MSH2	P43246	404	8	-22	5	7	1.1	0.8	1.0	1.1
LDHB	P07195	294	7	0	5	19	1.1	1.0	1.0	1.2
SEPT1	Q8WYJ6	102	7	-3	5	7	1.1	1.0	1.0	1.1
COPA	P53621	585	7	-5	5	-3	1.1	1.0	1.0	1.0
HK3	P52790	915	7	-6	5	0	1.1	0.9	1.0	1.0
TUBB2B	Q9BVA1	239	7	-6	5	-5	1.1	0.9	1.0	1.0
TDRD7	Q8NHU6	798	7	-7	5	-7	1.1	0.9	1.0	0.9
TBCE	Q15813	228	7	-13	5	7	1.1	0.9	1.0	1.1
XAB2	Q9HCS7	66	7	2	5	9	1.1	1.0	1.0	1.1
XRCC4	Q13426	128	6	-1	5	7	1.1	1.0	1.0	1.1
CKAP5	Q14008	1768	6	-9	5	4	1.1	0.9	1.0	1.0
MTMR3	Q13615	915	6	3	5	-3	1.1	1.0	1.0	1.0
RPL7	P18124	186	6	-1	5	15	1.1	1.0	1.0	1.2
INPP1	P49441	97	6	-4	5	-2	1.1	1.0	1.0	1.0
FES	P07332	372	6	-13	5	-11	1.1	0.9	1.0	0.9
TES	Q9UGI8	29	6	-17	5	-8	1.1	0.9	1.0	0.9
RGS14	O43566	305	5	3	5	1	1.1	1.0	1.0	1.0
SMCHD1	A6NHR9	897	5	-2	5	7	1.1	1.0	1.0	1.1
ORC5	O43913	237	5	-5	5	-19	1.1	1.0	1.0	0.8
SHMT2	P34897	80	5	-12	5	-1	1.1	0.9	1.0	1.0
PXN	P49023	535	5	-6	5	-3	1.0	0.9	1.0	1.0
POLR2B	P30876	892	5	-9	5	-15	1.0	0.9	1.0	0.9
RAB29	O14966	127	5	-16	5	-13	1.0	0.9	1.0	0.9
YARS	P54577	424	5	-20	5	9	1.0	0.8	1.0	1.1
PDLIM5	Q96HC4	467	4	-1	5	10	1.0	1.0	1.0	1.1
RNF113A	O15541	15	4	-1	5	6	1.0	1.0	1.0	1.1
AP2A1	O95782	970	4	-7	5	7	1.0	0.9	1.0	1.1
IRF2BP1	Q8IU81	280	4	-8	5	-9	1.0	0.9	1.0	0.9
ARFGAP3	Q9NP61	312	4	4	5	7	1.0	1.0	1.0	1.1
RNF213	Q63HN8	2620	4	3	5	-2	1.0	1.0	1.0	1.0
TEP1	Q99973	149	4	-8	5	-1	1.0	0.9	1.0	1.0

RPP30	P78346	87	4	-8	5	-12	1.0	0.9	1.0	0.9
DCTN1	Q14203	1252	4	-9	5	-9	1.0	0.9	1.0	0.9
NFATC2	Q13469	569	4	-16	5	-18	1.0	0.9	1.0	0.8
IPO4	Q8TEX9	735	3	-3	5	-3	1.0	1.0	1.0	1.0
USP28	Q96RU2	1058	3	-6	5	10	1.0	0.9	1.0	1.1
WARS	P23381	274	3	-6	5	3	1.0	0.9	1.0	1.0
ARPC1A	Q92747	279	3	-7	5	1	1.0	0.9	1.0	1.0
CHD9	Q3L8U1	784	3	-7	5	-16	1.0	0.9	1.0	0.9
CRYZL1	O95825	302	2	7	5	5	1.0	1.1	1.0	1.1
AP2A2	O94973	282	2	-16	5	7	1.0	0.9	1.0	1.1
KLC1	Q07866	456	2	-2	5	-7	1.0	1.0	1.0	0.9
ALDH9A1	P49189	484	2	-4	5	3	1.0	1.0	1.0	1.0
TDP1	Q9NUW8	135	1	-1	5	6	1.0	1.0	1.0	1.1
TACC3	Q9Y6A5	426	1	-23	5	-20	1.0	0.8	1.0	0.8
HNRNPLL	Q8WVV9	84	1	2	5	-2	1.0	1.0	1.0	1.0
SUFU	Q9UMX1	292	1	-4	5	1	1.0	1.0	1.0	1.0
GSK3A	P49840	281	1	-8	5	-6	1.0	0.9	1.0	0.9
GSK3B	P49841	218	1	-8	5	-6	1.0	0.9	1.0	0.9
SGTA	O43765	129	1	-9	5	-3	1.0	0.9	1.0	1.0
PARP10	Q53GL7	124	1	-17	5	1	1.0	0.9	1.0	1.0
DYSF	O75923	1245	1	-25	5	-7	1.0	0.8	1.0	0.9
GCN1	Q92616	55	-1	-2	5	17	1.0	1.0	1.0	1.2
CDC37	Q16543	64	-1	-6	5	18	1.0	0.9	1.0	1.2
ABCB8	Q9NUT2	454	-1	-10	5	-15	1.0	0.9	1.0	0.9
KPNB1	Q14974	345	-1	-8	5	-2	1.0	0.9	1.0	1.0
TASP1	Q9H6P5	351	-1	-15	5	-9	1.0	0.9	1.0	0.9
TAF5L	O75529	15	-2	6	5	4	1.0	1.1	1.0	1.0
RMND5A	Q9H871	109	-2	-26	5	-22	1.0	0.8	1.0	0.8
SWAP70	Q9UH65	40	-2	-28	5	-11	1.0	0.8	1.0	0.9
ACTB	P60709	272	-2	9	5	4	1.0	1.1	1.0	1.0
SARS2	Q9NP81	66	-2	-1	5	6	1.0	1.0	1.0	1.1
IFT74	Q96LB3	53	-2	-3	5	-5	1.0	1.0	1.0	1.0
DOK1	Q99704	308	-2	-3	5	-3	1.0	1.0	1.0	1.0
RALGAPB	Q86X10	344	-2	-8	5	8	1.0	0.9	1.0	1.1
CRTC2	Q53ET0	515	-3	2	5	-6	1.0	1.0	1.0	0.9
IQSEC1	Q6DN90	744	-3	-1	5	31	1.0	1.0	1.0	1.4
TNFAIP3	P21580	627	-3	-6	5	-3	1.0	0.9	1.0	1.0
TLN1	Q9Y490	1478	-3	-17	5	-11	1.0	0.9	1.0	0.9
HAUS5	O94927	12	-4	-7	5	-15	1.0	0.9	1.0	0.9
CANX	P27824	194	-4	1	5	-20	1.0	1.0	1.0	0.8
ALDH7A1	P49419	478	-4	-14	5	5	1.0	0.9	1.0	1.1
SOS2	Q07890	1064	-5	14	5	-13	1.0	1.2	1.0	0.9
CASS4	Q9NQ75	741	-5	6	5	-7	1.0	1.1	1.0	0.9
SURF2	Q15527	127	-5	1	5	-9	1.0	1.0	1.0	0.9
NUP153	P49790	667	-5	-15	5	-11	1.0	0.9	1.0	0.9
RBM22	Q9NW64	269	-5	-7	5	-5	1.0	0.9	1.0	1.0
NLRC4	Q9NPP4	165	-6	-8	5	12	0.9	0.9	1.0	1.1
GPD2	P43304	285	-6	-11	5	7	0.9	0.9	1.0	1.1
ZC3HC1	Q86WB0	429	-8	-26	5	-22	0.9	0.8	1.0	0.8
USP48	Q86UV5	39	-9	-4	5	-13	0.9	1.0	1.0	0.9
SEPT9	Q9UHD8	375	-9	-9	5	-20	0.9	0.9	1.0	0.8
FSCN1	Q16658	89	-9	-63	5	-8	0.9	0.6	1.0	0.9
TLN1	Q9Y490	1506	-10	6	5	0	0.9	1.1	1.0	1.0

NIP7	Q9Y221	36	-10	-11	5	-9	0.9	0.9	1.0	0.9
CMSS1	Q9BQ75	55	-10	-27	5	-10	0.9	0.8	1.0	0.9
WRNIP1	Q96S55	39	-12	-25	5	-35	0.9	0.8	1.0	0.7
TLR1	Q15399	528	-15	-28	5	-14	0.9	0.8	1.0	0.9
TXN	P10599	73	-56	-28	5	-3	0.6	0.8	1.0	1.0
MARS	P56192	66	28	-5	4	28	1.4	1.0	1.0	1.4
JAK3	P52333	162	26	6	4	7	1.3	1.1	1.0	1.1
AP4B1	Q9Y6B7	144	24	-2	4	2	1.3	1.0	1.0	1.0
MYO1F	O00160	402	22	-11	4	8	1.3	0.9	1.0	1.1
MESDC1	Q9H1K6	331	21	-4	4	-7	1.3	1.0	1.0	0.9
LYST	Q99698	2965	20	-15	4	8	1.3	0.9	1.0	1.1
L2HGDH	Q9H9P8	124	20	-8	4	-7	1.2	0.9	1.0	0.9
YWHAZ	P63104	25	19	5	4	12	1.2	1.1	1.0	1.1
TNPO1	Q92973	620	19	-21	4	-3	1.2	0.8	1.0	1.0
ANXA2	P07355	335	18	-1	4	15	1.2	1.0	1.0	1.2
FLNA	P21333	2102	18	-2	4	6	1.2	1.0	1.0	1.1
ATXN10	Q9UBB4	134	17	-8	4	10	1.2	0.9	1.0	1.1
GSAP	A4D1B5	424	17	-17	4	8	1.2	0.9	1.0	1.1
SCAI	Q8N9R8	63	17	-2	4	8	1.2	1.0	1.0	1.1
DHX40	Q8IX18	225	16	-9	4	-6	1.2	0.9	1.0	0.9
CCDC93	Q567U6	282	16	-12	4	11	1.2	0.9	1.0	1.1
UBE4A	Q14139	450	16	-17	4	2	1.2	0.9	1.0	1.0
WDFY4	Q6ZS81	1260	16	11	4	1	1.2	1.1	1.0	1.0
BTK	Q06187	464	16	-3	4	1	1.2	1.0	1.0	1.0
TP53BP1	Q12888	1703	16	-5	4	4	1.2	1.0	1.0	1.0
NEK9	Q8TD19	556	16	-6	4	3	1.2	0.9	1.0	1.0
CS	O75390	211	15	3	4	21	1.2	1.0	1.0	1.3
MROH1	Q8NDA8	1376	15	1	4	11	1.2	1.0	1.0	1.1
KMT5A	Q9NQR1	343	15	-2	4	1	1.2	1.0	1.0	1.0
MRPS12	O15235	93	15	-8	4	-12	1.2	0.9	1.0	0.9
NARS	O43776	438	15	-1	4	-1	1.2	1.0	1.0	1.0
CYFIP1	Q7L576	179	14	7	4	21	1.2	1.1	1.0	1.3
CYFIP2	Q96F07	179	14	7	4	21	1.2	1.1	1.0	1.3
NUP160	Q12769	1309	14	-3	4	22	1.2	1.0	1.0	1.3
PRRC2C	Q9Y520	177	14	-14	4	-17	1.2	0.9	1.0	0.9
MYBBP1A	Q9BQG0	614	14	-4	4	-4	1.2	1.0	1.0	1.0
ALDH9A1	P49189	355	14	-12	4	4	1.2	0.9	1.0	1.0
AASS	Q9UDR5	87	13	13	4	4	1.1	1.1	1.0	1.0
DGCR8	Q8WYQ5	128	13	9	4	-9	1.1	1.1	1.0	0.9
HSPA4	P34932	380	13	-10	4	2	1.1	0.9	1.0	1.0
HSPH1	Q92598	380	13	-10	4	2	1.1	0.9	1.0	1.0
COPB2	P35606	56	12	-2	4	-4	1.1	1.0	1.0	1.0
PADI2	Q9Y2J8	647	12	-3	4	6	1.1	1.0	1.0	1.1
SYMPK	Q92797	686	12	-5	4	-2	1.1	1.0	1.0	1.0
MYO1F	O00160	577	12	-32	4	6	1.1	0.8	1.0	1.1
PPP2R4	Q15257	202	12	7	4	4	1.1	1.1	1.0	1.0
BIRC6	Q9NR09	2892	12	5	4	4	1.1	1.1	1.0	1.0
MTMR1	Q13613	117	11	2	4	-7	1.1	1.0	1.0	0.9
SNX5	Q9Y5X3	347	11	1	4	5	1.1	1.0	1.0	1.0
FAM208A	Q9UK61	371	11	-5	4	1	1.1	1.0	1.0	1.0
TRIM14	Q14142	291	11	-16	4	-8	1.1	0.9	1.0	0.9
KARS	Q15046	427	11	-18	4	4	1.1	0.9	1.0	1.0
MGLL	Q99685	32	11	-20	4	7	1.1	0.8	1.0	1.1

PRPF6	O94906	513	11	-25	4	-13	1.1	0.8	1.0	0.9
NDUFA10	E7ESZ7	183	11	-5	4	-1	1.1	1.0	1.0	1.0
EHBP1L1	Q8N3D4	23	11	-8	4	0	1.1	0.9	1.0	1.0
LGALS3	P17931	173	11	-13	4	16	1.1	0.9	1.0	1.2
YDJC	A8MPS7	182	10	3	4	6	1.1	1.0	1.0	1.1
CHORDC1	Q9UHD1	211	10	-2	4	-15	1.1	1.0	1.0	0.9
PPP1R14A	Q96A00	100	10	-6	4	0	1.1	0.9	1.0	1.0
RPS2	P15880	188	10	-13	4	-2	1.1	0.9	1.0	1.0
RPUSD3	Q6P087	147	10	-3	4	2	1.1	1.0	1.0	1.0
EXOC6	Q8TAG9	532	10	-4	4	14	1.1	1.0	1.0	1.2
POLRMT	O00411	726	10	-5	4	-8	1.1	1.0	1.0	0.9
CMPK2	Q5EBM0	287	9	4	4	2	1.1	1.0	1.0	1.0
ADD1	P35611	68	9	2	4	0	1.1	1.0	1.0	1.0
RNF31	Q96EP0	702	9	-3	4	-2	1.1	1.0	1.0	1.0
PACS2	Q86VP3	831	9	-6	4	7	1.1	0.9	1.0	1.1
MLYCD	O95822	68	9	8	4	10	1.1	1.1	1.0	1.1
TES	Q9UGI8	416	9	4	4	2	1.1	1.0	1.0	1.0
RNH1	P13489	159	9	1	4	-5	1.1	1.0	1.0	1.0
PIK3CB	P42338	745	8	2	4	-1	1.1	1.0	1.0	1.0
ELAC2	Q9BQ52	670	8	1	4	5	1.1	1.0	1.0	1.0
TMEM63A	O94886	370	8	-1	4	25	1.1	1.0	1.0	1.3
SPATS2	Q86XZ4	61	8	-4	4	-5	1.1	1.0	1.0	1.0
FAM208B	Q5VWN6	111	8	-6	4	8	1.1	0.9	1.0	1.1
DPP3	Q9NY33	176	8	-11	4	4	1.1	0.9	1.0	1.0
DOCK8	Q8NF50	121	8	-12	4	-1	1.1	0.9	1.0	1.0
NUP160	Q12769	1042	7	4	4	2	1.1	1.0	1.0	1.0
SEPHS1	P49903	31	7	1	4	-1	1.1	1.0	1.0	1.0
TRABD	Q9H4I3	287	7	1	4	-3	1.1	1.0	1.0	1.0
IGHA1	P01876	192	7	-14	4	1	1.1	0.9	1.0	1.0
FAM65B	Q9Y4F9	983	7	-15	4	-15	1.1	0.9	1.0	0.9
NUB1	Q9Y5A7	299	7	-18	4	-10	1.1	0.8	1.0	0.9
NLRC5	Q86WI3	1838	7	5	4	1	1.1	1.0	1.0	1.0
IFRD1	O00458	178	7	-2	4	7	1.1	1.0	1.0	1.1
PUS1	Q9Y606	125	7	-8	4	17	1.1	0.9	1.0	1.2
SSNA1	O43805	23	6	4	4	1	1.1	1.0	1.0	1.0
FAM160B1	Q5W0V3	304	6	3	4	11	1.1	1.0	1.0	1.1
CUL2	Q13617	103	6	3	4	8	1.1	1.0	1.0	1.1
PARP9	Q8IXQ6	82	6	-3	4	3	1.1	1.0	1.0	1.0
ROCK1	Q13464	1070	6	-7	4	4	1.1	0.9	1.0	1.0
TLN1	Q9Y490	243	6	-7	4	4	1.1	0.9	1.0	1.0
MAP3K2	Q9Y2U5	512	6	6	4	1	1.1	1.1	1.0	1.0
FRMD8	Q9BZ67	191	6	4	4	4	1.1	1.0	1.0	1.0
FAM168B	A1KXE4	63	6	1	4	-2	1.1	1.0	1.0	1.0
PLEKHO2	Q8TD55	295	6	-1	4	-8	1.1	1.0	1.0	0.9
OTULIN	Q96BN8	347	6	-2	4	-1	1.1	1.0	1.0	1.0
ABCF1	Q8NE71	758	6	-3	4	5	1.1	1.0	1.0	1.0
C15orf41	Q9Y2V0	277	6	-9	4	-6	1.1	0.9	1.0	0.9
DYNC1H1	Q14204	3147	5	0	4	-1	1.1	1.0	1.0	1.0
ZNF600	Q6ZNG1	481	5	-3	4	-4	1.1	1.0	1.0	1.0
RANBP6	O60518	48	5	-5	4	-5	1.1	1.0	1.0	1.0
COPB1	P53618	616	5	-6	4	1	1.1	0.9	1.0	1.0
MYO1G	B0I1T2	655	5	2	4	2	1.0	1.0	1.0	1.0
PAPPA2	Q9BXP8	50	5	1	4	0	1.0	1.0	1.0	1.0

MIOS	Q9NXC5	148	5	-5	4	2	1.0	1.0	1.0	1.0
LAP3	P28838	445	5	-5	4	1	1.0	1.0	1.0	1.0
TMPO	P42167	363	5	-5	4	-7	1.0	1.0	1.0	0.9
RBM14-RBM	A0A0A0MSL8	90	5	-6	4	-2	1.0	0.9	1.0	1.0
RALGAPB	Q86X10	327	5	-6	4	-8	1.0	0.9	1.0	0.9
IARS	P41252	87	5	-8	4	-4	1.0	0.9	1.0	1.0
AAMP	Q13685	428	5	-11	4	1	1.0	0.9	1.0	1.0
KDM5A	P29375	340	5	-13	4	-13	1.0	0.9	1.0	0.9
POLD1	P28340	360	5	-14	4	-11	1.0	0.9	1.0	0.9
SH3BP1	Q9Y3L3	338	4	4	4	3	1.0	1.0	1.0	1.0
KDM3B	Q7LBC6	1124	4	2	4	14	1.0	1.0	1.0	1.2
ZNFX1	Q9P2E3	1076	4	-5	4	3	1.0	1.0	1.0	1.0
GFPT1	Q06210	638	4	-5	4	-1	1.0	1.0	1.0	1.0
RPS6KB1	P23443	90	4	-9	4	2	1.0	0.9	1.0	1.0
TLN1	Q9Y490	1202	4	-11	4	4	1.0	0.9	1.0	1.0
GNL3	Q9BVP2	234	4	-13	4	-13	1.0	0.9	1.0	0.9
CARD11	Q9BXL7	1052	4	-16	4	4	1.0	0.9	1.0	1.0
RBM47	A0AV96	145	4	0	4	-3	1.0	1.0	1.0	1.0
BAZ1B	Q9UIG0	1395	4	-9	4	-5	1.0	0.9	1.0	1.0
DHRS13	Q6UX07	28	4	-9	4	9	1.0	0.9	1.0	1.1
CLEC16A	Q2KHT3	657	4	-16	4	-5	1.0	0.9	1.0	1.0
LSM4	Q9Y4Z0	59	4	-17	4	11	1.0	0.9	1.0	1.1
CHD2	O14647	415	3	-13	4	8	1.0	0.9	1.0	1.1
PSMC4	P43686	210	3	-1	4	-1	1.0	1.0	1.0	1.0
TBCK	Q8TEA7	702	3	-1	4	-4	1.0	1.0	1.0	1.0
LTBP1	Q14766	702	3	-2	4	-2	1.0	1.0	1.0	1.0
PDS5A	Q29RF7	1084	3	-6	4	-2	1.0	0.9	1.0	1.0
SUGP2	Q8IX01	656	3	-6	4	-4	1.0	0.9	1.0	1.0
MDN1	Q9NU22	309	3	-8	4	-1	1.0	0.9	1.0	1.0
PDLIM7	Q9NR12	332	3	-11	4	3	1.0	0.9	1.0	1.0
GORASP2	Q9H8Y8	173	3	-14	4	3	1.0	0.9	1.0	1.0
DOCK10	Q96BY6	1310	2	-6	4	-11	1.0	0.9	1.0	0.9
DOK3	Q7L591	175	2	-12	4	1	1.0	0.9	1.0	1.0
GNPNAT1	Q96EK6	113	2	-17	4	20	1.0	0.9	1.0	1.3
FAM117B	Q6P1L5	198	2	-4	4	-8	1.0	1.0	1.0	0.9
FAM168A	Q92567	75	2	-4	4	-8	1.0	1.0	1.0	0.9
CD36	P16671	313	2	-4	4	-14	1.0	1.0	1.0	0.9
TSSC1	Q53HC9	251	2	-9	4	7	1.0	0.9	1.0	1.1
NSUN5	Q96P11	404	2	-12	4	-20	1.0	0.9	1.0	0.8
DCXR	Q7Z4W1	244	1	0	4	-11	1.0	1.0	1.0	0.9
EHBP1L1	Q8N3D4	1141	1	-1	4	5	1.0	1.0	1.0	1.0
NCOA3	Q9Y6Q9	31	1	-4	4	-2	1.0	1.0	1.0	1.0
RPS3A	P61247	201	1	-5	4	5	1.0	1.0	1.0	1.1
PNKP	Q96T60	516	1	-8	4	7	1.0	0.9	1.0	1.1
GSK3A	P49840	241	1	-15	4	0	1.0	0.9	1.0	1.0
MGME1	Q9BQP7	79	1	3	4	-13	1.0	1.0	1.0	0.9
PGK1	P00558	379	1	-3	4	8	1.0	1.0	1.0	1.1
ZC3H11A	O75152	22	1	-3	4	14	1.0	1.0	1.0	1.2
GOLGA4	Q13439	1085	1	-3	4	-6	1.0	1.0	1.0	0.9
BLOC1S4	Q9NUP1	211	1	-8	4	6	1.0	0.9	1.0	1.1
PPOX	P50336	258	1	-9	4	14	1.0	0.9	1.0	1.2
EXOSC9	Q06265	45	1	-10	4	4	1.0	0.9	1.0	1.0
TRAF3IP3	Q9Y228	105	1	-12	4	-21	1.0	0.9	1.0	0.8

IL7R	P16871	287	0	-4	4	-6	1.0	1.0	1.0	0.9
SP4	Q02446	630	-1	3	4	-4	1.0	1.0	1.0	1.0
ACTR5	Q9H9F9	48	-1	-4	4	-5	1.0	1.0	1.0	1.0
SART3	Q15020	729	-1	-14	4	-1	1.0	0.9	1.0	1.0
HUWE1	Q7Z6Z7	2721	-2	3	4	-9	1.0	1.0	1.0	0.9
PRKCH	P24723	185	-2	2	4	7	1.0	1.0	1.0	1.1
IMPDH1	P20839	215	-2	-4	4	-8	1.0	1.0	1.0	0.9
AHNAK	Q09666	5382	-3	-5	4	-7	1.0	1.0	1.0	0.9
ZNF676	Q8N7Q3	367	-3	-5	4	12	1.0	1.0	1.0	1.1
NAA10	P41227	194	-3	-6	4	-9	1.0	0.9	1.0	0.9
EEA1	Q15075	1134	-3	2	4	1	1.0	1.0	1.0	1.0
UBR5	O95071	2314	-3	-6	4	-3	1.0	0.9	1.0	1.0
PSME4	Q14997	1001	-3	-8	4	-12	1.0	0.9	1.0	0.9
ERCC5	P28715	529	-4	11	4	6	1.0	1.1	1.0	1.1
ARID2	Q68CP9	1271	-4	-14	4	-14	1.0	0.9	1.0	0.9
GAPDHS	O14556	319	-4	-20	4	9	1.0	0.8	1.0	1.1
BANP	Q8N9N5	435	-5	4	4	0	1.0	1.0	1.0	1.0
RIN3	Q8TB24	484	-5	2	4	1	1.0	1.0	1.0	1.0
VRK1	Q99986	50	-5	-2	4	-6	1.0	1.0	1.0	0.9
AIMP1	Q12904	161	-5	-7	4	-6	1.0	0.9	1.0	0.9
RAB3GAP2	Q9H2M9	1336	-6	-19	4	-14	0.9	0.8	1.0	0.9
RPS8	P62241	182	-6	-5	4	-15	0.9	1.0	1.0	0.9
ZNF428	Q96B54	115	-7	-16	4	-7	0.9	0.9	1.0	0.9
FLNB	O75369	604	-7	-22	4	-13	0.9	0.8	1.0	0.9
RIC1	Q4ADV7	818	-8	-14	4	-10	0.9	0.9	1.0	0.9
KIF21A	Q7Z4S6	299	-9	3	4	20	0.9	1.0	1.0	1.2
UBN1	Q9NPG3	711	-9	1	4	-19	0.9	1.0	1.0	0.8
TRAFD1	O14545	32	-10	-6	4	-14	0.9	0.9	1.0	0.9
HCFC2	Q9Y5Z7	501	-11	15	4	-7	0.9	1.2	1.0	0.9
CDKN1B	P46527	148	-11	-15	4	-26	0.9	0.9	1.0	0.8
SHOC2	Q9UQ13	342	-12	-2	4	-21	0.9	1.0	1.0	0.8
CLU	P10909	285	-12	-11	4	-9	0.9	0.9	1.0	0.9
AP1G1	O43747	31	-12	-3	4	-13	0.9	1.0	1.0	0.9
LYPLA2	O95372	213	-13	10	4	-6	0.9	1.1	1.0	0.9
TMEM30A	Q9NV96	17	-13	-18	4	-27	0.9	0.9	1.0	0.8
TAPBP	O15533	91	-13	-11	4	-9	0.9	0.9	1.0	0.9
ABHD5	Q8WTS1	299	-14	-3	4	-7	0.9	1.0	1.0	0.9
PGGT1B	P53609	369	-17	9	4	-6	0.9	1.1	1.0	0.9
PPP2R5C	Q13362	334	-19	-22	4	-10	0.8	0.8	1.0	0.9
PPP2R5D	Q14738	410	-19	-22	4	-10	0.8	0.8	1.0	0.9
PPP2R5E	Q16537	351	-19	-22	4	-10	0.8	0.8	1.0	0.9
CDC42BPB	Q9Y5S2	784	-23	-1	4	-30	0.8	1.0	1.0	0.8
DYNC1H1	Q14204	2142	31	18	4	-8	1.4	1.2	1.0	0.9
PSMD12	O00232	193	24	-9	4	11	1.3	0.9	1.0	1.1
STK24	Q9Y6E0	375	23	13	4	17	1.3	1.1	1.0	1.2
SMARCC2	Q8TAQ2	80	21	-22	4	7	1.3	0.8	1.0	1.1
BCOR	Q6W2J9	1311	21	10	4	6	1.3	1.1	1.0	1.1
PDIA5	Q14554	426	20	17	4	20	1.2	1.2	1.0	1.2
METTL2A	Q96IZ6	241	20	-21	4	4	1.2	0.8	1.0	1.0
IKBKAP	O95163	1012	19	-2	4	9	1.2	1.0	1.0	1.1
THEMIS2	Q5TEJ8	146	19	-4	4	-4	1.2	1.0	1.0	1.0
VPS35	Q96QK1	653	18	-12	4	6	1.2	0.9	1.0	1.1
SURF4	O15260	32	17	-1	4	21	1.2	1.0	1.0	1.3

TSTD1	Q8NFU3	79	17	-1	4	9	1.2	1.0	1.0	1.1
MOSPD2	Q8NHP6	254	17	-2	4	4	1.2	1.0	1.0	1.0
GMFB	P60983	96	16	2	4	-22	1.2	1.0	1.0	0.8
WIZ	O95785	1279	16	-7	4	-14	1.2	0.9	1.0	0.9
MMS19	Q96T76	760	16	-8	4	-2	1.2	0.9	1.0	1.0
SRSF9	Q13242	138	16	-4	4	1	1.2	1.0	1.0	1.0
ST13	P50502	209	16	-6	4	7	1.2	0.9	1.0	1.1
RANBP2	P49792	536	16	-9	4	-5	1.2	0.9	1.0	1.0
HMHA1	Q92619	781	16	-12	4	-4	1.2	0.9	1.0	1.0
UBR4	Q5T4S7	4487	15	4	4	6	1.2	1.0	1.0	1.1
SRP68	Q9UHB9	525	14	-2	4	-9	1.2	1.0	1.0	0.9
RIC1	Q4ADV7	434	14	-17	4	-4	1.2	0.9	1.0	1.0
GOLGA3	Q08378	1431	14	5	4	2	1.2	1.1	1.0	1.0
DDX24	Q9GZR7	639	14	-4	4	-4	1.2	1.0	1.0	1.0
UBXN7	O94888	160	13	-2	4	0	1.1	1.0	1.0	1.0
DYNC1H1	Q14204	3389	13	-4	4	3	1.1	1.0	1.0	1.0
STK39	Q9UEW8	317	13	-21	4	-18	1.1	0.8	1.0	0.8
MMRN1	Q13201	245	13	8	4	-5	1.1	1.1	1.0	1.0
PGP	A6NDG6	217	13	-7	4	4	1.1	0.9	1.0	1.0
IDH3B	O43837	232	13	-10	4	5	1.1	0.9	1.0	1.1
ARFGEF2	Q9Y6D5	1617	12	-1	4	15	1.1	1.0	1.0	1.2
SRRT	Q9BXP5	421	12	-6	4	3	1.1	0.9	1.0	1.0
PCCB	P05166	516	12	-2	4	-3	1.1	1.0	1.0	1.0
LRRC41	Q15345	607	12	-2	4	2	1.1	1.0	1.0	1.0
RANBP2	P49792	1787	12	-7	4	2	1.1	0.9	1.0	1.0
RANGAP1	P46060	141	12	-9	4	3	1.1	0.9	1.0	1.0
HNRNPD	Q14103	226	12	-15	4	5	1.1	0.9	1.0	1.0
LRPPRC	P42704	208	12	-29	4	8	1.1	0.8	1.0	1.1
ERCC3	P19447	108	11	6	4	6	1.1	1.1	1.0	1.1
KIAA0430	Q9Y4F3	194	11	5	4	2	1.1	1.0	1.0	1.0
NDUFA7	O95182	55	11	-15	4	6	1.1	0.9	1.0	1.1
FAM65B	Q9Y4F9	218	11	-1	4	-1	1.1	1.0	1.0	1.0
ARHGAP30	Q7Z6I6	94	11	-5	4	3	1.1	1.0	1.0	1.0
TNFAIP2	Q03169	429	10	-1	4	-3	1.1	1.0	1.0	1.0
DDX24	Q9GZR7	620	10	-2	4	-3	1.1	1.0	1.0	1.0
SCYL2	Q6P3W7	511	10	-3	4	18	1.1	1.0	1.0	1.2
KCTD18	Q6PI47	21	10	-13	4	0	1.1	0.9	1.0	1.0
ARHGAP30	Q7Z6I6	20	10	-15	4	5	1.1	0.9	1.0	1.1
AGFG2	O95081	107	10	5	4	-6	1.1	1.0	1.0	0.9
MCM3	P25205	123	10	3	4	4	1.1	1.0	1.0	1.0
RELA	Q04206	160	10	-3	4	9	1.1	1.0	1.0	1.1
TBC1D1	Q86TI0	96	10	-4	4	13	1.1	1.0	1.0	1.1
HUWE1	Q7Z6Z7	19	10	-8	4	5	1.1	0.9	1.0	1.1
LCK	P06239	217	9	1	4	14	1.1	1.0	1.0	1.2
TNPO1	Q92973	153	9	0	4	11	1.1	1.0	1.0	1.1
PURB	Q96QR8	238	9	2	4	-3	1.1	1.0	1.0	1.0
DBNL	Q9UJU6	127	9	1	4	5	1.1	1.0	1.0	1.1
RCSD1	Q6JBY9	155	9	-3	4	-5	1.1	1.0	1.0	1.0
CYLD	Q9NQC7	526	9	-7	4	8	1.1	0.9	1.0	1.1
NUP155	O75694	917	9	-28	4	-6	1.1	0.8	1.0	0.9
LMF2	Q9BU23	659	8	3	4	14	1.1	1.0	1.0	1.2
RPS4Y1	P22090	41	8	-1	4	-6	1.1	1.0	1.0	0.9
GPX1	P07203	156	8	-3	4	-6	1.1	1.0	1.0	0.9

UPF1	Q92900	188	8	-5	4	4	1.1	1.0	1.0	1.0
EDC3	Q96F86	413	8	-7	4	1	1.1	0.9	1.0	1.0
UAP1L1	Q3KQV9	57	8	-8	4	0	1.1	0.9	1.0	1.0
ATXN10	Q9UBB4	382	8	-15	4	-4	1.1	0.9	1.0	1.0
FAM21C	Q9Y4E1	826	8	5	4	-10	1.1	1.1	1.0	0.9
ZCCHC6	Q5VYS8	1413	8	-3	4	-5	1.1	1.0	1.0	1.0
HSPA6	P17066	624	8	-4	4	-8	1.1	1.0	1.0	0.9
ARFGEF2	Q9Y6D5	76	8	-4	4	1	1.1	1.0	1.0	1.0
SUCLA2	Q9P2R7	152	8	-5	4	7	1.1	1.0	1.0	1.1
HMHA1	Q92619	136	8	-20	4	-5	1.1	0.8	1.0	1.0
IRF9	Q00978	329	7	12	4	10	1.1	1.1	1.0	1.1
NUBP1	P53384	25	7	7	4	-12	1.1	1.1	1.0	0.9
NPLOC4	Q8TAT6	188	7	-6	4	7	1.1	0.9	1.0	1.1
CSRP1	P21291	61	7	-6	4	-1	1.1	0.9	1.0	1.0
PLCG2	P16885	791	7	-7	4	9	1.1	0.9	1.0	1.1
IDH2	P48735	418	7	-11	4	7	1.1	0.9	1.0	1.1
SP140	Q13342	696	7	-21	4	-19	1.1	0.8	1.0	0.8
SP140L	Q9H930	409	7	-21	4	-19	1.1	0.8	1.0	0.8
UBR1	Q8IWV7	477	7	6	4	4	1.1	1.1	1.0	1.0
FAF2	Q96CS3	349	7	2	4	8	1.1	1.0	1.0	1.1
PHGDH	O43175	48	7	-1	4	11	1.1	1.0	1.0	1.1
ALDH1A1	P00352	370	7	-2	4	7	1.1	1.0	1.0	1.1
ZWILCH	Q9H900	7	6	2	4	0	1.1	1.0	1.0	1.0
SPR	P35270	234	6	-1	4	7	1.1	1.0	1.0	1.1
MAF1	Q9H063	34	6	-1	4	1	1.1	1.0	1.0	1.0
PRPF6	O94906	429	6	-3	4	-4	1.1	1.0	1.0	1.0
AGTPBP1	Q9UPW5	179	6	-5	4	11	1.1	1.0	1.0	1.1
SMARCA5	O60264	259	6	-7	4	-3	1.1	0.9	1.0	1.0
SLX4IP	Q5VYV7	108	6	-9	4	-4	1.1	0.9	1.0	1.0
MCTP2	Q6DN12	497	6	-18	4	13	1.1	0.9	1.0	1.1
SP140	Q13342	686	6	1	4	-9	1.1	1.0	1.0	0.9
SP140L	Q9H930	399	6	1	4	-9	1.1	1.0	1.0	0.9
YLPM1	P49750	1772	6	1	4	-7	1.1	1.0	1.0	0.9
VPS53	Q5VIR6	471	6	0	4	-7	1.1	1.0	1.0	0.9
NUP98	P52948	1027	6	-2	4	-3	1.1	1.0	1.0	1.0
MX2	P20592	164	6	-2	4	-7	1.1	1.0	1.0	0.9
LRRK2	Q5S007	1123	6	-3	4	-6	1.1	1.0	1.0	0.9
Uncharacteri	G3V599	682	6	-5	4	1	1.1	1.0	1.0	1.0
DDX1	Q92499	631	6	-6	4	-3	1.1	0.9	1.0	1.0
DYNC1H1	Q14204	3940	6	-6	4	-1	1.1	0.9	1.0	1.0
CPNE3	O75131	385	6	-10	4	2	1.1	0.9	1.0	1.0
PDLIM5	Q96HC4	213	5	6	4	-1	1.1	1.1	1.0	1.0
HECTD1	Q9ULT8	1995	5	0	4	3	1.1	1.0	1.0	1.0
DSCR3	O14972	221	5	-2	4	9	1.1	1.0	1.0	1.1
DYNC1H1	Q14204	4510	5	-2	4	8	1.1	1.0	1.0	1.1
SGF29	Q96ES7	287	5	-3	4	3	1.1	1.0	1.0	1.0
CARD11	Q9BXL7	427	5	-3	4	1	1.1	1.0	1.0	1.0
SEC31A	O94979	689	5	-7	4	-1	1.1	0.9	1.0	1.0
LGALS1	P09382	61	5	-7	4	2	1.1	0.9	1.0	1.0
PSMD5	Q16401	412	5	-10	4	11	1.1	0.9	1.0	1.1
FLNB	O75369	1326	5	-11	4	-5	1.1	0.9	1.0	1.0
EPRS	P07814	1076	5	-3	4	6	1.0	1.0	1.0	1.1
TMPO	P42166	684	5	-8	4	-19	1.0	0.9	1.0	0.8

TBC1D10A	Q9BXI6	381	5	-11	4	-14	1.0	0.9	1.0	0.9
HCFC1	P51610	352	4	-3	4	3	1.0	1.0	1.0	1.0
ZNF512	Q96ME7	174	4	-10	4	-8	1.0	0.9	1.0	0.9
UBAP1	Q9NZ09	45	4	3	4	1	1.0	1.0	1.0	1.0
PLEK	P08567	160	4	-1	4	-1	1.0	1.0	1.0	1.0
ANKS1A	Q92625	965	4	-3	4	-11	1.0	1.0	1.0	0.9
COPB1	P53618	635	4	-7	4	-1	1.0	0.9	1.0	1.0
NT5DC3	Q86UY8	276	4	-38	4	-12	1.0	0.7	1.0	0.9
ACLY	P53396	633	3	-5	4	7	1.0	1.0	1.0	1.1
METAP2	P50579	121	3	-5	4	6	1.0	1.0	1.0	1.1
UBA6	A0AVT1	455	3	-6	4	17	1.0	0.9	1.0	1.2
FHL1	Q13642	36	3	-9	4	-2	1.0	0.9	1.0	1.0
CUL5	Q93034	404	3	-10	4	2	1.0	0.9	1.0	1.0
RPS5	P46782	155	3	-11	4	-8	1.0	0.9	1.0	0.9
GAK	O14976	87	3	-5	4	-8	1.0	1.0	1.0	0.9
RBM28	Q9NW13	97	3	-12	4	-16	1.0	0.9	1.0	0.9
CD300LF	Q8TDQ1	108	2	9	4	5	1.0	1.1	1.0	1.0
PCIF1	Q9H4Z3	626	2	2	4	9	1.0	1.0	1.0	1.1
PARP4	Q9UKK3	1293	2	-2	4	-1	1.0	1.0	1.0	1.0
MEPCE	Q7L2J0	244	2	-5	4	0	1.0	1.0	1.0	1.0
MGLL	Q99685	242	2	-5	4	23	1.0	1.0	1.0	1.3
FMNL1	O95466	69	2	-8	4	-6	1.0	0.9	1.0	0.9
RPL4	P36578	250	2	-10	4	8	1.0	0.9	1.0	1.1
SMAD2	Q15796	129	2	-17	4	3	1.0	0.9	1.0	1.0
SMAD1	Q15797	89	2	-17	4	3	1.0	0.9	1.0	1.0
SMAD5	Q99717	90	2	-17	4	3	1.0	0.9	1.0	1.0
SBF1	O95248	986	2	-32	4	-14	1.0	0.8	1.0	0.9
NPRL3	Q12980	141	2	-3	4	2	1.0	1.0	1.0	1.0
HNRNPU	Q00839	648	2	-3	4	-2	1.0	1.0	1.0	1.0
RANGAP1	P46060	573	2	-4	4	1	1.0	1.0	1.0	1.0
KIF1BP	Q96EK5	371	2	-5	4	-4	1.0	1.0	1.0	1.0
GGACT	Q9BVM4	84	2	-7	4	-4	1.0	0.9	1.0	1.0
SMCHD1	A6NHR9	492	2	-14	4	-9	1.0	0.9	1.0	0.9
PCBP1	Q15365	201	2	-25	4	-4	1.0	0.8	1.0	1.0
SSBP4	Q9BWG4	81	1	9	4	-4	1.0	1.1	1.0	1.0
RALGAPA2	Q2PPJ7	495	1	-2	4	-4	1.0	1.0	1.0	1.0
ACSL3	O95573	652	1	-3	4	-2	1.0	1.0	1.0	1.0
HAUS7	Q99871	130	1	-7	4	-10	1.0	0.9	1.0	0.9
PRKRA	O75569	163	1	-7	4	-4	1.0	0.9	1.0	1.0
MPHOSPH8	Q99549	753	1	-25	4	1	1.0	0.8	1.0	1.0
PPP1R21	Q6ZMI0	368	1	-3	4	6	1.0	1.0	1.0	1.1
TCEA2	Q15560	135	1	-6	4	-1	1.0	0.9	1.0	1.0
CCS	O14618	244	1	-7	4	-10	1.0	0.9	1.0	0.9
PRF1	P14222	497	0	-3	4	1	1.0	1.0	1.0	1.0
ANKS1A	Q92625	114	0	-4	4	6	1.0	1.0	1.0	1.1
TERF2	Q15554	207	0	-18	4	-3	1.0	0.8	1.0	1.0
Uncharacteri	A0A087WW\	72	-1	7	4	-15	1.0	1.1	1.0	0.9
AAMP	Q13685	208	-1	-13	4	-3	1.0	0.9	1.0	1.0
PITPNB	P48739	94	-1	-17	4	-10	1.0	0.9	1.0	0.9
PITPNA	Q00169	94	-1	-17	4	-10	1.0	0.9	1.0	0.9
UCHL5	Q9Y5K5	88	-1	-2	4	-6	1.0	1.0	1.0	0.9
ARIH1	Q9Y4X5	327	-1	-3	4	16	1.0	1.0	1.0	1.2
USP10	Q14694	40	-1	-3	4	-4	1.0	1.0	1.0	1.0

ECHDC1	Q9NTX5	133	-1	-17	4	13	1.0	0.9	1.0	1.1
SHARPIN	Q9H0F6	275	-2	1	4	5	1.0	1.0	1.0	1.1
TRAPPC10	P48553	696	-2	-4	4	-5	1.0	1.0	1.0	1.0
FAM98C	Q17RN3	98	-2	-11	4	-7	1.0	0.9	1.0	0.9
CHM	P24386	364	-2	-17	4	-12	1.0	0.9	1.0	0.9
USP48	Q86UV5	691	-2	3	4	6	1.0	1.0	1.0	1.1
VRK3	Q8IV63	191	-2	-8	4	-7	1.0	0.9	1.0	0.9
HNRNPK	P61978	185	-3	-2	4	-1	1.0	1.0	1.0	1.0
GGA2	Q9UJY4	429	-3	-13	4	-6	1.0	0.9	1.0	0.9
RASGRP2	Q7LDG7	186	-4	5	4	-8	1.0	1.1	1.0	0.9
CNBP	P62633	98	-4	-8	4	-7	1.0	0.9	1.0	0.9
RXRA	P19793	135	-4	-12	4	11	1.0	0.9	1.0	1.1
PDXP	Q96GD0	91	-5	-18	4	-17	1.0	0.8	1.0	0.9
NVL	O15381	431	-5	-14	4	-5	1.0	0.9	1.0	1.0
PXN	P49023	361	-5	-15	4	5	1.0	0.9	1.0	1.0
UBA1	P22314	494	-5	-28	4	-7	1.0	0.8	1.0	0.9
RFT1	Q96AA3	448	-6	7	4	14	0.9	1.1	1.0	1.2
NARFL	Q9H6Q4	300	-6	1	4	1	0.9	1.0	1.0	1.0
USP34	Q70CQ2	1380	-6	-4	4	-1	0.9	1.0	1.0	1.0
SSU72	Q9NP77	111	-6	-36	4	-24	0.9	0.7	1.0	0.8
PCBP2	Q15366	301	-7	-2	4	-7	0.9	1.0	1.0	0.9
ITPA	Q9BY32	146	-7	-8	4	-12	0.9	0.9	1.0	0.9
BSPRY	Q5W0U4	262	-8	-7	4	8	0.9	0.9	1.0	1.1
SYNE1	Q8NF91	7341	-9	-1	4	-10	0.9	1.0	1.0	0.9
NT5DC1	Q5TFE4	119	-10	-6	4	18	0.9	0.9	1.0	1.2
PAPSS1	O43252	53	-11	-12	4	-13	0.9	0.9	1.0	0.9
PAPSS2	O95340	43	-11	-12	4	-13	0.9	0.9	1.0	0.9
SENP1	Q9P0U3	60	-12	2	4	-16	0.9	1.0	1.0	0.9
IRAK3	Q9Y616	581	-12	-5	4	-30	0.9	1.0	1.0	0.8
HPCAL4	Q9UM19	187	-13	10	4	37	0.9	1.1	1.0	1.6
EIF2B4	Q9UI10	69	-13	-7	4	-12	0.9	0.9	1.0	0.9
CNBP	P62633	171	-17	-7	4	1	0.9	0.9	1.0	1.0
METTL18	O95568	48	25	-6	3	-8	1.3	0.9	1.0	0.9
ETFB	P38117	66	20	-4	3	8	1.2	1.0	1.0	1.1
ATP2A2	P16615	471	20	-30	3	3	1.2	0.8	1.0	1.0
KBTBD11	O94819	236	19	5	3	17	1.2	1.1	1.0	1.2
CCNL2	Q96S94	190	19	1	3	-3	1.2	1.0	1.0	1.0
UBR4	Q5T4S7	4916	19	-10	3	1	1.2	0.9	1.0	1.0
ATM	Q13315	786	18	-15	3	-1	1.2	0.9	1.0	1.0
ARHGAP6	O43182	417	18	6	3	-3	1.2	1.1	1.0	1.0
NEK9	Q8TD19	506	18	-2	3	3	1.2	1.0	1.0	1.0
VAV1	P15498	284	18	-6	3	9	1.2	0.9	1.0	1.1
PITPNC1	Q9UKF7	9	17	-9	3	0	1.2	0.9	1.0	1.0
LPP	Q93052	364	16	-6	3	-2	1.2	0.9	1.0	1.0
SYNE2	Q8WXH0	2212	16	-7	3	-1	1.2	0.9	1.0	1.0
H2AFY	O75367	286	15	-3	3	10	1.2	1.0	1.0	1.1
THOC5	Q13769	208	15	-4	3	2	1.2	1.0	1.0	1.0
EXOC8	Q8IYI6	172	15	-5	3	0	1.2	1.0	1.0	1.0
AKAP13	Q12802	65	15	-6	3	10	1.2	0.9	1.0	1.1
DAGLB	Q8NCG7	658	15	3	3	-13	1.2	1.0	1.0	0.9
RANGAP1	P46060	274	15	-4	3	1	1.2	1.0	1.0	1.0
HECTD4	Q9Y4D8	3985	14	1	3	7	1.2	1.0	1.0	1.1
IQGAP1	P46940	148	14	-3	3	-4	1.2	1.0	1.0	1.0

OSBP	P22059	343	14	-5	3	-3	1.2	1.0	1.0	1.0
PLCB2	Q00722	945	14	-7	3	-22	1.2	0.9	1.0	0.8
RNF213	Q63HN8	2536	14	2	3	1	1.2	1.0	1.0	1.0
DBNL	Q9UJU6	67	14	-24	3	2	1.2	0.8	1.0	1.0
ATP11A	P98196	625	13	11	3	12	1.1	1.1	1.0	1.1
SMAD2	Q15796	149	13	-1	3	4	1.1	1.0	1.0	1.0
CNN2	Q99439	61	13	-6	3	15	1.1	0.9	1.0	1.2
NTMT1	Q9BV86	184	13	-11	3	-7	1.1	0.9	1.0	0.9
NANS	Q9NR45	184	13	-14	3	-10	1.1	0.9	1.0	0.9
Uncharacteri	G3V4G9	95	12	20	3	18	1.1	1.3	1.0	1.2
PRKD2	Q9BZL6	861	12	10	3	-9	1.1	1.1	1.0	0.9
CAND1	Q86VP6	1153	12	-5	3	3	1.1	1.0	1.0	1.0
EHMT2	Q96KQ7	1115	12	0	3	4	1.1	1.0	1.0	1.0
HCK	P08631	234	12	-9	3	3	1.1	0.9	1.0	1.0
NMD3	Q96D46	214	11	-2	3	-2	1.1	1.0	1.0	1.0
SYNE1	Q8NF91	8380	11	-6	3	-5	1.1	0.9	1.0	1.0
STK39	Q9UEW8	99	11	-7	3	3	1.1	0.9	1.0	1.0
CRLF3	Q8IUI8	336	11	-17	3	2	1.1	0.9	1.0	1.0
TKFC	Q3LXA3	13	11	6	3	3	1.1	1.1	1.0	1.0
ZCCHC6	Q5VYS8	343	11	-2	3	-8	1.1	1.0	1.0	0.9
TBC1D9B	Q66K14	444	11	-2	3	-10	1.1	1.0	1.0	0.9
ARFGEF1	Q9Y6D6	102	11	-8	3	11	1.1	0.9	1.0	1.1
CWF19L1	Q69YN2	511	10	-2	3	6	1.1	1.0	1.0	1.1
ARMC8	Q8IUR7	661	10	-3	3	3	1.1	1.0	1.0	1.0
KEAP1	Q14145	288	10	-13	3	2	1.1	0.9	1.0	1.0
RABEP2	Q9H5N1	422	10	5	3	3	1.1	1.1	1.0	1.0
AGO2	Q9UKV8	564	10	3	3	4	1.1	1.0	1.0	1.0
Uncharacteri	V9GY48	86	10	-1	3	5	1.1	1.0	1.0	1.1
CCT4	P50991	252	10	-1	3	-2	1.1	1.0	1.0	1.0
RPL30	P62888	92	10	-6	3	8	1.1	0.9	1.0	1.1
MYO1F	O00160	856	10	-14	3	2	1.1	0.9	1.0	1.0
INTS2	Q9H0H0	33	10	-18	3	-14	1.1	0.8	1.0	0.9
TRIM25	Q14258	524	10	-20	3	-3	1.1	0.8	1.0	1.0
TAP1	Q03518	795	9	3	3	1	1.1	1.0	1.0	1.0
USP9Y	O00507	1238	9	2	3	-2	1.1	1.0	1.0	1.0
USP9X	Q93008	1237	9	2	3	-2	1.1	1.0	1.0	1.0
DGUOK	Q16854	87	9	-3	3	-14	1.1	1.0	1.0	0.9
AKAP13	Q12802	2142	9	-5	3	-3	1.1	1.0	1.0	1.0
DSCR3	O14972	137	9	-7	3	-8	1.1	0.9	1.0	0.9
ARFGEF1	Q9Y6D6	37	9	-12	3	-11	1.1	0.9	1.0	0.9
PDS5B	Q9NTI5	317	9	9	3	2	1.1	1.1	1.0	1.0
NT5DC1	Q5TFE4	179	9	-4	3	-20	1.1	1.0	1.0	0.8
MTMR1	Q13613	317	9	-7	3	12	1.1	0.9	1.0	1.1
DNM1L	O00429	644	9	-7	3	2	1.1	0.9	1.0	1.0
DPYSL2	Q16555	504	8	0	3	-5	1.1	1.0	1.0	1.0
EIF5	P55010	59	8	-3	3	4	1.1	1.0	1.0	1.0
GART	P22102	134	8	-3	3	3	1.1	1.0	1.0	1.0
GATAD2A	Q86YP4	417	8	-3	3	1	1.1	1.0	1.0	1.0
CMPK2	Q5EBM0	40	8	-2	3	-6	1.1	1.0	1.0	0.9
ACAP2	Q15057	468	8	-3	3	6	1.1	1.0	1.0	1.1
STRN	O43815	665	8	-3	3	1	1.1	1.0	1.0	1.0
VCL	P18206	85	8	-5	3	-3	1.1	1.0	1.0	1.0
CASP9	P55211	172	8	-7	3	-17	1.1	0.9	1.0	0.9

ARID4A	P29374	107	8	-8	3	-5	1.1	0.9	1.0	1.0
MROH1	Q8NDA8	505	8	-11	3	-7	1.1	0.9	1.0	0.9
PPP2CA	P67775	20	8	-22	3	10	1.1	0.8	1.0	1.1
VPS41	P49754	794	7	3	3	4	1.1	1.0	1.0	1.0
ACLY	P53396	845	7	2	3	-6	1.1	1.0	1.0	0.9
DNAJB1	P25685	267	7	1	3	2	1.1	1.0	1.0	1.0
AP3D1	O14617	574	7	0	3	5	1.1	1.0	1.0	1.0
SP110	Q9HB58	435	7	-1	3	-4	1.1	1.0	1.0	1.0
NEK9	Q8TD19	909	7	-2	3	-6	1.1	1.0	1.0	0.9
ATG12	O94817	134	7	-4	3	4	1.1	1.0	1.0	1.0
ADSL	P30566	27	7	-6	3	-3	1.1	0.9	1.0	1.0
BYSL	Q13895	300	7	-7	3	-4	1.1	0.9	1.0	1.0
ERO1A	Q96HE7	85	7	-9	3	9	1.1	0.9	1.0	1.1
RABGGTA	Q92696	516	7	4	3	8	1.1	1.0	1.0	1.1
SNX20	Q7Z614	222	7	-1	3	-10	1.1	1.0	1.0	0.9
RPL32	P62910	91	7	-2	3	-1	1.1	1.0	1.0	1.0
MTMR3	Q13615	188	7	-17	3	5	1.1	0.9	1.0	1.1
HNRNPU	Q00839	562	6	2	3	-3	1.1	1.0	1.0	1.0
LRCH1	Q9Y2L9	251	6	-2	3	-15	1.1	1.0	1.0	0.9
ZZEF1	O43149	1441	6	-2	3	1	1.1	1.0	1.0	1.0
STK11	Q15831	151	6	-4	3	-5	1.1	1.0	1.0	1.0
CACYBP	Q9HB71	154	6	6	3	-9	1.1	1.1	1.0	0.9
NCOR1	O75376	1274	6	0	3	-2	1.1	1.0	1.0	1.0
MYO1G	B0I1T2	788	6	-3	3	-5	1.1	1.0	1.0	1.0
TFAM	Q00059	246	6	-6	3	-6	1.1	0.9	1.0	0.9
THEMIS2	Q5TEJ8	62	6	-7	3	9	1.1	0.9	1.0	1.1
CCT3	P49368	455	6	-7	3	3	1.1	0.9	1.0	1.0
PDIA3	P30101	406	6	-8	3	-11	1.1	0.9	1.0	0.9
TYMP	P19971	361	6	-9	3	2	1.1	0.9	1.0	1.0
SMYD3	Q9H7B4	263	6	-12	3	-21	1.1	0.9	1.0	0.8
INPP5D	Q92835	819	6	-14	3	-3	1.1	0.9	1.0	1.0
HNRNPUL2-B	H3BQZ7	57	5	1	3	-4	1.1	1.0	1.0	1.0
SHOC2	Q9UQ13	260	5	-2	3	3	1.1	1.0	1.0	1.0
SNX6	Q9UNH7	264	5	-9	3	-3	1.1	0.9	1.0	1.0
ARHGAP9	Q9BRR9	548	5	-10	3	-2	1.1	0.9	1.0	1.0
COPB1	P53618	390	5	-17	3	24	1.1	0.9	1.0	1.3
MMRN1	Q13201	238	5	-5	3	-10	1.0	1.0	1.0	0.9
EXOC4	Q96A65	957	5	-8	3	-7	1.0	0.9	1.0	0.9
CARD9	Q9H257	454	5	-8	3	-13	1.0	0.9	1.0	0.9
NAIF1	Q69YI7	266	5	-8	3	-5	1.0	0.9	1.0	1.0
GALK1	P51570	182	4	12	3	-1	1.0	1.1	1.0	1.0
PPP2R2A	P63151	262	4	5	3	9	1.0	1.0	1.0	1.1
RANBP1	P43487	132	4	-1	3	-7	1.0	1.0	1.0	0.9
ZRANB2	O95218	71	4	-3	3	-8	1.0	1.0	1.0	0.9
POLR3E	Q9NVU0	70	4	-11	3	-10	1.0	0.9	1.0	0.9
PLEC	Q15149	1136	4	-4	3	3	1.0	1.0	1.0	1.0
APC	P25054	2664	4	-6	3	-2	1.0	0.9	1.0	1.0
PGRMC1	O00264	129	4	-7	3	-13	1.0	0.9	1.0	0.9
NOP56	O00567	142	4	-8	3	-5	1.0	0.9	1.0	1.0
PDXDC1	Q6P996	491	4	-17	3	-5	1.0	0.9	1.0	1.0
ZNF185	O15231	615	3	3	3	-8	1.0	1.0	1.0	0.9
MTHFD1	P11586	147	3	-5	3	-3	1.0	1.0	1.0	1.0
STRBP	Q96SI9	142	3	-8	3	-2	1.0	0.9	1.0	1.0

CSRP1	P21291	37	3	-9	3	-11	1.0	0.9	1.0	0.9
BOP1	Q14137	373	3	-10	3	7	1.0	0.9	1.0	1.1
DOCK10	Q96BY6	319	3	-10	3	-6	1.0	0.9	1.0	0.9
NIPBL	Q6KC79	279	3	-15	3	-11	1.0	0.9	1.0	0.9
PIK3R1	P27986	670	3	-35	3	0	1.0	0.7	1.0	1.0
VAC14	Q08AM6	516	3	-2	3	-9	1.0	1.0	1.0	0.9
KDM1B	Q8NB78	234	3	-5	3	8	1.0	1.0	1.0	1.1
SLC25A6	P12236	57	3	-8	3	-2	1.0	0.9	1.0	1.0
CEP131	Q9UPN4	218	3	-9	3	-8	1.0	0.9	1.0	0.9
SYTL3	Q4VX76	585	3	-17	3	-7	1.0	0.9	1.0	0.9
IGHG3	A0A087WVW	158	2	-5	3	-4	1.0	1.0	1.0	1.0
EXOSC7	Q15024	238	2	-6	3	-4	1.0	0.9	1.0	1.0
EML3	Q32P44	420	2	-8	3	4	1.0	0.9	1.0	1.0
RSBN1	Q5VWQ0	207	2	-8	3	-19	1.0	0.9	1.0	0.8
BCL11B	Q9C0K0	801	2	-9	3	1	1.0	0.9	1.0	1.0
PTGES2	Q9H7Z7	110	2	-5	3	13	1.0	1.0	1.0	1.1
GALE	Q14376	262	2	-6	3	12	1.0	0.9	1.0	1.1
COLGALT1	Q8NBJ5	412	2	-8	3	3	1.0	0.9	1.0	1.0
UNK	Q9C0B0	782	2	-9	3	-10	1.0	0.9	1.0	0.9
FNTA	P49354	341	2	-25	3	-7	1.0	0.8	1.0	0.9
GPRC6A	Q5T6X5	744	2	-29	3	-1	1.0	0.8	1.0	1.0
HEATR3	Q7Z4Q2	57	1	4	3	5	1.0	1.0	1.0	1.0
NSUN2	Q08J23	271	1	3	3	-1	1.0	1.0	1.0	1.0
FAHD2B	Q6P2I3	119	1	3	3	-2	1.0	1.0	1.0	1.0
EEF1A1	P68104	363	1	-3	3	-10	1.0	1.0	1.0	0.9
HECTD3	Q5T447	487	1	-4	3	-3	1.0	1.0	1.0	1.0
DPP3	Q9NY33	509	1	-9	3	-6	1.0	0.9	1.0	0.9
RAVER1	Q8IY67	297	1	-12	3	2	1.0	0.9	1.0	1.0
C15orf39	Q6ZRI6	831	1	5	3	7	1.0	1.0	1.0	1.1
SPTY2D1	Q68D10	535	1	-1	3	9	1.0	1.0	1.0	1.1
VPS50	Q96JG6	324	1	-2	3	10	1.0	1.0	1.0	1.1
RHOF	Q9HBH0	173	1	-3	3	10	1.0	1.0	1.0	1.1
EIF1AD	Q8N9N8	89	1	-5	3	0	1.0	1.0	1.0	1.0
METTL3	Q86U44	294	1	-7	3	11	1.0	0.9	1.0	1.1
S100A11	P31949	13	1	-10	3	-8	1.0	0.9	1.0	0.9
HECTD1	Q9ULT8	436	0	7	3	-3	1.0	1.1	1.0	1.0
AASS	Q9UDR5	342	0	-8	3	8	1.0	0.9	1.0	1.1
SCYL1	Q96KG9	241	-1	-7	3	-1	1.0	0.9	1.0	1.0
PPP6R2	O75170	366	-1	-8	3	-7	1.0	0.9	1.0	0.9
WDR26	Q9H7D7	656	-1	3	3	1	1.0	1.0	1.0	1.0
HIVEP2	P31629	641	-1	-15	3	-8	1.0	0.9	1.0	0.9
EXOC2	Q96KP1	638	-2	-6	3	-5	1.0	0.9	1.0	1.0
RNF213	Q63HN8	3794	-2	7	3	1	1.0	1.1	1.0	1.0
ACAP2	Q15057	691	-2	1	3	-6	1.0	1.0	1.0	0.9
EXOC7	Q9UPT5	83	-2	-3	3	-10	1.0	1.0	1.0	0.9
TTLL12	Q14166	612	-2	-8	3	-5	1.0	0.9	1.0	1.0
MED19	A0JLT2	62	-3	1	3	-7	1.0	1.0	1.0	0.9
GAPVD1	Q14C86	1129	-3	-5	3	6	1.0	1.0	1.0	1.1
UBR5	O95071	730	-3	-10	3	-1	1.0	0.9	1.0	1.0
USP16	Q9Y5T5	509	-3	-6	3	-17	1.0	0.9	1.0	0.9
ATG7	O95352	550	-3	-12	3	8	1.0	0.9	1.0	1.1
ZBTB40	Q9NUA8	234	-3	-18	3	-12	1.0	0.8	1.0	0.9
UBE2Z	Q9H832	154	-3	-33	3	-7	1.0	0.8	1.0	0.9

CASP7	P55210	186	-4	-6	3	3	1.0	0.9	1.0	1.0
IQSEC1	Q6DN90	359	-4	-8	3	-13	1.0	0.9	1.0	0.9
MAFK	O60675	68	-4	-9	3	-3	1.0	0.9	1.0	1.0
EIF2B3	Q9NR50	64	-4	-10	3	-2	1.0	0.9	1.0	1.0
SLC25A1	P53007	141	-4	6	3	3	1.0	1.1	1.0	1.0
BCL7B	Q9BQE9	189	-4	1	3	-1	1.0	1.0	1.0	1.0
OSGEP	Q9NPF4	265	-4	-1	3	-7	1.0	1.0	1.0	0.9
DGCR8	Q8WYQ5	514	-4	-12	3	-6	1.0	0.9	1.0	0.9
WDR70	Q9NW82	227	-4	-14	3	2	1.0	0.9	1.0	1.0
FN3KRP	Q9HA64	24	-5	-3	3	16	1.0	1.0	1.0	1.2
ADCK3	Q8NI60	406	-5	-17	3	-10	1.0	0.9	1.0	0.9
EPHX2	P34913	423	-5	-8	3	24	1.0	0.9	1.0	1.3
DYNC1LI2	O43237	191	-6	-5	3	-8	0.9	1.0	1.0	0.9
PRKCG	P05129	636	-6	-6	3	12	0.9	0.9	1.0	1.1
RGP1	Q92546	208	-6	-22	3	-32	0.9	0.8	1.0	0.8
RPL3	P39023	114	-7	2	3	-1	0.9	1.0	1.0	1.0
TRABD	Q9H4I3	184	-8	-21	3	-8	0.9	0.8	1.0	0.9
HBB	P68871	94	-9	-10	3	5	0.9	0.9	1.0	1.1
MCM3AP	O60318	1377	-10	-7	3	1	0.9	0.9	1.0	1.0
GSDMD	P57764	191	-11	5	3	-1	0.9	1.1	1.0	1.0
FTH1	P02794	91	-12	-2	3	4	0.9	1.0	1.0	1.0
IFNAR2	P48551	395	-12	-7	3	-14	0.9	0.9	1.0	0.9
ZNF148	Q9UQR1	14	-14	-5	3	-10	0.9	1.0	1.0	0.9
LPP	Q93052	468	-15	-18	3	-11	0.9	0.8	1.0	0.9
NAE1	Q13564	153	-16	-16	3	-2	0.9	0.9	1.0	1.0
PML	P29590	189	-18	-13	3	-21	0.8	0.9	1.0	0.8
KIAA1429	Q69YN4	1170	-18	-15	3	7	0.8	0.9	1.0	1.1
NIPBL	Q6KC79	304	-21	-12	3	-20	0.8	0.9	1.0	0.8
PRPF19	Q9UMS4	230	-21	-2	3	-17	0.8	1.0	1.0	0.9
EIF3D	O15371	258	29	-25	3	1	1.4	0.8	1.0	1.0
THOC2	Q8NI27	384	29	-4	3	-3	1.4	1.0	1.0	1.0
CDK5RAP3	Q96JB5	136	28	-4	3	14	1.4	1.0	1.0	1.2
COQ10B	Q9H8M1	152	25	0	3	2	1.3	1.0	1.0	1.0
TEX264	Q9Y6I9	94	24	0	3	11	1.3	1.0	1.0	1.1
EML4	Q9HC35	811	24	-17	3	3	1.3	0.9	1.0	1.0
CNOT1	A5YKK6	1420	22	3	3	6	1.3	1.0	1.0	1.1
TRRAP	Q9Y4A5	620	22	-2	3	5	1.3	1.0	1.0	1.0
FNDC3A	Q9Y2H6	369	22	-3	3	-3	1.3	1.0	1.0	1.0
TLE3	Q04726	26	22	-17	3	-31	1.3	0.9	1.0	0.8
ADD1	P35611	525	21	9	3	-9	1.3	1.1	1.0	0.9
WDR1	O75083	170	19	-9	3	9	1.2	0.9	1.0	1.1
CTU1	Q7Z7A3	290	19	-6	3	-1	1.2	0.9	1.0	1.0
PML	P29590	215	18	6	3	2	1.2	1.1	1.0	1.0
TPST2	O60704	156	18	6	3	-2	1.2	1.1	1.0	1.0
FBXL3	Q9UKT7	78	18	-7	3	-20	1.2	0.9	1.0	0.8
TBCB	Q99426	216	18	-23	3	-6	1.2	0.8	1.0	0.9
GFPT1	Q06210	620	18	3	3	4	1.2	1.0	1.0	1.0
PLEC	Q15149	4454	18	-2	3	7	1.2	1.0	1.0	1.1
POLD1	P28340	1011	18	-2	3	-2	1.2	1.0	1.0	1.0
SPATA13	Q96N96	542	17	-15	3	9	1.2	0.9	1.0	1.1
SNRNP200	O75643	133	17	-5	3	8	1.2	1.0	1.0	1.1
ACACA	Q13085	813	16	-1	3	6	1.2	1.0	1.0	1.1
CASP2	P42575	436	15	7	3	-13	1.2	1.1	1.0	0.9

MDN1	Q9NU22	2192	15	-7	3	-8	1.2	0.9	1.0	0.9
UBASH3B	Q8TF42	240	15	-9	3	7	1.2	0.9	1.0	1.1
DEK	P35659	161	15	-116	3	5	1.2	0.5	1.0	1.1
SLC25A46	Q96AG3	239	15	-9	3	-5	1.2	0.9	1.0	1.0
KIAA0391	O15091	568	14	6	3	-4	1.2	1.1	1.0	1.0
TMEM252	Q8N6L7	170	14	1	3	6	1.2	1.0	1.0	1.1
DARS2	Q6PI48	371	14	-1	3	7	1.2	1.0	1.0	1.1
COIL	P38432	126	14	-4	3	5	1.2	1.0	1.0	1.0
INPPL1	O15357	405	14	-7	3	21	1.2	0.9	1.0	1.3
DHRS1	Q96LJ7	235	14	-27	3	-4	1.2	0.8	1.0	1.0
UBE3B	Q7Z3V4	1036	13	-2	3	-9	1.1	1.0	1.0	0.9
MAP2K2	P36507	349	13	-9	3	-2	1.1	0.9	1.0	1.0
MAP2K1	Q02750	341	13	-9	3	-2	1.1	0.9	1.0	1.0
DCTN1	Q14203	759	13	-9	3	-3	1.1	0.9	1.0	1.0
TBCD	Q9BTW9	773	13	-10	3	6	1.1	0.9	1.0	1.1
COPS6	Q7L5N1	143	12	5	3	12	1.1	1.1	1.0	1.1
DARS	P14868	257	12	-5	3	2	1.1	1.0	1.0	1.0
LSS	P48449	609	12	-8	3	0	1.1	0.9	1.0	1.0
PAN2	Q504Q3	263	12	-8	3	-3	1.1	0.9	1.0	1.0
MYO1F	O00160	445	11	-27	3	16	1.1	0.8	1.0	1.2
EXOC7	Q9UPT5	550	11	-13	3	6	1.1	0.9	1.0	1.1
PARP12	Q9H0J9	474	10	8	3	6	1.1	1.1	1.0	1.1
HNRNPUL2-B	H3BQZ7	602	10	0	3	2	1.1	1.0	1.0	1.0
SUPT6H	Q7KZ85	1465	10	2	3	3	1.1	1.0	1.0	1.0
PLSCR1	O15162	237	10	2	3	-2	1.1	1.0	1.0	1.0
SENP3-EIF4A	A0A087X0R7	52	10	0	3	-7	1.1	1.0	1.0	0.9
RAD50	Q92878	221	10	-4	3	-1	1.1	1.0	1.0	1.0
CWC22	Q9HCG8	253	10	-4	3	9	1.1	1.0	1.0	1.1
LRRK2	Q5S007	1526	10	-4	3	6	1.1	1.0	1.0	1.1
ACTR3	P61158	12	10	-5	3	11	1.1	1.0	1.0	1.1
EIF5	P55010	138	10	-6	3	7	1.1	0.9	1.0	1.1
PIK3CD	O00329	438	10	-6	3	1	1.1	0.9	1.0	1.0
PTGES3	Q15185	75	10	-6	3	-1	1.1	0.9	1.0	1.0
EIF5	P55010	122	10	-7	3	12	1.1	0.9	1.0	1.1
RRBP1	Q9P2E9	1128	10	-13	3	1	1.1	0.9	1.0	1.0
GABPA	Q06546	69	10	-19	3	-8	1.1	0.8	1.0	0.9
ABCB7	O75027	750	9	14	3	-5	1.1	1.2	1.0	1.0
PKN1	Q16512	410	9	-9	3	5	1.1	0.9	1.0	1.1
LEF1	Q9UJU2	321	9	-11	3	-8	1.1	0.9	1.0	0.9
HDDC3	Q8N4P3	133	9	3	3	-4	1.1	1.0	1.0	1.0
SUCLA2	Q9P2R7	158	9	-4	3	2	1.1	1.0	1.0	1.0
NLRX1	Q86UT6	481	9	-5	3	-12	1.1	1.0	1.0	0.9
STAT6	P42226	272	9	-9	3	2	1.1	0.9	1.0	1.0
INTS3	Q68E01	80	9	-9	3	1	1.1	0.9	1.0	1.0
ALDH3B1	P43353	300	9	-13	3	2	1.1	0.9	1.0	1.0
TRPV2	Q9Y5S1	205	9	-14	3	16	1.1	0.9	1.0	1.2
NUP85	Q9BW27	230	9	-22	3	14	1.1	0.8	1.0	1.2
IPO5	O00410	972	9	-27	3	-6	1.1	0.8	1.0	0.9
SCAF8	Q9UPN6	1170	8	5	3	0	1.1	1.1	1.0	1.0
VPRBP	Q9Y4B6	784	8	-3	3	-12	1.1	1.0	1.0	0.9
RPRD2	Q5VT52	88	8	-3	3	9	1.1	1.0	1.0	1.1
CD14	P08571	217	8	-4	3	0	1.1	1.0	1.0	1.0
EIF2D	P41214	489	8	-5	3	-4	1.1	1.0	1.0	1.0

PDCD6IP	Q8WUM4	231	8	-9	3	1	1.1	0.9	1.0	1.0
CTR9	Q6PD62	231	8	-10	3	1	1.1	0.9	1.0	1.0
UBE3A	Q05086	310	8	-12	3	35	1.1	0.9	1.0	1.5
ANXA1	P04083	324	8	-1	3	-6	1.1	1.0	1.0	0.9
PPP1R3D	O95685	60	8	-2	3	4	1.1	1.0	1.0	1.0
PIKFYVE	Q9Y2I7	723	8	-2	3	-7	1.1	1.0	1.0	0.9
CUL2	Q13617	385	8	-4	3	8	1.1	1.0	1.0	1.1
ABCD1	P33897	39	8	-9	3	5	1.1	0.9	1.0	1.0
RANBP2	P49792	1296	8	-15	3	-22	1.1	0.9	1.0	0.8
DDX41	Q9UJV9	585	7	-9	3	-10	1.1	0.9	1.0	0.9
UBL5	Q9BZL1	6	7	-12	3	-4	1.1	0.9	1.0	1.0
PRKDC	P78527	1164	7	-16	3	4	1.1	0.9	1.0	1.0
HEATR5A	Q86XA9	350	7	8	3	10	1.1	1.1	1.0	1.1
GNB2	P62879	271	7	7	3	-11	1.1	1.1	1.0	0.9
MYO1G	B0I1T2	793	7	1	3	5	1.1	1.0	1.0	1.1
TOMM70	O94826	136	7	-1	3	8	1.1	1.0	1.0	1.1
VCPIP1	Q96JH7	816	7	-2	3	4	1.1	1.0	1.0	1.0
PARP14	Q460N5	1274	7	-3	3	-11	1.1	1.0	1.0	0.9
RALGAPA1	Q6GYQ0	257	7	-5	3	3	1.1	1.0	1.0	1.0
WDFY3	Q8IZQ1	1975	7	-6	3	9	1.1	0.9	1.0	1.1
CTR9	Q6PD62	817	7	-8	3	-3	1.1	0.9	1.0	1.0
AKAP13	Q12802	732	7	-11	3	-10	1.1	0.9	1.0	0.9
SRSF1	Q07955	148	7	-11	3	2	1.1	0.9	1.0	1.0
ARHGEF7	Q14155	448	7	-13	3	1	1.1	0.9	1.0	1.0
TCOF1	Q13428	1298	6	13	3	6	1.1	1.1	1.0	1.1
UPF3B	Q9BZI7	453	6	4	3	-5	1.1	1.0	1.0	1.0
FASTKD3	Q14CZ7	378	6	2	3	7	1.1	1.0	1.0	1.1
MYCBP2	O75592	3152	6	-1	3	-9	1.1	1.0	1.0	0.9
RBBP6	Q7Z6E9	1566	6	-4	3	2	1.1	1.0	1.0	1.0
U2AF1	Q01081	18	6	-8	3	0	1.1	0.9	1.0	1.0
AHCTF1	Q8WYP5	722	6	8	3	13	1.1	1.1	1.0	1.1
IPO7	O95373	757	6	2	3	8	1.1	1.0	1.0	1.1
OSBPL8	Q9BZF1	209	6	1	3	10	1.1	1.0	1.0	1.1
OSBPL5	Q9H0X9	187	6	1	3	10	1.1	1.0	1.0	1.1
HADHB	P55084	458	6	-5	3	6	1.1	1.0	1.0	1.1
FASN	P49327	1459	6	-6	3	1	1.1	0.9	1.0	1.0
EEF2	P13639	136	6	-11	3	-2	1.1	0.9	1.0	1.0
SPTB	P11277	1552	5	0	3	-7	1.1	1.0	1.0	0.9
ADCY7	P51828	261	5	-1	3	-1	1.1	1.0	1.0	1.0
PPA2	Q9H2U2	44	5	-3	3	-1	1.1	1.0	1.0	1.0
PLEK	P08567	295	5	-5	3	0	1.1	1.0	1.0	1.0
MRPS18B	Q9Y676	128	5	-5	3	-3	1.1	1.0	1.0	1.0
ARHGEF1	Q92888	892	5	-7	3	-5	1.1	0.9	1.0	1.0
HERC2	O95714	349	5	-8	3	-3	1.1	0.9	1.0	1.0
SPG20	Q8N0X7	562	5	6	3	-1	1.0	1.1	1.0	1.0
PDLIM1	O00151	73	5	-4	3	1	1.0	1.0	1.0	1.0
ZFAND6	Q6FIF0	163	5	-5	3	-2	1.0	1.0	1.0	1.0
HEATR3	Q7Z4Q2	655	5	-8	3	-5	1.0	0.9	1.0	1.0
SUCLA2	Q9P2R7	430	5	-16	3	-1	1.0	0.9	1.0	1.0
DOCK9	Q9BZ29	613	4	4	3	-1	1.0	1.0	1.0	1.0
DENND3	A2RUS2	104	4	3	3	-6	1.0	1.0	1.0	0.9
EIF3M	Q7L2H7	134	4	-1	3	-6	1.0	1.0	1.0	0.9
PLPP6	Q8IY26	55	4	-2	3	-5	1.0	1.0	1.0	1.0

HERPUD1	Q15011	68	4	0	3	4	1.0	1.0	1.0	1.0
ASPSCR1	Q9BZE9	224	4	0	3	-3	1.0	1.0	1.0	1.0
MAPKAPK3	Q16644	379	4	-2	3	-4	1.0	1.0	1.0	1.0
RAD50	Q92878	1302	4	-2	3	2	1.0	1.0	1.0	1.0
THEMIS2	Q5TEJ8	290	4	-3	3	4	1.0	1.0	1.0	1.0
SCAF8	Q9UPN6	102	4	-6	3	-2	1.0	0.9	1.0	1.0
LARS	Q9P2J5	113	4	-11	3	3	1.0	0.9	1.0	1.0
PNPLA6	Q8IY17	1212	4	-13	3	1	1.0	0.9	1.0	1.0
RAB27A	P51159	123	4	-20	3	-21	1.0	0.8	1.0	0.8
ATM	Q13315	1899	3	5	3	10	1.0	1.1	1.0	1.1
L2HGDH	Q9H9P8	179	3	-2	3	-7	1.0	1.0	1.0	0.9
OLA1	Q9NTK5	187	3	-4	3	6	1.0	1.0	1.0	1.1
WDR33	Q9C0J8	996	3	-5	3	-8	1.0	1.0	1.0	0.9
SUGT1	Q9Y2Z0	88	3	-6	3	1	1.0	0.9	1.0	1.0
PIK3CA	P42336	340	3	-8	3	-15	1.0	0.9	1.0	0.9
UBE2I	P63279	138	3	-9	3	-3	1.0	0.9	1.0	1.0
BRAF	P15056	748	3	-3	3	-9	1.0	1.0	1.0	0.9
RPS17	P08708	35	3	-8	3	11	1.0	0.9	1.0	1.1
WDR37	Q9Y2I8	417	2	3	3	4	1.0	1.0	1.0	1.0
ARV1	Q9H2C2	30	2	-4	3	-13	1.0	1.0	1.0	0.9
ZNF174	Q15697	232	2	-7	3	-11	1.0	0.9	1.0	0.9
SPATA5L1	Q9BVQ7	509	2	-7	3	26	1.0	0.9	1.0	1.3
SUV39H2	Q9H5I1	400	2	-8	3	-19	1.0	0.9	1.0	0.8
TRPV2	Q9Y5S1	332	2	-26	3	-3	1.0	0.8	1.0	1.0
HNRNPK	P61978	145	2	-11	3	3	1.0	0.9	1.0	1.0
BIRC6	Q9NR09	2123	1	5	3	2	1.0	1.0	1.0	1.0
ALDH16A1	Q8IZ83	28	1	2	3	7	1.0	1.0	1.0	1.1
GMPS	P49915	523	1	-2	3	-3	1.0	1.0	1.0	1.0
YY1AP1	Q9H869	266	1	-6	3	11	1.0	0.9	1.0	1.1
CDK11B	J3QR44	432	1	-8	3	-8	1.0	0.9	1.0	0.9
FASN	P49327	1448	1	-9	3	-5	1.0	0.9	1.0	1.0
DARS	P14868	349	1	-11	3	-10	1.0	0.9	1.0	0.9
SQSTM1	Q13501	154	1	-22	3	-8	1.0	0.8	1.0	0.9
AK2	P54819	232	1	1	3	2	1.0	1.0	1.0	1.0
ZFP64	Q9NPA5	147	1	-2	3	-12	1.0	1.0	1.0	0.9
NSUN5	Q96P11	362	1	-3	3	-4	1.0	1.0	1.0	1.0
RAB3GAP1	Q15042	218	1	-5	3	1	1.0	1.0	1.0	1.0
MYCBP2	O75592	4634	1	-9	3	-3	1.0	0.9	1.0	1.0
LIMS1	P48059	281	1	-9	3	-7	1.0	0.9	1.0	0.9
KPNA4	O00629	325	1	-15	3	-15	1.0	0.9	1.0	0.9
LRRC40	Q9H9A6	264	0	0	3	8	1.0	1.0	1.0	1.1
C10orf54	Q9H7M9	83	0	-2	3	-21	1.0	1.0	1.0	0.8
TRIP13	Q15645	14	0	-3	3	-9	1.0	1.0	1.0	0.9
DNMT1	P26358	1476	0	-7	3	-8	1.0	0.9	1.0	0.9
MOCS3	O95396	95	0	-13	3	4	1.0	0.9	1.0	1.0
HARS2	P49590	173	-1	8	3	1	1.0	1.1	1.0	1.0
LIMS1	P48059	272	-1	-1	3	3	1.0	1.0	1.0	1.0
GATAD2B	Q8WXI9	308	-1	-1	3	-12	1.0	1.0	1.0	0.9
VPS53	Q5VIR6	365	-1	-4	3	-3	1.0	1.0	1.0	1.0
TBXAS1	P24557	183	-1	-5	3	-14	1.0	1.0	1.0	0.9
PKNOX1	P55347	326	-1	-9	3	-16	1.0	0.9	1.0	0.9
RNF213	Q63HN8	1464	-1	-10	3	-3	1.0	0.9	1.0	1.0
DUSP3	P51452	124	-1	-11	3	4	1.0	0.9	1.0	1.0

GIMAP1-GIM	A0A087WTJ2	98	-1	-16	3	3	1.0	0.9	1.0	1.0
GIMAP1	Q8WWP7	98	-1	-16	3	3	1.0	0.9	1.0	1.0
FHL2	Q14192	132	-1	-10	3	-17	1.0	0.9	1.0	0.9
HNRNPR	O43390	99	-1	-14	3	5	1.0	0.9	1.0	1.0
SYNCRIP	O60506	96	-1	-14	3	5	1.0	0.9	1.0	1.0
GHDC	Q8N2G8	502	-2	-3	3	1	1.0	1.0	1.0	1.0
CCDC88B	A6NC98	1228	-2	-8	3	-2	1.0	0.9	1.0	1.0
TUBGCP5	A0A0G2JSA7	680	-2	-5	3	10	1.0	1.0	1.0	1.1
STIP1	P31948	403	-3	-1	3	9	1.0	1.0	1.0	1.1
VCPIP1	Q96JH7	219	-3	-4	3	-1	1.0	1.0	1.0	1.0
PDLIM5	Q96HC4	73	-3	-14	3	-3	1.0	0.9	1.0	1.0
AGTPBP1	Q9UPW5	971	-3	-19	3	-3	1.0	0.8	1.0	1.0
ZNF185	O15231	639	-3	-11	3	-11	1.0	0.9	1.0	0.9
ERI1	Q8IV48	243	-3	-26	3	-3	1.0	0.8	1.0	1.0
TPRKB	Q9Y3C4	167	-4	-2	3	4	1.0	1.0	1.0	1.0
BCS1L	Q9Y276	234	-4	-9	3	5	1.0	0.9	1.0	1.0
CKAP5	Q14008	1795	-4	-22	3	-15	1.0	0.8	1.0	0.9
PDCD11	Q14690	599	-4	-14	3	-1	1.0	0.9	1.0	1.0
RPGR	Q92834	914	-4	-16	3	-2	1.0	0.9	1.0	1.0
ZNF22	P17026	175	-5	11	3	4	1.0	1.1	1.0	1.0
AP1G1	O43747	539	-5	-2	3	-17	1.0	1.0	1.0	0.9
ACOT11	Q8WXI4	498	-5	-7	3	0	1.0	0.9	1.0	1.0
CARD8	Q9Y2G2	44	-5	-8	3	-21	1.0	0.9	1.0	0.8
SPTLC2	O15270	19	-6	1	3	-10	0.9	1.0	1.0	0.9
PSTPIP1	O43586	180	-7	-14	3	-3	0.9	0.9	1.0	1.0
LRCH4	O75427	179	-8	-6	3	-6	0.9	0.9	1.0	0.9
ATP2C1	P98194	162	-8	-7	3	-7	0.9	0.9	1.0	0.9
MYOF	Q9NZM1	1425	-9	-6	3	-18	0.9	0.9	1.0	0.9
FAM65A	Q6ZS17	987	-10	-17	3	-2	0.9	0.9	1.0	1.0
SH3BP5	O60239	438	-10	-5	3	-9	0.9	1.0	1.0	0.9
ERCC4	Q92889	176	-10	-7	3	-3	0.9	0.9	1.0	1.0
MKRN2	Q9H000	337	-10	-20	3	-23	0.9	0.8	1.0	0.8
RCC1	P18754	352	-10	-102	3	-19	0.9	0.5	1.0	0.8
RUFY1	Q96T51	667	-11	-23	3	-9	0.9	0.8	1.0	0.9
SP100	P23497	96	-11	-6	3	-10	0.9	0.9	1.0	0.9
NIT1	Q86X76	203	-14	-3	3	5	0.9	1.0	1.0	1.1
HNRNPH1	P31943	267	-28	-12	3	-17	0.8	0.9	1.0	0.9
HNRNPH2	P55795	267	-28	-12	3	-17	0.8	0.9	1.0	0.9
PAFAH1B2	P68402	188	-33	-23	3	-20	0.8	0.8	1.0	0.8
GNB2	P62879	121	23	11	2	13	1.3	1.1	1.0	1.1
SAMD4A	Q9UPU9	20	22	-7	2	6	1.3	0.9	1.0	1.1
HMHA1	Q92619	809	21	-2	2	5	1.3	1.0	1.0	1.0
IRGQ	Q8WZA9	373	21	1	2	-37	1.3	1.0	1.0	0.7
ANKRD44	Q8N8A2	854	21	-5	2	14	1.3	1.0	1.0	1.2
MTMR3	Q13615	11	20	-4	2	-6	1.3	1.0	1.0	0.9
NUP98	P52948	1636	20	-27	2	13	1.3	0.8	1.0	1.1
SETX	Q7Z333	133	18	7	2	-10	1.2	1.1	1.0	0.9
PGD	P52209	289	18	-6	2	19	1.2	0.9	1.0	1.2
PITRM1	Q5JRX3	627	18	-3	2	6	1.2	1.0	1.0	1.1
EXOC4	Q96A65	619	18	-8	2	-20	1.2	0.9	1.0	0.8
POLR2A	P24928	602	17	-1	2	4	1.2	1.0	1.0	1.0
DCXR	Q7Z4W1	150	17	3	2	14	1.2	1.0	1.0	1.2
USP15	Q9Y4E8	127	17	-10	2	5	1.2	0.9	1.0	1.0

SPECC1	Q5M775	598	16	6	2	3	1.2	1.1	1.0	1.0
PDCD11	Q14690	81	16	5	2	9	1.2	1.0	1.0	1.1
PIK3R4	Q99570	693	16	-9	2	-19	1.2	0.9	1.0	0.8
TBC1D15	Q8TC07	605	16	-1	2	-1	1.2	1.0	1.0	1.0
ARHGAP9	Q9BRR9	562	16	-24	2	5	1.2	0.8	1.0	1.1
PCYT2	Q99447	180	15	3	2	-5	1.2	1.0	1.0	1.0
GTF2H3	Q13889	142	15	-6	2	11	1.2	0.9	1.0	1.1
HSP90AA1	P07900	572	15	-8	2	14	1.2	0.9	1.0	1.2
GRK6	P43250	80	15	-20	2	-10	1.2	0.8	1.0	0.9
PLAA	Q9Y263	27	14	9	2	8	1.2	1.1	1.0	1.1
ORC5	O43913	409	14	-6	2	6	1.2	0.9	1.0	1.1
SPATA13	Q96N96	486	14	-14	2	-6	1.2	0.9	1.0	0.9
PKN1	Q16512	317	14	9	2	-5	1.2	1.1	1.0	1.0
COPA	P53621	1013	14	-13	2	13	1.2	0.9	1.0	1.1
NAA16	Q6N069	58	13	-14	2	-7	1.1	0.9	1.0	0.9
NAA15	Q9BXJ9	58	13	-14	2	-7	1.1	0.9	1.0	0.9
SDCBP	O00560	166	13	-20	2	9	1.1	0.8	1.0	1.1
CAPN15	O75808	101	13	6	2	-1	1.1	1.1	1.0	1.0
GLUD1	P00367	327	13	6	2	1	1.1	1.1	1.0	1.0
PKM	P14618	358	13	2	2	11	1.1	1.0	1.0	1.1
RALGAPA2	Q2PPJ7	775	13	-1	2	11	1.1	1.0	1.0	1.1
PCBP2	Q15366	158	13	-4	2	-1	1.1	1.0	1.0	1.0
COQ3	Q9NZJ6	155	13	-12	2	2	1.1	0.9	1.0	1.0
DOCK11	Q5JSL3	371	13	-26	2	3	1.1	0.8	1.0	1.0
RNF166	Q96A37	127	12	-1	2	-2	1.1	1.0	1.0	1.0
UBR4	Q5T4S7	260	12	-4	2	6	1.1	1.0	1.0	1.1
GIMAP1-GIM	A0A087WTJ2	389	12	-7	2	6	1.1	0.9	1.0	1.1
ZC3H11A	O75152	588	12	-8	2	-17	1.1	0.9	1.0	0.9
TYMP	P19971	280	12	-3	2	24	1.1	1.0	1.0	1.3
DAPP1	Q9UN19	227	12	-6	2	11	1.1	0.9	1.0	1.1
DDX19A	Q9NUU7	224	12	-11	2	-4	1.1	0.9	1.0	1.0
DDX19B	Q9UMR2	225	12	-11	2	-4	1.1	0.9	1.0	1.0
KDM5A	P29375	1409	11	1	2	-8	1.1	1.0	1.0	0.9
RASAL3	Q86YV0	1005	11	1	2	-8	1.1	1.0	1.0	0.9
UBE3C	Q15386	140	11	-8	2	13	1.1	0.9	1.0	1.1
LCK	P06239	378	11	-11	2	0	1.1	0.9	1.0	1.0
BLOC1S3	Q6QNY0	180	11	8	2	2	1.1	1.1	1.0	1.0
SP100	P23497	609	11	1	2	-11	1.1	1.0	1.0	0.9
UROD	P06132	294	11	-6	2	7	1.1	0.9	1.0	1.1
POLR2G	P62487	38	10	-3	2	8	1.1	1.0	1.0	1.1
RABEP1	Q15276	533	10	-4	2	10	1.1	1.0	1.0	1.1
PSMD8	P48556	173	10	-9	2	-7	1.1	0.9	1.0	0.9
STARD7	Q9NQZ5	362	10	8	2	3	1.1	1.1	1.0	1.0
UNC119	Q13432	77	10	1	2	0	1.1	1.0	1.0	1.0
STUB1	Q9UNE7	48	10	-1	2	9	1.1	1.0	1.0	1.1
COPB1	P53618	623	10	-5	2	1	1.1	1.0	1.0	1.0
NCF4	Q15080	242	10	-13	2	-1	1.1	0.9	1.0	1.0
NCF2	P19878	165	9	2	2	2	1.1	1.0	1.0	1.0
ANXA6	P08133	669	9	-6	2	12	1.1	0.9	1.0	1.1
FCHSD1	Q86WN1	471	9	2	2	-2	1.1	1.0	1.0	1.0
HNRNPU	Q00839	497	9	1	2	3	1.1	1.0	1.0	1.0
CXorf38	Q8TB03	77	9	-6	2	-5	1.1	0.9	1.0	1.0
BAX	Q07812	62	9	-15	2	-26	1.1	0.9	1.0	0.8

BMS1	Q14692	327	9	-17	2	-10	1.1	0.9	1.0	0.9
CSTF2T	Q9H0L4	441	8	0	2	-6	1.1	1.0	1.0	0.9
CNOT2	Q9NZN8	175	8	-1	2	-3	1.1	1.0	1.0	1.0
UTRN	P46939	2185	8	-1	2	-3	1.1	1.0	1.0	1.0
FES	P07332	781	8	-9	2	4	1.1	0.9	1.0	1.0
NBEAL2	Q6ZNJ1	712	8	-10	2	-12	1.1	0.9	1.0	0.9
AKR1B1	P15121	304	8	-11	2	12	1.1	0.9	1.0	1.1
RNF213	Q63HN8	2943	8	-13	2	-9	1.1	0.9	1.0	0.9
IRF8	Q02556	223	8	3	2	8	1.1	1.0	1.0	1.1
ARHGEF7	Q14155	721	8	0	2	6	1.1	1.0	1.0	1.1
NLRX1	Q86UT6	155	8	-2	2	-14	1.1	1.0	1.0	0.9
ATP6V1C1	P21283	15	8	-5	2	2	1.1	1.0	1.0	1.0
IKBKB	O14920	299	8	-7	2	-8	1.1	0.9	1.0	0.9
GMIP	Q9P107	335	8	-7	2	0	1.1	0.9	1.0	1.0
NAGK	Q9UJ70	211	8	-8	2	6	1.1	0.9	1.0	1.1
USF1	P22415	229	7	5	2	4	1.1	1.1	1.0	1.0
CASP4	P49662	258	7	2	2	6	1.1	1.0	1.0	1.1
FRY	Q5TBA9	2723	7	1	2	-7	1.1	1.0	1.0	0.9
RRBP1	Q9P2E9	1038	7	-1	2	-7	1.1	1.0	1.0	0.9
ATM	Q13315	633	7	-3	2	-9	1.1	1.0	1.0	0.9
ITPRIP	Q8IWB1	255	7	-4	2	-3	1.1	1.0	1.0	1.0
SDCBP	O00560	118	7	-4	2	3	1.1	1.0	1.0	1.0
GFM1	Q96RP9	514	7	-11	2	-3	1.1	0.9	1.0	1.0
DENND4B	O75064	815	7	-11	2	-19	1.1	0.9	1.0	0.8
ANKRD44	Q8N8A2	967	7	-1	2	-12	1.1	1.0	1.0	0.9
NFX1	Q12986	295	7	-2	2	-1	1.1	1.0	1.0	1.0
MED13	Q9UHV7	245	7	-3	2	5	1.1	1.0	1.0	1.1
TAF12	Q16514	143	7	-3	2	-8	1.1	1.0	1.0	0.9
ZMYM2	Q9UBW7	587	7	-4	2	-7	1.1	1.0	1.0	0.9
MAP3K3	Q99759	430	7	-5	2	-1	1.1	1.0	1.0	1.0
PRKCB	P05771	71	7	-6	2	4	1.1	0.9	1.0	1.0
PRKCA	P17252	71	7	-6	2	4	1.1	0.9	1.0	1.0
DTX3L	Q8TDB6	219	7	-12	2	-8	1.1	0.9	1.0	0.9
UBE3B	Q7Z3V4	979	7	-13	2	3	1.1	0.9	1.0	1.0
IDH2	P48735	113	7	-26	2	-1	1.1	0.8	1.0	1.0
AP3M1	Q9Y2T2	10	6	8	2	25	1.1	1.1	1.0	1.3
ERI3	O43414	285	6	-1	2	-2	1.1	1.0	1.0	1.0
TEP1	Q99973	2053	6	-1	2	1	1.1	1.0	1.0	1.0
LASP1	Q14847	35	6	-4	2	-5	1.1	1.0	1.0	1.0
WDR11	Q9BZH6	1071	6	-7	2	9	1.1	0.9	1.0	1.1
THNSL1	Q8IYQ7	324	6	2	2	-5	1.1	1.0	1.0	1.0
TRAF5	O00463	116	6	1	2	5	1.1	1.0	1.0	1.0
ERMP1	Q7Z2K6	43	6	-1	2	-1	1.1	1.0	1.0	1.0
MTHFD1	P11586	152	6	-3	2	6	1.1	1.0	1.0	1.1
PDCD6IP	Q8WUM4	40	6	-12	2	4	1.1	0.9	1.0	1.0
OPA3	Q9H6K4	164	5	8	2	2	1.1	1.1	1.0	1.0
HNRNPL	P14866	151	5	-4	2	-1	1.1	1.0	1.0	1.0
TGFB1I1	O43294	228	5	-4	2	-15	1.1	1.0	1.0	0.9
ZDHHC6	Q9H6R6	396	5	-6	2	-1	1.1	0.9	1.0	1.0
ETFA	P13804	155	5	-8	2	12	1.1	0.9	1.0	1.1
RFC3	P40938	32	5	-8	2	-8	1.1	0.9	1.0	0.9
ECHS1	P30084	111	5	-9	2	-2	1.1	0.9	1.0	1.0
SMCHD1	A6NHR9	505	5	-15	2	-5	1.1	0.9	1.0	1.0

ACTB	P60709	257	5	6	2	1	1.0	1.1	1.0	1.0
IFI16	Q16666	637	5	-2	2	-7	1.0	1.0	1.0	0.9
MTFR1L	Q9H019	37	5	-2	2	-4	1.0	1.0	1.0	1.0
ABL1	P00519	1100	5	-6	2	-15	1.0	0.9	1.0	0.9
RASA2	Q15283	314	5	-7	2	-8	1.0	0.9	1.0	0.9
NCK2	O43639	343	5	-8	2	-4	1.0	0.9	1.0	1.0
TRIM25	Q14258	176	5	-8	2	-6	1.0	0.9	1.0	0.9
NBEAL2	Q6ZNJ1	822	5	-12	2	-2	1.0	0.9	1.0	1.0
SP1	P08047	68	4	-1	2	-3	1.0	1.0	1.0	1.0
MTHFD1L	Q6UB35	779	4	-2	2	5	1.0	1.0	1.0	1.0
RNF213	Q63HN8	3554	4	-4	2	2	1.0	1.0	1.0	1.0
PHF5A	Q7RTV0	40	4	-8	2	0	1.0	0.9	1.0	1.0
VRK2	Q86Y07	194	4	-9	2	-14	1.0	0.9	1.0	0.9
DOCK8	Q8NF50	685	4	-11	2	-3	1.0	0.9	1.0	1.0
GMEB2	Q9UKD1	171	4	-13	2	-8	1.0	0.9	1.0	0.9
AP1B1	Q10567	57	4	-3	2	-5	1.0	1.0	1.0	1.0
DCXR	Q7Z4W1	138	4	-4	2	-2	1.0	1.0	1.0	1.0
SP1	P08047	604	4	-4	2	-3	1.0	1.0	1.0	1.0
IKBKAP	O95163	213	4	-5	2	-9	1.0	1.0	1.0	0.9
BRAP	Q7Z569	110	4	-6	2	-10	1.0	0.9	1.0	0.9
MATK	P42679	81	4	-9	2	-10	1.0	0.9	1.0	0.9
GNL3L	Q9NVN8	278	3	-8	2	5	1.0	0.9	1.0	1.0
SH3GLB2	Q9NR46	172	3	-10	2	-6	1.0	0.9	1.0	0.9
SMC6	Q96SB8	385	3	-12	2	-12	1.0	0.9	1.0	0.9
AGAP2	Q99490	579	3	-13	2	-5	1.0	0.9	1.0	1.0
MRI1	Q9BV20	168	3	-20	2	-5	1.0	0.8	1.0	1.0
USP10	Q14694	94	3	-1	2	-9	1.0	1.0	1.0	0.9
MVB12A	Q96EY5	33	3	-2	2	2	1.0	1.0	1.0	1.0
VBP1	P61758	8	3	-2	2	-10	1.0	1.0	1.0	0.9
PCIF1	Q9H4Z3	29	3	-11	2	-1	1.0	0.9	1.0	1.0
COPS4	Q9BT78	378	3	-13	2	-4	1.0	0.9	1.0	1.0
FADD	Q13158	98	2	8	2	-4	1.0	1.1	1.0	1.0
INPPL1	O15357	1187	2	-2	2	1	1.0	1.0	1.0	1.0
DOCK8	Q8NF50	939	2	-2	2	-7	1.0	1.0	1.0	0.9
BANK1	Q8NDB2	18	2	-2	2	-10	1.0	1.0	1.0	0.9
DDX49	Q9Y6V7	45	2	-3	2	0	1.0	1.0	1.0	1.0
NSF	P46459	264	2	-4	2	2	1.0	1.0	1.0	1.0
TRIM5	Q9C035	106	2	-11	2	-12	1.0	0.9	1.0	0.9
TMPO	P42166	330	2	-14	2	-20	1.0	0.9	1.0	0.8
FHL1	Q13642	153	2	5	2	-1	1.0	1.0	1.0	1.0
TYK2	P29597	278	2	-1	2	2	1.0	1.0	1.0	1.0
AGPS	O00116	214	2	-4	2	8	1.0	1.0	1.0	1.1
SETD1B	Q9UPS6	1007	1	8	2	14	1.0	1.1	1.0	1.2
PDE12	Q6L8Q7	268	1	0	2	3	1.0	1.0	1.0	1.0
UBE2N	P61088	87	1	-4	2	-3	1.0	1.0	1.0	1.0
ELMO1	Q92556	726	1	-5	2	-5	1.0	1.0	1.0	1.0
CNP	P09543	111	1	-5	2	-29	1.0	1.0	1.0	0.8
PPIL2	Q13356	15	1	-8	2	-3	1.0	0.9	1.0	1.0
OAS3	Q9Y6K5	83	1	-12	2	-2	1.0	0.9	1.0	1.0
THOP1	P52888	175	1	-3	2	9	1.0	1.0	1.0	1.1
CES1	P23141	390	1	-5	2	6	1.0	1.0	1.0	1.1
CRTC3	Q6UUV7	541	1	-6	2	-12	1.0	0.9	1.0	0.9
HNRNPM	P52272	114	1	-10	2	-8	1.0	0.9	1.0	0.9

RQCD1	Q92600	252	0	-8	2	1	1.0	0.9	1.0	1.0
CASP6	P55212	68	-1	-2	2	-13	1.0	1.0	1.0	0.9
TANGO2	Q6ICL3	168	-1	-3	2	2	1.0	1.0	1.0	1.0
MCMBP	Q9BTE3	636	-1	-4	2	-5	1.0	1.0	1.0	1.0
EXD2	Q9NVH0	423	-1	-9	2	-4	1.0	0.9	1.0	1.0
DDX58	O95786	490	-1	-16	2	-4	1.0	0.9	1.0	1.0
TMEM104	Q8NE00	455	-1	-4	2	-11	1.0	1.0	1.0	0.9
KMT2A	Q03164	2051	-1	-7	2	-29	1.0	0.9	1.0	0.8
KMT2B	Q9UMN6	1760	-1	-7	2	-29	1.0	0.9	1.0	0.8
RNF20	Q5VTR2	905	-1	-9	2	11	1.0	0.9	1.0	1.1
UBR3	Q6ZT12	1858	-2	-2	2	-1	1.0	1.0	1.0	1.0
HID1	Q8IV36	712	-2	-3	2	7	1.0	1.0	1.0	1.1
UBE3B	Q7Z3V4	998	-2	-9	2	-7	1.0	0.9	1.0	0.9
PHF14	O94880	874	-2	-16	2	-11	1.0	0.9	1.0	0.9
MRPL32	Q9BYC8	110	-2	-21	2	-11	1.0	0.8	1.0	0.9
XYLB	O75191	161	-2	-1	2	-12	1.0	1.0	1.0	0.9
ARHGEF40	Q8TER5	1233	-2	-5	2	6	1.0	1.0	1.0	1.1
ZNF587	Q96SQ5	366	-3	8	2	-1	1.0	1.1	1.0	1.0
HNRNPH2	P55795	290	-3	-9	2	11	1.0	0.9	1.0	1.1
SEPT1	Q8WYJ6	231	-3	-12	2	-1	1.0	0.9	1.0	1.0
ZDHHC4	Q9NPG8	337	-3	1	2	-4	1.0	1.0	1.0	1.0
PARG	Q86W56	963	-3	-3	2	-6	1.0	1.0	1.0	0.9
SPATA5	Q8NB90	672	-3	-4	2	-3	1.0	1.0	1.0	1.0
CTSC	P53634	448	-3	-14	2	-1	1.0	0.9	1.0	1.0
SP4	Q02446	625	-4	-10	2	-6	1.0	0.9	1.0	0.9
XPO5	Q9HAV4	714	-4	-10	2	-20	1.0	0.9	1.0	0.8
ORC4	O43929	421	-4	-12	2	-16	1.0	0.9	1.0	0.9
ATM	Q13315	1396	-5	-4	2	41	1.0	1.0	1.0	1.7
ABHD16A	O95870	258	-5	6	2	18	1.0	1.1	1.0	1.2
CTCF	P49711	504	-5	-1	2	1	1.0	1.0	1.0	1.0
CTNND1	O60716	692	-5	-3	2	-4	1.0	1.0	1.0	1.0
DNAJC13	O75165	1770	-5	-10	2	-1	1.0	0.9	1.0	1.0
SCRN1	Q12765	290	-5	-16	2	-3	1.0	0.9	1.0	1.0
GMPPA	Q96IJ6	389	-6	-4	2	1	0.9	1.0	1.0	1.0
LRRFIP1	Q32MZ4	644	-7	-3	2	-7	0.9	1.0	1.0	0.9
ZBTB34	Q8NCN2	436	-7	-12	2	-15	0.9	0.9	1.0	0.9
GAPDH	P04406	247	-7	-15	2	0	0.9	0.9	1.0	1.0
GSDMD	P57764	38	-7	-9	2	-9	0.9	0.9	1.0	0.9
CHURC1-FNT	B4DL54	333	-8	-6	2	37	0.9	0.9	1.0	1.6
PHC2	Q8IXK0	809	-8	-14	2	-8	0.9	0.9	1.0	0.9
UBE2O	Q9C0C9	400	-9	5	2	-15	0.9	1.0	1.0	0.9
POLR3B	Q9NW08	582	-9	-12	2	-1	0.9	0.9	1.0	1.0
AHDC1	Q5TGY3	147	-11	-17	2	-27	0.9	0.9	1.0	0.8
Uncharacteri	F5H5T6	83	-12	-3	2	-4	0.9	1.0	1.0	1.0
ITPRIP	Q8IWB1	257	-12	-10	2	-8	0.9	0.9	1.0	0.9
ISYNA1	Q9NPH2	485	-13	-9	2	5	0.9	0.9	1.0	1.0
PPP6R2	O75170	623	-13	-13	2	6	0.9	0.9	1.0	1.1
LYZ	P61626	95	-14	14	2	18	0.9	1.2	1.0	1.2
UTP14A	Q9BVJ6	380	-15	-5	2	-11	0.9	1.0	1.0	0.9
POLR3B	Q9NW08	1099	-15	2	2	14	0.9	1.0	1.0	1.2
CSE1L	P55060	939	-15	-10	2	6	0.9	0.9	1.0	1.1
CYFIP1	Q7L576	1241	-19	6	2	7	0.8	1.1	1.0	1.1
PRRC2B	Q5JSZ5	819	-24	-10	2	-22	0.8	0.9	1.0	0.8

EIF3H	Q15372	327	38	9	2	24	1.6	1.1	1.0	1.3
PRKAA1	Q13131	141	33	13	2	17	1.5	1.1	1.0	1.2
GDI2	P50395	335	25	-18	2	10	1.3	0.8	1.0	1.1
PML	P29590	676	23	-4	2	9	1.3	1.0	1.0	1.1
TWF2	Q6IBS0	275	22	-2	2	9	1.3	1.0	1.0	1.1
MCM2	P49736	522	22	5	2	25	1.3	1.1	1.0	1.3
SRRT	Q9BXP5	640	21	-13	2	7	1.3	0.9	1.0	1.1
HSPA4	P34932	245	20	-13	2	19	1.2	0.9	1.0	1.2
FAM49B	Q9NUQ9	223	19	-8	2	8	1.2	0.9	1.0	1.1
SPATA5L1	Q9BVQ7	580	18	17	2	-16	1.2	1.2	1.0	0.9
USP9Y	O00507	1773	18	-16	2	-9	1.2	0.9	1.0	0.9
USP9X	Q93008	1771	18	-16	2	-9	1.2	0.9	1.0	0.9
HEATR1	Q9H583	631	18	-6	2	-1	1.2	0.9	1.0	1.0
RAB34	Q9BZG1	19	17	9	2	0	1.2	1.1	1.0	1.0
HADHA	P40939	550	17	-7	2	12	1.2	0.9	1.0	1.1
COPS6	Q7L5N1	299	17	-8	2	10	1.2	0.9	1.0	1.1
PLEKHF2	Q9H8W4	129	15	-5	2	9	1.2	1.0	1.0	1.1
SCFD2	Q8WU76	79	15	-6	2	3	1.2	0.9	1.0	1.0
STAM	Q92783	85	15	-17	2	29	1.2	0.9	1.0	1.4
GNL3	Q9BVP2	131	14	10	2	24	1.2	1.1	1.0	1.3
BIN2	Q9UBW5	205	14	-7	2	10	1.2	0.9	1.0	1.1
TMEM104	Q8NE00	345	14	-21	2	-13	1.2	0.8	1.0	0.9
HCCS	P53701	46	13	4	2	1	1.1	1.0	1.0	1.0
RTF1	Q92541	599	13	-5	2	-4	1.1	1.0	1.0	1.0
PFKP	Q01813	360	13	9	2	-14	1.1	1.1	1.0	0.9
CEP104	O60308	445	13	6	2	0	1.1	1.1	1.0	1.0
GPS1	Q13098	192	13	-7	2	18	1.1	0.9	1.0	1.2
GDI2	P50395	302	13	-13	2	4	1.1	0.9	1.0	1.0
PPP2R5D	Q14738	484	13	-14	2	-4	1.1	0.9	1.0	1.0
LARS2	Q15031	578	13	-17	2	-9	1.1	0.9	1.0	0.9
CAND1	Q86VP6	802	13	-28	2	-1	1.1	0.8	1.0	1.0
AKNA	Q7Z591	289	12	16	2	13	1.1	1.2	1.0	1.1
GAPVD1	Q14C86	741	12	13	2	14	1.1	1.1	1.0	1.2
HNRNPU	Q00839	289	12	-1	2	3	1.1	1.0	1.0	1.0
RAD21	O60216	392	12	-7	2	-2	1.1	0.9	1.0	1.0
PDS5A	Q29RF7	92	12	-11	2	-2	1.1	0.9	1.0	1.0
PDS5B	Q9NTI5	82	12	-11	2	-2	1.1	0.9	1.0	1.0
ADRBK2	P35626	501	12	-4	2	-4	1.1	1.0	1.0	1.0
DICER1	Q9UPY3	699	12	-11	2	9	1.1	0.9	1.0	1.1
CD53	P19397	170	11	10	2	-6	1.1	1.1	1.0	0.9
MINA	Q8IUF8	103	11	8	2	10	1.1	1.1	1.0	1.1
CHMP6	Q96FZ7	110	11	6	2	-1	1.1	1.1	1.0	1.0
HEATR1	Q9H583	1138	11	-3	2	3	1.1	1.0	1.0	1.0
UFL1	O94874	143	11	-9	2	-13	1.1	0.9	1.0	0.9
YKT6	O15498	66	11	-10	2	-5	1.1	0.9	1.0	1.0
SUPV3L1	Q8IYB8	575	11	-12	2	-4	1.1	0.9	1.0	1.0
CPT2	P23786	639	11	-49	2	1	1.1	0.7	1.0	1.0
SEC23B	Q15437	180	11	-4	2	-9	1.1	1.0	1.0	0.9
MYO18A	Q92614	1022	11	-6	2	8	1.1	0.9	1.0	1.1
LRP1	Q07954	1812	11	-12	2	3	1.1	0.9	1.0	1.0
TAGLN2	P37802	63	11	-22	2	4	1.1	0.8	1.0	1.0
RPL10	P27635	49	10	13	2	-2	1.1	1.1	1.0	1.0
FKBP15	Q5T1M5	809	10	13	2	-16	1.1	1.1	1.0	0.9

MTPAP	Q9NVV4	299	10	3	2	9	1.1	1.0	1.0	1.1
ATP6V1B2	P21281	207	10	-3	2	3	1.1	1.0	1.0	1.0
CNDP2	Q96KP4	192	10	-5	2	-3	1.1	1.0	1.0	1.0
PTPN7	P35236	324	10	-6	2	1	1.1	0.9	1.0	1.0
ABHD10	Q9NUJ1	229	10	-7	2	17	1.1	0.9	1.0	1.2
CTPS1	P17812	218	10	-10	2	-6	1.1	0.9	1.0	0.9
CORO1C	Q9ULV4	343	10	-20	2	8	1.1	0.8	1.0	1.1
NSD1	Q96L73	2022	10	3	2	1	1.1	1.0	1.0	1.0
SEPT6	Q14141	301	10	-5	2	1	1.1	1.0	1.0	1.0
SEPT11	Q9NVA2	300	10	-5	2	1	1.1	1.0	1.0	1.0
DTYMK	P23919	117	10	-6	2	-61	1.1	0.9	1.0	0.6
CASP9	P55211	272	10	-9	2	-2	1.1	0.9	1.0	1.0
VCP	P55072	77	10	-16	2	3	1.1	0.9	1.0	1.0
XRCC5	P13010	235	10	-19	2	12	1.1	0.8	1.0	1.1
ATG2B	Q96BY7	1667	9	-2	2	2	1.1	1.0	1.0	1.0
DENND2D	Q9H6A0	162	9	-2	2	-16	1.1	1.0	1.0	0.9
ARRB1	P49407	140	9	-4	2	7	1.1	1.0	1.0	1.1
APOH	P02749	84	9	-5	2	7	1.1	1.0	1.0	1.1
MKNK1	Q9BUB5	101	9	-10	2	-3	1.1	0.9	1.0	1.0
MYH9	P35579	896	9	-1	2	4	1.1	1.0	1.0	1.0
BORCS7-ASM	A0A0B4J1R7	28	9	-7	2	-1	1.1	0.9	1.0	1.0
PLEC	Q15149	4245	9	-9	2	-8	1.1	0.9	1.0	0.9
NCKAP1L	P55160	612	9	-10	2	-1	1.1	0.9	1.0	1.0
UBE2M	P61081	65	9	-15	2	-13	1.1	0.9	1.0	0.9
CLCN7	P51798	637	8	4	2	-2	1.1	1.0	1.0	1.0
SP110	Q9HB58	520	8	-7	2	-7	1.1	0.9	1.0	0.9
SF3B1	O75533	796	8	-8	2	-5	1.1	0.9	1.0	1.0
SAAL1	Q96ER3	248	8	-3	2	5	1.1	1.0	1.0	1.1
ADNP	Q9H2P0	79	8	-7	2	24	1.1	0.9	1.0	1.3
EEF1G	P26641	266	7	4	2	2	1.1	1.0	1.0	1.0
SRPRA	P08240	253	7	4	2	-12	1.1	1.0	1.0	0.9
GUF1	Q8N442	129	7	-7	2	-2	1.1	0.9	1.0	1.0
GOLPH3	Q9H4A6	280	7	-11	2	9	1.1	0.9	1.0	1.1
AP2B1	P63010	123	7	-11	2	3	1.1	0.9	1.0	1.0
AP1B1	Q10567	123	7	-11	2	3	1.1	0.9	1.0	1.0
ATP6V1C1	P21283	376	7	-33	2	-5	1.1	0.8	1.0	1.0
FANCD2	Q9BXW9	1369	7	0	2	-6	1.1	1.0	1.0	0.9
INPP5D	Q92835	217	7	-3	2	4	1.1	1.0	1.0	1.0
GNLY	P22749	138	7	-3	2	-5	1.1	1.0	1.0	1.0
NCF2	P19878	291	7	-3	2	2	1.1	1.0	1.0	1.0
CNOT1	A5YKK6	1541	7	-4	2	-7	1.1	1.0	1.0	0.9
VPS51	Q9UID3	190	7	-6	2	7	1.1	0.9	1.0	1.1
PDIA3	P30101	92	7	-9	2	6	1.1	0.9	1.0	1.1
GTF2H1	P32780	246	7	-9	2	-1	1.1	0.9	1.0	1.0
SSH3	Q8TE77	413	7	-11	2	-7	1.1	0.9	1.0	0.9
MARS2	Q96GW9	114	7	-25	2	-10	1.1	0.8	1.0	0.9
TDRD7	Q8NHU6	673	6	3	2	7	1.1	1.0	1.0	1.1
COMMD6	Q7Z4G1	35	6	3	2	4	1.1	1.0	1.0	1.0
TLN2	Q9Y4G6	118	6	2	2	4	1.1	1.0	1.0	1.0
RARS	P54136	369	6	-5	2	0	1.1	1.0	1.0	1.0
ADAM17	P78536	365	6	-7	2	-5	1.1	0.9	1.0	1.0
HAAO	P46952	281	6	-11	2	5	1.1	0.9	1.0	1.1
MACF1	Q9UPN3	5742	6	-19	2	-27	1.1	0.8	1.0	0.8

ZMYM2	Q9UBW7	724	6	-29	2	-23	1.1	0.8	1.0	0.8
SPTLC1	O15269	133	6	1	2	5	1.1	1.0	1.0	1.1
IARS	P41252	120	6	1	2	3	1.1	1.0	1.0	1.0
RETSAT	Q6NUM9	534	6	1	2	-8	1.1	1.0	1.0	0.9
C2orf76	Q3KRA6	111	6	-1	2	-3	1.1	1.0	1.0	1.0
CNP	P09543	158	6	-3	2	10	1.1	1.0	1.0	1.1
ADH5	P11766	174	6	-4	2	-1	1.1	1.0	1.0	1.0
ARID1A	O14497	1983	6	-6	2	7	1.1	0.9	1.0	1.1
RASAL3	Q86YV0	614	6	-9	2	17	1.1	0.9	1.0	1.2
SUPT6H	Q7KZ85	1435	6	-9	2	1	1.1	0.9	1.0	1.0
GSTK1	Q9Y2Q3	176	6	-10	2	-5	1.1	0.9	1.0	1.0
TRAPPC11	Q7Z392	162	6	-11	2	4	1.1	0.9	1.0	1.0
CDYL	Q9Y232	515	6	-12	2	-8	1.1	0.9	1.0	0.9
ACTB	P60709	217	6	-16	2	3	1.1	0.9	1.0	1.0
POTEE	Q6S8J3	917	6	-16	2	3	1.1	0.9	1.0	1.0
ROCK2	O75116	428	5	7	2	-8	1.1	1.1	1.0	0.9
UBA7	P41226	599	5	6	2	8	1.1	1.1	1.0	1.1
TRIM22	Q8IYM9	441	5	0	2	1	1.1	1.0	1.0	1.0
C16orf13	Q96S19	118	5	0	2	-4	1.1	1.0	1.0	1.0
FDFT1	P37268	374	5	-5	2	-15	1.1	1.0	1.0	0.9
C1orf50	Q9BV19	144	5	-7	2	1	1.1	0.9	1.0	1.0
ITPR2	Q14571	1522	5	-28	2	-16	1.1	0.8	1.0	0.9
PIK3CG	P48736	219	5	4	2	2	1.0	1.0	1.0	1.0
VPS51	Q9UID3	421	5	-5	2	10	1.0	1.0	1.0	1.1
MYH9	P35579	1834	5	-5	2	5	1.0	1.0	1.0	1.0
MKLN1	Q9UL63	435	5	-6	2	-9	1.0	0.9	1.0	0.9
DHX30	Q7L2E3	461	5	-6	2	-4	1.0	0.9	1.0	1.0
SORD	Q00796	120	5	-18	2	4	1.0	0.9	1.0	1.0
EED	O75530	182	4	1	2	1	1.0	1.0	1.0	1.0
PHGDH	O43175	18	4	-2	2	1	1.0	1.0	1.0	1.0
SMAP2	Q8WU79	196	4	-2	2	-8	1.0	1.0	1.0	0.9
RAD21	O60216	585	4	-4	2	-17	1.0	1.0	1.0	0.9
PDIA4	P13667	555	4	-5	2	-8	1.0	1.0	1.0	0.9
HGS	O14964	190	4	-6	2	-4	1.0	0.9	1.0	1.0
SLC27A4	Q6P1M0	560	4	-7	2	-3	1.0	0.9	1.0	1.0
ANKHD1	Q8IWZ3	1716	4	-9	2	-14	1.0	0.9	1.0	0.9
STX16-NPEPL	H3BU86	279	4	-12	2	-11	1.0	0.9	1.0	0.9
ACO2	Q99798	126	4	-24	2	-18	1.0	0.8	1.0	0.8
PTPN23	Q9H3S7	1445	4	6	2	-8	1.0	1.1	1.0	0.9
APPL1	Q9UKG1	551	4	2	2	-2	1.0	1.0	1.0	1.0
BAZ2B	Q9UIF8	776	4	-1	2	-3	1.0	1.0	1.0	1.0
LIMS1	P48059	275	4	-1	2	5	1.0	1.0	1.0	1.1
GMPPB	Q9Y5P6	245	4	-5	2	3	1.0	1.0	1.0	1.0
STK11IP	Q8N1F8	133	4	-9	2	-6	1.0	0.9	1.0	0.9
NME2	P22392	145	4	-11	2	-6	1.0	0.9	1.0	0.9
DDX6	P26196	390	4	-12	2	-9	1.0	0.9	1.0	0.9
NXT1	Q9UKK6	14	4	-16	2	-3	1.0	0.9	1.0	1.0
SLC27A1	Q6PCB7	640	4	-21	2	-8	1.0	0.8	1.0	0.9
PRKDC	P78527	1128	4	-39	2	3	1.0	0.7	1.0	1.0
RBM22	Q9NW64	84	3	3	2	4	1.0	1.0	1.0	1.0
TET3	O43151	693	3	3	2	4	1.0	1.0	1.0	1.0
EPRS	P07814	856	3	3	2	1	1.0	1.0	1.0	1.0
UBE3C	Q15386	1051	3	1	2	-8	1.0	1.0	1.0	0.9

PDE12	Q6L8Q7	277	3	-3	2	15	1.0	1.0	1.0	1.2
PTPRC	P08575	398	3	-7	2	-5	1.0	0.9	1.0	1.0
MYSM1	Q5VVJ2	440	3	-13	2	-23	1.0	0.9	1.0	0.8
PRDX5	P30044	100	3	-2	2	4	1.0	1.0	1.0	1.0
MMRN1	Q13201	315	3	-2	2	-10	1.0	1.0	1.0	0.9
PSMD7	P51665	116	3	-4	2	1	1.0	1.0	1.0	1.0
DPYD	Q12882	684	3	-6	2	-3	1.0	0.9	1.0	1.0
FLNA	P21333	1912	3	-12	2	2	1.0	0.9	1.0	1.0
DAGLB	Q8NCG7	310	3	-27	2	2	1.0	0.8	1.0	1.0
KRIT1	O00522	134	2	2	2	-1	1.0	1.0	1.0	1.0
PPP6R2	O75170	511	2	1	2	-3	1.0	1.0	1.0	1.0
LSP1	P33241	283	2	-2	2	-2	1.0	1.0	1.0	1.0
KCNAB2	Q13303	248	2	-3	2	9	1.0	1.0	1.0	1.1
HUWE1	Q7Z6Z7	3658	2	-11	2	-10	1.0	0.9	1.0	0.9
SRBD1	Q8N5C6	502	2	-15	2	13	1.0	0.9	1.0	1.1
LRRC8D	Q7L1W4	786	2	-18	2	6	1.0	0.8	1.0	1.1
MAP2K1	Q02750	376	2	19	2	-3	1.0	1.2	1.0	1.0
Uncharacteri	U3KPZ7	785	2	3	2	2	1.0	1.0	1.0	1.0
RPS6KB2	Q9UBS0	66	2	-1	2	1	1.0	1.0	1.0	1.0
NSDHL	Q15738	86	2	-2	2	-2	1.0	1.0	1.0	1.0
DDX20	Q9UHI6	577	2	-4	2	-9	1.0	1.0	1.0	0.9
RABL6	H0Y4Z8	492	2	-6	2	-7	1.0	0.9	1.0	0.9
PRKCD	Q05655	393	2	-7	2	-5	1.0	0.9	1.0	1.0
KARS	Q15046	496	2	-10	2	8	1.0	0.9	1.0	1.1
ARFGEF2	Q9Y6D5	1661	2	-12	2	5	1.0	0.9	1.0	1.1
WDR81	Q562E7	1226	2	-12	2	-6	1.0	0.9	1.0	0.9
USP16	Q9Y5T5	286	1	-3	2	-3	1.0	1.0	1.0	1.0
CAPNS1	P04632	190	1	-4	2	-4	1.0	1.0	1.0	1.0
SDHAF3	Q9NRP4	80	1	-4	2	-3	1.0	1.0	1.0	1.0
ADRBK2	P35626	120	1	-5	2	15	1.0	1.0	1.0	1.2
DHRS4	Q9BTZ2	209	1	-5	2	-2	1.0	1.0	1.0	1.0
POLDIP3	Q9BY77	301	1	-6	2	2	1.0	0.9	1.0	1.0
CSTF2	P33240	150	1	-10	2	-5	1.0	0.9	1.0	1.0
RENBP	P51606	423	1	-1	2	-7	1.0	1.0	1.0	0.9
IMP3	Q9NV31	107	1	-6	2	-1	1.0	0.9	1.0	1.0
TRPM7	Q96QT4	29	1	-6	2	-10	1.0	0.9	1.0	0.9
PTPRE	P23469	132	1	-11	2	6	1.0	0.9	1.0	1.1
GCN1	Q92616	2179	1	-12	2	-8	1.0	0.9	1.0	0.9
ALDH5A1	P51649	110	1	-13	2	-1	1.0	0.9	1.0	1.0
NADSYN1	Q6IA69	686	1	-14	2	-1	1.0	0.9	1.0	1.0
ARHGAP30	Q7Z6I6	449	0	-4	2	-8	1.0	1.0	1.0	0.9
EPRS	P07814	1497	0	-5	2	6	1.0	1.0	1.0	1.1
ELF2	Q15723	470	0	-5	2	-10	1.0	1.0	1.0	0.9
PI4KA	P42356	19	0	-9	2	-8	1.0	0.9	1.0	0.9
PLXNB2	O15031	1408	0	-14	2	-4	1.0	0.9	1.0	1.0
INTS3	Q68E01	843	-1	1	2	-1	1.0	1.0	1.0	1.0
ATRX	P46100	1789	-1	0	2	-9	1.0	1.0	1.0	0.9
RAF1	P04049	95	-1	-2	2	-11	1.0	1.0	1.0	0.9
ACOT9	Q9Y305	299	-1	-5	2	-10	1.0	1.0	1.0	0.9
VWA8	A3KMH1	416	-1	-8	2	-13	1.0	0.9	1.0	0.9
CDK4	P11802	135	-1	-8	2	-13	1.0	0.9	1.0	0.9
TSN	Q15631	225	-1	-8	2	-4	1.0	0.9	1.0	1.0
OSBPL7	Q9BZF2	553	-1	-13	2	11	1.0	0.9	1.0	1.1

WDR7	Q9Y4E6	336	-1	-14	2	-3	1.0	0.9	1.0	1.0
GEMIN7	Q9H840	44	-1	-15	2	-11	1.0	0.9	1.0	0.9
ZC3H13	Q5T200	42	-1	-3	2	-5	1.0	1.0	1.0	1.0
SRSF5	Q13243	63	-1	-4	2	1	1.0	1.0	1.0	1.0
MKLN1	Q9UL63	411	-1	-7	2	-10	1.0	0.9	1.0	0.9
CXorf38	Q8TB03	12	-1	-8	2	-5	1.0	0.9	1.0	1.0
ZNF512	Q96ME7	413	-2	-1	2	7	1.0	1.0	1.0	1.1
FHL2	Q14192	150	-2	-4	2	-5	1.0	1.0	1.0	1.0
MBD1	Q9UIS9	136	-2	-9	2	1	1.0	0.9	1.0	1.0
DTX3L	Q8TDB6	175	-2	-11	2	-9	1.0	0.9	1.0	0.9
ADAT3	Q96EY9	216	-2	19	2	20	1.0	1.2	1.0	1.2
GMEB1	Q9Y692	274	-2	-2	2	-9	1.0	1.0	1.0	0.9
BIN2	Q9UBW5	496	-2	-5	2	-13	1.0	1.0	1.0	0.9
PSME1	Q06323	22	-2	-9	2	38	1.0	0.9	1.0	1.6
DAGLB	Q8NCG7	661	-2	-10	2	-10	1.0	0.9	1.0	0.9
SYNE1	Q8NF91	4879	-2	-19	2	-3	1.0	0.8	1.0	1.0
KDM3A	Q9Y4C1	953	-3	7	2	-4	1.0	1.1	1.0	1.0
TSC22D4	Q9Y3Q8	92	-3	-9	2	0	1.0	0.9	1.0	1.0
BIRC6	Q9NR09	396	-3	-16	2	9	1.0	0.9	1.0	1.1
PLSCR1	O15162	148	-3	-5	2	22	1.0	1.0	1.0	1.3
GNB1L	Q9BYB4	109	-3	-6	2	-8	1.0	0.9	1.0	0.9
MVD	P53602	160	-3	-13	2	5	1.0	0.9	1.0	1.1
TPM4	P67936	247	-4	2	2	-4	1.0	1.0	1.0	1.0
GLS	O94925	525	-4	-11	2	8	1.0	0.9	1.0	1.1
WDR70	Q9NW82	448	-4	-11	2	-6	1.0	0.9	1.0	0.9
TRMT2A	Q8IZ69	551	-4	10	2	4	1.0	1.1	1.0	1.0
APOBR	Q0VD83	129	-4	-12	2	-11	1.0	0.9	1.0	0.9
THAP11	Q96EK4	48	-5	1	2	-23	1.0	1.0	1.0	0.8
ATP13A1	Q9HD20	711	-5	-9	2	-5	1.0	0.9	1.0	1.0
MATR3	A8MXP9	230	-5	-12	2	-13	1.0	0.9	1.0	0.9
PDCD1	Q15116	93	-5	-5	2	-6	1.0	1.0	1.0	0.9
MIPEP	Q99797	142	-5	-14	2	-2	1.0	0.9	1.0	1.0
PARP4	Q9UKK3	1488	-6	1	2	-8	0.9	1.0	1.0	0.9
MXI1	P50539	100	-6	-1	2	-11	0.9	1.0	1.0	0.9
CUTC	Q9NTM9	174	-6	-10	2	22	0.9	0.9	1.0	1.3
TRMT13	Q9NUP7	59	-8	-3	2	1	0.9	1.0	1.0	1.0
NDOR1	Q9UHB4	486	-8	-5	2	1	0.9	1.0	1.0	1.0
SIGIRR	Q6IA17	238	-8	-4	2	18	0.9	1.0	1.0	1.2
EIF2B3	Q9NR50	281	-8	-9	2	-5	0.9	0.9	1.0	1.0
TUBA8	Q9NY65	295	-9	-7	2	18	0.9	0.9	1.0	1.2
TLK1	Q9UKI8	81	-10	-8	2	-11	0.9	0.9	1.0	0.9
PLEKHM1	Q9Y4G2	555	-10	-15	2	-4	0.9	0.9	1.0	1.0
DHX29	Q7Z478	1245	-11	-22	2	-7	0.9	0.8	1.0	0.9
ATG9A	Q7Z3C6	433	-11	-10	2	-10	0.9	0.9	1.0	0.9
PML	P29590	207	-12	0	2	5	0.9	1.0	1.0	1.0
MAP4K3	Q8IVH8	720	-13	-5	2	-4	0.9	1.0	1.0	1.0
AIM1	Q9Y4K1	1574	-13	-13	2	-13	0.9	0.9	1.0	0.9
SYNE1	Q8NF91	8703	-14	-4	2	-11	0.9	1.0	1.0	0.9
ZNF548	Q8NEK5	208	-14	-33	2	-7	0.9	0.8	1.0	0.9
AKR7A2	O43488	132	-16	-19	2	-16	0.9	0.8	1.0	0.9
PYURF	Q96I23	60	-18	-12	2	-1	0.8	0.9	1.0	1.0
CCT5	P48643	181	-19	-8	2	-11	0.8	0.9	1.0	0.9
BAG5	Q9UL15	360	-24	-38	2	-45	0.8	0.7	1.0	0.7

PNPO	Q9NVS9	156	-169	-32	2	-8	0.4	0.8	1.0	0.9
MCCC1	Q96RQ3	509	27	-22	1	8	1.4	0.8	1.0	1.1
PDHA1	P08559	273	26	-4	1	9	1.3	1.0	1.0	1.1
CCT4	P50991	295	22	-4	1	9	1.3	1.0	1.0	1.1
NDUFB9	Q9Y6M9	31	20	-7	1	8	1.3	0.9	1.0	1.1
GPS1	Q13098	251	19	-14	1	-8	1.2	0.9	1.0	0.9
TUBA3D	Q13748	316	19	13	1	-7	1.2	1.1	1.0	0.9
TUBA8	Q9NY65	316	19	13	1	-7	1.2	1.1	1.0	0.9
ATP1A1	P05023	463	19	-9	1	4	1.2	0.9	1.0	1.0
PDIA6	Q15084	291	18	-5	1	9	1.2	1.0	1.0	1.1
YWHAQ	P27348	94	18	-21	1	15	1.2	0.8	1.0	1.2
MYLK	Q15746	1524	18	-12	1	-1	1.2	0.9	1.0	1.0
MACF1	Q9UPN3	222	18	-13	1	-6	1.2	0.9	1.0	0.9
RFC1	P35251	752	17	-4	1	5	1.2	1.0	1.0	1.0
PDCD6IP	Q8WUM4	691	17	-27	1	19	1.2	0.8	1.0	1.2
RNH1	P13489	102	16	0	1	13	1.2	1.0	1.0	1.1
MTMR9	Q96QG7	380	16	-17	1	-4	1.2	0.9	1.0	1.0
BZW1	Q7L1Q6	96	16	10	1	8	1.2	1.1	1.0	1.1
RRP12	Q5JTH9	517	16	6	1	4	1.2	1.1	1.0	1.0
NBAS	A2RRP1	1594	15	-1	1	2	1.2	1.0	1.0	1.0
KPNA4	O00629	417	15	-16	1	7	1.2	0.9	1.0	1.1
PTBP1	P26599	251	14	2	1	6	1.2	1.0	1.0	1.1
FAM3C	Q92520	185	14	2	1	4	1.2	1.0	1.0	1.0
MARCH1	Q8TCQ1	241	14	-3	1	5	1.2	1.0	1.0	1.0
SLFN5	Q08AF3	601	14	10	1	3	1.2	1.1	1.0	1.0
NUP205	Q92621	141	14	-16	1	3	1.2	0.9	1.0	1.0
RENBP	P51606	249	13	-2	1	-3	1.1	1.0	1.0	1.0
TNPO1	Q92973	142	13	-3	1	1	1.1	1.0	1.0	1.0
ATP2B4	P23634	710	13	-14	1	11	1.1	0.9	1.0	1.1
TMEM120A	Q9BXJ8	47	13	-25	1	-4	1.1	0.8	1.0	1.0
CD99	P14209	154	13	3	1	-4	1.1	1.0	1.0	1.0
IVNS1ABP	Q9Y6Y0	575	12	-14	1	-7	1.1	0.9	1.0	0.9
PRKDC	P78527	457	12	-30	1	8	1.1	0.8	1.0	1.1
TRRAP	Q9Y4A5	2513	12	20	1	21	1.1	1.3	1.0	1.3
FAM129A	Q9BZQ8	516	12	-13	1	-4	1.1	0.9	1.0	1.0
DIS3	Q9Y2L1	194	11	-9	1	2	1.1	0.9	1.0	1.0
ADH5	P11766	170	11	-13	1	1	1.1	0.9	1.0	1.0
PRKDC	P78527	1742	11	-64	1	3	1.1	0.6	1.0	1.0
VARS	P26640	444	10	-6	1	-15	1.1	0.9	1.0	0.9
PARP10	Q53GL7	494	10	-15	1	1	1.1	0.9	1.0	1.0
GARS	P41250	616	10	-8	1	1	1.1	0.9	1.0	1.0
PAFAH2	Q99487	40	9	2	1	12	1.1	1.0	1.0	1.1
MAP3K3	Q99759	411	9	2	1	-14	1.1	1.0	1.0	0.9
SYNE2	Q8WXH0	90	9	-5	1	-13	1.1	1.0	1.0	0.9
SMCHD1	A6NHR9	61	9	-9	1	11	1.1	0.9	1.0	1.1
TRIM28	Q13263	65	9	-13	1	-6	1.1	0.9	1.0	0.9
SRRT	Q9BXP5	441	9	-17	1	-6	1.1	0.9	1.0	0.9
RPF1	Q9H9Y2	187	9	-18	1	-12	1.1	0.9	1.0	0.9
TAB1	Q15750	235	9	-1	1	5	1.1	1.0	1.0	1.0
RPL10	P27635	195	9	-2	1	-13	1.1	1.0	1.0	0.9
BCKDHB	P21953	235	9	-3	1	2	1.1	1.0	1.0	1.0
SUGP2	Q8IX01	381	9	-3	1	-13	1.1	1.0	1.0	0.9
TUBB1	Q9H4B7	315	9	-4	1	-1	1.1	1.0	1.0	1.0

YTHDC2	Q9H6S0	460	9	-5	1	-11	1.1	1.0	1.0	0.9
TLN1	Q9Y490	956	9	-8	1	2	1.1	0.9	1.0	1.0
PIK3R6	Q5UE93	593	8	-1	1	13	1.1	1.0	1.0	1.1
PIK3CD	O00329	991	8	-1	1	-5	1.1	1.0	1.0	1.0
MARCH7	Q9H992	604	8	-4	1	1	1.1	1.0	1.0	1.0
XPNPEP1	Q9NQW7	316	8	-6	1	-3	1.1	0.9	1.0	1.0
PIK3AP1	Q6ZUJ8	147	8	-9	1	-14	1.1	0.9	1.0	0.9
DENND3	A2RUS2	71	8	-11	1	-5	1.1	0.9	1.0	1.0
PTPN12	Q05209	164	8	-6	1	-2	1.1	0.9	1.0	1.0
AGTPBP1	Q9UPW5	558	8	-6	1	-16	1.1	0.9	1.0	0.9
NCKAP1L	P55160	1032	8	-7	1	9	1.1	0.9	1.0	1.1
COQ5	Q5HYK3	244	8	-8	1	2	1.1	0.9	1.0	1.0
SAFB2	Q14151	449	7	10	1	-8	1.1	1.1	1.0	0.9
TBC1D13	Q9NVG8	131	7	-4	1	-1	1.1	1.0	1.0	1.0
SBF1	O95248	1374	7	-5	1	-4	1.1	1.0	1.0	1.0
ZAP70	P43403	249	7	-7	1	-6	1.1	0.9	1.0	0.9
GNB1	P62873	148	7	-14	1	11	1.1	0.9	1.0	1.1
PRKDC	P78527	3014	7	-51	1	5	1.1	0.7	1.0	1.0
EPC2	Q52LR7	446	7	6	1	-2	1.1	1.1	1.0	1.0
EPC1	Q9H2F5	451	7	6	1	-2	1.1	1.1	1.0	1.0
RNH1	P13489	220	7	0	1	3	1.1	1.0	1.0	1.0
UBR4	Q5T4S7	3765	7	-1	1	21	1.1	1.0	1.0	1.3
UBE4B	O95155	829	7	-2	1	-7	1.1	1.0	1.0	0.9
MACF1	Q9UPN3	3085	7	-3	1	-5	1.1	1.0	1.0	1.0
TRMT10C	Q7L0Y3	78	7	-4	1	-16	1.1	1.0	1.0	0.9
GNB2	P62879	148	7	-5	1	-2	1.1	1.0	1.0	1.0
GNB4	Q9HAV0	148	7	-5	1	-2	1.1	1.0	1.0	1.0
CDK2AP1	O14519	105	7	-8	1	5	1.1	0.9	1.0	1.0
PARP14	Q460N5	509	7	-8	1	-2	1.1	0.9	1.0	1.0
CSRP2BP	Q9H8E8	547	7	-11	1	-11	1.1	0.9	1.0	0.9
EVI5L	Q96CN4	342	7	-14	1	-2	1.1	0.9	1.0	1.0
HTATSF1	O43719	512	7	-16	1	-28	1.1	0.9	1.0	0.8
GTF2H2	Q13888	299	6	-1	1	-1	1.1	1.0	1.0	1.0
ATAD2B	Q9ULI0	53	6	-2	1	2	1.1	1.0	1.0	1.0
POLR2D	O15514	104	6	-3	1	-1	1.1	1.0	1.0	1.0
RBM39	Q14498	303	6	-5	1	1	1.1	1.0	1.0	1.0
PIK3CB	P42338	100	6	-6	1	12	1.1	0.9	1.0	1.1
UBE2R2	Q712K3	191	6	-7	1	-7	1.1	0.9	1.0	0.9
FLNA	P21333	2293	6	-8	1	1	1.1	0.9	1.0	1.0
UPF2	Q9HAU5	578	6	-10	1	-5	1.1	0.9	1.0	1.0
UBE2O	Q9C0C9	375	6	3	1	-11	1.1	1.0	1.0	0.9
CPT1A	P50416	608	6	-8	1	-2	1.1	0.9	1.0	1.0
SECISBP2L	Q93073	683	6	-11	1	-11	1.1	0.9	1.0	0.9
ARIH1	Q9Y4X5	304	6	-13	1	-3	1.1	0.9	1.0	1.0
CUL4A	Q13619	94	6	-15	1	-5	1.1	0.9	1.0	1.0
CUL5	Q93034	112	5	-2	1	-8	1.1	1.0	1.0	0.9
SYNE2	Q8WXH0	1235	5	-5	1	-1	1.1	1.0	1.0	1.0
ZNF490	Q9ULM2	317	5	-7	1	0	1.1	0.9	1.0	1.0
FGD3	Q5JSP0	541	5	-7	1	-3	1.1	0.9	1.0	1.0
NCF4	Q15080	84	5	-7	1	-6	1.1	0.9	1.0	0.9
IARS2	Q9NSE4	883	5	-10	1	0	1.1	0.9	1.0	1.0
PLS3	P13797	143	5	-12	1	7	1.1	0.9	1.0	1.1
LCP1	P13796	140	5	-12	1	7	1.1	0.9	1.0	1.1

RHOG	P84095	22	5	-17	1	-1	1.1	0.9	1.0	1.0
MCM3	P25205	119	5	13	1	5	1.0	1.1	1.0	1.1
CASP3	P42574	170	5	-2	1	-9	1.0	1.0	1.0	0.9
ANAPC7	Q9UJX3	259	5	-2	1	2	1.0	1.0	1.0	1.0
ATP13A1	Q9HD20	723	5	-2	1	-1	1.0	1.0	1.0	1.0
PLCG1	P19174	1088	5	-6	1	5	1.0	0.9	1.0	1.1
GBP2	P32456	394	5	-7	1	-2	1.0	0.9	1.0	1.0
KTN1	Q86UP2	1105	5	-9	1	-13	1.0	0.9	1.0	0.9
ZNF185	O15231	681	5	-10	1	-2	1.0	0.9	1.0	1.0
KLHL36	Q8N4N3	308	4	3	1	9	1.0	1.0	1.0	1.1
C1orf198	Q9H425	73	4	2	1	-6	1.0	1.0	1.0	0.9
DDX24	Q9GZR7	380	4	-5	1	-2	1.0	1.0	1.0	1.0
PDCD6IP	Q8WUM4	512	4	-6	1	-4	1.0	0.9	1.0	1.0
PTK2B	Q14289	972	4	-6	1	13	1.0	0.9	1.0	1.1
DARS	P14868	259	4	-7	1	-1	1.0	0.9	1.0	1.0
BRAT1	Q6PJG6	487	4	-7	1	-2	1.0	0.9	1.0	1.0
RAPGEF1	Q13905	474	4	-10	1	-13	1.0	0.9	1.0	0.9
SURF2	Q15527	29	4	-12	1	3	1.0	0.9	1.0	1.0
ACADS	P16219	246	4	-13	1	7	1.0	0.9	1.0	1.1
CUTA	O60888	96	4	6	1	-3	1.0	1.1	1.0	1.0
ADO	Q96SZ5	239	4	2	1	-7	1.0	1.0	1.0	0.9
FUK	Q8N0W3	779	4	-2	1	-1	1.0	1.0	1.0	1.0
NF1	P21359	124	4	-2	1	-10	1.0	1.0	1.0	0.9
ANXA1	P04083	343	4	-4	1	-3	1.0	1.0	1.0	1.0
LYPLA1	O75608	169	4	-5	1	5	1.0	1.0	1.0	1.0
Uncharacteri	E9PCH4	1541	4	-5	1	-2	1.0	1.0	1.0	1.0
KALRN	O60229	2083	4	-8	1	-1	1.0	0.9	1.0	1.0
CDYL2	Q8N8U2	288	4	-8	1	-11	1.0	0.9	1.0	0.9
CDK5	Q00535	94	4	-13	1	4	1.0	0.9	1.0	1.0
PRMT1	Q99873	240	4	-13	1	-8	1.0	0.9	1.0	0.9
PLEC	Q15149	1098	3	5	1	12	1.0	1.1	1.0	1.1
DNM2	P50570	607	3	2	1	0	1.0	1.0	1.0	1.0
MECR	Q9BV79	263	3	0	1	3	1.0	1.0	1.0	1.0
DGKA	P23743	95	3	-2	1	-12	1.0	1.0	1.0	0.9
PCK2	Q16822	306	3	-3	1	1	1.0	1.0	1.0	1.0
PAF1	Q8N7H5	31	3	-4	1	3	1.0	1.0	1.0	1.0
CORO7-PAM:	A0A0A6YYL4	505	3	-6	1	3	1.0	0.9	1.0	1.0
UBA1	P22314	632	3	-6	1	-3	1.0	0.9	1.0	1.0
DCTD	P32321	83	3	-6	1	3	1.0	0.9	1.0	1.0
DUS2	Q9NX74	116	3	-6	1	-8	1.0	0.9	1.0	0.9
TCEA1	P23193	212	3	-7	1	-3	1.0	0.9	1.0	1.0
FUK	Q8N0W3	582	3	-7	1	-17	1.0	0.9	1.0	0.9
PDS5A	Q29RF7	583	3	-8	1	2	1.0	0.9	1.0	1.0
ZNF598	Q86UK7	32	3	-14	1	2	1.0	0.9	1.0	1.0
PNPLA2	Q96AD5	412	3	-14	1	-18	1.0	0.9	1.0	0.8
UIMC1	Q96RL1	601	3	2	1	1	1.0	1.0	1.0	1.0
KDM8	Q8N371	164	3	-3	1	-9	1.0	1.0	1.0	0.9
PAPOLA	P51003	36	3	-3	1	9	1.0	1.0	1.0	1.1
MAU2	Q9Y6X3	252	3	-4	1	15	1.0	1.0	1.0	1.2
OTUD6B	Q8N6M0	292	3	-5	1	-14	1.0	1.0	1.0	0.9
MROH1	Q8NDA8	1394	3	-6	1	1	1.0	0.9	1.0	1.0
LANCL1	O43813	108	3	-8	1	-12	1.0	0.9	1.0	0.9
USP4	Q13107	624	3	-9	1	-6	1.0	0.9	1.0	0.9

BIRC6	Q9NR09	381	3	-17	1	21	1.0	0.9	1.0	1.3
SNX22	Q96L94	33	2	6	1	0	1.0	1.1	1.0	1.0
VPS39	Q96JC1	681	2	-1	1	-5	1.0	1.0	1.0	1.0
PPP1R18	Q6NYC8	611	2	-4	1	-7	1.0	1.0	1.0	0.9
PABPN1	Q86U42	205	2	-5	1	3	1.0	1.0	1.0	1.0
GAB2	Q9UQC2	303	2	-7	1	-13	1.0	0.9	1.0	0.9
CYBB	P04839	85	2	-7	1	3	1.0	0.9	1.0	1.0
STIP1	P31948	461	2	-8	1	-3	1.0	0.9	1.0	1.0
SCLY	Q96I15	22	2	-10	1	-10	1.0	0.9	1.0	0.9
PIK3CG	P48736	817	2	-23	1	-8	1.0	0.8	1.0	0.9
CNTRL	Q7Z7A1	1781	2	1	1	-2	1.0	1.0	1.0	1.0
CDK5	Q00535	290	2	1	1	-4	1.0	1.0	1.0	1.0
APOBR	Q0VD83	257	2	0	1	3	1.0	1.0	1.0	1.0
DHX29	Q7Z478	670	2	0	1	3	1.0	1.0	1.0	1.0
GFPT1	Q06210	55	2	-1	1	6	1.0	1.0	1.0	1.1
GFPT1	Q06210	254	2	-1	1	-14	1.0	1.0	1.0	0.9
RPS3	P23396	97	2	-6	1	2	1.0	0.9	1.0	1.0
MMAB	Q96EY8	132	2	-6	1	0	1.0	0.9	1.0	1.0
BCAS3	Q9H6U6	258	2	-7	1	0	1.0	0.9	1.0	1.0
PNKD	Q8N490	209	2	-7	1	-14	1.0	0.9	1.0	0.9
VAT1	Q99536	86	2	-7	1	3	1.0	0.9	1.0	1.0
GOLPH3L	Q9H4A5	70	2	-9	1	-3	1.0	0.9	1.0	1.0
GOLPH3	Q9H4A6	84	2	-9	1	-3	1.0	0.9	1.0	1.0
AGPS	O00116	565	2	-9	1	-4	1.0	0.9	1.0	1.0
ALDH2	P05091	386	2	-9	1	7	1.0	0.9	1.0	1.1
KIAA1429	Q69YN4	353	2	-11	1	-10	1.0	0.9	1.0	0.9
ILK	Q13418	422	1	0	1	0	1.0	1.0	1.0	1.0
ARFRP1	Q13795	125	1	-1	1	-1	1.0	1.0	1.0	1.0
FNDC3A	Q9Y2H6	1005	1	-3	1	-4	1.0	1.0	1.0	1.0
PSMC4	P43686	379	1	-4	1	-12	1.0	1.0	1.0	0.9
SYMPK	Q92797	578	1	-5	1	-8	1.0	1.0	1.0	0.9
TES	Q9UGI8	164	1	-7	1	2	1.0	0.9	1.0	1.0
DOCK9	Q9BZ29	94	1	-12	1	-15	1.0	0.9	1.0	0.9
WDR81	Q562E7	207	1	1	1	-9	1.0	1.0	1.0	0.9
MAP4K1	Q92918	484	1	1	1	9	1.0	1.0	1.0	1.1
C18orf32	Q8TCD1	71	1	-2	1	-5	1.0	1.0	1.0	1.0
IPO9	Q96P70	606	1	-6	1	7	1.0	0.9	1.0	1.1
PFKL	P17858	653	1	-6	1	2	1.0	0.9	1.0	1.0
WDFY4	Q6ZS81	1696	1	-8	1	-11	1.0	0.9	1.0	0.9
DST	Q03001	3653	1	-11	1	-7	1.0	0.9	1.0	0.9
BOLA1	Q9Y3E2	126	1	-14	1	-9	1.0	0.9	1.0	0.9
NCOR2	Q9Y618	1511	0	-1	1	4	1.0	1.0	1.0	1.0
NEK9	Q8TD19	892	0	-2	1	-1	1.0	1.0	1.0	1.0
ATE1	O95260	429	0	-2	1	4	1.0	1.0	1.0	1.0
TSEN15	Q8WW01	13	0	-6	1	-6	1.0	0.9	1.0	0.9
NUP62	P37198	506	0	-8	1	-2	1.0	0.9	1.0	1.0
FYCO1	Q9BQS8	1206	-1	1	1	-10	1.0	1.0	1.0	0.9
MGA	Q8IWI9	1056	-1	-3	1	-5	1.0	1.0	1.0	1.0
CAPN7	Q9Y6W3	242	-1	-4	1	3	1.0	1.0	1.0	1.0
MROH1	Q8NDA8	637	-1	-5	1	-4	1.0	1.0	1.0	1.0
NUDT8	Q8WV74	224	-1	-6	1	-18	1.0	0.9	1.0	0.9
PIK3AP1	Q6ZUJ8	358	-1	-7	1	1	1.0	0.9	1.0	1.0
C10orf12	Q8N655	1236	-1	-7	1	4	1.0	0.9	1.0	1.0

SNAP23	O00161	79	-1	-8	1	8	1.0	0.9	1.0	1.1
SNAPIN	O95295	66	-1	-9	1	-3	1.0	0.9	1.0	1.0
CARS	P49589	182	-1	-15	1	-5	1.0	0.9	1.0	1.0
GEMIN5	Q8TEQ6	240	-1	-1	1	11	1.0	1.0	1.0	1.1
IBA57	Q5T440	170	-1	-2	1	-6	1.0	1.0	1.0	0.9
GALK1	P51570	243	-1	-4	1	6	1.0	1.0	1.0	1.1
PNPLA8	Q9NP80	714	-1	-4	1	-2	1.0	1.0	1.0	1.0
CSTF2T	Q9H0L4	150	-1	-5	1	1	1.0	1.0	1.0	1.0
ZMYM2	Q9UBW7	750	-1	-6	1	11	1.0	0.9	1.0	1.1
STK4	Q13043	80	-1	-7	1	-17	1.0	0.9	1.0	0.9
FMNL2	Q96PY5	65	-1	-11	1	-7	1.0	0.9	1.0	0.9
EXOC2	Q96KP1	586	-1	-12	1	-14	1.0	0.9	1.0	0.9
FMR1	Q06787	77	-2	-4	1	-1	1.0	1.0	1.0	1.0
GOLGB1	Q14789	2664	-2	-5	1	-11	1.0	1.0	1.0	0.9
WDTC1	Q8N5D0	431	-2	-8	1	-6	1.0	0.9	1.0	0.9
ZNF845	Q96IR2	24	-2	-9	1	-7	1.0	0.9	1.0	0.9
AHCYL1	O43865	293	-2	-11	1	4	1.0	0.9	1.0	1.0
ZNF618	Q5T7W0	619	-2	-14	1	-16	1.0	0.9	1.0	0.9
MAPK3	P27361	82	-2	4	1	-3	1.0	1.0	1.0	1.0
MAPK1	P28482	65	-2	4	1	-3	1.0	1.0	1.0	1.0
ATG4C	Q96DT6	111	-2	-1	1	-2	1.0	1.0	1.0	1.0
UFC1	Q9Y3C8	116	-2	-3	1	-3	1.0	1.0	1.0	1.0
DOCK8	Q8NF50	259	-2	-4	1	-1	1.0	1.0	1.0	1.0
MDN1	Q9NU22	1011	-2	-13	1	-14	1.0	0.9	1.0	0.9
SP2	Q02086	384	-3	-2	1	4	1.0	1.0	1.0	1.0
MBD1	Q9UIS9	57	-3	-21	1	-5	1.0	0.8	1.0	1.0
CWF19L1	Q69YN2	87	-3	-23	1	-5	1.0	0.8	1.0	1.0
OGT	O15294	323	-3	-2	1	-7	1.0	1.0	1.0	0.9
ANKRD10	Q9NXR5	73	-3	-5	1	-10	1.0	1.0	1.0	0.9
TEP1	Q99973	360	-3	-9	1	-14	1.0	0.9	1.0	0.9
DGKA	P23743	238	-3	-10	1	-2	1.0	0.9	1.0	1.0
MTMR12	Q9C0I1	236	-3	-16	1	-7	1.0	0.9	1.0	0.9
DGKA	P23743	222	-4	-5	1	-6	1.0	1.0	1.0	0.9
TRAP1	Q12931	573	-4	-13	1	-24	1.0	0.9	1.0	0.8
CTSB	P07858	211	-4	-16	1	-8	1.0	0.9	1.0	0.9
RPS2	P15880	229	-5	-8	1	3	1.0	0.9	1.0	1.0
KIF13B	Q9NQT8	1234	-6	-11	1	3	0.9	0.9	1.0	1.0
CMPK2	Q5EBM0	153	-6	-11	1	34	0.9	0.9	1.0	1.5
SYNE1	Q8NF91	1074	-6	-13	1	-8	0.9	0.9	1.0	0.9
HADHB	P55084	435	-7	-12	1	11	0.9	0.9	1.0	1.1
NUP188	Q5SRE5	1433	-7	-19	1	8	0.9	0.8	1.0	1.1
SYNE2	Q8WXH0	6462	-7	-26	1	-31	0.9	0.8	1.0	0.8
BACH1	O14867	621	-7	-7	1	-6	0.9	0.9	1.0	0.9
CPSF1	Q10570	1044	-7	-10	1	-6	0.9	0.9	1.0	0.9
AKNA	Q7Z591	1078	-8	4	1	-8	0.9	1.0	1.0	0.9
PARG	Q86W56	397	-9	-7	1	-13	0.9	0.9	1.0	0.9
TRAPPC12	Q8WVT3	160	-9	6	1	-11	0.9	1.1	1.0	0.9
ATG16L1	Q676U5	145	-9	-23	1	-27	0.9	0.8	1.0	0.8
PMM1	Q92871	57	-10	-11	1	0	0.9	0.9	1.0	1.0
C19orf35	Q6ZS72	318	-10	3	1	-4	0.9	1.0	1.0	1.0
TRANK1	O15050	383	-11	2	1	-22	0.9	1.0	1.0	0.8
NUMA1	Q14980	80	-11	0	1	-13	0.9	1.0	1.0	0.9
TRMT2A	Q8IZ69	260	-13	-22	1	-2	0.9	0.8	1.0	1.0

CNBP	P62633	97	-14	-9	1	-10	0.9	0.9	1.0	0.9
CLIC3	O95833	22	-15	-2	1	-7	0.9	1.0	1.0	0.9
CTTN	Q14247	112	-15	-5	1	-17	0.9	1.0	1.0	0.9
TNIP1	Q15025	171	-17	-19	1	-5	0.9	0.8	1.0	1.0
XPO4	Q9C0E2	1149	-19	-17	1	-16	0.8	0.9	1.0	0.9
FBXO11	Q86XK2	581	-20	-15	1	5	0.8	0.9	1.0	1.0
RFC1	P35251	665	-27	-14	1	-24	0.8	0.9	1.0	0.8
SMAD2	Q15796	312	32	-13	1	-2	1.5	0.9	1.0	1.0
SMAD1	Q15797	310	32	-13	1	-2	1.5	0.9	1.0	1.0
SMAD5	Q99717	310	32	-13	1	-2	1.5	0.9	1.0	1.0
CCDC109B	Q9NWR8	324	23	-9	1	6	1.3	0.9	1.0	1.1
INTS4	Q96HW7	926	22	4	1	-14	1.3	1.0	1.0	0.9
RECQL	P46063	447	22	-7	1	3	1.3	0.9	1.0	1.0
GLS	O94925	203	22	-18	1	10	1.3	0.9	1.0	1.1
UVSSA	Q2YD98	360	20	2	1	-2	1.3	1.0	1.0	1.0
AP2B1	P63010	57	19	-4	1	1	1.2	1.0	1.0	1.0
DDX41	Q9UJV9	540	19	-7	1	6	1.2	0.9	1.0	1.1
BPNT1	O95861	206	18	14	1	-3	1.2	1.2	1.0	1.0
PSTPIP2	Q9H939	213	17	-10	1	5	1.2	0.9	1.0	1.1
PEX1	O43933	1002	17	-2	1	1	1.2	1.0	1.0	1.0
MNDA	P41218	61	16	-13	1	9	1.2	0.9	1.0	1.1
GSK3A	P49840	170	15	-1	1	10	1.2	1.0	1.0	1.1
GSK3B	P49841	107	15	-1	1	10	1.2	1.0	1.0	1.1
GBF1	Q92538	1414	15	-2	1	9	1.2	1.0	1.0	1.1
TTC37	Q6PGP7	329	15	-11	1	-2	1.2	0.9	1.0	1.0
AHCYL1	O43865	373	15	-12	1	9	1.2	0.9	1.0	1.1
DGKA	P23743	277	14	-3	1	5	1.2	1.0	1.0	1.1
ILKAP	Q9H0C8	190	14	-3	1	4	1.2	1.0	1.0	1.0
BPNT1	O95861	59	14	-9	1	7	1.2	0.9	1.0	1.1
CHD3	Q12873	879	14	-9	1	4	1.2	0.9	1.0	1.0
LCP2	Q13094	32	14	-20	1	-11	1.2	0.8	1.0	0.9
PA2G4	Q9UQ80	296	14	-21	1	4	1.2	0.8	1.0	1.0
LACTB2	Q53H82	58	13	6	1	6	1.1	1.1	1.0	1.1
THOP1	P52888	248	13	-8	1	-1	1.1	0.9	1.0	1.0
PHF6	Q8IWS0	283	13	-12	1	-6	1.1	0.9	1.0	0.9
ZNF808	Q8N4W9	276	13	-3	1	-24	1.1	1.0	1.0	0.8
SH3GL1	Q99961	147	13	-10	1	-9	1.1	0.9	1.0	0.9
NACC1	Q96RE7	416	13	-12	1	-17	1.1	0.9	1.0	0.9
AP2A2	O94973	171	13	-18	1	5	1.1	0.9	1.0	1.0
AP2A1	O95782	171	13	-18	1	5	1.1	0.9	1.0	1.0
BCAT2	O15382	135	13	-29	1	27	1.1	0.8	1.0	1.4
LASP1	Q14847	53	12	-2	1	5	1.1	1.0	1.0	1.1
SUCLG1	P53597	60	12	-8	1	3	1.1	0.9	1.0	1.0
SSRP1	Q08945	340	12	-2	1	5	1.1	1.0	1.0	1.1
WDR1	O75083	225	12	-9	1	4	1.1	0.9	1.0	1.0
OSBPL5	Q9H0X9	233	12	-12	1	3	1.1	0.9	1.0	1.0
XPO5	Q9HAV4	736	11	-8	1	-3	1.1	0.9	1.0	1.0
ABCF2	Q9UG63	486	11	-18	1	-17	1.1	0.9	1.0	0.9
TNFSF14	O43557	154	11	4	1	9	1.1	1.0	1.0	1.1
AGL	P35573	767	11	-4	1	9	1.1	1.0	1.0	1.1
CYTIP	O60759	284	10	2	1	1	1.1	1.0	1.0	1.0
SP110	Q9HB58	327	10	-3	1	-6	1.1	1.0	1.0	0.9
XPO1	O14980	119	10	-6	1	-6	1.1	0.9	1.0	0.9

HNRNPL	P14866	452	10	-9	1	-1	1.1	0.9	1.0	1.0
NUB1	Q9Y5A7	317	10	-11	1	-6	1.1	0.9	1.0	0.9
SMYD4	Q8IYR2	572	10	-4	1	0	1.1	1.0	1.0	1.0
CCDC127	Q96BQ5	144	10	-8	1	-8	1.1	0.9	1.0	0.9
HSPH1	Q92598	650	10	-11	1	1	1.1	0.9	1.0	1.0
PLEC	Q15149	3299	9	-7	1	2	1.1	0.9	1.0	1.0
RANBP2	P49792	220	9	-7	1	1	1.1	0.9	1.0	1.0
MAP2K4	P45985	266	9	-10	1	9	1.1	0.9	1.0	1.1
HMGCL	P35914	307	9	-11	1	2	1.1	0.9	1.0	1.0
COPS7B	Q9H9Q2	240	9	0	1	-4	1.1	1.0	1.0	1.0
KIAA1211	Q6ZU35	1228	9	-5	1	-11	1.1	1.0	1.0	0.9
LRPPRC	P42704	113	9	-11	1	6	1.1	0.9	1.0	1.1
TBCE	Q15813	422	9	-14	1	-6	1.1	0.9	1.0	0.9
TP53BP1	Q12888	986	8	-1	1	-15	1.1	1.0	1.0	0.9
FRYL	O94915	2369	8	-2	1	-5	1.1	1.0	1.0	1.0
EIF3CL	B5ME19	620	8	-7	1	3	1.1	0.9	1.0	1.0
ACSF2	Q96CM8	122	8	-11	1	-5	1.1	0.9	1.0	1.0
Uncharacteri	K7ELQ4	398	8	4	1	10	1.1	1.0	1.0	1.1
RNF213	Q63HN8	2424	8	0	1	4	1.1	1.0	1.0	1.0
EIF3D	O15371	195	8	-1	1	-1	1.1	1.0	1.0	1.0
GNL3L	Q9NVN8	152	8	-2	1	-5	1.1	1.0	1.0	1.0
C11orf68	Q9H3H3	199	8	-7	1	5	1.1	0.9	1.0	1.0
C12orf57	Q99622	73	8	-10	1	-5	1.1	0.9	1.0	1.0
PLEC	Q15149	1156	8	-10	1	-5	1.1	0.9	1.0	1.0
SPOP	O43791	361	8	-11	1	-3	1.1	0.9	1.0	1.0
UBE3A	Q05086	140	8	-24	1	-12	1.1	0.8	1.0	0.9
PCCA	P05165	111	7	1	1	8	1.1	1.0	1.0	1.1
MCM6	Q14566	302	7	-6	1	-6	1.1	0.9	1.0	0.9
INPP4A	Q96PE3	367	7	-6	1	3	1.1	0.9	1.0	1.0
DOCK5	Q9H7D0	841	7	-7	1	-2	1.1	0.9	1.0	1.0
SENP7	Q9BQF6	316	7	-9	1	-18	1.1	0.9	1.0	0.8
PML	P29590	204	7	3	1	-3	1.1	1.0	1.0	1.0
GBF1	Q92538	1110	7	-3	1	-5	1.1	1.0	1.0	1.0
SFXN4	Q6P4A7	70	7	-4	1	3	1.1	1.0	1.0	1.0
FAM204A	Q9H8W3	192	7	-6	1	-11	1.1	0.9	1.0	0.9
PROSER2	Q86WR7	367	7	-7	1	-6	1.1	0.9	1.0	0.9
POLRMT	O00411	413	7	-13	1	-15	1.1	0.9	1.0	0.9
LCP1	P13796	164	7	-14	1	11	1.1	0.9	1.0	1.1
ADRBK1	P25098	340	6	-2	1	15	1.1	1.0	1.0	1.2
ADRBK2	P35626	340	6	-2	1	15	1.1	1.0	1.0	1.2
STXBP3	O00186	49	6	-5	1	6	1.1	1.0	1.0	1.1
U2SURP	O15042	624	6	-5	1	13	1.1	1.0	1.0	1.1
GAK	O14976	145	6	-6	1	-4	1.1	0.9	1.0	1.0
ARHGAP4	P98171	569	6	-7	1	-7	1.1	0.9	1.0	0.9
RABL6	H0Y4Z8	172	6	-8	1	-3	1.1	0.9	1.0	1.0
CFL1	P23528	147	6	-11	1	-9	1.1	0.9	1.0	0.9
CSRP1	P21291	58	6	-14	1	2	1.1	0.9	1.0	1.0
CCR7	P32248	358	6	-17	1	-17	1.1	0.9	1.0	0.9
KIF21B	O75037	1398	6	-18	1	-16	1.1	0.8	1.0	0.9
TAOK3	Q9H2K8	612	6	-20	1	-12	1.1	0.8	1.0	0.9
PSTPIP2	Q9H939	148	6	-30	1	13	1.1	0.8	1.0	1.1
CASP9	P55211	287	6	1	1	1	1.1	1.0	1.0	1.0
LARP4B	Q92615	590	6	-1	1	-10	1.1	1.0	1.0	0.9

MED13	Q9UHV7	1756	6	-1	1	2	1.1	1.0	1.0	1.0
UBR4	Q5T4S7	2554	6	-4	1	4	1.1	1.0	1.0	1.0
NR3C1	P04150	367	6	-6	1	-4	1.1	0.9	1.0	1.0
SSRP1	Q08945	200	6	-8	1	13	1.1	0.9	1.0	1.1
EAF2	Q96CJ1	29	6	-8	1	-7	1.1	0.9	1.0	0.9
SCFD1	Q8WVM8	485	6	-9	1	0	1.1	0.9	1.0	1.0
MRPL11	Q9Y3B7	50	6	-9	1	-2	1.1	0.9	1.0	1.0
PNPLA6	Q8IY17	199	6	-12	1	-7	1.1	0.9	1.0	0.9
VPS11	Q9H270	231	6	-12	1	-7	1.1	0.9	1.0	0.9
OAS3	Q9Y6K5	648	5	5	1	-21	1.1	1.1	1.0	0.8
UTP20	O75691	618	5	-2	1	1	1.1	1.0	1.0	1.0
ZMYM4	Q5VZL5	531	5	-2	1	-4	1.1	1.0	1.0	1.0
FUK	Q8N0W3	787	5	-4	1	-3	1.1	1.0	1.0	1.0
MYOF	Q9NZM1	1574	5	-4	1	8	1.1	1.0	1.0	1.1
BABAM1	Q9NWV8	222	5	-4	1	-1	1.1	1.0	1.0	1.0
SPTLC1	O15269	192	5	-5	1	5	1.1	1.0	1.0	1.1
CTC1	Q2NKJ3	584	5	-5	1	-4	1.1	1.0	1.0	1.0
PARP14	Q460N5	557	5	-5	1	-12	1.1	1.0	1.0	0.9
GCN1	Q92616	1527	5	-7	1	-8	1.1	0.9	1.0	0.9
AGO1	Q9UL18	270	5	-10	1	-5	1.1	0.9	1.0	1.0
PPP4R2	Q9NY27	30	5	-11	1	4	1.1	0.9	1.0	1.0
ARIH2	O95376	252	5	-11	1	-11	1.1	0.9	1.0	0.9
AHCTF1	Q8WYP5	693	5	-15	1	-6	1.1	0.9	1.0	0.9
CTSZ	Q9UBR2	170	5	-37	1	-13	1.1	0.7	1.0	0.9
PFAS	O15067	1287	5	-2	1	-1	1.0	1.0	1.0	1.0
TRRAP	Q9Y4A5	1879	5	-2	1	-3	1.0	1.0	1.0	1.0
MGEA5	O60502	596	5	-6	1	-6	1.0	0.9	1.0	0.9
MAP2K3	P46734	305	5	-6	1	-8	1.0	0.9	1.0	0.9
GMPR2	Q9P2T1	186	5	-7	1	2	1.0	0.9	1.0	1.0
PLEKHF2	Q9H8W4	186	5	-7	1	-4	1.0	0.9	1.0	1.0
SNX6	Q9UNH7	149	5	-7	1	-3	1.0	0.9	1.0	1.0
EVI5	O60447	641	5	-9	1	-6	1.0	0.9	1.0	0.9
PAXIP1	Q6ZW49	985	4	7	1	-6	1.0	1.1	1.0	0.9
CRYL1	Q9Y2S2	125	4	2	1	3	1.0	1.0	1.0	1.0
PTPN6	P29350	171	4	-2	1	1	1.0	1.0	1.0	1.0
MCM3AP	O60318	981	4	-7	1	-1	1.0	0.9	1.0	1.0
RPS4X	P62701	41	4	-7	1	-18	1.0	0.9	1.0	0.8
SPCS2	Q15005	17	4	-9	1	-10	1.0	0.9	1.0	0.9
AAAS	Q9NRG9	368	4	-10	1	-6	1.0	0.9	1.0	0.9
PDHA1	P08559	261	4	-21	1	7	1.0	0.8	1.0	1.1
ARIH1	Q9Y4X5	362	4	4	1	18	1.0	1.0	1.0	1.2
DNMT3A	Q9Y6K1	710	4	-1	1	5	1.0	1.0	1.0	1.1
ANK3	Q12955	1409	4	-2	1	9	1.0	1.0	1.0	1.1
UBA6	A0AVT1	546	4	-2	1	-1	1.0	1.0	1.0	1.0
IDH3G	P51553	148	4	-4	1	-2	1.0	1.0	1.0	1.0
SLC9A1	P19634	561	4	-6	1	-1	1.0	0.9	1.0	1.0
RSL1D1	O76021	47	4	-6	1	4	1.0	0.9	1.0	1.0
DICER1	Q9UPY3	199	4	-9	1	-5	1.0	0.9	1.0	1.0
TPK1	Q9H3S4	88	4	-9	1	11	1.0	0.9	1.0	1.1
NFKBIB	Q15653	121	4	-10	1	-5	1.0	0.9	1.0	1.0
TRANK1	O15050	2766	3	4	1	6	1.0	1.0	1.0	1.1
STARD7	Q9NQZ5	302	3	2	1	-8	1.0	1.0	1.0	0.9
NBEAL1	Q6ZS30	2129	3	-1	1	-2	1.0	1.0	1.0	1.0

RNF213	Q63HN8	4570	3	-4	1	0	1.0	1.0	1.0	1.0
MICAL2	O94851	703	3	-5	1	-8	1.0	1.0	1.0	0.9
DDX58	O95786	268	3	-6	1	-10	1.0	0.9	1.0	0.9
PSMG2	Q969U7	168	3	-7	1	-9	1.0	0.9	1.0	0.9
GTF2I	P78347	121	3	-8	1	4	1.0	0.9	1.0	1.0
NUP133	Q8WUM0	641	3	-9	1	-3	1.0	0.9	1.0	1.0
AP4M1	O00189	235	3	-11	1	-4	1.0	0.9	1.0	1.0
CASS4	Q9NQ75	608	3	-15	1	-8	1.0	0.9	1.0	0.9
RNF103-CHM	A0A140T963	167	3	-17	1	-4	1.0	0.9	1.0	1.0
SLC25A40	Q8TBP6	314	3	-1	1	-2	1.0	1.0	1.0	1.0
Uncharacteri	F5H5P2	231	3	-2	1	7	1.0	1.0	1.0	1.1
MED23	Q9ULK4	222	3	-5	1	1	1.0	1.0	1.0	1.0
TYK2	P29597	536	3	-8	1	-8	1.0	0.9	1.0	0.9
LATS2	Q9NRM7	813	3	-9	1	-1	1.0	0.9	1.0	1.0
MKRN2	Q9H000	216	3	-21	1	19	1.0	0.8	1.0	1.2
HIRA	P54198	1007	3	-22	1	-7	1.0	0.8	1.0	0.9
RNF213	Q63HN8	3979	2	3	1	-1	1.0	1.0	1.0	1.0
NLRC4	Q9NPP4	517	2	2	1	-8	1.0	1.0	1.0	0.9
CAAP1	Q9H8G2	152	2	1	1	-13	1.0	1.0	1.0	0.9
ZZEF1	O43149	69	2	-2	1	-4	1.0	1.0	1.0	1.0
MYH9	P35579	917	2	-3	1	5	1.0	1.0	1.0	1.1
CYBB	P04839	86	2	-6	1	-3	1.0	0.9	1.0	1.0
PES1	O00541	391	2	-13	1	-5	1.0	0.9	1.0	1.0
AKAP13	Q12802	2813	2	-23	1	-22	1.0	0.8	1.0	0.8
RABGAP1	Q9Y3P9	433	2	1	1	-20	1.0	1.0	1.0	0.8
PRG2	P13727	147	2	0	1	-6	1.0	1.0	1.0	0.9
UIMC1	Q96RL1	121	2	-2	1	-19	1.0	1.0	1.0	0.8
LRRFIP1	Q32MZ4	334	2	-4	1	-39	1.0	1.0	1.0	0.7
MYO1A	Q9UBC5	122	2	-5	1	1	1.0	1.0	1.0	1.0
EEFSEC	P57772	289	2	-7	1	5	1.0	0.9	1.0	1.1
MED15	Q96RN5	660	2	-9	1	-11	1.0	0.9	1.0	0.9
NDUFA5	Q16718	17	2	-11	1	-2	1.0	0.9	1.0	1.0
ZC3HAV1	Q7Z2W4	527	2	-11	1	-6	1.0	0.9	1.0	0.9
ARHGAP31	Q2M1Z3	572	1	9	1	-12	1.0	1.1	1.0	0.9
CEP57	Q86XR8	285	1	-4	1	-1	1.0	1.0	1.0	1.0
CERK	Q8TCT0	50	1	-6	1	-2	1.0	0.9	1.0	1.0
AAK1	Q2M2I8	319	1	-7	1	1	1.0	0.9	1.0	1.0
DNPH1	O43598	117	1	-10	1	0	1.0	0.9	1.0	1.0
NEK1	Q96PY6	1223	1	-15	1	24	1.0	0.9	1.0	1.3
MT1E	P04732	33	1	-17	1	5	1.0	0.9	1.0	1.0
NCKAP1	Q9Y2A7	622	1	4	1	-14	1.0	1.0	1.0	0.9
IKBKG	Q9Y6K9	95	1	1	1	0	1.0	1.0	1.0	1.0
FAM192A	Q9GZU8	44	1	-5	1	1	1.0	1.0	1.0	1.0
RPS27L	Q71UM5	77	1	-6	1	-3	1.0	0.9	1.0	1.0
MYO1G	B0I1T2	831	1	-6	1	-4	1.0	0.9	1.0	1.0
SMAD4	Q13485	115	1	-7	1	-4	1.0	0.9	1.0	1.0
CCDC22	O60826	441	1	-8	1	-22	1.0	0.9	1.0	0.8
NCF2	P19878	514	1	-8	1	-14	1.0	0.9	1.0	0.9
ANXA7	P20073	363	1	-9	1	-1	1.0	0.9	1.0	1.0
SNF8	Q96H20	72	1	-11	1	10	1.0	0.9	1.0	1.1
BCL10	O95999	57	1	-16	1	-14	1.0	0.9	1.0	0.9
FBXO30	Q8TB52	725	0	4	1	-5	1.0	1.0	1.0	1.0
USP7	Q93009	702	0	-6	1	4	1.0	0.9	1.0	1.0

NKIRAS2	Q9NYR9	122	0	-11	1	1	1.0	0.9	1.0	1.0
FBL	P22087	268	0	-17	1	-1	1.0	0.9	1.0	1.0
COMMD7	Q86VX2	200	-1	7	1	4	1.0	1.1	1.0	1.0
MYOF	Q9NZM1	275	-1	-1	1	-4	1.0	1.0	1.0	1.0
THOC2	Q8NI27	743	-1	-36	1	2	1.0	0.7	1.0	1.0
OAS1	P00973	54	-1	-11	1	-7	1.0	0.9	1.0	0.9
SYVN1	Q86TM6	329	-1	-12	1	-6	1.0	0.9	1.0	0.9
TBC1D5	Q92609	676	-1	-13	1	-19	1.0	0.9	1.0	0.8
NBEAL2	Q6ZNJ1	121	-2	3	1	-12	1.0	1.0	1.0	0.9
TRIM38	O00635	58	-2	-9	1	-4	1.0	0.9	1.0	1.0
GMEB2	Q9UKD1	323	-2	-9	1	-26	1.0	0.9	1.0	0.8
SARS	P49591	300	-2	-10	1	-2	1.0	0.9	1.0	1.0
TTC27	Q6P3X3	514	-2	-10	1	-23	1.0	0.9	1.0	0.8
CASP10	Q92851	16	-2	-2	1	-7	1.0	1.0	1.0	0.9
POGZ	Q7Z3K3	987	-2	-3	1	-9	1.0	1.0	1.0	0.9
CORO7-PAM:	A0A0A6YYL4	34	-2	-5	1	-1	1.0	1.0	1.0	1.0
FNDC3B	Q53EP0	1025	-2	-10	1	-8	1.0	0.9	1.0	0.9
PSMD2	Q13200	779	-2	-10	1	-6	1.0	0.9	1.0	0.9
DRAP1	Q14919	73	-2	-10	1	-11	1.0	0.9	1.0	0.9
MTOR	P42345	1214	-3	-1	1	3	1.0	1.0	1.0	1.0
LPIN1	Q14693	843	-3	-7	1	-14	1.0	0.9	1.0	0.9
NUP205	Q92621	922	-3	-7	1	2	1.0	0.9	1.0	1.0
STBD1	O95210	168	-3	-8	1	-12	1.0	0.9	1.0	0.9
SIN3A	Q96ST3	551	-3	-8	1	-16	1.0	0.9	1.0	0.9
FAAP100	Q0VG06	860	-3	-10	1	-6	1.0	0.9	1.0	0.9
TAP2	Q03519	353	-3	-3	1	-5	1.0	1.0	1.0	1.0
AHCTF1	Q8WYP5	521	-3	-9	1	-11	1.0	0.9	1.0	0.9
SEPT11	Q9NVA2	41	-3	-11	1	-2	1.0	0.9	1.0	1.0
LAS1L	Q9Y4W2	173	-3	-21	1	3	1.0	0.8	1.0	1.0
APOOL	Q6UXV4	74	-3	-24	1	-7	1.0	0.8	1.0	0.9
VPS13C	Q709C8	2906	-3	-35	1	-8	1.0	0.7	1.0	0.9
USP4	Q13107	702	-4	-9	1	-8	1.0	0.9	1.0	0.9
IRF4	Q15306	343	-4	-11	1	-8	1.0	0.9	1.0	0.9
HNRNPDL	O14979	177	-4	-22	1	5	1.0	0.8	1.0	1.1
DNTTIP2	Q5QJE6	618	-5	-3	1	-11	1.0	1.0	1.0	0.9
WDFY4	Q6ZS81	1963	-5	-9	1	18	1.0	0.9	1.0	1.2
DNPEP	Q9ULA0	327	-5	-2	1	-5	1.0	1.0	1.0	1.0
PCID2	Q5JVF3	25	-6	-29	1	-2	0.9	0.8	1.0	1.0
NADK2	Q4G0N4	58	-6	4	1	-12	0.9	1.0	1.0	0.9
MRPL17	Q9NRX2	129	-6	-6	1	-17	0.9	0.9	1.0	0.9
HPS5	Q9UPZ3	727	-7	14	1	14	0.9	1.2	1.0	1.2
GOLGB1	Q14789	681	-7	-4	1	-13	0.9	1.0	1.0	0.9
CTNNA1	P35221	324	-7	-6	1	10	0.9	0.9	1.0	1.1
FLNA	P21333	2107	-7	-12	1	-4	0.9	0.9	1.0	1.0
EHBP1L1	Q8N3D4	1364	-7	-7	1	-1	0.9	0.9	1.0	1.0
QARS	P47897	298	-7	-11	1	6	0.9	0.9	1.0	1.1
TRAFD1	O14545	90	-7	-18	1	-25	0.9	0.8	1.0	0.8
MTG2	Q9H4K7	175	-7	-22	1	-15	0.9	0.8	1.0	0.9
SYNJ1	O43426	799	-7	-25	1	-6	0.9	0.8	1.0	0.9
DAXX	Q9UER7	720	-8	7	1	0	0.9	1.1	1.0	1.0
ANKRD13A	Q8IZ07	146	-8	-22	1	-3	0.9	0.8	1.0	1.0
RNF114	Q9Y508	56	-9	-5	1	-15	0.9	1.0	1.0	0.9
GPATCH4	Q5T3I0	370	-10	-7	1	-13	0.9	0.9	1.0	0.9

MLLT4	P55196	324	-10	-26	1	-10	0.9	0.8	1.0	0.9
ESYT1	Q9BSJ8	635	-11	-2	1	-14	0.9	1.0	1.0	0.9
ZYX	Q15942	433	-11	-13	1	1	0.9	0.9	1.0	1.0
FMNL1	O95466	1090	-12	7	1	-13	0.9	1.1	1.0	0.9
C20orf27	Q9GZN8	156	-12	-19	1	-27	0.9	0.8	1.0	0.8
ALDOA	P04075	240	-13	-15	1	-16	0.9	0.9	1.0	0.9
GIMAP4	Q9NUV9	106	-13	-39	1	15	0.9	0.7	1.0	1.2
DIS3L2	Q8IYB7	378	-15	-14	1	6	0.9	0.9	1.0	1.1
GOLGA3	Q08378	455	-19	1	1	1	0.8	1.0	1.0	1.0
UNC13D	Q70J99	276	-35	-17	1	-8	0.7	0.9	1.0	0.9
DSP	P15924	1069	29	-7	0	-7	1.4	0.9	1.0	0.9
FEN1	P39748	163	26	-2	0	26	1.4	1.0	1.0	1.4
CPT1A	P50416	659	21	-22	0	14	1.3	0.8	1.0	1.2
NNT	Q13423	308	21	-56	0	8	1.3	0.6	1.0	1.1
UBA2	Q9UBT2	185	20	-14	0	3	1.3	0.9	1.0	1.0
SCML4	Q8N228	403	20	4	0	-12	1.2	1.0	1.0	0.9
PLAA	Q9Y263	664	19	-8	0	-7	1.2	0.9	1.0	0.9
PLCG2	P16885	496	18	-8	0	4	1.2	0.9	1.0	1.0
CAND1	Q86VP6	71	17	-26	0	4	1.2	0.8	1.0	1.0
NEK7	Q8TDX7	79	16	-8	0	2	1.2	0.9	1.0	1.0
RPA1	P27694	323	16	-28	0	5	1.2	0.8	1.0	1.1
SETDB1	Q15047	1226	15	9	0	3	1.2	1.1	1.0	1.0
APBB1IP	Q7Z5R6	304	15	-1	0	2	1.2	1.0	1.0	1.0
LTBP1	Q14766	407	15	-8	0	8	1.2	0.9	1.0	1.1
HEATR5A	Q86XA9	1635	15	-17	0	-6	1.2	0.9	1.0	0.9
SNX5	Q9Y5X3	346	15	-19	0	-3	1.2	0.8	1.0	1.0
SPTAN1	Q13813	2441	15	-10	0	5	1.2	0.9	1.0	1.0
MMS22L	Q6ZRQ5	597	15	-16	0	7	1.2	0.9	1.0	1.1
RAB3GAP1	Q15042	783	14	-6	0	7	1.2	0.9	1.0	1.1
ELAC2	Q9BQ52	609	14	-13	0	20	1.2	0.9	1.0	1.2
OAS2	P29728	108	14	-16	0	3	1.2	0.9	1.0	1.0
ITPR2	Q14571	1929	14	-4	0	4	1.2	1.0	1.0	1.0
ITPR3	Q14573	1881	14	-4	0	4	1.2	1.0	1.0	1.0
ITPR1	Q14643	1985	14	-4	0	4	1.2	1.0	1.0	1.0
THADA	Q6YHU6	1452	14	-4	0	15	1.2	1.0	1.0	1.2
EXOSC7	Q15024	29	14	-7	0	6	1.2	0.9	1.0	1.1
RIN1	Q13671	639	14	-9	0	-15	1.2	0.9	1.0	0.9
HSP90AA1	P07900	481	14	-10	0	9	1.2	0.9	1.0	1.1
DIAPH1	O60610	1005	14	-15	0	-8	1.2	0.9	1.0	0.9
LRRK2	Q5S007	746	13	-6	0	-11	1.1	0.9	1.0	0.9
TUBA1A	Q71U36	129	13	-10	0	-27	1.1	0.9	1.0	0.8
NLRC3	Q7RTR2	224	13	-18	0	-10	1.1	0.9	1.0	0.9
CFL1	P23528	39	13	-18	0	2	1.1	0.8	1.0	1.0
NAPRT	Q6XQN6	477	13	6	0	25	1.1	1.1	1.0	1.3
COPB1	P53618	248	13	-12	0	6	1.1	0.9	1.0	1.1
ARHGAP4	P98171	855	12	-7	0	-9	1.1	0.9	1.0	0.9
SH3KBP1	Q96B97	103	11	-10	0	6	1.1	0.9	1.0	1.1
WDR43	Q15061	291	11	-17	0	-4	1.1	0.9	1.0	1.0
PDIA3	P30101	60	11	4	0	11	1.1	1.0	1.0	1.1
TRMT1L	Q7Z2T5	419	11	3	0	-10	1.1	1.0	1.0	0.9
THUMPD3	Q9BV44	239	11	1	0	10	1.1	1.0	1.0	1.1
HNRNPL	P14866	261	11	-5	0	4	1.1	1.0	1.0	1.0
NUP188	Q5SRE5	1152	11	-9	0	7	1.1	0.9	1.0	1.1

TLN1	Q9Y490	1927	11	-9	0	-5	1.1	0.9	1.0	1.0
ACOT2	P49753	76	11	-10	0	0	1.1	0.9	1.0	1.0
COG7	P83436	419	11	-10	0	11	1.1	0.9	1.0	1.1
DAPP1	Q9UN19	54	10	-3	0	4	1.1	1.0	1.0	1.0
SYK	P43405	259	10	-4	0	5	1.1	1.0	1.0	1.0
PDS5A	Q29RF7	430	10	-4	0	-3	1.1	1.0	1.0	1.0
UPP1	Q16831	57	10	5	0	9	1.1	1.1	1.0	1.1
VPS13C	Q709C8	1094	10	5	0	4	1.1	1.1	1.0	1.0
ANKMY2	Q8IV38	277	10	4	0	-5	1.1	1.0	1.0	1.0
CLPB	Q9H078	572	10	-3	0	2	1.1	1.0	1.0	1.0
CTBP2	P56545	60	10	-4	0	5	1.1	1.0	1.0	1.1
OARD1	Q9Y530	24	10	-4	0	7	1.1	1.0	1.0	1.1
ZZEF1	O43149	728	10	-5	0	-6	1.1	1.0	1.0	0.9
ISCU	Q9H1K1	130	10	-5	0	3	1.1	1.0	1.0	1.0
PREX1	Q8TCU6	1543	10	-21	0	-9	1.1	0.8	1.0	0.9
SLC9A3R1	O14745	16	9	-6	0	1	1.1	0.9	1.0	1.0
ALDH1A1	P00352	186	9	-7	0	3	1.1	0.9	1.0	1.0
CCAR1	Q8IX12	579	9	-9	0	14	1.1	0.9	1.0	1.2
USP16	Q9Y5T5	726	9	-15	0	-8	1.1	0.9	1.0	0.9
PFKFB2	O60825	105	9	-1	0	17	1.1	1.0	1.0	1.2
BRCC3	P46736	228	9	-7	0	-5	1.1	0.9	1.0	1.0
IPO9	Q96P70	545	9	-8	0	-11	1.1	0.9	1.0	0.9
PEX11B	O96011	153	9	-8	0	-7	1.1	0.9	1.0	0.9
EXOSC1	Q9Y3B2	73	9	-9	0	5	1.1	0.9	1.0	1.0
LIMK1	P53667	297	8	19	0	-21	1.1	1.2	1.0	0.8
GP1BB	P13224	118	8	2	0	9	1.1	1.0	1.0	1.1
LRP1	Q07954	2660	8	-4	0	-12	1.1	1.0	1.0	0.9
TYMP	P19971	138	8	-4	0	13	1.1	1.0	1.0	1.1
DDX46	Q7L014	501	8	-7	0	1	1.1	0.9	1.0	1.0
JAK3	P52333	934	8	-8	0	-3	1.1	0.9	1.0	1.0
XRN2	Q9H0D6	557	8	-9	0	10	1.1	0.9	1.0	1.1
DCP2	Q8IU60	158	8	-12	0	6	1.1	0.9	1.0	1.1
FAM120A	Q9NZB2	53	8	-13	0	4	1.1	0.9	1.0	1.0
NUP93	Q8N1F7	397	8	-20	0	7	1.1	0.8	1.0	1.1
KDM3A	Q9Y4C1	479	8	7	0	-11	1.1	1.1	1.0	0.9
POM121	Q96HA1	217	8	7	0	-6	1.1	1.1	1.0	0.9
LRCH3	Q96II8	676	8	1	0	-7	1.1	1.0	1.0	0.9
RASA2	Q15283	567	8	-1	0	-2	1.1	1.0	1.0	1.0
URB1	O60287	1900	8	-2	0	-7	1.1	1.0	1.0	0.9
ACAP2	Q15057	415	8	-3	0	3	1.1	1.0	1.0	1.0
XPO1	O14980	164	8	-6	0	-7	1.1	0.9	1.0	0.9
SMG1	Q96Q15	1373	8	-7	0	-12	1.1	0.9	1.0	0.9
PML	P29590	479	8	-11	0	-12	1.1	0.9	1.0	0.9
CLIC1	O00299	223	8	-12	0	6	1.1	0.9	1.0	1.1
TBXAS1	P24557	418	8	-18	0	7	1.1	0.9	1.0	1.1
DNM2	P50570	427	7	-5	0	13	1.1	1.0	1.0	1.1
RILPL2	Q969X0	161	7	-7	0	1	1.1	0.9	1.0	1.0
XPOT	O43592	38	7	-7	0	4	1.1	0.9	1.0	1.0
DSTN	P60981	46	7	-8	0	-4	1.1	0.9	1.0	1.0
SKIV2L	Q15477	1014	7	-12	0	-1	1.1	0.9	1.0	1.0
RANBP2	P49792	1749	7	-13	0	-8	1.1	0.9	1.0	0.9
NAT10	Q9H0A0	194	7	-13	0	13	1.1	0.9	1.0	1.1
SMAD4	Q13485	123	7	-14	0	13	1.1	0.9	1.0	1.1

GP1BA	P07359	81	7	-16	0	4	1.1	0.9	1.0	1.0
LRRK2	Q5S007	271	7	4	0	30	1.1	1.0	1.0	1.4
RPS6KA5	O75582	631	7	4	0	-6	1.1	1.0	1.0	0.9
GMPS	P49915	137	7	1	0	7	1.1	1.0	1.0	1.1
IDH1	O75874	379	7	-4	0	1	1.1	1.0	1.0	1.0
RAB34	Q9BZG1	40	7	-6	0	3	1.1	0.9	1.0	1.0
SYNE2	Q8WXH0	5348	7	-7	0	0	1.1	0.9	1.0	1.0
FBXW11	Q9UKB1	145	7	-8	0	1	1.1	0.9	1.0	1.0
RDH13	Q8NBN7	30	7	-9	0	-1	1.1	0.9	1.0	1.0
RFC4	P35249	177	7	-9	0	-4	1.1	0.9	1.0	1.0
KDM5C	P41229	1213	7	-10	0	-24	1.1	0.9	1.0	0.8
Uncharacteri	K7ESF4	129	7	-11	0	4	1.1	0.9	1.0	1.0
PDCD2L	Q9BRP1	100	7	-11	0	4	1.1	0.9	1.0	1.0
HLTF	Q14527	461	7	-12	0	-9	1.1	0.9	1.0	0.9
CBFB	Q13951	124	7	-17	0	2	1.1	0.9	1.0	1.0
METTL3	Q86U44	375	6	4	0	12	1.1	1.0	1.0	1.1
HSP90AB1	P08238	366	6	3	0	2	1.1	1.0	1.0	1.0
CMPK2	Q5EBM0	85	6	-2	0	-1	1.1	1.0	1.0	1.0
CRYBG3	Q68DQ2	2946	6	-2	0	-9	1.1	1.0	1.0	0.9
HSP90AA1	P07900	374	6	-5	0	-3	1.1	1.0	1.0	1.0
NADSYN1	Q6IA69	627	6	-5	0	5	1.1	1.0	1.0	1.1
GBF1	Q92538	1707	6	-6	0	6	1.1	0.9	1.0	1.1
BAZ2A	Q9UIF9	1658	6	-6	0	5	1.1	0.9	1.0	1.1
TGFB1I1	O43294	455	6	-7	0	-4	1.1	0.9	1.0	1.0
CASP10	Q92851	341	6	-16	0	-5	1.1	0.9	1.0	1.0
METAP2	P50579	290	6	-17	0	-9	1.1	0.9	1.0	0.9
TTC38	Q5R3I4	53	6	-19	0	5	1.1	0.8	1.0	1.1
MTR	Q99707	798	6	-2	0	4	1.1	1.0	1.0	1.0
ARAF	P10398	58	6	-2	0	-7	1.1	1.0	1.0	0.9
DDX6	P26196	341	6	-6	0	-2	1.1	0.9	1.0	1.0
ME2	P23368	441	6	-9	0	9	1.1	0.9	1.0	1.1
ANKFY1	Q9P2R3	843	6	-11	0	8	1.1	0.9	1.0	1.1
PDS5A	Q29RF7	742	6	-12	0	-14	1.1	0.9	1.0	0.9
EDC3	Q96F86	137	6	-13	0	7	1.1	0.9	1.0	1.1
PDS5B	Q9NTI5	732	6	-13	0	-7	1.1	0.9	1.0	0.9
ACLY	P53396	764	6	-13	0	-6	1.1	0.9	1.0	0.9
VPS13C	Q709C8	2588	6	-14	0	1	1.1	0.9	1.0	1.0
BIRC3	Q13489	294	6	-17	0	1	1.1	0.9	1.0	1.0
BIRC2	Q13490	308	6	-17	0	1	1.1	0.9	1.0	1.0
NEK9	Q8TD19	890	5	1	0	-3	1.1	1.0	1.0	1.0
RPS28	P62857	27	5	-1	0	5	1.1	1.0	1.0	1.1
RUFY1	Q96T51	184	5	-3	0	-4	1.1	1.0	1.0	1.0
VPS8	Q8N3P4	1371	5	-4	0	-4	1.1	1.0	1.0	1.0
ARHGEF6	Q15052	553	5	-7	0	-7	1.1	0.9	1.0	0.9
ANKRD13A	Q8IZ07	540	5	-7	0	-26	1.1	0.9	1.0	0.8
HIBCH	Q6NVY1	45	5	-8	0	-2	1.1	0.9	1.0	1.0
LRRC47	Q8N1G4	390	5	-12	0	-6	1.1	0.9	1.0	0.9
IPO8	O15397	749	5	-14	0	3	1.1	0.9	1.0	1.0
IPO7	O95373	749	5	-14	0	3	1.1	0.9	1.0	1.0
AP1G2	O75843	354	5	-16	0	-7	1.1	0.9	1.0	0.9
GUCY1B3	Q02153	292	5	-20	0	-12	1.1	0.8	1.0	0.9
TCEA3	O75764	318	5	-38	0	-8	1.1	0.7	1.0	0.9
ARHGEF2	Q92974	342	5	3	0	-11	1.0	1.0	1.0	0.9

NCF2	P19878	499	5	1	0	-6	1.0	1.0	1.0	0.9
PDHA1	P08559	222	5	-2	0	8	1.0	1.0	1.0	1.1
XIAP	P98170	202	5	-3	0	1	1.0	1.0	1.0	1.0
CLIP1	P30622	1022	5	-7	0	-5	1.0	0.9	1.0	1.0
NFKB2	Q00653	114	5	-13	0	-3	1.0	0.9	1.0	1.0
SLC38A7	Q9NVC3	30	5	-15	0	-11	1.0	0.9	1.0	0.9
MYH9	P35579	1379	4	-3	0	-3	1.0	1.0	1.0	1.0
CAPN2	P17655	82	4	-4	0	-2	1.0	1.0	1.0	1.0
TPP2	P29144	150	4	-6	0	3	1.0	0.9	1.0	1.0
AKAP9	Q99996	882	4	-7	0	-18	1.0	0.9	1.0	0.9
TFIP11	Q9UBB9	445	4	-8	0	-3	1.0	0.9	1.0	1.0
ARHGAP30	Q7Z6I6	89	4	-9	0	-4	1.0	0.9	1.0	1.0
WDFY3	Q8IZQ1	3027	4	-9	0	-6	1.0	0.9	1.0	0.9
DENND1C	Q8IV53	174	4	-9	0	-9	1.0	0.9	1.0	0.9
TRRAP	Q9Y4A5	2203	4	-14	0	-9	1.0	0.9	1.0	0.9
IQSEC1	Q6DN90	636	4	4	0	8	1.0	1.0	1.0	1.1
PREX1	Q8TCU6	37	4	2	0	-5	1.0	1.0	1.0	1.0
NELFCD	Q8IXH7	398	4	1	0	-6	1.0	1.0	1.0	0.9
DENND1B	Q6P3S1	412	4	-1	0	-9	1.0	1.0	1.0	0.9
ODF2	Q5BJF6	243	4	-2	0	-7	1.0	1.0	1.0	0.9
DAXX	Q9UER7	131	4	-3	0	26	1.0	1.0	1.0	1.4
PITPNB	P48739	187	4	-6	0	-6	1.0	0.9	1.0	0.9
VPS37A	Q8NEZ2	373	4	-7	0	1	1.0	0.9	1.0	1.0
UBE2L3	P68036	86	4	-7	0	-1	1.0	0.9	1.0	1.0
PANK4	Q9NVE7	537	4	-10	0	-1	1.0	0.9	1.0	1.0
LIMD2	Q9BT23	88	4	-11	0	-2	1.0	0.9	1.0	1.0
PMS2	P54278	202	4	-19	0	-17	1.0	0.8	1.0	0.9
NLRC3	Q7RTR2	195	3	15	0	15	1.0	1.2	1.0	1.2
PRKG1	Q13976	175	3	13	0	-14	1.0	1.1	1.0	0.9
ALPK1	Q96QP1	512	3	5	0	5	1.0	1.1	1.0	1.0
ELP2	Q6IA86	204	3	5	0	-2	1.0	1.1	1.0	1.0
METAP1	P53582	40	3	5	0	10	1.0	1.0	1.0	1.1
DOCK2	Q92608	730	3	3	0	4	1.0	1.0	1.0	1.0
ITSN2	Q9NZM3	907	3	2	0	4	1.0	1.0	1.0	1.0
EIF2AK2	P19525	121	3	0	0	-7	1.0	1.0	1.0	0.9
MED14	O60244	635	3	-1	0	3	1.0	1.0	1.0	1.0
STAT6	P42226	228	3	-4	0	-5	1.0	1.0	1.0	1.0
PREX1	Q8TCU6	963	3	-5	0	-5	1.0	1.0	1.0	1.0
GIMAP4	Q9NUV9	187	3	-6	0	-4	1.0	0.9	1.0	1.0
GNL3	Q9BVP2	251	3	-6	0	-4	1.0	0.9	1.0	1.0
COMT	P21964	207	3	-8	0	8	1.0	0.9	1.0	1.1
ITK	Q08881	289	3	-16	0	-2	1.0	0.9	1.0	1.0
BLM	P54132	120	3	4	0	7	1.0	1.0	1.0	1.1
BOD1L1	Q8NFC6	1170	3	2	0	-6	1.0	1.0	1.0	0.9
WDR44	Q5JSH3	640	3	1	0	-15	1.0	1.0	1.0	0.9
RPS6	P62753	12	3	-1	0	-3	1.0	1.0	1.0	1.0
MECP2	P51608	429	3	-2	0	-10	1.0	1.0	1.0	0.9
GOLGA4	Q13439	1340	3	-2	0	-6	1.0	1.0	1.0	0.9
VCAN	P13611	3296	3	-5	0	0	1.0	1.0	1.0	1.0
UBE2F	Q969M7	50	3	-9	0	-5	1.0	0.9	1.0	1.0
TRIM8	Q9BZR9	262	3	-11	0	-18	1.0	0.9	1.0	0.9
NUP88	Q99567	561	3	-12	0	-3	1.0	0.9	1.0	1.0
CUL3	Q13618	298	3	-15	0	-1	1.0	0.9	1.0	1.0

PRKCB	P05771	502	3	-15	0	-4	1.0	0.9	1.0	1.0
PRKCG	P05129	516	3	-15	0	-4	1.0	0.9	1.0	1.0
PRKCA	P17252	499	3	-15	0	-4	1.0	0.9	1.0	1.0
PIK3C2A	O00443	418	2	-3	0	-13	1.0	1.0	1.0	0.9
COMTD1	Q86VU5	45	2	-5	0	3	1.0	1.0	1.0	1.0
PTPN6	P29350	102	2	-5	0	-5	1.0	1.0	1.0	1.0
GAK	O14976	1142	2	-6	0	-5	1.0	0.9	1.0	1.0
DIAPH1	O60610	1227	2	-7	0	-9	1.0	0.9	1.0	0.9
FLNB	O75369	706	2	-8	0	-14	1.0	0.9	1.0	0.9
RANBP3	Q9H6Z4	384	2	-9	0	-2	1.0	0.9	1.0	1.0
ZNF207	O43670	54	2	-11	0	2	1.0	0.9	1.0	1.0
TRIM56	Q9BRZ2	101	2	-21	0	5	1.0	0.8	1.0	1.1
DSTN	P60981	163	2	-2	0	-5	1.0	1.0	1.0	1.0
UPF1	Q92900	683	2	-4	0	-10	1.0	1.0	1.0	0.9
RPRD2	Q5VT52	1071	2	-5	0	-14	1.0	1.0	1.0	0.9
MBNL1	Q9NR56	27	2	-6	0	-3	1.0	0.9	1.0	1.0
AQR	O60306	28	2	-6	0	-1	1.0	0.9	1.0	1.0
VAC14	Q08AM6	620	2	-7	0	4	1.0	0.9	1.0	1.0
GCN1	Q92616	1535	2	-8	0	1	1.0	0.9	1.0	1.0
FOCAD	Q5VW36	56	2	-15	0	-12	1.0	0.9	1.0	0.9
METTL13	Q8N6R0	226	2	-18	0	-17	1.0	0.8	1.0	0.9
TBC1D2	Q9BYX2	419	1	-3	0	-12	1.0	1.0	1.0	0.9
KDM5A	P29375	1248	1	-4	0	-7	1.0	1.0	1.0	0.9
KDM5C	P41229	1280	1	-4	0	-7	1.0	1.0	1.0	0.9
KDM5D	Q9BY66	1267	1	-4	0	-7	1.0	1.0	1.0	0.9
ALDH7A1	P49419	522	1	-4	0	1	1.0	1.0	1.0	1.0
ANXA11	P50995	294	1	-5	0	2	1.0	1.0	1.0	1.0
EXOSC9	Q06265	122	1	-5	0	-3	1.0	1.0	1.0	1.0
NCOA5	Q9HCD5	137	1	-5	0	-5	1.0	1.0	1.0	1.0
TAB2	Q9NYJ8	608	1	-5	0	-4	1.0	1.0	1.0	1.0
ARL6IP4	Q66PJ3	410	1	-7	0	-12	1.0	0.9	1.0	0.9
PSMD6	Q15008	112	1	-9	0	-1	1.0	0.9	1.0	1.0
IRAK3	Q9Y616	572	1	-9	0	-13	1.0	0.9	1.0	0.9
ABCD3	P28288	472	1	-11	0	-1	1.0	0.9	1.0	1.0
TMPO	P42166	518	1	4	0	-3	1.0	1.0	1.0	1.0
FAM129C	Q86XR2	204	1	0	0	10	1.0	1.0	1.0	1.1
CTDP1	Q9Y5B0	73	1	-1	0	-11	1.0	1.0	1.0	0.9
IAH1	Q2TAA2	145	1	-4	0	-4	1.0	1.0	1.0	1.0
MAP1S	Q66K74	440	1	-5	0	-3	1.0	1.0	1.0	1.0
CBR1	P16152	227	1	-6	0	1	1.0	0.9	1.0	1.0
GBF1	Q92538	685	1	-6	0	-18	1.0	0.9	1.0	0.9
CBX4	O00257	185	1	-6	0	-3	1.0	0.9	1.0	1.0
NELFA	Q9H3P2	471	1	-7	0	-5	1.0	0.9	1.0	1.0
FSCN1	Q16658	80	1	-16	0	-9	1.0	0.9	1.0	0.9
PREP	P48147	601	0	0	0	10	1.0	1.0	1.0	1.1
PTPN11	Q06124	259	0	-4	0	-3	1.0	1.0	1.0	1.0
CAND1	Q86VP6	1134	0	-7	0	-8	1.0	0.9	1.0	0.9
SART1	O43290	560	0	-8	0	-8	1.0	0.9	1.0	0.9
PPP2R5C	Q13362	513	0	-9	0	-15	1.0	0.9	1.0	0.9
CHD3	Q12873	1131	0	-10	0	-3	1.0	0.9	1.0	1.0
CHD4	Q14839	1121	0	-10	0	-3	1.0	0.9	1.0	1.0
FAM65A	Q6ZS17	945	0	-31	0	-24	1.0	0.8	1.0	0.8
STK10	O94804	138	-1	2	0	1	1.0	1.0	1.0	1.0

USP48	Q86UV5	290	-1	-3	0	-4	1.0	1.0	1.0	1.0
MCM3AP	O60318	1238	-1	-6	0	4	1.0	0.9	1.0	1.0
GNPAT	O15228	66	-1	-8	0	-2	1.0	0.9	1.0	1.0
LDAH	Q9H6V9	261	-1	-9	0	-17	1.0	0.9	1.0	0.9
NCKIPSD	Q9NZQ3	376	-1	-11	0	-29	1.0	0.9	1.0	0.8
HDAC4	P56524	700	-1	-14	0	-3	1.0	0.9	1.0	1.0
IQGAP2	Q13576	1449	-1	-2	0	-2	1.0	1.0	1.0	1.0
AIM1	Q9Y4K1	976	-1	-4	0	-5	1.0	1.0	1.0	1.0
E4F1	Q66K89	361	-1	-5	0	-10	1.0	1.0	1.0	0.9
EIF6	P56537	15	-1	-8	0	-2	1.0	0.9	1.0	1.0
DENND4C	Q5VZ89	850	-1	-9	0	-9	1.0	0.9	1.0	0.9
SIRT7	Q9NRC8	266	-1	-12	0	8	1.0	0.9	1.0	1.1
NFRKB	Q6P4R8	487	-1	-13	0	-18	1.0	0.9	1.0	0.8
DOCK7	Q96N67	193	-2	-2	0	-6	1.0	1.0	1.0	0.9
TSR2	Q969E8	17	-2	-3	0	-7	1.0	1.0	1.0	0.9
RNF213	Q63HN8	876	-2	-6	0	13	1.0	0.9	1.0	1.1
NOP9	Q86U38	242	-2	-7	0	-8	1.0	0.9	1.0	0.9
SS18L1	O75177	47	-2	-14	0	2	1.0	0.9	1.0	1.0
HLA-C	P10321	188	-2	-17	0	-11	1.0	0.9	1.0	0.9
HLA-A	P16188	188	-2	-17	0	-11	1.0	0.9	1.0	0.9
HLA-A	P16190	188	-2	-17	0	-11	1.0	0.9	1.0	0.9
HLA-C	P30510	188	-2	-17	0	-11	1.0	0.9	1.0	0.9
HLA-B	P30479	188	-2	-17	0	-11	1.0	0.9	1.0	0.9
HLA-B	P30462	188	-2	-17	0	-11	1.0	0.9	1.0	0.9
SEC24C	P53992	78	-2	3	0	-15	1.0	1.0	1.0	0.9
ATM	Q13315	1509	-2	-4	0	1	1.0	1.0	1.0	1.0
NSUN6	Q8TEA1	415	-2	-9	0	-13	1.0	0.9	1.0	0.9
NCBP2-AS2	Q69YL0	63	-2	-14	0	-4	1.0	0.9	1.0	1.0
FAM105A	Q9NUU6	177	-2	-15	0	-10	1.0	0.9	1.0	0.9
C20orf27	Q9GZN8	124	-3	-5	0	-4	1.0	1.0	1.0	1.0
EFHD2	Q96C19	172	-3	-6	0	-5	1.0	0.9	1.0	1.0
GMPR	P36959	186	-3	-9	0	2	1.0	0.9	1.0	1.0
LRRK2	Q5S007	1187	-3	-9	0	-5	1.0	0.9	1.0	1.0
NOL9	Q5SY16	648	-3	-11	0	-2	1.0	0.9	1.0	1.0
CHM	P24386	276	-3	1	0	-5	1.0	1.0	1.0	1.0
CHML	P26374	278	-3	1	0	-5	1.0	1.0	1.0	1.0
RUNX3	Q13761	22	-3	-3	0	-12	1.0	1.0	1.0	0.9
HMOX2	P30519	265	-3	-7	0	25	1.0	0.9	1.0	1.3
DSN1	Q9H410	287	-3	-8	0	24	1.0	0.9	1.0	1.3
IGLC2	A0A075B6K9	105	-3	-10	0	-7	1.0	0.9	1.0	0.9
ATP6V1G1	O75348	69	-4	-5	0	-5	1.0	1.0	1.0	1.0
MCM3	P25205	360	-4	-7	0	1	1.0	0.9	1.0	1.0
DMXL1	Q9Y485	2916	-4	-16	0	1	1.0	0.9	1.0	1.0
FAM213B	Q8TBF2	44	-4	-21	0	-18	1.0	0.8	1.0	0.8
SVIL	O95425	26	-4	6	0	-8	1.0	1.1	1.0	0.9
APBA2	Q99767	654	-4	-14	0	-13	1.0	0.9	1.0	0.9
STX7	O15400	28	-4	-17	0	-11	1.0	0.9	1.0	0.9
BUD31	P41223	134	-4	-19	0	-8	1.0	0.8	1.0	0.9
SYNE2	Q8WXH0	5212	-4	-24	0	-23	1.0	0.8	1.0	0.8
DENND5B	Q6ZUT9	733	-5	-2	0	11	1.0	1.0	1.0	1.1
COL4A3BP	Q9Y5P4	70	-5	-2	0	-1	1.0	1.0	1.0	1.0
ARL2BP	Q9Y2Y0	149	-5	-8	0	-9	1.0	0.9	1.0	0.9
GPX1	P07203	202	-5	-9	0	-13	1.0	0.9	1.0	0.9

ZW10	O43264	568	-5	-14	0	-17	1.0	0.9	1.0	0.9
TNIP2	Q8NFZ5	21	-5	-7	0	-9	1.0	0.9	1.0	0.9
THOC2	Q8NI27	1016	-5	-13	0	-8	1.0	0.9	1.0	0.9
GAB2	Q9UQC2	406	-6	-5	0	-26	0.9	1.0	1.0	0.8
EPRS	P07814	1148	-6	-8	0	3	0.9	0.9	1.0	1.0
PLA2G16	P53816	113	-6	-8	0	-14	0.9	0.9	1.0	0.9
FAM96A	Q9H5X1	90	-7	8	0	-14	0.9	1.1	1.0	0.9
LYZ	P61626	48	-7	-2	0	8	0.9	1.0	1.0	1.1
IMPDH2	P12268	339	-7	-2	0	-4	0.9	1.0	1.0	1.0
UBASH3A	P57075	607	-7	-4	0	-8	0.9	1.0	1.0	0.9
CAPZB	P47756	147	-8	-8	0	-9	0.9	0.9	1.0	0.9
RPS5	P46782	66	-8	-9	0	13	0.9	0.9	1.0	1.1
CCP110	O43303	600	-10	-9	0	-1	0.9	0.9	1.0	1.0
NDUFB9	Q9Y6M9	42	-11	-24	0	-13	0.9	0.8	1.0	0.9
RPS11	P62280	116	-12	6	0	-6	0.9	1.1	1.0	0.9
RNF7	Q9UBF6	61	-14	-3	0	-3	0.9	1.0	1.0	1.0
LAT2	Q9GZY6	142	-14	-9	0	-13	0.9	0.9	1.0	0.9
AARS	P49588	901	-21	-21	0	-1	0.8	0.8	1.0	1.0
CHD4	Q14839	1827	-22	-33	0	-51	0.8	0.8	1.0	0.7
XPNPEP1	Q9NQW7	502	-24	-23	0	-24	0.8	0.8	1.0	0.8
MPO	P05164	309	-30	-22	0	5	0.8	0.8	1.0	1.0
HSP90AB1	P08238	589	-31	-10	0	2	0.8	0.9	1.0	1.0
ALOX5	P09917	100	-50	-15	0	12	0.7	0.9	1.0	1.1
FADD	Q13158	168	35	-9	-1	-2	1.5	0.9	1.0	1.0
GP5	P40197	427	25	-6	-1	-18	1.3	0.9	1.0	0.9
IRF2BP1	Q8IU81	239	22	1	-1	-1	1.3	1.0	1.0	1.0
PRKDC	P78527	491	22	-43	-1	6	1.3	0.7	1.0	1.1
F13A1	P00488	696	21	13	-1	4	1.3	1.1	1.0	1.0
VARS	P26640	682	21	-3	-1	2	1.3	1.0	1.0	1.0
GALK1	P51570	170	19	3	-1	2	1.2	1.0	1.0	1.0
RRP12	Q5JTH9	673	19	-19	-1	11	1.2	0.8	1.0	1.1
SPATA13	Q96N96	361	18	-19	-1	-6	1.2	0.8	1.0	0.9
GNAI2	P04899	255	18	-36	-1	20	1.2	0.7	1.0	1.2
GNAI3	P08754	254	18	-36	-1	20	1.2	0.7	1.0	1.2
GNAI1	P63096	254	18	-36	-1	20	1.2	0.7	1.0	1.2
MSH6	P52701	1337	18	14	-1	-19	1.2	1.2	1.0	0.8
PFAS	O15067	1338	17	-4	-1	-5	1.2	1.0	1.0	1.0
MYH9	P35579	816	17	-13	-1	-1	1.2	0.9	1.0	1.0
MYH10	P35580	823	17	-13	-1	-1	1.2	0.9	1.0	1.0
FGD2	Q7Z6J4	89	16	-6	-1	-12	1.2	0.9	1.0	0.9
CUL4B	Q13620	248	16	-8	-1	3	1.2	0.9	1.0	1.0
KDM3B	Q7LBC6	1126	16	5	-1	9	1.2	1.0	1.0	1.1
TOR1AIP1	Q5JTV8	424	16	4	-1	20	1.2	1.0	1.0	1.3
CABIN1	Q9Y6J0	717	15	10	-1	8	1.2	1.1	1.0	1.1
DDX17	Q92841	431	15	-8	-1	9	1.2	0.9	1.0	1.1
XPO5	Q9HAV4	1044	15	-9	-1	-8	1.2	0.9	1.0	0.9
CLTC	Q00610	1102	15	-37	-1	1	1.2	0.7	1.0	1.0
RAB3GAP1	Q15042	858	15	-1	-1	-8	1.2	1.0	1.0	0.9
TANK	Q92844	20	14	2	-1	5	1.2	1.0	1.0	1.0
TRAPPC5	Q8IUR0	139	14	5	-1	9	1.2	1.1	1.0	1.1
LRRK1	Q38SD2	1670	14	-6	-1	1	1.2	0.9	1.0	1.0
CLASP1	Q7Z460	453	14	-10	-1	-7	1.2	0.9	1.0	0.9
ZNF276	Q8N554	533	13	2	-1	4	1.1	1.0	1.0	1.0

TXNIP	Q9H3M7	36	13	-6	-1	4	1.1	0.9	1.0	1.0
VAV1	P15498	31	13	4	-1	8	1.1	1.0	1.0	1.1
RFC2	P35250	171	13	1	-1	-6	1.1	1.0	1.0	0.9
LDHA	P00338	293	13	-5	-1	7	1.1	1.0	1.0	1.1
SACM1L	Q9NTJ5	344	13	-10	-1	5	1.1	0.9	1.0	1.0
SRP68	Q9UHB9	173	13	-14	-1	-1	1.1	0.9	1.0	1.0
ADK	P55263	106	12	-24	-1	7	1.1	0.8	1.0	1.1
EHMT1	Q9H9B1	1045	12	-2	-1	-8	1.1	1.0	1.0	0.9
ZNF808	Q8N4W9	466	12	-6	-1	0	1.1	0.9	1.0	1.0
WDR6	Q9NNW5	341	12	-15	-1	-9	1.1	0.9	1.0	0.9
MRPS27	Q92552	49	11	-1	-1	-1	1.1	1.0	1.0	1.0
CDK5RAP3	Q96JB5	150	11	-3	-1	-10	1.1	1.0	1.0	0.9
DDX42	Q86XP3	281	11	-7	-1	-4	1.1	0.9	1.0	1.0
ALDH2	P05091	66	11	-7	-1	13	1.1	0.9	1.0	1.1
PPA2	Q9H2U2	302	11	-19	-1	-1	1.1	0.8	1.0	1.0
AKT2	P31751	124	11	2	-1	-7	1.1	1.0	1.0	0.9
DPM3	Q9P2X0	67	11	-21	-1	2	1.1	0.8	1.0	1.0
CLPB	Q9H078	267	10	-2	-1	2	1.1	1.0	1.0	1.0
CARS2	Q9HA77	387	10	-2	-1	-1	1.1	1.0	1.0	1.0
SCRIB	A0A0G2JPP5	864	10	-10	-1	-2	1.1	0.9	1.0	1.0
GDI1	P31150	282	10	-20	-1	1	1.1	0.8	1.0	1.0
JAK1	P23458	543	9	-3	-1	-13	1.1	1.0	1.0	0.9
TLN1	Q9Y490	236	9	-6	-1	4	1.1	0.9	1.0	1.0
PHB	P35232	69	9	-6	-1	-2	1.1	0.9	1.0	1.0
AP1G2	O75843	539	9	-6	-1	-1	1.1	0.9	1.0	1.0
JMJD7	P0C870	47	9	-11	-1	3	1.1	0.9	1.0	1.0
KARS	Q15046	209	9	13	-1	13	1.1	1.1	1.0	1.1
CD48	P09326	148	9	-1	-1	-1	1.1	1.0	1.0	1.0
ACSL4	O60488	363	9	-5	-1	4	1.1	1.0	1.0	1.0
DOCK6	Q96HP0	1853	9	-5	-1	-6	1.1	1.0	1.0	0.9
GM2A	P17900	138	9	-8	-1	21	1.1	0.9	1.0	1.3
PMVK	Q15126	92	9	-8	-1	4	1.1	0.9	1.0	1.0
OSGEP	Q9NPF4	160	9	-9	-1	1	1.1	0.9	1.0	1.0
PSMD13	Q9UNM6	182	9	-20	-1	-3	1.1	0.8	1.0	1.0
RAB40B	Q12829	77	9	-23	-1	-5	1.1	0.8	1.0	1.0
APAF1	O14727	8	9	-23	-1	-9	1.1	0.8	1.0	0.9
PPOX	P50336	183	8	-3	-1	9	1.1	1.0	1.0	1.1
CCDC88B	A6NC98	238	8	-7	-1	-16	1.1	0.9	1.0	0.9
SLFN5	Q08AF3	610	8	-9	-1	1	1.1	0.9	1.0	1.0
HNRNPLL	Q8WVV9	235	8	-12	-1	-4	1.1	0.9	1.0	1.0
PSMB10	P40306	82	8	-17	-1	6	1.1	0.9	1.0	1.1
EIF1	P41567	94	8	-5	-1	-1	1.1	1.0	1.0	1.0
STK26	Q9P289	410	8	-5	-1	-4	1.1	1.0	1.0	1.0
CAP1	Q01518	93	8	-8	-1	-1	1.1	0.9	1.0	1.0
MAP4K1	Q92918	334	8	-11	-1	-8	1.1	0.9	1.0	0.9
CYBB	P04839	428	8	-14	-1	-10	1.1	0.9	1.0	0.9
MED9	Q9NWA0	139	7	2	-1	-9	1.1	1.0	1.0	0.9
HCFC1	P51610	227	7	-1	-1	0	1.1	1.0	1.0	1.0
HMHA1	Q92619	731	7	-2	-1	-2	1.1	1.0	1.0	1.0
TNK2	Q07912	843	7	-3	-1	-4	1.1	1.0	1.0	1.0
SUGP2	Q8IX01	970	7	-3	-1	-20	1.1	1.0	1.0	0.8
PLCB2	Q00722	1162	7	-5	-1	-13	1.1	1.0	1.0	0.9
COMMD5	Q9GZQ3	64	7	-8	-1	-3	1.1	0.9	1.0	1.0

UFL1	Q94874	708	7	-12	-1	-11	1.1	0.9	1.0	0.9
FLNA	P21333	205	7	-13	-1	1	1.1	0.9	1.0	1.0
RANBP2	P49792	1747	7	-17	-1	-17	1.1	0.9	1.0	0.9
RBM15	Q96T37	926	7	-1	-1	2	1.1	1.0	1.0	1.0
DDX39A	O00148	223	7	-4	-1	-3	1.1	1.0	1.0	1.0
ATP6V1B2	P21281	112	7	-5	-1	-5	1.1	1.0	1.0	1.0
EPS15	P42566	419	7	-6	-1	-10	1.1	0.9	1.0	0.9
PSME1	Q06323	106	7	-6	-1	-33	1.1	0.9	1.0	0.8
ECI2	O75521	312	7	-7	-1	-3	1.1	0.9	1.0	1.0
MICALL1	Q8N3F8	597	7	-9	-1	-14	1.1	0.9	1.0	0.9
MATN2	O00339	618	6	3	-1	-2	1.1	1.0	1.0	1.0
GTF3C1	Q12789	1520	6	3	-1	-8	1.1	1.0	1.0	0.9
GNL1	P36915	410	6	-3	-1	17	1.1	1.0	1.0	1.2
LPXN	O60711	376	6	-5	-1	1	1.1	1.0	1.0	1.0
STAT3	P40763	542	6	-5	-1	2	1.1	1.0	1.0	1.0
TSSC1	Q53HC9	95	6	-6	-1	3	1.1	0.9	1.0	1.0
DBN1	Q16643	96	6	-8	-1	-1	1.1	0.9	1.0	1.0
RASAL3	Q86YV0	539	6	-9	-1	-4	1.1	0.9	1.0	1.0
ATM	Q13315	465	6	-14	-1	6	1.1	0.9	1.0	1.1
GRK5	P34947	138	6	-16	-1	-13	1.1	0.9	1.0	0.9
PPP2R2A	P63151	398	6	-2	-1	4	1.1	1.0	1.0	1.0
SYMPK	Q92797	969	6	-2	-1	-2	1.1	1.0	1.0	1.0
NECAP1	Q8NC96	162	6	-4	-1	-10	1.1	1.0	1.0	0.9
XRCC5	P13010	157	6	-5	-1	-2	1.1	1.0	1.0	1.0
PCBP2	Q15366	163	6	-7	-1	-12	1.1	0.9	1.0	0.9
PTPN11	Q06124	104	6	-9	-1	2	1.1	0.9	1.0	1.0
CLTC	Q00610	1205	6	-12	-1	3	1.1	0.9	1.0	1.0
PSMC6	P62333	347	6	-19	-1	-2	1.1	0.8	1.0	1.0
INO80E	Q8NBZ0	179	5	0	-1	5	1.1	1.0	1.0	1.1
DOCK9	Q9BZ29	69	5	-1	-1	-7	1.1	1.0	1.0	0.9
HTATSF1	O43719	480	5	-2	-1	-18	1.1	1.0	1.0	0.8
LRRC58	Q96CX6	309	5	-4	-1	-6	1.1	1.0	1.0	0.9
ACAP2	Q15057	321	5	-5	-1	2	1.1	1.0	1.0	1.0
SDAD1	Q9NVU7	307	5	-7	-1	-4	1.1	0.9	1.0	1.0
OSBPL11	Q9BXB4	34	5	-7	-1	-7	1.1	0.9	1.0	0.9
SHTN1	A0MZ66	442	5	-8	-1	-9	1.1	0.9	1.0	0.9
MAPK7	Q13164	194	5	-10	-1	-8	1.1	0.9	1.0	0.9
MAPRE1	Q15691	228	5	-11	-1	-7	1.1	0.9	1.0	0.9
RPS14	P62263	85	5	-12	-1	-6	1.1	0.9	1.0	0.9
C14orf159	Q7Z3D6	316	5	-14	-1	7	1.1	0.9	1.0	1.1
MT-CYB	P00156	93	5	-15	-1	13	1.1	0.9	1.0	1.1
PHF12	Q96QT6	613	5	-16	-1	-8	1.1	0.9	1.0	0.9
SCFD2	Q8WU76	404	5	1	-1	-4	1.0	1.0	1.0	1.0
EPHX2	P34913	74	5	1	-1	10	1.0	1.0	1.0	1.1
VPS13B	Q7Z7G8	2907	5	0	-1	3	1.0	1.0	1.0	1.0
MPP1	Q00013	242	5	-2	-1	-11	1.0	1.0	1.0	0.9
MAPKAPK2	P49137	98	5	-3	-1	5	1.0	1.0	1.0	1.1
FAM160B1	Q5W0V3	574	5	-6	-1	1	1.0	0.9	1.0	1.0
HNRNPL	P14866	260	5	-6	-1	-1	1.0	0.9	1.0	1.0
SRPK2	P78362	320	5	-6	-1	-9	1.0	0.9	1.0	0.9
GRPEL1	Q9HAV7	124	5	-7	-1	7	1.0	0.9	1.0	1.1
EIF4G3	O43432	1411	5	-7	-1	-7	1.0	0.9	1.0	0.9
MFN1	Q8IWA4	418	5	-12	-1	-21	1.0	0.9	1.0	0.8

CLIC4	Q9Y696	234	5	-12	-1	7	1.0	0.9	1.0	1.1
KIAA0196	Q12768	969	5	-12	-1	-3	1.0	0.9	1.0	1.0
SEC23A	Q15436	180	5	-13	-1	-8	1.0	0.9	1.0	0.9
ITGB2	P05107	191	4	5	-1	-3	1.0	1.0	1.0	1.0
ASH2L	Q9UBL3	120	4	2	-1	-1	1.0	1.0	1.0	1.0
SRPK1	Q96SB4	455	4	1	-1	-11	1.0	1.0	1.0	0.9
CASP8	Q14790	236	4	-3	-1	-5	1.0	1.0	1.0	1.0
PPIP5K2	O43314	900	4	-4	-1	3	1.0	1.0	1.0	1.0
SCAF11	Q99590	450	4	3	-1	-15	1.0	1.0	1.0	0.9
ZHX2	Q9Y6X8	11	4	0	-1	-9	1.0	1.0	1.0	0.9
METTL6	Q8TCB7	256	4	-1	-1	3	1.0	1.0	1.0	1.0
ANAPC5	Q9UJX4	86	4	-1	-1	-8	1.0	1.0	1.0	0.9
KIF1B	O60333	1771	4	-7	-1	-12	1.0	0.9	1.0	0.9
PLEKHF1	Q96S99	186	4	-9	-1	-1	1.0	0.9	1.0	1.0
ALKBH8	Q96BT7	442	4	-27	-1	-10	1.0	0.8	1.0	0.9
TBC1D9B	Q66K14	289	3	6	-1	4	1.0	1.1	1.0	1.0
SGK3	Q96BR1	308	3	-2	-1	4	1.0	1.0	1.0	1.0
SEC23B	Q15437	767	3	-4	-1	-7	1.0	1.0	1.0	0.9
INTS2	Q9H0H0	1164	3	-4	-1	-9	1.0	1.0	1.0	0.9
ARHGEF1	Q92888	911	3	-6	-1	-9	1.0	0.9	1.0	0.9
ATRIP	Q8WXE1	585	3	-7	-1	-2	1.0	0.9	1.0	1.0
ACIN1	Q9UKV3	546	3	-9	-1	-11	1.0	0.9	1.0	0.9
FTO	Q9C0B1	346	3	-10	-1	2	1.0	0.9	1.0	1.0
EP400	Q96L91	694	3	-16	-1	-13	1.0	0.9	1.0	0.9
RAB44	Q7Z6P3	653	3	6	-1	-17	1.0	1.1	1.0	0.9
UBN1	Q9NPG3	420	3	-1	-1	-5	1.0	1.0	1.0	1.0
TDP1	Q9NUW8	48	3	-2	-1	-8	1.0	1.0	1.0	0.9
NBEAL2	Q6ZNJ1	1661	3	-6	-1	-3	1.0	0.9	1.0	1.0
ILKAP	Q9H0C8	312	3	-11	-1	-3	1.0	0.9	1.0	1.0
WDR6	Q9NNW5	26	3	-15	-1	4	1.0	0.9	1.0	1.0
L2HGDH	Q9H9P8	258	3	-19	-1	-23	1.0	0.8	1.0	0.8
CCR6	P51684	348	3	-29	-1	-6	1.0	0.8	1.0	0.9
TAP2	Q03519	213	2	6	-1	26	1.0	1.1	1.0	1.3
HAT1	O14929	168	2	6	-1	-2	1.0	1.1	1.0	1.0
VPS52	Q8N1B4	220	2	1	-1	1	1.0	1.0	1.0	1.0
MYLK	Q15746	1384	2	-1	-1	9	1.0	1.0	1.0	1.1
RNF213	Q63HN8	1429	2	-2	-1	-5	1.0	1.0	1.0	1.0
KLHL36	Q8N4N3	263	2	-4	-1	-4	1.0	1.0	1.0	1.0
CLIC2	O15247	114	2	-4	-1	-5	1.0	1.0	1.0	1.0
HAT1	O14929	299	2	-5	-1	-3	1.0	1.0	1.0	1.0
CEBPB	P17676	248	2	-6	-1	-11	1.0	0.9	1.0	0.9
CTPS2	Q9NRF8	216	2	-6	-1	5	1.0	0.9	1.0	1.1
NAA25	Q14CX7	381	2	-7	-1	16	1.0	0.9	1.0	1.2
FLNA	P21333	478	2	-8	-1	1	1.0	0.9	1.0	1.0
FUBP3	Q96I24	109	2	-10	-1	6	1.0	0.9	1.0	1.1
VAV1	P15498	794	2	-10	-1	-5	1.0	0.9	1.0	1.0
VAV3	Q9UKW4	800	2	-10	-1	-5	1.0	0.9	1.0	1.0
HNRNPL	P14866	472	2	-5	-1	-3	1.0	1.0	1.0	1.0
PSMC6	P62333	170	2	-5	-1	-4	1.0	1.0	1.0	1.0
PSTPIP2	Q9H939	221	2	-5	-1	-1	1.0	1.0	1.0	1.0
XPOT	O43592	650	2	-6	-1	-5	1.0	0.9	1.0	1.0
ERBB2IP	Q96RT1	464	2	-6	-1	-14	1.0	0.9	1.0	0.9
ALDOA	P04075	339	2	-7	-1	2	1.0	0.9	1.0	1.0

RNGTT	O60942	375	2	-7	-1	-7	1.0	0.9	1.0	0.9
HNRNPM	P52272	653	2	-8	-1	-7	1.0	0.9	1.0	0.9
SUPT16H	Q9Y5B9	323	2	-11	-1	11	1.0	0.9	1.0	1.1
CSRP1	P21291	167	2	-13	-1	-2	1.0	0.9	1.0	1.0
BIN1	O00499	47	1	-2	-1	-6	1.0	1.0	1.0	0.9
ABCE1	P61221	88	1	-5	-1	0	1.0	1.0	1.0	1.0
GRSF1	Q12849	476	1	-5	-1	-1	1.0	1.0	1.0	1.0
NUP107	P57740	78	1	-5	-1	-5	1.0	1.0	1.0	1.0
ACSL1	P33121	108	1	-5	-1	-1	1.0	1.0	1.0	1.0
ARHGEF1	Q92888	815	1	-7	-1	-9	1.0	0.9	1.0	0.9
PIP5K1C	O60331	306	1	-9	-1	-13	1.0	0.9	1.0	0.9
RANBP1	P43487	158	1	-14	-1	3	1.0	0.9	1.0	1.0
SPTBN1	Q01082	964	1	-15	-1	-4	1.0	0.9	1.0	1.0
MCCC2	Q9HCC0	267	1	-1	-1	-3	1.0	1.0	1.0	1.0
MAP3K5	Q99683	928	1	-1	-1	-1	1.0	1.0	1.0	1.0
TBC1D13	Q9NVG8	36	1	-2	-1	-6	1.0	1.0	1.0	0.9
RSBN1	Q5VWQ0	282	1	-3	-1	-14	1.0	1.0	1.0	0.9
KBTBD11	O94819	99	1	-6	-1	-8	1.0	0.9	1.0	0.9
INPP4A	Q96PE3	464	1	-7	-1	1	1.0	0.9	1.0	1.0
SYNE1	Q8NF91	6090	1	-9	-1	2	1.0	0.9	1.0	1.0
MYBBP1A	Q9BQG0	884	1	-9	-1	-3	1.0	0.9	1.0	1.0
SZT2	Q5T011	2453	1	-10	-1	-13	1.0	0.9	1.0	0.9
PACS2	Q86VP3	519	1	-14	-1	-14	1.0	0.9	1.0	0.9
SYDE1	Q6ZW31	290	1	-15	-1	-10	1.0	0.9	1.0	0.9
RASA2	Q15283	354	1	-15	-1	7	1.0	0.9	1.0	1.1
WDR26	Q9H7D7	355	0	-8	-1	-3	1.0	0.9	1.0	1.0
TRRAP	Q9Y4A5	567	0	-8	-1	-3	1.0	0.9	1.0	1.0
TBC1D31	Q96DN5	1004	0	-9	-1	-11	1.0	0.9	1.0	0.9
TBXAS1	P24557	479	0	-12	-1	2	1.0	0.9	1.0	1.0
PPP1CA	P62136	158	0	-12	-1	5	1.0	0.9	1.0	1.0
SLFN5	Q08AF3	495	0	-14	-1	3	1.0	0.9	1.0	1.0
SRC	P12931	188	0	-17	-1	-6	1.0	0.9	1.0	0.9
NPAT	Q14207	978	-1	5	-1	-3	1.0	1.1	1.0	1.0
RPL37A	P61513	57	-1	5	-1	8	1.0	1.0	1.0	1.1
ACAD9	Q9H845	327	-1	-1	-1	4	1.0	1.0	1.0	1.0
ITGB2	P05107	559	-1	-2	-1	-11	1.0	1.0	1.0	0.9
MCMBP	Q9BTE3	108	-1	-5	-1	11	1.0	1.0	1.0	1.1
BZW1	Q7L1Q6	35	-1	-6	-1	-7	1.0	0.9	1.0	0.9
SLC12A9	Q9BXP2	911	-1	-8	-1	-4	1.0	0.9	1.0	1.0
TGFB1	P01137	285	-1	-12	-1	-10	1.0	0.9	1.0	0.9
SMARCA2	P51531	906	-1	-19	-1	-8	1.0	0.8	1.0	0.9
NAIP	Q13075	609	-1	0	-1	7	1.0	1.0	1.0	1.1
KDM3B	Q7LBC6	1470	-1	-2	-1	-7	1.0	1.0	1.0	0.9
pk	D4Q8H0	231	-1	-5	-1	-2	1.0	1.0	1.0	1.0
SERPINA1	A0A024R6I7	256	-1	-5	-1	-4	1.0	1.0	1.0	1.0
SEPT9	Q9UHD8	248	-1	-5	-1	-19	1.0	1.0	1.0	0.8
PYHIN1	Q6K0P9	119	-1	-6	-1	-10	1.0	0.9	1.0	0.9
SPAST	Q9UBP0	171	-1	-8	-1	-10	1.0	0.9	1.0	0.9
EEA1	Q15075	346	-1	-15	-1	-7	1.0	0.9	1.0	0.9
SENP6	Q9GZR1	607	-1	-15	-1	-11	1.0	0.9	1.0	0.9
EPHX4	Q8IUS5	287	-1	-17	-1	-4	1.0	0.9	1.0	1.0
RAVER1	Q8IY67	255	-2	3	-1	7	1.0	1.0	1.0	1.1
DIS3	Q9Y2L1	213	-2	0	-1	-6	1.0	1.0	1.0	0.9

INF2	Q27J81	971	-2	-3	-1	-13	1.0	1.0	1.0	0.9
METAP1	P53582	36	-2	-4	-1	0	1.0	1.0	1.0	1.0
PSMD10	O75832	107	-2	-7	-1	2	1.0	0.9	1.0	1.0
CPSF4	O95639	41	-2	-8	-1	-4	1.0	0.9	1.0	1.0
NDRG3	Q9UGV2	30	-2	-11	-1	-2	1.0	0.9	1.0	1.0
CBR3	O75828	122	-2	-12	-1	10	1.0	0.9	1.0	1.1
UBE2A	P49459	152	-2	-13	-1	-6	1.0	0.9	1.0	0.9
SASH3	O75995	152	-2	-14	-1	-8	1.0	0.9	1.0	0.9
ORC1	Q13415	469	-2	-19	-1	-17	1.0	0.8	1.0	0.9
PIAS4	Q8N2W9	164	-2	0	-1	-5	1.0	1.0	1.0	1.0
AREL1	O15033	382	-2	-2	-1	-4	1.0	1.0	1.0	1.0
INTS4	Q96HW7	44	-2	-2	-1	-8	1.0	1.0	1.0	0.9
MCM3AP	O60318	1285	-2	-3	-1	-3	1.0	1.0	1.0	1.0
NF1	P21359	454	-2	-6	-1	-6	1.0	0.9	1.0	0.9
TRIP11	Q15643	726	-2	-9	-1	-9	1.0	0.9	1.0	0.9
EML2	O95834	53	-2	-15	-1	-5	1.0	0.9	1.0	1.0
UFD1L	Q92890	185	-2	-16	-1	-9	1.0	0.9	1.0	0.9
VCP	P55072	209	-3	-5	-1	-2	1.0	1.0	1.0	1.0
DCUN1D4	Q92564	253	-3	-9	-1	-13	1.0	0.9	1.0	0.9
SSFA2	P28290	305	-3	-9	-1	-14	1.0	0.9	1.0	0.9
PLBD1	Q6P4A8	475	-3	-13	-1	-10	1.0	0.9	1.0	0.9
PUM3	Q15397	576	-3	-14	-1	-12	1.0	0.9	1.0	0.9
RAC2	P15153	6	-3	-15	-1	8	1.0	0.9	1.0	1.1
CDC42	P60953	6	-3	-15	-1	8	1.0	0.9	1.0	1.1
RAC1	P63000	6	-3	-15	-1	8	1.0	0.9	1.0	1.1
RHOG	P84095	6	-3	-15	-1	8	1.0	0.9	1.0	1.1
METTL13	Q8N6R0	256	-3	-16	-1	-18	1.0	0.9	1.0	0.8
RNF213	Q63HN8	4407	-3	-1	-1	2	1.0	1.0	1.0	1.0
APAF1	O14727	317	-3	-2	-1	-17	1.0	1.0	1.0	0.9
ILKAP	Q9H0C8	325	-3	-7	-1	-2	1.0	0.9	1.0	1.0
NEK1	Q96PY6	276	-3	-8	-1	-7	1.0	0.9	1.0	0.9
HNRNPH1	P31943	22	-3	-9	-1	-2	1.0	0.9	1.0	1.0
GPATCH8	Q9UKJ3	569	-3	-13	-1	-11	1.0	0.9	1.0	0.9
ARHGEF1	Q92888	595	-4	-11	-1	-4	1.0	0.9	1.0	1.0
PUSL1	Q8N0Z8	292	-4	13	-1	13	1.0	1.1	1.0	1.1
ARHGEF10L	Q9HCE6	767	-4	-22	-1	-1	1.0	0.8	1.0	1.0
KIAA1033	Q2M389	1031	-4	-30	-1	1	1.0	0.8	1.0	1.0
PTBP1	P26599	23	-5	-4	-1	-3	1.0	1.0	1.0	1.0
CYLD	Q9NQC7	129	-5	-9	-1	-10	1.0	0.9	1.0	0.9
CENPB	P07199	259	-5	-11	-1	-19	1.0	0.9	1.0	0.8
RANBP9	Q96S59	610	-5	-12	-1	-27	1.0	0.9	1.0	0.8
USP9Y	O00507	1729	-5	-4	-1	-17	1.0	1.0	1.0	0.9
USP9X	Q93008	1727	-5	-4	-1	-17	1.0	1.0	1.0	0.9
CDKN2AIP	Q9NXV6	178	-5	-6	-1	-12	1.0	0.9	1.0	0.9
FBXO4	Q9UKT5	147	-5	-21	-1	-2	1.0	0.8	1.0	1.0
MVB12B	Q9H7P6	267	-6	1	-1	5	0.9	1.0	1.0	1.0
DNAJC2	Q99543	276	-6	-8	-1	-1	0.9	0.9	1.0	1.0
ASAP1	Q9ULH1	740	-6	15	-1	-1	0.9	1.2	1.0	1.0
RXRA	P19793	171	-6	-11	-1	4	0.9	0.9	1.0	1.0
RNF14	Q9UBS8	404	-6	-13	-1	-9	0.9	0.9	1.0	0.9
LSM7	Q9UK45	85	-7	-11	-1	-3	0.9	0.9	1.0	1.0
KIAA1109	Q2LD37	1053	-7	-12	-1	-9	0.9	0.9	1.0	0.9
CMPK2	Q5EBM0	119	-7	-16	-1	4	0.9	0.9	1.0	1.0

CSK	P41240	31	-8	-4	-1	-7	0.9	1.0	1.0	0.9
C9orf142	Q9BUH6	80	-8	-5	-1	-4	0.9	1.0	1.0	1.0
ANKRD54	Q6NXT1	185	-8	-7	-1	-2	0.9	0.9	1.0	1.0
ARHGEF12	Q9NZN5	1128	-8	-13	-1	-3	0.9	0.9	1.0	1.0
KIAA0513	O60268	149	-8	-25	-1	-11	0.9	0.8	1.0	0.9
TBCCD1	Q9NVR7	298	-9	-1	-1	-10	0.9	1.0	1.0	0.9
NR3C2	P08235	910	-9	-11	-1	5	0.9	0.9	1.0	1.0
RASA3	Q14644	709	-9	7	-1	6	0.9	1.1	1.0	1.1
MRPS12	O15235	64	-9	-13	-1	-35	0.9	0.9	1.0	0.7
ITGAM	P11215	123	-10	3	-1	1	0.9	1.0	1.0	1.0
MMS19	Q96T76	746	-10	2	-1	-5	0.9	1.0	1.0	1.0
DGKZ	Q13574	876	-10	-21	-1	-7	0.9	0.8	1.0	0.9
CASP8	Q14790	409	-11	-7	-1	-20	0.9	0.9	1.0	0.8
SYNE1	Q8NF91	5692	-11	-13	-1	-15	0.9	0.9	1.0	0.9
MYO18A	Q92614	1199	-11	-15	-1	-16	0.9	0.9	1.0	0.9
DDX10	Q13206	196	-11	-17	-1	9	0.9	0.9	1.0	1.1
ZBTB17	Q13105	108	-11	-13	-1	-4	0.9	0.9	1.0	1.0
GARS	P41250	471	-12	-8	-1	-17	0.9	0.9	1.0	0.9
G6PC3	Q9BUM1	269	-13	-20	-1	15	0.9	0.8	1.0	1.2
NEPRO	Q6NW34	316	-16	-16	-1	-14	0.9	0.9	1.0	0.9
CPSF4	O95639	55	-17	-31	-1	-1	0.9	0.8	1.0	1.0
PRKDC	P78527	1904	-22	1	-1	-15	0.8	1.0	1.0	0.9
UHRF1	Q96T88	316	-24	-32	-1	-29	0.8	0.8	1.0	0.8
RNH1	P13489	85	-25	-19	-1	1	0.8	0.8	1.0	1.0
APOOL	Q6UXV4	79	31	-6	-1	11	1.4	0.9	1.0	1.1
PDCD6IP	Q8WUM4	127	23	-11	-1	2	1.3	0.9	1.0	1.0
SUPT16H	Q9Y5B9	283	23	-30	-1	6	1.3	0.8	1.0	1.1
IKZF3	Q9UKT9	281	21	2	-1	9	1.3	1.0	1.0	1.1
DDX19A	Q9NUU7	313	20	-12	-1	-8	1.3	0.9	1.0	0.9
DDX19B	Q9UMR2	314	20	-12	-1	-8	1.3	0.9	1.0	0.9
EPG5	Q9HCE0	1538	20	-13	-1	2	1.2	0.9	1.0	1.0
LRBA	P50851	256	19	-13	-1	-8	1.2	0.9	1.0	0.9
DCAF7	P61962	109	17	-19	-1	18	1.2	0.8	1.0	1.2
ANXA4	P09525	315	16	4	-1	25	1.2	1.0	1.0	1.3
ANKRD44	Q8N8A2	368	15	-5	-1	8	1.2	1.0	1.0	1.1
ATM	Q13315	2753	15	-14	-1	-7	1.2	0.9	1.0	0.9
Uncharacteri	E9PCH4	1329	15	-4	-1	2	1.2	1.0	1.0	1.0
GLYR1	Q49A26	486	15	-4	-1	1	1.2	1.0	1.0	1.0
DPP7	Q9UHL4	216	15	-15	-1	4	1.2	0.9	1.0	1.0
PSMC2	P35998	389	14	0	-1	9	1.2	1.0	1.0	1.1
TMEM55A	Q8N4L2	61	14	-4	-1	11	1.2	1.0	1.0	1.1
EXOSC1	Q9Y3B2	15	14	-14	-1	8	1.2	0.9	1.0	1.1
LYN	P07948	219	14	-20	-1	11	1.2	0.8	1.0	1.1
EXOC6	Q8TAG9	28	14	14	-1	1	1.2	1.2	1.0	1.0
OSTF1	Q92882	185	14	1	-1	-6	1.2	1.0	1.0	0.9
DNAJC2	Q99543	140	14	-2	-1	5	1.2	1.0	1.0	1.1
HUWE1	Q7Z6Z7	1567	14	-5	-1	-8	1.2	1.0	1.0	0.9
ATP5H	O75947	101	14	-22	-1	9	1.2	0.8	1.0	1.1
DDB1	Q16531	173	13	7	-1	10	1.1	1.1	1.0	1.1
EDC4	Q6P2E9	510	13	-6	-1	12	1.1	0.9	1.0	1.1
OSBPL9	Q96SU4	173	13	-2	-1	0	1.1	1.0	1.0	1.0
SUGP2	Q8IX01	183	13	-10	-1	-4	1.1	0.9	1.0	1.0
CCT3	P49368	366	13	-12	-1	4	1.1	0.9	1.0	1.0

DSTN	P60981	39	13	-23	-1	0	1.1	0.8	1.0	1.0
UPF1	Q92900	374	12	-19	-1	-3	1.1	0.8	1.0	1.0
GP5	P40197	472	12	-25	-1	-16	1.1	0.8	1.0	0.9
CAMK4	Q16566	208	12	4	-1	6	1.1	1.0	1.0	1.1
CPSF3	Q9UKF6	527	12	-12	-1	-6	1.1	0.9	1.0	0.9
SZT2	Q5T011	3012	12	-12	-1	-18	1.1	0.9	1.0	0.8
POLD1	P28340	1061	12	-14	-1	-21	1.1	0.9	1.0	0.8
SFSWAP	Q12872	220	12	-15	-1	-2	1.1	0.9	1.0	1.0
SEC13	P55735	234	12	-20	-1	1	1.1	0.8	1.0	1.0
CAMK2G	Q13555	453	12	-23	-1	6	1.1	0.8	1.0	1.1
FAM117B	Q6P1L5	513	11	-14	-1	16	1.1	0.9	1.0	1.2
CASP1	P29466	169	11	-21	-1	1	1.1	0.8	1.0	1.0
EEF1D	P29692	217	11	1	-1	-1	1.1	1.0	1.0	1.0
RNH1	P13489	362	11	-17	-1	-1	1.1	0.9	1.0	1.0
KIAA0907	Q7Z7F0	280	10	-1	-1	-1	1.1	1.0	1.0	1.0
HUWE1	Q7Z6Z7	1879	10	-12	-1	-5	1.1	0.9	1.0	1.0
ATG3	Q9NT62	81	10	-18	-1	-12	1.1	0.9	1.0	0.9
ZFYVE26	Q68DK2	2407	10	-18	-1	1	1.1	0.8	1.0	1.0
MYBBP1A	Q9BQG0	1046	10	-20	-1	-1	1.1	0.8	1.0	1.0
PKM	P14618	326	10	0	-1	1	1.1	1.0	1.0	1.0
GCN1	Q92616	2015	10	-1	-1	-2	1.1	1.0	1.0	1.0
SDE2	Q6IQ49	406	10	-5	-1	-7	1.1	1.0	1.0	0.9
MRPL37	Q9BZE1	104	10	-17	-1	2	1.1	0.9	1.0	1.0
LIMS1	P48059	181	9	3	-1	-8	1.1	1.0	1.0	0.9
LIMS2	Q7Z4I7	186	9	3	-1	-8	1.1	1.0	1.0	0.9
ZCCHC11	Q5TAX3	20	9	-3	-1	-5	1.1	1.0	1.0	1.0
AIMP2	Q13155	143	9	-3	-1	-5	1.1	1.0	1.0	1.0
DHX32	Q7L7V1	566	9	-5	-1	-10	1.1	1.0	1.0	0.9
AP3B1	O00203	893	9	-7	-1	4	1.1	0.9	1.0	1.0
MYH9	P35579	91	9	-9	-1	-2	1.1	0.9	1.0	1.0
CORO1A	P31146	24	9	-16	-1	6	1.1	0.9	1.0	1.1
SORBS3	O60504	521	9	-17	-1	2	1.1	0.9	1.0	1.0
FHOD1	Q9Y613	502	9	3	-1	-3	1.1	1.0	1.0	1.0
RPRD1B	Q9NQG5	234	9	0	-1	-1	1.1	1.0	1.0	1.0
VPS4B	O75351	240	9	-4	-1	-5	1.1	1.0	1.0	1.0
WDR59	Q6PJI9	917	9	-4	-1	-7	1.1	1.0	1.0	0.9
PLCB2	Q00722	1001	9	-5	-1	-1	1.1	1.0	1.0	1.0
DENND4A	Q7Z401	35	9	-6	-1	0	1.1	0.9	1.0	1.0
CFL1	P23528	80	9	-9	-1	4	1.1	0.9	1.0	1.0
IPO7	O95373	90	9	-9	-1	3	1.1	0.9	1.0	1.0
TAF5	Q15542	632	9	-11	-1	-5	1.1	0.9	1.0	1.0
AP1G1	O43747	400	9	-15	-1	1	1.1	0.9	1.0	1.0
PUM3	Q15397	610	9	-15	-1	-8	1.1	0.9	1.0	0.9
CCT5	P48643	302	8	8	-1	8	1.1	1.1	1.0	1.1
SON	P18583	2070	8	-2	-1	-8	1.1	1.0	1.0	0.9
CTSD	P07339	117	8	-2	-1	-28	1.1	1.0	1.0	0.8
AIMP2	Q13155	306	8	-10	-1	-1	1.1	0.9	1.0	1.0
GCN1	Q92616	1595	8	-11	-1	1	1.1	0.9	1.0	1.0
PLXNC1	O60486	1217	8	-12	-1	-5	1.1	0.9	1.0	1.0
METTL16	Q86W50	480	8	-12	-1	-6	1.1	0.9	1.0	0.9
PIK3C2A	O00443	541	8	-14	-1	-15	1.1	0.9	1.0	0.9
PAPSS1	O43252	360	8	-2	-1	-6	1.1	1.0	1.0	0.9
CPSF2	Q9P2I0	577	8	-8	-1	-2	1.1	0.9	1.0	1.0

DUSP3	P51452	171	8	-9	-1	9	1.1	0.9	1.0	1.1
FHL1	Q13642	188	8	-9	-1	-1	1.1	0.9	1.0	1.0
HGH1	Q9BTY7	228	8	-15	-1	-6	1.1	0.9	1.0	0.9
PDIA3	P30101	57	7	2	-1	10	1.1	1.0	1.0	1.1
MOB3A	Q96BX8	88	7	2	-1	-1	1.1	1.0	1.0	1.0
FERMT3	Q86UX7	235	7	-3	-1	-5	1.1	1.0	1.0	1.0
RHOH	Q15669	7	7	-4	-1	1	1.1	1.0	1.0	1.0
MYH9	P35579	790	7	-6	-1	8	1.1	0.9	1.0	1.1
DEF6	Q9H4E7	267	7	-7	-1	2	1.1	0.9	1.0	1.0
DEF6	Q9H4E7	279	7	-7	-1	-2	1.1	0.9	1.0	1.0
FOXP4	Q8IVH2	342	7	-12	-1	11	1.1	0.9	1.0	1.1
STOM	P27105	87	7	-13	-1	19	1.1	0.9	1.0	1.2
SCFD2	Q8WU76	54	7	-2	-1	3	1.1	1.0	1.0	1.0
NUB1	Q9Y5A7	52	7	-2	-1	-33	1.1	1.0	1.0	0.8
CPSF4	O95639	110	7	-4	-1	-4	1.1	1.0	1.0	1.0
MNDA	P41218	274	7	-5	-1	-4	1.1	1.0	1.0	1.0
ARFGEF1	Q9Y6D6	1729	7	-7	-1	-3	1.1	0.9	1.0	1.0
FNBP1	Q96RU3	70	7	-8	-1	3	1.1	0.9	1.0	1.0
USP9Y	O00507	684	7	-10	-1	1	1.1	0.9	1.0	1.0
USP9X	Q93008	683	7	-10	-1	1	1.1	0.9	1.0	1.0
ACLY	P53396	229	7	-19	-1	0	1.1	0.8	1.0	1.0
ARHGEF1	Q92888	594	6	-4	-1	6	1.1	1.0	1.0	1.1
PTPRJ	Q12913	1039	6	-6	-1	-10	1.1	0.9	1.0	0.9
LRBA	P50851	2740	6	-7	-1	2	1.1	0.9	1.0	1.0
NMD3	Q96D46	250	6	-7	-1	1	1.1	0.9	1.0	1.0
PSMA4	P25789	163	6	-7	-1	0	1.1	0.9	1.0	1.0
ACSL4	O60488	420	6	-7	-1	-2	1.1	0.9	1.0	1.0
ARL8B	Q9NVJ2	164	6	-14	-1	9	1.1	0.9	1.0	1.1
CDC5L	Q99459	96	6	-32	-1	-7	1.1	0.8	1.0	0.9
EHD4	Q9H223	175	6	-4	-1	12	1.1	1.0	1.0	1.1
GZMB	P10144	142	6	-8	-1	-12	1.1	0.9	1.0	0.9
PABPC1	P11940	339	6	-8	-1	2	1.1	0.9	1.0	1.0
USP9Y	O00507	674	6	-10	-1	-12	1.1	0.9	1.0	0.9
USP9X	Q93008	673	6	-10	-1	-12	1.1	0.9	1.0	0.9
PMPCB	O75439	485	6	-11	-1	-1	1.1	0.9	1.0	1.0
GIMAP1	Q8WWP7	186	6	-11	-1	4	1.1	0.9	1.0	1.0
CAND1	Q86VP6	954	6	-30	-1	-11	1.1	0.8	1.0	0.9
GNB1	P62873	271	5	3	-1	7	1.1	1.0	1.0	1.1
ZNF644	Q9H582	62	5	0	-1	-12	1.1	1.0	1.0	0.9
FLI1	Q01543	83	5	-4	-1	-10	1.1	1.0	1.0	0.9
FAM60A	Q9NP50	19	5	-5	-1	3	1.1	1.0	1.0	1.0
SOS1	Q07889	980	5	-8	-1	-2	1.1	0.9	1.0	1.0
SOS2	Q07890	978	5	-8	-1	-2	1.1	0.9	1.0	1.0
KLHL36	Q8N4N3	254	5	-10	-1	-11	1.1	0.9	1.0	0.9
XPO7	Q9UIA9	606	5	-13	-1	-2	1.1	0.9	1.0	1.0
VPS54	Q9P1Q0	785	5	3	-1	-9	1.0	1.0	1.0	0.9
NIPBL	Q6KC79	52	5	0	-1	-2	1.0	1.0	1.0	1.0
STAU2	Q9NUL3	11	5	-2	-1	-2	1.0	1.0	1.0	1.0
ZNF318	Q5VUA4	1860	5	-4	-1	-9	1.0	1.0	1.0	0.9
RFC1	P35251	607	5	-5	-1	8	1.0	1.0	1.0	1.1
INPP5D	Q92835	4	5	-6	-1	-17	1.0	0.9	1.0	0.9
NLRC3	Q7RTR2	936	5	-8	-1	-14	1.0	0.9	1.0	0.9
CSDE1	O75534	464	5	-9	-1	-11	1.0	0.9	1.0	0.9

CDC42	P60953	18	5	-10	-1	-3	1.0	0.9	1.0	1.0
ZNF189	O75820	385	5	-10	-1	-5	1.0	0.9	1.0	1.0
ARPC4-TTLL3	A0A0A6YYG9	21	5	-14	-1	-3	1.0	0.9	1.0	1.0
EIF3M	Q7L2H7	175	4	-5	-1	-2	1.0	1.0	1.0	1.0
CNOT10	Q9H9A5	327	4	-5	-1	-6	1.0	1.0	1.0	0.9
ESD	P10768	56	4	-9	-1	0	1.0	0.9	1.0	1.0
EVI5	O60447	479	4	-9	-1	-13	1.0	0.9	1.0	0.9
TBC1D1	Q86TI0	766	4	-9	-1	-9	1.0	0.9	1.0	0.9
MTERF4	Q7Z6M4	76	4	-10	-1	-4	1.0	0.9	1.0	1.0
SPTBN2	O15020	186	4	-13	-1	-5	1.0	0.9	1.0	1.0
SPTB	P11277	183	4	-13	-1	-5	1.0	0.9	1.0	1.0
SPTBN1	Q01082	183	4	-13	-1	-5	1.0	0.9	1.0	1.0
PRPF4	O43172	399	4	-18	-1	8	1.0	0.8	1.0	1.1
TTC4	O95801	110	4	-19	-1	-4	1.0	0.8	1.0	1.0
HP1BP3	Q5SSJ5	412	4	-26	-1	1	1.0	0.8	1.0	1.0
SUPV3L1	Q8IYB8	587	4	-27	-1	9	1.0	0.8	1.0	1.1
PTK2B	Q14289	677	4	1	-1	-12	1.0	1.0	1.0	0.9
AHCTF1	Q8WYP5	937	4	-2	-1	3	1.0	1.0	1.0	1.0
USP5	P45974	195	4	-3	-1	-7	1.0	1.0	1.0	0.9
SAMHD1	Q9Y3Z3	573	4	-15	-1	-5	1.0	0.9	1.0	1.0
PRKCA	P17252	583	4	-17	-1	2	1.0	0.9	1.0	1.0
MGST1	P10620	50	4	-18	-1	-9	1.0	0.8	1.0	0.9
CD48	P09326	193	4	-23	-1	-18	1.0	0.8	1.0	0.9
GLRX	P35754	26	3	2	-1	5	1.0	1.0	1.0	1.0
AHCYL1	O43865	272	3	0	-1	1	1.0	1.0	1.0	1.0
ZNF740	Q8NDX6	182	3	-2	-1	-6	1.0	1.0	1.0	0.9
EIF1B	O60739	69	3	-4	-1	2	1.0	1.0	1.0	1.0
EIF1	P41567	69	3	-4	-1	2	1.0	1.0	1.0	1.0
METTL7A	Q9H8H3	79	3	-5	-1	5	1.0	1.0	1.0	1.0
RNF213	Q63HN8	1510	3	-5	-1	-1	1.0	1.0	1.0	1.0
CEBPB	P17676	184	3	-5	-1	-15	1.0	1.0	1.0	0.9
ATP2A3	Q93084	581	3	-7	-1	-5	1.0	0.9	1.0	1.0
EIF5B	O60841	635	3	-9	-1	-8	1.0	0.9	1.0	0.9
MRPL3	P09001	78	3	-9	-1	-8	1.0	0.9	1.0	0.9
TEP1	Q99973	2275	3	-12	-1	1	1.0	0.9	1.0	1.0
RALBP1	Q15311	411	3	-13	-1	-5	1.0	0.9	1.0	1.0
RXRB	P28702	241	3	-15	-1	-27	1.0	0.9	1.0	0.8
COPG1	Q9Y678	446	3	-16	-1	11	1.0	0.9	1.0	1.1
OSBPL5	Q9H0X9	253	3	-21	-1	-22	1.0	0.8	1.0	0.8
LANCL2	Q9NS86	187	3	3	-1	-4	1.0	1.0	1.0	1.0
DCTN1	Q14203	888	3	1	-1	-2	1.0	1.0	1.0	1.0
TRAF3IP3	Q9Y228	42	3	1	-1	-3	1.0	1.0	1.0	1.0
FASTKD5	Q7L8L6	525	3	-5	-1	8	1.0	1.0	1.0	1.1
RAC1	P63000	178	3	-6	-1	-7	1.0	0.9	1.0	0.9
RASSF1	Q9NS23	102	3	-6	-1	-11	1.0	0.9	1.0	0.9
BMF	Q96LC9	139	3	-7	-1	-12	1.0	0.9	1.0	0.9
ECM29	Q5VYK3	1257	3	-8	-1	3	1.0	0.9	1.0	1.0
CNDP2	Q96KP4	205	3	-9	-1	-1	1.0	0.9	1.0	1.0
MED25	Q71SY5	429	3	-11	-1	-3	1.0	0.9	1.0	1.0
DUT	P33316	166	3	-11	-1	0	1.0	0.9	1.0	1.0
DGKQ	P52824	666	3	-18	-1	-15	1.0	0.8	1.0	0.9
ZZEF1	O43149	2312	2	-3	-1	-2	1.0	1.0	1.0	1.0
HNRNPA3	P51991	85	2	-3	-1	-5	1.0	1.0	1.0	1.0

GTF3C4	Q9UKN8	257	2	-4	-1	13	1.0	1.0	1.0	1.1
IRF4	Q15306	250	2	-5	-1	1	1.0	1.0	1.0	1.0
GMPS	P49915	456	2	-5	-1	-5	1.0	1.0	1.0	1.0
SPAG9	O60271	1155	2	-5	-1	-15	1.0	1.0	1.0	0.9
RANBP2	P49792	2577	2	-7	-1	-5	1.0	0.9	1.0	1.0
CRACR2A	Q9BSW2	272	2	-7	-1	-5	1.0	0.9	1.0	1.0
RAP1A	P62834	48	2	-8	-1	17	1.0	0.9	1.0	1.2
EFL1	Q7Z2Z2	474	2	-11	-1	-16	1.0	0.9	1.0	0.9
MYL12B	O14950	109	2	-12	-1	-5	1.0	0.9	1.0	1.0
ALOX12	P18054	508	2	-12	-1	-10	1.0	0.9	1.0	0.9
NPRL3	Q12980	278	2	-13	-1	-9	1.0	0.9	1.0	0.9
TBC1D15	Q8TC07	686	2	-17	-1	-21	1.0	0.9	1.0	0.8
PHAX	Q9H814	51	2	4	-1	3	1.0	1.0	1.0	1.0
DCK	P27707	45	2	2	-1	-5	1.0	1.0	1.0	1.0
PPME1	Q9Y570	347	2	-2	-1	-13	1.0	1.0	1.0	0.9
PYHIN1	Q6K0P9	163	2	-5	-1	-15	1.0	1.0	1.0	0.9
BLK	P51451	373	2	-9	-1	-1	1.0	0.9	1.0	1.0
LTA4H	P09960	147	2	-12	-1	9	1.0	0.9	1.0	1.1
L3HYPDH	Q96EM0	139	1	1	-1	6	1.0	1.0	1.0	1.1
MTHFD1	P11586	408	1	1	-1	2	1.0	1.0	1.0	1.0
NUP93	Q8N1F7	522	1	-1	-1	-2	1.0	1.0	1.0	1.0
PANK4	Q9NVE7	150	1	-3	-1	8	1.0	1.0	1.0	1.1
CENPW	Q5EE01	63	1	-7	-1	-4	1.0	0.9	1.0	1.0
BMX	P51813	137	1	-10	-1	-16	1.0	0.9	1.0	0.9
NUP160	Q12769	1124	1	-11	-1	10	1.0	0.9	1.0	1.1
ADO	Q96SZ5	18	1	-12	-1	-11	1.0	0.9	1.0	0.9
NOL11	Q9H8H0	333	1	-25	-1	-14	1.0	0.8	1.0	0.9
KAT7	O95251	602	1	2	-1	6	1.0	1.0	1.0	1.1
TLN1	Q9Y490	1023	1	-4	-1	7	1.0	1.0	1.0	1.1
RNF123	Q5XPI4	1180	1	-6	-1	-1	1.0	0.9	1.0	1.0
LIMS1	P48059	255	1	-7	-1	-2	1.0	0.9	1.0	1.0
SP140	Q13342	773	1	-7	-1	-5	1.0	0.9	1.0	1.0
SP140L	Q9H930	486	1	-7	-1	-5	1.0	0.9	1.0	1.0
WDSUB1	Q8N9V3	316	1	-10	-1	-16	1.0	0.9	1.0	0.9
NUP205	Q92621	698	1	-10	-1	-17	1.0	0.9	1.0	0.9
USP24	Q9UPU5	1859	1	-14	-1	-15	1.0	0.9	1.0	0.9
SEPT1	Q8WYJ6	99	0	-4	-1	7	1.0	1.0	1.0	1.1
KDM2A	Q9Y2K7	840	0	-5	-1	-6	1.0	1.0	1.0	0.9
FAM98B	Q52LJ0	216	0	-6	-1	3	1.0	0.9	1.0	1.0
NMRK1	Q9NWW6	125	0	-7	-1	-6	1.0	0.9	1.0	0.9
DHX57	Q6P158	453	0	-8	-1	-7	1.0	0.9	1.0	0.9
COG1	Q8WTW3	513	0	-9	-1	0	1.0	0.9	1.0	1.0
SMCHD1	A6NHR9	1018	0	-9	-1	-2	1.0	0.9	1.0	1.0
PARK7	Q99497	106	-1	-4	-1	-7	1.0	1.0	1.0	0.9
IMPDH1	P20839	331	-1	-6	-1	0	1.0	0.9	1.0	1.0
MYBBP1A	Q9BQG0	623	-1	-6	-1	-1	1.0	0.9	1.0	1.0
ARFGEF1	Q9Y6D6	1050	-1	-7	-1	-5	1.0	0.9	1.0	1.0
WDR33	Q9C0J8	120	-1	-14	-1	-1	1.0	0.9	1.0	1.0
SKIV2L	Q15477	913	-1	-18	-1	-9	1.0	0.9	1.0	0.9
PSMD1	Q99460	898	-1	-5	-1	-7	1.0	1.0	1.0	0.9
RAC2	P15153	178	-1	-8	-1	-7	1.0	0.9	1.0	0.9
VAPA	Q9P0L0	60	-1	-8	-1	-7	1.0	0.9	1.0	0.9
UBE2O	Q9C0C9	182	-1	-8	-1	-9	1.0	0.9	1.0	0.9

RUVBL1	Q9Y265	141	-1	-14	-1	-2	1.0	0.9	1.0	1.0
RHOF	Q9HBH0	121	-1	-15	-1	-8	1.0	0.9	1.0	0.9
UPF1	Q92900	209	-2	-4	-1	-4	1.0	1.0	1.0	1.0
MYO1G	B0I1T2	942	-2	-6	-1	3	1.0	0.9	1.0	1.0
IRF3	Q14653	222	-2	-6	-1	-3	1.0	0.9	1.0	1.0
RRBP1	Q9P2E9	933	-2	-7	-1	-5	1.0	0.9	1.0	1.0
PDCD11	Q14690	333	-2	-7	-1	-4	1.0	0.9	1.0	1.0
ARID2	Q68CP9	1512	-2	-7	-1	-15	1.0	0.9	1.0	0.9
WDFY1	Q8IWB7	142	-2	-11	-1	-6	1.0	0.9	1.0	0.9
PCF11	O94913	1518	-2	-26	-1	-26	1.0	0.8	1.0	0.8
RPL10	P27635	105	-2	0	-1	-5	1.0	1.0	1.0	1.0
SESTD1	Q86VW0	265	-2	-1	-1	-8	1.0	1.0	1.0	0.9
FCGR2B	P31994	261	-2	-4	-1	-6	1.0	1.0	1.0	0.9
MLLT4	P55196	1684	-2	-6	-1	1	1.0	0.9	1.0	1.0
PPM1G	O15355	241	-2	-7	-1	-13	1.0	0.9	1.0	0.9
RBM28	Q9NW13	71	-2	-9	-1	-11	1.0	0.9	1.0	0.9
CCDC25	Q86WR0	83	-2	-10	-1	-7	1.0	0.9	1.0	0.9
UNC45A	Q9H3U1	426	-2	-11	-1	-11	1.0	0.9	1.0	0.9
UTP18	Q9Y5J1	507	-2	-14	-1	30	1.0	0.9	1.0	1.4
ALKBH4	Q9NXW9	126	-2	-19	-1	2	1.0	0.8	1.0	1.0
ZNF687	Q8N1G0	1140	-2	-21	-1	-19	1.0	0.8	1.0	0.8
WDR11	Q9BZH6	843	-3	3	-1	28	1.0	1.0	1.0	1.4
MTHFD1	P11586	863	-3	-7	-1	10	1.0	0.9	1.0	1.1
ADAR	P55265	622	-3	-7	-1	-3	1.0	0.9	1.0	1.0
ATP6V1A	P38606	277	-3	-9	-1	-3	1.0	0.9	1.0	1.0
GIMAP1-GIM	A0A087WTJ2	442	-3	-10	-1	-12	1.0	0.9	1.0	0.9
LRBA	P50851	2675	-3	-12	-1	-7	1.0	0.9	1.0	0.9
GRHPR	Q9UBQ7	29	-3	-16	-1	-6	1.0	0.9	1.0	0.9
PDS5A	Q29RF7	589	-3	-19	-1	0	1.0	0.8	1.0	1.0
ORC3	Q9UBD5	483	-3	2	-1	-15	1.0	1.0	1.0	0.9
MTCH2	Q9Y6C9	49	-3	-5	-1	-9	1.0	1.0	1.0	0.9
NUMA1	Q14980	65	-3	-6	-1	1	1.0	0.9	1.0	1.0
SEPT7	Q16181	280	-3	-8	-1	-3	1.0	0.9	1.0	1.0
RNF213	Q63HN8	4012	-3	-12	-1	-6	1.0	0.9	1.0	0.9
SURF2	Q15527	111	-3	-19	-1	-28	1.0	0.8	1.0	0.8
ZFYVE19	Q96K21	104	-4	-7	-1	-11	1.0	0.9	1.0	0.9
AP1G2	O75843	180	-4	-8	-1	11	1.0	0.9	1.0	1.1
TBCE	Q15813	152	-4	-8	-1	4	1.0	0.9	1.0	1.0
POLR2B	P30876	945	-4	-10	-1	-4	1.0	0.9	1.0	1.0
FBXL12	Q9NXK8	212	-4	-10	-1	5	1.0	0.9	1.0	1.0
HLA-C	P10321	345	-4	-11	-1	-13	1.0	0.9	1.0	0.9
EIF5A2	Q9GZV4	73	-4	-6	-1	5	1.0	0.9	1.0	1.0
RPS3	P23396	134	-4	-10	-1	4	1.0	0.9	1.0	1.0
TMEM173	Q86WV6	91	-4	-11	-1	25	1.0	0.9	1.0	1.3
TRRAP	Q9Y4A5	2449	-4	-12	-1	-9	1.0	0.9	1.0	0.9
ZZEF1	O43149	2084	-5	-6	-1	-6	1.0	0.9	1.0	0.9
ASPSCR1	Q9BZE9	127	-5	-7	-1	-19	1.0	0.9	1.0	0.8
ZYX	Q15942	447	-5	-10	-1	-1	1.0	0.9	1.0	1.0
SEC23IP	Q9Y6Y8	814	-5	-19	-1	1	1.0	0.8	1.0	1.0
TRIM65	Q6PJ69	500	-5	-19	-1	-8	1.0	0.8	1.0	0.9
MDN1	Q9NU22	2890	-5	1	-1	-12	1.0	1.0	1.0	0.9
THBS1	P07996	894	-5	-2	-1	-12	1.0	1.0	1.0	0.9
NMT2	O60551	104	-5	-3	-1	-11	1.0	1.0	1.0	0.9

EXOSC7	Q15024	85	-5	-16	-1	-4	1.0	0.9	1.0	1.0
BIRC6	Q9NR09	777	-6	-10	-1	-19	0.9	0.9	1.0	0.8
SNX27	Q96L92	354	-6	-14	-1	-9	0.9	0.9	1.0	0.9
IFIT5	Q13325	476	-6	-14	-1	-19	0.9	0.9	1.0	0.8
DST	Q03001	5080	-6	-14	-1	-7	0.9	0.9	1.0	0.9
ACTR6	Q9GZN1	28	-6	-15	-1	-9	0.9	0.9	1.0	0.9
PISD	Q9UG56	181	-7	2	-1	-11	0.9	1.0	1.0	0.9
ZFYVE21	Q9BQ24	88	-7	-3	-1	-6	0.9	1.0	1.0	0.9
RPA3	P35244	26	-7	-19	-1	-10	0.9	0.8	1.0	0.9
CLIC4	Q9Y696	35	-7	-20	-1	-12	0.9	0.8	1.0	0.9
RING1	Q06587	69	-8	-8	-1	-2	0.9	0.9	1.0	1.0
KMT2B	Q9UMN6	353	-8	-10	-1	-8	0.9	0.9	1.0	0.9
TTC14	Q96N46	371	-8	-10	-1	-9	0.9	0.9	1.0	0.9
ATXN10	Q9UBB4	356	-8	-11	-1	-1	0.9	0.9	1.0	1.0
TNKS1BP1	Q9C0C2	136	-8	-16	-1	-14	0.9	0.9	1.0	0.9
PRAF2	O60831	28	-9	-5	-1	6	0.9	1.0	1.0	1.1
NLRC5	Q86WI3	741	-9	-7	-1	5	0.9	0.9	1.0	1.0
ADNP	Q9H2P0	170	-9	-9	-1	-5	0.9	0.9	1.0	1.0
ZNF451	Q9Y4E5	722	-9	-12	-1	-5	0.9	0.9	1.0	1.0
BAZ1B	Q9UIG0	1231	-9	-2	-1	9	0.9	1.0	1.0	1.1
UBA2	Q9UBT2	441	-10	-6	-1	-5	0.9	0.9	1.0	1.0
UTP14A	Q9BVJ6	522	-10	-21	-1	-25	0.9	0.8	1.0	0.8
NAGK	Q9UJ70	45	-10	-2	-1	-2	0.9	1.0	1.0	1.0
HSPA8	P11142	17	-10	-10	-1	3	0.9	0.9	1.0	1.0
ELANE	P08246	187	-10	-17	-1	5	0.9	0.9	1.0	1.0
ZYX	Q15942	507	-11	3	-1	-20	0.9	1.0	1.0	0.8
ZC3H12D	A2A288	177	-12	-7	-1	-17	0.9	0.9	1.0	0.9
F8A3	P23610	110	-13	2	-1	4	0.9	1.0	1.0	1.0
LMO7	Q8WWI1	1646	-13	-5	-1	-39	0.9	1.0	1.0	0.7
GLTSCR1L	Q6AI39	618	-13	-12	-1	-25	0.9	0.9	1.0	0.8
GPD2	P43304	270	-13	-18	-1	-10	0.9	0.9	1.0	0.9
KLHL6	Q8WZ60	252	-14	-15	-1	-7	0.9	0.9	1.0	0.9
JAML	Q86YT9	332	-16	-7	-1	-20	0.9	0.9	1.0	0.8
SUPT5H	O00267	740	-16	-1	-1	-18	0.9	1.0	1.0	0.9
PIAS4	Q8N2W9	299	21	-2	-2	6	1.3	1.0	1.0	1.1
ARHGAP25	P42331	183	20	-34	-2	-4	1.3	0.7	1.0	1.0
ZMYM2	Q9UBW7	734	19	-9	-2	-2	1.2	0.9	1.0	1.0
DTX3L	Q8TDB6	130	19	1	-2	-17	1.2	1.0	1.0	0.9
LAP3	P28838	129	19	-12	-2	10	1.2	0.9	1.0	1.1
API5	Q9BZZ5	234	18	-10	-2	12	1.2	0.9	1.0	1.1
ETFA	P13804	60	17	-4	-2	-3	1.2	1.0	1.0	1.0
ITSN2	Q9NZM3	1307	16	-17	-2	10	1.2	0.9	1.0	1.1
CRYZ	Q08257	145	16	-2	-2	9	1.2	1.0	1.0	1.1
PRDX4	Q13162	51	16	-7	-2	-10	1.2	0.9	1.0	0.9
VPS53	Q5VIR6	242	15	-4	-2	3	1.2	1.0	1.0	1.0
ABHD10	Q9NUJ1	100	15	-7	-2	1	1.2	0.9	1.0	1.0
DHRS4	Q9BTZ2	248	15	1	-2	9	1.2	1.0	1.0	1.1
TRRAP	Q9Y4A5	2279	14	-7	-2	7	1.2	0.9	1.0	1.1
PREX1	Q8TCU6	234	14	-15	-2	1	1.2	0.9	1.0	1.0
CAPN15	O75808	360	14	-15	-2	-31	1.2	0.9	1.0	0.8
PSMG2	Q969U7	178	14	-7	-2	-1	1.2	0.9	1.0	1.0
RANGAP1	P46060	152	13	-7	-2	3	1.1	0.9	1.0	1.0
JAK3	P52333	227	13	-11	-2	10	1.1	0.9	1.0	1.1

TOP1	P11387	733	13	-39	-2	8	1.1	0.7	1.0	1.1
LARP4B	Q92615	679	13	15	-2	-9	1.1	1.2	1.0	0.9
GOT2	P00505	295	13	3	-2	10	1.1	1.0	1.0	1.1
SUPT6H	Q7KZ85	1463	13	-12	-2	3	1.1	0.9	1.0	1.0
CTCF	P49711	268	12	-4	-2	-3	1.1	1.0	1.0	1.0
NUP58	Q9BVL2	252	12	-7	-2	5	1.1	0.9	1.0	1.0
C9orf114	Q5T280	151	11	-2	-2	13	1.1	1.0	1.0	1.1
CORO7-PAM1	A0A0A6YYL4	325	11	-15	-2	3	1.1	0.9	1.0	1.0
SMG1	Q96Q15	254	11	-5	-2	2	1.1	1.0	1.0	1.0
KDELR2	P33947	29	11	-9	-2	-2	1.1	0.9	1.0	1.0
WDR11	Q9BZH6	364	11	-20	-2	-12	1.1	0.8	1.0	0.9
RCC2	Q9P258	271	10	0	-2	-7	1.1	1.0	1.0	0.9
GTF3C2	Q8WUA4	608	10	-6	-2	6	1.1	0.9	1.0	1.1
TRIP12	Q14669	1538	10	-7	-2	6	1.1	0.9	1.0	1.1
HSD17B12	Q53GQ0	215	10	-7	-2	0	1.1	0.9	1.0	1.0
ING3	Q9NXR8	187	10	-9	-2	-14	1.1	0.9	1.0	0.9
AVL9	Q8NBF6	119	10	-17	-2	-4	1.1	0.9	1.0	1.0
ADCY7	P51828	1050	10	11	-2	-3	1.1	1.1	1.0	1.0
EFL1	Q7Z2Z2	646	10	11	-2	-4	1.1	1.1	1.0	1.0
LPXN	O60711	340	10	-6	-2	0	1.1	0.9	1.0	1.0
PDS5A	Q29RF7	350	10	-9	-2	-1	1.1	0.9	1.0	1.0
PDS5B	Q9NTI5	340	10	-9	-2	-1	1.1	0.9	1.0	1.0
RIF1	Q5UIP0	2450	10	-10	-2	-8	1.1	0.9	1.0	0.9
CTC1	Q2NKJ3	632	10	-12	-2	10	1.1	0.9	1.0	1.1
ACSL3	O95573	85	10	-16	-2	4	1.1	0.9	1.0	1.0
MROH1	Q8NDA8	1263	9	-4	-2	3	1.1	1.0	1.0	1.0
CUL3	Q13618	464	9	-5	-2	1	1.1	1.0	1.0	1.0
DVL2	O14641	354	9	-8	-2	-2	1.1	0.9	1.0	1.0
XPO7	Q9UIA9	123	9	-10	-2	-1	1.1	0.9	1.0	1.0
SYTL4	Q96C24	88	9	-5	-2	-2	1.1	1.0	1.0	1.0
MAT2B	Q9NZL9	297	9	-10	-2	0	1.1	0.9	1.0	1.0
RSL1D1	O76021	211	9	-13	-2	-2	1.1	0.9	1.0	1.0
EPS15L1	Q9UBC2	470	9	-15	-2	4	1.1	0.9	1.0	1.0
TBC1D9B	Q66K14	494	9	-23	-2	-9	1.1	0.8	1.0	0.9
LAGE3	Q14657	23	8	0	-2	2	1.1	1.0	1.0	1.0
TAF10	Q12962	174	8	-3	-2	-1	1.1	1.0	1.0	1.0
PSTPIP1	O43586	13	8	-9	-2	-1	1.1	0.9	1.0	1.0
GCDH	Q92947	228	8	-10	-2	9	1.1	0.9	1.0	1.1
CCT4	P50991	379	8	-12	-2	-4	1.1	0.9	1.0	1.0
KIAA0391	O15091	367	8	-14	-2	-15	1.1	0.9	1.0	0.9
DLAT	P10515	488	8	-16	-2	3	1.1	0.9	1.0	1.0
PITPNM1	O00562	889	8	7	-2	-4	1.1	1.1	1.0	1.0
DYSF	O75923	1522	8	-4	-2	1	1.1	1.0	1.0	1.0
HSPA4	P34932	34	8	-9	-2	4	1.1	0.9	1.0	1.0
EIF4A2	Q14240	135	7	3	-2	-3	1.1	1.0	1.0	1.0
MRPL50	Q8N5N7	52	7	-6	-2	1	1.1	0.9	1.0	1.0
NHEJ1	Q9H9Q4	74	7	-10	-2	-6	1.1	0.9	1.0	0.9
C10orf11	Q9H2I8	106	7	-13	-2	-5	1.1	0.9	1.0	1.0
BTK	Q06187	63	7	-19	-2	-6	1.1	0.8	1.0	0.9
ZNF106	Q9H2Y7	1069	7	6	-2	-2	1.1	1.1	1.0	1.0
CEP192	Q8TEP8	286	7	4	-2	14	1.1	1.0	1.0	1.2
AHCY	P23526	421	7	-2	-2	-3	1.1	1.0	1.0	1.0
LIG4	P49917	855	7	-3	-2	-11	1.1	1.0	1.0	0.9

PIP5K1A	Q99755	312	7	-5	-2	-10	1.1	1.0	1.0	0.9
NT5C3A	Q9H0P0	73	7	-6	-2	1	1.1	0.9	1.0	1.0
MTMR2	Q13614	95	7	-8	-2	8	1.1	0.9	1.0	1.1
PDXDC1	Q6P996	361	7	-9	-2	7	1.1	0.9	1.0	1.1
CYTH1	Q15438	94	7	-10	-2	-6	1.1	0.9	1.0	0.9
IP6K1	Q92551	248	7	-11	-2	-9	1.1	0.9	1.0	0.9
RNF213	Q63HN8	2412	7	-18	-2	-5	1.1	0.9	1.0	1.0
PRKCH	P24723	399	6	20	-2	9	1.1	1.3	1.0	1.1
PPP2R4	Q15257	199	6	4	-2	11	1.1	1.0	1.0	1.1
RREB1	Q92766	329	6	2	-2	1	1.1	1.0	1.0	1.0
SMARCA2	P51531	91	6	2	-2	-9	1.1	1.0	1.0	0.9
ACTN1	P12814	370	6	-3	-2	-3	1.1	1.0	1.0	1.0
DYNC1H1	Q14204	1888	6	-4	-2	-2	1.1	1.0	1.0	1.0
GABPA	Q06546	421	6	-5	-2	5	1.1	1.0	1.0	1.1
GOLGB1	Q14789	1569	6	-6	-2	-5	1.1	0.9	1.0	1.0
GATB	O75879	170	6	-6	-2	-15	1.1	0.9	1.0	0.9
NEK7	Q8TDX7	247	6	-9	-2	-34	1.1	0.9	1.0	0.7
ADK	P55263	160	6	-11	-2	4	1.1	0.9	1.0	1.0
EEF2	P13639	651	6	-12	-2	1	1.1	0.9	1.0	1.0
PARP9	Q8IXQ6	91	6	-13	-2	9	1.1	0.9	1.0	1.1
IKBKG	Q9Y6K9	131	6	-13	-2	-7	1.1	0.9	1.0	0.9
ACOT7	O00154	288	6	-17	-2	-6	1.1	0.9	1.0	0.9
HAUS6	Q7Z4H7	323	6	-2	-2	-3	1.1	1.0	1.0	1.0
SHKBP1	Q8TBC3	550	6	-2	-2	7	1.1	1.0	1.0	1.1
RPLP1	P05386	61	6	-7	-2	-8	1.1	0.9	1.0	0.9
RABIF	P47224	106	6	-9	-2	-9	1.1	0.9	1.0	0.9
DPAGT1	Q9H3H5	42	6	-14	-2	-14	1.1	0.9	1.0	0.9
RPS12	P25398	108	6	-15	-2	-7	1.1	0.9	1.0	0.9
CBX1	P83916	156	6	-28	-2	-10	1.1	0.8	1.0	0.9
XRCC5	P13010	493	6	-38	-2	16	1.1	0.7	1.0	1.2
UNK	Q9C0B0	791	5	1	-2	-31	1.1	1.0	1.0	0.8
IDNK	Q5T6J7	63	5	-2	-2	9	1.1	1.0	1.0	1.1
SMARCAD1	Q9H4L7	772	5	-2	-2	0	1.1	1.0	1.0	1.0
FBXO38	Q6PIJ6	401	5	-3	-2	-3	1.1	1.0	1.0	1.0
KBTBD11	O94819	485	5	-4	-2	-4	1.1	1.0	1.0	1.0
RAP1A	P62834	141	5	-10	-2	2	1.1	0.9	1.0	1.0
RNF34	Q969K3	137	5	-10	-2	-19	1.1	0.9	1.0	0.8
LRRC59	Q96AG4	48	5	-13	-2	5	1.1	0.9	1.0	1.0
SYNE1	Q8NF91	7251	5	-17	-2	-17	1.1	0.9	1.0	0.9
CACTIN	Q8WUQ7	699	5	4	-2	-1	1.0	1.0	1.0	1.0
C4orf27	Q9NWY4	17	5	2	-2	4	1.0	1.0	1.0	1.0
ZNF687	Q8N1G0	91	5	1	-2	-18	1.0	1.0	1.0	0.8
XPO5	Q9HAV4	706	5	0	-2	5	1.0	1.0	1.0	1.0
DCUN1D1	Q96GG9	115	5	-3	-2	-8	1.0	1.0	1.0	0.9
TXNIP	Q9H3M7	170	5	-6	-2	-5	1.0	0.9	1.0	1.0
NNT	Q13423	936	5	-8	-2	5	1.0	0.9	1.0	1.1
SART3	Q15020	472	5	-9	-2	10	1.0	0.9	1.0	1.1
STK17B	O94768	73	5	-11	-2	-17	1.0	0.9	1.0	0.9
PRKCB	P05771	586	5	-15	-2	3	1.0	0.9	1.0	1.0
DUS2	Q9NX74	154	4	-1	-2	20	1.0	1.0	1.0	1.2
RPL3	P39023	157	4	-3	-2	20	1.0	1.0	1.0	1.3
LIMS1	P48059	240	4	-4	-2	5	1.0	1.0	1.0	1.0
TLN1	Q9Y490	1353	4	-5	-2	-2	1.0	1.0	1.0	1.0

PRMT1	Q99873	354	4	-6	-2	1	1.0	0.9	1.0	1.0
GAPVD1	Q14C86	71	4	-6	-2	-5	1.0	0.9	1.0	1.0
NUP88	Q99567	454	4	-6	-2	-5	1.0	0.9	1.0	1.0
DIAPH2	O60879	862	4	-11	-2	5	1.0	0.9	1.0	1.0
ANKRD44	Q8N8A2	704	4	-11	-2	-9	1.0	0.9	1.0	0.9
UROD	P06132	59	4	-12	-2	-5	1.0	0.9	1.0	1.0
SPG11	Q96JI7	838	4	-15	-2	-12	1.0	0.9	1.0	0.9
NCAPD3	P42695	330	4	-1	-2	-1	1.0	1.0	1.0	1.0
PLCL2	Q9UPR0	90	4	-3	-2	-10	1.0	1.0	1.0	0.9
EIF2B4	Q9UI10	465	4	-4	-2	-1	1.0	1.0	1.0	1.0
NSA2	O95478	193	4	-6	-2	-1	1.0	0.9	1.0	1.0
ZNF185	O15231	655	4	-8	-2	-2	1.0	0.9	1.0	1.0
COG1	Q8WTW3	631	4	-8	-2	-7	1.0	0.9	1.0	0.9
PDIA3	P30101	85	4	-11	-2	12	1.0	0.9	1.0	1.1
PLA2G4A	P47712	726	4	-14	-2	-9	1.0	0.9	1.0	0.9
HTT	P42858	1961	4	-14	-2	-24	1.0	0.9	1.0	0.8
TBC1D1	Q86TI0	67	4	-20	-2	-2	1.0	0.8	1.0	1.0
RPA1	P27694	486	3	-1	-2	0	1.0	1.0	1.0	1.0
ZMYM3	Q14202	636	3	-1	-2	-3	1.0	1.0	1.0	1.0
ATP6V1G1	O75348	104	3	-2	-2	-11	1.0	1.0	1.0	0.9
NUMA1	Q14980	658	3	-3	-2	-3	1.0	1.0	1.0	1.0
ORC3	Q9UBD5	561	3	-3	-2	5	1.0	1.0	1.0	1.0
AK2	P54819	40	3	-4	-2	-7	1.0	1.0	1.0	0.9
HSPH1	Q92598	658	3	-4	-2	-4	1.0	1.0	1.0	1.0
DNAJB1	P25685	179	3	-5	-2	4	1.0	1.0	1.0	1.0
INPP5D	Q92835	385	3	-5	-2	6	1.0	1.0	1.0	1.1
SCFD2	Q8WU76	507	3	-6	-2	9	1.0	0.9	1.0	1.1
ABCF2	Q9UG63	388	3	-7	-2	-10	1.0	0.9	1.0	0.9
SMARCD1	Q96GM5	492	3	-8	-2	-19	1.0	0.9	1.0	0.8
SLFN5	Q08AF3	14	3	-10	-2	-1	1.0	0.9	1.0	1.0
INTS9	Q9NV88	471	3	-10	-2	22	1.0	0.9	1.0	1.3
MDC1	Q14676	1742	3	-12	-2	-11	1.0	0.9	1.0	0.9
CPT1A	P50416	304	3	-13	-2	5	1.0	0.9	1.0	1.0
KIAA1211	Q6ZU35	860	3	-20	-2	-14	1.0	0.8	1.0	0.9
LIN37	Q96GY3	28	3	-21	-2	-6	1.0	0.8	1.0	0.9
NAPRT	Q6XQN6	484	3	-3	-2	3	1.0	1.0	1.0	1.0
NCAPD2	Q15021	596	3	-3	-2	-16	1.0	1.0	1.0	0.9
FMNL1	O95466	939	3	-4	-2	4	1.0	1.0	1.0	1.0
FOXN3	O00409	469	3	-5	-2	-10	1.0	1.0	1.0	0.9
TMEM248	Q9NWD8	305	3	-5	-2	-12	1.0	1.0	1.0	0.9
ASH2L	Q9UBL3	581	3	-7	-2	-6	1.0	0.9	1.0	0.9
MDN1	Q9NU22	1394	3	-7	-2	-7	1.0	0.9	1.0	0.9
AKAP9	Q99996	1156	3	-8	-2	-1	1.0	0.9	1.0	1.0
UBN1	Q9NPG3	548	3	-11	-2	-7	1.0	0.9	1.0	0.9
TLN1	Q9Y490	2161	2	-5	-2	1	1.0	1.0	1.0	1.0
GNB1	P62873	149	2	-5	-2	-4	1.0	1.0	1.0	1.0
ENOSF1	Q7L5Y1	67	2	-5	-2	-7	1.0	1.0	1.0	0.9
PDCD4	Q53EL6	288	2	-7	-2	-5	1.0	0.9	1.0	1.0
SWAP70	Q9UH65	261	2	-7	-2	-6	1.0	0.9	1.0	0.9
PURB	Q96QR8	17	2	-8	-2	-17	1.0	0.9	1.0	0.9
ABI3	Q9P2A4	145	2	-9	-2	-3	1.0	0.9	1.0	1.0
ARHGEF2	Q92974	715	2	-9	-2	-14	1.0	0.9	1.0	0.9
GRK5	P34947	201	2	-9	-2	-1	1.0	0.9	1.0	1.0

GRK6	P43250	201	2	-9	-2	-1	1.0	0.9	1.0	1.0
EP300	Q09472	1250	2	-9	-2	-5	1.0	0.9	1.0	1.0
SETX	Q7Z333	1277	2	-11	-2	-15	1.0	0.9	1.0	0.9
WRNIP1	Q96S55	502	2	-12	-2	-4	1.0	0.9	1.0	1.0
TBL1XR1	Q9BZK7	434	2	-19	-2	-7	1.0	0.8	1.0	0.9
ANKRD44	Q8N8A2	834	2	5	-2	-8	1.0	1.0	1.0	0.9
NUMA1	Q14980	160	2	-2	-2	-6	1.0	1.0	1.0	0.9
RAD54L2	Q9Y4B4	820	2	-5	-2	-1	1.0	1.0	1.0	1.0
MEPCE	Q7L2J0	419	2	-5	-2	-6	1.0	1.0	1.0	0.9
DIDO1	Q9BTC0	455	2	-7	-2	-1	1.0	0.9	1.0	1.0
NDRG3	Q9UGV2	166	2	-10	-2	-1	1.0	0.9	1.0	1.0
ERAP1	Q9NZ08	193	2	-11	-2	-8	1.0	0.9	1.0	0.9
CORO7-PAM:	A0A0A6YYL4	389	2	-13	-2	-7	1.0	0.9	1.0	0.9
CD2BP2	O95400	234	1	-3	-2	-7	1.0	1.0	1.0	0.9
EP300	Q09472	1790	1	-3	-2	2	1.0	1.0	1.0	1.0
CREBBP	Q92793	1827	1	-3	-2	2	1.0	1.0	1.0	1.0
USP7	Q93009	315	1	-3	-2	-3	1.0	1.0	1.0	1.0
SLC9A3R1	O14745	206	1	-5	-2	-7	1.0	1.0	1.0	0.9
IDH3B	O43837	185	1	-5	-2	-2	1.0	1.0	1.0	1.0
SIDT2	Q8NBJ9	335	1	-5	-2	-11	1.0	1.0	1.0	0.9
STAT1	P42224	155	1	-6	-2	-7	1.0	0.9	1.0	0.9
OSBPL1A	Q9BXW6	387	1	-7	-2	-2	1.0	0.9	1.0	1.0
TDRD7	Q8NHU6	692	1	-13	-2	-14	1.0	0.9	1.0	0.9
C7orf43	Q8WVR3	302	1	-14	-2	1	1.0	0.9	1.0	1.0
ENTHD2	Q96N21	31	1	-16	-2	1	1.0	0.9	1.0	1.0
NASP	P49321	254	1	-17	-2	-12	1.0	0.9	1.0	0.9
FAM49B	Q9NUQ9	253	1	-28	-2	-10	1.0	0.8	1.0	0.9
ZRSR2	Q15696	302	1	-4	-2	2	1.0	1.0	1.0	1.0
RNF213	Q63HN8	1614	1	-5	-2	-1	1.0	1.0	1.0	1.0
PI4KA	P42356	1131	1	-5	-2	-5	1.0	1.0	1.0	1.0
MDN1	Q9NU22	1332	1	-6	-2	2	1.0	0.9	1.0	1.0
SLC25A40	Q8TBP6	142	1	-8	-2	8	1.0	0.9	1.0	1.1
DDX31	Q9H8H2	660	1	-9	-2	-4	1.0	0.9	1.0	1.0
NSF	P46459	250	1	-10	-2	-4	1.0	0.9	1.0	1.0
RPS26	P62854	26	1	-10	-2	-8	1.0	0.9	1.0	0.9
SPECC1	Q5M775	578	1	-11	-2	-12	1.0	0.9	1.0	0.9
TGFB1I1	O43294	416	1	-12	-2	-5	1.0	0.9	1.0	1.0
EIF3K	Q9UBQ5	190	1	-17	-2	-17	1.0	0.9	1.0	0.9
ETS1	P14921	99	0	1	-2	-5	1.0	1.0	1.0	1.0
DNAJA1	P31689	149	0	-4	-2	-9	1.0	1.0	1.0	0.9
DRAP1	Q14919	54	0	-6	-2	-3	1.0	0.9	1.0	1.0
PRKDC	P78527	1507	0	-6	-2	4	1.0	0.9	1.0	1.0
COG1	Q8WTW3	625	0	-8	-2	13	1.0	0.9	1.0	1.1
NARFL	Q9H6Q4	179	0	-8	-2	-11	1.0	0.9	1.0	0.9
C16orf62	Q7Z3J2	588	0	-11	-2	-11	1.0	0.9	1.0	0.9
KDM3A	Q9Y4C1	390	0	-11	-2	-18	1.0	0.9	1.0	0.8
KIFC1	Q9BW19	144	0	-12	-2	-12	1.0	0.9	1.0	0.9
GTF3C1	Q12789	431	0	-13	-2	-15	1.0	0.9	1.0	0.9
CAPZA1	P52907	124	0	-18	-2	9	1.0	0.8	1.0	1.1
UPF2	Q9HAU5	687	0	-38	-2	-2	1.0	0.7	1.0	1.0
MTHFSD	Q2M296	361	-1	4	-2	3	1.0	1.0	1.0	1.0
NOL7	Q9UMY1	149	-1	-5	-2	-10	1.0	1.0	1.0	0.9
TPR	P12270	75	-1	-8	-2	-4	1.0	0.9	1.0	1.0

ACBD6	Q9BR61	267	-1	-8	-2	-7	1.0	0.9	1.0	0.9
INPPL1	O15357	926	-1	-11	-2	1	1.0	0.9	1.0	1.0
TLK2	Q86UE8	737	-1	-16	-2	-15	1.0	0.9	1.0	0.9
MTMR14	Q8NCE2	252	-1	2	-2	4	1.0	1.0	1.0	1.0
WDFY4	Q6ZS81	1569	-1	2	-2	-3	1.0	1.0	1.0	1.0
FASN	P49327	1118	-1	-3	-2	-7	1.0	1.0	1.0	0.9
LACTB2	Q53H82	100	-1	-5	-2	-8	1.0	1.0	1.0	0.9
USP7	Q93009	711	-1	-5	-2	-1	1.0	1.0	1.0	1.0
STAU2	Q9NUL3	491	-1	-6	-2	-15	1.0	0.9	1.0	0.9
RPS16	P62249	25	-1	-8	-2	4	1.0	0.9	1.0	1.0
PDS5A	Q29RF7	327	-1	-11	-2	13	1.0	0.9	1.0	1.1
CREBBP	Q92793	380	-1	-16	-2	-8	1.0	0.9	1.0	0.9
PLEC	Q15149	4494	-2	-6	-2	-4	1.0	0.9	1.0	1.0
FAM98C	Q17RN3	36	-2	-6	-2	-4	1.0	0.9	1.0	1.0
ATM	Q13315	532	-2	-6	-2	-12	1.0	0.9	1.0	0.9
PRDX3	P30048	229	-2	-8	-2	1	1.0	0.9	1.0	1.0
Uncharacteri	G3V599	1114	-2	-9	-2	-14	1.0	0.9	1.0	0.9
IKBKG	Q9Y6K9	76	-2	-12	-2	-20	1.0	0.9	1.0	0.8
POGZ	Q7Z3K3	496	-2	-14	-2	5	1.0	0.9	1.0	1.0
LACE1	Q8WV93	72	-2	-15	-2	-3	1.0	0.9	1.0	1.0
PIK3C2B	O00750	384	-2	-17	-2	-2	1.0	0.9	1.0	1.0
GMIP	Q9P107	537	-2	-21	-2	1	1.0	0.8	1.0	1.0
ADAM10	O14672	173	-2	-27	-2	-1	1.0	0.8	1.0	1.0
TTC9	Q92623	67	-2	-1	-2	5	1.0	1.0	1.0	1.0
CAPN1	P07384	677	-2	-3	-2	4	1.0	1.0	1.0	1.0
VDAC2	P45880	13	-2	-4	-2	-8	1.0	1.0	1.0	0.9
NANS	Q9NR45	287	-2	-4	-2	-7	1.0	1.0	1.0	0.9
SIPA1	Q96FS4	811	-2	-5	-2	-2	1.0	1.0	1.0	1.0
SP100	P23497	309	-2	-5	-2	-12	1.0	1.0	1.0	0.9
MGMT	P16455	145	-2	-6	-2	-4	1.0	0.9	1.0	1.0
PAN2	Q504Q3	575	-2	-6	-2	-9	1.0	0.9	1.0	0.9
C9orf114	Q5T280	139	-2	-7	-2	-2	1.0	0.9	1.0	1.0
CMPK2	Q5EBM0	243	-2	-16	-2	-7	1.0	0.9	1.0	0.9
WDR11	Q9BZH6	1029	-3	0	-2	4	1.0	1.0	1.0	1.0
RB1	P06400	853	-3	0	-2	-11	1.0	1.0	1.0	0.9
PSME3	P61289	92	-3	-4	-2	-10	1.0	1.0	1.0	0.9
CUL9	Q8IWT3	2177	-3	-7	-2	-15	1.0	0.9	1.0	0.9
RAP1GDS1	P52306	29	-3	-7	-2	1	1.0	0.9	1.0	1.0
VPS13C	Q709C8	3707	-3	-9	-2	-5	1.0	0.9	1.0	1.0
PPAT	Q06203	100	-3	-11	-2	-3	1.0	0.9	1.0	1.0
CAND1	Q86VP6	571	-3	-16	-2	3	1.0	0.9	1.0	1.0
SP140	Q13342	97	-3	-3	-2	-13	1.0	1.0	1.0	0.9
IPO4	Q8TEX9	269	-3	-5	-2	1	1.0	1.0	1.0	1.0
ALKBH8	Q96BT7	296	-3	-6	-2	-12	1.0	0.9	1.0	0.9
CASP8	Q14790	360	-3	-7	-2	-13	1.0	0.9	1.0	0.9
PFN1	P07737	71	-3	-10	-2	4	1.0	0.9	1.0	1.0
NOP56	O00567	211	-3	-14	-2	6	1.0	0.9	1.0	1.1
TBCK	Q8TEA7	309	-3	-20	-2	7	1.0	0.8	1.0	1.1
ZBTB18	Q99592	13	-3	-22	-2	5	1.0	0.8	1.0	1.0
DUS1L	Q6P1R4	265	-4	3	-2	7	1.0	1.0	1.0	1.1
TBC1D9B	Q66K14	839	-4	-4	-2	-11	1.0	1.0	1.0	0.9
UXS1	Q8NBZ7	230	-4	-6	-2	-9	1.0	0.9	1.0	0.9
ACSL3	O95573	503	-4	-12	-2	-9	1.0	0.9	1.0	0.9

ARHGEF11	O15085	1047	-4	-12	-2	-19	1.0	0.9	1.0	0.8
N4BP1	O75113	454	-4	-14	-2	-21	1.0	0.9	1.0	0.8
ARHGEF40	Q8TER5	183	-4	-3	-2	-5	1.0	1.0	1.0	1.0
UBA3	Q8TBC4	28	-4	-6	-2	-15	1.0	0.9	1.0	0.9
MBNL1	Q9NR56	193	-4	-11	-2	-1	1.0	0.9	1.0	1.0
AHCTF1	Q8WYP5	313	-4	-13	-2	-14	1.0	0.9	1.0	0.9
IKBKB	O14920	114	-4	-19	-2	-21	1.0	0.8	1.0	0.8
SEPT2	Q15019	114	-5	-7	-2	-16	1.0	0.9	1.0	0.9
EIF3CL	B5ME19	444	-5	-11	-2	-7	1.0	0.9	1.0	0.9
TBCE	Q15813	522	-5	-15	-2	4	1.0	0.9	1.0	1.0
RGP1	Q92546	314	-5	-17	-2	-6	1.0	0.9	1.0	0.9
SLC25A20	O43772	155	-5	-18	-2	15	1.0	0.9	1.0	1.2
TTC38	Q5R3I4	28	-5	-25	-2	-1	1.0	0.8	1.0	1.0
DALRD3	Q5D0E6	468	-5	0	-2	-7	1.0	1.0	1.0	0.9
PAXBP1	Q9Y5B6	457	-5	-3	-2	-4	1.0	1.0	1.0	1.0
UNC13D	Q70J99	112	-5	-15	-2	13	1.0	0.9	1.0	1.1
NUDCD1	Q96RS6	376	-6	-9	-2	-11	0.9	0.9	1.0	0.9
ATG2A	Q2TAZ0	483	-6	-11	-2	-1	0.9	0.9	1.0	1.0
FER	P16591	593	-6	-13	-2	-10	0.9	0.9	1.0	0.9
HECTD1	Q9ULT8	2071	-6	-21	-2	1	0.9	0.8	1.0	1.0
N4BP1	O75113	56	-6	-13	-2	-7	0.9	0.9	1.0	0.9
HMGCS1	Q01581	224	-6	-15	-2	2	0.9	0.9	1.0	1.0
MAT2B	Q9NZL9	17	-7	-1	-2	2	0.9	1.0	1.0	1.0
GCFC2	P16383	595	-7	-4	-2	-10	0.9	1.0	1.0	0.9
SP110	Q9HB58	468	-7	-5	-2	-8	0.9	1.0	1.0	0.9
TNS3	Q68CZ2	1251	-7	-7	-2	-15	0.9	0.9	1.0	0.9
ZZEF1	O43149	716	-7	-9	-2	-2	0.9	0.9	1.0	1.0
UBE2O	Q9C0C9	585	-7	-10	-2	9	0.9	0.9	1.0	1.1
HELZ2	Q9BYK8	2131	-7	-5	-2	-14	0.9	1.0	1.0	0.9
TRIM33	Q9UPN9	150	-7	-7	-2	-3	0.9	0.9	1.0	1.0
CFAP97	Q9P2B7	104	-7	-11	-2	-11	0.9	0.9	1.0	0.9
ARAP1	Q96P48	376	-7	-12	-2	-11	0.9	0.9	1.0	0.9
L3MBTL2	Q969R5	114	-8	-2	-2	-1	0.9	1.0	1.0	1.0
HYAL1	Q12794	429	-8	-10	-2	12	0.9	0.9	1.0	1.1
HYAL1	Q12794	420	-8	-10	-2	12	0.9	0.9	1.0	1.1
ANKLE2	Q86XL3	91	-8	-25	-2	-7	0.9	0.8	1.0	0.9
DNM1L	O00429	367	-8	-2	-2	6	0.9	1.0	1.0	1.1
PIAS1	O75925	335	-8	-10	-2	1	0.9	0.9	1.0	1.0
OPLAH	O14841	25	-8	-13	-2	-6	0.9	0.9	1.0	0.9
U2AF1	Q01081	169	-9	-8	-2	3	0.9	0.9	1.0	1.0
RASA1	P20936	576	-9	-15	-2	-15	0.9	0.9	1.0	0.9
CCAR2	Q8N163	399	-9	-35	-2	-19	0.9	0.7	1.0	0.8
PTPN9	P43378	506	-11	1	-2	-14	0.9	1.0	1.0	0.9
RAD17	O75943	540	-11	-10	-2	-8	0.9	0.9	1.0	0.9
PDP1	Q9P0J1	132	-11	-22	-2	-9	0.9	0.8	1.0	0.9
ZZEF1	O43149	1287	-11	-22	-2	-17	0.9	0.8	1.0	0.9
MRPS16	Q9Y3D3	26	-12	-5	-2	-6	0.9	1.0	1.0	0.9
TNK2	Q07912	434	-12	-8	-2	11	0.9	0.9	1.0	1.1
SMYD5	Q6GMV2	194	-13	-12	-2	-12	0.9	0.9	1.0	0.9
POLDIP2	Q9Y2S7	143	-14	-6	-2	-7	0.9	0.9	1.0	0.9
TRIM27	P14373	365	-22	-16	-2	-17	0.8	0.9	1.0	0.9
ANKS3	Q6ZW76	636	30	-4	-2	-22	1.4	1.0	1.0	0.8
EML2	O95834	301	25	-23	-2	7	1.3	0.8	1.0	1.1

FAM91A1	Q658Y4	191	20	-2	-2	20	1.2	1.0	1.0	1.2
HGH1	Q9BTY7	227	19	-14	-2	-8	1.2	0.9	1.0	0.9
CORO7-PAM1	A0A0A6YYL4	691	18	-11	-2	2	1.2	0.9	1.0	1.0
ABR	Q12979	346	17	-5	-2	12	1.2	1.0	1.0	1.1
ACTN4	O43707	60	17	-15	-2	0	1.2	0.9	1.0	1.0
ACTN1	P12814	41	17	-15	-2	0	1.2	0.9	1.0	1.0
CCT7	Q99832	370	16	-6	-2	11	1.2	0.9	1.0	1.1
SMU1	Q2TAY7	448	16	-11	-2	4	1.2	0.9	1.0	1.0
ISY1	Q9ULR0	36	16	5	-2	15	1.2	1.1	1.0	1.2
HNRNPR	O43390	226	16	-7	-2	5	1.2	0.9	1.0	1.1
PPP2R1A	P30153	329	15	-5	-2	7	1.2	1.0	1.0	1.1
RNF113A	O15541	282	15	-1	-2	8	1.2	1.0	1.0	1.1
TRAPPC8	Q9Y2L5	339	15	-11	-2	-6	1.2	0.9	1.0	0.9
KLC1	Q07866	320	14	12	-2	24	1.2	1.1	1.0	1.3
NBAS	A2RRP1	256	14	5	-2	-3	1.2	1.0	1.0	1.0
AP3B1	O00203	215	14	3	-2	1	1.2	1.0	1.0	1.0
NCKAP1L	P55160	632	14	-5	-2	8	1.2	1.0	1.0	1.1
SUCLG2	Q96I99	255	14	-8	-2	-2	1.2	0.9	1.0	1.0
FERMT3	Q86UX7	452	14	-12	-2	3	1.2	0.9	1.0	1.0
AP2A1	O95782	397	14	-13	-2	11	1.2	0.9	1.0	1.1
DCUN1D1	Q96GG9	29	13	-12	-2	-10	1.1	0.9	1.0	0.9
PRPF38A	Q8NAV1	158	12	-4	-2	2	1.1	1.0	1.0	1.0
TXNIP	Q9H3M7	267	12	-12	-2	-5	1.1	0.9	1.0	1.0
LRMP	Q12912	264	12	-14	-2	-4	1.1	0.9	1.0	1.0
RP2	O75695	131	12	-15	-2	-1	1.1	0.9	1.0	1.0
CWF19L1	Q69YN2	160	12	-11	-2	-8	1.1	0.9	1.0	0.9
PRIM1	P49642	122	12	-17	-2	-2	1.1	0.9	1.0	1.0
COG1	Q8WTW3	577	12	-18	-2	3	1.1	0.9	1.0	1.0
CYBB	P04839	371	11	-4	-2	3	1.1	1.0	1.0	1.0
CAND1	Q86VP6	131	11	-7	-2	8	1.1	0.9	1.0	1.1
SSH2	Q76I76	388	11	-10	-2	9	1.1	0.9	1.0	1.1
SSH1	Q8WYL5	389	11	-10	-2	9	1.1	0.9	1.0	1.1
ABR	Q12979	201	11	-14	-2	-11	1.1	0.9	1.0	0.9
EOGT	Q5NDL2	67	11	-15	-2	-7	1.1	0.9	1.0	0.9
FECH	P22830	395	11	1	-2	14	1.1	1.0	1.0	1.2
TRIP12	Q14669	1276	11	-1	-2	5	1.1	1.0	1.0	1.1
MYCBP2	O75592	4537	11	-8	-2	4	1.1	0.9	1.0	1.0
OSGEP	Q9NPF4	277	10	14	-2	-1	1.1	1.2	1.0	1.0
ELMO1	Q92556	438	10	6	-2	6	1.1	1.1	1.0	1.1
SEC24A	O95486	388	10	-7	-2	17	1.1	0.9	1.0	1.2
RABGEF1	Q9UJ41	621	10	-7	-2	-4	1.1	0.9	1.0	1.0
OAS3	Q9Y6K5	582	10	-15	-2	-13	1.1	0.9	1.0	0.9
LYRM7	Q5U5X0	97	10	5	-2	-17	1.1	1.0	1.0	0.9
TRIM28	Q13263	91	10	-1	-2	5	1.1	1.0	1.0	1.0
GAR1	Q9NY12	80	10	-11	-2	-5	1.1	0.9	1.0	1.0
PSMD13	Q9UNM6	357	9	-5	-2	-4	1.1	1.0	1.0	1.0
ATXN7	O15265	839	9	-8	-2	-18	1.1	0.9	1.0	0.8
ZC3HAV1	Q7Z2W4	15	9	-9	-2	9	1.1	0.9	1.0	1.1
LIMS2	Q7Z4I7	105	9	-14	-2	7	1.1	0.9	1.0	1.1
ACAA2	P42765	103	9	-2	-2	4	1.1	1.0	1.0	1.0
PGLS	O95336	33	9	-16	-2	-6	1.1	0.9	1.0	0.9
Uncharacteri	H3BMM5	121	9	-17	-2	-3	1.1	0.9	1.0	1.0
ILVBL	A1L0T0	608	9	-20	-2	16	1.1	0.8	1.0	1.2

ABCC1	P33527	730	9	-22	-2	-12	1.1	0.8	1.0	0.9
H2AFY	O75367	297	9	-76	-2	1	1.1	0.6	1.0	1.0
EXOSC1	Q9Y3B2	40	8	-3	-2	-5	1.1	1.0	1.0	1.0
SMARCA4	P51532	936	8	1	-2	25	1.1	1.0	1.0	1.3
DIAPH2	O60879	484	8	-3	-2	-4	1.1	1.0	1.0	1.0
SLFN5	Q08AF3	258	8	-10	-2	-12	1.1	0.9	1.0	0.9
EP400	Q96L91	1820	8	-11	-2	-20	1.1	0.9	1.0	0.8
MYO1F	O00160	517	8	-11	-2	3	1.1	0.9	1.0	1.0
ELP4	Q96EB1	218	7	-3	-2	-1	1.1	1.0	1.0	1.0
FCHSD2	O94868	200	7	-5	-2	5	1.1	1.0	1.0	1.0
ZNF800	Q2TB10	49	7	-6	-2	-12	1.1	0.9	1.0	0.9
ATP5EP2	Q5VTU8	19	7	-8	-2	-3	1.1	0.9	1.0	1.0
CSNK1G1	Q9HCP0	110	7	-8	-2	-3	1.1	0.9	1.0	1.0
VAV1	P15498	753	7	-8	-2	-8	1.1	0.9	1.0	0.9
NCF1	P14598	111	7	-9	-2	-2	1.1	0.9	1.0	1.0
MAPKAPK3	Q16644	203	7	1	-2	-1	1.1	1.0	1.0	1.0
RASGRP2	Q7LDG7	11	7	0	-2	4	1.1	1.0	1.0	1.0
SSH1	Q8WYL5	564	7	0	-2	-4	1.1	1.0	1.0	1.0
SYNE2	Q8WXH0	2355	7	-2	-2	-9	1.1	1.0	1.0	0.9
SELPLG	Q14242	364	7	-3	-2	-23	1.1	1.0	1.0	0.8
COPS7B	Q9H9Q2	110	7	-9	-2	1	1.1	0.9	1.0	1.0
ZMYM2	Q9UBW7	494	7	-9	-2	-8	1.1	0.9	1.0	0.9
EMILIN2	Q9BXX0	691	7	-10	-2	-16	1.1	0.9	1.0	0.9
DOCK11	Q5JSL3	615	7	-11	-2	-1	1.1	0.9	1.0	1.0
MT1E	P04732	37	7	-12	-2	-14	1.1	0.9	1.0	0.9
SMARCC1	Q92922	164	7	-15	-2	1	1.1	0.9	1.0	1.0
MED12	Q93074	1188	6	1	-2	6	1.1	1.0	1.0	1.1
MSH6	P52701	88	6	1	-2	2	1.1	1.0	1.0	1.0
ZC3H13	Q5T200	1592	6	-2	-2	4	1.1	1.0	1.0	1.0
WDR45	Q9Y484	148	6	-4	-2	10	1.1	1.0	1.0	1.1
NLRC5	Q86WI3	698	6	-4	-2	-5	1.1	1.0	1.0	1.0
TAF15	Q92804	365	6	-7	-2	9	1.1	0.9	1.0	1.1
OSBPL8	Q9BZF1	617	6	-9	-2	-5	1.1	0.9	1.0	1.0
LUC7L3	O95232	40	6	-9	-2	-3	1.1	0.9	1.0	1.0
PRMT3	O60678	417	6	-12	-2	-6	1.1	0.9	1.0	0.9
SIPA1	Q96FS4	632	6	-12	-2	-7	1.1	0.9	1.0	0.9
RPL28	P46779	13	6	-13	-2	-5	1.1	0.9	1.0	1.0
FYN	P06241	404	6	-15	-2	-6	1.1	0.9	1.0	0.9
GPD2	P43304	188	6	-17	-2	6	1.1	0.9	1.0	1.1
ZFP82	Q8N141	321	6	-17	-2	2	1.1	0.9	1.0	1.0
ACD	Q96AP0	224	6	-22	-2	-5	1.1	0.8	1.0	1.0
ECHS1	P30084	62	6	-2	-2	4	1.1	1.0	1.0	1.0
CHD1L	Q86WJ1	135	6	-5	-2	-6	1.1	1.0	1.0	0.9
PPAT	Q06203	348	6	-5	-2	-7	1.1	1.0	1.0	0.9
S100A8	P05109	42	6	-6	-2	5	1.1	0.9	1.0	1.1
TRA2B	P62995	118	6	-7	-2	1	1.1	0.9	1.0	1.0
ZNF836	Q6ZNA1	70	6	-10	-2	10	1.1	0.9	1.0	1.1
ATM	Q13315	536	5	4	-2	-2	1.1	1.0	1.0	1.0
SMPD4	Q9NXE4	182	5	1	-2	-19	1.1	1.0	1.0	0.8
LASP1	Q14847	29	5	-7	-2	9	1.1	0.9	1.0	1.1
ANO6	Q4KMQ2	250	5	-8	-2	-3	1.1	0.9	1.0	1.0
MYH9	P35579	172	5	-8	-2	-7	1.1	0.9	1.0	0.9
SMG5	Q9UPR3	109	5	-8	-2	3	1.1	0.9	1.0	1.0

XPNPEP1	Q9NQW7	309	5	-8	-2	-7	1.1	0.9	1.0	0.9
DDI2	Q5TDH0	361	5	-10	-2	-6	1.1	0.9	1.0	0.9
XRN1	Q8IZH2	657	5	-11	-2	-11	1.1	0.9	1.0	0.9
OAS3	Q9Y6K5	781	5	-14	-2	5	1.1	0.9	1.0	1.1
KDM3B	Q7LBC6	904	5	-18	-2	-5	1.1	0.9	1.0	1.0
CMAS	Q8NFW8	364	5	2	-2	-10	1.0	1.0	1.0	0.9
CNTRL	Q7Z7A1	169	5	-4	-2	-11	1.0	1.0	1.0	0.9
PARVG	Q9HBI0	314	5	-5	-2	-1	1.0	1.0	1.0	1.0
EEFSEC	P57772	406	5	-6	-2	-1	1.0	0.9	1.0	1.0
CAD	P27708	1636	5	-8	-2	7	1.0	0.9	1.0	1.1
RNF213	Q63HN8	1677	5	-8	-2	-2	1.0	0.9	1.0	1.0
PSMB1	P20618	82	5	-10	-2	0	1.0	0.9	1.0	1.0
PRMT1	Q99873	262	5	-12	-2	0	1.0	0.9	1.0	1.0
SAMHD1	Q9Y3Z3	51	5	-16	-2	-3	1.0	0.9	1.0	1.0
NHLRC2	Q8NBF2	609	4	-2	-2	2	1.0	1.0	1.0	1.0
PTK2B	Q14289	298	4	-2	-2	5	1.0	1.0	1.0	1.1
DR1	Q01658	94	4	-3	-2	5	1.0	1.0	1.0	1.0
CCS	O14618	246	4	-3	-2	-13	1.0	1.0	1.0	0.9
TBC1D5	Q92609	96	4	-3	-2	-6	1.0	1.0	1.0	0.9
STAT6	P42226	355	4	-5	-2	9	1.0	1.0	1.0	1.1
USF1	P22415	248	4	-5	-2	16	1.0	1.0	1.0	1.2
POLR2B	P30876	177	4	-6	-2	23	1.0	0.9	1.0	1.3
RNF213	Q63HN8	4258	4	-8	-2	-8	1.0	0.9	1.0	0.9
ROCK2	O75116	804	4	-8	-2	-9	1.0	0.9	1.0	0.9
BANP	Q8N9N5	74	4	-9	-2	-9	1.0	0.9	1.0	0.9
C19orf54	Q5BKX5	226	4	10	-2	7	1.0	1.1	1.0	1.1
HSPA9	P38646	317	4	-1	-2	1	1.0	1.0	1.0	1.0
XPO5	Q9HAV4	1131	4	-3	-2	-5	1.0	1.0	1.0	1.0
ADSL	P30566	483	4	-3	-2	2	1.0	1.0	1.0	1.0
SSNA1	O43805	18	4	-4	-2	-9	1.0	1.0	1.0	0.9
NAIP	Q13075	1335	4	-5	-2	-7	1.0	1.0	1.0	0.9
AKAP17A	Q02040	579	4	-6	-2	-9	1.0	0.9	1.0	0.9
DHRS4	Q9BTZ2	88	4	-6	-2	6	1.0	0.9	1.0	1.1
UBE3B	Q7Z3V4	399	4	-7	-2	-2	1.0	0.9	1.0	1.0
ODR4	Q5SWX8	231	4	-7	-2	-16	1.0	0.9	1.0	0.9
DNTTIP2	Q5QJE6	116	4	-9	-2	1	1.0	0.9	1.0	1.0
METAP2	P50579	448	4	-9	-2	-3	1.0	0.9	1.0	1.0
PARP16	Q8N5Y8	52	4	-10	-2	-12	1.0	0.9	1.0	0.9
FXR2	P51116	270	4	-10	-2	-19	1.0	0.9	1.0	0.8
PPP4R2	Q9NY27	22	4	-15	-2	-7	1.0	0.9	1.0	0.9
PCNA	P12004	162	4	-18	-2	9	1.0	0.9	1.0	1.1
PPP6R3	Q5H9R7	172	3	2	-2	-16	1.0	1.0	1.0	0.9
SNAP23	O00161	112	3	-2	-2	-3	1.0	1.0	1.0	1.0
NEURL4	Q96JN8	57	3	-2	-2	-7	1.0	1.0	1.0	0.9
MED14	O60244	729	3	-3	-2	-1	1.0	1.0	1.0	1.0
RARS2	Q5T160	576	3	-3	-2	-2	1.0	1.0	1.0	1.0
PDCD6IP	Q8WUM4	90	3	-6	-2	-9	1.0	0.9	1.0	0.9
SYK	P43405	206	3	-6	-2	-12	1.0	0.9	1.0	0.9
SMCHD1	A6NHR9	1899	3	-6	-2	1	1.0	0.9	1.0	1.0
EEF2	P13639	751	3	-7	-2	-1	1.0	0.9	1.0	1.0
SEC13	P55735	299	3	-8	-2	4	1.0	0.9	1.0	1.0
ANXA6	P08133	59	3	-8	-2	-2	1.0	0.9	1.0	1.0
R3HCC1L	Q7Z5L2	665	3	-8	-2	-19	1.0	0.9	1.0	0.8

NSUN4	Q96CB9	258	3	-8	-2	7	1.0	0.9	1.0	1.1
EML2	O95834	395	3	-14	-2	1	1.0	0.9	1.0	1.0
CTCF	P49711	497	3	-15	-2	-6	1.0	0.9	1.0	0.9
NUP155	O75694	844	3	-25	-2	-11	1.0	0.8	1.0	0.9
PLEKHF2	Q9H8W4	219	3	9	-2	9	1.0	1.1	1.0	1.1
INPP5K	Q9BT40	173	3	3	-2	-3	1.0	1.0	1.0	1.0
WDR44	Q5JSH3	581	3	2	-2	-6	1.0	1.0	1.0	0.9
RASGRP4	Q8TDF6	502	3	1	-2	-7	1.0	1.0	1.0	0.9
ACO2	Q99798	451	3	-1	-2	-2	1.0	1.0	1.0	1.0
RPS6KA3	P51812	579	3	-4	-2	3	1.0	1.0	1.0	1.0
RPS6KA1	Q15418	575	3	-4	-2	3	1.0	1.0	1.0	1.0
BCL7C	Q8WUZ0	211	3	-5	-2	-11	1.0	1.0	1.0	0.9
PIK3CD	O00329	219	3	-6	-2	-6	1.0	0.9	1.0	0.9
MVD	P53602	108	3	-6	-2	-3	1.0	0.9	1.0	1.0
SRSF3	P84103	6	3	-6	-2	-13	1.0	0.9	1.0	0.9
LPXN	O60711	379	3	-7	-2	-1	1.0	0.9	1.0	1.0
WDFY3	Q8IZQ1	1600	3	-7	-2	-7	1.0	0.9	1.0	0.9
STK39	Q9UEW8	82	3	-9	-2	1	1.0	0.9	1.0	1.0
DOK2	O60496	36	3	-9	-2	-11	1.0	0.9	1.0	0.9
ZFR	Q96KR1	525	3	-22	-2	-15	1.0	0.8	1.0	0.9
FMNL1	O95466	787	2	5	-2	-8	1.0	1.1	1.0	0.9
NFX1	Q12986	947	2	3	-2	6	1.0	1.0	1.0	1.1
SETD1B	Q9UPS6	1954	2	2	-2	-5	1.0	1.0	1.0	1.0
ELMOD2	Q8IZ81	285	2	-4	-2	-4	1.0	1.0	1.0	1.0
GIGYF2	Q6Y7W6	573	2	-7	-2	1	1.0	0.9	1.0	1.0
HNRNPM	P52272	694	2	-7	-2	-5	1.0	0.9	1.0	1.0
NOP56	O00567	112	2	-8	-2	6	1.0	0.9	1.0	1.1
SP4	Q02446	55	2	-8	-2	-10	1.0	0.9	1.0	0.9
RAP1GAP2	Q684P5	228	2	-10	-2	-7	1.0	0.9	1.0	0.9
PPP2R1A	P30153	390	2	-10	-2	-5	1.0	0.9	1.0	1.0
PPP2R1B	P30154	402	2	-10	-2	-5	1.0	0.9	1.0	1.0
ZZEF1	O43149	1786	2	-12	-2	7	1.0	0.9	1.0	1.1
GFM1	Q96RP9	516	2	-14	-2	2	1.0	0.9	1.0	1.0
TAGLN2	P37802	124	2	-15	-2	-7	1.0	0.9	1.0	0.9
BAG1	Q99933	272	2	2	-2	7	1.0	1.0	1.0	1.1
NDUFB11	Q9NX14	141	2	0	-2	0	1.0	1.0	1.0	1.0
BOLA3	Q53S33	59	2	-4	-2	-6	1.0	1.0	1.0	0.9
DSTN	P60981	147	2	-4	-2	-3	1.0	1.0	1.0	1.0
STK39	Q9UEW8	525	2	-4	-2	-7	1.0	1.0	1.0	0.9
PYCARD	Q9ULZ3	173	2	-5	-2	-2	1.0	1.0	1.0	1.0
AHNAK	Q09666	2806	2	-5	-2	-5	1.0	1.0	1.0	1.0
NUP160	Q12769	65	2	-6	-2	-6	1.0	0.9	1.0	0.9
PBRM1	Q86U86	951	2	-6	-2	-10	1.0	0.9	1.0	0.9
EFL1	Q7Z2Z2	953	2	-7	-2	-7	1.0	0.9	1.0	0.9
UTP15	Q8TED0	385	2	-8	-2	-1	1.0	0.9	1.0	1.0
APAF1	O14727	203	2	-8	-2	-13	1.0	0.9	1.0	0.9
HK2	P52789	158	2	-9	-2	-9	1.0	0.9	1.0	0.9
GMEB1	Q9Y692	174	2	-11	-2	1	1.0	0.9	1.0	1.0
TCEB3	Q14241	568	2	-12	-2	-16	1.0	0.9	1.0	0.9
TUBGCP3	Q96CW5	18	2	-21	-2	-4	1.0	0.8	1.0	1.0
CLCN3	P51790	733	1	-1	-2	2	1.0	1.0	1.0	1.0
GYS1	P13807	699	1	-2	-2	-7	1.0	1.0	1.0	0.9
OGT	O15294	620	1	-5	-2	-1	1.0	1.0	1.0	1.0

LIPT1	Q9Y234	337	1	-6	-2	-9	1.0	0.9	1.0	0.9
CREBRF	Q8IUR6	265	1	-6	-2	-12	1.0	0.9	1.0	0.9
PFKP	Q01813	112	1	-7	-2	-1	1.0	0.9	1.0	1.0
FN3K	Q9H479	24	1	-8	-2	-12	1.0	0.9	1.0	0.9
RANBP2	P49792	2659	1	-16	-2	-6	1.0	0.9	1.0	0.9
GBF1	Q92538	661	1	-19	-2	-13	1.0	0.8	1.0	0.9
PSMB1	P20618	224	1	-27	-2	-9	1.0	0.8	1.0	0.9
EFHD2	Q96C19	53	1	-2	-2	-7	1.0	1.0	1.0	0.9
RPL11	P62913	21	1	-4	-2	3	1.0	1.0	1.0	1.0
ACAA2	P42765	92	1	-6	-2	-1	1.0	0.9	1.0	1.0
NUMA1	Q14980	1136	1	-6	-2	-4	1.0	0.9	1.0	1.0
MATR3	A8MXP9	854	1	-14	-2	1	1.0	0.9	1.0	1.0
SMAP2	Q8WU79	28	1	-21	-2	-2	1.0	0.8	1.0	1.0
XPO4	Q9C0E2	723	0	5	-2	-6	1.0	1.0	1.0	0.9
CCDC88C	Q9P219	1467	0	4	-2	-10	1.0	1.0	1.0	0.9
CDK11B	J3QR44	440	0	-3	-2	-10	1.0	1.0	1.0	0.9
TRPV2	Q9Y5S1	349	0	-4	-2	-9	1.0	1.0	1.0	0.9
ALDH18A1	P54886	88	0	-6	-2	-8	1.0	0.9	1.0	0.9
NLRC3	Q7RTR2	57	0	-6	-2	-11	1.0	0.9	1.0	0.9
SRRM2	Q9UQ35	1480	0	-7	-2	-11	1.0	0.9	1.0	0.9
CHERP	Q8IWX8	69	0	-7	-2	-18	1.0	0.9	1.0	0.9
GNB2	P62879	149	0	-8	-2	-2	1.0	0.9	1.0	1.0
GNB4	Q9HAV0	149	0	-8	-2	-2	1.0	0.9	1.0	1.0
AIM2	O14862	153	0	-8	-2	-29	1.0	0.9	1.0	0.8
MTA2	O94776	209	0	-9	-2	5	1.0	0.9	1.0	1.1
SRSF9	Q13242	80	0	-12	-2	-4	1.0	0.9	1.0	1.0
TP53BP1	Q12888	1178	0	-12	-2	-18	1.0	0.9	1.0	0.8
TKT	P29401	133	0	-15	-2	-9	1.0	0.9	1.0	0.9
NEDD9	Q14511	281	0	-20	-2	-16	1.0	0.8	1.0	0.9
TBRG4	Q969Z0	335	0	-28	-2	-8	1.0	0.8	1.0	0.9
DENND4A	Q7Z401	1137	-1	1	-2	-6	1.0	1.0	1.0	0.9
BOD1L1	Q8NFC6	2386	-1	-1	-2	-23	1.0	1.0	1.0	0.8
TNPO3	Q9Y5L0	312	-1	-1	-2	-6	1.0	1.0	1.0	0.9
TXNIP	Q9H3M7	120	-1	-3	-2	-2	1.0	1.0	1.0	1.0
SIK3	Q9Y2K2	597	-1	-9	-2	-7	1.0	0.9	1.0	0.9
INO80B-WBP	J3KQ70	334	-1	-14	-2	-4	1.0	0.9	1.0	1.0
TYK2	P29597	1151	-1	-25	-2	-12	1.0	0.8	1.0	0.9
SERPINB2	P05120	79	-1	3	-2	-1	1.0	1.0	1.0	1.0
NRBP2	Q9NSY0	285	-1	-2	-2	6	1.0	1.0	1.0	1.1
RB1	P06400	438	-1	-3	-2	-12	1.0	1.0	1.0	0.9
EIF4E3	Q8N5X7	69	-1	-7	-2	-11	1.0	0.9	1.0	0.9
TUBGCP2	Q9BSJ2	469	-1	-9	-2	11	1.0	0.9	1.0	1.1
CD180	Q99467	419	-1	-12	-2	-7	1.0	0.9	1.0	0.9
UBE2G2	P60604	89	-2	1	-2	2	1.0	1.0	1.0	1.0
UBR4	Q5T4S7	2619	-2	0	-2	-9	1.0	1.0	1.0	0.9
CARD11	Q9BXL7	971	-2	-5	-2	-2	1.0	1.0	1.0	1.0
KAT6A	Q92794	393	-2	-6	-2	-17	1.0	0.9	1.0	0.9
GANC	Q8TET4	114	-2	-7	-2	-5	1.0	0.9	1.0	1.0
MMRN1	Q13201	1067	-2	-8	-2	9	1.0	0.9	1.0	1.1
NBEAL2	Q6ZNJ1	1491	-2	-8	-2	-8	1.0	0.9	1.0	0.9
NBAS	A2RRP1	1777	-2	-9	-2	-8	1.0	0.9	1.0	0.9
RASSF1	Q9NS23	341	-2	-10	-2	-3	1.0	0.9	1.0	1.0
UBE2Z	Q9H832	100	-2	-13	-2	0	1.0	0.9	1.0	1.0

DENR	O43583	132	-2	-13	-2	-2	1.0	0.9	1.0	1.0
DAXX	Q9UER7	245	-2	-15	-2	8	1.0	0.9	1.0	1.1
PREX1	Q8TCU6	1651	-2	-16	-2	-9	1.0	0.9	1.0	0.9
SMAD2	Q15796	374	-2	-18	-2	-7	1.0	0.9	1.0	0.9
NIT2	Q9NQR4	153	-2	-4	-2	-5	1.0	1.0	1.0	1.0
EPS15	P42566	586	-2	-4	-2	-12	1.0	1.0	1.0	0.9
CELF2	O95319	174	-2	-8	-2	-5	1.0	0.9	1.0	1.0
BOP1	Q14137	404	-2	-8	-2	-9	1.0	0.9	1.0	0.9
COPB1	P53618	888	-2	-11	-2	-8	1.0	0.9	1.0	0.9
NAA16	Q6N069	322	-2	-13	-2	-11	1.0	0.9	1.0	0.9
CNN2	Q99439	215	-2	-14	-2	-13	1.0	0.9	1.0	0.9
SAMD1	E9PIW9	277	-3	2	-2	-13	1.0	1.0	1.0	0.9
TMEM259	Q4ZIN3	129	-3	-5	-2	3	1.0	1.0	1.0	1.0
L2HGDH	Q9H9P8	113	-3	-5	-2	0	1.0	1.0	1.0	1.0
DCK	P27707	59	-3	-7	-2	-6	1.0	0.9	1.0	0.9
HERC1	Q15751	56	-3	-8	-2	-2	1.0	0.9	1.0	1.0
RPL32	P62910	96	-3	-9	-2	-3	1.0	0.9	1.0	1.0
RIPK3	Q9Y572	234	-3	-11	-2	-11	1.0	0.9	1.0	0.9
TRANK1	O15050	1042	-3	-15	-2	-3	1.0	0.9	1.0	1.0
TRMU	O75648	101	-3	4	-2	-5	1.0	1.0	1.0	1.0
CNOT4	O95628	175	-3	-12	-2	5	1.0	0.9	1.0	1.1
POLR3B	Q9NW08	161	-4	4	-2	18	1.0	1.0	1.0	1.2
FAM101B	Q8N5W9	186	-4	-10	-2	-8	1.0	0.9	1.0	0.9
TRAF6	Q9Y4K3	139	-4	-11	-2	-7	1.0	0.9	1.0	0.9
PGK1	P00558	380	-4	-12	-2	-2	1.0	0.9	1.0	1.0
RPS26	P62854	23	-4	-14	-2	-12	1.0	0.9	1.0	0.9
EXOC2	Q96KP1	719	-4	-22	-2	-12	1.0	0.8	1.0	0.9
PML	P29590	213	-4	-2	-2	-8	1.0	1.0	1.0	0.9
USP36	Q9P275	975	-4	-4	-2	-9	1.0	1.0	1.0	0.9
PAG1	Q9NWQ8	355	-4	-6	-2	-7	1.0	0.9	1.0	0.9
LIMD2	Q9BT23	64	-4	-7	-2	1	1.0	0.9	1.0	1.0
PPIG	Q13427	310	-4	-8	-2	-10	1.0	0.9	1.0	0.9
FHL1	Q13642	126	-4	-10	-2	3	1.0	0.9	1.0	1.0
NLRP1	Q9C000	837	-4	-22	-2	-12	1.0	0.8	1.0	0.9
RNF31	Q96EP0	930	-5	10	-2	-10	1.0	1.1	1.0	0.9
SCRN1	Q12765	324	-5	-3	-2	6	1.0	1.0	1.0	1.1
NUDC	Q9Y266	188	-5	-10	-2	-18	1.0	0.9	1.0	0.8
KIF21B	O75037	298	-5	-16	-2	-8	1.0	0.9	1.0	0.9
IRAK3	Q9Y616	410	-5	-19	-2	0	1.0	0.8	1.0	1.0
ARHGAP21	Q5T5U3	118	-5	4	-2	-19	1.0	1.0	1.0	0.8
UBE3C	Q15386	252	-5	-1	-2	-11	1.0	1.0	1.0	0.9
XPO6	Q96QU8	299	-5	-4	-2	-10	1.0	1.0	1.0	0.9
TIPRL	O75663	14	-5	-7	-2	-5	1.0	0.9	1.0	1.0
INADL	Q8NI35	744	-5	-8	-2	-3	1.0	0.9	1.0	1.0
MMS19	Q96T76	549	-5	-8	-2	-6	1.0	0.9	1.0	0.9
L3HYPDH	Q96EM0	39	-5	-10	-2	0	1.0	0.9	1.0	1.0
CTSB	P07858	105	-5	-11	-2	11	1.0	0.9	1.0	1.1
HUWE1	Q7Z6Z7	29	-5	-24	-2	3	1.0	0.8	1.0	1.0
RPS21	P63220	17	-5	-25	-2	-17	1.0	0.8	1.0	0.9
RACK1	P63244	182	-6	-12	-2	5	0.9	0.9	1.0	1.0
B4GALNT1	Q00973	412	-6	-13	-2	-15	0.9	0.9	1.0	0.9
UBASH3B	Q8TF42	367	-6	-17	-2	-15	0.9	0.9	1.0	0.9
CHMP5	Q9NZZ3	20	-6	-13	-2	-10	0.9	0.9	1.0	0.9

DARS	P14868	203	-7	-7	-2	2	0.9	0.9	1.0	1.0
ZMYM1	Q5SVZ6	989	-7	-21	-2	-33	0.9	0.8	1.0	0.8
E2F4	Q16254	88	-7	-6	-2	-9	0.9	0.9	1.0	0.9
ZC3H12A	Q5D1E8	223	-7	-13	-2	-4	0.9	0.9	1.0	1.0
PREX1	Q8TCU6	1266	-8	-7	-2	1	0.9	0.9	1.0	1.0
HGH1	Q9BTY7	199	-8	-9	-2	-6	0.9	0.9	1.0	0.9
CBLL1	Q75N03	73	-8	-10	-2	-16	0.9	0.9	1.0	0.9
INPP5B	P32019	362	-10	-12	-2	-18	0.9	0.9	1.0	0.8
ATG7	O95352	524	-10	-6	-2	-10	0.9	0.9	1.0	0.9
KDM4B	O94953	694	-10	-19	-2	-28	0.9	0.8	1.0	0.8
LSM14A	Q8ND56	85	-11	2	-2	-21	0.9	1.0	1.0	0.8
HIBCH	Q6NVY1	95	-11	-23	-2	-3	0.9	0.8	1.0	1.0
STK11IP	Q8N1F8	149	-12	-16	-2	-4	0.9	0.9	1.0	1.0
MEMO1	Q9Y316	55	-13	-9	-2	-3	0.9	0.9	1.0	1.0
PFKFB4	Q16877	159	-14	-11	-2	-11	0.9	0.9	1.0	0.9
MCM3	P25205	148	-14	-3	-2	21	0.9	1.0	1.0	1.3
RASGRP4	Q8TDF6	237	-15	-22	-2	-12	0.9	0.8	1.0	0.9
ADCK1	Q86TW2	94	-16	-42	-2	-8	0.9	0.7	1.0	0.9
GNB1L	Q9BYB4	29	-17	-9	-2	-24	0.9	0.9	1.0	0.8
ALAD	P13716	124	-18	-9	-2	-15	0.8	0.9	1.0	0.9
PREB	Q9HCU5	280	-18	-16	-2	-8	0.8	0.9	1.0	0.9
PHKG2	P15735	353	35	4	-3	16	1.5	1.0	1.0	1.2
MAPK14	Q16539	39	29	-1	-3	9	1.4	1.0	1.0	1.1
TRNAU1AP	Q9NX07	123	27	-9	-3	16	1.4	0.9	1.0	1.2
HSP90AA1	P07900	529	23	9	-3	22	1.3	1.1	1.0	1.3
C9orf64	Q5T6V5	170	23	-16	-3	-3	1.3	0.9	1.0	1.0
NPLOC4	Q8TAT6	355	20	-2	-3	9	1.3	1.0	1.0	1.1
ANKRD44	Q8N8A2	122	19	-13	-3	-2	1.2	0.9	1.0	1.0
SLC15A5	A6NIM6	68	18	2	-3	15	1.2	1.0	1.0	1.2
CA8	P35219	200	18	-15	-3	-16	1.2	0.9	1.0	0.9
PDCD4	Q53EL6	350	16	-9	-3	-10	1.2	0.9	1.0	0.9
PSMD11	O00231	289	15	-10	-3	10	1.2	0.9	1.0	1.1
BRAT1	Q6PJG6	228	15	-25	-3	-10	1.2	0.8	1.0	0.9
UCHL5	Q9Y5K5	191	14	-6	-3	5	1.2	0.9	1.0	1.1
UFL1	O94874	32	14	-13	-3	-8	1.2	0.9	1.0	0.9
PRPF31	Q8WWY3	149	14	-16	-3	-5	1.2	0.9	1.0	1.0
YDJC	A8MPS7	310	14	10	-3	12	1.2	1.1	1.0	1.1
SLC25A4	P12235	257	14	-14	-3	2	1.2	0.9	1.0	1.0
SLC25A6	P12236	257	14	-14	-3	2	1.2	0.9	1.0	1.0
CDC123	O75794	289	13	4	-3	1	1.1	1.0	1.0	1.0
RASSF5	Q8WWW0	225	13	-3	-3	-17	1.1	1.0	1.0	0.9
THG1L	Q9NWX6	279	13	-6	-3	1	1.1	0.9	1.0	1.0
PDHA1	P08559	145	13	-6	-3	6	1.1	0.9	1.0	1.1
THOP1	P52888	644	13	-11	-3	-3	1.1	0.9	1.0	1.0
MMS19	Q96T76	331	13	-14	-3	1	1.1	0.9	1.0	1.0
AGGF1	Q8N302	494	13	-1	-3	-5	1.1	1.0	1.0	1.0
THOC2	Q8NI27	986	13	-7	-3	8	1.1	0.9	1.0	1.1
DDX39B	Q13838	239	13	-9	-3	-11	1.1	0.9	1.0	0.9
TARS2	Q9BW92	240	12	2	-3	1	1.1	1.0	1.0	1.0
ITGAM	P11215	494	12	-4	-3	5	1.1	1.0	1.0	1.0
ITGAX	P20702	495	12	-4	-3	5	1.1	1.0	1.0	1.0
BRD1	O95696	937	12	-7	-3	10	1.1	0.9	1.0	1.1
RNF213	Q63HN8	2633	12	-7	-3	3	1.1	0.9	1.0	1.0

MMS19	Q96T76	386	12	-12	-3	4	1.1	0.9	1.0	1.0
NAE1	Q13564	294	12	-22	-3	9	1.1	0.8	1.0	1.1
SLC25A24	Q6NUK1	330	12	-8	-3	4	1.1	0.9	1.0	1.0
TROVE2	P10155	102	11	-3	-3	2	1.1	1.0	1.0	1.0
SAMD9	Q5K651	1538	11	-7	-3	2	1.1	0.9	1.0	1.0
TCAF1	Q9Y4C2	488	11	-11	-3	7	1.1	0.9	1.0	1.1
DHX9	Q08211	12	11	-25	-3	-1	1.1	0.8	1.0	1.0
ZNFX1	Q9P2E3	1264	10	2	-3	-1	1.1	1.0	1.0	1.0
COPB1	P53618	143	10	-4	-3	10	1.1	1.0	1.0	1.1
PDLIM7	Q9NR12	282	10	-9	-3	-7	1.1	0.9	1.0	0.9
WDTC1	Q8N5D0	236	10	-12	-3	-14	1.1	0.9	1.0	0.9
ZFYVE26	Q68DK2	1320	10	-15	-3	14	1.1	0.9	1.0	1.2
RXRA	P19793	432	10	-17	-3	4	1.1	0.9	1.0	1.0
RXRB	P28702	503	10	-17	-3	4	1.1	0.9	1.0	1.0
FNBP1	Q96RU3	248	10	-19	-3	6	1.1	0.8	1.0	1.1
PRKDC	P78527	3347	10	-19	-3	3	1.1	0.8	1.0	1.0
CORO1A	P31146	51	10	-20	-3	8	1.1	0.8	1.0	1.1
ATP2A2	P16615	447	10	-29	-3	-6	1.1	0.8	1.0	0.9
ATP2A3	Q93084	447	10	-29	-3	-6	1.1	0.8	1.0	0.9
PDIA3	P30101	409	10	7	-3	-8	1.1	1.1	1.0	0.9
MED30	Q96HR3	34	10	3	-3	-2	1.1	1.0	1.0	1.0
RNH1	P13489	96	10	-4	-3	5	1.1	1.0	1.0	1.0
RRP1B	Q14684	583	10	-4	-3	-17	1.1	1.0	1.0	0.9
ANXA7	P20073	285	10	-7	-3	5	1.1	0.9	1.0	1.0
RAD50	Q92878	990	10	-7	-3	10	1.1	0.9	1.0	1.1
SART1	O43290	674	10	-7	-3	-7	1.1	0.9	1.0	0.9
RNF213	Q63HN8	3330	10	-8	-3	7	1.1	0.9	1.0	1.1
RBM39	Q14498	157	10	-11	-3	-2	1.1	0.9	1.0	1.0
RECQL	P46063	321	10	-12	-3	-2	1.1	0.9	1.0	1.0
TRMT10C	Q7L0Y3	246	10	-14	-3	0	1.1	0.9	1.0	1.0
UBTF	P17480	328	10	-22	-3	-9	1.1	0.8	1.0	0.9
NR2C2	P49116	204	9	-3	-3	-16	1.1	1.0	1.0	0.9
EED	O75530	324	9	-4	-3	9	1.1	1.0	1.0	1.1
FAM129B	Q96TA1	466	9	-4	-3	6	1.1	1.0	1.0	1.1
TCEB1	Q15369	11	9	-9	-3	0	1.1	0.9	1.0	1.0
RBM4B	Q9BQ04	31	9	-24	-3	-1	1.1	0.8	1.0	1.0
RBM4	Q9BWF3	31	9	-24	-3	-1	1.1	0.8	1.0	1.0
GSTO1	P78417	112	9	4	-3	9	1.1	1.0	1.0	1.1
VAV2	P52735	201	9	-1	-3	-8	1.1	1.0	1.0	0.9
RAD9A	Q99638	114	9	-4	-3	5	1.1	1.0	1.0	1.1
PRKAR1A	P10644	362	9	-6	-3	10	1.1	0.9	1.0	1.1
MAPK12	P53778	165	9	-8	-3	-1	1.1	0.9	1.0	1.0
HDAC4	P56524	777	9	-9	-3	-30	1.1	0.9	1.0	0.8
OGDH	Q02218	395	9	-11	-3	7	1.1	0.9	1.0	1.1
PPBP	P02775	89	9	-11	-3	31	1.1	0.9	1.0	1.4
TGM2	P21980	545	9	-17	-3	3	1.1	0.9	1.0	1.0
DDX21	Q9NR30	291	9	-21	-3	1	1.1	0.8	1.0	1.0
ATP2A2	P16615	349	8	4	-3	2	1.1	1.0	1.0	1.0
ATP2A3	Q93084	349	8	4	-3	2	1.1	1.0	1.0	1.0
GTF3C3	Q9Y5Q9	574	8	3	-3	-10	1.1	1.0	1.0	0.9
DEF8	Q6ZN54	424	8	1	-3	-3	1.1	1.0	1.0	1.0
HSPD1	P10809	447	8	-5	-3	-2	1.1	1.0	1.0	1.0
NAA15	Q9BXJ9	817	8	-9	-3	-5	1.1	0.9	1.0	1.0

UACA	Q9BZF9	994	8	-14	-3	-18	1.1	0.9	1.0	0.8
NUP160	Q12769	529	8	-17	-3	-7	1.1	0.9	1.0	0.9
AKAP13	Q12802	418	8	4	-3	-11	1.1	1.0	1.0	0.9
RANBP2	P49792	2791	8	-3	-3	-15	1.1	1.0	1.0	0.9
THEMIS	Q8N1K5	450	8	-7	-3	3	1.1	0.9	1.0	1.0
MIA3	Q5JRA6	1486	8	-9	-3	-4	1.1	0.9	1.0	1.0
PRKDC	P78527	1364	8	-9	-3	-4	1.1	0.9	1.0	1.0
ZC2HC1A	Q96GY0	141	8	-13	-3	-17	1.1	0.9	1.0	0.9
LNPEP	Q9UIQ6	103	8	-15	-3	2	1.1	0.9	1.0	1.0
VPRBP	Q9Y4B6	1113	8	-16	-3	-15	1.1	0.9	1.0	0.9
NXF1	Q9UBU9	328	7	3	-3	-10	1.1	1.0	1.0	0.9
ZNF451	Q9Y4E5	730	7	-3	-3	-11	1.1	1.0	1.0	0.9
PGAM5	Q96HS1	168	7	-6	-3	-1	1.1	0.9	1.0	1.0
ABAT	P80404	467	7	-6	-3	-3	1.1	0.9	1.0	1.0
VPS28	Q9UK41	103	7	-8	-3	7	1.1	0.9	1.0	1.1
JAK3	P52333	839	7	-8	-3	-10	1.1	0.9	1.0	0.9
WDR11	Q9BZH6	1080	7	-10	-3	17	1.1	0.9	1.0	1.2
PRR14L	Q5THK1	1640	7	-13	-3	-4	1.1	0.9	1.0	1.0
AKAP11	Q9UKA4	1257	7	15	-3	4	1.1	1.2	1.0	1.0
GMDS	O60547	237	7	2	-3	-2	1.1	1.0	1.0	1.0
ZMYM2	Q9UBW7	823	7	1	-3	-9	1.1	1.0	1.0	0.9
ELP2	Q6IA86	68	7	-4	-3	-1	1.1	1.0	1.0	1.0
NPL	Q9BXD5	185	7	-8	-3	5	1.1	0.9	1.0	1.0
DNAJC13	O75165	1354	7	-11	-3	-5	1.1	0.9	1.0	1.0
PIK3CB	P42338	765	7	-12	-3	0	1.1	0.9	1.0	1.0
USP24	Q9UPU5	1362	6	6	-3	-14	1.1	1.1	1.0	0.9
RNF31	Q96EP0	722	6	4	-3	5	1.1	1.0	1.0	1.1
LRRK2	Q5S007	272	6	-2	-3	5	1.1	1.0	1.0	1.0
ARRB1	P49407	269	6	-3	-3	-19	1.1	1.0	1.0	0.8
AFTPH	Q6ULP2	250	6	-4	-3	-11	1.1	1.0	1.0	0.9
MICAL1	Q8TDZ2	95	6	-5	-3	-3	1.1	1.0	1.0	1.0
TRANK1	O15050	1514	6	-5	-3	-1	1.1	1.0	1.0	1.0
PTK2B	Q14289	562	6	-6	-3	5	1.1	0.9	1.0	1.1
MTMR6	Q9Y217	569	6	-6	-3	-7	1.1	0.9	1.0	0.9
SND1	Q7KZF4	560	6	-8	-3	2	1.1	0.9	1.0	1.0
H2AFY	O75367	276	6	-10	-3	7	1.1	0.9	1.0	1.1
RIPK1	Q13546	256	6	-13	-3	3	1.1	0.9	1.0	1.0
SLTM	Q9NWH9	311	6	-6	-3	-11	1.1	0.9	1.0	0.9
TFCP2	Q12800	395	6	-7	-3	3	1.1	0.9	1.0	1.0
SCAF1	Q9H7N4	826	6	-8	-3	-15	1.1	0.9	1.0	0.9
ZNF561	Q8N587	395	6	-14	-3	-2	1.1	0.9	1.0	1.0
WDR1	O75083	325	6	-18	-3	7	1.1	0.8	1.0	1.1
DECR1	Q16698	86	6	-22	-3	10	1.1	0.8	1.0	1.1
AKAP10	O43572	466	5	-4	-3	-1	1.1	1.0	1.0	1.0
PARP14	Q460N5	906	5	-4	-3	-11	1.1	1.0	1.0	0.9
GTF2E2	P29084	208	5	-5	-3	-4	1.1	1.0	1.0	1.0
RCSD1	Q6JBY9	49	5	-6	-3	-12	1.1	0.9	1.0	0.9
WBP2	Q969T9	80	5	-6	-3	-4	1.1	0.9	1.0	1.0
CHAMP1	Q96JM3	119	5	-6	-3	-13	1.1	0.9	1.0	0.9
CMAS	Q8NFW8	422	5	-10	-3	-9	1.1	0.9	1.0	0.9
PHGDH	O43175	19	5	-11	-3	6	1.1	0.9	1.0	1.1
RBM17	Q96I25	385	5	-12	-3	-1	1.1	0.9	1.0	1.0
FHL1	Q13642	191	5	-13	-3	-7	1.1	0.9	1.0	0.9

CD8A	P01732	54	5	-19	-3	5	1.1	0.8	1.0	1.0
SSH2	Q76I76	1054	5	3	-3	-4	1.0	1.0	1.0	1.0
FUBP3	Q96I24	460	5	-2	-3	-19	1.0	1.0	1.0	0.8
PML	P29590	389	5	-5	-3	-7	1.0	1.0	1.0	0.9
SLFN14	P0C7P3	431	5	-6	-3	-9	1.0	0.9	1.0	0.9
PLEC	Q15149	950	5	-8	-3	-1	1.0	0.9	1.0	1.0
ADCY7	P51828	1068	5	-8	-3	0	1.0	0.9	1.0	1.0
SQSTM1	Q13501	131	5	-8	-3	-9	1.0	0.9	1.0	0.9
PTBP3	O95758	248	5	-10	-3	-6	1.0	0.9	1.0	0.9
CORO1C	Q9ULV4	456	5	-11	-3	1	1.0	0.9	1.0	1.0
RRP12	Q5JTH9	261	5	-11	-3	-4	1.0	0.9	1.0	1.0
LTN1	O94822	981	5	-12	-3	-6	1.0	0.9	1.0	0.9
UBA6	A0AVT1	449	5	-13	-3	-4	1.0	0.9	1.0	1.0
ZFP64	Q9NPA5	352	5	-19	-3	-15	1.0	0.8	1.0	0.9
CCDC84	Q86UT8	16	4	16	-3	17	1.0	1.2	1.0	1.2
DNAJC2	Q99543	394	4	3	-3	-7	1.0	1.0	1.0	0.9
UQCRC1	P31930	69	4	2	-3	30	1.0	1.0	1.0	1.4
UBA2	Q9UBT2	444	4	-2	-3	-2	1.0	1.0	1.0	1.0
AFAP1	Q8N556	622	4	-2	-3	-4	1.0	1.0	1.0	1.0
GCN1	Q92616	932	4	-2	-3	-8	1.0	1.0	1.0	0.9
COBLL1	Q53SF7	261	4	-2	-3	-10	1.0	1.0	1.0	0.9
TES	Q9UGI8	22	4	-4	-3	-1	1.0	1.0	1.0	1.0
FGG	P02679	179	4	-4	-3	-6	1.0	1.0	1.0	0.9
TBC1D1	Q86TI0	161	4	-5	-3	-3	1.0	1.0	1.0	1.0
ARHGEF1	Q92888	752	4	-5	-3	-6	1.0	1.0	1.0	0.9
GINS3	Q9BRX5	188	4	-7	-3	-7	1.0	0.9	1.0	0.9
HDLBP	Q00341	940	4	-9	-3	-12	1.0	0.9	1.0	0.9
FAM91A1	Q658Y4	750	4	-11	-3	1	1.0	0.9	1.0	1.0
EPB41L2	O43491	232	4	-12	-3	1	1.0	0.9	1.0	1.0
RBBP5	Q15291	258	4	-13	-3	-5	1.0	0.9	1.0	1.0
DENND1B	Q6P3S1	52	4	-15	-3	-9	1.0	0.9	1.0	0.9
DENND1C	Q8IV53	52	4	-15	-3	-9	1.0	0.9	1.0	0.9
RRBP1	Q9P2E9	1323	4	-5	-3	-8	1.0	1.0	1.0	0.9
UBE2O	Q9C0C9	1040	4	-6	-3	-9	1.0	0.9	1.0	0.9
KDM3B	Q7LBC6	1357	4	-8	-3	-13	1.0	0.9	1.0	0.9
RRP8	O43159	451	4	-9	-3	-16	1.0	0.9	1.0	0.9
BCAS3	Q9H6U6	417	4	-10	-3	3	1.0	0.9	1.0	1.0
KIAA0513	O60268	317	4	-11	-3	-13	1.0	0.9	1.0	0.9
LIMD2	Q9BT23	40	4	-20	-3	3	1.0	0.8	1.0	1.0
ZFAND5	O76080	76	3	5	-3	4	1.0	1.1	1.0	1.0
LYST	Q99698	1827	3	3	-3	2	1.0	1.0	1.0	1.0
HNRNPK	P61978	132	3	-2	-3	-5	1.0	1.0	1.0	1.0
LRSAM1	Q6UWE0	418	3	-3	-3	-15	1.0	1.0	1.0	0.9
STX11	O75558	102	3	-4	-3	0	1.0	1.0	1.0	1.0
AES	Q08117	26	3	-4	-3	-2	1.0	1.0	1.0	1.0
EIF3J	O75822	207	3	-4	-3	-2	1.0	1.0	1.0	1.0
GBE1	Q04446	221	3	-5	-3	0	1.0	1.0	1.0	1.0
CLTC	Q00610	753	3	-6	-3	-3	1.0	0.9	1.0	1.0
IMPA1	P29218	24	3	-7	-3	-8	1.0	0.9	1.0	0.9
TCAF2	A6NFQ2	576	3	-7	-3	8	1.0	0.9	1.0	1.1
HNRNPA3	P51991	94	3	-7	-3	-8	1.0	0.9	1.0	0.9
BRE	Q9NXR7	44	3	-10	-3	-4	1.0	0.9	1.0	1.0
RNF213	Q63HN8	3609	3	-11	-3	-4	1.0	0.9	1.0	1.0

CDC73	Q6P1J9	145	3	-11	-3	-10	1.0	0.9	1.0	0.9
ARAP1	Q96P48	984	3	-14	-3	1	1.0	0.9	1.0	1.0
IRF9	Q00978	241	3	-16	-3	-14	1.0	0.9	1.0	0.9
PPP2R2D	Q66LE6	268	3	3	-3	3	1.0	1.0	1.0	1.0
SRD5A3	Q9H8P0	238	3	2	-3	-13	1.0	1.0	1.0	0.9
RIF1	Q5UIP0	312	3	-3	-3	13	1.0	1.0	1.0	1.1
SLFN5	Q08AF3	114	3	-4	-3	3	1.0	1.0	1.0	1.0
SYNE2	Q8WXH0	5601	3	-6	-3	23	1.0	0.9	1.0	1.3
PFKP	Q01813	664	3	-6	-3	-5	1.0	0.9	1.0	1.0
NPRL2	Q8WTW4	96	3	-6	-3	-17	1.0	0.9	1.0	0.9
RIN3	Q8TB24	881	3	-8	-3	-3	1.0	0.9	1.0	1.0
TRAPPC8	Q9Y2L5	1330	3	-11	-3	-37	1.0	0.9	1.0	0.7
TOP1	P11387	630	3	-13	-3	-1	1.0	0.9	1.0	1.0
DIDO1	Q9BTC0	1196	2	1	-3	-4	1.0	1.0	1.0	1.0
TAF6	P49848	130	2	-1	-3	3	1.0	1.0	1.0	1.0
ZCCHC11	Q5TAX3	994	2	-3	-3	-2	1.0	1.0	1.0	1.0
SGF29	Q96ES7	74	2	-8	-3	-10	1.0	0.9	1.0	0.9
RHOC	P08134	159	2	-10	-3	-2	1.0	0.9	1.0	1.0
BRAP	Q7Z569	47	2	-10	-3	-16	1.0	0.9	1.0	0.9
MACF1	Q9UPN3	877	2	-11	-3	-6	1.0	0.9	1.0	0.9
HUWE1	Q7Z6Z7	1891	2	-12	-3	-3	1.0	0.9	1.0	1.0
FGD3	Q5JSP0	566	2	-15	-3	-36	1.0	0.9	1.0	0.7
BRD4	O60885	674	2	-28	-3	-3	1.0	0.8	1.0	1.0
TLN1	Q9Y490	1486	2	-3	-3	-6	1.0	1.0	1.0	0.9
JAK1	P23458	763	2	-7	-3	-13	1.0	0.9	1.0	0.9
pk	D4Q8H0	22	2	-11	-3	12	1.0	0.9	1.0	1.1
TDRD7	Q8NHU6	714	2	-15	-3	-12	1.0	0.9	1.0	0.9
RRP12	Q5JTH9	839	2	-17	-3	18	1.0	0.9	1.0	1.2
PPP4R1	Q8TF05	284	2	-18	-3	-11	1.0	0.8	1.0	0.9
ZFYVE26	Q68DK2	715	1	-1	-3	-9	1.0	1.0	1.0	0.9
EXOSC9	Q06265	61	1	-3	-3	9	1.0	1.0	1.0	1.1
TBCB	Q99426	83	1	-4	-3	-7	1.0	1.0	1.0	0.9
HNRNPUL2-B	H3BQZ7	308	1	-5	-3	-2	1.0	1.0	1.0	1.0
GAPVD1	Q14C86	374	1	-5	-3	9	1.0	1.0	1.0	1.1
AASDH	Q4L235	369	1	-6	-3	-20	1.0	0.9	1.0	0.8
VIM	P08670	328	1	-7	-3	-7	1.0	0.9	1.0	0.9
PABPC1	P11940	132	1	-7	-3	-8	1.0	0.9	1.0	0.9
QTRTD1	Q9H974	151	1	-7	-3	-11	1.0	0.9	1.0	0.9
OPA1	O60313	375	1	-9	-3	-8	1.0	0.9	1.0	0.9
ACAA1	P09110	123	1	-11	-3	-8	1.0	0.9	1.0	0.9
MTIF2	P46199	646	1	-12	-3	-6	1.0	0.9	1.0	0.9
TDRD7	Q8NHU6	662	1	-17	-3	-8	1.0	0.9	1.0	0.9
GNB2	P62879	204	1	-22	-3	-9	1.0	0.8	1.0	0.9
CAD	P27708	1889	1	6	-3	26	1.0	1.1	1.0	1.3
BCKDK	O14874	111	1	1	-3	1	1.0	1.0	1.0	1.0
APEX2	Q9UBZ4	27	1	1	-3	-2	1.0	1.0	1.0	1.0
KIN	O60870	212	1	-3	-3	-9	1.0	1.0	1.0	0.9
ETFB	P38117	71	1	-3	-3	0	1.0	1.0	1.0	1.0
SATB1	Q01826	529	1	-5	-3	-16	1.0	1.0	1.0	0.9
HGD	Q93099	418	1	-6	-3	3	1.0	0.9	1.0	1.0
ZMYM4	Q5VZL5	756	1	-8	-3	-10	1.0	0.9	1.0	0.9
AP1B1	Q10567	866	1	-9	-3	2	1.0	0.9	1.0	1.0
PISD	Q9UG56	300	1	-10	-3	0	1.0	0.9	1.0	1.0

PCBP1	Q15365	109	1	-10	-3	-10	1.0	0.9	1.0	0.9
CLIC1	O00299	24	1	-11	-3	-4	1.0	0.9	1.0	1.0
CBL	P22681	508	1	-12	-3	-13	1.0	0.9	1.0	0.9
LARS	Q9P2J5	554	1	-14	-3	-14	1.0	0.9	1.0	0.9
RABL3	Q5HYI8	180	0	-1	-3	1	1.0	1.0	1.0	1.0
TRIM24	O15164	246	0	-4	-3	-1	1.0	1.0	1.0	1.0
TRIM33	Q9UPN9	299	0	-4	-3	-1	1.0	1.0	1.0	1.0
PHIP	Q8WWQ0	1612	0	-7	-3	-8	1.0	0.9	1.0	0.9
RPAP2	Q8IXW5	105	0	-8	-3	-2	1.0	0.9	1.0	1.0
TTC39C	Q8N584	517	0	-9	-3	-10	1.0	0.9	1.0	0.9
MDM1	Q8TC05	563	0	-10	-3	-13	1.0	0.9	1.0	0.9
FHL1	Q13642	212	0	-14	-3	-5	1.0	0.9	1.0	1.0
GCA	P28676	181	0	-16	-3	-9	1.0	0.9	1.0	0.9
EPB41	P11171	224	0	-18	-3	-5	1.0	0.8	1.0	1.0
SMAD2	Q15796	70	-1	-2	-3	-5	1.0	1.0	1.0	1.0
ROCK1	Q13464	1206	-1	-3	-3	-8	1.0	1.0	1.0	0.9
UHRF1BP1L	A0JNW5	744	-1	-3	-3	-12	1.0	1.0	1.0	0.9
SIPA1	Q96FS4	307	-1	-5	-3	-4	1.0	1.0	1.0	1.0
SND1	Q7KZF4	31	-1	-5	-3	-7	1.0	1.0	1.0	0.9
MCM3AP	O60318	439	-1	-7	-3	-5	1.0	0.9	1.0	1.0
TRAF2	Q12933	112	-1	-7	-3	-1	1.0	0.9	1.0	1.0
GTF3C1	Q12789	544	-1	-7	-3	-9	1.0	0.9	1.0	0.9
TRIP12	Q14669	35	-1	-9	-3	-8	1.0	0.9	1.0	0.9
FASTKD2	Q9NYY8	377	-1	-18	-3	-3	1.0	0.9	1.0	1.0
USP48	Q86UV5	841	-1	3	-3	-2	1.0	1.0	1.0	1.0
HERC5	Q9UII4	994	-1	-5	-3	-6	1.0	1.0	1.0	0.9
RNASEH2A	O75792	24	-1	-7	-3	-2	1.0	0.9	1.0	1.0
TUBA4A	P68366	347	-1	-8	-3	-8	1.0	0.9	1.0	0.9
DGKA	P23743	645	-1	-9	-3	-5	1.0	0.9	1.0	1.0
CPSF6	Q16630	159	-1	-9	-3	-6	1.0	0.9	1.0	0.9
HMHA1	Q92619	1020	-1	-10	-3	-17	1.0	0.9	1.0	0.9
PML	P29590	60	-1	-12	-3	-14	1.0	0.9	1.0	0.9
SYNE2	Q8WXH0	2520	-1	-13	-3	-7	1.0	0.9	1.0	0.9
NUDT3	O95989	68	-1	-16	-3	-17	1.0	0.9	1.0	0.9
AGPS	O00116	226	-2	-3	-3	-5	1.0	1.0	1.0	1.0
USP4	Q13107	500	-2	-3	-3	-5	1.0	1.0	1.0	1.0
AATK	Q6ZMQ8	277	-2	-6	-3	1	1.0	0.9	1.0	1.0
QKI	Q96PU8	35	-2	-6	-3	-6	1.0	0.9	1.0	0.9
ENOSF1	Q7L5Y1	330	-2	-7	-3	-7	1.0	0.9	1.0	0.9
EIF4ENIF1	Q9NRA8	767	-2	-7	-3	-10	1.0	0.9	1.0	0.9
CASP1	P29466	285	-2	-9	-3	-2	1.0	0.9	1.0	1.0
VCP	P55072	522	-2	-9	-3	1	1.0	0.9	1.0	1.0
DNAAF5	Q86Y56	173	-2	-10	-3	-10	1.0	0.9	1.0	0.9
PLEKHA2	Q9HB19	205	-2	-13	-3	-5	1.0	0.9	1.0	1.0
HCFC1	P51610	1886	-2	-14	-3	-13	1.0	0.9	1.0	0.9
NUMA1	Q14980	735	-2	-15	-3	-11	1.0	0.9	1.0	0.9
MOSPD2	Q8NHP6	471	-2	-20	-3	3	1.0	0.8	1.0	1.0
PRKDC	P78527	373	-2	-20	-3	1	1.0	0.8	1.0	1.0
NOTCH4	A0A140T9R5	233	-2	1	-3	-3	1.0	1.0	1.0	1.0
NOTCH4	A0A140T9R5	213	-2	1	-3	-3	1.0	1.0	1.0	1.0
SIPA1L3	O60292	620	-2	-2	-3	-2	1.0	1.0	1.0	1.0
SRP68	Q9UHB9	562	-2	-7	-3	-1	1.0	0.9	1.0	1.0
NELFCD	Q8IXH7	169	-2	-8	-3	19	1.0	0.9	1.0	1.2

CCT3	P49368	213	-2	-10	-3	-5	1.0	0.9	1.0	1.0
RABEPK	Q7Z6M1	29	-2	-12	-3	-8	1.0	0.9	1.0	0.9
UBR3	Q6ZT12	288	-2	-16	-3	-14	1.0	0.9	1.0	0.9
RAVER1	Q8IY67	224	-2	-17	-3	6	1.0	0.9	1.0	1.1
SMURF2	Q9HAU4	706	-3	-4	-3	-8	1.0	1.0	1.0	0.9
ATXN2	Q99700	556	-3	-7	-3	-5	1.0	0.9	1.0	1.0
GLOD4	Q9HC38	221	-3	-11	-3	5	1.0	0.9	1.0	1.0
NCKAP1L	P55160	617	-3	-13	-3	2	1.0	0.9	1.0	1.0
FAM76A	Q8TAV0	88	-3	-24	-3	-12	1.0	0.8	1.0	0.9
KBTBD4	Q9NVX7	68	-3	1	-3	-13	1.0	1.0	1.0	0.9
SLFN5	Q08AF3	489	-3	-2	-3	-3	1.0	1.0	1.0	1.0
ESD	P10768	181	-3	-7	-3	-2	1.0	0.9	1.0	1.0
NMRAL1	Q9HBL8	154	-3	-7	-3	-8	1.0	0.9	1.0	0.9
MCMBP	Q9BTE3	396	-3	-7	-3	2	1.0	0.9	1.0	1.0
GMPS	P49915	631	-3	-8	-3	-15	1.0	0.9	1.0	0.9
POC5	Q8NA72	208	-3	-9	-3	-8	1.0	0.9	1.0	0.9
CYFIP1	Q7L576	428	-3	-10	-3	-11	1.0	0.9	1.0	0.9
RCCD1	A6NED2	42	-3	-11	-3	-6	1.0	0.9	1.0	0.9
SLFN5	Q08AF3	302	-3	-13	-3	-5	1.0	0.9	1.0	1.0
UROS	P10746	241	-3	-16	-3	-19	1.0	0.9	1.0	0.8
DHX30	Q7L2E3	786	-4	-7	-3	-2	1.0	0.9	1.0	1.0
DOPEY2	Q9Y3R5	1329	-4	-8	-3	-2	1.0	0.9	1.0	1.0
ZNF598	Q86UK7	33	-4	-10	-3	-10	1.0	0.9	1.0	0.9
MARK2	Q7KZI7	553	-4	-10	-3	-7	1.0	0.9	1.0	0.9
MYCBP2	O75592	2662	-4	-12	-3	-18	1.0	0.9	1.0	0.8
TMEM33	P57088	224	-4	2	-3	2	1.0	1.0	1.0	1.0
RBM28	Q9NW13	490	-4	-9	-3	-9	1.0	0.9	1.0	0.9
ABAT	P80404	224	-4	-33	-3	-9	1.0	0.8	1.0	0.9
MYBBP1A	Q9BQG0	942	-5	-5	-3	-8	1.0	1.0	1.0	0.9
AARS	P49588	903	-5	-19	-3	4	1.0	0.8	1.0	1.0
NAMPT	P43490	401	-5	-47	-3	-16	1.0	0.7	1.0	0.9
OAS1	P00973	38	-5	-2	-3	-4	1.0	1.0	1.0	1.0
SLFN5	Q08AF3	207	-5	-5	-3	-14	1.0	1.0	1.0	0.9
AKAP17A	Q02040	563	-5	-7	-3	13	1.0	0.9	1.0	1.1
DLG1	Q12959	378	-5	-9	-3	-7	1.0	0.9	1.0	0.9
WDFY4	Q6ZS81	582	-5	-9	-3	-14	1.0	0.9	1.0	0.9
CRNKL1	Q9BZJ0	640	-5	-13	-3	1	1.0	0.9	1.0	1.0
TCP1	P17987	397	-6	-3	-3	25	0.9	1.0	1.0	1.3
EEFSEC	P57772	55	-6	-4	-3	11	0.9	1.0	1.0	1.1
DDX5	P17844	221	-6	-6	-3	-1	0.9	0.9	1.0	1.0
DDX17	Q92841	298	-6	-6	-3	-1	0.9	0.9	1.0	1.0
KHSRP	Q92945	176	-6	-9	-3	-4	0.9	0.9	1.0	1.0
GOLGB1	Q14789	2279	-6	-15	-3	-2	0.9	0.9	1.0	1.0
PI4KA	P42356	1898	-6	-17	-3	-18	0.9	0.9	1.0	0.9
UROS	P10746	143	-6	-12	-3	-6	0.9	0.9	1.0	0.9
LY9	Q9HBG7	627	-7	-4	-3	-12	0.9	1.0	1.0	0.9
MCM9	Q9NXL9	106	-7	-7	-3	2	0.9	0.9	1.0	1.0
NF1	P21359	1711	-7	-13	-3	-5	0.9	0.9	1.0	1.0
HNRNPK	P61978	184	-7	-6	-3	-1	0.9	0.9	1.0	1.0
TBL1X	O60907	571	-7	-27	-3	-13	0.9	0.8	1.0	0.9
TBL1XR1	Q9BZK7	508	-7	-27	-3	-13	0.9	0.8	1.0	0.9
DDHD2	O94830	207	-8	-5	-3	-2	0.9	1.0	1.0	1.0
PARP10	Q53GL7	50	-8	-10	-3	-7	0.9	0.9	1.0	0.9

COL4A3BP	Q9Y5P4	172	-8	-13	-3	-3	0.9	0.9	1.0	1.0
ITPR3	Q14573	1511	-8	-2	-3	-1	0.9	1.0	1.0	1.0
PRMT1	Q99873	350	-8	-11	-3	-17	0.9	0.9	1.0	0.9
POLG	P54098	647	-9	-13	-3	9	0.9	0.9	1.0	1.1
RPP38	P78345	80	-9	-15	-3	-14	0.9	0.9	1.0	0.9
NUB1	Q9Y5A7	400	-9	-11	-3	-2	0.9	0.9	1.0	1.0
UBN2	Q6ZU65	966	-9	-19	-3	-13	0.9	0.8	1.0	0.9
PLEKHO2	Q8TD55	44	-10	-8	-3	-23	0.9	0.9	1.0	0.8
ALDOA	P04075	73	-10	-8	-3	11	0.9	0.9	1.0	1.1
IL17RA	Q96F46	632	-10	10	-3	-13	0.9	1.1	1.0	0.9
ATP2B4	P23634	537	-10	-23	-3	3	0.9	0.8	1.0	1.0
PLAA	Q9Y263	193	-12	-3	-3	-11	0.9	1.0	1.0	0.9
BLOC1S3	Q6QNY0	168	-12	-8	-3	-6	0.9	0.9	1.0	0.9
WHSC1L1	Q9BZ95	173	-12	-12	-3	-13	0.9	0.9	1.0	0.9
TERF2IP	Q9NYB0	54	-12	-14	-3	-8	0.9	0.9	1.0	0.9
DTWD2	Q8NBA8	226	-12	-18	-3	5	0.9	0.8	1.0	1.0
HSP90AB1	P08238	590	-13	-11	-3	-7	0.9	0.9	1.0	0.9
APAF1	O14727	633	-13	-21	-3	26	0.9	0.8	1.0	1.3
TEFM	Q96QE5	135	-14	1	-3	-23	0.9	1.0	1.0	0.8
NUP210	Q8TEM1	76	-15	-26	-3	5	0.9	0.8	1.0	1.0
DOCK9	Q9BZ29	1682	-16	-4	-3	-17	0.9	1.0	1.0	0.9
ATIC	P31939	325	-16	-29	-3	-15	0.9	0.8	1.0	0.9
GTPBP6	H0Y2S1	305	-16	-11	-3	-17	0.9	0.9	1.0	0.9
PGM1	P36871	374	-17	-8	-3	-19	0.9	0.9	1.0	0.8
TECPR1	Q7Z6L1	736	-20	-8	-3	2	0.8	0.9	1.0	1.0
PABPN1	Q86U42	195	-21	-17	-3	-8	0.8	0.9	1.0	0.9
BDH2	Q9BUT1	145	22	-11	-3	10	1.3	0.9	1.0	1.1
TNPO1	Q92973	683	21	8	-3	14	1.3	1.1	1.0	1.2
PPP1R3D	O95685	291	21	8	-3	2	1.3	1.1	1.0	1.0
PHF1	O43189	442	20	-19	-3	-14	1.3	0.8	1.0	0.9
ARAP3	Q8WWN8	1051	20	4	-3	1	1.2	1.0	1.0	1.0
KMT2B	Q9UMN6	2655	20	3	-3	9	1.2	1.0	1.0	1.1
ZAP70	P43403	618	20	-3	-3	-5	1.2	1.0	1.0	1.0
CLIC3	O95833	174	20	-12	-3	13	1.2	0.9	1.0	1.1
SPG11	Q96JI7	2291	19	-6	-3	-19	1.2	0.9	1.0	0.8
ANKRD44	Q8N8A2	547	19	-15	-3	1	1.2	0.9	1.0	1.0
RNMT	O43148	356	17	-5	-3	19	1.2	1.0	1.0	1.2
RAB3GAP2	Q9H2M9	608	15	-14	-3	9	1.2	0.9	1.0	1.1
F8A3	P23610	76	14	-13	-3	1	1.2	0.9	1.0	1.0
DSTN	P60981	135	14	-8	-3	8	1.2	0.9	1.0	1.1
TRIM22	Q8IYM9	347	13	-5	-3	5	1.1	1.0	1.0	1.1
RCAN1	P53805	83	13	-13	-3	6	1.1	0.9	1.0	1.1
IDH3A	P50213	359	13	-15	-3	4	1.1	0.9	1.0	1.0
CTNNA1	P35221	793	13	-2	-3	-2	1.1	1.0	1.0	1.0
GLOD4	Q9HC38	197	13	-22	-3	4	1.1	0.8	1.0	1.0
RASA3	Q14644	154	13	-27	-3	0	1.1	0.8	1.0	1.0
PRKDC	P78527	1791	12	-8	-3	-3	1.1	0.9	1.0	1.0
MX1	P20591	336	12	0	-3	-10	1.1	1.0	1.0	0.9
NLRP1	Q9C000	1310	12	-8	-3	-15	1.1	0.9	1.0	0.9
EPG5	Q9HCE0	1201	12	-11	-3	-5	1.1	0.9	1.0	1.0
ASPHD2	Q6ICH7	200	11	-2	-3	-9	1.1	1.0	1.0	0.9
FKBP8	Q14318	295	11	-8	-3	-2	1.1	0.9	1.0	1.0
ZYX	Q15942	565	11	-16	-3	31	1.1	0.9	1.0	1.4

GEMIN5	Q8TEQ6	706	11	5	-3	13	1.1	1.1	1.0	1.1
CBL	P22681	567	11	2	-3	-2	1.1	1.0	1.0	1.0
ANXA2	P07355	133	11	-4	-3	-3	1.1	1.0	1.0	1.0
DDAH2	O95865	262	11	-5	-3	10	1.1	1.0	1.0	1.1
DOCK8	Q8NF50	391	11	-6	-3	9	1.1	0.9	1.0	1.1
GIMAP4	Q9NUV9	220	11	-9	-3	-3	1.1	0.9	1.0	1.0
TRIM4	Q9C037	293	11	-11	-3	3	1.1	0.9	1.0	1.0
PRPF6	O94906	913	10	-1	-3	13	1.1	1.0	1.0	1.1
APPL2	Q8NEU8	606	10	-4	-3	1	1.1	1.0	1.0	1.0
MVB12A	Q96EY5	231	10	-6	-3	-2	1.1	0.9	1.0	1.0
ASAP1	Q9ULH1	557	10	-9	-3	-3	1.1	0.9	1.0	1.0
STAT5B	P51692	688	10	-13	-3	-21	1.1	0.9	1.0	0.8
DARS2	Q6PI48	152	10	-14	-3	3	1.1	0.9	1.0	1.0
MED8	Q96G25	31	10	-1	-3	-5	1.1	1.0	1.0	1.0
SETD2	Q9BYW2	495	9	6	-3	-11	1.1	1.1	1.0	0.9
PNPLA6	Q8IY17	482	9	3	-3	-3	1.1	1.0	1.0	1.0
SYNE3	Q6ZMZ3	74	9	-1	-3	-5	1.1	1.0	1.0	1.0
INPP5D	Q92835	672	9	-2	-3	6	1.1	1.0	1.0	1.1
AP2B1	P63010	95	9	-17	-3	8	1.1	0.9	1.0	1.1
BAG5	Q9UL15	157	9	-5	-3	-6	1.1	1.0	1.0	0.9
MICAL3	Q7RTP6	1760	9	-6	-3	-5	1.1	0.9	1.0	1.0
LARP7	Q4G0J3	148	9	-8	-3	-1	1.1	0.9	1.0	1.0
MORC3	Q14149	307	9	-11	-3	-2	1.1	0.9	1.0	1.0
MYH10	P35580	95	9	-11	-3	-7	1.1	0.9	1.0	0.9
AGO1	Q9UL18	64	9	-11	-3	-13	1.1	0.9	1.0	0.9
TRIM33	Q9UPN9	241	9	-12	-3	6	1.1	0.9	1.0	1.1
WDR3	Q9UNX4	44	9	-20	-3	2	1.1	0.8	1.0	1.0
AOAH	P28039	249	9	-23	-3	-6	1.1	0.8	1.0	0.9
FRYL	O94915	1107	9	-27	-3	-5	1.1	0.8	1.0	1.0
SYNE2	Q8WXH0	3513	8	7	-3	-16	1.1	1.1	1.0	0.9
UBR4	Q5T4S7	4839	8	1	-3	3	1.1	1.0	1.0	1.0
OTUB1	Q96FW1	23	8	1	-3	-16	1.1	1.0	1.0	0.9
THEMIS2	Q5TEJ8	410	8	-11	-3	-11	1.1	0.9	1.0	0.9
TALDO1	P37837	250	8	-21	-3	3	1.1	0.8	1.0	1.0
CORO1C	Q9ULV4	190	8	-30	-3	-1	1.1	0.8	1.0	1.0
PAICS	P22234	81	8	0	-3	0	1.1	1.0	1.0	1.0
SIN3A	Q96ST3	1167	8	-3	-3	-6	1.1	1.0	1.0	0.9
DDX27	Q96GQ7	259	8	-7	-3	-5	1.1	0.9	1.0	1.0
KLHL36	Q8N4N3	523	8	-7	-3	-8	1.1	0.9	1.0	0.9
CPSF1	Q10570	324	8	-10	-3	-20	1.1	0.9	1.0	0.8
GNL3L	Q9NVN8	244	8	-12	-3	-20	1.1	0.9	1.0	0.8
PPFIBP2	Q8ND30	699	8	-17	-3	-1	1.1	0.9	1.0	1.0
INPP4B	O15327	534	7	5	-3	2	1.1	1.1	1.0	1.0
PRRC2C	Q9Y520	620	7	3	-3	-13	1.1	1.0	1.0	0.9
HEATR5B	Q9P2D3	518	7	1	-3	3	1.1	1.0	1.0	1.0
RNF213	Q63HN8	3766	7	-2	-3	-1	1.1	1.0	1.0	1.0
STK24	Q9Y6E0	394	7	-4	-3	-6	1.1	1.0	1.0	0.9
TTC39C	Q8N584	529	7	-8	-3	-13	1.1	0.9	1.0	0.9
EDC3	Q96F86	47	7	-11	-3	-7	1.1	0.9	1.0	0.9
TRAPPC13	A5PLN9	127	7	-15	-3	-7	1.1	0.9	1.0	0.9
KBTBD11	O94819	537	7	-18	-3	3	1.1	0.8	1.0	1.0
MYO1G	B0I1T2	143	7	-18	-3	-8	1.1	0.8	1.0	0.9
PDPR	Q8NCN5	357	7	-1	-3	2	1.1	1.0	1.0	1.0

SUCLG1	P53597	181	7	-4	-3	2	1.1	1.0	1.0	1.0
TNPO3	Q9Y5L0	511	7	-6	-3	-2	1.1	0.9	1.0	1.0
JAK2	O60674	243	7	-8	-3	5	1.1	0.9	1.0	1.1
COPG1	Q9Y678	169	7	-10	-3	-2	1.1	0.9	1.0	1.0
CASP1	P29466	136	7	-13	-3	-4	1.1	0.9	1.0	1.0
FAM129A	Q9BZQ8	41	7	-13	-3	-7	1.1	0.9	1.0	0.9
UCHL3	P15374	95	6	-5	-3	-2	1.1	1.0	1.0	1.0
GCLC	P48506	553	6	-6	-3	-5	1.1	0.9	1.0	1.0
C7orf26	Q96N11	157	6	-8	-3	-14	1.1	0.9	1.0	0.9
CEP162	Q5TB80	1014	6	-15	-3	-5	1.1	0.9	1.0	1.0
VAPA	Q9P0L0	128	6	-15	-3	-3	1.1	0.9	1.0	1.0
SYTL2	Q9HCH5	468	6	6	-3	11	1.1	1.1	1.0	1.1
NUMA1	Q14980	1673	6	-1	-3	0	1.1	1.0	1.0	1.0
DMXL1	Q9Y485	551	6	-1	-3	-6	1.1	1.0	1.0	0.9
INO80	Q9ULG1	1001	6	-2	-3	-4	1.1	1.0	1.0	1.0
YWHAE	P62258	97	6	-9	-3	5	1.1	0.9	1.0	1.1
POGZ	Q7Z3K3	1267	6	-9	-3	-3	1.1	0.9	1.0	1.0
APTX	Q7Z2E3	161	6	-11	-3	-13	1.1	0.9	1.0	0.9
CEP41	Q9BYV8	171	6	-12	-3	7	1.1	0.9	1.0	1.1
CNDP2	Q96KP4	222	6	-16	-3	-12	1.1	0.9	1.0	0.9
PSMD8	P48556	112	6	-18	-3	-10	1.1	0.9	1.0	0.9
AMPD2	Q01433	107	5	2	-3	-5	1.1	1.0	1.0	1.0
ZC3H13	Q5T200	1591	5	-6	-3	-1	1.1	0.9	1.0	1.0
STAT5A	P42229	126	5	-8	-3	-12	1.1	0.9	1.0	0.9
DOCK7	Q96N67	1944	5	7	-3	3	1.0	1.1	1.0	1.0
NHSL2	Q5HYW2	679	5	1	-3	-2	1.0	1.0	1.0	1.0
THNSL1	Q8IYQ7	277	5	-2	-3	-8	1.0	1.0	1.0	0.9
EEFSEC	P57772	130	5	-3	-3	-7	1.0	1.0	1.0	0.9
ARHGEF6	Q15052	25	5	-4	-3	-13	1.0	1.0	1.0	0.9
SNX1	Q13596	318	5	-5	-3	-7	1.0	1.0	1.0	0.9
PIBF1	Q8WXW3	199	5	-7	-3	-4	1.0	0.9	1.0	1.0
SNU13	P55769	93	5	-9	-3	0	1.0	0.9	1.0	1.0
DHX30	Q7L2E3	1183	5	-12	-3	-4	1.0	0.9	1.0	1.0
IQGAP1	P46940	45	5	-18	-3	-1	1.0	0.9	1.0	1.0
FLAD1	Q8NFF5	561	5	-23	-3	4	1.0	0.8	1.0	1.0
HNRNPU	Q00839	391	4	-3	-3	-4	1.0	1.0	1.0	1.0
FGD3	Q5JSP0	558	4	-4	-3	-6	1.0	1.0	1.0	0.9
CDK19	Q9BWU1	25	4	-5	-3	5	1.0	1.0	1.0	1.0
PDS5A	Q29RF7	1116	4	-5	-3	-11	1.0	1.0	1.0	0.9
LLGL1	Q15334	505	4	-6	-3	-2	1.0	0.9	1.0	1.0
ATP11C	Q8NB49	200	4	-6	-3	-11	1.0	0.9	1.0	0.9
CLIP2	Q9UDT6	132	4	-6	-3	-2	1.0	0.9	1.0	1.0
IMPDH2	P12268	140	4	-7	-3	-1	1.0	0.9	1.0	1.0
IFI16	Q16666	679	4	-10	-3	3	1.0	0.9	1.0	1.0
TUBB6	Q9BUF5	12	4	-11	-3	-5	1.0	0.9	1.0	1.0
PRPF4B	Q13523	962	4	-11	-3	-10	1.0	0.9	1.0	0.9
IDH2	P48735	308	4	-12	-3	6	1.0	0.9	1.0	1.1
PREPL	Q4J6C6	127	4	-12	-3	-6	1.0	0.9	1.0	0.9
SLFN14	P0C7P3	883	4	-17	-3	-39	1.0	0.9	1.0	0.7
USP48	Q86UV5	850	4	4	-3	5	1.0	1.0	1.0	1.1
NAPRT	Q6XQN6	463	4	3	-3	-1	1.0	1.0	1.0	1.0
NAA15	Q9BXJ9	721	4	-2	-3	-6	1.0	1.0	1.0	0.9
EP300	Q09472	1738	4	-3	-3	-4	1.0	1.0	1.0	1.0

CREBBP	Q92793	1775	4	-3	-3	-4	1.0	1.0	1.0	1.0
MKRN1	Q9UHC7	214	4	-3	-3	-5	1.0	1.0	1.0	1.0
DIS3	Q9Y2L1	799	4	-5	-3	-14	1.0	1.0	1.0	0.9
TSTA3	Q13630	116	4	-6	-3	0	1.0	0.9	1.0	1.0
THYN1	Q9P016	118	4	-6	-3	-6	1.0	0.9	1.0	0.9
FGD3	Q5JSP0	608	4	-6	-3	-5	1.0	0.9	1.0	1.0
LGALS1	P09382	89	4	-8	-3	7	1.0	0.9	1.0	1.1
DTYMK	P23919	31	4	-8	-3	-1	1.0	0.9	1.0	1.0
HSD17B11	Q8NBQ5	215	4	-10	-3	-4	1.0	0.9	1.0	1.0
AARSD1	Q9BTE6	22	4	-11	-3	-12	1.0	0.9	1.0	0.9
NOP9	Q86U38	123	4	-13	-3	20	1.0	0.9	1.0	1.2
CORO1A	P31146	40	4	-14	-3	-2	1.0	0.9	1.0	1.0
CTNNA1	P35221	772	4	-25	-3	-1	1.0	0.8	1.0	1.0
RANBP2	P49792	2696	4	-27	-3	-23	1.0	0.8	1.0	0.8
NUP210	Q8TEM1	767	3	-1	-3	7	1.0	1.0	1.0	1.1
XPC	Q01831	60	3	-2	-3	-14	1.0	1.0	1.0	0.9
RPA1	P27694	476	3	-3	-3	-2	1.0	1.0	1.0	1.0
CCT5	P48643	253	3	-7	-3	3	1.0	0.9	1.0	1.0
TBC1D2B	Q9UPU7	38	3	-7	-3	-7	1.0	0.9	1.0	0.9
GIMAP2	Q9UG22	261	3	-8	-3	16	1.0	0.9	1.0	1.2
ANP32A	P39687	123	3	-10	-3	4	1.0	0.9	1.0	1.0
THUMPD3	Q9BV44	391	3	-11	-3	-16	1.0	0.9	1.0	0.9
ABRACL	Q9P1F3	39	3	-12	-3	-5	1.0	0.9	1.0	1.0
MBD2	Q9UBB5	359	3	-12	-3	-19	1.0	0.9	1.0	0.8
PIK3CD	O00329	718	3	-16	-3	1	1.0	0.9	1.0	1.0
GCN1	Q92616	1275	3	-5	-3	-8	1.0	1.0	1.0	0.9
SPR	P35270	159	3	-7	-3	13	1.0	0.9	1.0	1.1
MGEA5	O60502	896	3	-10	-3	-6	1.0	0.9	1.0	0.9
ILF3	Q12906	116	3	-13	-3	6	1.0	0.9	1.0	1.1
GGNBP2	Q9H3C7	236	3	-13	-3	-4	1.0	0.9	1.0	1.0
MGMT	P16455	24	3	-14	-3	-16	1.0	0.9	1.0	0.9
ACAA2	P42765	287	3	-15	-3	2	1.0	0.9	1.0	1.0
RARS	P54136	150	3	-19	-3	17	1.0	0.8	1.0	1.2
TSEN54	Q7Z6J9	338	3	-23	-3	-14	1.0	0.8	1.0	0.9
YPEL4	Q96NS1	90	2	-4	-3	0	1.0	1.0	1.0	1.0
PRKDC	P78527	4045	2	-5	-3	1	1.0	1.0	1.0	1.0
NSMCE2	Q96MF7	210	2	-5	-3	-7	1.0	1.0	1.0	0.9
OAS2	P29728	668	2	-6	-3	0	1.0	0.9	1.0	1.0
TNIP2	Q8NFZ5	427	2	-9	-3	-12	1.0	0.9	1.0	0.9
TSC2	P49815	253	2	-9	-3	-5	1.0	0.9	1.0	1.0
KLHL4	Q9C0H6	639	2	-10	-3	-3	1.0	0.9	1.0	1.0
MYO1G	B0I1T2	979	2	-12	-3	7	1.0	0.9	1.0	1.1
TLN1	Q9Y490	2196	2	-13	-3	-1	1.0	0.9	1.0	1.0
TLN2	Q9Y4G6	2197	2	-13	-3	-1	1.0	0.9	1.0	1.0
FAM102A	Q5T9C2	119	2	-14	-3	-10	1.0	0.9	1.0	0.9
CKAP5	Q14008	592	2	-32	-3	3	1.0	0.8	1.0	1.0
TLR7	Q9NYK1	445	2	7	-3	6	1.0	1.1	1.0	1.1
DDX60L	Q5H9U9	1031	2	-2	-3	0	1.0	1.0	1.0	1.0
CXorf38	Q8TB03	300	2	-3	-3	0	1.0	1.0	1.0	1.0
PSMC6	P62333	193	2	-4	-3	6	1.0	1.0	1.0	1.1
WDR81	Q562E7	198	2	-5	-3	-10	1.0	1.0	1.0	0.9
CYLD	Q9NQC7	934	2	-8	-3	-5	1.0	0.9	1.0	1.0
UBR4	Q5T4S7	934	2	-9	-3	-5	1.0	0.9	1.0	1.0

CPSF3	Q9UKF6	498	2	-10	-3	-13	1.0	0.9	1.0	0.9
GCN1	Q92616	1984	2	-10	-3	-7	1.0	0.9	1.0	0.9
MED16	Q9Y2X0	801	2	-11	-3	-6	1.0	0.9	1.0	0.9
PIP4K2C	Q8TBX8	104	2	-12	-3	6	1.0	0.9	1.0	1.1
SYNE1	Q8NF91	7984	2	-12	-3	-8	1.0	0.9	1.0	0.9
SYNE2	Q8WXH0	6121	2	-12	-3	-8	1.0	0.9	1.0	0.9
GORAB	Q5T7V8	156	2	-17	-3	-22	1.0	0.9	1.0	0.8
SNX27	Q96L92	519	1	1	-3	-4	1.0	1.0	1.0	1.0
CARS	P49589	27	1	-3	-3	-7	1.0	1.0	1.0	0.9
CHMP2B	Q9UQN3	58	1	-5	-3	-8	1.0	1.0	1.0	0.9
NUP205	Q92621	925	1	-5	-3	-3	1.0	1.0	1.0	1.0
TSR2	Q969E8	114	1	-6	-3	-14	1.0	0.9	1.0	0.9
INTS1	Q8N201	1534	1	-7	-3	3	1.0	0.9	1.0	1.0
ANAPC7	Q9UJX3	329	1	-9	-3	-5	1.0	0.9	1.0	1.0
IDH1	O75874	269	1	-11	-3	-11	1.0	0.9	1.0	0.9
DCUN1D4	Q92564	219	1	-12	-3	13	1.0	0.9	1.0	1.1
RAP1GDS1	P52306	144	1	-12	-3	-11	1.0	0.9	1.0	0.9
CDK4	P11802	215	1	-17	-3	-5	1.0	0.9	1.0	1.0
COPA	P53621	580	1	-17	-3	-9	1.0	0.9	1.0	0.9
ACSL1	P33121	311	1	5	-3	-9	1.0	1.1	1.0	0.9
ACAT1	P24752	126	1	-4	-3	-4	1.0	1.0	1.0	1.0
FARS2	O95363	334	1	-4	-3	-6	1.0	1.0	1.0	0.9
SF1	Q15637	279	1	-5	-3	-5	1.0	1.0	1.0	1.0
NOP58	Q9Y2X3	439	1	-14	-3	-11	1.0	0.9	1.0	0.9
HMCES	Q96FZ2	131	1	-17	-3	-7	1.0	0.9	1.0	0.9
PPIA	P62937	52	0	4	-3	-3	1.0	1.0	1.0	1.0
FTO	Q9C0B1	171	0	-2	-3	-13	1.0	1.0	1.0	0.9
LIMK1	P53667	349	0	-2	-3	1	1.0	1.0	1.0	1.0
ATP13A1	Q9HD20	969	0	-3	-3	8	1.0	1.0	1.0	1.1
UBE2F	Q969M7	116	0	-4	-3	24	1.0	1.0	1.0	1.3
GCN1	Q92616	1781	0	-4	-3	6	1.0	1.0	1.0	1.1
CHCHD3	Q9NX63	112	0	-6	-3	-2	1.0	0.9	1.0	1.0
ATF7IP	Q6VMQ6	955	0	-7	-3	-6	1.0	0.9	1.0	0.9
HUWE1	Q7Z6Z7	790	0	-8	-3	-5	1.0	0.9	1.0	1.0
FPGT	O14772	395	0	-9	-3	-19	1.0	0.9	1.0	0.8
SRSF7	Q16629	109	0	-11	-3	-6	1.0	0.9	1.0	0.9
PRPF19	Q9UMS4	114	0	-12	-3	-1	1.0	0.9	1.0	1.0
HMHA1	Q92619	732	0	-12	-3	-6	1.0	0.9	1.0	0.9
FAM208A	Q9UK61	1421	0	-17	-3	-17	1.0	0.9	1.0	0.9
DNAJC13	O75165	1432	0	-32	-3	-25	1.0	0.8	1.0	0.8
LIMD2	Q9BT23	43	-1	0	-3	10	1.0	1.0	1.0	1.1
RPL37A	P61513	39	-1	-4	-3	0	1.0	1.0	1.0	1.0
GRAP2	O75791	244	-1	-6	-3	-9	1.0	0.9	1.0	0.9
RNF213	Q63HN8	438	-1	-7	-3	-7	1.0	0.9	1.0	0.9
UBR4	Q5T4S7	779	-1	-8	-3	7	1.0	0.9	1.0	1.1
C8orf82	Q6P1X6	107	-1	-10	-3	18	1.0	0.9	1.0	1.2
NUBP2	Q9Y5Y2	54	-1	-10	-3	-16	1.0	0.9	1.0	0.9
RPL11	P62913	25	-1	-11	-3	-3	1.0	0.9	1.0	1.0
ANKRD44	Q8N8A2	914	-1	-28	-3	-24	1.0	0.8	1.0	0.8
MKL1	Q969V6	326	-1	2	-3	-6	1.0	1.0	1.0	0.9
COG7	P83436	505	-1	-1	-3	1	1.0	1.0	1.0	1.0
MAP2K4	P45985	379	-1	-3	-3	-4	1.0	1.0	1.0	1.0
ARAP1	Q96P48	900	-1	-4	-3	-4	1.0	1.0	1.0	1.0

PRKRA	O75569	77	-1	-5	-3	-5	1.0	1.0	1.0	1.0
CXorf21	Q9HAI6	117	-1	-8	-3	-14	1.0	0.9	1.0	0.9
RNF213	Q63HN8	2918	-1	-8	-3	-7	1.0	0.9	1.0	0.9
RBBP4	Q09028	138	-1	-10	-3	-3	1.0	0.9	1.0	1.0
ACADSB	P45954	175	-1	-11	-3	-1	1.0	0.9	1.0	1.0
BAG1	Q99933	330	-1	-22	-3	-8	1.0	0.8	1.0	0.9
ZBTB4	Q9P1Z0	770	-1	-24	-3	-18	1.0	0.8	1.0	0.8
GLG1	Q92896	515	-2	-4	-3	-2	1.0	1.0	1.0	1.0
EPX	P11678	455	-2	-5	-3	-14	1.0	1.0	1.0	0.9
MTM1	Q13496	191	-2	-8	-3	-14	1.0	0.9	1.0	0.9
DIS3	Q9Y2L1	533	-2	-9	-3	-8	1.0	0.9	1.0	0.9
MTCH1	Q9NZJ7	385	-2	-9	-3	-10	1.0	0.9	1.0	0.9
FNBP1	Q96RU3	130	-2	-9	-3	-11	1.0	0.9	1.0	0.9
ATP2B4	P23634	632	-2	-9	-3	-10	1.0	0.9	1.0	0.9
LCP1	P13796	336	-2	-10	-3	4	1.0	0.9	1.0	1.0
PRDX6	P30041	91	-2	-11	-3	-8	1.0	0.9	1.0	0.9
DFNA5	O60443	417	-2	-14	-3	-4	1.0	0.9	1.0	1.0
CMAS	Q8NFW8	432	-2	0	-3	-7	1.0	1.0	1.0	0.9
ICE1	Q9Y2F5	2094	-2	-2	-3	-11	1.0	1.0	1.0	0.9
FXR1	P51114	157	-2	-3	-3	6	1.0	1.0	1.0	1.1
CBX3	Q13185	177	-2	-3	-3	-11	1.0	1.0	1.0	0.9
CRBN	Q96SW2	287	-2	-7	-3	-5	1.0	0.9	1.0	1.0
MVK	Q03426	339	-2	-7	-3	-10	1.0	0.9	1.0	0.9
MBNL2	Q5VZF2	43	-2	-9	-3	-1	1.0	0.9	1.0	1.0
MBNL1	Q9NR56	43	-2	-9	-3	-1	1.0	0.9	1.0	1.0
DDX5	P17844	234	-2	-10	-3	-6	1.0	0.9	1.0	0.9
WRNIP1	Q96S55	272	-2	-12	-3	-5	1.0	0.9	1.0	1.0
TARSL2	A2RTX5	62	-3	-5	-3	-11	1.0	1.0	1.0	0.9
RASA3	Q14644	662	-3	-5	-3	-8	1.0	1.0	1.0	0.9
IGKC	A0A087X1V9	239	-3	-5	-3	-13	1.0	1.0	1.0	0.9
DOCK8	Q8NF50	1076	-3	-6	-3	-6	1.0	0.9	1.0	0.9
MYH9	P35579	988	-3	-8	-3	-9	1.0	0.9	1.0	0.9
AKR1B1	P15121	299	-3	-13	-3	2	1.0	0.9	1.0	1.0
SUPT16H	Q9Y5B9	574	-3	-13	-3	-5	1.0	0.9	1.0	1.0
TMEM189-UI	I3L0A0	367	-3	-14	-3	-6	1.0	0.9	1.0	0.9
GCLC	P48506	613	-3	-14	-3	-16	1.0	0.9	1.0	0.9
TMCO6	Q96DC7	14	-3	-15	-3	-1	1.0	0.9	1.0	1.0
PPP2R3C	Q969Q6	17	-3	-2	-3	-2	1.0	1.0	1.0	1.0
CCT7	Q99832	29	-3	-3	-3	-11	1.0	1.0	1.0	0.9
DIS3L	Q8TF46	482	-3	-4	-3	3	1.0	1.0	1.0	1.0
WDR7	Q9Y4E6	1103	-3	-5	-3	-6	1.0	1.0	1.0	0.9
GNL2	Q13823	336	-3	-14	-3	-11	1.0	0.9	1.0	0.9
SLC7A6OS	Q96CW6	27	-3	-15	-3	-18	1.0	0.9	1.0	0.9
MORC3	Q14149	15	-4	-5	-3	-7	1.0	1.0	1.0	0.9
LBP	P18428	198	-4	-12	-3	8	1.0	0.9	1.0	1.1
MAD2L1BP	Q15013	247	-4	-14	-3	-53	1.0	0.9	1.0	0.7
PDGFRB	P09619	835	-4	-16	-3	-13	1.0	0.9	1.0	0.9
WIPI2	Q9Y4P8	334	-4	-17	-3	-4	1.0	0.9	1.0	1.0
GTF2H3	Q13889	257	-4	-20	-3	2	1.0	0.8	1.0	1.0
SEC16A	O15027	1115	-4	-2	-3	-17	1.0	1.0	1.0	0.9
TRIM28	Q13263	221	-4	-6	-3	-5	1.0	0.9	1.0	1.0
DNAJA2	O60884	308	-4	-12	-3	2	1.0	0.9	1.0	1.0
EDC3	Q96F86	499	-4	-13	-3	-6	1.0	0.9	1.0	0.9

RPS20	P60866	70	-4	-15	-3	1	1.0	0.9	1.0	1.0
ARL1	P40616	80	-4	-24	-3	-14	1.0	0.8	1.0	0.9
CCT5	P48643	440	-5	2	-3	-6	1.0	1.0	1.0	0.9
TGFB1I1	O43294	275	-5	-2	-3	-4	1.0	1.0	1.0	1.0
HNRNPUL1	Q9BUJ2	391	-5	-10	-3	-6	1.0	0.9	1.0	0.9
WDR81	Q562E7	215	-5	-10	-3	-5	1.0	0.9	1.0	1.0
THTPA	Q9BU02	228	-5	-11	-3	-13	1.0	0.9	1.0	0.9
ANO6	Q4KMQ2	261	-5	-4	-3	-1	1.0	1.0	1.0	1.0
NFU1	Q9UMS0	213	-5	-6	-3	-1	1.0	0.9	1.0	1.0
NLRP3	Q96P20	770	-5	-8	-3	3	1.0	0.9	1.0	1.0
SLC25A20	O43772	58	-5	-9	-3	3	1.0	0.9	1.0	1.0
RSF1	Q96T23	562	-5	-9	-3	-2	1.0	0.9	1.0	1.0
JOSD1	Q15040	36	-5	-13	-3	-10	1.0	0.9	1.0	0.9
TEX264	Q9Y6I9	182	-5	-28	-3	-13	1.0	0.8	1.0	0.9
C15orf39	Q6ZRI6	367	-6	-2	-3	-6	0.9	1.0	1.0	0.9
ITPRIP	Q8IWB1	268	-6	-7	-3	-12	0.9	0.9	1.0	0.9
IMMT	Q16891	603	-6	-9	-3	-6	0.9	0.9	1.0	0.9
SGTA	O43765	153	-6	-9	-3	-5	0.9	0.9	1.0	1.0
XPO6	Q96QU8	159	-6	-11	-3	-1	0.9	0.9	1.0	1.0
AUP1	Q9Y679	391	-6	-19	-3	5	0.9	0.8	1.0	1.1
YARS	P54577	442	-6	-29	-3	15	0.9	0.8	1.0	1.2
CHURC1-FNT	B4DL54	17	-6	-5	-3	5	0.9	1.0	1.0	1.0
TBC1D2	Q9BYX2	686	-6	-5	-3	-13	0.9	1.0	1.0	0.9
CHAMP1	Q96JM3	194	-6	-7	-3	1	0.9	0.9	1.0	1.0
NCDN	Q9UBB6	98	-6	-11	-3	23	0.9	0.9	1.0	1.3
NFE2	Q16621	283	-7	9	-3	11	0.9	1.1	1.0	1.1
AHNAK	Q09666	2162	-7	-7	-3	-17	0.9	0.9	1.0	0.9
FLII	Q13045	560	-7	-7	-3	2	0.9	0.9	1.0	1.0
YLPM1	P49750	1035	-7	-10	-3	-9	0.9	0.9	1.0	0.9
SDAD1	Q9NVU7	87	-8	-3	-3	-12	0.9	1.0	1.0	0.9
CLUH	O75153	200	-8	-7	-3	-5	0.9	0.9	1.0	1.0
HCFC1	P51610	1139	-8	-11	-3	-18	0.9	0.9	1.0	0.8
POLD1	P28340	1029	-8	-24	-3	-3	0.9	0.8	1.0	1.0
IRF2BP1	Q8IU81	363	-8	-12	-3	-36	0.9	0.9	1.0	0.7
SEC24D	O94855	1022	-9	-13	-3	-4	0.9	0.9	1.0	1.0
BUD31	P41223	119	-9	-23	-3	-12	0.9	0.8	1.0	0.9
FCHO1	O14526	84	-10	-12	-3	-7	0.9	0.9	1.0	0.9
VPS13A	Q96RL7	1608	-10	-20	-3	-5	0.9	0.8	1.0	1.0
CCDC91	Q7Z6B0	424	-11	-11	-3	-9	0.9	0.9	1.0	0.9
RPL18A	Q02543	109	-11	-27	-3	5	0.9	0.8	1.0	1.0
COG3	Q96JB2	349	-11	-15	-3	-2	0.9	0.9	1.0	1.0
NAGA	P17050	343	-11	-19	-3	2	0.9	0.8	1.0	1.0
PSMB10	P40306	17	-12	-11	-3	-23	0.9	0.9	1.0	0.8
PHF3	Q92576	1631	-12	-20	-3	-19	0.9	0.8	1.0	0.8
ABCB8	Q9NUT2	483	-12	-4	-3	-17	0.9	1.0	1.0	0.9
GTF3C1	Q12789	1091	-12	-10	-3	-29	0.9	0.9	1.0	0.8
ARAF	P10398	59	-13	-6	-3	1	0.9	0.9	1.0	1.0
BAZ2A	Q9UIF9	583	-13	-17	-3	-16	0.9	0.9	1.0	0.9
POGK	Q9P215	310	-15	-9	-3	-23	0.9	0.9	1.0	0.8
DDT	P30046	24	-16	-31	-3	-8	0.9	0.8	1.0	0.9
SHMT1	P34896	110	-17	-6	-3	1	0.9	0.9	1.0	1.0
BPTF	Q12830	1548	-19	-18	-3	-39	0.8	0.8	1.0	0.7
UBR4	Q5T4S7	3430	-21	-19	-3	-15	0.8	0.8	1.0	0.9

CPPED1	Q9BRF8	54	-22	-15	-3	-11	0.8	0.9	1.0	0.9
MTA2	O94776	44	-22	-19	-3	4	0.8	0.8	1.0	1.0
CIB1	Q99828	134	-23	-27	-3	-5	0.8	0.8	1.0	1.0
PDCD2L	Q9BRP1	278	-24	-16	-3	-25	0.8	0.9	1.0	0.8
FLNA	P21333	444	-25	-17	-3	2	0.8	0.9	1.0	1.0
ENO1	P06733	119	-30	-36	-3	3	0.8	0.7	1.0	1.0
CELF2	O95319	86	-40	-31	-3	-7	0.7	0.8	1.0	0.9
CELF1	Q92879	62	-40	-31	-3	-7	0.7	0.8	1.0	0.9
STX11	O75558	157	27	-6	-4	6	1.4	0.9	1.0	1.1
XPOT	O43592	845	25	-30	-4	2	1.3	0.8	1.0	1.0
MTMR6	Q9Y217	347	24	-7	-4	1	1.3	0.9	1.0	1.0
CUL1	Q13616	149	21	9	-4	-2	1.3	1.1	1.0	1.0
CARD6	Q9BX69	975	21	-4	-4	-7	1.3	1.0	1.0	0.9
GYS1	P13807	214	20	-2	-4	1	1.3	1.0	1.0	1.0
CHD6	Q8TD26	811	19	1	-4	-7	1.2	1.0	1.0	0.9
CHD8	Q9HCK8	1161	19	1	-4	-7	1.2	1.0	1.0	0.9
CHD7	Q9P2D1	1318	19	1	-4	-7	1.2	1.0	1.0	0.9
NFKB1	P19838	118	18	-4	-4	15	1.2	1.0	1.0	1.2
CAND1	Q86VP6	942	17	3	-4	15	1.2	1.0	1.0	1.2
GMPPB	Q9Y5P6	285	17	-13	-4	-1	1.2	0.9	1.0	1.0
DYNC1I2	Q13409	460	17	-23	-4	-19	1.2	0.8	1.0	0.8
AHCYL1	O43865	106	16	2	-4	1	1.2	1.0	1.0	1.0
XRN1	Q8IZH2	1047	16	-19	-4	-11	1.2	0.8	1.0	0.9
IKBKB	O14920	524	15	5	-4	5	1.2	1.1	1.0	1.1
TTC27	Q6P3X3	188	15	-2	-4	6	1.2	1.0	1.0	1.1
CCT7	Q99832	158	14	-15	-4	-5	1.2	0.9	1.0	1.0
CLTC	Q00610	1260	14	-13	-4	5	1.2	0.9	1.0	1.0
PMF1-BGLAP	U3KQ54	61	14	-32	-4	12	1.2	0.8	1.0	1.1
DOCK10	Q96BY6	1510	13	2	-4	-4	1.1	1.0	1.0	1.0
INF2	Q27J81	332	12	0	-4	-6	1.1	1.0	1.0	0.9
TRMT112	Q9UI30	33	11	2	-4	4	1.1	1.0	1.0	1.0
PDXDC1	Q6P996	425	11	-5	-4	9	1.1	1.0	1.0	1.1
EIF3D	O15371	196	11	-9	-4	4	1.1	0.9	1.0	1.0
HMHA1	Q92619	469	11	-12	-4	1	1.1	0.9	1.0	1.0
GRK6	P43250	474	11	-15	-4	-4	1.1	0.9	1.0	1.0
RFC4	P35249	141	11	1	-4	11	1.1	1.0	1.0	1.1
IKBKAP	O95163	341	11	-3	-4	-12	1.1	1.0	1.0	0.9
MSH2	P43246	843	11	-4	-4	2	1.1	1.0	1.0	1.0
RPS4Y1	P22090	181	11	-17	-4	0	1.1	0.9	1.0	1.0
UPP1	Q16831	162	10	-2	-4	3	1.1	1.0	1.0	1.0
NOP9	Q86U38	557	10	-20	-4	-10	1.1	0.8	1.0	0.9
NR2C2	P49116	414	10	6	-4	-2	1.1	1.1	1.0	1.0
SMARCD2	Q92925	355	10	5	-4	12	1.1	1.0	1.0	1.1
DYSF	O75923	1420	10	1	-4	-4	1.1	1.0	1.0	1.0
JARID2	Q92833	206	10	0	-4	8	1.1	1.0	1.0	1.1
UPP1	Q16831	17	10	-1	-4	5	1.1	1.0	1.0	1.0
TMC6	Q7Z403	751	10	-1	-4	1	1.1	1.0	1.0	1.0
ROCK1	Q13464	1300	10	-3	-4	-3	1.1	1.0	1.0	1.0
THEMIS	Q8N1K5	413	10	-4	-4	4	1.1	1.0	1.0	1.0
DOCK8	Q8NF50	361	10	-8	-4	5	1.1	0.9	1.0	1.0
HCK	P08631	241	9	3	-4	1	1.1	1.0	1.0	1.0
SMARCA4	P51532	1359	9	0	-4	-1	1.1	1.0	1.0	1.0
CBL	P22681	840	9	-1	-4	-7	1.1	1.0	1.0	0.9

NCBP2	P52298	81	9	-7	-4	-7	1.1	0.9	1.0	0.9
LRBA	P50851	2655	9	-9	-4	-5	1.1	0.9	1.0	1.0
CD2AP	Q9Y5K6	540	9	-12	-4	-5	1.1	0.9	1.0	1.0
MYH10	P35580	678	9	-14	-4	3	1.1	0.9	1.0	1.0
SAR1A	Q9NR31	102	9	-15	-4	3	1.1	0.9	1.0	1.0
ZBTB1	Q9Y2K1	532	9	6	-4	-8	1.1	1.1	1.0	0.9
EXOC6B	Q9Y2D4	32	9	0	-4	-2	1.1	1.0	1.0	1.0
SYNJ2	O15056	795	8	-4	-4	-6	1.1	1.0	1.0	0.9
RNF25	Q96BH1	360	8	-5	-4	-7	1.1	1.0	1.0	0.9
SMG9	Q9H0W8	380	8	-12	-4	-3	1.1	0.9	1.0	1.0
PLCB3	Q01970	892	8	3	-4	-6	1.1	1.0	1.0	0.9
DOCK8	Q8NF50	846	8	1	-4	-2	1.1	1.0	1.0	1.0
XRCC2	O43543	217	8	-5	-4	9	1.1	1.0	1.0	1.1
TRAPPC4	Q9Y296	195	8	-5	-4	-1	1.1	1.0	1.0	1.0
CTBP1	Q13363	54	8	-6	-4	-7	1.1	0.9	1.0	0.9
TAPT1	Q6NXT6	474	8	-7	-4	-10	1.1	0.9	1.0	0.9
CHD2	O14647	365	8	-10	-4	-6	1.1	0.9	1.0	0.9
ZNF318	Q5VUA4	1559	8	-13	-4	-5	1.1	0.9	1.0	1.0
SBF1	O95248	600	8	-16	-4	-8	1.1	0.9	1.0	0.9
KIF2A	O00139	199	8	-18	-4	-2	1.1	0.8	1.0	1.0
UBA2	Q9UBT2	173	7	-6	-4	-1	1.1	0.9	1.0	1.0
PPA2	Q9H2U2	283	7	-11	-4	1	1.1	0.9	1.0	1.0
FBXW5	Q969U6	277	7	-23	-4	-1	1.1	0.8	1.0	1.0
EPX	P11678	291	7	3	-4	-10	1.1	1.0	1.0	0.9
FBXO30	Q8TB52	687	7	-2	-4	9	1.1	1.0	1.0	1.1
MTMR6	Q9Y217	214	7	-7	-4	-2	1.1	0.9	1.0	1.0
IFT27	Q9BW83	166	7	-7	-4	-9	1.1	0.9	1.0	0.9
ZAP70	P43403	222	7	-8	-4	1	1.1	0.9	1.0	1.0
GLYR1	Q49A26	303	7	-10	-4	-6	1.1	0.9	1.0	0.9
TRIM27	P14373	359	7	-11	-4	-7	1.1	0.9	1.0	0.9
NUMB	P49757	37	7	-13	-4	-6	1.1	0.9	1.0	0.9
ALG2	Q9H553	70	7	-14	-4	-6	1.1	0.9	1.0	0.9
ERCC6	Q03468	662	7	-23	-4	-12	1.1	0.8	1.0	0.9
ZNF276	Q8N554	425	6	-3	-4	-15	1.1	1.0	1.0	0.9
PNP	P00491	31	6	-4	-4	-2	1.1	1.0	1.0	1.0
HEATR5B	Q9P2D3	1256	6	-4	-4	-3	1.1	1.0	1.0	1.0
DHX29	Q7Z478	627	6	-9	-4	-4	1.1	0.9	1.0	1.0
LIMS1	P48059	59	6	-9	-4	-10	1.1	0.9	1.0	0.9
POLR1A	O95602	185	6	-10	-4	-13	1.1	0.9	1.0	0.9
PIK3IP1	Q96FE7	209	6	-11	-4	31	1.1	0.9	1.0	1.4
MYCBP2	O75592	4520	6	-11	-4	-9	1.1	0.9	1.0	0.9
OGT	O15294	845	6	-18	-4	5	1.1	0.8	1.0	1.0
PSMD12	O00232	255	6	0	-4	4	1.1	1.0	1.0	1.0
BTAF1	O14981	1542	6	-2	-4	1	1.1	1.0	1.0	1.0
RNF146	Q9NTX7	185	6	-5	-4	-25	1.1	1.0	1.0	0.8
GPCPD1	Q9NPB8	205	6	-5	-4	3	1.1	1.0	1.0	1.0
MAD1L1	Q9Y6D9	201	6	-8	-4	1	1.1	0.9	1.0	1.0
HNRNPLL	Q8WVV9	405	6	-12	-4	-3	1.1	0.9	1.0	1.0
PARP9	Q8IXQ6	646	6	-13	-4	-7	1.1	0.9	1.0	0.9
BBX	Q8WY36	938	5	8	-4	-3	1.1	1.1	1.0	1.0
DHX36	Q9H2U1	284	5	0	-4	-10	1.1	1.0	1.0	0.9
TAF2	Q6P1X5	1093	5	-2	-4	6	1.1	1.0	1.0	1.1
TP53BP1	Q12888	1796	5	-2	-4	-7	1.1	1.0	1.0	0.9

MOB3A	Q96BX8	186	5	-3	-4	-8	1.1	1.0	1.0	0.9
LYN	P07948	468	5	-5	-4	7	1.1	1.0	1.0	1.1
GIMAP1-GIM	A0A087WTJ2	66	5	-5	-4	1	1.1	1.0	1.0	1.0
GIMAP1	Q8WWP7	66	5	-5	-4	1	1.1	1.0	1.0	1.0
MARC2	Q969Z3	299	5	-14	-4	-10	1.1	0.9	1.0	0.9
TP53	P04637	275	5	-3	-4	14	1.0	1.0	1.0	1.2
IRF2BP1	Q8IU81	207	5	-4	-4	-5	1.0	1.0	1.0	1.0
TRIM38	O00635	335	5	-4	-4	7	1.0	1.0	1.0	1.1
STOML2	Q9UJZ1	167	5	-6	-4	-6	1.0	0.9	1.0	0.9
TNFAIP3	P21580	612	5	-7	-4	-5	1.0	0.9	1.0	1.0
DYNC1H1	Q14204	2712	5	-7	-4	-5	1.0	0.9	1.0	1.0
PITRM1	Q5JRX3	556	5	-10	-4	-13	1.0	0.9	1.0	0.9
DOCK11	Q5JSL3	1906	5	-16	-4	-10	1.0	0.9	1.0	0.9
PRKDC	P78527	4061	5	-40	-4	1	1.0	0.7	1.0	1.0
NUP188	Q5SRE5	585	4	2	-4	-8	1.0	1.0	1.0	0.9
TLN1	Q9Y490	1392	4	0	-4	5	1.0	1.0	1.0	1.1
SMCHD1	A6NHR9	1982	4	0	-4	-3	1.0	1.0	1.0	1.0
PIGS	Q96S52	408	4	-6	-4	-7	1.0	0.9	1.0	0.9
METTL1	Q9UBP6	136	4	-8	-4	4	1.0	0.9	1.0	1.0
PTPRF	P10586	298	4	-9	-4	-8	1.0	0.9	1.0	0.9
PDS5A	Q29RF7	1093	4	-10	-4	-11	1.0	0.9	1.0	0.9
ZAP70	P43403	346	4	-10	-4	-11	1.0	0.9	1.0	0.9
DENND3	A2RUS2	499	4	-11	-4	-4	1.0	0.9	1.0	1.0
TRIP4	Q15650	86	4	-12	-4	-12	1.0	0.9	1.0	0.9
MX1	P20591	42	4	-14	-4	-14	1.0	0.9	1.0	0.9
NIPBL	Q6KC79	1971	4	7	-4	-4	1.0	1.1	1.0	1.0
PLEC	Q15149	730	4	3	-4	-4	1.0	1.0	1.0	1.0
NIPBL	Q6KC79	2374	4	-2	-4	-12	1.0	1.0	1.0	0.9
AHNAK	Q09666	1967	4	-3	-4	-8	1.0	1.0	1.0	0.9
PARP9	Q8IXQ6	728	4	-4	-4	-2	1.0	1.0	1.0	1.0
NUP153	P49790	753	4	-5	-4	-2	1.0	1.0	1.0	1.0
STAT1	P42224	324	4	-6	-4	-1	1.0	0.9	1.0	1.0
STAT4	Q14765	323	4	-6	-4	-1	1.0	0.9	1.0	1.0
MAP7D1	Q3KQU3	373	4	-6	-4	-17	1.0	0.9	1.0	0.9
LAS1L	Q9Y4W2	306	4	-8	-4	-8	1.0	0.9	1.0	0.9
CSTF3	Q12996	536	4	-9	-4	9	1.0	0.9	1.0	1.1
CIAPIN1	Q6FI81	92	4	-10	-4	4	1.0	0.9	1.0	1.0
OAS2	P29728	381	4	-20	-4	-3	1.0	0.8	1.0	1.0
TAB3	Q8N5C8	590	3	6	-4	-15	1.0	1.1	1.0	0.9
SEC31A	O94979	704	3	-2	-4	1	1.0	1.0	1.0	1.0
MICAL1	Q8TDZ2	711	3	-4	-4	-7	1.0	1.0	1.0	0.9
RAD17	O75943	141	3	-4	-4	-1	1.0	1.0	1.0	1.0
PHKB	Q93100	76	3	-7	-4	1	1.0	0.9	1.0	1.0
KLC1	Q07866	559	3	-7	-4	-18	1.0	0.9	1.0	0.8
RPL9P9	P32969	134	3	-9	-4	-3	1.0	0.9	1.0	1.0
HCFC1	P51610	1872	3	-10	-4	3	1.0	0.9	1.0	1.0
EML3	Q32P44	816	3	-19	-4	-7	1.0	0.8	1.0	0.9
ABLIM3	O94929	128	3	-21	-4	-4	1.0	0.8	1.0	1.0
LENG8	Q96PV6	547	3	-6	-4	-18	1.0	0.9	1.0	0.8
OTUB1	Q96FW1	212	3	-8	-4	-6	1.0	0.9	1.0	0.9
THOC6	Q86W42	314	3	-8	-4	-1	1.0	0.9	1.0	1.0
LRRK2	Q5S007	1770	3	-9	-4	-1	1.0	0.9	1.0	1.0
AAMP	Q13685	216	3	-9	-4	0	1.0	0.9	1.0	1.0

STRN	O43815	765	3	-9	-4	-6	1.0	0.9	1.0	0.9
RBM5	P52756	766	3	-9	-4	-6	1.0	0.9	1.0	0.9
CENPB	P07199	65	3	-11	-4	-11	1.0	0.9	1.0	0.9
CAPN1	P07384	384	3	-12	-4	-3	1.0	0.9	1.0	1.0
CAPN2	P17655	374	3	-12	-4	-3	1.0	0.9	1.0	1.0
KIF3A	Q9Y496	12	3	-14	-4	-10	1.0	0.9	1.0	0.9
CDKN2AIP	Q9NXV6	503	3	-21	-4	-13	1.0	0.8	1.0	0.9
RASSF4	Q9H2L5	236	2	2	-4	-2	1.0	1.0	1.0	1.0
VPS16	Q9H269	490	2	-4	-4	4	1.0	1.0	1.0	1.0
PCMT1	P22061	102	2	-4	-4	-8	1.0	1.0	1.0	0.9
GAPVD1	Q14C86	293	2	-6	-4	-9	1.0	0.9	1.0	0.9
MCMBP	Q9BTE3	200	2	-7	-4	-15	1.0	0.9	1.0	0.9
RPS12	P25398	106	2	-9	-4	6	1.0	0.9	1.0	1.1
SCP2	P22307	71	2	-9	-4	-2	1.0	0.9	1.0	1.0
ORC3	Q9UBD5	621	2	-9	-4	-16	1.0	0.9	1.0	0.9
CPNE3	O75131	54	2	-10	-4	-4	1.0	0.9	1.0	1.0
TRAPPC13	A5PLN9	233	2	-10	-4	-18	1.0	0.9	1.0	0.9
KANSL1	A0A0G2JNT7	84	2	-11	-4	-13	1.0	0.9	1.0	0.9
APOBEC3C	Q9NRW3	130	2	-11	-4	-16	1.0	0.9	1.0	0.9
NIPBL	Q6KC79	661	2	-12	-4	-10	1.0	0.9	1.0	0.9
ITPR1	Q14643	530	2	-14	-4	-6	1.0	0.9	1.0	0.9
ALDH9A1	P49189	45	2	-19	-4	2	1.0	0.8	1.0	1.0
FAM65A	Q6ZS17	896	2	4	-4	-7	1.0	1.0	1.0	0.9
FGD3	Q5JSP0	284	2	3	-4	-8	1.0	1.0	1.0	0.9
IK	Q13123	263	2	-1	-4	-5	1.0	1.0	1.0	1.0
NFKB2	Q00653	891	2	-7	-4	-7	1.0	0.9	1.0	0.9
WDR81	Q562E7	765	2	-9	-4	-3	1.0	0.9	1.0	1.0
DNAJC13	O75165	12	2	-10	-4	-1	1.0	0.9	1.0	1.0
RARS2	Q5T160	74	2	-10	-4	-4	1.0	0.9	1.0	1.0
KBTBD8	Q8NFY9	490	2	-10	-4	-8	1.0	0.9	1.0	0.9
RPL27A	P46776	70	2	-12	-4	-4	1.0	0.9	1.0	1.0
BANF1	O75531	85	2	-15	-4	12	1.0	0.9	1.0	1.1
ARID1B	Q8NFD5	1141	2	-23	-4	-15	1.0	0.8	1.0	0.9
HNRNPD	Q14103	126	2	-26	-4	3	1.0	0.8	1.0	1.0
PRRC2C	Q9Y520	481	1	0	-4	-3	1.0	1.0	1.0	1.0
FAM175A	Q6UWZ7	186	1	-4	-4	-1	1.0	1.0	1.0	1.0
TBCEL	Q5QJ74	14	1	-5	-4	-10	1.0	1.0	1.0	0.9
CPSF2	Q9P2I0	621	1	-5	-4	8	1.0	1.0	1.0	1.1
RARS2	Q5T160	11	1	-6	-4	-10	1.0	0.9	1.0	0.9
SRP9	P49458	48	1	-7	-4	2	1.0	0.9	1.0	1.0
GIT2	Q14161	122	1	-10	-4	-7	1.0	0.9	1.0	0.9
TNPO3	Q9Y5L0	912	1	-10	-4	-5	1.0	0.9	1.0	1.0
RBM34	P42696	196	1	-11	-4	-12	1.0	0.9	1.0	0.9
TCERG1	O14776	1062	1	-12	-4	-5	1.0	0.9	1.0	1.0
TUBA4A	P68366	316	1	-13	-4	-14	1.0	0.9	1.0	0.9
TUBA1A	Q71U36	316	1	-13	-4	-14	1.0	0.9	1.0	0.9
HPRT1	P00492	206	1	-18	-4	5	1.0	0.9	1.0	1.1
TBL2	Q9Y4P3	367	1	-18	-4	2	1.0	0.9	1.0	1.0
TMLHE	Q9NVH6	85	1	1	-4	17	1.0	1.0	1.0	1.2
ATRX	P46100	1531	1	-6	-4	-7	1.0	0.9	1.0	0.9
ZC3HC1	Q86WB0	125	1	-6	-4	-12	1.0	0.9	1.0	0.9
LIMS1	P48059	184	1	-6	-4	-1	1.0	0.9	1.0	1.0
LIMS2	Q7Z4I7	189	1	-6	-4	-1	1.0	0.9	1.0	1.0

TBC1D15	Q8TC07	197	1	-7	-4	-13	1.0	0.9	1.0	0.9
PTRH2	Q9Y3E5	111	1	-8	-4	-14	1.0	0.9	1.0	0.9
DDX19A	Q9NUU7	166	1	-8	-4	-3	1.0	0.9	1.0	1.0
SATB1	Q01826	173	1	-8	-4	-6	1.0	0.9	1.0	0.9
LDHA	P00338	131	1	-9	-4	3	1.0	0.9	1.0	1.0
ATM	Q13315	74	1	-9	-4	2	1.0	0.9	1.0	1.0
PC	P11498	622	1	-10	-4	-11	1.0	0.9	1.0	0.9
PCNT	O95613	430	1	-11	-4	-15	1.0	0.9	1.0	0.9
TEX10	Q9NXF1	774	1	-12	-4	7	1.0	0.9	1.0	1.1
RGS14	O43566	183	1	-17	-4	-3	1.0	0.9	1.0	1.0
SNX19	Q92543	91	1	-20	-4	-11	1.0	0.8	1.0	0.9
GNAI2	P04899	140	1	-21	-4	5	1.0	0.8	1.0	1.0
CTDP1	Q9Y5B0	700	0	1	-4	-3	1.0	1.0	1.0	1.0
ZZEF1	O43149	2546	0	-2	-4	-6	1.0	1.0	1.0	0.9
STAT4	Q14765	378	0	-4	-4	-7	1.0	1.0	1.0	0.9
INTS6	Q9UL03	659	0	-4	-4	-7	1.0	1.0	1.0	0.9
PPP2R1A	P30153	294	0	-5	-4	-3	1.0	1.0	1.0	1.0
MTMR14	Q8NCE2	429	0	-8	-4	-15	1.0	0.9	1.0	0.9
PADI4	Q9UM07	434	0	-9	-4	-1	1.0	0.9	1.0	1.0
NRDC	O43847	673	0	-10	-4	6	1.0	0.9	1.0	1.1
MMTAG2	Q9BU76	58	0	-10	-4	-16	1.0	0.9	1.0	0.9
AIP	O00170	208	0	-11	-4	4	1.0	0.9	1.0	1.0
DDX5	P17844	191	0	-11	-4	4	1.0	0.9	1.0	1.0
ADA	P00813	75	0	-11	-4	-11	1.0	0.9	1.0	0.9
CCDC66	A2RUB6	104	0	-13	-4	-4	1.0	0.9	1.0	1.0
TAZ	Q16635	122	0	-16	-4	-21	1.0	0.9	1.0	0.8
BLK	P51451	213	0	-18	-4	2	1.0	0.8	1.0	1.0
Uncharacteri	A0A087WZG	300	-1	-5	-4	-4	1.0	1.0	1.0	1.0
PI4KB	Q9UBF8	52	-1	-5	-4	-16	1.0	1.0	1.0	0.9
RNH1	P13489	75	-1	-5	-4	-1	1.0	1.0	1.0	1.0
THOC7	Q6I9Y2	90	-1	-6	-4	-8	1.0	0.9	1.0	0.9
METTL25	Q8N6Q8	96	-1	-7	-4	-4	1.0	0.9	1.0	1.0
TUBA4A	P68366	54	-1	-7	-4	-7	1.0	0.9	1.0	0.9
PTK2B	Q14289	899	-1	-7	-4	-4	1.0	0.9	1.0	1.0
RBBP9	O75884	163	-1	-9	-4	-3	1.0	0.9	1.0	1.0
NUDT16L1	Q9BRJ7	88	-1	-11	-4	-6	1.0	0.9	1.0	0.9
XPO1	O14980	723	-1	-13	-4	-6	1.0	0.9	1.0	0.9
GLRX	P35754	83	-1	-14	-4	-2	1.0	0.9	1.0	1.0
CAD	P27708	183	-1	-16	-4	-1	1.0	0.9	1.0	1.0
MCM5	P33992	221	-1	-17	-4	-1	1.0	0.9	1.0	1.0
UGP2	Q16851	123	-1	-5	-4	-5	1.0	1.0	1.0	1.0
TRANK1	O15050	924	-1	-6	-4	-7	1.0	0.9	1.0	0.9
ROGDI	Q9GZN7	66	-1	-7	-4	-6	1.0	0.9	1.0	0.9
SYNE2	Q8WXH0	1151	-1	-8	-4	-3	1.0	0.9	1.0	1.0
EP300	Q09472	364	-1	-8	-4	-3	1.0	0.9	1.0	1.0
MRPL44	Q9H9J2	53	-1	-8	-4	-9	1.0	0.9	1.0	0.9
GBF1	Q92538	613	-1	-8	-4	-10	1.0	0.9	1.0	0.9
PPOX	P50336	26	-1	-8	-4	-14	1.0	0.9	1.0	0.9
RAB11FIP1	Q6WKZ4	318	-1	-8	-4	-11	1.0	0.9	1.0	0.9
UBE2O	Q9C0C9	406	-1	-13	-4	-5	1.0	0.9	1.0	1.0
SUPT6H	Q7KZ85	1281	-1	-17	-4	-8	1.0	0.9	1.0	0.9
IPO5	O00410	110	-1	-20	-4	9	1.0	0.8	1.0	1.1
UBR4	Q5T4S7	3414	-1	-21	-4	-5	1.0	0.8	1.0	1.0

LRRC57	Q8N9N7	82	-1	-22	-4	-5	1.0	0.8	1.0	1.0
RHOT1	Q8IXI2	350	-2	1	-4	-4	1.0	1.0	1.0	1.0
JAK3	P52333	909	-2	-1	-4	-1	1.0	1.0	1.0	1.0
DOK2	O60496	217	-2	-2	-4	5	1.0	1.0	1.0	1.1
ATAD1	Q8NBU5	310	-2	-2	-4	-1	1.0	1.0	1.0	1.0
EGLN1	Q9GZT9	127	-2	-6	-4	-14	1.0	0.9	1.0	0.9
LTBP1	Q14766	665	-2	-6	-4	0	1.0	0.9	1.0	1.0
TAF6	P49848	235	-2	-8	-4	-11	1.0	0.9	1.0	0.9
HNRNPU	Q00839	335	-2	-9	-4	-4	1.0	0.9	1.0	1.0
LRRC41	Q15345	123	-2	-9	-4	-12	1.0	0.9	1.0	0.9
DHX15	O43143	307	-2	-10	-4	3	1.0	0.9	1.0	1.0
FLNA	P21333	649	-2	4	-4	-3	1.0	1.0	1.0	1.0
GMPS	P49915	489	-2	1	-4	5	1.0	1.0	1.0	1.0
LYN	P07948	203	-2	0	-4	-9	1.0	1.0	1.0	0.9
C16orf70	Q9BSU1	222	-2	-6	-4	-8	1.0	0.9	1.0	0.9
GTF2B	Q00403	223	-2	-6	-4	-1	1.0	0.9	1.0	1.0
GTPBP4	Q9BZE4	174	-2	-7	-4	-8	1.0	0.9	1.0	0.9
OSBPL1A	Q9BXW6	412	-2	-8	-4	4	1.0	0.9	1.0	1.0
CTSW	P56202	352	-2	-8	-4	-10	1.0	0.9	1.0	0.9
ZFAND1	Q8TCF1	184	-2	-9	-4	-10	1.0	0.9	1.0	0.9
STAT1	P42224	492	-2	-10	-4	-14	1.0	0.9	1.0	0.9
POLA2	Q14181	198	-2	-12	-4	-8	1.0	0.9	1.0	0.9
SLC27A3	Q5K4L6	579	-2	-15	-4	-3	1.0	0.9	1.0	1.0
MDC1	Q14676	26	-2	-16	-4	-21	1.0	0.9	1.0	0.8
PTPN12	Q05209	470	-3	-4	-4	-15	1.0	1.0	1.0	0.9
LYSMD2	Q8IV50	106	-3	-8	-4	-17	1.0	0.9	1.0	0.9
GDPD1	Q8N9F7	124	-3	-9	-4	-21	1.0	0.9	1.0	0.8
TBCK	Q8TEA7	737	-3	-10	-4	-15	1.0	0.9	1.0	0.9
ATG5	Q9H1Y0	19	-3	-11	-4	4	1.0	0.9	1.0	1.0
ZYX	Q15942	540	-3	-14	-4	8	1.0	0.9	1.0	1.1
LTBP1	Q14766	421	-3	-14	-4	-4	1.0	0.9	1.0	1.0
BYSL	Q13895	295	-3	-15	-4	5	1.0	0.9	1.0	1.1
ISCU	Q9H1K1	69	-3	-16	-4	-15	1.0	0.9	1.0	0.9
EIF2B3	Q9NR50	379	-3	-16	-4	27	1.0	0.9	1.0	1.4
RPL10	P27635	80	-3	-4	-4	1	1.0	1.0	1.0	1.0
TUBA4A	P68366	376	-3	-9	-4	-7	1.0	0.9	1.0	0.9
TUBA3D	Q13748	376	-3	-9	-4	-7	1.0	0.9	1.0	0.9
TUBA1A	Q71U36	376	-3	-9	-4	-7	1.0	0.9	1.0	0.9
TUBA8	Q9NY65	376	-3	-9	-4	-7	1.0	0.9	1.0	0.9
ZNF512B	Q96KM6	129	-3	-10	-4	-16	1.0	0.9	1.0	0.9
TARBP2	Q15633	282	-3	-11	-4	-15	1.0	0.9	1.0	0.9
DCPS	Q96C86	37	-3	-12	-4	-8	1.0	0.9	1.0	0.9
SP4	Q02446	627	-3	-13	-4	-5	1.0	0.9	1.0	1.0
IFIT5	Q13325	142	-3	-16	-4	-13	1.0	0.9	1.0	0.9
KIAA1109	Q2LD37	1842	-3	-26	-4	-7	1.0	0.8	1.0	0.9
IPO5	O00410	687	-4	-2	-4	-12	1.0	1.0	1.0	0.9
KDM3A	Q9Y4C1	753	-4	-8	-4	-8	1.0	0.9	1.0	0.9
AHNAK	Q09666	1833	-4	-9	-4	-12	1.0	0.9	1.0	0.9
TRAF6	Q9Y4K3	85	-4	-11	-4	-7	1.0	0.9	1.0	0.9
KLF2	Q9Y5W3	159	-4	-21	-4	-30	1.0	0.8	1.0	0.8
ZMYM2	Q9UBW7	608	-4	-7	-4	3	1.0	0.9	1.0	1.0
RPL23	P62829	28	-4	-10	-4	-14	1.0	0.9	1.0	0.9
LIMD1	Q9UGP4	585	-4	-11	-4	-2	1.0	0.9	1.0	1.0

PTP4A2	Q12974	101	-4	-13	-4	-5	1.0	0.9	1.0	1.0
ZC3HAV1	Q7Z2W4	521	-4	-19	-4	-10	1.0	0.8	1.0	0.9
FGD2	Q7Z6J4	467	-4	-24	-4	-9	1.0	0.8	1.0	0.9
AKAP13	Q12802	972	-5	-7	-4	-19	1.0	0.9	1.0	0.8
PKHD1L1	Q86WI1	1236	-5	-14	-4	-5	1.0	0.9	1.0	1.0
MBD1	Q9UIS9	525	-5	-9	-4	-23	1.0	0.9	1.0	0.8
BCL2A1	Q16548	55	-6	5	-4	6	0.9	1.0	1.0	1.1
TGFBR2	P37173	483	-6	-1	-4	-15	0.9	1.0	1.0	0.9
ARHGAP12	Q8IWW6	199	-6	-2	-4	-12	0.9	1.0	1.0	0.9
GOLPH3	Q9H4A6	108	-6	-5	-4	1	0.9	1.0	1.0	1.0
TMEM55B	Q86T03	125	-6	-11	-4	3	0.9	0.9	1.0	1.0
TMEM55A	Q8N4L2	99	-6	-11	-4	3	0.9	0.9	1.0	1.0
TTC37	Q6PGP7	305	-6	-19	-4	-13	0.9	0.8	1.0	0.9
TBC1D24	Q9ULP9	29	-6	-3	-4	5	0.9	1.0	1.0	1.1
FXR2	P51116	87	-6	-7	-4	-6	0.9	0.9	1.0	0.9
PPTC7	Q8NI37	124	-7	-17	-4	8	0.9	0.9	1.0	1.1
KSR1	Q8IVT5	41	-7	-30	-4	-6	0.9	0.8	1.0	0.9
DTX3	Q8N9I9	272	-8	-7	-4	-13	0.9	0.9	1.0	0.9
ERCC2	P18074	489	-9	-16	-4	1	0.9	0.9	1.0	1.0
AKAP8L	Q9ULX6	128	-10	-7	-4	-13	0.9	0.9	1.0	0.9
HTT	P42858	1708	-10	-19	-4	-12	0.9	0.8	1.0	0.9
CTSC	P53634	331	-10	-22	-4	-5	0.9	0.8	1.0	1.0
CCDC51	Q96ER9	316	-10	-18	-4	3	0.9	0.8	1.0	1.0
ACADVL	P49748	477	-11	-4	-4	-11	0.9	1.0	1.0	0.9
RING1	Q06587	72	-11	-12	-4	-11	0.9	0.9	1.0	0.9
EMSY	Q7Z589	194	-11	-13	-4	-3	0.9	0.9	1.0	1.0
C1orf123	Q9NWV4	102	-11	-26	-4	1	0.9	0.8	1.0	1.0
TASP1	Q9H6P5	148	-11	-15	-4	1	0.9	0.9	1.0	1.0
SLC25A22	Q9H936	111	-11	-19	-4	-32	0.9	0.8	1.0	0.8
RHOH	Q15669	130	-12	-8	-4	-4	0.9	0.9	1.0	1.0
HOPX	Q9BPY8	68	-12	-12	-4	-12	0.9	0.9	1.0	0.9
RABGEF1	Q9UJ41	359	-13	-17	-4	-22	0.9	0.9	1.0	0.8
CARD6	Q9BX69	872	-13	-18	-4	-32	0.9	0.9	1.0	0.8
PRKD3	O94806	100	-13	-22	-4	-28	0.9	0.8	1.0	0.8
VRK2	Q86Y07	373	-14	-2	-4	1	0.9	1.0	1.0	1.0
NARF	Q9UHQ1	99	-15	-16	-4	-22	0.9	0.9	1.0	0.8
SP100	P23497	289	-16	-4	-4	-31	0.9	1.0	1.0	0.8
ALDOC	P09972	240	-17	-27	-4	-8	0.9	0.8	1.0	0.9
ACO2	Q99798	592	-17	-27	-4	-15	0.9	0.8	1.0	0.9
MPI	P34949	11	-17	-16	-4	0	0.9	0.9	1.0	1.0
ZFAND3	Q9H8U3	118	-17	-33	-4	-44	0.9	0.8	1.0	0.7
AP3M1	Q9Y2T2	236	-18	-8	-4	-5	0.9	0.9	1.0	1.0
ISCU	Q9H1K1	95	-18	-15	-4	-16	0.9	0.9	1.0	0.9
GCLM	P48507	35	-20	-13	-4	-24	0.8	0.9	1.0	0.8
RNF31	Q96EP0	504	-21	-12	-4	-22	0.8	0.9	1.0	0.8
CTC1	Q2NKJ3	424	-21	-26	-4	-14	0.8	0.8	1.0	0.9
SEC31A	O94979	173	-22	-11	-4	-10	0.8	0.9	1.0	0.9
THEMIS	Q8N1K5	401	-27	6	-4	-13	0.8	1.1	1.0	0.9
PIGQ	Q9BRB3	69	-29	-26	-4	5	0.8	0.8	1.0	1.0
DDX1	Q92499	571	-51	-53	-4	-31	0.7	0.7	1.0	0.8
INO80	Q9ULG1	1011	28	-8	-4	7	1.4	0.9	1.0	1.1
MON2	Q7Z3U7	86	22	-6	-4	-4	1.3	0.9	1.0	1.0
DIAPH2	O60879	884	22	-21	-4	13	1.3	0.8	1.0	1.1

OXCT1	P55809	456	19	-16	-4	1	1.2	0.9	1.0	1.0
HSD17B10	Q99714	91	17	16	-4	6	1.2	1.2	1.0	1.1
CHD9	Q3L8U1	1257	15	1	-4	11	1.2	1.0	1.0	1.1
CHD8	Q9HCK8	1208	15	1	-4	11	1.2	1.0	1.0	1.1
CHD7	Q9P2D1	1365	15	1	-4	11	1.2	1.0	1.0	1.1
CNST	Q6PJW8	408	15	-11	-4	-13	1.2	0.9	1.0	0.9
USP5	P45974	532	14	-14	-4	14	1.2	0.9	1.0	1.2
VPS53	Q5VIR6	321	14	-1	-4	9	1.2	1.0	1.0	1.1
PSMD1	Q99460	703	14	-6	-4	10	1.2	0.9	1.0	1.1
DHX15	O43143	750	14	-18	-4	2	1.2	0.8	1.0	1.0
FBXO18	Q8NFZ0	302	13	-8	-4	-15	1.1	0.9	1.0	0.9
NCOA5	Q9HCD5	301	13	-9	-4	-6	1.1	0.9	1.0	0.9
COPG1	Q9Y678	258	13	-13	-4	-9	1.1	0.9	1.0	0.9
ZBTB11	O95625	197	12	-11	-4	-5	1.1	0.9	1.0	1.0
AKR1B1	P15121	200	12	-10	-4	3	1.1	0.9	1.0	1.0
NOL6	Q9H6R4	256	11	-22	-4	13	1.1	0.8	1.0	1.1
SSH3	Q8TE77	634	11	5	-4	5	1.1	1.1	1.0	1.0
SRPRB	Q9Y5M8	246	11	-2	-4	-10	1.1	1.0	1.0	0.9
TBC1D24	Q9ULP9	161	11	-10	-4	-2	1.1	0.9	1.0	1.0
CHD1	O14646	411	11	-10	-4	-7	1.1	0.9	1.0	0.9
PKM	P14618	423	11	-13	-4	-3	1.1	0.9	1.0	1.0
SDHB	P21912	68	11	-41	-4	-6	1.1	0.7	1.0	0.9
CIAO1	O76071	212	10	-6	-4	-9	1.1	0.9	1.0	0.9
PUS7L	Q9H0K6	626	10	-10	-4	7	1.1	0.9	1.0	1.1
TLN1	Q9Y490	719	10	-16	-4	2	1.1	0.9	1.0	1.0
TTC39C	Q8N584	463	10	-5	-4	3	1.1	1.0	1.0	1.0
HNRNPM	P52272	676	10	-7	-4	-2	1.1	0.9	1.0	1.0
PFKFB2	O60825	430	10	-9	-4	-29	1.1	0.9	1.0	0.8
PFKFB4	Q16877	430	10	-9	-4	-29	1.1	0.9	1.0	0.8
LARP4B	Q92615	654	10	-12	-4	1	1.1	0.9	1.0	1.0
ZNF165	P49910	36	10	-15	-4	-6	1.1	0.9	1.0	0.9
FEM1B	Q9UK73	465	9	-9	-4	-2	1.1	0.9	1.0	1.0
MAST3	O60307	762	9	-26	-4	-24	1.1	0.8	1.0	0.8
NLRC5	Q86WI3	917	9	1	-4	-12	1.1	1.0	1.0	0.9
GLRX3	O76003	229	9	-7	-4	-1	1.1	0.9	1.0	1.0
TMEM57	Q8N5G2	8	9	-31	-4	-4	1.1	0.8	1.0	1.0
MYD88	Q99836	203	8	1	-4	-4	1.1	1.0	1.0	1.0
SLC4A2	P04920	1215	8	-5	-4	-4	1.1	1.0	1.0	1.0
OPA1	O60313	551	8	-10	-4	7	1.1	0.9	1.0	1.1
POLR3E	Q9NVU0	456	8	-10	-4	-14	1.1	0.9	1.0	0.9
MYO1C	O00159	161	8	5	-4	-8	1.1	1.0	1.0	0.9
CSK	P41240	411	8	1	-4	6	1.1	1.0	1.0	1.1
RLF	Q13129	78	8	-2	-4	3	1.1	1.0	1.0	1.0
SPTAN1	Q13813	315	8	-9	-4	-7	1.1	0.9	1.0	0.9
IREB2	P48200	467	8	-19	-4	-1	1.1	0.8	1.0	1.0
SQRDL	Q9Y6N5	337	7	3	-4	3	1.1	1.0	1.0	1.0
PDLIM7	Q9NR12	431	7	-3	-4	-9	1.1	1.0	1.0	0.9
PDLIM7	Q9NR12	428	7	-3	-4	-9	1.1	1.0	1.0	0.9
ARL8B	Q9NVJ2	158	7	-5	-4	11	1.1	1.0	1.0	1.1
SH3BP1	Q9Y3L3	205	7	-5	-4	-7	1.1	1.0	1.0	0.9
LYPLA2	O95372	56	7	-8	-4	-3	1.1	0.9	1.0	1.0
CWF19L1	Q69YN2	176	7	-14	-4	-11	1.1	0.9	1.0	0.9
CLTC	Q00610	1257	7	-15	-4	-12	1.1	0.9	1.0	0.9

CAPN2	P17655	240	7	-26	-4	7	1.1	0.8	1.0	1.1
SLC25A1	P53007	262	7	-4	-4	2	1.1	1.0	1.0	1.0
HERC1	Q15751	2525	7	-6	-4	-4	1.1	0.9	1.0	1.0
ZAP70	P43403	102	7	-9	-4	1	1.1	0.9	1.0	1.0
C1QBP	Q07021	186	7	-9	-4	-7	1.1	0.9	1.0	0.9
DHX37	Q8IY37	1083	7	-11	-4	-10	1.1	0.9	1.0	0.9
EML4	Q9HC35	610	7	-12	-4	-4	1.1	0.9	1.0	1.0
ERO1A	Q96HE7	35	7	-14	-4	-27	1.1	0.9	1.0	0.8
ATP5A1	P25705	244	7	-15	-4	9	1.1	0.9	1.0	1.1
NUP205	Q92621	1275	7	-15	-4	-3	1.1	0.9	1.0	1.0
STXBP2	Q15833	311	7	-16	-4	1	1.1	0.9	1.0	1.0
ATL3	Q6DD88	388	6	1	-4	-3	1.1	1.0	1.0	1.0
DNTTIP1	Q9H147	192	6	1	-4	1	1.1	1.0	1.0	1.0
RPS3A	P61247	111	6	-1	-4	5	1.1	1.0	1.0	1.0
TARDBP	G3V162	50	6	-2	-4	-7	1.1	1.0	1.0	0.9
UTRN	P46939	447	6	-2	-4	-1	1.1	1.0	1.0	1.0
PTEN	A0A0U1RR63	244	6	-5	-4	0	1.1	1.0	1.0	1.0
PTRH1	Q86Y79	20	6	-8	-4	-12	1.1	0.9	1.0	0.9
FPGT	O14772	401	6	-15	-4	-16	1.1	0.9	1.0	0.9
RAD54L2	Q9Y4B4	601	6	0	-4	-14	1.1	1.0	1.0	0.9
RASSF4	Q9H2L5	33	6	-3	-4	1	1.1	1.0	1.0	1.0
PSME2	Q9UL46	91	6	-11	-4	-16	1.1	0.9	1.0	0.9
SLC25A12	O75746	563	6	-12	-4	3	1.1	0.9	1.0	1.0
SLC25A13	Q9UJS0	565	6	-12	-4	3	1.1	0.9	1.0	1.0
ROCK1	Q13464	231	6	-12	-4	-1	1.1	0.9	1.0	1.0
RNF213	Q63HN8	1064	6	-12	-4	-9	1.1	0.9	1.0	0.9
INF2	Q27J81	745	6	-13	-4	-8	1.1	0.9	1.0	0.9
SMC6	Q96SB8	854	6	-21	-4	-1	1.1	0.8	1.0	1.0
EML4	Q9HC35	314	6	-25	-4	-8	1.1	0.8	1.0	0.9
SMC1A	Q14683	933	6	-27	-4	-6	1.1	0.8	1.0	0.9
RNF146	Q9NTX7	57	5	-5	-4	-6	1.1	1.0	1.0	0.9
PSME4	Q14997	1100	5	-8	-4	9	1.1	0.9	1.0	1.1
NXF1	Q9UBU9	252	5	-11	-4	-8	1.1	0.9	1.0	0.9
FAM65B	Q9Y4F9	147	5	-14	-4	-3	1.1	0.9	1.0	1.0
SEC24C	P53992	881	5	-16	-4	2	1.1	0.9	1.0	1.0
PAICS	P22234	185	5	-16	-4	-4	1.1	0.9	1.0	1.0
EGLN1	Q9GZT9	201	5	-4	-4	1	1.0	1.0	1.0	1.0
EIF4G1	Q04637	662	5	-5	-4	-10	1.0	1.0	1.0	0.9
BOD1L1	Q8NFC6	2570	5	-7	-4	-4	1.0	0.9	1.0	1.0
SKA3	Q8IX90	8	5	-9	-4	-2	1.0	0.9	1.0	1.0
POLR1A	O95602	613	5	-10	-4	5	1.0	0.9	1.0	1.0
NSUN2	Q08J23	93	5	-10	-4	0	1.0	0.9	1.0	1.0
RBPJ	Q06330	252	5	-11	-4	-8	1.0	0.9	1.0	0.9
EPRS	P07814	1487	5	-11	-4	8	1.0	0.9	1.0	1.1
ATP2A3	Q93084	636	5	-11	-4	-1	1.0	0.9	1.0	1.0
RAPGEF1	Q13905	817	5	-13	-4	-9	1.0	0.9	1.0	0.9
GLE1	Q53GS7	528	5	-14	-4	4	1.0	0.9	1.0	1.0
RPS2	P15880	182	5	-14	-4	2	1.0	0.9	1.0	1.0
GIMAP7	Q8NHV1	165	5	-14	-4	-7	1.0	0.9	1.0	0.9
SLFN5	Q08AF3	48	5	-16	-4	7	1.0	0.9	1.0	1.1
HMHA1	Q92619	747	5	-16	-4	-10	1.0	0.9	1.0	0.9
NAP1L1	P55209	88	5	-25	-4	-15	1.0	0.8	1.0	0.9
ERCC6	Q03468	61	4	6	-4	-15	1.0	1.1	1.0	0.9

FAM101B	Q8N5W9	81	4	3	-4	-6	1.0	1.0	1.0	0.9
SON	P18583	92	4	-5	-4	-10	1.0	1.0	1.0	0.9
PKN1	Q16512	762	4	-9	-4	13	1.0	0.9	1.0	1.1
PKN2	Q16513	804	4	-9	-4	13	1.0	0.9	1.0	1.1
DHX36	Q9H2U1	234	4	-11	-4	-3	1.0	0.9	1.0	1.0
SURF2	Q15527	93	4	-12	-4	-14	1.0	0.9	1.0	0.9
PRPF8	Q6P2Q9	1896	4	-13	-4	-4	1.0	0.9	1.0	1.0
CLTC	Q00610	918	4	-17	-4	5	1.0	0.9	1.0	1.0
MACF1	Q9UPN3	7133	4	-2	-4	8	1.0	1.0	1.0	1.1
INTS8	Q75QN2	385	4	-5	-4	-10	1.0	1.0	1.0	0.9
PCID2	Q5JVF3	152	4	-6	-4	6	1.0	0.9	1.0	1.1
P4HB	P07237	53	4	-6	-4	-11	1.0	0.9	1.0	0.9
SAMM50	Q9Y512	237	4	-7	-4	2	1.0	0.9	1.0	1.0
CRYZL1	O95825	184	4	-7	-4	1	1.0	0.9	1.0	1.0
PPFIA2	O75334	143	4	-7	-4	-3	1.0	0.9	1.0	1.0
AGO3	Q9H9G7	491	4	-7	-4	-3	1.0	0.9	1.0	1.0
AGO2	Q9UKV8	490	4	-7	-4	-3	1.0	0.9	1.0	1.0
AGO1	Q9UL18	488	4	-7	-4	-3	1.0	0.9	1.0	1.0
EPC1	Q9H2F5	797	4	-7	-4	-14	1.0	0.9	1.0	0.9
PARK7	Q99497	53	4	-10	-4	8	1.0	0.9	1.0	1.1
SEC31A	O94979	458	4	-12	-4	-1	1.0	0.9	1.0	1.0
LRRC40	Q9H9A6	438	4	-12	-4	-9	1.0	0.9	1.0	0.9
APEH	P13798	312	4	-16	-4	-1	1.0	0.9	1.0	1.0
ALDH16A1	Q8IZ83	249	4	-18	-4	-1	1.0	0.8	1.0	1.0
CORO7-PAM:	A0A0A6YYL4	75	4	-23	-4	19	1.0	0.8	1.0	1.2
HSPA4	P34932	270	4	-28	-4	9	1.0	0.8	1.0	1.1
RBM33	Q96EV2	285	3	-1	-4	-13	1.0	1.0	1.0	0.9
CD7	P09564	219	3	-2	-4	-9	1.0	1.0	1.0	0.9
MPHOSPH8	Q99549	799	3	-4	-4	-14	1.0	1.0	1.0	0.9
WDHD1	O75717	1008	3	-8	-4	6	1.0	0.9	1.0	1.1
MBNL2	Q5VZF2	53	3	-8	-4	2	1.0	0.9	1.0	1.0
MBNL1	Q9NR56	53	3	-8	-4	2	1.0	0.9	1.0	1.0
ABCB1	P08183	1074	3	-10	-4	-1	1.0	0.9	1.0	1.0
PRKCQ	Q04759	322	3	-11	-4	-12	1.0	0.9	1.0	0.9
FHL1	Q13642	209	3	-11	-4	-8	1.0	0.9	1.0	0.9
CYTH4	Q9UIA0	321	3	-12	-4	-4	1.0	0.9	1.0	1.0
WWP2	O00308	802	3	-12	-4	-7	1.0	0.9	1.0	0.9
ITCH	Q96J02	835	3	-12	-4	-7	1.0	0.9	1.0	0.9
TRIP12	Q14669	625	3	-16	-4	1	1.0	0.9	1.0	1.0
SETD2	Q9BYW2	1125	3	-16	-4	-20	1.0	0.9	1.0	0.8
SH3GLB2	Q9NR46	252	3	-19	-4	28	1.0	0.8	1.0	1.4
HEATR6	Q6AI08	476	3	-27	-4	-1	1.0	0.8	1.0	1.0
AK5	Q9Y6K8	245	3	1	-4	-6	1.0	1.0	1.0	0.9
NSMCE2	Q96MF7	215	3	-3	-4	-3	1.0	1.0	1.0	1.0
G6PD	P11413	385	3	-6	-4	-7	1.0	0.9	1.0	0.9
NFXL1	Q6ZNB6	775	3	-7	-4	-5	1.0	0.9	1.0	1.0
CMTR1	Q8N1G2	9	3	-7	-4	-17	1.0	0.9	1.0	0.9
RPL18A	Q02543	16	3	-11	-4	-4	1.0	0.9	1.0	1.0
NPEPPS	P55786	66	3	-19	-4	-8	1.0	0.8	1.0	0.9
MED6	O75586	45	3	-24	-4	-13	1.0	0.8	1.0	0.9
SETD1A	O15047	1638	2	2	-4	21	1.0	1.0	1.0	1.3
TBL3	Q12788	696	2	-1	-4	6	1.0	1.0	1.0	1.1
PI4KA	P42356	1312	2	-2	-4	-7	1.0	1.0	1.0	0.9

SAP30	O75446	184	2	-2	-4	-4	1.0	1.0	1.0	1.0
ZC3H11A	O75152	209	2	-4	-4	-5	1.0	1.0	1.0	1.0
DDX27	Q96GQ7	321	2	-4	-4	-7	1.0	1.0	1.0	0.9
KLHDC4	Q8TBB5	373	2	-4	-4	-13	1.0	1.0	1.0	0.9
DDX58	O95786	680	2	-5	-4	-15	1.0	1.0	1.0	0.9
ZC3HAV1	Q7Z2W4	82	2	-6	-4	7	1.0	0.9	1.0	1.1
CPNE2	Q96FN4	116	2	-8	-4	-13	1.0	0.9	1.0	0.9
SAP18	O00422	26	2	-9	-4	-3	1.0	0.9	1.0	1.0
SRGAP2	A2RUF3	1020	2	-11	-4	-16	1.0	0.9	1.0	0.9
ELAC2	Q9BQ52	136	2	-12	-4	-4	1.0	0.9	1.0	1.0
EIF3A	Q14152	185	2	-12	-4	-9	1.0	0.9	1.0	0.9
PRKDC	P78527	3293	2	-18	-4	-1	1.0	0.8	1.0	1.0
MAPK14	Q16539	162	2	-18	-4	-15	1.0	0.8	1.0	0.9
DOCK11	Q5JSL3	592	2	-5	-4	-1	1.0	1.0	1.0	1.0
SYNRG	Q9UMZ2	208	2	-5	-4	-19	1.0	1.0	1.0	0.8
CAMK2D	Q13557	273	2	-5	-4	-8	1.0	1.0	1.0	0.9
TDRKH	Q9Y2W6	109	2	-6	-4	-14	1.0	0.9	1.0	0.9
ZC3H15	Q8WU90	105	2	-6	-4	-2	1.0	0.9	1.0	1.0
ZBTB1	Q9Y2K1	448	2	-6	-4	-7	1.0	0.9	1.0	0.9
NAA15	Q9BXJ9	238	2	-7	-4	-9	1.0	0.9	1.0	0.9
UBA6	A0AVT1	178	2	-8	-4	-7	1.0	0.9	1.0	0.9
CNTRL	Q7Z7A1	1535	2	-10	-4	6	1.0	0.9	1.0	1.1
ADAR	P55265	1224	2	-10	-4	-15	1.0	0.9	1.0	0.9
NPLOC4	Q8TAT6	403	2	-11	-4	-2	1.0	0.9	1.0	1.0
HCK	P08631	12	2	-15	-4	-12	1.0	0.9	1.0	0.9
TRRAP	Q9Y4A5	1868	1	5	-4	-2	1.0	1.0	1.0	1.0
HSD17B12	Q53GQ0	166	1	2	-4	-14	1.0	1.0	1.0	0.9
ERI1	Q8IV48	75	1	-3	-4	-3	1.0	1.0	1.0	1.0
GMIP	Q9P107	599	1	-5	-4	2	1.0	1.0	1.0	1.0
IL16	Q14005	975	1	-5	-4	-10	1.0	1.0	1.0	0.9
RSBN1L	Q6PCB5	595	1	-5	-4	9	1.0	1.0	1.0	1.1
ACOT2	P49753	401	1	-7	-4	-7	1.0	0.9	1.0	0.9
SYNE1	Q8NF91	2649	1	-7	-4	-7	1.0	0.9	1.0	0.9
EPM2AIP1	Q7L775	337	1	-7	-4	0	1.0	0.9	1.0	1.0
ZFP41	A0A0G2JH32	155	1	-8	-4	-2	1.0	0.9	1.0	1.0
CEP128	Q6ZU80	784	1	-8	-4	-10	1.0	0.9	1.0	0.9
EXOC1	Q9NV70	27	1	-11	-4	-5	1.0	0.9	1.0	1.0
ELMSAN1	Q6PJG2	16	1	-11	-4	-6	1.0	0.9	1.0	0.9
EHBP1L1	Q8N3D4	346	1	-11	-4	-13	1.0	0.9	1.0	0.9
ZNF346	Q9UL40	68	1	-12	-4	-11	1.0	0.9	1.0	0.9
DDX51	Q8N8A6	402	1	-14	-4	-7	1.0	0.9	1.0	0.9
ARFGEF2	Q9Y6D5	566	1	-17	-4	-11	1.0	0.9	1.0	0.9
ARFGEF1	Q9Y6D6	615	1	-17	-4	-11	1.0	0.9	1.0	0.9
UBIAD1	Q9Y5Z9	31	1	-22	-4	-20	1.0	0.8	1.0	0.8
CTSB	P07858	319	1	-23	-4	-4	1.0	0.8	1.0	1.0
CTSC	P53634	258	1	1	-4	-7	1.0	1.0	1.0	0.9
CASP1	P29466	364	1	0	-4	2	1.0	1.0	1.0	1.0
MAP2K3	P46734	29	1	-1	-4	-13	1.0	1.0	1.0	0.9
KDM6B	O15054	1602	1	-3	-4	-15	1.0	1.0	1.0	0.9
VPS11	Q9H270	317	1	-5	-4	-7	1.0	1.0	1.0	0.9
UBQLN4	Q9NRR5	29	1	-6	-4	-14	1.0	0.9	1.0	0.9
LYST	Q99698	20	1	-8	-4	-1	1.0	0.9	1.0	1.0
IMPACT	Q9P2X3	195	1	-8	-4	-17	1.0	0.9	1.0	0.9

OTULIN	Q96BN8	129	1	-8	-4	-4	1.0	0.9	1.0	1.0
DNM1L	O00429	361	1	-8	-4	-6	1.0	0.9	1.0	0.9
CNN2	Q99439	164	1	-8	-4	-8	1.0	0.9	1.0	0.9
WIPF1	O43516	446	1	-8	-4	-12	1.0	0.9	1.0	0.9
CACUL1	Q86Y37	362	1	-9	-4	-5	1.0	0.9	1.0	1.0
CIC	Q96RK0	336	1	-9	-4	-10	1.0	0.9	1.0	0.9
NEK7	Q8TDX7	53	1	-9	-4	-4	1.0	0.9	1.0	1.0
TRIM25	Q14258	498	1	-10	-4	-7	1.0	0.9	1.0	0.9
IKBKB	O14920	115	1	-11	-4	-8	1.0	0.9	1.0	0.9
FCF1	Q9Y324	134	1	-12	-4	-1	1.0	0.9	1.0	1.0
EXOC2	Q96KP1	541	1	-18	-4	-13	1.0	0.8	1.0	0.9
NELFB	Q8WX92	141	0	-1	-4	-5	1.0	1.0	1.0	1.0
CCT8	P50990	244	0	-4	-4	10	1.0	1.0	1.0	1.1
LDLRAP1	Q5SW96	199	0	-6	-4	-9	1.0	0.9	1.0	0.9
CAST	P20810	413	0	-6	-4	-16	1.0	0.9	1.0	0.9
PSAT1	Q9Y617	224	0	-7	-4	9	1.0	0.9	1.0	1.1
SH2D3C	Q8N5H7	452	0	-7	-4	-7	1.0	0.9	1.0	0.9
APPL2	Q8NEU8	412	0	-7	-4	-10	1.0	0.9	1.0	0.9
CAPZA2	P47755	111	0	-8	-4	-4	1.0	0.9	1.0	1.0
NUDCD1	Q96RS6	111	0	-9	-4	-6	1.0	0.9	1.0	0.9
TBCK	Q8TEA7	386	0	-10	-4	-21	1.0	0.9	1.0	0.8
CD37	P11049	230	0	-10	-4	-7	1.0	0.9	1.0	0.9
ARAP1	Q96P48	774	0	-13	-4	5	1.0	0.9	1.0	1.0
UBA7	P41226	680	0	-28	-4	-1	1.0	0.8	1.0	1.0
NOP56	O00567	52	-1	1	-4	-3	1.0	1.0	1.0	1.0
SAFB2	Q14151	672	-1	-4	-4	-9	1.0	1.0	1.0	0.9
FLNA	P21333	717	-1	-7	-4	-2	1.0	0.9	1.0	1.0
LST1	O00453	90	-1	-8	-4	-10	1.0	0.9	1.0	0.9
EEF1A1	P68104	370	-1	-8	-4	-17	1.0	0.9	1.0	0.9
UBR2	Q8IWV8	1717	-1	-9	-4	-4	1.0	0.9	1.0	1.0
DOCK2	Q92608	41	-1	-10	-4	-8	1.0	0.9	1.0	0.9
HECTD3	Q5T447	823	-1	-10	-4	-12	1.0	0.9	1.0	0.9
SLU7	O95391	469	-1	-10	-4	-17	1.0	0.9	1.0	0.9
RPL34	P49207	49	-1	-11	-4	-10	1.0	0.9	1.0	0.9
JAK1	P23458	944	-1	-12	-4	-19	1.0	0.9	1.0	0.8
ANAPC2	Q9UJX6	221	-1	-16	-4	-10	1.0	0.9	1.0	0.9
MTMR12	Q9C0I1	152	-1	5	-4	-31	1.0	1.0	1.0	0.8
PRKCB	P05771	50	-1	4	-4	4	1.0	1.0	1.0	1.0
PRKCA	P17252	50	-1	4	-4	4	1.0	1.0	1.0	1.0
CDK4	P11802	78	-1	1	-4	4	1.0	1.0	1.0	1.0
NFKB2	Q00653	57	-1	0	-4	-4	1.0	1.0	1.0	1.0
SAMSN1	Q9NSI8	196	-1	-5	-4	18	1.0	1.0	1.0	1.2
CRKL	P46109	249	-1	-7	-4	-9	1.0	0.9	1.0	0.9
EXOSC9	Q06265	9	-1	-7	-4	-4	1.0	0.9	1.0	1.0
ATP6V1A	P38606	254	-1	-10	-4	3	1.0	0.9	1.0	1.0
KDSR	Q06136	245	-1	-14	-4	-9	1.0	0.9	1.0	0.9
VPS72	Q15906	292	-1	-27	-4	-14	1.0	0.8	1.0	0.9
WDR89	Q96FK6	16	-1	-27	-4	-12	1.0	0.8	1.0	0.9
EPRS	P07814	692	-2	-4	-4	-8	1.0	1.0	1.0	0.9
OTUD4	Q01804	686	-2	-7	-4	-9	1.0	0.9	1.0	0.9
MORC3	Q14149	694	-2	-8	-4	2	1.0	0.9	1.0	1.0
PAN2	Q504Q3	798	-2	-13	-4	-17	1.0	0.9	1.0	0.9
RNF114	Q9Y508	64	-2	-14	-4	-7	1.0	0.9	1.0	0.9

ARAP3	Q8WWN8	1022	-2	-21	-4	-9	1.0	0.8	1.0	0.9
IVNS1ABP	Q9Y6Y0	143	-2	-22	-4	-19	1.0	0.8	1.0	0.8
GCOM1	H8Y6P7	662	-2	10	-4	13	1.0	1.1	1.0	1.1
ZZEF1	O43149	1304	-2	1	-4	-8	1.0	1.0	1.0	0.9
RAF1	P04049	637	-2	-3	-4	10	1.0	1.0	1.0	1.1
LAS1L	Q9Y4W2	690	-2	-5	-4	-17	1.0	1.0	1.0	0.9
HSPD1	P10809	442	-2	-8	-4	-4	1.0	0.9	1.0	1.0
RBBP7	Q16576	116	-2	-8	-4	1	1.0	0.9	1.0	1.0
CHAMP1	Q96JM3	770	-2	-23	-4	-17	1.0	0.8	1.0	0.9
NUP214	P35658	728	-3	2	-4	-1	1.0	1.0	1.0	1.0
CHML	P26374	582	-3	1	-4	8	1.0	1.0	1.0	1.1
PLOD1	Q02809	680	-3	-2	-4	1	1.0	1.0	1.0	1.0
CPSF2	Q9P2I0	763	-3	-4	-4	4	1.0	1.0	1.0	1.0
MAPRE3	Q9UPY8	182	-3	-5	-4	-4	1.0	1.0	1.0	1.0
CMTM7	Q96FZ5	12	-3	-9	-4	-15	1.0	0.9	1.0	0.9
CCDC25	Q86WR0	150	-3	-10	-4	-6	1.0	0.9	1.0	0.9
TMEM174	Q8WUU8	101	-3	-15	-4	-7	1.0	0.9	1.0	0.9
RAB10	P61026	24	-3	-18	-4	-6	1.0	0.9	1.0	0.9
NLRC4	Q9NPP4	283	-3	-18	-4	-11	1.0	0.8	1.0	0.9
DMXL2	Q8TDJ6	1556	-3	-31	-4	-20	1.0	0.8	1.0	0.8
SDCCAG8	Q86SQ7	604	-3	-6	-4	0	1.0	0.9	1.0	1.0
NEK9	Q8TD19	623	-3	-7	-4	-5	1.0	0.9	1.0	1.0
FHL1	Q13642	150	-3	-8	-4	3	1.0	0.9	1.0	1.0
UNC93B1	Q9H1C4	583	-3	-8	-4	-7	1.0	0.9	1.0	0.9
IWS1	Q96ST2	749	-3	-8	-4	-12	1.0	0.9	1.0	0.9
EEF2	P13639	812	-3	-12	-4	-2	1.0	0.9	1.0	1.0
PCM1	Q15154	1717	-3	-13	-4	-17	1.0	0.9	1.0	0.9
FUBP1	Q96AE4	148	-3	-17	-4	-8	1.0	0.9	1.0	0.9
THOC2	Q8NI27	1236	-4	-4	-4	-13	1.0	1.0	1.0	0.9
TRIP6	Q15654	328	-4	-7	-4	-3	1.0	0.9	1.0	1.0
ARAF	P10398	597	-4	-8	-4	-15	1.0	0.9	1.0	0.9
TLN2	Q9Y4G6	1954	-4	-10	-4	-4	1.0	0.9	1.0	1.0
TRIM26	Q12899	353	-4	-13	-4	-6	1.0	0.9	1.0	0.9
TINF2	Q9BSI4	332	-4	-13	-4	-14	1.0	0.9	1.0	0.9
RNH1	P13489	38	-4	-14	-4	2	1.0	0.9	1.0	1.0
SPATA5	Q8NB90	574	-4	-14	-4	-8	1.0	0.9	1.0	0.9
TNIP2	Q8NFZ5	428	-4	1	-4	1	1.0	1.0	1.0	1.0
PDCD7	Q8N8D1	339	-4	-2	-4	4	1.0	1.0	1.0	1.0
EPPK1	A0A087X1U6	1151	-4	-3	-4	3	1.0	1.0	1.0	1.0
ZC3HAV1	Q7Z2W4	168	-4	-4	-4	8	1.0	1.0	1.0	1.1
USP47	Q96K76	856	-4	-7	-4	-10	1.0	0.9	1.0	0.9
CELF1	Q92879	150	-4	-8	-4	1	1.0	0.9	1.0	1.0
GPX4	P36969	93	-4	-8	-4	-13	1.0	0.9	1.0	0.9
COASY	Q13057	140	-4	-10	-4	-5	1.0	0.9	1.0	1.0
CLUH	O75153	1196	-4	-11	-4	-8	1.0	0.9	1.0	0.9
TP53BP1	Q12888	1040	-5	3	-4	-12	1.0	1.0	1.0	0.9
SLC27A1	Q6PCB7	392	-5	-9	-4	-11	1.0	0.9	1.0	0.9
RAD1	O60671	239	-5	-11	-4	-2	1.0	0.9	1.0	1.0
GOLGA3	Q08378	769	-5	-14	-4	-8	1.0	0.9	1.0	0.9
MACF1	Q9UPN3	443	-5	-17	-4	-17	1.0	0.9	1.0	0.9
NIT2	Q9NQR4	146	-5	-21	-4	-1	1.0	0.8	1.0	1.0
ACSL4	O60488	602	-5	-21	-4	-19	1.0	0.8	1.0	0.8
JAK3	P52333	1066	-5	-4	-4	-10	1.0	1.0	1.0	0.9

RANBP6	O60518	31	-5	-7	-4	-11	1.0	0.9	1.0	0.9
TEC	P42680	625	-5	-8	-4	-9	1.0	0.9	1.0	0.9
ZMYM4	Q5VZL5	467	-5	-9	-4	-3	1.0	0.9	1.0	1.0
BLNK	Q8WV28	271	-5	-10	-4	-16	1.0	0.9	1.0	0.9
SH3GL1	Q99961	277	-5	-11	-4	-13	1.0	0.9	1.0	0.9
NUP153	P49790	1065	-5	-12	-4	-11	1.0	0.9	1.0	0.9
CNOT1	A5YKK6	219	-5	-16	-4	-2	1.0	0.9	1.0	1.0
DHX9	Q08211	415	-5	-19	-4	-10	1.0	0.8	1.0	0.9
COPG2	Q9UBF2	440	-5	-25	-4	-8	1.0	0.8	1.0	0.9
MCM6	Q14566	721	-6	-10	-4	-3	0.9	0.9	1.0	1.0
PIK3R6	Q5UE93	713	-6	-10	-4	-8	0.9	0.9	1.0	0.9
MTA1	Q13330	68	-6	-16	-4	-14	0.9	0.9	1.0	0.9
ACSL3	O95573	504	-6	-20	-4	-14	0.9	0.8	1.0	0.9
VPS29	Q9UBQ0	36	-6	-25	-4	5	0.9	0.8	1.0	1.1
SGK3	Q96BR1	13	-6	2	-4	-10	0.9	1.0	1.0	0.9
CCT2	P78371	395	-6	-10	-4	27	0.9	0.9	1.0	1.4
MIB2	Q96AX9	972	-7	1	-4	-17	0.9	1.0	1.0	0.9
ARID2	Q68CP9	711	-7	-8	-4	-6	0.9	0.9	1.0	0.9
VPS13B	Q7Z7G8	1891	-7	-17	-4	-21	0.9	0.9	1.0	0.8
ZRSR2	Q15696	172	-7	10	-4	12	0.9	1.1	1.0	1.1
MALT1	Q9UDY8	71	-7	-2	-4	-8	0.9	1.0	1.0	0.9
RAD51C	O43502	68	-7	-4	-4	-28	0.9	1.0	1.0	0.8
CAMSAP1	Q5T5Y3	848	-7	-6	-4	-1	0.9	0.9	1.0	1.0
XPO5	Q9HAV4	44	-7	-11	-4	18	0.9	0.9	1.0	1.2
TRMT2A	Q8IZ69	538	-7	-14	-4	-15	0.9	0.9	1.0	0.9
KIAA0355	O15063	502	-7	-15	-4	-13	0.9	0.9	1.0	0.9
KAT6B	Q8WYB5	837	-7	-16	-4	20	0.9	0.9	1.0	1.3
KAT6A	Q92794	626	-7	-16	-4	20	0.9	0.9	1.0	1.3
ACAP1	Q15027	320	-7	-17	-4	-1	0.9	0.9	1.0	1.0
MRM3	Q9HC36	290	-8	-5	-4	-17	0.9	1.0	1.0	0.9
AKR1C3	P42330	242	-8	-5	-4	0	0.9	1.0	1.0	1.0
ARAP1	Q96P48	1357	-8	-5	-4	-12	0.9	1.0	1.0	0.9
EXOSC6	Q5RKV6	117	-8	-10	-4	-1	0.9	0.9	1.0	1.0
NHP2	Q9NX24	18	-8	-12	-4	-13	0.9	0.9	1.0	0.9
ACAA1	P09110	218	-8	-21	-4	-15	0.9	0.8	1.0	0.9
NUP62	P37198	475	-9	-8	-4	-15	0.9	0.9	1.0	0.9
ANKRD17	O75179	1158	-9	-9	-4	12	0.9	0.9	1.0	1.1
ANKHD1	Q8IWZ3	1130	-9	-9	-4	12	0.9	0.9	1.0	1.1
MBNL1	Q9NR56	200	-9	-9	-4	-8	0.9	0.9	1.0	0.9
HSD17B8	Q92506	147	-9	-8	-4	-12	0.9	0.9	1.0	0.9
HMOX2	P30519	127	-9	-18	-4	-13	0.9	0.9	1.0	0.9
FLNA	P21333	1353	-9	-19	-4	-5	0.9	0.8	1.0	1.0
VAC14	Q08AM6	719	-10	-11	-4	-1	0.9	0.9	1.0	1.0
NPC1	O15118	1261	-10	-11	-4	-8	0.9	0.9	1.0	0.9
EPB41	P11171	306	-10	-29	-4	-2	0.9	0.8	1.0	1.0
ACO1	P21399	118	-11	-4	-4	6	0.9	1.0	1.0	1.1
PDDC1	Q8NB37	216	-11	-20	-4	-27	0.9	0.8	1.0	0.8
PDP1	Q9P0J1	149	-11	-7	-4	-4	0.9	0.9	1.0	1.0
MAP3K4	Q9Y6R4	1484	-12	-9	-4	-4	0.9	0.9	1.0	1.0
CRKL	P46109	44	-12	-11	-4	-9	0.9	0.9	1.0	0.9
ERN1	O75460	605	-12	-17	-4	-27	0.9	0.9	1.0	0.8
UBE3A	Q05086	83	-12	-3	-4	-3	0.9	1.0	1.0	1.0
WDR13	Q9H1Z4	413	-12	-4	-4	0	0.9	1.0	1.0	1.0

FAM8A1	Q9UBU6	38	-12	-13	-4	-13	0.9	0.9	1.0	0.9
CBL	P22681	372	-13	-14	-4	-14	0.9	0.9	1.0	0.9
CBLB	Q13191	364	-13	-14	-4	-14	0.9	0.9	1.0	0.9
Integrin	H3BM21	510	-14	-7	-4	-9	0.9	0.9	1.0	0.9
SNRPN	P63162	19	-16	-26	-4	-14	0.9	0.8	1.0	0.9
WARS	P23381	305	-17	-21	-4	-5	0.9	0.8	1.0	1.0
TADA3	O75528	255	-19	-7	-4	1	0.8	0.9	1.0	1.0
CRYZ	Q08257	166	-19	-10	-4	5	0.8	0.9	1.0	1.0
C1orf174	Q8IYL3	124	-20	-16	-4	-21	0.8	0.9	1.0	0.8
TRIM33	Q9UPN9	145	-24	-1	-4	-19	0.8	1.0	1.0	0.8
KMT2B	Q9UMN6	1279	-26	-22	-4	2	0.8	0.8	1.0	1.0
VPS41	P49754	125	-32	18	-4	-9	0.8	1.2	1.0	0.9
CPNE3	O75131	100	-74	-44	-4	-8	0.6	0.7	1.0	0.9
PEPD	P12955	58	-145	-41	-4	-26	0.4	0.7	1.0	0.8
C17orf75	Q9HAS0	127	30	3	-5	16	1.4	1.0	1.0	1.2
MRI1	Q9BV20	199	24	-21	-5	20	1.3	0.8	1.0	1.3
SERPINB9	P50453	108	22	-13	-5	14	1.3	0.9	1.0	1.2
NR2C1	P13056	130	21	-13	-5	2	1.3	0.9	1.0	1.0
PHF1	O43189	237	20	-48	-5	-8	1.3	0.7	1.0	0.9
ZNFX1	Q9P2E3	1302	20	11	-5	-3	1.2	1.1	1.0	1.0
HEATR5B	Q9P2D3	870	19	-15	-5	-8	1.2	0.9	1.0	0.9
MYO1F	O00160	584	18	-6	-5	18	1.2	0.9	1.0	1.2
AP3D1	O14617	60	16	-1	-5	11	1.2	1.0	1.0	1.1
STAG2	Q8N3U4	632	16	-8	-5	-5	1.2	0.9	1.0	1.0
PRMT9	Q6P2P2	747	15	-18	-5	14	1.2	0.9	1.0	1.2
PTK2B	Q14289	463	15	-18	-5	-32	1.2	0.9	1.0	0.8
PXN	P49023	505	15	4	-5	-10	1.2	1.0	1.0	0.9
NLRP3	Q96P20	196	15	-2	-5	-15	1.2	1.0	1.0	0.9
UBR4	Q5T4S7	4049	15	-9	-5	-8	1.2	0.9	1.0	0.9
IQGAP2	Q13576	628	14	-8	-5	2	1.2	0.9	1.0	1.0
ZNF512B	Q96KM6	112	13	5	-5	10	1.1	1.1	1.0	1.1
AMPD3	Q01432	694	13	-1	-5	-12	1.1	1.0	1.0	0.9
EIF3K	Q9UBQ5	87	12	-12	-5	2	1.1	0.9	1.0	1.0
CUL1	Q13616	496	12	-17	-5	4	1.1	0.9	1.0	1.0
NUMA1	Q14980	1907	12	-4	-5	-4	1.1	1.0	1.0	1.0
MAP7D3	Q8IWC1	572	12	-9	-5	-10	1.1	0.9	1.0	0.9
HARS	P12081	235	12	-15	-5	-8	1.1	0.9	1.0	0.9
PRPF4	O43172	299	12	-26	-5	-3	1.1	0.8	1.0	1.0
SPATA5L1	Q9BVQ7	732	11	5	-5	0	1.1	1.0	1.0	1.0
SH3BGRL2	Q9UJC5	76	11	-20	-5	-1	1.1	0.8	1.0	1.0
NFATC3	Q12968	358	11	-4	-5	-16	1.1	1.0	1.0	0.9
MACROD1	Q9BQ69	199	11	-5	-5	-3	1.1	1.0	1.0	1.0
L2HGDH	Q9H9P8	252	11	-12	-5	-5	1.1	0.9	1.0	1.0
MYD88	Q99836	233	11	-15	-5	14	1.1	0.9	1.0	1.2
ATP6V1C1	P21283	225	11	-18	-5	-8	1.1	0.9	1.0	0.9
VPS35	Q96QK1	286	11	-29	-5	1	1.1	0.8	1.0	1.0
RNF34	Q969K3	65	10	-12	-5	-12	1.1	0.9	1.0	0.9
RPS6KA4	O75676	257	9	6	-5	5	1.1	1.1	1.0	1.1
ALMS1	Q8TCU4	2276	9	3	-5	-10	1.1	1.0	1.0	0.9
NOP58	Q9Y2X3	205	9	-32	-5	4	1.1	0.8	1.0	1.0
UPF3A	Q9H1J1	315	9	-36	-5	-31	1.1	0.7	1.0	0.8
THADA	Q6YHU6	635	8	-2	-5	10	1.1	1.0	1.0	1.1
GAB3	Q8WWW8	336	8	-2	-5	-6	1.1	1.0	1.0	0.9

SPG20	Q8N0X7	405	8	-3	-5	-11	1.1	1.0	1.0	0.9
POM121	Q96HA1	307	8	-8	-5	-8	1.1	0.9	1.0	0.9
SETD1B	Q9UPS6	1812	8	-11	-5	-9	1.1	0.9	1.0	0.9
MYH9	P35579	671	8	-16	-5	3	1.1	0.9	1.0	1.0
RGS14	O43566	332	8	-17	-5	-1	1.1	0.9	1.0	1.0
FKBP4	Q02790	396	8	-19	-5	-5	1.1	0.8	1.0	1.0
TLE3	Q04726	528	8	-45	-5	13	1.1	0.7	1.0	1.1
METAP2	P50579	135	7	-3	-5	-3	1.1	1.0	1.0	1.0
PRPS1	P60891	41	7	-3	-5	4	1.1	1.0	1.0	1.0
TRMT2A	Q8IZ69	270	7	-4	-5	-9	1.1	1.0	1.0	0.9
KLC4	Q9NSK0	318	7	-13	-5	-3	1.1	0.9	1.0	1.0
ACAP2	Q15057	329	7	-15	-5	-4	1.1	0.9	1.0	1.0
SMC5	Q8IY18	176	7	-17	-5	-5	1.1	0.9	1.0	1.0
ARRB2	P32121	243	7	-22	-5	-8	1.1	0.8	1.0	0.9
MAP7D1	Q3KQU3	382	7	-2	-5	-12	1.1	1.0	1.0	0.9
TUBB6	Q9BUF5	303	7	-5	-5	-16	1.1	1.0	1.0	0.9
TUBB2B	Q9BVA1	303	7	-5	-5	-16	1.1	1.0	1.0	0.9
GIMAP7	Q8NHV1	164	7	-7	-5	-4	1.1	0.9	1.0	1.0
SEC63	Q9UGP8	490	7	-8	-5	-1	1.1	0.9	1.0	1.0
CD8A	P01732	217	7	-10	-5	-5	1.1	0.9	1.0	1.0
CORO7-PAM:	A0A0A6YYL4	187	7	-12	-5	3	1.1	0.9	1.0	1.0
RINT1	Q6NUQ1	720	7	-12	-5	-5	1.1	0.9	1.0	1.0
CAMKK2	Q96RR4	397	7	-15	-5	-31	1.1	0.9	1.0	0.8
CARMIL2	Q6F5E8	823	7	-16	-5	0	1.1	0.9	1.0	1.0
HINT3	Q9NQE9	73	7	-17	-5	-13	1.1	0.9	1.0	0.9
UIMC1	Q96RL1	298	7	-19	-5	-12	1.1	0.8	1.0	0.9
KBTBD4	Q9NVX7	455	6	13	-5	-11	1.1	1.1	1.0	0.9
TRMT1L	Q7Z2T5	320	6	-7	-5	-17	1.1	0.9	1.0	0.9
Uncharacteri	H0YHG0	333	6	-9	-5	2	1.1	0.9	1.0	1.0
cDNA	B4DLN1	395	6	-11	-5	10	1.1	0.9	1.0	1.1
AP1G1	O43747	353	6	-18	-5	-8	1.1	0.8	1.0	0.9
RHOT2	Q8IXI1	185	6	-20	-5	0	1.1	0.8	1.0	1.0
CCT8	P50990	430	6	-1	-5	6	1.1	1.0	1.0	1.1
TCAF2	A6NFQ2	278	6	-4	-5	-2	1.1	1.0	1.0	1.0
HGS	O14964	185	6	-4	-5	-2	1.1	1.0	1.0	1.0
ZC3H11A	O75152	431	6	-5	-5	-22	1.1	1.0	1.0	0.8
UPP1	Q16831	71	6	-7	-5	1	1.1	0.9	1.0	1.0
YWHAE	P62258	98	6	-9	-5	0	1.1	0.9	1.0	1.0
CREBRF	Q8IUR6	186	6	-9	-5	-11	1.1	0.9	1.0	0.9
PRKAR2B	P31323	205	6	-10	-5	-5	1.1	0.9	1.0	1.0
RNF213	Q63HN8	3365	6	-24	-5	-3	1.1	0.8	1.0	1.0
ATP2A2	P16615	344	5	-5	-5	4	1.1	1.0	1.0	1.0
ATP2A3	Q93084	344	5	-5	-5	4	1.1	1.0	1.0	1.0
MPP1	Q00013	179	5	-5	-5	-3	1.1	1.0	1.0	1.0
MBOAT7	Q96N66	304	5	-6	-5	-9	1.1	0.9	1.0	0.9
HECTD1	Q9ULT8	1855	5	-7	-5	0	1.1	0.9	1.0	1.0
FKBP4	Q02790	328	5	-9	-5	5	1.1	0.9	1.0	1.0
TEP1	Q99973	692	5	-11	-5	-8	1.1	0.9	1.0	0.9
SERPINB6	P35237	350	5	-11	-5	3	1.1	0.9	1.0	1.0
NAPA	P54920	66	5	-12	-5	3	1.1	0.9	1.0	1.0
DDX5	P17844	200	5	-12	-5	-3	1.1	0.9	1.0	1.0
DDX17	Q92841	277	5	-12	-5	-3	1.1	0.9	1.0	1.0
DIAPH1	O60610	964	5	-12	-5	-9	1.1	0.9	1.0	0.9

BICD2	Q8TD16	754	5	-18	-5	-7	1.1	0.9	1.0	0.9
ANKRD44	Q8N8A2	334	5	-20	-5	0	1.1	0.8	1.0	1.0
SH3GLB1	Q9Y371	121	5	-20	-5	-12	1.1	0.8	1.0	0.9
TMX1	Q9H3N1	106	5	-27	-5	-6	1.1	0.8	1.0	0.9
NUBP1	P53384	31	5	-3	-5	-6	1.0	1.0	1.0	0.9
KHSRP	Q92945	296	5	-4	-5	1	1.0	1.0	1.0	1.0
CSE1L	P55060	85	5	-7	-5	1	1.0	0.9	1.0	1.0
STAT2	P52630	676	5	-11	-5	-11	1.0	0.9	1.0	0.9
CKAP5	Q14008	1946	5	-12	-5	-15	1.0	0.9	1.0	0.9
MYOF	Q9NZM1	1467	5	-12	-5	-6	1.0	0.9	1.0	0.9
RAP1GAP2	Q684P5	443	5	-13	-5	-2	1.0	0.9	1.0	1.0
NUP98	P52948	1735	5	-15	-5	-1	1.0	0.9	1.0	1.0
CASP1	P29466	397	5	-16	-5	-10	1.0	0.9	1.0	0.9
JAK3	P52333	1105	4	2	-5	-9	1.0	1.0	1.0	0.9
DDX17	Q92841	447	4	-2	-5	5	1.0	1.0	1.0	1.0
POLR3A	O14802	261	4	-3	-5	7	1.0	1.0	1.0	1.1
VAV1	P15498	83	4	-5	-5	4	1.0	1.0	1.0	1.0
PHF3	Q92576	885	4	-5	-5	-8	1.0	1.0	1.0	0.9
FLII	Q13045	46	4	-6	-5	-7	1.0	0.9	1.0	0.9
ZMYM3	Q14202	450	4	-10	-5	4	1.0	0.9	1.0	1.0
SYNE2	Q8WXH0	5758	4	-15	-5	-6	1.0	0.9	1.0	0.9
EWSR1	Q01844	384	4	-17	-5	-2	1.0	0.9	1.0	1.0
EML4	Q9HC35	82	4	-1	-5	-4	1.0	1.0	1.0	1.0
PPP5C	P53041	11	4	-5	-5	-11	1.0	1.0	1.0	0.9
AIP	O00170	90	4	-7	-5	5	1.0	0.9	1.0	1.1
ITK	Q08881	143	4	-8	-5	-15	1.0	0.9	1.0	0.9
MYO1F	O00160	101	4	-9	-5	-12	1.0	0.9	1.0	0.9
GNPAT	O15228	544	4	-11	-5	-8	1.0	0.9	1.0	0.9
DOK1	Q99704	224	4	-11	-5	-13	1.0	0.9	1.0	0.9
PSMC5	P62195	112	4	-12	-5	-5	1.0	0.9	1.0	1.0
PPP1CA	P62136	140	4	-18	-5	5	1.0	0.9	1.0	1.1
ZFPL1	O95159	56	4	-18	-5	-5	1.0	0.8	1.0	1.0
ROCK1	Q13464	714	3	-2	-5	-2	1.0	1.0	1.0	1.0
NCK2	O43639	297	3	-4	-5	-7	1.0	1.0	1.0	0.9
BRAT1	Q6PJG6	366	3	-4	-5	-10	1.0	1.0	1.0	0.9
PPIL2	Q13356	387	3	-8	-5	-6	1.0	0.9	1.0	0.9
PIKFYVE	Q9Y2I7	1970	3	-8	-5	-6	1.0	0.9	1.0	0.9
FLNB	O75369	1081	3	-10	-5	-4	1.0	0.9	1.0	1.0
FLNC	Q14315	1103	3	-10	-5	-4	1.0	0.9	1.0	1.0
PARP1	P09874	256	3	-12	-5	4	1.0	0.9	1.0	1.0
PLEC	Q15149	4071	3	-12	-5	2	1.0	0.9	1.0	1.0
HLA-C	P10321	364	3	-16	-5	-10	1.0	0.9	1.0	0.9
NUP155	O75694	974	3	-17	-5	-5	1.0	0.9	1.0	1.0
VPS35	Q96QK1	673	3	-18	-5	-7	1.0	0.8	1.0	0.9
PTK2B	Q14289	310	3	-19	-5	0	1.0	0.8	1.0	1.0
TMEM173	Q86WV6	309	3	-22	-5	-3	1.0	0.8	1.0	1.0
PRKDC	P78527	630	3	-176	-5	8	1.0	0.4	1.0	1.1
TMEM256-PI	I3L3X5	125	3	0	-5	25	1.0	1.0	1.0	1.3
TRIM5	Q9C035	482	3	-7	-5	-3	1.0	0.9	1.0	1.0
PRPSAP2	O60256	135	3	-8	-5	-1	1.0	0.9	1.0	1.0
FAM8A1	Q9UBU6	93	3	-8	-5	-17	1.0	0.9	1.0	0.9
C5orf51	A6NDU8	179	3	-10	-5	-14	1.0	0.9	1.0	0.9
PRKRA	O75569	54	3	-10	-5	-9	1.0	0.9	1.0	0.9

ZC3HAV1	Q7Z2W4	721	3	-11	-5	-23	1.0	0.9	1.0	0.8
SEC13	P55735	245	3	-13	-5	-11	1.0	0.9	1.0	0.9
SYNE2	Q8WXH0	5586	3	-23	-5	-9	1.0	0.8	1.0	0.9
SBDS	Q9Y3A5	31	3	-26	-5	3	1.0	0.8	1.0	1.0
PCCB	P05166	517	2	-3	-5	2	1.0	1.0	1.0	1.0
RECQL	P46063	49	2	-3	-5	-4	1.0	1.0	1.0	1.0
ALKBH4	Q9NXW9	267	2	-6	-5	-6	1.0	0.9	1.0	0.9
PIK3CD	O00329	132	2	-7	-5	36	1.0	0.9	1.0	1.6
TES	Q9UGI8	196	2	-7	-5	-8	1.0	0.9	1.0	0.9
MAP2K2	P36507	384	2	-9	-5	-3	1.0	0.9	1.0	1.0
IL16	Q14005	1004	2	-9	-5	-6	1.0	0.9	1.0	0.9
NLRP1	Q9C000	264	2	-9	-5	-13	1.0	0.9	1.0	0.9
RPA2	P15927	219	2	-10	-5	-7	1.0	0.9	1.0	0.9
POTEE	Q6S8J3	957	2	-11	-5	-2	1.0	0.9	1.0	1.0
GLIPR2	Q9H4G4	32	2	-11	-5	2	1.0	0.9	1.0	1.0
QRICH1	Q2TAL8	701	2	-13	-5	-1	1.0	0.9	1.0	1.0
PKM	P14618	474	2	-13	-5	-3	1.0	0.9	1.0	1.0
GNLY	P22749	43	2	-13	-5	-9	1.0	0.9	1.0	0.9
ARFRP1	Q13795	154	2	-25	-5	9	1.0	0.8	1.0	1.1
MYH9	P35579	569	2	-28	-5	-1	1.0	0.8	1.0	1.0
MYH10	P35580	576	2	-28	-5	-1	1.0	0.8	1.0	1.0
AFTPH	Q6ULP2	511	2	1	-5	-12	1.0	1.0	1.0	0.9
WIPI2	Q9Y4P8	70	2	-5	-5	-5	1.0	1.0	1.0	1.0
AGPS	O00116	541	2	-8	-5	-5	1.0	0.9	1.0	1.0
HUWE1	Q7Z6Z7	3635	2	-9	-5	-5	1.0	0.9	1.0	1.0
AVEN	Q9NQS1	193	2	-10	-5	-4	1.0	0.9	1.0	1.0
IPO9	Q96P70	90	2	-11	-5	0	1.0	0.9	1.0	1.0
PTPRE	P23469	186	2	-14	-5	-7	1.0	0.9	1.0	0.9
NDUFV1	P49821	238	1	4	-5	2	1.0	1.0	1.0	1.0
NUB1	Q9Y5A7	270	1	-3	-5	-8	1.0	1.0	1.0	0.9
FLNA	P21333	1686	1	-4	-5	-3	1.0	1.0	1.0	1.0
PHF1	O43189	139	1	-4	-5	1	1.0	1.0	1.0	1.0
FGR	P09769	273	1	-4	-5	-5	1.0	1.0	1.0	1.0
MAP4	P27816	535	1	-6	-5	-9	1.0	0.9	1.0	0.9
AGAP2	Q99490	811	1	-7	-5	-10	1.0	0.9	1.0	0.9
ALDH6A1	Q02252	317	1	-7	-5	2	1.0	0.9	1.0	1.0
GUCY1A3	Q02108	79	1	-7	-5	-5	1.0	0.9	1.0	1.0
HSDL1	Q3SXM5	209	1	-8	-5	12	1.0	0.9	1.0	1.1
ATG14	Q6ZNE5	330	1	-8	-5	-6	1.0	0.9	1.0	0.9
CDA	P32320	65	1	-9	-5	1	1.0	0.9	1.0	1.0
TACC1	O75410	715	1	-9	-5	-16	1.0	0.9	1.0	0.9
HERC1	Q15751	3262	1	-9	-5	-17	1.0	0.9	1.0	0.9
PXN	P49023	108	1	-10	-5	-17	1.0	0.9	1.0	0.9
PARP14	Q460N5	849	1	-11	-5	-10	1.0	0.9	1.0	0.9
PELP1	Q8IZL8	237	1	-15	-5	21	1.0	0.9	1.0	1.3
DAGLB	Q8NCG7	519	1	-18	-5	-2	1.0	0.9	1.0	1.0
L3MBTL2	Q969R5	408	1	-19	-5	-10	1.0	0.8	1.0	0.9
DIEXF	Q68CQ4	642	1	3	-5	-4	1.0	1.0	1.0	1.0
MYO9B	Q13459	1701	1	-2	-5	-8	1.0	1.0	1.0	0.9
MMRN1	Q13201	765	1	-2	-5	-11	1.0	1.0	1.0	0.9
CCDC50	Q8IVM0	85	1	-2	-5	-3	1.0	1.0	1.0	1.0
GBF1	Q92538	1430	1	-5	-5	-7	1.0	1.0	1.0	0.9
ADCK4	Q96D53	415	1	-7	-5	-5	1.0	0.9	1.0	1.0

SNU13	P55769	30	1	-8	-5	-3	1.0	0.9	1.0	1.0
VPS11	Q9H270	890	1	-9	-5	-12	1.0	0.9	1.0	0.9
FLNA	P21333	2160	1	-10	-5	-15	1.0	0.9	1.0	0.9
FAM120A	Q9NZB2	14	1	-16	-5	-8	1.0	0.9	1.0	0.9
SAMD9	Q5K651	1376	1	-19	-5	3	1.0	0.8	1.0	1.0
NOP58	Q9Y2X3	106	0	-3	-5	-7	1.0	1.0	1.0	0.9
METAP1	P53582	25	0	-4	-5	-6	1.0	1.0	1.0	0.9
ARID1A	O14497	1105	0	-6	-5	-6	1.0	0.9	1.0	0.9
PTPRE	P23469	278	0	-6	-5	-25	1.0	0.9	1.0	0.8
PHKA2	P46019	681	0	-7	-5	-5	1.0	0.9	1.0	1.0
EIF2D	P41214	528	0	-22	-5	-14	1.0	0.8	1.0	0.9
GOLGA4	Q13439	1862	-1	6	-5	-7	1.0	1.1	1.0	0.9
RRP1B	Q14684	155	-1	-2	-5	14	1.0	1.0	1.0	1.2
TARS	P26639	656	-1	-3	-5	-3	1.0	1.0	1.0	1.0
PDCD11	Q14690	361	-1	-5	-5	-3	1.0	1.0	1.0	1.0
ZNF638	Q14966	747	-1	-6	-5	-4	1.0	0.9	1.0	1.0
BECN1	Q14457	391	-1	-9	-5	-8	1.0	0.9	1.0	0.9
HNRNPU	Q00839	594	-1	-10	-5	-13	1.0	0.9	1.0	0.9
PARP16	Q8N5Y8	169	-1	-10	-5	-2	1.0	0.9	1.0	1.0
Uncharacteri	H3BRB1	415	-1	-11	-5	2	1.0	0.9	1.0	1.0
GEMIN2	O14893	63	-1	-11	-5	-17	1.0	0.9	1.0	0.9
MYCBP2	O75592	2243	-1	-14	-5	-10	1.0	0.9	1.0	0.9
ZNF407	Q9C0G0	1753	-1	-17	-5	-10	1.0	0.9	1.0	0.9
SPG21	Q9NZD8	204	-1	-22	-5	-9	1.0	0.8	1.0	0.9
OPTN	Q96CV9	239	-1	-2	-5	-1	1.0	1.0	1.0	1.0
ZC3HAV1	Q7Z2W4	78	-1	-3	-5	-5	1.0	1.0	1.0	1.0
ZMYM3	Q14202	432	-1	-5	-5	2	1.0	1.0	1.0	1.0
CAST	P20810	241	-1	-6	-5	-11	1.0	0.9	1.0	0.9
TMA16	Q96EY4	162	-1	-8	-5	-5	1.0	0.9	1.0	1.0
RPAP2	Q8IXW5	358	-1	-9	-5	-11	1.0	0.9	1.0	0.9
TNPO3	Q9Y5L0	908	-1	-9	-5	-6	1.0	0.9	1.0	0.9
EXOC3	O60645	161	-1	-11	-5	-5	1.0	0.9	1.0	1.0
STK25	O00506	357	-1	-12	-5	-6	1.0	0.9	1.0	0.9
MFN1	Q8IWA4	411	-1	-15	-5	-6	1.0	0.9	1.0	0.9
DENND2D	Q9H6A0	159	-1	-15	-5	-11	1.0	0.9	1.0	0.9
SPTAN1	Q13813	1622	-1	-16	-5	-5	1.0	0.9	1.0	1.0
PFKFB2	O60825	415	-1	-18	-5	1	1.0	0.8	1.0	1.0
BAZ1B	Q9UIG0	497	-1	-30	-5	-23	1.0	0.8	1.0	0.8
BECN1	Q14457	353	-2	-5	-5	8	1.0	1.0	1.0	1.1
APPL1	Q9UKG1	570	-2	-7	-5	-12	1.0	0.9	1.0	0.9
GZMA	P12544	105	-2	-8	-5	-19	1.0	0.9	1.0	0.8
DDX59	Q5T1V6	453	-2	-8	-5	-13	1.0	0.9	1.0	0.9
BRAT1	Q6PJG6	28	-2	-9	-5	2	1.0	0.9	1.0	1.0
LTBP1	Q14766	568	-2	-10	-5	-14	1.0	0.9	1.0	0.9
HADHA	P40939	349	-2	-10	-5	1	1.0	0.9	1.0	1.0
AGFG2	O95081	30	-2	-12	-5	-13	1.0	0.9	1.0	0.9
ANXA6	P08133	114	-2	-13	-5	-3	1.0	0.9	1.0	1.0
BPHL	Q86WA6	234	-2	-13	-5	-9	1.0	0.9	1.0	0.9
FIS1	Q9Y3D6	41	-2	-15	-5	-9	1.0	0.9	1.0	0.9
STAT3	P40763	251	-2	-17	-5	5	1.0	0.9	1.0	1.1
GNPAT	O15228	54	-2	-23	-5	9	1.0	0.8	1.0	1.1
ZKSCAN1	P17029	63	-2	-4	-5	-10	1.0	1.0	1.0	0.9
CHD4	Q14839	495	-2	-9	-5	-10	1.0	0.9	1.0	0.9

OASL	Q15646	188	-2	-10	-5	0	1.0	0.9	1.0	1.0
ESYT1	Q9BSJ8	370	-2	-11	-5	1	1.0	0.9	1.0	1.0
CTSW	P56202	284	-2	-12	-5	-11	1.0	0.9	1.0	0.9
HNRNPU	Q00839	453	-2	-14	-5	1	1.0	0.9	1.0	1.0
RARRES3	Q9UL19	73	-2	-14	-5	0	1.0	0.9	1.0	1.0
RIPK1	Q13546	325	-3	-4	-5	-18	1.0	1.0	1.0	0.8
UBE4A	Q14139	79	-3	-4	-5	-8	1.0	1.0	1.0	0.9
ERLIN1	O75477	310	-3	-5	-5	-4	1.0	1.0	1.0	1.0
PIGQ	Q9BRB3	107	-3	-7	-5	2	1.0	0.9	1.0	1.0
AHCYL1	O43865	292	-3	-9	-5	-5	1.0	0.9	1.0	1.0
PPIL1	Q9Y3C6	133	-3	-9	-5	-9	1.0	0.9	1.0	0.9
EIF2S2	P20042	302	-3	-10	-5	2	1.0	0.9	1.0	1.0
HDGF	P51858	108	-3	-12	-5	-33	1.0	0.9	1.0	0.8
DDX52	Q9Y2R4	536	-3	-13	-5	-3	1.0	0.9	1.0	1.0
MRPS9	P82933	330	-3	-13	-5	-14	1.0	0.9	1.0	0.9
GGPS1	O95749	205	-3	-13	-5	-7	1.0	0.9	1.0	0.9
RAB30	Q15771	168	-3	-16	-5	-8	1.0	0.9	1.0	0.9
STAT2	P52630	74	-3	-20	-5	-9	1.0	0.8	1.0	0.9
TAOK3	Q9H2K8	795	-3	-3	-5	0	1.0	1.0	1.0	1.0
ATP13A1	Q9HD20	971	-3	-5	-5	15	1.0	1.0	1.0	1.2
VPS18	Q9P253	22	-3	-6	-5	-10	1.0	0.9	1.0	0.9
SBF1	O95248	1550	-3	-7	-5	-9	1.0	0.9	1.0	0.9
AARS2	Q5JTZ9	609	-3	-8	-5	-18	1.0	0.9	1.0	0.9
LRRK2	Q5S007	2025	-3	-8	-5	7	1.0	0.9	1.0	1.1
MTMR1	Q13613	170	-3	-8	-5	-8	1.0	0.9	1.0	0.9
MCRS1	Q96EZ8	84	-3	-11	-5	-18	1.0	0.9	1.0	0.9
OPTN	Q96CV9	334	-3	-14	-5	-12	1.0	0.9	1.0	0.9
MCU	Q8NE86	191	-3	-16	-5	-8	1.0	0.9	1.0	0.9
ZMYND11	Q15326	73	-3	-16	-5	-5	1.0	0.9	1.0	1.0
GLIPR2	Q9H4G4	63	-3	-20	-5	-8	1.0	0.8	1.0	0.9
SEPT5	Q99719	193	-3	-29	-5	1	1.0	0.8	1.0	1.0
ALMS1	Q8TCU4	436	-4	3	-5	1	1.0	1.0	1.0	1.0
NABP2	Q9BQ15	99	-4	-2	-5	-3	1.0	1.0	1.0	1.0
TRNT1	Q96Q11	373	-4	-7	-5	-8	1.0	0.9	1.0	0.9
UNC45A	Q9H3U1	853	-4	-9	-5	-8	1.0	0.9	1.0	0.9
APOBR	Q0VD83	844	-4	-10	-5	-15	1.0	0.9	1.0	0.9
HSPA4	P34932	417	-4	-17	-5	-9	1.0	0.9	1.0	0.9
TRAFD1	O14545	476	-4	-20	-5	-24	1.0	0.8	1.0	0.8
PELP1	Q8IZL8	71	-4	-6	-5	-4	1.0	0.9	1.0	1.0
MAP2K6	P52564	38	-4	-7	-5	-4	1.0	0.9	1.0	1.0
UPP1	Q16831	89	-4	-10	-5	-4	1.0	0.9	1.0	1.0
DUS1L	Q6P1R4	213	-4	-11	-5	-8	1.0	0.9	1.0	0.9
USP3	Q9Y6I4	157	-4	-13	-5	-4	1.0	0.9	1.0	1.0
ANKRD54	Q6NXT1	230	-4	-19	-5	-15	1.0	0.8	1.0	0.9
PECR	Q9BY49	191	-5	-3	-5	-8	1.0	1.0	1.0	0.9
MTA2	O94776	391	-5	-4	-5	-4	1.0	1.0	1.0	1.0
DOK3	Q7L591	493	-5	-7	-5	-13	1.0	0.9	1.0	0.9
CDC37L1	Q7L3B6	124	-5	-8	-5	-20	1.0	0.9	1.0	0.8
TXNDC12	O95881	66	-5	-9	-5	-2	1.0	0.9	1.0	1.0
ANKFY1	Q9P2R3	742	-5	-13	-5	4	1.0	0.9	1.0	1.0
PCBP1	Q15365	293	-5	-13	-5	-16	1.0	0.9	1.0	0.9
TCEB2	Q15370	89	-5	-14	-5	5	1.0	0.9	1.0	1.1
TROVE2	P10155	71	-5	-18	-5	-14	1.0	0.9	1.0	0.9

RBM22	Q9NW64	48	-5	-7	-5	-13	1.0	0.9	1.0	0.9
SRRM2	Q9UQ35	1016	-5	-10	-5	-18	1.0	0.9	1.0	0.8
METAP1	P53582	22	-5	-12	-5	2	1.0	0.9	1.0	1.0
HTT	P42858	777	-5	-15	-5	-7	1.0	0.9	1.0	0.9
PSMD4	P55036	37	-5	-20	-5	-8	1.0	0.8	1.0	0.9
ITPR2	Q14571	1879	-6	-6	-5	3	0.9	0.9	1.0	1.0
PDK3	Q15120	41	-6	-7	-5	-11	0.9	0.9	1.0	0.9
RNF169	Q8NCN4	337	-6	-11	-5	-18	0.9	0.9	1.0	0.9
SSB	P05455	245	-6	-15	-5	-18	0.9	0.9	1.0	0.8
SRPRB	Q9Y5M8	179	-6	-19	-5	-19	0.9	0.8	1.0	0.8
FTSJ3	Q8IY81	577	-6	-11	-5	-8	0.9	0.9	1.0	0.9
BMS1	Q14692	556	-6	-11	-5	-21	0.9	0.9	1.0	0.8
DDX21	Q9NR30	378	-6	-15	-5	1	0.9	0.9	1.0	1.0
U2SURP	O15042	65	-7	-5	-5	-13	0.9	1.0	1.0	0.9
CAST	P20810	381	-7	-8	-5	-15	0.9	0.9	1.0	0.9
MBNL3	Q9NUK0	195	-7	-11	-5	-5	0.9	0.9	1.0	1.0
RBKS	Q9H477	59	-7	-16	-5	-8	0.9	0.9	1.0	0.9
CARS	P49589	405	-7	-8	-5	-8	0.9	0.9	1.0	0.9
RELB	Q01201	389	-7	-13	-5	-2	0.9	0.9	1.0	1.0
SCCPDH	Q8NBX0	238	-7	-14	-5	-20	0.9	0.9	1.0	0.8
SEH1L	Q96EE3	30	-8	-5	-5	-8	0.9	1.0	1.0	0.9
PRDX1	Q06830	83	-8	-8	-5	-18	0.9	0.9	1.0	0.8
RPRD2	Q5VT52	903	-8	-8	-5	-25	0.9	0.9	1.0	0.8
ZC3H4	Q9UPT8	433	-8	-13	-5	-16	0.9	0.9	1.0	0.9
PIK3IP1	Q96FE7	228	-8	-11	-5	-13	0.9	0.9	1.0	0.9
BTF3	P20290	22	-8	-12	-5	-18	0.9	0.9	1.0	0.8
DIAPH2	O60879	655	-8	-14	-5	-6	0.9	0.9	1.0	0.9
NUMA1	Q14980	1729	-8	-21	-5	-29	0.9	0.8	1.0	0.8
C16orf62	Q7Z3J2	315	-8	-22	-5	-12	0.9	0.8	1.0	0.9
GDI1	P31150	302	-9	-34	-5	9	0.9	0.7	1.0	1.1
STRAP	Q9Y3F4	340	-10	-10	-5	-12	0.9	0.9	1.0	0.9
TAB3	Q8N5C8	564	-10	-10	-5	-14	0.9	0.9	1.0	0.9
MACF1	Q9UPN3	3407	-10	-9	-5	-7	0.9	0.9	1.0	0.9
STIP1	P31948	26	-10	-11	-5	-6	0.9	0.9	1.0	0.9
DNMT1	P26358	62	-10	-13	-5	-25	0.9	0.9	1.0	0.8
TEC	P42680	449	-11	-15	-5	5	0.9	0.9	1.0	1.1
RSBN1L	Q6PCB5	280	-11	-7	-5	-15	0.9	0.9	1.0	0.9
MTMR14	Q8NCE2	131	-11	-10	-5	-20	0.9	0.9	1.0	0.8
SLC25A22	Q9H936	246	-11	-20	-5	-1	0.9	0.8	1.0	1.0
BRAF	P15056	173	-11	-20	-5	-13	0.9	0.8	1.0	0.9
PPP1R12A	O14974	553	-12	-11	-5	-10	0.9	0.9	1.0	0.9
GCLM	P48507	72	-12	-12	-5	4	0.9	0.9	1.0	1.0
KLHDC3	Q9BQ90	33	-12	-28	-5	-12	0.9	0.8	1.0	0.9
MTMR14	Q8NCE2	182	-13	-23	-5	-15	0.9	0.8	1.0	0.9
CYFIP2	Q96F07	1265	-15	3	-5	6	0.9	1.0	1.0	1.1
ARID4B	Q4LE39	833	-15	-24	-5	-17	0.9	0.8	1.0	0.9
TBCD	Q9BTW9	279	-16	-27	-5	-31	0.9	0.8	1.0	0.8
KIAA0922	A2VDJ0	32	-17	-47	-5	-26	0.9	0.7	1.0	0.8
PDPR	Q8NCN5	85	-19	-21	-5	-1	0.8	0.8	1.0	1.0
ZMYM2	Q9UBW7	448	-21	-7	-5	-11	0.8	0.9	1.0	0.9
TRIM22	Q8IYM9	30	-22	-21	-5	-11	0.8	0.8	1.0	0.9
LIMS1	P48059	196	-23	-16	-5	-11	0.8	0.9	1.0	0.9
LIMS2	Q7Z4I7	201	-23	-16	-5	-11	0.8	0.9	1.0	0.9

NSMCE2	Q96MF7	140	-24	-17	-5	-18	0.8	0.9	1.0	0.8
DMXL1	Q9Y485	1760	-25	-8	-5	-31	0.8	0.9	1.0	0.8
GIT2	Q14161	672	-46	2	-5	-15	0.7	1.0	1.0	0.9
SUN2	Q9UH99	705	30	10	-5	34	1.4	1.1	1.0	1.5
XPOT	O43592	900	26	-5	-5	-21	1.3	1.0	1.0	0.8
NT5C3A	Q9H0P0	103	17	-4	-5	2	1.2	1.0	1.0	1.0
GPD1L	Q8N335	216	17	-9	-5	2	1.2	0.9	1.0	1.0
RTCA	O00442	28	16	-5	-5	-4	1.2	1.0	1.0	1.0
ATP2A2	P16615	669	16	-7	-5	11	1.2	0.9	1.0	1.1
TRPV2	Q9Y5S1	218	15	4	-5	19	1.2	1.0	1.0	1.2
SYK	P43405	593	14	5	-5	3	1.2	1.0	1.0	1.0
CCR4	P51679	232	13	-5	-5	2	1.1	1.0	1.0	1.0
INPP4A	Q96PE3	884	12	-12	-5	8	1.1	0.9	1.0	1.1
NR3C1	P04150	431	12	-23	-5	-13	1.1	0.8	1.0	0.9
ARID4A	P29374	25	12	-27	-5	-7	1.1	0.8	1.0	0.9
ARID4B	Q4LE39	25	12	-27	-5	-7	1.1	0.8	1.0	0.9
SYNE2	Q8WXH0	923	11	-14	-5	14	1.1	0.9	1.0	1.2
MDH1	P40925	137	11	-9	-5	6	1.1	0.9	1.0	1.1
GAR1	Q9NY12	88	10	-12	-5	-1	1.1	0.9	1.0	1.0
PCK2	Q16822	325	10	-15	-5	9	1.1	0.9	1.0	1.1
NEK9	Q8TD19	569	10	2	-5	-6	1.1	1.0	1.0	0.9
OAS1	P00973	189	10	-6	-5	8	1.1	0.9	1.0	1.1
BANK1	Q8NDB2	349	10	-11	-5	5	1.1	0.9	1.0	1.1
DOCK2	Q92608	1203	10	-17	-5	-4	1.1	0.9	1.0	1.0
PML	P29590	484	10	-18	-5	-8	1.1	0.8	1.0	0.9
SPTAN1	Q13813	1930	9	-18	-5	1	1.1	0.8	1.0	1.0
HSP90AB1	P08238	564	9	-19	-5	-9	1.1	0.8	1.0	0.9
TDG	Q13569	276	9	-12	-5	-10	1.1	0.9	1.0	0.9
CLTC	Q00610	778	9	-26	-5	1	1.1	0.8	1.0	1.0
ILVBL	A1L0T0	315	8	-2	-5	10	1.1	1.0	1.0	1.1
CROCC	Q5TZA2	1759	8	-10	-5	-5	1.1	0.9	1.0	1.0
PPA1	Q15181	254	8	-12	-5	-6	1.1	0.9	1.0	0.9
GIMAP8	Q8ND71	166	8	-23	-5	-2	1.1	0.8	1.0	1.0
TCP11L1	Q9NUJ3	115	8	1	-5	-6	1.1	1.0	1.0	0.9
THOC1	Q96FV9	156	8	-13	-5	-7	1.1	0.9	1.0	0.9
DNAJC13	O75165	149	8	-16	-5	-3	1.1	0.9	1.0	1.0
CD27	P26842	258	7	1	-5	-17	1.1	1.0	1.0	0.9
MDN1	Q9NU22	1867	7	-9	-5	-5	1.1	0.9	1.0	1.0
INPPL1	O15357	689	7	-11	-5	-11	1.1	0.9	1.0	0.9
PDE12	Q6L8Q7	321	7	-14	-5	12	1.1	0.9	1.0	1.1
UTRN	P46939	3094	7	4	-5	-8	1.1	1.0	1.0	0.9
IKBKG	Q9Y6K9	347	7	4	-5	7	1.1	1.0	1.0	1.1
HK2	P52789	813	7	-4	-5	-2	1.1	1.0	1.0	1.0
UHRF2	Q96PU4	42	7	-4	-5	-8	1.1	1.0	1.0	0.9
MBOAT7	Q96N66	280	7	-7	-5	2	1.1	0.9	1.0	1.0
BUD31	P41223	139	7	-8	-5	-2	1.1	0.9	1.0	1.0
PLIN3	O60664	60	7	-8	-5	-6	1.1	0.9	1.0	0.9
ZC3H12D	A2A288	517	7	-8	-5	-10	1.1	0.9	1.0	0.9
TMEM94	Q12767	381	7	-14	-5	1	1.1	0.9	1.0	1.0
CUL3	Q13618	636	7	-15	-5	1	1.1	0.9	1.0	1.0
TBC1D2	Q9BYX2	496	7	-56	-5	-13	1.1	0.6	1.0	0.9
RNPEP	Q9H4A4	254	6	-5	-5	-6	1.1	1.0	1.0	0.9
CEP192	Q8TEP8	966	6	-6	-5	-15	1.1	0.9	1.0	0.9

MTM1	Q13496	113	6	-8	-5	-2	1.1	0.9	1.0	1.0
EEF2	P13639	369	6	-10	-5	2	1.1	0.9	1.0	1.0
Uncharacteri	H0YIZ8	34	6	-11	-5	9	1.1	0.9	1.0	1.1
SRSF3	P84103	10	6	-14	-5	-13	1.1	0.9	1.0	0.9
MPP7	Q5T2T1	462	6	-2	-5	-6	1.1	1.0	1.0	0.9
MRPS12	O15235	132	6	-9	-5	-5	1.1	0.9	1.0	1.0
UBE3C	Q15386	1018	6	-11	-5	-8	1.1	0.9	1.0	0.9
GBP3	Q9H0R5	394	6	-11	-5	-2	1.1	0.9	1.0	1.0
RBBP9	O75884	39	6	-12	-5	2	1.1	0.9	1.0	1.0
ACSL4	O60488	221	6	-19	-5	-8	1.1	0.8	1.0	0.9
XPO1	O14980	99	6	-21	-5	-3	1.1	0.8	1.0	1.0
LRRC16A	Q5VZK9	1181	5	3	-5	1	1.1	1.0	1.0	1.0
VCPKMT	Q9H867	168	5	1	-5	-14	1.1	1.0	1.0	0.9
STAT5A	P42229	508	5	-2	-5	7	1.1	1.0	1.0	1.1
STAT5B	P51692	508	5	-2	-5	7	1.1	1.0	1.0	1.1
ODF2	Q5BJF6	136	5	-5	-5	-8	1.1	1.0	1.0	0.9
TBC1D2	Q9BYX2	504	5	-7	-5	-3	1.1	0.9	1.0	1.0
RRP1	P56182	198	5	-8	-5	-8	1.1	0.9	1.0	0.9
CSK	P41240	223	5	-9	-5	-12	1.1	0.9	1.0	0.9
TCF7	P36402	291	5	-12	-5	-10	1.1	0.9	1.0	0.9
ABR	Q12979	158	5	-13	-5	0	1.1	0.9	1.0	1.0
TMX2	Q9Y320	186	5	-27	-5	-17	1.1	0.8	1.0	0.9
F13A1	P00488	315	5	5	-5	8	1.0	1.0	1.0	1.1
HAT1	O14929	101	5	-2	-5	-3	1.0	1.0	1.0	1.0
CPSF6	Q16630	476	5	-3	-5	-4	1.0	1.0	1.0	1.0
DHX57	Q6P158	1369	5	-6	-5	-2	1.0	0.9	1.0	1.0
HS1BP3	Q53T59	118	5	-8	-5	1	1.0	0.9	1.0	1.0
PIK3CB	P42338	654	5	-9	-5	3	1.0	0.9	1.0	1.0
AHSA1	O95433	301	5	-10	-5	-7	1.0	0.9	1.0	0.9
DHX36	Q9H2U1	261	5	-10	-5	-25	1.0	0.9	1.0	0.8
DENND1C	Q8IV53	594	5	-11	-5	-12	1.0	0.9	1.0	0.9
MAVS	Q7Z434	283	5	-12	-5	-9	1.0	0.9	1.0	0.9
STIP1	P31948	339	5	-15	-5	4	1.0	0.9	1.0	1.0
ADD1	P35611	430	5	-15	-5	-8	1.0	0.9	1.0	0.9
ATP6V1B2	P21281	289	5	-20	-5	-14	1.0	0.8	1.0	0.9
XPO1	O14980	1070	4	-2	-5	-2	1.0	1.0	1.0	1.0
SKP1	P63208	160	4	-2	-5	-2	1.0	1.0	1.0	1.0
SRRT	Q9BXP5	490	4	-5	-5	-2	1.0	1.0	1.0	1.0
C14orf166	Q9Y224	19	4	-6	-5	-1	1.0	0.9	1.0	1.0
DOCK8	Q8NF50	521	4	-8	-5	-14	1.0	0.9	1.0	0.9
ACAP1	Q15027	64	4	-8	-5	-1	1.0	0.9	1.0	1.0
CDK9	P50750	13	4	-9	-5	3	1.0	0.9	1.0	1.0
LRBA	P50851	431	4	-9	-5	-3	1.0	0.9	1.0	1.0
NUMA1	Q14980	2009	4	-9	-5	-12	1.0	0.9	1.0	0.9
WDR91	A4D1P6	366	4	-11	-5	-4	1.0	0.9	1.0	1.0
NSUN5	Q96P11	146	4	-12	-5	-4	1.0	0.9	1.0	1.0
RAB31	Q13636	9	4	-19	-5	-3	1.0	0.8	1.0	1.0
RAB22A	Q9UL26	9	4	-19	-5	-3	1.0	0.8	1.0	1.0
ZC3H15	Q8WU90	180	4	-21	-5	-28	1.0	0.8	1.0	0.8
SNURF	Q9Y675	38	4	-5	-5	-5	1.0	1.0	1.0	1.0
ANK1	P16157	476	4	-6	-5	10	1.0	0.9	1.0	1.1
SCAF1	Q9H7N4	675	4	-7	-5	-10	1.0	0.9	1.0	0.9
AP2M1	Q96CW1	212	4	-8	-5	-5	1.0	0.9	1.0	1.0

SMARCAD1	Q9H4L7	861	4	-8	-5	-7	1.0	0.9	1.0	0.9
EWSR1	Q01844	540	4	-9	-5	-1	1.0	0.9	1.0	1.0
RIPK1	Q13546	34	4	-9	-5	-8	1.0	0.9	1.0	0.9
MED28	Q9H204	93	4	-10	-5	-2	1.0	0.9	1.0	1.0
DDX60	Q8IY21	517	4	-11	-5	-5	1.0	0.9	1.0	1.0
SUN1	O94901	657	4	-14	-5	-13	1.0	0.9	1.0	0.9
SUN2	Q9UH99	563	4	-14	-5	-13	1.0	0.9	1.0	0.9
EHMT2	Q96KQ7	126	4	-15	-5	-3	1.0	0.9	1.0	1.0
PRKDC	P78527	25	4	-20	-5	-7	1.0	0.8	1.0	0.9
MORF4L2	Q15014	165	4	-20	-5	-8	1.0	0.8	1.0	0.9
NGLY1	Q96IV0	484	3	-2	-5	4	1.0	1.0	1.0	1.0
L3MBTL3	Q96JM7	233	3	-3	-5	-7	1.0	1.0	1.0	0.9
NUMA1	Q14980	299	3	-4	-5	-2	1.0	1.0	1.0	1.0
DDX24	Q9GZR7	599	3	-4	-5	-8	1.0	1.0	1.0	0.9
C7orf50	Q9BRJ6	107	3	-7	-5	-4	1.0	0.9	1.0	1.0
MARS	P56192	38	3	-7	-5	-5	1.0	0.9	1.0	1.0
DMXL2	Q8TDJ6	1788	3	-8	-5	-10	1.0	0.9	1.0	0.9
SMCHD1	A6NHR9	59	3	-10	-5	-4	1.0	0.9	1.0	1.0
KHSRP	Q92945	436	3	-11	-5	-6	1.0	0.9	1.0	0.9
LMNB1	P20700	110	3	-17	-5	-8	1.0	0.9	1.0	0.9
ASMTL	O95671	441	3	-22	-5	-10	1.0	0.8	1.0	0.9
DPF2	Q92785	276	3	-1	-5	-7	1.0	1.0	1.0	0.9
HNRNPLL	Q8WVV9	464	3	-5	-5	-6	1.0	1.0	1.0	0.9
PTPRC	P08575	1259	3	-6	-5	-9	1.0	0.9	1.0	0.9
EXOSC10	Q01780	852	3	-7	-5	-12	1.0	0.9	1.0	0.9
GPCPD1	Q9NPB8	83	3	-9	-5	-3	1.0	0.9	1.0	1.0
BAG5	Q9UL15	191	3	-9	-5	-7	1.0	0.9	1.0	0.9
POLR2A	P24928	1245	3	-12	-5	0	1.0	0.9	1.0	1.0
UPF1	Q92900	237	3	-15	-5	-5	1.0	0.9	1.0	1.0
ARFGAP3	Q9NP61	28	2	3	-5	5	1.0	1.0	1.0	1.0
OSBPL8	Q9BZF1	255	2	-2	-5	0	1.0	1.0	1.0	1.0
ZNF324B	Q6AW86	198	2	-4	-5	-10	1.0	1.0	1.0	0.9
PLAA	Q9Y263	26	2	-5	-5	-3	1.0	1.0	1.0	1.0
PDE12	Q6L8Q7	180	2	-6	-5	2	1.0	0.9	1.0	1.0
PPP6R2	O75170	82	2	-9	-5	3	1.0	0.9	1.0	1.0
ACAT1	P24752	413	2	-9	-5	-12	1.0	0.9	1.0	0.9
ZFP91	Q96JP5	182	2	-10	-5	-18	1.0	0.9	1.0	0.8
JAK2	O60674	125	2	-12	-5	-19	1.0	0.9	1.0	0.8
PLCB2	Q00722	1164	2	-14	-5	-11	1.0	0.9	1.0	0.9
PADI2	Q9Y2J8	338	2	-16	-5	-1	1.0	0.9	1.0	1.0
HLA-B	Q95365	188	2	-20	-5	5	1.0	0.8	1.0	1.0
JAK2	O60674	961	2	-28	-5	-16	1.0	0.8	1.0	0.9
HAUS4	Q9H6D7	27	2	1	-5	3	1.0	1.0	1.0	1.0
SARG	Q9BW04	338	2	-4	-5	4	1.0	1.0	1.0	1.0
RIN3	Q8TB24	942	2	-7	-5	-14	1.0	0.9	1.0	0.9
USP24	Q9UPU5	802	2	-8	-5	-7	1.0	0.9	1.0	0.9
AARS	P49588	773	2	-9	-5	-7	1.0	0.9	1.0	0.9
STAT2	P52630	284	2	-9	-5	-18	1.0	0.9	1.0	0.9
MYH9	P35579	1437	2	-10	-5	-8	1.0	0.9	1.0	0.9
PDXK	O00764	273	2	-12	-5	-8	1.0	0.9	1.0	0.9
MDH2	P40926	89	2	-13	-5	-2	1.0	0.9	1.0	1.0
MAEA	Q7L5Y9	61	2	-19	-5	-7	1.0	0.8	1.0	0.9
RRP1B	Q14684	197	1	-2	-5	-9	1.0	1.0	1.0	0.9

NCBP1	Q09161	483	1	-4	-5	2	1.0	1.0	1.0	1.0
NUBP2	Q9Y5Y2	269	1	-5	-5	-9	1.0	1.0	1.0	0.9
UBE4A	Q14139	710	1	-8	-5	-18	1.0	0.9	1.0	0.9
RIOK3	O14730	287	1	-8	-5	-10	1.0	0.9	1.0	0.9
TRIM22	Q8IYM9	416	1	-9	-5	-10	1.0	0.9	1.0	0.9
SRPRB	Q9Y5M8	73	1	-11	-5	-9	1.0	0.9	1.0	0.9
CREB1	P16220	337	1	-3	-5	-11	1.0	1.0	1.0	0.9
RAB34	Q9BZG1	38	1	-4	-5	-9	1.0	1.0	1.0	0.9
RNF213	Q63HN8	981	1	-5	-5	-9	1.0	1.0	1.0	0.9
IQGAP1	P46940	494	1	-6	-5	-4	1.0	0.9	1.0	1.0
GNL3L	Q9NVN8	89	1	-6	-5	-22	1.0	0.9	1.0	0.8
SOST	Q9BQB4	134	1	-7	-5	-4	1.0	0.9	1.0	1.0
SPRYD3	Q8NCJ5	229	1	-8	-5	-3	1.0	0.9	1.0	1.0
DDX23	Q9BUQ8	692	1	-8	-5	-7	1.0	0.9	1.0	0.9
TPR	P12270	1068	1	-8	-5	-5	1.0	0.9	1.0	1.0
PES1	O00541	272	1	-8	-5	-11	1.0	0.9	1.0	0.9
RPL27A	P46776	144	1	-10	-5	-9	1.0	0.9	1.0	0.9
GCLC	P48506	501	1	-10	-5	-24	1.0	0.9	1.0	0.8
Uncharacteri	G3V3G9	427	1	-13	-5	-16	1.0	0.9	1.0	0.9
TRIM28	Q13263	232	1	-13	-5	-6	1.0	0.9	1.0	0.9
PLEC	Q15149	992	1	-16	-5	-13	1.0	0.9	1.0	0.9
PDLIM1	O00151	263	1	-18	-5	-9	1.0	0.8	1.0	0.9
GLIPR1	P48060	192	1	-27	-5	-13	1.0	0.8	1.0	0.9
TBC1D13	Q9NVG8	282	0	-5	-5	-1	1.0	1.0	1.0	1.0
LRRC59	Q96AG4	59	0	-7	-5	-1	1.0	0.9	1.0	1.0
PDE4DIP	Q5VU43	617	0	-11	-5	-17	1.0	0.9	1.0	0.9
EGLN1	Q9GZT9	43	0	-13	-5	-11	1.0	0.9	1.0	0.9
S100A10	P60903	62	-1	-6	-5	-17	1.0	0.9	1.0	0.9
HPS3	Q969F9	707	-1	-6	-5	-6	1.0	0.9	1.0	0.9
NAT10	Q9H0A0	489	-1	-7	-5	-9	1.0	0.9	1.0	0.9
RANBP2	P49792	348	-1	-9	-5	-12	1.0	0.9	1.0	0.9
GIT2	Q14161	235	-1	-10	-5	-2	1.0	0.9	1.0	1.0
GIT1	Q9Y2X7	235	-1	-10	-5	-2	1.0	0.9	1.0	1.0
WDR1	O75083	382	-1	-11	-5	-1	1.0	0.9	1.0	1.0
DNTTIP1	Q9H147	156	-1	-11	-5	-9	1.0	0.9	1.0	0.9
CTCF	P49711	500	-1	-31	-5	-18	1.0	0.8	1.0	0.8
KAT2A	Q92830	695	-1	-6	-5	-1	1.0	0.9	1.0	1.0
KAT2B	Q92831	690	-1	-6	-5	-1	1.0	0.9	1.0	1.0
HUWE1	Q7Z6Z7	1832	-1	-8	-5	-6	1.0	0.9	1.0	0.9
DHX8	Q14562	123	-1	-8	-5	-12	1.0	0.9	1.0	0.9
KBTBD2	Q8IY47	349	-1	-10	-5	-5	1.0	0.9	1.0	1.0
DNMT3A	Q9Y6K1	340	-1	-14	-5	-13	1.0	0.9	1.0	0.9
Uncharacteri	E7EUB6	68	-1	-39	-5	-6	1.0	0.7	1.0	0.9
AAR2	Q9Y312	156	-2	-3	-5	-12	1.0	1.0	1.0	0.9
TIAM1	Q13009	1061	-2	-4	-5	-1	1.0	1.0	1.0	1.0
PTAR1	Q7Z6K3	381	-2	-6	-5	-8	1.0	0.9	1.0	0.9
CCT3	P49368	372	-2	-7	-5	11	1.0	0.9	1.0	1.1
FLNA	P21333	2543	-2	-8	-5	-11	1.0	0.9	1.0	0.9
GCN1	Q92616	2255	-2	-9	-5	14	1.0	0.9	1.0	1.2
PIN1	Q13526	113	-2	-9	-5	-10	1.0	0.9	1.0	0.9
EIF2S3	P41091	105	-2	-12	-5	-5	1.0	0.9	1.0	1.0
RIPK2	O43353	414	-2	-14	-5	-26	1.0	0.9	1.0	0.8
LOC1027241!	A0A0B4J2E5	716	-2	-16	-5	-16	1.0	0.9	1.0	0.9

CISD3	P0C7P0	109	-2	-22	-5	-7	1.0	0.8	1.0	0.9
IL12A	P29459	96	-2	-6	-5	-6	1.0	0.9	1.0	0.9
IL12A	P29459	110	-2	-6	-5	-6	1.0	0.9	1.0	0.9
MMS19	Q96T76	819	-2	-7	-5	17	1.0	0.9	1.0	1.2
TACO1	Q9BSH4	256	-2	-8	-5	4	1.0	0.9	1.0	1.0
DFFA	O00273	165	-2	-8	-5	-15	1.0	0.9	1.0	0.9
HMGCL	P35914	323	-2	-8	-5	-8	1.0	0.9	1.0	0.9
WNK1	Q9H4A3	459	-2	-8	-5	-21	1.0	0.9	1.0	0.8
TMEM63A	O94886	372	-2	-11	-5	6	1.0	0.9	1.0	1.1
TUBB1	Q9H4B7	12	-2	-11	-5	-8	1.0	0.9	1.0	0.9
NUP160	Q12769	929	-2	-11	-5	-9	1.0	0.9	1.0	0.9
MDH1	P40925	154	-2	-12	-5	-2	1.0	0.9	1.0	1.0
ZMYM2	Q9UBW7	667	-2	-14	-5	-7	1.0	0.9	1.0	0.9
MSH2	P43246	199	-2	-17	-5	-9	1.0	0.9	1.0	0.9
KIAA1524	Q8TCG1	337	-2	-18	-5	-13	1.0	0.8	1.0	0.9
SHMT2	P34897	412	-2	-21	-5	-8	1.0	0.8	1.0	0.9
ACAP1	Q15027	10	-2	-21	-5	-12	1.0	0.8	1.0	0.9
AKAP8	O43823	631	-2	-21	-5	-21	1.0	0.8	1.0	0.8
NCKAP1L	P55160	942	-3	4	-5	13	1.0	1.0	1.0	1.1
RNF130	Q86XS8	320	-3	-1	-5	-8	1.0	1.0	1.0	0.9
SPECC1	Q5M775	493	-3	-4	-5	-13	1.0	1.0	1.0	0.9
LIG3	P49916	929	-3	-6	-5	-6	1.0	0.9	1.0	0.9
SHTN1	A0MZ66	565	-3	-8	-5	-16	1.0	0.9	1.0	0.9
IBA57	Q5T440	259	-3	-11	-5	-17	1.0	0.9	1.0	0.9
ABCB1	P08183	431	-3	-12	-5	-14	1.0	0.9	1.0	0.9
RASA3	Q14644	58	-3	-13	-5	2	1.0	0.9	1.0	1.0
ZNF516	Q92618	1016	-3	-13	-5	-21	1.0	0.9	1.0	0.8
GLYR1	Q49A26	416	-3	-16	-5	-7	1.0	0.9	1.0	0.9
SGF29	Q96ES7	243	-3	-17	-5	-14	1.0	0.9	1.0	0.9
DNM1L	O00429	505	-3	-5	-5	4	1.0	1.0	1.0	1.0
NOL8	Q76FK4	578	-3	-7	-5	-20	1.0	0.9	1.0	0.8
TANGO6	Q9C0B7	15	-3	-8	-5	-6	1.0	0.9	1.0	0.9
SIPA1L3	O60292	18	-3	-9	-5	-7	1.0	0.9	1.0	0.9
CDK12	Q9NYV4	1009	-3	-9	-5	-10	1.0	0.9	1.0	0.9
DHX37	Q8IY37	1026	-3	-9	-5	-15	1.0	0.9	1.0	0.9
AKR1C3	P42330	188	-3	-22	-5	-17	1.0	0.8	1.0	0.9
LEMD3	Q9Y2U8	346	-4	-7	-5	-34	1.0	0.9	1.0	0.7
SPG11	Q96JI7	1890	-4	-7	-5	-15	1.0	0.9	1.0	0.9
CD36	P16671	272	-4	-14	-5	-7	1.0	0.9	1.0	0.9
OSBPL3	Q9H4L5	520	-4	-15	-5	7	1.0	0.9	1.0	1.1
RNPEP	Q9H4A4	85	-4	-17	-5	1	1.0	0.9	1.0	1.0
AIFM1	O95831	256	-4	-31	-5	-17	1.0	0.8	1.0	0.9
GNAI2	P04899	112	-4	-6	-5	-10	1.0	0.9	1.0	0.9
FECH	P22830	196	-4	-8	-5	-1	1.0	0.9	1.0	1.0
ODR4	Q5SWX8	141	-4	-11	-5	-12	1.0	0.9	1.0	0.9
LGALSL	Q3ZCW2	101	-4	-16	-5	-10	1.0	0.9	1.0	0.9
FLII	Q13045	808	-4	-17	-5	-1	1.0	0.9	1.0	1.0
RPS12	P25398	92	-4	-21	-5	-4	1.0	0.8	1.0	1.0
NFIC	P08651	88	-5	2	-5	3	1.0	1.0	1.0	1.0
GTF2H1	P32780	506	-5	-5	-5	-2	1.0	1.0	1.0	1.0
C9orf114	Q5T280	239	-5	-7	-5	-11	1.0	0.9	1.0	0.9
FGD3	Q5JSP0	563	-5	-16	-5	-15	1.0	0.9	1.0	0.9
ATM	Q13315	2021	-5	-7	-5	-5	1.0	0.9	1.0	1.0

RAPH1	Q70E73	1045	-5	-9	-5	-19	1.0	0.9	1.0	0.8
DGKA	P23743	235	-5	-10	-5	-11	1.0	0.9	1.0	0.9
NEURL4	Q96JN8	1368	-5	-11	-5	-7	1.0	0.9	1.0	0.9
TRRAP	Q9Y4A5	3555	-5	-11	-5	3	1.0	0.9	1.0	1.0
TGM2	P21980	230	-6	-7	-5	-21	0.9	0.9	1.0	0.8
ALDH9A1	P49189	288	-6	-9	-5	-11	0.9	0.9	1.0	0.9
SRRM2	Q9UQ35	956	-6	1	-5	-4	0.9	1.0	1.0	1.0
GLUD1	P00367	112	-6	-6	-5	-4	0.9	0.9	1.0	1.0
TDRD7	Q8NHU6	77	-6	-6	-5	-6	0.9	0.9	1.0	0.9
HAGH	Q16775	219	-6	-9	-5	-2	0.9	0.9	1.0	1.0
PDE5A	O76074	447	-6	-9	-5	-14	0.9	0.9	1.0	0.9
PTPN6	P29350	382	-6	-13	-5	-4	0.9	0.9	1.0	1.0
RANBP2	P49792	1375	-6	-18	-5	-12	0.9	0.9	1.0	0.9
PAX2	Q02962	52	-7	-9	-5	-6	0.9	0.9	1.0	0.9
TLN1	Q9Y490	116	-7	-9	-5	-8	0.9	0.9	1.0	0.9
SEC24D	O94855	371	-7	-8	-5	-1	0.9	0.9	1.0	1.0
SEC24C	P53992	433	-7	-8	-5	-1	0.9	0.9	1.0	1.0
ZC3H12D	A2A288	64	-7	-8	-5	-20	0.9	0.9	1.0	0.8
PCNX1	Q96RV3	1621	-7	-13	-5	-8	0.9	0.9	1.0	0.9
EIF2S3	P41091	348	-7	-16	-5	9	0.9	0.9	1.0	1.1
QTRTD1	Q9H974	174	-7	-18	-5	-6	0.9	0.8	1.0	0.9
PXN	P49023	405	-7	-20	-5	-8	0.9	0.8	1.0	0.9
COMT	P21964	119	-8	-15	-5	-4	0.9	0.9	1.0	1.0
PRKDC	P78527	4106	-8	-50	-5	-14	0.9	0.7	1.0	0.9
PPP1R21	Q6ZMI0	351	-8	-2	-5	-5	0.9	1.0	1.0	1.0
FLNB	O75369	183	-8	-10	-5	-13	0.9	0.9	1.0	0.9
RB1CC1	Q8TDY2	1236	-8	-21	-5	-26	0.9	0.8	1.0	0.8
SETD7	Q8WTS6	200	-9	-9	-5	-15	0.9	0.9	1.0	0.9
RIF1	Q5UIP0	2169	-9	-4	-5	-2	0.9	1.0	1.0	1.0
CMIP	Q8IY22	280	-9	-13	-5	3	0.9	0.9	1.0	1.0
CORO7-PAM1	A0A0A6YYL4	303	-9	-15	-5	-2	0.9	0.9	1.0	1.0
CHST11	Q9NPF2	128	-9	-16	-5	-3	0.9	0.9	1.0	1.0
LEMD3	Q9Y2U8	754	-9	-20	-5	-13	0.9	0.8	1.0	0.9
KRIT1	O00522	297	-9	-26	-5	-1	0.9	0.8	1.0	1.0
ABI1	Q8IZP0	488	-9	-34	-5	4	0.9	0.7	1.0	1.0
TACC1	O75410	78	-10	-13	-5	-20	0.9	0.9	1.0	0.8
MBNL2	Q5VZF2	197	-10	-20	-5	-5	0.9	0.8	1.0	1.0
METAP1	P53582	14	-10	-9	-5	-11	0.9	0.9	1.0	0.9
TCP1	P17987	76	-10	-29	-5	-22	0.9	0.8	1.0	0.8
ZAP70	P43403	596	-11	-20	-5	-11	0.9	0.8	1.0	0.9
XIAP	P98170	12	-12	-13	-5	-9	0.9	0.9	1.0	0.9
EPRS	P07814	697	-12	-15	-5	3	0.9	0.9	1.0	1.0
MACF1	Q9UPN3	892	-12	-16	-5	-15	0.9	0.9	1.0	0.9
ARHGAP6	O43182	819	-13	-9	-5	-20	0.9	0.9	1.0	0.8
FRY	Q5TBA9	1193	-13	-2	-5	4	0.9	1.0	1.0	1.0
SNIP1	Q8TAD8	299	-13	-5	-5	-4	0.9	1.0	1.0	1.0
DHX37	Q8IY37	766	-14	-9	-5	-6	0.9	0.9	1.0	0.9
ZC3H12A	Q5D1E8	306	-14	-17	-5	-15	0.9	0.9	1.0	0.9
UBE4B	O95155	1164	-14	-17	-5	-24	0.9	0.9	1.0	0.8
VCL	P18206	325	-16	-10	-5	3	0.9	0.9	1.0	1.0
PF4V1	P10720	70	-19	-18	-5	5	0.8	0.9	1.0	1.1
TXN	P10599	32	-20	-21	-5	9	0.8	0.8	1.0	1.1
SPARC	P09486	155	-23	-11	-5	-12	0.8	0.9	1.0	0.9

GNG11	P61952	36	21	-17	-6	6	1.3	0.9	0.9	1.1
HSPA4	P34932	167	18	-5	-6	-2	1.2	1.0	0.9	1.0
TNK2	Q07912	159	17	-4	-6	2	1.2	1.0	0.9	1.0
ILF3	Q12906	278	17	9	-6	1	1.2	1.1	0.9	1.0
ITGA2B	P08514	96	17	0	-6	-3	1.2	1.0	0.9	1.0
PPP5C	P53041	77	17	-14	-6	0	1.2	0.9	0.9	1.0
PPP2R1A	P30153	154	16	-10	-6	6	1.2	0.9	0.9	1.1
PPP2R1B	P30154	166	16	-10	-6	6	1.2	0.9	0.9	1.1
ANKFY1	Q9P2R3	675	15	-11	-6	4	1.2	0.9	0.9	1.0
AGFG1	P52594	89	15	-18	-6	3	1.2	0.8	0.9	1.0
MAU2	Q9Y6X3	367	14	-4	-6	-8	1.2	1.0	0.9	0.9
IQGAP2	Q13576	575	14	-10	-6	4	1.2	0.9	0.9	1.0
EML3	Q32P44	615	14	-10	-6	-6	1.2	0.9	0.9	0.9
SLAIN2	Q9P270	152	13	6	-6	-2	1.1	1.1	0.9	1.0
GOLGA4	Q13439	1771	13	0	-6	3	1.1	1.0	0.9	1.0
DOCK7	Q96N67	608	13	-12	-6	-6	1.1	0.9	0.9	0.9
ACTR3B	Q9P1U1	408	13	-14	-6	-15	1.1	0.9	0.9	0.9
FAF2	Q96CS3	194	13	-45	-6	-7	1.1	0.7	0.9	0.9
NUDT12	Q9BQG2	117	13	-3	-6	-6	1.1	1.0	0.9	0.9
Uncharacteri	A0A0C4DFX4	848	13	-9	-6	-6	1.1	0.9	0.9	0.9
SKIV2L	Q15477	877	13	-10	-6	1	1.1	0.9	0.9	1.0
CEP97	Q8IW35	34	12	-4	-6	-12	1.1	1.0	0.9	0.9
TBC1D2	Q9BYX2	528	12	-6	-6	-10	1.1	0.9	0.9	0.9
TBL2	Q9Y4P3	247	12	-34	-6	-4	1.1	0.7	0.9	1.0
DCTN6	O00399	18	12	-1	-6	-6	1.1	1.0	0.9	0.9
LRMP	Q12912	355	12	-4	-6	-12	1.1	1.0	0.9	0.9
WDR4	P57081	137	12	-6	-6	0	1.1	0.9	0.9	1.0
P2RY8	Q86VZ1	310	11	-3	-6	1	1.1	1.0	0.9	1.0
MAP2K7	O14733	280	11	-3	-6	-12	1.1	1.0	0.9	0.9
DHX15	O43143	774	11	-4	-6	3	1.1	1.0	0.9	1.0
AIP	O00170	122	11	-4	-6	-3	1.1	1.0	0.9	1.0
TRAPPC8	Q9Y2L5	505	11	-30	-6	-1	1.1	0.8	0.9	1.0
RNH1	P13489	95	11	1	-6	1	1.1	1.0	0.9	1.0
SAMD9L	Q8IVG5	1246	11	-18	-6	-1	1.1	0.9	0.9	1.0
UIMC1	Q96RL1	257	10	-8	-6	-7	1.1	0.9	0.9	0.9
EPPK1	A0A087X1U6	2225	10	-9	-6	-7	1.1	0.9	0.9	0.9
PAPSS1	O43252	83	10	-22	-6	3	1.1	0.8	0.9	1.0
METTL7B	Q6UX53	203	10	-10	-6	1	1.1	0.9	0.9	1.0
ESF1	Q9H501	462	10	-11	-6	13	1.1	0.9	0.9	1.1
VPS16	Q9H269	259	9	-5	-6	-9	1.1	1.0	0.9	0.9
TLN1	Q9Y490	2442	9	-7	-6	-4	1.1	0.9	0.9	1.0
TLN2	Q9Y4G6	2443	9	-7	-6	-4	1.1	0.9	0.9	1.0
GAPVD1	Q14C86	70	9	-10	-6	-13	1.1	0.9	0.9	0.9
PYHIN1	Q6K0P9	361	9	-11	-6	4	1.1	0.9	0.9	1.0
MEMO1	Q9Y316	88	9	-14	-6	-11	1.1	0.9	0.9	0.9
MYO1G	B0I1T2	618	9	-16	-6	-4	1.1	0.9	0.9	1.0
COPG1	Q9Y678	44	9	-17	-6	3	1.1	0.9	0.9	1.0
XRCC5	P13010	339	9	-20	-6	-6	1.1	0.8	0.9	0.9
PPP4C	P60510	130	9	-22	-6	0	1.1	0.8	0.9	1.0
PPP2CB	P62714	133	9	-22	-6	0	1.1	0.8	0.9	1.0
PPP2CA	P67775	133	9	-22	-6	0	1.1	0.8	0.9	1.0
EHHADH	Q08426	566	8	11	-6	10	1.1	1.1	0.9	1.1
NDUFAB1	O14561	140	8	-3	-6	-3	1.1	1.0	0.9	1.0

ABI3	Q9P2A4	33	8	-8	-6	-6	1.1	0.9	0.9	0.9
ZBTB24	O43167	481	8	-1	-6	-3	1.1	1.0	0.9	1.0
SPEN	Q96T58	598	8	-3	-6	-9	1.1	1.0	0.9	0.9
NIPBL	Q6KC79	1795	8	-10	-6	-14	1.1	0.9	0.9	0.9
ZNF330	Q9Y3S2	186	8	-11	-6	-6	1.1	0.9	0.9	0.9
RASGRP4	Q8TDF6	532	7	-2	-6	-12	1.1	1.0	0.9	0.9
C5orf51	A6NDU8	244	7	-4	-6	-33	1.1	1.0	0.9	0.8
GIGYF1	O75420	177	7	-12	-6	-18	1.1	0.9	0.9	0.8
SERPINB9	P50453	370	7	-22	-6	-1	1.1	0.8	0.9	1.0
CPT2	P23786	84	7	-57	-6	-9	1.1	0.6	0.9	0.9
PRUNE	Q86TP1	303	7	13	-6	5	1.1	1.1	0.9	1.0
IRS2	Q9Y4H2	97	7	0	-6	-5	1.1	1.0	0.9	1.0
KBTBD11	O94819	421	7	-5	-6	-4	1.1	1.0	0.9	1.0
SPECC1	Q5M775	740	7	-8	-6	-13	1.1	0.9	0.9	0.9
CSDE1	O75534	680	7	-9	-6	-12	1.1	0.9	0.9	0.9
TPMT	P51580	165	7	-10	-6	-5	1.1	0.9	0.9	1.0
NUDT16L1	Q9BRJ7	171	7	-11	-6	1	1.1	0.9	0.9	1.0
MYO18A	Q92614	811	7	-13	-6	-5	1.1	0.9	0.9	1.0
AKR1B1	P15121	81	7	-14	-6	4	1.1	0.9	0.9	1.0
CORO1A	P31146	345	7	-14	-6	-4	1.1	0.9	0.9	1.0
CYP51A1	Q16850	449	7	-22	-6	-20	1.1	0.8	0.9	0.8
HEATR6	Q6AI08	846	6	-6	-6	1	1.1	0.9	0.9	1.0
DDX42	Q86XP3	339	6	-7	-6	3	1.1	0.9	0.9	1.0
RPA1	P27694	481	6	-11	-6	-4	1.1	0.9	0.9	1.0
SYNE2	Q8WXH0	5359	6	-16	-6	-12	1.1	0.9	0.9	0.9
CLIP1	P30622	752	6	-16	-6	-16	1.1	0.9	0.9	0.9
PARP1	P09874	429	6	-35	-6	5	1.1	0.7	0.9	1.0
ALDH9A1	P49189	376	6	2	-6	20	1.1	1.0	0.9	1.3
RBPJ	Q06330	397	6	-7	-6	-10	1.1	0.9	0.9	0.9
CNOT1	A5YKK6	1706	6	-8	-6	-5	1.1	0.9	0.9	1.0
TLR2	O60603	713	6	-8	-6	-6	1.1	0.9	0.9	0.9
PRPF8	Q6P2Q9	2116	6	-18	-6	-4	1.1	0.8	0.9	1.0
ARHGEF2	Q92974	335	6	-19	-6	7	1.1	0.8	0.9	1.1
ZNF318	Q5VUA4	1363	5	0	-6	-9	1.1	1.0	0.9	0.9
ARHGAP18	Q8N392	323	5	-5	-6	-7	1.1	1.0	0.9	0.9
HK3	P52790	190	5	-5	-6	-10	1.1	1.0	0.9	0.9
SPG21	Q9NZD8	44	5	-6	-6	-6	1.1	0.9	0.9	0.9
KCMF1	Q9P0J7	12	5	-6	-6	-1	1.1	0.9	0.9	1.0
HADHA	P40939	97	5	-7	-6	-1	1.1	0.9	0.9	1.0
NTPCR	Q9BSD7	110	5	-8	-6	-10	1.1	0.9	0.9	0.9
HUS1	O60921	44	5	-9	-6	2	1.1	0.9	0.9	1.0
PAK2	Q13177	390	5	-10	-6	3	1.1	0.9	0.9	1.0
RACK1	P63244	138	5	-11	-6	4	1.1	0.9	0.9	1.0
LGALS1	P09382	43	5	-14	-6	-3	1.1	0.9	0.9	1.0
PTBP3	O95758	500	5	-19	-6	5	1.1	0.8	0.9	1.0
MX2	P20592	407	5	-22	-6	-23	1.1	0.8	0.9	0.8
DPYD	Q12882	385	5	-30	-6	3	1.1	0.8	0.9	1.0
KDM2B	Q8NHM5	770	5	2	-6	-16	1.0	1.0	0.9	0.9
RPS20	P60866	36	5	-3	-6	0	1.0	1.0	0.9	1.0
FAM98B	Q52LJ0	220	5	-5	-6	12	1.0	1.0	0.9	1.1
CSRP1	P21291	40	5	-5	-6	-5	1.0	1.0	0.9	1.0
GTF3C1	Q12789	42	5	-6	-6	-1	1.0	0.9	0.9	1.0
BTN3A3	O00478	412	5	-6	-6	12	1.0	0.9	0.9	1.1

BTN3A1	O00481	412	5	-6	-6	12	1.0	0.9	0.9	1.1
DHX57	Q6P158	1349	5	-10	-6	-8	1.0	0.9	0.9	0.9
BAZ1A	Q9NRL2	970	5	-10	-6	-9	1.0	0.9	0.9	0.9
PUS1	Q9Y606	260	5	-13	-6	-7	1.0	0.9	0.9	0.9
SEC14L1	Q92503	258	5	-14	-6	-6	1.0	0.9	0.9	0.9
CRY2	Q49AN0	334	5	-27	-6	-11	1.0	0.8	0.9	0.9
RHOC	P08134	20	4	-10	-6	7	1.0	0.9	0.9	1.1
RHOA	P61586	20	4	-10	-6	7	1.0	0.9	0.9	1.1
Uncharacteri	U3KQV3	184	4	-10	-6	7	1.0	0.9	0.9	1.1
SART3	Q15020	812	4	-10	-6	-8	1.0	0.9	0.9	0.9
EIF3L	Q9Y262	417	4	-13	-6	-4	1.0	0.9	0.9	1.0
ARHGAP4	P98171	34	4	-13	-6	-7	1.0	0.9	0.9	0.9
RAB43	Q86YS6	157	4	-21	-6	-7	1.0	0.8	0.9	0.9
TSR3	Q9UJK0	283	4	12	-6	-13	1.0	1.1	0.9	0.9
SETX	Q7Z333	1915	4	1	-6	2	1.0	1.0	0.9	1.0
FAM98B	Q52LJ0	93	4	-5	-6	1	1.0	1.0	0.9	1.0
ZNF609	O15014	178	4	-11	-6	-14	1.0	0.9	0.9	0.9
SLF2	Q8IX21	244	4	-13	-6	-15	1.0	0.9	0.9	0.9
TMPO	P42166	629	4	-17	-6	6	1.0	0.9	0.9	1.1
CLTC	Q00610	736	4	-20	-6	-8	1.0	0.8	0.9	0.9
ANKFY1	Q9P2R3	1105	4	-22	-6	-8	1.0	0.8	0.9	0.9
EIF2S2	P20042	226	4	-24	-6	-1	1.0	0.8	0.9	1.0
IKZF3	Q9UKT9	434	3	-7	-6	-11	1.0	0.9	0.9	0.9
ZNF638	Q14966	652	3	-8	-6	-6	1.0	0.9	0.9	0.9
MTO1	Q9Y2Z2	315	3	-8	-6	-8	1.0	0.9	0.9	0.9
SPG11	Q96JI7	526	3	-8	-6	-9	1.0	0.9	0.9	0.9
DOK3	Q7L591	323	3	-8	-6	-13	1.0	0.9	0.9	0.9
COASY	Q13057	144	3	-9	-6	-4	1.0	0.9	0.9	1.0
NUP50	Q9UKX7	151	3	-10	-6	-10	1.0	0.9	0.9	0.9
TRRAP	Q9Y4A5	1874	3	-11	-6	-8	1.0	0.9	0.9	0.9
DIS3L	Q8TF46	918	3	-13	-6	-19	1.0	0.9	0.9	0.8
ELF2	Q15723	348	3	-17	-6	-17	1.0	0.9	0.9	0.9
UBR4	Q5T4S7	3703	3	-17	-6	-10	1.0	0.9	0.9	0.9
NAPA	P54920	103	3	-20	-6	8	1.0	0.8	0.9	1.1
ATP2C1	P98194	139	3	-10	-6	-1	1.0	0.9	0.9	1.0
DYNC1H1	Q14204	633	3	-10	-6	-5	1.0	0.9	0.9	1.0
GSTO1	P78417	192	3	-14	-6	-2	1.0	0.9	0.9	1.0
C14orf159	Q7Z3D6	265	3	-16	-6	-8	1.0	0.9	0.9	0.9
RABIF	P47224	23	2	23	-6	15	1.0	1.3	0.9	1.2
FCHSD1	Q86WN1	88	2	-1	-6	6	1.0	1.0	0.9	1.1
LAMTOR2	Q9Y2Q5	76	2	-3	-6	-1	1.0	1.0	0.9	1.0
RPAP3	Q9H6T3	519	2	-4	-6	-7	1.0	1.0	0.9	0.9
RNF213	Q63HN8	4000	2	-5	-6	-3	1.0	1.0	0.9	1.0
ASPSCR1	Q9BZE9	48	2	-5	-6	-11	1.0	1.0	0.9	0.9
SRD5A3	Q9H8P0	57	2	-8	-6	15	1.0	0.9	0.9	1.2
THADA	Q6YHU6	1853	2	-9	-6	-3	1.0	0.9	0.9	1.0
KIF5B	P33176	421	2	-11	-6	-10	1.0	0.9	0.9	0.9
MGEA5	O60502	663	2	-12	-6	-14	1.0	0.9	0.9	0.9
INTS10	Q9NVR2	122	2	-12	-6	-7	1.0	0.9	0.9	0.9
EGLN1	Q9GZT9	42	2	-19	-6	-10	1.0	0.8	0.9	0.9
SMARCE1	Q969G3	274	2	-21	-6	-8	1.0	0.8	0.9	0.9
CIAPIN1	Q6FI81	249	2	-23	-6	-12	1.0	0.8	0.9	0.9
TRIP6	Q15654	310	2	-30	-6	36	1.0	0.8	0.9	1.6

TRIM22	Q8IYM9	97	2	0	-6	1	1.0	1.0	0.9	1.0
ASUN	Q9NVM9	120	2	-2	-6	-1	1.0	1.0	0.9	1.0
PGD	P52209	171	2	-3	-6	9	1.0	1.0	0.9	1.1
MTM1	Q13496	53	2	-3	-6	0	1.0	1.0	0.9	1.0
CREBBP	Q92793	1212	2	-4	-6	-6	1.0	1.0	0.9	0.9
HADHA	P40939	470	2	-6	-6	5	1.0	0.9	0.9	1.1
MAP1S	Q66K74	342	2	-8	-6	-8	1.0	0.9	0.9	0.9
PLEC	Q15149	3336	2	-14	-6	-11	1.0	0.9	0.9	0.9
INPP5B	P32019	678	2	-17	-6	-8	1.0	0.9	0.9	0.9
FARSB	Q9NSD9	195	1	-4	-6	-9	1.0	1.0	0.9	0.9
RBPJ	Q06330	313	1	-5	-6	0	1.0	1.0	0.9	1.0
HGS	O14964	215	1	-5	-6	-5	1.0	1.0	0.9	1.0
ZNF318	Q5VUA4	2168	1	-5	-6	-7	1.0	1.0	0.9	0.9
GLTSCR1	Q9NZM4	1382	1	-5	-6	-11	1.0	1.0	0.9	0.9
UTP6	Q9NYH9	370	1	-5	-6	-17	1.0	1.0	0.9	0.9
BRE	Q9NXR7	34	1	-7	-6	-9	1.0	0.9	0.9	0.9
NAT10	Q9H0A0	499	1	-8	-6	-13	1.0	0.9	0.9	0.9
HK1	P19367	813	1	-8	-6	-3	1.0	0.9	0.9	1.0
MCTS1	Q9ULC4	14	1	-9	-6	-5	1.0	0.9	0.9	1.0
DDX46	Q7L014	590	1	-9	-6	-7	1.0	0.9	0.9	0.9
OGFR	Q9NZT2	443	1	-9	-6	-8	1.0	0.9	0.9	0.9
RAD9A	Q99638	98	1	-11	-6	-10	1.0	0.9	0.9	0.9
TUBB6	Q9BUF5	354	1	-12	-6	-14	1.0	0.9	0.9	0.9
TUBB1	Q9H4B7	354	1	-12	-6	-14	1.0	0.9	0.9	0.9
PRKAA1	Q13131	185	1	-12	-6	-20	1.0	0.9	0.9	0.8
ATXN2	Q99700	301	1	-12	-6	1	1.0	0.9	0.9	1.0
CAND1	Q86VP6	413	1	-13	-6	-1	1.0	0.9	0.9	1.0
ACAP1	Q15027	328	1	-13	-6	-6	1.0	0.9	0.9	0.9
DBT	P11182	333	1	-15	-6	-4	1.0	0.9	0.9	1.0
SULT1A3	P0DMM9	70	1	-23	-6	-4	1.0	0.8	0.9	1.0
MACF1	Q9UPN3	5131	1	1	-6	-21	1.0	1.0	0.9	0.8
TRIM25	Q14258	475	1	-4	-6	-8	1.0	1.0	0.9	0.9
ACTN4	O43707	173	1	-4	-6	-1	1.0	1.0	0.9	1.0
ACTN1	P12814	154	1	-4	-6	-1	1.0	1.0	0.9	1.0
HEATR5A	Q86XA9	354	1	-5	-6	-9	1.0	1.0	0.9	0.9
HEATR5B	Q9P2D3	356	1	-5	-6	-9	1.0	1.0	0.9	0.9
DMXL2	Q8TDJ6	920	1	-6	-6	1	1.0	0.9	0.9	1.0
DOCK8	Q8NF50	186	1	-7	-6	-1	1.0	0.9	0.9	1.0
PIK3CD	O00329	366	1	-7	-6	-4	1.0	0.9	0.9	1.0
WWP2	O00308	186	1	-8	-6	-15	1.0	0.9	0.9	0.9
CHD4	Q14839	1594	1	-8	-6	-19	1.0	0.9	0.9	0.8
UBR1	Q8IWV7	180	1	-11	-6	-7	1.0	0.9	0.9	0.9
PRPF6	O94906	604	1	-12	-6	2	1.0	0.9	0.9	1.0
SEC24C	P53992	383	1	-18	-6	-7	1.0	0.8	0.9	0.9
GRIN3A	Q8TCU5	580	1	-22	-6	-12	1.0	0.8	0.9	0.9
COMMD3-BN	R4GMX3	101	1	-26	-6	-8	1.0	0.8	0.9	0.9
MPI	P34949	289	0	-1	-6	0	1.0	1.0	0.9	1.0
PRPF38B	Q5VTL8	113	0	-6	-6	-7	1.0	0.9	0.9	0.9
MILR1	Q7Z6M3	297	0	-7	-6	-18	1.0	0.9	0.9	0.9
UBE2O	Q9C0C9	341	0	-8	-6	-4	1.0	0.9	0.9	1.0
RPS6KA3	P51812	229	0	-9	-6	-8	1.0	0.9	0.9	0.9
RPS6KA1	Q15418	223	0	-9	-6	-8	1.0	0.9	0.9	0.9
DNAJC13	O75165	2181	0	-10	-6	-3	1.0	0.9	0.9	1.0

PSMB2	P49721	91	0	-11	-6	-7	1.0	0.9	0.9	0.9
FHOD1	Q9Y613	31	0	-13	-6	-18	1.0	0.9	0.9	0.8
TBC1D4	O60343	74	0	-16	-6	-11	1.0	0.9	0.9	0.9
IPO7	O95373	736	0	-19	-6	-8	1.0	0.8	0.9	0.9
CIAO1	O76071	234	-1	1	-6	-3	1.0	1.0	0.9	1.0
LRRC59	Q96AG4	131	-1	-1	-6	16	1.0	1.0	0.9	1.2
EVI2B	P34910	253	-1	-2	-6	-8	1.0	1.0	0.9	0.9
BTAF1	O14981	914	-1	-13	-6	-1	1.0	0.9	0.9	1.0
Uncharacteri	H0Y980	125	-1	-19	-6	-5	1.0	0.8	0.9	1.0
SAMD9	Q5K651	1364	-1	-20	-6	-10	1.0	0.8	0.9	0.9
FABP5	Q01469	120	-1	-22	-6	-11	1.0	0.8	0.9	0.9
PCM1	Q15154	582	-1	-3	-6	3	1.0	1.0	0.9	1.0
DNMT1	P26358	896	-1	-8	-6	-3	1.0	0.9	0.9	1.0
HTATIP2	Q9BUP3	172	-1	-9	-6	-8	1.0	0.9	0.9	0.9
ZMYM3	Q14202	633	-1	-9	-6	-15	1.0	0.9	0.9	0.9
ATG7	O95352	368	-1	-10	-6	4	1.0	0.9	0.9	1.0
ARRB1	P49407	150	-1	-12	-6	-5	1.0	0.9	0.9	1.0
GLG1	Q92896	235	-1	-15	-6	-1	1.0	0.9	0.9	1.0
EIF4A3	P38919	137	-2	-2	-6	2	1.0	1.0	0.9	1.0
PDS5A	Q29RF7	14	-2	-3	-6	-8	1.0	1.0	0.9	0.9
HGH1	Q9BTY7	51	-2	-3	-6	-8	1.0	1.0	0.9	0.9
EEFSEC	P57772	442	-2	-6	-6	-4	1.0	0.9	0.9	1.0
SUGT1	Q9Y2Z0	62	-2	-7	-6	-8	1.0	0.9	0.9	0.9
ILK	Q13418	346	-2	-8	-6	-4	1.0	0.9	0.9	1.0
PLEKHF1	Q96S99	21	-2	-9	-6	-10	1.0	0.9	0.9	0.9
COMT	P21964	223	-2	-9	-6	-10	1.0	0.9	0.9	0.9
CDC42EP3	Q9UKI2	145	-2	-9	-6	-19	1.0	0.9	0.9	0.8
APAF1	O14727	258	-2	-12	-6	-13	1.0	0.9	0.9	0.9
SNX8	Q9Y5X2	200	-2	-12	-6	-8	1.0	0.9	0.9	0.9
SMTN	P53814	410	-2	-12	-6	-11	1.0	0.9	0.9	0.9
ZMYM3	Q14202	484	-2	-13	-6	-8	1.0	0.9	0.9	0.9
WDR7	Q9Y4E6	234	-2	-13	-6	-8	1.0	0.9	0.9	0.9
TLN1	Q9Y490	1953	-2	-14	-6	-6	1.0	0.9	0.9	0.9
SKIV2L	Q15477	579	-2	-14	-6	-10	1.0	0.9	0.9	0.9
ORC3	Q9UBD5	158	-2	-26	-6	18	1.0	0.8	0.9	1.2
INTS9	Q9NV88	578	-2	-2	-6	-13	1.0	1.0	0.9	0.9
FMNL1	O95466	45	-2	-3	-6	-21	1.0	1.0	0.9	0.8
PARP1	P09874	845	-2	-6	-6	-3	1.0	0.9	0.9	1.0
UTRN	P46939	1627	-2	-7	-6	-8	1.0	0.9	0.9	0.9
FLNA	P21333	574	-2	-7	-6	-9	1.0	0.9	0.9	0.9
GAPDH	P04406	152	-2	-8	-6	-4	1.0	0.9	0.9	1.0
HNRNPA0	Q13151	49	-2	-9	-6	3	1.0	0.9	0.9	1.0
SEC24C	P53992	1083	-2	-9	-6	-4	1.0	0.9	0.9	1.0
MTOR	P42345	300	-2	-10	-6	-5	1.0	0.9	0.9	1.0
EDC4	Q6P2E9	838	-2	-11	-6	-14	1.0	0.9	0.9	0.9
YWHAQ	P27348	134	-2	-13	-6	-7	1.0	0.9	0.9	0.9
TARS	P26639	107	-2	-23	-6	-14	1.0	0.8	0.9	0.9
TIGAR	Q9NQ88	114	-3	-6	-6	-5	1.0	0.9	0.9	1.0
RPL4	P36578	208	-3	-12	-6	3	1.0	0.9	0.9	1.0
LRRC16A	Q5VZK9	51	-3	-12	-6	-29	1.0	0.9	0.9	0.8
SMARCC2	Q8TAQ2	495	-3	-13	-6	-7	1.0	0.9	0.9	0.9
COPG1	Q9Y678	706	-3	-14	-6	8	1.0	0.9	0.9	1.1
ASPSCR1	Q9BZE9	174	-3	-14	-6	4	1.0	0.9	0.9	1.0

ETF1	P62495	335	-3	-17	-6	-5	1.0	0.9	0.9	1.0
CEP350	Q5VT06	413	-3	-19	-6	-17	1.0	0.8	0.9	0.9
USP34	Q70CQ2	1486	-3	-40	-6	-18	1.0	0.7	0.9	0.9
PUS7L	Q9H0K6	640	-3	-2	-6	-1	1.0	1.0	0.9	1.0
URI1	O94763	44	-3	-4	-6	-18	1.0	1.0	0.9	0.8
CCDC88B	A6NC98	1222	-3	-7	-6	-5	1.0	0.9	0.9	1.0
TRAPPC8	Q9Y2L5	978	-3	-8	-6	3	1.0	0.9	0.9	1.0
SORBS3	O60504	482	-3	-9	-6	1	1.0	0.9	0.9	1.0
SIPA1	Q96FS4	755	-3	-9	-6	-6	1.0	0.9	0.9	0.9
AHNAK	Q09666	1900	-3	-9	-6	-23	1.0	0.9	0.9	0.8
PYROXD1	Q8WU10	122	-3	-9	-6	-5	1.0	0.9	0.9	1.0
STAT3	P40763	259	-3	-10	-6	-9	1.0	0.9	0.9	0.9
POLR2B	P30876	837	-3	-12	-6	-10	1.0	0.9	0.9	0.9
RPS23	P62266	90	-3	-13	-6	-22	1.0	0.9	0.9	0.8
ANXA2	P07355	262	-3	-16	-6	0	1.0	0.9	0.9	1.0
WDR1	O75083	438	-3	-17	-6	-6	1.0	0.9	0.9	0.9
CTNNA1	P35221	116	-4	-4	-6	-17	1.0	1.0	0.9	0.9
NCAPH	Q15003	255	-4	-7	-6	-8	1.0	0.9	0.9	0.9
WDR6	Q9NNW5	688	-4	-8	-6	-10	1.0	0.9	0.9	0.9
DICER1	Q9UPY3	196	-4	-9	-6	-5	1.0	0.9	0.9	1.0
CTBP1	Q13363	237	-4	-11	-6	-5	1.0	0.9	0.9	1.0
GIT1	Q9Y2X7	576	-4	-12	-6	-14	1.0	0.9	0.9	0.9
QTRTD1	Q9H974	324	-4	-12	-6	-8	1.0	0.9	0.9	0.9
MACF1	Q9UPN3	5346	-4	-13	-6	-16	1.0	0.9	0.9	0.9
LRRC57	Q8N9N7	128	-4	-13	-6	-7	1.0	0.9	0.9	0.9
FAM129B	Q96TA1	154	-4	-14	-6	-1	1.0	0.9	0.9	1.0
FLNA	P21333	2582	-4	-16	-6	-2	1.0	0.9	0.9	1.0
TRIP12	Q14669	1411	-4	-16	-6	-15	1.0	0.9	0.9	0.9
CHD6	Q8TD26	1307	-4	-16	-6	-9	1.0	0.9	0.9	0.9
CHD8	Q9HCK8	1655	-4	-16	-6	-9	1.0	0.9	0.9	0.9
TRIM28	Q13263	229	-4	-17	-6	-5	1.0	0.9	0.9	1.0
TANGO2	Q6ICL3	228	-4	-22	-6	-3	1.0	0.8	0.9	1.0
AGO3	Q9H9G7	58	-4	-23	-6	-7	1.0	0.8	0.9	0.9
CTSS	P25774	126	-4	-30	-6	-3	1.0	0.8	0.9	1.0
DUSP6	Q16828	218	-4	-3	-6	-3	1.0	1.0	0.9	1.0
TTLL12	Q14166	528	-4	-8	-6	26	1.0	0.9	0.9	1.3
FRMD4B	Q9Y2L6	527	-4	-10	-6	-9	1.0	0.9	0.9	0.9
SOS2	Q07890	403	-4	-13	-6	3	1.0	0.9	0.9	1.0
BCAT2	O15382	342	-4	-13	-6	2	1.0	0.9	0.9	1.0
FAM160B1	Q5W0V3	306	-4	-13	-6	3	1.0	0.9	0.9	1.0
SRM	P19623	209	-4	-18	-6	1	1.0	0.9	0.9	1.0
TRANK1	O15050	1319	-5	3	-6	-4	1.0	1.0	0.9	1.0
NUP50	Q9UKX7	165	-5	-6	-6	-3	1.0	0.9	0.9	1.0
PIK3CA	P42336	242	-5	-6	-6	-4	1.0	0.9	0.9	1.0
RPS8	P62241	72	-5	-7	-6	-1	1.0	0.9	0.9	1.0
CTSD	P07339	329	-5	-8	-6	-12	1.0	0.9	0.9	0.9
ROCK2	O75116	649	-5	-10	-6	-10	1.0	0.9	0.9	0.9
COPB2	P35606	190	-5	-19	-6	1	1.0	0.8	0.9	1.0
BPNT1	O95861	42	-5	-23	-6	-12	1.0	0.8	0.9	0.9
DOCK11	Q5JSL3	1204	-5	-2	-6	-3	1.0	1.0	0.9	1.0
PARP14	Q460N5	1455	-5	-10	-6	-10	1.0	0.9	0.9	0.9
ALB	P02768	416	-5	-11	-6	7	1.0	0.9	0.9	1.1
CORO1C	Q9ULV4	330	-5	-12	-6	-8	1.0	0.9	0.9	0.9

SKIV2L2	P42285	650	-5	-12	-6	-6	1.0	0.9	0.9	0.9
AHCTF1	Q8WYP5	1261	-6	-4	-6	-8	0.9	1.0	0.9	0.9
ARMC1	Q9NVT9	69	-6	-6	-6	-7	0.9	0.9	0.9	0.9
TRIM25	Q14258	110	-6	-8	-6	-2	0.9	0.9	0.9	1.0
SF3A1	Q15459	244	-6	-11	-6	-8	0.9	0.9	0.9	0.9
KRAS	P01116	180	-6	-12	-6	-5	0.9	0.9	0.9	1.0
POLR1B	Q9H9Y6	307	-6	-12	-6	-4	0.9	0.9	0.9	1.0
TARS2	Q9BW92	506	-6	-12	-6	-16	0.9	0.9	0.9	0.9
LMNA	P02545	522	-6	-14	-6	-12	0.9	0.9	0.9	0.9
BRAT1	Q6PJG6	326	-6	-16	-6	-7	0.9	0.9	0.9	0.9
AKAP12	Q02952	1479	-6	-13	-6	-25	0.9	0.9	0.9	0.8
MVP	Q14764	59	-6	-17	-6	-2	0.9	0.9	0.9	1.0
DAPK1	P53355	1142	-7	-10	-6	16	0.9	0.9	0.9	1.2
RECQL	P46063	223	-7	-11	-6	-10	0.9	0.9	0.9	0.9
RPS6KC1	Q96S38	905	-7	-12	-6	-9	0.9	0.9	0.9	0.9
PTK2B	Q14289	89	-7	-16	-6	-9	0.9	0.9	0.9	0.9
PTK2B	Q14289	661	-7	-17	-6	3	0.9	0.9	0.9	1.0
PHF5A	Q7RTV0	61	-7	-18	-6	-15	0.9	0.8	0.9	0.9
STK26	Q9P289	254	-7	-19	-6	-10	0.9	0.8	0.9	0.9
CCAR1	Q8IX12	465	-7	-23	-6	-2	0.9	0.8	0.9	1.0
SETD1B	Q9UPS6	1961	-7	-27	-6	-17	0.9	0.8	0.9	0.9
ZC3HAV1	Q7Z2W4	272	-8	-8	-6	-19	0.9	0.9	0.9	0.8
RETSAT	Q6NUM9	547	-8	-8	-6	14	0.9	0.9	0.9	1.2
PRRC2C	Q9Y520	2340	-8	-13	-6	-18	0.9	0.9	0.9	0.8
TRAFD1	O14545	93	-8	-17	-6	-16	0.9	0.9	0.9	0.9
TNFAIP3	P21580	57	-8	-18	-6	-5	0.9	0.9	0.9	1.0
MAML2	Q8IZL2	275	-8	-7	-6	-7	0.9	0.9	0.9	0.9
DOCK10	Q96BY6	2164	-8	-10	-6	-22	0.9	0.9	0.9	0.8
PELO	Q9BRX2	258	-8	-15	-6	-16	0.9	0.9	0.9	0.9
SPAST	Q9UBP0	220	-8	-18	-6	-24	0.9	0.9	0.9	0.8
DDX58	O95786	829	-9	-10	-6	-14	0.9	0.9	0.9	0.9
DPH2	Q9BQC3	251	-9	-16	-6	-5	0.9	0.9	0.9	1.0
COMT	P21964	238	-10	-12	-6	-21	0.9	0.9	0.9	0.8
ZYX	Q15942	384	-10	-12	-6	-8	0.9	0.9	0.9	0.9
TRIM65	Q6PJ69	320	-10	-14	-6	-10	0.9	0.9	0.9	0.9
RBM25	P49756	132	-10	-15	-6	-8	0.9	0.9	0.9	0.9
BLMH	Q13867	164	-10	-18	-6	-3	0.9	0.9	0.9	1.0
GBF1	Q92538	403	-11	-10	-6	4	0.9	0.9	0.9	1.0
MCM3AP	O60318	1269	-11	-22	-6	-12	0.9	0.8	0.9	0.9
PROS1	P07225	113	-11	-14	-6	-24	0.9	0.9	0.9	0.8
LIPE	Q05469	1033	-11	-27	-6	16	0.9	0.8	0.9	1.2
PHF14	O94880	463	-12	-24	-6	-23	0.9	0.8	0.9	0.8
GTF3C2	Q8WUA4	622	-12	-1	-6	-9	0.9	1.0	0.9	0.9
RADIL	Q96JH8	699	-12	-9	-6	-6	0.9	0.9	0.9	0.9
FAF2	Q96CS3	189	-13	-8	-6	1	0.9	0.9	0.9	1.0
ACAD9	Q9H845	271	-14	-11	-6	6	0.9	0.9	0.9	1.1
VPS41	P49754	638	-16	-7	-6	5	0.9	0.9	0.9	1.0
BOD1L1	Q8NFC6	2599	-16	-19	-6	-28	0.9	0.8	0.9	0.8
AP5B1	Q2VPB7	800	-17	-17	-6	9	0.9	0.9	0.9	1.1
SIPA1L1	O43166	105	-18	-15	-6	-20	0.9	0.9	0.9	0.8
PRF1	P14222	408	-19	-1	-6	2	0.8	1.0	0.9	1.0
ELP5	Q8TE02	135	-23	0	-6	-13	0.8	1.0	0.9	0.9
PARP10	Q53GL7	981	-25	-14	-6	-16	0.8	0.9	0.9	0.9

CHD3	Q12873	1838	-25	-53	-6	-53	0.8	0.7	0.9	0.7
PTGDS	P41222	65	-65	-50	-6	4	0.6	0.7	0.9	1.0
TBCB	Q99426	51	21	1	-6	7	1.3	1.0	0.9	1.1
AAAS	Q9NRG9	153	20	24	-6	1	1.3	1.3	0.9	1.0
NSMCE2	Q96MF7	169	19	8	-6	7	1.2	1.1	0.9	1.1
SLA	Q13239	76	19	-7	-6	4	1.2	0.9	0.9	1.0
MTMR3	Q13615	564	18	-29	-6	-3	1.2	0.8	0.9	1.0
RTFDC1	Q9BY42	262	16	-10	-6	4	1.2	0.9	0.9	1.0
POLR2B	P30876	224	16	-16	-6	-3	1.2	0.9	0.9	1.0
LTA4H	P09960	26	16	-28	-6	-1	1.2	0.8	0.9	1.0
RAB44	Q7Z6P3	584	15	-1	-6	5	1.2	1.0	0.9	1.0
ADAMTSL3	P82987	1499	15	-16	-6	-3	1.2	0.9	0.9	1.0
CCT7	Q99832	450	15	-12	-6	0	1.2	0.9	0.9	1.0
ACO2	Q99798	385	14	-15	-6	-3	1.2	0.9	0.9	1.0
LPCAT2	Q7L5N7	223	14	-7	-6	-3	1.2	0.9	0.9	1.0
MED1	Q15648	443	13	-5	-6	1	1.1	1.0	0.9	1.0
CNOT1	A5YKK6	890	13	-8	-6	11	1.1	0.9	0.9	1.1
TNPO2	O14787	287	13	-16	-6	18	1.1	0.9	0.9	1.2
DYSF	O75923	933	12	0	-6	-12	1.1	1.0	0.9	0.9
FLII	Q13045	1067	12	-24	-6	-7	1.1	0.8	0.9	0.9
RSF1	Q96T23	1311	12	-24	-6	-18	1.1	0.8	0.9	0.8
ATP1A1	P05023	428	12	-47	-6	5	1.1	0.7	0.9	1.0
GDI1	P31150	317	12	-14	-6	1	1.1	0.9	0.9	1.0
GDI2	P50395	317	12	-14	-6	1	1.1	0.9	0.9	1.0
FHL1	Q13642	185	11	-18	-6	-5	1.1	0.9	0.9	1.0
DDX51	Q8N8A6	419	11	-1	-6	-19	1.1	1.0	0.9	0.8
CLTC	Q00610	934	11	-3	-6	-2	1.1	1.0	0.9	1.0
TPT1	P13693	172	11	-19	-6	6	1.1	0.8	0.9	1.1
SCAF4	O95104	102	10	-6	-6	-5	1.1	0.9	0.9	1.0
PMPCB	O75439	419	10	-18	-6	0	1.1	0.9	0.9	1.0
GIMAP2	Q9UG22	175	10	-50	-6	-2	1.1	0.7	0.9	1.0
MACF1	Q9UPN3	1025	10	-24	-6	-15	1.1	0.8	0.9	0.9
DGKZ	Q13574	713	9	3	-6	10	1.1	1.0	0.9	1.1
CHD4	Q14839	493	9	-10	-6	-11	1.1	0.9	0.9	0.9
STIP1	P31948	420	9	-13	-6	-6	1.1	0.9	0.9	0.9
FBXL15	Q9H469	139	9	-7	-6	3	1.1	0.9	0.9	1.0
YWHAH	Q04917	112	9	-10	-6	-6	1.1	0.9	0.9	0.9
STRN4	Q9NRL3	713	9	-20	-6	-2	1.1	0.8	0.9	1.0
PLEC	Q15149	317	8	-9	-6	2	1.1	0.9	0.9	1.0
GCFC2	P16383	320	8	-12	-6	-6	1.1	0.9	0.9	0.9
PLCG2	P16885	937	8	-17	-6	-14	1.1	0.9	0.9	0.9
SAFB	Q15424	448	8	2	-6	-4	1.1	1.0	0.9	1.0
GAR1	Q9NY12	86	8	1	-6	-2	1.1	1.0	0.9	1.0
AGO3	Q9H9G7	493	8	-1	-6	-2	1.1	1.0	0.9	1.0
AGO2	Q9UKV8	492	8	-1	-6	-2	1.1	1.0	0.9	1.0
AGO1	Q9UL18	490	8	-1	-6	-2	1.1	1.0	0.9	1.0
PARP1	P09874	298	8	-12	-6	8	1.1	0.9	0.9	1.1
DIAPH1	O60610	314	8	-13	-6	-3	1.1	0.9	0.9	1.0
PREX1	Q8TCU6	1173	7	3	-6	-8	1.1	1.0	0.9	0.9
FCHO1	O14526	371	7	-6	-6	-7	1.1	0.9	0.9	0.9
IMPDH2	P12268	173	7	-9	-6	-2	1.1	0.9	0.9	1.0
BAG5	Q9UL15	327	7	-12	-6	-4	1.1	0.9	0.9	1.0
CLNS1A	P54105	73	7	2	-6	-8	1.1	1.0	0.9	0.9

TRIM28	Q13263	224	7	-2	-6	0	1.1	1.0	0.9	1.0
DDX55	Q8NHQ9	437	7	-2	-6	-6	1.1	1.0	0.9	0.9
NUDCD1	Q96RS6	501	7	-15	-6	-3	1.1	0.9	0.9	1.0
WDR41	Q9HAD4	99	6	-1	-6	-10	1.1	1.0	0.9	0.9
RANBP2	P49792	206	6	-2	-6	2	1.1	1.0	0.9	1.0
PPP1R17	O96001	24	6	-4	-6	-3	1.1	1.0	0.9	1.0
SUCLG1	P53597	172	6	-5	-6	-3	1.1	1.0	0.9	1.0
GIMAP1-GIM	A0A087WTJ2	375	6	-7	-6	-8	1.1	0.9	0.9	0.9
PCK2	Q16822	63	6	-16	-6	-13	1.1	0.9	0.9	0.9
RABGGTA	Q92696	114	6	-19	-6	-7	1.1	0.8	0.9	0.9
COPB2	P35606	741	6	-23	-6	0	1.1	0.8	0.9	1.0
NLK	Q9UBE8	369	6	-4	-6	4	1.1	1.0	0.9	1.0
CEP192	Q8TEP8	1805	6	-6	-6	-12	1.1	0.9	0.9	0.9
ZNF561	Q8N587	17	6	-7	-6	-15	1.1	0.9	0.9	0.9
PKM	P14618	49	6	-8	-6	5	1.1	0.9	0.9	1.1
MICU1	Q9BPX6	275	6	-10	-6	12	1.1	0.9	0.9	1.1
YES1	P07947	410	6	-11	-6	-1	1.1	0.9	0.9	1.0
SRC	P12931	403	6	-11	-6	-1	1.1	0.9	0.9	1.0
SKIV2L2	P42285	1008	6	-18	-6	8	1.1	0.9	0.9	1.1
EML2	O95834	163	6	-21	-6	2	1.1	0.8	0.9	1.0
TMEM55B	Q86T03	127	5	-5	-6	4	1.1	1.0	0.9	1.0
TMEM55A	Q8N4L2	101	5	-5	-6	4	1.1	1.0	0.9	1.0
PRDX6	P30041	47	5	-7	-6	1	1.1	0.9	0.9	1.0
RHOF	Q9HBH0	162	5	-9	-6	0	1.1	0.9	0.9	1.0
GUK1	Q16774	98	5	-14	-6	-4	1.1	0.9	0.9	1.0
PRKDC	P78527	3683	5	-30	-6	-1	1.1	0.8	0.9	1.0
SP140	Q13342	792	5	1	-6	-5	1.0	1.0	0.9	1.0
SP140L	Q9H930	505	5	1	-6	-5	1.0	1.0	0.9	1.0
RBM4B	Q9BQ04	175	5	-3	-6	7	1.0	1.0	0.9	1.1
UBE3B	Q7Z3V4	75	5	-7	-6	-9	1.0	0.9	0.9	0.9
BOD1L1	Q8NFC6	686	5	-8	-6	-16	1.0	0.9	0.9	0.9
UTP20	O75691	2571	5	-8	-6	-4	1.0	0.9	0.9	1.0
HNRNPH1	P31943	122	5	-9	-6	1	1.0	0.9	0.9	1.0
HNRNPH2	P55795	122	5	-9	-6	1	1.0	0.9	0.9	1.0
PTPN23	Q9H3S7	1466	5	-9	-6	-19	1.0	0.9	0.9	0.8
LARS	Q9P2J5	546	5	-9	-6	-1	1.0	0.9	0.9	1.0
ANKRD13D	Q6ZTN6	160	5	-9	-6	-13	1.0	0.9	0.9	0.9
AKR1A1	P14550	200	5	-11	-6	2	1.0	0.9	0.9	1.0
FBXW11	Q9UKB1	484	5	-16	-6	11	1.0	0.9	0.9	1.1
GNB4	Q9HAV0	204	5	-17	-6	1	1.0	0.9	0.9	1.0
RAB37	Q96AX2	44	5	-17	-6	-5	1.0	0.9	0.9	1.0
FTSJ1	Q9UET6	295	5	-17	-6	-8	1.0	0.9	0.9	0.9
WDR11	Q9BZH6	297	5	-20	-6	0	1.0	0.8	0.9	1.0
FAM65B	Q9Y4F9	35	4	-8	-6	-15	1.0	0.9	0.9	0.9
DDX55	Q8NHQ9	389	4	-11	-6	0	1.0	0.9	0.9	1.0
NSUN2	Q08J23	221	4	-13	-6	-11	1.0	0.9	0.9	0.9
GLE1	Q53GS7	131	4	-14	-6	-23	1.0	0.9	0.9	0.8
STXBP5	Q5T5C0	933	4	-5	-6	-4	1.0	1.0	0.9	1.0
TMPO	P42166	341	4	-6	-6	-10	1.0	0.9	0.9	0.9
WAC	Q9BTA9	150	4	-7	-6	-10	1.0	0.9	0.9	0.9
CD44	P16070	77	4	-7	-6	-11	1.0	0.9	0.9	0.9
HSPE1-MOB4	S4R3N1	170	4	-8	-6	1	1.0	0.9	0.9	1.0
C4orf27	Q9NWY4	152	4	-10	-6	-11	1.0	0.9	0.9	0.9

SVIL	Q95425	671	4	-11	-6	-12	1.0	0.9	0.9	0.9
ILVBL	A1L0T0	354	4	-12	-6	0	1.0	0.9	0.9	1.0
KATNB1	Q9BVA0	250	4	-16	-6	-2	1.0	0.9	0.9	1.0
EIF2S2	P20042	281	4	-17	-6	-8	1.0	0.9	0.9	0.9
PFKFB3	Q16875	155	3	-1	-6	-8	1.0	1.0	0.9	0.9
PAN3	Q58A45	519	3	-5	-6	3	1.0	1.0	0.9	1.0
SNW1	Q13573	250	3	-6	-6	-5	1.0	0.9	0.9	1.0
THOC2	Q8NI27	1044	3	-8	-6	-4	1.0	0.9	0.9	1.0
VPS18	Q9P253	421	3	-9	-6	-5	1.0	0.9	0.9	1.0
LRRK2	Q5S007	749	3	-10	-6	-15	1.0	0.9	0.9	0.9
FLII	Q13045	576	3	-10	-6	3	1.0	0.9	0.9	1.0
PPM1A	P35813	314	3	-10	-6	-8	1.0	0.9	0.9	0.9
VPS13A	Q96RL7	1848	3	-12	-6	-10	1.0	0.9	0.9	0.9
ANKRD27	Q96NW4	39	3	-13	-6	-11	1.0	0.9	0.9	0.9
HAUS4	Q9H6D7	213	3	-24	-6	-13	1.0	0.8	0.9	0.9
MTG2	Q9H4K7	206	3	-6	-6	-1	1.0	0.9	0.9	1.0
EEF1A1	P68104	411	3	-6	-6	-2	1.0	0.9	0.9	1.0
PTPRE	P23469	287	3	-6	-6	-10	1.0	0.9	0.9	0.9
HSPA4	P34932	146	3	-7	-6	3	1.0	0.9	0.9	1.0
RARA	P10276	265	3	-8	-6	-13	1.0	0.9	0.9	0.9
SCAF11	Q99590	506	3	-9	-6	-10	1.0	0.9	0.9	0.9
PLEC	Q15149	3821	3	-10	-6	-7	1.0	0.9	0.9	0.9
TRIP12	Q14669	332	3	-11	-6	-16	1.0	0.9	0.9	0.9
TRIM22	Q8IYM9	140	3	-12	-6	-8	1.0	0.9	0.9	0.9
FCHO1	O14526	571	3	-12	-6	-10	1.0	0.9	0.9	0.9
ANAPC7	Q9UJX3	131	3	-13	-6	-10	1.0	0.9	0.9	0.9
GPATCH11	Q8N954	69	3	-13	-6	-12	1.0	0.9	0.9	0.9
TKT	P29401	206	3	-14	-6	-3	1.0	0.9	0.9	1.0
PPP2CB	P62714	266	3	-24	-6	4	1.0	0.8	0.9	1.0
PPP2CA	P67775	266	3	-24	-6	4	1.0	0.8	0.9	1.0
KDM1B	Q8NB78	278	2	1	-6	-4	1.0	1.0	0.9	1.0
ZNF512	Q96ME7	192	2	-3	-6	4	1.0	1.0	0.9	1.0
VPS13C	Q709C8	2925	2	-3	-6	-1	1.0	1.0	0.9	1.0
KLK8	O60259	173	2	-4	-6	10	1.0	1.0	0.9	1.1
DTX3L	Q8TDB6	250	2	-4	-6	2	1.0	1.0	0.9	1.0
MDN1	Q9NU22	2158	2	-4	-6	-3	1.0	1.0	0.9	1.0
MED23	Q9ULK4	238	2	-5	-6	2	1.0	1.0	0.9	1.0
FAAP100	Q0VG06	15	2	-5	-6	-6	1.0	1.0	0.9	0.9
THEMIS2	Q5TEJ8	453	2	-6	-6	6	1.0	0.9	0.9	1.1
VPS13C	Q709C8	2184	2	-7	-6	-7	1.0	0.9	0.9	0.9
ALDH9A1	P49189	267	2	-8	-6	1	1.0	0.9	0.9	1.0
HSPA1B	P0DMV9	17	2	-11	-6	0	1.0	0.9	0.9	1.0
GMIP	Q9P107	958	2	-11	-6	-10	1.0	0.9	0.9	0.9
MPO	P05164	180	2	-12	-6	-10	1.0	0.9	0.9	0.9
POLR3E	Q9NVU0	676	2	-17	-6	-18	1.0	0.9	0.9	0.8
SEC24C	P53992	635	2	-19	-6	-6	1.0	0.8	0.9	0.9
RAB3D	O95716	184	2	-23	-6	-3	1.0	0.8	0.9	1.0
SPTBN1	Q01082	2262	2	-34	-6	-6	1.0	0.7	0.9	0.9
ESYT2	A0FGR8	611	2	-6	-6	-2	1.0	0.9	0.9	1.0
SP110	Q9HB58	283	2	-7	-6	-5	1.0	0.9	0.9	1.0
POLR3A	O14802	960	2	-7	-6	-6	1.0	0.9	0.9	0.9
NUP88	Q99567	713	2	-8	-6	-11	1.0	0.9	0.9	0.9
MCM6	Q14566	91	2	-9	-6	-38	1.0	0.9	0.9	0.7

MICAL1	Q8TDZ2	837	2	-10	-6	-15	1.0	0.9	0.9	0.9
DFFB	O76075	306	2	-11	-6	13	1.0	0.9	0.9	1.1
CCT8	P50990	36	2	-13	-6	-15	1.0	0.9	0.9	0.9
RENBP	P51606	390	2	-18	-6	-9	1.0	0.8	0.9	0.9
ANK3	Q12955	4321	1	6	-6	-6	1.0	1.1	0.9	0.9
XPOT	O43592	522	1	-3	-6	-8	1.0	1.0	0.9	0.9
DNAJC7	Q99615	58	1	-4	-6	7	1.0	1.0	0.9	1.1
TCEA1	P23193	263	1	-5	-6	-9	1.0	1.0	0.9	0.9
TCEA2	Q15560	261	1	-5	-6	-9	1.0	1.0	0.9	0.9
HLA-DRB1	P04229	108	1	-9	-6	-9	1.0	0.9	0.9	0.9
PHC3	Q8NDX5	816	1	-12	-6	-16	1.0	0.9	0.9	0.9
APEX1	P27695	99	1	-12	-6	0	1.0	0.9	0.9	1.0
ANP32B	Q92688	123	1	-15	-6	8	1.0	0.9	0.9	1.1
AKAP11	Q9UKA4	1232	1	7	-6	-8	1.0	1.1	0.9	0.9
FBXO7	Q9Y3I1	36	1	1	-6	-10	1.0	1.0	0.9	0.9
TRAPPC10	P48553	1130	1	1	-6	-11	1.0	1.0	0.9	0.9
MAPK9	P45984	177	1	-3	-6	-11	1.0	1.0	0.9	0.9
RTFDC1	Q9BY42	121	1	-9	-6	-13	1.0	0.9	0.9	0.9
C9orf142	Q9BUH6	180	1	-9	-6	-11	1.0	0.9	0.9	0.9
DOCK8	Q8NF50	2091	1	-9	-6	-15	1.0	0.9	0.9	0.9
HADHA	P40939	322	1	-10	-6	-3	1.0	0.9	0.9	1.0
PPM1M	Q96MI6	9	1	-12	-6	-10	1.0	0.9	0.9	0.9
PYCRL	Q53H96	49	1	-12	-6	-13	1.0	0.9	0.9	0.9
NUMA1	Q14980	375	1	-13	-6	-10	1.0	0.9	0.9	0.9
ARPC1B	O15143	70	1	-14	-6	-3	1.0	0.9	0.9	1.0
FGR	P09769	127	1	-17	-6	-5	1.0	0.9	0.9	1.0
CYBB	P04839	537	1	-18	-6	-12	1.0	0.8	0.9	0.9
APOBEC3C	Q9NRW3	76	1	-22	-6	-5	1.0	0.8	0.9	1.0
CAPG	P40121	77	1	-24	-6	-4	1.0	0.8	0.9	1.0
YEATS4	O95619	210	0	-3	-6	3	1.0	1.0	0.9	1.0
RAB21	Q9UL25	29	0	-3	-6	1	1.0	1.0	0.9	1.0
DMTN	Q08495	336	0	-5	-6	-13	1.0	1.0	0.9	0.9
POTEI	P0CG38	965	0	-5	-6	-3	1.0	1.0	0.9	1.0
POTEE	Q6S8J3	965	0	-5	-6	-3	1.0	1.0	0.9	1.0
KDM3A	Q9Y4C1	1140	0	-5	-6	-12	1.0	1.0	0.9	0.9
P4HB	P07237	397	0	-6	-6	-17	1.0	0.9	0.9	0.9
IKBKB	O14920	412	0	-7	-6	-13	1.0	0.9	0.9	0.9
ZCCHC8	Q6NZY4	393	0	-8	-6	-2	1.0	0.9	0.9	1.0
HDAC1	Q13547	408	0	-9	-6	-14	1.0	0.9	0.9	0.9
MCM5	P33992	207	0	-10	-6	-8	1.0	0.9	0.9	0.9
HNRNPU	Q00839	607	0	-10	-6	-8	1.0	0.9	0.9	0.9
TOR4A	Q9NXH8	413	0	-12	-6	-4	1.0	0.9	0.9	1.0
PARP14	Q460N5	1318	0	-12	-6	-9	1.0	0.9	0.9	0.9
HNMT	P50135	82	0	-14	-6	-4	1.0	0.9	0.9	1.0
PSMC6	P62333	83	0	-15	-6	-8	1.0	0.9	0.9	0.9
TMPO	P42166	280	0	-18	-6	-18	1.0	0.8	0.9	0.9
RACK1	P63244	240	0	-23	-6	9	1.0	0.8	0.9	1.1
RNF213	Q63HN8	3008	-1	-8	-6	-14	1.0	0.9	0.9	0.9
SNRPN	P63162	43	-1	-9	-6	11	1.0	0.9	0.9	1.1
NECAP2	Q9NVZ3	133	-1	-10	-6	-8	1.0	0.9	0.9	0.9
CCT2	P78371	535	-1	-10	-6	-15	1.0	0.9	0.9	0.9
HSD17B10	Q99714	214	-1	-11	-6	-12	1.0	0.9	0.9	0.9
KCTD12	Q96CX2	50	-1	-12	-6	-11	1.0	0.9	0.9	0.9

RPLP0	P05388	119	-1	-16	-6	-3	1.0	0.9	0.9	1.0
COG7	P83436	174	-1	-18	-6	4	1.0	0.9	0.9	1.0
CBFB	Q13951	48	-1	-25	-6	6	1.0	0.8	0.9	1.1
MTAP	Q13126	55	-1	-27	-6	-14	1.0	0.8	0.9	0.9
TRRAP	Q9Y4A5	3535	-1	-2	-6	-3	1.0	1.0	0.9	1.0
DNMBP	Q6XZF7	691	-1	-2	-6	-7	1.0	1.0	0.9	0.9
PRPSAP2	O60256	31	-1	-4	-6	-4	1.0	1.0	0.9	1.0
TCEA3	O75764	310	-1	-6	-6	-13	1.0	0.9	0.9	0.9
CNTRL	Q7Z7A1	1784	-1	-8	-6	-11	1.0	0.9	0.9	0.9
VCP	P55072	535	-1	-9	-6	7	1.0	0.9	0.9	1.1
CDKAL1	Q5VV42	138	-1	-9	-6	-13	1.0	0.9	0.9	0.9
RPS11	P62280	60	-1	-11	-6	1	1.0	0.9	0.9	1.0
LRCH1	Q9Y2L9	292	-1	-11	-6	-13	1.0	0.9	0.9	0.9
MRPL39	Q9NYK5	133	-1	-13	-6	-7	1.0	0.9	0.9	0.9
SMCHD1	A6NHR9	1235	-1	-14	-6	-6	1.0	0.9	0.9	0.9
EIF2S2	P20042	309	-1	-15	-6	2	1.0	0.9	0.9	1.0
AHCTF1	Q8WYP5	354	-1	-16	-6	-14	1.0	0.9	0.9	0.9
KDM1A	O60341	573	-1	-18	-6	-10	1.0	0.9	0.9	0.9
MLH1	P40692	142	-1	-23	-6	-19	1.0	0.8	0.9	0.8
POLR2A	P24928	981	-2	2	-6	5	1.0	1.0	0.9	1.1
LRSAM1	Q6UWE0	75	-2	0	-6	-1	1.0	1.0	0.9	1.0
GRIPAP1	Q4V328	744	-2	-5	-6	-6	1.0	1.0	0.9	0.9
USP15	Q9Y4E8	809	-2	-7	-6	5	1.0	0.9	0.9	1.0
TAP1	Q03518	239	-2	-10	-6	-2	1.0	0.9	0.9	1.0
UHRF2	Q96PU4	704	-2	-11	-6	-10	1.0	0.9	0.9	0.9
FOXK1	P85037	665	-2	-12	-6	-10	1.0	0.9	0.9	0.9
ARHGEF6	Q15052	182	-2	-12	-6	-11	1.0	0.9	0.9	0.9
CTBP1	Q13363	232	-2	-24	-6	-8	1.0	0.8	0.9	0.9
EDC4	Q6P2E9	90	-2	-27	-6	-4	1.0	0.8	0.9	1.0
FAM49B	Q9NUQ9	10	-2	-3	-6	-11	1.0	1.0	0.9	0.9
TBC1D2B	Q9UPU7	536	-2	-4	-6	-5	1.0	1.0	0.9	1.0
FCHSD2	O94868	354	-2	-6	-6	-4	1.0	0.9	0.9	1.0
ANXA11	P50995	226	-2	-8	-6	-3	1.0	0.9	0.9	1.0
GIT1	Q9Y2X7	122	-2	-10	-6	-7	1.0	0.9	0.9	0.9
DFFA	O00273	47	-2	-10	-6	-7	1.0	0.9	0.9	0.9
SPAG1	Q07617	657	-3	2	-6	-20	1.0	1.0	0.9	0.8
UBA3	Q8TBC4	139	-3	-4	-6	0	1.0	1.0	0.9	1.0
BTAF1	O14981	109	-3	-6	-6	-8	1.0	0.9	0.9	0.9
ARID1A	O14497	2163	-3	-7	-6	3	1.0	0.9	0.9	1.0
ARID1B	Q8NFD5	2114	-3	-7	-6	3	1.0	0.9	0.9	1.0
PCBP2	Q15366	54	-3	-8	-6	-5	1.0	0.9	0.9	1.0
ARHGEF2	Q92974	306	-3	-9	-6	-6	1.0	0.9	0.9	0.9
GTPBP2	Q9BX10	450	-3	-9	-6	-7	1.0	0.9	0.9	0.9
PHF1	O43189	93	-3	-9	-6	-15	1.0	0.9	0.9	0.9
TIAM1	Q13009	1046	-3	-12	-6	-4	1.0	0.9	0.9	1.0
PIK3R4	Q99570	899	-3	-14	-6	-18	1.0	0.9	0.9	0.8
STAT2	P52630	529	-3	-16	-6	-8	1.0	0.9	0.9	0.9
TRIM25	Q14258	179	-3	-16	-6	-4	1.0	0.9	0.9	1.0
ZNF106	Q9H2Y7	643	-3	-22	-6	-25	1.0	0.8	0.9	0.8
PRKDC	P78527	458	-3	-25	-6	-6	1.0	0.8	0.9	0.9
PPM1B	O75688	172	-3	-8	-6	-21	1.0	0.9	0.9	0.8
PRKDC	P78527	2342	-3	-9	-6	-5	1.0	0.9	0.9	1.0
ZFYVE16	Q7Z3T8	269	-3	-9	-6	-14	1.0	0.9	0.9	0.9

HPRT1	P00492	106	-3	-10	-6	-15	1.0	0.9	0.9	0.9
NFXL1	Q6ZNB6	823	-3	-12	-6	-7	1.0	0.9	0.9	0.9
ZFYVE27	Q5T4F4	165	-3	-13	-6	-1	1.0	0.9	0.9	1.0
RASGRP4	Q8TDF6	590	-3	-13	-6	-14	1.0	0.9	0.9	0.9
PHF23	Q9BUL5	381	-3	-16	-6	-11	1.0	0.9	0.9	0.9
SPTBN1	Q01082	604	-3	-18	-6	-10	1.0	0.8	0.9	0.9
TRMT61A	Q96FX7	209	-4	-1	-6	-5	1.0	1.0	0.9	1.0
NFKB1	P19838	925	-4	-5	-6	-17	1.0	1.0	0.9	0.9
ELMSAN1	Q6PJG2	442	-4	-6	-6	-14	1.0	0.9	0.9	0.9
GATAD2A	Q86YP4	420	-4	-9	-6	-8	1.0	0.9	0.9	0.9
BOP1	Q14137	108	-4	-10	-6	-10	1.0	0.9	0.9	0.9
SMC1A	Q14683	1210	-4	-12	-6	-2	1.0	0.9	0.9	1.0
CHD8	Q9HCK8	1780	-4	-12	-6	-11	1.0	0.9	0.9	0.9
UPF2	Q9HAU5	25	-4	-13	-6	-7	1.0	0.9	0.9	0.9
GMPR2	Q9P2T1	348	-4	-13	-6	-9	1.0	0.9	0.9	0.9
PI4KA	P42356	246	-4	-6	-6	-12	1.0	0.9	0.9	0.9
NR2C2	P49116	159	-4	-6	-6	3	1.0	0.9	0.9	1.0
PGLS	O95336	32	-4	-10	-6	-15	1.0	0.9	0.9	0.9
RRP8	O43159	320	-4	-13	-6	-9	1.0	0.9	0.9	0.9
SEC23A	Q15436	444	-4	-16	-6	-4	1.0	0.9	0.9	1.0
CMAS	Q8NFW8	394	-4	-23	-6	-17	1.0	0.8	0.9	0.9
PSMD9	O00233	59	-5	-7	-6	-14	1.0	0.9	0.9	0.9
UHRF2	Q96PU4	671	-5	-9	-6	-20	1.0	0.9	0.9	0.8
MDN1	Q9NU22	3308	-5	-13	-6	-11	1.0	0.9	0.9	0.9
RGS14	O43566	496	-5	-13	-6	-18	1.0	0.9	0.9	0.8
TRAF6	Q9Y4K3	349	-5	-14	-6	-2	1.0	0.9	0.9	1.0
CSRP2BP	Q9H8E8	478	-5	-18	-6	8	1.0	0.8	0.9	1.1
COG8	Q96MW5	595	-5	-19	-6	-39	1.0	0.8	0.9	0.7
AGO3	Q9H9G7	627	-5	-21	-6	-8	1.0	0.8	0.9	0.9
AGO2	Q9UKV8	626	-5	-21	-6	-8	1.0	0.8	0.9	0.9
AGO1	Q9UL18	624	-5	-21	-6	-8	1.0	0.8	0.9	0.9
UBE3A	Q05086	843	-5	0	-6	-1	1.0	1.0	0.9	1.0
ACAA1	P09110	177	-5	-5	-6	-11	1.0	1.0	0.9	0.9
IAH1	Q2TAA2	150	-5	-7	-6	-13	1.0	0.9	0.9	0.9
DGKZ	Q13574	265	-5	-8	-6	-12	1.0	0.9	0.9	0.9
DLD	P09622	80	-5	-14	-6	-10	1.0	0.9	0.9	0.9
TRIM33	Q9UPN9	291	-5	-18	-6	-17	1.0	0.8	0.9	0.9
POLR3E	Q9NVU0	378	-5	-19	-6	-11	1.0	0.8	0.9	0.9
UBA6	A0AVT1	347	-6	-4	-6	-8	0.9	1.0	0.9	0.9
MRPL33	O75394	29	-6	-6	-6	-13	0.9	0.9	0.9	0.9
KDM5C	P41229	1047	-6	-2	-6	-3	0.9	1.0	0.9	1.0
ZNF136	P52737	291	-6	-9	-6	-18	0.9	0.9	0.9	0.9
DLD	P09622	69	-6	-10	-6	3	0.9	0.9	0.9	1.0
SIPA1	Q96FS4	446	-6	-10	-6	-2	0.9	0.9	0.9	1.0
PYCRL	Q53H96	266	-6	-10	-6	-16	0.9	0.9	0.9	0.9
CAPZB	P47756	206	-6	-10	-6	-19	0.9	0.9	0.9	0.8
FNBP1	Q96RU3	511	-6	-15	-6	-18	0.9	0.9	0.9	0.8
NCBP1	Q09161	44	-6	-18	-6	-18	0.9	0.9	0.9	0.8
IL16	Q14005	1011	-7	-6	-6	-15	0.9	0.9	0.9	0.9
MTHFD1L	Q6UB35	906	-7	-8	-6	-7	0.9	0.9	0.9	0.9
RNPC3	Q96LT9	420	-7	-9	-6	-11	0.9	0.9	0.9	0.9
LARS2	Q15031	167	-7	-13	-6	-8	0.9	0.9	0.9	0.9
SASH3	O75995	351	-7	4	-6	-13	0.9	1.0	0.9	0.9

WDR73	Q6P4I2	106	-7	-9	-6	-22	0.9	0.9	0.9	0.8
ITPKC	Q96DU7	434	-7	-12	-6	-17	0.9	0.9	0.9	0.9
PPP2R1B	P30154	306	-7	-13	-6	-8	0.9	0.9	0.9	0.9
MIF4GD	A9UHW6	49	-7	-14	-6	-12	0.9	0.9	0.9	0.9
FGA	P02671	47	-8	-1	-6	-18	0.9	1.0	0.9	0.8
TMEM53	Q6P2H8	46	-8	-12	-6	-12	0.9	0.9	0.9	0.9
CARMIL2	Q6F5E8	1339	-8	-25	-6	-6	0.9	0.8	0.9	0.9
MYO9A	B2RTY4	81	-8	-27	-6	-5	0.9	0.8	0.9	1.0
LRRK2	Q5S007	2024	-8	-6	-6	1	0.9	0.9	0.9	1.0
CLIC5	Q9NZA1	191	-8	-9	-6	-7	0.9	0.9	0.9	0.9
ARHGEF6	Q15052	119	-8	-10	-6	-18	0.9	0.9	0.9	0.8
MYLK	Q15746	83	-8	-11	-6	-7	0.9	0.9	0.9	0.9
PPP6R3	Q5H9R7	605	-9	3	-6	20	0.9	1.0	0.9	1.3
TRPV2	Q9Y5S1	281	-9	-15	-6	2	0.9	0.9	0.9	1.0
EMD	P50402	147	-9	-16	-6	-13	0.9	0.9	0.9	0.9
RBCK1	Q9BYM8	323	-9	-22	-6	-23	0.9	0.8	0.9	0.8
TRRAP	Q9Y4A5	2691	-9	-5	-6	-14	0.9	1.0	0.9	0.9
APOBEC3G	Q9HC16	261	-9	-7	-6	15	0.9	0.9	0.9	1.2
RPP40	O75818	219	-9	-26	-6	0	0.9	0.8	0.9	1.0
VARS2	A0A0G2JL52	260	-10	-5	-6	-27	0.9	1.0	0.9	0.8
WDR48	Q8TAF3	342	-10	-13	-6	-18	0.9	0.9	0.9	0.9
SNX27	Q96L92	432	-10	-19	-6	-11	0.9	0.8	0.9	0.9
PARP14	Q460N5	1265	-10	-19	-6	-16	0.9	0.8	0.9	0.9
RPL11	P62913	72	-10	-6	-6	2	0.9	0.9	0.9	1.0
PLEKHO2	Q8TD55	135	-10	-16	-6	-9	0.9	0.9	0.9	0.9
RGL2	O15211	508	-10	-16	-6	-25	0.9	0.9	0.9	0.8
SMARCAL1	Q9NZC9	508	-11	-9	-6	-20	0.9	0.9	0.9	0.8
SCRIB	A0A0G2JPP5	496	-11	-20	-6	-24	0.9	0.8	0.9	0.8
DMD	P11532	3207	-11	-28	-6	-2	0.9	0.8	0.9	1.0
APRT	P07741	83	-12	-25	-6	-13	0.9	0.8	0.9	0.9
SNTB2	Q13425	391	-12	-4	-6	6	0.9	1.0	0.9	1.1
PWP1	Q13610	329	-12	-10	-6	-16	0.9	0.9	0.9	0.9
TRABD	Q9H4I3	108	-13	-16	-6	-5	0.9	0.9	0.9	1.0
NAA50	Q9GZZ1	60	-14	-40	-6	-16	0.9	0.7	0.9	0.9
FRMD4B	Q9Y2L6	929	-15	-8	-6	-6	0.9	0.9	0.9	0.9
SNTB2	Q13425	374	-15	1	-6	6	0.9	1.0	0.9	1.1
WDR81	Q562E7	464	-16	-9	-6	-5	0.9	0.9	0.9	1.0
SPECC1	Q5M775	633	-17	-6	-6	-28	0.9	0.9	0.9	0.8
HNRNPUL1	Q9BUJ2	487	-17	-17	-6	-4	0.9	0.9	0.9	1.0
NUAK2	Q9H093	351	-18	-10	-6	-7	0.9	0.9	0.9	0.9
FNDC3A	Q9Y2H6	1124	-21	-12	-6	-10	0.8	0.9	0.9	0.9
PRF1	P14222	395	-26	16	-6	7	0.8	1.2	0.9	1.1
RAVER2	Q9HCJ3	362	-29	-7	-6	-23	0.8	0.9	0.9	0.8
GP9	P14770	24	-38	-16	-6	-16	0.7	0.9	0.9	0.9
CAND1	Q86VP6	940	35	13	-7	31	1.5	1.1	0.9	1.4
RABEPK	Q7Z6M1	80	24	-15	-7	4	1.3	0.9	0.9	1.0
UNC13D	Q70J99	255	20	-11	-7	-3	1.2	0.9	0.9	1.0
ZMYM2	Q9UBW7	708	18	-2	-7	11	1.2	1.0	0.9	1.1
SH3BP1	Q9Y3L3	56	18	-4	-7	-11	1.2	1.0	0.9	0.9
DLGAP4	Q9Y2H0	736	14	-10	-7	7	1.2	0.9	0.9	1.1
PAK2	Q13177	352	14	-10	-7	-1	1.2	0.9	0.9	1.0
TLN1	Q9Y490	286	14	-15	-7	-3	1.2	0.9	0.9	1.0
ALKBH4	Q9NXW9	159	13	-5	-7	6	1.1	1.0	0.9	1.1

TNPO1	Q92973	103	13	-11	-7	-10	1.1	0.9	0.9	0.9
LYPLAL1	Q5VWZ2	12	13	0	-7	4	1.1	1.0	0.9	1.0
HNMT	P50135	196	13	-1	-7	-5	1.1	1.0	0.9	1.0
EFTUD2	Q15029	166	13	-8	-7	-1	1.1	0.9	0.9	1.0
HARS2	P49590	456	13	-8	-7	-1	1.1	0.9	0.9	1.0
ITCH	Q96J02	57	12	-6	-7	5	1.1	0.9	0.9	1.1
INPP4A	Q96PE3	845	12	-2	-7	-5	1.1	1.0	0.9	1.0
SRP54	P61011	136	12	-16	-7	-9	1.1	0.9	0.9	0.9
NUP205	Q92621	1032	12	-19	-7	5	1.1	0.8	0.9	1.0
FTSJ3	Q8IY81	52	11	1	-7	-5	1.1	1.0	0.9	1.0
PIKFYVE	Q9Y2I7	192	11	-12	-7	-11	1.1	0.9	0.9	0.9
SF1	Q15637	282	11	-5	-7	-1	1.1	1.0	0.9	1.0
ACAA2	P42765	107	11	-5	-7	-6	1.1	1.0	0.9	0.9
ZMYND8	Q9ULU4	274	10	0	-7	-5	1.1	1.0	0.9	1.0
RRAGA	Q7L523	219	10	-2	-7	-16	1.1	1.0	0.9	0.9
COPE	O14579	34	10	-34	-7	4	1.1	0.7	0.9	1.0
AP4M1	O00189	431	10	-9	-7	-5	1.1	0.9	0.9	1.0
GSKIP	Q9P0R6	59	10	-11	-7	11	1.1	0.9	0.9	1.1
ATP6V0D1	P61421	335	10	-24	-7	-11	1.1	0.8	0.9	0.9
TAF1	P21675	280	9	-15	-7	-7	1.1	0.9	0.9	0.9
ERAL1	O75616	150	9	-24	-7	-10	1.1	0.8	0.9	0.9
RCC1	P18754	93	9	-32	-7	-15	1.1	0.8	0.9	0.9
ZBTB1	Q9Y2K1	227	9	6	-7	5	1.1	1.1	0.9	1.1
ACAP3	Q96P50	753	9	-8	-7	-11	1.1	0.9	0.9	0.9
PML	P29590	227	9	-30	-7	-16	1.1	0.8	0.9	0.9
COPS5	Q92905	218	8	-11	-7	2	1.1	0.9	0.9	1.0
RPS3	P23396	119	8	-14	-7	0	1.1	0.9	0.9	1.0
SYNE1	Q8NF91	3049	8	2	-7	-5	1.1	1.0	0.9	1.0
FKBP15	Q5T1M5	845	8	-6	-7	-14	1.1	0.9	0.9	0.9
BACH1	O14867	140	8	-12	-7	-11	1.1	0.9	0.9	0.9
GID4	Q8IVV7	90	8	-13	-7	-30	1.1	0.9	0.9	0.8
VPS35	Q96QK1	640	8	-14	-7	-2	1.1	0.9	0.9	1.0
PKM	P14618	152	8	-22	-7	5	1.1	0.8	0.9	1.1
CYFIP2	Q96F07	423	7	3	-7	4	1.1	1.0	0.9	1.0
SAFB2	Q14151	361	7	-4	-7	-10	1.1	1.0	0.9	0.9
SAFB	Q15424	362	7	-4	-7	-10	1.1	1.0	0.9	0.9
HLA-DRB1	P04229	202	7	-8	-7	-9	1.1	0.9	0.9	0.9
IDH2	P48735	154	7	-15	-7	-3	1.1	0.9	0.9	1.0
ALDH16A1	Q8IZ83	39	7	-16	-7	-8	1.1	0.9	0.9	0.9
CNBP	P62633	140	7	-16	-7	-9	1.1	0.9	0.9	0.9
DICER1	Q9UPY3	214	7	-5	-7	6	1.1	1.0	0.9	1.1
SART3	Q15020	486	7	-9	-7	4	1.1	0.9	0.9	1.0
CD3EAP	O15446	150	7	-9	-7	-8	1.1	0.9	0.9	0.9
CPNE3	O75131	202	7	-10	-7	-5	1.1	0.9	0.9	1.0
RAB18	Q9NP72	110	7	-11	-7	3	1.1	0.9	0.9	1.0
PCIF1	Q9H4Z3	404	7	-17	-7	0	1.1	0.9	0.9	1.0
PRPF19	Q9UMS4	351	6	2	-7	-4	1.1	1.0	0.9	1.0
RRS1	Q15050	52	6	-7	-7	-2	1.1	0.9	0.9	1.0
THEMIS2	Q5TEJ8	545	6	-7	-7	-11	1.1	0.9	0.9	0.9
GNL3L	Q9NVN8	490	6	-7	-7	-22	1.1	0.9	0.9	0.8
PTPN23	Q9H3S7	628	6	-10	-7	-12	1.1	0.9	0.9	0.9
SMAD2	Q15796	81	6	-18	-7	-20	1.1	0.9	0.9	0.8
POLR3E	Q9NVU0	227	6	-25	-7	-10	1.1	0.8	0.9	0.9

OXR1	Q8N573	299	5	-1	-7	-10	1.1	1.0	0.9	0.9
MAP3K2	Q9Y2U5	405	5	-4	-7	-3	1.1	1.0	0.9	1.0
COQ5	Q5HYK3	170	5	-4	-7	-11	1.1	1.0	0.9	0.9
XRCC1	P18887	362	5	-6	-7	-3	1.1	0.9	0.9	1.0
ATP2C1	P98194	49	5	-7	-7	-16	1.1	0.9	0.9	0.9
TBCC	Q15814	184	5	-8	-7	1	1.1	0.9	0.9	1.0
RANBP2	P49792	2982	5	-11	-7	-6	1.1	0.9	0.9	0.9
AFAP1	Q8N556	713	5	-12	-7	-8	1.1	0.9	0.9	0.9
DDX39B	Q13838	165	5	-14	-7	-3	1.1	0.9	0.9	1.0
RPS8	P62241	100	5	-16	-7	-15	1.1	0.9	0.9	0.9
TLN1	Q9Y490	750	5	-18	-7	-10	1.1	0.9	0.9	0.9
NARS	O43776	255	5	-23	-7	2	1.1	0.8	0.9	1.0
TRIM21	P19474	359	5	3	-7	-4	1.0	1.0	0.9	1.0
PMVK	Q15126	38	5	-4	-7	5	1.0	1.0	0.9	1.1
ZNF346	Q9UL40	79	5	-4	-7	-13	1.0	1.0	0.9	0.9
RDH13	Q8NBN7	201	5	-11	-7	-10	1.0	0.9	0.9	0.9
PSMB3	P49720	19	5	-12	-7	-7	1.0	0.9	0.9	0.9
SPTB	P11277	603	5	-13	-7	-7	1.0	0.9	0.9	0.9
PAFAH1B1	P43034	356	5	-15	-7	-6	1.0	0.9	0.9	0.9
EXOSC7	Q15024	189	5	-15	-7	-12	1.0	0.9	0.9	0.9
ARHGDIB	P52566	76	5	-16	-7	4	1.0	0.9	0.9	1.0
KDM4B	O94953	309	5	-18	-7	-16	1.0	0.9	0.9	0.9
CTIF	O43310	501	4	3	-7	12	1.0	1.0	0.9	1.1
RIPK3	Q9Y572	365	4	2	-7	-1	1.0	1.0	0.9	1.0
FAM160B1	Q5W0V3	156	4	-9	-7	-13	1.0	0.9	0.9	0.9
MYO1C	O00159	802	4	-10	-7	-2	1.0	0.9	0.9	1.0
PSMD3	O43242	483	4	-10	-7	-10	1.0	0.9	0.9	0.9
NEK9	Q8TD19	878	4	-10	-7	-17	1.0	0.9	0.9	0.9
CTDSPL	O15194	197	4	-14	-7	5	1.0	0.9	0.9	1.1
MRPL37	Q9BZE1	366	4	-30	-7	1	1.0	0.8	0.9	1.0
SMU1	Q2TAY7	298	4	2	-7	6	1.0	1.0	0.9	1.1
VPS11	Q9H270	44	4	-1	-7	0	1.0	1.0	0.9	1.0
FAM126B	Q8IXS8	7	4	-3	-7	-9	1.0	1.0	0.9	0.9
TES	Q9UGI8	412	4	-5	-7	-9	1.0	1.0	0.9	0.9
COL4A3BP	Q9Y5P4	185	4	-7	-7	-1	1.0	0.9	0.9	1.0
RAB2B	Q8WUD1	154	4	-7	-7	-4	1.0	0.9	0.9	1.0
UBA6	A0AVT1	721	4	-7	-7	-9	1.0	0.9	0.9	0.9
RIPK1	Q13546	53	4	-8	-7	-9	1.0	0.9	0.9	0.9
PAICS	P22234	295	4	-10	-7	-3	1.0	0.9	0.9	1.0
OAS1	P00973	109	4	-10	-7	-13	1.0	0.9	0.9	0.9
DENND1A	Q8TEH3	338	4	-14	-7	-8	1.0	0.9	0.9	0.9
DHX9	Q08211	438	4	-20	-7	0	1.0	0.8	0.9	1.0
PHC2	Q8IXK0	673	3	-6	-7	-6	1.0	0.9	0.9	0.9
ATG7	O95352	572	3	-6	-7	-11	1.0	0.9	0.9	0.9
POLDIP3	Q9BY77	303	3	-7	-7	-6	1.0	0.9	0.9	0.9
ZZEF1	O43149	466	3	-8	-7	-2	1.0	0.9	0.9	1.0
ZC3H7B	Q9UGR2	956	3	-9	-7	-7	1.0	0.9	0.9	0.9
SMAD2	Q15796	380	3	-12	-7	-10	1.0	0.9	0.9	0.9
DCTN3	O75935	140	3	-12	-7	-10	1.0	0.9	0.9	0.9
TCEA1	P23193	271	3	-13	-7	-17	1.0	0.9	0.9	0.9
TCEA2	Q15560	269	3	-13	-7	-17	1.0	0.9	0.9	0.9
DDB1	Q16531	128	3	-20	-7	-3	1.0	0.8	0.9	1.0
ACOX3	O15254	220	3	-52	-7	-4	1.0	0.7	0.9	1.0

CTSC	P53634	355	3	13	-7	16	1.0	1.1	0.9	1.2
PDS5A	Q29RF7	532	3	-3	-7	-3	1.0	1.0	0.9	1.0
NAXE	Q8NCW5	283	3	-5	-7	-6	1.0	1.0	0.9	0.9
FHL1	Q13642	10	3	-6	-7	-1	1.0	0.9	0.9	1.0
LIMS1	P48059	70	3	-7	-7	-8	1.0	0.9	0.9	0.9
USO1	O60763	303	3	-9	-7	-6	1.0	0.9	0.9	0.9
DDX6	P26196	102	3	-12	-7	-12	1.0	0.9	0.9	0.9
SIRT1	Q96EB6	380	3	-14	-7	-8	1.0	0.9	0.9	0.9
DGCR8	Q8WYQ5	430	3	-14	-7	-3	1.0	0.9	0.9	1.0
CDC40	O60508	378	3	-15	-7	5	1.0	0.9	0.9	1.1
ARMC6	Q6NXE6	121	3	-19	-7	6	1.0	0.8	0.9	1.1
DYSF	O75923	1685	3	-19	-7	4	1.0	0.8	0.9	1.0
CHERP	Q8IWX8	190	3	-23	-7	-15	1.0	0.8	0.9	0.9
TRIM65	Q6PJ69	114	3	-23	-7	-24	1.0	0.8	0.9	0.8
ADPGK	Q9BRR6	40	2	-1	-7	2	1.0	1.0	0.9	1.0
NUP54	Q7Z3B4	180	2	-3	-7	-5	1.0	1.0	0.9	1.0
DHRS7	Q9Y394	147	2	-8	-7	-11	1.0	0.9	0.9	0.9
PPCDC	Q96CD2	166	2	-10	-7	-4	1.0	0.9	0.9	1.0
SLFN13	Q68D06	104	2	-10	-7	-3	1.0	0.9	0.9	1.0
SLFN11	Q7Z7L1	102	2	-10	-7	-3	1.0	0.9	0.9	1.0
DYSF	O75923	1621	2	-11	-7	-2	1.0	0.9	0.9	1.0
KIF4A	O95239	977	2	-11	-7	-10	1.0	0.9	0.9	0.9
KDM3B	Q7LBC6	1451	2	-13	-7	-13	1.0	0.9	0.9	0.9
HIP1	O00291	220	2	-16	-7	2	1.0	0.9	0.9	1.0
AKR1A1	P14550	134	2	-17	-7	0	1.0	0.9	0.9	1.0
PTPRJ	Q12913	1149	2	-26	-7	-8	1.0	0.8	0.9	0.9
PLEKHF2	Q9H8W4	21	2	-34	-7	-1	1.0	0.7	0.9	1.0
ANAPC16	Q96DE5	55	2	-3	-7	-3	1.0	1.0	0.9	1.0
PSMC1	P62191	58	2	-4	-7	1	1.0	1.0	0.9	1.0
VARS	P26640	381	2	-5	-7	-9	1.0	1.0	0.9	0.9
PGRMC2	O15173	159	2	-7	-7	-11	1.0	0.9	0.9	0.9
ARL8B	Q9NVJ2	159	2	-11	-7	3	1.0	0.9	0.9	1.0
ZW10	O43264	404	2	-13	-7	3	1.0	0.9	0.9	1.0
RHOF	Q9HBH0	30	2	-15	-7	9	1.0	0.9	0.9	1.1
ATG3	Q9NT62	182	2	-15	-7	-9	1.0	0.9	0.9	0.9
CCT3	P49368	173	2	-21	-7	-6	1.0	0.8	0.9	0.9
HMGB1	P09429	23	2	-33	-7	-7	1.0	0.8	0.9	0.9
HMGB2	P26583	23	2	-33	-7	-7	1.0	0.8	0.9	0.9
KDM5A	P29375	962	1	-3	-7	3	1.0	1.0	0.9	1.0
TP53BP1	Q12888	1375	1	-6	-7	-16	1.0	0.9	0.9	0.9
FNBP1	Q96RU3	609	1	-9	-7	-6	1.0	0.9	0.9	0.9
ZMYND8	Q9ULU4	138	1	-12	-7	-10	1.0	0.9	0.9	0.9
CAMK2G	Q13555	273	1	-13	-7	-9	1.0	0.9	0.9	0.9
HERC3	Q15034	552	1	-14	-7	-15	1.0	0.9	0.9	0.9
WDFY1	Q8IWB7	290	1	-18	-7	-8	1.0	0.8	0.9	0.9
ZFAND3	Q9H8U3	20	1	-23	-7	2	1.0	0.8	0.9	1.0
ZNF709	Q8N972	375	1	-2	-7	11	1.0	1.0	0.9	1.1
LMNA	P02545	591	1	-2	-7	-10	1.0	1.0	0.9	0.9
ALKBH4	Q9NXW9	26	1	-2	-7	0	1.0	1.0	0.9	1.0
FLNB	O75369	2501	1	-8	-7	-8	1.0	0.9	0.9	0.9
HMHA1	Q92619	783	1	-9	-7	-9	1.0	0.9	0.9	0.9
USO1	O60763	678	1	-12	-7	-12	1.0	0.9	0.9	0.9
CSTF3	Q12996	646	1	-14	-7	-11	1.0	0.9	0.9	0.9

TEP1	Q99973	2524	1	-15	-7	-23	1.0	0.9	0.9	0.8
PDE5A	O76074	81	1	-17	-7	13	1.0	0.9	0.9	1.1
WDR44	Q5JSH3	701	1	-18	-7	-5	1.0	0.9	0.9	1.0
PIK3R1	P27986	146	0	-6	-7	-7	1.0	0.9	0.9	0.9
CCZ1B	P86790	358	0	-7	-7	-1	1.0	0.9	0.9	1.0
DOCK10	Q96BY6	1092	0	-9	-7	1	1.0	0.9	0.9	1.0
LCP1	P13796	101	0	-10	-7	-15	1.0	0.9	0.9	0.9
MYO18A	Q92614	1699	0	-10	-7	-22	1.0	0.9	0.9	0.8
PRDM10	Q9NQV6	558	0	-10	-7	-29	1.0	0.9	0.9	0.8
RREB1	Q92766	1251	0	-12	-7	-13	1.0	0.9	0.9	0.9
SEPT6	Q14141	269	0	-14	-7	-10	1.0	0.9	0.9	0.9
EP300	Q09472	1753	0	-15	-7	-4	1.0	0.9	0.9	1.0
CREBBP	Q92793	1790	0	-15	-7	-4	1.0	0.9	0.9	1.0
EIF3E	P60228	345	0	-36	-7	4	1.0	0.7	0.9	1.0
ALKBH4	Q9NXW9	204	-1	7	-7	-4	1.0	1.1	0.9	1.0
NUP153	P49790	593	-1	-2	-7	-19	1.0	1.0	0.9	0.8
LRRC8D	Q7L1W4	218	-1	-4	-7	-3	1.0	1.0	0.9	1.0
DCLRE1B	Q9H816	303	-1	-8	-7	-8	1.0	0.9	0.9	0.9
EPG5	Q9HCE0	472	-1	-11	-7	-5	1.0	0.9	0.9	1.0
ZNHIT1	O43257	140	-1	-12	-7	-5	1.0	0.9	0.9	1.0
DOCK9	Q9BZ29	628	-1	-13	-7	-13	1.0	0.9	0.9	0.9
ROCK2	O75116	607	-1	-16	-7	-9	1.0	0.9	0.9	0.9
TLE4	Q04727	529	-1	-19	-7	-15	1.0	0.8	0.9	0.9
MGMT	P16455	150	-1	-6	-7	-8	1.0	0.9	0.9	0.9
NASP	P49321	708	-1	-7	-7	-15	1.0	0.9	0.9	0.9
CNTN6	Q9UQ52	249	-1	-9	-7	-13	1.0	0.9	0.9	0.9
SYNJ2	O15056	1182	-1	-9	-7	-17	1.0	0.9	0.9	0.9
TANK	Q92844	218	-1	-11	-7	-15	1.0	0.9	0.9	0.9
TARS	P26639	630	-1	-14	-7	-6	1.0	0.9	0.9	0.9
XPNPEP1	Q9NQW7	279	-1	-26	-7	1	1.0	0.8	0.9	1.0
CCAR2	Q8N163	644	-2	9	-7	-3	1.0	1.1	0.9	1.0
MCM3AP	O60318	274	-2	-3	-7	-13	1.0	1.0	0.9	0.9
DOCK11	Q5JSL3	797	-2	-6	-7	-14	1.0	0.9	0.9	0.9
PDK1	Q15118	71	-2	-6	-7	-7	1.0	0.9	0.9	0.9
ARHGEF2	Q92974	478	-2	-7	-7	-1	1.0	0.9	0.9	1.0
GNB2	P62879	25	-2	-9	-7	-1	1.0	0.9	0.9	1.0
NBAS	A2RRP1	1255	-2	-10	-7	-5	1.0	0.9	0.9	1.0
KPNA1	P52294	141	-2	-10	-7	-7	1.0	0.9	0.9	0.9
ACADSB	P45954	261	-2	-11	-7	-2	1.0	0.9	0.9	1.0
NUBP2	Q9Y5Y2	72	-2	-11	-7	7	1.0	0.9	0.9	1.1
SUPT6H	Q7KZ85	336	-2	-12	-7	-13	1.0	0.9	0.9	0.9
SCAF4	O95104	545	-2	-14	-7	-13	1.0	0.9	0.9	0.9
ADSS	P30520	58	-2	-15	-7	-6	1.0	0.9	0.9	0.9
EEF2	P13639	591	-2	-16	-7	-1	1.0	0.9	0.9	1.0
DOCK2	Q92608	465	-2	-18	-7	-4	1.0	0.8	0.9	1.0
PIK3C2B	O00750	1373	-2	0	-7	12	1.0	1.0	0.9	1.1
PTGES3	Q15185	76	-2	-4	-7	2	1.0	1.0	0.9	1.0
NBN	O60934	119	-2	-9	-7	-10	1.0	0.9	0.9	0.9
VDAC1	P21796	232	-2	-9	-7	-5	1.0	0.9	0.9	1.0
HCFC1	P51610	1895	-2	-9	-7	-11	1.0	0.9	0.9	0.9
LTBP1	Q14766	361	-2	-11	-7	-15	1.0	0.9	0.9	0.9
DMXL1	Q9Y485	165	-2	-12	-7	-7	1.0	0.9	0.9	0.9
TUBA3D	Q13748	347	-2	-12	-7	-16	1.0	0.9	0.9	0.9

TUBA1A	Q71U36	347	-2	-12	-7	-16	1.0	0.9	0.9	0.9
TUBA8	Q9NY65	347	-2	-12	-7	-16	1.0	0.9	0.9	0.9
PHF5A	Q7RTV0	49	-2	-14	-7	9	1.0	0.9	0.9	1.1
SRPRB	Q9Y5M8	100	-3	-7	-7	-3	1.0	0.9	0.9	1.0
PCBP1	Q15365	54	-3	-8	-7	-6	1.0	0.9	0.9	0.9
ZAP70	P43403	117	-3	-9	-7	-9	1.0	0.9	0.9	0.9
KPNA6	O60684	253	-3	-10	-7	4	1.0	0.9	0.9	1.0
RAP1A	P62834	139	-3	-14	-7	-9	1.0	0.9	0.9	0.9
ACSS1	Q9NUB1	422	-3	-15	-7	-11	1.0	0.9	0.9	0.9
ITGB2	P05107	420	-3	-18	-7	-25	1.0	0.8	0.9	0.8
ABCF2	Q9UG63	411	-3	-21	-7	-12	1.0	0.8	0.9	0.9
NUDT2	P50583	123	-3	-25	-7	2	1.0	0.8	0.9	1.0
MGA	Q8IWI9	2974	-3	-8	-7	-6	1.0	0.9	0.9	0.9
TCF12	Q99081	129	-3	-10	-7	-15	1.0	0.9	0.9	0.9
TRAF3IP3	Q9Y228	315	-3	-10	-7	-2	1.0	0.9	0.9	1.0
SEPT2	Q15019	111	-3	-11	-7	-10	1.0	0.9	0.9	0.9
NMRK1	Q9NWW6	194	-3	-13	-7	-12	1.0	0.9	0.9	0.9
ACP1	P24666	13	-3	-30	-7	-8	1.0	0.8	0.9	0.9
FBXL18	Q96ME1	383	-4	-4	-7	-2	1.0	1.0	0.9	1.0
NOL6	Q9H6R4	265	-4	-7	-7	-3	1.0	0.9	0.9	1.0
SUGP2	Q8IX01	540	-4	-9	-7	-6	1.0	0.9	0.9	0.9
ABCF3	Q9NUQ8	102	-4	-9	-7	-16	1.0	0.9	0.9	0.9
ICAM3	P32942	407	-4	-10	-7	9	1.0	0.9	0.9	1.1
AP4E1	Q9UPM8	1119	-4	-12	-7	-10	1.0	0.9	0.9	0.9
PGLS	O95336	237	-4	-13	-7	-3	1.0	0.9	0.9	1.0
CHM	P24386	580	-4	-14	-7	-11	1.0	0.9	0.9	0.9
RASSF2	P50749	251	-4	-14	-7	-13	1.0	0.9	0.9	0.9
PPA2	Q9H2U2	180	-4	-36	-7	-3	1.0	0.7	0.9	1.0
VDAC3	Q9Y277	36	-4	1	-7	-4	1.0	1.0	0.9	1.0
MACF1	Q9UPN3	3227	-4	-5	-7	-16	1.0	1.0	0.9	0.9
GSTZ1	O43708	205	-4	-9	-7	-1	1.0	0.9	0.9	1.0
NSUN2	Q08J23	502	-4	-10	-7	-10	1.0	0.9	0.9	0.9
BTBD11	A6QL63	844	-4	-10	-7	-10	1.0	0.9	0.9	0.9
OAS1	P00973	25	-4	-12	-7	-16	1.0	0.9	0.9	0.9
CHORDC1	Q9UHD1	86	-4	-13	-7	-15	1.0	0.9	0.9	0.9
ZNF318	Q5VUA4	1681	-4	-13	-7	-17	1.0	0.9	0.9	0.9
PIK3R1	P27986	167	-4	-14	-7	-15	1.0	0.9	0.9	0.9
IVNS1ABP	Q9Y6Y0	454	-4	-14	-7	-23	1.0	0.9	0.9	0.8
INPP5B	P32019	990	-4	-16	-7	-11	1.0	0.9	0.9	0.9
SRI	P30626	163	-4	-17	-7	3	1.0	0.9	0.9	1.0
NDUFA4	O00483	44	-4	-17	-7	-11	1.0	0.9	0.9	0.9
GATM	P50440	50	-4	-20	-7	-29	1.0	0.8	0.9	0.8
TTC4	O95801	238	-5	-1	-7	-2	1.0	1.0	0.9	1.0
ANKLE2	Q86XL3	350	-5	-7	-7	-8	1.0	0.9	0.9	0.9
FLNA	P21333	623	-5	-13	-7	-12	1.0	0.9	0.9	0.9
FLNC	Q14315	618	-5	-13	-7	-12	1.0	0.9	0.9	0.9
ZYX	Q15942	562	-5	-14	-7	3	1.0	0.9	0.9	1.0
ATM	Q13315	2991	-5	-19	-7	-18	1.0	0.8	0.9	0.9
RNF141	Q8WVD5	174	-5	-25	-7	-14	1.0	0.8	0.9	0.9
ATAD2	Q6PL18	1388	-5	-1	-7	10	1.0	1.0	0.9	1.1
MED15	Q96RN5	618	-5	-5	-7	1	1.0	1.0	0.9	1.0
ITPR2	Q14571	379	-5	-5	-7	-8	1.0	1.0	0.9	0.9
AIM1	Q9Y4K1	1404	-5	-9	-7	-8	1.0	0.9	0.9	0.9

SEC63	Q9UGP8	295	-5	-16	-7	-11	1.0	0.9	0.9	0.9
NDUFS8	O00217	117	-5	-20	-7	-2	1.0	0.8	0.9	1.0
SYNE1	Q8NF91	4436	-6	-1	-7	-1	0.9	1.0	0.9	1.0
TPP2	P29144	582	-6	-4	-7	0	0.9	1.0	0.9	1.0
TPP2	P29144	592	-6	-4	-7	0	0.9	1.0	0.9	1.0
FAM98B	Q52LJ0	63	-6	-4	-7	-6	0.9	1.0	0.9	0.9
SCAF1	Q9H7N4	362	-6	-6	-7	-12	0.9	0.9	0.9	0.9
RAE1	P78406	106	-6	-8	-7	-3	0.9	0.9	0.9	1.0
ABI3	Q9P2A4	137	-6	-8	-7	-13	0.9	0.9	0.9	0.9
ANXA4	P09525	108	-6	-9	-7	0	0.9	0.9	0.9	1.0
ARIH2	O95376	161	-6	-11	-7	-6	0.9	0.9	0.9	0.9
IFI35	P80217	193	-6	-13	-7	2	0.9	0.9	0.9	1.0
PMS1	P54277	725	-6	-19	-7	-10	0.9	0.8	0.9	0.9
PGPEP1	Q9NXJ5	149	-6	-4	-7	-10	0.9	1.0	0.9	0.9
FAM185A	Q8N0U4	106	-6	-6	-7	-2	0.9	0.9	0.9	1.0
UBR4	Q5T4S7	4272	-6	-6	-7	-7	0.9	0.9	0.9	0.9
C15orf39	Q6ZRI6	892	-6	-8	-7	-4	0.9	0.9	0.9	1.0
PDIA3	P30101	244	-6	-11	-7	-7	0.9	0.9	0.9	0.9
RAB29	O14966	120	-6	-14	-7	-1	0.9	0.9	0.9	1.0
PTPRC	P08575	432	-6	-20	-7	-27	0.9	0.8	0.9	0.8
GIMAP2	Q9UG22	227	-6	-21	-7	-15	0.9	0.8	0.9	0.9
PDCD11	Q14690	557	-6	-25	-7	-9	0.9	0.8	0.9	0.9
SORD	Q00796	106	-7	-5	-7	-11	0.9	1.0	0.9	0.9
KDM3B	Q7LBC6	569	-7	-12	-7	-18	0.9	0.9	0.9	0.8
DNMBP	Q6XZF7	75	-7	-13	-7	-20	0.9	0.9	0.9	0.8
ACD	Q96AP0	508	-7	-15	-7	-15	0.9	0.9	0.9	0.9
EZR	P15311	284	-7	-17	-7	-27	0.9	0.9	0.9	0.8
ILVBL	A1L0T0	568	-7	-18	-7	-10	0.9	0.9	0.9	0.9
SLMAP	Q14BN4	37	-7	-18	-7	-24	0.9	0.8	0.9	0.8
SETX	Q7Z333	138	-7	-6	-7	-17	0.9	0.9	0.9	0.9
ALDH4A1	P30038	315	-7	-15	-7	4	0.9	0.9	0.9	1.0
AARS	P49588	671	-7	-19	-7	6	0.9	0.8	0.9	1.1
RRP1B	Q14684	286	-8	-7	-7	-20	0.9	0.9	0.9	0.8
KDM3A	Q9Y4C1	304	-8	-9	-7	-5	0.9	0.9	0.9	1.0
MTR	Q99707	812	-8	-9	-7	-17	0.9	0.9	0.9	0.9
ARID4B	Q4LE39	107	-8	-23	-7	-22	0.9	0.8	0.9	0.8
WAPL	Q7Z5K2	160	-8	-14	-7	-16	0.9	0.9	0.9	0.9
JAK3	P52333	1048	-8	-16	-7	-30	0.9	0.9	0.9	0.8
GIMAP8	Q8ND71	214	-8	-18	-7	-13	0.9	0.8	0.9	0.9
RHOC	P08134	83	-8	-26	-7	-19	0.9	0.8	0.9	0.8
RHOA	P61586	83	-8	-26	-7	-19	0.9	0.8	0.9	0.8
Uncharacteri	U3KQV3	247	-8	-26	-7	-19	0.9	0.8	0.9	0.8
RAD9A	Q99638	3	-9	-8	-7	-1	0.9	0.9	0.9	1.0
RIOK3	O14730	346	-9	-11	-7	-13	0.9	0.9	0.9	0.9
DARS2	Q6PI48	590	-9	-4	-7	-8	0.9	1.0	0.9	0.9
PLA2G15	Q8NCC3	355	-9	-10	-7	-5	0.9	0.9	0.9	1.0
PMPCB	O75439	62	-9	-15	-7	-12	0.9	0.9	0.9	0.9
NUP205	Q92621	1028	-10	-9	-7	-9	0.9	0.9	0.9	0.9
DNPEP	Q9ULA0	144	-10	-15	-7	-16	0.9	0.9	0.9	0.9
ANKRD13A	Q8IZ07	141	-10	-16	-7	-5	0.9	0.9	0.9	1.0
IMPDH2	P12268	331	-10	-10	-7	-8	0.9	0.9	0.9	0.9
XPOT	O43592	693	-11	-24	-7	-6	0.9	0.8	0.9	0.9
CLIC2	O15247	229	-12	-7	-7	-13	0.9	0.9	0.9	0.9

USP16	Q9Y5T5	618	-12	-9	-7	-9	0.9	0.9	0.9	0.9
GSPT1	P15170	381	-12	-19	-7	-3	0.9	0.8	0.9	1.0
DUSP12	Q9UNI6	115	-12	-22	-7	-8	0.9	0.8	0.9	0.9
AUP1	Q9Y679	212	-12	-22	-7	-11	0.9	0.8	0.9	0.9
FLNA	P21333	631	-13	-18	-7	-17	0.9	0.8	0.9	0.9
FLNC	Q14315	626	-13	-18	-7	-17	0.9	0.8	0.9	0.9
HSP90B1	P14625	645	-15	-15	-7	-6	0.9	0.9	0.9	0.9
SETDB1	Q15047	1279	-16	-9	-7	-36	0.9	0.9	0.9	0.7
WDR55	Q9H6Y2	306	-18	-16	-7	-20	0.8	0.9	0.9	0.8
MAN2C1	Q9NTJ4	793	-19	-18	-7	-1	0.8	0.9	0.9	1.0
TADA2A	O75478	405	-19	-55	-7	-53	0.8	0.6	0.9	0.7
RNF114	Q9Y508	8	-26	-20	-7	-17	0.8	0.8	0.9	0.9
RPL23	P62829	125	-30	-14	-7	-14	0.8	0.9	0.9	0.9
BRPF1	P55201	852	-33	-26	-7	-12	0.8	0.8	0.9	0.9
ETHE1	O95571	219	-54	-41	-7	-13	0.7	0.7	0.9	0.9
RAP1GDS1	P52306	26	29	17	-7	2	1.4	1.2	0.9	1.0
ADH5	P11766	282	28	-7	-7	9	1.4	0.9	0.9	1.1
CHD4	Q14839	1468	20	-4	-7	16	1.3	1.0	0.9	1.2
ATP13A1	Q9HD20	410	19	0	-7	19	1.2	1.0	0.9	1.2
SAFB	Q15424	463	18	-6	-7	5	1.2	0.9	0.9	1.1
SPHK1	Q9NYA1	316	17	2	-7	-2	1.2	1.0	0.9	1.0
NCBP1	Q09161	332	17	-13	-7	-2	1.2	0.9	0.9	1.0
EIF4G3	O43432	982	16	-10	-7	-5	1.2	0.9	0.9	1.0
PRPF3	O43395	630	15	-27	-7	-10	1.2	0.8	0.9	0.9
SNX17	Q15036	277	14	-10	-7	-15	1.2	0.9	0.9	0.9
MED24	O75448	218	14	-12	-7	-15	1.2	0.9	0.9	0.9
CHD4	Q14839	1019	13	-15	-7	-1	1.1	0.9	0.9	1.0
ABHD5	Q8WTS1	140	13	-17	-7	-16	1.1	0.9	0.9	0.9
SLFN14	P0C7P3	176	13	1	-7	-2	1.1	1.0	0.9	1.0
XPC	Q01831	670	13	-63	-7	12	1.1	0.6	0.9	1.1
LASP1	Q14847	32	12	-9	-7	-14	1.1	0.9	0.9	0.9
GIGYF1	O75420	583	12	-7	-7	2	1.1	0.9	0.9	1.0
PLEKHG3	A1L390	359	12	-7	-7	-5	1.1	0.9	0.9	1.0
WDR82	Q6UXN9	195	11	-13	-7	-3	1.1	0.9	0.9	1.0
ADAT2	Q7Z6V5	15	11	-9	-7	-6	1.1	0.9	0.9	0.9
NDUFA10	E7ESZ7	67	10	-11	-7	2	1.1	0.9	0.9	1.0
NDRG1	Q92597	289	10	-12	-7	7	1.1	0.9	0.9	1.1
NAA15	Q9BXJ9	214	9	-9	-7	-3	1.1	0.9	0.9	1.0
UBASH3A	P57075	435	9	-2	-7	-4	1.1	1.0	0.9	1.0
HSDL2	Q6YN16	71	9	-10	-7	-2	1.1	0.9	0.9	1.0
CSK	P41240	122	9	-14	-7	2	1.1	0.9	0.9	1.0
PKM	P14618	424	8	-10	-7	-7	1.1	0.9	0.9	0.9
DOK1	Q99704	70	8	-18	-7	-13	1.1	0.9	0.9	0.9
PPP4R1	Q8TF05	663	8	-18	-7	-4	1.1	0.8	0.9	1.0
LACTB	P83111	476	8	-9	-7	4	1.1	0.9	0.9	1.0
LRBA	P50851	2017	8	-10	-7	-8	1.1	0.9	0.9	0.9
NLRX1	Q86UT6	331	7	-1	-7	-12	1.1	1.0	0.9	0.9
TRIM28	Q13263	209	7	-2	-7	2	1.1	1.0	0.9	1.0
OXCT1	P55809	504	7	-10	-7	3	1.1	0.9	0.9	1.0
AP2B1	P63010	112	7	-18	-7	4	1.1	0.8	0.9	1.0
AP1B1	Q10567	112	7	-18	-7	4	1.1	0.8	0.9	1.0
GDI2	P50395	282	7	-21	-7	2	1.1	0.8	0.9	1.0
VPS39	Q96JC1	844	7	-21	-7	1	1.1	0.8	0.9	1.0

SERPINB10	P48595	68	7	-7	-7	-9	1.1	0.9	0.9	0.9
THAP4	Q8WY91	269	7	-10	-7	-25	1.1	0.9	0.9	0.8
HMHA1	Q92619	739	7	-19	-7	-3	1.1	0.8	0.9	1.0
CLTC	Q00610	491	7	-19	-7	-11	1.1	0.8	0.9	0.9
MTR	Q99707	324	6	-1	-7	11	1.1	1.0	0.9	1.1
MAPK10	P53779	283	6	-3	-7	-12	1.1	1.0	0.9	0.9
TLN1	Q9Y490	1045	6	-7	-7	-7	1.1	0.9	0.9	0.9
G3BP1	Q13283	73	6	-8	-7	-4	1.1	0.9	0.9	1.0
ATRX	P46100	450	6	-11	-7	-10	1.1	0.9	0.9	0.9
KDM6A	O15550	402	6	-17	-7	-16	1.1	0.9	0.9	0.9
ANKFY1	Q9P2R3	499	6	-24	-7	3	1.1	0.8	0.9	1.0
SETX	Q7Z333	688	6	-5	-7	-2	1.1	1.0	0.9	1.0
EPG5	Q9HCE0	53	6	-9	-7	-13	1.1	0.9	0.9	0.9
CNOT10	Q9H9A5	633	6	-11	-7	-4	1.1	0.9	0.9	1.0
APAF1	O14727	450	6	-11	-7	-8	1.1	0.9	0.9	0.9
KMT2D	O14686	5477	6	-11	-7	-13	1.1	0.9	0.9	0.9
NT5C3B	Q969T7	56	6	-14	-7	2	1.1	0.9	0.9	1.0
SFXN4	Q6P4A7	159	6	-15	-7	-2	1.1	0.9	0.9	1.0
ANXA7	P20073	298	6	-15	-7	-4	1.1	0.9	0.9	1.0
IREB2	P48200	137	6	-19	-7	-19	1.1	0.8	0.9	0.8
ANKIB1	Q9P2G1	359	5	6	-7	-2	1.1	1.1	0.9	1.0
NDUFS8	O00217	121	5	2	-7	-1	1.1	1.0	0.9	1.0
HSPBP1	Q9NZL4	204	5	-5	-7	-13	1.1	1.0	0.9	0.9
STAT2	P52630	174	5	-6	-7	-18	1.1	0.9	0.9	0.8
PTRH2	Q9Y3E5	36	5	-9	-7	3	1.1	0.9	0.9	1.0
U2SURP	O15042	320	5	-10	-7	-8	1.1	0.9	0.9	0.9
UTRN	P46939	3023	5	-16	-7	-14	1.1	0.9	0.9	0.9
PATL1	Q86TB9	563	5	-2	-7	-8	1.0	1.0	0.9	0.9
CTNNBL1	Q8WYA6	370	5	-3	-7	6	1.0	1.0	0.9	1.1
MTM1	Q13496	482	5	-7	-7	-1	1.0	0.9	0.9	1.0
PRKD2	Q9BZL6	84	5	-9	-7	-6	1.0	0.9	0.9	0.9
LSS	P48449	471	5	-11	-7	-4	1.0	0.9	0.9	1.0
IFIT5	Q13325	137	5	-13	-7	-16	1.0	0.9	0.9	0.9
SNRNP40	Q96DI7	99	5	-15	-7	-3	1.0	0.9	0.9	1.0
ALOX12	P18054	656	5	-23	-7	4	1.0	0.8	0.9	1.0
RB1CC1	Q8TDY2	603	4	-8	-7	-2	1.0	0.9	0.9	1.0
COPA	P53621	522	4	-12	-7	-3	1.0	0.9	0.9	1.0
MCTS1	Q9ULC4	113	4	-12	-7	8	1.0	0.9	0.9	1.1
TSFM	P43897	71	4	-13	-7	-9	1.0	0.9	0.9	0.9
DDX24	Q9GZR7	467	4	-14	-7	-6	1.0	0.9	0.9	0.9
DRG1	Q9Y295	243	4	-15	-7	3	1.0	0.9	0.9	1.0
DMXL2	Q8TDJ6	1374	4	-16	-7	11	1.0	0.9	0.9	1.1
SSRP1	Q08945	139	4	-28	-7	-7	1.0	0.8	0.9	0.9
ADRM1	Q16186	88	4	-3	-7	-1	1.0	1.0	0.9	1.0
METTL2A	Q96IZ6	171	4	-5	-7	10	1.0	1.0	0.9	1.1
SLC27A4	Q6P1M0	232	4	-8	-7	-4	1.0	0.9	0.9	1.0
MLH1	P40692	233	4	-8	-7	-13	1.0	0.9	0.9	0.9
PLD4	Q96BZ4	313	4	-11	-7	3	1.0	0.9	0.9	1.0
LRPPRC	P42704	571	4	-13	-7	-3	1.0	0.9	0.9	1.0
TOP2B	Q02880	271	4	-15	-7	-4	1.0	0.9	0.9	1.0
PTK2	Q05397	459	4	-18	-7	-14	1.0	0.8	0.9	0.9
MDC1	Q14676	928	4	-24	-7	-19	1.0	0.8	0.9	0.8
PHF3	Q92576	561	3	-2	-7	-2	1.0	1.0	0.9	1.0

PCF11	Q94913	102	3	-10	-7	-3	1.0	0.9	0.9	1.0
FGD3	Q5JSP0	655	3	-10	-7	-8	1.0	0.9	0.9	0.9
MYBBP1A	Q9BQG0	1031	3	-11	-7	10	1.0	0.9	0.9	1.1
PIK3R1	P27986	656	3	-11	-7	-2	1.0	0.9	0.9	1.0
HMHA1	Q92619	278	3	-12	-7	-8	1.0	0.9	0.9	0.9
RAB34	Q9BZG1	116	3	-17	-7	-9	1.0	0.9	0.9	0.9
TBCE	Q15813	371	3	-6	-7	-5	1.0	0.9	0.9	1.0
SYNE2	Q8WXH0	1980	3	-6	-7	-8	1.0	0.9	0.9	0.9
NOSIP	Q9Y314	250	3	-7	-7	-9	1.0	0.9	0.9	0.9
SYNE2	Q8WXH0	5930	3	-8	-7	-11	1.0	0.9	0.9	0.9
IPCEF1	Q8WWN9	368	3	-12	-7	-4	1.0	0.9	0.9	1.0
AMPD3	Q01432	47	3	-14	-7	-9	1.0	0.9	0.9	0.9
MLH1	P40692	77	3	-15	-7	-20	1.0	0.9	0.9	0.8
OARD1	Q9Y530	33	3	-20	-7	7	1.0	0.8	0.9	1.1
TOP2B	Q02880	426	3	-31	-7	-5	1.0	0.8	0.9	1.0
CUL4B	Q13620	292	3	-32	-7	-6	1.0	0.8	0.9	0.9
VPS26B	Q4G0F5	334	2	-6	-7	-5	1.0	0.9	0.9	1.0
PPA1	Q15181	274	2	-7	-7	-11	1.0	0.9	0.9	0.9
EME2	A4GXA9	271	2	-10	-7	1	1.0	0.9	0.9	1.0
BOD1L1	Q8NFC6	285	2	-11	-7	-11	1.0	0.9	0.9	0.9
RPN1	P04843	545	2	-11	-7	-2	1.0	0.9	0.9	1.0
LIMS1	P48059	100	2	-11	-7	-4	1.0	0.9	0.9	1.0
HNRNPUL2-B	H3BQZ7	538	2	-12	-7	-5	1.0	0.9	0.9	1.0
NCBP1	Q09161	320	2	-13	-7	4	1.0	0.9	0.9	1.0
SOAT1	P35610	92	2	-13	-7	-22	1.0	0.9	0.9	0.8
MAPKAP1	Q9BPZ7	149	2	-16	-7	-8	1.0	0.9	0.9	0.9
HACE1	Q8IYU2	481	2	-16	-7	-12	1.0	0.9	0.9	0.9
STAT3	P40763	687	2	-21	-7	-18	1.0	0.8	0.9	0.8
TNS3	Q68CZ2	1241	2	-4	-7	-4	1.0	1.0	0.9	1.0
TNFAIP3	P21580	158	2	-5	-7	-5	1.0	1.0	0.9	1.0
UROS	P10746	14	2	-7	-7	-15	1.0	0.9	0.9	0.9
STK26	Q9P289	392	2	-10	-7	-6	1.0	0.9	0.9	0.9
TUBB2B	Q9BVA1	354	2	-11	-7	-17	1.0	0.9	0.9	0.9
SRGAP2	A2RUF3	34	2	-12	-7	14	1.0	0.9	0.9	1.2
TRAF3IP3	Q9Y228	388	2	-14	-7	-23	1.0	0.9	0.9	0.8
DPP7	Q9UHL4	338	2	-20	-7	22	1.0	0.8	0.9	1.3
BRIX1	Q8TDN6	52	1	-3	-7	3	1.0	1.0	0.9	1.0
SH2D3C	Q8N5H7	317	1	-4	-7	-6	1.0	1.0	0.9	0.9
PIK3CB	P42338	116	1	-5	-7	3	1.0	1.0	0.9	1.0
CTCF	P49711	155	1	-5	-7	-14	1.0	1.0	0.9	0.9
MACF1	Q9UPN3	5049	1	-6	-7	-5	1.0	0.9	0.9	1.0
ASNA1	O43681	53	1	-10	-7	-2	1.0	0.9	0.9	1.0
ALG2	Q9H553	59	1	-11	-7	-3	1.0	0.9	0.9	1.0
PARP10	Q53GL7	434	1	-11	-7	-17	1.0	0.9	0.9	0.9
GTF3C5	Q9Y5Q8	432	1	-13	-7	-6	1.0	0.9	0.9	0.9
PDXDC1	Q6P996	135	1	-14	-7	-10	1.0	0.9	0.9	0.9
POTEI	P0CG38	957	1	-16	-7	-3	1.0	0.9	0.9	1.0
CASP6	P55212	264	1	-18	-7	-14	1.0	0.8	0.9	0.9
KDM3A	Q9Y4C1	695	1	-22	-7	-25	1.0	0.8	0.9	0.8
RANBP2	P49792	815	1	-3	-7	-5	1.0	1.0	0.9	1.0
KIF2A	O00139	406	1	-5	-7	5	1.0	1.0	0.9	1.1
FYCO1	Q9BQS8	293	1	-5	-7	-7	1.0	1.0	0.9	0.9
C2orf49	Q9BVC5	10	1	-6	-7	-3	1.0	0.9	0.9	1.0

EPM2AIP1	Q7L775	465	1	-7	-7	-13	1.0	0.9	0.9	0.9
FAM208A	Q9UK61	149	1	-10	-7	-2	1.0	0.9	0.9	1.0
MCM6	Q14566	180	1	-10	-7	-11	1.0	0.9	0.9	0.9
TUBA4A	P68366	295	1	-15	-7	13	1.0	0.9	0.9	1.1
TUBA3D	Q13748	295	1	-15	-7	13	1.0	0.9	0.9	1.1
TUBA1A	Q71U36	295	1	-15	-7	13	1.0	0.9	0.9	1.1
MAPK9	P45984	6	1	-16	-7	-8	1.0	0.9	0.9	0.9
DNAJA2	O60884	146	1	-18	-7	-7	1.0	0.9	0.9	0.9
NUP205	Q92621	1297	0	2	-7	2	1.0	1.0	0.9	1.0
RAD54L2	Q9Y4B4	453	0	-2	-7	-7	1.0	1.0	0.9	0.9
GCC2	Q8IWJ2	399	0	-5	-7	-6	1.0	1.0	0.9	0.9
DUSP22	Q9NRW4	124	0	-9	-7	-2	1.0	0.9	0.9	1.0
TXNRD1	Q16881	339	0	-9	-7	-5	1.0	0.9	0.9	1.0
BRE	Q9NXR7	53	0	-10	-7	-6	1.0	0.9	0.9	0.9
CASP3	P42574	264	0	-11	-7	-10	1.0	0.9	0.9	0.9
OSBPL9	Q96SU4	147	0	-18	-7	-4	1.0	0.8	0.9	1.0
EIF2A	Q9BY44	119	0	-19	-7	12	1.0	0.8	0.9	1.1
ZMYM3	Q14202	527	-1	1	-7	-3	1.0	1.0	0.9	1.0
PES1	O00541	361	-1	-5	-7	-6	1.0	1.0	0.9	0.9
GTF2I	P78347	215	-1	-9	-7	-14	1.0	0.9	0.9	0.9
MED23	Q9ULK4	1090	-1	-9	-7	-3	1.0	0.9	0.9	1.0
DIDO1	Q9BTC0	1079	-1	-9	-7	-6	1.0	0.9	0.9	0.9
MAPK9	P45984	222	-1	-9	-7	-12	1.0	0.9	0.9	0.9
MRPS6	P82932	105	-1	-10	-7	-12	1.0	0.9	0.9	0.9
SMAD2	Q15796	41	-1	-11	-7	-5	1.0	0.9	0.9	1.0
HERC6	Q8IVU3	698	-1	-13	-7	-5	1.0	0.9	0.9	1.0
PMS2	P54278	653	-1	-13	-7	-22	1.0	0.9	0.9	0.8
PDCD11	Q14690	1754	-1	-17	-7	-4	1.0	0.9	0.9	1.0
AP2B1	P63010	241	-1	-17	-7	-5	1.0	0.9	0.9	1.0
AP1B1	Q10567	241	-1	-17	-7	-5	1.0	0.9	0.9	1.0
PARP1	P09874	295	-1	-17	-7	0	1.0	0.9	0.9	1.0
NCBP1	Q09161	36	-1	-21	-7	-6	1.0	0.8	0.9	0.9
UBE2L6	O14933	86	-1	-9	-7	31	1.0	0.9	0.9	1.4
KMT2C	Q8NEZ4	4643	-1	-10	-7	-13	1.0	0.9	0.9	0.9
VPS13C	Q709C8	2159	-1	-11	-7	16	1.0	0.9	0.9	1.2
SLC2A9	Q9NRM0	36	-1	-13	-7	-12	1.0	0.9	0.9	0.9
RAB31	Q13636	49	-1	-14	-7	-8	1.0	0.9	0.9	0.9
DDB2	Q92466	322	-1	-15	-7	-4	1.0	0.9	0.9	1.0
NOA1	Q8NC60	505	-1	-15	-7	-8	1.0	0.9	0.9	0.9
KLRB1	Q12918	74	-1	-15	-7	-15	1.0	0.9	0.9	0.9
DPYSL2	Q16555	133	-1	-33	-7	-22	1.0	0.8	0.9	0.8
TENM4	Q6N022	601	-2	2	-7	15	1.0	1.0	0.9	1.2
AKAP9	Q99996	3085	-2	-4	-7	-24	1.0	1.0	0.9	0.8
PXN	P49023	585	-2	-5	-7	2	1.0	1.0	0.9	1.0
SETD1A	O15047	1649	-2	-8	-7	-6	1.0	0.9	0.9	0.9
FAM120A	Q9NZB2	531	-2	-9	-7	-23	1.0	0.9	0.9	0.8
MED14	O60244	1433	-2	-9	-7	11	1.0	0.9	0.9	1.1
CDC16	Q13042	194	-2	-10	-7	-10	1.0	0.9	0.9	0.9
MSH2	P43246	822	-2	-10	-7	-11	1.0	0.9	0.9	0.9
LRCH4	O75427	105	-2	-10	-7	-14	1.0	0.9	0.9	0.9
SRXN1	Q9BYN0	99	-2	-14	-7	-11	1.0	0.9	0.9	0.9
VPRBP	Q9Y4B6	1070	-2	-16	-7	-9	1.0	0.9	0.9	0.9
RAP1B	P61224	141	-2	-16	-7	-5	1.0	0.9	0.9	1.0

TAOK1	Q7L7X3	798	-2	-16	-7	-11	1.0	0.9	0.9	0.9
EEF1A1	P68104	234	-2	-18	-7	-4	1.0	0.9	0.9	1.0
UNC45A	Q9H3U1	535	-2	-18	-7	-8	1.0	0.9	0.9	0.9
RSPRY1	Q96DX4	190	-2	-23	-7	-4	1.0	0.8	0.9	1.0
BRMS1	Q9HCU9	126	-2	-27	-7	-17	1.0	0.8	0.9	0.9
NIN	Q8N4C6	647	-2	-5	-7	-3	1.0	1.0	0.9	1.0
HIVEP2	P31629	1500	-2	-5	-7	-21	1.0	1.0	0.9	0.8
PCMTD2	Q9NV79	223	-2	-8	-7	-17	1.0	0.9	0.9	0.9
DMXL1	Q9Y485	191	-2	-10	-7	-8	1.0	0.9	0.9	0.9
CSK	P41240	290	-2	-10	-7	-9	1.0	0.9	0.9	0.9
TDRD3	Q9H7E2	98	-2	-12	-7	-9	1.0	0.9	0.9	0.9
DHX37	Q8IY37	680	-2	-12	-7	-4	1.0	0.9	0.9	1.0
ZCCHC3	Q9NUD5	179	-2	-13	-7	-2	1.0	0.9	0.9	1.0
GIMAP8	Q8ND71	599	-2	-13	-7	-18	1.0	0.9	0.9	0.8
MON2	Q7Z3U7	1453	-2	-14	-7	3	1.0	0.9	0.9	1.0
MFN1	Q8IWA4	681	-2	-15	-7	-18	1.0	0.9	0.9	0.9
ZNF280D	Q6N043	487	-2	-17	-7	-14	1.0	0.9	0.9	0.9
ZC3HAV1	Q7Z2W4	162	-3	-4	-7	-14	1.0	1.0	0.9	0.9
IKBKE	Q14164	546	-3	-9	-7	-13	1.0	0.9	0.9	0.9
DOCK11	Q5JSL3	1878	-3	-9	-7	-10	1.0	0.9	0.9	0.9
TRAPPC8	Q9Y2L5	943	-3	-12	-7	-7	1.0	0.9	0.9	0.9
ROCK1	Q13464	1281	-3	-14	-7	-12	1.0	0.9	0.9	0.9
SSSCA1	O60232	53	-3	-15	-7	-13	1.0	0.9	0.9	0.9
DCP2	Q8IU60	140	-3	-16	-7	-35	1.0	0.9	0.9	0.7
MYLK	Q15746	1339	-3	-18	-7	-17	1.0	0.9	0.9	0.9
KNTC1	P50748	683	-3	-21	-7	-15	1.0	0.8	0.9	0.9
TANGO2	Q6ICL3	231	-3	-22	-7	5	1.0	0.8	0.9	1.0
SDPR	O95810	136	-3	-7	-7	-5	1.0	0.9	0.9	1.0
SRM	P19623	25	-3	-8	-7	-15	1.0	0.9	0.9	0.9
MED12	Q93074	997	-3	-10	-7	-9	1.0	0.9	0.9	0.9
MPI	P34949	309	-3	-12	-7	11	1.0	0.9	0.9	1.1
ZC3H7B	Q9UGR2	142	-3	-12	-7	-7	1.0	0.9	0.9	0.9
USP7	Q93009	90	-3	-12	-7	-1	1.0	0.9	0.9	1.0
CRACR2A	Q9BSW2	341	-3	-14	-7	-16	1.0	0.9	0.9	0.9
PHF20L1	A8MW92	472	-3	-14	-7	-26	1.0	0.9	0.9	0.8
RPL3	P39023	253	-4	-7	-7	-23	1.0	0.9	0.9	0.8
SART3	Q15020	780	-4	-12	-7	-4	1.0	0.9	0.9	1.0
TNS3	Q68CZ2	615	-4	-15	-7	-12	1.0	0.9	0.9	0.9
PIK3CD	O00329	474	-4	-5	-7	-6	1.0	1.0	0.9	0.9
ARHGAP26	Q9UNA1	441	-4	-7	-7	-9	1.0	0.9	0.9	0.9
MEFV	O15553	639	-4	-11	-7	-8	1.0	0.9	0.9	0.9
C2CD5	Q86YS7	449	-4	-12	-7	-7	1.0	0.9	0.9	0.9
ACTR3	P61158	34	-4	-21	-7	3	1.0	0.8	0.9	1.0
GMIP	Q9P107	957	-5	-1	-7	-20	1.0	1.0	0.9	0.8
MTAP	Q13126	145	-5	-13	-7	1	1.0	0.9	0.9	1.0
RNF40	O75150	890	-5	-13	-7	-9	1.0	0.9	0.9	0.9
RNF14	Q9UBS8	417	-5	-17	-7	-20	1.0	0.9	0.9	0.8
TRAF3IP3	Q9Y228	35	-5	-23	-7	-13	1.0	0.8	0.9	0.9
ATE1	O95260	138	-5	-8	-7	-14	1.0	0.9	0.9	0.9
PIAS4	Q8N2W9	326	-5	-9	-7	-14	1.0	0.9	0.9	0.9
CD48	P09326	196	-5	-9	-7	-12	1.0	0.9	0.9	0.9
NUP133	Q8WUM0	530	-5	-12	-7	-4	1.0	0.9	0.9	1.0
ASMTL	O95671	274	-5	-14	-7	-16	1.0	0.9	0.9	0.9

CAPN1	P07384	351	-5	-25	-7	-3	1.0	0.8	0.9	1.0
ZC3HAV1	Q7Z2W4	581	-5	-32	-7	4	1.0	0.8	0.9	1.0
NAIP	Q13075	1259	-6	2	-7	-24	0.9	1.0	0.9	0.8
ATG9A	Q7Z3C6	764	-6	-4	-7	-16	0.9	1.0	0.9	0.9
SEPT6	Q14141	14	-6	-17	-7	-1	0.9	0.9	0.9	1.0
TYSND1	Q2T9J0	110	-6	1	-7	-13	0.9	1.0	0.9	0.9
UBE2D2	P62837	85	-6	-10	-7	22	0.9	0.9	0.9	1.3
DOCK10	Q96BY6	1997	-6	-10	-7	-4	0.9	0.9	0.9	1.0
RCSD1	Q6JBY9	181	-6	-11	-7	-13	0.9	0.9	0.9	0.9
RPS3A	P61247	139	-6	-15	-7	3	0.9	0.9	0.9	1.0
NUAK2	Q9H093	277	-7	-31	-7	-12	0.9	0.8	0.9	0.9
VRK1	Q99986	205	-7	-35	-7	-2	0.9	0.7	0.9	1.0
AIM1	Q9Y4K1	541	-8	-4	-7	-11	0.9	1.0	0.9	0.9
USP19	O94966	817	-8	-10	-7	-9	0.9	0.9	0.9	0.9
PANK4	Q9NVE7	699	-8	-16	-7	8	0.9	0.9	0.9	1.1
DNMT3A	Q9Y6K1	554	-8	-17	-7	-9	0.9	0.9	0.9	0.9
RUVBL2	Q9Y230	227	-8	-27	-7	-9	0.9	0.8	0.9	0.9
VPS18	Q9P253	445	-8	-18	-7	-7	0.9	0.9	0.9	0.9
LRWD1	Q9UFC0	449	-9	-5	-7	-14	0.9	1.0	0.9	0.9
PLEC	Q15149	530	-9	-14	-7	-16	0.9	0.9	0.9	0.9
RAI1	Q7Z5J4	1015	-9	-9	-7	-17	0.9	0.9	0.9	0.9
NUP93	Q8N1F7	422	-9	-11	-7	-18	0.9	0.9	0.9	0.8
RAI1	Q7Z5J4	594	-9	-16	-7	-27	0.9	0.9	0.9	0.8
ACAT2	Q9BWD1	65	-9	-19	-7	-4	0.9	0.8	0.9	1.0
INTS2	Q9H0H0	988	-9	-38	-7	2	0.9	0.7	0.9	1.0
TRDMT1	O14717	287	-10	-7	-7	-25	0.9	0.9	0.9	0.8
BRMS1L	Q5PSV4	127	-10	-8	-7	-12	0.9	0.9	0.9	0.9
MTMR6	Q9Y217	72	-10	-8	-7	-4	0.9	0.9	0.9	1.0
OTUB1	Q96FW1	91	-10	-15	-7	-7	0.9	0.9	0.9	0.9
VARS	P26640	917	-10	-15	-7	-2	0.9	0.9	0.9	1.0
ARHGAP31	Q2M1Z3	76	-11	5	-7	-4	0.9	1.0	0.9	1.0
FN3KRP	Q9HA64	11	-11	-7	-7	3	0.9	0.9	0.9	1.0
INPP1	P49441	10	-11	-11	-7	-7	0.9	0.9	0.9	0.9
NSA2	O95478	253	-11	-16	-7	-19	0.9	0.9	0.9	0.8
DUSP6	Q16828	353	-12	-16	-7	-21	0.9	0.9	0.9	0.8
ADD2	P35612	210	-12	-21	-7	-11	0.9	0.8	0.9	0.9
HPS4	Q9NQG7	535	-12	-10	-7	-25	0.9	0.9	0.9	0.8
DLAT	P10515	291	-14	-15	-7	6	0.9	0.9	0.9	1.1
TWF2	Q6IBS0	67	-14	-20	-7	4	0.9	0.8	0.9	1.0
XPO7	Q9UIA9	244	-16	-16	-7	25	0.9	0.9	0.9	1.3
TRIM65	Q6PJ69	174	-16	-21	-7	-18	0.9	0.8	0.9	0.8
ALPK1	Q96QP1	439	-17	-15	-7	-18	0.9	0.9	0.9	0.9
GFER	P55789	165	-21	-5	-7	-14	0.8	1.0	0.9	0.9
PTDSS1	P48651	347	-24	-29	-7	-29	0.8	0.8	0.9	0.8
ITPR3	Q14573	1726	-25	-21	-7	-16	0.8	0.8	0.9	0.9
PITHD1	Q9GZP4	14	-27	-25	-7	-16	0.8	0.8	0.9	0.9
MARS	P56192	441	-32	-5	-7	1	0.8	1.0	0.9	1.0
PREP	P48147	532	-33	-27	-7	-11	0.8	0.8	0.9	0.9
FLNA	P21333	483	-35	-20	-7	-8	0.7	0.8	0.9	0.9
PREP	P48147	255	-42	-30	-7	-7	0.7	0.8	0.9	0.9
CARD11	Q9BXL7	1060	17	-23	-8	19	1.2	0.8	0.9	1.2
FBXW11	Q9UKB1	151	16	-10	-8	6	1.2	0.9	0.9	1.1
FAM160B1	Q5W0V3	624	16	-12	-8	2	1.2	0.9	0.9	1.0

SLC25A4	P12235	129	16	-13	-8	9	1.2	0.9	0.9	1.1
SLC25A6	P12236	129	16	-13	-8	9	1.2	0.9	0.9	1.1
IFIT3	O14879	365	15	-10	-8	-6	1.2	0.9	0.9	0.9
UBR4	Q5T4S7	953	14	-14	-8	-6	1.2	0.9	0.9	0.9
RABGAP1	Q9Y3P9	250	13	-22	-8	6	1.1	0.8	0.9	1.1
NUBP2	Q9Y5Y2	177	12	-3	-8	-8	1.1	1.0	0.9	0.9
NCKAP1L	P55160	36	12	-8	-8	3	1.1	0.9	0.9	1.0
ARID4A	P29374	849	12	-9	-8	-2	1.1	0.9	0.9	1.0
NPEPPS	P55786	537	12	-2	-8	6	1.1	1.0	0.9	1.1
SMARCA2	P51531	1175	12	-18	-8	-10	1.1	0.9	0.9	0.9
SMARCA4	P51532	1205	12	-18	-8	-10	1.1	0.9	0.9	0.9
ARFRP1	Q13795	168	11	-3	-8	-12	1.1	1.0	0.9	0.9
PLEC	Q15149	3493	11	-14	-8	-3	1.1	0.9	0.9	1.0
ATP2A2	P16615	498	11	-18	-8	5	1.1	0.9	0.9	1.0
KAT6B	Q8WYB5	751	11	-21	-8	-9	1.1	0.8	0.9	0.9
KAT6A	Q92794	540	11	-21	-8	-9	1.1	0.8	0.9	0.9
TTF1	Q15361	708	10	14	-8	-2	1.1	1.2	0.9	1.0
PPIH	O43447	131	10	-7	-8	9	1.1	0.9	0.9	1.1
MRPL44	Q9H9J2	276	10	-11	-8	-20	1.1	0.9	0.9	0.8
NUP93	Q8N1F7	569	10	-49	-8	-11	1.1	0.7	0.9	0.9
MED17	Q9NVC6	363	9	-6	-8	0	1.1	0.9	0.9	1.0
EHD3	Q9NZN3	138	9	-7	-8	-12	1.1	0.9	0.9	0.9
RNH1	P13489	216	9	-8	-8	-5	1.1	0.9	0.9	1.0
PXN	P49023	538	9	-8	-8	-4	1.1	0.9	0.9	1.0
PRKCH	P24723	493	9	-14	-8	0	1.1	0.9	0.9	1.0
YWHAG	P61981	112	9	-14	-8	-5	1.1	0.9	0.9	1.0
EIF3A	Q14152	78	9	-20	-8	-2	1.1	0.8	0.9	1.0
CASP9	P55211	403	8	-2	-8	-4	1.1	1.0	0.9	1.0
PHF6	Q8IWS0	292	8	-15	-8	2	1.1	0.9	0.9	1.0
RAI1	Q7Z5J4	8	8	-23	-8	-18	1.1	0.8	0.9	0.8
USP9X	Q93008	540	8	-2	-8	0	1.1	1.0	0.9	1.0
GAPVD1	Q14C86	275	8	-6	-8	-16	1.1	0.9	0.9	0.9
SPTAN1	Q13813	466	8	-10	-8	-5	1.1	0.9	0.9	1.0
VPS13C	Q709C8	1617	8	-12	-8	-3	1.1	0.9	0.9	1.0
DDX60	Q8IY21	1224	8	-18	-8	-11	1.1	0.8	0.9	0.9
RBM22	Q9NW64	179	7	-4	-8	1	1.1	1.0	0.9	1.0
C2CD5	Q86YS7	867	7	-5	-8	-5	1.1	1.0	0.9	1.0
SUPV3L1	Q8IYB8	230	7	-10	-8	1	1.1	0.9	0.9	1.0
RPP30	P78346	225	7	-16	-8	-8	1.1	0.9	0.9	0.9
AIMP2	Q13155	23	7	3	-8	-17	1.1	1.0	0.9	0.9
ATP13A1	Q9HD20	648	7	-7	-8	1	1.1	0.9	0.9	1.0
PSMA7	O14818	63	7	-8	-8	1	1.1	0.9	0.9	1.0
SF3A3	Q12874	274	7	-14	-8	-11	1.1	0.9	0.9	0.9
SYNE2	Q8WXH0	1509	7	-27	-8	-21	1.1	0.8	0.9	0.8
LSM14A	Q8ND56	375	6	-6	-8	-6	1.1	0.9	0.9	0.9
XPC	Q01831	377	6	-9	-8	-20	1.1	0.9	0.9	0.8
SHMT1	P34896	204	6	-10	-8	4	1.1	0.9	0.9	1.0
PRKCD	Q05655	127	6	-11	-8	-12	1.1	0.9	0.9	0.9
EARS2	Q5JPH6	341	6	-1	-8	7	1.1	1.0	0.9	1.1
CUL7	Q14999	1569	6	-1	-8	-4	1.1	1.0	0.9	1.0
HSPBP1	Q9NZL4	22	6	-1	-8	-4	1.1	1.0	0.9	1.0
TLN1	Q9Y490	1363	6	-3	-8	-9	1.1	1.0	0.9	0.9
EPB41L2	O43491	424	6	-7	-8	-13	1.1	0.9	0.9	0.9

IRF1	P10914	53	6	-8	-8	6	1.1	0.9	0.9	1.1
MAP4	P27816	635	6	-8	-8	-15	1.1	0.9	0.9	0.9
TACC1	O75410	219	6	-11	-8	-3	1.1	0.9	0.9	1.0
TYW3	Q6IPR3	49	6	-19	-8	-19	1.1	0.8	0.9	0.8
LPCAT2	Q7L5N7	326	5	-5	-8	-8	1.1	1.0	0.9	0.9
PITPNC1	Q9UKF7	142	5	-6	-8	0	1.1	0.9	0.9	1.0
XRN2	Q9H0D6	230	5	-8	-8	-10	1.1	0.9	0.9	0.9
PITPNA	Q00169	187	5	-11	-8	-5	1.1	0.9	0.9	1.0
HK3	P52790	892	5	-13	-8	2	1.1	0.9	0.9	1.0
WDR37	Q9Y2I8	146	5	-13	-8	-16	1.1	0.9	0.9	0.9
NDUFA2	O43678	58	5	-27	-8	-5	1.1	0.8	0.9	1.0
SUMO1	P63165	52	5	-4	-8	3	1.0	1.0	0.9	1.0
SACM1L	Q9NTJ5	23	5	-4	-8	2	1.0	1.0	0.9	1.0
CLTC	Q00610	824	5	-9	-8	-12	1.0	0.9	0.9	0.9
TUBB1	Q9H4B7	303	5	-12	-8	-17	1.0	0.9	0.9	0.9
SNRNP200	O75643	238	5	-13	-8	-12	1.0	0.9	0.9	0.9
FAM208A	Q9UK61	538	5	-14	-8	-20	1.0	0.9	0.9	0.8
DSTN	P60981	23	5	-15	-8	-17	1.0	0.9	0.9	0.9
CLASP1	Q7Z460	1212	5	-22	-8	-20	1.0	0.8	0.9	0.8
PRKDC	P78527	1629	5	-31	-8	-6	1.0	0.8	0.9	0.9
MAPK9	P45984	163	4	0	-8	-6	1.0	1.0	0.9	0.9
MAPK10	P53779	201	4	0	-8	-6	1.0	1.0	0.9	0.9
RQCD1	Q92600	99	4	-12	-8	1	1.0	0.9	0.9	1.0
FUBP3	Q96I24	366	4	-12	-8	-9	1.0	0.9	0.9	0.9
SND1	Q7KZF4	736	4	-15	-8	3	1.0	0.9	0.9	1.0
ATP5SL	Q9NW81	184	4	-18	-8	-16	1.0	0.8	0.9	0.9
CBWD1	Q9BRT8	109	4	-29	-8	-31	1.0	0.8	0.9	0.8
UHRF1BP1L	A0JNW5	476	4	5	-8	-11	1.0	1.0	0.9	0.9
UQCRC1	P31930	453	4	2	-8	11	1.0	1.0	0.9	1.1
RNPEPL1	Q9HAU8	51	4	-5	-8	-11	1.0	1.0	0.9	0.9
SLC6A4	P31645	622	4	-6	-8	1	1.0	0.9	0.9	1.0
PRMT1	Q99873	109	4	-6	-8	-11	1.0	0.9	0.9	0.9
INPPL1	O15357	1121	4	-7	-8	-9	1.0	0.9	0.9	0.9
IQGAP1	P46940	1534	4	-9	-8	-4	1.0	0.9	0.9	1.0
ATRX	P46100	223	4	-11	-8	-16	1.0	0.9	0.9	0.9
TBXAS1	P24557	505	4	-13	-8	0	1.0	0.9	0.9	1.0
HSPA4	P34932	38	4	-14	-8	-6	1.0	0.9	0.9	0.9
RB1CC1	Q8TDY2	81	4	-15	-8	-5	1.0	0.9	0.9	1.0
PLEC	Q15149	3667	4	-20	-8	-5	1.0	0.8	0.9	1.0
pk	D4Q8H0	150	3	6	-8	11	1.0	1.1	0.9	1.1
ABHD14B	Q96IU4	190	3	-4	-8	-17	1.0	1.0	0.9	0.9
TBCK	Q8TEA7	772	3	-5	-8	-7	1.0	1.0	0.9	0.9
UBR2	Q8IWV8	1360	3	-7	-8	-2	1.0	0.9	0.9	1.0
RANBP2	P49792	1424	3	-10	-8	-10	1.0	0.9	0.9	0.9
MAP3K3	Q99759	379	3	-12	-8	-17	1.0	0.9	0.9	0.9
MAP3K2	Q9Y2U5	373	3	-12	-8	-17	1.0	0.9	0.9	0.9
ILKAP	Q9H0C8	301	3	-13	-8	-7	1.0	0.9	0.9	0.9
MRPS9	P82933	233	3	-17	-8	-11	1.0	0.9	0.9	0.9
GALE	Q14376	264	3	-23	-8	-4	1.0	0.8	0.9	1.0
IPO13	O94829	838	3	7	-8	4	1.0	1.1	0.9	1.0
XPO1	O14980	34	3	-9	-8	-6	1.0	0.9	0.9	0.9
ZFAND3	Q9H8U3	35	3	-9	-8	-8	1.0	0.9	0.9	0.9
KIAA1109	Q2LD37	1314	3	-10	-8	-24	1.0	0.9	0.9	0.8

SYK	P43405	597	3	-10	-8	0	1.0	0.9	0.9	1.0
CORO7-PAM:	A0A0A6YYL4	656	3	-11	-8	-3	1.0	0.9	0.9	1.0
CLTC	Q00610	1528	3	-11	-8	-5	1.0	0.9	0.9	1.0
CABIN1	Q9Y6J0	1679	3	-11	-8	-14	1.0	0.9	0.9	0.9
PTPN6	P29350	361	3	-11	-8	-18	1.0	0.9	0.9	0.9
CBWD1	Q9BRT8	310	3	-14	-8	-2	1.0	0.9	0.9	1.0
MRPL36	Q9P0J6	76	3	-15	-8	6	1.0	0.9	0.9	1.1
SBF2	Q86WG5	1540	3	-23	-8	-17	1.0	0.8	0.9	0.9
DNAAF5	Q86Y56	138	2	-7	-8	-6	1.0	0.9	0.9	0.9
MDH2	P40926	275	2	-11	-8	3	1.0	0.9	0.9	1.0
HTATSF1	O43719	186	2	-12	-8	1	1.0	0.9	0.9	1.0
TRIM56	Q9BRZ2	131	2	-12	-8	-4	1.0	0.9	0.9	1.0
PCK2	Q16822	151	2	-12	-8	-16	1.0	0.9	0.9	0.9
PSMD2	Q13200	251	2	-21	-8	0	1.0	0.8	0.9	1.0
ATM	Q13315	669	2	-5	-8	-9	1.0	1.0	0.9	0.9
WDFY4	Q6ZS81	2510	2	-7	-8	1	1.0	0.9	0.9	1.0
CLTC	Q00610	926	2	-9	-8	2	1.0	0.9	0.9	1.0
LMNA	P02545	588	2	-9	-8	-17	1.0	0.9	0.9	0.9
DHX37	Q8IY37	903	2	-10	-8	4	1.0	0.9	0.9	1.0
CXXC1	Q9P0U4	420	2	-10	-8	-7	1.0	0.9	0.9	0.9
MINA	Q8IUF8	19	2	-10	-8	-19	1.0	0.9	0.9	0.8
FLII	Q13045	289	2	-13	-8	-1	1.0	0.9	0.9	1.0
RANBP2	P49792	50	2	-13	-8	-8	1.0	0.9	0.9	0.9
WAPL	Q7Z5K2	1170	2	-15	-8	-2	1.0	0.9	0.9	1.0
POLR2B	P30876	357	1	2	-8	3	1.0	1.0	0.9	1.0
PRPF40A	O75400	39	1	-3	-8	-1	1.0	1.0	0.9	1.0
STK39	Q9UEW8	237	1	-4	-8	-6	1.0	1.0	0.9	0.9
TRIO	O75962	1207	1	-7	-8	-10	1.0	0.9	0.9	0.9
EXOSC4	Q9NPD3	97	1	-10	-8	-6	1.0	0.9	0.9	0.9
EFTUD2	Q15029	780	1	-12	-8	-4	1.0	0.9	0.9	1.0
ARHGAP25	P42331	448	1	-13	-8	-19	1.0	0.9	0.9	0.8
CDA	P32320	59	1	-13	-8	-16	1.0	0.9	0.9	0.9
GANAB	Q14697	502	1	-15	-8	-10	1.0	0.9	0.9	0.9
LYN	P07948	419	1	-16	-8	-23	1.0	0.9	0.9	0.8
ACAD9	Q9H845	613	1	-12	-8	1	1.0	0.9	0.9	1.0
PPA2	Q9H2U2	290	1	-14	-8	1	1.0	0.9	0.9	1.0
SH2D3C	Q8N5H7	599	0	5	-8	-16	1.0	1.0	0.9	0.9
CAMLG	P49069	123	0	-3	-8	33	1.0	1.0	0.9	1.5
PDDC1	Q8NB37	154	0	-7	-8	2	1.0	0.9	0.9	1.0
PPME1	Q9Y570	238	0	-9	-8	-2	1.0	0.9	0.9	1.0
GIMAP7	Q8NHV1	195	0	-9	-8	-3	1.0	0.9	0.9	1.0
FAS	P25445	304	0	-12	-8	-21	1.0	0.9	0.9	0.8
DDX39A	O00148	164	0	-13	-8	-10	1.0	0.9	0.9	0.9
HECTD1	Q9ULT8	2579	0	-14	-8	-12	1.0	0.9	0.9	0.9
PGM2	Q96G03	573	0	-15	-8	-8	1.0	0.9	0.9	0.9
NPEPPS	P55786	190	0	-16	-8	-3	1.0	0.9	0.9	1.0
GRK5	P34947	54	0	-21	-8	-7	1.0	0.8	0.9	0.9
APOA2	P02652	29	-1	8	-8	-6	1.0	1.1	0.9	0.9
CIAPIN1	Q6FI81	237	-1	-2	-8	-6	1.0	1.0	0.9	0.9
BIRC6	Q9NR09	752	-1	-4	-8	-12	1.0	1.0	0.9	0.9
VDAC2	P45880	47	-1	-6	-8	-7	1.0	0.9	0.9	0.9
NFKB1	P19838	261	-1	-7	-8	-8	1.0	0.9	0.9	0.9
TXNDC5	Q8NBS9	350	-1	-7	-8	-13	1.0	0.9	0.9	0.9

ALKBH3	Q96Q83	201	-1	-8	-8	-13	1.0	0.9	0.9	0.9
CCDC22	O60826	369	-1	-9	-8	-8	1.0	0.9	0.9	0.9
ORC5	O43913	171	-1	-11	-8	-8	1.0	0.9	0.9	0.9
CMIP	Q8IY22	738	-1	-11	-8	0	1.0	0.9	0.9	1.0
PHKB	Q93100	434	-1	-11	-8	-12	1.0	0.9	0.9	0.9
SAMHD1	Q9Y3Z3	80	-1	-13	-8	-15	1.0	0.9	0.9	0.9
MDH2	P40926	93	-1	-14	-8	9	1.0	0.9	0.9	1.1
KAT6A	Q92794	723	-1	-2	-8	-9	1.0	1.0	0.9	0.9
WASF2	Q9Y6W5	27	-1	-3	-8	-12	1.0	1.0	0.9	0.9
ZNF14	P17017	234	-1	-9	-8	-13	1.0	0.9	0.9	0.9
ABCF1	Q8NE71	741	-1	-9	-8	-6	1.0	0.9	0.9	0.9
ACTR3	P61158	408	-1	-11	-8	-3	1.0	0.9	0.9	1.0
FAM98B	Q52LJ0	295	-1	-13	-8	-12	1.0	0.9	0.9	0.9
FAM98A	Q8NCA5	293	-1	-13	-8	-12	1.0	0.9	0.9	0.9
FGB	P02675	231	-1	-13	-8	-7	1.0	0.9	0.9	0.9
PPP3R1	P63098	154	-1	-13	-8	-12	1.0	0.9	0.9	0.9
ACTR3	P61158	235	-1	-15	-8	-2	1.0	0.9	0.9	1.0
DPYD	Q12882	1025	-1	-15	-8	-17	1.0	0.9	0.9	0.9
SNX9	Q9Y5X1	427	-1	-16	-8	-8	1.0	0.9	0.9	0.9
AKAP11	Q9UKA4	576	-2	-1	-8	-6	1.0	1.0	0.9	0.9
CNOT1	A5YKK6	2359	-2	-5	-8	-7	1.0	1.0	0.9	0.9
ALB	P02768	58	-2	-6	-8	-8	1.0	0.9	0.9	0.9
PAF1	Q8N7H5	36	-2	-7	-8	-6	1.0	0.9	0.9	0.9
KIFC1	Q9BW19	509	-2	-7	-8	-23	1.0	0.9	0.9	0.8
STRAP	Q9Y3F4	305	-2	-9	-8	-9	1.0	0.9	0.9	0.9
SNRNP200	O75643	428	-2	-9	-8	-11	1.0	0.9	0.9	0.9
VAV1	P15498	652	-2	-9	-8	-7	1.0	0.9	0.9	0.9
RNF169	Q8NCN4	293	-2	-9	-8	-11	1.0	0.9	0.9	0.9
EMSY	Q7Z589	971	-2	-10	-8	-20	1.0	0.9	0.9	0.8
CSDE1	O75534	506	-2	-10	-8	-8	1.0	0.9	0.9	0.9
ILK	Q13418	239	-2	-12	-8	6	1.0	0.9	0.9	1.1
LRRC45	Q96CN5	385	-2	-15	-8	-23	1.0	0.9	0.9	0.8
ACTR2	P61160	20	-2	-17	-8	-8	1.0	0.9	0.9	0.9
MAT2B	Q9NZL9	58	-2	-18	-8	1	1.0	0.9	0.9	1.0
SAFB	Q15424	225	-2	-5	-8	-10	1.0	1.0	0.9	0.9
SUCLG2	Q96I99	162	-2	-7	-8	-7	1.0	0.9	0.9	0.9
CLCC1	Q96S66	550	-2	-8	-8	-21	1.0	0.9	0.9	0.8
CGGBP1	Q9UFW8	92	-2	-10	-8	-22	1.0	0.9	0.9	0.8
RBL2	Q08999	274	-2	-10	-8	-6	1.0	0.9	0.9	0.9
DUS2	Q9NX74	475	-2	-10	-8	-11	1.0	0.9	0.9	0.9
RBL2	Q08999	579	-2	-11	-8	3	1.0	0.9	0.9	1.0
ARHGEF1	Q92888	537	-2	-11	-8	-6	1.0	0.9	0.9	0.9
PDCD4	Q53EL6	275	-2	-11	-8	-12	1.0	0.9	0.9	0.9
CXorf56	Q9H5V9	11	-2	-12	-8	-21	1.0	0.9	0.9	0.8
DPYD	Q12882	52	-2	-13	-8	-21	1.0	0.9	0.9	0.8
COG1	Q8WTW3	72	-2	-16	-8	-14	1.0	0.9	0.9	0.9
PRMT9	Q6P2P2	290	-2	-16	-8	-24	1.0	0.9	0.9	0.8
DNMT3A	Q9Y6K1	666	-2	-16	-8	-6	1.0	0.9	0.9	0.9
ZMIZ1	Q9ULJ6	742	-2	-18	-8	-5	1.0	0.8	0.9	1.0
CAD	P27708	2092	-2	-21	-8	5	1.0	0.8	0.9	1.0
FLNB	O75369	2537	-2	-31	-8	-19	1.0	0.8	0.9	0.8
CDC42BPB	Q9Y5S2	1517	-3	-9	-8	-9	1.0	0.9	0.9	0.9
GTF3C1	Q12789	286	-3	-10	-8	-5	1.0	0.9	0.9	1.0

ARF4	P18085	62	-3	-10	-8	-6	1.0	0.9	0.9	0.9
PPIP5K2	O43314	488	-3	-11	-8	-6	1.0	0.9	0.9	0.9
SYNE1	Q8NF91	8076	-3	-11	-8	-10	1.0	0.9	0.9	0.9
Uncharacteri	A0A087WZG	664	-3	-12	-8	-17	1.0	0.9	0.9	0.9
ACP1	P24666	18	-3	-14	-8	-13	1.0	0.9	0.9	0.9
THADA	Q6YHU6	1393	-3	-16	-8	-19	1.0	0.9	0.9	0.8
DMXL1	Q9Y485	543	-3	-17	-8	-11	1.0	0.9	0.9	0.9
PIK3R1	P27986	498	-3	-25	-8	-8	1.0	0.8	0.9	0.9
ZNF101	Q8IZC7	321	-3	-6	-8	-7	1.0	0.9	0.9	0.9
HDHD3	Q9BSH5	243	-3	-6	-8	-11	1.0	0.9	0.9	0.9
ZNF106	Q9H2Y7	1472	-3	-7	-8	-1	1.0	0.9	0.9	1.0
GET4	Q7L5D6	160	-3	-7	-8	-12	1.0	0.9	0.9	0.9
CHML	P26374	320	-3	-10	-8	-13	1.0	0.9	0.9	0.9
CNTRL	Q7Z7A1	1375	-3	-11	-8	-8	1.0	0.9	0.9	0.9
KDM5A	P29375	692	-3	-12	-8	-5	1.0	0.9	0.9	1.0
DDX5	P17844	170	-3	-15	-8	-6	1.0	0.9	0.9	0.9
DDX17	Q92841	247	-3	-15	-8	-6	1.0	0.9	0.9	0.9
RABEP2	Q9H5N1	482	-3	-16	-8	-16	1.0	0.9	0.9	0.9
APIP	Q96GX9	32	-3	-29	-8	-1	1.0	0.8	0.9	1.0
FYCO1	Q9BQS8	928	-4	-6	-8	3	1.0	0.9	0.9	1.0
PPP1CA	P62136	172	-4	-6	-8	-5	1.0	0.9	0.9	1.0
PIGH	Q14442	11	-4	-7	-8	-16	1.0	0.9	0.9	0.9
TLR2	O60603	640	-4	-8	-8	-11	1.0	0.9	0.9	0.9
PRKD2	Q9BZL6	217	-4	-7	-8	-14	1.0	0.9	0.9	0.9
KIAA0196	Q12768	385	-4	-8	-8	-5	1.0	0.9	0.9	1.0
SEC13	P55735	187	-4	-13	-8	-11	1.0	0.9	0.9	0.9
PHF14	O94880	441	-4	-16	-8	4	1.0	0.9	0.9	1.0
RNPC3	Q96LT9	156	-4	-16	-8	-23	1.0	0.9	0.9	0.8
HK3	P52790	612	-4	-17	-8	-3	1.0	0.9	0.9	1.0
UBR5	O95071	769	-4	-32	-8	-4	1.0	0.8	0.9	1.0
UTP11	Q9Y3A2	201	-5	-7	-8	-1	1.0	0.9	0.9	1.0
GTF3C1	Q12789	853	-5	-9	-8	-16	1.0	0.9	0.9	0.9
SLC25A3	Q00325	237	-5	-17	-8	-2	1.0	0.9	0.9	1.0
GIMAP2	Q9UG22	88	-5	-7	-8	-6	1.0	0.9	0.9	0.9
CAD	P27708	897	-5	-13	-8	-1	1.0	0.9	0.9	1.0
RNPEP	Q9H4A4	151	-5	-13	-8	-1	1.0	0.9	0.9	1.0
IARS	P41252	400	-5	-14	-8	1	1.0	0.9	0.9	1.0
PLEC	Q15149	4574	-5	-15	-8	-7	1.0	0.9	0.9	0.9
MTFR1L	Q9H019	52	-6	-6	-8	-12	0.9	0.9	0.9	0.9
INO80	Q9ULG1	965	-6	-7	-8	26	0.9	0.9	0.9	1.4
TOR4A	Q9NXH8	239	-6	-9	-8	-3	0.9	0.9	0.9	1.0
RFC4	P35249	48	-6	-9	-8	-4	0.9	0.9	0.9	1.0
TMEM209	Q96SK2	295	-6	-11	-8	-5	0.9	0.9	0.9	1.0
CDA	P32320	21	-6	-17	-8	-14	0.9	0.9	0.9	0.9
SPTLC1	O15269	419	-6	-23	-8	3	0.9	0.8	0.9	1.0
RPL14	P50914	54	-6	-26	-8	3	0.9	0.8	0.9	1.0
CMPK1	P30085	20	-6	-5	-8	1	0.9	1.0	0.9	1.0
GTF3C2	Q8WUA4	828	-6	-6	-8	-19	0.9	0.9	0.9	0.8
PLSCR1	O15162	254	-6	-8	-8	-13	0.9	0.9	0.9	0.9
LRRK2	Q5S007	2492	-6	-9	-8	-6	0.9	0.9	0.9	0.9
RASAL3	Q86YV0	546	-6	-11	-8	-8	0.9	0.9	0.9	0.9
SMAD1	Q15797	57	-6	-16	-8	13	0.9	0.9	0.9	1.1
SLK	Q9H2G2	1212	-6	-17	-8	-9	0.9	0.9	0.9	0.9

MVD	P53602	386	-7	-2	-8	-22	0.9	1.0	0.9	0.8
PMPCA	Q10713	466	-7	-9	-8	0	0.9	0.9	0.9	1.0
TRMT2A	Q8IZ69	243	-7	-13	-8	-12	0.9	0.9	0.9	0.9
THG1L	Q9NWX6	47	-7	-13	-8	-16	0.9	0.9	0.9	0.9
GDI1	P31150	202	-7	-16	-8	-4	0.9	0.9	0.9	1.0
MAD1L1	Q9Y6D9	662	-7	-21	-8	-15	0.9	0.8	0.9	0.9
WDR1	O75083	507	-7	-25	-8	6	0.9	0.8	0.9	1.1
SAMM50	Q9Y512	457	-7	-10	-8	11	0.9	0.9	0.9	1.1
PSTPIP2	Q9H939	90	-7	-14	-8	-5	0.9	0.9	0.9	1.0
WARS	P23381	309	-7	-15	-8	-5	0.9	0.9	0.9	1.0
UBE4B	O95155	581	-8	-5	-8	-8	0.9	1.0	0.9	0.9
PPP1CA	P62136	171	-8	-10	-8	-9	0.9	0.9	0.9	0.9
ACSF2	Q96CM8	64	-8	-13	-8	-12	0.9	0.9	0.9	0.9
ITPK1	Q13572	156	-8	-19	-8	16	0.9	0.8	0.9	1.2
PDXDC1	Q6P996	126	-8	-22	-8	-20	0.9	0.8	0.9	0.8
STAMBPL1	Q96FJ0	38	-8	-14	-8	-24	0.9	0.9	0.9	0.8
TMEM55B	Q86T03	129	-8	-15	-8	-14	0.9	0.9	0.9	0.9
TMEM55A	Q8N4L2	103	-8	-15	-8	-14	0.9	0.9	0.9	0.9
PTGS1	P23219	574	-9	-15	-8	-11	0.9	0.9	0.9	0.9
Uncharacteri	A0A0G2JPG4	111	-9	-4	-8	-11	0.9	1.0	0.9	0.9
QARS	P47897	358	-9	-6	-8	-6	0.9	0.9	0.9	0.9
NUP88	Q99567	608	-9	-8	-8	-13	0.9	0.9	0.9	0.9
PCNT	O95613	2628	-9	-9	-8	-15	0.9	0.9	0.9	0.9
SEC62	Q99442	32	-9	-13	-8	-5	0.9	0.9	0.9	1.0
ATG16L2	Q8NAA4	568	-9	-16	-8	-14	0.9	0.9	0.9	0.9
TERF1	P54274	118	-9	-23	-8	-18	0.9	0.8	0.9	0.8
LAS1L	Q9Y4W2	256	-10	-11	-8	-21	0.9	0.9	0.9	0.8
BLOC1S4	Q9NUP1	210	-10	-14	-8	3	0.9	0.9	0.9	1.0
NARF	Q9UHQ1	192	-10	-13	-8	-7	0.9	0.9	0.9	0.9
PXN	P49023	546	-10	-15	-8	-14	0.9	0.9	0.9	0.9
NLN	Q9BYT8	256	-10	-17	-8	-13	0.9	0.9	0.9	0.9
VPS28	Q9UK41	96	-11	-21	-8	-14	0.9	0.8	0.9	0.9
ITSN2	Q9NZM3	1389	-11	-22	-8	-28	0.9	0.8	0.9	0.8
ZNF652	Q9Y2D9	239	-12	-21	-8	-26	0.9	0.8	0.9	0.8
NIPBL	Q6KC79	56	-12	-29	-8	-5	0.9	0.8	0.9	1.0
XPO7	Q9UIA9	43	-12	-10	-8	5	0.9	0.9	0.9	1.1
RNH1	P13489	387	-14	-19	-8	-20	0.9	0.8	0.9	0.8
ERCC4	Q92889	560	-14	-11	-8	-17	0.9	0.9	0.9	0.9
ZNF638	Q14966	1023	-15	-14	-8	15	0.9	0.9	0.9	1.2
ATXN2	Q99700	892	-15	-22	-8	-21	0.9	0.8	0.9	0.8
HK3	P52790	171	-16	-2	-8	-4	0.9	1.0	0.9	1.0
RBX1	P62877	94	-17	-17	-8	-1	0.9	0.9	0.9	1.0
HLA-C	P04222	125	-17	-24	-8	-13	0.9	0.8	0.9	0.9
HLA-A	P30457	125	-17	-24	-8	-13	0.9	0.8	0.9	0.9
HLA-B	P30479	125	-17	-24	-8	-13	0.9	0.8	0.9	0.9
HLA-B	Q95365	125	-17	-24	-8	-13	0.9	0.8	0.9	0.9
METTL16	Q86W50	57	-17	-29	-8	-20	0.9	0.8	0.9	0.8
SYPL1	Q16563	193	-18	-26	-8	-5	0.8	0.8	0.9	1.0
ADPRH	P54922	129	-19	-13	-8	-5	0.8	0.9	0.9	1.0
TGM2	P21980	371	-19	-14	-8	-13	0.8	0.9	0.9	0.9
CLIC1	O00299	89	-35	-21	-8	-26	0.7	0.8	0.9	0.8
RAN	P62826	112	-36	-30	-8	-7	0.7	0.8	0.9	0.9
PITHD1	Q9GZP4	42	-40	-46	-8	-17	0.7	0.7	0.9	0.9

HSP90AA1	P07900	598	-41	-18	-8	-5	0.7	0.9	0.9	1.0
GTPBP2	Q9BX10	342	21	-3	-8	-2	1.3	1.0	0.9	1.0
AP3M1	Q9Y2T2	29	18	-14	-8	1	1.2	0.9	0.9	1.0
SERPINB9	P50453	98	15	-9	-8	5	1.2	0.9	0.9	1.1
PYCRL	Q53H96	235	14	-5	-8	3	1.2	1.0	0.9	1.0
LYST	Q99698	1354	14	-6	-8	-3	1.2	0.9	0.9	1.0
C17orf80	Q9BSJ5	602	14	-17	-8	9	1.2	0.9	0.9	1.1
ALMS1	Q8TCU4	2878	14	-38	-8	-50	1.2	0.7	0.9	0.7
TLN1	Q9Y490	1558	13	-4	-8	4	1.1	1.0	0.9	1.0
ATP6V1H	Q9UI12	73	13	-6	-8	-6	1.1	0.9	0.9	0.9
OCIAD2	Q56VL3	130	13	-9	-8	-15	1.1	0.9	0.9	0.9
AKAP2	Q9Y2D5	205	12	-9	-8	2	1.1	0.9	0.9	1.0
RASGRP2	Q7LDG7	532	12	-1	-8	11	1.1	1.0	0.9	1.1
FBXW4	P57775	64	11	6	-8	-13	1.1	1.1	0.9	0.9
TECR	Q9NZ01	18	11	-6	-8	-13	1.1	0.9	0.9	0.9
LRPPRC	P42704	930	11	-41	-8	-3	1.1	0.7	0.9	1.0
UBE2Z	Q9H832	263	11	-15	-8	-4	1.1	0.9	0.9	1.0
NEK6	Q9HC98	90	11	-23	-8	-13	1.1	0.8	0.9	0.9
SPAG5	Q96R06	704	10	-12	-8	-20	1.1	0.9	0.9	0.8
VARS	P26640	479	10	-23	-8	-10	1.1	0.8	0.9	0.9
HDHD2	Q9H0R4	244	9	-9	-8	5	1.1	0.9	0.9	1.0
NLE1	Q9NVX2	175	9	-15	-8	8	1.1	0.9	0.9	1.1
RANGAP1	P46060	303	9	-15	-8	-10	1.1	0.9	0.9	0.9
YLPM1	P49750	580	8	-9	-8	-3	1.1	0.9	0.9	1.0
NUDT2	P50583	143	8	-14	-8	-10	1.1	0.9	0.9	0.9
NSUN5	Q96P11	359	8	-5	-8	2	1.1	1.0	0.9	1.0
LIMS1	P48059	13	8	-20	-8	-10	1.1	0.8	0.9	0.9
GCN1	Q92616	907	7	1	-8	7	1.1	1.0	0.9	1.1
NBAS	A2RRP1	406	7	-11	-8	-16	1.1	0.9	0.9	0.9
DDX21	Q9NR30	682	7	-25	-8	-11	1.1	0.8	0.9	0.9
SYNE1	Q8NF91	7793	7	-7	-8	-5	1.1	0.9	0.9	1.0
POLDIP3	Q9BY77	338	7	-7	-8	-13	1.1	0.9	0.9	0.9
TBC1D2	Q9BYX2	102	7	-11	-8	2	1.1	0.9	0.9	1.0
PSMA6	P60900	161	7	-15	-8	-1	1.1	0.9	0.9	1.0
CEP70	Q8NHQ1	215	7	-26	-8	-11	1.1	0.8	0.9	0.9
RPP40	O75818	49	6	-6	-8	-12	1.1	0.9	0.9	0.9
BAG5	Q9UL15	166	6	-6	-8	-19	1.1	0.9	0.9	0.8
CBLB	Q13191	345	6	-8	-8	-4	1.1	0.9	0.9	1.0
SNX6	Q9UNH7	348	6	-16	-8	-2	1.1	0.9	0.9	1.0
PRKDC	P78527	2435	6	-135	-8	-1	1.1	0.4	0.9	1.0
MCM3AP	O60318	1945	6	-3	-8	-2	1.1	1.0	0.9	1.0
HK1	P19367	628	6	-4	-8	5	1.1	1.0	0.9	1.0
MROH1	Q8NDA8	976	6	-5	-8	7	1.1	1.0	0.9	1.1
NUMA1	Q14980	1930	6	-6	-8	-6	1.1	0.9	0.9	0.9
GLUD1	P00367	376	6	-9	-8	-1	1.1	0.9	0.9	1.0
METTL9	Q9H1A3	215	6	-9	-8	-1	1.1	0.9	0.9	1.0
FRY	Q5TBA9	1809	6	-9	-8	-6	1.1	0.9	0.9	0.9
FAM76A	Q8TAV0	108	6	-11	-8	2	1.1	0.9	0.9	1.0
APOBEC3B	Q9UH17	247	6	-17	-8	-11	1.1	0.9	0.9	0.9
INF2	Q27J81	172	6	-17	-8	-13	1.1	0.9	0.9	0.9
MYO5A	Q9Y4I1	535	6	-20	-8	-13	1.1	0.8	0.9	0.9
NMT1	P30419	483	5	-15	-8	-4	1.1	0.9	0.9	1.0
UBASH3B	Q8TF42	385	5	-16	-8	-4	1.1	0.9	0.9	1.0

CDC42	P60953	157	5	-22	-8	15	1.1	0.8	0.9	1.2
PSMA6	P60900	47	5	-26	-8	2	1.1	0.8	0.9	1.0
LRBA	P50851	1843	5	-6	-8	-8	1.0	0.9	0.9	0.9
ZC3H11A	O75152	636	5	-9	-8	-15	1.0	0.9	0.9	0.9
TACC3	Q9Y6A5	662	5	-9	-8	-31	1.0	0.9	0.9	0.8
COPA	P53621	514	5	-13	-8	-7	1.0	0.9	0.9	0.9
METTL3	Q86U44	376	4	1	-8	-18	1.0	1.0	0.9	0.9
UBA2	Q9UBT2	30	4	-12	-8	-1	1.0	0.9	0.9	1.0
LRBA	P50851	289	4	-12	-8	-2	1.0	0.9	0.9	1.0
ANKFY1	Q9P2R3	34	4	-16	-8	-10	1.0	0.9	0.9	0.9
MFN2	O95140	348	4	-21	-8	-12	1.0	0.8	0.9	0.9
CCDC88C	Q9P219	1871	4	5	-8	14	1.0	1.1	0.9	1.2
ANAPC1	Q9H1A4	988	4	2	-8	-3	1.0	1.0	0.9	1.0
NBR1	Q14596	433	4	-1	-8	-19	1.0	1.0	0.9	0.8
EMC8	O43402	136	4	-9	-8	-6	1.0	0.9	0.9	0.9
ZFPL1	O95159	230	4	-11	-8	4	1.0	0.9	0.9	1.0
VPS4A	Q9UN37	403	4	-14	-8	-1	1.0	0.9	0.9	1.0
HNRNPUL2-B	H3BQZ7	405	4	-21	-8	-10	1.0	0.8	0.9	0.9
WHSC1L1	Q9BZ95	495	3	3	-8	-15	1.0	1.0	0.9	0.9
PLCG1	P19174	646	3	-3	-8	-11	1.0	1.0	0.9	0.9
RHOH	Q15669	108	3	-5	-8	-12	1.0	1.0	0.9	0.9
PSMB8	P28062	160	3	-6	-8	-5	1.0	0.9	0.9	1.0
POLR2C	P19387	107	3	-7	-8	1	1.0	0.9	0.9	1.0
RANBP2	P49792	1196	3	-9	-8	0	1.0	0.9	0.9	1.0
TACC3	Q9Y6A5	828	3	-10	-8	4	1.0	0.9	0.9	1.0
PBRM1	Q86U86	1163	3	-13	-8	-2	1.0	0.9	0.9	1.0
THOP1	P52888	253	3	-13	-8	-5	1.0	0.9	0.9	1.0
ACP1	P24666	63	3	-13	-8	-7	1.0	0.9	0.9	0.9
AGTPBP1	Q9UPW5	305	3	-14	-8	9	1.0	0.9	0.9	1.1
COPG2	Q9UBF2	44	3	-15	-8	12	1.0	0.9	0.9	1.1
TTC38	Q5R3I4	367	3	-18	-8	-1	1.0	0.8	0.9	1.0
PHF6	Q8IWS0	20	3	-35	-8	-10	1.0	0.7	0.9	0.9
TDG	Q13569	233	3	4	-8	-5	1.0	1.0	0.9	1.0
CAMK4	Q16566	202	3	-5	-8	-7	1.0	1.0	0.9	0.9
ATP13A1	Q9HD20	531	3	-8	-8	-3	1.0	0.9	0.9	1.0
PEX6	Q13608	309	3	-8	-8	-17	1.0	0.9	0.9	0.9
TMLHE	Q9NVH6	103	3	-10	-8	-3	1.0	0.9	0.9	1.0
SKIL	P12757	73	3	-11	-8	-16	1.0	0.9	0.9	0.9
TOP2B	Q02880	476	3	-13	-8	-11	1.0	0.9	0.9	0.9
COG2	Q14746	287	3	-15	-8	-13	1.0	0.9	0.9	0.9
APEH	P13798	292	3	-34	-8	-11	1.0	0.7	0.9	0.9
EZH2	Q15910	503	2	-2	-8	-8	1.0	1.0	0.9	0.9
EZH1	Q92800	504	2	-2	-8	-8	1.0	1.0	0.9	0.9
PFAS	O15067	606	2	-3	-8	-3	1.0	1.0	0.9	1.0
JAK1	P23458	424	2	-6	-8	-10	1.0	0.9	0.9	0.9
DHX9	Q08211	773	2	-7	-8	-4	1.0	0.9	0.9	1.0
COPB1	P53618	684	2	-7	-8	-7	1.0	0.9	0.9	0.9
BCL2L11	O43521	12	2	-8	-8	-8	1.0	0.9	0.9	0.9
TNRC6B	Q9UPQ9	600	2	-11	-8	-15	1.0	0.9	0.9	0.9
THUMPD1	Q9NXG2	31	2	-14	-8	-9	1.0	0.9	0.9	0.9
INPP5D	Q92835	138	2	-16	-8	-14	1.0	0.9	0.9	0.9
HSPH1	Q92598	34	2	-19	-8	-11	1.0	0.8	0.9	0.9
THOC2	Q8NI27	931	2	-23	-8	-9	1.0	0.8	0.9	0.9

TTC27	Q6P3X3	299	2	-5	-8	-2	1.0	1.0	0.9	1.0
PPP2R1A	P30153	377	2	-7	-8	-5	1.0	0.9	0.9	1.0
ACTR1A	P61163	222	2	-9	-8	-3	1.0	0.9	0.9	1.0
TCP1	P17987	147	2	-11	-8	0	1.0	0.9	0.9	1.0
FYB	O15117	519	2	-11	-8	-9	1.0	0.9	0.9	0.9
KTN1	Q86UP2	1136	2	-12	-8	-13	1.0	0.9	0.9	0.9
WDR45B	Q5MNZ6	63	2	-13	-8	-14	1.0	0.9	0.9	0.9
SPATA13	Q96N96	419	2	-13	-8	-17	1.0	0.9	0.9	0.9
NCL	P19338	543	2	-15	-8	-9	1.0	0.9	0.9	0.9
UBR5	O95071	2267	2	-17	-8	-9	1.0	0.9	0.9	0.9
SMC2	O95347	326	2	-17	-8	-13	1.0	0.9	0.9	0.9
CAD	P27708	758	1	0	-8	4	1.0	1.0	0.9	1.0
RALBP1	Q15311	451	1	-9	-8	-5	1.0	0.9	0.9	1.0
POLR2B	P30876	984	1	-9	-8	-16	1.0	0.9	0.9	0.9
HSD17B10	Q99714	58	1	-12	-8	-6	1.0	0.9	0.9	0.9
RCC2	Q9P258	144	1	-13	-8	-6	1.0	0.9	0.9	0.9
ACTR3	P61158	8	1	-14	-8	-21	1.0	0.9	0.9	0.8
RBM22	Q9NW64	71	1	-17	-8	-6	1.0	0.9	0.9	0.9
ATG7	O95352	298	1	-17	-8	-16	1.0	0.9	0.9	0.9
MTMR3	Q13615	81	1	-18	-8	-11	1.0	0.9	0.9	0.9
ACBD3	Q9H3P7	129	1	-4	-8	-13	1.0	1.0	0.9	0.9
Uncharacteri	A0A087WZG	633	1	-4	-8	-6	1.0	1.0	0.9	0.9
APOBEC3G	Q9HC16	321	1	-9	-8	3	1.0	0.9	0.9	1.0
EPRS	P07814	1377	1	-10	-8	-4	1.0	0.9	0.9	1.0
FTSJ1	Q9UET6	238	1	-10	-8	-3	1.0	0.9	0.9	1.0
ALDH18A1	P54886	612	1	-11	-8	-7	1.0	0.9	0.9	0.9
ATG16L2	Q8NAA4	267	1	-11	-8	-10	1.0	0.9	0.9	0.9
CAD	P27708	1374	1	-16	-8	-21	1.0	0.9	0.9	0.8
COMT	P21964	145	1	-17	-8	-18	1.0	0.9	0.9	0.8
DGKA	P23743	246	1	-20	-8	-17	1.0	0.8	0.9	0.9
ZFYVE28	Q9HCC9	49	1	-22	-8	-18	1.0	0.8	0.9	0.9
CHRAC1	Q9NRG0	55	0	-8	-8	4	1.0	0.9	0.9	1.0
LRSAM1	Q6UWE0	193	0	-9	-8	1	1.0	0.9	0.9	1.0
AKAP10	O43572	110	0	-11	-8	-15	1.0	0.9	0.9	0.9
SACM1L	Q9NTJ5	392	0	-12	-8	-4	1.0	0.9	0.9	1.0
RPL11	P62913	150	0	-13	-8	-8	1.0	0.9	0.9	0.9
AP3M1	Q9Y2T2	288	0	-13	-8	-14	1.0	0.9	0.9	0.9
NADSYN1	Q6IA69	428	0	-16	-8	-13	1.0	0.9	0.9	0.9
TAF6L	Q9Y6J9	230	0	-18	-8	6	1.0	0.9	0.9	1.1
MYOF	Q9NZM1	409	-1	-6	-8	-15	1.0	0.9	0.9	0.9
PLS3	P13797	33	-1	-7	-8	-8	1.0	0.9	0.9	0.9
EEF2	P13639	41	-1	-9	-8	-20	1.0	0.9	0.9	0.8
SH2D1A	O60880	124	-1	-10	-8	-13	1.0	0.9	0.9	0.9
RPS15A	P62244	30	-1	-11	-8	6	1.0	0.9	0.9	1.1
PCNT	O95613	2614	-1	-11	-8	-13	1.0	0.9	0.9	0.9
EXOC1	Q9NV70	650	-1	-11	-8	-2	1.0	0.9	0.9	1.0
PCNA	P12004	81	-1	-12	-8	-5	1.0	0.9	0.9	1.0
INCENP	Q9NQS7	163	-1	-12	-8	-12	1.0	0.9	0.9	0.9
AKAP17A	Q02040	95	-1	-13	-8	-14	1.0	0.9	0.9	0.9
NIPSNAP1	Q9BPW8	138	-1	-13	-8	4	1.0	0.9	0.9	1.0
GNAI3	P08754	351	-1	-15	-8	-10	1.0	0.9	0.9	0.9
GNPNAT1	Q96EK6	157	-1	-17	-8	-12	1.0	0.9	0.9	0.9
RIPK3	Q9Y572	279	-1	-4	-8	-6	1.0	1.0	0.9	0.9

NMI	Q13287	230	-1	-6	-8	-6	1.0	0.9	0.9	0.9
RSF1	Q96T23	1436	-1	-6	-8	-6	1.0	0.9	0.9	0.9
TAF4	O00268	867	-1	-6	-8	-18	1.0	0.9	0.9	0.8
MBNL3	Q9NUK0	35	-1	-10	-8	-13	1.0	0.9	0.9	0.9
C15orf38-AP:	A0A0A6YYH1	183	-1	-15	-8	-21	1.0	0.9	0.9	0.8
DUS4L	O95620	116	-2	6	-8	-11	1.0	1.1	0.9	0.9
NME3	Q13232	158	-2	-4	-8	-11	1.0	1.0	0.9	0.9
PLEKHA2	Q9HB19	191	-2	-8	-8	-11	1.0	0.9	0.9	0.9
SYNE2	Q8WXH0	568	-2	-9	-8	-8	1.0	0.9	0.9	0.9
NUDT4	Q9NZJ9	131	-2	-12	-8	-19	1.0	0.9	0.9	0.8
HNRNPH1	P31943	34	-2	-17	-8	-3	1.0	0.9	0.9	1.0
HNRNPH2	P55795	34	-2	-17	-8	-3	1.0	0.9	0.9	1.0
TRIM28	Q13263	628	-2	-17	-8	-15	1.0	0.9	0.9	0.9
PAFAH1B1	P43034	252	-2	-21	-8	-4	1.0	0.8	0.9	1.0
MYH9	P35579	694	-2	-31	-8	-4	1.0	0.8	0.9	1.0
MYH10	P35580	701	-2	-31	-8	-4	1.0	0.8	0.9	1.0
UBA3	Q8TBC4	367	-2	7	-8	14	1.0	1.1	0.9	1.2
RASGRP2	Q7LDG7	548	-2	-4	-8	-14	1.0	1.0	0.9	0.9
MALT1	Q9UDY8	441	-2	-6	-8	-3	1.0	0.9	0.9	1.0
DPYD	Q12882	671	-2	-8	-8	-4	1.0	0.9	0.9	1.0
STK10	O94804	947	-2	-9	-8	-30	1.0	0.9	0.9	0.8
TBC1D13	Q9NVG8	387	-2	-13	-8	-8	1.0	0.9	0.9	0.9
UNC45A	Q9H3U1	384	-2	-13	-8	8	1.0	0.9	0.9	1.1
UFL1	O94874	372	-2	-14	-8	-14	1.0	0.9	0.9	0.9
GNB1	P62873	25	-2	-16	-8	0	1.0	0.9	0.9	1.0
PTPRC	P08575	309	-2	-29	-8	-18	1.0	0.8	0.9	0.8
LRSAM1	Q6UWE0	397	-3	8	-8	-10	1.0	1.1	0.9	0.9
CDA	P32320	8	-3	-2	-8	-7	1.0	1.0	0.9	0.9
PDS5A	Q29RF7	581	-3	-5	-8	4	1.0	1.0	0.9	1.0
MAP3K4	Q9Y6R4	193	-3	-8	-8	-28	1.0	0.9	0.9	0.8
RAB14	P61106	40	-3	-10	-8	-8	1.0	0.9	0.9	0.9
UBE2L6	O14933	102	-3	-13	-8	-15	1.0	0.9	0.9	0.9
MYO1G	B0I1T2	965	-3	-18	-8	-8	1.0	0.8	0.9	0.9
NUMA1	Q14980	961	-3	-4	-8	-13	1.0	1.0	0.9	0.9
IRF2BP2	Q7Z5L9	19	-3	-7	-8	-11	1.0	0.9	0.9	0.9
IRF2BPL	Q9H1B7	17	-3	-7	-8	-11	1.0	0.9	0.9	0.9
EPRS	P07814	744	-3	-7	-8	-14	1.0	0.9	0.9	0.9
VARS	P26640	41	-3	-8	-8	-6	1.0	0.9	0.9	0.9
ADAR	P55265	392	-3	-10	-8	-15	1.0	0.9	0.9	0.9
CDA	P32320	14	-3	-12	-8	-11	1.0	0.9	0.9	0.9
OAS1	P00973	331	-3	-13	-8	-4	1.0	0.9	0.9	1.0
FAM185A	Q8N0U4	284	-3	-14	-8	-11	1.0	0.9	0.9	0.9
ARMC6	Q6NXE6	484	-3	-16	-8	-9	1.0	0.9	0.9	0.9
WDR12	Q9GZL7	309	-3	-22	-8	2	1.0	0.8	0.9	1.0
NEDD9	Q14511	18	-4	-8	-8	-9	1.0	0.9	0.9	0.9
MTCH2	Q9Y6C9	296	-4	-8	-8	-14	1.0	0.9	0.9	0.9
PRKDC	P78527	3837	-4	-10	-8	-7	1.0	0.9	0.9	0.9
ELP2	Q6IA86	746	-4	-12	-8	-18	1.0	0.9	0.9	0.9
PNO1	Q9NRX1	64	-4	-14	-8	-12	1.0	0.9	0.9	0.9
RNASEH2A	O75792	29	-4	-15	-8	-3	1.0	0.9	0.9	1.0
SULT1B1	O43704	22	-4	-16	-8	11	1.0	0.9	0.9	1.1
OXSM	Q9NWU1	86	-4	-18	-8	-12	1.0	0.8	0.9	0.9
ACTN4	O43707	879	-4	-19	-8	-6	1.0	0.8	0.9	0.9

ACTN1	P12814	860	-4	-19	-8	-6	1.0	0.8	0.9	0.9
STRBP	Q96SI9	83	-4	-20	-8	-18	1.0	0.8	0.9	0.9
BAZ2A	Q9UIF9	795	-4	-21	-8	-9	1.0	0.8	0.9	0.9
ZNF850	A8MQ14	90	-4	-2	-8	12	1.0	1.0	0.9	1.1
CNN2	Q99439	175	-4	-5	-8	-17	1.0	1.0	0.9	0.9
SUN1	O94901	63	-4	-6	-8	-18	1.0	0.9	0.9	0.9
VAC14	Q08AM6	619	-4	-7	-8	3	1.0	0.9	0.9	1.0
PAXBP1	Q9Y5B6	597	-4	-7	-8	-2	1.0	0.9	0.9	1.0
LIG1	P18858	895	-4	-7	-8	-9	1.0	0.9	0.9	0.9
MCCC2	Q9HCC0	167	-4	-9	-8	-6	1.0	0.9	0.9	0.9
PSMA4	P25789	115	-4	-10	-8	-7	1.0	0.9	0.9	0.9
POLG2	Q9UHN1	377	-4	-10	-8	-9	1.0	0.9	0.9	0.9
PRMT9	Q6P2P2	683	-4	-13	-8	-16	1.0	0.9	0.9	0.9
KIF1B	O60333	1447	-4	-15	-8	-2	1.0	0.9	0.9	1.0
RNPEP	Q9H4A4	130	-4	-16	-8	-6	1.0	0.9	0.9	0.9
ITGB2	P05107	497	-4	-17	-8	-21	1.0	0.9	0.9	0.8
RWDD2B	P57060	27	-5	0	-8	12	1.0	1.0	0.9	1.1
ATM	Q13315	2770	-5	-9	-8	-17	1.0	0.9	0.9	0.9
MYO1G	B0I1T2	503	-5	-10	-8	-6	1.0	0.9	0.9	0.9
KMT2B	Q9UMN6	1447	-5	-12	-8	3	1.0	0.9	0.9	1.0
GPD2	P43304	385	-5	-12	-8	-19	1.0	0.9	0.9	0.8
TFCP2	Q12800	453	-5	-14	-8	-15	1.0	0.9	0.9	0.9
PIP4K2A	P48426	94	-5	-15	-8	1	1.0	0.9	0.9	1.0
PIP4K2B	P78356	99	-5	-15	-8	1	1.0	0.9	0.9	1.0
TES	Q9UGI8	239	-5	-19	-8	-3	1.0	0.8	0.9	1.0
PLAA	Q9Y263	584	-5	-19	-8	-11	1.0	0.8	0.9	0.9
PTPRC	P08575	608	-5	-7	-8	-15	1.0	0.9	0.9	0.9
EP300	Q09472	369	-5	-11	-8	-16	1.0	0.9	0.9	0.9
EPRS	P07814	1453	-5	-12	-8	-5	1.0	0.9	0.9	1.0
NIPBL	Q6KC79	2151	-5	-14	-8	-10	1.0	0.9	0.9	0.9
GNAQ	P50148	144	-5	-18	-8	-7	1.0	0.9	0.9	0.9
SAMM50	Q9Y512	421	-5	-21	-8	2	1.0	0.8	0.9	1.0
MED13	Q9UHV7	681	-5	-25	-8	-21	1.0	0.8	0.9	0.8
UBR4	Q5T4S7	3075	-6	-6	-8	-6	0.9	0.9	0.9	0.9
RABGGTA	Q92696	186	-6	-7	-8	8	0.9	0.9	0.9	1.1
THNSL1	Q8IYQ7	81	-6	-12	-8	-7	0.9	0.9	0.9	0.9
SLFN5	Q08AF3	875	-6	-14	-8	-19	0.9	0.9	0.9	0.8
HNRNPUL1	Q9BUJ2	258	-6	-4	-8	-6	0.9	1.0	0.9	0.9
CAPN2	P17655	640	-6	-6	-8	3	0.9	0.9	0.9	1.0
CEP128	Q6ZU80	915	-6	-14	-8	-12	0.9	0.9	0.9	0.9
DTNB	O60941	246	-6	-18	-8	24	0.9	0.9	0.9	1.3
SMARCC2	Q8TAQ2	136	-6	-20	-8	-8	0.9	0.8	0.9	0.9
PPA2	Q9H2U2	171	-6	-23	-8	-7	0.9	0.8	0.9	0.9
ATIC	P31939	434	-6	-24	-8	-4	0.9	0.8	0.9	1.0
ALG11	Q2TAA5	282	-7	-6	-8	13	0.9	0.9	0.9	1.1
NUFIP2	Q7Z417	234	-7	-7	-8	-10	0.9	0.9	0.9	0.9
TBC1D5	Q92609	706	-7	-8	-8	-19	0.9	0.9	0.9	0.8
LUC7L2	A0A0A6YYJ8	256	-7	-10	-8	-1	0.9	0.9	0.9	1.0
LUC7L	Q9NQ29	190	-7	-10	-8	-1	0.9	0.9	0.9	1.0
TREML1	Q86YW5	59	-7	-12	-8	-7	0.9	0.9	0.9	0.9
LSM4	Q9Y4Z0	45	-7	-13	-8	5	0.9	0.9	0.9	1.1
SH3GLB2	Q9NR46	267	-7	-15	-8	-11	0.9	0.9	0.9	0.9
U2AF2	P26368	464	-7	-17	-8	-3	0.9	0.9	0.9	1.0

MRPL10	Q7Z7H8	180	-7	-19	-8	-2	0.9	0.8	0.9	1.0
OSBPL9	Q96SU4	581	-7	-32	-8	-1	0.9	0.8	0.9	1.0
TUFM	P49411	222	-7	-9	-8	-7	0.9	0.9	0.9	0.9
HCFC1	P51610	89	-7	-12	-8	-7	0.9	0.9	0.9	0.9
ZNF830	Q96NB3	314	-7	-15	-8	-22	0.9	0.9	0.9	0.8
NUDCD1	Q96RS6	513	-7	-16	-8	-13	0.9	0.9	0.9	0.9
CTPS1	P17812	491	-8	-8	-8	-9	0.9	0.9	0.9	0.9
AGAP2	Q99490	548	-8	-11	-8	-7	0.9	0.9	0.9	0.9
NUP160	Q12769	480	-8	-11	-8	-9	0.9	0.9	0.9	0.9
SLAIN2	Q9P270	165	-8	-12	-8	-23	0.9	0.9	0.9	0.8
PDLIM2	Q96JY6	160	-8	-12	-8	-17	0.9	0.9	0.9	0.9
CCR7	P32248	346	-8	-15	-8	-6	0.9	0.9	0.9	0.9
PFKL	P17858	708	-8	-17	-8	-15	0.9	0.9	0.9	0.9
ZNF800	Q2TB10	293	-8	-23	-8	-23	0.9	0.8	0.9	0.8
EDC3	Q96F86	272	-8	-31	-8	-31	0.9	0.8	0.9	0.8
SYNE1	Q8NF91	6415	-9	-10	-8	-13	0.9	0.9	0.9	0.9
EIF3G	O75821	139	-9	-12	-8	-7	0.9	0.9	0.9	0.9
GOLGA1	Q92805	450	-9	-14	-8	-18	0.9	0.9	0.9	0.9
NAPRT	Q6XQN6	48	-9	-15	-8	-5	0.9	0.9	0.9	1.0
ELL	P55199	121	-9	-22	-8	-16	0.9	0.8	0.9	0.9
CORO1A	P31146	332	-9	-22	-8	-9	0.9	0.8	0.9	0.9
TRIM41	Q8WV44	180	-9	3	-8	-12	0.9	1.0	0.9	0.9
YWHAQ	P27348	237	-9	-7	-8	-29	0.9	0.9	0.9	0.8
CPSF2	Q9P2I0	335	-9	-9	-8	-2	0.9	0.9	0.9	1.0
PIK3R6	Q5UE93	496	-10	-5	-8	-1	0.9	1.0	0.9	1.0
PTPN7	P35236	204	-10	-18	-8	10	0.9	0.9	0.9	1.1
VWF	P04275	1972	-10	-11	-8	-17	0.9	0.9	0.9	0.9
GLRX5	Q86SX6	67	-10	-14	-8	-10	0.9	0.9	0.9	0.9
APPL1	Q9UKG1	462	-11	-22	-8	-10	0.9	0.8	0.9	0.9
PAPSS1	O43252	207	-11	-20	-8	-16	0.9	0.8	0.9	0.9
NEPRO	Q6NW34	492	-12	-7	-8	28	0.9	0.9	0.9	1.4
ALDH1A1	P00352	302	-12	-8	-8	-25	0.9	0.9	0.9	0.8
MRI1	Q9BV20	127	-13	-20	-8	-12	0.9	0.8	0.9	0.9
ATAD2B	Q9ULI0	1430	-13	-21	-8	25	0.9	0.8	0.9	1.3
AHNAK	Q09666	108	-14	-15	-8	-16	0.9	0.9	0.9	0.9
AP2M1	Q96CW1	251	-14	-18	-8	-3	0.9	0.9	0.9	1.0
IKBKG	Q9Y6K9	54	-14	-18	-8	-34	0.9	0.8	0.9	0.7
NLRX1	Q86UT6	644	-15	-13	-8	-4	0.9	0.9	0.9	1.0
DNM1L	O00429	446	-15	-4	-8	-4	0.9	1.0	0.9	1.0
SELO	A0A087X0Q9	629	-15	-22	-8	-23	0.9	0.8	0.9	0.8
DUSP7	Q16829	54	-16	-13	-8	-4	0.9	0.9	0.9	1.0
IRF9	Q00978	319	-16	-19	-8	-3	0.9	0.8	0.9	1.0
CAPN7	Q9Y6W3	361	-16	-15	-8	-21	0.9	0.9	0.9	0.8
CBX5	P45973	133	-18	-15	-8	-9	0.8	0.9	0.9	0.9
MED13L	Q71F56	1256	-22	-20	-8	-23	0.8	0.8	0.9	0.8
SUMF2	Q8NBJ7	290	-27	-41	-8	-7	0.8	0.7	0.9	0.9
WDSUB1	Q8N9V3	410	-30	-7	-8	-10	0.8	0.9	0.9	0.9
GDI2	P50395	414	25	-13	-9	18	1.3	0.9	0.9	1.2
UBA6	A0AVT1	174	24	-20	-9	11	1.3	0.8	0.9	1.1
PPIP5K2	O43314	615	22	4	-9	7	1.3	1.0	0.9	1.1
AP2B1	P63010	380	17	-31	-9	-5	1.2	0.8	0.9	1.0
AP1B1	Q10567	380	17	-31	-9	-5	1.2	0.8	0.9	1.0
F8A3	P23610	62	16	1	-9	-5	1.2	1.0	0.9	1.0

SNRNP200	O75643	1702	15	-21	-9	-12	1.2	0.8	0.9	0.9
LATS2	Q9NRM7	868	14	8	-9	-21	1.2	1.1	0.9	0.8
MCM2	P49736	584	14	-7	-9	-7	1.2	0.9	0.9	0.9
EIF1B	O60739	94	14	-17	-9	1	1.2	0.9	0.9	1.0
WDFY1	Q8IWB7	322	14	-39	-9	-13	1.2	0.7	0.9	0.9
HIST1H3J	P68431	111	13	-9	-9	-26	1.1	0.9	0.9	0.8
GMIP	Q9P107	571	13	-16	-9	-6	1.1	0.9	0.9	0.9
IRF2	P14316	83	13	-12	-9	-11	1.1	0.9	0.9	0.9
WAC	Q9BTA9	615	13	-26	-9	-23	1.1	0.8	0.9	0.8
RAD50	Q92878	633	12	-10	-9	-1	1.1	0.9	0.9	1.0
POLD3	Q15054	398	12	-20	-9	-15	1.1	0.8	0.9	0.9
PACS1	Q6VY07	616	12	2	-9	-1	1.1	1.0	0.9	1.0
GNAS	P63092	365	11	25	-9	16	1.1	1.3	0.9	1.2
NEDD1	Q8NHV4	66	11	-16	-9	-20	1.1	0.9	0.9	0.8
TINF2	Q9BSI4	27	11	-5	-9	-18	1.1	1.0	0.9	0.9
GGPS1	O95749	138	10	-16	-9	1	1.1	0.9	0.9	1.0
SEC23B	Q15437	40	9	-5	-9	2	1.1	1.0	0.9	1.0
FASN	P49327	1127	9	-9	-9	-7	1.1	0.9	0.9	0.9
ZYG11B	Q9C0D3	735	9	-11	-9	-12	1.1	0.9	0.9	0.9
ZFYVE19	Q96K21	80	9	-19	-9	-24	1.1	0.8	0.9	0.8
RBL2	Q08999	120	9	-21	-9	-9	1.1	0.8	0.9	0.9
MAP4K4	O95819	883	8	2	-9	-10	1.1	1.0	0.9	0.9
HDGFRP2	A0A087WT5‹	16	8	-6	-9	3	1.1	0.9	0.9	1.0
FERMT3	Q86UX7	439	8	-21	-9	13	1.1	0.8	0.9	1.1
TNPO1	Q92973	285	7	-2	-9	16	1.1	1.0	0.9	1.2
HTATSF1	O43719	187	7	-11	-9	-16	1.1	0.9	0.9	0.9
PRKDC	P78527	123	7	-20	-9	3	1.1	0.8	0.9	1.0
SOD1	P00441	112	7	14	-9	9	1.1	1.2	0.9	1.1
PI4K2B	Q8TCG2	446	7	1	-9	8	1.1	1.0	0.9	1.1
ACVR1	Q04771	395	7	0	-9	-34	1.1	1.0	0.9	0.7
Uncharacteri	G3V599	1163	7	-5	-9	-8	1.1	1.0	0.9	0.9
ME2	P23368	185	7	-6	-9	4	1.1	0.9	0.9	1.0
CCNL2	Q96S94	52	7	-7	-9	-40	1.1	0.9	0.9	0.7
POLR1E	Q9GZS1	143	7	-8	-9	-35	1.1	0.9	0.9	0.7
RPL10A	P62906	74	7	-9	-9	-1	1.1	0.9	0.9	1.0
KPNA6	O60684	467	7	-15	-9	-4	1.1	0.9	0.9	1.0
GOT1	P17174	46	7	-22	-9	-7	1.1	0.8	0.9	0.9
SF3A3	Q12874	145	7	-27	-9	-1	1.1	0.8	0.9	1.0
ZNF350	Q9GZX5	332	6	-3	-9	-10	1.1	1.0	0.9	0.9
RFC2	P35250	255	6	-11	-9	13	1.1	0.9	0.9	1.1
KPNA3	O00505	417	6	-13	-9	-1	1.1	0.9	0.9	1.0
SMU1	Q2TAY7	312	6	-34	-9	1	1.1	0.7	0.9	1.0
SDR39U1	Q9NRG7	78	6	-3	-9	-8	1.1	1.0	0.9	0.9
ADD3	Q9UEY8	73	6	-6	-9	-6	1.1	0.9	0.9	0.9
SF1	Q15637	292	6	-9	-9	2	1.1	0.9	0.9	1.0
MIC13	Q5XKP0	60	6	-10	-9	-4	1.1	0.9	0.9	1.0
RANBP2	P49792	1335	6	-10	-9	-8	1.1	0.9	0.9	0.9
CAND1	Q86VP6	264	6	-11	-9	7	1.1	0.9	0.9	1.1
SPTLC2	O15270	188	6	-22	-9	-8	1.1	0.8	0.9	0.9
PDS5B	Q9NTI5	573	5	-9	-9	-3	1.1	0.9	0.9	1.0
TLE4	Q04727	32	5	-15	-9	-15	1.1	0.9	0.9	0.9
HSH2D	Q96JZ2	125	5	-20	-9	-7	1.1	0.8	0.9	0.9
WDR43	Q15061	307	5	-8	-9	12	1.0	0.9	0.9	1.1

VARS	P26640	663	5	-9	-9	3	1.0	0.9	0.9	1.0
HEXIM2	Q96MH2	80	5	-11	-9	-8	1.0	0.9	0.9	0.9
NBAS	A2RRP1	1453	5	-14	-9	-13	1.0	0.9	0.9	0.9
RHOC	P08134	107	5	-15	-9	-2	1.0	0.9	0.9	1.0
RHOA	P61586	107	5	-15	-9	-2	1.0	0.9	0.9	1.0
CRYL1	Q9Y2S2	182	5	-16	-9	-5	1.0	0.9	0.9	1.0
ADRBK1	P25098	120	4	-7	-9	-10	1.0	0.9	0.9	0.9
RNF213	Q63HN8	1736	4	-9	-9	-6	1.0	0.9	0.9	0.9
ANP32E	Q9BTT0	123	4	-19	-9	-8	1.0	0.8	0.9	0.9
DOCK11	Q5JSL3	633	4	-23	-9	-13	1.0	0.8	0.9	0.9
ERLIN2	O94905	262	4	-4	-9	-1	1.0	1.0	0.9	1.0
ASMTL	O95671	333	4	-6	-9	-11	1.0	0.9	0.9	0.9
ACOX1	Q15067	199	4	-13	-9	-5	1.0	0.9	0.9	1.0
BAG6	P46379	349	4	-14	-9	-8	1.0	0.9	0.9	0.9
TRIM65	Q6PJ69	106	4	-16	-9	-21	1.0	0.9	0.9	0.8
NARS	O43776	452	4	-17	-9	-1	1.0	0.9	0.9	1.0
KDM1B	Q8NB78	600	3	-4	-9	-8	1.0	1.0	0.9	0.9
ZC3H11A	O75152	559	3	-5	-9	-12	1.0	1.0	0.9	0.9
IQCB1	Q15051	125	3	-7	-9	1	1.0	0.9	0.9	1.0
PDE5A	O76074	220	3	-10	-9	0	1.0	0.9	0.9	1.0
MARS	P56192	12	3	-11	-9	-1	1.0	0.9	0.9	1.0
TUFM	P49411	290	3	-12	-9	-3	1.0	0.9	0.9	1.0
PPP1CA	P62136	62	3	-22	-9	1	1.0	0.8	0.9	1.0
CARD11	Q9BXL7	797	3	-25	-9	16	1.0	0.8	0.9	1.2
DCUN1D3	Q8IWE4	299	3	-3	-9	2	1.0	1.0	0.9	1.0
GATM	P50440	252	3	-14	-9	-10	1.0	0.9	0.9	0.9
SMC1A	Q14683	1180	3	-14	-9	-4	1.0	0.9	0.9	1.0
ZNFX1	Q9P2E3	1292	2	-6	-9	13	1.0	0.9	0.9	1.1
CEP250	Q9BV73	1910	2	-8	-9	-8	1.0	0.9	0.9	0.9
ROCK2	O75116	766	2	-10	-9	-5	1.0	0.9	0.9	1.0
ZNF217	O75362	641	2	-12	-9	-14	1.0	0.9	0.9	0.9
KTN1	Q86UP2	736	2	-12	-9	-13	1.0	0.9	0.9	0.9
AKAP13	Q12802	400	2	2	-9	-5	1.0	1.0	0.9	1.0
MPRIP	Q6WCQ1	831	2	-2	-9	-14	1.0	1.0	0.9	0.9
HIRA	P54198	763	2	-6	-9	-11	1.0	0.9	0.9	0.9
LRRK2	Q5S007	2384	2	-12	-9	-10	1.0	0.9	0.9	0.9
CELF1	Q92879	119	2	-13	-9	-6	1.0	0.9	0.9	0.9
CSRP1	P21291	122	2	-20	-9	-7	1.0	0.8	0.9	0.9
HGS	O14964	212	1	-2	-9	-7	1.0	1.0	0.9	0.9
XRN1	Q8IZH2	893	1	-7	-9	-6	1.0	0.9	0.9	0.9
RCC2	Q9P258	337	1	-19	-9	-8	1.0	0.8	0.9	0.9
CAPZB	P47756	62	1	-21	-9	-8	1.0	0.8	0.9	0.9
RRP12	Q5JTH9	102	1	-2	-9	-7	1.0	1.0	0.9	0.9
LUC7L2	A0A0A6YYJ8	259	1	-5	-9	-1	1.0	1.0	0.9	1.0
LUC7L	Q9NQ29	193	1	-5	-9	-1	1.0	1.0	0.9	1.0
POLR1B	Q9H9Y6	103	1	-11	-9	-1	1.0	0.9	0.9	1.0
ATP2A2	P16615	997	1	-12	-9	-14	1.0	0.9	0.9	0.9
ALOX12	P18054	439	1	-13	-9	-26	1.0	0.9	0.9	0.8
THBS1	P07996	657	1	-14	-9	-8	1.0	0.9	0.9	0.9
SPAG7	O75391	191	1	-14	-9	-21	1.0	0.9	0.9	0.8
ASNA1	O43681	55	1	-20	-9	-4	1.0	0.8	0.9	1.0
MAT2A	P31153	56	1	-23	-9	-8	1.0	0.8	0.9	0.9
FLAD1	Q8NFF5	409	0	-7	-9	-3	1.0	0.9	0.9	1.0

LIMD1	Q9UGP4	305	0	-11	-9	-21	1.0	0.9	0.9	0.8
ARFGEF1	Q9Y6D6	1685	0	-12	-9	6	1.0	0.9	0.9	1.1
RFTN1	Q14699	551	0	-12	-9	-20	1.0	0.9	0.9	0.8
STEAP3	Q658P3	168	0	-12	-9	-5	1.0	0.9	0.9	1.0
ACADVL	P49748	237	0	-13	-9	-9	1.0	0.9	0.9	0.9
LRBA	P50851	2820	0	-13	-9	-25	1.0	0.9	0.9	0.8
PRKAR2B	P31323	211	0	-16	-9	-5	1.0	0.9	0.9	1.0
pk	D4Q8H0	285	0	-16	-9	-10	1.0	0.9	0.9	0.9
DTX3L	Q8TDB6	309	0	-22	-9	-15	1.0	0.8	0.9	0.9
NLRC5	Q86WI3	1362	-1	1	-9	-7	1.0	1.0	0.9	0.9
TRIM6-TRIM3	B2RNG4	593	-1	-7	-9	-6	1.0	0.9	0.9	0.9
RSBN1L	Q6PCB5	495	-1	-9	-9	-15	1.0	0.9	0.9	0.9
BACH1	O14867	184	-1	-15	-9	-19	1.0	0.9	0.9	0.8
LAP3	P28838	376	-1	-15	-9	2	1.0	0.9	0.9	1.0
GOLGA2	Q08379	427	-1	-16	-9	-14	1.0	0.9	0.9	0.9
TTC27	Q6P3X3	549	-1	-1	-9	-7	1.0	1.0	0.9	0.9
DHX30	Q7L2E3	346	-1	-7	-9	-2	1.0	0.9	0.9	1.0
CAMK2G	Q13555	290	-1	-10	-9	-3	1.0	0.9	0.9	1.0
SYNRG	Q9UMZ2	1194	-1	-12	-9	-3	1.0	0.9	0.9	1.0
RPS29	P62273	39	-1	-12	-9	1	1.0	0.9	0.9	1.0
DOK2	O60496	65	-1	-13	-9	6	1.0	0.9	0.9	1.1
IKBKAP	O95163	1033	-1	-14	-9	-9	1.0	0.9	0.9	0.9
NUP88	Q99567	460	-1	-16	-9	-10	1.0	0.9	0.9	0.9
MAP3K6	O95382	232	-1	-19	-9	1	1.0	0.8	0.9	1.0
TMF1	P82094	518	-1	-19	-9	-17	1.0	0.8	0.9	0.9
EIF4EBP2	Q13542	35	-1	-19	-9	-25	1.0	0.8	0.9	0.8
PML	P29590	57	-1	-20	-9	-13	1.0	0.8	0.9	0.9
TOP2A	P11388	170	-1	-30	-9	-2	1.0	0.8	0.9	1.0
TOP2B	Q02880	191	-1	-30	-9	-2	1.0	0.8	0.9	1.0
TPT1	P13693	28	-1	-40	-9	-6	1.0	0.7	0.9	0.9
PSTPIP1	O43586	305	-2	-1	-9	-10	1.0	1.0	0.9	0.9
CLP1	Q92989	311	-2	-8	-9	-9	1.0	0.9	0.9	0.9
AKAP13	Q12802	2685	-2	-10	-9	-20	1.0	0.9	0.9	0.8
ITGB2	P05107	198	-2	-12	-9	-10	1.0	0.9	0.9	0.9
APOBR	Q0VD83	149	-2	-12	-9	-14	1.0	0.9	0.9	0.9
REL	Q04864	250	-2	-13	-9	-4	1.0	0.9	0.9	1.0
SETD1B	Q9UPS6	931	-2	-13	-9	-8	1.0	0.9	0.9	0.9
CAMK2D	Q13557	290	-2	-18	-9	-21	1.0	0.9	0.9	0.8
HNRNPF	P52597	122	-2	-21	-9	-3	1.0	0.8	0.9	1.0
RABGAP1	Q9Y3P9	941	-2	-1	-9	-8	1.0	1.0	0.9	0.9
KIF13B	Q9NQT8	651	-2	-3	-9	-10	1.0	1.0	0.9	0.9
HPS5	Q9UPZ3	707	-2	-6	-9	-7	1.0	0.9	0.9	0.9
TMEM55B	Q86T03	147	-2	-7	-9	-2	1.0	0.9	0.9	1.0
ZCCHC8	Q6NZY4	157	-2	-7	-9	-8	1.0	0.9	0.9	0.9
AKR7A2	O43488	214	-2	-10	-9	-3	1.0	0.9	0.9	1.0
TTLL12	Q14166	98	-2	-10	-9	-6	1.0	0.9	0.9	0.9
WDR5	P61964	205	-2	-12	-9	-1	1.0	0.9	0.9	1.0
LIMS1	P48059	97	-2	-17	-9	-9	1.0	0.9	0.9	0.9
PKDREJ	Q9NTG1	1119	-3	-6	-9	-9	1.0	0.9	0.9	0.9
GRB2	P62993	32	-3	-7	-9	-7	1.0	0.9	0.9	0.9
NOP9	Q86U38	141	-3	-8	-9	1	1.0	0.9	0.9	1.0
VWA9	Q96SY0	193	-3	-9	-9	1	1.0	0.9	0.9	1.0
PRKAR1A	P10644	18	-3	-10	-9	-11	1.0	0.9	0.9	0.9

BCCIP	Q9P287	275	-3	-11	-9	-1	1.0	0.9	0.9	1.0
LPCAT2	Q7L5N7	4	-3	-11	-9	-28	1.0	0.9	0.9	0.8
KMT2D	O14686	4424	-3	-12	-9	-9	1.0	0.9	0.9	0.9
AKAP9	Q99996	3067	-3	-14	-9	-27	1.0	0.9	0.9	0.8
EEF1G	P26641	194	-3	-15	-9	-2	1.0	0.9	0.9	1.0
ACSS2	Q9NR19	554	-3	-17	-9	-11	1.0	0.9	0.9	0.9
PFAS	O15067	270	-3	-18	-9	43	1.0	0.8	0.9	1.8
PFAS	O15067	587	-3	-29	-9	-8	1.0	0.8	0.9	0.9
HTT	P42858	1441	-3	-9	-9	-8	1.0	0.9	0.9	0.9
NUMA1	Q14980	1367	-3	-10	-9	-9	1.0	0.9	0.9	0.9
TAOK2	Q9UL54	623	-3	-10	-9	-12	1.0	0.9	0.9	0.9
CEP85	Q6P2H3	281	-3	-10	-9	-20	1.0	0.9	0.9	0.8
GLYR1	Q49A26	330	-3	-13	-9	-7	1.0	0.9	0.9	0.9
BTN3A3	O00478	512	-3	-13	-9	-8	1.0	0.9	0.9	0.9
PSIP1	O75475	204	-3	-16	-9	-15	1.0	0.9	0.9	0.9
CCT2	P78371	289	-3	-17	-9	1	1.0	0.9	0.9	1.0
ALAD	P13716	223	-3	-17	-9	-10	1.0	0.9	0.9	0.9
Uncharacteri	A0A087WZG	534	-3	-18	-9	-11	1.0	0.9	0.9	0.9
PRRC2A	P48634	486	-4	-3	-9	-11	1.0	1.0	0.9	0.9
TWISTNB	Q3B726	193	-4	-10	-9	1	1.0	0.9	0.9	1.0
SARM1	Q6SZW1	311	-4	-11	-9	-3	1.0	0.9	0.9	1.0
LRMP	Q12912	72	-4	-11	-9	-15	1.0	0.9	0.9	0.9
SEC23IP	Q9Y6Y8	457	-4	-15	-9	-5	1.0	0.9	0.9	1.0
ISCA2	Q86U28	146	-4	-18	-9	-8	1.0	0.8	0.9	0.9
SP3	Q02447	600	-4	-8	-9	-5	1.0	0.9	0.9	1.0
TPP2	P29144	28	-4	-13	-9	-13	1.0	0.9	0.9	0.9
ZBTB2	Q8N680	296	-4	-19	-9	-22	1.0	0.8	0.9	0.8
RPS19BP1	Q86WX3	104	-5	-1	-9	0	1.0	1.0	0.9	1.0
HPS6	Q86YV9	408	-5	-8	-9	-3	1.0	0.9	0.9	1.0
LYPD6	Q86Y78	96	-5	-8	-9	-13	1.0	0.9	0.9	0.9
LYPD6	Q86Y78	107	-5	-8	-9	-13	1.0	0.9	0.9	0.9
CSNK1G2	P78368	54	-5	-10	-9	-13	1.0	0.9	0.9	0.9
CSNK1G1	Q9HCP0	52	-5	-10	-9	-13	1.0	0.9	0.9	0.9
DIP2B	Q9P265	592	-5	-11	-9	8	1.0	0.9	0.9	1.1
PIK3C2A	O00443	669	-5	-14	-9	-12	1.0	0.9	0.9	0.9
BRD1	O95696	393	-5	-14	-9	-14	1.0	0.9	0.9	0.9
INF2	Q27J81	1029	-5	-15	-9	-17	1.0	0.9	0.9	0.9
ZBED1	O96006	38	-5	-15	-9	-18	1.0	0.9	0.9	0.9
JRK	Q86XJ5	428	-5	-16	-9	-11	1.0	0.9	0.9	0.9
OAS2	P29728	652	-5	-17	-9	-12	1.0	0.9	0.9	0.9
DGKZ	Q13574	793	-5	-17	-9	4	1.0	0.9	0.9	1.0
SRI	P30626	162	-5	-17	-9	-3	1.0	0.9	0.9	1.0
POLR2B	P30876	1093	-5	-23	-9	-18	1.0	0.8	0.9	0.9
SAFB2	Q14151	224	-5	-7	-9	-12	1.0	0.9	0.9	0.9
LRP1B	Q9NZR2	3560	-5	-8	-9	-5	1.0	0.9	0.9	1.0
UBR7	Q8N806	260	-5	-9	-9	-14	1.0	0.9	0.9	0.9
TNFAIP2	Q03169	45	-5	-12	-9	-28	1.0	0.9	0.9	0.8
ARFGEF1	Q9Y6D6	1503	-5	-13	-9	-6	1.0	0.9	0.9	0.9
PPP6R1	Q9UPN7	172	-5	-16	-9	-19	1.0	0.9	0.9	0.8
SLC27A3	Q5K4L6	426	-5	-23	-9	-2	1.0	0.8	0.9	1.0
MCCC2	Q9HCC0	392	-5	-23	-9	-3	1.0	0.8	0.9	1.0
HTT	P42858	942	-5	-25	-9	-10	1.0	0.8	0.9	0.9
CCT5	P48643	429	-6	-8	-9	16	0.9	0.9	0.9	1.2

BUB3	O43684	129	-6	-17	-9	-11	0.9	0.9	0.9	0.9
NUP155	O75694	1344	-6	-25	-9	-24	0.9	0.8	0.9	0.8
JOSD2	Q8TAC2	24	-6	-6	-9	-6	0.9	0.9	0.9	0.9
RPS11	P62280	131	-6	-9	-9	-6	0.9	0.9	0.9	0.9
LRCH3	Q96II8	227	-6	-17	-9	-18	0.9	0.9	0.9	0.9
ZNF292	O60281	430	-6	-17	-9	-27	0.9	0.9	0.9	0.8
LONP1	P36776	520	-6	-20	-9	-5	0.9	0.8	0.9	1.0
RAB3GAP1	Q15042	522	-6	-20	-9	-12	0.9	0.8	0.9	0.9
SIT1	Q9Y3P8	173	-7	-17	-9	-18	0.9	0.9	0.9	0.9
INTS9	Q9NV88	446	-7	-23	-9	-8	0.9	0.8	0.9	0.9
VCP	P55072	184	-7	-24	-9	-12	0.9	0.8	0.9	0.9
ASH2L	Q9UBL3	362	-7	-4	-9	-6	0.9	1.0	0.9	0.9
TXNRD1	Q16881	209	-7	-16	-9	-14	0.9	0.9	0.9	0.9
TXNRD2	Q9NNW7	86	-7	-16	-9	-14	0.9	0.9	0.9	0.9
NUDT3	O95989	25	-8	-11	-9	-4	0.9	0.9	0.9	1.0
NUDT4	Q9NZJ9	25	-8	-11	-9	-4	0.9	0.9	0.9	1.0
IDH3A	P50213	222	-8	-11	-9	-18	0.9	0.9	0.9	0.8
PRKCD	Q05655	344	-8	-24	-9	-10	0.9	0.8	0.9	0.9
COG4	Q9H9E3	25	-8	-29	-9	-4	0.9	0.8	0.9	1.0
ACIN1	Q9UKV3	733	-8	-7	-9	-19	0.9	0.9	0.9	0.8
TEP1	Q99973	2000	-8	-9	-9	-12	0.9	0.9	0.9	0.9
GIPC3	Q8TF64	209	-8	-12	-9	-5	0.9	0.9	0.9	1.0
INO80	Q9ULG1	963	-8	-25	-9	9	0.9	0.8	0.9	1.1
RARS	P54136	502	-8	-44	-9	5	0.9	0.7	0.9	1.1
DIDO1	Q9BTC0	1449	-9	-5	-9	-14	0.9	1.0	0.9	0.9
EPS15	P42566	274	-9	-7	-9	-17	0.9	0.9	0.9	0.9
EXOSC3	Q9NQT5	215	-9	-13	-9	-13	0.9	0.9	0.9	0.9
RAPGEF1	Q13905	328	-9	-17	-9	-25	0.9	0.9	0.9	0.8
BRF1	Q92994	175	-9	-28	-9	-2	0.9	0.8	0.9	1.0
ALDH9A1	P49189	289	-9	-5	-9	-4	0.9	1.0	0.9	1.0
TRIM28	Q13263	156	-9	-19	-9	-17	0.9	0.8	0.9	0.9
ARRB1	P49407	251	-10	-22	-9	-6	0.9	0.8	0.9	0.9
PRPF19	Q9UMS4	298	-10	-29	-9	-22	0.9	0.8	0.9	0.8
GAK	O14976	595	-11	-13	-9	-11	0.9	0.9	0.9	0.9
DARS	P14868	76	-11	-29	-9	-4	0.9	0.8	0.9	1.0
NSD1	Q96L73	2273	-11	-1	-9	-23	0.9	1.0	0.9	0.8
KYNU	Q16719	327	-11	-19	-9	0	0.9	0.8	0.9	1.0
TBC1D14	Q9P2M4	219	-11	-27	-9	8	0.9	0.8	0.9	1.1
PRRC2C	Q9Y520	2860	-12	-21	-9	-7	0.9	0.8	0.9	0.9
ZYX	Q15942	504	-12	-12	-9	-26	0.9	0.9	0.9	0.8
NUDCD1	Q96RS6	32	-12	-15	-9	6	0.9	0.9	0.9	1.1
SAMD9L	Q8IVG5	1313	-12	-16	-9	-21	0.9	0.9	0.9	0.8
EXOSC8	Q96B26	89	-12	-27	-9	-4	0.9	0.8	0.9	1.0
AKAP9	Q99996	2777	-13	-30	-9	-26	0.9	0.8	0.9	0.8
ATOX1	O00244	12	-13	2	-9	-24	0.9	1.0	0.9	0.8
SERPINB9	P50453	259	-13	-13	-9	-9	0.9	0.9	0.9	0.9
PRKD2	Q9BZL6	180	-14	-19	-9	-25	0.9	0.8	0.9	0.8
SERPINB8	P50452	364	-14	-13	-9	-18	0.9	0.9	0.9	0.9
AP1G1	O43747	160	-15	-29	-9	-14	0.9	0.8	0.9	0.9
RTCB	Q9Y3I0	255	-15	-19	-9	-9	0.9	0.8	0.9	0.9
PPP1R3D	O95685	41	-16	-28	-9	-14	0.9	0.8	0.9	0.9
CHEK2	O96017	539	-17	-10	-9	-27	0.9	0.9	0.9	0.8
CTSZ	Q9UBR2	92	-19	7	-9	56	0.8	1.1	0.9	2.3

ZNF512	Q96ME7	408	-20	-9	-9	-1	0.8	0.9	0.9	1.0
BLMH	Q13867	73	-23	-14	-9	-4	0.8	0.9	0.9	1.0
CSNK2B-LY6(	N0E472	109	-24	-17	-9	0	0.8	0.9	0.9	1.0
AGAP1	Q9UPQ3	211	-26	-8	-9	-21	0.8	0.9	0.9	0.8
NTPCR	Q9BSD7	184	-27	-16	-9	-20	0.8	0.9	0.9	0.8
PPP1R21	Q6ZMI0	200	-27	-11	-9	-20	0.8	0.9	0.9	0.8
RALY	Q9UKM9	51	-29	-14	-9	-5	0.8	0.9	0.9	1.0
FLNA	P21333	2601	-30	-22	-9	-7	0.8	0.8	0.9	0.9
FAM120A	Q9NZB2	919	-32	6	-9	-18	0.8	1.1	0.9	0.9
CTC1	Q2NKJ3	128	-37	-20	-9	-22	0.7	0.8	0.9	0.8
HSPE1-MOB4	S4R3N1	87	23	-2	-9	4	1.3	1.0	0.9	1.0
HUWE1	Q7Z6Z7	3361	17	-4	-9	-5	1.2	1.0	0.9	1.0
TRAFD1	O14545	135	16	-18	-9	13	1.2	0.9	0.9	1.1
FAM129B	Q96TA1	194	16	-2	-9	10	1.2	1.0	0.9	1.1
GOLGA7	Q7Z5G4	24	16	-13	-9	-4	1.2	0.9	0.9	1.0
ESD	P10768	176	16	-13	-9	-8	1.2	0.9	0.9	0.9
NAIP	Q13075	1349	15	2	-9	0	1.2	1.0	0.9	1.0
ATXN7	O15265	692	15	-9	-9	-3	1.2	0.9	0.9	1.0
ANKFY1	Q9P2R3	1156	14	3	-9	-1	1.2	1.0	0.9	1.0
LIPE	Q05469	1072	14	2	-9	3	1.2	1.0	0.9	1.0
RANBP2	P49792	3122	13	-15	-9	3	1.1	0.9	0.9	1.0
STRIP1	Q5VSL9	668	13	-22	-9	14	1.1	0.8	0.9	1.2
STRIP2	Q9ULQ0	664	13	-22	-9	14	1.1	0.8	0.9	1.2
HUWE1	Q7Z6Z7	3375	12	-6	-9	12	1.1	0.9	0.9	1.1
KMT2C	Q8NEZ4	4684	12	-7	-9	11	1.1	0.9	0.9	1.1
SEC16A	O15027	1514	12	-2	-9	-6	1.1	1.0	0.9	0.9
PRKDC	P78527	1312	11	-30	-9	-13	1.1	0.8	0.9	0.9
FKBP8	Q14318	227	11	-3	-9	-4	1.1	1.0	0.9	1.0
ANK1	P16157	1216	10	-20	-9	-5	1.1	0.8	0.9	1.0
FAM160B1	Q5W0V3	99	10	-4	-9	-3	1.1	1.0	0.9	1.0
PPP6C	O00743	192	10	-7	-9	2	1.1	0.9	0.9	1.0
ARHGEF12	Q9NZN5	784	9	-4	-9	-25	1.1	1.0	0.9	0.8
ANXA7	P20073	413	9	-5	-9	-16	1.1	1.0	0.9	0.9
KDSR	Q06136	121	9	-13	-9	-5	1.1	0.9	0.9	1.0
ACAP1	Q15027	338	9	-15	-9	5	1.1	0.9	0.9	1.0
LIMA1	Q9UHB6	316	8	-6	-9	-10	1.1	0.9	0.9	0.9
ACSF3	Q4G176	88	8	-9	-9	7	1.1	0.9	0.9	1.1
ECM29	Q5VYK3	449	8	-15	-9	0	1.1	0.9	0.9	1.0
ME2	P23368	202	8	-24	-9	-10	1.1	0.8	0.9	0.9
PA2G4	Q9UQ80	179	8	-23	-9	23	1.1	0.8	0.9	1.3
RBL2	Q08999	1118	7	-7	-9	-12	1.1	0.9	0.9	0.9
OGDH	Q02218	331	7	-15	-9	-5	1.1	0.9	0.9	1.0
NDRG1	Q92597	272	7	-16	-9	-11	1.1	0.9	0.9	0.9
DYNC1H1	Q14204	2076	7	5	-9	4	1.1	1.1	0.9	1.0
NR3C1	P04150	302	7	-4	-9	-34	1.1	1.0	0.9	0.7
KDM6A	O15550	384	7	-5	-9	-3	1.1	1.0	0.9	1.0
URB2	Q14146	931	7	-10	-9	-18	1.1	0.9	0.9	0.9
GNL3L	Q9NVN8	150	7	-14	-9	-10	1.1	0.9	0.9	0.9
MYH10	P35580	176	7	-17	-9	-8	1.1	0.9	0.9	0.9
CORO7-PAM:	A0A0A6YYL4	695	7	-36	-9	-7	1.1	0.7	0.9	0.9
AKNA	Q7Z591	1241	6	-1	-9	-19	1.1	1.0	0.9	0.8
DBR1	Q9UK59	339	6	-3	-9	-19	1.1	1.0	0.9	0.8
SYNE1	Q8NF91	1234	6	-12	-9	-14	1.1	0.9	0.9	0.9

PTRHD1	Q6GMV3	52	6	-14	-9	7	1.1	0.9	0.9	1.1
ANAPC7	Q9UJX3	509	6	-28	-9	-18	1.1	0.8	0.9	0.9
TBC1D2	Q9BYX2	49	6	-13	-9	-4	1.1	0.9	0.9	1.0
CENPB	P07199	135	6	-15	-9	-14	1.1	0.9	0.9	0.9
SPATA2	Q9UM82	41	5	-5	-9	-3	1.1	1.0	0.9	1.0
NIN	Q8N4C6	1912	5	-14	-9	9	1.1	0.9	0.9	1.1
USP9Y	O00507	1735	5	1	-9	-2	1.0	1.0	0.9	1.0
USP9X	Q93008	1733	5	1	-9	-2	1.0	1.0	0.9	1.0
RASA2	Q15283	129	5	-5	-9	-1	1.0	1.0	0.9	1.0
TP53BP2	Q13625	290	5	-9	-9	-10	1.0	0.9	0.9	0.9
BPNT1	O95861	243	5	-14	-9	1	1.0	0.9	0.9	1.0
C19orf54	Q5BKX5	338	5	-14	-9	-29	1.0	0.9	0.9	0.8
RNF14	Q9UBS8	453	5	-16	-9	-6	1.0	0.9	0.9	0.9
PSMD9	O00233	216	5	-19	-9	-13	1.0	0.8	0.9	0.9
HSD17B4	P51659	189	4	0	-9	-3	1.0	1.0	0.9	1.0
OSBPL3	Q9H4L5	515	4	-2	-9	-20	1.0	1.0	0.9	0.8
COPS7B	Q9H9Q2	157	4	-3	-9	16	1.0	1.0	0.9	1.2
ELMO2	Q96JJ3	615	4	-8	-9	-3	1.0	0.9	0.9	1.0
SNRNP200	O75643	502	4	-10	-9	-1	1.0	0.9	0.9	1.0
ANXA11	P50995	384	4	-10	-9	-1	1.0	0.9	0.9	1.0
TNRC6B	Q9UPQ9	97	4	-12	-9	-16	1.0	0.9	0.9	0.9
USP4	Q13107	464	4	-13	-9	-8	1.0	0.9	0.9	0.9
USP15	Q9Y4E8	451	4	-13	-9	-8	1.0	0.9	0.9	0.9
LUZP1	Q86V48	594	4	1	-9	3	1.0	1.0	0.9	1.0
PHC3	Q8NDX5	808	4	-1	-9	1	1.0	1.0	0.9	1.0
Uncharacteri	A0A087WZG	240	4	-7	-9	16	1.0	0.9	0.9	1.2
SNX29	Q8TEQ0	44	4	-8	-9	1	1.0	0.9	0.9	1.0
CLASP2	O75122	1184	4	-8	-9	-1	1.0	0.9	0.9	1.0
CLASP1	Q7Z460	1428	4	-8	-9	-1	1.0	0.9	0.9	1.0
TACC3	Q9Y6A5	749	4	-11	-9	-27	1.0	0.9	0.9	0.8
RXRA	P19793	138	4	-12	-9	-6	1.0	0.9	0.9	0.9
C19orf25	Q9UFG5	87	4	-12	-9	-10	1.0	0.9	0.9	0.9
LONP2	Q86WA8	405	4	-14	-9	-9	1.0	0.9	0.9	0.9
RPL37	P61927	37	4	-15	-9	-10	1.0	0.9	0.9	0.9
PLEK	P08567	102	4	-16	-9	-7	1.0	0.9	0.9	0.9
KAT6A	Q92794	508	4	-16	-9	-17	1.0	0.9	0.9	0.9
RAD50	Q92878	1296	4	-17	-9	-6	1.0	0.9	0.9	0.9
AARS	P49588	947	4	-19	-9	-15	1.0	0.8	0.9	0.9
ANAPC2	Q9UJX6	394	3	7	-9	14	1.0	1.1	0.9	1.2
GAPVD1	Q14C86	1253	3	3	-9	-19	1.0	1.0	0.9	0.8
TCP11L2	Q8N4U5	451	3	-3	-9	-10	1.0	1.0	0.9	0.9
DAZAP1	Q96EP5	63	3	-11	-9	4	1.0	0.9	0.9	1.0
PPM1B	O75688	149	3	-14	-9	5	1.0	0.9	0.9	1.1
GAK	O14976	691	3	-15	-9	-11	1.0	0.9	0.9	0.9
PGM3	O95394	200	3	-7	-9	-16	1.0	0.9	0.9	0.9
LANCL2	Q9NS86	169	3	-7	-9	-17	1.0	0.9	0.9	0.9
SEC24B	O95487	562	3	-8	-9	-2	1.0	0.9	0.9	1.0
MX2	P20592	707	3	-8	-9	-7	1.0	0.9	0.9	0.9
GLRX	P35754	23	3	-8	-9	-22	1.0	0.9	0.9	0.8
Uncharacteri	A0A087WW	135	3	-9	-9	-9	1.0	0.9	0.9	0.9
HK2	P52789	794	3	-9	-9	-25	1.0	0.9	0.9	0.8
ARMC8	Q8IUR7	283	3	-13	-9	5	1.0	0.9	0.9	1.0
DENND1C	Q8IV53	412	3	-14	-9	-15	1.0	0.9	0.9	0.9

SAMHD1	Q9Y3Z3	15	3	-16	-9	-13	1.0	0.9	0.9	0.9
WDR82	Q6UXN9	287	3	-21	-9	-2	1.0	0.8	0.9	1.0
HELQ	Q8TDG4	149	3	-26	-9	-27	1.0	0.8	0.9	0.8
SEMA4B	Q9NPR2	180	3	-27	-9	-8	1.0	0.8	0.9	0.9
NIF3L1	Q9GZT8	213	2	-8	-9	6	1.0	0.9	0.9	1.1
MRRF	Q96E11	7	2	-16	-9	-25	1.0	0.9	0.9	0.8
ILVBL	A1L0T0	173	2	-18	-9	1	1.0	0.9	0.9	1.0
LRCH4	O75427	134	2	-24	-9	-6	1.0	0.8	0.9	0.9
RAB3GAP1	Q15042	873	2	-4	-9	-7	1.0	1.0	0.9	0.9
ARHGEF2	Q92974	68	2	-5	-9	-9	1.0	1.0	0.9	0.9
TOPBP1	Q92547	1099	2	-6	-9	-16	1.0	0.9	0.9	0.9
HUWE1	Q7Z6Z7	1252	2	-7	-9	-7	1.0	0.9	0.9	0.9
HIRA	P54198	745	2	-7	-9	-7	1.0	0.9	0.9	0.9
DYRK1B	Q9Y463	264	2	-10	-9	-1	1.0	0.9	0.9	1.0
FLNA	P21333	210	2	-11	-9	-4	1.0	0.9	0.9	1.0
GSDMD	P57764	56	2	-12	-9	-5	1.0	0.9	0.9	1.0
PDS5B	Q9NTI5	333	2	-24	-9	11	1.0	0.8	0.9	1.1
FOXK1	P85037	404	1	-4	-9	11	1.0	1.0	0.9	1.1
SART3	Q15020	375	1	-5	-9	-10	1.0	1.0	0.9	0.9
TPP2	P29144	787	1	-12	-9	6	1.0	0.9	0.9	1.1
SERPINB8	P50452	348	1	-14	-9	1	1.0	0.9	0.9	1.0
SERPINB9	P50453	350	1	-14	-9	1	1.0	0.9	0.9	1.0
SMCHD1	A6NHR9	1710	1	-19	-9	-12	1.0	0.8	0.9	0.9
INPP5A	Q14642	159	1	-6	-9	-11	1.0	0.9	0.9	0.9
NFS1	Q9Y697	158	1	-10	-9	-10	1.0	0.9	0.9	0.9
PSMB10	P40306	70	1	-10	-9	-2	1.0	0.9	0.9	1.0
MKRN1	Q9UHC7	186	1	-11	-9	-16	1.0	0.9	0.9	0.9
ACTR2	P61160	11	1	-12	-9	-1	1.0	0.9	0.9	1.0
TAF2	Q6P1X5	732	1	-12	-9	-6	1.0	0.9	0.9	0.9
TRAF2	Q12933	171	1	-12	-9	-19	1.0	0.9	0.9	0.8
LARS	Q9P2J5	573	1	-14	-9	-8	1.0	0.9	0.9	0.9
TCF25	Q9BQ70	216	1	-20	-9	-15	1.0	0.8	0.9	0.9
ARF6	P62330	155	1	-23	-9	-16	1.0	0.8	0.9	0.9
PPP1R18	Q6NYC8	276	0	-5	-9	-10	1.0	1.0	0.9	0.9
NCAPH	Q15003	239	0	-9	-9	-11	1.0	0.9	0.9	0.9
PRDM2	Q13029	1204	0	-10	-9	-7	1.0	0.9	0.9	0.9
VPS13C	Q709C8	2872	0	-16	-9	-13	1.0	0.9	0.9	0.9
CBL	P22681	353	0	-16	-9	-14	1.0	0.9	0.9	0.9
FAM102A	Q5T9C2	175	0	-16	-9	-17	1.0	0.9	0.9	0.9
RPS27A	P62979	145	0	-22	-9	2	1.0	0.8	0.9	1.0
NPEPL1	Q8NDH3	189	-1	-5	-9	3	1.0	1.0	0.9	1.0
BLOC1S5-TXN	H3BN57	113	-1	-8	-9	-8	1.0	0.9	0.9	0.9
MYH9	P35579	789	-1	-10	-9	5	1.0	0.9	0.9	1.1
PREX1	Q8TCU6	52	-1	-10	-9	-11	1.0	0.9	0.9	0.9
POLI	Q9UNA4	91	-1	-10	-9	-8	1.0	0.9	0.9	0.9
BRWD3	Q6RI45	202	-1	-13	-9	7	1.0	0.9	0.9	1.1
PHIP	Q8WWQ0	205	-1	-13	-9	7	1.0	0.9	0.9	1.1
BRWD1	Q9NSI6	208	-1	-13	-9	7	1.0	0.9	0.9	1.1
MYO9B	Q13459	1857	-1	-15	-9	-20	1.0	0.9	0.9	0.8
CDC5L	Q99459	769	-1	-16	-9	-13	1.0	0.9	0.9	0.9
TOMM70	O94826	144	-1	-17	-9	0	1.0	0.9	0.9	1.0
DCAF10	Q5QP82	290	-1	-18	-9	2	1.0	0.9	0.9	1.0
TPRG1L	Q5T0D9	207	-1	-21	-9	1	1.0	0.8	0.9	1.0

ZNF330	Q9Y3S2	192	-1	-21	-9	-4	1.0	0.8	0.9	1.0
ARFGAP2	Q8N6H7	29	-1	-21	-9	-6	1.0	0.8	0.9	0.9
ZNF600	Q6ZNG1	164	-1	-5	-9	-13	1.0	1.0	0.9	0.9
ZNF845	Q96IR2	217	-1	-5	-9	-13	1.0	1.0	0.9	0.9
ZW10	O43264	124	-1	-8	-9	-8	1.0	0.9	0.9	0.9
PHF11	Q9UIL8	311	-1	-8	-9	-11	1.0	0.9	0.9	0.9
RGS3	P49796	1135	-1	-8	-9	-13	1.0	0.9	0.9	0.9
MACF1	Q9UPN3	4825	-1	-21	-9	-20	1.0	0.8	0.9	0.8
BCKDHB	P21953	316	-1	-22	-9	-19	1.0	0.8	0.9	0.8
FLOT2	Q14254	88	-1	-27	-9	-12	1.0	0.8	0.9	0.9
PHGDH	O43175	295	-2	-6	-9	-7	1.0	0.9	0.9	0.9
GUSB	P08236	644	-2	-8	-9	-15	1.0	0.9	0.9	0.9
ZNFX1	Q9P2E3	1822	-2	-9	-9	-15	1.0	0.9	0.9	0.9
ACTN1	P12814	180	-2	-10	-9	-1	1.0	0.9	0.9	1.0
OSBPL1A	Q9BXW6	297	-2	-12	-9	6	1.0	0.9	0.9	1.1
TOE1	Q96GM8	80	-2	-12	-9	-6	1.0	0.9	0.9	0.9
AHNAK	Q09666	5502	-2	-12	-9	-17	1.0	0.9	0.9	0.9
GNAI3	P08754	139	-2	-15	-9	-7	1.0	0.9	0.9	0.9
FHOD1	Q9Y613	43	-2	-16	-9	-8	1.0	0.9	0.9	0.9
PPM1G	O15355	13	-2	-20	-9	-10	1.0	0.8	0.9	0.9
ZC3H10	Q96K80	56	-2	-26	-9	-7	1.0	0.8	0.9	0.9
ZMIZ1	Q9ULJ6	657	-2	-27	-9	-5	1.0	0.8	0.9	1.0
PTRH1	Q86Y79	147	-2	-4	-9	-11	1.0	1.0	0.9	0.9
UBR1	Q8IWV7	350	-2	-7	-9	-4	1.0	0.9	0.9	1.0
HMHA1	Q92619	599	-2	-7	-9	-10	1.0	0.9	0.9	0.9
ESYT2	A0FGR8	181	-2	-9	-9	8	1.0	0.9	0.9	1.1
TBC1D2	Q9BYX2	469	-2	-13	-9	-17	1.0	0.9	0.9	0.9
LPP	Q93052	593	-2	-14	-9	12	1.0	0.9	0.9	1.1
HERC3	Q15034	333	-2	-15	-9	-16	1.0	0.9	0.9	0.9
MARK3	P27448	510	-2	-16	-9	-11	1.0	0.9	0.9	0.9
KCMF1	Q9P0J7	33	-2	-16	-9	-14	1.0	0.9	0.9	0.9
MVB12B	Q9H7P6	313	-2	-17	-9	-15	1.0	0.9	0.9	0.9
RAB37	Q96AX2	22	-2	-17	-9	-27	1.0	0.9	0.9	0.8
MYO1D	O94832	353	-2	-19	-9	-1	1.0	0.8	0.9	1.0
RACK1	P63244	153	-2	-32	-9	-2	1.0	0.8	0.9	1.0
TCAF2	A6NFQ2	275	-3	-6	-9	-18	1.0	0.9	0.9	0.9
SRSF3	P84103	72	-3	-6	-9	7	1.0	0.9	0.9	1.1
PRKDC	P78527	1499	-3	-7	-9	-1	1.0	0.9	0.9	1.0
ATM	Q13315	2246	-3	-7	-9	-3	1.0	0.9	0.9	1.0
LNP	Q9C0E8	149	-3	-7	-9	-14	1.0	0.9	0.9	0.9
GLS	O94925	283	-3	-7	-9	4	1.0	0.9	0.9	1.0
CDKN2AIP	Q9NXV6	516	-3	-9	-9	-3	1.0	0.9	0.9	1.0
SMARCAD1	Q9H4L7	951	-3	-9	-9	-15	1.0	0.9	0.9	0.9
TXNDC5	Q8NBS9	217	-3	-12	-9	-14	1.0	0.9	0.9	0.9
CHUK	O15111	30	-3	-13	-9	-26	1.0	0.9	0.9	0.8
CTSG	P08311	142	-3	-19	-9	-5	1.0	0.8	0.9	1.0
HCCS	P53701	178	-3	-19	-9	-14	1.0	0.8	0.9	0.9
ATXN7L3B	Q96GX2	75	-3	-22	-9	-14	1.0	0.8	0.9	0.9
ACLY	P53396	20	-3	-13	-9	-9	1.0	0.9	0.9	0.9
SUGT1	Q9Y2Z0	49	-3	-15	-9	-7	1.0	0.9	0.9	0.9
FPGT	O14772	411	-3	-15	-9	-9	1.0	0.9	0.9	0.9
IFIT2	P09913	289	-3	-19	-9	-9	1.0	0.8	0.9	0.9
IPCEF1	Q8WWN9	44	-3	-20	-9	6	1.0	0.8	0.9	1.1

TRAPPC10	P48553	916	-3	-26	-9	-25	1.0	0.8	0.9	0.8
HNRNPUL2-B	H3BQZ7	518	-3	-26	-9	-6	1.0	0.8	0.9	0.9
LRCH4	O75427	224	-3	-26	-9	-8	1.0	0.8	0.9	0.9
TTI2	Q6NXR4	36	-4	-9	-9	17	1.0	0.9	0.9	1.2
MBNL1	Q9NR56	185	-4	-10	-9	-2	1.0	0.9	0.9	1.0
OCIAD2	Q56VL3	27	-4	-11	-9	-6	1.0	0.9	0.9	0.9
GPX4	P36969	134	-4	-11	-9	-15	1.0	0.9	0.9	0.9
BCR	P11274	609	-4	-25	-9	-5	1.0	0.8	0.9	1.0
ZC3H4	Q9UPT8	439	-4	6	-9	2	1.0	1.1	0.9	1.0
STIM2	Q9P246	426	-4	-11	-9	-1	1.0	0.9	0.9	1.0
ANKMY2	Q8IV38	341	-4	-11	-9	-12	1.0	0.9	0.9	0.9
PGM2L1	Q6PCE3	348	-4	-12	-9	-12	1.0	0.9	0.9	0.9
TEX10	Q9NXF1	454	-4	-14	-9	-10	1.0	0.9	0.9	0.9
KHNYN	O15037	157	-4	-20	-9	-16	1.0	0.8	0.9	0.9
CEP76	Q8TAP6	624	-5	0	-9	-9	1.0	1.0	0.9	0.9
HBP1	O60381	383	-5	-5	-9	-6	1.0	1.0	0.9	0.9
UBE3A	Q05086	108	-5	-6	-9	-21	1.0	0.9	0.9	0.8
TRMT2A	Q8IZ69	225	-5	-11	-9	-6	1.0	0.9	0.9	0.9
VCPIP1	Q96JH7	160	-5	-11	-9	-22	1.0	0.9	0.9	0.8
YY1	P25490	298	-5	-14	-9	-7	1.0	0.9	0.9	0.9
TEX2	Q8IWB9	1114	-5	-15	-9	-14	1.0	0.9	0.9	0.9
RBM25	P49756	83	-5	-16	-9	-1	1.0	0.9	0.9	1.0
NUP205	Q92621	921	-5	-16	-9	-3	1.0	0.9	0.9	1.0
PPP1R11	O60927	60	-5	-17	-9	-10	1.0	0.9	0.9	0.9
TGFBRAP1	Q8WUH2	33	-5	-20	-9	-5	1.0	0.8	0.9	1.0
BCL11B	Q9C0K0	88	-5	-20	-9	-20	1.0	0.8	0.9	0.8
DPYD	Q12882	49	-5	-20	-9	-20	1.0	0.8	0.9	0.8
RECQL	P46063	606	-5	-3	-9	-15	1.0	1.0	0.9	0.9
MYOF	Q9NZM1	1553	-5	-9	-9	-2	1.0	0.9	0.9	1.0
PDCL3	Q9H2J4	154	-5	-10	-9	1	1.0	0.9	0.9	1.0
RETSAT	Q6NUM9	105	-5	-11	-9	1	1.0	0.9	0.9	1.0
SUPT5H	O00267	626	-5	-11	-9	-10	1.0	0.9	0.9	0.9
INPP4A	Q96PE3	230	-5	-11	-9	-5	1.0	0.9	0.9	1.0
VEZF1	Q14119	327	-5	-12	-9	-17	1.0	0.9	0.9	0.9
SRC	P12931	248	-5	-15	-9	-7	1.0	0.9	0.9	0.9
OPA1	O60313	786	-5	-19	-9	-17	1.0	0.8	0.9	0.9
NCAPD2	Q15021	286	-5	-21	-9	-17	1.0	0.8	0.9	0.9
DYNC1H1	Q14204	1977	-6	-8	-9	-14	0.9	0.9	0.9	0.9
MRPL47	Q9HD33	87	-6	-11	-9	-12	0.9	0.9	0.9	0.9
FES	P07332	785	-6	-14	-9	1	0.9	0.9	0.9	1.0
EPS15	P42566	657	-6	-14	-9	-20	0.9	0.9	0.9	0.8
SUPT4H1	P63272	19	-6	-15	-9	-9	0.9	0.9	0.9	0.9
C19orf68	Q86XI8	403	-6	-15	-9	9	0.9	0.9	0.9	1.1
GSS	P48637	409	-6	-16	-9	14	0.9	0.9	0.9	1.2
SLFN5	Q08AF3	627	-6	-16	-9	-20	0.9	0.9	0.9	0.8
ELL	P55199	14	-6	-21	-9	-15	0.9	0.8	0.9	0.9
TIPRL	O75663	87	-6	-8	-9	-15	0.9	0.9	0.9	0.9
ACAP2	Q15057	407	-6	-12	-9	-4	0.9	0.9	0.9	1.0
ABCD3	P28288	477	-6	-14	-9	-17	0.9	0.9	0.9	0.9
SRRT	Q9BXP5	715	-7	-12	-9	1	0.9	0.9	0.9	1.0
POLE4	Q9NR33	84	-7	-14	-9	-7	0.9	0.9	0.9	0.9
TRIM56	Q9BRZ2	104	-7	8	-9	-6	0.9	1.1	0.9	0.9
NBEAL2	Q6ZNJ1	390	-7	-8	-9	-16	0.9	0.9	0.9	0.9

SYNE1	Q8NF91	3027	-7	-10	-9	-8	0.9	0.9	0.9	0.9
ACAT2	Q9BWD1	92	-7	-11	-9	-13	0.9	0.9	0.9	0.9
MTA2	O94776	495	-7	-12	-9	-7	0.9	0.9	0.9	0.9
WDR92	Q96MX6	29	-7	-12	-9	-12	0.9	0.9	0.9	0.9
GART	P22102	41	-7	-14	-9	-10	0.9	0.9	0.9	0.9
METTL7A	Q9H8H3	92	-7	-20	-9	-8	0.9	0.8	0.9	0.9
SNRNP200	O75643	1127	-8	-8	-9	-12	0.9	0.9	0.9	0.9
PSMC5	P62195	209	-8	-8	-9	-5	0.9	0.9	0.9	1.0
ESD	P10768	45	-8	-17	-9	-3	0.9	0.9	0.9	1.0
NSMCE2	Q96MF7	185	-8	-18	-9	-16	0.9	0.9	0.9	0.9
ALDH3A2	P51648	220	-8	-23	-9	-12	0.9	0.8	0.9	0.9
ARHGAP4	P98171	330	-9	3	-9	-6	0.9	1.0	0.9	0.9
VPS39	Q96JC1	235	-9	-8	-9	-23	0.9	0.9	0.9	0.8
PHKB	Q93100	734	-9	-10	-9	8	0.9	0.9	0.9	1.1
CALCOCO1	Q9P1Z2	57	-9	-12	-9	-13	0.9	0.9	0.9	0.9
PSMB8	P28062	120	-9	-14	-9	-9	0.9	0.9	0.9	0.9
RASA3	Q14644	712	-9	-15	-9	-9	0.9	0.9	0.9	0.9
RALGAPA2	Q2PPJ7	169	-9	-23	-9	-16	0.9	0.8	0.9	0.9
TREML1	Q86YW5	196	-9	-11	-9	15	0.9	0.9	0.9	1.2
LANCL1	O43813	264	-9	-17	-9	-8	0.9	0.9	0.9	0.9
EIF2S2	P20042	305	-9	-17	-9	-9	0.9	0.9	0.9	0.9
IREB2	P48200	620	-9	-18	-9	12	0.9	0.8	0.9	1.1
MARS2	Q96GW9	425	-10	-2	-9	-4	0.9	1.0	0.9	1.0
FKBP8	Q14318	178	-10	-12	-9	-13	0.9	0.9	0.9	0.9
HAUS8	Q9BT25	354	-10	-12	-9	-23	0.9	0.9	0.9	0.8
CLP1	Q92989	338	-10	-16	-9	-27	0.9	0.9	0.9	0.8
PAK1IP1	Q9NWT1	288	-10	-17	-9	-6	0.9	0.9	0.9	0.9
BOD1L1	Q8NFC6	2164	-10	-22	-9	-23	0.9	0.8	0.9	0.8
ILF2	Q12905	291	-10	-22	-9	-15	0.9	0.8	0.9	0.9
FUBP3	Q96I24	125	-11	-19	-9	-6	0.9	0.8	0.9	0.9
PAPSS1	O43252	212	-11	-24	-9	-22	0.9	0.8	0.9	0.8
DUSP23	Q9BVJ7	53	-11	-5	-9	-3	0.9	1.0	0.9	1.0
MTG1	Q9BT17	262	-11	-19	-9	-9	0.9	0.8	0.9	0.9
RAD18	Q9NS91	64	-12	-19	-9	-23	0.9	0.8	0.9	0.8
ACTR10	Q9NZ32	27	-13	-3	-9	-16	0.9	1.0	0.9	0.9
RDH14	Q9HBH5	97	-13	-8	-9	-6	0.9	0.9	0.9	0.9
RDH14	Q9HBH5	148	-13	7	-9	11	0.9	1.1	0.9	1.1
VPS13C	Q709C8	1098	-13	-13	-9	-12	0.9	0.9	0.9	0.9
ACSL4	O60488	595	-13	-23	-9	1	0.9	0.8	0.9	1.0
RACK1	P63244	168	-14	-19	-9	-7	0.9	0.8	0.9	0.9
DDX59	Q5T1V6	414	-14	-20	-9	-15	0.9	0.8	0.9	0.9
TIGAR	Q9NQ88	161	-15	-18	-9	-7	0.9	0.9	0.9	0.9
PDS5A	Q29RF7	1079	-15	-27	-9	-6	0.9	0.8	0.9	0.9
TRIM32	Q13049	464	-16	-16	-9	-11	0.9	0.9	0.9	0.9
ZNF837	Q96EG3	179	-18	-4	-9	-27	0.9	1.0	0.9	0.8
ZNF837	Q96EG3	167	-18	-4	-9	-27	0.9	1.0	0.9	0.8
ZNF837	Q96EG3	161	-18	-4	-9	-27	0.9	1.0	0.9	0.8
KLHL22	Q53GT1	22	-20	-15	-9	-13	0.8	0.9	0.9	0.9
MAPKAPK2	P49137	114	-23	-5	-9	-6	0.8	1.0	0.9	0.9
EEF1G	P26641	68	-26	-22	-9	-12	0.8	0.8	0.9	0.9
FAH	P16930	408	-37	-72	-9	-1	0.7	0.6	0.9	1.0
MED29	Q9NX70	170	-38	-44	-9	-83	0.7	0.7	0.9	0.5
TRNAU1AP	Q9NX07	48	-51	-26	-9	-7	0.7	0.8	0.9	0.9

CUL1	Q13616	170	29	-18	-10	7	1.4	0.8	0.9	1.1
RANBP2	P49792	1499	16	2	-10	-9	1.2	1.0	0.9	0.9
DDX5	P17844	354	15	-14	-10	-5	1.2	0.9	0.9	1.0
EIF4H	Q15056	85	13	-18	-10	3	1.1	0.8	0.9	1.0
SP140	Q13342	763	13	-37	-10	-5	1.1	0.7	0.9	1.0
SP140L	Q9H930	476	13	-37	-10	-5	1.1	0.7	0.9	1.0
MTOR	P42345	1498	13	1	-10	-2	1.1	1.0	0.9	1.0
ABAT	P80404	440	13	-19	-10	2	1.1	0.8	0.9	1.0
IPO13	O94829	810	12	-5	-10	-6	1.1	1.0	0.9	0.9
ZC3H7B	Q9UGR2	501	12	-5	-10	-2	1.1	1.0	0.9	1.0
LRCH3	Q96II8	740	12	-12	-10	5	1.1	0.9	0.9	1.0
DET1	Q7L5Y6	482	11	4	-10	14	1.1	1.0	0.9	1.2
TRIM38	O00635	339	11	-9	-10	-12	1.1	0.9	0.9	0.9
TTC27	Q6P3X3	749	11	-15	-10	-13	1.1	0.9	0.9	0.9
EIF4G1	Q04637	1265	10	3	-10	1	1.1	1.0	0.9	1.0
ZNHIT2	Q9UHR6	188	10	-2	-10	-14	1.1	1.0	0.9	0.9
ADNP	Q9H2P0	624	10	-6	-10	8	1.1	0.9	0.9	1.1
UBR2	Q8IWV8	1619	10	-7	-10	5	1.1	0.9	0.9	1.0
RXRA	P19793	269	9	-3	-10	11	1.1	1.0	0.9	1.1
SEC24C	P53992	842	9	-14	-10	-8	1.1	0.9	0.9	0.9
HECTD1	Q9ULT8	1944	9	-26	-10	-19	1.1	0.8	0.9	0.8
ARMC6	Q6NXE6	118	8	-11	-10	12	1.1	0.9	0.9	1.1
MMS22L	Q6ZRQ5	121	8	2	-10	10	1.1	1.0	0.9	1.1
MED17	Q9NVC6	304	8	-28	-10	-6	1.1	0.8	0.9	0.9
PLEC	Q15149	965	7	-1	-10	-2	1.1	1.0	0.9	1.0
PA2G4	Q9UQ80	149	7	-38	-10	27	1.1	0.7	0.9	1.4
USP7	Q93009	961	7	-15	-10	-11	1.1	0.9	0.9	0.9
WDR26	Q9H7D7	238	7	-17	-10	-20	1.1	0.9	0.9	0.8
RBCK1	Q9BYM8	30	7	-23	-10	-17	1.1	0.8	0.9	0.9
ARHGAP30	Q7Z6I6	40	7	-24	-10	3	1.1	0.8	0.9	1.0
ZZEF1	O43149	162	7	-32	-10	-18	1.1	0.8	0.9	0.9
THOC2	Q8NI27	28	7	-36	-10	-2	1.1	0.7	0.9	1.0
STAT5B	P51692	101	6	-8	-10	8	1.1	0.9	0.9	1.1
ANKLE2	Q86XL3	286	6	-9	-10	-5	1.1	0.9	0.9	1.0
USP14	P54578	257	6	-16	-10	-1	1.1	0.9	0.9	1.0
WDFY4	Q6ZS81	2226	6	-23	-10	-9	1.1	0.8	0.9	0.9
GRK6	P43250	425	6	-5	-10	4	1.1	1.0	0.9	1.0
WTAP	Q15007	270	6	-8	-10	-11	1.1	0.9	0.9	0.9
ACTL6A	O96019	206	6	-28	-10	-7	1.1	0.8	0.9	0.9
HEATR3	Q7Z4Q2	59	5	-4	-10	-3	1.1	1.0	0.9	1.0
FBL	P22087	99	5	-8	-10	-6	1.1	0.9	0.9	0.9
AP2A2	O94973	932	5	-14	-10	11	1.1	0.9	0.9	1.1
OGDH	Q02218	283	5	-5	-10	6	1.0	1.0	0.9	1.1
DLG4	P78352	445	5	-9	-10	-13	1.0	0.9	0.9	0.9
KIF13B	Q9NQT8	26	5	-17	-10	-24	1.0	0.9	0.9	0.8
PRKDC	P78527	795	5	-23	-10	-3	1.0	0.8	0.9	1.0
TTN	Q8WZ42	23581	5	-23	-10	-24	1.0	0.8	0.9	0.8
RAB7A	P51149	83	5	-33	-10	4	1.0	0.8	0.9	1.0
L3MBTL3	Q96JM7	682	4	10	-10	-14	1.0	1.1	0.9	0.9
EIF2AK4	Q9P2K8	1255	4	-4	-10	-4	1.0	1.0	0.9	1.0
PPM1G	O15355	164	4	-9	-10	-12	1.0	0.9	0.9	0.9
RAD50	Q92878	133	4	-17	-10	-20	1.0	0.9	0.9	0.8
GPD2	P43304	45	4	-18	-10	-18	1.0	0.8	0.9	0.8

SON	P18583	1551	4	1	-10	-13	1.0	1.0	0.9	0.9
WDR7	Q9Y4E6	74	4	-4	-10	1	1.0	1.0	0.9	1.0
EIF2S3	P41091	96	4	-12	-10	-6	1.0	0.9	0.9	0.9
ARRB2	P32121	270	3	-4	-10	-24	1.0	1.0	0.9	0.8
IVD	P26440	352	3	-5	-10	7	1.0	1.0	0.9	1.1
KLC4	Q9NSK0	113	3	-7	-10	-9	1.0	0.9	0.9	0.9
USP8	P40818	575	3	-9	-10	-19	1.0	0.9	0.9	0.8
THBS1	P07996	930	3	-11	-10	-27	1.0	0.9	0.9	0.8
PHGDH	O43175	369	3	-12	-10	-5	1.0	0.9	0.9	1.0
TRAF3IP3	Q9Y228	256	3	-13	-10	-9	1.0	0.9	0.9	0.9
SEC23B	Q15437	425	3	-25	-10	-7	1.0	0.8	0.9	0.9
THADA	Q6YHU6	1126	3	-8	-10	-2	1.0	0.9	0.9	1.0
CCDC88B	A6NC98	1382	3	-8	-10	-6	1.0	0.9	0.9	0.9
DCTN2	Q13561	256	3	-14	-10	-1	1.0	0.9	0.9	1.0
NUP35	Q8NFH5	255	3	-17	-10	-10	1.0	0.9	0.9	0.9
KIAA1551	Q9HCM1	798	3	-17	-10	-16	1.0	0.9	0.9	0.9
NELFE	P18615	297	3	-18	-10	-15	1.0	0.8	0.9	0.9
IDH3A	P50213	127	3	-23	-10	-1	1.0	0.8	0.9	1.0
UBR4	Q5T4S7	3700	2	-10	-10	-10	1.0	0.9	0.9	0.9
TLN1	Q9Y490	1939	2	-15	-10	-9	1.0	0.9	0.9	0.9
SLC15A4	Q8N697	299	2	-17	-10	-22	1.0	0.9	0.9	0.8
ZNF189	O75820	610	2	-3	-10	-3	1.0	1.0	0.9	1.0
OGFR	Q9NZT2	330	2	-7	-10	-10	1.0	0.9	0.9	0.9
ACTR2	P61160	221	2	-11	-10	-5	1.0	0.9	0.9	1.0
PDLIM7	Q9NR12	311	2	-12	-10	-11	1.0	0.9	0.9	0.9
CLIC1	O00299	59	2	-13	-10	4	1.0	0.9	0.9	1.0
OAS2	P29728	523	2	-17	-10	0	1.0	0.9	0.9	1.0
TBL2	Q9Y4P3	44	2	-19	-10	-18	1.0	0.8	0.9	0.8
TXNRD2	Q9NNW7	54	2	-26	-10	9	1.0	0.8	0.9	1.1
ARFGEF2	Q9Y6D5	1147	1	-1	-10	-14	1.0	1.0	0.9	0.9
ARFGEF1	Q9Y6D6	1200	1	-1	-10	-14	1.0	1.0	0.9	0.9
PRPS1	P60891	265	1	-9	-10	-7	1.0	0.9	0.9	0.9
SLFN5	Q08AF3	362	1	-12	-10	-12	1.0	0.9	0.9	0.9
TBC1D1	Q86TI0	1160	1	-15	-10	-22	1.0	0.9	0.9	0.8
CDK2	P24941	177	1	-17	-10	-17	1.0	0.9	0.9	0.9
UQCRC2	P22695	192	1	-18	-10	-3	1.0	0.9	0.9	1.0
GLCCI1	Q86VQ1	207	1	-18	-10	-25	1.0	0.9	0.9	0.8
GPR17	Q13304	341	1	-32	-10	-19	1.0	0.8	0.9	0.8
KAT6B	Q8WYB5	252	1	-8	-10	-14	1.0	0.9	0.9	0.9
XPA	P23025	153	1	-12	-10	-8	1.0	0.9	0.9	0.9
OTUD6B	Q8N6M0	172	1	-12	-10	-12	1.0	0.9	0.9	0.9
ACTL6A	O96019	423	1	-19	-10	-13	1.0	0.8	0.9	0.9
RAB27B	O00194	123	1	-22	-10	-35	1.0	0.8	0.9	0.7
WNK1	Q9H4A3	547	0	-3	-10	-5	1.0	1.0	0.9	1.0
PPP2R1A	P30153	317	0	-6	-10	-3	1.0	0.9	0.9	1.0
FBXO38	Q6PIJ6	303	0	-9	-10	1	1.0	0.9	0.9	1.0
GNA13	Q14344	318	0	-9	-10	-10	1.0	0.9	0.9	0.9
BIRC2	Q13490	45	0	-10	-10	-13	1.0	0.9	0.9	0.9
MRPL20	Q9BYC9	93	0	-11	-10	-13	1.0	0.9	0.9	0.9
GMEB2	Q9UKD1	110	0	-13	-10	-3	1.0	0.9	0.9	1.0
GMEB1	Q9Y692	113	0	-13	-10	-3	1.0	0.9	0.9	1.0
MSH3	P20585	763	0	-13	-10	-11	1.0	0.9	0.9	0.9
TAOK1	Q7L7X3	730	0	-15	-10	-4	1.0	0.9	0.9	1.0

TAOK3	Q9H2K8	727	0	-15	-10	-4	1.0	0.9	0.9	1.0
UBN1	Q9NPG3	59	0	-40	-10	-9	1.0	0.7	0.9	0.9
MFN2	O95140	521	-1	-2	-10	-8	1.0	1.0	0.9	0.9
RNF213	Q63HN8	1007	-1	-3	-10	1	1.0	1.0	0.9	1.0
USP19	O94966	659	-1	-8	-10	-7	1.0	0.9	0.9	0.9
NUP50	Q9UKX7	181	-1	-8	-10	-13	1.0	0.9	0.9	0.9
MEPCE	Q7L2J0	463	-1	-9	-10	-20	1.0	0.9	0.9	0.8
VBP1	P61758	113	-1	-13	-10	-11	1.0	0.9	0.9	0.9
RRAGA	Q7L523	124	-1	-14	-10	-2	1.0	0.9	0.9	1.0
CELF2	O95319	143	-1	-14	-10	-11	1.0	0.9	0.9	0.9
MYO1F	O00160	807	-1	-14	-10	-7	1.0	0.9	0.9	0.9
SETD1B	Q9UPS6	1908	-1	-15	-10	-8	1.0	0.9	0.9	0.9
CPSF3L	Q5TA45	542	-1	-2	-10	-1	1.0	1.0	0.9	1.0
ASPH	Q12797	384	-1	-5	-10	2	1.0	1.0	0.9	1.0
GNB1	P62873	114	-1	-5	-10	-2	1.0	1.0	0.9	1.0
GNB4	Q9HAV0	114	-1	-5	-10	-2	1.0	1.0	0.9	1.0
MACROD1	Q9BQ69	186	-1	-9	-10	-4	1.0	0.9	0.9	1.0
SQSTM1	Q13501	27	-1	-11	-10	-7	1.0	0.9	0.9	0.9
MFN2	O95140	8	-1	-13	-10	-19	1.0	0.9	0.9	0.8
PRPS2	P11908	230	-1	-16	-10	-5	1.0	0.9	0.9	1.0
PRPS1	P60891	230	-1	-16	-10	-5	1.0	0.9	0.9	1.0
RNMT	O43148	73	-1	-16	-10	-14	1.0	0.9	0.9	0.9
NXF1	Q9UBU9	373	-1	-17	-10	-2	1.0	0.9	0.9	1.0
SQSTM1	Q13501	151	-1	-17	-10	-17	1.0	0.9	0.9	0.9
RUFY1	Q96T51	351	-2	-5	-10	-14	1.0	1.0	0.9	0.9
MIB1	Q86YT6	88	-2	-8	-10	-5	1.0	0.9	0.9	1.0
AGFG2	O95081	50	-2	-15	-10	-7	1.0	0.9	0.9	0.9
BAG5	Q9UL15	420	-2	-15	-10	-8	1.0	0.9	0.9	0.9
UBE2Z	Q9H832	243	-2	-25	-10	-20	1.0	0.8	0.9	0.8
TRRAP	Q9Y4A5	2241	-2	-4	-10	-6	1.0	1.0	0.9	0.9
MRGBP	Q9NV56	170	-2	-5	-10	-6	1.0	1.0	0.9	0.9
INTS1	Q8N201	1770	-2	-7	-10	-5	1.0	0.9	0.9	1.0
FAM208A	Q9UK61	690	-2	-7	-10	-37	1.0	0.9	0.9	0.7
RRM1	P23921	492	-2	-8	-10	-19	1.0	0.9	0.9	0.8
LUC7L2	A0A0A6YYJ8	414	-2	-9	-10	-9	1.0	0.9	0.9	0.9
TTLL12	Q14166	572	-2	-11	-10	-5	1.0	0.9	0.9	1.0
CPNE1	Q99829	53	-2	-13	-10	-9	1.0	0.9	0.9	0.9
FRYL	O94915	888	-2	-14	-10	-9	1.0	0.9	0.9	0.9
ZNF185	O15231	317	-2	-16	-10	-16	1.0	0.9	0.9	0.9
RASSF1	Q9NS23	106	-2	-16	-10	-12	1.0	0.9	0.9	0.9
LUC7L2	A0A0A6YYJ8	102	-2	-17	-10	-10	1.0	0.9	0.9	0.9
QRICH1	Q2TAL8	713	-2	-17	-10	-9	1.0	0.9	0.9	0.9
KANSL1	A0A0G2JNT7	633	-2	-23	-10	-20	1.0	0.8	0.9	0.8
KANSL1	A0A0G2JNT7	627	-2	-23	-10	-20	1.0	0.8	0.9	0.8
LANCL1	O43813	363	-3	-6	-10	-10	1.0	0.9	0.9	0.9
TXN2	Q99757	90	-3	-8	-10	-1	1.0	0.9	0.9	1.0
TBCD	Q9BTW9	1192	-3	-9	-10	2	1.0	0.9	0.9	1.0
NPEPPS	P55786	339	-3	-9	-10	-10	1.0	0.9	0.9	0.9
PRPSAP1	Q14558	19	-3	-10	-10	-8	1.0	0.9	0.9	0.9
ATP2A3	Q93084	404	-3	-14	-10	-4	1.0	0.9	0.9	1.0
UBA2	Q9UBT2	432	-3	-14	-10	-5	1.0	0.9	0.9	1.0
ZAP70	P43403	510	-3	-15	-10	-15	1.0	0.9	0.9	0.9
DCTN4	Q9UJW0	33	-3	-13	-10	-11	1.0	0.9	0.9	0.9

ARAP1	Q96P48	1310	-3	-13	-10	-14	1.0	0.9	0.9	0.9
PPP3CB	P16298	477	-3	-16	-10	-12	1.0	0.9	0.9	0.9
EIF5B	O60841	1092	-3	-17	-10	1	1.0	0.9	0.9	1.0
TMED8	Q6PL24	161	-4	-12	-10	-1	1.0	0.9	0.9	1.0
SMCHD1	A6NHR9	1656	-4	-13	-10	-9	1.0	0.9	0.9	0.9
ENOSF1	Q7L5Y1	307	-4	-15	-10	-10	1.0	0.9	0.9	0.9
WDR81	Q562E7	974	-4	-15	-10	-22	1.0	0.9	0.9	0.8
ANXA1	P04083	270	-4	-19	-10	-3	1.0	0.8	0.9	1.0
TRRAP	Q9Y4A5	429	-4	-22	-10	0	1.0	0.8	0.9	1.0
RAVER1	Q8IY67	161	-4	-31	-10	-16	1.0	0.8	0.9	0.9
PSMA1	P25786	85	-4	-8	-10	4	1.0	0.9	0.9	1.0
FIZ1	Q96SL8	455	-4	-13	-10	-12	1.0	0.9	0.9	0.9
HELZ	P42694	256	-4	-15	-10	-12	1.0	0.9	0.9	0.9
NUDT6	P53370	44	-4	-16	-10	0	1.0	0.9	0.9	1.0
SAMM50	Q9Y512	65	-4	-24	-10	4	1.0	0.8	0.9	1.0
MCM5	P33992	219	-5	-11	-10	-24	1.0	0.9	0.9	0.8
GGPS1	O95749	247	-5	-11	-10	-12	1.0	0.9	0.9	0.9
ARHGAP6	O43182	769	-5	-12	-10	-22	1.0	0.9	0.9	0.8
NRDC	O43847	60	-5	-12	-10	-5	1.0	0.9	0.9	1.0
RPTOR	Q8N122	283	-5	-15	-10	-8	1.0	0.9	0.9	0.9
SIPA1	Q96FS4	732	-5	-15	-10	-13	1.0	0.9	0.9	0.9
CD4	P01730	445	-5	-16	-10	-12	1.0	0.9	0.9	0.9
SF3A3	Q12874	437	-5	-22	-10	-17	1.0	0.8	0.9	0.9
SMC5	Q8IY18	393	-5	-12	-10	-13	1.0	0.9	0.9	0.9
CRBN	Q96SW2	318	-5	-17	-10	2	1.0	0.9	0.9	1.0
ABCF2	Q9UG63	586	-5	-17	-10	-27	1.0	0.9	0.9	0.8
RNH1	P13489	313	-5	-21	-10	-7	1.0	0.8	0.9	0.9
MSL3	Q8N5Y2	358	-5	-29	-10	-8	1.0	0.8	0.9	0.9
WAPL	Q7Z5K2	906	-6	-14	-10	-8	0.9	0.9	0.9	0.9
PHKB	Q93100	736	-6	-15	-10	-5	0.9	0.9	0.9	1.0
VPS13D	Q5THJ4	3795	-6	-15	-10	-16	0.9	0.9	0.9	0.9
RFTN1	Q14699	433	-6	-18	-10	-7	0.9	0.9	0.9	0.9
ACSL5	Q9ULC5	93	-6	-18	-10	-8	0.9	0.8	0.9	0.9
RBM22	Q9NW64	74	-6	-21	-10	-5	0.9	0.8	0.9	1.0
RNF40	O75150	69	-6	-10	-10	-9	0.9	0.9	0.9	0.9
UQCRC2	P22695	125	-6	-17	-10	-16	0.9	0.9	0.9	0.9
SEC61B	P60468	39	-6	-18	-10	-13	0.9	0.8	0.9	0.9
IAH1	Q2TAA2	137	-7	-7	-10	-15	0.9	0.9	0.9	0.9
DOCK8	Q8NF50	522	-7	-9	-10	-7	0.9	0.9	0.9	0.9
HIVEP2	P31629	2329	-7	-10	-10	-18	0.9	0.9	0.9	0.8
NOSIP	Q9Y314	185	-7	-10	-10	-5	0.9	0.9	0.9	1.0
CSNK2A2	P19784	336	-7	-11	-10	-15	0.9	0.9	0.9	0.9
UBA1	P22314	278	-7	-21	-10	-7	0.9	0.8	0.9	0.9
HDAC10	Q969S8	145	-7	-10	-10	-10	0.9	0.9	0.9	0.9
CECR1	Q9NZK5	408	-7	-17	-10	-8	0.9	0.9	0.9	0.9
FGD2	Q7Z6J4	567	-7	-32	-10	-13	0.9	0.8	0.9	0.9
SETD2	Q9BYW2	1685	-8	-2	-10	-21	0.9	1.0	0.9	0.8
L3MBTL2	Q969R5	110	-8	-9	-10	8	0.9	0.9	0.9	1.1
RAD21	O60216	78	-8	-12	-10	-13	0.9	0.9	0.9	0.9
NT5C2	P49902	336	-8	-20	-10	-4	0.9	0.8	0.9	1.0
ACSF3	Q4G176	399	-8	-7	-10	-19	0.9	0.9	0.9	0.8
RABGGTA	Q92696	532	-8	-13	-10	-1	0.9	0.9	0.9	1.0
NCK1	P16333	340	-8	-15	-10	4	0.9	0.9	0.9	1.0

PLEK	P08567	59	-8	-16	-10	-4	0.9	0.9	0.9	1.0
MSL3	Q8N5Y2	188	-9	-9	-10	-11	0.9	0.9	0.9	0.9
ARHGEF6	Q15052	738	-9	-10	-10	-11	0.9	0.9	0.9	0.9
FLYWCH2	Q96CP2	132	-9	-16	-10	-18	0.9	0.9	0.9	0.9
PFN1	P07737	128	-9	-22	-10	-11	0.9	0.8	0.9	0.9
AKT1S1	Q96B36	44	-9	-16	-10	-14	0.9	0.9	0.9	0.9
RSBN1	Q5VWQ0	646	-9	-16	-10	-15	0.9	0.9	0.9	0.9
NTMT1	Q9BV86	64	-9	-25	-10	-8	0.9	0.8	0.9	0.9
JADE1	Q6IE81	258	-9	-27	-10	-6	0.9	0.8	0.9	0.9
ZNF512B	Q96KM6	786	-9	-27	-10	-6	0.9	0.8	0.9	0.9
CNST	Q6PJW8	439	-10	-10	-10	-18	0.9	0.9	0.9	0.9
ACSL3	O95573	573	-10	-22	-10	-17	0.9	0.8	0.9	0.9
SMAD4	Q13485	391	-10	-11	-10	-12	0.9	0.9	0.9	0.9
PTK2B	Q14289	650	-10	-11	-10	-8	0.9	0.9	0.9	0.9
PCYT1A	P49585	73	-10	-13	-10	-13	0.9	0.9	0.9	0.9
DECR2	Q9NUI1	22	-11	-20	-10	-13	0.9	0.8	0.9	0.9
GTPBP1	O00178	313	-11	-12	-10	-4	0.9	0.9	0.9	1.0
TANGO6	Q9C0B7	360	-11	-25	-10	-12	0.9	0.8	0.9	0.9
TXNL1	O43396	137	-11	-28	-10	-15	0.9	0.8	0.9	0.9
RAB11FIP1	Q6WKZ4	1007	-12	-12	-10	-30	0.9	0.9	0.9	0.8
ZYX	Q15942	553	-12	-24	-10	6	0.9	0.8	0.9	1.1
HMG20B	Q9P0W2	177	-12	5	-10	-6	0.9	1.0	0.9	0.9
STIM2	Q9P246	313	-13	-21	-10	-25	0.9	0.8	0.9	0.8
TRAPPC11	Q7Z392	615	-14	-24	-10	-15	0.9	0.8	0.9	0.9
RXRA	P19793	152	-14	-11	-10	8	0.9	0.9	0.9	1.1
RXRB	P28702	222	-14	-11	-10	8	0.9	0.9	0.9	1.1
EMC8	O43402	24	-15	-6	-10	-3	0.9	0.9	0.9	1.0
EEF2	P13639	466	-15	-17	-10	3	0.9	0.9	0.9	1.0
GARS	P41250	466	-15	-17	-10	-20	0.9	0.9	0.9	0.8
USP10	Q14694	456	-15	-22	-10	-19	0.9	0.8	0.9	0.8
ERP29	P30040	157	-16	-12	-10	-9	0.9	0.9	0.9	0.9
ARPC1B	O15143	227	-17	-3	-10	-3	0.9	1.0	0.9	1.0
SYNE3	Q6ZMZ3	651	-17	-16	-10	-22	0.9	0.9	0.9	0.8
GNAI2	P04899	326	-17	-19	-10	-3	0.9	0.8	0.9	1.0
GNAI3	P08754	325	-17	-19	-10	-3	0.9	0.8	0.9	1.0
GNAI1	P63096	325	-17	-19	-10	-3	0.9	0.8	0.9	1.0
ZNF302	Q9NR11	200	-18	-29	-10	-41	0.9	0.8	0.9	0.7
DEPDC5	O75140	952	-19	-11	-10	-2	0.8	0.9	0.9	1.0
KIDINS220	Q9ULH0	1670	-20	-14	-10	-10	0.8	0.9	0.9	0.9
POR	P16435	629	-21	-12	-10	2	0.8	0.9	0.9	1.0
BOLA2B	Q9H3K6	59	-30	-25	-10	-9	0.8	0.8	0.9	0.9
NT5C3A	Q9H0P0	106	20	-4	-10	3	1.2	1.0	0.9	1.0
PARP14	Q460N5	1618	16	-12	-10	-8	1.2	0.9	0.9	0.9
EIF4A2	Q14240	132	15	1	-10	-4	1.2	1.0	0.9	1.0
IDH3A	P50213	215	15	1	-10	-1	1.2	1.0	0.9	1.0
DPYD	Q12882	622	15	-15	-10	-1	1.2	0.9	0.9	1.0
POLR2B	P30876	958	15	-18	-10	7	1.2	0.8	0.9	1.1
PSMD3	O43242	210	15	-41	-10	-8	1.2	0.7	0.9	0.9
MBOAT2	Q6ZWT7	505	14	-14	-10	7	1.2	0.9	0.9	1.1
PFKL	P17858	114	12	-10	-10	-3	1.1	0.9	0.9	1.0
MRPL38	Q96DV4	143	12	-15	-10	3	1.1	0.9	0.9	1.0
ZFC3H1	O60293	1068	11	-4	-10	-3	1.1	1.0	0.9	1.0
USP24	Q9UPU5	1377	10	5	-10	-11	1.1	1.0	0.9	0.9

NCBP1	Q09161	477	10	3	-10	24	1.1	1.0	0.9	1.3
CHD3	Q12873	420	9	-3	-10	-15	1.1	1.0	0.9	0.9
CHD4	Q14839	411	9	-3	-10	-15	1.1	1.0	0.9	0.9
TRMT1L	Q7Z2T5	132	9	-6	-10	17	1.1	0.9	0.9	1.2
CDK5	Q00535	117	9	-19	-10	-2	1.1	0.8	0.9	1.0
TRAP1	Q12931	261	9	-20	-10	-5	1.1	0.8	0.9	1.0
CTR9	Q6PD62	171	9	-23	-10	-9	1.1	0.8	0.9	0.9
ALAD	P13716	203	9	-4	-10	-5	1.1	1.0	0.9	1.0
NSF	P46459	11	9	-7	-10	1	1.1	0.9	0.9	1.0
CWC27	Q6UX04	164	9	-29	-10	3	1.1	0.8	0.9	1.0
C12orf4	Q9NQ89	25	8	-9	-10	0	1.1	0.9	0.9	1.0
ALG1	Q9BT22	396	8	-40	-10	18	1.1	0.7	0.9	1.2
IRF2BP2	Q7Z5L9	533	7	1	-10	8	1.1	1.0	0.9	1.1
IRF2BP1	Q8IU81	530	7	1	-10	8	1.1	1.0	0.9	1.1
IRF2BPL	Q9H1B7	742	7	1	-10	8	1.1	1.0	0.9	1.1
PRAM1	Q96QH2	677	7	-6	-10	1	1.1	0.9	0.9	1.0
TOP2A	P11388	733	7	-14	-10	-7	1.1	0.9	0.9	0.9
TOP2B	Q02880	754	7	-14	-10	-7	1.1	0.9	0.9	0.9
DOCK7	Q96N67	457	7	-17	-10	2	1.1	0.9	0.9	1.0
UBA2	Q9UBT2	161	7	-12	-10	-2	1.1	0.9	0.9	1.0
COPA	P53621	1191	7	-13	-10	-6	1.1	0.9	0.9	0.9
CKAP5	Q14008	695	7	-20	-10	-6	1.1	0.8	0.9	0.9
ETF1	P62495	302	6	-14	-10	4	1.1	0.9	0.9	1.0
ARPC2	O15144	120	6	-27	-10	-5	1.1	0.8	0.9	1.0
CORO1B	Q9BR76	41	6	-3	-10	4	1.1	1.0	0.9	1.0
PRKCB	P05771	386	6	-5	-10	1	1.1	1.0	0.9	1.0
PRKCA	P17252	383	6	-5	-10	1	1.1	1.0	0.9	1.0
TRAPPC8	Q9Y2L5	1074	6	-8	-10	-1	1.1	0.9	0.9	1.0
PLCG2	P16885	1082	5	-8	-10	5	1.1	0.9	0.9	1.0
OAS2	P29728	361	5	-8	-10	-3	1.1	0.9	0.9	1.0
BMS1	Q14692	725	5	-10	-10	-11	1.1	0.9	0.9	0.9
RPA1	P27694	503	5	-11	-10	-4	1.1	0.9	0.9	1.0
LUZP1	Q86V48	969	5	-15	-10	-8	1.1	0.9	0.9	0.9
WAPL	Q7Z5K2	675	5	-23	-10	-14	1.1	0.8	0.9	0.9
PACS1	Q6VY07	116	5	-7	-10	-10	1.0	0.9	0.9	0.9
MRPS14	O60783	83	5	-9	-10	8	1.0	0.9	0.9	1.1
ARF5	P84085	62	5	-9	-10	1	1.0	0.9	0.9	1.0
GTF3A	Q92664	62	5	-17	-10	-6	1.0	0.9	0.9	0.9
LUZP1	Q86V48	138	4	3	-10	-15	1.0	1.0	0.9	0.9
FIG4	Q92562	489	4	2	-10	-10	1.0	1.0	0.9	0.9
VWF	P04275	1940	4	-2	-10	-8	1.0	1.0	0.9	0.9
APOBEC3C	Q9NRW3	70	4	-7	-10	-9	1.0	0.9	0.9	0.9
GOLGB1	Q14789	395	4	-9	-10	-12	1.0	0.9	0.9	0.9
DNAJC1	Q96KC8	380	4	-13	-10	-16	1.0	0.9	0.9	0.9
FAM98C	Q17RN3	92	4	-14	-10	18	1.0	0.9	0.9	1.2
TMEM201	Q5SNT2	164	4	-18	-10	-5	1.0	0.9	0.9	1.0
BOD1L1	Q8NFC6	74	4	-6	-10	-14	1.0	0.9	0.9	0.9
DLGAP4	Q9Y2H0	823	3	-4	-10	-5	1.0	1.0	0.9	1.0
MAST3	O60307	541	3	-8	-10	-9	1.0	0.9	0.9	0.9
AMPD2	Q01433	263	3	-10	-10	-6	1.0	0.9	0.9	0.9
FGR	P09769	415	3	-10	-10	-24	1.0	0.9	0.9	0.8
NAMPT	P43490	39	3	-12	-10	0	1.0	0.9	0.9	1.0
SEPT11	Q9NVA2	268	3	-12	-10	-4	1.0	0.9	0.9	1.0

SEPT10	Q9P0V9	293	3	-12	-10	-4	1.0	0.9	0.9	1.0
WDFY4	Q6ZS81	1536	3	-3	-10	-31	1.0	1.0	0.9	0.8
EIF4G1	Q04637	1516	3	-10	-10	-10	1.0	0.9	0.9	0.9
FHL1	Q13642	92	3	-14	-10	-11	1.0	0.9	0.9	0.9
TMEM126A	Q9H061	101	2	-5	-10	-10	1.0	1.0	0.9	0.9
MTERF4	Q7Z6M4	69	2	-8	-10	-20	1.0	0.9	0.9	0.8
RPS27A	P62979	121	2	-15	-10	-8	1.0	0.9	0.9	0.9
ACTL6A	O96019	32	2	-17	-10	-2	1.0	0.9	0.9	1.0
EFTUD2	Q15029	148	2	-19	-10	-10	1.0	0.8	0.9	0.9
CSNK1G2	P78368	111	2	22	-10	25	1.0	1.3	0.9	1.3
LARS	Q9P2J5	248	2	-5	-10	6	1.0	1.0	0.9	1.1
PLEKHA3	Q9HB20	94	2	-8	-10	-14	1.0	0.9	0.9	0.9
CFAP20	Q9Y6A4	160	2	-9	-10	-9	1.0	0.9	0.9	0.9
SRRT	Q9BXP5	412	2	-11	-10	-7	1.0	0.9	0.9	0.9
USP9X	Q93008	842	2	-12	-10	-22	1.0	0.9	0.9	0.8
PLAA	Q9Y263	605	2	-15	-10	-5	1.0	0.9	0.9	1.0
SND1	Q7KZF4	152	2	-16	-10	-5	1.0	0.9	0.9	1.0
HSDL2	Q6YN16	11	2	-17	-10	-6	1.0	0.9	0.9	0.9
DTX3L	Q8TDB6	417	2	-18	-10	-17	1.0	0.9	0.9	0.9
PRKDC	P78527	3187	2	-38	-10	3	1.0	0.7	0.9	1.0
IRF8	Q02556	306	1	-8	-10	-13	1.0	0.9	0.9	0.9
CLIC3	O95833	219	1	-12	-10	10	1.0	0.9	0.9	1.1
TSSC4	Q9Y5U2	99	1	-19	-10	-13	1.0	0.8	0.9	0.9
NAA60	Q9H7X0	19	1	-22	-10	3	1.0	0.8	0.9	1.0
ACSS1	Q9NUB1	151	1	-26	-10	-2	1.0	0.8	0.9	1.0
RPL5	P46777	62	1	-30	-10	-7	1.0	0.8	0.9	0.9
USP8	P40818	809	1	-4	-10	-34	1.0	1.0	0.9	0.7
RPN1	P04843	477	1	-8	-10	-11	1.0	0.9	0.9	0.9
TBC1D22B	Q9NU19	144	1	-9	-10	-4	1.0	0.9	0.9	1.0
RBMXL1	Q96E39	338	1	-10	-10	-10	1.0	0.9	0.9	0.9
GBAS	O75323	85	1	-12	-10	0	1.0	0.9	0.9	1.0
JAK1	P23458	988	1	-13	-10	-16	1.0	0.9	0.9	0.9
CNTRL	Q7Z7A1	2100	1	-14	-10	-16	1.0	0.9	0.9	0.9
ATP2A2	P16615	635	1	-22	-10	-9	1.0	0.8	0.9	0.9
RNH1	P13489	380	1	-23	-10	-2	1.0	0.8	0.9	1.0
DDX20	Q9UHI6	98	0	-9	-10	-8	1.0	0.9	0.9	0.9
LRRC58	Q96CX6	296	0	-11	-10	-18	1.0	0.9	0.9	0.9
GPAT3	Q53EU6	266	0	-14	-10	2	1.0	0.9	0.9	1.0
RARS	P54136	32	0	-14	-10	-20	1.0	0.9	0.9	0.8
LNPEP	Q9UIQ6	305	0	-15	-10	-1	1.0	0.9	0.9	1.0
CTSG	P08311	186	0	-18	-10	-16	1.0	0.9	0.9	0.9
PPP2R5D	Q14738	17	0	-20	-10	-19	1.0	0.8	0.9	0.8
IGHG3	A0A087WVW	255	-1	-7	-10	-15	1.0	0.9	0.9	0.9
IKBKB	O14920	464	-1	-12	-10	-6	1.0	0.9	0.9	0.9
CERS5	Q8N5B7	124	-1	-13	-10	-9	1.0	0.9	0.9	0.9
NRF1	Q16656	229	-1	-14	-10	-10	1.0	0.9	0.9	0.9
HECTD3	Q5T447	112	-1	-14	-10	-11	1.0	0.9	0.9	0.9
SAC3D1	A6NKF1	84	-1	-14	-10	-13	1.0	0.9	0.9	0.9
PHF1	O43189	327	-1	-16	-10	-9	1.0	0.9	0.9	0.9
RPS15A	P62244	72	-1	-17	-10	5	1.0	0.9	0.9	1.1
SLFN5	Q08AF3	369	-1	-18	-10	-18	1.0	0.8	0.9	0.8
MMAA	Q8IVH4	184	-1	-22	-10	-1	1.0	0.8	0.9	1.0
ZMYM3	Q14202	412	-1	-24	-10	-6	1.0	0.8	0.9	0.9

CTR9	Q6PD62	533	-1	-27	-10	-12	1.0	0.8	0.9	0.9
XPC	Q01831	514	-1	4	-10	-9	1.0	1.0	0.9	0.9
CERS5	Q8N5B7	369	-1	-5	-10	-5	1.0	1.0	0.9	1.0
SEC22A	Q96IW7	111	-1	-7	-10	-5	1.0	0.9	0.9	1.0
DDX24	Q9GZR7	15	-1	-7	-10	-21	1.0	0.9	0.9	0.8
PRPF3	O43395	651	-1	-8	-10	-3	1.0	0.9	0.9	1.0
RNF169	Q8NCN4	103	-1	-9	-10	-8	1.0	0.9	0.9	0.9
ADD3	Q9UEY8	286	-1	-11	-10	3	1.0	0.9	0.9	1.0
ITPR2	Q14571	2087	-1	-11	-10	-2	1.0	0.9	0.9	1.0
HMGCL	P35914	141	-1	-15	-10	-7	1.0	0.9	0.9	0.9
DMXL1	Q9Y485	259	-1	-16	-10	-4	1.0	0.9	0.9	1.0
C18orf8	Q96DM3	25	-1	-17	-10	-12	1.0	0.9	0.9	0.9
RNF41	Q9H4P4	238	-2	4	-10	4	1.0	1.0	0.9	1.0
CCND3	P30281	47	-2	-5	-10	-10	1.0	1.0	0.9	0.9
DDX60	Q8IY21	1051	-2	-6	-10	-7	1.0	0.9	0.9	0.9
PXK	Q7Z7A4	196	-2	-7	-10	-12	1.0	0.9	0.9	0.9
NPEPPS	P55786	265	-2	-11	-10	1	1.0	0.9	0.9	1.0
AGK	Q53H12	72	-2	-11	-10	-11	1.0	0.9	0.9	0.9
NUMB	P49757	117	-2	-14	-10	-2	1.0	0.9	0.9	1.0
ACTR10	Q9NZ32	37	-2	-14	-10	-4	1.0	0.9	0.9	1.0
SART3	Q15020	670	-2	-16	-10	-24	1.0	0.9	0.9	0.8
FLII	Q13045	232	-2	-16	-10	1	1.0	0.9	0.9	1.0
USP15	Q9Y4E8	264	-2	-19	-10	-19	1.0	0.8	0.9	0.8
CORO1C	Q9ULV4	420	-2	-19	-10	-15	1.0	0.8	0.9	0.9
SNRNP40	Q96DI7	168	-2	-20	-10	-11	1.0	0.8	0.9	0.9
ZNF22	P17026	147	-2	-6	-10	-3	1.0	0.9	0.9	1.0
OAS3	Q9Y6K5	73	-2	-10	-10	-6	1.0	0.9	0.9	0.9
ATAD2	Q6PL18	463	-2	-15	-10	-6	1.0	0.9	0.9	0.9
ATAD2B	Q9ULI0	437	-2	-15	-10	-6	1.0	0.9	0.9	0.9
COPA	P53621	453	-2	-17	-10	-11	1.0	0.9	0.9	0.9
SDHA	P31040	654	-2	-23	-10	-17	1.0	0.8	0.9	0.9
MAP2K6	P52564	216	-3	-7	-10	-7	1.0	0.9	0.9	0.9
PSMG1	O95456	169	-3	-9	-10	-5	1.0	0.9	0.9	1.0
PLCG2	P16885	624	-3	-11	-10	-13	1.0	0.9	0.9	0.9
UMODL1	Q5DID0	154	-3	-13	-10	-13	1.0	0.9	0.9	0.9
FCMR	O60667	318	-3	-17	-10	-22	1.0	0.9	0.9	0.8
ACTR3B	Q9P1U1	34	-3	-28	-10	-18	1.0	0.8	0.9	0.9
EFNB1	P98172	314	-3	-40	-10	-53	1.0	0.7	0.9	0.7
ZC3HAV1	Q7Z2W4	88	-3	-10	-10	-9	1.0	0.9	0.9	0.9
MYO5A	Q9Y4I1	1008	-3	-10	-10	-14	1.0	0.9	0.9	0.9
LRRK2	Q5S007	1618	-3	-11	-10	-18	1.0	0.9	0.9	0.8
ATM	Q13315	384	-3	-11	-10	-16	1.0	0.9	0.9	0.9
PYCR2	Q96C36	262	-3	-18	-10	-19	1.0	0.9	0.9	0.8
RNF13	O43567	264	-3	-21	-10	-19	1.0	0.8	0.9	0.8
RAD50	Q92878	1201	-3	-22	-10	-18	1.0	0.8	0.9	0.9
HMGB1	P09429	106	-3	-56	-10	-5	1.0	0.6	0.9	1.0
MARCH6	O60337	52	-4	-11	-10	-6	1.0	0.9	0.9	0.9
PSMD1	Q99460	571	-4	-11	-10	-25	1.0	0.9	0.9	0.8
URI1	O94763	167	-4	-11	-10	-6	1.0	0.9	0.9	0.9
GRK6	P43250	138	-4	-12	-10	-23	1.0	0.9	0.9	0.8
ARNTL	O00327	269	-4	-14	-10	-18	1.0	0.9	0.9	0.8
XPO5	Q9HAV4	419	-4	-14	-10	-13	1.0	0.9	0.9	0.9
ZMYM3	Q14202	570	-4	-14	-10	-19	1.0	0.9	0.9	0.8

THEM6	Q8WUY1	168	-4	-18	-10	-9	1.0	0.8	0.9	0.9
NUP210	Q8TEM1	543	-4	-3	-10	-1	1.0	1.0	0.9	1.0
SMARCC2	Q8TAQ2	145	-4	-8	-10	-11	1.0	0.9	0.9	0.9
THADA	Q6YHU6	1397	-4	-10	-10	-15	1.0	0.9	0.9	0.9
FGG	P02679	34	-4	-15	-10	-16	1.0	0.9	0.9	0.9
RAD1	O60671	148	-4	-21	-10	-16	1.0	0.8	0.9	0.9
MED29	Q9NX70	131	-5	-1	-10	23	1.0	1.0	0.9	1.3
PIK3C2A	O00443	514	-5	-1	-10	-17	1.0	1.0	0.9	0.9
ATG14	Q6ZNE5	17	-5	-5	-10	-9	1.0	1.0	0.9	0.9
Uncharacteri	A0A087WZG	394	-5	-13	-10	-5	1.0	0.9	0.9	1.0
HNRNPU	Q00839	295	-5	-13	-10	-9	1.0	0.9	0.9	0.9
GNB1	P62873	204	-5	-14	-10	3	1.0	0.9	0.9	1.0
LAS1L	Q9Y4W2	456	-5	-14	-10	-21	1.0	0.9	0.9	0.8
PLEKHO1	Q53GL0	269	-5	-16	-10	-15	1.0	0.9	0.9	0.9
APRT	P07741	140	-5	-16	-10	4	1.0	0.9	0.9	1.0
HPCAL1	P37235	38	-5	-19	-10	-10	1.0	0.8	0.9	0.9
EPM2A	O95278	169	-5	1	-10	-19	1.0	1.0	0.9	0.8
POGZ	Q7Z3K3	782	-5	-8	-10	-15	1.0	0.9	0.9	0.9
OSBPL10	Q9BXB5	615	-5	-11	-10	-12	1.0	0.9	0.9	0.9
NMNAT3	Q96T66	134	-5	-12	-10	-1	1.0	0.9	0.9	1.0
GPX4	P36969	102	-5	-13	-10	1	1.0	0.9	0.9	1.0
DHX8	Q14562	637	-5	-13	-10	-23	1.0	0.9	0.9	0.8
RPS21	P63220	56	-5	-14	-10	-8	1.0	0.9	0.9	0.9
CD7	P09564	230	-5	-14	-10	-10	1.0	0.9	0.9	0.9
BAZ2A	Q9UIF9	917	-5	-17	-10	-5	1.0	0.9	0.9	1.0
CEP44	Q9C0F1	28	-6	-7	-10	-6	0.9	0.9	0.9	0.9
CYFIP2	Q96F07	427	-6	-10	-10	-15	0.9	0.9	0.9	0.9
C14orf166	Q9Y224	69	-6	-11	-10	-4	0.9	0.9	0.9	1.0
SMARCC2	Q8TAQ2	486	-6	-15	-10	-10	0.9	0.9	0.9	0.9
SMARCC1	Q92922	511	-6	-15	-10	-10	0.9	0.9	0.9	0.9
PGM1	P36871	101	-6	-15	-10	-1	0.9	0.9	0.9	1.0
CUTC	Q9NTM9	248	-6	-18	-10	-12	0.9	0.9	0.9	0.9
HINT1	P49773	38	-6	-32	-10	-8	0.9	0.8	0.9	0.9
GDPD1	Q8N9F7	107	-6	-8	-10	-1	0.9	0.9	0.9	1.0
PDLIM5	Q96HC4	479	-6	-19	-10	-5	0.9	0.8	0.9	1.0
KIAA1033	Q2M389	604	-6	-20	-10	-16	0.9	0.8	0.9	0.9
MLST8	Q9BVC4	121	-7	6	-10	-8	0.9	1.1	0.9	0.9
MADD	Q8WXG6	1489	-7	-8	-10	-2	0.9	0.9	0.9	1.0
CENPC	Q03188	800	-7	-9	-10	-22	0.9	0.9	0.9	0.8
IQSEC1	Q6DN90	260	-7	-14	-10	-20	0.9	0.9	0.9	0.8
CIAO1	O76071	52	-7	-15	-10	-5	0.9	0.9	0.9	1.0
TMOD3	Q9NYL9	231	-7	-15	-10	-10	0.9	0.9	0.9	0.9
STK38L	Q9Y2H1	235	-7	-16	-10	4	0.9	0.9	0.9	1.0
ZMYM3	Q14202	743	-7	-18	-10	-11	0.9	0.9	0.9	0.9
RAB14	P61106	26	-7	-19	-10	-1	0.9	0.8	0.9	1.0
RPL22	P35268	25	-7	5	-10	-5	0.9	1.1	0.9	1.0
PPP1R37	O75864	375	-7	-5	-10	-8	0.9	1.0	0.9	0.9
POLG	P54098	1078	-7	-7	-10	4	0.9	0.9	0.9	1.0
CCDC94	Q9BW85	275	-7	-13	-10	-17	0.9	0.9	0.9	0.9
DHX36	Q9H2U1	611	-8	-16	-10	4	0.9	0.9	0.9	1.0
TECPR2	O15040	226	-8	-16	-10	-9	0.9	0.9	0.9	0.9
LEMD2	Q8NC56	261	-8	-8	-10	-9	0.9	0.9	0.9	0.9
CASP1	P29466	362	-8	-9	-10	-13	0.9	0.9	0.9	0.9

PGM2L1	Q6PCE3	303	-8	-10	-10	-13	0.9	0.9	0.9	0.9
EHHADH	Q08426	559	-8	-16	-10	-2	0.9	0.9	0.9	1.0
ZDHHC18	Q9NUE0	156	-9	-8	-10	-22	0.9	0.9	0.9	0.8
ANO6	Q4KMQ2	105	-9	-11	-10	-15	0.9	0.9	0.9	0.9
NFATC1	O95644	228	-9	-13	-10	-25	0.9	0.9	0.9	0.8
LRRC47	Q8N1G4	224	-9	-8	-10	-6	0.9	0.9	0.9	0.9
L2HGDH	Q9H9P8	376	-9	-20	-10	-14	0.9	0.8	0.9	0.9
FARSB	Q9NSD9	255	-9	-42	-10	3	0.9	0.7	0.9	1.0
SRC	P12931	280	-10	-5	-10	-7	0.9	1.0	0.9	0.9
RNF213	Q63HN8	614	-10	-19	-10	7	0.9	0.8	0.9	1.1
ESPL1	Q14674	250	-10	-42	-10	-23	0.9	0.7	0.9	0.8
FBXW2	Q9UKT8	327	-10	10	-10	5	0.9	1.1	0.9	1.1
PSMD1	Q99460	633	-10	-9	-10	-10	0.9	0.9	0.9	0.9
ZMYM2	Q9UBW7	612	-10	-13	-10	1	0.9	0.9	0.9	1.0
GTF2B	Q00403	15	-10	-19	-10	-7	0.9	0.8	0.9	0.9
KMT2D	O14686	58	-11	0	-10	-11	0.9	1.0	0.9	0.9
DNPEP	Q9ULA0	413	-11	-24	-10	-10	0.9	0.8	0.9	0.9
TLN1	Q9Y490	1509	-12	3	-10	2	0.9	1.0	0.9	1.0
DNAJA4	Q8WW22	368	-12	-5	-10	-18	0.9	1.0	0.9	0.8
DIP2A	Q14689	536	-12	-14	-10	-9	0.9	0.9	0.9	0.9
NBEAL2	Q6ZNJ1	2335	-12	-16	-10	-8	0.9	0.9	0.9	0.9
NR2C2	P49116	153	-12	-24	-10	-22	0.9	0.8	0.9	0.8
AKAP9	Q99996	1794	-13	-10	-10	-16	0.9	0.9	0.9	0.9
DOPEY1	Q5JWR5	2388	-13	-17	-10	-11	0.9	0.9	0.9	0.9
CNBP	P62633	57	-14	-16	-10	-9	0.9	0.9	0.9	0.9
IMMP1L	Q96LU5	128	-14	-17	-10	-25	0.9	0.9	0.9	0.8
USP28	Q96RU2	203	-14	-23	-10	-12	0.9	0.8	0.9	0.9
AMBP	P02760	53	-14	-14	-10	-8	0.9	0.9	0.9	0.9
DOCK11	Q5JSL3	1883	-14	-24	-10	-14	0.9	0.8	0.9	0.9
FTO	Q9C0B1	104	-15	-23	-10	-5	0.9	0.8	0.9	1.0
CREBBP	Q92793	409	-16	-8	-10	-14	0.9	0.9	0.9	0.9
VCP	P55072	691	-18	-24	-10	-14	0.9	0.8	0.9	0.9
AKAP9	Q99996	1493	-18	-51	-10	-18	0.9	0.7	0.9	0.8
TBXAS1	P24557	220	-20	-16	-10	-8	0.8	0.9	0.9	0.9
IARS	P41252	350	-22	-19	-10	-11	0.8	0.8	0.9	0.9
TSC22D2	O75157	63	-22	-21	-10	-31	0.8	0.8	0.9	0.8
CDC42BPB	Q9Y5S2	1174	-23	-27	-10	-14	0.8	0.8	0.9	0.9
OAS3	Q9Y6K5	350	-28	-4	-10	-1	0.8	1.0	0.9	1.0
CPNE3	O75131	249	-28	-36	-10	-20	0.8	0.7	0.9	0.8
ORC5	O43913	63	27	7	-11	20	1.4	1.1	0.9	1.3
NELFCD	Q8IXH7	417	25	-19	-11	5	1.3	0.8	0.9	1.1
KIF13B	Q9NQT8	127	24	4	-11	18	1.3	1.0	0.9	1.2
MYCBP2	O75592	114	20	4	-11	-1	1.3	1.0	0.9	1.0
PRMT1	Q99873	93	20	-18	-11	12	1.3	0.8	0.9	1.1
VPS13C	Q709C8	3723	17	-8	-11	8	1.2	0.9	0.9	1.1
DDOST	P39656	145	15	-14	-11	-9	1.2	0.9	0.9	0.9
IPO7	O95373	477	15	-13	-11	9	1.2	0.9	0.9	1.1
POLD1	P28340	1015	14	-9	-11	8	1.2	0.9	0.9	1.1
ETF1	P62495	127	14	-15	-11	4	1.2	0.9	0.9	1.0
MYO9B	Q13459	1662	13	-15	-11	-7	1.1	0.9	0.9	0.9
SPTLC1	O15269	370	13	-35	-11	6	1.1	0.7	0.9	1.1
INTS3	Q68E01	959	12	-12	-11	4	1.1	0.9	0.9	1.0
CARD9	Q9H257	168	11	-21	-11	-12	1.1	0.8	0.9	0.9

BACH1	O14867	646	10	-12	-11	-14	1.1	0.9	0.9	0.9
COMMD3-BM	R4GMX3	90	9	-13	-11	-6	1.1	0.9	0.9	0.9
TLDC1	Q6P9B6	13	9	-9	-11	-13	1.1	0.9	0.9	0.9
HSD17B4	P51659	277	9	-15	-11	21	1.1	0.9	0.9	1.3
CNOT10	Q9H9A5	504	9	-26	-11	-7	1.1	0.8	0.9	0.9
AP1B1	Q10567	95	8	-17	-11	-9	1.1	0.9	0.9	0.9
FYCO1	Q9BQS8	1425	8	-9	-11	-7	1.1	0.9	0.9	0.9
PRKCQ	Q04759	281	8	-13	-11	-14	1.1	0.9	0.9	0.9
PRKCD	Q05655	280	8	-13	-11	-14	1.1	0.9	0.9	0.9
CDC23	Q9UJX2	532	8	-19	-11	1	1.1	0.8	0.9	1.0
CDKN1B	P46527	29	7	3	-11	-1	1.1	1.0	0.9	1.0
CORO1B	Q9BR76	25	7	-14	-11	7	1.1	0.9	0.9	1.1
TXNL1	O43396	149	7	-14	-11	-3	1.1	0.9	0.9	1.0
AGO3	Q9H9G7	328	7	-10	-11	-5	1.1	0.9	0.9	1.0
AGO2	Q9UKV8	327	7	-10	-11	-5	1.1	0.9	0.9	1.0
AGO1	Q9UL18	325	7	-10	-11	-5	1.1	0.9	0.9	1.0
ANK1	P16157	278	7	-15	-11	-10	1.1	0.9	0.9	0.9
ARFGEF1	Q9Y6D6	88	6	-13	-11	-8	1.1	0.9	0.9	0.9
SYNE2	Q8WXH0	3096	6	-15	-11	-16	1.1	0.9	0.9	0.9
EXOSC7	Q15024	199	6	-18	-11	-12	1.1	0.9	0.9	0.9
TRANK1	O15050	867	6	-19	-11	-11	1.1	0.8	0.9	0.9
PYGB	P11216	373	6	-32	-11	-4	1.1	0.8	0.9	1.0
APPL1	Q9UKG1	616	6	-10	-11	4	1.1	0.9	0.9	1.0
NEMP1	O14524	434	6	-16	-11	-17	1.1	0.9	0.9	0.9
FXR1	P51114	211	5	-11	-11	-9	1.1	0.9	0.9	0.9
RELA	Q04206	109	5	-13	-11	-2	1.1	0.9	0.9	1.0
UBR4	Q5T4S7	3804	5	-18	-11	-13	1.1	0.8	0.9	0.9
PPP2CB	P62714	269	5	-27	-11	-6	1.1	0.8	0.9	0.9
PPP2CA	P67775	269	5	-27	-11	-6	1.1	0.8	0.9	0.9
HTATSF1	O43719	295	5	-8	-11	0	1.0	0.9	0.9	1.0
BAZ2A	Q9UIF9	726	5	-8	-11	-10	1.0	0.9	0.9	0.9
PRDX1	Q06830	173	5	-9	-11	-4	1.0	0.9	0.9	1.0
SRP9	P49458	39	5	-12	-11	3	1.0	0.9	0.9	1.0
GALK2	Q01415	303	4	-9	-11	2	1.0	0.9	0.9	1.0
CPSF1	Q10570	1020	4	-9	-11	-6	1.0	0.9	0.9	0.9
EEF1B2	P24534	50	4	-10	-11	-6	1.0	0.9	0.9	0.9
NBEAL2	Q6ZNJ1	1814	4	-13	-11	-11	1.0	0.9	0.9	0.9
LBR	Q14739	591	4	-16	-11	2	1.0	0.9	0.9	1.0
GLMN	Q92990	218	4	-17	-11	7	1.0	0.9	0.9	1.1
HNRNPLL	Q8WVV9	505	4	-9	-11	-9	1.0	0.9	0.9	0.9
SEPT1	Q8WYJ6	293	4	-11	-11	-10	1.0	0.9	0.9	0.9
C5orf45	Q6NTE8	300	4	-14	-11	16	1.0	0.9	0.9	1.2
PARG	Q86W56	943	4	-20	-11	-15	1.0	0.8	0.9	0.9
MYO9B	Q13459	1169	3	-7	-11	-7	1.0	0.9	0.9	0.9
SMARCA2	P51531	1296	3	-18	-11	-17	1.0	0.9	0.9	0.9
PSMD1	Q99460	806	3	-19	-11	-17	1.0	0.8	0.9	0.9
LGALS12	Q96DT0	212	3	-5	-11	9	1.0	1.0	0.9	1.1
EPPK1	A0A087X1U6	1888	3	-11	-11	-15	1.0	0.9	0.9	0.9
RNPEP	Q9H4A4	181	3	-13	-11	-1	1.0	0.9	0.9	1.0
GIMAP8	Q8ND71	75	3	-15	-11	-16	1.0	0.9	0.9	0.9
GPX1	P07203	115	3	-18	-11	-13	1.0	0.8	0.9	0.9
CCT2	P78371	412	2	6	-11	2	1.0	1.1	0.9	1.0
CECR1	Q9NZK5	134	2	-9	-11	5	1.0	0.9	0.9	1.0

EPPK1	A0A087X1U6	927	2	-11	-11	-19	1.0	0.9	0.9	0.8
STAG2	Q8N3U4	176	2	-14	-11	13	1.0	0.9	0.9	1.1
SQRDL	Q9Y6N5	201	2	-17	-11	-17	1.0	0.9	0.9	0.9
PPM1M	Q96MI6	7	2	-23	-11	-22	1.0	0.8	0.9	0.8
OPA1	O60313	801	2	-6	-11	-3	1.0	0.9	0.9	1.0
CHUK	O15111	379	2	-6	-11	-1	1.0	0.9	0.9	1.0
CTTN	Q14247	246	2	-9	-11	-5	1.0	0.9	0.9	1.0
Uncharacteri	G3V3G9	426	2	-9	-11	-12	1.0	0.9	0.9	0.9
HARS	P12081	507	2	-10	-11	-25	1.0	0.9	0.9	0.8
ITPR3	Q14573	420	2	-13	-11	-13	1.0	0.9	0.9	0.9
RANBP2	P49792	1791	2	-13	-11	-17	1.0	0.9	0.9	0.9
WAPL	Q7Z5K2	891	2	-19	-11	-5	1.0	0.8	0.9	1.0
GTF2I	P78347	903	2	-19	-11	3	1.0	0.8	0.9	1.0
TCF20	Q9UGU0	1328	2	-21	-11	-19	1.0	0.8	0.9	0.8
HCK	P08631	395	2	-25	-11	-11	1.0	0.8	0.9	0.9
EPC2	Q52LR7	626	1	-9	-11	-14	1.0	0.9	0.9	0.9
RSF1	Q96T23	118	1	-11	-11	-3	1.0	0.9	0.9	1.0
HTT	P42858	1808	1	-12	-11	-13	1.0	0.9	0.9	0.9
TTC5	Q8N0Z6	439	1	-12	-11	-11	1.0	0.9	0.9	0.9
RPS6KA2	Q15349	429	1	-12	-11	-16	1.0	0.9	0.9	0.9
POLR2G	P62487	106	1	-16	-11	6	1.0	0.9	0.9	1.1
KLC1	Q07866	114	1	-7	-11	-9	1.0	0.9	0.9	0.9
SARS	P49591	438	1	-12	-11	-4	1.0	0.9	0.9	1.0
PREX1	Q8TCU6	936	1	-12	-11	-6	1.0	0.9	0.9	0.9
SPATA13	Q96N96	599	1	-12	-11	-11	1.0	0.9	0.9	0.9
POC1B	Q8TC44	306	1	-16	-11	0	1.0	0.9	0.9	1.0
UAP1L1	Q3KQV9	254	1	-24	-11	-5	1.0	0.8	0.9	1.0
XPO4	Q9C0E2	492	1	-30	-11	6	1.0	0.8	0.9	1.1
CMPK2	Q5EBM0	189	0	-1	-11	3	1.0	1.0	0.9	1.0
HMHA1	Q92619	728	0	-7	-11	-3	1.0	0.9	0.9	1.0
MON1B	Q7L1V2	297	0	-12	-11	1	1.0	0.9	0.9	1.0
MTF1	Q14872	129	0	-13	-11	-10	1.0	0.9	0.9	0.9
UROD	P06132	308	0	-20	-11	-1	1.0	0.8	0.9	1.0
VAPB	O95292	121	0	-23	-11	-5	1.0	0.8	0.9	1.0
GTF3C2	Q8WUA4	476	0	-24	-11	-1	1.0	0.8	0.9	1.0
DDX60L	Q5H9U9	284	-1	-4	-11	-4	1.0	1.0	0.9	1.0
SMCHD1	A6NHR9	1433	-1	-16	-11	-1	1.0	0.9	0.9	1.0
NRDE2	Q9H7Z3	781	-1	-18	-11	-18	1.0	0.9	0.9	0.9
UTP6	Q9NYH9	310	-1	-18	-11	-9	1.0	0.8	0.9	0.9
OXSM	Q9NWU1	415	-1	-19	-11	-13	1.0	0.8	0.9	0.9
ZZEF1	O43149	2553	-1	-19	-11	-11	1.0	0.8	0.9	0.9
CTSC	P53634	136	-1	-22	-11	-10	1.0	0.8	0.9	0.9
MLKL	Q8NB16	437	-1	-29	-11	-16	1.0	0.8	0.9	0.9
PGD	P52209	170	-1	-9	-11	-7	1.0	0.9	0.9	0.9
MCM4	P33991	212	-1	-11	-11	-3	1.0	0.9	0.9	1.0
MDM1	Q8TC05	405	-1	-18	-11	-41	1.0	0.9	0.9	0.7
SEPT7	Q16181	204	-1	-19	-11	-7	1.0	0.8	0.9	0.9
EIF4G3	O43432	928	-1	-27	-11	-5	1.0	0.8	0.9	1.0
EIF4G1	Q04637	934	-1	-27	-11	-5	1.0	0.8	0.9	1.0
RPL7A	P62424	182	-2	-3	-11	1	1.0	1.0	0.9	1.0
STAP1	Q9ULZ2	269	-2	-8	-11	-20	1.0	0.9	0.9	0.8
LENG8	Q96PV6	529	-2	-11	-11	-16	1.0	0.9	0.9	0.9
MACF1	Q9UPN3	666	-2	-11	-11	-16	1.0	0.9	0.9	0.9

LRWD1	Q9UFC0	454	-2	-13	-11	-8	1.0	0.9	0.9	0.9
MYO9B	Q13459	62	-2	-13	-11	-6	1.0	0.9	0.9	0.9
RAD50	Q92878	48	-2	-13	-11	-29	1.0	0.9	0.9	0.8
VDAC3	Q9Y277	65	-2	-14	-11	-10	1.0	0.9	0.9	0.9
USP9Y	O00507	148	-2	-16	-11	-10	1.0	0.9	0.9	0.9
USP9X	Q93008	147	-2	-16	-11	-10	1.0	0.9	0.9	0.9
LDHA	P00338	163	-2	-17	-11	-5	1.0	0.9	0.9	1.0
LDHB	P07195	164	-2	-17	-11	-5	1.0	0.9	0.9	1.0
HAUS1	Q96CS2	156	-2	-21	-11	-22	1.0	0.8	0.9	0.8
TEX264	Q9Y6I9	165	-2	-4	-11	-11	1.0	1.0	0.9	0.9
NTHL1	P78549	118	-2	-12	-11	-9	1.0	0.9	0.9	0.9
ARMC8	Q8IUR7	275	-2	-13	-11	4	1.0	0.9	0.9	1.0
SPG21	Q9NZD8	260	-2	-20	-11	-15	1.0	0.8	0.9	0.9
HLA-A	P16188	363	-3	-6	-11	-12	1.0	0.9	0.9	0.9
CASS4	Q9NQ75	26	-3	-9	-11	-20	1.0	0.9	0.9	0.8
STRN4	Q9NRL3	337	-3	-15	-11	-26	1.0	0.9	0.9	0.8
MYCBP2	O75592	3225	-3	-27	-11	23	1.0	0.8	0.9	1.3
IQGAP1	P46940	781	-3	-8	-11	-15	1.0	0.9	0.9	0.9
TBCE	Q15813	141	-3	-10	-11	-16	1.0	0.9	0.9	0.9
SMC1A	Q14683	1073	-3	-11	-11	-3	1.0	0.9	0.9	1.0
SNRPF	P62306	66	-3	-13	-11	-9	1.0	0.9	0.9	0.9
PTPN11	Q06124	333	-3	-14	-11	-4	1.0	0.9	0.9	1.0
FHOD1	Q9Y613	650	-3	-15	-11	-13	1.0	0.9	0.9	0.9
INO80E	Q8NBZ0	31	-3	-18	-11	-12	1.0	0.8	0.9	0.9
MTMR12	Q9C0I1	433	-3	-20	-11	-1	1.0	0.8	0.9	1.0
TMEM256-PL	I3L3X5	103	-3	-22	-11	-15	1.0	0.8	0.9	0.9
HADHA	P40939	110	-4	-8	-11	-4	1.0	0.9	0.9	1.0
WDFY4	Q6ZS81	1665	-4	-12	-11	-3	1.0	0.9	0.9	1.0
PREB	Q9HCU5	128	-4	-12	-11	-17	1.0	0.9	0.9	0.9
LTBP1	Q14766	559	-4	-16	-11	-19	1.0	0.9	0.9	0.8
CORO1A	P31146	192	-4	-18	-11	-2	1.0	0.9	0.9	1.0
CSDE1	O75534	730	-4	-20	-11	-16	1.0	0.8	0.9	0.9
ZYX	Q15942	444	-4	-22	-11	-22	1.0	0.8	0.9	0.8
TBC1D15	Q8TC07	184	-4	-26	-11	-15	1.0	0.8	0.9	0.9
PRKCQ	Q04759	17	-4	-1	-11	3	1.0	1.0	0.9	1.0
L2HGDH	Q9H9P8	187	-4	-5	-11	-6	1.0	1.0	0.9	0.9
HLA-C	P04222	345	-4	-11	-11	-12	1.0	0.9	0.9	0.9
HLA-C	P30510	345	-4	-11	-11	-12	1.0	0.9	0.9	0.9
ZMYM3	Q14202	1073	-4	-13	-11	-10	1.0	0.9	0.9	0.9
MALT1	Q9UDY8	91	-4	-15	-11	-16	1.0	0.9	0.9	0.9
SLC37A1	P57057	250	-4	-15	-11	-10	1.0	0.9	0.9	0.9
CLIC1	O00299	178	-4	-18	-11	3	1.0	0.8	0.9	1.0
ACTR5	Q9H9F9	265	-4	-18	-11	-13	1.0	0.8	0.9	0.9
ADSL	P30566	304	-4	-25	-11	-6	1.0	0.8	0.9	0.9
CXorf38	Q8TB03	24	-4	-39	-11	31	1.0	0.7	0.9	1.4
PRKDC	P78527	1135	-5	-7	-11	-6	1.0	0.9	0.9	0.9
MYO1C	O00159	625	-5	-8	-11	-6	1.0	0.9	0.9	0.9
LIMA1	Q9UHB6	164	-5	-11	-11	-11	1.0	0.9	0.9	0.9
ATG7	O95352	406	-5	-11	-11	-19	1.0	0.9	0.9	0.8
Uncharacteri	V9GY48	263	-5	-16	-11	-11	1.0	0.9	0.9	0.9
LRCH3	Q96II8	531	-5	-17	-11	-17	1.0	0.9	0.9	0.9
HMGCL	P35914	174	-5	-18	-11	0	1.0	0.9	0.9	1.0
WDR91	A4D1P6	246	-5	-20	-11	-5	1.0	0.8	0.9	1.0

DOK3	Q7L591	144	-5	-21	-11	2	1.0	0.8	0.9	1.0
KIAA1524	Q8TCG1	92	-5	-22	-11	-5	1.0	0.8	0.9	1.0
LENG9	Q96B70	219	-5	-3	-11	-11	1.0	1.0	0.9	0.9
ARRB2	P32121	141	-5	-10	-11	-10	1.0	0.9	0.9	0.9
RCC2	Q9P258	428	-5	-17	-11	-22	1.0	0.9	0.9	0.8
RPS3A	P61247	96	-5	-21	-11	-13	1.0	0.8	0.9	0.9
PHGDH	O43175	281	-6	-9	-11	-4	0.9	0.9	0.9	1.0
HTT	P42858	2971	-6	-12	-11	5	0.9	0.9	0.9	1.0
HMCES	Q96FZ2	39	-6	-13	-11	-10	0.9	0.9	0.9	0.9
PARP10	Q53GL7	768	-6	-14	-11	-18	0.9	0.9	0.9	0.9
UBASH3A	P57075	456	-6	-11	-11	-4	0.9	0.9	0.9	1.0
DAZAP1	Q96EP5	124	-6	-13	-11	-3	0.9	0.9	0.9	1.0
ADH5	P11766	100	-6	-14	-11	-2	0.9	0.9	0.9	1.0
KDM3A	Q9Y4C1	251	-6	-15	-11	-25	0.9	0.9	0.9	0.8
QKI	Q96PU8	119	-6	-17	-11	-15	0.9	0.9	0.9	0.9
LTA4H	P09960	136	-6	-19	-11	9	0.9	0.8	0.9	1.1
HMGCL	P35914	170	-6	-20	-11	-1	0.9	0.8	0.9	1.0
WDR81	Q562E7	76	-6	-21	-11	-8	0.9	0.8	0.9	0.9
DNAJC11	Q9NVH1	494	-7	-12	-11	-5	0.9	0.9	0.9	1.0
IVNS1ABP	Q9Y6Y0	274	-7	-20	-11	-20	0.9	0.8	0.9	0.8
USP47	Q96K76	1138	-7	-22	-11	-12	0.9	0.8	0.9	0.9
TRMT1L	Q7Z2T5	656	-7	-22	-11	-15	0.9	0.8	0.9	0.9
GEMIN5	Q8TEQ6	572	-7	-25	-11	4	0.9	0.8	0.9	1.0
MTHFD1	P11586	691	-7	-10	-11	-9	0.9	0.9	0.9	0.9
RPS12	P25398	69	-7	-18	-11	-2	0.9	0.9	0.9	1.0
IMPACT	Q9P2X3	226	-7	-20	-11	-30	0.9	0.8	0.9	0.8
PPM1G	O15355	351	-8	-19	-11	-2	0.9	0.8	0.9	1.0
NLRC3	Q7RTR2	561	-8	-19	-11	-3	0.9	0.8	0.9	1.0
CENPV	Q7Z7K6	216	-8	-21	-11	7	0.9	0.8	0.9	1.1
ELP5	Q8TE02	151	-8	-23	-11	1	0.9	0.8	0.9	1.0
PCBD2	Q9H0N5	109	-8	-23	-11	-13	0.9	0.8	0.9	0.9
STRN	O43815	587	-8	-23	-11	-10	0.9	0.8	0.9	0.9
HNRNPU	Q00839	450	-8	-24	-11	1	0.9	0.8	0.9	1.0
PHF10	Q8WUB8	134	-8	-12	-11	-4	0.9	0.9	0.9	1.0
PPL	O60437	660	-8	-12	-11	-11	0.9	0.9	0.9	0.9
SARS	P49591	398	-8	-14	-11	0	0.9	0.9	0.9	1.0
PFKP	Q01813	411	-8	-23	-11	-10	0.9	0.8	0.9	0.9
LIMD2	Q9BT23	67	-9	-1	-11	-1	0.9	1.0	0.9	1.0
CNTRL	Q7Z7A1	1451	-9	-13	-11	-10	0.9	0.9	0.9	0.9
RNH1	P13489	209	-9	-19	-11	-7	0.9	0.8	0.9	0.9
GCOM1	H8Y6P7	479	-9	-20	-11	-14	0.9	0.8	0.9	0.9
KDM2A	Q9Y2K7	582	-9	-27	-11	-24	0.9	0.8	0.9	0.8
RFC3	P40938	11	-9	-12	-11	-7	0.9	0.9	0.9	0.9
KRI1	Q8N9T8	531	-9	-16	-11	-18	0.9	0.9	0.9	0.8
OPTN	Q96CV9	472	-9	-17	-11	-20	0.9	0.9	0.9	0.8
AKAP11	Q9UKA4	1587	-10	-17	-11	-18	0.9	0.9	0.9	0.8
ASCC3	Q8N3C0	1535	-10	-4	-11	-23	0.9	1.0	0.9	0.8
ADAR	P55265	909	-10	-21	-11	-12	0.9	0.8	0.9	0.9
ATP6V1A	P38606	138	-11	-16	-11	-12	0.9	0.9	0.9	0.9
PDF	Q9HBH1	108	-11	-21	-11	-19	0.9	0.8	0.9	0.8
UPP1	Q16831	80	-12	-12	-11	-22	0.9	0.9	0.9	0.8
SEC23A	Q15436	61	-12	-21	-11	-3	0.9	0.8	0.9	1.0
RAP1GAP2	Q684P5	185	-13	-2	-11	-8	0.9	1.0	0.9	0.9

GDAP2	Q9NXN4	348	-13	-19	-11	-18	0.9	0.8	0.9	0.9
ADSL	P30566	305	-13	-25	-11	-7	0.9	0.8	0.9	0.9
MYO18A	Q92614	1080	-13	-7	-11	-10	0.9	0.9	0.9	0.9
ECHS1	P30084	225	-13	-18	-11	-19	0.9	0.8	0.9	0.8
WDFY4	Q6ZS81	545	-14	-9	-11	-12	0.9	0.9	0.9	0.9
NRDE2	Q9H7Z3	1119	-14	-22	-11	0	0.9	0.8	0.9	1.0
CAD	P27708	73	-15	-16	-11	-12	0.9	0.9	0.9	0.9
PSMC2	P35998	377	-15	-17	-11	-12	0.9	0.9	0.9	0.9
NCOA7	Q8NI08	439	-15	-8	-11	-32	0.9	0.9	0.9	0.8
NOCT	Q9UK39	302	-15	-13	-11	-11	0.9	0.9	0.9	0.9
FAM129C	Q86XR2	120	-15	-15	-11	-22	0.9	0.9	0.9	0.8
DNAJC30	Q96LL9	73	-16	-2	-11	-6	0.9	1.0	0.9	0.9
INPP4A	Q96PE3	854	-16	-11	-11	-27	0.9	0.9	0.9	0.8
F5	P12259	1725	-16	-21	-11	-20	0.9	0.8	0.9	0.8
PREP	P48147	343	-19	-67	-11	-8	0.8	0.6	0.9	0.9
NOSIP	Q9Y314	236	-19	-21	-11	-11	0.8	0.8	0.9	0.9
RBCK1	Q9BYM8	332	-21	-21	-11	-8	0.8	0.8	0.9	0.9
FLNB	O75369	1952	-21	-24	-11	10	0.8	0.8	0.9	1.1
CASP6	P55212	277	-21	-25	-11	-11	0.8	0.8	0.9	0.9
KPNA5	O15131	253	-21	-25	-11	-8	0.8	0.8	0.9	0.9
DDX39A	O00148	86	-21	-22	-11	-49	0.8	0.8	0.9	0.7
DDX39B	Q13838	87	-21	-22	-11	-49	0.8	0.8	0.9	0.7
SVIL	O95425	178	-22	-15	-11	-32	0.8	0.9	0.9	0.8
MED23	Q9ULK4	40	-26	-18	-11	-19	0.8	0.9	0.9	0.8
POLD1	P28340	713	-36	-18	-11	-9	0.7	0.9	0.9	0.9
UBA6	A0AVT1	924	27	-13	-11	-22	1.4	0.9	0.9	0.8
RPAP1	Q9BWH6	586	23	-20	-11	-1	1.3	0.8	0.9	1.0
ARL15	Q9NXU5	53	22	-12	-11	2	1.3	0.9	0.9	1.0
GP5	P40197	451	16	17	-11	-4	1.2	1.2	0.9	1.0
SF3B5	Q9BWJ5	76	16	-6	-11	-5	1.2	0.9	0.9	1.0
ATP2A2	P16615	560	16	-12	-11	10	1.2	0.9	0.9	1.1
PREB	Q9HCU5	90	14	-3	-11	-5	1.2	1.0	0.9	1.0
MNDA	P41218	371	13	-26	-11	6	1.1	0.8	0.9	1.1
DHRS7	Q9Y394	233	13	-11	-11	1	1.1	0.9	0.9	1.0
Uncharacteri	E7EQ34	18	12	-28	-11	14	1.1	0.8	0.9	1.2
SPTAN1	Q13813	1314	11	-16	-11	-8	1.1	0.9	0.9	0.9
SLIRP	Q9GZT3	48	10	-15	-11	-3	1.1	0.9	0.9	1.0
PMPCA	Q10713	140	9	-2	-11	6	1.1	1.0	0.9	1.1
TGFB1I1	O43294	91	9	-3	-11	-5	1.1	1.0	0.9	1.0
AHCYL1	O43865	327	9	-14	-11	2	1.1	0.9	0.9	1.0
ESRRA	P11474	46	8	-2	-11	8	1.1	1.0	0.9	1.1
BCOR	Q6W2J9	1488	8	-5	-11	15	1.1	1.0	0.9	1.2
GLRX3	O76003	146	8	-13	-11	22	1.1	0.9	0.9	1.3
DHX38	Q92620	865	8	-35	-11	-4	1.1	0.7	0.9	1.0
MFSD5	Q6N075	33	8	-9	-11	-2	1.1	0.9	0.9	1.0
UGP2	Q16851	276	8	-12	-11	-7	1.1	0.9	0.9	0.9
GNL3L	Q9NVN8	322	8	-13	-11	-19	1.1	0.9	0.9	0.8
SMC1A	Q14683	556	8	-14	-11	-6	1.1	0.9	0.9	0.9
NDUFV1	P49821	206	8	-16	-11	-4	1.1	0.9	0.9	1.0
FABP5	Q01469	47	8	-20	-11	-5	1.1	0.8	0.9	1.0
FNBP4	Q8N3X1	673	7	-14	-11	-8	1.1	0.9	0.9	0.9
TROVE2	P10155	38	7	-20	-11	2	1.1	0.8	0.9	1.0
MPG	P29372	222	7	-10	-11	-10	1.1	0.9	0.9	0.9

RABEP1	Q15276	536	7	-18	-11	-5	1.1	0.8	0.9	1.0
RAB3GAP1	Q15042	693	6	-7	-11	-3	1.1	0.9	0.9	1.0
PLAA	Q9Y263	263	6	-17	-11	0	1.1	0.9	0.9	1.0
PNKP	Q96T60	445	6	-18	-11	-3	1.1	0.9	0.9	1.0
LRRK2	Q5S007	925	6	-20	-11	-6	1.1	0.8	0.9	0.9
SLC27A1	Q6PCB7	442	6	0	-11	-9	1.1	1.0	0.9	0.9
SACS	Q9NZJ4	3655	6	-3	-11	5	1.1	1.0	0.9	1.1
RALGAPA2	Q2PPJ7	1662	6	-8	-11	-8	1.1	0.9	0.9	0.9
CCAR2	Q8N163	754	6	-14	-11	-9	1.1	0.9	0.9	0.9
CAPN1	P07384	417	6	-21	-11	-5	1.1	0.8	0.9	1.0
TRIM38	O00635	237	6	-21	-11	-11	1.1	0.8	0.9	0.9
AGO2	Q9UKV8	462	5	-3	-11	1	1.1	1.0	0.9	1.0
HK1	P19367	886	5	-24	-11	-8	1.1	0.8	0.9	0.9
RSL1D1	O76021	197	5	-40	-11	-8	1.1	0.7	0.9	0.9
RFC2	P35250	88	5	-10	-11	-23	1.0	0.9	0.9	0.8
TRIM21	P19474	285	5	-10	-11	2	1.0	0.9	0.9	1.0
RNF213	Q63HN8	1156	5	-16	-11	-2	1.0	0.9	0.9	1.0
FLNB	O75369	26	5	-23	-11	-2	1.0	0.8	0.9	1.0
FLNA	P21333	53	5	-23	-11	-2	1.0	0.8	0.9	1.0
FLNC	Q14315	46	5	-23	-11	-2	1.0	0.8	0.9	1.0
ASUN	Q9NVM9	349	4	-8	-11	-4	1.0	0.9	0.9	1.0
TRIM25	Q14258	506	4	-14	-11	1	1.0	0.9	0.9	1.0
GLS	O94925	266	4	-15	-11	-12	1.0	0.9	0.9	0.9
TXNRD2	Q9NNW7	168	4	-16	-11	-7	1.0	0.9	0.9	0.9
CBLB	Q13191	686	4	-18	-11	-19	1.0	0.8	0.9	0.8
GYS1	P13807	564	4	-5	-11	9	1.0	1.0	0.9	1.1
RNMT	O43148	206	4	-13	-11	1	1.0	0.9	0.9	1.0
DMAP1	Q9NPF5	199	4	-15	-11	21	1.0	0.9	0.9	1.3
PUS3	Q9BZE2	420	4	-16	-11	-18	1.0	0.9	0.9	0.8
GLE1	Q53GS7	7	4	-18	-11	-9	1.0	0.8	0.9	0.9
MTCH2	Q9Y6C9	297	3	-12	-11	-9	1.0	0.9	0.9	0.9
MEFV	O15553	355	3	-7	-11	-4	1.0	0.9	0.9	1.0
ARHGAP31	Q2M1Z3	122	3	-10	-11	-10	1.0	0.9	0.9	0.9
PISD	Q9UG56	203	3	-15	-11	-24	1.0	0.9	0.9	0.8
RETSAT	Q6NUM9	106	3	-16	-11	-3	1.0	0.9	0.9	1.0
TRANK1	O15050	1041	2	-1	-11	10	1.0	1.0	0.9	1.1
APBA2	Q99767	483	2	-6	-11	-21	1.0	0.9	0.9	0.8
SMARCD2	Q92925	365	2	-7	-11	6	1.0	0.9	0.9	1.1
DIS3L	Q8TF46	124	2	-10	-11	-6	1.0	0.9	0.9	0.9
RPL9P9	P32969	74	2	-11	-11	2	1.0	0.9	0.9	1.0
RTEL1-TNFRS	F6WH68	20	2	-17	-11	-15	1.0	0.9	0.9	0.9
MORC3	Q14149	671	2	-17	-11	-19	1.0	0.9	0.9	0.8
CXCR1	P25024	277	2	-18	-11	-27	1.0	0.9	0.9	0.8
NNT	Q13423	1073	2	-26	-11	-8	1.0	0.8	0.9	0.9
RFX1	P22670	461	2	-10	-11	-16	1.0	0.9	0.9	0.9
CHEK2	O96017	231	2	-16	-11	-28	1.0	0.9	0.9	0.8
CLTC	Q00610	909	2	-28	-11	5	1.0	0.8	0.9	1.1
TARBP1	Q13395	1556	1	-10	-11	-4	1.0	0.9	0.9	1.0
SMYD3	Q9H7B4	41	1	-13	-11	-2	1.0	0.9	0.9	1.0
RPL34	P49207	46	1	-14	-11	-9	1.0	0.9	0.9	0.9
GSTM3	P21266	39	1	-14	-11	-11	1.0	0.9	0.9	0.9
SFI1	A8K8P3	77	1	-24	-11	27	1.0	0.8	0.9	1.4
UQCRC1	P31930	154	1	-49	-11	-12	1.0	0.7	0.9	0.9

MLKL	Q8NB16	184	1	-5	-11	-22	1.0	1.0	0.9	0.8
FAM192A	Q9GZU8	187	1	-7	-11	-18	1.0	0.9	0.9	0.9
ATRX	P46100	618	1	-14	-11	-13	1.0	0.9	0.9	0.9
TRA2A	Q13595	120	1	-18	-11	5	1.0	0.8	0.9	1.0
LRP1	Q07954	3119	0	-11	-11	1	1.0	0.9	0.9	1.0
STX11	O75558	275	0	-14	-11	-6	1.0	0.9	0.9	0.9
RASA3	Q14644	206	0	-14	-11	-18	1.0	0.9	0.9	0.9
MIA3	Q5JRA6	1899	0	-18	-11	-18	1.0	0.9	0.9	0.9
ARID1A	O14497	1827	0	-18	-11	-11	1.0	0.8	0.9	0.9
DBT	P11182	279	-1	-1	-11	3	1.0	1.0	0.9	1.0
TYK2	P29597	1140	-1	-4	-11	0	1.0	1.0	0.9	1.0
DENND1B	Q6P3S1	356	-1	-5	-11	-5	1.0	1.0	0.9	1.0
FRY	Q5TBA9	336	-1	-9	-11	-9	1.0	0.9	0.9	0.9
DNTTIP1	Q9H147	286	-1	-9	-11	-16	1.0	0.9	0.9	0.9
NRBF2	Q96F24	155	-1	-11	-11	-13	1.0	0.9	0.9	0.9
PTBP1	P26599	250	-1	-11	-11	-5	1.0	0.9	0.9	1.0
KDM1A	O60341	623	-1	-30	-11	-28	1.0	0.8	0.9	0.8
DCTN1	Q14203	636	-1	-14	-11	-4	1.0	0.9	0.9	1.0
FSD1L	Q9BXM9	250	-1	-17	-11	-22	1.0	0.9	0.9	0.8
HPS3	Q969F9	52	-2	-2	-11	-11	1.0	1.0	0.9	0.9
ERC1	X6RLX0	258	-2	-13	-11	-9	1.0	0.9	0.9	0.9
HK3	P52790	18	-2	-18	-11	-25	1.0	0.9	0.9	0.8
PRPF38A	Q8NAV1	72	-2	-21	-11	-5	1.0	0.8	0.9	1.0
LRRK1	Q38SD2	835	-2	-9	-11	-18	1.0	0.9	0.9	0.9
SEC23IP	Q9Y6Y8	604	-2	-11	-11	-20	1.0	0.9	0.9	0.8
HSD17B11	Q8NBQ5	217	-2	-12	-11	-2	1.0	0.9	0.9	1.0
PPTC7	Q8NI37	57	-2	-13	-11	-35	1.0	0.9	0.9	0.7
HLA-C	P10321	350	-2	-14	-11	-19	1.0	0.9	0.9	0.8
PUS7	Q96PZ0	421	-2	-20	-11	-26	1.0	0.8	0.9	0.8
PTPN7	P35236	228	-3	-7	-11	5	1.0	0.9	0.9	1.1
RPP14	O95059	31	-3	-9	-11	-14	1.0	0.9	0.9	0.9
PDK2	Q15119	45	-3	-12	-11	-15	1.0	0.9	0.9	0.9
MICU2	Q8IYU8	144	-3	-12	-11	-20	1.0	0.9	0.9	0.8
SPTLC1	O15269	438	-3	-16	-11	-8	1.0	0.9	0.9	0.9
HPS5	Q9UPZ3	228	-3	-16	-11	-16	1.0	0.9	0.9	0.9
UBXN6	Q9BZV1	210	-3	-7	-11	-11	1.0	0.9	0.9	0.9
UPF2	Q9HAU5	944	-3	-8	-11	-4	1.0	0.9	0.9	1.0
VPS51	Q9UID3	316	-3	-13	-11	-5	1.0	0.9	0.9	1.0
UGDH	O60701	276	-3	-14	-11	-23	1.0	0.9	0.9	0.8
FLNA	P21333	810	-3	-24	-11	-6	1.0	0.8	0.9	0.9
SMU1	Q2TAY7	416	-3	-26	-11	-3	1.0	0.8	0.9	1.0
RFFL	Q8WZ73	50	-4	-2	-11	-3	1.0	1.0	0.9	1.0
DENND4A	Q7Z401	117	-4	-8	-11	-12	1.0	0.9	0.9	0.9
MAN2C1	Q9NTJ4	322	-4	-9	-11	-9	1.0	0.9	0.9	0.9
Uncharacteri	K7ESF4	46	-4	-11	-11	-2	1.0	0.9	0.9	1.0
VDAC3	Q9Y277	229	-4	-11	-11	-13	1.0	0.9	0.9	0.9
FAM21A	Q641Q2	594	-4	-11	-11	-15	1.0	0.9	0.9	0.9
ZC3HAV1	Q7Z2W4	96	-4	-15	-11	8	1.0	0.9	0.9	1.1
TRIM21	P19474	463	-4	-17	-11	-9	1.0	0.9	0.9	0.9
FHL1	Q13642	43	-4	-17	-11	-15	1.0	0.9	0.9	0.9
DISC1	Q9NRI5	684	-4	-24	-11	-11	1.0	0.8	0.9	0.9
BTN3A3	O00478	166	-4	6	-11	-3	1.0	1.1	0.9	1.0
PPP1R9B	Q96SB3	270	-4	-12	-11	-16	1.0	0.9	0.9	0.9

ESYT1	Q9BSJ8	522	-4	-12	-11	-4	1.0	0.9	0.9	1.0
MEPCE	Q7L2J0	54	-4	-17	-11	-7	1.0	0.9	0.9	0.9
IFI16	Q16666	191	-4	-17	-11	-20	1.0	0.9	0.9	0.8
ADAR	P55265	851	-4	-19	-11	-11	1.0	0.8	0.9	0.9
TRIP12	Q14669	1959	-5	-4	-11	-13	1.0	1.0	0.9	0.9
DHX16	O60231	714	-5	-7	-11	-7	1.0	0.9	0.9	0.9
EPRS	P07814	337	-5	-10	-11	-3	1.0	0.9	0.9	1.0
LYAR	Q9NX58	51	-5	-13	-11	-16	1.0	0.9	0.9	0.9
CHD6	Q8TD26	1555	-5	-18	-11	-10	1.0	0.8	0.9	0.9
EHBP1L1	Q8N3D4	1135	-5	-8	-11	-7	1.0	0.9	0.9	0.9
ACOX1	Q15067	449	-5	-12	-11	-9	1.0	0.9	0.9	0.9
WAPL	Q7Z5K2	344	-5	-14	-11	-15	1.0	0.9	0.9	0.9
TBCK	Q8TEA7	871	-5	-16	-11	-5	1.0	0.9	0.9	1.0
MYO9A	B2RTY4	152	-5	-18	-11	3	1.0	0.9	0.9	1.0
ZNF512B	Q96KM6	353	-5	-18	-11	-4	1.0	0.9	0.9	1.0
HERC4	Q5GLZ8	392	-5	-22	-11	-12	1.0	0.8	0.9	0.9
IRF2BP2	Q7Z5L9	530	-6	-2	-11	-11	0.9	1.0	0.9	0.9
IRF2BP1	Q8IU81	527	-6	-2	-11	-11	0.9	1.0	0.9	0.9
IRF2BPL	Q9H1B7	739	-6	-2	-11	-11	0.9	1.0	0.9	0.9
PDS5B	Q9NTI5	571	-6	-8	-11	-11	0.9	0.9	0.9	0.9
ZNF189	O75820	525	-6	-15	-11	-18	0.9	0.9	0.9	0.9
RAP1GAP2	Q684P5	584	-6	-20	-11	-24	0.9	0.8	0.9	0.8
FLNB	O75369	660	-6	-21	-11	-12	0.9	0.8	0.9	0.9
LRCH4	O75427	247	-6	-22	-11	-15	0.9	0.8	0.9	0.9
SNRNP200	O75643	1359	-6	-26	-11	-7	0.9	0.8	0.9	0.9
TST	Q16762	248	-6	-34	-11	-2	0.9	0.7	0.9	1.0
PPIG	Q13427	549	-6	-7	-11	-12	0.9	0.9	0.9	0.9
SUN1	O94901	526	-6	-19	-11	4	0.9	0.8	0.9	1.0
PLP2	Q04941	12	-6	-20	-11	-7	0.9	0.8	0.9	0.9
TRAF2	Q12933	287	-7	-9	-11	-9	0.9	0.9	0.9	0.9
Uncharacteri	E9PAM4	290	-7	-11	-11	-20	0.9	0.9	0.9	0.8
RPL4	P36578	96	-7	-12	-11	-23	0.9	0.9	0.9	0.8
GCC2	Q8IWJ2	416	-7	-14	-11	-15	0.9	0.9	0.9	0.9
LPXN	O60711	240	-7	-6	-11	-5	0.9	0.9	0.9	1.0
PSMD11	O00231	202	-7	-18	-11	1	0.9	0.8	0.9	1.0
RNH1	P13489	152	-7	-22	-11	-3	0.9	0.8	0.9	1.0
FBXO42	Q6P3S6	302	-7	-22	-11	-5	0.9	0.8	0.9	1.0
MMAB	Q96EY8	119	-8	-14	-11	-13	0.9	0.9	0.9	0.9
ZNF12	P17014	587	-8	-21	-11	-1	0.9	0.8	0.9	1.0
ITPA	Q9BY32	33	-9	-6	-11	4	0.9	0.9	0.9	1.0
ZFYVE16	Q7Z3T8	66	-9	-7	-11	-7	0.9	0.9	0.9	0.9
NONO	Q15233	145	-9	-10	-11	-9	0.9	0.9	0.9	0.9
FHOD1	Q9Y613	373	-9	-10	-11	-16	0.9	0.9	0.9	0.9
ELMO1	Q92556	561	-9	-11	-11	-7	0.9	0.9	0.9	0.9
TLN1	Q9Y490	732	-9	-13	-11	-8	0.9	0.9	0.9	0.9
NPLOC4	Q8TAT6	130	-9	-13	-11	-11	0.9	0.9	0.9	0.9
NUDT5	Q9UKK9	76	-9	-31	-11	24	0.9	0.8	0.9	1.3
RASA3	Q14644	67	-9	-5	-11	-8	0.9	1.0	0.9	0.9
ATAD1	Q8NBU5	359	-9	-8	-11	-29	0.9	0.9	0.9	0.8
GTF2B	Q00403	37	-9	-16	-11	-9	0.9	0.9	0.9	0.9
TM9SF3	Q9HD45	428	-9	-18	-11	-7	0.9	0.8	0.9	0.9
KHNYN	O15037	46	-10	-12	-11	-20	0.9	0.9	0.9	0.8
FAM98C	Q17RN3	301	-10	-12	-11	-17	0.9	0.9	0.9	0.9

RPP25	Q9BUL9	70	-11	-30	-11	-27	0.9	0.8	0.9	0.8
ATG2B	Q96BY7	243	-11	-10	-11	-6	0.9	0.9	0.9	0.9
GSK3A	P49840	262	-11	-13	-11	1	0.9	0.9	0.9	1.0
GSK3B	P49841	199	-11	-13	-11	1	0.9	0.9	0.9	1.0
FLNA	P21333	2476	-11	-20	-11	-15	0.9	0.8	0.9	0.9
PSME4	Q14997	1000	-12	-13	-11	-9	0.9	0.9	0.9	0.9
NBEAL2	Q6ZNJ1	482	-13	-8	-11	-8	0.9	0.9	0.9	0.9
C3orf38	Q5JPI3	308	-13	-19	-11	-32	0.9	0.8	0.9	0.8
PRKCB	P05771	217	-13	-20	-11	-11	0.9	0.8	0.9	0.9
TAPBP	O15533	115	-13	-18	-11	-10	0.9	0.8	0.9	0.9
NTPCR	Q9BSD7	101	-13	-32	-11	-3	0.9	0.8	0.9	1.0
LRWD1	Q9UFC0	249	-14	-20	-11	-23	0.9	0.8	0.9	0.8
FAM65A	Q6ZS17	997	-14	-25	-11	-14	0.9	0.8	0.9	0.9
AKAP11	Q9UKA4	1391	-14	-17	-11	-10	0.9	0.9	0.9	0.9
PLCL2	Q9UPR0	425	-15	-5	-11	-10	0.9	1.0	0.9	0.9
UBR3	Q6ZT12	1289	-15	-17	-11	-12	0.9	0.9	0.9	0.9
FBXO11	Q86XK2	113	-15	-32	-11	-9	0.9	0.8	0.9	0.9
FBP1	P09467	39	-15	-32	-11	2	0.9	0.8	0.9	1.0
PYGO2	Q9BRQ0	346	-17	-14	-11	-12	0.9	0.9	0.9	0.9
HCK	P08631	7	-17	-35	-11	-33	0.9	0.7	0.9	0.8
CNBP	P62633	161	-26	-18	-11	-26	0.8	0.8	0.9	0.8
PLEKHA2	Q9HB19	81	-30	-17	-11	-11	0.8	0.9	0.9	0.9
TCEAL3	Q969E4	44	-37	-15	-11	-29	0.7	0.9	0.9	0.8
DENND2C	Q68D51	599	22	-12	-12	-10	1.3	0.9	0.9	0.9
ANXA6	P08133	552	22	-18	-12	6	1.3	0.9	0.9	1.1
ARID2	Q68CP9	82	21	-1	-12	-3	1.3	1.0	0.9	1.0
PLCE1	Q9P212	557	18	-14	-12	-15	1.2	0.9	0.9	0.9
PLCE1	Q9P212	574	18	-14	-12	-15	1.2	0.9	0.9	0.9
ETFDH	Q16134	101	16	-28	-12	-18	1.2	0.8	0.9	0.9
PLEC	Q15149	1405	16	-4	-12	8	1.2	1.0	0.9	1.1
EIF3D	O15371	438	16	-21	-12	-3	1.2	0.8	0.9	1.0
SETDB1	Q15047	1281	15	-5	-12	7	1.2	1.0	0.9	1.1
CPSF3	Q9UKF6	223	14	-17	-12	-7	1.2	0.9	0.9	0.9
PCK2	Q16822	92	12	-14	-12	6	1.1	0.9	0.9	1.1
UBN1	Q9NPG3	467	11	2	-12	2	1.1	1.0	0.9	1.0
UTRN	P46939	2098	11	6	-12	19	1.1	1.1	0.9	1.2
TOP2A	P11388	392	11	-6	-12	1	1.1	0.9	0.9	1.0
RASAL3	Q86YV0	896	9	-8	-12	-7	1.1	0.9	0.9	0.9
MICALL2	Q8IY33	676	9	-11	-12	-32	1.1	0.9	0.9	0.8
PRKDC	P78527	703	9	-30	-12	10	1.1	0.8	0.9	1.1
UBR4	Q5T4S7	2222	9	-12	-12	-18	1.1	0.9	0.9	0.9
DHX32	Q7L7V1	316	8	-14	-12	-14	1.1	0.9	0.9	0.9
ZNF644	Q9H582	507	7	-5	-12	-11	1.1	1.0	0.9	0.9
TARBP2	Q15633	294	7	-11	-12	-18	1.1	0.9	0.9	0.9
SAMD9	Q5K651	975	7	-12	-12	-8	1.1	0.9	0.9	0.9
SARS2	Q9NP81	425	7	-13	-12	-11	1.1	0.9	0.9	0.9
RECQL	P46063	414	7	-2	-12	4	1.1	1.0	0.9	1.0
ZFR	Q96KR1	856	7	-11	-12	-7	1.1	0.9	0.9	0.9
RPL10A	P62906	66	6	-11	-12	-8	1.1	0.9	0.9	0.9
UPP1	Q16831	225	6	-12	-12	-12	1.1	0.9	0.9	0.9
HCFC2	Q9Y5Z7	345	6	-15	-12	-8	1.1	0.9	0.9	0.9
RPS27A	P62979	126	6	-17	-12	9	1.1	0.9	0.9	1.1
XRN1	Q8IZH2	16	6	-19	-12	-2	1.1	0.8	0.9	1.0

DHX15	O43143	226	6	-22	-12	-1	1.1	0.8	0.9	1.0
BCL11B	Q9C0K0	457	5	-4	-12	1	1.1	1.0	0.9	1.0
COG5	Q9UP83	173	5	-10	-12	9	1.1	0.9	0.9	1.1
DGKQ	P52824	123	5	-19	-12	-20	1.1	0.8	0.9	0.8
RAB3GAP1	Q15042	511	5	-21	-12	-5	1.1	0.8	0.9	1.0
MYLK	Q15746	1515	5	-9	-12	-21	1.0	0.9	0.9	0.8
COG5	Q9UP83	664	5	-11	-12	6	1.0	0.9	0.9	1.1
DDX46	Q7L014	478	5	-24	-12	-8	1.0	0.8	0.9	0.9
HINT1	P49773	84	5	-30	-12	-11	1.0	0.8	0.9	0.9
PPBP	P02775	105	4	-4	-12	-10	1.0	1.0	0.9	0.9
AMPD2	Q01433	123	4	-11	-12	-6	1.0	0.9	0.9	0.9
NIPBL	Q6KC79	2035	4	-12	-12	-19	1.0	0.9	0.9	0.8
ELMO1	Q92556	622	4	-7	-12	-2	1.0	0.9	0.9	1.0
CAST	P20810	328	4	-9	-12	-7	1.0	0.9	0.9	0.9
DHX34	Q14147	628	4	-14	-12	-14	1.0	0.9	0.9	0.9
SLFN5	Q08AF3	342	4	-17	-12	-22	1.0	0.9	0.9	0.8
MLLT1	Q03111	42	4	-19	-12	-13	1.0	0.8	0.9	0.9
NGLY1	Q96IV0	309	3	-8	-12	-6	1.0	0.9	0.9	0.9
GDI2	P50395	202	3	-12	-12	1	1.0	0.9	0.9	1.0
BPTF	Q12830	1357	3	-14	-12	-23	1.0	0.9	0.9	0.8
PML	P29590	151	3	-23	-12	-16	1.0	0.8	0.9	0.9
CAPNS1	P04632	232	3	-28	-12	13	1.0	0.8	0.9	1.1
CS	O75390	101	3	-14	-12	0	1.0	0.9	0.9	1.0
SMC2	O95347	132	3	-15	-12	2	1.0	0.9	0.9	1.0
TPR	P12270	224	3	-19	-12	-26	1.0	0.8	0.9	0.8
KLHDC3	Q9BQ90	102	3	-21	-12	-7	1.0	0.8	0.9	0.9
CAD	P27708	379	2	-11	-12	-6	1.0	0.9	0.9	0.9
POGZ	Q7Z3K3	649	2	-12	-12	-12	1.0	0.9	0.9	0.9
OSGEPL1	Q9H4B0	390	2	-15	-12	-10	1.0	0.9	0.9	0.9
PYGB	P11216	326	2	-7	-12	-22	1.0	0.9	0.9	0.8
ITPR2	Q14571	420	2	-10	-12	0	1.0	0.9	0.9	1.0
ZC3HAV1	Q7Z2W4	645	2	-11	-12	-14	1.0	0.9	0.9	0.9
MTCH2	Q9Y6C9	79	2	-13	-12	-2	1.0	0.9	0.9	1.0
MDN1	Q9NU22	4409	2	-19	-12	-19	1.0	0.8	0.9	0.8
HDAC7	Q8WUI4	904	1	-5	-12	-6	1.0	1.0	0.9	0.9
IGHMBP2	P38935	191	1	-9	-12	-9	1.0	0.9	0.9	0.9
DNMT1	P26358	41	1	-10	-12	-15	1.0	0.9	0.9	0.9
LRMP	Q12912	436	1	-14	-12	-21	1.0	0.9	0.9	0.8
DQX1	Q8TE96	136	1	-18	-12	-5	1.0	0.9	0.9	1.0
NFKBIB	Q15653	240	1	-19	-12	-2	1.0	0.8	0.9	1.0
TANGO6	Q9C0B7	184	1	2	-12	-10	1.0	1.0	0.9	0.9
ASAP2	O43150	88	1	-1	-12	-21	1.0	1.0	0.9	0.8
THOC6	Q86W42	279	1	-16	-12	-16	1.0	0.9	0.9	0.9
FBXW11	Q9UKB1	432	0	-6	-12	2	1.0	0.9	0.9	1.0
BRD8	Q9H0E9	142	0	-14	-12	-22	1.0	0.9	0.9	0.8
GSPT1	P15170	387	0	-17	-12	-1	1.0	0.9	0.9	1.0
PPIG	Q13427	33	0	-17	-12	-5	1.0	0.9	0.9	1.0
SEPT6	Q14141	42	0	-22	-12	-14	1.0	0.8	0.9	0.9
RIN3	Q8TB24	931	0	-25	-12	-10	1.0	0.8	0.9	0.9
CAD	P27708	868	0	-29	-12	-13	1.0	0.8	0.9	0.9
RBM12B	Q8IXT5	204	-1	-8	-12	-8	1.0	0.9	0.9	0.9
RRP12	Q5JTH9	317	-1	-18	-12	1	1.0	0.9	0.9	1.0
PIK3CB	P42338	287	-1	-5	-12	-15	1.0	1.0	0.9	0.9

ATM	Q13315	1821	-1	-16	-12	-7	1.0	0.9	0.9	0.9
ZADH2	Q8N4Q0	218	-1	-20	-12	-2	1.0	0.8	0.9	1.0
IRAK4	Q9NWZ3	13	-1	-20	-12	-19	1.0	0.8	0.9	0.8
CHD3	Q12873	502	-1	-26	-12	-10	1.0	0.8	0.9	0.9
SYNE1	Q8NF91	5598	-2	-10	-12	-11	1.0	0.9	0.9	0.9
OLA1	Q9NTK5	55	-2	-11	-12	-12	1.0	0.9	0.9	0.9
EXOSC1	Q9Y3B2	8	-2	-19	-12	5	1.0	0.8	0.9	1.0
MACF1	Q9UPN3	3173	-2	-4	-12	-14	1.0	1.0	0.9	0.9
DNMT1	P26358	1478	-2	-7	-12	-7	1.0	0.9	0.9	0.9
SMCHD1	A6NHR9	458	-2	-10	-12	-16	1.0	0.9	0.9	0.9
SYNE1	Q8NF91	5472	-2	-11	-12	-4	1.0	0.9	0.9	1.0
ATF2	P15336	79	-2	-11	-12	-27	1.0	0.9	0.9	0.8
CAP1	Q01518	375	-2	-22	-12	-3	1.0	0.8	0.9	1.0
XAB2	Q9HCS7	260	-2	-40	-12	8	1.0	0.7	0.9	1.1
PTPN2	P17706	330	-3	-9	-12	-15	1.0	0.9	0.9	0.9
NDUFS6	O75380	87	-3	-19	-12	-2	1.0	0.8	0.9	1.0
POLR1A	O95602	1332	-3	-20	-12	-8	1.0	0.8	0.9	0.9
FRG1	Q14331	205	-3	-6	-12	-17	1.0	0.9	0.9	0.9
FNDC3B	Q53EP0	558	-3	-10	-12	-16	1.0	0.9	0.9	0.9
TTC33	Q6PID6	55	-3	-15	-12	-16	1.0	0.9	0.9	0.9
RHOG	P84095	157	-3	-21	-12	1	1.0	0.8	0.9	1.0
UHRF1BP1L	A0JNW5	1235	-4	-7	-12	-12	1.0	0.9	0.9	0.9
DNMT1	P26358	1125	-4	-7	-12	-19	1.0	0.9	0.9	0.8
SRBD1	Q8N5C6	912	-4	-10	-12	-7	1.0	0.9	0.9	0.9
TARDBP	G3V162	39	-4	-10	-12	-9	1.0	0.9	0.9	0.9
ARHGAP25	P42331	154	-4	-10	-12	-13	1.0	0.9	0.9	0.9
PARN	O95453	169	-4	-12	-12	-26	1.0	0.9	0.9	0.8
HSPD1	P10809	237	-4	-15	-12	-2	1.0	0.9	0.9	1.0
C8orf82	Q6P1X6	98	-4	-15	-12	-12	1.0	0.9	0.9	0.9
PIK3R5	Q8WYR1	813	-4	-22	-12	-10	1.0	0.8	0.9	0.9
DENND6A	Q8IWF6	108	-4	-24	-12	-20	1.0	0.8	0.9	0.8
FXR1	P51114	77	-4	-11	-12	2	1.0	0.9	0.9	1.0
HDAC1	Q13547	273	-4	-14	-12	-7	1.0	0.9	0.9	0.9
PGP	A6NDG6	297	-4	-17	-12	-15	1.0	0.9	0.9	0.9
CXXC1	Q9P0U4	566	-5	-16	-12	-2	1.0	0.9	0.9	1.0
RAB7A	P51149	84	-5	-18	-12	6	1.0	0.9	0.9	1.1
ANXA5	P08758	316	-5	-27	-12	3	1.0	0.8	0.9	1.0
ARMC10	Q8N2F6	59	-5	-29	-12	-14	1.0	0.8	0.9	0.9
ACTR1B	P42025	34	-5	-12	-12	-12	1.0	0.9	0.9	0.9
ACTR1A	P61163	34	-5	-12	-12	-12	1.0	0.9	0.9	0.9
ZC3H7A	Q8IWR0	123	-5	-12	-12	-12	1.0	0.9	0.9	0.9
ATRIP	Q8WXE1	682	-5	-13	-12	-16	1.0	0.9	0.9	0.9
TRAPPC10	P48553	833	-5	-18	-12	14	1.0	0.8	0.9	1.2
CORO1C	Q9ULV4	39	-5	-21	-12	-6	1.0	0.8	0.9	0.9
GABPB1	Q06547	331	-6	-10	-12	-24	0.9	0.9	0.9	0.8
RSRC2	Q7L4I2	382	-6	-11	-12	2	0.9	0.9	0.9	1.0
ERO1A	Q96HE7	241	-6	-11	-12	-8	0.9	0.9	0.9	0.9
CCNH	P51946	244	-6	-20	-12	-21	0.9	0.8	0.9	0.8
PPM1F	P49593	315	-6	-26	-12	0	0.9	0.8	0.9	1.0
IMPA1	P29218	184	-6	-28	-12	-13	0.9	0.8	0.9	0.9
NUP205	Q92621	1216	-6	-18	-12	-11	0.9	0.9	0.9	0.9
BRAF	P15056	195	-6	-34	-12	-12	0.9	0.7	0.9	0.9
SRR	Q9GZT4	217	-7	-12	-12	-1	0.9	0.9	0.9	1.0

GOLGA2	Q08379	934	-7	-28	-12	-22	0.9	0.8	0.9	0.8
ZNF799	Q96GE5	348	-7	-18	-12	-11	0.9	0.9	0.9	0.9
CRNKL1	Q9BZJ0	603	-8	-2	-12	-3	0.9	1.0	0.9	1.0
BOLA1	Q9Y3E2	20	-8	-12	-12	-4	0.9	0.9	0.9	1.0
PIK3CD	O00329	90	-8	-15	-12	-9	0.9	0.9	0.9	0.9
DNAJA1	P31689	302	-8	-22	-12	-11	0.9	0.8	0.9	0.9
MAP1S	Q66K74	51	-8	-25	-12	1	0.9	0.8	0.9	1.0
ZMYND8	Q9ULU4	1062	-8	-30	-12	-27	0.9	0.8	0.9	0.8
TRAPPC11	Q7Z392	481	-8	-10	-12	-7	0.9	0.9	0.9	0.9
DCXR	Q7Z4W1	58	-8	-18	-12	-6	0.9	0.9	0.9	0.9
USP7	Q93009	799	-8	-21	-12	-1	0.9	0.8	0.9	1.0
H3F3B	P84243	111	-9	-4	-12	6	0.9	1.0	0.9	1.1
SYNE1	Q8NF91	2598	-9	-9	-12	-8	0.9	0.9	0.9	0.9
NTMT1	Q9BV86	195	-9	-10	-12	-19	0.9	0.9	0.9	0.8
ZBP1	Q9H171	327	-9	-15	-12	-11	0.9	0.9	0.9	0.9
FBXO30	Q8TB52	592	-9	-17	-12	-13	0.9	0.9	0.9	0.9
YDJC	A8MPS7	18	-9	-22	-12	0	0.9	0.8	0.9	1.0
Integrin	H3BM21	199	-9	-33	-12	-11	0.9	0.8	0.9	0.9
PDK4	Q16654	49	-9	-13	-12	-19	0.9	0.9	0.9	0.8
CCT4	P50991	221	-9	-16	-12	-1	0.9	0.9	0.9	1.0
COPA	P53621	245	-9	-20	-12	-7	0.9	0.8	0.9	0.9
PNPLA6	Q8IY17	1221	-9	-21	-12	-10	0.9	0.8	0.9	0.9
MYOF	Q9NZM1	1167	-9	-34	-12	-27	0.9	0.7	0.9	0.8
DDX28	Q9NUL7	170	-10	-16	-12	-19	0.9	0.9	0.9	0.8
MED20	Q9H944	120	-10	-22	-12	-20	0.9	0.8	0.9	0.8
SLC15A4	Q8N697	133	-10	-29	-12	-9	0.9	0.8	0.9	0.9
PRKAR1B	P31321	362	-11	-10	-12	-13	0.9	0.9	0.9	0.9
EPRS	P07814	92	-11	-19	-12	-22	0.9	0.8	0.9	0.8
RSBN1	Q5VWQ0	736	-11	-10	-12	-17	0.9	0.9	0.9	0.9
HAUS6	Q7Z4H7	926	-11	-15	-12	-35	0.9	0.9	0.9	0.7
AP5Z1	O43299	637	-11	-16	-12	-12	0.9	0.9	0.9	0.9
GLB1	P16278	393	-11	-21	-12	7	0.9	0.8	0.9	1.1
TRIM25	Q14258	168	-12	-16	-12	-4	0.9	0.9	0.9	1.0
KYAT3	Q6YP21	337	-12	-31	-12	-21	0.9	0.8	0.9	0.8
CCZ1B	P86790	65	-12	-29	-12	-14	0.9	0.8	0.9	0.9
PASK	Q96RG2	991	-13	-15	-12	-1	0.9	0.9	0.9	1.0
NUP155	O75694	704	-14	-17	-12	-15	0.9	0.9	0.9	0.9
RPL14	P50914	42	-17	-24	-12	-2	0.9	0.8	0.9	1.0
THOC2	Q8NI27	804	-18	-37	-12	-22	0.9	0.7	0.9	0.8
FLNA	P21333	2479	-18	-17	-12	2	0.8	0.9	0.9	1.0
SQRDL	Q9Y6N5	379	-20	-12	-12	4	0.8	0.9	0.9	1.0
ACSS2	Q9NR19	75	-20	-18	-12	-6	0.8	0.8	0.9	0.9
NAT1	P18440	148	-21	-23	-12	-3	0.8	0.8	0.9	1.0
ZW10	O43264	588	-23	-39	-12	-15	0.8	0.7	0.9	0.9
UBR3	Q6ZT12	1598	-24	-36	-12	-35	0.8	0.7	0.9	0.7
NUP160	Q12769	916	-28	-55	-12	-8	0.8	0.6	0.9	0.9
ACD	Q96AP0	406	-29	-19	-12	-29	0.8	0.8	0.9	0.8
PPME1	Q9Y570	312	-30	-17	-12	-11	0.8	0.9	0.9	0.9
MPO	P05164	398	-33	-27	-12	3	0.8	0.8	0.9	1.0
EPX	P11678	370	-33	-27	-12	3	0.8	0.8	0.9	1.0
PSIP1	O75475	373	24	-42	-12	1	1.3	0.7	0.9	1.0
HEATR5A	Q86XA9	516	20	-2	-12	13	1.2	1.0	0.9	1.1
RABGAP1L	Q5R372	665	20	-5	-12	12	1.2	1.0	0.9	1.1

AKR1A1	P14550	46	19	4	-12	23	1.2	1.0	0.9	1.3
PTK2B	Q14289	61	19	-11	-12	-7	1.2	0.9	0.9	0.9
GBF1	Q92538	1392	14	-4	-12	6	1.2	1.0	0.9	1.1
SYNE2	Q8WXH0	601	14	-7	-12	-11	1.2	0.9	0.9	0.9
GFM1	Q96RP9	723	13	-13	-12	7	1.1	0.9	0.9	1.1
CNOT3	O75175	600	12	-3	-12	1	1.1	1.0	0.9	1.0
NDUFS1	P28331	176	12	-15	-12	-7	1.1	0.9	0.9	0.9
RAB8B	Q92930	123	11	-5	-12	2	1.1	1.0	0.9	1.0
KMT2A	Q03164	2065	10	-9	-12	-9	1.1	0.9	0.9	0.9
ACAP2	Q15057	417	9	-6	-12	12	1.1	0.9	0.9	1.1
PLEC	Q15149	4254	9	-16	-12	-9	1.1	0.9	0.9	0.9
ACAP2	Q15057	339	8	-8	-12	-5	1.1	0.9	0.9	1.0
ACAP3	Q96P50	341	8	-8	-12	-5	1.1	0.9	0.9	1.0
CCDC9	Q9Y3X0	233	8	-8	-12	-5	1.1	0.9	0.9	1.0
RNMT	O43148	225	8	-13	-12	-6	1.1	0.9	0.9	0.9
SRP72	O76094	50	8	-16	-12	0	1.1	0.9	0.9	1.0
SAE1	Q9UBE0	134	8	1	-12	7	1.1	1.0	0.9	1.1
MRPL24	Q96A35	190	8	-12	-12	-5	1.1	0.9	0.9	1.0
RAB39A	Q14964	129	8	-18	-12	-13	1.1	0.8	0.9	0.9
MMS19	Q96T76	502	8	-20	-12	-21	1.1	0.8	0.9	0.8
ZMYM3	Q14202	524	7	-8	-12	-8	1.1	0.9	0.9	0.9
SRRM1	Q8IYB3	33	7	-14	-12	-5	1.1	0.9	0.9	1.0
UBASH3B	Q8TF42	474	7	-17	-12	-5	1.1	0.9	0.9	1.0
NLN	Q9BYT8	153	7	-17	-12	27	1.1	0.9	0.9	1.4
MCU	Q8NE86	97	7	-10	-12	-4	1.1	0.9	0.9	1.0
SLTM	Q9NWH9	441	7	-14	-12	1	1.1	0.9	0.9	1.0
BCLAF1	Q9NYF8	688	7	-15	-12	-5	1.1	0.9	0.9	1.0
HK2	P52789	368	7	-24	-12	-7	1.1	0.8	0.9	0.9
TRMT61B	Q9BVS5	376	6	-5	-12	-4	1.1	1.0	0.9	1.0
ASB8	Q9H765	237	6	-16	-12	10	1.1	0.9	0.9	1.1
MYCBP2	O75592	273	6	-16	-12	-25	1.1	0.9	0.9	0.8
SCYL1	Q96KG9	88	6	-19	-12	22	1.1	0.8	0.9	1.3
RPS27A	P62979	149	6	-10	-12	-10	1.1	0.9	0.9	0.9
FAM126A	Q9BYI3	300	6	-11	-12	15	1.1	0.9	0.9	1.2
ARFGEF1	Q9Y6D6	1526	6	-12	-12	-9	1.1	0.9	0.9	0.9
PHF5A	Q7RTV0	85	5	-8	-12	-1	1.1	0.9	0.9	1.0
TXN2	Q99757	93	5	-13	-12	2	1.1	0.9	0.9	1.0
AIFM1	O95831	441	5	-25	-12	-4	1.0	0.8	0.9	1.0
RNH1	P13489	266	5	-41	-12	-12	1.0	0.7	0.9	0.9
FUK	Q8N0W3	484	4	-9	-12	-9	1.0	0.9	0.9	0.9
AMPD2	Q01433	230	4	-11	-12	-4	1.0	0.9	0.9	1.0
TPP2	P29144	342	4	-12	-12	-4	1.0	0.9	0.9	1.0
EPB41L2	O43491	314	4	-14	-12	-12	1.0	0.9	0.9	0.9
MAST4	O15021	1512	4	-15	-12	5	1.0	0.9	0.9	1.0
PPP1R11	O60927	61	4	-16	-12	-17	1.0	0.9	0.9	0.9
NUMB	P49757	176	4	-5	-12	-11	1.0	1.0	0.9	0.9
PFAS	O15067	318	4	-7	-12	2	1.0	0.9	0.9	1.0
RELB	Q01201	144	3	-12	-12	-18	1.0	0.9	0.9	0.8
HSPA1B	P0DMV9	603	3	-27	-12	5	1.0	0.8	0.9	1.0
RLF	Q13129	453	3	-28	-12	-28	1.0	0.8	0.9	0.8
GCN1	Q92616	939	3	-3	-12	-18	1.0	1.0	0.9	0.9
IL16	Q14005	1016	3	-5	-12	-18	1.0	1.0	0.9	0.9
PRRC2B	Q5JSZ5	1121	3	-12	-12	-19	1.0	0.9	0.9	0.8

RAP1GDS1	P52306	117	3	-16	-12	-11	1.0	0.9	0.9	0.9
DHX9	Q08211	242	3	-22	-12	3	1.0	0.8	0.9	1.0
AGPS	O00116	413	3	-22	-12	-4	1.0	0.8	0.9	1.0
SLFN14	P0C7P3	668	2	2	-12	-5	1.0	1.0	0.9	1.0
C19orf35	Q6ZS72	165	2	-5	-12	-12	1.0	1.0	0.9	0.9
PTPN1	P18031	344	2	-12	-12	-8	1.0	0.9	0.9	0.9
WDR81	Q562E7	1256	2	2	-12	-2	1.0	1.0	0.9	1.0
BANF1	O75531	67	2	-10	-12	-2	1.0	0.9	0.9	1.0
SMYD5	Q6GMV2	119	2	-14	-12	-4	1.0	0.9	0.9	1.0
RFXAP	O00287	185	2	-17	-12	-10	1.0	0.9	0.9	0.9
RABGAP1L	Q5R372	248	2	-18	-12	-17	1.0	0.9	0.9	0.9
AGL	P35573	502	1	-2	-12	2	1.0	1.0	0.9	1.0
DTX3L	Q8TDB6	406	1	-7	-12	-14	1.0	0.9	0.9	0.9
FABP5	Q01469	127	1	-12	-12	-5	1.0	0.9	0.9	1.0
RHOT2	Q8IXI1	543	1	-21	-12	-3	1.0	0.8	0.9	1.0
ACOT7	O00154	100	1	-25	-12	-7	1.0	0.8	0.9	0.9
LANCL2	Q9NS86	121	1	-5	-12	-18	1.0	1.0	0.9	0.8
NT5C2	P49902	181	1	-10	-12	-12	1.0	0.9	0.9	0.9
GART	P22102	298	1	-15	-12	2	1.0	0.9	0.9	1.0
IVNS1ABP	Q9Y6Y0	52	1	-20	-12	-26	1.0	0.8	0.9	0.8
BAZ1B	Q9UIG0	338	0	-9	-12	-12	1.0	0.9	0.9	0.9
DDX19A	Q9NUU7	392	0	-11	-12	-6	1.0	0.9	0.9	0.9
DDX19B	Q9UMR2	393	0	-11	-12	-6	1.0	0.9	0.9	0.9
CAPNS1	P04632	144	0	-13	-12	0	1.0	0.9	0.9	1.0
PRPS2	P11908	91	0	-17	-12	-4	1.0	0.9	0.9	1.0
PRPS1	P60891	91	0	-17	-12	-4	1.0	0.9	0.9	1.0
KANSL3	Q9P2N6	301	0	-18	-12	6	1.0	0.8	0.9	1.1
MYD88	Q99836	280	0	-21	-12	-20	1.0	0.8	0.9	0.8
SBNO2	Q9Y2G9	501	-1	-9	-12	-16	1.0	0.9	0.9	0.9
ZMIZ1	Q9ULJ6	17	-1	-13	-12	-10	1.0	0.9	0.9	0.9
NCF1	P14598	378	-1	-13	-12	-18	1.0	0.9	0.9	0.9
INF2	Q27J81	898	-1	-18	-12	-14	1.0	0.8	0.9	0.9
IQGAP1	P46940	660	-1	-20	-12	-8	1.0	0.8	0.9	0.9
COPS7A	Q9UBW8	110	-1	-25	-12	-3	1.0	0.8	0.9	1.0
SCRN2	Q96FV2	277	-1	-10	-12	-4	1.0	0.9	0.9	1.0
SDHC	Q99643	107	-1	-17	-12	-5	1.0	0.9	0.9	1.0
BLVRA	P53004	204	-2	-10	-12	-12	1.0	0.9	0.9	0.9
GSS	P48637	294	-2	-13	-12	-8	1.0	0.9	0.9	0.9
DRG1	Q9Y295	195	-2	-14	-12	-10	1.0	0.9	0.9	0.9
ECI1	P42126	173	-2	-14	-12	-20	1.0	0.9	0.9	0.8
RUFY1	Q96T51	81	-2	-17	-12	-15	1.0	0.9	0.9	0.9
GOLGB1	Q14789	3070	-2	-22	-12	-22	1.0	0.8	0.9	0.8
POP1	Q99575	530	-2	-24	-12	-1	1.0	0.8	0.9	1.0
UROS	P10746	119	-2	-30	-12	-28	1.0	0.8	0.9	0.8
SMARCC2	Q8TAQ2	635	-2	-35	-12	4	1.0	0.7	0.9	1.0
SMARCC1	Q92922	657	-2	-35	-12	4	1.0	0.7	0.9	1.0
ARAF	P10398	538	-2	-6	-12	-5	1.0	0.9	0.9	1.0
C3orf38	Q5JPI3	259	-2	-10	-12	-10	1.0	0.9	0.9	0.9
ZFP36	P26651	109	-2	-12	-12	-8	1.0	0.9	0.9	0.9
SARS	P49591	395	-2	-17	-12	-2	1.0	0.9	0.9	1.0
HK3	P52790	634	-2	-18	-12	13	1.0	0.8	0.9	1.1
MIF	P14174	81	-2	-20	-12	-2	1.0	0.8	0.9	1.0
CYFIP1	Q7L576	519	-2	-23	-12	7	1.0	0.8	0.9	1.1

SERPINB2	P05120	161	-3	-7	-12	-16	1.0	0.9	0.9	0.9
SMUG1	Q53HV7	131	-3	-9	-12	-10	1.0	0.9	0.9	0.9
ASUN	Q9NVM9	406	-3	-9	-12	-5	1.0	0.9	0.9	1.0
ZBTB7A	O95365	123	-3	-13	-12	-20	1.0	0.9	0.9	0.8
POC5	Q8NA72	494	-3	-16	-12	-7	1.0	0.9	0.9	0.9
NBAS	A2RRP1	1634	-3	-17	-12	27	1.0	0.9	0.9	1.4
PYGB	P11216	808	-3	-24	-12	-2	1.0	0.8	0.9	1.0
ADH5	P11766	111	-3	-8	-12	5	1.0	0.9	0.9	1.0
GAMT	Q14353	91	-3	-10	-12	-9	1.0	0.9	0.9	0.9
CORO1B	Q9BR76	332	-3	-12	-12	-3	1.0	0.9	0.9	1.0
PLEKHO2	Q8TD55	388	-3	-15	-12	-17	1.0	0.9	0.9	0.9
LIMS1	P48059	222	-3	-20	-12	-15	1.0	0.8	0.9	0.9
LIMS2	Q7Z4I7	227	-3	-20	-12	-15	1.0	0.8	0.9	0.9
RECQL5	O94762	434	-3	-23	-12	6	1.0	0.8	0.9	1.1
GCDH	Q92947	232	-3	-25	-12	2	1.0	0.8	0.9	1.0
LRCH1	Q9Y2L9	276	-3	-32	-12	-6	1.0	0.8	0.9	0.9
POLD2	P49005	83	-4	-12	-12	-11	1.0	0.9	0.9	0.9
CPOX	P36551	198	-4	-14	-12	-10	1.0	0.9	0.9	0.9
GMDS	O60547	142	-4	-15	-12	-10	1.0	0.9	0.9	0.9
TLN1	Q9Y490	1199	-4	-19	-12	-4	1.0	0.8	0.9	1.0
DHX38	Q92620	618	-4	-11	-12	-12	1.0	0.9	0.9	0.9
ZNF276	Q8N554	529	-4	-13	-12	-3	1.0	0.9	0.9	1.0
COMT	P21964	241	-4	-14	-12	-7	1.0	0.9	0.9	0.9
DHX36	Q9H2U1	135	-4	-14	-12	-16	1.0	0.9	0.9	0.9
ABTB1	Q969K4	62	-4	-18	-12	-32	1.0	0.9	0.9	0.8
GFPT1	Q06210	264	-4	-22	-12	-19	1.0	0.8	0.9	0.8
RRP7A	Q9Y3A4	70	-4	-33	-12	-9	1.0	0.8	0.9	0.9
AKT2	P31751	311	-5	-11	-12	-22	1.0	0.9	0.9	0.8
MKI67	P46013	1285	-5	-11	-12	-25	1.0	0.9	0.9	0.8
ADCY7	P51828	904	-5	-14	-12	-10	1.0	0.9	0.9	0.9
SF3B1	O75533	1244	-5	-16	-12	5	1.0	0.9	0.9	1.0
UBA7	P41226	145	-5	-19	-12	-5	1.0	0.8	0.9	1.0
WDR70	Q9NW82	229	-5	-11	-12	-8	1.0	0.9	0.9	0.9
PCM1	Q15154	1585	-6	-7	-12	-6	0.9	0.9	0.9	0.9
FRYL	O94915	372	-6	-8	-12	-8	0.9	0.9	0.9	0.9
PRF1	P14222	377	-6	-11	-12	-11	0.9	0.9	0.9	0.9
HDAC6	Q9UBN7	572	-6	-12	-12	-7	0.9	0.9	0.9	0.9
ZFYVE26	Q68DK2	60	-6	-13	-12	-9	0.9	0.9	0.9	0.9
PSMC3	P17980	396	-6	-19	-12	3	0.9	0.8	0.9	1.0
PFAS	O15067	584	-6	-23	-12	-7	0.9	0.8	0.9	0.9
TMEM63A	O94886	791	-6	-9	-12	-6	0.9	0.9	0.9	0.9
XRCC6	P12956	398	-6	-21	-12	-1	0.9	0.8	0.9	1.0
MAVS	Q7Z434	46	-7	-3	-12	-6	0.9	1.0	0.9	0.9
RELA	Q04206	105	-7	-15	-12	-9	0.9	0.9	0.9	0.9
ANKRD54	Q6NXT1	265	-7	-16	-12	-7	0.9	0.9	0.9	0.9
DARS	P14868	334	-7	-18	-12	-7	0.9	0.9	0.9	0.9
GNAI2	P04899	66	-7	-24	-12	13	0.9	0.8	0.9	1.1
DDX56	Q9NY93	185	-7	-10	-12	-3	0.9	0.9	0.9	1.0
Uncharacteri	A0A0J9YVX5	171	-7	-10	-12	-15	0.9	0.9	0.9	0.9
SIGIRR	Q6IA17	174	-7	-12	-12	7	0.9	0.9	0.9	1.1
CORO1A	P31146	195	-7	-15	-12	-3	0.9	0.9	0.9	1.0
UTY	O14607	511	-7	-15	-12	-19	0.9	0.9	0.9	0.8
MCMBP	Q9BTE3	325	-7	-17	-12	23	0.9	0.9	0.9	1.3

AP3M1	Q9Y2T2	209	-7	-24	-12	-10	0.9	0.8	0.9	0.9
CYLD	Q9NQC7	909	-8	-13	-12	-10	0.9	0.9	0.9	0.9
SYNE2	Q8WXH0	2993	-8	1	-12	2	0.9	1.0	0.9	1.0
EDC4	Q6P2E9	1130	-8	-19	-12	-10	0.9	0.8	0.9	0.9
SUN2	Q9UH99	601	-8	-20	-12	-10	0.9	0.8	0.9	0.9
SUPT20H	Q8NEM7	298	-8	-22	-12	-16	0.9	0.8	0.9	0.9
SH3BP5L	Q7L8J4	201	-9	-5	-12	-15	0.9	1.0	0.9	0.9
DOCK2	Q92608	1117	-9	-10	-12	-7	0.9	0.9	0.9	0.9
UBE4A	Q14139	398	-9	-6	-12	-21	0.9	0.9	0.9	0.8
NRDE2	Q9H7Z3	890	-9	-12	-12	-15	0.9	0.9	0.9	0.9
DDX1	Q92499	122	-9	-25	-12	-11	0.9	0.8	0.9	0.9
ERH	P84090	28	-9	-28	-12	-1	0.9	0.8	0.9	1.0
RNF213	Q63HN8	4017	-10	8	-12	6	0.9	1.1	0.9	1.1
TRMT1	Q9NXH9	621	-10	-12	-12	-6	0.9	0.9	0.9	0.9
LTN1	O94822	723	-10	-13	-12	-15	0.9	0.9	0.9	0.9
FAM188A	Q9H8M7	27	-10	-14	-12	-8	0.9	0.9	0.9	0.9
PAPSS1	O43252	165	-10	-6	-12	5	0.9	0.9	0.9	1.1
RNF126	Q9BV68	15	-10	-13	-12	-12	0.9	0.9	0.9	0.9
PPWD1	Q96BP3	512	-10	-16	-12	4	0.9	0.9	0.9	1.0
SPEN	Q96T58	1066	-10	-20	-12	-32	0.9	0.8	0.9	0.8
BLMH	Q13867	326	-10	-25	-12	-12	0.9	0.8	0.9	0.9
GPX1	P07203	78	-11	-18	-12	-9	0.9	0.8	0.9	0.9
FLNB	O75369	991	-11	-28	-12	-14	0.9	0.8	0.9	0.9
ARCN1	P48444	479	-11	-36	-12	-9	0.9	0.7	0.9	0.9
VPS13C	Q709C8	1613	-11	-12	-12	-17	0.9	0.9	0.9	0.9
HMGB2	P26583	106	-12	-168	-12	-1	0.9	0.4	0.9	1.0
RABGGTA	Q92696	342	-12	-10	-12	-15	0.9	0.9	0.9	0.9
ATP5O	P48047	141	-12	-13	-12	-9	0.9	0.9	0.9	0.9
TNFAIP3	P21580	54	-12	-19	-12	-16	0.9	0.8	0.9	0.9
RBM6	P78332	559	-12	-41	-12	-25	0.9	0.7	0.9	0.8
FAM120A	Q9NZB2	669	-13	-15	-12	-9	0.9	0.9	0.9	0.9
EDC4	Q6P2E9	1054	-13	-15	-12	8	0.9	0.9	0.9	1.1
PHKB	Q93100	280	-13	-16	-12	-11	0.9	0.9	0.9	0.9
ADI1	Q9BV57	70	-13	-25	-12	-34	0.9	0.8	0.9	0.7
KAT8	Q9H7Z6	416	-13	-26	-12	8	0.9	0.8	0.9	1.1
GPS1	Q13098	67	-13	-16	-12	-6	0.9	0.9	0.9	0.9
TRIO	O75962	1717	-13	-18	-12	-16	0.9	0.8	0.9	0.9
DENND2C	Q68D51	657	-13	-26	-12	-11	0.9	0.8	0.9	0.9
ALKBH5	Q6P6C2	378	-14	-18	-12	-25	0.9	0.9	0.9	0.8
ZBTB14	O43829	124	-14	-21	-12	-21	0.9	0.8	0.9	0.8
PARN	O95453	205	-14	-23	-12	-6	0.9	0.8	0.9	0.9
TNFAIP3	P21580	767	-15	-17	-12	-36	0.9	0.9	0.9	0.7
ZNF563	Q8TA94	42	-15	-1	-12	-11	0.9	1.0	0.9	0.9
ACSL4	O60488	564	-16	-16	-12	-36	0.9	0.9	0.9	0.7
IL17RA	Q96F46	703	-18	-8	-12	-17	0.8	0.9	0.9	0.9
CPSF2	Q9P2I0	765	-18	-10	-12	-15	0.8	0.9	0.9	0.9
EPPK1	A0A087X1U6	406	-20	-14	-12	-15	0.8	0.9	0.9	0.9
ZYX	Q15942	473	-22	-18	-12	-1	0.8	0.9	0.9	1.0
IBTK	Q9P2D0	522	-22	-32	-12	-7	0.8	0.8	0.9	0.9
PPP6R1	Q9UPN7	795	-24	-22	-12	-24	0.8	0.8	0.9	0.8
ZNF48	Q96MX3	288	31	25	-13	19	1.4	1.3	0.9	1.2
GEMIN5	Q8TEQ6	1051	16	-19	-13	-9	1.2	0.8	0.9	0.9
NGLY1	Q96IV0	393	14	-4	-13	-10	1.2	1.0	0.9	0.9

WDR7	Q9Y4E6	1387	13	-7	-13	-3	1.1	0.9	0.9	1.0
RRP12	Q5JTH9	662	13	-9	-13	13	1.1	0.9	0.9	1.1
AP2B1	P63010	391	13	-25	-13	-1	1.1	0.8	0.9	1.0
AP1B1	Q10567	391	13	-25	-13	-1	1.1	0.8	0.9	1.0
VWA8	A3KMH1	162	11	-9	-13	-5	1.1	0.9	0.9	1.0
PAXIP1	Q6ZW49	740	11	3	-13	-10	1.1	1.0	0.9	0.9
INPP4A	Q96PE3	286	11	0	-13	2	1.1	1.0	0.9	1.0
PDHA1	P08559	41	10	-16	-13	1	1.1	0.9	0.9	1.0
POR	P16435	363	9	8	-13	-5	1.1	1.1	0.9	1.0
HSPA9	P38646	487	9	-7	-13	3	1.1	0.9	0.9	1.0
TBC1D1	Q86TI0	75	9	-7	-13	1	1.1	0.9	0.9	1.0
MYL9	P24844	109	9	-22	-13	-2	1.1	0.8	0.9	1.0
AKAP9	Q99996	2137	9	-5	-13	-5	1.1	1.0	0.9	1.0
NAIF1	Q69YI7	61	9	-9	-13	6	1.1	0.9	0.9	1.1
KDM5A	P29375	598	9	-10	-13	-16	1.1	0.9	0.9	0.9
RBM12	Q9NTZ6	545	9	-13	-13	-4	1.1	0.9	0.9	1.0
NFX1	Q12986	593	8	-22	-13	-3	1.1	0.8	0.9	1.0
OGT	O15294	962	8	-26	-13	-7	1.1	0.8	0.9	0.9
ZFYVE26	Q68DK2	262	7	-23	-13	-16	1.1	0.8	0.9	0.9
RPAP1	Q9BWH6	1039	7	-7	-13	-8	1.1	0.9	0.9	0.9
GSE1	Q14687	630	7	-12	-13	-8	1.1	0.9	0.9	0.9
LARP1	Q6PKG0	238	6	-12	-13	-8	1.1	0.9	0.9	0.9
PBRM1	Q86U86	1047	6	-12	-13	-20	1.1	0.9	0.9	0.8
KLHDC3	Q9BQ90	213	6	4	-13	0	1.1	1.0	0.9	1.0
ACTB	P60709	285	6	-26	-13	-8	1.1	0.8	0.9	0.9
TRANK1	O15050	882	5	-11	-13	-15	1.1	0.9	0.9	0.9
DNAJC13	O75165	116	5	-15	-13	4	1.1	0.9	0.9	1.0
PSMA5	P28066	76	5	-19	-13	-7	1.1	0.8	0.9	0.9
USP31	Q70CQ4	465	5	-16	-13	-3	1.0	0.9	0.9	1.0
CTNND1	O60716	394	5	-19	-13	-12	1.0	0.8	0.9	0.9
AP2A2	O94973	491	4	-9	-13	-4	1.0	0.9	0.9	1.0
CWF19L1	Q69YN2	11	4	-18	-13	-10	1.0	0.8	0.9	0.9
RTCB	Q9Y3I0	485	4	-13	-13	-23	1.0	0.9	0.9	0.8
HSP90AA1	P07900	420	4	-58	-13	-9	1.0	0.6	0.9	0.9
ZMYM4	Q5VZL5	752	3	-4	-13	-8	1.0	1.0	0.9	0.9
PARP14	Q460N5	522	3	-9	-13	-10	1.0	0.9	0.9	0.9
UBL4A	P11441	13	3	-13	-13	-1	1.0	0.9	0.9	1.0
TRIM38	O00635	188	3	-19	-13	-2	1.0	0.8	0.9	1.0
STAG2	Q8N3U4	535	3	-11	-13	0	1.0	0.9	0.9	1.0
OCIAD2	Q56VL3	134	3	-12	-13	-5	1.0	0.9	0.9	1.0
GSR	P00390	107	3	-18	-13	-1	1.0	0.8	0.9	1.0
GTF2E1	P29083	42	3	-19	-13	-15	1.0	0.8	0.9	0.9
FES	P07332	689	3	-36	-13	-14	1.0	0.7	0.9	0.9
SUPT16H	Q9Y5B9	153	3	-51	-13	-14	1.0	0.7	0.9	0.9
PPIA	P62937	62	2	-19	-13	-12	1.0	0.8	0.9	0.9
PPIAL4A	Q9Y536	62	2	-19	-13	-12	1.0	0.8	0.9	0.9
LARP1	Q6PKG0	1054	2	-20	-13	-27	1.0	0.8	0.9	0.8
SMARCA4	P51532	423	2	-10	-13	-13	1.0	0.9	0.9	0.9
NUP153	P49790	404	2	-14	-13	-16	1.0	0.9	0.9	0.9
PYHIN1	Q6K0P9	366	2	-15	-13	-2	1.0	0.9	0.9	1.0
RNH1	P13489	30	2	-17	-13	4	1.0	0.9	0.9	1.0
ROCK2	O75116	330	2	-19	-13	-13	1.0	0.8	0.9	0.9
ROCK1	Q13464	314	2	-19	-13	-13	1.0	0.8	0.9	0.9

PTPN9	P43378	338	2	-26	-13	1	1.0	0.8	0.9	1.0
PRKCSH	P14314	471	1	-7	-13	-7	1.0	0.9	0.9	0.9
CPT2	P23786	489	1	-31	-13	10	1.0	0.8	0.9	1.1
XRN2	Q9H0D6	736	1	0	-13	1	1.0	1.0	0.9	1.0
CBL	P22681	572	1	-6	-13	-2	1.0	0.9	0.9	1.0
PYGB	P11216	437	1	-8	-13	-8	1.0	0.9	0.9	0.9
FRYL	O94915	288	1	-16	-13	-2	1.0	0.9	0.9	1.0
TARS	P26639	343	1	-20	-13	-5	1.0	0.8	0.9	1.0
PARP1	P09874	311	1	-23	-13	-6	1.0	0.8	0.9	0.9
RPL17-C18or	A0A0A6YYL6	57	0	-5	-13	-14	1.0	1.0	0.9	0.9
ZCCHC6	Q5VYS8	417	0	-10	-13	-9	1.0	0.9	0.9	0.9
METTL16	Q86W50	253	0	-15	-13	-14	1.0	0.9	0.9	0.9
MMS22L	Q6ZRQ5	1068	0	-19	-13	3	1.0	0.8	0.9	1.0
STXBP3	O00186	90	-1	-9	-13	-3	1.0	0.9	0.9	1.0
ARHGAP25	P42331	302	-1	-15	-13	22	1.0	0.9	0.9	1.3
IMPA1	P29218	141	-1	-17	-13	-9	1.0	0.9	0.9	0.9
ICAM3	P32942	423	-1	-18	-13	-10	1.0	0.8	0.9	0.9
NOL11	Q9H8H0	578	-1	-20	-13	-19	1.0	0.8	0.9	0.8
GPATCH8	Q9UKJ3	795	-1	-9	-13	3	1.0	0.9	0.9	1.0
SRSF11	Q05519	455	-1	-10	-13	-10	1.0	0.9	0.9	0.9
GSR	P00390	102	-1	-22	-13	-15	1.0	0.8	0.9	0.9
ATL3	Q6DD88	429	-1	-32	-13	-9	1.0	0.8	0.9	0.9
PAXIP1	Q6ZW49	136	-1	-36	-13	15	1.0	0.7	0.9	1.2
DGKA	P23743	432	-2	1	-13	-24	1.0	1.0	0.9	0.8
NCEH1	Q6PIU2	186	-2	-12	-13	-5	1.0	0.9	0.9	1.0
BAZ1B	Q9UIG0	1420	-2	-13	-13	-9	1.0	0.9	0.9	0.9
HSPA9	P38646	66	-2	-20	-13	-7	1.0	0.8	0.9	0.9
ING1	Q9UK53	216	-2	-18	-13	4	1.0	0.9	0.9	1.0
STAG2	Q8N3U4	711	-2	-19	-13	-12	1.0	0.8	0.9	0.9
SDHA	P31040	357	-2	-20	-13	-7	1.0	0.8	0.9	0.9
RAP1GAP2	Q684P5	444	-2	-21	-13	-16	1.0	0.8	0.9	0.9
MED1	Q15648	373	-2	-24	-13	-32	1.0	0.8	0.9	0.8
NUP88	Q99567	391	-3	-6	-13	2	1.0	0.9	0.9	1.0
NFKB1	P19838	61	-3	-8	-13	-11	1.0	0.9	0.9	0.9
MARCH6	O60337	28	-3	-11	-13	-12	1.0	0.9	0.9	0.9
SYAP1	Q96A49	283	-3	-12	-13	-7	1.0	0.9	0.9	0.9
IQGAP2	Q13576	276	-3	-13	-13	-16	1.0	0.9	0.9	0.9
ADAR	P55265	773	-3	-16	-13	-15	1.0	0.9	0.9	0.9
NBAS	A2RRP1	862	-3	-21	-13	-2	1.0	0.8	0.9	1.0
SEC22A	Q96IW7	33	-3	-25	-13	-2	1.0	0.8	0.9	1.0
HPS1	Q92902	561	-4	-11	-13	-10	1.0	0.9	0.9	0.9
FLNA	P21333	1920	-4	-13	-13	-10	1.0	0.9	0.9	0.9
HMHA1	Q92619	324	-4	-19	-13	16	1.0	0.8	0.9	1.2
AKAP9	Q99996	1966	-4	-20	-13	-13	1.0	0.8	0.9	0.9
RPL5	P46777	100	-4	-21	-13	9	1.0	0.8	0.9	1.1
AIMP2	Q13155	205	-4	-10	-13	-9	1.0	0.9	0.9	0.9
PDE4DIP	Q5VU43	603	-4	-13	-13	-11	1.0	0.9	0.9	0.9
ECM29	Q5VYK3	1503	-4	-15	-13	-13	1.0	0.9	0.9	0.9
TRANK1	O15050	646	-4	-15	-13	-20	1.0	0.9	0.9	0.8
GTPBP2	Q9BX10	457	-5	-6	-13	-13	1.0	0.9	0.9	0.9
CISD2	Q8N5K1	92	-5	-11	-13	-16	1.0	0.9	0.9	0.9
FAM193A	P78312	1237	-5	-13	-13	-20	1.0	0.9	0.9	0.8
THOC1	Q96FV9	49	-5	-17	-13	-22	1.0	0.9	0.9	0.8

GART	P22102	93	-5	-18	-13	-6	1.0	0.8	0.9	0.9
DICER1	Q9UPY3	1621	-5	-22	-13	-35	1.0	0.8	0.9	0.7
MRPL35	Q9NZE8	150	-5	-29	-13	-22	1.0	0.8	0.9	0.8
RHOT1	Q8IXI2	522	-5	-10	-13	2	1.0	0.9	0.9	1.0
C7orf43	Q8WVR3	418	-5	-13	-13	-14	1.0	0.9	0.9	0.9
VDAC2	P45880	76	-5	-13	-13	-12	1.0	0.9	0.9	0.9
LPXN	O60711	199	-5	-17	-13	-14	1.0	0.9	0.9	0.9
ATXN10	Q9UBB4	95	-5	-19	-13	-5	1.0	0.8	0.9	1.0
NCOA5	Q9HCD5	200	-5	-21	-13	-4	1.0	0.8	0.9	1.0
SGPP1	Q9BX95	399	-5	-21	-13	-10	1.0	0.8	0.9	0.9
CBX5	P45973	59	-5	-22	-13	-9	1.0	0.8	0.9	0.9
SMPD4	Q9NXE4	737	-5	-55	-13	-15	1.0	0.6	0.9	0.9
SERGEF	Q9UGK8	157	-6	-17	-13	-18	0.9	0.9	0.9	0.8
PPP1R35	Q8TAP8	142	-6	-18	-13	-11	0.9	0.9	0.9	0.9
EARS2	Q5JPH6	386	-6	-9	-13	-17	0.9	0.9	0.9	0.9
RAPGEF1	Q13905	958	-6	-10	-13	-4	0.9	0.9	0.9	1.0
RBBP5	Q15291	233	-6	-15	-13	-6	0.9	0.9	0.9	0.9
USP24	Q9UPU5	2160	-6	-17	-13	0	0.9	0.9	0.9	1.0
TDRD7	Q8NHU6	568	-6	-19	-13	-17	0.9	0.8	0.9	0.9
AGPS	O00116	349	-7	-13	-13	-5	0.9	0.9	0.9	1.0
MYCBP2	O75592	4280	-7	-15	-13	-8	0.9	0.9	0.9	0.9
NDUFS1	P28331	554	-7	-17	-13	-17	0.9	0.9	0.9	0.9
VPS13C	Q709C8	2177	-7	-31	-13	-42	0.9	0.8	0.9	0.7
FAM102A	Q5T9C2	151	-7	-1	-13	-14	0.9	1.0	0.9	0.9
SSC5D	A1L4H1	815	-7	-8	-13	-23	0.9	0.9	0.9	0.8
SSC5D	A1L4H1	810	-7	-8	-13	-23	0.9	0.9	0.9	0.8
PAK1IP1	Q9NWT1	298	-7	-22	-13	-5	0.9	0.8	0.9	1.0
CCNT2	O60583	26	-7	-30	-13	-17	0.9	0.8	0.9	0.9
LIN37	Q96GY3	176	-7	-32	-13	-18	0.9	0.8	0.9	0.9
RABGAP1	Q9Y3P9	155	-8	-10	-13	-5	0.9	0.9	0.9	1.0
MANBA	O00462	748	-8	-19	-13	-8	0.9	0.8	0.9	0.9
SAV1	Q9H4B6	266	-8	3	-13	8	0.9	1.0	0.9	1.1
BMS1	Q14692	101	-8	-28	-13	-24	0.9	0.8	0.9	0.8
KPNB1	Q14974	228	-9	-11	-13	-6	0.9	0.9	0.9	0.9
MDN1	Q9NU22	979	-9	-19	-13	-11	0.9	0.8	0.9	0.9
RAI1	Q7Z5J4	239	-9	-4	-13	-26	0.9	1.0	0.9	0.8
ZNHIT6	Q9NWK9	211	-9	-45	-13	-25	0.9	0.7	0.9	0.8
CARD6	Q9BX69	185	-10	-19	-13	-9	0.9	0.8	0.9	0.9
KAT6A	Q92794	1096	-10	-20	-13	-22	0.9	0.8	0.9	0.8
USP40	Q9NVE5	500	-10	-20	-13	-22	0.9	0.8	0.9	0.8
HLA-A	P16188	283	-10	-26	-13	-23	0.9	0.8	0.9	0.8
HLA-A	P16190	283	-10	-26	-13	-23	0.9	0.8	0.9	0.8
HLA-B	P30462	283	-10	-26	-13	-23	0.9	0.8	0.9	0.8
HLA-A	P30457	283	-10	-26	-13	-23	0.9	0.8	0.9	0.8
HLA-B	P30479	283	-10	-26	-13	-23	0.9	0.8	0.9	0.8
HLA-B	Q95365	283	-10	-26	-13	-23	0.9	0.8	0.9	0.8
SQSTM1	Q13501	26	-10	-9	-13	-26	0.9	0.9	0.9	0.8
RCSD1	Q6JBY9	296	-11	-18	-13	-32	0.9	0.8	0.9	0.8
TBL3	Q12788	358	-11	-19	-13	3	0.9	0.8	0.9	1.0
ADCY7	P51828	895	-12	-16	-13	-14	0.9	0.9	0.9	0.9
UBR1	Q8IWV7	1369	-12	-19	-13	-14	0.9	0.8	0.9	0.9
IRF5	Q13568	272	-12	-23	-13	-14	0.9	0.8	0.9	0.9
LRRC16A	Q5VZK9	1106	-12	-15	-13	-31	0.9	0.9	0.9	0.8

GNPNAT1	Q96EK6	128	-12	-32	-13	-21	0.9	0.8	0.9	0.8
DCTN4	Q9UJW0	30	-13	-5	-13	1	0.9	1.0	0.9	1.0
DDX3X	O00571	298	-13	-15	-13	-4	0.9	0.9	0.9	1.0
DDX3Y	O15523	296	-13	-15	-13	-4	0.9	0.9	0.9	1.0
NSUN5	Q96P11	343	-13	-18	-13	-9	0.9	0.9	0.9	0.9
RNF31	Q96EP0	473	-13	-13	-13	-30	0.9	0.9	0.9	0.8
POGZ	Q7Z3K3	547	-13	-20	-13	-27	0.9	0.8	0.9	0.8
UTY	O14607	381	-13	-30	-13	-28	0.9	0.8	0.9	0.8
ZFR	Q96KR1	844	-14	-23	-13	-10	0.9	0.8	0.9	0.9
NPM1	P06748	275	-14	-29	-13	-18	0.9	0.8	0.9	0.8
MARCH5	Q9NX47	46	-14	-7	-13	12	0.9	0.9	0.9	1.1
TRAPPC1	Q9Y5R8	115	-15	-6	-13	-18	0.9	0.9	0.9	0.8
EP300	Q09472	1176	-15	-13	-13	-18	0.9	0.9	0.9	0.9
LARP7	Q4G0J3	438	-15	-26	-13	-30	0.9	0.8	0.9	0.8
HSPE1-MOB4	S4R3N1	155	-16	-15	-13	-7	0.9	0.9	0.9	0.9
PPIAL4A	Q9Y536	52	-16	-18	-13	39	0.9	0.9	0.9	1.6
SEC16A	O15027	1619	-16	-20	-13	-15	0.9	0.8	0.9	0.9
ABTB1	Q969K4	72	-16	-24	-13	-24	0.9	0.8	0.9	0.8
STEAP3	Q658P3	140	-16	-16	-13	-3	0.9	0.9	0.9	1.0
MAP3K5	Q99683	835	-16	-30	-13	-12	0.9	0.8	0.9	0.9
PHF3	Q92576	1673	-17	-23	-13	-22	0.9	0.8	0.9	0.8
CLIC2	O15247	30	-18	-18	-13	-11	0.9	0.8	0.9	0.9
TSC22D1	Q15714	426	-18	-29	-13	-18	0.8	0.8	0.9	0.9
IMPDH1	P20839	468	-18	-49	-13	-2	0.8	0.7	0.9	1.0
PGLS	O95336	78	-19	-35	-13	-13	0.8	0.7	0.9	0.9
NAT10	Q9H0A0	658	-19	-17	-13	-13	0.8	0.9	0.9	0.9
LSP1	P33241	36	-21	-11	-13	-12	0.8	0.9	0.9	0.9
SIPA1	Q96FS4	340	-22	-28	-13	-18	0.8	0.8	0.9	0.9
TOMM70	O94826	544	-22	-48	-13	-32	0.8	0.7	0.9	0.8
FTH1	P02794	131	-23	-28	-13	-2	0.8	0.8	0.9	1.0
YTHDC2	Q9H6S0	788	-24	-27	-13	3	0.8	0.8	0.9	1.0
SH2D2A	Q9NP31	116	-39	-32	-13	-16	0.7	0.8	0.9	0.9
PROSC	O94903	86	-53	-35	-13	-6	0.7	0.7	0.9	0.9
ACYP2	P14621	22	-119	-54	-13	-10	0.5	0.6	0.9	0.9
PEPD	P12955	482	-199	-36	-13	-15	0.3	0.7	0.9	0.9
MED10	Q9BTT4	54	23	-11	-13	-24	1.3	0.9	0.9	0.8
PTRH2	Q9Y3E5	86	20	-19	-13	1	1.3	0.8	0.9	1.0
CROT	Q9UKG9	210	14	4	-13	9	1.2	1.0	0.9	1.1
ACTR3	P61158	189	14	-15	-13	-7	1.2	0.9	0.9	0.9
UBR2	Q8IWV8	280	12	-13	-13	9	1.1	0.9	0.9	1.1
CCT6A	P40227	282	12	-20	-13	-8	1.1	0.8	0.9	0.9
ZMYND11	Q15326	126	11	-12	-13	-6	1.1	0.9	0.9	0.9
VPS11	Q9H270	266	11	-21	-13	-15	1.1	0.8	0.9	0.9
VPS18	Q9P253	780	10	1	-13	27	1.1	1.0	0.9	1.4
FES	P07332	221	10	-3	-13	-3	1.1	1.0	0.9	1.0
RNH1	P13489	409	9	-6	-13	-4	1.1	0.9	0.9	1.0
CHID1	Q9BWS9	68	9	-26	-13	15	1.1	0.8	0.9	1.2
IRF4	Q15306	194	8	8	-13	9	1.1	1.1	0.9	1.1
ELMSAN1	Q6PJG2	502	8	-6	-13	-1	1.1	0.9	0.9	1.0
DBR1	Q9UK59	9	8	-4	-13	-11	1.1	1.0	0.9	0.9
DUS3L	Q96G46	124	8	-12	-13	3	1.1	0.9	0.9	1.0
PNPLA6	Q8IY17	409	8	-13	-13	-19	1.1	0.9	0.9	0.8
HMHA1	Q92619	413	8	-23	-13	-2	1.1	0.8	0.9	1.0

MAP2K5	Q13163	300	7	-22	-13	9	1.1	0.8	0.9	1.1
PUM1	Q14671	1179	7	-41	-13	-10	1.1	0.7	0.9	0.9
SPAG9	O60271	443	6	-9	-13	-7	1.1	0.9	0.9	0.9
UVSSA	Q2YD98	585	5	-15	-13	0	1.1	0.9	0.9	1.0
DYNC1H1	Q14204	2639	5	-27	-13	1	1.1	0.8	0.9	1.0
NUTF2	P61970	114	5	-38	-13	-8	1.1	0.7	0.9	0.9
STK39	Q9UEW8	450	5	6	-13	-14	1.0	1.1	0.9	0.9
EP300	Q09472	1796	5	-3	-13	1	1.0	1.0	0.9	1.0
CREBBP	Q92793	1833	5	-3	-13	1	1.0	1.0	0.9	1.0
SHOC2	Q9UQ13	238	5	-3	-13	-2	1.0	1.0	0.9	1.0
DENR	O43583	44	4	-15	-13	-6	1.0	0.9	0.9	0.9
LIMS1	P48059	300	4	-10	-13	-9	1.0	0.9	0.9	0.9
LIMS2	Q7Z4I7	305	4	-10	-13	-9	1.0	0.9	0.9	0.9
GLTSCR1	Q9NZM4	1083	4	-11	-13	1	1.0	0.9	0.9	1.0
CLASP2	O75122	129	4	-12	-13	-2	1.0	0.9	0.9	1.0
BDH1	Q02338	115	4	-22	-13	-1	1.0	0.8	0.9	1.0
FLII	Q13045	241	3	-10	-13	-7	1.0	0.9	0.9	0.9
MYO9B	Q13459	34	3	-15	-13	-23	1.0	0.9	0.9	0.8
AAMP	Q13685	220	3	-13	-13	-11	1.0	0.9	0.9	0.9
GLE1	Q53GS7	39	3	-21	-13	-16	1.0	0.8	0.9	0.9
F13A1	P00488	410	3	-30	-13	-4	1.0	0.8	0.9	1.0
CKAP5	Q14008	637	2	-16	-13	-4	1.0	0.9	0.9	1.0
GAPDH	P04406	156	2	-16	-13	-6	1.0	0.9	0.9	0.9
FLNA	P21333	1165	2	-27	-13	-7	1.0	0.8	0.9	0.9
SNUPN	O95149	199	2	2	-13	-2	1.0	1.0	0.9	1.0
ERO1A	Q96HE7	99	2	-2	-13	-23	1.0	1.0	0.9	0.8
DIAPH2	O60879	159	2	-10	-13	-3	1.0	0.9	0.9	1.0
SLK	Q9H2G2	358	2	-12	-13	-12	1.0	0.9	0.9	0.9
IDH1	O75874	73	2	-15	-13	-6	1.0	0.9	0.9	0.9
QDPR	P09417	26	2	-18	-13	-2	1.0	0.8	0.9	1.0
NT5C3B	Q969T7	210	2	-20	-13	-21	1.0	0.8	0.9	0.8
SF3B1	O75533	933	2	-21	-13	-8	1.0	0.8	0.9	0.9
NR3C1	P04150	463	2	-26	-13	-12	1.0	0.8	0.9	0.9
NR3C2	P08235	645	2	-26	-13	-12	1.0	0.8	0.9	0.9
DDX3X	O00571	317	2	-38	-13	29	1.0	0.7	0.9	1.4
DDX3Y	O15523	315	2	-38	-13	29	1.0	0.7	0.9	1.4
ADK	P55263	353	1	-14	-13	-3	1.0	0.9	0.9	1.0
TTC5	Q8N0Z6	133	1	-15	-13	1	1.0	0.9	0.9	1.0
MINOS1	Q5TGZ0	13	1	-20	-13	-15	1.0	0.8	0.9	0.9
WDR61	Q9GZS3	303	1	-21	-13	1	1.0	0.8	0.9	1.0
FKBP8	Q14318	274	1	-22	-13	-3	1.0	0.8	0.9	1.0
TES	Q9UGI8	46	1	-8	-13	-4	1.0	0.9	0.9	1.0
GZMH	P20718	170	1	-12	-13	-13	1.0	0.9	0.9	0.9
FER	P16591	344	1	-14	-13	-18	1.0	0.9	0.9	0.9
GABARAPL2	P60520	15	1	-26	-13	-15	1.0	0.8	0.9	0.9
cDNA	B4DLN1	336	0	-4	-13	1	1.0	1.0	0.9	1.0
TKFC	Q3LXA3	155	0	-10	-13	-6	1.0	0.9	0.9	0.9
ME2	P23368	274	0	-18	-13	-2	1.0	0.8	0.9	1.0
AKNA	Q7Z591	818	0	-25	-13	-31	1.0	0.8	0.9	0.8
BPTF	Q12830	1746	-1	-3	-13	-29	1.0	1.0	0.9	0.8
WDFY1	Q8IWB7	401	-1	-8	-13	-7	1.0	0.9	0.9	0.9
CASK	O14936	146	-1	-11	-13	-7	1.0	0.9	0.9	0.9
CBR1	P16152	150	-1	-14	-13	-4	1.0	0.9	0.9	1.0

THBS1	P07996	813	-1	-14	-13	-25	1.0	0.9	0.9	0.8
PPP2R1A	P30153	174	-1	-22	-13	-11	1.0	0.8	0.9	0.9
BANF1	O75531	80	-1	-23	-13	-4	1.0	0.8	0.9	1.0
PRPF4	O43172	342	-1	-25	-13	-9	1.0	0.8	0.9	0.9
ZWILCH	Q9H900	354	-1	0	-13	-16	1.0	1.0	0.9	0.9
HADH	Q16836	211	-1	-20	-13	-3	1.0	0.8	0.9	1.0
PSMA6	P60900	154	-1	-21	-13	-11	1.0	0.8	0.9	0.9
CSRP1	P21291	13	-1	-26	-13	-22	1.0	0.8	0.9	0.8
ATXN10	Q9UBB4	283	-2	-10	-13	0	1.0	0.9	0.9	1.0
METTL7A	Q9H8H3	96	-2	-12	-13	1	1.0	0.9	0.9	1.0
GNL3	Q9BVP2	158	-2	-15	-13	-19	1.0	0.9	0.9	0.8
PTPRC	P08575	1167	-2	-17	-13	-6	1.0	0.9	0.9	0.9
SLFN11	Q7Z7L1	51	-2	-10	-13	-14	1.0	0.9	0.9	0.9
TRMU	O75648	48	-2	-18	-13	-22	1.0	0.9	0.9	0.8
PGK1	P00558	316	-2	-20	-13	-7	1.0	0.8	0.9	0.9
FLNB	O75369	2556	-2	-28	-13	2	1.0	0.8	0.9	1.0
DOCK2	Q92608	1408	-2	-32	-13	-3	1.0	0.8	0.9	1.0
CNKSR1	Q969H4	42	-3	-14	-13	-10	1.0	0.9	0.9	0.9
USP32	Q8NFA0	66	-3	-17	-13	-14	1.0	0.9	0.9	0.9
NDUFV1	P49821	255	-3	-24	-13	-5	1.0	0.8	0.9	1.0
ACTN4	O43707	351	-3	-24	-13	-14	1.0	0.8	0.9	0.9
ACTN1	P12814	332	-3	-24	-13	-14	1.0	0.8	0.9	0.9
SLC3A2	P08195	431	-3	-35	-13	-11	1.0	0.7	0.9	0.9
ZC3H13	Q5T200	51	-3	-10	-13	-11	1.0	0.9	0.9	0.9
TRAPPC9	Q96Q05	317	-3	-11	-13	-12	1.0	0.9	0.9	0.9
HNRNPR	O43390	292	-3	-15	-13	-4	1.0	0.9	0.9	1.0
SYNCRIP	O60506	289	-3	-15	-13	-4	1.0	0.9	0.9	1.0
INPP5D	Q92835	1050	-3	-15	-13	-19	1.0	0.9	0.9	0.8
RPP30	P78346	180	-3	-17	-13	13	1.0	0.9	0.9	1.1
APOBEC3G	Q9HC16	243	-3	-17	-13	-6	1.0	0.9	0.9	0.9
PPIG	Q13427	174	-3	-17	-13	-1	1.0	0.9	0.9	1.0
BPNT1	O95861	249	-3	-23	-13	-16	1.0	0.8	0.9	0.9
TARS2	Q9BW92	322	-4	-3	-13	-6	1.0	1.0	0.9	0.9
COG4	Q9H9E3	163	-4	-19	-13	-8	1.0	0.8	0.9	0.9
LRMP	Q12912	223	-4	-20	-13	-20	1.0	0.8	0.9	0.8
EIF3I	Q13347	99	-4	-23	-13	7	1.0	0.8	0.9	1.1
OLA1	Q9NTK5	75	-4	-27	-13	-8	1.0	0.8	0.9	0.9
GBF1	Q92538	1766	-4	-10	-13	-13	1.0	0.9	0.9	0.9
GPATCH2	Q9NW75	59	-4	-17	-13	-12	1.0	0.9	0.9	0.9
TRIM65	Q6PJ69	15	-4	-18	-13	-14	1.0	0.9	0.9	0.9
CHMP1A	Q9HD42	44	-4	-19	-13	-25	1.0	0.8	0.9	0.8
MYO9B	Q13459	152	-5	2	-13	-24	1.0	1.0	0.9	0.8
EHBP1L1	Q8N3D4	422	-5	-5	-13	-18	1.0	1.0	0.9	0.9
MON2	Q7Z3U7	276	-5	-7	-13	-16	1.0	0.9	0.9	0.9
ERI3	O43414	245	-5	-12	-13	-18	1.0	0.9	0.9	0.9
SAMHD1	Q9Y3Z3	341	-5	-13	-13	-19	1.0	0.9	0.9	0.8
ALDH1B1	P30837	169	-5	-15	-13	-36	1.0	0.9	0.9	0.7
ITPR1	Q14643	61	-5	-5	-13	-9	1.0	1.0	0.9	0.9
FGFR1OP2	Q9NVK5	198	-5	-14	-13	-17	1.0	0.9	0.9	0.9
VWA5A	O00534	624	-5	-23	-13	-32	1.0	0.8	0.9	0.8
DCK	P27707	9	-6	-8	-13	-11	0.9	0.9	0.9	0.9
LCMT1	Q9UIC8	250	-6	-15	-13	-1	0.9	0.9	0.9	1.0
CMIP	Q8IY22	247	-6	-13	-13	-12	0.9	0.9	0.9	0.9

EIF5	P55010	102	-6	-14	-13	-6	0.9	0.9	0.9	0.9
TOR1A	O14656	50	-6	-17	-13	-14	0.9	0.9	0.9	0.9
PRKD3	O94806	719	-6	-22	-13	11	0.9	0.8	0.9	1.1
PRKD2	Q9BZL6	694	-6	-22	-13	11	0.9	0.8	0.9	1.1
PLCE1	Q9P212	106	-6	-23	-13	13	0.9	0.8	0.9	1.1
LGALSL	Q3ZCW2	41	-7	-31	-13	-13	0.9	0.8	0.9	0.9
SPTBN1	Q01082	1900	-7	-12	-13	-6	0.9	0.9	0.9	0.9
TMEM175	Q9BSA9	32	-7	-16	-13	-18	0.9	0.9	0.9	0.9
HABP4	Q5JVS0	236	-7	-21	-13	-3	0.9	0.8	0.9	1.0
FLNA	P21333	1260	-7	-30	-13	-3	0.9	0.8	0.9	1.0
RRAGA	Q7L523	157	-8	-11	-13	-6	0.9	0.9	0.9	0.9
PRKCD	Q05655	28	-8	-13	-13	-7	0.9	0.9	0.9	0.9
THOP1	P52888	350	-8	-19	-13	-7	0.9	0.8	0.9	0.9
DSN1	Q9H410	65	-8	-24	-13	-30	0.9	0.8	0.9	0.8
PXN	P49023	290	-8	-13	-13	-3	0.9	0.9	0.9	1.0
GIGYF2	Q6Y7W6	938	-8	-16	-13	-14	0.9	0.9	0.9	0.9
BTAF1	O14981	936	-8	-17	-13	-11	0.9	0.9	0.9	0.9
MAST2	Q6P0Q8	930	-8	-22	-13	-20	0.9	0.8	0.9	0.8
MATR3	A8MXP9	851	-8	-25	-13	-9	0.9	0.8	0.9	0.9
ACAP2	Q15057	414	-9	-11	-13	-12	0.9	0.9	0.9	0.9
NOC2L	Q9Y3T9	585	-9	-12	-13	-16	0.9	0.9	0.9	0.9
GRAP	Q13588	161	-9	-15	-13	-17	0.9	0.9	0.9	0.9
HDLBP	Q00341	948	-9	-15	-13	-6	0.9	0.9	0.9	0.9
PSMD4	P55036	87	-9	-13	-13	-3	0.9	0.9	0.9	1.0
ABCE1	P61221	227	-10	-18	-13	-13	0.9	0.9	0.9	0.9
TBCD	Q9BTW9	905	-10	-38	-13	-15	0.9	0.7	0.9	0.9
NUP155	O75694	373	-11	-12	-13	-6	0.9	0.9	0.9	0.9
AK2	P54819	92	-11	-25	-13	-6	0.9	0.8	0.9	0.9
C17orf62	Q9BQA9	165	-12	-8	-13	-19	0.9	0.9	0.9	0.8
PRPF4	O43172	263	-12	-13	-13	-16	0.9	0.9	0.9	0.9
PAFAH1B3	Q15102	205	-12	-18	-13	1	0.9	0.8	0.9	1.0
ZC3H12A	Q5D1E8	328	-12	-16	-13	13	0.9	0.9	0.9	1.1
GTF3C1	Q12789	179	-12	-30	-13	-15	0.9	0.8	0.9	0.9
NCAPD3	P42695	867	-13	-7	-13	1	0.9	0.9	0.9	1.0
ZMYM3	Q14202	682	-13	-21	-13	-17	0.9	0.8	0.9	0.9
GBP2	P32456	233	-13	-22	-13	1	0.9	0.8	0.9	1.0
DICER1	Q9UPY3	251	-14	-16	-13	-17	0.9	0.9	0.9	0.9
OGT	O15294	297	-14	-23	-13	-8	0.9	0.8	0.9	0.9
HNRNPL	P14866	404	-14	-34	-13	-6	0.9	0.7	0.9	0.9
AFG3L2	Q9Y4W6	402	-14	-8	-13	-3	0.9	0.9	0.9	1.0
VCL	P18206	313	-15	-16	-13	-1	0.9	0.9	0.9	1.0
CEP135	Q66GS9	615	-16	-23	-13	-30	0.9	0.8	0.9	0.8
ACP2	P11117	349	-17	-22	-13	-4	0.9	0.8	0.9	1.0
USP34	Q70CQ2	2364	-17	-10	-13	7	0.9	0.9	0.9	1.1
MIOS	Q9NXC5	276	-21	-16	-13	-5	0.8	0.9	0.9	1.0
RNF113A	O15541	285	-21	-39	-13	-18	0.8	0.7	0.9	0.9
CAT	P04040	377	-22	-27	-13	-7	0.8	0.8	0.9	0.9
AMMECR1	Q9Y4X0	175	-35	-14	-13	-34	0.7	0.9	0.9	0.7
TPI1	P60174	124	-41	-36	-13	-5	0.7	0.7	0.9	1.0
ETHE1	O95571	34	-42	-25	-13	-8	0.7	0.8	0.9	0.9
LIN7C	Q9NUP9	47	-48	-20	-13	-33	0.7	0.8	0.9	0.8
LONP1	P36776	637	24	-22	-14	16	1.3	0.8	0.9	1.2
GALM	Q96C23	305	23	-15	-14	-2	1.3	0.9	0.9	1.0

GPAT3	Q53EU6	306	20	9	-14	1	1.2	1.1	0.9	1.0
GFRA2	O00451	40	17	-8	-14	4	1.2	0.9	0.9	1.0
GTF3C1	Q12789	320	14	-13	-14	-9	1.2	0.9	0.9	0.9
SKAP2	O75563	185	12	-7	-14	-2	1.1	0.9	0.9	1.0
HPCAL4	Q9UM19	87	12	-10	-14	8	1.1	0.9	0.9	1.1
SMAD2	Q15796	161	11	-23	-14	-6	1.1	0.8	0.9	0.9
PARP9	Q8IXQ6	251	10	-23	-14	-5	1.1	0.8	0.9	1.0
URB2	Q14146	702	10	-10	-14	-6	1.1	0.9	0.9	0.9
H6PD	O95479	378	8	-25	-14	-11	1.1	0.8	0.9	0.9
MTAP	Q13126	136	8	-9	-14	4	1.1	0.9	0.9	1.0
FASN	P49327	1558	8	-16	-14	-8	1.1	0.9	0.9	0.9
PML	P29590	338	7	-8	-14	-4	1.1	0.9	0.9	1.0
TBL1X	O60907	401	7	-12	-14	-8	1.1	0.9	0.9	0.9
SLFN11	Q7Z7L1	150	6	6	-14	9	1.1	1.1	0.9	1.1
GZMH	P20718	49	6	-6	-14	3	1.1	0.9	0.9	1.0
STRN	O43815	740	6	-19	-14	-10	1.1	0.8	0.9	0.9
SRP72	O76094	54	6	-22	-14	-3	1.1	0.8	0.9	1.0
CORO1C	Q9ULV4	23	6	-24	-14	-3	1.1	0.8	0.9	1.0
THOC6	Q86W42	324	5	-19	-14	-8	1.1	0.8	0.9	0.9
NFXL1	Q6ZNB6	688	5	-25	-14	-23	1.1	0.8	0.9	0.8
RAB33B	Q9H082	48	5	-15	-14	-2	1.0	0.9	0.9	1.0
TBC1D9	Q6ZT07	1033	4	-15	-14	-3	1.0	0.9	0.9	1.0
DNAJC2	Q99543	240	4	-19	-14	-23	1.0	0.8	0.9	0.8
SCYL2	Q6P3W7	552	4	-14	-14	-13	1.0	0.9	0.9	0.9
LOC1027240	A0A0B4J2D5	153	4	-24	-14	-16	1.0	0.8	0.9	0.9
POLR2A	P24928	451	3	-4	-14	14	1.0	1.0	0.9	1.2
PTPRC	P08575	342	3	-12	-14	-11	1.0	0.9	0.9	0.9
KSR1	Q8IVT5	62	3	-12	-14	-14	1.0	0.9	0.9	0.9
MAP2K3	P46734	207	3	-13	-14	22	1.0	0.9	0.9	1.3
MAP2K6	P52564	196	3	-13	-14	22	1.0	0.9	0.9	1.3
YTHDF2	Q9Y5A9	482	2	-8	-14	-15	1.0	0.9	0.9	0.9
FUK	Q8N0W3	536	2	-18	-14	-7	1.0	0.9	0.9	0.9
OSBPL8	Q9BZF1	266	2	-18	-14	-13	1.0	0.9	0.9	0.9
SCYL1	Q96KG9	309	2	-19	-14	-20	1.0	0.8	0.9	0.8
UBXN6	Q9BZV1	347	2	-19	-14	-23	1.0	0.8	0.9	0.8
CASP1	P29466	270	1	-19	-14	-8	1.0	0.8	0.9	0.9
VWA5A	O00534	743	1	-28	-14	-22	1.0	0.8	0.9	0.8
ATP6V1H	Q9UI12	85	1	-5	-14	-19	1.0	1.0	0.9	0.8
GSTO1	P78417	32	1	-12	-14	-2	1.0	0.9	0.9	1.0
CHD1	O14646	376	1	-16	-14	-15	1.0	0.9	0.9	0.9
ILF3	Q12906	203	1	-18	-14	-7	1.0	0.8	0.9	0.9
XPO6	Q96QU8	324	0	-7	-14	-13	1.0	0.9	0.9	0.9
MEPCE	Q7L2J0	522	0	-10	-14	-18	1.0	0.9	0.9	0.9
CFL1	P23528	139	0	-11	-14	-8	1.0	0.9	0.9	0.9
VPS11	Q9H270	660	0	-13	-14	11	1.0	0.9	0.9	1.1
JMJD1C	Q15652	508	0	-13	-14	-29	1.0	0.9	0.9	0.8
TBCA	O75347	67	0	-15	-14	-7	1.0	0.9	0.9	0.9
ADRM1	Q16186	80	0	-19	-14	-6	1.0	0.8	0.9	0.9
TARBP2	Q15633	263	-1	-2	-14	-6	1.0	1.0	0.9	0.9
LYZ	P61626	83	-1	-8	-14	4	1.0	0.9	0.9	1.0
NUP88	Q99567	447	-1	-13	-14	-6	1.0	0.9	0.9	0.9
SEC24B	O95487	555	-1	-23	-14	-13	1.0	0.8	0.9	0.9
KIF2A	O00139	334	-1	-26	-14	2	1.0	0.8	0.9	1.0

EIF2S3	P41091	434	-1	-20	-14	-4	1.0	0.8	0.9	1.0
RBM15B	Q8NDT2	323	-2	-6	-14	-28	1.0	0.9	0.9	0.8
MKI67	P46013	903	-2	-13	-14	-18	1.0	0.9	0.9	0.8
MED17	Q9NVC6	15	-2	-17	-14	-28	1.0	0.9	0.9	0.8
PAPOLA	P51003	677	-2	0	-14	2	1.0	1.0	0.9	1.0
RHOC	P08134	16	-2	-17	-14	-7	1.0	0.9	0.9	0.9
RHOA	P61586	16	-2	-17	-14	-7	1.0	0.9	0.9	0.9
Uncharacteri	U3KQV3	180	-2	-17	-14	-7	1.0	0.9	0.9	0.9
STAT5A	P42229	101	-2	-18	-14	0	1.0	0.8	0.9	1.0
ZNF318	Q5VUA4	158	-3	-7	-14	-3	1.0	0.9	0.9	1.0
PTCD3	Q96EY7	139	-3	-8	-14	-11	1.0	0.9	0.9	0.9
NUP214	P35658	1003	-3	-13	-14	-11	1.0	0.9	0.9	0.9
MSH3	P20585	252	-3	-16	-14	20	1.0	0.9	0.9	1.2
ITPRIP	Q8IWB1	228	-3	-16	-14	8	1.0	0.9	0.9	1.1
TOMM40	O96008	86	-3	-17	-14	-8	1.0	0.9	0.9	0.9
UBA6	A0AVT1	699	-3	-39	-14	-13	1.0	0.7	0.9	0.9
CRTC1	Q6UUV9	131	-3	-1	-14	18	1.0	1.0	0.9	1.2
SMARCAL1	Q9NZC9	108	-3	-11	-14	-10	1.0	0.9	0.9	0.9
PRMT2	P55345	75	-3	-13	-14	-6	1.0	0.9	0.9	0.9
INTS1	Q8N201	1755	-3	-14	-14	8	1.0	0.9	0.9	1.1
PFKP	Q01813	641	-3	-17	-14	-23	1.0	0.9	0.9	0.8
ATP1A1	P05023	374	-3	-18	-14	-7	1.0	0.9	0.9	0.9
DDX18	Q9NVP1	407	-3	-18	-14	-7	1.0	0.9	0.9	0.9
ADSL	P30566	399	-3	-26	-14	1	1.0	0.8	0.9	1.0
DDX1	Q92499	231	-3	-37	-14	-8	1.0	0.7	0.9	0.9
GRB2	P62993	198	-4	-10	-14	-12	1.0	0.9	0.9	0.9
PARP4	Q9UKK3	1019	-4	-18	-14	-5	1.0	0.8	0.9	1.0
VPS13C	Q709C8	1372	-4	-22	-14	-25	1.0	0.8	0.9	0.8
FBXO30	Q8TB52	570	-4	-17	-14	-18	1.0	0.9	0.9	0.9
PGM1	P36871	160	-4	-23	-14	-6	1.0	0.8	0.9	0.9
FYCO1	Q9BQS8	1025	-4	-24	-14	0	1.0	0.8	0.9	1.0
PPP1R3E	Q9H7J1	131	-5	-18	-14	-16	1.0	0.8	0.9	0.9
IRF2BP1	Q8IU81	15	-5	-10	-14	-9	1.0	0.9	0.9	0.9
G6PD	P11413	13	-5	-12	-14	-19	1.0	0.9	0.9	0.8
PPP1R3D	O95685	97	-5	-14	-14	-20	1.0	0.9	0.9	0.8
CCDC88C	Q9P219	1321	-6	-8	-14	-12	0.9	0.9	0.9	0.9
PHKB	Q93100	559	-6	-10	-14	2	0.9	0.9	0.9	1.0
HAT1	O14929	120	-6	-11	-14	3	0.9	0.9	0.9	1.0
RPS6KA1	Q15418	432	-6	-15	-14	-14	0.9	0.9	0.9	0.9
LIMK2	P53671	173	-6	-15	-14	-17	0.9	0.9	0.9	0.9
EEF2KMT	Q96G04	72	-6	-15	-14	-19	0.9	0.9	0.9	0.8
HAUS3	Q68CZ6	271	-6	-16	-14	-15	0.9	0.9	0.9	0.9
MARK3	P27448	213	-6	-11	-14	-22	0.9	0.9	0.9	0.8
MARK2	Q7KZI7	210	-6	-11	-14	-22	0.9	0.9	0.9	0.8
MARK4	Q96L34	216	-6	-11	-14	-22	0.9	0.9	0.9	0.8
DEF6	Q9H4E7	253	-6	-19	-14	-22	0.9	0.8	0.9	0.8
FYCO1	Q9BQS8	1110	-7	-11	-14	-3	0.9	0.9	0.9	1.0
RPP38	P78345	159	-7	-15	-14	-7	0.9	0.9	0.9	0.9
IL32	P24001	227	-7	-17	-14	-25	0.9	0.9	0.9	0.8
RPL12	P30050	141	-7	-19	-14	-3	0.9	0.8	0.9	1.0
TUBB1	Q9H4B7	340	-7	-21	-14	-16	0.9	0.8	0.9	0.9
ACAD8	Q9UKU7	366	-7	-26	-14	-17	0.9	0.8	0.9	0.9
APBB1IP	Q7Z5R6	400	-7	-28	-14	-18	0.9	0.8	0.9	0.8

PCMT1	P22061	95	-7	-30	-14	-6	0.9	0.8	0.9	0.9
RAB30	Q15771	93	-8	-10	-14	-19	0.9	0.9	0.9	0.8
ATP6V0A1	Q93050	247	-8	-19	-14	-11	0.9	0.8	0.9	0.9
RING1	Q06587	84	-8	-27	-14	-14	0.9	0.8	0.9	0.9
RNF2	Q99496	87	-8	-27	-14	-14	0.9	0.8	0.9	0.9
MAP2K4	P45985	246	-9	-10	-14	9	0.9	0.9	0.9	1.1
SRPK1	Q96SB4	188	-9	-14	-14	-3	0.9	0.9	0.9	1.0
PPA2	Q9H2U2	161	-9	-19	-14	-17	0.9	0.8	0.9	0.9
RAB27A	P51159	188	-9	-21	-14	-17	0.9	0.8	0.9	0.9
TEX264	Q9Y6I9	68	-9	-16	-14	-7	0.9	0.9	0.9	0.9
ANP32B	Q92688	27	-9	-18	-14	-14	0.9	0.9	0.9	0.9
CACYBP	Q9HB71	173	-9	-19	-14	-19	0.9	0.8	0.9	0.8
ITGA2B	P08514	87	-9	-26	-14	-8	0.9	0.8	0.9	0.9
Uncharacteri	A0A0C4DFX4	671	-10	-22	-14	-13	0.9	0.8	0.9	0.9
ADCY6	O43306	1145	-10	-27	-14	-15	0.9	0.8	0.9	0.9
ZDHHC24	Q6UX98	242	-10	-38	-14	4	0.9	0.7	0.9	1.0
ZC3H14	Q6PJT7	261	-10	-9	-14	-6	0.9	0.9	0.9	0.9
ARHGAP35	Q9NRY4	559	-10	-16	-14	-12	0.9	0.9	0.9	0.9
CENPV	Q7Z7K6	240	-11	-17	-14	7	0.9	0.9	0.9	1.1
HK1	P19367	158	-12	-15	-14	-4	0.9	0.9	0.9	1.0
EEF2	P13639	290	-12	-23	-14	-5	0.9	0.8	0.9	1.0
NEXN	Q0ZGT2	224	-12	-10	-14	-35	0.9	0.9	0.9	0.7
INTS7	Q9NVH2	374	-12	-14	-14	-16	0.9	0.9	0.9	0.9
ENOPH1	Q9UHY7	202	-12	-19	-14	2	0.9	0.8	0.9	1.0
DGKZ	Q13574	905	-12	-21	-14	-15	0.9	0.8	0.9	0.9
RAB39A	Q14964	41	-13	-22	-14	-25	0.9	0.8	0.9	0.8
PRKCB	P05771	67	-13	-26	-14	-13	0.9	0.8	0.9	0.9
PRKCA	P17252	67	-13	-26	-14	-13	0.9	0.8	0.9	0.9
ACTN1	P12814	774	-13	-45	-14	-12	0.9	0.7	0.9	0.9
FBXL6	Q8N531	219	-14	-27	-14	-15	0.9	0.8	0.9	0.9
BRAT1	Q6PJG6	720	-15	-35	-14	17	0.9	0.7	0.9	1.2
SNX27	Q96L92	30	-15	-2	-14	-25	0.9	1.0	0.9	0.8
NOP2	P46087	463	-15	-11	-14	-4	0.9	0.9	0.9	1.0
ARL6IP4	Q66PJ3	220	-15	-36	-14	-26	0.9	0.7	0.9	0.8
KDM8	Q8N371	232	-16	-9	-14	-30	0.9	0.9	0.9	0.8
QSER1	Q2KHR3	283	-17	-12	-14	-22	0.9	0.9	0.9	0.8
TKT	P29401	376	-17	-7	-14	-26	0.9	0.9	0.9	0.8
HERC1	Q15751	1941	-18	-28	-14	-17	0.9	0.8	0.9	0.9
PIK3CD	O00329	815	-18	-39	-14	-26	0.9	0.7	0.9	0.8
SND1	Q7KZF4	440	-19	-19	-14	-12	0.8	0.8	0.9	0.9
EIF5B	O60841	749	-20	-10	-14	-2	0.8	0.9	0.9	1.0
NOP14	P78316	580	-21	-22	-14	-21	0.8	0.8	0.9	0.8
CAT	P04040	460	-22	-37	-14	-1	0.8	0.7	0.9	1.0
CDC123	O75794	170	-23	-11	-14	-31	0.8	0.9	0.9	0.8
PHF5A	Q7RTV0	72	-23	-18	-14	-47	0.8	0.8	0.9	0.7
APEX1	P27695	93	-25	-77	-14	-4	0.8	0.6	0.9	1.0
NLRP3	Q96P20	150	-38	-26	-14	-34	0.7	0.8	0.9	0.7
PMPCA	Q10713	142	15	-8	-14	3	1.2	0.9	0.9	1.0
MTHFSD	Q2M296	113	15	-37	-14	-17	1.2	0.7	0.9	0.9
ENTHD2	Q96N21	493	13	14	-14	-15	1.1	1.2	0.9	0.9
SHMT2	P34897	91	11	-29	-14	2	1.1	0.8	0.9	1.0
DNAJC7	Q99615	337	10	-16	-14	7	1.1	0.9	0.9	1.1
EPB41	P11171	416	10	-30	-14	-21	1.1	0.8	0.9	0.8

IDNK	Q5T6J7	105	9	-14	-14	-16	1.1	0.9	0.9	0.9
PRKDC	P78527	974	9	-70	-14	-7	1.1	0.6	0.9	0.9
CHD1	O14646	1242	8	-36	-14	-9	1.1	0.7	0.9	0.9
SDHA	P31040	189	8	-24	-14	-2	1.1	0.8	0.9	1.0
EFTUD2	Q15029	144	7	-9	-14	-10	1.1	0.9	0.9	0.9
NARS	O43776	511	7	-14	-14	-4	1.1	0.9	0.9	1.0
RABGGTA	Q92696	354	7	-12	-14	-5	1.1	0.9	0.9	1.0
IFIH1	Q9BYX4	951	6	-29	-14	-5	1.1	0.8	0.9	1.0
PRPF8	Q6P2Q9	1626	6	-4	-14	-5	1.1	1.0	0.9	1.0
GNPAT	O15228	73	6	-9	-14	-6	1.1	0.9	0.9	0.9
NSUN2	Q08J23	599	6	-15	-14	-4	1.1	0.9	0.9	1.0
UBE2D2	P62837	111	5	-7	-14	3	1.1	0.9	0.9	1.0
RLF	Q13129	400	5	-9	-14	2	1.1	0.9	0.9	1.0
PTPRC	P08575	760	5	-10	-14	-19	1.1	0.9	0.9	0.8
SMARCAD1	Q9H4L7	407	5	-15	-14	11	1.0	0.9	0.9	1.1
RCBTB2	O95199	201	4	-11	-14	-12	1.0	0.9	0.9	0.9
LHPP	Q9H008	180	4	-28	-14	-10	1.0	0.8	0.9	0.9
ACAP1	Q15027	501	3	-5	-14	-7	1.0	1.0	0.9	0.9
OGFR	Q9NZT2	87	3	-7	-14	1	1.0	0.9	0.9	1.0
ALOX12	P18054	89	3	-13	-14	-17	1.0	0.9	0.9	0.9
FCF1	Q9Y324	154	3	-13	-14	-33	1.0	0.9	0.9	0.8
SCAF11	Q99590	1020	3	-19	-14	-24	1.0	0.8	0.9	0.8
SCML4	Q8N228	324	3	-21	-14	-12	1.0	0.8	0.9	0.9
PCCB	P05166	365	3	-19	-14	-3	1.0	0.8	0.9	1.0
EXOC1	Q9NV70	114	3	-20	-14	-17	1.0	0.8	0.9	0.9
LIMS1	P48059	303	3	-24	-14	-9	1.0	0.8	0.9	0.9
LIMS2	Q7Z4I7	308	3	-24	-14	-9	1.0	0.8	0.9	0.9
MED17	Q9NVC6	488	3	-24	-14	-36	1.0	0.8	0.9	0.7
DDB1	Q16531	378	3	-25	-14	-11	1.0	0.8	0.9	0.9
EXOC2	Q96KP1	859	3	-27	-14	-17	1.0	0.8	0.9	0.9
METTL16	Q86W50	276	2	-8	-14	-2	1.0	0.9	0.9	1.0
PFAS	O15067	1027	2	-17	-14	0	1.0	0.9	0.9	1.0
DOCK8	Q8NF50	1836	2	-23	-14	-8	1.0	0.8	0.9	0.9
C1orf174	Q8IYL3	61	2	-25	-14	-15	1.0	0.8	0.9	0.9
RGS19	P49795	73	2	-10	-14	-24	1.0	0.9	0.9	0.8
SNX29	Q8TEQ0	121	2	-16	-14	-13	1.0	0.9	0.9	0.9
DGKA	P23743	682	2	-24	-14	-9	1.0	0.8	0.9	0.9
NR2C2	P49116	120	2	-24	-14	-26	1.0	0.8	0.9	0.8
FMNL1	O95466	300	2	-39	-14	12	1.0	0.7	0.9	1.1
TAF7	Q15545	72	1	4	-14	10	1.0	1.0	0.9	1.1
GTPBP1	O00178	531	1	-4	-14	3	1.0	1.0	0.9	1.0
HPS1	Q92902	180	1	-6	-14	5	1.0	0.9	0.9	1.0
DDX19A	Q9NUU7	164	1	-9	-14	-13	1.0	0.9	0.9	0.9
PI4KA	P42356	2081	1	-10	-14	3	1.0	0.9	0.9	1.0
PLXND1	Q9Y4D7	1363	1	-21	-14	-15	1.0	0.8	0.9	0.9
SNRNP200	O75643	2011	1	-32	-14	-3	1.0	0.8	0.9	1.0
ATXN3	P54252	172	1	-32	-14	-30	1.0	0.8	0.9	0.8
TES	Q9UGI8	238	1	-13	-14	-3	1.0	0.9	0.9	1.0
LRPPRC	P42704	130	1	-21	-14	-27	1.0	0.8	0.9	0.8
TGFBI	Q15582	65	0	-11	-14	-17	1.0	0.9	0.9	0.9
MTG1	Q9BT17	23	0	-28	-14	-23	1.0	0.8	0.9	0.8
ECH1	Q13011	187	-1	-6	-14	4	1.0	0.9	0.9	1.0
COPG1	Q9Y678	813	-1	-9	-14	-4	1.0	0.9	0.9	1.0

HLCS	P50747	289	-1	-15	-14	-9	1.0	0.9	0.9	0.9
XRCC5	P13010	296	-1	-80	-14	-16	1.0	0.6	0.9	0.9
TGFBRAP1	Q8WUH2	826	-1	-4	-14	-14	1.0	1.0	0.9	0.9
KPNB1	Q14974	223	-1	-16	-14	-10	1.0	0.9	0.9	0.9
URB2	Q14146	1280	-1	-16	-14	-3	1.0	0.9	0.9	1.0
VKORC1L1	Q8N0U8	58	-1	-16	-14	-10	1.0	0.9	0.9	0.9
PCNT	O95613	2576	-2	-15	-14	-12	1.0	0.9	0.9	0.9
CDC123	O75794	212	-2	-17	-14	-4	1.0	0.9	0.9	1.0
RIC8A	Q9NPQ8	79	-2	-18	-14	-17	1.0	0.9	0.9	0.9
KDM2A	Q9Y2K7	675	-2	-19	-14	-15	1.0	0.8	0.9	0.9
ANKRD44	Q8N8A2	420	-2	-25	-14	-40	1.0	0.8	0.9	0.7
MORC2	Q9Y6X9	962	-2	-30	-14	-26	1.0	0.8	0.9	0.8
NRDC	O43847	965	-2	-20	-14	-2	1.0	0.8	0.9	1.0
GBE1	Q04446	81	-2	-24	-14	4	1.0	0.8	0.9	1.0
TMCO6	Q96DC7	309	-3	2	-14	7	1.0	1.0	0.9	1.1
PAPSS2	O95340	350	-3	-7	-14	-7	1.0	0.9	0.9	0.9
ATP2A3	Q93084	614	-3	-15	-14	-10	1.0	0.9	0.9	0.9
NLRP1	Q9C000	649	-3	-16	-14	15	1.0	0.9	0.9	1.2
MLST8	Q9BVC4	298	-3	-16	-14	-14	1.0	0.9	0.9	0.9
WDFY1	Q8IWB7	344	-3	-20	-14	8	1.0	0.8	0.9	1.1
TPM4	P67936	154	-3	-23	-14	-7	1.0	0.8	0.9	0.9
GPATCH11	Q8N954	169	-3	-27	-14	-15	1.0	0.8	0.9	0.9
GSDMB	Q8TAX9	49	-3	-28	-14	-16	1.0	0.8	0.9	0.9
METTL13	Q8N6R0	90	-3	-2	-14	-5	1.0	1.0	0.9	1.0
CCT8	P50990	136	-3	-11	-14	-4	1.0	0.9	0.9	1.0
ANXA6	P08133	96	-3	-11	-14	-9	1.0	0.9	0.9	0.9
DCXR	Q7Z4W1	51	-3	-15	-14	2	1.0	0.9	0.9	1.0
DOCK2	Q92608	607	-3	-23	-14	-10	1.0	0.8	0.9	0.9
LARS	Q9P2J5	1093	-4	-17	-14	6	1.0	0.9	0.9	1.1
DERA	Q9Y315	107	-4	-18	-14	7	1.0	0.9	0.9	1.1
PXN	P49023	564	-4	-30	-14	18	1.0	0.8	0.9	1.2
CARMIL2	Q6F5E8	562	-4	-18	-14	-19	1.0	0.8	0.9	0.8
KLC1	Q07866	390	-4	-22	-14	-15	1.0	0.8	0.9	0.9
KLC4	Q9NSK0	388	-4	-22	-14	-15	1.0	0.8	0.9	0.9
CAPG	P40121	290	-5	-14	-14	13	1.0	0.9	0.9	1.1
RPL36A-HNRI	H7BZ11	72	-5	-15	-14	-30	1.0	0.9	0.9	0.8
ZMYM3	Q14202	656	-5	-13	-14	1	1.0	0.9	0.9	1.0
RPL36	Q9Y3U8	48	-5	-13	-14	-6	1.0	0.9	0.9	0.9
CNOT7	Q9UIV1	67	-5	-13	-14	-11	1.0	0.9	0.9	0.9
LRCH4	O75427	283	-5	-20	-14	-21	1.0	0.8	0.9	0.8
VPS8	Q8N3P4	286	-5	-25	-14	-18	1.0	0.8	0.9	0.8
TDRKH	Q9Y2W6	160	-6	-14	-14	-13	0.9	0.9	0.9	0.9
TJAP1	Q5JTD0	350	-6	-18	-14	-19	0.9	0.8	0.9	0.8
ARPC1B	O15143	26	-6	-20	-14	1	0.9	0.8	0.9	1.0
PELP1	Q8IZL8	594	-6	-24	-14	-20	0.9	0.8	0.9	0.8
CAND2	O75155	828	-6	-10	-14	-7	0.9	0.9	0.9	0.9
HK2	P52789	438	-6	-16	-14	-23	0.9	0.9	0.9	0.8
CASS4	Q9NQ75	23	-6	-19	-14	-22	0.9	0.8	0.9	0.8
TADA2A	O75478	298	-6	-22	-14	-29	0.9	0.8	0.9	0.8
TOE1	Q96GM8	371	-6	-27	-14	-21	0.9	0.8	0.9	0.8
ATG4B	Q9Y4P1	74	-7	-11	-14	-11	0.9	0.9	0.9	0.9
NOP14	P78316	755	-7	-12	-14	-9	0.9	0.9	0.9	0.9
ZMYND8	Q9ULU4	567	-7	-18	-14	-18	0.9	0.8	0.9	0.8

ATP6V0D1	P61421	244	-7	-17	-14	-5	0.9	0.9	0.9	1.0
TRIM33	Q9UPN9	786	-7	-17	-14	-24	0.9	0.9	0.9	0.8
PFKFB4	Q16877	106	-8	-8	-14	-15	0.9	0.9	0.9	0.9
PFKFB3	Q16875	102	-8	-8	-14	-15	0.9	0.9	0.9	0.9
PPP2CB	P62714	50	-8	-16	-14	-3	0.9	0.9	0.9	1.0
PPP2CA	P67775	50	-8	-16	-14	-3	0.9	0.9	0.9	1.0
PARP1	P09874	24	-8	-22	-14	-16	0.9	0.8	0.9	0.9
NANS	Q9NR45	180	-8	-15	-14	-9	0.9	0.9	0.9	0.9
ACTN4	O43707	499	-8	-16	-14	5	0.9	0.9	0.9	1.0
SMARCB1	Q12824	167	-8	-17	-14	-14	0.9	0.9	0.9	0.9
ZC3HC1	Q86WB0	406	-8	-20	-14	-11	0.9	0.8	0.9	0.9
NOC3L	Q8WTT2	426	-8	-20	-14	-19	0.9	0.8	0.9	0.8
ACBD5	Q5T8D3	83	-8	-21	-14	-5	0.9	0.8	0.9	1.0
VAV3	Q9UKW4	660	-8	-21	-14	-15	0.9	0.8	0.9	0.9
GDPD1	Q8N9F7	126	-9	-9	-14	2	0.9	0.9	0.9	1.0
TRIP11	Q15643	1722	-9	-15	-14	-11	0.9	0.9	0.9	0.9
CRIP1	P50238	7	-9	-18	-14	-11	0.9	0.9	0.9	0.9
MBNL3	Q9NUK0	20	-9	-24	-14	-19	0.9	0.8	0.9	0.8
MBNL2	Q5VZF2	19	-9	-24	-14	-19	0.9	0.8	0.9	0.8
MBNL1	Q9NR56	19	-9	-24	-14	-19	0.9	0.8	0.9	0.8
NSMCE1	Q8WV22	40	-9	-16	-14	-2	0.9	0.9	0.9	1.0
ARHGEF6	Q15052	763	-9	-17	-14	-5	0.9	0.9	0.9	1.0
DHX9	Q08211	1029	-9	-20	-14	-6	0.9	0.8	0.9	0.9
GTPBP6	H0Y2S1	394	-10	-13	-14	-16	0.9	0.9	0.9	0.9
CNST	Q6PJW8	268	-10	-16	-14	-19	0.9	0.9	0.9	0.8
NUBP1	P53384	22	-10	-18	-14	-29	0.9	0.9	0.9	0.8
GTF2A2	P52657	98	-10	-27	-14	-19	0.9	0.8	0.9	0.8
WWC3	Q9ULE0	988	-10	-13	-14	-14	0.9	0.9	0.9	0.9
RASA3	Q14644	144	-10	-21	-14	-22	0.9	0.8	0.9	0.8
HDAC2	Q92769	274	-11	-21	-14	-7	0.9	0.8	0.9	0.9
RPS8	P62241	174	-12	-16	-14	-14	0.9	0.9	0.9	0.9
CAPN1	P07384	590	-12	-31	-14	-15	0.9	0.8	0.9	0.9
SRP72	O76094	349	-13	-16	-14	-18	0.9	0.9	0.9	0.9
ARFGAP3	Q9NP61	241	-13	-13	-14	-24	0.9	0.9	0.9	0.8
USP9Y	O00507	843	-13	-17	-14	-30	0.9	0.9	0.9	0.8
ACAD8	Q9UKU7	150	-13	-22	-14	-16	0.9	0.8	0.9	0.9
RTCA	O00442	153	-14	-23	-14	-29	0.9	0.8	0.9	0.8
MYCBP2	O75592	1131	-14	-32	-14	-17	0.9	0.8	0.9	0.9
PDSS2	Q86YH6	71	-15	-15	-14	-11	0.9	0.9	0.9	0.9
MTPAP	Q9NVV4	82	-15	-41	-14	-21	0.9	0.7	0.9	0.8
DOPEY2	Q9Y3R5	2199	-16	-13	-14	-24	0.9	0.9	0.9	0.8
DCTPP1	Q9H773	162	-17	-16	-14	-30	0.9	0.9	0.9	0.8
HPS5	Q9UPZ3	431	-17	-18	-14	-23	0.9	0.8	0.9	0.8
PMPCA	Q10713	225	-17	-15	-14	-5	0.9	0.9	0.9	1.0
ZMYND8	Q9ULU4	106	-17	-20	-14	4	0.9	0.8	0.9	1.0
WDR75	Q8IWA0	444	-20	-17	-14	-18	0.8	0.9	0.9	0.8
RPS12	P25398	50	-21	-17	-14	32	0.8	0.9	0.9	1.5
RANBP9	Q96S59	719	-21	-42	-14	-19	0.8	0.7	0.9	0.8
LSP1	P33241	170	-24	-16	-14	-30	0.8	0.9	0.9	0.8
PRF1	P14222	397	-25	-5	-14	-13	0.8	1.0	0.9	0.9
RBM22	Q9NW64	58	-26	-18	-14	-8	0.8	0.8	0.9	0.9
HDAC1	Q13547	100	-27	-19	-14	-5	0.8	0.8	0.9	1.0
RCHY1	Q96PM5	136	-31	-26	-14	-6	0.8	0.8	0.9	0.9

NARS2	Q96I59	291	16	-6	-15	11	1.2	0.9	0.9	1.1
OSBPL2	Q9H1P3	83	15	-24	-15	-24	1.2	0.8	0.9	0.8
SMS	P52788	318	15	-16	-15	8	1.2	0.9	0.9	1.1
FDFT1	P37268	6	13	-16	-15	5	1.1	0.9	0.9	1.0
VCPIP1	Q96JH7	397	11	-14	-15	3	1.1	0.9	0.9	1.0
MRPL24	Q96A35	58	11	-22	-15	-25	1.1	0.8	0.9	0.8
NFATC2	Q13469	355	10	-2	-15	-9	1.1	1.0	0.9	0.9
RAD23B	P54727	390	10	-30	-15	-5	1.1	0.8	0.9	1.0
TNFAIP2	Q03169	512	10	-6	-15	0	1.1	0.9	0.9	1.0
ARAP1	Q96P48	977	9	-17	-15	-1	1.1	0.9	0.9	1.0
AKAP13	Q12802	2101	9	-21	-15	-7	1.1	0.8	0.9	0.9
CKAP5	Q14008	604	9	-12	-15	2	1.1	0.9	0.9	1.0
SEC31A	O94979	1130	7	-38	-15	-5	1.1	0.7	0.9	1.0
RASGRP2	Q7LDG7	296	6	-32	-15	-6	1.1	0.8	0.9	0.9
RSAD1	Q9HA92	53	5	-2	-15	-11	1.1	1.0	0.9	0.9
LCP1	P13796	206	4	-13	-15	11	1.0	0.9	0.9	1.1
RB1CC1	Q8TDY2	853	4	-15	-15	-16	1.0	0.9	0.9	0.9
MAGOHB	Q96A72	133	4	-19	-15	-20	1.0	0.8	0.9	0.8
PARP10	Q53GL7	123	4	-14	-15	-3	1.0	0.9	0.9	1.0
TRRAP	Q9Y4A5	350	3	-13	-15	-5	1.0	0.9	0.9	1.0
DARS	P14868	130	3	-14	-15	-3	1.0	0.9	0.9	1.0
SHMT1	P34896	384	3	-29	-15	-2	1.0	0.8	0.9	1.0
OGFR	Q9NZT2	417	3	-12	-15	-10	1.0	0.9	0.9	0.9
CCDC186	Q7Z3E2	507	3	-13	-15	-8	1.0	0.9	0.9	0.9
ZC3H13	Q5T200	57	2	-12	-15	-11	1.0	0.9	0.9	0.9
ZNF740	Q8NDX6	103	2	-13	-15	-10	1.0	0.9	0.9	0.9
HLTF	Q14527	360	2	-17	-15	-21	1.0	0.9	0.9	0.8
MAP3K7	O43318	588	2	-28	-15	-23	1.0	0.8	0.9	0.8
RPL7A	P62424	199	1	-15	-15	1	1.0	0.9	0.9	1.0
UTRN	P46939	3076	1	-16	-15	-5	1.0	0.9	0.9	1.0
PARP4	Q9UKK3	1687	1	-16	-15	-13	1.0	0.9	0.9	0.9
TARS	P26639	261	1	-26	-15	-10	1.0	0.8	0.9	0.9
TMEM63B	Q5T3F8	275	1	-8	-15	6	1.0	0.9	0.9	1.1
GUCY1A3	Q02108	460	1	-9	-15	-3	1.0	0.9	0.9	1.0
EIF2S1	P05198	199	1	-17	-15	-4	1.0	0.9	0.9	1.0
SLFN5	Q08AF3	439	0	-14	-15	3	1.0	0.9	0.9	1.0
PRKAR2A	P13861	101	-1	-16	-15	-14	1.0	0.9	0.9	0.9
RB1CC1	Q8TDY2	831	-1	-17	-15	-5	1.0	0.9	0.9	1.0
SBF1	O95248	209	-1	-20	-15	-11	1.0	0.8	0.9	0.9
GNB1	P62873	294	-1	-26	-15	-8	1.0	0.8	0.9	0.9
HNRNPUL2-B	H3BQZ7	293	-1	-19	-15	-13	1.0	0.8	0.9	0.9
SMC5	Q8IY18	881	-2	-13	-15	-19	1.0	0.9	0.9	0.8
UBR4	Q5T4S7	1080	-2	-14	-15	-5	1.0	0.9	0.9	1.0
LTA4H	P09960	141	-2	-20	-15	10	1.0	0.8	0.9	1.1
CDK9	P50750	85	-2	-9	-15	-21	1.0	0.9	0.9	0.8
GNPAT	O15228	650	-2	-13	-15	-20	1.0	0.9	0.9	0.8
RNF121	Q9H920	264	-2	-20	-15	-20	1.0	0.8	0.9	0.8
BPGM	P07738	23	-3	-6	-15	-10	1.0	0.9	0.9	0.9
REL	Q04864	195	-3	-8	-15	-4	1.0	0.9	0.9	1.0
USP19	O94966	362	-3	-1	-15	-26	1.0	1.0	0.9	0.8
USP48	Q86UV5	557	-3	-7	-15	-6	1.0	0.9	0.9	0.9
SMCHD1	A6NHR9	1286	-3	-11	-15	-11	1.0	0.9	0.9	0.9
SPHKAP	Q2M3C7	259	-3	-12	-15	-5	1.0	0.9	0.9	1.0

BCL11B	Q9C0K0	808	-3	-16	-15	-25	1.0	0.9	0.9	0.8
PTPRC	P08575	787	-3	-18	-15	-10	1.0	0.8	0.9	0.9
IRAK3	Q9Y616	420	-3	-21	-15	-24	1.0	0.8	0.9	0.8
PGK1	P00558	50	-4	-24	-15	-7	1.0	0.8	0.9	0.9
IREB2	P48200	178	-4	-9	-15	-12	1.0	0.9	0.9	0.9
MIPEP	Q99797	448	-4	-10	-15	-5	1.0	0.9	0.9	1.0
KDM1A	O60341	360	-4	-17	-15	-5	1.0	0.9	0.9	1.0
ALOX5	P09917	419	-4	-20	-15	-15	1.0	0.8	0.9	0.9
PDIA5	Q14554	231	-4	-22	-15	-1	1.0	0.8	0.9	1.0
LAPTM5	Q13571	218	-5	-7	-15	-19	1.0	0.9	0.9	0.8
CPT1A	P50416	742	-5	-16	-15	-2	1.0	0.9	0.9	1.0
PNN	Q9H307	249	-5	-16	-15	-20	1.0	0.9	0.9	0.8
MACF1	Q9UPN3	6754	-5	-36	-15	-33	1.0	0.7	0.9	0.8
DPH6	Q7L8W6	88	-5	-14	-15	-6	1.0	0.9	0.9	0.9
BOLA2B	Q9H3K6	31	-5	-18	-15	-20	1.0	0.8	0.9	0.8
LHPP	Q9H008	88	-5	-20	-15	-5	1.0	0.8	0.9	1.0
PRF1	P14222	73	-6	-3	-15	-4	0.9	1.0	0.9	1.0
TBC1D2B	Q9UPU7	554	-6	-7	-15	-22	0.9	0.9	0.9	0.8
PPP2R1B	P30154	389	-6	-14	-15	-14	0.9	0.9	0.9	0.9
ASRGL1	Q7L266	176	-6	-21	-15	-6	0.9	0.8	0.9	0.9
SCYL1	Q96KG9	446	-6	-22	-15	-7	0.9	0.8	0.9	0.9
DIS3L2	Q8IYB7	739	-6	-24	-15	-7	0.9	0.8	0.9	0.9
PTBP3	O95758	68	-6	-9	-15	-21	0.9	0.9	0.9	0.8
RPL24	P83731	36	-6	-13	-15	-15	0.9	0.9	0.9	0.9
TRAF3IP3	Q9Y228	380	-6	-15	-15	-15	0.9	0.9	0.9	0.9
BIRC6	Q9NR09	4750	-6	-18	-15	-12	0.9	0.9	0.9	0.9
ETFDH	Q16134	561	-6	-27	-15	2	0.9	0.8	0.9	1.0
KIAA0100	Q14667	1979	-7	-17	-15	-17	0.9	0.9	0.9	0.9
DOCK8	Q8NF50	143	-7	-19	-15	-18	0.9	0.8	0.9	0.8
DHX15	O43143	190	-7	-22	-15	-10	0.9	0.8	0.9	0.9
ILVBL	A1L0T0	276	-7	-53	-15	-2	0.9	0.7	0.9	1.0
ELAC2	Q9BQ52	421	-8	-11	-15	-6	0.9	0.9	0.9	0.9
KMT2D	O14686	2249	-8	-17	-15	-26	0.9	0.9	0.9	0.8
PTPN7	P35236	62	-8	-10	-15	-11	0.9	0.9	0.9	0.9
FASN	P49327	1828	-8	-14	-15	-11	0.9	0.9	0.9	0.9
UBA1	P22314	1039	-8	-15	-15	-16	0.9	0.9	0.9	0.9
CYLD	Q9NQC7	751	-8	-18	-15	-18	0.9	0.8	0.9	0.9
IPO4	Q8TEX9	708	-8	-24	-15	-20	0.9	0.8	0.9	0.8
KRR1	Q13601	142	-8	-29	-15	-7	0.9	0.8	0.9	0.9
GEMIN4	P57678	927	-9	-8	-15	-9	0.9	0.9	0.9	0.9
PHRF1	Q9P1Y6	878	-9	-11	-15	-23	0.9	0.9	0.9	0.8
APPL1	Q9UKG1	549	-10	-10	-15	-2	0.9	0.9	0.9	1.0
ALDH5A1	P51649	340	-10	-19	-15	-19	0.9	0.8	0.9	0.8
GART	P22102	646	-11	-22	-15	-10	0.9	0.8	0.9	0.9
GFM2	Q969S9	248	-11	-25	-15	-29	0.9	0.8	0.9	0.8
SDE2	Q6IQ49	415	-11	-19	-15	-13	0.9	0.8	0.9	0.9
DESI1	Q6ICB0	108	-12	-5	-15	13	0.9	1.0	0.9	1.1
CTBP1	Q13363	134	-13	-10	-15	-9	0.9	0.9	0.9	0.9
NABP2	Q9BQ15	81	-13	-11	-15	7	0.9	0.9	0.9	1.1
PPP6R1	Q9UPN7	455	-14	-14	-15	-22	0.9	0.9	0.9	0.8
UBR1	Q8IWV7	1603	-14	-23	-15	-19	0.9	0.8	0.9	0.8
UNC119B	A6NIH7	117	-15	-9	-15	-7	0.9	0.9	0.9	0.9
COPS3	Q9UNS2	25	-16	-15	-15	-2	0.9	0.9	0.9	1.0

DYNC1H1	Q14204	3573	-16	-20	-15	-15	0.9	0.8	0.9	0.9
AAR2	Q9Y312	181	-17	-21	-15	-34	0.9	0.8	0.9	0.7
NOP56	O00567	384	-17	-22	-15	-27	0.9	0.8	0.9	0.8
NMI	Q13287	295	-17	-23	-15	-5	0.9	0.8	0.9	1.0
PADI2	Q9Y2J8	629	-18	-6	-15	-12	0.9	0.9	0.9	0.9
SPCS2	Q15005	26	-18	-15	-15	-13	0.8	0.9	0.9	0.9
SMS	P52788	337	-32	-18	-15	-7	0.8	0.8	0.9	0.9
HNRNPA0	Q13151	36	21	-51	-15	-5	1.3	0.7	0.9	1.0
QDPR	P09417	104	15	-20	-15	19	1.2	0.8	0.9	1.2
AQP3	Q92482	11	13	-10	-15	-7	1.1	0.9	0.9	0.9
MYO18A	Q92614	1155	13	-15	-15	-3	1.1	0.9	0.9	1.0
PPP4R1	Q8TF05	645	12	-1	-15	-7	1.1	1.0	0.9	0.9
RASGRP4	Q8TDF6	574	12	-5	-15	-15	1.1	1.0	0.9	0.9
BANF1	O75531	77	12	-23	-15	-22	1.1	0.8	0.9	0.8
XPC	Q01831	680	11	-31	-15	-11	1.1	0.8	0.9	0.9
DMXL1	Q9Y485	2618	9	-12	-15	-24	1.1	0.9	0.9	0.8
RNF213	Q63HN8	4111	9	-14	-15	4	1.1	0.9	0.9	1.0
SYNE2	Q8WXH0	6161	8	-16	-15	-12	1.1	0.9	0.9	0.9
LYN	P07948	381	8	-31	-15	-10	1.1	0.8	0.9	0.9
CBLL1	Q75N03	133	7	-1	-15	8	1.1	1.0	0.9	1.1
ATIC	P31939	575	7	-12	-15	8	1.1	0.9	0.9	1.1
RNF150	Q9ULK6	102	7	-21	-15	-23	1.1	0.8	0.9	0.8
BBX	Q8WY36	606	7	-28	-15	-14	1.1	0.8	0.9	0.9
NLN	Q9BYT8	272	6	3	-15	-6	1.1	1.0	0.9	0.9
APAF1	O14727	158	6	-14	-15	-8	1.1	0.9	0.9	0.9
TRAPPC11	Q7Z392	658	6	-12	-15	-1	1.1	0.9	0.9	1.0
PRKRA	O75569	106	5	-1	-15	-28	1.1	1.0	0.9	0.8
CYFIP1	Q7L576	993	5	-37	-15	-4	1.1	0.7	0.9	1.0
RPE	Q96AT9	217	5	-24	-15	-33	1.0	0.8	0.9	0.8
ROCK2	O75116	314	4	-11	-15	-2	1.0	0.9	0.9	1.0
BBX	Q8WY36	712	4	-10	-15	-20	1.0	0.9	0.9	0.8
ATP6V1A	P38606	394	4	-19	-15	-6	1.0	0.8	0.9	0.9
NDUFV1	P49821	425	3	-17	-15	-5	1.0	0.9	0.9	1.0
UHRF1BP1L	A0JNW5	81	3	-19	-15	5	1.0	0.8	0.9	1.0
FBP1	P09467	93	3	-23	-15	-6	1.0	0.8	0.9	0.9
CCM2	Q9BSQ5	170	2	-13	-15	-10	1.0	0.9	0.9	0.9
RAB10	P61026	124	2	-11	-15	-9	1.0	0.9	0.9	0.9
PIKFYVE	Q9Y2I7	1663	2	-16	-15	-9	1.0	0.9	0.9	0.9
IDH2	P48735	336	2	-25	-15	18	1.0	0.8	0.9	1.2
PGM2	Q96G03	510	2	-28	-15	-8	1.0	0.8	0.9	0.9
INO80	Q9ULG1	108	1	-14	-15	-20	1.0	0.9	0.9	0.8
DPP4	P27487	551	1	-17	-15	1	1.0	0.9	0.9	1.0
MCM5	P33992	482	1	-17	-15	-33	1.0	0.9	0.9	0.8
FPGT	O14772	531	1	-19	-15	-19	1.0	0.8	0.9	0.8
SMG8	Q8ND04	248	1	-19	-15	-6	1.0	0.8	0.9	0.9
PGK1	P00558	99	1	-21	-15	-4	1.0	0.8	0.9	1.0
TRIO	O75962	2909	1	-28	-15	15	1.0	0.8	0.9	1.2
PDIA4	P13667	209	0	-1	-15	14	1.0	1.0	0.9	1.2
WDR33	Q9C0J8	220	0	-11	-15	-18	1.0	0.9	0.9	0.9
PRPF8	Q6P2Q9	1194	0	-19	-15	-9	1.0	0.8	0.9	0.9
KAT6B	Q8WYB5	266	0	-20	-15	-14	1.0	0.8	0.9	0.9
DPF2	Q92785	324	0	-20	-15	-14	1.0	0.8	0.9	0.9
KAT6A	Q92794	259	0	-20	-15	-14	1.0	0.8	0.9	0.9

RPUSD3	Q6P087	345	-1	-16	-15	-15	1.0	0.9	0.9	0.9
MPG	P29372	215	-1	-14	-15	-8	1.0	0.9	0.9	0.9
MRPL14	Q6P1L8	57	-1	-16	-15	4	1.0	0.9	0.9	1.0
THUMPD2	Q9BTF0	161	-1	-21	-15	-22	1.0	0.8	0.9	0.8
PNKP	Q96T60	405	-1	-24	-15	-10	1.0	0.8	0.9	0.9
MDH2	P40926	285	-1	-24	-15	-10	1.0	0.8	0.9	0.9
TOP1	P11387	300	-1	-26	-15	-4	1.0	0.8	0.9	1.0
PPIA	P62937	161	-1	-29	-15	-6	1.0	0.8	0.9	0.9
LUC7L3	O95232	58	-2	-10	-15	-8	1.0	0.9	0.9	0.9
C2orf69	Q8N8R5	43	-2	-22	-15	-12	1.0	0.8	0.9	0.9
SRRT	Q9BXP5	628	-2	4	-15	-2	1.0	1.0	0.9	1.0
SQRDL	Q9Y6N5	127	-2	-12	-15	-4	1.0	0.9	0.9	1.0
PCK2	Q16822	230	-2	-14	-15	34	1.0	0.9	0.9	1.5
ABTB1	Q969K4	291	-3	-5	-15	23	1.0	1.0	0.9	1.3
SYNE1	Q8NF91	8052	-3	-14	-15	1	1.0	0.9	0.9	1.0
CD14	P08571	306	-3	-14	-15	-1	1.0	0.9	0.9	1.0
SRSF6	Q13247	121	-3	-21	-15	-14	1.0	0.8	0.9	0.9
PPIA	P62937	115	-3	-31	-15	-6	1.0	0.8	0.9	0.9
DYSF	O75923	1398	-3	-36	-15	-18	1.0	0.7	0.9	0.8
CYFIP1	Q7L576	98	-4	-24	-15	-26	1.0	0.8	0.9	0.8
CYFIP2	Q96F07	98	-4	-24	-15	-26	1.0	0.8	0.9	0.8
LMNB1	P20700	198	-4	-29	-15	-20	1.0	0.8	0.9	0.8
LCP1	P13796	283	-4	-16	-15	-2	1.0	0.9	0.9	1.0
POT1	Q9NUX5	329	-4	-20	-15	-10	1.0	0.8	0.9	0.9
ATP13A2	Q9NQ11	365	-4	-21	-15	-19	1.0	0.8	0.9	0.8
SUGP2	Q8IX01	417	-4	-23	-15	-15	1.0	0.8	0.9	0.9
PML	P29590	140	-4	-23	-15	-18	1.0	0.8	0.9	0.9
CMAS	Q8NFW8	405	-4	-24	-15	-11	1.0	0.8	0.9	0.9
CASP4	P49662	109	-5	-14	-15	-9	1.0	0.9	0.9	0.9
OAS3	Q9Y6K5	850	-5	-23	-15	-19	1.0	0.8	0.9	0.8
CBX8	Q9HC52	261	-5	-10	-15	-12	1.0	0.9	0.9	0.9
IMMT	Q16891	697	-5	-14	-15	-7	1.0	0.9	0.9	0.9
IMPA1	P29218	125	-5	-26	-15	-3	1.0	0.8	0.9	1.0
KIF21B	O75037	697	-6	-12	-15	-2	0.9	0.9	0.9	1.0
FAM98C	Q17RN3	287	-6	-15	-15	-18	0.9	0.9	0.9	0.8
MND1	Q9BWT6	115	-6	-6	-15	-24	0.9	0.9	0.9	0.8
SEPT9	Q9UHD8	531	-6	-11	-15	-14	0.9	0.9	0.9	0.9
SMARCA2	P51531	1401	-6	-25	-15	-33	0.9	0.8	0.9	0.8
MTMR3	Q13615	1152	-7	-23	-15	-23	0.9	0.8	0.9	0.8
PRDX4	Q13162	245	-7	-27	-15	-6	0.9	0.8	0.9	0.9
Uncharacteri	K7ESF4	111	-8	-15	-15	2	0.9	0.9	0.9	1.0
PDCD2L	Q9BRP1	82	-8	-15	-15	2	0.9	0.9	0.9	1.0
OFD1	O75665	503	-8	-4	-15	-21	0.9	1.0	0.9	0.8
PITPNC1	Q9UKF7	136	-8	-8	-15	-7	0.9	0.9	0.9	0.9
PRDX1	Q06830	71	-8	-8	-15	-11	0.9	0.9	0.9	0.9
OSTC	Q9NRP0	14	-8	-12	-15	-3	0.9	0.9	0.9	1.0
GPCPD1	Q9NPB8	642	-8	-18	-15	-13	0.9	0.8	0.9	0.9
HNRNPA2B1	P22626	50	-8	-30	-15	3	0.9	0.8	0.9	1.0
MYO1G	B0I1T2	97	-8	-36	-15	8	0.9	0.7	0.9	1.1
DHX9	Q08211	777	-9	-17	-15	-11	0.9	0.9	0.9	0.9
NBN	O60934	487	-9	-17	-15	-22	0.9	0.9	0.9	0.8
MAP2K2	P36507	211	-9	-21	-15	4	0.9	0.8	0.9	1.0
MAP2K1	Q02750	207	-9	-21	-15	4	0.9	0.8	0.9	1.0

PHF20	Q9BVI0	59	-9	-18	-15	-37	0.9	0.9	0.9	0.7
PFKFB3	Q16875	412	-10	-12	-15	-8	0.9	0.9	0.9	0.9
PFKFB4	Q16877	415	-10	-12	-15	-8	0.9	0.9	0.9	0.9
TUBA4A	P68366	315	-10	-16	-15	-10	0.9	0.9	0.9	0.9
TUBA1A	Q71U36	315	-10	-16	-15	-10	0.9	0.9	0.9	0.9
ADAP1	O75689	101	-10	-18	-15	2	0.9	0.8	0.9	1.0
ACAA1	P09110	381	-10	-21	-15	-6	0.9	0.8	0.9	0.9
ABCC1	P33527	1047	-10	-26	-15	11	0.9	0.8	0.9	1.1
PSMD10	O75832	180	-10	-29	-15	-12	0.9	0.8	0.9	0.9
LPXN	O60711	332	-12	-13	-15	-16	0.9	0.9	0.9	0.9
ADH5	P11766	97	-12	-21	-15	-6	0.9	0.8	0.9	0.9
HIP1R	O75146	650	-12	-12	-15	-36	0.9	0.9	0.9	0.7
NEDD1	Q8NHV4	64	-12	-15	-15	-6	0.9	0.9	0.9	0.9
TRIM58	Q8NG06	278	-12	-16	-15	-2	0.9	0.9	0.9	1.0
TRAPPC9	Q96Q05	935	-13	-22	-15	-4	0.9	0.8	0.9	1.0
GMPR	P36959	316	-13	-26	-15	-8	0.9	0.8	0.9	0.9
GMPR2	Q9P2T1	316	-13	-26	-15	-8	0.9	0.8	0.9	0.9
OGT	O15294	315	-14	-17	-15	-17	0.9	0.9	0.9	0.9
WDR1	O75083	194	-14	-25	-15	-2	0.9	0.8	0.9	1.0
GATAD2A	Q86YP4	426	-14	-6	-15	-10	0.9	0.9	0.9	0.9
PPA1	Q15181	270	-15	-8	-15	-23	0.9	0.9	0.9	0.8
SEPHS1	P49903	337	-15	-24	-15	-14	0.9	0.8	0.9	0.9
PPIL4	Q8WUA2	25	-15	-28	-15	-10	0.9	0.8	0.9	0.9
COL4A3BP	Q9Y5P4	65	-15	-32	-15	-8	0.9	0.8	0.9	0.9
NUP85	Q9BW27	51	-16	-15	-15	-3	0.9	0.9	0.9	1.0
TRIM27	P14373	393	-16	-22	-15	-35	0.9	0.8	0.9	0.7
NLRC5	Q86WI3	1113	-16	-4	-15	-9	0.9	1.0	0.9	0.9
PFKFB3	Q16875	440	-17	-40	-15	-13	0.9	0.7	0.9	0.9
TOMM40	O96008	76	-18	-5	-15	-29	0.9	1.0	0.9	0.8
UTP23	Q9BRU9	91	-21	-26	-15	-6	0.8	0.8	0.9	0.9
SMG7	Q92540	345	-22	-31	-15	3	0.8	0.8	0.9	1.0
PRKDC	P78527	2469	-23	-13	-15	-6	0.8	0.9	0.9	0.9
RCSD1	Q6JBY9	381	-23	-40	-15	-40	0.8	0.7	0.9	0.7
RBM47	A0AV96	273	-25	-17	-15	-10	0.8	0.9	0.9	0.9
COPB1	P53618	189	-26	-42	-15	-3	0.8	0.7	0.9	1.0
ANKHD1	Q8IWZ3	643	-27	-22	-15	-19	0.8	0.8	0.9	0.8
TRAPPC10	P48553	373	-38	-29	-15	-37	0.7	0.8	0.9	0.7
PREP	P48147	57	-39	-30	-15	-7	0.7	0.8	0.9	0.9
ETHE1	O95571	98	-49	-47	-15	-5	0.7	0.7	0.9	1.0
RANBP2	P49792	581	12	-2	-16	15	1.1	1.0	0.9	1.2
ECH1	Q13011	159	12	-8	-16	-5	1.1	0.9	0.9	1.0
AGO3	Q9H9G7	345	10	-11	-16	-6	1.1	0.9	0.9	0.9
AGO2	Q9UKV8	344	10	-11	-16	-6	1.1	0.9	0.9	0.9
AGO1	Q9UL18	342	10	-11	-16	-6	1.1	0.9	0.9	0.9
TMEM154	Q6P9G4	164	9	-19	-16	-21	1.1	0.8	0.9	0.8
STX17	P56962	290	9	-5	-16	-24	1.1	1.0	0.9	0.8
MAVS	Q7Z434	20	9	-14	-16	-14	1.1	0.9	0.9	0.9
PGK1	P00558	367	7	-18	-16	-4	1.1	0.8	0.9	1.0
PRKD2	Q9BZL6	425	7	-20	-16	1	1.1	0.8	0.9	1.0
SIRT7	Q9NRC8	79	6	-2	-16	-1	1.1	1.0	0.9	1.0
TOLLIP	Q9H0E2	229	5	-14	-16	-19	1.1	0.9	0.9	0.8
HDGF	P51858	12	5	-21	-16	-19	1.1	0.8	0.9	0.8
USP39	Q53GS9	105	5	-5	-16	-1	1.0	1.0	0.9	1.0

CCDC109B	Q9NWR8	147	5	-28	-16	-11	1.0	0.8	0.9	0.9
PRIMPOL	Q96LW4	69	4	-4	-16	20	1.0	1.0	0.9	1.3
PSMA6	P60900	78	4	-10	-16	-7	1.0	0.9	0.9	0.9
ZNF20	P17024	378	4	-15	-16	-13	1.0	0.9	0.9	0.9
MNDA	P41218	385	4	-20	-16	-9	1.0	0.8	0.9	0.9
RAP1B	P61224	118	4	-20	-16	-3	1.0	0.8	0.9	1.0
NLRC5	Q86WI3	672	4	8	-16	-8	1.0	1.1	0.9	0.9
ITPR2	Q14571	2169	4	6	-16	-5	1.0	1.1	0.9	1.0
PYGL	P06737	79	4	-10	-16	-7	1.0	0.9	0.9	0.9
ARHGAP9	Q9BRR9	239	4	-31	-16	-21	1.0	0.8	0.9	0.8
DENND4C	Q5VZ89	809	3	3	-16	-13	1.0	1.0	0.9	0.9
SAMHD1	Q9Y3Z3	177	3	-18	-16	17	1.0	0.8	0.9	1.2
XRCC5	P13010	346	2	-27	-16	-7	1.0	0.8	0.9	0.9
CAD	P27708	252	2	-8	-16	3	1.0	0.9	0.9	1.0
APOBR	Q0VD83	895	2	-15	-16	-8	1.0	0.9	0.9	0.9
ZMYM3	Q14202	686	2	-16	-16	-8	1.0	0.9	0.9	0.9
MCM3	P25205	446	2	-26	-16	-23	1.0	0.8	0.9	0.8
DOCK9	Q9BZ29	1292	1	-15	-16	-10	1.0	0.9	0.9	0.9
CRBN	Q96SW2	205	1	-21	-16	-23	1.0	0.8	0.9	0.8
CKAP5	Q14008	619	1	-31	-16	-15	1.0	0.8	0.9	0.9
IRF2BP2	Q7Z5L9	16	1	-7	-16	-1	1.0	0.9	0.9	1.0
IRF2BPL	Q9H1B7	14	1	-7	-16	-1	1.0	0.9	0.9	1.0
GANAB	Q14697	41	1	-19	-16	-6	1.0	0.8	0.9	0.9
DEF6	Q9H4E7	246	1	-25	-16	-15	1.0	0.8	0.9	0.9
DHX16	O60231	932	0	-12	-16	-25	1.0	0.9	0.9	0.8
PGD	P52209	402	-1	-9	-16	7	1.0	0.9	0.9	1.1
Uncharacteri	F8W031	28	-1	-10	-16	-3	1.0	0.9	0.9	1.0
STT3A	P46977	637	-1	-48	-16	-11	1.0	0.7	0.9	0.9
PLXNC1	O60486	1061	-1	-7	-16	-18	1.0	0.9	0.9	0.9
DEPDC5	O75140	481	-1	-14	-16	-13	1.0	0.9	0.9	0.9
RARS	P54136	34	-1	-20	-16	-22	1.0	0.8	0.9	0.8
STK11	Q15831	418	-2	-12	-16	-15	1.0	0.9	0.9	0.9
GLUD1	P00367	172	-2	-13	-16	-6	1.0	0.9	0.9	0.9
GNB1L	Q9BYB4	116	-2	-14	-16	-12	1.0	0.9	0.9	0.9
GCN1	Q92616	648	-2	-10	-16	-14	1.0	0.9	0.9	0.9
EP400	Q96L91	2013	-2	-19	-16	-2	1.0	0.8	0.9	1.0
RPS6KA3	P51812	436	-2	-23	-16	-14	1.0	0.8	0.9	0.9
ATP2B4	P23634	721	-2	-24	-16	-14	1.0	0.8	0.9	0.9
ZNF276	Q8N554	18	-3	-5	-16	-29	1.0	1.0	0.9	0.8
TXNDC17	Q9BRA2	43	-3	-6	-16	-21	1.0	0.9	0.9	0.8
ETHE1	O95571	247	-3	-12	-16	-16	1.0	0.9	0.9	0.9
LUC7L3	O95232	43	-3	-15	-16	-5	1.0	0.9	0.9	1.0
PIK3CG	P48736	395	-3	-20	-16	-7	1.0	0.8	0.9	0.9
PHIP	Q8WWQ0	28	-3	-19	-16	-16	1.0	0.8	0.9	0.9
SOS1	Q07889	405	-3	-23	-16	0	1.0	0.8	0.9	1.0
RASA2	Q15283	691	-4	-10	-16	-19	1.0	0.9	0.9	0.8
HCFC1	P51610	353	-4	-18	-16	-10	1.0	0.8	0.9	0.9
C20orf27	Q9GZN8	131	-4	-23	-16	-24	1.0	0.8	0.9	0.8
ACSL5	Q9ULC5	69	-4	-28	-16	-7	1.0	0.8	0.9	0.9
CCAR1	Q8IX12	974	-4	-9	-16	1	1.0	0.9	0.9	1.0
EXO5	Q9H790	245	-4	-17	-16	-19	1.0	0.9	0.9	0.8
LPXN	O60711	211	-4	-20	-16	-8	1.0	0.8	0.9	0.9
FLNA	P21333	1453	-4	-26	-16	-8	1.0	0.8	0.9	0.9

CNST	Q6PJW8	317	-5	-11	-16	-27	1.0	0.9	0.9	0.8
DPYD	Q12882	82	-5	-29	-16	-7	1.0	0.8	0.9	0.9
APEH	P13798	30	-5	-9	-16	-14	1.0	0.9	0.9	0.9
UQCRC1	P31930	268	-5	-11	-16	-7	1.0	0.9	0.9	0.9
ACTR3B	Q9P1U1	235	-5	-12	-16	-13	1.0	0.9	0.9	0.9
PDLIM2	Q96JY6	310	-5	-15	-16	-2	1.0	0.9	0.9	1.0
LAGE3	Q14657	113	-5	-23	-16	-22	1.0	0.8	0.9	0.8
GCC2	Q8IWJ2	1306	-5	-24	-16	-29	1.0	0.8	0.9	0.8
SASH1	O94885	1120	-6	-8	-16	-12	0.9	0.9	0.9	0.9
DYNC1H1	Q14204	4570	-6	-20	-16	-11	0.9	0.8	0.9	0.9
MBD1	Q9UIS9	344	-6	20	-16	-10	0.9	1.2	0.9	0.9
FLNA	P21333	1997	-6	-17	-16	-9	0.9	0.9	0.9	0.9
AKAP9	Q99996	3525	-7	-10	-16	-23	0.9	0.9	0.9	0.8
USP48	Q86UV5	658	-7	-15	-16	-20	0.9	0.9	0.9	0.8
EP400	Q96L91	879	-7	-18	-16	-6	0.9	0.9	0.9	0.9
CBX3	Q13185	69	-7	-18	-16	-11	0.9	0.9	0.9	0.9
ANP32E	Q9BTT0	87	-8	-18	-16	-2	0.9	0.9	0.9	1.0
GARS	P41250	461	-8	-15	-16	8	0.9	0.9	0.9	1.1
USP47	Q96K76	767	-8	-23	-16	-17	0.9	0.8	0.9	0.9
YTHDC2	Q9H6S0	25	-9	-20	-16	-4	0.9	0.8	0.9	1.0
B3GNT5	Q9BYG0	83	-9	-22	-16	-36	0.9	0.8	0.9	0.7
SF3B3	Q15393	1156	-10	-19	-16	16	0.9	0.8	0.9	1.2
EXOC5	O00471	71	-10	-36	-16	-23	0.9	0.7	0.9	0.8
LAS1L	Q9Y4W2	316	-10	-14	-16	4	0.9	0.9	0.9	1.0
NBN	O60934	478	-10	-18	-16	-4	0.9	0.9	0.9	1.0
RAVER1	Q8IY67	87	-10	-30	-16	-9	0.9	0.8	0.9	0.9
AHCTF1	Q8WYP5	1628	-11	-13	-16	-32	0.9	0.9	0.9	0.8
TMLHE	Q9NVH6	51	-11	-20	-16	-6	0.9	0.8	0.9	0.9
SURF6	O75683	189	-11	-24	-16	-24	0.9	0.8	0.9	0.8
MEFV	O15553	773	-11	-32	-16	-15	0.9	0.8	0.9	0.9
SLFN5	Q08AF3	268	-12	-20	-16	-9	0.9	0.8	0.9	0.9
RPUSD2	Q8IZ73	246	-12	-10	-16	-44	0.9	0.9	0.9	0.7
FAAH	O00519	166	-13	-14	-16	-2	0.9	0.9	0.9	1.0
CAPN2	P17655	301	-13	-23	-16	-2	0.9	0.8	0.9	1.0
DTWD2	Q8NBA8	220	-13	-25	-16	-8	0.9	0.8	0.9	0.9
LAS1L	Q9Y4W2	474	-13	-14	-16	-3	0.9	0.9	0.9	1.0
TPMT	P51580	70	-13	-16	-16	-10	0.9	0.9	0.9	0.9
EIF3I	Q13347	81	-13	-32	-16	1	0.9	0.8	0.9	1.0
MTMR12	Q9C0I1	67	-14	-26	-16	-23	0.9	0.8	0.9	0.8
CASP10	Q92851	169	-14	-20	-16	-28	0.9	0.8	0.9	0.8
DOCK5	Q9H7D0	508	-14	-24	-16	-22	0.9	0.8	0.9	0.8
EIF3B	P55884	420	-20	-20	-16	-19	0.8	0.8	0.9	0.8
ZNF592	Q92610	463	-20	-22	-16	-10	0.8	0.8	0.9	0.9
SNAPC4	Q5SXM2	1309	-21	-22	-16	-20	0.8	0.8	0.9	0.8
BAZ2B	Q9UIF8	212	-21	-32	-16	-25	0.8	0.8	0.9	0.8
DOCK10	Q96BY6	238	-22	-24	-16	-12	0.8	0.8	0.9	0.9
MUL1	Q969V5	113	-23	-36	-16	6	0.8	0.7	0.9	1.1
C14orf93	Q9H972	287	-25	-18	-16	-6	0.8	0.8	0.9	0.9
PNPLA6	Q8IY17	1199	-25	-30	-16	-16	0.8	0.8	0.9	0.9
ITGA4	P13612	198	-32	-20	-16	-20	0.8	0.8	0.9	0.8
PPIL3	Q9H2H8	18	-43	-64	-16	-26	0.7	0.6	0.9	0.8
RAN	P62826	120	-47	-39	-16	-7	0.7	0.7	0.9	0.9
PLBD2	Q8NHP8	342	-55	-41	-16	-2	0.6	0.7	0.9	1.0

HDAC3	O15379	268	28	4	-16	18	1.4	1.0	0.9	1.2
HK1	P19367	823	20	-26	-16	14	1.2	0.8	0.9	1.2
CBLB	Q13191	895	17	-7	-16	0	1.2	0.9	0.9	1.0
PA2G4	Q9UQ80	49	14	-12	-16	-4	1.2	0.9	0.9	1.0
VAMP7	P51809	21	14	5	-16	-7	1.2	1.0	0.9	0.9
CALR	P27797	105	11	-28	-16	-4	1.1	0.8	0.9	1.0
THADA	Q6YHU6	588	10	-7	-16	-2	1.1	0.9	0.9	1.0
ATP2A2	P16615	364	10	-29	-16	-4	1.1	0.8	0.9	1.0
ATP2A3	Q93084	364	10	-29	-16	-4	1.1	0.8	0.9	1.0
RBM17	Q96I25	339	10	-20	-16	-2	1.1	0.8	0.9	1.0
TTC37	Q6PGP7	352	9	-24	-16	-15	1.1	0.8	0.9	0.9
DGCR14	Q96DF8	263	9	-12	-16	-13	1.1	0.9	0.9	0.9
SND1	Q7KZF4	549	8	-14	-16	-5	1.1	0.9	0.9	1.0
PTPRC	P08575	1070	7	-10	-16	0	1.1	0.9	0.9	1.0
SART3	Q15020	537	7	-12	-16	-3	1.1	0.9	0.9	1.0
ABCF1	Q8NE71	655	7	-23	-16	-5	1.1	0.8	0.9	1.0
RASA3	Q14644	751	5	-16	-16	-9	1.1	0.9	0.9	0.9
CCT6A	P40227	406	5	-4	-16	-7	1.0	1.0	0.9	0.9
AKR1B1	P15121	187	5	-13	-16	-2	1.0	0.9	0.9	1.0
ABCE1	P61221	38	5	-19	-16	-13	1.0	0.8	0.9	0.9
STX5	Q13190	68	5	-30	-16	-21	1.0	0.8	0.9	0.8
C9orf78	Q9NZ63	145	4	-13	-16	0	1.0	0.9	0.9	1.0
OXCT1	P55809	235	4	-24	-16	-10	1.0	0.8	0.9	0.9
FLII	Q13045	119	3	-10	-16	-11	1.0	0.9	0.9	0.9
TTC37	Q6PGP7	1162	3	-12	-16	-11	1.0	0.9	0.9	0.9
FLOT2	Q14254	56	2	-20	-16	6	1.0	0.8	0.9	1.1
ARHGDIA	P52565	79	2	-40	-16	-9	1.0	0.7	0.9	0.9
RRP36	Q96EU6	87	2	-8	-16	-14	1.0	0.9	0.9	0.9
ACTR6	Q9GZN1	161	1	-19	-16	-7	1.0	0.8	0.9	0.9
BRAT1	Q6PJG6	308	1	-15	-16	11	1.0	0.9	0.9	1.1
AGPS	O00116	404	0	-22	-16	2	1.0	0.8	0.9	1.0
HEATR1	Q9H583	1010	-1	-17	-16	-1	1.0	0.9	0.9	1.0
RCC2	Q9P258	305	-1	-21	-16	-14	1.0	0.8	0.9	0.9
RHOG	P84095	18	-1	-35	-16	-4	1.0	0.7	0.9	1.0
GSPT1	P15170	453	-1	-14	-16	-14	1.0	0.9	0.9	0.9
GSPT2	Q8IYD1	582	-1	-14	-16	-14	1.0	0.9	0.9	0.9
ADRBK1	P25098	208	-1	-15	-16	-7	1.0	0.9	0.9	0.9
ADRBK2	P35626	208	-1	-15	-16	-7	1.0	0.9	0.9	0.9
MICAL1	Q8TDZ2	82	-1	-17	-16	3	1.0	0.9	0.9	1.0
ZFYVE26	Q68DK2	66	-2	-23	-16	-14	1.0	0.8	0.9	0.9
DMXL2	Q8TDJ6	1833	-2	-14	-16	9	1.0	0.9	0.9	1.1
OCIAD2	Q56VL3	137	-2	-15	-16	-5	1.0	0.9	0.9	1.0
NCK2	O43639	144	-2	-29	-16	3	1.0	0.8	0.9	1.0
NCK1	P16333	139	-2	-29	-16	3	1.0	0.8	0.9	1.0
MSL1	Q68DK7	221	-3	-8	-16	-14	1.0	0.9	0.9	0.9
DOCK8	Q8NF50	197	-3	-9	-16	-3	1.0	0.9	0.9	1.0
ILF3	Q12906	295	-3	-13	-16	-1	1.0	0.9	0.9	1.0
TRIM56	Q9BRZ2	514	-3	-24	-16	-6	1.0	0.8	0.9	0.9
CDK13	Q14004	701	-3	-9	-16	5	1.0	0.9	0.9	1.0
CDK12	Q9NYV4	723	-3	-9	-16	5	1.0	0.9	0.9	1.0
SMYD5	Q6GMV2	101	-3	-9	-16	-3	1.0	0.9	0.9	1.0
TCIRG1	Q13488	238	-3	-12	-16	-6	1.0	0.9	0.9	0.9
RAB7A	P51149	143	-3	-21	-16	-11	1.0	0.8	0.9	0.9

HUWE1	Q7Z6Z7	1892	-3	-22	-16	-13	1.0	0.8	0.9	0.9
TKT	P29401	225	-3	-23	-16	-6	1.0	0.8	0.9	0.9
USP14	P54578	277	-3	-24	-16	-12	1.0	0.8	0.9	0.9
PLEKHA2	Q9HB19	232	-4	-9	-16	-19	1.0	0.9	0.9	0.8
IGHD	A0A0A0MS0[illegible]	161	-4	-17	-16	2	1.0	0.9	0.9	1.0
HUWE1	Q7Z6Z7	1401	-4	-18	-16	-19	1.0	0.8	0.9	0.8
DIP2A	Q14689	252	-4	-19	-16	-29	1.0	0.8	0.9	0.8
ANKFY1	Q9P2R3	716	-5	-4	-16	-2	1.0	1.0	0.9	1.0
IFI16	Q16666	727	-5	-18	-16	-6	1.0	0.9	0.9	0.9
PHPT1	Q9NRX4	69	-5	-25	-16	-9	1.0	0.8	0.9	0.9
DOCK11	Q5JSL3	160	-5	-14	-16	-12	1.0	0.9	0.9	0.9
XAB2	Q9HCS7	86	-5	-19	-16	-10	1.0	0.8	0.9	0.9
FBXL18	Q96ME1	423	-5	-21	-16	-13	1.0	0.8	0.9	0.9
TBX21	Q9UL17	207	-5	-29	-16	-28	1.0	0.8	0.9	0.8
ALDH9A1	P49189	443	-5	-31	-16	-14	1.0	0.8	0.9	0.9
UQCRC1	P31930	380	-5	-41	-16	-11	1.0	0.7	0.9	0.9
GBP5	Q96PP8	233	-6	-21	-16	-4	0.9	0.8	0.9	1.0
USP3	Q9Y6I4	362	-6	-21	-16	-7	0.9	0.8	0.9	0.9
BRE	Q9NXR7	129	-6	-30	-16	-17	0.9	0.8	0.9	0.9
RCAN3	Q9UKA8	239	-6	0	-16	-11	0.9	1.0	0.9	0.9
AGL	P35573	126	-6	-11	-16	5	0.9	0.9	0.9	1.0
MYLK	Q15746	1307	-6	-15	-16	16	0.9	0.9	0.9	1.2
DDB1	Q16531	732	-7	-28	-16	-6	0.9	0.8	0.9	0.9
ERP44	Q9BS26	92	-7	-38	-16	-5	0.9	0.7	0.9	1.0
RNF13	O43567	258	-7	-8	-16	-16	0.9	0.9	0.9	0.9
SYMPK	Q92797	859	-7	-24	-16	11	0.9	0.8	0.9	1.1
ARCN1	P48444	441	-7	-35	-16	-4	0.9	0.7	0.9	1.0
TBC1D2	Q9BYX2	651	-8	-17	-16	30	0.9	0.9	0.9	1.4
ANP32A	P39687	87	-8	-23	-16	-9	0.9	0.8	0.9	0.9
NUP153	P49790	66	-8	-13	-16	-14	0.9	0.9	0.9	0.9
GNA13	Q14344	37	-8	-17	-16	-12	0.9	0.9	0.9	0.9
COPA	P53621	1185	-8	-18	-16	-11	0.9	0.9	0.9	0.9
PLEC	Q15149	1122	-8	-21	-16	-19	0.9	0.8	0.9	0.8
ZDHHC6	Q9H6R6	398	-8	-22	-16	-29	0.9	0.8	0.9	0.8
GEMIN6	Q8WXD5	91	-9	-11	-16	17	0.9	0.9	0.9	1.2
CYLD	Q9NQC7	106	-9	-9	-16	-4	0.9	0.9	0.9	1.0
DAGLB	Q8NCG7	86	-9	-13	-16	-8	0.9	0.9	0.9	0.9
CAPRIN1	Q14444	226	-9	-21	-16	-16	0.9	0.8	0.9	0.9
RIT1	Q92963	158	-10	-19	-16	-12	0.9	0.8	0.9	0.9
POU2F2	P09086	346	-10	-10	-16	-7	0.9	0.9	0.9	0.9
CHD6	Q8TD26	172	-10	-27	-16	-28	0.9	0.8	0.9	0.8
GAPVD1	Q14C86	568	-11	-12	-16	-11	0.9	0.9	0.9	0.9
TOMM40	O96008	74	-11	-19	-16	-11	0.9	0.8	0.9	0.9
VCP	P55072	105	-11	-23	-16	1	0.9	0.8	0.9	1.0
VPS29	Q9UBQ0	41	-11	-31	-16	18	0.9	0.8	0.9	1.2
EIF2AK4	Q9P2K8	1245	-11	-8	-16	-4	0.9	0.9	0.9	1.0
JAK1	P23458	440	-11	-13	-16	-13	0.9	0.9	0.9	0.9
DDX1	Q92499	638	-12	-14	-16	2	0.9	0.9	0.9	1.0
UBR2	Q8IWV8	602	-12	-17	-16	-22	0.9	0.9	0.9	0.8
SCAF1	Q9H7N4	950	-12	-16	-16	-30	0.9	0.9	0.9	0.8
MSH6	P52701	615	-13	-16	-16	-19	0.9	0.9	0.9	0.8
ERH	P84090	33	-14	-24	-16	-11	0.9	0.8	0.9	0.9
AP5M1	Q9H0R1	18	-14	-42	-16	-29	0.9	0.7	0.9	0.8

UTP4	Q969X6	21	-14	-62	-16	-23	0.9	0.6	0.9	0.8
TRIM28	Q13263	83	-15	-21	-16	-16	0.9	0.8	0.9	0.9
CSDE1	O75534	42	-16	-22	-16	-9	0.9	0.8	0.9	0.9
TXLNA	P40222	523	-16	-8	-16	-24	0.9	0.9	0.9	0.8
PHIP	Q8WWQ0	954	-16	-9	-16	-2	0.9	0.9	0.9	1.0
PSME1	Q06323	101	-16	-19	-16	-23	0.9	0.8	0.9	0.8
MED23	Q9ULK4	1043	-17	-26	-16	-32	0.9	0.8	0.9	0.8
SP140	Q13342	745	-19	-22	-16	-43	0.8	0.8	0.9	0.7
SP140L	Q9H930	458	-19	-22	-16	-43	0.8	0.8	0.9	0.7
DFFB	O76075	194	-20	-13	-16	-12	0.8	0.9	0.9	0.9
STX12	Q86Y82	29	-21	-23	-16	-14	0.8	0.8	0.9	0.9
TAP1	Q03518	722	-21	-52	-16	-2	0.8	0.7	0.9	1.0
TMEM62	Q0P6H9	325	-22	-21	-16	-1	0.8	0.8	0.9	1.0
RPSA	P08865	163	-22	-18	-16	3	0.8	0.9	0.9	1.0
CETP	P11597	30	-22	-25	-16	-6	0.8	0.8	0.9	0.9
MORC3	Q14149	446	-23	-23	-16	-35	0.8	0.8	0.9	0.7
ANKRD17	O75179	210	-23	-19	-16	-37	0.8	0.8	0.9	0.7
ANKHD1	Q8IWZ3	181	-23	-19	-16	-37	0.8	0.8	0.9	0.7
ALKBH2	Q6NS38	192	-27	-27	-16	-23	0.8	0.8	0.9	0.8
ETHE1	O95571	80	-28	-30	-16	-23	0.8	0.8	0.9	0.8
APEX1	P27695	310	-61	-21	-16	0	0.6	0.8	0.9	1.0
HSP90AA1	P07900	597	-69	-32	-16	-18	0.6	0.8	0.9	0.9
NPC2	P61916	99	-70	-40	-16	-13	0.6	0.7	0.9	0.9
NFKBIE	O00221	345	19	-18	-17	-12	1.2	0.8	0.9	0.9
ATPAF1	Q5TC12	321	18	-30	-17	-13	1.2	0.8	0.9	0.9
COPG1	Q9Y678	129	16	-16	-17	-3	1.2	0.9	0.9	1.0
FIBP	O43427	262	13	0	-17	7	1.1	1.0	0.9	1.1
KRI1	Q8N9T8	521	11	-4	-17	-10	1.1	1.0	0.9	0.9
GPHN	Q9NQX3	26	9	-8	-17	-16	1.1	0.9	0.9	0.9
RBM33	Q96EV2	726	8	-22	-17	-24	1.1	0.8	0.9	0.8
NEK9	Q8TD19	375	8	-10	-17	-4	1.1	0.9	0.9	1.0
DDX50	Q9BQ39	417	7	-12	-17	-17	1.1	0.9	0.9	0.9
TRIM26	Q12899	334	7	-22	-17	-14	1.1	0.8	0.9	0.9
ARFGEF2	Q9Y6D5	166	7	-16	-17	-8	1.1	0.9	0.9	0.9
ARFGEF1	Q9Y6D6	178	7	-16	-17	-8	1.1	0.9	0.9	0.9
EIF2S3	P41091	101	6	-17	-17	-9	1.1	0.9	0.9	0.9
ITK	Q08881	339	6	-20	-17	-10	1.1	0.8	0.9	0.9
UBR4	Q5T4S7	1962	5	-4	-17	-31	1.0	1.0	0.9	0.8
ERO1A	Q96HE7	394	5	-15	-17	-11	1.0	0.9	0.9	0.9
MAVS	Q7Z434	33	5	-15	-17	4	1.0	0.9	0.9	1.0
RBM39	Q14498	478	4	-20	-17	-16	1.0	0.8	0.9	0.9
ZC3H7A	Q8IWR0	506	3	-15	-17	-16	1.0	0.9	0.9	0.9
SPTLC2	O15270	204	3	-23	-17	-5	1.0	0.8	0.9	1.0
IPO5	O00410	1057	3	-19	-17	-2	1.0	0.8	0.9	1.0
GSTM2	P28161	174	3	-32	-17	-8	1.0	0.8	0.9	0.9
DFFA	O00273	289	2	-26	-17	-2	1.0	0.8	0.9	1.0
SPTAN1	Q13813	2120	2	-27	-17	-15	1.0	0.8	0.9	0.9
BRMS1	Q9HCU9	136	1	-13	-17	-2	1.0	0.9	0.9	1.0
NMT2	O60551	485	1	-6	-17	-4	1.0	0.9	0.9	1.0
MAP3K8	P41279	398	1	-8	-17	-18	1.0	0.9	0.9	0.8
NUMB	P49757	611	0	-8	-17	-9	1.0	0.9	0.9	0.9
DEF6	Q9H4E7	244	0	-17	-17	-20	1.0	0.9	0.9	0.8
CYP20A1	Q6UW02	409	0	-19	-17	-15	1.0	0.8	0.9	0.9

CTR9	Q6PD62	196	0	-21	-17	-24	1.0	0.8	0.9	0.8
ABCE1	P61221	201	0	-24	-17	-17	1.0	0.8	0.9	0.9
NANS	Q9NR45	50	-1	-23	-17	-9	1.0	0.8	0.9	0.9
MCMBP	Q9BTE3	249	-2	3	-17	4	1.0	1.0	0.9	1.0
PIK3CD	O00329	416	-2	-6	-17	7	1.0	0.9	0.9	1.1
TBC1D1	Q86TI0	676	-2	-35	-17	-15	1.0	0.7	0.9	0.9
NIT2	Q9NQR4	44	-2	-15	-17	-1	1.0	0.9	0.9	1.0
DNAJC11	Q9NVH1	518	-2	-22	-17	-3	1.0	0.8	0.9	1.0
RIN3	Q8TB24	60	-3	5	-17	-1	1.0	1.1	0.9	1.0
CD99L2	Q8TCZ2	236	-3	-12	-17	-10	1.0	0.9	0.9	0.9
RHOA	P61586	159	-3	-15	-17	-1	1.0	0.9	0.9	1.0
POLR1A	O95602	1289	-3	-21	-17	-12	1.0	0.8	0.9	0.9
VPS35	Q96QK1	156	-3	-23	-17	-25	1.0	0.8	0.9	0.8
MED16	Q9Y2X0	539	-3	4	-17	2	1.0	1.0	0.9	1.0
RAB1A	P62820	126	-3	-25	-17	-8	1.0	0.8	0.9	0.9
VPS13C	Q709C8	2395	-3	-26	-17	-12	1.0	0.8	0.9	0.9
PPP6C	O00743	265	-3	-32	-17	-20	1.0	0.8	0.9	0.8
SFPQ	P23246	431	-4	-11	-17	-14	1.0	0.9	0.9	0.9
CD93	Q9NPY3	104	-4	-23	-17	-26	1.0	0.8	0.9	0.8
CSAD	Q9Y600	356	-4	-25	-17	-17	1.0	0.8	0.9	0.9
SYNE1	Q8NF91	2036	-4	-10	-17	-25	1.0	0.9	0.9	0.8
CD4	P01730	447	-4	-13	-17	-10	1.0	0.9	0.9	0.9
PRKCQ	Q04759	303	-5	-21	-17	-11	1.0	0.8	0.9	0.9
CTU1	Q7Z7A3	143	-6	-10	-17	-17	0.9	0.9	0.9	0.9
EIF5B	O60841	1126	-6	-17	-17	-4	0.9	0.9	0.9	1.0
RASA2	Q15283	170	-6	-19	-17	-14	0.9	0.8	0.9	0.9
CDC23	Q9UJX2	537	-6	-23	-17	-9	0.9	0.8	0.9	0.9
RAC2	P15153	157	-6	-25	-17	3	0.9	0.8	0.9	1.0
RAC1	P63000	157	-6	-25	-17	3	0.9	0.8	0.9	1.0
FAHD2B	Q6P2I3	215	-6	-29	-17	9	0.9	0.8	0.9	1.1
GCSH	P23434	138	-6	-36	-17	-15	0.9	0.7	0.9	0.9
PPIG	Q13427	10	-6	-14	-17	-8	0.9	0.9	0.9	0.9
NONO	Q15233	208	-6	-14	-17	-21	0.9	0.9	0.9	0.8
ADSS	P30520	182	-6	-25	-17	11	0.9	0.8	0.9	1.1
RBM26	Q5T8P6	25	-7	-1	-17	-7	0.9	1.0	0.9	0.9
BTK	Q06187	481	-7	-9	-17	-11	0.9	0.9	0.9	0.9
KCNAB2	Q13303	212	-7	-16	-17	-14	0.9	0.9	0.9	0.9
AMPD2	Q01433	795	-7	-20	-17	-13	0.9	0.8	0.9	0.9
BCAT2	O15382	345	-7	-16	-17	-5	0.9	0.9	0.9	1.0
ROCK2	O75116	887	-7	-21	-17	5	0.9	0.8	0.9	1.0
ANKRD50	Q9ULJ7	122	-7	-22	-17	-7	0.9	0.8	0.9	0.9
CNOT1	A5YKK6	1932	-7	-24	-17	18	0.9	0.8	0.9	1.2
MRPS35	P82673	212	-7	-27	-17	-14	0.9	0.8	0.9	0.9
DYSF	O75923	1884	-8	-15	-17	1	0.9	0.9	0.9	1.0
ALDOA	P04075	290	-8	-22	-17	-8	0.9	0.8	0.9	0.9
TRRAP	Q9Y4A5	555	-9	-11	-17	-5	0.9	0.9	0.9	1.0
AP2M1	Q96CW1	246	-9	-21	-17	-7	0.9	0.8	0.9	0.9
PREX1	Q8TCU6	558	-9	-4	-17	-4	0.9	1.0	0.9	1.0
RUVBL2	Q9Y230	413	-9	-23	-17	-25	0.9	0.8	0.9	0.8
MAEA	Q7L5Y9	195	-10	-13	-17	-14	0.9	0.9	0.9	0.9
NISCH	Q9Y2I1	185	-10	-24	-17	-5	0.9	0.8	0.9	1.0
SNRNP200	O75643	1278	-10	-23	-17	-18	0.9	0.8	0.9	0.9
RSL1D1	O76021	96	-10	-24	-17	-16	0.9	0.8	0.9	0.9

SYTL3	Q4VX76	66	-10	-42	-17	-30	0.9	0.7	0.9	0.8
COX20	Q5RI15	29	-11	-10	-17	1	0.9	0.9	0.9	1.0
PTPN23	Q9H3S7	218	-12	-19	-17	1	0.9	0.8	0.9	1.0
ECI2	O75521	380	-12	-12	-17	-22	0.9	0.9	0.9	0.8
TRIM25	Q14258	186	-13	-17	-17	-12	0.9	0.9	0.9	0.9
ANKMY2	Q8IV38	390	-14	-24	-17	-25	0.9	0.8	0.9	0.8
ABCE1	P61221	65	-14	-20	-17	-7	0.9	0.8	0.9	0.9
TCAF1	Q9Y4C2	487	-14	-20	-17	-18	0.9	0.8	0.9	0.9
ABHD10	Q9NUJ1	239	-16	-24	-17	-17	0.9	0.8	0.9	0.9
HDLBP	Q00341	960	-16	-5	-17	-7	0.9	1.0	0.9	0.9
HCCS	P53701	66	-16	-16	-17	-17	0.9	0.9	0.9	0.9
PPP6C	O00743	129	-16	-23	-17	-14	0.9	0.8	0.9	0.9
ARFGAP1	Q8N6T3	351	-17	-20	-17	-18	0.9	0.8	0.9	0.8
SKAP2	O75563	142	-18	-23	-17	-14	0.9	0.8	0.9	0.9
LYZ	P61626	146	-18	-25	-17	4	0.9	0.8	0.9	1.0
UBR5	O95071	1208	-19	-22	-17	-34	0.8	0.8	0.9	0.7
XRCC5	P13010	249	-19	-27	-17	-11	0.8	0.8	0.9	0.9
PPP4C	P60510	52	-20	-33	-17	-15	0.8	0.8	0.9	0.9
FLNB	O75369	455	-23	-21	-17	-19	0.8	0.8	0.9	0.8
APAF1	O14727	115	-26	-37	-17	-26	0.8	0.7	0.9	0.8
MRPS30	Q9NP92	293	-26	-13	-17	3	0.8	0.9	0.9	1.0
STAG1	Q8WVM7	644	-27	-46	-17	-24	0.8	0.7	0.9	0.8
LRRC8A	Q8IWT6	776	-29	-23	-17	-13	0.8	0.8	0.9	0.9
TTLL12	Q14166	361	-31	-26	-17	-12	0.8	0.8	0.9	0.9
ENO1	P06733	337	-63	-61	-17	-5	0.6	0.6	0.9	1.0
ZMYM3	Q14202	747	14	-7	-17	4	1.2	0.9	0.9	1.0
BMP2K	Q9NSY1	32	11	-20	-17	-21	1.1	0.8	0.9	0.8
EXOSC4	Q9NPD3	74	9	1	-17	-3	1.1	1.0	0.9	1.0
ETFB	P38117	42	9	-13	-17	-5	1.1	0.9	0.9	1.0
KDM3B	Q7LBC6	295	9	-21	-17	-22	1.1	0.8	0.9	0.8
PTPN11	Q06124	318	9	-27	-17	-23	1.1	0.8	0.9	0.8
SOS2	Q07890	1255	9	-28	-17	-33	1.1	0.8	0.9	0.8
DRAM2	Q6UX65	252	8	-16	-17	-14	1.1	0.9	0.9	0.9
PTPN1	P18031	121	7	-22	-17	-17	1.1	0.8	0.9	0.9
PIN4	Q9Y237	45	7	-6	-17	3	1.1	0.9	0.9	1.0
NAGK	Q9UJ70	268	6	-28	-17	2	1.1	0.8	0.9	1.0
RAB22A	Q9UL26	120	6	-30	-17	-4	1.1	0.8	0.9	1.0
ACAA2	P42765	179	5	-17	-17	-9	1.1	0.9	0.9	0.9
ECD	O95905	267	5	-33	-17	-3	1.1	0.8	0.9	1.0
LZTR1	Q8N653	560	4	-10	-17	2	1.0	0.9	0.9	1.0
DDB1	Q16531	725	4	-25	-17	-4	1.0	0.8	0.9	1.0
RIF1	Q5UIP0	1594	4	-32	-17	-24	1.0	0.8	0.9	0.8
RBL2	Q08999	368	3	-14	-17	-9	1.0	0.9	0.9	0.9
NAPA	P54920	84	3	-23	-17	-9	1.0	0.8	0.9	0.9
SENP7	Q9BQF6	934	3	-27	-17	-9	1.0	0.8	0.9	0.9
EIF2S1	P05198	98	3	-28	-17	-8	1.0	0.8	0.9	0.9
AARSD1	Q9BTE6	266	2	-6	-17	-21	1.0	0.9	0.9	0.8
PRPF18	Q99633	336	2	-12	-17	-11	1.0	0.9	0.9	0.9
MAP3K4	Q9Y6R4	326	2	-15	-17	-21	1.0	0.9	0.9	0.8
MAP7D3	Q8IWC1	264	2	-18	-17	-17	1.0	0.9	0.9	0.9
CDK9	P50750	10	1	-12	-17	-6	1.0	0.9	0.9	0.9
PSMB10	P40306	83	1	-16	-17	-13	1.0	0.9	0.9	0.9
PIK3CD	O00329	382	1	-21	-17	-6	1.0	0.8	0.9	0.9

SMCHD1	A6NHR9	444	1	-20	-17	0	1.0	0.8	0.9	1.0
GSR	P00390	377	1	-29	-17	-21	1.0	0.8	0.9	0.8
ARID1A	O14497	1874	0	-12	-17	-12	1.0	0.9	0.9	0.9
HUWE1	Q7Z6Z7	471	0	-14	-17	1	1.0	0.9	0.9	1.0
UNK	Q9C0B0	308	0	-16	-17	-21	1.0	0.9	0.9	0.8
FBXO3	Q9UK99	37	0	-25	-17	-15	1.0	0.8	0.9	0.9
XPO6	Q96QU8	547	-1	-11	-17	-13	1.0	0.9	0.9	0.9
RAPGEF1	Q13905	1024	-1	-13	-17	-13	1.0	0.9	0.9	0.9
GMIP	Q9P107	529	-2	-7	-17	-2	1.0	0.9	0.9	1.0
EEF2	P13639	728	-2	-21	-17	-4	1.0	0.8	0.9	1.0
TCP1	P17987	357	-2	-18	-17	5	1.0	0.8	0.9	1.0
ATP2B4	P23634	55	-3	-8	-17	-3	1.0	0.9	0.9	1.0
AGK	Q53H12	408	-3	-18	-17	-21	1.0	0.9	0.9	0.8
DNMT1	P26358	1001	-3	-20	-17	-9	1.0	0.8	0.9	0.9
SLC15A4	Q8N697	37	-3	-23	-17	-12	1.0	0.8	0.9	0.9
SYNE2	Q8WXH0	2994	-3	-26	-17	-20	1.0	0.8	0.9	0.8
TXNDC5	Q8NBS9	254	-3	-30	-17	-2	1.0	0.8	0.9	1.0
CECR5	Q9BXW7	362	-3	-31	-17	-16	1.0	0.8	0.9	0.9
GBA2	Q9HCG7	867	-3	-7	-17	4	1.0	0.9	0.9	1.0
RFWD3	Q6PCD5	450	-3	-23	-17	-31	1.0	0.8	0.9	0.8
CYFIP1	Q7L576	346	-4	-11	-17	-19	1.0	0.9	0.9	0.8
CAD	P27708	1296	-4	-20	-17	-18	1.0	0.8	0.9	0.8
HP1BP3	Q5SSJ5	359	-4	-21	-17	-10	1.0	0.8	0.9	0.9
ATE1	O95260	205	-4	-27	-17	-6	1.0	0.8	0.9	0.9
SBF1	O95248	895	-4	-16	-17	-12	1.0	0.9	0.9	0.9
CHUK	O15111	406	-4	-24	-17	-11	1.0	0.8	0.9	0.9
CHD3	Q12873	405	-4	-31	-17	29	1.0	0.8	0.9	1.4
TSC2	P49815	414	-5	-20	-17	-5	1.0	0.8	0.9	1.0
CACUL1	Q86Y37	94	-5	-29	-17	-4	1.0	0.8	0.9	1.0
ANKHD1	Q8IWZ3	615	-5	-19	-17	17	1.0	0.8	0.9	1.2
CENPH	Q9H3R5	35	-5	-23	-17	-27	1.0	0.8	0.9	0.8
TRIM33	Q9UPN9	943	-5	-25	-17	-19	1.0	0.8	0.9	0.8
SMAD4	Q13485	523	-6	-23	-17	-4	0.9	0.8	0.9	1.0
APIP	Q96GX9	147	-6	-19	-17	-14	0.9	0.8	0.9	0.9
MRPL53	Q96EL3	21	-7	-7	-17	-5	0.9	0.9	0.9	1.0
NANS	Q9NR45	283	-7	-18	-17	-12	0.9	0.9	0.9	0.9
PITRM1	Q5JRX3	780	-7	7	-17	-11	0.9	1.1	0.9	0.9
LGALS12	Q96DT0	87	-8	-1	-17	-2	0.9	1.0	0.9	1.0
FKBP15	Q5T1M5	1031	-8	-6	-17	-13	0.9	0.9	0.9	0.9
SREK1	Q8WXA9	494	-8	-22	-17	-11	0.9	0.8	0.9	0.9
UBE3A	Q05086	480	-8	-19	-17	-13	0.9	0.8	0.9	0.9
TRAPPC10	P48553	162	-9	-18	-17	-14	0.9	0.9	0.9	0.9
MRPS11	P82912	112	-9	-20	-17	22	0.9	0.8	0.9	1.3
HCFC2	Q9Y5Z7	199	-10	-27	-17	21	0.9	0.8	0.9	1.3
PARP9	Q8IXQ6	190	-10	-32	-17	-6	0.9	0.8	0.9	0.9
SDCCAG8	Q86SQ7	649	-10	-15	-17	-14	0.9	0.9	0.9	0.9
ODR4	Q5SWX8	275	-10	-16	-17	-13	0.9	0.9	0.9	0.9
SMG1	Q96Q15	3186	-10	-35	-17	-14	0.9	0.7	0.9	0.9
ATP13A1	Q9HD20	336	-12	-18	-17	-14	0.9	0.9	0.9	0.9
SAP30BP	Q9UHR5	172	-12	-36	-17	-14	0.9	0.7	0.9	0.9
CMIP	Q8IY22	243	-12	-5	-17	1	0.9	1.0	0.9	1.0
HUWE1	Q7Z6Z7	3239	-12	-18	-17	-24	0.9	0.8	0.9	0.8
MUS81	Q96NY9	451	-13	-33	-17	8	0.9	0.8	0.9	1.1

PAFAH2	Q99487	72	-14	-27	-17	17	0.9	0.8	0.9	1.2
ZNF668	Q96K58	126	-14	-42	-17	-48	0.9	0.7	0.9	0.7
SMTN	P53814	296	-15	-3	-17	-11	0.9	1.0	0.9	0.9
FAM65B	Q9Y4F9	284	-15	-24	-17	-5	0.9	0.8	0.9	1.0
RACK1	P63244	249	-17	-27	-17	-2	0.9	0.8	0.9	1.0
DGKA	P23743	707	-20	-26	-17	-12	0.8	0.8	0.9	0.9
Uncharacteri	E9PCH4	116	-21	-17	-17	-36	0.8	0.9	0.9	0.7
KDM3B	Q7LBC6	1212	-25	-12	-17	-7	0.8	0.9	0.9	0.9
TUBGCP5	A0A0G2JSA7	670	-25	-18	-17	1	0.8	0.8	0.9	1.0
SBSPON	Q8IVN8	75	-27	-6	-17	-3	0.8	0.9	0.9	1.0
NBEAL1	Q6ZS30	2634	-29	-70	-17	-48	0.8	0.6	0.9	0.7
APEX1	P27695	208	-37	-73	-17	-13	0.7	0.6	0.9	0.9
FHL3	Q13643	150	-41	-28	-17	19	0.7	0.8	0.9	1.2
MIB1	Q86YT6	869	-42	-38	-17	1	0.7	0.7	0.9	1.0
ERO1A	Q96HE7	166	22	8	-18	18	1.3	1.1	0.9	1.2
AP2A2	O94973	903	17	-11	-18	-17	1.2	0.9	0.9	0.9
PELO	Q9BRX2	204	14	-20	-18	2	1.2	0.8	0.9	1.0
VAV3	Q9UKW4	669	11	-7	-18	6	1.1	0.9	0.9	1.1
CARS	P49589	506	11	-44	-18	5	1.1	0.7	0.9	1.0
DPYD	Q12882	322	9	-13	-18	-2	1.1	0.9	0.9	1.0
SNRNP200	O75643	1706	9	-19	-18	-21	1.1	0.8	0.9	0.8
GPD1L	Q8N335	104	9	-42	-18	2	1.1	0.7	0.9	1.0
CTC1	Q2NKJ3	736	8	-1	-18	5	1.1	1.0	0.9	1.1
ZNF501	Q96CX3	148	8	-15	-18	3	1.1	0.9	0.9	1.0
PTPRA	P18433	442	8	-7	-18	9	1.1	0.9	0.9	1.1
GSKIP	Q9P0R6	77	8	-19	-18	8	1.1	0.8	0.9	1.1
GNE	Q9Y223	17	5	1	-18	-5	1.0	1.0	0.9	1.0
TRAPPC6B	Q86SZ2	32	5	-15	-18	-3	1.0	0.9	0.9	1.0
ATP11B	Q9Y2G3	605	4	-28	-18	-17	1.0	0.8	0.9	0.9
ALDH16A1	Q8IZ83	467	3	-8	-18	-12	1.0	0.9	0.9	0.9
ARHGAP26	Q9UNA1	596	3	-23	-18	-5	1.0	0.8	0.9	1.0
EEF2	P13639	567	2	-14	-18	-10	1.0	0.9	0.9	0.9
HLA-B	P30462	91	2	-24	-18	-3	1.0	0.8	0.9	1.0
HLA-B	Q95365	91	2	-24	-18	-3	1.0	0.8	0.9	1.0
DHX29	Q7Z478	648	2	-7	-18	4	1.0	0.9	0.9	1.0
SWAP70	Q9UH65	98	1	-1	-18	1	1.0	1.0	0.9	1.0
UBA1	P22314	179	0	-9	-18	-14	1.0	0.9	0.9	0.9
TRIM56	Q9BRZ2	184	0	-23	-18	-19	1.0	0.8	0.9	0.8
RABEP2	Q9H5N1	423	-1	-25	-18	-39	1.0	0.8	0.9	0.7
CFAP20	Q9Y6A4	61	-1	-26	-18	1	1.0	0.8	0.9	1.0
TBCK	Q8TEA7	353	-1	-30	-18	-19	1.0	0.8	0.9	0.8
RRAGC	Q9HB90	358	-1	-12	-18	-4	1.0	0.9	0.9	1.0
RBM26	Q5T8P6	294	-2	-22	-18	9	1.0	0.8	0.9	1.1
FDPS	P14324	333	-2	-33	-18	-15	1.0	0.8	0.9	0.9
GRAMD1A	Q96CP6	480	-2	-10	-18	10	1.0	0.9	0.9	1.1
MICU2	Q8IYU8	381	-3	-21	-18	-7	1.0	0.8	0.9	0.9
ZC3H7A	Q8IWR0	907	-3	-7	-18	-13	1.0	0.9	0.9	0.9
CLUH	O75153	732	-3	-22	-18	-6	1.0	0.8	0.9	0.9
ZRSR2	Q15696	326	-4	-15	-18	-13	1.0	0.9	0.9	0.9
XAB2	Q9HCS7	98	-4	-20	-18	-4	1.0	0.8	0.9	1.0
LAS1L	Q9Y4W2	140	-5	-15	-18	-14	1.0	0.9	0.9	0.9
ZZEF1	O43149	719	-5	-21	-18	-16	1.0	0.8	0.9	0.9
GSN	P06396	331	-5	-32	-18	-2	1.0	0.8	0.9	1.0

TRMT2A	Q8IZ69	463	-6	-15	-18	-20	0.9	0.9	0.9	0.8
PSMC2	P35998	270	-6	-6	-18	-7	0.9	0.9	0.9	0.9
UTP20	O75691	313	-6	-15	-18	-6	0.9	0.9	0.9	0.9
FAM208A	Q9UK61	1302	-7	-15	-18	-7	0.9	0.9	0.9	0.9
CDYL2	Q8N8U2	423	-7	-17	-18	-11	0.9	0.9	0.9	0.9
CD86	P42081	322	-7	-32	-18	-18	0.9	0.8	0.9	0.8
SLC9A1	P19634	794	-8	-14	-18	-17	0.9	0.9	0.9	0.9
CEP41	Q9BYV8	274	-8	-10	-18	-17	0.9	0.9	0.9	0.9
NLRP1	Q9C000	11	-8	-36	-18	-30	0.9	0.7	0.9	0.8
AKAP9	Q99996	1907	-9	-21	-18	-28	0.9	0.8	0.9	0.8
SLC26A6	Q9BXS9	628	-10	-11	-18	-37	0.9	0.9	0.9	0.7
NFKB1	P19838	87	-10	-20	-18	-6	0.9	0.8	0.9	0.9
RNH1	P13489	199	-10	-15	-18	-14	0.9	0.9	0.9	0.9
ACSL5	Q9ULC5	70	-10	-20	-18	-8	0.9	0.8	0.9	0.9
SDHA	P31040	89	-11	-35	-18	-10	0.9	0.7	0.9	0.9
LRBA	P50851	1455	-12	-1	-18	-24	0.9	1.0	0.9	0.8
QTRT1	Q9BXR0	23	-12	-17	-18	8	0.9	0.9	0.9	1.1
AHCYL1	O43865	317	-12	-22	-18	-23	0.9	0.8	0.9	0.8
LRCH3	Q96II8	236	-12	-20	-18	-16	0.9	0.8	0.9	0.9
ZMYM3	Q14202	715	-13	-23	-18	-18	0.9	0.8	0.9	0.9
MYCBP2	O75592	4181	-13	-33	-18	-13	0.9	0.8	0.9	0.9
FAM91A1	Q658Y4	490	-13	-13	-18	-8	0.9	0.9	0.9	0.9
DDX42	Q86XP3	333	-14	-18	-18	-6	0.9	0.9	0.9	0.9
RANBP10	Q6VN20	610	-14	-19	-18	-1	0.9	0.8	0.9	1.0
KBTBD11	O94819	555	-14	-29	-18	-11	0.9	0.8	0.9	0.9
ATG4A	Q8WYN0	217	-16	-4	-18	-28	0.9	1.0	0.9	0.8
EIF2S1	P05198	218	-18	-19	-18	-7	0.8	0.8	0.9	0.9
MLST8	Q9BVC4	34	-18	-31	-18	-48	0.8	0.8	0.9	0.7
UPF1	Q92900	186	-19	-32	-18	-44	0.8	0.8	0.9	0.7
EZH2	Q15910	695	-19	-35	-18	-26	0.8	0.7	0.9	0.8
EZH1	Q92800	696	-19	-35	-18	-26	0.8	0.7	0.9	0.8
PPP1CA	P62136	105	-22	-27	-18	-15	0.8	0.8	0.9	0.9
DST	Q03001	5610	-29	-30	-18	-18	0.8	0.8	0.9	0.8
PDLIM7	Q9NR12	329	-31	-31	-18	-3	0.8	0.8	0.9	1.0
LAMTOR3	Q9UHA4	73	-34	-30	-18	-3	0.7	0.8	0.9	1.0
RALYL	Q86SE5	51	-35	-18	-18	-9	0.7	0.9	0.9	0.9
WDR81	Q562E7	643	14	11	-18	-10	1.2	1.1	0.8	0.9
ZNF672	Q499Z4	350	13	8	-18	-2	1.1	1.1	0.8	1.0
CORO7-PAM:	A0A0A6YYL4	42	10	-27	-18	-15	1.1	0.8	0.8	0.9
GALNT6	Q8NCL4	141	8	-34	-18	-11	1.1	0.7	0.8	0.9
PSMA4	P25789	74	7	-31	-18	-8	1.1	0.8	0.8	0.9
ECH1	Q13011	182	6	-16	-18	-9	1.1	0.9	0.8	0.9
TLN1	Q9Y490	709	5	-11	-18	6	1.1	0.9	0.8	1.1
CALR	P27797	163	5	-23	-18	-6	1.1	0.8	0.8	0.9
PPWD1	Q96BP3	180	5	-26	-18	-13	1.1	0.8	0.8	0.9
PRRC2C	Q9Y520	223	4	-22	-18	-22	1.0	0.8	0.8	0.8
SMARCB1	Q12824	350	3	-14	-18	-2	1.0	0.9	0.8	1.0
ZMYM4	Q5VZL5	775	3	1	-18	3	1.0	1.0	0.8	1.0
NAPG	Q99747	193	3	-21	-18	-13	1.0	0.8	0.8	0.9
ISOC2	Q96AB3	21	3	-28	-18	15	1.0	0.8	0.8	1.2
ARL14EP	Q8N8R7	17	2	-20	-18	-2	1.0	0.8	0.8	1.0
GDE1	Q9NZC3	127	2	-30	-18	-1	1.0	0.8	0.8	1.0
FLNA	P21333	1410	1	-10	-18	-10	1.0	0.9	0.8	0.9

TAF2	Q6P1X5	127	1	-23	-18	-5	1.0	0.8	0.8	1.0
ISG20L2	Q9H9L3	168	1	-24	-18	-25	1.0	0.8	0.8	0.8
LANCL1	O43813	300	1	-3	-18	-15	1.0	1.0	0.8	0.9
CDK12	Q9NYV4	862	1	-4	-18	-30	1.0	1.0	0.8	0.8
BANK1	Q8NDB2	332	1	-12	-18	-12	1.0	0.9	0.8	0.9
GNPDA1	P46926	48	0	-22	-18	-4	1.0	0.8	0.8	1.0
AP2B1	P63010	918	0	-30	-18	1	1.0	0.8	0.8	1.0
PIK3R6	Q5UE93	446	-1	-25	-18	-16	1.0	0.8	0.8	0.9
AP1S1	P61966	47	-1	1	-18	-5	1.0	1.0	0.8	1.0
ODF2	Q5BJF6	206	-1	-8	-18	-3	1.0	0.9	0.8	1.0
CDK5RAP2	Q96SN8	434	-1	-15	-18	-17	1.0	0.9	0.8	0.9
ALKBH1	Q13686	371	-2	1	-18	2	1.0	1.0	0.8	1.0
DUS3L	Q96G46	315	-2	-12	-18	4	1.0	0.9	0.8	1.0
DHTKD1	Q96HY7	64	-2	-57	-18	-31	1.0	0.6	0.8	0.8
OSBPL10	Q9BXB5	33	-2	-7	-18	-18	1.0	0.9	0.8	0.9
PLEKHG3	A1L390	967	-2	-24	-18	-24	1.0	0.8	0.8	0.8
EPRS	P07814	350	-3	-9	-18	-1	1.0	0.9	0.8	1.0
NCOA4	Q13772	62	-3	-16	-18	-13	1.0	0.9	0.8	0.9
SMC1A	Q14683	1115	-3	-21	-18	-18	1.0	0.8	0.8	0.9
CIZ1	Q9ULV3	252	-3	-22	-18	-26	1.0	0.8	0.8	0.8
PDHB	P11177	169	-3	-3	-18	-8	1.0	1.0	0.8	0.9
Uncharacteri	A0A087WW\	34	-3	-13	-18	16	1.0	0.9	0.8	1.2
MYCBP2	O75592	1868	-4	-11	-18	-11	1.0	0.9	0.8	0.9
NDUFV1	P49821	125	-4	-21	-18	-11	1.0	0.8	0.8	0.9
JRKL	Q9Y4A0	102	-4	-14	-18	-3	1.0	0.9	0.8	1.0
AKT2	P31751	77	-4	-15	-18	-15	1.0	0.9	0.8	0.9
PRKCQ	Q04759	193	-5	-21	-18	-17	1.0	0.8	0.8	0.9
PRKCD	Q05655	192	-5	-21	-18	-17	1.0	0.8	0.8	0.9
ADPRHL2	Q9NX46	132	-5	-22	-18	5	1.0	0.8	0.8	1.0
ZNF292	O60281	55	-5	13	-18	-3	1.0	1.1	0.8	1.0
RPA1	P27694	500	-5	-13	-18	-22	1.0	0.9	0.8	0.8
NAPRT	Q6XQN6	385	-5	-16	-18	-9	1.0	0.9	0.8	0.9
GRIPAP1	Q4V328	104	-6	-8	-18	-5	0.9	0.9	0.8	1.0
STRIP1	Q5VSL9	100	-6	-29	-18	-5	0.9	0.8	0.8	1.0
INPP5K	Q9BT40	213	-6	-22	-18	-26	0.9	0.8	0.8	0.8
BCCIP	Q9P287	141	-6	-23	-18	-10	0.9	0.8	0.8	0.9
STXBP2	Q15833	110	-7	-15	-18	29	0.9	0.9	0.8	1.4
NIPBL	Q6KC79	573	-7	-27	-18	-8	0.9	0.8	0.8	0.9
WDR12	Q9GZL7	205	-7	-28	-18	-18	0.9	0.8	0.8	0.8
FRY	Q5TBA9	2009	-7	-21	-18	-27	0.9	0.8	0.8	0.8
SIRT2	Q8IXJ6	221	-7	-33	-18	-22	0.9	0.8	0.8	0.8
FECH	P22830	360	-8	-9	-18	15	0.9	0.9	0.8	1.2
STK11IP	Q8N1F8	633	-8	-19	-18	-17	0.9	0.8	0.8	0.9
FANCB	Q8NB91	78	-8	-20	-18	-21	0.9	0.8	0.8	0.8
VAV1	P15498	71	-9	-11	-18	-8	0.9	0.9	0.8	0.9
NELFA	Q9H3P2	131	-9	-13	-18	-20	0.9	0.9	0.8	0.8
DOLPP1	Q86YN1	86	-9	-20	-18	-15	0.9	0.8	0.8	0.9
ADAM10	O14672	435	-9	-17	-18	-19	0.9	0.9	0.8	0.8
HSP90B1	P14625	576	-9	-23	-18	0	0.9	0.8	0.8	1.0
ZYX	Q15942	467	-9	-30	-18	12	0.9	0.8	0.8	1.1
MBNL2	Q5VZF2	34	-9	-31	-18	-12	0.9	0.8	0.8	0.9
PANK4	Q9NVE7	451	-10	-13	-18	-22	0.9	0.9	0.8	0.8
RNF114	Q9Y508	67	-10	-23	-18	-4	0.9	0.8	0.8	1.0

SETDB1	Q15047	92	-10	-22	-18	-25	0.9	0.8	0.8	0.8
GRK6	P43250	420	-10	-26	-18	-19	0.9	0.8	0.8	0.8
DHX58	Q96C10	322	-10	-32	-18	-14	0.9	0.8	0.8	0.9
EZH2	Q15910	14	-11	-26	-18	-28	0.9	0.8	0.8	0.8
ISCA2	Q86U28	144	-12	-17	-18	-27	0.9	0.9	0.8	0.8
KMT5C	Q86Y97	111	-13	-26	-18	-20	0.9	0.8	0.8	0.8
BCL11B	Q9C0K0	460	-13	-27	-18	-12	0.9	0.8	0.8	0.9
BUB3	O43684	270	-14	-25	-18	-6	0.9	0.8	0.8	0.9
ACSL3	O95573	429	-14	-25	-18	-28	0.9	0.8	0.8	0.8
CARD19	Q96LW7	63	-14	-14	-18	-8	0.9	0.9	0.8	0.9
DDX41	Q9UJV9	264	-14	-21	-18	0	0.9	0.8	0.8	1.0
SMCHD1	A6NHR9	1856	-14	-25	-18	11	0.9	0.8	0.8	1.1
CLMN	Q96JQ2	353	-15	-14	-18	-25	0.9	0.9	0.8	0.8
POP1	Q99575	705	-15	-19	-18	-16	0.9	0.8	0.8	0.9
USP4	Q13107	110	-15	-39	-18	-15	0.9	0.7	0.8	0.9
ANO6	Q4KMQ2	355	-16	-18	-18	5	0.9	0.8	0.8	1.0
BTN3A3	O00478	387	-16	-29	-18	-16	0.9	0.8	0.8	0.9
ALK	Q9UM73	1237	-18	-26	-18	-17	0.9	0.8	0.8	0.9
PRPF8	Q6P2Q9	772	-24	-24	-18	-24	0.8	0.8	0.8	0.8
PGD	P52209	422	-30	-18	-18	-18	0.8	0.9	0.8	0.8
R3HCC1	Q9Y3T6	134	-42	-42	-18	-23	0.7	0.7	0.8	0.8
FARSB	Q9NSD9	76	-44	-51	-18	-22	0.7	0.7	0.8	0.8
APEX1	P27695	65	-51	-64	-18	-8	0.7	0.6	0.8	0.9
LCK	P06239	465	19	-10	-19	-16	1.2	0.9	0.8	0.9
KANSL1	A0A0G2JNT7	599	12	-18	-19	-2	1.1	0.9	0.8	1.0
FABP5	Q01469	67	10	-23	-19	-13	1.1	0.8	0.8	0.9
GNB4	Q9HAV0	294	8	-25	-19	-12	1.1	0.8	0.8	0.9
SDHA	P31040	191	8	-17	-19	-2	1.1	0.9	0.8	1.0
ASMTL	O95671	461	5	-7	-19	-7	1.1	0.9	0.8	0.9
ECH1	Q13011	171	5	-27	-19	-26	1.0	0.8	0.8	0.8
PSMD9	O00233	81	4	-29	-19	1	1.0	0.8	0.8	1.0
PDCD7	Q8N8D1	281	4	-38	-19	-16	1.0	0.7	0.8	0.9
RPP38	P78345	135	3	-16	-19	-13	1.0	0.9	0.8	0.9
ZSWIM8	A7E2V4	190	3	-1	-19	11	1.0	1.0	0.8	1.1
SPATA20	Q8TB22	671	3	-10	-19	-21	1.0	0.9	0.8	0.8
PTPN1	P18031	32	3	-17	-19	-3	1.0	0.9	0.8	1.0
SNX30	Q5VWJ9	103	3	-19	-19	-10	1.0	0.8	0.8	0.9
FHL1	Q13642	71	2	-27	-19	16	1.0	0.8	0.8	1.2
JAK1	P23458	1131	2	-15	-19	-22	1.0	0.9	0.8	0.8
PHPT1	Q9NRX4	71	1	-17	-19	-1	1.0	0.9	0.8	1.0
RABGEF1	Q9UJ41	507	1	-15	-19	-4	1.0	0.9	0.8	1.0
HAT1	O14929	27	1	-28	-19	-15	1.0	0.8	0.8	0.9
CYLD	Q9NQC7	712	0	-12	-19	-7	1.0	0.9	0.8	0.9
GPKOW	Q92917	394	-1	2	-19	4	1.0	1.0	0.8	1.0
UROD	P06132	66	-1	-13	-19	-29	1.0	0.9	0.8	0.8
ITK	Q08881	74	-1	-15	-19	-1	1.0	0.9	0.8	1.0
TRAPPC9	Q96Q05	800	-2	-4	-19	14	1.0	1.0	0.8	1.2
PRF1	P14222	381	-2	-11	-19	3	1.0	0.9	0.8	1.0
URB2	Q14146	570	-2	-20	-19	-35	1.0	0.8	0.8	0.7
HERC4	Q5GLZ8	175	-3	-11	-19	-16	1.0	0.9	0.8	0.9
FMNL2	Q96PY5	735	-3	-14	-19	-14	1.0	0.9	0.8	0.9
POLR2B	P30876	221	-3	-19	-19	-14	1.0	0.8	0.8	0.9
CTSH	P09668	138	-4	-16	-19	-15	1.0	0.9	0.8	0.9

RBBP5	Q15291	212	-4	-25	-19	-13	1.0	0.8	0.8	0.9
NOSIP	Q9Y314	8	-4	-25	-19	-32	1.0	0.8	0.8	0.8
EXD3	Q8N9H8	138	-4	-11	-19	-7	1.0	0.9	0.8	0.9
PSMA7	O14818	70	-4	-19	-19	-4	1.0	0.8	0.8	1.0
PIK3R4	Q99570	195	-4	-22	-19	-21	1.0	0.8	0.8	0.8
PSMA1	P25786	92	-4	-27	-19	-9	1.0	0.8	0.8	0.9
DNMT1	P26358	1339	-4	-29	-19	-10	1.0	0.8	0.8	0.9
APPBP2	Q92624	54	-5	1	-19	-24	1.0	1.0	0.8	0.8
ATIC	P31939	101	-5	-20	-19	3	1.0	0.8	0.8	1.0
UBE2L6	O14933	98	-5	-21	-19	17	1.0	0.8	0.8	1.2
PXN	P49023	561	-5	-29	-19	24	1.0	0.8	0.8	1.3
HNRNPA3	P51991	64	-6	-22	-19	-8	0.9	0.8	0.8	0.9
IARS2	Q9NSE4	819	-6	-12	-19	-2	0.9	0.9	0.8	1.0
RASSF4	Q9H2L5	249	-6	-28	-19	27	0.9	0.8	0.8	1.4
TATDN2	Q93075	721	-6	-33	-19	-38	0.9	0.8	0.8	0.7
IFIT3	O14879	39	-7	-14	-19	-17	0.9	0.9	0.8	0.9
SMPD4	Q9NXE4	125	-7	-35	-19	-19	0.9	0.7	0.8	0.8
CACNA2D2	Q9NY47	1062	-7	-53	-19	-50	0.9	0.7	0.8	0.7
SEPT1	Q8WYJ6	260	-8	-5	-19	-9	0.9	1.0	0.8	0.9
STXBP5	Q5T5C0	510	-8	-17	-19	13	0.9	0.9	0.8	1.1
SGTA	O43765	148	-9	-43	-19	-22	0.9	0.7	0.8	0.8
ZMYM2	Q9UBW7	704	-10	-24	-19	-13	0.9	0.8	0.8	0.9
ABI1	Q8IZP0	33	-10	-25	-19	-14	0.9	0.8	0.8	0.9
LEMD2	Q8NC56	253	-10	-13	-19	-33	0.9	0.9	0.8	0.8
PREX1	Q8TCU6	879	-10	-22	-19	-12	0.9	0.8	0.8	0.9
RNF14	Q9UBS8	350	-11	-19	-19	-6	0.9	0.8	0.8	0.9
TAPBP	O15533	440	-11	-15	-19	-16	0.9	0.9	0.8	0.9
DPYSL2	Q16555	179	-12	-21	-19	-13	0.9	0.8	0.8	0.9
PLRG1	O43660	264	-13	-27	-19	-15	0.9	0.8	0.8	0.9
UPF1	Q92900	852	-13	-32	-19	-4	0.9	0.8	0.8	1.0
ENTHD2	Q96N21	302	-15	-5	-19	-11	0.9	1.0	0.8	0.9
ARPC1B	O15143	162	-15	-16	-19	-21	0.9	0.9	0.8	0.8
ARPC1A	Q92747	162	-15	-16	-19	-21	0.9	0.9	0.8	0.8
KCNAB2	Q13303	301	-15	-25	-19	-23	0.9	0.8	0.8	0.8
ACAT1	P24752	119	-16	-20	-19	-17	0.9	0.8	0.8	0.9
FTL	P02792	127	-16	-32	-19	4	0.9	0.8	0.8	1.0
SPG20	Q8N0X7	504	-17	-30	-19	-23	0.9	0.8	0.8	0.8
PTP4A2	Q12974	96	-18	-26	-19	-11	0.9	0.8	0.8	0.9
ADAR	P55265	649	-22	-12	-19	-25	0.8	0.9	0.8	0.8
APEX1	P27695	296	-30	-135	-19	-2	0.8	0.4	0.8	1.0
GHDC	Q8N2G8	377	-33	-23	-19	-9	0.8	0.8	0.8	0.9
ETHE1	O95571	170	-80	-37	-19	-11	0.6	0.7	0.8	0.9
GORASP2	Q9H8Y8	192	17	-38	-19	4	1.2	0.7	0.8	1.0
WDR43	Q15061	372	14	-3	-19	-17	1.2	1.0	0.8	0.9
CCT7	Q99832	345	8	-48	-19	-14	1.1	0.7	0.8	0.9
ZNF316	A6NFI3	431	7	1	-19	-6	1.1	1.0	0.8	0.9
DDX23	Q9BUQ8	677	7	-12	-19	-14	1.1	0.9	0.8	0.9
ATM	Q13315	2801	7	-19	-19	-6	1.1	0.8	0.8	0.9
EXOSC9	Q06265	46	6	10	-19	6	1.1	1.1	0.8	1.1
MRPL28	Q13084	154	6	-24	-19	-20	1.1	0.8	0.8	0.8
TRIM3	O75382	25	5	-11	-19	-4	1.1	0.9	0.8	1.0
RNF213	Q63HN8	2868	5	-28	-19	16	1.1	0.8	0.8	1.2
CNBP	P62633	111	5	-16	-19	3	1.0	0.9	0.8	1.0

CAP1	Q01518	416	5	-25	-19	3	1.0	0.8	0.8	1.0
RASGRP1	O95267	237	4	-17	-19	-16	1.0	0.9	0.8	0.9
Uncharacteri	E9PLN8	43	4	-22	-19	-2	1.0	0.8	0.8	1.0
ELAC2	Q9BQ52	753	4	-43	-19	-22	1.0	0.7	0.8	0.8
USP5	P45974	838	3	-20	-19	-12	1.0	0.8	0.8	0.9
ALDOC	P09972	135	2	-31	-19	-8	1.0	0.8	0.8	0.9
U2AF2	P26368	442	1	-12	-19	-3	1.0	0.9	0.8	1.0
STAG1	Q8WVM7	179	0	-14	-19	-5	1.0	0.9	0.8	1.0
CXCR2	P25025	286	0	-15	-19	-16	1.0	0.9	0.8	0.9
AP2A1	O95782	225	0	-25	-19	-1	1.0	0.8	0.8	1.0
MGEA5	O60502	166	-1	-12	-19	-1	1.0	0.9	0.8	1.0
IFIT3	O14879	239	-1	-3	-19	-11	1.0	1.0	0.8	0.9
MICU1	Q9BPX6	83	-1	-22	-19	-21	1.0	0.8	0.8	0.8
RPLP0	P05388	27	-1	-23	-19	-5	1.0	0.8	0.8	1.0
UPF1	Q92900	213	-2	-11	-19	-7	1.0	0.9	0.8	0.9
GNL3L	Q9NVN8	213	-2	-16	-19	-22	1.0	0.9	0.8	0.8
RAB32	Q13637	145	-2	-26	-19	-10	1.0	0.8	0.8	0.9
TXLNA	P40222	245	-4	-13	-19	-14	1.0	0.9	0.8	0.9
TXLNG	Q9NUQ3	212	-4	-13	-19	-14	1.0	0.9	0.8	0.9
ARAP1	Q96P48	995	-4	-18	-19	-23	1.0	0.8	0.8	0.8
MRPL37	Q9BZE1	177	-4	-18	-19	-6	1.0	0.8	0.8	0.9
GANAB	Q14697	47	-4	-24	-19	-13	1.0	0.8	0.8	0.9
RNF169	Q8NCN4	28	-5	-28	-19	-33	1.0	0.8	0.8	0.8
EIF4G3	O43432	461	-5	-11	-19	-22	1.0	0.9	0.8	0.8
ACTN1	P12814	480	-5	-18	-19	3	1.0	0.9	0.8	1.0
MIS12	Q9H081	104	-6	-23	-19	-17	0.9	0.8	0.8	0.9
CAMSAP1	Q5T5Y3	395	-6	-13	-19	-26	0.9	0.9	0.8	0.8
PELP1	Q8IZL8	600	-6	-16	-19	-16	0.9	0.9	0.8	0.9
SARS	P49591	162	-7	-21	-19	-11	0.9	0.8	0.8	0.9
IMPDH2	P12268	468	-7	-26	-19	-8	0.9	0.8	0.8	0.9
NUP188	Q5SRE5	285	-7	-35	-19	-9	0.9	0.7	0.8	0.9
KDM5A	P29375	558	-8	-22	-19	-12	0.9	0.8	0.8	0.9
ZFC3H1	O60293	1379	-8	-7	-19	-13	0.9	0.9	0.8	0.9
SRI	P30626	75	-8	-28	-19	-7	0.9	0.8	0.8	0.9
BTK	Q06187	145	-8	-37	-19	-9	0.9	0.7	0.8	0.9
CARD11	Q9BXL7	718	-9	-24	-19	10	0.9	0.8	0.8	1.1
PPWD1	Q96BP3	98	-9	-25	-19	-12	0.9	0.8	0.8	0.9
WDCP	Q9H6R7	125	-9	-28	-19	-25	0.9	0.8	0.8	0.8
C6orf106	Q9H6K1	18	-10	-12	-19	-23	0.9	0.9	0.8	0.8
TRIM28	Q13263	68	-11	-19	-19	-6	0.9	0.8	0.8	0.9
U2AF1	Q01081	163	-11	-21	-19	-7	0.9	0.8	0.8	0.9
DENND3	A2RUS2	61	-11	-9	-19	-9	0.9	0.9	0.8	0.9
ANXA4	P09525	198	-11	-22	-19	-13	0.9	0.8	0.8	0.9
SPATA5	Q8NB90	318	-12	-24	-19	-16	0.9	0.8	0.8	0.9
DPYSL2	Q16555	132	-12	-29	-19	-4	0.9	0.8	0.8	1.0
HECTD4	Q9Y4D8	1488	-13	-15	-19	-9	0.9	0.9	0.8	0.9
THAP12	O43422	644	-16	-18	-19	-19	0.9	0.8	0.8	0.8
GUCY1A3	Q02108	15	-16	-33	-19	-17	0.9	0.8	0.8	0.9
SCRIB	A0A0G2JPP5	1637	-16	-18	-19	-23	0.9	0.9	0.8	0.8
MORF4L1	Q9UBU8	19	-16	-36	-19	-12	0.9	0.7	0.8	0.9
NPEPL1	Q8NDH3	357	-17	-32	-19	-35	0.9	0.8	0.8	0.7
RAB11FIP1	Q6WKZ4	711	-17	-36	-19	-27	0.9	0.7	0.8	0.8
NAIP	Q13075	179	-18	-31	-19	-33	0.9	0.8	0.8	0.8

TACC1	O75410	174	-19	-15	-19	3	0.8	0.9	0.8	1.0
SENP7	Q9BQF6	221	-19	-19	-19	-25	0.8	0.8	0.8	0.8
KDM5C	P41229	724	-20	-10	-19	-18	0.8	0.9	0.8	0.8
KDM5D	Q9BY66	714	-20	-10	-19	-18	0.8	0.9	0.8	0.8
EEFSEC	P57772	426	-21	-54	-19	-12	0.8	0.6	0.8	0.9
RNF40	O75150	950	-27	-2	-19	2	0.8	1.0	0.8	1.0
PEAK1	Q9H792	1468	-28	-39	-19	-16	0.8	0.7	0.8	0.9
STAT4	Q14765	680	-30	6	-19	-12	0.8	1.1	0.8	0.9
HELZ2	Q9BYK8	745	17	-13	-20	20	1.2	0.9	0.8	1.2
CORO1A	P31146	285	16	-15	-20	22	1.2	0.9	0.8	1.3
ATP2A3	Q93084	471	14	2	-20	17	1.2	1.0	0.8	1.2
FIG4	Q92562	455	11	1	-20	-6	1.1	1.0	0.8	0.9
PPM1A	P35813	275	10	-57	-20	-21	1.1	0.6	0.8	0.8
NSL1	Q96IY1	175	6	-15	-20	-9	1.1	0.9	0.8	0.9
ASB6	Q9NWX5	313	6	7	-20	-4	1.1	1.1	0.8	1.0
SMPD2	O60906	304	4	-4	-20	-32	1.0	1.0	0.8	0.8
EPC2	Q52LR7	420	4	-20	-20	8	1.0	0.8	0.8	1.1
OSBPL2	Q9H1P3	122	4	-23	-20	-10	1.0	0.8	0.8	0.9
SCAF11	Q99590	566	3	-17	-20	-23	1.0	0.9	0.8	0.8
HEATR3	Q7Z4Q2	585	3	-21	-20	-23	1.0	0.8	0.8	0.8
PPP6R2	O75170	870	3	-8	-20	-3	1.0	0.9	0.8	1.0
XPNPEP3	Q9NQH7	492	2	-21	-20	1	1.0	0.8	0.8	1.0
UBL5	Q9BZL1	18	2	-38	-20	-6	1.0	0.7	0.8	0.9
PSMA7	O14818	91	1	-16	-20	-4	1.0	0.9	0.8	1.0
LCMT1	Q9UIC8	258	1	-24	-20	-4	1.0	0.8	0.8	1.0
NOV	P48745	292	0	-16	-20	-13	1.0	0.9	0.8	0.9
EIF3B	P55884	515	-1	-22	-20	-13	1.0	0.8	0.8	0.9
ALOX5	P09917	301	-1	-23	-20	-22	1.0	0.8	0.8	0.8
NPAT	Q14207	551	-1	-16	-20	-20	1.0	0.9	0.8	0.8
TBL2	Q9Y4P3	335	-1	-21	-20	-7	1.0	0.8	0.8	0.9
ABCC1	P33527	1439	-1	-24	-20	-9	1.0	0.8	0.8	0.9
SRBD1	Q8N5C6	571	-2	-14	-20	-15	1.0	0.9	0.8	0.9
SETD1A	O15047	1514	-2	-25	-20	-12	1.0	0.8	0.8	0.9
SLC7A6OS	Q96CW6	62	-3	-16	-20	-15	1.0	0.9	0.8	0.9
HSPA8	P11142	603	-3	-46	-20	-4	1.0	0.7	0.8	1.0
PGK1	P00558	108	-3	-16	-20	-4	1.0	0.9	0.8	1.0
RPS27A	P62979	144	-3	-28	-20	-19	1.0	0.8	0.8	0.8
ZCCHC11	Q5TAX3	204	-4	-7	-20	-25	1.0	0.9	0.8	0.8
TRIM58	Q8NG06	60	-4	-20	-20	-14	1.0	0.8	0.8	0.9
TRMT44	Q8IYL2	490	-4	-20	-20	-26	1.0	0.8	0.8	0.8
PNMA3	Q9UL41	130	-4	-39	-20	-8	1.0	0.7	0.8	0.9
NAT10	Q9H0A0	505	-5	-26	-20	-16	1.0	0.8	0.8	0.9
SP140L	Q9H930	191	-5	-28	-20	-3	1.0	0.8	0.8	1.0
TCP1	P17987	296	-5	-13	-20	-15	1.0	0.9	0.8	0.9
NAXD	Q8IW45	82	-5	-25	-20	-14	1.0	0.8	0.8	0.9
IRF2BP2	Q7Z5L9	521	-6	-11	-20	-11	0.9	0.9	0.8	0.9
IRF2BPL	Q9H1B7	730	-6	-11	-20	-11	0.9	0.9	0.8	0.9
ERN1	O75460	109	-6	-30	-20	-12	0.9	0.8	0.8	0.9
RPL36A-HNR	H7BZ11	77	-7	-14	-20	-11	0.9	0.9	0.8	0.9
COX11	Q9Y6N1	219	-7	-17	-20	-25	0.9	0.9	0.8	0.8
CAP1	Q01518	356	-7	-28	-20	-2	0.9	0.8	0.8	1.0
CDA	P32320	31	-7	-13	-20	-4	0.9	0.9	0.8	1.0
HELZ2	Q9BYK8	1545	-7	-17	-20	-33	0.9	0.9	0.8	0.8

PLCD1	P51178	367	-7	-18	-20	-14	0.9	0.8	0.8	0.9
MDN1	Q9NU22	4517	-7	-21	-20	-18	0.9	0.8	0.8	0.9
MYO9B	Q13459	1279	-8	-19	-20	-31	0.9	0.8	0.8	0.8
PCYT1A	P49585	346	-8	-24	-20	-12	0.9	0.8	0.8	0.9
ZFP62	Q8NB50	321	-8	-5	-20	-13	0.9	1.0	0.8	0.9
ELANE	P08246	254	-9	-19	-20	-11	0.9	0.8	0.8	0.9
SCRN3	Q0VDG4	143	-9	-21	-20	-57	0.9	0.8	0.8	0.6
GMDS	O60547	336	-11	-11	-20	12	0.9	0.9	0.8	1.1
MTSS1	O43312	274	-11	-27	-20	-30	0.9	0.8	0.8	0.8
ARPC5	O15511	45	-11	-22	-20	-25	0.9	0.8	0.8	0.8
NAA25	Q14CX7	365	-12	-25	-20	4	0.9	0.8	0.8	1.0
VCL	P18206	545	-16	-38	-20	-8	0.9	0.7	0.8	0.9
EEFSEC	P57772	422	-19	-22	-20	-10	0.8	0.8	0.8	0.9
HADHA	P40939	747	-19	-17	-20	-8	0.8	0.9	0.8	0.9
NXF1	Q9UBU9	528	-20	-23	-20	-19	0.8	0.8	0.8	0.8
ARHGEF7	Q14155	490	-21	-16	-20	-8	0.8	0.9	0.8	0.9
ATP6V1A	P38606	532	-25	-21	-20	-5	0.8	0.8	0.8	1.0
SKI	P12755	303	-28	-44	-20	-33	0.8	0.7	0.8	0.8
ZNF331	Q9NQX6	366	-29	-18	-20	-33	0.8	0.8	0.8	0.8
PM20D2	Q8IYS1	30	-35	-52	-20	-40	0.7	0.7	0.8	0.7
ACIN1	Q9UKV3	1223	-37	-14	-20	-23	0.7	0.9	0.8	0.8
FKBP2	P26885	42	-59	-51	-20	-11	0.6	0.7	0.8	0.9
ENO1	P06733	399	-78	-55	-20	-15	0.6	0.6	0.8	0.9
FADD	Q13158	105	26	14	-20	15	1.3	1.2	0.8	1.2
PARP14	Q460N5	1327	17	-13	-20	-6	1.2	0.9	0.8	0.9
UTP20	O75691	2058	12	-15	-20	1	1.1	0.9	0.8	1.0
COTL1	Q14019	52	12	-24	-20	3	1.1	0.8	0.8	1.0
ARFGAP3	Q9NP61	25	11	-30	-20	-24	1.1	0.8	0.8	0.8
TFIP11	Q9UBB9	391	10	5	-20	-2	1.1	1.1	0.8	1.0
TNPO3	Q9Y5L0	634	9	-7	-20	12	1.1	0.9	0.8	1.1
DHX29	Q7Z478	749	8	-4	-20	3	1.1	1.0	0.8	1.0
MESDC2	Q14696	180	7	-22	-20	0	1.1	0.8	0.8	1.0
C7orf26	Q96N11	145	7	-30	-20	-10	1.1	0.8	0.8	0.9
SAE1	Q9UBE0	146	7	-18	-20	3	1.1	0.8	0.8	1.0
FAM65B	Q9Y4F9	820	7	-28	-20	-12	1.1	0.8	0.8	0.9
TECPR2	O15040	974	5	-26	-20	-10	1.1	0.8	0.8	0.9
PPP6R2	O75170	762	2	-20	-20	-15	1.0	0.8	0.8	0.9
SHPK	Q9UHJ6	132	2	-29	-20	-11	1.0	0.8	0.8	0.9
RAB28	P51157	31	1	-18	-20	-30	1.0	0.8	0.8	0.8
PLEC	Q15149	545	1	-20	-20	-68	1.0	0.8	0.8	0.6
TXNRD1	Q16881	515	1	-28	-20	-10	1.0	0.8	0.8	0.9
GSK3B	P49841	76	0	-26	-20	-7	1.0	0.8	0.8	0.9
PDLIM2	Q96JY6	289	-1	-22	-20	-24	1.0	0.8	0.8	0.8
XRCC5	P13010	418	-2	-10	-20	-5	1.0	0.9	0.8	1.0
TFB2M	Q9H5Q4	383	-3	-7	-20	8	1.0	0.9	0.8	1.1
NUP160	Q12769	473	-3	-29	-20	8	1.0	0.8	0.8	1.1
UBR4	Q5T4S7	140	-3	-19	-20	-16	1.0	0.8	0.8	0.9
LYST	Q99698	548	-3	-32	-20	-25	1.0	0.8	0.8	0.8
SRR	Q9GZT4	269	-3	-48	-20	-12	1.0	0.7	0.8	0.9
KDM1B	Q8NB78	62	-4	-9	-20	-4	1.0	0.9	0.8	1.0
L2HGDH	Q9H9P8	272	-4	-23	-20	-20	1.0	0.8	0.8	0.8
CAP1	Q01518	427	-4	-26	-20	-9	1.0	0.8	0.8	0.9
CHPT1	Q8WUD6	386	-5	-22	-20	-9	1.0	0.8	0.8	0.9

CPT1A	P50416	613	-5	-12	-20	-9	1.0	0.9	0.8	0.9
KIF2A	O00139	420	-5	-18	-20	-28	1.0	0.9	0.8	0.8
ISCA2	Q86U28	56	-6	-21	-20	-8	0.9	0.8	0.8	0.9
PIP4K2B	P78356	30	-6	-19	-20	-21	0.9	0.8	0.8	0.8
HDAC3	O15379	123	-7	-14	-20	-6	0.9	0.9	0.8	0.9
PGAM1	P18669	153	-7	-27	-20	-6	0.9	0.8	0.8	0.9
USP22	Q9UPT9	171	-7	-29	-20	-19	0.9	0.8	0.8	0.8
GPCPD1	Q9NPB8	640	-7	-12	-20	-16	0.9	0.9	0.8	0.9
LANCL1	O43813	98	-8	-21	-20	-12	0.9	0.8	0.8	0.9
MSN	P26038	284	-8	-24	-20	-4	0.9	0.8	0.8	1.0
RCBTB2	O95199	488	-8	-24	-20	-19	0.9	0.8	0.8	0.8
CMIP	Q8IY22	363	-8	-14	-20	-38	0.9	0.9	0.8	0.7
MPRIP	Q6WCQ1	957	-8	-19	-20	-27	0.9	0.8	0.8	0.8
ADCK4	Q96D53	147	-9	-19	-20	-11	0.9	0.8	0.8	0.9
ABHD10	Q9NUJ1	107	-9	-18	-20	-19	0.9	0.9	0.8	0.8
AKR1A1	P14550	260	-9	-19	-20	-6	0.9	0.8	0.8	0.9
FAM175A	Q6UWZ7	67	-11	-17	-20	-20	0.9	0.9	0.8	0.8
PPP2R5A	Q15172	359	-11	-19	-20	1	0.9	0.8	0.8	1.0
SPG7	Q9UQ90	353	-11	-61	-20	-23	0.9	0.6	0.8	0.8
SBF1	O95248	1638	-12	-22	-20	-4	0.9	0.8	0.8	1.0
GLCCI1	Q86VQ1	461	-13	-15	-20	-13	0.9	0.9	0.8	0.9
SAT2	Q96F10	14	-14	-16	-20	-8	0.9	0.9	0.8	0.9
TMEM23	D3DWC4	50	-15	-19	-20	-11	0.9	0.8	0.8	0.9
GUCY1B3	Q02153	122	-17	-27	-20	-13	0.9	0.8	0.8	0.9
EIF3D	O15371	19	-17	-20	-20	-2	0.9	0.8	0.8	1.0
TGFB1I1	O43294	405	-18	-8	-20	-24	0.9	0.9	0.8	0.8
RARRES3	Q9UL19	113	-19	-14	-20	-37	0.8	0.9	0.8	0.7
REXO2	Q9Y3B8	137	-22	-10	-20	-9	0.8	0.9	0.8	0.9
THNSL1	Q8IYQ7	532	-25	-31	-20	-18	0.8	0.8	0.8	0.9
RAVER2	Q9HCJ3	595	-26	-13	-20	-27	0.8	0.9	0.8	0.8
GPKOW	Q92917	137	-30	-19	-20	-35	0.8	0.8	0.8	0.7
ZBED6	P86452	463	8	-4	-21	-12	1.1	1.0	0.8	0.9
NELFCD	Q8IXH7	397	7	-1	-21	-16	1.1	1.0	0.8	0.9
XRCC1	P18887	20	6	-2	-21	17	1.1	1.0	0.8	1.2
ANK3	Q12955	1014	6	-22	-21	-9	1.1	0.8	0.8	0.9
RAB24	Q969Q5	118	4	-17	-21	-8	1.0	0.9	0.8	0.9
PRPF8	Q6P2Q9	547	4	-52	-21	-9	1.0	0.7	0.8	0.9
CEP170	Q5SW79	967	3	-16	-21	-22	1.0	0.9	0.8	0.8
DPP8	Q6V1X1	211	3	-18	-21	-15	1.0	0.8	0.8	0.9
Uncharacteri	G3V4G9	75	3	-15	-21	-2	1.0	0.9	0.8	1.0
CDKN2AIP	Q9NXV6	24	2	-29	-21	-6	1.0	0.8	0.8	0.9
WDR44	Q5JSH3	716	1	-24	-21	-2	1.0	0.8	0.8	1.0
FBXO42	Q6P3S6	403	1	-27	-21	-14	1.0	0.8	0.8	0.9
PARP4	Q9UKK3	1499	-1	-15	-21	-3	1.0	0.9	0.8	1.0
NUP205	Q92621	1662	-1	-21	-21	2	1.0	0.8	0.8	1.0
MYOF	Q9NZM1	1392	-3	-21	-21	4	1.0	0.8	0.8	1.0
PSMB1	P20618	89	-3	-21	-21	-6	1.0	0.8	0.8	0.9
SRRM2	Q9UQ35	1036	-3	-23	-21	-2	1.0	0.8	0.8	1.0
AHCY	P23526	195	-3	-23	-21	-10	1.0	0.8	0.8	0.9
GSPT1	P15170	464	-3	-28	-21	-12	1.0	0.8	0.8	0.9
GSPT2	Q8IYD1	593	-3	-28	-21	-12	1.0	0.8	0.8	0.9
DARS2	Q6PI48	108	-3	-12	-21	-16	1.0	0.9	0.8	0.9
CBX5	P45973	160	-3	-21	-21	-11	1.0	0.8	0.8	0.9

CBX3	Q13185	160	-3	-21	-21	-11	1.0	0.8	0.8	0.9
PRPF4	O43172	441	-3	-29	-21	-11	1.0	0.8	0.8	0.9
HUWE1	Q7Z6Z7	3213	-4	-26	-21	-11	1.0	0.8	0.8	0.9
MIS12	Q9H081	69	-4	-14	-21	-3	1.0	0.9	0.8	1.0
RNH1	P13489	191	-4	-18	-21	-7	1.0	0.8	0.8	0.9
ARHGAP35	Q9NRY4	924	-6	-17	-21	-4	0.9	0.9	0.8	1.0
RNF213	Q63HN8	1985	-6	-27	-21	-33	0.9	0.8	0.8	0.8
HS3ST1	O14792	265	-7	-24	-21	27	0.9	0.8	0.8	1.4
IKBKAP	O95163	456	-7	-28	-21	-14	0.9	0.8	0.8	0.9
CMPK2	Q5EBM0	117	-8	-24	-21	-13	0.9	0.8	0.8	0.9
TRAF6	Q9Y4K3	105	-8	-27	-21	-14	0.9	0.8	0.8	0.9
MAP2K7	O14733	260	-9	-35	-21	-3	0.9	0.7	0.8	1.0
CPNE8	Q86YQ8	155	-10	-26	-21	-22	0.9	0.8	0.8	0.8
TXNRD1	Q16881	577	-11	-24	-21	-4	0.9	0.8	0.8	1.0
FLCN	Q8NFG4	215	-12	-8	-21	-16	0.9	0.9	0.8	0.9
CCDC106	Q9BWC9	184	-12	-29	-21	-22	0.9	0.8	0.8	0.8
RUVBL1	Q9Y265	94	-12	-18	-21	-16	0.9	0.9	0.8	0.9
PCYOX1	Q9UHG3	445	-13	-11	-21	-15	0.9	0.9	0.8	0.9
SUPV3L1	Q8IYB8	418	-14	-16	-21	0	0.9	0.9	0.8	1.0
SEC31A	O94979	669	-14	-18	-21	2	0.9	0.9	0.8	1.0
TES	Q9UGI8	301	-14	-33	-21	-14	0.9	0.8	0.8	0.9
MAP4K1	Q92918	494	-15	-15	-21	1	0.9	0.9	0.8	1.0
ACADVL	P49748	156	-15	-19	-21	-18	0.9	0.8	0.8	0.9
ZC3H13	Q5T200	1643	-16	-9	-21	4	0.9	0.9	0.8	1.0
SLAMF7	Q9NQ25	275	-21	-24	-21	-27	0.8	0.8	0.8	0.8
PROSC	O94903	261	-77	-38	-21	-24	0.6	0.7	0.8	0.8
PI4KA	P42356	1824	14	-20	-21	-26	1.2	0.8	0.8	0.8
IFI16	Q16666	356	11	-7	-21	2	1.1	0.9	0.8	1.0
EED	O75530	126	10	5	-21	-21	1.1	1.1	0.8	0.8
TOMM34	Q15785	236	9	-7	-21	14	1.1	0.9	0.8	1.2
USP7	Q93009	917	8	-22	-21	-12	1.1	0.8	0.8	0.9
THNSL1	Q8IYQ7	242	6	-3	-21	4	1.1	1.0	0.8	1.0
TAF9B	Q9HBM6	121	6	-19	-21	-30	1.1	0.8	0.8	0.8
BANP	Q8N9N5	48	4	-19	-21	-29	1.0	0.8	0.8	0.8
DHRS1	Q96LJ7	177	2	-41	-21	-17	1.0	0.7	0.8	0.9
BLMH	Q13867	40	2	-35	-21	1	1.0	0.7	0.8	1.0
PRKCQ	Q04759	661	0	-21	-21	-21	1.0	0.8	0.8	0.8
TRMT2A	Q8IZ69	486	-1	-22	-21	-10	1.0	0.8	0.8	0.9
RAP1A	P62834	118	-1	-25	-21	-5	1.0	0.8	0.8	1.0
RBMS1	P29558	221	-1	-17	-21	-11	1.0	0.9	0.8	0.9
EPPK1	A0A087X1U6	1465	-1	-40	-21	-12	1.0	0.7	0.8	0.9
ZBTB11	O95625	39	-2	-8	-21	-14	1.0	0.9	0.8	0.9
ZNF280C	Q8ND82	734	-2	-13	-21	-11	1.0	0.9	0.8	0.9
DMXL1	Q9Y485	2532	-2	-20	-21	-2	1.0	0.8	0.8	1.0
TBL3	Q12788	43	-2	-27	-21	-18	1.0	0.8	0.8	0.8
RNASEL	Q05823	407	-2	-1	-21	-7	1.0	1.0	0.8	0.9
ALDOA	P04075	202	-2	-25	-21	-6	1.0	0.8	0.8	0.9
ALDOC	P09972	202	-2	-25	-21	-6	1.0	0.8	0.8	0.9
FCHO1	O14526	647	-2	-29	-21	7	1.0	0.8	0.8	1.1
UQCRC1	P31930	410	-3	-24	-21	-18	1.0	0.8	0.8	0.9
TAGLN2	P37802	38	-3	-31	-21	5	1.0	0.8	0.8	1.0
NLRC5	Q86WI3	984	-3	-41	-21	17	1.0	0.7	0.8	1.2
NADK	O95544	402	-4	-10	-21	-14	1.0	0.9	0.8	0.9

MICAL2	O94851	97	-4	-16	-21	-24	1.0	0.9	0.8	0.8
RPL12	P30050	17	-4	-29	-21	-13	1.0	0.8	0.8	0.9
PPP4C	P60510	266	-5	-22	-21	-13	1.0	0.8	0.8	0.9
WDR61	Q9GZS3	266	-5	-29	-21	6	1.0	0.8	0.8	1.1
MYLK	Q15746	1305	-6	-22	-21	5	0.9	0.8	0.8	1.1
PIK3CA	P42336	862	-6	-23	-21	11	0.9	0.8	0.8	1.1
GNAI3	P08754	66	-6	-17	-21	4	0.9	0.9	0.8	1.0
TAF2	Q6P1X5	767	-6	-32	-21	30	0.9	0.8	0.8	1.4
USP5	P45974	219	-7	-33	-21	-12	0.9	0.8	0.8	0.9
TARBP2	Q15633	73	-7	-37	-21	-33	0.9	0.7	0.8	0.8
CDK17	Q00537	233	-8	-21	-21	12	0.9	0.8	0.8	1.1
RCC2	Q9P258	209	-8	-22	-21	-9	0.9	0.8	0.8	0.9
VCP	P55072	572	-9	-26	-21	-4	0.9	0.8	0.8	1.0
ATP6V1A	P38606	240	-9	-20	-21	-1	0.9	0.8	0.8	1.0
ARHGEF40	Q8TER5	1148	-9	-24	-21	-11	0.9	0.8	0.8	0.9
PSMG1	O95456	157	-10	-11	-21	-17	0.9	0.9	0.8	0.9
TCP1	P17987	236	-10	-17	-21	-11	0.9	0.9	0.8	0.9
PURA	Q00577	272	-10	-27	-21	18	0.9	0.8	0.8	1.2
SENP3-EIF4A	A0A087X0R7	462	-10	-32	-21	-30	0.9	0.8	0.8	0.8
KPNA4	O00629	57	-10	-17	-21	-27	0.9	0.9	0.8	0.8
SIRT5	Q9NXA8	181	-11	-19	-21	-6	0.9	0.8	0.8	0.9
SEPT6	Q14141	47	-11	-22	-21	2	0.9	0.8	0.8	1.0
ADAR	P55265	630	-11	-26	-21	-19	0.9	0.8	0.8	0.8
CD97	P48960	802	-11	-29	-21	-15	0.9	0.8	0.8	0.9
PRRC2A	P48634	437	-15	-34	-21	-32	0.9	0.7	0.8	0.8
CLIP2	Q9UDT6	390	-15	-6	-21	-18	0.9	0.9	0.8	0.8
ALDOA	P04075	178	-16	-50	-21	-14	0.9	0.7	0.8	0.9
ALDOC	P09972	178	-16	-50	-21	-14	0.9	0.7	0.8	0.9
PNKD	Q8N490	329	-18	-24	-21	-2	0.9	0.8	0.8	1.0
EHMT1	Q9H9B1	1203	-18	-34	-21	-10	0.8	0.7	0.8	0.9
TMEM214	Q6NUQ4	529	-18	-44	-21	-25	0.8	0.7	0.8	0.8
KDM5A	P29375	683	-23	-21	-21	-30	0.8	0.8	0.8	0.8
DDX3Y	O15523	62	-23	-25	-21	19	0.8	0.8	0.8	1.2
POLD1	P28340	837	-27	-36	-21	-14	0.8	0.7	0.8	0.9
FASN	P49327	1881	-30	-18	-21	-4	0.8	0.9	0.8	1.0
LCN2	P80188	107	-46	-45	-21	-15	0.7	0.7	0.8	0.9
HTT	P42858	823	12	4	-22	-2	1.1	1.0	0.8	1.0
GRK6	P43250	71	10	-40	-22	2	1.1	0.7	0.8	1.0
WDR59	Q6PJI9	888	6	-14	-22	-11	1.1	0.9	0.8	0.9
CHD3	Q12873	1464	5	-9	-22	-4	1.1	0.9	0.8	1.0
EHHADH	Q08426	227	4	-25	-22	-11	1.0	0.8	0.8	0.9
MAP2K3	P46734	227	3	-8	-22	-8	1.0	0.9	0.8	0.9
GTF2A2	P52657	68	3	-11	-22	8	1.0	0.9	0.8	1.1
CDC16	Q13042	544	2	-13	-22	12	1.0	0.9	0.8	1.1
MGLL	Q99685	208	1	-11	-22	-71	1.0	0.9	0.8	0.6
U2AF2	P26368	429	1	-20	-22	1	1.0	0.8	0.8	1.0
C16orf58	Q96GQ5	201	0	-15	-22	-12	1.0	0.9	0.8	0.9
GBP3	Q9H0R5	405	0	-25	-22	-15	1.0	0.8	0.8	0.9
DGKQ	P52824	357	-2	-7	-22	-16	1.0	0.9	0.8	0.9
PSMA4	P25789	107	-4	-20	-22	-10	1.0	0.8	0.8	0.9
CAD	P27708	736	-4	-26	-22	-12	1.0	0.8	0.8	0.9
LMNB2	Q03252	212	-5	-14	-22	-9	1.0	0.9	0.8	0.9
Uncharacteri	B8ZZF3	279	-5	-14	-22	-18	1.0	0.9	0.8	0.9

ARHGAP24	Q8N264	327	-5	-20	-22	-24	1.0	0.8	0.8	0.8
ACACA	Q13085	1396	-5	-48	-22	-7	1.0	0.7	0.8	0.9
PELO	Q9BRX2	68	-5	-3	-22	-27	1.0	1.0	0.8	0.8
EXOSC7	Q15024	34	-5	-20	-22	-8	1.0	0.8	0.8	0.9
LHPP	Q9H008	53	-5	-30	-22	-12	1.0	0.8	0.8	0.9
PTPRA	P18433	743	-5	-31	-22	-21	1.0	0.8	0.8	0.8
SDHA	P31040	536	-5	-31	-22	-6	1.0	0.8	0.8	0.9
ZNF101	Q8IZC7	352	-6	-17	-22	-32	0.9	0.9	0.8	0.8
AKAP17A	Q02040	522	-7	-13	-22	-21	0.9	0.9	0.8	0.8
MRPS14	O60783	91	-7	-28	-22	-39	0.9	0.8	0.8	0.7
AHCY	P23526	228	-8	-38	-22	-13	0.9	0.7	0.8	0.9
PQLC3	Q8N755	157	-8	-27	-22	-59	0.9	0.8	0.8	0.6
METAP1	P53582	174	-9	-24	-22	-7	0.9	0.8	0.8	0.9
CORO2A	Q92828	25	-9	-34	-22	-4	0.9	0.7	0.8	1.0
KMT2D	O14686	5142	-10	-18	-22	-9	0.9	0.8	0.8	0.9
TDP1	Q9NUW8	413	-10	-23	-22	-27	0.9	0.8	0.8	0.8
MARS	P56192	392	-10	-27	-22	4	0.9	0.8	0.8	1.0
ASTE1	Q2TB18	173	-11	-31	-22	-13	0.9	0.8	0.8	0.9
PECR	Q9BY49	79	-12	-42	-22	-32	0.9	0.7	0.8	0.8
TBCD	Q9BTW9	850	-12	-10	-22	-23	0.9	0.9	0.8	0.8
RNASEL	Q05823	301	-12	-13	-22	-46	0.9	0.9	0.8	0.7
IDE	P14735	171	-12	-18	-22	-10	0.9	0.9	0.8	0.9
MICALL2	Q8IY33	37	-14	-59	-22	-42	0.9	0.6	0.8	0.7
MICALL1	Q8N3F8	38	-14	-59	-22	-42	0.9	0.6	0.8	0.7
C2CD2L	O14523	385	-15	-28	-22	-11	0.9	0.8	0.8	0.9
CDC42	P60953	105	-16	-25	-22	-2	0.9	0.8	0.8	1.0
EEF1E1	O43324	147	-17	-31	-22	-25	0.9	0.8	0.8	0.8
VPS29	Q9UBQ0	15	-17	-41	-22	-30	0.9	0.7	0.8	0.8
RABEP1	Q15276	263	-18	-29	-22	-2	0.9	0.8	0.8	1.0
CEP350	Q5VT06	2716	-19	-12	-22	-7	0.8	0.9	0.8	0.9
HELZ2	Q9BYK8	2617	-20	-19	-22	-26	0.8	0.8	0.8	0.8
ELMO1	Q92556	411	-21	-22	-22	-9	0.8	0.8	0.8	0.9
ELMO2	Q96JJ3	404	-21	-22	-22	-9	0.8	0.8	0.8	0.9
NOL8	Q76FK4	259	-27	-48	-22	-6	0.8	0.7	0.8	0.9
PRKDC	P78527	1183	-28	-30	-22	-23	0.8	0.8	0.8	0.8
EML4	Q9HC35	311	17	-4	-22	6	1.2	1.0	0.8	1.1
BAZ1B	Q9UIG0	1045	11	-25	-22	20	1.1	0.8	0.8	1.2
CPOX	P36551	373	9	-16	-22	15	1.1	0.9	0.8	1.2
ERO1A	Q96HE7	131	6	-16	-22	-30	1.1	0.9	0.8	0.8
SDHA	P31040	238	5	-44	-22	-23	1.1	0.7	0.8	0.8
PGM2L1	Q6PCE3	125	4	-29	-22	-15	1.0	0.8	0.8	0.9
HCFC1	P51610	135	2	-19	-22	-13	1.0	0.8	0.8	0.9
CDK6	Q00534	83	2	-26	-22	-8	1.0	0.8	0.8	0.9
BAHD1	Q8TBE0	703	0	-32	-22	-10	1.0	0.8	0.8	0.9
RPS2	P15880	222	-1	-19	-22	-9	1.0	0.8	0.8	0.9
COQ4	Q9Y3A0	231	-2	-14	-22	-21	1.0	0.9	0.8	0.8
PIK3R5	Q8WYR1	460	-4	-16	-22	-14	1.0	0.9	0.8	0.9
XRN2	Q9H0D6	296	-4	-21	-22	8	1.0	0.8	0.8	1.1
KMT2C	Q8NEZ4	473	-4	-27	-22	8	1.0	0.8	0.8	1.1
LPP	Q93052	566	-5	-28	-22	-20	1.0	0.8	0.8	0.8
MAP2K4	P45985	158	-6	-25	-22	10	0.9	0.8	0.8	1.1
TECPR1	Q7Z6L1	84	-7	-23	-22	-35	0.9	0.8	0.8	0.7
DTX3L	Q8TDB6	582	-7	-29	-22	18	0.9	0.8	0.8	1.2

NIPBL	Q6KC79	1940	-7	-34	-22	-18	0.9	0.7	0.8	0.8
AK2	P54819	42	-8	-14	-22	-7	0.9	0.9	0.8	0.9
RAB4A	P20338	28	-9	-25	-22	-13	0.9	0.8	0.8	0.9
TLK2	Q86UE8	606	-9	-32	-22	-30	0.9	0.8	0.8	0.8
DUS3L	Q96G46	396	-10	-11	-22	-10	0.9	0.9	0.8	0.9
ARFGEF2	Q9Y6D5	1450	-10	-15	-22	-9	0.9	0.9	0.8	0.9
TYK2	P29597	965	-10	-27	-22	-35	0.9	0.8	0.8	0.7
ERO1A	Q96HE7	37	-11	-19	-22	-37	0.9	0.8	0.8	0.7
DYNLL1	P63167	56	-11	-22	-22	-10	0.9	0.8	0.8	0.9
BRF1	Q92994	184	-11	-22	-22	-11	0.9	0.8	0.8	0.9
PARP14	Q460N5	1530	-11	-25	-22	-40	0.9	0.8	0.8	0.7
BDH1	Q02338	63	-12	-18	-22	-22	0.9	0.9	0.8	0.8
TATDN1	Q6P1N9	123	-12	-24	-22	-23	0.9	0.8	0.8	0.8
HPS5	Q9UPZ3	709	-12	-46	-22	-21	0.9	0.7	0.8	0.8
ALDH5A1	P51649	531	-13	-22	-22	-15	0.9	0.8	0.8	0.9
XPR1	Q9UBH6	188	-13	-19	-22	14	0.9	0.8	0.8	1.2
GPAM	Q9HCL2	813	-13	-21	-22	-10	0.9	0.8	0.8	0.9
RAE1	P78406	68	-14	-32	-22	-11	0.9	0.8	0.8	0.9
FSCN1	Q16658	397	-15	-21	-22	18	0.9	0.8	0.8	1.2
EIF5	P55010	99	-16	-20	-22	-14	0.9	0.8	0.8	0.9
LEMD3	Q9Y2U8	785	-16	-32	-22	-17	0.9	0.8	0.8	0.9
CARS	P49589	335	-17	-33	-22	-13	0.9	0.8	0.8	0.9
SEC24D	O94855	1021	-20	-22	-22	-19	0.8	0.8	0.8	0.8
SPATA20	Q8TB22	778	-21	-40	-22	-29	0.8	0.7	0.8	0.8
GRWD1	Q9BQ67	66	-22	-25	-22	-11	0.8	0.8	0.8	0.9
ACACA	Q13085	1297	-26	-12	-22	-28	0.8	0.9	0.8	0.8
VHL	P40337	77	-33	-18	-22	-25	0.8	0.9	0.8	0.8
PRF1	P14222	407	-39	-3	-22	-17	0.7	1.0	0.8	0.9
TPI1	P60174	104	-48	-47	-22	-4	0.7	0.7	0.8	1.0
TARS	P26639	254	8	-30	-23	-15	1.1	0.8	0.8	0.9
CUL3	Q13618	522	8	-13	-23	-1	1.1	0.9	0.8	1.0
RIOK3	O14730	171	7	-24	-23	1	1.1	0.8	0.8	1.0
COPG1	Q9Y678	280	7	-27	-23	23	1.1	0.8	0.8	1.3
VDAC2	P45880	103	7	-41	-23	-12	1.1	0.7	0.8	0.9
USP16	Q9Y5T5	205	5	-10	-23	-44	1.1	0.9	0.8	0.7
DNAJC13	O75165	1480	3	-19	-23	-10	1.0	0.8	0.8	0.9
GAS7	O60861	318	2	-26	-23	5	1.0	0.8	0.8	1.0
PRKCQ	Q04759	14	1	-18	-23	-17	1.0	0.9	0.8	0.9
RANBP2	P49792	3032	-1	-26	-23	-35	1.0	0.8	0.8	0.7
GNPDA1	P46926	239	-2	-23	-23	-9	1.0	0.8	0.8	0.9
UBR1	Q8IWV7	1569	-3	-26	-23	-35	1.0	0.8	0.8	0.7
PTPRC	P08575	752	-5	-25	-23	-11	1.0	0.8	0.8	0.9
PHPT1	Q9NRX4	73	-5	-31	-23	-15	1.0	0.8	0.8	0.9
HUS1	O60921	200	-7	-27	-23	-19	0.9	0.8	0.8	0.8
CIR1	Q86X95	10	-9	-7	-23	-29	0.9	0.9	0.8	0.8
SIRT6	Q8N6T7	18	-10	-15	-23	-6	0.9	0.9	0.8	0.9
INPP5D	Q92835	506	-10	-32	-23	14	0.9	0.8	0.8	1.2
MYSM1	Q5VVJ2	822	-11	-30	-23	-44	0.9	0.8	0.8	0.7
HDHD3	Q9BSH5	109	-12	-14	-23	-19	0.9	0.9	0.8	0.8
STK17B	O94768	190	-12	-25	-23	-4	0.9	0.8	0.8	1.0
NCOA7	Q8NI08	487	-13	-2	-23	-26	0.9	1.0	0.8	0.8
EIF3I	Q13347	76	-13	-28	-23	-6	0.9	0.8	0.8	0.9
NEK4	P51957	466	-15	-9	-23	-20	0.9	0.9	0.8	0.8

ARPC3	O15145	162	-15	-25	-23	-15	0.9	0.8	0.8	0.9
SLFN11	Q7Z7L1	884	-16	-23	-23	-19	0.9	0.8	0.8	0.8
SYNE2	Q8WXH0	2935	-17	-37	-23	-18	0.9	0.7	0.8	0.8
RAP2C	Q9Y3L5	140	-18	-16	-23	-12	0.8	0.9	0.8	0.9
MSH6	P52701	1117	-19	-36	-23	-32	0.8	0.7	0.8	0.8
BZW2	Q9Y6E2	270	-20	-20	-23	-10	0.8	0.8	0.8	0.9
ARRB2	P32121	252	-25	-22	-23	-6	0.8	0.8	0.8	0.9
FBXL15	Q9H469	244	-54	-25	-23	-23	0.6	0.8	0.8	0.8
PSMD1	Q99460	141	12	-22	-23	3	1.1	0.8	0.8	1.0
MSH2	P43246	641	11	-6	-23	7	1.1	0.9	0.8	1.1
TMEM209	Q96SK2	422	11	-16	-23	-9	1.1	0.9	0.8	0.9
FASN	P49327	2010	11	-19	-23	-16	1.1	0.8	0.8	0.9
ACSS1	Q9NUB1	242	10	-15	-23	16	1.1	0.9	0.8	1.2
KMT5B	Q4FZB7	156	7	-17	-23	-16	1.1	0.9	0.8	0.9
MED16	Q9Y2X0	545	1	-25	-23	13	1.0	0.8	0.8	1.1
THAP12	O43422	230	1	-10	-23	-11	1.0	0.9	0.8	0.9
CRACR2A	Q9BSW2	29	0	-28	-23	-13	1.0	0.8	0.8	0.9
HIP1	O00291	249	0	-32	-23	-8	1.0	0.8	0.8	0.9
HIP1R	O75146	240	0	-32	-23	-8	1.0	0.8	0.8	0.9
NANS	Q9NR45	46	-1	-28	-23	-10	1.0	0.8	0.8	0.9
TARDBP	G3V162	173	-2	-26	-23	9	1.0	0.8	0.8	1.1
ARHGAP30	Q7Z6I6	1021	-3	2	-23	-9	1.0	1.0	0.8	0.9
TUBGCP3	Q96CW5	844	-3	-16	-23	5	1.0	0.9	0.8	1.1
KMT2B	Q9UMN6	268	-3	-32	-23	-41	1.0	0.8	0.8	0.7
SF1	Q15637	171	-4	-21	-23	-14	1.0	0.8	0.8	0.9
ZMPSTE24	O75844	324	-6	-24	-23	-14	0.9	0.8	0.8	0.9
ZNF425	Q6IV72	315	-7	-8	-23	-19	0.9	0.9	0.8	0.8
WDR20	Q8TBZ3	514	-7	-7	-23	-1	0.9	0.9	0.8	1.0
CHST14	Q8NCH0	159	-9	-22	-23	-25	0.9	0.8	0.8	0.8
ESD	P10768	11	-9	-36	-23	-9	0.9	0.7	0.8	0.9
METTL13	Q8N6R0	391	-10	-20	-23	-4	0.9	0.8	0.8	1.0
CTDP1	Q9Y5B0	95	-11	-24	-23	-28	0.9	0.8	0.8	0.8
TEP1	Q99973	1638	-11	-24	-23	-12	0.9	0.8	0.8	0.9
VPS8	Q8N3P4	974	-11	-11	-23	14	0.9	0.9	0.8	1.2
FLII	Q13045	1265	-11	-31	-23	15	0.9	0.8	0.8	1.2
CPSF6	Q16630	466	-11	-42	-23	-16	0.9	0.7	0.8	0.9
WDR12	Q9GZL7	185	-12	-27	-23	14	0.9	0.8	0.8	1.2
SEH1L	Q96EE3	165	-17	-37	-23	-13	0.9	0.7	0.8	0.9
COG5	Q9UP83	64	-18	-17	-23	-30	0.8	0.9	0.8	0.8
PPP2R4	Q15257	103	-19	-34	-23	18	0.8	0.7	0.8	1.2
TPRKB	Q9Y3C4	13	-22	-16	-23	-2	0.8	0.9	0.8	1.0
WIPI1	Q5MNZ9	95	-24	-20	-23	-23	0.8	0.8	0.8	0.8
USP15	Q9Y4E8	462	-25	-25	-23	-7	0.8	0.8	0.8	0.9
PPAT	Q06203	207	-30	-28	-23	-24	0.8	0.8	0.8	0.8
ASCC3	Q8N3C0	1315	15	-1	-24	-1	1.2	1.0	0.8	1.0
EIF3B	P55884	700	14	-32	-24	-14	1.2	0.8	0.8	0.9
GAN	Q9H2C0	30	13	-15	-24	3	1.1	0.9	0.8	1.0
TGM1	P22735	377	8	-4	-24	-13	1.1	1.0	0.8	0.9
TRMT1L	Q7Z2T5	239	5	-22	-24	-14	1.0	0.8	0.8	0.9
ZNF7	P17097	558	4	-27	-24	-15	1.0	0.8	0.8	0.9
ZNF181	Q2M3W8	522	4	-27	-24	-15	1.0	0.8	0.8	0.9
ZNF836	Q6ZNA1	251	4	-27	-24	-15	1.0	0.8	0.8	0.9
ZNF600	Q6ZNG1	223	4	-27	-24	-15	1.0	0.8	0.8	0.9

ZNF808	Q8N4W9	320	4	-27	-24	-15	1.0	0.8	0.8	0.9
ZFP62	Q8NB50	845	4	-27	-24	-15	1.0	0.8	0.8	0.9
ZNF845	Q96IR2	332	4	-27	-24	-15	1.0	0.8	0.8	0.9
ZNF501	Q96CX3	111	4	-27	-24	-15	1.0	0.8	0.8	0.9
NDUFS1	P28331	367	3	-21	-24	-9	1.0	0.8	0.8	0.9
ZMYM3	Q14202	498	2	-15	-24	6	1.0	0.9	0.8	1.1
RING1	Q06587	87	1	-20	-24	-18	1.0	0.8	0.8	0.9
RNF2	Q99496	90	1	-20	-24	-18	1.0	0.8	0.8	0.9
KDM5A	P29375	690	0	-18	-24	-2	1.0	0.9	0.8	1.0
ALDH3A2	P51648	214	-3	-22	-24	-12	1.0	0.8	0.8	0.9
PABPC1	P11940	128	-3	-41	-24	-3	1.0	0.7	0.8	1.0
KHSRP	Q92945	379	-4	-26	-24	19	1.0	0.8	0.8	1.2
SRSF7	Q16629	106	-4	-33	-24	-28	1.0	0.8	0.8	0.8
TATDN1	Q6P1N9	81	-6	-27	-24	-25	0.9	0.8	0.8	0.8
TYW1B	A0A087WZB:	169	-7	-23	-24	-18	0.9	0.8	0.8	0.9
VWA8	A3KMH1	858	-7	-26	-24	27	0.9	0.8	0.8	1.4
WDFY4	Q6ZS81	2218	-8	-26	-24	-33	0.9	0.8	0.8	0.8
NANS	Q9NR45	19	-9	-21	-24	-10	0.9	0.8	0.8	0.9
DDX3X	O00571	128	-9	-27	-24	-5	0.9	0.8	0.8	1.0
BCR	P11274	753	-9	-34	-24	-30	0.9	0.7	0.8	0.8
FARS2	O95363	344	-9	-35	-24	-30	0.9	0.7	0.8	0.8
ARF5	P84085	159	-10	-40	-24	-11	0.9	0.7	0.8	0.9
CAD	P27708	2161	-11	-16	-24	-3	0.9	0.9	0.8	1.0
MATR3	A8MXP9	869	-14	-20	-24	8	0.9	0.8	0.8	1.1
NUMB	P49757	165	-14	-21	-24	23	0.9	0.8	0.8	1.3
RANBP2	P49792	2407	-15	-13	-24	-17	0.9	0.9	0.8	0.9
RAP2B	P61225	140	-17	-23	-24	-15	0.9	0.8	0.8	0.9
TKT	P29401	386	-17	-35	-24	-9	0.9	0.7	0.8	0.9
STAT3	P40763	718	-17	-20	-24	-16	0.9	0.8	0.8	0.9
Uncharacteri	G3V3G9	531	-17	-22	-24	-17	0.9	0.8	0.8	0.9
PHF5A	Q7RTV0	46	-17	-30	-24	-8	0.9	0.8	0.8	0.9
ADAP2	Q9NPF8	282	-19	-20	-24	-27	0.8	0.8	0.8	0.8
UBXN7	O94888	478	-21	-27	-24	15	0.8	0.8	0.8	1.2
DLD	P09622	85	-21	-37	-24	-29	0.8	0.7	0.8	0.8
ACSL4	O60488	552	-22	-8	-24	-26	0.8	0.9	0.8	0.8
TRMT11	Q7Z4G4	161	-22	0	-24	17	0.8	1.0	0.8	1.2
EPC1	Q9H2F5	22	-23	-23	-24	-4	0.8	0.8	0.8	1.0
SNX2	O60749	332	-32	-9	-24	15	0.8	0.9	0.8	1.2
MAPKAPK3	Q16644	61	-37	-34	-24	-14	0.7	0.7	0.8	0.9
P4HB	P07237	312	-41	-31	-24	-17	0.7	0.8	0.8	0.9
ME2	P23368	198	19	-9	-24	-5	1.2	0.9	0.8	1.0
CTNND1	O60716	450	10	-22	-24	6	1.1	0.8	0.8	1.1
FLYWCH1	Q4VC44	478	9	-24	-24	-10	1.1	0.8	0.8	0.9
UTY	O14607	76	6	-19	-24	2	1.1	0.8	0.8	1.0
KDM6A	O15550	79	6	-19	-24	2	1.1	0.8	0.8	1.0
ACLY	P53396	1040	5	-22	-24	-7	1.0	0.8	0.8	0.9
ADAR	P55265	499	3	-23	-24	-37	1.0	0.8	0.8	0.7
SF3B3	Q15393	1179	3	-32	-24	-23	1.0	0.8	0.8	0.8
GTF3C1	Q12789	1363	3	-14	-24	-11	1.0	0.9	0.8	0.9
CUX1	P39880	802	2	-3	-24	-3	1.0	1.0	0.8	1.0
CHKB	Q9Y259	28	2	-25	-24	-33	1.0	0.8	0.8	0.8
FRMD3	A2A2Y4	596	1	-11	-24	-16	1.0	0.9	0.8	0.9
GMPS	P49915	213	1	-21	-24	-6	1.0	0.8	0.8	0.9

CKAP5	Q14008	1113	1	-15	-24	-14	1.0	0.9	0.8	0.9
LDAH	Q9H6V9	274	-2	-23	-24	-9	1.0	0.8	0.8	0.9
RENBP	P51606	114	-4	-21	-24	-19	1.0	0.8	0.8	0.8
SMG8	Q8ND04	261	-5	-18	-24	-22	1.0	0.9	0.8	0.8
DFFA	O00273	38	-5	-28	-24	7	1.0	0.8	0.8	1.1
TRAF2	Q12933	129	-6	-27	-24	-35	0.9	0.8	0.8	0.7
TEP1	Q99973	531	-6	-39	-24	-48	0.9	0.7	0.8	0.7
SPTBN1	Q01082	624	-9	-22	-24	25	0.9	0.8	0.8	1.3
COG6	Q9Y2V7	621	-9	-20	-24	-14	0.9	0.8	0.8	0.9
GPHN	Q9NQX3	419	-9	-27	-24	-32	0.9	0.8	0.8	0.8
IL6R	P08887	25	-10	-15	-24	-3	0.9	0.9	0.8	1.0
RCC2	Q9P258	158	-10	-26	-24	-18	0.9	0.8	0.8	0.9
SCLY	Q96I15	377	-10	-29	-24	-9	0.9	0.8	0.8	0.9
RNGTT	O60942	419	-11	-22	-24	-35	0.9	0.8	0.8	0.7
SART3	Q15020	749	-11	-29	-24	-35	0.9	0.8	0.8	0.7
CEP164	Q9UPV0	626	-11	-41	-24	-10	0.9	0.7	0.8	0.9
CWF19L1	Q69YN2	288	-11	-20	-24	-25	0.9	0.8	0.8	0.8
MAP2K6	P52564	109	-12	-11	-24	-2	0.9	0.9	0.8	1.0
ANKRD17	O75179	644	-12	-25	-24	15	0.9	0.8	0.8	1.2
RARA	P10276	203	-12	-32	-24	37	0.9	0.8	0.8	1.6
CAPN1	P07384	49	-12	-35	-24	-6	0.9	0.7	0.8	0.9
PNPLA8	Q9NP80	559	-13	-27	-24	-13	0.9	0.8	0.8	0.9
SPG11	Q96JI7	2435	-17	-32	-24	-17	0.9	0.8	0.8	0.9
EPRS	P07814	381	-17	-40	-24	-16	0.9	0.7	0.8	0.9
RB1CC1	Q8TDY2	60	-22	-28	-24	-42	0.8	0.8	0.8	0.7
ZNF830	Q96NB3	53	-26	-50	-24	-22	0.8	0.7	0.8	0.8
PDCL	Q13371	81	-27	-40	-24	-60	0.8	0.7	0.8	0.6
ZNF451	Q9Y4E5	133	-27	-35	-24	-43	0.8	0.7	0.8	0.7
MRPL38	Q96DV4	290	-55	-48	-24	-6	0.6	0.7	0.8	0.9
WAS	P42768	73	18	-30	-25	-9	1.2	0.8	0.8	0.9
AKR1C3	P42330	193	16	-6	-25	9	1.2	0.9	0.8	1.1
NSF	P46459	21	9	-26	-25	-22	1.1	0.8	0.8	0.8
PFAS	O15067	336	7	-6	-25	10	1.1	0.9	0.8	1.1
PEX11B	O96011	216	5	-24	-25	-15	1.1	0.8	0.8	0.9
IPO5	O00410	229	4	-19	-25	-14	1.0	0.8	0.8	0.9
PIKFYVE	Q9Y2I7	384	4	-36	-25	5	1.0	0.7	0.8	1.1
AFG3L2	Q9Y4W6	313	4	-24	-25	-11	1.0	0.8	0.8	0.9
ALDOA	P04075	135	1	-37	-25	-12	1.0	0.7	0.8	0.9
GNAI2	P04899	287	1	-44	-25	18	1.0	0.7	0.8	1.2
DDAH2	O95865	11	-1	-36	-25	27	1.0	0.7	0.8	1.4
GSR	P00390	278	-2	-24	-25	17	1.0	0.8	0.8	1.2
HMHA1	Q92619	717	-2	-6	-25	-7	1.0	0.9	0.8	0.9
RAB3D	O95716	137	-3	-23	-25	-7	1.0	0.8	0.8	0.9
HDAC1	Q13547	284	-3	-30	-25	-13	1.0	0.8	0.8	0.9
TUBGCP6	Q96RT7	487	-4	-17	-25	-22	1.0	0.9	0.8	0.8
THUMPD1	Q9NXG2	50	-4	-26	-25	-4	1.0	0.8	0.8	1.0
TBL1XR1	Q9BZK7	383	-4	-27	-25	-6	1.0	0.8	0.8	0.9
PSMA6	P60900	115	-5	-36	-25	-22	1.0	0.7	0.8	0.8
RRAGA	Q7L523	159	-6	-17	-25	-19	0.9	0.9	0.8	0.8
SMC1A	Q14683	619	-7	-39	-25	-23	0.9	0.7	0.8	0.8
ARL11	Q969Q4	180	-9	-22	-25	-25	0.9	0.8	0.8	0.8
RNF213	Q63HN8	1134	-12	-8	-25	-26	0.9	0.9	0.8	0.8
NUTF2	P61970	38	-15	-22	-25	-14	0.9	0.8	0.8	0.9

AARS	P49588	723	-16	-28	-25	-1	0.9	0.8	0.8	1.0
EPRS	P07814	1480	-23	-26	-25	-16	0.8	0.8	0.8	0.9
WDFY1	Q8IWB7	347	17	-10	-25	4	1.2	0.9	0.8	1.0
MLC1	Q15049	171	12	-34	-25	-17	1.1	0.7	0.8	0.9
LTN1	O94822	1131	4	-25	-25	-16	1.0	0.8	0.8	0.9
CTC1	Q2NKJ3	28	4	-20	-25	-26	1.0	0.8	0.8	0.8
NUBP1	P53384	277	3	-29	-25	1	1.0	0.8	0.8	1.0
FLOT1	O75955	34	1	-38	-25	-35	1.0	0.7	0.8	0.7
MAP2K1	Q02750	277	-1	-30	-25	-22	1.0	0.8	0.8	0.8
TIAM1	Q13009	1006	-2	-16	-25	-42	1.0	0.9	0.8	0.7
KIAA1551	Q9HCM1	717	-3	-7	-25	-12	1.0	0.9	0.8	0.9
KBTBD2	Q8IY47	145	-5	-8	-25	-15	1.0	0.9	0.8	0.9
IARS2	Q9NSE4	91	-6	-27	-25	-23	0.9	0.8	0.8	0.8
MTHFD1	P11586	785	-6	-46	-25	27	0.9	0.7	0.8	1.4
UROD	P06132	65	-7	-15	-25	-6	0.9	0.9	0.8	0.9
ARRB2	P32121	126	-8	-28	-25	1	0.9	0.8	0.8	1.0
GOT2	P00505	106	-8	-110	-25	-15	0.9	0.5	0.8	0.9
ACAP2	Q15057	53	-9	2	-25	-4	0.9	1.0	0.8	1.0
THOC2	Q8NI27	1518	-10	-6	-25	-31	0.9	0.9	0.8	0.8
BRAT1	Q6PJG6	513	-10	-19	-25	8	0.9	0.8	0.8	1.1
ALG13	Q9NP73	86	-12	-5	-25	-35	0.9	1.0	0.8	0.7
GIMAP2	Q9UG22	180	-12	-30	-25	-24	0.9	0.8	0.8	0.8
SYNE1	Q8NF91	4575	-12	-32	-25	21	0.9	0.8	0.8	1.3
ERCC3	P19447	180	-15	-10	-25	-26	0.9	0.9	0.8	0.8
RPS12	P25398	56	-15	-24	-25	18	0.9	0.8	0.8	1.2
FAM96B	Q9Y3D0	158	-16	-17	-25	-22	0.9	0.9	0.8	0.8
BLMH	Q13867	269	-18	-30	-25	-13	0.9	0.8	0.8	0.9
ZNF280C	Q8ND82	278	-19	-29	-25	-18	0.8	0.8	0.8	0.9
PRKCH	P24723	203	-21	-37	-25	-16	0.8	0.7	0.8	0.9
GTPBP1	O00178	360	-24	-20	-25	-23	0.8	0.8	0.8	0.8
DDX51	Q8N8A6	187	-28	-25	-25	-38	0.8	0.8	0.8	0.7
MTOR	P42345	2243	-30	-51	-25	-38	0.8	0.7	0.8	0.7
CTBS	Q01459	93	-32	-25	-25	-11	0.8	0.8	0.8	0.9
ZNF512B	Q96KM6	142	-72	-41	-25	-56	0.6	0.7	0.8	0.6
SNRPD2	P62316	46	-78	-45	-25	-3	0.6	0.7	0.8	1.0
NOL10	Q9BSC4	79	10	-30	-26	-1	1.1	0.8	0.8	1.0
ZMYM2	Q9UBW7	791	3	-14	-26	11	1.0	0.9	0.8	1.1
TLR1	Q15399	530	2	-18	-26	-7	1.0	0.9	0.8	0.9
DDX50	Q9BQ39	488	1	-1	-26	-4	1.0	1.0	0.8	1.0
TPBGL	P0DKB5	43	0	-19	-26	-15	1.0	0.8	0.8	0.9
ARFGAP2	Q8N6H7	26	0	-20	-26	2	1.0	0.8	0.8	1.0
PI4KA	P42356	1774	-1	-23	-26	-17	1.0	0.8	0.8	0.9
NT5DC1	Q5TFE4	197	-1	-35	-26	13	1.0	0.7	0.8	1.1
UBE2M	P61081	47	-1	-35	-26	8	1.0	0.7	0.8	1.1
OGDH	Q02218	507	-3	-35	-26	-1	1.0	0.7	0.8	1.0
TAP2	Q03519	362	-3	-55	-26	-28	1.0	0.6	0.8	0.8
BRPF1	P55201	570	-4	-38	-26	0	1.0	0.7	0.8	1.0
SYTL4	Q96C24	477	-6	-31	-26	-3	0.9	0.8	0.8	1.0
CLK1	P49759	200	-7	-19	-26	-21	0.9	0.8	0.8	0.8
LRBA	P50851	832	-8	-18	-26	-15	0.9	0.8	0.8	0.9
INTS1	Q8N201	867	-8	-32	-26	24	0.9	0.8	0.8	1.3
TRIM56	Q9BRZ2	338	-9	-24	-26	-26	0.9	0.8	0.8	0.8
NSUN2	Q08J23	235	-9	-23	-26	-10	0.9	0.8	0.8	0.9

EHMT2	Q96KQ7	842	-10	-21	-26	-12	0.9	0.8	0.8	0.9
AGFG1	P52594	29	-10	-28	-26	7	0.9	0.8	0.8	1.1
STXBP3	O00186	114	-12	-20	-26	1	0.9	0.8	0.8	1.0
PRKDC	P78527	1176	-12	-21	-26	-21	0.9	0.8	0.8	0.8
SCYL3	Q8IZE3	184	-12	-28	-26	-22	0.9	0.8	0.8	0.8
BOD1L1	Q8NFC6	1554	-12	-34	-26	-39	0.9	0.7	0.8	0.7
UBE2A	P49459	88	-13	-11	-26	25	0.9	0.9	0.8	1.3
ISG15	P05161	78	-13	-16	-26	-24	0.9	0.9	0.8	0.8
OAS3	Q9Y6K5	449	-13	-24	-26	28	0.9	0.8	0.8	1.4
SAE1	Q9UBE0	214	-14	-21	-26	-14	0.9	0.8	0.8	0.9
AIMP1	Q12904	284	-31	-11	-26	-24	0.8	0.9	0.8	0.8
CCT7	Q99832	364	-32	-26	-26	-13	0.8	0.8	0.8	0.9
SIMC1	Q8NDZ2	430	-66	-45	-26	-30	0.6	0.7	0.8	0.8
ENO1	P06733	389	-68	-68	-26	-13	0.6	0.6	0.8	0.9
RRN3	Q9NYV6	224	23	-16	-26	-6	1.3	0.9	0.8	0.9
SEC24C	P53992	816	7	-1	-26	-5	1.1	1.0	0.8	1.0
ARHGAP21	Q5T5U3	1167	5	-34	-26	-10	1.1	0.7	0.8	0.9
SETD2	Q9BYW2	594	5	-14	-26	27	1.0	0.9	0.8	1.4
ZC3HAV1	Q7Z2W4	831	3	-25	-26	-3	1.0	0.8	0.8	1.0
ERAP2	Q6P179	210	-3	-28	-26	-17	1.0	0.8	0.8	0.9
MSN	P26038	117	-3	-29	-26	-17	1.0	0.8	0.8	0.9
GRHPR	Q9UBQ7	216	-6	-33	-26	-13	0.9	0.8	0.8	0.9
CTCF	P49711	557	-6	-48	-26	-10	0.9	0.7	0.8	0.9
OSBPL9	Q96SU4	720	-8	-20	-26	16	0.9	0.8	0.8	1.2
RTTN	Q86VV8	1502	-9	-19	-26	-28	0.9	0.8	0.8	0.8
RUVBL1	Q9Y265	206	-9	-20	-26	-22	0.9	0.8	0.8	0.8
RALGAPA2	Q2PPJ7	378	-9	-25	-26	-24	0.9	0.8	0.8	0.8
MDH2	P40926	212	-10	-46	-26	-18	0.9	0.7	0.8	0.9
MARS2	Q96GW9	562	-11	-16	-26	-17	0.9	0.9	0.8	0.9
LRRK2	Q5S007	249	-11	-41	-26	-29	0.9	0.7	0.8	0.8
MRPL44	Q9H9J2	96	-15	-39	-26	-32	0.9	0.7	0.8	0.8
USP15	Q9Y4E8	570	-18	-40	-26	-20	0.9	0.7	0.8	0.8
VCP	P55072	174	-24	-23	-26	-5	0.8	0.8	0.8	1.0
CHFR	Q96EP1	601	-25	-28	-26	-21	0.8	0.8	0.8	0.8
CRYZL1	O95825	177	-29	-33	-26	-31	0.8	0.8	0.8	0.8
PHF12	Q96QT6	534	-31	-46	-26	-45	0.8	0.7	0.8	0.7
HERC4	Q5GLZ8	352	-36	-22	-26	-45	0.7	0.8	0.8	0.7
PROSC	O94903	15	-44	-34	-26	-3	0.7	0.7	0.8	1.0
FGD2	Q7Z6J4	43	25	14	-27	-30	1.3	1.2	0.8	0.8
UPF2	Q9HAU5	1107	21	-1	-27	16	1.3	1.0	0.8	1.2
GRK6	P43250	57	10	-56	-27	33	1.1	0.6	0.8	1.5
PADI4	Q9UM07	645	5	-13	-27	12	1.0	0.9	0.8	1.1
GOT2	P00505	187	2	-37	-27	-7	1.0	0.7	0.8	0.9
SIRT1	Q96EB6	574	1	-34	-27	-28	1.0	0.7	0.8	0.8
CRYZL1	O95825	40	1	-49	-27	4	1.0	0.7	0.8	1.0
MXD1	Q05195	111	-1	-37	-27	-15	1.0	0.7	0.8	0.9
IDH3A	P50213	351	-3	-33	-27	2	1.0	0.8	0.8	1.0
HCFC1	P51610	149	-4	-33	-27	-1	1.0	0.8	0.8	1.0
TNPO1	Q92973	297	-7	-33	-27	-7	0.9	0.8	0.8	0.9
LAMP1	P11279	338	-8	-28	-27	-13	0.9	0.8	0.8	0.9
PDE3B	Q13370	35	-10	-14	-27	-5	0.9	0.9	0.8	1.0
TSC1	Q92574	822	-11	-29	-27	0	0.9	0.8	0.8	1.0
ELMO2	Q96JJ3	550	-12	-27	-27	-14	0.9	0.8	0.8	0.9

CASP9	P55211	187	-13	-26	-27	-11	0.9	0.8	0.8	0.9
AGL	P35573	957	-14	-34	-27	-19	0.9	0.7	0.8	0.8
PPIH	O43447	47	-14	-46	-27	-9	0.9	0.7	0.8	0.9
TRAF1	Q13077	37	-15	-16	-27	-36	0.9	0.9	0.8	0.7
NLRC5	Q86WI3	1002	-16	-1	-27	-2	0.9	1.0	0.8	1.0
SRRD	Q9UH36	152	-16	-37	-27	-23	0.9	0.7	0.8	0.8
PITPNB	P48739	13	-24	-24	-27	-14	0.8	0.8	0.8	0.9
ASUN	Q9NVM9	17	-30	-26	-27	-10	0.8	0.8	0.8	0.9
DPP9	Q86TI2	844	-30	-30	-27	-14	0.8	0.8	0.8	0.9
MED16	Q9Y2X0	30	-31	-9	-27	33	0.8	0.9	0.8	1.5
TOP1	P11387	504	-33	-42	-27	-22	0.8	0.7	0.8	0.8
DPYSL2	Q16555	248	-38	-32	-27	-15	0.7	0.8	0.8	0.9
TPI1	P60174	79	-46	-50	-27	-2	0.7	0.7	0.8	1.0
BRIX1	Q8TDN6	115	5	-26	-27	-16	1.0	0.8	0.8	0.9
LASP1	Q14847	20	-2	-5	-27	22	1.0	1.0	0.8	1.3
NELFCD	Q8IXH7	582	-2	-41	-27	-22	1.0	0.7	0.8	0.8
NDUFV2	P19404	225	-5	-13	-27	-13	1.0	0.9	0.8	0.9
DDX47	Q9H0S4	226	-5	-21	-27	-5	1.0	0.8	0.8	1.0
NO66	Q9H6W3	613	-8	-25	-27	-12	0.9	0.8	0.8	0.9
CCDC91	Q7Z6B0	247	-9	-37	-27	17	0.9	0.7	0.8	1.2
PGM2	Q96G03	86	-9	-47	-27	-4	0.9	0.7	0.8	1.0
MTPAP	Q9NVV4	324	-13	-2	-27	-20	0.9	1.0	0.8	0.8
PASK	Q96RG2	228	-13	-7	-27	-18	0.9	0.9	0.8	0.8
AKAP8	O43823	395	-14	-18	-27	15	0.9	0.8	0.8	1.2
FRY	Q5TBA9	2719	-16	-24	-27	-15	0.9	0.8	0.8	0.9
FAHD1	Q6P587	129	-122	-44	-27	-3	0.5	0.7	0.8	1.0
ELP2	Q6IA86	127	13	-13	-28	-3	1.1	0.9	0.8	1.0
LGMN	Q99538	50	9	6	-28	30	1.1	1.1	0.8	1.4
FBXL8	Q96CD0	228	8	-20	-28	-11	1.1	0.8	0.8	0.9
EFTUD2	Q15029	674	1	-38	-28	-29	1.0	0.7	0.8	0.8
BCCIP	Q9P287	216	-2	-22	-28	-33	1.0	0.8	0.8	0.8
KMT2E	Q8IZD2	815	-3	-40	-28	-39	1.0	0.7	0.8	0.7
BOP1	Q14137	532	-4	-23	-28	12	1.0	0.8	0.8	1.1
FKBP15	Q5T1M5	828	-4	-30	-28	13	1.0	0.8	0.8	1.1
TLN1	Q9Y490	1087	-4	-32	-28	-21	1.0	0.8	0.8	0.8
GSPT1	P15170	327	-4	-27	-28	-24	1.0	0.8	0.8	0.8
ECHDC2	Q86YB7	180	-5	-39	-28	-27	1.0	0.7	0.8	0.8
LSM7	Q9UK45	76	-9	-26	-28	-15	0.9	0.8	0.8	0.9
SRBD1	Q8N5C6	120	-10	-29	-28	-28	0.9	0.8	0.8	0.8
METAP1	P53582	194	-11	-10	-28	-1	0.9	0.9	0.8	1.0
PRMT5	O14744	22	-12	-25	-28	-5	0.9	0.8	0.8	1.0
GNPAT	O15228	122	-14	-35	-28	-21	0.9	0.7	0.8	0.8
DNPEP	Q9ULA0	280	-16	-49	-28	-4	0.9	0.7	0.8	1.0
ANKFY1	Q9P2R3	724	-17	1	-28	15	0.9	1.0	0.8	1.2
MACROD1	Q9BQ69	246	-17	-35	-28	-39	0.9	0.7	0.8	0.7
FCGR1B	Q92637	259	-18	-44	-28	-29	0.8	0.7	0.8	0.8
AXIN1	O15169	833	-20	-24	-28	-10	0.8	0.8	0.8	0.9
ASCC3	Q8N3C0	1840	-25	-44	-28	-16	0.8	0.7	0.8	0.9
TPI1	P60174	255	-39	-54	-28	-16	0.7	0.7	0.8	0.9
ERP44	Q9BS26	318	-52	-26	-28	8	0.7	0.8	0.8	1.1
DDX39A	O00148	197	17	13	-28	16	1.2	1.1	0.8	1.2
GSTCD	Q8NEC7	140	4	-1	-28	-19	1.0	1.0	0.8	0.8
MYBBP1A	Q9BQG0	890	0	-38	-28	6	1.0	0.7	0.8	1.1

CEP170	Q5SW79	776	-1	-34	-28	-29	1.0	0.7	0.8	0.8
RECQL5	O94762	457	-2	-28	-28	-25	1.0	0.8	0.8	0.8
HSD17B4	P51659	373	-2	-40	-28	-14	1.0	0.7	0.8	0.9
COPB1	P53618	235	-2	-43	-28	28	1.0	0.7	0.8	1.4
FAAP100	Q0VG06	342	-3	-22	-28	-10	1.0	0.8	0.8	0.9
ADSS	P30520	338	-4	-21	-28	-15	1.0	0.8	0.8	0.9
CARS2	Q9HA77	526	-9	-11	-28	-8	0.9	0.9	0.8	0.9
SETMAR	Q53H47	394	-13	-44	-28	-42	0.9	0.7	0.8	0.7
ZRANB2	O95218	74	-14	-28	-28	-50	0.9	0.8	0.8	0.7
SPTBN1	Q01082	619	-17	-46	-28	31	0.9	0.7	0.8	1.4
RAB3IP	Q96QF0	395	-22	-23	-28	-30	0.8	0.8	0.8	0.8
DCAF13	Q9NV06	87	9	-15	-29	-17	1.1	0.9	0.8	0.9
MAP1A	P78559	160	-1	-21	-29	-25	1.0	0.8	0.8	0.8
WDR36	Q8NI36	470	-1	-36	-29	-19	1.0	0.7	0.8	0.8
ANKRD54	Q6NXT1	152	-4	-2	-29	-8	1.0	1.0	0.8	0.9
CBWD1	Q9BRT8	107	-5	-23	-29	-19	1.0	0.8	0.8	0.8
AP2B1	P63010	857	-6	-49	-29	22	0.9	0.7	0.8	1.3
TES	Q9UGI8	294	-7	-26	-29	-13	0.9	0.8	0.8	0.9
IGFBPL1	Q8WX77	151	-7	-27	-29	-15	0.9	0.8	0.8	0.9
KMT2A	Q03164	2074	-8	-13	-29	-13	0.9	0.9	0.8	0.9
HDLBP	Q00341	683	-12	-13	-29	-34	0.9	0.9	0.8	0.7
ERCC2	P18074	588	-13	-21	-29	-34	0.9	0.8	0.8	0.7
HSPE1-MOB4	S4R3N1	224	-14	-47	-29	-13	0.9	0.7	0.8	0.9
LSM2	Q9Y333	26	-15	-32	-29	-10	0.9	0.8	0.8	0.9
NFKBIL1	Q9UBC1	82	-16	-35	-29	-13	0.9	0.7	0.8	0.9
HCFC1	P51610	326	-18	-39	-29	16	0.9	0.7	0.8	1.2
SGPP1	Q9BX95	76	-19	-39	-29	-27	0.8	0.7	0.8	0.8
RRP8	O43159	317	-22	-27	-29	-30	0.8	0.8	0.8	0.8
LEO1	Q8WVC0	530	-27	-21	-29	-32	0.8	0.8	0.8	0.8
C5orf51	A6NDU8	120	8	-16	-29	10	1.1	0.9	0.8	1.1
KIF13B	Q9NQT8	1754	5	-39	-29	-27	1.0	0.7	0.8	0.8
C11orf54	Q9H0W9	187	3	-43	-29	-15	1.0	0.7	0.8	0.9
RAB18	Q9NP72	160	3	-36	-29	7	1.0	0.7	0.8	1.1
ALDH3A2	P51648	425	0	-12	-29	18	1.0	0.9	0.8	1.2
NEK6	Q9HC98	64	0	-17	-29	-33	1.0	0.9	0.8	0.8
POLR2A	P24928	109	-2	-45	-29	-14	1.0	0.7	0.8	0.9
POP4	O95707	147	-3	-12	-29	3	1.0	0.9	0.8	1.0
MYH9	P35579	931	-3	-29	-29	10	1.0	0.8	0.8	1.1
CNOT1	A5YKK6	624	-4	-24	-29	-13	1.0	0.8	0.8	0.9
PDLIM5	Q96HC4	470	-5	-14	-29	-38	1.0	0.9	0.8	0.7
EIF2S2	P20042	284	-6	-24	-29	-5	0.9	0.8	0.8	1.0
RAB39B	Q96DA2	125	-6	-14	-29	-121	0.9	0.9	0.8	0.5
TXNRD1	Q16881	214	-9	-31	-29	-26	0.9	0.8	0.8	0.8
TXNRD2	Q9NNW7	91	-9	-31	-29	-26	0.9	0.8	0.8	0.8
PNP	P00491	206	-11	-22	-29	-19	0.9	0.8	0.8	0.8
LTN1	O94822	237	-11	-19	-29	-25	0.9	0.8	0.8	0.8
HELZ	P42694	858	-13	-43	-29	-14	0.9	0.7	0.8	0.9
DDX58	O95786	738	-14	-29	-29	-22	0.9	0.8	0.8	0.8
LIMS1	P48059	219	-15	-39	-29	7	0.9	0.7	0.8	1.1
LIMS2	Q7Z4I7	224	-15	-39	-29	7	0.9	0.7	0.8	1.1
JAK1	P23458	731	-16	-26	-29	-35	0.9	0.8	0.8	0.7
FAM129C	Q86XR2	62	-18	-38	-29	-25	0.9	0.7	0.8	0.8
WDR26	Q9H7D7	345	-20	-23	-29	-20	0.8	0.8	0.8	0.8

GHDC	Q8N2G8	82	-39	-19	-29	-19	0.7	0.8	0.8	0.8
RNH1	P13489	248	-48	-14	-29	-12	0.7	0.9	0.8	0.9
DAGLB	Q8NCG7	465	4	-19	-30	-4	1.0	0.8	0.8	1.0
CYC1	P08574	271	0	-49	-30	-9	1.0	0.7	0.8	0.9
TOMM70	O94826	502	-5	-51	-30	-8	1.0	0.7	0.8	0.9
EEF2	P13639	693	-9	-35	-30	17	0.9	0.7	0.8	1.2
TATDN2	Q93075	602	-9	-50	-30	-15	0.9	0.7	0.8	0.9
TMEM68	Q96MH6	183	-10	-4	-30	-17	0.9	1.0	0.8	0.9
PRPS2	P11908	165	-13	-49	-30	-22	0.9	0.7	0.8	0.8
GSDMD	P57764	268	-14	-26	-30	22	0.9	0.8	0.8	1.3
EARS2	Q5JPH6	411	-14	-39	-30	19	0.9	0.7	0.8	1.2
PAX2	Q02962	64	-16	-30	-30	12	0.9	0.8	0.8	1.1
OSBPL2	Q9H1P3	273	-21	-56	-30	-38	0.8	0.6	0.8	0.7
CAPN15	O75808	271	-25	-91	-30	-39	0.8	0.5	0.8	0.7
SDHA	P31040	305	-30	-31	-30	-20	0.8	0.8	0.8	0.8
SKI	P12755	449	-31	-36	-30	-12	0.8	0.7	0.8	0.9
WDFY4	Q6ZS81	234	13	-4	-30	-6	1.1	1.0	0.8	0.9
FAM206A	Q9NX38	74	2	-15	-30	-13	1.0	0.9	0.8	0.9
GRIPAP1	Q4V328	581	1	-23	-30	-12	1.0	0.8	0.8	0.9
ATM	Q13315	489	-1	15	-30	-21	1.0	1.2	0.8	0.8
UBA52	P62987	115	-5	-34	-30	25	1.0	0.7	0.8	1.3
TRIM24	O15164	78	-8	-18	-30	-3	0.9	0.9	0.8	1.0
UTRN	P46939	1218	-9	-23	-30	-8	0.9	0.8	0.8	0.9
PAPSS1	O43252	78	-9	-40	-30	-46	0.9	0.7	0.8	0.7
RINL	Q6ZS11	421	-13	-52	-30	21	0.9	0.7	0.8	1.3
DNM1	Q05193	424	-18	-41	-30	-34	0.9	0.7	0.8	0.7
THOP1	P52888	434	-21	-44	-30	12	0.8	0.7	0.8	1.1
SENP7	Q9BQF6	274	-22	-21	-30	-23	0.8	0.8	0.8	0.8
RPL30	P62888	52	-99	-47	-30	-11	0.5	0.7	0.8	0.9
PBDC1	Q9BVG4	125	4	-24	-31	-25	1.0	0.8	0.8	0.8
TRIM4	Q9C037	368	-6	-32	-31	22	0.9	0.8	0.8	1.3
SMARCC1	Q92922	520	-9	-25	-31	-14	0.9	0.8	0.8	0.9
UBE2Q1	Q7Z7E8	65	-9	-17	-31	-1	0.9	0.9	0.8	1.0
APOBEC3D	Q96AK3	261	-9	-21	-31	-2	0.9	0.8	0.8	1.0
ABCF2	Q9UG63	186	-10	-32	-31	23	0.9	0.8	0.8	1.3
BIRC6	Q9NR09	410	-10	-55	-31	-28	0.9	0.6	0.8	0.8
USP48	Q86UV5	986	-31	-20	-31	-43	0.8	0.8	0.8	0.7
CDC40	O60508	29	-43	-19	-31	-41	0.7	0.8	0.8	0.7
FAM129A	Q9BZQ8	177	14	1	-31	21	1.2	1.0	0.8	1.3
CASP2	P42575	244	7	-23	-31	-29	1.1	0.8	0.8	0.8
DNAJC13	O75165	2082	6	9	-31	16	1.1	1.1	0.8	1.2
C2orf88	Q9BSF0	71	3	-15	-31	-23	1.0	0.9	0.8	0.8
LRP1	Q07954	2404	-3	-21	-31	-1	1.0	0.8	0.8	1.0
THOP1	P52888	231	-13	-39	-31	24	0.9	0.7	0.8	1.3
PDE3B	Q13370	394	-17	-19	-31	-14	0.9	0.8	0.8	0.9
JMJD1C	Q15652	1722	-17	-29	-31	-34	0.9	0.8	0.8	0.7
COPA	P53621	975	-18	-15	-31	-19	0.9	0.9	0.8	0.8
TXNDC17	Q9BRA2	46	-24	-41	-31	-18	0.8	0.7	0.8	0.9
ZNF638	Q14966	1654	-32	-41	-31	-61	0.8	0.7	0.8	0.6
RNF31	Q96EP0	237	-40	-51	-31	-46	0.7	0.7	0.8	0.7
NUP98	P52948	1711	13	-6	-32	5	1.1	0.9	0.8	1.0
DALRD3	Q5D0E6	156	8	-37	-32	4	1.1	0.7	0.8	1.0
GLOD4	Q9HC38	171	7	-24	-32	-29	1.1	0.8	0.8	0.8

AAK1	Q2M2I8	193	6	-39	-32	-4	1.1	0.7	0.8	1.0
RPL8	P62917	90	5	-29	-32	-5	1.0	0.8	0.8	1.0
RELA	Q04206	206	1	-29	-32	-25	1.0	0.8	0.8	0.8
GTF3C3	Q9Y5Q9	810	-5	-11	-32	6	1.0	0.9	0.8	1.1
CCT6A	P40227	343	-6	-16	-32	2	0.9	0.9	0.8	1.0
ELP3	Q9H9T3	398	-7	-9	-32	-7	0.9	0.9	0.8	0.9
HLA-DRB1	P04229	59	-28	-62	-32	-19	0.8	0.6	0.8	0.8
CBLL1	Q75N03	130	10	-13	-32	4	1.1	0.9	0.8	1.0
MYCBP2	O75592	3932	5	-6	-32	-6	1.1	0.9	0.8	0.9
ADRM1	Q16186	121	3	-21	-32	10	1.0	0.8	0.8	1.1
SDE2	Q6IQ49	298	2	-25	-32	-21	1.0	0.8	0.8	0.8
NCAPG2	Q86XI2	564	-4	-22	-32	-21	1.0	0.8	0.8	0.8
MMS22L	Q6ZRQ5	467	-11	-20	-32	-3	0.9	0.8	0.8	1.0
FRYL	O94915	560	-18	-39	-32	-52	0.8	0.7	0.8	0.7
CASP8	Q14790	345	-19	-39	-32	-12	0.8	0.7	0.8	0.9
CEBPD	P49716	268	-20	-15	-32	-19	0.8	0.9	0.8	0.8
TOP2B	Q02880	237	-27	-53	-32	-3	0.8	0.7	0.8	1.0
LAP3	P28838	145	-27	-54	-32	-7	0.8	0.7	0.8	0.9
NUTF2	P61970	80	-30	-42	-32	19	0.8	0.7	0.8	1.2
NUDCD3	Q8IVD9	260	-30	-63	-32	-43	0.8	0.6	0.8	0.7
NUP85	Q9BW27	175	9	-45	-33	-14	1.1	0.7	0.8	0.9
MRPS2	Q9Y399	162	6	-36	-33	0	1.1	0.7	0.8	1.0
NRAS	P01111	118	2	-7	-33	0	1.0	0.9	0.8	1.0
IFI16	Q16666	351	-1	-19	-33	-12	1.0	0.8	0.8	0.9
PNKP	Q96T60	308	-1	-26	-33	6	1.0	0.8	0.8	1.1
MAVS	Q7Z434	133	-8	-27	-33	-6	0.9	0.8	0.8	0.9
MAU2	Q9Y6X3	364	-11	-11	-33	-28	0.9	0.9	0.8	0.8
INTS7	Q9NVH2	352	-12	-248	-33	-155	0.9	0.3	0.8	0.4
EIF3B	P55884	384	-20	-34	-33	19	0.8	0.7	0.8	1.2
ZNF609	O15014	1120	-23	-21	-33	-32	0.8	0.8	0.8	0.8
CFH	P08603	931	-28	-41	-33	-44	0.8	0.7	0.8	0.7
TPI1	P60174	164	-51	-67	-33	29	0.7	0.6	0.8	1.4
PUS7L	Q9H0K6	649	-1	-5	-33	7	1.0	1.0	0.8	1.1
N4BP2	Q86UW6	1438	-8	-25	-33	-37	0.9	0.8	0.8	0.7
NAP1L4	Q99733	77	-8	-38	-33	17	0.9	0.7	0.8	1.2
NUMB	P49757	160	-13	-33	-33	17	0.9	0.8	0.8	1.2
CASP8	Q14790	426	-13	-33	-33	-14	0.9	0.8	0.8	0.9
SNX27	Q96L92	434	-21	-11	-33	-21	0.8	0.9	0.8	0.8
CHD2	O14647	458	-24	-38	-33	-13	0.8	0.7	0.8	0.9
ECHS1	P30084	213	-35	-29	-33	-19	0.7	0.8	0.8	0.8
ENO1	P06733	339	-93	-85	-33	-12	0.5	0.5	0.8	0.9
LAP3	P28838	313	10	-25	-34	-20	1.1	0.8	0.7	0.8
NAXE	Q8NCW5	115	2	-12	-34	-19	1.0	0.9	0.7	0.8
SDHA	P31040	190	1	-40	-34	-12	1.0	0.7	0.7	0.9
CHUK	O15111	114	1	1	-34	-2	1.0	1.0	0.7	1.0
PSMA3	P25788	42	-3	-46	-34	-8	1.0	0.7	0.7	0.9
APBA3	O96018	500	-13	-44	-34	-32	0.9	0.7	0.7	0.8
TRAPPC6B	Q86SZ2	123	-22	-35	-34	-15	0.8	0.7	0.7	0.9
PRG2	P13727	201	-22	-41	-34	12	0.8	0.7	0.7	1.1
PAPOLG	Q9BWT3	604	-3	-6	-34	-3	1.0	0.9	0.7	1.0
NLRP1	Q9C000	894	-5	-27	-34	-8	1.0	0.8	0.7	0.9
TYK2	P29597	736	-6	-36	-34	-32	0.9	0.7	0.7	0.8
CEP135	Q66GS9	316	-7	-18	-34	-23	0.9	0.9	0.7	0.8

SCRN2	Q96FV2	59	-7	-36	-34	-16	0.9	0.7	0.7	0.9
PI4KA	P42356	1902	-10	-11	-34	-12	0.9	0.9	0.7	0.9
DAXX	Q9UER7	664	-12	-21	-34	-46	0.9	0.8	0.7	0.7
DOCK2	Q92608	297	-13	-32	-34	-14	0.9	0.8	0.7	0.9
FGD2	Q7Z6J4	510	-20	-30	-34	-21	0.8	0.8	0.7	0.8
HDHD3	Q9BSH5	196	-26	-38	-34	8	0.8	0.7	0.7	1.1
CTCF	P49711	577	2	-10	-35	18	1.0	0.9	0.7	1.2
LARP4B	Q92615	633	-26	-41	-35	-14	0.8	0.7	0.7	0.9
MAP3K7	O43318	527	-27	-32	-35	-43	0.8	0.8	0.7	0.7
ETHE1	O95571	189	-123	-65	-35	-23	0.4	0.6	0.7	0.8
FGD3	Q5JSP0	184	18	-53	-35	9	1.2	0.7	0.7	1.1
GAPVD1	Q14C86	1160	1	-11	-35	4	1.0	0.9	0.7	1.0
PDHA1	P08559	94	-4	-25	-35	-4	1.0	0.8	0.7	1.0
KSR1	Q8IVT5	777	-11	-29	-35	18	0.9	0.8	0.7	1.2
DDX51	Q8N8A6	280	-12	-32	-35	-18	0.9	0.8	0.7	0.9
UHRF1BP1L	A0JNW5	647	-14	-42	-35	-3	0.9	0.7	0.7	1.0
F13A1	P00488	328	-14	-82	-35	-17	0.9	0.5	0.7	0.9
AKAP13	Q12802	1666	-34	-29	-35	-22	0.7	0.8	0.7	0.8
CARMIL2	Q6F5E8	502	-7	-32	-36	24	0.9	0.8	0.7	1.3
TRIM14	Q14142	42	-25	-6	-36	2	0.8	0.9	0.7	1.0
UMPS	P11172	174	18	-40	-36	-4	1.2	0.7	0.7	1.0
CYC1	P08574	219	-2	-25	-36	-10	1.0	0.8	0.7	0.9
ANKFY1	Q9P2R3	749	-7	-18	-36	13	0.9	0.9	0.7	1.1
RPL36A-HNRI	H7BZ11	88	-13	-43	-36	8	0.9	0.7	0.7	1.1
IFIT5	Q13325	429	-16	-44	-36	17	0.9	0.7	0.7	1.2
MKI67	P46013	3014	-22	-27	-36	-7	0.8	0.8	0.7	0.9
ANKFY1	Q9P2R3	460	-31	-50	-36	21	0.8	0.7	0.7	1.3
WDFY4	Q6ZS81	242	11	-37	-37	-5	1.1	0.7	0.7	1.0
PGAM1	P18669	55	-11	-43	-37	-7	0.9	0.7	0.7	0.9
CAPN15	O75808	392	-12	-57	-37	-34	0.9	0.6	0.7	0.7
RPA2	P15927	49	-19	-36	-37	18	0.8	0.7	0.7	1.2
NANP	Q8TBE9	67	-28	-51	-37	-55	0.8	0.7	0.7	0.6
ARL11	Q969Q4	178	-34	16	-37	-77	0.7	1.2	0.7	0.6
DKC1	O60832	74	-48	-50	-37	35	0.7	0.7	0.7	1.5
MAPK1	P28482	254	12	-12	-37	29	1.1	0.9	0.7	1.4
BIRC6	Q9NR09	4183	6	-44	-37	-5	1.1	0.7	0.7	1.0
SECISBP2	Q96T21	291	-2	-22	-37	-30	1.0	0.8	0.7	0.8
CAD	P27708	280	-7	-28	-37	5	0.9	0.8	0.7	1.1
PDXDC1	Q6P996	456	-11	-37	-37	-36	0.9	0.7	0.7	0.7
GFOD2	Q3B7J2	377	-12	-18	-37	-21	0.9	0.8	0.7	0.8
PPP1CA	P62136	245	-16	-48	-37	11	0.9	0.7	0.7	1.1
NMNAT1	Q9HAN9	111	-54	-48	-37	-35	0.7	0.7	0.7	0.7
FAM118B	Q9BPY3	93	2	-38	-38	8	1.0	0.7	0.7	1.1
RPL12	P30050	162	-5	-19	-38	12	1.0	0.8	0.7	1.1
TRAPPC10	P48553	1080	-33	-28	-38	-27	0.8	0.8	0.7	0.8
STRIP1	Q5VSL9	769	-48	-25	-38	-6	0.7	0.8	0.7	0.9
OSBPL3	Q9H4L5	337	1	-5	-38	-15	1.0	1.0	0.7	0.9
GALK1	P51570	203	1	-58	-38	-21	1.0	0.6	0.7	0.8
BIRC6	Q9NR09	3830	-9	-14	-38	-17	0.9	0.9	0.7	0.9
SQSTM1	Q13501	142	-31	-33	-38	-41	0.8	0.8	0.7	0.7
FASN	P49327	1548	-35	-44	-38	-7	0.7	0.7	0.7	0.9
ZBTB33	Q86T24	505	8	-2	-39	-61	1.1	1.0	0.7	0.6
CCDC88A	Q3V6T2	1244	-1	-17	-39	-18	1.0	0.9	0.7	0.9

ACADVL	P49748	215	-6	-26	-39	-22	0.9	0.8	0.7	0.8
TNFAIP2	Q03169	356	1	-44	-39	-6	1.0	0.7	0.7	0.9
MUT	P22033	471	-10	-37	-39	-17	0.9	0.7	0.7	0.9
PARP1	P09874	162	-17	-30	-39	3	0.9	0.8	0.7	1.0
GBP4	Q96PP9	172	-25	-30	-39	-38	0.8	0.8	0.7	0.7
PDLIM5	Q96HC4	449	-13	-47	-40	-19	0.9	0.7	0.7	0.8
RABGEF1	Q9UJ41	442	5	-9	-40	-27	1.0	0.9	0.7	0.8
TUFM	P49411	127	-3	-20	-40	-7	1.0	0.8	0.7	0.9
UNC13D	Q70J99	992	-17	-56	-40	25	0.9	0.6	0.7	1.3
FUBP3	Q96I24	310	-28	-63	-40	10	0.8	0.6	0.7	1.1
RB1CC1	Q8TDY2	839	5	-42	-41	-11	1.1	0.7	0.7	0.9
KIF1B	O60333	1810	-3	-35	-41	-28	1.0	0.7	0.7	0.8
QRSL1	Q9H0R6	203	-11	-47	-41	-21	0.9	0.7	0.7	0.8
TMEM240	Q5SV17	135	25	-20	-41	-2	1.3	0.8	0.7	1.0
NUP205	Q92621	1486	4	-9	-41	15	1.0	0.9	0.7	1.2
NUP188	Q5SRE5	1270	3	-15	-41	6	1.0	0.9	0.7	1.1
HDAC3	O15379	279	1	-45	-41	-4	1.0	0.7	0.7	1.0
SQSTM1	Q13501	113	-4	-31	-41	2	1.0	0.8	0.7	1.0
SYNE2	Q8WXH0	3204	-6	-9	-41	3	0.9	0.9	0.7	1.0
RBBP7	Q16576	166	-8	-37	-41	-20	0.9	0.7	0.7	0.8
SERPINB6	P35237	100	-11	-46	-41	3	0.9	0.7	0.7	1.0
OGFOD3	Q6PK18	90	-12	-24	-41	13	0.9	0.8	0.7	1.1
OGFOD3	Q6PK18	103	-12	-24	-41	13	0.9	0.8	0.7	1.1
SEC14L1	Q92503	644	18	-34	-42	-6	1.2	0.7	0.7	0.9
TREM1	Q9NP99	163	15	14	-42	-43	1.2	1.2	0.7	0.7
PIN1	Q13526	57	-16	-43	-42	19	0.9	0.7	0.7	1.2
LIMS1	P48059	164	9	-5	-42	7	1.1	1.0	0.7	1.1
SERPINB8	P50452	98	-5	-47	-42	8	1.0	0.7	0.7	1.1
FBXL3	Q9UKT7	63	-15	-55	-42	-27	0.9	0.6	0.7	0.8
IGHG3	A0A087WVW	264	-20	-35	-42	-3	0.8	0.7	0.7	1.0
BRAT1	Q6PJG6	510	-22	-47	-42	19	0.8	0.7	0.7	1.2
PREX1	Q8TCU6	923	-25	-36	-42	-53	0.8	0.7	0.7	0.7
C16orf62	Q7Z3J2	925	-2	-36	-43	-21	1.0	0.7	0.7	0.8
RAB18	Q9NP72	155	-16	-38	-43	2	0.9	0.7	0.7	1.0
MBNL2	Q5VZF2	231	-1	-24	-43	-36	1.0	0.8	0.7	0.7
MBNL1	Q9NR56	234	-1	-24	-43	-36	1.0	0.8	0.7	0.7
TRIO	O75962	2136	-8	-32	-43	-14	0.9	0.8	0.7	0.9
RECQL	P46063	478	-15	-41	-43	-4	0.9	0.7	0.7	1.0
CEBPZ	Q03701	411	-17	-52	-43	21	0.9	0.7	0.7	1.3
EFTUD2	Q15029	135	-4	-40	-44	-21	1.0	0.7	0.7	0.8
HUWE1	Q7Z6Z7	3259	-1	-30	-44	-50	1.0	0.8	0.7	0.7
RGS2	P41220	199	-10	-41	-44	-36	0.9	0.7	0.7	0.7
PPP1CA	P62136	127	-27	-47	-44	8	0.8	0.7	0.7	1.1
DYNLT3	P51808	8	9	-9	-45	8	1.1	0.9	0.7	1.1
NFRKB	Q6P4R8	637	8	-60	-45	-9	1.1	0.6	0.7	0.9
CDR2	Q01850	331	-4	-54	-45	-25	1.0	0.7	0.7	0.8
PREX1	Q8TCU6	968	-17	-40	-45	-5	0.9	0.7	0.7	1.0
BIRC6	Q9NR09	566	-29	-23	-45	-4	0.8	0.8	0.7	1.0
RNF214	Q8ND24	655	-31	-36	-45	25	0.8	0.7	0.7	1.3
NFKBIE	O00221	335	5	-19	-46	16	1.1	0.8	0.7	1.2
CDKAL1	Q5VV42	556	3	-60	-47	46	1.0	0.6	0.7	1.8
PXK	Q7Z7A4	570	-3	-36	-47	-6	1.0	0.7	0.7	0.9
VPS33B	Q9H267	82	-10	-29	-47	-13	0.9	0.8	0.7	0.9

SAMD9	Q5K651	420	13	-45	-47	6	1.1	0.7	0.7	1.1
DYNC1H1	Q14204	1999	5	-60	-47	-15	1.1	0.6	0.7	0.9
PTGES3	Q15185	58	3	-8	-47	9	1.0	0.9	0.7	1.1
SSH2	Q76I76	890	-10	-33	-47	-50	0.9	0.8	0.7	0.7
KIAA1468	Q9P260	917	-14	-2	-47	19	0.9	1.0	0.7	1.2
IRF5	Q13568	44	-14	-33	-47	-29	0.9	0.8	0.7	0.8
Integrin	H3BM21	619	-22	-31	-47	-54	0.8	0.8	0.7	0.7
ZNF174	Q15697	45	-29	-28	-47	-45	0.8	0.8	0.7	0.7
SZT2	Q5T011	3172	18	-1	-48	9	1.2	1.0	0.7	1.1
CCT8	P50990	149	-2	-54	-48	3	1.0	0.6	0.7	1.0
MYOF	Q9NZM1	610	-6	-43	-48	-59	0.9	0.7	0.7	0.6
EML3	Q32P44	307	10	-51	-49	-6	1.1	0.7	0.7	0.9
ZBTB9	Q96C00	452	9	-56	-49	-19	1.1	0.6	0.7	0.8
CWC22	Q9HCG8	496	3	-50	-49	-20	1.0	0.7	0.7	0.8
TGM2	P21980	277	-47	-56	-49	-88	0.7	0.6	0.7	0.5
LANCL1	O43813	252	14	-49	-49	13	1.2	0.7	0.7	1.1
EXOC5	O00471	111	-11	-59	-49	3	0.9	0.6	0.7	1.0
EIF5B	O60841	853	-34	-77	-49	5	0.7	0.6	0.7	1.0
TNPO1	Q92973	205	13	-89	-50	-17	1.1	0.5	0.7	0.9
TSR1	Q2NL82	126	1	-23	-50	-9	1.0	0.8	0.7	0.9
HAUS8	Q9BT25	182	-10	-37	-50	-25	0.9	0.7	0.7	0.8
ARPC1B	O15143	134	-15	-25	-50	-20	0.9	0.8	0.7	0.8
CAPN7	Q9Y6W3	197	5	-47	-50	-12	1.1	0.7	0.7	0.9
RBM4	Q9BWF3	89	-6	-57	-50	1	0.9	0.6	0.7	1.0
PRICKLE1	Q96MT3	346	16	-83	-51	-32	1.2	0.5	0.7	0.8
CASP1	P29466	331	11	-63	-51	-6	1.1	0.6	0.7	0.9
NDUFA10	E7ESZ7	112	3	-12	-51	21	1.0	0.9	0.7	1.3
ASPHD2	Q6ICH7	116	-18	-48	-51	15	0.8	0.7	0.7	1.2
DNAJB6	O75190	275	-12	-10	-52	-14	0.9	0.9	0.7	0.9
WDFY4	Q6ZS81	233	11	-53	-52	-26	1.1	0.7	0.7	0.8
PIK3CD	O00329	500	6	-12	-53	-1	1.1	0.9	0.7	1.0
NAA25	Q14CX7	46	-11	-69	-53	-13	0.9	0.6	0.7	0.9
CPVL	Q9H3G5	271	-18	-66	-53	12	0.8	0.6	0.7	1.1
CDC27	P30260	71	11	-49	-53	-16	1.1	0.7	0.7	0.9
CD5	P06127	342	-21	-25	-53	-32	0.8	0.8	0.7	0.8
RABEP2	Q9H5N1	372	-22	-61	-53	11	0.8	0.6	0.7	1.1
THEMIS	Q8N1K5	116	-17	-50	-54	8	0.9	0.7	0.7	1.1
SNRPD3	P62318	20	-108	-43	-54	39	0.5	0.7	0.7	1.6
MCM4	P33991	328	-67	-75	-54	-57	0.6	0.6	0.6	0.6
APAF1	O14727	1129	0	-66	-55	-25	1.0	0.6	0.6	0.8
CTR9	Q6PD62	363	24	11	-56	-7	1.3	1.1	0.6	0.9
ADSL	P30566	99	7	-7	-56	8	1.1	0.9	0.6	1.1
SRC	P12931	241	-3	-32	-56	19	1.0	0.8	0.6	1.2
DNM2	P50570	86	-15	-86	-56	4	0.9	0.5	0.6	1.0
RANBP2	P49792	188	1	-26	-57	2	1.0	0.8	0.6	1.0
TELO2	Q9Y4R8	568	-12	-34	-57	-9	0.9	0.7	0.6	0.9
PXN	P49023	446	-12	-65	-57	-25	0.9	0.6	0.6	0.8
ITGAL	P20701	119	-21	-42	-57	-25	0.8	0.7	0.6	0.8
MYBBP1A	Q9BQG0	338	16	-9	-58	26	1.2	0.9	0.6	1.3
CLIC1	O00299	191	-5	-89	-58	3	1.0	0.5	0.6	1.0
PRF1	P14222	510	11	-3	-59	23	1.1	1.0	0.6	1.3
THEM6	Q8WUY1	104	8	-67	-59	10	1.1	0.6	0.6	1.1
THOC6	Q86W42	35	-1	-53	-59	-10	1.0	0.7	0.6	0.9

STRIP1	Q5VSL9	798	-3	-54	-59	-6	1.0	0.6	0.6	0.9
TXNIP	Q9H3M7	247	-39	-47	-59	9	0.7	0.7	0.6	1.1
CYFIP1	Q7L576	144	-19	-67	-60	21	0.8	0.6	0.6	1.3
CYFIP2	Q96F07	144	-19	-67	-60	21	0.8	0.6	0.6	1.3
ANKRD44	Q8N8A2	615	11	-74	-61	-10	1.1	0.6	0.6	0.9
SYTL1	Q8IYJ3	290	2	-67	-61	-15	1.0	0.6	0.6	0.9
EIF3CL	B5ME19	753	-23	-106	-62	5	0.8	0.5	0.6	1.1
MRPS30	Q9NP92	204	-38	-34	-62	-32	0.7	0.7	0.6	0.8
LPXN	O60711	152	21	-74	-63	-19	1.3	0.6	0.6	0.8
MED1	Q15648	135	-38	-97	-63	-12	0.7	0.5	0.6	0.9
PHF6	Q8IWS0	107	-42	-127	-63	-32	0.7	0.4	0.6	0.8
APOBEC3C	Q9NRW3	65	-7	-34	-63	-1	0.9	0.7	0.6	1.0
FARS2	O95363	413	-43	-72	-63	3	0.7	0.6	0.6	1.0
DDX6	P26196	324	-1	-61	-64	-17	1.0	0.6	0.6	0.9
POLR2A	P24928	184	8	-59	-65	-9	1.1	0.6	0.6	0.9
MRPL46	Q9H2W6	210	-11	-71	-65	-35	0.9	0.6	0.6	0.7
HN1L	Q9H910	118	-19	-84	-65	-33	0.8	0.5	0.6	0.8
SKI	P12755	10	-15	-89	-66	-51	0.9	0.5	0.6	0.7
CAD	P27708	1455	5	-64	-66	-16	1.0	0.6	0.6	0.9
CLIP4	Q8N3C7	259	1	-17	-66	-42	1.0	0.9	0.6	0.7
TAF6	P49848	141	-5	-68	-67	-16	1.0	0.6	0.6	0.9
MX1	P20591	533	14	-51	-68	-3	1.2	0.7	0.6	1.0
TOR4A	Q9NXH8	62	1	-35	-68	-105	1.0	0.7	0.6	0.5
FUBP1	Q96AE4	332	-26	-88	-68	13	0.8	0.5	0.6	1.1
ARHGAP30	Q7Z6I6	965	3	-70	-69	-19	1.0	0.6	0.6	0.8
SMU1	Q2TAY7	219	9	-92	-69	-10	1.1	0.5	0.6	0.9
DDX5	P17844	89	5	-72	-69	-13	1.1	0.6	0.6	0.9
ZC3HAV1	Q7Z2W4	219	-23	-79	-69	-41	0.8	0.6	0.6	0.7
CCT8	P50990	148	5	-63	-70	-4	1.0	0.6	0.6	1.0
LPXN	O60711	155	8	-68	-71	-4	1.1	0.6	0.6	1.0
FLNA	P21333	1157	4	-71	-71	-15	1.0	0.6	0.6	0.9
MED25	Q71SY5	506	-6	-16	-71	-21	0.9	0.9	0.6	0.8
SSB	P05455	18	-35	-122	-71	17	0.7	0.5	0.6	1.2
EIF2B4	Q9UI10	444	3	-64	-72	-12	1.0	0.6	0.6	0.9
ARAF	P10398	192	-35	-88	-73	-15	0.7	0.5	0.6	0.9
SAMHD1	Q9Y3Z3	320	-24	-148	-74	-43	0.8	0.4	0.6	0.7
SEPT5	Q99719	311	12	-68	-74	-13	1.1	0.6	0.6	0.9
MRPL35	Q9NZE8	119	-7	-92	-74	-16	0.9	0.5	0.6	0.9
ADCK4	Q96D53	479	10	10	-75	-43	1.1	1.1	0.6	0.7
VEZF1	Q14119	289	-4	-16	-75	-75	1.0	0.9	0.6	0.6
APOL3	O95236	399	-7	-9	-76	7	0.9	0.9	0.6	1.1
EML2	O95834	605	1	-81	-78	-19	1.0	0.6	0.6	0.8
EIF2B4	Q9UI10	438	3	-77	-79	-19	1.0	0.6	0.6	0.8
GIMAP8	Q8ND71	321	2	-70	-79	-40	1.0	0.6	0.6	0.7
PDLIM1	O00151	307	-5	-81	-79	-29	1.0	0.6	0.6	0.8
GYG1	P46976	89	-8	-114	-79	-25	0.9	0.5	0.6	0.8
APMAP	Q9HDC9	149	-70	-68	-79	-36	0.6	0.6	0.6	0.7
MAST4	O15021	310	-17	-21	-79	-11	0.9	0.8	0.6	0.9
MAST2	Q6P0Q8	252	-17	-21	-79	-11	0.9	0.8	0.6	0.9
ZMYM2	Q9UBW7	663	17	-101	-80	-22	1.2	0.5	0.6	0.8
DOK3	Q7L591	295	-31	-111	-80	2	0.8	0.5	0.6	1.0
CTR9	Q6PD62	207	-83	-110	-81	-86	0.5	0.5	0.6	0.5
TBL2	Q9Y4P3	375	5	-83	-81	-20	1.1	0.5	0.6	0.8

MTMR12	Q9C0I1	694	-20	-75	-81	-2	0.8	0.6	0.6	1.0
SEPT1	Q8WYJ6	136	2	-84	-82	-20	1.0	0.5	0.6	0.8
UBA6	A0AVT1	625	-9	-154	-82	-65	0.9	0.4	0.5	0.6
ZBED1	O96006	76	-3	-83	-83	-28	1.0	0.5	0.5	0.8
FLAD1	Q8NFF5	236	2	-78	-85	-24	1.0	0.6	0.5	0.8
PDE5A	O76074	68	10	-93	-85	-23	1.1	0.5	0.5	0.8
FAM120A	Q9NZB2	279	5	-86	-85	-20	1.1	0.5	0.5	0.8
ZSWIM8	A7E2V4	215	-6	-84	-86	-26	0.9	0.5	0.5	0.8
NOP2	P46087	487	-9	-54	-86	-34	0.9	0.6	0.5	0.7
VPS13C	Q709C8	3636	20	-92	-88	-28	1.3	0.5	0.5	0.8
PLEC	Q15149	3295	13	-106	-88	-29	1.1	0.5	0.5	0.8
AGO3	Q9H9G7	283	-4	-90	-88	-39	1.0	0.5	0.5	0.7
AGO2	Q9UKV8	282	-4	-90	-88	-39	1.0	0.5	0.5	0.7
AGO1	Q9UL18	280	-4	-90	-88	-39	1.0	0.5	0.5	0.7
GIMAP1-GIM	A0A087WTJ2	104	-7	-106	-89	-23	0.9	0.5	0.5	0.8
GIMAP1	Q8WWP7	104	-7	-106	-89	-23	0.9	0.5	0.5	0.8
ZNF512B	Q96KM6	757	-2	-81	-90	-32	1.0	0.6	0.5	0.8
HSD17B11	Q8NBQ5	94	1	-100	-90	-21	1.0	0.5	0.5	0.8
NEDD4	P46934	1286	3	-93	-91	-25	1.0	0.5	0.5	0.8
SMURF2	Q9HAU4	716	3	-93	-91	-25	1.0	0.5	0.5	0.8
YY1	P25490	330	-2	-92	-93	-26	1.0	0.5	0.5	0.8
CCDC88C	Q9P219	1194	12	-72	-94	-16	1.1	0.6	0.5	0.9
SEPT1	Q8WYJ6	137	21	-84	-95	-18	1.3	0.5	0.5	0.8
HERC4	Q5GLZ8	60	8	-8	-95	23	1.1	0.9	0.5	1.3
MED13	Q9UHV7	1878	-40	-115	-95	-27	0.7	0.5	0.5	0.8
DPYD	Q12882	324	2	-95	-95	-40	1.0	0.5	0.5	0.7
ARFGAP2	Q8N6H7	97	-25	-88	-97	-35	0.8	0.5	0.5	0.7
MFN1	Q8IWA4	498	10	-116	-98	-43	1.1	0.5	0.5	0.7
FARSB	Q9NSD9	151	18	-113	-98	-22	1.2	0.5	0.5	0.8
RAD21	O60216	35	7	-97	-98	-25	1.1	0.5	0.5	0.8
NCOR2	Q9Y618	2179	5	-65	-98	-10	1.0	0.6	0.5	0.9
HERC3	Q15034	321	14	-126	-99	-45	1.2	0.4	0.5	0.7
RPS26	P62854	77	4	-105	-99	-37	1.0	0.5	0.5	0.7
AKR1B1	P15121	45	10	-8	-101	6	1.1	0.9	0.5	1.1
NEK7	Q8TDX7	298	2	-105	-101	-28	1.0	0.5	0.5	0.8
ZMYM2	Q9UBW7	681	20	-93	-103	-40	1.2	0.5	0.5	0.7
EML3	Q32P44	825	9	-120	-104	-26	1.1	0.5	0.5	0.8
ADCK4	Q96D53	508	1	-94	-105	-24	1.0	0.5	0.5	0.8
RAC2	P15153	105	-12	-62	-105	-9	0.9	0.6	0.5	0.9
PHF5A	Q7RTV0	11	-8	-73	-107	-27	0.9	0.6	0.5	0.8
CTDSPL2	Q05D32	381	8	-116	-108	-32	1.1	0.5	0.5	0.8
CRYZ	Q08257	45	14	-108	-109	-14	1.2	0.5	0.5	0.9
DYNC1H1	Q14204	4121	-5	-80	-110	-45	1.0	0.6	0.5	0.7
ZNF512	Q96ME7	430	5	-129	-111	-38	1.1	0.4	0.5	0.7
PASK	Q96RG2	89	2	-78	-111	-62	1.0	0.6	0.5	0.6
CLTC	Q00610	617	10	-143	-112	-34	1.1	0.4	0.5	0.7
TRERF1	Q96PN7	876	-15	-96	-112	-50	0.9	0.5	0.5	0.7
COG3	Q96JB2	363	-11	-131	-112	-52	0.9	0.4	0.5	0.7
RPS26	P62854	74	-2	-121	-114	-32	1.0	0.5	0.5	0.8
IFI30	P13284	117	-39	-164	-114	-35	0.7	0.4	0.5	0.7
IL4I1	Q96RQ9	252	-14	-110	-115	-29	0.9	0.5	0.5	0.8
VEZF1	Q14119	316	10	-109	-116	-10	1.1	0.5	0.5	0.9
MED16	Q9Y2X0	790	-5	-106	-116	-41	1.0	0.5	0.5	0.7

HIST2H3PS2	Q5TEC6	111	-8	-72	-116	-8	0.9	0.6	0.5	0.9
TRRAP	Q9Y4A5	3075	1	-110	-117	-33	1.0	0.5	0.5	0.8
IRF2BPL	Q9H1B7	63	2	-123	-117	-38	1.0	0.4	0.5	0.7
HSDL2	Q6YN16	166	16	-133	-118	-28	1.2	0.4	0.5	0.8
DDA1	Q9BW61	25	11	-104	-120	-27	1.1	0.5	0.5	0.8
ZNF512B	Q96KM6	774	10	-117	-120	-25	1.1	0.5	0.5	0.8
SYNE2	Q8WXH0	6739	-24	-146	-121	-29	0.8	0.4	0.5	0.8
DCP1A	Q9NPI6	39	-5	-125	-121	-37	1.0	0.4	0.5	0.7
PES1	O00541	153	9	-126	-122	-28	1.1	0.4	0.5	0.8
FLAD1	Q8NFF5	136	-2	-120	-126	-27	1.0	0.5	0.4	0.8
NUP160	Q12769	1166	-55	-160	-128	-35	0.6	0.4	0.4	0.7
COPA	P53621	254	-9	-142	-128	-51	0.9	0.4	0.4	0.7
HMHA1	Q92619	153	-1	-140	-131	-46	1.0	0.4	0.4	0.7
GMPR	P36959	224	1	-100	-132	-40	1.0	0.5	0.4	0.7
NUP155	O75694	561	5	-107	-134	-27	1.0	0.5	0.4	0.8
EIF3E	P60228	350	2	-136	-134	-81	1.0	0.4	0.4	0.6
PRPF8	Q6P2Q9	435	4	-124	-134	-35	1.0	0.4	0.4	0.7
LPXN	O60711	358	-1	-138	-138	-54	1.0	0.4	0.4	0.6
SNRNP70	P08621	39	-15	-64	-140	-5	0.9	0.6	0.4	1.0
WAS	P42768	69	10	-147	-141	-42	1.1	0.4	0.4	0.7
IRF2BP2	Q7Z5L9	65	-6	-142	-142	-48	0.9	0.4	0.4	0.7
HCLS1	P14317	470	12	-143	-145	-25	1.1	0.4	0.4	0.8
LRPPRC	P42704	863	14	-190	-146	-52	1.2	0.3	0.4	0.7
NCKAP1L	P55160	338	-15	-157	-147	-75	0.9	0.4	0.4	0.6
GMPR2	Q9P2T1	224	-16	-148	-147	-49	0.9	0.4	0.4	0.7
ATP1A1	P05023	705	-24	-39	-148	-33	0.8	0.7	0.4	0.8
PDDC1	Q8NB37	94	1	-167	-153	-54	1.0	0.4	0.4	0.6
GTF2H4	Q92759	16	8	-154	-153	-52	1.1	0.4	0.4	0.7
IFI30	P13284	124	-59	-194	-153	-37	0.6	0.3	0.4	0.7
EML4	Q9HC35	820	13	-149	-154	-42	1.1	0.4	0.4	0.7
INTS1	Q8N201	1433	4	-166	-154	1	1.0	0.4	0.4	1.0
ZBTB7A	O95365	471	2	-162	-155	-61	1.0	0.4	0.4	0.6
YY1	P25490	385	-2	-150	-157	-45	1.0	0.4	0.4	0.7
NLE1	Q9NVX2	280	-3	-148	-158	-55	1.0	0.4	0.4	0.6
HELZ2	Q9BYK8	1867	-7	-103	-159	-71	0.9	0.5	0.4	0.6
MED19	A0JLT2	163	5	-159	-161	-46	1.1	0.4	0.4	0.7
TPR	P12270	1127	0	-142	-161	-76	1.0	0.4	0.4	0.6
EXOC1	Q9NV70	566	16	-151	-162	-69	1.2	0.4	0.4	0.6
ZAP70	P43403	78	-7	-157	-164	-65	0.9	0.4	0.4	0.6
IFI35	P80217	107	-11	-151	-165	-58	0.9	0.4	0.4	0.6
LTN1	O94822	869	6	-145	-165	-81	1.1	0.4	0.4	0.6
DNM1L	O00429	470	-5	-167	-167	-58	1.0	0.4	0.4	0.6
IARS2	Q9NSE4	155	2	-164	-168	-55	1.0	0.4	0.4	0.6
INPP4A	Q96PE3	565	-3	-155	-168	-77	1.0	0.4	0.4	0.6
RPL18A	Q02543	22	16	-149	-168	-47	1.2	0.4	0.4	0.7
HNRNPDL	O14979	303	19	-181	-169	-58	1.2	0.4	0.4	0.6
C11orf68	Q9H3H3	222	-33	-178	-172	-82	0.8	0.4	0.4	0.6
FGD3	Q5JSP0	43	-20	-160	-173	-74	0.8	0.4	0.4	0.6
HIP1R	O75146	105	-21	-178	-173	-72	0.8	0.4	0.4	0.6
NDUFA2	O43678	24	8	-180	-174	-66	1.1	0.4	0.4	0.6
IFI35	P80217	74	-36	-162	-180	-39	0.7	0.4	0.4	0.7
STAU2	Q9NUL3	140	8	-164	-182	-91	1.1	0.4	0.4	0.5
TRIM33	Q9UPN9	461	16	-185	-184	-72	1.2	0.4	0.4	0.6

RASGRP4	Q8TDF6	14	2	-163	-185	-68	1.0	0.4	0.4	0.6
CRBN	Q96SW2	234	-7	-179	-188	-90	0.9	0.4	0.3	0.5
ACIN1	Q9UKV3	1052	22	-236	-191	-60	1.3	0.3	0.3	0.6
ZNF414	Q96IQ9	134	0	-172	-192	-94	1.0	0.4	0.3	0.5
GAS7	O60861	424	10	-200	-193	-74	1.1	0.3	0.3	0.6
ABHD16A	O95870	416	1	-228	-193	-68	1.0	0.3	0.3	0.6
MAT2A	P31153	214	-13	-184	-195	-95	0.9	0.4	0.3	0.5
DOK3	Q7L591	300	-28	-138	-195	-34	0.8	0.4	0.3	0.7
ZBTB7A	O95365	468	-19	-190	-196	-76	0.8	0.3	0.3	0.6
APBB1IP	Q7Z5R6	220	-4	-189	-196	-89	1.0	0.3	0.3	0.5
TRANK1	O15050	1930	-10	-186	-198	-79	0.9	0.4	0.3	0.6
SDHB	P21912	243	1	-177	-198	-65	1.0	0.4	0.3	0.6
ZBTB7A	O95365	472	-14	-202	-201	-98	0.9	0.3	0.3	0.5
SEPT9	Q9UHD8	411	15	-147	-201	-45	1.2	0.4	0.3	0.7
DNAJC13	O75165	884	13	-137	-201	-39	1.1	0.4	0.3	0.7
FEN1	P39748	141	7	-231	-202	-93	1.1	0.3	0.3	0.5
ZAP70	P43403	96	-4	-168	-204	-64	1.0	0.4	0.3	0.6
C9orf85	Q96MD7	45	0	-184	-206	-84	1.0	0.4	0.3	0.5
SRSF7	Q16629	119	7	-194	-207	-68	1.1	0.3	0.3	0.6
RHOG	P84095	102	-10	-215	-211	-66	0.9	0.3	0.3	0.6
TRIM21	P19474	103	4	-200	-211	-74	1.0	0.3	0.3	0.6
CNTRL	Q7Z7A1	1423	-1	-90	-211	-27	1.0	0.5	0.3	0.8
SEPT9	Q9UHD8	410	1	-203	-213	-86	1.0	0.3	0.3	0.5
C11orf54	Q9H0W9	154	7	-245	-214	-73	1.1	0.3	0.3	0.6
RNF213	Q63HN8	856	1	-200	-214	-86	1.0	0.3	0.3	0.5
ACAT1	P24752	196	-11	-204	-215	-88	0.9	0.3	0.3	0.5
RAC1	P63000	105	-13	-113	-225	-7	0.9	0.5	0.3	0.9
VPS39	Q96JC1	855	-18	-14	-232	-340	0.9	0.9	0.3	0.2
VPS39	Q96JC1	852	-18	-14	-232	-340	0.9	0.9	0.3	0.2
ZNF574	Q6ZN55	394	-3	-218	-233	-73	1.0	0.3	0.3	0.6
SP1	P08047	633	-1	-234	-234	-95	1.0	0.3	0.3	0.5
NFKB2	Q00653	738	-29	-232	-235	-114	0.8	0.3	0.3	0.5
ZNF740	Q8NDX6	159	-12	-190	-236	-74	0.9	0.3	0.3	0.6
SPTBN2	O15020	115	2	-202	-239	-47	1.0	0.3	0.3	0.7
SPTB	P11277	112	2	-202	-239	-47	1.0	0.3	0.3	0.7
SPTBN1	Q01082	112	2	-202	-239	-47	1.0	0.3	0.3	0.7
ZYX	Q15942	387	8	-243	-241	-93	1.1	0.3	0.3	0.5
UBOX5	O94941	6	-2	-91	-242	-7	1.0	0.5	0.3	0.9
LRRC47	Q8N1G4	367	-7	-239	-243	-77	0.9	0.3	0.3	0.6
ZAP70	P43403	84	-16	-265	-251	-84	0.9	0.3	0.3	0.5
CCDC79	Q8NA31	280	-20	-172	-258	-255	0.8	0.4	0.3	0.3
PRKCH	P24723	224	11	-237	-260	-114	1.1	0.3	0.3	0.5
WWP2	O00308	838	-1	-238	-264	-93	1.0	0.3	0.3	0.5
ITCH	Q96J02	871	-1	-238	-264	-93	1.0	0.3	0.3	0.5
EP300	Q09472	1201	4	-248	-270	-104	1.0	0.3	0.3	0.5
CREBBP	Q92793	1237	4	-248	-270	-104	1.0	0.3	0.3	0.5
SCLY	Q96I15	359	-13	-25	-274	-240	0.9	0.8	0.3	0.3
SMPD4	Q9NXE4	540	2	-283	-296	-115	1.0	0.3	0.3	0.5
IKZF1	Q13422	175	12	-254	-302	-103	1.1	0.3	0.2	0.5
MNDA	P41218	361	8	-268	-308	-121	1.1	0.3	0.2	0.5
LIMS1	P48059	138	6	-236	-310	-80	1.1	0.3	0.2	0.6
RPL37	P61927	19	9	-263	-315	-120	1.1	0.3	0.2	0.5
ZNF22	P17026	144	-26	-263	-323	-137	0.8	0.3	0.2	0.4

TAF5	Q15542	273	3	-291	-346	-126	1.0	0.3	0.2	0.4
ALB	P02768	269	-27	-365	-354	-215	0.8	0.2	0.2	0.3
GIMAP1-GIM	A0A087WTJ2	76	4	-325	-359	-133	1.0	0.2	0.2	0.4
GIMAP1	Q8WWP7	76	4	-325	-359	-133	1.0	0.2	0.2	0.4
NSUN5	Q96P11	308	-40	-313	-371	-169	0.7	0.2	0.2	0.4
NSMCE3	Q96MG7	283	-6	-326	-374	-193	0.9	0.2	0.2	0.3
SNX20	Q7Z614	155	-12	-317	-380	-188	0.9	0.2	0.2	0.3
TGFB1I1	O43294	434	-42	-382	-389	-166	0.7	0.2	0.2	0.4
SIPA1	Q96FS4	467	-23	-373	-411	-122	0.8	0.2	0.2	0.5
PDE7A	Q13946	343	-12	-341	-416	-196	0.9	0.2	0.2	0.3
FEM1B	Q9UK73	186	-32	-425	-437	-240	0.8	0.2	0.2	0.3
EXOC5	O00471	194	-3	-357	-460	-201	1.0	0.2	0.2	0.3
ENGASE	Q8NFI3	454	-34	-383	-472	-210	0.7	0.2	0.2	0.3
HNRNPA3	P51991	196	-37	-411	-483	-215	0.7	0.2	0.2	0.3
RNF213	Q63HN8	310	-17	-345	-522	-170	0.9	0.2	0.2	0.4
FAM129A	Q9BZQ8	891	-28	-431	-524	-284	0.8	0.2	0.2	0.3
STK10	O94804	888	-9	-281	-547	-170	0.9	0.3	0.2	0.4
SIRT5	Q9NXA8	303	-35	-447	-548	-236	0.7	0.2	0.2	0.3
MRPL15	Q9P015	257	-11	-391	-611	-247	0.9	0.2	0.1	0.3
ZC3HAV1	Q7Z2W4	174	-22	-665	-637	-311	0.8	0.1	0.1	0.2
CLTC	Q00610	870	-39	-556	-638	-336	0.7	0.2	0.1	0.2
HNRNPA1	P09651	175	-52	-777	-863	-405	0.7	0.1	0.1	0.2
PMPCB	O75439	265	-24	-683	-918	-399	0.8	0.1	0.1	0.2
SLK	Q9H2G2	1153	-26	-580	-1103	-348	0.8	0.1	0.1	0.2
