# Supplementary material for: Selective inhibitors of JAK1 targeting an isoform-restricted allosteric cysteine
Source: Nat Chem Biol. Author manuscript; Available in PMC 2023 Jul 18. (PMC7614775; doi:10.1038/s41589-022-01098-0)
Supplement: Proteomics Table 3 [file EMS178742-supplement-Proteomics_Table_3.pdf]

Protein	Uniprot Accession	Cysteine Number	%Engagement				Engagement Ratio (DMSO/VVD-118313)			
			0.01 µM	0.1 µM	1 µM	10 µM	0.01 µM	0.1 µM	1 µM	10 µM
Jak1	B1ASP2	816	93	97	98	98	13.3	20.0	20.0	20.0
Bach1	P97302	438	2	76	73	79	1.0	4.2	3.6	4.7
Snrpd3	P62320	20	27	4	70	-164	1.4	1.0	3.3	0.4
Trappc10	F8VQF9	915	44	31	64	-7	1.8	1.4	2.7	0.9
Slfn2	Q9Z0I6	114	-8	21	62	70	0.9	1.3	2.6	3.3
Top2b	Q64511	225	27	13	61	-16	1.4	1.1	2.5	0.9
Znf569	Q80W31	517	7	-23	60	85	1.1	0.8	2.5	6.5
Nit1	Q8VDK1	161	35	28	59	-11	1.5	1.4	2.4	0.9
Naa15	G3X8Y3	720	27	10	55	-15	1.4	1.1	2.2	0.9
Ppp1cb	P62141	126	15	9	55	-49	1.2	1.1	2.2	0.7
Ppp1cc	P63087	127	15	9	55	-49	1.2	1.1	2.2	0.7
Ppat	Q8CIH9	247	23	-5	54	-19	1.3	1.0	2.2	0.8
Taf2	B9EJX5	767	13	-4	53	-10	1.1	1.0	2.1	0.9
Hk2	O08528	368	36	30	51	33	1.6	1.4	2.0	1.5
Psme1	P97371	22	-11	0	50	95	0.9	1.0	2.0	18.2
Akr1b10	G5E895	299	-19	-3	49	90	0.8	1.0	2.0	10.0
Hmha1	Q3TBD2	193	16	8	48	-19	1.2	1.1	1.9	0.8
Ints1	Q6P4S8	869	14	-7	48	-7	1.2	0.9	1.9	0.9
Ctcf	Q61164	577	35	8	46	45	1.5	1.1	1.9	1.8
Hvcn1	Q3U2S8	245	21	9	46	-7	1.3	1.1	1.8	0.9
Pura	P42669	271	16	6	46	-18	1.2	1.1	1.8	0.9
Nup205	A0A0J9YUD5	1539	40	27	45	30	1.7	1.4	1.8	1.4
Trio	Q0KL02	2712	9	27	45	44	1.1	1.4	1.8	1.8
Spg11	Q3UHA3	1589	-16	-4	44	94	0.9	1.0	1.8	15.4
Ttc1	Q91Z38	41	7	4	42	-46	1.1	1.0	1.7	0.7
Usp4	P35123	475	-26	-7	41	90	0.8	0.9	1.7	9.5
Glod4	Q9CPV4	41	23	-7	40	17	1.3	0.9	1.7	1.2
Dmxl1	Q6PNC0	679	18	11	40	-16	1.2	1.1	1.7	0.9
Dis3	Q9CSH3	483	15	22	39	38	1.2	1.3	1.6	1.6
Snx12	Q3V2H3	141	16	4	38	22	1.2	1.0	1.6	1.3
Hcfc1	Q61191	326	-6	-6	38	4	0.9	0.9	1.6	1.0
Hsdl1	Q8BTX9	265	-7	-10	38	70	0.9	0.9	1.6	3.3
Gtf3c5	Q8R2T8	71	-30	57	37	74	0.8	2.3	1.6	3.8
Wdr1	O88342	382	41	43	37	32	1.7	1.7	1.6	1.5
Unc119b	Q8C4B4	201	12	15	36	15	1.1	1.2	1.6	1.2
Fam168a	Q8BGZ2	75	9	-5	36	17	1.1	1.0	1.6	1.2
Gripap1	Q8VD04	709	12	8	36	-8	1.1	1.1	1.6	0.9
Vps18	Q8R307	806	-7	-8	35	87	0.9	0.9	1.5	7.7
Hspbp1	Q99P31	308	24	36	35	40	1.3	1.6	1.5	1.7
Pebp1	P70296	133	9	5	35	-34	1.1	1.0	1.5	0.7
Prpf6	Q91YR7	913	-3	-6	35	82	1.0	0.9	1.5	5.6
Cyp4v2	Q9DBW0	260	-9	-8	34	65	0.9	0.9	1.5	2.8
Syne3	Q4FZC9	333	3	5	34	92	1.0	1.0	1.5	11.8
Hcfc2	G5E837	199	9	-4	34	-13	1.1	1.0	1.5	0.9
Coro1a	O89053	285	25	9	33	38	1.3	1.1	1.5	1.6
Ctsb	P10605	108	-2	8	33	27	1.0	1.1	1.5	1.4
Snx3	Q78ZM0	140	10	10	33	24	1.1	1.1	1.5	1.3
Son	Q9QX47	1702	10	8	33	-34	1.1	1.1	1.5	0.7
Atad1	Q9D5T0	303	29	2	32	3	1.4	1.0	1.5	1.0

Tuba3b	P05214	295	9	3	31	-32	1.1	1.0	1.4	0.8
Tuba4a	P68368	295	9	3	31	-32	1.1	1.0	1.4	0.8
Tuba1a	P68369	295	9	3	31	-32	1.1	1.0	1.4	0.8
Adam10	O35598	173	-13	29	31	38	0.9	1.4	1.4	1.6
Ncdn	Q9Z0E0	98	-20	2	31	92	0.8	1.0	1.4	12.5
Dkc1	Q9ESX5	74	-14	-15	31	-67	0.9	0.9	1.4	0.6
Prepl	Q8C167	402	29	35	30	41	1.4	1.5	1.4	1.7
Uvrag	Q8K245	238	-1	8	30	91	1.0	1.1	1.4	11.1
Ufsp1	Q9CZP0	119	7	0	30	-19	1.1	1.0	1.4	0.8
Nap1l4	Q78ZA7	77	-1	-20	30	9	1.0	0.8	1.4	1.1
Gmfg	Q9ERL7	96	32	31	30	24	1.5	1.4	1.4	1.3
Trip12	G5E870	1444	-16	10	30	31	0.9	1.1	1.4	1.4
Plec	Q9QXS1	1413	16	10	30	24	1.2	1.1	1.4	1.3
Ctc1	Q5SUQ9	533	20	-7	30	33	1.3	0.9	1.4	1.5
Rac1	P63001	105	62	-24	29	20	2.6	0.8	1.4	1.2
Cep72	Q9D3R3	10	32	28	29	36	1.5	1.4	1.4	1.6
Akap11	E9Q777	1581	27	13	29	9	1.4	1.1	1.4	1.1
Vrk3	Q8K3G5	65	10	-8	29	-47	1.1	0.9	1.4	0.7
Plekhm1	Q7TSI1	132	23	26	28	31	1.3	1.3	1.4	1.4
Prpf6	Q91YR7	429	12	13	28	28	1.1	1.1	1.4	1.4
Atox1	O08997	12	43	10	28	22	1.8	1.1	1.4	1.3
Srp68	Q8BMA6	343	-2	7	28	35	1.0	1.1	1.4	1.5
Klhl25	Q8R2P1	31	16	3	28	-3	1.2	1.0	1.4	1.0
Lasp1	Q61792	20	25	0	28	28	1.3	1.0	1.4	1.4
Trmt2a	Q8BNV1	341	-21	-4	28	-1	0.8	1.0	1.4	1.0
Pmpca	Q9DC61	141	2	15	28	23	1.0	1.2	1.4	1.3
Khsrp	Q3U0V1	380	9	10	28	9	1.1	1.1	1.4	1.1
Mri1	Q9CQT1	199	11	1	28	5	1.1	1.0	1.4	1.1
Gapvd1	Q6PAR5	1140	24	-2	28	26	1.3	1.0	1.4	1.3
Akr1b7	P21300	299	-11	-3	28	82	0.9	1.0	1.4	5.4
Pck2	Q8BH04	230	-17	-14	28	-50	0.9	0.9	1.4	0.7
Upf2	A2AT37	1104	24	3	27	34	1.3	1.0	1.4	1.5
Ankrd17	Q99NH0	640	-1	-2	27	-21	1.0	1.0	1.4	0.8
Cep192	E9Q4Y4	2043	-21	-17	27	4	0.8	0.9	1.4	1.0
Ccdc88c	Q6VGS5	1171	26	18	27	21	1.4	1.2	1.4	1.3
Birc6	O88738	1381	-5	3	27	87	1.0	1.0	1.4	7.4
Inpp5d	Q9ES52	509	5	2	27	-8	1.1	1.0	1.4	0.9
Nvl	Q9DBY8	625	-23	-16	27	68	0.8	0.9	1.4	3.1
Cdan1	Q8CC12	405	11	9	26	25	1.1	1.1	1.4	1.3
Ankhd1	E9PUR0	618	9	9	26	-21	1.1	1.1	1.4	0.8
Vps18	Q8R307	776	16	1	26	16	1.2	1.0	1.4	1.2
Syne1	Q6ZWR6	2080	4	17	26	26	1.0	1.2	1.3	1.3
Tkfc	Q8VC30	155	14	15	26	57	1.2	1.2	1.3	2.3
Ercc3	P49135	343	-3	-1	26	68	1.0	1.0	1.3	3.1
Unc13d	B2RUP2	991	-8	-7	26	-9	0.9	0.9	1.3	0.9
Psmd10	Q9Z2X2	107	-12	-20	26	-38	0.9	0.8	1.3	0.7
Ube3a	O08759	47	-3	-26	26	-10	1.0	0.8	1.3	0.9
Pak1ip1	Q9DCE5	239	-1	30	25	16	1.0	1.4	1.3	1.2
Ivns1abp	Q920Q8	39	9	11	25	27	1.1	1.1	1.3	1.4
Zmym3	Q9JLM4	456	-1	-7	25	16	1.0	0.9	1.3	1.2
Hdgfrp2	Q3UMU9	64	-1	12	25	14	1.0	1.1	1.3	1.2

Kpna4	O35343	228	-6	8	25	28	0.9	1.1	1.3	1.4
Card11	Q8CIS0	723	4	-2	25	-9	1.0	1.0	1.3	0.9
Mrto4	Q9D0I8	176	14	32	24	34	1.2	1.5	1.3	1.5
Znf598	Q80YR4	58	-4	12	24	-8	1.0	1.1	1.3	0.9
Ercc2	O08811	663	15	-3	24	30	1.2	1.0	1.3	1.4
Dmxl1	Q6PNC0	858	6	-4	24	-9	1.1	1.0	1.3	0.9
Stt3b	Q3TDQ1	712	3	-13	24	25	1.0	0.9	1.3	1.3
Gm27029	A2A4P4	397	11	-18	24	31	1.1	0.9	1.3	1.4
Ubn2	Q80WC1	501	12	-23	24	18	1.1	0.8	1.3	1.2
Ints4	Q8CIM8	474	7	20	24	33	1.1	1.2	1.3	1.5
Aftph	Q80WT5	509	13	14	24	30	1.1	1.2	1.3	1.4
Wdr24	Q8CFJ9	457	-3	2	24	2	1.0	1.0	1.3	1.0
Eif3b	Q8JZQ9	373	1	-6	24	-11	1.0	0.9	1.3	0.9
Rnf113a2	Q14B01	282	7	-7	24	2	1.1	0.9	1.3	1.0
Eif3c	Q8R1B4	79	20	-10	24	30	1.2	0.9	1.3	1.4
Krtap13	O88375	86	14	29	23	32	1.2	1.4	1.3	1.5
Krtap13	O88375	76	14	29	23	32	1.2	1.4	1.3	1.5
Krtap13	O88375	96	14	29	23	32	1.2	1.4	1.3	1.5
Fscn1	Q61553	397	8	11	23	8	1.1	1.1	1.3	1.1
Xrn2	Q9DBR1	296	12	6	23	-29	1.1	1.1	1.3	0.8
Dtx3l	Q3UIR3	585	1	-5	23	4	1.0	1.0	1.3	1.0
Rapgef1	Q3UHC1	964	8	-15	23	-29	1.1	0.9	1.3	0.8
Sp100	O35892	24	19	25	23	39	1.2	1.3	1.3	1.6
Txnip	Q8BG60	171	4	20	23	48	1.0	1.3	1.3	1.9
Arid1a	A2BH40	1966	17	6	23	33	1.2	1.1	1.3	1.5
Rabggtb	P53612	48	8	-1	23	5	1.1	1.0	1.3	1.0
Eml4	Q3UMY5	322	20	-2	23	14	1.3	1.0	1.3	1.2
Cbl	P22682	565	9	-8	23	1	1.1	0.9	1.3	1.0
Diaph1	O08808	305	27	23	22	37	1.4	1.3	1.3	1.6
Kat8	Q9D1P2	416	10	13	22	-8	1.1	1.1	1.3	0.9
Ralgapa2	A3KGS3	1155	11	3	22	-12	1.1	1.0	1.3	0.9
Fen1	Q91Z50	163	15	-11	22	45	1.2	0.9	1.3	1.8
Chrnd	P02716	388	-53	51	22	55	0.7	2.0	1.3	2.2
Sp140	Q6NSQ5	373	-9	7	22	33	0.9	1.1	1.3	1.5
Agpat5	Q9D1E8	253	0	1	22	1	1.0	1.0	1.3	1.0
Suv39h1	O54864	246	9	-1	22	21	1.1	1.0	1.3	1.3
Nxf1	Q99JX7	32	9	-2	22	-5	1.1	1.0	1.3	1.0
Pax5	Q02650	64	-3	-5	22	-10	1.0	1.0	1.3	0.9
Gm14569	E9Q0C6	439	5	-6	22	28	1.0	0.9	1.3	1.4
Taf2	B9EJX5	94	17	22	21	15	1.2	1.3	1.3	1.2
Cbx1	P83917	60	5	11	21	30	1.1	1.1	1.3	1.4
Ube2a	Q9Z255	88	3	7	21	-12	1.0	1.1	1.3	0.9
Cnbp	P53996	112	23	1	21	17	1.3	1.0	1.3	1.2
Rps12-ps3	Q6ZWZ6	50	-1	6	21	2	1.0	1.1	1.3	1.0
Polr2a	P08775	451	19	4	21	20	1.2	1.0	1.3	1.3
Smc5	Q8CG46	1042	7	2	21	4	1.1	1.0	1.3	1.0
Xpo5	Q924C1	44	-4	-1	21	93	1.0	1.0	1.3	14.3
Alkbh3	Q8K1E6	110	4	-6	21	21	1.0	0.9	1.3	1.3
Themis2	Q91YX0	354	2	-6	21	-11	1.0	0.9	1.3	0.9
Capza2	P47754	157	9	-6	21	36	1.1	0.9	1.3	1.6
Adi1	Q99JT9	70	2	-7	21	-54	1.0	0.9	1.3	0.7

Pebp1	P70296	168	-24	-22	21	-49	0.8	0.8	1.3	0.7
Git2	Q9JLQ2	621	19	28	20	23	1.2	1.4	1.3	1.3
Kidins220	E9Q9B7	779	-6	16	20	75	0.9	1.2	1.3	3.9
Irak3	Q8K4B2	423	-4	10	20	25	1.0	1.1	1.3	1.3
D1Ertd622	Q8VEB3	156	15	6	20	25	1.2	1.1	1.3	1.3
Bag5	Q8CI32	213	-3	5	20	-8	1.0	1.0	1.3	0.9
Aldh9a1	Q9JLJ2	376	5	-1	20	18	1.0	1.0	1.3	1.2
Rnf213	E9Q555	1188	-5	-1	20	-5	1.0	1.0	1.3	1.0
Prep	Q9QUR6	532	-21	-21	20	-73	0.8	0.8	1.3	0.6
Fntb	Q8K2I1	299	-11	-22	20	92	0.9	0.8	1.3	11.8
Calr	P14211	163	14	-26	20	25	1.2	0.8	1.3	1.3
Rnf213	E9Q555	2829	20	-30	20	-63	1.3	0.8	1.3	0.6
Xpo1	Q6P5F9	1070	2	10	20	10	1.0	1.1	1.2	1.1
Lpxn	Q99N69	332	-18	8	20	14	0.8	1.1	1.2	1.2
Gpd2	Q64521	84	12	3	20	28	1.1	1.0	1.2	1.4
Ncapd2	Q8K2Z4	1042	7	-5	20	38	1.1	1.0	1.2	1.6
Arhgap25	Q8BYW1	303	1	-5	20	-26	1.0	1.0	1.2	0.8
Tes	Q921W7	329	24	-6	20	32	1.3	0.9	1.2	1.5
Helz2	E9QAM5	100	16	-7	20	25	1.2	0.9	1.2	1.3
Urb2	E9Q7L1	1067	14	-10	20	10	1.2	0.9	1.2	1.1
Gsdmdc1	Q9D8T2	265	55	-31	20	10	2.2	0.8	1.2	1.1
Thada	A8C756	626	18	11	19	26	1.2	1.1	1.2	1.3
Usp16	Q99LG0	619	-5	9	19	18	1.0	1.1	1.2	1.2
Macrod1	Q922B1	114	21	8	19	-7	1.3	1.1	1.2	0.9
Krr1	Q8BGA5	101	2	7	19	25	1.0	1.1	1.2	1.3
Znf280c	Q6P3Y5	719	-1	5	19	6	1.0	1.1	1.2	1.1
Traf3ip3	G3X949	297	-10	2	19	42	0.9	1.0	1.2	1.7
Rps12-ps3	Q6ZWZ6	56	0	1	19	-5	1.0	1.0	1.2	1.0
Parp3	Q3ULW8	66	14	-1	19	34	1.2	1.0	1.2	1.5
Tln1	P26039	116	4	-3	19	18	1.0	1.0	1.2	1.2
Tes	Q921W7	25	3	-5	19	1	1.0	1.0	1.2	1.0
Phf14	Q9D4H9	381	-2	-13	19	10	1.0	0.9	1.2	1.1
Snrnp70	Q62376	39	46	-21	19	9	1.8	0.8	1.2	1.1
Khsrp	Q3U0V1	297	19	31	19	31	1.2	1.4	1.2	1.4
Myg1	Q9JK81	61	27	12	19	27	1.4	1.1	1.2	1.4
Sdha	Q8K2B3	190	11	8	19	13	1.1	1.1	1.2	1.1
Cyc1	Q9D0M3	139	2	6	19	24	1.0	1.1	1.2	1.3
Hnrnpul2	Q00PI9	322	-23	4	19	-8	0.8	1.0	1.2	0.9
Dffb	O54788	20	3	1	19	-2	1.0	1.0	1.2	1.0
Pfkfb4	Q6DTY7	159	1	-1	19	67	1.0	1.0	1.2	3.0
Trim12a	Q99PQ1	58	7	-4	19	13	1.1	1.0	1.2	1.1
Alg5	Q9DB25	99	5	-7	19	-1	1.0	0.9	1.2	1.0
Syne2	Q6ZWQ0	4882	-14	-8	19	32	0.9	0.9	1.2	1.5
Thop1	Q8C1A5	434	-5	-9	19	-13	1.0	0.9	1.2	0.9
Wdr12	Q9JJA4	309	-48	24	18	19	0.7	1.3	1.2	1.2
Stim2	P83093	313	-9	17	18	12	0.9	1.2	1.2	1.1
Top3b	Q9Z321	161	6	10	18	24	1.1	1.1	1.2	1.3
Cnbp	P53996	57	-6	6	18	-10	0.9	1.1	1.2	0.9
Ifngr1	P15261	203	26	0	18	4	1.4	1.0	1.2	1.0
Ifngr1	P15261	208	26	0	18	4	1.4	1.0	1.2	1.0
Kbtbd11	Q8BNW9	489	-7	-1	18	17	0.9	1.0	1.2	1.2

Ncapd3	K4DI67	608	-3	-1	18	26	1.0	1.0	1.2	1.3
Ikzf3	O08900	280	-2	-3	18	-8	1.0	1.0	1.2	0.9
Metap1	Q8BP48	292	-1	-5	18	6	1.0	1.0	1.2	1.1
Nop58	Q6DFW4	514	15	-7	18	1	1.2	0.9	1.2	1.0
Fmnl1	Q9JL26	299	-21	-9	18	-48	0.8	0.9	1.2	0.7
Pin1rt1	Q3ULQ2	53	3	-36	18	-24	1.0	0.7	1.2	0.8
Dhx34	Q9DBV3	59	11	19	18	26	1.1	1.2	1.2	1.3
Vmn2r112	L7N221	106	21	14	18	20	1.3	1.2	1.2	1.2
Pa2g4	P50580	179	-6	5	18	13	0.9	1.0	1.2	1.1
Gemin6	Q9CX53	90	0	3	18	5	1.0	1.0	1.2	1.0
Trim24	Q64127	69	-21	1	18	44	0.8	1.0	1.2	1.8
Sart3	Q9JLI8	473	7	0	18	9	1.1	1.0	1.2	1.1
Ppp2r2d	Q925E7	268	-3	-3	18	14	1.0	1.0	1.2	1.2
Madd	Q80U28	201	-13	-5	18	-3	0.9	1.0	1.2	1.0
Dhrs1	Q99L04	256	-8	-12	18	68	0.9	0.9	1.2	3.1
Ikzf3	O08900	150	46	-45	18	-8	1.9	0.7	1.2	0.9
Ikzf1	Q5SWT9	170	46	-45	18	-8	1.9	0.7	1.2	0.9
Kdm5a	Q3UXZ9	690	13	15	17	37	1.1	1.2	1.2	1.6
Tbcd	Q8BYA0	908	-1	-5	17	-11	1.0	1.0	1.2	0.9
Fam208a	Q69ZR9	533	11	-5	17	13	1.1	1.0	1.2	1.1
Coq8b	Q566J8	294	1	-7	17	37	1.0	0.9	1.2	1.6
Spg20	Q8R1X6	409	6	-7	17	-6	1.1	0.9	1.2	0.9
Chd4	Q6PDQ2	1461	14	-9	17	22	1.2	0.9	1.2	1.3
Sf3b3	Q921M3	1156	2	-12	17	-100	1.0	0.9	1.2	0.5
Ttll12	Q3UDE2	356	1	-13	17	7	1.0	0.9	1.2	1.1
Apobec3	Q99J72	254	13	-13	17	6	1.1	0.9	1.2	1.1
Bop1	P97452	518	-12	-25	17	-36	0.9	0.8	1.2	0.7
Lynx1	Q9WVC2	39	-2	18	17	30	1.0	1.2	1.2	1.4
March7	Q9WV66	606	15	12	17	19	1.2	1.1	1.2	1.2
Prkce	P16054	352	21	11	17	-9	1.3	1.1	1.2	0.9
Rpl12	P35979	162	9	2	17	6	1.1	1.0	1.2	1.1
Pds5b	Q4VA53	317	-6	1	17	11	0.9	1.0	1.2	1.1
Pccb	Q99MN9	271	6	1	17	8	1.1	1.0	1.2	1.1
Tes	Q921W7	410	-17	-9	17	10	0.9	0.9	1.2	1.1
Xpo7	Q9EPK7	244	-5	-13	17	-16	1.0	0.9	1.2	0.9
Prep	Q9QUR6	255	-2	-15	17	-61	1.0	0.9	1.2	0.6
Ptk2	P34152	497	-7	-22	17	-12	0.9	0.8	1.2	0.9
Wdr3	Q8BHB4	44	17	23	16	31	1.2	1.3	1.2	1.4
Trim56	Q80VI1	493	-2	12	16	31	1.0	1.1	1.2	1.4
Gm15446	D3Z5Y8	282	-5	11	16	11	1.0	1.1	1.2	1.1
Kbtbd11	Q8BNW9	545	-4	-1	16	24	1.0	1.0	1.2	1.3
Banf1	O54962	77	-7	-2	16	-13	0.9	1.0	1.2	0.9
Ddx50	Q99MJ9	485	13	-8	16	3	1.1	0.9	1.2	1.0
Ppp1cb	P62141	170	0	-9	16	3	1.0	0.9	1.2	1.0
Ppp1cc	P63087	171	0	-9	16	3	1.0	0.9	1.2	1.0
Dnmt1	P13864	765	10	23	16	40	1.1	1.3	1.2	1.7
Opa3	Q505D7	164	9	14	16	2	1.1	1.2	1.2	1.0
Mtr	A6H5Y3	800	6	8	16	26	1.1	1.1	1.2	1.4
Aldh18a1	Q9Z110	546	9	6	16	2	1.1	1.1	1.2	1.0
Glrx	Q9QUH0	83	7	5	16	9	1.1	1.1	1.2	1.1
Unc13d	B2RUP2	447	10	5	16	41	1.1	1.0	1.2	1.7

Glyr1	Q922P9	406	7	3	16	23	1.1	1.0	1.2	1.3
Gca	Q8VC88	185	1	2	16	17	1.0	1.0	1.2	1.2
Cdk5rap2	Q8K389	1534	13	-8	16	-1	1.1	0.9	1.2	1.0
Hsph1	Q61699	245	-7	-10	16	23	0.9	0.9	1.2	1.3
Sympk	Q80X82	859	3	-12	16	-24	1.0	0.9	1.2	0.8
Fhl3	Q9R059	150	2	-13	16	14	1.0	0.9	1.2	1.2
Usp19	Q3UJD6	999	12	18	15	-3	1.1	1.2	1.2	1.0
Dennd5a	Q6PAL8	679	-4	17	15	7	1.0	1.2	1.2	1.1
N4bp2	F8VQG7	520	9	13	15	9	1.1	1.1	1.2	1.1
Fndc3a	Q8BX90	245	14	13	15	7	1.2	1.1	1.2	1.1
UPF0317	Q8BH86	252	7	10	15	15	1.1	1.1	1.2	1.2
Naa50	Q6PGB6	79	7	9	15	17	1.1	1.1	1.2	1.2
Prpf19	Q99KP6	351	10	8	15	10	1.1	1.1	1.2	1.1
Usp4	P35123	110	-4	6	15	25	1.0	1.1	1.2	1.3
Dscr3	O35075	221	-3	5	15	61	1.0	1.1	1.2	2.5
Cad	B2RQC6	1889	12	5	15	13	1.1	1.1	1.2	1.1
Phf6	Q9D4J7	212	-13	5	15	35	0.9	1.0	1.2	1.5
Abhd17b	Q7M759	39	-1	3	15	0	1.0	1.0	1.2	1.0
Osbpl5	Q9ER64	233	-14	1	15	8	0.9	1.0	1.2	1.1
Akap9	Q70FJ1	2974	-21	-2	15	38	0.8	1.0	1.2	1.6
Rbm27	Q5SFM8	49	12	-2	15	7	1.1	1.0	1.2	1.1
Celf1	P28659	150	-1	-3	15	1	1.0	1.0	1.2	1.0
Dfna5	Q9Z2D3	312	6	-4	15	19	1.1	1.0	1.2	1.2
Hectd1	Q69ZR2	1373	-1	-6	15	8	1.0	0.9	1.2	1.1
Cog5	Q8C0L8	654	19	-9	15	9	1.2	0.9	1.2	1.1
As3mt	Q91WU5	251	-30	-11	15	12	0.8	0.9	1.2	1.1
Dcaf13	Q6PAC3	87	-30	-19	15	5	0.8	0.8	1.2	1.1
Rac2	Q05144	105	31	-24	15	3	1.4	0.8	1.2	1.0
Isyna1	Q9JHU9	485	0	20	15	37	1.0	1.2	1.2	1.6
Ogt	Q8CGY8	417	-4	17	15	40	1.0	1.2	1.2	1.7
Eif2s3y	Q9Z0N2	101	22	15	15	13	1.3	1.2	1.2	1.1
Mis18bp1	Q80WQ8	406	-14	13	15	4	0.9	1.1	1.2	1.0
Scyl1	Q9EQC5	88	7	11	15	-17	1.1	1.1	1.2	0.9
Cstf2t	Q8C7E9	450	8	10	15	18	1.1	1.1	1.2	1.2
Dpf2	Q61103	273	15	9	15	20	1.2	1.1	1.2	1.2
Col4a3bp	Q9EQG9	172	-18	8	15	32	0.8	1.1	1.2	1.5
Ripk1	Q60855	641	-2	7	15	17	1.0	1.1	1.2	1.2
Ankrd44	B2RXR6	453	15	7	15	13	1.2	1.1	1.2	1.1
Tgm2	P21981	230	9	2	15	-11	1.1	1.0	1.2	0.9
Tes	Q921W7	350	2	-1	15	-20	1.0	1.0	1.2	0.8
Ankrd16	A2AS55	231	-4	-2	15	-27	1.0	1.0	1.2	0.8
Plekhf2	Q91WB4	219	5	-5	15	1	1.0	1.0	1.2	1.0
Fhl3	Q9R059	185	-10	-17	15	8	0.9	0.9	1.2	1.1
Nop2	E9QN31	472	-2	-19	15	-3	1.0	0.8	1.2	1.0
Pabpc1	P29341	128	-20	-21	15	3	0.8	0.8	1.2	1.0
Pabpc4	Q6PHQ9	128	-20	-21	15	3	0.8	0.8	1.2	1.0
Arih1	Q9Z1K5	267	-25	-28	15	-24	0.8	0.8	1.2	0.8
Gca	Q8VC88	184	22	26	14	46	1.3	1.3	1.2	1.9
Xpnpep1	Q6P1B1	279	-11	16	14	5	0.9	1.2	1.2	1.0
G3bp2	P97379	73	2	13	14	15	1.0	1.1	1.2	1.2
Rpap2	Q8VC34	306	8	13	14	11	1.1	1.1	1.2	1.1

Cbx5	Q61686	133	3	13	14	4	1.0	1.1	1.2	1.0
Atrx	Q61687	1580	-8	8	14	24	0.9	1.1	1.2	1.3
Atp2a3	Q64518	498	-14	7	14	28	0.9	1.1	1.2	1.4
Rsbn1	Q80T69	671	11	7	14	17	1.1	1.1	1.2	1.2
Ctsc	P97821	257	3	6	14	19	1.0	1.1	1.2	1.2
Fmo5	P97872	468	5	6	14	13	1.1	1.1	1.2	1.1
Aurkb	O70126	196	4	5	14	28	1.0	1.0	1.2	1.4
Ddx39a	Q8VDW0	197	3	2	14	13	1.0	1.0	1.2	1.1
Gm15800	E9Q2E4	765	-6	2	14	80	0.9	1.0	1.2	4.9
Gcn1	E9PVA8	2179	13	-1	14	-8	1.1	1.0	1.2	0.9
Dbt	P53395	333	-14	-1	14	-8	0.9	1.0	1.2	0.9
Retnlg	Q8K426	51	3	-3	14	38	1.0	1.0	1.2	1.6
Sipa1	E9Q0Y4	628	-5	-4	14	21	1.0	1.0	1.2	1.3
Nup98	Q6PFD9	1026	-6	-4	14	-22	0.9	1.0	1.2	0.8
Snrpn	P63163	43	7	-5	14	6	1.1	1.0	1.2	1.1
Usp7	E9PXY8	958	1	-9	14	8	1.0	0.9	1.2	1.1
Hirip3	Q8BLH7	464	-6	-10	14	-14	0.9	0.9	1.2	0.9
Ppp1cb	P62141	244	-1	-25	14	-8	1.0	0.8	1.2	0.9
Ppp1cc	P63087	245	-1	-25	14	-8	1.0	0.8	1.2	0.9
Psma7	Q9Z2U0	63	-6	27	14	27	0.9	1.4	1.2	1.4
Pgk1	P09411	108	7	25	14	38	1.1	1.3	1.2	1.6
Alg2	Q9DBE8	59	14	18	14	-10	1.2	1.2	1.2	0.9
Tcp1	P11983	385	10	16	14	13	1.1	1.2	1.2	1.1
Pwp1	Q99LL5	328	-18	15	14	0	0.8	1.2	1.2	1.0
Csk	P41241	223	2	13	14	7	1.0	1.1	1.2	1.1
Psmb10	O35955	82	5	12	14	14	1.0	1.1	1.2	1.2
Ttk	P35761	410	3	10	14	13	1.0	1.1	1.2	1.1
Cyfip2	Q5SQX6	423	4	7	14	-27	1.0	1.1	1.2	0.8
Tmem214	Q8BM55	377	5	6	14	33	1.0	1.1	1.2	1.5
Usp38	Q8BW70	834	10	5	14	6	1.1	1.1	1.2	1.1
Eprs	Q8CGC7	1252	2	4	14	23	1.0	1.0	1.2	1.3
Xpo5	Q924C1	47	-6	4	14	75	0.9	1.0	1.2	4.0
Ube2s	Q921J4	95	7	1	14	-11	1.1	1.0	1.2	0.9
Casp7	P97864	290	15	-1	14	59	1.2	1.0	1.2	2.4
Btaf1	E9QAE3	1309	-5	-3	14	11	1.0	1.0	1.2	1.1
Macf1	E9PVY8	6603	-14	-6	14	-5	0.9	0.9	1.2	1.0
Det1	Q9D0A0	482	13	-7	14	5	1.1	0.9	1.2	1.0
Il21r	Q9JHX3	428	1	-7	14	12	1.0	0.9	1.2	1.1
Pdlim2	Q8R1G6	307	-11	-7	14	2	0.9	0.9	1.2	1.0
Zw10	O54692	39	7	-9	14	36	1.1	0.9	1.2	1.6
Psmd9	Q9CR00	81	-4	-13	14	-3	1.0	0.9	1.2	1.0
Prpf31	Q8CCF0	247	18	12	13	25	1.2	1.1	1.1	1.3
Ccdc88a	Q5SNZ0	1731	-20	11	13	26	0.8	1.1	1.1	1.3
Irak3	Q8K4B2	522	11	8	13	2	1.1	1.1	1.1	1.0
Brd1	E9PZ26	274	-1	5	13	35	1.0	1.0	1.1	1.5
Pex1	Q5BL07	772	-11	1	13	32	0.9	1.0	1.1	1.5
Akap10	O88845	475	-17	-1	13	21	0.9	1.0	1.1	1.3
Zrsr2	B1B0E8	330	9	-4	13	3	1.1	1.0	1.1	1.0
Fam98c	E9PYD1	83	-5	-4	13	1	1.0	1.0	1.1	1.0
Nfkb1	P25799	121	4	-6	13	19	1.0	0.9	1.1	1.2
Cnot1	Q6ZQ08	219	-12	-6	13	11	0.9	0.9	1.1	1.1

Bmp2k	Q91Z96	320	-25	-8	13	19	0.8	0.9	1.1	1.2
Nup210	Q9QY81	746	24	-8	13	11	1.3	0.9	1.1	1.1
Zmym3	Q9JLM4	572	-11	-9	13	13	0.9	0.9	1.1	1.1
Nans	Q99J77	19	24	-9	13	7	1.3	0.9	1.1	1.1
Prkcq	Q02111	14	-45	-9	13	4	0.7	0.9	1.1	1.0
Rhot2	Q8JZN7	185	-9	-10	13	17	0.9	0.9	1.1	1.2
Mgll	O35678	201	-2	-11	13	31	1.0	0.9	1.1	1.4
Vps52	Q8C754	220	-3	-12	13	18	1.0	0.9	1.1	1.2
Kpnb1	P70168	345	-13	-13	13	14	0.9	0.9	1.1	1.2
Farsb	Q9WUA2	255	3	-22	13	3	1.0	0.8	1.1	1.0
Rnf130	Q8VEM1	320	1	-33	13	-30	1.0	0.8	1.1	0.8
Acap2	Q6ZQK5	414	-3	23	13	28	1.0	1.3	1.1	1.4
Gtf3c1	Q8K284	179	1	19	13	46	1.0	1.2	1.1	1.9
Ccar1	Q8CH18	370	2	15	13	21	1.0	1.2	1.1	1.3
Ankfy1	Q810B6	1156	4	14	13	20	1.0	1.2	1.1	1.3
Klf4	Q60793	282	17	12	13	17	1.2	1.1	1.1	1.2
Lancl1	O89112	98	-3	7	13	-12	1.0	1.1	1.1	0.9
Wac	Q924H7	614	13	4	13	20	1.1	1.0	1.1	1.2
Nfxl1	E9Q8I7	782	-2	4	13	9	1.0	1.0	1.1	1.1
Snrnp200	Q6P4T2	1580	13	3	13	22	1.1	1.0	1.1	1.3
Birc6	O88738	2915	4	1	13	23	1.0	1.0	1.1	1.3
Ak2	Q9WTP6	232	-4	-1	13	15	1.0	1.0	1.1	1.2
Med14	A2ABV5	735	-2	-2	13	27	1.0	1.0	1.1	1.4
Hnrnpdl	Q9Z130	158	-17	-2	13	3	0.9	1.0	1.1	1.0
Akr1b1	P45376	299	-3	-3	13	61	1.0	1.0	1.1	2.5
Flnb	Q80X90	1952	-18	-3	13	-26	0.8	1.0	1.1	0.8
Hps5	P59438	722	-4	-4	13	11	1.0	1.0	1.1	1.1
Map1s	Q8C052	443	-4	-4	13	9	1.0	1.0	1.1	1.1
Arih1	Q9Z1K5	325	7	-5	13	-36	1.1	1.0	1.1	0.7
Acsl1	P41216	626	2	-7	13	0	1.0	0.9	1.1	1.0
Prpf8	Q99PV0	1626	-6	-8	13	30	0.9	0.9	1.1	1.4
Mroh1	E0CZ22	1328	-17	-9	13	60	0.9	0.9	1.1	2.5
Eno1	P17182	337	-4	-9	13	-46	1.0	0.9	1.1	0.7
Zranb2	Q9R020	15	25	-9	13	12	1.3	0.9	1.1	1.1
Afg3l2	Q8JZQ2	401	-19	-9	13	-20	0.8	0.9	1.1	0.8
Samhd1	Q60710	178	-6	-10	13	-14	0.9	0.9	1.1	0.9
Parp14	Q2EMV9	885	-2	-11	13	14	1.0	0.9	1.1	1.2
Map3k4	O08648	587	1	-11	13	14	1.0	0.9	1.1	1.2
Ncald	Q91X97	185	-10	-27	13	-28	0.9	0.8	1.1	0.8
Heatr6	Q6P1G0	849	9	17	12	21	1.1	1.2	1.1	1.3
Phf1	Q9Z1B8	139	-6	15	12	19	0.9	1.2	1.1	1.2
Triobp	Q99KW3	1952	8	13	12	17	1.1	1.1	1.1	1.2
Rdh14	Q9ERI6	75	-1	10	12	39	1.0	1.1	1.1	1.6
Ampd2	Q9DBT5	183	-4	9	12	19	1.0	1.1	1.1	1.2
Samm50	Q8BGH2	421	6	9	12	-8	1.1	1.1	1.1	0.9
Peli1	Q8C669	282	-4	9	12	60	1.0	1.1	1.1	2.5
Wdr7	Q920I9	289	3	9	12	9	1.0	1.1	1.1	1.1
Rabl6	Q5U3K5	172	-2	5	12	21	1.0	1.0	1.1	1.3
Fem1b	Q9Z2G0	465	16	5	12	15	1.2	1.0	1.1	1.2
Parn	Q8VDG3	310	-7	4	12	6	0.9	1.0	1.1	1.1
Ppp6r3	Q922D4	172	0	3	12	-9	1.0	1.0	1.1	0.9

Appl1	Q8K3H0	615	13	2	12	6	1.1	1.0	1.1	1.1
Pank2	Q3U4S0	233	-8	1	12	22	0.9	1.0	1.1	1.3
Klhdc3	Q8VEM9	213	4	1	12	-5	1.0	1.0	1.1	1.0
Pck2	Q8BH04	151	-9	-1	12	10	0.9	1.0	1.1	1.1
Acy1	Q99JW2	23	-4	-1	12	4	1.0	1.0	1.1	1.0
Nup93	Q8BJ71	392	7	-2	12	6	1.1	1.0	1.1	1.1
Ctsz	Q9WUU7	156	9	-7	12	-4	1.1	0.9	1.1	1.0
Glipr2	Q9CYL5	32	-2	-7	12	-46	1.0	0.9	1.1	0.7
Vav1	P27870	557	0	-8	12	9	1.0	0.9	1.1	1.1
Pml	Q60953	361	-15	-11	12	77	0.9	0.9	1.1	4.3
Phf10	K4DI61	382	-3	-14	12	13	1.0	0.9	1.1	1.1
Top2b	Q64511	309	0	-15	12	12	1.0	0.9	1.1	1.1
Cpsf1	Q9EPU4	1133	15	26	12	10	1.2	1.3	1.1	1.1
Rasgrp2	Q9QUG9	548	12	20	12	9	1.1	1.3	1.1	1.1
Ctps2	P70303	216	21	19	12	15	1.3	1.2	1.1	1.2
Fbxo30	Q8BJL1	726	6	16	12	13	1.1	1.2	1.1	1.1
Ccdc93	Q7TQK5	282	12	14	12	12	1.1	1.2	1.1	1.1
Metap2	O08663	468	-11	11	12	18	0.9	1.1	1.1	1.2
Ift140	E9PY46	918	8	9	12	-5	1.1	1.1	1.1	1.0
Padi4	Q9Z183	311	5	8	12	20	1.1	1.1	1.1	1.2
Eno1	P17182	119	7	7	12	-13	1.1	1.1	1.1	0.9
Ppp1cb	P62141	171	13	5	12	8	1.1	1.0	1.1	1.1
Ppp1cc	P63087	172	13	5	12	8	1.1	1.0	1.1	1.1
Specc1	Q5SXY1	740	14	4	12	-12	1.2	1.0	1.1	0.9
Mum1	Q6DID5	528	-7	4	12	-13	0.9	1.0	1.1	0.9
Cdc42bpg	Q80UW5	514	24	-1	12	19	1.3	1.0	1.1	1.2
Papola	Q61183	671	2	-2	12	-3	1.0	1.0	1.1	1.0
Fam169b	Q8CHT6	331	-8	-2	12	-8	0.9	1.0	1.1	0.9
Trap1	Q9CQN1	503	-5	-3	12	21	1.0	1.0	1.1	1.3
Baz2b	A2AUY4	48	-1	-4	12	-11	1.0	1.0	1.1	0.9
Sh2b1	Q91ZM2	323	-5	-7	12	-14	1.0	0.9	1.1	0.9
Ncoa4	Q5U4H9	378	4	-14	12	4	1.0	0.9	1.1	1.0
Srgap2	Q91Z67	486	-3	-15	12	4	1.0	0.9	1.1	1.0
LRWD1	Q8BUI3	178	-6	-15	12	3	0.9	0.9	1.1	1.0
Narf	Q9CYQ7	234	-22	-17	12	2	0.8	0.9	1.1	1.0
Sun1	Q9D666	635	-1	-29	12	53	1.0	0.8	1.1	2.1
Txn	P10639	73	-20	-29	12	-34	0.8	0.8	1.1	0.7
Upf1	Q9EPU0	652	-1	19	11	32	1.0	1.2	1.1	1.5
Tmed8	Q3UHI4	162	-14	18	11	0	0.9	1.2	1.1	1.0
Chd8	Q09XV5	1915	29	15	11	34	1.4	1.2	1.1	1.5
Wdr45b	Q9CR39	333	9	15	11	9	1.1	1.2	1.1	1.1
As3mt	Q91WU5	62	3	11	11	12	1.0	1.1	1.1	1.1
Gmppb	Q8BTZ7	285	-8	10	11	18	0.9	1.1	1.1	1.2
Hnrnpu	Q8VEK3	473	11	8	11	15	1.1	1.1	1.1	1.2
Tbcel	Q8C5W3	62	15	8	11	9	1.2	1.1	1.1	1.1
Cap1	P40124	415	-10	6	11	11	0.9	1.1	1.1	1.1
Npepl1	Q6NSR8	504	13	6	11	-10	1.1	1.1	1.1	0.9
Macf1	E9PVY8	777	5	5	11	47	1.0	1.1	1.1	1.9
Golga4	Q91VW5	1355	-5	5	11	7	1.0	1.1	1.1	1.1
Sbf1	Q6ZPE2	209	14	5	11	6	1.2	1.0	1.1	1.1
Mob4	Q6PEB6	188	-2	3	11	22	1.0	1.0	1.1	1.3

Cct8	P42932	244	-5	2	11	73	1.0	1.0	1.1	3.7
Ggact	Q923B0	84	8	2	11	2	1.1	1.0	1.1	1.0
Eefsec	Q9JHW4	116	4	2	11	29	1.0	1.0	1.1	1.4
Kpna1	Q60960	141	4	2	11	-1	1.0	1.0	1.1	1.0
Txnrd1	Q9JMH6	178	-16	0	11	11	0.9	1.0	1.1	1.1
Adh5	P28474	97	-2	-1	11	27	1.0	1.0	1.1	1.4
Flii	Q9JJ28	1226	2	-2	11	27	1.0	1.0	1.1	1.4
Ints4	Q8CIM8	171	-20	-3	11	-5	0.8	1.0	1.1	1.0
Ints1	Q6P4S8	1773	4	-5	11	24	1.0	1.0	1.1	1.3
Sept1	P42209	102	-5	-6	11	20	1.0	0.9	1.1	1.3
Igf2bp3	Q9CPN8	194	-15	-7	11	-6	0.9	0.9	1.1	0.9
Trim33	Q99PP7	257	10	-10	11	-11	1.1	0.9	1.1	0.9
Det1	Q9D0A0	60	-2	-11	11	-8	1.0	0.9	1.1	0.9
Pus7	B7ZNL8	116	-3	-13	11	-3	1.0	0.9	1.1	1.0
Eprs	Q8CGC7	1487	-22	17	11	12	0.8	1.2	1.1	1.1
Rasgrp2	Q9QUG9	11	-4	16	11	14	1.0	1.2	1.1	1.2
Uncharact	Q6DI92	127	7	14	11	24	1.1	1.2	1.1	1.3
Cnn2	Q08093	61	-1	12	11	30	1.0	1.1	1.1	1.4
Sacs	Q9JLC8	2978	-3	11	11	35	1.0	1.1	1.1	1.5
Rad50	Q5SV02	633	13	11	11	14	1.1	1.1	1.1	1.2
Prrc2c	Q3TLH4	870	10	11	11	-11	1.1	1.1	1.1	0.9
Gm18025	E9Q1N8	201	9	10	11	-3	1.1	1.1	1.1	1.0
Acap1	Q8K2H4	392	-25	9	11	-12	0.8	1.1	1.1	0.9
Setx	A2AKX3	1891	2	9	11	25	1.0	1.1	1.1	1.3
Prkcb	P68404	386	0	7	11	-11	1.0	1.1	1.1	0.9
Bbip1	J3QMK2	29	3	7	11	37	1.0	1.1	1.1	1.6
Ipo5	Q8BKC5	348	3	7	11	24	1.0	1.1	1.1	1.3
Atad2	G3X963	628	0	6	11	26	1.0	1.1	1.1	1.3
Decr1	Q9CQ62	268	5	5	11	5	1.0	1.0	1.1	1.1
Capn2	O08529	405	-5	3	11	16	1.0	1.0	1.1	1.2
Aldh16a1	Q571I9	28	-4	3	11	10	1.0	1.0	1.1	1.1
FAM120A	Q6A0A9	823	9	2	11	5	1.1	1.0	1.1	1.1
Znfx1	Q8R151	1211	4	2	11	12	1.0	1.0	1.1	1.1
Dennd4b	Q3U1Y4	180	13	1	11	14	1.1	1.0	1.1	1.2
Tbc1d2	B1AVH7	680	-15	0	11	19	0.9	1.0	1.1	1.2
Rcl1	Q9JJT0	346	4	0	11	1	1.0	1.0	1.1	1.0
Plcg1	Q62077	1088	9	-2	11	11	1.1	1.0	1.1	1.1
Pacs1	Q8K212	114	-18	-5	11	3	0.9	1.0	1.1	1.0
Sbf1	Q6ZPE2	1374	-10	-5	11	-4	0.9	1.0	1.1	1.0
Ube2f	Q9CY34	116	-3	-8	11	-16	1.0	0.9	1.1	0.9
Itpr1	P11881	2610	3	-9	11	11	1.0	0.9	1.1	1.1
Itpr3	P70227	2537	3	-9	11	11	1.0	0.9	1.1	1.1
Itpr2	Q9Z329	2562	3	-9	11	11	1.0	0.9	1.1	1.1
Prune	Q8BIW1	303	-3	-10	11	9	1.0	0.9	1.1	1.1
Wisp2	A2AHD1	64	-19	-10	11	-9	0.8	0.9	1.1	0.9
Trip12	G5E870	1309	-5	-11	11	2	1.0	0.9	1.1	1.0
Ep400	Q8CHI8	2028	-7	-13	11	-23	0.9	0.9	1.1	0.8
Hus1	Q8BQY8	154	4	-19	11	22	1.0	0.8	1.1	1.3
Arhgef2	Q60875	714	-4	-22	11	4	1.0	0.8	1.1	1.0
Cntrl	A2AL36	1784	4	-24	11	-4	1.0	0.8	1.1	1.0
Zbtb41	Q811F1	672	-24	-25	11	-30	0.8	0.8	1.1	0.8

Gm5580	A0A0N4SVP8	140	-2	24	10	11	1.0	1.3	1.1	1.1
Cwc22	Q8C5N3	495	-6	19	10	29	0.9	1.2	1.1	1.4
Mut	P16332	469	1	15	10	23	1.0	1.2	1.1	1.3
Gtf2b	P62915	181	3	10	10	12	1.0	1.1	1.1	1.1
Alb	P07724	77	18	7	10	25	1.2	1.1	1.1	1.3
Zfyve26	Q5DU37	1949	-19	5	10	20	0.8	1.1	1.1	1.2
Psma4	Q9R1P0	163	-13	4	10	24	0.9	1.0	1.1	1.3
Lims1	Q99JW4	281	1	3	10	20	1.0	1.0	1.1	1.2
Dscr3	O35075	243	-7	2	10	16	0.9	1.0	1.1	1.2
Cdkal1	Q91WE6	217	10	1	10	19	1.1	1.0	1.1	1.2
Lrif1	Q8CDD9	548	5	1	10	8	1.0	1.0	1.1	1.1
Eml4	Q3UMY5	822	9	-2	10	25	1.1	1.0	1.1	1.3
Gnai3	Q9DC51	66	-16	-2	10	-1	0.9	1.0	1.1	1.0
Prune	Q8BIW1	438	-10	-2	10	-18	0.9	1.0	1.1	0.9
Lrif1	Q8CDD9	104	4	-3	10	26	1.0	1.0	1.1	1.3
Fam208a	Q69ZR9	441	-2	-4	10	39	1.0	1.0	1.1	1.6
Eif2b1	Q99LC8	218	0	-4	10	9	1.0	1.0	1.1	1.1
Ppia	P17742	161	-1	-5	10	-16	1.0	1.0	1.1	0.9
Asun	Q8QZV7	556	-36	-5	10	11	0.7	1.0	1.1	1.1
Usp3	Q91W36	408	-7	-6	10	3	0.9	0.9	1.1	1.0
Fars2	Q99M01	185	26	-7	10	19	1.4	0.9	1.1	1.2
Ggct	Q9D7X8	42	-1	-7	10	16	1.0	0.9	1.1	1.2
Hgh1	Q8C3I8	235	-16	-7	10	25	0.9	0.9	1.1	1.3
Gpcpd1	Q8C0L9	208	5	-7	10	2	1.1	0.9	1.1	1.0
Dld	O08749	312	-1	-8	10	-13	1.0	0.9	1.1	0.9
Akap9	Q70FJ1	2111	-22	-8	10	-11	0.8	0.9	1.1	0.9
Cbfa2t3	O54972	294	-2	-9	10	-5	1.0	0.9	1.1	1.0
Znf638	Q61464	1023	-9	-10	10	58	0.9	0.9	1.1	2.4
Gm20390	E9PZF0	109	7	-11	10	17	1.1	0.9	1.1	1.2
Nme1	P15532	109	7	-11	10	17	1.1	0.9	1.1	1.2
Numa1	E9Q7G0	65	-1	-11	10	15	1.0	0.9	1.1	1.2
Dock2	Q8C3J5	1407	-6	-11	10	-2	0.9	0.9	1.1	1.0
Aars	Q8BGQ7	901	-2	-12	10	21	1.0	0.9	1.1	1.3
Prex1	Q69ZK0	725	-21	-13	10	11	0.8	0.9	1.1	1.1
Smurf2	A2A5Z6	743	5	-13	10	-1	1.1	0.9	1.1	1.0
U2af1	Q9D883	102	-1	-14	10	20	1.0	0.9	1.1	1.2
Cdk19	Q8BWD8	25	-9	-17	10	32	0.9	0.9	1.1	1.5
Cdk8	Q8R3L8	25	-9	-17	10	32	0.9	0.9	1.1	1.5
F13a1	Q8BH61	153	-9	-21	10	-17	0.9	0.8	1.1	0.9
Usp4	P35123	500	4	23	10	22	1.0	1.3	1.1	1.3
Mkln1	O89050	446	30	22	10	15	1.4	1.3	1.1	1.2
Lrch3	Q8BVU0	733	-4	19	10	30	1.0	1.2	1.1	1.4
Metap2	O08663	380	10	15	10	10	1.1	1.2	1.1	1.1
Golga4	Q91VW5	1548	-7	13	10	3	0.9	1.1	1.1	1.0
Rffl	Q6ZQM0	64	-13	11	10	-31	0.9	1.1	1.1	0.8
Dnmt1	P13864	1229	3	7	10	25	1.0	1.1	1.1	1.3
Acly	Q91V92	718	2	5	10	12	1.0	1.1	1.1	1.1
Herc4	Q6PAV2	352	1	5	10	-10	1.0	1.1	1.1	0.9
Rangap1	P46061	152	-4	5	10	24	1.0	1.0	1.1	1.3
Tmpo	Q61033	407	15	3	10	22	1.2	1.0	1.1	1.3
Hint3	Q9CPS6	49	-7	3	10	-2	0.9	1.0	1.1	1.0

Gm29609	Q3UU56	604	-5	3	10	23	1.0	1.0	1.1	1.3
Rasgrp2	Q9QUG9	186	-3	2	10	63	1.0	1.0	1.1	2.7
Cyld	Q80TQ2	849	2	2	10	22	1.0	1.0	1.1	1.3
Arhgap9	Q8QZW8	416	-7	1	10	17	0.9	1.0	1.1	1.2
Cxxc1	Q9CWW7	584	2	1	10	4	1.0	1.0	1.1	1.0
Stat5a	P42230	101	1	-1	10	-11	1.0	1.0	1.1	0.9
Cpeb2	Q812E0	459	-7	-1	10	21	0.9	1.0	1.1	1.3
Cpeb2	Q812E0	462	-7	-1	10	21	0.9	1.0	1.1	1.3
Myo1g	Q5SUA5	971	-26	-2	10	10	0.8	1.0	1.1	1.1
Cdk19	Q8BWD8	64	5	-2	10	-19	1.0	1.0	1.1	0.8
Cdk8	Q8R3L8	64	5	-2	10	-19	1.0	1.0	1.1	0.8
Mcmbp	Q8R3C0	325	-6	-5	10	13	0.9	1.0	1.1	1.1
Cpsf2	O35218	577	2	-5	10	8	1.0	1.0	1.1	1.1
Prpf38b	Q80SY5	197	-12	-5	10	22	0.9	1.0	1.1	1.3
Dnmt1	P13864	897	7	-6	10	23	1.1	0.9	1.1	1.3
Pgp	Q8CHP8	193	-8	-6	10	-1	0.9	0.9	1.1	1.0
Sptbn1	Q62261	1970	3	-8	10	15	1.0	0.9	1.1	1.2
Scrn2	Q8VCA8	250	3	-8	10	7	1.0	0.9	1.1	1.1
Phf6	Q9D4J7	280	-18	-9	10	43	0.9	0.9	1.1	1.7
Trmu	Q9DAT5	222	1	-11	10	4	1.0	0.9	1.1	1.0
Papd4	Q91YI6	225	21	-17	10	39	1.3	0.9	1.1	1.6
Fam160b1	Q8CDM8	304	-19	-18	10	70	0.8	0.8	1.1	3.3
Scyl1	Q9EQC5	446	-1	-20	10	17	1.0	0.8	1.1	1.2
Rreb1	Q3UH06	1405	-22	-27	10	-17	0.8	0.8	1.1	0.9
Pdcd4	Q61823	227	-6	19	9	23	0.9	1.2	1.1	1.3
Exosc7	Q9D0M0	238	17	19	9	37	1.2	1.2	1.1	1.6
Fpgs	P48760	111	-12	9	9	36	0.9	1.1	1.1	1.6
Gimap8	Q75N62	205	-2	8	9	-2	1.0	1.1	1.1	1.0
Fam76b	Q80XP8	110	15	6	9	18	1.2	1.1	1.1	1.2
Fam76a	Q922G2	108	15	6	9	18	1.2	1.1	1.1	1.2
Mapk14	P47811	39	2	5	9	14	1.0	1.1	1.1	1.2
Mms19	Q9D071	820	6	1	9	82	1.1	1.0	1.1	5.4
Dync1h1	Q9JHU4	2710	3	0	9	17	1.0	1.0	1.1	1.2
Usp39	Q3TIX9	104	-11	-1	9	27	0.9	1.0	1.1	1.4
Apeh	Q8R146	292	-30	-1	9	18	0.8	1.0	1.1	1.2
Pycrl	Q9DCC4	129	-10	-1	9	4	0.9	1.0	1.1	1.0
Xrcc5	P27641	152	15	-2	9	8	1.2	1.0	1.1	1.1
Ints1	Q6P4S8	971	-11	-3	9	44	0.9	1.0	1.1	1.8
Ppat	Q8CIH9	105	-5	-3	9	27	1.0	1.0	1.1	1.4
Kcmf1	Q80UY2	12	-5	-3	9	2	1.0	1.0	1.1	1.0
Dnajc13	D4AFX7	149	-6	-4	9	24	0.9	1.0	1.1	1.3
Sptbn1	Q62261	1900	1	-5	9	19	1.0	1.0	1.1	1.2
Supt6h	Q62383	1169	-3	-5	9	8	1.0	1.0	1.1	1.1
Ercc4	Q9QZD4	143	2	-6	9	8	1.0	0.9	1.1	1.1
Pelp1	Q9DBD5	71	-7	-7	9	69	0.9	0.9	1.1	3.2
Pdxdc1	Q99K01	425	-1	-7	9	41	1.0	0.9	1.1	1.7
Ap1g2	O88512	555	6	-7	9	3	1.1	0.9	1.1	1.0
Pom121	Q8K3Z9	243	4	-8	9	4	1.0	0.9	1.1	1.0
Wdhd1	P59328	998	4	-9	9	-22	1.0	0.9	1.1	0.8
Srsf1	Q6PDM2	16	-13	-9	9	-4	0.9	0.9	1.1	1.0
Araf	P04627	547	-14	-10	9	15	0.9	0.9	1.1	1.2

Fam26f	Q8C9E8	247	-10	-10	9	-14	0.9	0.9	1.1	0.9
Smc1a	Q9CU62	933	-10	-12	9	5	0.9	0.9	1.1	1.0
Coro1a	O89053	24	-4	-13	9	21	1.0	0.9	1.1	1.3
Maml2	F6U238	104	-10	-13	9	5	0.9	0.9	1.1	1.1
Des	P31001	332	-10	-20	9	-5	0.9	0.8	1.1	1.0
Ddx50	Q99MJ9	414	-17	-24	9	1	0.9	0.8	1.1	1.0
Cybb	Q61093	257	-3	-25	9	6	1.0	0.8	1.1	1.1
Rpl13a	P19253	38	-10	-27	9	-18	0.9	0.8	1.1	0.9
Polr2i	P60898	52	11	-42	9	-15	1.1	0.7	1.1	0.9
Fscn1	Q61553	481	6	24	9	-3	1.1	1.3	1.1	1.0
Sept5	Q9Z2Q6	300	21	19	9	43	1.3	1.2	1.1	1.8
Ndufaf3	Q9JKL4	158	19	16	9	30	1.2	1.2	1.1	1.4
Nadk	P58058	79	-3	16	9	-13	1.0	1.2	1.1	0.9
Vps4a	Q8VEJ9	403	7	15	9	4	1.1	1.2	1.1	1.0
Myo9b	E9PZW8	1673	12	13	9	-11	1.1	1.1	1.1	0.9
Cd79a	P11911	50	7	12	9	32	1.1	1.1	1.1	1.5
Pdcd4	Q61823	150	-7	12	9	12	0.9	1.1	1.1	1.1
Dcun1d2	Q8BZJ7	18	28	11	9	-29	1.4	1.1	1.1	0.8
Pml	Q60953	193	1	9	9	6	1.0	1.1	1.1	1.1
Ppp2r2a	Q6P1F6	398	1	9	9	6	1.0	1.1	1.1	1.1
Aldh1b1	Q9CZS1	388	-4	7	9	36	1.0	1.1	1.1	1.6
Sp1	O89090	754	11	7	9	19	1.1	1.1	1.1	1.2
Polr2a	P08775	641	-2	6	9	1	1.0	1.1	1.1	1.0
Usp9x	Q4FE56	1727	-18	6	9	15	0.8	1.1	1.1	1.2
Scml4	Q80VG1	318	-5	6	9	-12	1.0	1.1	1.1	0.9
Zcchc8	Q9CYA6	395	-3	4	9	51	1.0	1.0	1.1	2.0
Tpt1	P63028	172	4	4	9	3	1.0	1.0	1.1	1.0
Mdn1	A2ANY6	309	13	4	9	-7	1.1	1.0	1.1	0.9
Asna1	O54984	53	-22	3	9	23	0.8	1.0	1.1	1.3
Hgh1	Q8C3I8	145	1	3	9	31	1.0	1.0	1.1	1.4
Ubr4	A2AN08	3801	-14	3	9	18	0.9	1.0	1.1	1.2
Wdfy4	E9Q2M9	716	8	2	9	41	1.1	1.0	1.1	1.7
Cdk9	Q99J95	85	14	2	9	25	1.2	1.0	1.1	1.3
Lta4h	P24527	147	-2	2	9	17	1.0	1.0	1.1	1.2
Samd9l	E9PX59	1238	-9	-1	9	67	0.9	1.0	1.1	3.0
Slc28a2	O88627	18	-1	-1	9	-6	1.0	1.0	1.1	0.9
Wdr91	Q7TMQ7	247	-1	-2	9	10	1.0	1.0	1.1	1.1
Dapk3	O54784	445	-4	-2	9	-5	1.0	1.0	1.1	1.0
Ush2A	Q2QI47	3272	-11	-3	9	12	0.9	1.0	1.1	1.1
Ush2A	Q2QI47	3258	-11	-3	9	12	0.9	1.0	1.1	1.1
Ush2A	Q2QI47	3273	-11	-3	9	12	0.9	1.0	1.1	1.1
Polr2c	P97760	94	-20	-7	9	16	0.8	0.9	1.1	1.2
Ptpn1	P35821	32	2	-7	9	11	1.0	0.9	1.1	1.1
Rbm26	Q6NZN0	53	-5	-7	9	10	1.0	0.9	1.1	1.1
Tdp2	Q9JJX7	303	3	-9	9	8	1.0	0.9	1.1	1.1
Arhgdia	Q99PT1	79	-11	-9	9	3	0.9	0.9	1.1	1.0
Dock8	Q8C147	121	1	-9	9	-5	1.0	0.9	1.1	1.0
Tmem33	Q9CR67	224	-10	-10	9	8	0.9	0.9	1.1	1.1
Scly	Q9JLI6	101	-9	-10	9	-15	0.9	0.9	1.1	0.9
Tceb3	Q8CB77	583	-2	-12	9	17	1.0	0.9	1.1	1.2
Kntc1	Q8C3Y4	1786	-11	-13	9	42	0.9	0.9	1.1	1.7

Pfas	Q5SUR0	270	0	-13	9	65	1.0	0.9	1.1	2.8
Ascc3	E9PZJ8	1316	19	-13	9	14	1.2	0.9	1.1	1.2
Jak2	G5E852	243	-10	-14	9	58	0.9	0.9	1.1	2.4
Vezf1	Q5SXC4	263	2	-14	9	15	1.0	0.9	1.1	1.2
Fhl3	Q9R059	191	-8	-14	9	15	0.9	0.9	1.1	1.2
Rb1	P13405	153	-14	-14	9	4	0.9	0.9	1.1	1.0
Prpf19	Q99KP6	298	-27	-14	9	2	0.8	0.9	1.1	1.0
Dmap1	Q9JI44	199	3	-19	9	-12	1.0	0.8	1.1	0.9
Nol10	Q5RJG1	216	-25	-19	9	0	0.8	0.8	1.1	1.0
Chordc1	Q9D1P4	59	3	-21	9	9	1.0	0.8	1.1	1.1
Padi2	Q08642	346	22	-24	9	19	1.3	0.8	1.1	1.2
Fhl3	Q9R059	10	-12	-25	9	8	0.9	0.8	1.1	1.1
Snx2	Q9CWK8	332	-19	28	8	12	0.8	1.4	1.1	1.1
Casp1	P29452	362	13	16	8	15	1.1	1.2	1.1	1.2
Cluh	Q5SW19	755	-1	15	8	15	1.0	1.2	1.1	1.2
Rnf123	Q5XPI3	1180	-2	13	8	12	1.0	1.1	1.1	1.1
Pck2	Q8BH04	325	-3	12	8	-6	1.0	1.1	1.1	0.9
Vars	Q9Z1Q9	662	1	11	8	12	1.0	1.1	1.1	1.1
H2-DMb2	Q31099	97	9	10	8	14	1.1	1.1	1.1	1.2
Actl6a	Q9Z2N8	423	-7	10	8	3	0.9	1.1	1.1	1.0
Actb	P60710	217	5	8	8	1	1.1	1.1	1.1	1.0
Dffb	O54788	307	-10	8	8	-16	0.9	1.1	1.1	0.9
Psmc1	P62192	58	5	8	8	26	1.0	1.1	1.1	1.4
Syne1	Q6ZWR6	5430	12	8	8	-4	1.1	1.1	1.1	1.0
Abcf3	Q8K268	102	4	7	8	14	1.0	1.1	1.1	1.2
Pax5	Q02650	124	13	7	8	16	1.1	1.1	1.1	1.2
Tcp1	P11983	397	-6	6	8	-82	0.9	1.1	1.1	0.5
Lsg1	Q3UM18	112	8	6	8	14	1.1	1.1	1.1	1.2
Prkd2	Q8BZ03	616	5	4	8	15	1.1	1.0	1.1	1.2
Kiaa0196	Q8C2E7	1110	0	4	8	6	1.0	1.0	1.1	1.1
Rap1a	P62835	141	3	4	8	5	1.0	1.0	1.1	1.1
Exosc10	P56960	93	-5	3	8	24	1.0	1.0	1.1	1.3
Ftsj1	Q8CBC7	278	19	2	8	-12	1.2	1.0	1.1	0.9
Tbcel	Q8C5W3	14	-14	1	8	-8	0.9	1.0	1.1	0.9
Atad3	Q925I1	471	9	1	8	-1	1.1	1.0	1.1	1.0
Fam193a	Q8CGI1	1203	0	1	8	-15	1.0	1.0	1.1	0.9
Fbxl15	Q91W61	139	-7	0	8	16	0.9	1.0	1.1	1.2
Kars	Q99MN1	432	-7	0	8	1	0.9	1.0	1.1	1.0
Akr1b1	P45376	304	-18	-1	8	54	0.9	1.0	1.1	2.2
Eif2s2	Q99L45	300	-6	-2	8	27	0.9	1.0	1.1	1.4
Kdm3b	B9EKS2	905	2	-2	8	20	1.0	1.0	1.1	1.2
Mcm6	P97311	301	-4	-5	8	42	1.0	1.0	1.1	1.7
Cdc27	A2A6Q5	478	5	-6	8	1	1.1	0.9	1.1	1.0
Nudt2	P56380	123	8	-7	8	7	1.1	0.9	1.1	1.1
Coq8b	Q566J8	148	-15	-7	8	32	0.9	0.9	1.1	1.5
Fbxl8	Q8CIG9	228	-1	-7	8	-3	1.0	0.9	1.1	1.0
Trrap	A0A1D5RLL4	950	-2	-8	8	15	1.0	0.9	1.1	1.2
Tradd	Q3U0V2	102	13	-9	8	3	1.1	0.9	1.1	1.0
Pold2	O35654	92	-13	-10	8	14	0.9	0.9	1.1	1.2
Sh3pxd2a	O89032	541	15	-10	8	-10	1.2	0.9	1.1	0.9
Fam160b1	Q8CDM8	298	-19	-11	8	74	0.8	0.9	1.1	3.8

Ptar1	E9QAB6	37	-9	-13	8	-8	0.9	0.9	1.1	0.9
Bcas3	Q8CCN5	836	-21	-13	8	-19	0.8	0.9	1.1	0.8
Tmpo	Q61033	628	-5	-15	8	-11	1.0	0.9	1.1	0.9
Ran	P62827	112	1	-19	8	-47	1.0	0.8	1.1	0.7
Gtf2h3	Q8VD76	256	-1	-20	8	24	1.0	0.8	1.1	1.3
Cutc	Q9D8X1	173	-30	-28	8	-46	0.8	0.8	1.1	0.7
Eif3e	P60229	345	3	26	8	16	1.0	1.4	1.1	1.2
Lyz2	P08905	113	17	23	8	45	1.2	1.3	1.1	1.8
Rraga	Q80X95	157	14	18	8	31	1.2	1.2	1.1	1.4
Pgm2	Q7TSV4	94	6	16	8	-3	1.1	1.2	1.1	1.0
Zfp273	Q7M6W8	344	3	13	8	30	1.0	1.1	1.1	1.4
Ap1g1	P22892	392	17	10	8	24	1.2	1.1	1.1	1.3
Gmeb1	Q9JL60	264	10	10	8	14	1.1	1.1	1.1	1.2
Atic	Q9CWJ9	325	-7	9	8	19	0.9	1.1	1.1	1.2
Rasa4	Q6PFQ7	301	-1	9	8	10	1.0	1.1	1.1	1.1
Stk11ip	Q3TAA7	138	-20	8	8	-2	0.8	1.1	1.1	1.0
Inpp5d	Q9ES52	388	-7	7	8	33	0.9	1.1	1.1	1.5
Polr2a	P08775	1287	-7	6	8	8	0.9	1.1	1.1	1.1
Fnbp4	Q6ZQ03	112	8	6	8	4	1.1	1.1	1.1	1.0
Hk3	Q3TRM8	169	-15	6	8	-6	0.9	1.1	1.1	0.9
Parg	O88622	596	-10	5	8	4	0.9	1.1	1.1	1.0
Celf2	Q9Z0H4	161	-7	3	8	13	0.9	1.0	1.1	1.1
Rack1	P68040	240	-3	3	8	13	1.0	1.0	1.1	1.1
Luc7l2	Q7TNC4	190	-9	2	8	30	0.9	1.0	1.1	1.4
Wdr37	Q8CBE3	146	-2	2	8	27	1.0	1.0	1.1	1.4
Trim5	E9PV98	48	4	2	8	5	1.0	1.0	1.1	1.0
Trim12c	D3Z3L3	48	4	2	8	5	1.0	1.0	1.1	1.0
Cct2	P80314	395	-11	2	8	-20	0.9	1.0	1.1	0.8
Mblac2	Q8BL86	66	0	1	8	-13	1.0	1.0	1.1	0.9
Pitpnm2	Q6ZPQ6	888	10	0	8	-3	1.1	1.0	1.1	1.0
Cog1	Q9Z160	510	-9	-1	8	68	0.9	1.0	1.1	3.1
Utp18	Q5SSI6	423	0	-2	8	6	1.0	1.0	1.1	1.1
Sp140	Q6NSQ5	361	6	-3	8	28	1.1	1.0	1.1	1.4
Pitpnm1	O35954	888	-5	-3	8	2	1.0	1.0	1.1	1.0
Impdh2	P24547	140	-7	-3	8	55	0.9	1.0	1.1	2.2
Mars	Q68FL6	670	-6	-3	8	-2	0.9	1.0	1.1	1.0
Inpp5b	Q8K337	149	9	-4	8	5	1.1	1.0	1.1	1.0
Nosip	Q9D6T0	250	-18	-5	8	36	0.8	1.0	1.1	1.6
Ddx1	Q91VR5	122	-16	-6	8	7	0.9	0.9	1.1	1.1
Dnajc1	Q61712	533	-9	-6	8	-4	0.9	0.9	1.1	1.0
Pola1	P33609	1358	1	-7	8	16	1.0	0.9	1.1	1.2
Atp2b1	G5E829	60	19	-8	8	-3	1.2	0.9	1.1	1.0
Ppia	P17742	115	-1	-8	8	-16	1.0	0.9	1.1	0.9
Znf706	Q9D115	41	-8	-9	8	-9	0.9	0.9	1.1	0.9
Flna	Q8BTM8	1312	-16	-9	8	-12	0.9	0.9	1.1	0.9
Prg2	Q61878	192	-22	-10	8	-17	0.8	0.9	1.1	0.9
Zfp433	A0A0J9YUD4	89	2	-10	8	24	1.0	0.9	1.1	1.3
Rbl1	Q64701	247	-12	-12	8	-5	0.9	0.9	1.1	1.0
Rnf40	Q3U319	986	-18	-12	8	-8	0.8	0.9	1.1	0.9
Copb1	Q9JIF7	390	-28	-13	8	-13	0.8	0.9	1.1	0.9
Ube2d2	P62838	85	-11	-13	8	-26	0.9	0.9	1.1	0.8

Bdh1	Q80XN0	288	-13	-15	8	-15	0.9	0.9	1.1	0.9
Adat1	Q9JHI2	258	-3	-18	8	-1	1.0	0.9	1.1	1.0
Smchd1	Q6P5D8	1857	-23	-20	8	4	0.8	0.8	1.1	1.0
Alms1	Q8K4E0	2713	-1	-20	8	-26	1.0	0.8	1.1	0.8
Txn	P10639	46	-16	-22	8	-27	0.9	0.8	1.1	0.8
Trappc8	E9PWG2	1074	9	19	7	11	1.1	1.2	1.1	1.1
Sart3	Q9JLI8	342	5	16	7	10	1.0	1.2	1.1	1.1
Sympk	Q80X82	848	-4	15	7	10	1.0	1.2	1.1	1.1
Urod	P70697	65	10	10	7	15	1.1	1.1	1.1	1.2
Dhx15	O35286	774	-3	9	7	18	1.0	1.1	1.1	1.2
Dpm3	Q9D1Q4	67	5	9	7	15	1.0	1.1	1.1	1.2
Morc3	F7BJB9	307	-13	7	7	-8	0.9	1.1	1.1	0.9
Fes	P16879	785	-11	6	7	35	0.9	1.1	1.1	1.5
Uncharact	Q8C5K5	60	-20	5	7	3	0.8	1.1	1.1	1.0
Nup205	A0A0J9YUD5	1350	-4	5	7	5	1.0	1.0	1.1	1.1
Ipo5	Q8BKC5	733	-8	4	7	33	0.9	1.0	1.1	1.5
Stk39	Q9Z1W9	249	1	4	7	16	1.0	1.0	1.1	1.2
Card19	Q9D1I2	106	-19	4	7	14	0.8	1.0	1.1	1.2
Rab19	P35294	146	15	4	7	8	1.2	1.0	1.1	1.1
Rnf114	Q9ET26	68	-25	4	7	1	0.8	1.0	1.1	1.0
Usp10	P52479	450	-8	4	7	1	0.9	1.0	1.1	1.0
Polr2b	Q8CFI7	1122	6	3	7	8	1.1	1.0	1.1	1.1
Nubp1	Q9R060	256	4	3	7	12	1.0	1.0	1.1	1.1
Rp2	Q9EPK2	127	-17	2	7	29	0.9	1.0	1.1	1.4
Cad	B2RQC6	2161	-10	2	7	4	0.9	1.0	1.1	1.0
Casp8	O89110	211	-3	2	7	-1	1.0	1.0	1.1	1.0
Atad3	Q925I1	514	-4	1	7	-4	1.0	1.0	1.1	1.0
Dnmt3a	O88508	706	-16	1	7	14	0.9	1.0	1.1	1.2
Fn3krp	Q8K274	24	-6	0	7	47	0.9	1.0	1.1	1.9
Srsf1	Q6PDM2	148	1	-1	7	5	1.0	1.0	1.1	1.0
Uba1	Q02053	179	-1	-1	7	13	1.0	1.0	1.1	1.1
Vdac2	Q60930	228	-7	-2	7	44	0.9	1.0	1.1	1.8
Dpf2	Q61103	295	-9	-2	7	14	0.9	1.0	1.1	1.2
Arhgef7	Q9ES28	469	-13	-3	7	56	0.9	1.0	1.1	2.2
Zfp84	Q9D654	456	-3	-3	7	16	1.0	1.0	1.1	1.2
Zmat2	Q9CPW7	87	2	-4	7	-1	1.0	1.0	1.1	1.0
Ranbp2	Q9ERU9	2244	-2	-4	7	6	1.0	1.0	1.1	1.1
Copb1	Q9JIF7	635	-4	-5	7	24	1.0	1.0	1.1	1.3
Bloc1s4	Q8VED2	209	10	-6	7	-6	1.1	0.9	1.1	0.9
Nprl2	Q9WUE4	145	-2	-6	7	-23	1.0	0.9	1.1	0.8
Nt5dc1	Q8C5P5	117	-14	-6	7	53	0.9	0.9	1.1	2.1
Cytip	Q91VY6	103	-14	-6	7	12	0.9	0.9	1.1	1.1
Acot7	Q91V12	289	-9	-7	7	7	0.9	0.9	1.1	1.1
Apobr	Q8VBT6	873	-8	-7	7	-18	0.9	0.9	1.1	0.8
Pdlim5	Q8CI51	474	-8	-8	7	11	0.9	0.9	1.1	1.1
Setd2	E9Q5F9	1494	5	-8	7	3	1.1	0.9	1.1	1.0
Plcg2	Q8CIH5	937	-9	-9	7	4	0.9	0.9	1.1	1.0
Mrps16	Q9CPX7	26	-37	-9	7	-4	0.7	0.9	1.1	1.0
Fhl3	Q9R059	209	-5	-12	7	20	1.0	0.9	1.1	1.3
Mycbp2	E9PUJ6	3464	4	-13	7	17	1.0	0.9	1.1	1.2
Topbp1	Q6ZQF0	22	1	-15	7	16	1.0	0.9	1.1	1.2

Taf5l	Q91WQ5	188	-9	-16	7	55	0.9	0.9	1.1	2.2
Trappc10	F8VQF9	695	-19	-17	7	-10	0.8	0.9	1.1	0.9
Mbnl2	Q8C181	19	-14	-18	7	1	0.9	0.9	1.1	1.0
Mbnl3	Q8R003	20	-14	-18	7	1	0.9	0.9	1.1	1.0
Mbnl1	Q9JKP5	19	-14	-18	7	1	0.9	0.9	1.1	1.0
Cnbp	P53996	98	-3	-24	7	-25	1.0	0.8	1.1	0.8
Ppil4	Q9CXG3	294	9	18	7	19	1.1	1.2	1.1	1.2
Pml	Q60953	266	11	17	7	14	1.1	1.2	1.1	1.2
Nkrf	Q8BY02	453	20	16	7	1	1.3	1.2	1.1	1.0
Fam65b	Q80U16	993	16	14	7	10	1.2	1.2	1.1	1.1
Gpn2	Q8VEJ1	262	8	13	7	10	1.1	1.1	1.1	1.1
Tlk1	Q8C0V0	600	3	13	7	-10	1.0	1.1	1.1	0.9
Slc12a4	Q9JIS8	51	22	12	7	27	1.3	1.1	1.1	1.4
Ptk2b	Q9QVP9	828	9	11	7	13	1.1	1.1	1.1	1.1
Pnn	Q3TUQ5	439	2	10	7	-4	1.0	1.1	1.1	1.0
Zcchc8	Q9CYA6	160	6	9	7	0	1.1	1.1	1.1	1.0
Ptk2b	Q9QVP9	545	-20	8	7	32	0.8	1.1	1.1	1.5
Plcg1	Q62077	1109	-20	8	7	0	0.8	1.1	1.1	1.0
Akap12	Q9WTQ5	1403	10	7	7	11	1.1	1.1	1.1	1.1
Tln1	P26039	1661	5	6	7	44	1.1	1.1	1.1	1.8
Eml2	Q7TNG5	301	23	6	7	9	1.3	1.1	1.1	1.1
Ptbp3	Q8BHD7	219	5	5	7	6	1.0	1.1	1.1	1.1
Sri	Q6P069	163	-5	3	7	22	1.0	1.0	1.1	1.3
Pdcd11	Q6NS46	330	-6	3	7	10	0.9	1.0	1.1	1.1
Mocs3	A2BDX3	316	7	2	7	-4	1.1	1.0	1.1	1.0
Efl1	Q8C0D5	831	-8	2	7	11	0.9	1.0	1.1	1.1
Ilkap	Q8R0F6	367	-4	1	7	9	1.0	1.0	1.1	1.1
Polr2g	P62488	106	-3	1	7	7	1.0	1.0	1.1	1.1
Dfna5	Q9Z2D3	235	-2	0	7	-4	1.0	1.0	1.1	1.0
Ppp2cb	P62715	266	6	-1	7	7	1.1	1.0	1.1	1.1
Ppp2ca	P63330	266	6	-1	7	7	1.1	1.0	1.1	1.1
Ipo8	Q7TMY7	749	0	-2	7	6	1.0	1.0	1.1	1.1
Ipo7	Q9EPL8	749	0	-2	7	6	1.0	1.0	1.1	1.1
Lemd3	D3YU56	910	-2	-2	7	24	1.0	1.0	1.1	1.3
Nfatc2	Q60591	357	5	-2	7	-15	1.1	1.0	1.1	0.9
Parp1	Q921K2	312	-9	-3	7	22	0.9	1.0	1.1	1.3
Uhrf2	Q7TMI3	772	-14	-3	7	21	0.9	1.0	1.1	1.3
Uhrf1	Q8VDF2	751	-14	-3	7	21	0.9	1.0	1.1	1.3
Lrrc8a	Q80WG5	776	-3	-3	7	-8	1.0	1.0	1.1	0.9
Rbbp4	Q60972	138	-6	-3	7	2	0.9	1.0	1.1	1.0
Apaf1	O88879	258	-3	-5	7	13	1.0	1.0	1.1	1.1
Rhoc	Q62159	20	-7	-6	7	35	0.9	0.9	1.1	1.5
Rhoa	Q9QUI0	20	-7	-6	7	35	0.9	0.9	1.1	1.5
Ndufv1	Q91YT0	238	-14	-6	7	15	0.9	0.9	1.1	1.2
Mtmr12	Q80TA6	153	0	-6	7	-4	1.0	0.9	1.1	1.0
Osbpl7	A2A716	374	3	-6	7	-5	1.0	0.9	1.1	1.0
Nln	Q91YP2	153	-3	-6	7	-8	1.0	0.9	1.1	0.9
Tkt	P40142	206	4	-7	7	13	1.0	0.9	1.1	1.1
Ehbp1l1	E9QP49	23	-28	-7	7	-11	0.8	0.9	1.1	0.9
Klrd1	O54707	166	-11	-7	7	8	0.9	0.9	1.1	1.1
Nop56	Q9D6Z1	52	-15	-8	7	-9	0.9	0.9	1.1	0.9

Kpna6	O35345	253	-11	-8	7	27	0.9	0.9	1.1	1.4
Nol10	Q5RJG1	16	-12	-9	7	64	0.9	0.9	1.1	2.8
Htt	G3X9H5	923	3	-9	7	9	1.0	0.9	1.1	1.1
Sh3gl1	Q62419	96	-12	-10	7	19	0.9	0.9	1.1	1.2
Phkb	Q7TSH2	272	-6	-10	7	1	0.9	0.9	1.1	1.0
Znf638	Q61464	1020	-9	-12	7	55	0.9	0.9	1.1	2.2
Acp2	P24638	349	-9	-12	7	-5	0.9	0.9	1.1	1.0
Dusp22	Q99N11	124	-10	-14	7	54	0.9	0.9	1.1	2.2
Ptpn7	Q8BUM3	204	-14	-14	7	-26	0.9	0.9	1.1	0.8
Nmi	O35309	296	-9	-16	7	12	0.9	0.9	1.1	1.1
Zc3h14	Q8BJ05	613	12	-19	7	3	1.1	0.8	1.1	1.0
Zyx	Q62523	436	-19	-20	7	-2	0.8	0.8	1.1	1.0
Lgals9	O08573	258	-8	-21	7	15	0.9	0.8	1.1	1.2
Hdac3	O88895	218	-9	-25	7	15	0.9	0.8	1.1	1.2
Ankle2	Q6P1H6	715	-13	-38	7	-12	0.9	0.7	1.1	0.9
Txnl1	Q8CDN6	135	-8	18	6	12	0.9	1.2	1.1	1.1
Pcbp1	P60335	194	8	14	6	12	1.1	1.2	1.1	1.1
Acaa1a	Q921H8	26	5	10	6	4	1.1	1.1	1.1	1.0
Pag1	Q3U1F9	181	-1	10	6	-11	1.0	1.1	1.1	0.9
Acaa1a	Q921H8	177	12	9	6	13	1.1	1.1	1.1	1.1
Tsr1	Q5SWD9	126	6	9	6	17	1.1	1.1	1.1	1.2
Prpf4	Q9DAW6	298	-22	8	6	1	0.8	1.1	1.1	1.0
Prmt5	Q8CIG8	22	-4	8	6	-6	1.0	1.1	1.1	0.9
Sp2	Q8C5J0	505	1	7	6	14	1.0	1.1	1.1	1.2
Slc9a3r1	P70441	201	2	7	6	7	1.0	1.1	1.1	1.1
Mettl15	Q9DCL4	52	-3	7	6	-8	1.0	1.1	1.1	0.9
Usp7	E9PXY8	762	-10	6	6	15	0.9	1.1	1.1	1.2
Ddx58	Q6Q899	264	2	6	6	13	1.0	1.1	1.1	1.1
Med23	Q80YQ2	1319	0	6	6	12	1.0	1.1	1.1	1.1
Chtf8	P0CG14	173	11	6	6	9	1.1	1.1	1.1	1.1
Psma5	Q9Z2U1	76	-4	5	6	16	1.0	1.0	1.1	1.2
Pdha1	P35486	222	-5	5	6	14	1.0	1.0	1.1	1.2
Noc2l	J3QK52	571	-6	5	6	4	0.9	1.0	1.1	1.0
Hnrnpu	Q8VEK3	538	-4	3	6	32	1.0	1.0	1.1	1.5
Hps1	O08983	180	-4	3	6	15	1.0	1.0	1.1	1.2
Kifc5b	E9PUA5	270	-3	2	6	-2	1.0	1.0	1.1	1.0
Kifc1	Q9QWT9	272	-3	2	6	-2	1.0	1.0	1.1	1.0
Cnot1	Q6ZQ08	2359	0	1	6	23	1.0	1.0	1.1	1.3
Crot	Q9DC50	584	3	1	6	0	1.0	1.0	1.1	1.0
Mthfd1	Q922D8	195	-11	1	6	-2	0.9	1.0	1.1	1.0
Atp5a1	Q03265	294	-8	-1	6	9	0.9	1.0	1.1	1.1
Mapkapk2	P49138	126	-5	-1	6	1	1.0	1.0	1.1	1.0
Tyk2	E9QJS1	1160	-7	-1	6	35	0.9	1.0	1.1	1.5
Itpkc	Q7TS72	429	-2	-1	6	18	1.0	1.0	1.1	1.2
Coro2a	Q8C0P5	335	-4	-1	6	-1	1.0	1.0	1.1	1.0
Rttn	Q8R4Y8	755	-5	-2	6	49	1.0	1.0	1.1	1.9
Tut1	Q8R3F9	569	-1	-2	6	21	1.0	1.0	1.1	1.3
Kmt2c	Q8BRH4	4367	8	-2	6	14	1.1	1.0	1.1	1.2
Crebbp	F8VPR5	408	-3	-2	6	6	1.0	1.0	1.1	1.1
Mki67	E9PVX6	2116	5	-2	6	-7	1.0	1.0	1.1	0.9
Pou2f1	P25425	506	-3	-2	6	15	1.0	1.0	1.1	1.2

Zc3h15	Q3TIV5	105	-2	-4	6	10	1.0	1.0	1.1	1.1
Mccc2	Q3ULD5	167	-7	-4	6	9	0.9	1.0	1.1	1.1
Gfm2	Q8R2Q4	601	2	-5	6	-1	1.0	1.0	1.1	1.0
Pdlim1	O70400	284	6	-5	6	-3	1.1	1.0	1.1	1.0
Ube2d2	P62838	111	-1	-5	6	-7	1.0	1.0	1.1	0.9
Pom121	Q8K3Z9	192	8	-6	6	18	1.1	0.9	1.1	1.2
Thumpd3	P97770	241	-19	-7	6	10	0.8	0.9	1.1	1.1
Eif3i	Q9QZD9	76	-31	-8	6	-2	0.8	0.9	1.1	1.0
Hmbs	P22907	120	-1	-8	6	9	1.0	0.9	1.1	1.1
Sp4	K4DI62	625	-2	-9	6	2	1.0	0.9	1.1	1.0
Arid1a	A2BH40	1106	-5	-10	6	-3	1.0	0.9	1.1	1.0
Pde3b	E9QLQ3	299	5	-12	6	27	1.0	0.9	1.1	1.4
Tcea1	P10711	263	-10	-12	6	17	0.9	0.9	1.1	1.2
Nub1	P54729	316	-4	-14	6	-13	1.0	0.9	1.1	0.9
Pfkfb2	A0A087WRM	107	4	-15	6	-3	1.0	0.9	1.1	1.0
Irf1	P15314	53	-1	-16	6	15	1.0	0.9	1.1	1.2
Sp140	Q6NSQ5	439	-10	-16	6	-8	0.9	0.9	1.1	0.9
Copg2	Q9QXK3	44	-1	-17	6	9	1.0	0.9	1.1	1.1
Mta2	Q9R190	44	-11	-22	6	-7	0.9	0.8	1.1	0.9
Ngly1	Q9JI78	250	-6	-24	6	-12	0.9	0.8	1.1	0.9
Znf768	Q8R0T2	409	-28	-34	6	-17	0.8	0.7	1.1	0.9
Cyfip2	Q5SQX6	1086	14	27	6	26	1.2	1.4	1.1	1.3
Cyfip1	Q7TMB8	1087	14	27	6	26	1.2	1.4	1.1	1.3
Tnpo1	Q8BFY9	164	-9	20	6	2	0.9	1.3	1.1	1.0
Limd2	Q8BGB5	65	-23	18	6	10	0.8	1.2	1.1	1.1
Lrba	E9Q3Y4	289	-2	15	6	17	1.0	1.2	1.1	1.2
Ncapd2	Q8K2Z4	806	-14	14	6	36	0.9	1.2	1.1	1.6
Pde12	Q3TIU4	267	4	12	6	13	1.0	1.1	1.1	1.1
Suox	Q8R086	300	10	11	6	-6	1.1	1.1	1.1	0.9
Pdia6	Q3TML0	296	-6	9	6	13	0.9	1.1	1.1	1.1
Utp4	Q8R2N2	401	-4	7	6	2	1.0	1.1	1.1	1.0
Pes1	Q9EQ61	360	-2	6	6	19	1.0	1.1	1.1	1.2
Hspa2	P17156	191	11	6	6	3	1.1	1.1	1.1	1.0
Fcho1	Q8K285	631	25	6	6	2	1.3	1.1	1.1	1.0
Rps4x	P62702	181	-8	5	6	16	0.9	1.1	1.1	1.2
Sdha	Q8K2B3	536	-7	5	6	6	0.9	1.1	1.1	1.1
Gnpnat1	Q9JK38	113	13	5	6	-24	1.1	1.1	1.1	0.8
Golga4	Q91VW5	873	-7	5	6	3	0.9	1.0	1.1	1.0
Uba1	Q02053	494	12	4	6	22	1.1	1.0	1.1	1.3
Fxr1	Q61584	157	-6	4	6	36	0.9	1.0	1.1	1.6
Gpi	P06745	404	-8	3	6	13	0.9	1.0	1.1	1.1
Rufy1	Q8BIJ7	548	-4	3	6	-11	1.0	1.0	1.1	0.9
Irf2bp1	Q8R3Y8	239	5	2	6	72	1.1	1.0	1.1	3.5
Ahcyl2	Q68FL4	376	-8	2	6	19	0.9	1.0	1.1	1.2
Ahcyl1	Q80SW1	293	-8	2	6	19	0.9	1.0	1.1	1.2
Arid1b	E9Q4N7	1927	8	2	6	9	1.1	1.0	1.1	1.1
Casp3	P70677	264	-8	1	6	26	0.9	1.0	1.1	1.4
Gstp2	P46425	48	-16	-1	6	46	0.9	1.0	1.1	1.8
Sirt5	Q8K2C6	181	-3	-1	6	17	1.0	1.0	1.1	1.2
Cenpc	P49452	430	-4	-1	6	-6	1.0	1.0	1.1	0.9
Commd5	Q8R395	64	-8	-2	6	17	0.9	1.0	1.1	1.2

Rap1b	Q99JI6	141	-23	-2	6	4	0.8	1.0	1.1	1.0
Eef2	P58252	466	-11	-2	6	13	0.9	1.0	1.1	1.1
Smc4	Q8CG47	940	-1	-2	6	9	1.0	1.0	1.1	1.1
Pdcd11	Q6NS46	599	3	-3	6	-2	1.0	1.0	1.1	1.0
Acaa2	Q8BWT1	128	-11	-3	6	20	0.9	1.0	1.1	1.3
Pafah1b3	Q61205	206	-4	-3	6	-12	1.0	1.0	1.1	0.9
Rnf213	E9Q555	2385	10	-4	6	18	1.1	1.0	1.1	1.2
Psmd10	Q9Z2X2	180	-17	-4	6	22	0.9	1.0	1.1	1.3
Dnajc7	Q9QYI3	58	-7	-5	6	8	0.9	1.0	1.1	1.1
Ctc1	Q5SUQ9	434	4	-5	6	17	1.0	1.0	1.1	1.2
Ptk2b	Q9QVP9	298	3	-6	6	17	1.0	0.9	1.1	1.2
Ripk1	Q60855	257	2	-6	6	12	1.0	0.9	1.1	1.1
Rnf20	Q5DTM8	344	7	-6	6	-5	1.1	0.9	1.1	1.0
Mpi	Q924M7	289	-3	-8	6	-1	1.0	0.9	1.1	1.0
Dync1h1	Q9JHU4	3938	-17	-8	6	8	0.9	0.9	1.1	1.1
Hat1	Q8BY71	24	6	-9	6	22	1.1	0.9	1.1	1.3
Drg2	Q9QXB9	270	6	-11	6	27	1.1	0.9	1.1	1.4
Cxxc1	Q9CWW7	424	-9	-11	6	10	0.9	0.9	1.1	1.1
Exosc7	Q9D0M0	199	-9	-11	6	10	0.9	0.9	1.1	1.1
Bzw2	Q91VK1	270	-18	-11	6	2	0.8	0.9	1.1	1.0
Mcm3ap	Q9WUU9	1277	-15	-12	6	14	0.9	0.9	1.1	1.2
Mical1	Q8VDP3	697	-1	-12	6	4	1.0	0.9	1.1	1.0
Jade2	Q6ZQF7	361	4	-13	6	-9	1.0	0.9	1.1	0.9
Exosc6	Q8BTW3	259	-17	-15	6	48	0.9	0.9	1.1	1.9
Nr2c2	P49117	153	-16	-16	6	-1	0.9	0.9	1.1	1.0
Hsp90aa1	P07901	599	-23	-19	6	4	0.8	0.8	1.1	1.0
Cand1	Q6ZQ38	942	1	-20	6	-13	1.0	0.8	1.1	0.9
Abcg3	Q99P81	18	7	-22	6	-22	1.1	0.8	1.1	0.8
Znf148	Q61624	14	1	-27	6	-1	1.0	0.8	1.1	1.0
Dnpep	Q9Z2W0	411	-13	-31	6	-24	0.9	0.8	1.1	0.8
Svil	Q8K4L3	1542	-47	-41	6	-3	0.7	0.7	1.1	1.0
Sdha	Q8K2B3	654	-19	15	5	18	0.8	1.2	1.1	1.2
Rab17	P35292	212	8	13	5	-2	1.1	1.1	1.1	1.0
Ttc4	Q8R3H9	375	15	10	5	20	1.2	1.1	1.1	1.2
Aldh9a1	Q9JLJ2	267	5	9	5	22	1.0	1.1	1.1	1.3
Gphn	Q8BUV3	26	3	8	5	12	1.0	1.1	1.1	1.1
Mtap	Q9CQ65	145	-9	7	5	17	0.9	1.1	1.1	1.2
Pola2	P33611	392	14	6	5	21	1.2	1.1	1.1	1.3
Plekha2	Q9ERS5	11	-17	6	5	4	0.9	1.1	1.1	1.0
Brd1	E9PZ26	393	-3	6	5	-6	1.0	1.1	1.1	0.9
Wasf2	Q8BH43	55	-5	4	5	-1	1.0	1.0	1.1	1.0
Ssrp1	Q08943	207	-15	3	5	20	0.9	1.0	1.1	1.3
Nmd3	Q99L48	55	-2	2	5	-1	1.0	1.0	1.1	1.0
Ncapg2	Q6DFV1	1032	4	2	5	-4	1.0	1.0	1.1	1.0
Myh9	Q8VDD5	789	-3	1	5	31	1.0	1.0	1.1	1.4
Sars	P26638	398	-3	1	5	18	1.0	1.0	1.1	1.2
Ankrd44	B2RXR6	122	-6	0	5	14	0.9	1.0	1.1	1.2
Arhgap4	B1AUY3	351	5	0	5	-4	1.0	1.0	1.1	1.0
Dhx34	Q9DBV3	39	-11	-1	5	-7	0.9	1.0	1.1	0.9
Sephs1	Q8BH69	71	-7	-1	5	37	0.9	1.0	1.1	1.6
Arhgef2	Q60875	384	5	-2	5	15	1.0	1.0	1.1	1.2

Themis2	Q91YX0	289	-16	-2	5	2	0.9	1.0	1.1	1.0
Rbmx2	Q8R0F5	186	-7	-2	5	-5	0.9	1.0	1.1	1.0
Cdt1	Q8R4E9	26	-11	-2	5	-19	0.9	1.0	1.1	0.8
Kctd14	A0A0U1RNG7	251	-7	-2	5	4	0.9	1.0	1.1	1.0
Inpp5b	Q8K337	597	4	-2	5	-6	1.0	1.0	1.1	0.9
Baz1a	O88379	548	3	-3	5	11	1.0	1.0	1.1	1.1
Psmg1	Q9JK23	140	-29	-3	5	-13	0.8	1.0	1.1	0.9
Phip	F8VQ93	954	2	-3	5	41	1.0	1.0	1.1	1.7
Prdx3	P20108	230	-4	-3	5	36	1.0	1.0	1.1	1.6
Man2c1	Q91W89	29	-8	-4	5	47	0.9	1.0	1.1	1.9
Mcm9	Q2KHI9	106	-1	-4	5	44	1.0	1.0	1.1	1.8
Abhd5	Q9DBL9	301	-10	-5	5	54	0.9	1.0	1.1	2.2
Sord	Q64442	106	-4	-5	5	15	1.0	1.0	1.1	1.2
Cenph	Q9QYM8	16	-1	-5	5	-9	1.0	1.0	1.1	0.9
Gtpbp2	Q3UJK4	321	-23	-6	5	-8	0.8	0.9	1.1	0.9
Nr3c1	E9PYV1	654	-8	-6	5	58	0.9	0.9	1.1	2.4
Ranbp2	Q9ERU9	2900	-2	-6	5	3	1.0	0.9	1.1	1.0
Tbca	P48428	67	-1	-6	5	-7	1.0	0.9	1.1	0.9
Cdc40	Q9DC48	378	-6	-7	5	11	0.9	0.9	1.1	1.1
Med16	Q6PGF3	571	-8	-7	5	-25	0.9	0.9	1.1	0.8
Nop14	Q8R3N1	583	-13	-7	5	17	0.9	0.9	1.1	1.2
Map2k4	P47809	380	-7	-7	5	1	0.9	0.9	1.1	1.0
Nvl	Q9DBY8	686	6	-8	5	63	1.1	0.9	1.1	2.7
Nup85	Q8R480	226	7	-8	5	-2	1.1	0.9	1.1	1.0
Ap1g1	P22892	106	-4	-8	5	-3	1.0	0.9	1.1	1.0
Ubr5	E9Q2H1	2084	-10	-9	5	41	0.9	0.9	1.1	1.7
Srsf9	Q9D0B0	139	-2	-9	5	0	1.0	0.9	1.1	1.0
Msn	P26041	284	-12	-10	5	5	0.9	0.9	1.1	1.0
Rdx	P26043	284	-12	-10	5	5	0.9	0.9	1.1	1.0
Zfand3	Q497H0	118	4	-10	5	2	1.0	0.9	1.1	1.0
Tank	P70347	210	2	-10	5	-12	1.0	0.9	1.1	0.9
Cxxc1	Q9CWW7	570	-12	-10	5	26	0.9	0.9	1.1	1.4
Mdn1	A2ANY6	4032	4	-10	5	-3	1.0	0.9	1.1	1.0
Ctr9	Q62018	817	-4	-10	5	-5	1.0	0.9	1.1	1.0
Trafd1	Q3UDK1	109	-7	-11	5	11	0.9	0.9	1.1	1.1
Ccdc22	Q9JIG7	222	-4	-12	5	1	1.0	0.9	1.1	1.0
Prkcq	Q02111	661	1	-12	5	-10	1.0	0.9	1.1	0.9
Rock2	F8VPK5	247	-7	-14	5	3	0.9	0.9	1.1	1.0
Shmt2	Q9CZN7	119	-15	-14	5	20	0.9	0.9	1.1	1.2
Nmt2	O70311	135	-15	-14	5	-7	0.9	0.9	1.1	0.9
Znf280d	Q68FE8	499	-14	-15	5	-18	0.9	0.9	1.1	0.8
Ubxn1	Q922Y1	214	-9	-16	5	-7	0.9	0.9	1.1	0.9
Nsd1	E9QAE4	2274	2	-17	5	-4	1.0	0.9	1.1	1.0
Pepd	Q11136	158	-28	-21	5	-1	0.8	0.8	1.1	1.0
Usp3	Q91W36	98	-11	-27	5	8	0.9	0.8	1.1	1.1
Zfp85	B8JJY3	497	-16	-28	5	-39	0.9	0.8	1.1	0.7
Znf592	Q8BHZ4	769	2	24	5	32	1.0	1.3	1.0	1.5
Ctbp1	O88712	38	-5	17	5	13	1.0	1.2	1.0	1.1
Ctbp2	P56546	44	-5	17	5	13	1.0	1.2	1.0	1.1
Erp29	P57759	159	7	12	5	19	1.1	1.1	1.0	1.2
Hdac10	Q6P3E7	145	-5	11	5	11	1.0	1.1	1.0	1.1

Acad9	Q8JZN5	331	-2	10	5	27	1.0	1.1	1.0	1.4
Tk1	P04184	156	0	10	5	50	1.0	1.1	1.0	2.0
Syk	P48025	587	-5	9	5	34	1.0	1.1	1.0	1.5
Trrap	A0A1D5RLL4	3555	22	9	5	18	1.3	1.1	1.0	1.2
Cwc25	Q9DBF7	107	6	9	5	12	1.1	1.1	1.0	1.1
Mpp1	P70290	94	0	9	5	-25	1.0	1.1	1.0	0.8
Ehmt2	Q9Z148	481	-8	8	5	3	0.9	1.1	1.0	1.0
Nbeal2	Q6ZQA0	389	11	7	5	20	1.1	1.1	1.0	1.3
Trim56	Q80VI1	41	8	6	5	4	1.1	1.1	1.0	1.0
Rabif	Q91X96	106	9	5	5	-4	1.1	1.1	1.0	1.0
Inpp5f	Q8CDA1	893	-11	5	5	19	0.9	1.0	1.0	1.2
Eri3	Q8C460	285	-10	4	5	2	0.9	1.0	1.0	1.0
Ap3d1	O54774	1149	10	4	5	17	1.1	1.0	1.0	1.2
Nlrc3	Q5DU56	858	17	3	5	12	1.2	1.0	1.0	1.1
Rabepk	Q8VCH5	31	5	2	5	29	1.1	1.0	1.0	1.4
Kcnab2	P62482	248	-5	2	5	36	1.0	1.0	1.0	1.6
Lrrk2	Q5S006	804	-1	2	5	4	1.0	1.0	1.0	1.0
Uba3	Q8C878	139	-7	1	5	-6	0.9	1.0	1.0	0.9
Ipo5	Q8BKC5	972	-3	1	5	30	1.0	1.0	1.0	1.4
Gtpbp2	Q3UJK4	450	-9	1	5	-1	0.9	1.0	1.0	1.0
Herc4	Q6PAV2	392	17	-1	5	-5	1.2	1.0	1.0	1.0
Sash1	F8VQK5	1103	-4	-1	5	2	1.0	1.0	1.0	1.0
Mia3	J3KMH5	364	4	-2	5	8	1.0	1.0	1.0	1.1
Prkar1a	Q9DBC7	39	-5	-2	5	6	1.0	1.0	1.0	1.1
Smg6	P61406	97	-7	-2	5	2	0.9	1.0	1.0	1.0
Itpr2	Q9Z329	433	-4	-2	5	-10	1.0	1.0	1.0	0.9
Bmx	P97504	518	-8	-2	5	8	0.9	1.0	1.0	1.1
Copa	Q8CIE6	245	-17	-2	5	-7	0.9	1.0	1.0	0.9
Dync1h1	Q9JHU4	4119	1	-2	5	-14	1.0	1.0	1.0	0.9
Ctcf	Q61164	500	-1	-4	5	13	1.0	1.0	1.0	1.1
Kmt2b	O08550	1312	-7	-4	5	11	0.9	1.0	1.0	1.1
Trafd1	Q3UDK1	461	-7	-4	5	-12	0.9	1.0	1.0	0.9
Bud31	Q6PGH1	78	-8	-4	5	26	0.9	1.0	1.0	1.4
Nup160	Q9Z0W3	571	-7	-4	5	23	0.9	1.0	1.0	1.3
Zbtb1	Q91VL9	450	-4	-4	5	-1	1.0	1.0	1.0	1.0
Dnm1l	Q8K1M6	373	-22	-5	5	61	0.8	1.0	1.0	2.6
Trim28	Q62318	125	-7	-5	5	4	0.9	1.0	1.0	1.0
Jak3	Q62137	835	-1	-5	5	-5	1.0	1.0	1.0	1.0
Cse1l	Q9ERK4	939	-8	-5	5	14	0.9	1.0	1.0	1.2
Cep135	Q6P5D4	356	6	-6	5	10	1.1	0.9	1.0	1.1
Arih1	Q9Z1K5	302	-9	-6	5	12	0.9	0.9	1.0	1.1
Pmvk	Q9D1G2	38	-8	-7	5	31	0.9	0.9	1.0	1.4
Nat10	Q8K224	194	2	-7	5	29	1.0	0.9	1.0	1.4
Nbeal2	Q6ZQA0	1615	-35	-8	5	-5	0.7	0.9	1.0	1.0
Malt1	Q2TBA3	449	-8	-8	5	4	0.9	0.9	1.0	1.0
Trabd	Q99JY4	107	-10	-9	5	18	0.9	0.9	1.0	1.2
Slc9a1	Q61165	799	-2	-10	5	-31	1.0	0.9	1.0	0.8
Mettl6	Q8BVH9	256	-7	-10	5	17	0.9	0.9	1.0	1.2
Spata6	Q3U6K5	352	2	-10	5	-5	1.0	0.9	1.0	1.0
Pcyt2	Q922E4	324	-1	-11	5	12	1.0	0.9	1.0	1.1
Otud4	B2RRE7	45	-4	-12	5	13	1.0	0.9	1.0	1.1

Knstrn	Q9D9Z1	214	-12	-13	5	-4	0.9	0.9	1.0	1.0
Col4a5	Q63ZW6	1515	25	-16	5	17	1.3	0.9	1.0	1.2
Fam45a	Q9D8N2	135	-5	-19	5	11	1.0	0.8	1.0	1.1
Abtb2	Q7TQI7	361	-35	-21	5	80	0.7	0.8	1.0	4.9
Gars	Q9CZD3	362	-20	-26	5	-17	0.8	0.8	1.0	0.9
Stxbp3	Q60770	114	-9	21	4	14	0.9	1.3	1.0	1.2
Spg20	Q8R1X6	508	16	20	4	49	1.2	1.3	1.0	2.0
Mcm6	P97311	721	-8	16	4	11	0.9	1.2	1.0	1.1
Srp54	P14576	36	7	14	4	-14	1.1	1.2	1.0	0.9
Baz2a	E9Q374	907	-3	11	4	3	1.0	1.1	1.0	1.0
Gtpbp3	Q923K4	419	-7	10	4	-21	0.9	1.1	1.0	0.8
Pfkl	P12382	351	3	10	4	18	1.0	1.1	1.0	1.2
Hus1	Q8BQY8	159	1	6	4	5	1.0	1.1	1.0	1.0
Gm45140	A0A0N4SUM8	28	-1	6	4	-2	1.0	1.1	1.0	1.0
Wrnip1	Q91XU0	267	-9	4	4	23	0.9	1.0	1.0	1.3
Fcho1	Q8K285	555	-11	4	4	2	0.9	1.0	1.0	1.0
Stambpl1	Q76N33	268	-6	4	4	-12	0.9	1.0	1.0	0.9
Ppp1r12c	Q3UMT1	61	6	4	4	12	1.1	1.0	1.0	1.1
Wdr77	Q99J09	26	6	3	4	5	1.1	1.0	1.0	1.1
Scaper	F8VQ70	71	4	3	4	-9	1.0	1.0	1.0	0.9
Lrmp	G5E880	271	-4	3	4	-2	1.0	1.0	1.0	1.0
Gphn	Q8BUV3	502	2	3	4	-10	1.0	1.0	1.0	0.9
Mtx1	F7C846	375	-28	2	4	-27	0.8	1.0	1.0	0.8
Hadha	Q8BMS1	156	9	2	4	38	1.1	1.0	1.0	1.6
G6pdx	Q00612	232	-19	2	4	-3	0.8	1.0	1.0	1.0
Polr1a	O35134	185	12	1	4	15	1.1	1.0	1.0	1.2
Dync1h1	Q9JHU4	3387	6	0	4	28	1.1	1.0	1.0	1.4
Lasp1	Q61792	32	-3	0	4	27	1.0	1.0	1.0	1.4
Otulin	Q3UCV8	347	-8	0	4	-3	0.9	1.0	1.0	1.0
Ppa2	Q91VM9	156	9	-1	4	8	1.1	1.0	1.0	1.1
Srd5a3	Q9WUP4	250	-7	-2	4	16	0.9	1.0	1.0	1.2
Hat1	Q8BY71	165	-1	-2	4	12	1.0	1.0	1.0	1.1
Usp4	P35123	855	-8	-2	4	9	0.9	1.0	1.0	1.1
Rassf1	Q99MK9	337	-9	-2	4	19	0.9	1.0	1.0	1.2
Phf10	K4DI61	379	8	-2	4	8	1.1	1.0	1.0	1.1
Ahctf1	Q8CJF7	937	2	-3	4	56	1.0	1.0	1.0	2.2
Hnrnpul2	Q00PI9	600	-4	-3	4	14	1.0	1.0	1.0	1.2
Vcpkmt	Q8C436	167	9	-3	4	7	1.1	1.0	1.0	1.1
Cnnm1	Q0GA42	492	6	-3	4	6	1.1	1.0	1.0	1.1
Setd6	Q9CWY3	20	-3	-3	4	2	1.0	1.0	1.0	1.0
Dgka	O88673	220	-17	-3	4	-22	0.9	1.0	1.0	0.8
Safb	D3YXK2	470	-13	-3	4	-4	0.9	1.0	1.0	1.0
Tns3	Q5SSZ5	85	-7	-4	4	14	0.9	1.0	1.0	1.2
Lasp1	Q61792	29	-14	-5	4	13	0.9	1.0	1.0	1.1
Smchd1	Q6P5D8	61	4	-6	4	2	1.0	0.9	1.0	1.0
Ewsr1	Q61545	539	-4	-6	4	21	1.0	0.9	1.0	1.3
Znf706	Q9D115	44	-6	-7	4	4	0.9	0.9	1.0	1.0
Tcf25	Q8R3L2	216	-6	-7	4	-4	0.9	0.9	1.0	1.0
Marc2	Q922Q1	160	-17	-8	4	8	0.9	0.9	1.0	1.1
Adnp	Q9Z103	79	-7	-8	4	34	0.9	0.9	1.0	1.5
Uncharact	Q3TQI7	145	-3	-9	4	5	1.0	0.9	1.0	1.0

Otud6b	Q8K2H2	293	-15	-9	4	-18	0.9	0.9	1.0	0.8
Mrps6	P58064	105	2	-9	4	-10	1.0	0.9	1.0	0.9
Eml2	Q7TNG5	203	-21	-10	4	7	0.8	0.9	1.0	1.1
Golga4	Q91VW5	1160	3	-10	4	-11	1.0	0.9	1.0	0.9
Plcg1	Q62077	646	-2	-11	4	-1	1.0	0.9	1.0	1.0
Syne2	Q6ZWQ0	90	-37	-11	4	-5	0.7	0.9	1.0	1.0
U2af1	Q9D883	169	-11	-11	4	-5	0.9	0.9	1.0	1.0
Cntrl	A2AL36	1787	-8	-11	4	-12	0.9	0.9	1.0	0.9
Yars2	Q8BYL4	131	-10	-11	4	-17	0.9	0.9	1.0	0.9
Bckdhb	Q6P3A8	175	-3	-12	4	24	1.0	0.9	1.0	1.3
Osbpl9	A2A8Z1	173	-1	-12	4	7	1.0	0.9	1.0	1.1
Gbf1	Q6DFZ1	1709	3	-13	4	0	1.0	0.9	1.0	1.0
Acox3	Q9EPL9	334	15	-14	4	1	1.2	0.9	1.0	1.0
Cyb5r4	Q3TDX8	356	-15	-14	4	-14	0.9	0.9	1.0	0.9
Dbnl	Q62418	67	-13	-15	4	3	0.9	0.9	1.0	1.0
Apbb1ip	Q8R5A3	358	-8	-17	4	-10	0.9	0.9	1.0	0.9
Ganc	A2AQJ8	855	-16	-18	4	-5	0.9	0.9	1.0	1.0
Mib2	Q8R516	94	-8	-18	4	8	0.9	0.8	1.0	1.1
Tnfaip3	Q60769	57	-22	-22	4	50	0.8	0.8	1.0	2.0
Dock10	E9QM99	1997	-7	-27	4	16	0.9	0.8	1.0	1.2
Themis	Q8BGW0	413	5	19	4	27	1.0	1.2	1.0	1.4
Itk	Q03526	74	2	16	4	31	1.0	1.2	1.0	1.4
Ccnk	Q3U3M5	98	3	13	4	38	1.0	1.1	1.0	1.6
Ces1a	E9PYP1	407	-1	13	4	5	1.0	1.1	1.0	1.0
Map4k2	Q61161	599	5	12	4	32	1.0	1.1	1.0	1.5
Pi4ka	E9Q3L2	1901	17	10	4	11	1.2	1.1	1.0	1.1
Ppp6r3	Q922D4	467	-6	10	4	-5	0.9	1.1	1.0	1.0
Gmpr	Q9DCZ1	186	13	9	4	18	1.1	1.1	1.0	1.2
Trappc11	B2RXC1	615	-1	9	4	11	1.0	1.1	1.0	1.1
Eml4	Q3UMY5	621	8	8	4	25	1.1	1.1	1.0	1.3
Kif21b	E9Q0A4	698	9	8	4	14	1.1	1.1	1.0	1.2
Fam65b	Q80U16	243	4	7	4	15	1.0	1.1	1.0	1.2
Pold1	P52431	824	-15	7	4	0	0.9	1.1	1.0	1.0
Fam63a	Q76LS9	94	-8	7	4	-2	0.9	1.1	1.0	1.0
Tsc2	Q7TT21	52	-5	7	4	11	1.0	1.1	1.0	1.1
Hbp1	Q8R316	243	-2	7	4	-19	1.0	1.1	1.0	0.8
Lypla2	Q9WTL7	171	4	5	4	38	1.0	1.1	1.0	1.6
Srp68	Q8BMA6	86	8	5	4	33	1.1	1.1	1.0	1.5
Ftsj3	Q9DBE9	153	2	4	4	14	1.0	1.0	1.0	1.2
Dnmbp	Q6TXD4	1500	0	4	4	12	1.0	1.0	1.0	1.1
Dync1h1	Q9JHU4	865	3	3	4	26	1.0	1.0	1.0	1.4
Ubr7	Q8BU04	123	-1	3	4	13	1.0	1.0	1.0	1.1
Sp100	O35892	432	6	2	4	10	1.1	1.0	1.0	1.1
Tbc1d15	Q9CXF4	65	-16	2	4	-23	0.9	1.0	1.0	0.8
Ckb	Q04447	254	8	2	4	21	1.1	1.0	1.0	1.3
Lta4h	P24527	543	11	1	4	23	1.1	1.0	1.0	1.3
Slk	O54988	1210	-6	1	4	12	0.9	1.0	1.0	1.1
Irf2bp2	E9Q1P8	513	1	1	4	11	1.0	1.0	1.0	1.1
Irf2bpl	Q8K3X4	718	1	1	4	11	1.0	1.0	1.0	1.1
Irf2bp1	Q8R3Y8	527	1	1	4	11	1.0	1.0	1.0	1.1
Ctdp1	Q7TSG2	95	7	1	4	18	1.1	1.0	1.0	1.2

Sbno2	Q7TNB8	1208	-15	1	4	12	0.9	1.0	1.0	1.1
Prkcd	P28867	28	-8	1	4	7	0.9	1.0	1.0	1.1
Ahcyl2	Q68FL4	456	-12	1	4	3	0.9	1.0	1.0	1.0
Ddx3y	Q62095	297	-1	0	4	16	1.0	1.0	1.0	1.2
Hira	Q61666	761	-5	-1	4	18	1.0	1.0	1.0	1.2
Myo9b	E9PZW8	1999	-3	-1	4	-6	1.0	1.0	1.0	0.9
Il16	O54824	1225	-12	-1	4	1	0.9	1.0	1.0	1.0
Znf512	Q69Z99	513	-2	-1	4	-5	1.0	1.0	1.0	1.0
Itpr3	P70227	1511	-16	-2	4	25	0.9	1.0	1.0	1.3
Psmd13	Q9WVJ2	182	-9	-2	4	8	0.9	1.0	1.0	1.1
Tmem173	Q3TBT3	65	-20	-2	4	-7	0.8	1.0	1.0	0.9
Ncoa5	Q91W39	200	-2	-3	4	12	1.0	1.0	1.0	1.1
Timm50	Q9D880	249	-11	-3	4	-11	0.9	1.0	1.0	0.9
Stag1	Q9D3E6	636	8	-4	4	11	1.1	1.0	1.0	1.1
Samhd1	Q60710	104	-2	-4	4	7	1.0	1.0	1.0	1.1
Ube4a	E9Q735	465	-5	-4	4	-7	1.0	1.0	1.0	0.9
Sphk2	Q9JIA7	81	1	-4	4	-83	1.0	1.0	1.0	0.5
Gtf2b	P62915	163	-8	-4	4	1	0.9	1.0	1.0	1.0
Lpxn	Q99N69	329	-9	-4	4	-5	0.9	1.0	1.0	1.0
Atxn3	Q9CVD2	265	-8	-5	4	14	0.9	1.0	1.0	1.2
Imp3	Q921Y2	107	2	-5	4	5	1.0	1.0	1.0	1.0
Foxp3	Q99JB6	168	-4	-5	4	19	1.0	1.0	1.0	1.2
Zc3h15	Q3TIV5	180	-16	-6	4	31	0.9	0.9	1.0	1.4
Ankfy1	Q810B6	995	-3	-6	4	15	1.0	0.9	1.0	1.2
Tbcd	Q8BYA0	805	-3	-6	4	6	1.0	0.9	1.0	1.1
Dnaja2	Q9QYJ0	143	-3	-6	4	-3	1.0	0.9	1.0	1.0
Nipsnap1	O55125	83	-8	-6	4	-14	0.9	0.9	1.0	0.9
Cpsf2	O35218	763	-12	-6	4	28	0.9	0.9	1.0	1.4
Phip	F8VQ93	612	17	-6	4	-7	1.2	0.9	1.0	0.9
Nhej1	Q3KNJ2	97	-6	-7	4	30	0.9	0.9	1.0	1.4
Haus7	Q8BKT8	39	-20	-7	4	30	0.8	0.9	1.0	1.4
Uhrf2	Q7TMI3	433	-11	-7	4	-15	0.9	0.9	1.0	0.9
Uhrf1	Q8VDF2	408	-11	-7	4	-15	0.9	0.9	1.0	0.9
Polr2a	P08775	981	-15	-8	4	23	0.9	0.9	1.0	1.3
Dock10	E9QM99	238	-14	-9	4	-2	0.9	0.9	1.0	1.0
Sf3b3	Q921M3	547	-19	-10	4	-1	0.8	0.9	1.0	1.0
Cad	B2RQC6	868	0	-10	4	28	1.0	0.9	1.0	1.4
Ngly1	Q9JI78	306	-4	-11	4	31	1.0	0.9	1.0	1.4
Ece2	Q80Z60	57	-4	-11	4	-13	1.0	0.9	1.0	0.9
Acot6	Q32Q92	278	-20	-11	4	17	0.8	0.9	1.0	1.2
Erh	P84089	33	14	-12	4	17	1.2	0.9	1.0	1.2
Cmpk1	Q9DBP5	20	-13	-12	4	-15	0.9	0.9	1.0	0.9
Zfp472	B0V2W5	516	-29	-13	4	-8	0.8	0.9	1.0	0.9
Baz1b	Q9Z277	156	1	-13	4	-11	1.0	0.9	1.0	0.9
Zmiz1	Q6P1E1	17	-23	-13	4	-3	0.8	0.9	1.0	1.0
Trappc4	Q9ES56	195	-5	-14	4	52	1.0	0.9	1.0	2.1
Aagab	Q8R2R3	118	-11	-14	4	-30	0.9	0.9	1.0	0.8
Usp7	E9PXY8	131	-16	-15	4	2	0.9	0.9	1.0	1.0
Wdr43	Q6ZQL4	306	-1	-16	4	13	1.0	0.9	1.0	1.1
Pla2g15	Q8VEB4	8	-4	-16	4	-34	1.0	0.9	1.0	0.7
Dnmt1	P13864	420	-6	-17	4	27	0.9	0.9	1.0	1.4

Nthl1	O35980	23	-14	-19	4	-24	0.9	0.8	1.0	0.8
Rad50	Q5SV02	1296	6	-19	4	17	1.1	0.8	1.0	1.2
Exosc7	Q9D0M0	189	-8	-19	4	-8	0.9	0.8	1.0	0.9
Slf1	Q8R3P9	734	-23	-20	4	-9	0.8	0.8	1.0	0.9
Ahctf1	Q8CJF7	1471	-14	-27	4	-10	0.9	0.8	1.0	0.9
Prep	Q9QUR6	25	-7	-33	4	-65	0.9	0.8	1.0	0.6
Mtr	A6H5Y3	786	4	18	3	44	1.0	1.2	1.0	1.8
Agpat4	Q8K4X7	228	-5	17	3	15	1.0	1.2	1.0	1.2
Sart3	Q9JLI8	538	8	15	3	16	1.1	1.2	1.0	1.2
Sptan1	P16546	956	8	14	3	4	1.1	1.2	1.0	1.0
Cbfa2t2	O70374	252	-1	12	3	26	1.0	1.1	1.0	1.4
Fam96b	Q9D187	93	11	11	3	20	1.1	1.1	1.0	1.2
Rasgrp2	Q9QUG9	536	18	8	3	4	1.2	1.1	1.0	1.0
Arhgef11	Q68FM7	1194	4	7	3	-15	1.0	1.1	1.0	0.9
Cltc	Q68FD5	1528	4	6	3	14	1.0	1.1	1.0	1.2
Gar1	Q9CY66	91	-4	6	3	12	1.0	1.1	1.0	1.1
Fbxo4	Q8CHQ0	134	-1	6	3	11	1.0	1.1	1.0	1.1
Uncharact	Q8BGA7	157	2	6	3	12	1.0	1.1	1.0	1.1
Znf598	Q80YR4	30	-11	6	3	6	0.9	1.1	1.0	1.1
Hnrnpk	P61979	145	-2	5	3	15	1.0	1.1	1.0	1.2
Cltc	Q68FD5	824	1	5	3	13	1.0	1.1	1.0	1.1
Gabpa	Q00422	22	2	5	3	-14	1.0	1.1	1.0	0.9
Dcaf7	P61963	61	-3	4	3	12	1.0	1.0	1.0	1.1
Mic1	Q8VC42	333	-17	4	3	-4	0.9	1.0	1.0	1.0
Dok3	Q9QZK7	77	-4	3	3	12	1.0	1.0	1.0	1.1
Aldh9a1	Q9JLJ2	288	-15	3	3	37	0.9	1.0	1.0	1.6
Ahnak	E9Q616	406	-3	3	3	6	1.0	1.0	1.0	1.1
Aqr	Q8CFQ3	1148	2	2	3	38	1.0	1.0	1.0	1.6
Pgk1	P09411	380	-6	2	3	31	0.9	1.0	1.0	1.4
Lacc1	Q8BZT9	47	8	2	3	3	1.1	1.0	1.0	1.0
Tbc1d2	B1AVH7	656	-6	2	3	-9	0.9	1.0	1.0	0.9
Jak3	Q62137	905	-3	1	3	48	1.0	1.0	1.0	1.9
Ifit3	Q64345	283	17	1	3	9	1.2	1.0	1.0	1.1
Nit2	Q9JHW2	25	4	1	3	6	1.0	1.0	1.0	1.1
Dnaja2	Q9QYJ0	308	-5	1	3	22	1.0	1.0	1.0	1.3
Myh9	Q8VDD5	91	-3	1	3	17	1.0	1.0	1.0	1.2
Mtx1	F7C846	299	-5	1	3	8	1.0	1.0	1.0	1.1
Eif3c	Q8R1B4	441	-5	1	3	8	1.0	1.0	1.0	1.1
Nup98	Q6PFD9	1726	-1	1	3	4	1.0	1.0	1.0	1.0
Fech	P22315	320	-15	0	3	36	0.9	1.0	1.0	1.6
Ago2	Q8CJG0	493	-7	0	3	7	0.9	1.0	1.0	1.1
Ago1	Q8CJG1	490	-7	0	3	7	0.9	1.0	1.0	1.1
Copa	Q8CIE6	514	-1	-1	3	17	1.0	1.0	1.0	1.2
Zwilch	Q8R060	261	-8	-2	3	61	0.9	1.0	1.0	2.6
Gsto1	O09131	236	-8	-2	3	25	0.9	1.0	1.0	1.3
Phgdh	Q61753	19	-8	-2	3	23	0.9	1.0	1.0	1.3
Exosc8	Q9D753	89	-4	-2	3	5	1.0	1.0	1.0	1.0
Atp6v0d1	P51863	335	2	-2	3	4	1.0	1.0	1.0	1.0
Cd74	P04441	196	15	-2	3	-3	1.2	1.0	1.0	1.0
Trim28	Q62318	233	-2	-3	3	17	1.0	1.0	1.0	1.2
Gm7075	D3Z497	60	-9	-3	3	-7	0.9	1.0	1.0	0.9

Capg	Q99LB4	77	-1	-3	3	10	1.0	1.0	1.0	1.1
Scaf4	Q6PFF0	610	-10	-3	3	-7	0.9	1.0	1.0	0.9
Ascc3	E9PZJ8	1907	-9	-4	3	-8	0.9	1.0	1.0	0.9
Nup133	Q8R0G9	676	-4	-4	3	9	1.0	1.0	1.0	1.1
Nbas	E9Q411	543	0	-4	3	-5	1.0	1.0	1.0	1.0
Card11	Q8CIS0	427	-2	-5	3	-2	1.0	1.0	1.0	1.0
Pfkp	Q9WUA3	80	-19	-5	3	86	0.8	1.0	1.0	7.1
Parp1	Q921K2	296	-8	-5	3	25	0.9	1.0	1.0	1.3
Shkbp1	Q6P7W2	550	-10	-6	3	21	0.9	0.9	1.0	1.3
Hnrnpa2b:	O88569	50	-6	-6	3	11	0.9	0.9	1.0	1.1
Brcc3	P46737	215	-4	-7	3	11	1.0	0.9	1.0	1.1
Thop1	Q8C1A5	687	9	-7	3	-6	1.1	0.9	1.0	0.9
Macf1	E9PVY8	4796	-6	-7	3	6	0.9	0.9	1.0	1.1
Osgep	Q8BWU5	73	2	-7	3	-1	1.0	0.9	1.0	1.0
Ttll12	Q3UDE2	607	-4	-9	3	26	1.0	0.9	1.0	1.3
Dhx37	Q6NZL1	673	-12	-9	3	16	0.9	0.9	1.0	1.2
Polr1a	O35134	208	-12	-10	3	20	0.9	0.9	1.0	1.2
Nrg4	Q9WTX4	96	-8	-10	3	5	0.9	0.9	1.0	1.1
Ilf3	Q9Z1X4	295	-8	-10	3	34	0.9	0.9	1.0	1.5
Pold1	P52431	835	-11	-10	3	21	0.9	0.9	1.0	1.3
Eif4g1	Q6NZJ6	666	-3	-10	3	5	1.0	0.9	1.0	1.1
Pdzd8	B9EJ80	700	-1	-10	3	0	1.0	0.9	1.0	1.0
Mcm5	Q52KC3	570	-8	-11	3	7	0.9	0.9	1.0	1.1
Mat2b	Q99LB6	297	-4	-11	3	7	1.0	0.9	1.0	1.1
Phf6	Q9D4J7	292	-12	-12	3	27	0.9	0.9	1.0	1.4
Dhx9	E9QNN1	615	8	-12	3	23	1.1	0.9	1.0	1.3
Nup210	Q9QY81	543	-22	-12	3	-13	0.8	0.9	1.0	0.9
Eea1	Q8BL66	704	-4	-12	3	-25	1.0	0.9	1.0	0.8
Nt5dc1	Q8C5P5	207	-4	-13	3	7	1.0	0.9	1.0	1.1
Atp13a1	Q9EPE9	333	-27	-14	3	-14	0.8	0.9	1.0	0.9
Rsf1	E9PWW9	627	-9	-14	3	-24	0.9	0.9	1.0	0.8
Arfgef1	G3X9K3	1682	-20	-14	3	9	0.8	0.9	1.0	1.1
Ncapg2	Q6DFV1	962	-15	-15	3	81	0.9	0.9	1.0	5.3
Dok1	P97465	70	1	-15	3	-37	1.0	0.9	1.0	0.7
Calr	P14211	105	3	-16	3	15	1.0	0.9	1.0	1.2
Caap1	Q8VDY9	132	-10	-16	3	13	0.9	0.9	1.0	1.1
Helz2	E9QAM5	34	-21	-16	3	-9	0.8	0.9	1.0	0.9
Ncapg2	Q6DFV1	771	11	-17	3	17	1.1	0.9	1.0	1.2
Lss	Q8BLN5	428	-10	-17	3	7	0.9	0.9	1.0	1.1
Ptpn11	P35235	104	0	-17	3	-7	1.0	0.9	1.0	0.9
Rtn4	Q99P72	404	9	-18	3	-12	1.1	0.9	1.0	0.9
Fasn	P19096	180	-25	-20	3	11	0.8	0.8	1.0	1.1
Pdlim4	P70271	302	-12	-20	3	6	0.9	0.8	1.0	1.1
Atxn2	E9QM77	271	-8	-31	3	-1	0.9	0.8	1.0	1.0
Wdr59	Q8C0M0	906	6	-37	3	3	1.1	0.7	1.0	1.0
Med26	Q7TN02	356	-58	20	3	27	0.6	1.3	1.0	1.4
Por	P37040	566	6	15	3	23	1.1	1.2	1.0	1.3
Kbtbd4	Q8R179	471	5	15	3	1	1.0	1.2	1.0	1.0
Pdcd6ip	Q9WU78	250	-5	13	3	-3	1.0	1.1	1.0	1.0
Xpo1	Q6P5F9	34	-6	12	3	5	0.9	1.1	1.0	1.1
U2af1l4	Q8BGJ9	163	-19	12	3	-4	0.8	1.1	1.0	1.0

U2af1	Q9D883	163	-19	12	3	-4	0.8	1.1	1.0	1.0
Gar1	Q9CY66	97	-9	11	3	21	0.9	1.1	1.0	1.3
Dcaf8	Q8N7N5	250	18	11	3	-1	1.2	1.1	1.0	1.0
Ddx17	Q501J6	219	-2	11	3	23	1.0	1.1	1.0	1.3
Ddx5	Q8BTS0	221	-2	11	3	23	1.0	1.1	1.0	1.3
Ankrd27	Q3UMR0	51	-8	8	3	17	0.9	1.1	1.0	1.2
Dtx3l	Q3UIR3	572	7	8	3	5	1.1	1.1	1.0	1.1
Smarcc1	P97496	510	-13	6	3	11	0.9	1.1	1.0	1.1
Smarcc2	Q6PDG5	486	-13	6	3	11	0.9	1.1	1.0	1.1
Psmc6	P62334	193	4	6	3	-4	1.0	1.1	1.0	1.0
Ddx46	Q569Z5	501	-2	5	3	17	1.0	1.1	1.0	1.2
Polr1d	P97304	39	1	5	3	16	1.0	1.1	1.0	1.2
Mapkapk3	Q3UMW7	63	-7	5	3	7	0.9	1.1	1.0	1.1
Abhd14b	E9QN99	138	-9	5	3	0	0.9	1.1	1.0	1.0
Itpripl1	A2ASA8	277	10	5	3	-16	1.1	1.0	1.0	0.9
Tes	Q921W7	347	2	2	3	14	1.0	1.0	1.0	1.2
Ifi207	E9Q3L4	852	-2	2	3	-1	1.0	1.0	1.0	1.0
Ifi204	P0DOV2	493	-2	2	3	-1	1.0	1.0	1.0	1.0
Zbtb44	Q8R0A2	272	-1	2	3	-15	1.0	1.0	1.0	0.9
Mybbp1a	Q7TPV4	1028	-4	1	3	30	1.0	1.0	1.0	1.4
Tln1	P26039	236	-2	1	3	26	1.0	1.0	1.0	1.3
Ddx3y	Q62095	222	7	1	3	17	1.1	1.0	1.0	1.2
Ddx3x	Q62167	223	7	1	3	17	1.1	1.0	1.0	1.2
Uty	G3X9F2	379	-2	1	3	17	1.0	1.0	1.0	1.2
Lin28a	Q8K3Y3	117	9	1	3	12	1.1	1.0	1.0	1.1
Vprbp	Q80TR8	1112	-1	1	3	10	1.0	1.0	1.0	1.1
Lancl1	O89112	300	4	1	3	-5	1.0	1.0	1.0	1.0
Psmd12	Q9D8W5	255	2	0	3	9	1.0	1.0	1.0	1.1
Zap70	P43404	345	-8	-1	3	4	0.9	1.0	1.0	1.0
Dstn	Q9R0P5	80	0	-1	3	10	1.0	1.0	1.0	1.1
Gimap1	P70224	157	-11	-1	3	-22	0.9	1.0	1.0	0.8
Lef1	P27782	319	-4	-2	3	11	1.0	1.0	1.0	1.1
Chd3	B1AR17	473	-2	-2	3	8	1.0	1.0	1.0	1.1
Chd4	Q6PDQ2	404	-2	-2	3	8	1.0	1.0	1.0	1.1
Pik3cb	Q8BTI9	739	-3	-2	3	3	1.0	1.0	1.0	1.0
Exoc1	Q8R3S6	27	-20	-2	3	-1	0.8	1.0	1.0	1.0
Plcg2	Q8CIH5	791	-3	-3	3	23	1.0	1.0	1.0	1.3
Fam129a	Q3UW53	408	-13	-3	3	12	0.9	1.0	1.0	1.1
Copb1	Q9JIF7	248	-5	-3	3	20	1.0	1.0	1.0	1.2
Wrnip1	Q91XU0	39	-7	-3	3	14	0.9	1.0	1.0	1.2
Zfp868	Q3UTQ6	194	11	-3	3	-8	1.1	1.0	1.0	0.9
Dopey2	Q3UHQ6	2269	-13	-4	3	-13	0.9	1.0	1.0	0.9
Setx	A2AKX3	1250	-5	-4	3	10	1.0	1.0	1.0	1.1
Pdia3	P27773	60	3	-4	3	8	1.0	1.0	1.0	1.1
Coro1c	Q9WUM4	39	-8	-4	3	7	0.9	1.0	1.0	1.1
Ptk2b	Q9QVP9	972	-11	-5	3	53	0.9	1.0	1.0	2.1
Idh3g	P70404	81	15	-6	3	-9	1.2	0.9	1.0	0.9
Map2k7	Q8CE90	276	9	-7	3	10	1.1	0.9	1.0	1.1
Slc27a4	Q91VE0	624	-5	-7	3	-9	1.0	0.9	1.0	0.9
Flna	Q8BTM8	1453	-8	-7	3	5	0.9	0.9	1.0	1.1
Ctu1	Q99J10	144	0	-8	3	13	1.0	0.9	1.0	1.1

Rabggta	Q9JHK4	532	1	-8	3	-4	1.0	0.9	1.0	1.0
Ganc	A2AQJ8	113	-3	-8	3	-7	1.0	0.9	1.0	0.9
Jak3	Q62137	471	0	-8	3	8	1.0	0.9	1.0	1.1
Micu1	Q8VCX5	277	-16	-9	3	18	0.9	0.9	1.0	1.2
Shtn1	Q8K2Q9	550	2	-9	3	-29	1.0	0.9	1.0	0.8
Egln1	Q91YE3	58	3	-9	3	-2	1.0	0.9	1.0	1.0
Pds5a	E9QPI5	588	-15	-10	3	5	0.9	0.9	1.0	1.1
Gm9774	A0A0A6YVU8	80	-11	-10	3	-2	0.9	0.9	1.0	1.0
Rsf1	E9PWW9	408	1	-10	3	1	1.0	0.9	1.0	1.0
Zcchc17	Q9ESX4	133	-21	-10	3	-10	0.8	0.9	1.0	0.9
Tpx2	A2APB8	623	-6	-11	3	13	0.9	0.9	1.0	1.1
Nxf1	Q99JX7	372	-7	-11	3	7	0.9	0.9	1.0	1.1
Mic1	Q8VC42	25	-10	-11	3	4	0.9	0.9	1.0	1.0
Ndufs1	Q91VD9	463	-8	-11	3	4	0.9	0.9	1.0	1.0
Ctcf	Q61164	497	-4	-11	3	11	1.0	0.9	1.0	1.1
Sec23a	Q01405	74	-15	-11	3	9	0.9	0.9	1.0	1.1
Plec	Q9QXS1	4461	12	-12	3	5	1.1	0.9	1.0	1.0
Skiv2l2	Q9CZU3	295	-1	-13	3	12	1.0	0.9	1.0	1.1
Smurf2	A2A5Z6	151	2	-13	3	3	1.0	0.9	1.0	1.0
Agap2	Q3UHD9	530	-7	-15	3	-7	0.9	0.9	1.0	0.9
Ciapin1	Q8WTY4	274	-15	-16	3	-5	0.9	0.9	1.0	1.0
Pfkfb3	A7UAK5	412	-23	-18	3	6	0.8	0.8	1.0	1.1
Pfkfb4	Q6DTY7	415	-23	-18	3	6	0.8	0.8	1.0	1.1
Atp2a2	O55143	447	-3	-19	3	1	1.0	0.8	1.0	1.0
Atp2a3	Q64518	447	-3	-19	3	1	1.0	0.8	1.0	1.0
Nit1	Q8VDK1	199	-10	-19	3	-25	0.9	0.8	1.0	0.8
Isyna1	Q9JHU9	235	-11	-21	3	-7	0.9	0.8	1.0	0.9
Psmd4	O35226	58	5	-22	3	1	1.0	0.8	1.0	1.0
Fam76b	Q80XP8	57	6	-22	3	0	1.1	0.8	1.0	1.0
Fam76a	Q922G2	55	6	-22	3	0	1.1	0.8	1.0	1.0
Cep131	Q62036	967	-4	-22	3	-14	1.0	0.8	1.0	0.9
Ccdc71l	E9Q4T4	99	-24	-24	3	-5	0.8	0.8	1.0	1.0
Cobll1	Q3UMF0	650	-5	-25	3	-17	1.0	0.8	1.0	0.9
Mthfd1l	Q3V3R1	813	3	14	2	18	1.0	1.2	1.0	1.2
Lgals9	O08573	101	6	13	2	13	1.1	1.1	1.0	1.1
Umps	P13439	255	-14	12	2	8	0.9	1.1	1.0	1.1
Prex1	Q69ZK0	1642	-19	12	2	2	0.8	1.1	1.0	1.0
Wdfy4	E9Q2M9	482	0	11	2	47	1.0	1.1	1.0	1.9
Traf5	E9QMA6	395	9	9	2	4	1.1	1.1	1.0	1.0
Srsf3	P84104	10	-1	9	2	1	1.0	1.1	1.0	1.0
Hmha1	Q3TBD2	806	-3	8	2	27	1.0	1.1	1.0	1.4
Eif4enif1	Q9EST3	49	11	6	2	-13	1.1	1.1	1.0	0.9
Sac3d1	G5E8Q7	166	-1	6	2	-3	1.0	1.1	1.0	1.0
Metap2	O08663	263	-10	4	2	22	0.9	1.0	1.0	1.3
Srbd1	F8WGW3	834	6	4	2	-2	1.1	1.0	1.0	1.0
Trim25	Q61510	109	-1	4	2	13	1.0	1.0	1.0	1.1
Hnrnpul1	Q8VDM6	259	-6	3	2	13	0.9	1.0	1.0	1.1
Casp8	O89110	347	-20	3	2	12	0.8	1.0	1.0	1.1
Sept1	P42209	231	8	3	2	0	1.1	1.0	1.0	1.0
Rnf213	E9Q555	951	-12	3	2	25	0.9	1.0	1.0	1.3
Pdcd11	Q6NS46	1229	0	3	2	19	1.0	1.0	1.0	1.2

Sin3a	Q60520	552	-5	3	2	6	1.0	1.0	1.0	1.1
Card11	Q8CIS0	1009	3	2	2	20	1.0	1.0	1.0	1.2
Emg1	O35130	134	6	2	2	11	1.1	1.0	1.0	1.1
Med25	Q8VCB2	429	-3	2	2	4	1.0	1.0	1.0	1.0
Xpo5	Q924C1	765	-2	2	2	-6	1.0	1.0	1.0	0.9
Itprip	Q3TNL8	466	-8	1	2	42	0.9	1.0	1.0	1.7
Znf598	Q80YR4	31	-7	1	2	-7	0.9	1.0	1.0	0.9
Akr7a2	Q8CG76	222	-6	1	2	23	0.9	1.0	1.0	1.3
Smarce1	O54941	274	-2	1	2	17	1.0	1.0	1.0	1.2
Scyl1	Q9EQC5	309	-7	1	2	9	0.9	1.0	1.0	1.1
Lyst	G5E8Q0	1791	-6	1	2	8	0.9	1.0	1.0	1.1
Ubr4	A2AN08	2552	-4	0	2	21	1.0	1.0	1.0	1.3
Parp1	Q921K2	257	-3	0	2	16	1.0	1.0	1.0	1.2
Casp8	O89110	362	-2	0	2	-4	1.0	1.0	1.0	1.0
Sec24d	Q6NXL1	573	-1	-1	2	12	1.0	1.0	1.0	1.1
Camk1d	Q8BW96	182	-5	-1	2	12	1.0	1.0	1.0	1.1
Smchd1	Q6P5D8	883	-11	-1	2	6	0.9	1.0	1.0	1.1
Stip1	Q60864	62	-4	-1	2	-7	1.0	1.0	1.0	0.9
Ctc1	Q5SUQ9	398	-5	-2	2	6	1.0	1.0	1.0	1.1
Ltn1	Q6A009	868	-5	-2	2	-1	1.0	1.0	1.0	1.0
Map2k1	P31938	277	-2	-2	2	0	1.0	1.0	1.0	1.0
Ifi47	Q61635	218	-3	-2	2	-5	1.0	1.0	1.0	1.0
Trappc11	B2RXC1	162	-10	-2	2	-9	0.9	1.0	1.0	0.9
Parp3	Q3ULW8	50	-13	-3	2	18	0.9	1.0	1.0	1.2
Bud31	Q6PGH1	98	-16	-3	2	14	0.9	1.0	1.0	1.2
Sde2	Q8K1J5	403	1	-3	2	5	1.0	1.0	1.0	1.0
Dars	Q922B2	349	0	-3	2	-6	1.0	1.0	1.0	0.9
Asnsd1	Q8BFS9	257	-3	-3	2	-15	1.0	1.0	1.0	0.9
Fam46c	Q5SSF7	114	-1	-4	2	25	1.0	1.0	1.0	1.3
Naa15	G3X8Y3	816	-4	-4	2	9	1.0	1.0	1.0	1.1
Stat3	P42227	367	-6	-4	2	5	0.9	1.0	1.0	1.0
Rpl34	Q9D1R9	83	-4	-4	2	2	1.0	1.0	1.0	1.0
Asun	Q8QZV7	560	-12	-4	2	17	0.9	1.0	1.0	1.2
Zc3h14	Q8BJ05	692	-4	-5	2	1	1.0	1.0	1.0	1.0
Sec24c	G3X972	883	1	-5	2	19	1.0	1.0	1.0	1.2
Arhgef6	Q8K4I3	57	-13	-6	2	-2	0.9	0.9	1.0	1.0
Abcb7	Q61102	750	-3	-6	2	-2	1.0	0.9	1.0	1.0
Prrc2c	Q3TLH4	179	-23	-6	2	-9	0.8	0.9	1.0	0.9
Hspa4	Q3U2G2	417	-16	-7	2	0	0.9	0.9	1.0	1.0
Adprh	P54923	134	-4	-7	2	-3	1.0	0.9	1.0	1.0
Rbm34	Q8C5L7	26	-5	-7	2	-9	1.0	0.9	1.0	0.9
Cdc37	Q61081	64	-8	-7	2	18	0.9	0.9	1.0	1.2
Arf4	P61750	62	-4	-7	2	-2	1.0	0.9	1.0	1.0
Malt1	Q2TBA3	472	-12	-8	2	-14	0.9	0.9	1.0	0.9
Kiaa1551	Q5DTW7	1196	1	-8	2	-19	1.0	0.9	1.0	0.8
Slc15a4	Q91W98	300	4	-8	2	-31	1.0	0.9	1.0	0.8
Fam160b1	Q8CDM8	306	-16	-8	2	78	0.9	0.9	1.0	4.4
Akap13	E9Q394	2105	-6	-8	2	14	0.9	0.9	1.0	1.2
Dok3	Q9QZK7	200	-15	-8	2	2	0.9	0.9	1.0	1.0
Kdm5c	P41230	1280	-5	-9	2	-4	1.0	0.9	1.0	1.0
Kdm5a	Q3UXZ9	1248	-5	-9	2	-4	1.0	0.9	1.0	1.0

Cog7	Q3UM29	174	-5	-10	2	-3	1.0	0.9	1.0	1.0
Ap2a2	P17427	282	-7	-10	2	15	0.9	0.9	1.0	1.2
Aifm2	Q8BUE4	187	-13	-11	2	36	0.9	0.9	1.0	1.6
UbiE2	Q76I24	96	-14	-11	2	24	0.9	0.9	1.0	1.3
Dhx30	Q99PU8	629	-23	-11	2	15	0.8	0.9	1.0	1.2
Mcm4	P49717	508	-12	-11	2	3	0.9	0.9	1.0	1.0
Pik3c2b	E9QAN8	993	-1	-11	2	-7	1.0	0.9	1.0	0.9
Ltn1	Q6A009	237	-2	-12	2	-4	1.0	0.9	1.0	1.0
Mtm1	Q9Z2C5	191	-9	-12	2	-6	0.9	0.9	1.0	0.9
Stag1	Q9D3E6	644	-14	-12	2	-6	0.9	0.9	1.0	0.9
Nup205	A0A0J9YUD5	975	-3	-13	2	9	1.0	0.9	1.0	1.1
Mrpl37	Q921S7	104	-10	-13	2	4	0.9	0.9	1.0	1.0
Gpx4	O70325	102	-3	-13	2	-1	1.0	0.9	1.0	1.0
Cwf19l1	Q8CI33	288	-4	-13	2	10	1.0	0.9	1.0	1.1
Fasn	P19096	2461	-24	-13	2	7	0.8	0.9	1.0	1.1
Ethe1	Q9DCM0	219	-11	-14	2	-14	0.9	0.9	1.0	0.9
Rae1	Q8C570	173	-6	-16	2	-2	0.9	0.9	1.0	1.0
Orc1	Q9Z1N2	585	-15	-16	2	-7	0.9	0.9	1.0	0.9
Mrpl19	Q9D338	212	-20	-17	2	-4	0.8	0.9	1.0	1.0
Dcp1b	Q3U564	290	-15	-18	2	23	0.9	0.9	1.0	1.3
Ptp4a2	O70274	95	-24	-18	2	13	0.8	0.8	1.0	1.1
Mbnl3	Q8R003	180	0	-19	2	8	1.0	0.8	1.0	1.1
Mbnl1	Q9JKP5	184	0	-19	2	8	1.0	0.8	1.0	1.1
Pitrm1	Q8K411	34	-2	-20	2	0	1.0	0.8	1.0	1.0
Cep192	E9Q4Y4	403	7	-22	2	-14	1.1	0.8	1.0	0.9
Rpl30	P62889	52	-17	-32	2	-41	0.9	0.8	1.0	0.7
Ndufs1	Q91VD9	75	19	16	2	25	1.2	1.2	1.0	1.3
Patl1	Q3TC46	563	8	13	2	13	1.1	1.1	1.0	1.1
Trp53bp1	A2AU91	1923	-3	13	2	21	1.0	1.1	1.0	1.3
Kifc1	Q9QWT9	98	-9	12	2	-3	0.9	1.1	1.0	1.0
Pkm	P52480	326	-5	11	2	18	1.0	1.1	1.0	1.2
Btk	P35991	464	9	11	2	13	1.1	1.1	1.0	1.1
Smg9	Q9DB90	380	1	9	2	35	1.0	1.1	1.0	1.5
Dnpep	Q9Z2W0	445	-4	9	2	7	1.0	1.1	1.0	1.1
Mcm3ap	Q9WUU9	1261	4	8	2	16	1.0	1.1	1.0	1.2
Vps35	Q9EQH3	699	-5	8	2	17	1.0	1.1	1.0	1.2
Ctu1	Q99J10	409	4	8	2	-7	1.0	1.1	1.0	0.9
Sars2	Q9JJL8	66	3	7	2	3	1.0	1.1	1.0	1.0
Serinc3	Q9QZI9	452	-1	7	2	-7	1.0	1.1	1.0	0.9
Ydjc	Q14BV6	18	8	6	2	14	1.1	1.1	1.0	1.2
Ankrd44	B2RXR6	704	-7	5	2	22	0.9	1.1	1.0	1.3
Fam129b	Q8R1F1	466	7	5	2	11	1.1	1.1	1.0	1.1
Hnrnpu	Q8VEK3	311	-2	5	2	10	1.0	1.1	1.0	1.1
Itpr1	P11881	530	-3	5	2	2	1.0	1.1	1.0	1.0
Acsl5	Q8JZR0	93	-9	5	2	5	0.9	1.0	1.0	1.1
Zbtb11	G5E8B9	940	-4	5	2	-1	1.0	1.0	1.0	1.0
Mina	Q8CD15	432	-14	5	2	-11	0.9	1.0	1.0	0.9
Spg11	Q3UHA3	351	-1	4	2	14	1.0	1.0	1.0	1.2
Gnai2	P08752	287	6	4	2	23	1.1	1.0	1.0	1.3
Dgka	O88673	278	1	3	2	14	1.0	1.0	1.0	1.2
Trim30a	P15533	157	-15	3	2	-15	0.9	1.0	1.0	0.9

Bag1	Q60739	223	-8	2	2	3	0.9	1.0	1.0	1.0
Ppp2r1b	Q7TNP2	306	-1	2	2	-3	1.0	1.0	1.0	1.0
Trim33	Q99PP7	598	9	1	2	6	1.1	1.0	1.0	1.1
Ranbp2	Q9ERU9	1355	-2	1	2	11	1.0	1.0	1.0	1.1
Ttc27	Q8CD92	303	5	1	2	4	1.0	1.0	1.0	1.0
Sap30bp	Q02614	172	-9	1	2	-7	0.9	1.0	1.0	0.9
Gnl3	Q8CI11	309	-2	0	2	22	1.0	1.0	1.0	1.3
Pstk	Q8BP74	135	18	0	2	13	1.2	1.0	1.0	1.1
Zmym2	Q9CU65	333	-1	0	2	5	1.0	1.0	1.0	1.1
Wrn	O09053	266	6	-1	2	32	1.1	1.0	1.0	1.5
Gatad2b	Q8VHR5	421	-1	-1	2	14	1.0	1.0	1.0	1.2
Gnl3l	Q6PGG6	238	4	-1	2	1	1.0	1.0	1.0	1.0
Myh9	Q8VDD5	740	-12	-2	2	25	0.9	1.0	1.0	1.3
Hcfc1	Q61191	89	-11	-2	2	6	0.9	1.0	1.0	1.1
Rnf2	Q9CQJ4	75	-6	-2	2	1	0.9	1.0	1.0	1.0
Snx30	Q8CE50	103	2	-2	2	-7	1.0	1.0	1.0	0.9
Mars	Q68FL6	568	0	-2	2	15	1.0	1.0	1.0	1.2
Ppp4r3b	Q922R5	90	-22	-2	2	12	0.8	1.0	1.0	1.1
Psmd13	Q9WVJ2	357	-8	-2	2	9	0.9	1.0	1.0	1.1
Pycrl	Q9DCC4	266	0	-2	2	-2	1.0	1.0	1.0	1.0
Spg11	Q3UHA3	978	-5	-3	2	5	1.0	1.0	1.0	1.0
Sec23b	Q9D662	425	-2	-3	2	12	1.0	1.0	1.0	1.1
Atg2b	Q80XK6	243	-13	-3	2	-18	0.9	1.0	1.0	0.9
Lasp1	Q61792	35	-9	-4	2	17	0.9	1.0	1.0	1.2
Sepw1	P63300	87	-6	-4	2	-10	0.9	1.0	1.0	0.9
Uncharact	Q8C5K5	77	-14	-5	2	27	0.9	1.0	1.0	1.4
Nup160	Q9Z0W3	1275	-4	-5	2	27	1.0	1.0	1.0	1.4
Tnpo1	Q8BFY9	153	-15	-5	2	21	0.9	1.0	1.0	1.3
Trim24	Q64127	74	-16	-5	2	1	0.9	1.0	1.0	1.0
Alg2	Q9DBE8	70	-4	-5	2	-21	1.0	1.0	1.0	0.8
Hadha	Q8BMS1	145	-23	-5	2	38	0.8	1.0	1.0	1.6
Gspt1	Q8R050	601	-14	-5	2	16	0.9	1.0	1.0	1.2
Rps6ka1	P18653	541	-9	-5	2	8	0.9	1.0	1.0	1.1
Atr	E9QPK4	416	-12	-6	2	5	0.9	0.9	1.0	1.0
Pik3cd	O35904	219	1	-6	2	-1	1.0	0.9	1.0	1.0
Lsm7	Q9CQQ8	76	-15	-6	2	-21	0.9	0.9	1.0	0.8
Hadha	Q8BMS1	349	-12	-6	2	28	0.9	0.9	1.0	1.4
Macf1	E9PVY8	7094	-8	-6	2	17	0.9	0.9	1.0	1.2
Copa	Q8CIE6	580	-11	-6	2	12	0.9	0.9	1.0	1.1
Rraga	Q80X95	159	8	-7	2	12	1.1	0.9	1.0	1.1
Tbl2	Q9R099	255	-8	-7	2	-12	0.9	0.9	1.0	0.9
Sf1	Q64213	279	-9	-8	2	17	0.9	0.9	1.0	1.2
Dnajc2	P54103	140	1	-8	2	8	1.0	0.9	1.0	1.1
Rps27a	P62983	121	-15	-8	2	1	0.9	0.9	1.0	1.0
Tagln2	Q9WVA4	124	-11	-8	2	-3	0.9	0.9	1.0	1.0
Pikfyve	Q9Z1T6	1969	-6	-9	2	30	0.9	0.9	1.0	1.4
Mocs3	A2BDX3	324	11	-9	2	19	1.1	0.9	1.0	1.2
Pdcd11	Q6NS46	510	-12	-9	2	-3	0.9	0.9	1.0	1.0
Tyk2	E9QJS1	559	-3	-9	2	-20	1.0	0.9	1.0	0.8
Elof1	P60003	29	-12	-9	2	24	0.9	0.9	1.0	1.3
Gemin4	Q6P6L6	481	-8	-9	2	24	0.9	0.9	1.0	1.3

Ethe1	Q9DCM0	98	0	-9	2	-7	1.0	0.9	1.0	0.9
Bptf	A2A654	1970	-9	-9	2	-9	0.9	0.9	1.0	0.9
Ccdc69	Q3TCJ8	60	-5	-9	2	-22	1.0	0.9	1.0	0.8
Acsf2	Q8VCW8	254	-10	-10	2	19	0.9	0.9	1.0	1.2
Polr1a	O35134	360	-6	-10	2	-12	0.9	0.9	1.0	0.9
Phf5a	P83870	46	-4	-11	2	9	1.0	0.9	1.0	1.1
Smc1a	Q9CU62	987	-9	-11	2	19	0.9	0.9	1.0	1.2
Rsf1	E9PWW9	123	-9	-12	2	-7	0.9	0.9	1.0	0.9
Dhrs11	Q3U0B3	153	-4	-12	2	10	1.0	0.9	1.0	1.1
Morc3	F7BJB9	790	-15	-12	2	-2	0.9	0.9	1.0	1.0
Rbm33	Q9CXK9	1154	-4	-12	2	-16	1.0	0.9	1.0	0.9
Thyn1	Q91YJ3	90	-13	-13	2	8	0.9	0.9	1.0	1.1
Ezr	P26040	117	-13	-13	2	6	0.9	0.9	1.0	1.1
Mgll	O35678	208	-16	-13	2	-36	0.9	0.9	1.0	0.7
Ect2	Q07139	256	-15	-14	2	10	0.9	0.9	1.0	1.1
Sqrdl	Q9R112	379	-14	-15	2	60	0.9	0.9	1.0	2.5
Psmc3	O88685	399	-2	-15	2	3	1.0	0.9	1.0	1.0
Arfgef2	A2A5R2	573	-6	-15	2	26	0.9	0.9	1.0	1.4
Arfgef1	G3X9K3	612	-6	-15	2	26	0.9	0.9	1.0	1.4
Themis2	Q91YX0	394	-18	-15	2	-4	0.8	0.9	1.0	1.0
2310022A	G5E8E3	112	2	-15	2	-12	1.0	0.9	1.0	0.9
Dock9	F8VPN7	623	-4	-16	2	-7	1.0	0.9	1.0	0.9
Aldh6a1	Q9EQ20	317	-17	-17	2	74	0.9	0.9	1.0	3.8
Tapt1	Q4VBD2	452	-8	-18	2	-36	0.9	0.9	1.0	0.7
Psmd5	Q8BJY1	64	-4	-18	2	33	1.0	0.8	1.0	1.5
Dync1h1	Q9JHU4	4568	-28	-19	2	0	0.8	0.8	1.0	1.0
Capn1	O35350	49	-15	-22	2	14	0.9	0.8	1.0	1.2
Dmxl1	Q6PNC0	2518	1	-22	2	8	1.0	0.8	1.0	1.1
Fus	P56959	437	-5	-27	2	12	1.0	0.8	1.0	1.1
Gimap6	Q8K349	254	-3	-31	2	-3	1.0	0.8	1.0	1.0
Pepd	Q11136	482	-37	-41	2	-45	0.7	0.7	1.0	0.7
Mrps25	Q9D125	149	-6	14	1	9	0.9	1.2	1.0	1.1
Ctsa	P16675	438	-5	11	1	-30	1.0	1.1	1.0	0.8
Vars	Q9Z1Q9	478	-3	11	1	19	1.0	1.1	1.0	1.2
Psme4	Q5SSW2	1000	-9	10	1	32	0.9	1.1	1.0	1.5
Coro7	Q9D2V7	394	4	9	1	0	1.0	1.1	1.0	1.0
Mbd1	Q9Z2E2	209	-13	9	1	18	0.9	1.1	1.0	1.2
Stat1	A0A087WSP5	324	12	9	1	13	1.1	1.1	1.0	1.1
Coro1b	Q9WUM3	345	1	8	1	22	1.0	1.1	1.0	1.3
Shmt1	P50431	198	-2	8	1	17	1.0	1.1	1.0	1.2
Kmt2d	Q6PDK2	5528	14	8	1	-7	1.2	1.1	1.0	0.9
Stk10	O55098	138	0	7	1	33	1.0	1.1	1.0	1.5
Parp14	Q2EMV9	1285	4	7	1	29	1.0	1.1	1.0	1.4
Ndufs8	Q8K3J1	123	-9	7	1	1	0.9	1.1	1.0	1.0
Tle3	Q08122	528	-2	6	1	19	1.0	1.1	1.0	1.2
Inpp4b	Q6P1Y8	517	-16	6	1	4	0.9	1.1	1.0	1.0
Cars	Q9ER72	110	6	5	1	14	1.1	1.1	1.0	1.2
Aim2	Q91VJ1	113	-1	5	1	-12	1.0	1.1	1.0	0.9
Lap3	Q9CPY7	129	11	5	1	14	1.1	1.0	1.0	1.2
Idh3b	Q91VA7	231	-10	5	1	9	0.9	1.0	1.0	1.1
Dgka	O88673	427	-14	3	1	19	0.9	1.0	1.0	1.2

UPF0600	Q8BR90	179	-5	3	1	6	1.0	1.0	1.0	1.1
Crbn	Q8C7D2	191	-13	3	1	-32	0.9	1.0	1.0	0.8
Lyn	P25911	203	-8	3	1	-5	0.9	1.0	1.0	1.0
Dock2	Q8C3J5	465	-5	2	1	10	1.0	1.0	1.0	1.1
Uba6	Q8C7R4	449	-3	2	1	10	1.0	1.0	1.0	1.1
Spata5	Q3UMC0	672	-4	1	1	15	1.0	1.0	1.0	1.2
Tbc1d5	Q80XQ2	192	-13	1	1	-3	0.9	1.0	1.0	1.0
Phrf1	A6H619	971	2	1	1	-12	1.0	1.0	1.0	0.9
Plxnc1	Q9QZC2	1223	-7	1	1	-14	0.9	1.0	1.0	0.9
Btk	P35991	63	-3	1	1	14	1.0	1.0	1.0	1.2
Foxk1	P42128	651	-7	1	1	2	0.9	1.0	1.0	1.0
Pum3	Q8BKS9	575	-9	0	1	15	0.9	1.0	1.0	1.2
Zc3h4	E9Q8K8	1254	12	0	1	2	1.1	1.0	1.0	1.0
Ap2a1	P17426	283	-11	-1	1	25	0.9	1.0	1.0	1.3
Nup160	Q9Z0W3	1008	-11	-1	1	-4	0.9	1.0	1.0	1.0
Trim14	Q8BVW3	22	-23	-2	1	14	0.8	1.0	1.0	1.2
Zc3h18	Q0P678	234	-8	-2	1	10	0.9	1.0	1.0	1.1
Kif4	P33174	896	-13	-2	1	9	0.9	1.0	1.0	1.1
Pdhb	Q9D051	263	-1	-2	1	7	1.0	1.0	1.0	1.1
Fasn	P19096	2352	4	-2	1	4	1.0	1.0	1.0	1.0
Birc6	O88738	2137	3	-3	1	14	1.0	1.0	1.0	1.2
Ero1a	Q8R180	37	7	-3	1	8	1.1	1.0	1.0	1.1
RUS1	Q91W34	12	-19	-3	1	-4	0.8	1.0	1.0	1.0
Lpp	Q8BFW7	594	-11	-3	1	3	0.9	1.0	1.0	1.0
Dock8	Q8C147	198	-2	-3	1	2	1.0	1.0	1.0	1.0
Rnf166	Q3U9F6	127	-7	-3	1	2	0.9	1.0	1.0	1.0
Ppil1	Q9D0W5	133	-7	-3	1	-3	0.9	1.0	1.0	1.0
Sucla2	Q9Z2I9	430	-6	-3	1	-7	0.9	1.0	1.0	0.9
Ly9	Q01965	626	0	-3	1	-13	1.0	1.0	1.0	0.9
Ints1	Q6P4S8	1536	-7	-4	1	44	0.9	1.0	1.0	1.8
Parn	Q8VDG3	198	-5	-4	1	-4	1.0	1.0	1.0	1.0
Ankfy1	Q810B6	34	-9	-5	1	38	0.9	1.0	1.0	1.6
Hnrnpll	Q921F4	283	-3	-5	1	3	1.0	1.0	1.0	1.0
Uncharact	Q3UTZ3	369	-13	-5	1	16	0.9	1.0	1.0	1.2
Ppa1	Q9D819	254	3	-5	1	-2	1.0	1.0	1.0	1.0
Becn1	O88597	351	-4	-5	1	-2	1.0	1.0	1.0	1.0
Ear6	Q923L7	141	-7	-6	1	45	0.9	0.9	1.0	1.8
Srp72	F8VQC1	54	-13	-6	1	23	0.9	0.9	1.0	1.3
Polr2a	P08775	1245	-11	-6	1	16	0.9	0.9	1.0	1.2
Mtmr2	Q9Z2D1	635	4	-6	1	-3	1.0	0.9	1.0	1.0
Ep400	Q8CHI8	1430	5	-6	1	-3	1.0	0.9	1.0	1.0
Eprs	Q8CGC7	1497	-7	-6	1	-5	0.9	0.9	1.0	1.0
Bloc1s5	Q8R015	115	-9	-6	1	-24	0.9	0.9	1.0	0.8
Ampd3	O08739	690	-4	-7	1	7	1.0	0.9	1.0	1.1
Polr1b	P70700	307	-6	-7	1	-10	0.9	0.9	1.0	0.9
Kdm3b	B9EKS2	296	-3	-8	1	-1	1.0	0.9	1.0	1.0
Abcf2	Q99LE6	591	-6	-8	1	-7	0.9	0.9	1.0	0.9
Pcnt	F8VPV0	2184	-23	-8	1	-14	0.8	0.9	1.0	0.9
Pgrmc2	Q80UU9	75	-13	-8	1	24	0.9	0.9	1.0	1.3
Arrb1	Q8BWG8	242	-20	-8	1	14	0.8	0.9	1.0	1.2
Tubgcp3	P58854	18	1	-8	1	6	1.0	0.9	1.0	1.1

Armc8	Q9DBR3	661	-8	-8	1	-6	0.9	0.9	1.0	0.9
Cdk12	Q14AX6	472	-5	-8	1	-10	1.0	0.9	1.0	0.9
Stip1	Q60864	403	-11	-9	1	17	0.9	0.9	1.0	1.2
Gm29609	Q3UU56	499	-12	-10	1	27	0.9	0.9	1.0	1.4
Txn	P10639	62	-17	-10	1	10	0.9	0.9	1.0	1.1
E4f1	Q8CCE9	62	-17	-10	1	-12	0.9	0.9	1.0	0.9
Pias3	O54714	327	-14	-11	1	7	0.9	0.9	1.0	1.1
Pias2	Q8C5D8	346	-14	-11	1	7	0.9	0.9	1.0	1.1
Hk1	P17710	662	-22	-11	1	8	0.8	0.9	1.0	1.1
Plekhf2	Q91WB4	21	-14	-11	1	-2	0.9	0.9	1.0	1.0
Gtf2b	P62915	15	-14	-12	1	14	0.9	0.9	1.0	1.2
Usp4	P35123	624	-21	-12	1	-6	0.8	0.9	1.0	0.9
Mcm3ap	Q9WUU9	634	-4	-12	1	37	1.0	0.9	1.0	1.6
Luc7l3	Q5SUF2	40	-20	-12	1	7	0.8	0.9	1.0	1.1
Pld4	Q8BG07	311	4	-13	1	16	1.0	0.9	1.0	1.2
Ncoa5	Q91W39	301	-14	-14	1	13	0.9	0.9	1.0	1.1
Sf1	Q64213	282	-7	-14	1	2	0.9	0.9	1.0	1.0
Eipr1	Q8K0G5	95	-3	-14	1	-11	1.0	0.9	1.0	0.9
Ddx54	Q8K4L0	72	-1	-14	1	-26	1.0	0.9	1.0	0.8
Fam175b	Q3TCJ1	237	0	-14	1	-6	1.0	0.9	1.0	0.9
Rnh1	Q91VI7	204	-2	-15	1	-2	1.0	0.9	1.0	1.0
Arrb1	Q8BWG8	269	9	-15	1	-14	1.1	0.9	1.0	0.9
Champ1	Q8K327	183	-14	-15	1	-11	0.9	0.9	1.0	0.9
Pan3	Q640Q5	465	-8	-16	1	26	0.9	0.9	1.0	1.3
Nipbl	Q6KCD5	573	-3	-16	1	-5	1.0	0.9	1.0	1.0
Flna	Q8BTM8	2479	-3	-17	1	-14	1.0	0.9	1.0	0.9
Tex15	F8VPN2	1920	-21	-18	1	12	0.8	0.8	1.0	1.1
Ppih	Q9D868	131	-2	-20	1	1	1.0	0.8	1.0	1.0
Sec24c	G3X972	912	-17	-21	1	31	0.9	0.8	1.0	1.4
Srpk2	O54781	528	-6	-21	1	1	0.9	0.8	1.0	1.0
Srpk1	O70551	495	-6	-21	1	1	0.9	0.8	1.0	1.0
Rpl18a	P62717	109	-24	-25	1	-19	0.8	0.8	1.0	0.8
Mmp9	P41245	100	-25	-42	1	-42	0.8	0.7	1.0	0.7
Mic1	Q8VC42	106	15	20	1	17	1.2	1.3	1.0	1.2
Abcb7	Q61102	747	-5	15	1	6	1.0	1.2	1.0	1.1
Clcn7	O70496	635	-2	13	1	8	1.0	1.1	1.0	1.1
Ubl4a	P21126	13	-12	12	1	9	0.9	1.1	1.0	1.1
Tpp1	O89023	364	1	11	1	16	1.0	1.1	1.0	1.2
Ddx55	Q6ZPL9	249	14	10	1	16	1.2	1.1	1.0	1.2
Bpnt1	Q9Z0S1	249	7	9	1	19	1.1	1.1	1.0	1.2
Crocc	Q8CJ40	810	-12	8	1	-11	0.9	1.1	1.0	0.9
Evi2b	Q8VD58	254	4	8	1	-15	1.0	1.1	1.0	0.9
Hnrnpu	Q8VEK3	367	-8	6	1	-1	0.9	1.1	1.0	1.0
Nrde2	Q80XC6	211	-1	5	1	8	1.0	1.1	1.0	1.1
Mcmbp	Q8R3C0	108	-10	5	1	1	0.9	1.1	1.0	1.0
Crbn	Q8C7D2	237	-6	5	1	-53	0.9	1.1	1.0	0.7
Tomm34	Q9CYG7	67	-5	4	1	20	1.0	1.0	1.0	1.2
Polr3e	Q9CZT4	378	6	4	1	16	1.1	1.0	1.0	1.2
Kif5b	Q61768	174	12	4	1	2	1.1	1.0	1.0	1.0
Mtch1	Q791T5	385	-12	4	1	-3	0.9	1.0	1.0	1.0
Fahd2	Q3TC72	119	-2	4	1	-8	1.0	1.0	1.0	0.9

Farsa	Q8C0C7	493	-3	3	1	27	1.0	1.0	1.0	1.4
Ddx41	Q91VN6	585	-6	3	1	8	0.9	1.0	1.0	1.1
Usp28	Q5I043	203	-14	3	1	-5	0.9	1.0	1.0	1.0
Myo1b	P46735	129	1	3	1	8	1.0	1.0	1.0	1.1
Rps6ka5	Q8C050	695	-2	2	1	31	1.0	1.0	1.0	1.4
Hnrnpa3	Q8BG05	94	-2	2	1	5	1.0	1.0	1.0	1.1
Aldh5a1	Q8BWF0	330	11	2	1	-9	1.1	1.0	1.0	0.9
Wrnip1	Q91XU0	342	6	2	1	-10	1.1	1.0	1.0	0.9
Irf2bp1	Q8R3Y8	363	-7	2	1	-13	0.9	1.0	1.0	0.9
Cnn2	Q08093	164	-5	2	1	13	1.0	1.0	1.0	1.1
Lcp1	Q61233	42	-4	2	1	-12	1.0	1.0	1.0	0.9
Ahctf1	Q8CJF7	1419	-18	2	1	-27	0.9	1.0	1.0	0.8
Btla	Q7TSA3	303	-5	2	1	-31	1.0	1.0	1.0	0.8
Fus	P56959	426	-3	1	1	13	1.0	1.0	1.0	1.1
Acp1	Q9D358	18	3	1	1	14	1.0	1.0	1.0	1.2
Eef2	P58252	136	3	1	1	8	1.0	1.0	1.0	1.1
Elac2	Q80Y81	666	-1	1	1	4	1.0	1.0	1.0	1.0
Mnda	P0DOV1	317	-3	1	1	-8	1.0	1.0	1.0	0.9
Ifi205a	Q8CGE8	296	-3	1	1	-8	1.0	1.0	1.0	0.9
Hsh2d	Q6VYH9	186	-5	1	1	-12	1.0	1.0	1.0	0.9
Stk40	Q7TNL3	218	-3	0	1	13	1.0	1.0	1.0	1.1
G6pdx	Q00612	158	-1	0	1	10	1.0	1.0	1.0	1.1
Fasn	P19096	1441	-19	0	1	8	0.8	1.0	1.0	1.1
Map2k4	P47809	264	-6	0	1	7	0.9	1.0	1.0	1.1
Nr2c1	Q505F1	104	6	0	1	7	1.1	1.0	1.0	1.1
Ctsb	P10605	93	-11	-1	1	-25	0.9	1.0	1.0	0.8
Sh2d3c	Q9QZS8	593	2	-1	1	25	1.0	1.0	1.0	1.3
Usp16	Q99LG0	125	5	-1	1	6	1.0	1.0	1.0	1.1
Vrk1	Q80X41	50	-8	-1	1	1	0.9	1.0	1.0	1.0
Capn2	O08529	82	-7	-1	1	-4	0.9	1.0	1.0	1.0
Dnajc7	Q9QYI3	175	-13	-2	1	21	0.9	1.0	1.0	1.3
Aven	Q9D9K3	157	6	-2	1	21	1.1	1.0	1.0	1.3
Tcf20	Q9EPQ8	1669	1	-2	1	2	1.0	1.0	1.0	1.0
Prpf18	Q8BM39	336	-6	-2	1	-8	0.9	1.0	1.0	0.9
Syne2	Q6ZWQ0	5334	-8	-3	1	32	0.9	1.0	1.0	1.5
Unc45a	Q99KD5	663	5	-3	1	8	1.0	1.0	1.0	1.1
Senp1	P59110	207	-10	-3	1	-2	0.9	1.0	1.0	1.0
Fubp1	Q91WJ8	144	-8	-3	1	13	0.9	1.0	1.0	1.1
Wdr75	Q3U821	396	-13	-3	1	12	0.9	1.0	1.0	1.1
Srbd1	F8WGW3	289	-8	-4	1	-5	0.9	1.0	1.0	1.0
Nup107	Q8BH74	622	-3	-4	1	19	1.0	1.0	1.0	1.2
Cisd2	Q9CQB5	92	-7	-4	1	-11	0.9	1.0	1.0	0.9
Ep400	Q8CHI8	1704	-11	-5	1	44	0.9	1.0	1.0	1.8
Luc7l2	Q7TNC4	193	4	-5	1	24	1.0	1.0	1.0	1.3
Phgdh	Q61753	18	-10	-5	1	14	0.9	1.0	1.0	1.2
Vwa9	Q8R3P6	193	-9	-6	1	5	0.9	0.9	1.0	1.1
Galt	Q03249	75	-12	-6	1	3	0.9	0.9	1.0	1.0
Gimap6	Q8K349	259	-3	-6	1	-1	1.0	0.9	1.0	1.0
Strip1	Q8C079	769	9	-6	1	42	1.1	0.9	1.0	1.7
Inpp5d	Q9ES52	220	-18	-6	1	34	0.9	0.9	1.0	1.5
Kif15	Q6P9L6	346	-2	-6	1	9	1.0	0.9	1.0	1.1

Map2k3	O09110	207	8	-6	1	8	1.1	0.9	1.0	1.1
Map2k6	P70236	196	8	-6	1	8	1.1	0.9	1.0	1.1
Toe1	Q9D2E2	285	-1	-7	1	-7	1.0	0.9	1.0	0.9
Ddx42	Q810A7	382	-3	-7	1	11	1.0	0.9	1.0	1.1
Nfic	P70255	88	-11	-7	1	3	0.9	0.9	1.0	1.0
Pak1ip1	Q9DCE5	237	0	-8	1	9	1.0	0.9	1.0	1.1
Ankrd13a	Q80UP5	141	-20	-9	1	5	0.8	0.9	1.0	1.0
Tmem55a	Q9CZX7	99	-7	-9	1	-2	0.9	0.9	1.0	1.0
Rbl2	Q64700	117	1	-9	1	2	1.0	0.9	1.0	1.0
Zc3h14	Q8BJ05	681	-14	-9	1	-17	0.9	0.9	1.0	0.9
Nudt7	Q99P30	72	-13	-10	1	-6	0.9	0.9	1.0	0.9
Rnf31	Q924T7	879	-37	-10	1	-13	0.7	0.9	1.0	0.9
Kifc5b	E9PUA5	556	-16	-10	1	-14	0.9	0.9	1.0	0.9
Kifc1	Q9QWT9	558	-16	-10	1	-14	0.9	0.9	1.0	0.9
Ptk2b	Q9QVP9	677	12	-10	1	-7	1.1	0.9	1.0	0.9
Nr2c2	P49117	204	-4	-10	1	-14	1.0	0.9	1.0	0.9
Mob4	Q6PEB6	134	-4	-11	1	2	1.0	0.9	1.0	1.0
Itpr3	P70227	1726	-15	-11	1	-2	0.9	0.9	1.0	1.0
Dmxl1	Q6PNC0	1870	-29	-11	1	12	0.8	0.9	1.0	1.1
Vps35	Q9EQH3	640	1	-11	1	7	1.0	0.9	1.0	1.1
Ctdp1	Q7TSG2	277	-10	-11	1	-4	0.9	0.9	1.0	1.0
Utrn	E9Q6R7	2283	-9	-12	1	2	0.9	0.9	1.0	1.0
Rpl18a	P62717	64	-12	-12	1	-6	0.9	0.9	1.0	0.9
Cdc42	P60766	157	-12	-12	1	-11	0.9	0.9	1.0	0.9
Syne1	Q6ZWR6	5596	-4	-12	1	-12	1.0	0.9	1.0	0.9
Flii	Q9JJ28	1069	-17	-12	1	14	0.9	0.9	1.0	1.2
Abce1	P61222	475	10	-12	1	10	1.1	0.9	1.0	1.1
Ig	P01837	106	1	-13	1	-25	1.0	0.9	1.0	0.8
Hsp90ab1	P11499	589	-25	-14	1	9	0.8	0.9	1.0	1.1
Supv3l1	Q80YD1	587	-1	-15	1	18	1.0	0.9	1.0	1.2
Snd1	Q78PY7	31	-5	-15	1	3	1.0	0.9	1.0	1.0
Tollip	Q9QZ06	229	-4	-15	1	-29	1.0	0.9	1.0	0.8
Ilkap	Q8R0F6	190	-8	-16	1	6	0.9	0.9	1.0	1.1
Ctr9	Q62018	196	-13	-16	1	20	0.9	0.9	1.0	1.3
Ipo5	Q8BKC5	110	-3	-17	1	37	1.0	0.9	1.0	1.6
Inpp5d	Q9ES52	46	-7	-17	1	5	0.9	0.9	1.0	1.0
Mrps31	Q61733	345	-2	-19	1	-23	1.0	0.8	1.0	0.8
Dffa	O54786	78	-2	-20	1	-1	1.0	0.8	1.0	1.0
Ube2o	Q6ZPJ3	593	2	-21	1	-30	1.0	0.8	1.0	0.8
Eprs	Q8CGC7	381	-32	-22	1	3	0.8	0.8	1.0	1.0
Supt6h	Q62383	1463	-16	-22	1	14	0.9	0.8	1.0	1.2
UPF0505	Q8BWQ6	925	-13	-23	1	-3	0.9	0.8	1.0	1.0
Flii	Q9JJ28	576	-26	-27	1	-8	0.8	0.8	1.0	0.9
Zc3hc1	Q80YV2	450	-24	-27	1	-22	0.8	0.8	1.0	0.8
Kif5b	Q61768	858	-17	-27	1	1	0.9	0.8	1.0	1.0
Cdca5	Q9CPY3	182	-27	-37	1	-20	0.8	0.7	1.0	0.8
Tcf25	Q8R3L2	138	3	18	0	-8	1.0	1.2	1.0	0.9
Ppp2r1a	Q76MZ3	329	2	16	0	31	1.0	1.2	1.0	1.4
Ddx1	Q91VR5	110	-1	15	0	-1	1.0	1.2	1.0	1.0
Trim28	Q62318	89	-18	13	0	27	0.8	1.1	1.0	1.4
Bckdhb	Q6P3A8	233	-21	13	0	10	0.8	1.1	1.0	1.1

Iars	Q8BU30	350	-10	12	0	13	0.9	1.1	1.0	1.1
Kif1bp	Q6ZPU9	367	-1	11	0	-11	1.0	1.1	1.0	0.9
Gnb4	P29387	148	-10	11	0	15	0.9	1.1	1.0	1.2
Gnb2	P62880	148	-10	11	0	15	0.9	1.1	1.0	1.2
Zzef1	Q5SSH7	2509	-1	9	0	10	1.0	1.1	1.0	1.1
Rhot2	Q8JZN7	216	19	9	0	10	1.2	1.1	1.0	1.1
Rnf126	Q91YL2	29	-14	7	0	5	0.9	1.1	1.0	1.0
Zfp429	Q7M6Y0	379	-5	6	0	7	1.0	1.1	1.0	1.1
Usf1	Q61069	229	-1	5	0	1	1.0	1.0	1.0	1.0
Psmg1	Q9JK23	107	6	5	0	-1	1.1	1.0	1.0	1.0
Golga2	Q921M4	981	-8	5	0	-4	0.9	1.0	1.0	1.0
Aldh9a1	Q9JLJ2	289	-6	4	0	32	0.9	1.0	1.0	1.5
Rabepk	Q8VCH5	37	11	4	0	13	1.1	1.0	1.0	1.1
Znf592	Q8BHZ4	199	5	4	0	-5	1.1	1.0	1.0	1.0
Ppp1r12c	Q3UMT1	650	4	4	0	-8	1.0	1.0	1.0	0.9
Rpl9	P51410	134	-3	3	0	-6	1.0	1.0	1.0	0.9
Plekhm1	Q7TSI1	464	10	3	0	3	1.1	1.0	1.0	1.0
Actr5	Q80US4	48	-7	3	0	2	0.9	1.0	1.0	1.0
Ctu1	Q99J10	299	-4	3	0	2	1.0	1.0	1.0	1.0
Aff3	D3YYI6	212	4	2	0	19	1.0	1.0	1.0	1.2
Luc7l2	Q7TNC4	59	-5	2	0	8	1.0	1.0	1.0	1.1
Supt16	G3X956	574	-12	1	0	21	0.9	1.0	1.0	1.3
Ptk2b	Q9QVP9	89	3	1	0	16	1.0	1.0	1.0	1.2
Snapin	Q9Z266	66	3	1	0	18	1.0	1.0	1.0	1.2
Ccar2	Q8VDP4	643	-3	1	0	-1	1.0	1.0	1.0	1.0
Nsf	P46460	11	2	1	0	-2	1.0	1.0	1.0	1.0
Cars2	Q8BYM8	470	1	1	0	-5	1.0	1.0	1.0	1.0
Hira	Q61666	750	2	0	0	10	1.0	1.0	1.0	1.1
Rasa3	Q60790	678	-3	0	0	1	1.0	1.0	1.0	1.0
Tln1	P26039	732	-12	-1	0	11	0.9	1.0	1.0	1.1
Mocs3	A2BDX3	180	-3	-1	0	8	1.0	1.0	1.0	1.1
Rbm22	Q8BHS3	84	3	-1	0	6	1.0	1.0	1.0	1.1
Hk3	Q3TRM8	914	-4	-1	0	5	1.0	1.0	1.0	1.1
Trip13	Q3UA06	14	9	-1	0	-12	1.1	1.0	1.0	0.9
Vat1	Q62465	99	-12	-1	0	14	0.9	1.0	1.0	1.2
Cpt2	P52825	639	-9	-1	0	14	0.9	1.0	1.0	1.2
Nck1	Q99M51	340	-13	-1	0	-2	0.9	1.0	1.0	1.0
Rps3	P62908	97	-10	-2	0	59	0.9	1.0	1.0	2.4
Fam168b	Q80XQ8	62	-5	-2	0	33	1.0	1.0	1.0	1.5
Map2k1	P31938	207	7	-2	0	3	1.1	1.0	1.0	1.0
Map2k2	Q63932	211	7	-2	0	3	1.1	1.0	1.0	1.0
Gnpda2	Q9CRC9	239	-8	-2	0	0	0.9	1.0	1.0	1.0
Gtf3c3	Q3TMP1	806	-18	-2	0	-12	0.9	1.0	1.0	0.9
Cse1l	Q9ERK4	842	-7	-2	0	37	0.9	1.0	1.0	1.6
Stag2	O35638	176	12	-2	0	30	1.1	1.0	1.0	1.4
Psmb7	P70195	74	-8	-2	0	13	0.9	1.0	1.0	1.1
Psmd1	Q3TXS7	898	-23	-2	0	5	0.8	1.0	1.0	1.1
Cfap20	Q8BTU1	160	-14	-2	0	2	0.9	1.0	1.0	1.0
Camsap2	Q8C1B1	649	-3	-3	0	-27	1.0	1.0	1.0	0.8
Tec	P24604	448	-10	-3	0	56	0.9	1.0	1.0	2.3
Flna	Q8BTM8	1723	-7	-3	0	-3	0.9	1.0	1.0	1.0

Mipep	A6H611	140	-3	-4	0	15	1.0	1.0	1.0	1.2
Iqsec1	Q8R0S2	904	-14	-4	0	-13	0.9	1.0	1.0	0.9
Hdac7	Q8C2B3	891	-10	-4	0	15	0.9	1.0	1.0	1.2
Zscan26	Q5RJ54	42	-8	-4	0	12	0.9	1.0	1.0	1.1
AK157302	I3ITR1	57	-16	-4	0	7	0.9	1.0	1.0	1.1
Gtf2b	P62915	37	-2	-4	0	2	1.0	1.0	1.0	1.0
Rps8	P62242	72	-6	-4	0	-2	0.9	1.0	1.0	1.0
Nt5dc1	Q8C5P5	189	0	-4	0	-6	1.0	1.0	1.0	0.9
Bysl	O54825	299	-8	-5	0	16	0.9	1.0	1.0	1.2
Maea	Q4VC33	378	-12	-5	0	1	0.9	1.0	1.0	1.0
Nosip	Q9D6T0	236	-19	-5	0	13	0.8	1.0	1.0	1.1
Hectd1	Q69ZR2	2000	-14	-5	0	-4	0.9	1.0	1.0	1.0
Wdhd1	P59328	591	-14	-6	0	13	0.9	0.9	1.0	1.1
Phf14	Q9D4H9	867	-15	-6	0	10	0.9	0.9	1.0	1.1
Iars	Q8BU30	336	-4	-6	0	7	1.0	0.9	1.0	1.1
Nxf1	Q99JX7	527	-18	-6	0	-10	0.8	0.9	1.0	0.9
Pigq	Q9QYT7	10	-32	-6	0	-68	0.8	0.9	1.0	0.6
Dennd4a	E9Q8V6	1290	-13	-7	0	39	0.9	0.9	1.0	1.6
Rbbp7	Q60973	166	-7	-7	0	3	0.9	0.9	1.0	1.0
Tssc4	Q9JHE7	205	-12	-7	0	-15	0.9	0.9	1.0	0.9
Ccar1	Q8CH18	462	-11	-7	0	11	0.9	0.9	1.0	1.1
Hnrnph2	P70333	22	-13	-7	0	9	0.9	0.9	1.0	1.1
Prpf38a	Q4FK66	158	-4	-7	0	7	1.0	0.9	1.0	1.1
Smarca5	Q91ZW3	164	-13	-7	0	-2	0.9	0.9	1.0	1.0
Flii	Q9JJ28	241	-9	-8	0	31	0.9	0.9	1.0	1.4
Sec24d	Q6NXL1	1028	-1	-8	0	4	1.0	0.9	1.0	1.0
Btk	P35991	337	-6	-8	0	-8	0.9	0.9	1.0	0.9
Cand1	Q6ZQ38	571	-9	-8	0	23	0.9	0.9	1.0	1.3
Hace1	Q3U0D9	481	-8	-8	0	-16	0.9	0.9	1.0	0.9
Raver1	Q9CW46	161	-32	-9	0	28	0.8	0.9	1.0	1.4
Scrn3	Q3TMH2	143	-12	-9	0	23	0.9	0.9	1.0	1.3
Setdb1	D3YYC3	53	-13	-9	0	14	0.9	0.9	1.0	1.2
Huwe1	Q7TMY8	1832	-4	-9	0	10	1.0	0.9	1.0	1.1
Ighm	A0A075B6A0	353	-8	-9	0	4	0.9	0.9	1.0	1.0
Ciapin1	Q8WTY4	92	1	-9	0	-4	1.0	0.9	1.0	1.0
Ring1	O35730	84	-7	-9	0	-5	0.9	0.9	1.0	1.0
Rnf2	Q9CQJ4	87	-7	-9	0	-5	0.9	0.9	1.0	1.0
Whsc1l1	Q6P2L6	1187	22	-9	0	-7	1.3	0.9	1.0	0.9
Stk24	Q99KH8	382	-7	-9	0	-5	0.9	0.9	1.0	1.0
Prdx4	O08807	54	-8	-9	0	-25	0.9	0.9	1.0	0.8
Gsr	P47791	80	-13	-10	0	49	0.9	0.9	1.0	1.9
Dock11	A2AF47	150	-11	-10	0	16	0.9	0.9	1.0	1.2
Srsf6	Q3TWW8	121	-8	-10	0	8	0.9	0.9	1.0	1.1
Tada2a	Q8CHV6	298	-4	-10	0	-4	1.0	0.9	1.0	1.0
Suz12	Q80U70	533	-7	-11	0	-19	0.9	0.9	1.0	0.8
Cnn3	Q9DAW9	59	-6	-11	0	30	0.9	0.9	1.0	1.4
Hectd1	Q69ZR2	2420	1	-11	0	13	1.0	0.9	1.0	1.1
Sbf2	E9PXF8	1563	-10	-11	0	-1	0.9	0.9	1.0	1.0
Ltf	P08071	665	-9	-11	0	-8	0.9	0.9	1.0	0.9
Usp10	P52479	251	5	-11	0	-22	1.0	0.9	1.0	0.8
Znrf2	Q71FD5	213	-16	-12	0	-3	0.9	0.9	1.0	1.0

Nbeal2	Q6ZQA0	1802	-6	-12	0	14	0.9	0.9	1.0	1.2
Nelfa	Q8BG30	131	-8	-12	0	2	0.9	0.9	1.0	1.0
Zc3hc1	Q80YV2	156	-10	-13	0	2	0.9	0.9	1.0	1.0
Bcat2	O35855	346	-14	-14	0	4	0.9	0.9	1.0	1.0
Jade2	Q6ZQF7	222	-17	-14	0	-6	0.9	0.9	1.0	0.9
Gart	Q64737	134	-18	-14	0	-11	0.9	0.9	1.0	0.9
Ctr9	Q62018	533	4	-15	0	6	1.0	0.9	1.0	1.1
Lipe	P54310	163	-34	-15	0	-7	0.7	0.9	1.0	0.9
Arid1a	A2BH40	1981	-18	-15	0	27	0.8	0.9	1.0	1.4
Pelp1	Q9DBD5	237	-13	-15	0	12	0.9	0.9	1.0	1.1
Ubash3a	Q3V3E1	224	-14	-16	0	-11	0.9	0.9	1.0	0.9
Tbk1	Q9WUN2	426	-30	-16	0	13	0.8	0.9	1.0	1.1
Ube4b	Q9ES00	319	-17	-16	0	1	0.9	0.9	1.0	1.0
Appl1	Q8K3H0	548	-14	-16	0	-10	0.9	0.9	1.0	0.9
Abcg3	Q99P81	119	-8	-16	0	-12	0.9	0.9	1.0	0.9
Syne1	Q6ZWR6	6621	-17	-17	0	-24	0.9	0.9	1.0	0.8
Ewsr1	Q61545	528	-4	-18	0	2	1.0	0.9	1.0	1.0
Pip4k2c	Q91XU3	104	-10	-18	0	-4	0.9	0.9	1.0	1.0
Ncbp1	Q3UYV9	44	-13	-18	0	-22	0.9	0.9	1.0	0.8
Tbl2	Q9R099	43	-12	-18	0	-35	0.9	0.9	1.0	0.7
Park7	Q99LX0	53	-3	-18	0	1	1.0	0.8	1.0	1.0
Ctsb	P10605	122	-17	-19	0	-41	0.9	0.8	1.0	0.7
Thoc1	Q8R3N6	156	0	-24	0	27	1.0	0.8	1.0	1.4
Znf740	Q6NZQ6	90	-2	-24	0	6	1.0	0.8	1.0	1.1
Ranbp2	Q9ERU9	2951	-23	-25	0	0	0.8	0.8	1.0	1.0
Tma16	Q9CR02	162	-3	-26	0	-10	1.0	0.8	1.0	0.9
Iars2	Q8BIJ6	91	-18	-27	0	-2	0.8	0.8	1.0	1.0
Nudcd3	Q8R1N4	262	-22	-30	0	-11	0.8	0.8	1.0	0.9
Rnh1	Q91VI7	40	-17	-34	0	5	0.9	0.7	1.0	1.1
Mospd1	Q8VEL0	74	-7	-36	0	-25	0.9	0.7	1.0	0.8
Mcmbp	Q8R3C0	334	-6	16	-1	14	0.9	1.2	1.0	1.2
Exosc10	P56960	555	15	14	-1	25	1.2	1.2	1.0	1.3
Pgk1	P09411	367	1	12	-1	19	1.0	1.1	1.0	1.2
Gvin1	L7N451	2289	-10	11	-1	8	0.9	1.1	1.0	1.1
Rbm28	Q8CGC6	368	-15	11	-1	5	0.9	1.1	1.0	1.1
Unc13d	B2RUP2	1022	11	10	-1	-11	1.1	1.1	1.0	0.9
Cpsf4	Q8BQZ5	130	0	9	-1	7	1.0	1.1	1.0	1.1
Cd5l	Q9QWK4	194	-11	9	-1	-7	0.9	1.1	1.0	0.9
Dhx36	Q8VHK9	604	7	8	-1	11	1.1	1.1	1.0	1.1
Rrp8	Q9DB85	321	5	8	-1	-6	1.1	1.1	1.0	0.9
Psmd12	Q9D8W5	31	-6	7	-1	26	0.9	1.1	1.0	1.4
Fam169b	Q8CHT6	240	2	7	-1	13	1.0	1.1	1.0	1.1
Suz12	Q80U70	496	3	7	-1	-6	1.0	1.1	1.0	0.9
Anxa11	P97384	224	-9	6	-1	29	0.9	1.1	1.0	1.4
Actr1a	P61164	222	-8	6	-1	14	0.9	1.1	1.0	1.2
Shmt2	Q9CZN7	80	-5	5	-1	15	1.0	1.1	1.0	1.2
Lime1	Q9EQR5	188	11	5	-1	-13	1.1	1.1	1.0	0.9
Nosip	Q9D6T0	185	-2	5	-1	45	1.0	1.0	1.0	1.8
Ipo5	Q8BKC5	687	-6	4	-1	12	0.9	1.0	1.0	1.1
Fam98b	Q80VD1	93	-8	4	-1	23	0.9	1.0	1.0	1.3
Flnb	Q80X90	1434	1	4	-1	-1	1.0	1.0	1.0	1.0

Trex1	Q91XB0	152	-17	3	-1	23	0.9	1.0	1.0	1.3
Cryz	P47199	250	-11	3	-1	5	0.9	1.0	1.0	1.0
Limd1	Q9QXD8	303	-2	3	-1	1	1.0	1.0	1.0	1.0
Syk	P48025	205	5	3	-1	4	1.1	1.0	1.0	1.0
Rpl32	P62911	91	-3	2	-1	-3	1.0	1.0	1.0	1.0
Rhog	P84096	22	-10	2	-1	22	0.9	1.0	1.0	1.3
Lcp1	Q61233	164	4	2	-1	18	1.0	1.0	1.0	1.2
Rtcb	Q99LF4	193	-1	2	-1	15	1.0	1.0	1.0	1.2
Ldha	P06151	163	-11	2	-1	8	0.9	1.0	1.0	1.1
Ldhb	P16125	164	-11	2	-1	8	0.9	1.0	1.0	1.1
Hnrnpa3	Q8BG05	85	1	2	-1	-1	1.0	1.0	1.0	1.0
Dok3	Q9QZK7	267	-3	2	-1	-11	1.0	1.0	1.0	0.9
Acsf2	Q8VCW8	555	-7	1	-1	13	0.9	1.0	1.0	1.1
Kifc5b	E9PUA5	218	2	1	-1	4	1.0	1.0	1.0	1.0
Kifc1	Q9QWT9	220	2	1	-1	4	1.0	1.0	1.0	1.0
Pik3r1	P26450	498	-5	1	-1	2	1.0	1.0	1.0	1.0
Arhgef18	Q6P9R4	542	-6	1	-1	-12	0.9	1.0	1.0	0.9
Tmem209	Q8BRG8	367	9	1	-1	20	1.1	1.0	1.0	1.2
Iqcb1	Q8BP00	299	0	1	-1	16	1.0	1.0	1.0	1.2
Tbc1d1	Q60949	96	-19	1	-1	14	0.8	1.0	1.0	1.2
Erap1	Q9EQH2	182	-8	1	-1	9	0.9	1.0	1.0	1.1
Dhx9	E9QNN1	245	-14	1	-1	8	0.9	1.0	1.0	1.1
Ubn2	Q80WC1	934	-1	1	-1	-8	1.0	1.0	1.0	0.9
Ring1	O35730	72	7	0	-1	15	1.1	1.0	1.0	1.2
Zfp292	Q9Z2U2	16	-1	0	-1	9	1.0	1.0	1.0	1.1
Ddx1	Q91VR5	111	-11	0	-1	7	0.9	1.0	1.0	1.1
Kif1c	O35071	974	-10	0	-1	2	0.9	1.0	1.0	1.0
Flnb	Q80X90	1326	-3	0	-1	1	1.0	1.0	1.0	1.0
Cep192	E9Q4Y4	79	10	-1	-1	4	1.1	1.0	1.0	1.0
Wdfy4	E9Q2M9	2186	1	-1	-1	-14	1.0	1.0	1.0	0.9
Armc10	Q9D0L7	146	5	-1	-1	33	1.0	1.0	1.0	1.5
Senp7	Q8BUH8	921	-11	-1	-1	11	0.9	1.0	1.0	1.1
Znf330	Q922H9	186	6	-2	-1	17	1.1	1.0	1.0	1.2
Sp140	Q6NSQ5	376	-12	-2	-1	13	0.9	1.0	1.0	1.1
Rnf114	Q9ET26	144	-3	-2	-1	13	1.0	1.0	1.0	1.1
Dnmt1	P13864	1481	-7	-2	-1	3	0.9	1.0	1.0	1.0
Macf1	E9PVY8	5009	4	-2	-1	-2	1.0	1.0	1.0	1.0
Mtmr2	Q9Z2D1	95	0	-2	-1	-3	1.0	1.0	1.0	1.0
Hnrnpul2	Q00PI9	516	-9	-2	-1	35	0.9	1.0	1.0	1.5
Sf3b1	G5E866	1244	-4	-2	-1	21	1.0	1.0	1.0	1.3
U2af1l4	Q8BGJ9	18	-2	-2	-1	4	1.0	1.0	1.0	1.0
U2af1	Q9D883	18	-2	-2	-1	4	1.0	1.0	1.0	1.0
Ftsj1	Q8CBC7	179	1	-3	-1	23	1.0	1.0	1.0	1.3
Hsp90ab1	P11499	590	-17	-3	-1	6	0.9	1.0	1.0	1.1
Rragc	Q99K70	357	0	-3	-1	3	1.0	1.0	1.0	1.0
Sept6	Q9R1T4	42	-16	-3	-1	2	0.9	1.0	1.0	1.0
Rubcn	Q80U62	45	-13	-3	-1	-3	0.9	1.0	1.0	1.0
Zc3h12d	E9QNR7	180	-9	-3	-1	-15	0.9	1.0	1.0	0.9
Raver1	Q9CW46	222	-9	-3	-1	18	0.9	1.0	1.0	1.2
Narfl	Q7TMW6	270	-18	-3	-1	13	0.9	1.0	1.0	1.1
Rab7a	P51150	83	-8	-3	-1	9	0.9	1.0	1.0	1.1

Cul4a	Q3TCH7	241	-2	-3	-1	2	1.0	1.0	1.0	1.0
Prdm2	A2A7B5	629	-2	-3	-1	-27	1.0	1.0	1.0	0.8
Cdk19	Q8BWD8	349	-19	-4	-1	7	0.8	1.0	1.0	1.1
Xiap	Q60989	213	-10	-4	-1	3	0.9	1.0	1.0	1.0
Baz1b	Q9Z277	1416	-7	-4	-1	-3	0.9	1.0	1.0	1.0
Xdh	Q00519	538	-4	-4	-1	-10	1.0	1.0	1.0	0.9
Arhgef1	Q61210	593	-11	-4	-1	40	0.9	1.0	1.0	1.7
Unc45a	Q99KD5	650	-6	-4	-1	23	0.9	1.0	1.0	1.3
Ap3b1	Q9Z1T1	508	-9	-4	-1	6	0.9	1.0	1.0	1.1
Gigyf2	Q6Y7W8	574	-10	-4	-1	-1	0.9	1.0	1.0	1.0
Zhx3	Q8C0Q2	331	-4	-4	-1	-8	1.0	1.0	1.0	0.9
Morc2a	Q69ZX6	960	-17	-4	-1	-16	0.9	1.0	1.0	0.9
Arhgef1	Q61210	594	-17	-5	-1	37	0.9	1.0	1.0	1.6
Zc3hav1	Q3UPF5	168	-12	-5	-1	6	0.9	1.0	1.0	1.1
Impa1	Q924B0	183	-9	-5	-1	6	0.9	1.0	1.0	1.1
Ahcy	P50247	79	-26	-5	-1	-3	0.8	1.0	1.0	1.0
Abce1	P61222	231	-2	-5	-1	26	1.0	1.0	1.0	1.3
Themis	Q8BGW0	454	-19	-5	-1	9	0.8	1.0	1.0	1.1
Poc5	Q9DBS8	296	-7	-5	-1	4	0.9	1.0	1.0	1.0
Usp5	P56399	219	-9	-5	-1	-9	0.9	1.0	1.0	0.9
Rtfdc1	Q99K95	51	9	-5	-1	-13	1.1	1.0	1.0	0.9
Arrb1	Q8BWG8	140	-6	-6	-1	-1	0.9	0.9	1.0	1.0
Nploc4	P60670	188	-8	-6	-1	10	0.9	0.9	1.0	1.1
Vasp	P70460	64	-5	-6	-1	4	1.0	0.9	1.0	1.0
Mia2	H3BJS0	686	-8	-6	-1	-5	0.9	0.9	1.0	1.0
Sf3a3	Q9D554	145	-7	-7	-1	20	0.9	0.9	1.0	1.3
Pogz	Q8BZH4	1263	-5	-7	-1	10	1.0	0.9	1.0	1.1
Sirt7	Q8BKJ9	267	-12	-7	-1	-3	0.9	0.9	1.0	1.0
Cpsf1	Q9EPU4	914	-8	-7	-1	-15	0.9	0.9	1.0	0.9
Ccdc88b	Q4QRL3	1233	1	-7	-1	4	1.0	0.9	1.0	1.0
Arid2	E9Q7E2	1265	1	-7	-1	-11	1.0	0.9	1.0	0.9
Gmip	Q6PGG2	528	-14	-8	-1	28	0.9	0.9	1.0	1.4
Elmo1	Q8BPU7	561	-11	-8	-1	7	0.9	0.9	1.0	1.1
Cyth4	Q80YW0	172	-21	-8	-1	6	0.8	0.9	1.0	1.1
Nckipsd	Q9ESJ4	368	6	-8	-1	-7	1.1	0.9	1.0	0.9
Asun	Q8QZV7	120	-20	-8	-1	-14	0.8	0.9	1.0	0.9
Srp54	P14576	118	-29	-8	-1	6	0.8	0.9	1.0	1.1
Mrps35	Q8BJZ4	209	-3	-9	-1	6	1.0	0.9	1.0	1.1
Pan2	Q8BGF7	667	-23	-9	-1	4	0.8	0.9	1.0	1.0
Arid2	E9Q7E2	711	-9	-9	-1	-10	0.9	0.9	1.0	0.9
Ahsa2	Q8N9S3	269	-1	-9	-1	40	1.0	0.9	1.0	1.7
Fkbp5	Q64378	394	-7	-9	-1	7	0.9	0.9	1.0	1.1
Nt5c3b	Q3UFY7	294	-5	-9	-1	-7	1.0	0.9	1.0	0.9
Nup98	Q6PFD9	1491	-11	-10	-1	-12	0.9	0.9	1.0	0.9
Grk6	O70293	71	-19	-10	-1	13	0.8	0.9	1.0	1.1
Kmt2a	P55200	1535	1	-10	-1	-3	1.0	0.9	1.0	1.0
Flna	Q8BTM8	1165	-6	-11	-1	1	0.9	0.9	1.0	1.0
UPF0183	Q922R1	82	-10	-11	-1	-5	0.9	0.9	1.0	1.0
Nxf1	Q99JX7	251	-3	-11	-1	-5	1.0	0.9	1.0	1.0
Ing1	Q9QXV3	73	-14	-11	-1	-7	0.9	0.9	1.0	0.9
Pogz	Q8BZH4	983	6	-11	-1	-13	1.1	0.9	1.0	0.9

Elk4	P41158	105	7	-11	-1	8	1.1	0.9	1.0	1.1
Mthfd1	Q922D8	863	-11	-12	-1	48	0.9	0.9	1.0	1.9
Hdlbp	Q8VDJ3	948	-8	-12	-1	5	0.9	0.9	1.0	1.1
Themis2	Q91YX0	382	-6	-12	-1	-8	0.9	0.9	1.0	0.9
Slc4a1	P04919	330	-23	-14	-1	8	0.8	0.9	1.0	1.1
Eno1	P17182	399	-7	-14	-1	-15	0.9	0.9	1.0	0.9
Pdcd4	Q61823	447	-26	-14	-1	26	0.8	0.9	1.0	1.3
Atp2a3	Q64518	377	-29	-15	-1	1	0.8	0.9	1.0	1.0
Napa	Q9DB05	84	-9	-15	-1	12	0.9	0.9	1.0	1.1
Smyd3	Q9CWR2	41	-11	-15	-1	-3	0.9	0.9	1.0	1.0
Ltf	P08071	643	-23	-16	-1	4	0.8	0.9	1.0	1.0
Por	P37040	363	-14	-16	-1	2	0.9	0.9	1.0	1.0
Kif21b	E9Q0A4	239	1	-16	-1	-10	1.0	0.9	1.0	0.9
Sf1	Q64213	292	-10	-17	-1	13	0.9	0.9	1.0	1.1
Naglu	O88325	674	-21	-17	-1	-3	0.8	0.9	1.0	1.0
Protein	Q9CYI0	182	-6	-17	-1	-13	0.9	0.9	1.0	0.9
Ran	P62827	120	-10	-17	-1	-16	0.9	0.9	1.0	0.9
Cnbp	P53996	162	-19	-18	-1	-17	0.8	0.9	1.0	0.9
Rfx1	P48377	780	-6	-18	-1	27	0.9	0.8	1.0	1.4
Tars2	Q3UQ84	585	-23	-19	-1	42	0.8	0.8	1.0	1.7
Ctnna3	Q65CL1	225	-4	-19	-1	-32	1.0	0.8	1.0	0.8
Ctnna3	Q65CL1	222	-4	-19	-1	-32	1.0	0.8	1.0	0.8
Ankle2	Q6P1H6	93	-12	-20	-1	-25	0.9	0.8	1.0	0.8
Exosc1	Q9DAA6	8	-18	-20	-1	-13	0.9	0.8	1.0	0.9
Sqstm1	Q64337	142	-5	-25	-1	10	1.0	0.8	1.0	1.1
Ctcf	Q61164	472	-3	-26	-1	2	1.0	0.8	1.0	1.0
Sri	Q6P069	57	-23	-35	-1	22	0.8	0.7	1.0	1.3
Ppm1d	Q9QZ67	97	9	20	-1	24	1.1	1.2	1.0	1.3
Dcp1b	Q3U564	238	-10	14	-1	14	0.9	1.2	1.0	1.2
Rcc2	Q8BK67	156	-20	13	-1	3	0.8	1.1	1.0	1.0
Kmt2b	O08550	2538	-11	10	-1	10	0.9	1.1	1.0	1.1
Zzef1	Q5SSH7	1304	3	10	-1	-1	1.0	1.1	1.0	1.0
Rbm47	Q91WT8	146	-9	9	-1	-5	0.9	1.1	1.0	1.0
Pcbp2	Q61990	163	4	7	-1	7	1.0	1.1	1.0	1.1
Actb	P60710	272	-10	7	-1	4	0.9	1.1	1.0	1.0
Dhx30	Q99PU8	369	4	6	-1	-2	1.0	1.1	1.0	1.0
Ywhaz	P63101	25	-1	5	-1	19	1.0	1.1	1.0	1.2
Nfkbib	Q60778	240	-2	5	-1	16	1.0	1.1	1.0	1.2
Numa1	E9Q7G0	80	-32	5	-1	6	0.8	1.1	1.0	1.1
Rbms1	Q91W59	221	-12	5	-1	20	0.9	1.0	1.0	1.2
Atxn7l3b	Q3UD01	75	-11	4	-1	30	0.9	1.0	1.0	1.4
Mccc2	Q3ULD5	267	2	4	-1	8	1.0	1.0	1.0	1.1
Flna	Q8BTM8	649	-1	4	-1	2	1.0	1.0	1.0	1.0
Trim33	Q99PP7	292	-40	4	-1	11	0.7	1.0	1.0	1.1
Prmt1	Q9JIF0	360	-7	4	-1	-9	0.9	1.0	1.0	0.9
Anxa7	Q07076	302	-10	3	-1	19	0.9	1.0	1.0	1.2
Ptpn6	P29351	171	-9	3	-1	4	0.9	1.0	1.0	1.0
Psmd9	Q9CR00	59	-6	3	-1	2	0.9	1.0	1.0	1.0
Prpf3	Q922U1	651	-12	2	-1	9	0.9	1.0	1.0	1.1
Sbf1	Q6ZPE2	552	-12	2	-1	-2	0.9	1.0	1.0	1.0
Phf3	B2RQG2	1755	-9	2	-1	-8	0.9	1.0	1.0	0.9

Cep250	Q60952	1477	13	2	-1	-8	1.1	1.0	1.0	0.9
Nelfcd	Q922L6	294	-7	2	-1	23	0.9	1.0	1.0	1.3
Rassf2	Q8BMS9	251	-11	2	-1	6	0.9	1.0	1.0	1.1
Ptpn6	P29351	102	-6	2	-1	-1	0.9	1.0	1.0	1.0
Rragb	Q6NTA4	280	2	2	-1	-4	1.0	1.0	1.0	1.0
Rraga	Q80X95	219	2	2	-1	-4	1.0	1.0	1.0	1.0
Nans	Q99J77	283	-9	1	-1	19	0.9	1.0	1.0	1.2
Smchd1	Q6P5D8	897	-8	1	-1	5	0.9	1.0	1.0	1.0
Zc3h14	Q8BJ05	628	1	1	-1	4	1.0	1.0	1.0	1.0
Anxa11	P97384	292	-22	1	-1	31	0.8	1.0	1.0	1.4
Tcp1	P11983	236	-9	1	-1	9	0.9	1.0	1.0	1.1
Heatr3	Q8BQM4	654	-2	1	-1	8	1.0	1.0	1.0	1.1
Kansl3	A2RSY1	161	-4	1	-1	3	1.0	1.0	1.0	1.0
Bank1	Q80VH0	279	-11	1	-1	2	0.9	1.0	1.0	1.0
Otulin	Q3UCV8	129	-5	0	-1	12	1.0	1.0	1.0	1.1
Pdia4	P08003	199	9	-1	-1	14	1.1	1.0	1.0	1.2
Numa1	E9Q7G0	1991	5	-1	-1	-6	1.0	1.0	1.0	0.9
Lap3	Q9CPY7	376	-3	-1	-1	4	1.0	1.0	1.0	1.0
Dctn3	Q9Z0Y1	140	-13	-1	-1	3	0.9	1.0	1.0	1.0
Kctd20	Q8CDD8	369	-5	-1	-1	2	1.0	1.0	1.0	1.0
Dock7	A2A9M4	457	-9	-1	-1	2	0.9	1.0	1.0	1.0
Casp3	P70677	170	-5	-1	-1	-1	1.0	1.0	1.0	1.0
Flad1	Q8R123	466	-12	-1	-1	-18	0.9	1.0	1.0	0.9
Grk6	O70293	201	-8	-2	-1	-1	0.9	1.0	1.0	1.0
Mrpl15	Q9CPR5	8	-5	-2	-1	-13	1.0	1.0	1.0	0.9
Ptbp1	Q922I7	23	-1	-2	-1	9	1.0	1.0	1.0	1.1
Igha	A0A0A6YXW6	183	9	-2	-1	2	1.1	1.0	1.0	1.0
Ddx58	Q6Q899	681	-6	-2	-1	-3	0.9	1.0	1.0	1.0
Pold2	O35654	83	-7	-2	-1	-4	0.9	1.0	1.0	1.0
Tln1	P26039	1023	-2	-3	-1	16	1.0	1.0	1.0	1.2
Traf3ip3	G3X949	257	-2	-3	-1	15	1.0	1.0	1.0	1.2
Pde2a	F7D3W5	369	-8	-3	-1	12	0.9	1.0	1.0	1.1
Shprh	Q7TPQ3	817	-8	-3	-1	12	0.9	1.0	1.0	1.1
Eftud2	O08810	779	-2	-3	-1	11	1.0	1.0	1.0	1.1
Tnpo3	Q6P2B1	511	-12	-3	-1	14	0.9	1.0	1.0	1.2
Brms1	Q99N20	136	-24	-3	-1	3	0.8	1.0	1.0	1.0
Setd7	Q8VHL1	200	1	-3	-1	-5	1.0	1.0	1.0	1.0
Gigyf1	Q99MR1	585	-1	-4	-1	57	1.0	1.0	1.0	2.3
Kdm2a	F6YRW4	585	-15	-4	-1	6	0.9	1.0	1.0	1.1
Ik	Q9Z1M8	263	-2	-4	-1	-4	1.0	1.0	1.0	1.0
Raver1	Q9CW46	224	-15	-4	-1	23	0.9	1.0	1.0	1.3
Lrch4	Q921G6	213	-3	-4	-1	5	1.0	1.0	1.0	1.1
Nampt	Q99KQ4	39	-27	-4	-1	-6	0.8	1.0	1.0	0.9
Pde12	Q3TIU4	41	-5	-5	-1	1	1.0	1.0	1.0	1.0
Snrnp40	Q6PE01	169	-11	-5	-1	-2	0.9	1.0	1.0	1.0
Ttc27	Q8CD92	753	8	-5	-1	-8	1.1	1.0	1.0	0.9
Otub1	Q7TQI3	212	-9	-5	-1	-4	0.9	1.0	1.0	1.0
Edc4	Q3UJB9	90	2	-6	-1	10	1.0	0.9	1.0	1.1
Mex3c	Q05A36	79	-14	-6	-1	-1	0.9	0.9	1.0	1.0
Gab3	Q8BSM5	473	-5	-6	-1	-3	1.0	0.9	1.0	1.0
Kifc5b	E9PUA5	484	-9	-6	-1	-3	0.9	0.9	1.0	1.0

Kifc1	Q9QWT9	486	-9	-6	-1	-3	0.9	0.9	1.0	1.0
Actr10	Q9QZB7	37	-15	-6	-1	6	0.9	0.9	1.0	1.1
Rps12-ps3	Q6ZWZ6	69	-6	-7	-1	2	0.9	0.9	1.0	1.0
Ppcs	Q8VDG5	308	-5	-7	-1	2	1.0	0.9	1.0	1.0
Rbck1	Q9WUB0	330	-3	-7	-1	25	1.0	0.9	1.0	1.3
Ctcf	Q61164	271	-16	-7	-1	14	0.9	0.9	1.0	1.2
Dgkz	Q80UP3	525	2	-7	-1	13	1.0	0.9	1.0	1.1
Rasal3	Q8C2K5	677	-4	-7	-1	3	1.0	0.9	1.0	1.0
Dgkz	Q80UP3	717	-11	-7	-1	0	0.9	0.9	1.0	1.0
Kat6b	Q8BRB7	619	-9	-8	-1	34	0.9	0.9	1.0	1.5
Kat8	Q9D1P2	260	-9	-8	-1	34	0.9	0.9	1.0	1.5
Rcc1	Q8VE37	352	-10	-8	-1	32	0.9	0.9	1.0	1.5
Nelfb	Q8C4Y3	235	-7	-8	-1	25	0.9	0.9	1.0	1.3
Supt16	G3X956	323	4	-8	-1	22	1.0	0.9	1.0	1.3
U2af2	P26369	429	-10	-8	-1	10	0.9	0.9	1.0	1.1
Pik3r4	Q8VD65	358	13	-8	-1	7	1.1	0.9	1.0	1.1
Ppm1g	Q61074	13	-2	-8	-1	-3	1.0	0.9	1.0	1.0
Prf1	P10820	406	5	-8	-1	4	1.1	0.9	1.0	1.0
Lims1	Q99JW4	181	-6	-8	-1	-5	0.9	0.9	1.0	1.0
Trim56	Q80VI1	24	-12	-8	-1	-7	0.9	0.9	1.0	0.9
Polr2j	Q6PI63	31	-6	-9	-1	4	0.9	0.9	1.0	1.0
Sdha	Q8K2B3	238	-25	-9	-1	-11	0.8	0.9	1.0	0.9
Cnbp	P53996	141	-7	-10	-1	10	0.9	0.9	1.0	1.1
Fxr1	Q61584	77	-6	-10	-1	6	0.9	0.9	1.0	1.1
Tnks1bp1	P58871	136	-22	-10	-1	-16	0.8	0.9	1.0	0.9
Fgd4	Q91ZT5	234	-10	-10	-1	-22	0.9	0.9	1.0	0.8
Nsf	P46460	599	-6	-10	-1	-28	0.9	0.9	1.0	0.8
Top1	Q04750	507	-20	-11	-1	12	0.8	0.9	1.0	1.1
Macf1	E9PVY8	5306	-3	-11	-1	4	1.0	0.9	1.0	1.0
Cdc23	Q8BGZ4	500	-2	-11	-1	-4	1.0	0.9	1.0	1.0
Zyx	Q62523	376	-6	-11	-1	-8	0.9	0.9	1.0	0.9
Hcfc2	G5E837	491	1	-11	-1	-29	1.0	0.9	1.0	0.8
Wapl	Q65Z40	1180	-14	-12	-1	23	0.9	0.9	1.0	1.3
Nhsl2	B1AXH1	796	-11	-12	-1	2	0.9	0.9	1.0	1.0
Wdr81	Q5ND34	1219	-14	-13	-1	14	0.9	0.9	1.0	1.2
Cul2	Q9D4H8	385	-16	-13	-1	14	0.9	0.9	1.0	1.2
Dstyk	Q6XUX1	840	-4	-13	-1	5	1.0	0.9	1.0	1.1
Plaa	P27612	263	-10	-13	-1	10	0.9	0.9	1.0	1.1
Eif3d	O70194	438	-12	-14	-1	16	0.9	0.9	1.0	1.2
Cpsf6	Q6NVF9	476	-12	-14	-1	-2	0.9	0.9	1.0	1.0
Rbbp5	Q8BX09	233	-6	-14	-1	-2	0.9	0.9	1.0	1.0
Sdcbp	O08992	119	-18	-14	-1	5	0.8	0.9	1.0	1.0
Xpo6	Q924Z6	324	8	-14	-1	5	1.1	0.9	1.0	1.0
Pkn2	Q8BWW9	118	-3	-14	-1	-8	1.0	0.9	1.0	0.9
Zfp318	B0V2M3	1592	-14	-15	-1	5	0.9	0.9	1.0	1.0
Tkt	P40142	225	-15	-15	-1	-3	0.9	0.9	1.0	1.0
Elmo3	Q8BYZ7	398	3	-15	-1	15	1.0	0.9	1.0	1.2
Isoc2a	P85094	206	-14	-15	-1	3	0.9	0.9	1.0	1.0
Ears2	Q9CXJ1	341	-14	-15	-1	-26	0.9	0.9	1.0	0.8
Syne3	Q4FZC9	75	-16	-16	-1	0	0.9	0.9	1.0	1.0
Csnk2b	P67871	109	-33	-16	-1	-18	0.8	0.9	1.0	0.8

Hopx	Q8R1H0	68	-6	-17	-1	18	0.9	0.9	1.0	1.2
Lims1	Q99JW4	184	-5	-17	-1	-7	1.0	0.9	1.0	0.9
Lsm7	Q9CQQ8	85	3	-18	-1	12	1.0	0.9	1.0	1.1
Pan3	Q640Q5	584	-24	-18	-1	-4	0.8	0.8	1.0	1.0
Ovca2	Q9D7E3	152	-13	-18	-1	-10	0.9	0.8	1.0	0.9
2610044O	A0A087WRJ1	133	-12	-19	-1	-18	0.9	0.8	1.0	0.9
Cdc37	Q61081	337	-8	-20	-1	-6	0.9	0.8	1.0	0.9
Osbpl8	B9EJ86	255	-13	-20	-1	-7	0.9	0.8	1.0	0.9
Phf5a	P83870	11	20	-25	-1	-8	1.3	0.8	1.0	0.9
Kpna6	O35345	238	-9	-25	-1	-8	0.9	0.8	1.0	0.9
Kpna1	Q60960	240	-9	-25	-1	-8	0.9	0.8	1.0	0.9
Plec	Q9QXS1	1000	-14	-27	-1	9	0.9	0.8	1.0	1.1
Tars	Q9D0R2	106	-1	-28	-1	11	1.0	0.8	1.0	1.1
Ppp1cb	P62141	157	-14	-29	-1	11	0.9	0.8	1.0	1.1
Ppp1cc	P63087	158	-14	-29	-1	11	0.9	0.8	1.0	1.1
Trmt2a	Q8BNV1	101	-26	-30	-1	1	0.8	0.8	1.0	1.0
Csnk1a1	Q8BK63	150	-30	-33	-1	19	0.8	0.8	1.0	1.2
Apex1	P28352	64	-27	-45	-1	-46	0.8	0.7	1.0	0.7
Dhx8	A2A4P0	1086	16	25	-2	26	1.2	1.3	1.0	1.3
Gbp9	Q8BTS3	308	-17	15	-2	14	0.9	1.2	1.0	1.2
Mcm2	P97310	584	5	14	-2	24	1.1	1.2	1.0	1.3
Dpp4	P28843	545	6	13	-2	13	1.1	1.1	1.0	1.1
Znfx1	Q8R151	1538	8	13	-2	10	1.1	1.1	1.0	1.1
Pstpip1	P97814	148	34	12	-2	22	1.5	1.1	1.0	1.3
Slc27a4	Q91VE0	560	-2	10	-2	4	1.0	1.1	1.0	1.0
Ddx39a	Q8VDW0	223	-6	10	-2	0	0.9	1.1	1.0	1.0
Ptpre	P49446	286	7	9	-2	5	1.1	1.1	1.0	1.1
Supt6h	Q62383	575	-9	8	-2	6	0.9	1.1	1.0	1.1
Arhgap30	Q640N3	447	-5	7	-2	11	1.0	1.1	1.0	1.1
Pdha1	P35486	218	0	7	-2	6	1.0	1.1	1.0	1.1
Zfyve19	Q9DAZ9	29	-4	6	-2	3	1.0	1.1	1.0	1.0
Copg1	Q9QZE5	129	-1	6	-2	3	1.0	1.1	1.0	1.0
Rnf31	Q924T7	716	-5	5	-2	25	1.0	1.0	1.0	1.3
Usp5	P56399	532	-1	5	-2	19	1.0	1.0	1.0	1.2
Stx8	O88983	126	-5	5	-2	3	1.0	1.0	1.0	1.0
Mcm3	P25206	148	-10	5	-2	-5	0.9	1.0	1.0	1.0
Tatdn1	Q6P8M1	123	-3	4	-2	18	1.0	1.0	1.0	1.2
Mcm7	Q61881	378	-5	4	-2	18	1.0	1.0	1.0	1.2
Exoc3	Q6KAR6	531	-8	4	-2	10	0.9	1.0	1.0	1.1
Tnks1bp1	P58871	1346	25	4	-2	9	1.3	1.0	1.0	1.1
Exosc3	Q7TQK4	183	-6	4	-2	7	0.9	1.0	1.0	1.1
Msh3	A0A087WQ16	166	10	4	-2	1	1.1	1.0	1.0	1.0
Ears2	Q9CXJ1	116	-3	3	-2	27	1.0	1.0	1.0	1.4
Gprasp1	Q5U4C1	1094	12	3	-2	23	1.1	1.0	1.0	1.3
Cwc27	Q3TKY6	164	-3	3	-2	9	1.0	1.0	1.0	1.1
Ppp4r1	E9QPR5	224	-8	3	-2	-6	0.9	1.0	1.0	0.9
Atg2b	Q80XK6	1595	8	3	-2	17	1.1	1.0	1.0	1.2
Myh9	Q8VDD5	790	-2	2	-2	42	1.0	1.0	1.0	1.7
Adrbk2	Q3UYH7	340	-4	2	-2	10	1.0	1.0	1.0	1.1
Grk2	Q99MK8	340	-4	2	-2	10	1.0	1.0	1.0	1.1
Uncharact	Q3UTZ3	418	1	2	-2	9	1.0	1.0	1.0	1.1

Raly	Q64012	124	-8	2	-2	8	0.9	1.0	1.0	1.1
Vrk1	Q80X41	68	-7	2	-2	7	0.9	1.0	1.0	1.1
Kbtbd4	Q8R179	84	13	2	-2	2	1.1	1.0	1.0	1.0
Dtx3l	Q3UIR3	188	-16	2	-2	-8	0.9	1.0	1.0	0.9
Glt28d2	Q8BML3	86	-2	1	-2	22	1.0	1.0	1.0	1.3
Syk	P48025	591	-14	1	-2	20	0.9	1.0	1.0	1.2
Coro1a	O89053	40	-6	1	-2	15	0.9	1.0	1.0	1.2
Limk1	P53668	349	-8	1	-2	40	0.9	1.0	1.0	1.7
Chd7	A2AJK6	1308	-1	1	-2	26	1.0	1.0	1.0	1.3
Chd6	A3KFM7	810	-1	1	-2	26	1.0	1.0	1.0	1.3
Chd8	Q09XV5	1163	-1	1	-2	26	1.0	1.0	1.0	1.3
Lrrk2	Q5S006	1770	-7	1	-2	12	0.9	1.0	1.0	1.1
Ube4b	Q9ES00	113	8	1	-2	-16	1.1	1.0	1.0	0.9
Ifi47	Q61635	317	-3	0	-2	4	1.0	1.0	1.0	1.0
Smg1	Q8BKX6	252	2	-1	-2	-7	1.0	1.0	1.0	0.9
Iqgap1	Q9JKF1	781	1	-1	-2	18	1.0	1.0	1.0	1.2
Dcun1d1	Q9QZ73	115	-8	-1	-2	3	0.9	1.0	1.0	1.0
Ccdc22	Q9JIG7	369	-7	-1	-2	-2	0.9	1.0	1.0	1.0
Dock8	Q8C147	392	-1	-2	-2	15	1.0	1.0	1.0	1.2
Ndufv2	Q9D6J6	223	-8	-2	-2	1	0.9	1.0	1.0	1.0
Zwint	Q9CQU5	118	-2	-2	-2	-3	1.0	1.0	1.0	1.0
Mlh1	Q9JK91	498	5	-2	-2	-3	1.0	1.0	1.0	1.0
Nup205	A0A0J9YUD5	978	-8	-2	-2	6	0.9	1.0	1.0	1.1
Lsp1	P19973	10	24	-2	-2	0	1.3	1.0	1.0	1.0
Flna	Q8BTM8	717	-3	-2	-2	-2	1.0	1.0	1.0	1.0
Psma7	Q9Z2U0	91	-11	-3	-2	6	0.9	1.0	1.0	1.1
Zfp433	A0A0J9YUD4	370	7	-3	-2	-6	1.1	1.0	1.0	0.9
Rnf213	E9Q555	1947	-10	-3	-2	-6	0.9	1.0	1.0	0.9
Itpr3	P70227	393	-1	-3	-2	-18	1.0	1.0	1.0	0.9
Akap9	Q70FJ1	2681	5	-3	-2	-32	1.0	1.0	1.0	0.8
Gapvd1	Q6PAR5	176	-2	-3	-2	38	1.0	1.0	1.0	1.6
Gtf3c1	Q8K284	1051	-5	-3	-2	38	1.0	1.0	1.0	1.6
Grk6	O70293	203	-2	-3	-2	17	1.0	1.0	1.0	1.2
Crebbp	F8VPR5	379	3	-3	-2	13	1.0	1.0	1.0	1.1
Map2k2	Q63932	385	-4	-3	-2	11	1.0	1.0	1.0	1.1
Anapc2	Q8BZQ7	246	-7	-4	-2	55	0.9	1.0	1.0	2.2
As3mt	Q91WU5	86	2	-4	-2	18	1.0	1.0	1.0	1.2
Tars	Q9D0R2	655	-4	-4	-2	2	1.0	1.0	1.0	1.0
Mmtag2	Q99LX5	58	2	-4	-2	-5	1.0	1.0	1.0	1.0
Dock9	F8VPN7	2075	-8	-4	-2	32	0.9	1.0	1.0	1.5
Hsd11b1	P50172	241	-13	-4	-2	6	0.9	1.0	1.0	1.1
Golph3	Q9CRA5	108	-15	-4	-2	-7	0.9	1.0	1.0	0.9
Pds5a	E9QPI5	91	-22	-4	-2	-11	0.8	1.0	1.0	0.9
Pds5b	Q4VA53	82	-22	-4	-2	-11	0.8	1.0	1.0	0.9
Macf1	E9PVY8	5091	-10	-5	-2	-1	0.9	1.0	1.0	1.0
Rps29	P62274	39	-7	-5	-2	-15	0.9	1.0	1.0	0.9
Rab44	Q8CB87	141	-2	-5	-2	10	1.0	1.0	1.0	1.1
Mycbp2	E9PUJ6	3262	3	-5	-2	2	1.0	1.0	1.0	1.0
Dffa	O54786	47	-6	-5	-2	-2	0.9	1.0	1.0	1.0
Gripap1	Q8VD04	515	-1	-5	-2	-9	1.0	1.0	1.0	0.9
Npepl1	Q6NSR8	284	-16	-5	-2	-16	0.9	1.0	1.0	0.9

Acd	Q5EE38	359	15	-6	-2	-11	1.2	0.9	1.0	0.9
Sp3	O70494	602	-10	-6	-2	14	0.9	0.9	1.0	1.2
Txlna	Q6PAM1	531	-13	-6	-2	2	0.9	0.9	1.0	1.0
Arfip1	G5E8V9	76	-7	-6	-2	-7	0.9	0.9	1.0	0.9
Nfam1	Q8R4V1	228	-1	-6	-2	-10	1.0	0.9	1.0	0.9
Cep128	Q8BI22	532	-10	-7	-2	40	0.9	0.9	1.0	1.7
Pik3cb	Q8BTI9	238	-13	-7	-2	15	0.9	0.9	1.0	1.2
Dennd1c	Q8CFK6	412	-3	-7	-2	9	1.0	0.9	1.0	1.1
Zbtb18	Q9WUK6	13	-8	-7	-2	8	0.9	0.9	1.0	1.1
Eci2	Q9WUR2	365	-12	-7	-2	4	0.9	0.9	1.0	1.0
D2Wsu81e	Q3UHX9	240	-6	-7	-2	-9	0.9	0.9	1.0	0.9
Abl1	P00520	1093	-8	-7	-2	-5	0.9	0.9	1.0	1.0
Aspscr1	Q8VBT9	109	1	-7	-2	-12	1.0	0.9	1.0	0.9
Cse1l	Q9ERK4	272	-13	-8	-2	21	0.9	0.9	1.0	1.3
Tgm2	P21981	370	-8	-8	-2	7	0.9	0.9	1.0	1.1
Sucla2	Q9Z2I9	152	-12	-8	-2	2	0.9	0.9	1.0	1.0
Polr3a	B2RXC6	961	8	-8	-2	4	1.1	0.9	1.0	1.0
Rufy1	Q8BIJ7	355	-11	-8	-2	4	0.9	0.9	1.0	1.0
Nrdc	Q8BHG1	685	-4	-8	-2	0	1.0	0.9	1.0	1.0
Hdac9	Q99N13	532	-3	-8	-2	-17	1.0	0.9	1.0	0.9
Nol11	Q8BJW5	286	-21	-9	-2	2	0.8	0.9	1.0	1.0
Rfc1	G3UWX1	749	-6	-9	-2	-6	0.9	0.9	1.0	0.9
Cct3	P80318	213	-15	-9	-2	12	0.9	0.9	1.0	1.1
Limd2	Q8BGB5	41	-11	-9	-2	6	0.9	0.9	1.0	1.1
Srp68	Q8BMA6	172	-4	-9	-2	-4	1.0	0.9	1.0	1.0
Parp1	Q921K2	162	-18	-9	-2	-15	0.9	0.9	1.0	0.9
Gmip	Q6PGG2	570	-6	-10	-2	28	0.9	0.9	1.0	1.4
Guk1	Q64520	98	-9	-10	-2	15	0.9	0.9	1.0	1.2
Gipc3	Q8R5M0	194	-31	-10	-2	2	0.8	0.9	1.0	1.0
Top2a	Q01320	1205	-3	-10	-2	-2	1.0	0.9	1.0	1.0
Leo1	Q5XJE5	531	-5	-10	-2	-12	1.0	0.9	1.0	0.9
Gba2	Q69ZF3	858	-11	-10	-2	41	0.9	0.9	1.0	1.7
Kmt2d	Q6PDK2	5365	-7	-10	-2	16	0.9	0.9	1.0	1.2
Dgkd	E9PUQ8	508	-23	-10	-2	11	0.8	0.9	1.0	1.1
Msh3	A0A087WQ16	721	-4	-10	-2	9	1.0	0.9	1.0	1.1
Pitpnc1	Q8K4R4	327	-4	-11	-2	15	1.0	0.9	1.0	1.2
Nup188	Q6ZQH8	1540	-13	-11	-2	1	0.9	0.9	1.0	1.0
Map3k4	O08648	1593	-3	-11	-2	-2	1.0	0.9	1.0	1.0
Zc3h12d	E9QNR7	418	-12	-11	-2	23	0.9	0.9	1.0	1.3
Fdxr	Q61578	398	-20	-12	-2	9	0.8	0.9	1.0	1.1
Naip2	Q9QUK4	1379	-4	-12	-2	8	1.0	0.9	1.0	1.1
Znhit3	Q9CQK1	34	-11	-12	-2	3	0.9	0.9	1.0	1.0
Utp20	E9QK83	2059	-8	-12	-2	-3	0.9	0.9	1.0	1.0
Eif2ak4	Q9QZ05	1244	-11	-12	-2	33	0.9	0.9	1.0	1.5
Myo1c	Q9WTI7	480	-15	-13	-2	12	0.9	0.9	1.0	1.1
Nsa2	Q9CR47	154	-15	-13	-2	-12	0.9	0.9	1.0	0.9
Galnt6	Q8C7U7	141	-24	-13	-2	-35	0.8	0.9	1.0	0.7
Pip4k2a	O70172	94	-4	-13	-2	15	1.0	0.9	1.0	1.2
Pip4k2b	Q80XI4	99	-4	-13	-2	15	1.0	0.9	1.0	1.2
Lcmt1	A2RTH5	256	9	-13	-2	-3	1.1	0.9	1.0	1.0
Nup133	Q8R0G9	1036	-13	-14	-2	-6	0.9	0.9	1.0	0.9

Cep44	Q5HZK1	145	1	-14	-2	-13	1.0	0.9	1.0	0.9
Flii	Q9JJ28	808	-14	-14	-2	13	0.9	0.9	1.0	1.1
Smarcb1	Q9Z0H3	350	-11	-14	-2	11	0.9	0.9	1.0	1.1
Msh6	P54276	615	-21	-14	-2	9	0.8	0.9	1.0	1.1
Mtnd3	P03899	39	-15	-15	-2	19	0.9	0.9	1.0	1.2
Glul	P15105	269	-18	-15	-2	-3	0.9	0.9	1.0	1.0
Nedd9	O35177	783	-7	-16	-2	16	0.9	0.9	1.0	1.2
Arid5a	Q3U108	125	-18	-16	-2	6	0.8	0.9	1.0	1.1
Asrgl1	Q8C0M9	132	-6	-16	-2	-3	0.9	0.9	1.0	1.0
Pus7l	Q8CE46	561	-7	-16	-2	-16	0.9	0.9	1.0	0.9
Adh5	P28474	100	-16	-17	-2	20	0.9	0.9	1.0	1.2
Dcun1d1	Q9QZ73	29	-14	-17	-2	7	0.9	0.9	1.0	1.1
Dennd1b	Q3U1T9	392	-5	-18	-2	13	1.0	0.9	1.0	1.1
Epg5	Q80TA9	1195	4	-18	-2	2	1.0	0.9	1.0	1.0
Lrrc14	Q8VC16	15	-7	-18	-2	-2	0.9	0.9	1.0	1.0
Sdha	Q8K2B3	189	-12	-18	-2	1	0.9	0.8	1.0	1.0
Copb1	Q9JIF7	102	-17	-18	-2	-13	0.9	0.8	1.0	0.9
Pias1	O88907	399	-13	-19	-2	-33	0.9	0.8	1.0	0.8
Polr3b	P59470	161	-4	-19	-2	55	1.0	0.8	1.0	2.2
Golgb1	E9PVZ8	828	-9	-19	-2	-7	0.9	0.8	1.0	0.9
Eif5	P59325	122	-31	-22	-2	33	0.8	0.8	1.0	1.5
Nudt9	Q8BVU5	195	-11	-24	-2	15	0.9	0.8	1.0	1.2
Sh2d2a	Q9QXK9	137	-21	-24	-2	-6	0.8	0.8	1.0	0.9
Uhrf1	Q8VDF2	166	15	-27	-2	-5	1.2	0.8	1.0	1.0
Ftl1-ps1	Q9CPX4	127	-14	-27	-2	-47	0.9	0.8	1.0	0.7
Eif2s3x	Q9Z0N1	348	-25	-31	-2	-36	0.8	0.8	1.0	0.7
Eif2s3y	Q9Z0N2	348	-25	-31	-2	-36	0.8	0.8	1.0	0.7
Ndor1	A2AI05	487	-23	-37	-2	-1	0.8	0.7	1.0	1.0
Ptpn7	Q8BUM3	299	-27	-43	-2	-4	0.8	0.7	1.0	1.0
Tradd	Q3U0V2	197	3	21	-2	-13	1.0	1.3	1.0	0.9
Nme7	Q3UMG6	4	16	20	-2	15	1.2	1.3	1.0	1.2
Hmbs	P22907	114	-10	19	-2	30	0.9	1.2	1.0	1.4
Pcf11	G3X9Z4	102	20	19	-2	-12	1.2	1.2	1.0	0.9
Emg1	O35130	66	-10	17	-2	2	0.9	1.2	1.0	1.0
Ppp6r3	Q922D4	575	15	12	-2	21	1.2	1.1	1.0	1.3
Pitpnc1	Q8K4R4	136	3	11	-2	10	1.0	1.1	1.0	1.1
Txnip	Q8BG60	36	4	9	-2	13	1.0	1.1	1.0	1.1
Polr2b	Q8CFI7	221	-12	9	-2	13	0.9	1.1	1.0	1.1
Nop58	Q6DFW4	139	-7	8	-2	11	0.9	1.1	1.0	1.1
Fig4	Q91WF7	839	-1	8	-2	-33	1.0	1.1	1.0	0.8
Oard1	Q8R5F3	24	-5	7	-2	21	1.0	1.1	1.0	1.3
Snx4	Q91YJ2	172	-2	7	-2	6	1.0	1.1	1.0	1.1
Lax1	Q8BHB3	283	-3	7	-2	5	1.0	1.1	1.0	1.1
Tpm4	Q6IRU2	247	6	6	-2	-2	1.1	1.1	1.0	1.0
Rcbtb2	Q99LJ7	201	-17	6	-2	-18	0.9	1.1	1.0	0.9
Samm50	Q8BGH2	237	-7	5	-2	20	0.9	1.1	1.0	1.2
Elp4	Q9ER73	359	-7	5	-2	4	0.9	1.1	1.0	1.0
Setd2	E9Q5F9	1445	1	5	-2	0	1.0	1.1	1.0	1.0
Srrt	Q99MR6	440	1	5	-2	18	1.0	1.0	1.0	1.2
Zc3h3	Q8CHP0	773	-13	4	-2	13	0.9	1.0	1.0	1.1
Nat10	Q8K224	505	-11	4	-2	9	0.9	1.0	1.0	1.1

Syne2	Q6ZWQ0	5722	7	3	-2	8	1.1	1.0	1.0	1.1
Aip	O08915	90	-7	3	-2	21	0.9	1.0	1.0	1.3
Pcyt1a	P49586	73	5	2	-2	19	1.0	1.0	1.0	1.2
Tbrg1	Q3UB74	227	7	2	-2	9	1.1	1.0	1.0	1.1
Dbnl	Q62418	127	-2	2	-2	8	1.0	1.0	1.0	1.1
Rasal3	Q8C2K5	875	6	2	-2	-3	1.1	1.0	1.0	1.0
Gnb4	P29387	149	-15	2	-2	8	0.9	1.0	1.0	1.1
Gnb2	P62880	149	-15	2	-2	8	0.9	1.0	1.0	1.1
Lims1	Q99JW4	255	-5	2	-2	-3	1.0	1.0	1.0	1.0
Aldh1b1	Q9CZS1	321	-16	1	-2	54	0.9	1.0	1.0	2.2
Smug1	Q6P5C5	167	-4	1	-2	45	1.0	1.0	1.0	1.8
Dnmt1	P13864	1479	-19	1	-2	4	0.8	1.0	1.0	1.0
Fam49a	Q8BHZ0	83	-5	1	-2	4	1.0	1.0	1.0	1.0
Aldh5a1	Q8BWF0	328	4	1	-2	-6	1.0	1.0	1.0	0.9
Pcca	Q91ZA3	107	-2	1	-2	-8	1.0	1.0	1.0	0.9
Ipo5	Q8BKC5	915	9	1	-2	10	1.1	1.0	1.0	1.1
Wdr7	Q920I9	74	-6	1	-2	5	0.9	1.0	1.0	1.0
Gtf3c1	Q8K284	1330	-8	1	-2	2	0.9	1.0	1.0	1.0
Myo9b	E9PZW8	1829	3	1	-2	-14	1.0	1.0	1.0	0.9
Slc25a4	P48962	160	-9	0	-2	20	0.9	1.0	1.0	1.2
Polr2a	P08775	1407	-6	0	-2	9	0.9	1.0	1.0	1.1
Ppme1	Q8BVQ5	238	-6	0	-2	5	0.9	1.0	1.0	1.0
Tnpo1	Q8BFY9	862	-2	-1	-2	4	1.0	1.0	1.0	1.0
Trappc8	E9PWG2	306	5	-1	-2	3	1.1	1.0	1.0	1.0
Ap3m1	Q9JKC8	288	-9	-1	-2	-4	0.9	1.0	1.0	1.0
Akap13	E9Q394	2649	-20	-1	-2	-8	0.8	1.0	1.0	0.9
Rasa4	Q6PFQ7	223	-14	-1	-2	-10	0.9	1.0	1.0	0.9
Sri	Q6P069	162	-9	-1	-2	17	0.9	1.0	1.0	1.2
Tbl1xr1	Q8BHJ5	383	5	-1	-2	14	1.0	1.0	1.0	1.2
Cdc5l	Q6A068	769	-3	-1	-2	3	1.0	1.0	1.0	1.0
Ncf2	O70145	513	-4	-1	-2	-8	1.0	1.0	1.0	0.9
Uimc1	Q5U5Q9	611	-10	-1	-2	-13	0.9	1.0	1.0	0.9
Borcs7	Q9CRC6	27	-8	-2	-2	29	0.9	1.0	1.0	1.4
Smad2	Q62432	41	-15	-2	-2	-4	0.9	1.0	1.0	1.0
Uncharact	Q3U4G0	277	-8	-2	-2	-5	0.9	1.0	1.0	1.0
Sike1	Q9CPR7	164	-11	-2	-2	-5	0.9	1.0	1.0	1.0
Mcm2	P97310	522	-11	-2	-2	20	0.9	1.0	1.0	1.2
Eef1a1	P10126	411	2	-2	-2	17	1.0	1.0	1.0	1.2
Cad	B2RQC6	179	-12	-2	-2	16	0.9	1.0	1.0	1.2
Dpysl2	O08553	248	-20	-2	-2	15	0.8	1.0	1.0	1.2
Zfp644	E9QA22	508	1	-2	-2	8	1.0	1.0	1.0	1.1
Uqcrfs1	Q9CR68	51	-19	-2	-2	-6	0.8	1.0	1.0	0.9
Adcy6	F8VQ52	62	0	-2	-2	-8	1.0	1.0	1.0	0.9
Tefm	Q5SSK3	53	-8	-2	-2	-11	0.9	1.0	1.0	0.9
Hnrnpl	Q8R081	449	-11	-3	-2	5	0.9	1.0	1.0	1.0
Parp10	Q8CIE4	47	-9	-3	-2	0	0.9	1.0	1.0	1.0
Sept6	Q9R1T4	388	-7	-3	-2	-7	0.9	1.0	1.0	0.9
Oas3	Q8VI93	524	-23	-3	-2	12	0.8	1.0	1.0	1.1
Hnrnpul2	Q00PI9	406	-3	-3	-2	3	1.0	1.0	1.0	1.0
Ylpm1	Q9R0I7	1207	-15	-3	-2	-1	0.9	1.0	1.0	1.0
Soat1	Q61263	61	-3	-3	-2	-13	1.0	1.0	1.0	0.9

Ehbp1l1	E9QP49	1559	6	-4	-2	4	1.1	1.0	1.0	1.0
Nt5c2	Q3V1L4	181	-3	-4	-2	1	1.0	1.0	1.0	1.0
Tha1	Q6XPS7	198	-22	-4	-2	-5	0.8	1.0	1.0	1.0
Shcbp1	Q9Z179	148	-4	-4	-2	6	1.0	1.0	1.0	1.1
Heatr1	G3X9B1	182	-10	-4	-2	-1	0.9	1.0	1.0	1.0
Numa1	E9Q7G0	652	2	-4	-2	-3	1.0	1.0	1.0	1.0
Nup214	Q80U93	722	-11	-4	-2	-5	0.9	1.0	1.0	1.0
Myh9	Q8VDD5	988	-7	-4	-2	-7	0.9	1.0	1.0	0.9
Kbtbd11	Q8BNW9	427	-12	-4	-2	-11	0.9	1.0	1.0	0.9
Syne2	Q6ZWQ0	3429	-20	-5	-2	37	0.8	1.0	1.0	1.6
Ric8a	Q3TIR3	329	-10	-5	-2	6	0.9	1.0	1.0	1.1
Serpinb6b	O08804	62	-16	-5	-2	5	0.9	1.0	1.0	1.1
Mcmbp	Q8R3C0	249	-7	-5	-2	-6	0.9	1.0	1.0	0.9
Itprip	Q3TNL8	254	-11	-5	-2	14	0.9	1.0	1.0	1.2
Hspa4	Q3U2G2	245	7	-5	-2	9	1.1	1.0	1.0	1.1
Plcb2	A3KGF7	1104	-14	-5	-2	-11	0.9	1.0	1.0	0.9
Zadh2	Q8BGC4	330	-18	-6	-2	18	0.8	0.9	1.0	1.2
Trrap	A0A1D5RLL4	2876	-4	-6	-2	11	1.0	0.9	1.0	1.1
Ddx23	D3Z0M9	691	-3	-6	-2	5	1.0	0.9	1.0	1.0
Pdia3	P27773	244	-15	-6	-2	5	0.9	0.9	1.0	1.0
H2-K1	P01901	142	-1	-6	-2	3	1.0	0.9	1.0	1.0
Epc2	Q8C0I4	627	6	-6	-2	-10	1.1	0.9	1.0	0.9
Plec	Q9QXS1	3117	-4	-6	-2	-2	1.0	0.9	1.0	1.0
Elk3	P41971	170	-14	-6	-2	-19	0.9	0.9	1.0	0.8
Rpl36	Q6ZWZ4	48	-13	-7	-2	-1	0.9	0.9	1.0	1.0
Asrgl1	Q8C0M9	317	-8	-7	-2	-19	0.9	0.9	1.0	0.8
Pdlim1	O70400	73	-6	-7	-2	-1	0.9	0.9	1.0	1.0
Cyld	Q80TQ2	852	-1	-8	-2	4	1.0	0.9	1.0	1.0
Prkcd	P28867	280	-5	-8	-2	-4	1.0	0.9	1.0	1.0
Prkcq	Q02111	281	-5	-8	-2	-4	1.0	0.9	1.0	1.0
Hexim1	Q8R409	79	0	-8	-2	-15	1.0	0.9	1.0	0.9
Fam76b	Q80XP8	28	4	-8	-2	-20	1.0	0.9	1.0	0.8
Chd9	Q8BYH8	2271	-20	-8	-2	20	0.8	0.9	1.0	1.2
Plcg2	Q8CIH5	849	8	-8	-2	18	1.1	0.9	1.0	1.2
Mul1	Q8VCM5	326	-5	-8	-2	17	1.0	0.9	1.0	1.2
Eml2	Q7TNG5	395	-9	-8	-2	10	0.9	0.9	1.0	1.1
Rhog	P84096	18	-17	-8	-2	8	0.9	0.9	1.0	1.1
Prkdc	P97313	1497	-16	-9	-2	40	0.9	0.9	1.0	1.7
Gtf2i	Q9ESZ8	215	-1	-9	-2	-7	1.0	0.9	1.0	0.9
Ranbp2	Q9ERU9	3046	-12	-9	-2	-8	0.9	0.9	1.0	0.9
Pdcd11	Q6NS46	333	-17	-9	-2	-11	0.9	0.9	1.0	0.9
Myo9a	Q8C170	81	-33	-9	-2	21	0.8	0.9	1.0	1.3
Dnmt1	P13864	927	-17	-10	-2	11	0.9	0.9	1.0	1.1
Srsf4	Q542V3	115	-13	-10	-2	5	0.9	0.9	1.0	1.1
Dus3l	Q91XI1	124	-8	-10	-2	-2	0.9	0.9	1.0	1.0
Gnpat	P98192	543	1	-10	-2	-3	1.0	0.9	1.0	1.0
Gabpb1	Q00420	329	-9	-10	-2	-34	0.9	0.9	1.0	0.7
Gltscr1	F8VPZ9	1093	-3	-10	-2	1	1.0	0.9	1.0	1.0
Rars2	Q3U186	11	-18	-10	-2	-11	0.9	0.9	1.0	0.9
Ppp2r4	P58389	164	2	-11	-2	27	1.0	0.9	1.0	1.4
Lrba	E9Q3Y4	1455	-20	-11	-2	0	0.8	0.9	1.0	1.0

Pex5	O09012	11	-2	-12	-2	7	1.0	0.9	1.0	1.1
Dnpep	Q9Z2W0	142	-14	-12	-2	-12	0.9	0.9	1.0	0.9
Hace1	Q3U0D9	99	-1	-12	-2	-17	1.0	0.9	1.0	0.9
Lypla1	P97823	173	0	-12	-2	14	1.0	0.9	1.0	1.2
Ruvbl1	P60122	94	-13	-12	-2	7	0.9	0.9	1.0	1.1
Nlrc5	C3VPR6	701	-2	-12	-2	2	1.0	0.9	1.0	1.0
Stk26	Q99JT2	410	-11	-12	-2	-2	0.9	0.9	1.0	1.0
Map4k1	P70218	345	-19	-12	-2	-3	0.8	0.9	1.0	1.0
Srbd1	F8WGW3	392	-6	-12	-2	-6	0.9	0.9	1.0	0.9
Syne1	Q6ZWR6	6099	-13	-12	-2	-9	0.9	0.9	1.0	0.9
Ranbp2	Q9ERU9	1190	-1	-12	-2	-10	1.0	0.9	1.0	0.9
Ddx55	Q6ZPL9	389	-22	-13	-2	21	0.8	0.9	1.0	1.3
Rap2c	Q8BU31	140	-17	-13	-2	7	0.9	0.9	1.0	1.1
Xiap	Q60989	202	6	-13	-2	6	1.1	0.9	1.0	1.1
Zbtb20	Q8K0L9	120	-6	-13	-2	12	0.9	0.9	1.0	1.1
Rock2	F8VPK5	766	-4	-13	-2	5	1.0	0.9	1.0	1.1
Lpgat1	Q91YX5	108	-16	-13	-2	3	0.9	0.9	1.0	1.0
Cnot10	Q8BH15	633	-6	-13	-2	0	0.9	0.9	1.0	1.0
Spta1	P08032	473	-13	-14	-2	3	0.9	0.9	1.0	1.0
Gatad2a	Q8CHY6	425	-10	-14	-2	-6	0.9	0.9	1.0	0.9
Hmgb2	P30681	23	-6	-15	-2	-1	0.9	0.9	1.0	1.0
Hmgb1	P63158	23	-6	-15	-2	-1	0.9	0.9	1.0	1.0
Neurl3	Q8CJC5	73	-5	-16	-2	4	1.0	0.9	1.0	1.0
Znf652	Q5DU09	238	17	-17	-2	14	1.2	0.9	1.0	1.2
Ino80	Q6ZPV2	675	-13	-18	-2	25	0.9	0.9	1.0	1.3
Polr2i	P60898	119	-21	-18	-2	17	0.8	0.8	1.0	1.2
Copa	Q8CIE6	522	-11	-18	-2	-8	0.9	0.8	1.0	0.9
Stx7	Q8BH40	28	-6	-18	-2	-9	0.9	0.8	1.0	0.9
Copb1	Q9JIF7	616	-12	-19	-2	-8	0.9	0.8	1.0	0.9
Ahctf1	Q8CJF7	313	-12	-19	-2	-9	0.9	0.8	1.0	0.9
Myo18a	Q9JMH9	1076	2	-20	-2	-22	1.0	0.8	1.0	0.8
Abcd1	P48410	511	-2	-20	-2	4	1.0	0.8	1.0	1.0
Gpx1	P11352	113	-20	-21	-2	15	0.8	0.8	1.0	1.2
Synj2bp	Q9D6K5	90	-6	-21	-2	-32	0.9	0.8	1.0	0.8
Bles03	Q8VD62	222	-20	-21	-2	22	0.8	0.8	1.0	1.3
Rpl14	Q9CR57	54	-12	-21	-2	-6	0.9	0.8	1.0	0.9
Rnf213	E9Q555	4216	-3	-22	-2	4	1.0	0.8	1.0	1.0
Rad17	Q6NXW6	251	-25	-22	-2	-2	0.8	0.8	1.0	1.0
Pold1	P52431	1009	-12	-23	-2	-13	0.9	0.8	1.0	0.9
Alas2	P08680	471	-9	-25	-2	6	0.9	0.8	1.0	1.1
Ndufa9	Q9DC69	86	-8	-26	-2	-9	0.9	0.8	1.0	0.9
Wdr91	Q7TMQ7	369	2	20	-3	24	1.0	1.2	1.0	1.3
Neil1	Q8K4Q6	136	-23	12	-3	15	0.8	1.1	1.0	1.2
Tln1	P26039	1486	-10	10	-3	23	0.9	1.1	1.0	1.3
Asah1	Q9WV54	291	-6	10	-3	16	0.9	1.1	1.0	1.2
Hip1r	Q9JKY5	211	-13	9	-3	27	0.9	1.1	1.0	1.4
Slx4	Q6P1D7	1071	11	9	-3	-1	1.1	1.1	1.0	1.0
Mad2l1bp	Q9DCX1	77	5	9	-3	51	1.0	1.1	1.0	2.0
Csrp1	P97315	40	7	9	-3	17	1.1	1.1	1.0	1.2
Hsp90ab1	P11499	521	-10	9	-3	16	0.9	1.1	1.0	1.2
Mtmr1	Q9Z2C4	174	-4	9	-3	7	1.0	1.1	1.0	1.1

Casp8	O89110	238	-17	8	-3	4	0.9	1.1	1.0	1.0
Exoc8	Q6PGF7	512	1	8	-3	-1	1.0	1.1	1.0	1.0
Ezh2	Q61188	536	16	6	-3	27	1.2	1.1	1.0	1.4
Safb2	Q80YR5	714	-7	5	-3	7	0.9	1.0	1.0	1.1
Dennd1b	Q3U1T9	653	-2	4	-3	-3	1.0	1.0	1.0	1.0
Ube3a	O08759	193	-13	4	-3	-4	0.9	1.0	1.0	1.0
Pus3	Q9JI38	471	1	4	-3	-32	1.0	1.0	1.0	0.8
Phgdh	Q61753	234	-10	4	-3	30	0.9	1.0	1.0	1.4
Pnn	Q3TUQ5	249	-4	4	-3	15	1.0	1.0	1.0	1.2
Apobec3	Q99J72	45	-15	4	-3	3	0.9	1.0	1.0	1.0
Nin	Q61043	1249	0	4	-3	-6	1.0	1.0	1.0	0.9
Ddx19a	Q61655	392	-6	3	-3	5	0.9	1.0	1.0	1.1
Ddx19b	Q8BZY3	408	-6	3	-3	5	0.9	1.0	1.0	1.1
Acadl	P51174	166	-3	3	-3	10	1.0	1.0	1.0	1.1
Ywhaz	P63101	189	-1	3	-3	9	1.0	1.0	1.0	1.1
Arhgef2	Q60875	68	-16	2	-3	21	0.9	1.0	1.0	1.3
Zmiz1	Q6P1E1	749	-7	2	-3	21	0.9	1.0	1.0	1.3
Zmiz2	Q8CIE2	600	-7	2	-3	21	0.9	1.0	1.0	1.3
Mcm4	P49717	161	-6	2	-3	20	0.9	1.0	1.0	1.3
Tln1	P26039	243	-4	2	-3	20	1.0	1.0	1.0	1.2
Rnh1	Q91VI7	137	-8	2	-3	10	0.9	1.0	1.0	1.1
Uba6	Q8C7R4	156	-6	2	-3	8	0.9	1.0	1.0	1.1
Zyx	Q62523	484	-6	2	-3	-5	0.9	1.0	1.0	1.0
Ubap1	Q8BH48	45	3	1	-3	13	1.0	1.0	1.0	1.1
Eftud2	O08810	143	-1	1	-3	12	1.0	1.0	1.0	1.1
Agl	F8VPN4	1053	-6	1	-3	10	0.9	1.0	1.0	1.1
Gimap8	Q75N62	636	-8	1	-3	-4	0.9	1.0	1.0	1.0
Nemp1	Q6ZQE4	369	-13	1	-3	-7	0.9	1.0	1.0	0.9
Mthfd1	Q922D8	143	-7	1	-3	11	0.9	1.0	1.0	1.1
Pcbp2	Q61990	158	-16	1	-3	-2	0.9	1.0	1.0	1.0
Samd9l	E9PX59	500	-10	1	-3	-5	0.9	1.0	1.0	1.0
Akap8l	Q5RL57	212	-3	0	-3	34	1.0	1.0	1.0	1.5
Gda	Q9R111	218	1	0	-3	33	1.0	1.0	1.0	1.5
Rhoa	Q9QUI0	159	-9	0	-3	15	0.9	1.0	1.0	1.2
Med17	Q8VCD5	15	-6	0	-3	11	0.9	1.0	1.0	1.1
Ifi203	O35368	332	1	0	-3	-20	1.0	1.0	1.0	0.8
Spg21	Q9CQC8	204	-3	-1	-3	10	1.0	1.0	1.0	1.1
Luc7l3	Q5SUF2	43	-21	-1	-3	7	0.8	1.0	1.0	1.1
Cops5	O35864	218	-1	-1	-3	4	1.0	1.0	1.0	1.0
Abca7	Q91V24	1028	-4	-1	-3	-9	1.0	1.0	1.0	0.9
Rufy1	Q8BIJ7	577	-8	-1	-3	-11	0.9	1.0	1.0	0.9
Pds5a	E9QPI5	531	-2	-1	-3	19	1.0	1.0	1.0	1.2
Dus3l	Q91XI1	113	-11	-1	-3	8	0.9	1.0	1.0	1.1
Erlin2	Q8BFZ9	262	-6	-1	-3	7	0.9	1.0	1.0	1.1
Hinfp	Q8K1K9	153	8	-1	-3	7	1.1	1.0	1.0	1.1
Ralgps2	Q9ERD6	76	-8	-2	-3	24	0.9	1.0	1.0	1.3
Smchd1	Q6P5D8	1019	-10	-2	-3	7	0.9	1.0	1.0	1.1
Copb1	Q9JIF7	888	-10	-2	-3	7	0.9	1.0	1.0	1.1
Ipo7	Q9EPL8	90	-5	-2	-3	-8	1.0	1.0	1.0	0.9
Sp100	O35892	213	-7	-2	-3	-13	0.9	1.0	1.0	0.9
Rps12-ps3	Q6ZWZ6	106	-8	-2	-3	19	0.9	1.0	1.0	1.2

Dnajb1	Q9QYJ3	179	-11	-2	-3	6	0.9	1.0	1.0	1.1
Pcif1	P59114	246	-5	-2	-3	4	1.0	1.0	1.0	1.0
Flna	Q8BTM8	1018	-8	-2	-3	2	0.9	1.0	1.0	1.0
Zzef1	Q5SSH7	2076	-5	-2	-3	0	1.0	1.0	1.0	1.0
Tcp11l2	Q8K1H7	35	-5	-2	-3	-1	1.0	1.0	1.0	1.0
Trp53inp1	Q9QXE4	233	-11	-2	-3	-26	0.9	1.0	1.0	0.8
Supt6h	Q62383	1465	-6	-3	-3	17	0.9	1.0	1.0	1.2
Cbx3	Q9DCC5	69	-5	-3	-3	13	1.0	1.0	1.0	1.1
Trex1	Q91XB0	77	-11	-3	-3	1	0.9	1.0	1.0	1.0
Chmp5	Q9D7S9	20	-1	-3	-3	-5	1.0	1.0	1.0	1.0
Pef1	Q8BFY6	233	-13	-3	-3	12	0.9	1.0	1.0	1.1
Iqgap1	Q9JKF1	660	-12	-3	-3	7	0.9	1.0	1.0	1.1
Lrrc8a	Q80WG5	768	-8	-3	-3	-7	0.9	1.0	1.0	0.9
Hspa4	Q3U2G2	270	-11	-4	-3	30	0.9	1.0	1.0	1.4
Zc3hav1	Q3UPF5	612	14	-4	-3	24	1.2	1.0	1.0	1.3
Capza1	Q5RKN9	124	-7	-4	-3	14	0.9	1.0	1.0	1.2
Usp3	Q91W36	157	-9	-4	-3	6	0.9	1.0	1.0	1.1
Bcl7c	O08664	211	-7	-4	-3	-2	0.9	1.0	1.0	1.0
Ddx11	Q6AXC6	608	-4	-4	-3	-9	1.0	1.0	1.0	0.9
Eml4	Q3UMY5	988	-7	-4	-3	-20	0.9	1.0	1.0	0.8
Nup160	Q9Z0W3	1090	-17	-4	-3	41	0.9	1.0	1.0	1.7
Nisch	Q80TM9	405	-10	-4	-3	20	0.9	1.0	1.0	1.3
Kansl1	Q80TG1	585	-22	-4	-3	-16	0.8	1.0	1.0	0.9
Gbf1	Q6DFZ1	403	-9	-5	-3	27	0.9	1.0	1.0	1.4
Sec13	Q9D1M0	234	0	-5	-3	27	1.0	1.0	1.0	1.4
Mgmt	P26187	149	-5	-5	-3	13	1.0	1.0	1.0	1.1
Psma4	Q9R1P0	115	-24	-5	-3	12	0.8	1.0	1.0	1.1
Hnrnpu	Q8VEK3	449	-13	-5	-3	1	0.9	1.0	1.0	1.0
Arhgef6	Q8K4I3	666	-9	-5	-3	-4	0.9	1.0	1.0	1.0
Sp100	O35892	235	-7	-5	-3	-8	0.9	1.0	1.0	0.9
Lgals9	O08573	314	-2	-5	-3	4	1.0	1.0	1.0	1.0
Flna	Q8BTM8	1997	-16	-5	-3	3	0.9	1.0	1.0	1.0
Spr	Q91XH5	235	-7	-5	-3	1	0.9	1.0	1.0	1.0
Son	Q9QX47	2088	-2	-5	-3	-5	1.0	1.0	1.0	1.0
Parp9	Q8CAS9	192	-9	-6	-3	14	0.9	0.9	1.0	1.2
Psmd7	P26516	116	-4	-6	-3	1	1.0	0.9	1.0	1.0
Herc4	Q6PAV2	175	-9	-6	-3	-3	0.9	0.9	1.0	1.0
Snx8	Q8CFD4	195	-7	-6	-3	53	0.9	0.9	1.0	2.1
Ncor1	Q5RIM6	475	0	-6	-3	16	1.0	0.9	1.0	1.2
Nup205	A0A0J9YUD5	1081	-12	-6	-3	13	0.9	0.9	1.0	1.1
Dock2	Q8C3J5	607	-7	-6	-3	11	0.9	0.9	1.0	1.1
Ung	P97931	96	-8	-6	-3	4	0.9	0.9	1.0	1.0
Dtymk	P97930	31	-11	-6	-3	-10	0.9	0.9	1.0	0.9
Abr	Q5SSL4	346	-4	-7	-3	33	1.0	0.9	1.0	1.5
Ddx5	Q8BTS0	191	-11	-7	-3	14	0.9	0.9	1.0	1.2
Cyfip2	Q5SQX6	1087	-1	-7	-3	-7	1.0	0.9	1.0	0.9
Cyfip1	Q7TMB8	1088	-1	-7	-3	-7	1.0	0.9	1.0	0.9
Grcc10	O35127	44	-13	-7	-3	-26	0.9	0.9	1.0	0.8
Pias4	Q9JM05	292	3	-7	-3	18	1.0	0.9	1.0	1.2
Srrm2	Q8BTI8	1144	-5	-7	-3	-9	1.0	0.9	1.0	0.9
Phgdh	Q61753	295	-1	-8	-3	17	1.0	0.9	1.0	1.2

Ttll12	Q3UDE2	93	-3	-8	-3	8	1.0	0.9	1.0	1.1
Anp32a	O35381	87	-16	-8	-3	7	0.9	0.9	1.0	1.1
Dpysl2	O08553	504	-6	-8	-3	-13	0.9	0.9	1.0	0.9
Lyn	P25911	419	-12	-8	-3	-23	0.9	0.9	1.0	0.8
Cct5	P80316	253	-10	-8	-3	17	0.9	0.9	1.0	1.2
Prkar1a	Q9DBC7	362	-5	-8	-3	16	1.0	0.9	1.0	1.2
Aldh3a2	P47740	425	-19	-8	-3	2	0.8	0.9	1.0	1.0
Cnot2	Q8C5L3	175	-11	-8	-3	1	0.9	0.9	1.0	1.0
Tle4	Q62441	529	-6	-8	-3	-1	0.9	0.9	1.0	1.0
Nup160	Q9Z0W3	895	-1	-8	-3	-1	1.0	0.9	1.0	1.0
Ccdc22	Q9JIG7	441	-3	-9	-3	-13	1.0	0.9	1.0	0.9
Znf592	Q8BHZ4	790	-10	-9	-3	2	0.9	0.9	1.0	1.0
Sgta	Q8BJU0	130	-12	-9	-3	1	0.9	0.9	1.0	1.0
Esyt1	Q3U7R1	983	-22	-9	-3	-11	0.8	0.9	1.0	0.9
Rbm22	Q8BHS3	71	-12	-10	-3	8	0.9	0.9	1.0	1.1
Cntrl	A2AL36	584	-4	-10	-3	0	1.0	0.9	1.0	1.0
Nf1	Q04690	491	-17	-10	-3	50	0.9	0.9	1.0	2.0
Arap1	Q4LDD4	380	-12	-10	-3	-1	0.9	0.9	1.0	1.0
Cmas	Q99KK2	392	-5	-10	-3	-3	1.0	0.9	1.0	1.0
Ddx28	Q9CWT6	170	-5	-11	-3	8	1.0	0.9	1.0	1.1
Cct3	P80318	372	-11	-11	-3	15	0.9	0.9	1.0	1.2
Alox15	P39654	247	-13	-11	-3	1	0.9	0.9	1.0	1.0
Ahsa2	Q8N9S3	303	-17	-11	-3	-1	0.9	0.9	1.0	1.0
Irf2bp2	E9Q1P8	516	-10	-12	-3	-4	0.9	0.9	1.0	1.0
Irf2bpl	Q8K3X4	721	-10	-12	-3	-4	0.9	0.9	1.0	1.0
Irf2bp1	Q8R3Y8	530	-10	-12	-3	-4	0.9	0.9	1.0	1.0
Kiaa0226l	Q3TD16	318	-12	-12	-3	10	0.9	0.9	1.0	1.1
Nup214	Q80U93	1188	-3	-12	-3	1	1.0	0.9	1.0	1.0
Agfg1	Q8K2K6	32	-6	-12	-3	-7	0.9	0.9	1.0	0.9
Rad9a	Q9Z0F6	3	-9	-13	-3	8	0.9	0.9	1.0	1.1
Ruvbl1	P60122	141	-12	-13	-3	-15	0.9	0.9	1.0	0.9
Nthl1	O35980	106	-14	-13	-3	7	0.9	0.9	1.0	1.1
Sptan1	P16546	2441	-9	-13	-3	-6	0.9	0.9	1.0	0.9
Zcchc7	B1AX39	261	-23	-14	-3	-20	0.8	0.9	1.0	0.8
Dhrs1	Q99L04	177	-28	-15	-3	35	0.8	0.9	1.0	1.5
Bub3	Q9WVA3	270	-12	-15	-3	-10	0.9	0.9	1.0	0.9
Pcnt	F8VPV0	174	-11	-15	-3	-23	0.9	0.9	1.0	0.8
Dnmt1	P13864	1342	-13	-15	-3	2	0.9	0.9	1.0	1.0
Hspa4l	P48722	417	-8	-15	-3	0	0.9	0.9	1.0	1.0
Rap1b	Q99JI6	118	-15	-16	-3	-1	0.9	0.9	1.0	1.0
Kiaa1429	A2AIV2	353	-12	-16	-3	-2	0.9	0.9	1.0	1.0
Ahctf1	Q8CJF7	1145	-16	-16	-3	27	0.9	0.9	1.0	1.4
Hnrnpm	Q9D0E1	675	-3	-16	-3	-6	1.0	0.9	1.0	0.9
Tnks1bp1	P58871	779	-8	-16	-3	-19	0.9	0.9	1.0	0.8
Got2	P05202	274	-23	-17	-3	20	0.8	0.9	1.0	1.3
Uap1l1	Q3TW96	85	-13	-17	-3	-10	0.9	0.9	1.0	0.9
Plrg1	Q922V4	263	-19	-17	-3	-10	0.8	0.9	1.0	0.9
Naa15	G3X8Y3	322	-12	-17	-3	-6	0.9	0.9	1.0	0.9
Tmem154	Q8C4Q9	162	-3	-17	-3	-6	1.0	0.9	1.0	0.9
Hmha1	Q3TBD2	744	-11	-18	-3	-2	0.9	0.9	1.0	1.0
Nudcd1	Q6PIP5	469	-6	-18	-3	-17	0.9	0.9	1.0	0.9

Ddb1	Q3U1J4	128	-16	-18	-3	12	0.9	0.8	1.0	1.1
Rttn	Q8R4Y8	235	-3	-18	-3	5	1.0	0.8	1.0	1.0
Syne1	Q6ZWR6	7796	-6	-19	-3	-1	0.9	0.8	1.0	1.0
Trrap	A0A1D5RLL4	2279	-8	-19	-3	-3	0.9	0.8	1.0	1.0
Vrk3	Q8K3G5	372	-14	-19	-3	-21	0.9	0.8	1.0	0.8
Prep	Q9QUR6	57	-12	-20	-3	-37	0.9	0.8	1.0	0.7
Atad5	Q4QY64	1441	-14	-20	-3	-12	0.9	0.8	1.0	0.9
Atad5	Q4QY64	1671	-2	-21	-3	-11	1.0	0.8	1.0	0.9
Pik3r6	Q3U6Q4	268	0	-21	-3	-31	1.0	0.8	1.0	0.8
Smc5	Q8CG46	582	-3	-22	-3	-23	1.0	0.8	1.0	0.8
Tln1	P26039	1199	-4	-22	-3	18	1.0	0.8	1.0	1.2
Prkdc	P97313	2916	2	-23	-3	-15	1.0	0.8	1.0	0.9
Recql5	Q8VID5	665	-16	-28	-3	-18	0.9	0.8	1.0	0.8
Ester	Q91V76	226	69	-29	-3	-74	3.2	0.8	1.0	0.6
Rnf213	E9Q555	4251	-25	-37	-3	-23	0.8	0.7	1.0	0.8
Hsp90aa1	P07901	598	-34	-45	-3	-11	0.7	0.7	1.0	0.9
Cbfa2t2	O70374	563	-13	28	-3	31	0.9	1.4	1.0	1.4
Tbl1xr1	Q8BHJ5	434	-17	16	-3	-12	0.9	1.2	1.0	0.9
Zc3h12a	Q5D1E7	96	-10	12	-3	-11	0.9	1.1	1.0	0.9
Hirip3	Q8BLH7	18	4	11	-3	-1	1.0	1.1	1.0	1.0
Rpa1	Q8VEE4	485	-6	9	-3	19	0.9	1.1	1.0	1.2
Npepps	Q11011	340	3	9	-3	-5	1.0	1.1	1.0	1.0
Dctn3	Q9Z0Y1	173	-4	9	-3	-7	1.0	1.1	1.0	0.9
Cltc	Q68FD5	1205	-5	8	-3	18	1.0	1.1	1.0	1.2
Elmo1	Q8BPU7	438	4	8	-3	7	1.0	1.1	1.0	1.1
Lrrk1	Q3UHC2	147	-3	8	-3	3	1.0	1.1	1.0	1.0
Ogfr	Q99PG2	416	6	8	-3	-19	1.1	1.1	1.0	0.8
Naa40	Q8VE10	26	2	7	-3	11	1.0	1.1	1.0	1.1
UPF0687	Q9D1K7	131	3	7	-3	-7	1.0	1.1	1.0	0.9
Cpne1	Q8C166	199	-23	7	-3	-12	0.8	1.1	1.0	0.9
Ptbp3	Q8BHD7	220	-4	5	-3	24	1.0	1.0	1.0	1.3
Usp5	P56399	838	3	5	-3	3	1.0	1.0	1.0	1.0
Dhx16	G3X8X0	717	-11	5	-3	-2	0.9	1.0	1.0	1.0
Mphosph8	Q3TYA6	708	-4	4	-3	20	1.0	1.0	1.0	1.2
Crot	Q9DC50	210	-8	4	-3	4	0.9	1.0	1.0	1.0
Dlat	Q8BMF4	290	-14	3	-3	19	0.9	1.0	1.0	1.2
Flna	Q8BTM8	1912	-5	3	-3	3	1.0	1.0	1.0	1.0
Sirt7	Q8BKJ9	283	-5	3	-3	-9	1.0	1.0	1.0	0.9
Brf1	G3X8S2	429	-5	3	-3	-17	1.0	1.0	1.0	0.9
Dytn	A2CI98	231	-10	3	-3	-36	0.9	1.0	1.0	0.7
Ppp6r2	G3X9K4	513	-7	3	-3	4	0.9	1.0	1.0	1.0
Ncoa2	Q61026	22	-1	3	-3	-3	1.0	1.0	1.0	1.0
Rrbp1	Q99PL5	1327	4	3	-3	-12	1.0	1.0	1.0	0.9
Scaf11	E9PZM7	492	-11	2	-3	-19	0.9	1.0	1.0	0.8
Arhgap9	Q8QZW8	389	-11	2	-3	5	0.9	1.0	1.0	1.0
Git1	Q68FF6	235	-16	2	-3	-7	0.9	1.0	1.0	0.9
Git2	Q9JLQ2	235	-16	2	-3	-7	0.9	1.0	1.0	0.9
Limd2	Q8BGB5	44	-6	1	-3	16	0.9	1.0	1.0	1.2
Mib1	Q80SY4	88	-7	1	-3	2	0.9	1.0	1.0	1.0
Cops3	O88543	25	13	1	-3	17	1.1	1.0	1.0	1.2
Atg2b	Q80XK6	1697	-5	1	-3	3	1.0	1.0	1.0	1.0

Ddx17	Q501J6	368	-5	1	-3	2	1.0	1.0	1.0	1.0
Qars	Q8BML9	657	-3	1	-3	2	1.0	1.0	1.0	1.0
Map7d1	A2AJI0	88	-6	0	-3	-8	0.9	1.0	1.0	0.9
Thoc2	B1AZI6	986	8	-1	-3	48	1.1	1.0	1.0	1.9
Pak1ip1	Q9DCE5	288	-12	-1	-3	8	0.9	1.0	1.0	1.1
Coq3	Q8BMS4	156	-1	-1	-3	-7	1.0	1.0	1.0	0.9
Tiprl	Q8BH58	75	-1	-1	-3	19	1.0	1.0	1.0	1.2
Kpnb1	P70168	228	-11	-1	-3	14	0.9	1.0	1.0	1.2
Rapgef1	Q3UHC1	1105	-16	-1	-3	7	0.9	1.0	1.0	1.1
Opa1	P58281	856	-1	-2	-3	26	1.0	1.0	1.0	1.3
Wdr7	Q920I9	234	7	-2	-3	1	1.1	1.0	1.0	1.0
Cep83	Q9D5R3	284	-1	-2	-3	-9	1.0	1.0	1.0	0.9
Dicer1	F8VQ54	189	11	-2	-3	25	1.1	1.0	1.0	1.3
Adk	P55264	105	-5	-2	-3	22	1.0	1.0	1.0	1.3
Hpcal1	P62748	38	-11	-2	-3	-1	0.9	1.0	1.0	1.0
Gm20431	E9PY39	368	-6	-2	-3	-1	0.9	1.0	1.0	1.0
Top2b	Q64511	113	-4	-2	-3	-3	1.0	1.0	1.0	1.0
Ddx19a	Q61655	164	-5	-3	-3	23	1.0	1.0	1.0	1.3
Denr	Q9CQJ6	132	-14	-3	-3	10	0.9	1.0	1.0	1.1
Glrx3	Q9CQM9	231	-4	-3	-3	7	1.0	1.0	1.0	1.1
Gpsm1	Q6IR34	495	9	-3	-3	6	1.1	1.0	1.0	1.1
Fpgt	G5E8F4	404	-23	-3	-3	4	0.8	1.0	1.0	1.0
Sec62	Q8BU14	55	-12	-3	-3	4	0.9	1.0	1.0	1.0
Sptb	Q3UGX2	603	-7	-3	-3	-6	0.9	1.0	1.0	0.9
Prox2	Q8BII1	77	3	-3	-3	-7	1.0	1.0	1.0	0.9
Hdac1	O09106	273	-10	-3	-3	9	0.9	1.0	1.0	1.1
Cops4	O88544	378	-3	-3	-3	8	1.0	1.0	1.0	1.1
Pygo2	Q80V76	349	-10	-3	-3	6	0.9	1.0	1.0	1.1
Rps6ka1	P18653	564	-4	-3	-3	3	1.0	1.0	1.0	1.0
Rps6ka3	P18654	579	-4	-3	-3	3	1.0	1.0	1.0	1.0
Rps6ka2	Q9WUT3	572	-4	-3	-3	3	1.0	1.0	1.0	1.0
Rilpl2	Q99LE1	150	-6	-3	-3	1	0.9	1.0	1.0	1.0
Psmc4	P54775	379	-3	-3	-3	-2	1.0	1.0	1.0	1.0
Kif13b	E9Q4K7	26	-1	-3	-3	-14	1.0	1.0	1.0	0.9
Kpna6	O35345	357	-16	-4	-3	7	0.9	1.0	1.0	1.1
Kpna1	Q60960	359	-16	-4	-3	7	0.9	1.0	1.0	1.1
Smarcad1	Q04692	296	-4	-4	-3	-5	1.0	1.0	1.0	1.0
Hspa9	P38647	66	-6	-4	-3	-21	0.9	1.0	1.0	0.8
Sms	P97355	318	1	-4	-3	-21	1.0	1.0	1.0	0.8
Fxr2	Q6P5B5	270	10	-4	-3	-27	1.1	1.0	1.0	0.8
Scyl3	Q9DBQ7	327	1	-4	-3	27	1.0	1.0	1.0	1.4
Cul5	Q9D5V5	404	15	-4	-3	14	1.2	1.0	1.0	1.2
Hnrnpul2	Q00PI9	403	-12	-4	-3	9	0.9	1.0	1.0	1.1
Dhx58	Q99J87	221	-11	-4	-3	1	0.9	1.0	1.0	1.0
Plec	Q9QXS1	541	-9	-4	-3	1	0.9	1.0	1.0	1.0
Trim24	Q64127	134	-13	-5	-3	16	0.9	1.0	1.0	1.2
Nelfb	Q8C4Y3	141	-6	-5	-3	12	0.9	1.0	1.0	1.1
Tax1bp1	Q3UKC1	271	9	-5	-3	-3	1.1	1.0	1.0	1.0
Rbm25	B2RY56	83	-5	-5	-3	-8	1.0	1.0	1.0	0.9
Crebrf	Q8CDG5	266	2	-5	-3	2	1.0	1.0	1.0	1.0
Ociad1	Q9CRD0	42	-2	-5	-3	-26	1.0	1.0	1.0	0.8

Usp9x	Q4FE56	1237	-8	-6	-3	3	0.9	0.9	1.0	1.0
Mta1	Q8K4B0	229	-7	-6	-3	1	0.9	0.9	1.0	1.0
Mta3	Q924K8	211	-7	-6	-3	1	0.9	0.9	1.0	1.0
Psmb8	P28063	120	-16	-6	-3	-6	0.9	0.9	1.0	0.9
UPF0585	Q9DCS2	162	-16	-6	-3	-12	0.9	0.9	1.0	0.9
Itprip	Q3TNL8	256	-2	-6	-3	15	1.0	0.9	1.0	1.2
Ehbp1l1	E9QP49	1345	-5	-6	-3	-5	1.0	0.9	1.0	1.0
Gbp7	Q91Z40	568	-5	-6	-3	-8	1.0	0.9	1.0	0.9
Upf1	Q9EPU0	183	-9	-6	-3	-9	0.9	0.9	1.0	0.9
Ptprc	P06800	1109	-5	-7	-3	10	1.0	0.9	1.0	1.1
Kiaa1429	A2AIV2	906	-15	-7	-3	12	0.9	0.9	1.0	1.1
Eprs	Q8CGC7	856	-9	-7	-3	-4	0.9	0.9	1.0	1.0
Ranbp2	Q9ERU9	2626	-1	-7	-3	-19	1.0	0.9	1.0	0.8
Arhgap4	B1AUY3	931	-4	-7	-3	-28	1.0	0.9	1.0	0.8
Hdac3	O88895	268	-22	-8	-3	10	0.8	0.9	1.0	1.1
Gbas	Q7TMG8	80	-13	-8	-3	1	0.9	0.9	1.0	1.0
Tcp11l2	Q8K1H7	476	-6	-8	-3	-2	0.9	0.9	1.0	1.0
Brpf1	A0A0N4SUT9	514	-10	-8	-3	-16	0.9	0.9	1.0	0.9
Sipa1l3	G3X9J0	614	-15	-8	-3	7	0.9	0.9	1.0	1.1
Mbd1	Q9Z2E2	70	-7	-8	-3	-2	0.9	0.9	1.0	1.0
Eif3k	Q9DBZ5	190	-3	-8	-3	-7	1.0	0.9	1.0	0.9
Irf2	P23906	83	-9	-8	-3	-14	0.9	0.9	1.0	0.9
Numa1	E9Q7G0	905	-14	-9	-3	9	0.9	0.9	1.0	1.1
Hk3	Q3TRM8	188	-6	-9	-3	6	0.9	0.9	1.0	1.1
Nup88	Q8CEC0	567	-8	-9	-3	2	0.9	0.9	1.0	1.0
Bptf	A2A654	457	9	-9	-3	-3	1.1	0.9	1.0	1.0
Senp1	P59110	60	-8	-9	-3	-3	0.9	0.9	1.0	1.0
Myh9	Q8VDD5	896	-9	-9	-3	-6	0.9	0.9	1.0	0.9
Ist1	Q9CX00	125	-7	-9	-3	22	0.9	0.9	1.0	1.3
Srbd1	F8WGW3	160	12	-9	-3	5	1.1	0.9	1.0	1.0
Sae1	Q9R1T2	134	-4	-9	-3	0	1.0	0.9	1.0	1.0
Nars	Q8BP47	125	-5	-9	-3	-3	1.0	0.9	1.0	1.0
Ubr2	Q6WKZ8	19	2	-9	-3	-3	1.0	0.9	1.0	1.0
Mapkapk2	P49138	84	-4	-10	-3	19	1.0	0.9	1.0	1.2
Adh5	P28474	111	-20	-10	-3	18	0.8	0.9	1.0	1.2
Tln1	P26039	750	-19	-10	-3	16	0.8	0.9	1.0	1.2
Adrbk2	Q3UYH7	501	-3	-10	-3	-10	1.0	0.9	1.0	0.9
Nsd1	E9QAE4	1007	-7	-10	-3	-19	0.9	0.9	1.0	0.8
Cdc123	Q8CII2	212	-13	-10	-3	10	0.9	0.9	1.0	1.1
Ppig	A2AR02	33	-16	-11	-3	0	0.9	0.9	1.0	1.0
Tepsin	Q3U3N6	369	-7	-11	-3	0	0.9	0.9	1.0	1.0
Strn	O55106	765	-14	-11	-3	-1	0.9	0.9	1.0	1.0
Mdh2	P08249	93	-6	-11	-3	5	0.9	0.9	1.0	1.0
Taf8	Q9EQH4	78	-9	-11	-3	0	0.9	0.9	1.0	1.0
Phf3	B2RQG2	275	-9	-11	-3	-19	0.9	0.9	1.0	0.8
Hadha	Q8BMS1	110	-14	-12	-3	25	0.9	0.9	1.0	1.3
Ogdh	Q60597	604	-20	-12	-3	5	0.8	0.9	1.0	1.0
Acaa1a	Q921H8	123	-12	-12	-3	-2	0.9	0.9	1.0	1.0
Smad2	Q62432	380	0	-12	-3	7	1.0	0.9	1.0	1.1
Smarcc1	P97496	656	0	-12	-3	2	1.0	0.9	1.0	1.0
Smarcc2	Q6PDG5	635	0	-12	-3	2	1.0	0.9	1.0	1.0

H2-DMb2	Q31099	64	-19	-13	-3	2	0.8	0.9	1.0	1.0
Slfn5	Q8CBA2	365	-4	-13	-3	2	1.0	0.9	1.0	1.0
Rttn	Q8R4Y8	597	2	-13	-3	-3	1.0	0.9	1.0	1.0
Banp	Q8VBU8	82	-6	-14	-3	-15	0.9	0.9	1.0	0.9
Dpf2	Q61103	24	-17	-15	-3	19	0.9	0.9	1.0	1.2
Nrde2	Q80XC6	1127	-17	-15	-3	14	0.9	0.9	1.0	1.2
Iglc3	A0A0G2JGQ8	28	-5	-15	-3	21	1.0	0.9	1.0	1.3
Zgpat	Q8VDM1	357	-7	-15	-3	-1	0.9	0.9	1.0	1.0
Add1	Q9QYC0	525	-16	-16	-3	31	0.9	0.9	1.0	1.4
Noct	O35710	300	-12	-16	-3	26	0.9	0.9	1.0	1.3
Slfn9	B1ARD6	404	-20	-16	-3	7	0.8	0.9	1.0	1.1
Slfn8	B1ARD8	404	-20	-16	-3	7	0.8	0.9	1.0	1.1
Gtf3c5	Q8R2T8	242	-17	-16	-3	5	0.9	0.9	1.0	1.1
Gusb	P12265	640	-6	-17	-3	4	0.9	0.9	1.0	1.0
Rbm22	Q8BHS3	179	-20	-18	-3	7	0.8	0.8	1.0	1.1
Mtmr6	Q8VE11	214	-6	-19	-3	-6	0.9	0.8	1.0	0.9
Ube2o	Q6ZPJ3	365	-16	-19	-3	1	0.9	0.8	1.0	1.0
Epg5	Q80TA9	467	-10	-19	-3	-7	0.9	0.8	1.0	0.9
Wdr12	Q9JJA4	205	-16	-19	-3	-8	0.9	0.8	1.0	0.9
Pcca	Q91ZA3	72	-36	-20	-3	3	0.7	0.8	1.0	1.0
Mccc1	Q99MR8	58	-36	-20	-3	3	0.7	0.8	1.0	1.0
Pfkfb3	A7UAK5	102	-15	-20	-3	2	0.9	0.8	1.0	1.0
Setd1a	E9PYH6	1647	-13	-21	-3	9	0.9	0.8	1.0	1.1
Dennd5a	Q6PAL8	629	6	-21	-3	3	1.1	0.8	1.0	1.0
Uba1	Q02053	234	-16	-21	-3	-15	0.9	0.8	1.0	0.9
Ggps1	Q9WTN0	247	-16	-21	-3	-23	0.9	0.8	1.0	0.8
Traf7	Q922B6	350	-16	-23	-3	-25	0.9	0.8	1.0	0.8
Mki67	E9PVX6	3101	-8	-23	-3	-37	0.9	0.8	1.0	0.7
Copg1	Q9QZE5	44	-11	-24	-3	3	0.9	0.8	1.0	1.0
Eed	Q921E6	311	-16	-28	-3	-6	0.9	0.8	1.0	0.9
Arcn1	Q5XJY5	286	18	14	-4	14	1.2	1.2	1.0	1.2
Vps54	Q5SPW0	886	-2	11	-4	16	1.0	1.1	1.0	1.2
Smu1	Q3UKJ7	447	-5	11	-4	15	1.0	1.1	1.0	1.2
Wnk1	P83741	547	-2	10	-4	12	1.0	1.1	1.0	1.1
Nbeal2	Q6ZQA0	2003	2	8	-4	1	1.0	1.1	1.0	1.0
Ttc38	A3KMP2	363	-12	6	-4	5	0.9	1.1	1.0	1.0
Myof	Q69ZN7	275	-2	6	-4	-16	1.0	1.1	1.0	0.9
Gimap9	G3X987	78	-2	6	-4	17	1.0	1.1	1.0	1.2
Kcnab2	P62482	212	-6	6	-4	12	0.9	1.1	1.0	1.1
Fntb	Q8K2I1	426	1	6	-4	8	1.0	1.1	1.0	1.1
Fyb	O35601	507	-10	6	-4	-6	0.9	1.1	1.0	0.9
Pyurf	Q9D1C3	58	-6	5	-4	54	0.9	1.1	1.0	2.2
Dicer1	F8VQ54	1632	3	5	-4	7	1.0	1.1	1.0	1.1
Cdan1	Q8CC12	1083	2	5	-4	44	1.0	1.0	1.0	1.8
Ptk2b	Q9QVP9	180	6	4	-4	28	1.1	1.0	1.0	1.4
Dars	Q922B2	257	-9	4	-4	13	0.9	1.0	1.0	1.1
Rbbp9	O88851	39	-3	4	-4	12	1.0	1.0	1.0	1.1
Zfand6	Q9DCH6	178	1	4	-4	24	1.0	1.0	1.0	1.3
Zbtb11	G5E8B9	39	-5	2	-4	14	1.0	1.0	1.0	1.2
Tbc1d13	Q8R3D1	131	3	2	-4	6	1.0	1.0	1.0	1.1
Ppp2r5a	Q6PD03	72	-7	2	-4	-1	0.9	1.0	1.0	1.0

Wdr81	Q5ND34	74	1	2	-4	15	1.0	1.0	1.0	1.2
Ncl	P09405	544	3	2	-4	12	1.0	1.0	1.0	1.1
Ptpn6	P29351	480	-7	2	-4	9	0.9	1.0	1.0	1.1
Fen1	Q91Z50	311	-4	1	-4	34	1.0	1.0	1.0	1.5
Acp1	Q9D358	13	-6	1	-4	19	0.9	1.0	1.0	1.2
Dmxl1	Q6PNC0	549	-1	1	-4	13	1.0	1.0	1.0	1.1
Eef2	P58252	651	-8	1	-4	7	0.9	1.0	1.0	1.1
Stk39	Q9Z1W9	111	-3	1	-4	-1	1.0	1.0	1.0	1.0
Huwe1	Q7TMY8	471	-2	1	-4	20	1.0	1.0	1.0	1.3
Dnaja1	P63037	394	2	1	-4	6	1.0	1.0	1.0	1.1
Rhof	Q8BYP3	30	-6	1	-4	-10	0.9	1.0	1.0	0.9
Usp47	Q8BY87	856	-3	0	-4	3	1.0	1.0	1.0	1.0
Caprin1	Q60865	224	-3	0	-4	2	1.0	1.0	1.0	1.0
Mif4gd	Q3UBZ5	49	1	0	-4	-34	1.0	1.0	1.0	0.7
Ndufv1	Q91YT0	255	-14	-1	-4	16	0.9	1.0	1.0	1.2
Tubb2a	Q7TMM9	129	-3	-1	-4	-5	1.0	1.0	1.0	1.0
Sptan1	P16546	1622	-9	-1	-4	6	0.9	1.0	1.0	1.1
Rpl24	Q8BP67	6	-6	-1	-4	1	0.9	1.0	1.0	1.0
Thap12	Q9CUX1	68	-8	-1	-4	-3	0.9	1.0	1.0	1.0
Capn2	O08529	374	-3	-2	-4	14	1.0	1.0	1.0	1.2
Capn1	O35350	384	-3	-2	-4	14	1.0	1.0	1.0	1.2
Capn11	Q6J756	385	-3	-2	-4	14	1.0	1.0	1.0	1.2
Atic	Q9CWJ9	363	-1	-2	-4	14	1.0	1.0	1.0	1.2
Actn1	Q7TPR4	180	-8	-2	-4	12	0.9	1.0	1.0	1.1
Plekha3	Q9ERS4	173	-5	-2	-4	9	1.0	1.0	1.0	1.1
Zmym2	Q9CU65	538	-6	-2	-4	2	0.9	1.0	1.0	1.0
Acads	Q07417	246	-10	-2	-4	2	0.9	1.0	1.0	1.0
Cdk4	P30285	78	-4	-2	-4	1	1.0	1.0	1.0	1.0
Zc3h13	E9Q784	1652	-11	-2	-4	20	0.9	1.0	1.0	1.3
Pank4	Q80YV4	680	-5	-2	-4	0	1.0	1.0	1.0	1.0
Glcci1	Q8K3I9	451	-13	-2	-4	-5	0.9	1.0	1.0	1.0
Abcc1	O35379	1505	-16	-2	-4	-24	0.9	1.0	1.0	0.8
Pkn1	P70268	766	-10	-3	-4	25	0.9	1.0	1.0	1.3
Pkn2	Q8BWW9	803	-10	-3	-4	25	0.9	1.0	1.0	1.3
Snd1	Q78PY7	736	-8	-3	-4	21	0.9	1.0	1.0	1.3
Gclm	O09172	72	-11	-3	-4	19	0.9	1.0	1.0	1.2
Wdr37	Q8CBE3	419	-4	-3	-4	4	1.0	1.0	1.0	1.0
Tln1	P26039	1506	-29	-3	-4	-5	0.8	1.0	1.0	1.0
Ubash3a	Q3V3E1	570	3	-3	-4	-5	1.0	1.0	1.0	1.0
Hnrnpm	Q9D0E1	113	-9	-3	-4	4	0.9	1.0	1.0	1.0
Shmt2	Q9CZN7	91	-15	-4	-4	15	0.9	1.0	1.0	1.2
Rb1cc1	Q9ESK9	830	-2	-4	-4	9	1.0	1.0	1.0	1.1
Tln1	P26039	1353	-1	-4	-4	2	1.0	1.0	1.0	1.0
Shc1	P98083	248	-17	-4	-4	-5	0.9	1.0	1.0	1.0
Swap70	Q6A028	40	-5	-4	-4	25	1.0	1.0	1.0	1.3
Uncharact	A0A1B0GSH8	185	-13	-4	-4	22	0.9	1.0	1.0	1.3
Mrpl10	Q3TBW2	180	-12	-4	-4	10	0.9	1.0	1.0	1.1
Gspt1	Q8R050	464	-9	-4	-4	-1	0.9	1.0	1.0	1.0
Cep128	Q8BI22	331	-6	-4	-4	-2	0.9	1.0	1.0	1.0
Psma6	Q9QUM9	47	-10	-4	-4	-3	0.9	1.0	1.0	1.0
Adat3	Q6PAT0	182	-13	-4	-4	-6	0.9	1.0	1.0	0.9

Tepsin	Q3U3N6	204	-9	-4	-4	-12	0.9	1.0	1.0	0.9
L3mbtl3	Q8BLB7	583	-4	-4	-4	-15	1.0	1.0	1.0	0.9
Cnot1	Q6ZQ08	624	-15	-5	-4	23	0.9	1.0	1.0	1.3
Dnajb1	Q9QYJ3	267	-6	-5	-4	12	0.9	1.0	1.0	1.1
Zmynd8	A2A484	110	-17	-5	-4	-5	0.9	1.0	1.0	1.0
Eprs	Q8CGC7	744	-5	-5	-4	-8	1.0	1.0	1.0	0.9
Syne1	Q6ZWR6	1623	-8	-5	-4	-9	0.9	1.0	1.0	0.9
Mrps18a	Q5U5I3	186	-8	-5	-4	-11	0.9	1.0	1.0	0.9
Zmynd8	A2A484	142	-10	-5	-4	-13	0.9	1.0	1.0	0.9
Tln1	P26039	286	-3	-5	-4	19	1.0	1.0	1.0	1.2
Ccar1	Q8CH18	970	-11	-5	-4	18	0.9	1.0	1.0	1.2
Rbm43	Q99J64	34	-4	-5	-4	13	1.0	1.0	1.0	1.1
Luc7l2	Q7TNC4	36	-12	-6	-4	17	0.9	0.9	1.0	1.2
Casp6	O08738	70	-21	-6	-4	15	0.8	0.9	1.0	1.2
Eef1a1	P10126	234	-11	-6	-4	10	0.9	0.9	1.0	1.1
Cstf2	Q8BIQ5	150	-12	-6	-4	32	0.9	0.9	1.0	1.5
Dhx9	E9QNN1	1032	-11	-6	-4	29	0.9	0.9	1.0	1.4
Gm9774	A0A0A6YVU8	88	-9	-6	-4	24	0.9	0.9	1.0	1.3
Gba2	Q69ZF3	143	-17	-6	-4	17	0.9	0.9	1.0	1.2
Haao	Q78JT3	23	-13	-6	-4	-4	0.9	0.9	1.0	1.0
Pdcl3	Q8BVF2	155	-6	-6	-4	-7	0.9	0.9	1.0	0.9
Mmtag2	Q99LX5	103	1	-6	-4	-9	1.0	0.9	1.0	0.9
Dock11	A2AF47	592	2	-6	-4	-11	1.0	0.9	1.0	0.9
Rpl37a	P61514	42	-13	-6	-4	-13	0.9	0.9	1.0	0.9
Gm20425	E9Q035	780	-5	-7	-4	32	1.0	0.9	1.0	1.5
Syne1	Q6ZWR6	1234	-6	-7	-4	2	0.9	0.9	1.0	1.0
Noc2l	J3QK52	221	-7	-7	-4	0	0.9	0.9	1.0	1.0
Pax5	Q02650	52	-11	-7	-4	-2	0.9	0.9	1.0	1.0
Tox4	Q8BU11	575	-2	-7	-4	-4	1.0	0.9	1.0	1.0
Ksr1	Q61097	164	-13	-7	-4	29	0.9	0.9	1.0	1.4
Baz2a	E9Q374	1682	-11	-7	-4	19	0.9	0.9	1.0	1.2
Faf2	Q3TDN2	349	-6	-7	-4	-8	0.9	0.9	1.0	0.9
Ncapg	E9PWG6	177	-27	-7	-4	-8	0.8	0.9	1.0	0.9
Ado	Q6PDY2	225	-9	-7	-4	-11	0.9	0.9	1.0	0.9
Rpa3	Q9CQ71	81	0	-7	-4	-18	1.0	0.9	1.0	0.9
Gtf3c1	Q8K284	1053	-1	-8	-4	31	1.0	0.9	1.0	1.4
Dars	Q922B2	259	-10	-8	-4	-1	0.9	0.9	1.0	1.0
Rac1	P63001	157	-10	-8	-4	-4	0.9	0.9	1.0	1.0
Rac2	Q05144	157	-10	-8	-4	-4	0.9	0.9	1.0	1.0
Rac1	P63001	178	-6	-8	-4	-6	0.9	0.9	1.0	0.9
Cog5	Q8C0L8	142	-14	-8	-4	-12	0.9	0.9	1.0	0.9
Ranbp10	Q6VN19	501	-11	-8	-4	-20	0.9	0.9	1.0	0.8
Nr2c2	P49117	120	-15	-8	-4	5	0.9	0.9	1.0	1.0
Rnf213	E9Q555	3499	-12	-8	-4	2	0.9	0.9	1.0	1.0
Nup160	Q9Z0W3	446	-11	-8	-4	-1	0.9	0.9	1.0	1.0
Thumpd2	Q9CZB3	55	-6	-8	-4	-3	0.9	0.9	1.0	1.0
Sipa1	E9Q0Y4	728	-14	-8	-4	-5	0.9	0.9	1.0	1.0
Sec24c	G3X972	727	-2	-8	-4	-7	1.0	0.9	1.0	0.9
Nfkb1	P25799	471	-17	-8	-4	-8	0.9	0.9	1.0	0.9
Ppa1	Q9D819	270	-3	-8	-4	-10	1.0	0.9	1.0	0.9
Ech1	O35459	186	7	-8	-4	-28	1.1	0.9	1.0	0.8

Rabgef1	Q9JM13	404	-11	-9	-4	-3	0.9	0.9	1.0	1.0
Hmbs	P22907	261	-1	-9	-4	15	1.0	0.9	1.0	1.2
Mb21d1	Q8C6L5	385	-21	-9	-4	5	0.8	0.9	1.0	1.0
Mrps12	O35680	132	-10	-9	-4	2	0.9	0.9	1.0	1.0
Sugt1	Q9CX34	49	-5	-9	-4	-11	1.0	0.9	1.0	0.9
Shprh	Q7TPQ3	1016	-7	-9	-4	-13	0.9	0.9	1.0	0.9
Gvin1	L7N451	601	-4	-10	-4	6	1.0	0.9	1.0	1.1
Msh2	P43247	822	-11	-10	-4	0	0.9	0.9	1.0	1.0
Lrrc20	Q8CI70	32	-12	-10	-4	12	0.9	0.9	1.0	1.1
Farsb	Q9WUA2	548	-16	-10	-4	8	0.9	0.9	1.0	1.1
Fbl	P35550	105	2	-10	-4	-1	1.0	0.9	1.0	1.0
Agps	Q8C0I1	201	-9	-11	-4	-9	0.9	0.9	1.0	0.9
Rragb	Q6NTA4	370	-19	-11	-4	5	0.8	0.9	1.0	1.1
Nup93	Q8BJ71	422	-13	-11	-4	3	0.9	0.9	1.0	1.0
Ppp6r1	Q7TSI3	775	-15	-11	-4	-16	0.9	0.9	1.0	0.9
Gm20517	G3UVU6	120	-7	-12	-4	-16	0.9	0.9	1.0	0.9
Aftph	Q80WT5	250	-10	-12	-4	2	0.9	0.9	1.0	1.0
Nup133	Q8R0G9	640	-11	-13	-4	22	0.9	0.9	1.0	1.3
Med13	Q5SWW4	1273	-3	-13	-4	-8	1.0	0.9	1.0	0.9
Nubp2	Q9R061	58	-8	-14	-4	-10	0.9	0.9	1.0	0.9
Rab40c	Q8VHQ4	77	-26	-14	-4	42	0.8	0.9	1.0	1.7
Mcm6	P97311	180	-11	-14	-4	12	0.9	0.9	1.0	1.1
Rab22a	P35285	120	-14	-14	-4	1	0.9	0.9	1.0	1.0
Ppia	P17742	62	-4	-14	-4	-6	1.0	0.9	1.0	0.9
Pcna	P17918	162	-7	-14	-4	-9	0.9	0.9	1.0	0.9
Atf2	P16951	61	-6	-15	-4	-2	0.9	0.9	1.0	1.0
Mga	A2AWL7	1142	-2	-15	-4	-15	1.0	0.9	1.0	0.9
Fam126a	Q6P9N1	484	-17	-15	-4	-19	0.9	0.9	1.0	0.8
Ppox	P51175	183	-17	-16	-4	28	0.9	0.9	1.0	1.4
Araf	P04627	595	-10	-16	-4	23	0.9	0.9	1.0	1.3
Sptan1	P16546	466	-15	-16	-4	15	0.9	0.9	1.0	1.2
Mtmr9	Q9Z2D0	214	-11	-16	-4	-5	0.9	0.9	1.0	1.0
Tfg	Q9Z1A1	80	-6	-16	-4	-23	0.9	0.9	1.0	0.8
Dnajc13	D4AFX7	2115	-12	-17	-4	39	0.9	0.9	1.0	1.6
Rpl14	Q9CR57	42	-12	-17	-4	-14	0.9	0.9	1.0	0.9
Sap30	O88574	199	-3	-17	-4	0	1.0	0.9	1.0	1.0
Dnm1l	Q8K1M6	650	-34	-17	-4	-2	0.7	0.9	1.0	1.0
Plec	Q9QXS1	328	4	-17	-4	-7	1.0	0.9	1.0	0.9
Polr3e	Q9CZT4	678	-16	-18	-4	2	0.9	0.9	1.0	1.0
Nemf	Q8CCP0	394	-18	-18	-4	-3	0.8	0.8	1.0	1.0
Dnajc13	D4AFX7	12	-8	-19	-4	18	0.9	0.8	1.0	1.2
Crip1	P63254	7	-5	-19	-4	12	1.0	0.8	1.0	1.1
Phf6	Q9D4J7	28	-10	-19	-4	11	0.9	0.8	1.0	1.1
Cblb	B9EKI5	345	-3	-19	-4	-4	1.0	0.8	1.0	1.0
Taf6l	Q8R2K4	230	-16	-21	-4	25	0.9	0.8	1.0	1.3
Thap11	Q9JJD0	48	13	-22	-4	-12	1.1	0.8	1.0	0.9
Noa1	Q9JJG9	395	-22	-23	-4	40	0.8	0.8	1.0	1.7
Mtmr3	Q8K296	11	-11	-24	-4	-22	0.9	0.8	1.0	0.8
Atrx	Q61687	1703	-7	-25	-4	4	0.9	0.8	1.0	1.0
Inpp5d	Q9ES52	52	-14	-26	-4	3	0.9	0.8	1.0	1.0
Hyou1	Q9JKR6	805	-12	-27	-4	-16	0.9	0.8	1.0	0.9

Banf1	O54962	85	-8	-30	-4	-23	0.9	0.8	1.0	0.8
Phf11a	Q8BVM9	279	-31	-30	-4	-34	0.8	0.8	1.0	0.7
Syne2	Q6ZWQ0	986	-10	-30	-4	-35	0.9	0.8	1.0	0.7
Vcp	Q01853	174	-43	-33	-4	-19	0.7	0.8	1.0	0.8
Recql5	Q8VID5	435	-15	-36	-4	-12	0.9	0.7	1.0	0.9
Pus3	Q9JI38	454	-23	-38	-4	-44	0.8	0.7	1.0	0.7
Fh	P97807	431	24	35	-4	43	1.3	1.5	1.0	1.8
Hdlbp	Q8VDJ3	53	-13	19	-4	4	0.9	1.2	1.0	1.0
Irf3	P70671	364	3	17	-4	42	1.0	1.2	1.0	1.7
Zmym2	Q9CU65	646	-12	17	-4	1	0.9	1.2	1.0	1.0
Eprs	Q8CGC7	1148	7	13	-4	11	1.1	1.1	1.0	1.1
Polm	Q9JIW4	82	-5	12	-4	29	1.0	1.1	1.0	1.4
Gcc2	B2RSU7	412	-3	9	-4	2	1.0	1.1	1.0	1.0
Gfm1	Q8K0D5	516	-4	9	-4	31	1.0	1.1	1.0	1.4
Dhrs1	Q99L04	58	-3	8	-4	13	1.0	1.1	1.0	1.1
Arl8b	Q9CQW2	164	-11	7	-4	46	0.9	1.1	1.0	1.8
Ahctf1	Q8CJF7	610	-6	7	-4	21	0.9	1.1	1.0	1.3
Matr3	Q8K310	552	-8	7	-4	13	0.9	1.1	1.0	1.1
Mgea5	Q9EQQ9	596	-1	7	-4	5	1.0	1.1	1.0	1.0
Sash3	Q8K352	116	-5	6	-4	-9	1.0	1.1	1.0	0.9
Hsp90ab1	P11499	366	-13	6	-4	13	0.9	1.1	1.0	1.1
Bckdha	Q3U3J1	198	-2	6	-4	2	1.0	1.1	1.0	1.0
Hectd1	Q69ZR2	2553	4	4	-4	3	1.0	1.0	1.0	1.0
Dhx9	E9QNN1	776	-6	4	-4	17	0.9	1.0	1.0	1.2
Tomm34	Q9CYG7	60	-1	4	-4	15	1.0	1.0	1.0	1.2
Zranb2	Q9R020	88	2	4	-4	1	1.0	1.0	1.0	1.0
Dmxl1	Q6PNC0	257	-2	3	-4	33	1.0	1.0	1.0	1.5
Ipo7	Q9EPL8	736	-11	3	-4	28	0.9	1.0	1.0	1.4
Gnb1	P62874	148	-15	3	-4	19	0.9	1.0	1.0	1.2
Kdm2a	F6YRW4	840	-1	3	-4	1	1.0	1.0	1.0	1.0
Gmip	Q6PGG2	920	-7	3	-4	-1	0.9	1.0	1.0	1.0
Wnk1	P83741	459	10	3	-4	28	1.1	1.0	1.0	1.4
Ywhah	P68510	97	11	3	-4	27	1.1	1.0	1.0	1.4
Tubb1	A2AQ07	12	-7	3	-4	15	0.9	1.0	1.0	1.2
Mllt1	Q9ERL0	42	-4	3	-4	10	1.0	1.0	1.0	1.1
Snd1	Q78PY7	96	1	3	-4	9	1.0	1.0	1.0	1.1
Cd180	Q62192	419	-13	3	-4	4	0.9	1.0	1.0	1.0
Strn4	P58404	271	-20	3	-4	-23	0.8	1.0	1.0	0.8
Prpf6	Q91YR7	522	-16	2	-4	26	0.9	1.0	1.0	1.3
Uba7	Q9DBK7	586	0	2	-4	25	1.0	1.0	1.0	1.3
Ap2a1	P17426	331	-7	2	-4	13	0.9	1.0	1.0	1.1
Ap2a2	P17427	330	-7	2	-4	13	0.9	1.0	1.0	1.1
Kif21b	E9Q0A4	299	1	2	-4	12	1.0	1.0	1.0	1.1
Celf2	Q9Z0H4	174	-33	2	-4	-7	0.8	1.0	1.0	0.9
Pdcd11	Q6NS46	1165	-8	1	-4	27	0.9	1.0	1.0	1.4
Gpam	Q61586	812	1	1	-4	-7	1.0	1.0	1.0	0.9
Map2k4	P47809	156	14	1	-4	-9	1.2	1.0	1.0	0.9
Eif3g	Q9Z1D1	139	5	1	-4	-12	1.0	1.0	1.0	0.9
Atrx	Q61687	1774	6	1	-4	26	1.1	1.0	1.0	1.3
Pcbp1	P60335	158	-14	1	-4	5	0.9	1.0	1.0	1.0
Nipbl	Q6KCD5	2368	-6	1	-4	-3	0.9	1.0	1.0	1.0

Rasal3	Q8C2K5	922	-2	1	-4	-6	1.0	1.0	1.0	0.9
Psmc4	P54775	210	-11	0	-4	33	0.9	1.0	1.0	1.5
Ap1g1	P22892	353	-13	0	-4	17	0.9	1.0	1.0	1.2
Cnot1	Q6ZQ08	2358	-2	0	-4	17	1.0	1.0	1.0	1.2
Gtf2b	P62915	168	-5	0	-4	8	1.0	1.0	1.0	1.1
Dlgap4	B1AZP2	823	2	0	-4	1	1.0	1.0	1.0	1.0
Med9	Q8VCS6	135	-8	-1	-4	26	0.9	1.0	1.0	1.4
Atp5a1	Q03265	244	-11	-1	-4	10	0.9	1.0	1.0	1.1
Trim35	Q8C006	121	-9	-1	-4	-1	0.9	1.0	1.0	1.0
Tpr	F6ZDS4	1223	-8	-1	-4	-13	0.9	1.0	1.0	0.9
Cdpf1	Q8R3A2	72	-5	-1	-4	0	1.0	1.0	1.0	1.0
Fto	Q8BGW1	323	-3	-2	-4	7	1.0	1.0	1.0	1.1
Fanci	Q8K368	146	-7	-2	-4	-1	0.9	1.0	1.0	1.0
Nop56	Q9D6Z1	384	-9	-2	-4	-1	0.9	1.0	1.0	1.0
Bag5	Q8CI32	191	-11	-2	-4	-22	0.9	1.0	1.0	0.8
Tomm34	Q9CYG7	222	-12	-2	-4	1	0.9	1.0	1.0	1.0
Diaph1	O08808	988	-2	-3	-4	11	1.0	1.0	1.0	1.1
Ubac1	Q8VDI7	134	2	-3	-4	6	1.0	1.0	1.0	1.1
Npat	Q8BMA5	1371	-9	-3	-4	-16	0.9	1.0	1.0	0.9
Cog1	Q9Z160	634	-6	-3	-4	8	0.9	1.0	1.0	1.1
Hnrnpl	Q8R081	469	-8	-3	-4	1	0.9	1.0	1.0	1.0
Ilk	O55222	239	-5	-3	-4	-1	1.0	1.0	1.0	1.0
Wdr33	Q8K4P0	220	-13	-4	-4	8	0.9	1.0	1.0	1.1
Syne2	Q6ZWQ0	4549	-5	-4	-4	4	1.0	1.0	1.0	1.0
Khsrp	Q3U0V1	177	-9	-4	-4	3	0.9	1.0	1.0	1.0
Actl6a	Q9Z2N8	32	-11	-4	-4	1	0.9	1.0	1.0	1.0
Cdc73	Q8JZM7	145	1	-4	-4	-2	1.0	1.0	1.0	1.0
Flna	Q8BTM8	478	-9	-4	-4	-6	0.9	1.0	1.0	0.9
Eef2	P58252	591	-4	-4	-4	7	1.0	1.0	1.0	1.1
Gpd1l	Q3ULJ0	104	-1	-4	-4	7	1.0	1.0	1.0	1.1
Sucla2	Q9Z2I9	384	-1	-4	-4	5	1.0	1.0	1.0	1.1
Vps39	Q8R5L3	59	-3	-4	-4	1	1.0	1.0	1.0	1.0
Mprip	P97434	723	4	-4	-4	-11	1.0	1.0	1.0	0.9
Znf280d	Q68FE8	741	-24	-4	-4	-14	0.8	1.0	1.0	0.9
Polr2a	P08775	1050	-15	-5	-4	20	0.9	1.0	1.0	1.2
Supt4h1a	P63271	19	-14	-5	-4	13	0.9	1.0	1.0	1.1
Pcbp2	Q61990	54	-11	-5	-4	-6	0.9	1.0	1.0	0.9
Rhoc	Q62159	16	-12	-5	-4	19	0.9	1.0	1.0	1.2
Rhoa	Q9QUI0	16	-12	-5	-4	19	0.9	1.0	1.0	1.2
Irf2bp2	E9Q1P8	504	-10	-5	-4	10	0.9	1.0	1.0	1.1
Irf2bpl	Q8K3X4	709	-10	-5	-4	10	0.9	1.0	1.0	1.1
Upf1	Q9EPU0	208	-4	-5	-4	4	1.0	1.0	1.0	1.0
Ncf2	O70145	291	-13	-5	-4	-2	0.9	1.0	1.0	1.0
Cltc	Q68FD5	753	-5	-5	-4	-2	1.0	1.0	1.0	1.0
Pik3cg	Q9JHG7	436	-9	-5	-4	-16	0.9	1.0	1.0	0.9
Arih1	Q9Z1K5	159	-6	-6	-4	16	0.9	0.9	1.0	1.2
Wdhd1	P59328	766	-8	-6	-4	12	0.9	0.9	1.0	1.1
Glyr1	Q922P9	41	-14	-6	-4	10	0.9	0.9	1.0	1.1
Ppp4r1	E9QPR5	385	-2	-6	-4	-20	1.0	0.9	1.0	0.8
Pom121	Q8K3Z9	282	-15	-6	-4	-20	0.9	0.9	1.0	0.8
Map3k4	O08648	1473	-9	-6	-4	52	0.9	0.9	1.0	2.1

Cmas	Q99KK2	403	-9	-6	-4	10	0.9	0.9	1.0	1.1
Alox15	P39654	292	-8	-6	-4	6	0.9	0.9	1.0	1.1
Utrn	E9Q6R7	870	-10	-7	-4	21	0.9	0.9	1.0	1.3
Rnf213	E9Q555	1041	-16	-7	-4	4	0.9	0.9	1.0	1.0
Rbm28	Q8CGC6	71	-6	-7	-4	-6	0.9	0.9	1.0	0.9
Tbcd	Q8BYA0	665	-16	-7	-4	-8	0.9	0.9	1.0	0.9
Gtf2h3	Q8VD76	143	-13	-7	-4	25	0.9	0.9	1.0	1.3
Aven	Q9D9K3	231	-5	-7	-4	6	1.0	0.9	1.0	1.1
Rpl12	P35979	141	-8	-7	-4	-1	0.9	0.9	1.0	1.0
Arhgef6	Q8K4I3	181	-6	-7	-4	-6	0.9	0.9	1.0	0.9
Rbsn	Q80Y56	181	-2	-7	-4	-7	1.0	0.9	1.0	0.9
Trabd	Q99JY4	286	-8	-7	-4	-7	0.9	0.9	1.0	0.9
Gcc2	B2RSU7	93	-5	-7	-4	-8	1.0	0.9	1.0	0.9
Gcn1	E9PVA8	1535	-2	-7	-4	-9	1.0	0.9	1.0	0.9
Zmym4	A2A791	752	-20	-7	-4	-10	0.8	0.9	1.0	0.9
Srm	Q64674	25	-12	-7	-4	-16	0.9	0.9	1.0	0.9
Nup85	Q8R480	51	-12	-8	-4	9	0.9	0.9	1.0	1.1
Gm29609	Q3UU56	824	-19	-8	-4	0	0.8	0.9	1.0	1.0
Mgme1	Q9CXC3	98	-21	-8	-4	-1	0.8	0.9	1.0	1.0
Pfkfb3	A7UAK5	193	-16	-8	-4	-44	0.9	0.9	1.0	0.7
Irf4	Q64287	194	-14	-8	-4	-1	0.9	0.9	1.0	1.0
Eprs	Q8CGC7	697	-17	-8	-4	-16	0.9	0.9	1.0	0.9
Syk	P48025	537	-16	-9	-4	18	0.9	0.9	1.0	1.2
Rangap1	P46061	141	-13	-9	-4	17	0.9	0.9	1.0	1.2
Rsbn1	Q80T69	646	-24	-9	-4	10	0.8	0.9	1.0	1.1
Ddx27	Q921N6	225	-5	-9	-4	4	1.0	0.9	1.0	1.0
Wars	P32921	66	-1	-9	-4	-12	1.0	0.9	1.0	0.9
Dtwd2	Q9D0U1	207	-2	-9	-4	9	1.0	0.9	1.0	1.1
Cxxc1	Q9CWW7	384	-8	-9	-4	-12	0.9	0.9	1.0	0.9
Ranbp2	Q9ERU9	1727	0	-9	-4	-12	1.0	0.9	1.0	0.9
Atg7	Q9D906	402	-4	-9	-4	-14	1.0	0.9	1.0	0.9
Trim14	Q8BVW3	76	-10	-10	-4	-2	0.9	0.9	1.0	1.0
Snx6	Q6P8X1	149	-10	-10	-4	-3	0.9	0.9	1.0	1.0
Acot6	Q32Q92	14	-12	-10	-4	-8	0.9	0.9	1.0	0.9
Mrps11	Q3U8Y1	109	-12	-10	-4	-13	0.9	0.9	1.0	0.9
Rab7a	P51150	143	-19	-10	-4	5	0.8	0.9	1.0	1.0
Hspa4	Q3U2G2	167	-14	-11	-4	10	0.9	0.9	1.0	1.1
Ddb1	Q3U1J4	378	-14	-11	-4	7	0.9	0.9	1.0	1.1
Ranbp3	Q9CT10	308	-11	-11	-4	-1	0.9	0.9	1.0	1.0
Psma6	Q9QUM9	154	-7	-12	-4	5	0.9	0.9	1.0	1.0
Adsl	P54822	305	-13	-12	-4	3	0.9	0.9	1.0	1.0
Mtor	Q9JLN9	1498	3	-12	-4	-10	1.0	0.9	1.0	0.9
Carmil2	Q3V3V9	1227	-13	-12	-4	-11	0.9	0.9	1.0	0.9
Rfc4	Q99J62	48	-12	-13	-4	-9	0.9	0.9	1.0	0.9
Cluh	Q5SW19	734	-17	-13	-4	-18	0.9	0.9	1.0	0.9
Cap1	P40124	426	-18	-13	-4	4	0.9	0.9	1.0	1.0
Nsmce4a	G3XA30	251	-7	-13	-4	1	0.9	0.9	1.0	1.0
Ankrd54	Q91WK7	136	-22	-13	-4	-13	0.8	0.9	1.0	0.9
Rhof	Q8BYP3	162	-7	-14	-4	-26	0.9	0.9	1.0	0.8
Ccdc51	Q3URS9	311	-12	-14	-4	-2	0.9	0.9	1.0	1.0
Spop	Q6ZWS8	361	-15	-16	-4	27	0.9	0.9	1.0	1.4

Dnaja2	Q9QYJ0	146	-13	-16	-4	8	0.9	0.9	1.0	1.1
Zfp446	Q8C9M8	133	-2	-16	-4	4	1.0	0.9	1.0	1.0
Brd4	Q9ESU6	675	-8	-16	-4	-11	0.9	0.9	1.0	0.9
Ikbkg	O88522	131	-8	-16	-4	-16	0.9	0.9	1.0	0.9
H2-T23	P06339	338	-15	-16	-4	-21	0.9	0.9	1.0	0.8
Oxct1	Q9D0K2	235	-10	-17	-4	8	0.9	0.9	1.0	1.1
Ticrr	Q8BQ33	177	-21	-17	-4	-10	0.8	0.9	1.0	0.9
Mga	A2AWL7	696	-4	-19	-4	-14	1.0	0.8	1.0	0.9
Irf3	P70671	216	-29	-19	-4	10	0.8	0.8	1.0	1.1
Smc2	Q8CG48	132	-13	-20	-4	2	0.9	0.8	1.0	1.0
Irf8	P23611	120	-16	-21	-4	-6	0.9	0.8	1.0	0.9
S100a9	P31725	91	-12	-21	-4	-54	0.9	0.8	1.0	0.6
Sms	P97355	337	-28	-24	-4	-5	0.8	0.8	1.0	1.0
Dus1l	Q8C2P3	213	-15	-24	-4	-5	0.9	0.8	1.0	1.0
Whsc1	Q8BVE8	1193	-28	-25	-4	-20	0.8	0.8	1.0	0.8
Sptlc2	P97363	186	-18	-25	-4	2	0.9	0.8	1.0	1.0
Nisch	Q80TM9	186	-6	-25	-4	-16	0.9	0.8	1.0	0.9
Mrpl46	Q9EQI8	208	-23	-27	-4	-26	0.8	0.8	1.0	0.8
Ppie	Q9QZH3	174	-16	-27	-4	-14	0.9	0.8	1.0	0.9
Dcaf12	Q8BGZ3	380	-14	-29	-4	-14	0.9	0.8	1.0	0.9
Lrpprc	Q6PB66	926	-20	-37	-4	9	0.8	0.7	1.0	1.1
Alox5	P48999	100	-46	-63	-4	-107	0.7	0.6	1.0	0.5
Apex1	P28352	309	-62	-88	-4	-142	0.6	0.5	1.0	0.4
Rnh1	Q91VI7	97	1	13	-5	21	1.0	1.1	1.0	1.3
Anxa2	P07356	335	-3	11	-5	18	1.0	1.1	1.0	1.2
Myo1c	Q9WTI7	161	4	10	-5	-1	1.0	1.1	1.0	1.0
Lyst	G5E8Q0	1133	-9	10	-5	16	0.9	1.1	1.0	1.2
Prex1	Q69ZK0	1164	7	10	-5	-19	1.1	1.1	1.0	0.8
Ldha	P06151	293	-6	9	-5	15	0.9	1.1	1.0	1.2
Cad	B2RQC6	1374	1	9	-5	-4	1.0	1.1	1.0	1.0
Pcm1	Q9R0L6	607	-5	8	-5	-6	1.0	1.1	1.0	0.9
Dis3	Q9CSH3	194	-10	6	-5	20	0.9	1.1	1.0	1.2
Gamt	O35969	91	-11	6	-5	5	0.9	1.1	1.0	1.0
Ppp1r3d	A2AJW4	77	-2	5	-5	2	1.0	1.1	1.0	1.0
Arhgef6	Q8K4I3	552	-2	5	-5	0	1.0	1.1	1.0	1.0
Acly	Q91V92	835	4	4	-5	-22	1.0	1.0	1.0	0.8
D10Wsu1(	Q9CX66	20	0	4	-5	-26	1.0	1.0	1.0	0.8
Ampd3	O08739	47	4	4	-5	12	1.0	1.0	1.0	1.1
Rnf20	Q5DTM8	922	-1	4	-5	6	1.0	1.0	1.0	1.1
Cast	Q8CE80	702	3	3	-5	-11	1.0	1.0	1.0	0.9
Pop5	Q9DB28	146	-15	3	-5	59	0.9	1.0	1.0	2.4
Capn7	Q9R1S8	242	-8	3	-5	5	0.9	1.0	1.0	1.1
Znf512	Q69Z99	177	-14	3	-5	-9	0.9	1.0	1.0	0.9
Tcof1	O08784	580	-13	3	-5	-29	0.9	1.0	1.0	0.8
Myo1f	Q8CG29	517	-15	2	-5	15	0.9	1.0	1.0	1.2
Ubxn7	G5E8R8	160	3	2	-5	12	1.0	1.0	1.0	1.1
Hsd3b7	Q9EQC1	161	-7	2	-5	5	0.9	1.0	1.0	1.0
Cactin	Q9CS00	713	2	2	-5	-4	1.0	1.0	1.0	1.0
Wasl	Q91YD9	427	9	2	-5	-8	1.1	1.0	1.0	0.9
Eml4	Q3UMY5	379	-8	2	-5	25	0.9	1.0	1.0	1.3
Arhgap24	Q8C4V1	73	0	2	-5	-14	1.0	1.0	1.0	0.9

Vps51	Q3UVL4	226	-6	1	-5	6	0.9	1.0	1.0	1.1
Prpsap2	Q8R574	135	-9	1	-5	1	0.9	1.0	1.0	1.0
Limk2	O54785	15	-8	1	-5	-6	0.9	1.0	1.0	0.9
Coq8a	Q60936	638	-10	1	-5	-7	0.9	1.0	1.0	0.9
Crip1	P63254	28	-15	1	-5	24	0.9	1.0	1.0	1.3
Ppp2r5e	Q61151	106	-4	1	-5	6	1.0	1.0	1.0	1.1
Nup133	Q8R0G9	529	-13	1	-5	1	0.9	1.0	1.0	1.0
Ccdc88b	Q4QRL3	154	-29	0	-5	17	0.8	1.0	1.0	1.2
Cdk5rap3	Q99LM2	165	5	0	-5	10	1.0	1.0	1.0	1.1
Acap1	Q8K2H4	64	-8	0	-5	7	0.9	1.0	1.0	1.1
Pitpnc1	Q8K4R4	142	-6	-1	-5	-1	0.9	1.0	1.0	1.0
Ywhae	P62259	97	-7	-1	-5	-6	0.9	1.0	1.0	0.9
Ncbp1	Q3UYV9	320	-3	-1	-5	21	1.0	1.0	1.0	1.3
Rangap1	P46061	169	-12	-1	-5	14	0.9	1.0	1.0	1.2
Eefsec	Q9JHW4	429	-7	-1	-5	11	0.9	1.0	1.0	1.1
Trp53rkb	Q543M9	129	-3	-1	-5	1	1.0	1.0	1.0	1.0
Helb	Q6NVF4	714	-7	-1	-5	-3	0.9	1.0	1.0	1.0
Tcp11l1	Q8BTG3	495	-4	-2	-5	3	1.0	1.0	1.0	1.0
Mbnl2	Q8C181	43	-6	-2	-5	2	0.9	1.0	1.0	1.0
Mbnl1	Q9JKP5	43	-6	-2	-5	2	0.9	1.0	1.0	1.0
Gpatch3	Q8BIY1	517	-6	-2	-5	-18	0.9	1.0	1.0	0.9
Diaph1	O08808	779	-2	-2	-5	22	1.0	1.0	1.0	1.3
Nat10	Q8K224	658	0	-2	-5	0	1.0	1.0	1.0	1.0
Zzef1	Q5SSH7	466	-3	-3	-5	37	1.0	1.0	1.0	1.6
Ap1b1	O35643	863	-15	-3	-5	16	0.9	1.0	1.0	1.2
Zc3hav1	Q3UPF5	15	-7	-3	-5	12	0.9	1.0	1.0	1.1
Gm21992	F7BGR7	90	-9	-3	-5	4	0.9	1.0	1.0	1.0
Rbm14	Q8C2Q3	90	-9	-3	-5	4	0.9	1.0	1.0	1.0
Zbp1	Q9QY24	206	-5	-3	-5	2	1.0	1.0	1.0	1.0
Pxn	Q8VI36	405	-1	-3	-5	-9	1.0	1.0	1.0	0.9
Lyar	Q08288	51	-1	-3	-5	11	1.0	1.0	1.0	1.1
Pdpk1	Q9Z2A0	21	-10	-3	-5	7	0.9	1.0	1.0	1.1
Zfp119b	Q8K0G9	354	-23	-3	-5	-2	0.8	1.0	1.0	1.0
Hgs	Q99LI8	190	-8	-3	-5	-5	0.9	1.0	1.0	1.0
Dcaf5	Q80T85	711	-7	-3	-5	-10	0.9	1.0	1.0	0.9
Tbl3	Q8C4J7	43	-2	-4	-5	6	1.0	1.0	1.0	1.1
Pds5a	E9QPI5	326	-4	-4	-5	5	1.0	1.0	1.0	1.1
Ostc	Q78XF5	14	-16	-4	-5	1	0.9	1.0	1.0	1.0
Stat1	A0A087WSP5	155	-2	-4	-5	1	1.0	1.0	1.0	1.0
Urod	P70697	59	-7	-4	-5	-5	0.9	1.0	1.0	1.0
Rnf187	Q8BFX1	108	-10	-4	-5	-7	0.9	1.0	1.0	0.9
Npepl1	Q6NSR8	102	-7	-4	-5	-13	0.9	1.0	1.0	0.9
Ankrd54	Q91WK7	264	-4	-4	-5	10	1.0	1.0	1.0	1.1
Lcp1	Q61233	336	-15	-4	-5	7	0.9	1.0	1.0	1.1
Crebbp	F8VPR5	384	-12	-4	-5	7	0.9	1.0	1.0	1.1
Impdh2	P24547	331	-12	-5	-5	10	0.9	1.0	1.0	1.1
Ubr5	E9Q2H1	730	-9	-5	-5	1	0.9	1.0	1.0	1.0
Map1s	Q8C052	350	-9	-5	-5	-1	0.9	1.0	1.0	1.0
Hgs	Q99LI8	185	-8	-5	-5	-1	0.9	1.0	1.0	1.0
Dxo	O70348	244	-9	-5	-5	-6	0.9	1.0	1.0	0.9
Ecm29	Q6PDI5	1385	-20	-5	-5	43	0.8	1.0	1.0	1.8

Rab3gap1	Q80UJ7	218	-4	-5	-5	12	1.0	1.0	1.0	1.1
Zc3hav1	Q3UPF5	604	-18	-5	-5	11	0.8	1.0	1.0	1.1
Anapc5	Q8BTZ4	74	-15	-5	-5	6	0.9	1.0	1.0	1.1
Bop1	P97452	390	-5	-5	-5	1	1.0	1.0	1.0	1.0
Rprd1b	Q9CSU0	234	-9	-5	-5	-2	0.9	1.0	1.0	1.0
Xpo7	Q9EPK7	123	-31	-5	-5	-37	0.8	1.0	1.0	0.7
Nt5dc1	Q8C5P5	111	-11	-5	-5	-74	0.9	1.0	1.0	0.6
Eif3c	Q8R1B4	617	-9	-6	-5	17	0.9	0.9	1.0	1.2
Rap1gds1	E9Q912	29	-2	-6	-5	1	1.0	0.9	1.0	1.0
Gnb1	P62874	271	-12	-6	-5	-17	0.9	0.9	1.0	0.9
Spr	Q91XH5	160	-13	-6	-5	25	0.9	0.9	1.0	1.3
Phf1	Q9Z1B8	510	-3	-6	-5	20	1.0	0.9	1.0	1.3
Nfatc1	B5B2N2	384	-12	-6	-5	-1	0.9	0.9	1.0	1.0
Smad2	Q62432	70	-4	-6	-5	-1	1.0	0.9	1.0	1.0
Csde1	Q91W50	645	-11	-6	-5	-8	0.9	0.9	1.0	0.9
Uncharact	Q9CZL2	19	3	-6	-5	-24	1.0	0.9	1.0	0.8
Polr2b	Q8CFI7	622	-14	-7	-5	23	0.9	0.9	1.0	1.3
Traf2	P39429	287	-8	-7	-5	11	0.9	0.9	1.0	1.1
Znf740	Q6NZQ6	166	-4	-7	-5	8	1.0	0.9	1.0	1.1
Rogdi	Q3TDK6	66	-5	-7	-5	-3	1.0	0.9	1.0	1.0
Anxa6	P14824	59	-10	-7	-5	-4	0.9	0.9	1.0	1.0
Phospho2	Q9D9M5	185	-8	-7	-5	-6	0.9	0.9	1.0	0.9
Tnks1bp1	P58871	159	-9	-7	-5	-7	0.9	0.9	1.0	0.9
Irak4	Q8R4K2	204	-12	-7	-5	-11	0.9	0.9	1.0	0.9
Elp2	Q91WG4	699	-4	-7	-5	-14	1.0	0.9	1.0	0.9
Rps5	Q91V55	66	-10	-7	-5	31	0.9	0.9	1.0	1.4
Fuk	Q7TMC8	552	-4	-7	-5	7	1.0	0.9	1.0	1.1
Eif3j2	Q66JS6	212	-7	-7	-5	4	0.9	0.9	1.0	1.0
Ddx51	Q6P9R1	374	-12	-7	-5	4	0.9	0.9	1.0	1.0
H2-Eb1	O78196	106	-13	-7	-5	-3	0.9	0.9	1.0	1.0
Exoc4	O35382	958	-8	-7	-5	-4	0.9	0.9	1.0	1.0
Acads	Q07417	289	-14	-7	-5	-33	0.9	0.9	1.0	0.8
Dnm1l	Q8K1M6	367	-8	-8	-5	20	0.9	0.9	1.0	1.2
Vapa	Q9WV55	128	4	-8	-5	13	1.0	0.9	1.0	1.1
Trim30a	P15533	460	-21	-8	-5	6	0.8	0.9	1.0	1.1
Tpx2	A2APB8	535	-4	-8	-5	5	1.0	0.9	1.0	1.0
Fiz1	Q9WTJ4	459	-1	-8	-5	3	1.0	0.9	1.0	1.0
Lap3	Q9CPY7	445	-3	-8	-5	1	1.0	0.9	1.0	1.0
Flnb	Q80X90	1155	-10	-8	-5	-9	0.9	0.9	1.0	0.9
Hnrnpll	Q921F4	454	-8	-8	-5	-9	0.9	0.9	1.0	0.9
Cpsf6	Q6NVF9	159	-9	-8	-5	-13	0.9	0.9	1.0	0.9
Otulin	Q3UCV8	47	-11	-8	-5	-24	0.9	0.9	1.0	0.8
Celf1	P28659	62	-30	-8	-5	11	0.8	0.9	1.0	1.1
Celf2	Q9Z0H4	86	-30	-8	-5	11	0.8	0.9	1.0	1.1
Ltf	P08071	175	-19	-8	-5	5	0.8	0.9	1.0	1.0
Asun	Q8QZV7	406	-6	-8	-5	2	0.9	0.9	1.0	1.0
Copb1	Q9JIF7	684	-15	-8	-5	-4	0.9	0.9	1.0	1.0
Thoc5	Q8BKT7	613	-6	-8	-5	-8	0.9	0.9	1.0	0.9
Nfx1	B1AY10	941	-5	-8	-5	-9	1.0	0.9	1.0	0.9
Prps2	Q9CS42	91	-16	-9	-5	5	0.9	0.9	1.0	1.0
Prps1	Q9D7G0	91	-16	-9	-5	5	0.9	0.9	1.0	1.0

Ahnak	E9Q616	2012	-5	-9	-5	-7	1.0	0.9	1.0	0.9
Nlrc5	C3VPR6	1329	3	-9	-5	9	1.0	0.9	1.0	1.1
Mob4	Q6PEB6	119	-26	-9	-5	1	0.8	0.9	1.0	1.0
Dctn1	O08788	888	-12	-9	-5	1	0.9	0.9	1.0	1.0
Pabpc1	P29341	339	-10	-9	-5	0	0.9	0.9	1.0	1.0
Pabpc4	Q6PHQ9	339	-10	-9	-5	0	0.9	0.9	1.0	1.0
Pabpc6	Q9D4E6	349	-10	-9	-5	0	0.9	0.9	1.0	1.0
Gfpt1	P47856	636	-9	-10	-5	10	0.9	0.9	1.0	1.1
Ighm	A0A075B6A0	89	-6	-10	-5	4	0.9	0.9	1.0	1.0
Cct3	P80318	455	-17	-10	-5	3	0.9	0.9	1.0	1.0
Syvn1	Q9DBY1	329	-24	-10	-5	2	0.8	0.9	1.0	1.0
Aqr	Q8CFQ3	28	-6	-10	-5	-7	0.9	0.9	1.0	0.9
Phf10	K4DI61	133	-15	-10	-5	-17	0.9	0.9	1.0	0.9
Rpl4	Q9D8E6	250	-6	-10	-5	-19	0.9	0.9	1.0	0.8
Acad9	Q8JZN5	617	-24	-10	-5	22	0.8	0.9	1.0	1.3
Atxn10	P28658	382	5	-10	-5	18	1.0	0.9	1.0	1.2
Mad1l1	Q9WTX8	116	-5	-10	-5	-8	1.0	0.9	1.0	0.9
Numa1	E9Q7G0	1655	-12	-11	-5	-13	0.9	0.9	1.0	0.9
Lpin1	Q91ZP3	877	-6	-11	-5	-16	0.9	0.9	1.0	0.9
Atxn10	P28658	134	-10	-11	-5	21	0.9	0.9	1.0	1.3
Txlna	Q6PAM1	245	-12	-11	-5	13	0.9	0.9	1.0	1.1
Txlng	Q8BHN1	212	-12	-11	-5	13	0.9	0.9	1.0	1.1
Ranbp2	Q9ERU9	1921	0	-11	-5	5	1.0	0.9	1.0	1.1
Hnrnph1	O35737	22	-13	-11	-5	4	0.9	0.9	1.0	1.0
Gak	Q99KY4	145	-8	-11	-5	1	0.9	0.9	1.0	1.0
Bag1	Q60739	282	-9	-11	-5	-2	0.9	0.9	1.0	1.0
Wdr44	Q6NVE8	703	-12	-11	-5	-8	0.9	0.9	1.0	0.9
Pds5a	E9QPI5	1083	-7	-11	-5	-19	0.9	0.9	1.0	0.8
Myo1g	Q5SUA5	985	-7	-11	-5	-20	0.9	0.9	1.0	0.8
Vps37a	Q8CHS8	375	-12	-12	-5	23	0.9	0.9	1.0	1.3
Rps6kb2	Q9Z1M4	66	-7	-12	-5	12	0.9	0.9	1.0	1.1
Adnp2	Q8CHC8	765	-13	-12	-5	-7	0.9	0.9	1.0	0.9
Atp13a1	Q9EPE9	720	-8	-12	-5	38	0.9	0.9	1.0	1.6
Acsl5	Q8JZR0	185	-20	-13	-5	5	0.8	0.9	1.0	1.0
Galk2	Q68FH4	303	-3	-13	-5	2	1.0	0.9	1.0	1.0
Znf281	Q99LI5	364	-8	-13	-5	1	0.9	0.9	1.0	1.0
Odf2	A3KGV1	136	-4	-13	-5	-3	1.0	0.9	1.0	1.0
Vars2	Q3U2A8	550	-10	-13	-5	-28	0.9	0.9	1.0	0.8
Ercc4	Q9QZD4	418	-22	-13	-5	-5	0.8	0.9	1.0	1.0
Dld	O08749	85	-18	-13	-5	-16	0.8	0.9	1.0	0.9
Bod1l	E9Q6J5	1597	-1	-13	-5	-37	1.0	0.9	1.0	0.7
Prkdc	P97313	3660	-3	-14	-5	-6	1.0	0.9	1.0	0.9
Pxn	Q8VI36	546	-13	-14	-5	-6	0.9	0.9	1.0	0.9
Ranbp2	Q9ERU9	1290	-6	-15	-5	-11	0.9	0.9	1.0	0.9
Mki67	E9PVX6	2523	-9	-15	-5	-18	0.9	0.9	1.0	0.9
Lmna	P48678	590	-15	-15	-5	-21	0.9	0.9	1.0	0.8
Orc4	O88708	418	-15	-15	-5	11	0.9	0.9	1.0	1.1
Sec23b	Q9D662	74	-24	-15	-5	-2	0.8	0.9	1.0	1.0
Plekha2	Q9ERS5	81	-21	-15	-5	-3	0.8	0.9	1.0	1.0
Dnmt1	P13864	62	-19	-15	-5	-20	0.8	0.9	1.0	0.8
Nudt15	Q8BG93	22	-13	-16	-5	-3	0.9	0.9	1.0	1.0

Acat1	Q8QZT1	410	-24	-16	-5	-28	0.8	0.9	1.0	0.8
Gimap7	Q8R379	78	-5	-16	-5	11	1.0	0.9	1.0	1.1
Aamp	J3QN89	218	-13	-16	-5	-16	0.9	0.9	1.0	0.9
Cwf19l2	Q8BG79	342	-8	-16	-5	-24	0.9	0.9	1.0	0.8
Ep300	B2RWS6	1752	-10	-17	-5	13	0.9	0.9	1.0	1.1
Crebbp	F8VPR5	1791	-10	-17	-5	13	0.9	0.9	1.0	1.1
Atp2a2	O55143	364	-12	-17	-5	5	0.9	0.9	1.0	1.1
Atp2a3	Q64518	364	-12	-17	-5	5	0.9	0.9	1.0	1.1
Psmb3	Q9R1P1	19	-30	-17	-5	-19	0.8	0.9	1.0	0.8
Thoc1	Q8R3N6	396	-5	-18	-5	11	1.0	0.9	1.0	1.1
Cd300lf	Q6SJQ7	240	-3	-18	-5	6	1.0	0.9	1.0	1.1
Ddx21	Q9JIK5	609	-17	-18	-5	23	0.9	0.8	1.0	1.3
Lipe	P54310	733	-34	-18	-5	9	0.7	0.8	1.0	1.1
Eif4e	P63073	170	-20	-18	-5	8	0.8	0.8	1.0	1.1
Traf5	E9QMA6	65	-16	-19	-5	5	0.9	0.8	1.0	1.1
Pdcd11	Q6NS46	977	-14	-19	-5	2	0.9	0.8	1.0	1.0
Rps15a	P62245	72	-26	-21	-5	36	0.8	0.8	1.0	1.6
Rchy1	Q9CR50	136	-12	-21	-5	-2	0.9	0.8	1.0	1.0
Dhrs11	Q3U0B3	195	-5	-22	-5	-52	1.0	0.8	1.0	0.7
Cramp1	Q6PG95	97	-33	-23	-5	-20	0.8	0.8	1.0	0.8
Poc1b	Q8BHD1	30	-15	-23	-5	-18	0.9	0.8	1.0	0.8
Scaper	F8VQ70	346	-12	-24	-5	-21	0.9	0.8	1.0	0.8
Uba7	Q9DBK7	29	-27	-24	-5	7	0.8	0.8	1.0	1.1
Zfyve1	Q810J8	103	-2	-24	-5	-11	1.0	0.8	1.0	0.9
Prkcb	P68404	217	-27	-25	-5	15	0.8	0.8	1.0	1.2
Rbm26	Q6NZN0	25	-9	-25	-5	-9	0.9	0.8	1.0	0.9
Gstm5	P48774	177	-9	-26	-5	30	0.9	0.8	1.0	1.4
Eif3i	Q9QZD9	81	-25	-27	-5	-31	0.8	0.8	1.0	0.8
Lancl2	Q9JJK2	248	-14	-28	-5	-3	0.9	0.8	1.0	1.0
Rab2b	P59279	21	-20	-35	-5	9	0.8	0.7	1.0	1.1
Ppm1g	Q61074	241	-38	42	-5	92	0.7	1.7	1.0	12.5
S100a8	P27005	42	9	24	-5	49	1.1	1.3	1.0	1.9
Aldh2	P47738	388	7	24	-5	-24	1.1	1.3	1.0	0.8
Alox15	P39654	97	17	20	-5	25	1.2	1.2	1.0	1.3
Xpo5	Q924C1	1044	5	15	-5	-18	1.1	1.2	1.0	0.8
Parp14	Q2EMV9	736	-5	10	-5	20	1.0	1.1	1.0	1.3
Mcoln1	Q99J21	544	6	10	-5	0	1.1	1.1	1.0	1.0
Dhx9	E9QNN1	418	-7	10	-5	16	0.9	1.1	1.0	1.2
Cmas	Q99KK2	378	-7	9	-5	12	0.9	1.1	1.0	1.1
Ddx24	Q9ESV0	49	-21	8	-5	11	0.8	1.1	1.0	1.1
Pdcd4	Q61823	350	-9	8	-5	14	0.9	1.1	1.0	1.2
Polr1a	O35134	1311	-4	8	-5	2	1.0	1.1	1.0	1.0
Mcm6	P97311	242	2	7	-5	30	1.0	1.1	1.0	1.4
Lap3	Q9CPY7	335	-6	7	-5	11	0.9	1.1	1.0	1.1
Parp14	Q2EMV9	648	1	7	-5	1	1.0	1.1	1.0	1.0
Paics	Q9DCL9	81	-1	6	-5	10	1.0	1.1	1.0	1.1
Zmynd8	A2A484	278	2	6	-5	25	1.0	1.1	1.0	1.3
Cul3	Q9JLV5	319	-9	6	-5	5	0.9	1.1	1.0	1.0
Spag9	Q58A65	1155	2	5	-5	-7	1.0	1.1	1.0	0.9
Msl1	Q6PDM1	126	-10	5	-5	-11	0.9	1.1	1.0	0.9
Arhgap9	Q8QZW8	271	0	5	-5	27	1.0	1.0	1.0	1.4

Plcb2	A3KGF7	188	-10	4	-5	7	0.9	1.0	1.0	1.1
Ankrd50	F7BE84	85	-7	4	-5	-5	0.9	1.0	1.0	1.0
Ptpn22	P29352	653	-1	4	-5	8	1.0	1.0	1.0	1.1
Gnl3l	Q6PGG6	224	-8	3	-5	2	0.9	1.0	1.0	1.0
Vps36	Q91XD6	271	-13	3	-5	18	0.9	1.0	1.0	1.2
Npepps	Q11011	191	-8	2	-5	23	0.9	1.0	1.0	1.3
Rfc2	Q9WUK4	83	-1	2	-5	10	1.0	1.0	1.0	1.1
Sin3a	Q60520	1168	1	2	-5	-3	1.0	1.0	1.0	1.0
Mlh1	Q9JK91	233	3	2	-5	-8	1.0	1.0	1.0	0.9
Srp54	P14576	133	-12	2	-5	25	0.9	1.0	1.0	1.3
Ncoa3	Q05BA5	708	7	2	-5	-12	1.1	1.0	1.0	0.9
Adss	P46664	58	-15	1	-5	2	0.9	1.0	1.0	1.0
Vps4b	P46467	240	-8	1	-5	-2	0.9	1.0	1.0	1.0
Kat2a	Q9JHD2	808	9	1	-5	14	1.1	1.0	1.0	1.2
Map3k2	G5E8L8	405	-6	1	-5	-4	0.9	1.0	1.0	1.0
Pml	Q60953	386	-6	0	-5	16	0.9	1.0	1.0	1.2
Acaca	Q5SWU9	1341	-4	0	-5	1	1.0	1.0	1.0	1.0
Gvin1	L7N451	1654	4	0	-5	0	1.0	1.0	1.0	1.0
Rin3	P59729	971	-5	0	-5	-2	1.0	1.0	1.0	1.0
Eif3m	Q99JX4	134	2	-1	-5	7	1.0	1.0	1.0	1.1
Tcirg1	Q9JHF5	239	-15	-1	-5	-6	0.9	1.0	1.0	0.9
Cntrl	A2AL36	2103	-8	-1	-5	-9	0.9	1.0	1.0	0.9
Ubr4	A2AN08	260	1	-1	-5	6	1.0	1.0	1.0	1.1
Ptgr2	Q8VDQ1	187	4	-1	-5	1	1.0	1.0	1.0	1.0
Sclt1	G5E861	292	-3	-1	-5	-3	1.0	1.0	1.0	1.0
Ddx20	Q9JJY4	578	-11	-2	-5	13	0.9	1.0	1.0	1.1
Fbxw11	Q5SRY7	145	-4	-2	-5	10	1.0	1.0	1.0	1.1
Coro7	Q9D2V7	187	-12	-2	-5	10	0.9	1.0	1.0	1.1
Zzef1	Q5SSH7	719	-3	-2	-5	9	1.0	1.0	1.0	1.1
Kdm3a	Q6PCM1	753	-15	-2	-5	8	0.9	1.0	1.0	1.1
Bcat2	O35855	343	-8	-2	-5	1	0.9	1.0	1.0	1.0
Oasl1	Q8VI94	164	2	-2	-5	-3	1.0	1.0	1.0	1.0
Chek2	Q9Z265	542	-12	-2	-5	-8	0.9	1.0	1.0	0.9
Mcm7	Q61881	566	0	-2	-5	13	1.0	1.0	1.0	1.1
Hmha1	Q3TBD2	135	-11	-2	-5	5	0.9	1.0	1.0	1.0
Pik3cd	O35904	990	1	-2	-5	-4	1.0	1.0	1.0	1.0
Pml	Q60953	96	-12	-3	-5	34	0.9	1.0	1.0	1.5
Gsr	P47791	85	-4	-3	-5	14	1.0	1.0	1.0	1.2
Tbc1d13	Q8R3D1	145	-6	-3	-5	-2	0.9	1.0	1.0	1.0
Vprbp	Q80TR8	1069	-4	-3	-5	-7	1.0	1.0	1.0	0.9
Ehmt2	Q9Z148	179	-19	-3	-5	-11	0.8	1.0	1.0	0.9
Acsf2	Q8VCW8	194	-12	-3	-5	21	0.9	1.0	1.0	1.3
Vps18	Q8R307	421	-6	-3	-5	2	0.9	1.0	1.0	1.0
Ubr5	E9Q2H1	2314	-6	-3	-5	-1	0.9	1.0	1.0	1.0
Zc3hc1	Q80YV2	405	-12	-3	-5	-3	0.9	1.0	1.0	1.0
Sec23b	Q9D662	180	-3	-3	-5	-5	1.0	1.0	1.0	1.0
Zfp62	Q8C827	807	-3	-3	-5	-6	1.0	1.0	1.0	0.9
Dmtn	Q9WV69	84	-4	-3	-5	-17	1.0	1.0	1.0	0.9
Mcm2	P97310	315	3	-4	-5	22	1.0	1.0	1.0	1.3
Parg	O88622	153	-4	-4	-5	1	1.0	1.0	1.0	1.0
Ncbp1	Q3UYV9	36	5	-4	-5	1	1.0	1.0	1.0	1.0

Papd4	Q91YI6	431	2	-4	-5	-3	1.0	1.0	1.0	1.0
Nckap1l	Q8K1X4	617	-17	-4	-5	2	0.9	1.0	1.0	1.0
Cep192	E9Q4Y4	687	5	-4	-5	0	1.0	1.0	1.0	1.0
Pml	Q60953	219	-12	-5	-5	19	0.9	1.0	1.0	1.2
Lgals9	O08573	325	-11	-5	-5	15	0.9	1.0	1.0	1.2
Sec31a	Q3UPL0	643	-14	-5	-5	10	0.9	1.0	1.0	1.1
Sec31a	Q3UPL0	703	-13	-5	-5	9	0.9	1.0	1.0	1.1
Pyhin1	Q8BV49	383	-15	-5	-5	9	0.9	1.0	1.0	1.1
Lars	Q8BMJ2	556	-6	-5	-5	6	0.9	1.0	1.0	1.1
Itgb3bp	Q9CQ82	129	-17	-5	-5	3	0.9	1.0	1.0	1.0
Tln1	P26039	1939	-5	-5	-5	-1	1.0	1.0	1.0	1.0
Fam188a	Q9CV28	27	-14	-5	-5	-5	0.9	1.0	1.0	1.0
Gm5678	A0A1B0GRG8	227	-10	-5	-5	-5	0.9	1.0	1.0	1.0
Cpsf1	Q9EPU4	1042	-6	-5	-5	11	0.9	1.0	1.0	1.1
Snrnp200	Q6P4T2	238	-11	-5	-5	7	0.9	1.0	1.0	1.1
Exosc4	Q921I9	97	-15	-5	-5	-2	0.9	1.0	1.0	1.0
Arl8b	Q9CQW2	158	-9	-6	-5	26	0.9	0.9	1.0	1.3
Raver1	Q9CW46	297	-6	-6	-5	12	0.9	0.9	1.0	1.1
Cct5	P80316	493	-6	-6	-5	-1	0.9	0.9	1.0	1.0
Eif2ak3	Q9Z2B5	594	-10	-6	-5	-15	0.9	0.9	1.0	0.9
Pi4ka	E9Q3L2	1835	-5	-6	-5	-21	1.0	0.9	1.0	0.8
Ap3d1	O54774	574	-17	-6	-5	25	0.9	0.9	1.0	1.3
Nfkb2	Q9WTK5	120	-10	-6	-5	8	0.9	0.9	1.0	1.1
Cct3	P80318	366	-12	-6	-5	5	0.9	0.9	1.0	1.1
Pdlim2	Q8R1G6	286	-8	-6	-5	2	0.9	0.9	1.0	1.0
Dgka	O88673	223	-5	-6	-5	1	1.0	0.9	1.0	1.0
Prpsap1	Q9D0M1	19	-4	-6	-5	-2	1.0	0.9	1.0	1.0
UPF0505	Q8BWQ6	588	-10	-6	-5	-6	0.9	0.9	1.0	0.9
Nln	Q91YP2	256	-11	-6	-5	-15	0.9	0.9	1.0	0.9
Reep4	Q8K072	106	0	-6	-5	-19	1.0	0.9	1.0	0.8
Hpcal1	P62748	185	-7	-7	-5	55	0.9	0.9	1.0	2.2
Tardbp	Q921F2	50	-9	-7	-5	3	0.9	0.9	1.0	1.0
Cntrl	A2AL36	1538	-4	-7	-5	-8	1.0	0.9	1.0	0.9
Gtf2h1	E9QKD9	251	2	-7	-5	-10	1.0	0.9	1.0	0.9
Bud31	Q6PGH1	60	-12	-7	-5	24	0.9	0.9	1.0	1.3
Capn1	O35350	250	-12	-7	-5	11	0.9	0.9	1.0	1.1
Fam98b	Q80VD1	183	-11	-7	-5	-4	0.9	0.9	1.0	1.0
Top2a	Q01320	732	-6	-8	-5	17	0.9	0.9	1.0	1.2
Top2b	Q64511	742	-6	-8	-5	17	0.9	0.9	1.0	1.2
Zc3h4	E9Q8K8	389	-5	-8	-5	2	1.0	0.9	1.0	1.0
Nek7	Q9ES74	79	-16	-8	-5	-5	0.9	0.9	1.0	1.0
Vps13a	Q5H8C4	870	-12	-8	-5	-7	0.9	0.9	1.0	0.9
Tbck	Q8BM85	737	-7	-8	-5	-7	0.9	0.9	1.0	0.9
Nars2	Q8BGV0	121	-6	-8	-5	-37	0.9	0.9	1.0	0.7
Rps6kb1	Q8BSK8	90	-5	-8	-5	24	1.0	0.9	1.0	1.3
Nfe2l3	Q9WTM4	558	-10	-8	-5	21	0.9	0.9	1.0	1.3
Itpr3	P70227	420	-17	-8	-5	7	0.9	0.9	1.0	1.1
Mrpl14	Q9D1I6	57	-9	-8	-5	-25	0.9	0.9	1.0	0.8
Birc6	O88738	1425	-12	-9	-5	40	0.9	0.9	1.0	1.7
Arrb2	Q91YI4	244	-1	-9	-5	5	1.0	0.9	1.0	1.0
Zmym2	Q9CU65	1267	-5	-9	-5	-1	1.0	0.9	1.0	1.0

Sptbn1	Q62261	964	-20	-9	-5	-1	0.8	0.9	1.0	1.0
Vps8	Q0P5W1	38	-10	-9	-5	-4	0.9	0.9	1.0	1.0
Cntrl	A2AL36	1454	-17	-9	-5	-13	0.9	0.9	1.0	0.9
Myh9	Q8VDD5	569	-17	-9	-5	7	0.9	0.9	1.0	1.1
Fan1	Q69ZT1	513	-2	-9	-5	5	1.0	0.9	1.0	1.1
Etfdh	Q921G7	247	-27	-9	-5	-1	0.8	0.9	1.0	1.0
Fam160b1	Q8CDM8	99	-11	-9	-5	-5	0.9	0.9	1.0	1.0
Phf11a	Q8BVM9	227	0	-9	-5	-14	1.0	0.9	1.0	0.9
Gpkow	Q56A08	404	7	-10	-5	18	1.1	0.9	1.0	1.2
Wdfy4	E9Q2M9	1655	-1	-10	-5	9	1.0	0.9	1.0	1.1
Cdkn1b	P46414	29	-6	-10	-5	9	0.9	0.9	1.0	1.1
Rap1gap2	Q5SVL6	425	-9	-10	-5	2	0.9	0.9	1.0	1.0
Cpsf2	O35218	621	-6	-10	-5	2	0.9	0.9	1.0	1.0
Zfp456	B2RUK9	400	-2	-10	-5	5	1.0	0.9	1.0	1.0
Vwa5a	Q99KC8	645	-1	-10	-5	-15	1.0	0.9	1.0	0.9
Mdn1	A2ANY6	4507	-19	-11	-5	21	0.8	0.9	1.0	1.3
Pgp	Q8CHP8	272	-9	-11	-5	10	0.9	0.9	1.0	1.1
Rnh1	Q91VI7	90	-19	-11	-5	6	0.8	0.9	1.0	1.1
Srsf7	Q8BL97	138	-9	-11	-5	5	0.9	0.9	1.0	1.1
Stag2	O35638	535	-14	-11	-5	-6	0.9	0.9	1.0	0.9
Clic5	Q8BXK9	32	-20	-11	-5	-14	0.8	0.9	1.0	0.9
Ganc	A2AQJ8	889	-12	-11	-5	-28	0.9	0.9	1.0	0.8
Sema4b	Q62179	172	-17	-11	-5	18	0.9	0.9	1.0	1.2
Arhgap26	Q6ZQ82	596	2	-11	-5	0	1.0	0.9	1.0	1.0
Phip	F8VQ93	1692	-9	-11	-5	-5	0.9	0.9	1.0	1.0
Pank4	Q80YV4	150	-8	-12	-5	46	0.9	0.9	1.0	1.9
Trmt61a	Q80XC2	209	-15	-12	-5	34	0.9	0.9	1.0	1.5
Eif5b	Q05D44	1088	-13	-12	-5	3	0.9	0.9	1.0	1.0
Trim34a	Q99PP6	96	-15	-12	-5	-2	0.9	0.9	1.0	1.0
Tfpt	Q3U1J1	89	-21	-12	-5	25	0.8	0.9	1.0	1.3
Traf6	P70196	172	-13	-12	-5	-4	0.9	0.9	1.0	1.0
Wapl	Q65Z40	974	-27	-13	-5	4	0.8	0.9	1.0	1.0
Mcts1	Q9DB27	113	-22	-13	-5	2	0.8	0.9	1.0	1.0
Pbrm1	Q8BSQ9	799	-13	-13	-5	-2	0.9	0.9	1.0	1.0
Hps4	Q99KG7	651	-8	-13	-5	-2	0.9	0.9	1.0	1.0
Ptpn9	O35239	338	-1	-13	-5	-17	1.0	0.9	1.0	0.9
Med12	A2AGH6	1652	-6	-14	-5	-16	0.9	0.9	1.0	0.9
Hsp90b1	P08113	645	-28	-14	-5	-2	0.8	0.9	1.0	1.0
Adssl1	P28650	183	-7	-14	-5	-4	0.9	0.9	1.0	1.0
Cstf1	Q99LC2	296	-15	-14	-5	-4	0.9	0.9	1.0	1.0
Rpp30	O88796	87	-13	-14	-5	-8	0.9	0.9	1.0	0.9
Pole	Q9WVF7	1076	-9	-14	-5	-14	0.9	0.9	1.0	0.9
Zc3h13	E9Q784	1653	-10	-15	-5	7	0.9	0.9	1.0	1.1
Mcub	Q810S1	305	-7	-15	-5	-34	0.9	0.9	1.0	0.7
Brd2	Q7JJ13	424	-18	-15	-5	8	0.8	0.9	1.0	1.1
Brd4	Q9ESU6	430	-18	-15	-5	8	0.8	0.9	1.0	1.1
Supv3l1	Q80YD1	575	-12	-15	-5	7	0.9	0.9	1.0	1.1
Xdh	Q00519	152	-8	-15	-5	-12	0.9	0.9	1.0	0.9
Pde12	Q3TIU4	237	-17	-15	-5	-15	0.9	0.9	1.0	0.9
Pinx1	Q9CZX5	145	-15	-16	-5	-11	0.9	0.9	1.0	0.9
Nck2	O55033	297	-9	-16	-5	-13	0.9	0.9	1.0	0.9

Akap9	Q70FJ1	1970	-7	-17	-5	-7	0.9	0.9	1.0	0.9
Ttc4	Q8R3H9	110	-5	-17	-5	-19	1.0	0.9	1.0	0.8
Ppil2	Q9D787	15	-20	-17	-5	16	0.8	0.9	1.0	1.2
Rnf169	E9Q7F2	261	-9	-17	-5	-13	0.9	0.9	1.0	0.9
Lmo2	P25801	130	-8	-17	-5	-19	0.9	0.9	1.0	0.8
Dhx38	Q80X98	866	-21	-18	-5	12	0.8	0.9	1.0	1.1
Eif5b	Q05D44	745	-20	-18	-5	36	0.8	0.8	1.0	1.6
Gdi2	Q61598	282	-11	-18	-5	7	0.9	0.8	1.0	1.1
Ubxn6	Q99PL6	210	3	-18	-5	-2	1.0	0.8	1.0	1.0
Ltf	P08071	648	-32	-18	-5	-8	0.8	0.8	1.0	0.9
Csde1	Q91W50	730	-15	-19	-5	4	0.9	0.8	1.0	1.0
Numa1	E9Q7G0	1912	-4	-19	-5	-7	1.0	0.8	1.0	0.9
Tomm34	Q9CYG7	70	-9	-19	-5	28	0.9	0.8	1.0	1.4
Keap1	Q9Z2X8	288	-5	-19	-5	21	1.0	0.8	1.0	1.3
Chd3	B1AR17	1183	-20	-19	-5	6	0.8	0.8	1.0	1.1
Chd4	Q6PDQ2	1114	-20	-19	-5	6	0.8	0.8	1.0	1.1
Eif2s2	Q99L45	307	-12	-21	-5	12	0.9	0.8	1.0	1.1
Samm50	Q8BGH2	65	-11	-22	-5	-8	0.9	0.8	1.0	0.9
F13a1	Q8BH61	410	-11	-22	-5	-10	0.9	0.8	1.0	0.9
Nat10	Q8K224	105	-13	-22	-5	-31	0.9	0.8	1.0	0.8
Baz1b	Q9Z277	120	-16	-23	-5	14	0.9	0.8	1.0	1.2
Nup98	Q6PFD9	760	-11	-23	-5	7	0.9	0.8	1.0	1.1
Irf5	P56477	271	-32	-24	-5	3	0.8	0.8	1.0	1.0
Mbd1	Q9Z2E2	57	-15	-24	-5	-7	0.9	0.8	1.0	0.9
Phf3	B2RQG2	360	-3	-24	-5	-13	1.0	0.8	1.0	0.9
Lrch1	P62046	151	-16	-26	-5	16	0.9	0.8	1.0	1.2
Acaa2	Q8BWT1	179	-11	-28	-5	10	0.9	0.8	1.0	1.1
Lrmp	G5E880	248	-12	-28	-5	12	0.9	0.8	1.0	1.1
Arhgap21	B7ZCJ1	477	-9	-28	-5	-13	0.9	0.8	1.0	0.9
Vps29	Q9QZ88	15	-12	-30	-5	-22	0.9	0.8	1.0	0.8
Pdcd4	Q61823	433	-14	-30	-5	-3	0.9	0.8	1.0	1.0
Nudt3	Q9JI46	68	-11	-31	-5	-31	0.9	0.8	1.0	0.8
Pgm2l1	Q8CAA7	125	-3	17	-6	14	1.0	1.2	0.9	1.2
Papss2	O88428	191	-6	13	-6	11	0.9	1.1	0.9	1.1
Gmip	Q6PGG2	894	-3	12	-6	-17	1.0	1.1	0.9	0.9
Arhgef2	Q60875	695	9	11	-6	-15	1.1	1.1	0.9	0.9
Mcm4	P49717	604	12	10	-6	-2	1.1	1.1	0.9	1.0
Brd7	O88665	107	8	10	-6	-13	1.1	1.1	0.9	0.9
Lypla2	Q9WTL7	213	-8	9	-6	13	0.9	1.1	0.9	1.1
Eif2s3x	Q9Z0N1	101	2	8	-6	18	1.0	1.1	0.9	1.2
Gm43738	A0A0G2JEA5	756	5	8	-6	3	1.0	1.1	0.9	1.0
Stat5a	P42230	126	-1	8	-6	-6	1.0	1.1	0.9	0.9
Tmpo	Q61029	362	-2	6	-6	8	1.0	1.1	0.9	1.1
Dhx29	Q6PGC1	647	-2	6	-6	8	1.0	1.1	0.9	1.1
Ogt	Q8CGY8	962	-12	5	-6	24	0.9	1.1	0.9	1.3
Orc3	Q9JK30	446	-13	4	-6	2	0.9	1.0	0.9	1.0
Wdr59	Q8C0M0	654	-16	4	-6	1	0.9	1.0	0.9	1.0
Mcm3ap	Q9WUU9	1232	-4	4	-6	15	1.0	1.0	0.9	1.2
Nup88	Q8CEC0	157	-23	4	-6	12	0.8	1.0	0.9	1.1
Iqgap1	Q9JKF1	1534	9	3	-6	-8	1.1	1.0	0.9	0.9
Ikbke	Q9R0T8	548	-5	3	-6	0	1.0	1.0	0.9	1.0

Samhd1	Q60710	342	-7	2	-6	-6	0.9	1.0	0.9	0.9
Leng8	Q8CBY3	567	-4	2	-6	4	1.0	1.0	0.9	1.0
Dok3	Q9QZK7	441	-7	2	-6	-2	0.9	1.0	0.9	1.0
Ppm1h	Q3UYC0	238	-5	1	-6	17	1.0	1.0	0.9	1.2
Dhx8	A2A4P0	123	-2	1	-6	-3	1.0	1.0	0.9	1.0
Zdhhc18	Q5Y5T2	148	-5	1	-6	-8	1.0	1.0	0.9	0.9
Pop1	Q8K205	781	-5	1	-6	-12	1.0	1.0	0.9	0.9
Anxa1	P10107	343	-3	1	-6	-21	1.0	1.0	0.9	0.8
Mmaa	Q8C7H1	97	-9	1	-6	-32	0.9	1.0	0.9	0.8
Kpna4	O35343	417	-12	1	-6	24	0.9	1.0	0.9	1.3
Rps12-ps3	Q6ZWZ6	108	-7	1	-6	12	0.9	1.0	0.9	1.1
Trmt2a	Q8BNV1	215	-3	1	-6	6	1.0	1.0	0.9	1.1
Snx20	Q9D2Y5	19	-4	1	-6	-8	1.0	1.0	0.9	0.9
Rqcd1	Q9JKY0	91	-16	0	-6	26	0.9	1.0	0.9	1.4
Ilf3	Q9Z1X4	116	-8	0	-6	3	0.9	1.0	0.9	1.0
Preb	Q9WUQ2	161	2	0	-6	-12	1.0	1.0	0.9	0.9
FAM120A	Q6A0A9	667	-9	-1	-6	19	0.9	1.0	0.9	1.2
Vwa5a	Q99KC8	279	-2	-1	-6	10	1.0	1.0	0.9	1.1
Cdk2	P97377	177	1	-1	-6	6	1.0	1.0	0.9	1.1
Kmt5b	Q3U8K7	276	-13	-1	-6	4	0.9	1.0	0.9	1.0
Suclg1	Q9WUM5	181	-10	-1	-6	13	0.9	1.0	0.9	1.1
Ap3b1	Q9Z1T1	904	-6	-1	-6	12	0.9	1.0	0.9	1.1
Gatad2a	Q8CHY6	419	-7	-1	-6	3	0.9	1.0	0.9	1.0
Ptp4a2	O70274	101	-3	-1	-6	-6	1.0	1.0	0.9	0.9
Tnpo1	Q8BFY9	103	-8	-2	-6	28	0.9	1.0	0.9	1.4
Rsf1	E9PWW9	947	-10	-2	-6	12	0.9	1.0	0.9	1.1
Hcfc1	Q61191	227	-9	-2	-6	5	0.9	1.0	0.9	1.1
Ppip5k2	Q6ZQB6	494	-7	-2	-6	-1	0.9	1.0	0.9	1.0
Ugp2	Q91ZJ5	123	-6	-2	-6	-3	0.9	1.0	0.9	1.0
Pip5k1c	O70161	581	-17	-2	-6	-35	0.9	1.0	0.9	0.7
Iba57	Q8CAK1	260	-12	-2	-6	24	0.9	1.0	0.9	1.3
Myh9	Q8VDD5	172	-14	-2	-6	6	0.9	1.0	0.9	1.1
Dhx34	Q9DBV3	34	-5	-2	-6	5	1.0	1.0	0.9	1.0
Ywhaq	P68254	134	-10	-2	-6	-9	0.9	1.0	0.9	0.9
Papola	Q61183	36	-8	-3	-6	12	0.9	1.0	0.9	1.1
Arid1b	E9Q4N7	1149	-8	-3	-6	9	0.9	1.0	0.9	1.1
Atm	Q62388	2098	-7	-3	-6	7	0.9	1.0	0.9	1.1
Pml	Q60953	211	-10	-3	-6	5	0.9	1.0	0.9	1.0
Aldh9a1	Q9JLJ2	484	1	-3	-6	3	1.0	1.0	0.9	1.0
Vps11	Q91W86	231	0	-3	-6	15	1.0	1.0	0.9	1.2
Nfkbil1	O88995	83	7	-3	-6	12	1.1	1.0	0.9	1.1
Uhrf1	Q8VDF2	326	-25	-3	-6	6	0.8	1.0	0.9	1.1
Prkcb	P68404	586	-12	-3	-6	1	0.9	1.0	0.9	1.0
Rgs19	Q9CX84	76	-2	-3	-6	-11	1.0	1.0	0.9	0.9
Gigyf1	Q99MR1	516	14	-3	-6	-12	1.2	1.0	0.9	0.9
Zfp407	G3UVV3	1385	0	-3	-6	-16	1.0	1.0	0.9	0.9
Noc4l	Q8BHY2	514	-5	-4	-6	2	1.0	1.0	0.9	1.0
Ubn1	Q4G0F8	59	-1	-4	-6	-1	1.0	1.0	0.9	1.0
Cct4	P80315	252	-4	-4	-6	-2	1.0	1.0	0.9	1.0
Siah1b	A2AHZ2	71	-3	-4	-6	-5	1.0	1.0	0.9	1.0
Nob1	Q8BW10	276	-14	-4	-6	-6	0.9	1.0	0.9	0.9

Nxt1	Q9QZV9	14	0	-4	-6	-10	1.0	1.0	0.9	0.9
Ipo5	Q8BKC5	266	0	-4	-6	13	1.0	1.0	0.9	1.1
Hnrnpl	Q8R081	257	-10	-4	-6	4	0.9	1.0	0.9	1.0
Msh2	P43247	863	-10	-4	-6	-16	0.9	1.0	0.9	0.9
Polr1a	O35134	620	-11	-4	-6	-19	0.9	1.0	0.9	0.8
Hsp90aa1	P07901	573	-4	-5	-6	21	1.0	1.0	0.9	1.3
Nmd3	Q99L48	250	-14	-5	-6	10	0.9	1.0	0.9	1.1
Ppid	Q9CR16	52	-6	-5	-6	6	0.9	1.0	0.9	1.1
Ranbp2	Q9ERU9	2929	-6	-5	-6	6	0.9	1.0	0.9	1.1
Hmha1	Q3TBD2	323	-9	-5	-6	4	0.9	1.0	0.9	1.0
Hectd1	Q69ZR2	254	-7	-5	-6	-4	0.9	1.0	0.9	1.0
Sumo1	P63166	52	-7	-5	-6	15	0.9	1.0	0.9	1.2
Elmo2	Q8BHL5	627	-5	-5	-6	9	1.0	1.0	0.9	1.1
Ttpal	Q9D3D0	106	-14	-5	-6	8	0.9	1.0	0.9	1.1
Xpo1	Q6P5F9	164	-6	-5	-6	3	0.9	1.0	0.9	1.0
Gemin5	Q8BX17	726	-4	-5	-6	-4	1.0	1.0	0.9	1.0
Dhx40	Q6PE54	42	-10	-5	-6	-22	0.9	1.0	0.9	0.8
Spata13	Q5DU57	423	2	-6	-6	11	1.0	0.9	0.9	1.1
Hadha	Q8BMS1	97	-11	-6	-6	10	0.9	0.9	0.9	1.1
Dgka	O88673	239	-8	-6	-6	9	0.9	0.9	0.9	1.1
Lars2	Q8VDC0	166	-10	-6	-6	5	0.9	0.9	0.9	1.0
Nsf	P46460	250	-11	-6	-6	3	0.9	0.9	0.9	1.0
Tubgcp2	Q921G8	469	-15	-6	-6	2	0.9	0.9	0.9	1.0
Gne	Q91WG8	357	-9	-6	-6	1	0.9	0.9	0.9	1.0
Rars	Q9D0I9	86	-12	-6	-6	-8	0.9	0.9	0.9	0.9
Hnrnpc	Q9Z204	46	-12	-6	-6	-9	0.9	0.9	0.9	0.9
Spg7	Q3ULF4	353	-14	-6	-6	-24	0.9	0.9	0.9	0.8
Xpo5	Q924C1	50	-24	-6	-6	73	0.8	0.9	0.9	3.6
Bank1	Q80VH0	98	-14	-6	-6	22	0.9	0.9	0.9	1.3
Atm	Q62388	2460	-9	-6	-6	20	0.9	0.9	0.9	1.2
Ice1	E9Q286	2059	-1	-6	-6	14	1.0	0.9	0.9	1.2
Cbfb	Q08024	48	-9	-6	-6	11	0.9	0.9	0.9	1.1
Blmh	Q8R016	40	-3	-6	-6	8	1.0	0.9	0.9	1.1
Las1l	A2BE28	290	-9	-6	-6	-3	0.9	0.9	0.9	1.0
Smarcb1	Q9Z0H3	167	-7	-6	-6	-6	0.9	0.9	0.9	0.9
Atf7ip	Q7TT18	489	-12	-6	-6	-8	0.9	0.9	0.9	0.9
Blk	P16277	214	-19	-6	-6	-10	0.8	0.9	0.9	0.9
Skp1	Q9WTX5	120	-10	-7	-6	8	0.9	0.9	0.9	1.1
Smap2	Q7TN29	196	-5	-7	-6	7	1.0	0.9	0.9	1.1
Lsp1	P19973	274	-8	-7	-6	-2	0.9	0.9	0.9	1.0
Naa16	Q9DBB4	322	-4	-7	-6	-6	1.0	0.9	0.9	0.9
Inpp5d	Q9ES52	1049	-5	-7	-6	-12	1.0	0.9	0.9	0.9
Stim2	P83093	426	-12	-7	-6	30	0.9	0.9	0.9	1.4
Dock2	Q8C3J5	1202	-12	-7	-6	25	0.9	0.9	0.9	1.3
Cse1l	Q9ERK4	387	-11	-7	-6	18	0.9	0.9	0.9	1.2
Prpf4	Q9DAW6	398	-3	-7	-6	12	1.0	0.9	0.9	1.1
Dhrs7	Q9CXR1	172	-21	-7	-6	10	0.8	0.9	0.9	1.1
Trappc11	B2RXC1	658	-5	-7	-6	-1	1.0	0.9	0.9	1.0
Myo1g	Q5SUA5	149	-7	-7	-6	-10	0.9	0.9	0.9	0.9
Kiaa1429	A2AIV2	1559	-3	-8	-6	11	1.0	0.9	0.9	1.1
Smarcc2	Q6PDG5	495	-18	-8	-6	10	0.8	0.9	0.9	1.1

Zfr	O88532	844	-6	-8	-6	7	0.9	0.9	0.9	1.1
Rad1	Q9QWZ1	239	-6	-8	-6	1	0.9	0.9	0.9	1.0
Cpsf2	O35218	335	-17	-8	-6	11	0.9	0.9	0.9	1.1
Ggnbp2	Q5SV77	243	4	-8	-6	2	1.0	0.9	0.9	1.0
Sptb	Q3UGX2	1167	-13	-8	-6	-1	0.9	0.9	0.9	1.0
rp9	P97762	112	1	-8	-6	-2	1.0	0.9	0.9	1.0
Birc6	O88738	399	-6	-8	-6	-7	0.9	0.9	0.9	0.9
Gfpt1	P47856	252	-7	-8	-6	-24	0.9	0.9	0.9	0.8
Arfgap2	Q99K28	26	-22	-9	-6	25	0.8	0.9	0.9	1.3
Rpl8	P62918	115	0	-9	-6	9	1.0	0.9	0.9	1.1
Arhgap27	A2AB59	810	-14	-9	-6	1	0.9	0.9	0.9	1.0
Eefsec	Q9JHW4	275	-7	-9	-6	-1	0.9	0.9	0.9	1.0
Pyhin1	Q8BV49	350	-11	-9	-6	-5	0.9	0.9	0.9	1.0
Acin1	Q9JIX8	1222	1	-9	-6	-11	1.0	0.9	0.9	0.9
Ubr1	O70481	477	-8	-9	-6	-14	0.9	0.9	0.9	0.9
Ezh1	P70351	696	-22	-9	-6	28	0.8	0.9	0.9	1.4
Ezh2	Q61188	695	-22	-9	-6	28	0.8	0.9	0.9	1.4
Bap1	Q99PU7	542	-2	-9	-6	8	1.0	0.9	0.9	1.1
Pola1	P33609	1397	-10	-9	-6	2	0.9	0.9	0.9	1.0
Fkbp8	O35465	251	-6	-9	-6	-21	0.9	0.9	0.9	0.8
Psme4	Q5SSW2	1001	-4	-10	-6	8	1.0	0.9	0.9	1.1
Sptan1	P16546	1930	-1	-10	-6	2	1.0	0.9	0.9	1.0
Dis3	Q9CSH3	799	-2	-10	-6	-2	1.0	0.9	0.9	1.0
Csad	Q9DBE0	356	-11	-10	-6	-5	0.9	0.9	0.9	1.0
Pcm1	Q9R0L6	1780	-21	-10	-6	-12	0.8	0.9	0.9	0.9
Tcerg1	Q8CGF7	1064	-11	-10	-6	-2	0.9	0.9	0.9	1.0
Fmnl1	Q9JL26	934	-11	-10	-6	-2	0.9	0.9	0.9	1.0
Immt	Q8CAQ8	602	-8	-10	-6	-7	0.9	0.9	0.9	0.9
Atg12	Q9CQY1	135	-15	-10	-6	-7	0.9	0.9	0.9	0.9
Ywhae	P62259	98	-9	-10	-6	-9	0.9	0.9	0.9	0.9
Eea1	Q8BL66	421	-4	-10	-6	-42	1.0	0.9	0.9	0.7
Lpcat1	Q3TFD2	330	8	-11	-6	18	1.1	0.9	0.9	1.2
Zak	Q9ESL4	285	-2	-11	-6	8	1.0	0.9	0.9	1.1
Crmp1	P97427	248	-21	-11	-6	3	0.8	0.9	0.9	1.0
Rplp0	P14869	119	-17	-11	-6	-5	0.9	0.9	0.9	1.0
Rabl6	Q5U3K5	501	-6	-11	-6	-8	0.9	0.9	0.9	0.9
Usp9x	Q4FE56	1061	-17	-11	-6	-11	0.9	0.9	0.9	0.9
Nop58	Q6DFW4	106	-19	-11	-6	-11	0.8	0.9	0.9	0.9
Kif5b	Q61768	294	-17	-11	-6	-2	0.9	0.9	0.9	1.0
Ripk1	Q60855	234	-1	-11	-6	-27	1.0	0.9	0.9	0.8
Arhgef6	Q8K4I3	318	-15	-12	-6	34	0.9	0.9	0.9	1.5
Smu1	Q3UKJ7	416	-11	-12	-6	22	0.9	0.9	0.9	1.3
Trim28	Q62318	69	-21	-12	-6	-13	0.8	0.9	0.9	0.9
Ubr4	A2AN08	3700	14	-12	-6	28	1.2	0.9	0.9	1.4
Zfand2b	Q91X58	84	-14	-12	-6	16	0.9	0.9	0.9	1.2
Marc2	Q922Q1	78	-9	-12	-6	-1	0.9	0.9	0.9	1.0
Exosc3	Q7TQK4	214	-16	-12	-6	-13	0.9	0.9	0.9	0.9
Tbc1d1	Q60949	384	-18	-13	-6	3	0.8	0.9	0.9	1.0
Las1l	A2BE28	532	-4	-13	-6	-14	1.0	0.9	0.9	0.9
Sipa1	E9Q0Y4	442	-8	-13	-6	-17	0.9	0.9	0.9	0.9
Nmt1	O70310	483	-12	-13	-6	6	0.9	0.9	0.9	1.1

Kmt5a	D3YXI6	157	-3	-13	-6	-1	1.0	0.9	0.9	1.0
Pck2	Q8BH04	92	-7	-13	-6	-16	0.9	0.9	0.9	0.9
Prkd2	Q8BZ03	188	-8	-14	-6	11	0.9	0.9	0.9	1.1
Bpnt1	Q9Z0S1	28	-16	-14	-6	-1	0.9	0.9	0.9	1.0
Kiaa0196	Q8C2E7	385	-7	-14	-6	-4	0.9	0.9	0.9	1.0
Ago2	Q8CJG0	189	-8	-14	-6	-4	0.9	0.9	0.9	1.0
Dock11	A2AF47	615	1	-14	-6	-6	1.0	0.9	0.9	0.9
Fam76b	Q80XP8	106	-22	-14	-6	-9	0.8	0.9	0.9	0.9
Yy1	Q00899	298	-4	-14	-6	4	1.0	0.9	0.9	1.0
Smchd1	Q6P5D8	1711	-12	-14	-6	3	0.9	0.9	0.9	1.0
Oas1g	Q8K469	39	-7	-14	-6	-9	0.9	0.9	0.9	0.9
Nars2	Q8BGV0	43	-13	-14	-6	-36	0.9	0.9	0.9	0.7
Stxbp2	Q64324	311	-7	-15	-6	15	0.9	0.9	0.9	1.2
Nudcd2	Q9CQ48	45	-17	-15	-6	10	0.9	0.9	0.9	1.1
Med16	Q6PGF3	827	-5	-15	-6	2	1.0	0.9	0.9	1.0
Frg1	P97376	155	-20	-15	-6	-5	0.8	0.9	0.9	1.0
Gtf3c1	Q8K284	317	-10	-15	-6	-20	0.9	0.9	0.9	0.8
Dock8	Q8C147	847	-5	-16	-6	-5	1.0	0.9	0.9	1.0
Fxr1	Q61584	211	-12	-17	-6	-2	0.9	0.9	0.9	1.0
Ep400	Q8CHI8	1367	-4	-17	-6	-10	1.0	0.9	0.9	0.9
Zak	Q9ESL4	22	-18	-17	-6	28	0.8	0.9	0.9	1.4
Hars	Q61035	379	-21	-17	-6	2	0.8	0.9	0.9	1.0
Rnf213	E9Q555	586	-15	-18	-6	29	0.9	0.9	0.9	1.4
Tubb1	A2AQ07	340	-18	-18	-6	2	0.9	0.9	0.9	1.0
Supt6h	Q62383	939	3	-18	-6	13	1.0	0.8	0.9	1.1
Nup205	A0A0J9YUD5	751	-16	-18	-6	2	0.9	0.8	0.9	1.0
Ercc6l	Q8BHK9	647	-16	-18	-6	-13	0.9	0.8	0.9	0.9
Mcm5	Q52KC3	355	-19	-19	-6	11	0.8	0.8	0.9	1.1
Banf1	O54962	80	-16	-19	-6	-7	0.9	0.8	0.9	0.9
Brpf1	A0A0N4SUT9	1099	-16	-22	-6	-4	0.9	0.8	0.9	1.0
Flii	Q9JJ28	289	-17	-24	-6	22	0.9	0.8	0.9	1.3
Lrrc57	Q8JZX5	129	-15	-27	-6	-19	0.9	0.8	0.9	0.8
Bcl10	Q9Z0H7	119	-6	-33	-6	-10	0.9	0.8	0.9	0.9
Wasf2	Q8BH43	127	-37	-46	-6	-14	0.7	0.7	0.9	0.9
Cpne3	Q8BT60	249	-38	-48	-6	-28	0.7	0.7	0.9	0.8
Tbc1d15	Q9CXF4	588	-11	13	-6	7	0.9	1.1	0.9	1.1
Sae1	Q9R1T2	138	-4	10	-6	5	1.0	1.1	0.9	1.0
Dgke	Q9R1C6	132	-2	9	-6	-13	1.0	1.1	0.9	0.9
Dhx9	E9QNN1	780	-3	8	-6	36	1.0	1.1	0.9	1.6
Ndufs1	Q91VD9	92	-3	8	-6	-3	1.0	1.1	0.9	1.0
Pitpna	P53810	188	-7	7	-6	18	0.9	1.1	0.9	1.2
Tyk2	E9QJS1	645	-15	7	-6	-27	0.9	1.1	0.9	0.8
Hibch	Q8QZS1	44	-12	6	-6	20	0.9	1.1	0.9	1.3
Mettl3	Q8C3P7	336	-5	6	-6	-24	1.0	1.1	0.9	0.8
Pi4kb	Q8BKC8	52	-2	6	-6	-21	1.0	1.1	0.9	0.8
Coro1b	Q9WUM3	41	-9	5	-6	12	0.9	1.1	0.9	1.1
Kat6a	G3X940	722	-9	5	-6	6	0.9	1.1	0.9	1.1
Zadh2	Q8BGC4	55	-9	4	-6	10	0.9	1.0	0.9	1.1
Arl8b	Q9CQW2	159	-12	3	-6	27	0.9	1.0	0.9	1.4
Ints1	Q6P4S8	1838	-7	3	-6	32	0.9	1.0	0.9	1.5
Prpf6	Q91YR7	604	-9	3	-6	19	0.9	1.0	0.9	1.2

Snapc4	Q8BP86	1139	-15	3	-6	-6	0.9	1.0	0.9	0.9
Blmh	Q8R016	73	-8	2	-6	6	0.9	1.0	0.9	1.1
Lrrk1	Q3UHC2	1670	0	2	-6	20	1.0	1.0	0.9	1.3
Nubp2	Q9R061	181	-6	2	-6	13	0.9	1.0	0.9	1.1
Ostf1	Q62422	185	-1	2	-6	5	1.0	1.0	0.9	1.1
Tubb5	P99024	127	2	2	-6	2	1.0	1.0	0.9	1.0
Ogdh	Q60597	331	1	2	-6	-5	1.0	1.0	0.9	1.0
Ranbp2	Q9ERU9	386	-12	1	-6	27	0.9	1.0	0.9	1.4
Ogt	Q8CGY8	315	-15	1	-6	8	0.9	1.0	0.9	1.1
Actn1	Q7TPR4	370	4	1	-6	-2	1.0	1.0	0.9	1.0
Dph1	Q5NCQ5	403	-6	1	-6	-10	0.9	1.0	0.9	0.9
Eif3l	Q8QZY1	417	-5	0	-6	23	1.0	1.0	0.9	1.3
Eprs	Q8CGC7	1309	7	0	-6	18	1.1	1.0	0.9	1.2
Unk	Q8BL48	782	8	0	-6	10	1.1	1.0	0.9	1.1
Acsl5	Q8JZR0	322	-5	0	-6	5	1.0	1.0	0.9	1.0
Hnrnph1	O35737	267	-14	0	-6	-7	0.9	1.0	0.9	0.9
Hnrnph2	P70333	267	-14	0	-6	-7	0.9	1.0	0.9	0.9
Ube2l3	P68037	86	-3	0	-6	-8	1.0	1.0	0.9	0.9
Babam1	Q3UI43	95	-14	0	-6	-8	0.9	1.0	0.9	0.9
Zc3h11a	Q6NZF1	407	-3	0	-6	-9	1.0	1.0	0.9	0.9
Nom1	Q3UFM5	65	-9	0	-6	-10	0.9	1.0	0.9	0.9
Cct4	P80315	379	-16	-1	-6	18	0.9	1.0	0.9	1.2
Rabgef1	Q9JM13	225	3	-1	-6	17	1.0	1.0	0.9	1.2
Kdm1a	Q6ZQ88	574	-16	-1	-6	-7	0.9	1.0	0.9	0.9
Rpl4	Q9D8E6	208	-10	-1	-6	-9	0.9	1.0	0.9	0.9
Thap12	Q9CUX1	641	-17	-1	-6	-17	0.9	1.0	0.9	0.9
Polr2b	Q8CFI7	1155	-7	-1	-6	25	0.9	1.0	0.9	1.3
Adh5	P28474	240	-15	-1	-6	12	0.9	1.0	0.9	1.1
Phc3	Q8CHP6	806	-2	-1	-6	7	1.0	1.0	0.9	1.1
Mob3a	Q8BSU7	186	-16	-1	-6	4	0.9	1.0	0.9	1.0
Zc3h11a	Q6NZF1	499	-1	-1	-6	-3	1.0	1.0	0.9	1.0
Mdh1	P14152	137	-8	-2	-6	16	0.9	1.0	0.9	1.2
Zw10	O54692	451	-6	-2	-6	9	0.9	1.0	0.9	1.1
Mdh2	P08249	89	-15	-2	-6	9	0.9	1.0	0.9	1.1
Ifi47	Q61635	376	1	-2	-6	9	1.0	1.0	0.9	1.1
Prpf8	Q99PV0	1896	-9	-2	-6	23	0.9	1.0	0.9	1.3
Lrrc59	Q922Q8	131	-10	-2	-6	16	0.9	1.0	0.9	1.2
Pik3c2a	F8VPL2	256	-1	-2	-6	-2	1.0	1.0	0.9	1.0
Taf6	Q62311	235	-13	-2	-6	-2	0.9	1.0	0.9	1.0
Prpf8	Q99PV0	2116	-11	-3	-6	27	0.9	1.0	0.9	1.4
Adal	Q80SY6	354	4	-3	-6	16	1.0	1.0	0.9	1.2
Tbc1d22b	Q80VE5	144	-1	-3	-6	13	1.0	1.0	0.9	1.1
Anapc2	Q8BZQ7	236	-7	-3	-6	13	0.9	1.0	0.9	1.1
Ptprc	P06800	776	-15	-3	-6	12	0.9	1.0	0.9	1.1
Klhdc3	Q8VEM9	33	-11	-3	-6	-2	0.9	1.0	0.9	1.0
Mroh1	E0CZ22	834	0	-3	-6	-4	1.0	1.0	0.9	1.0
Smarca4	Q3TKT4	423	-5	-3	-6	-5	1.0	1.0	0.9	1.0
Slc12a9	Q99MR3	911	-16	-3	-6	26	0.9	1.0	0.9	1.3
Yeats4	Q9CR11	210	-6	-3	-6	7	0.9	1.0	0.9	1.1
Dctn1	O08788	1255	-15	-3	-6	4	0.9	1.0	0.9	1.0
Kdm3b	B9EKS2	570	-2	-3	-6	-2	1.0	1.0	0.9	1.0

Taf6	Q62311	318	-12	-3	-6	-2	0.9	1.0	0.9	1.0
Champ1	Q8K327	462	-7	-4	-6	-1	0.9	1.0	0.9	1.0
Ppp6r1	Q7TSI3	172	2	-4	-6	-2	1.0	1.0	0.9	1.0
Klhl14	Q69ZK5	595	-4	-4	-6	-5	1.0	1.0	0.9	1.0
Rasgrp2	Q9QUG9	532	-11	-4	-6	-5	0.9	1.0	0.9	1.0
Chd3	B1AR17	2052	0	-4	-6	-7	1.0	1.0	0.9	0.9
Ppa2	Q91VM9	67	-2	-4	-6	-13	1.0	1.0	0.9	0.9
Hnrnpa0	Q9CX86	49	-15	-4	-6	28	0.9	1.0	0.9	1.4
Brat1	Q8C3R1	28	-5	-4	-6	25	1.0	1.0	0.9	1.3
Htt	G3X9H5	2949	-10	-4	-6	13	0.9	1.0	0.9	1.1
Med23	Q80YQ2	1090	-11	-4	-6	3	0.9	1.0	0.9	1.0
Hectd1	Q69ZR2	1860	-7	-4	-6	1	0.9	1.0	0.9	1.0
Cdyl2	Q9D5D8	285	-10	-4	-6	0	0.9	1.0	0.9	1.0
Lin37	Q9D8N6	176	-1	-4	-6	-1	1.0	1.0	0.9	1.0
Cep170	Q6A065	211	-3	-4	-6	-10	1.0	1.0	0.9	0.9
Arhgef1	Q61210	536	-6	-5	-6	-4	0.9	1.0	0.9	1.0
Hdlbp	Q8VDJ3	636	-18	-5	-6	-4	0.9	1.0	0.9	1.0
Pcbp1	P60335	54	-12	-5	-6	-6	0.9	1.0	0.9	0.9
Cep97	Q9CZ62	34	-13	-5	-6	-6	0.9	1.0	0.9	0.9
Pggt1b	Q8BUY9	369	-5	-5	-6	-10	1.0	1.0	0.9	0.9
Prkd2	Q8BZ03	217	-9	-5	-6	-20	0.9	1.0	0.9	0.8
Api5	O35841	234	-10	-5	-6	22	0.9	1.0	0.9	1.3
Chd6	A3KFM7	1306	-12	-5	-6	13	0.9	1.0	0.9	1.1
Chd8	Q09XV5	1657	-12	-5	-6	13	0.9	1.0	0.9	1.1
Vdac2	Q60930	48	-5	-5	-6	7	1.0	1.0	0.9	1.1
Rpl5	P47962	62	-18	-5	-6	2	0.8	1.0	0.9	1.0
Tax1bp1	Q3UKC1	328	-7	-5	-6	-5	0.9	1.0	0.9	1.0
Drg1	P32233	195	-14	-5	-6	-6	0.9	1.0	0.9	0.9
Otulin	Q3UCV8	17	-3	-5	-6	-7	1.0	1.0	0.9	0.9
Slfn5	Q8CBA2	237	-11	-5	-6	-7	0.9	1.0	0.9	0.9
Zfp106	R4GML0	1478	-9	-5	-6	-15	0.9	1.0	0.9	0.9
Pias4	Q9JM05	157	-11	-6	-6	20	0.9	0.9	0.9	1.3
Ilk	O55222	346	-6	-6	-6	8	0.9	0.9	0.9	1.1
Exoc2	Q9D4H1	719	3	-6	-6	3	1.0	0.9	0.9	1.0
Dpp3	Q99KK7	654	-1	-6	-6	2	1.0	0.9	0.9	1.0
Cltc	Q68FD5	778	-1	-6	-6	2	1.0	0.9	0.9	1.0
Sars	P26638	438	-8	-6	-6	-10	0.9	0.9	0.9	0.9
Gk2	Q9WU65	381	-23	-6	-6	16	0.8	0.9	0.9	1.2
Tsr1	Q5SWD9	617	-8	-6	-6	13	0.9	0.9	0.9	1.1
Tceb1	P83940	11	-19	-6	-6	11	0.8	0.9	0.9	1.1
Senp7	Q8BUH8	528	-13	-6	-6	-9	0.9	0.9	0.9	0.9
Tbc1d5	Q80XQ2	96	-11	-6	-6	-9	0.9	0.9	0.9	0.9
Macf1	E9PVY8	3316	-11	-6	-6	-12	0.9	0.9	0.9	0.9
Ubr5	E9Q2H1	2267	-3	-6	-6	-15	1.0	0.9	0.9	0.9
Ifi207	E9Q3L4	16	-14	-6	-6	-19	0.9	0.9	0.9	0.8
Mnda	P0DOV1	16	-14	-6	-6	-19	0.9	0.9	0.9	0.8
Ifi204	P0DOV2	16	-14	-6	-6	-19	0.9	0.9	0.9	0.8
Pi4ka	E9Q3L2	1858	-5	-6	-6	-26	1.0	0.9	0.9	0.8
Nans	Q99J77	184	-8	-7	-6	6	0.9	0.9	0.9	1.1
Hdlbp	Q8VDJ3	855	-12	-7	-6	1	0.9	0.9	0.9	1.0
Bin2	S4R270	205	-9	-7	-6	16	0.9	0.9	0.9	1.2

Taf7	Q9R1C0	72	-13	-7	-6	12	0.9	0.9	0.9	1.1
Cand1	Q6ZQ38	1007	-6	-7	-6	11	0.9	0.9	0.9	1.1
Rfk	Q8CFV9	8	-4	-7	-6	5	1.0	0.9	0.9	1.1
Herc1	E9PZP8	1388	-22	-7	-6	1	0.8	0.9	0.9	1.0
Clip2	Q9Z0H8	391	-12	-7	-6	-5	0.9	0.9	0.9	1.0
Akap8	Q9DBR0	348	-2	-7	-6	-21	1.0	0.9	0.9	0.8
Uba7	Q9DBK7	130	-15	-8	-6	12	0.9	0.9	0.9	1.1
Thop1	Q8C1A5	246	-12	-8	-6	1	0.9	0.9	0.9	1.0
Kdm5a	Q3UXZ9	345	-15	-8	-6	-4	0.9	0.9	0.9	1.0
Ncbp2	Q9CQ49	81	-10	-8	-6	-8	0.9	0.9	0.9	0.9
Ddx21	Q9JIK5	531	-12	-8	-6	-10	0.9	0.9	0.9	0.9
Msrb2	Q78J03	132	-7	-8	-6	-10	0.9	0.9	0.9	0.9
Rlf	A2A7F4	1875	-13	-8	-6	-12	0.9	0.9	0.9	0.9
Ptgr1	Q91YR9	239	-13	-8	-6	14	0.9	0.9	0.9	1.2
Ccdc94	Q9D6J3	248	-10	-8	-6	11	0.9	0.9	0.9	1.1
Prmt1	Q9JIF0	272	-5	-8	-6	-2	1.0	0.9	0.9	1.0
Parp9	Q8CAS9	83	-11	-8	-6	-5	0.9	0.9	0.9	1.0
Baz1a	O88379	966	-11	-8	-6	-10	0.9	0.9	0.9	0.9
Hus1	Q8BQY8	253	-10	-8	-6	-11	0.9	0.9	0.9	0.9
Sacm1l	Q9EP69	84	-11	-9	-6	36	0.9	0.9	0.9	1.6
Ambra1	A2AH22	502	-7	-9	-6	35	0.9	0.9	0.9	1.5
Ecm29	Q6PDI5	444	-2	-9	-6	1	1.0	0.9	0.9	1.0
Rangap1	P46061	506	-7	-9	-6	-10	0.9	0.9	0.9	0.9
Cad	B2RQC6	379	-17	-9	-6	-16	0.9	0.9	0.9	0.9
Frmd6	Q8C0V9	306	3	-9	-6	19	1.0	0.9	0.9	1.2
Rpsa	P14206	163	-12	-9	-6	11	0.9	0.9	0.9	1.1
Mapk9	Q9WTU6	177	-7	-9	-6	8	0.9	0.9	0.9	1.1
Talpid3	E9PV87	207	-18	-9	-6	4	0.8	0.9	0.9	1.0
Aldh3a2	P47740	214	-21	-9	-6	1	0.8	0.9	0.9	1.0
Pdcd4	Q61823	288	-7	-9	-6	-1	0.9	0.9	0.9	1.0
Thop1	Q8C1A5	175	-12	-9	-6	-3	0.9	0.9	0.9	1.0
Nipbl	Q6KCD5	56	-7	-9	-6	-13	0.9	0.9	0.9	0.9
Znf800	Q0VEE6	294	-7	-9	-6	-19	0.9	0.9	0.9	0.8
Cstf2t	Q8C7E9	150	-21	-10	-6	21	0.8	0.9	0.9	1.3
Anp32b	Q9EST5	123	-9	-10	-6	10	0.9	0.9	0.9	1.1
Plcg2	Q8CIH5	1082	-4	-10	-6	18	1.0	0.9	0.9	1.2
Ndufs1	Q91VD9	554	-4	-10	-6	13	1.0	0.9	0.9	1.1
Rnasel	Q05921	410	-1	-10	-6	2	1.0	0.9	0.9	1.0
Rps27a	P62983	144	-4	-10	-6	1	1.0	0.9	0.9	1.0
Pop1	Q8K205	736	-9	-10	-6	-2	0.9	0.9	0.9	1.0
Wdr6	Q99ME2	523	-8	-10	-6	-6	0.9	0.9	0.9	0.9
Nr3c1	E9PYV1	304	-4	-10	-6	-13	1.0	0.9	0.9	0.9
Lig1	Q3U4X8	82	-13	-10	-6	-14	0.9	0.9	0.9	0.9
Myl12b	Q3THE2	109	-8	-11	-6	12	0.9	0.9	0.9	1.1
Ncf4	P97369	122	-22	-11	-6	-7	0.8	0.9	0.9	0.9
Pitpnm1	O35954	259	-5	-11	-6	-8	1.0	0.9	0.9	0.9
Gpatch11	Q3UFS4	69	-3	-11	-6	-9	1.0	0.9	0.9	0.9
Psip1	Q99JF8	203	-5	-11	-6	-10	1.0	0.9	0.9	0.9
Smarcc2	Q6PDG5	136	-5	-11	-6	23	1.0	0.9	0.9	1.3
Hmha1	Q3TBD2	152	-23	-11	-6	18	0.8	0.9	0.9	1.2
Pnpla2	Q8BJ56	314	-14	-11	-6	3	0.9	0.9	0.9	1.0

Cnn2	Q08093	175	-4	-11	-6	2	1.0	0.9	0.9	1.0
Rxrb	P28704	234	-15	-11	-6	-1	0.9	0.9	0.9	1.0
Wdr82	Q8BFQ4	287	-17	-12	-6	8	0.9	0.9	0.9	1.1
Tomm40	Q9QYA2	86	-13	-12	-6	8	0.9	0.9	0.9	1.1
Rcc2	Q8BK67	303	-10	-12	-6	-3	0.9	0.9	0.9	1.0
Agfg2	Q80WC7	50	-15	-12	-6	-5	0.9	0.9	0.9	1.0
Fam63a	Q76LS9	115	-8	-12	-6	-14	0.9	0.9	0.9	0.9
Inpp5d	Q9ES52	822	-7	-12	-6	-13	0.9	0.9	0.9	0.9
Zfp318	B0V2M3	1915	-5	-13	-6	1	1.0	0.9	0.9	1.0
Flna	Q8BTM8	2476	-8	-13	-6	-33	0.9	0.9	0.9	0.8
Senp7	Q8BUH8	266	3	-13	-6	-32	1.0	0.9	0.9	0.8
Gtpbp1	O08582	360	-25	-14	-6	5	0.8	0.9	0.9	1.1
Ppp2r1a	Q76MZ3	310	-13	-14	-6	4	0.9	0.9	0.9	1.0
Ice2	Q3UZ18	257	-3	-14	-6	-14	1.0	0.9	0.9	0.9
Aff3	D3YYI6	403	-12	-14	-6	-19	0.9	0.9	0.9	0.8
Eif2s2	Q99L45	303	-17	-14	-6	8	0.9	0.9	0.9	1.1
Cnot4	Q8BT14	175	-17	-14	-6	-7	0.9	0.9	0.9	0.9
Tubb1	A2AQ07	315	-11	-14	-6	-13	0.9	0.9	0.9	0.9
Ccdc88b	Q4QRL3	93	-10	-14	-6	-15	0.9	0.9	0.9	0.9
Mdm1	Q9D067	476	-4	-14	-6	-17	1.0	0.9	0.9	0.9
Map3k2	G5E8L8	424	-17	-15	-6	-8	0.9	0.9	0.9	0.9
Map3k3	Q61084	430	-17	-15	-6	-8	0.9	0.9	0.9	0.9
Herpud1	Q9JJK5	86	-8	-15	-6	-20	0.9	0.9	0.9	0.8
Atxn2l	Q7TQH0	58	-26	-15	-6	-26	0.8	0.9	0.9	0.8
Ppp2r2d	Q925E7	404	6	-15	-6	-38	1.1	0.9	0.9	0.7
Abce1	P61222	65	-8	-15	-6	14	0.9	0.9	0.9	1.2
Rap1a	P62835	118	-12	-15	-6	-3	0.9	0.9	0.9	1.0
Adk	P55264	352	-16	-16	-6	25	0.9	0.9	0.9	1.3
Nop9	Q8BMC4	242	-12	-16	-6	-5	0.9	0.9	0.9	1.0
Epb41l2	O70318	307	-10	-16	-6	18	0.9	0.9	0.9	1.2
Phpt1	Q9DAK9	72	-6	-16	-6	-4	0.9	0.9	0.9	1.0
Nvl	Q9DBY8	430	-10	-16	-6	-5	0.9	0.9	0.9	1.0
Dstn	Q9R0P5	46	-8	-16	-6	-8	0.9	0.9	0.9	0.9
Mxi1	P50540	100	-22	-16	-6	-17	0.8	0.9	0.9	0.9
Gsk3a	Q2NL51	262	-2	-17	-6	14	1.0	0.9	0.9	1.2
Gsk3b	Q9WV60	199	-2	-17	-6	14	1.0	0.9	0.9	1.2
Plec	Q9QXS1	1144	-14	-17	-6	2	0.9	0.9	0.9	1.0
Zfyve1	Q810J8	217	-14	-17	-6	-5	0.9	0.9	0.9	1.0
Ppp1r7	Q3UM45	113	-13	-17	-6	-11	0.9	0.9	0.9	0.9
Pds5b	Q4VA53	333	2	-18	-6	32	1.0	0.8	0.9	1.5
Lrba	E9Q3Y4	832	-8	-18	-6	-8	0.9	0.8	0.9	0.9
Taf4	E9QAP7	979	-8	-18	-6	-10	0.9	0.8	0.9	0.9
Mapk7	Q9WVS8	194	-16	-20	-6	10	0.9	0.8	0.9	1.1
Faf2	Q3TDN2	194	-13	-20	-6	10	0.9	0.8	0.9	1.1
Csrp2	P97314	167	-3	-20	-6	3	1.0	0.8	0.9	1.0
Elmo2	Q8BHL5	416	-16	-20	-6	1	0.9	0.8	0.9	1.0
Elmo1	Q8BPU7	411	-16	-20	-6	1	0.9	0.8	0.9	1.0
Znf346	Q9R0B7	68	-14	-22	-6	28	0.9	0.8	0.9	1.4
Hyls1	Q9CXX0	45	-11	-22	-6	5	0.9	0.8	0.9	1.0
Myh9	Q8VDD5	816	-20	-24	-6	10	0.8	0.8	0.9	1.1
Usp24	B1AY13	1577	-13	-24	-6	-6	0.9	0.8	0.9	0.9

Pus3	Q9JI38	421	-12	-25	-6	-11	0.9	0.8	0.9	0.9
Uncharact	Q3TEI4	359	-25	-26	-6	32	0.8	0.8	0.9	1.5
Gm5449	D3Z5N9	46	-50	-96	-6	-124	0.7	0.5	0.9	0.4
Tbc1d21	Q9D9D3	98	-1	20	-7	-4	1.0	1.2	0.9	1.0
Uncharact	Q9D727	82	8	18	-7	39	1.1	1.2	0.9	1.6
Ap2a1	P17426	941	3	13	-7	17	1.0	1.1	0.9	1.2
Unc13d	B2RUP2	566	5	13	-7	-4	1.1	1.1	0.9	1.0
Tcea2	Q9QVN7	135	-1	13	-7	-11	1.0	1.1	0.9	0.9
Rbm22	Q8BHS3	269	-9	12	-7	10	0.9	1.1	0.9	1.1
Dync1h1	Q9JHU4	976	4	10	-7	25	1.0	1.1	0.9	1.3
Adprh	P54923	108	10	9	-7	-23	1.1	1.1	0.9	0.8
Eef2	P58252	693	-15	7	-7	15	0.9	1.1	0.9	1.2
Trim34a	Q99PP6	15	-5	7	-7	14	1.0	1.1	0.9	1.2
Phf1	Q9Z1B8	93	-8	6	-7	19	0.9	1.1	0.9	1.2
Commd9	Q8K2Q0	36	-4	6	-7	8	1.0	1.1	0.9	1.1
Upf1	Q9EPU0	678	-9	5	-7	1	0.9	1.1	0.9	1.0
Smchd1	Q6P5D8	505	-9	5	-7	31	0.9	1.0	0.9	1.4
Gmip	Q6PGG2	340	-1	5	-7	6	1.0	1.0	0.9	1.1
Gimap9	G3X987	160	-7	5	-7	5	0.9	1.0	0.9	1.1
Aco1	P28271	118	-16	5	-7	2	0.9	1.0	0.9	1.0
Syne2	Q6ZWQ0	491	9	5	-7	-14	1.1	1.0	0.9	0.9
Ap1g1	P22892	400	-4	4	-7	28	1.0	1.0	0.9	1.4
Ip6k1	Q6PD10	248	-3	4	-7	-13	1.0	1.0	0.9	0.9
Anxa6	P14824	552	-14	4	-7	7	0.9	1.0	0.9	1.1
Rnf14	Q9JI90	405	-4	4	-7	-15	1.0	1.0	0.9	0.9
Ap1b1	O35643	112	-11	3	-7	12	0.9	1.0	0.9	1.1
Ap2b1	Q9DBG3	112	-11	3	-7	12	0.9	1.0	0.9	1.1
Trappc10	F8VQF9	1129	-10	3	-7	12	0.9	1.0	0.9	1.1
Yrdc	Q3U5F4	106	-4	3	-7	3	1.0	1.0	0.9	1.0
Sp110	Q8BVK9	386	-10	2	-7	15	0.9	1.0	0.9	1.2
Rqcd1	Q9JKY0	252	2	2	-7	0	1.0	1.0	0.9	1.0
Ccdc88b	Q4QRL3	1387	-5	2	-7	-2	1.0	1.0	0.9	1.0
Edc4	Q3UJB9	981	2	2	-7	-5	1.0	1.0	0.9	1.0
Eif3d	O70194	196	-13	2	-7	25	0.9	1.0	0.9	1.3
Tpi1	P17751	117	-8	2	-7	10	0.9	1.0	0.9	1.1
Git1	Q68FF6	585	-7	2	-7	-19	0.9	1.0	0.9	0.8
Pdcd11	Q6NS46	557	3	1	-7	26	1.0	1.0	0.9	1.3
Eef2	P58252	369	-11	1	-7	11	0.9	1.0	0.9	1.1
Prmt7	Q922X9	571	-25	1	-7	10	0.8	1.0	0.9	1.1
Zc3hc1	Q80YV2	112	-8	1	-7	7	0.9	1.0	0.9	1.1
Gadd45gip	Q9CR59	124	-11	1	-7	1	0.9	1.0	0.9	1.0
Sipa1l1	Q8C0T5	105	-16	1	-7	-10	0.9	1.0	0.9	0.9
Helb	Q6NVF4	808	13	1	-7	-51	1.1	1.0	0.9	0.7
Tbck	Q8BM85	702	-7	1	-7	19	0.9	1.0	0.9	1.2
Mtr	A6H5Y3	311	-19	1	-7	15	0.8	1.0	0.9	1.2
Zfp646	Q6NV66	1286	-5	1	-7	-3	1.0	1.0	0.9	1.0
Dnm1l	Q8K1M6	306	-7	0	-7	1	0.9	1.0	0.9	1.0
Huwe1	Q7TMY8	3659	-6	0	-7	-6	0.9	1.0	0.9	0.9
Eif6	O55135	15	-9	0	-7	-11	0.9	1.0	0.9	0.9
Gpd1l	Q3ULJ0	202	-2	-1	-7	19	1.0	1.0	0.9	1.2
Lrba	E9Q3Y4	1223	-3	-1	-7	-4	1.0	1.0	0.9	1.0

Bod1l	E9Q6J5	1261	-10	-1	-7	-14	0.9	1.0	0.9	0.9
Ppp2r1a	Q76MZ3	154	3	-1	-7	21	1.0	1.0	0.9	1.3
Ppp2r1b	Q7TNP2	166	3	-1	-7	21	1.0	1.0	0.9	1.3
Aimp2	Q8R010	205	-11	-1	-7	16	0.9	1.0	0.9	1.2
Wdfy3	Q6VNB8	1975	-11	-1	-7	-7	0.9	1.0	0.9	0.9
Trim14	Q8BVW3	149	-1	-1	-7	-9	1.0	1.0	0.9	0.9
Polr1e	Q8K202	379	-19	-2	-7	7	0.8	1.0	0.9	1.1
Gm43796	A0A0J9YV32	29	-6	-2	-7	6	0.9	1.0	0.9	1.1
Pus7l	Q8CE46	381	6	-2	-7	3	1.1	1.0	0.9	1.0
Vcl	Q64727	85	-8	-2	-7	-7	0.9	1.0	0.9	0.9
Dhx38	Q80X98	27	-18	-2	-7	-8	0.9	1.0	0.9	0.9
Calcoco1	Q8CGU1	57	-11	-2	-7	-14	0.9	1.0	0.9	0.9
Hdac3	O88895	123	4	-2	-7	12	1.0	1.0	0.9	1.1
Polr2a	P08775	602	-16	-2	-7	12	0.9	1.0	0.9	1.1
Coro7	Q9D2V7	502	-19	-2	-7	5	0.8	1.0	0.9	1.1
Zfp36	P22893	101	-7	-2	-7	1	0.9	1.0	0.9	1.0
Zfp36l2	P23949	161	-7	-2	-7	1	0.9	1.0	0.9	1.0
Rps6ka1	P18653	223	-6	-3	-7	4	0.9	1.0	0.9	1.0
Rps6ka3	P18654	229	-6	-3	-7	4	0.9	1.0	0.9	1.0
Tbc1d2b	Q3U0J8	653	-4	-3	-7	-15	1.0	1.0	0.9	0.9
Syne2	Q6ZWQ0	6041	1	-3	-7	-18	1.0	1.0	0.9	0.8
Lrrk2	Q5S006	2024	-4	-3	-7	46	1.0	1.0	0.9	1.9
Hnrnpl	Q8R081	258	-9	-3	-7	17	0.9	1.0	0.9	1.2
Rab4b	Q91ZR1	119	-11	-3	-7	16	0.9	1.0	0.9	1.2
Fgfr1op	Q66JX5	244	-6	-3	-7	2	0.9	1.0	0.9	1.0
Gcn1	E9PVA8	2657	-6	-3	-7	-1	0.9	1.0	0.9	1.0
Gcn1	E9PVA8	648	-9	-4	-7	39	0.9	1.0	0.9	1.6
Setd1b	Q8CFT2	922	-18	-4	-7	17	0.8	1.0	0.9	1.2
Mecr	Q9DCS3	169	-11	-4	-7	12	0.9	1.0	0.9	1.1
Eed	Q921E6	182	-4	-4	-7	5	1.0	1.0	0.9	1.1
Rnaseh2a	Q9CWY8	29	-4	-4	-7	4	1.0	1.0	0.9	1.0
Snrnp200	Q6P4T2	502	-13	-4	-7	-2	0.9	1.0	0.9	1.0
Rps25	P62852	59	-9	-4	-7	-4	0.9	1.0	0.9	1.0
Ppwd1	Q8CEC6	520	-15	-4	-7	-4	0.9	1.0	0.9	1.0
Mndal	D0QMC3	266	-4	-4	-7	-11	1.0	1.0	0.9	0.9
Ifi204	P0DOV2	180	-4	-4	-7	-11	1.0	1.0	0.9	0.9
Cep152	A2AUM9	1294	0	-4	-7	-14	1.0	1.0	0.9	0.9
Rps6ka3	P18654	436	4	-4	-7	4	1.0	1.0	0.9	1.0
Znf512	Q69Z99	174	-8	-4	-7	-5	0.9	1.0	0.9	1.0
Pdlim7	Q3TJD7	332	2	-4	-7	-8	1.0	1.0	0.9	0.9
Malt1	Q2TBA3	577	-12	-4	-7	-9	0.9	1.0	0.9	0.9
Elmo1	Q8BPU7	622	-6	-5	-7	10	0.9	1.0	0.9	1.1
Orc3	Q9JK30	166	-10	-5	-7	6	0.9	1.0	0.9	1.1
Zmym2	Q9CU65	497	-8	-5	-7	6	0.9	1.0	0.9	1.1
Polr1a	O35134	846	-5	-5	-7	1	1.0	1.0	0.9	1.0
Fam122a	Q9DB52	203	-1	-5	-7	1	1.0	1.0	0.9	1.0
Jak3	Q62137	227	14	-5	-7	-4	1.2	1.0	0.9	1.0
Fmr1	E9QAS8	77	-12	-5	-7	-4	0.9	1.0	0.9	1.0
Kdm3b	B9EKS2	1125	-10	-5	-7	27	0.9	1.0	0.9	1.4
Cbx5	Q61686	160	-11	-5	-7	7	0.9	1.0	0.9	1.1
Cbx3	Q9DCC5	160	-11	-5	-7	7	0.9	1.0	0.9	1.1

Hk2	O08528	628	-10	-5	-7	-3	0.9	1.0	0.9	1.0
Rac2	Q05144	178	-7	-5	-7	-3	0.9	1.0	0.9	1.0
Nsun2	Q1HFZ0	271	-3	-5	-7	-10	1.0	1.0	0.9	0.9
Zc3h12a	Q5D1E7	223	-7	-5	-7	-11	0.9	1.0	0.9	0.9
Rhof	Q8BYP3	131	-16	-5	-7	-51	0.9	1.0	0.9	0.7
Fanci	Q8K368	359	10	-6	-7	22	1.1	0.9	0.9	1.3
Snrnp200	Q6P4T2	769	-9	-6	-7	14	0.9	0.9	0.9	1.2
UPF0568	Q9CQE8	69	-15	-6	-7	10	0.9	0.9	0.9	1.1
Rab4b	Q91ZR1	23	-11	-6	-7	3	0.9	0.9	0.9	1.0
Sptan1	P16546	2233	-13	-6	-7	-1	0.9	0.9	0.9	1.0
Vps11	Q91W86	317	3	-6	-7	-1	1.0	0.9	0.9	1.0
Rrm1	P07742	254	-11	-6	-7	-4	0.9	0.9	0.9	1.0
Eif4enif1	Q9EST3	765	-14	-6	-7	-7	0.9	0.9	0.9	0.9
Snx6	Q6P8X1	264	-9	-6	-7	11	0.9	0.9	0.9	1.1
Chmp3	Q9CQ10	60	-8	-6	-7	7	0.9	0.9	0.9	1.1
Samhd1	Q60710	584	-15	-6	-7	3	0.9	0.9	0.9	1.0
Syne1	Q6ZWR6	2097	-25	-6	-7	3	0.8	0.9	0.9	1.0
Kmt5b	Q3U8K7	157	-7	-6	-7	1	0.9	0.9	0.9	1.0
Hnrnpul2	Q00PI9	306	-9	-6	-7	1	0.9	0.9	0.9	1.0
Nup50	Q9JIH2	180	-7	-6	-7	-2	0.9	0.9	0.9	1.0
Pik3r1	P26450	656	-5	-6	-7	-4	1.0	0.9	0.9	1.0
Ppp2r1a	Q76MZ3	390	-11	-6	-7	-7	0.9	0.9	0.9	0.9
Ppp2r1b	Q7TNP2	402	-11	-6	-7	-7	0.9	0.9	0.9	0.9
Prkcq	Q02111	322	-11	-6	-7	-10	0.9	0.9	0.9	0.9
Nt5dc1	Q8C5P5	130	-9	-7	-7	55	0.9	0.9	0.9	2.2
Rnmt	Q9D0L8	195	-10	-7	-7	6	0.9	0.9	0.9	1.1
Syncrip	Q7TMK9	96	-8	-7	-7	3	0.9	0.9	0.9	1.0
Hnrnpr	Q8VHM5	99	-8	-7	-7	3	0.9	0.9	0.9	1.0
Tcerg1	Q8CGF7	537	-9	-7	-7	2	0.9	0.9	0.9	1.0
Nbeal2	Q6ZQA0	481	-5	-7	-7	0	1.0	0.9	0.9	1.0
Xaf1	Q5NBU8	242	-13	-7	-7	-3	0.9	0.9	0.9	1.0
Ddx17	Q501J6	168	-25	-7	-7	-5	0.8	0.9	0.9	1.0
Ddx5	Q8BTS0	170	-25	-7	-7	-5	0.8	0.9	0.9	1.0
Ttn	A2ASS6	30082	-7	-7	-7	-19	0.9	0.9	0.9	0.8
Mecr	Q9DCS3	263	-8	-7	-7	-24	0.9	0.9	0.9	0.8
Brk1	Q91VR8	43	-16	-7	-7	3	0.9	0.9	0.9	1.0
Mvp	Q9EQK5	59	-21	-7	-7	-1	0.8	0.9	0.9	1.0
Crebrf	Q8CDG5	187	-17	-7	-7	-2	0.9	0.9	0.9	1.0
Gsdmdc1	Q9D8T2	57	-1	-7	-7	-9	1.0	0.9	0.9	0.9
Fam98b	Q80VD1	216	-7	-7	-7	-12	0.9	0.9	0.9	0.9
Akr1b1	P45376	45	-3	-8	-7	8	1.0	0.9	0.9	1.1
Nt5c3a	Q9D020	68	-5	-8	-7	-1	1.0	0.9	0.9	1.0
Rpl4	Q9D8E6	96	-8	-8	-7	-5	0.9	0.9	0.9	1.0
Nhp2	Q9CRB2	18	-11	-8	-7	-16	0.9	0.9	0.9	0.9
Riok3	Q9DBU3	287	-10	-8	-7	17	0.9	0.9	0.9	1.2
Sod1	P08228	147	-19	-8	-7	13	0.8	0.9	0.9	1.1
Nop9	Q8BMC4	557	1	-8	-7	8	1.0	0.9	0.9	1.1
Asrgl1	Q8C0M9	179	-10	-8	-7	-19	0.9	0.9	0.9	0.8
Abcf2	Q99LE6	416	-8	-9	-7	18	0.9	0.9	0.9	1.2
Tcf20	Q9EPQ8	897	-11	-9	-7	-5	0.9	0.9	0.9	1.0
Zcchc11	B2RX14	94	-6	-9	-7	-6	0.9	0.9	0.9	0.9

Map2k4	P47809	377	-12	-9	-7	-7	0.9	0.9	0.9	0.9
Tapt1	Q4VBD2	559	-10	-9	-7	-29	0.9	0.9	0.9	0.8
Dus3l	Q91XI1	383	-17	-9	-7	15	0.9	0.9	0.9	1.2
Cdkal1	Q91WE6	137	-6	-9	-7	9	0.9	0.9	0.9	1.1
Aco2	Q99KI0	385	-12	-9	-7	5	0.9	0.9	0.9	1.1
Cfl1	P18760	139	-1	-9	-7	-1	1.0	0.9	0.9	1.0
As3mt	Q91WU5	370	-1	-9	-7	-7	1.0	0.9	0.9	0.9
Dgka	O88673	254	-8	-9	-7	-7	0.9	0.9	0.9	0.9
Kdsr	Q6GV12	245	-8	-9	-7	-11	0.9	0.9	0.9	0.9
Hmha1	Q3TBD2	412	-1	-9	-7	-21	1.0	0.9	0.9	0.8
Gimap1	P70224	162	-2	-9	-7	-24	1.0	0.9	0.9	0.8
Zbtb1	Q91VL9	218	-5	-10	-7	7	1.0	0.9	0.9	1.1
Ubr5	E9Q2H1	739	-6	-10	-7	-1	0.9	0.9	0.9	1.0
Lims1	Q99JW4	272	-14	-10	-7	-4	0.9	0.9	0.9	1.0
Txlna	Q6PAM1	68	-8	-10	-7	-8	0.9	0.9	0.9	0.9
Tbl1x	Q9QXE7	351	-13	-10	-7	-10	0.9	0.9	0.9	0.9
Tnks1bp1	P58871	259	-7	-10	-7	-10	0.9	0.9	0.9	0.9
Oas1g	Q8K469	46	-22	-10	-7	-12	0.8	0.9	0.9	0.9
Ube2z	Q3UE37	265	-4	-10	-7	22	1.0	0.9	0.9	1.3
Bag5	Q8CI32	327	-11	-10	-7	4	0.9	0.9	0.9	1.0
Hsd17b11	Q9EQ06	94	-18	-10	-7	4	0.9	0.9	0.9	1.0
Pnp	Q543K9	77	-11	-10	-7	-5	0.9	0.9	0.9	1.0
Rrp12	Q6P5B0	102	-5	-11	-7	15	1.0	0.9	0.9	1.2
Fech	P22315	193	-19	-11	-7	13	0.8	0.9	0.9	1.1
Gbf1	Q6DFZ1	1110	-15	-11	-7	-2	0.9	0.9	0.9	1.0
Utp23	Q9CX11	208	-9	-11	-7	-8	0.9	0.9	0.9	0.9
Ankfy1	Q810B6	742	-19	-12	-7	6	0.8	0.9	0.9	1.1
Srrm1	Q52KI8	33	-8	-12	-7	-5	0.9	0.9	0.9	1.0
Utp11	Q9CZJ1	201	-8	-12	-7	-7	0.9	0.9	0.9	0.9
Xab2	Q9DCD2	98	2	-12	-7	-16	1.0	0.9	0.9	0.9
Ibtk	Q6ZPR6	523	-9	-12	-7	3	0.9	0.9	0.9	1.0
Cat	P24270	377	-12	-12	-7	-7	0.9	0.9	0.9	0.9
Cblb	B9EKI5	523	-4	-13	-7	-22	1.0	0.9	0.9	0.8
Uba7	Q9DBK7	794	-18	-13	-7	3	0.9	0.9	0.9	1.0
Baz1b	Q9Z277	1205	-9	-13	-7	-2	0.9	0.9	0.9	1.0
Diaph2	O70566	483	-16	-13	-7	-4	0.9	0.9	0.9	1.0
Brox	Q8K2Q7	288	-16	-13	-7	-6	0.9	0.9	0.9	0.9
Pik3cd	O35904	90	-10	-13	-7	-25	0.9	0.9	0.9	0.8
Kat6a	G3X940	262	-12	-14	-7	7	0.9	0.9	0.9	1.1
Dpf2	Q61103	327	-12	-14	-7	7	0.9	0.9	0.9	1.1
Kat6b	Q8BRB7	270	-12	-14	-7	7	0.9	0.9	0.9	1.1
Wdr81	Q5ND34	128	-12	-14	-7	-5	0.9	0.9	0.9	1.0
Med16	Q6PGF3	413	-15	-14	-7	-10	0.9	0.9	0.9	0.9
Riok3	Q9DBU3	346	-9	-14	-7	6	0.9	0.9	0.9	1.1
Ivns1abp	Q920Q8	454	-9	-14	-7	-1	0.9	0.9	0.9	1.0
Pogz	Q8BZH4	377	-11	-14	-7	-4	0.9	0.9	0.9	1.0
Bub3	Q9WVA3	129	-13	-14	-7	-16	0.9	0.9	0.9	0.9
Lpp	Q8BFW7	365	-8	-14	-7	-23	0.9	0.9	0.9	0.8
Tbc1d10c	Q8C9V1	303	-20	-15	-7	20	0.8	0.9	0.9	1.2
Urod	P70697	308	3	-15	-7	6	1.0	0.9	0.9	1.1
Nlrx1	Q3TL44	644	-23	-15	-7	-3	0.8	0.9	0.9	1.0

Mocos	Q14CH1	623	-13	-15	-7	-8	0.9	0.9	0.9	0.9
Spc24	Q9D083	96	-15	-15	-7	-1	0.9	0.9	0.9	1.0
Lpxn	Q99N69	199	-15	-15	-7	-3	0.9	0.9	0.9	1.0
Gnpat	P98192	65	-23	-15	-7	-5	0.8	0.9	0.9	1.0
Rsl1d1	Q8BVY0	347	-15	-15	-7	-21	0.9	0.9	0.9	0.8
Metap1	Q8BP48	22	-4	-15	-7	-31	1.0	0.9	0.9	0.8
Nup155	Q99P88	917	-21	-16	-7	4	0.8	0.9	0.9	1.0
Kmt2b	O08550	1453	-18	-16	-7	-27	0.9	0.9	0.9	0.8
Got1	P05201	391	-17	-16	-7	-3	0.9	0.9	0.9	1.0
Cnbp	P53996	172	-18	-16	-7	-16	0.9	0.9	0.9	0.9
Ufl1	Q8CCJ3	672	-9	-17	-7	16	0.9	0.9	0.9	1.2
Wapl	Q65Z40	231	-6	-17	-7	-13	0.9	0.9	0.9	0.9
Stk11	Q9WTK7	151	3	-17	-7	-26	1.0	0.9	0.9	0.8
Fto	Q8BGW1	104	-18	-17	-7	4	0.9	0.9	0.9	1.0
Trim28	Q62318	210	-7	-18	-7	0	0.9	0.8	0.9	1.0
Gtf2h1	E9QKD9	511	-10	-18	-7	-5	0.9	0.8	0.9	1.0
FAM120A	Q6A0A9	14	-21	-19	-7	-6	0.8	0.8	0.9	0.9
Hk1	P17710	669	2	-19	-7	-2	1.0	0.8	0.9	1.0
Mrpl37	Q921S7	177	-4	-20	-7	25	1.0	0.8	0.9	1.3
Crbn	Q8C7D2	224	-14	-20	-7	1	0.9	0.8	0.9	1.0
Vat1	Q62465	63	-18	-21	-7	28	0.8	0.8	0.9	1.4
Mon2	Q80TL7	1451	-22	-21	-7	-21	0.8	0.8	0.9	0.8
Tln1	P26039	1558	-28	-21	-7	6	0.8	0.8	0.9	1.1
Map3k1	F8VQ72	125	-12	-21	-7	-14	0.9	0.8	0.9	0.9
Nmi	O35309	183	-5	-21	-7	-16	1.0	0.8	0.9	0.9
Rars2	Q3U186	74	-10	-21	-7	-24	0.9	0.8	0.9	0.8
Mtch2	Q791V5	49	-23	-22	-7	16	0.8	0.8	0.9	1.2
Itpr3	P70227	1236	-17	-22	-7	8	0.9	0.8	0.9	1.1
Akap12	Q9WTQ5	1280	-16	-22	-7	-11	0.9	0.8	0.9	0.9
Gpatch8	A2A6A1	565	-12	-22	-7	-14	0.9	0.8	0.9	0.9
Lyn	P25911	219	-5	-23	-7	-13	1.0	0.8	0.9	0.9
Padi4	Q9Z183	613	-20	-24	-7	14	0.8	0.8	0.9	1.2
Rbck1	Q9WUB0	303	-13	-24	-7	-11	0.9	0.8	0.9	0.9
Adprhl2	Q8CG72	138	1	-24	-7	-13	1.0	0.8	0.9	0.9
Asrgl1	Q8C0M9	193	-12	-24	-7	-14	0.9	0.8	0.9	0.9
Sdha	Q8K2B3	89	-20	-26	-7	-17	0.8	0.8	0.9	0.9
Lima1	Q9ERG0	342	-11	-27	-7	-29	0.9	0.8	0.9	0.8
Znf592	Q8BHZ4	463	-1	-34	-7	-44	1.0	0.7	0.9	0.7
Ddx39a	Q8VDW0	238	3	20	-7	11	1.0	1.2	0.9	1.1
Ddx39b	Q9Z1N5	239	3	20	-7	11	1.0	1.2	0.9	1.1
Uba7	Q9DBK7	432	-16	16	-7	-7	0.9	1.2	0.9	0.9
Ube2z	Q3UE37	102	1	13	-7	24	1.0	1.1	0.9	1.3
Rnh1	Q91VI7	215	-2	10	-7	24	1.0	1.1	0.9	1.3
Inpp4a	Q9EPW0	541	-1	9	-7	8	1.0	1.1	0.9	1.1
Pml	Q60953	217	-6	7	-7	-9	0.9	1.1	0.9	0.9
RtcA	Q9D7H3	28	3	7	-7	-6	1.0	1.1	0.9	0.9
Spice1	Q8C804	633	-5	7	-7	-17	1.0	1.1	0.9	0.9
Nabp1	Q8BGW5	85	-7	6	-7	3	0.9	1.1	0.9	1.0
Nabp2	Q8R2Y9	81	-7	6	-7	3	0.9	1.1	0.9	1.0
Lpp	Q8BFW7	567	-8	6	-7	-11	0.9	1.1	0.9	0.9
Dstn	Q9R0P5	39	-8	5	-7	20	0.9	1.1	0.9	1.3

Rbpj	P31266	278	-8	5	-7	25	0.9	1.0	0.9	1.3
Smu1	Q3UKJ7	448	-2	5	-7	9	1.0	1.0	0.9	1.1
Trip12	G5E870	1681	3	4	-7	7	1.0	1.0	0.9	1.1
Rufy1	Q8BIJ7	668	4	4	-7	-2	1.0	1.0	0.9	1.0
Rpl11	Q9CXW4	72	-15	4	-7	-23	0.9	1.0	0.9	0.8
Gpatch8	A2A6A1	402	-4	3	-7	9	1.0	1.0	0.9	1.1
D10Jhu81e	Q9D172	174	-2	3	-7	8	1.0	1.0	0.9	1.1
Stip1	Q60864	370	-6	3	-7	5	0.9	1.0	0.9	1.1
Rab3gap2	E9QKE4	791	-9	3	-7	1	0.9	1.0	0.9	1.0
Lcp1	Q61233	140	-9	3	-7	11	0.9	1.0	0.9	1.1
Pls3	Q99K51	143	-9	3	-7	11	0.9	1.0	0.9	1.1
Smad4	P97471	522	-22	2	-7	8	0.8	1.0	0.9	1.1
Prkdc	P97313	3994	1	2	-7	18	1.0	1.0	0.9	1.2
Ankrd44	B2RXR6	334	-11	2	-7	-7	0.9	1.0	0.9	0.9
Tmem201	A2A8U2	167	-7	2	-7	-7	0.9	1.0	0.9	0.9
Brat1	Q8C3R1	422	-6	1	-7	28	0.9	1.0	0.9	1.4
Eif1ad	Q3THJ3	89	-1	1	-7	3	1.0	1.0	0.9	1.0
Syne2	Q6ZWQ0	4897	-4	1	-7	-9	1.0	1.0	0.9	0.9
Pdia3	P27773	85	-7	1	-7	2	0.9	1.0	0.9	1.0
Trmt5	Q9D0C4	401	2	0	-7	8	1.0	1.0	0.9	1.1
Mapk14	P47811	162	-9	0	-7	7	0.9	1.0	0.9	1.1
Nit2	Q9JHW2	153	-8	0	-7	5	0.9	1.0	0.9	1.1
Tbl1x	Q9QXE7	447	-11	0	-7	5	0.9	1.0	0.9	1.1
Nsmce3	Q9CPR8	257	-7	0	-7	2	0.9	1.0	0.9	1.0
Rabgap1l	A6H6A9	248	-2	0	-7	-1	1.0	1.0	0.9	1.0
Xrn1	F8VQ87	1301	-7	0	-7	-6	0.9	1.0	0.9	0.9
Cct4	P80315	450	-4	-1	-7	4	1.0	1.0	0.9	1.0
Lrrc59	Q922Q8	48	-8	-1	-7	1	0.9	1.0	0.9	1.0
Vwa5a	Q99KC8	112	3	-1	-7	-10	1.0	1.0	0.9	0.9
Cad	B2RQC6	758	-2	-1	-7	23	1.0	1.0	0.9	1.3
Hdac4	Q6NZM9	769	-8	-1	-7	19	0.9	1.0	0.9	1.2
Anxa7	Q07076	260	-11	-1	-7	15	0.9	1.0	0.9	1.2
Cnot6l	Q8VEG6	449	-5	-2	-7	9	1.0	1.0	0.9	1.1
Ndufv1	Q91YT0	125	-12	-2	-7	-1	0.9	1.0	0.9	1.0
Qki	Q9QYS9	119	-3	-2	-7	12	1.0	1.0	0.9	1.1
Rab3gap1	Q80UJ7	678	-7	-2	-7	4	0.9	1.0	0.9	1.0
Anxa6	P14824	358	-10	-2	-7	-11	0.9	1.0	0.9	0.9
Iqsec1	Q8R0S2	258	-9	-2	-7	-12	0.9	1.0	0.9	0.9
Dars	Q922B2	334	-19	-3	-7	12	0.8	1.0	0.9	1.1
Got2	P05202	272	-18	-3	-7	10	0.9	1.0	0.9	1.1
Scaf8	Q6DID3	514	-14	-3	-7	-11	0.9	1.0	0.9	0.9
Ap4e1	Q80V94	960	-9	-3	-7	-15	0.9	1.0	0.9	0.9
Syne1	Q6ZWR6	7327	8	-3	-7	22	1.1	1.0	0.9	1.3
Rnh1	Q91VI7	129	-19	-3	-7	18	0.8	1.0	0.9	1.2
Dlat	Q8BMF4	581	1	-3	-7	15	1.0	1.0	0.9	1.2
Arhgdib	Q61599	75	-9	-3	-7	13	0.9	1.0	0.9	1.1
Nelfcd	Q922L6	583	-5	-3	-7	-2	1.0	1.0	0.9	1.0
Polr3k	Q9CQZ7	28	-25	-4	-7	11	0.8	1.0	0.9	1.1
Psmd2	Q8VDM4	779	-12	-4	-7	9	0.9	1.0	0.9	1.1
Slfn1	Q9Z0I7	45	-18	-4	-7	4	0.8	1.0	0.9	1.0
Glod4	Q9CPV4	206	-19	-4	-7	1	0.8	1.0	0.9	1.0

Fbxo38	Q8BMI0	401	-4	-4	-7	-2	1.0	1.0	0.9	1.0
Gnai2	P08752	326	-22	-4	-7	-7	0.8	1.0	0.9	0.9
Tln1	P26039	1202	-15	-4	-7	12	0.9	1.0	0.9	1.1
Add3	Q9QYB5	286	-13	-4	-7	10	0.9	1.0	0.9	1.1
Cct7	P80313	450	-7	-4	-7	8	0.9	1.0	0.9	1.1
Itpr1	P11881	1458	-8	-4	-7	-5	0.9	1.0	0.9	1.0
Chd4	Q6PDQ2	1587	10	-4	-7	-6	1.1	1.0	0.9	0.9
Pcnt	F8VPV0	2564	-8	-4	-7	-16	0.9	1.0	0.9	0.9
Aldh3a2	P47740	220	-20	-5	-7	13	0.8	1.0	0.9	1.1
Lcn2	P11672	98	-8	-5	-7	10	0.9	1.0	0.9	1.1
Zmym2	Q9CU65	607	-2	-5	-7	8	1.0	1.0	0.9	1.1
Cryl1	Q99KP3	125	-9	-5	-7	7	0.9	1.0	0.9	1.1
Cbll1	Q9JIY2	158	-14	-5	-7	5	0.9	1.0	0.9	1.1
Wdr6	Q99ME2	688	-14	-5	-7	-3	0.9	1.0	0.9	1.0
Hgs	Q99LI8	212	-12	-5	-7	-3	0.9	1.0	0.9	1.0
Syne1	Q6ZWR6	3047	-5	-5	-7	-5	1.0	1.0	0.9	1.0
Irf8	P23611	408	-22	-5	-7	-18	0.8	1.0	0.9	0.8
Arhgap1	Q5FWK3	91	-18	-5	-7	24	0.9	1.0	0.9	1.3
Ugdh	O70475	276	-12	-5	-7	17	0.9	1.0	0.9	1.2
Gpatch1	Q9DBM1	837	-7	-5	-7	3	0.9	1.0	0.9	1.0
Chd8	Q09XV5	1904	0	-5	-7	-1	1.0	1.0	0.9	1.0
Pak1	G5E884	410	-3	-5	-7	-3	1.0	1.0	0.9	1.0
Camkk2	Q8C078	397	-9	-5	-7	-4	0.9	1.0	0.9	1.0
Vac14	Q80WQ2	731	-11	-5	-7	-10	0.9	1.0	0.9	0.9
Mecp2	Q9Z2D6	427	-5	-5	-7	-17	1.0	1.0	0.9	0.9
Shc1	P98083	196	-9	-6	-7	7	0.9	0.9	0.9	1.1
Tbc1d1	Q60949	853	-10	-6	-7	3	0.9	0.9	0.9	1.0
Gsn	P13020	329	-4	-6	-7	-2	1.0	0.9	0.9	1.0
Hira	Q61666	1005	-8	-6	-7	-2	0.9	0.9	0.9	1.0
Rbm28	Q8CGC6	480	-8	-6	-7	-2	0.9	0.9	0.9	1.0
Gm20822	J3KMI7	23	-22	-6	-7	-5	0.8	0.9	0.9	1.0
Hmgcs2	P54869	261	-18	-6	-7	-8	0.8	0.9	0.9	0.9
Tsn	Q62348	225	-7	-6	-7	-11	0.9	0.9	0.9	0.9
Hnrnpu	Q8VEK3	624	-16	-6	-7	-13	0.9	0.9	0.9	0.9
Parp14	Q2EMV9	1324	-19	-6	-7	-15	0.8	0.9	0.9	0.9
LRWD1	Q8BUI3	485	-3	-6	-7	-17	1.0	0.9	0.9	0.9
Rpl34	Q9D1R9	49	-8	-6	-7	7	0.9	0.9	0.9	1.1
Synj1	D3Z656	1252	-4	-6	-7	3	1.0	0.9	0.9	1.0
Anapc7	Q9WVM3	295	-12	-6	-7	1	0.9	0.9	0.9	1.0
Usp9x	Q4FE56	673	-5	-6	-7	-1	1.0	0.9	0.9	1.0
Arhgap4	B1AUY3	590	-11	-6	-7	-5	0.9	0.9	0.9	1.0
Rps3a	P97351	111	-9	-6	-7	-8	0.9	0.9	0.9	0.9
Yrdc	Q3U5F4	279	-15	-6	-7	-9	0.9	0.9	0.9	0.9
Rbm19	Q8R3C6	77	-9	-6	-7	-9	0.9	0.9	0.9	0.9
Acsf3	Q3URE1	77	-8	-6	-7	-10	0.9	0.9	0.9	0.9
Numa1	E9Q7G0	728	-7	-6	-7	-13	0.9	0.9	0.9	0.9
Pds5a	E9QPI5	582	-10	-7	-7	28	0.9	0.9	0.9	1.4
Snw1	A0A0B4J1E2	250	-5	-7	-7	22	1.0	0.9	0.9	1.3
Usp4	P35123	649	-10	-7	-7	2	0.9	0.9	0.9	1.0
Mtmr14	Q8VEL2	427	-12	-7	-7	-1	0.9	0.9	0.9	1.0
Sec13	Q9D1M0	299	-5	-7	-7	-12	1.0	0.9	0.9	0.9

Pcbp1	P60335	109	-1	-7	-7	-19	1.0	0.9	0.9	0.8
Bud31	Q6PGH1	61	-16	-7	-7	21	0.9	0.9	0.9	1.3
Eif5	P59325	138	-13	-7	-7	18	0.9	0.9	0.9	1.2
Zfp871	G5E905	372	-17	-7	-7	9	0.9	0.9	0.9	1.1
Trip12	G5E870	35	-1	-7	-7	5	1.0	0.9	0.9	1.1
Ola1	Q9CZ30	187	-17	-7	-7	-1	0.9	0.9	0.9	1.0
Sipa1	E9Q0Y4	751	-9	-7	-7	-3	0.9	0.9	0.9	1.0
Rpa2	Q3TE40	219	-10	-7	-7	-4	0.9	0.9	0.9	1.0
Clic1	Q9Z1Q5	24	-13	-7	-7	-4	0.9	0.9	0.9	1.0
Rfc1	G3UWX1	913	4	-7	-7	-5	1.0	0.9	0.9	1.0
Psme3	P61290	92	-8	-7	-7	-9	0.9	0.9	0.9	0.9
Rab18	P35293	110	-23	-7	-7	-11	0.8	0.9	0.9	0.9
Cep44	Q5HZK1	228	4	-7	-7	-18	1.0	0.9	0.9	0.8
Copg2	Q9QXK3	296	-10	-8	-7	15	0.9	0.9	0.9	1.2
Ddx17	Q501J6	240	-12	-8	-7	11	0.9	0.9	0.9	1.1
Slfn5	Q8CBA2	244	-2	-8	-7	1	1.0	0.9	0.9	1.0
Wdr5	P61965	205	-7	-8	-7	-5	0.9	0.9	0.9	1.0
Upf1	Q9EPU0	204	-9	-8	-7	-7	0.9	0.9	0.9	0.9
Psmg2	Q9EST4	168	5	-8	-7	-16	1.1	0.9	0.9	0.9
Trim30a	P15533	96	5	-8	-7	-20	1.0	0.9	0.9	0.8
Atp5o	Q9DB20	141	-20	-8	-7	7	0.8	0.9	0.9	1.1
Gnai2	P08752	112	-7	-8	-7	-5	0.9	0.9	0.9	1.0
Fam60a	Q8C8M1	173	-14	-8	-7	-16	0.9	0.9	0.9	0.9
Chd1	P40201	1324	-11	-8	-7	-26	0.9	0.9	0.9	0.8
Taok2	Q6ZQ29	630	6	-9	-7	-5	1.1	0.9	0.9	1.0
Rif1	Q6PR54	1692	-7	-9	-7	-7	0.9	0.9	0.9	0.9
Nt5dc1	Q8C5P5	129	-14	-9	-7	53	0.9	0.9	0.9	2.1
Slfn5	Q8CBA2	353	-12	-9	-7	2	0.9	0.9	0.9	1.0
Plod3	Q9R0E1	694	-11	-9	-7	2	0.9	0.9	0.9	1.0
Pdia3	P27773	57	-5	-9	-7	-1	1.0	0.9	0.9	1.0
Tnk2	O54967	434	-10	-9	-7	-8	0.9	0.9	0.9	0.9
Atrx	Q61687	323	-9	-9	-7	-9	0.9	0.9	0.9	0.9
Tufm	Q8BFR5	222	-13	-9	-7	-9	0.9	0.9	0.9	0.9
Itsn2	E9QNG1	1350	-9	-9	-7	-16	0.9	0.9	0.9	0.9
Sipa1	E9Q0Y4	961	-12	-9	-7	-17	0.9	0.9	0.9	0.9
Fasn	P19096	779	-12	-10	-7	15	0.9	0.9	0.9	1.2
Ipo4	Q8VI75	709	-5	-10	-7	8	1.0	0.9	0.9	1.1
Mdn1	A2ANY6	1390	-6	-10	-7	7	0.9	0.9	0.9	1.1
Atf7ip	Q7TT18	642	-6	-10	-7	6	0.9	0.9	0.9	1.1
Stk11ip	Q3TAA7	124	-17	-10	-7	1	0.9	0.9	0.9	1.0
Usp15	Q8R5H1	570	-15	-10	-7	-2	0.9	0.9	0.9	1.0
Themis2	Q91YX0	587	-8	-10	-7	-4	0.9	0.9	0.9	1.0
Ppp4r2	Q0VGB7	22	-14	-10	-7	-10	0.9	0.9	0.9	0.9
Zfc3h1	B2RT41	1144	-25	-10	-7	42	0.8	0.9	0.9	1.7
Rpl34	Q9D1R9	46	-9	-10	-7	7	0.9	0.9	0.9	1.1
Xpo5	Q924C1	1131	-6	-10	-7	0	0.9	0.9	0.9	1.0
Lancl2	Q9JJK2	51	-8	-10	-7	-4	0.9	0.9	0.9	1.0
Ubr7	Q8BU04	260	-6	-10	-7	-5	0.9	0.9	0.9	1.0
Ahnak	E9Q616	5210	-2	-10	-7	-18	1.0	0.9	0.9	0.8
Pdcl	Q9DBX2	81	4	-11	-7	0	1.0	0.9	0.9	1.0
Tpr	F6ZDS4	1201	-7	-11	-7	-11	0.9	0.9	0.9	0.9

Ttr	P07309	30	-10	-11	-7	1	0.9	0.9	0.9	1.0
Inpp4b	Q6P1Y8	261	-13	-11	-7	-2	0.9	0.9	0.9	1.0
Cherp	Q8CGZ0	69	-12	-11	-7	-7	0.9	0.9	0.9	0.9
Dennd1c	Q8CFK6	597	-14	-11	-7	-14	0.9	0.9	0.9	0.9
Cmc4	Q61908	12	-18	-12	-7	17	0.8	0.9	0.9	1.2
Zfp106	R4GML0	444	-14	-12	-7	7	0.9	0.9	0.9	1.1
Dis3l	Q8C0S1	646	-15	-12	-7	-8	0.9	0.9	0.9	0.9
Stip1	Q60864	461	-7	-12	-7	-8	0.9	0.9	0.9	0.9
Creb1	Q01147	337	-7	-12	-7	-11	0.9	0.9	0.9	0.9
Msh6	P54276	88	-7	-12	-7	-14	0.9	0.9	0.9	0.9
Pmpca	Q9DC61	465	-15	-12	-7	10	0.9	0.9	0.9	1.1
Ilkap	Q8R0F6	301	-6	-12	-7	8	0.9	0.9	0.9	1.1
Adar	Q99MU3	583	2	-12	-7	2	1.0	0.9	0.9	1.0
Gart	Q64737	466	-14	-12	-7	-3	0.9	0.9	0.9	1.0
Sptb	Q3UGX2	960	-11	-12	-7	-15	0.9	0.9	0.9	0.9
Usp25	P57080	1015	-10	-12	-7	-26	0.9	0.9	0.9	0.8
Rap1gap2	Q5SVL6	209	-12	-13	-7	16	0.9	0.9	0.9	1.2
Pgk1	P09411	316	-6	-13	-7	11	0.9	0.9	0.9	1.1
Zrsr2	B1B0E8	306	-12	-13	-7	8	0.9	0.9	0.9	1.1
Abcd1	P48410	571	-20	-13	-7	-3	0.8	0.9	0.9	1.0
Myd88	P22366	233	0	-13	-7	-9	1.0	0.9	0.9	0.9
Dalrd3	Q6PJN8	66	-8	-13	-7	1	0.9	0.9	0.9	1.0
Ppp2cb	P62715	269	-18	-13	-7	-12	0.8	0.9	0.9	0.9
Ppp2ca	P63330	269	-18	-13	-7	-12	0.8	0.9	0.9	0.9
Ipo9	E9QKZ2	606	-17	-14	-7	19	0.9	0.9	0.9	1.2
Akap11	E9Q777	1595	3	-14	-7	-3	1.0	0.9	0.9	1.0
Rab3d	P35276	184	-7	-14	-7	-12	0.9	0.9	0.9	0.9
Srp9	P49962	39	-14	-14	-7	-17	0.9	0.9	0.9	0.9
Ranbp2	Q9ERU9	815	-7	-14	-7	17	0.9	0.9	0.9	1.2
Gpr63	Q9EQQ3	418	-15	-14	-7	-2	0.9	0.9	0.9	1.0
Usp34	Q6ZQ93	1417	-9	-15	-7	4	0.9	0.9	0.9	1.0
Itpr2	Q9Z329	379	-7	-15	-7	-1	0.9	0.9	0.9	1.0
Smpd4	Q6ZPR5	723	-9	-15	-7	-23	0.9	0.9	0.9	0.8
Tmlhe	Q91ZE0	103	-17	-15	-7	-21	0.9	0.9	0.9	0.8
Slfn8	B1ARD8	756	-24	-16	-7	-16	0.8	0.9	0.9	0.9
Ctcf	Q61164	268	-5	-17	-7	11	1.0	0.9	0.9	1.1
Il16	O54824	997	-1	-17	-7	-16	1.0	0.9	0.9	0.9
Pdcd6ip	Q9WU78	90	-17	-18	-7	1	0.9	0.9	0.9	1.0
Med12	A2AGH6	998	-12	-18	-7	-4	0.9	0.9	0.9	1.0
Map3k8	Q07174	398	-19	-18	-7	17	0.8	0.8	0.9	1.2
Rfc4	Q99J62	141	-12	-18	-7	-6	0.9	0.8	0.9	0.9
Arid5a	Q3U108	232	-5	-19	-7	23	1.0	0.8	0.9	1.3
P4hb	P09103	314	-29	-19	-7	3	0.8	0.8	0.9	1.0
Uba6	Q8C7R4	455	-24	-19	-7	-10	0.8	0.8	0.9	0.9
Bend3	Q6PAL0	260	-21	-20	-7	-16	0.8	0.8	0.9	0.9
Man2c1	Q91W89	792	-17	-20	-7	9	0.9	0.8	0.9	1.1
Fubp3	Q3TIX6	366	-16	-20	-7	-10	0.9	0.8	0.9	0.9
Brd1	E9PZ26	1068	-14	-21	-7	4	0.9	0.8	0.9	1.0
Srbd1	F8WGW3	497	-16	-22	-7	17	0.9	0.8	0.9	1.2
Rab1A	P62821	126	-11	-22	-7	-15	0.9	0.8	0.9	0.9
Phc2	Q9QWH1	657	-8	-22	-7	-6	0.9	0.8	0.9	0.9

Zmynd11	Q8R5C8	126	-23	-24	-7	6	0.8	0.8	0.9	1.1
Pum1	Q80U78	1182	-15	-24	-7	-10	0.9	0.8	0.9	0.9
Fndc3a	Q8BX90	465	5	-25	-7	0	1.1	0.8	0.9	1.0
Setx	A2AKX3	1836	-6	-26	-7	21	0.9	0.8	0.9	1.3
Magohb	Q9CQL1	131	-13	-26	-7	-41	0.9	0.8	0.9	0.7
Eefsec	Q9JHW4	413	-27	-28	-7	11	0.8	0.8	0.9	1.1
H2-M3	Q31093	60	-8	-28	-7	11	0.9	0.8	0.9	1.1
Cltc	Q68FD5	1102	-24	-29	-7	4	0.8	0.8	0.9	1.0
Dus3l	Q91XI1	151	-21	-29	-7	8	0.8	0.8	0.9	1.1
Tars	Q9D0R2	342	-4	-29	-7	-1	1.0	0.8	0.9	1.0
Zfp280b	Q505F4	351	-16	-34	-7	-20	0.9	0.7	0.9	0.8
Wac	Q924H7	150	-28	-39	-7	-28	0.8	0.7	0.9	0.8
Slc25a51	Q5HZI9	167	64	-51	-7	-63	2.7	0.7	0.9	0.6
Vps35	Q9EQH3	673	-2	24	-8	11	1.0	1.3	0.9	1.1
Twf2	Q9Z0P5	275	3	16	-8	18	1.0	1.2	0.9	1.2
Rbbp5	Q8BX09	126	-2	13	-8	31	1.0	1.1	0.9	1.4
Nars	Q8BP47	277	16	13	-8	3	1.2	1.1	0.9	1.0
Bach2	P97303	675	10	12	-8	-3	1.1	1.1	0.9	1.0
Slc25a5	P51881	257	-2	11	-8	24	1.0	1.1	0.9	1.3
Birc2	Q62210	45	-12	8	-8	21	0.9	1.1	0.9	1.3
Actr3	Q99JY9	12	-6	7	-8	20	0.9	1.1	0.9	1.3
Pold1	P52431	1013	3	6	-8	-1	1.0	1.1	0.9	1.0
Bod1l	E9Q6J5	1176	7	6	-8	-26	1.1	1.1	0.9	0.8
Dazap2	Q9DCP9	132	-4	5	-8	44	1.0	1.1	0.9	1.8
Sae1	Q9R1T2	150	-8	4	-8	5	0.9	1.0	0.9	1.1
Otud6b	Q8K2H2	173	-11	3	-8	-16	0.9	1.0	0.9	0.9
Sart3	Q9JLI8	821	2	3	-8	25	1.0	1.0	0.9	1.3
Adrbk2	Q3UYH7	208	-9	3	-8	20	0.9	1.0	0.9	1.2
Grk2	Q99MK8	208	-9	3	-8	20	0.9	1.0	0.9	1.2
Pstpip2	Q99M15	221	-4	3	-8	7	1.0	1.0	0.9	1.1
Cct4	P80315	295	-1	2	-8	29	1.0	1.0	0.9	1.4
Trrap	A0A1D5RLL4	2240	-6	2	-8	5	0.9	1.0	0.9	1.1
Pelo	Q80X73	258	2	2	-8	-14	1.0	1.0	0.9	0.9
Ube2z	Q3UE37	263	-6	2	-8	29	0.9	1.0	0.9	1.4
Rnf14	Q9JI90	224	-9	2	-8	-10	0.9	1.0	0.9	0.9
Pyhin1	Q8BV49	335	-20	1	-8	13	0.8	1.0	0.9	1.1
Tor1aip1	Q921T2	195	12	1	-8	9	1.1	1.0	0.9	1.1
Eif4g1	Q6NZJ6	1516	3	1	-8	1	1.0	1.0	0.9	1.0
Kdm3b	B9EKS2	1471	-1	1	-8	-9	1.0	1.0	0.9	0.9
Hnrnpf	Q9Z2X1	267	-3	1	-8	-9	1.0	1.0	0.9	0.9
Gsap	Q3TCV3	71	4	0	-8	12	1.0	1.0	0.9	1.1
Ankfy1	Q810B6	675	-4	0	-8	11	1.0	1.0	0.9	1.1
P33monox	Q9DBN4	27	-10	-1	-8	-6	0.9	1.0	0.9	0.9
Pml	Q60953	134	-11	-1	-8	-13	0.9	1.0	0.9	0.9
Map4	P27546	595	-3	-1	-8	-22	1.0	1.0	0.9	0.8
Nfrkb	Q6PIJ4	487	1	-1	-8	8	1.0	1.0	0.9	1.1
Dus4l	Q32M08	11	-1	-1	-8	4	1.0	1.0	0.9	1.0
Ankrd26	D3Z482	761	12	-1	-8	-8	1.1	1.0	0.9	0.9
Tomm34	Q9CYG7	236	-4	-2	-8	8	1.0	1.0	0.9	1.1
Sp1	O89090	607	-8	-2	-8	7	0.9	1.0	0.9	1.1
Actn4	P57780	880	-14	-2	-8	8	0.9	1.0	0.9	1.1

Actn1	Q7TPR4	860	-14	-2	-8	8	0.9	1.0	0.9	1.1
Ipo7	Q9EPL8	757	-1	-2	-8	3	1.0	1.0	0.9	1.0
Gnl2	Q99LH1	514	0	-2	-8	-16	1.0	1.0	0.9	0.9
Rnf213	E9Q555	2594	-9	-3	-8	6	0.9	1.0	0.9	1.1
Sp1	O89090	605	-13	-3	-8	6	0.9	1.0	0.9	1.1
Trmt2a	Q8BNV1	127	-15	-3	-8	-7	0.9	1.0	0.9	0.9
Msn	P26041	117	-17	-4	-8	10	0.9	1.0	0.9	1.1
Sart1	Q9Z315	566	-8	-4	-8	3	0.9	1.0	0.9	1.0
Arhgef1	Q61210	282	-12	-4	-8	-6	0.9	1.0	0.9	0.9
Ppa1	Q9D819	242	-9	-4	-8	-12	0.9	1.0	0.9	0.9
Pde3b	E9QLQ3	38	-2	-4	-8	-26	1.0	1.0	0.9	0.8
Rras2	P62071	183	-13	-4	-8	-29	0.9	1.0	0.9	0.8
Acat1	Q8QZT1	123	-7	-4	-8	0	0.9	1.0	0.9	1.0
Tln1	P26039	956	-17	-5	-8	12	0.9	1.0	0.9	1.1
Atp1a3	Q6PIC6	695	-25	-5	-8	5	0.8	1.0	0.9	1.1
Atp1a1	Q8VDN2	705	-25	-5	-8	5	0.8	1.0	0.9	1.1
Maea	Q4VC33	195	-3	-5	-8	-1	1.0	1.0	0.9	1.0
Gmps	Q3THK7	631	-15	-5	-8	-2	0.9	1.0	0.9	1.0
Zcchc6	E9PUA2	689	0	-5	-8	-18	1.0	1.0	0.9	0.9
Syne2	Q6ZWQ0	2951	-12	-5	-8	8	0.9	1.0	0.9	1.1
Inpp5b	Q8K337	678	-2	-5	-8	7	1.0	1.0	0.9	1.1
Rnf213	E9Q555	955	-11	-5	-8	6	0.9	1.0	0.9	1.1
Zmym2	Q9CU65	707	-6	-5	-8	1	0.9	1.0	0.9	1.0
Esyt3	Q5DTI8	619	-7	-5	-8	1	0.9	1.0	0.9	1.0
Sept11	Q8C1B7	41	-20	-5	-8	-13	0.8	1.0	0.9	0.9
Trappc1	Q5NCF2	115	-13	-6	-8	15	0.9	0.9	0.9	1.2
Ak2	Q9WTP6	40	-11	-6	-8	-3	0.9	0.9	0.9	1.0
Vcp	Q01853	105	-9	-6	-8	-4	0.9	0.9	0.9	1.0
Bola1	Q9D8S9	126	-7	-6	-8	-6	0.9	0.9	0.9	0.9
Zfp869	Q9DC47	120	-7	-6	-8	-15	0.9	0.9	0.9	0.9
Cep350	E9Q309	408	-14	-6	-8	-17	0.9	0.9	0.9	0.9
Agpat4	Q8K4X7	175	-2	-6	-8	-24	1.0	0.9	0.9	0.8
Milr1	Q3TB92	204	-14	-6	-8	-39	0.9	0.9	0.9	0.7
Cep63	Q3UPP8	312	-23	-6	-8	-46	0.8	0.9	0.9	0.7
Plec	Q9QXS1	4078	-21	-6	-8	31	0.8	0.9	0.9	1.4
Srrt	Q99MR6	714	-8	-6	-8	12	0.9	0.9	0.9	1.1
C2cd3	Q52KB6	1923	-7	-6	-8	6	0.9	0.9	0.9	1.1
Fam129c	D3YZB0	209	-9	-6	-8	5	0.9	0.9	0.9	1.0
Metap2	O08663	448	-11	-6	-8	4	0.9	0.9	0.9	1.0
Wdr11	G5E8J3	390	-1	-6	-8	0	1.0	0.9	0.9	1.0
Gm45233	A0A0N4SVE0	71	-12	-6	-8	-4	0.9	0.9	0.9	1.0
Cpsf4	Q8BQZ5	55	-6	-6	-8	-8	0.9	0.9	0.9	0.9
Ifi35	Q9D8C4	107	-14	-6	-8	-8	0.9	0.9	0.9	0.9
Pycrl	Q9DCC4	49	-12	-6	-8	-9	0.9	0.9	0.9	0.9
Polr3c	Q9D483	160	-12	-7	-8	18	0.9	0.9	0.9	1.2
Dhx15	O35286	190	-15	-7	-8	5	0.9	0.9	0.9	1.0
Ppwd1	Q8CEC6	512	-24	-7	-8	5	0.8	0.9	0.9	1.0
Sec24d	Q6NXL1	1021	-5	-7	-8	4	1.0	0.9	0.9	1.0
Sf3a1	Q8K4Z5	244	-4	-7	-8	-3	1.0	0.9	0.9	1.0
Wdr73	Q9CWR1	143	-7	-7	-8	-13	0.9	0.9	0.9	0.9
Rai1	Q61818	998	-5	-7	-8	-19	1.0	0.9	0.9	0.8

Wdhd1	P59328	156	-5	-7	-8	18	1.0	0.9	0.9	1.2
Zmynd8	A2A484	107	1	-7	-8	11	1.0	0.9	0.9	1.1
Zfc3h1	B2RT41	1734	-6	-7	-8	7	0.9	0.9	0.9	1.1
Zmym2	Q9CU65	749	-5	-7	-8	7	1.0	0.9	0.9	1.1
Idh2	P54071	418	-11	-7	-8	-4	0.9	0.9	0.9	1.0
Cdc16	Q8R349	194	-8	-7	-8	-6	0.9	0.9	0.9	0.9
Rnf146	Q9CZW6	186	-16	-7	-8	-28	0.9	0.9	0.9	0.8
Brcc3	P46737	203	-9	-8	-8	15	0.9	0.9	0.9	1.2
Mdn1	A2ANY6	2885	-9	-8	-8	6	0.9	0.9	0.9	1.1
Pex19	Q8VCI5	128	-6	-8	-8	0	0.9	0.9	0.9	1.0
Phf20l1	Q8CCJ9	301	4	-8	-8	-4	1.0	0.9	0.9	1.0
Ccdc50	Q810U5	85	-1	-8	-8	-6	1.0	0.9	0.9	0.9
Ap3b1	Q9Z1T1	898	-16	-8	-8	-9	0.9	0.9	0.9	0.9
Nudt3	Q9JI46	131	-10	-8	-8	-23	0.9	0.9	0.9	0.8
Plcg2	Q8CIH5	496	-2	-8	-8	17	1.0	0.9	0.9	1.2
Mtfr1l	Q9CWE0	49	-7	-8	-8	11	0.9	0.9	0.9	1.1
Asun	Q8QZV7	349	-17	-8	-8	8	0.9	0.9	0.9	1.1
Akap10	O88845	110	-12	-8	-8	-2	0.9	0.9	0.9	1.0
Aar2	Q9D2V5	181	-10	-8	-8	-6	0.9	0.9	0.9	0.9
Tra2b	P62996	118	-11	-8	-8	-7	0.9	0.9	0.9	0.9
Cep135	Q6P5D4	316	-14	-8	-8	-12	0.9	0.9	0.9	0.9
Hspd1	P63038	237	-20	-9	-8	15	0.8	0.9	0.9	1.2
Ano6	Q6P9J9	262	-8	-9	-8	8	0.9	0.9	0.9	1.1
Med13	Q5SWW4	926	-8	-9	-8	3	0.9	0.9	0.9	1.0
Opa1	P58281	375	-13	-9	-8	-5	0.9	0.9	0.9	1.0
Ktn1	F8VQC7	1081	-3	-9	-8	-7	1.0	0.9	0.9	0.9
Neil1	Q8K4Q6	26	-5	-9	-8	-9	1.0	0.9	0.9	0.9
Taok3	Q8BYC6	727	-15	-9	-8	-20	0.9	0.9	0.9	0.8
Bcar3	Q9QZK2	316	-18	-9	-8	-36	0.9	0.9	0.9	0.7
Bach2	P97303	369	-9	-9	-8	3	0.9	0.9	0.9	1.0
Ccdc25	Q78PG9	83	-13	-9	-8	1	0.9	0.9	0.9	1.0
Vars	Q9Z1Q9	681	-13	-10	-8	17	0.9	0.9	0.9	1.2
Rsbn1l	D3Z0K6	92	-13	-10	-8	-1	0.9	0.9	0.9	1.0
Rps3a	P97351	201	-10	-10	-8	-7	0.9	0.9	0.9	0.9
Ccsap	Q8QZT2	141	-10	-10	-8	-15	0.9	0.9	0.9	0.9
Ftsj3	Q9DBE9	293	-14	-10	-8	15	0.9	0.9	0.9	1.2
Hnrnpm	Q9D0E1	693	-12	-10	-8	5	0.9	0.9	0.9	1.0
Rpl23	P62830	125	-13	-10	-8	2	0.9	0.9	0.9	1.0
Rbck1	Q9WUB0	283	-12	-10	-8	-4	0.9	0.9	0.9	1.0
Rngtt	O55236	375	-13	-10	-8	-5	0.9	0.9	0.9	1.0
Prmt9	Q3U3W5	290	-9	-10	-8	-12	0.9	0.9	0.9	0.9
Glcci1	Q8K3I9	287	-12	-10	-8	-30	0.9	0.9	0.9	0.8
Rps6	P62754	12	-9	-11	-8	-6	0.9	0.9	0.9	0.9
Foxk1	P42128	425	-7	-11	-8	-10	0.9	0.9	0.9	0.9
Hmgcl	P38060	170	-18	-11	-8	-12	0.8	0.9	0.9	0.9
Fxr2	Q6P5B5	87	-12	-11	-8	-13	0.9	0.9	0.9	0.9
Scaf1	Q5U4C3	897	-11	-11	-8	-24	0.9	0.9	0.9	0.8
Coro1c	Q9WUM4	23	-16	-11	-8	17	0.9	0.9	0.9	1.2
Ptgr1	Q91YR9	298	-2	-11	-8	-1	1.0	0.9	0.9	1.0
Eif2s3x	Q9Z0N1	105	-13	-11	-8	-7	0.9	0.9	0.9	0.9
Rbm14	Q8C2Q3	108	-6	-11	-8	-10	0.9	0.9	0.9	0.9

Pglyrp1	O88593	141	12	-11	-8	-11	1.1	0.9	0.9	0.9
Ddx20	Q9JJY4	538	-21	-11	-8	-14	0.8	0.9	0.9	0.9
Pitrm1	Q8K411	556	-2	-11	-8	-24	1.0	0.9	0.9	0.8
Ptpn12	P35831	641	-23	-11	-8	-28	0.8	0.9	0.9	0.8
Nfkbie	O54910	199	-22	-12	-8	11	0.8	0.9	0.9	1.1
Erp44	Q9D1Q6	92	-18	-12	-8	10	0.9	0.9	0.9	1.1
Tbce	Q8CIV8	152	-6	-12	-8	6	0.9	0.9	0.9	1.1
Klhl25	Q8R2P1	340	-16	-12	-8	-11	0.9	0.9	0.9	0.9
Fkbp8	O35465	185	-31	-12	-8	-12	0.8	0.9	0.9	0.9
Pstpip2	Q99M15	90	-6	-12	-8	3	0.9	0.9	0.9	1.0
Rbm22	Q8BHS3	48	-15	-12	-8	-3	0.9	0.9	0.9	1.0
Helb	Q6NVF4	963	-16	-12	-8	-9	0.9	0.9	0.9	0.9
Akap13	E9Q394	1633	-18	-12	-8	-18	0.8	0.9	0.9	0.9
Sumf1	Q8R0F3	48	-5	-12	-8	-23	1.0	0.9	0.9	0.8
Dscr3	O35075	137	-12	-13	-8	3	0.9	0.9	0.9	1.0
Cbx1	P83917	156	-13	-13	-8	-1	0.9	0.9	0.9	1.0
Sp100	O35892	68	-8	-13	-8	-10	0.9	0.9	0.9	0.9
Actn1	Q7TPR4	480	-6	-13	-8	-11	0.9	0.9	0.9	0.9
Fchsd2	Q3USJ8	354	-9	-13	-8	-13	0.9	0.9	0.9	0.9
Trim5	E9PV98	469	-12	-13	-8	-14	0.9	0.9	0.9	0.9
Trim12c	D3Z3L3	469	-12	-13	-8	-14	0.9	0.9	0.9	0.9
Fryl	F8VQ05	889	-6	-13	-8	14	0.9	0.9	0.9	1.2
Sqstm1	Q64337	26	-13	-13	-8	4	0.9	0.9	0.9	1.0
Cnn3	Q9DAW9	173	-7	-13	-8	3	0.9	0.9	0.9	1.0
Atg2b	Q80XK6	1748	-12	-13	-8	-21	0.9	0.9	0.9	0.8
Slc38a7	Q8BWH0	30	-15	-13	-8	-29	0.9	0.9	0.9	0.8
Mif	P34884	81	-23	-14	-8	7	0.8	0.9	0.9	1.1
Hnrnpa3	Q8BG05	64	-9	-14	-8	7	0.9	0.9	0.9	1.1
Cdca7l	Q922M5	344	-25	-14	-8	4	0.8	0.9	0.9	1.0
Guk1	Q64520	116	-2	-14	-8	-5	1.0	0.9	0.9	1.0
Mccc1	Q99MR8	591	-15	-14	-8	-5	0.9	0.9	0.9	1.0
Ncaph2	Q8BSP2	168	-14	-14	-8	-7	0.9	0.9	0.9	0.9
Scrn2	Q8VCA8	277	-4	-14	-8	37	1.0	0.9	0.9	1.6
Helz2	E9QAM5	1362	-16	-14	-8	31	0.9	0.9	0.9	1.4
Psmc5	P62196	112	-20	-15	-8	4	0.8	0.9	0.9	1.0
Hnrnph2	P70333	290	-11	-15	-8	-6	0.9	0.9	0.9	0.9
Gmps	Q3THK7	213	-18	-15	-8	-6	0.9	0.9	0.9	0.9
Vps39	Q8R5L3	681	-6	-15	-8	-12	0.9	0.9	0.9	0.9
Qrich1	G3X8R5	714	-15	-15	-8	12	0.9	0.9	0.9	1.1
Hps6	Q8BLY7	294	-16	-15	-8	-7	0.9	0.9	0.9	0.9
Ccar2	Q8VDP4	514	-12	-16	-8	16	0.9	0.9	0.9	1.2
Mndal	D0QMC3	476	7	-16	-8	11	1.1	0.9	0.9	1.1
Mnda	P0DOV1	390	7	-16	-8	11	1.1	0.9	0.9	1.1
Ifi204	P0DOV2	390	7	-16	-8	11	1.1	0.9	0.9	1.1
Ifi205a	Q8CGE8	369	7	-16	-8	11	1.1	0.9	0.9	1.1
Nup160	Q9Z0W3	495	-10	-16	-8	6	0.9	0.9	0.9	1.1
Gdi2	Q61598	302	-11	-16	-8	-1	0.9	0.9	0.9	1.0
Aldh18a1	Q9Z110	606	-5	-16	-8	2	1.0	0.9	0.9	1.0
Sirt4	Q8R216	216	-7	-16	-8	1	0.9	0.9	0.9	1.0
Zc3h4	E9Q8K8	1050	-4	-16	-8	-21	1.0	0.9	0.9	0.8
Esd	Q9R0P3	11	-17	-17	-8	10	0.9	0.9	0.9	1.1

Rnf113a1	Q8R3P8	280	-17	-17	-8	-6	0.9	0.9	0.9	0.9
Ifit3	Q64345	383	-4	-17	-8	-13	1.0	0.9	0.9	0.9
Mki67	E9PVX6	1688	-8	-18	-8	-2	0.9	0.9	0.9	1.0
Tbc1d2b	Q3U0J8	38	-24	-18	-8	-4	0.8	0.8	0.9	1.0
Srsf11	E9Q6E5	483	-19	-18	-8	-13	0.8	0.8	0.9	0.9
Rabep1	O35551	533	3	-19	-8	-12	1.0	0.8	0.9	0.9
Larp1	Q6ZQ58	211	-12	-19	-8	-22	0.9	0.8	0.9	0.8
Bcl11b	Q99PV8	87	-3	-19	-8	11	1.0	0.8	0.9	1.1
Znf865	Q3U3I9	796	-14	-19	-8	0	0.9	0.8	0.9	1.0
Snrpn	P63163	19	-17	-19	-8	-14	0.9	0.8	0.9	0.9
Fam134a	Q6NS82	417	-12	-19	-8	-17	0.9	0.8	0.9	0.9
Phb	P67778	69	-14	-20	-8	-3	0.9	0.8	0.9	1.0
Tigd2	Q0VBL1	102	-10	-20	-8	-27	0.9	0.8	0.9	0.8
Psph	Q99LS3	38	-7	-20	-8	1	0.9	0.8	0.9	1.0
Ctcf	Q61164	504	-8	-21	-8	17	0.9	0.8	0.9	1.2
Gtpbp1	O08582	313	-19	-21	-8	3	0.8	0.8	0.9	1.0
Smc1a	Q9CU62	1115	-13	-21	-8	-15	0.9	0.8	0.9	0.9
Utp23	Q9CX11	35	-8	-22	-8	14	0.9	0.8	0.9	1.2
Brpf1	A0A0N4SUT9	332	-9	-22	-8	6	0.9	0.8	0.9	1.1
Rab3d	P35276	137	-13	-22	-8	-12	0.9	0.8	0.9	0.9
Haus6	Q6NV99	288	-12	-22	-8	-17	0.9	0.8	0.9	0.9
Smarca5	Q91ZW3	975	-19	-23	-8	-4	0.8	0.8	0.9	1.0
Nploc4	P60670	403	2	-23	-8	-12	1.0	0.8	0.9	0.9
Dnmt1	P13864	1194	-11	-24	-8	17	0.9	0.8	0.9	1.2
Crlf3	Q9Z2L7	313	-2	-26	-8	-17	1.0	0.8	0.9	0.9
Pcnt	F8VPV0	2364	-14	-28	-8	-3	0.9	0.8	0.9	1.0
Baz1b	Q9Z277	1045	8	-28	-8	-15	1.1	0.8	0.9	0.9
Fkbp5	Q64378	183	-10	-29	-8	-14	0.9	0.8	0.9	0.9
Myl12a	Q6ZWQ9	109	-25	-30	-8	9	0.8	0.8	0.9	1.1
Cwf19l1	Q8CI33	510	9	-32	-8	0	1.1	0.8	0.9	1.0
Slbp	P97440	72	11	-45	-8	-19	1.1	0.7	0.9	0.8
Pkm	P52480	358	-5	18	-8	5	1.0	1.2	0.9	1.1
Usp24	B1AY13	169	4	14	-8	35	1.0	1.2	0.9	1.5
Park7	Q99LX0	121	-10	12	-8	11	0.9	1.1	0.9	1.1
Scml4	Q80VG1	268	-1	12	-8	-7	1.0	1.1	0.9	0.9
Tbc1d2	B1AVH7	35	12	9	-8	-5	1.1	1.1	0.9	1.0
Pgp	Q8CHP8	217	0	7	-8	10	1.0	1.1	0.9	1.1
Marc2	Q922Q1	271	-7	7	-8	-3	0.9	1.1	0.9	1.0
Bop1	P97452	94	6	6	-8	-15	1.1	1.1	0.9	0.9
Tln1	P26039	29	-8	5	-8	18	0.9	1.1	0.9	1.2
Zyx	Q62523	143	7	4	-8	20	1.1	1.0	0.9	1.2
Dr1	Q91WV0	42	10	4	-8	35	1.1	1.0	0.9	1.5
Mtif2	Q91YJ5	646	10	4	-8	7	1.1	1.0	0.9	1.1
Klhl40	Q9D783	145	-9	3	-8	15	0.9	1.0	0.9	1.2
Actr2	P61161	11	-4	3	-8	11	1.0	1.0	0.9	1.1
Usp34	Q6ZQ93	767	10	3	-8	-2	1.1	1.0	0.9	1.0
Rassf4	Q8CB96	237	-7	3	-8	-2	0.9	1.0	0.9	1.0
Srr	Q9QZX7	217	-19	3	-8	-12	0.8	1.0	0.9	0.9
Uba1	Q02053	719	1	2	-8	5	1.0	1.0	0.9	1.0
Cfl1	P18760	147	-5	2	-8	-5	1.0	1.0	0.9	1.0
Eif2b5	Q8CHW4	567	3	2	-8	-3	1.0	1.0	0.9	1.0

Ssbp4	Q3U4B1	9	2	1	-8	2	1.0	1.0	0.9	1.0
Ago2	Q8CJG0	273	3	1	-8	-3	1.0	1.0	0.9	1.0
Vwa8	Q8CC88	415	-6	1	-8	-4	0.9	1.0	0.9	1.0
Rnmt	Q9D0L8	345	-6	1	-8	15	0.9	1.0	0.9	1.2
Slc25a5	P51881	160	-12	1	-8	12	0.9	1.0	0.9	1.1
Crtc2	Q3U182	514	-9	1	-8	9	0.9	1.0	0.9	1.1
Tigar	Q8BZA9	114	-6	0	-8	5	0.9	1.0	0.9	1.0
Ifi205a	Q8CGE8	16	-8	0	-8	-12	0.9	1.0	0.9	0.9
Psmd5	Q8BJY1	412	-17	-1	-8	19	0.9	1.0	0.9	1.2
Mcm3	P25206	119	-1	-1	-8	13	1.0	1.0	0.9	1.1
Pus7l	Q8CE46	639	-3	-1	-8	7	1.0	1.0	0.9	1.1
Sympk	Q80X82	578	0	-1	-8	4	1.0	1.0	0.9	1.0
Eif2d	Q61211	446	-12	-1	-8	-1	0.9	1.0	0.9	1.0
Atp13a1	Q9EPE9	645	-5	-1	-8	-3	1.0	1.0	0.9	1.0
Bag4	Q8CI61	443	-6	-1	-8	-14	0.9	1.0	0.9	0.9
Rasgrp2	Q9QUG9	398	-11	-2	-8	6	0.9	1.0	0.9	1.1
Dcaf8	Q8N7N5	266	-8	-2	-8	-9	0.9	1.0	0.9	0.9
Saraf	Q8R3Q0	313	-7	-2	-8	-9	0.9	1.0	0.9	0.9
Rps6ka5	Q8C050	213	-3	-2	-8	30	1.0	1.0	0.9	1.4
Kiaa0196	Q8C2E7	969	-22	-2	-8	16	0.8	1.0	0.9	1.2
Ifi47	Q61635	115	-4	-2	-8	6	1.0	1.0	0.9	1.1
Dnajb4	Q9D832	175	-10	-2	-8	1	0.9	1.0	0.9	1.0
Bola3	Q8CEI1	62	-5	-2	-8	-1	1.0	1.0	0.9	1.0
Kiaa0907	Q3TCX3	282	-12	-2	-8	-9	0.9	1.0	0.9	0.9
Eif2b4	Q61749	466	-6	-2	-8	-17	0.9	1.0	0.9	0.9
Trrap	A0A1D5RLL4	1869	-1	-3	-8	23	1.0	1.0	0.9	1.3
Pcgf2	P23798	233	-10	-3	-8	16	0.9	1.0	0.9	1.2
Aldh6a1	Q9EQ20	368	-11	-3	-8	12	0.9	1.0	0.9	1.1
Paics	Q9DCL9	295	-8	-3	-8	12	0.9	1.0	0.9	1.1
Eif6	O55135	11	-6	-3	-8	6	0.9	1.0	0.9	1.1
Thada	A8C756	1922	-8	-3	-8	-16	0.9	1.0	0.9	0.9
Atxn2	E9QM77	863	-11	-3	-8	-23	0.9	1.0	0.9	0.8
Gimap7	Q8R379	47	-8	-3	-8	-3	0.9	1.0	0.9	1.0
Swap70	Q6A028	260	-10	-3	-8	-9	0.9	1.0	0.9	0.9
Atad1	Q9D5T0	310	-1	-3	-8	-12	1.0	1.0	0.9	0.9
Madd	Q80U28	1485	-1	-4	-8	22	1.0	1.0	0.9	1.3
Eif5	P59325	102	-16	-4	-8	7	0.9	1.0	0.9	1.1
Rdh13	Q8CEE7	30	-2	-4	-8	3	1.0	1.0	0.9	1.0
Idh1	O88844	245	-7	-4	-8	0	0.9	1.0	0.9	1.0
Mthfd1	Q922D8	147	-9	-4	-8	-2	0.9	1.0	0.9	1.0
Eif3m	Q99JX4	175	-6	-4	-8	-7	0.9	1.0	0.9	0.9
Crebbp	F8VPR5	1213	-3	-4	-8	-9	1.0	1.0	0.9	0.9
Gcn1	E9PVA8	1362	-20	-4	-8	-23	0.8	1.0	0.9	0.8
Hsd17b12	O70503	166	-7	-4	-8	15	0.9	1.0	0.9	1.2
Rabgap1	A2AWA9	150	-11	-4	-8	7	0.9	1.0	0.9	1.1
Pdia3	P27773	406	-3	-4	-8	7	1.0	1.0	0.9	1.1
Pitrm1	Q8K411	119	-7	-4	-8	6	0.9	1.0	0.9	1.1
Sh3gl1	Q62419	277	-10	-4	-8	-6	0.9	1.0	0.9	0.9
Cog4	Q8R1U1	494	-6	-4	-8	-7	0.9	1.0	0.9	0.9
Vamp5	Q9Z2P8	9	2	-4	-8	-11	1.0	1.0	0.9	0.9
Heatr5b	Q8C547	1895	-5	-4	-8	-13	1.0	1.0	0.9	0.9

Prc1	Q99K43	14	-13	-5	-8	17	0.9	1.0	0.9	1.2
FAM120A	Q6A0A9	53	-4	-5	-8	11	1.0	1.0	0.9	1.1
Pigq	Q9QYT7	67	-9	-5	-8	11	0.9	1.0	0.9	1.1
Sec23ip	G3X928	812	-13	-5	-8	9	0.9	1.0	0.9	1.1
Pdcd6ip	Q9WU78	76	-14	-5	-8	3	0.9	1.0	0.9	1.0
Ncor1	Q5RIM6	1286	-4	-5	-8	-7	1.0	1.0	0.9	0.9
Nup50	Q9JIH2	164	-10	-5	-8	-8	0.9	1.0	0.9	0.9
Wipi2	Q80W47	310	-8	-5	-8	-20	0.9	1.0	0.9	0.8
Eif5b	Q05D44	631	-3	-5	-8	1	1.0	1.0	0.9	1.0
Pdk3	Q922H2	41	-5	-5	-8	1	1.0	1.0	0.9	1.0
Smap2	Q7TN29	31	-4	-5	-8	-1	1.0	1.0	0.9	1.0
Wdr4	Q9EP82	311	-9	-5	-8	-1	0.9	1.0	0.9	1.0
Gtf3c5	Q8R2T8	75	-17	-6	-8	7	0.9	0.9	0.9	1.1
Anapc5	Q8BTZ4	297	-10	-6	-8	4	0.9	0.9	0.9	1.0
Dync1h1	Q9JHU4	1975	-8	-6	-8	1	0.9	0.9	0.9	1.0
Bloc1s3	Q5U5M8	161	-14	-6	-8	-1	0.9	0.9	0.9	1.0
Gdi2	Q61598	203	-5	-6	-8	-1	1.0	0.9	0.9	1.0
Usp5	P56399	195	-7	-6	-8	-4	0.9	0.9	0.9	1.0
Nmnat3	Q99JR6	133	-9	-6	-8	-10	0.9	0.9	0.9	0.9
Ints3	Q7TPD0	79	-19	-6	-8	-12	0.8	0.9	0.9	0.9
Thg1l	Q9CY52	279	-21	-6	-8	-14	0.8	0.9	0.9	0.9
Cic	Q924A2	1243	-6	-6	-8	-15	0.9	0.9	0.9	0.9
Rab43	Q8CG50	155	-24	-6	-8	-16	0.8	0.9	0.9	0.9
Sf3b1	G5E866	1035	-14	-6	-8	17	0.9	0.9	0.9	1.2
Usp4	P35123	714	-6	-6	-8	5	0.9	0.9	0.9	1.0
Mcm3	P25206	360	-14	-6	-8	-1	0.9	0.9	0.9	1.0
Metap1	Q8BP48	40	-7	-6	-8	-3	0.9	0.9	0.9	1.0
Rapgef1	Q3UHC1	107	-4	-6	-8	-4	1.0	0.9	0.9	1.0
Araf	P04627	58	-7	-6	-8	-6	0.9	0.9	0.9	0.9
Sec16a	A2AIX1	1560	-14	-7	-8	11	0.9	0.9	0.9	1.1
Ppm1g	Q61074	348	-15	-7	-8	8	0.9	0.9	0.9	1.1
Dgkz	Q80UP3	605	-14	-7	-8	5	0.9	0.9	0.9	1.1
Rad21	Q61550	78	-10	-7	-8	5	0.9	0.9	0.9	1.0
Pde2a	F7D3W5	118	-13	-7	-8	1	0.9	0.9	0.9	1.0
Tmpo	Q61033	328	-6	-7	-8	-7	0.9	0.9	0.9	0.9
Acly	Q91V92	20	-9	-7	-8	-8	0.9	0.9	0.9	0.9
Setx	A2AKX3	138	-10	-7	-8	-20	0.9	0.9	0.9	0.8
Rrm1	P07742	238	2	-7	-8	10	1.0	0.9	0.9	1.1
Flna	Q8BTM8	2293	-14	-7	-8	7	0.9	0.9	0.9	1.1
Aimp1	P31230	90	-12	-7	-8	-5	0.9	0.9	0.9	1.0
Apeh	Q8R146	312	-15	-7	-8	-9	0.9	0.9	0.9	0.9
Iqgap2	Q3UQ44	246	-5	-7	-8	-10	1.0	0.9	0.9	0.9
Eif4g3	Q80XI3	1473	-7	-7	-8	-14	0.9	0.9	0.9	0.9
Rif1	Q6PR54	312	-6	-8	-8	28	0.9	0.9	0.9	1.4
Hnrnpd	Q60668	226	-1	-8	-8	14	1.0	0.9	0.9	1.2
Eif5	P59325	59	-11	-8	-8	6	0.9	0.9	0.9	1.1
Arap3	Q8R5G7	1018	-10	-8	-8	5	0.9	0.9	0.9	1.0
Surf2	P09926	127	-6	-8	-8	-4	0.9	0.9	0.9	1.0
Tmc8	Q7TN58	680	-2	-8	-8	-6	1.0	0.9	0.9	0.9
Casp4	P70343	254	-3	-8	-8	-15	1.0	0.9	0.9	0.9
Coro7	Q9D2V7	34	-7	-8	-8	6	0.9	0.9	0.9	1.1

Stip1	Q60864	26	-5	-8	-8	4	1.0	0.9	0.9	1.0
Pan3	Q640Q5	452	-8	-8	-8	1	0.9	0.9	0.9	1.0
Fbxo22	Q78JE5	132	-2	-8	-8	-6	1.0	0.9	0.9	0.9
Ptbp1	Q922I7	249	-13	-8	-8	-14	0.9	0.9	0.9	0.9
Ripk2	P58801	413	-3	-8	-8	-27	1.0	0.9	0.9	0.8
Mcrip1	Q3UGS4	61	-14	-9	-8	10	0.9	0.9	0.9	1.1
Inpp4a	Q9EPW0	816	-12	-9	-8	7	0.9	0.9	0.9	1.1
Ptpn1	P35821	121	-8	-9	-8	5	0.9	0.9	0.9	1.1
Riok3	Q9DBU3	171	-2	-9	-8	3	1.0	0.9	0.9	1.0
Rock1	P70335	1300	-10	-9	-8	-2	0.9	0.9	0.9	1.0
Tes	Q921W7	236	-9	-9	-8	-4	0.9	0.9	0.9	1.0
Sept2	P42208	111	-10	-9	-8	-10	0.9	0.9	0.9	0.9
Prkdc	P97313	1505	-9	-9	-8	50	0.9	0.9	0.9	2.0
Ptges2	Q8BWM0	109	-13	-9	-8	31	0.9	0.9	0.9	1.4
Actn4	P57780	352	-12	-9	-8	9	0.9	0.9	0.9	1.1
Actn1	Q7TPR4	332	-12	-9	-8	9	0.9	0.9	0.9	1.1
Casp1	P29452	135	-20	-9	-8	5	0.8	0.9	0.9	1.1
Paxx	Q8K0Y7	181	-5	-9	-8	2	1.0	0.9	0.9	1.0
Capn1	O35350	209	2	-9	-8	-1	1.0	0.9	0.9	1.0
Aco1	P28271	165	-9	-9	-8	-3	0.9	0.9	0.9	1.0
Smc5	Q8CG46	393	-10	-9	-8	-6	0.9	0.9	0.9	0.9
Hcfc1	Q61191	1882	-12	-9	-8	-12	0.9	0.9	0.9	0.9
Zfp41	Q02526	89	-3	-10	-8	-1	1.0	0.9	0.9	1.0
Dcun1d2	Q8BZJ7	115	-12	-10	-8	-3	0.9	0.9	0.9	1.0
Rnpep	Q8VCT3	181	-14	-10	-8	-3	0.9	0.9	0.9	1.0
Nusap1	Q9ERH4	174	-12	-10	-8	-9	0.9	0.9	0.9	0.9
Cfl1	P18760	39	-6	-10	-8	-11	0.9	0.9	0.9	0.9
Zbtb1	Q91VL9	448	-5	-10	-8	-11	1.0	0.9	0.9	0.9
Setd1a	E9PYH6	1523	-2	-10	-8	-15	1.0	0.9	0.9	0.9
Dut	Q8VCG1	114	-11	-10	-8	4	0.9	0.9	0.9	1.0
Thoc1	Q8R3N6	49	-6	-10	-8	-3	0.9	0.9	0.9	1.0
Ints5	Q8CHT3	797	-8	-10	-8	-4	0.9	0.9	0.9	1.0
Capzb	P47757	62	-17	-11	-8	5	0.9	0.9	0.9	1.1
Eif2s3y	Q9Z0N2	105	-8	-11	-8	2	0.9	0.9	0.9	1.0
Synj1	D3Z656	839	-2	-11	-8	-4	1.0	0.9	0.9	1.0
Eps15l1	Q60902	533	-9	-11	-8	-5	0.9	0.9	0.9	1.0
Ndufaf7	Q9CWG8	250	-9	-11	-8	-17	0.9	0.9	0.9	0.9
Bod1l	E9Q6J5	605	-10	-11	-8	-19	0.9	0.9	0.9	0.8
Ddx56	Q9D0R4	185	-6	-11	-8	-19	0.9	0.9	0.9	0.8
Sik3	E9PU87	1226	-15	-11	-8	-21	0.9	0.9	0.9	0.8
Stk38	Q91VJ4	234	-6	-11	-8	21	0.9	0.9	0.9	1.3
Mbnl2	Q8C181	53	-16	-11	-8	9	0.9	0.9	0.9	1.1
Mbnl1	Q9JKP5	53	-16	-11	-8	9	0.9	0.9	0.9	1.1
Cptp	Q8BS40	165	10	-11	-8	4	1.1	0.9	0.9	1.0
Cct3	P80318	40	-21	-11	-8	3	0.8	0.9	0.9	1.0
Sap30	O88574	184	-14	-11	-8	-3	0.9	0.9	0.9	1.0
Dis3	Q9CSH3	533	-10	-11	-8	-6	0.9	0.9	0.9	0.9
Vcp	Q01853	535	-10	-11	-8	-7	0.9	0.9	0.9	0.9
Utrn	E9Q6R7	859	-11	-11	-8	-11	0.9	0.9	0.9	0.9
Zap70	P43404	117	-7	-12	-8	1	0.9	0.9	0.9	1.0
Tdrd7	Q8K1H1	786	-9	-12	-8	-14	0.9	0.9	0.9	0.9

Sept10	Q8C650	400	-14	-12	-8	-14	0.9	0.9	0.9	0.9
Mrps12	O35680	93	-14	-12	-8	-20	0.9	0.9	0.9	0.8
Fcf1	Q9CTH6	144	-27	-12	-8	0	0.8	0.9	0.9	1.0
Dph6	Q9CQ28	88	-4	-12	-8	-5	1.0	0.9	0.9	1.0
Fgd3	O88842	537	-14	-12	-8	-7	0.9	0.9	0.9	0.9
Vim	P20152	328	-12	-12	-8	-9	0.9	0.9	0.9	0.9
Ercc1	P07903	33	-13	-12	-8	-17	0.9	0.9	0.9	0.9
Mtmr14	Q8VEL2	130	-27	-13	-8	12	0.8	0.9	0.9	1.1
Ralgps2	Q9ERD6	246	-20	-13	-8	8	0.8	0.9	0.9	1.1
Atp6v1c1	Q9Z1G3	15	-10	-13	-8	4	0.9	0.9	0.9	1.0
Dennd3	A2RT67	438	-11	-13	-8	0	0.9	0.9	0.9	1.0
Usp36	B1AQJ2	840	-9	-13	-8	-27	0.9	0.9	0.9	0.8
Birc6	O88738	755	-7	-13	-8	-2	0.9	0.9	0.9	1.0
Aim1	A0A0G2JG52	1748	-13	-13	-8	-3	0.9	0.9	0.9	1.0
Mbd1	Q9Z2E2	212	3	-13	-8	-5	1.0	0.9	0.9	1.0
Nfs1	Q9Z1J3	224	-16	-13	-8	-13	0.9	0.9	0.9	0.9
Ncoa4	Q5U4H9	244	-31	-14	-8	16	0.8	0.9	0.9	1.2
Chd1	P40201	409	-18	-14	-8	13	0.9	0.9	0.9	1.1
Gsn	P13020	670	-19	-14	-8	0	0.8	0.9	0.9	1.0
Stip1	Q60864	339	-6	-14	-8	-5	0.9	0.9	0.9	1.0
Uso1	Q9Z1Z0	802	-8	-14	-8	-10	0.9	0.9	0.9	0.9
Mbd1	Q9Z2E2	226	-14	-14	-8	-12	0.9	0.9	0.9	0.9
Smarcd2	Q99JR8	365	-12	-14	-8	9	0.9	0.9	0.9	1.1
Dennd1c	Q8CFK6	159	-13	-14	-8	1	0.9	0.9	0.9	1.0
Cct2	P80314	289	-24	-14	-8	1	0.8	0.9	0.9	1.0
Wdr82	Q8BFQ4	195	-12	-14	-8	-4	0.9	0.9	0.9	1.0
Nfkb1	P25799	259	-17	-14	-8	-6	0.9	0.9	0.9	0.9
Rsbn1	Q80T69	318	-4	-14	-8	-8	1.0	0.9	0.9	0.9
Nub1	P54729	115	-10	-14	-8	-9	0.9	0.9	0.9	0.9
Cpt1a	P97742	304	-22	-15	-8	26	0.8	0.9	0.9	1.3
Smc2	Q8CG48	585	-18	-15	-8	7	0.8	0.9	0.9	1.1
Pml	Q60953	65	-11	-15	-8	3	0.9	0.9	0.9	1.0
Lta4h	P24527	26	-25	-15	-8	-10	0.8	0.9	0.9	0.9
Herpud1	Q9JJK5	68	-10	-15	-8	8	0.9	0.9	0.9	1.1
Rps3a	P97351	96	-11	-15	-8	-14	0.9	0.9	0.9	0.9
Kiaa0907	Q3TCX3	118	-14	-15	-8	-15	0.9	0.9	0.9	0.9
Ahsa1	Q8BK64	186	-16	-16	-8	8	0.9	0.9	0.9	1.1
Psma3	O70435	42	-5	-16	-8	1	1.0	0.9	0.9	1.0
Rsrc2	A2RTL5	324	-9	-16	-8	-10	0.9	0.9	0.9	0.9
Rcc2	Q8BK67	207	-13	-16	-8	10	0.9	0.9	0.9	1.1
Slc7a6os	Q7TPE5	27	-9	-16	-8	-1	0.9	0.9	0.9	1.0
Ap2b1	Q9DBG3	857	-9	-16	-8	-2	0.9	0.9	0.9	1.0
Isoc2a	P85094	84	-13	-17	-8	13	0.9	0.9	0.9	1.1
Snrnp40	Q6PE01	292	-25	-17	-8	4	0.8	0.9	0.9	1.0
Phip	F8VQ93	1165	-9	-17	-8	9	0.9	0.9	0.9	1.1
Cep192	E9Q4Y4	589	1	-17	-8	3	1.0	0.9	0.9	1.0
Edc4	Q3UJB9	510	-10	-17	-8	3	0.9	0.9	0.9	1.0
Tex11	Q14AT2	757	-9	-17	-8	-4	0.9	0.9	0.9	1.0
Cep170	Q6A065	625	-15	-17	-8	-12	0.9	0.9	0.9	0.9
Cltc	Q68FD5	909	-27	-18	-8	8	0.8	0.9	0.9	1.1
Rack1	P68040	249	-23	-18	-8	-6	0.8	0.9	0.9	0.9

Dcaf8	Q8N7N5	371	-8	-18	-8	-7	0.9	0.9	0.9	0.9
Hcfc1	Q61191	149	-10	-18	-8	8	0.9	0.8	0.9	1.1
Sun1	Q9D666	759	-13	-18	-8	-1	0.9	0.8	0.9	1.0
Zbtb33	Q8BN78	429	-23	-18	-8	-17	0.8	0.8	0.9	0.9
Pias1	O88907	335	-21	-19	-8	5	0.8	0.8	0.9	1.0
Csrp1	P97315	122	-8	-19	-8	1	0.9	0.8	0.9	1.0
Ppig	A2AR02	10	-21	-19	-8	-19	0.8	0.8	0.9	0.8
Chdh	Q8BJ64	244	-13	-20	-8	21	0.9	0.8	0.9	1.3
Znf638	Q61464	1929	-17	-20	-8	-2	0.9	0.8	0.9	1.0
Mcm6	P97311	185	-20	-20	-8	-3	0.8	0.8	0.9	1.0
Wash1	Q8VDD8	8	-11	-20	-8	-9	0.9	0.8	0.9	0.9
Got2	P05202	187	-11	-21	-8	2	0.9	0.8	0.9	1.0
Rasal3	Q8C2K5	1031	-12	-22	-8	-25	0.9	0.8	0.9	0.8
Trrap	A0A1D5RLL4	1880	-12	-22	-8	-1	0.9	0.8	0.9	1.0
Rbx1	P62878	94	-20	-23	-8	3	0.8	0.8	0.9	1.0
Lcmt1	A2RTH5	248	-8	-25	-8	-20	0.9	0.8	0.9	0.8
Rpl30	P62889	92	-17	-27	-8	-27	0.9	0.8	0.9	0.8
Pithd1	Q8BWR2	14	-16	-27	-8	-64	0.9	0.8	0.9	0.6
Cdc23	Q8BGZ4	532	16	-28	-8	7	1.2	0.8	0.9	1.1
Klc1	Q8CD76	55	-12	-28	-8	-26	0.9	0.8	0.9	0.8
Gvin1	L7N451	368	-12	-29	-8	-5	0.9	0.8	0.9	1.0
Nop58	Q6DFW4	205	-5	-31	-8	4	1.0	0.8	0.9	1.0
Akt2	Q60823	77	-13	-31	-8	-20	0.9	0.8	0.9	0.8
Lemd3	D3YU56	814	-8	-31	-8	4	0.9	0.8	0.9	1.0
Hmgb2	P30681	106	-11	-32	-8	-15	0.9	0.8	0.9	0.9
Ipcef1	Q5DU31	341	-7	-33	-8	-4	0.9	0.8	0.9	1.0
Pold4	Q9CWP8	61	-20	-34	-8	-9	0.8	0.7	0.9	0.9
Oxsm	Q9D404	372	56	-54	-8	-62	2.2	0.6	0.9	0.6
Blk	P16277	368	0	11	-9	17	1.0	1.1	0.9	1.2
Ctnnd1	P30999	618	0	10	-9	8	1.0	1.1	0.9	1.1
Fry	E9Q8I9	2726	0	9	-9	14	1.0	1.1	0.9	1.2
Rps27a	P62983	126	-10	7	-9	-8	0.9	1.1	0.9	0.9
Eml4	Q3UMY5	82	-2	7	-9	-3	1.0	1.1	0.9	1.0
Rbfa	Q6P3B9	261	-25	6	-9	-6	0.8	1.1	0.9	0.9
Fv1	P70213	117	-4	6	-9	-19	1.0	1.1	0.9	0.8
Hdac2	P70288	274	-11	5	-9	14	0.9	1.1	0.9	1.2
Hk3	Q3TRM8	18	-20	5	-9	-1	0.8	1.0	0.9	1.0
Lars	Q8BMJ2	783	-8	4	-9	-6	0.9	1.0	0.9	0.9
Sept8	Q8CHH9	400	-2	4	-9	-20	1.0	1.0	0.9	0.8
Pcsk5	Q04592	1416	-6	4	-9	-8	0.9	1.0	0.9	0.9
Zc3h12d	E9QNR7	114	-16	3	-9	18	0.9	1.0	0.9	1.2
Rangap1	P46061	274	-32	3	-9	8	0.8	1.0	0.9	1.1
Ube2o	Q6ZPJ3	370	-6	3	-9	1	0.9	1.0	0.9	1.0
Syne2	Q6ZWQ0	3117	-16	3	-9	-2	0.9	1.0	0.9	1.0
Atp13a1	Q9EPE9	859	-5	3	-9	20	1.0	1.0	0.9	1.3
Lrrk1	Q3UHC2	319	-15	3	-9	-5	0.9	1.0	0.9	1.0
Erap1	Q9EQH2	896	-3	2	-9	17	1.0	1.0	0.9	1.2
Klhl7	Q8BUL5	158	-8	2	-9	6	0.9	1.0	0.9	1.1
Fnbp1	Q80TY0	70	-9	2	-9	44	0.9	1.0	0.9	1.8
Arfgap1	Q9EPJ9	350	1	2	-9	-191	1.0	1.0	0.9	0.3
Hnrnpl	Q8R081	148	1	1	-9	-27	1.0	1.0	0.9	0.8

Tsta3	P23591	116	-5	1	-9	25	1.0	1.0	0.9	1.3
Ivd	Q9JHI5	252	-17	1	-9	5	0.9	1.0	0.9	1.0
Ccdc88c	Q6VGS5	460	-16	1	-9	1	0.9	1.0	0.9	1.0
Dhx57	Q6P5D3	1351	-2	1	-9	-6	1.0	1.0	0.9	0.9
Tardbp	Q921F2	173	3	0	-9	16	1.0	1.0	0.9	1.2
Prpf4b	Q61136	962	-4	0	-9	4	1.0	1.0	0.9	1.0
Rpl10l	P86048	49	-6	0	-9	2	0.9	1.0	0.9	1.0
Rpl10l	P86048	105	-5	-1	-9	-4	1.0	1.0	0.9	1.0
Vps51	Q3UVL4	316	-10	-1	-9	20	0.9	1.0	0.9	1.3
Fam102a	Q78T81	151	9	-1	-9	9	1.1	1.0	0.9	1.1
Prmt1	Q9JIF0	119	-3	-1	-9	0	1.0	1.0	0.9	1.0
Arl3	Q9WUL7	118	-4	-1	-9	-7	1.0	1.0	0.9	0.9
Sars	P26638	300	-13	-1	-9	-17	0.9	1.0	0.9	0.9
Ddx60	E9PZQ1	1050	-9	-2	-9	3	0.9	1.0	0.9	1.0
Rnf40	Q3U319	890	-11	-2	-9	-3	0.9	1.0	0.9	1.0
Lck	P06240	378	-7	-2	-9	-5	0.9	1.0	0.9	1.0
Stub1	Q9WUD1	200	-7	-2	-9	-6	0.9	1.0	0.9	0.9
Kifc5b	E9PUA5	143	-11	-2	-9	-12	0.9	1.0	0.9	0.9
Kifc1	Q9QWT9	145	-11	-2	-9	-12	0.9	1.0	0.9	0.9
Elmod2	Q8BGF6	285	-12	-2	-9	-31	0.9	1.0	0.9	0.8
Zc3h3	Q8CHP0	298	-13	-2	-9	-3	0.9	1.0	0.9	1.0
Trip12	G5E870	1992	-9	-2	-9	-7	0.9	1.0	0.9	0.9
Eif2s3y	Q9Z0N2	96	-11	-2	-9	-11	0.9	1.0	0.9	0.9
Rsbn1	Q80T69	206	1	-2	-9	-16	1.0	1.0	0.9	0.9
Wdr1	O88342	225	-8	-3	-9	10	0.9	1.0	0.9	1.1
Alox15	P39654	459	-10	-3	-9	9	0.9	1.0	0.9	1.1
Tnpo3	Q6P2B1	908	-3	-3	-9	3	1.0	1.0	0.9	1.0
Slc27a1	Q60714	80	3	-3	-9	-2	1.0	1.0	0.9	1.0
Pygb	Q8CI94	437	-5	-3	-9	-3	1.0	1.0	0.9	1.0
Ppp1r18	Q8BQ30	592	-4	-3	-9	-10	1.0	1.0	0.9	0.9
Slk	O54988	614	-11	-3	-9	-11	0.9	1.0	0.9	0.9
Tsc22d2	E9Q7M2	189	4	-3	-9	-13	1.0	1.0	0.9	0.9
Gfpt1	P47856	55	-7	-3	-9	-15	0.9	1.0	0.9	0.9
Rhoh	Q9D3G9	165	-11	-3	-9	-17	0.9	1.0	0.9	0.9
Rfc1	G3UWX1	321	-1	-3	-9	-19	1.0	1.0	0.9	0.8
Dars	Q922B2	130	-13	-3	-9	6	0.9	1.0	0.9	1.1
Gnai3	Q9DC51	325	-10	-3	-9	0	0.9	1.0	0.9	1.0
Grap	Q9CX99	161	-8	-3	-9	-13	0.9	1.0	0.9	0.9
Csde1	Q91W50	680	-7	-3	-9	-19	0.9	1.0	0.9	0.8
Pik3ca	P42337	242	-5	-4	-9	19	1.0	1.0	0.9	1.2
Nfkbia	Q9Z1E3	135	-20	-4	-9	18	0.8	1.0	0.9	1.2
Syk	P48025	258	-14	-4	-9	10	0.9	1.0	0.9	1.1
Znf512	Q69Z99	415	-13	-4	-9	6	0.9	1.0	0.9	1.1
Ranbp1	P34022	132	-5	-4	-9	-1	1.0	1.0	0.9	1.0
Dhx57	Q6P5D3	1371	-5	-4	-9	-1	1.0	1.0	0.9	1.0
Wdr43	Q6ZQL4	379	-4	-4	-9	-2	1.0	1.0	0.9	1.0
Eif5a2	Q8BGY2	73	-15	-4	-9	-6	0.9	1.0	0.9	0.9
Kpnb1	P70168	543	-5	-4	-9	-8	1.0	1.0	0.9	0.9
Ankrd44	B2RXR6	967	-6	-4	-9	-10	0.9	1.0	0.9	0.9
Nfx1	B1AY10	289	-9	-4	-9	-16	0.9	1.0	0.9	0.9
Nasp	Q99MD9	694	-10	-4	-9	-20	0.9	1.0	0.9	0.8

Ogt	Q8CGY8	620	-8	-4	-9	20	0.9	1.0	0.9	1.3
Coro1a	O89053	195	-12	-4	-9	16	0.9	1.0	0.9	1.2
Rttn	Q8R4Y8	1724	-4	-4	-9	5	1.0	1.0	0.9	1.0
Cul3	Q9JLV5	636	-6	-4	-9	4	0.9	1.0	0.9	1.0
Parp14	Q2EMV9	717	-1	-4	-9	-1	1.0	1.0	0.9	1.0
Sfr1	Q8BP27	199	-1	-4	-9	-1	1.0	1.0	0.9	1.0
Ciz1	Q8VEH2	796	-21	-4	-9	-5	0.8	1.0	0.9	1.0
Rab21	P35282	27	-7	-4	-9	-6	0.9	1.0	0.9	0.9
Cybb	Q61093	428	6	-4	-9	-13	1.1	1.0	0.9	0.9
Dnajc2	P54103	548	-7	-4	-9	-14	0.9	1.0	0.9	0.9
Malt1	Q2TBA3	738	-2	-4	-9	-17	1.0	1.0	0.9	0.9
Dnaja2	Q9QYJ0	280	-18	-5	-9	17	0.9	1.0	0.9	1.2
Anp32b	Q9EST5	27	-2	-5	-9	3	1.0	1.0	0.9	1.0
Med14	A2ABV5	641	9	-5	-9	1	1.1	1.0	0.9	1.0
Stk4	Q9JI11	80	0	-5	-9	-3	1.0	1.0	0.9	1.0
Marc2	Q922Q1	74	-10	-5	-9	-8	0.9	1.0	0.9	0.9
Zfp869	Q9DC47	123	-12	-5	-9	-9	0.9	1.0	0.9	0.9
Adsl	P54822	27	-4	-5	-9	-10	1.0	1.0	0.9	0.9
Hnrnpll	Q921F4	284	-18	-5	-9	32	0.9	1.0	0.9	1.5
Znf771	Q8BJ90	189	-7	-5	-9	6	0.9	1.0	0.9	1.1
Trmt2a	Q8BNV1	453	-6	-5	-9	3	0.9	1.0	0.9	1.0
Wdr48	Q8BH57	342	3	-5	-9	0	1.0	1.0	0.9	1.0
Abi3	Q8BYZ1	148	-5	-5	-9	-6	1.0	1.0	0.9	0.9
Prkcd	P28867	127	-4	-5	-9	-8	1.0	1.0	0.9	0.9
Ctc1	Q5SUQ9	903	-12	-5	-9	-10	0.9	1.0	0.9	0.9
Sp140	Q6NSQ5	481	10	-5	-9	-22	1.1	1.0	0.9	0.8
Lrrk2	Q5S006	2025	-5	-6	-9	50	1.0	0.9	0.9	2.0
Gpx4	O70325	55	-9	-6	-9	0	0.9	0.9	0.9	1.0
Pabpc4	Q6PHQ9	132	-9	-6	-9	-5	0.9	0.9	0.9	1.0
Pabpc6	Q9D4E6	132	-9	-6	-9	-5	0.9	0.9	0.9	1.0
Elmo1	Q8BPU7	726	-13	-6	-9	-10	0.9	0.9	0.9	0.9
Gm3839	S4R1W1	245	-4	-6	-9	-15	1.0	0.9	0.9	0.9
Klhl9	Q6ZPT1	559	-10	-6	-9	23	0.9	0.9	0.9	1.3
Nol6	Q8R5K4	1040	-10	-6	-9	23	0.9	0.9	0.9	1.3
Ivd	Q9JHI5	348	6	-6	-9	11	1.1	0.9	0.9	1.1
Anp32a	O35381	123	-9	-6	-9	10	0.9	0.9	0.9	1.1
Mybbp1a	Q7TPV4	477	15	-6	-9	6	1.2	0.9	0.9	1.1
Samhd1	Q60710	52	-3	-6	-9	5	1.0	0.9	0.9	1.1
Trim33	Q99PP7	463	9	-6	-9	-2	1.1	0.9	0.9	1.0
Ddx24	Q9ESV0	374	-9	-6	-9	-4	0.9	0.9	0.9	1.0
Prrc2c	Q3TLH4	482	-8	-6	-9	-6	0.9	0.9	0.9	0.9
Ube2r2	Q6ZWZ2	191	-4	-6	-9	-6	1.0	0.9	0.9	0.9
Gapdhs	Q64467	351	-4	-6	-9	-15	1.0	0.9	0.9	0.9
Sbf1	Q6ZPE2	1550	-7	-7	-9	11	0.9	0.9	0.9	1.1
Tbc1d13	Q8R3D1	36	-10	-7	-9	3	0.9	0.9	0.9	1.0
Dgkd	E9PUQ8	121	-19	-7	-9	-2	0.8	0.9	0.9	1.0
Pibf1	E9Q6K3	199	-7	-7	-9	-5	0.9	0.9	0.9	1.0
Hspd1	P63038	442	-11	-7	-9	-10	0.9	0.9	0.9	0.9
Rb1	P13405	846	-13	-7	-9	-14	0.9	0.9	0.9	0.9
Txn2	P97493	90	-11	-7	-9	32	0.9	0.9	0.9	1.5
Kbtbd11	Q8BNW9	301	-7	-7	-9	7	0.9	0.9	0.9	1.1

Celf1	P28659	61	-25	-7	-9	6	0.8	0.9	0.9	1.1
Trim14	Q8BVW3	235	-4	-7	-9	6	1.0	0.9	0.9	1.1
Celf2	Q9Z0H4	85	-25	-7	-9	6	0.8	0.9	0.9	1.1
Mtap	Q9CQ65	55	-1	-7	-9	2	1.0	0.9	0.9	1.0
Pola1	P33609	1407	-7	-7	-9	-2	0.9	0.9	0.9	1.0
Rpl9	P51410	74	-15	-7	-9	-2	0.9	0.9	0.9	1.0
Atrx	Q61687	1516	-9	-7	-9	-5	0.9	0.9	0.9	1.0
Tulp3	O88413	325	-17	-7	-9	-8	0.9	0.9	0.9	0.9
Ptpn2	Q06180	301	-5	-7	-9	-27	1.0	0.9	0.9	0.8
Smarcd1	Q61466	492	-18	-8	-9	13	0.8	0.9	0.9	1.1
Gnl3	Q8CI11	275	-13	-8	-9	13	0.9	0.9	0.9	1.1
Gtf3c1	Q8K284	2033	-7	-8	-9	6	0.9	0.9	0.9	1.1
Aftph	Q80WT5	725	-8	-8	-9	4	0.9	0.9	0.9	1.0
Aatk	B1AZF3	287	-4	-8	-9	3	1.0	0.9	0.9	1.0
Actr1a	P61164	34	-10	-8	-9	-4	0.9	0.9	0.9	1.0
Actr1b	Q8R5C5	34	-10	-8	-9	-4	0.9	0.9	0.9	1.0
Numb	Q9QZS3	176	-8	-8	-9	-24	0.9	0.9	0.9	0.8
Ap1g2	O88512	180	-12	-8	-9	14	0.9	0.9	0.9	1.2
Fermt3	Q8K1B8	235	-2	-8	-9	3	1.0	0.9	0.9	1.0
Ets1	P27577	99	-11	-8	-9	-2	0.9	0.9	0.9	1.0
Epb41l2	O70318	417	-15	-8	-9	-6	0.9	0.9	0.9	0.9
Ppp2r1a	Q76MZ3	294	-8	-8	-9	-8	0.9	0.9	0.9	0.9
Tln1	P26039	2161	-10	-8	-9	-9	0.9	0.9	0.9	0.9
Fam120c	Q8C3F2	14	13	-8	-9	-9	1.1	0.9	0.9	0.9
Zdhhc13	Q9CWU2	51	-6	-8	-9	-12	0.9	0.9	0.9	0.9
Atad2b	E9Q166	55	-8	-8	-9	-14	0.9	0.9	0.9	0.9
Myo1f	Q8CG29	445	-13	-9	-9	2	0.9	0.9	0.9	1.0
Cse1l	Q9ERK4	85	-18	-9	-9	1	0.8	0.9	0.9	1.0
Psmd4	O35226	37	-16	-9	-9	-1	0.9	0.9	0.9	1.0
Cnp	P16330	157	-13	-9	-9	-3	0.9	0.9	0.9	1.0
Gpx4	O70325	134	-15	-9	-9	-5	0.9	0.9	0.9	1.0
Gart	Q64737	41	-13	-9	-9	-7	0.9	0.9	0.9	0.9
Tecr	Q9CY27	18	-14	-9	-9	-30	0.9	0.9	0.9	0.8
Nelfcd	Q922L6	399	-6	-9	-9	11	0.9	0.9	0.9	1.1
Elk3	P41971	112	-4	-9	-9	-8	1.0	0.9	0.9	0.9
Htt	G3X9H5	645	-8	-9	-9	-10	0.9	0.9	0.9	0.9
Cmpk2	Q3U5Q7	85	-13	-9	-9	-10	0.9	0.9	0.9	0.9
Bcas3	Q8CCN5	139	-8	-9	-9	-14	0.9	0.9	0.9	0.9
Rras2	P62071	55	-14	-9	-9	-35	0.9	0.9	0.9	0.7
Rnaseh2a	Q9CWY8	281	-6	-10	-9	2	0.9	0.9	0.9	1.0
Zfc3h1	B2RT41	245	-10	-10	-9	-2	0.9	0.9	0.9	1.0
Eprs	Q8CGC7	1377	-9	-10	-9	-6	0.9	0.9	0.9	0.9
Lrch1	P62046	241	-11	-10	-9	-6	0.9	0.9	0.9	0.9
Tbc1d2b	Q3U0J8	538	-15	-10	-9	-10	0.9	0.9	0.9	0.9
Capn1	O35350	351	-14	-10	-9	17	0.9	0.9	0.9	1.2
Nmral1	Q8K2T1	227	-30	-10	-9	4	0.8	0.9	0.9	1.0
Dock2	Q8C3J5	730	-7	-10	-9	-2	0.9	0.9	0.9	1.0
Grap	Q9CX99	149	-9	-10	-9	-10	0.9	0.9	0.9	0.9
Sass6	Q80UK7	304	-18	-10	-9	-13	0.8	0.9	0.9	0.9
Gm10320	E9PW43	56	-10	-10	-9	-18	0.9	0.9	0.9	0.9
Larp4b	Q6A0A2	592	-9	-10	-9	-20	0.9	0.9	0.9	0.8

Prr14l	E9Q7C4	1223	-6	-10	-9	-23	0.9	0.9	0.9	0.8
Sae1	Q9R1T2	218	-14	-11	-9	9	0.9	0.9	0.9	1.1
Rack1	P68040	182	-16	-11	-9	6	0.9	0.9	0.9	1.1
Ncaph	Q8C156	233	-9	-11	-9	5	0.9	0.9	0.9	1.1
Lcn2	P11672	197	-7	-11	-9	1	0.9	0.9	0.9	1.0
Atm	Q62388	2027	-18	-11	-9	-1	0.8	0.9	0.9	1.0
Supv3l1	Q80YD1	230	-21	-11	-9	-4	0.8	0.9	0.9	1.0
Fam160b1	Q8CDM8	573	-13	-11	-9	-16	0.9	0.9	0.9	0.9
Rhoh	Q9D3G9	130	-8	-11	-9	-24	0.9	0.9	0.9	0.8
U2af2	P26369	442	-22	-11	-9	20	0.8	0.9	0.9	1.2
Fem1aa	Q9Z2G1	626	-15	-11	-9	8	0.9	0.9	0.9	1.1
Sept6	Q9R1T4	14	-8	-11	-9	-3	0.9	0.9	0.9	1.0
Cbll1	Q9JIY2	73	-15	-11	-9	-5	0.9	0.9	0.9	1.0
Limk2	O54785	365	-7	-11	-9	-7	0.9	0.9	0.9	0.9
Myh9	Q8VDD5	1379	-10	-11	-9	-7	0.9	0.9	0.9	0.9
Plekha2	Q9ERS5	205	-3	-11	-9	-11	1.0	0.9	0.9	0.9
Sntb2	Q61235	371	-18	-11	-9	-27	0.9	0.9	0.9	0.8
Cd3eap	Q76KJ5	107	-10	-12	-9	26	0.9	0.9	0.9	1.3
Dnajc2	P54103	276	-3	-12	-9	2	1.0	0.9	0.9	1.0
Eif2d	Q61211	197	1	-12	-9	-11	1.0	0.9	0.9	0.9
Cyfip2	Q5SQX6	346	-1	-12	-9	-13	1.0	0.9	0.9	0.9
Sp110	Q8BVK9	240	-5	-12	-9	-16	1.0	0.9	0.9	0.9
Hspa9	P38647	317	-8	-12	-9	1	0.9	0.9	0.9	1.0
Lrpprc	Q6PB66	112	-9	-12	-9	-2	0.9	0.9	0.9	1.0
Gm21992	F7BGR7	108	-8	-12	-9	-13	0.9	0.9	0.9	0.9
Pold3	Q9EQ28	394	-4	-12	-9	-14	1.0	0.9	0.9	0.9
Pelp1	Q9DBD5	595	-10	-13	-9	10	0.9	0.9	0.9	1.1
Idh3g	P70404	236	-11	-13	-9	5	0.9	0.9	0.9	1.0
Dock8	Q8C147	1472	-21	-13	-9	2	0.8	0.9	0.9	1.0
Gart	Q64737	237	-20	-13	-9	-5	0.8	0.9	0.9	1.0
Sssca1	P56873	70	-10	-13	-9	12	0.9	0.9	0.9	1.1
Cdk9	Q99J95	10	-2	-13	-9	5	1.0	0.9	0.9	1.1
UPF0547	Q9CR55	27	-3	-13	-9	1	1.0	0.9	0.9	1.0
Ank1	Q02357	375	-15	-13	-9	-2	0.9	0.9	0.9	1.0
Bnip1	Q6QD59	12	-10	-13	-9	-11	0.9	0.9	0.9	0.9
Skap1	Q3UUV5	252	-13	-13	-9	-15	0.9	0.9	0.9	0.9
Nlk	O54949	369	-14	-14	-9	45	0.9	0.9	0.9	1.8
Wdyhv1	Q80WB5	30	-16	-14	-9	-5	0.9	0.9	0.9	1.0
Usp19	Q3UJD6	863	-12	-14	-9	-8	0.9	0.9	0.9	0.9
Gale	Q8R059	261	-25	-14	-9	-13	0.8	0.9	0.9	0.9
Gemin4	Q6P6L6	784	-14	-14	-9	24	0.9	0.9	0.9	1.3
Srrt	Q99MR6	420	-20	-14	-9	19	0.8	0.9	0.9	1.2
Gem	P55041	208	-12	-14	-9	-5	0.9	0.9	0.9	1.0
Cars2	Q8BYM8	458	-9	-15	-9	2	0.9	0.9	0.9	1.0
Gabpa	Q00422	338	-17	-15	-9	-13	0.9	0.9	0.9	0.9
Kat6a	G3X940	772	-8	-15	-9	4	0.9	0.9	0.9	1.0
Pcna	P17918	81	-10	-15	-9	-5	0.9	0.9	0.9	1.0
Ddx60	E9PZQ1	1223	-8	-15	-9	-12	0.9	0.9	0.9	0.9
Ptar1	E9QAB6	403	-16	-15	-9	-18	0.9	0.9	0.9	0.9
Armc8	Q9DBR3	275	-10	-16	-9	-7	0.9	0.9	0.9	0.9
Tpt1	P63028	28	-10	-16	-9	9	0.9	0.9	0.9	1.1

Itpr3	P70227	254	-9	-16	-9	6	0.9	0.9	0.9	1.1
Rasa1	E9PYG6	567	-24	-16	-9	3	0.8	0.9	0.9	1.0
Ralgapa2	A3KGS3	495	-6	-16	-9	-2	0.9	0.9	0.9	1.0
Ddx46	Q569Z5	590	-18	-17	-9	-17	0.9	0.9	0.9	0.9
Mettl10	Q9D853	192	-16	-17	-9	-20	0.9	0.9	0.9	0.8
Trim14	Q8BVW3	42	-18	-18	-9	9	0.8	0.9	0.9	1.1
Chst11	Q9JME2	128	-5	-18	-9	4	1.0	0.9	0.9	1.0
Parp1	Q921K2	24	-10	-19	-9	3	0.9	0.8	0.9	1.0
Recql5	Q8VID5	458	-15	-19	-9	-10	0.9	0.8	0.9	0.9
Lims1	Q99JW4	100	-16	-20	-9	3	0.9	0.8	0.9	1.0
Acaa1a	Q921H8	381	-11	-20	-9	-7	0.9	0.8	0.9	0.9
Hgs	Q99LI8	166	-16	-20	-9	-20	0.9	0.8	0.9	0.8
Vrk3	Q8K3G5	397	-8	-20	-9	4	0.9	0.8	0.9	1.0
Rela	Q04207	206	-12	-21	-9	-1	0.9	0.8	0.9	1.0
Rpl10l	P86048	80	-9	-21	-9	-17	0.9	0.8	0.9	0.9
Celf2	Q9Z0H4	143	-2	-21	-9	-1	1.0	0.8	0.9	1.0
Mcm3ap	Q9WUU9	1230	-7	-22	-9	22	0.9	0.8	0.9	1.3
Akap17b	A2A3V1	168	-24	-23	-9	-6	0.8	0.8	0.9	0.9
Rps8	P62242	100	-16	-23	-9	-14	0.9	0.8	0.9	0.9
Rpl7a-ps5	A0A140T8L3	201	-13	-23	-9	-5	0.9	0.8	0.9	1.0
Rgp1	Q8BHT7	308	-34	-24	-9	37	0.7	0.8	0.9	1.6
Rbl2	Q64700	365	-20	-24	-9	-1	0.8	0.8	0.9	1.0
Rpa1	Q8VEE4	512	-18	-24	-9	-9	0.9	0.8	0.9	0.9
Vrk3	Q8K3G5	261	-16	-26	-9	19	0.9	0.8	0.9	1.2
Kmt2b	O08550	1004	-6	-26	-9	-4	0.9	0.8	0.9	1.0
Parp3	Q3ULW8	182	-13	-26	-9	-11	0.9	0.8	0.9	0.9
Terf2ip	Q91VL8	357	-26	-27	-9	-25	0.8	0.8	0.9	0.8
Aplf	Q9D842	62	-17	-28	-9	17	0.9	0.8	0.9	1.2
Utp6	Q8VCY6	311	-11	-29	-9	-2	0.9	0.8	0.9	1.0
Dgkz	Q80UP3	688	-23	-33	-9	-10	0.8	0.8	0.9	0.9
Phf1	Q9Z1B8	237	-31	-35	-9	-5	0.8	0.7	0.9	1.0
Rnf135	Q9CWS1	59	-22	16	-9	2	0.8	1.2	0.9	1.0
Arih1	Q9Z1K5	355	8	14	-9	38	1.1	1.2	0.9	1.6
Heatr6	Q6P1G0	429	-13	13	-9	29	0.9	1.1	0.9	1.4
Mob3a	Q8BSU7	88	1	11	-9	14	1.0	1.1	0.9	1.2
Sec16a	A2AIX1	1707	4	9	-9	42	1.0	1.1	0.9	1.7
Exoc8	Q6PGF7	415	-14	9	-9	22	0.9	1.1	0.9	1.3
Ankhd1	E9PUR0	216	4	8	-9	7	1.0	1.1	0.9	1.1
Cep152	A2AUM9	878	1	7	-9	18	1.0	1.1	0.9	1.2
Eif2s3x	Q9Z0N1	96	-4	4	-9	2	1.0	1.0	0.9	1.0
Srm	Q64674	205	-4	4	-9	-5	1.0	1.0	0.9	1.0
Tsc2	Q7TT21	648	-4	3	-9	-2	1.0	1.0	0.9	1.0
Mapk11	Q9WUI1	162	-8	3	-9	2	0.9	1.0	0.9	1.0
Pml	Q60953	77	-9	2	-9	26	0.9	1.0	0.9	1.4
Crlf3	Q9Z2L7	336	-7	2	-9	18	0.9	1.0	0.9	1.2
Arhgef2	Q60875	674	7	2	-9	-11	1.1	1.0	0.9	0.9
Als2	Q920R0	1448	-9	2	-9	18	0.9	1.0	0.9	1.2
Tle3	Q08122	26	1	2	-9	15	1.0	1.0	0.9	1.2
Hk3	Q3TRM8	570	-6	2	-9	-7	0.9	1.0	0.9	0.9
Gtf2e1	Q9D0D5	42	-42	2	-9	-8	0.7	1.0	0.9	0.9
Map4k1	P70218	148	-6	1	-9	-8	0.9	1.0	0.9	0.9

Whsc1	Q8BVE8	207	-12	1	-9	-13	0.9	1.0	0.9	0.9
Pgd	Q9DCD0	402	-13	1	-9	16	0.9	1.0	0.9	1.2
Dync1h1	Q9JHU4	3710	-8	1	-9	14	0.9	1.0	0.9	1.2
Plcg2	Q8CIH5	551	-3	1	-9	1	1.0	1.0	0.9	1.0
Tcp11l2	Q8K1H7	449	-5	1	-9	0	1.0	1.0	0.9	1.0
Ipcef1	Q5DU31	168	-8	1	-9	-9	0.9	1.0	0.9	0.9
Stat3	P42227	687	-9	1	-9	-10	0.9	1.0	0.9	0.9
Synrg	Q5SV85	619	-3	1	-9	-17	1.0	1.0	0.9	0.9
Ncbp1	Q3UYV9	616	7	0	-9	11	1.1	1.0	0.9	1.1
Ruvbl1	P60122	49	-5	0	-9	10	1.0	1.0	0.9	1.1
Arpc4	P59999	21	-4	0	-9	7	1.0	1.0	0.9	1.1
Pdpk1	Q9Z2A0	184	-1	0	-9	-1	1.0	1.0	0.9	1.0
Med15	G3X8S4	589	-10	0	-9	-13	0.9	1.0	0.9	0.9
Fam63b	Q6PDI6	120	-7	0	-9	-21	0.9	1.0	0.9	0.8
Ago2	Q8CJG0	491	3	-1	-9	20	1.0	1.0	0.9	1.2
Ago1	Q8CJG1	488	3	-1	-9	20	1.0	1.0	0.9	1.2
Adar	Q99MU3	129	-2	-1	-9	2	1.0	1.0	0.9	1.0
Zfp119b	Q8K0G9	410	-5	-1	-9	-4	1.0	1.0	0.9	1.0
Acly	Q91V92	229	-10	-1	-9	9	0.9	1.0	0.9	1.1
Acd	Q5EE38	185	-6	-2	-9	-13	0.9	1.0	0.9	0.9
Dtx3l	Q3UIR3	171	-12	-2	-9	12	0.9	1.0	0.9	1.1
Stat3	P42227	542	-5	-2	-9	11	1.0	1.0	0.9	1.1
Sp1	O89090	610	-17	-2	-9	8	0.9	1.0	0.9	1.1
Mycbp2	E9PUJ6	4643	-13	-2	-9	-7	0.9	1.0	0.9	0.9
Rabgap1	A2AWA9	1021	-5	-2	-9	-20	1.0	1.0	0.9	0.8
Iqcb1	Q8BP00	125	-5	-3	-9	17	1.0	1.0	0.9	1.2
Inpp5d	Q9ES52	1093	-6	-3	-9	-4	0.9	1.0	0.9	1.0
Kif1bp	Q6ZPU9	400	-8	-3	-9	-8	0.9	1.0	0.9	0.9
Card11	Q8CIS0	1057	-1	-3	-9	17	1.0	1.0	0.9	1.2
Klc4	Q9DBS5	113	-10	-3	-9	0	0.9	1.0	0.9	1.0
Traf2	P39429	112	-13	-3	-9	-1	0.9	1.0	0.9	1.0
Rbm34	Q8C5L7	200	-9	-3	-9	-1	0.9	1.0	0.9	1.0
Dffa	O54786	90	-6	-3	-9	-9	0.9	1.0	0.9	0.9
Pld3	O35405	364	-32	-3	-9	-30	0.8	1.0	0.9	0.8
Coro7	Q9D2V7	653	-12	-4	-9	24	0.9	1.0	0.9	1.3
Parp10	Q8CIE4	929	-7	-4	-9	5	0.9	1.0	0.9	1.0
Trim34a	Q99PP6	35	-5	-4	-9	-1	1.0	1.0	0.9	1.0
Rpl37a	P61514	60	-21	-4	-9	-4	0.8	1.0	0.9	1.0
Exosc10	P56960	288	1	-4	-9	20	1.0	1.0	0.9	1.3
Mcm3ap	Q9WUU9	1420	-11	-4	-9	12	0.9	1.0	0.9	1.1
Wbp2	P97765	80	-10	-4	-9	9	0.9	1.0	0.9	1.1
Zcchc11	B2RX14	143	-11	-4	-9	-4	0.9	1.0	0.9	1.0
Ola1	Q9CZ30	55	-2	-4	-9	-6	1.0	1.0	0.9	0.9
Vav2	Q60992	386	-23	-4	-9	-12	0.8	1.0	0.9	0.9
Dennd4c	A6H8H2	1444	-3	-4	-9	-23	1.0	1.0	0.9	0.8
Tnrc6b	Q8BKI2	637	-3	-5	-9	11	1.0	1.0	0.9	1.1
Traf2	P39429	303	-28	-5	-9	9	0.8	1.0	0.9	1.1
Uba2	Q9Z1F9	442	-8	-5	-9	-11	0.9	1.0	0.9	0.9
Tcp1	P11983	357	3	-5	-9	-12	1.0	1.0	0.9	0.9
Map1s	Q8C052	373	-10	-5	-9	-17	0.9	1.0	0.9	0.9
Sp100	O35892	392	-12	-5	-9	12	0.9	1.0	0.9	1.1

Bpnt1	Q9Z0S1	243	-10	-5	-9	10	0.9	1.0	0.9	1.1
Aldh3a2	P47740	226	-17	-5	-9	9	0.9	1.0	0.9	1.1
Top3a	O70157	705	-4	-5	-9	8	1.0	1.0	0.9	1.1
Sirt2	Q8VDQ8	148	-13	-5	-9	8	0.9	1.0	0.9	1.1
Ppp2r4	P58389	167	-5	-5	-9	0	1.0	1.0	0.9	1.0
Sp110	Q8BVK9	186	-11	-5	-9	-3	0.9	1.0	0.9	1.0
Vars	Q9Z1Q9	41	-8	-5	-9	-4	0.9	1.0	0.9	1.0
Fam177a1	Q8BR63	7	-5	-5	-9	-31	1.0	1.0	0.9	0.8
Nup58	Q8R332	240	-16	-6	-9	9	0.9	0.9	0.9	1.1
Tnpo1	Q8BFY9	142	-16	-6	-9	8	0.9	0.9	0.9	1.1
Nek9	Q8K1R7	626	-5	-6	-9	8	1.0	0.9	0.9	1.1
Eif2s2	Q99L45	279	-9	-6	-9	8	0.9	0.9	0.9	1.1
U2surp	Q6NV83	624	-7	-6	-9	2	0.9	0.9	0.9	1.0
Irf8	P23611	297	-12	-6	-9	1	0.9	0.9	0.9	1.0
Atp13a1	Q9EPE9	708	-7	-6	-9	-3	0.9	0.9	0.9	1.0
Ppp2r1a	Q76MZ3	317	-9	-6	-9	-4	0.9	0.9	0.9	1.0
Gmps	Q3THK7	489	-5	-6	-9	-6	1.0	0.9	0.9	0.9
Ldha	P06151	84	-8	-6	-9	-8	0.9	0.9	0.9	0.9
Rbpj	P31266	339	-13	-6	-9	-9	0.9	0.9	0.9	0.9
Phactr4	Q501J7	177	-1	-6	-9	-21	1.0	0.9	0.9	0.8
Hmha1	Q3TBD2	468	-15	-6	-9	19	0.9	0.9	0.9	1.2
Ctdp1	Q7TSG2	690	-15	-6	-9	13	0.9	0.9	0.9	1.1
Iars2	Q8BIJ6	819	-11	-6	-9	10	0.9	0.9	0.9	1.1
Rnpep	Q8VCT3	151	-17	-6	-9	9	0.9	0.9	0.9	1.1
Trim65	Q8BFW4	323	-7	-6	-9	9	0.9	0.9	0.9	1.1
Bin1	O08539	47	-6	-6	-9	-5	0.9	0.9	0.9	1.0
Ssb	P32067	244	-13	-6	-9	-16	0.9	0.9	0.9	0.9
Csnk1g1	Q8BTH8	52	-6	-6	-9	-20	0.9	0.9	0.9	0.8
Csnk1g2	Q8BVP5	54	-6	-6	-9	-20	0.9	0.9	0.9	0.8
Lcp1	Q61233	460	-16	-7	-9	17	0.9	0.9	0.9	1.2
Copg1	Q9QZE5	296	-13	-7	-9	11	0.9	0.9	0.9	1.1
Copg1	Q9QZE5	169	-6	-7	-9	4	0.9	0.9	0.9	1.0
Map2k6	P70236	216	-5	-7	-9	-2	1.0	0.9	0.9	1.0
Hemgn	Q9ERZ0	345	-9	-7	-9	-5	0.9	0.9	0.9	1.0
Rsbn1l	D3Z0K6	259	-14	-7	-9	32	0.9	0.9	0.9	1.5
Snu13	Q9D0T1	93	-15	-7	-9	3	0.9	0.9	0.9	1.0
Mtmr12	Q80TA6	201	-2	-7	-9	0	1.0	0.9	0.9	1.0
Slc7a1	Q09143	21	-14	-7	-9	-7	0.9	0.9	0.9	0.9
Noa1	Q9JJG9	506	-13	-7	-9	-7	0.9	0.9	0.9	0.9
Pabpc1	P29341	132	-13	-7	-9	-9	0.9	0.9	0.9	0.9
Ighmbp2	A0A0B4J1E3	821	-14	-7	-9	-16	0.9	0.9	0.9	0.9
Xpo5	Q924C1	37	-14	-8	-9	30	0.9	0.9	0.9	1.4
Tpi1	P17751	268	-5	-8	-9	16	1.0	0.9	0.9	1.2
Nufip2	Q5F2E7	235	-12	-8	-9	3	0.9	0.9	0.9	1.0
Wars	P32921	313	-15	-8	-9	2	0.9	0.9	0.9	1.0
Dnaja1	P63037	153	-13	-8	-9	2	0.9	0.9	0.9	1.0
Pde4dip	Q80YT7	267	-7	-8	-9	-1	0.9	0.9	0.9	1.0
Git2	Q9JLQ2	122	-11	-8	-9	-5	0.9	0.9	0.9	1.0
Nup188	Q6ZQH8	585	-5	-8	-9	-5	1.0	0.9	0.9	1.0
Parp3	Q3ULW8	502	-15	-8	-9	-7	0.9	0.9	0.9	0.9
Xpr1	Q9Z0U0	188	-9	-8	-9	-9	0.9	0.9	0.9	0.9

Gvin1	L7N451	2023	-4	-8	-9	-21	1.0	0.9	0.9	0.8
Aldh18a1	Q9Z110	612	-9	-8	-9	-29	0.9	0.9	0.9	0.8
Arhgap9	Q8QZW8	265	-9	-8	-9	13	0.9	0.9	0.9	1.1
Glud1	P26443	376	-12	-8	-9	12	0.9	0.9	0.9	1.1
Hnrnpf	Q9Z2X1	122	-20	-8	-9	6	0.8	0.9	0.9	1.1
Wiz	O88286	1312	1	-8	-9	-2	1.0	0.9	0.9	1.0
UPF0235	Q9CRC3	77	-10	-8	-9	-3	0.9	0.9	0.9	1.0
Rock1	P70335	1206	-6	-8	-9	-7	0.9	0.9	0.9	0.9
Limd1	Q9QXD8	423	-5	-8	-9	-8	1.0	0.9	0.9	0.9
Ahnak	E9Q616	1590	-9	-8	-9	-14	0.9	0.9	0.9	0.9
Hsp90aa1	P07901	482	-1	-9	-9	8	1.0	0.9	0.9	1.1
Iscu	Q9D7P6	131	-7	-9	-9	-18	0.9	0.9	0.9	0.8
Sbk1	Q8QZX0	220	-13	-9	-9	-22	0.9	0.9	0.9	0.8
Morc3	F7BJB9	15	-13	-9	-9	23	0.9	0.9	0.9	1.3
Mad2l1bp	Q9DCX1	188	-17	-9	-9	14	0.9	0.9	0.9	1.2
Ogdh	Q60597	507	-10	-9	-9	13	0.9	0.9	0.9	1.1
Map1s	Q8C052	102	-12	-9	-9	12	0.9	0.9	0.9	1.1
Acly	Q91V92	623	-8	-9	-9	6	0.9	0.9	0.9	1.1
Hgh1	Q8C3I8	206	-18	-9	-9	2	0.9	0.9	0.9	1.0
Itk	Q03526	294	-13	-9	-9	-9	0.9	0.9	0.9	0.9
Kdm3b	B9EKS2	1358	-9	-9	-9	-10	0.9	0.9	0.9	0.9
Mllt3	A2AM29	321	-18	-10	-9	27	0.9	0.9	0.9	1.4
Gorasp2	Q99JX3	173	-14	-10	-9	5	0.9	0.9	0.9	1.1
Hnrnpu	Q8VEK3	271	-5	-10	-9	2	1.0	0.9	0.9	1.0
Mto1	G5E889	314	-1	-10	-9	-1	1.0	0.9	0.9	1.0
Pkm	P52480	424	-12	-10	-9	-1	0.9	0.9	0.9	1.0
Mars	Q68FL6	38	-8	-10	-9	-3	0.9	0.9	0.9	1.0
Macf1	E9PVY8	4058	-8	-10	-9	-7	0.9	0.9	0.9	0.9
Vdac1	Q60932	245	-10	-10	-9	-9	0.9	0.9	0.9	0.9
Anxa1	P10107	324	-9	-10	-9	-11	0.9	0.9	0.9	0.9
Znf638	Q61464	711	-8	-10	-9	-15	0.9	0.9	0.9	0.9
Arhgap25	Q8BYW1	89	-2	-10	-9	-15	1.0	0.9	0.9	0.9
Zranb2	Q9R020	71	-16	-10	-9	-31	0.9	0.9	0.9	0.8
Casp2	P29594	30	2	-10	-9	5	1.0	0.9	0.9	1.1
Pola1	P33609	1314	-13	-10	-9	4	0.9	0.9	0.9	1.0
Flna	Q8BTM8	2582	-16	-10	-9	-8	0.9	0.9	0.9	0.9
Flna	Q8BTM8	574	-16	-10	-9	-11	0.9	0.9	0.9	0.9
Kdm3b	B9EKS2	217	-25	-10	-9	-11	0.8	0.9	0.9	0.9
Clk3	O35492	321	-13	-10	-9	-12	0.9	0.9	0.9	0.9
Tufm	Q8BFR5	290	-10	-10	-9	-14	0.9	0.9	0.9	0.9
Agfg2	Q80WC7	30	-26	-10	-9	-58	0.8	0.9	0.9	0.6
Smc1a	Q9CU62	619	-14	-11	-9	15	0.9	0.9	0.9	1.2
Ep300	B2RWS6	1789	-6	-11	-9	8	0.9	0.9	0.9	1.1
Crebbp	F8VPR5	1828	-6	-11	-9	8	0.9	0.9	0.9	1.1
Suv39h1	O54864	30	-20	-11	-9	6	0.8	0.9	0.9	1.1
Smchd1	Q6P5D8	1434	-10	-11	-9	3	0.9	0.9	0.9	1.0
Gnb2	P62880	25	-20	-11	-9	-2	0.8	0.9	0.9	1.0
Ints2	Q80UK8	982	-5	-11	-9	-3	1.0	0.9	0.9	1.0
Pds5a	E9QPI5	1115	-10	-11	-9	-8	0.9	0.9	0.9	0.9
Wdr33	Q8K4P0	1156	-8	-11	-9	-11	0.9	0.9	0.9	0.9
Nasp	Q99MD9	236	-2	-11	-9	-12	1.0	0.9	0.9	0.9

Atp1a3	Q6PIC6	49	-12	-11	-9	-13	0.9	0.9	0.9	0.9
Hsd17b10	A2AFQ2	58	-8	-11	-9	12	0.9	0.9	0.9	1.1
Rpl12	P35979	17	-17	-11	-9	9	0.9	0.9	0.9	1.1
Eefsec	Q9JHW4	409	-22	-11	-9	8	0.8	0.9	0.9	1.1
Rps23	P62267	90	-15	-11	-9	7	0.9	0.9	0.9	1.1
Rnf114	Q9ET26	65	-11	-11	-9	6	0.9	0.9	0.9	1.1
Pml	Q60953	322	-5	-11	-9	-12	1.0	0.9	0.9	0.9
Cog2	Q921L5	287	-20	-12	-9	12	0.8	0.9	0.9	1.1
Thoc2	B1AZI6	1064	-15	-12	-9	9	0.9	0.9	0.9	1.1
UPF0690	Q9CWU4	102	-10	-12	-9	9	0.9	0.9	0.9	1.1
Cad	B2RQC6	897	-5	-12	-9	8	1.0	0.9	0.9	1.1
Nup93	Q8BJ71	522	-9	-12	-9	6	0.9	0.9	0.9	1.1
Fig4	Q91WF7	427	-4	-12	-9	2	1.0	0.9	0.9	1.0
Cir1	Q9DA19	10	-13	-12	-9	-5	0.9	0.9	0.9	1.0
Ppp6c	Q9CQR6	129	-16	-12	-9	-15	0.9	0.9	0.9	0.9
Eef1g	Q9D8N0	266	-9	-12	-9	-15	0.9	0.9	0.9	0.9
Gak	Q99KY4	689	0	-12	-9	-19	1.0	0.9	0.9	0.8
Tln1	P26039	709	-3	-12	-9	17	1.0	0.9	0.9	1.2
Ets1	P27577	31	-9	-12	-9	10	0.9	0.9	0.9	1.1
Ddx21	Q9JIK5	450	-15	-12	-9	-1	0.9	0.9	0.9	1.0
Fgd3	O88842	148	-11	-12	-9	-4	0.9	0.9	0.9	1.0
Ddx39a	Q8VDW0	164	-10	-12	-9	-5	0.9	0.9	0.9	1.0
Recql5	Q8VID5	432	-1	-12	-9	-8	1.0	0.9	0.9	0.9
Trim59	Q922Y2	172	-19	-12	-9	-17	0.8	0.9	0.9	0.9
Pik3ap1	Q9EQ32	148	-18	-12	-9	-25	0.8	0.9	0.9	0.8
Zeb2	Q9R0G7	539	1	-13	-9	14	1.0	0.9	0.9	1.2
Fam126a	Q6P9N1	300	-9	-13	-9	0	0.9	0.9	0.9	1.0
Il16	O54824	1009	-3	-13	-9	-6	1.0	0.9	0.9	0.9
Ptp4a2	O70274	46	2	-13	-9	3	1.0	0.9	0.9	1.0
Vac14	Q80WQ2	619	-11	-13	-9	-4	0.9	0.9	0.9	1.0
Lin37	Q9D8N6	28	-7	-13	-9	-10	0.9	0.9	0.9	0.9
Safb	D3YXK2	384	-7	-13	-9	-26	0.9	0.9	0.9	0.8
Safb2	Q80YR5	405	-7	-13	-9	-26	0.9	0.9	0.9	0.8
Rad50	Q5SV02	221	-18	-14	-9	-5	0.8	0.9	0.9	1.0
Mthfd1l	Q3V3R1	197	-13	-14	-9	-7	0.9	0.9	0.9	0.9
Ptpra	P18052	242	-4	-14	-9	-12	1.0	0.9	0.9	0.9
Hcfc1	Q61191	135	-7	-14	-9	14	0.9	0.9	0.9	1.2
Rara	P11416	174	-10	-14	-9	4	0.9	0.9	0.9	1.0
Fhod1	Q6P9Q4	31	-11	-14	-9	-5	0.9	0.9	0.9	1.0
Prpf19	Q99KP6	114	-9	-14	-9	-6	0.9	0.9	0.9	0.9
Dock11	A2AF47	1204	-13	-14	-9	-7	0.9	0.9	0.9	0.9
Selenbp2	Q63836	371	-5	-14	-9	-8	1.0	0.9	0.9	0.9
Exosc9	Q9JHI7	122	5	-14	-9	-25	1.0	0.9	0.9	0.8
Eprs	Q8CGC7	92	-12	-15	-9	23	0.9	0.9	0.9	1.3
Kiaa0226l	Q3TD16	397	-15	-15	-9	13	0.9	0.9	0.9	1.1
Atp1a1	Q8VDN2	249	-8	-15	-9	13	0.9	0.9	0.9	1.1
Pola1	P33609	501	-29	-15	-9	12	0.8	0.9	0.9	1.1
Grcc10	O35127	73	-18	-15	-9	-8	0.8	0.9	0.9	0.9
Cluh	Q5SW19	1198	-12	-15	-9	-15	0.9	0.9	0.9	0.9
Kmt2a	P55200	454	-4	-15	-9	-24	1.0	0.9	0.9	0.8
Snrnp200	Q6P4T2	1359	-22	-15	-9	14	0.8	0.9	0.9	1.2

Trappc6b	Q9D289	32	-10	-15	-9	-4	0.9	0.9	0.9	1.0
Kdm2a	F6YRW4	675	-12	-15	-9	-15	0.9	0.9	0.9	0.9
Lsm12	Q9D0R8	20	-15	-16	-9	12	0.9	0.9	0.9	1.1
Phgdh	Q61753	369	-10	-16	-9	2	0.9	0.9	0.9	1.0
Rars	Q9D0I9	34	-4	-16	-9	0	1.0	0.9	0.9	1.0
Med16	Q6PGF3	788	-10	-16	-9	-4	0.9	0.9	0.9	1.0
Tpr	F6ZDS4	149	-18	-16	-9	-9	0.9	0.9	0.9	0.9
Zc3hc1	Q80YV2	102	-19	-16	-9	-17	0.8	0.9	0.9	0.9
Clasp2	F7DCH5	710	-8	-17	-9	12	0.9	0.9	0.9	1.1
Clasp1	Q80TV8	895	-8	-17	-9	12	0.9	0.9	0.9	1.1
Cdk12	Q14AX6	858	-7	-17	-9	-1	0.9	0.9	0.9	1.0
Cdca8	Q8BHX3	282	-14	-17	-9	-14	0.9	0.9	0.9	0.9
Helz2	E9QAM5	1584	-17	-17	-9	-12	0.9	0.9	0.9	0.9
Psma1	Q9R1P4	85	-16	-17	-9	-15	0.9	0.9	0.9	0.9
Kmt5c	Q6Q783	111	-20	-17	-9	-23	0.8	0.9	0.9	0.8
Arhgap17	Q3UIA2	53	-31	-18	-9	-6	0.8	0.9	0.9	0.9
Napepld	Q8BH82	372	1	-18	-9	-18	1.0	0.9	0.9	0.9
Hmgcl	P38060	323	-6	-18	-9	-27	0.9	0.9	0.9	0.8
Hnrnpd	Q60668	126	-13	-19	-9	14	0.9	0.8	0.9	1.2
Vars	Q9Z1Q9	562	-21	-19	-9	-9	0.8	0.8	0.9	0.9
Cxxc5	Q91WA4	273	-10	-19	-9	1	0.9	0.8	0.9	1.0
Isca2	Q9DCB8	144	-9	-19	-9	-13	0.9	0.8	0.9	0.9
Rbm10	Q99KG3	222	-14	-20	-9	10	0.9	0.8	0.9	1.1
Prkd2	Q8BZ03	180	-16	-20	-9	-5	0.9	0.8	0.9	1.0
Alb	P07724	58	-15	-20	-9	-15	0.9	0.8	0.9	0.9
Flna	Q8BTM8	2601	-22	-20	-9	-17	0.8	0.8	0.9	0.9
Ank1	Q02357	274	-6	-21	-9	4	0.9	0.8	0.9	1.0
Flnb	Q80X90	660	-12	-21	-9	-6	0.9	0.8	0.9	0.9
Xaf1	Q5NBU8	154	-14	-21	-9	-6	0.9	0.8	0.9	0.9
Ctsh	P49935	139	-11	-22	-9	-10	0.9	0.8	0.9	0.9
Lims1	Q99JW4	222	-14	-22	-9	-7	0.9	0.8	0.9	0.9
Gimap8	Q75N62	113	-9	-23	-9	4	0.9	0.8	0.9	1.0
Prdm10	Q3UTQ7	548	-10	-24	-9	-13	0.9	0.8	0.9	0.9
Zkscan8	Q8BSL0	59	-10	-24	-9	-13	0.9	0.8	0.9	0.9
Trpv2	Q9WTR1	329	-5	-25	-9	-9	1.0	0.8	0.9	0.9
Rbm27	Q5SFM8	279	-10	-25	-9	-12	0.9	0.8	0.9	0.9
Dhrs1	Q99L04	63	-3	-26	-9	13	1.0	0.8	0.9	1.1
Exoc1	Q8R3S6	650	-17	-26	-9	5	0.9	0.8	0.9	1.1
Mbnl2	Q8C181	34	-7	-26	-9	-9	0.9	0.8	0.9	0.9
Oas2	E9Q9A9	196	-5	-29	-9	-20	1.0	0.8	0.9	0.8
Ddx1	Q91VR5	231	-16	-30	-9	-6	0.9	0.8	0.9	0.9
Sp140	Q6NSQ5	178	-26	-30	-9	-16	0.8	0.8	0.9	0.9
Tdrkh	Q80VL1	160	-16	-31	-9	5	0.9	0.8	0.9	1.0
Setx	A2AKX3	1545	-7	-31	-9	-19	0.9	0.8	0.9	0.8
Inpp5b	Q8K337	499	-1	-31	-9	-19	1.0	0.8	0.9	0.8
Peak1	Q69Z38	1536	-33	-33	-9	27	0.8	0.8	0.9	1.4
Dnmt3b	O88509	500	-1	-48	-9	-4	1.0	0.7	0.9	1.0
Rad1	Q9QWZ1	148	-9	21	-10	0	0.9	1.3	0.9	1.0
Ap1b1	O35643	57	-8	16	-10	21	0.9	1.2	0.9	1.3
Dnajc7	Q9QYI3	250	-4	11	-10	12	1.0	1.1	0.9	1.1
Insrr	Q9WTL4	249	-4	11	-10	12	1.0	1.1	0.9	1.1

Lims1	Q99JW4	275	-3	11	-10	18	1.0	1.1	0.9	1.2
Hnrnpr	Q8VHM5	214	6	10	-10	25	1.1	1.1	0.9	1.3
Clasp1	Q80TV8	453	4	10	-10	8	1.0	1.1	0.9	1.1
Hsd11b2	P51661	128	-10	8	-10	26	0.9	1.1	0.9	1.4
Copb2	O55029	190	-17	7	-10	11	0.9	1.1	0.9	1.1
Flcn	Q8QZS3	215	-12	7	-10	-2	0.9	1.1	0.9	1.0
Hnrnpl	Q8R081	578	-18	6	-10	-19	0.8	1.1	0.9	0.8
Galk1	Q9R0N0	203	-13	5	-10	-66	0.9	1.1	0.9	0.6
Chd8	Q09XV5	438	-4	4	-10	-4	1.0	1.0	0.9	1.0
Map4k1	P70218	334	-10	3	-10	-16	0.9	1.0	0.9	0.9
Sptb	Q3UGX2	1892	-3	3	-10	12	1.0	1.0	0.9	1.1
Flna	Q8BTM8	210	-13	3	-10	8	0.9	1.0	0.9	1.1
Taco1	Q8K0Z7	109	1	3	-10	8	1.0	1.0	0.9	1.1
Ranbp9	E9Q5D6	591	-7	3	-10	-5	0.9	1.0	0.9	1.0
Hdac6	Q9Z2V5	571	-9	2	-10	5	0.9	1.0	0.9	1.0
Dnajb1	Q9QYJ3	269	2	2	-10	-2	1.0	1.0	0.9	1.0
Pdcd6ip	Q9WU78	40	-12	1	-10	6	0.9	1.0	0.9	1.1
Kif2a	P28740	419	-19	1	-10	4	0.8	1.0	0.9	1.0
Edc4	Q3UJB9	384	6	0	-10	-5	1.1	1.0	0.9	1.0
Sptan1	P16546	315	0	-1	-10	-11	1.0	1.0	0.9	0.9
Rbm17	Q8JZX4	389	-10	-1	-10	-3	0.9	1.0	0.9	1.0
Dph2	Q9CR25	431	-16	-1	-10	-4	0.9	1.0	0.9	1.0
Prex1	Q69ZK0	47	-2	-1	-10	-10	1.0	1.0	0.9	0.9
Hspa4l	P48722	380	-17	-2	-10	15	0.9	1.0	0.9	1.2
Hspa4	Q3U2G2	380	-17	-2	-10	15	0.9	1.0	0.9	1.2
Hsph1	Q61699	380	-17	-2	-10	15	0.9	1.0	0.9	1.2
Cnot1	Q6ZQ08	1540	-14	-2	-10	11	0.9	1.0	0.9	1.1
Mybbp1a	Q7TPV4	361	6	-2	-10	2	1.1	1.0	0.9	1.0
Mki67	E9PVX6	435	-4	-2	-10	-3	1.0	1.0	0.9	1.0
Dennd3	A2RT67	581	1	-2	-10	-3	1.0	1.0	0.9	1.0
Smc4	Q8CG47	108	-16	-2	-10	-18	0.9	1.0	0.9	0.9
Tmem201	A2A8U2	121	-11	-3	-10	-6	0.9	1.0	0.9	0.9
Hk1	P17710	869	-9	-3	-10	7	0.9	1.0	0.9	1.1
Dgka	O88673	247	0	-3	-10	-8	1.0	1.0	0.9	0.9
Rpl11	Q9CXW4	21	-10	-3	-10	-9	0.9	1.0	0.9	0.9
Gmds	Q8K0C9	237	-3	-4	-10	-3	1.0	1.0	0.9	1.0
Ahnak	E9Q616	1945	-6	-4	-10	-7	0.9	1.0	0.9	0.9
Ddhd2	Q80Y98	77	-17	-4	-10	-7	0.9	1.0	0.9	0.9
Plek	Q9JHK5	59	-16	-4	-10	-8	0.9	1.0	0.9	0.9
Rprd2	Q6NXI6	922	-7	-4	-10	-10	0.9	1.0	0.9	0.9
Iqgap2	Q3UQ44	628	-13	-4	-10	-17	0.9	1.0	0.9	0.9
Rpl27a	P14115	144	-6	-4	-10	-17	0.9	1.0	0.9	0.9
Hltf	Q6PCN7	381	-6	-4	-10	0	0.9	1.0	0.9	1.0
Cobll1	Q3UMF0	761	-6	-4	-10	-1	0.9	1.0	0.9	1.0
Btk	P35991	145	-6	-4	-10	-2	0.9	1.0	0.9	1.0
Xpo6	Q924Z6	159	-10	-5	-10	2	0.9	1.0	0.9	1.0
Cap1	P40124	92	-10	-5	-10	-1	0.9	1.0	0.9	1.0
Mrpl44	Q9CY73	53	-4	-5	-10	-6	1.0	1.0	0.9	0.9
Pgm3	Q9CYR6	200	-8	-5	-10	-6	0.9	1.0	0.9	0.9
Rgp1	Q8BHT7	90	-6	-5	-10	-9	0.9	1.0	0.9	0.9
Rbl2	Q64700	271	-14	-5	-10	-10	0.9	1.0	0.9	0.9

Ccdc88b	Q4QRL3	1227	-13	-5	-10	-12	0.9	1.0	0.9	0.9
Dock11	A2AF47	1878	-12	-5	-10	-14	0.9	1.0	0.9	0.9
Pfkp	Q9WUA3	722	-9	-5	-10	-36	0.9	1.0	0.9	0.7
Ddx3y	Q62095	340	-9	-5	-10	19	0.9	1.0	0.9	1.2
Ddx3x	Q62167	341	-9	-5	-10	19	0.9	1.0	0.9	1.2
Crbn	Q8C7D2	290	2	-5	-10	12	1.0	1.0	0.9	1.1
Satb1	Q60611	173	-10	-5	-10	-4	0.9	1.0	0.9	1.0
Gda	Q9R111	59	-5	-5	-10	-5	1.0	1.0	0.9	1.0
Zmpste24	Q80W54	324	-15	-6	-10	8	0.9	0.9	0.9	1.1
Cdc14b	Q6PFY9	347	-5	-6	-10	6	1.0	0.9	0.9	1.1
Lyst	G5E8Q0	1347	-5	-6	-10	-5	1.0	0.9	0.9	1.0
Hnrnpll	Q921F4	133	-7	-6	-10	-8	0.9	0.9	0.9	0.9
Cd300lf	Q6SJQ7	308	-9	-6	-10	-34	0.9	0.9	0.9	0.7
Vcpip1	Q8CDG3	218	-12	-6	-10	10	0.9	0.9	0.9	1.1
Pkm	P52480	423	-5	-6	-10	9	1.0	0.9	0.9	1.1
Hid1	Q8R1F6	712	-16	-6	-10	4	0.9	0.9	0.9	1.0
Prkd2	Q8BZ03	74	-18	-6	-10	3	0.8	0.9	0.9	1.0
Ubqln4	Q99NB8	29	-9	-6	-10	2	0.9	0.9	0.9	1.0
Irak4	Q8R4K2	127	-5	-6	-10	-6	1.0	0.9	0.9	0.9
Ercc3	P49135	287	-17	-6	-10	-8	0.9	0.9	0.9	0.9
Gramd3	Q9D2W5	85	-11	-6	-10	-10	0.9	0.9	0.9	0.9
Ddx52	Q8K301	59	-11	-6	-10	-17	0.9	0.9	0.9	0.9
Gatsl3	Q9CWQ8	35	-11	-7	-10	23	0.9	0.9	0.9	1.3
Eif5	P59325	99	-14	-7	-10	19	0.9	0.9	0.9	1.2
Eif1	P48024	94	3	-7	-10	10	1.0	0.9	0.9	1.1
Zmiz1	Q6P1E1	712	-19	-7	-10	9	0.8	0.9	0.9	1.1
Zmiz2	Q8CIE2	564	-19	-7	-10	9	0.8	0.9	0.9	1.1
Akap8	Q9DBR0	392	-11	-7	-10	5	0.9	0.9	0.9	1.1
Syne1	Q6ZWR6	7344	-17	-7	-10	0	0.9	0.9	0.9	1.0
Arrb1	Q8BWG8	150	-12	-7	-10	-7	0.9	0.9	0.9	0.9
Rpl11	Q9CXW4	25	-6	-7	-10	-7	0.9	0.9	0.9	0.9
Gmeb2	P58929	323	-11	-7	-10	-10	0.9	0.9	0.9	0.9
Ddx21	Q9JIK5	585	-10	-7	-10	-10	0.9	0.9	0.9	0.9
Dnajc7	Q9QYI3	225	-12	-7	-10	-10	0.9	0.9	0.9	0.9
Pxk	Q8BX57	574	-11	-7	-10	-11	0.9	0.9	0.9	0.9
Fcmr	A1KXC4	334	-11	-7	-10	-20	0.9	0.9	0.9	0.8
Pfkfb3	A7UAK5	155	-23	-7	-10	9	0.8	0.9	0.9	1.1
Psmb10	O35955	70	-8	-7	-10	5	0.9	0.9	0.9	1.1
Aldh18a1	Q9Z110	88	-18	-7	-10	1	0.8	0.9	0.9	1.0
Nckap1l	Q8K1X4	632	-12	-7	-10	-3	0.9	0.9	0.9	1.0
Iws1	Q8C1D8	696	-19	-7	-10	-4	0.8	0.9	0.9	1.0
Mis18bp1	Q80WQ8	597	-16	-7	-10	-12	0.9	0.9	0.9	0.9
Fmnl3	Q6ZPF4	623	-9	-7	-10	-12	0.9	0.9	0.9	0.9
Nbas	E9Q411	1254	-6	-8	-10	9	0.9	0.9	0.9	1.1
Cct4	P80315	221	-17	-8	-10	6	0.9	0.9	0.9	1.1
Rpa1	Q8VEE4	495	-5	-8	-10	5	1.0	0.9	0.9	1.0
Smchd1	Q6P5D8	1236	-12	-8	-10	3	0.9	0.9	0.9	1.0
Appl2	Q8K3G9	412	-8	-8	-10	-1	0.9	0.9	0.9	1.0
Xpo1	Q6P5F9	119	-2	-8	-10	-2	1.0	0.9	0.9	1.0
Pdlim5	Q8CI51	462	-10	-8	-10	-3	0.9	0.9	0.9	1.0
Mdn1	A2ANY6	1007	-11	-8	-10	-6	0.9	0.9	0.9	0.9

Gvin1	L7N451	2268	-10	-8	-10	-7	0.9	0.9	0.9	0.9
Smc5	Q8CG46	881	-15	-8	-10	-12	0.9	0.9	0.9	0.9
Wasf2	Q8BH43	27	-10	-8	-10	-12	0.9	0.9	0.9	0.9
Nsrp1	Q5NCR9	231	-13	-8	-10	-13	0.9	0.9	0.9	0.9
Malt1	Q2TBA3	97	-5	-8	-10	-17	1.0	0.9	0.9	0.9
Dock10	E9QM99	1310	-10	-8	-10	-18	0.9	0.9	0.9	0.8
Rsbn1	Q80T69	282	-17	-8	-10	17	0.9	0.9	0.9	1.2
Top2b	Q64511	192	-13	-8	-10	8	0.9	0.9	0.9	1.1
Psmf1	Q8BHL8	203	-14	-8	-10	-2	0.9	0.9	0.9	1.0
Wdfy4	E9Q2M9	2984	-11	-8	-10	-3	0.9	0.9	0.9	1.0
Skiv2l	Q6NZR5	874	-10	-8	-10	-4	0.9	0.9	0.9	1.0
Rars2	Q3U186	110	-6	-8	-10	-5	0.9	0.9	0.9	1.0
Purb	O35295	285	-13	-8	-10	-5	0.9	0.9	0.9	1.0
Smc1a	Q9CU62	1180	1	-8	-10	-6	1.0	0.9	0.9	0.9
Syncrip	Q7TMK9	289	-17	-9	-10	11	0.9	0.9	0.9	1.1
Hnrnpr	Q8VHM5	292	-17	-9	-10	11	0.9	0.9	0.9	1.1
Ppat	Q8CIH9	100	-13	-9	-10	8	0.9	0.9	0.9	1.1
Nup98	Q6PFD9	1734	-13	-9	-10	6	0.9	0.9	0.9	1.1
Smc1a	Q9CU62	1210	-15	-9	-10	0	0.9	0.9	0.9	1.0
Metap1	Q8BP48	14	-13	-9	-10	-1	0.9	0.9	0.9	1.0
Camk2d	Q6PHZ2	481	-4	-9	-10	-2	1.0	0.9	0.9	1.0
Nup205	A0A0J9YUD5	1269	-13	-9	-10	-3	0.9	0.9	0.9	1.0
Plec	Q9QXS1	4501	-11	-9	-10	-6	0.9	0.9	0.9	0.9
Pml	Q60953	216	-12	-9	-10	15	0.9	0.9	0.9	1.2
Ppp2ca	P63330	20	-4	-9	-10	8	1.0	0.9	0.9	1.1
Hk1	P17710	684	-15	-9	-10	5	0.9	0.9	0.9	1.0
Ctps2	P70303	362	-7	-9	-10	1	0.9	0.9	0.9	1.0
Anxa4	P97429	198	-17	-9	-10	1	0.9	0.9	0.9	1.0
Pum3	Q8BKS9	609	-15	-9	-10	-15	0.9	0.9	0.9	0.9
Golph3	Q9CRA5	280	-17	-10	-10	16	0.9	0.9	0.9	1.2
Vps28	Q9D1C8	103	-15	-10	-10	14	0.9	0.9	0.9	1.2
Ints10	Q8K2A7	555	-18	-10	-10	8	0.8	0.9	0.9	1.1
Ddx58	Q6Q899	269	-12	-10	-10	4	0.9	0.9	0.9	1.0
Pml	Q60953	153	-14	-10	-10	2	0.9	0.9	0.9	1.0
Nifk	Q91VE6	260	-10	-10	-10	-8	0.9	0.9	0.9	0.9
Atp2c1	Q80XR2	162	-19	-10	-10	-13	0.8	0.9	0.9	0.9
Mcm5	Q52KC3	207	-3	-10	-10	18	1.0	0.9	0.9	1.2
Kyat1	Q8BTY1	416	-15	-10	-10	4	0.9	0.9	0.9	1.0
Atic	Q9CWJ9	101	-7	-10	-10	2	0.9	0.9	0.9	1.0
Atxn2l	Q7TQH0	466	-15	-10	-10	-2	0.9	0.9	0.9	1.0
Nr3c1	E9PYV1	384	1	-10	-10	-3	1.0	0.9	0.9	1.0
Arcn1	Q5XJY5	94	-15	-10	-10	-5	0.9	0.9	0.9	1.0
Smchd1	Q6P5D8	25	-8	-10	-10	-7	0.9	0.9	0.9	0.9
Atp2a3	Q64518	581	-4	-10	-10	-14	1.0	0.9	0.9	0.9
Ghdc	Q99J23	508	-26	-10	-10	-19	0.8	0.9	0.9	0.8
Safb	D3YXK2	81	-11	-10	-10	-23	0.9	0.9	0.9	0.8
Mettl2	Q8BMK1	63	-8	-11	-10	11	0.9	0.9	0.9	1.1
Nup98	Q6PFD9	1710	-5	-11	-10	-1	1.0	0.9	0.9	1.0
Pgm2l1	Q8CAA7	303	-11	-11	-10	-1	0.9	0.9	0.9	1.0
Ilkap	Q8R0F6	325	-11	-11	-10	-3	0.9	0.9	0.9	1.0
Dgka	O88673	43	-6	-11	-10	-4	0.9	0.9	0.9	1.0

Uba7	Q9DBK7	933	-1	-11	-10	-9	1.0	0.9	0.9	0.9
Nedd1	P33215	516	-30	-11	-10	-12	0.8	0.9	0.9	0.9
Znf638	Q61464	1473	-13	-11	-10	-23	0.9	0.9	0.9	0.8
Sqrdl	Q9R112	127	-14	-11	-10	-31	0.9	0.9	0.9	0.8
Lrrc57	Q8JZX5	82	-11	-11	-10	13	0.9	0.9	0.9	1.1
Nup153	E9Q3G8	674	-8	-11	-10	6	0.9	0.9	0.9	1.1
Drg1	P32233	243	-26	-11	-10	4	0.8	0.9	0.9	1.0
Fnbp1	Q80TY0	130	-1	-11	-10	2	1.0	0.9	0.9	1.0
Rpl37a	P61514	39	-16	-11	-10	2	0.9	0.9	0.9	1.0
Gfm2	Q8R2Q4	611	-16	-11	-10	-19	0.9	0.9	0.9	0.8
Cmas	Q99KK2	430	-11	-11	-10	-29	0.9	0.9	0.9	0.8
Med12	A2AGH6	1189	-16	-12	-10	11	0.9	0.9	0.9	1.1
Echs1	Q8BH95	111	-9	-12	-10	10	0.9	0.9	0.9	1.1
Dnaja1	P63037	150	-4	-12	-10	9	1.0	0.9	0.9	1.1
Setdb2	Q8C267	329	-14	-12	-10	7	0.9	0.9	0.9	1.1
Zc3h13	E9Q784	57	-12	-12	-10	-2	0.9	0.9	0.9	1.0
Naa30	Q8CES0	197	-7	-12	-10	-6	0.9	0.9	0.9	0.9
Dnmt3a	O88508	662	-25	-12	-10	20	0.8	0.9	0.9	1.2
Banp	Q8VBU8	48	-15	-12	-10	3	0.9	0.9	0.9	1.0
Gtf3c1	Q8K284	434	-4	-12	-10	2	1.0	0.9	0.9	1.0
Bre	Q8K3W0	53	-11	-12	-10	-1	0.9	0.9	0.9	1.0
Trim27	Q62158	393	-21	-12	-10	-7	0.8	0.9	0.9	0.9
Dnph1	Q80VJ3	164	-13	-12	-10	-15	0.9	0.9	0.9	0.9
Kdm1a	Q6ZQ88	361	-19	-13	-10	14	0.8	0.9	0.9	1.2
Prpf38a	Q4FK66	72	-21	-13	-10	8	0.8	0.9	0.9	1.1
Pikfyve	Q9Z1T6	1676	-17	-13	-10	5	0.9	0.9	0.9	1.0
Ddx3x	Q62167	298	-26	-13	-10	2	0.8	0.9	0.9	1.0
Dxo	O70348	301	-17	-13	-10	-8	0.9	0.9	0.9	0.9
Coro7	Q9D2V7	787	-15	-13	-10	5	0.9	0.9	0.9	1.0
Pafah1b1	P63005	356	-20	-13	-10	-13	0.8	0.9	0.9	0.9
Trmt6	Q8CE96	244	-8	-13	-10	-21	0.9	0.9	0.9	0.8
Mtus1	Q5HZI1	102	-27	-14	-10	1	0.8	0.9	0.9	1.0
Lars	Q8BMJ2	115	-13	-14	-10	-3	0.9	0.9	0.9	1.0
Gnl3l	Q6PGG6	272	-13	-14	-10	-10	0.9	0.9	0.9	0.9
Dok1	P97465	464	-18	-14	-10	-15	0.9	0.9	0.9	0.9
Map2k6	P70236	38	-15	-14	-10	2	0.9	0.9	0.9	1.0
Rbbp7	Q60973	116	-20	-14	-10	2	0.8	0.9	0.9	1.0
Lrrc45	Q8CIM1	523	-8	-14	-10	-3	0.9	0.9	0.9	1.0
Ctcf	Q61164	275	-15	-14	-10	-3	0.9	0.9	0.9	1.0
Baz1b	Q9Z277	498	-14	-14	-10	-12	0.9	0.9	0.9	0.9
Pik3cb	Q8BTI9	110	-8	-15	-10	-16	0.9	0.9	0.9	0.9
Hk2	O08528	909	-22	-15	-10	-30	0.8	0.9	0.9	0.8
Uba2	Q9Z1F9	30	-6	-15	-10	11	0.9	0.9	0.9	1.1
Epsti1	Q8VDI1	204	-12	-15	-10	9	0.9	0.9	0.9	1.1
Syne3	Q4FZC9	626	-10	-15	-10	-10	0.9	0.9	0.9	0.9
Ddi2	A2ADY9	361	-22	-15	-10	-15	0.8	0.9	0.9	0.9
Cap1	P40124	355	-10	-15	-10	-15	0.9	0.9	0.9	0.9
Zmat2	Q9CPW7	82	-25	-16	-10	32	0.8	0.9	0.9	1.5
Fbxl6	Q9QXW0	319	-14	-16	-10	5	0.9	0.9	0.9	1.1
Chd3	B1AR17	554	-26	-16	-10	3	0.8	0.9	0.9	1.0
Etf1	Q8BWY3	127	-8	-16	-10	1	0.9	0.9	0.9	1.0

Tgm2	P21981	553	-11	-16	-10	-1	0.9	0.9	0.9	1.0
Dnttip1	Q99LB0	121	-4	-16	-10	-5	1.0	0.9	0.9	1.0
Hk3	Q3TRM8	780	-5	-16	-10	5	1.0	0.9	0.9	1.1
Usp7	E9PXY8	840	-15	-16	-10	2	0.9	0.9	0.9	1.0
Abce1	P61222	29	-15	-16	-10	-2	0.9	0.9	0.9	1.0
Mars	Q68FL6	443	-15	-16	-10	-2	0.9	0.9	0.9	1.0
Cdc34b	A0A140T8I4	191	-2	-16	-10	-2	1.0	0.9	0.9	1.0
Heatr3	Q8BQM4	57	-21	-16	-10	-6	0.8	0.9	0.9	0.9
Otub1	Q7TQI3	91	-16	-16	-10	-7	0.9	0.9	0.9	0.9
Golgb1	E9PVZ8	388	-6	-16	-10	-17	0.9	0.9	0.9	0.9
Ddx20	Q9JJY4	99	-10	-17	-10	-2	0.9	0.9	0.9	1.0
Ehhadh	Q9DBM2	17	-14	-17	-10	-10	0.9	0.9	0.9	0.9
Tpi1	P17751	77	-7	-17	-10	10	0.9	0.9	0.9	1.1
Arfgap2	Q99K28	29	-15	-17	-10	5	0.9	0.9	0.9	1.0
Bach2	P97303	339	-19	-17	-10	-3	0.8	0.9	0.9	1.0
Tarsl2	Q8BLY2	26	-17	-17	-10	-18	0.9	0.9	0.9	0.8
Eif3b	Q8JZQ9	504	-15	-18	-10	7	0.9	0.9	0.9	1.1
Pnkp	G5E8N7	446	-8	-18	-10	-3	0.9	0.9	0.9	1.0
Mtm1	Q9Z2C5	482	-15	-18	-10	-9	0.9	0.9	0.9	0.9
Pou2f2	Q00196	171	-3	-18	-10	-10	1.0	0.9	0.9	0.9
Slc25a19	Q9DAM5	51	-8	-18	-10	-12	0.9	0.9	0.9	0.9
Spata5	Q3UMC0	568	-20	-18	-10	-16	0.8	0.9	0.9	0.9
Nfatc2	Q60591	258	-17	-18	-10	-13	0.9	0.8	0.9	0.9
Wapl	Q65Z40	160	-23	-18	-10	-18	0.8	0.8	0.9	0.8
Ywhah	P68510	112	-2	-19	-10	-9	1.0	0.8	0.9	0.9
Trim33	Q99PP7	169	-13	-19	-10	-24	0.9	0.8	0.9	0.8
Pram1	Q6BCL1	278	2	-20	-10	-2	1.0	0.8	0.9	1.0
Lsm11	Q8BUV6	52	-28	-20	-10	6	0.8	0.8	0.9	1.1
Wdr91	Q7TMQ7	488	-26	-20	-10	-20	0.8	0.8	0.9	0.8
Myo1e	E9Q634	447	-29	-21	-10	1	0.8	0.8	0.9	1.0
Mthfd1l	Q3V3R1	450	-30	-21	-10	-15	0.8	0.8	0.9	0.9
Ciapin1	Q8WTY4	116	-14	-21	-10	-20	0.9	0.8	0.9	0.8
Trim28	Q62318	66	-29	-22	-10	8	0.8	0.8	0.9	1.1
Rab3il1	Q8VDV3	297	-8	-22	-10	-14	0.9	0.8	0.9	0.9
Tada3	Q8R0L9	7	5	-23	-10	-10	1.1	0.8	0.9	0.9
Ppme1	Q8BVQ5	312	-32	-23	-10	-5	0.8	0.8	0.9	1.0
Samm50	Q8BGH2	457	-2	-24	-10	10	1.0	0.8	0.9	1.1
Josd1	Q9DBJ6	36	-11	-24	-10	-30	0.9	0.8	0.9	0.8
Bccip	Q9CWI3	219	-10	-24	-10	-8	0.9	0.8	0.9	0.9
Cct7	P80313	364	-28	-25	-10	4	0.8	0.8	0.9	1.0
Sdha	Q8K2B3	191	-25	-25	-10	-12	0.8	0.8	0.9	0.9
Cmpk2	Q3U5Q7	243	-26	-25	-10	-1	0.8	0.8	0.9	1.0
Med16	Q6PGF3	233	-23	-25	-10	-32	0.8	0.8	0.9	0.8
Ecd	Q9CS74	267	-21	-28	-10	5	0.8	0.8	0.9	1.1
Zfp263	Q8CF60	473	-24	-28	-10	-12	0.8	0.8	0.9	0.9
Epb41	P48193	225	-15	-30	-10	-10	0.9	0.8	0.9	0.9
Phf14	Q9D4H9	511	1	-32	-10	-5	1.0	0.8	0.9	1.0
Wdr43	Q6ZQL4	340	-19	-41	-10	-12	0.8	0.7	0.9	0.9
Dld	O08749	69	-37	-42	-10	-24	0.7	0.7	0.9	0.8
Med16	Q6PGF3	532	10	-47	-10	-16	1.1	0.7	0.9	0.9
Plpp6	Q9D4F2	52	2	10	-10	-14	1.0	1.1	0.9	0.9

Matr3	Q8K310	230	-1	10	-10	-26	1.0	1.1	0.9	0.8
Syne1	Q6ZWR6	8383	-17	9	-10	3	0.9	1.1	0.9	1.0
Usp14	Q9JMA1	277	16	8	-10	15	1.2	1.1	0.9	1.2
Hadhb	Q99JY0	459	-7	7	-10	34	0.9	1.1	0.9	1.5
Ddx39b	Q9Z1N5	300	-13	7	-10	18	0.9	1.1	0.9	1.2
Naxe	Q8K4Z3	277	-5	4	-10	17	1.0	1.0	0.9	1.2
Galk1	Q9R0N0	243	9	4	-10	-10	1.1	1.0	0.9	0.9
Snd1	Q78PY7	152	-13	3	-10	10	0.9	1.0	0.9	1.1
Prdx5	P99029	200	-10	3	-10	11	0.9	1.0	0.9	1.1
Peak1	Q69Z38	682	1	3	-10	-23	1.0	1.0	0.9	0.8
Srrt	Q99MR6	489	-9	2	-10	1	0.9	1.0	0.9	1.0
Rab3gap1	Q80UJ7	322	-2	2	-10	27	1.0	1.0	0.9	1.4
Cltc	Q68FD5	1260	-5	2	-10	15	1.0	1.0	0.9	1.2
Ago1	Q8CJG1	64	-5	2	-10	10	1.0	1.0	0.9	1.1
Plk1	Q07832	573	-2	2	-10	8	1.0	1.0	0.9	1.1
Clic1	Q9Z1Q5	178	-14	0	-10	8	0.9	1.0	0.9	1.1
Tomm70	Q9CZW5	147	-2	0	-10	4	1.0	1.0	0.9	1.0
Prps2	Q9CS42	230	-6	0	-10	2	0.9	1.0	0.9	1.0
Prps1	Q9D7G0	230	-6	0	-10	2	0.9	1.0	0.9	1.0
Ogfr	Q99PG2	400	-7	0	-10	-10	0.9	1.0	0.9	0.9
Carmil2	Q3V3V9	737	-16	0	-10	-34	0.9	1.0	0.9	0.7
Mybbp1a	Q7TPV4	1004	-2	-1	-10	21	1.0	1.0	0.9	1.3
Glrx	Q9QUH0	26	-5	-1	-10	14	1.0	1.0	0.9	1.2
Ak2	Q9WTP6	42	-10	-1	-10	6	0.9	1.0	0.9	1.1
Gabpa	Q00422	388	-2	-1	-10	2	1.0	1.0	0.9	1.0
Zcchc9	Q8R1J3	213	-18	-1	-10	-11	0.8	1.0	0.9	0.9
Rnf139	Q7TMV1	563	-14	-2	-10	4	0.9	1.0	0.9	1.0
Rab30	Q923S9	93	-8	-2	-10	1	0.9	1.0	0.9	1.0
Unc45a	Q99KD5	426	-1	-2	-10	-3	1.0	1.0	0.9	1.0
Cnp	P16330	49	-7	-2	-10	-26	0.9	1.0	0.9	0.8
Lrrk1	Q3UHC2	835	-7	-2	-10	13	0.9	1.0	0.9	1.1
Pot1	Q91WC1	286	-8	-2	-10	4	0.9	1.0	0.9	1.0
Polr2g	P62488	38	-10	-2	-10	2	0.9	1.0	0.9	1.0
Vhl	P40338	43	-2	-2	-10	1	1.0	1.0	0.9	1.0
Tatdn2	B7ZNL9	215	-5	-2	-10	-5	1.0	1.0	0.9	1.0
Ifi207	E9Q3L4	863	-4	-3	-10	13	1.0	1.0	0.9	1.1
Ifi204	P0DOV2	504	-4	-3	-10	13	1.0	1.0	0.9	1.1
Arfgef2	A2A5R2	76	-19	-3	-10	11	0.8	1.0	0.9	1.1
Ctu1	Q99J10	413	-9	-3	-10	8	0.9	1.0	0.9	1.1
Gpat4	Q8K2C8	285	-13	-3	-10	6	0.9	1.0	0.9	1.1
Smarcad1	Q04692	856	-6	-3	-10	2	0.9	1.0	0.9	1.0
E2f4	Q8R0K9	88	-10	-3	-10	-3	0.9	1.0	0.9	1.0
Klc1	Q8CD76	114	-11	-3	-10	-9	0.9	1.0	0.9	0.9
Rfc2	Q9WUK4	166	2	-3	-10	-27	1.0	1.0	0.9	0.8
Swap70	Q6A028	261	-9	-3	-10	-12	0.9	1.0	0.9	0.9
Ctu1	Q99J10	205	-7	-3	-10	-12	0.9	1.0	0.9	0.9
Mad2l1	Q9Z1B5	106	-7	-4	-10	-6	0.9	1.0	0.9	0.9
Larp7	Q05CL8	243	-13	-4	-10	-16	0.9	1.0	0.9	0.9
Top2a	Q01320	169	-17	-4	-10	18	0.9	1.0	0.9	1.2
Top2b	Q64511	179	-17	-4	-10	18	0.9	1.0	0.9	1.2
Ago2	Q8CJG0	345	-18	-4	-10	0	0.8	1.0	0.9	1.0

Ago1	Q8CJG1	342	-18	-4	-10	0	0.8	1.0	0.9	1.0
UPF0769	Q8BL95	264	-9	-4	-10	-4	0.9	1.0	0.9	1.0
Vps28	Q9D1C8	60	4	-4	-10	-6	1.0	1.0	0.9	0.9
Trp53bp1	A2AU91	1775	-4	-4	-10	-12	1.0	1.0	0.9	0.9
Ndufs6	P52503	79	-13	-5	-10	8	0.9	1.0	0.9	1.1
Golgb1	E9PVZ8	3123	-11	-5	-10	-2	0.9	1.0	0.9	1.0
Arl2bp	Q9D385	149	-10	-5	-10	-3	0.9	1.0	0.9	1.0
Irf2bp2	E9Q1P8	19	-11	-5	-10	-4	0.9	1.0	0.9	1.0
Irf2bpl	Q8K3X4	17	-11	-5	-10	-4	0.9	1.0	0.9	1.0
Zc3h13	E9Q784	42	-11	-5	-10	-6	0.9	1.0	0.9	0.9
Ammecr1l	Q8JZZ6	18	-2	-5	-10	-7	1.0	1.0	0.9	0.9
Nat10	Q8K224	254	2	-5	-10	-42	1.0	1.0	0.9	0.7
Ppid	Q9CR16	282	-20	-5	-10	13	0.8	1.0	0.9	1.1
Pi4ka	E9Q3L2	1134	-2	-5	-10	8	1.0	1.0	0.9	1.1
Ap1b1	O35643	123	-7	-5	-10	7	0.9	1.0	0.9	1.1
Ap2b1	Q9DBG3	123	-7	-5	-10	7	0.9	1.0	0.9	1.1
Snd1	Q78PY7	560	-7	-5	-10	4	0.9	1.0	0.9	1.0
Stk26	Q99JT2	392	-7	-5	-10	-1	0.9	1.0	0.9	1.0
Actr3	Q99JY9	34	-9	-5	-10	-1	0.9	1.0	0.9	1.0
Ppig	A2AR02	308	-3	-5	-10	-8	1.0	1.0	0.9	0.9
Pram1	Q6BCL1	337	-7	-5	-10	-9	0.9	1.0	0.9	0.9
Cebpz	P53569	560	-8	-6	-10	8	0.9	0.9	0.9	1.1
Nek9	Q8K1R7	623	-7	-6	-10	6	0.9	0.9	0.9	1.1
Znf32	Q80V23	231	-15	-6	-10	2	0.9	0.9	0.9	1.0
Hmha1	Q3TBD2	729	1	-6	-10	-2	1.0	0.9	0.9	1.0
Bcl10	Q9Z0H7	57	-2	-6	-10	-4	1.0	0.9	0.9	1.0
Rps6	P62754	100	-9	-6	-10	-13	0.9	0.9	0.9	0.9
Foxred1	Q3TQB2	187	-19	-6	-10	-22	0.8	0.9	0.9	0.8
Wdr81	Q5ND34	213	-5	-6	-10	15	1.0	0.9	0.9	1.2
Mocs3	A2BDX3	331	-13	-6	-10	7	0.9	0.9	0.9	1.1
Mettl13	Q91YR5	605	-18	-6	-10	7	0.8	0.9	0.9	1.1
Rnf31	Q924T7	696	-6	-6	-10	2	0.9	0.9	0.9	1.0
Psmg1	Q9JK23	170	-10	-6	-10	-1	0.9	0.9	0.9	1.0
Ikbkg	O88522	95	-9	-6	-10	-2	0.9	0.9	0.9	1.0
Vps28	Q9D1C8	96	-2	-6	-10	-10	1.0	0.9	0.9	0.9
Ddx42	Q810A7	281	-8	-7	-10	19	0.9	0.9	0.9	1.2
Ddx46	Q569Z5	478	-8	-7	-10	7	0.9	0.9	0.9	1.1
Ogdh	Q60597	283	-23	-7	-10	6	0.8	0.9	0.9	1.1
Pdcd6ip	Q9WU78	231	-13	-7	-10	4	0.9	0.9	0.9	1.0
Sptb	Q3UGX2	183	-21	-7	-10	3	0.8	0.9	0.9	1.0
Sptbn1	Q62261	183	-21	-7	-10	3	0.8	0.9	0.9	1.0
Rpusd3	Q14AI6	140	-8	-7	-10	-2	0.9	0.9	0.9	1.0
Acot11	Q8VHQ9	498	-6	-7	-10	-8	0.9	0.9	0.9	0.9
Pde4dip	Q80YT7	617	-1	-7	-10	-16	1.0	0.9	0.9	0.9
Zfyve16	Q80U44	851	-1	-7	-10	-18	1.0	0.9	0.9	0.8
Tubb6	Q922F4	12	-10	-7	-10	22	0.9	0.9	0.9	1.3
Cbx4	O55187	185	-11	-7	-10	-4	0.9	0.9	0.9	1.0
Papolg	Q6PCL9	607	-9	-7	-10	-10	0.9	0.9	0.9	0.9
Ddx10	Q80Y44	709	-10	-7	-10	-10	0.9	0.9	0.9	0.9
Dock8	Q8C147	187	-10	-7	-10	-13	0.9	0.9	0.9	0.9
UPF0538	Q9CRW3	111	-11	-7	-10	-16	0.9	0.9	0.9	0.9

Uri1	Q3TLD5	42	-11	-7	-10	-21	0.9	0.9	0.9	0.8
Psmd11	Q8BG32	289	-11	-8	-10	1	0.9	0.9	0.9	1.0
Nup205	A0A0J9YUD5	974	-17	-8	-10	0	0.9	0.9	0.9	1.0
Med29	Q9DB91	169	-15	-8	-10	-7	0.9	0.9	0.9	0.9
Eprs	Q8CGC7	910	-8	-8	-10	-9	0.9	0.9	0.9	0.9
Casp7	P97864	186	-19	-8	-10	-15	0.8	0.9	0.9	0.9
Nfu1	Q9QZ23	210	-5	-8	-10	24	1.0	0.9	0.9	1.3
Qki	Q9QYS9	35	-1	-8	-10	5	1.0	0.9	0.9	1.0
Eif2b3	B1AUN2	64	-17	-8	-10	2	0.9	0.9	0.9	1.0
Map4k1	P70218	333	-7	-8	-10	-3	0.9	0.9	0.9	1.0
Prdx5	P99029	96	-14	-8	-10	-3	0.9	0.9	0.9	1.0
Arrb2	Q91YI4	17	-15	-8	-10	-9	0.9	0.9	0.9	0.9
Nlrc5	C3VPR6	957	-10	-8	-10	-21	0.9	0.9	0.9	0.8
Thoc7	Q7TMY4	90	-12	-8	-10	-23	0.9	0.9	0.9	0.8
Lgals3	Q8C253	187	-15	-9	-10	9	0.9	0.9	0.9	1.1
Pxn	Q8VI36	582	-11	-9	-10	6	0.9	0.9	0.9	1.1
Vars	Q9Z1Q9	916	-13	-9	-10	5	0.9	0.9	0.9	1.1
Ckb	Q04447	283	-8	-9	-10	4	0.9	0.9	0.9	1.0
Ltn1	Q6A009	980	-12	-9	-10	-11	0.9	0.9	0.9	0.9
Tut1	Q8R3F9	354	-19	-9	-10	-11	0.8	0.9	0.9	0.9
Nudt13	Q8JZU0	347	-11	-9	-10	-16	0.9	0.9	0.9	0.9
Crtc1	Q68ED7	131	-10	-9	-10	18	0.9	0.9	0.9	1.2
Myd88	P22366	274	-12	-9	-10	2	0.9	0.9	0.9	1.0
Ncoa3	Q05BA5	23	-10	-9	-10	-3	0.9	0.9	0.9	1.0
Ctsd	P18242	117	-11	-9	-10	-5	0.9	0.9	0.9	1.0
Med14	A2ABV5	627	-9	-9	-10	-13	0.9	0.9	0.9	0.9
Wdr81	Q5ND34	638	-12	-9	-10	-22	0.9	0.9	0.9	0.8
Matr3	Q8K310	805	-17	-10	-10	0	0.9	0.9	0.9	1.0
Pef1	Q8BFY6	238	-15	-10	-10	-2	0.9	0.9	0.9	1.0
Gtf2h2	Q9JIB4	300	-9	-10	-10	-6	0.9	0.9	0.9	0.9
Fam98c	E9PYD1	293	-5	-10	-10	-11	1.0	0.9	0.9	0.9
Lrpprc	Q6PB66	499	-4	-10	-10	-11	1.0	0.9	0.9	0.9
Dapp1	Q9QXT1	137	-22	-10	-10	-11	0.8	0.9	0.9	0.9
Prrc2c	Q3TLH4	224	-11	-10	-10	-15	0.9	0.9	0.9	0.9
Snx20	Q9D2Y5	219	-20	-10	-10	-26	0.8	0.9	0.9	0.8
Yipf5	Q9EQQ2	42	-9	-10	-10	-26	0.9	0.9	0.9	0.8
Casp1	P29452	284	-15	-10	-10	3	0.9	0.9	0.9	1.0
Syne2	Q6ZWQ0	6148	-20	-10	-10	3	0.8	0.9	0.9	1.0
Bri3bp	Q8BXV2	218	-12	-10	-10	3	0.9	0.9	0.9	1.0
Ywhaz	P63101	94	-1	-10	-10	0	1.0	0.9	0.9	1.0
Card11	Q8CIS0	736	-8	-10	-10	-2	0.9	0.9	0.9	1.0
Rhoc	Q62159	107	-12	-10	-10	-2	0.9	0.9	0.9	1.0
Rhoa	Q9QUI0	107	-12	-10	-10	-2	0.9	0.9	0.9	1.0
Hnrnpu	Q8VEK3	583	-7	-10	-10	-6	0.9	0.9	0.9	0.9
Myo1e	E9Q634	960	-17	-10	-10	-7	0.9	0.9	0.9	0.9
Anapc7	Q9WVM3	225	-8	-10	-10	-7	0.9	0.9	0.9	0.9
Wdr53	Q9DB94	295	-15	-10	-10	-10	0.9	0.9	0.9	0.9
Npepl1	Q6NSR8	357	-7	-10	-10	-11	0.9	0.9	0.9	0.9
Tbc1d10c	Q8C9V1	51	-13	-10	-10	-11	0.9	0.9	0.9	0.9
Ctsb	P10605	319	-7	-10	-10	-27	0.9	0.9	0.9	0.8
Epb41l2	O70318	225	-19	-11	-10	10	0.8	0.9	0.9	1.1

Elmsan1	E9Q2I4	16	-14	-11	-10	6	0.9	0.9	0.9	1.1
Znf830	Q8R1N0	305	-12	-11	-10	-5	0.9	0.9	0.9	1.0
Gstk1	Q9DCM2	176	-9	-11	-10	-14	0.9	0.9	0.9	0.9
Ranbp2	Q9ERU9	2301	-10	-11	-10	-15	0.9	0.9	0.9	0.9
Pds5a	E9QPI5	1092	-12	-11	-10	-21	0.9	0.9	0.9	0.8
Pik3c2b	E9QAN8	705	-12	-11	-10	-24	0.9	0.9	0.9	0.8
Napg	Q9CWZ7	193	-19	-11	-10	10	0.8	0.9	0.9	1.1
Rbm12b2	Q66JV4	203	-13	-11	-10	-6	0.9	0.9	0.9	0.9
Trim34a	Q99PP6	173	-11	-11	-10	-6	0.9	0.9	0.9	0.9
Slfn9	B1ARD6	133	-11	-11	-10	-9	0.9	0.9	0.9	0.9
Dnajc7	Q9QYI3	317	-27	-12	-10	6	0.8	0.9	0.9	1.1
Nme1	P15532	145	-10	-12	-10	-2	0.9	0.9	0.9	1.0
DSCC1	Q14AI0	104	-12	-12	-10	-6	0.9	0.9	0.9	0.9
Eed	Q921E6	324	-14	-12	-10	-9	0.9	0.9	0.9	0.9
Ubr3	Q5U430	288	-18	-12	-10	-13	0.8	0.9	0.9	0.9
Nop2	E9QN31	448	-11	-12	-10	-14	0.9	0.9	0.9	0.9
Ctu1	Q99J10	290	-5	-12	-10	-14	1.0	0.9	0.9	0.9
Hmgcl	P38060	141	-13	-12	-10	-23	0.9	0.9	0.9	0.8
Bcl11b	Q99PV8	819	-24	-12	-10	-3	0.8	0.9	0.9	1.0
Dock11	A2AF47	1190	-10	-12	-10	-5	0.9	0.9	0.9	1.0
Sp140	Q6NSQ5	71	-6	-12	-10	-5	0.9	0.9	0.9	1.0
Actr3	Q99JY9	235	-15	-13	-10	15	0.9	0.9	0.9	1.2
Ddx58	Q6Q899	739	-17	-13	-10	14	0.9	0.9	0.9	1.2
Hdac10	Q6P3E7	102	-15	-13	-10	7	0.9	0.9	0.9	1.1
Tinf2	Q8K1K3	50	-2	-13	-10	6	1.0	0.9	0.9	1.1
Trim27	Q62158	365	-30	-13	-10	6	0.8	0.9	0.9	1.1
L3mbtl2	P59178	110	-10	-13	-10	-7	0.9	0.9	0.9	0.9
Eef2	P58252	567	2	-13	-10	-8	1.0	0.9	0.9	0.9
Gabpa	Q00422	421	-9	-13	-10	-11	0.9	0.9	0.9	0.9
Tyms	P07607	189	-13	-13	-10	17	0.9	0.9	0.9	1.2
Cox5b	Q9D881	116	-22	-13	-10	14	0.8	0.9	0.9	1.2
Cotl1	Q9CQI6	10	-6	-13	-10	10	0.9	0.9	0.9	1.1
Serpinb9	O08797	98	-28	-13	-10	4	0.8	0.9	0.9	1.0
Serpinb9b	Q9DAV6	98	-28	-13	-10	4	0.8	0.9	0.9	1.0
Sept9	Q80UG5	246	-1	-13	-10	-12	1.0	0.9	0.9	0.9
Zbp1	Q9QY24	312	-15	-13	-10	-13	0.9	0.9	0.9	0.9
Evi5l	H3BKQ3	653	-13	-13	-10	-16	0.9	0.9	0.9	0.9
Ahnak	E9Q616	4201	-11	-13	-10	-18	0.9	0.9	0.9	0.9
Hibch	Q8QZS1	94	-16	-14	-10	12	0.9	0.9	0.9	1.1
Wdfy4	E9Q2M9	889	-20	-14	-10	10	0.8	0.9	0.9	1.1
Syne1	Q6ZWR6	8630	-9	-14	-10	10	0.9	0.9	0.9	1.1
Zbtb11	G5E8B9	197	-11	-14	-10	1	0.9	0.9	0.9	1.0
Swsap1	Q8VCI7	221	-10	-14	-10	-2	0.9	0.9	0.9	1.0
Iqgap1	Q9JKF1	45	-14	-14	-10	-12	0.9	0.9	0.9	0.9
Pafah1b1	P63005	184	-11	-14	-10	-13	0.9	0.9	0.9	0.9
Rack1	P68040	168	-19	-14	-10	-35	0.8	0.9	0.9	0.7
Vps33a	Q9D2N9	121	-13	-14	-10	13	0.9	0.9	0.9	1.1
Ptpn11	P35235	259	-14	-14	-10	1	0.9	0.9	0.9	1.0
Acap1	Q8K2H4	328	-13	-14	-10	-7	0.9	0.9	0.9	0.9
Znf280d	Q68FE8	239	-7	-14	-10	-16	0.9	0.9	0.9	0.9
Rps8	P62242	174	-12	-14	-10	-19	0.9	0.9	0.9	0.8

Csrp2	P97314	61	-5	-15	-10	6	1.0	0.9	0.9	1.1
Csrp1	P97315	61	-5	-15	-10	6	1.0	0.9	0.9	1.1
Jak1	B1ASP2	1130	-15	-15	-10	-36	0.9	0.9	0.9	0.7
Cd79a	P11911	113	-15	-15	-10	19	0.9	0.9	0.9	1.2
Mvb12a	Q78HU3	229	-13	-15	-10	-8	0.9	0.9	0.9	0.9
Acat1	Q8QZT1	116	-20	-15	-10	-16	0.8	0.9	0.9	0.9
Smarca5	Q91ZW3	526	-19	-16	-10	13	0.8	0.9	0.9	1.1
Copg1	Q9QZE5	813	-7	-16	-10	4	0.9	0.9	0.9	1.0
Casp6	O08738	259	-21	-16	-10	-3	0.8	0.9	0.9	1.0
Lcp1	Q61233	283	-14	-16	-10	-4	0.9	0.9	0.9	1.0
Mad1l1	Q9WTX8	233	-4	-16	-10	-6	1.0	0.9	0.9	0.9
Ints12	Q9D168	378	7	-16	-10	-7	1.1	0.9	0.9	0.9
Tubb5	P99024	303	-7	-16	-10	-33	0.9	0.9	0.9	0.8
Tubb2a	Q7TMM9	303	-7	-16	-10	-33	0.9	0.9	0.9	0.8
Tubb6	Q922F4	303	-7	-16	-10	-33	0.9	0.9	0.9	0.8
Rxrb	P28704	462	-5	-16	-10	6	1.0	0.9	0.9	1.1
Eif4a2	P10630	135	-4	-16	-10	-8	1.0	0.9	0.9	0.9
Cbwd1	Q8VEH6	390	-21	-17	-10	-11	0.8	0.9	0.9	0.9
Cbr3	Q8K354	227	-11	-17	-10	-15	0.9	0.9	0.9	0.9
Ezh1	P70351	504	-15	-17	-10	-17	0.9	0.9	0.9	0.9
Ezh2	Q61188	503	-15	-17	-10	-17	0.9	0.9	0.9	0.9
Fbxl8	Q8CIG9	247	-15	-17	-10	-21	0.9	0.9	0.9	0.8
Irgm1	Q60766	257	-17	-17	-10	16	0.9	0.9	0.9	1.2
Phip	F8VQ93	28	-22	-17	-10	14	0.8	0.9	0.9	1.2
Cpsf1	Q9EPU4	1018	-2	-17	-10	4	1.0	0.9	0.9	1.0
Msh2	P43247	843	-10	-17	-10	2	0.9	0.9	0.9	1.0
Morc4	Q8BMD7	426	-6	-17	-10	-18	0.9	0.9	0.9	0.9
Opa1	P58281	786	-10	-17	-10	-20	0.9	0.9	0.9	0.8
Cttn	Q60598	246	-5	-18	-10	2	1.0	0.9	0.9	1.0
Gcn1	E9PVA8	756	-4	-18	-10	-16	1.0	0.9	0.9	0.9
Asap1	Q9QWY8	572	-13	-18	-10	-16	0.9	0.9	0.9	0.9
Sgf29	Q9DA08	287	2	-18	-10	-23	1.0	0.9	0.9	0.8
Ap2m1	P84091	246	-22	-18	-10	-4	0.8	0.8	0.9	1.0
Tmf1	B9EKI3	446	5	-18	-10	-13	1.1	0.8	0.9	0.9
Zfp84	Q9D654	485	-7	-18	-10	-15	0.9	0.8	0.9	0.9
Brms1l	Q3U1T3	137	1	-19	-10	-7	1.0	0.8	0.9	0.9
Ggnbp2	Q5SV77	356	-5	-19	-10	-13	1.0	0.8	0.9	0.9
Dnm1l	Q8K1M6	351	-15	-19	-10	-2	0.9	0.8	0.9	1.0
Inpp4a	Q9EPW0	465	-18	-19	-10	-13	0.9	0.8	0.9	0.9
Dlg1	Q811D0	378	-36	-20	-10	2	0.7	0.8	0.9	1.0
Atp2a3	Q64518	404	-19	-20	-10	-3	0.8	0.8	0.9	1.0
Nup93	Q8BJ71	569	-19	-20	-10	4	0.8	0.8	0.9	1.0
Plcl1	Q3USB7	951	-10	-20	-10	4	0.9	0.8	0.9	1.0
Ano6	Q6P9J9	251	-15	-20	-10	4	0.9	0.8	0.9	1.0
Smarca5	Q91ZW3	956	5	-20	-10	2	1.0	0.8	0.9	1.0
Rpp40	Q8R1F9	219	-22	-21	-10	-21	0.8	0.8	0.9	0.8
N4bp2	F8VQG7	1485	2	-21	-10	-24	1.0	0.8	0.9	0.8
Ints9	Q8K114	446	-28	-21	-10	11	0.8	0.8	0.9	1.1
Ddx5	Q8BTS0	354	-6	-22	-10	22	0.9	0.8	0.9	1.3
Rabgap1	A2AWA9	471	-13	-22	-10	-10	0.9	0.8	0.9	0.9
Suox	Q8R086	311	-1	-22	-10	-24	1.0	0.8	0.9	0.8

Trim34a	Q99PP6	414	-19	-22	-10	-25	0.8	0.8	0.9	0.8
Cdt1	Q8R4E9	12	-11	-22	-10	-25	0.9	0.8	0.9	0.8
Utp23	Q9CX11	60	13	-24	-10	4	1.1	0.8	0.9	1.0
Ube3c	Q80U95	158	-5	-26	-10	-8	1.0	0.8	0.9	0.9
Trafd1	Q3UDK1	135	34	-26	-10	-20	1.5	0.8	0.9	0.8
Tkt	P40142	386	-20	-26	-10	-23	0.8	0.8	0.9	0.8
Lmnb1	P14733	199	-20	-27	-10	0	0.8	0.8	0.9	1.0
Dhcr7	O88455	376	-5	-27	-10	-28	1.0	0.8	0.9	0.8
Heatr1	G3X9B1	1222	-9	-27	-10	-8	0.9	0.8	0.9	0.9
Ppil2	Q9D787	304	-20	-29	-10	2	0.8	0.8	0.9	1.0
Arhgap12	Q8C0D4	197	7	-29	-10	-25	1.1	0.8	0.9	0.8
Slc9a3r1	P70441	16	-5	-30	-10	-9	1.0	0.8	0.9	0.9
Hnrnpdl	Q9Z130	58	-28	-32	-10	6	0.8	0.8	0.9	1.1
Ampd2	Q9DBT5	42	0	-33	-10	-38	1.0	0.8	0.9	0.7
Ndufs1	Q91VD9	176	-46	-37	-10	-29	0.7	0.7	0.9	0.8
Thumpd1	Q99J36	169	0	11	-11	30	1.0	1.1	0.9	1.4
A430078G	E9Q7Y4	182	7	10	-11	11	1.1	1.1	0.9	1.1
Dbr1	Q923B1	8	-3	7	-11	20	1.0	1.1	0.9	1.2
Cltc	Q68FD5	926	-1	7	-11	27	1.0	1.1	0.9	1.4
Apobec3	Q99J72	279	-11	6	-11	15	0.9	1.1	0.9	1.2
Ap1b1	O35643	241	-7	6	-11	9	0.9	1.1	0.9	1.1
Ap2b1	Q9DBG3	241	-7	6	-11	9	0.9	1.1	0.9	1.1
G3bp1	P97855	73	-2	6	-11	4	1.0	1.1	0.9	1.0
Sit1	Q8C503	157	-5	5	-11	-9	1.0	1.0	0.9	0.9
Stk11ip	Q3TAA7	766	3	4	-11	-14	1.0	1.0	0.9	0.9
Fam208a	Q69ZR9	190	-3	3	-11	13	1.0	1.0	0.9	1.1
Naa15	G3X8Y3	58	-7	3	-11	12	0.9	1.0	0.9	1.1
Get4	Q9D1H7	160	-16	3	-11	11	0.9	1.0	0.9	1.1
Sf3b1	G5E866	796	-5	3	-11	13	1.0	1.0	0.9	1.1
Stat5b	P42232	101	-10	2	-11	3	0.9	1.0	0.9	1.0
Ahnak	E9Q616	3249	-3	2	-11	-9	1.0	1.0	0.9	0.9
Cdkn2aip	Q8BI72	302	-8	2	-11	-25	0.9	1.0	0.9	0.8
Snta1	Q61234	319	-2	1	-11	19	1.0	1.0	0.9	1.2
Hnmt	Q91VF2	82	-8	1	-11	-3	0.9	1.0	0.9	1.0
Rae1	Q8C570	106	-11	1	-11	13	0.9	1.0	0.9	1.1
Rpa1	Q8VEE4	332	-13	0	-11	23	0.9	1.0	0.9	1.3
Ecm29	Q6PDI5	1454	-8	0	-11	16	0.9	1.0	0.9	1.2
Narf	Q9CYQ7	105	-9	0	-11	6	0.9	1.0	0.9	1.1
Cryzl1	Q921W4	40	-7	-1	-11	32	0.9	1.0	0.9	1.5
Usp24	B1AY13	2157	0	-1	-11	-2	1.0	1.0	0.9	1.0
Macf1	E9PVY8	666	-3	-1	-11	-4	1.0	1.0	0.9	1.0
Srrm2	Q8BTI8	70	7	-2	-11	11	1.1	1.0	0.9	1.1
Zbtb33	Q8BN78	503	-3	-2	-11	7	1.0	1.0	0.9	1.1
Mri1	Q9CQT1	168	-10	-2	-11	-6	0.9	1.0	0.9	0.9
Wdfy4	E9Q2M9	857	-12	-2	-11	44	0.9	1.0	0.9	1.8
Anxa6	P14824	96	-4	-2	-11	7	1.0	1.0	0.9	1.1
Flna	Q8BTM8	841	-8	-2	-11	-2	0.9	1.0	0.9	1.0
Gm3839	S4R1W1	154	-4	-2	-11	-4	1.0	1.0	0.9	1.0
Nfx1	B1AY10	227	-9	-2	-11	-8	0.9	1.0	0.9	0.9
Uso1	Q9Z1Z0	303	-8	-3	-11	29	0.9	1.0	0.9	1.4
Apbb1ip	Q8R5A3	404	-20	-3	-11	16	0.8	1.0	0.9	1.2

Paics	Q9DCL9	185	-25	-3	-11	8	0.8	1.0	0.9	1.1
Actr3	Q99JY9	408	-13	-3	-11	3	0.9	1.0	0.9	1.0
Gimap1	P70224	144	-5	-3	-11	-11	1.0	1.0	0.9	0.9
Gtpbp1	O08582	661	-5	-3	-11	-26	1.0	1.0	0.9	0.8
Lypla1	P97823	144	-28	-3	-11	31	0.8	1.0	0.9	1.4
Foxp4	Q9DBY0	439	27	-3	-11	13	1.4	1.0	0.9	1.1
Blk	P16277	367	-8	-3	-11	-3	0.9	1.0	0.9	1.0
Eri1	Q7TMF2	71	-5	-3	-11	-8	1.0	1.0	0.9	0.9
Cct5	P80316	181	-17	-3	-11	-30	0.9	1.0	0.9	0.8
Rps6ka1	P18653	421	-6	-4	-11	12	0.9	1.0	0.9	1.1
Stau1	Q9Z108	185	-13	-4	-11	-9	0.9	1.0	0.9	0.9
Ssh1	Q76I79	510	-8	-4	-11	-15	0.9	1.0	0.9	0.9
Oat	P29758	150	-4	-4	-11	-6	1.0	1.0	0.9	0.9
Flii	Q9JJ28	560	-18	-4	-11	-10	0.8	1.0	0.9	0.9
Atp13a1	Q9EPE9	527	-13	-5	-11	28	0.9	1.0	0.9	1.4
Cand1	Q6ZQ38	1134	-16	-5	-11	19	0.9	1.0	0.9	1.2
Ipo9	E9QKZ2	90	-4	-5	-11	11	1.0	1.0	0.9	1.1
Daxx	Q3UKR0	251	6	-5	-11	6	1.1	1.0	0.9	1.1
Cabin1	G3X8Q1	717	-14	-5	-11	2	0.9	1.0	0.9	1.0
Strn	O55106	665	-1	-5	-11	-4	1.0	1.0	0.9	1.0
Trmt2a	Q8BNV1	260	-4	-5	-11	-10	1.0	1.0	0.9	0.9
Wdr3	Q8BHB4	240	-10	-5	-11	-15	0.9	1.0	0.9	0.9
Cep350	E9Q309	2428	-9	-5	-11	-26	0.9	1.0	0.9	0.8
Dck	P43346	59	-9	-5	-11	17	0.9	1.0	0.9	1.2
Dnmbp	Q6TXD4	690	-9	-5	-11	17	0.9	1.0	0.9	1.2
Phf5a	P83870	49	-6	-5	-11	11	0.9	1.0	0.9	1.1
Als2cl	Q60I26	320	-1	-5	-11	9	1.0	1.0	0.9	1.1
Btaf1	E9QAE3	1541	-5	-5	-11	9	1.0	1.0	0.9	1.1
Flnb	Q80X90	1081	-5	-5	-11	5	1.0	1.0	0.9	1.1
Flnc	Q8VHX6	1104	-5	-5	-11	5	1.0	1.0	0.9	1.1
Hnrnph1	O35737	122	-18	-5	-11	4	0.9	1.0	0.9	1.0
Hnrnph2	P70333	122	-18	-5	-11	4	0.9	1.0	0.9	1.0
Tbc1d4	Q8BYJ6	1286	-12	-5	-11	1	0.9	1.0	0.9	1.0
Abcb8	Q9CXJ4	465	-11	-5	-11	-1	0.9	1.0	0.9	1.0
Gatad2b	Q8VHR5	424	-17	-5	-11	-3	0.9	1.0	0.9	1.0
Rnf40	Q3U319	950	-9	-5	-11	-4	0.9	1.0	0.9	1.0
Acap1	Q8K2H4	501	-6	-5	-11	-8	0.9	1.0	0.9	0.9
Nfatc1	B5B2N2	265	-22	-5	-11	-10	0.8	1.0	0.9	0.9
Wbscr27	Q8BGM4	27	-14	-5	-11	-11	0.9	1.0	0.9	0.9
Wdr91	Q7TMQ7	354	-21	-5	-11	-18	0.8	1.0	0.9	0.9
Mapre1	Q61166	228	-12	-6	-11	11	0.9	0.9	0.9	1.1
Itsn2	E9QNG1	861	-9	-6	-11	-7	0.9	0.9	0.9	0.9
Tcof1	O08784	465	-9	-6	-11	-17	0.9	0.9	0.9	0.9
Crocc	Q8CJ40	692	3	-6	-11	-18	1.0	0.9	0.9	0.9
Scfd1	Q8BRF7	482	-8	-6	-11	8	0.9	0.9	0.9	1.1
Daglb	Q91WC9	574	-1	-6	-11	-13	1.0	0.9	0.9	0.9
Ago2	Q8CJG0	328	-18	-7	-11	9	0.8	0.9	0.9	1.1
Ago1	Q8CJG1	325	-18	-7	-11	9	0.8	0.9	0.9	1.1
Pds5b	Q4VA53	573	-10	-7	-11	8	0.9	0.9	0.9	1.1
Mtor	Q9JLN9	1214	-7	-7	-11	2	0.9	0.9	0.9	1.0
Rin3	P59729	878	-13	-7	-11	-2	0.9	0.9	0.9	1.0

Cdk11b	P24788	429	-10	-7	-11	-6	0.9	0.9	0.9	0.9
Apbb1ip	Q8R5A3	384	-10	-7	-11	5	0.9	0.9	0.9	1.1
Trim25	Q61510	228	-7	-7	-11	0	0.9	0.9	0.9	1.0
Hspa8	P63017	17	-19	-7	-11	-2	0.8	0.9	0.9	1.0
Rbm39	Q8VH51	303	-14	-7	-11	-7	0.9	0.9	0.9	0.9
Fuk	Q7TMC8	1080	-10	-7	-11	-7	0.9	0.9	0.9	0.9
Cnot6	Q8K3P5	451	5	-7	-11	-9	1.0	0.9	0.9	0.9
Taf12	Q8VE65	143	-11	-7	-11	-13	0.9	0.9	0.9	0.9
Herc1	E9PZP8	56	-5	-8	-11	19	1.0	0.9	0.9	1.2
Taf5l	Q91WQ5	15	-8	-8	-11	2	0.9	0.9	0.9	1.0
Pola1	P33609	600	-2	-8	-11	-6	1.0	0.9	0.9	0.9
Cnp	P16330	111	-12	-8	-11	-25	0.9	0.9	0.9	0.8
Aldh4a1	Q8CHT0	347	-9	-8	-11	-59	0.9	0.9	0.9	0.6
Got1	P05201	160	-9	-8	-11	9	0.9	0.9	0.9	1.1
Upf3b	Q3ULL6	26	-10	-8	-11	-5	0.9	0.9	0.9	1.0
Aptx	Q7TQC5	310	-10	-8	-11	-5	0.9	0.9	0.9	1.0
Pik3r1	P26450	146	-14	-8	-11	-8	0.9	0.9	0.9	0.9
Taf6	Q62311	130	-10	-8	-11	-8	0.9	0.9	0.9	0.9
C2cd5	Q7TPS5	884	-9	-8	-11	-12	0.9	0.9	0.9	0.9
Psmb1	O09061	223	-15	-8	-11	-16	0.9	0.9	0.9	0.9
Chuk	E9QNL4	406	-8	-9	-11	11	0.9	0.9	0.9	1.1
Slc25a1	Q8JZU2	70	-18	-9	-11	3	0.8	0.9	0.9	1.0
Gltscr1	F8VPZ9	1396	-8	-9	-11	-5	0.9	0.9	0.9	1.0
Rab3gap1	Q80UJ7	693	-21	-9	-11	-12	0.8	0.9	0.9	0.9
Ddx51	Q6P9R1	225	-14	-9	-11	-21	0.9	0.9	0.9	0.8
Ddx21	Q9JIK5	363	-8	-9	-11	17	0.9	0.9	0.9	1.2
Gpsm3	Q3U1Z5	115	-15	-9	-11	17	0.9	0.9	0.9	1.2
Scaf8	Q6DID3	102	-24	-9	-11	17	0.8	0.9	0.9	1.2
Kat6a	G3X940	392	-13	-9	-11	4	0.9	0.9	0.9	1.0
Myo1f	Q8CG29	101	-14	-9	-11	2	0.9	0.9	0.9	1.0
Trim25	Q61510	70	-16	-9	-11	-1	0.9	0.9	0.9	1.0
Cep128	Q8BI22	561	-15	-9	-11	-9	0.9	0.9	0.9	0.9
Gse1	Q3U3C9	629	-6	-9	-11	-14	0.9	0.9	0.9	0.9
Ep300	B2RWS6	1672	-11	-10	-11	5	0.9	0.9	0.9	1.1
Crebbp	F8VPR5	1711	-11	-10	-11	5	0.9	0.9	0.9	1.1
Btaf1	E9QAE3	108	-14	-10	-11	-7	0.9	0.9	0.9	0.9
Celf1	P28659	119	-12	-10	-11	-9	0.9	0.9	0.9	0.9
Sp2	Q8C5J0	81	-10	-10	-11	-3	0.9	0.9	0.9	1.0
Zmym2	Q9CU65	611	-17	-10	-11	-5	0.9	0.9	0.9	1.0
Rif1	Q6PR54	1641	2	-10	-11	-13	1.0	0.9	0.9	0.9
Apobr	Q8VBT6	258	-14	-10	-11	-16	0.9	0.9	0.9	0.9
Mndal	D0QMC3	267	-15	-10	-11	-19	0.9	0.9	0.9	0.8
Ifi204	P0DOV2	181	-15	-10	-11	-19	0.9	0.9	0.9	0.8
Acss1	Q99NB1	144	-27	-11	-11	29	0.8	0.9	0.9	1.4
Pitrm1	Q8K411	241	-11	-11	-11	-5	0.9	0.9	0.9	1.0
Setd1a	E9PYH6	1657	-19	-11	-11	-9	0.8	0.9	0.9	0.9
Rrbp1	Q99PL5	845	-2	-11	-11	-20	1.0	0.9	0.9	0.8
Mthfd1l	Q3V3R1	905	-12	-11	-11	-26	0.9	0.9	0.9	0.8
Adh5	P28474	174	-11	-11	-11	-6	0.9	0.9	0.9	0.9
Usp9x	Q4FE56	787	-12	-11	-11	-9	0.9	0.9	0.9	0.9
Vps53	Q8CCB4	764	-10	-11	-11	-9	0.9	0.9	0.9	0.9

Hspa8	P63017	603	-20	-11	-11	-11	0.8	0.9	0.9	0.9
Arhgef1	Q61210	891	-14	-11	-11	-15	0.9	0.9	0.9	0.9
Osgep	Q8BWU5	265	-10	-12	-11	-1	0.9	0.9	0.9	1.0
Txnrd1	Q9JMH6	173	-12	-12	-11	-4	0.9	0.9	0.9	1.0
Syne1	Q6ZWR6	4512	-14	-12	-11	-7	0.9	0.9	0.9	0.9
Zak	Q9ESL4	150	1	-12	-11	-10	1.0	0.9	0.9	0.9
Gimap1	P70224	74	-7	-12	-11	-16	0.9	0.9	0.9	0.9
Prorsd1	Q9D820	105	-9	-12	-11	-3	0.9	0.9	0.9	1.0
Ado	Q6PDY2	18	-13	-12	-11	-3	0.9	0.9	0.9	1.0
Uba1	Q02053	632	-10	-12	-11	-5	0.9	0.9	0.9	1.0
Rab22a	P35285	9	-6	-12	-11	-6	0.9	0.9	0.9	0.9
Nfatc2	Q60591	571	-33	-12	-11	-6	0.8	0.9	0.9	0.9
Rab31	Q921E2	9	-6	-12	-11	-6	0.9	0.9	0.9	0.9
Ankle2	Q6P1H6	358	-13	-12	-11	-7	0.9	0.9	0.9	0.9
Tmpo	Q61033	517	-8	-12	-11	-12	0.9	0.9	0.9	0.9
Trim33	Q99PP7	864	-9	-12	-11	-18	0.9	0.9	0.9	0.9
Gpd2	Q64521	270	-12	-13	-11	10	0.9	0.9	0.9	1.1
Lin54	Q571G4	700	-6	-13	-11	3	0.9	0.9	0.9	1.0
Pml	Q60953	393	-9	-13	-11	-9	0.9	0.9	0.9	0.9
Top2b	Q64511	259	-15	-13	-11	3	0.9	0.9	0.9	1.0
Tnfaip8l2	Q9D8Y7	78	-20	-13	-11	-1	0.8	0.9	0.9	1.0
Ppp2cb	P62715	133	-25	-13	-11	-3	0.8	0.9	0.9	1.0
Ppp2ca	P63330	133	-25	-13	-11	-3	0.8	0.9	0.9	1.0
Ppp4c	P97470	130	-25	-13	-11	-3	0.8	0.9	0.9	1.0
Fry	E9Q8I9	1809	-16	-13	-11	-4	0.9	0.9	0.9	1.0
Ksr1	Q61097	60	-14	-13	-11	-19	0.9	0.9	0.9	0.8
Smarca2	Q6DIC0	1301	-5	-14	-11	-17	1.0	0.9	0.9	0.9
Lrrfip1	Q3UZ39	354	-10	-14	-11	-27	0.9	0.9	0.9	0.8
Etfa	Q99LC5	155	-13	-14	-11	-27	0.9	0.9	0.9	0.8
Sqrdl	Q9R112	361	-5	-14	-11	-44	1.0	0.9	0.9	0.7
Supt4h1a	P63271	16	-18	-14	-11	4	0.9	0.9	0.9	1.0
Acsl3	Q9CZW4	450	-15	-14	-11	2	0.9	0.9	0.9	1.0
Myo1g	Q5SUA5	509	-18	-14	-11	-3	0.9	0.9	0.9	1.0
Ahctf1	Q8CJF7	1777	-18	-14	-11	-12	0.9	0.9	0.9	0.9
Prps1	Q9D7G0	265	-14	-14	-11	-13	0.9	0.9	0.9	0.9
Flna	Q8BTM8	631	-19	-14	-11	-14	0.8	0.9	0.9	0.9
Flnc	Q8VHX6	627	-19	-14	-11	-14	0.8	0.9	0.9	0.9
Scml4	Q80VG1	397	-8	-14	-11	-16	0.9	0.9	0.9	0.9
Tbc1d2	B1AVH7	645	-6	-14	-11	-17	0.9	0.9	0.9	0.9
Mdh2	P08249	285	-15	-15	-11	11	0.9	0.9	0.9	1.1
Numa1	E9Q7G0	1717	-7	-15	-11	-17	0.9	0.9	0.9	0.9
Hcfc2	G5E837	345	-16	-15	-11	-19	0.9	0.9	0.9	0.8
Parg	O88622	704	-15	-15	-11	-19	0.9	0.9	0.9	0.8
Tpm3-rs7	D3Z2H9	233	1	-15	-11	-72	1.0	0.9	0.9	0.6
Lrpprc	Q6PB66	129	-15	-15	-11	14	0.9	0.9	0.9	1.2
Rnft1	Q9DCN7	379	-14	-15	-11	-4	0.9	0.9	0.9	1.0
Cep350	E9Q309	80	-1	-15	-11	-5	1.0	0.9	0.9	1.0
Tasp1	Q8R1G1	36	-6	-15	-11	-9	0.9	0.9	0.9	0.9
Fam120c	Q8C3F2	1050	-14	-15	-11	-15	0.9	0.9	0.9	0.9
Polr1a	O35134	1339	-20	-15	-11	-16	0.8	0.9	0.9	0.9
Pdf	S4R2K0	96	-28	-15	-11	-28	0.8	0.9	0.9	0.8

Arhgap21	B7ZCJ1	1839	-11	-16	-11	17	0.9	0.9	0.9	1.2
Rnpep	Q8VCT3	69	-19	-16	-11	-5	0.8	0.9	0.9	1.0
Ada	P03958	75	-11	-16	-11	-9	0.9	0.9	0.9	0.9
Adcy7	P51829	914	1	-16	-11	5	1.0	0.9	0.9	1.1
Nop56	Q9D6Z1	471	-15	-16	-11	-16	0.9	0.9	0.9	0.9
Orc1	Q9Z1N2	256	-5	-16	-11	-22	1.0	0.9	0.9	0.8
Prpf8	Q99PV0	1228	-26	-17	-11	4	0.8	0.9	0.9	1.0
Mast3	Q3U214	563	-15	-17	-11	-3	0.9	0.9	0.9	1.0
Nhlrc2	Q8BZW8	715	-16	-17	-11	-5	0.9	0.9	0.9	1.0
Pcyox1	Q9CQF9	445	-24	-17	-11	-13	0.8	0.9	0.9	0.9
Xpc	P51612	500	-18	-17	-11	-15	0.9	0.9	0.9	0.9
Xpc	P51612	59	-9	-17	-11	-18	0.9	0.9	0.9	0.9
Nup155	Q99P88	561	74	-17	-11	-37	3.8	0.9	0.9	0.7
Chd4	Q6PDQ2	486	-20	-17	-11	3	0.8	0.9	0.9	1.0
Hnrnpul2	Q00PI9	291	-13	-17	-11	0	0.9	0.9	0.9	1.0
Golgb1	E9PVZ8	2645	-9	-17	-11	-11	0.9	0.9	0.9	0.9
Adcy7	P51829	1087	-17	-17	-11	-16	0.9	0.9	0.9	0.9
Ttc33	Q9D6K7	55	-12	-17	-11	-21	0.9	0.9	0.9	0.8
Dstn	Q9R0P5	147	-17	-18	-11	-7	0.9	0.8	0.9	0.9
Cstf1	Q99LC2	380	-16	-19	-11	9	0.9	0.8	0.9	1.1
Ncf1	Q09014	111	-10	-19	-11	-6	0.9	0.8	0.9	0.9
Gnai2	P08752	140	-26	-19	-11	1	0.8	0.8	0.9	1.0
Smc6	Q924W5	391	-14	-19	-11	-2	0.9	0.8	0.9	1.0
Ltf	P08071	188	-19	-20	-11	-2	0.8	0.8	0.9	1.0
Mettl25	Q6NXH8	484	-18	-20	-11	-18	0.9	0.8	0.9	0.8
Pop1	Q8K205	441	-31	-20	-11	47	0.8	0.8	0.9	1.9
Zmym1	Q3TJB1	830	-16	-20	-11	-3	0.9	0.8	0.9	1.0
Csrp1	P97315	58	-13	-21	-11	-10	0.9	0.8	0.9	0.9
Cdkn2aip	Q8BI72	486	-37	-21	-11	1	0.7	0.8	0.9	1.0
Lrrc41	Q8K1C9	602	-14	-22	-11	10	0.9	0.8	0.9	1.1
Kiaa0196	Q8C2E7	21	-26	-22	-11	-19	0.8	0.8	0.9	0.8
Angel2	Q8K1C0	274	-2	-22	-11	-20	1.0	0.8	0.9	0.8
Rps6ka4	Q9Z2B9	257	-15	-22	-11	-1	0.9	0.8	0.9	1.0
Nsun2	Q1HFZ0	501	3	-22	-11	-5	1.0	0.8	0.9	1.0
Atad2	G3X963	366	-9	-22	-11	-12	0.9	0.8	0.9	0.9
Cabin1	G3X8Q1	690	-13	-23	-11	-8	0.9	0.8	0.9	0.9
Fbxl8	Q8CIG9	190	-27	-23	-11	-14	0.8	0.8	0.9	0.9
Rfc1	G3UWX1	605	-15	-23	-11	11	0.9	0.8	0.9	1.1
Rock1	P70335	231	-8	-23	-11	-13	0.9	0.8	0.9	0.9
Cebpb	P28033	201	-8	-24	-11	-15	0.9	0.8	0.9	0.9
Fhl2	O70433	150	-6	-27	-11	-28	0.9	0.8	0.9	0.8
Clptm1	Q8VBZ3	454	-9	-28	-11	-27	0.9	0.8	0.9	0.8
Ube2z	Q3UE37	156	-31	-30	-11	1	0.8	0.8	0.9	1.0
Usp48	Q3V0C5	124	-8	-31	-11	-3	0.9	0.8	0.9	1.0
Cdk4	P30285	135	-25	-32	-11	-27	0.8	0.8	0.9	0.8
Smpd4	Q6ZPR5	182	-8	-33	-11	-29	0.9	0.8	0.9	0.8
Cdc16	Q8R349	544	16	-42	-11	-10	1.2	0.7	0.9	0.9
Wdfy3	Q6VNB8	3009	2	23	-11	13	1.0	1.3	0.9	1.1
Hspa9	P38647	487	-6	9	-11	-4	0.9	1.1	0.9	1.0
Elane	Q3UP87	54	-22	9	-11	-6	0.8	1.1	0.9	0.9
Hook1	Q8BIL5	652	6	8	-11	11	1.1	1.1	0.9	1.1

Nasp	Q99MD9	84	9	7	-11	28	1.1	1.1	0.9	1.4
Atp5c1	Q91VR2	103	-18	6	-11	13	0.9	1.1	0.9	1.1
Sh2d1a	O88890	122	-2	5	-11	9	1.0	1.0	0.9	1.1
Ogfr	Q99PG2	181	2	3	-11	5	1.0	1.0	0.9	1.0
Ago1	Q8CJG1	270	-2	3	-11	-4	1.0	1.0	0.9	1.0
Lxn	P70202	20	3	2	-11	-1	1.0	1.0	0.9	1.0
Vars	Q9Z1Q9	380	-3	2	-11	-4	1.0	1.0	0.9	1.0
Braf	P28028	178	2	1	-11	-4	1.0	1.0	0.9	1.0
Sec13	Q9D1M0	31	-1	0	-11	-10	1.0	1.0	0.9	0.9
Ccs	Q9WU84	244	-13	-1	-11	-3	0.9	1.0	0.9	1.0
Tbc1d13	Q8R3D1	387	-16	-1	-11	-5	0.9	1.0	0.9	1.0
Serpinb6	Q60854	102	2	-1	-11	10	1.0	1.0	0.9	1.1
Letm1	Q9Z2I0	378	-4	-1	-11	7	1.0	1.0	0.9	1.1
Iqgap1	Q9JKF1	494	-7	-1	-11	-12	0.9	1.0	0.9	0.9
Rpl10a	Q5XJF6	74	-8	-2	-11	14	0.9	1.0	0.9	1.2
Csl	Q80X68	101	-12	-2	-11	8	0.9	1.0	0.9	1.1
Glcci1	Q8K3I9	435	-8	-2	-11	-7	0.9	1.0	0.9	0.9
Brms1	Q99N20	126	-13	-2	-11	2	0.9	1.0	0.9	1.0
Sssca1	P56873	53	-8	-2	-11	-1	0.9	1.0	0.9	1.0
Rai1	Q61818	1437	-9	-2	-11	-5	0.9	1.0	0.9	1.0
Zmym2	Q9CU65	822	-8	-2	-11	-8	0.9	1.0	0.9	0.9
Helz2	E9QAM5	1331	-9	-2	-11	-18	0.9	1.0	0.9	0.9
Ciapin1	Q8WTY4	271	-11	-2	-11	-18	0.9	1.0	0.9	0.8
Hmgcs1	Q8JZK9	224	-15	-3	-11	31	0.9	1.0	0.9	1.4
Arid1a	A2BH40	2161	2	-3	-11	-4	1.0	1.0	0.9	1.0
Arid1b	E9Q4N7	2122	2	-3	-11	-4	1.0	1.0	0.9	1.0
UPF0711	Q5XFZ0	18	-5	-3	-11	-17	1.0	1.0	0.9	0.9
Mettl1	Q9Z120	130	-6	-3	-11	16	0.9	1.0	0.9	1.2
Atg4b	Q8BGE6	74	-10	-3	-11	5	0.9	1.0	0.9	1.0
Vps13c	Q8BX70	363	4	-3	-11	-9	1.0	1.0	0.9	0.9
Ints2	Q80UK8	1158	-11	-4	-11	8	0.9	1.0	0.9	1.1
Hccs	P53702	39	-6	-4	-11	-6	0.9	1.0	0.9	0.9
Llgl2	Q3TJ91	170	-7	-4	-11	-31	0.9	1.0	0.9	0.8
Hnrnpu	Q8VEK3	426	-14	-4	-11	10	0.9	1.0	0.9	1.1
Exosc9	Q9JHI7	61	-5	-4	-11	9	1.0	1.0	0.9	1.1
Mfn1	Q811U4	411	-9	-4	-11	-9	0.9	1.0	0.9	0.9
Rbl1	Q64701	1042	-3	-4	-11	-20	1.0	1.0	0.9	0.8
Wdfy4	E9Q2M9	582	-6	-4	-11	-23	0.9	1.0	0.9	0.8
Elf2	Q9JHC9	472	-14	-5	-11	-1	0.9	1.0	0.9	1.0
Vezf1	Q5SXC4	289	-15	-5	-11	-6	0.9	1.0	0.9	0.9
Hcfc2	G5E837	346	-10	-5	-11	-12	0.9	1.0	0.9	0.9
Pds5a	E9QPI5	1078	-13	-5	-11	8	0.9	1.0	0.9	1.1
Ddx1	Q91VR5	631	-24	-5	-11	5	0.8	1.0	0.9	1.1
Tmpo	Q61033	254	-7	-5	-11	-2	0.9	1.0	0.9	1.0
Cul3	Q9JLV5	464	-3	-5	-11	-2	1.0	1.0	0.9	1.0
Csrp1	P97315	167	-13	-5	-11	-3	0.9	1.0	0.9	1.0
Ptbp3	Q8BHD7	26	-5	-6	-11	-1	1.0	0.9	0.9	1.0
Grhpr	Q91Z53	57	-22	-6	-11	-4	0.8	0.9	0.9	1.0
Ints10	Q8K2A7	281	-23	-6	-11	14	0.8	0.9	0.9	1.2
Glyr1	Q922P9	317	-14	-6	-11	10	0.9	0.9	0.9	1.1
Tln1	P26039	2196	-12	-6	-11	8	0.9	0.9	0.9	1.1

Gnb1	P62874	204	-10	-7	-11	8	0.9	0.9	0.9	1.1
Ints7	Q7TQK1	374	-6	-7	-11	-2	0.9	0.9	0.9	1.0
Slc25a23	Q6GQS1	320	-6	-7	-11	-5	0.9	0.9	0.9	1.0
Kdm6a	O70546	404	-12	-7	-11	-8	0.9	0.9	0.9	0.9
Ocrl	Q6NVF0	427	-11	-7	-11	4	0.9	0.9	0.9	1.0
Pfas	Q5SUR0	606	-9	-7	-11	-7	0.9	0.9	0.9	0.9
Utp20	E9QK83	1758	-11	-7	-11	-7	0.9	0.9	0.9	0.9
Eea1	Q8BL66	783	-7	-7	-11	-13	0.9	0.9	0.9	0.9
Arhgap17	Q3UIA2	364	-11	-8	-11	14	0.9	0.9	0.9	1.2
Lasp1	Q61792	53	-8	-8	-11	10	0.9	0.9	0.9	1.1
Usp19	Q3UJD6	859	-8	-8	-11	9	0.9	0.9	0.9	1.1
Rufy1	Q8BIJ7	188	-15	-8	-11	4	0.9	0.9	0.9	1.0
Parp1	Q921K2	456	-7	-8	-11	-4	0.9	0.9	0.9	1.0
Anp32a	O35381	27	-7	-8	-11	4	0.9	0.9	0.9	1.0
Gcn1	E9PVA8	2563	-16	-8	-11	2	0.9	0.9	0.9	1.0
Uncharact	A0A1B0GSH8	250	-14	-8	-11	-2	0.9	0.9	0.9	1.0
Ikbke	Q9R0T8	524	-17	-8	-11	-5	0.9	0.9	0.9	1.0
Rgs19	Q9CX84	73	1	-8	-11	-6	1.0	0.9	0.9	0.9
Kmt2c	Q8BRH4	4635	-23	-8	-11	-9	0.8	0.9	0.9	0.9
Rnf113a2	Q14B01	15	-6	-8	-11	-13	0.9	0.9	0.9	0.9
Mmaa	Q8C7H1	181	-10	-8	-11	-14	0.9	0.9	0.9	0.9
Cdc42bpb	Q7TT50	1519	-9	-8	-11	-25	0.9	0.9	0.9	0.8
Tln1	P26039	1671	-6	-9	-11	12	0.9	0.9	0.9	1.1
Mms19	Q9D071	582	-9	-9	-11	2	0.9	0.9	0.9	1.0
Csk	P41241	290	-8	-9	-11	-7	0.9	0.9	0.9	0.9
Dctn1	O08788	636	-16	-9	-11	-11	0.9	0.9	0.9	0.9
Hck	P08103	12	-16	-9	-11	-11	0.9	0.9	0.9	0.9
Aars	Q8BGQ7	403	-11	-9	-11	-11	0.9	0.9	0.9	0.9
Ttc5	Q99LG4	439	-8	-9	-11	-11	0.9	0.9	0.9	0.9
Golgb1	E9PVZ8	1451	-9	-9	-11	-20	0.9	0.9	0.9	0.8
Gfpt1	P47856	262	-6	-9	-11	-26	0.9	0.9	0.9	0.8
Arhgef1	Q61210	751	-7	-9	-11	7	0.9	0.9	0.9	1.1
Nat10	Q8K224	499	-10	-9	-11	6	0.9	0.9	0.9	1.1
Actn4	P57780	174	-13	-9	-11	2	0.9	0.9	0.9	1.0
Actn1	Q7TPR4	154	-13	-9	-11	2	0.9	0.9	0.9	1.0
Sh3bgrl	Q9JJU8	25	-9	-9	-11	0	0.9	0.9	0.9	1.0
Ddx55	Q6ZPL9	437	-13	-9	-11	-2	0.9	0.9	0.9	1.0
Pi4ka	E9Q3L2	1846	-9	-9	-11	-4	0.9	0.9	0.9	1.0
Trnau1ap	Q80VC6	165	-7	-9	-11	-4	0.9	0.9	0.9	1.0
Cyfip2	Q5SQX6	427	-4	-9	-11	-11	1.0	0.9	0.9	0.9
Acap2	Q6ZQK5	339	-22	-10	-11	10	0.8	0.9	0.9	1.1
Dnajc11	Q5U458	494	-13	-10	-11	5	0.9	0.9	0.9	1.0
Prps1	Q9D7G0	41	-11	-10	-11	-8	0.9	0.9	0.9	0.9
Rps17	P63276	35	-8	-10	-11	-11	0.9	0.9	0.9	0.9
Kdm4b	Q91VY5	309	-15	-10	-11	-15	0.9	0.9	0.9	0.9
Atp6v1b2	P62814	112	-15	-10	-11	-19	0.9	0.9	0.9	0.8
Stk39	Q9Z1W9	536	-4	-10	-11	-21	1.0	0.9	0.9	0.8
Dock8	Q8C147	2092	-13	-10	-11	-25	0.9	0.9	0.9	0.8
Cpsf2	O35218	765	-11	-10	-11	20	0.9	0.9	0.9	1.3
Acaa2	Q8BWT1	92	-11	-10	-11	9	0.9	0.9	0.9	1.1
Arhgap9	Q8QZW8	225	-12	-10	-11	-2	0.9	0.9	0.9	1.0

Mbip	Q99LQ1	241	-6	-10	-11	-6	0.9	0.9	0.9	0.9
Rgs14	P97492	465	-3	-11	-11	-2	1.0	0.9	0.9	1.0
C1qbp	Q8R5L1	183	-17	-11	-11	-7	0.9	0.9	0.9	0.9
Sp140	Q6NSQ5	440	-13	-11	-11	-7	0.9	0.9	0.9	0.9
Cep350	E9Q309	1426	-17	-11	-11	-12	0.9	0.9	0.9	0.9
Renbp	P82343	250	-7	-11	-11	-13	0.9	0.9	0.9	0.9
Ube2f	Q9CY34	50	-11	-11	-11	-13	0.9	0.9	0.9	0.9
Uhrf2	Q7TMI3	705	-6	-11	-11	-17	0.9	0.9	0.9	0.9
Map3k3	Q61084	411	-27	-11	-11	-20	0.8	0.9	0.9	0.8
Ruvbl2	Q9WTM5	227	-24	-11	-11	2	0.8	0.9	0.9	1.0
Utp6	Q8VCY6	175	16	-11	-11	-1	1.2	0.9	0.9	1.0
Vav1	P27870	369	-7	-11	-11	-6	0.9	0.9	0.9	0.9
Tcf7	Q00417	326	-11	-11	-11	-8	0.9	0.9	0.9	0.9
Fam208a	Q69ZR9	823	-12	-11	-11	-15	0.9	0.9	0.9	0.9
Cct3	P80318	398	-6	-12	-11	3	0.9	0.9	0.9	1.0
Mrps14	Q9CR88	91	-18	-12	-11	-6	0.8	0.9	0.9	0.9
Rcc2	Q8BK67	142	-13	-12	-11	-8	0.9	0.9	0.9	0.9
2010005H	Q9D8D6	63	-19	-12	-11	-10	0.8	0.9	0.9	0.9
Nup133	Q8R0G9	199	-8	-12	-11	-13	0.9	0.9	0.9	0.9
Zmynd11	Q8R5C8	73	-10	-12	-11	-14	0.9	0.9	0.9	0.9
Top2a	Q01320	391	-11	-12	-11	43	0.9	0.9	0.9	1.7
Dhrs1	Q99L04	235	-31	-12	-11	10	0.8	0.9	0.9	1.1
Ddx5	Q8BTS0	234	-10	-12	-11	-11	0.9	0.9	0.9	0.9
Ankmy2	Q3TPE9	277	-1	-12	-11	-13	1.0	0.9	0.9	0.9
UPF0183	Q922R1	222	-16	-12	-11	-16	0.9	0.9	0.9	0.9
Hsph1	Q61699	651	-10	-12	-11	-19	0.9	0.9	0.9	0.8
Cc2d1b	Q8BRN9	216	-12	-12	-11	-20	0.9	0.9	0.9	0.8
Noc2l	J3QK52	199	-6	-13	-11	-1	0.9	0.9	0.9	1.0
Ddx21	Q9JIK5	517	-10	-13	-11	-1	0.9	0.9	0.9	1.0
Supt20h	Q7TT00	298	-9	-13	-11	-7	0.9	0.9	0.9	0.9
Ruvbl2	Q9WTM5	413	-25	-13	-11	-10	0.8	0.9	0.9	0.9
Kif1b	Q60575	600	-8	-13	-11	-10	0.9	0.9	0.9	0.9
Tln1	P26039	1087	-13	-13	-11	10	0.9	0.9	0.9	1.1
Nelfa	Q8BG30	473	-11	-13	-11	-2	0.9	0.9	0.9	1.0
Sept11	Q8C1B7	268	-18	-14	-11	11	0.8	0.9	0.9	1.1
Sept8	Q8CHH9	271	-18	-14	-11	11	0.8	0.9	0.9	1.1
Cenpv	Q9CXS4	217	-16	-14	-11	-2	0.9	0.9	0.9	1.0
Glg1	Q61543	231	-6	-14	-11	-5	0.9	0.9	0.9	1.0
Coro1c	Q9WUM4	456	-12	-14	-11	-12	0.9	0.9	0.9	0.9
Stxbp6	Q3TYA4	120	-7	-14	-11	-13	0.9	0.9	0.9	0.9
Usf2	Q64705	265	-10	-14	-11	-13	0.9	0.9	0.9	0.9
Fra10ac1	Q8BP78	240	-17	-14	-11	-20	0.9	0.9	0.9	0.8
Ankrd40	Q5SUE8	205	-23	-14	-11	-27	0.8	0.9	0.9	0.8
Ubr4	A2AN08	779	-24	-14	-11	24	0.8	0.9	0.9	1.3
Qars	Q8BML9	694	-19	-14	-11	5	0.8	0.9	0.9	1.1
Usp9x	Q4FE56	147	-21	-14	-11	1	0.8	0.9	0.9	1.0
Psma4	Q9R1P0	107	-18	-14	-11	0	0.8	0.9	0.9	1.0
Urb1	Q571H0	2269	-12	-14	-11	-5	0.9	0.9	0.9	1.0
Kansl1	Q80TG1	596	-14	-14	-11	-7	0.9	0.9	0.9	0.9
Pml	Q60953	62	-16	-14	-11	-11	0.9	0.9	0.9	0.9
Fam49b	Q921M7	223	-21	-15	-11	2	0.8	0.9	0.9	1.0

Ppt1	O88531	210	-10	-15	-11	-1	0.9	0.9	0.9	1.0
Smarca5	Q91ZW3	258	-15	-15	-11	-5	0.9	0.9	0.9	1.0
U2af2	P26369	464	-4	-15	-11	-11	1.0	0.9	0.9	0.9
Gtf2b	P62915	223	-14	-15	-11	-12	0.9	0.9	0.9	0.9
Smarca4	Q3TKT4	1326	-6	-15	-11	-24	0.9	0.9	0.9	0.8
Gphn	Q8BUV3	284	-7	-15	-11	-29	0.9	0.9	0.9	0.8
Tdg	P56581	287	-8	-15	-11	5	0.9	0.9	0.9	1.1
Dok3	Q9QZK7	226	-5	-15	-11	5	1.0	0.9	0.9	1.1
Rbm15	Q0VBL3	927	-11	-15	-11	-9	0.9	0.9	0.9	0.9
Tbl3	Q8C4J7	793	-14	-15	-11	-12	0.9	0.9	0.9	0.9
Ssh2	Q5SW75	1332	-12	-15	-11	-27	0.9	0.9	0.9	0.8
Ppp1r21	Q3TDD9	200	-18	-15	-11	-30	0.9	0.9	0.9	0.8
Fdxr	Q61578	108	-2	-16	-11	27	1.0	0.9	0.9	1.4
Atad2b	E9Q166	436	-24	-16	-11	-3	0.8	0.9	0.9	1.0
Atad2	G3X963	442	-24	-16	-11	-3	0.8	0.9	0.9	1.0
Ino80b	Q99PT3	353	-19	-16	-11	-7	0.8	0.9	0.9	0.9
Tpp2	Q64514	28	-17	-16	-11	-12	0.9	0.9	0.9	0.9
Gltscr1l	Q8CHH5	616	-11	-16	-11	-16	0.9	0.9	0.9	0.9
UPF0688	Q80WR5	92	-14	-16	-11	-17	0.9	0.9	0.9	0.9
Fndc3a	Q8BX90	1005	-13	-16	-11	-19	0.9	0.9	0.9	0.8
Oas3	Q8VI93	82	-11	-16	-11	15	0.9	0.9	0.9	1.2
Ccar2	Q8VDP4	753	-16	-16	-11	4	0.9	0.9	0.9	1.0
Mdh1	P14152	154	-24	-16	-11	3	0.8	0.9	0.9	1.0
Rlf	A2A7F4	453	-2	-16	-11	-1	1.0	0.9	0.9	1.0
Dnm2	P39054	607	-9	-16	-11	-9	0.9	0.9	0.9	0.9
Fiz1	Q9WTJ4	87	-14	-16	-11	-23	0.9	0.9	0.9	0.8
Usp22	Q5DU02	171	-16	-17	-11	11	0.9	0.9	0.9	1.1
Cyp4f3	Q99N16	401	-21	-17	-11	1	0.8	0.9	0.9	1.0
Cdk12	Q14AX6	719	-2	-17	-11	-1	1.0	0.9	0.9	1.0
Cdk13	Q69ZA1	701	-2	-17	-11	-1	1.0	0.9	0.9	1.0
Srsf3	P84104	74	-10	-17	-11	-9	0.9	0.9	0.9	0.9
Syne1	Q6ZWR6	2647	-5	-17	-11	-12	1.0	0.9	0.9	0.9
Hps5	P59438	228	-14	-17	-11	-10	0.9	0.9	0.9	0.9
Rrbp1	Q99PL5	1415	-18	-18	-11	-10	0.9	0.9	0.9	0.9
Prrc2a	Q7TSC1	484	-6	-18	-11	-14	0.9	0.9	0.9	0.9
Lgals1	P16045	61	-8	-18	-11	-18	0.9	0.9	0.9	0.9
Akap13	E9Q394	1644	-25	-18	-11	-22	0.8	0.9	0.9	0.8
Dtx3l	Q3UIR3	421	-15	-18	-11	0	0.9	0.8	0.9	1.0
Clic4	Q9QYB1	35	-16	-18	-11	-23	0.9	0.8	0.9	0.8
Ndufa5	Q9CPP6	17	-9	-18	-11	-31	0.9	0.8	0.9	0.8
Gspt1	Q8R050	524	-19	-19	-11	7	0.8	0.8	0.9	1.1
Rnf213	E9Q555	1427	-29	-19	-11	4	0.8	0.8	0.9	1.0
Tbce	Q8CIV8	368	-7	-19	-11	-3	0.9	0.8	0.9	1.0
Arid1a	A2BH40	1825	-8	-19	-11	-6	0.9	0.8	0.9	0.9
Zfp655	Q9CZP3	394	-16	-19	-11	-22	0.9	0.8	0.9	0.8
Irgm1	Q60766	258	-16	-19	-11	27	0.9	0.8	0.9	1.4
Psmb6	Q60692	77	-11	-19	-11	5	0.9	0.8	0.9	1.0
Cyth1	Q9QX11	94	-16	-19	-11	-8	0.9	0.8	0.9	0.9
Shmt1	P50431	383	-7	-20	-11	-9	0.9	0.8	0.9	0.9
Fkbp4	P30416	202	-9	-20	-11	-15	0.9	0.8	0.9	0.9
Actn4	P57780	500	-21	-20	-11	-45	0.8	0.8	0.9	0.7

Kmt2d	Q6PDK2	270	-12	-20	-11	-4	0.9	0.8	0.9	1.0
Actr1b	Q8R5C5	222	-20	-20	-11	-6	0.8	0.8	0.9	0.9
Chkb	O55229	28	-18	-20	-11	-10	0.8	0.8	0.9	0.9
Kiaa1524	Q8BWY9	801	-20	-21	-11	-18	0.8	0.8	0.9	0.8
Top1	Q04750	506	-26	-21	-11	9	0.8	0.8	0.9	1.1
Coro1a	O89053	332	-23	-21	-11	-2	0.8	0.8	0.9	1.0
Strn	O55106	316	-6	-22	-11	0	0.9	0.8	0.9	1.0
Pycr1	Q922W5	262	-21	-22	-11	0	0.8	0.8	0.9	1.0
Malt1	Q2TBA3	804	-16	-22	-11	-6	0.9	0.8	0.9	0.9
Macf1	E9PVY8	443	-11	-22	-11	5	0.9	0.8	0.9	1.0
Arpc1b	Q9WV32	26	-16	-22	-11	-21	0.9	0.8	0.9	0.8
Nup88	Q8CEC0	455	-12	-23	-11	10	0.9	0.8	0.9	1.1
Serpinb9b	Q9DAV6	131	-16	-24	-11	-28	0.9	0.8	0.9	0.8
Phf1	Q9Z1B8	435	-26	-25	-11	-16	0.8	0.8	0.9	0.9
Cops7b	Q8BV13	110	-7	-26	-11	6	0.9	0.8	0.9	1.1
Rreb1	Q3UH06	1247	-12	-26	-11	-15	0.9	0.8	0.9	0.9
Tprn	A2AI08	148	-30	-26	-11	-33	0.8	0.8	0.9	0.8
Zeb2	Q9R0G7	140	-14	-29	-11	-7	0.9	0.8	0.9	0.9
Myh9	Q8VDD5	694	-25	-31	-11	7	0.8	0.8	0.9	1.1
Ddx41	Q91VN6	264	-17	-36	-11	-2	0.9	0.7	0.9	1.0
Dek	Q7TNV0	165	-19	-39	-11	9	0.8	0.7	0.9	1.1
Cog3	E9QL65	363	51	-49	-11	-46	2.0	0.7	0.9	0.7
Mast1	Q9R1L5	1547	-14	-51	-11	-13	0.9	0.7	0.9	0.9
Syncrip	Q7TMK9	211	19	26	-12	32	1.2	1.4	0.9	1.5
Tln1	P26039	1045	9	15	-12	8	1.1	1.2	0.9	1.1
Actb	P60710	257	-15	12	-12	1	0.9	1.1	0.9	1.0
Hk3	Q3TRM8	839	1	10	-12	3	1.0	1.1	0.9	1.0
Chchd6	Q91VN4	98	-8	8	-12	5	0.9	1.1	0.9	1.1
Nabp2	Q8R2Y9	99	-22	4	-12	-24	0.8	1.0	0.9	0.8
Ywhaq	P68254	94	5	4	-12	6	1.1	1.0	0.9	1.1
Paxbp1	P58501	459	-17	3	-12	20	0.9	1.0	0.9	1.3
Uba3	Q8C878	28	-7	3	-12	-20	0.9	1.0	0.9	0.8
Plekhf1	Q3TB82	21	-1	2	-12	7	1.0	1.0	0.9	1.1
Taf10	Q8K0H5	174	-14	2	-12	2	0.9	1.0	0.9	1.0
Rasgrp1	Q9Z1S3	237	-12	2	-12	10	0.9	1.0	0.9	1.1
Rps6ka5	Q8C050	795	-1	2	-12	-7	1.0	1.0	0.9	0.9
Stat4	Q3V157	378	-5	2	-12	-11	1.0	1.0	0.9	0.9
Psmc6	P62334	83	-14	1	-12	20	0.9	1.0	0.9	1.2
Vars	Q9Z1Q9	443	-2	1	-12	-53	1.0	1.0	0.9	0.7
Zfp84	Q9D654	400	2	0	-12	14	1.0	1.0	0.9	1.2
Hrsp12	P52760	71	-12	0	-12	13	0.9	1.0	0.9	1.1
Camkk2	Q8C078	223	-8	0	-12	-12	0.9	1.0	0.9	0.9
Map3k3	Q61084	518	1	-1	-12	6	1.0	1.0	0.9	1.1
Gna13	P27601	318	1	-1	-12	-21	1.0	1.0	0.9	0.8
Gm45233	A0A0N4SVE0	66	-6	-1	-12	-2	0.9	1.0	0.9	1.0
Gvin1	L7N451	1251	-13	-1	-12	-75	0.9	1.0	0.9	0.6
Pde2a	F7D3W5	412	-11	-2	-12	9	0.9	1.0	0.9	1.1
Copa	Q8CIE6	453	-10	-2	-12	2	0.9	1.0	0.9	1.0
Nub1	P54729	267	-11	-2	-12	-1	0.9	1.0	0.9	1.0
Mettl16	Q9CQG2	276	-5	-2	-12	-17	1.0	1.0	0.9	0.9
Tut1	Q8R3F9	725	-7	-2	-12	-17	0.9	1.0	0.9	0.9

Akap13	E9Q394	973	1	-2	-12	-20	1.0	1.0	0.9	0.8
Smc2	Q8CG48	303	4	-3	-12	1	1.0	1.0	0.9	1.0
Klhl14	Q69ZK5	625	-4	-3	-12	-3	1.0	1.0	0.9	1.0
Lysmd2	G3X915	106	-10	-3	-12	-7	0.9	1.0	0.9	0.9
Adat2	Q6P6J0	126	-15	-3	-12	-8	0.9	1.0	0.9	0.9
Rps6ka5	Q8C050	474	-5	-3	-12	-16	1.0	1.0	0.9	0.9
Nudcd1	Q6PIP5	375	3	-4	-12	12	1.0	1.0	0.9	1.1
Herc2	Q4U2R1	1411	-4	-4	-12	-8	1.0	1.0	0.9	0.9
Prdm2	A2A7B5	714	-10	-4	-12	-18	0.9	1.0	0.9	0.9
Jak1	B1ASP2	762	-6	-4	-12	-30	0.9	1.0	0.9	0.8
Eif3b	Q8JZQ9	291	-6	-4	-12	2	0.9	1.0	0.9	1.0
Akap8l	Q5RL57	394	-6	-4	-12	-7	0.9	1.0	0.9	0.9
Dok3	Q9QZK7	244	-17	-4	-12	-21	0.9	1.0	0.9	0.8
Ctsg	P28293	142	-3	-4	-12	-30	1.0	1.0	0.9	0.8
Ptges3	Q9R0Q7	76	-17	-5	-12	6	0.9	1.0	0.9	1.1
G6pdx	Q00612	385	-8	-5	-12	-37	0.9	1.0	0.9	0.7
Hspa4l	P48722	376	-12	-5	-12	20	0.9	1.0	0.9	1.2
Hspa4	Q3U2G2	376	-12	-5	-12	20	0.9	1.0	0.9	1.2
Hsph1	Q61699	376	-12	-5	-12	20	0.9	1.0	0.9	1.2
Wdr1	O88342	438	-12	-5	-12	3	0.9	1.0	0.9	1.0
Tcea1	P10711	212	-8	-5	-12	-6	0.9	1.0	0.9	0.9
Akap8	Q9DBR0	626	-18	-5	-12	-11	0.8	1.0	0.9	0.9
Cic	Q924A2	1569	-5	-5	-12	-12	1.0	1.0	0.9	0.9
Tcp1	P11983	147	-12	-6	-12	15	0.9	0.9	0.9	1.2
Tbc1d1	Q60949	161	-3	-6	-12	-1	1.0	0.9	0.9	1.0
Rps8	P62242	182	-8	-6	-12	-2	0.9	0.9	0.9	1.0
Serpinb9b	Q9DAV6	286	-10	-6	-12	-2	0.9	0.9	0.9	1.0
Slc25a11	Q9CR62	184	-18	-6	-12	-5	0.9	0.9	0.9	1.0
Gapvd1	Q6PAR5	71	-14	-6	-12	-7	0.9	0.9	0.9	0.9
Nup50	Q9JIH2	331	7	-6	-12	-25	1.1	0.9	0.9	0.8
Mdn1	A2ANY6	645	-12	-6	-12	39	0.9	0.9	0.9	1.6
Mlh1	Q9JK91	77	-6	-6	-12	-2	0.9	0.9	0.9	1.0
Snx1	Q6NZD2	317	-11	-6	-12	-18	0.9	0.9	0.9	0.8
Prkcb	P68404	572	-2	-7	-12	1	1.0	0.9	0.9	1.0
Cmss1	Q9CZT6	51	-13	-7	-12	-4	0.9	0.9	0.9	1.0
Gm3839	S4R1W1	22	-7	-7	-12	3	0.9	0.9	0.9	1.0
Map3k5	O35099	935	-12	-7	-12	-1	0.9	0.9	0.9	1.0
Zcchc11	B2RX14	1243	-6	-7	-12	-3	0.9	0.9	0.9	1.0
Atp5s	Q9CRA7	65	-12	-7	-12	-21	0.9	0.9	0.9	0.8
Ets1	P27577	237	-6	-7	-12	-22	0.9	0.9	0.9	0.8
Herc1	E9PZP8	1939	5	-8	-12	16	1.1	0.9	0.9	1.2
Helz2	E9QAM5	350	-15	-8	-12	1	0.9	0.9	0.9	1.0
Flnb	Q80X90	2501	-13	-8	-12	-6	0.9	0.9	0.9	0.9
Psmc3	O88685	390	-7	-8	-12	-7	0.9	0.9	0.9	0.9
Dpp3	Q99KK7	701	-14	-8	-12	-8	0.9	0.9	0.9	0.9
Eef2	P58252	41	-8	-8	-12	-10	0.9	0.9	0.9	0.9
Nsf	P46460	264	-16	-8	-12	-14	0.9	0.9	0.9	0.9
Rcsd1	Q3UZA1	379	-15	-8	-12	-29	0.9	0.9	0.9	0.8
Vcp	Q01853	522	-15	-8	-12	-1	0.9	0.9	0.9	1.0
Sbds	P70122	84	-21	-8	-12	-3	0.8	0.9	0.9	1.0
Cyfip2	Q5SQX6	1240	-13	-8	-12	-5	0.9	0.9	0.9	1.0

Gm4951	Q3UED7	235	-11	-8	-12	-7	0.9	0.9	0.9	0.9
Abcb1a	P21447	1070	-12	-8	-12	-8	0.9	0.9	0.9	0.9
Tuba3b	P05214	376	-8	-8	-12	-10	0.9	0.9	0.9	0.9
Tuba4a	P68368	376	-8	-8	-12	-10	0.9	0.9	0.9	0.9
Tuba1a	P68369	376	-8	-8	-12	-10	0.9	0.9	0.9	0.9
Naa10	Q9QY36	194	-10	-8	-12	-15	0.9	0.9	0.9	0.9
Mcm3	P25206	250	-9	-8	-12	-20	0.9	0.9	0.9	0.8
Immt	Q8CAQ8	172	-18	-8	-12	-24	0.9	0.9	0.9	0.8
Spata5	Q3UMC0	80	-2	-9	-12	38	1.0	0.9	0.9	1.6
Kdm3b	B9EKS2	1127	-9	-9	-12	8	0.9	0.9	0.9	1.1
Ubn1	Q4G0F8	548	-11	-9	-12	5	0.9	0.9	0.9	1.1
Gnl3l	Q6PGG6	145	-7	-9	-12	3	0.9	0.9	0.9	1.0
Uba6	Q8C7R4	546	-11	-9	-12	-1	0.9	0.9	0.9	1.0
Pus1	Q9WU56	234	2	-9	-12	-5	1.0	0.9	0.9	1.0
Rnmt	Q9D0L8	37	-9	-9	-12	-13	0.9	0.9	0.9	0.9
Nisch	Q80TM9	894	-9	-9	-12	19	0.9	0.9	0.9	1.2
Coro1a	O89053	345	-4	-9	-12	14	1.0	0.9	0.9	1.2
Grk6	O70293	138	-12	-9	-12	10	0.9	0.9	0.9	1.1
Mybbp1a	Q7TPV4	612	-26	-9	-12	4	0.8	0.9	0.9	1.0
Zfp958	Q99LG7	226	2	-9	-12	3	1.0	0.9	0.9	1.0
Anapc4	Q91W96	7	-17	-9	-12	-7	0.9	0.9	0.9	0.9
Actn1	Q7TPR4	690	-6	-9	-12	-7	0.9	0.9	0.9	0.9
Gpx4	O70325	195	-14	-9	-12	-10	0.9	0.9	0.9	0.9
Rps20	P60867	36	-8	-9	-12	-14	0.9	0.9	0.9	0.9
Klhl6	Q6V595	250	-18	-10	-12	4	0.9	0.9	0.9	1.0
Epb41l2	O70318	756	-2	-10	-12	-2	1.0	0.9	0.9	1.0
Arhgef6	Q8K4I3	733	-11	-10	-12	-3	0.9	0.9	0.9	1.0
Zfp729b	Q80VN4	142	-11	-10	-12	-5	0.9	0.9	0.9	1.0
Bahd1	Z4YJL0	695	3	-10	-12	-8	1.0	0.9	0.9	0.9
Ranbp2	Q9ERU9	2415	-7	-10	-12	-14	0.9	0.9	0.9	0.9
Rpl36a	P83882	72	-9	-10	-12	-16	0.9	0.9	0.9	0.9
Pi4ka	E9Q3L2	1777	-3	-10	-12	-17	1.0	0.9	0.9	0.9
Xpo1	Q6P5F9	528	-5	-10	-12	-18	1.0	0.9	0.9	0.8
Uso1	Q9Z1Z0	816	-13	-10	-12	-18	0.9	0.9	0.9	0.8
Safb	D3YXK2	225	-14	-10	-12	-23	0.9	0.9	0.9	0.8
Safb2	Q80YR5	219	-14	-10	-12	-23	0.9	0.9	0.9	0.8
Lrpprc	Q6PB66	626	-14	-10	-12	7	0.9	0.9	0.9	1.1
Prkar1a	Q9DBC7	18	-10	-10	-12	7	0.9	0.9	0.9	1.1
Akr1b1	P45376	81	-15	-10	-12	3	0.9	0.9	0.9	1.0
Mthfd1l	Q3V3R1	778	-3	-10	-12	-2	1.0	0.9	0.9	1.0
Gcn1	E9PVA8	932	-13	-10	-12	-19	0.9	0.9	0.9	0.8
Nop56	Q9D6Z1	142	-14	-11	-12	0	0.9	0.9	0.9	1.0
Uchl3	Q9JKB1	209	4	-11	-12	-2	1.0	0.9	0.9	1.0
Usp7	E9PXY8	356	-13	-11	-12	-10	0.9	0.9	0.9	0.9
Rhoh	Q9D3G9	108	-11	-11	-12	-14	0.9	0.9	0.9	0.9
Zap70	P43404	617	-11	-11	-12	-15	0.9	0.9	0.9	0.9
Nol8	Q3UHX0	628	-8	-11	-12	-16	0.9	0.9	0.9	0.9
Suz12	Q80U70	46	-7	-11	-12	-19	0.9	0.9	0.9	0.8
Rps15a	P62245	30	-21	-12	-12	15	0.8	0.9	0.9	1.2
Zw10	O54692	82	-8	-12	-12	-9	0.9	0.9	0.9	0.9
Fkbp4	P30416	396	-20	-12	-12	4	0.8	0.9	0.9	1.0

Fgd2	Q8BY35	484	-11	-12	-12	1	0.9	0.9	0.9	1.0
Ifi30	Q9ESY9	72	-18	-12	-12	-7	0.9	0.9	0.9	0.9
Gmip	Q6PGG2	369	-15	-12	-12	-9	0.9	0.9	0.9	0.9
Lsm4	Q9QXA5	45	-24	-12	-12	-10	0.8	0.9	0.9	0.9
Nsun6	Q7TS68	120	-15	-12	-12	-15	0.9	0.9	0.9	0.9
Zfp292	Q9Z2U2	375	-15	-13	-12	7	0.9	0.9	0.9	1.1
Urb2	E9Q7L1	570	-13	-13	-12	-11	0.9	0.9	0.9	0.9
Fastk	Q8C725	208	-9	-13	-12	27	0.9	0.9	0.9	1.4
Pygl	Q9ET01	79	-6	-13	-12	9	0.9	0.9	0.9	1.1
Psmb2	Q9R1P3	91	-10	-13	-12	-2	0.9	0.9	0.9	1.0
Dmxl1	Q6PNC0	541	-9	-13	-12	-21	0.9	0.9	0.9	0.8
Traf5	E9QMA6	110	-4	-13	-12	-28	1.0	0.9	0.9	0.8
Stk11ip	Q3TAA7	612	-8	-14	-12	36	0.9	0.9	0.9	1.6
Mtf1	Q07243	128	-4	-14	-12	-4	1.0	0.9	0.9	1.0
Lig3	P97386	935	-10	-14	-12	-14	0.9	0.9	0.9	0.9
Mad1l1	Q9WTX8	494	-35	-14	-12	-18	0.7	0.9	0.9	0.8
Atrx	Q61687	308	-5	-14	-12	-25	1.0	0.9	0.9	0.8
Cd6	Q61003	136	-7	-14	-12	-50	0.9	0.9	0.9	0.7
Hnrnph1	O35737	34	-25	-14	-12	7	0.8	0.9	0.9	1.1
Rplp0	P14869	27	-19	-14	-12	7	0.8	0.9	0.9	1.1
Hnrnph2	P70333	34	-25	-14	-12	7	0.8	0.9	0.9	1.1
Mcm3	P25206	263	-11	-14	-12	-2	0.9	0.9	0.9	1.0
Cdk5	P49615	290	-8	-14	-12	-12	0.9	0.9	0.9	0.9
Luc7l3	Q5SUF2	58	-13	-14	-12	-13	0.9	0.9	0.9	0.9
Hps3	Q91VB4	56	-22	-15	-12	8	0.8	0.9	0.9	1.1
D10Jhu81e	Q9D172	151	-16	-15	-12	7	0.9	0.9	0.9	1.1
Mcm4	P49717	171	-17	-15	-12	4	0.9	0.9	0.9	1.0
Lonp1	Q8CGK3	509	-23	-15	-12	2	0.8	0.9	0.9	1.0
Rrp12	Q6P5B0	261	-10	-15	-12	-9	0.9	0.9	0.9	0.9
Mta3	Q924K8	529	-4	-15	-12	-11	1.0	0.9	0.9	0.9
Ms4a6b	Q99N09	234	-11	-15	-12	-49	0.9	0.9	0.9	0.7
Txnrd1	Q9JMH6	303	-17	-15	-12	3	0.9	0.9	0.9	1.0
Cabin1	G3X8Q1	207	-28	-15	-12	2	0.8	0.9	0.9	1.0
Ecm29	Q6PDI5	1589	-9	-15	-12	-2	0.9	0.9	0.9	1.0
Gm20696	H3BJN7	52	-20	-15	-12	-6	0.8	0.9	0.9	0.9
Zc3h4	E9Q8K8	383	-9	-15	-12	-9	0.9	0.9	0.9	0.9
Pitpnb	P53811	187	-9	-15	-12	-10	0.9	0.9	0.9	0.9
Prdm2	A2A7B5	308	-7	-15	-12	-19	0.9	0.9	0.9	0.8
Dapk2	Q8VDF3	347	-11	-15	-12	-35	0.9	0.9	0.9	0.7
Slfn1	Q9Z0I7	253	-13	-16	-12	5	0.9	0.9	0.9	1.0
Zc3hc1	Q80YV2	428	-16	-16	-12	0	0.9	0.9	0.9	1.0
Dpp3	Q99KK7	509	-23	-16	-12	-5	0.8	0.9	0.9	1.0
Pms1	Q8K119	533	-16	-16	-12	-18	0.9	0.9	0.9	0.8
Map2k6	P70236	109	-33	-16	-12	-5	0.8	0.9	0.9	1.0
Grpel1	Q99LP6	47	-14	-16	-12	-8	0.9	0.9	0.9	0.9
Ddx21	Q9JIK5	754	-24	-17	-12	9	0.8	0.9	0.9	1.1
Trmt1l	A2RSY6	652	-15	-17	-12	6	0.9	0.9	0.9	1.1
Slc25a12	Q8BH59	505	-27	-17	-12	17	0.8	0.9	0.9	1.2
Acap2	Q6ZQK5	468	-11	-17	-12	-5	0.9	0.9	0.9	1.0
Dbr1	Q923B1	499	-8	-17	-12	-11	0.9	0.9	0.9	0.9
Rbm28	Q8CGC6	33	-18	-17	-12	-13	0.8	0.9	0.9	0.9

Itpr2	Q9Z329	1879	3	-17	-12	-19	1.0	0.9	0.9	0.8
Ddb2	Q99J79	6	-11	-17	-12	-25	0.9	0.9	0.9	0.8
Myo9b	E9PZW8	133	-8	-18	-12	20	0.9	0.9	0.9	1.3
Lysmd1	Q9D0E3	32	-24	-18	-12	-4	0.8	0.9	0.9	1.0
Rfx1	P48377	778	-21	-18	-12	-8	0.8	0.9	0.9	0.9
Gmps	Q3THK7	523	-16	-18	-12	-11	0.9	0.9	0.9	0.9
Nup153	E9Q3G8	589	-11	-18	-12	-12	0.9	0.9	0.9	0.9
Hccs	P53702	70	-21	-18	-12	-16	0.8	0.9	0.9	0.9
Hp1bp3	Q3TEA8	359	-27	-18	-12	2	0.8	0.8	0.9	1.0
Apip	Q9WVQ5	146	-13	-18	-12	-5	0.9	0.8	0.9	1.0
Ppp1cc	P63087	62	-12	-19	-12	-16	0.9	0.8	0.9	0.9
Trim10	Q9WUH5	358	-8	-19	-12	-19	0.9	0.8	0.9	0.8
Map3k19	E9Q3S4	1190	-15	-20	-12	-4	0.9	0.8	0.9	1.0
Zfp292	Q9Z2U2	2637	-12	-20	-12	-16	0.9	0.8	0.9	0.9
Lrch3	Q8BVU0	236	-13	-20	-12	-5	0.9	0.8	0.9	1.0
Aldoa	P05064	135	-15	-21	-12	13	0.9	0.8	0.9	1.1
Srpk1	O70551	188	-15	-21	-12	3	0.9	0.8	0.9	1.0
Irf2bp1	Q8R3Y8	280	-13	-21	-12	-12	0.9	0.8	0.9	0.9
Acsl4	Q9QUJ7	542	-12	-21	-12	-27	0.9	0.8	0.9	0.8
Gm20521	D3Z5F7	222	-16	-21	-12	-8	0.9	0.8	0.9	0.9
Steap3	Q8CI59	168	-1	-21	-12	-26	1.0	0.8	0.9	0.8
Nrde2	Q80XC6	1096	-10	-22	-12	18	0.9	0.8	0.9	1.2
Inpp5d	Q9ES52	986	-21	-22	-12	-5	0.8	0.8	0.9	1.0
Ski	B1AUF1	448	-13	-22	-12	-17	0.9	0.8	0.9	0.9
Kbtbd11	Q8BNW9	563	-19	-22	-12	-24	0.8	0.8	0.9	0.8
Eps15l1	Q60902	468	-7	-22	-12	-15	0.9	0.8	0.9	0.9
Mms19	Q9D071	503	-21	-23	-12	-20	0.8	0.8	0.9	0.8
Dhx36	Q8VHK9	633	-30	-23	-12	-8	0.8	0.8	0.9	0.9
Zbtb44	Q8R0A2	167	-29	-24	-12	-27	0.8	0.8	0.9	0.8
Nbas	E9Q411	405	-14	-25	-12	-9	0.9	0.8	0.9	0.9
Cpne3	Q8BT60	385	-13	-25	-12	-36	0.9	0.8	0.9	0.7
Acad8	Q9D7B6	32	5	-28	-12	-29	1.0	0.8	0.9	0.8
Dennd4c	A6H8H2	1042	-38	-28	-12	-34	0.7	0.8	0.9	0.7
Grk6	O70293	80	-7	-29	-12	-10	0.9	0.8	0.9	0.9
Prkdc	P97313	25	-30	-33	-12	-20	0.8	0.8	0.9	0.8
Ethe1	Q9DCM0	170	-27	-36	-12	-25	0.8	0.7	0.9	0.8
Fech	P22315	357	-23	-43	-12	-11	0.8	0.7	0.9	0.9
Cdc5l	Q6A068	96	-20	-44	-12	-12	0.8	0.7	0.9	0.9
Arhgef3	Q91X46	17	-20	25	-12	15	0.8	1.3	0.9	1.2
Dhrs1	Q99L04	28	-5	20	-12	24	1.0	1.2	0.9	1.3
Uncharact	Q8VCE4	73	-6	9	-12	22	0.9	1.1	0.9	1.3
Uba1	Q02053	481	6	8	-12	28	1.1	1.1	0.9	1.4
Zxdb	A2CE44	814	-7	8	-12	15	0.9	1.1	0.9	1.2
Ube2e1	P52482	67	-9	7	-12	10	0.9	1.1	0.9	1.1
Ppid	Q9CR16	275	-8	6	-12	26	0.9	1.1	0.9	1.3
Atox1	O08997	41	-21	6	-12	-9	0.8	1.1	0.9	0.9
Uba1	Q02053	278	-10	6	-12	8	0.9	1.1	0.9	1.1
Pxdc1	Q8JZU6	115	-10	4	-12	1	0.9	1.0	0.9	1.0
Usp7	E9PXY8	743	-11	3	-12	16	0.9	1.0	0.9	1.2
Aldh9a1	Q9JLJ2	45	-5	3	-12	7	1.0	1.0	0.9	1.1
Diexf	Q8BTT6	658	-4	3	-12	0	1.0	1.0	0.9	1.0

Riok2	Q9CQS5	488	-5	3	-12	-1	1.0	1.0	0.9	1.0
Nfatc1	B5B2N2	230	-20	3	-12	-4	0.8	1.0	0.9	1.0
Rbm6	S4R1W5	277	-8	3	-12	-5	0.9	1.0	0.9	1.0
Ppa1	Q9D819	274	-3	3	-12	-11	1.0	1.0	0.9	0.9
Tbcd	Q8BYA0	459	-3	3	-12	-24	1.0	1.0	0.9	0.8
Uchl5	Q9WUP7	191	6	2	-12	9	1.1	1.0	0.9	1.1
Hnrnpk	P61979	185	-19	2	-12	7	0.8	1.0	0.9	1.1
UPF0317	Q8BH86	161	-4	2	-12	-5	1.0	1.0	0.9	1.0
Rpl10a	Q5XJF6	66	-7	2	-12	18	0.9	1.0	0.9	1.2
Tbck	Q8BM85	309	6	1	-12	6	1.1	1.0	0.9	1.1
Ripk3	Q9QZL0	334	-7	1	-12	-8	0.9	1.0	0.9	0.9
Pcmt1	P23506	95	-18	0	-12	5	0.9	1.0	0.9	1.1
Paxip1	Q6NZQ4	883	0	0	-12	1	1.0	1.0	0.9	1.0
Supt6h	Q62383	1281	0	0	-12	0	1.0	1.0	0.9	1.0
Strn	O55106	740	-13	0	-12	-5	0.9	1.0	0.9	1.0
Mroh1	E0CZ22	104	-10	-1	-12	31	0.9	1.0	0.9	1.4
Pds5a	E9QPI5	13	-12	-1	-12	-4	0.9	1.0	0.9	1.0
Uhrf2	Q7TMI3	42	-18	-1	-12	-7	0.9	1.0	0.9	0.9
Myo1g	Q5SUA5	837	-6	-1	-12	-23	0.9	1.0	0.9	0.8
Vps13b	E9QKX5	2880	-4	-2	-12	41	1.0	1.0	0.9	1.7
Clec16a	Q80U30	641	-10	-2	-12	-4	0.9	1.0	0.9	1.0
Sucla2	Q9Z2I9	158	-21	-2	-12	-6	0.8	1.0	0.9	0.9
Ehbp1l1	E9QP49	1231	-9	-2	-12	11	0.9	1.0	0.9	1.1
Tubb5	P99024	129	-10	-2	-12	-12	0.9	1.0	0.9	0.9
Eps15	P42567	657	-5	-3	-12	-18	1.0	1.0	0.9	0.9
Coro1a	O89053	192	-13	-3	-12	15	0.9	1.0	0.9	1.2
Kars	Q99MN1	494	-8	-3	-12	9	0.9	1.0	0.9	1.1
Ranbp2	Q9ERU9	1329	-12	-3	-12	-13	0.9	1.0	0.9	0.9
Hnrnpll	Q921F4	584	-51	-3	-12	-20	0.7	1.0	0.9	0.8
Ranbp2	Q9ERU9	1593	-8	-3	-12	-20	0.9	1.0	0.9	0.8
Nfu1	Q9QZ23	213	-10	-4	-12	20	0.9	1.0	0.9	1.2
Cand1	Q6ZQ38	954	-8	-4	-12	8	0.9	1.0	0.9	1.1
Sp140	Q6NSQ5	183	-13	-4	-12	5	0.9	1.0	0.9	1.0
Stambpl1	Q76N33	38	-5	-4	-12	-5	1.0	1.0	0.9	1.0
Prkcb	P68404	71	-3	-4	-12	-30	1.0	1.0	0.9	0.8
UPF0317	Q8BH86	86	-14	-4	-12	17	0.9	1.0	0.9	1.2
Rab29	E9QLQ7	120	-15	-4	-12	11	0.9	1.0	0.9	1.1
Zyx	Q62523	532	6	-4	-12	-1	1.1	1.0	0.9	1.0
Vav1	P27870	31	-7	-4	-12	-9	0.9	1.0	0.9	0.9
Arhgef6	Q8K4I3	119	-10	-4	-12	-11	0.9	1.0	0.9	0.9
Scai	Q8C8N2	63	-16	-4	-12	-18	0.9	1.0	0.9	0.8
Paxip1	Q6NZQ4	727	-15	-5	-12	17	0.9	1.0	0.9	1.2
Actr3	Q99JY9	189	-10	-5	-12	2	0.9	1.0	0.9	1.0
Vmac	Q8BP01	46	-15	-5	-12	-8	0.9	1.0	0.9	0.9
Nans	Q99J77	287	1	-5	-12	1	1.0	1.0	0.9	1.0
Dtx3	Q80V91	272	-14	-5	-12	-5	0.9	1.0	0.9	1.0
Rbbp5	Q8BX09	212	-15	-5	-12	-9	0.9	1.0	0.9	0.9
Mina	Q8CD15	19	-6	-5	-12	-11	0.9	1.0	0.9	0.9
Usp31	E9Q6Y8	458	-14	-5	-12	-12	0.9	1.0	0.9	0.9
Ercc4	Q9QZD4	176	-4	-5	-12	-14	1.0	1.0	0.9	0.9
Nsa2	Q9CR47	193	-9	-5	-12	-17	0.9	1.0	0.9	0.9

Kbtbd2	G3X9X1	524	-4	-6	-12	8	1.0	0.9	0.9	1.1
Oas3	Q8VI93	495	-11	-6	-12	-3	0.9	0.9	0.9	1.0
Atp6v1b2	P62814	289	-14	-6	-12	-4	0.9	0.9	0.9	1.0
Cebpb	P28033	143	-24	-6	-12	-9	0.8	0.9	0.9	0.9
Psmc2	P46471	389	-5	-6	-12	-16	1.0	0.9	0.9	0.9
Ppp2r2a	Q6P1F6	262	-10	-6	-12	-23	0.9	0.9	0.9	0.8
Elp5	Q99L85	187	-15	-6	-12	-22	0.9	0.9	0.9	0.8
Rhot1	Q8BG51	188	-17	-6	-12	-31	0.9	0.9	0.9	0.8
Nans	Q99J77	46	-14	-7	-12	7	0.9	0.9	0.9	1.1
Fermt3	Q8K1B8	128	-18	-7	-12	2	0.9	0.9	0.9	1.0
Herc1	E9PZP8	4809	-1	-7	-12	-4	1.0	0.9	0.9	1.0
Exoc2	Q9D4H1	638	-9	-7	-12	-5	0.9	0.9	0.9	1.0
Hmgcl	P38060	307	-12	-7	-12	-16	0.9	0.9	0.9	0.9
Eif1	P48024	69	-13	-7	-12	-3	0.9	0.9	0.9	1.0
Eif1b	Q9CXU9	69	-13	-7	-12	-3	0.9	0.9	0.9	1.0
Gm5786	J3QMG5	182	-10	-7	-12	-5	0.9	0.9	0.9	1.0
Ighm	A0A075B6A0	293	-14	-7	-12	-8	0.9	0.9	0.9	0.9
Alkbh8	Q80Y20	442	-14	-7	-12	-8	0.9	0.9	0.9	0.9
Tpr	F6ZDS4	1142	-9	-7	-12	-11	0.9	0.9	0.9	0.9
Cacul1	Q8R0X2	370	-7	-8	-12	13	0.9	0.9	0.9	1.1
Wdr70	G3X934	230	-10	-8	-12	8	0.9	0.9	0.9	1.1
Map3k11	Q80XI6	328	-17	-8	-12	2	0.9	0.9	0.9	1.0
Rbm5	Q91YE7	187	-6	-8	-12	-2	0.9	0.9	0.9	1.0
Fam98b	Q80VD1	295	-11	-8	-12	-3	0.9	0.9	0.9	1.0
Tardbp	Q921F2	39	-9	-8	-12	-7	0.9	0.9	0.9	0.9
Galt	Q03249	367	-13	-8	-12	-9	0.9	0.9	0.9	0.9
Clpx	Q9JHS4	539	-11	-8	-12	-12	0.9	0.9	0.9	0.9
Tsc1	Q9EP53	583	-11	-8	-12	-15	0.9	0.9	0.9	0.9
Ilvbl	Q8BU33	568	-12	-8	-12	-21	0.9	0.9	0.9	0.8
Parp1	Q921K2	299	-15	-8	-12	13	0.9	0.9	0.9	1.1
Ate1	Q9Z2A5	427	-10	-8	-12	7	0.9	0.9	0.9	1.1
Sept7	O55131	203	-9	-8	-12	-6	0.9	0.9	0.9	0.9
Utp20	E9QK83	618	-7	-8	-12	-10	0.9	0.9	0.9	0.9
Myd88	P22366	280	-13	-8	-12	-10	0.9	0.9	0.9	0.9
Eif4b	Q8BGD9	543	-4	-8	-12	-18	1.0	0.9	0.9	0.9
Srr	Q9QZX7	113	1	-8	-12	-33	1.0	0.9	0.9	0.8
Casp8	O89110	26	-27	-9	-12	15	0.8	0.9	0.9	1.2
Gak	Q99KY4	87	-12	-9	-12	-4	0.9	0.9	0.9	1.0
Vwa5a	Q99KC8	374	-9	-9	-12	-6	0.9	0.9	0.9	0.9
Zfyve26	Q5DU37	637	-8	-9	-12	-9	0.9	0.9	0.9	0.9
Plekha2	Q9ERS5	191	-17	-9	-12	-15	0.9	0.9	0.9	0.9
Atg16l1	Q8C0J2	145	-6	-9	-12	-21	0.9	0.9	0.9	0.8
Eif2b4	Q61749	130	-13	-9	-12	-28	0.9	0.9	0.9	0.8
Gpd2	Q64521	285	-15	-9	-12	19	0.9	0.9	0.9	1.2
Nfkb1	P25799	85	2	-9	-12	14	1.0	0.9	0.9	1.2
Pgm1	Q9D0F9	160	-18	-9	-12	3	0.9	0.9	0.9	1.0
Bank1	Q80VH0	778	-12	-9	-12	2	0.9	0.9	0.9	1.0
Brms1l	Q3U1T3	275	-5	-9	-12	-13	1.0	0.9	0.9	0.9
Zmym2	Q9CU65	754	-23	-9	-12	-15	0.8	0.9	0.9	0.9
Smad2	Q62432	149	-6	-10	-12	9	0.9	0.9	0.9	1.1
Sfn	O70456	38	-11	-10	-12	-11	0.9	0.9	0.9	0.9

Rps12-ps3	Q6ZWZ6	92	-8	-10	-12	-12	0.9	0.9	0.9	0.9
Rfc5	Q9D0F6	237	-6	-10	-12	-18	0.9	0.9	0.9	0.9
Lrrfip1	Q3UZ39	444	-15	-10	-12	-23	0.9	0.9	0.9	0.8
Hint1	P70349	84	-4	-10	-12	9	1.0	0.9	0.9	1.1
Ddx39b	Q9Z1N5	165	-9	-10	-12	1	0.9	0.9	0.9	1.0
Heatr5a	Q5PRF0	693	-6	-10	-12	-5	0.9	0.9	0.9	1.0
Mndal	D0QMC3	490	-10	-10	-12	-6	0.9	0.9	0.9	0.9
Mnda	P0DOV1	404	-10	-10	-12	-6	0.9	0.9	0.9	0.9
Ifi205a	Q8CGE8	383	-10	-10	-12	-6	0.9	0.9	0.9	0.9
Snu13	Q9D0T1	30	-10	-10	-12	-9	0.9	0.9	0.9	0.9
Pi4ka	E9Q3L2	249	-15	-10	-12	-11	0.9	0.9	0.9	0.9
Rapgef1	Q3UHC1	484	-10	-10	-12	-26	0.9	0.9	0.9	0.8
Tmpo	Q61033	560	-10	-10	-12	-28	0.9	0.9	0.9	0.8
Arih1	Q9Z1K5	279	-13	-11	-12	12	0.9	0.9	0.9	1.1
Zc3h11a	Q6NZF1	209	4	-11	-12	3	1.0	0.9	0.9	1.0
Smndc1	Q8BGT7	214	-8	-11	-12	-1	0.9	0.9	0.9	1.0
Vbp1	P61759	8	-7	-11	-12	-5	0.9	0.9	0.9	1.0
Tmem209	Q8BRG8	301	-16	-11	-12	-8	0.9	0.9	0.9	0.9
Epc1	Q8C9X6	774	-14	-11	-12	-12	0.9	0.9	0.9	0.9
Kctd12	A0A0R4J2B2	50	-12	-11	-12	-13	0.9	0.9	0.9	0.9
Fhod1	Q6P9Q4	539	-14	-11	-12	-16	0.9	0.9	0.9	0.9
Ptpn1	P35821	226	-29	-12	-12	15	0.8	0.9	0.9	1.2
Atic	Q9CWJ9	434	-19	-12	-12	9	0.8	0.9	0.9	1.1
Bloc1s5	Q8R015	98	-11	-12	-12	4	0.9	0.9	0.9	1.0
Rsad2	Q8CBB9	314	-19	-12	-12	1	0.8	0.9	0.9	1.0
Fgd2	Q8BY35	88	-12	-12	-12	-13	0.9	0.9	0.9	0.9
Inpp5d	Q9ES52	675	-14	-12	-12	4	0.9	0.9	0.9	1.0
Rassf4	Q8CB96	146	-9	-12	-12	2	0.9	0.9	0.9	1.0
Exosc9	Q9JHI7	45	-20	-12	-12	1	0.8	0.9	0.9	1.0
Pcnt	F8VPV0	1720	-13	-12	-12	-5	0.9	0.9	0.9	1.0
Setdb2	Q8C267	111	-9	-12	-12	-10	0.9	0.9	0.9	0.9
Rbl1	Q64701	353	-19	-12	-12	-11	0.8	0.9	0.9	0.9
Arhgap30	Q640N3	1021	-12	-12	-12	-19	0.9	0.9	0.9	0.8
Nol8	Q3UHX0	555	-12	-12	-12	-21	0.9	0.9	0.9	0.8
Trim56	Q80VI1	104	-21	-13	-12	3	0.8	0.9	0.9	1.0
Ddx59	Q9DBN9	414	-18	-13	-12	1	0.9	0.9	0.9	1.0
Mrm1	Q99J25	56	-42	-13	-12	-9	0.7	0.9	0.9	0.9
Echdc1	Q9D9V3	4	-18	-13	-12	-14	0.8	0.9	0.9	0.9
Esyt1	Q3U7R1	360	-12	-13	-12	-10	0.9	0.9	0.9	0.9
Map4k4	E9PVG7	932	-13	-13	-12	-11	0.9	0.9	0.9	0.9
Zfp407	G3UVV3	322	-8	-13	-12	-14	0.9	0.9	0.9	0.9
Pikfyve	Q9Z1T6	1662	-6	-14	-12	-5	0.9	0.9	0.9	1.0
Anapc7	Q9WVM3	475	-5	-14	-12	-12	1.0	0.9	0.9	0.9
Nop2	E9QN31	725	-10	-14	-12	-14	0.9	0.9	0.9	0.9
Etf1	Q8BWY3	335	-11	-14	-12	-17	0.9	0.9	0.9	0.9
Afg3l2	Q8JZQ2	312	-10	-14	-12	-25	0.9	0.9	0.9	0.8
Zrsr2	B1B0E8	176	-14	-14	-12	7	0.9	0.9	0.9	1.1
Sirt1	Q923E4	372	-12	-14	-12	2	0.9	0.9	0.9	1.0
Rsl1d1	Q8BVY0	196	-11	-14	-12	-3	0.9	0.9	0.9	1.0
Ewsr1	Q61545	383	-16	-14	-12	-4	0.9	0.9	0.9	1.0
Exosc7	Q9D0M0	34	-14	-14	-12	-11	0.9	0.9	0.9	0.9

Satb1	Q60611	529	-9	-14	-12	-12	0.9	0.9	0.9	0.9
Akna	Q80VW7	1244	-22	-14	-12	-18	0.8	0.9	0.9	0.8
Thumpd3	P97770	162	-6	-14	-12	-27	0.9	0.9	0.9	0.8
Rars	Q9D0I9	32	-10	-15	-12	-3	0.9	0.9	0.9	1.0
Metap2	O08663	121	-15	-15	-12	-3	0.9	0.9	0.9	1.0
Abce1	P61222	88	-13	-15	-12	-5	0.9	0.9	0.9	1.0
Hivep2	Q3UHF7	2313	5	-15	-12	-9	1.0	0.9	0.9	0.9
Zfp512b	Q6PHP4	744	0	-15	-12	-21	1.0	0.9	0.9	0.8
Acsf2	Q8VCW8	503	-4	-15	-12	-54	1.0	0.9	0.9	0.6
Rela	Q04207	105	-14	-15	-12	-4	0.9	0.9	0.9	1.0
Smchd1	Q6P5D8	1657	-11	-15	-12	-21	0.9	0.9	0.9	0.8
Mapkap1	Q8BKH7	149	-14	-16	-12	11	0.9	0.9	0.9	1.1
Xdh	Q00519	970	-16	-16	-12	4	0.9	0.9	0.9	1.0
Phkg2	Q9DB30	294	-12	-16	-12	0	0.9	0.9	0.9	1.0
Agk	Q9ESW4	72	-13	-16	-12	-27	0.9	0.9	0.9	0.8
Dnmt1	P13864	694	-16	-16	-12	14	0.9	0.9	0.9	1.2
Dennd4c	A6H8H2	126	-9	-16	-12	9	0.9	0.9	0.9	1.1
Akap13	E9Q394	1801	-19	-16	-12	-7	0.8	0.9	0.9	0.9
Nlrx1	Q3TL44	310	-23	-16	-12	-21	0.8	0.9	0.9	0.8
Tbl2	Q9R099	243	-11	-17	-12	17	0.9	0.9	0.9	1.2
Rab33b	O35963	48	7	-17	-12	-1	1.1	0.9	0.9	1.0
Pus7	B7ZNL8	448	-14	-17	-12	-7	0.9	0.9	0.9	0.9
Gna13	P27601	37	-19	-17	-12	-13	0.8	0.9	0.9	0.9
Uqcrc1	Q9CZ13	268	-23	-18	-12	10	0.8	0.9	0.9	1.1
Rfc4	Q99J62	177	-12	-18	-12	8	0.9	0.9	0.9	1.1
Hpf1	Q8CFE2	152	-10	-18	-12	-13	0.9	0.9	0.9	0.9
Specc1	Q5SXY1	527	-23	-18	-12	-17	0.8	0.9	0.9	0.9
Sde2	Q8K1J5	150	-11	-18	-12	-23	0.9	0.9	0.9	0.8
Dcp2	Q9CYC6	140	-22	-18	-12	-37	0.8	0.9	0.9	0.7
Kntc1	Q8C3Y4	1095	-11	-18	-12	-3	0.9	0.8	0.9	1.0
Trrap	A0A1D5RLL4	2139	-9	-18	-12	-7	0.9	0.8	0.9	0.9
Ube2z	Q3UE37	245	-5	-19	-12	23	1.0	0.8	0.9	1.3
Cyld	Q80TQ2	651	-14	-19	-12	10	0.9	0.8	0.9	1.1
Cdk5rap3	Q99LM2	136	-9	-19	-12	-10	0.9	0.8	0.9	0.9
Pcnt	F8VPV0	477	-7	-19	-12	-16	0.9	0.8	0.9	0.9
Ash2l	Q91X20	115	-19	-19	-12	-17	0.8	0.8	0.9	0.9
Lpcat2	Q8BYI6	4	-3	-19	-12	-32	1.0	0.8	0.9	0.8
Pkm	P52480	152	-12	-19	-12	26	0.9	0.8	0.9	1.4
Xpnpep1	Q6P1B1	309	-13	-19	-12	-8	0.9	0.8	0.9	0.9
Ufl1	Q8CCJ3	707	-21	-20	-12	-1	0.8	0.8	0.9	1.0
Zc3h11a	Q6NZF1	618	-21	-20	-12	-6	0.8	0.8	0.9	0.9
Znf628	Q8CJ78	929	-12	-20	-12	-22	0.9	0.8	0.9	0.8
Gnai2	P08752	66	-19	-20	-12	13	0.8	0.8	0.9	1.1
Dnajc9	Q91WN1	243	-14	-20	-12	0	0.9	0.8	0.9	1.0
Rragb	Q6NTA4	185	-25	-20	-12	-5	0.8	0.8	0.9	1.0
Rraga	Q80X95	124	-25	-20	-12	-5	0.8	0.8	0.9	1.0
Smad1	P70340	89	-17	-20	-12	-7	0.9	0.8	0.9	0.9
Smad2	Q62432	129	-17	-20	-12	-7	0.9	0.8	0.9	0.9
Fkbp8	O35465	285	-10	-21	-12	-12	0.9	0.8	0.9	0.9
Gripap1	Q8VD04	792	-18	-21	-12	-27	0.9	0.8	0.9	0.8
Ppp6c	Q9CQR6	265	-5	-21	-12	-37	1.0	0.8	0.9	0.7

Vapa	Q9WV55	60	-9	-21	-12	-27	0.9	0.8	0.9	0.8
Cast	Q8CE80	288	3	-22	-12	-14	1.0	0.8	0.9	0.9
H2afy	Q9QZQ8	297	-12	-22	-12	10	0.9	0.8	0.9	1.1
Inf2	E9QLA5	1002	-13	-22	-12	-6	0.9	0.8	0.9	0.9
Acss1	Q99NB1	41	-31	-23	-12	-28	0.8	0.8	0.9	0.8
Gatm	Q9D964	50	-14	-23	-12	3	0.9	0.8	0.9	1.0
Mki67	E9PVX6	549	-13	-23	-12	-20	0.9	0.8	0.9	0.8
Cblb	B9EKI5	376	-16	-23	-12	-22	0.9	0.8	0.9	0.8
Cbl	P22682	382	-16	-23	-12	-22	0.9	0.8	0.9	0.8
Pde2a	F7D3W5	657	-10	-24	-12	8	0.9	0.8	0.9	1.1
Flnb	Q80X90	991	-3	-24	-12	-31	1.0	0.8	0.9	0.8
Foxn3	Q499D0	436	-12	-24	-12	-8	0.9	0.8	0.9	0.9
Srsf9	Q9D0B0	81	-17	-24	-12	-11	0.9	0.8	0.9	0.9
Fbxo6	Q9QZN4	60	-8	-24	-12	-18	0.9	0.8	0.9	0.9
Nsmce1	Q9D720	40	-24	-25	-12	-30	0.8	0.8	0.9	0.8
Trafd1	Q3UDK1	116	-11	-25	-12	-12	0.9	0.8	0.9	0.9
Pold1	P52431	711	-41	-26	-12	-8	0.7	0.8	0.9	0.9
Dido1	Q8C9B9	410	3	-26	-12	-2	1.0	0.8	0.9	1.0
Nup205	A0A0J9YUD5	1085	-32	-27	-12	22	0.8	0.8	0.9	1.3
Zfp217	Q3U0X6	349	-2	-28	-12	-4	1.0	0.8	0.9	1.0
Flna	Q8BTM8	483	-12	-28	-12	-34	0.9	0.8	0.9	0.7
Hmmr	Q00547	243	-15	-29	-12	-22	0.9	0.8	0.9	0.8
Usp38	Q8BW70	432	-24	-34	-12	-21	0.8	0.7	0.9	0.8
Cct8	P42932	149	34	-42	-12	-37	1.5	0.7	0.9	0.7
Thumpd3	P97770	393	-23	-43	-12	-20	0.8	0.7	0.9	0.8
Fbxo42	Q6PDJ6	302	-16	13	-13	24	0.9	1.1	0.9	1.3
Ddx24	Q9ESV0	639	-17	9	-13	1	0.9	1.1	0.9	1.0
Edc3	Q8K2D3	353	-18	7	-13	-11	0.8	1.1	0.9	0.9
Gm10073	E9Q3T0	61	-9	5	-13	4	0.9	1.1	0.9	1.0
Pdcd6ip	Q9WU78	122	-6	5	-13	38	0.9	1.0	0.9	1.6
Park7	Q99LX0	46	-2	4	-13	10	1.0	1.0	0.9	1.1
Pdhx	Q8BKZ9	170	-12	4	-13	-14	0.9	1.0	0.9	0.9
Slfn5	Q8CBA2	621	2	3	-13	5	1.0	1.0	0.9	1.0
Rps6ka2	Q9WUT3	429	2	2	-13	5	1.0	1.0	0.9	1.1
Acap2	Q6ZQK5	407	1	2	-13	-1	1.0	1.0	0.9	1.0
Tomm40	Q9QYA2	76	2	2	-13	-24	1.0	1.0	0.9	0.8
Fam206a	Q80ZQ9	45	-22	2	-13	-27	0.8	1.0	0.9	0.8
Ehd4	Q9EQP2	141	-11	2	-13	1	0.9	1.0	0.9	1.0
Cotl1	Q9CQI6	52	3	1	-13	16	1.0	1.0	0.9	1.2
Gvin1	L7N451	2122	-16	1	-13	13	0.9	1.0	0.9	1.1
Kif21b	E9Q0A4	1153	-14	1	-13	-12	0.9	1.0	0.9	0.9
Tbc1d8b	A3KGB4	875	-2	0	-13	9	1.0	1.0	0.9	1.1
Letm1	Q9Z2I0	551	-13	-1	-13	-21	0.9	1.0	0.9	0.8
Cd44	A2APM2	762	0	-1	-13	-32	1.0	1.0	0.9	0.8
Snx19	Q6P4T1	91	-2	-1	-13	20	1.0	1.0	0.9	1.2
Rnh1	Q91VI7	33	-7	-1	-13	10	0.9	1.0	0.9	1.1
Kiaa0196	Q8C2E7	1064	-28	-1	-13	7	0.8	1.0	0.9	1.1
Rlf	A2A7F4	227	9	-1	-13	5	1.1	1.0	0.9	1.0
Nedd9	O35177	474	-11	-2	-13	10	0.9	1.0	0.9	1.1
Sept7	O55131	279	-8	-2	-13	28	0.9	1.0	0.9	1.4
Hgh1	Q8C3I8	234	-9	-2	-13	20	0.9	1.0	0.9	1.3

Card6	E9PWH2	857	3	-2	-13	-7	1.0	1.0	0.9	0.9
Parn	Q8VDG3	162	-9	-3	-13	3	0.9	1.0	0.9	1.0
Smc2	Q8CG48	279	-5	-3	-13	3	1.0	1.0	0.9	1.0
Idh3b	Q91VA7	184	-7	-3	-13	-7	0.9	1.0	0.9	0.9
Tdp1	B8JJC1	565	-6	-3	-13	-21	0.9	1.0	0.9	0.8
Rnf213	E9Q555	3918	4	-3	-13	-3	1.0	1.0	0.9	1.0
Rps6ka5	Q8C050	439	7	-3	-13	-4	1.1	1.0	0.9	1.0
Lacc1	Q8BZT9	83	-15	-4	-13	-5	0.9	1.0	0.9	1.0
Ddx59	Q9DBN9	453	-12	-4	-13	-10	0.9	1.0	0.9	0.9
N6amt1	Q6SKR2	198	-8	-4	-13	-10	0.9	1.0	0.9	0.9
Kbtbd11	Q8BNW9	491	-9	-4	-13	-16	0.9	1.0	0.9	0.9
Nars	Q8BP47	449	-9	-4	-13	11	0.9	1.0	0.9	1.1
Isg20l2	Q3U1G5	310	-12	-4	-13	8	0.9	1.0	0.9	1.1
Dennd4a	E9Q8V6	117	-7	-4	-13	6	0.9	1.0	0.9	1.1
Fbxo22	Q78JE5	227	-3	-4	-13	-4	1.0	1.0	0.9	1.0
Clk2	O35491	480	-13	-4	-13	-7	0.9	1.0	0.9	0.9
Uba2	Q9Z1F9	439	-18	-4	-13	-12	0.9	1.0	0.9	0.9
Chmp1a	Q921W0	44	-8	-5	-13	-16	0.9	1.0	0.9	0.9
Gmip	Q6PGG2	816	-7	-5	-13	-17	0.9	1.0	0.9	0.9
Ska1	Q9CPV1	158	5	-5	-13	-21	1.0	1.0	0.9	0.8
Htatip2	Q9Z2G9	172	-14	-5	-13	14	0.9	1.0	0.9	1.2
Itpr3	P70227	2667	-7	-5	-13	6	0.9	1.0	0.9	1.1
Uchl3	Q9JKB1	95	-9	-5	-13	-13	0.9	1.0	0.9	0.9
Man2c1	Q91W89	321	-11	-5	-13	-17	0.9	1.0	0.9	0.9
Vdac3-ps1	A0A140T8V3	36	-16	-5	-13	-18	0.9	1.0	0.9	0.9
Smarcc1	P97496	118	3	-6	-13	32	1.0	0.9	0.9	1.5
Gm18025	E9Q1N8	208	-13	-6	-13	-2	0.9	0.9	0.9	1.0
Gm5786	J3QMG5	229	-13	-6	-13	-2	0.9	0.9	0.9	1.0
Dock8	Q8C147	940	-13	-6	-13	-19	0.9	0.9	0.9	0.8
Bod1l	E9Q6J5	283	-5	-6	-13	-22	1.0	0.9	0.9	0.8
Alkbh4	Q9D8F1	265	-8	-6	-13	3	0.9	0.9	0.9	1.0
Cndp2	Q9D1A2	300	-5	-6	-13	-5	1.0	0.9	0.9	1.0
Camk2g	Q923T9	273	-7	-6	-13	-10	0.9	0.9	0.9	0.9
Impa1	Q924B0	125	-6	-7	-13	4	0.9	0.9	0.9	1.0
Ift22	Q9DAI2	12	-14	-7	-13	-2	0.9	0.9	0.9	1.0
Csk	P41241	31	-8	-7	-13	-12	0.9	0.9	0.9	0.9
D330038O	Q6PGD2	257	-19	-7	-13	-12	0.8	0.9	0.9	0.9
Themis2	Q91YX0	571	-13	-7	-13	-13	0.9	0.9	0.9	0.9
Mmp9	P41245	692	-22	-7	-13	11	0.8	0.9	0.9	1.1
Vps16	G3X8X7	490	-15	-7	-13	-3	0.9	0.9	0.9	1.0
Ctc1	Q5SUQ9	1176	-11	-7	-13	-11	0.9	0.9	0.9	0.9
Huwe1	Q7TMY8	3239	-15	-7	-13	-22	0.9	0.9	0.9	0.8
Psmb10	O35955	17	-7	-7	-13	-28	0.9	0.9	0.9	0.8
Aim2	Q91VJ1	264	-12	-8	-13	-7	0.9	0.9	0.9	0.9
Zc3h3	Q8CHP0	638	-13	-8	-13	-9	0.9	0.9	0.9	0.9
Dnmt3a	O88508	336	-13	-8	-13	12	0.9	0.9	0.9	1.1
Ntpcr	Q9CQA9	76	-10	-8	-13	-3	0.9	0.9	0.9	1.0
Wdr46	Q9Z0H1	514	-11	-8	-13	-3	0.9	0.9	0.9	1.0
Ranbp2	Q9ERU9	2811	-11	-8	-13	-9	0.9	0.9	0.9	0.9
Sec16a	A2AIX1	663	-14	-8	-13	-10	0.9	0.9	0.9	0.9
Nek9	Q8K1R7	885	-4	-8	-13	-13	1.0	0.9	0.9	0.9

Strn4	P58404	337	-14	-8	-13	-33	0.9	0.9	0.9	0.8
Cand2	Q6ZQ73	827	-12	-9	-13	14	0.9	0.9	0.9	1.2
Hspa14	Q99M31	440	-3	-9	-13	3	1.0	0.9	0.9	1.0
Scfd2	Q8BTY8	54	-16	-9	-13	-1	0.9	0.9	0.9	1.0
Mbnl2	Q8C181	197	2	-9	-13	-2	1.0	0.9	0.9	1.0
Cep250	Q60952	2261	-9	-9	-13	-10	0.9	0.9	0.9	0.9
Rab14	Q91V41	40	-4	-9	-13	-12	1.0	0.9	0.9	0.9
Zmym2	Q9CU65	586	-14	-9	-13	-14	0.9	0.9	0.9	0.9
Kmt5c	Q6Q783	185	-15	-9	-13	-17	0.9	0.9	0.9	0.9
Bach1	P97302	649	-16	-10	-13	19	0.9	0.9	0.9	1.2
Vrk2	Q8BN21	285	-19	-10	-13	2	0.8	0.9	0.9	1.0
Brd8	Q8R3B7	941	-7	-10	-13	0	0.9	0.9	0.9	1.0
Ifi204	P0DOV2	404	-11	-10	-13	-4	0.9	0.9	0.9	1.0
Mlycd	Q99J39	205	-14	-10	-13	-7	0.9	0.9	0.9	0.9
Gmps	Q3THK7	456	-11	-10	-13	-9	0.9	0.9	0.9	0.9
Hprt1	P00493	106	-10	-10	-13	-9	0.9	0.9	0.9	0.9
Naa15	G3X8Y3	238	-9	-10	-13	-12	0.9	0.9	0.9	0.9
Glb1	P23780	394	-6	-10	-13	-15	0.9	0.9	0.9	0.9
Pcnt	F8VPV0	1755	-12	-10	-13	-18	0.9	0.9	0.9	0.8
Ctdp1	Q7TSG2	73	-11	-10	-13	-19	0.9	0.9	0.9	0.8
Rps27a	P62983	149	-4	-10	-13	-6	1.0	0.9	0.9	0.9
Rreb1	Q3UH06	1253	-10	-10	-13	-12	0.9	0.9	0.9	0.9
Psmc2	P46471	377	-43	-10	-13	-13	0.7	0.9	0.9	0.9
Ppm1g	Q61074	164	-9	-10	-13	-16	0.9	0.9	0.9	0.9
Gimap1	P70224	209	-4	-10	-13	-18	1.0	0.9	0.9	0.9
Fgr	P14234	261	-13	-11	-13	38	0.9	0.9	0.9	1.6
Pdk1	Q8BFP9	71	-10	-11	-13	-1	0.9	0.9	0.9	1.0
Rictor	Q6QI06	1316	-15	-11	-13	-10	0.9	0.9	0.9	0.9
Numa1	E9Q7G0	1132	-15	-11	-13	-11	0.9	0.9	0.9	0.9
Dnmt1	P13864	41	-16	-11	-13	-14	0.9	0.9	0.9	0.9
Ddx17	Q501J6	505	-10	-11	-13	-14	0.9	0.9	0.9	0.9
Ints4	Q8CIM8	45	-19	-11	-13	-16	0.8	0.9	0.9	0.9
Rasal3	Q8C2K5	473	-9	-11	-13	-38	0.9	0.9	0.9	0.7
Sec13	Q9D1M0	187	-13	-11	-13	1	0.9	0.9	0.9	1.0
Foxo1	Q9R1E0	454	-9	-11	-13	-8	0.9	0.9	0.9	0.9
Mgst1	Q91VS7	50	-12	-11	-13	-9	0.9	0.9	0.9	0.9
Kiaa1551	Q5DTW7	1413	-7	-11	-13	-10	0.9	0.9	0.9	0.9
Fam206a	Q80ZQ9	86	-18	-11	-13	-15	0.9	0.9	0.9	0.9
Cbx3	Q9DCC5	177	-14	-11	-13	-20	0.9	0.9	0.9	0.8
Cope	O89079	34	-17	-12	-13	6	0.9	0.9	0.9	1.1
Hk1	P17710	214	-21	-12	-13	-1	0.8	0.9	0.9	1.0
Camk2d	Q6PHZ2	273	-13	-12	-13	-8	0.9	0.9	0.9	0.9
Mapre3	Q6PER3	182	-12	-12	-13	-16	0.9	0.9	0.9	0.9
Dut	Q8VCG1	170	-13	-12	-13	-18	0.9	0.9	0.9	0.8
Ppp6r3	Q922D4	815	-10	-12	-13	-18	0.9	0.9	0.9	0.8
Fhod1	Q6P9Q4	936	-12	-12	-13	-4	0.9	0.9	0.9	1.0
Smarcc2	Q6PDG5	145	-12	-12	-13	-5	0.9	0.9	0.9	1.0
Heatr1	G3X9B1	689	-11	-12	-13	-8	0.9	0.9	0.9	0.9
Ppp2r5c	Q60996	513	-12	-12	-13	-13	0.9	0.9	0.9	0.9
Trrap	A0A1D5RLL4	2449	-18	-13	-13	1	0.8	0.9	0.9	1.0
Dbt	P53395	279	-7	-13	-13	0	0.9	0.9	0.9	1.0

Rars	Q9D0I9	369	-18	-13	-13	-1	0.8	0.9	0.9	1.0
Mgea5	Q9EQQ9	896	-13	-13	-13	-3	0.9	0.9	0.9	1.0
Kif15	Q6P9L6	1017	-11	-13	-13	-10	0.9	0.9	0.9	0.9
Gnl2	Q99LH1	336	-16	-13	-13	-12	0.9	0.9	0.9	0.9
Raf1	Q99N57	588	-9	-13	-13	-12	0.9	0.9	0.9	0.9
Kat6b	Q8BRB7	100	-30	-13	-13	-21	0.8	0.9	0.9	0.8
Top1	Q04750	632	-14	-13	-13	-22	0.9	0.9	0.9	0.8
Apbb1ip	Q8R5A3	403	-20	-13	-13	15	0.8	0.9	0.9	1.2
Wdhd1	P59328	709	-21	-13	-13	11	0.8	0.9	0.9	1.1
Cluh	Q5SW19	200	-15	-13	-13	-5	0.9	0.9	0.9	1.0
Uck1	P52623	66	-14	-13	-13	-6	0.9	0.9	0.9	0.9
Cdc42	P60766	18	-13	-13	-13	-13	0.9	0.9	0.9	0.9
Gmppa	Q922H4	389	-15	-14	-13	12	0.9	0.9	0.9	1.1
Wdr6	Q99ME2	26	-26	-14	-13	5	0.8	0.9	0.9	1.1
Ddx27	Q921N6	287	-15	-14	-13	-7	0.9	0.9	0.9	0.9
Kat6a	G3X940	135	-14	-14	-13	-10	0.9	0.9	0.9	0.9
Nr3c1	E9PYV1	480	-30	-14	-13	-12	0.8	0.9	0.9	0.9
Als2cl	Q60I26	42	-17	-14	-13	35	0.9	0.9	0.9	1.5
Pgm1	Q9D0F9	101	-12	-14	-13	12	0.9	0.9	0.9	1.1
Ndufa7	Q9Z1P6	55	-13	-14	-13	3	0.9	0.9	0.9	1.0
Fyttd1	Q91Z49	241	-12	-14	-13	-17	0.9	0.9	0.9	0.9
Casp6	O08738	246	-6	-14	-13	-22	0.9	0.9	0.9	0.8
Gpd2	Q64521	188	-6	-14	-13	-27	0.9	0.9	0.9	0.8
Plec	Q9QXS1	958	10	-15	-13	26	1.1	0.9	0.9	1.4
Cnn2	Q08093	274	1	-15	-13	7	1.0	0.9	0.9	1.1
Ilvbl	Q8BU33	354	-17	-15	-13	6	0.9	0.9	0.9	1.1
Kiaa1551	Q5DTW7	277	-7	-15	-13	-5	0.9	0.9	0.9	1.0
Ubash3a	Q3V3E1	331	-4	-15	-13	-9	1.0	0.9	0.9	0.9
Ankrd44	B2RXR6	921	-20	-15	-13	27	0.8	0.9	0.9	1.4
Ssrp1	Q08943	200	-18	-15	-13	18	0.8	0.9	0.9	1.2
Oxct1	Q9D0K2	456	-16	-15	-13	8	0.9	0.9	0.9	1.1
Tbc1d1	Q60949	90	-12	-16	-13	-7	0.9	0.9	0.9	0.9
Rel	A4QPD3	496	-34	-16	-13	-24	0.7	0.9	0.9	0.8
Trmt6	Q8CE96	473	-4	-16	-13	-38	1.0	0.9	0.9	0.7
Card11	Q8CIS0	1041	-12	-16	-13	-10	0.9	0.9	0.9	0.9
Nup205	A0A0J9YUD5	1328	-16	-17	-13	17	0.9	0.9	0.9	1.2
Rps14	P62264	85	-8	-17	-13	7	0.9	0.9	0.9	1.1
Brwd3	A2AHJ4	202	-27	-17	-13	0	0.8	0.9	0.9	1.0
Phip	F8VQ93	205	-27	-17	-13	0	0.8	0.9	0.9	1.0
Brwd1	Q921C3	208	-27	-17	-13	0	0.8	0.9	0.9	1.0
Kat6a	G3X940	1097	8	-17	-13	-12	1.1	0.9	0.9	0.9
Gmeb2	P58929	117	-18	-17	-13	-19	0.8	0.9	0.9	0.8
Arid5a	Q3U108	277	-3	-17	-13	4	1.0	0.9	0.9	1.0
Gnl3	Q8CI11	246	-7	-17	-13	-18	0.9	0.9	0.9	0.8
Gm5786	J3QMG5	188	-12	-18	-13	-8	0.9	0.9	0.9	0.9
Rabep2	Q91WG2	467	-8	-18	-13	-38	0.9	0.9	0.9	0.7
Znf319	Q9ERR8	372	-16	-18	-13	-19	0.9	0.8	0.9	0.8
Las1l	A2BE28	118	-13	-19	-13	34	0.9	0.8	0.9	1.5
Stxbp3	Q60770	501	-14	-19	-13	-12	0.9	0.8	0.9	0.9
Usp8	Q80U87	771	-6	-19	-13	-19	0.9	0.8	0.9	0.8
Ctnnbl1	Q9CWL8	370	-29	-19	-13	4	0.8	0.8	0.9	1.0

Nt5c3b	Q3UFY7	210	-17	-19	-13	-19	0.9	0.8	0.9	0.8
Tceb3	Q8CB77	541	-21	-20	-13	-1	0.8	0.8	0.9	1.0
Gcn1	E9PVA8	1595	-22	-20	-13	-7	0.8	0.8	0.9	0.9
Nras	Q9D091	118	0	-20	-13	-10	1.0	0.8	0.9	0.9
Lrch3	Q8BVU0	478	1	-20	-13	-19	1.0	0.8	0.9	0.8
Nat10	Q8K224	517	-6	-20	-13	-49	0.9	0.8	0.9	0.7
Iars	Q8BU30	400	-20	-20	-13	-9	0.8	0.8	0.9	0.9
Itpr3	P70227	638	-5	-20	-13	-16	1.0	0.8	0.9	0.9
2210013O	A0A0N4SUI7	28	-19	-21	-13	45	0.8	0.8	0.9	1.8
Rab14	Q91V41	26	-25	-21	-13	-8	0.8	0.8	0.9	0.9
Rad21	Q61550	392	-15	-21	-13	9	0.9	0.8	0.9	1.1
Tomm70	Q9CZW5	478	-17	-21	-13	-7	0.9	0.8	0.9	0.9
Ubtf	A2AWT5	328	-10	-21	-13	-16	0.9	0.8	0.9	0.9
Abcd1	P48410	631	-4	-22	-13	-3	1.0	0.8	0.9	1.0
Pds5a	E9QPI5	507	-4	-22	-13	-5	1.0	0.8	0.9	1.0
Trappc8	E9PWG2	1343	-17	-23	-13	-43	0.9	0.8	0.9	0.7
Sptan1	P16546	2120	-20	-23	-13	4	0.8	0.8	0.9	1.0
Cmss1	Q9CZT6	118	-7	-23	-13	4	0.9	0.8	0.9	1.0
Ctnna1	P26231	116	-18	-23	-13	-27	0.8	0.8	0.9	0.8
Cstf3	Q99LI7	646	-12	-24	-13	-3	0.9	0.8	0.9	1.0
Capg	Q99LB4	283	-30	-24	-13	-14	0.8	0.8	0.9	0.9
Vps13a	Q5H8C4	1601	-34	-24	-13	5	0.7	0.8	0.9	1.1
Rpn1	Q91YQ5	546	-17	-25	-13	-21	0.9	0.8	0.9	0.8
Rbm22	Q8BHS3	58	-9	-25	-13	3	0.9	0.8	0.9	1.0
Jak3	Q62137	807	-12	-27	-13	-11	0.9	0.8	0.9	0.9
Xiap	Q60989	161	-18	-27	-13	-4	0.8	0.8	0.9	1.0
Khsrp	Q3U0V1	437	-16	-29	-13	-14	0.9	0.8	0.9	0.9
Dnmt1	P13864	444	-22	-30	-13	-1	0.8	0.8	0.9	1.0
Fasn	P19096	1874	-37	-30	-13	-3	0.7	0.8	0.9	1.0
Slmap	Q3URD3	37	-27	-32	-13	-28	0.8	0.8	0.9	0.8
Pitrm1	Q8K411	627	-28	-32	-13	-34	0.8	0.8	0.9	0.7
Rnf14	Q9JI90	351	-8	-35	-13	-3	0.9	0.7	0.9	1.0
Ranbp1	P34022	158	-11	-37	-13	-6	0.9	0.7	0.9	0.9
Snx27	Q3UHD6	352	-23	-38	-13	-6	0.8	0.7	0.9	0.9
Pdlim2	Q8R1G6	331	25	-40	-13	-33	1.3	0.7	0.9	0.8
Prkcd	P28867	391	-4	20	-13	-5	1.0	1.3	0.9	1.0
Abcf1	Q6P542	750	1	9	-13	7	1.0	1.1	0.9	1.1
Khnyn	Q80U38	328	-3	7	-13	-22	1.0	1.1	0.9	0.8
Rsf1	E9PWW9	566	-2	3	-13	-20	1.0	1.0	0.9	0.8
Zc3h3	Q8CHP0	907	-14	2	-13	-1	0.9	1.0	0.9	1.0
Rhof	Q8BYP3	173	-20	2	-13	-48	0.8	1.0	0.9	0.7
Exosc9	Q9JHI7	9	-3	2	-13	7	1.0	1.0	0.9	1.1
Rrp8	Q9DB85	318	-5	1	-13	1	1.0	1.0	0.9	1.0
Cd3g	P11942	87	-6	0	-13	7	0.9	1.0	0.9	1.1
Rpp14	Q9CQH8	76	3	0	-13	1	1.0	1.0	0.9	1.0
Pik3r4	Q8VD65	899	-10	-1	-13	-8	0.9	1.0	0.9	0.9
Ndufv2	Q9D6J6	224	-21	-1	-13	9	0.8	1.0	0.9	1.1
Atp2a2	O55143	344	-4	-1	-13	5	1.0	1.0	0.9	1.0
Atp2a3	Q64518	344	-4	-1	-13	5	1.0	1.0	0.9	1.0
Col4a3bp	Q9EQG9	70	-12	-1	-13	-4	0.9	1.0	0.9	1.0
Nol9	Q3TZX8	259	1	-1	-13	-5	1.0	1.0	0.9	1.0

Ruvbl1	P60122	206	-26	-2	-13	8	0.8	1.0	0.9	1.1
Psmc1	P62192	399	-8	-2	-13	-16	0.9	1.0	0.9	0.9
Xrn1	F8VQ87	893	6	-2	-13	-4	1.1	1.0	0.9	1.0
Plaa	P27612	26	-5	-2	-13	-4	1.0	1.0	0.9	1.0
Lgals1	P16045	43	-9	-2	-13	-5	0.9	1.0	0.9	1.0
Smchd1	Q6P5D8	84	-6	-2	-13	-15	0.9	1.0	0.9	0.9
Mrpl58	Q8R035	82	-6	-2	-13	-21	0.9	1.0	0.9	0.8
Xab2	Q9DCD2	66	-15	-3	-13	13	0.9	1.0	0.9	1.1
Gtf3c1	Q8K284	1986	-8	-3	-13	12	0.9	1.0	0.9	1.1
Ankrd44	B2RXR6	408	-3	-3	-13	1	1.0	1.0	0.9	1.0
Fam208a	Q69ZR9	144	-8	-3	-13	-3	0.9	1.0	0.9	1.0
Cep250	Q60952	1732	-9	-3	-13	-15	0.9	1.0	0.9	0.9
Fmnl1	Q9JL26	45	-8	-3	-13	-20	0.9	1.0	0.9	0.8
Helz2	E9QAM5	1938	-27	-3	-13	24	0.8	1.0	0.9	1.3
UPF0711	Q5XFZ0	178	-19	-3	-13	-19	0.8	1.0	0.9	0.8
Scaf11	E9PZM7	1112	-10	-4	-13	-7	0.9	1.0	0.9	0.9
Rangap1	P46061	303	-11	-4	-13	10	0.9	1.0	0.9	1.1
Ythdf2	Q91YT7	482	-4	-4	-13	9	1.0	1.0	0.9	1.1
Eif2s3x	Q9Z0N1	434	-10	-4	-13	0	0.9	1.0	0.9	1.0
Eif2s3y	Q9Z0N2	434	-10	-4	-13	0	0.9	1.0	0.9	1.0
Gtf3c1	Q8K284	320	-6	-4	-13	-15	0.9	1.0	0.9	0.9
Hmha1	Q3TBD2	780	-14	-4	-13	-19	0.9	1.0	0.9	0.8
Mlycd	Q99J39	214	-15	-4	-13	-46	0.9	1.0	0.9	0.7
Aldoa	P05064	339	-7	-5	-13	13	0.9	1.0	0.9	1.1
Rock1	P70335	1070	-9	-5	-13	2	0.9	1.0	0.9	1.0
Llgl1	Q80Y17	504	-5	-5	-13	-18	1.0	1.0	0.9	0.9
Ddx58	Q6Q899	288	-13	-5	-13	-23	0.9	1.0	0.9	0.8
Hadha	Q8BMS1	470	-20	-5	-13	20	0.8	1.0	0.9	1.2
Atad1	Q9D5T0	137	-11	-5	-13	13	0.9	1.0	0.9	1.1
Smu1	Q3UKJ7	383	-14	-5	-13	9	0.9	1.0	0.9	1.1
Zzef1	Q5SSH7	787	-10	-5	-13	6	0.9	1.0	0.9	1.1
Usp9x	Q4FE56	692	-15	-5	-13	-3	0.9	1.0	0.9	1.0
Exosc1	Q9DAA6	15	-5	-5	-13	-3	1.0	1.0	0.9	1.0
Ccdc88b	Q4QRL3	769	-13	-5	-13	-5	0.9	1.0	0.9	1.0
Ints3	Q7TPD0	496	-5	-5	-13	-11	1.0	1.0	0.9	0.9
Nemf	Q8CCP0	820	-2	-5	-13	-15	1.0	1.0	0.9	0.9
Znf622	Q91VY9	304	-33	-6	-13	9	0.8	0.9	0.9	1.1
Ppox	P51175	258	-14	-6	-13	-9	0.9	0.9	0.9	0.9
Fgd3	O88842	280	-5	-6	-13	-11	1.0	0.9	0.9	0.9
Pml	Q60953	19	-13	-6	-13	-21	0.9	0.9	0.9	0.8
Ankzf1	J3QM81	74	-6	-6	-13	7	0.9	0.9	0.9	1.1
Ddb2	Q99J79	200	-14	-6	-13	5	0.9	0.9	0.9	1.1
Ankrd44	B2RXR6	547	-24	-6	-13	-1	0.8	0.9	0.9	1.0
Dgcr8	Q9EQM6	430	1	-6	-13	-2	1.0	0.9	0.9	1.0
Myo18a	Q9JMH9	809	-16	-6	-13	-25	0.9	0.9	0.9	0.8
Akr1b1	P45376	187	-9	-7	-13	3	0.9	0.9	0.9	1.0
Pum2	Q80U58	983	-15	-7	-13	-8	0.9	0.9	0.9	0.9
Grsf1	Q8C5Q4	475	-14	-7	-13	-8	0.9	0.9	0.9	0.9
Srrm2	Q8BTI8	1436	-6	-7	-13	-20	0.9	0.9	0.9	0.8
Lyst	G5E8Q0	20	-6	-7	-13	5	0.9	0.9	0.9	1.0
Maff	O54791	68	-14	-7	-13	-1	0.9	0.9	0.9	1.0

Pik3cd	O35904	366	-6	-7	-13	-4	0.9	0.9	0.9	1.0
Mapkapk3	Q3UMW7	205	-7	-7	-13	-8	0.9	0.9	0.9	0.9
Scaf4	Q6PFF0	102	-16	-8	-13	14	0.9	0.9	0.9	1.2
Pon2	Q62086	42	-16	-8	-13	9	0.9	0.9	0.9	1.1
Naprt	Q8CC86	385	-8	-8	-13	9	0.9	0.9	0.9	1.1
Vps35	Q9EQH3	653	1	-8	-13	-4	1.0	0.9	0.9	1.0
Smu1	Q3UKJ7	298	-10	-8	-13	-4	0.9	0.9	0.9	1.0
Cdk2ap2	Q9CPY4	117	-1	-8	-13	-12	1.0	0.9	0.9	0.9
Pccb	Q99MN9	518	-6	-8	-13	-15	0.9	0.9	0.9	0.9
Got2	P05202	295	-11	-8	-13	14	0.9	0.9	0.9	1.2
Ddx19a	Q61655	224	-10	-8	-13	8	0.9	0.9	0.9	1.1
Ddx19b	Q8BZY3	240	-10	-8	-13	8	0.9	0.9	0.9	1.1
Parp14	Q2EMV9	1740	-12	-8	-13	-1	0.9	0.9	0.9	1.0
Bre	Q8K3W0	34	-7	-8	-13	-4	0.9	0.9	0.9	1.0
Cmpk2	Q3U5Q7	189	-12	-8	-13	-6	0.9	0.9	0.9	0.9
Rrp36	Q3UFY0	72	-11	-8	-13	-7	0.9	0.9	0.9	0.9
Dennd3	A2RT67	72	-17	-8	-13	-8	0.9	0.9	0.9	0.9
Trrap	A0A1D5RLL4	3535	-15	-9	-13	8	0.9	0.9	0.9	1.1
Glrx5	Q80Y14	63	-15	-9	-13	-1	0.9	0.9	0.9	1.0
Skil	Q60665	492	-8	-9	-13	-4	0.9	0.9	0.9	1.0
Usp4	P35123	926	-10	-9	-13	-8	0.9	0.9	0.9	0.9
Adar	Q99MU3	722	-19	-9	-13	-12	0.8	0.9	0.9	0.9
Ranbp2	Q9ERU9	141	-4	-9	-13	-15	1.0	0.9	0.9	0.9
Shc1	P98083	212	-19	-9	-13	-17	0.8	0.9	0.9	0.9
Sart1	Q9Z315	651	-11	-9	-13	-18	0.9	0.9	0.9	0.8
Dok3	Q9QZK7	329	-6	-9	-13	-7	0.9	0.9	0.9	0.9
Znfx1	Q8R151	392	-7	-9	-13	-12	0.9	0.9	0.9	0.9
Pde3b	E9QLQ3	388	6	-9	-13	-15	1.1	0.9	0.9	0.9
Lrch1	P62046	282	-10	-9	-13	-16	0.9	0.9	0.9	0.9
Trp53bp1	A2AU91	871	-19	-9	-13	-20	0.8	0.9	0.9	0.8
Cct4	P80315	337	-10	-10	-13	26	0.9	0.9	0.9	1.3
Prkd2	Q8BZ03	84	-12	-10	-13	2	0.9	0.9	0.9	1.0
Rgl2	Q61193	508	-8	-10	-13	1	0.9	0.9	0.9	1.0
Cnot8	Q9D8X5	276	-7	-10	-13	-6	0.9	0.9	0.9	0.9
Idh3a	Q9D6R2	222	-9	-10	-13	-8	0.9	0.9	0.9	0.9
Plekhj1	Q9D240	155	-13	-10	-13	-8	0.9	0.9	0.9	0.9
Sigirr	Q9JLZ8	173	-14	-10	-13	-8	0.9	0.9	0.9	0.9
Nf1	Q04690	1713	-1	-10	-13	-16	1.0	0.9	0.9	0.9
Thoc2	B1AZI6	1044	-12	-10	-13	11	0.9	0.9	0.9	1.1
Zcchc11	B2RX14	1011	-9	-10	-13	-5	0.9	0.9	0.9	1.0
Ufc1	Q9CR09	116	-9	-10	-13	-8	0.9	0.9	0.9	0.9
Rpl10l	P86048	195	-10	-10	-13	-9	0.9	0.9	0.9	0.9
Ahnak	E9Q616	5282	-13	-10	-13	-13	0.9	0.9	0.9	0.9
Trp53bp1	A2AU91	596	-11	-10	-13	-25	0.9	0.9	0.9	0.8
Dock11	A2AF47	492	-19	-11	-13	1	0.8	0.9	0.9	1.0
Metap1	Q8BP48	36	-13	-11	-13	-6	0.9	0.9	0.9	0.9
Nbeal2	Q6ZQA0	913	-12	-11	-13	-9	0.9	0.9	0.9	0.9
Pura	P42669	291	-18	-11	-13	-18	0.9	0.9	0.9	0.8
Gtpbp6	Q3U6U5	88	2	-11	-13	-20	1.0	0.9	0.9	0.8
Trmt2a	Q8BNV1	233	-5	-11	-13	3	1.0	0.9	0.9	1.0
Lcp1	Q61233	31	-11	-11	-13	-7	0.9	0.9	0.9	0.9

Hars	Q61035	235	-17	-11	-13	-8	0.9	0.9	0.9	0.9
Kbtbd11	Q8BNW9	514	-14	-11	-13	-23	0.9	0.9	0.9	0.8
Sec22a	Q8BH47	111	-22	-11	-13	-44	0.8	0.9	0.9	0.7
Cnn2	Q08093	215	-15	-12	-13	9	0.9	0.9	0.9	1.1
Mcm5	Q52KC3	249	-26	-12	-13	8	0.8	0.9	0.9	1.1
Brix1	Q9DCA5	115	-11	-12	-13	1	0.9	0.9	0.9	1.0
Sec31a	Q3UPL0	173	-35	-12	-13	-6	0.7	0.9	0.9	0.9
Blk	P16277	313	-16	-12	-13	-7	0.9	0.9	0.9	0.9
Rnf40	Q3U319	69	-19	-12	-13	-9	0.8	0.9	0.9	0.9
Il16	O54824	968	-10	-12	-13	-20	0.9	0.9	0.9	0.8
Anxa7	Q07076	338	-16	-12	-13	7	0.9	0.9	0.9	1.1
Hcfc1	Q61191	1905	-15	-12	-13	-5	0.9	0.9	0.9	1.0
Akr1c13	Q8VC28	39	-8	-12	-13	-11	0.9	0.9	0.9	0.9
Cdt1	Q8R4E9	176	-11	-12	-13	-16	0.9	0.9	0.9	0.9
Kmt2d	Q6PDK2	5532	12	-12	-13	-18	1.1	0.9	0.9	0.8
Pmpcb	Q9CXT8	248	-16	-12	-13	-21	0.9	0.9	0.9	0.8
Ube2i	P63280	93	-15	-13	-13	-2	0.9	0.9	0.9	1.0
Lage3	Q9CR70	23	-8	-13	-13	-4	0.9	0.9	0.9	1.0
Trp53bp1	A2AU91	1700	-5	-13	-13	-11	1.0	0.9	0.9	0.9
Lta4h	P24527	136	-4	-13	-13	-2	1.0	0.9	0.9	1.0
Lnpep	Q8C129	305	-24	-13	-13	-2	0.8	0.9	0.9	1.0
Ppp2r1a	Q76MZ3	377	-14	-13	-13	-10	0.9	0.9	0.9	0.9
Hnrnpm	Q9D0E1	26	-7	-13	-13	-11	0.9	0.9	0.9	0.9
Dnm2	P39054	86	-14	-14	-13	1	0.9	0.9	0.9	1.0
Lrch1	P62046	677	-10	-14	-13	-4	0.9	0.9	0.9	1.0
Phf5a	P83870	40	-16	-14	-13	-7	0.9	0.9	0.9	0.9
Lactb2	Q99KR3	100	-12	-14	-13	-8	0.9	0.9	0.9	0.9
D10Jhu81e	Q9D172	175	-7	-14	-13	-11	0.9	0.9	0.9	0.9
Manba	Q8K2I4	153	-11	-14	-13	-17	0.9	0.9	0.9	0.9
Numa1	E9Q7G0	439	-20	-14	-13	-19	0.8	0.9	0.9	0.8
Prrc2c	Q3TLH4	2292	-10	-14	-13	-20	0.9	0.9	0.9	0.8
Chuk	E9QNL4	371	-17	-14	-13	5	0.9	0.9	0.9	1.0
Hadh	Q61425	201	-21	-14	-13	-6	0.8	0.9	0.9	0.9
Whsc1l1	Q6P2L6	1295	-11	-14	-13	-36	0.9	0.9	0.9	0.7
Pde12	Q3TIU4	179	-17	-15	-13	1	0.9	0.9	0.9	1.0
Bmx	P97504	202	-13	-15	-13	-1	0.9	0.9	0.9	1.0
Golph3l	Q8R088	70	-15	-15	-13	-13	0.9	0.9	0.9	0.9
Golph3	Q9CRA5	84	-15	-15	-13	-13	0.9	0.9	0.9	0.9
Kiaa0391	Q8JZY4	504	-1	-15	-13	-3	1.0	0.9	0.9	1.0
Uba6	Q8C7R4	721	-23	-15	-13	-17	0.8	0.9	0.9	0.9
Eprs	Q8CGC7	1076	-15	-16	-13	-6	0.9	0.9	0.9	0.9
Pds5a	E9QPI5	349	-8	-16	-13	17	0.9	0.9	0.9	1.2
Pds5b	Q4VA53	340	-8	-16	-13	17	0.9	0.9	0.9	1.2
Dock11	A2AF47	160	-6	-16	-13	7	0.9	0.9	0.9	1.1
Pdlim1	O70400	261	-9	-16	-13	5	0.9	0.9	0.9	1.0
Cisd3	B1AR13	108	-28	-16	-13	-8	0.8	0.9	0.9	0.9
Uvrag	Q8K245	185	-15	-16	-13	-11	0.9	0.9	0.9	0.9
Card19	Q9D1I2	63	4	-16	-13	-21	1.0	0.9	0.9	0.8
Gba2	Q69ZF3	80	-19	-16	-13	-58	0.8	0.9	0.9	0.6
Iars	Q8BU30	526	-14	-17	-13	12	0.9	0.9	0.9	1.1
Ccdc91	Q9D8L5	425	-13	-17	-13	7	0.9	0.9	0.9	1.1

Fbxl20	Q9CZV8	127	-8	-17	-13	7	0.9	0.9	0.9	1.1
Ncf4	P97369	242	-23	-17	-13	6	0.8	0.9	0.9	1.1
Gimap5	Q8BWF2	184	-21	-17	-13	-2	0.8	0.9	0.9	1.0
Gimap3	Q99MI6	180	-21	-17	-13	-2	0.8	0.9	0.9	1.0
Mis18a	Q9CZJ6	86	-17	-17	-13	-5	0.9	0.9	0.9	1.0
Tada2a	Q8CHV6	405	-7	-17	-13	-12	0.9	0.9	0.9	0.9
Nipbl	Q6KCD5	304	-16	-17	-13	-22	0.9	0.9	0.9	0.8
Flnb	Q80X90	1438	-20	-17	-13	-23	0.8	0.9	0.9	0.8
Anapc5	Q8BTZ4	188	-10	-17	-13	8	0.9	0.9	0.9	1.1
Cenpl	Q3U3S3	53	-9	-17	-13	2	0.9	0.9	0.9	1.0
Nup153	E9Q3G8	797	-5	-17	-13	-16	1.0	0.9	0.9	0.9
Cap1	P40124	374	-17	-17	-13	-31	0.9	0.9	0.9	0.8
H2-D1	P01899	188	-18	-18	-13	16	0.9	0.9	0.9	1.2
Trip11	E9Q512	813	-12	-18	-13	-13	0.9	0.9	0.9	0.9
Zbtb43	Q9DAI4	59	-23	-18	-13	-13	0.8	0.9	0.9	0.9
Tpp2	Q64514	787	-17	-18	-13	-13	0.9	0.9	0.9	0.9
Cdc42	P60766	105	-16	-18	-13	-23	0.9	0.9	0.9	0.8
Cisd3	B1AR13	110	-21	-18	-13	-9	0.8	0.8	0.9	0.9
Whsc1l1	Q6P2L6	495	-9	-18	-13	-10	0.9	0.8	0.9	0.9
Noc3l	Q8VI84	779	-22	-18	-13	-14	0.8	0.8	0.9	0.9
Arid4a	F8VPQ2	356	-18	-18	-13	-19	0.8	0.8	0.9	0.8
Wdr1	O88342	507	-31	-19	-13	-12	0.8	0.8	0.9	0.9
Kif2c	Q922S8	437	-40	-19	-13	-25	0.7	0.8	0.9	0.8
Med13	Q5SWW4	823	-11	-19	-13	-29	0.9	0.8	0.9	0.8
Glud1	P26443	172	-17	-19	-13	5	0.9	0.8	0.9	1.1
Fry	E9Q8I9	336	-11	-19	-13	-3	0.9	0.8	0.9	1.0
Ahcyl2	Q68FL4	375	-27	-19	-13	-3	0.8	0.8	0.9	1.0
Ahcyl1	Q80SW1	292	-27	-19	-13	-3	0.8	0.8	0.9	1.0
Dync1h1	Q9JHU4	4508	-7	-19	-13	-14	0.9	0.8	0.9	0.9
Prkcd	P28867	342	-8	-20	-13	-1	0.9	0.8	0.9	1.0
Ahcyl2	Q68FL4	355	-10	-20	-13	-15	0.9	0.8	0.9	0.9
Ahcyl1	Q80SW1	272	-10	-20	-13	-15	0.9	0.8	0.9	0.9
Bop1	P97452	359	-13	-20	-13	-13	0.9	0.8	0.9	0.9
Kdm4c	Q8VCD7	873	-13	-20	-13	-13	0.9	0.8	0.9	0.9
Hmgb1	P63158	106	-11	-20	-13	-17	0.9	0.8	0.9	0.9
Mepce	Q8K3A9	396	-14	-21	-13	-5	0.9	0.8	0.9	1.0
Scaf1	Q5U4C3	355	-19	-21	-13	-22	0.8	0.8	0.9	0.8
Kat6a	G3X940	1189	-28	-21	-13	-37	0.8	0.8	0.9	0.7
Gphn	Q8BUV3	452	-23	-21	-13	10	0.8	0.8	0.9	1.1
Ptprc	P06800	382	-20	-21	-13	-20	0.8	0.8	0.9	0.8
Cast	Q8CE80	374	-10	-21	-13	-20	0.9	0.8	0.9	0.8
Mcmbp	Q8R3C0	200	-18	-21	-13	-24	0.9	0.8	0.9	0.8
Nipbl	Q6KCD5	1934	-36	-22	-13	-30	0.7	0.8	0.9	0.8
Brat1	Q8C3R1	326	-10	-22	-13	38	0.9	0.8	0.9	1.6
Rbpj	P31266	423	-17	-22	-13	-9	0.9	0.8	0.9	0.9
Casp4	P70343	273	-21	-22	-13	-14	0.8	0.8	0.9	0.9
Nars	Q8BP47	548	-19	-23	-13	-46	0.8	0.8	0.9	0.7
Myg1	Q9JK81	119	-16	-23	-13	-13	0.9	0.8	0.9	0.9
Ilkap	Q8R0F6	312	-13	-24	-13	-3	0.9	0.8	0.9	1.0
Uncharact	Q8K039	109	-8	-24	-13	-4	0.9	0.8	0.9	1.0
Rttn	Q8R4Y8	2013	-42	-24	-13	-7	0.7	0.8	0.9	0.9

Gpx1	P11352	76	-23	-24	-13	-31	0.8	0.8	0.9	0.8
Ddx47	Q9CWX9	226	-24	-24	-13	-8	0.8	0.8	0.9	0.9
Nfatc2ip	O09130	225	-21	-25	-13	-14	0.8	0.8	0.9	0.9
Mthfd1	Q922D8	408	-29	-25	-13	4	0.8	0.8	0.9	1.0
Mlxip	G5E8D8	579	-3	-25	-13	-11	1.0	0.8	0.9	0.9
Pla2g4a	P47713	725	-13	-26	-13	-15	0.9	0.8	0.9	0.9
Cyth4	Q80YW0	174	-30	-26	-13	-5	0.8	0.8	0.9	1.0
Syne2	Q6ZWQ0	2667	-13	-27	-13	-5	0.9	0.8	0.9	1.0
Mdn1	A2ANY6	1540	-14	-27	-13	-24	0.9	0.8	0.9	0.8
Uhrf2	Q7TMI3	769	-26	-28	-13	17	0.8	0.8	0.9	1.2
Uhrf1	Q8VDF2	748	-26	-28	-13	17	0.8	0.8	0.9	1.2
Esco1	Q69Z69	72	-13	-32	-13	-10	0.9	0.8	0.9	0.9
Med13l	A0A0J9YUA8	2130	-5	-34	-13	8	1.0	0.7	0.9	1.1
Tradd	Q3U0V2	135	-15	-34	-13	-27	0.9	0.7	0.9	0.8
Fcho2	Q3UQN2	620	-4	-43	-13	-25	1.0	0.7	0.9	0.8
Ankfy1	Q810B6	1113	-9	13	-14	-3	0.9	1.1	0.9	1.0
Nab1	Q61122	378	-19	12	-14	15	0.8	1.1	0.9	1.2
Lrba	E9Q3Y4	2646	21	11	-14	-5	1.3	1.1	0.9	1.0
Mtr	A6H5Y3	107	-12	10	-14	-3	0.9	1.1	0.9	1.0
Khnyn	Q80U38	64	-3	8	-14	7	1.0	1.1	0.9	1.1
Tm6sf1	P58749	353	-16	8	-14	-1	0.9	1.1	0.9	1.0
Aff3	D3YYI6	126	-11	8	-14	-4	0.9	1.1	0.9	1.0
Stk17b	Q8BG48	330	7	5	-14	-12	1.1	1.0	0.9	0.9
Ubr3	Q5U430	1859	-10	4	-14	4	0.9	1.0	0.9	1.0
Uvssa	Q9D479	479	-2	4	-14	-5	1.0	1.0	0.9	1.0
Rrbp1	Q99PL5	1136	-16	4	-14	-5	0.9	1.0	0.9	1.0
Dock2	Q8C3J5	1257	9	1	-14	-1	1.1	1.0	0.9	1.0
Itpr3	P70227	1558	-9	1	-14	-11	0.9	1.0	0.9	0.9
Rin3	P59729	518	-7	0	-14	-2	0.9	1.0	0.9	1.0
Zc3h7b	F8VPP8	941	-13	-1	-14	12	0.9	1.0	0.9	1.1
Trim56	Q80VI1	532	-19	-1	-14	7	0.8	1.0	0.9	1.1
Bmx	P97504	137	-2	-1	-14	3	1.0	1.0	0.9	1.0
Pik3c2a	F8VPL2	515	-9	-1	-14	-4	0.9	1.0	0.9	1.0
Tardbp	Q921F2	244	-8	-3	-14	-10	0.9	1.0	0.9	0.9
Clcc1	Q99LI2	538	0	-3	-14	-47	1.0	1.0	0.9	0.7
Prpf4	Q9DAW6	440	-12	-4	-14	8	0.9	1.0	0.9	1.1
Parp1	Q921K2	430	-12	-4	-14	-2	0.9	1.0	0.9	1.0
Plxnb2	B2RXS4	1412	-19	-4	-14	-5	0.8	1.0	0.9	1.0
Rab3gap1	Q80UJ7	873	-20	-4	-14	-19	0.8	1.0	0.9	0.8
Pfkl	P12382	708	-17	-4	-14	12	0.9	1.0	0.9	1.1
Tln1	P26039	1953	1	-4	-14	-8	1.0	1.0	0.9	0.9
Hnrnpk	P61979	184	-12	-4	-14	-9	0.9	1.0	0.9	0.9
Syne2	Q6ZWQ0	328	-10	-4	-14	-11	0.9	1.0	0.9	0.9
Slc25a53	A2AF28	190	-9	-4	-14	-13	0.9	1.0	0.9	0.9
Tbce	Q8CIV8	102	-3	-5	-14	7	1.0	1.0	0.9	1.1
Hcfc1	Q61191	353	-7	-5	-14	-3	0.9	1.0	0.9	1.0
Vps72	Q62481	163	-10	-5	-14	-17	0.9	1.0	0.9	0.9
Dph2	Q9CR25	251	-4	-5	-14	-3	1.0	1.0	0.9	1.0
Rpap2	Q8VC34	105	-14	-5	-14	-4	0.9	1.0	0.9	1.0
Brd7	O88665	338	-22	-5	-14	-17	0.8	1.0	0.9	0.9
Siva1	O54926	6	-8	-6	-14	29	0.9	0.9	0.9	1.4

Ino80e	A0A0U1RP99	127	-2	-6	-14	1	1.0	0.9	0.9	1.0
Gm28042	B7ZCM9	40	6	-6	-14	-2	1.1	0.9	0.9	1.0
Capn2	O08529	350	-5	-6	-14	-15	1.0	0.9	0.9	0.9
Wars	P32921	278	-11	-6	-14	-16	0.9	0.9	0.9	0.9
Pcmtd2	Q8BHD8	223	-12	-6	-14	-19	0.9	0.9	0.9	0.8
Ddx49	Q4FZF3	45	-3	-6	-14	7	1.0	0.9	0.9	1.1
Birc3	O08863	26	-16	-6	-14	-2	0.9	0.9	0.9	1.0
Gtf3c1	Q8K284	286	-6	-6	-14	-4	0.9	0.9	0.9	1.0
Flad1	Q8R123	280	-12	-6	-14	-8	0.9	0.9	0.9	0.9
Rpn1	Q91YQ5	478	-8	-6	-14	-8	0.9	0.9	0.9	0.9
Nbas	E9Q411	2175	-7	-6	-14	-10	0.9	0.9	0.9	0.9
Senp7	Q8BUH8	69	-12	-6	-14	-15	0.9	0.9	0.9	0.9
Ssh2	Q5SW75	1210	-5	-6	-14	-18	1.0	0.9	0.9	0.9
Acap1	Q8K2H4	44	-18	-6	-14	-19	0.9	0.9	0.9	0.8
Lars	Q8BMJ2	1055	-26	-7	-14	3	0.8	0.9	0.9	1.0
Gbp2	Q9Z0E6	281	-10	-7	-14	1	0.9	0.9	0.9	1.0
Akap13	E9Q394	2437	-10	-7	-14	-15	0.9	0.9	0.9	0.9
Plec	Q9QXS1	4581	-20	-7	-14	4	0.8	0.9	0.9	1.0
Gbf1	Q6DFZ1	1432	-5	-7	-14	-2	1.0	0.9	0.9	1.0
Pafah2	Q8VDG7	46	-6	-7	-14	-4	0.9	0.9	0.9	1.0
Eif2s1	Q6ZWX6	199	-1	-7	-14	-9	1.0	0.9	0.9	0.9
Ranbp2	Q9ERU9	2596	-6	-7	-14	-10	0.9	0.9	0.9	0.9
Prex1	Q69ZK0	32	-14	-7	-14	-25	0.9	0.9	0.9	0.8
Itpkb	B2RXC2	485	-6	-7	-14	-28	0.9	0.9	0.9	0.8
Pik3r6	Q3U6Q4	716	-13	-8	-14	10	0.9	0.9	0.9	1.1
Hnrnpl	Q8R081	401	-18	-8	-14	9	0.8	0.9	0.9	1.1
Hnrnpul2	Q00PI9	536	-13	-8	-14	8	0.9	0.9	0.9	1.1
Pla2g6	P97819	332	-9	-8	-14	-2	0.9	0.9	0.9	1.0
Zfyve26	Q5DU37	705	-16	-8	-14	-8	0.9	0.9	0.9	0.9
Parp3	Q3ULW8	324	-5	-8	-14	-15	1.0	0.9	0.9	0.9
Rock2	F8VPK5	428	-11	-8	-14	-26	0.9	0.9	0.9	0.8
Lsm14a	Q8K2F8	374	-6	-8	-14	-27	0.9	0.9	0.9	0.8
Cybb	Q61093	537	-8	-8	-14	-55	0.9	0.9	0.9	0.6
Fgr	P14234	272	-16	-8	-14	29	0.9	0.9	0.9	1.4
Hmha1	Q3TBD2	736	-14	-8	-14	8	0.9	0.9	0.9	1.1
Sympk	Q80X82	969	-22	-8	-14	7	0.8	0.9	0.9	1.1
Kctd12	A0A0R4J2B2	241	-2	-8	-14	1	1.0	0.9	0.9	1.0
Pdia4	P08003	548	-8	-8	-14	1	0.9	0.9	0.9	1.0
Vps39	Q8R5L3	235	-12	-8	-14	-3	0.9	0.9	0.9	1.0
Gsdmdc1	Q9D8T2	39	-7	-8	-14	-3	0.9	0.9	0.9	1.0
Ntpcr	Q9CQA9	110	-7	-8	-14	-4	0.9	0.9	0.9	1.0
Atg3	Q9CPX6	182	-7	-8	-14	-9	0.9	0.9	0.9	0.9
Irak2	Q8CFA1	174	-15	-8	-14	-14	0.9	0.9	0.9	0.9
Polk	Q9QUG2	578	0	-8	-14	-20	1.0	0.9	0.9	0.8
Pgk1	P09411	50	-10	-9	-14	10	0.9	0.9	0.9	1.1
Kat6a	G3X940	507	-6	-9	-14	1	0.9	0.9	0.9	1.0
Acin1	Q9JIX8	513	-10	-9	-14	-13	0.9	0.9	0.9	0.9
Atrx	Q61687	597	-5	-9	-14	-23	1.0	0.9	0.9	0.8
Dgka	O88673	618	-12	-9	-14	-26	0.9	0.9	0.9	0.8
Suv39h2	Q9EQQ0	74	-9	-9	-14	-7	0.9	0.9	0.9	0.9
Parvg	Q9ERD8	186	-7	-9	-14	-7	0.9	0.9	0.9	0.9

Rnf2	Q9CQJ4	72	-14	-9	-14	-9	0.9	0.9	0.9	0.9
Jak1	B1ASP2	715	-17	-9	-14	-19	0.9	0.9	0.9	0.8
Trmt44	Q9D2Q2	522	-12	-9	-14	-21	0.9	0.9	0.9	0.8
Ddx17	Q501J6	198	-18	-10	-14	17	0.8	0.9	0.9	1.2
Ddx5	Q8BTS0	200	-18	-10	-14	17	0.8	0.9	0.9	1.2
Gle1	Q8R322	39	-4	-10	-14	10	1.0	0.9	0.9	1.1
Poc1b	Q8BHD1	387	-10	-10	-14	2	0.9	0.9	0.9	1.0
Rnpep	Q8VCT3	254	-13	-10	-14	-8	0.9	0.9	0.9	0.9
Eif4g3	Q80XI3	1405	-15	-10	-14	-13	0.9	0.9	0.9	0.9
Dlat	Q8BMF4	483	-9	-10	-14	-18	0.9	0.9	0.9	0.8
Kl	O35082	972	-21	-10	-14	-22	0.8	0.9	0.9	0.8
Kl	O35082	965	-21	-10	-14	-22	0.8	0.9	0.9	0.8
Ube2k	P61087	170	-32	-10	-14	11	0.8	0.9	0.9	1.1
Edc3	Q8K2D3	499	-20	-10	-14	9	0.8	0.9	0.9	1.1
Nsun2	Q1HFZ0	93	-11	-10	-14	8	0.9	0.9	0.9	1.1
Rel	A4QPD3	307	-14	-10	-14	5	0.9	0.9	0.9	1.1
Mybbp1a	Q7TPV4	621	-12	-10	-14	1	0.9	0.9	0.9	1.0
Usp9x	Q4FE56	540	-11	-10	-14	-10	0.9	0.9	0.9	0.9
Clcn3	P51791	733	-12	-10	-14	-13	0.9	0.9	0.9	0.9
Sqrdl	Q9R112	337	-17	-10	-14	-33	0.9	0.9	0.9	0.8
Arid1b	E9Q4N7	2082	-14	-11	-14	4	0.9	0.9	0.9	1.0
Mepce	Q8K3A9	499	-14	-11	-14	3	0.9	0.9	0.9	1.0
Triobp	Q99KW3	1549	9	-11	-14	-8	1.1	0.9	0.9	0.9
Tarbp1	E9Q368	443	-14	-11	-14	7	0.9	0.9	0.9	1.1
Kdm6b	Q5NCY0	1600	-11	-11	-14	-2	0.9	0.9	0.9	1.0
Camk2d	Q6PHZ2	290	-15	-11	-14	-6	0.9	0.9	0.9	0.9
Twistnb	Q78WZ7	28	-6	-11	-14	-8	0.9	0.9	0.9	0.9
Inpp5b	Q8K337	990	-9	-11	-14	-8	0.9	0.9	0.9	0.9
Tuba4a	P68368	54	-12	-11	-14	-18	0.9	0.9	0.9	0.8
Polr1b	P70700	1061	-12	-12	-14	20	0.9	0.9	0.9	1.2
Cse1l	Q9ERK4	344	-21	-12	-14	13	0.8	0.9	0.9	1.1
Psat1	Q99K85	224	-14	-12	-14	4	0.9	0.9	0.9	1.0
Nedd1	P33215	66	-4	-12	-14	-4	1.0	0.9	0.9	1.0
Pdcd4	Q61823	275	-11	-12	-14	-12	0.9	0.9	0.9	0.9
Rap1gds1	E9Q912	117	-12	-12	-14	-18	0.9	0.9	0.9	0.9
Scyl3	Q9DBQ7	36	-13	-12	-14	-20	0.9	0.9	0.9	0.8
Wipf1	Q8K1I7	436	-7	-12	-14	-22	0.9	0.9	0.9	0.8
Ctdspl2	Q8BG15	131	-15	-12	-14	-34	0.9	0.9	0.9	0.7
Papola	Q61183	293	-17	-12	-14	-2	0.9	0.9	0.9	1.0
Mepce	Q8K3A9	406	-11	-12	-14	-10	0.9	0.9	0.9	0.9
Frg1	P97376	205	-11	-12	-14	-12	0.9	0.9	0.9	0.9
Myh9	Q8VDD5	1942	-7	-12	-14	-26	0.9	0.9	0.9	0.8
Fam101b	Q5SVD0	88	-17	-13	-14	6	0.9	0.9	0.9	1.1
Ccm2	Q8K2Y9	170	-12	-13	-14	-11	0.9	0.9	0.9	0.9
Lnx2	Q91XL2	458	-13	-13	-14	-13	0.9	0.9	0.9	0.9
Map7d1	A2AJI0	375	-6	-13	-14	-23	0.9	0.9	0.9	0.8
Ctbp1	O88712	134	-36	-13	-14	27	0.7	0.9	0.9	1.4
Kdm5a	Q3UXZ9	692	-24	-13	-14	4	0.8	0.9	0.9	1.0
Med6	Q921D4	45	-14	-13	-14	-1	0.9	0.9	0.9	1.0
Ago2	Q8CJG0	463	-4	-13	-14	-3	1.0	0.9	0.9	1.0
Capg	Q99LB4	291	-8	-13	-14	-11	0.9	0.9	0.9	0.9

Tradd	Q3U0V2	160	-16	-13	-14	-16	0.9	0.9	0.9	0.9
Haus8	Q99L00	129	-14	-13	-14	-16	0.9	0.9	0.9	0.9
Lrrfip1	Q3UZ39	571	-3	-13	-14	-22	1.0	0.9	0.9	0.8
Tuba4a	P68368	316	-28	-13	-14	-27	0.8	0.9	0.9	0.8
Tuba1a	P68369	316	-28	-13	-14	-27	0.8	0.9	0.9	0.8
Psma6	Q9QUM9	161	-15	-14	-14	2	0.9	0.9	0.9	1.0
Tusc3	Q8BTV1	123	-13	-14	-14	-7	0.9	0.9	0.9	0.9
Rps8	P62242	71	-5	-14	-14	-9	1.0	0.9	0.9	0.9
Wdr41	Q3UDP0	131	-13	-14	-14	-10	0.9	0.9	0.9	0.9
Ints9	Q8K114	578	-10	-14	-14	-16	0.9	0.9	0.9	0.9
Clp1	Q99LI9	311	-16	-14	-14	-20	0.9	0.9	0.9	0.8
Gapvd1	Q6PAR5	293	-14	-14	-14	-4	0.9	0.9	0.9	1.0
Rin3	P59729	928	-13	-14	-14	-5	0.9	0.9	0.9	1.0
Uncharact	Q9D4A5	51	-11	-14	-14	-10	0.9	0.9	0.9	0.9
Cd27	P41272	222	-22	-14	-14	-24	0.8	0.9	0.9	0.8
Atp6v1a	P50516	254	-15	-15	-14	12	0.9	0.9	0.9	1.1
Ivns1abp	Q920Q8	143	-17	-15	-14	9	0.9	0.9	0.9	1.1
Idh3g	P70404	148	-13	-15	-14	-5	0.9	0.9	0.9	1.0
Ddx24	Q9ESV0	599	-7	-15	-14	-6	0.9	0.9	0.9	0.9
Kmt2d	Q6PDK2	414	-15	-15	-14	-32	0.9	0.9	0.9	0.8
Etfa	Q99LC5	68	-14	-15	-14	-34	0.9	0.9	0.9	0.7
Ass1	P16460	132	-4	-15	-14	-49	1.0	0.9	0.9	0.7
Adpgk	Q8VDL4	178	-19	-15	-14	-8	0.8	0.9	0.9	0.9
Trim39	G3UWI0	170	-2	-15	-14	-8	1.0	0.9	0.9	0.9
Cltc	Q68FD5	491	-15	-15	-14	-8	0.9	0.9	0.9	0.9
Gcat	O88986	26	-11	-15	-14	-11	0.9	0.9	0.9	0.9
Gcn1	E9PVA8	246	-16	-15	-14	-15	0.9	0.9	0.9	0.9
Phkb	Q7TSH2	68	-11	-15	-14	-19	0.9	0.9	0.9	0.8
Gtpbp6	Q3U6U5	420	-13	-15	-14	-25	0.9	0.9	0.9	0.8
Pias2	Q8C5D8	231	-5	-16	-14	-11	1.0	0.9	0.9	0.9
Sqstm1	Q64337	145	-23	-16	-14	-15	0.8	0.9	0.9	0.9
Thoc2	B1AZI6	1518	2	-16	-14	-27	1.0	0.9	0.9	0.8
Apobec3	Q99J72	144	-15	-16	-14	3	0.9	0.9	0.9	1.0
Ywhag	P61982	112	-13	-16	-14	3	0.9	0.9	0.9	1.0
Nudcd1	Q6PIP5	31	-14	-16	-14	3	0.9	0.9	0.9	1.0
Cdyl2	Q9D5D8	420	-16	-16	-14	-3	0.9	0.9	0.9	1.0
Abi1	Q8CBW3	33	-12	-16	-14	-7	0.9	0.9	0.9	0.9
Ttc27	Q8CD92	314	-19	-17	-14	9	0.8	0.9	0.9	1.1
Cmtr1	Q9DBC3	793	-16	-17	-14	8	0.9	0.9	0.9	1.1
Aco1	P28271	369	-22	-17	-14	-12	0.8	0.9	0.9	0.9
Nrdc	Q8BHG1	61	-10	-17	-14	-12	0.9	0.9	0.9	0.9
Ankrd27	Q3UMR0	39	-17	-17	-14	-14	0.9	0.9	0.9	0.9
Flnb	Q80X90	26	-10	-17	-14	-17	0.9	0.9	0.9	0.9
Flna	Q8BTM8	53	-10	-17	-14	-17	0.9	0.9	0.9	0.9
Flnc	Q8VHX6	47	-10	-17	-14	-17	0.9	0.9	0.9	0.9
S100a10	P08207	62	-21	-17	-14	-27	0.8	0.9	0.9	0.8
Uncharact	Q3UUX7	86	-37	-17	-14	17	0.7	0.9	0.9	1.2
Pes1	Q9EQ61	400	-22	-17	-14	3	0.8	0.9	0.9	1.0
Gart	Q64737	168	-11	-17	-14	1	0.9	0.9	0.9	1.0
Anapc1	P53995	988	-29	-17	-14	-10	0.8	0.9	0.9	0.9
Smc1a	Q9CU62	1073	-14	-17	-14	-18	0.9	0.9	0.9	0.8

Numa1	E9Q7G0	1363	-17	-18	-14	-10	0.9	0.9	0.9	0.9
Triobp	Q99KW3	1921	-5	-18	-14	-15	1.0	0.9	0.9	0.9
Hsph1	Q61699	659	-11	-18	-14	-16	0.9	0.9	0.9	0.9
Rpp38	A2AJG0	154	-34	-18	-14	-24	0.7	0.9	0.9	0.8
Wdr33	Q8K4P0	120	-29	-18	-14	12	0.8	0.8	0.9	1.1
Ssna1	Q9JJ94	18	-14	-19	-14	20	0.9	0.8	0.9	1.2
Thoc6	Q5U4D9	314	-29	-19	-14	15	0.8	0.8	0.9	1.2
Ifrd1	P19182	176	-23	-19	-14	-14	0.8	0.8	0.9	0.9
Nemp1	Q6ZQE4	364	-21	-19	-14	-25	0.8	0.8	0.9	0.8
Rps3a	P97351	139	-21	-19	-14	-6	0.8	0.8	0.9	0.9
Uqcrc1	Q9CZ13	453	-19	-19	-14	-18	0.8	0.8	0.9	0.8
Uba6	Q8C7R4	23	-21	-19	-14	-28	0.8	0.8	0.9	0.8
Gdi1	P50396	282	-16	-20	-14	-3	0.9	0.8	0.9	1.0
Dnttip2	Q8R2M2	620	5	-20	-14	-16	1.1	0.8	0.9	0.9
Vrk1	Q80X41	205	-31	-21	-14	5	0.8	0.8	0.9	1.0
Rangap1	P46061	575	-15	-21	-14	4	0.9	0.8	0.9	1.0
Tpm4	Q6IRU2	154	-12	-21	-14	-4	0.9	0.8	0.9	1.0
Xrcc1	Q60596	613	-22	-21	-14	-15	0.8	0.8	0.9	0.9
Aspscr1	Q8VBT9	175	-2	-21	-14	-27	1.0	0.8	0.9	0.8
Dhx37	Q6NZL1	759	-30	-21	-14	-5	0.8	0.8	0.9	1.0
Ddx18	Q8K363	138	-7	-21	-14	-33	0.9	0.8	0.9	0.8
Chd4	Q6PDQ2	488	-18	-22	-14	4	0.8	0.8	0.9	1.0
Helz2	E9QAM5	25	-18	-22	-14	-20	0.9	0.8	0.9	0.8
Usp48	Q3V0C5	290	-28	-23	-14	-57	0.8	0.8	0.9	0.6
Lpxn	Q99N69	211	-20	-23	-14	-7	0.8	0.8	0.9	0.9
Mcts1	Q9DB27	14	3	-24	-14	-8	1.0	0.8	0.9	0.9
Klc3	Q91W40	314	-24	-24	-14	-18	0.8	0.8	0.9	0.9
Fgd2	Q8BY35	467	-16	-24	-14	-26	0.9	0.8	0.9	0.8
Tex11	Q14AT2	303	-12	-25	-14	-7	0.9	0.8	0.9	0.9
Dido1	Q8C9B9	212	-16	-25	-14	-24	0.9	0.8	0.9	0.8
Cyp4f3	Q99N16	50	-24	-25	-14	-39	0.8	0.8	0.9	0.7
Sh2d3c	Q9QZS8	372	-16	-26	-14	-16	0.9	0.8	0.9	0.9
Gpx1	P11352	154	-23	-26	-14	14	0.8	0.8	0.9	1.2
Bcs1l	Q9CZP5	234	-26	-27	-14	-3	0.8	0.8	0.9	1.0
Cpsf3	Q9QXK7	498	-3	-27	-14	-4	1.0	0.8	0.9	1.0
Adsl	P54822	304	-9	-29	-14	20	0.9	0.8	0.9	1.2
Mtpap	Q9D0D3	82	-34	-30	-14	-18	0.7	0.8	0.9	0.8
Gvin1	L7N451	2389	-58	-30	-14	-1	0.6	0.8	0.9	1.0
Wdfy3	Q6VNB8	1600	-8	-31	-14	-7	0.9	0.8	0.9	0.9
Polr1e	Q8K202	96	-20	-33	-14	10	0.8	0.8	0.9	1.1
Dph7	Q9CYU6	417	-20	-35	-14	-45	0.8	0.7	0.9	0.7
Idh3a	Q9D6R2	351	-11	-35	-14	-20	0.9	0.7	0.9	0.8
Hmox2	O70252	126	-21	-40	-14	17	0.8	0.7	0.9	1.2
Ecm29	Q6PDI5	1707	-10	-41	-14	8	0.9	0.7	0.9	1.1
Mpi	Q924M7	29	-32	-43	-14	-10	0.8	0.7	0.9	0.9
Pa2g4	P50580	49	7	23	-14	17	1.1	1.3	0.9	1.2
Nt5c3a	Q9D020	98	12	19	-14	31	1.1	1.2	0.9	1.4
Mbd1	Q9Z2E2	259	-11	11	-14	-3	0.9	1.1	0.9	1.0
Dnm2	P39054	27	-20	10	-14	-2	0.8	1.1	0.9	1.0
Slx4	Q6P1D7	475	-14	6	-14	-11	0.9	1.1	0.9	0.9
Ikzf1	Q5SWT9	273	11	5	-14	-10	1.1	1.1	0.9	0.9

Polr2d	Q9D7M8	104	5	4	-14	14	1.1	1.0	0.9	1.2
Sfr1	Q8BP27	145	-35	4	-14	-22	0.7	1.0	0.9	0.8
Vdac2	Q60930	14	-1	3	-14	-40	1.0	1.0	0.9	0.7
Mob4	Q6PEB6	51	0	2	-14	11	1.0	1.0	0.9	1.1
Vprbp	Q80TR8	783	-8	2	-14	-14	0.9	1.0	0.9	0.9
Metap1	Q8BP48	65	-6	1	-14	3	0.9	1.0	0.9	1.0
Eef2k	O08796	481	3	1	-14	-20	1.0	1.0	0.9	0.8
Znf865	Q3U3I9	225	-6	1	-14	10	0.9	1.0	0.9	1.1
Toe1	Q9D2E2	80	-2	1	-14	6	1.0	1.0	0.9	1.1
Fam129a	Q3UW53	41	-16	-1	-14	9	0.9	1.0	0.9	1.1
Rock2	F8VPK5	607	-2	-1	-14	-14	1.0	1.0	0.9	0.9
Thada	A8C756	1910	11	-1	-14	-146	1.1	1.0	0.9	0.4
Glipr2	Q9CYL5	63	-12	-2	-14	-3	0.9	1.0	0.9	1.0
Gtf2e2	Q9D902	209	-7	-2	-14	11	0.9	1.0	0.9	1.1
Ppa2	Q91VM9	157	8	-2	-14	5	1.1	1.0	0.9	1.0
Trpm7	Q923J1	29	-5	-2	-14	-12	1.0	1.0	0.9	0.9
Bag1	Q60739	340	-1	-3	-14	-7	1.0	1.0	0.9	0.9
Rere	Q80TZ9	41	-14	-3	-14	-19	0.9	1.0	0.9	0.8
Pkm	P52480	474	-14	-3	-14	18	0.9	1.0	0.9	1.2
Leng8	Q8CBY3	696	-7	-3	-14	8	0.9	1.0	0.9	1.1
Spr	Q91XH5	262	2	-3	-14	-4	1.0	1.0	0.9	1.0
Ep300	B2RWS6	1175	-10	-4	-14	-1	0.9	1.0	0.9	1.0
Cyth4	Q80YW0	66	-10	-4	-14	-11	0.9	1.0	0.9	0.9
Cwf19l2	Q8BG79	412	-9	-4	-14	-25	0.9	1.0	0.9	0.8
Idh1	O88844	269	-9	-4	-14	-86	0.9	1.0	0.9	0.5
Rangrf	Q9JIB0	89	-10	-4	-14	18	0.9	1.0	0.9	1.2
Anxa5	P48036	314	-6	-4	-14	13	0.9	1.0	0.9	1.1
Myo1e	E9Q634	790	7	-5	-14	-4	1.1	1.0	0.9	1.0
Cdk18	Q04899	162	-11	-5	-14	8	0.9	1.0	0.9	1.1
Uap1l1	Q3TW96	254	-14	-5	-14	-30	0.9	1.0	0.9	0.8
Pold1	P52431	358	-20	-6	-14	-4	0.8	0.9	0.9	1.0
N4bp1	Q6A037	82	-9	-6	-14	-6	0.9	0.9	0.9	0.9
Dhx8	A2A4P0	661	-9	-6	-14	-14	0.9	0.9	0.9	0.9
Prg3	Q9JL95	108	-3	-6	-14	-20	1.0	0.9	0.9	0.8
Fam76a	Q922G2	104	-38	-6	-14	11	0.7	0.9	0.9	1.1
Prkdc	P97313	3229	-7	-6	-14	8	0.9	0.9	0.9	1.1
Nbeal1	E9PYP2	810	-10	-6	-14	2	0.9	0.9	0.9	1.0
Hus1	Q8BQY8	44	-14	-6	-14	-5	0.9	0.9	0.9	1.0
Mta2	Q9R190	209	-5	-6	-14	-5	1.0	0.9	0.9	1.0
Lcp1	Q61233	101	-16	-6	-14	-37	0.9	0.9	0.9	0.7
Nprl3	Q8VIJ8	141	-11	-7	-14	7	0.9	0.9	0.9	1.1
Gdi2	Q61598	202	-7	-7	-14	4	0.9	0.9	0.9	1.0
Ndufa10	Q99LC3	67	-19	-7	-14	4	0.8	0.9	0.9	1.0
Zfp512b	Q6PHP4	346	-7	-7	-14	-8	0.9	0.9	0.9	0.9
Pop1	Q8K205	802	-7	-7	-14	-16	0.9	0.9	0.9	0.9
Rdh14	Q9ERI6	93	-14	-7	-14	-18	0.9	0.9	0.9	0.9
Irf8	P23611	227	-15	-7	-14	-31	0.9	0.9	0.9	0.8
Macf1	E9PVY8	3743	-18	-7	-14	4	0.9	0.9	0.9	1.0
Adnp	Q9Z103	219	-10	-7	-14	-10	0.9	0.9	0.9	0.9
Samd9l	E9PX59	629	-9	-7	-14	-11	0.9	0.9	0.9	0.9
Edc3	Q8K2D3	413	4	-7	-14	-14	1.0	0.9	0.9	0.9

Pias4	Q9JM05	50	1	-7	-14	-14	1.0	0.9	0.9	0.9
Gsk3b	Q9WV60	14	-6	-8	-14	10	0.9	0.9	0.9	1.1
Glud1	P26443	112	-19	-8	-14	5	0.8	0.9	0.9	1.0
Zmym2	Q9CU65	493	-7	-8	-14	-5	0.9	0.9	0.9	1.0
Kdm2b	Q6P1G2	743	-21	-8	-14	-25	0.8	0.9	0.9	0.8
Paf1	Q8K2T8	36	-7	-8	-14	3	0.9	0.9	0.9	1.0
Fam98b	Q80VD1	147	-5	-8	-14	-5	1.0	0.9	0.9	1.0
Gnb1	P62874	25	-21	-8	-14	-6	0.8	0.9	0.9	0.9
Znf592	Q8BHZ4	282	-16	-8	-14	-6	0.9	0.9	0.9	0.9
Slirp	Q9D8T7	48	-11	-9	-14	1	0.9	0.9	0.9	1.0
Kdm5b	Q80Y84	725	-6	-9	-14	-1	0.9	0.9	0.9	1.0
Sp110	Q8BVK9	55	-11	-9	-14	-1	0.9	0.9	0.9	1.0
Myo1f	Q8CG29	788	-19	-9	-14	-2	0.8	0.9	0.9	1.0
Atm	Q62388	383	-7	-9	-14	-4	0.9	0.9	0.9	1.0
Xab2	Q9DCD2	86	-11	-9	-14	-16	0.9	0.9	0.9	0.9
Ankle2	Q6P1H6	261	-11	-9	-14	-24	0.9	0.9	0.9	0.8
Cog1	Q9Z160	72	-14	-9	-14	7	0.9	0.9	0.9	1.1
Ptbp1	Q922I7	250	-29	-9	-14	3	0.8	0.9	0.9	1.0
Cul1	Q9WTX6	594	-16	-9	-14	-1	0.9	0.9	0.9	1.0
Plec	Q9QXS1	1386	-16	-9	-14	-9	0.9	0.9	0.9	0.9
Rnh1	Q91VI7	243	-23	-10	-14	2	0.8	0.9	0.9	1.0
Hcfc1	Q61191	352	-10	-10	-14	-6	0.9	0.9	0.9	0.9
Myh9	Q8VDD5	917	-17	-10	-14	-8	0.9	0.9	0.9	0.9
Diaph1	O08808	1210	-13	-10	-14	-9	0.9	0.9	0.9	0.9
Neil1	Q8K4Q6	204	-13	-10	-14	-12	0.9	0.9	0.9	0.9
Arhgef2	Q60875	479	-10	-10	-14	-20	0.9	0.9	0.9	0.8
Nipbl	Q6KCD5	1789	-15	-10	-14	10	0.9	0.9	0.9	1.1
Dok3	Q9QZK7	88	-15	-10	-14	8	0.9	0.9	0.9	1.1
Crkl	P47941	44	-8	-10	-14	-7	0.9	0.9	0.9	0.9
Trmu	Q9DAT5	101	2	-10	-14	-24	1.0	0.9	0.9	0.8
Ampd2	Q9DBT5	26	-6	-10	-14	-27	0.9	0.9	0.9	0.8
Pde12	Q3TIU4	320	2	-11	-14	11	1.0	0.9	0.9	1.1
Ccdc85b	Q6PDY0	82	-19	-11	-14	2	0.8	0.9	0.9	1.0
Spag9	Q58A65	443	-10	-11	-14	-4	0.9	0.9	0.9	1.0
Ahdc1	Q6PAL7	934	-11	-11	-14	-9	0.9	0.9	0.9	0.9
Chrac1	Q9JKP8	59	-11	-11	-14	-10	0.9	0.9	0.9	0.9
Atm	Q62388	563	0	-11	-14	-18	1.0	0.9	0.9	0.8
Mtch2	Q791V5	291	-9	-11	-14	-19	0.9	0.9	0.9	0.8
Lrrfip1	Q3UZ39	278	-1	-11	-14	-20	1.0	0.9	0.9	0.8
Herc4	Q6PAV2	1025	-16	-11	-14	-38	0.9	0.9	0.9	0.7
Recql5	Q8VID5	533	-14	-11	-14	11	0.9	0.9	0.9	1.1
Dis3l	Q8C0S1	482	-15	-11	-14	-2	0.9	0.9	0.9	1.0
Lrrc40	Q9CRC8	329	-20	-11	-14	-5	0.8	0.9	0.9	1.0
Cbl	P22682	570	9	-11	-14	-6	1.1	0.9	0.9	0.9
Hpf1	Q8CFE2	17	-16	-11	-14	-8	0.9	0.9	0.9	0.9
Rpl23	P62830	28	-12	-11	-14	-11	0.9	0.9	0.9	0.9
Phip	F8VQ93	1612	-12	-11	-14	-14	0.9	0.9	0.9	0.9
Son	Q9QX47	1610	-13	-11	-14	-20	0.9	0.9	0.9	0.8
Metap2	O08663	290	-13	-12	-14	0	0.9	0.9	0.9	1.0
Smyd5	Q3TYX3	193	-16	-12	-14	-7	0.9	0.9	0.9	0.9
Lig1	Q3U4X8	219	-14	-12	-14	-8	0.9	0.9	0.9	0.9

Akap8	Q9DBR0	173	-17	-12	-14	-12	0.9	0.9	0.9	0.9
Akap13	E9Q394	609	-11	-12	-14	-19	0.9	0.9	0.9	0.8
Gpatch8	A2A6A1	506	-8	-12	-14	-21	0.9	0.9	0.9	0.8
Fam49b	Q921M7	10	-6	-12	-14	-25	0.9	0.9	0.9	0.8
Oas3	Q8VI93	496	-16	-12	-14	8	0.9	0.9	0.9	1.1
Oxsm	Q9D404	86	-8	-12	-14	8	0.9	0.9	0.9	1.1
Ube2m	P61082	65	-9	-12	-14	-5	0.9	0.9	0.9	1.0
Pgam1	Q9DBJ1	153	-8	-12	-14	-8	0.9	0.9	0.9	0.9
Esyt2	Q3TZZ7	535	-10	-12	-14	-10	0.9	0.9	0.9	0.9
Ppp1r18	Q8BQ30	459	-12	-12	-14	-12	0.9	0.9	0.9	0.9
Erbin	Q80TH2	464	-14	-12	-14	-21	0.9	0.9	0.9	0.8
Slain2	Q8CI08	165	-8	-12	-14	-25	0.9	0.9	0.9	0.8
Hgs	Q99LI8	215	-16	-13	-14	-9	0.9	0.9	0.9	0.9
Cacybp	Q9CXW3	174	-19	-13	-14	-10	0.8	0.9	0.9	0.9
Ranbp2	Q9ERU9	2496	-23	-13	-14	-13	0.8	0.9	0.9	0.9
Mrpl46	Q9EQI8	282	-8	-13	-14	-20	0.9	0.9	0.9	0.8
Bloc1s3	Q5U5M8	173	-5	-13	-14	-31	1.0	0.9	0.9	0.8
Lmnb2	P21619	190	-16	-13	-14	-1	0.9	0.9	0.9	1.0
Rabep2	Q91WG2	66	-10	-13	-14	-9	0.9	0.9	0.9	0.9
Sugt1	Q9CX34	79	-24	-14	-14	-9	0.8	0.9	0.9	0.9
Setd1b	Q8CFT2	998	9	-14	-14	-36	1.1	0.9	0.9	0.7
Abcd3	P55096	472	-7	-14	-14	16	0.9	0.9	0.9	1.2
Zfp715	G3X9T1	565	-14	-14	-14	-2	0.9	0.9	0.9	1.0
Ash2l	Q91X20	357	-10	-14	-14	-2	0.9	0.9	0.9	1.0
Hnrnpm	Q9D0E1	652	-17	-14	-14	-4	0.9	0.9	0.9	1.0
Arid1a	A2BH40	1872	-10	-14	-14	-5	0.9	0.9	0.9	1.0
Tuba4a	P68368	315	-13	-14	-14	-7	0.9	0.9	0.9	0.9
Tuba1a	P68369	315	-13	-14	-14	-7	0.9	0.9	0.9	0.9
Plek	Q9JHK5	102	-16	-14	-14	-9	0.9	0.9	0.9	0.9
Pdcd11	Q6NS46	535	-10	-14	-14	-18	0.9	0.9	0.9	0.9
Pi4ka	E9Q3L2	1827	-6	-14	-14	-24	0.9	0.9	0.9	0.8
Phax	Q9JJT9	42	-13	-15	-14	-5	0.9	0.9	0.9	1.0
Cwc22	Q8C5N3	871	0	-15	-14	-14	1.0	0.9	0.9	0.9
Sipa1	E9Q0Y4	807	-15	-15	-14	-17	0.9	0.9	0.9	0.9
Pnkp	G5E8N7	408	-22	-15	-14	-19	0.8	0.9	0.9	0.8
Fmnl2	A2APV2	65	-15	-15	-14	-21	0.9	0.9	0.9	0.8
Fmnl1	Q9JL26	69	-15	-15	-14	-21	0.9	0.9	0.9	0.8
Pfdn5	Q9WU28	49	-26	-15	-14	-22	0.8	0.9	0.9	0.8
Tbl3	Q8C4J7	696	-12	-15	-14	15	0.9	0.9	0.9	1.2
Gdi1	P50396	202	-15	-15	-14	0	0.9	0.9	0.9	1.0
Syne1	Q6ZWR6	6418	-19	-15	-14	-6	0.8	0.9	0.9	0.9
Nrdc	Q8BHG1	59	-6	-15	-14	-7	0.9	0.9	0.9	0.9
Usp8	Q80U87	567	-11	-15	-14	-11	0.9	0.9	0.9	0.9
Med16	Q6PGF3	18	-2	-15	-14	-15	1.0	0.9	0.9	0.9
Pold1	P52431	317	-10	-16	-14	12	0.9	0.9	0.9	1.1
Blnk	Q9QUN3	272	-11	-16	-14	-5	0.9	0.9	0.9	1.0
Cat	P24270	425	-15	-16	-14	-12	0.9	0.9	0.9	0.9
Lrch4	Q921G6	283	-14	-16	-14	-21	0.9	0.9	0.9	0.8
Tbck	Q8BM85	386	-7	-16	-14	-24	0.9	0.9	0.9	0.8
Capns1	O88456	145	-17	-16	-14	8	0.9	0.9	0.9	1.1
Clasp2	F7DCH5	989	-7	-16	-14	-18	0.9	0.9	0.9	0.8

Rtfdc1	Q99K95	208	-13	-16	-14	-21	0.9	0.9	0.9	0.8
Ogdh	Q60597	395	-14	-17	-14	-15	0.9	0.9	0.9	0.9
Nmnat1	Q9EPA7	111	-14	-17	-14	-26	0.9	0.9	0.9	0.8
Klc4	Q9DBS5	318	-23	-17	-14	-27	0.8	0.9	0.9	0.8
Ctsc	P97821	447	-12	-17	-14	10	0.9	0.9	0.9	1.1
Ncf1	Q09014	378	-13	-17	-14	-3	0.9	0.9	0.9	1.0
Syne2	Q6ZWQ0	3322	-16	-17	-14	-11	0.9	0.9	0.9	0.9
Pla2g16	Q8R3U1	66	-12	-17	-14	-11	0.9	0.9	0.9	0.9
Anxa11	P97384	382	-27	-18	-14	22	0.8	0.9	0.9	1.3
Kif5b	Q61768	421	-8	-18	-14	-8	0.9	0.9	0.9	0.9
Dnmt1	P13864	1487	-11	-18	-14	-27	0.9	0.9	0.9	0.8
Hmha1	Q3TBD2	277	-12	-18	-14	-11	0.9	0.8	0.9	0.9
Dmxl1	Q6PNC0	2604	-13	-18	-14	-15	0.9	0.8	0.9	0.9
Mki67	E9PVX6	1053	-14	-18	-14	-18	0.9	0.8	0.9	0.8
Clasp2	F7DCH5	227	-7	-19	-14	5	0.9	0.8	0.9	1.1
Xpo1	Q6P5F9	723	-10	-19	-14	-9	0.9	0.8	0.9	0.9
Gorasp2	Q99JX3	434	-8	-19	-14	-28	0.9	0.8	0.9	0.8
Zfr	O88532	525	-13	-19	-14	-29	0.9	0.8	0.9	0.8
Trim34a	Q99PP6	18	-21	-19	-14	-4	0.8	0.8	0.9	1.0
Rpl27a	P14115	70	-12	-19	-14	-10	0.9	0.8	0.9	0.9
Rasal3	Q8C2K5	957	-14	-19	-14	-13	0.9	0.8	0.9	0.9
Uvssa	Q9D479	349	-22	-19	-14	-29	0.8	0.8	0.9	0.8
Pag1	Q3U1F9	423	-1	-19	-14	-41	1.0	0.8	0.9	0.7
Uqcrc1	Q9CZ13	410	-13	-20	-14	3	0.9	0.8	0.9	1.0
Smchd1	Q6P5D8	458	-6	-20	-14	-16	0.9	0.8	0.9	0.9
Flna	Q8BTM8	623	-19	-20	-14	-16	0.8	0.8	0.9	0.9
Flnc	Q8VHX6	619	-19	-20	-14	-16	0.8	0.8	0.9	0.9
Nup205	A0A0J9YUD5	1715	-1	-20	-14	-2	1.0	0.8	0.9	1.0
Terf2ip	Q91VL8	235	-4	-20	-14	-16	1.0	0.8	0.9	0.9
Dennd5b	A2RSQ0	488	-33	-20	-14	-24	0.8	0.8	0.9	0.8
Rif1	Q6PR54	1605	-13	-20	-14	-26	0.9	0.8	0.9	0.8
Kdm3a	Q6PCM1	391	-15	-21	-14	-18	0.9	0.8	0.9	0.8
Comt	O88587	200	-34	-21	-14	-9	0.7	0.8	0.9	0.9
Rpp40	Q8R1F9	49	-11	-21	-14	-12	0.9	0.8	0.9	0.9
Ppm1b	P36993	209	-15	-21	-14	-16	0.9	0.8	0.9	0.9
Hira	Q61666	743	-19	-22	-14	-4	0.8	0.8	0.9	1.0
Ring1	O35730	69	-17	-22	-14	-21	0.9	0.8	0.9	0.8
Apbb1ip	Q8R5A3	535	1	-22	-14	-29	1.0	0.8	0.9	0.8
Acbd5	Q5XG73	86	-22	-22	-14	-2	0.8	0.8	0.9	1.0
Zyx	Q62523	379	-11	-22	-14	-22	0.9	0.8	0.9	0.8
Aim2	Q91VJ1	308	-21	-23	-14	-9	0.8	0.8	0.9	0.9
Rngtt	O55236	565	-6	-23	-14	-11	0.9	0.8	0.9	0.9
Sart3	Q9JLI8	376	-20	-24	-14	8	0.8	0.8	0.9	1.1
Pds5a	E9QPI5	741	-14	-24	-14	1	0.9	0.8	0.9	1.0
Iars2	Q8BIJ6	465	-20	-24	-14	-3	0.8	0.8	0.9	1.0
Cand2	Q6ZQ73	953	-14	-24	-14	-11	0.9	0.8	0.9	0.9
Hadha	Q8BMS1	747	-28	-24	-14	-1	0.8	0.8	0.9	1.0
Tdrd3	Q91W18	191	-13	-25	-14	-7	0.9	0.8	0.9	0.9
Dhx32	Q8BZS9	281	0	-25	-14	-11	1.0	0.8	0.9	0.9
Sf3a3	Q9D554	274	-5	-25	-14	-11	1.0	0.8	0.9	0.9
Iars	Q8BU30	433	11	-25	-14	-69	1.1	0.8	0.9	0.6

Il4i1	O09046	251	52	-26	-14	-1	2.1	0.8	0.9	1.0
Zfp352	A2AML7	280	-7	-26	-14	-17	0.9	0.8	0.9	0.9
Spata6	Q3U6K5	46	-31	-27	-14	-27	0.8	0.8	0.9	0.8
Kif2a	P28740	333	-8	-28	-14	11	0.9	0.8	0.9	1.1
Sptbn1	Q62261	831	-12	-29	-14	-10	0.9	0.8	0.9	0.9
Psmd4	O35226	87	15	-29	-14	-16	1.2	0.8	0.9	0.9
Pml	Q60953	156	-19	-29	-14	-6	0.8	0.8	0.9	0.9
Mad2l1bp	Q9DCX1	126	-23	-29	-14	-39	0.8	0.8	0.9	0.7
Sbf1	Q6ZPE2	895	-19	-30	-14	-17	0.8	0.8	0.9	0.9
Telo2	Q9DC40	449	-25	-34	-14	-42	0.8	0.7	0.9	0.7
Nol11	Q8BJW5	333	-20	-34	-14	-9	0.8	0.7	0.9	0.9
Elac2	Q80Y81	128	-23	-35	-14	-13	0.8	0.7	0.9	0.9
Rpl35a	O55142	47	-33	-53	-14	-27	0.8	0.7	0.9	0.8
Phip	F8VQ93	130	-7	18	-15	-4	0.9	1.2	0.9	1.0
Me2	Q99KE1	190	-8	12	-15	29	0.9	1.1	0.9	1.4
Tbrg1	Q3UB74	55	-2	8	-15	6	1.0	1.1	0.9	1.1
Ncapg	E9PWG6	841	-15	5	-15	-4	0.9	1.0	0.9	1.0
Tln1	P26039	2243	-16	3	-15	23	0.9	1.0	0.9	1.3
Sin3b	Q62141	385	-6	2	-15	8	0.9	1.0	0.9	1.1
Nol6	Q8R5K4	274	-7	1	-15	-7	0.9	1.0	0.9	0.9
Tubb5	P99024	239	-5	1	-15	-21	1.0	1.0	0.9	0.8
Tubb2a	Q7TMM9	239	-5	1	-15	-21	1.0	1.0	0.9	0.8
Ntn1	O09118	70	-7	0	-15	3	0.9	1.0	0.9	1.0
Ntn1	O09118	78	-7	0	-15	3	0.9	1.0	0.9	1.0
Ephb4	P54761	754	-8	0	-15	-2	0.9	1.0	0.9	1.0
Ctps2	P70303	313	-25	-1	-15	1	0.8	1.0	0.9	1.0
Srbd1	F8WGW3	898	-17	-1	-15	-1	0.9	1.0	0.9	1.0
Rsl1d1	Q8BVY0	210	-16	-1	-15	-16	0.9	1.0	0.9	0.9
Ammecr1l	Q8JZZ6	153	-15	-2	-15	5	0.9	1.0	0.9	1.1
Rhoq	Q8R527	144	-2	-2	-15	-17	1.0	1.0	0.9	0.9
Bdh1	Q80XN0	221	-4	-2	-15	-16	1.0	1.0	0.9	0.9
Srrt	Q99MR6	411	-3	-3	-15	-12	1.0	1.0	0.9	0.9
Mettl13	Q91YR5	256	-24	-4	-15	59	0.8	1.0	0.9	2.4
Dock2	Q8C3J5	453	-9	-4	-15	23	0.9	1.0	0.9	1.3
Prkcd	P28867	117	-17	-4	-15	0	0.9	1.0	0.9	1.0
U2surp	Q6NV83	65	-16	-4	-15	-13	0.9	1.0	0.9	0.9
Smarca4	Q3TKT4	1205	-15	-5	-15	7	0.9	1.0	0.9	1.1
Smarca2	Q6DIC0	1180	-15	-5	-15	7	0.9	1.0	0.9	1.1
Ncaph	Q8C156	451	-4	-5	-15	-18	1.0	1.0	0.9	0.8
Ctcf	Q61164	155	1	-5	-15	-21	1.0	1.0	0.9	0.8
Pced1b	Q8BGX1	41	-14	-5	-15	-4	0.9	1.0	0.9	1.0
Exo1	Q9QZ11	645	-1	-6	-15	18	1.0	0.9	0.9	1.2
Zfp62	Q8C827	311	-9	-6	-15	-15	0.9	0.9	0.9	0.9
Ciapin1	Q8WTY4	235	-11	-6	-15	-16	0.9	0.9	0.9	0.9
Ipo4	Q8VI75	269	-4	-6	-15	-18	1.0	0.9	0.9	0.9
Farsb	Q9WUA2	195	-9	-6	-15	-8	0.9	0.9	0.9	0.9
Thoc5	Q8BKT7	208	-4	-7	-15	1	1.0	0.9	0.9	1.0
Prdm2	A2A7B5	1196	-15	-7	-15	-8	0.9	0.9	0.9	0.9
Ap2a2	P17427	491	-14	-7	-15	15	0.9	0.9	0.9	1.2
Ralbp1	Q62172	411	-21	-7	-15	-33	0.8	0.9	0.9	0.8
Mtm1	Q9Z2C5	53	-9	-8	-15	-9	0.9	0.9	0.9	0.9

Gem	P55041	214	-12	-8	-15	-14	0.9	0.9	0.9	0.9
Adk	P55264	159	-9	-8	-15	10	0.9	0.9	0.9	1.1
Nars	Q8BP47	522	-14	-8	-15	-12	0.9	0.9	0.9	0.9
Arpc2	Q9CVB6	120	-13	-9	-15	11	0.9	0.9	0.9	1.1
Ehmt1	Q5DW34	1032	-14	-9	-15	9	0.9	0.9	0.9	1.1
Nit2	Q9JHW2	146	-19	-9	-15	-4	0.8	0.9	0.9	1.0
Trim56	Q80VI1	347	-10	-9	-15	-7	0.9	0.9	0.9	0.9
Pccb	Q99MN9	519	-12	-9	-15	-8	0.9	0.9	0.9	0.9
Arhgef1	Q61210	814	-15	-9	-15	-13	0.9	0.9	0.9	0.9
Recql5	Q8VID5	894	-11	-9	-15	-31	0.9	0.9	0.9	0.8
Trim5	E9PV98	422	-14	-9	-15	22	0.9	0.9	0.9	1.3
Trim12c	D3Z3L3	422	-14	-9	-15	22	0.9	0.9	0.9	1.3
Ctu1	Q99J10	147	-12	-9	-15	15	0.9	0.9	0.9	1.2
Cnot1	Q6ZQ08	1705	-9	-9	-15	5	0.9	0.9	0.9	1.0
Dennd4c	A6H8H2	115	-16	-9	-15	-4	0.9	0.9	0.9	1.0
Chtf18	Q8BIW9	209	-18	-9	-15	-8	0.8	0.9	0.9	0.9
Usp48	Q3V0C5	867	-10	-9	-15	-10	0.9	0.9	0.9	0.9
Wdfy4	E9Q2M9	88	-18	-9	-15	-12	0.9	0.9	0.9	0.9
Map3k4	O08648	186	-11	-9	-15	-23	0.9	0.9	0.9	0.8
Bdh1	Q80XN0	115	-18	-9	-15	-32	0.9	0.9	0.9	0.8
Raf1	Q99N57	95	-14	-10	-15	10	0.9	0.9	0.9	1.1
Heatr1	G3X9B1	1010	-24	-10	-15	5	0.8	0.9	0.9	1.1
Nek7	Q9ES74	53	-14	-10	-15	-3	0.9	0.9	0.9	1.0
Ddb1	Q3U1J4	732	-31	-10	-15	-4	0.8	0.9	0.9	1.0
Mcm3ap	Q9WUU9	939	-14	-10	-15	-6	0.9	0.9	0.9	0.9
Arap1	Q4LDD4	1312	-16	-10	-15	-7	0.9	0.9	0.9	0.9
Arih2	Q9Z1K6	160	-20	-10	-15	-13	0.8	0.9	0.9	0.9
Nckap1l	Q8K1X4	612	-7	-10	-15	21	0.9	0.9	0.9	1.3
Eprs	Q8CGC7	1448	-17	-10	-15	6	0.9	0.9	0.9	1.1
Synj1	D3Z656	414	-35	-10	-15	0	0.7	0.9	0.9	1.0
Larp1	Q6ZQ58	841	1	-10	-15	-20	1.0	0.9	0.9	0.8
Gcn1	E9PVA8	2246	-6	-10	-15	-20	0.9	0.9	0.9	0.8
Prr14	Q7TPN9	610	-20	-10	-15	-27	0.8	0.9	0.9	0.8
Rbm48	Q8K2X2	262	-14	-11	-15	9	0.9	0.9	0.9	1.1
Dhrs4	Q99LB2	210	-17	-11	-15	-2	0.9	0.9	0.9	1.0
Xpo6	Q924Z6	547	-13	-11	-15	-3	0.9	0.9	0.9	1.0
Gemin5	Q8BX17	972	-12	-11	-15	-8	0.9	0.9	0.9	0.9
Grb2	Q60631	32	-8	-11	-15	-24	0.9	0.9	0.9	0.8
Gm20425	E9Q035	953	-11	-11	-15	8	0.9	0.9	0.9	1.1
Ctps2	P70303	277	-16	-11	-15	-18	0.9	0.9	0.9	0.8
Stk11	Q9WTK7	276	1	-11	-15	-28	1.0	0.9	0.9	0.8
Rpp38	A2AJG0	130	-7	-11	-15	-46	0.9	0.9	0.9	0.7
Ppp4r1	E9QPR5	78	-15	-12	-15	10	0.9	0.9	0.9	1.1
Tardbp	Q921F2	175	-8	-12	-15	7	0.9	0.9	0.9	1.1
Jak1	B1ASP2	169	-10	-12	-15	-5	0.9	0.9	0.9	1.0
Tns3	Q5SSZ5	1236	-15	-12	-15	-13	0.9	0.9	0.9	0.9
Pla2g16	Q8R3U1	113	-12	-12	-15	-14	0.9	0.9	0.9	0.9
Xpo4	Q9ESJ0	723	-5	-12	-15	-2	1.0	0.9	0.9	1.0
Snx6	Q6P8X1	348	-3	-12	-15	-4	1.0	0.9	0.9	1.0
Arhgap30	Q640N3	20	-20	-12	-15	-6	0.8	0.9	0.9	0.9
Amdhd2	Q8JZV7	6	-17	-12	-15	-6	0.9	0.9	0.9	0.9

Lrsam1	Q80ZI6	75	-17	-12	-15	-7	0.9	0.9	0.9	0.9
Fryl	F8VQ05	288	-10	-13	-15	-2	0.9	0.9	0.9	1.0
Aip	O08915	208	-16	-13	-15	-6	0.9	0.9	0.9	0.9
Coro1c	Q9WUM4	424	-15	-13	-15	-8	0.9	0.9	0.9	0.9
Strbp	Q91WM1	254	-19	-13	-15	-10	0.8	0.9	0.9	0.9
Exo5	Q9CXP9	245	-16	-13	-15	-15	0.9	0.9	0.9	0.9
Ppcdc	Q8BZB2	7	-17	-13	-15	-17	0.9	0.9	0.9	0.9
Zbtb1	Q91VL9	238	-10	-13	-15	-21	0.9	0.9	0.9	0.8
Usp8	Q80U87	710	3	-13	-15	1	1.0	0.9	0.9	1.0
Atg7	Q9D906	520	-15	-13	-15	-2	0.9	0.9	0.9	1.0
Shprh	Q7TPQ3	349	-14	-13	-15	-4	0.9	0.9	0.9	1.0
Wdr1	O88342	194	-28	-13	-15	-5	0.8	0.9	0.9	1.0
Smchd1	Q6P5D8	1334	-13	-13	-15	-11	0.9	0.9	0.9	0.9
Stambp	Q9CQ26	264	-15	-14	-15	-11	0.9	0.9	0.9	0.9
Rsf1	E9PWW9	1436	-17	-14	-15	-16	0.9	0.9	0.9	0.9
Syne2	Q6ZWQ0	1150	-18	-14	-15	-17	0.9	0.9	0.9	0.9
Cers4	Q9D6J1	384	-11	-14	-15	-18	0.9	0.9	0.9	0.8
Svil	Q8K4L3	1193	-21	-14	-15	-25	0.8	0.9	0.9	0.8
Git2	Q9JLQ2	546	-19	-14	-15	-27	0.8	0.9	0.9	0.8
Rps27l	Q6ZWY3	77	-13	-14	-15	-9	0.9	0.9	0.9	0.9
Nhlrc2	Q8BZW8	571	-13	-14	-15	-10	0.9	0.9	0.9	0.9
Ccnh	Q61458	244	-18	-15	-15	9	0.8	0.9	0.9	1.1
Vps72	Q62481	296	-18	-15	-15	-1	0.9	0.9	0.9	1.0
Fkbp15	Q6P9Q6	753	-13	-15	-15	-15	0.9	0.9	0.9	0.9
Dync1h1	Q9JHU4	1886	-12	-15	-15	-32	0.9	0.9	0.9	0.8
Raver1	Q9CW46	255	-16	-15	-15	3	0.9	0.9	0.9	1.0
Lrrc20	Q8CI70	40	-11	-15	-15	2	0.9	0.9	0.9	1.0
Fam102a	Q78T81	119	-15	-15	-15	-5	0.9	0.9	0.9	1.0
Anxa1	P10107	189	-11	-15	-15	-9	0.9	0.9	0.9	0.9
Drap1	Q9D6N5	54	-16	-16	-15	-11	0.9	0.9	0.9	0.9
Tpm3-rs7	D3Z2H9	170	-10	-16	-15	-13	0.9	0.9	0.9	0.9
Trmt2a	Q8BNV1	489	-14	-16	-15	-13	0.9	0.9	0.9	0.9
Arhgef6	Q8K4I3	25	-11	-16	-15	-24	0.9	0.9	0.9	0.8
Rasa3	Q60790	144	-11	-16	-15	-36	0.9	0.9	0.9	0.7
Fbl	P35550	274	-17	-17	-15	-9	0.9	0.9	0.9	0.9
Rpl36a	P83882	77	-12	-17	-15	-12	0.9	0.9	0.9	0.9
Purb	O35295	250	-14	-17	-15	-14	0.9	0.9	0.9	0.9
Wtap	Q9ER69	270	-13	-17	-15	-21	0.9	0.9	0.9	0.8
Rap1a	P62835	139	-11	-17	-15	-22	0.9	0.9	0.9	0.8
Atad5	Q4QY64	1417	-13	-18	-15	2	0.9	0.9	0.9	1.0
Kdm6a	O70546	386	-23	-18	-15	1	0.8	0.9	0.9	1.0
Myh9	Q8VDD5	931	-15	-18	-15	-5	0.9	0.9	0.9	1.0
Hsph1	Q61699	34	-13	-18	-15	-10	0.9	0.9	0.9	0.9
Rps11	P62281	60	-15	-18	-15	9	0.9	0.8	0.9	1.1
Prmt9	Q3U3W5	684	-11	-18	-15	-8	0.9	0.8	0.9	0.9
Ankfy1	Q810B6	389	-11	-18	-15	-12	0.9	0.8	0.9	0.9
Ugp2	Q91ZJ5	276	-23	-19	-15	10	0.8	0.8	0.9	1.1
Gstm3	P19639	174	-22	-19	-15	-2	0.8	0.8	0.9	1.0
Parp9	Q8CAS9	98	-19	-19	-15	-11	0.8	0.8	0.9	0.9
Zbtb7a	O88939	123	-21	-19	-15	-13	0.8	0.8	0.9	0.9
Jade2	Q6ZQF7	516	-19	-19	-15	-21	0.8	0.8	0.9	0.8

Git1	Q68FF6	122	-15	-19	-15	-23	0.9	0.8	0.9	0.8
Setd1b	Q8CFT2	1831	-14	-20	-15	-15	0.9	0.8	0.9	0.9
Tacc1	Q6Y685	512	-3	-20	-15	-41	1.0	0.8	0.9	0.7
Sugp2	Q8CH09	407	-8	-21	-15	3	0.9	0.8	0.9	1.0
Tpp2	Q64514	150	-16	-21	-15	-15	0.9	0.8	0.9	0.9
Cyfip2	Q5SQX6	98	-15	-21	-15	-28	0.9	0.8	0.9	0.8
Cyfip1	Q7TMB8	98	-15	-21	-15	-28	0.9	0.8	0.9	0.8
Cdca7l	Q922M5	410	-7	-22	-15	4	0.9	0.8	0.9	1.0
Nol11	Q8BJW5	513	-15	-22	-15	-9	0.9	0.8	0.9	0.9
Rplp0	P14869	226	-18	-22	-15	-12	0.9	0.8	0.9	0.9
Mybbp1a	Q7TPV4	884	-20	-22	-15	30	0.8	0.8	0.9	1.4
Gvin1	L7N451	1972	-10	-22	-15	-18	0.9	0.8	0.9	0.8
Pdxdc1	Q99K01	135	-12	-22	-15	-27	0.9	0.8	0.9	0.8
Copb2	O55029	56	-18	-23	-15	-9	0.8	0.8	0.9	0.9
Sptb	Q3UGX2	1552	-19	-23	-15	-13	0.8	0.8	0.9	0.9
Nap1l1	P28656	88	-18	-23	-15	11	0.9	0.8	0.9	1.1
Snx9	Q91VH2	427	-12	-23	-15	9	0.9	0.8	0.9	1.1
Atp6v1a	P50516	532	-30	-23	-15	-5	0.8	0.8	0.9	1.0
Pml	Q60953	208	-22	-23	-15	-44	0.8	0.8	0.9	0.7
Fam208a	Q69ZR9	496	-12	-24	-15	8	0.9	0.8	0.9	1.1
Kif23	E9Q5G3	447	-20	-24	-15	0	0.8	0.8	0.9	1.0
Ndufaf3	Q9JKL4	72	-27	-25	-15	-16	0.8	0.8	0.9	0.9
Bag6	Q9Z1R2	350	-14	-26	-15	-10	0.9	0.8	0.9	0.9
Macrod1	Q922B1	203	-16	-27	-15	6	0.9	0.8	0.9	1.1
Plec	Q9QXS1	3343	-21	-27	-15	-7	0.8	0.8	0.9	0.9
Shprh	Q7TPQ3	679	-17	-27	-15	-14	0.9	0.8	0.9	0.9
Sept1	P42209	292	-20	-27	-15	-16	0.8	0.8	0.9	0.9
Txn	P10639	32	9	-27	-15	-41	1.1	0.8	0.9	0.7
Eed	Q921E6	401	-15	-29	-15	-9	0.9	0.8	0.9	0.9
Znf276	Q8CE64	425	-8	-30	-15	-20	0.9	0.8	0.9	0.8
Zfp91	Q62511	184	-13	-30	-15	-44	0.9	0.8	0.9	0.7
Mrpl23	O35972	137	-17	-32	-15	-5	0.9	0.8	0.9	1.0
Atp6v1c1	Q9Z1G3	376	-15	-35	-15	2	0.9	0.7	0.9	1.0
Mettl9	Q9EPL4	215	-12	-35	-15	-6	0.9	0.7	0.9	0.9
Dock10	E9QM99	1042	-18	-36	-15	-16	0.8	0.7	0.9	0.9
Hebp1	Q9R257	36	-30	-38	-15	19	0.8	0.7	0.9	1.2
Stxbp3	Q60770	90	-43	-47	-15	-25	0.7	0.7	0.9	0.8
Ywhag	P61982	194	7	8	-15	12	1.1	1.1	0.9	1.1
Snx5	Q9D8U8	346	3	6	-15	5	1.0	1.1	0.9	1.1
Hus1	Q8BQY8	200	4	6	-15	-10	1.0	1.1	0.9	0.9
Ddx6	P54823	184	-9	4	-15	4	0.9	1.0	0.9	1.0
Atp2a2	O55143	349	-18	4	-15	-4	0.9	1.0	0.9	1.0
Atp2a3	Q64518	349	-18	4	-15	-4	0.9	1.0	0.9	1.0
Arid1b	E9Q4N7	1942	-12	3	-15	31	0.9	1.0	0.9	1.4
Snx5	Q9D8U8	347	-5	3	-15	-4	1.0	1.0	0.9	1.0
Xntrpc	E9PVR2	37	-13	1	-15	-2	0.9	1.0	0.9	1.0
Dars	Q922B2	203	-19	1	-15	-16	0.8	1.0	0.9	0.9
Wdfy4	E9Q2M9	1219	10	1	-15	2	1.1	1.0	0.9	1.0
Uros	P51163	241	-9	0	-15	0	0.9	1.0	0.9	1.0
Gsdmdc1	Q9D8T2	77	-6	0	-15	-6	0.9	1.0	0.9	0.9
Prpf8	Q99PV0	2223	-10	-1	-15	21	0.9	1.0	0.9	1.3

Pan2	Q8BGF7	790	-15	-1	-15	-3	0.9	1.0	0.9	1.0
Dennd4c	A6H8H2	1083	-19	-1	-15	-3	0.8	1.0	0.9	1.0
Arfgap3	Q9D8S3	312	-11	-2	-15	7	0.9	1.0	0.9	1.1
Acap1	Q8K2H4	320	-18	-2	-15	-1	0.9	1.0	0.9	1.0
Gimap1	P70224	42	-10	-2	-15	-9	0.9	1.0	0.9	0.9
S100a11	P50543	8	-10	-3	-15	24	0.9	1.0	0.9	1.3
Ubr4	A2AN08	140	-10	-3	-15	-16	0.9	1.0	0.9	0.9
Park7	Q99LX0	106	-10	-4	-15	-5	0.9	1.0	0.9	1.0
Trim10	Q9WUH5	481	-10	-4	-15	-10	0.9	1.0	0.9	0.9
Msh6	P54276	1035	-12	-4	-15	-16	0.9	1.0	0.9	0.9
Uncharact	Q8K2W9	288	8	-4	-15	-16	1.1	1.0	0.9	0.9
Osbpl11	G5E8A0	43	-7	-4	-15	12	0.9	1.0	0.9	1.1
Marc2	Q922Q1	211	-8	-4	-15	3	0.9	1.0	0.9	1.0
Arap1	Q4LDD4	997	-12	-4	-15	-1	0.9	1.0	0.9	1.0
Ikbkap	Q7TT37	213	-7	-5	-15	-1	0.9	1.0	0.9	1.0
Rpl17	Q6ZWZ7	57	3	-5	-15	-12	1.0	1.0	0.9	0.9
Naa35	Q6PHQ8	409	-16	-5	-15	14	0.9	1.0	0.9	1.2
Eftud2	O08810	165	-23	-5	-15	-2	0.8	1.0	0.9	1.0
Dhx40	Q6PE54	641	-1	-5	-15	-8	1.0	1.0	0.9	0.9
Pcbp1	P60335	163	-18	-5	-15	-27	0.8	1.0	0.9	0.8
Capn2	O08529	640	1	-6	-15	14	1.0	0.9	0.9	1.2
UPF0688	Q80WR5	117	-6	-6	-15	-3	0.9	0.9	0.9	1.0
Ncapd2	Q8K2Z4	286	-14	-6	-15	-6	0.9	0.9	0.9	0.9
Acsl4	Q9QUJ7	133	-1	-6	-15	-8	1.0	0.9	0.9	0.9
Frmd8	Q3UFK8	191	-9	-6	-15	-37	0.9	0.9	0.9	0.7
Matk	P41242	264	-7	-6	-15	17	0.9	0.9	0.9	1.2
Dock2	Q8C3J5	41	-12	-6	-15	4	0.9	0.9	0.9	1.0
Rnf146	Q9CZW6	58	4	-6	-15	0	1.0	0.9	0.9	1.0
Pold1	P52431	1024	-24	-6	-15	-18	0.8	0.9	0.9	0.8
Nvl	Q9DBY8	308	-11	-7	-15	6	0.9	0.9	0.9	1.1
Arpc1a	Q9R0Q6	279	-8	-7	-15	-6	0.9	0.9	0.9	0.9
Dnaja1	P63037	149	-7	-7	-15	-8	0.9	0.9	0.9	0.9
Hmgcl	P38060	174	-17	-7	-15	-9	0.9	0.9	0.9	0.9
Ascc3	E9PZJ8	324	-9	-7	-15	-18	0.9	0.9	0.9	0.9
Gatm	Q9D964	252	-7	-7	-15	9	0.9	0.9	0.9	1.1
Sp110	Q8BVK9	418	-24	-7	-15	5	0.8	0.9	0.9	1.1
Adsl	P54822	399	-12	-7	-15	4	0.9	0.9	0.9	1.0
Phgdh	Q61753	281	-20	-7	-15	3	0.8	0.9	0.9	1.0
Hsp90aa1	P07901	375	-15	-7	-15	-3	0.9	0.9	0.9	1.0
Syne2	Q6ZWQ0	3691	-15	-7	-15	-5	0.9	0.9	0.9	1.0
Grk6	O70293	474	-17	-7	-15	-8	0.9	0.9	0.9	0.9
Med12	A2AGH6	445	-4	-8	-15	24	1.0	0.9	0.9	1.3
Pi4ka	E9Q3L2	1905	-17	-8	-15	-2	0.9	0.9	0.9	1.0
Tln1	P26039	1434	-15	-8	-15	-7	0.9	0.9	0.9	0.9
Ap2m1	P84091	251	-36	-8	-15	-11	0.7	0.9	0.9	0.9
Larp1	Q6ZQ58	1030	-12	-8	-15	-20	0.9	0.9	0.9	0.8
Larp4b	Q6A0A2	682	-5	-8	-15	-40	1.0	0.9	0.9	0.7
Trim21	Q3U7K7	269	-22	-8	-15	10	0.8	0.9	0.9	1.1
Nono	Q99K48	147	-15	-8	-15	0	0.9	0.9	0.9	1.0
Psmd1	Q3TXS7	806	-10	-8	-15	-4	0.9	0.9	0.9	1.0
Gspt1	Q8R050	590	-25	-8	-15	-10	0.8	0.9	0.9	0.9

Casp9	Q8C3Q9	325	-8	-9	-15	1	0.9	0.9	0.9	1.0
Frmd8	Q3UFK8	412	-23	-9	-15	-15	0.8	0.9	0.9	0.9
Arhgap4	B1AUY3	824	-10	-9	-15	-28	0.9	0.9	0.9	0.8
Phf5a	P83870	85	-11	-9	-15	-2	0.9	0.9	0.9	1.0
Nfkb1	P25799	59	-9	-9	-15	-15	0.9	0.9	0.9	0.9
Ppp2r4	P58389	299	-2	-10	-15	23	1.0	0.9	0.9	1.3
Ppp1r11	A5A4Y9	65	-3	-10	-15	5	1.0	0.9	0.9	1.1
Stat5b	P42232	688	-16	-10	-15	-26	0.9	0.9	0.9	0.8
Mon2	Q80TL7	275	-13	-10	-15	25	0.9	0.9	0.9	1.3
Polr1a	O35134	1296	-7	-10	-15	-1	0.9	0.9	0.9	1.0
Iah1	Q9DB29	137	-12	-10	-15	-7	0.9	0.9	0.9	0.9
Pdk2	Q9JK42	45	-12	-10	-15	-9	0.9	0.9	0.9	0.9
Rrbp1	Q99PL5	1198	-14	-10	-15	-15	0.9	0.9	0.9	0.9
Ncoa5	Q91W39	137	-13	-10	-15	-31	0.9	0.9	0.9	0.8
Trmt2a	Q8BNV1	250	-8	-11	-15	3	0.9	0.9	0.9	1.0
Rack1	P68040	138	-15	-11	-15	-2	0.9	0.9	0.9	1.0
Gls	D3Z7P3	271	-11	-11	-15	15	0.9	0.9	0.9	1.2
Hnrnpk	P61979	132	-12	-11	-15	-5	0.9	0.9	0.9	1.0
Nsun5	Q8K4F6	343	-7	-11	-15	-10	0.9	0.9	0.9	0.9
Col4a3bp	Q9EQG9	185	-15	-11	-15	-13	0.9	0.9	0.9	0.9
Was	P70315	322	-13	-11	-15	-21	0.9	0.9	0.9	0.8
Ptprc	P06800	1246	-7	-11	-15	-29	0.9	0.9	0.9	0.8
Prg2	Q61878	118	-10	-12	-15	-14	0.9	0.9	0.9	0.9
Dennd4b	Q3U1Y4	1220	0	-12	-15	-18	1.0	0.9	0.9	0.9
Gtf2i	Q9ESZ8	80	-1	-12	-15	-23	1.0	0.9	0.9	0.8
Mipep	A6H611	412	1	-12	-15	-26	1.0	0.9	0.9	0.8
Armc5	Q5EBP3	891	-19	-12	-15	15	0.8	0.9	0.9	1.2
Rad17	Q6NXW6	140	-18	-12	-15	8	0.9	0.9	0.9	1.1
Dync1h1	Q9JHU4	631	-10	-12	-15	-7	0.9	0.9	0.9	0.9
Tnpo1	Q8BFY9	620	-18	-12	-15	-8	0.9	0.9	0.9	0.9
Gga3	Q8BMI3	392	-19	-12	-15	-10	0.8	0.9	0.9	0.9
Vdac3-ps1	A0A140T8V3	65	-10	-12	-15	-11	0.9	0.9	0.9	0.9
Bcat2	O35855	136	-16	-12	-15	-16	0.9	0.9	0.9	0.9
Nab2	Q61127	499	-14	-12	-15	-16	0.9	0.9	0.9	0.9
Trp53bp1	A2AU91	1028	-8	-12	-15	-29	0.9	0.9	0.9	0.8
Vcl	Q64727	985	-16	-13	-15	14	0.9	0.9	0.9	1.2
Fbxl4	Q8BH70	547	-10	-13	-15	-11	0.9	0.9	0.9	0.9
Sirt7	Q8BKJ9	170	-11	-13	-15	1	0.9	0.9	0.9	1.0
Lyrm7	Q9DA03	97	-6	-13	-15	-18	0.9	0.9	0.9	0.9
Sympk	Q80X82	1180	-11	-13	-15	-30	0.9	0.9	0.9	0.8
Pptc7	Q6NVE9	63	-12	-13	-15	-40	0.9	0.9	0.9	0.7
Ranbp2	Q9ERU9	1433	-15	-14	-15	10	0.9	0.9	0.9	1.1
Banp	Q8VBU8	65	-13	-14	-15	10	0.9	0.9	0.9	1.1
Snrnp200	Q6P4T2	576	-8	-14	-15	-1	0.9	0.9	0.9	1.0
Pnkp	G5E8N7	307	-14	-14	-15	-11	0.9	0.9	0.9	0.9
Pgls	Q9CQ60	33	-16	-14	-15	-13	0.9	0.9	0.9	0.9
Pcbp2	Q61990	109	-6	-14	-15	-3	0.9	0.9	0.9	1.0
Zzef1	Q5SSH7	69	-15	-14	-15	-6	0.9	0.9	0.9	0.9
Arhgef2	Q60875	307	-20	-14	-15	-15	0.8	0.9	0.9	0.9
Mcm3ap	Q9WUU9	974	-30	-14	-15	-16	0.8	0.9	0.9	0.9
Nup188	Q6ZQH8	1433	-16	-15	-15	13	0.9	0.9	0.9	1.1

Dock8	Q8C147	1837	-20	-15	-15	-4	0.8	0.9	0.9	1.0
Emc8	O70378	24	-32	-15	-15	-7	0.8	0.9	0.9	0.9
Myo1g	Q5SUA5	624	-12	-15	-15	-12	0.9	0.9	0.9	0.9
Fermt3	Q8K1B8	403	-22	-15	-15	4	0.8	0.9	0.9	1.0
Sptbn1	Q62261	604	-17	-15	-15	2	0.9	0.9	0.9	1.0
Grk2	Q99MK8	120	-12	-15	-15	-13	0.9	0.9	0.9	0.9
Twistnb	Q78WZ7	193	-12	-15	-15	-20	0.9	0.9	0.9	0.8
Tcf25	Q8R3L2	83	-10	-15	-15	-33	0.9	0.9	0.9	0.8
Slc6a4	Q60857	622	-6	-15	-15	-41	0.9	0.9	0.9	0.7
Hells	Q60848	478	-12	-16	-15	7	0.9	0.9	0.9	1.1
Ldb1	P70662	273	-16	-16	-15	-7	0.9	0.9	0.9	0.9
Dstn	Q9R0P5	23	-13	-16	-15	-11	0.9	0.9	0.9	0.9
Dync1h1	Q9JHU4	4214	-14	-16	-15	-17	0.9	0.9	0.9	0.9
U2surp	Q6NV83	918	-1	-16	-15	-24	1.0	0.9	0.9	0.8
Uhrf1bp1l	A2RSJ4	81	-14	-17	-15	-6	0.9	0.9	0.9	0.9
Appl1	Q8K3H0	569	-17	-17	-15	-17	0.9	0.9	0.9	0.9
Sqrdl	Q9R112	201	-10	-17	-15	6	0.9	0.9	0.9	1.1
Cd2	P08920	332	-14	-17	-15	-40	0.9	0.9	0.9	0.7
Sos2	Q02384	404	-23	-18	-15	32	0.8	0.9	0.9	1.5
Skp1	Q9WTX5	160	-10	-18	-15	-2	0.9	0.9	0.9	1.0
Phf5a	P83870	61	-15	-18	-15	-3	0.9	0.9	0.9	1.0
Slfn5	Q8CBA2	122	-11	-18	-15	-8	0.9	0.9	0.9	0.9
Ppil2	Q9D787	387	-22	-18	-15	-9	0.8	0.9	0.9	0.9
Dnmt1	P13864	900	-12	-18	-15	-16	0.9	0.9	0.9	0.9
Lpcat2	Q8BYI6	223	-10	-18	-15	-40	0.9	0.9	0.9	0.7
Adh5	P28474	170	1	-18	-15	21	1.0	0.8	0.9	1.3
Psmd11	Q8BG32	202	-17	-18	-15	2	0.9	0.8	0.9	1.0
Plcg2	Q8CIH5	624	-4	-18	-15	-5	1.0	0.8	0.9	1.0
Sptlc1	O35704	438	-17	-18	-15	-8	0.9	0.8	0.9	0.9
Fry	E9Q8I9	2017	-15	-18	-15	-20	0.9	0.8	0.9	0.8
Pank4	Q80YV4	537	-6	-19	-15	-5	0.9	0.8	0.9	1.0
Znf672	Q99LH4	353	-5	-19	-15	-14	1.0	0.8	0.9	0.9
Hltf	Q6PCN7	372	-17	-19	-15	-13	0.9	0.8	0.9	0.9
Stx2	Q80W45	11	-8	-19	-15	-22	0.9	0.8	0.9	0.8
Ppp1r12a	Q9DBR7	565	-15	-19	-15	-31	0.9	0.8	0.9	0.8
Dpf2	Q61103	298	-21	-20	-15	0	0.8	0.8	0.9	1.0
Cops3	O88543	383	-12	-20	-15	-5	0.9	0.8	0.9	1.0
Son	Q9QX47	92	-15	-20	-15	-6	0.9	0.8	0.9	0.9
Rnf126	Q91YL2	15	-20	-20	-15	-11	0.8	0.8	0.9	0.9
Clgn	P52194	185	2	-20	-15	-14	1.0	0.8	0.9	0.9
Flna	Q8BTM8	8	-11	-20	-15	-17	0.9	0.8	0.9	0.9
Dap3	Q9ER88	379	-8	-20	-15	-20	0.9	0.8	0.9	0.8
Atad2b	E9Q166	608	-15	-21	-15	-1	0.9	0.8	0.9	1.0
Gnai2	P08752	352	-16	-21	-15	-32	0.9	0.8	0.9	0.8
Copa	Q8CIE6	1185	-8	-22	-15	6	0.9	0.8	0.9	1.1
Bcl11a	Q9QYE3	607	1	-22	-15	-31	1.0	0.8	0.9	0.8
Supt6h	Q62383	336	-13	-22	-15	-12	0.9	0.8	0.9	0.9
Stx5	Q8K1E0	91	-9	-22	-15	-17	0.9	0.8	0.9	0.9
Rbm22	Q8BHS3	74	-27	-23	-15	8	0.8	0.8	0.9	1.1
Stat2	E9QJX9	675	-24	-23	-15	-12	0.8	0.8	0.9	0.9
Acin1	Q9JIX8	1082	-20	-23	-15	-22	0.8	0.8	0.9	0.8

Spg11	Q3UHA3	389	-10	-23	-15	-7	0.9	0.8	0.9	0.9
Dennd6a	Q8BH65	105	-20	-23	-15	-16	0.8	0.8	0.9	0.9
Zc3h15	Q3TIV5	113	-28	-23	-15	-17	0.8	0.8	0.9	0.9
Hmox2	O70252	264	-11	-24	-15	25	0.9	0.8	0.9	1.3
Mrps9	Q9D7N3	227	-12	-24	-15	-9	0.9	0.8	0.9	0.9
Clic1	Q9Z1Q5	59	-26	-24	-15	-21	0.8	0.8	0.9	0.8
Fcho1	Q8K285	417	-12	-24	-15	-22	0.9	0.8	0.9	0.8
Zgpat	Q8VDM1	179	-14	-25	-15	-4	0.9	0.8	0.9	1.0
Gne	Q91WG8	211	7	-25	-15	-16	1.1	0.8	0.9	0.9
Sfswap	Q3USH5	220	-29	-25	-15	-19	0.8	0.8	0.9	0.8
Zfand5	O88878	76	-31	-25	-15	-44	0.8	0.8	0.9	0.7
Rxra	P28700	437	-10	-27	-15	16	0.9	0.8	0.9	1.2
Rxrb	P28704	490	-10	-27	-15	16	0.9	0.8	0.9	1.2
Trim25	Q61510	185	-18	-27	-15	-6	0.9	0.8	0.9	0.9
Itpripl1	A2ASA8	540	-21	-27	-15	-18	0.8	0.8	0.9	0.8
Brwd3	A2AHJ4	781	-6	-27	-15	-34	0.9	0.8	0.9	0.7
Rnf213	E9Q555	1710	-4	-28	-15	-9	1.0	0.8	0.9	0.9
Mrm1	Q99J25	180	-22	-28	-15	-11	0.8	0.8	0.9	0.9
Ufl1	Q8CCJ3	32	-12	-30	-15	-3	0.9	0.8	0.9	1.0
Sugp2	Q8CH09	955	-15	-30	-15	-26	0.9	0.8	0.9	0.8
Vps8	Q0P5W1	284	-22	-30	-15	-10	0.8	0.8	0.9	0.9
Ints7	Q7TQK1	851	-11	-30	-15	-23	0.9	0.8	0.9	0.8
Sfpq	Q8VIJ6	423	-18	-31	-15	-5	0.9	0.8	0.9	1.0
Rab18	P35293	155	11	-31	-15	1	1.1	0.8	0.9	1.0
Ctsh	P49935	136	-17	-34	-15	-23	0.9	0.7	0.9	0.8
Prkdc	P97313	3188	-16	-38	-15	2	0.9	0.7	0.9	1.0
Mcm3	P25206	134	-31	-53	-15	-29	0.8	0.7	0.9	0.8
Wdr1	O88342	170	7	18	-16	15	1.1	1.2	0.9	1.2
Pdha1	P35486	273	-4	7	-16	21	1.0	1.1	0.9	1.3
Itpa	Q9D892	33	5	5	-16	6	1.1	1.1	0.9	1.1
Ints9	Q8K114	471	-23	5	-16	19	0.8	1.0	0.9	1.2
Rtcb	Q99LF4	485	-4	4	-16	6	1.0	1.0	0.9	1.1
Cltc	Q68FD5	934	-5	3	-16	16	1.0	1.0	0.9	1.2
Chil3	O35744	394	-6	3	-16	-23	0.9	1.0	0.9	0.8
Csl	Q80X68	211	-9	3	-16	25	0.9	1.0	0.9	1.3
Fam98c	E9PYD1	28	-11	1	-16	4	0.9	1.0	0.9	1.0
Ndufaf7	Q9CWG8	418	-14	0	-16	-14	0.9	1.0	0.9	0.9
Rnh1	Q91VI7	194	-10	-1	-16	14	0.9	1.0	0.9	1.2
Ddx58	Q6Q899	158	-13	-1	-16	-13	0.9	1.0	0.9	0.9
Glmn	Q8BZM1	351	1	-1	-16	10	1.0	1.0	0.9	1.1
Pfas	Q5SUR0	587	-14	-1	-16	7	0.9	1.0	0.9	1.1
Zfp334	A2A4U6	573	-27	-1	-16	-2	0.8	1.0	0.9	1.0
Ifi35	Q9D8C4	74	-4	-2	-16	-10	1.0	1.0	0.9	0.9
Raly	Q64012	255	6	-2	-16	-15	1.1	1.0	0.9	0.9
Cplx2	P84086	90	-29	-2	-16	4	0.8	1.0	0.9	1.0
Nubp1	Q9R060	25	-6	-2	-16	-25	0.9	1.0	0.9	0.8
Sgf29	Q9DA08	243	-25	-3	-16	2	0.8	1.0	0.9	1.0
Itpr1	P11881	1976	-11	-3	-16	10	0.9	1.0	0.9	1.1
Itpr3	P70227	1880	-11	-3	-16	10	0.9	1.0	0.9	1.1
Itpr2	Q9Z329	1929	-11	-3	-16	10	0.9	1.0	0.9	1.1
Stard5	Q9EPQ7	58	-14	-3	-16	-13	0.9	1.0	0.9	0.9

Exosc10	P56960	554	-10	-4	-16	19	0.9	1.0	0.9	1.2
Ldha	P06151	131	-19	-4	-16	-3	0.8	1.0	0.9	1.0
Sarnp	Q9D1J3	20	-7	-4	-16	-11	0.9	1.0	0.9	0.9
Bola2	Q8BGS2	59	-4	-4	-16	12	1.0	1.0	0.9	1.1
Tpi1	P17751	92	-4	-5	-16	28	1.0	1.0	0.9	1.4
Pcbp2	Q61990	298	-20	-5	-16	-13	0.8	1.0	0.9	0.9
Rasa3	Q60790	662	-6	-5	-16	-17	0.9	1.0	0.9	0.9
Alkbh3	Q8K1E6	50	-6	-5	-16	-17	0.9	1.0	0.9	0.9
Arid2	E9Q7E2	82	-14	-5	-16	16	0.9	1.0	0.9	1.2
Ppp5c	Q60676	404	-12	-5	-16	15	0.9	1.0	0.9	1.2
Ipo5	Q8BKC5	473	-16	-5	-16	6	0.9	1.0	0.9	1.1
Aldh16a1	Q571I9	467	-15	-5	-16	5	0.9	1.0	0.9	1.0
Tpp2	Q64514	342	-8	-6	-16	-37	0.9	0.9	0.9	0.7
Hat1	Q8BY71	98	-10	-6	-16	4	0.9	0.9	0.9	1.0
Cad	B2RQC6	1636	-19	-6	-16	-1	0.8	0.9	0.9	1.0
Hdc	P23738	179	-17	-6	-16	-13	0.9	0.9	0.9	0.9
Trim30d	E9PWL0	372	2	-6	-16	-19	1.0	0.9	0.9	0.8
Trim30a	P15533	371	2	-6	-16	-19	1.0	0.9	0.9	0.8
Eif2s2	Q99L45	224	-1	-7	-16	5	1.0	0.9	0.9	1.0
Anks1a	P59672	110	-11	-7	-16	-14	0.9	0.9	0.9	0.9
Eprs	Q8CGC7	692	-12	-7	-16	-17	0.9	0.9	0.9	0.9
Aak1	Q3UHJ0	288	-17	-7	-16	22	0.9	0.9	0.9	1.3
Ube2c	Q9D1C1	23	-12	-7	-16	5	0.9	0.9	0.9	1.0
Gimap5	Q8BWF2	26	-25	-7	-16	-7	0.8	0.9	0.9	0.9
Rrp8	Q9DB85	452	-16	-8	-16	1	0.9	0.9	0.9	1.0
Akap13	E9Q394	2064	-9	-8	-16	-10	0.9	0.9	0.9	0.9
Cggbp1	Q8BHG9	92	-10	-8	-16	-24	0.9	0.9	0.9	0.8
Nt5c3b	Q3UFY7	114	41	-8	-16	-41	1.7	0.9	0.9	0.7
Ptpn6	P29351	361	-15	-8	-16	7	0.9	0.9	0.9	1.1
Mtmr12	Q80TA6	67	-9	-8	-16	-7	0.9	0.9	0.9	0.9
Fam208b	Q5DTT3	381	-8	-8	-16	-20	0.9	0.9	0.9	0.8
Akap12	Q9WTQ5	1248	-15	-8	-16	-33	0.9	0.9	0.9	0.8
Kbtbd2	G3X9X1	349	10	-9	-16	4	1.1	0.9	0.9	1.0
Ncaph	Q8C156	249	-34	-9	-16	2	0.7	0.9	0.9	1.0
Dennd1c	Q8CFK6	688	-10	-9	-16	-14	0.9	0.9	0.9	0.9
Irak2	Q8CFA1	438	-16	-9	-16	4	0.9	0.9	0.9	1.0
Macrod1	Q922B1	184	-15	-9	-16	2	0.9	0.9	0.9	1.0
Casp9	Q8C3Q9	441	-4	-9	-16	-10	1.0	0.9	0.9	0.9
Stat3	P42227	259	-9	-10	-16	19	0.9	0.9	0.9	1.2
Exoc7	O35250	512	-14	-10	-16	16	0.9	0.9	0.9	1.2
Ptbp3	Q8BHD7	39	-10	-10	-16	-7	0.9	0.9	0.9	0.9
Mdm1	Q9D067	598	-18	-10	-16	-8	0.8	0.9	0.9	0.9
Bard1	O70445	545	-16	-10	-16	-10	0.9	0.9	0.9	0.9
Rnf214	Q8BFU3	620	-12	-10	-16	-18	0.9	0.9	0.9	0.8
Gstm5	P48774	38	-12	-10	-16	1	0.9	0.9	0.9	1.0
Rpl3	P27659	336	-12	-10	-16	-10	0.9	0.9	0.9	0.9
Gfap	P03995	291	-12	-10	-16	-11	0.9	0.9	0.9	0.9
Acadvl	P50544	238	-11	-10	-16	-21	0.9	0.9	0.9	0.8
Sphk2	Q9JIA7	65	-6	-10	-16	-60	0.9	0.9	0.9	0.6
Pik3r5	Q5SW28	753	-12	-11	-16	5	0.9	0.9	0.9	1.1
Cmpk2	Q3U5Q7	287	-22	-11	-16	-10	0.8	0.9	0.9	0.9

Irf8	P23611	249	-17	-11	-16	-18	0.9	0.9	0.9	0.9
Akap13	E9Q394	573	-9	-11	-16	-21	0.9	0.9	0.9	0.8
Atrx	Q61687	870	-13	-11	-16	-21	0.9	0.9	0.9	0.8
Mdn1	A2ANY6	1328	-10	-11	-16	-22	0.9	0.9	0.9	0.8
Nup160	Q9Z0W3	439	-10	-11	-16	22	0.9	0.9	0.9	1.3
Plec	Q9QXS1	3500	-20	-11	-16	11	0.8	0.9	0.9	1.1
Dazap1	Q9JII5	124	-20	-11	-16	4	0.8	0.9	0.9	1.0
Map1s	Q8C052	54	-14	-11	-16	-4	0.9	0.9	0.9	1.0
Idh2	P54071	308	-14	-11	-16	-10	0.9	0.9	0.9	0.9
Nfatc1	B5B2N2	680	-12	-11	-16	-11	0.9	0.9	0.9	0.9
Arhgap4	B1AUY3	730	-23	-11	-16	-14	0.8	0.9	0.9	0.9
Lrmp	G5E880	339	-11	-11	-16	-27	0.9	0.9	0.9	0.8
Helz2	E9QAM5	1415	-3	-12	-16	-2	1.0	0.9	0.9	1.0
Glud1	P26443	327	-7	-12	-16	-3	0.9	0.9	0.9	1.0
Stk38	Q91VJ4	362	-4	-12	-16	-19	1.0	0.9	0.9	0.8
Nfxl1	E9Q8I7	830	-6	-12	-16	-19	0.9	0.9	0.9	0.8
Vps16	G3X8X7	529	-8	-12	-16	-40	0.9	0.9	0.9	0.7
Ppm1d	Q9QZ67	471	-8	-12	-16	-8	0.9	0.9	0.9	0.9
Mrpl38	Q8K2M0	143	-4	-12	-16	-12	1.0	0.9	0.9	0.9
Ddx10	Q80Y44	323	-17	-13	-16	-11	0.9	0.9	0.9	0.9
Syne1	Q6ZWR6	8079	-10	-13	-16	-11	0.9	0.9	0.9	0.9
Lrch3	Q8BVU0	277	-20	-13	-16	-28	0.8	0.9	0.9	0.8
Txndc17	Q9CQM5	43	-10	-13	-16	-104	0.9	0.9	0.9	0.5
Smg5	Q6ZPY2	109	-16	-13	-16	13	0.9	0.9	0.9	1.1
Aldh9a1	Q9JLJ2	355	-12	-13	-16	5	0.9	0.9	0.9	1.0
Vps53	Q8CCB4	365	-9	-13	-16	-7	0.9	0.9	0.9	0.9
Pom121	Q8K3Z9	112	-16	-13	-16	-11	0.9	0.9	0.9	0.9
Wdr73	Q9CWR1	221	-19	-13	-16	-13	0.8	0.9	0.9	0.9
Arap2	E9QP44	1122	-15	-14	-16	17	0.9	0.9	0.9	1.2
Ipo4	Q8VI75	933	-14	-14	-16	-10	0.9	0.9	0.9	0.9
Myof	Q69ZN7	1412	-10	-14	-16	-12	0.9	0.9	0.9	0.9
Cluh	Q5SW19	665	10	-14	-16	-18	1.1	0.9	0.9	0.8
Itm2b	O89051	38	-16	-14	-16	-24	0.9	0.9	0.9	0.8
Phkb	Q7TSH2	411	-9	-14	-16	-8	0.9	0.9	0.9	0.9
Glo1	Q9CPU0	139	-15	-14	-16	-23	0.9	0.9	0.9	0.8
Cand1	Q6ZQ38	131	-21	-15	-16	4	0.8	0.9	0.9	1.0
Ptk2b	Q9QVP9	562	-16	-15	-16	-5	0.9	0.9	0.9	1.0
Myo1c	Q9WTI7	802	-17	-15	-16	-10	0.9	0.9	0.9	0.9
Vwa5a	Q99KC8	659	-13	-15	-16	-16	0.9	0.9	0.9	0.9
Hmces	Q8R1M0	39	-10	-15	-16	-21	0.9	0.9	0.9	0.8
Ppp4r2	Q0VGB7	377	-15	-15	-16	-25	0.9	0.9	0.9	0.8
Abcd3	P55096	477	-16	-15	-16	-2	0.9	0.9	0.9	1.0
Zfp444	Q3TDV8	257	-25	-15	-16	-13	0.8	0.9	0.9	0.9
Spty2d1	Q68FG3	534	-15	-15	-16	-18	0.9	0.9	0.9	0.8
Ptges3	Q9R0Q7	75	-10	-16	-16	25	0.9	0.9	0.9	1.3
Eif3k	Q9DBZ5	85	-20	-16	-16	14	0.8	0.9	0.9	1.2
Surf6	P70279	19	-11	-16	-16	-17	0.9	0.9	0.9	0.9
Map2k4	P47809	244	-11	-16	-16	6	0.9	0.9	0.9	1.1
Strap	Q9Z1Z2	305	-14	-16	-16	-4	0.9	0.9	0.9	1.0
Nudt16	Q6P3D0	68	1	-16	-16	-4	1.0	0.9	0.9	1.0
Rpl3	P27659	253	-10	-16	-16	-8	0.9	0.9	0.9	0.9

Nfatc2	Q60591	72	-18	-16	-16	-10	0.8	0.9	0.9	0.9
Fdxr	Q61578	44	-17	-16	-16	-23	0.9	0.9	0.9	0.8
Mfn2	Q80U63	521	-3	-16	-16	-42	1.0	0.9	0.9	0.7
Bod1l	E9Q6J5	72	-15	-17	-16	20	0.9	0.9	0.9	1.2
Wash1	Q8VDD8	145	-12	-17	-16	-8	0.9	0.9	0.9	0.9
Pop7	Q9DCH2	9	-10	-17	-16	-11	0.9	0.9	0.9	0.9
Phkb	Q7TSH2	426	-18	-17	-16	-27	0.8	0.9	0.9	0.8
Akap13	E9Q394	1392	-17	-17	-16	-29	0.9	0.9	0.9	0.8
Lage3	Q9CR70	107	-5	-17	-16	-5	1.0	0.9	0.9	1.0
Cad	B2RQC6	1455	51	-17	-16	-12	2.0	0.9	0.9	0.9
Ranbp2	Q9ERU9	2545	-17	-17	-16	-23	0.9	0.9	0.9	0.8
UPF0428	Q8VDP2	11	-8	-17	-16	-24	0.9	0.9	0.9	0.8
Rnf34	Q99KR6	149	-13	-17	-16	-24	0.9	0.9	0.9	0.8
Edc4	Q3UJB9	843	-20	-17	-16	-25	0.8	0.9	0.9	0.8
Atg7	Q9D906	364	-11	-18	-16	34	0.9	0.9	0.9	1.5
Cad	B2RQC6	183	-12	-18	-16	9	0.9	0.9	0.9	1.1
Mdp1	Q9D967	138	-17	-18	-16	4	0.9	0.9	0.9	1.0
UPF0598	Q8VE95	100	-18	-18	-16	-24	0.9	0.9	0.9	0.8
Lemd3	D3YU56	783	-20	-18	-16	-28	0.8	0.9	0.9	0.8
Strbp	Q91WM1	142	-21	-18	-16	-17	0.8	0.8	0.9	0.9
Trip12	G5E870	332	-14	-18	-16	-27	0.9	0.8	0.9	0.8
Sept9	Q80UG5	529	-25	-19	-16	26	0.8	0.8	0.9	1.3
Eef2	P58252	728	-21	-19	-16	-2	0.8	0.8	0.9	1.0
Mbnl3	Q8R003	35	-18	-19	-16	-6	0.9	0.8	0.9	0.9
Macf1	E9PVY8	4785	-15	-19	-16	-14	0.9	0.8	0.9	0.9
Prkaa1	Q5EG47	185	-11	-19	-16	-17	0.9	0.8	0.9	0.9
Ahdc1	Q6PAL7	788	-14	-19	-16	-22	0.9	0.8	0.9	0.8
Ak2	Q9WTP6	92	-16	-20	-16	5	0.9	0.8	0.9	1.0
Mcrs1	Q99L90	84	-16	-20	-16	-26	0.9	0.8	0.9	0.8
Tapbp	Q9R233	118	-24	-20	-16	-8	0.8	0.8	0.9	0.9
Zcchc11	B2RX14	20	-11	-20	-16	-13	0.9	0.8	0.9	0.9
Akap2	O54931	416	-11	-20	-16	-34	0.9	0.8	0.9	0.7
Stat6	P52633	355	9	-21	-16	17	1.1	0.8	0.9	1.2
Uqcrc2	Q9DB77	192	-22	-21	-16	2	0.8	0.8	0.9	1.0
Sec24b	Q80ZX0	545	-21	-21	-16	-3	0.8	0.8	0.9	1.0
Lactb	Q9EP89	480	-10	-21	-16	-17	0.9	0.8	0.9	0.9
Wdfy4	E9Q2M9	3144	-27	-21	-16	-20	0.8	0.8	0.9	0.8
Actb	P60710	285	-10	-21	-16	-25	0.9	0.8	0.9	0.8
Kiaa0226l	Q3TD16	31	-24	-21	-16	-27	0.8	0.8	0.9	0.8
Trrap	A0A1D5RLL4	429	-16	-21	-16	1	0.9	0.8	0.9	1.0
Orc3	Q9JK30	521	-11	-21	-16	-19	0.9	0.8	0.9	0.8
Mtmr3	Q8K296	913	-11	-21	-16	-20	0.9	0.8	0.9	0.8
Ttf2	Q5NC05	445	-23	-21	-16	-29	0.8	0.8	0.9	0.8
Baz1a	O88379	27	-15	-22	-16	-9	0.9	0.8	0.9	0.9
Klhl36	Q8R124	521	-11	-22	-16	-17	0.9	0.8	0.9	0.9
Btk	P35991	527	-14	-22	-16	-24	0.9	0.8	0.9	0.8
Esyt2	Q3TZZ7	105	-8	-22	-16	-28	0.9	0.8	0.9	0.8
Ank1	Q02357	1212	-28	-22	-16	22	0.8	0.8	0.9	1.3
Ddx54	Q8K4L0	388	-8	-22	-16	9	0.9	0.8	0.9	1.1
Fam129a	Q3UW53	802	-22	-22	-16	-26	0.8	0.8	0.9	0.8
Hnrnpul1	Q8VDM6	488	-30	-23	-16	35	0.8	0.8	0.9	1.5

Dhx29	Q6PGC1	617	-15	-23	-16	-15	0.9	0.8	0.9	0.9
Svil	Q8K4L3	26	-22	-23	-16	-35	0.8	0.8	0.9	0.7
Ankrd44	B2RXR6	7	-16	-23	-16	-11	0.9	0.8	0.9	0.9
Phf12	Q5SPL2	335	-5	-23	-16	-14	1.0	0.8	0.9	0.9
Gdap2	Q9DBL2	349	-14	-23	-16	-25	0.9	0.8	0.9	0.8
Taok3	Q8BYC6	795	-20	-23	-16	-32	0.8	0.8	0.9	0.8
Pmpca	Q9DC61	224	-32	-24	-16	1	0.8	0.8	0.9	1.0
Cdk6	Q64261	15	-20	-24	-16	-4	0.8	0.8	0.9	1.0
Cdc42	P60766	6	-20	-24	-16	-11	0.8	0.8	0.9	0.9
Rac1	P63001	6	-20	-24	-16	-11	0.8	0.8	0.9	0.9
Rhog	P84096	6	-20	-24	-16	-11	0.8	0.8	0.9	0.9
Rac2	Q05144	6	-20	-24	-16	-11	0.8	0.8	0.9	0.9
Rhoq	Q8R527	12	-20	-24	-16	-11	0.8	0.8	0.9	0.9
Ndufs1	Q91VD9	64	-21	-24	-16	-15	0.8	0.8	0.9	0.9
Arcn1	Q5XJY5	441	-25	-25	-16	5	0.8	0.8	0.9	1.1
Dock10	E9QM99	244	-32	-25	-16	0	0.8	0.8	0.9	1.0
Paf1	Q8K2T8	31	-28	-25	-16	-5	0.8	0.8	0.9	1.0
Znf524	Q9D0B1	227	-14	-25	-16	-27	0.9	0.8	0.9	0.8
Ddx17	Q501J6	352	-26	-26	-16	-3	0.8	0.8	0.9	1.0
Fbxo30	Q8BJL1	563	-19	-27	-16	3	0.8	0.8	0.9	1.0
Huwe1	Q7TMY8	1891	-15	-28	-16	-3	0.9	0.8	0.9	1.0
Phf20l1	Q8CCJ9	405	-15	-29	-16	-35	0.9	0.8	0.9	0.7
Osbpl8	B9EJ86	266	-17	-30	-16	6	0.9	0.8	0.9	1.1
Rnh1	Q91VI7	80	-37	-30	-16	-7	0.7	0.8	0.9	0.9
Gbp2	Q9Z0E6	233	-29	-30	-16	-1	0.8	0.8	0.9	1.0
Tsr3	Q5HZH2	160	-25	-33	-16	4	0.8	0.8	0.9	1.0
Epc1	Q8C9X6	22	-20	-36	-16	-21	0.8	0.7	0.9	0.8
Hsp90aa1	P07901	421	-31	-42	-16	8	0.8	0.7	0.9	1.1
Apex1	P28352	92	-35	-63	-16	-69	0.7	0.6	0.9	0.6
Cep55	Q8BT07	441	-16	11	-16	-6	0.9	1.1	0.9	0.9
Thumpd1	Q99J36	248	-1	9	-16	24	1.0	1.1	0.9	1.3
Uba6	Q8C7R4	682	-2	7	-16	7	1.0	1.1	0.9	1.1
Pabpc4	Q6PHQ9	488	-1	5	-16	-11	1.0	1.1	0.9	0.9
Limd1	Q9QXD8	217	11	5	-16	-25	1.1	1.1	0.9	0.8
Taldo1	Q93092	250	-3	3	-16	18	1.0	1.0	0.9	1.2
Sacs	Q9JLC8	2516	-25	1	-16	1	0.8	1.0	0.9	1.0
Gm20503	G3UZK1	126	-9	0	-16	2	0.9	1.0	0.9	1.0
Iars2	Q8BIJ6	883	-3	-1	-16	5	1.0	1.0	0.9	1.0
Rnasel	Q05921	300	-16	-1	-16	4	0.9	1.0	0.9	1.0
Tnfaip3	Q60769	612	-20	-1	-16	-12	0.8	1.0	0.9	0.9
Srek1ip1	Q4V9W2	28	-5	-1	-16	-8	1.0	1.0	0.9	0.9
Rfx7	F8VPJ6	63	-8	-1	-16	-11	0.9	1.0	0.9	0.9
Pgd	Q9DCD0	171	-17	-2	-16	12	0.9	1.0	0.9	1.1
Lrrfip1	Q3UZ39	652	-9	-2	-16	-28	0.9	1.0	0.9	0.8
Cand1	Q6ZQ38	802	-8	-3	-16	24	0.9	1.0	0.9	1.3
Osgep	Q8BWU5	160	-14	-3	-16	15	0.9	1.0	0.9	1.2
Crot	Q9DC50	188	-14	-3	-16	2	0.9	1.0	0.9	1.0
Arid4a	F8VPQ2	243	-10	-3	-16	-13	0.9	1.0	0.9	0.9
Pgap1	Q3UUQ7	805	-4	-3	-16	-25	1.0	1.0	0.9	0.8
Ppp6r3	Q922D4	576	-18	-4	-16	18	0.9	1.0	0.9	1.2
Vps13c	Q8BX70	1096	-6	-4	-16	9	0.9	1.0	0.9	1.1

Ankrd17	Q99NH0	50	-16	-5	-16	-5	0.9	1.0	0.9	1.0
Zcchc6	E9PUA2	1409	-12	-5	-16	-21	0.9	1.0	0.9	0.8
Phf10	K4DI61	476	-15	-6	-16	1	0.9	0.9	0.9	1.0
Poc1b	Q8BHD1	399	-3	-6	-16	-4	1.0	0.9	0.9	1.0
Cdk6	Q64261	83	-8	-6	-16	-18	0.9	0.9	0.9	0.9
Tbc1d1	Q60949	788	-29	-6	-16	-38	0.8	0.9	0.9	0.7
Rnf213	E9Q555	2904	-7	-6	-16	-3	0.9	0.9	0.9	1.0
Arid4b	A2CG63	1115	-10	-6	-16	-15	0.9	0.9	0.9	0.9
Fam98c	E9PYD1	89	-11	-7	-16	-4	0.9	0.9	0.9	1.0
Ndufs2	Q91WD5	326	-16	-7	-16	27	0.9	0.9	0.9	1.4
Nanp	Q9CPT3	67	-14	-7	-16	-11	0.9	0.9	0.9	0.9
Specc1	Q5SXY1	492	-19	-8	-16	1	0.8	0.9	0.9	1.0
Tbc1d1	Q60949	54	-9	-8	-16	-10	0.9	0.9	0.9	0.9
Rab3ip	Q68EF0	375	-21	-8	-16	-13	0.8	0.9	0.9	0.9
Csnk2a2	O54833	336	-14	-8	-16	-24	0.9	0.9	0.9	0.8
Zfyve26	Q5DU37	2397	-14	-8	-16	26	0.9	0.9	0.9	1.3
Gsto1	O09131	32	-12	-8	-16	-1	0.9	0.9	0.9	1.0
Dhx9	E9QNN1	943	-17	-9	-16	-5	0.9	0.9	0.9	1.0
Wdfy4	E9Q2M9	1528	-10	-9	-16	-6	0.9	0.9	0.9	0.9
Mdh2	P08249	275	-28	-9	-16	11	0.8	0.9	0.9	1.1
Cstf3	Q99LI7	536	-13	-9	-16	-3	0.9	0.9	0.9	1.0
Bmper	Q8CJ69	646	-3	-9	-16	-13	1.0	0.9	0.9	0.9
Mycbp2	E9PUJ6	4626	-1	-9	-16	-15	1.0	0.9	0.9	0.9
Akr1b1	P45376	200	-5	-10	-16	6	1.0	0.9	0.9	1.1
Smc6	Q924W5	417	-7	-10	-16	-6	0.9	0.9	0.9	0.9
Kiaa0513	Q8R0A7	313	-14	-10	-16	-10	0.9	0.9	0.9	0.9
Ptprc	P06800	749	-13	-10	-16	7	0.9	0.9	0.9	1.1
Dido1	Q8C9B9	258	-1	-10	-16	-11	1.0	0.9	0.9	0.9
Tapbpl	Q8VD31	119	2	-10	-16	-13	1.0	0.9	0.9	0.9
Ptpn11	P35235	318	5	-10	-16	-13	1.1	0.9	0.9	0.9
Lypla2	Q9WTL7	56	-15	-11	-16	11	0.9	0.9	0.9	1.1
Gucd1	Q8BZI6	199	-12	-11	-16	-7	0.9	0.9	0.9	0.9
Hspbp1	Q99P31	22	-9	-11	-16	-11	0.9	0.9	0.9	0.9
Gimap4	Q99JY3	187	-20	-11	-16	4	0.8	0.9	0.9	1.0
Sirt1	Q923E4	494	-26	-11	-16	0	0.8	0.9	0.9	1.0
Hnrnpul2	Q00PI9	57	-17	-11	-16	-7	0.9	0.9	0.9	0.9
Lrch4	Q921G6	454	-11	-11	-16	-7	0.9	0.9	0.9	0.9
Rasa3	Q60790	206	-10	-11	-16	-11	0.9	0.9	0.9	0.9
Trappc12	Q8K2L8	793	-3	-11	-16	-12	1.0	0.9	0.9	0.9
Bcl7b	Q921K9	189	-15	-11	-16	-13	0.9	0.9	0.9	0.9
Srsf3	P84104	6	-8	-11	-16	-20	0.9	0.9	0.9	0.8
Ifi30	Q9ESY9	69	-11	-11	-16	-27	0.9	0.9	0.9	0.8
Dnmt1	P13864	232	-23	-12	-16	-10	0.8	0.9	0.9	0.9
Casp8	O89110	435	-5	-12	-16	-11	1.0	0.9	0.9	0.9
Fmnl1	Q9JL26	684	-13	-12	-16	-27	0.9	0.9	0.9	0.8
Uncharact	Q8C5K5	12	-15	-12	-16	3	0.9	0.9	0.9	1.0
Ints3	Q7TPD0	841	-11	-12	-16	-7	0.9	0.9	0.9	0.9
Sqstm1	Q64337	219	-7	-12	-16	-16	0.9	0.9	0.9	0.9
Il16	O54824	1004	-21	-13	-16	-19	0.8	0.9	0.9	0.8
Cdkn2aip	Q8BI72	499	-15	-13	-16	2	0.9	0.9	0.9	1.0
Agps	Q8C0I1	213	-10	-13	-16	1	0.9	0.9	0.9	1.0

Lrrc59	Q922Q8	59	-25	-13	-16	0	0.8	0.9	0.9	1.0
Pin1rt1	Q3ULQ2	109	-12	-13	-16	-8	0.9	0.9	0.9	0.9
Tuba4a	P68368	347	-15	-13	-16	-19	0.9	0.9	0.9	0.8
Lace1	Q3V384	72	-13	-13	-16	-19	0.9	0.9	0.9	0.8
Abce1	P61222	227	-7	-14	-16	15	0.9	0.9	0.9	1.2
Ppp4r2	Q0VGB7	136	2	-14	-16	3	1.0	0.9	0.9	1.0
Alkbh7	Q9D6Z0	15	-16	-14	-16	-5	0.9	0.9	0.9	1.0
Pcgf5	Q3UK78	145	-15	-14	-16	-9	0.9	0.9	0.9	0.9
Nccrp1	G3X9C2	149	-22	-14	-16	-11	0.8	0.9	0.9	0.9
Trip11	E9Q512	212	-14	-14	-16	-12	0.9	0.9	0.9	0.9
Ddx6	P54823	102	-10	-14	-16	-18	0.9	0.9	0.9	0.8
Ppp1r37	Q8BKR5	381	-13	-14	-16	-18	0.9	0.9	0.9	0.8
Adk	P55264	139	-17	-14	-16	33	0.9	0.9	0.9	1.5
Smg8	Q8VE18	248	-13	-14	-16	15	0.9	0.9	0.9	1.2
Ndrg3	Q9QYF9	166	-7	-14	-16	4	0.9	0.9	0.9	1.0
Cacybp	Q9CXW3	155	-21	-14	-16	1	0.8	0.9	0.9	1.0
Tmc6	Q7TN60	750	-7	-14	-16	-1	0.9	0.9	0.9	1.0
Trim25	Q61510	503	-12	-14	-16	-11	0.9	0.9	0.9	0.9
Grpel1	Q99LP6	108	-13	-15	-16	26	0.9	0.9	0.9	1.4
Ppip5k2	Q6ZQB6	906	-20	-15	-16	-18	0.8	0.9	0.9	0.9
Ddx24	Q9ESV0	134	-15	-15	-16	-24	0.9	0.9	0.9	0.8
Vdac2	Q60930	77	-13	-15	-16	-32	0.9	0.9	0.9	0.8
Tnfaip3	Q60769	575	-11	-15	-16	-15	0.9	0.9	0.9	0.9
Rpl11	Q9CXW4	150	-13	-15	-16	-21	0.9	0.9	0.9	0.8
Prpf4	Q9DAW6	262	-26	-16	-16	2	0.8	0.9	0.9	1.0
Pop1	Q8K205	735	-9	-16	-16	-8	0.9	0.9	0.9	0.9
Zfp236	S4R299	1215	-9	-16	-16	-14	0.9	0.9	0.9	0.9
Fam114a2	Q8VE88	490	-21	-16	-16	-14	0.8	0.9	0.9	0.9
Eif3a	P23116	78	-9	-16	-16	14	0.9	0.9	0.9	1.2
Cpsf2	O35218	294	-9	-16	-16	5	0.9	0.9	0.9	1.1
Rps11	P62281	131	-13	-16	-16	-16	0.9	0.9	0.9	0.9
Lrrk2	Q5S006	1123	-20	-16	-16	-28	0.8	0.9	0.9	0.8
Ubr4	A2AN08	3703	-22	-17	-16	2	0.8	0.9	0.9	1.0
Trim34a	Q99PP6	333	-5	-17	-16	1	1.0	0.9	0.9	1.0
Flnb	Q80X90	2289	-9	-17	-16	-6	0.9	0.9	0.9	0.9
Gm10263	J3QNN8	27	-13	-17	-16	-15	0.9	0.9	0.9	0.9
Ikbkb	O88351	299	-16	-17	-16	-16	0.9	0.9	0.9	0.9
Rpl28	P41105	13	-15	-17	-16	-23	0.9	0.9	0.9	0.8
Gnb2	P62880	204	-22	-18	-16	0	0.8	0.9	0.9	1.0
Ttc38	A3KMP2	49	-18	-18	-16	-2	0.9	0.9	0.9	1.0
Casp6	O08738	271	18	-18	-16	-15	1.2	0.9	0.9	0.9
Rtf1	A2AQ19	604	-20	-18	-16	-9	0.8	0.8	0.9	0.9
Ube2a	Q9Z255	152	-12	-18	-16	-14	0.9	0.8	0.9	0.9
Rdh13	Q8CEE7	201	-15	-18	-16	-17	0.9	0.8	0.9	0.9
Nedd9	O35177	18	-16	-18	-16	-24	0.9	0.8	0.9	0.8
Usp24	B1AY13	799	-15	-19	-16	-3	0.9	0.8	0.9	1.0
Pmpcb	Q9CXT8	62	-7	-19	-16	-10	0.9	0.8	0.9	0.9
Pus7l	Q8CE46	625	-16	-19	-16	-16	0.9	0.8	0.9	0.9
Mysm1	Q69Z66	436	-12	-19	-16	-5	0.9	0.8	0.9	1.0
Ivd	Q9JHI5	259	-27	-19	-16	-5	0.8	0.8	0.9	1.0
Spen	A2ADB0	900	-11	-19	-16	-15	0.9	0.8	0.9	0.9

Ppp4r2	Q0VGB7	30	-18	-20	-16	-11	0.9	0.8	0.9	0.9
Tacc1	Q6Y685	684	-10	-20	-16	-13	0.9	0.8	0.9	0.9
Ckap5	A2AGT5	1768	-22	-20	-16	-17	0.8	0.8	0.9	0.9
Ccz1	Q8C1Y8	356	-22	-21	-16	-7	0.8	0.8	0.9	0.9
Ncoa6	Q5XJV5	2006	-25	-22	-16	-13	0.8	0.8	0.9	0.9
Numa1	E9Q7G0	373	-12	-22	-16	-14	0.9	0.8	0.9	0.9
Tomm40	Q9QYA2	74	-25	-22	-16	-22	0.8	0.8	0.9	0.8
Plekhg3	Q4VAC9	929	-9	-22	-16	-6	0.9	0.8	0.9	0.9
Rasal3	Q8C2K5	557	-8	-22	-16	-12	0.9	0.8	0.9	0.9
9930111J2	Q5SVP0	625	-14	-22	-16	-18	0.9	0.8	0.9	0.8
Eef1g	Q9D8N0	194	-25	-23	-16	-1	0.8	0.8	0.9	1.0
Zfp362	B1ASA5	230	-17	-23	-16	2	0.9	0.8	0.9	1.0
Tigar	Q8BZA9	215	-12	-23	-16	-10	0.9	0.8	0.9	0.9
Itprip	Q3TNL8	148	-16	-24	-16	-108	0.9	0.8	0.9	0.5
Mki67	E9PVX6	130	-9	-25	-16	-22	0.9	0.8	0.9	0.8
Xdh	Q00519	995	-13	-26	-16	0	0.9	0.8	0.9	1.0
Ifi203	O35368	365	-25	-26	-16	-3	0.8	0.8	0.9	1.0
Rasa4	Q6PFQ7	760	-31	-27	-16	-25	0.8	0.8	0.9	0.8
Anp32e	P97822	87	-23	-28	-16	-9	0.8	0.8	0.9	0.9
Ppp6r3	Q922D4	801	-15	-28	-16	-37	0.9	0.8	0.9	0.7
Kif15	Q6P9L6	798	-12	-29	-16	-18	0.9	0.8	0.9	0.9
Sh3bp5	Q9Z131	441	-10	-29	-16	-40	0.9	0.8	0.9	0.7
Coq8b	Q566J8	318	-44	-30	-16	-13	0.7	0.8	0.9	0.9
Rad18	Q9QXK2	64	-22	-31	-16	-20	0.8	0.8	0.9	0.8
Cep192	E9Q4Y4	787	-29	-32	-16	-27	0.8	0.8	0.9	0.8
Mpp1	P70290	179	-18	-33	-16	27	0.8	0.8	0.9	1.4
Txn2	P97493	58	-3	-40	-16	-19	1.0	0.7	0.9	0.8
Apool	Q78IK4	152	-18	7	-17	4	0.8	1.1	0.9	1.0
Csnk1d	Q9DC28	41	0	5	-17	-2	1.0	1.1	0.9	1.0
Ppfia2	Q8BSS9	143	-5	1	-17	3	1.0	1.0	0.9	1.0
Ifit1bl2	Q3U687	264	-15	-1	-17	9	0.9	1.0	0.9	1.1
Nup160	Q9Z0W3	31	-7	-1	-17	-2	0.9	1.0	0.9	1.0
Lrrfip1	Q3UZ39	14	-5	-1	-17	-15	1.0	1.0	0.9	0.9
Psmd9	Q9CR00	215	-16	-2	-17	5	0.9	1.0	0.9	1.0
Ptk2b	Q9QVP9	650	4	-2	-17	12	1.0	1.0	0.9	1.1
Ap1b1	O35643	144	0	-3	-17	-1	1.0	1.0	0.9	1.0
Ap2b1	Q9DBG3	144	0	-3	-17	-1	1.0	1.0	0.9	1.0
Golga4	Q91VW5	1512	-16	-3	-17	-14	0.9	1.0	0.9	0.9
Sars	P26638	162	-9	-3	-17	1	0.9	1.0	0.9	1.0
Zap70	P43404	102	-15	-4	-17	5	0.9	1.0	0.9	1.1
Dffb	O54788	197	-9	-4	-17	4	0.9	1.0	0.9	1.0
Pdlim5	Q8CI51	213	-5	-4	-17	-4	1.0	1.0	0.9	1.0
Rnf146	Q9CZW6	354	-9	-4	-17	-10	0.9	1.0	0.9	0.9
Hsdl1	Q8BTX9	328	-5	-4	-17	-29	1.0	1.0	0.9	0.8
Ctps1	P70698	216	-18	-5	-17	-24	0.9	1.0	0.9	0.8
Ptgr2	Q8VDQ1	344	-10	-5	-17	-14	0.9	1.0	0.9	0.9
Dennd2d	Q91VV4	162	-7	-5	-17	-28	0.9	1.0	0.9	0.8
Msh6	P54276	1335	-3	-6	-17	6	1.0	0.9	0.9	1.1
Gm29609	Q3UU56	648	-20	-6	-17	-8	0.8	0.9	0.9	0.9
Map1s	Q8C052	552	-11	-6	-17	-9	0.9	0.9	0.9	0.9
Smarca2	Q6DIC0	91	-12	-6	-17	-13	0.9	0.9	0.9	0.9

Uba2	Q9Z1F9	161	-13	-6	-17	-16	0.9	0.9	0.9	0.9
D3Ertd751	Q8BGN2	26	-13	-6	-17	-21	0.9	0.9	0.9	0.8
Psmc3	O88685	243	-7	-6	-17	-45	0.9	0.9	0.9	0.7
Psmg4	P0C7N9	55	-19	-7	-17	-13	0.8	0.9	0.9	0.9
Rel	A4QPD3	143	-22	-7	-17	-2	0.8	0.9	0.9	1.0
Wdfy4	E9Q2M9	242	-30	-9	-17	-5	0.8	0.9	0.9	1.0
Mrps21	P58059	49	6	-9	-17	-10	1.1	0.9	0.9	0.9
Zzef1	Q5SSH7	2192	3	-9	-17	-18	1.0	0.9	0.9	0.8
Actr5	Q80US4	265	-14	-9	-17	10	0.9	0.9	0.9	1.1
Txndc12	Q9CQU0	64	-16	-9	-17	8	0.9	0.9	0.9	1.1
Engase	Q8BX80	406	-10	-9	-17	6	0.9	0.9	0.9	1.1
Cfl1	P18760	80	-5	-9	-17	-4	1.0	0.9	0.9	1.0
Dcaf8	Q8N7N5	267	-9	-9	-17	-11	0.9	0.9	0.9	0.9
Cct2	P80314	535	-11	-9	-17	-21	0.9	0.9	0.9	0.8
Wapl	Q65Z40	352	-11	-9	-17	-21	0.9	0.9	0.9	0.8
Vcpip1	Q8CDG3	396	-14	-10	-17	8	0.9	0.9	0.9	1.1
Sp140	Q6NSQ5	180	-15	-10	-17	-7	0.9	0.9	0.9	0.9
Kdm5b	Q80Y84	1395	-15	-10	-17	-9	0.9	0.9	0.9	0.9
Cd19	P25918	169	-16	-10	-17	-17	0.9	0.9	0.9	0.9
Cep170	Q6A065	959	-8	-11	-17	-22	0.9	0.9	0.9	0.8
Mdh2	P08249	212	-20	-11	-17	2	0.8	0.9	0.9	1.0
Atf7ip	Q7TT18	646	-10	-11	-17	2	0.9	0.9	0.9	1.0
Esd	Q9R0P3	181	-12	-11	-17	2	0.9	0.9	0.9	1.0
Rhbdf2	Q80WQ6	151	-11	-11	-17	-31	0.9	0.9	0.9	0.8
Idh3g	P70404	235	-17	-12	-17	14	0.9	0.9	0.9	1.2
Dock7	A2A9M4	193	-18	-12	-17	-7	0.9	0.9	0.9	0.9
Psmg2	Q9EST4	146	-25	-12	-17	-1	0.8	0.9	0.9	1.0
Rasa4	Q6PFQ7	654	-10	-12	-17	-8	0.9	0.9	0.9	0.9
Ankle2	Q6P1H6	629	-6	-12	-17	-13	0.9	0.9	0.9	0.9
Slfn5	Q8CBA2	115	-6	-12	-17	-13	0.9	0.9	0.9	0.9
Iqsec1	Q8R0S2	357	-12	-12	-17	-36	0.9	0.9	0.9	0.7
Lrba	E9Q3Y4	950	-7	-13	-17	-19	0.9	0.9	0.9	0.8
Hnrnpr	Q8VHM5	226	4	-13	-17	-4	1.0	0.9	0.9	1.0
Fhod1	Q6P9Q4	674	-18	-13	-17	-6	0.9	0.9	0.9	0.9
Dalrd3	Q6PJN8	59	-21	-13	-17	-7	0.8	0.9	0.9	0.9
Syne1	Q6ZWR6	4019	-7	-13	-17	-10	0.9	0.9	0.9	0.9
Acox1	Q9R0H0	199	-18	-13	-17	-23	0.8	0.9	0.9	0.8
Necap2	Q9D1J1	133	-3	-14	-17	1	1.0	0.9	0.9	1.0
Arhgap26	Q6ZQ82	441	-16	-14	-17	-4	0.9	0.9	0.9	1.0
Utp18	Q5SSI6	84	-15	-14	-17	-15	0.9	0.9	0.9	0.9
Mycbp2	E9PUJ6	4740	-17	-14	-17	-16	0.9	0.9	0.9	0.9
Tpr	F6ZDS4	112	-14	-14	-17	-19	0.9	0.9	0.9	0.8
Armc10	Q9D0L7	289	-11	-14	-17	-21	0.9	0.9	0.9	0.8
Cep70	Q6IQY5	243	-29	-14	-17	-22	0.8	0.9	0.9	0.8
Arpc5l	Q9D898	42	-7	-14	-17	-27	0.9	0.9	0.9	0.8
Hsp90ab1	P11499	564	-18	-14	-17	14	0.8	0.9	0.9	1.2
Bphl	Q8R164	225	-11	-14	-17	14	0.9	0.9	0.9	1.2
Hadh	Q61425	211	-20	-14	-17	-2	0.8	0.9	0.9	1.0
Colgalt1	Q8K297	407	-16	-14	-17	-21	0.9	0.9	0.9	0.8
Vezf1	Q5SXC4	327	-15	-14	-17	-29	0.9	0.9	0.9	0.8
Psmd2	Q8VDM4	251	-10	-15	-17	0	0.9	0.9	0.9	1.0

H2-Ab1	P14483	106	-16	-15	-17	-5	0.9	0.9	0.9	1.0
Gtpbp6	Q3U6U5	157	-23	-15	-17	-6	0.8	0.9	0.9	0.9
Helz2	E9QAM5	338	-12	-15	-17	-8	0.9	0.9	0.9	0.9
Bank1	Q80VH0	779	-18	-15	-17	-9	0.9	0.9	0.9	0.9
Htt	G3X9H5	1675	-12	-15	-17	-9	0.9	0.9	0.9	0.9
Kifc5b	E9PUA5	508	-13	-15	-17	-11	0.9	0.9	0.9	0.9
Macrod1	Q922B1	197	-12	-15	-17	-11	0.9	0.9	0.9	0.9
Kifc1	Q9QWT9	510	-13	-15	-17	-11	0.9	0.9	0.9	0.9
Dnmt3a	O88508	132	-14	-15	-17	-17	0.9	0.9	0.9	0.9
Rrp8	Q9DB85	333	-27	-15	-17	-39	0.8	0.9	0.9	0.7
Cbl	P22682	351	-12	-16	-17	-6	0.9	0.9	0.9	0.9
Ubr4	A2AN08	3427	-16	-16	-17	-8	0.9	0.9	0.9	0.9
Plaa	P27612	193	-17	-16	-17	-11	0.9	0.9	0.9	0.9
Nipbl	Q6KCD5	52	-12	-16	-17	-16	0.9	0.9	0.9	0.9
Them6	Q80ZW2	168	-11	-16	-17	-21	0.9	0.9	0.9	0.8
Rngtt	O55236	13	-37	-17	-17	9	0.7	0.9	0.9	1.1
Bcor	Q8CGN4	1492	-6	-17	-17	-8	0.9	0.9	0.9	0.9
Gmeb2	P58929	110	-11	-17	-17	-11	0.9	0.9	0.9	0.9
Gmeb1	Q9JL60	103	-11	-17	-17	-11	0.9	0.9	0.9	0.9
Ammecr1l	Q8JZZ6	6	-6	-17	-17	-11	0.9	0.9	0.9	0.9
Aldh9a1	Q9JLJ2	220	-17	-17	-17	-12	0.9	0.9	0.9	0.9
Galk2	Q68FH4	175	7	-17	-17	-305	1.1	0.9	0.9	0.2
Polr2b	Q8CFI7	945	-13	-18	-17	12	0.9	0.9	0.9	1.1
Ireb2	Q811J3	138	-9	-18	-17	5	0.9	0.9	0.9	1.1
Rhoc	Q62159	159	-1	-18	-17	-22	1.0	0.9	0.9	0.8
Kmt2d	Q6PDK2	4475	-10	-18	-17	-26	0.9	0.9	0.9	0.8
Sfxn2	Q925N2	18	-18	-18	-17	-4	0.8	0.8	0.9	1.0
Dalrd3	Q6PJN8	194	-13	-18	-17	-6	0.9	0.8	0.9	0.9
Ppp2r1a	Q76MZ3	148	-17	-19	-17	11	0.9	0.8	0.9	1.1
Ppp2r1b	Q7TNP2	160	-17	-19	-17	11	0.9	0.8	0.9	1.1
Tigd2	Q0VBL1	200	-13	-19	-17	8	0.9	0.8	0.9	1.1
Rcc2	Q8BK67	335	-11	-19	-17	8	0.9	0.8	0.9	1.1
Prkar2b	P31324	209	-21	-19	-17	-3	0.8	0.8	0.9	1.0
Uhrf1bp1l	A2RSJ4	476	-15	-19	-17	-26	0.9	0.8	0.9	0.8
Nup88	Q8CEC0	725	-15	-20	-17	-4	0.9	0.8	0.9	1.0
Dhx37	Q6NZL1	493	-8	-20	-17	-15	0.9	0.8	0.9	0.9
Zfp809	G3X9G7	338	-9	-20	-17	-11	0.9	0.8	0.9	0.9
Sri	Q6P069	75	-26	-21	-17	4	0.8	0.8	0.9	1.0
Ptpro	E9Q612	865	-5	-21	-17	-24	1.0	0.8	0.9	0.8
Casp8	O89110	411	-3	-21	-17	-32	1.0	0.8	0.9	0.8
Kdelr1	Q99JH8	29	-21	-21	-17	-11	0.8	0.8	0.9	0.9
Larp7	Q05CL8	333	-23	-21	-17	-20	0.8	0.8	0.9	0.8
Utrn	E9Q6R7	3074	-39	-21	-17	-23	0.7	0.8	0.9	0.8
Mepce	Q8K3A9	127	-7	-21	-17	-29	0.9	0.8	0.9	0.8
Gga2	Q6P5E6	384	-11	-22	-17	-2	0.9	0.8	0.9	1.0
Phf3	B2RQG2	1594	-12	-22	-17	-25	0.9	0.8	0.9	0.8
Mdn1	A2ANY6	975	-23	-22	-17	-20	0.8	0.8	0.9	0.8
Slk	O54988	358	-14	-22	-17	-30	0.9	0.8	0.9	0.8
Tpm3-rs7	D3Z2H9	226	-16	-22	-17	-55	0.9	0.8	0.9	0.6
Uba2	Q9Z1F9	173	-25	-23	-17	-13	0.8	0.8	0.9	0.9
Stat1	A0A087WSP5	174	-29	-23	-17	-2	0.8	0.8	0.9	1.0

Ctps1	P70698	218	-21	-24	-17	-9	0.8	0.8	0.9	0.9
Cdyl	Q9WTK2	510	-21	-24	-17	-8	0.8	0.8	0.9	0.9
Trappc8	E9PWG2	978	-10	-24	-17	-17	0.9	0.8	0.9	0.9
Akap11	E9Q777	1280	-15	-25	-17	-21	0.9	0.8	0.9	0.8
Cnst	Q8CBC4	446	-3	-25	-17	-28	1.0	0.8	0.9	0.8
Mtmr4	Q91XS1	1139	1	-26	-17	-3	1.0	0.8	0.9	1.0
Usp19	Q3UJD6	402	-17	-26	-17	-19	0.9	0.8	0.9	0.8
Ppm1h	Q3UYC0	56	-19	-26	-17	-31	0.8	0.8	0.9	0.8
Mdn1	A2ANY6	1485	-14	-26	-17	-18	0.9	0.8	0.9	0.9
Rnf169	E9Q7F2	176	-15	-26	-17	-23	0.9	0.8	0.9	0.8
Ptk2b	Q9QVP9	661	-18	-27	-17	1	0.8	0.8	0.9	1.0
Wwp2	Q9DBH0	186	-18	-27	-17	-28	0.9	0.8	0.9	0.8
Pi4ka	E9Q3L2	1764	-34	-27	-17	-38	0.7	0.8	0.9	0.7
Znf800	Q0VEE6	49	-17	-27	-17	-6	0.9	0.8	0.9	0.9
Pnpla6	Q3TRM4	1210	-8	-28	-17	-31	0.9	0.8	0.9	0.8
Glyr1	Q922P9	479	-15	-30	-17	4	0.9	0.8	0.9	1.0
Pigs	Q6PD26	408	-32	-30	-17	-7	0.8	0.8	0.9	0.9
Gcn1	E9PVA8	1781	-31	-30	-17	-8	0.8	0.8	0.9	0.9
Polr2a	P08775	1159	-7	-32	-17	1	0.9	0.8	0.9	1.0
Pold2	O35654	91	-24	-38	-17	-12	0.8	0.7	0.9	0.9
Rab18	P35293	160	-5	-39	-17	-33	1.0	0.7	0.9	0.8
Rtfdc1	Q99K95	139	-16	-39	-17	-29	0.9	0.7	0.9	0.8
Znf687	Q9D2D7	1140	-28	-40	-17	34	0.8	0.7	0.9	1.5
Xpo1	Q6P5F9	99	-24	-44	-17	-7	0.8	0.7	0.9	0.9
Otof	Q9ESF1	1064	-1	10	-17	15	1.0	1.1	0.9	1.2
Pqlc3	Q8C6U2	157	4	7	-17	18	1.0	1.1	0.9	1.2
Myo5a	Q99104	894	3	7	-17	-22	1.0	1.1	0.9	0.8
Mrpl11	Q9CQF0	122	-14	6	-17	-31	0.9	1.1	0.9	0.8
Bckdha	Q3U3J1	179	14	6	-17	-20	1.2	1.1	0.9	0.8
Pak1ip1	Q9DCE5	320	-11	5	-17	9	0.9	1.0	0.9	1.1
Gm21994	F6T2D2	227	-6	5	-17	-12	0.9	1.0	0.9	0.9
Cntrob	Q8CB62	322	-32	3	-17	-23	0.8	1.0	0.9	0.8
Dnttip1	Q99LB0	191	7	2	-17	-2	1.1	1.0	0.9	1.0
Ggnbp2	Q5SV77	236	-12	2	-17	-17	0.9	1.0	0.9	0.9
Gm20390	E9PZF0	260	-12	1	-17	-3	0.9	1.0	0.9	1.0
Ncor2	Q9WU42	1473	-6	0	-17	-16	0.9	1.0	0.9	0.9
Naa15	G3X8Y3	214	-5	-1	-17	-6	1.0	1.0	0.9	0.9
Zufsp	Q3T9Z9	164	-6	-1	-17	-5	0.9	1.0	0.9	1.0
Rpl10a	Q5XJF6	164	-16	-2	-17	6	0.9	1.0	0.9	1.1
Dusp12	Q9D0T2	308	-9	-2	-17	-15	0.9	1.0	0.9	0.9
Kmt5b	Q3U8K7	201	7	-2	-17	-27	1.1	1.0	0.9	0.8
Tln1	P26039	1927	-23	-3	-17	13	0.8	1.0	0.9	1.1
Anxa2	P07356	133	-22	-4	-17	11	0.8	1.0	0.9	1.1
Klhl6	Q6V595	337	-29	-4	-17	-38	0.8	1.0	0.9	0.7
Gmip	Q6PGG2	339	-7	-5	-17	-8	0.9	1.0	0.9	0.9
Ppp1cb	P62141	139	-4	-5	-17	-11	1.0	1.0	0.9	0.9
Ppp1cc	P63087	140	-4	-5	-17	-11	1.0	1.0	0.9	0.9
Bdh1	Q80XN0	63	-20	-5	-17	10	0.8	1.0	0.9	1.1
Lyn	P25911	381	-6	-5	-17	8	0.9	1.0	0.9	1.1
Hsp90b1	P08113	576	-22	-5	-17	1	0.8	1.0	0.9	1.0
Agpat1	O35083	273	-9	-6	-17	-21	0.9	0.9	0.9	0.8

Sephs1	Q8BH69	31	-10	-6	-17	-31	0.9	0.9	0.9	0.8
Tacc3	Q99LH8	89	-11	-6	-17	1	0.9	0.9	0.9	1.0
Brix1	Q9DCA5	52	-5	-6	-17	-8	1.0	0.9	0.9	0.9
Prpf40a	Q9R1C7	39	-14	-7	-17	6	0.9	0.9	0.9	1.1
Dars	Q922B2	76	-20	-7	-17	-3	0.8	0.9	0.9	1.0
Slc25a3	Q8VEM8	63	-13	-7	-17	-6	0.9	0.9	0.9	0.9
Fhod1	Q6P9Q4	376	-8	-7	-17	-15	0.9	0.9	0.9	0.9
Mphosph8	Q3TYA6	800	-5	-7	-17	23	1.0	0.9	0.9	1.3
Mast3	Q3U214	385	-15	-7	-17	-15	0.9	0.9	0.9	0.9
Ttf1	Q62187	246	-12	-7	-17	-19	0.9	0.9	0.9	0.8
Cuta	Q9CQ89	94	-28	-7	-17	-21	0.8	0.9	0.9	0.8
Bzw1	Q9CQC6	35	-3	-8	-17	-1	1.0	0.9	0.9	1.0
Atp6v1a	P50516	277	-11	-8	-17	-3	0.9	0.9	0.9	1.0
Lrrc47	Q505F5	544	-14	-8	-17	-13	0.9	0.9	0.9	0.9
Znf622	Q91VY9	157	-10	-8	-17	-23	0.9	0.9	0.9	0.8
Cops4	O88544	255	-8	-8	-17	19	0.9	0.9	0.9	1.2
Dffa	O54786	21	-15	-8	-17	1	0.9	0.9	0.9	1.0
Gstt3	Q99L20	238	-9	-8	-17	-7	0.9	0.9	0.9	0.9
A630001G	Q3UTB2	58	-3	-8	-17	-15	1.0	0.9	0.9	0.9
Zap70	P43404	609	-10	-8	-17	-16	0.9	0.9	0.9	0.9
Rftn1	Q6A0D4	496	-12	-8	-17	-26	0.9	0.9	0.9	0.8
Gda	Q9R111	63	-10	-8	-17	-30	0.9	0.9	0.9	0.8
Klhl6	Q6V595	254	-17	-9	-17	11	0.9	0.9	0.9	1.1
Anxa6	P14824	114	-21	-9	-17	3	0.8	0.9	0.9	1.0
Zbtb1	Q91VL9	227	-14	-9	-17	-4	0.9	0.9	0.9	1.0
Helz2	E9QAM5	2416	-15	-9	-17	-8	0.9	0.9	0.9	0.9
Rad18	Q9QXK2	144	-11	-9	-17	-17	0.9	0.9	0.9	0.9
Ap2a2	P17427	931	-31	-9	-17	18	0.8	0.9	0.9	1.2
Rnf213	E9Q555	2196	-11	-10	-17	7	0.9	0.9	0.9	1.1
Mus81	Q91ZJ0	16	-1	-10	-17	-3	1.0	0.9	0.9	1.0
Gm3839	S4R1W1	150	-7	-10	-17	-11	0.9	0.9	0.9	0.9
Epc2	Q8C0I4	544	-14	-10	-17	-19	0.9	0.9	0.9	0.8
Srpra	Q9DBG7	252	-10	-10	-17	-26	0.9	0.9	0.9	0.8
Clptm1	Q8VBZ3	645	-5	-10	-17	-29	1.0	0.9	0.9	0.8
Polg	Q75WC0	628	-19	-10	-17	15	0.8	0.9	0.9	1.2
Mdn1	A2ANY6	1801	-20	-10	-17	9	0.8	0.9	0.9	1.1
Wdfy4	E9Q2M9	2178	-8	-10	-17	-1	0.9	0.9	0.9	1.0
Fgfr1op2	Q9CRA9	198	-9	-10	-17	-14	0.9	0.9	0.9	0.9
Rcsd1	Q3UZA1	49	-1	-10	-17	-18	1.0	0.9	0.9	0.8
Tbc1d13	Q8R3D1	282	-11	-10	-17	-21	0.9	0.9	0.9	0.8
Gm7293	D3YYI5	153	-9	-11	-17	-11	0.9	0.9	0.9	0.9
Eri1	Q7TMF2	23	-5	-11	-17	-12	1.0	0.9	0.9	0.9
Rnf113a1	Q8R3P8	15	-15	-11	-17	-8	0.9	0.9	0.9	0.9
Memo1	Q91VH6	88	-28	-11	-17	-14	0.8	0.9	0.9	0.9
Fmnl2	A2APV2	734	-11	-12	-17	-59	0.9	0.9	0.9	0.6
Ankfy1	Q810B6	460	-16	-12	-17	17	0.9	0.9	0.9	1.2
Nop56	Q9D6Z1	112	-21	-12	-17	12	0.8	0.9	0.9	1.1
Hspa4	Q3U2G2	38	-18	-12	-17	7	0.8	0.9	0.9	1.1
Jmjd1c	Q69ZK6	378	-17	-12	-17	-22	0.9	0.9	0.9	0.8
Srp68	Q8BMA6	560	-12	-13	-17	-2	0.9	0.9	0.9	1.0
Thop1	Q8C1A5	253	-10	-13	-17	-9	0.9	0.9	0.9	0.9

Gdpd1	Q9CRY7	107	-18	-13	-17	-14	0.9	0.9	0.9	0.9
UbiE2	Q76I24	79	-9	-13	-17	-14	0.9	0.9	0.9	0.9
Akap12	Q9WTQ5	374	-3	-13	-17	-18	1.0	0.9	0.9	0.9
Srsf5	O35326	63	-11	-14	-17	-16	0.9	0.9	0.9	0.9
Uso1	Q9Z1Z0	678	-8	-14	-17	-24	0.9	0.9	0.9	0.8
Sfxn2	Q925N2	205	-12	-14	-17	19	0.9	0.9	0.9	1.2
Pole4	Q9CQ36	85	-17	-14	-17	-1	0.9	0.9	0.9	1.0
Eefsec	Q9JHW4	367	-15	-14	-17	-8	0.9	0.9	0.9	0.9
Dcaf13	Q6PAC3	190	-26	-14	-17	-10	0.8	0.9	0.9	0.9
Rcsd1	Q3UZA1	181	-18	-14	-17	-14	0.8	0.9	0.9	0.9
Gtpbp1	O08582	608	-8	-14	-17	-16	0.9	0.9	0.9	0.9
Irgq	Q8VIM9	370	-14	-14	-17	-22	0.9	0.9	0.9	0.8
Dhrs11	Q3U0B3	55	-8	-14	-17	-27	0.9	0.9	0.9	0.8
Carmil2	Q3V3V9	756	-15	-15	-17	23	0.9	0.9	0.9	1.3
Kat2a	Q9JHD2	688	-16	-15	-17	-5	0.9	0.9	0.9	1.0
Kat2b	Q9JHD1	671	-16	-15	-17	-5	0.9	0.9	0.9	1.0
Tpp2	Q64514	967	-10	-15	-17	-10	0.9	0.9	0.9	0.9
Lrch4	Q921G6	224	-24	-16	-17	0	0.8	0.9	0.9	1.0
Rnf31	Q924T7	58	-13	-16	-17	-4	0.9	0.9	0.9	1.0
Gpx4	O70325	93	-20	-16	-17	-19	0.8	0.9	0.9	0.8
Flnb	Q80X90	2537	-17	-16	-17	-19	0.9	0.9	0.9	0.8
Dicer1	F8VQ54	241	-14	-16	-17	-31	0.9	0.9	0.9	0.8
Rchy1	Q9CR50	164	-23	-16	-17	9	0.8	0.9	0.9	1.1
Rgs3	Q9DC04	903	-12	-16	-17	-36	0.9	0.9	0.9	0.7
Sema4d	O09126	707	-13	-17	-17	-4	0.9	0.9	0.9	1.0
Hdac1	O09106	408	-17	-17	-17	-26	0.9	0.9	0.9	0.8
Larp4	G3X9Q6	594	-17	-17	-17	-32	0.9	0.9	0.9	0.8
Znfx1	Q8R151	1813	-19	-17	-17	-5	0.8	0.9	0.9	1.0
Atad2b	E9Q166	99	-13	-17	-17	-29	0.9	0.9	0.9	0.8
Farsb	Q9WUA2	498	-8	-18	-17	18	0.9	0.9	0.9	1.2
Sfswap	Q3USH5	718	-13	-18	-17	7	0.9	0.9	0.9	1.1
Esco1	Q69Z69	379	-13	-18	-17	-11	0.9	0.9	0.9	0.9
Osbpl5	Q9ER64	423	-12	-18	-17	-23	0.9	0.9	0.9	0.8
Arfgef2	A2A5R2	620	8	-19	-17	-4	1.1	0.8	0.9	1.0
Rab27a	Q9ERI2	188	-19	-19	-17	-11	0.8	0.8	0.9	0.9
Dock7	A2A9M4	2115	-35	-19	-17	-12	0.7	0.8	0.9	0.9
Nup98	Q6PFD9	1067	-15	-19	-17	-16	0.9	0.8	0.9	0.9
Isg20l2	Q3U1G5	183	-9	-19	-17	-19	0.9	0.8	0.9	0.8
Atpaf1	Q811I0	26	-20	-19	-17	7	0.8	0.8	0.9	1.1
Def6	Q8C2K1	246	-8	-19	-17	-6	0.9	0.8	0.9	0.9
Ktn1	F8VQC7	955	-16	-19	-17	-11	0.9	0.8	0.9	0.9
Trim34a	Q99PP6	342	-13	-19	-17	-12	0.9	0.8	0.9	0.9
Xrn2	Q9DBR1	451	-14	-19	-17	-29	0.9	0.8	0.9	0.8
Wdr7	Q920I9	326	-22	-20	-17	-6	0.8	0.8	0.9	0.9
Top1	Q04750	735	-12	-20	-17	5	0.9	0.8	0.9	1.1
Nrde2	Q80XC6	789	-14	-20	-17	-6	0.9	0.8	0.9	0.9
Brd4	Q9ESU6	357	-14	-20	-17	-8	0.9	0.8	0.9	0.9
Orc1	Q9Z1N2	712	-21	-20	-17	-28	0.8	0.8	0.9	0.8
Acsl4	Q9QUJ7	420	-11	-21	-17	-5	0.9	0.8	0.9	1.0
Inpp4b	Q6P1Y8	130	-18	-21	-17	-13	0.9	0.8	0.9	0.9
Ubr1	O70481	1373	-27	-21	-17	7	0.8	0.8	0.9	1.1

Nipbl	Q6KCD5	2145	-17	-21	-17	3	0.9	0.8	0.9	1.0
Pml	Q60953	137	-9	-21	-17	2	0.9	0.8	0.9	1.0
Cmpk2	Q3U5Q7	226	-9	-21	-17	1	0.9	0.8	0.9	1.0
Gimap7	Q8R379	160	-35	-21	-17	-2	0.7	0.8	0.9	1.0
Cyfip1	Q7TMB8	346	11	-21	-17	-21	1.1	0.8	0.9	0.8
Hp1bp3	Q3TEA8	391	-19	-22	-17	-8	0.8	0.8	0.9	0.9
Cpne1	Q8C166	52	-20	-22	-17	-12	0.8	0.8	0.9	0.9
Vav1	P27870	794	-8	-22	-17	-15	0.9	0.8	0.9	0.9
Vav3	Q9R0C8	800	-8	-22	-17	-15	0.9	0.8	0.9	0.9
Samhd1	Q60710	624	-22	-22	-17	-29	0.8	0.8	0.9	0.8
Lats2	Q7TSJ6	771	-20	-23	-17	-19	0.8	0.8	0.9	0.8
Rbmx2	Q8R0F5	243	-7	-23	-17	-30	0.9	0.8	0.9	0.8
Pag1	Q3U1F9	352	-14	-23	-17	-32	0.9	0.8	0.9	0.8
Rps5	Q91V55	155	-20	-24	-17	-59	0.8	0.8	0.9	0.6
Trmt1l	A2RSY6	651	-29	-25	-17	-31	0.8	0.8	0.9	0.8
Dynll2	Q9D0M5	56	-20	-26	-17	-14	0.8	0.8	0.9	0.9
Sf1	Q64213	171	-29	-27	-17	1	0.8	0.8	0.9	1.0
Prep	Q9QUR6	601	-12	-27	-17	-68	0.9	0.8	0.9	0.6
Gnl3	Q8CI11	154	-18	-28	-17	-27	0.9	0.8	0.9	0.8
Lysmd3	Q99LE3	173	7	-29	-17	-26	1.1	0.8	0.9	0.8
Lrrc41	Q8K1C9	123	-29	-31	-17	-11	0.8	0.8	0.9	0.9
Bptf	A2A654	1503	-4	-31	-17	-32	1.0	0.8	0.9	0.8
Epm2aip1	Q8VEH5	338	-26	-32	-17	20	0.8	0.8	0.9	1.3
Ubr7	Q8BU04	374	-6	-32	-17	-9	0.9	0.8	0.9	0.9
Vwa8	Q8CC88	723	-27	-33	-17	-3	0.8	0.8	0.9	1.0
Phf23	Q8BSN5	379	-24	-33	-17	-11	0.8	0.8	0.9	0.9
Kmt5c	Q6Q783	355	-41	-34	-17	-29	0.7	0.7	0.9	0.8
Nop14	Q8R3N1	460	-6	-62	-17	-72	0.9	0.6	0.9	0.6
Mat2a	Q3THS6	56	7	12	-18	-1	1.1	1.1	0.9	1.0
Fam65b	Q80U16	172	-6	7	-18	-1	0.9	1.1	0.9	1.0
Stag1	Q9D3E6	179	-3	5	-18	24	1.0	1.0	0.9	1.3
Sacm1l	Q9EP69	392	-6	3	-18	0	0.9	1.0	0.9	1.0
Ilvbl	Q8BU33	315	-10	2	-18	3	0.9	1.0	0.9	1.0
Sf3b6	P59708	83	-21	1	-18	14	0.8	1.0	0.9	1.2
Casp2	P29594	305	2	0	-18	22	1.0	1.0	0.9	1.3
Uros	P51163	143	-10	-1	-18	9	0.9	1.0	0.9	1.1
Kif11	Q6P9P6	910	-6	-2	-18	-4	0.9	1.0	0.9	1.0
Rars2	Q3U186	576	-8	-2	-18	-13	0.9	1.0	0.9	0.9
Mad2l1	Q9Z1B5	178	-9	-3	-18	-8	0.9	1.0	0.9	0.9
Gys1	Q9Z1E4	699	-11	-3	-18	-12	0.9	1.0	0.9	0.9
Lrrc47	Q505F5	226	-23	-3	-18	3	0.8	1.0	0.9	1.0
N4bp2	F8VQG7	575	-15	-3	-18	-19	0.9	1.0	0.9	0.8
Fam213a	Q9CYH2	77	-3	-3	-18	-41	1.0	1.0	0.9	0.7
Ube2v2	Q9D2M8	69	-18	-4	-18	16	0.8	1.0	0.9	1.2
Zranb3	Q6NZP1	958	-14	-4	-18	-1	0.9	1.0	0.9	1.0
Sh3pxd2a	O89032	746	-17	-4	-18	-14	0.9	1.0	0.9	0.9
Grap2	O89100	238	-21	-5	-18	-12	0.8	1.0	0.9	0.9
Copb1	Q9JIF7	284	-15	-5	-18	18	0.9	1.0	0.9	1.2
Pcbd2	Q9CZL5	115	-20	-6	-18	-20	0.8	0.9	0.9	0.8
Dgka	O88673	357	10	-7	-18	-16	1.1	0.9	0.9	0.9
Ciao1	Q99KN2	234	-13	-7	-18	-19	0.9	0.9	0.9	0.8

Gart	Q64737	298	-16	-7	-18	7	0.9	0.9	0.9	1.1
Ranbp2	Q9ERU9	220	-6	-7	-18	-10	0.9	0.9	0.9	0.9
Wdr33	Q8K4P0	990	-11	-7	-18	-19	0.9	0.9	0.9	0.8
Mtch2	Q791V5	79	-19	-8	-18	-6	0.8	0.9	0.9	0.9
March6	Q6ZQ89	28	-19	-8	-18	-9	0.8	0.9	0.9	0.9
Lyst	G5E8Q0	1712	-23	-8	-18	-2	0.8	0.9	0.9	1.0
Fuk	Q7TMC8	787	-12	-8	-18	-7	0.9	0.9	0.9	0.9
Prpf38b	Q80SY5	114	-12	-8	-18	-7	0.9	0.9	0.9	0.9
Epx	P49290	456	-16	-8	-18	-18	0.9	0.9	0.9	0.8
Cox18	Q8VC74	130	-13	-8	-18	-47	0.9	0.9	0.9	0.7
Gon4l	K4DI71	2098	-10	-9	-18	-24	0.9	0.9	0.9	0.8
Otud4	B2RRE7	681	-8	-9	-18	2	0.9	0.9	0.9	1.0
Gvin1	L7N451	1289	-9	-9	-18	-10	0.9	0.9	0.9	0.9
Dhx8	A2A4P0	675	4	-10	-18	-1	1.0	0.9	0.9	1.0
Vcp	Q01853	184	-7	-10	-18	-7	0.9	0.9	0.9	0.9
Gimap9	G3X987	39	-14	-10	-18	-13	0.9	0.9	0.9	0.9
Pxk	Q8BX57	196	-10	-10	-18	-14	0.9	0.9	0.9	0.9
Ice2	Q3UZ18	969	-24	-10	-18	-19	0.8	0.9	0.9	0.8
Ptpn2	Q06180	16	-6	-10	-18	-32	0.9	0.9	0.9	0.8
Med28	Q920D3	93	-14	-10	-18	13	0.9	0.9	0.9	1.1
Hk3	Q3TRM8	449	-5	-10	-18	5	1.0	0.9	0.9	1.0
Obfc1	Q8K2X3	158	-18	-10	-18	-6	0.9	0.9	0.9	0.9
Taf9	Q8VI33	121	-21	-10	-18	-16	0.8	0.9	0.9	0.9
Dusp3	Q9D7X3	171	-9	-11	-18	5	0.9	0.9	0.9	1.0
Mepce	Q8K3A9	219	-6	-11	-18	-2	0.9	0.9	0.9	1.0
Ankhd1	E9PUR0	184	-11	-11	-18	-21	0.9	0.9	0.9	0.8
Ankrd17	Q99NH0	206	-11	-11	-18	-21	0.9	0.9	0.9	0.8
Sart1	Q9Z315	680	-11	-11	-18	-21	0.9	0.9	0.9	0.8
Wdr44	Q6NVE8	718	-8	-11	-18	12	0.9	0.9	0.9	1.1
Atp13a2	Q9CTG6	360	-6	-11	-18	1	0.9	0.9	0.9	1.0
Ube2o	Q6ZPJ3	336	-11	-11	-18	-10	0.9	0.9	0.9	0.9
Vps8	Q0P5W1	1394	-13	-11	-18	-22	0.9	0.9	0.9	0.8
Tnfaip3	Q60769	662	-5	-11	-18	-26	1.0	0.9	0.9	0.8
Fam175a	Q8BPZ8	186	-5	-12	-18	-3	1.0	0.9	0.9	1.0
Iars	Q8BU30	27	-1	-12	-18	-11	1.0	0.9	0.9	0.9
Nfkb2	Q9WTK5	57	-23	-12	-18	-27	0.8	0.9	0.9	0.8
Pms2	B9EJ22	650	-7	-12	-18	-11	0.9	0.9	0.9	0.9
Ano6	Q6P9J9	107	-7	-13	-18	-4	0.9	0.9	0.9	1.0
Trim33	Q99PP7	166	-22	-13	-18	-4	0.8	0.9	0.9	1.0
Ubr2	Q6WKZ8	1717	-17	-13	-18	-18	0.9	0.9	0.9	0.8
Agap2	Q3UHD9	542	-30	-13	-18	-8	0.8	0.9	0.9	0.9
Zfp263	Q8CF60	285	-3	-13	-18	-20	1.0	0.9	0.9	0.8
Pacs2	E9Q7E9	861	-12	-14	-18	2	0.9	0.9	0.9	1.0
Med15	G3X8S4	661	-8	-14	-18	-13	0.9	0.9	0.9	0.9
Ilk	O55222	422	-21	-14	-18	-16	0.8	0.9	0.9	0.9
Nup155	Q99P88	1344	-10	-14	-18	-17	0.9	0.9	0.9	0.9
Abcf3	Q8K268	522	-11	-14	-18	-45	0.9	0.9	0.9	0.7
Dus1l	Q8C2P3	265	-11	-14	-18	9	0.9	0.9	0.9	1.1
Uba6	Q8C7R4	347	-15	-14	-18	-8	0.9	0.9	0.9	0.9
Usp7	E9PXY8	752	-21	-14	-18	-12	0.8	0.9	0.9	0.9
Tbc1d15	Q9CXF4	666	3	-14	-18	-18	1.0	0.9	0.9	0.9

Rnf169	E9Q7F2	191	-2	-14	-18	-27	1.0	0.9	0.9	0.8
Uba2	Q9Z1F9	158	-20	-15	-18	-14	0.8	0.9	0.9	0.9
Atad1	Q9D5T0	359	-12	-15	-18	-36	0.9	0.9	0.9	0.7
Zmym3	Q9JLM4	486	3	-15	-18	-11	1.0	0.9	0.9	0.9
Arid4b	A2CG63	25	-13	-15	-18	-15	0.9	0.9	0.9	0.9
Arid4a	F8VPQ2	25	-13	-15	-18	-15	0.9	0.9	0.9	0.9
Rgs14	P97492	236	-14	-15	-18	-19	0.9	0.9	0.9	0.8
Nsd1	E9QAE4	943	-24	-15	-18	-27	0.8	0.9	0.9	0.8
Rps5	Q91V55	172	-10	-15	-18	-73	0.9	0.9	0.9	0.6
Def6	Q8C2K1	267	-12	-16	-18	-23	0.9	0.9	0.9	0.8
Irf9	Q61179	313	-22	-16	-18	15	0.8	0.9	0.9	1.2
Sacm1l	Q9EP69	344	-18	-16	-18	-10	0.9	0.9	0.9	0.9
Strn4	P58404	720	-8	-16	-18	-22	0.9	0.9	0.9	0.8
Oas3	Q8VI93	470	-17	-16	-18	-24	0.9	0.9	0.9	0.8
Gnai3	Q9DC51	139	-20	-17	-18	3	0.8	0.9	0.9	1.0
Cdk11b	P24788	421	-17	-17	-18	-14	0.9	0.9	0.9	0.9
Pom121	Q8K3Z9	435	-13	-17	-18	-29	0.9	0.9	0.9	0.8
Cdk13	Q69ZA1	987	-16	-17	-18	18	0.9	0.9	0.9	1.2
Ppp1r35	Q9D8C8	149	-16	-17	-18	-7	0.9	0.9	0.9	0.9
Nup85	Q8R480	614	-18	-17	-18	-14	0.8	0.9	0.9	0.9
Pfkfb4	Q6DTY7	106	-18	-17	-18	-16	0.9	0.9	0.9	0.9
Sla	Q60898	76	-38	-18	-18	-1	0.7	0.9	0.9	1.0
Dync1h1	Q9JHU4	1997	24	-18	-18	-14	1.3	0.9	0.9	0.9
Usp47	Q8BY87	1139	-15	-18	-18	-20	0.9	0.9	0.9	0.8
Tcf20	Q9EPQ8	1438	-19	-18	-18	-21	0.8	0.9	0.9	0.8
Birc6	O88738	4775	-15	-18	-18	8	0.9	0.8	0.9	1.1
Plekhf2	Q91WB4	186	-12	-18	-18	-6	0.9	0.8	0.9	0.9
Prr14l	E9Q7C4	1389	-4	-18	-18	-9	1.0	0.8	0.9	0.9
Ep300	B2RWS6	1182	-31	-18	-18	-17	0.8	0.8	0.9	0.9
Crebbp	F8VPR5	1220	-31	-18	-18	-17	0.8	0.8	0.9	0.9
Cdca7	Q9D0M2	350	-13	-19	-18	-2	0.9	0.8	0.9	1.0
Cyld	Q80TQ2	930	-18	-19	-18	-4	0.9	0.8	0.9	1.0
Gnl3	Q8CI11	303	-6	-19	-18	-18	0.9	0.8	0.9	0.8
Safb	D3YXK2	303	-15	-19	-18	-22	0.9	0.8	0.9	0.8
Safb2	Q80YR5	297	-15	-19	-18	-22	0.9	0.8	0.9	0.8
Ldah	Q8BVA5	275	-25	-19	-18	6	0.8	0.8	0.9	1.1
Gart	Q64737	93	-22	-19	-18	3	0.8	0.8	0.9	1.0
Arhgap25	Q8BYW1	449	-11	-19	-18	-8	0.9	0.8	0.9	0.9
Maf1	Q9D0U6	133	-13	-19	-18	-11	0.9	0.8	0.9	0.9
Zbtb11	G5E8B9	796	-19	-19	-18	-18	0.8	0.8	0.9	0.9
Kiaa1109	A2AAE1	1053	-38	-19	-18	-21	0.7	0.8	0.9	0.8
Bcat2	O35855	229	-22	-19	-18	-21	0.8	0.8	0.9	0.8
Msh3	A0A087WQ16	132	-10	-19	-18	-22	0.9	0.8	0.9	0.8
Dock8	Q8C147	143	-11	-20	-18	-9	0.9	0.8	0.9	0.9
Lage3	Q9CR70	22	-11	-20	-18	-12	0.9	0.8	0.9	0.9
Rpl3	P27659	157	-24	-20	-18	-36	0.8	0.8	0.9	0.7
Nisch	Q80TM9	169	1	-20	-18	-5	1.0	0.8	0.9	1.0
Bola2	Q8BGS2	31	-13	-20	-18	-7	0.9	0.8	0.9	0.9
Itch	Q8C863	57	-2	-20	-18	-7	1.0	0.8	0.9	0.9
Pxk	Q8BX57	425	-13	-20	-18	-11	0.9	0.8	0.9	0.9
Mprip	P97434	956	8	-20	-18	-20	1.1	0.8	0.9	0.8

Ganc	A2AQJ8	836	-16	-20	-18	-34	0.9	0.8	0.9	0.7
Fhod1	Q6P9Q4	438	-18	-21	-18	-17	0.9	0.8	0.9	0.9
Tmem62	Q8BXJ9	325	-19	-21	-18	-29	0.8	0.8	0.9	0.8
Pik3ap1	Q9EQ32	359	-19	-21	-18	-1	0.8	0.8	0.9	1.0
Ring1	O35730	87	-3	-21	-18	-11	1.0	0.8	0.9	0.9
Rnf2	Q9CQJ4	90	-3	-21	-18	-11	1.0	0.8	0.9	0.9
Ddx3x	Q62167	128	1	-21	-18	-18	1.0	0.8	0.9	0.8
Aars	Q8BGQ7	947	-8	-22	-18	-7	0.9	0.8	0.9	0.9
Psmd8	Q9CX56	176	-13	-22	-18	-17	0.9	0.8	0.9	0.9
Usp34	Q6ZQ93	1523	-15	-22	-18	-22	0.9	0.8	0.9	0.8
Rhof	Q8BYP3	121	-14	-22	-18	-39	0.9	0.8	0.9	0.7
Anapc1	P53995	575	-21	-22	-18	-40	0.8	0.8	0.9	0.7
Myh9	Q8VDD5	671	-22	-23	-18	3	0.8	0.8	0.9	1.0
Atp13a1	Q9EPE9	528	-11	-24	-18	4	0.9	0.8	0.9	1.0
Gnl2	Q99LH1	257	-19	-24	-18	0	0.8	0.8	0.9	1.0
Tm9sf3	Q9ET30	426	-16	-24	-18	-20	0.9	0.8	0.9	0.8
Clec16a	Q80U30	364	-17	-24	-18	-7	0.9	0.8	0.9	0.9
Rnf213	E9Q555	3469	-9	-24	-18	-28	0.9	0.8	0.9	0.8
Abcb1a	P21447	427	-17	-25	-18	-11	0.9	0.8	0.9	0.9
Rpl37	Q9D823	37	-19	-25	-18	-14	0.8	0.8	0.9	0.9
Akna	Q80VW7	226	-15	-25	-18	-22	0.9	0.8	0.9	0.8
Gle1	Q8R322	289	-29	-26	-18	0	0.8	0.8	0.9	1.0
Dhx15	O35286	750	-25	-26	-18	-3	0.8	0.8	0.9	1.0
Phf1	Q9Z1B8	327	-21	-26	-18	-18	0.8	0.8	0.9	0.9
Zfp41	Q02526	185	-22	-26	-18	-31	0.8	0.8	0.9	0.8
Sirt7	Q8BKJ9	80	-19	-27	-18	-36	0.8	0.8	0.9	0.7
Ptprcap	Q64697	79	-15	-27	-18	-43	0.9	0.8	0.9	0.7
Nup205	A0A0J9YUD5	1280	-13	-28	-18	5	0.9	0.8	0.9	1.0
Klhl6	Q6V595	282	-33	-28	-18	3	0.8	0.8	0.9	1.0
St13	Q99L47	208	-8	-28	-18	-3	0.9	0.8	0.9	1.0
Prps1	Q9D7G0	165	-23	-31	-18	3	0.8	0.8	0.9	1.0
Top2a	Q01320	454	-25	-31	-18	0	0.8	0.8	0.9	1.0
Sirt6	P59941	18	-23	-31	-18	-4	0.8	0.8	0.9	1.0
Mrpl27	Q99N92	88	-36	-33	-18	-40	0.7	0.8	0.9	0.7
Pikfyve	Q9Z1T6	723	5	-34	-18	-4	1.1	0.7	0.9	1.0
Gbe1	Q9D6Y9	81	2	-35	-18	-13	1.0	0.7	0.9	0.9
Mkln1	O89050	411	-8	-42	-18	-13	0.9	0.7	0.9	0.9
Lancl1	O89112	264	-14	11	-18	0	0.9	1.1	0.8	1.0
Idh3b	Q91VA7	232	-24	7	-18	4	0.8	1.1	0.8	1.0
Ppp1r21	Q3TDD9	468	-24	6	-18	1	0.8	1.1	0.8	1.0
Ankrd27	Q3UMR0	166	-4	6	-18	-14	1.0	1.1	0.8	0.9
Bles03	Q8VD62	173	-2	4	-18	-17	1.0	1.0	0.8	0.9
Prdx1	P35700	71	-16	4	-18	18	0.9	1.0	0.8	1.2
Me2	Q99KE1	185	5	2	-18	6	1.1	1.0	0.8	1.1
Lrrc25	Q8K1T1	274	0	2	-18	-22	1.0	1.0	0.8	0.8
Nbeal2	Q6ZQA0	1487	-9	1	-18	3	0.9	1.0	0.8	1.0
Cnnm3	Q32NY4	628	-20	0	-18	-12	0.8	1.0	0.8	0.9
Flad1	Q8R123	314	-10	-3	-18	-21	0.9	1.0	0.8	0.8
Ncoa3	Q05BA5	762	-14	-4	-18	26	0.9	1.0	0.8	1.4
Rpp25l	Q99JH1	131	6	-4	-18	-4	1.1	1.0	0.8	1.0
Hip1	Q8VD75	220	-6	-4	-18	-19	0.9	1.0	0.8	0.8

Chtf18	Q8BIW9	368	-15	-5	-18	-13	0.9	1.0	0.8	0.9
Cyld	Q80TQ2	905	-18	-5	-18	-14	0.9	1.0	0.8	0.9
Sema4b	Q62179	807	-13	-6	-18	-21	0.9	0.9	0.8	0.8
Chm	A2AD03	175	-19	-6	-18	-21	0.8	0.9	0.8	0.8
Actr2	P61161	221	-12	-6	-18	9	0.9	0.9	0.8	1.1
Arfgef2	A2A5R2	1457	-17	-7	-18	22	0.9	0.9	0.8	1.3
Recql5	Q8VID5	823	-12	-7	-18	-21	0.9	0.9	0.8	0.8
Arfgef1	G3X9K3	1523	-8	-7	-18	12	0.9	0.9	0.8	1.1
Actn4	P57780	61	-4	-8	-18	6	1.0	0.9	0.8	1.1
Actn1	Q7TPR4	41	-4	-8	-18	6	1.0	0.9	0.8	1.1
Ddx6	P54823	390	-15	-8	-18	14	0.9	0.9	0.8	1.2
Zmym2	Q9CU65	447	-21	-8	-18	-4	0.8	0.9	0.8	1.0
Ubr2	Q6WKZ8	1360	-13	-8	-18	-9	0.9	0.9	0.8	0.9
Birc6	O88738	780	-7	-8	-18	-15	0.9	0.9	0.8	0.9
Tap1	P21958	463	-13	-8	-18	-18	0.9	0.9	0.8	0.8
Vps13d	B1ART2	3796	-4	-8	-18	-26	1.0	0.9	0.8	0.8
Gm20671	F6TVX7	467	-7	-8	-18	-49	0.9	0.9	0.8	0.7
Vav1	P27870	71	-38	-9	-18	3	0.7	0.9	0.8	1.0
Pfas	Q5SUR0	294	-14	-9	-18	-3	0.9	0.9	0.8	1.0
Ppm1b	P36993	172	-12	-9	-18	-8	0.9	0.9	0.8	0.9
Wdr81	Q5ND34	196	-9	-9	-18	-17	0.9	0.9	0.8	0.9
Aldoa	P05064	202	-16	-9	-18	13	0.9	0.9	0.8	1.1
Prr12	E9PYL2	414	-4	-9	-18	-2	1.0	0.9	0.8	1.0
Gnpnat1	Q9JK38	128	-41	-9	-18	-15	0.7	0.9	0.8	0.9
Ahnak	E9Q616	518	-17	-9	-18	-19	0.9	0.9	0.8	0.8
Mapk10	E9QN59	313	-13	-10	-18	-15	0.9	0.9	0.8	0.9
Crkl	P47941	249	-12	-10	-18	-18	0.9	0.9	0.8	0.9
Fxr2	Q6P5B5	282	-18	-10	-18	-26	0.9	0.9	0.8	0.8
Pde2a	F7D3W5	143	-28	-10	-18	-2	0.8	0.9	0.8	1.0
Gm7293	D3YYI5	157	-8	-10	-18	-9	0.9	0.9	0.8	0.9
Armt1	A6H630	82	-13	-10	-18	-17	0.9	0.9	0.8	0.9
Acbd6	Q9D061	267	-10	-10	-18	-19	0.9	0.9	0.8	0.8
Aldh2	P47738	68	-12	-10	-18	-29	0.9	0.9	0.8	0.8
Vps11	Q91W86	890	-16	-11	-18	7	0.9	0.9	0.8	1.1
Odr4	Q4PJX1	231	-7	-11	-18	-11	0.9	0.9	0.8	0.9
Grb2	Q60631	198	-23	-11	-18	-17	0.8	0.9	0.8	0.9
Rps16	P14131	25	-8	-11	-18	-27	0.9	0.9	0.8	0.8
Gne	Q91WG8	183	-10	-11	-18	-43	0.9	0.9	0.8	0.7
RUS1	Q91W34	355	-15	-12	-18	-10	0.9	0.9	0.8	0.9
Unc93b1	Q8VCW4	584	-16	-12	-18	-11	0.9	0.9	0.8	0.9
Plec	Q9QXS1	3015	-25	-12	-18	-12	0.8	0.9	0.8	0.9
Trim27	Q62158	359	-18	-12	-18	-23	0.9	0.9	0.8	0.8
Dnaaf5	B9EJR8	136	-12	-12	-18	-6	0.9	0.9	0.8	0.9
Utp14a	Q640M1	736	-10	-12	-18	-14	0.9	0.9	0.8	0.9
Kat6b	Q8BRB7	253	-10	-12	-18	-16	0.9	0.9	0.8	0.9
Zcchc11	B2RX14	983	-11	-12	-18	-34	0.9	0.9	0.8	0.7
Paip1	Q8VE62	391	-8	-13	-18	-12	0.9	0.9	0.8	0.9
Zcchc11	B2RX14	766	-18	-13	-18	-16	0.8	0.9	0.8	0.9
Rps20	P60867	70	-14	-13	-18	-21	0.9	0.9	0.8	0.8
Gcn1	E9PVA8	2647	-27	-13	-18	6	0.8	0.9	0.8	1.1
L3mbtl2	P59178	408	-3	-13	-18	-1	1.0	0.9	0.8	1.0

Pgm2	Q7TSV4	581	-13	-13	-18	-8	0.9	0.9	0.8	0.9
Phka2	Q8BWJ3	681	-14	-13	-18	-10	0.9	0.9	0.8	0.9
Cdk12	Q14AX6	846	-15	-14	-18	9	0.9	0.9	0.8	1.1
Cdk13	Q69ZA1	828	-15	-14	-18	9	0.9	0.9	0.8	1.1
UPF0585	Q9DCS2	63	-4	-14	-18	-1	1.0	0.9	0.8	1.0
Trappc11	B2RXC1	41	-23	-14	-18	-19	0.8	0.9	0.8	0.8
Mtmr12	Q80TA6	332	-16	-14	-18	-8	0.9	0.9	0.8	0.9
Glutaredo:	Q9CWB7	40	-10	-14	-18	-97	0.9	0.9	0.8	0.5
Acot9	Q9R0X4	299	-21	-15	-18	9	0.8	0.9	0.8	1.1
Arid4b	A2CG63	107	-22	-15	-18	-7	0.8	0.9	0.8	0.9
Gnl3	Q8CI11	156	-14	-15	-18	-16	0.9	0.9	0.8	0.9
Stx5	Q8K1E0	68	-15	-15	-18	-29	0.9	0.9	0.8	0.8
Limk2	O54785	329	-11	-15	-18	5	0.9	0.9	0.8	1.1
Pml	Q60953	489	-7	-15	-18	-17	0.9	0.9	0.8	0.9
Atp5e	P56382	19	-29	-15	-18	-23	0.8	0.9	0.8	0.8
Casp1	P29452	220	-15	-16	-18	-9	0.9	0.9	0.8	0.9
Idh3a	Q9D6R2	127	-18	-16	-18	-18	0.9	0.9	0.8	0.9
Psmd13	Q9WVJ2	253	-7	-16	-18	-18	0.9	0.9	0.8	0.9
Tmem209	Q8BRG8	295	-18	-17	-18	-4	0.8	0.9	0.8	1.0
Polr2b	Q8CFI7	892	-7	-17	-18	0	0.9	0.9	0.8	1.0
Hk3	Q3TRM8	54	-17	-17	-18	-15	0.9	0.9	0.8	0.9
Dhx37	Q6NZL1	1076	-8	-17	-18	-17	0.9	0.9	0.8	0.9
Ralbp1	Q62172	622	-20	-17	-18	-33	0.8	0.9	0.8	0.8
Uba2	Q9Z1F9	185	-4	-18	-18	6	1.0	0.9	0.8	1.1
Tango2	Q8BTN3	231	-11	-18	-18	-3	0.9	0.9	0.8	1.0
Zfp960	L7N267	389	-26	-18	-18	-1	0.8	0.8	0.8	1.0
Mon2	Q80TL7	211	-15	-18	-18	-11	0.9	0.8	0.8	0.9
Ccdc117	Q6PB51	256	-15	-18	-18	-13	0.9	0.8	0.8	0.9
Setd2	E9Q5F9	1100	-4	-18	-18	-15	1.0	0.8	0.8	0.9
Kmt2e	Q3UG20	723	-12	-18	-18	-20	0.9	0.8	0.8	0.8
Ccdc85b	Q6PDY0	81	-25	-19	-18	3	0.8	0.8	0.8	1.0
Rrp12	Q6P5B0	289	-14	-19	-18	-15	0.9	0.8	0.8	0.9
Ep400	Q8CHI8	1929	-7	-19	-18	5	0.9	0.8	0.8	1.0
Mcm5	Q52KC3	197	-7	-19	-18	-9	0.9	0.8	0.8	0.9
Rufy1	Q8BIJ7	87	-17	-19	-18	-28	0.9	0.8	0.8	0.8
Ddx51	Q6P9R1	310	-24	-20	-18	-7	0.8	0.8	0.8	0.9
Cep135	Q6P5D4	473	-9	-20	-18	-21	0.9	0.8	0.8	0.8
Brwd1	Q921C3	1938	-17	-20	-18	-41	0.9	0.8	0.8	0.7
Znf280d	Q68FE8	667	-11	-20	-18	2	0.9	0.8	0.8	1.0
Zfp512b	Q6PHP4	502	-17	-20	-18	-28	0.9	0.8	0.8	0.8
Cops6	O88545	261	-5	-21	-18	-4	1.0	0.8	0.8	1.0
Ate1	Q9Z2A5	239	-13	-21	-18	-9	0.9	0.8	0.8	0.9
Bcl11b	Q99PV8	628	-8	-21	-18	-27	0.9	0.8	0.8	0.8
Wapl	Q65Z40	133	-15	-21	-18	-27	0.9	0.8	0.8	0.8
Vav1	P27870	652	-24	-22	-18	-9	0.8	0.8	0.8	0.9
Coq5	Q9CXI0	244	-13	-22	-18	-22	0.9	0.8	0.8	0.8
Cblb	B9EKI5	685	-13	-22	-18	-22	0.9	0.8	0.8	0.8
Ep300	B2RWS6	365	-19	-23	-18	6	0.8	0.8	0.8	1.1
Alad	P10518	223	-16	-23	-18	-2	0.9	0.8	0.8	1.0
Mau2	Q9D2X5	258	-4	-23	-18	-5	1.0	0.8	0.8	1.0
Mars	Q68FL6	335	-10	-23	-18	-36	0.9	0.8	0.8	0.7

Hspa4	Q3U2G2	34	-15	-24	-18	-18	0.9	0.8	0.8	0.8
Thnsl1	Q8BH55	82	-12	-24	-18	-22	0.9	0.8	0.8	0.8
D17Wsu92	Q3TT38	18	-28	-25	-18	-19	0.8	0.8	0.8	0.8
Sart3	Q9JLI8	670	-14	-25	-18	-25	0.9	0.8	0.8	0.8
Nubp1	Q9R060	277	14	-25	-18	-32	1.2	0.8	0.8	0.8
Prmt1	Q9JIF0	250	-13	-25	-18	-57	0.9	0.8	0.8	0.6
Mib2	Q8R516	265	-11	-25	-18	6	0.9	0.8	0.8	1.1
Bclaf1	Q8K019	686	-14	-25	-18	-22	0.9	0.8	0.8	0.8
Dpp6	Q9Z218	493	-16	-27	-18	37	0.9	0.8	0.8	1.6
Sept1	P42209	259	-21	-27	-18	-19	0.8	0.8	0.8	0.8
Syne1	Q6ZWR6	8705	-18	-27	-18	-26	0.9	0.8	0.8	0.8
Lap3	Q9CPY7	462	-26	-28	-18	-25	0.8	0.8	0.8	0.8
Mcm7	Q61881	482	-5	-29	-18	2	1.0	0.8	0.8	1.0
Ncf4	P97369	84	-17	-29	-18	-8	0.9	0.8	0.8	0.9
Ppp1r11	A5A4Y9	66	-18	-29	-18	-11	0.8	0.8	0.8	0.9
Nsun2	Q1HFZ0	221	-9	-29	-18	-42	0.9	0.8	0.8	0.7
Stk19	Q9JHN8	121	-5	-30	-18	-14	1.0	0.8	0.8	0.9
Ddx54	Q8K4L0	466	-23	-30	-18	-19	0.8	0.8	0.8	0.8
Parn	Q8VDG3	400	-7	-30	-18	-32	0.9	0.8	0.8	0.8
Gtf3c1	Q8K284	1086	-15	-31	-18	-18	0.9	0.8	0.8	0.8
Stk10	O55098	721	-18	-31	-18	-2	0.8	0.8	0.8	1.0
Gclc	P97494	613	-26	-32	-18	-11	0.8	0.8	0.8	0.9
Arl6ip6	Q8BH07	48	-18	-32	-18	-27	0.9	0.8	0.8	0.8
Zc3h14	Q8BJ05	261	-28	-37	-18	-37	0.8	0.7	0.8	0.7
Znf513	Q6PD29	183	-11	-40	-18	-22	0.9	0.7	0.8	0.8
Rock2	F8VPK5	314	-26	-43	-18	-14	0.8	0.7	0.8	0.9
Lrrfip1	Q3UZ39	381	-5	4	-19	-11	1.0	1.0	0.8	0.9
Fam213b	Q9DB60	12	-11	2	-19	5	0.9	1.0	0.8	1.0
Rubcn	Q80U62	733	-15	2	-19	-26	0.9	1.0	0.8	0.8
Myo9b	E9PZW8	863	-5	0	-19	-60	1.0	1.0	0.8	0.6
Ube2z	Q3UE37	288	-18	-1	-19	8	0.8	1.0	0.8	1.1
Fasn	P19096	223	-9	-2	-19	-13	0.9	1.0	0.8	0.9
Suclg1	Q9WUM5	60	-8	-2	-19	-16	0.9	1.0	0.8	0.9
Hmces	Q8R1M0	265	-20	-2	-19	-16	0.8	1.0	0.8	0.9
Rpl3	P27659	114	-21	-3	-19	-12	0.8	1.0	0.8	0.9
Gsdmdc1	Q9D8T2	192	-7	-4	-19	-34	0.9	1.0	0.8	0.7
H2-Eb1	O78196	38	-2	-5	-19	-29	1.0	1.0	0.8	0.8
Ets1	P27577	106	-24	-6	-19	4	0.8	0.9	0.8	1.0
Ep300	B2RWS6	1737	-4	-6	-19	3	1.0	0.9	0.8	1.0
Crebbp	F8VPR5	1776	-4	-6	-19	3	1.0	0.9	0.8	1.0
Tubgcp6	G5E8P0	302	-19	-6	-19	-13	0.8	0.9	0.8	0.9
Supt5h	O55201	734	-34	-6	-19	-20	0.7	0.9	0.8	0.8
Smarcad1	Q04692	82	-14	-6	-19	-33	0.9	0.9	0.8	0.8
Leng8	Q8CBY3	585	-1	-6	-19	5	1.0	0.9	0.8	1.0
Parp4	E9PYK3	1265	-5	-8	-19	-11	1.0	0.9	0.8	0.9
Polr1e	Q8K202	201	-3	-8	-19	-12	1.0	0.9	0.8	0.9
Camk2g	Q923T9	385	-16	-8	-19	-16	0.9	0.9	0.8	0.9
Hk3	Q3TRM8	34	-14	-8	-19	-22	0.9	0.9	0.8	0.8
H2-K1	P01901	358	-12	-8	-19	-33	0.9	0.9	0.8	0.8
Acot7	Q91V12	101	-18	-9	-19	8	0.8	0.9	0.8	1.1
UPF0505	Q8BWQ6	29	-15	-9	-19	-1	0.9	0.9	0.8	1.0

Zfp85	B8JJY3	188	-8	-9	-19	-5	0.9	0.9	0.8	1.0
Zfp273	Q7M6W8	148	-8	-9	-19	-5	0.9	0.9	0.8	1.0
Cand1	Q6ZQ38	356	-7	-9	-19	14	0.9	0.9	0.8	1.2
Gpd1l	Q3ULJ0	267	-17	-9	-19	5	0.9	0.9	0.8	1.1
Tars	Q9D0R2	629	-7	-9	-19	-8	0.9	0.9	0.8	0.9
UPF0568	Q9CQE8	19	-10	-9	-19	-9	0.9	0.9	0.8	0.9
Ap3d1	O54774	1199	-13	-9	-19	-19	0.9	0.9	0.8	0.8
Poldip3	Q8BG81	303	-22	-9	-19	-19	0.8	0.9	0.8	0.8
Sh3bgrl2	Q8BG73	10	-11	-10	-19	-16	0.9	0.9	0.8	0.9
Psmg1	Q9JK23	183	-22	-10	-19	-20	0.8	0.9	0.8	0.8
Kpna2	P52293	237	-9	-10	-19	10	0.9	0.9	0.8	1.1
Vbp1	P61759	112	-15	-10	-19	-3	0.9	0.9	0.8	1.0
Itch	Q8C863	796	-12	-10	-19	-13	0.9	0.9	0.8	0.9
Wwp2	Q9DBH0	802	-12	-10	-19	-13	0.9	0.9	0.8	0.9
Ppfibp2	O35711	145	-10	-11	-19	-18	0.9	0.9	0.8	0.8
Sec24d	Q6NXL1	1022	-2	-12	-19	14	1.0	0.9	0.8	1.2
Suclg1	Q9WUM5	172	-18	-12	-19	1	0.9	0.9	0.8	1.0
Camsap1	A2AHC3	1053	2	-12	-19	-2	1.0	0.9	0.8	1.0
Usp48	Q3V0C5	39	-18	-12	-19	-3	0.8	0.9	0.8	1.0
Nfkb2	Q9WTK5	83	-15	-12	-19	-3	0.9	0.9	0.8	1.0
Farsa	Q8C0C7	60	-40	-12	-19	-4	0.7	0.9	0.8	1.0
Rapgef2	Q8CHG7	419	-8	-12	-19	-7	0.9	0.9	0.8	0.9
Nrde2	Q80XC6	141	-18	-12	-19	-16	0.9	0.9	0.8	0.9
Acox3	Q9EPL9	534	-11	-12	-19	-21	0.9	0.9	0.8	0.8
Wdr81	Q5ND34	1443	-14	-12	-19	-24	0.9	0.9	0.8	0.8
Kmt2b	O08550	1755	1	-12	-19	7	1.0	0.9	0.8	1.1
Fhod1	Q6P9Q4	43	-10	-12	-19	4	0.9	0.9	0.8	1.0
Spast	Q9QYY8	218	-18	-12	-19	-12	0.9	0.9	0.8	0.9
Kif2a	P28740	405	-19	-12	-19	-21	0.8	0.9	0.8	0.8
Kmt2a	P55200	2779	-11	-12	-19	-47	0.9	0.9	0.8	0.7
Galk1	Q9R0N0	170	-15	-12	-19	-56	0.9	0.9	0.8	0.6
Uba2	Q9Z1F9	430	-17	-13	-19	-8	0.9	0.9	0.8	0.9
Rasal1	Q9Z268	767	-13	-13	-19	-11	0.9	0.9	0.8	0.9
Rab30	Q923S9	168	-13	-13	-19	-15	0.9	0.9	0.8	0.9
Rnh1	Q91VI7	251	-25	-14	-19	2	0.8	0.9	0.8	1.0
Atrx	Q61687	222	-22	-14	-19	-2	0.8	0.9	0.8	1.0
Zfp472	B0V2W5	294	-15	-14	-19	-9	0.9	0.9	0.8	0.9
Ubash3a	Q3V3E1	513	-12	-14	-19	-9	0.9	0.9	0.8	0.9
Eef1b	O70251	50	-9	-14	-19	-23	0.9	0.9	0.8	0.8
Hnrnpll	Q921F4	554	-15	-15	-19	-19	0.9	0.9	0.8	0.8
Arpc1b	Q9WV32	70	-10	-15	-19	8	0.9	0.9	0.8	1.1
Mpnd	Q3TV65	133	-9	-15	-19	5	0.9	0.9	0.8	1.1
Dock11	A2AF47	1663	-15	-15	-19	3	0.9	0.9	0.8	1.0
Rabep2	Q91WG2	357	-18	-15	-19	-12	0.9	0.9	0.8	0.9
Uvrag	Q8K245	166	-9	-15	-19	-14	0.9	0.9	0.8	0.9
Sec24c	G3X972	1085	-7	-16	-19	21	0.9	0.9	0.8	1.3
Hectd1	Q69ZR2	2229	-29	-16	-19	-8	0.8	0.9	0.8	0.9
Zmym4	A2A791	557	-13	-16	-19	-5	0.9	0.9	0.8	1.0
Nrf1	Q9WU00	134	-15	-16	-19	-7	0.9	0.9	0.8	0.9
Tlk1	Q8C0V0	81	-17	-16	-19	-13	0.9	0.9	0.8	0.9
Cltc	Q68FD5	459	-18	-17	-19	6	0.8	0.9	0.8	1.1

Pkm	P52480	49	-18	-17	-19	5	0.8	0.9	0.8	1.0
Gnpnat1	Q9JK38	157	-10	-17	-19	-16	0.9	0.9	0.8	0.9
Ahctf1	Q8CJF7	1266	-4	-17	-19	3	1.0	0.9	0.8	1.0
Gm28043	E0CXC2	233	-13	-17	-19	-18	0.9	0.9	0.8	0.9
Rfc1	G3UWX1	561	-2	-18	-19	-34	1.0	0.9	0.8	0.7
Git1	Q68FF6	149	-34	-18	-19	10	0.7	0.8	0.8	1.1
Git2	Q9JLQ2	149	-34	-18	-19	10	0.7	0.8	0.8	1.1
Itgb2	Q542I8	191	-28	-18	-19	-29	0.8	0.8	0.8	0.8
Snrnp200	Q6P4T2	428	-17	-19	-19	-4	0.9	0.8	0.8	1.0
Cog1	Q9Z160	693	-9	-19	-19	20	0.9	0.8	0.8	1.3
Ncbp1	Q3UYV9	332	-9	-19	-19	7	0.9	0.8	0.8	1.1
Hint1	P70349	38	-19	-19	-19	-7	0.8	0.8	0.8	0.9
Pip4k2b	Q80XI4	30	-10	-19	-19	-28	0.9	0.8	0.8	0.8
Ppm1m	E9Q2I3	172	-14	-20	-19	-17	0.9	0.8	0.8	0.9
Gripap1	Q8VD04	104	-23	-20	-19	-19	0.8	0.8	0.8	0.8
Pbrm1	Q8BSQ9	1163	-19	-21	-19	-7	0.8	0.8	0.8	0.9
Pmm2	Q9Z2M7	132	-17	-21	-19	-15	0.9	0.8	0.8	0.9
Morf4l2	Q9R0Q4	165	-13	-21	-19	-21	0.9	0.8	0.8	0.8
Ctu1	Q99J10	210	-16	-22	-19	-6	0.9	0.8	0.8	0.9
Fam65b	Q80U16	60	-14	-22	-19	-18	0.9	0.8	0.8	0.8
Ptprcap	Q64697	133	-7	-22	-19	-39	0.9	0.8	0.8	0.7
Smarcc1	P97496	163	-21	-23	-19	-9	0.8	0.8	0.8	0.9
Alox15	P39654	197	-21	-24	-19	-10	0.8	0.8	0.8	0.9
Usp7	E9PXY8	1002	-14	-24	-19	-22	0.9	0.8	0.8	0.8
Nfkbie	O54910	208	-31	-24	-19	-25	0.8	0.8	0.8	0.8
Sec63	Q8VHE0	295	-7	-24	-19	-29	0.9	0.8	0.8	0.8
Gle1	Q8R322	529	-24	-24	-19	20	0.8	0.8	0.8	1.2
Zwint	Q9CQU5	222	-13	-24	-19	-4	0.9	0.8	0.8	1.0
Znf638	Q61464	1912	-23	-25	-19	4	0.8	0.8	0.8	1.0
Macf1	E9PVY8	5716	-22	-25	-19	-15	0.8	0.8	0.8	0.9
Spata5	Q3UMC0	290	-13	-25	-19	-17	0.9	0.8	0.8	0.9
Mgea5	Q9EQQ9	663	-29	-25	-19	-35	0.8	0.8	0.8	0.7
Dis3l2	Q8CI75	376	-27	-26	-19	9	0.8	0.8	0.8	1.1
Fkbp8	O35465	264	-14	-27	-19	1	0.9	0.8	0.8	1.0
Rpl24	Q8BP67	36	-16	-27	-19	-8	0.9	0.8	0.8	0.9
Pdlim2	Q8R1G6	133	-10	-30	-19	-14	0.9	0.8	0.8	0.9
Dock6	E9QPN7	1917	-24	-31	-19	-18	0.8	0.8	0.8	0.9
Ap1m2	Q9WVP1	241	-8	-34	-19	-6	0.9	0.7	0.8	0.9
Chd4	Q6PDQ2	1012	-15	-37	-19	32	0.9	0.7	0.8	1.5
Rbm17	Q8JZX4	306	-27	-39	-19	-17	0.8	0.7	0.8	0.9
Zfp512b	Q6PHP4	773	-31	-39	-19	-1	0.8	0.7	0.8	1.0
Jade2	Q6ZQF7	254	-31	-39	-19	-1	0.8	0.7	0.8	1.0
Impa1	Q924B0	141	-17	5	-19	8	0.9	1.0	0.8	1.1
Smg1	Q8BKX6	2736	-26	2	-19	3	0.8	1.0	0.8	1.0
Nub1	P54729	51	-8	-1	-19	-16	0.9	1.0	0.8	0.9
Cltc	Q68FD5	736	-21	-3	-19	8	0.8	1.0	0.8	1.1
Poll	Q9QXE2	444	-22	-3	-19	-6	0.8	1.0	0.8	0.9
Sae1	Q9R1T2	346	-16	-3	-19	-69	0.9	1.0	0.8	0.6
Cdt1	Q8R4E9	266	-2	-4	-19	-6	1.0	1.0	0.8	0.9
Zfp62	Q8C827	805	-4	-4	-19	-15	1.0	1.0	0.8	0.9
Ptcd2	Q8R3K3	230	-16	-5	-19	-22	0.9	1.0	0.8	0.8

Rsbn1	Q80T69	520	-15	-5	-19	-6	0.9	1.0	0.8	0.9
Ldah	Q8BVA5	295	-7	-5	-19	-42	0.9	1.0	0.8	0.7
Toe1	Q9D2E2	292	4	-6	-19	-22	1.0	0.9	0.8	0.8
Nubp1	Q9R060	31	-13	-6	-19	-28	0.9	0.9	0.8	0.8
Tmx1	Q8VBT0	106	-25	-6	-19	-43	0.8	0.9	0.8	0.7
Pik3cd	O35904	147	-14	-7	-19	-5	0.9	0.9	0.8	1.0
Wdr1	O88342	325	-19	-7	-19	5	0.8	0.9	0.8	1.0
Il4r	P16382	673	-7	-8	-19	-15	0.9	0.9	0.8	0.9
Tbc1d15	Q9CXF4	197	-14	-8	-19	9	0.9	0.9	0.8	1.1
Srsf3	P84104	72	-23	-8	-19	-3	0.8	0.9	0.8	1.0
Eif3d	O70194	19	-34	-8	-19	-5	0.7	0.9	0.8	1.0
Setd1b	Q8CFT2	1386	-7	-8	-19	-15	0.9	0.9	0.8	0.9
Sec31a	Q3UPL0	690	-8	-9	-19	23	0.9	0.9	0.8	1.3
Arhgef7	Q9ES28	427	-15	-9	-19	11	0.9	0.9	0.8	1.1
Nsun2	Q1HFZ0	598	-11	-9	-19	-5	0.9	0.9	0.8	1.0
Zcchc7	B1AX39	260	-21	-9	-19	-11	0.8	0.9	0.8	0.9
Rreb1	Q3UH06	1256	-9	-9	-19	-12	0.9	0.9	0.8	0.9
Uncharact	A0A1B0GS18	113	-18	-9	-19	-10	0.9	0.9	0.8	0.9
Gcn1	E9PVA8	939	-10	-9	-19	-74	0.9	0.9	0.8	0.6
Snx9	Q91VH2	502	-16	-10	-19	-4	0.9	0.9	0.8	1.0
Elane	Q3UP87	187	-13	-10	-19	-32	0.9	0.9	0.8	0.8
Supt6h	Q62383	1435	-11	-11	-19	-4	0.9	0.9	0.8	1.0
Pdcd7	Q3U2X6	338	-5	-11	-19	2	1.0	0.9	0.8	1.0
Znf639	Q99KZ6	419	-17	-11	-19	1	0.9	0.9	0.8	1.0
Ahcy	P50247	195	-18	-11	-19	-4	0.8	0.9	0.8	1.0
Phgdh	Q61753	48	-15	-11	-19	-10	0.9	0.9	0.8	0.9
Dok2	O70469	39	-16	-11	-19	-18	0.9	0.9	0.8	0.9
Nnt	Q8BGK0	685	-14	-11	-19	-24	0.9	0.9	0.8	0.8
Hmha1	Q3TBD2	778	-16	-12	-19	22	0.9	0.9	0.8	1.3
Fam134a	Q6NS82	182	-13	-12	-19	10	0.9	0.9	0.8	1.1
Parn	Q8VDG3	128	-24	-12	-19	3	0.8	0.9	0.8	1.0
Hk3	Q3TRM8	611	-18	-12	-19	-28	0.8	0.9	0.8	0.8
Map2k3	O09110	29	-15	-12	-19	1	0.9	0.9	0.8	1.0
Cand1	Q6ZQ38	237	-31	-13	-19	11	0.8	0.9	0.8	1.1
Trim34a	Q99PP6	332	-14	-13	-19	-2	0.9	0.9	0.8	1.0
Ubn1	Q4G0F8	711	-11	-13	-19	-19	0.9	0.9	0.8	0.8
Mpi	Q924M7	81	-13	-14	-19	1	0.9	0.9	0.8	1.0
Mlxip	G5E8D8	107	-19	-14	-19	-10	0.8	0.9	0.8	0.9
Fam63b	Q6PDI6	197	-11	-14	-19	-14	0.9	0.9	0.8	0.9
Arhgap11a	Q80Y19	115	-24	-14	-19	-18	0.8	0.9	0.8	0.8
Cmtr1	Q9DBC3	9	-11	-14	-19	-31	0.9	0.9	0.8	0.8
Ppp5c	Q60676	11	-14	-15	-19	-17	0.9	0.9	0.8	0.9
Aimp2	Q8R010	168	-5	-15	-19	-10	1.0	0.9	0.8	0.9
Mrpl16	Q99N93	167	-10	-15	-19	-14	0.9	0.9	0.8	0.9
Arhgap17	Q3UIA2	305	-23	-15	-19	-18	0.8	0.9	0.8	0.9
Ppm1e	Q80TL0	154	-32	-16	-19	-6	0.8	0.9	0.8	0.9
Pms1	Q8K119	794	-24	-16	-19	-22	0.8	0.9	0.8	0.8
Ankrd50	F7BE84	1290	-1	-16	-19	12	1.0	0.9	0.8	1.1
Hdgfrp2	Q3UMU9	492	-8	-16	-19	-9	0.9	0.9	0.8	0.9
Pycard	Q9EPB4	171	-14	-16	-19	-15	0.9	0.9	0.8	0.9
Samhd1	Q60710	614	-14	-16	-19	-19	0.9	0.9	0.8	0.8

Abracl	E9QMV2	39	-8	-17	-19	-9	0.9	0.9	0.8	0.9
Kif16b	B1AVY7	814	12	-17	-19	-19	1.1	0.9	0.8	0.8
Trub1	Q8C0D0	320	-16	-17	-19	-27	0.9	0.9	0.8	0.8
Cct8	P42932	430	-18	-17	-19	-7	0.9	0.9	0.8	0.9
Sept6	Q9R1T4	269	-20	-18	-19	12	0.8	0.9	0.8	1.1
Tbc1d15	Q9CXF4	184	-14	-18	-19	-24	0.9	0.9	0.8	0.8
Utrn	E9Q6R7	1086	-23	-19	-19	-17	0.8	0.8	0.8	0.9
Phf5a	P83870	72	-14	-20	-19	-10	0.9	0.8	0.8	0.9
Brap	Q99MP8	47	-17	-20	-19	-16	0.9	0.8	0.8	0.9
Sptan1	P16546	1314	-15	-20	-19	-10	0.9	0.8	0.8	0.9
Chaf1a	Q9QWF0	17	-13	-20	-19	-12	0.9	0.8	0.8	0.9
Acss2	Q9QXG4	75	-22	-21	-19	-2	0.8	0.8	0.8	1.0
Sqstm1	Q64337	131	-26	-21	-19	-6	0.8	0.8	0.8	0.9
Rbm33	Q9CXK9	795	-14	-21	-19	-19	0.9	0.8	0.8	0.8
Keap1	Q9Z2X8	257	-10	-21	-19	-22	0.9	0.8	0.8	0.8
Plekho1	Q9JIY0	268	-12	-21	-19	-32	0.9	0.8	0.8	0.8
Acbd3	Q8BMP6	166	-19	-22	-19	-13	0.8	0.8	0.8	0.9
Krit1	Q6S5J6	134	-14	-22	-19	-17	0.9	0.8	0.8	0.9
Ppwd1	Q8CEC6	287	-19	-23	-19	-7	0.8	0.8	0.8	0.9
Ppp2r1b	Q7TNP2	389	-18	-24	-19	-24	0.8	0.8	0.8	0.8
Slc25a1	Q8JZU2	262	-25	-24	-19	-11	0.8	0.8	0.8	0.9
Zfp407	G3UVV3	1279	-21	-24	-19	-47	0.8	0.8	0.8	0.7
Hcfc2	G5E837	602	-17	-26	-19	-17	0.9	0.8	0.8	0.9
Ddx24	Q9ESV0	832	-19	-26	-19	-23	0.8	0.8	0.8	0.8
Bach2	P97303	237	-17	-26	-19	-23	0.9	0.8	0.8	0.8
Rsf1	E9PWW9	270	-17	-26	-19	-31	0.9	0.8	0.8	0.8
Nxf1	Q99JX7	142	-22	-27	-19	-6	0.8	0.8	0.8	0.9
Epb42	P49222	399	-9	-27	-19	-10	0.9	0.8	0.8	0.9
Gramd1a	Q8VEF1	482	0	-28	-19	-5	1.0	0.8	0.8	1.0
Eef2	P58252	290	-28	-31	-19	-10	0.8	0.8	0.8	0.9
Edc3	Q8K2D3	137	-15	-31	-19	-55	0.9	0.8	0.8	0.6
Mybbp1a	Q7TPV4	890	-17	-32	-19	19	0.9	0.8	0.8	1.2
Vps11	Q91W86	660	-1	-32	-19	-5	1.0	0.8	0.8	1.0
Pfn1	P62962	71	-42	-33	-19	-2	0.7	0.8	0.8	1.0
Arhgap15	Q811M1	205	-33	-56	-19	-45	0.8	0.6	0.8	0.7
Ube2q1	Q7TSS2	65	-6	23	-20	9	0.9	1.3	0.8	1.1
Ifit1bl1	D3Z6F0	325	-15	10	-20	-7	0.9	1.1	0.8	0.9
Sec24c	G3X972	495	-12	8	-20	-4	0.9	1.1	0.8	1.0
Anxa4	P97429	108	-30	4	-20	2	0.8	1.0	0.8	1.0
Tbc1d9b	Q5SVR0	289	-26	2	-20	28	0.8	1.0	0.8	1.4
Mapk10	E9QN59	231	-6	2	-20	-1	0.9	1.0	0.8	1.0
Mapk9	Q9WTU6	163	-6	2	-20	-1	0.9	1.0	0.8	1.0
Rps11	P62281	116	-17	1	-20	-14	0.9	1.0	0.8	0.9
Cltc	Q68FD5	918	-21	-2	-20	1	0.8	1.0	0.8	1.0
Lrch4	Q921G6	179	-12	-4	-20	7	0.9	1.0	0.8	1.1
Gnpda1	O88958	239	1	-5	-20	-1	1.0	1.0	0.8	1.0
Pitpna	P53810	95	-13	-5	-20	-9	0.9	1.0	0.8	0.9
Pitpnb	P53811	94	-13	-5	-20	-9	0.9	1.0	0.8	0.9
Arrb2	Q91YI4	271	-3	-5	-20	-9	1.0	1.0	0.8	0.9
Rack1	P68040	153	-27	-5	-20	-36	0.8	1.0	0.8	0.7
Tlr7	P58681	445	-27	-5	-20	13	0.8	1.0	0.8	1.1

Wdr4	Q9EP82	139	-12	-5	-20	-19	0.9	1.0	0.8	0.8
Sp140	Q6NSQ5	262	-11	-6	-20	3	0.9	0.9	0.8	1.0
Irf8	P23611	304	-13	-6	-20	-24	0.9	0.9	0.8	0.8
Ireb2	Q811J3	618	1	-7	-20	0	1.0	0.9	0.8	1.0
Tdg	P56581	244	-13	-7	-20	-15	0.9	0.9	0.8	0.9
Gsr	P47791	355	-9	-7	-20	-4	0.9	0.9	0.8	1.0
Dctn4	Q8CBY8	114	-38	-8	-20	-3	0.7	0.9	0.8	1.0
Foxo1	Q9R1E0	87	-13	-8	-20	-28	0.9	0.9	0.8	0.8
Skap2	Q3UND0	190	-13	-9	-20	-12	0.9	0.9	0.8	0.9
Gimap4	Q99JY3	61	-12	-9	-20	-22	0.9	0.9	0.8	0.8
Idh3a	Q9D6R2	331	-21	-9	-20	-22	0.8	0.9	0.8	0.8
Sh3bgrl	Q9JJU8	73	-9	-9	-20	-25	0.9	0.9	0.8	0.8
Lonp1	Q8CGK3	671	-11	-9	-20	-28	0.9	0.9	0.8	0.8
Heatr5a	Q5PRF0	1771	-9	-10	-20	5	0.9	0.9	0.8	1.1
Skiv2l	Q6NZR5	911	-14	-10	-20	1	0.9	0.9	0.8	1.0
Mlst8	Q9DCJ1	298	-23	-10	-20	-10	0.8	0.9	0.8	0.9
Bcas3	Q8CCN5	307	-13	-10	-20	-11	0.9	0.9	0.8	0.9
Cltc	Q68FD5	1257	-16	-11	-20	8	0.9	0.9	0.8	1.1
Arhgef10l	A2AWP8	765	-30	-11	-20	-2	0.8	0.9	0.8	1.0
Ptges3	Q9R0Q7	40	-2	-11	-20	-6	1.0	0.9	0.8	0.9
Jak3	Q62137	115	-30	-11	-20	-6	0.8	0.9	0.8	0.9
Khnyn	Q80U38	46	-12	-11	-20	-13	0.9	0.9	0.8	0.9
Dync1li1	Q8R1Q8	51	-17	-11	-20	10	0.9	0.9	0.8	1.1
Plcb2	A3KGF7	958	-10	-11	-20	-11	0.9	0.9	0.8	0.9
Nfkbie	O54910	16	-4	-11	-20	-17	1.0	0.9	0.8	0.9
Ppat	Q8CIH9	348	-14	-12	-20	17	0.9	0.9	0.8	1.2
Rnf213	E9Q555	980	-37	-12	-20	33	0.7	0.9	0.8	1.5
Fam83f	Q3UKU4	28	-18	-12	-20	-12	0.9	0.9	0.8	0.9
Dcaf8	Q8N7N5	168	-20	-13	-20	9	0.8	0.9	0.8	1.1
Rasgrp1	Q9Z1S3	334	-13	-14	-20	8	0.9	0.9	0.8	1.1
Kiaa1033	Q3UMB9	99	-11	-14	-20	-18	0.9	0.9	0.8	0.9
Atp2a2	O55143	998	-9	-14	-20	-32	0.9	0.9	0.8	0.8
Prtn3	Q61096	226	-17	-14	-20	-41	0.9	0.9	0.8	0.7
Recql	Q9Z129	577	-11	-14	-20	-6	0.9	0.9	0.8	0.9
Aak1	Q3UHJ0	193	28	-14	-20	-13	1.4	0.9	0.8	0.9
Mkl1	Q8K4J6	545	-17	-15	-20	-3	0.9	0.9	0.8	1.0
Dnttip1	Q99LB0	285	-19	-15	-20	-16	0.8	0.9	0.8	0.9
Ubn1	Q4G0F8	837	-21	-15	-20	-7	0.8	0.9	0.8	0.9
Nrbf2	Q8VCQ3	155	-14	-15	-20	-17	0.9	0.9	0.8	0.9
Ttf1	Q62187	303	-9	-15	-20	-22	0.9	0.9	0.8	0.8
Adar	Q99MU3	345	-31	-15	-20	-22	0.8	0.9	0.8	0.8
Taf4	E9QAP7	824	-19	-16	-20	-10	0.8	0.9	0.8	0.9
Kntc1	Q8C3Y4	1107	-6	-16	-20	-10	0.9	0.9	0.8	0.9
Ank1	Q02357	939	-20	-17	-20	7	0.8	0.9	0.8	1.1
Pck2	Q8BH04	306	-15	-17	-20	-14	0.9	0.9	0.8	0.9
Rad21	Q61550	589	-11	-17	-20	-14	0.9	0.9	0.8	0.9
Hsph1	Q61699	845	-3	-17	-20	-18	1.0	0.9	0.8	0.9
Runx3	Q64131	22	-15	-17	-20	-18	0.9	0.9	0.8	0.8
Pnkp	G5E8N7	404	-25	-17	-20	-16	0.8	0.9	0.8	0.9
Itsn2	E9QNG1	771	-20	-17	-20	-16	0.8	0.9	0.8	0.9
Usp48	Q3V0C5	690	-27	-18	-20	6	0.8	0.9	0.8	1.1

Dna2	Q6ZQJ5	493	-3	-18	-20	-6	1.0	0.9	0.8	0.9
Ptprc	P06800	741	-8	-18	-20	-10	0.9	0.9	0.8	0.9
Hemgn	Q9ERZ0	284	-19	-18	-20	-18	0.8	0.9	0.8	0.8
Pot1	Q91WC1	335	-10	-18	-20	-7	0.9	0.8	0.8	0.9
Smchd1	Q6P5D8	1983	-12	-18	-20	-26	0.9	0.8	0.8	0.8
Pold2	O35654	398	-14	-19	-20	7	0.9	0.8	0.8	1.1
Trim59	Q922Y2	208	-21	-19	-20	-4	0.8	0.8	0.8	1.0
Ints1	Q6P4S8	1974	-15	-19	-20	-19	0.9	0.8	0.8	0.8
Nr1h2	Q60644	149	-4	-20	-20	-20	1.0	0.8	0.8	0.8
Mia3	J3KMH5	391	-18	-20	-20	-21	0.8	0.8	0.8	0.8
Zmym4	A2A791	155	-9	-20	-20	-29	0.9	0.8	0.8	0.8
Upf2	A2AT37	942	-7	-21	-20	5	0.9	0.8	0.8	1.1
Bag5	Q8CI32	360	-4	-21	-20	-19	1.0	0.8	0.8	0.8
Tradd	Q3U0V2	166	-14	-21	-20	-36	0.9	0.8	0.8	0.7
Adss	P46664	338	-25	-22	-20	-6	0.8	0.8	0.8	0.9
Igha	A0A0A6YXW6	171	-17	-22	-20	-16	0.9	0.8	0.8	0.9
Abr	Q5SSL4	201	-23	-23	-20	-21	0.8	0.8	0.8	0.8
Rab3il1	Q8VDV3	339	-15	-24	-20	-9	0.9	0.8	0.8	0.9
Cbx8	Q9QXV1	139	-11	-25	-20	-12	0.9	0.8	0.8	0.9
Rb1	P13405	432	-9	-25	-20	-13	0.9	0.8	0.8	0.9
Cabin1	G3X8Q1	1679	-9	-25	-20	-38	0.9	0.8	0.8	0.7
Trim24	Q64127	246	-12	-25	-20	-18	0.9	0.8	0.8	0.8
Trim33	Q99PP7	315	-12	-25	-20	-18	0.9	0.8	0.8	0.8
Fam98b	Q80VD1	63	-29	-26	-20	-17	0.8	0.8	0.8	0.9
Trim56	Q80VI1	131	-17	-26	-20	-17	0.9	0.8	0.8	0.9
Tpr	F6ZDS4	298	-12	-26	-20	-18	0.9	0.8	0.8	0.8
Protein	Q3UJP5	118	-5	-28	-20	-12	1.0	0.8	0.8	0.9
Dhx9	E9QNN1	12	-15	-28	-20	4	0.9	0.8	0.8	1.0
Psmd1	Q3TXS7	633	-38	-28	-20	-29	0.7	0.8	0.8	0.8
Rbm6	S4R1W5	407	-13	-29	-20	-30	0.9	0.8	0.8	0.8
Xdh	Q00519	1328	-17	-30	-20	-25	0.9	0.8	0.8	0.8
Mycbp2	E9PUJ6	1169	-20	-31	-20	-1	0.8	0.8	0.8	1.0
Top2b	Q64511	464	-31	-31	-20	-11	0.8	0.8	0.8	0.9
Kiaa0513	Q8R0A7	149	-18	-34	-20	-5	0.9	0.7	0.8	1.0
Xpo5	Q924C1	1052	-34	-35	-20	-56	0.7	0.7	0.8	0.6
Supt16	G3X956	153	-13	-38	-20	1	0.9	0.7	0.8	1.0
Kdm3b	B9EKS2	1452	-19	-39	-20	-28	0.8	0.7	0.8	0.8
Kpna4	O35343	57	-12	-40	-20	-26	0.9	0.7	0.8	0.8
Mark2	Q05512	550	-23	-42	-20	-30	0.8	0.7	0.8	0.8
Nanp	Q9CPT3	60	-30	12	-20	12	0.8	1.1	0.8	1.1
Incenp	Q9WU62	457	-14	7	-20	-10	0.9	1.1	0.8	0.9
Xdh	Q00519	150	2	1	-20	-11	1.0	1.0	0.8	0.9
Mphosph1	Q810V0	27	-6	-2	-20	-3	0.9	1.0	0.8	1.0
Pfas	Q5SUR0	108	-4	-2	-20	19	1.0	1.0	0.8	1.2
Rif1	Q6PR54	1413	-19	-2	-20	-27	0.8	1.0	0.8	0.8
Twf2	Q9Z0P5	67	-7	-3	-20	-5	0.9	1.0	0.8	1.0
Aldoa	P05064	178	-15	-3	-20	10	0.9	1.0	0.8	1.1
Fgd2	Q8BY35	510	-16	-4	-20	-10	0.9	1.0	0.8	0.9
Madd	Q80U28	763	-3	-4	-20	-33	1.0	1.0	0.8	0.8
Pgd	Q9DCD0	170	-6	-5	-20	9	0.9	1.0	0.8	1.1
Ctsb	P10605	211	-16	-5	-20	-56	0.9	1.0	0.8	0.6

Epb42	P49222	398	-6	-6	-20	-2	0.9	0.9	0.8	1.0
Cct8	P42932	136	-27	-7	-20	-11	0.8	0.9	0.8	0.9
Npl	Q9DCJ9	185	0	-7	-20	-24	1.0	0.9	0.8	0.8
Ap3m1	Q9JKC8	236	-41	-7	-20	-3	0.7	0.9	0.8	1.0
Zmym3	Q9JLM4	526	-5	-7	-20	-19	1.0	0.9	0.8	0.8
Kdm3a	Q6PCM1	251	-33	-7	-20	-27	0.8	0.9	0.8	0.8
Pml	Q60953	93	-16	-8	-20	6	0.9	0.9	0.8	1.1
Zfp292	Q9Z2U2	53	-17	-8	-20	6	0.9	0.9	0.8	1.1
Capzb	P47757	147	-21	-8	-20	-18	0.8	0.9	0.8	0.8
Rnf169	E9Q7F2	131	-12	-8	-20	-22	0.9	0.9	0.8	0.8
Ppp1r18	Q8BQ30	276	-3	-8	-20	-26	1.0	0.9	0.8	0.8
Rexo2	Q9D8S4	97	-7	-8	-20	-28	0.9	0.9	0.8	0.8
Rasa2	P58069	128	-5	-8	-20	-14	1.0	0.9	0.8	0.9
Atad2b	E9Q166	474	-6	-9	-20	4	0.9	0.9	0.8	1.0
Atad2	G3X963	480	-6	-9	-20	4	0.9	0.9	0.8	1.0
Ppp3cb	P48453	478	-11	-9	-20	-3	0.9	0.9	0.8	1.0
Ahnak	E9Q616	1743	-4	-10	-20	-18	1.0	0.9	0.8	0.8
Nktr	P30415	393	-10	-10	-20	-25	0.9	0.9	0.8	0.8
Ppp2r1a	Q76MZ3	174	-19	-10	-20	6	0.8	0.9	0.8	1.1
Naa16	Q9DBB4	334	-9	-10	-20	-32	0.9	0.9	0.8	0.8
Aimp2	Q8R010	306	3	-11	-20	4	1.0	0.9	0.8	1.0
Gimap7	Q8R379	164	-21	-11	-20	3	0.8	0.9	0.8	1.0
Plod1	Q9R0E2	681	-12	-12	-20	8	0.9	0.9	0.8	1.1
Gm20695	H3BLK7	19	-8	-12	-20	-5	0.9	0.9	0.8	1.0
Kpna3	O35344	417	-21	-12	-20	14	0.8	0.9	0.8	1.2
Fnbp1	Q80TY0	248	-7	-12	-20	9	0.9	0.9	0.8	1.1
Usp11	Q99K46	653	-16	-13	-20	13	0.9	0.9	0.8	1.1
Rps6kc1	Q8BLK9	895	-16	-13	-20	-3	0.9	0.9	0.8	1.0
Cmpk2	Q3U5Q7	137	-16	-13	-20	-27	0.9	0.9	0.8	0.8
Btbd11	Q6GQW0	849	-8	-14	-20	-4	0.9	0.9	0.8	1.0
Mettl22	Q8R1C6	263	-4	-14	-20	-12	1.0	0.9	0.8	0.9
Dnajc7	Q9QYI3	247	-21	-14	-20	-29	0.8	0.9	0.8	0.8
Insrr	Q9WTL4	246	-21	-14	-20	-29	0.8	0.9	0.8	0.8
Ikbkb	O88351	115	-14	-15	-20	-6	0.9	0.9	0.8	0.9
Apobr	Q8VBT6	669	-19	-15	-20	-8	0.8	0.9	0.8	0.9
Phrf1	A6H619	878	-10	-15	-20	-19	0.9	0.9	0.8	0.8
Orc4	O88708	252	12	-15	-20	-35	1.1	0.9	0.8	0.7
Atp6v0d1	P51863	244	-23	-16	-20	2	0.8	0.9	0.8	1.0
Rfc4	Q99J62	180	-16	-16	-20	-7	0.9	0.9	0.8	0.9
Mon2	Q80TL7	1221	-10	-16	-20	-18	0.9	0.9	0.8	0.8
Zmym2	Q9CU65	723	-22	-17	-20	-22	0.8	0.9	0.8	0.8
Zfp62	Q8C827	47	-8	-17	-20	-25	0.9	0.9	0.8	0.8
Dusp16	Q6PCP3	170	-20	-17	-20	-4	0.8	0.9	0.8	1.0
Ptpn7	Q8BUM3	244	-20	-18	-20	-9	0.8	0.9	0.8	0.9
Tiprl	Q8BH58	87	-26	-18	-20	-18	0.8	0.9	0.8	0.8
Myo1e	E9Q634	579	-24	-18	-20	8	0.8	0.8	0.8	1.1
Myo1f	Q8CG29	577	-24	-18	-20	8	0.8	0.8	0.8	1.1
Ddx60	E9PZQ1	704	-20	-18	-20	8	0.8	0.8	0.8	1.1
Snd1	Q78PY7	440	-19	-18	-20	5	0.8	0.8	0.8	1.0
Nck1	Q99M51	266	-14	-18	-20	-7	0.9	0.8	0.8	0.9
Uba7	Q9DBK7	619	-29	-18	-20	-20	0.8	0.8	0.8	0.8

Ak3	Q9WTP7	85	-22	-18	-20	-21	0.8	0.8	0.8	0.8
Vps13c	Q8BX70	826	-21	-18	-20	-36	0.8	0.8	0.8	0.7
Msh6	P54276	1115	-12	-19	-20	-17	0.9	0.8	0.8	0.9
Mtmr2	Q9Z2D1	7	-8	-19	-20	-22	0.9	0.8	0.8	0.8
Nup50	Q9JIH2	150	-17	-20	-20	-15	0.9	0.8	0.8	0.9
Aldh9a1	Q9JLJ2	443	-21	-21	-20	-13	0.8	0.8	0.8	0.9
Ankib1	Q6ZPS6	21	-13	-21	-20	3	0.9	0.8	0.8	1.0
Dock4	P59764	860	-18	-21	-20	-4	0.8	0.8	0.8	1.0
Hdac3	O88895	279	36	-21	-20	-17	1.6	0.8	0.8	0.9
Ell	O08856	121	-20	-21	-20	-28	0.8	0.8	0.8	0.8
Hdac3	O88895	94	-38	-22	-20	33	0.7	0.8	0.8	1.5
Fam129a	Q3UW53	193	-21	-22	-20	-10	0.8	0.8	0.8	0.9
Rpl32	P62911	96	-22	-22	-20	-20	0.8	0.8	0.8	0.8
L3mbtl3	Q8BLB7	233	-23	-22	-20	-22	0.8	0.8	0.8	0.8
Prkdc	P97313	2171	-35	-22	-20	-6	0.7	0.8	0.8	0.9
Arhgap25	Q8BYW1	293	-21	-23	-20	12	0.8	0.8	0.8	1.1
2210016F:	G3X8U3	168	5	-24	-20	4	1.0	0.8	0.8	1.0
Gatad2b	Q8VHR5	410	-21	-24	-20	-16	0.8	0.8	0.8	0.9
Dnajc14	Q921R4	423	-23	-24	-20	-16	0.8	0.8	0.8	0.9
Rcsd1	Q3UZA1	244	-17	-24	-20	-28	0.9	0.8	0.8	0.8
Zfp446	Q8C9M8	395	-19	-26	-20	-20	0.8	0.8	0.8	0.8
Kmt2a	P55200	987	-14	-26	-20	-30	0.9	0.8	0.8	0.8
Slc27a4	Q91VE0	403	-3	-26	-20	-8	1.0	0.8	0.8	0.9
Cep97	Q9CZ62	481	-18	-26	-20	-9	0.9	0.8	0.8	0.9
Mtfr1l	Q9CWE0	34	-26	-28	-20	-33	0.8	0.8	0.8	0.8
Ighg2c	F6TQW2	14	-33	-29	-20	-18	0.8	0.8	0.8	0.9
Gclc	P97494	152	-29	-29	-20	-19	0.8	0.8	0.8	0.8
Fam126a	Q6P9N1	504	-19	-31	-20	-10	0.8	0.8	0.8	0.9
Smarcc2	Q6PDG5	80	-9	-32	-20	17	0.9	0.8	0.8	1.2
Hectd1	Q69ZR2	2076	-36	-34	-20	13	0.7	0.7	0.8	1.1
Tufm	Q8BFR5	127	8	-35	-20	1	1.1	0.7	0.8	1.0
Gm20425	E9Q035	886	-18	-36	-20	-28	0.9	0.7	0.8	0.8
Lrch4	Q921G6	134	-29	-40	-20	-12	0.8	0.7	0.8	0.9
Secisbp2	Q3U1C4	47	-26	-51	-20	-25	0.8	0.7	0.8	0.8
Tomm70	Q9CZW5	445	-45	-69	-20	-2	0.7	0.6	0.8	1.0
Otub2	Q9CQX0	51	2	5	-21	-11	1.0	1.0	0.8	0.9
Ddx20	Q9JJY4	759	-16	-1	-21	3	0.9	1.0	0.8	1.0
Dennd1b	Q3U1T9	336	-9	-4	-21	-12	0.9	1.0	0.8	0.9
Mcm6	P97311	393	-10	-4	-21	-18	0.9	1.0	0.8	0.9
Gsk3b	Q9WV60	76	-6	-5	-21	-4	0.9	1.0	0.8	1.0
Padi2	Q08642	175	8	-5	-21	-8	1.1	1.0	0.8	0.9
Cep120	Q7TSG1	436	-16	-6	-21	-30	0.9	0.9	0.8	0.8
Nol8	Q3UHX0	1054	-15	-7	-21	7	0.9	0.9	0.8	1.1
Pias4	Q9JM05	319	-14	-7	-21	-6	0.9	0.9	0.8	0.9
Ube2g2	P60605	89	-2	-7	-21	-10	1.0	0.9	0.8	0.9
Huwe1	Q7TMY8	1401	-16	-7	-21	-15	0.9	0.9	0.8	0.9
Rbm6	S4R1W5	1052	-13	-9	-21	-13	0.9	0.9	0.8	0.9
Arrb2	Q91YI4	141	-24	-9	-21	-20	0.8	0.9	0.8	0.8
Nup54	Q8BTS4	183	-19	-10	-21	17	0.8	0.9	0.8	1.2
Abr	Q5SSL4	158	-6	-10	-21	-2	0.9	0.9	0.8	1.0
Shc1	P98083	412	-31	-10	-21	-5	0.8	0.9	0.8	1.0

Plaa	P27612	584	-8	-10	-21	-18	0.9	0.9	0.8	0.9
Eif2b3	B1AUN2	281	-13	-11	-21	-1	0.9	0.9	0.8	1.0
Coro1b	Q9WUM3	332	-18	-11	-21	8	0.8	0.9	0.8	1.1
Ankrd13a	Q80UP5	538	-12	-11	-21	-8	0.9	0.9	0.8	0.9
Rab31	Q921E2	49	-18	-11	-21	-12	0.8	0.9	0.8	0.9
Avl9	Q80U56	13	1	-11	-21	-12	1.0	0.9	0.8	0.9
Psmc6	P62334	170	-13	-11	-21	-23	0.9	0.9	0.8	0.8
Akap13	E9Q394	2613	-14	-12	-21	-23	0.9	0.9	0.8	0.8
Ahctf1	Q8CJF7	1131	-22	-12	-21	-4	0.8	0.9	0.8	1.0
Aco2	Q99KI0	451	-15	-12	-21	-7	0.9	0.9	0.8	0.9
Xpo5	Q924C1	706	-7	-12	-21	-12	0.9	0.9	0.8	0.9
Setx	A2AKX3	904	-13	-12	-21	-24	0.9	0.9	0.8	0.8
Pfkfb2	A0A087WRM[illegible]	432	-15	-13	-21	-33	0.9	0.9	0.8	0.8
Pfkfb4	Q6DTY7	430	-15	-13	-21	-33	0.9	0.9	0.8	0.8
Ints8	Q80V86	701	-20	-14	-21	12	0.8	0.9	0.8	1.1
Gapvd1	Q6PAR5	568	-14	-14	-21	-14	0.9	0.9	0.8	0.9
Isca2	Q9DCB8	146	-20	-14	-21	-17	0.8	0.9	0.8	0.9
Wdr53	Q9DB94	208	-41	-15	-21	16	0.7	0.9	0.8	1.2
Hnrnpll	Q921F4	513	-19	-15	-21	-4	0.8	0.9	0.8	1.0
Mbnl3	Q8R003	54	-9	-15	-21	1	0.9	0.9	0.8	1.0
Gcc2	B2RSU7	411	-3	-15	-21	-11	1.0	0.9	0.8	0.9
Zfc3h1	B2RT41	1732	-13	-15	-21	-14	0.9	0.9	0.8	0.9
Gnl3l	Q6PGG6	484	-33	-15	-21	-15	0.8	0.9	0.8	0.9
Themis2	Q91YX0	454	-19	-16	-21	-7	0.8	0.9	0.8	0.9
Chkb	O55229	72	-32	-16	-21	-14	0.8	0.9	0.8	0.9
Ptgr1	Q91YR9	251	-22	-16	-21	-22	0.8	0.9	0.8	0.8
Prdm10	Q3UTQ7	243	7	-16	-21	-6	1.1	0.9	0.8	0.9
Ep300	B2RWS6	1249	-29	-16	-21	-15	0.8	0.9	0.8	0.9
Rbm5	Q91YE7	766	-12	-17	-21	-10	0.9	0.9	0.8	0.9
Topaz1	E5FYH1	947	-20	-17	-21	-25	0.8	0.9	0.8	0.8
Heatr1	G3X9B1	631	-28	-17	-21	-15	0.8	0.9	0.8	0.9
Chd8	Q09XV5	1782	-15	-18	-21	-21	0.9	0.9	0.8	0.8
Gvin1	L7N451	6	-16	-18	-21	-23	0.9	0.9	0.8	0.8
Papss1	Q60967	360	-15	-18	-21	3	0.9	0.8	0.8	1.0
Limd1	Q9QXD8	577	-22	-18	-21	-23	0.8	0.8	0.8	0.8
Golgb1	E9PVZ8	1894	-21	-19	-21	-1	0.8	0.8	0.8	1.0
Slc25a3	Q8VEM8	71	-22	-19	-21	4	0.8	0.8	0.8	1.0
Smchd1	Q6P5D8	444	15	-19	-21	-16	1.2	0.8	0.8	0.9
Pcnt	F8VPV0	2217	-21	-20	-21	-16	0.8	0.8	0.8	0.9
Thyn1	Q91YJ3	119	-19	-20	-21	-21	0.8	0.8	0.8	0.8
Ankhd1	E9PUR0	1629	-17	-20	-21	-22	0.9	0.8	0.8	0.8
Gbf1	Q6DFZ1	685	-15	-20	-21	-13	0.9	0.8	0.8	0.9
Pxn	Q8VI36	108	-26	-20	-21	-14	0.8	0.8	0.8	0.9
Tnfaip3	Q60769	752	-17	-20	-21	-86	0.9	0.8	0.8	0.5
Rhog	P84096	157	-27	-21	-21	10	0.8	0.8	0.8	1.1
Tdrkh	Q80VL1	109	-21	-21	-21	-20	0.8	0.8	0.8	0.8
Kdm6a	O70546	4	-18	-21	-21	-21	0.8	0.8	0.8	0.8
Rap1gap2	Q5SVL6	565	-15	-21	-21	-25	0.9	0.8	0.8	0.8
Larp4b	Q6A0A2	656	-15	-21	-21	-39	0.9	0.8	0.8	0.7
Ppp1r12a	Q9DBR7	553	-28	-21	-21	-39	0.8	0.8	0.8	0.7
Marc2	Q922Q1	274	-17	-22	-21	8	0.9	0.8	0.8	1.1

Gm9923	J3KMK0	133	-12	-22	-21	-14	0.9	0.8	0.8	0.9
Lipt1	Q8VCM4	337	-28	-23	-21	22	0.8	0.8	0.8	1.3
Epb41	P48193	181	-6	-23	-21	-24	0.9	0.8	0.8	0.8
Srgap2	Q91Z67	1020	-35	-24	-21	-11	0.7	0.8	0.8	0.9
Shprh	Q7TPQ3	411	-19	-24	-21	-18	0.8	0.8	0.8	0.8
Gclc	P97494	553	-16	-24	-21	-11	0.9	0.8	0.8	0.9
Atp1a3	Q6PIC6	364	-24	-24	-21	-19	0.8	0.8	0.8	0.8
Atp1a1	Q8VDN2	374	-24	-24	-21	-19	0.8	0.8	0.8	0.8
Mki67	E9PVX6	3169	-16	-24	-21	-29	0.9	0.8	0.8	0.8
Dnajc13	D4AFX7	1461	-44	-25	-21	-12	0.7	0.8	0.8	0.9
Rrp7a	Q9D1C9	81	-13	-25	-21	-21	0.9	0.8	0.8	0.8
Lrmp	G5E880	207	-15	-26	-21	-5	0.9	0.8	0.8	1.0
Rnh1	Q91VI7	325	-29	-26	-21	-1	0.8	0.8	0.8	1.0
C2cd5	Q7TPS5	449	-25	-26	-21	-28	0.8	0.8	0.8	0.8
Sugt1	Q9CX34	54	-21	-27	-21	-4	0.8	0.8	0.8	1.0
Kynu	Q9CXF0	45	-19	-27	-21	-14	0.8	0.8	0.8	0.9
Macf1	E9PVY8	6032	-33	-28	-21	-4	0.8	0.8	0.8	1.0
Atad2	G3X963	1361	-24	-28	-21	-19	0.8	0.8	0.8	0.8
Pdlim2	Q8R1G6	122	-27	-29	-21	-39	0.8	0.8	0.8	0.7
Ddx23	D3Z0M9	408	-21	-31	-21	-8	0.8	0.8	0.8	0.9
Tes	Q921W7	237	-14	-32	-21	-5	0.9	0.8	0.8	1.0
Wdr26	Q8C6G8	218	-14	-33	-21	-45	0.9	0.8	0.8	0.7
Rnf169	E9Q7F2	280	-27	-33	-21	-54	0.8	0.8	0.8	0.7
Spg11	Q3UHA3	519	-28	-33	-21	-17	0.8	0.8	0.8	0.9
Xrcc5	P27641	493	-25	-36	-21	6	0.8	0.7	0.8	1.1
Ccdc47	Q9D024	283	-23	-37	-21	-13	0.8	0.7	0.8	0.9
Got2	P05202	106	-29	-62	-21	-22	0.8	0.6	0.8	0.8
Mrm1	Q99J25	225	-5	22	-21	-6	1.0	1.3	0.8	0.9
Rrp1	P56183	198	9	5	-21	11	1.1	1.1	0.8	1.1
Synj1	D3Z656	1539	-6	5	-21	-4	0.9	1.0	0.8	1.0
Map4k1	P70218	704	-16	4	-21	24	0.9	1.0	0.8	1.3
Parp14	Q2EMV9	860	-29	1	-21	-10	0.8	1.0	0.8	0.9
Polr3e	Q9CZT4	70	-9	-2	-21	-5	0.9	1.0	0.8	1.0
Inpp4a	Q9EPW0	286	-13	-3	-21	15	0.9	1.0	0.8	1.2
Smarcc2	Q6PDG5	91	-13	-4	-21	18	0.9	1.0	0.8	1.2
Ogt	Q8CGY8	323	-22	-5	-21	-19	0.8	1.0	0.8	0.8
Rab7a	P51150	84	-25	-6	-21	1	0.8	0.9	0.8	1.0
Hspa4	Q3U2G2	213	-10	-6	-21	7	0.9	0.9	0.8	1.1
Aco2	Q99KI0	592	-3	-6	-21	3	1.0	0.9	0.8	1.0
Fam129b	Q8R1F1	334	-18	-8	-21	0	0.8	0.9	0.8	1.0
Dock8	Q8C147	170	-17	-8	-21	-15	0.9	0.9	0.8	0.9
Bap18	Q9DCT6	66	-16	-10	-21	-33	0.9	0.9	0.8	0.8
Rqcd1	Q9JKY0	99	-20	-10	-21	-8	0.8	0.9	0.8	0.9
Ddb1	Q3U1J4	903	-18	-12	-21	3	0.8	0.9	0.8	1.0
Snrnp200	Q6P4T2	1278	-25	-12	-21	11	0.8	0.9	0.8	1.1
Kdm1b	Q8CIG3	284	-10	-12	-21	-13	0.9	0.9	0.8	0.9
Cobll1	Q3UMF0	1148	-11	-12	-21	-13	0.9	0.9	0.8	0.9
Coq5	Q9CXI0	170	-19	-13	-21	-21	0.8	0.9	0.8	0.8
Rapgef6	Q5NCJ1	778	-33	-13	-21	-37	0.8	0.9	0.8	0.7
Braf	P28028	785	-15	-13	-21	-7	0.9	0.9	0.8	0.9
Daxx	Q3UKR0	137	-10	-14	-21	-9	0.9	0.9	0.8	0.9

Dennd4b	Q3U1Y4	1044	-10	-14	-21	-14	0.9	0.9	0.8	0.9
Rel	A4QPD3	250	-16	-14	-21	-15	0.9	0.9	0.8	0.9
Pgp	Q8CHP8	297	-15	-14	-21	-31	0.9	0.9	0.8	0.8
Brat1	Q8C3R1	513	-21	-15	-21	29	0.8	0.9	0.8	1.4
Wdr76	A6PWY4	212	-2	-15	-21	-7	1.0	0.9	0.8	0.9
Copa	Q8CIE6	1191	-14	-15	-21	10	0.9	0.9	0.8	1.1
Smurf2	A2A5Z6	706	-16	-15	-21	-21	0.9	0.9	0.8	0.8
Rcc2	Q8BK67	426	-14	-15	-21	-33	0.9	0.9	0.8	0.8
Sdhaf3	Q8BQU3	80	-16	-16	-21	-24	0.9	0.9	0.8	0.8
Arhgap17	Q3UIA2	697	-12	-17	-21	-10	0.9	0.9	0.8	0.9
Zc3hav1	Q3UPF5	162	-17	-17	-21	13	0.9	0.9	0.8	1.1
Cyhr1	Q9QXA1	228	-14	-17	-21	-19	0.9	0.9	0.8	0.8
Prkar1a	Q9DBC7	67	-19	-17	-21	-20	0.8	0.9	0.8	0.8
Ppp1r3d	A2AJW4	73	-12	-18	-21	-13	0.9	0.9	0.8	0.9
Metap1	Q8BP48	174	-11	-18	-21	-14	0.9	0.9	0.8	0.9
Atg2b	Q80XK6	1665	-14	-18	-21	-15	0.9	0.9	0.8	0.9
Bre	Q8K3W0	44	-15	-18	-21	5	0.9	0.8	0.8	1.1
Flna	Q8BTM8	1920	-18	-18	-21	-22	0.8	0.8	0.8	0.8
Utrn	E9Q6R7	2098	-26	-18	-21	-23	0.8	0.8	0.8	0.8
Irf8	P23611	347	-21	-18	-21	-27	0.8	0.8	0.8	0.8
Eral1	Q9CZU4	150	-13	-18	-21	-36	0.9	0.8	0.8	0.7
Hcfc1	Q61191	1896	-18	-19	-21	-17	0.9	0.8	0.8	0.9
Def6	Q8C2K1	253	-17	-19	-21	-21	0.9	0.8	0.8	0.8
Gcn1	E9PVA8	103	-9	-19	-21	-22	0.9	0.8	0.8	0.8
Scaf11	E9PZM7	1045	-19	-19	-21	-23	0.8	0.8	0.8	0.8
Itgb1bp1	O35671	60	-19	-19	-21	-27	0.8	0.8	0.8	0.8
Jarid2	Q62315	857	-19	-20	-21	-15	0.8	0.8	0.8	0.9
Tnks1bp1	P58871	717	-23	-20	-21	-22	0.8	0.8	0.8	0.8
Trim65	Q8BFW4	176	-15	-21	-21	-26	0.9	0.8	0.8	0.8
Trim28	Q62318	225	-7	-21	-21	1	0.9	0.8	0.8	1.0
Ikzf3	O08900	432	-15	-22	-21	-5	0.9	0.8	0.8	1.0
Rbbp5	Q8BX09	258	-45	-23	-21	-3	0.7	0.8	0.8	1.0
Cyp2d22	Q9JKY7	491	-25	-23	-21	-14	0.8	0.8	0.8	0.9
Kpna3	O35344	228	-30	-24	-21	-12	0.8	0.8	0.8	0.9
Rexo4	Q6PAQ4	284	-19	-24	-21	-18	0.8	0.8	0.8	0.8
Gnl3l	Q6PGG6	207	-16	-24	-21	-26	0.9	0.8	0.8	0.8
Trim7	Q923T7	500	2	-24	-21	-39	1.0	0.8	0.8	0.7
G6pdx	Q00612	13	-22	-25	-21	-16	0.8	0.8	0.8	0.9
Ubn1	Q4G0F8	765	-26	-25	-21	-27	0.8	0.8	0.8	0.8
Ankrd10	Q99LW0	214	-15	-26	-21	-20	0.9	0.8	0.8	0.8
Arcn1	Q5XJY5	479	-9	-26	-21	4	0.9	0.8	0.8	1.0
Pikfyve	Q9Z1T6	384	8	-28	-21	-21	1.1	0.8	0.8	0.8
Haus3	Q8QZX2	420	-23	-30	-21	-16	0.8	0.8	0.8	0.9
Zmynd8	A2A484	1078	-27	-31	-21	-24	0.8	0.8	0.8	0.8
Dmxl2	Q8BPN8	921	-19	-31	-21	-19	0.8	0.8	0.8	0.8
Phc2	Q9QWH1	665	-19	-31	-21	-24	0.8	0.8	0.8	0.8
Tnrc6c	Q3UHC0	636	-22	-31	-21	-26	0.8	0.8	0.8	0.8
Bdp1	Q571C7	276	-36	-41	-21	-32	0.7	0.7	0.8	0.8
Zfp512b	Q6PHP4	761	51	-45	-21	-60	2.0	0.7	0.8	0.6
Mapk3	Q63844	83	-9	9	-22	-1	0.9	1.1	0.8	1.0
Top3b	Q9Z321	190	-1	7	-22	7	1.0	1.1	0.8	1.1

Lrp1b	A2API5	4369	9	6	-22	7	1.1	1.1	0.8	1.1
Ccdc77	Q9CZH8	55	-8	3	-22	-8	0.9	1.0	0.8	0.9
Anxa2	P07356	223	-22	1	-22	-31	0.8	1.0	0.8	0.8
Gm14781	A2A9Q9	129	-20	0	-22	-28	0.8	1.0	0.8	0.8
Eif3h	Q91WK2	327	-3	0	-22	-28	1.0	1.0	0.8	0.8
H2afy	Q9QZQ8	276	-17	-1	-22	15	0.9	1.0	0.8	1.2
Ift80	Q8K057	106	-9	-1	-22	-17	0.9	1.0	0.8	0.9
Sqstm1	Q64337	151	-12	-4	-22	1	0.9	1.0	0.8	1.0
Anapc16	Q9CPV2	55	-18	-4	-22	18	0.8	1.0	0.8	1.2
Cwf19l2	Q8BG79	294	-12	-4	-22	-5	0.9	1.0	0.8	1.0
Dctn1	O08788	791	-18	-5	-22	2	0.9	1.0	0.8	1.0
Rgs14	P97492	305	-3	-5	-22	-21	1.0	1.0	0.8	0.8
Ddx6	P54823	341	-38	-6	-22	-20	0.7	0.9	0.8	0.8
Mdn1	A2ANY6	2834	2	-7	-22	-21	1.0	0.9	0.8	0.8
Atg16l2	Q6KAU8	271	-17	-7	-22	-26	0.9	0.9	0.8	0.8
Tprg1l	Q9DBS2	139	-25	-7	-22	-10	0.8	0.9	0.8	0.9
Ube3b	Q9ES34	75	-10	-7	-22	-25	0.9	0.9	0.8	0.8
Kif4	P33174	28	-28	-8	-22	10	0.8	0.9	0.8	1.1
Xrn1	F8VQ87	657	-6	-8	-22	15	0.9	0.9	0.8	1.2
Fcho1	Q8K285	367	-7	-9	-22	-13	0.9	0.9	0.8	0.9
Smad2	Q62432	161	-5	-9	-22	3	1.0	0.9	0.8	1.0
Glyr1	Q922P9	241	-17	-9	-22	-15	0.9	0.9	0.8	0.9
Cops6	O88545	140	-20	-10	-22	15	0.8	0.9	0.8	1.2
Bin2	S4R270	455	-14	-10	-22	-14	0.9	0.9	0.8	0.9
Gimap8	Q75N62	204	-22	-11	-22	3	0.8	0.9	0.8	1.0
LRWD1	Q8BUI3	387	10	-11	-22	0	1.1	0.9	0.8	1.0
Eif3d	O70194	195	-11	-11	-22	-3	0.9	0.9	0.8	1.0
Nub1	P54729	258	-15	-11	-22	28	0.9	0.9	0.8	1.4
Hspd1	P63038	447	-12	-11	-22	-1	0.9	0.9	0.8	1.0
Fam213b	Q9DB60	34	-23	-11	-22	-8	0.8	0.9	0.8	0.9
Gcn1	E9PVA8	2255	-29	-11	-22	-12	0.8	0.9	0.8	0.9
Anxa1	P10107	263	-13	-12	-22	3	0.9	0.9	0.8	1.0
Supt5h	O55201	624	-18	-12	-22	-1	0.9	0.9	0.8	1.0
Akap12	Q9WTQ5	454	-18	-12	-22	-22	0.9	0.9	0.8	0.8
Zc3h13	E9Q784	51	-22	-13	-22	-15	0.8	0.9	0.8	0.9
Glrx	Q9QUH0	23	-8	-13	-22	-34	0.9	0.9	0.8	0.7
H2afy	Q9QZQ8	286	-14	-13	-22	1	0.9	0.9	0.8	1.0
Tradd	Q3U0V2	164	-9	-13	-22	-11	0.9	0.9	0.8	0.9
Gripap1	Q8VD04	239	-11	-13	-22	-13	0.9	0.9	0.8	0.9
Pak1ip1	Q9DCE5	298	-24	-14	-22	6	0.8	0.9	0.8	1.1
Ppp3r1	Q63810	154	-20	-14	-22	5	0.8	0.9	0.8	1.0
Cox11	Q6P8I6	216	-23	-14	-22	-11	0.8	0.9	0.8	0.9
Cdca2	Q14B71	105	-15	-14	-22	2	0.9	0.9	0.8	1.0
Gapvd1	Q6PAR5	1233	-19	-14	-22	0	0.8	0.9	0.8	1.0
Sypl1	O09117	64	-21	-15	-22	12	0.8	0.9	0.8	1.1
Dcaf13	Q6PAC3	120	-15	-15	-22	-19	0.9	0.9	0.8	0.8
Syne1	Q6ZWR6	6816	-13	-15	-22	-25	0.9	0.9	0.8	0.8
Rbm12b2	Q66JV4	368	-10	-15	-22	-33	0.9	0.9	0.8	0.8
Pik3cb	Q8BTI9	280	-10	-16	-22	14	0.9	0.9	0.8	1.2
Ctps1	P70698	491	-3	-16	-22	1	1.0	0.9	0.8	1.0
Serpinb2	P12388	79	-5	-16	-22	-31	1.0	0.9	0.8	0.8

Tbce	Q8CIV8	420	-15	-16	-22	-26	0.9	0.9	0.8	0.8
Sp110	Q8BVK9	366	-24	-17	-22	-9	0.8	0.9	0.8	0.9
Map4	P27546	636	-12	-17	-22	-34	0.9	0.9	0.8	0.7
Rsf1	E9PWW9	654	-26	-17	-22	-37	0.8	0.9	0.8	0.7
Pdia3	P27773	92	-16	-17	-22	0	0.9	0.9	0.8	1.0
Pptc7	Q6NVE9	47	-4	-17	-22	-11	1.0	0.9	0.8	0.9
Eif2d	Q61211	476	-23	-17	-22	-25	0.8	0.9	0.8	0.8
Rnf20	Q5DTM8	383	-7	-18	-22	11	0.9	0.8	0.8	1.1
Lta4h	P24527	141	-5	-18	-22	9	1.0	0.8	0.8	1.1
Efnb1	P52795	153	-27	-18	-22	-1	0.8	0.8	0.8	1.0
Mki67	E9PVX6	868	-13	-18	-22	-13	0.9	0.8	0.8	0.9
U2surp	Q6NV83	320	-22	-19	-22	-14	0.8	0.8	0.8	0.9
Nop58	Q6DFW4	439	-14	-19	-22	-33	0.9	0.8	0.8	0.8
Tubb5	P99024	354	-15	-19	-22	-39	0.9	0.8	0.8	0.7
Tubb2a	Q7TMM9	354	-15	-19	-22	-39	0.9	0.8	0.8	0.7
Tgfbr2	Q62312	508	-2	-19	-22	-28	1.0	0.8	0.8	0.8
Wdr81	Q5ND34	336	-14	-20	-22	15	0.9	0.8	0.8	1.2
Tagln2	Q9WVA4	63	-20	-20	-22	11	0.8	0.8	0.8	1.1
Nop56	Q9D6Z1	211	-26	-20	-22	-14	0.8	0.8	0.8	0.9
Nup107	Q8BH74	645	-22	-20	-22	-15	0.8	0.8	0.8	0.9
Mynn	Q99MD8	371	-29	-20	-22	-34	0.8	0.8	0.8	0.7
Ube2m	P61082	47	6	-21	-22	1	1.1	0.8	0.8	1.0
Dcp2	Q9CYC6	73	-20	-21	-22	-1	0.8	0.8	0.8	1.0
Fkbp8	O35465	217	-17	-23	-22	-20	0.9	0.8	0.8	0.8
Taf5	F8VPY2	633	-20	-24	-22	-10	0.8	0.8	0.8	0.9
Plekha1	Q8BUL6	311	-15	-24	-22	-11	0.9	0.8	0.8	0.9
Coro1c	Q9WUM4	330	-24	-24	-22	7	0.8	0.8	0.8	1.1
Rftn1	Q6A0D4	435	-25	-24	-22	1	0.8	0.8	0.8	1.0
Tmx1	Q8VBT0	268	-14	-24	-22	-27	0.9	0.8	0.8	0.8
Srebf2	Q3U1N2	821	-7	-25	-22	8	0.9	0.8	0.8	1.1
Lig4	Q8BTF7	735	-27	-25	-22	-11	0.8	0.8	0.8	0.9
Utp15	Q8C7V3	202	-24	-25	-22	-15	0.8	0.8	0.8	0.9
Atp2a2	O55143	560	-13	-25	-22	-3	0.9	0.8	0.8	1.0
Ptprc	P06800	463	-11	-26	-22	-8	0.9	0.8	0.8	0.9
Slc30a7	Q9JKN1	310	-29	-26	-22	-12	0.8	0.8	0.8	0.9
Nfatc2	Q60591	233	-19	-26	-22	-24	0.8	0.8	0.8	0.8
Mtmr6	Q8VE11	565	-26	-27	-22	-19	0.8	0.8	0.8	0.8
Rnf213	E9Q555	3471	11	-29	-22	-16	1.1	0.8	0.8	0.9
Dhx30	Q99PU8	809	-14	-33	-22	-22	0.9	0.8	0.8	0.8
Haus6	Q6NV99	907	-39	-49	-22	-23	0.7	0.7	0.8	0.8
Themis	Q8BGW0	117	27	-80	-22	-84	1.4	0.6	0.8	0.5
Pgd	Q9DCD0	289	23	29	-22	27	1.3	1.4	0.8	1.4
Gpd1l	Q3ULJ0	216	-3	1	-22	2	1.0	1.0	0.8	1.0
Ahcy	P50247	228	-11	0	-22	-7	0.9	1.0	0.8	0.9
Selo	Q9DBC0	385	-22	-2	-22	5	0.8	1.0	0.8	1.0
Ap1g1	P22892	539	-2	-4	-22	-9	1.0	1.0	0.8	0.9
Ranbp2	Q9ERU9	707	-5	-8	-22	-19	1.0	0.9	0.8	0.8
Nr3c1	E9PYV1	166	-18	-9	-22	-31	0.8	0.9	0.8	0.8
Ifi203	O35368	266	-8	-9	-22	-25	0.9	0.9	0.8	0.8
Nek7	Q9ES74	247	-11	-9	-22	-39	0.9	0.9	0.8	0.7
Elp2	Q91WG4	204	-12	-10	-22	-17	0.9	0.9	0.8	0.9

Ppp5c	Q60676	77	-26	-10	-22	-19	0.8	0.9	0.8	0.8
Klc4	Q9DBS5	489	-9	-10	-22	-34	0.9	0.9	0.8	0.7
Tdrd7	Q8K1H1	77	-13	-11	-22	-16	0.9	0.9	0.8	0.9
Zfp41	Q02526	154	-11	-11	-22	-21	0.9	0.9	0.8	0.8
Itsn2	E9QNG1	593	-19	-12	-22	5	0.8	0.9	0.8	1.0
Zmym3	Q9JLM4	337	-15	-12	-22	-4	0.9	0.9	0.8	1.0
Nsd1	E9QAE4	2023	-16	-12	-22	-7	0.9	0.9	0.8	0.9
Sbno1	B2RRI2	698	-18	-12	-22	-8	0.9	0.9	0.8	0.9
Sde2	Q8K1J5	312	-10	-12	-22	-22	0.9	0.9	0.8	0.8
Parp14	Q2EMV9	274	-15	-13	-22	-18	0.9	0.9	0.8	0.8
Aff1	E9Q921	1108	-14	-13	-22	-33	0.9	0.9	0.8	0.8
Pogz	Q8BZH4	777	-11	-13	-22	32	0.9	0.9	0.8	1.5
Thap12	Q9CUX1	226	-14	-13	-22	-7	0.9	0.9	0.8	0.9
Pacrgl	Q9D3X5	6	-5	-13	-22	-36	1.0	0.9	0.8	0.7
Mycbp2	E9PUJ6	4646	-11	-14	-22	-22	0.9	0.9	0.8	0.8
Hprt1	P00493	23	-11	-14	-22	-9	0.9	0.9	0.8	0.9
Hnrnpu	Q8VEK3	570	-19	-15	-22	-18	0.8	0.9	0.8	0.9
Iqgap2	Q3UQ44	276	-6	-15	-22	-21	0.9	0.9	0.8	0.8
Ube3c	Q80U95	1018	-20	-15	-22	-14	0.8	0.9	0.8	0.9
Lcp2	Q60787	532	-17	-16	-22	-16	0.9	0.9	0.8	0.9
Ttc37	F8VPK0	305	-27	-16	-22	-30	0.8	0.9	0.8	0.8
Anxa7	Q07076	388	-26	-17	-22	6	0.8	0.9	0.8	1.1
Hdgf	P51859	12	2	-17	-22	-82	1.0	0.9	0.8	0.6
Apaf1	O88879	317	-12	-18	-22	7	0.9	0.9	0.8	1.1
Lztr1	Q9CQ33	424	-13	-18	-22	-10	0.9	0.9	0.8	0.9
Bre	Q8K3W0	129	-21	-18	-22	-3	0.8	0.8	0.8	1.0
Zmym4	A2A791	467	-19	-18	-22	-6	0.8	0.8	0.8	0.9
Selo	Q9DBC0	82	-2	-18	-22	-26	1.0	0.8	0.8	0.8
Top1mt	Q8R4U6	443	-12	-19	-22	-35	0.9	0.8	0.8	0.7
Spcs2	Q9CYN2	26	-19	-19	-22	-40	0.8	0.8	0.8	0.7
Eprs	Q8CGC7	337	-13	-20	-22	-16	0.9	0.8	0.8	0.9
Ankle2	Q6P1H6	294	-17	-20	-22	-44	0.9	0.8	0.8	0.7
Frmd8	Q3UFK8	206	-21	-20	-22	-24	0.8	0.8	0.8	0.8
Map3k7	Q62073	51	-7	-20	-22	-29	0.9	0.8	0.8	0.8
Fbxo30	Q8BJL1	571	-12	-21	-22	-6	0.9	0.8	0.8	0.9
Myo1d	Q5SYD0	353	-22	-21	-22	-7	0.8	0.8	0.8	0.9
Dnmt3a	O88508	550	-16	-21	-22	-23	0.9	0.8	0.8	0.8
Pfn1	P62962	128	-19	-21	-22	3	0.8	0.8	0.8	1.0
Oas3	Q8VI93	839	-4	-22	-22	30	1.0	0.8	0.8	1.4
Efl1	Q8C0D5	670	-13	-22	-22	10	0.9	0.8	0.8	1.1
Cad	B2RQC6	834	-11	-22	-22	-7	0.9	0.8	0.8	0.9
Ints6	Q6PCM2	658	-23	-23	-22	-9	0.8	0.8	0.8	0.9
Erc1	Q99MI1	258	-30	-23	-22	-19	0.8	0.8	0.8	0.8
Uap1l1	Q3TW96	507	-24	-24	-22	-20	0.8	0.8	0.8	0.8
Msl1	Q6PDM1	324	-21	-24	-22	-12	0.8	0.8	0.8	0.9
Rai1	Q61818	1485	-21	-24	-22	-33	0.8	0.8	0.8	0.8
Adar	Q99MU3	1173	-19	-25	-22	4	0.8	0.8	0.8	1.0
Dnmt1	P13864	656	-11	-25	-22	4	0.9	0.8	0.8	1.0
Lmna	P48678	593	-25	-25	-22	-23	0.8	0.8	0.8	0.8
Ahsa1	Q8BK64	56	-14	-26	-22	-18	0.9	0.8	0.8	0.9
Anapc5	Q8BTZ4	691	-38	-26	-22	-10	0.7	0.8	0.8	0.9

Kmt2a	P55200	2289	-20	-26	-22	-26	0.8	0.8	0.8	0.8
Pelp1	Q9DBD5	523	-26	-27	-22	10	0.8	0.8	0.8	1.1
Gm15800	E9Q2E4	1911	-25	-27	-22	-2	0.8	0.8	0.8	1.0
Echdc1	Q9D9V3	27	-9	-27	-22	-9	0.9	0.8	0.8	0.9
Usp15	Q8R5H1	264	-12	-28	-22	-18	0.9	0.8	0.8	0.8
Carmil2	Q3V3V9	57	-16	-28	-22	-28	0.9	0.8	0.8	0.8
Skap2	Q3UND0	142	-48	-29	-22	-15	0.7	0.8	0.8	0.9
Ltf	P08071	199	-24	-29	-22	-6	0.8	0.8	0.8	0.9
Dus3l	Q91XI1	189	-3	-30	-22	-16	1.0	0.8	0.8	0.9
Fkbp4	P30416	342	-13	-31	-22	-2	0.9	0.8	0.8	1.0
Trrap	A0A1D5RLL4	568	-22	-32	-22	-20	0.8	0.8	0.8	0.8
Gcc2	B2RSU7	1051	-27	-32	-22	-25	0.8	0.8	0.8	0.8
Uap1l1	Q3TW96	50	-19	-36	-22	-65	0.8	0.7	0.8	0.6
Idh3a	Q9D6R2	359	2	-37	-22	-36	1.0	0.7	0.8	0.7
Sccpdh	Q8R127	218	-28	-38	-22	-45	0.8	0.7	0.8	0.7
Ccdc88c	Q6VGS5	1185	41	-44	-22	-37	1.7	0.7	0.8	0.7
Baz1b	Q9Z277	954	35	-72	-22	-92	1.5	0.6	0.8	0.5
Acsl1	P41216	109	-20	11	-23	0	0.8	1.1	0.8	1.0
Zcchc6	E9PUA2	828	-20	2	-23	1	0.8	1.0	0.8	1.0
Ect2	Q07139	254	-13	1	-23	10	0.9	1.0	0.8	1.1
Map4k1	P70218	444	-2	-2	-23	1	1.0	1.0	0.8	1.0
Rab24	P35290	51	-5	-4	-23	-1	1.0	1.0	0.8	1.0
Zbtb2	Q3V3W4	296	-17	-5	-23	-22	0.9	1.0	0.8	0.8
Lancl2	Q9JJK2	187	-8	-5	-23	-9	0.9	1.0	0.8	0.9
Slc25a16	Q8C0K5	311	-12	-6	-23	17	0.9	0.9	0.8	1.2
Map4k1	P70218	802	-18	-7	-23	6	0.8	0.9	0.8	1.1
4931406P:	Q8C5X1	498	-2	-7	-23	-1	1.0	0.9	0.8	1.0
Rars	Q9D0I9	502	-10	-8	-23	25	0.9	0.9	0.8	1.3
Dock11	A2AF47	459	-19	-8	-23	-6	0.8	0.9	0.8	0.9
Eif4g3	Q80XI3	977	-24	-8	-23	-6	0.8	0.9	0.8	0.9
Dip2b	Q3UH60	1017	-13	-10	-23	-5	0.9	0.9	0.8	1.0
Ubr4	A2AN08	4269	-1	-10	-23	-2	1.0	0.9	0.8	1.0
Ezh2	Q61188	14	-23	-10	-23	-15	0.8	0.9	0.8	0.9
Pcm1	Q9R0L6	768	-23	-11	-23	-5	0.8	0.9	0.8	1.0
Supt20h	Q7TT00	496	-11	-12	-23	-17	0.9	0.9	0.8	0.9
Larp4b	Q6A0A2	635	-16	-12	-23	-27	0.9	0.9	0.8	0.8
Upf3b	Q3ULL6	403	-13	-13	-23	-21	0.9	0.9	0.8	0.8
Gtpbp4	Q99ME9	174	-19	-13	-23	-31	0.8	0.9	0.8	0.8
Gvin1	L7N451	746	-7	-13	-23	15	0.9	0.9	0.8	1.2
Fubp1	Q91WJ8	128	-17	-13	-23	-9	0.9	0.9	0.8	0.9
Impa1	Q924B0	64	-11	-13	-23	-39	0.9	0.9	0.8	0.7
Nup155	Q99P88	974	-19	-14	-23	14	0.8	0.9	0.8	1.2
Rnf40	Q3U319	127	-14	-14	-23	-30	0.9	0.9	0.8	0.8
Slc7a1	Q09143	621	-5	-14	-23	-45	1.0	0.9	0.8	0.7
Stim2	P83093	653	-13	-14	-23	-24	0.9	0.9	0.8	0.8
Cnst	Q8CBC4	497	-34	-14	-23	-27	0.7	0.9	0.8	0.8
Birc3	O08863	292	-9	-15	-23	-4	0.9	0.9	0.8	1.0
Birc2	Q62210	301	-9	-15	-23	-4	0.9	0.9	0.8	1.0
Dnmt1	P13864	1501	-16	-15	-23	-7	0.9	0.9	0.8	0.9
Gimap8	Q75N62	462	-19	-15	-23	-9	0.8	0.9	0.8	0.9
Tmem173	Q3TBT3	205	-17	-16	-23	-3	0.9	0.9	0.8	1.0

Cep250	Q60952	2037	-14	-16	-23	-7	0.9	0.9	0.8	0.9
Tldc1	Q8K0P3	81	-20	-17	-23	-10	0.8	0.9	0.8	0.9
Trip11	E9Q512	1296	-6	-18	-23	-14	0.9	0.9	0.8	0.9
Gar1	Q9CY66	99	-15	-18	-23	-3	0.9	0.8	0.8	1.0
Chfr	Q810L3	603	-20	-18	-23	-18	0.8	0.8	0.8	0.8
Ppfibp2	O35711	555	-15	-18	-23	-28	0.9	0.8	0.8	0.8
Txndc5	Q91W90	203	-19	-19	-23	2	0.8	0.8	0.8	1.0
Rbm19	Q8R3C6	570	-27	-19	-23	-21	0.8	0.8	0.8	0.8
Ndufab1	Q9CR21	140	-18	-20	-23	3	0.8	0.8	0.8	1.0
Pds5a	E9QPI5	429	-18	-20	-23	-15	0.8	0.8	0.8	0.9
Nmrk1	Q91W63	125	-13	-21	-23	-24	0.9	0.8	0.8	0.8
Trip4	Q9QXN3	86	-8	-21	-23	-31	0.9	0.8	0.8	0.8
Gba2	Q69ZF3	711	-24	-22	-23	-14	0.8	0.8	0.8	0.9
Ldah	Q8BVA5	185	-8	-22	-23	-49	0.9	0.8	0.8	0.7
Fig4	Q91WF7	489	-19	-22	-23	-20	0.8	0.8	0.8	0.8
Rab10	P61027	24	-25	-23	-23	-1	0.8	0.8	0.8	1.0
Rnh1	Q91VI7	329	-19	-23	-23	-16	0.8	0.8	0.8	0.9
Senp7	Q8BUH8	119	-17	-23	-23	-33	0.9	0.8	0.8	0.8
Mtch2	Q791V5	56	-31	-24	-23	-3	0.8	0.8	0.8	1.0
Kiaa0391	Q8JZY4	567	-22	-24	-23	-17	0.8	0.8	0.8	0.9
Uap1l1	Q3TW96	401	-20	-24	-23	-22	0.8	0.8	0.8	0.8
Kat14	Q8CID0	544	-20	-26	-23	-25	0.8	0.8	0.8	0.8
Myo5a	Q99104	535	-16	-27	-23	-12	0.9	0.8	0.8	0.9
Eif3f	Q9DCH4	260	-21	-28	-23	25	0.8	0.8	0.8	1.3
Zfp292	Q9Z2U2	428	-36	-28	-23	-18	0.7	0.8	0.8	0.9
Bcl11b	Q99PV8	798	-10	-28	-23	-23	0.9	0.8	0.8	0.8
Incenp	Q9WU62	331	-28	-30	-23	2	0.8	0.8	0.8	1.0
Stk11ip	Q3TAA7	429	-10	-30	-23	-33	0.9	0.8	0.8	0.8
Cwf19l1	Q8CI33	176	-25	-31	-23	-8	0.8	0.8	0.8	0.9
Myo1e	E9Q634	586	7	-32	-23	-39	1.1	0.8	0.8	0.7
Myo1f	Q8CG29	584	7	-32	-23	-39	1.1	0.8	0.8	0.7
Tdrd7	Q8K1H1	680	-4	-33	-23	-22	1.0	0.8	0.8	0.8
Haus7	Q8BKT8	234	-18	-33	-23	-22	0.8	0.8	0.8	0.8
Psme4	Q5SSW2	1343	-33	-34	-23	-18	0.8	0.7	0.8	0.8
Vps8	Q0P5W1	1165	-27	-36	-23	22	0.8	0.7	0.8	1.3
Trim12c	D3Z3L3	334	-23	-37	-23	-24	0.8	0.7	0.8	0.8
Dido1	Q8C9B9	452	-19	-39	-23	-19	0.8	0.7	0.8	0.8
Khdrbs1	Q60749	19	-25	15	-23	-15	0.8	1.2	0.8	0.9
Psmd5	Q8BJY1	290	-11	9	-23	10	0.9	1.1	0.8	1.1
Znf48	Q3US17	259	-16	3	-23	-43	0.9	1.0	0.8	0.7
Gmip	Q6PGG2	959	-14	-2	-23	-17	0.9	1.0	0.8	0.9
Znf280d	Q68FE8	105	-9	-2	-23	-14	0.9	1.0	0.8	0.9
Atp5h	Q9DCX2	101	-22	-3	-23	17	0.8	1.0	0.8	1.2
Pcid2	Q8BFV2	152	-10	-4	-23	7	0.9	1.0	0.8	1.1
Mical1	Q8VDP3	82	-12	-5	-23	17	0.9	1.0	0.8	1.2
Csde1	Q91W50	506	-27	-5	-23	-6	0.8	1.0	0.8	0.9
Psmc5	P62196	209	-21	-5	-23	-17	0.8	1.0	0.8	0.9
Dmxl1	Q6PNC0	136	6	-6	-23	-9	1.1	0.9	0.8	0.9
Nme3	Q9WV85	158	-19	-7	-23	-16	0.8	0.9	0.8	0.9
Cdc40	Q9DC48	569	-18	-8	-23	8	0.8	0.9	0.8	1.1
Ctu1	Q99J10	302	-21	-8	-23	-4	0.8	0.9	0.8	1.0

Sept7	O55131	17	-3	-8	-23	-23	1.0	0.9	0.8	0.8
Dld	O08749	80	7	-8	-23	-16	1.1	0.9	0.8	0.9
Irf2bp1	Q8R3Y8	15	-16	-9	-23	-8	0.9	0.9	0.8	0.9
Mms19	Q9D071	335	-9	-10	-23	11	0.9	0.9	0.8	1.1
Pfkfb3	A7UAK5	206	-9	-10	-23	-17	0.9	0.9	0.8	0.9
Matr3	Q8K310	820	-14	-11	-23	27	0.9	0.9	0.8	1.4
Dfna5	Q9Z2D3	45	-13	-11	-23	17	0.9	0.9	0.8	1.2
Fam175a	Q8BPZ8	280	-22	-11	-23	-1	0.8	0.9	0.8	1.0
Hnrnpu	Q8VEK3	365	-8	-11	-23	6	0.9	0.9	0.8	1.1
Micu2	Q8CD10	141	-16	-12	-23	-11	0.9	0.9	0.8	0.9
Prkdc	P97313	2967	-18	-13	-23	-9	0.9	0.9	0.8	0.9
Toe1	Q9D2E2	301	-19	-13	-23	-22	0.8	0.9	0.8	0.8
Eif2ak1	Q9Z2R9	491	-10	-14	-23	-5	0.9	0.9	0.8	1.0
Zmym2	Q9CU65	703	-19	-14	-23	-12	0.8	0.9	0.8	0.9
Tab2	Q99K90	608	-19	-14	-23	-23	0.8	0.9	0.8	0.8
Polg2	Q9QZM2	351	-13	-14	-23	-24	0.9	0.9	0.8	0.8
Edc3	Q8K2D3	47	-11	-14	-23	-27	0.9	0.9	0.8	0.8
Ccnd3	P30282	47	-9	-15	-23	-13	0.9	0.9	0.8	0.9
Map2k5	Q9WVS7	300	-10	-15	-23	-21	0.9	0.9	0.8	0.8
Papolg	Q6PCL9	29	-35	-15	-23	-26	0.7	0.9	0.8	0.8
Rps4x	P62702	41	-11	-15	-23	-39	0.9	0.9	0.8	0.7
Nrf1	Q9WU00	229	-9	-16	-23	0	0.9	0.9	0.8	1.0
Riok1	Q922Q2	263	-17	-17	-23	-13	0.9	0.9	0.8	0.9
Wdr81	Q5ND34	970	-21	-17	-23	-6	0.8	0.9	0.8	0.9
Ntpcr	Q9CQA9	103	-8	-17	-23	-13	0.9	0.9	0.8	0.9
Abce1	P61222	201	-18	-18	-23	-19	0.9	0.9	0.8	0.8
Idh2	P54071	154	-12	-18	-23	4	0.9	0.8	0.8	1.0
Nono	Q99K48	210	-16	-18	-23	-13	0.9	0.8	0.8	0.9
Fam101b	Q5SVD0	188	-15	-19	-23	-12	0.9	0.8	0.8	0.9
P4hb	P09103	399	-13	-19	-23	13	0.9	0.8	0.8	1.1
Rab2b	P59279	154	-18	-19	-23	-24	0.8	0.8	0.8	0.8
Gabpa	Q00422	61	-1	-19	-23	-26	1.0	0.8	0.8	0.8
Fbxo30	Q8BJL1	593	-14	-20	-23	-17	0.9	0.8	0.8	0.9
UPF0688	Q80WR5	42	-19	-20	-23	-22	0.8	0.8	0.8	0.8
Mcm5	Q52KC3	482	-16	-20	-23	-29	0.9	0.8	0.8	0.8
Tpi1	P17751	71	-19	-20	-23	-1	0.8	0.8	0.8	1.0
Znf592	Q8BHZ4	351	-26	-20	-23	-22	0.8	0.8	0.8	0.8
Trp53bp1	A2AU91	1372	-5	-20	-23	-25	1.0	0.8	0.8	0.8
Gls	D3Z7P3	208	-26	-21	-23	26	0.8	0.8	0.8	1.4
Rab3gap1	Q80UJ7	919	-13	-21	-23	-42	0.9	0.8	0.8	0.7
Cmpk2	Q3U5Q7	44	-20	-22	-23	-29	0.8	0.8	0.8	0.8
Senp7	Q8BUH8	140	-13	-22	-23	-33	0.9	0.8	0.8	0.8
Kif4	P33174	966	-19	-26	-23	-28	0.8	0.8	0.8	0.8
Paf	Q9CQX4	53	-22	-26	-23	-39	0.8	0.8	0.8	0.7
Sar1a	Q99JZ4	102	-19	-26	-23	26	0.8	0.8	0.8	1.4
Mms19	Q9D071	761	-18	-26	-23	-24	0.8	0.8	0.8	0.8
Syne1	Q6ZWR6	7768	-10	-27	-23	-32	0.9	0.8	0.8	0.8
Nup43	P59235	208	-18	-27	-23	-22	0.8	0.8	0.8	0.8
Fam3c	Q91VU0	185	-16	-30	-23	2	0.9	0.8	0.8	1.0
H2-T23	P06339	134	-27	-30	-23	8	0.8	0.8	0.8	1.1
Ppa1	Q9D819	114	-27	-30	-23	-9	0.8	0.8	0.8	0.9

Fam105a	Q3TVP5	174	-10	-30	-23	-12	0.9	0.8	0.8	0.9
Neurl4	Q5NCX5	1369	-26	-36	-23	-21	0.8	0.7	0.8	0.8
Swap70	Q6A028	279	-38	-44	-23	-22	0.7	0.7	0.8	0.8
Ercc6	F8VPZ5	1452	6	-53	-23	4	1.1	0.7	0.8	1.0
Drap1	Q9D6N5	73	-24	6	-24	8	0.8	1.1	0.8	1.1
Gfm2	Q8R2Q4	329	-4	4	-24	-6	1.0	1.0	0.8	0.9
Pot1	Q91WC1	467	-23	2	-24	-7	0.8	1.0	0.8	0.9
Mum1	Q6DID5	319	-18	-4	-24	-30	0.9	1.0	0.8	0.8
Pfkp	Q9WUA3	111	-5	-5	-24	-57	1.0	1.0	0.8	0.6
Suclg2	Q9Z2I8	256	-7	-6	-24	-83	0.9	0.9	0.8	0.5
Gatb	Q99JT1	228	-20	-7	-24	-16	0.8	0.9	0.8	0.9
Pbrm1	Q8BSQ9	1047	-27	-9	-24	19	0.8	0.9	0.8	1.2
Msl1	Q6PDM1	223	-11	-10	-24	-9	0.9	0.9	0.8	0.9
Pfas	Q5SUR0	336	-8	-10	-24	3	0.9	0.9	0.8	1.0
Tes	Q921W7	46	-21	-11	-24	-7	0.8	0.9	0.8	0.9
Mbnl1	Q9JKP5	34	-11	-11	-24	-11	0.9	0.9	0.8	0.9
Csk	P41241	122	-15	-13	-24	4	0.9	0.9	0.8	1.0
Usp9x	Q4FE56	842	-11	-13	-24	-18	0.9	0.9	0.8	0.9
Haao	Q78JT3	245	-13	-14	-24	9	0.9	0.9	0.8	1.1
Inpp5b	Q8K337	362	-24	-15	-24	10	0.8	0.9	0.8	1.1
Smarcd2	Q99JR8	405	-24	-15	-24	-34	0.8	0.9	0.8	0.7
Irf8	P23611	223	-15	-15	-24	-37	0.9	0.9	0.8	0.7
Araf	P04627	536	-33	-16	-24	13	0.8	0.9	0.8	1.1
Zak	Q9ESL4	231	-29	-17	-24	-2	0.8	0.9	0.8	1.0
Ube3a	O08759	838	-20	-17	-24	-13	0.8	0.9	0.8	0.9
Hemgn	Q9ERZ0	296	-26	-17	-24	-16	0.8	0.9	0.8	0.9
Mybbp1a	Q7TPV4	896	-8	-17	-24	13	0.9	0.9	0.8	1.1
Apobec3	Q99J72	13	-13	-17	-24	-13	0.9	0.9	0.8	0.9
Gabpa	Q00422	37	-24	-17	-24	-22	0.8	0.9	0.8	0.8
Ralbp1	Q62172	451	-13	-17	-24	-24	0.9	0.9	0.8	0.8
Rnpep	Q8VCT3	130	-20	-18	-24	-15	0.8	0.9	0.8	0.9
Txlng	Q8BHN1	127	-9	-18	-24	-13	0.9	0.8	0.8	0.9
Paxbp1	P58501	168	-11	-19	-24	-14	0.9	0.8	0.8	0.9
Lrrk2	Q5S006	620	-17	-19	-24	-7	0.9	0.8	0.8	0.9
Orc2	Q60862	55	-12	-20	-24	-8	0.9	0.8	0.8	0.9
Hnrnpu	Q8VEK3	265	-14	-20	-24	-9	0.9	0.8	0.8	0.9
Dph1	Q5NCQ5	426	-31	-20	-24	-32	0.8	0.8	0.8	0.8
Il16	O54824	1313	-17	-20	-24	-29	0.9	0.8	0.8	0.8
Setd2	E9Q5F9	1214	-6	-21	-24	-26	0.9	0.8	0.8	0.8
Haus6	Q6NV99	693	-23	-22	-24	-26	0.8	0.8	0.8	0.8
Pgls	Q9CQ60	78	-36	-22	-24	-15	0.7	0.8	0.8	0.9
Mdn1	A2ANY6	374	-6	-22	-24	-20	0.9	0.8	0.8	0.8
Pde2a	F7D3W5	451	-33	-23	-24	-16	0.8	0.8	0.8	0.9
Zmym2	Q9CU65	800	-14	-24	-24	-17	0.9	0.8	0.8	0.9
Fasn	P19096	212	-18	-24	-24	-18	0.8	0.8	0.8	0.8
Dtx3l	Q3UIR3	314	-30	-24	-24	-22	0.8	0.8	0.8	0.8
A430078G	E9Q7Y4	144	-8	-24	-24	-23	0.9	0.8	0.8	0.8
Hccs	P53702	182	-24	-24	-24	-25	0.8	0.8	0.8	0.8
Rngtt	O55236	97	26	-24	-24	-28	1.3	0.8	0.8	0.8
Dnajc14	Q921R4	604	-43	-24	-24	-41	0.7	0.8	0.8	0.7
Rps21	Q9CQR2	17	-15	-24	-24	-68	0.9	0.8	0.8	0.6

Prdx6b	Q8BG37	47	5	-25	-24	-11	1.0	0.8	0.8	0.9
Mctp2	Q5RJH2	865	-34	-25	-24	-10	0.7	0.8	0.8	0.9
Cybb	Q61093	371	-17	-25	-24	-20	0.9	0.8	0.8	0.8
Irf9	Q61179	323	-14	-26	-24	-14	0.9	0.8	0.8	0.9
Rps26	P62855	26	-16	-26	-24	-24	0.9	0.8	0.8	0.8
Dock11	A2AF47	633	-18	-29	-24	-2	0.8	0.8	0.8	1.0
Cacul1	Q8R0X2	102	6	-30	-24	-56	1.1	0.8	0.8	0.6
Trim35	Q8C006	265	-22	-31	-24	-8	0.8	0.8	0.8	0.9
Aldoa	P05064	290	-25	-33	-24	-11	0.8	0.8	0.8	0.9
Pcx	E9QPD7	623	-36	-33	-24	-17	0.7	0.8	0.8	0.9
Mdc1	Q5PSV9	179	-28	-36	-24	-28	0.8	0.7	0.8	0.8
Cad	B2RQC6	1296	-21	-37	-24	34	0.8	0.7	0.8	1.5
Pds5a	E9QPI5	485	-25	-37	-24	13	0.8	0.7	0.8	1.1
Egfl8	Q6GUQ1	132	-34	-37	-24	-43	0.7	0.7	0.8	0.7
Egfl8	Q6GUQ1	130	-34	-37	-24	-43	0.7	0.7	0.8	0.7
Nrbp1	Q99J45	307	-23	-2	-24	-15	0.8	1.0	0.8	0.9
Kmt2d	Q6PDK2	5193	-21	-5	-24	-6	0.8	1.0	0.8	0.9
Dopey2	Q3UHQ6	1327	-17	-6	-24	5	0.9	0.9	0.8	1.1
Sp1	O89090	70	-1	-6	-24	-23	1.0	0.9	0.8	0.8
Rps3	P62908	119	-8	-7	-24	-86	0.9	0.9	0.8	0.5
Lsm14a	Q8K2F8	85	-12	-7	-24	-31	0.9	0.9	0.8	0.8
Pcnt	F8VPV0	31	-14	-9	-24	-19	0.9	0.9	0.8	0.8
Baz2a	E9Q374	574	-13	-10	-24	-10	0.9	0.9	0.8	0.9
Smyd5	Q3TYX3	101	-26	-11	-24	-10	0.8	0.9	0.8	0.9
Lrrk2	Q5S006	2384	-5	-11	-24	-10	1.0	0.9	0.8	0.9
Nrdc	Q8BHG1	977	-10	-11	-24	-3	0.9	0.9	0.8	1.0
Vac14	Q80WQ2	620	-23	-12	-24	5	0.8	0.9	0.8	1.1
Sympk	Q80X82	686	-12	-12	-24	-30	0.9	0.9	0.8	0.8
Nepro	Q8R2U2	427	-8	-13	-24	-17	0.9	0.9	0.8	0.9
Ankrd54	Q91WK7	229	-20	-14	-24	-8	0.8	0.9	0.8	0.9
Lonp2	Q9DBN5	137	-18	-14	-24	-15	0.9	0.9	0.8	0.9
Mical1	Q8VDP3	95	-15	-16	-24	13	0.9	0.9	0.8	1.1
Osbpl7	A2A716	587	-24	-16	-24	-11	0.8	0.9	0.8	0.9
Ifi47	Q61635	352	-19	-19	-24	25	0.8	0.8	0.8	1.3
Nosip	Q9D6T0	8	-12	-19	-24	-20	0.9	0.8	0.8	0.8
Ddx42	Q810A7	333	-26	-19	-24	-12	0.8	0.8	0.8	0.9
Ddx58	Q6Q899	474	-7	-19	-24	-12	0.9	0.8	0.8	0.9
Eif2d	Q61211	515	-17	-20	-24	-8	0.9	0.8	0.8	0.9
Carmil2	Q3V3V9	496	-13	-20	-24	-11	0.9	0.8	0.8	0.9
Noc3l	Q8VI84	793	-17	-20	-24	-16	0.9	0.8	0.8	0.9
Tnks1bp1	P58871	406	-28	-20	-24	-37	0.8	0.8	0.8	0.7
Zmym3	Q9JLM4	1074	-20	-21	-24	-11	0.8	0.8	0.8	0.9
As3mt	Q91WU5	33	-21	-21	-24	-16	0.8	0.8	0.8	0.9
Nipbl	Q6KCD5	279	-20	-21	-24	-20	0.8	0.8	0.8	0.8
Ube2s	Q921J4	118	-15	-22	-24	-9	0.9	0.8	0.8	0.9
Stoml2	Q99JB2	167	-32	-22	-24	-27	0.8	0.8	0.8	0.8
Ctdp1	Q7TSG2	24	-37	-25	-24	-18	0.7	0.8	0.8	0.8
Rpap2	Q8VC34	472	-22	-26	-24	-24	0.8	0.8	0.8	0.8
Xrcc5	P27641	235	-8	-26	-24	-30	0.9	0.8	0.8	0.8
Ubr5	E9Q2H1	1291	10	-27	-24	-19	1.1	0.8	0.8	0.8
Ccdc117	Q6PB51	79	-4	-27	-24	-33	1.0	0.8	0.8	0.8

Iars	Q8BU30	87	-8	-28	-24	-71	0.9	0.8	0.8	0.6
Znf638	Q61464	1648	-24	-28	-24	-15	0.8	0.8	0.8	0.9
Irs2	P81122	609	-22	-28	-24	-17	0.8	0.8	0.8	0.9
Filip1l	Q6P6L0	444	-25	-28	-24	-32	0.8	0.8	0.8	0.8
Gtf3c4	Q8BMQ2	112	-18	-29	-24	-17	0.8	0.8	0.8	0.9
Mphosph8	Q3TYA6	751	-37	-30	-24	1	0.7	0.8	0.8	1.0
Me2	Q99KE1	441	-22	-30	-24	-1	0.8	0.8	0.8	1.0
Tcf20	Q9EPQ8	1356	-16	-31	-24	-28	0.9	0.8	0.8	0.8
Gm45233	A0A0N4SVE0	42	-20	-33	-24	0	0.8	0.8	0.8	1.0
Lrrk2	Q5S006	1526	-8	-34	-24	4	0.9	0.7	0.8	1.0
Psmd2	Q8VDM4	448	-30	-37	-24	4	0.8	0.7	0.8	1.0
Lpp	Q8BFW7	525	-16	-37	-24	-35	0.9	0.7	0.8	0.7
Tyw1	Q8BJM7	409	-32	-38	-24	-9	0.8	0.7	0.8	0.9
Wdr81	Q5ND34	462	-15	-39	-24	-23	0.9	0.7	0.8	0.8
Fam21	Q6PGL7	814	-24	-44	-24	-22	0.8	0.7	0.8	0.8
Vta1	Q9CR26	155	-6	3	-25	10	0.9	1.0	0.8	1.1
Ercc4	Q9QZD4	516	-2	-4	-25	-14	1.0	1.0	0.8	0.9
Lancl2	Q9JJK2	169	-4	-5	-25	-21	1.0	1.0	0.8	0.8
Mark2	Q05512	210	-11	-5	-25	-43	0.9	1.0	0.8	0.7
Lgals9	O08573	138	-7	-7	-25	-17	0.9	0.9	0.8	0.9
Mtap	Q9CQ65	130	-39	-7	-25	-45	0.7	0.9	0.8	0.7
Lrrfip1	Q3UZ39	397	-15	-9	-25	-20	0.9	0.9	0.8	0.8
Babam1	Q3UI43	226	-18	-10	-25	1	0.8	0.9	0.8	1.0
Iqgap1	Q9JKF1	320	-16	-10	-25	-29	0.9	0.9	0.8	0.8
Dtymk	P97930	117	-3	-10	-25	-109	1.0	0.9	0.8	0.5
Ccar2	Q8VDP4	387	-8	-11	-25	7	0.9	0.9	0.8	1.1
Vta1	Q9CR26	87	-26	-11	-25	4	0.8	0.9	0.8	1.0
Rock2	F8VPK5	804	-15	-11	-25	-6	0.9	0.9	0.8	0.9
Tnks1bp1	P58871	1003	-7	-12	-25	-29	0.9	0.9	0.8	0.8
Ehbp1l1	E9QP49	1389	-9	-13	-25	-55	0.9	0.9	0.8	0.6
Zfc3h1	B2RT41	1382	-16	-13	-25	-8	0.9	0.9	0.8	0.9
Ipo9	E9QKZ2	545	-27	-14	-25	10	0.8	0.9	0.8	1.1
Scrn3	Q3TMH2	239	-12	-14	-25	-56	0.9	0.9	0.8	0.6
Sgpp1	Q9JI99	388	-31	-15	-25	-40	0.8	0.9	0.8	0.7
Cep57	Q8CEE0	252	-9	-15	-25	-32	0.9	0.9	0.8	0.8
Ighmbp2	A0A0B4J1E3	394	-11	-17	-25	13	0.9	0.9	0.8	1.1
Hk2	O08528	794	-8	-17	-25	-19	0.9	0.9	0.8	0.8
Ttpal	Q9D3D0	342	-11	-18	-25	-28	0.9	0.9	0.8	0.8
Pml	Q60953	82	-18	-18	-25	-1	0.9	0.8	0.8	1.0
Shmt2	Q9CZN7	412	-31	-18	-25	-4	0.8	0.8	0.8	1.0
Capn2	O08529	301	-7	-18	-25	-24	0.9	0.8	0.8	0.8
Acin1	Q9JIX8	733	-15	-19	-25	-31	0.9	0.8	0.8	0.8
Rnpepl1	G5E872	277	-18	-20	-25	-13	0.8	0.8	0.8	0.9
Tuba3b	P05214	347	-19	-20	-25	-28	0.8	0.8	0.8	0.8
Tuba1a	P68369	347	-19	-20	-25	-28	0.8	0.8	0.8	0.8
Macf1	E9PVY8	1722	-21	-21	-25	12	0.8	0.8	0.8	1.1
Ubr2	Q6WKZ8	280	-14	-21	-25	-4	0.9	0.8	0.8	1.0
Kntc1	Q8C3Y4	464	-13	-21	-25	-15	0.9	0.8	0.8	0.9
Samsn1	P57725	196	-18	-21	-25	-72	0.9	0.8	0.8	0.6
Kif21b	E9Q0A4	1400	-9	-22	-25	-21	0.9	0.8	0.8	0.8
Bmf	Q91ZE9	140	-21	-23	-25	-12	0.8	0.8	0.8	0.9

Psmd13	Q9WVJ2	114	-8	-24	-25	-7	0.9	0.8	0.8	0.9
Trim33	Q99PP7	161	-41	-24	-25	5	0.7	0.8	0.8	1.0
Pfkfb3	A7UAK5	427	-21	-24	-25	-37	0.8	0.8	0.8	0.7
Uhrf1bp1l	A2RSJ4	647	7	-28	-25	-26	1.1	0.8	0.8	0.8
Kdm3a	Q6PCM1	1142	-5	-29	-25	-6	1.0	0.8	0.8	0.9
Ttc37	F8VPK0	87	-19	-29	-25	-24	0.8	0.8	0.8	0.8
Ndc80	Q9D0F1	9	-9	-30	-25	-11	0.9	0.8	0.8	0.9
Rpl4	Q9D8E6	125	-17	-30	-25	-29	0.9	0.8	0.8	0.8
Smg7	Q5RJH6	536	-16	-30	-25	-24	0.9	0.8	0.8	0.8
Med17	Q8VCD5	486	-18	-31	-25	-7	0.8	0.8	0.8	0.9
Mdn1	A2ANY6	2535	-21	-31	-25	-21	0.8	0.8	0.8	0.8
Lrrfip1	Q3UZ39	311	-13	-33	-25	-42	0.9	0.8	0.8	0.7
Mcm5	Q52KC3	221	-16	-34	-25	-25	0.9	0.7	0.8	0.8
Fam129b	Q8R1F1	292	43	-35	-25	-33	1.7	0.7	0.8	0.8
Thop1	Q8C1A5	91	-18	2	-25	9	0.8	1.0	0.8	1.1
Vwa8	Q8CC88	161	-19	-2	-25	-2	0.8	1.0	0.8	1.0
Tgfbr2	Q62312	558	-11	-7	-25	-37	0.9	0.9	0.8	0.7
Ubr1	O70481	180	-28	-9	-25	-23	0.8	0.9	0.8	0.8
Kif2a	P28740	158	-31	-10	-25	-27	0.8	0.9	0.8	0.8
Cul5	Q9D5V5	112	-8	-10	-25	-11	0.9	0.9	0.8	0.9
Tada3	Q8R0L9	255	-28	-11	-25	14	0.8	0.9	0.8	1.2
Rbbp6	P97868	162	-32	-11	-25	-8	0.8	0.9	0.8	0.9
Bcl10	Q9Z0H7	215	-28	-11	-25	-38	0.8	0.9	0.8	0.7
Rab19	P35294	32	-10	-12	-25	-9	0.9	0.9	0.8	0.9
Akt2	Q60823	311	-17	-13	-25	-20	0.9	0.9	0.8	0.8
Cybb	Q61093	86	-25	-14	-25	-31	0.8	0.9	0.8	0.8
Lyar	Q08288	30	-12	-15	-25	-13	0.9	0.9	0.8	0.9
Hectd1	Q69ZR2	369	-38	-16	-25	-6	0.7	0.9	0.8	0.9
Dock10	E9QM99	1092	-9	-16	-25	-9	0.9	0.9	0.8	0.9
Mrpl11	Q9CQF0	50	-26	-16	-25	-57	0.8	0.9	0.8	0.6
Pgls	Q9CQ60	236	-25	-17	-25	-5	0.8	0.9	0.8	1.0
Hsd17b4	P51660	189	-40	-17	-25	-27	0.7	0.9	0.8	0.8
Ptpn9	O35239	67	-6	-18	-25	-7	0.9	0.9	0.8	0.9
Kdm3a	Q6PCM1	695	-1	-19	-25	-5	1.0	0.8	0.8	1.0
Txndc5	Q91W90	335	-22	-19	-25	-4	0.8	0.8	0.8	1.0
Casp8	O89110	267	-13	-19	-25	-13	0.9	0.8	0.8	0.9
Cobll1	Q3UMF0	267	-23	-19	-25	-30	0.8	0.8	0.8	0.8
Map2k3	O09110	305	-20	-20	-25	-37	0.8	0.8	0.8	0.7
Acap1	Q8K2H4	338	-32	-21	-25	23	0.8	0.8	0.8	1.3
Cpt2	P52825	489	-27	-22	-25	27	0.8	0.8	0.8	1.4
Psmd3	P14685	206	-14	-22	-25	10	0.9	0.8	0.8	1.1
Ywhaq	P68254	237	-6	-22	-25	-22	0.9	0.8	0.8	0.8
Zc3h13	E9Q784	1720	-28	-22	-25	-30	0.8	0.8	0.8	0.8
Srrt	Q99MR6	639	8	-23	-25	31	1.1	0.8	0.8	1.4
Map4k2	Q61161	677	-13	-23	-25	-8	0.9	0.8	0.8	0.9
Slfn5	Q8CBA2	48	-26	-24	-25	-5	0.8	0.8	0.8	1.0
Gnl3l	Q6PGG6	143	-18	-24	-25	-23	0.8	0.8	0.8	0.8
Lrch4	Q921G6	105	-15	-24	-25	-29	0.9	0.8	0.8	0.8
Anapc7	Q9WVM3	97	-29	-25	-25	-31	0.8	0.8	0.8	0.8
Snrpf	P62307	66	-13	-26	-25	-14	0.9	0.8	0.8	0.9
Rpl8	P62918	90	-15	-26	-25	-29	0.9	0.8	0.8	0.8

Zfp512b	Q6PHP4	314	-7	-26	-25	-32	0.9	0.8	0.8	0.8
Qars	Q8BML9	687	-27	-27	-25	-2	0.8	0.8	0.8	1.0
Hsd17b11	Q9EQ06	217	-26	-27	-25	-11	0.8	0.8	0.8	0.9
Stat2	E9QJX9	485	-19	-27	-25	4	0.8	0.8	0.8	1.0
Prkra	Q9WTX2	54	-32	-28	-25	-18	0.8	0.8	0.8	0.9
Nup155	Q99P88	373	-31	-29	-25	-5	0.8	0.8	0.8	1.0
Neu1	O35657	293	-18	-29	-25	-41	0.8	0.8	0.8	0.7
Rfc2	Q9WUK4	250	-5	-29	-25	-47	1.0	0.8	0.8	0.7
Psmd8	Q9CX56	115	-9	-30	-25	-8	0.9	0.8	0.8	0.9
Nudcd1	Q6PIP5	512	-35	-30	-25	-23	0.7	0.8	0.8	0.8
Utp23	Q9CX11	91	-30	-30	-25	-11	0.8	0.8	0.8	0.9
Nfx1	B1AY10	655	-22	-33	-25	-61	0.8	0.8	0.8	0.6
Ccdc88c	Q6VGS5	1458	-20	-34	-25	-21	0.8	0.7	0.8	0.8
Fasn	P19096	1752	-23	-37	-25	-4	0.8	0.7	0.8	1.0
Fgd3	O88842	180	63	-37	-25	-28	2.7	0.7	0.8	0.8
Rnf114	Q9ET26	111	29	7	-26	5	1.4	1.1	0.8	1.1
Rpap1	Q80TE0	1055	2	0	-26	13	1.0	1.0	0.8	1.1
Phkb	Q7TSH2	728	6	-1	-26	-3	1.1	1.0	0.8	1.0
Rps3	P62908	134	-9	-2	-26	-123	0.9	1.0	0.8	0.4
Crlf3	Q9Z2L7	218	-22	-4	-26	-6	0.8	1.0	0.8	0.9
Pnpla2	Q8BJ56	106	-3	-5	-26	-12	1.0	1.0	0.8	0.9
Plekho2	Q8K124	444	-27	-5	-26	-19	0.8	1.0	0.8	0.8
Dock8	Q8C147	1195	-23	-6	-26	-24	0.8	0.9	0.8	0.8
Abcg1	Q64343	209	-21	-7	-26	-25	0.8	0.9	0.8	0.8
Gpd1l	Q3ULJ0	164	-8	-8	-26	-15	0.9	0.9	0.8	0.9
Wdr45b	Q9CR39	63	4	-9	-26	21	1.0	0.9	0.8	1.3
Fnbp4	Q6ZQ03	663	-13	-10	-26	-19	0.9	0.9	0.8	0.8
Vav3	Q9R0C8	195	-16	-11	-26	-18	0.9	0.9	0.8	0.8
Eftud2	O08810	147	-6	-12	-26	4	0.9	0.9	0.8	1.0
Gpcpd1	Q8C0L9	83	-15	-12	-26	-33	0.9	0.9	0.8	0.8
Filip1l	Q6P6L0	988	-30	-13	-26	-24	0.8	0.9	0.8	0.8
Rrp12	Q6P5B0	800	-13	-13	-26	-1	0.9	0.9	0.8	1.0
March6	Q6ZQ89	52	-16	-13	-26	-6	0.9	0.9	0.8	0.9
Map3k5	O35099	842	-19	-13	-26	-24	0.8	0.9	0.8	0.8
Zbtb21	E9Q444	180	-22	-13	-26	-31	0.8	0.9	0.8	0.8
Aamp	J3QN89	430	-14	-14	-26	-1	0.9	0.9	0.8	1.0
Sp4	K4DI62	53	-18	-15	-26	-14	0.9	0.9	0.8	0.9
Prdm15	E9Q8T2	201	-20	-15	-26	-15	0.8	0.9	0.8	0.9
Prdm15	E9Q8T2	225	-20	-15	-26	-15	0.8	0.9	0.8	0.9
Ttf1	Q62187	32	-5	-18	-26	-19	1.0	0.9	0.8	0.8
Utrn	E9Q6R7	2190	-30	-18	-26	-29	0.8	0.8	0.8	0.8
Dhx29	Q6PGC1	1024	-18	-19	-26	-14	0.9	0.8	0.8	0.9
Cdkal1	Q91WE6	337	-22	-19	-26	-23	0.8	0.8	0.8	0.8
Tsc1	Q9EP53	614	-28	-19	-26	-27	0.8	0.8	0.8	0.8
Akap8	Q9DBR0	85	-18	-19	-26	-30	0.8	0.8	0.8	0.8
Kdm5b	Q80Y84	614	-19	-20	-26	-32	0.8	0.8	0.8	0.8
Ctu2	Q3U308	465	-26	-21	-26	-28	0.8	0.8	0.8	0.8
Alkbh2	Q6P6J4	170	-6	-21	-26	-30	0.9	0.8	0.8	0.8
Znf12	Q7TSI0	531	-43	-21	-26	-48	0.7	0.8	0.8	0.7
Sf3a3	Q9D554	437	-22	-21	-26	-8	0.8	0.8	0.8	0.9
Gpsm1	Q6IR34	513	-11	-22	-26	18	0.9	0.8	0.8	1.2

Rps27a	P62983	145	-16	-22	-26	-11	0.9	0.8	0.8	0.9
Trim65	Q8BFW4	108	-5	-23	-26	-23	1.0	0.8	0.8	0.8
Mki67	E9PVX6	1741	-16	-25	-26	-21	0.9	0.8	0.8	0.8
Bcas3	Q8CCN5	417	-29	-27	-26	-13	0.8	0.8	0.8	0.9
Iars2	Q8BIJ6	521	-30	-27	-26	-22	0.8	0.8	0.8	0.8
Ciz1	Q8VEH2	475	-18	-28	-26	-46	0.9	0.8	0.8	0.7
Phf5a	P83870	33	-25	-29	-26	-14	0.8	0.8	0.8	0.9
Fblim1	Q71FD7	35	-1	-31	-26	-16	1.0	0.8	0.8	0.9
Rai1	Q61818	577	-22	-31	-26	-23	0.8	0.8	0.8	0.8
Myo19	Q5SV80	515	-18	-31	-26	-11	0.9	0.8	0.8	0.9
Pde2a	F7D3W5	326	-18	-34	-26	3	0.9	0.7	0.8	1.0
Zc3hc1	Q80YV2	125	-22	-36	-26	-18	0.8	0.7	0.8	0.8
Fahd2	Q3TC72	214	-26	-36	-26	-29	0.8	0.7	0.8	0.8
Abcf1	Q6P542	647	-22	-54	-26	-7	0.8	0.6	0.8	0.9
Tceb1	P83940	74	-2	7	-26	15	1.0	1.1	0.8	1.2
Bag5	Q8CI32	166	-15	1	-26	-42	0.9	1.0	0.8	0.7
Eif2ak4	Q9QZ05	614	-13	-1	-26	14	0.9	1.0	0.8	1.2
Anxa6	P14824	669	-11	-1	-26	-6	0.9	1.0	0.8	0.9
Mcu	Q3UMR5	96	-12	-2	-26	-6	0.9	1.0	0.8	0.9
Arhgef18	Q6P9R4	443	-10	-3	-26	-8	0.9	1.0	0.8	0.9
Bptf	A2A654	1093	-35	-6	-26	5	0.7	0.9	0.8	1.0
Impact	O55091	225	-24	-6	-26	-31	0.8	0.9	0.8	0.8
Arhgef6	Q8K4I3	66	-9	-9	-26	23	0.9	0.9	0.8	1.3
Nisch	Q80TM9	959	-16	-9	-26	8	0.9	0.9	0.8	1.1
Prpf19	Q99KP6	230	-23	-9	-26	-23	0.8	0.9	0.8	0.8
Lyst	G5E8Q0	868	-24	-11	-26	-2	0.8	0.9	0.8	1.0
Bin2	S4R270	442	-4	-11	-26	-22	1.0	0.9	0.8	0.8
Add1	Q9QYC0	430	-22	-12	-26	-35	0.8	0.9	0.8	0.7
Mbd2	Q9Z2E1	362	-7	-13	-26	13	0.9	0.9	0.8	1.1
Lrrtm2	Q8BGA3	34	0	-13	-26	-21	1.0	0.9	0.8	0.8
Aars	Q8BGQ7	711	-5	-13	-26	-42	1.0	0.9	0.8	0.7
Hk1	P17710	879	-21	-14	-26	-9	0.8	0.9	0.8	0.9
Polr2b	Q8CFI7	1093	-13	-16	-26	-15	0.9	0.9	0.8	0.9
Snupn	Q80W37	201	-21	-16	-26	-21	0.8	0.9	0.8	0.8
Pygb	Q8CI94	326	-21	-17	-26	-30	0.8	0.9	0.8	0.8
Prrc2a	Q7TSC1	435	-15	-17	-26	-42	0.9	0.9	0.8	0.7
Nlrx1	Q3TL44	331	-13	-18	-26	-18	0.9	0.8	0.8	0.8
H2-K1	P01901	185	-37	-19	-26	4	0.7	0.8	0.8	1.0
Eng	Q63961	516	-23	-20	-26	-8	0.8	0.8	0.8	0.9
Hip1r	Q9JKY5	650	-25	-21	-26	-7	0.8	0.8	0.8	0.9
Fcmr	A1KXC4	80	-7	-22	-26	5	0.9	0.8	0.8	1.0
Idh1	O88844	73	-15	-23	-26	-43	0.9	0.8	0.8	0.7
Hbbt1	A8DUK4	94	-28	-23	-26	6	0.8	0.8	0.8	1.1
Ampd2	Q9DBT5	150	-16	-26	-26	-12	0.9	0.8	0.8	0.9
Naa35	Q6PHQ8	410	-15	-28	-26	-1	0.9	0.8	0.8	1.0
Arfgef1	G3X9K3	1500	-9	-29	-26	2	0.9	0.8	0.8	1.0
Cbx2	P30658	284	-6	-30	-26	3	0.9	0.8	0.8	1.0
Usp34	Q6ZQ93	132	-13	-30	-26	-27	0.9	0.8	0.8	0.8
Ezh1	P70351	11	-24	-31	-26	-9	0.8	0.8	0.8	0.9
Mcm3ap	Q9WUU9	924	-31	-38	-26	-30	0.8	0.7	0.8	0.8
Tceb2	P62869	60	-28	-39	-26	-18	0.8	0.7	0.8	0.8

Relb	Q04863	122	-23	-40	-26	-20	0.8	0.7	0.8	0.8
Spice1	Q8C804	455	-43	-41	-26	-29	0.7	0.7	0.8	0.8
Eif2s1	Q6ZWX6	98	-25	-43	-26	-1	0.8	0.7	0.8	1.0
Hsf1	P38532	103	-42	-51	-26	-12	0.7	0.7	0.8	0.9
Ears2	Q9CXJ1	140	-6	-62	-26	-40	0.9	0.6	0.8	0.7
Spr	Q91XH5	172	3	16	-27	6	1.0	1.2	0.8	1.1
Pfkp	Q9WUA3	359	6	11	-27	-189	1.1	1.1	0.8	0.3
Myo5a	Q99104	895	-17	9	-27	-65	0.9	1.1	0.8	0.6
Cltb	Q6IRU5	199	-10	2	-27	6	0.9	1.0	0.8	1.1
Isoc2a	P85094	21	-23	1	-27	23	0.8	1.0	0.8	1.3
Pdp1	Q3UV70	149	-11	-1	-27	-8	0.9	1.0	0.8	0.9
Sec23ip	G3X928	463	-15	-2	-27	-28	0.9	1.0	0.8	0.8
Hip1	Q8VD75	63	-14	-5	-27	15	0.9	1.0	0.8	1.2
Atg2a	Q6P4T0	1846	-27	-6	-27	-9	0.8	0.9	0.8	0.9
Hnrnph1	O35737	290	-21	-7	-27	-22	0.8	0.9	0.8	0.8
Nsd1	E9QAE4	1238	-16	-8	-27	-27	0.9	0.9	0.8	0.8
Pls3	Q99K51	33	-14	-10	-27	-34	0.9	0.9	0.8	0.7
Usp4	P35123	461	-7	-11	-27	-14	0.9	0.9	0.8	0.9
Usp15	Q8R5H1	448	-7	-11	-27	-14	0.9	0.9	0.8	0.9
Capzb	P47757	206	-17	-12	-27	-29	0.9	0.9	0.8	0.8
Ankrd11	E9Q4F7	2059	-20	-14	-27	-31	0.8	0.9	0.8	0.8
Tgm1	Q9JLF6	376	-17	-14	-27	-10	0.9	0.9	0.8	0.9
Zmynd8	A2A484	131	-5	-16	-27	10	1.0	0.9	0.8	1.1
Rfc1	G3UWX1	764	-20	-16	-27	-38	0.8	0.9	0.8	0.7
Prg2	Q61878	202	-15	-17	-27	-22	0.9	0.9	0.8	0.8
Epx	P49290	292	-18	-19	-27	-27	0.9	0.8	0.8	0.8
Pik3c2b	E9QAN8	1372	-11	-19	-27	-14	0.9	0.8	0.8	0.9
Ankrd17	Q99NH0	1618	-15	-20	-27	7	0.9	0.8	0.8	1.1
Rif1	Q6PR54	2118	-11	-20	-27	-8	0.9	0.8	0.8	0.9
Usp47	Q8BY87	767	-12	-21	-27	-19	0.9	0.8	0.8	0.8
Hspa9	P38647	366	-17	-21	-27	-22	0.9	0.8	0.8	0.8
Fbxo11	Q7TPD1	116	-14	-21	-27	-27	0.9	0.8	0.8	0.8
Apaf1	O88879	8	-21	-22	-27	-51	0.8	0.8	0.8	0.7
Ehbp1l1	E9QP49	221	-21	-23	-27	-25	0.8	0.8	0.8	0.8
Acot11	Q8VHQ9	369	-23	-23	-27	-33	0.8	0.8	0.8	0.8
Armt1	A6H630	193	-26	-23	-27	-42	0.8	0.8	0.8	0.7
Pom121	Q8K3Z9	375	-28	-24	-27	-33	0.8	0.8	0.8	0.8
Apaf1	O88879	434	-18	-25	-27	8	0.8	0.8	0.8	1.1
Rapgef1	Q3UHC1	1171	-15	-25	-27	-12	0.9	0.8	0.8	0.9
Dennd4a	E9Q8V6	35	-6	-26	-27	-25	0.9	0.8	0.8	0.8
Cops7b	Q8BV13	240	-38	-26	-27	-25	0.7	0.8	0.8	0.8
Rock2	F8VPK5	330	-13	-26	-27	2	0.9	0.8	0.8	1.0
Rock1	P70335	314	-13	-26	-27	2	0.9	0.8	0.8	1.0
Akt2	Q60823	124	-22	-27	-27	2	0.8	0.8	0.8	1.0
Pdha1	P35486	41	-39	-27	-27	6	0.7	0.8	0.8	1.1
Spag7	Q7TNE3	191	-18	-28	-27	-14	0.8	0.8	0.8	0.9
Cndp2	Q9D1A2	205	-15	-28	-27	-84	0.9	0.8	0.8	0.5
Chd2	E9PZM4	365	-42	-29	-27	-19	0.7	0.8	0.8	0.8
Klhdc3	Q8VEM9	229	-16	-29	-27	-17	0.9	0.8	0.8	0.9
Esf1	Q3V1V3	456	-31	-29	-27	-18	0.8	0.8	0.8	0.9
Prpf8	Q99PV0	547	-17	-33	-27	-6	0.9	0.8	0.8	0.9

Sirt4	Q8R216	160	-22	-35	-27	-25	0.8	0.7	0.8	0.8
Xrcc5	P27641	339	-12	-36	-27	-18	0.9	0.7	0.8	0.9
Trappc10	F8VQF9	728	-20	-39	-27	-31	0.8	0.7	0.8	0.8
Dhcr7	O88455	364	-28	-40	-27	-22	0.8	0.7	0.8	0.8
Ubn1	Q4G0F8	467	-33	-51	-27	-35	0.8	0.7	0.8	0.7
Rpl37a	P61514	57	-11	14	-27	-17	0.9	1.2	0.8	0.9
Uba3	Q8C878	82	9	0	-27	16	1.1	1.0	0.8	1.2
Rrp12	Q6P5B0	674	-16	-1	-27	2	0.9	1.0	0.8	1.0
Dtymk	P97930	163	-8	-2	-27	-94	0.9	1.0	0.8	0.5
Gmip	Q6PGG2	958	-28	-8	-27	-25	0.8	0.9	0.8	0.8
Rnf14	Q9JI90	407	-16	-8	-27	-3	0.9	0.9	0.8	1.0
Hnrnpf	Q9Z2X1	290	-12	-9	-27	-16	0.9	0.9	0.8	0.9
Serpinb8	O08800	154	-19	-9	-27	-39	0.8	0.9	0.8	0.7
Cdkn2aip	Q8BI72	206	-15	-10	-27	-37	0.9	0.9	0.8	0.7
Fgd3	O88842	701	-21	-11	-27	-2	0.8	0.9	0.8	1.0
Zc3hav1	Q3UPF5	82	-16	-12	-27	-5	0.9	0.9	0.8	1.0
Uncharact	Q8BGA7	145	1	-13	-27	-17	1.0	0.9	0.8	0.9
Rai1	Q61818	1013	-23	-16	-27	-26	0.8	0.9	0.8	0.8
Arpc1a	Q9R0Q6	162	-32	-16	-27	10	0.8	0.9	0.8	1.1
Arpc1b	Q9WV32	162	-32	-16	-27	10	0.8	0.9	0.8	1.1
Nudt4	Q8R2U6	130	-12	-16	-27	-20	0.9	0.9	0.8	0.8
Lrch3	Q8BVU0	669	-17	-17	-27	-26	0.9	0.9	0.8	0.8
Gmip	Q6PGG2	598	-15	-18	-27	11	0.9	0.9	0.8	1.1
Gopc	Q8BH60	409	-13	-19	-27	-23	0.9	0.8	0.8	0.8
Ncor1	Q5RIM6	2336	-21	-19	-27	-49	0.8	0.8	0.8	0.7
Arhgap30	Q640N3	40	-9	-20	-27	4	0.9	0.8	0.8	1.0
Nol8	Q3UHX0	729	-4	-21	-27	-32	1.0	0.8	0.8	0.8
Arpc3	Q9JM76	162	-30	-22	-27	-5	0.8	0.8	0.8	1.0
Espl1	P60330	67	-183	-23	-27	-47	0.4	0.8	0.8	0.7
Znf280c	Q6P3Y5	75	-19	-24	-27	-26	0.8	0.8	0.8	0.8
Flii	Q9JJ28	46	-27	-24	-27	-5	0.8	0.8	0.8	1.0
Cep131	Q62036	159	-28	-24	-27	-9	0.8	0.8	0.8	0.9
Lrrc41	Q8K1C9	297	-26	-25	-27	2	0.8	0.8	0.8	1.0
Dip2b	Q3UH60	590	-33	-26	-27	-28	0.8	0.8	0.8	0.8
Cbl	P22682	419	-23	-26	-27	-13	0.8	0.8	0.8	0.9
Actr2	P61161	20	-26	-27	-27	-13	0.8	0.8	0.8	0.9
Ssh2	Q5SW75	862	-21	-27	-27	-22	0.8	0.8	0.8	0.8
Uba6	Q8C7R4	298	-24	-28	-27	-17	0.8	0.8	0.8	0.9
Igfbp5	Q07079	99	-24	-30	-27	-38	0.8	0.8	0.8	0.7
Ogg1	O08760	28	-37	-32	-27	-4	0.7	0.8	0.8	1.0
Snx5	Q9D8U8	250	-25	-35	-27	-16	0.8	0.7	0.8	0.9
Senp1	P59110	164	-14	-35	-27	-30	0.9	0.7	0.8	0.8
Rnf213	E9Q555	3289	-30	-40	-27	-16	0.8	0.7	0.8	0.9
Eno1	P17182	339	-26	-40	-27	-22	0.8	0.7	0.8	0.8
Rtfdc1	Q99K95	263	-20	-42	-27	-36	0.8	0.7	0.8	0.7
Gyg1	Q9R062	89	31	-44	-27	-31	1.4	0.7	0.8	0.8
Sh2b3	O09039	235	-20	6	-28	-18	0.8	1.1	0.8	0.9
Eef1a1	P10126	363	-11	3	-28	1	0.9	1.0	0.8	1.0
Gtf3c1	Q8K284	1182	-8	1	-28	-12	0.9	1.0	0.8	0.9
Esco2	Q8CIB9	509	-23	-3	-28	-6	0.8	1.0	0.8	0.9
Gpatch8	A2A6A1	1113	-5	-9	-28	-10	1.0	0.9	0.8	0.9

Smug1	Q6P5C5	151	-22	-10	-28	14	0.8	0.9	0.8	1.2
Ap1b1	O35643	95	-12	-10	-28	-18	0.9	0.9	0.8	0.9
Ap2b1	Q9DBG3	95	-12	-10	-28	-18	0.9	0.9	0.8	0.9
Hectd1	Q69ZR2	2587	-2	-11	-28	-10	1.0	0.9	0.8	0.9
Hps5	P59438	431	-9	-12	-28	-12	0.9	0.9	0.8	0.9
Ttc28	Q80XJ3	672	-20	-12	-28	-15	0.8	0.9	0.8	0.9
Pgpep1	Q9ESW8	149	-2	-13	-28	5	1.0	0.9	0.8	1.0
Usp10	P52479	358	-13	-16	-28	-29	0.9	0.9	0.8	0.8
Rftn1	Q6A0D4	129	-23	-16	-28	-21	0.8	0.9	0.8	0.8
Ppme1	Q8BVQ5	347	-16	-16	-28	-58	0.9	0.9	0.8	0.6
Gars	Q9CZD3	456	-37	-17	-28	-19	0.7	0.9	0.8	0.8
Tex2	Q6ZPJ0	1115	-10	-19	-28	-25	0.9	0.8	0.8	0.8
Nhej1	Q3KNJ2	74	-39	-20	-28	-10	0.7	0.8	0.8	0.9
Rbl2	Q64700	1114	-25	-20	-28	-25	0.8	0.8	0.8	0.8
Polr2a	P08775	109	-20	-20	-28	-3	0.8	0.8	0.8	1.0
Dnajc13	D4AFX7	825	-7	-20	-28	-21	0.9	0.8	0.8	0.8
Anxa1	P10107	270	-28	-21	-28	-4	0.8	0.8	0.8	1.0
Mns1	Q61884	439	-21	-22	-28	-8	0.8	0.8	0.8	0.9
Rassf4	Q8CB96	33	-23	-22	-28	-16	0.8	0.8	0.8	0.9
Sh3kbp1	Q8R550	103	-1	-23	-28	-5	1.0	0.8	0.8	1.0
Paip1	Q8VE62	279	-36	-24	-28	-19	0.7	0.8	0.8	0.8
Tnfrsf18	O35714	55	-6	-24	-28	-53	0.9	0.8	0.8	0.7
Fbxo11	Q7TPD1	584	-16	-25	-28	-15	0.9	0.8	0.8	0.9
Rfc3	Q8R323	32	-22	-26	-28	-7	0.8	0.8	0.8	0.9
Gm29609	Q3UU56	618	-29	-27	-28	19	0.8	0.8	0.8	1.2
Jak1	B1ASP2	987	-24	-29	-28	-44	0.8	0.8	0.8	0.7
Stard3	Q61542	10	-34	-29	-28	-54	0.7	0.8	0.8	0.6
Nol8	Q3UHX0	1133	-15	-29	-28	-85	0.9	0.8	0.8	0.5
Crnkl1	P63154	479	-31	-30	-28	0	0.8	0.8	0.8	1.0
Unc45a	Q99KD5	384	-20	-33	-28	-23	0.8	0.8	0.8	0.8
Pdcd11	Q6NS46	738	-33	-35	-28	7	0.8	0.7	0.8	1.1
Heatr3	Q8BQM4	584	-19	-38	-28	-26	0.8	0.7	0.8	0.8
Sap18b	E9Q317	45	-27	-38	-28	-29	0.8	0.7	0.8	0.8
Clic1	Q9Z1Q5	191	35	-44	-28	-18	1.5	0.7	0.8	0.8
Cpsf3	Q9QXK7	223	-21	-46	-28	-22	0.8	0.7	0.8	0.8
Lars	Q8BMJ2	370	-15	2	-28	-9	0.9	1.0	0.8	0.9
Nfs1	Q9Z1J3	383	2	0	-28	-8	1.0	1.0	0.8	0.9
UPF0505	Q8BWQ6	253	-7	-4	-28	-92	0.9	1.0	0.8	0.5
Akap8l	Q5RL57	129	-1	-6	-28	-26	1.0	0.9	0.8	0.8
Atg5	Q99J83	19	-22	-8	-28	-37	0.8	0.9	0.8	0.7
Bptf	A2A654	1472	7	-11	-28	-26	1.1	0.9	0.8	0.8
Dazap1	Q9JII5	63	-12	-13	-28	-10	0.9	0.9	0.8	0.9
Zbtb7a	O88939	55	-32	-15	-28	-40	0.8	0.9	0.8	0.7
Kiaa0922	Q3U3D7	1104	-6	-16	-28	-3	0.9	0.9	0.8	1.0
Agap2	Q3UHD9	909	-21	-16	-28	-18	0.8	0.9	0.8	0.8
Wdr34	Q5U4F6	63	10	-17	-28	-17	1.1	0.9	0.8	0.9
Nup85	Q8R480	511	-37	-18	-28	-11	0.7	0.9	0.8	0.9
Banp	Q8VBU8	116	25	-19	-28	-26	1.3	0.8	0.8	0.8
Josd2	Q9CR30	24	-15	-19	-28	-12	0.9	0.8	0.8	0.9
Echs1	Q8BH95	225	-18	-20	-28	-21	0.8	0.8	0.8	0.8
Ric8a	Q3TIR3	79	-24	-21	-28	13	0.8	0.8	0.8	1.1

Vipas39	Q8BGQ1	168	-29	-22	-28	-11	0.8	0.8	0.8	0.9
Pias1	O88907	481	-23	-23	-28	-15	0.8	0.8	0.8	0.9
D2Wsu81e	Q3UHX9	140	-6	-23	-28	-23	0.9	0.8	0.8	0.8
Pelp1	Q9DBD5	601	-20	-24	-28	-12	0.8	0.8	0.8	0.9
Mettl16	Q9CQG2	253	-20	-26	-28	-16	0.8	0.8	0.8	0.9
Gpbp1	Q6NXH3	269	-21	-28	-28	-29	0.8	0.8	0.8	0.8
Hnrnpul1	Q8VDM6	378	-16	-29	-28	3	0.9	0.8	0.8	1.0
Capn7	Q9R1S8	159	-27	-29	-28	-21	0.8	0.8	0.8	0.8
Unc119b	Q8C4B4	85	-29	-30	-28	-25	0.8	0.8	0.8	0.8
Rprd2	Q6NXI6	88	-42	-30	-28	-48	0.7	0.8	0.8	0.7
Pcif1	P59114	403	-42	-31	-28	-21	0.7	0.8	0.8	0.8
Rint1	Q8BZ36	720	-20	-32	-28	1	0.8	0.8	0.8	1.0
Neu3	Q9JMH7	307	-19	-32	-28	-16	0.8	0.8	0.8	0.9
Eef1a1	P10126	370	-21	-32	-28	-17	0.8	0.8	0.8	0.9
Rbm25	B2RY56	132	-35	-34	-28	6	0.7	0.7	0.8	1.1
Gas7	Q3U432	364	-9	-35	-28	-11	0.9	0.7	0.8	0.9
Chaf1b	Q9D0N7	283	-27	-43	-28	-17	0.8	0.7	0.8	0.9
Ehmt2	Q9Z148	1168	-37	-43	-28	-34	0.7	0.7	0.8	0.7
Pik3c3	Q6PF93	336	-32	-56	-28	-1	0.8	0.6	0.8	1.0
Ino80b	Q99PT3	349	27	-118	-28	25	1.4	0.5	0.8	1.3
Nrde2	Q80XC6	248	-21	6	-29	-21	0.8	1.1	0.8	0.8
Dhx9	E9QNN1	472	-18	-3	-29	37	0.9	1.0	0.8	1.6
Phgdh	Q61753	254	-15	-6	-29	12	0.9	0.9	0.8	1.1
Ddx19b	Q8BZY3	182	-15	-8	-29	-15	0.9	0.9	0.8	0.9
Smug1	Q6P5C5	133	-20	-9	-29	-26	0.8	0.9	0.8	0.8
Tnpo3	Q6P2B1	912	-17	-10	-29	-24	0.9	0.9	0.8	0.8
Trim16	Q99PP9	170	-18	-11	-29	-17	0.9	0.9	0.8	0.9
Plekha2	Q9ERS5	232	-15	-12	-29	-15	0.9	0.9	0.8	0.9
Nup98	Q6PFD9	1609	-26	-14	-29	-18	0.8	0.9	0.8	0.9
Cdc14a	Q6GQT0	451	-25	-15	-29	-16	0.8	0.9	0.8	0.9
Cr1l	Q64735	455	-14	-15	-29	-47	0.9	0.9	0.8	0.7
Pbrm1	Q8BSQ9	951	-14	-16	-29	-4	0.9	0.9	0.8	1.0
Flywch1	Q8CI03	459	4	-20	-29	-38	1.0	0.8	0.8	0.7
Pfas	Q5SUR0	1055	-23	-20	-29	-46	0.8	0.8	0.8	0.7
Setx	A2AKX3	687	-17	-20	-29	-31	0.9	0.8	0.8	0.8
Engase	Q8BX80	547	-9	-21	-29	-33	0.9	0.8	0.8	0.8
Ckap5	A2AGT5	1946	-27	-21	-29	-28	0.8	0.8	0.8	0.8
Haus5	Q9D786	537	-6	-23	-29	-4	0.9	0.8	0.8	1.0
Tmx2	Q9D710	186	-29	-24	-29	-12	0.8	0.8	0.8	0.9
Vav1	P27870	83	-23	-24	-29	-14	0.8	0.8	0.8	0.9
Ptges3	Q9R0Q7	58	-25	-25	-29	-17	0.8	0.8	0.8	0.9
Abcf1	Q6P542	733	-24	-28	-29	-29	0.8	0.8	0.8	0.8
Irf7	P70434	412	-14	-29	-29	-28	0.9	0.8	0.8	0.8
Eef1e1	Q9D1M4	147	-36	-30	-29	-5	0.7	0.8	0.8	1.0
Eea1	Q8BL66	1364	-19	-32	-29	-25	0.8	0.8	0.8	0.8
Vapb	Q8BH80	121	-33	-34	-29	-19	0.8	0.7	0.8	0.8
Wdfy4	E9Q2M9	1136	-7	-35	-29	-3	0.9	0.7	0.8	1.0
Xrcc3	Q9CXE6	221	-32	-36	-29	-33	0.8	0.7	0.8	0.8
Fra10ac1	Q8BP78	179	-24	-36	-29	-18	0.8	0.7	0.8	0.8
Uros	P51163	265	-14	-37	-29	-22	0.9	0.7	0.8	0.8
Whsc1	Q8BVE8	730	-29	-38	-29	-36	0.8	0.7	0.8	0.7

Trrap	A0A1D5RLL4	556	-18	-39	-29	-24	0.9	0.7	0.8	0.8
Usf1	Q61069	248	-24	-40	-29	-41	0.8	0.7	0.8	0.7
Ckap5	A2AGT5	592	-30	-44	-29	-31	0.8	0.7	0.8	0.8
Pdlim1	O70400	305	51	-60	-29	-43	2.0	0.6	0.8	0.7
Mis18bp1	Q80WQ8	756	39	-60	-29	-72	1.6	0.6	0.8	0.6
Chuk	E9QNL4	330	-17	-1	-29	-16	0.9	1.0	0.8	0.9
Kti12	Q9D1R2	199	-14	-3	-29	5	0.9	1.0	0.8	1.0
Spg11	Q3UHA3	1629	-11	-7	-29	-32	0.9	0.9	0.8	0.8
Ide	F6RPJ9	872	-12	-8	-29	0	0.9	0.9	0.8	1.0
Ski	B1AUF1	368	-31	-12	-29	-20	0.8	0.9	0.8	0.8
Alas1	Q8VC19	110	-15	-13	-29	9	0.9	0.9	0.8	1.1
Gpatch8	A2A6A1	634	2	-14	-29	-37	1.0	0.9	0.8	0.7
Mepce	Q8K3A9	601	-13	-16	-29	-20	0.9	0.9	0.8	0.8
Helb	Q6NVF4	663	-21	-17	-29	-9	0.8	0.9	0.8	0.9
Gon4l	K4DI71	2142	-36	-19	-29	-29	0.7	0.8	0.8	0.8
Rsbn1l	D3Z0K6	613	-24	-21	-29	-20	0.8	0.8	0.8	0.8
Pds5b	Q4VA53	571	-30	-22	-29	2	0.8	0.8	0.8	1.0
Rsl1	Q7M6Y1	96	-22	-22	-29	-25	0.8	0.8	0.8	0.8
Lrrk2	Q5S006	766	-33	-23	-29	-18	0.8	0.8	0.8	0.9
Rps21	Q9CQR2	56	-25	-24	-29	-44	0.8	0.8	0.8	0.7
Tars	Q9D0R2	260	-24	-25	-29	7	0.8	0.8	0.8	1.1
Zranb2	Q9R020	74	-26	-25	-29	-29	0.8	0.8	0.8	0.8
Pot1	Q91WC1	355	-21	-25	-29	-22	0.8	0.8	0.8	0.8
Rbm39	Q8VH51	478	-36	-27	-29	3	0.7	0.8	0.8	1.0
Tatdn2	B7ZNL9	743	-15	-27	-29	-2	0.9	0.8	0.8	1.0
Cep295	Q8BQ48	521	-25	-28	-29	-19	0.8	0.8	0.8	0.8
Tpx2	A2APB8	100	-25	-28	-29	-44	0.8	0.8	0.8	0.7
Nacc2	Q9DCM7	391	-10	-31	-29	-40	0.9	0.8	0.8	0.7
Mki67	E9PVX6	2340	-28	-33	-29	-32	0.8	0.8	0.8	0.8
Fndc3a	Q8BX90	1124	-30	-34	-29	-25	0.8	0.7	0.8	0.8
Rnf31	Q924T7	628	-16	-34	-29	-26	0.9	0.7	0.8	0.8
Nvl	Q9DBY8	802	-8	-37	-29	-46	0.9	0.7	0.8	0.7
Tepsin	Q3U3N6	310	-28	-46	-29	-28	0.8	0.7	0.8	0.8
Cdk13	Q69ZA1	840	9	-2	-30	8	1.1	1.0	0.8	1.1
Rpap1	Q80TE0	185	-24	-5	-30	-25	0.8	1.0	0.8	0.8
Ero1a	Q8R180	237	2	-8	-30	-17	1.0	0.9	0.8	0.9
Rdh14	Q9ERI6	146	-20	-11	-30	2	0.8	0.9	0.8	1.0
Tamm41	G5E881	285	-5	-13	-30	-121	1.0	0.9	0.8	0.5
Rasgrp2	Q9QUG9	512	-31	-15	-30	-15	0.8	0.9	0.8	0.9
Agpat4	Q8K4X7	82	-23	-16	-30	-110	0.8	0.9	0.8	0.5
Mfn1	Q811U4	418	-20	-17	-30	-5	0.8	0.9	0.8	1.0
Rlf	A2A7F4	400	-13	-18	-30	-10	0.9	0.9	0.8	0.9
Rin3	P59729	60	-21	-19	-30	-5	0.8	0.8	0.8	1.0
Hectd3	Q3U487	487	-24	-23	-30	-9	0.8	0.8	0.8	0.9
Atp11c	Q9QZW0	197	-29	-23	-30	-48	0.8	0.8	0.8	0.7
Pex6	Q99LC9	565	-13	-23	-30	6	0.9	0.8	0.8	1.1
Eif4g3	Q80XI3	1239	-56	-28	-30	-10	0.6	0.8	0.8	0.9
Bles03	Q8VD62	199	-26	-28	-30	-19	0.8	0.8	0.8	0.8
Usp22	Q5DU02	494	-8	-31	-30	-52	0.9	0.8	0.8	0.7
Prr14	Q7TPN9	346	-25	-31	-30	-36	0.8	0.8	0.8	0.7
Atp6v1a	P50516	240	-50	-35	-30	-1	0.7	0.7	0.8	1.0

Iars2	Q8BIJ6	323	-25	-1	-30	-30	0.8	1.0	0.8	0.8
Alg11	Q3TZM9	345	-25	-7	-30	-19	0.8	0.9	0.8	0.8
Ecm29	Q6PDI5	1252	-17	-10	-30	14	0.9	0.9	0.8	1.2
Chek2	Q9Z265	198	-9	-10	-30	-6	0.9	0.9	0.8	0.9
Rnf213	E9Q555	2879	-20	-12	-30	-15	0.8	0.9	0.8	0.9
Tnfaip3	Q60769	54	-23	-15	-30	57	0.8	0.9	0.8	2.3
Flna	Q8BTM8	2160	-16	-16	-30	-30	0.9	0.9	0.8	0.8
Atp2b4	Q6Q477	538	-23	-16	-30	1	0.8	0.9	0.8	1.0
Tmem248	Q3TBN1	305	-14	-18	-30	-14	0.9	0.9	0.8	0.9
L3mbtl3	Q8BLB7	785	-16	-19	-30	-1	0.9	0.8	0.8	1.0
Vav1	P27870	311	-18	-20	-30	-24	0.8	0.8	0.8	0.8
Srp54	P14576	136	-21	-21	-30	11	0.8	0.8	0.8	1.1
Vdac3-ps1	A0A140T8V3	229	-21	-21	-30	-49	0.8	0.8	0.8	0.7
Gak	Q99KY4	542	-41	-23	-30	0	0.7	0.8	0.8	1.0
Arid4a	F8VPQ2	200	-15	-24	-30	-7	0.9	0.8	0.8	0.9
Plekho2	Q8K124	136	-38	-27	-30	-14	0.7	0.8	0.8	0.9
Pias3	O54714	184	-24	-28	-30	-16	0.8	0.8	0.8	0.9
Bpgm	P15327	145	-14	-28	-30	-9	0.9	0.8	0.8	0.9
Cad	B2RQC6	280	29	-28	-30	-15	1.4	0.8	0.8	0.9
Wdr73	Q9CWR1	259	-22	-31	-30	-17	0.8	0.8	0.8	0.9
Rad21	Q61550	35	47	-33	-30	-7	1.9	0.8	0.8	0.9
Iqgap2	Q3UQ44	1449	-23	-36	-30	-22	0.8	0.7	0.8	0.8
Cand2	Q6ZQ73	1138	-34	-38	-30	-21	0.7	0.7	0.8	0.8
Mis12	Q9CY25	69	-29	-39	-30	6	0.8	0.7	0.8	1.1
Brat1	Q8C3R1	674	-36	-43	-30	-12	0.7	0.7	0.8	0.9
Deaf1	Q9Z1T5	525	-7	-45	-30	-41	0.9	0.7	0.8	0.7
Tp53	P02340	176	-5	-46	-30	-36	1.0	0.7	0.8	0.7
Atp1a3	Q6PIC6	221	-24	-2	-31	-42	0.8	1.0	0.8	0.7
Mtmr3	Q8K296	1150	-20	-6	-31	-8	0.8	0.9	0.8	0.9
Fam208a	Q69ZR9	685	-40	-7	-31	-5	0.7	0.9	0.8	1.0
4930522L1	E9QAG4	226	-25	-19	-31	-13	0.8	0.8	0.8	0.9
Arap1	Q4LDD4	378	-21	-22	-31	-19	0.8	0.8	0.8	0.8
Gnl3l	Q6PGG6	316	-29	-22	-31	-20	0.8	0.8	0.8	0.8
Poldip3	Q8BG81	301	-37	-22	-31	-14	0.7	0.8	0.8	0.9
Dennd3	A2RT67	186	-12	-23	-31	-30	0.9	0.8	0.8	0.8
Ssh1	Q76I79	511	-16	-24	-31	-18	0.9	0.8	0.8	0.8
Ctu1	Q99J10	390	-16	-26	-31	-17	0.9	0.8	0.8	0.9
Gls	D3Z7P3	530	-26	-26	-31	-38	0.8	0.8	0.8	0.7
Safb	D3YXK2	485	-20	-32	-31	-16	0.8	0.8	0.8	0.9
Zfp472	B0V2W5	131	-14	-33	-31	-14	0.9	0.8	0.8	0.9
Dus3l	Q91XI1	302	-33	-43	-31	-23	0.8	0.7	0.8	0.8
Kyat3	Q71RI9	113	-13	0	-31	-19	0.9	1.0	0.8	0.8
Rnaseh2a	Q9CWY8	24	-7	-3	-31	15	0.9	1.0	0.8	1.2
Arhgap15	Q811M1	293	-20	-11	-31	-13	0.8	0.9	0.8	0.9
Pnkp	G5E8N7	436	-20	-11	-31	0	0.8	0.9	0.8	1.0
Ckmt2	Q6P8J7	317	-17	-11	-31	-13	0.9	0.9	0.8	0.9
Elp6	Q8BK75	218	-29	-16	-31	1	0.8	0.9	0.8	1.0
Trim28	Q62318	230	-14	-16	-31	-4	0.9	0.9	0.8	1.0
Nfs1	Q9Z1J3	160	-29	-18	-31	-59	0.8	0.9	0.8	0.6
Rubcn	Q80U62	896	-31	-19	-31	-16	0.8	0.8	0.8	0.9
Triobp	Q99KW3	1930	-19	-20	-31	-13	0.8	0.8	0.8	0.9

Ssh3	Q8K330	303	-45	-22	-31	-5	0.7	0.8	0.8	1.0
Akt1s1	Q9D1F4	44	-24	-24	-31	-27	0.8	0.8	0.8	0.8
Zfand3	Q497H0	20	-24	-25	-31	-3	0.8	0.8	0.8	1.0
Naxe	Q8K4Z3	121	-24	-25	-31	7	0.8	0.8	0.8	1.1
Ctps1	P70698	362	-30	-25	-31	-38	0.8	0.8	0.8	0.7
Mtmr3	Q8K296	926	-12	-25	-31	-45	0.9	0.8	0.8	0.7
Klhdc3	Q8VEM9	102	-13	-26	-31	-1	0.9	0.8	0.8	1.0
Zmynd11	Q8R5C8	347	-23	-26	-31	-76	0.8	0.8	0.8	0.6
Bptf	A2A654	1359	-18	-27	-31	-44	0.9	0.8	0.8	0.7
Ahctf1	Q8CJF7	521	-28	-27	-31	6	0.8	0.8	0.8	1.1
Taf1c	Q6PDZ2	678	-46	-27	-31	-23	0.7	0.8	0.8	0.8
Wdfy4	E9Q2M9	233	33	-28	-31	-29	1.5	0.8	0.8	0.8
Tpx2	A2APB8	462	-12	-28	-31	-33	0.9	0.8	0.8	0.8
Kctd1	Q5M956	140	-17	-30	-31	-6	0.9	0.8	0.8	0.9
Ctu2	Q3U308	31	-22	-30	-31	-12	0.8	0.8	0.8	0.9
Kif20a	P97329	807	-37	-32	-31	-15	0.7	0.8	0.8	0.9
Mboat7	Q8CHK3	310	-20	-33	-31	-29	0.8	0.8	0.8	0.8
Prtn3	Q61096	190	43	-45	-31	-61	1.8	0.7	0.8	0.6
Zap70	P43404	84	70	-63	-31	-120	3.3	0.6	0.8	0.5
Papolg	Q6PCL9	529	-13	3	-32	-41	0.9	1.0	0.8	0.7
Them6	Q80ZW2	104	-11	-5	-32	-88	0.9	1.0	0.8	0.5
Mrpl10	Q3TBW2	245	-18	-9	-32	-28	0.9	0.9	0.8	0.8
Fasn	P19096	2017	-22	-11	-32	-2	0.8	0.9	0.8	1.0
Tyk2	E9QJS1	986	-31	-11	-32	-12	0.8	0.9	0.8	0.9
Nup210	Q9QY81	76	-32	-15	-32	-7	0.8	0.9	0.8	0.9
Aqr	Q8CFQ3	449	-51	-16	-32	6	0.7	0.9	0.8	1.1
Uri1	Q3TLD5	268	-22	-17	-32	-26	0.8	0.9	0.8	0.8
Tirap	H3BKL1	162	-9	-21	-32	-21	0.9	0.8	0.8	0.8
Nsdhl	Q9R1J0	75	-19	-21	-32	10	0.8	0.8	0.8	1.1
Smad4	P97471	71	-31	-26	-32	5	0.8	0.8	0.8	1.1
Zfp407	G3UVV3	1744	-9	-26	-32	-7	0.9	0.8	0.8	0.9
Nbn	Q9R207	462	-25	-26	-32	-25	0.8	0.8	0.8	0.8
Dhx58	Q99J87	203	-20	-27	-32	-15	0.8	0.8	0.8	0.9
Rpa2	Q3TE40	250	-24	-28	-32	-14	0.8	0.8	0.8	0.9
Chd7	A2AJK6	1596	-23	-30	-32	3	0.8	0.8	0.8	1.0
Huwe1	Q7TMY8	2721	-21	-30	-32	-35	0.8	0.8	0.8	0.7
Pgrmc2	Q80UU9	153	-6	-31	-32	-48	0.9	0.8	0.8	0.7
Bank1	Q80VH0	364	-18	-31	-32	-49	0.8	0.8	0.8	0.7
Rbbp4	Q60972	167	-56	-32	-32	-34	0.6	0.8	0.8	0.7
Ethe1	Q9DCM0	80	-40	-39	-32	-62	0.7	0.7	0.8	0.6
Morc3	F7BJB9	446	-2	1	-32	2	1.0	1.0	0.8	1.0
Caap1	Q8VDY9	206	1	-2	-32	-44	1.0	1.0	0.8	0.7
Supv3l1	Q80YD1	175	-10	-7	-32	-32	0.9	0.9	0.8	0.8
Cdt1	Q8R4E9	302	-29	-11	-32	-9	0.8	0.9	0.8	0.9
Snx4	Q91YJ2	342	-22	-16	-32	-8	0.8	0.9	0.8	0.9
Riok2	Q9CQS5	367	-7	-20	-32	-7	0.9	0.8	0.8	0.9
Adat2	Q6P6J0	15	-39	-23	-32	-14	0.7	0.8	0.8	0.9
Thoc1	Q8R3N6	604	-7	-23	-32	-22	0.9	0.8	0.8	0.8
Mtch2	Q791V5	296	-27	-28	-32	-31	0.8	0.8	0.8	0.8
Flywch1	Q8CI03	638	-6	-30	-32	-32	0.9	0.8	0.8	0.8
Hsp90ab1	P11499	412	-3	-32	-32	1	1.0	0.8	0.8	1.0

Ppp1cb	P62141	154	-21	-33	-32	-2	0.8	0.8	0.8	1.0
Ppp1cc	P63087	155	-21	-33	-32	-2	0.8	0.8	0.8	1.0
Dcp1a	Q91YD3	59	65	-34	-32	-30	2.9	0.7	0.8	0.8
Clip2	Q9Z0H8	133	-50	-41	-32	-22	0.7	0.7	0.8	0.8
Eya3	P97480	455	18	-63	-32	-24	1.2	0.6	0.8	0.8
Tsen2	Q6P7W5	258	-14	2	-33	-15	0.9	1.0	0.8	0.9
Pbdc1	Q9D0B6	125	-21	-2	-33	3	0.8	1.0	0.8	1.0
Ubr4	A2AN08	2220	-28	-12	-33	-18	0.8	0.9	0.8	0.9
Rabl3	Q9D4V7	180	-35	-12	-33	-18	0.7	0.9	0.8	0.8
Rapgef6	Q5NCJ1	545	-29	-14	-33	-34	0.8	0.9	0.8	0.7
Pik3cg	Q9JHG7	395	-7	-14	-33	-22	0.9	0.9	0.8	0.8
Vta1	Q9CR26	38	-19	-16	-33	11	0.8	0.9	0.8	1.1
Uckl1	Q91YL3	317	-32	-17	-33	-25	0.8	0.9	0.8	0.8
Pus7	B7ZNL8	12	-27	-17	-33	-41	0.8	0.9	0.8	0.7
Cmtm7	Q9ESD6	12	-24	-18	-33	-68	0.8	0.8	0.8	0.6
Themis2	Q91YX0	146	-16	-19	-33	-14	0.9	0.8	0.8	0.9
Zfp873	A0A1B0GT64	451	-45	-19	-33	-22	0.7	0.8	0.8	0.8
Accs	A2AIG8	63	-24	-21	-33	-22	0.8	0.8	0.8	0.8
Uhrf2	Q7TMI3	119	-23	-27	-33	-25	0.8	0.8	0.8	0.8
Eif2d	Q61211	505	-28	-27	-33	-47	0.8	0.8	0.8	0.7
Cenpo	Q8K015	226	-29	-30	-33	-28	0.8	0.8	0.8	0.8
Rnf146	Q9CZW6	61	5	-30	-33	-29	1.1	0.8	0.8	0.8
Gart	Q64737	646	-44	-33	-33	-28	0.7	0.8	0.8	0.8
Xpa	Q64267	152	-28	-37	-33	-40	0.8	0.7	0.8	0.7
Unc13d	B2RUP2	1030	-86	-45	-33	-55	0.5	0.7	0.8	0.6
Tnfaip3	Q60769	475	-5	-1	-33	-24	1.0	1.0	0.8	0.8
Nepro	Q8R2U2	23	-28	-15	-33	-26	0.8	0.9	0.8	0.8
UPF0598	Q8VE95	132	-56	-16	-33	3	0.6	0.9	0.8	1.0
Cryzl1	Q921W4	301	-16	-17	-33	-11	0.9	0.9	0.8	0.9
Son	Q9QX47	1580	-15	-18	-33	-52	0.9	0.8	0.8	0.7
Med30	Q9CQI9	34	-30	-19	-33	31	0.8	0.8	0.8	1.4
Znf276	Q8CE64	137	-22	-19	-33	-8	0.8	0.8	0.8	0.9
Ncapd3	K4DI67	319	-17	-26	-33	-10	0.9	0.8	0.8	0.9
Eif3a	P23116	185	-36	-28	-33	-17	0.7	0.8	0.8	0.9
Abcf2	Q99LE6	393	-28	-29	-33	-29	0.8	0.8	0.8	0.8
Brd7	O88665	367	-10	-29	-33	-34	0.9	0.8	0.8	0.7
Nfe2l3	Q9WTM4	213	-32	-30	-33	-19	0.8	0.8	0.8	0.8
Rasa3	Q60790	67	-28	-30	-33	-26	0.8	0.8	0.8	0.8
Arfrp1	Q8BXL7	154	-13	-32	-33	-31	0.9	0.8	0.8	0.8
Nup205	A0A0J9YUD5	194	-36	-36	-33	-10	0.7	0.7	0.8	0.9
Prr12	E9PYL2	1121	-28	-38	-33	-24	0.8	0.7	0.8	0.8
Ankhd1	E9PUR0	1715	-27	-49	-33	-29	0.8	0.7	0.8	0.8
Pxn	Q8VI36	564	49	-89	-33	-101	2.0	0.5	0.8	0.5
Rbpj	P31266	149	-39	-8	-34	-13	0.7	0.9	0.7	0.9
Gars	Q9CZD3	461	-29	-11	-34	-56	0.8	0.9	0.7	0.6
Chaf1a	Q9QWF0	444	-6	-12	-34	-13	0.9	0.9	0.7	0.9
Alkbh5	Q3TSG4	379	-3	-14	-34	-27	1.0	0.9	0.7	0.8
Tsen15	G3X8S8	13	-22	-15	-34	-30	0.8	0.9	0.7	0.8
Ppp1r37	Q8BKR5	681	-24	-20	-34	-33	0.8	0.8	0.7	0.8
Bak1	O08734	14	-22	-22	-34	-47	0.8	0.8	0.7	0.7
Wrn	O09053	1332	-24	-22	-34	-32	0.8	0.8	0.7	0.8

Git2	Q9JLQ2	14	-29	-23	-34	10	0.8	0.8	0.7	1.1
Spns1	Q8R0G7	44	-21	-31	-34	-128	0.8	0.8	0.7	0.4
Heatr1	G3X9B1	795	-12	-33	-34	-12	0.9	0.8	0.7	0.9
Ifi207	E9Q3L4	515	-28	-37	-34	-14	0.8	0.7	0.7	0.9
Mphosph8	Q3TYA6	159	-24	-37	-34	-26	0.8	0.7	0.7	0.8
Dda1	Q9D9Z5	25	37	-49	-34	-47	1.6	0.7	0.7	0.7
Jmjd1c	Q69ZK6	1271	-11	-14	-34	-3	0.9	0.9	0.7	1.0
Sae1	Q9R1T2	137	-14	-23	-34	-35	0.9	0.8	0.7	0.7
Fam96b	Q9D187	158	-19	-23	-34	-65	0.8	0.8	0.7	0.6
Pphln1	G3X959	72	-24	-28	-34	-27	0.8	0.8	0.7	0.8
Nup50	Q9JIH2	133	-27	-30	-34	-41	0.8	0.8	0.7	0.7
Pfkfb4	Q6DTY7	197	-29	-31	-34	-29	0.8	0.8	0.7	0.8
Dopey2	Q3UHQ6	2236	49	-36	-34	-26	1.9	0.7	0.7	0.8
Cand1	Q6ZQ38	71	-39	-49	-34	-10	0.7	0.7	0.7	0.9
Zfp512b	Q6PHP4	739	58	-51	-34	-50	2.4	0.7	0.7	0.7
Yy1	Q00899	327	53	-72	-34	-35	2.1	0.6	0.7	0.7
Tyk2	E9QJS1	956	60	-86	-34	-70	2.5	0.5	0.7	0.6
Vwa5a	Q99KC8	750	-16	-5	-35	-27	0.9	1.0	0.7	0.8
Gapvd1	Q6PAR5	275	-7	-14	-35	-75	0.9	0.9	0.7	0.6
Tjp2	Q9Z0U1	581	-42	-18	-35	-33	0.7	0.9	0.7	0.8
Rpl5	P47962	100	-34	-22	-35	5	0.7	0.8	0.7	1.1
Ikbkb	O88351	464	-17	-23	-35	-6	0.9	0.8	0.7	0.9
Dnmt3a	O88508	551	-33	-25	-35	-34	0.8	0.8	0.7	0.7
Atg14	Q8CDJ3	330	-13	-27	-35	18	0.9	0.8	0.7	1.2
Fbxo38	Q8BMI0	676	-25	-27	-35	-19	0.8	0.8	0.7	0.8
Ccdc77	Q9CZH8	186	-13	-31	-35	-39	0.9	0.8	0.7	0.7
Sphk2	Q9JIA7	124	-17	-33	-35	-55	0.9	0.8	0.7	0.6
Emsy	Q8BMB0	921	-22	-34	-35	-11	0.8	0.7	0.7	0.9
Wdr33	Q8K4P0	249	-23	-42	-35	-24	0.8	0.7	0.7	0.8
Usp36	B1AQJ2	824	-36	-44	-35	-45	0.7	0.7	0.7	0.7
Snrnp48	Q9D361	132	-16	-46	-35	-51	0.9	0.7	0.7	0.7
Nbeal2	Q6ZQA0	1649	-28	-48	-35	-32	0.8	0.7	0.7	0.8
Ints6l	Q8BND4	745	-48	-58	-35	-35	0.7	0.6	0.7	0.7
Pi4ka	E9Q3L2	1896	-17	-6	-35	-13	0.9	0.9	0.7	0.9
Snx20	Q9D2Y5	152	-19	-17	-35	-21	0.8	0.9	0.7	0.8
Ngp	O08692	76	-28	-19	-35	2	0.8	0.8	0.7	1.0
Rasa4	Q6PFQ7	656	-14	-19	-35	-31	0.9	0.8	0.7	0.8
Steap3	Q8CI59	140	-35	-23	-35	-1	0.7	0.8	0.7	1.0
Tbx21	Q9JKD8	206	-21	-24	-35	-55	0.8	0.8	0.7	0.6
Srbd1	F8WGW3	566	-15	-28	-35	-29	0.9	0.8	0.7	0.8
Nudt6	Q8CH40	240	-26	-32	-35	-57	0.8	0.8	0.7	0.6
Hivep2	Q3UHF7	2039	-29	-33	-35	-43	0.8	0.8	0.7	0.7
Hemgn	Q9ERZ0	24	-7	-33	-35	-60	0.9	0.8	0.7	0.6
Ccsap	Q8QZT2	26	-33	-37	-35	-26	0.8	0.7	0.7	0.8
Dazap1	Q9JII5	85	-26	-53	-35	-33	0.8	0.7	0.7	0.8
Mat2b	Q99LB6	17	-1	5	-36	-9	1.0	1.1	0.7	0.9
Zbtb20	Q8K0L9	311	-8	1	-36	-16	0.9	1.0	0.7	0.9
Fabp5	Q05816	67	-6	-5	-36	5	0.9	1.0	0.7	1.0
Gmds	Q8K0C9	8	-12	-9	-36	-28	0.9	0.9	0.7	0.8
Pds5b	Q4VA53	732	-23	-15	-36	-2	0.8	0.9	0.7	1.0
Ago2	Q8CJG0	627	-41	-16	-36	-2	0.7	0.9	0.7	1.0

Ago1	Q8CJG1	624	-41	-16	-36	-2	0.7	0.9	0.7	1.0
Cct3	P80318	173	-27	-17	-36	10	0.8	0.9	0.7	1.1
Rnf213	E9Q555	3950	-21	-17	-36	-22	0.8	0.9	0.7	0.8
Hemgn	Q9ERZ0	144	-53	-19	-36	-21	0.7	0.8	0.7	0.8
Cdc123	Q8CII2	170	-32	-22	-36	-23	0.8	0.8	0.7	0.8
Fndc3b	Q6NWW9	1028	-17	-24	-36	-40	0.9	0.8	0.7	0.7
Rfx1	P48377	446	-19	-25	-36	-17	0.8	0.8	0.7	0.9
Lrrk2	Q5S006	1005	-22	-28	-36	-16	0.8	0.8	0.7	0.9
Inpp5f	Q8CDA1	1125	-11	-29	-36	-4	0.9	0.8	0.7	1.0
Pex1	Q5BL07	747	-24	-32	-36	-20	0.8	0.8	0.7	0.8
Sp1	O89090	629	67	-33	-36	-55	3.0	0.8	0.7	0.6
Megf9	Q8BH27	281	-28	-34	-36	-40	0.8	0.7	0.7	0.7
Mast4	Q811L6	2080	-16	-34	-36	-28	0.9	0.7	0.7	0.8
Slc25a12	Q8BH59	563	8	-40	-36	11	1.1	0.7	0.7	1.1
Fam69b	Q99ML4	84	-36	-44	-36	13	0.7	0.7	0.7	1.1
Znf512	Q69Z99	432	54	-46	-36	-43	2.2	0.7	0.7	0.7
Rnf213	E9Q555	1892	45	-47	-36	-7	1.8	0.7	0.7	0.9
Gvin1	L7N451	497	-22	-50	-36	-13	0.8	0.7	0.7	0.9
Elf2	Q9JHC9	348	-22	-16	-36	-28	0.8	0.9	0.7	0.8
Hsd17b12	O70503	215	-10	-20	-36	-6	0.9	0.8	0.7	0.9
Mndal	D0QMC3	166	-16	-26	-36	-26	0.9	0.8	0.7	0.8
Atp2a2	O55143	669	-36	-28	-36	-12	0.7	0.8	0.7	0.9
Rpl37	Q9D823	19	-25	-30	-36	-28	0.8	0.8	0.7	0.8
Znf821	Q6PD05	184	-18	-33	-36	-51	0.8	0.8	0.7	0.7
Ubr1	O70481	1611	-17	-34	-36	4	0.9	0.7	0.7	1.0
Ppp1r11	A5A4Y9	67	-41	-43	-36	-44	0.7	0.7	0.7	0.7
Topaz1	E5FYH1	782	-25	-47	-36	-27	0.8	0.7	0.7	0.8
Ccdc50	Q810U5	237	54	-49	-36	-46	2.2	0.7	0.7	0.7
Lars	Q8BMJ2	72	-18	-13	-37	-50	0.8	0.9	0.7	0.7
Mcm6	P97311	91	-15	-18	-37	-100	0.9	0.9	0.7	0.5
Nlrc5	C3VPR6	110	-26	-18	-37	-10	0.8	0.8	0.7	0.9
Hars	Q61035	509	-23	-20	-37	-55	0.8	0.8	0.7	0.6
Setd2	E9Q5F9	1652	-30	-22	-37	-13	0.8	0.8	0.7	0.9
Trio	Q0KL02	1717	-18	-28	-37	0	0.8	0.8	0.7	1.0
Ddx31	Q6NZQ2	323	-25	-32	-37	-70	0.8	0.8	0.7	0.6
Ralgapa1	Q6GYP7	257	-17	-36	-37	-28	0.9	0.7	0.7	0.8
Tor1aip2	Q8BYU6	414	6	-46	-37	-38	1.1	0.7	0.7	0.7
Tor1aip1	Q921T2	508	6	-46	-37	-38	1.1	0.7	0.7	0.7
Mrps30	Q9D0G0	196	-16	-50	-37	-14	0.9	0.7	0.7	0.9
Lpp	Q8BFW7	570	-11	-18	-37	-14	0.9	0.9	0.7	0.9
Ranbp2	Q9ERU9	1489	-26	-18	-37	-17	0.8	0.8	0.7	0.9
Pygb	Q8CI94	373	-34	-24	-37	1	0.7	0.8	0.7	1.0
Eif2b4	Q61749	445	47	-24	-37	-28	1.9	0.8	0.7	0.8
Exosc7	Q9D0M0	85	-32	-28	-37	-30	0.8	0.8	0.7	0.8
Ranbp2	Q9ERU9	503	1	-29	-37	6	1.0	0.8	0.7	1.1
Ptpn22	P29352	390	-30	-31	-37	-20	0.8	0.8	0.7	0.8
Ddx27	Q921N6	227	45	-44	-37	-56	1.8	0.7	0.7	0.6
Nfrkb	Q6PIJ4	636	41	-45	-37	-31	1.7	0.7	0.7	0.8
Traf3ip2	Q8N7N6	69	-25	-18	-38	-27	0.8	0.8	0.7	0.8
Znf668	Q8K2R5	126	-12	-28	-38	-13	0.9	0.8	0.7	0.9
Cic	Q924A2	442	-34	-29	-38	-46	0.7	0.8	0.7	0.7

Ctr9	Q62018	231	-29	-31	-38	19	0.8	0.8	0.7	1.2
Tlr9	Q9EQU3	1026	-24	-31	-38	-51	0.8	0.8	0.7	0.7
Itk	Q03526	138	-31	-32	-38	-47	0.8	0.8	0.7	0.7
Gemin5	Q8BX17	1044	-33	-33	-38	-42	0.8	0.8	0.7	0.7
Arhgap30	Q640N3	656	-62	-47	-38	-83	0.6	0.7	0.7	0.5
Dr1	Q91WV0	58	-31	-12	-38	16	0.8	0.9	0.7	1.2
Papd5	Q68ED3	535	-17	-19	-38	-38	0.9	0.8	0.7	0.7
Cars	Q9ER72	488	-27	-25	-38	-38	0.8	0.8	0.7	0.7
Smarcc1	P97496	519	-14	-32	-38	-10	0.9	0.8	0.7	0.9
Twf2	Q9Z0P5	141	-52	-9	-39	-28	0.7	0.9	0.7	0.8
Samd9l	E9PX59	987	-6	-12	-39	13	0.9	0.9	0.7	1.1
Pycr1	Q922W5	120	-30	-21	-39	-4	0.8	0.8	0.7	1.0
Btaf1	E9QAE3	935	-25	-23	-39	-10	0.8	0.8	0.7	0.9
Spg11	Q3UHA3	130	-12	-29	-39	-2	0.9	0.8	0.7	1.0
Smpd4	Q6ZPR5	207	-40	-35	-39	-14	0.7	0.7	0.7	0.9
Ddx5	Q8BTS0	89	35	-42	-39	-15	1.5	0.7	0.7	0.9
Fam129a	Q3UW53	756	29	-10	-39	-55	1.4	0.9	0.7	0.6
Uchl1	Q9R0P9	220	-10	-14	-39	-7	0.9	0.9	0.7	0.9
Uba3	Q8C878	249	-27	-18	-39	-24	0.8	0.9	0.7	0.8
Col1a1	P11087	83	-30	-20	-39	-52	0.8	0.8	0.7	0.7
Lars	Q8BMJ2	575	-22	-22	-39	-54	0.8	0.8	0.7	0.7
Cmpk2	Q3U5Q7	76	-33	-29	-39	-8	0.8	0.8	0.7	0.9
Mapkapk2	P49138	100	-24	-30	-39	-22	0.8	0.8	0.7	0.8
Ikbkap	Q7TT37	451	-39	-46	-39	-35	0.7	0.7	0.7	0.7
Lrrc47	Q505F5	366	53	-64	-39	-60	2.1	0.6	0.7	0.6
N4bp2	F8VQG7	1429	-22	-13	-40	-27	0.8	0.9	0.7	0.8
Klc1	Q8CD76	390	-8	-16	-40	17	0.9	0.9	0.7	1.2
Klc2	Q91YS4	375	-8	-16	-40	17	0.9	0.9	0.7	1.2
Klc4	Q9DBS5	388	-8	-16	-40	17	0.9	0.9	0.7	1.2
Aqr	Q8CFQ3	958	-6	-22	-40	-12	0.9	0.8	0.7	0.9
Ddx6	P54823	324	28	-25	-40	-29	1.4	0.8	0.7	0.8
Pogz	Q8BZH4	544	-35	-28	-40	-36	0.7	0.8	0.7	0.7
Elf2	Q9JHC9	591	-20	-29	-40	-27	0.8	0.8	0.7	0.8
Tmem94	Q7TSH8	678	-27	-29	-40	-29	0.8	0.8	0.7	0.8
Gvin1	L7N451	534	-32	-37	-40	-39	0.8	0.7	0.7	0.7
Polr1a	O35134	589	48	-41	-40	-67	1.9	0.7	0.7	0.6
Rptor	A2ACM0	283	-10	-43	-40	-15	0.9	0.7	0.7	0.9
Syne1	Q6ZWR6	7717	-57	-57	-40	-17	0.6	0.6	0.7	0.9
Tti2	Q8BGV4	137	-26	-18	-40	-14	0.8	0.9	0.7	0.9
Alkbh4	Q9D8F1	24	-13	-20	-40	-31	0.9	0.8	0.7	0.8
Madd	Q80U28	1492	-14	-21	-40	-23	0.9	0.8	0.7	0.8
Atm	Q62388	2780	-27	-22	-40	-12	0.8	0.8	0.7	0.9
Cenpv	Q9CXS4	196	-33	-37	-40	-13	0.8	0.7	0.7	0.9
Ctu2	Q3U308	266	42	-45	-40	-49	1.7	0.7	0.7	0.7
AY358078	Q6UY53	393	75	-106	-40	-45	3.9	0.5	0.7	0.7
Sap30bp	Q02614	127	-32	-13	-41	-33	0.8	0.9	0.7	0.8
Soat1	Q61263	82	-6	-21	-41	-13	0.9	0.8	0.7	0.9
Tubgcp5	Q8BKN5	580	-25	1	-41	-21	0.8	1.0	0.7	0.8
Nf1	Q04690	454	-26	-10	-41	-37	0.8	0.9	0.7	0.7
Nr3c1	E9PYV1	319	-6	-19	-41	-29	0.9	0.8	0.7	0.8
Prg2	Q61878	198	-14	-21	-41	-26	0.9	0.8	0.7	0.8

Kmt2a	P55200	2076	-34	-27	-41	-4	0.7	0.8	0.7	1.0
Zfp935	Q14DI0	80	-23	-37	-41	-23	0.8	0.7	0.7	0.8
Xpo5	Q924C1	714	-10	-6	-42	-63	0.9	0.9	0.7	0.6
Igtp	Q9DCE9	277	-27	-24	-42	-22	0.8	0.8	0.7	0.8
Prdx1	P35700	83	-25	-26	-42	-18	0.8	0.8	0.7	0.9
Trim33	Q99PP7	307	-21	-31	-42	-22	0.8	0.8	0.7	0.8
Srbd1	F8WGW3	596	-51	-34	-42	-18	0.7	0.7	0.7	0.9
Mboat7	Q8CHK3	280	-56	-39	-42	-21	0.6	0.7	0.7	0.8
Irf2bpl	Q8K3X4	63	59	-61	-42	-60	2.4	0.6	0.7	0.6
Rpl18a	P62717	22	71	-123	-42	-83	3.4	0.4	0.7	0.5
Med4	Q9CQA5	162	-16	-28	-42	-15	0.9	0.8	0.7	0.9
Tango6	Q8C3S2	542	-42	-46	-42	-36	0.7	0.7	0.7	0.7
Syne3	Q4FZC9	246	37	-76	-42	-58	1.6	0.6	0.7	0.6
Pfkl	P12382	653	-17	1	-43	-116	0.9	1.0	0.7	0.5
Pfkp	Q9WUA3	663	-17	1	-43	-116	0.9	1.0	0.7	0.5
Slc25a10	Q9QZD8	180	-23	-4	-43	-18	0.8	1.0	0.7	0.8
Paxip1	Q6NZQ4	972	-27	-31	-43	-3	0.8	0.8	0.7	1.0
Uba6	Q8C7R4	625	46	-40	-43	-57	1.9	0.7	0.7	0.6
FAM120A	Q6A0A9	279	49	-40	-43	-41	2.0	0.7	0.7	0.7
Fam120c	Q8C3F2	350	49	-40	-43	-41	2.0	0.7	0.7	0.7
Nle1	Q8VEJ4	280	43	-87	-43	-86	1.8	0.5	0.7	0.5
Rnf213	E9Q555	4455	-22	-14	-43	14	0.8	0.9	0.7	1.2
Gm12117	V9GXK0	18	-25	-20	-43	-23	0.8	0.8	0.7	0.8
Nfx1	B1AY10	32	-34	-29	-43	-43	0.7	0.8	0.7	0.7
Ric1	Q69ZJ7	434	-23	-33	-43	-34	0.8	0.8	0.7	0.7
Snx5	Q9D8U8	227	-14	-35	-43	-100	0.9	0.7	0.7	0.5
Psme1	P97371	101	-1	-9	-44	-16	1.0	0.9	0.7	0.9
Kpna6	O35345	467	-41	-28	-44	-5	0.7	0.8	0.7	1.0
Arhgap30	Q640N3	968	37	-45	-44	-60	1.6	0.7	0.7	0.6
Flna	Q8BTM8	1157	54	-56	-44	-60	2.2	0.6	0.7	0.6
Pacsin2	Q9WVE8	38	-40	-15	-44	-8	0.7	0.9	0.7	0.9
Nup155	Q99P88	874	-50	-42	-44	-44	0.7	0.7	0.7	0.7
Farsb	Q9WUA2	151	55	-52	-44	-55	2.2	0.7	0.7	0.6
Ctdspl2	Q8BG15	380	49	-86	-44	-75	1.9	0.5	0.7	0.6
Mcm4	P49717	325	-60	-14	-45	-28	0.6	0.9	0.7	0.8
Ankrd44	B2RXR6	420	-17	-21	-45	-81	0.9	0.8	0.7	0.6
Tmem201	A2A8U2	164	-8	-38	-45	-43	0.9	0.7	0.7	0.7
Nf1	Q04690	124	-18	-49	-45	-23	0.9	0.7	0.7	0.8
Acat1	Q8QZT1	193	34	-81	-45	-128	1.5	0.6	0.7	0.4
Suz12	Q80U70	203	-3	-13	-45	-37	1.0	0.9	0.7	0.7
Rab8b	P61028	123	-14	-28	-45	-9	0.9	0.8	0.7	0.9
Nvl	Q9DBY8	784	-28	-39	-45	-30	0.8	0.7	0.7	0.8
Prpf8	Q99PV0	435	52	-66	-45	-40	2.1	0.6	0.7	0.7
Tmem209	Q8BRG8	422	-32	-24	-46	-31	0.8	0.8	0.7	0.8
Zfp456	B2RUK9	198	-50	-55	-46	-66	0.7	0.6	0.7	0.6
Zfp429	Q7M6Y0	211	-50	-55	-46	-66	0.7	0.6	0.7	0.6
Casp1	P29452	329	35	-29	-46	-31	1.5	0.8	0.7	0.8
Eml4	Q3UMY5	527	1	-38	-46	-35	1.0	0.7	0.7	0.7
Mpp7	G5E8S8	59	43	-51	-46	-58	1.8	0.7	0.7	0.6
Cltc	Q68FD5	617	49	-61	-47	-36	1.9	0.6	0.7	0.7
Naxd	Q9CZ42	78	-49	-20	-47	4	0.7	0.8	0.7	1.0

Oat	P29758	93	-40	-33	-47	-31	0.7	0.8	0.7	0.8
Arid4a	F8VPQ2	839	-40	-33	-47	-70	0.7	0.8	0.7	0.6
Cdc37l1	Q9CZP7	189	-35	-40	-47	-58	0.7	0.7	0.7	0.6
Rnaseh2c	Q9CQ18	34	57	-43	-47	-44	2.3	0.7	0.7	0.7
Sept1	P42209	136	36	-66	-47	-38	1.6	0.6	0.7	0.7
Ctcf	Q61164	557	50	-73	-47	-28	2.0	0.6	0.7	0.8
Pcbp1	P60335	201	-30	-36	-48	-43	0.8	0.7	0.7	0.7
Asns	Q61024	158	-14	-14	-48	-160	0.9	0.9	0.7	0.4
Plin3	Q9DBG5	60	-19	-17	-48	-269	0.8	0.9	0.7	0.3
Ahdc1	Q6PAL7	146	-38	-33	-48	-45	0.7	0.8	0.7	0.7
Sumf1	Q8R0F3	50	-36	-37	-48	-22	0.7	0.7	0.7	0.8
Bfsp2	Q6NVD9	395	42	-43	-48	-5	1.7	0.7	0.7	1.0
Aifm1	Q9Z0X1	255	-48	-46	-48	-40	0.7	0.7	0.7	0.7
Samhd1	Q60710	533	31	-47	-48	-50	1.4	0.7	0.7	0.7
Nsun5	Q8K4F6	41	-50	-12	-49	-38	0.7	0.9	0.7	0.7
Acap1	Q8K2H4	657	-26	-26	-49	-46	0.8	0.8	0.7	0.7
Ints10	Q8K2A7	686	-12	-28	-49	-15	0.9	0.8	0.7	0.9
Zbtb14	Q08376	215	-21	-33	-49	-47	0.8	0.8	0.7	0.7
Snx27	Q3UHD6	517	-19	-42	-49	-43	0.8	0.7	0.7	0.7
Ski	B1AUF1	649	-6	-62	-49	-45	0.9	0.6	0.7	0.7
Irf2bp2	E9Q1P8	65	53	-71	-49	-69	2.1	0.6	0.7	0.6
Rps6kb2	Q9Z1M4	108	-16	-16	-49	-122	0.9	0.9	0.7	0.5
Hdgf	P51859	108	-11	-31	-49	-110	0.9	0.8	0.7	0.5
Fam208b	Q5DTT3	617	-45	-34	-49	-22	0.7	0.7	0.7	0.8
Rabggta	Q9JHK4	342	23	-41	-49	-57	1.3	0.7	0.7	0.6
Gmpr	Q9DCZ1	87	-25	-22	-50	-41	0.8	0.8	0.7	0.7
Rnf213	E9Q555	1592	47	-47	-50	-46	1.9	0.7	0.7	0.7
Zcchc7	B1AX39	273	45	-63	-50	-73	1.8	0.6	0.7	0.6
Ercc2	O08811	190	-52	-71	-50	-12	0.7	0.6	0.7	0.9
Ppp6r1	Q7TSI3	612	-18	-28	-51	5	0.8	0.8	0.7	1.1
Add1	Q9QYC0	253	-57	-36	-51	-43	0.6	0.7	0.7	0.7
Taf6	Q62311	141	45	-64	-51	-53	1.8	0.6	0.7	0.7
Asb3	Q9WV72	328	-3	-22	-51	-49	1.0	0.8	0.7	0.7
Pln	P61014	46	-22	-46	-51	-12	0.8	0.7	0.7	0.9
Pln	P61014	41	-22	-46	-51	-12	0.8	0.7	0.7	0.9
Rps26	P62855	77	42	-55	-51	-54	1.7	0.6	0.7	0.6
Zmym2	Q9CU65	683	65	-70	-52	-54	2.9	0.6	0.7	0.7
Plcl2	Q8K394	60	-15	-22	-52	-26	0.9	0.8	0.7	0.8
Cpsf3l	Q9CWS4	542	-24	-37	-52	-49	0.8	0.7	0.7	0.7
Hemgn	Q9ERZ0	134	-44	-40	-52	-45	0.7	0.7	0.7	0.7
Rasa2	P58069	552	-37	-15	-53	-81	0.7	0.9	0.7	0.6
Phospho1	Q8R2H9	168	-25	-25	-53	-42	0.8	0.8	0.7	0.7
Ikbkap	Q7TT37	1307	-22	-27	-53	-91	0.8	0.8	0.7	0.5
Pdcd11	Q6NS46	1344	-43	-38	-53	-24	0.7	0.7	0.7	0.8
Ripk3	Q9QZL0	360	56	-67	-53	-72	2.3	0.6	0.7	0.6
Fkbp5	Q64378	175	40	-75	-53	-68	1.7	0.6	0.7	0.6
Trim34a	Q99PP6	30	-50	-17	-54	-33	0.7	0.9	0.7	0.8
Esd	Q9R0P3	176	-41	-39	-54	-3	0.7	0.7	0.7	1.0
Rrp1b	Q91YK2	466	43	-50	-54	-35	1.7	0.7	0.7	0.7
Usp19	Q3UJD6	378	60	-80	-54	-73	2.5	0.6	0.7	0.6
Dnmt1	P13864	426	48	-84	-54	-51	1.9	0.5	0.6	0.7

Mfn1	Q811U4	498	33	-57	-55	-34	1.5	0.6	0.6	0.7
Mapk11	Q9WUI1	119	-33	-12	-56	-16	0.8	0.9	0.6	0.9
Arap1	Q4LDD4	1359	-25	-32	-56	-20	0.8	0.8	0.6	0.8
Insrr	Q9WTL4	317	-39	-51	-56	-27	0.7	0.7	0.6	0.8
Gbp7	Q91Z40	233	-36	-33	-56	1	0.7	0.8	0.6	1.0
Nfkbib	Q60778	144	30	-51	-56	-55	1.4	0.7	0.6	0.6
Cd37	Q61470	217	-26	-57	-56	-34	0.8	0.6	0.6	0.7
Thada	A8C756	387	30	-49	-57	-57	1.4	0.7	0.6	0.6
Oas3	Q8VI93	740	51	-63	-57	-66	2.0	0.6	0.6	0.6
Arhgap15	Q811M1	225	-26	-51	-57	-35	0.8	0.7	0.6	0.7
Ddx39a	Q8VDW0	86	-71	-6	-58	-25	0.6	0.9	0.6	0.8
Ddx39b	Q9Z1N5	87	-71	-6	-58	-25	0.6	0.9	0.6	0.8
Ranbp3	Q9CT10	175	-38	-25	-58	-68	0.7	0.8	0.6	0.6
Plekhm1	Q7TSI1	536	-18	-34	-58	-66	0.9	0.7	0.6	0.6
Trrap	A0A1D5RLL4	3075	55	-67	-59	-61	2.2	0.6	0.6	0.6
Rreb1	Q3UH06	1424	56	-83	-59	-125	2.2	0.5	0.6	0.4
Lemd3	D3YU56	713	76	-159	-59	-179	4.2	0.4	0.6	0.4
Neurl4	Q5NCX5	59	-12	-18	-59	-87	0.9	0.9	0.6	0.5
Apobec3	Q99J72	53	-34	-26	-59	-58	0.7	0.8	0.6	0.6
Rps26	P62855	74	49	-51	-60	-64	2.0	0.7	0.6	0.6
Dnm1l	Q8K1M6	476	54	-84	-60	-80	2.2	0.5	0.6	0.6
Aco2	Q99KI0	126	-41	-33	-61	-24	0.7	0.8	0.6	0.8
Znf592	Q8BHZ4	589	37	-74	-62	-76	1.6	0.6	0.6	0.6
Baz1a	O88379	232	33	-114	-62	-98	1.5	0.5	0.6	0.5
Ado	Q6PDY2	120	-23	-21	-62	-70	0.8	0.8	0.6	0.6
Cpox	P36552	362	39	-56	-62	-27	1.6	0.6	0.6	0.8
Iars2	Q8BIJ6	155	60	-89	-62	-53	2.5	0.5	0.6	0.7
Med19	Q8C1S0	163	59	-98	-62	-100	2.4	0.5	0.6	0.5
Ppm1b	P36993	71	-38	-24	-64	-67	0.7	0.8	0.6	0.6
Nckap1l	Q8K1X4	338	8	-48	-64	-61	1.1	0.7	0.6	0.6
Usp45	Q8K387	762	-21	-12	-65	-20	0.8	0.9	0.6	0.8
Cript	O70333	73	-50	-26	-65	-54	0.7	0.8	0.6	0.7
Anp32e	P97822	123	-32	-40	-66	1	0.8	0.7	0.6	1.0
Smurf2	A2A5Z6	716	42	-75	-66	-67	1.7	0.6	0.6	0.6
Nedd4l	E9PXB7	943	42	-75	-66	-67	1.7	0.6	0.6	0.6
Nedd4	P46935	854	42	-75	-66	-67	1.7	0.6	0.6	0.6
Zmym2	Q9CU65	662	54	-75	-67	-76	2.2	0.6	0.6	0.6
Alad	P10518	203	-54	-50	-67	-21	0.7	0.7	0.6	0.8
Mybbp1a	Q7TPV4	676	-53	-35	-68	-22	0.7	0.7	0.6	0.8
Znf574	Q8BY46	397	27	-85	-68	-107	1.4	0.5	0.6	0.5
Arl1	P61211	80	-28	-40	-68	-51	0.8	0.7	0.6	0.7
Gtf2h4	O70422	17	55	-81	-68	-72	2.2	0.6	0.6	0.6
Pex6	Q99LC9	501	-56	-26	-71	10	0.6	0.8	0.6	1.1
Pex6	Q99LC9	497	-56	-26	-71	10	0.6	0.8	0.6	1.1
Hspa4l	P48722	540	37	-87	-71	-93	1.6	0.5	0.6	0.5
Gmpr2	Q99L27	224	41	-58	-72	-93	1.7	0.6	0.6	0.5
Gps1	Q99LD4	155	33	-88	-73	-78	1.5	0.5	0.6	0.6
Uimc1	Q5U5Q9	27	-16	-15	-74	-41	0.9	0.9	0.6	0.7
Gm15800	E9Q2E4	1507	-18	-22	-74	-70	0.8	0.8	0.6	0.6
Thoc6	Q5U4D9	35	50	-47	-76	-88	2.0	0.7	0.6	0.5
Phpt1	Q9DAK9	39	55	-155	-81	-150	2.2	0.4	0.6	0.4

Cnn2	Q08093	204	47	-166	-89	-145	1.9	0.4	0.5	0.4
Brix1	Q9DCA5	158	-6	-14	-89	-12	0.9	0.9	0.5	0.9
Chd4	Q6PDQ2	1830	-60	-61	-90	-77	0.6	0.6	0.5	0.6
Gmds	Q8K0C9	92	65	-127	-90	-145	2.8	0.4	0.5	0.4
Mcm4	P49717	330	-38	-26	-92	-53	0.7	0.8	0.5	0.7
Flna	Q8BTM8	2378	54	-164	-94	-128	2.2	0.4	0.5	0.4
Flywch1	Q8CI03	292	25	-89	-102	-114	1.3	0.5	0.5	0.5
Lrrc14	Q8VC16	109	64	-160	-103	-177	2.8	0.4	0.5	0.4
Nfkb2	Q9WTK5	738	48	-155	-103	-195	1.9	0.4	0.5	0.3
Pmpcb	Q9CXT8	265	23	-208	-117	-303	1.3	0.3	0.5	0.2
Uncharact	Q9D735	162	60	-190	-118	-167	2.5	0.3	0.5	0.4
Psme2	P97372	91	-16	-33	-119	-352	0.9	0.8	0.5	0.2
Lpxn	Q99N69	358	58	-207	-123	-196	2.4	0.3	0.4	0.3
Hectd3	Q3U487	112	-4	-39	-130	7	1.0	0.7	0.4	1.1
Kdm5c	P41230	990	48	-55	-146	9	1.9	0.6	0.4	1.1
Fem1b	Q9Z2G0	186	38	-141	-153	-170	1.6	0.4	0.4	0.4
Scrib	Q80U72	1113	45	-239	-154	-384	1.8	0.3	0.4	0.2
Cltc	Q68FD5	870	60	-325	-158	-484	2.5	0.2	0.4	0.2
Tex264	E9Q137	68	19	-222	-175	-234	1.2	0.3	0.4	0.3
Srsf7	Q8BL97	148	9	-351	-189	-357	1.1	0.2	0.3	0.2
Hnrnpa3	Q8BG05	196	41	-368	-222	-473	1.7	0.2	0.3	0.2
Psme1	P97371	106	1	-26	-228	-370	1.0	0.8	0.3	0.2
Rbbp7	Q60973	97	43	-320	-235	-492	1.8	0.2	0.3	0.2
