# Supplementary material for: Selective inhibitors of JAK1 targeting an isoform-restricted allosteric cysteine
Source: Nat Chem Biol. Author manuscript; Available in PMC 2023 Jul 18. (PMC7614775; doi:10.1038/s41589-022-01098-0)
Supplement: Source data [file EMS178742-supplement-Source_data.pdf]

Source Data

Selective inhibitors of JAK1 targeting a subtype-restricted allosteric cysteine

Madeline E. Kavanagh[1,2], Benjamin D. Horning[1,3], Roli Khattri[3], Nilotpal Roy[3], Justine P. Lu[3], Landon R. Whitby[3], Jaclyn C. Brannon[3], Albert Parker[3], Joel M. Chick[3], Christie L. Eissler[3], Ashley Wong[3], Joe L. Rodriguez[3], Socorro Rodiles[3], Kim Masuda[2], John R. Teijaro[4], Gabriel M. Simon[3], Matthew P. Patricelli[3*], Benjamin F. Cravatt[2*]

[1]These authors contributed equally, [2]Department of Chemistry, Scripps Research, La Jolla, CA 92037, USA; [3]Vividion Therapeutics, 5820 Nancy Ridge Drive, San Diego, CA 92121, USA; [4]Department of Immunology and Microbial Science, The Scripps Research Institute, La Jolla, CA, 92307, USA.

*Correspondence – cravatt@scripps.edu; mattp@vividion.com

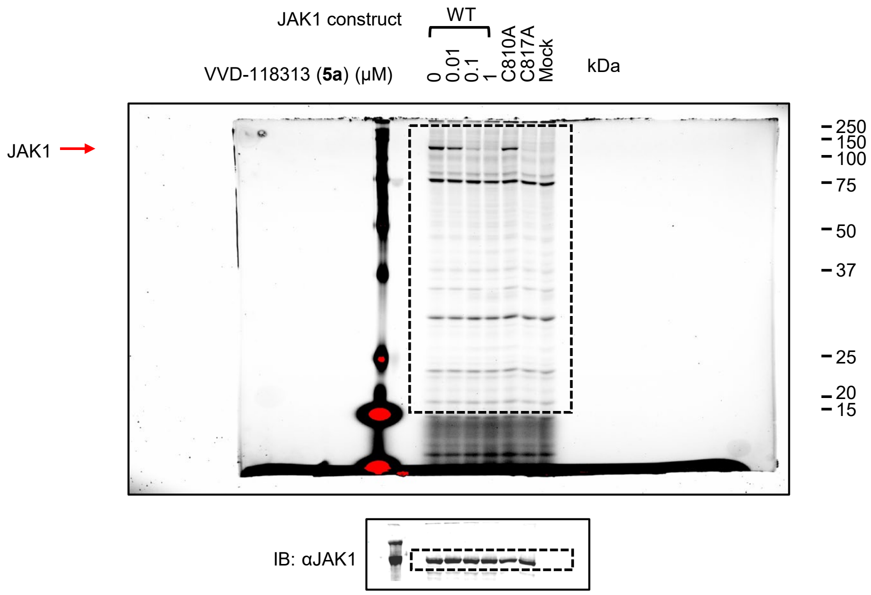

Source Data for Figure 2f. *Right*, gel-ABPP experiment showing labeling of recombinant WT-JAK1 and C810A-JAK1, but not C817A-JAK1, expressed in 22Rv1 cells with alkyne probe **6** (0.1 μM, 2 h, *in situ*). The labeling of WT-JAK1 is blocked by pretreatment with VVD-118313 (**5a**) (0.01-1 μM, 2 h, *in situ*). *Below,* western blot showing JAK1 expression in gel-ABPP experiment. Data are from a single experiment, representative of > two independent experiments.

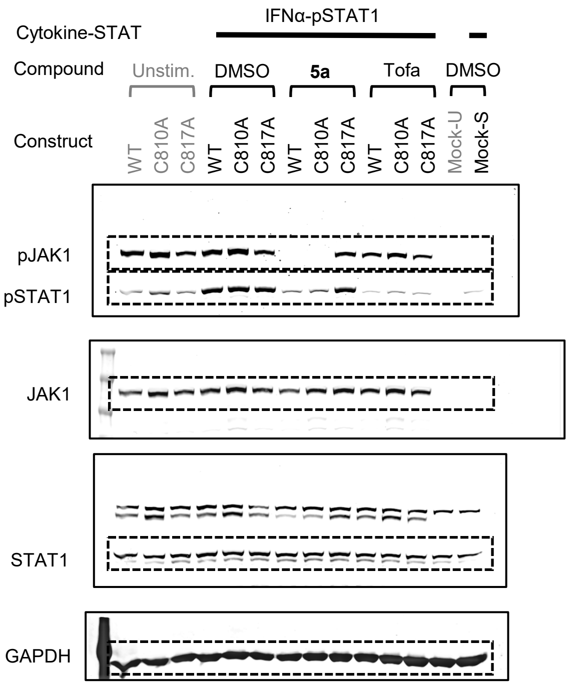

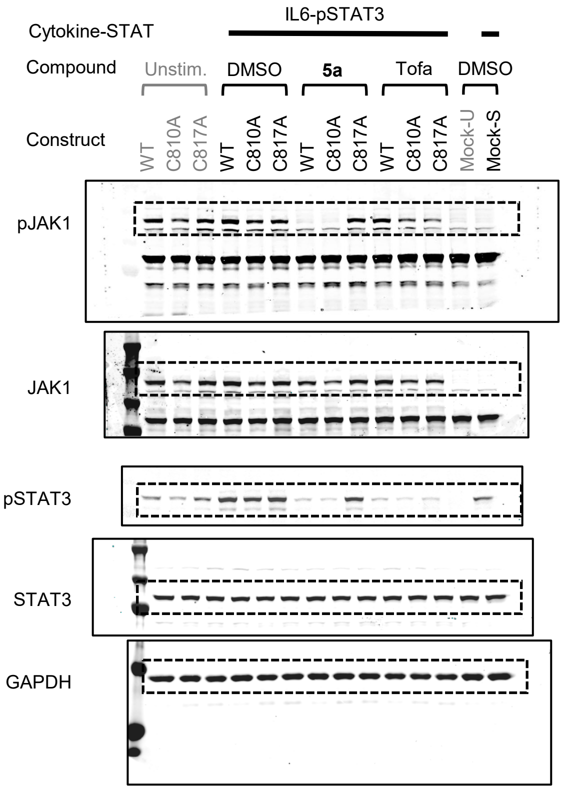

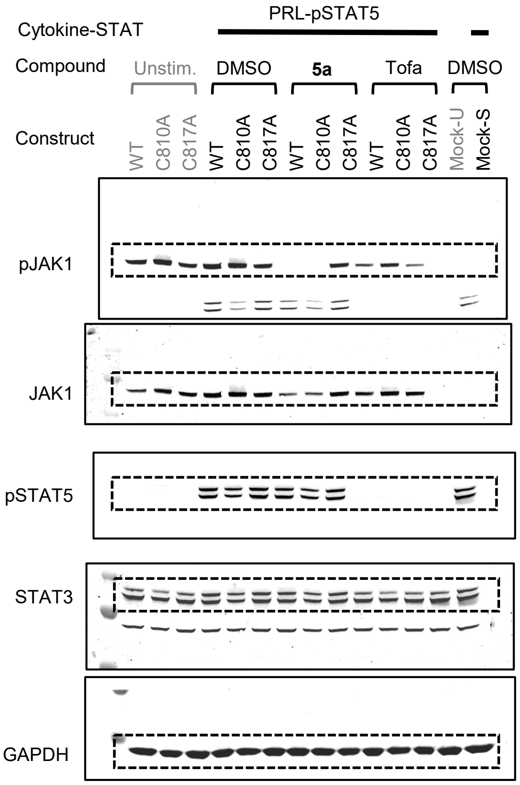

Source Data for Figure 3b, Western blots showing effects of VVD-118313 (**5a**) and the pan-JAK inhibitor tofacitinib (Tofa) on JAK1 phosphorylation (pJAK1) and IFNα-stimulated STAT1 (JAK1-dependent), IL-6-stimulated STAT3 (JAK1-dependent), and prolactin (PRL)-stimulated STAT5 (JAK2-dependent) phosphorylation in 22Rv1 cells expressing WT-, C810A-, or C817A-JAK1. Cells were treated with compounds (2 μM) for 2 h and then stimulated with IFNα (100 ng/mL, 30 min), IL-6 (50 ng/mL, 30 min) or PRL (15 ng/mL, 15 min) prior to analysis.

f

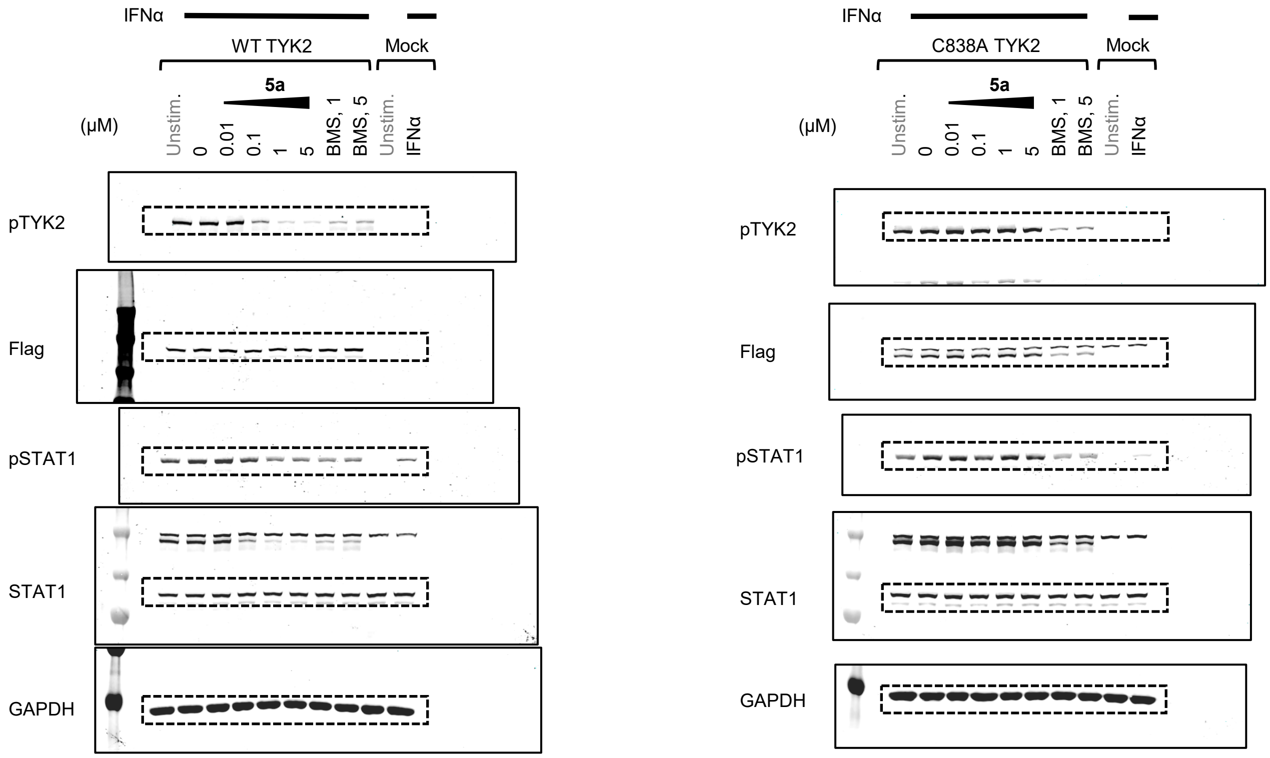

Source Data for Figure 3f, Concentration-dependent effects of VVD-118313 (**5a**; 0.01 – 5 μM, 2 h) and BMS- 986165 (BMS, 1 or 5 μM, 2 h) on TYK2 phosphorylation (pTYK2) and IFNα-stimulated STAT1 phosphorylation in 22Rv1 cells expressing recombinant WT-TYK2 or a C838A-TYK2 mutant.

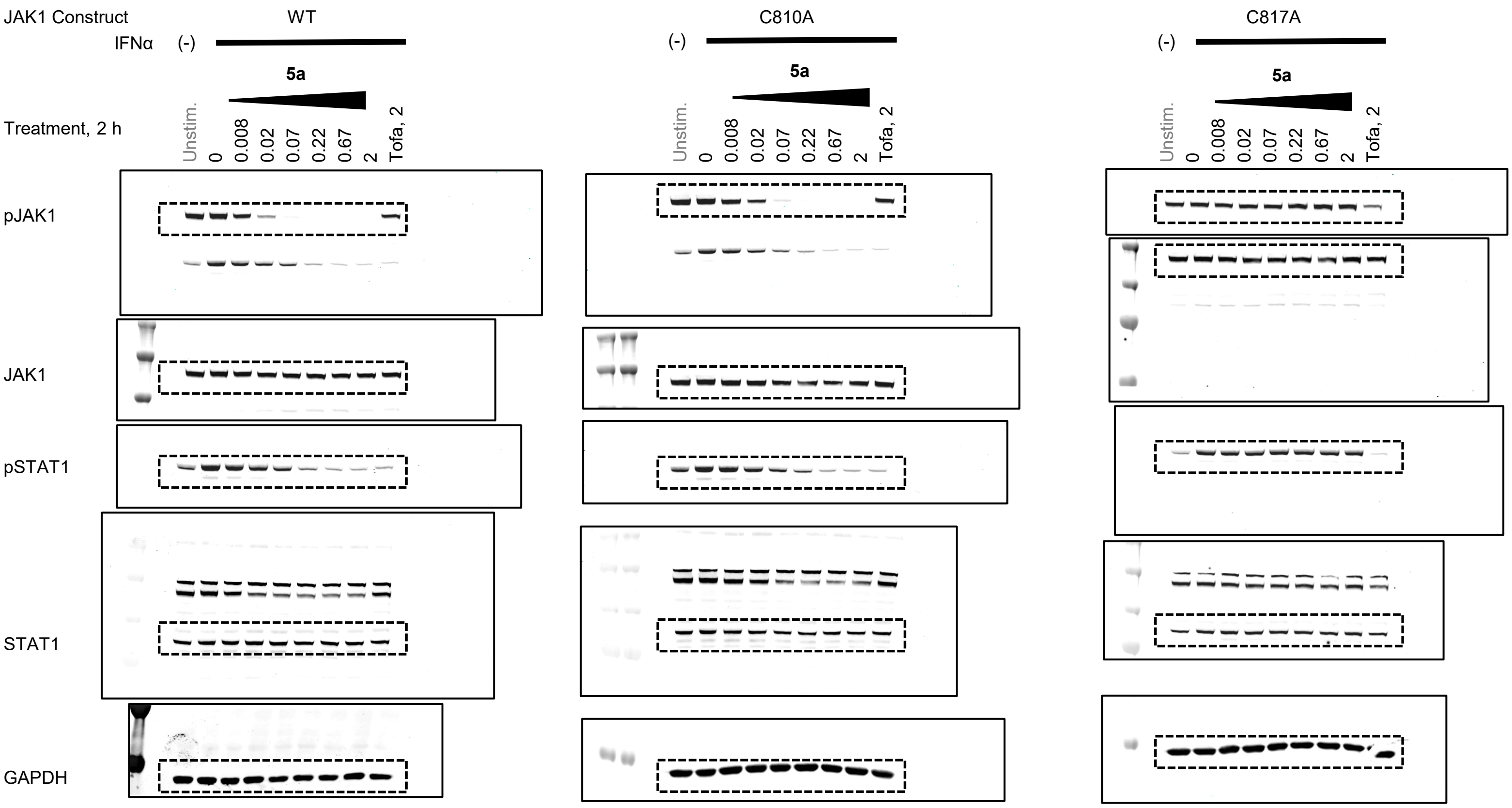

Source for Data Extended Data Fig. 3b Representative western blots showing concentration-dependent effects of VVD-118313 (**5a**) on IFNα-stimulated STAT1 phosphorylation in 22Rv1 cells expressing WT-, C810A-, or C817A-JAK1 variants.

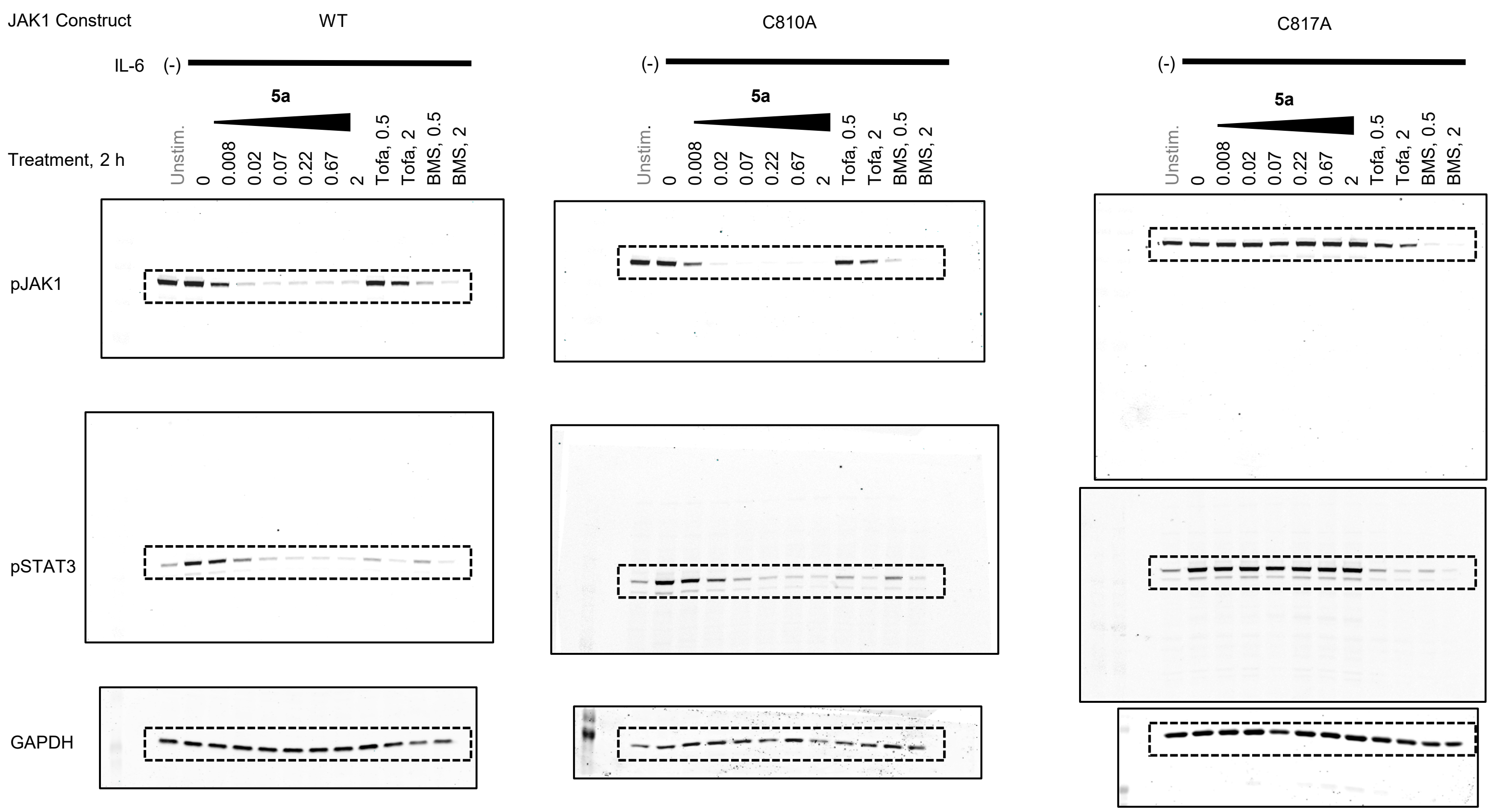

Source Data for Extended Data Fig. 3c Representative western blots showing concentration-dependent effects of VVD-118313 (**5a**) on IL-6-stimulated STAT3 phosphorylation in 22Rv1 cells expressing WT-, C810A-, or C817A-JAK1 variants.

a

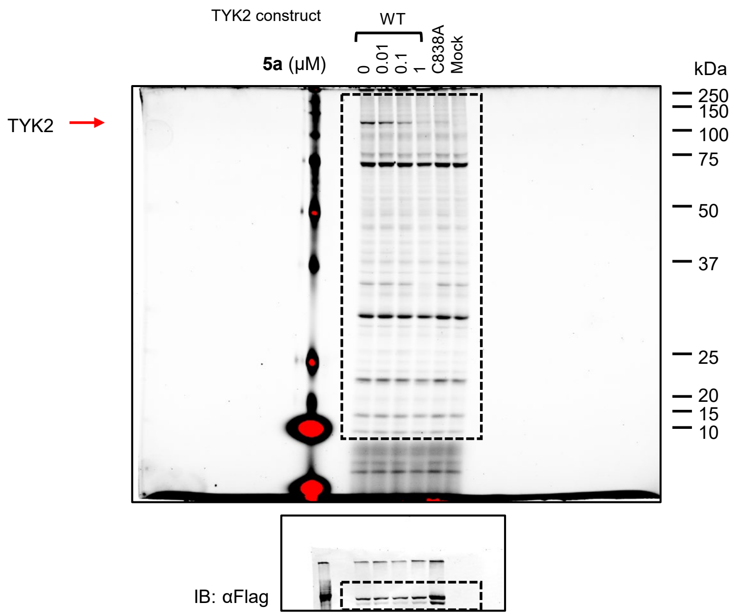

Source Data for Extended Data Fig. 4a, gel-ABPP experiment showing labeling of recombinant WT-TYK2, but not C838A-TYK2, expressed in 22Rv1 cells with alkyne probe **6** (0.1 μM, 2 h*, in situ*). The labeling of WT-TYK2 is blocked by pretreatment with VVD-118313 (**5a**) (0.01-1 μM, 2 h, *in situ*). We noted that the C838A-TYK2 mutant consistently expressed at higher levels than WT-TYK2, as revealed by the anti-TYK2 immunoblot (bottom). Data are from a single experiment representative of two independent experiments.

c

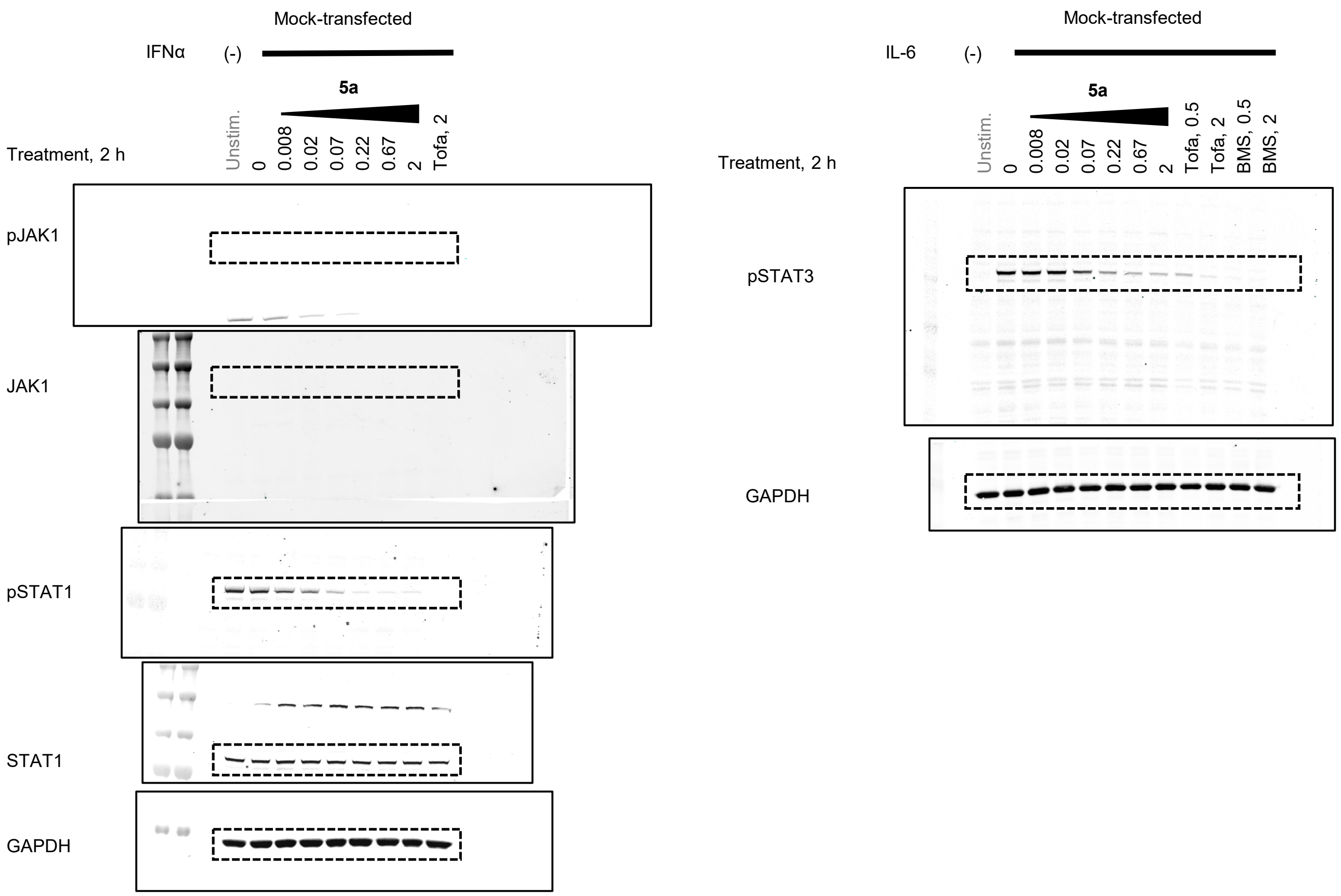

Source Data for Extended Data Fig. 4c, Western blots of the effect of VVD-118313 (**5a**), tofacitinib (Tofa), or BMS-986165 (BMS) on IFNα-stimulated STAT1 and IL-6-stimulated STAT3 phosphorylation in mock-transfected 22Rv1 cells, which lack JAK1

e

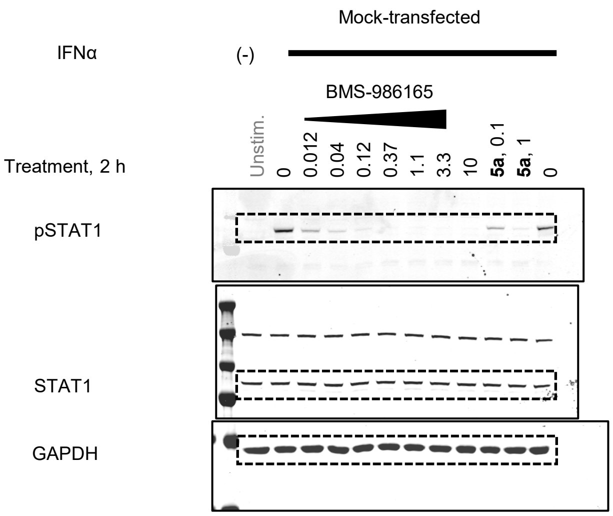

Source Data for Extended Data Fig. 4e, Western blots of the effect of VVD-118313 (**5a**) or BMS-986165 (BMS) on IFNα-stimulated STAT1 phosphorylation in mock-transfected 22Rv1 cells, which lack JAK1.

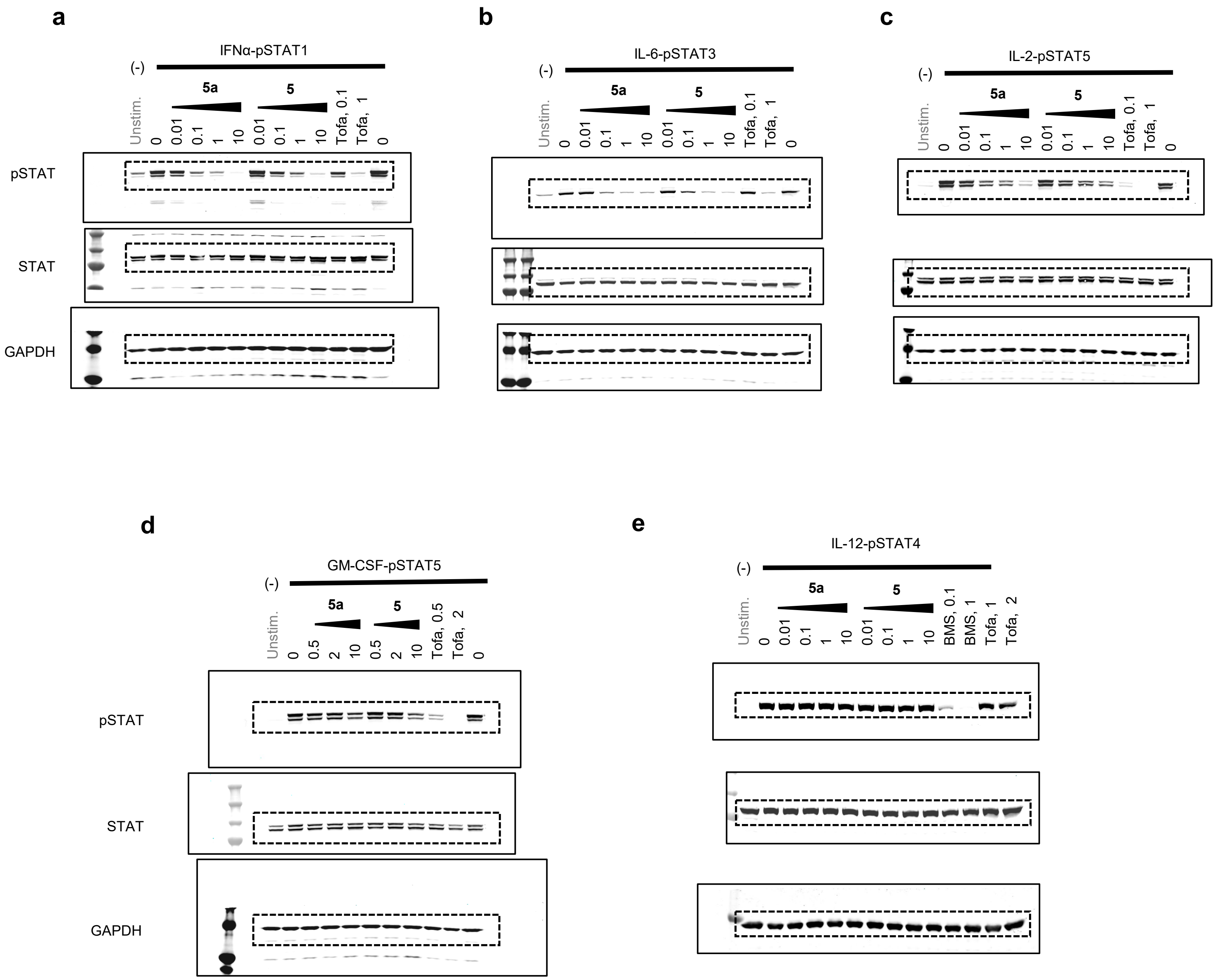

Source Data for Figure 4a-e, Effects of VVD-118313 (**5a**), racemate **5**, and tofacitinib (Tofa) on JAK-STAT signaling pathways in human PBMCs. PBMCs or PBMC-derived T-blast, were treated with compounds at the indicated concentrations for 2 h prior to stimulation with IFNα (**a**; 100 ng/mL, 30 min), IL-6 (**b**; 25 ng/mL, 30 min), IL-2 (**c**; 20 U/mL, 15 min), GM-CSF (**d**; 0.5 mg/mL, 15 min), or IL-12 **(e**; 12.5 ng/mL, 15 min)**.**

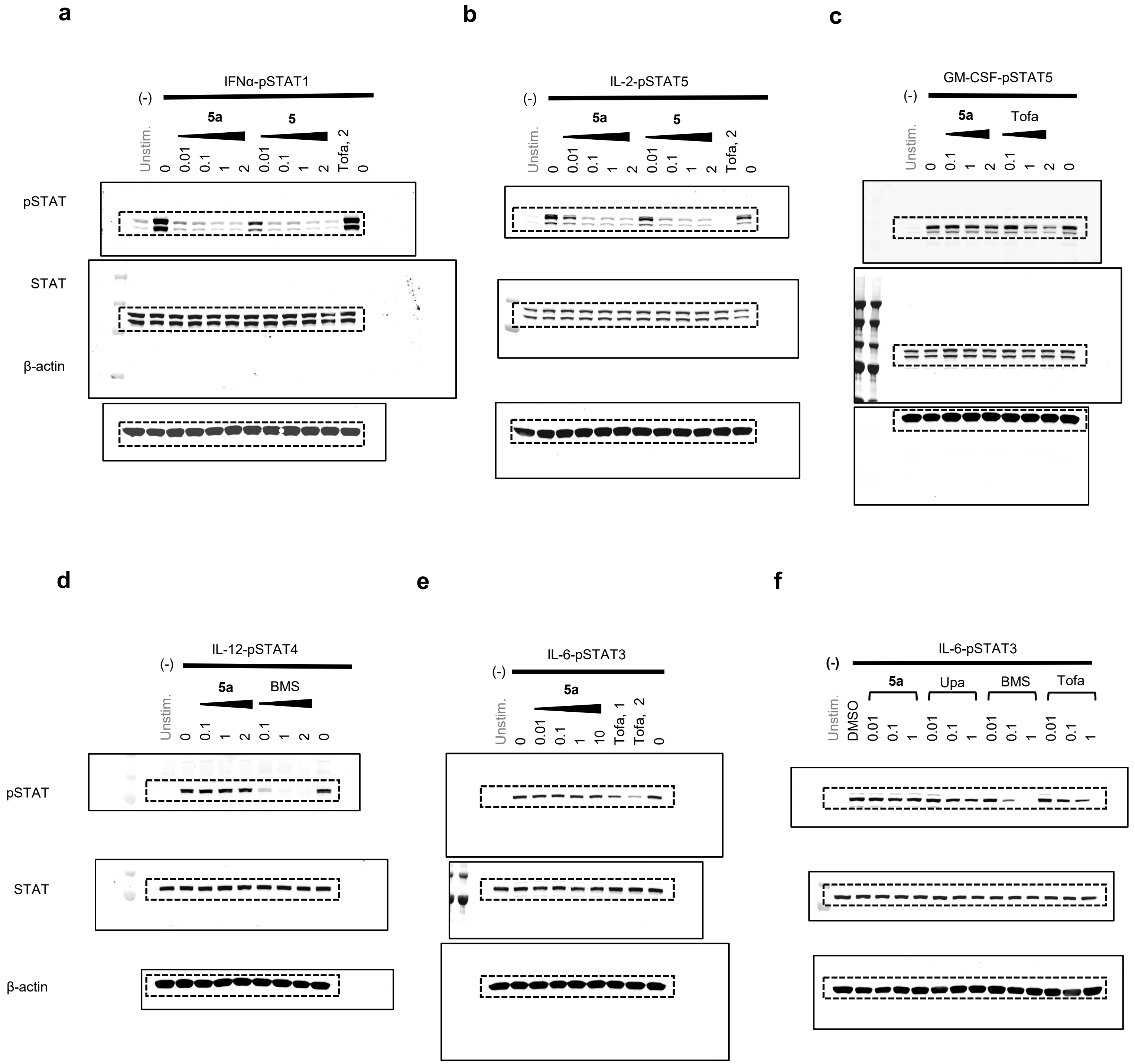

Extended Data for Fig. 6a-e, Western blots showing concentration-dependent effects of VVD-118313 (**5a**) and/or compound **5** on IFNα-stimulated STAT1 phosphorylation (**a**), IL-2-stimulated STAT5 phosphorylation (**b**), GM-CSF-stimulated STAT5 phosphorylation (**c**), IL-12-stimulated STAT4 phosphorylation (**d**), and IL-6-stimulated STAT3 phosphorylation (**e**) in mouse splenocytes. Tofacitinib (Tofa) and BMS-986165 were also tested where indicated. Splenocytes were treated with compounds at indicated concentration for 2 hours prior to stimulation with IFNα (100 ng/mL, 30 min), IL-6 (10 ng/mL, 30 min), IL-2 (20 U/mL, 15 min), GM-CSF (10 mg/mL, 15 min) or IL-12 (12.5 ng/mL, 15 min). **f**, Western blot showing effects of a panel of JAK inhibitors on IL-6-stimulated STAT3 phosphorylation in mouse splenocytes.

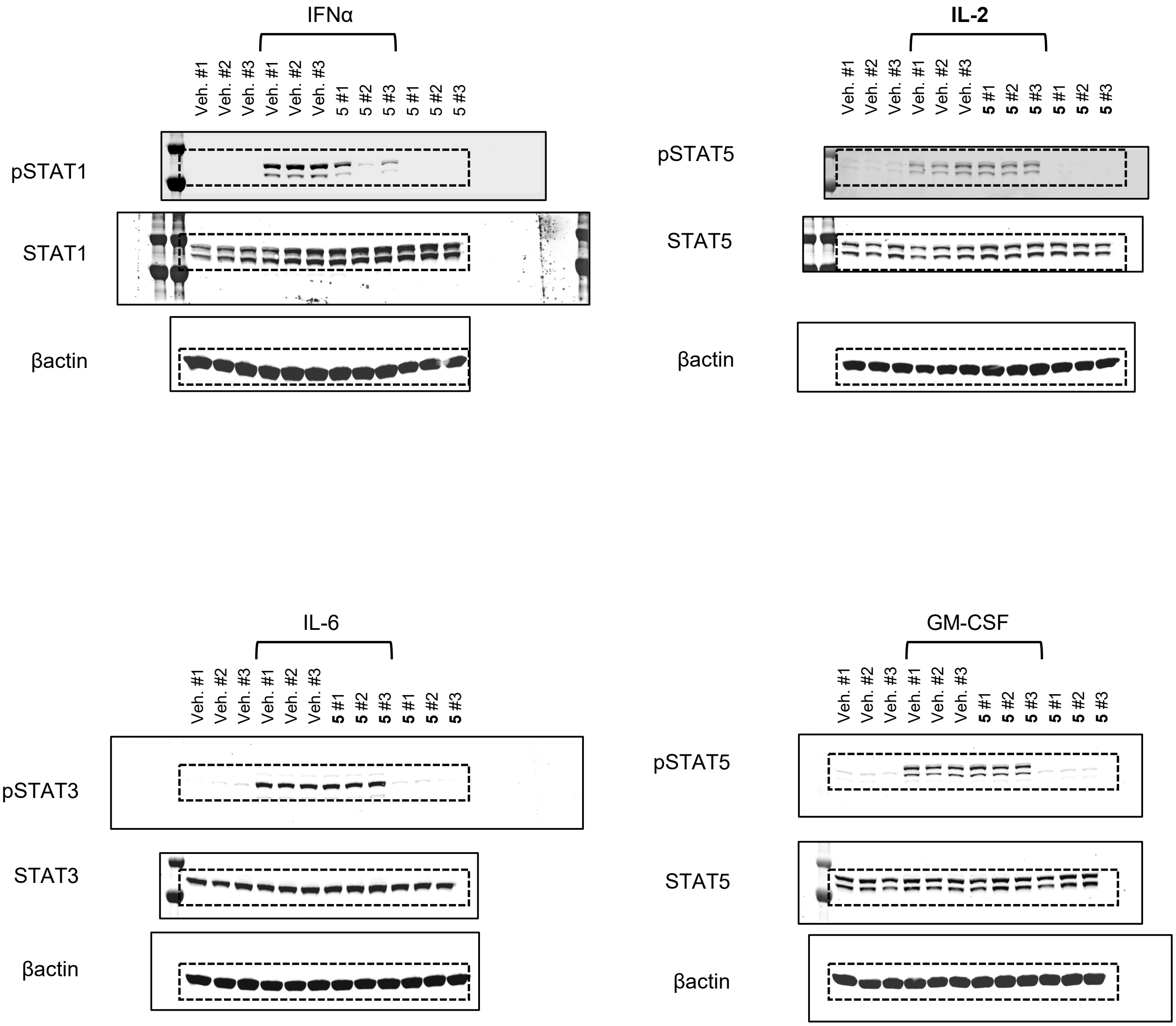

Source Data for Extended Data Fig. 7. Western blots containing the results quantified in **Fig. 4i**, which represents *ex vivo* cytokine-stimulated STAT phosphorylation in splenocytes from mice treated with vehicle or compound **5** (25 mg/kg, 2 x 4 h). Splenocytes were stimulated with IFNα (1000 U/mL, 30 min), IL-2 (20 U/mL, 15 min), IL-6 (10 ng/mL, 30 min) or GM-CSF (10 ng/mL) prior to analysis of indicated STAT phosphorylation signals. #1-3 correspond to three individual mice in each treatment groups.

b

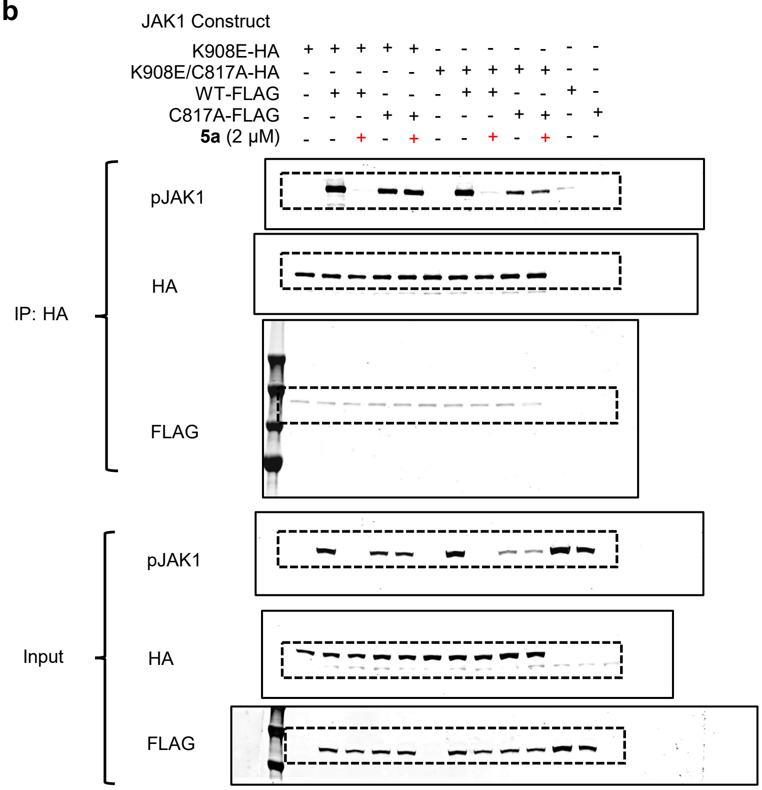

Source Data for Figure 5b, Western blots measuring JAK1 phosphorylation (pJAK1) from anti-HA immunoprecipitations (IPs) of HA-tagged kinase dead (K908E) JAK1 (WT or C817A mutant) expressed in 22Rv1 cells alongside catalytically active FLAG-tagged JAK1 (WT or C817A mutant).

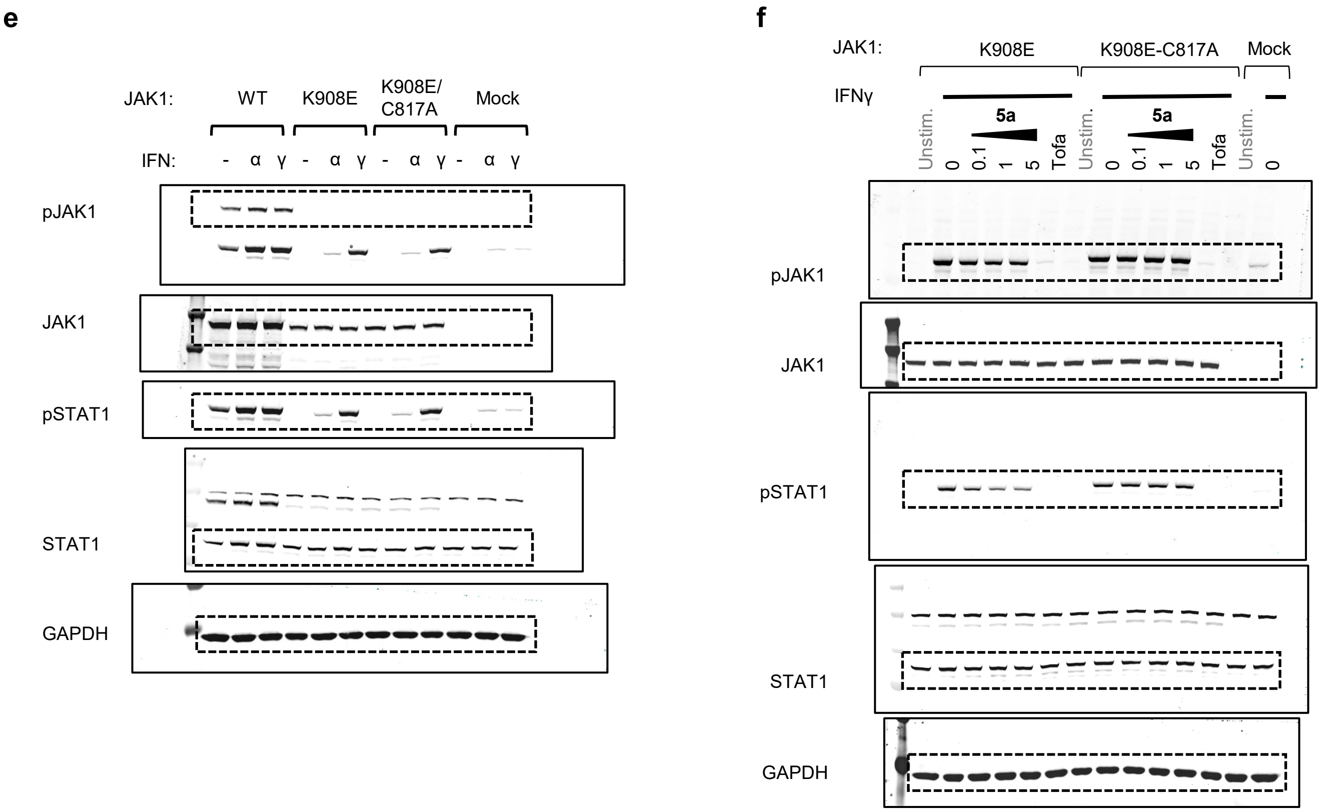

Source Data for Figure 5e and f. e, Western blots showing that both K908E- and K908E/C817A-JAK1 mutants support IFNγ-stimulated (50 ng/mL, 30 min), but not IFNα-stimulated (100 ng/mL, 30 min) STAT1 phosphorylation (pSTAT1) in 22Rv1 cells. WT-JAK1 supports both cytokine pathways. **f**, Western blots showing the effects of VVD-118313 (**5a**; 0.1-5 μM, 2 h) and tofacitinib (Tofa; 1 μM, 2 h) on IFNγ-dependent STAT1 phosphorylation (pSTAT1) in 22Rv1 cells expressing K908E-JAK1-HA or K908E/C817A-JAK1-HA.

a

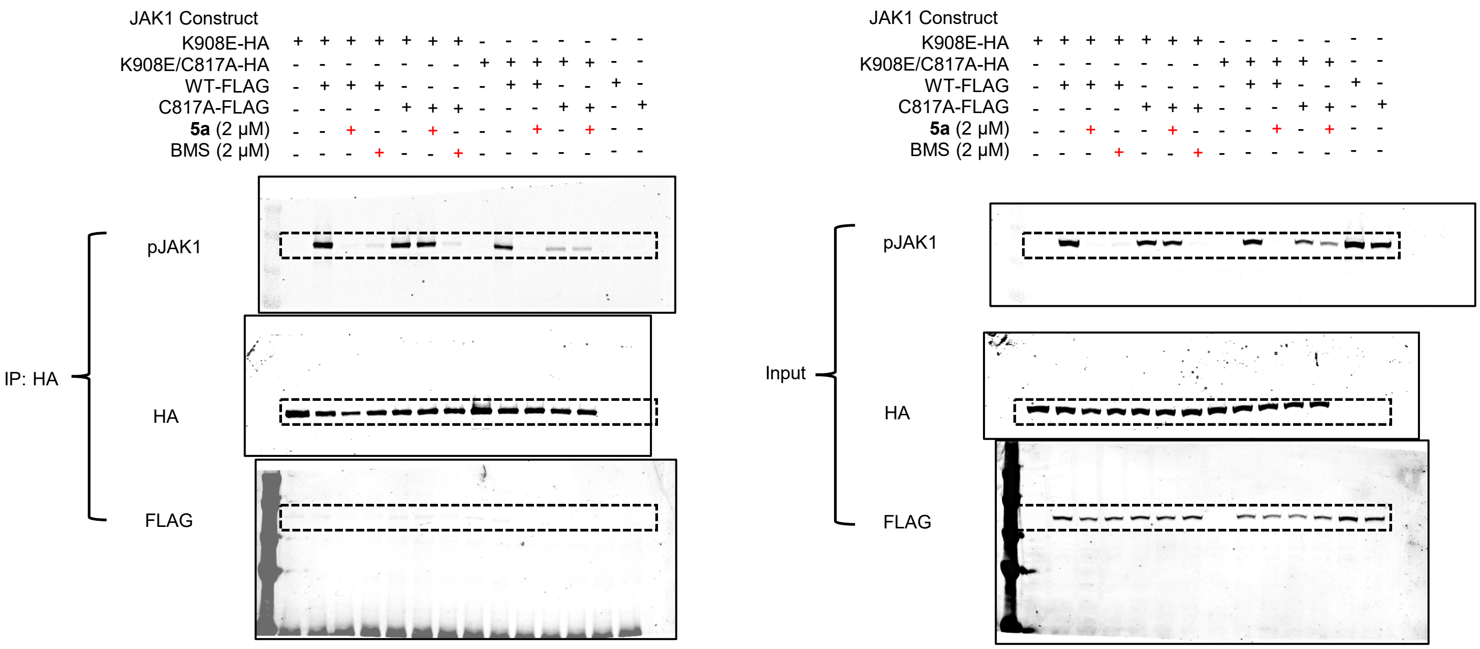

Source Data for Extended Data Figure 8a. Western blots measuring effects of VVD-118313 (**5a**) and BMS-986165 (BMS) (2 µM, 2 h) on JAK1 phosphorylation (pJAK1) from anti-HA immunoprecipitations (IPs) of HA-tagged kinase dead (K908E) JAK1 (WT or C817A mutant) expressed in 22Rv1 cells alongside catalytically active FLAG-tagged JAK1 (WT or C817A mutant).

b

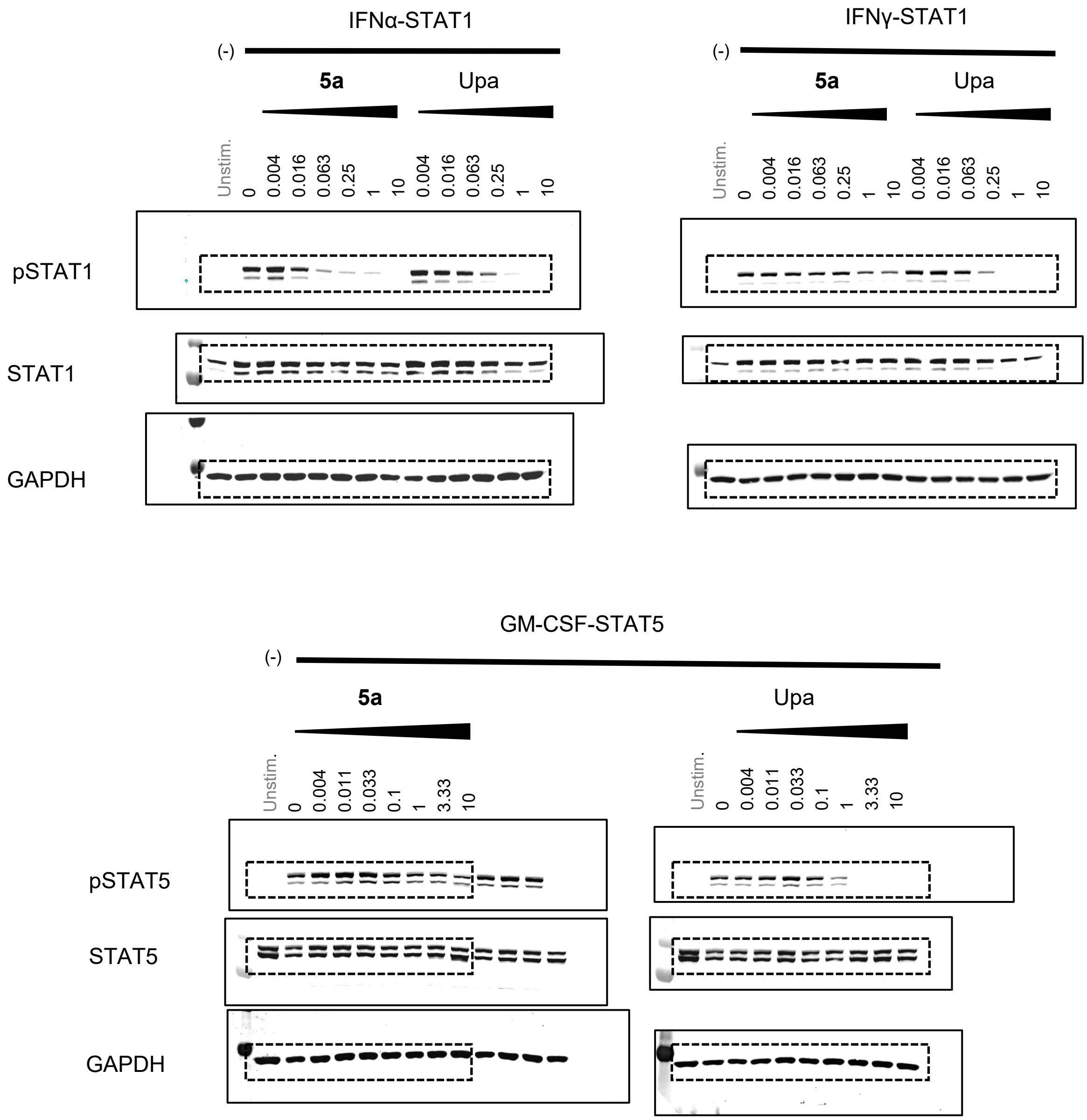

Source Data for Extended Data Figure 8b, Western blots related to **Fig. 5g** showing concentration-dependent effects of VVD-118313 (**5a**) and upadacitinib (Upa) in the indicated cytokine (IFNα, IFNγ or GM-CSF)-stimulated STAT (STAT1/5) phosphorylation pathways.

c

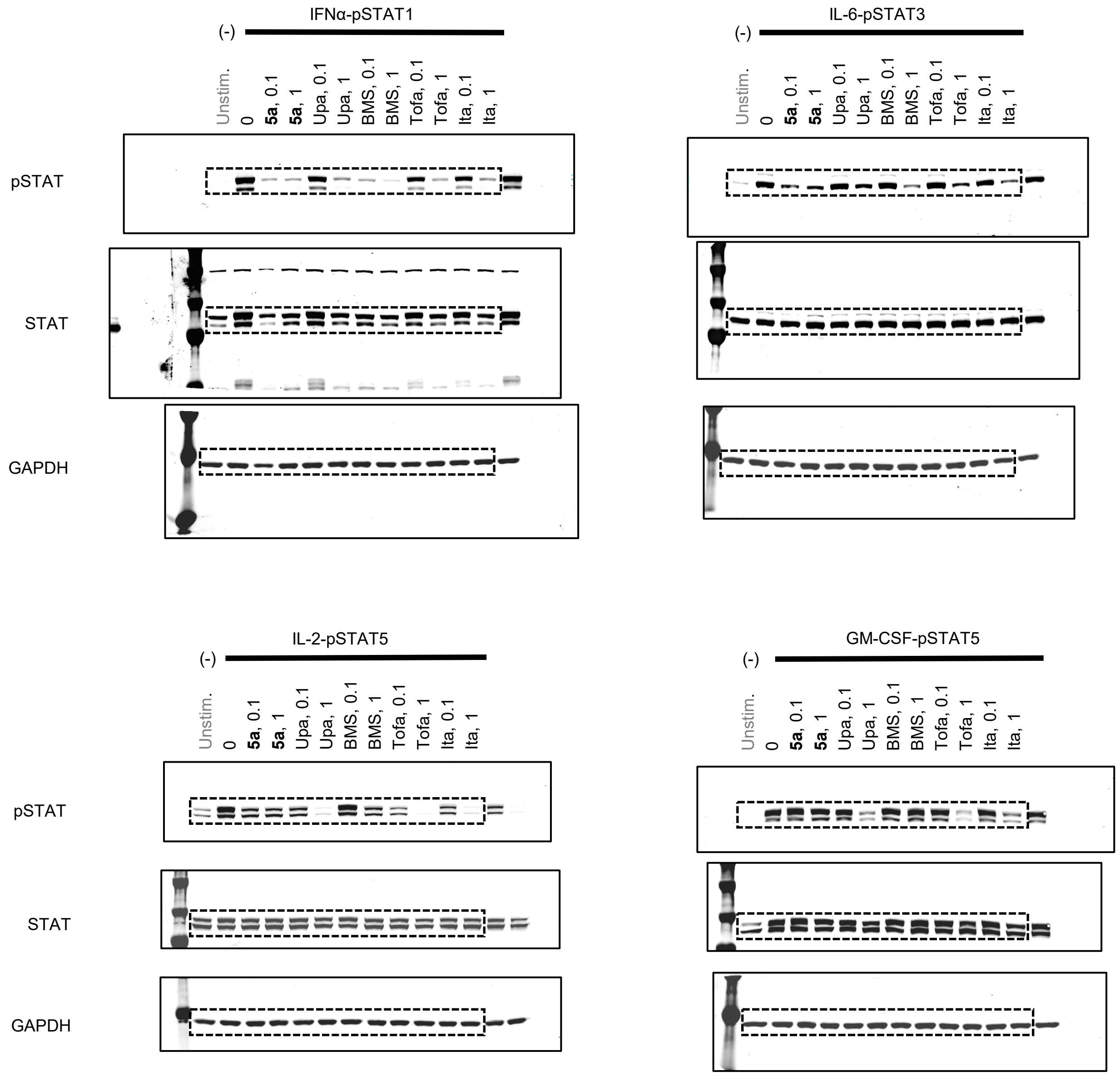

Extended Data for Figure 8c, Western blots related to **Fig. 5i** showing effects of the indicated JAK inhibitors on the indicated cytokine-STAT phosphorylation pathways. Human PBMCs were treated with the compounds – VVD-118313 (5a), upadactinib (Upa), BMS-986165 (BMS), tofacitinib (Tofa. and itacitinib (Ita) – at the indicated concentrations (µM) for 2 h and then stimulated with IFNα (100 ng/mL, 309 min), IL-6 (25 ng/mL, 30 min), IL-2 (20 U/mL< 15 min) or GM-CSF (0.5 ng/mL, 15 minutes).
