# Supplementary material for: Selective inhibitors of JAK1 targeting an isoform-restricted allosteric cysteine
Source: Nat Chem Biol. Author manuscript; Available in PMC 2023 Jul 18. (PMC7614775; doi:10.1038/s41589-022-01098-0)

checkCIF/PLATON report

Structure factors have been supplied for datablock(s) lib285-3-001

THIS REPORT IS FOR GUIDANCE ONLY. IF USED AS PART OF A REVIEW PROCEDURE FOR PUBLICATION, IT SHOULD NOT REPLACE THE EXPERTISE OF AN EXPERIENCED CRYSTALLOGRAPHIC REFEREE.

No syntax errors found. CIF dictionary Interpreting this report

Datablock: lib285-3-001

Bond precision:	C-C = 0.0086 A		Wavelength=1.54184
Cell:	a=9.8233(6)	b=7.3199(3)	c=10.3323(6)
	alpha=90	beta=117.889(8)	gamma=90
Temperature:	150 K		

	Calculated	Reported
Volume	656.66(8)	656.66(8)
Space group	P 21	P 1 21 1
Hall group	P 2yb	P 2yb
Moiety formula	C11 H14 Cl2 N, Cl	Cl, C11 H14 Cl2 N
Sum formula	C11 H14 Cl3 N	C11 H14 Cl3 N
Mr	266.58	266.58
Dx,g cm-3	1.348	1.348
Z	2	2
Mu (mm-1)	6.058	6.058
F000	276.0	276.0
F000'	278.65	
h,k,lmax	11,8,12	11,8,12
Nref	2316[1258]	2285
Tmin,Tmax	0.573,0.546	0.857,1.000
Tmin'	0.520	

Correction method= # Reported T Limits: Tmin=0.857 Tmax=1.000
AbsCorr = MULTI-SCAN

Data completeness= 1.82/0.99 Theta(max)= 66.566

R(reflections)= 0.0410(2241) wR2(reflections)= 0.1040(2285)

S = 1.099 Npar= 136

```
The following ALERTS were generated. Each ALERT has the format
       test-name_ALERT_alert-type_alert-level.
Click on the hyperlinks for more details of the test.
```

Alert level C

```
PLAT340_ALERT_3_C Low Bond Precision on  C-C Bonds ...............    0.00864 Ang.
PLAT911_ALERT_3_C Missing FCF Refl Between Thmin & STh/L=    0.595          5 Report
```

Alert level G

```
PLAT007_ALERT_5_G Number of Unrefined Donor-H Atoms .............          2 Report
PLAT012_ALERT_1_G No       _shelx_res_checksum Found in CIF ......     Please Check
PLAT042_ALERT_1_G Calc. and Reported Moiety Formula Strings Differ     Please Check
PLAT791_ALERT_4_G Model has Chirality at C4          (Sohnke SpGr)          S Verify
PLAT909_ALERT_3_G Percentage of I>2sig(I) Data at Theta(Max) Still        95% Note
PLAT933_ALERT_2_G Number of HKL-OMIT Records in Embedded .res File         10 Note
PLAT978_ALERT_2_G Number C-C Bonds with Positive Residual Density.          0 Info
```

```
   0 ALERT level A = Most likely a serious problem - resolve or explain
   0 ALERT level B = A potentially serious problem, consider carefully
   2 ALERT level C = Check. Ensure it is not caused by an omission or oversight
   7 ALERT level G = General information/check it is not something unexpected

   2 ALERT type 1 CIF construction/syntax error, inconsistent or missing data
   2 ALERT type 2 Indicator that the structure model may be wrong or deficient
   3 ALERT type 3 Indicator that the structure quality may be low
   1 ALERT type 4 Improvement, methodology, query or suggestion
   1 ALERT type 5 Informative message, check
```

checkCIF publication errors

Alert level A

```
PUBL002_ALERT_1_A  The contact author's address is missing,
            _publ_contact_author_address.
PUBL005_ALERT_1_A  _publ_contact_author_email, _publ_contact_author_fax and
            _publ_contact_author_phone are all missing.
            At least one of these should be present.
PUBL006_ALERT_1_A  _publ_requested_journal is missing
            e.g. 'Acta Crystallographica Section C'
PUBL008_ALERT_1_A  _publ_section_title is missing. Title of paper.
PUBL009_ALERT_1_A  _publ_author_name is missing. List of author(s) name(s).
PUBL010_ALERT_1_A  _publ_author_address is missing. Author(s) address(es).
PUBL012_ALERT_1_A  _publ_section_abstract is missing.
            Abstract of paper in English.
```

```
   7 ALERT level A = Data missing that is essential or data in wrong format
   0 ALERT level G = General alerts. Data that may be required is missing
```

Publication of your CIF

You should attempt to resolve as many as possible of the alerts in all categories. Often the minor alerts point to easily fixed oversights, errors and omissions in your CIF or refinement strategy, so attention to these fine details can be worthwhile. In order to resolve some of the more serious problems it may be necessary to carry out additional measurements or structure refinements. However, the nature of your study may justify the reported deviations from journal submission requirements and the more serious of these should be commented upon in the discussion or experimental section of a paper or in the "special_details" fields of the CIF. *checkCIF* was carefully designed to identify outliers and unusual parameters, but every test has its limitations and alerts that are not important in a particular case may appear. Conversely, the absence of alerts does not guarantee there are no aspects of the results needing attention. It is up to the individual to critically assess their own results and, if necessary, seek expert advice.

If level A alerts remain, which you believe to be justified deviations, and you intend to submit this CIF for publication in a journal, you should additionally insert an explanation in your CIF using the Validation Reply Form (VRF) below. This will allow your explanation to be considered as part of the review process.

Validation response form

Please find below a validation response form (VRF) that can be filled in and pasted into your CIF.

```
# start Validation Reply Form
_vrf_PUBL002_GLOBAL
;
PROBLEM: The contact author's address is missing,
RESPONSE: ...
;
_vrf_PUBL005_GLOBAL
;
PROBLEM: _publ_contact_author_email, _publ_contact_author_fax and
RESPONSE: ...
;
_vrf_PUBL006_GLOBAL
;
PROBLEM: _publ_requested_journal is missing
RESPONSE: ...
;
_vrf_PUBL008_GLOBAL
;
PROBLEM: _publ_section_title is missing. Title of paper.
RESPONSE: ...
;
_vrf_PUBL009_GLOBAL
;
PROBLEM: _publ_author_name is missing. List of author(s) name(s).
RESPONSE: ...
;
_vrf_PUBL010_GLOBAL
;
PROBLEM: _publ_author_address is missing. Author(s) address(es).
```

```
RESPONSE: ...
;
_vrf_PUBL012_GLOBAL
;
PROBLEM: _publ_section_abstract is missing.
RESPONSE: ...
;
# end Validation Reply Form
```

If you wish to submit your CIF for publication in Acta Crystallographica Section C or E, you should upload your CIF via the web. If you wish to submit your CIF for publication in IUCrData you should upload your CIF via the web. If your CIF is to form part of a submission to another IUCr journal, you will be asked, either during electronic submission or by the Co-editor handling your paper, to upload your CIF via our web site.

PLATON version of 19/02/2022; check.def file version of 19/01/2022

Datablock lib285-3-001 - ellipsoid plot

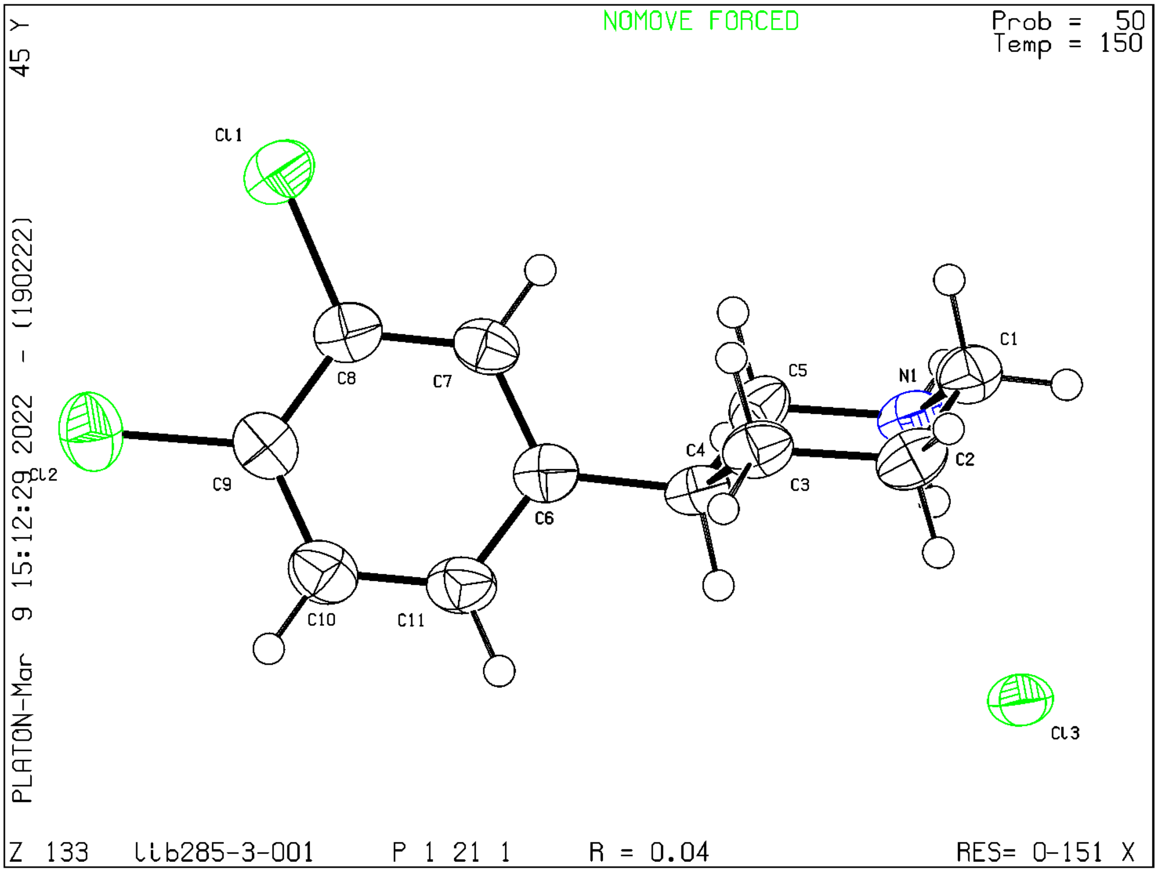

Supplement: Supplementary Data 2 [file EMS178742-supplement-Supplementary_Data_2.pdf]
